# Cancer progression models and fitness landscapes: a many-to-many relationship

**DOI:** 10.1093/bioinformatics/btx663

**Published:** 2017-10-18

**Authors:** Ramon Diaz-Uriarte

**Affiliations:** Department of Biochemistry, Universidad Autónoma de Madrid, Instituto de Investigaciones Biomédicas “Alberto Sols” (UAM-CSIC), Madrid, Spain

## Abstract

**Motivation:**

The identification of constraints, due to gene interactions, in the order of accumulation of mutations during cancer progression can allow us to single out therapeutic targets. Cancer progression models (CPMs) use genotype frequency data from cross-sectional samples to identify these constraints, and return Directed Acyclic Graphs (DAGs) of restrictions where arrows indicate dependencies or constraints. On the other hand, fitness landscapes, which map genotypes to fitness, contain all possible paths of tumor progression. Thus, we expect a correspondence between DAGs from CPMs and the fitness landscapes where evolution happened. But many fitness landscapes—e.g. those with reciprocal sign epistasis—cannot be represented by CPMs.

**Results:**

Using simulated data under 500 fitness landscapes, I show that CPMs’ performance (prediction of genotypes that can exist) degrades with reciprocal sign epistasis. There is large variability in the DAGs inferred from each landscape, which is also affected by mutation rate, detection regime and fitness landscape features, in ways that depend on CPM method. Using three cancer datasets, I show that these problems strongly affect the analysis of empirical data: fitness landscapes that are widely different from each other produce data similar to the empirically observed ones and lead to DAGs that infer very different restrictions. Because reciprocal sign epistasis can be common in cancer, these results question the use and interpretation of CPMs.

**Availability and implementation:**

Code available from Supplementary Material.

**Supplementary information:**

Supplementary data are available at *Bioinformatics* online.

## 1 Introduction

**Fig. 1.**
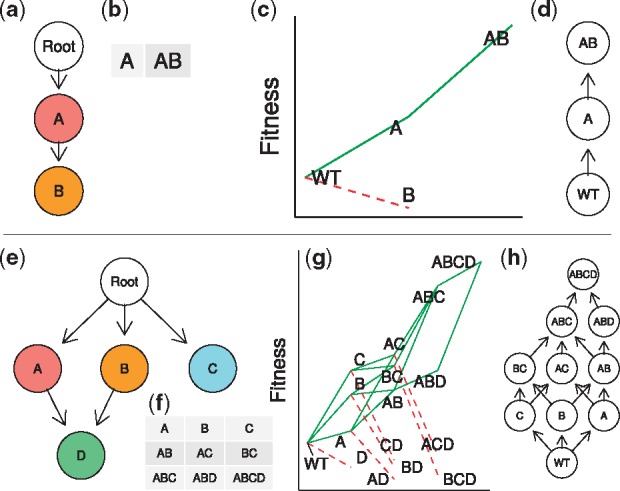
DAGs of restrictions from CPMs and representable fitness landscapes. (**a**) A DAG of restrictions, as obtained from a CPM, such as CBN or CAPRI, showing restrictions in the order of accumulation of mutations. This DAG shows genes—not genotypes—; an arrow (directed edge) from gene *i* to gene *j* indicates a direct dependency of a mutation in *j* on a mutation on *i*; a mutation in *j* cannot be observed unless *i* is mutated. Here a mutation in gene B can only be observed if A is mutated. (Conversely, the absence of an arrow between two genes indicates a lack of direct dependencies between the two genes; however, there could be indirect dependencies as in F → G → H where mutating H requires having F mutated because of H’s dependency on G which itself depends on F). Genes whose parent is Root do not depend on mutations in other genes. (**b**) Genotypes that fulfil the restrictions encoded in (**a**): these are the genotypes with mutations that can exist according to the DAG in (a), where the notation “A” means a genotype with gene A (but not B) mutated and “AB” a genotype with both genes A and B mutated. (**c**) A fitness landscape that can be represented by the DAG in (**a**) (fitness landscape representation based on Brouillet *et al.*, 2015); genotypes as in (**b**) (and WT denotes the initial “wild type” genotype—where we absorb all cancer initiation events in this genotype as explained in “Assumptions”). Green solid segments connect genotypes along accessible mutational paths and red dotted segments denote decreases in fitness. Genotypes along accessible paths will be called “accessible genotypes”; genotypes reachable through green segments are accessible. DAGs of restrictions are different from fitness graphs and graphs of mutational paths (Crona *et al.*, 2013; de Visser and Krug, 2014; Franke *et al.*, 2011; Greene *et al.*, 2014). In (**d**) the fitness graph for the accessible genotypes of fitness landscape (c) is shown. In fitness graphs nodes correspond to genotypes—not genes—and arrows point toward mutational neighbors of higher fitness. Thus, graph (**d**) also shows all the accessible mutational paths and adaptive walks that start from the WT genotype in fitness landscape (**c**). DAGs of restrictions and fitness graphs differ in what is represented in the nodes (genes versus genotypes) and in the meaning of the edges (dependencies of mutations versus possible adaptive transitions between genotypes). Note that the accessible genotypes in (**c**) are the same as the genotypes in (b). (**e–h**) As in (a–d) but for a fitness landscape with four genes. Genotypes “AB”, “AC”, “BC” and “ABC” fulfill the restrictions of DAG (**e**) and, thus, can exist because mutations in genes A, B, and C can happen independently of each other as there are no arrows connecting them in (**e**). This is also reflected in fitness graph (**h**): e.g., genotype “AB” can arise from a clone with genoytpe “A” or a clone with genotype “B”. In the graph of restrictions (**e**), nodes with multiple parents are given a logical AND—conjunction—interpretation: a mutation in gene D requires both genes A and B to be mutated; genotypes “ABD” and” “ABCD” can exist under DAG (**e**) because the restrictions (D can only appear if both A and B are mutated) are satisfied in those genotypes (and note that there are no additional restrictions relating genes C and D). Thus a mutation in gene D can only appear in genotypes “AB” or “ABC” leading to genotypes “ABC” and “ABCD”, as shown in (**h**). Therefore, a fitness landscapes is representable by a DAG of restrictions if the accessible genotypes in the fitness landscape are those genotypes that are predicted to exist under the DAG of restrictions

Epistatic interactions between genetic alterations constraint the order of accumulation of mutations during cancer progression (e.g. in colorectal cancer APC mutations precede KRAS mutations—Fearon and Vogelstein, 1990). Finding these constraints can single out therapeutic targets and disease markers and has lead to the development of cancer progression models (CPMs—Beerenwinkel *et al.*, 2015), such as CBN (Gerstung *et al.*, 2009, 2011) or CAPRI (Caravagna *et al.*, 2016; Ramazzotti *et al.*, 2015), that try to identify these constraints using genotype frequency data from cross-sectional samples. CPMs return directed acyclic graphs (DAGs) of restrictions where arrows between genes indicate direct dependencies or constraints in the order of accumulation of mutations (Fig. 1). Under the CPM model, only genotypes that fulfil the restrictions encoded by the arrows in the DAG can exist (Beerenwinkel *et al.*, 2006; Gerstung and Beerenwinkel, 2010).

Whereas DAGs of restrictions from CPMs do not contain information about the fitness of individual genotypes, fitness landscapes (or genotype-fitness maps) associate to each genotype its fitness value (de Visser and Krug, 2014). Thus, similarly to DAGs of restrictions, a fitness landscape, if we assume that populations will only move uphill in fitness, specifies what genotypes can be observed (Crona *et al.*, 2013; Franke *et al.*, 2011). Cross-sectional samples taken during tumor progression should contain only genotypes that are part of accessible mutational paths that start from the initial wild-type genotype (accessible mutational path: a trajectory through a collection of genotypes, where each genotype is separated from the preceding genotype by one mutation, along which fitness increases monotonically—Franke *et al.*, 2011). If we had detailed knowledge about the fitness landscape, we could predict the possible paths of tumor progression and identify genes that would block those paths (Greaves, 2015; Lipinski *et al.*, 2016). However, obtaining a complete picture of the cancer fitness landscape is not currently possible (Lipinski *et al.*, 2016; Sprouffske *et al.*, 2012). Here, CPMs can offer a feasible alternative: a model that identifies the key constraints in the order of accumulation of mutations might be enough to capture the possible tumor progression paths (Beerenwinkel *et al.*, 2016) and the genotypes that can and cannot exist.

But then, we should expect a close correspondence between DAGs of restrictions inferred by CPMs and the fitness landscapes where tumor evolution took place. DAGs of restrictions should provide accurate predictions about what genotypes can and cannot exist during tumor progression, and the same landscape should not lead to inferring widely different DAGs. Are these expectations justified? Do they hold with empirical data? And what are the consequences of using CPMs when these expectations do not hold?

With some fitness landscapes, that correspondence might hold (if sample sizes are sufficiently large to allow the estimation of the true DAG). For example, we will say that the fitness landscapes in Figure 1c and g are representable by the gene DAGs of restrictions from CPMs in Figure 1a and e, respectively: the DAGs and the landscapes make the same predictions about what genotypes we should observe. The landscapes are representable because the DAGs of restrictions capture the epistatic interactions that determine what genotypes are accessible. That a fitness landscape be representable by a DAG of restrictions depends only on the dependencies implied by the DAG—which determine the genotypes that can exist according to the DAG—and the genotypes that are accessible under the fitness landscape; it does not depend on the evolutionary dynamics as affected by population size and mutation rates—but see also Section 4. In particular, the constraints reflected in the DAGs of restrictions imply sign epistasis (Crona *et al.*, 2013; Weinreich *et al.*, 2005; see also Misra *et al.*, 2014), an interaction between genes where a mutation is beneficial or deleterious (i.e. can have different sign) depending on the genetic background or what other genes are mutated (this is the basis of the phenomenon of ‘oncogene addiction’; Sprouffske *et al.*, 2012). Figure 1a says that a mutation in B increases the fitness of a cell if A is already mutated but is detrimental otherwise; Figure 1e says that a mutation in D increases fitness if A and B are mutated but is detrimental otherwise.

For other fitness landscapes, however, the correspondence cannot hold. Although DAGs of restrictions represent sign epistasis, they cannot represent reciprocal sign epistasis, a genetic interaction where two mutations that individually increase fitness reduce it when combined (Crona *et al.*, 2013; Poelwijk *et al.*, 2007, 2011). CPMs assume that acquiring a mutation in one gene, say A in Figure 1e, does not decrease the probability of acquiring a mutation in another gene, say C (Misra *et al.*, 2014). The DAGs of restrictions can only say what mutations need to be present before another mutation is viable. Thus, neither the DAG in Figure 1e, nor any other DAG of restrictions, could represent the fitness landscape that would result if reciprocal sign epistasis between, say, genes A and C turned genotype AC into a low fitness or non-viable genotype. (See “Representable landscapes with reciprocal sign epistasis?” in Supplementary Material for apparent exceptions).

This is a potentially serious limitation of CPMs because reciprocal sign epistasis is probably common in cancer (Chiotti *et al.*, 2014), given the extent of synthetic lethality both in the human genome (Blomen *et al.*, 2015) and in cancer cells (Beijersbergen *et al.*, 2017; Leung *et al.*, 2016; synthetic lethality is an epistatic interaction where the combination of two mutations is lethal when each individual mutation is not; synthetic lethality between mutations that individually increase fitness constitutes reciprocal sign epistasis). Moreover, reciprocal sign epistasis is a key structural feature of fitness landscapes: it can lead to multiple peaks and affects ruggedness and predictability of the evolutionary process (Crona *et al.*, 2013; de Visser and Krug, 2014; Ferretti *et al.*, 2016; Poelwijk *et al.*, 2007), which itself affects our opportunities to block tumor progression (Greaves, 2015; Lipinski *et al.*, 2016). But the assessment of CPMs has used data simulated from generative models that are encoded by DAGs of restrictions (Gerstung *et al.*, 2009; Hainke *et al.*, 2012; Ramazzotti *et al.*, 2015; Szabo and Boucher, 2008), therefore assuming very restricted fitness landscapes. Two exceptions, none of which considered reciprocal sign epistasis explicitly, are Sprouffske *et al.* (2011), who conducted simulations using agent-based models with parameters tuned for colorectal cancer, and Diaz-Uriarte (2015), where the restrictions encoded in DAGs were embedded within evolutionary models that allowed to relax some of the constraints of the DAGs. Using fitness landscapes, as done in this article, is a more direct route to examine the consequences of different evolutionary scenarios for CPMs and it avoids the shortcomings of both agent-based models (Sprouffske *et al.*, 2011), where it can be hard to understand the model in terms of generalizable features, and of Diaz-Uriarte (2015), which could only use a limited subset of deviations from DAGs.

The main goal of this article is to understand the relationship between fitness landscapes and CPMs inferred from evolutionary process on those fitness landscapes. I first use evolutionary simulations on 500 fitness landscapes that include from none to extensive reciprocal sign epistasis, varying also mutation rate and time to tumor detection (as these factors affect what genotypes are observable from a landscape). Because the focus of this article is not method comparison *per se*, but to identify the limits that complex fitness landscape can impose on the use of CPMs, I use large sample sizes of *N* = 1000 genotypes and infer CPMs with CBN (Gerstung *et al.*, 2009, 2011) and CAPRI (Ramazzotti *et al.*, 2015), the two widely available state-of-the-art methods that accommodate multiple parents (convergent arrows) in DAGs (see Section 2.4 and ‘Cancer progression models and other software’ in Supplementary Material). I evaluate the effects of fitness landscape, mutation rate, tumor detection and reciprocal sign epistasis on the quality and variability of inferred DAGs. I find that method performance decreases with reciprocal sign epistasis and that there is a large variability in the identified restrictions even under the best conditions.

I then examine the practical consequences of variability and non-representability using three cancer datasets: I find that fitness landscapes that are widely different from each other can produce genotype frequencies similar to the empirically observed ones, and also lead to very different DAGs of restrictions (i.e. very different inferences about restrictions in the order of accumulation of mutations) when evolutionary processes run repeatedly on them. These results cast doubts on whether restrictions from DAGs can be used to capture the true restrictions in fitness landscapes.

### 1.1 Assumptions

Several assumptions were used in the arguments above and are taken for granted in the rest of this article. As is customary in the CPM literature (Beerenwinkel *et al.*, 2015; Misra *et al.*, 2014; Ramazzotti *et al.*, 2015) I assume that the DAGs of restrictions and fitness landscapes represent epistatic interactions between biallelic loci. Because we want to simulate data consistent with cross-sectional sampling, we absorb all the cancer initiation process in the root node; as in Attolini *et al.* (2010) all tumors start cancer progression without any of the mutations shown in the DAGs (though other mutations could already be present that caused the initial tumor growth). Also in agreement with common models in this field (Beerenwinkel *et al.*, 2007; Bozic *et al.*, 2010; McFarland *et al.*, 2013) back mutations are not allowed. Crossing valleys in the fitness landscape using a single multi-mutation step is not possible, but we do not need to exclude clonal interference (de Visser and Krug, 2014; Sniegowski and Gerrish, 2010). Finally, I assume that there are no observational errors and also assume that we know which are the driver genes (so we do not consider the need to remove passengers before CPM fitting; Cristea *et al.*, 2016; Diaz-Uriarte, 2015).

## 2 Materials and methods

### 2.1 Generating random fitness landscapes

All DAGs of restrictions and fitness landscapes used biallelic—mutated or not-mutated—loci, so the total number of possible genotypes is 2^*m*^, where *m* is the number of genes. All DAGs and fitness landscapes in the first section use seven genes. That is the number of genes in the pancreatic cancer dataset, the execution time of CBN increases steeply with number of genes beyond about seven genes (Diaz-Uriarte, 2015), seven genes is probably close to the upper limits of fitness landscapes that can be easily visualized and related to their true DAGs (see ‘Plots of fitness landscapes and inferred DAGs’ in Supplementary Material), and if number of genes has an effect on the problems reported in this article they are likely to become worse with increasing numbers of genes.

To generate **DAG-derived, representable** fitness landscapes, I first obtained the transitive reduction of random DAGs; then, I assigned fitness to genotypes using a multiplicative fitness model for the effects of genes with their dependencies satisfied (with the fitness effect of each gene drawn from a uniform distribution between 0.1 and 0.7); I set to 10^−^^9^ the fitness of any genotype that is not possible under the DAG (this makes it almost impossible to ever see a genotype of that kind—see ‘Paths through non-accessible genotypes’ in Supplementary Material). Generation of **DAG-derived, non-representable** fitness landscapes started by generating a representable DAG-derived fitness landscape as just described. Then, a randomly chosen subset of genotypes with two or more mutations and accessible under the DAG was made inaccessible. The 200 Rough Mount Fuji (**RMF**) non-representable fitness landscapes were obtained from an RMF model (Franke *et al.*, 2011; Neidhart *et al.*, 2014) where the reference genotype and the decrease in fitness of a genotype per each unit increase in Hamming distance from the reference genotype were randomly chosen (see ‘Random fitness landscapes’ in Supplementary Material). This gives a wide variety of fitness landscapes that encompass from close to additive to House of Cards models. All fitness landscapes were checked to ensure that all seven genes were present in at least one accessible genotype. See further details in Supplementary Material.

### 2.2 Evolutionary simulations

I simulated evolution on fitness landscapes using the model in McFarland *et al.* (2013), a continuous-time, logistic-like model where death rate depends on total population size, as implemented in OncoSimulR (Diaz-Uriarte, 2017). In addition to fitness (more precisely, birth rates) which are given by the fitness landscape, when using this model we need to specify initial population sizes, mutation rates and detection/stopping conditions. Initial population size was set at 2000, a value within ranges used previously that ensures that most simulations reach cancer (McFarland *et al.*, 2013). Three different conditions of mutation rates were used: common mutation rate of rate of 1e−5, common mutation rate of 1e−6, and variable, or gene-specific, mutation rates that have a geometric mean of 1e−5, with maximum spread between successive values, within the maximum and minimum of 5e−5 and 2e−6, respectively. These are mutation rates within ranges previously used in the literature (Bozic *et al.*, 2010; McFarland *et al.*, 2013), with a bias toward larger numbers (since we use only seven genes relevant for population growth and we could be modeling pathways, not individual genes). With these settings, crossing fitness valleys in the simulations was extremely unlikely (see ‘Paths through non-accessible genotypes’ in Supplementary Material). Each simulation was stopped when the tumor was detected and a whole tumor sample was then taken. The probability of tumor detection increased with total tumor size and two conditions were used: ‘fast’ and ‘slow’ that correspond to detection probabilities of 0.1 and 0.01 when tumor size was twice the initial population size (see ‘Simulations: parameters and detection’ in Supplementary Material); total tumor sizes at time of detection were very variable (mean, median, first, and third quartiles: 120 000, 23 000, 7000 and 103 000, respectively).

### 2.3 Cancer datasets: fitness landscapes and simulations

I repeatedly simulated data using a modified RMF random fitness landscape model where the observed genotype combinations in the empirical datasets were guaranteed to be accessible (see Section 2.1 and ‘Random fitness landscapes for the cancer datasets’ in Supplementary Material). Once the fitness landscape had been generated, I choose randomly selected parameters for mutation rates and detection regime (see ‘Simulations: parameters and detection’ in Supplementary Material). I simulated the same number of genotypes as genotypes with mutations were in the original datasets (90 for pancreatic and colorectal cancer and 67 for glioblastoma). I repeated this process 10 times. For each of the 10 times, I compared the genotype distributions of the observed and the simulated genotypes with a *χ*^2^ test. I kept those fitness landscapes (together with the mutation and detection settings), where in at least three of the 10 repetitions the simulated dataset fulfilled that the *P*-value was >0.6 (for both the reference *χ*^2^ distribution and a permutation test, because of possible cells of low counts) and all genes had been observed. Some of these thresholds are arbitrary, but the large *P*-value is used to ensure that no user would claim the genotype frequencies are different, and the requirement of the minimal of three cases is used to prevent occasionally achieving a large *P*-value from a fitness landscape that rarely produces data comparable with the observed one. Finally, for each fitness landscape that fulfilled the requirements, I simulated 20 000 evolutionary trajectories, which were analyzed as described in Section 3.2 (see scheme of the design in ‘Three cancer datasets: scheme’ in Supplementary Material).

### 2.4 CPMs: CBN and CAPRI

Detailed descriptions of CAPRI an CBN can be found in Ramazzotti *et al.* (2015), Caravagna *et al.* (2016) and Gerstung *et al.* (2009, 2011). Here, only a summary is provided. CPMs try to identify features of tumor progression that are common to a homogeneous type of cancer; they assume that each subject or individual sample is an independent realization of an evolutionary process where the same constraints hold for all tumors (Beerenwinkel *et al.*, 2015, 2016; Gerstung *et al.*, 2011). Both CBN and CAPRI can be regarded as extensions of oncogenetic trees (Desper *et al.*, 1999; Szabo and Boucher, 2008) that describe the accumulation of mutations with order constraints that can be represented as trees. CBN and CAPRI are the two widely available, state-of-the-art methods, that allow modeling the dependence of an event on more than one previous event, so the output of the model are graphs (DAGs) where some nodes have multiple parents, instead of a single parent (as in trees). As oncogenetic trees and other CPMs, CAPRI and CBN use cross-sectional data with information about genomic aberrations in a set of tumor samples. The input for both methods is a matrix of subjects or samples by driver alteration events, where each entry in the matrix is binary coded as mutated or not-mutated; the driver alteration events are referred in this article, generically, as ‘genes’ but they can actually be individual genes, specific parts or states of genes, or modules made from several genes; for example, Gerstung *et al.* (2011) model pathways, and Caravagna *et al.* (2016) model amplifications and deletions of genes. From that input, both methods return a DAG of restrictions. CBN models that mutations accumulate stochastically on each patient; the fixation of these mutations respect a set of order restrictions (the DAG of restrictions that CBN will try to infer). CAPRI tries to identify events (alterations) that constitute ‘selective advantage relationships’, where an alteration in one gene ‘selects’ for a later alteration in another gene (CAPRI’s algorithm builds upon Suppe’s probabilistic causation framework and the identification of selectivity relations requires establishing temporal priority—a mutation in one event occurs earlier—and probability rising—the probability of observing one event increases the probability of observing another). Both CBN and CAPRI can accommodate errors in the observational data (e.g. genotyping errors) and both assume that each genotype contains all alterations that appeared in its parent genotypes (i.e. there is no back mutation). CBN uses simulated annealing with a nested EM algorithm to estimate the parameters of the model: the error rate and rate of fixation of mutations and, of main interest to us, the best fitting DAG of restrictions. CAPRI uses Mann–Whitney *U*-tests (from bootstrap resamples of the input data) to examine temporal priority and probability rising and then optimization (e.g. using hill climbing) of the penalized (AIC or BIC) maximum likelihood fit to find the final DAG. I used CBN and CAPRI with their default settings (see ‘Cancer progression models and other software’ in Supplementary Material) to obtain inferred DAGs.

### 2.5 Measures of performance and variability

Reciprocal sign epistasis is the fraction of all pairs of mutations that had reciprocal sign epistasis. The distance between two DAGs of restrictions is the number of the edges that differ between the transitive reduction of the two DAGs. CAPRI can return DAGs that contain both direct and indirect edges between nodes and thus all comparisons involved the transitive reduction of the DAGs. The relative distance between DAGs is the distance between two DAGs divided by the total number of distinct edges in the two DAGs. PFD genotype mispredictions is the ratio of false positive genotype mispredictions (the DAG predicting that a non-accessible genotype should exist) over the total number of genotypes that can exist according to the DAG; this is equivalent to 1—precision or 1—positive predictive value. PND genotype mispredictions is the ratio of false negative genotype mispredictions (the DAG failing to predict that a genotype should exist) over the total number of genotypes that are accessible in a landscape. A genotype, even if accessible under a given landscape, might never be observable under small mutation rates and fast detection regimes. To avoid penalizing inferences for non-observable genotypes, I corrected the count of false negatives using, as reference, the subset of accessible genotypes that had been observed with a frequency larger than 5 in 1000 (measured on the 20 000 simulations) so that the probability of not observing the minimal frequency genotype in a sample of 1000 genotypes is <1%. PND is equivalent to 1—recall or 1—sensitivity. The relative pairwise difference of accessible genotypes is the ratio of the sum of the number of genotypes accessible under one landscape and not accessible under the other over the total number of distinct accessible genotypes in the two landscapes.

### 2.6 Linear mixed-effects models

**Fig. 2.**
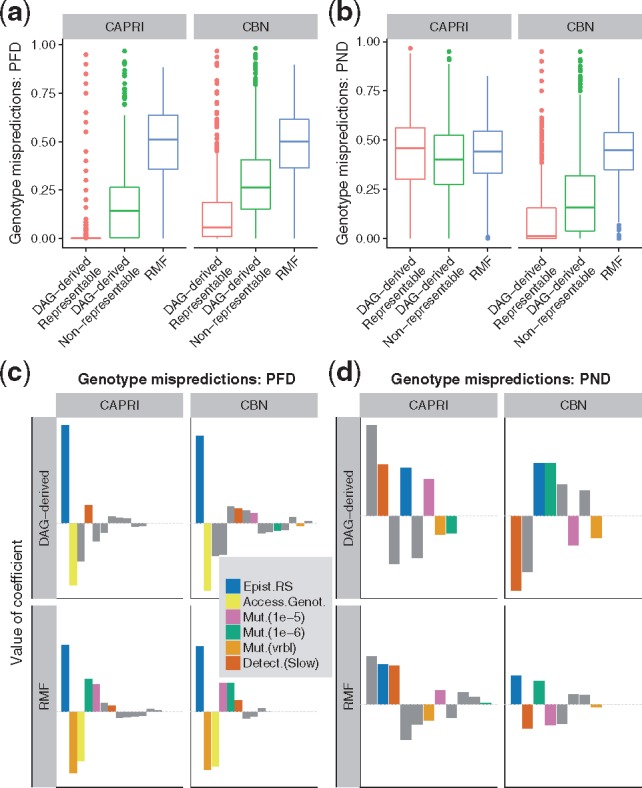
Genotype mispredictions: PFD and PND. (**a, b**) Boxplots of PFD and PND by method and type of fitness landscape. (**c, d**) Coefficients from linear mixed-effects models, with separate models fitted for each combination of method and type of fitness landscape. Within each panel, coefficients have been ordered from left to right according to decreasing absolute value of coefficient. The dotted horizontal gray line indicates 0 (i.e. no effect). Only coefficients that correspond to a term with a *P*-value <0.05 in Type II F (ANOVA) tests are shown. Coefficients involving landscape type “DAG-derived” and detection fast are not shown (they are minus the corresponding coefficient shown for the other value of the factor—see Section 2). Coefficients that correspond to main effects color coded as shown in the legend; the rest of the coefficients (“Other”) correspond to interaction terms; “Epist.RS”: fraction of pairs with reciprocal sign epistasis; “Mut.(vrbl)”: variable or gene-specific mutation

**Fig. 3.**
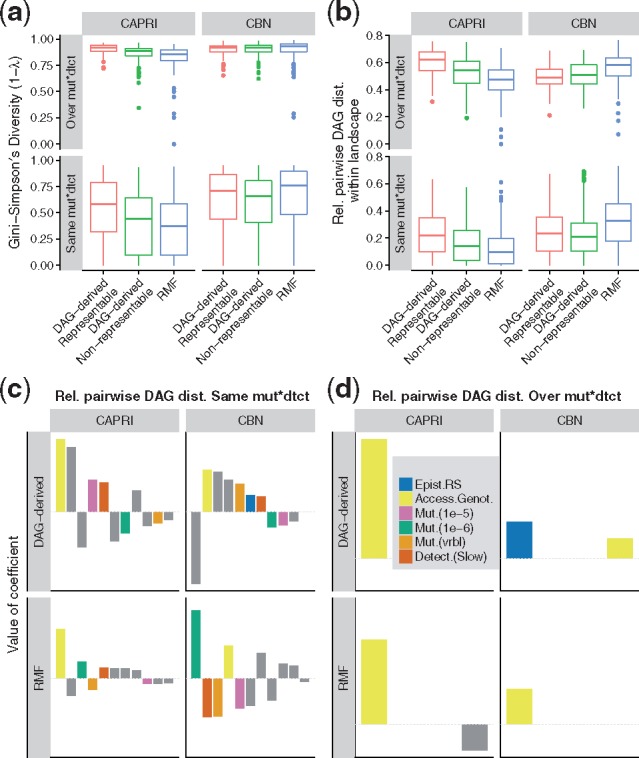
Variability of DAGs of restrictions inferred from a landscape. “Same mut×dtct”: values from the 20 replicates under each combination of landscape by mutation rate by detection. “Over mut×dtct”:20 × 6 replicates of a landscape over the 3 mutation by 2 detection regimes. (**a**) Gini-Simpson’s diversity index, the probability that two DAGs from the given scenario are different. (**b**) Relative pairwise DAG distance (see Section 2). The value shown for a landscape is the average relative pairwise DAG distance for all pairs of inferred DAGs for a landscape. (**c, d**) Coefficients from linear models; see legend of Figure 2. In panel (**d**) only accessible genotypes, reciprocal sign epistasis and their interaction could, by design, be examined

I used linear mixed-effects models to examine how type of landscape, mutation rate, detection scheme, reciprocal sign epistasis and number of accessible genotypes, affected PND, PFD and the two relative pairwise DAG distances (‘Linear mixed-effects models’ in Supplementary Material). Models used fitness landscape as random effect except for relative pairwise DAG distance over mutation and detection (because I averaged over all mutation × detection regimes only a single value per landscape was used). For ease of interpretation (to avoid presenting coefficients from models with four way interactions), Figures 2 and 3 present results from models for each method and landscape type separately, but I also fitted a single model to assess the interactions of method and landscape type with the other factors. To examine the significance of the differences in Figures 2a, b and 3b, I fitted models than only had landscape type and method as explanatory variables (these models are equivalent to multistratum models for split-plot designs: Pinheiro and Bates, 2000). In all cases, models were fitted using sum-to-zero contrasts (McCullagh and Nelder, 1989). Continuous regressors were scaled (mean 0, variance 1) for easier interpretation and so that the intercept term is interpreted as the predicted response at the average value of the regressors. See further details in Supplementary Material.

## 3 Results

### 3.1 Reciprocal sign epistasis, genotype mispredictions, DAG variability

#### 3.1.1 Steps of simulations and analysis

The following steps are used to understand the consequences of fitness landscape characteristics, tumor detection and mutation rates on the quality and variability of DAGs. Details of each step are provided below. (i) Generate 500 random fitness landscapes that differ in reciprocal sign epistasis and whether or not they are representable by DAGs of restrictions (Section 3.1.2). (ii) Simulate 20 000 genotypes under each one of the 3000 = 500 × 3 × 2 combinations of 500 fitness landscapes, 3 mutation rates and 2 detection regimes (Section 3.1.3). (iii) Split each set of 20 000 simulated genotypes into 20 sets of 1000 genotypes and run CBN and CAPRI on each set of 1000 genotypes to obtain DAGs of restrictions. (iv) For each inferred DAG of restrictions, measure the errors in the predictions of the genotypes by comparing against the accessible genotypes under the true fitness landscape (Section 3.1.4), where the true fitness landscape is the landscape generated in step (i) (and used for the simulations in (ii)). (v). Measure the variability of the DAGs inferred for each fitness landscape (Section 3.1.5).

#### 3.1.2 Fitness landscapes and simulation framework

I generated a total of 500 fitness landscapes. Of these, 100 were directly derived from DAGs of restrictions and are thus perfectly representable. These fitness landscapes, which contain no reciprocal sign epistasis, will be called ‘**DAG-derived, Representable’**.

Another 200 fitness landscapes were obtained by modifying representable fitness landscapes to turn them into non-representable fitness landscapes. I first generated a set of 200 representable fitness landscapes; then, for each landscape, randomly selected genotypes predicted to exist by the DAG of restrictions were made inaccessible—i.e., turned into synthetic lethals. Thus, I created DAG-derived, but non-representable, fitness landscapes with varying amounts of reciprocal sign epistasis (see ‘Fitness landscapes characteristics’ in Supplementary Material). These landscapes will be called ‘**DAG-derived, Non-representable’**.

Another set of 200 fitness landscapes were obtained from **RMF** models, which have been useful to model empirical fitness landscapes (de Visser and Krug, 2014; Neidhart *et al.*, 2014) and have very different characteristics from DAG-derived fitness landscapes (see ‘Fitness landscapes characteristics’ in Supplementary Material). All of the RMF models were non-representable. RMF landscapes had both more reciprocal sign epistasis and larger numbers of peaks than DAG-derived fitness landscapes (average fraction of pairs of loci with reciprocal sign epistasis; Ferretti *et al.*, 2016: 0.27 versus 0.02 for RMF- and non-representable DAG-derived landscapes; two-sample *t*-test, *t*_240_ = 45, *P* < 0.0001; mean number of peaks among the accessible genotypes: 11.5 and 2.8 in the RMF and DAG-derived, respectively; two-sample *t*-test, *t*_240_ = 24, *P* < 0.0001).

#### 3.1.3 Evolutionary simulations

Next, I simulated evolutionary processes on the 500 fitness landscapes using a logistic-like model, following McFarland *et al.* (2013), where death rate depends on total population size. I used a factorial design (see Section 2) where on each one of the 3000 combinations of 500 landscapes by three mutation rates by two tumor detection regimes, I run 20 000 evolutionary processes that resulted in 20 000 simulated sampled genotypes per condition. The 20 000 simulated genotypes were split into 20 sets of 1000 genotypes and each one of the 20 sets was analyzed with CBN and CAPRI (yielding, therefore, 20 DAGs of restrictions per method for each one of the 3000 combinations of landscape × mutation × detection). Plots of the 500 fitness landscapes and the modal inferred DAG are provided in the Supplementary Material.

#### 3.1.4 Genotype mispredictions in inferred DAGs

For each genotype, I compared the status accesible/non-accessible predicted by each DAG of restrictions with the true status from the fitness landscape. The proportion of false discoveries (PFD) is the number of genotypes that are erroneously predicted to exist relative to the number of genotypes predicted to exist by a DAG of restrictions. The proportion of negative discoveries (PND) is the number of accessible genotypes that a DAG fails to predict relative to the total number of accessible genotypes (the PND statistic corrects for genotypes that are not observable for a given combination of detection and mutation rates—see Section 2). Figure 2a and b shows the PFD and PND for each method and landscape type. Both PFD and PND differed between landscape type and method, and the effect of landscape was different for each method. PFD increased from representable, to DAG-derived non-representable, to RMF landscapes, but CBN and CAPRI were affected differently by landscape type, as PFD was larger with CBN than CAPRI in the two DAG-derived landscapes (interaction term method by landscape in linear mixed-effects model of PFD as dependent variable: Type II Wald *F* tests with Kenward–Roger d.f. adjustment: *F*_2,5493_ = 189.2, *P* < 0.0001). PND increased from representable, to DAG-derived non-representable, to RMF landscapes with CBN but showed no trend with CAPRI (interaction term method by landscape: *F*_2,5493_ = 524.20, *P* < 0.0001). Regardless of these interactions, PFD and PND were around 50% for both methods in the RMF landscapes.

I examined how PFD and PND were affected by mutation rate, detection regime, number of accessible genotypes, and reciprocal sign epistasis and their two-way interactions using linear mixed-effects models. Figure 2c and d shows the relative magnitude of the coefficients in the models (see ‘Coefficients of linear models’ in Supplementary Material for the complete set of coefficients). Mainly for PND, the effects of some predictors differed between CBN and CAPRI: in particular, the slow detection regime was associated with worse performance when using CAPRI but with better performance when using CBN, and the larger mutation rate was associated with worse performance when using CAPRI but better performance when using CBN (interactions between method and the effects of mutation and detection, *P* < 0.0001, from Type II Wald *F* tests with Kenward–Roger d.f. adjustment). For PND there were also other large interactions that involved mutation and detection regimes. Increasing reciprocal sign epistasis was associated with increasing PFD and PND for both methods and landscape types. Remarkably, the increase in mispredictions with reciprocal sign epistasis did not differ between CBN and CAPRI (we cannot reject the hypothesis that the increase in mispredictions with reciprocal sign epistasis is similar in CBN and CAPRI, *P* > 0.15). But both PFD and PND showed a faster increase with reciprocal sign epistasis in DAG-derived than in RMF landscapes (interactions between type of landscape and reciprocal sign epistasis: *P* < 0.0001).

#### 3.1.5 DAG variability from the same fitness landscape

To examine within-landscape variability in the inferred DAGs of restrictions I computed the average relative pairwise DAG distance between all possible DAGs of: (i) the 20 replicate inferences for each landscape by mutation by detection regime—i.e. same mutation and detection (‘Same’ in Fig. 3); (ii) the 20 × 6 replicates for each landscape over the six conditions of mutation by detection regime (‘Over’ in Fig. 3). Multiple, substantially different, DAGs of restrictions were inferred from the same landscape, even under the same mutation rates and detection regimes (Fig. 3a and b). Similar to what we saw in the previous section, the effect of landscape type on relative pairwise distance differed between methods, with CAPRI showing a decrease in relative distance from representable, to DAG-derived non-representable, to RMF landscapes (interactions, *P* < 0.0001, between method and landscape type in relative pairwise distance in both ‘Same’ and ‘Over’). Linear models (Fig. 3c and d) showed that increasing the number of accessible genotypes increased the variability of inferred DAGs. But, as we saw before, the relevance and sign of other terms depended on the method: mutation, detection, and landscape type had different effects in CAPRI and CBN (*P* < 0.0001 for all two-way interactions with method; *P* = 0.0009 for the interaction mutation by landscape type by method; and *P* < 0.0001 for the interaction mutation by detection by method). Increasing reciprocal sign epistasis was associated to increased variability when using CBN under the DAG-derived landscape but it had no effect with CAPRI nor with CBN under RMF (there was a three-way interaction method by landscape type by epistasis; *P* = 0.002 and *P* = 0.033 for ‘Same’ and ‘Over’, respectively). Note that an unavoidable consequence of the large variability between DAGs inferred from the same fitness landscape is that the same DAG of restrictions can be inferred from quite different fitness landscapes (see Supplementary Material, section ‘Inferring the same DAG from different fitness landscapes’).

### 3.2 Three cancer datasets: a many-to-many mapping between DAGs and landscapes

Using three cancer datasets I will show the implications that non-representability and variability have for the analysis of empirical data. These data, for pancreatic cancer, colorectal cancer and primary glioblastoma, are originally from Jones *et al.* (2008), Wood *et al.* (2007) and Parsons *et al.* (2008), respectively, and were analyzed by Gerstung *et al.* (2011) and have 90, 90 and 67 patients with at least one mutation, respectively, in the 7, 8 and 8 genes analyzed. Since we do not know the true fitness landscapes that generated those data we cannot assess the quality of the inferences. What I have done instead is find, for each cancer dataset, 150 random fitness landscapes (plus mutation rates and detection regimes) that can produce genotype frequencies similar to the observed ones (similar: in at least three out of 10 repetitions, all genes are observed mutated and a *χ*^2^ test comparing genotype frequencies of observed and simulated data has a *P*-value > 0.6—see Section 2). Next, from each one of those 150 fitness landscapes per dataset I generated 20 000 genotypes, and analyzed 20 subsets of 90 (or 67) genotypes and 20 subsets of 1000 genotypes with CAPRI and CBN. The 150 landscapes and the modal DAGs inferred with CBN and CAPRI for each of the datasets and sample sizes are provided in the Supplementary Material. I am not claiming any of these landscapes are the true fitness landscapes of these cancers: I am using them to examine the implications of the many-to-many problem for the analysis of empirical data.

The characteristics of the 150 fitness landscapes for each dataset are shown in the Supplementary Material; there was substantial reciprocal sign epistasis (medians: 0.09, 0.15, 0.1, for pancreas, glioblastoma and colon) and the fitness landscapes were multi-peaked (median number of peaks: 6, 15, 13, for pancreas, glioblastoma and colon). These results, *per se*, are not necessarily surprising given the way we searched for fitness landscapes, but these fitness landscapes are clearly not representable and yet they can consistently produce data similar to the empirically observed ones.

**Fig. 4.**
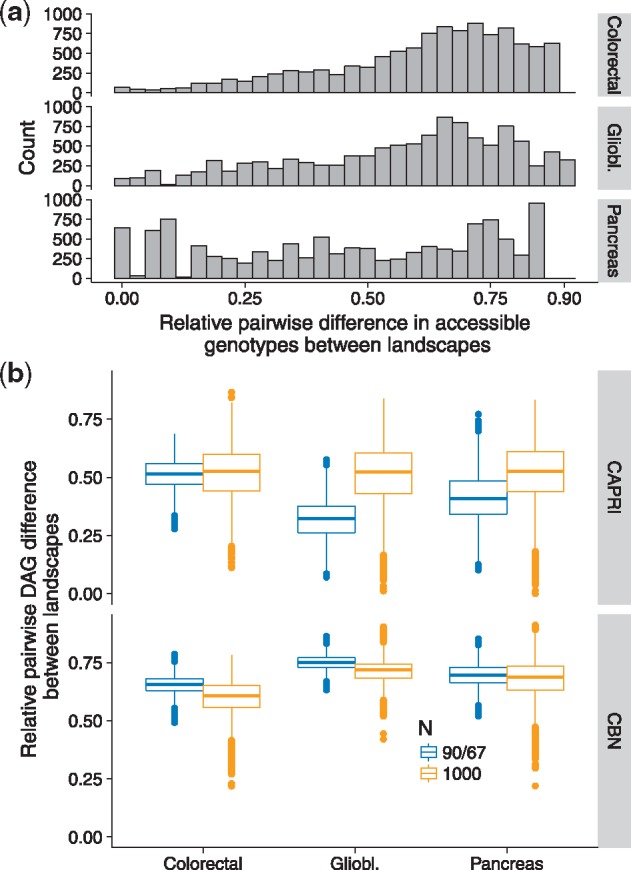
Fitness landscapes characteristics and DAG differences in three cancer datasets. (**a**) Histograms of relative pairwise differences in accessible genotypes over all possible 150 × 149/2 pairs of landscapes for each dataset. (**b**) Box-plots of relative pairwise differences between all pairs of DAGs inferred from different landscapes (each box-plot plot is based on 150 × 149/2 points; each point itself is the average of 400 (20 × 20) comparisons)

For each cancer dataset, the fitness landscapes were widely different among themselves, with median pairwise difference in accessible genotypes of 18, 46 and 87 genotypes for pancreas, glioblastoma and colon; relative to the number of distinct accessible genotypes, these were median pairwise differences of 47, 62 and 65%, respectively, as shown in Figure 4a.

As in Section 3.1.5, there was considerable DAG-to-DAG variation within landscape (see Supplementary Fig. S7). More relevant to us, however, are the differences between DAGs inferred from different landscapes. These are shown in Figure 4b: pairs of DAGs of restrictions differed in a median of more than 50% of their edges, probably an unavoidable consequence of the large differences between landscapes mentioned above (Fig. 4a).

Thus, these fitness landscapes, all of which can consistently produce genotype frequency data similar to the empirically observed data, not only were non-representable and showed large differences among themselves but, when evolutionary processes were run repeatedly on them, lead to very different inferences about the restrictions in the order of accumulation of mutations.

## 4 Discussion

CPMs assume restrictive fitness landscapes that, for instance, are devoid of reciprocal sign epistasis. Yet reciprocal sign epistasis may be common in cancer fitness landscapes (Chiotti *et al.*, 2014). What would be the consequences of using CPMs if tumors evolved on fitness landscapes that cannot be represented by DAGs of restrictions?

We saw (Fig. 2) lower performance of CPMs in RMF landscapes relative to representable landscapes, and decreasing performance with increasing reciprocal sign epistasis in both RMF and DAG-derived landscapes. CPMs assume that acquiring a mutation in one gene does not decrease the probability of acquiring a mutation in another gene (Misra *et al.*, 2014). CBN, Oncogenetic Trees (Desper *et al.*, 1999; Szabo and Boucher, 2008) and CAPRESE (Loohuis *et al.*, 2014) all share these features: even with unlimited data that faithfully represents the accessible genotypes in a landscape, they cannot fit non-representable fitness landscapes such as those that result from reciprocal sign epistasis. CAPRI can model XOR relationships (Caravagna *et al.*, 2016; Ramazzotti *et al.*, 2015) and, thus, for example, synthetic lethality, but it requires specifying the XOR hypothesis *a priori*; therefore, it is not suitable for automated usage in non-representable landscapes. Separating patients into subtypes prior to analysis (Caravagna *et al.*, 2016) cannot solve these problems as they are not the result of using CPMs on a collection of individuals with different underlying fitness landscapes; here all individuals have the same fitness landscape. Therefore, non-representability forces us to ask what is the meaning and how to interpret an inferred DAG of restrictions in the presence of reciprocal sign epistasis.

We also saw that the same fitness landscape produced genotype frequency data that lead to inferring widely different DAGs (Fig. 3). Even in representable landscapes with the same mutation and detection and sample sizes as large as *N* = 1000, DAGs of restrictions differed in about 20% of their edges (Fig. 3b, bottom row). Thus, even under the best conditions, currently available datasets are unlikely to be large enough to provide stable estimates of genotype frequencies that lead to stable, low-variability, inferences of DAGs of restrictions. Moreover, we can think of a DAG of restrictions as the output from applying a method on genotype frequency data that are the result of the function composition *Observed Genotype Frequencies *=* Detection*∘ *f* (*Mutation*, *PopulationSize*, *FitnessLandscape*), where *f* (*Mutation*, *PopulationSize*, *FitnessLandscape*) is a function that reflects the evolutionary dynamics and that depends non-additively on mutation rates, population sizes and fitness landscape. It is known that very different mutational paths can be observed from the same fitness landscape under different mutation rates and population sizes (de Visser and Krug, 2014); in our case, the same fitness landscape can lead to different observed genotype frequency data and, thus, different inferred DAGs if we change detection and mutation rates (see also Diaz-Uriarte, 2015). In fact, adding variation in detection and mutation rates made the problem worse and leads to inferred DAGs of restrictions that differed, on average, in 50% of their edges (Fig. 3b, top row). These results highlight the relevance of these two often neglected effects (see also Diaz-Uriarte, 2015). A practical consequence is that, because detection and mutation vary between tissues and cancer types (Hao *et al.*, 2016; Martincorena and Campbell, 2015), we could be inferring very different DAGs (and, thus, direct dependencies between mutational events) from similar underlying fitness landscapes.

We saw next that non-representability due to reciprocal sign epistasis and the difficulty of obtaining stable estimates of genotype frequencies, lead to a many-to-many relationship between fitness landscapes and DAGs of restrictions that affects the analysis of empirical data. In the three cancer datasets examined, genotype frequencies similar to the empirically observed ones were obtained under fitness landscapes that were very different from each other (Fig. 4a); these landscapes, when evolutionary processes run repeatedly on them, produced datasets that lead to very different and varied inferences about the restrictions in the order of mutations (Fig. 4b).

I have focused on the role of reciprocal sign epistatis. Other deviations from these models are possible in the absence of reciprocal sign epistasis, most notably disjunctive (OR) relationships. (With disjunctive relationships, an event can happen if at least one of its parents has occurred; for example, in Fig. 1e, gene D would need one of genes A or B to be mutated, not both.) Most common progression models that allow multiple incoming arrows interpret them as an AND, not an OR. This is what CBN does; CAPRI can examine ORs if provided as prior hypotheses but otherwise the convergent arrows should be interpreted as ANDs (noisy ANDs; see Ramazzotti *et al.*, 2015). Some of the issues caused by disjunctions might be solved if we used pathways or modules instead of individual genes (e.g. Cristea *et al.*, 2016; Diaz-Uriarte, 2017; Raphael and Vandin, 2015). And, compared with reciprocal sign epistasis, disjunctions do not violate that a mutation in a genes does not decrease the probability of another mutation so, in terms of relevance, assuming lack of reciprocal sign epistasis seems a more fundamental assumption of CPMs. But how much of a problem disjunctions are in landscapes with and without reciprocal sign epistasis is an open question.

Finally, CAPRI and CBN methods were affected differently by mutation rate, detection regime and even landscape type (Figs 2 and 3). CAPRI’s DAG variability (Fig. 3a and b) decreased in the RMF landscapes; this, however, does not mean that the quality of inferences was better in those landscapes, as it was not (see Fig. 2a and b), but simply that it was less variable around the error. The contrast in the behavior of CBN and CAPRI is probably caused by the different algorithms used by CAPRI and CBN. But there is an additional difference that affects interpretation. CBN (like Oncogenetic Trees and CAPRESE) should only place an arrow between two genes, *A* and *C*, if *C* cannot be observed without *A*. This is not necessarily the case for CAPRI. The arrows in CAPRI’s models (Ramazzotti *et al.*, 2015, p. 3017) “(…) imply ‘probability raising’ (…) [which] signifies that the presence of the earlier genomic alteration (…) increases the probability with which a subsequent advantageous genomic alteration (…) appears in the clonal evolution of the tumor.” Therefore, with CAPRI a simple interpretation of arrows as ‘needed for occurrence’ could be precluded (of course, CBN, as any other method, could make mistakes and add arrows when it should not—these comments refer to possible differences between methods in the **intended** meaning of the arrows). This difference in the intended meaning of the arrows is probably one of the reasons behind some of the behavior of CAPRI and its larger PNDs in the DAG-derived (both representable and non-representable) fitness landscapes. False negatives means failing to predict as possible an accessible genotype, and are the result of DAGs that encode too many restrictions, dependencies between genes that do not hold in the fitness landscapes.

## 5 Conclusion

How much of a problem are non-representable fitness landscapes and the many-to-many phenomenon for using the inferences obtained by CPMs, for instance to identify therapeutic targets to block the progress of disease? CPMs produce at best blurry maps of the underlying epistatic relationships in the fitness landscape. But differences in the underlying fitness landscape can have a large effect in the evolutionary dynamics of cancer and, thus, our opportunities for blocking the progress of disease. This raises the questions of whether we can asses from empirical data if landscapes are representable and, more importantly, what are the biomedical implications of errors and variability in the inferences of restrictions; even for representable landscapes, it might be extremely difficult to identify the correct dependency relationships between genes and, thus, the possible tumor progression paths, from cross-sectional data.

## Supplementary Material

Supplementary Data
