# Supplementary material for: Cancer progression models and fitness landscapes: a many-to-many relationship
Source: Bioinformatics. 2017 Oct 18;34(5):836–44. doi: 10.1093/bioinformatics/btx663 (PMC6031050; doi:10.1093/bioinformatics/btx663)
Supplement: Supplementary Data [file btx663_supp.zip › btx663-suppl_data/colon-landscapes-dags.pdf]

ID	p−value	Accessible Genot.
VUnqzXKgpKNSUEo	0.6	73

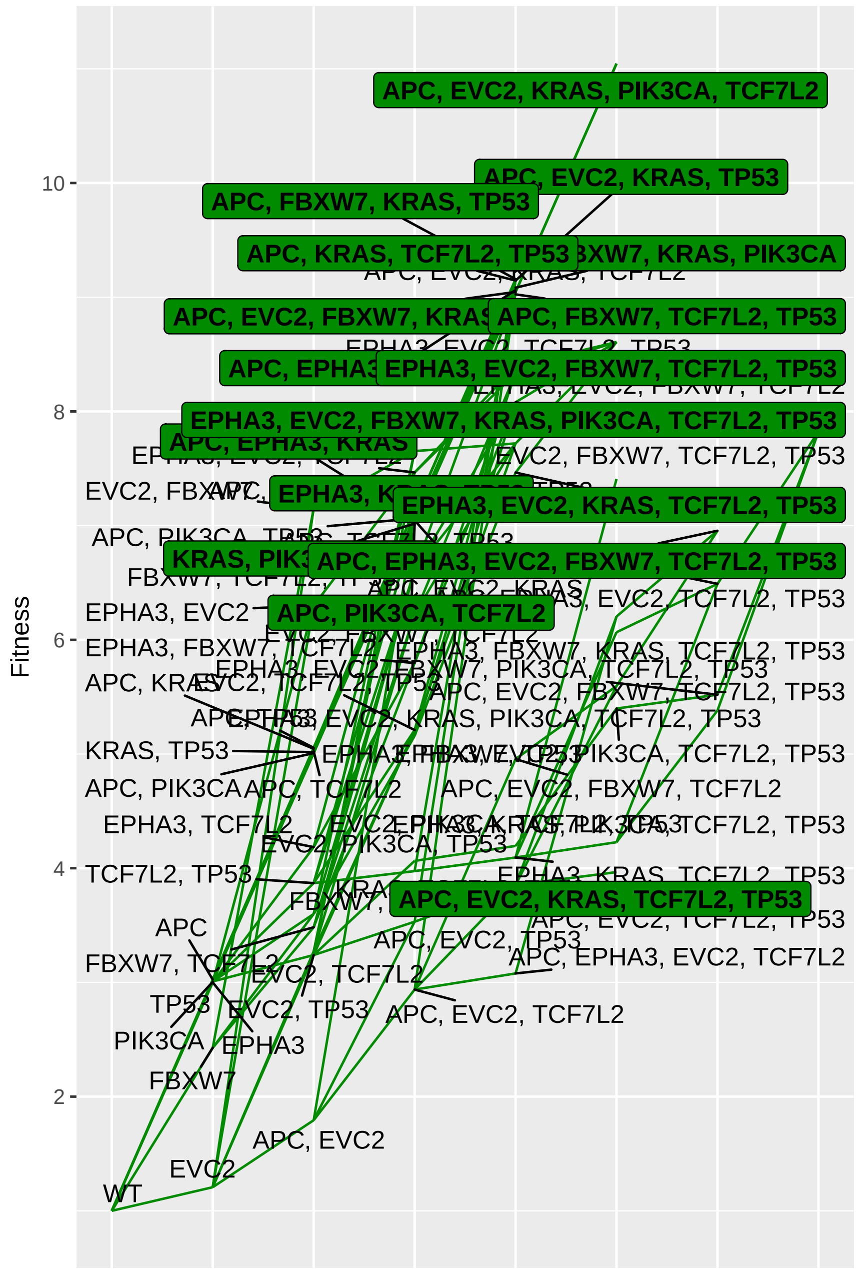

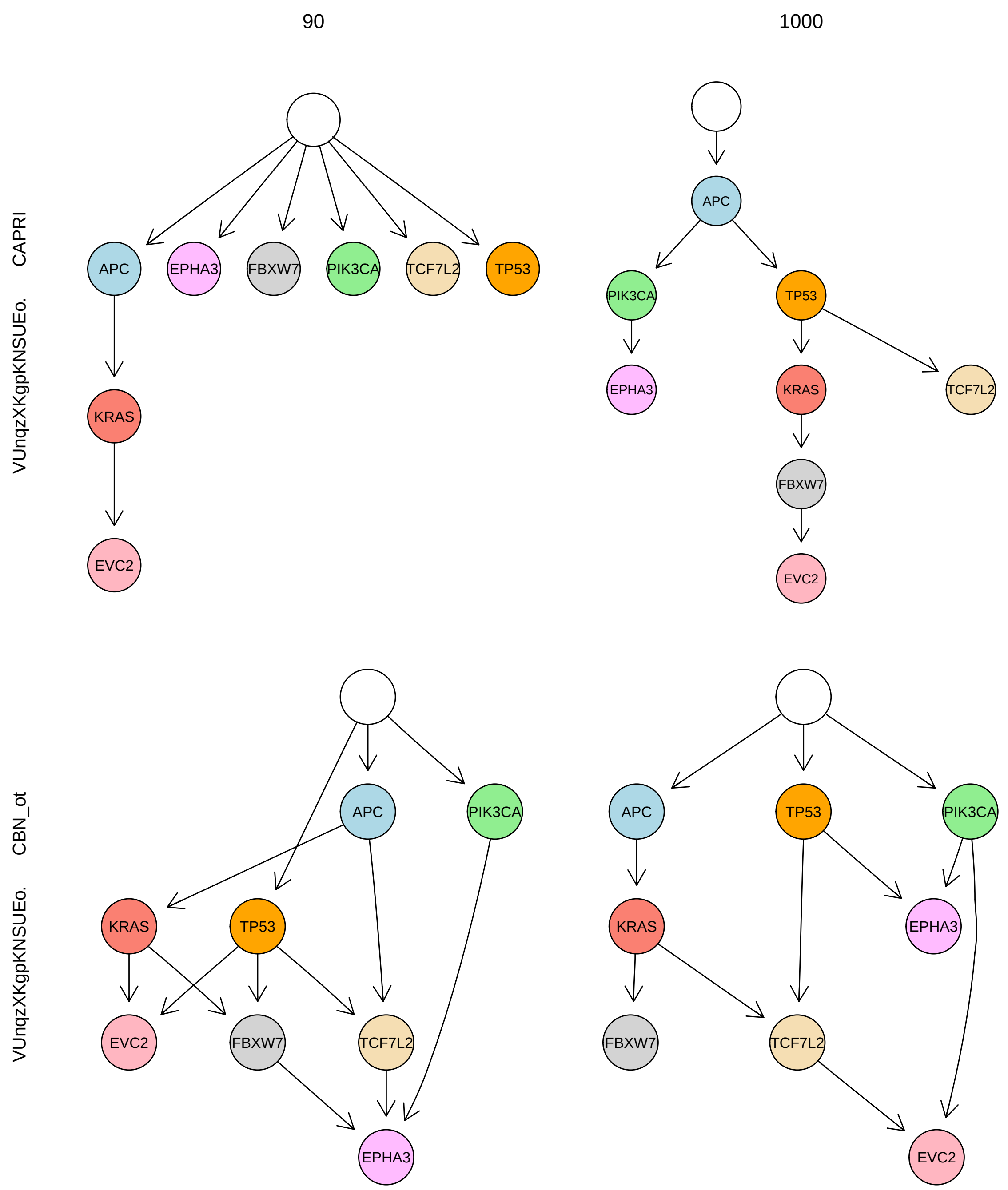

ID	p−value	Accessible Genot.
SMxHEqCvMMSoecs	0.602	72

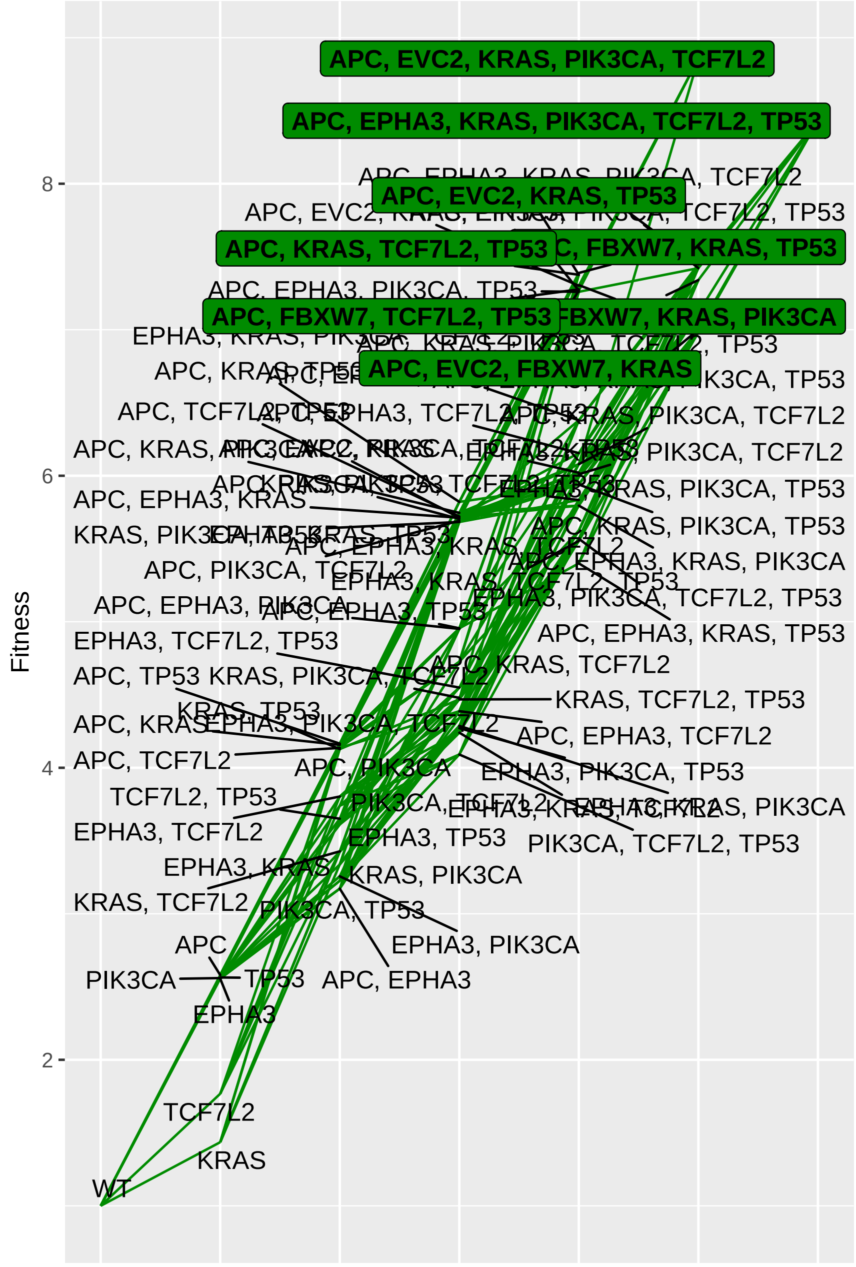

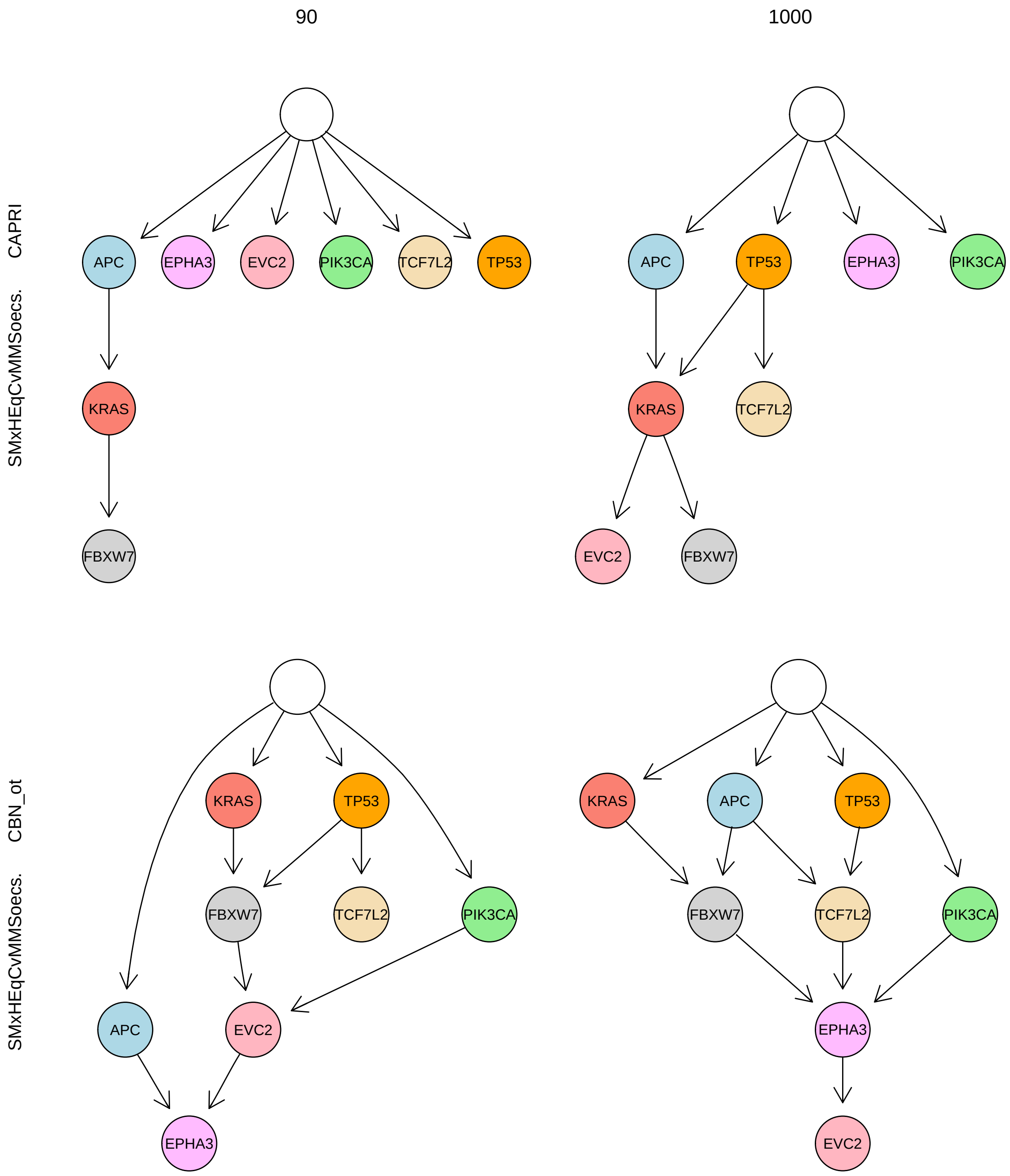

ID	p−value	Accessible Genot.
RbYzWbdlPplCfCw	0.602	174

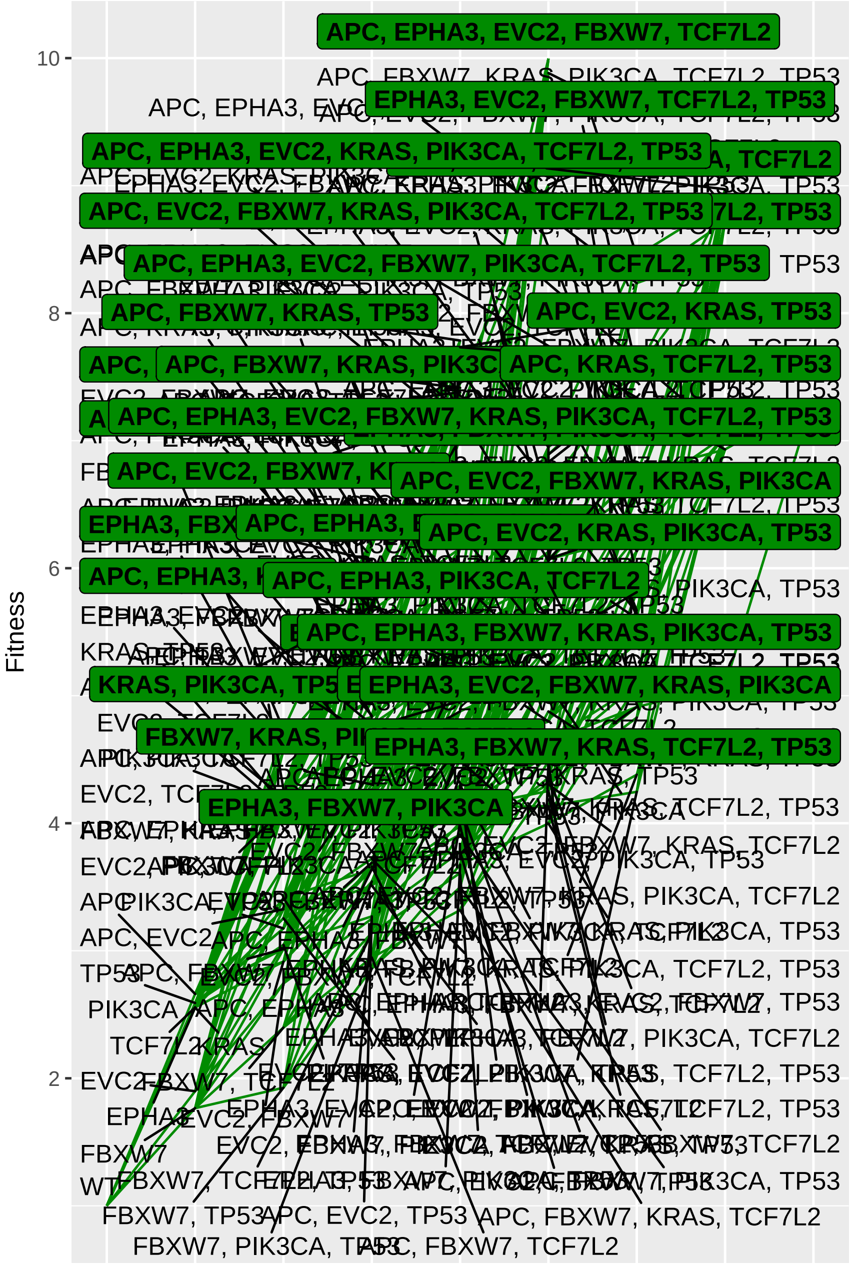

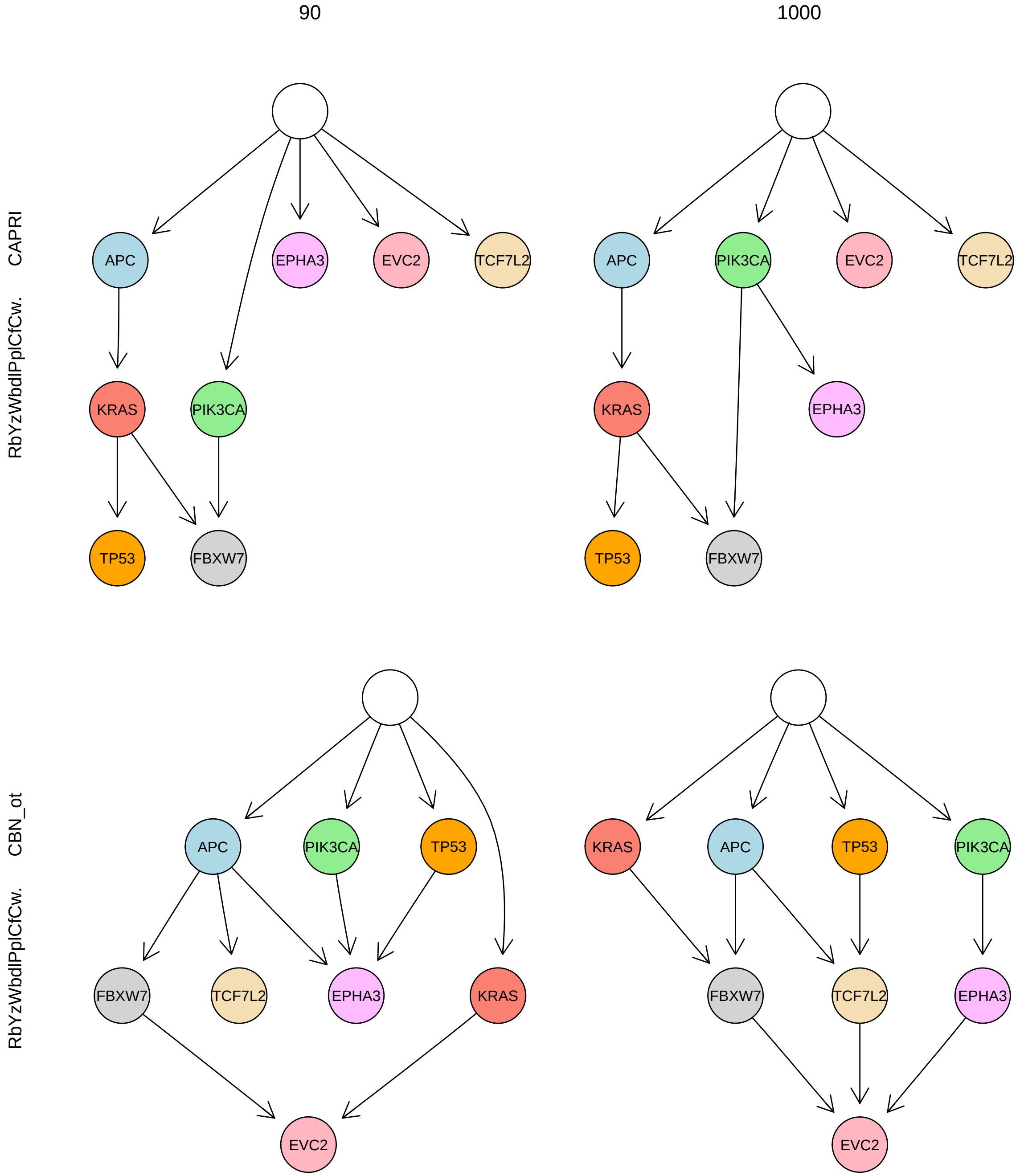

ID	p−value	Accessible Genot.
bSsgkGLOBspTsKL	0.602	255

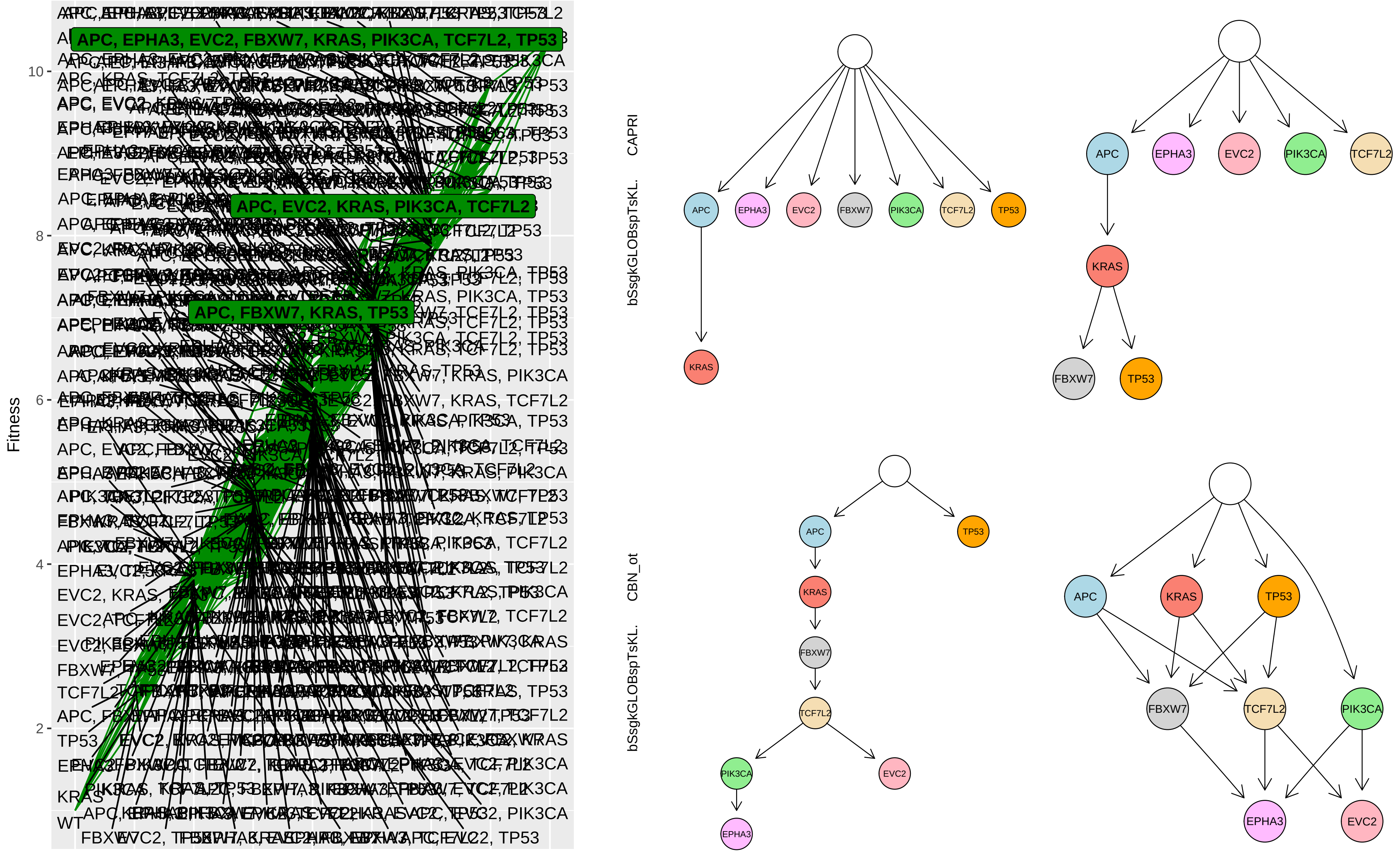

ID	p−value	Accessible Genot.
CledsZBCSaEkuVC	0.609	140

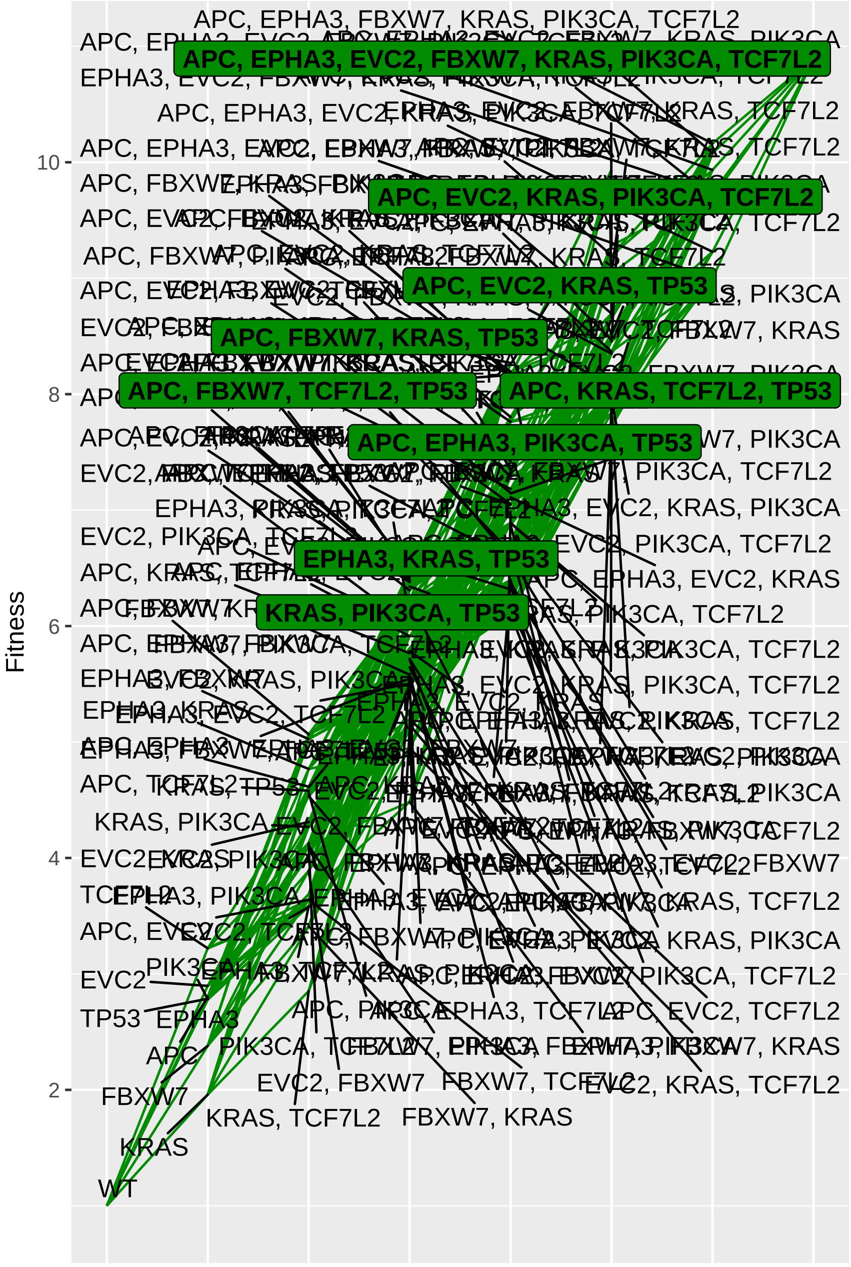

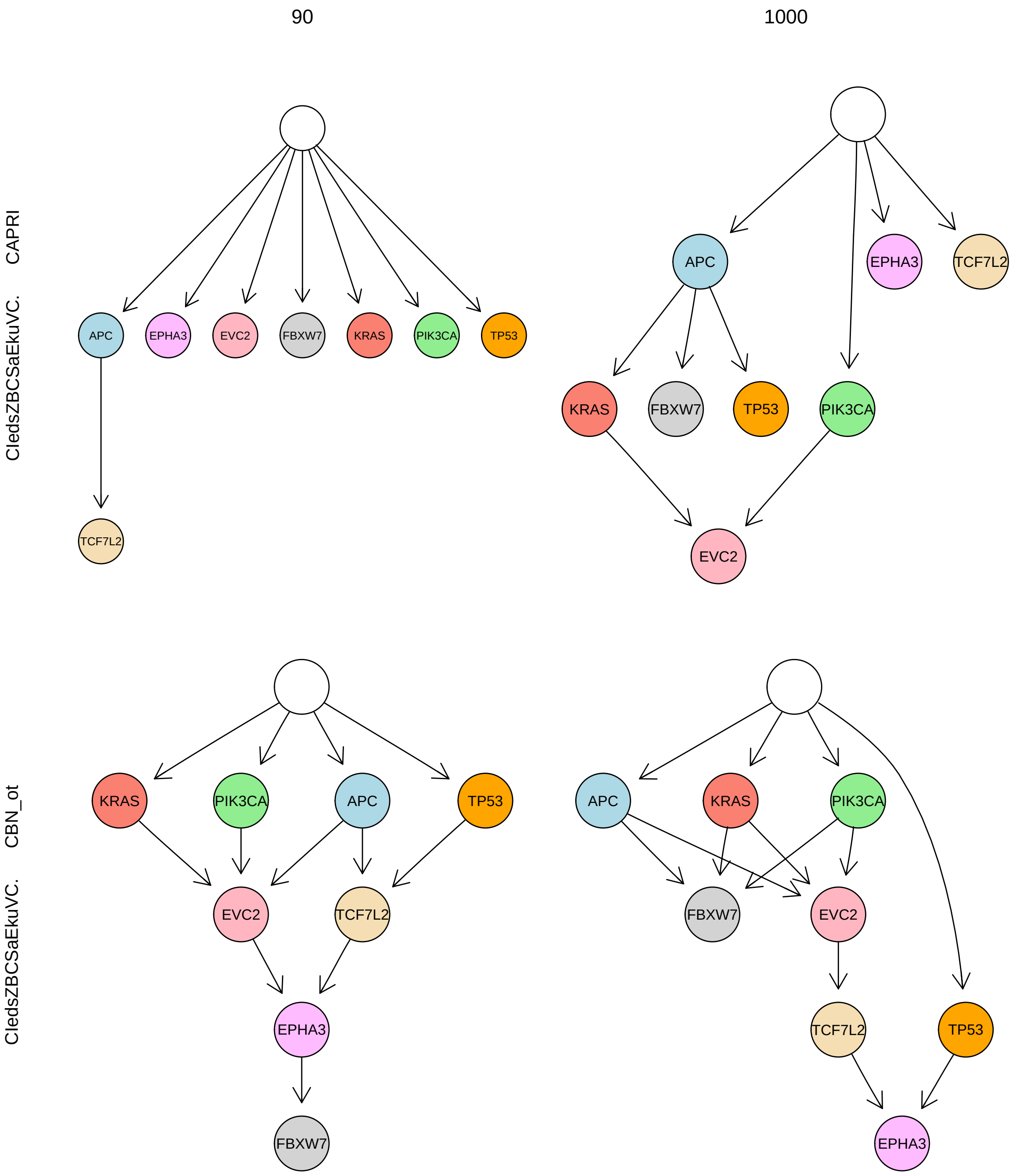

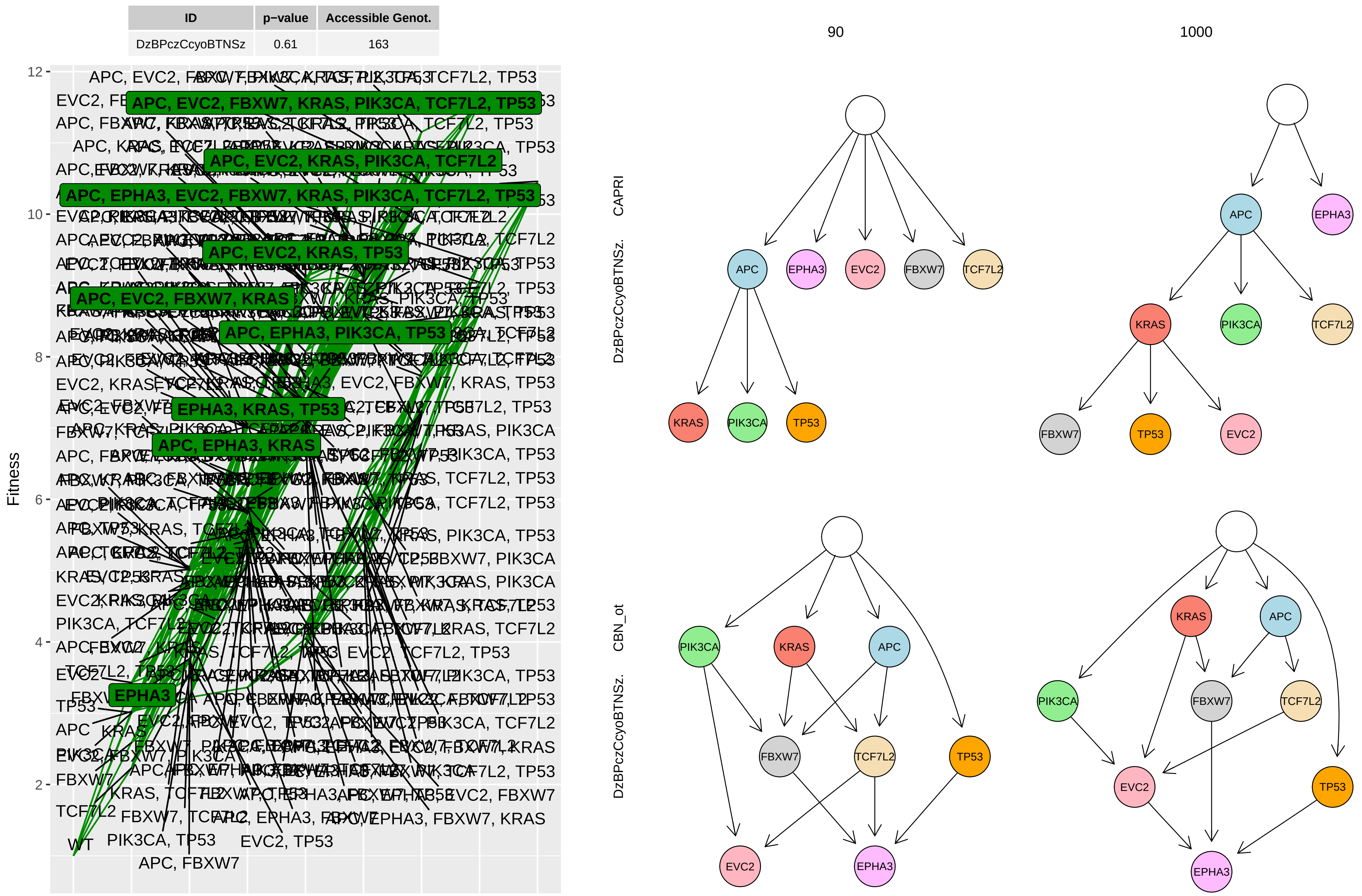
ID
p−value
Accessible Genot.
DzBPczCcyoBTNSz
0.61
163
Fitness
12
10
8
6
4
2
APC, EVC2, FBXW7, KRAS, PIK3CA, TCF7L2, TP53
APC, EVC2, KRAS, PIK3CA, TCF7L2
APC, EPHA3, EVC2, FBXW7, KRAS, PIK3CA, TCF7L2, TP53
APC, EVC2, KRAS, TP53
APC, EVC2, FBXW7, KRAS
APC, EPHA3, PIK3CA, TP53
EPHA3, KRAS, TP53
APC, EPHA3, KRAS
EPHA3
WT
PIK3CA, TP53
EVC2, TP53
APC, FBXW7
90
1000
DzBPczCcyoBTNSz. CAPRI
DzBPczCcyoBTNSz. CBN_ot
APC
EPHA3
EVC2
FBXW7
TCF7L2
KRAS
PIK3CA
TP53

ID	p−value	Accessible Genot.
xWRtABuUSnLvQlx	0.612	118

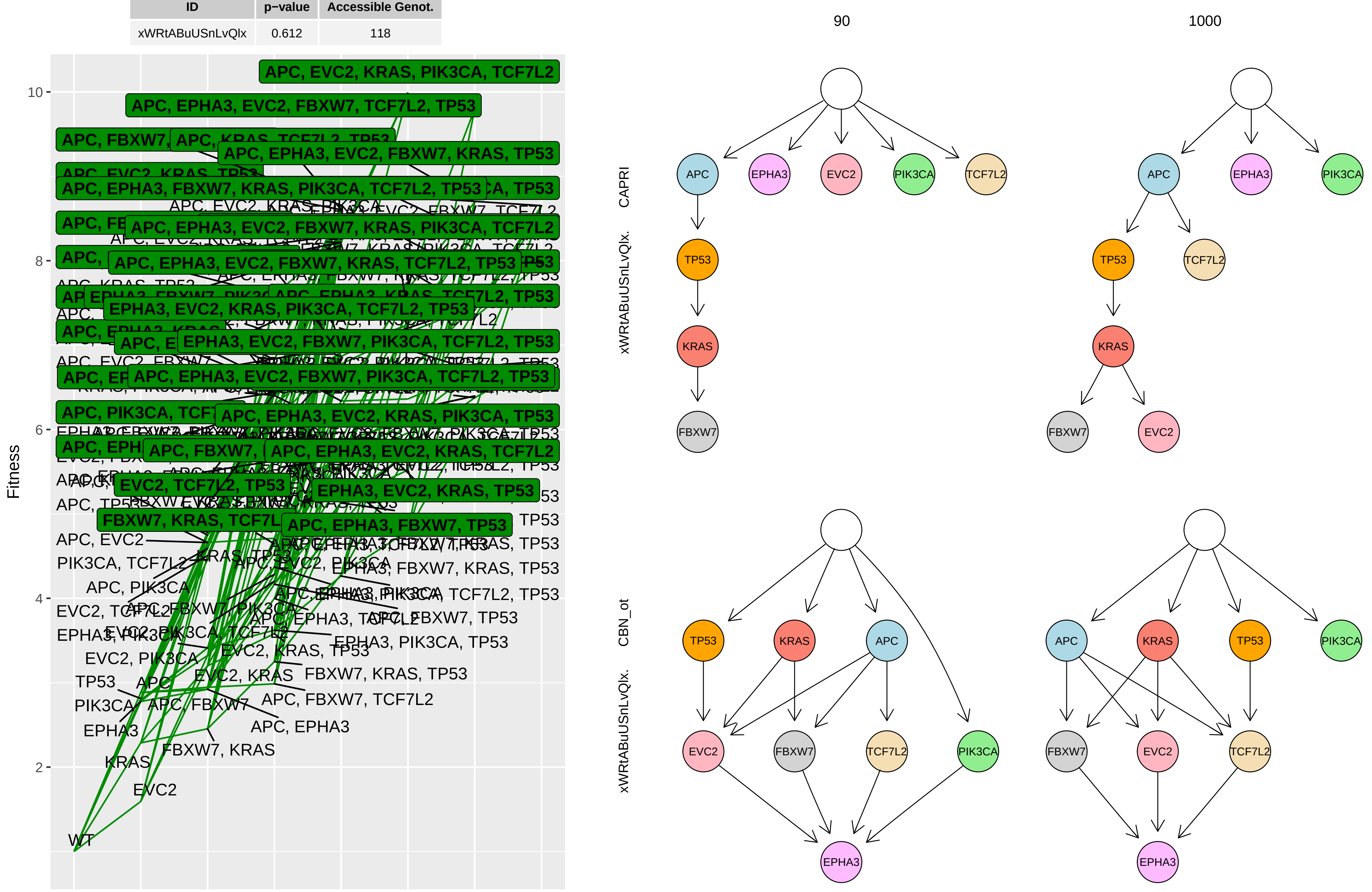

ID	p−value	Accessible Genot.
YXJWbdGvHzjyCdR	0.614	43

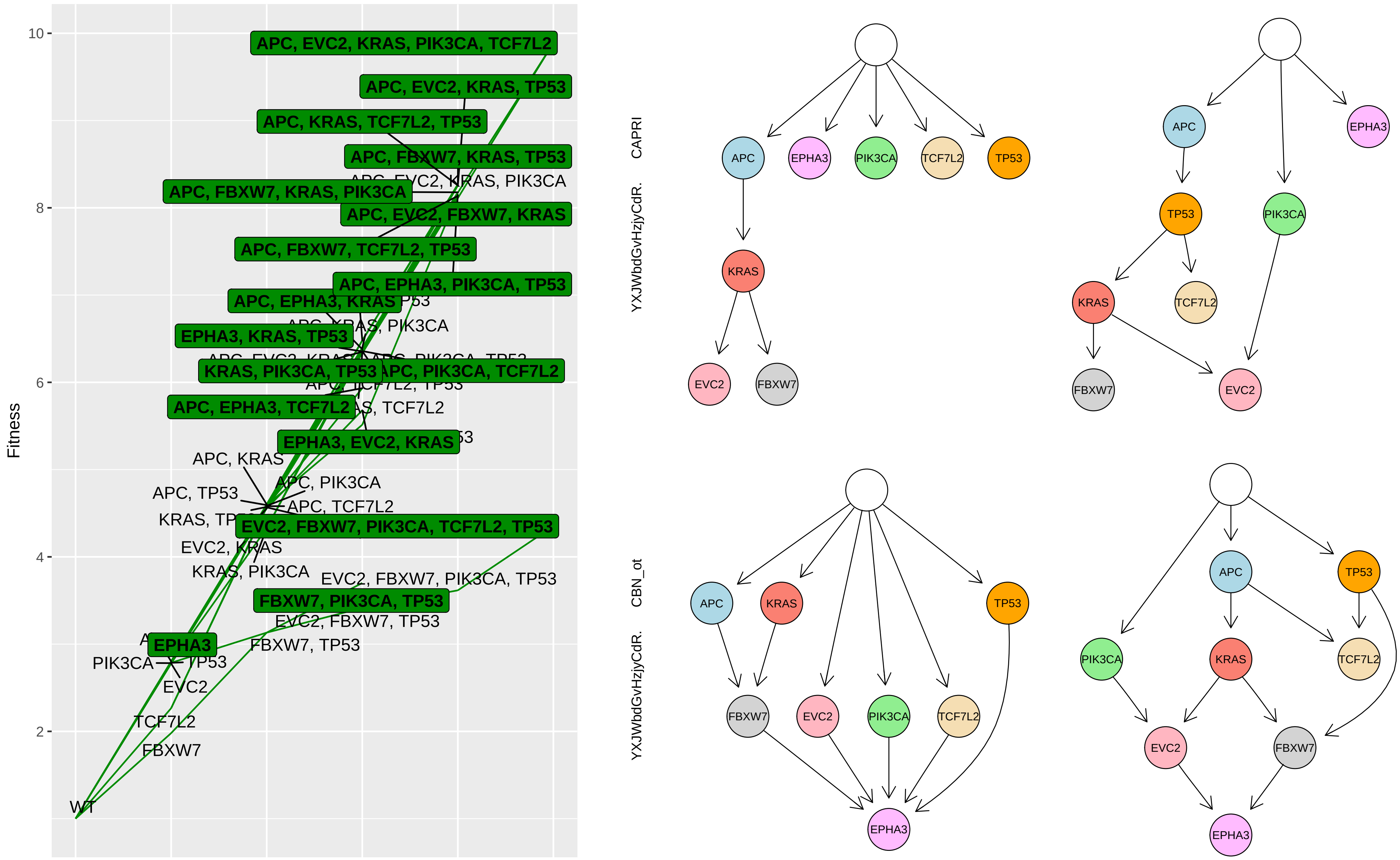

ID	p−value	Accessible Genot.
SZacVuyjyOoXhTa	0.615	133

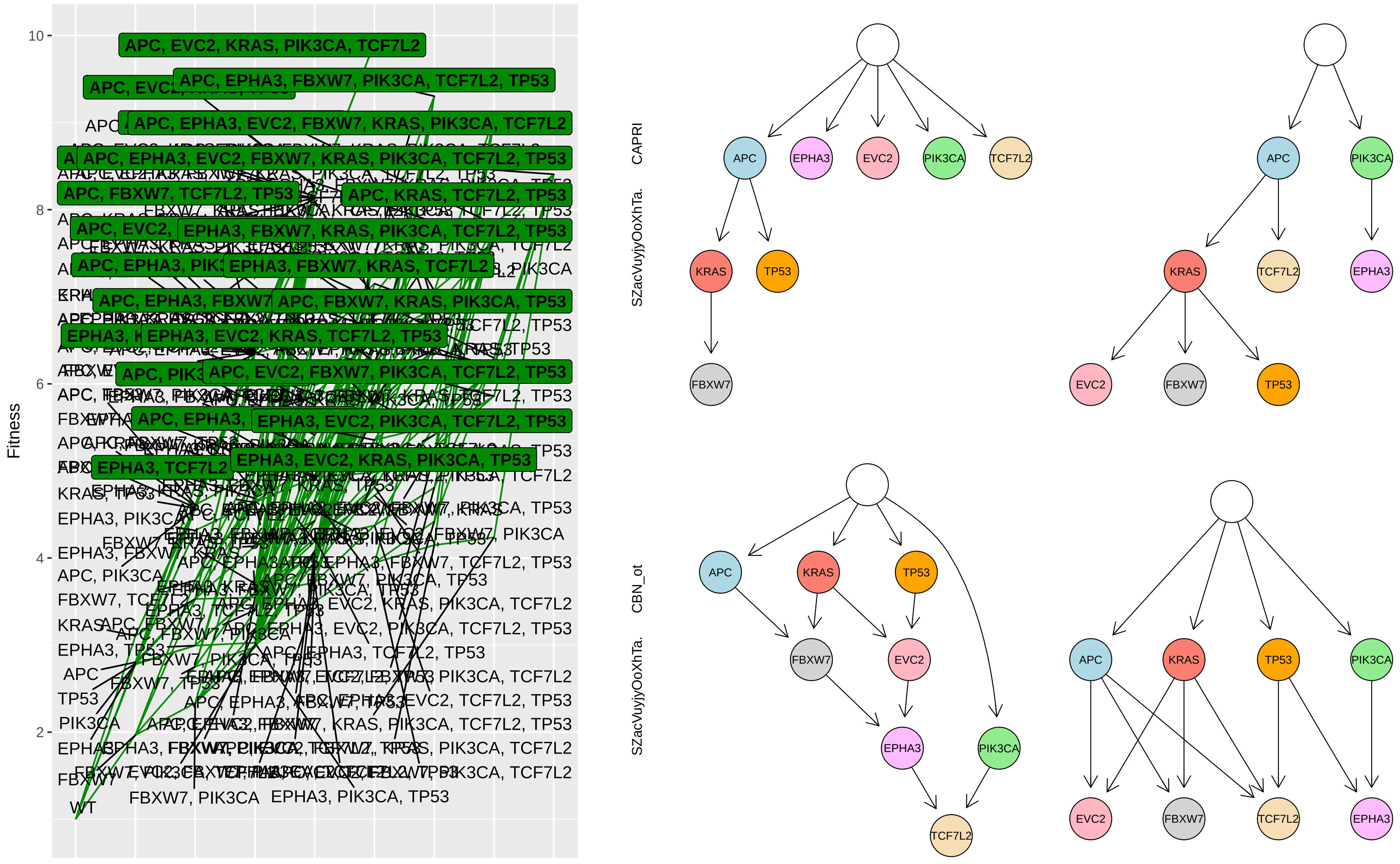

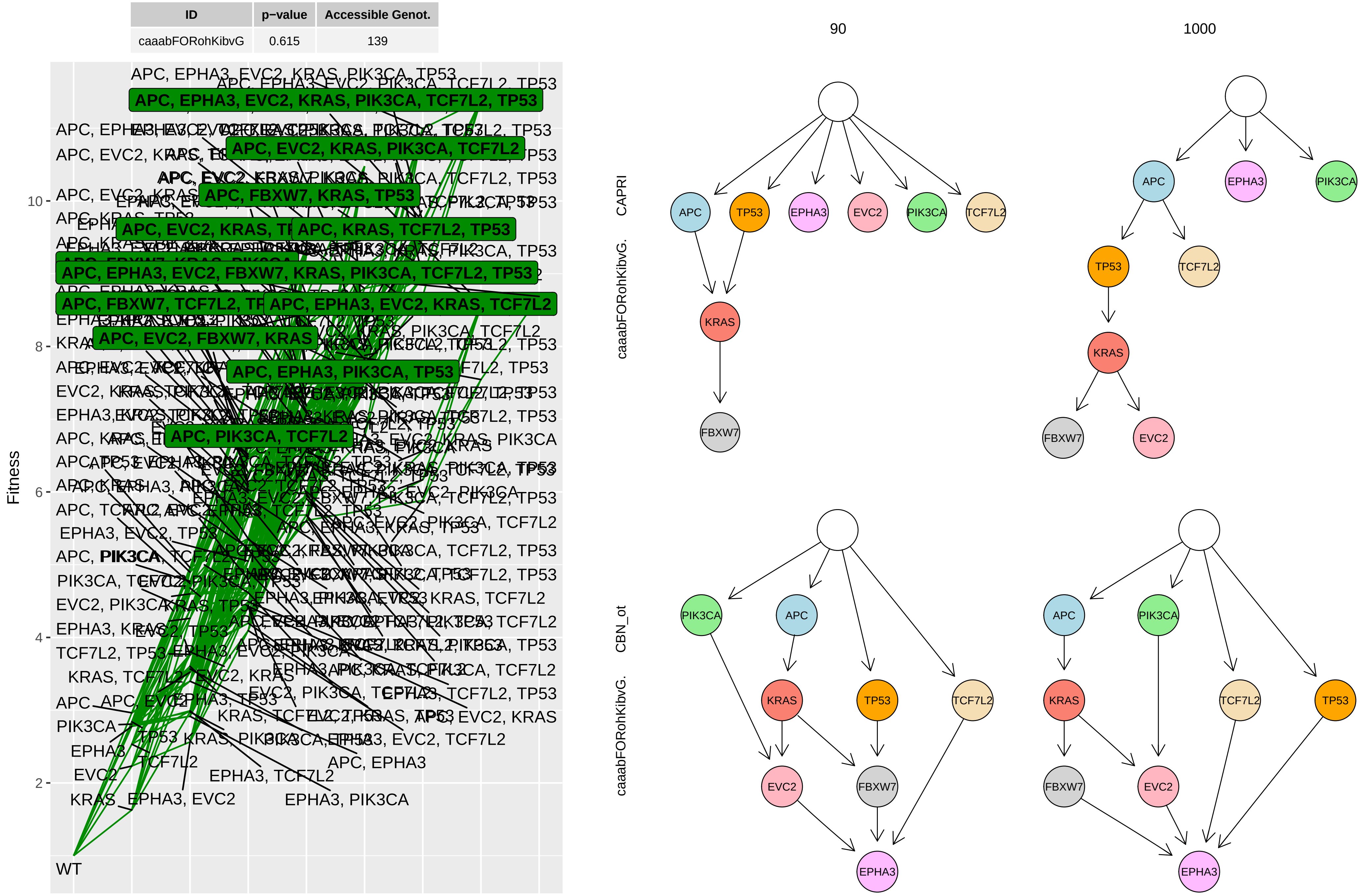
ID
p−value
Accessible Genot.
caaabFORohKibvG
0.615
139
Fitness
WT
90
1000
CAPRI
CBN_ot
caaabFORohKibvG.
APC
TP53
EPHA3
EVC2
PIK3CA
TCF7L2
KRAS
FBXW7

ID	p−value	Accessible Genot.
IGDYYidyLtlXYFy	0.616	37

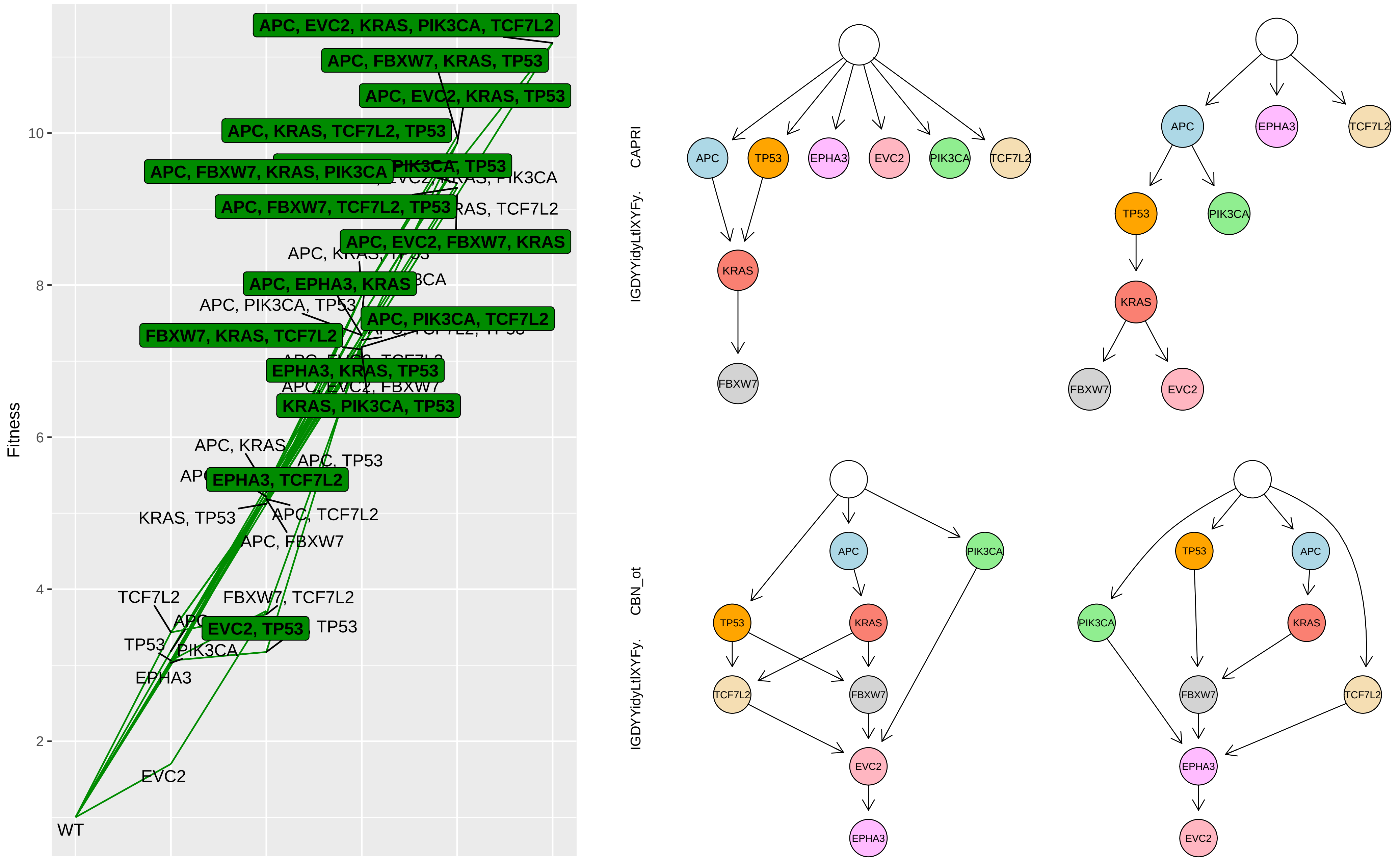

ID	p−value	Accessible Genot.
iMEZkHGtBJXsDnn	0.617	229

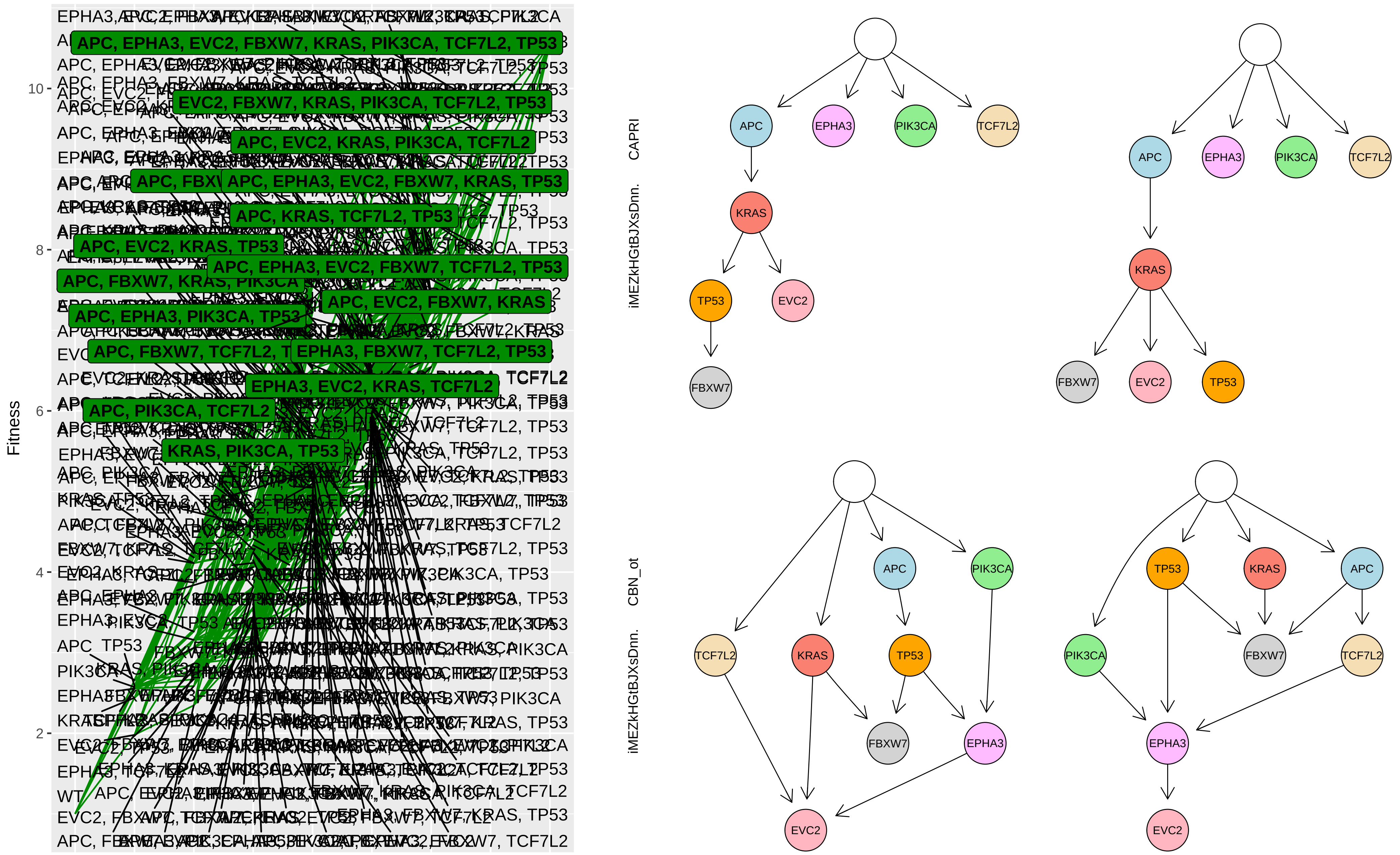

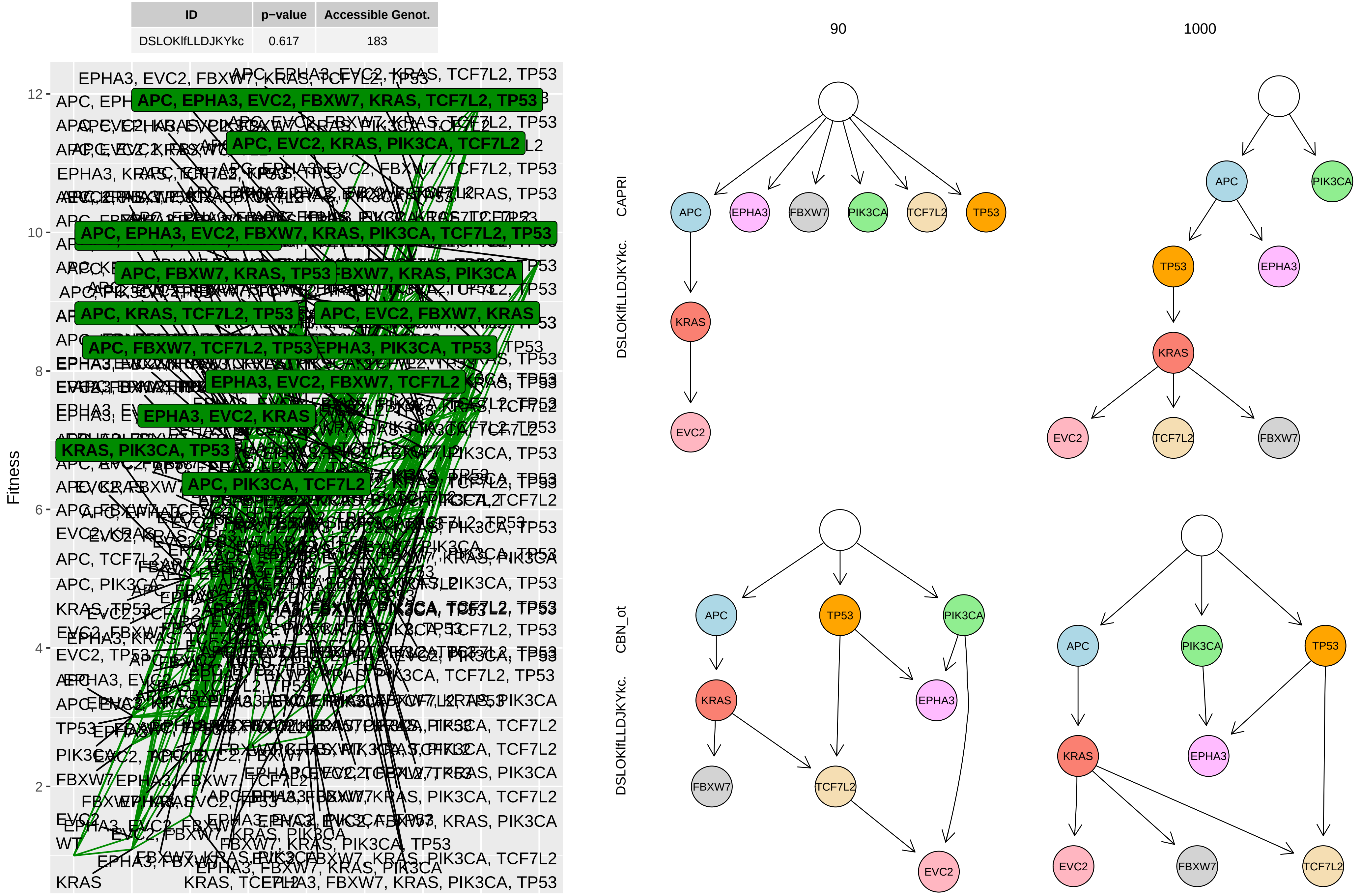

ID
p−value
Accessible Genot.
DSLOKIfLLDJKYkc
0.617
183
Fitness
12
10
8
6
4
2
APC, EPHA3, EVC2, FBXW7, KRAS, TCF7L2, TP53
APC, EVC2, KRAS, PIK3CA, TCF7L2
APC, EPHA3, EVC2, FBXW7, KRAS, PIK3CA, TCF7L2, TP53
APC, FBXW7, KRAS, TP53
APC, KRAS, TCF7L2, TP53
APC, EVC2, FBXW7, KRAS
APC, FBXW7, TCF7L2, TP53
EPHA3, PIK3CA, TP53
EPHA3, EVC2, FBXW7, TCF7L2
EPHA3, EVC2, KRAS
KRAS, PIK3CA, TP53
APC, PIK3CA, TCF7L2
WT
KRAS
90
1000
CAPRI
CBN_ot
DSLOKIfLLDJKYkc.
APC
EPHA3
FBXW7
PIK3CA
TCF7L2
TP53
KRAS
EVC2

ID	p−value	Accessible Genot.
vGIltCByrmZuzLS	0.617	253

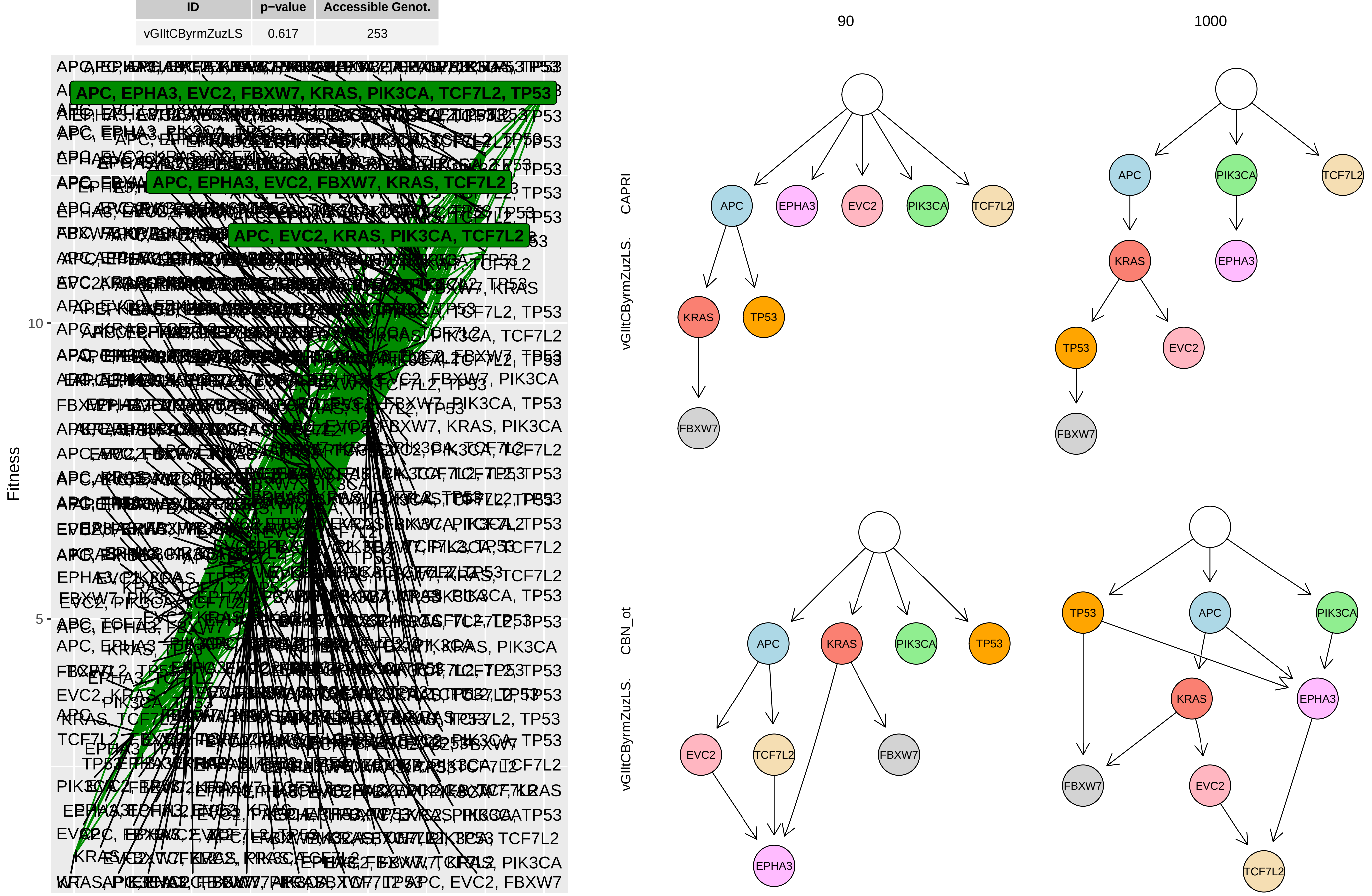

ID	p−value	Accessible Genot.
pCLTZWUjlSAcwWQ	0.618	87

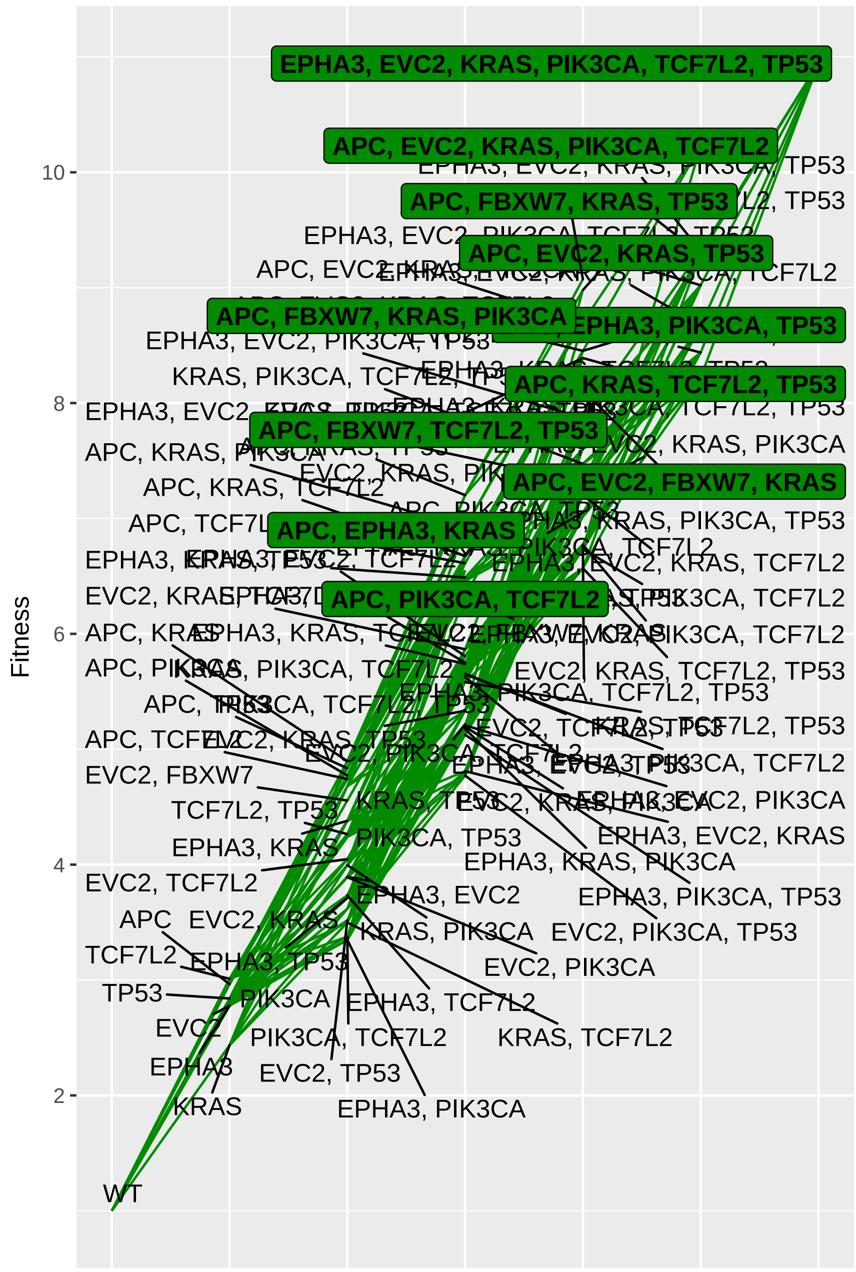

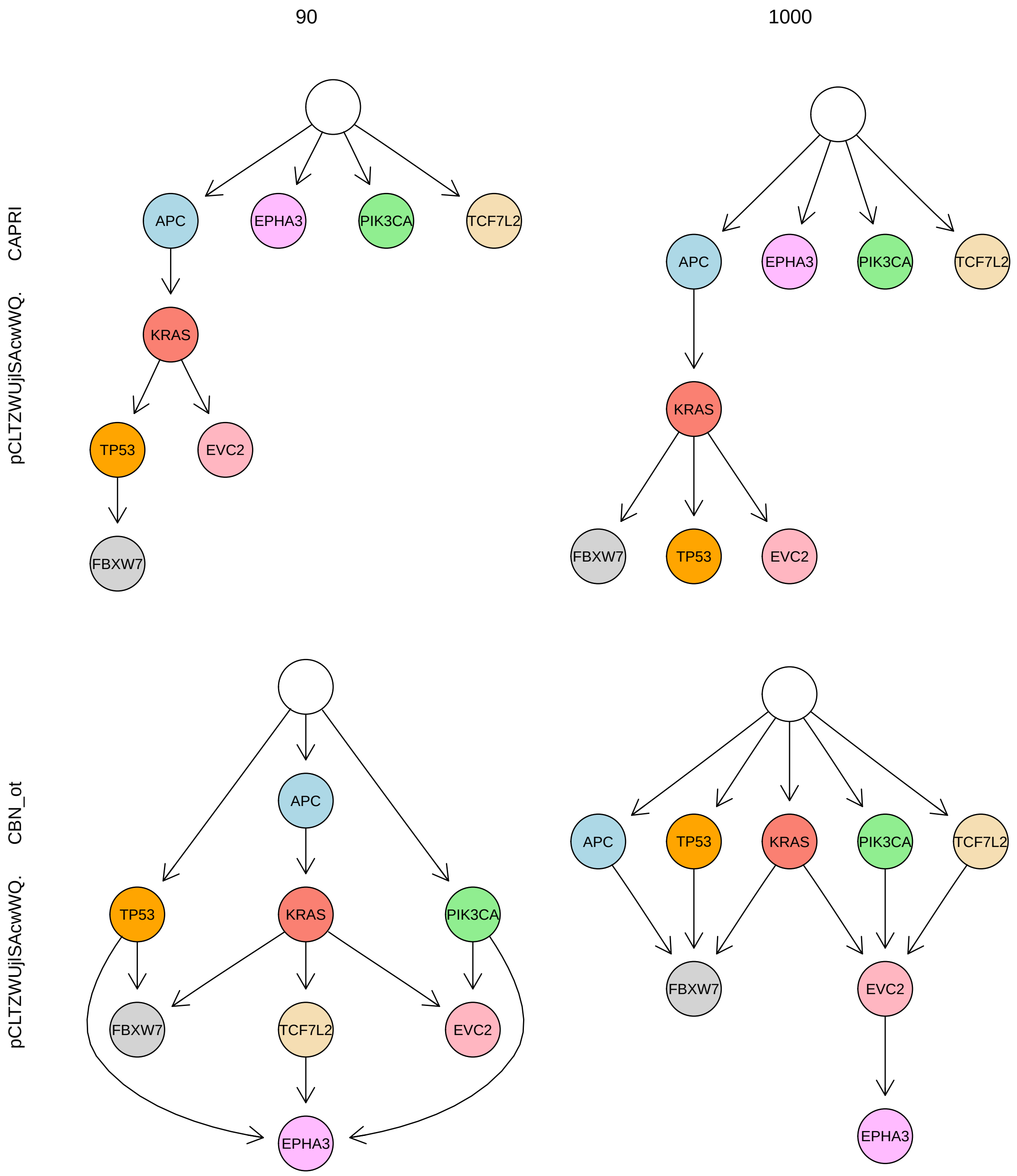

ID	p−value	Accessible Genot.
AUGMfEKPRmbsZXA	0.62	90

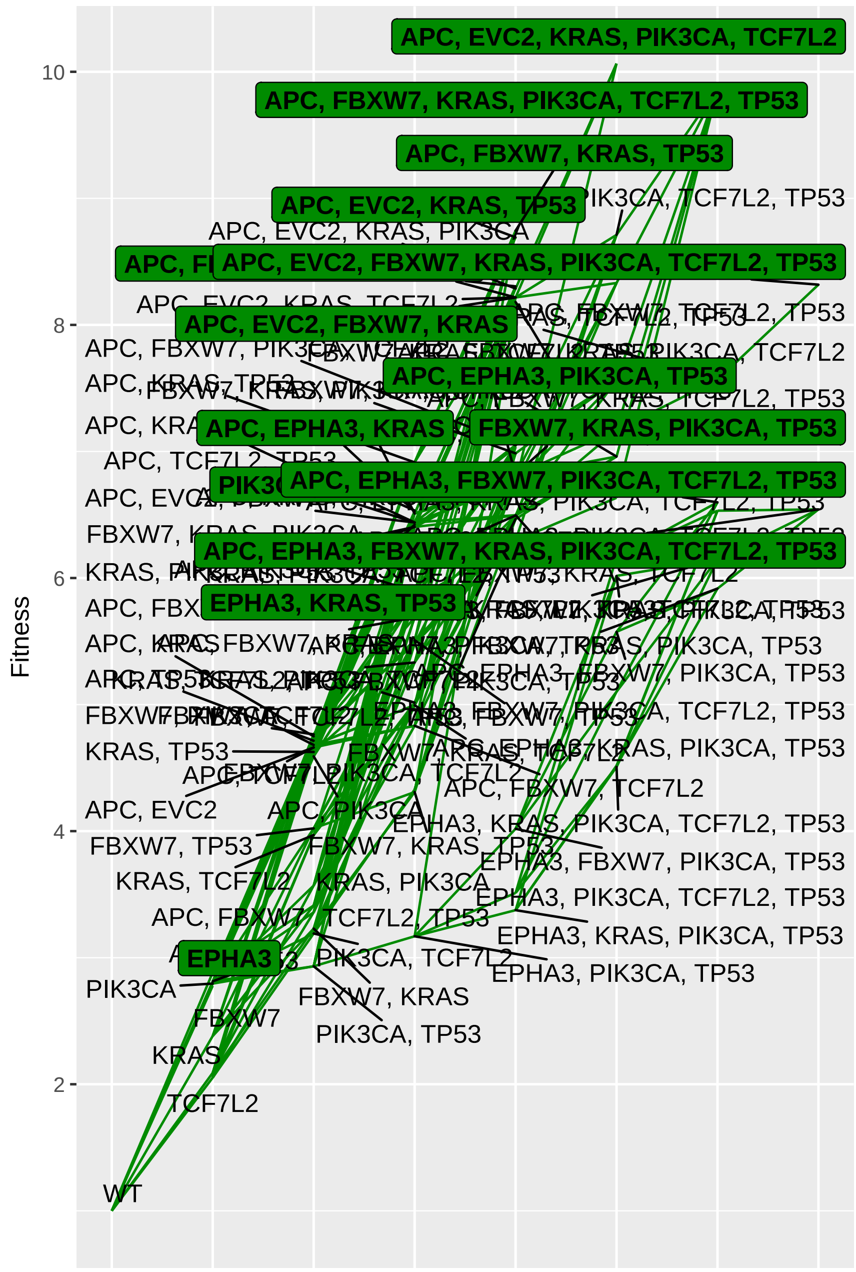

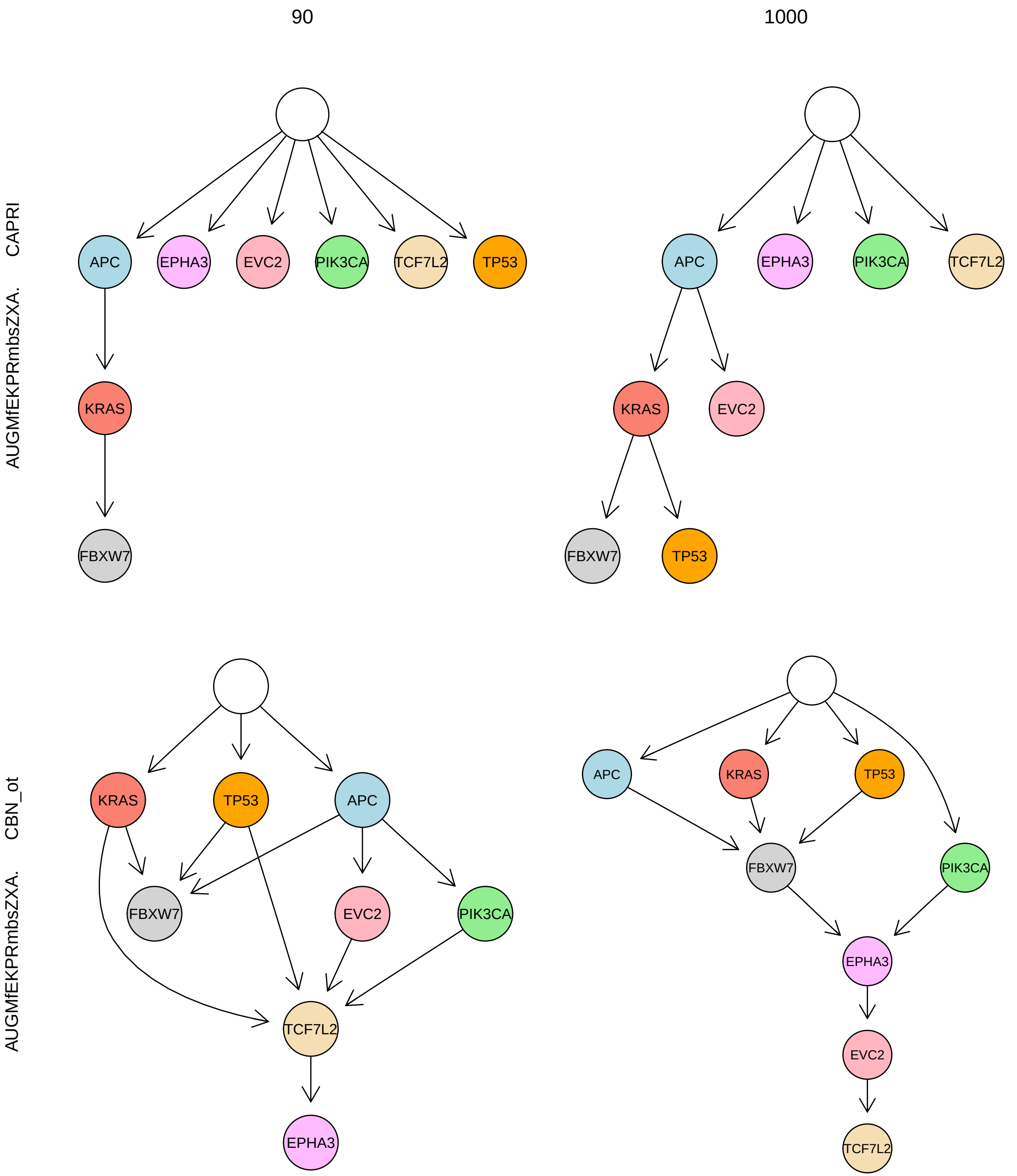

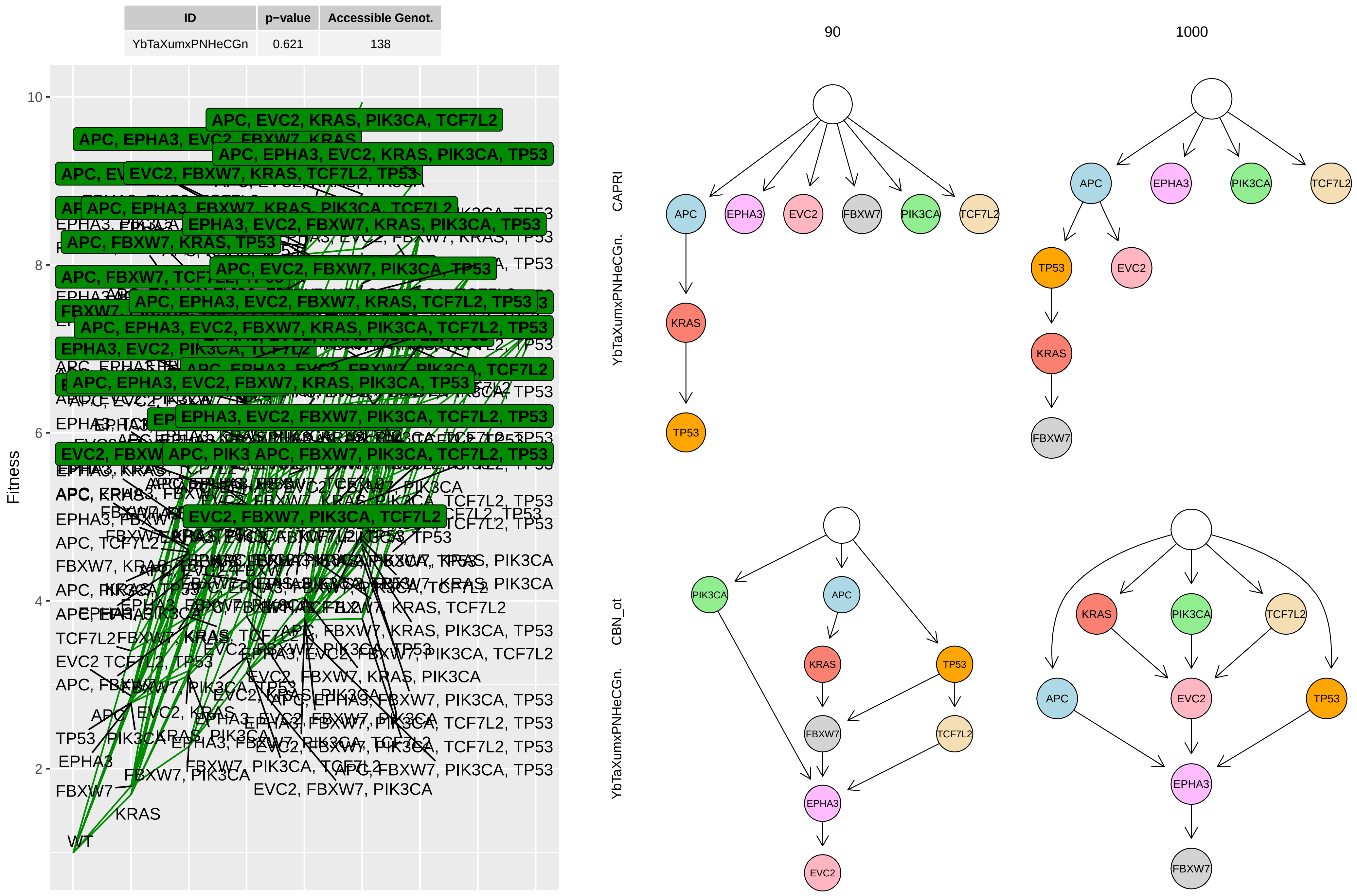

ID
p−value
Accessible Genot.
YbTaXumxPNHeCGn
0.621
138
Fitness
10
8
6
4
2
WT
KRAS
FBXW7
EPHA3
90
1000
CAPRI
CBN_ot
YbTaXumxPNHeCGn.
APC
EPHA3
EVC2
FBXW7
PIK3CA
TCF7L2
KRAS
TP53

ID	p−value	Accessible Genot.
BiAjpWzmICFOGUX	0.622	87

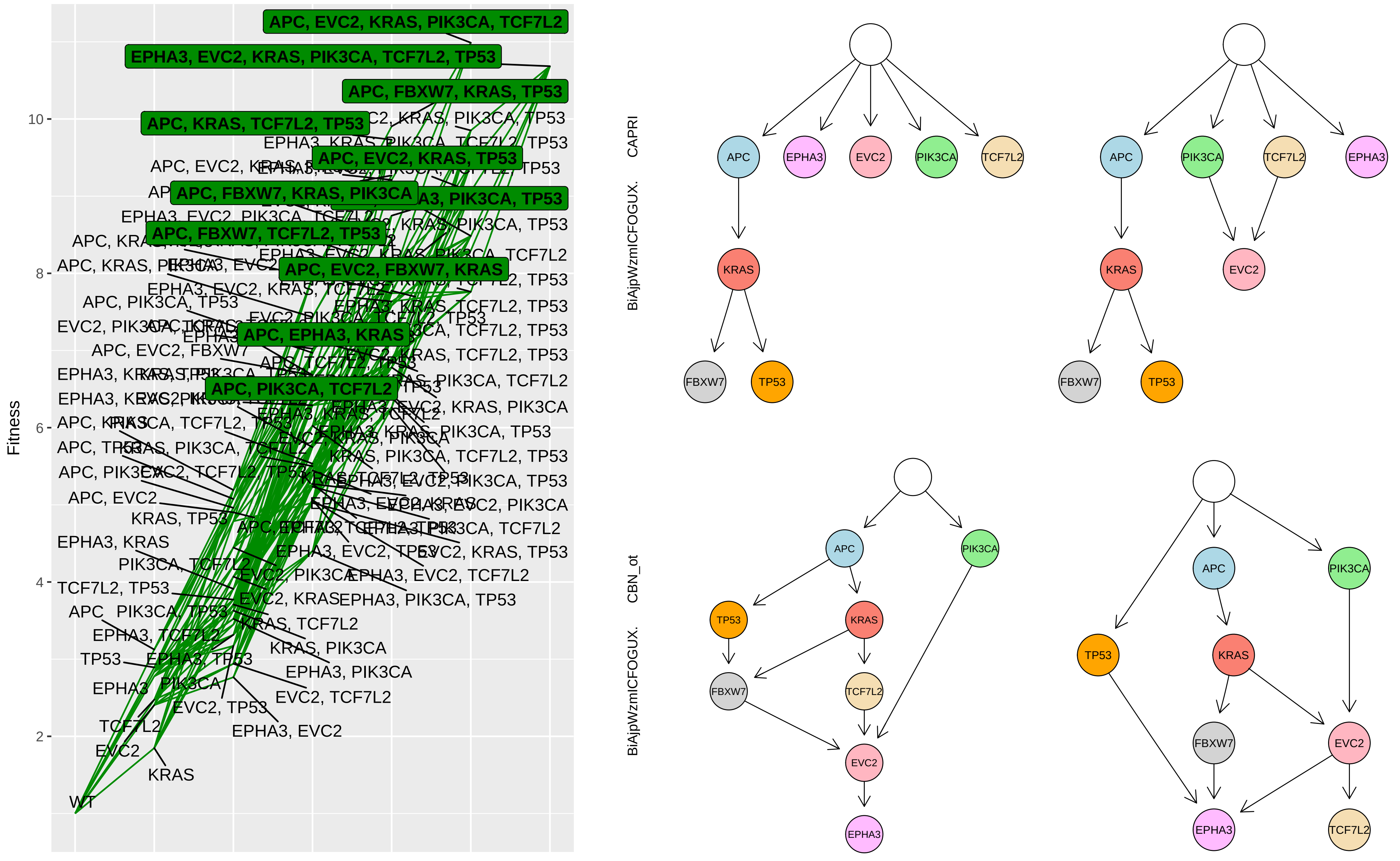

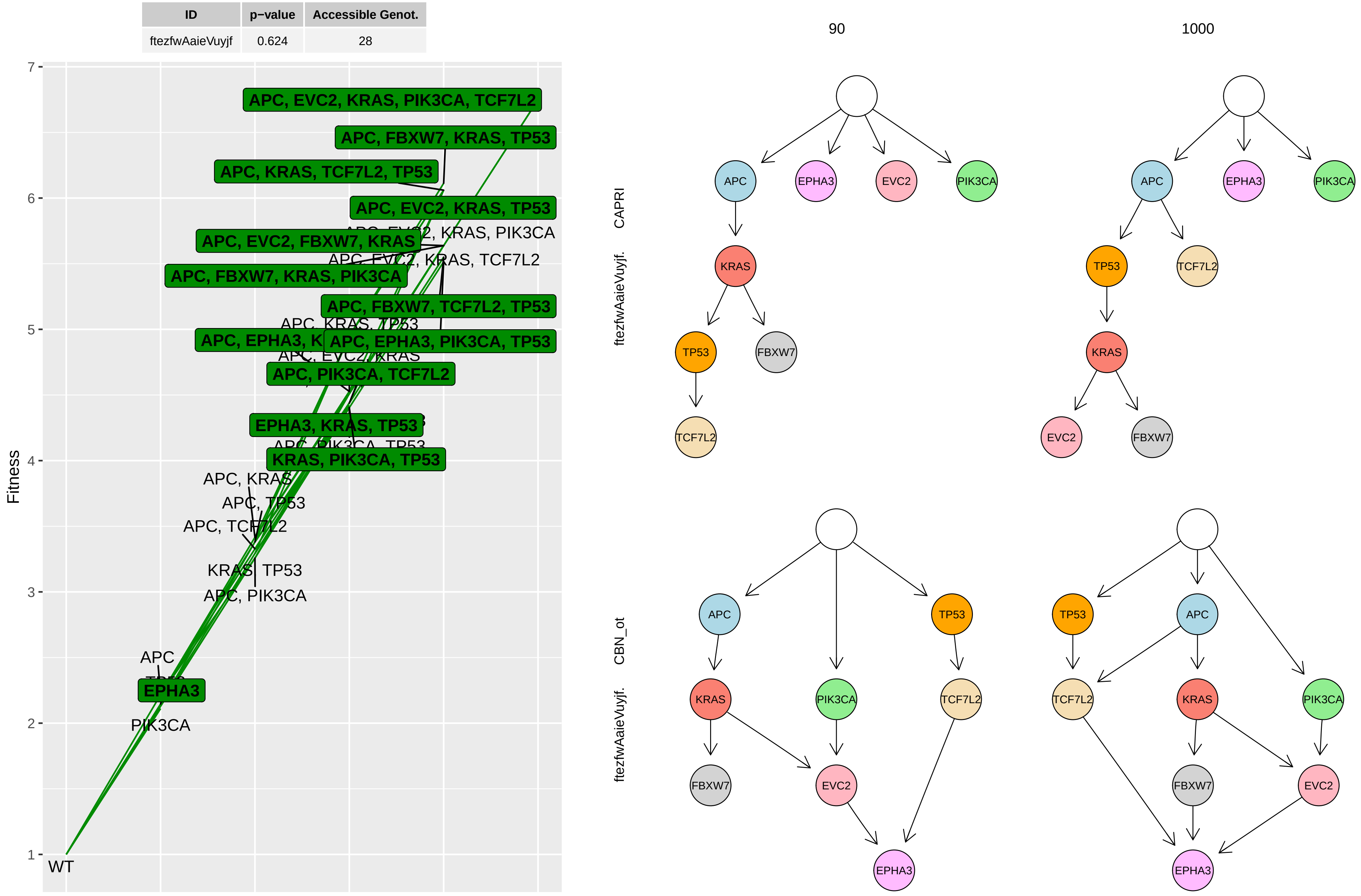
ID
p−value
Accessible Genot.
ftezfwAaieVuyjf
0.624
28
Fitness
APC, EVC2, KRAS, PIK3CA, TCF7L2
APC, FBXW7, KRAS, TP53
APC, KRAS, TCF7L2, TP53
APC, EVC2, KRAS, TP53
APC, EVC2, FBXW7, KRAS
APC, FBXW7, KRAS, PIK3CA
APC, FBXW7, TCF7L2, TP53
APC, EPHA3, PIK3CA, TP53
APC, PIK3CA, TCF7L2
EPHA3, KRAS, TP53
KRAS, PIK3CA, TP53
APC, KRAS
APC, TP53
APC, TCF7L2
KRAS
TP53
APC, PIK3CA
APC
EPHA3
PIK3CA
WT
90
1000
CAPRI
CBN_ot
ftezfwAaieVuyjf.
APC
EPHA3
EVC2
PIK3CA
KRAS
TP53
FBXW7
TCF7L2

ID	p−value	Accessible Genot.
NBjhTQTUvOWfNfH	0.626	178

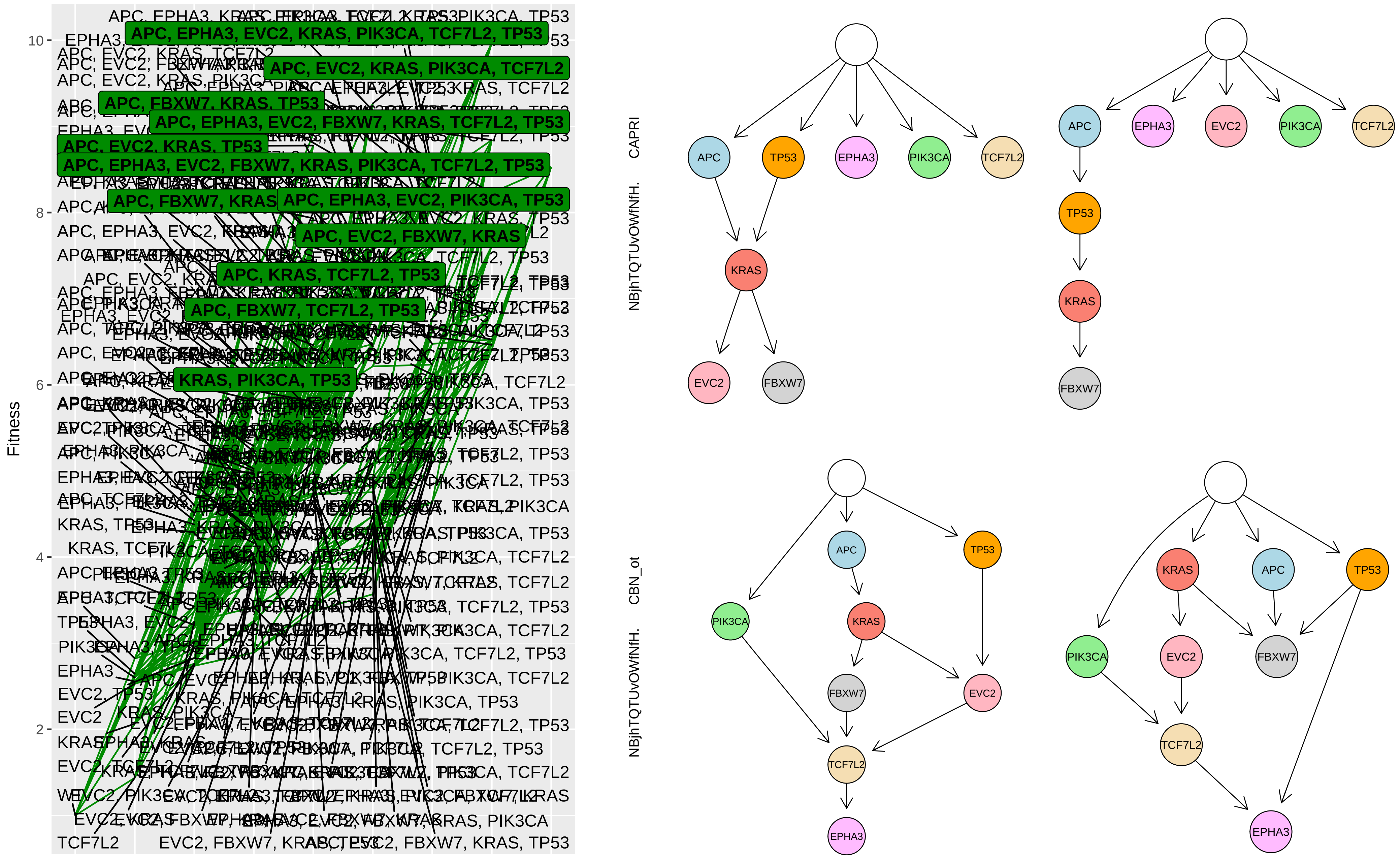

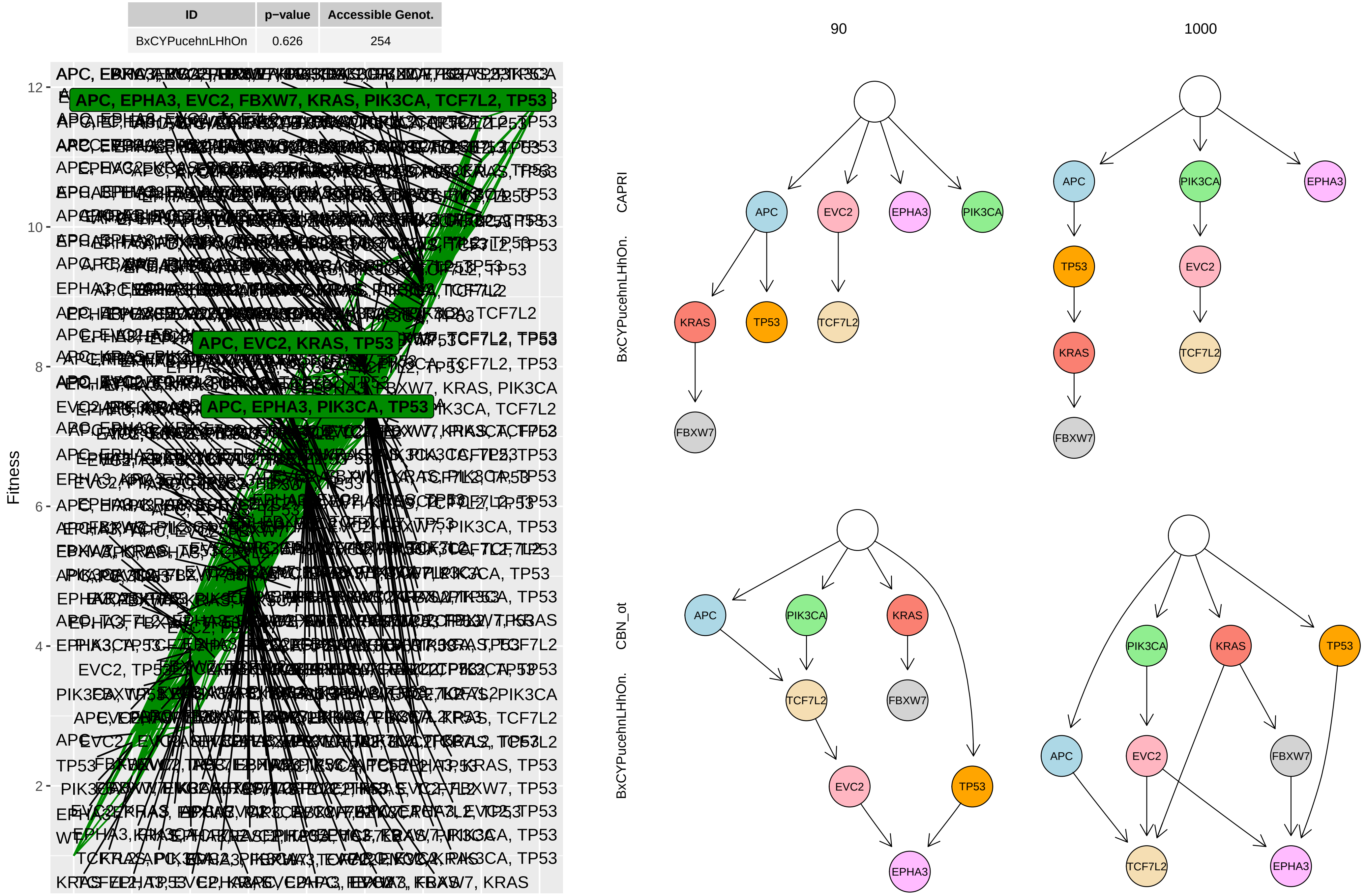

ID
p−value
Accessible Genot.
BxCYPucehnLHhOn
0.626
254
APC, EPHA3, EVC2, FBXW7, KRAS, PIK3CA, TCF7L2, TP53
APC, EVC2, KRAS, TP53
APC, EPHA3, PIK3CA, TP53
Fitness
12
10
8
6
4
2
90
1000
BxCYPucehnLHhOn. CAPRI
BxCYPucehnLHhOn. CBN_ot
APC
EVC2
EPHA3
PIK3CA
KRAS
TP53
TCF7L2
FBXW7

ID	p−value	Accessible Genot.
gPLOCqjxAYedeML	0.626	55

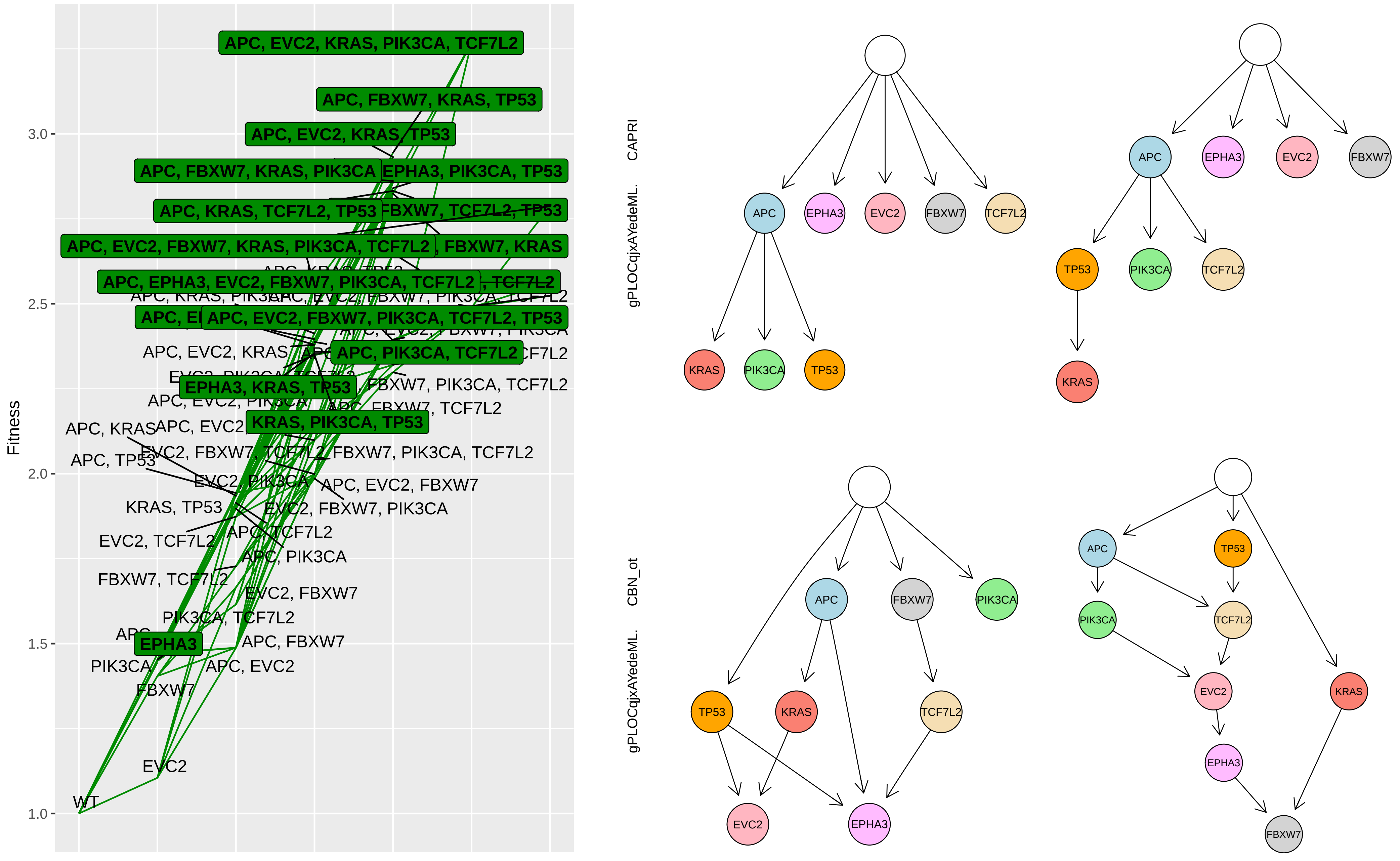

ID	p−value	Accessible Genot.
bDNUnRjuwkTwXbj	0.628	244

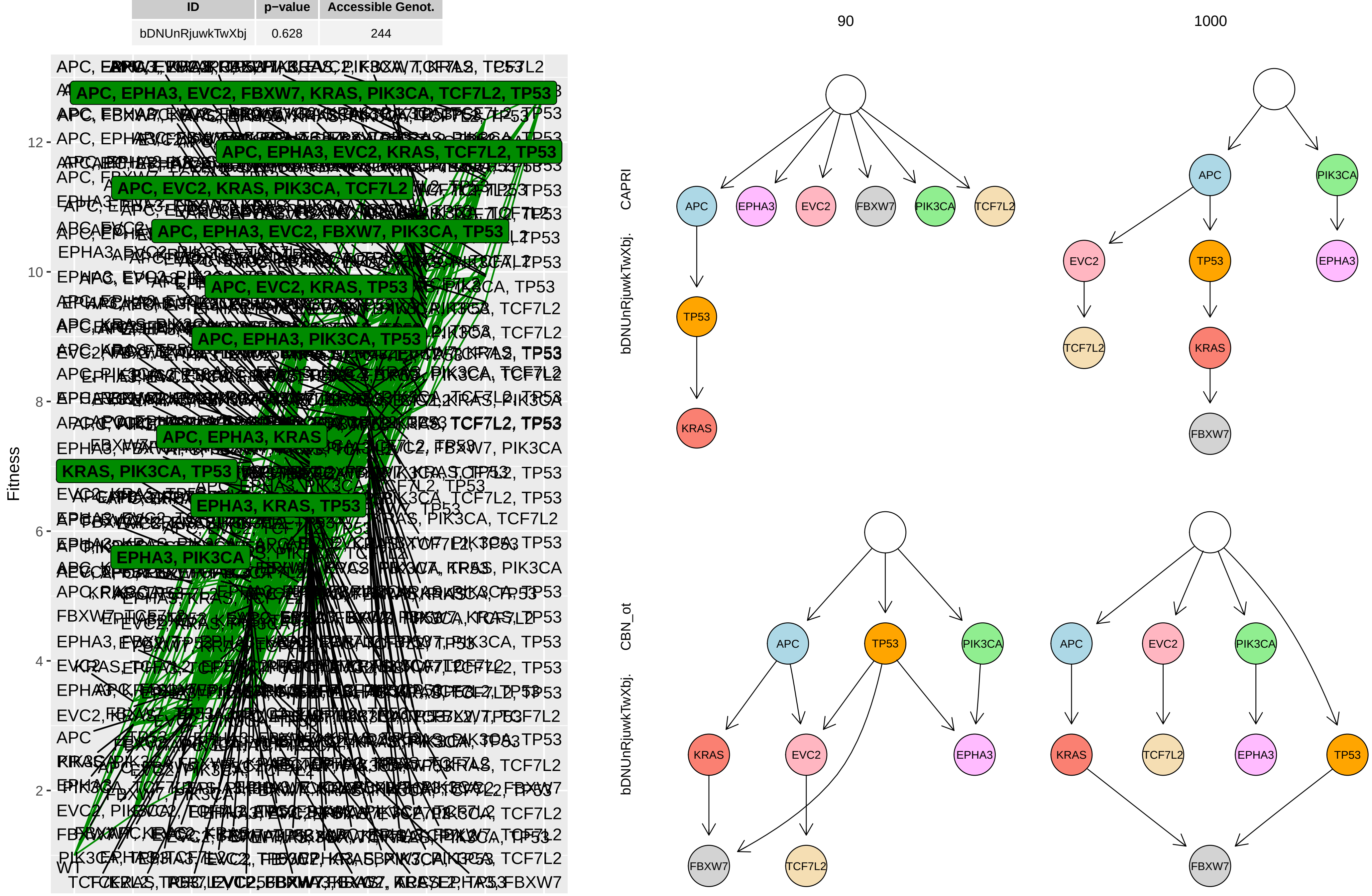

ID	p−value	Accessible Genot.
xAkyFmwvESuiOnV	0.63	36

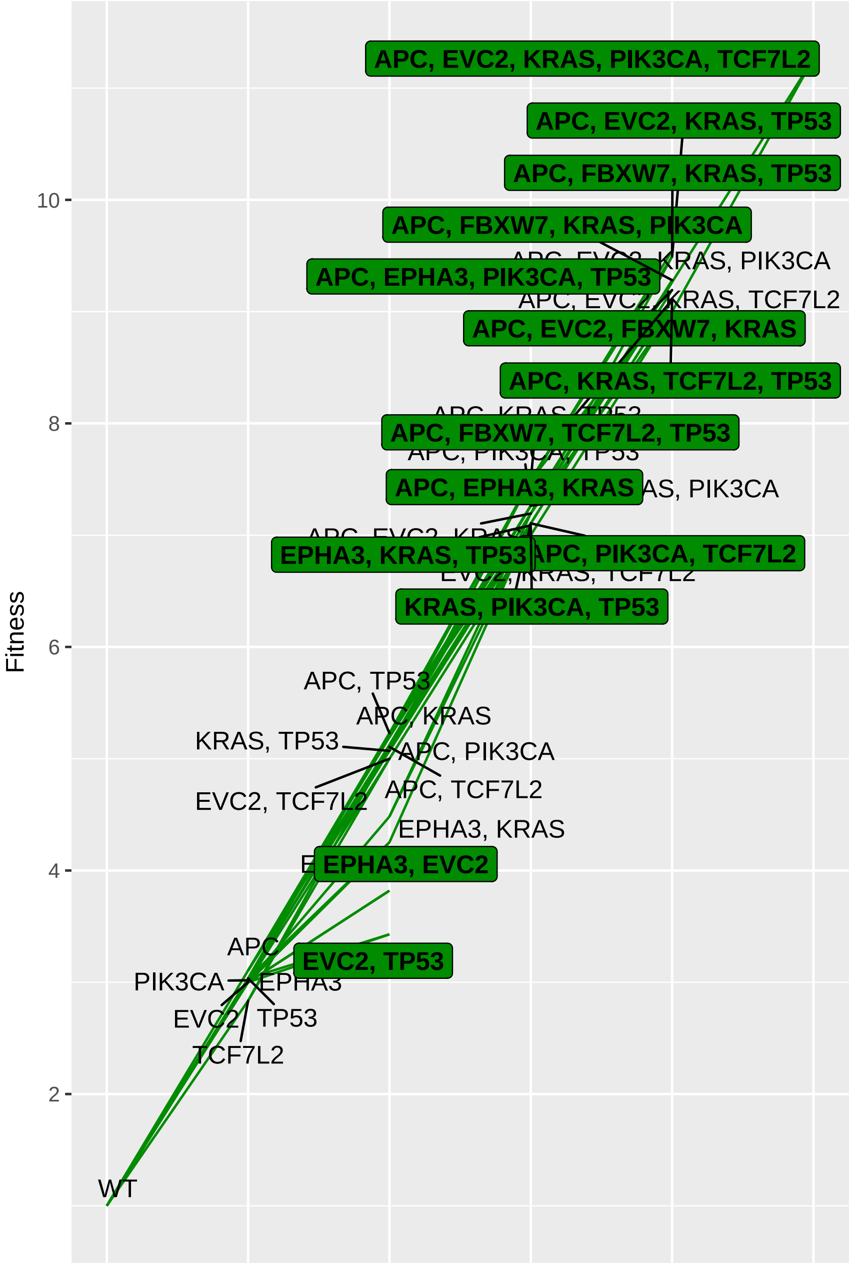

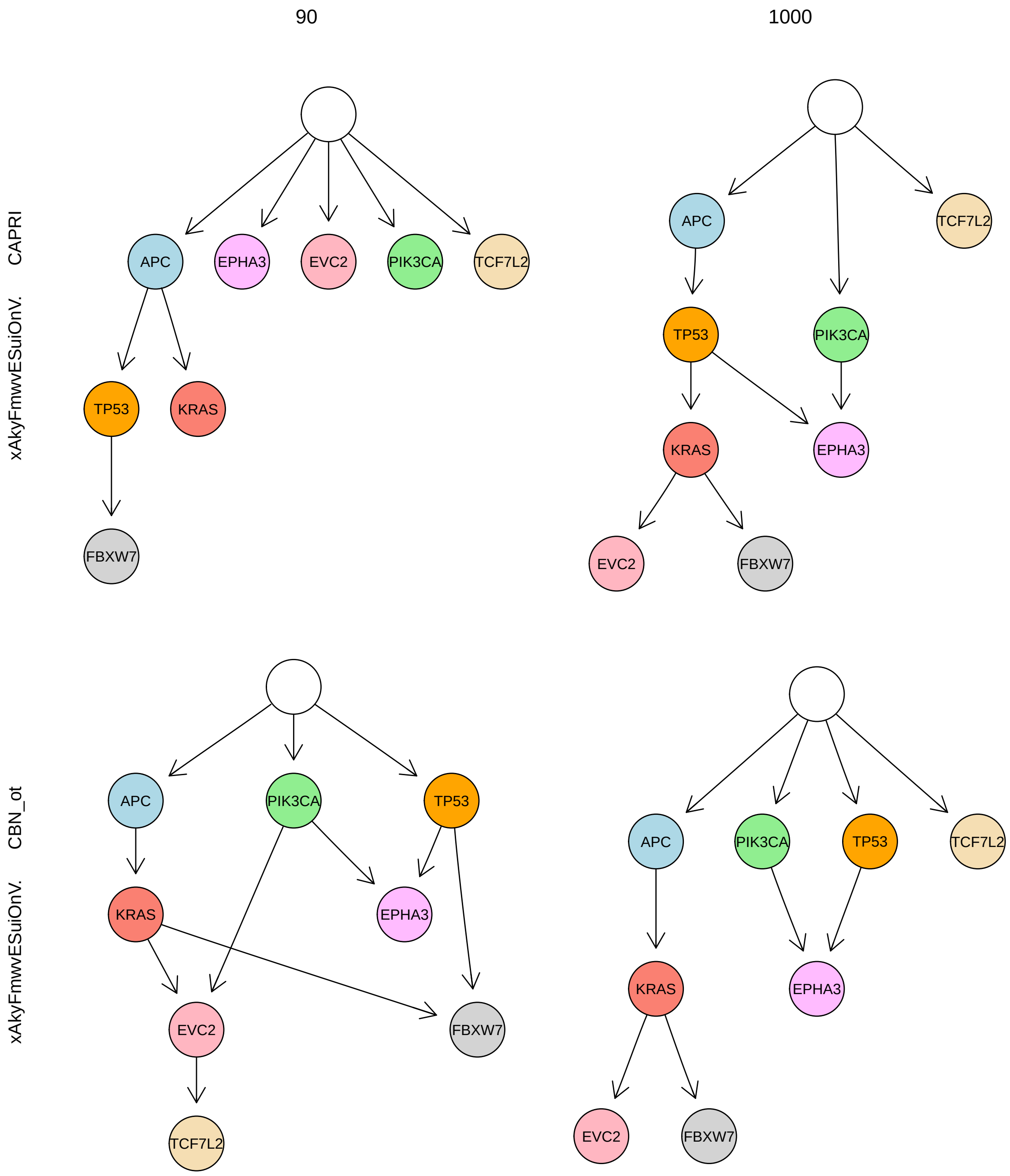

ID	p−value	Accessible Genot.
JIkWgmAqllbcRcw	0.631	35

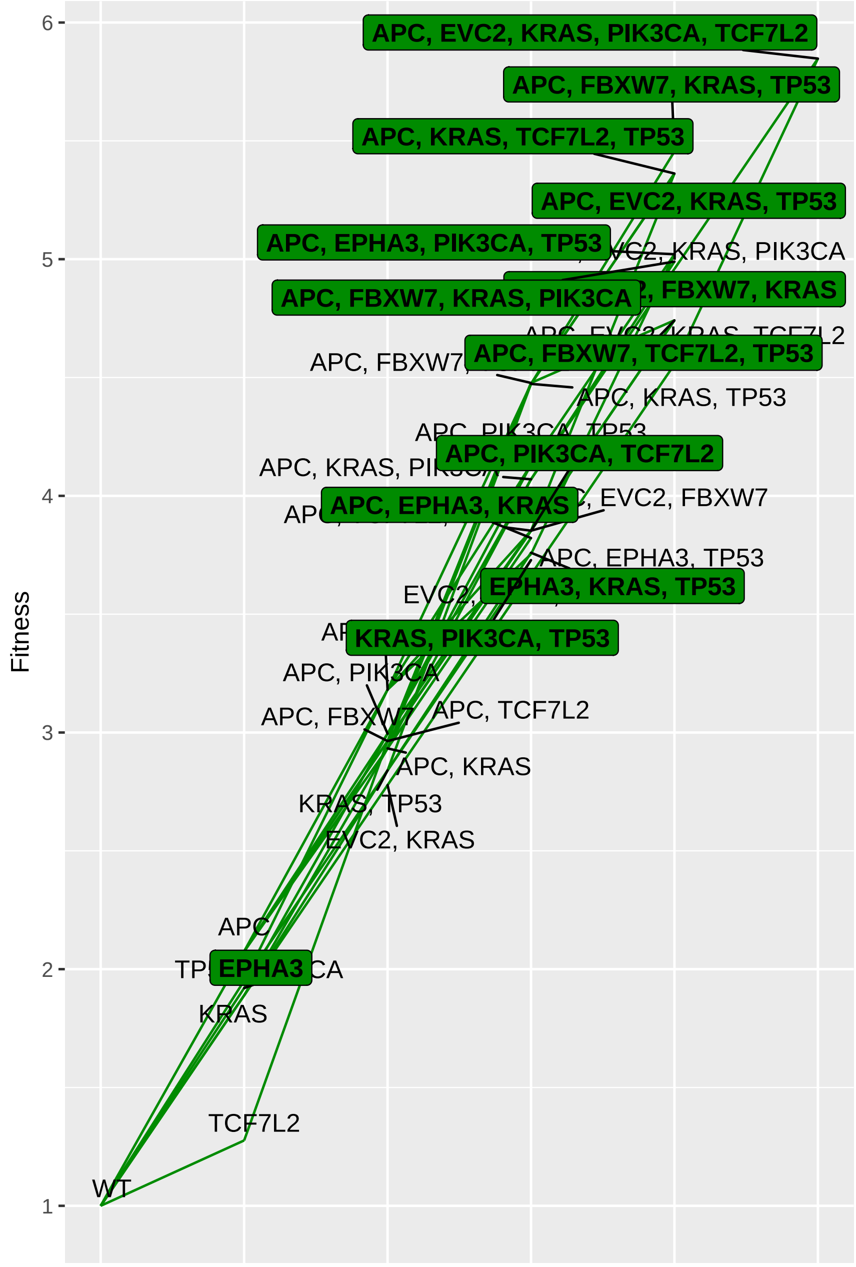

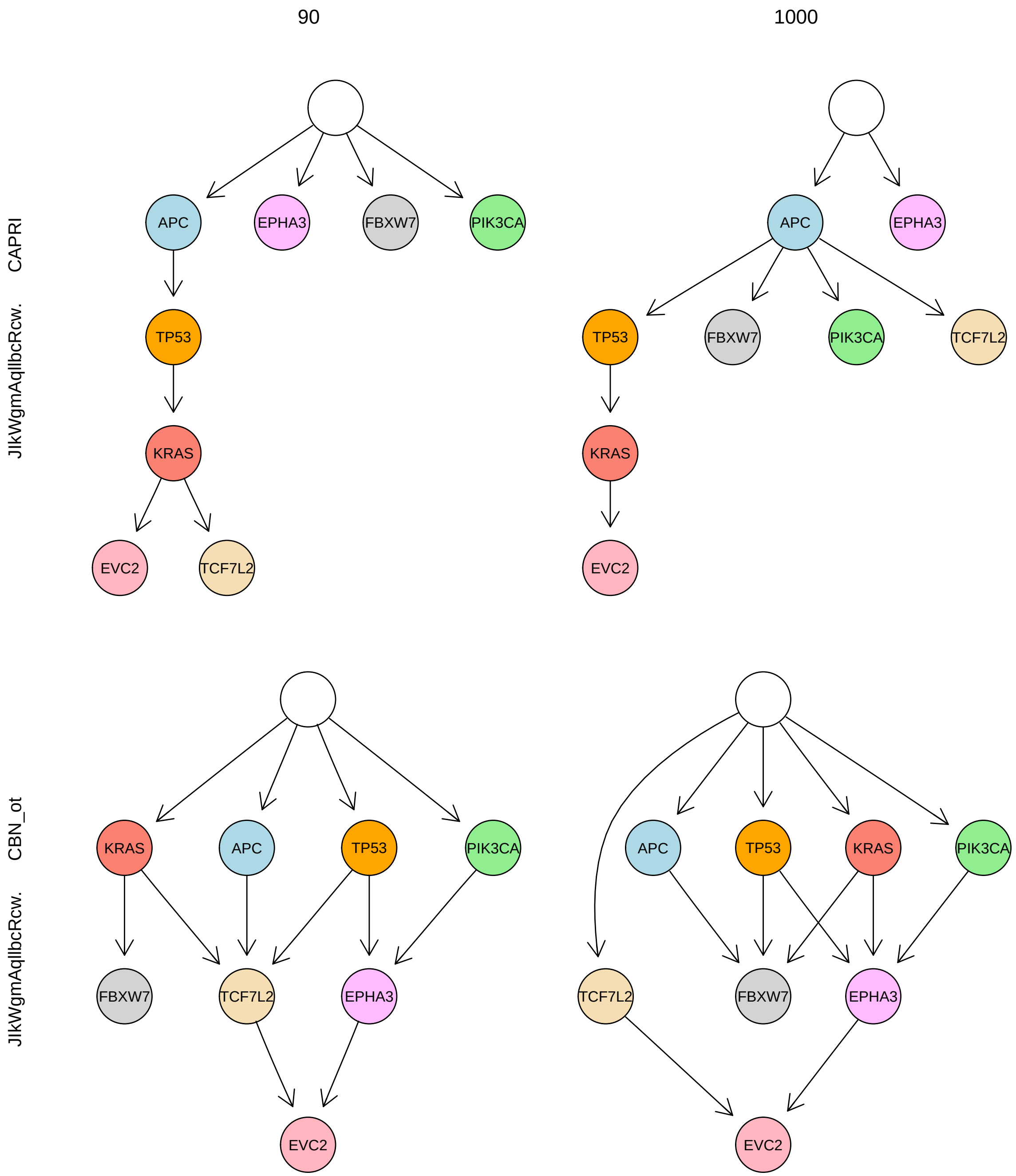

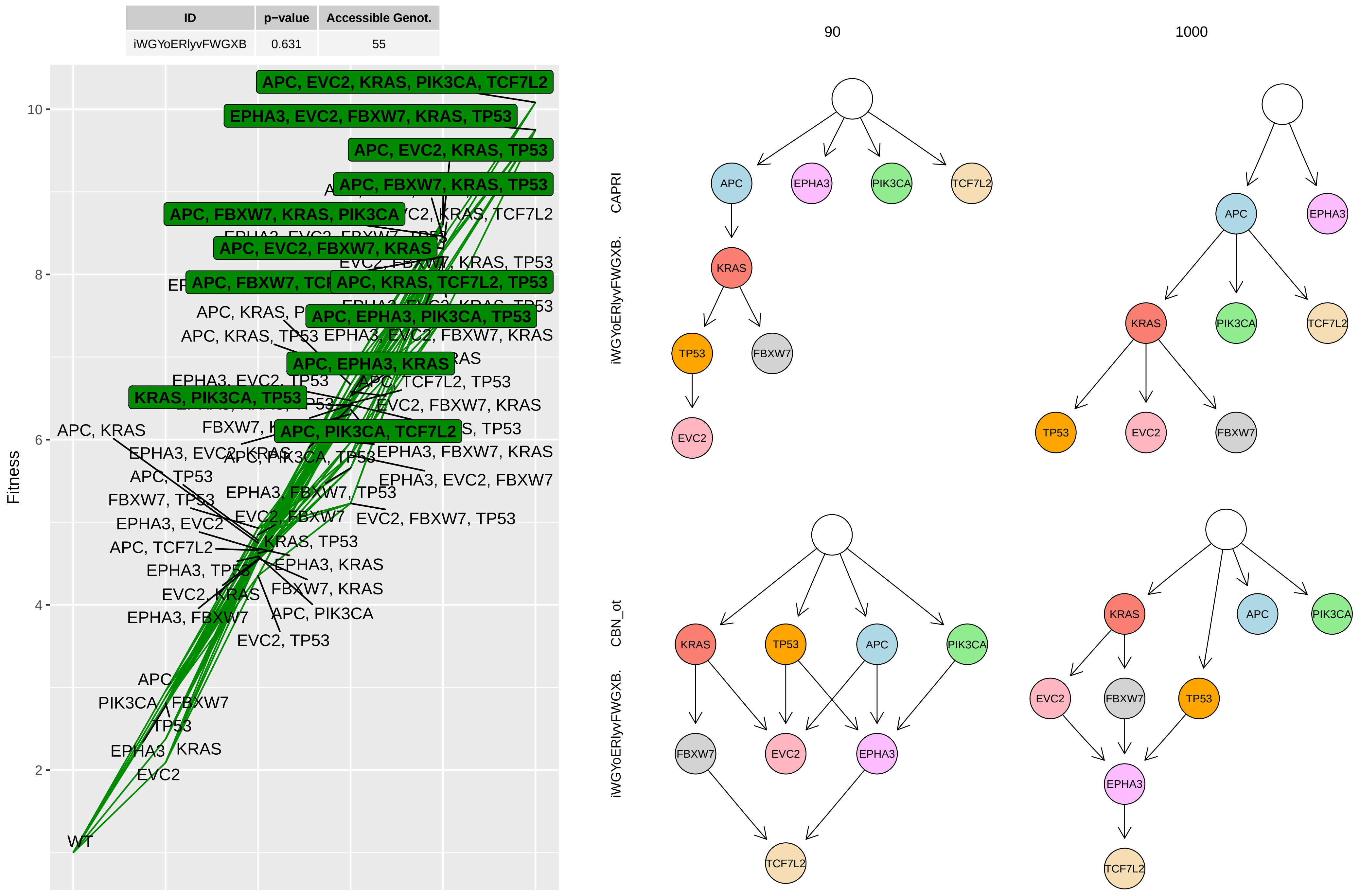

ID
p−value
Accessible Genot.
iWGYoERlyvFWGXB
0.631
55
Fitness
10
8
6
4
2
WT
APC
PIK3CA
FBXW7
TP53
EPHA3
KRAS
EVC2
APC, EVC2, KRAS, PIK3CA, TCF7L2
EPHA3, EVC2, FBXW7, KRAS, TP53
APC, EVC2, KRAS, TP53
APC, FBXW7, KRAS, TP53
APC, FBXW7, KRAS, PIK3CA
APC, EVC2, FBXW7, KRAS
APC, KRAS, TCF7L2, TP53
APC, EPHA3, PIK3CA, TP53
APC, EPHA3, KRAS
KRAS, PIK3CA, TP53
APC, PIK3CA, TCF7L2
APC, KRAS
90
1000
iWGYoERlyvFWGXB. CAPRI
iWGYoERlyvFWGXB. CBN_ot
APC
EPHA3
PIK3CA
TCF7L2
KRAS
TP53
FBXW7
EVC2

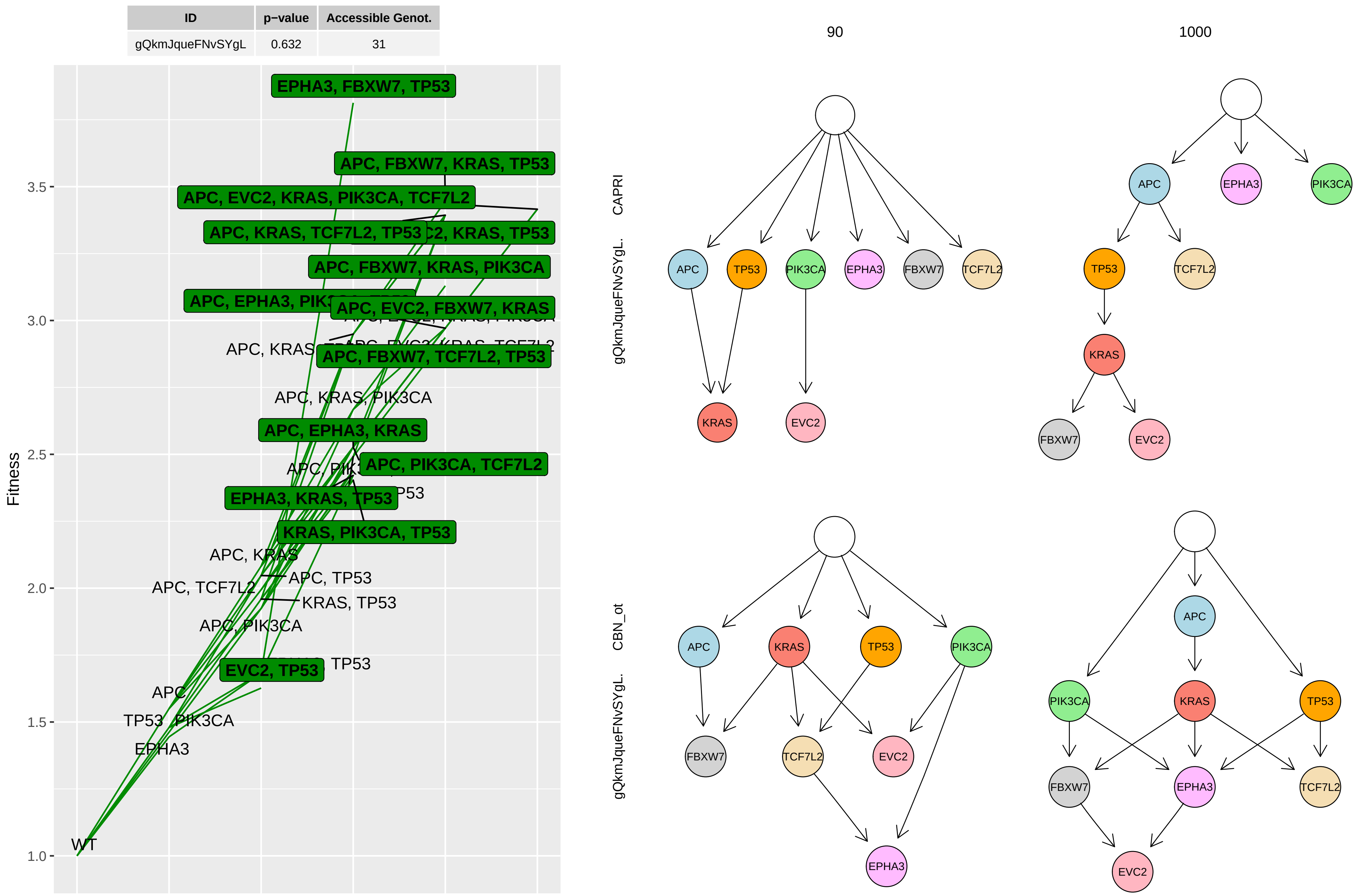

ID
p−value
Accessible Genot.
gQkmJqueFNvSYgL
0.632
31
Fitness
1.0
1.5
2.0
2.5
3.0
3.5
EPHA3, FBXW7, TP53
APC, FBXW7, KRAS, TP53
APC, EVC2, KRAS, PIK3CA, TCF7L2
APC, KRAS, TCF7L2, TP53
APC, FBXW7, KRAS, PIK3CA
APC, EVC2, FBXW7, KRAS
APC, FBXW7, TCF7L2, TP53
APC, KRAS, PIK3CA
APC, EPHA3, KRAS
APC, PIK3CA, TCF7L2
EPHA3, KRAS, TP53
KRAS, PIK3CA, TP53
APC, KRAS
APC, TP53
APC, TCF7L2
KRAS, TP53
APC, PIK3CA
EVC2, TP53
APC
TP53
PIK3CA
EPHA3
WT
90
1000
gQkmJqueFNvSYgL. CAPRI
gQkmJqueFNvSYgL. CBN_ot
APC
TP53
PIK3CA
EPHA3
FBXW7
TCF7L2
KRAS
EVC2

ID	p−value	Accessible Genot.
tNkbhTMENJNyVij	0.633	103

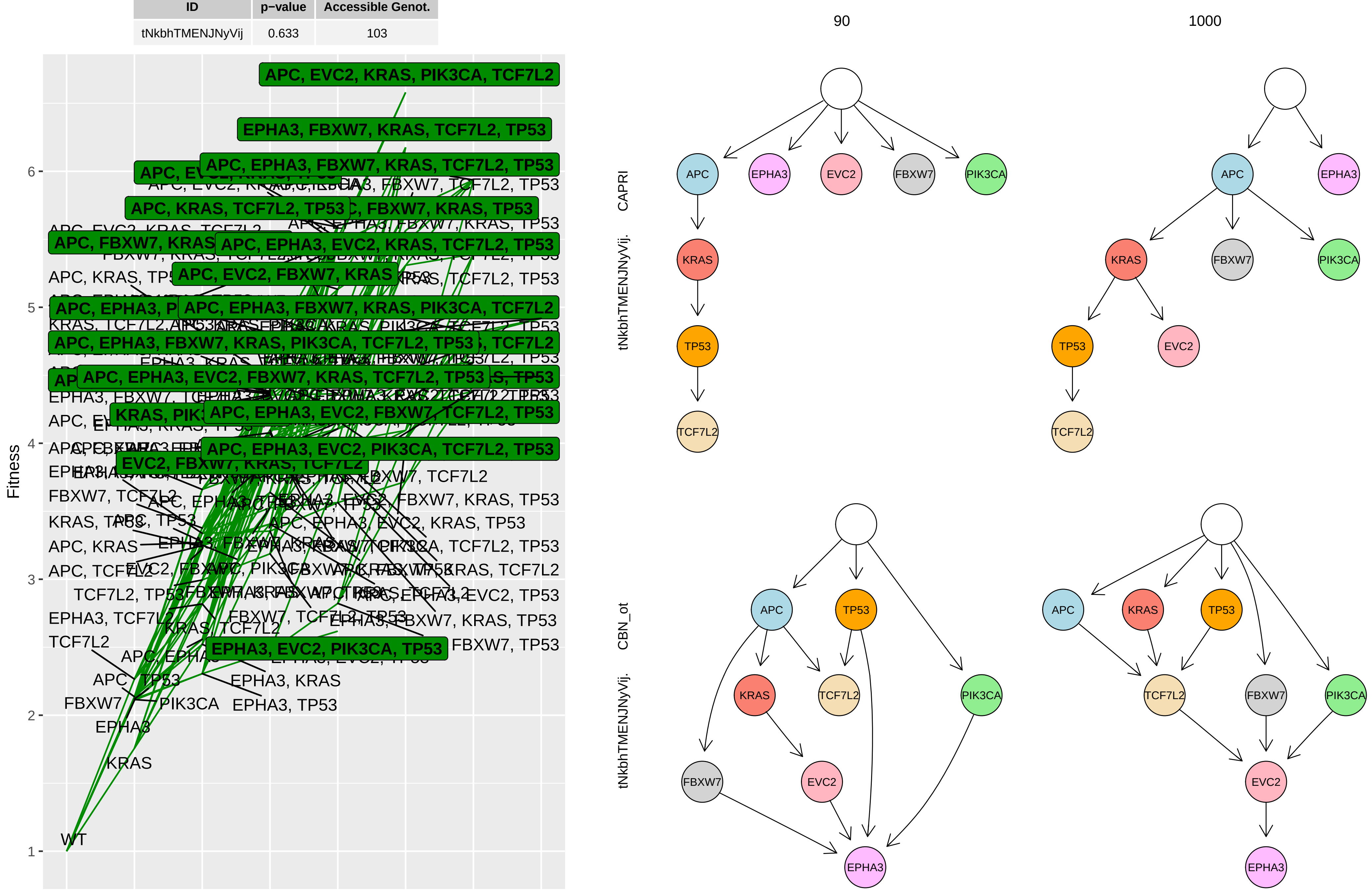

ID	p−value	Accessible Genot.
xutTkZcqJGYMzbu	0.634	103

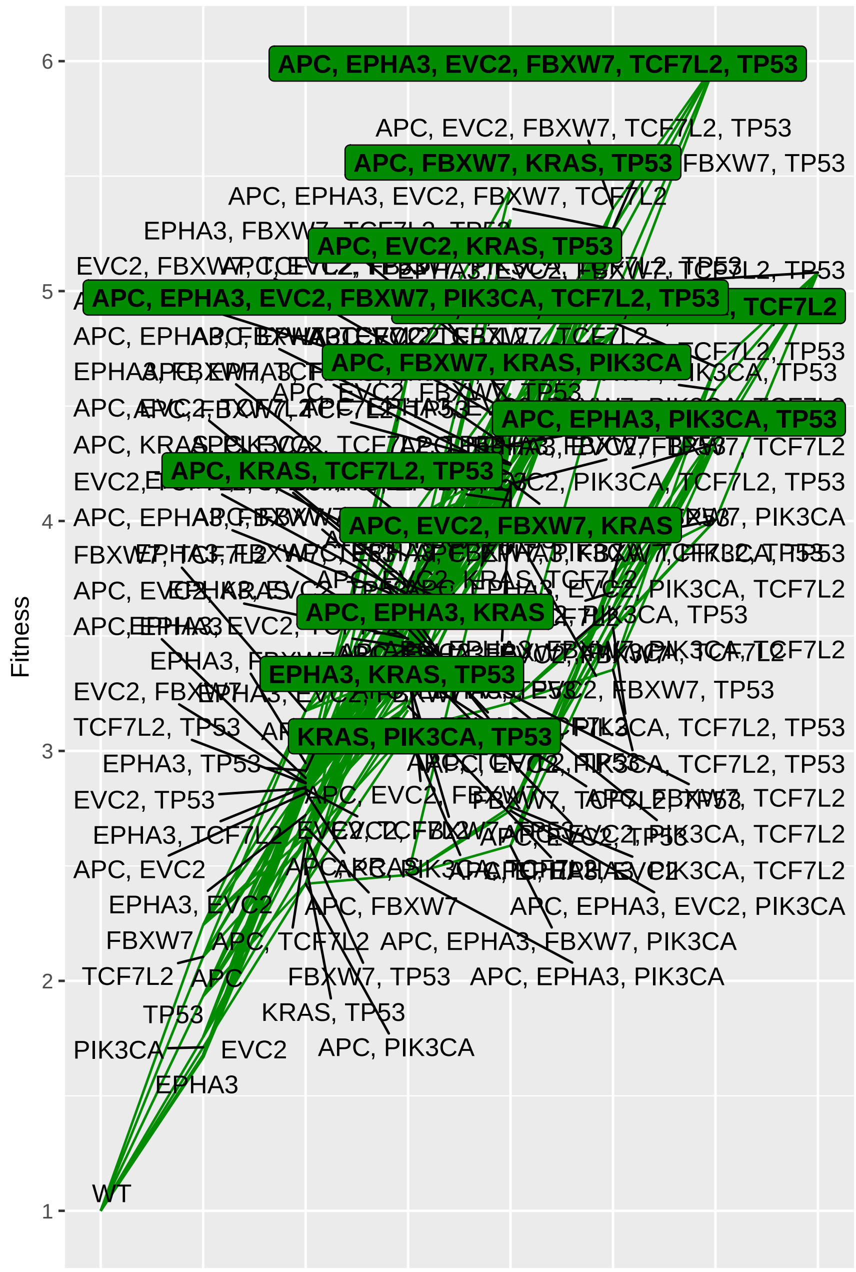

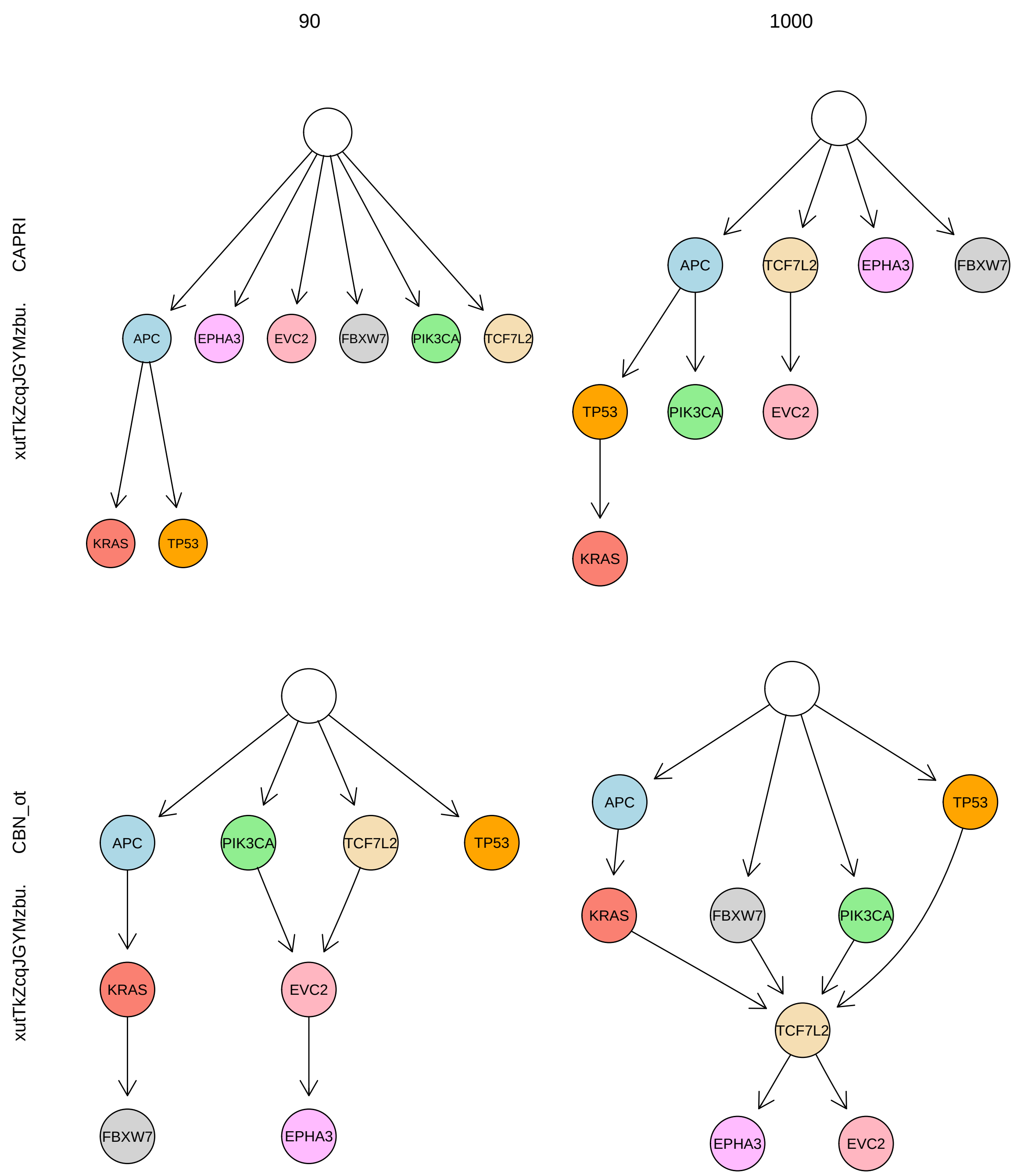

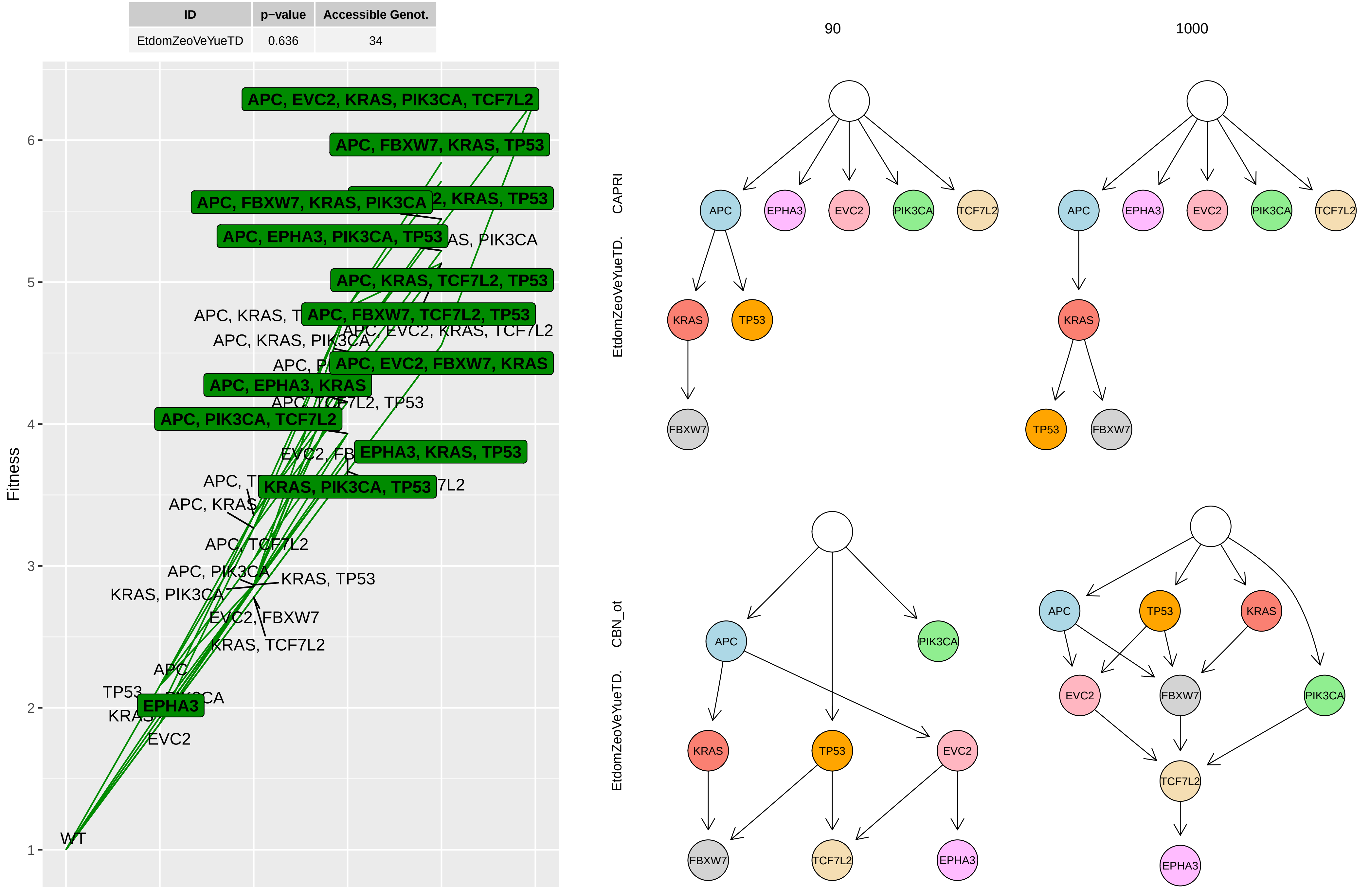

ID	p−value	Accessible Genot.
EtdomZeoVeYueTD	0.636	34

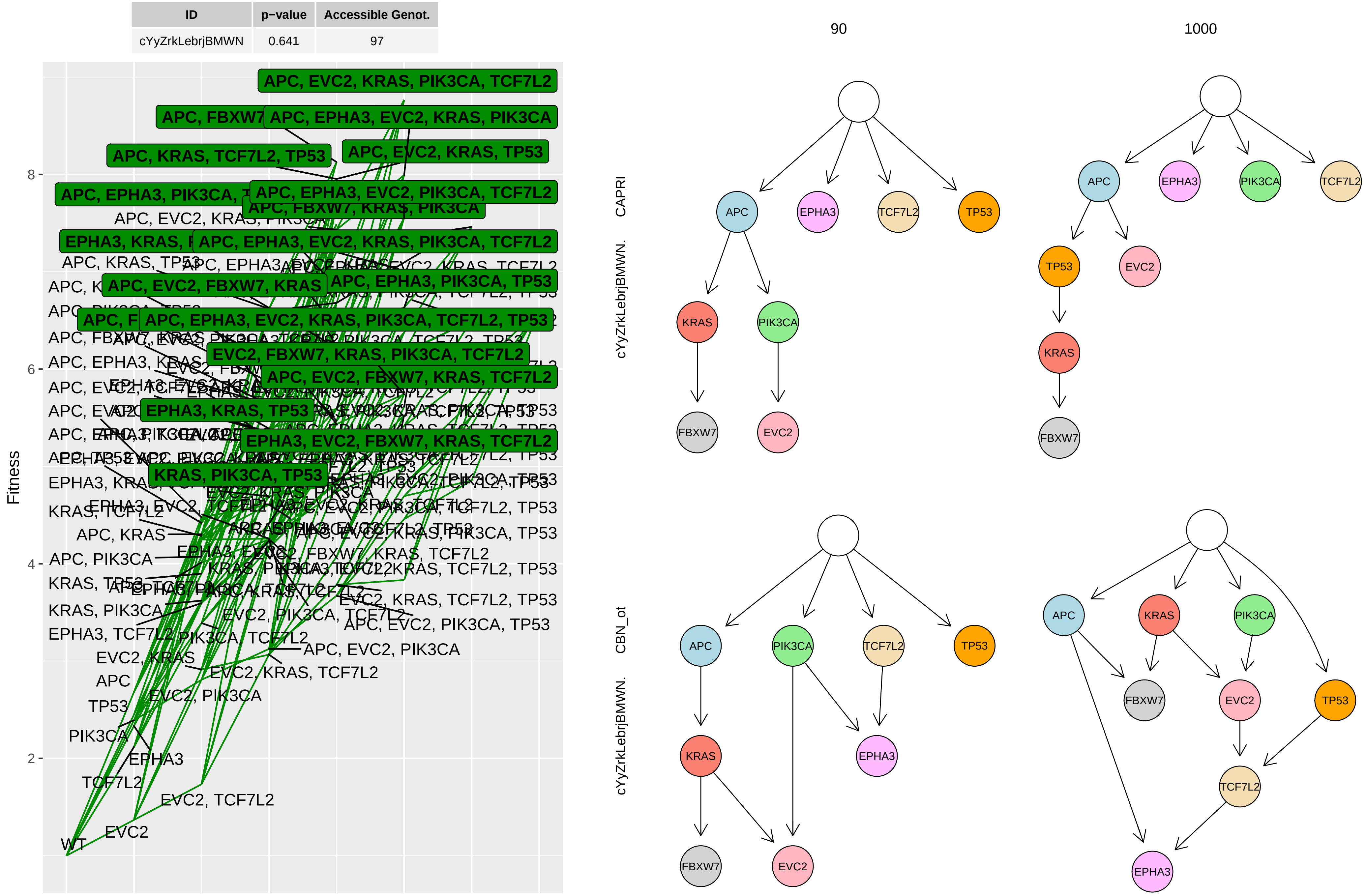
ID
p−value
Accessible Genot.
cYyZrkLebrjBMWN
0.641
97
Fitness
APC, EVC2, KRAS, PIK3CA, TCF7L2
APC, EPHA3, EVC2, KRAS, PIK3CA
APC, KRAS, TCF7L2, TP53
APC, EVC2, KRAS, TP53
APC, EPHA3, EVC2, PIK3CA, TCF7L2
APC, FBXW7, KRAS, PIK3CA
APC, EPHA3, EVC2, KRAS, PIK3CA, TCF7L2
APC, EVC2, FBXW7, KRAS
APC, EPHA3, PIK3CA, TP53
APC, EPHA3, EVC2, KRAS, PIK3CA, TCF7L2, TP53
EVC2, FBXW7, KRAS, PIK3CA, TCF7L2
APC, EVC2, FBXW7, KRAS, TCF7L2
EPHA3, KRAS, TP53
EPHA3, EVC2, FBXW7, KRAS, TCF7L2
KRAS, PIK3CA, TP53
APC, KRAS
APC, PIK3CA
KRAS, TP53
KRAS, PIK3CA
EPHA3, TCF7L2
EVC2, KRAS
APC
TP53
PIK3CA
EPHA3
TCF7L2
EVC2
WT
EVC2, TCF7L2
EVC2, PIK3CA
EVC2, KRAS, TCF7L2
APC, EVC2, PIK3CA
PIK3CA, TCF7L2
APC, EVC2, PIK3CA, TP53
EVC2, KRAS, TCF7L2, TP53
8
6
4
2
90
1000
CAPRI
cYyZrkLebrjBMWN.
CBN_ot
cYyZrkLebrjBMWN.
APC
EPHA3
TCF7L2
TP53
KRAS
PIK3CA
FBXW7
EVC2

ID	p−value	Accessible Genot.
LOAWpyoKPdANifz	0.641	30

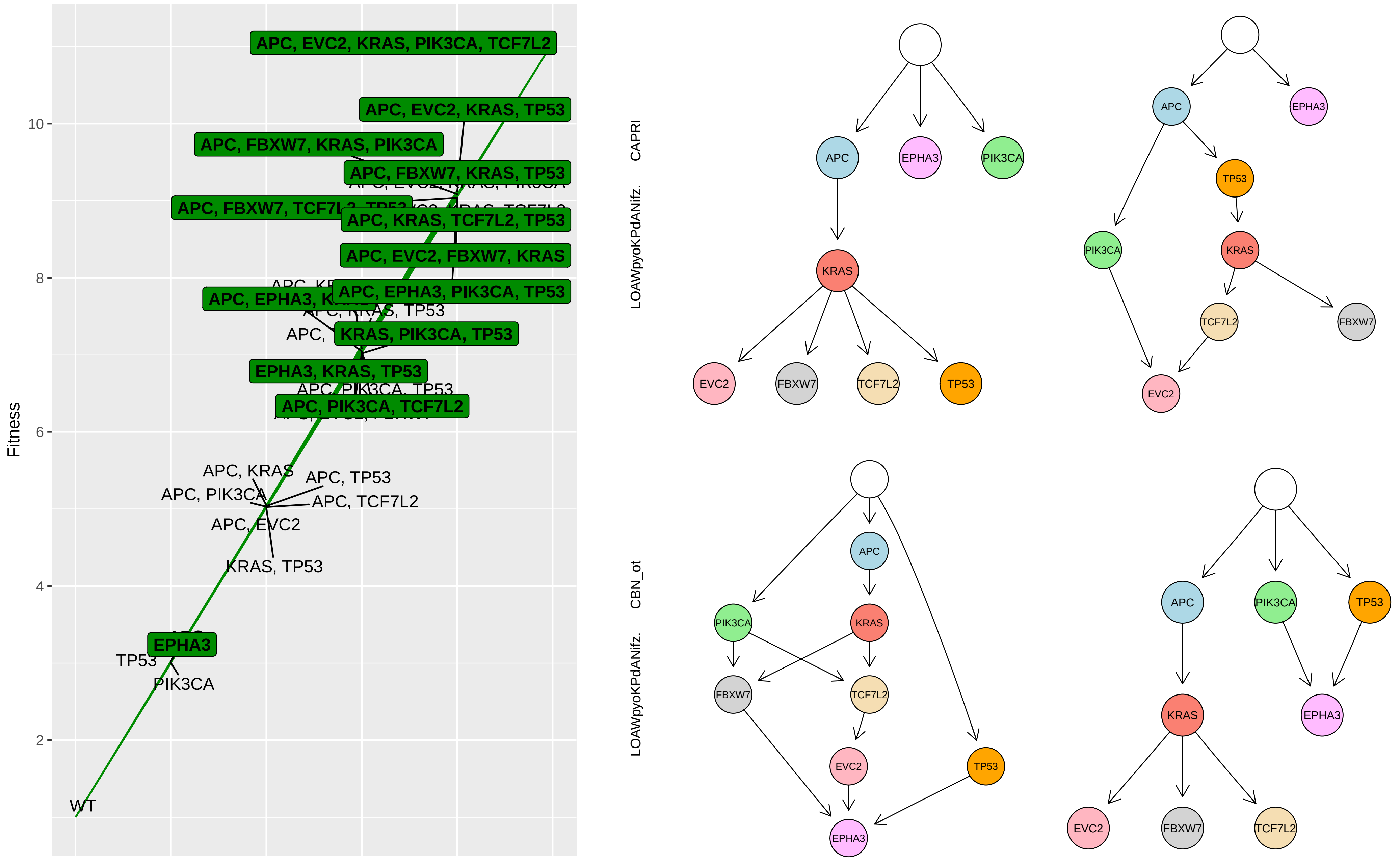

ID	p−value	Accessible Genot.
BeaXEBOFrAPkkyd	0.644	37

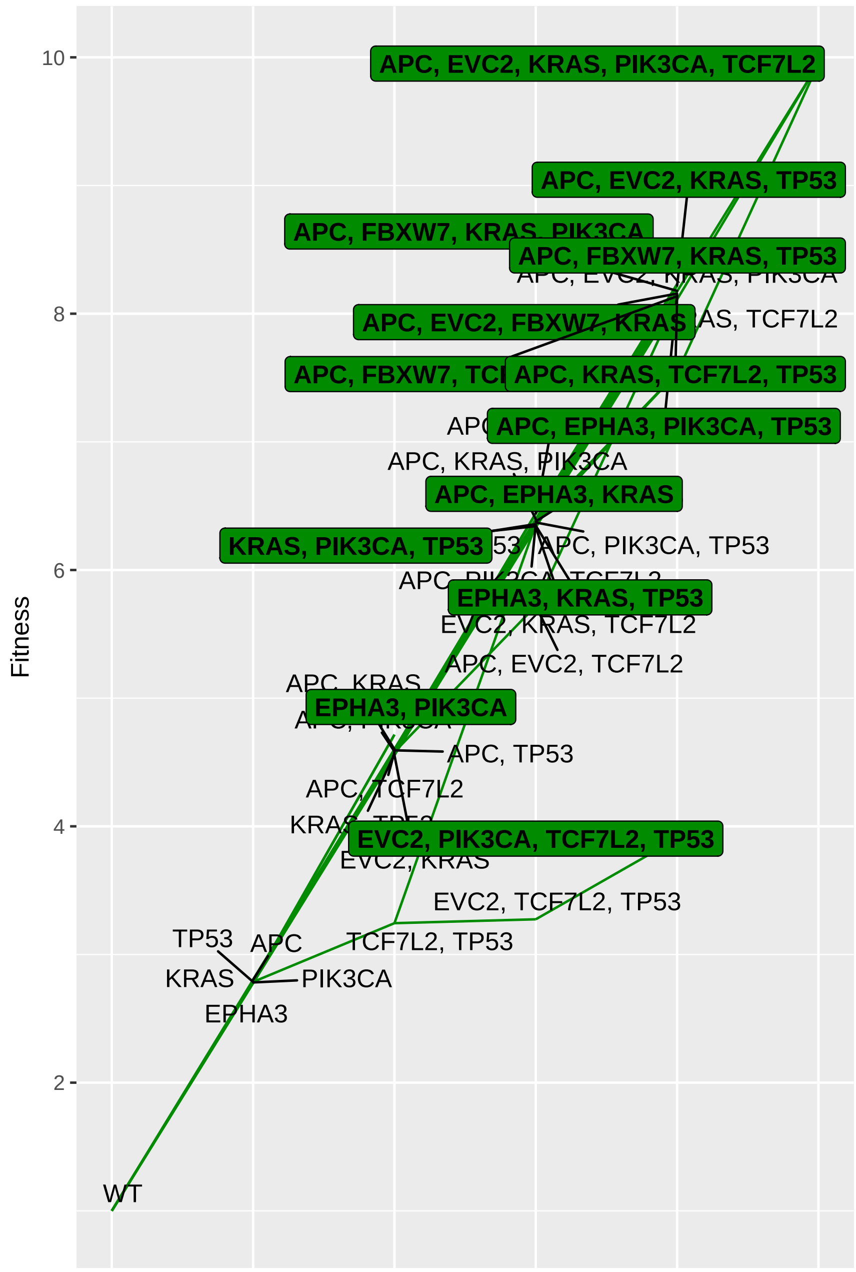

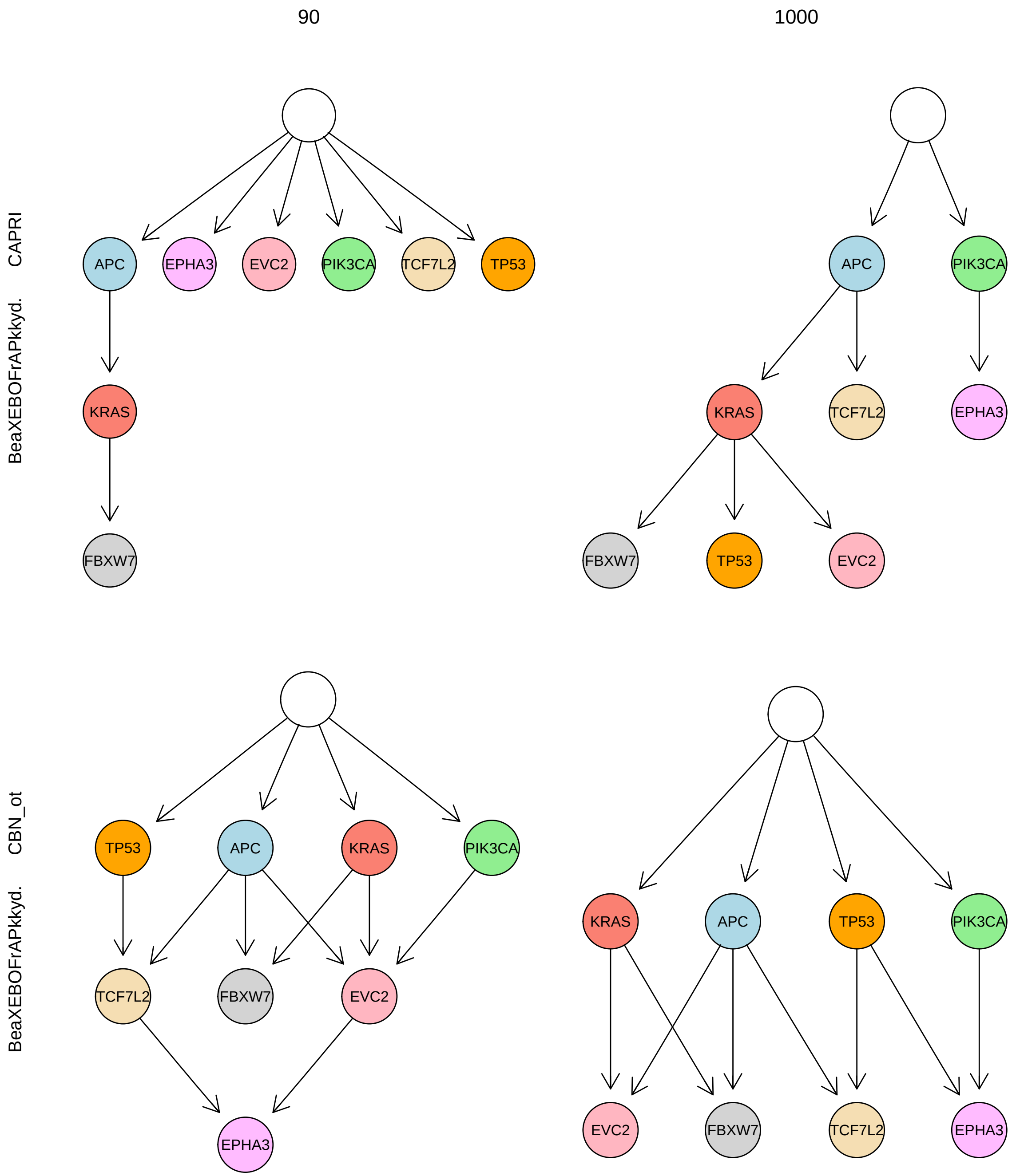

ID	p−value	Accessible Genot.
UIsGkeZYybJdjHH	0.65	30

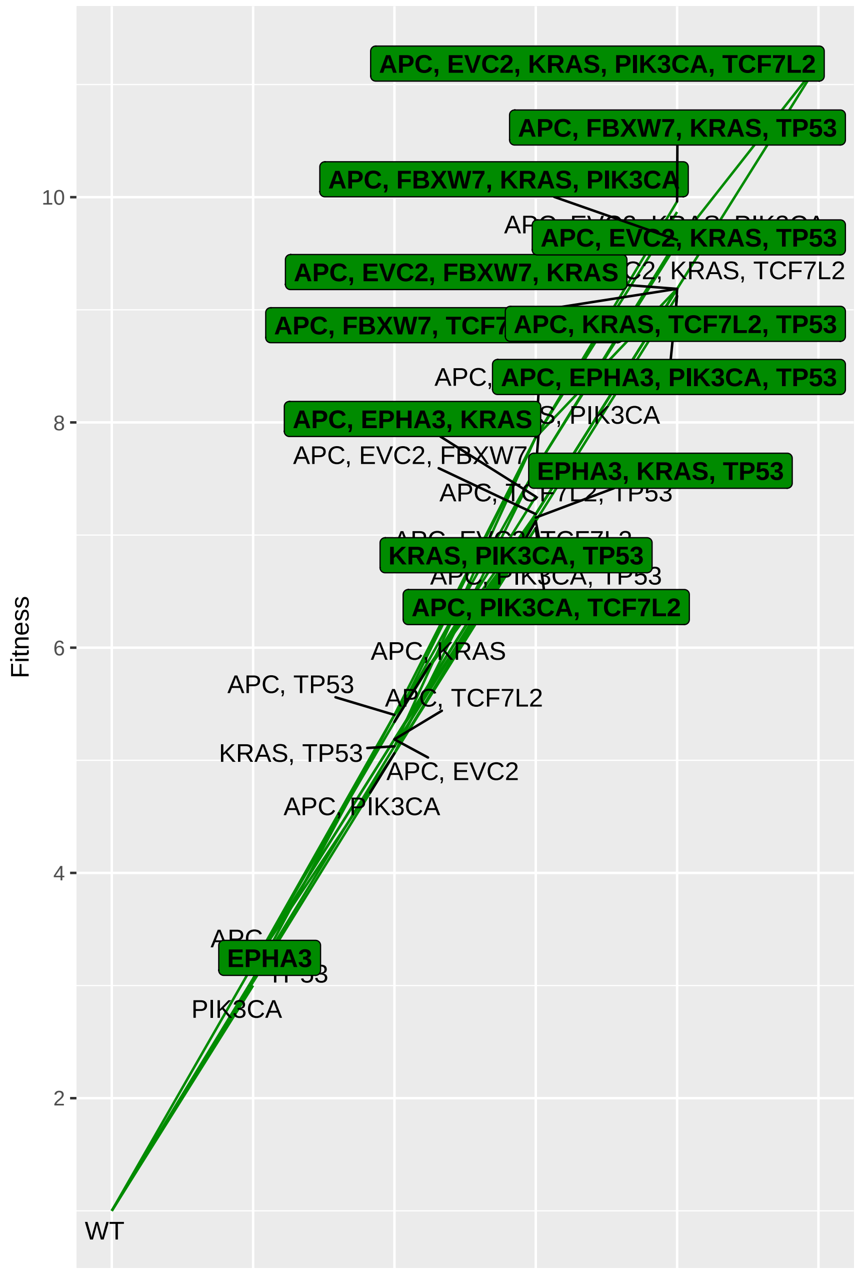

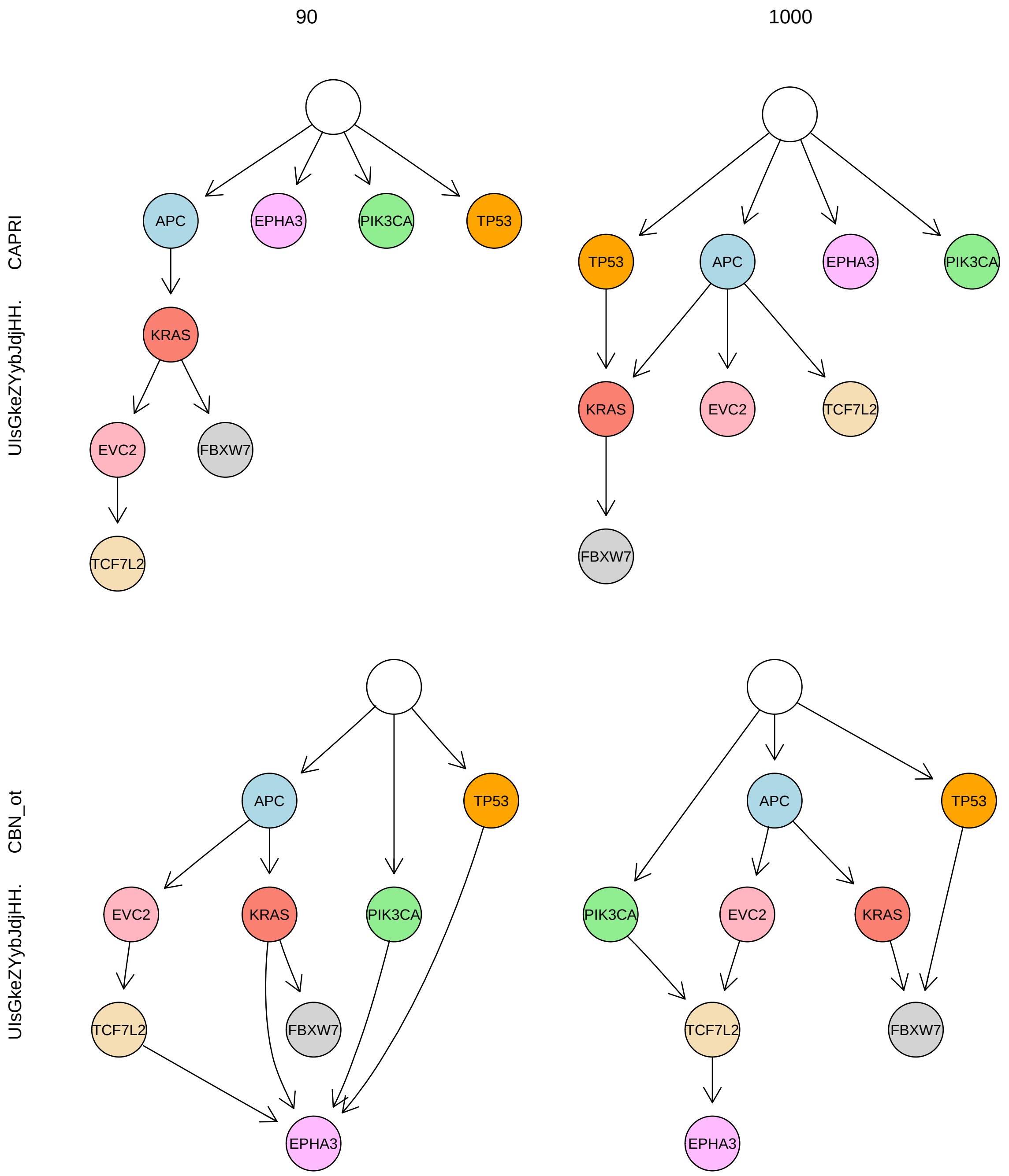

ID	p−value	Accessible Genot.
pydGFimSJnBnjbL	0.653	142

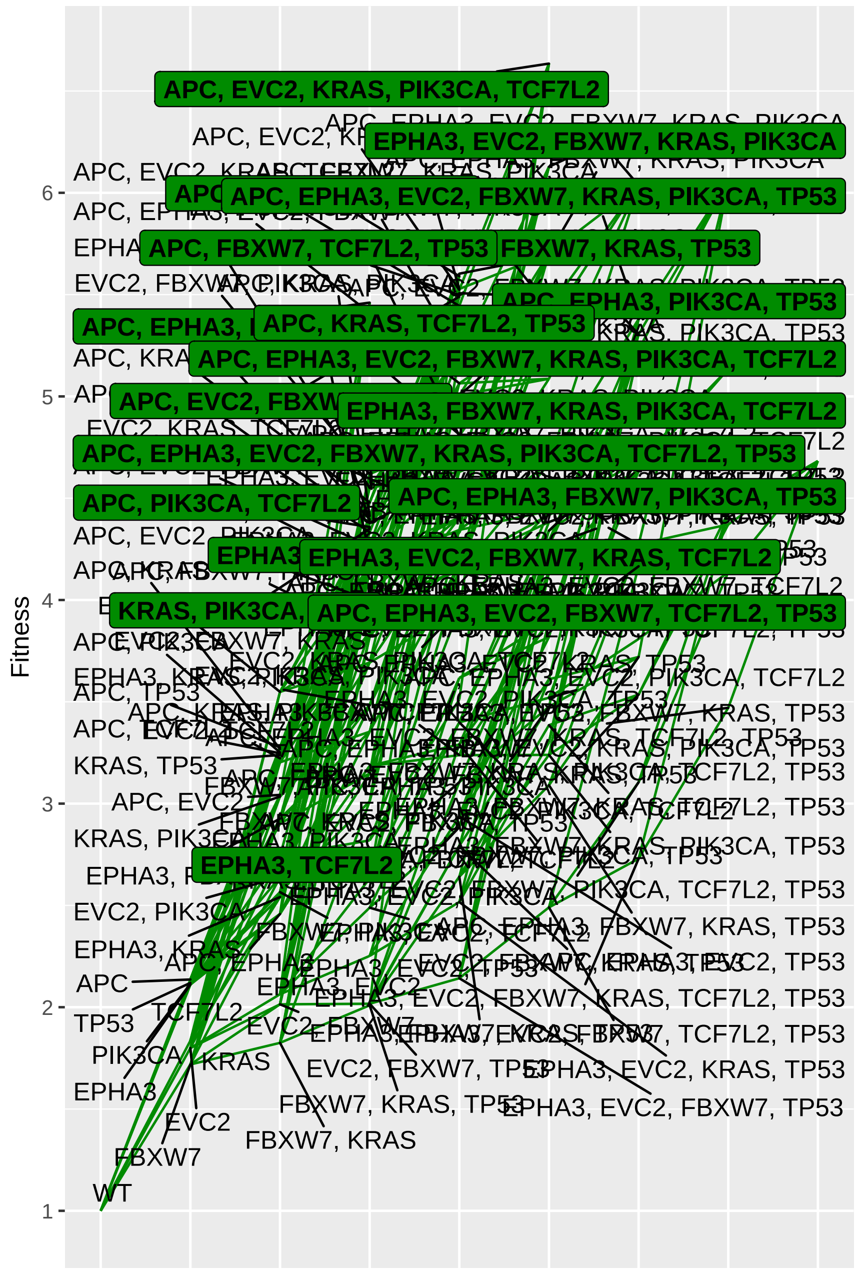

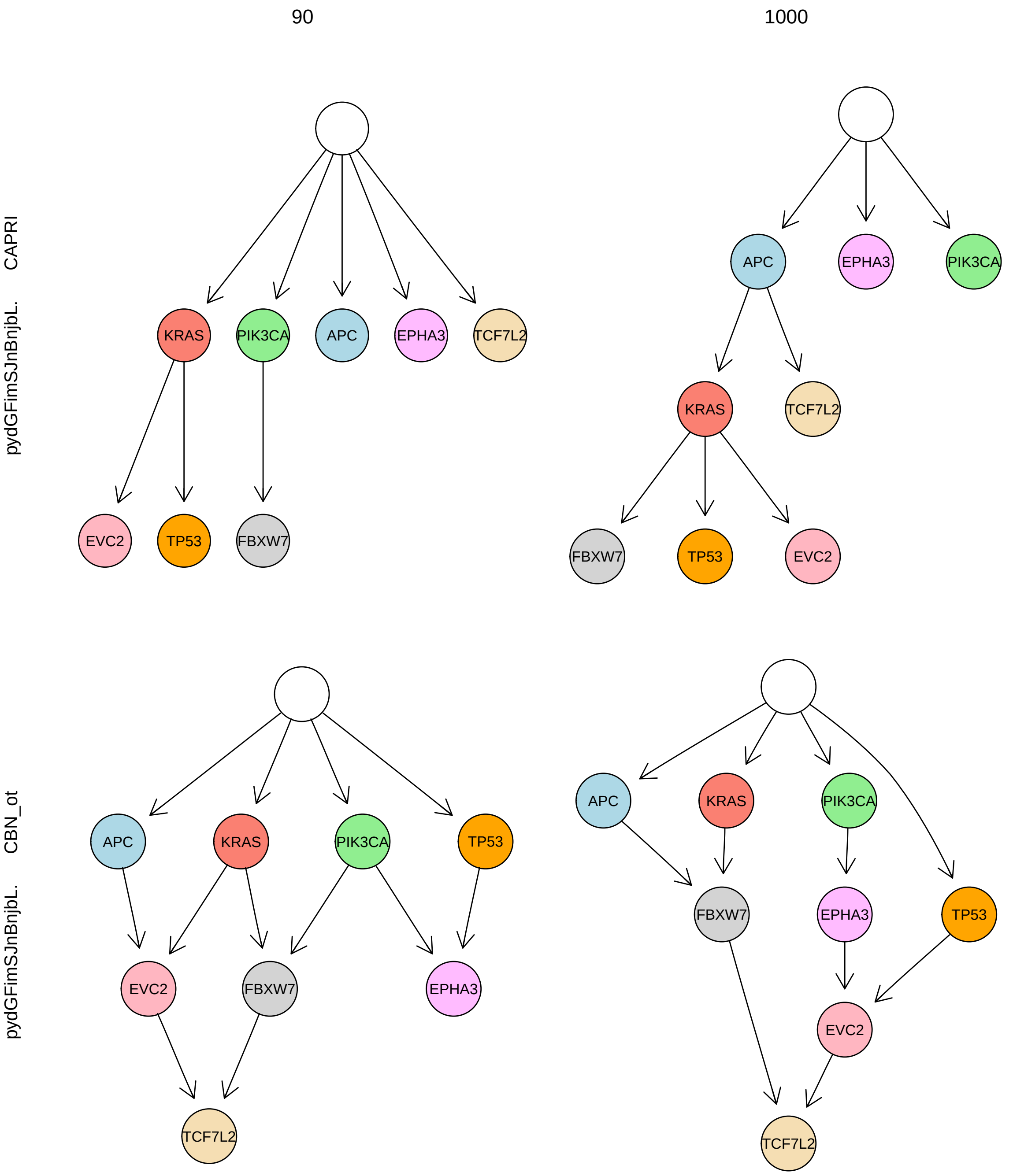

ID	p−value	Accessible Genot.
OhTsyORUccRCJuG	0.654	51

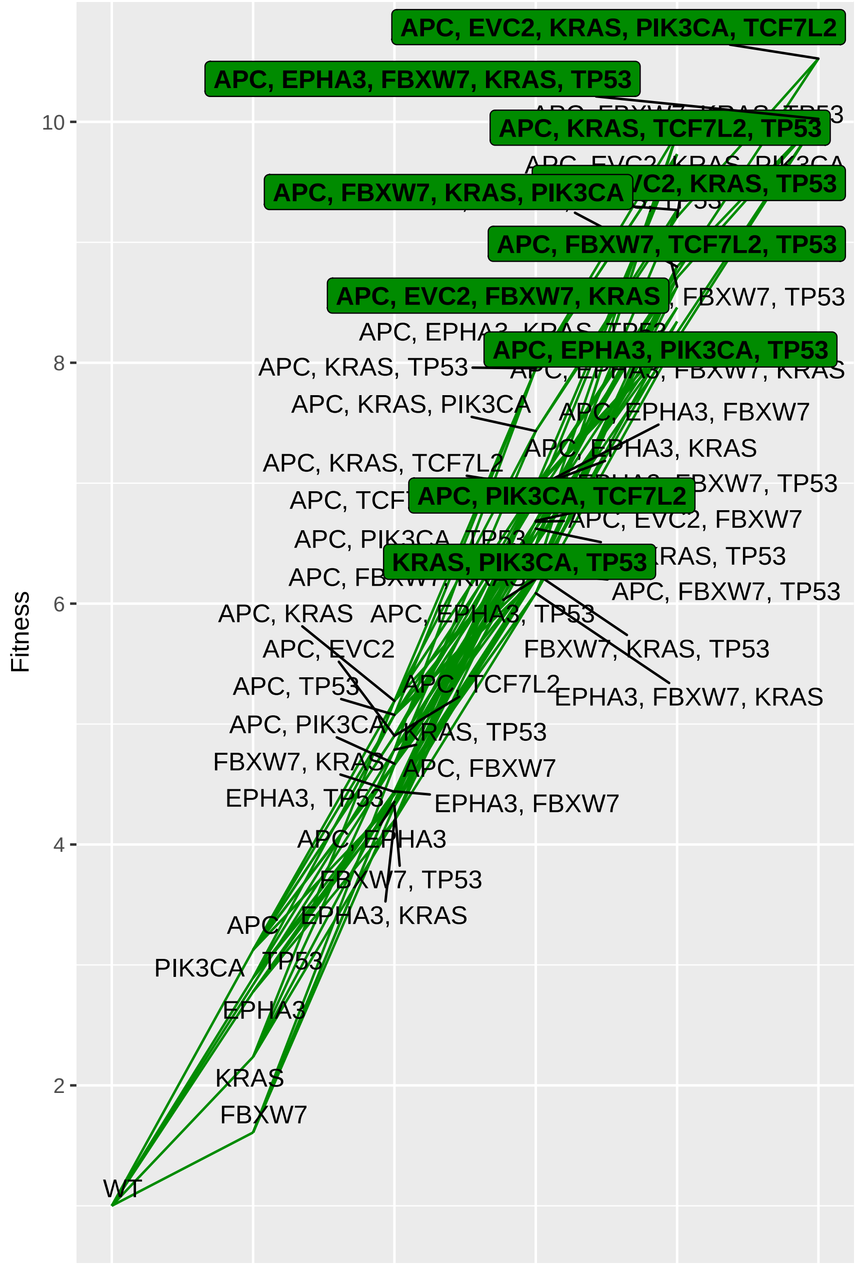

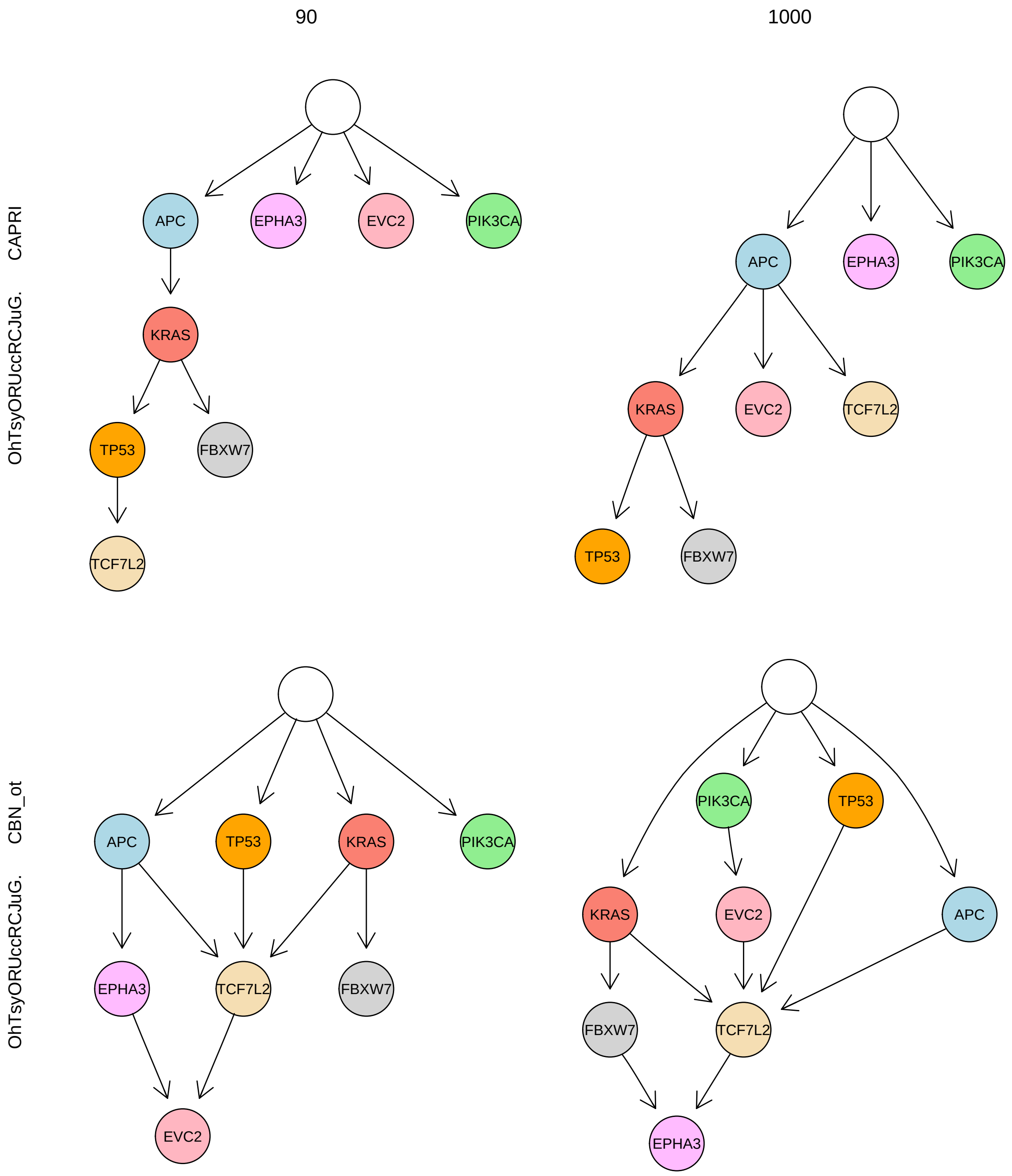

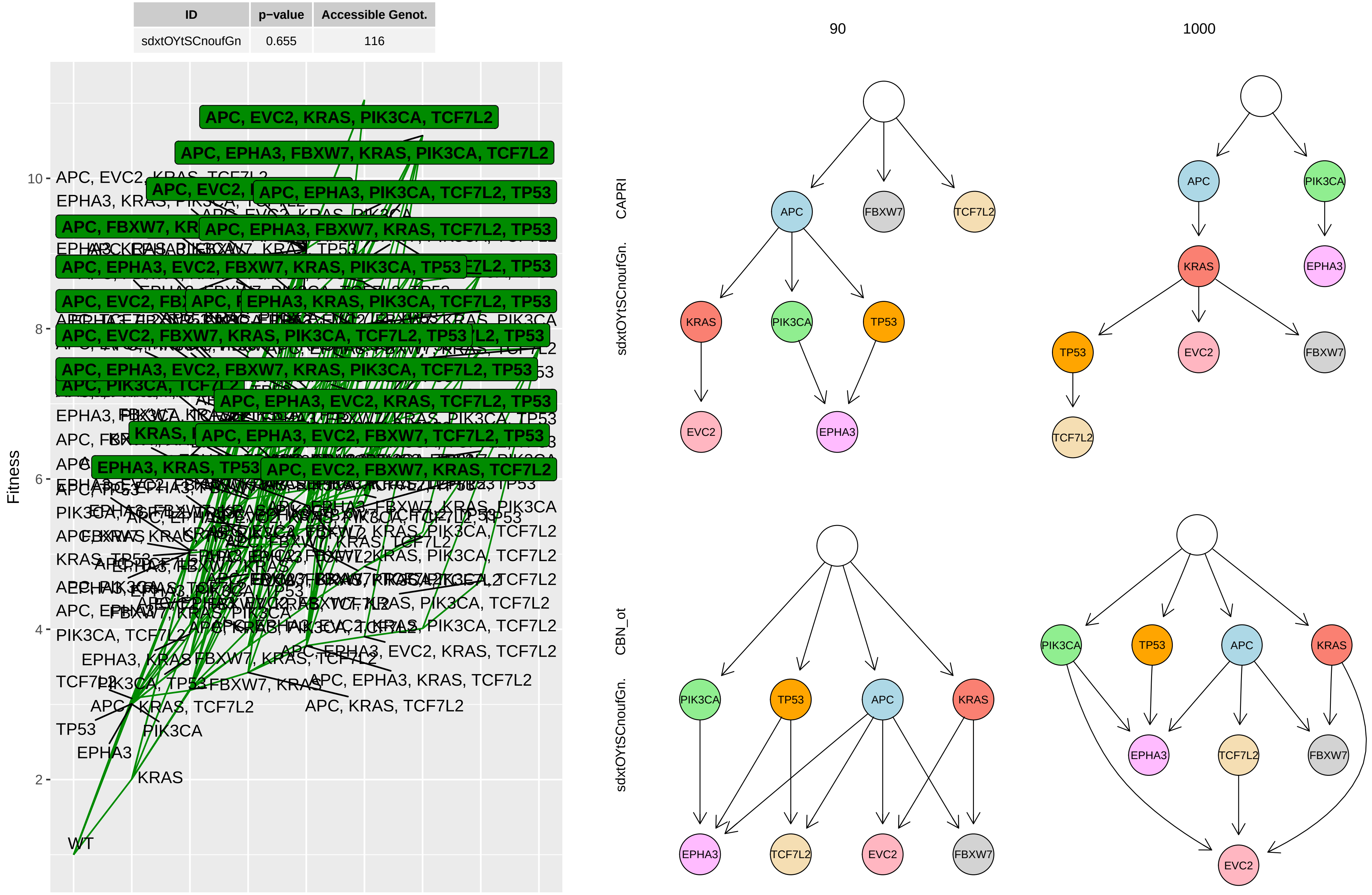

ID
p−value
Accessible Genot.
sdxtOYtSCnoufGn
0.655
116
Fitness
WT
KRAS
PIK3CA
EPHA3
TP53
APC, KRAS, TCF7L2
APC, EPHA3, KRAS, TCF7L2
APC, EPHA3, EVC2, KRAS, TCF7L2
APC, EVC2, KRAS, PIK3CA, TCF7L2
APC, EPHA3, FBXW7, KRAS, PIK3CA, TCF7L2
APC, EPHA3, PIK3CA, TCF7L2, TP53
APC, EPHA3, FBXW7, KRAS, TCF7L2, TP53
APC, EPHA3, EVC2, FBXW7, KRAS, PIK3CA, TP53
APC, EPHA3, KRAS, PIK3CA, TCF7L2, TP53
APC, EVC2, FBXW7, KRAS, PIK3CA, TCF7L2, TP53
APC, EPHA3, EVC2, FBXW7, KRAS, PIK3CA, TCF7L2, TP53
APC, EPHA3, EVC2, KRAS, TCF7L2, TP53
APC, EPHA3, EVC2, FBXW7, TCF7L2, TP53
APC, EVC2, FBXW7, KRAS, TCF7L2
90
1000
CAPRI
CBN_ot
sdxtOYtSCnoufGn.
APC
FBXW7
TCF7L2
KRAS
PIK3CA
TP53
EVC2
EPHA3

ID	p−value	Accessible Genot.
PegiAhgrtfjUyBM	0.656	53

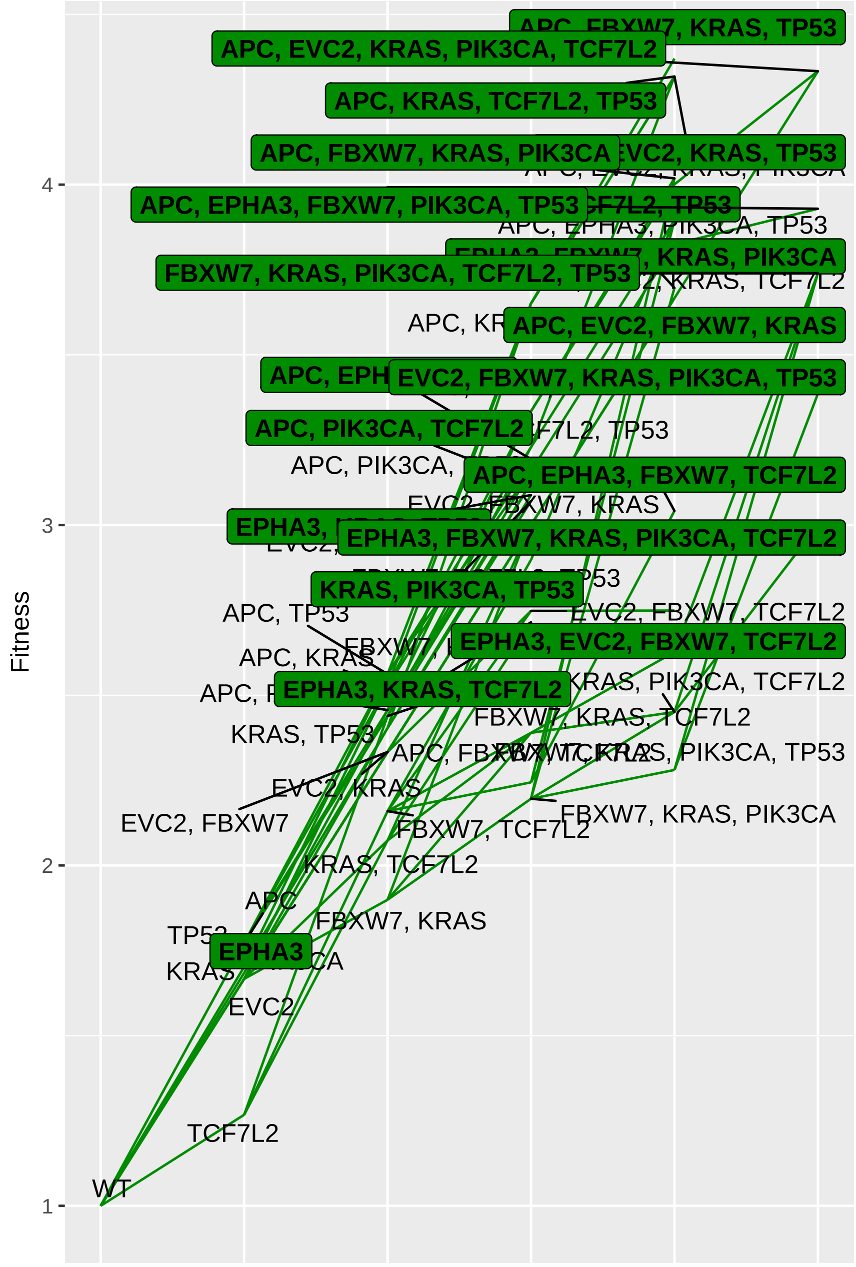

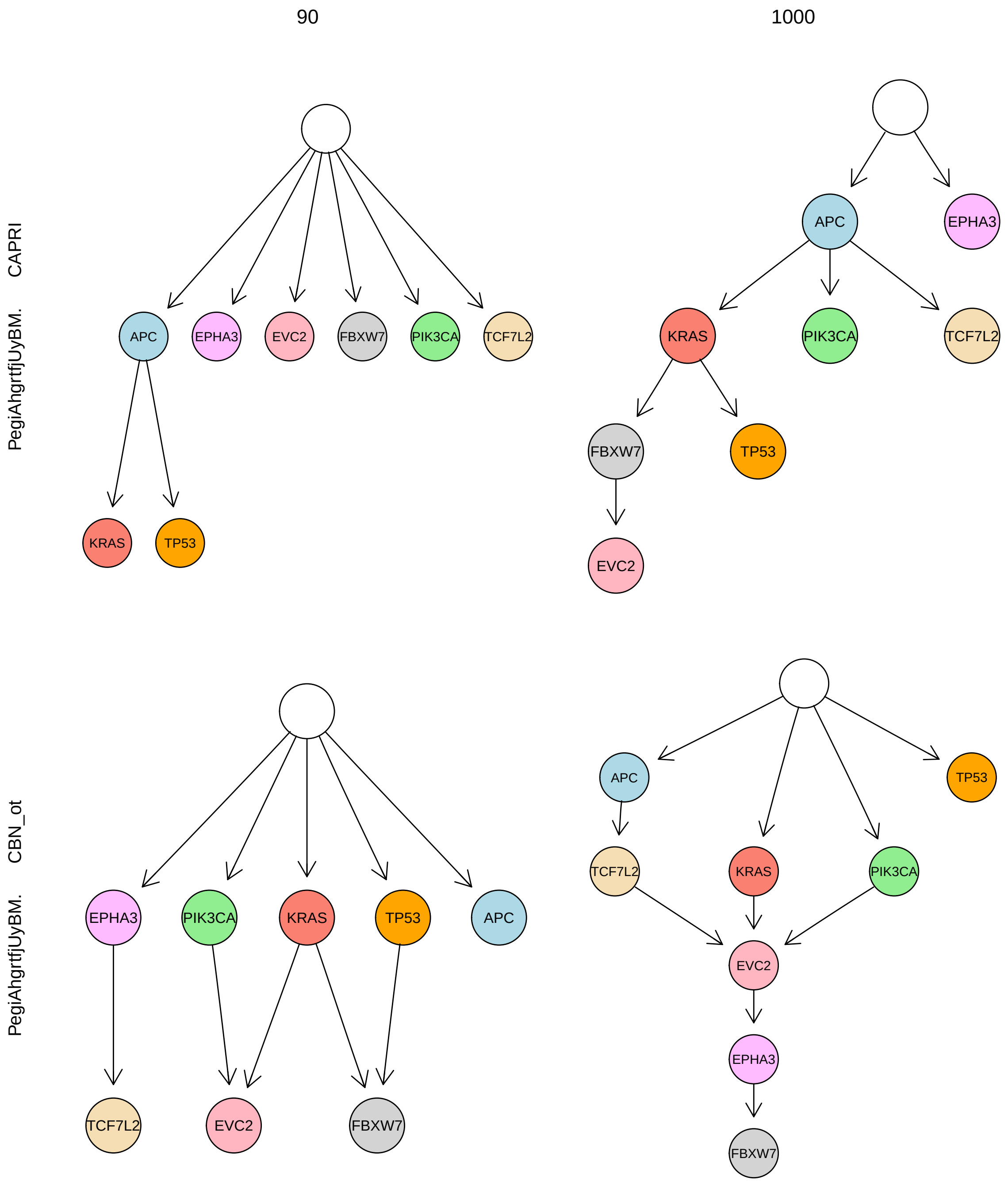

ID	p-value	Accessible Genot.
pSYfiBJKLagcznW	0.658	34

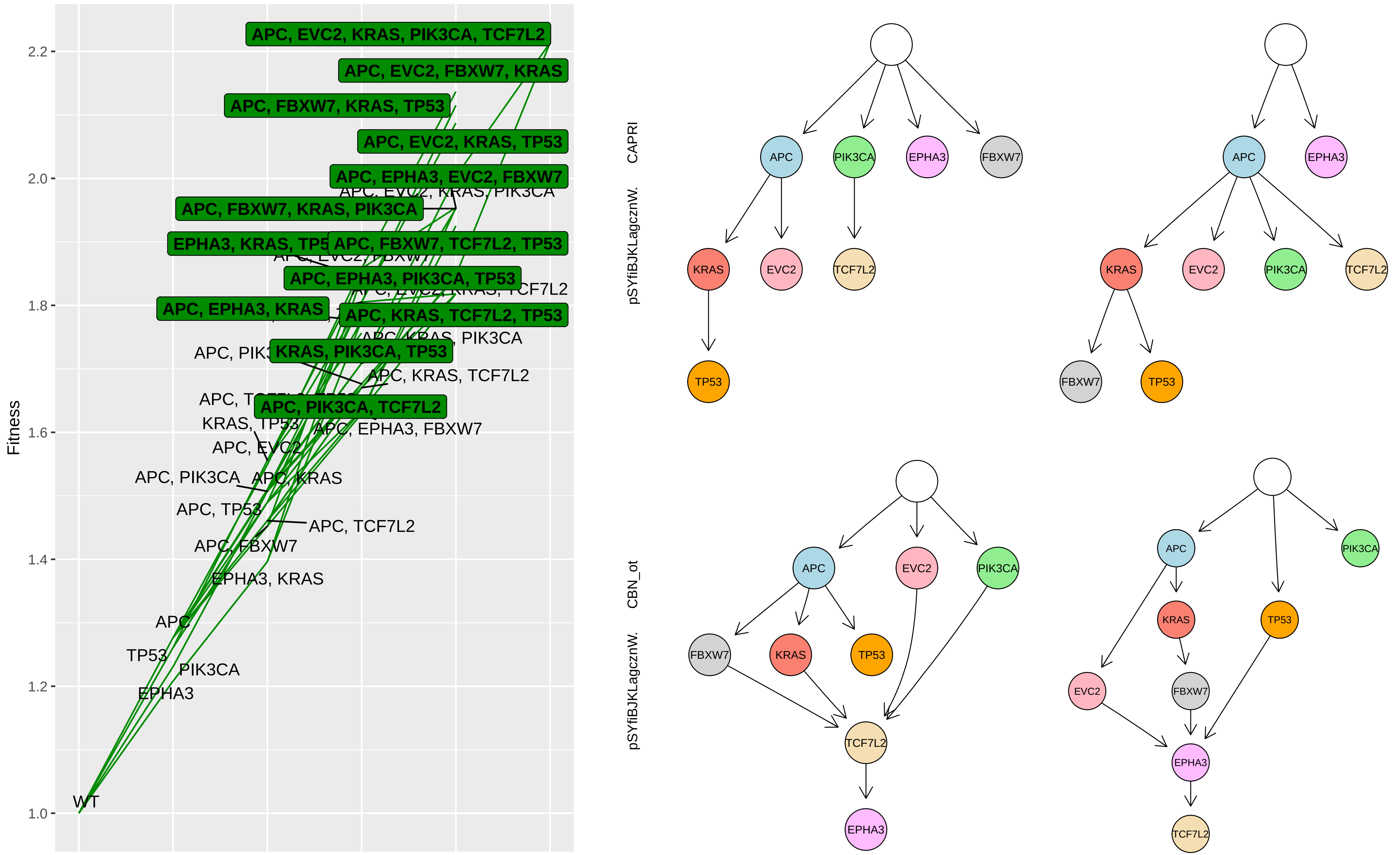

ID	p−value	Accessible Genot.
nMjMIkDEwZboBXs	0.658	138

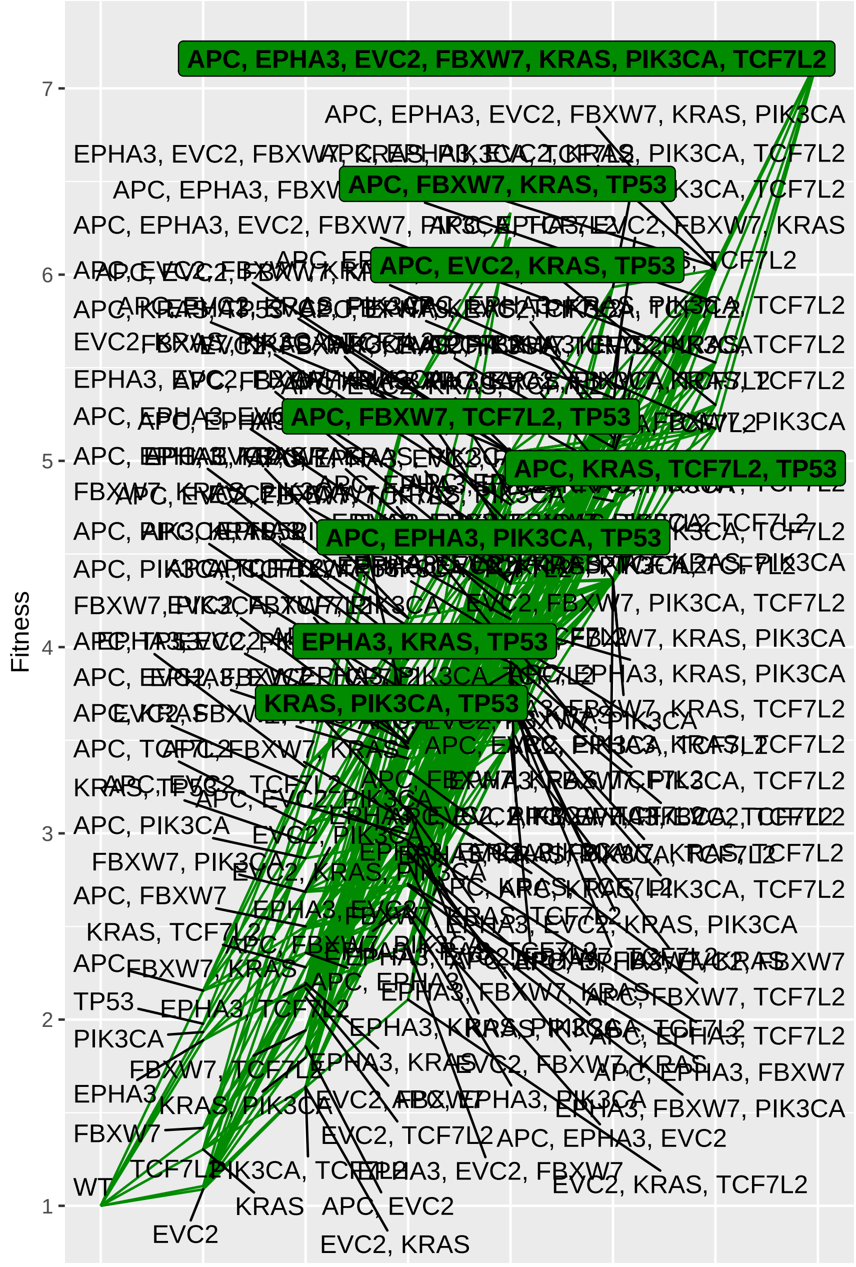

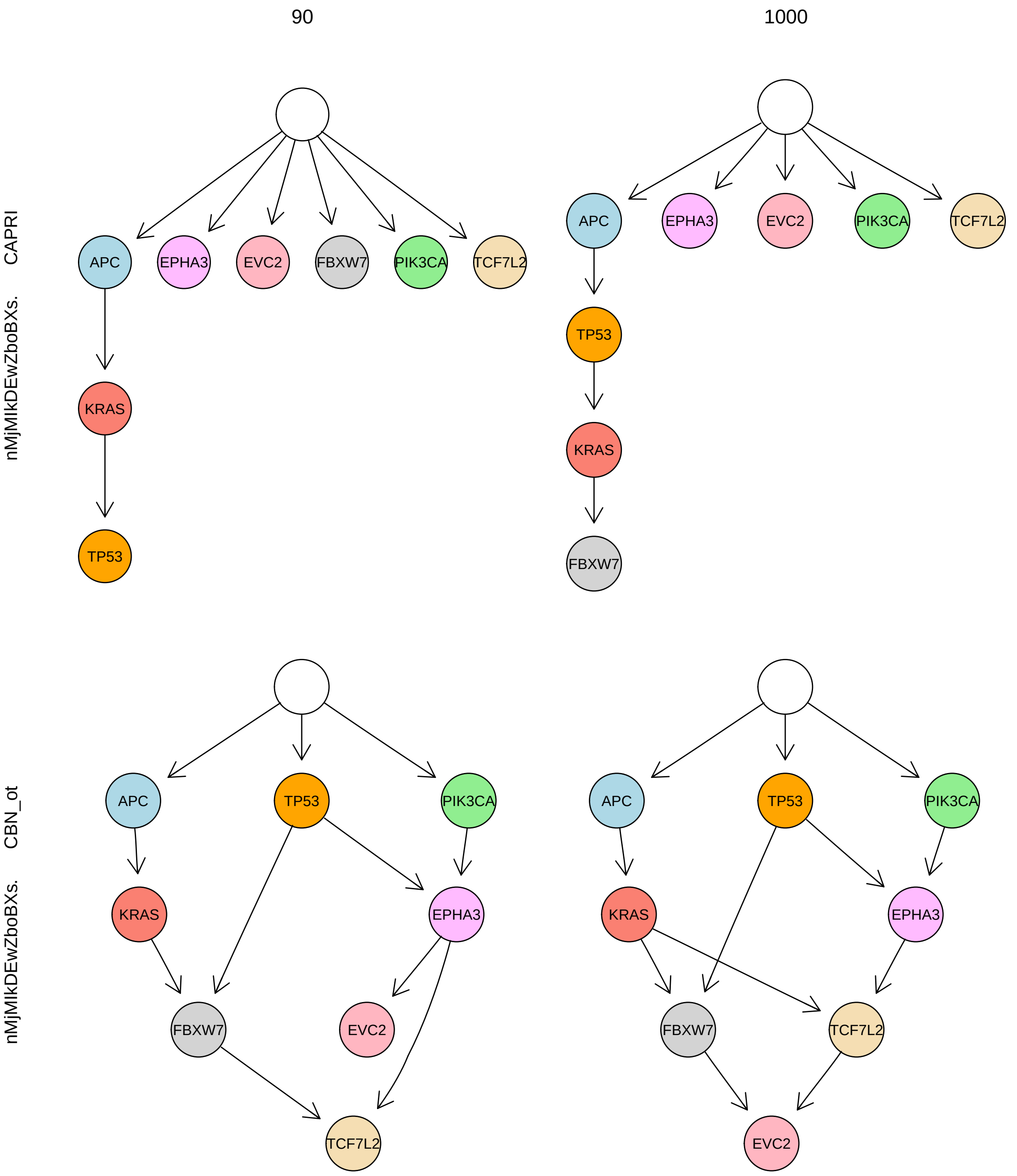

ID	p−value	Accessible Genot.
YdRwGMqRtpbmDfi	0.658	33

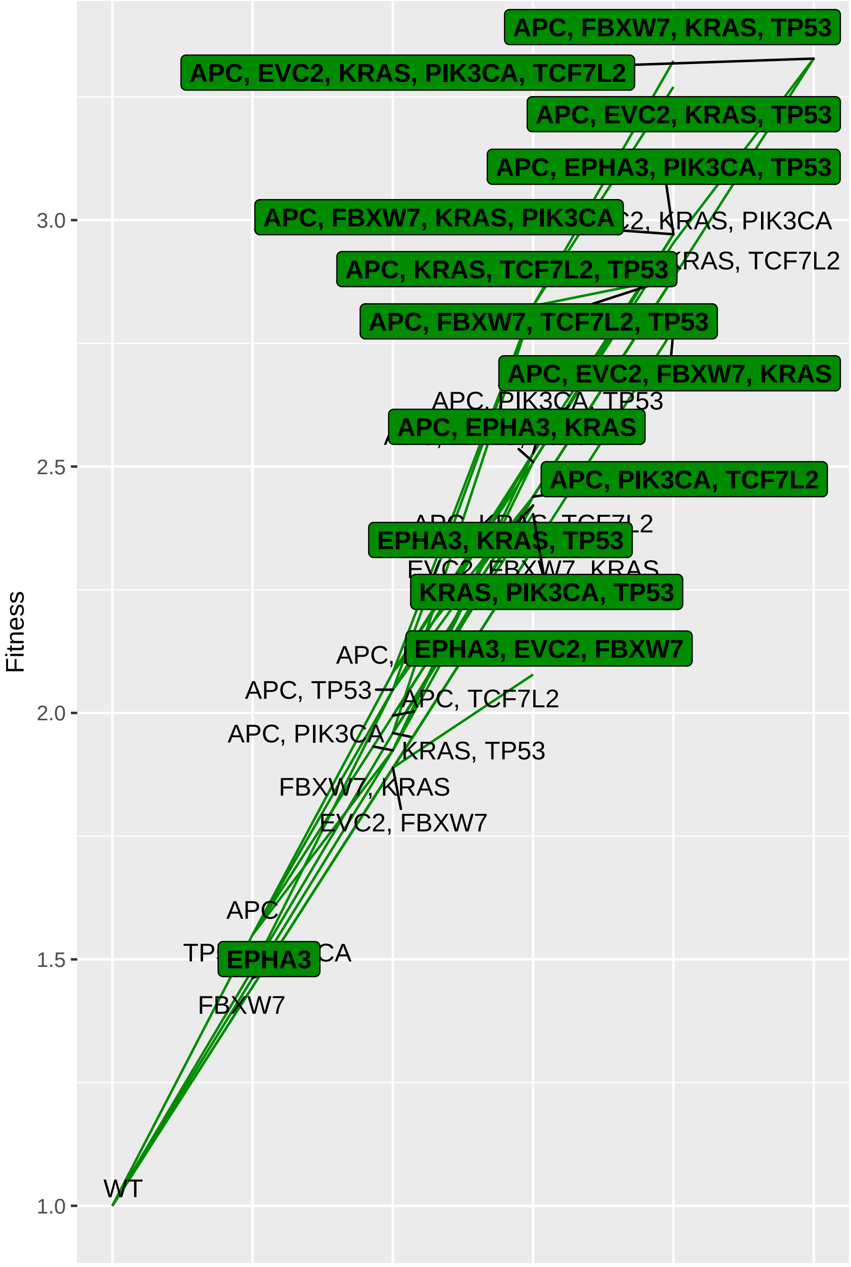

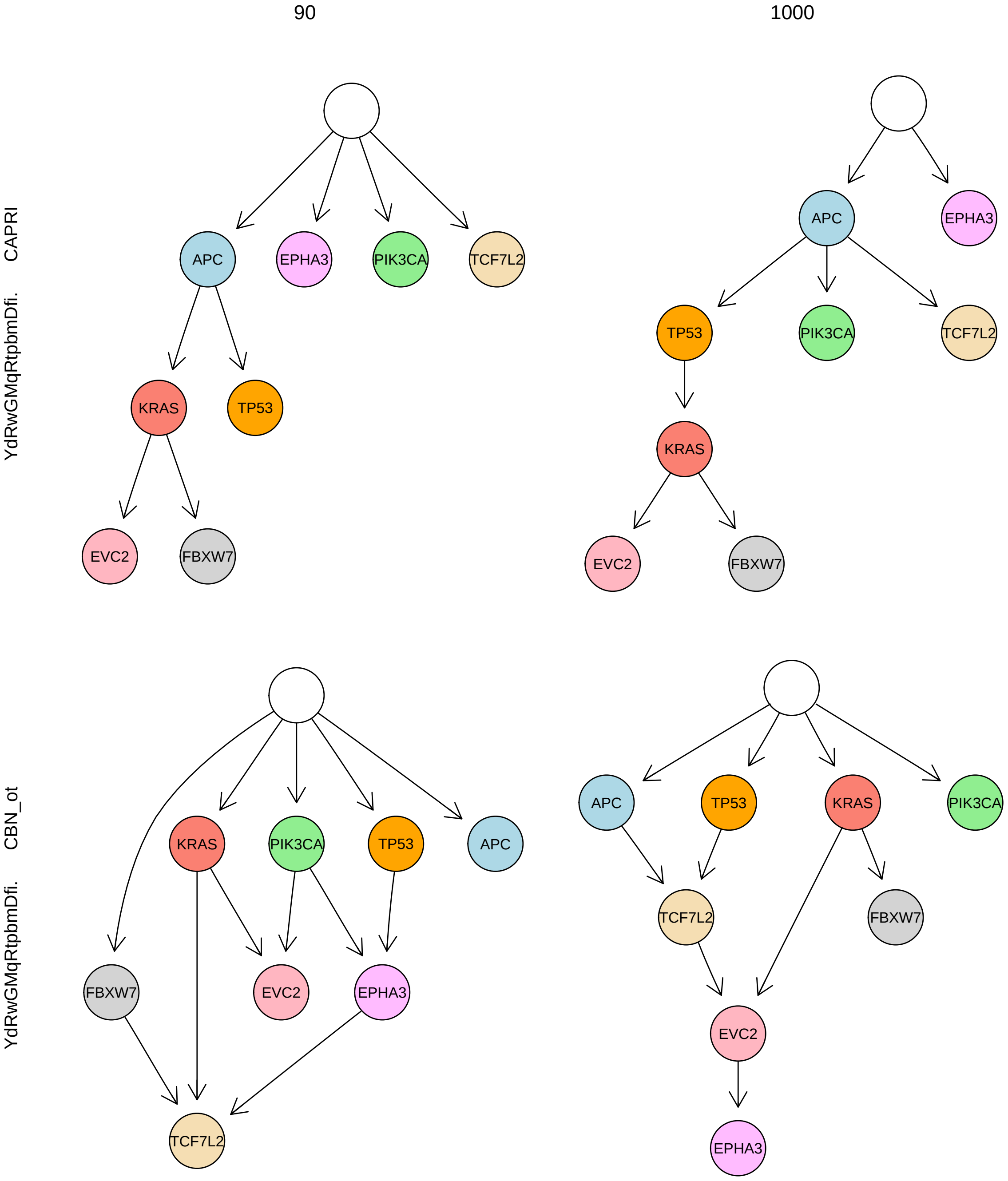

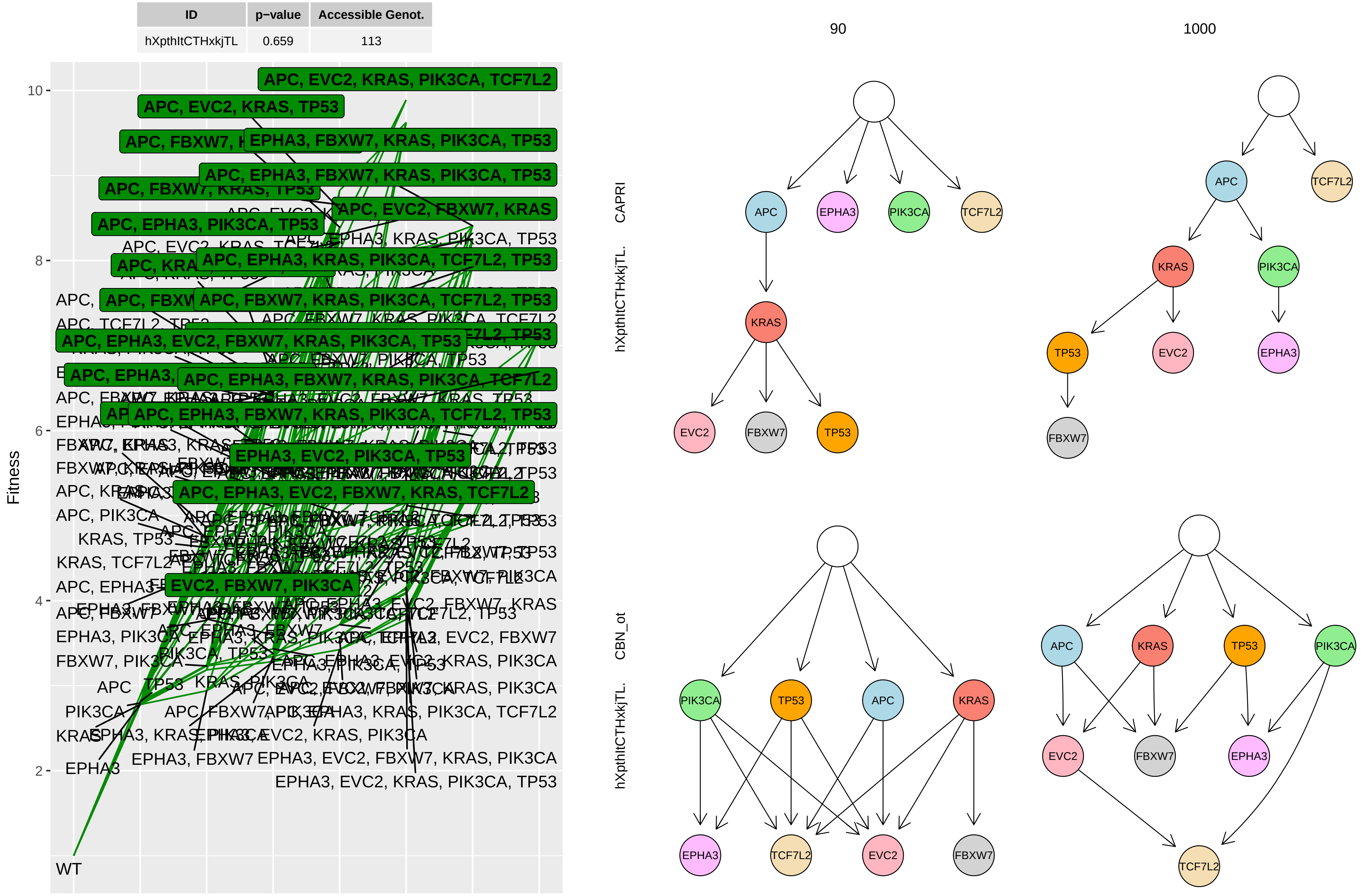

ID
p−value
Accessible Genot.
hXpthItCTHxkjTL
0.659
113
Fitness
WT
APC, EVC2, KRAS, PIK3CA, TCF7L2
APC, EVC2, KRAS, TP53
EPHA3, FBXW7, KRAS, PIK3CA, TP53
APC, EPHA3, FBXW7, KRAS, PIK3CA, TP53
APC, EVC2, FBXW7, KRAS
APC, EPHA3, PIK3CA, TP53
APC, EPHA3, KRAS, PIK3CA, TCF7L2, TP53
APC, FBXW7, KRAS, PIK3CA, TCF7L2, TP53
APC, EPHA3, EVC2, FBXW7, KRAS, PIK3CA, TP53
APC, EPHA3, FBXW7, KRAS, PIK3CA, TCF7L2
APC, EPHA3, FBXW7, KRAS, PIK3CA, TCF7L2, TP53
EPHA3, EVC2, PIK3CA, TP53
APC, EPHA3, EVC2, FBXW7, KRAS, TCF7L2
EVC2, FBXW7, PIK3CA
EPHA3, EVC2, FBXW7, KRAS, PIK3CA
EPHA3, EVC2, KRAS, PIK3CA, TP53
90
1000
CAPRI
CBN_ot
hXpthItCTHxkjTL.
APC
EPHA3
PIK3CA
TCF7L2
KRAS
EVC2
FBXW7
TP53

ID	p−value	Accessible Genot.
ovFZJBsQmflDgXQ	0.66	38

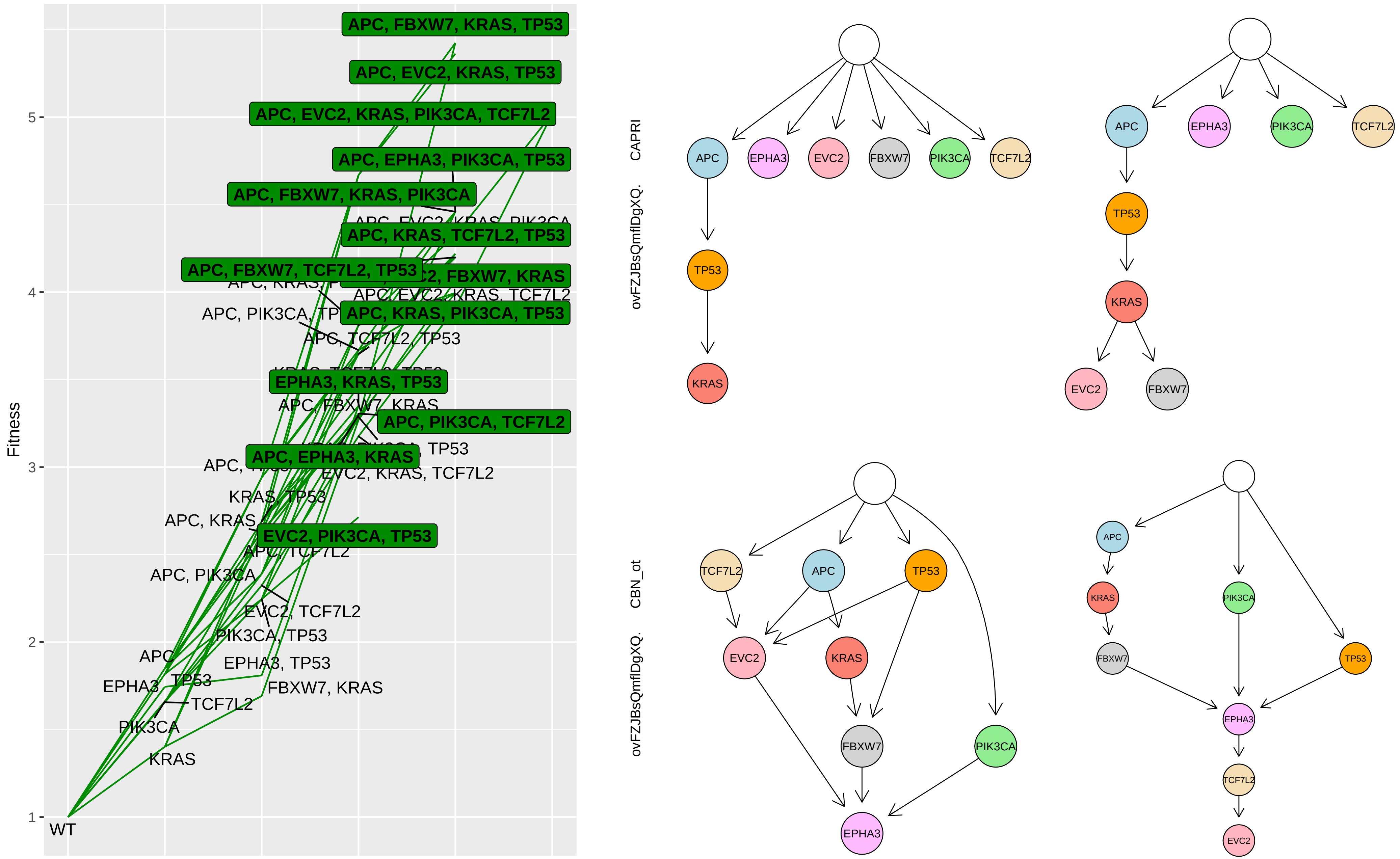

ID	p-value	Accessible Genot.
paiNMTqPZMILijY	0.661	65

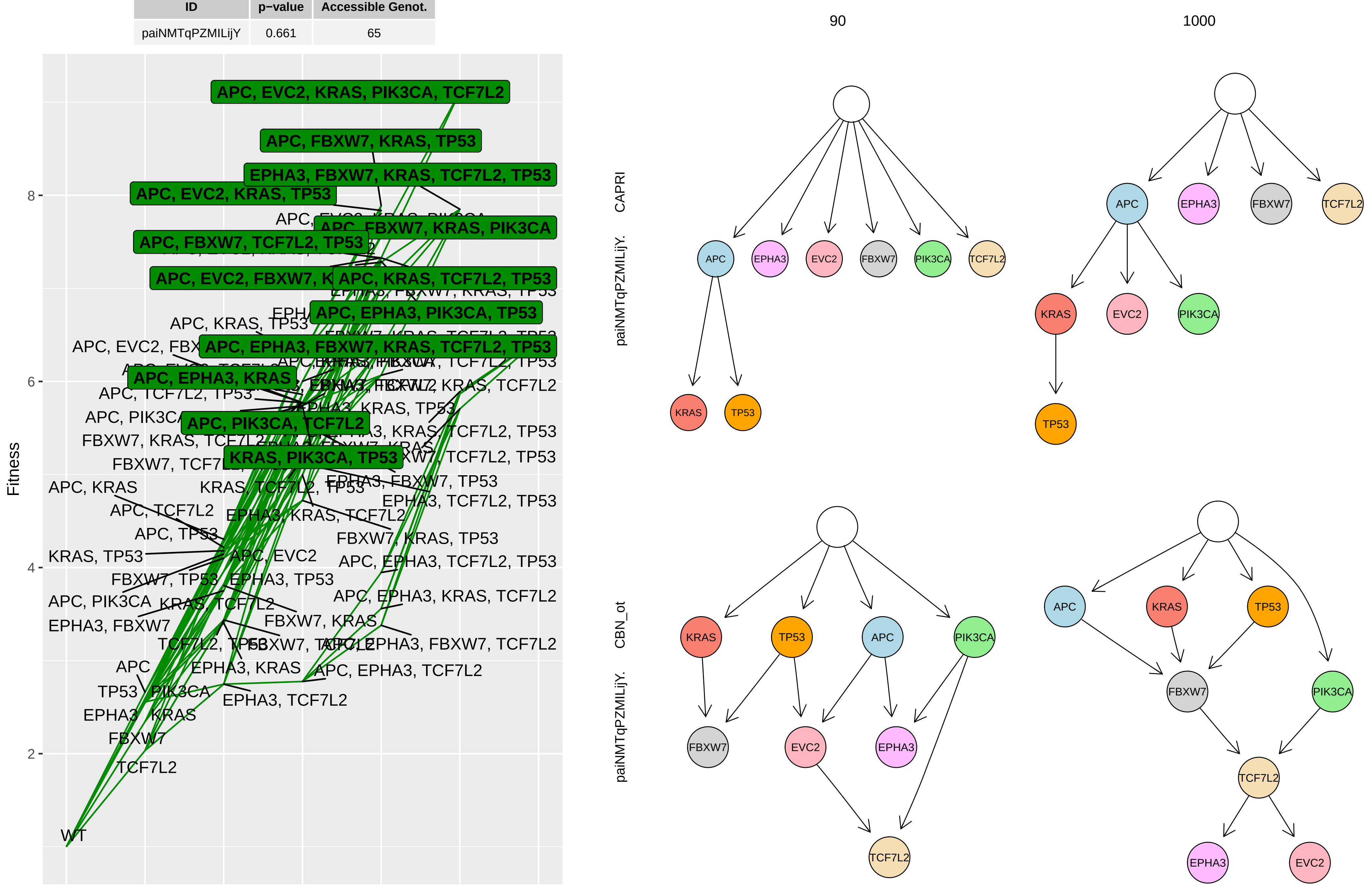

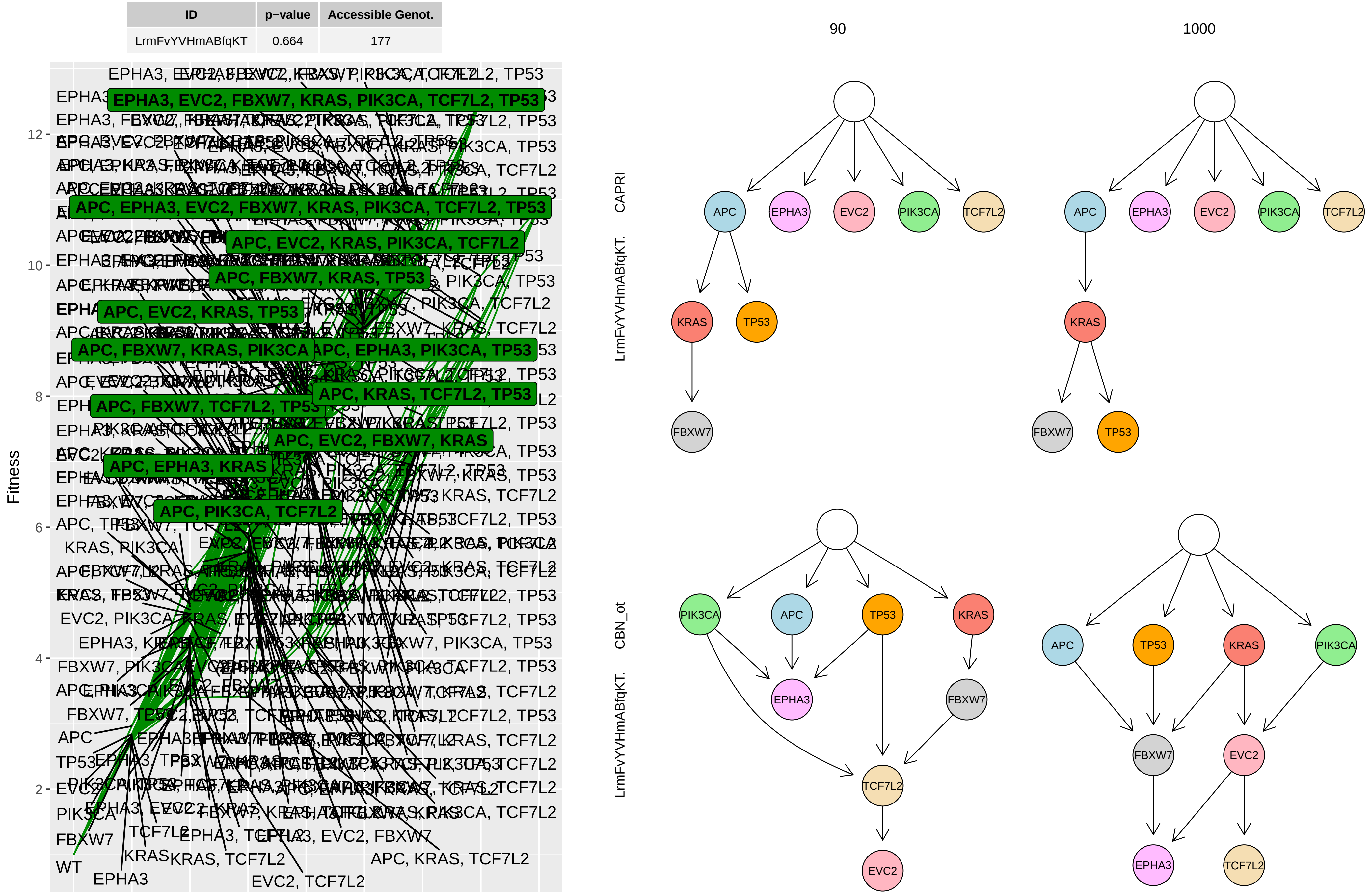

ID
p−value
Accessible Genot.
LrmFvYVHmABfqKT
0.664
177
90
1000
CAPRI
CBN_ot
LrmFvYVHmABfqKT.
Fitness
APC
EPHA3
EVC2
PIK3CA
TCF7L2
KRAS
TP53
FBXW7
EPHA3, EVC2, FBXW7, KRAS, PIK3CA, TCF7L2, TP53
APC, EPHA3, EVC2, FBXW7, KRAS, PIK3CA, TCF7L2, TP53
APC, EVC2, KRAS, PIK3CA, TCF7L2
APC, FBXW7, KRAS, TP53
APC, EVC2, KRAS, TP53
APC, FBXW7, KRAS, PIK3CA
APC, EPHA3, PIK3CA, TP53
APC, KRAS, TCF7L2, TP53
APC, FBXW7, TCF7L2, TP53
APC, EVC2, FBXW7, KRAS
APC, EPHA3, KRAS
APC, PIK3CA, TCF7L2
KRAS, PIK3CA
WT
EPHA3
KRAS
KRAS, TCF7L2
EVC2, TCF7L2
APC, KRAS, TCF7L2
EPHA3, EVC2, FBXW7

ID	p−value	Accessible Genot.
cuEBCNfQRiyzicO	0.669	53

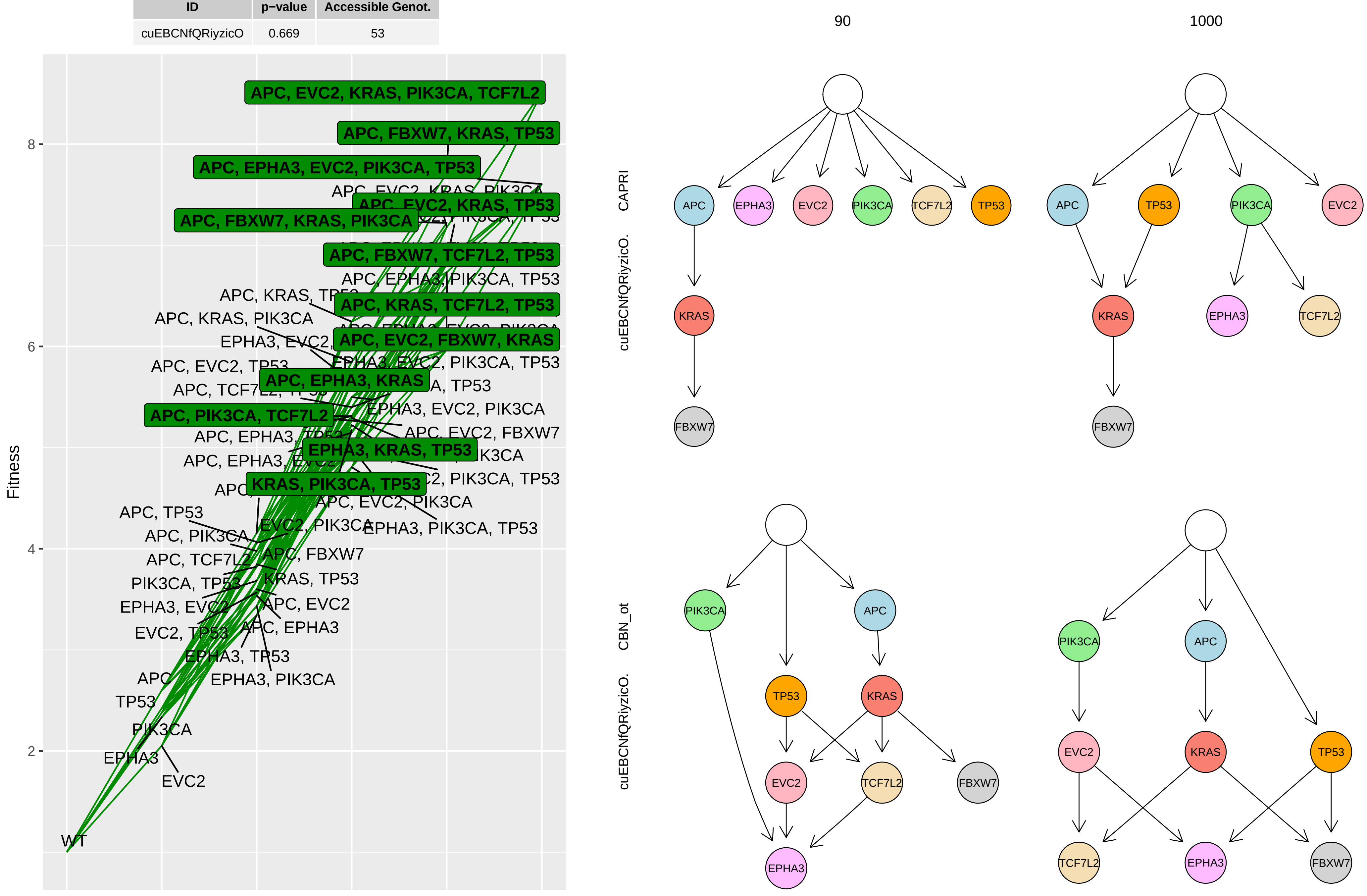

ID	p−value	Accessible Genot.
CVuORDVBWWINnNn	0.669	31

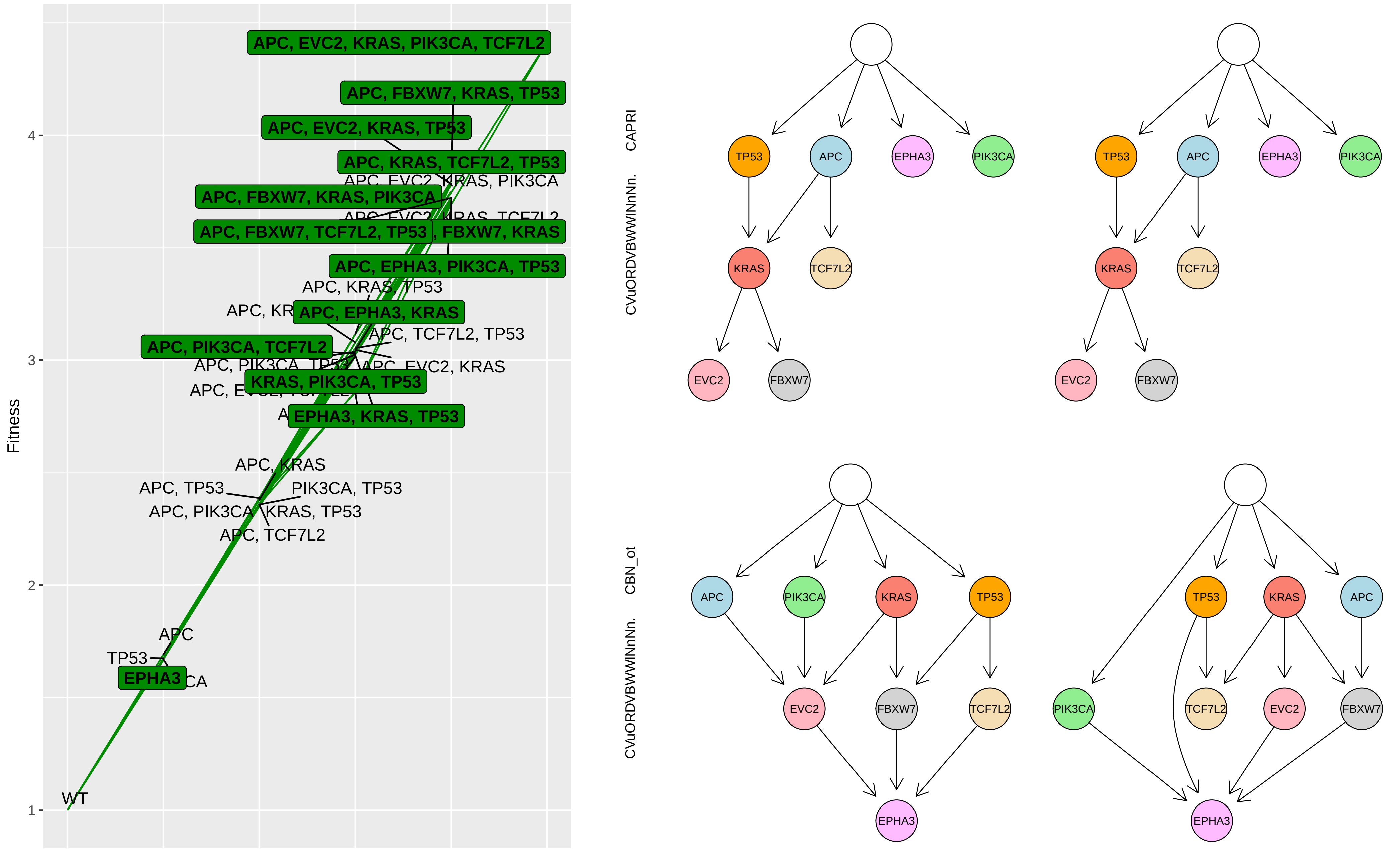

ID	p−value	Accessible Genot.
mcWUpkVqWFjxiyH	0.67	58

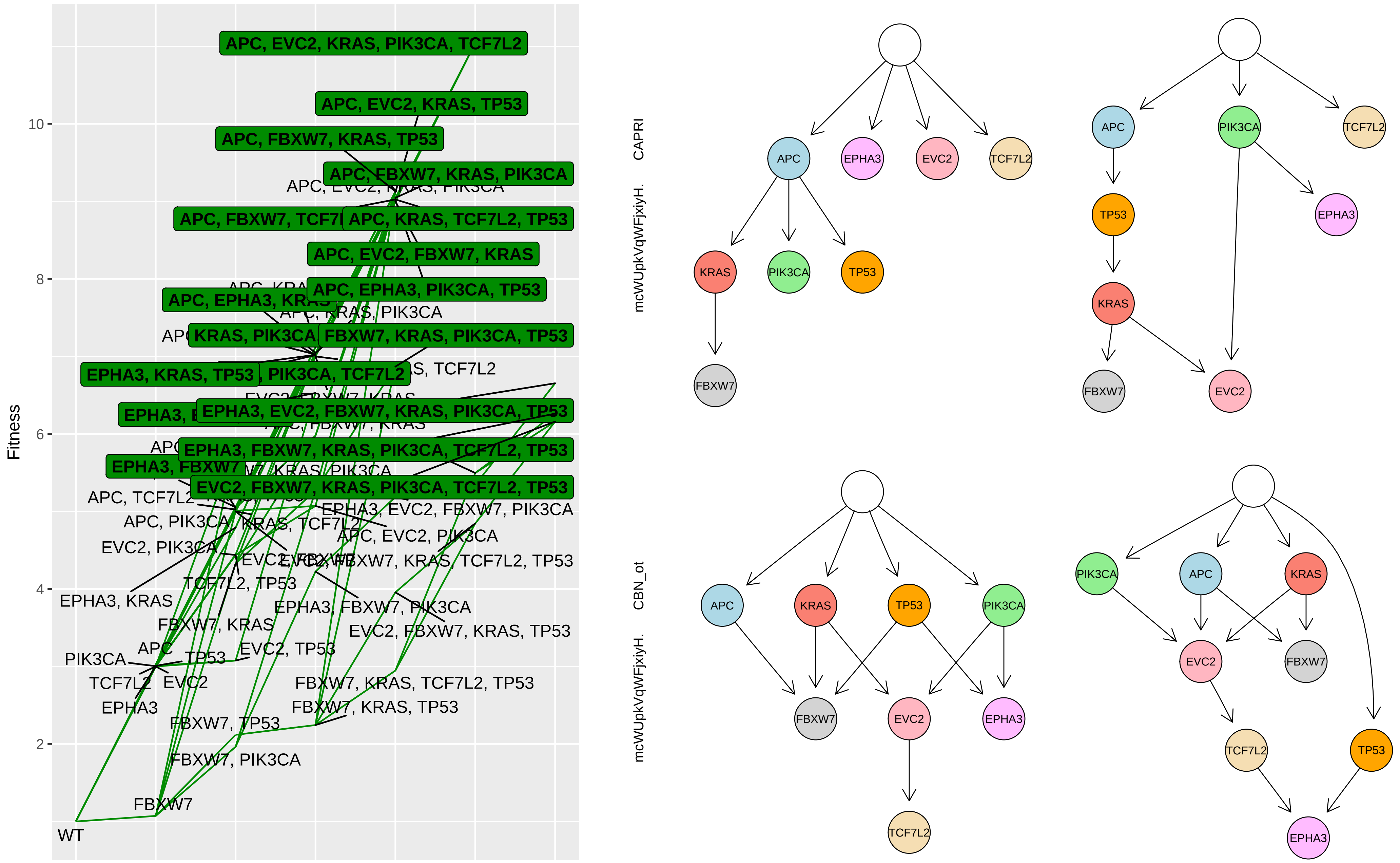

ID	p−value	Accessible Genot.
XMKSSQVlmvHhUEN	0.672	61

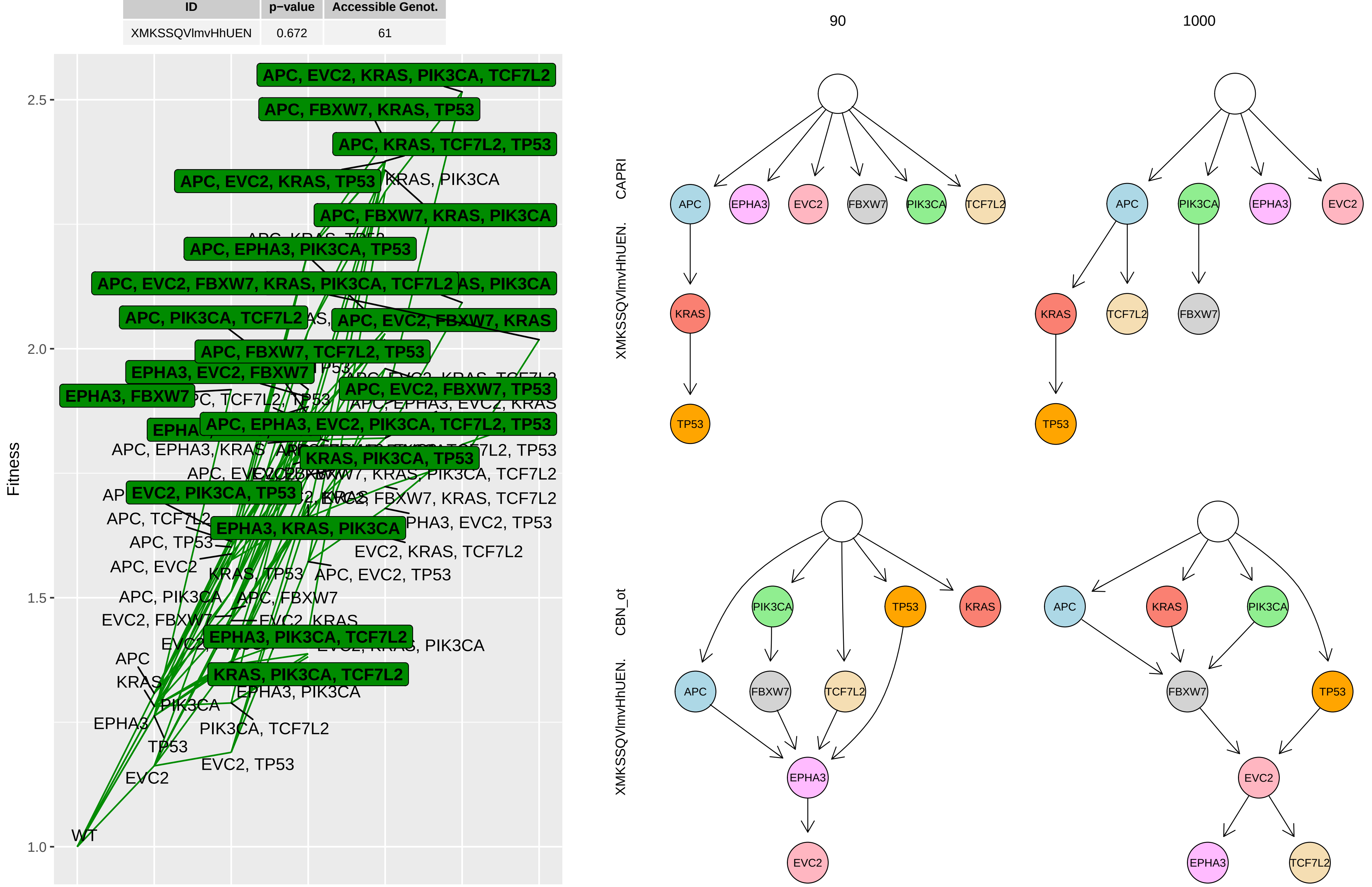

ID	p−value	Accessible Genot.
ClsPajYojshvbjs	0.672	79

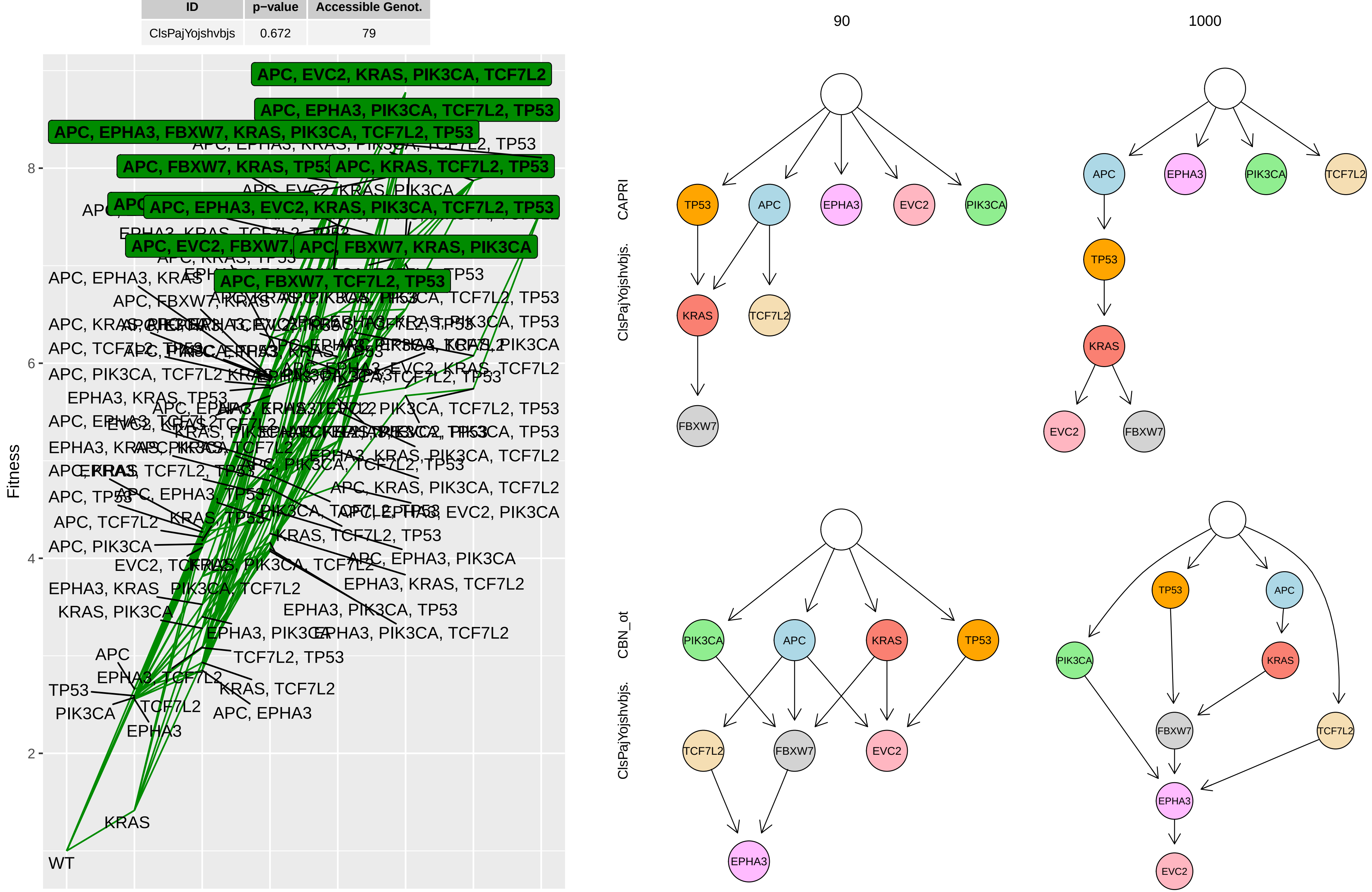

ID	p−value	Accessible Genot.
zrfpelittinYexz	0.674	155

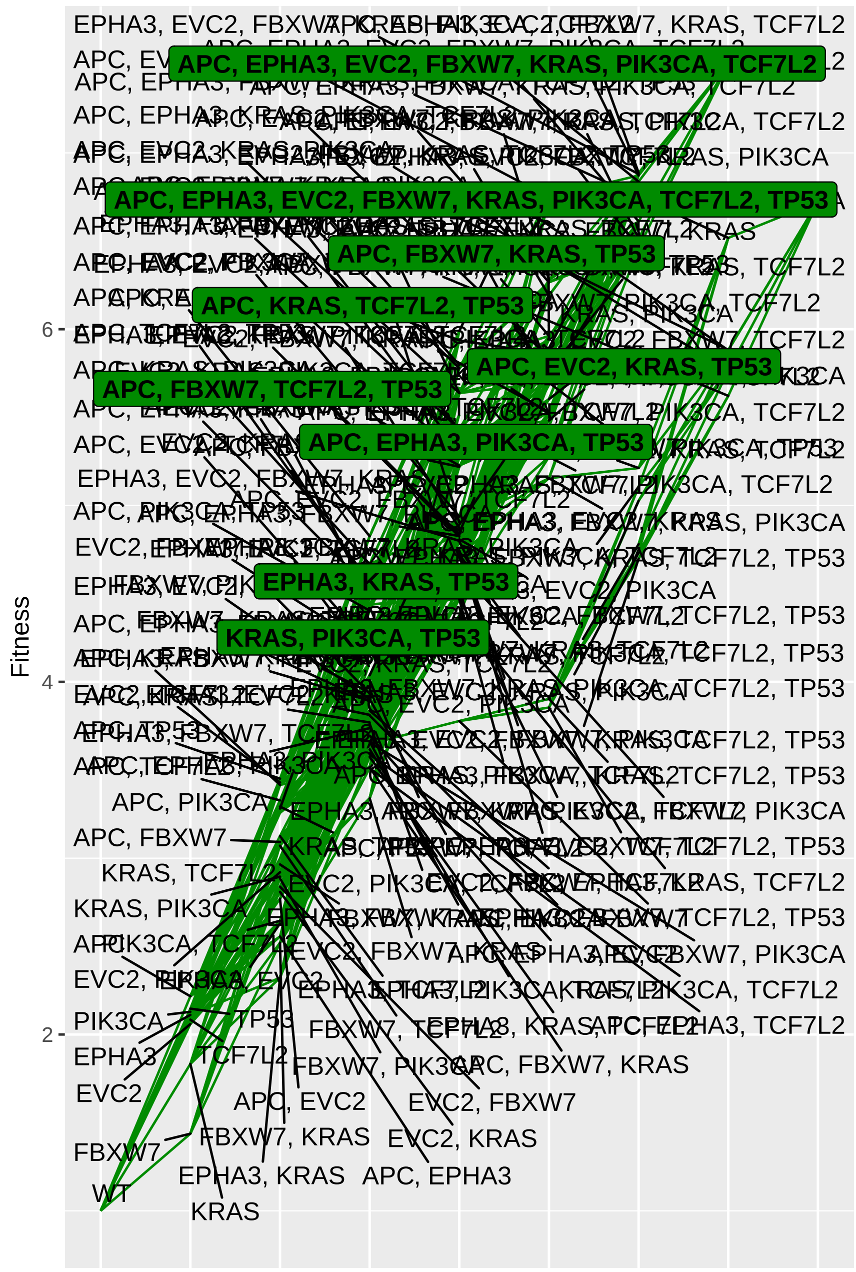

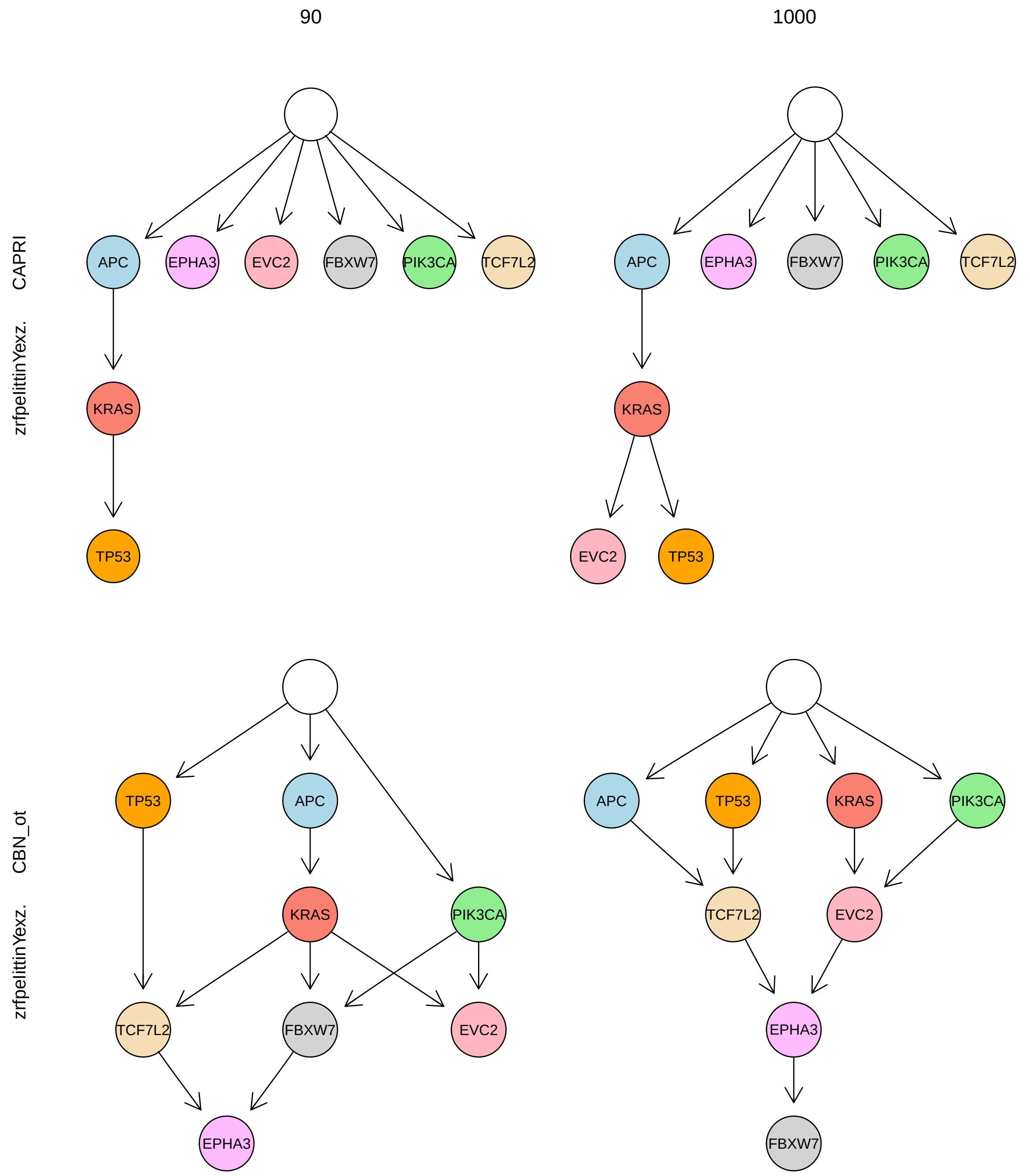

ID	p−value	Accessible Genot.
attOgpjeFRDuGJt	0.674	48

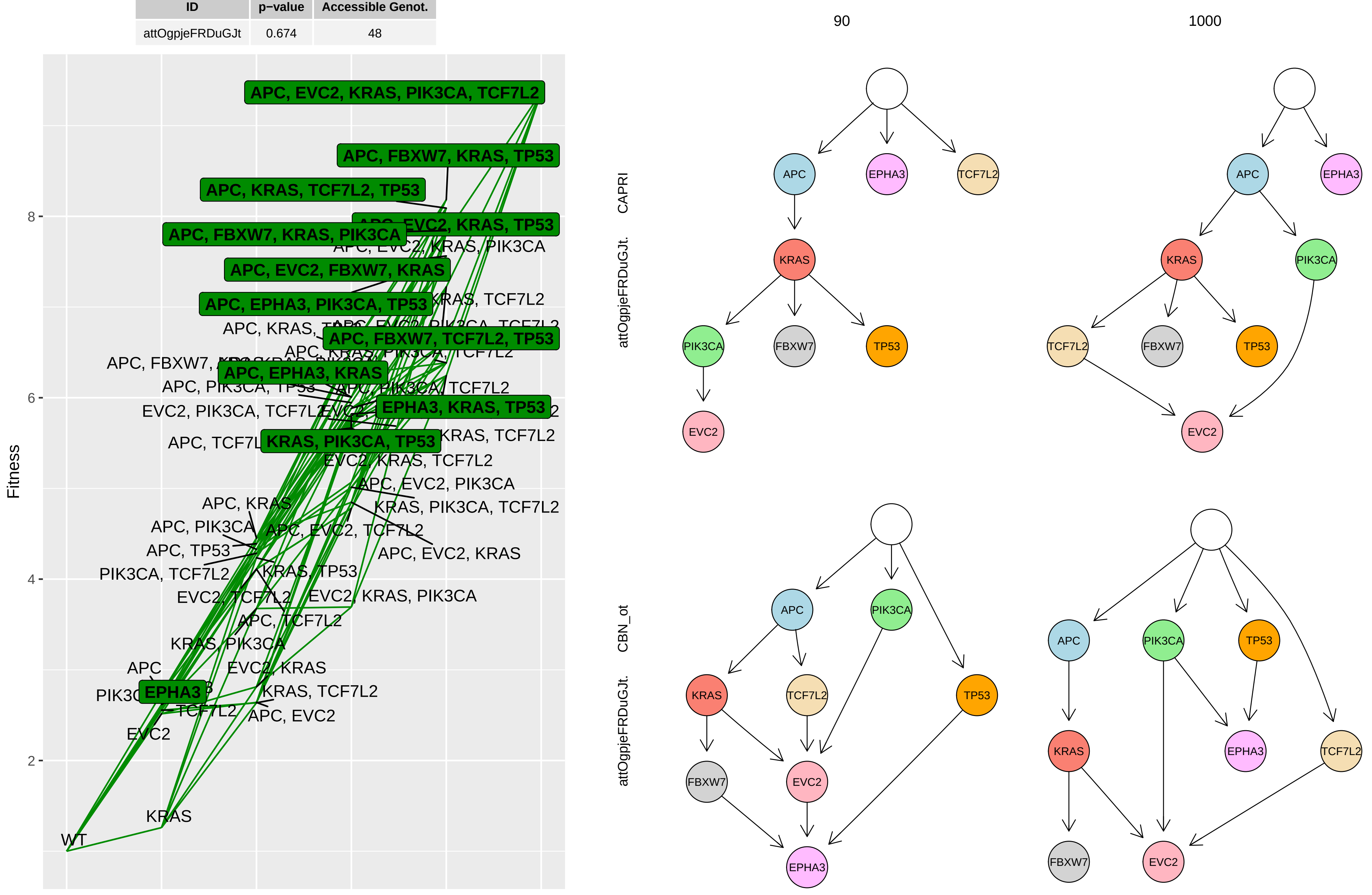

ID	p−value	Accessible Genot.
MizDyZXFPCNIBRa	0.675	69

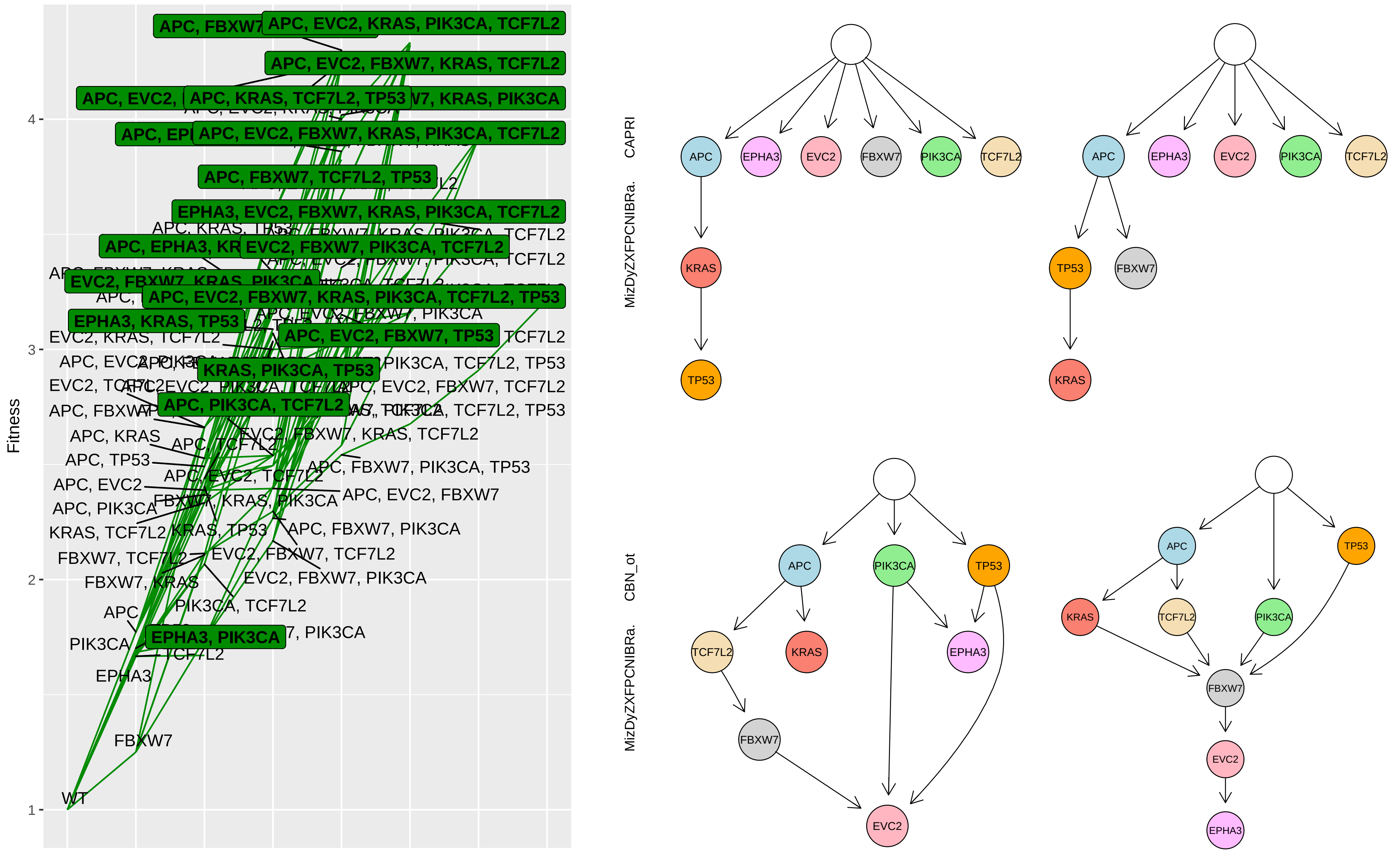

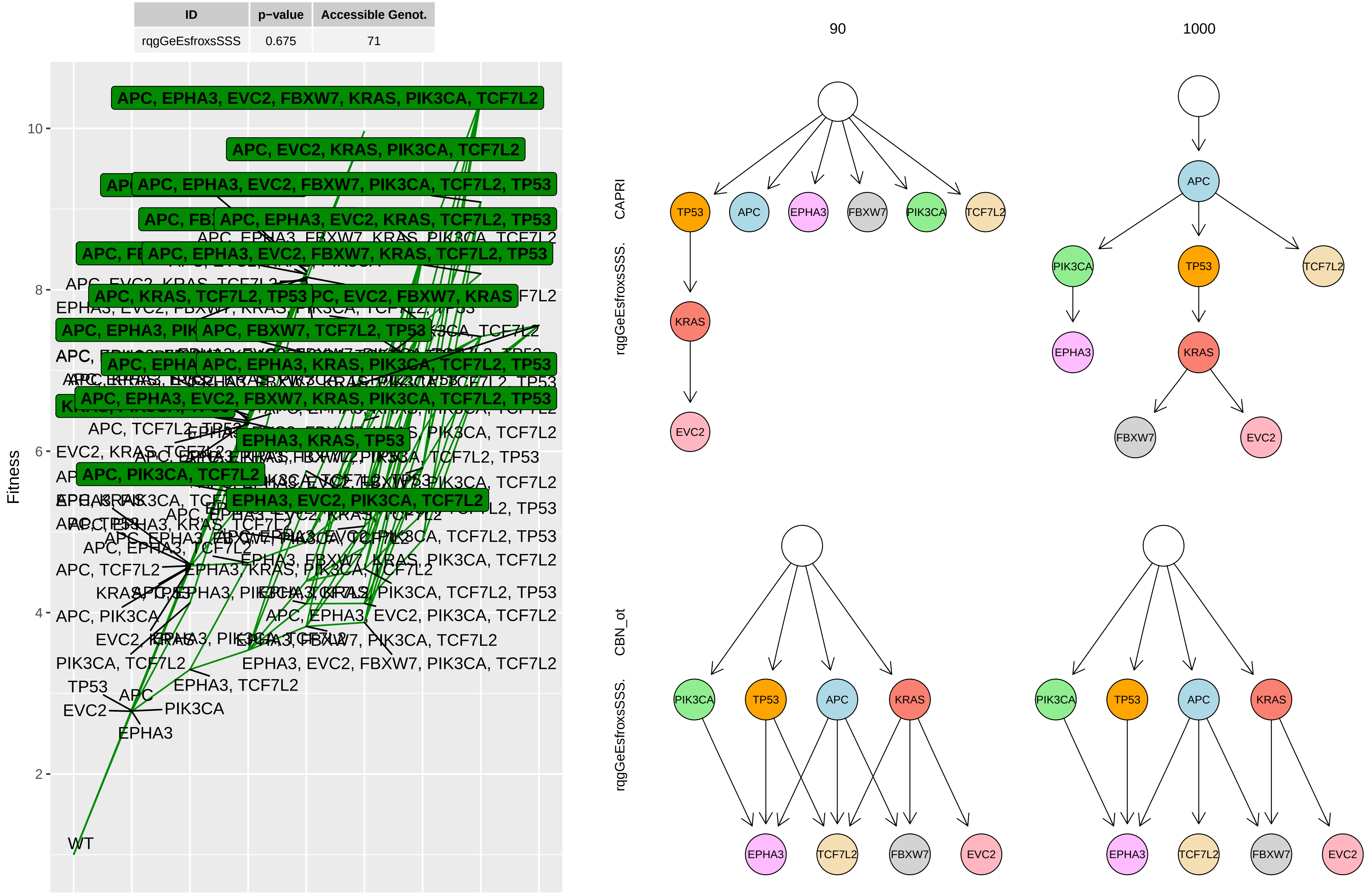

ID
p−value
Accessible Genot.
rqgGeEsfroxsSSS
0.675
71
Fitness
WT
APC, EPHA3, EVC2, FBXW7, KRAS, PIK3CA, TCF7L2
APC, EVC2, KRAS, PIK3CA, TCF7L2
APC, EPHA3, EVC2, FBXW7, PIK3CA, TCF7L2, TP53
APC, EPHA3, EVC2, KRAS, TCF7L2, TP53
APC, EPHA3, EVC2, FBXW7, KRAS, TCF7L2, TP53
APC, KRAS, TCF7L2, TP53
APC, FBXW7, TCF7L2, TP53
APC, EPHA3, KRAS, PIK3CA, TCF7L2, TP53
APC, EPHA3, EVC2, FBXW7, KRAS, PIK3CA, TCF7L2, TP53
EPHA3, KRAS, TP53
APC, PIK3CA, TCF7L2
EPHA3, EVC2, PIK3CA, TCF7L2
APC, TCF7L2
APC, PIK3CA
PIK3CA, TCF7L2
EPHA3, TCF7L2
TP53
APC
EVC2
PIK3CA
EPHA3
90
1000
CAPRI
CBN_ot
rqgGeEsfroxsSSS.
TP53
APC
EPHA3
FBXW7
PIK3CA
TCF7L2
KRAS
EVC2

ID	p−value	Accessible Genot.
kZwgAdeboKnwBqh	0.675	78

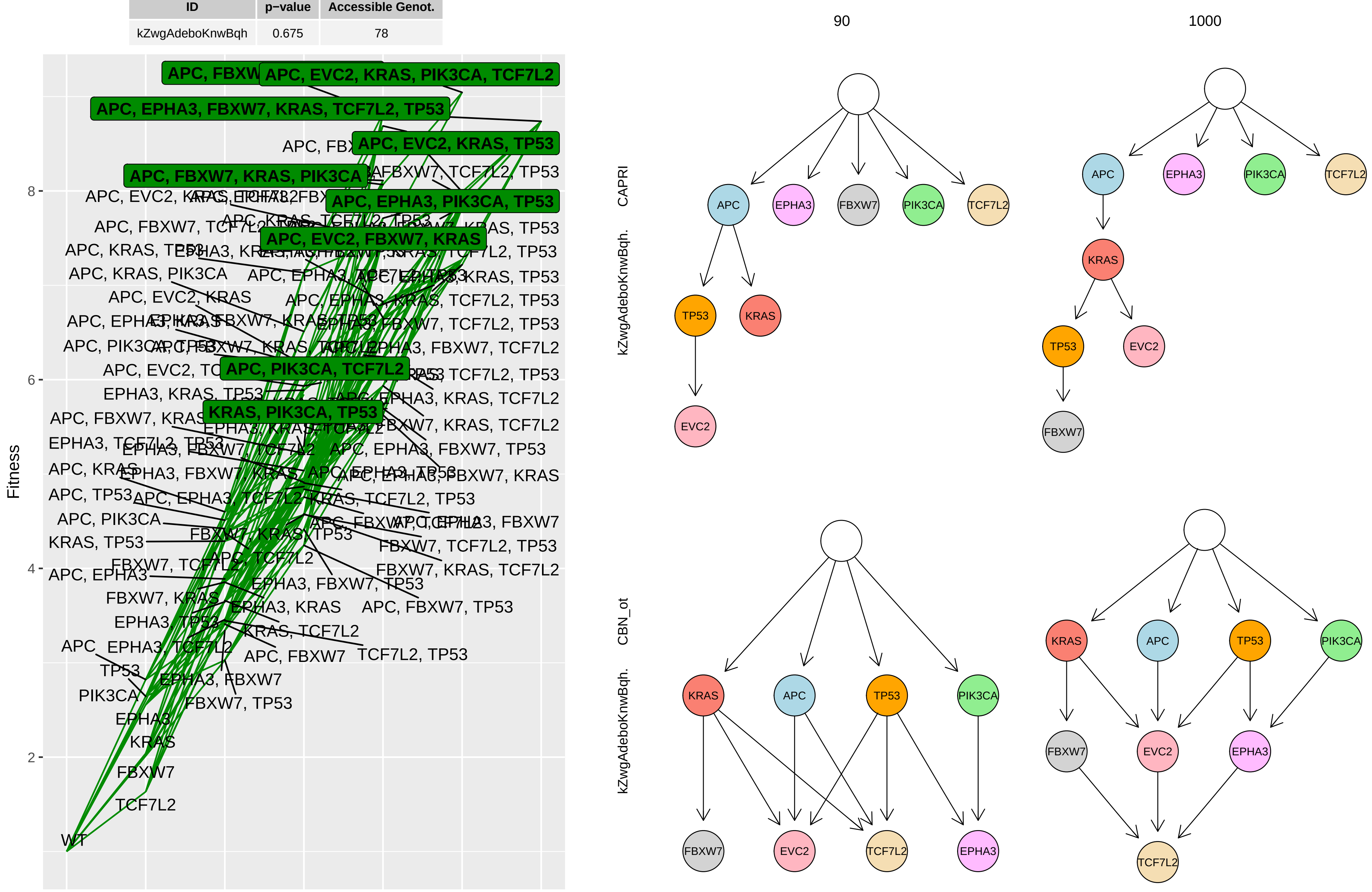

ID	p−value	Accessible Genot.
WSbKbPsCnDeJmNI	0.676	90

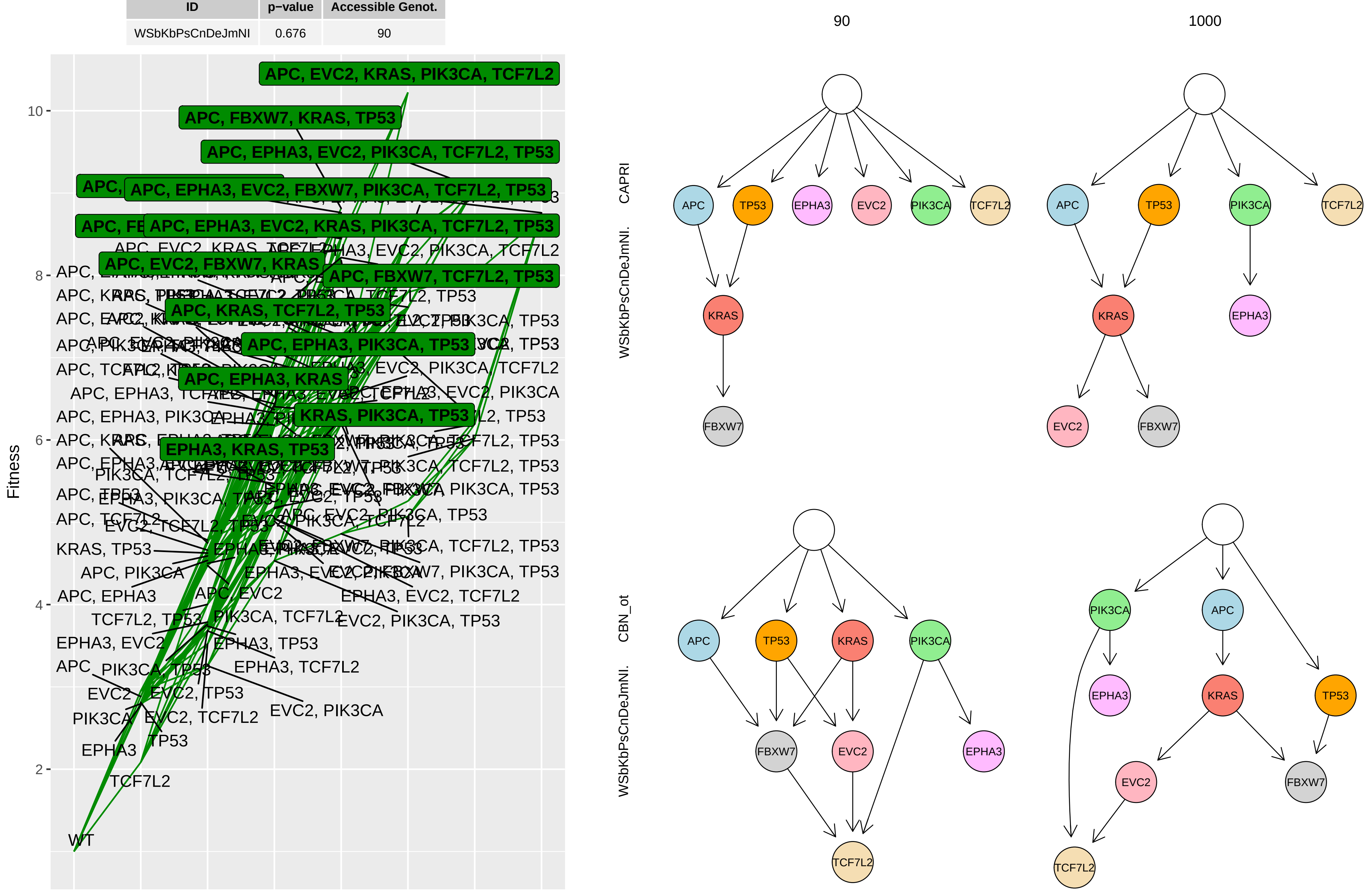

ID	p−value	Accessible Genot.
laeqhUuyJrtjNza	0.677	255

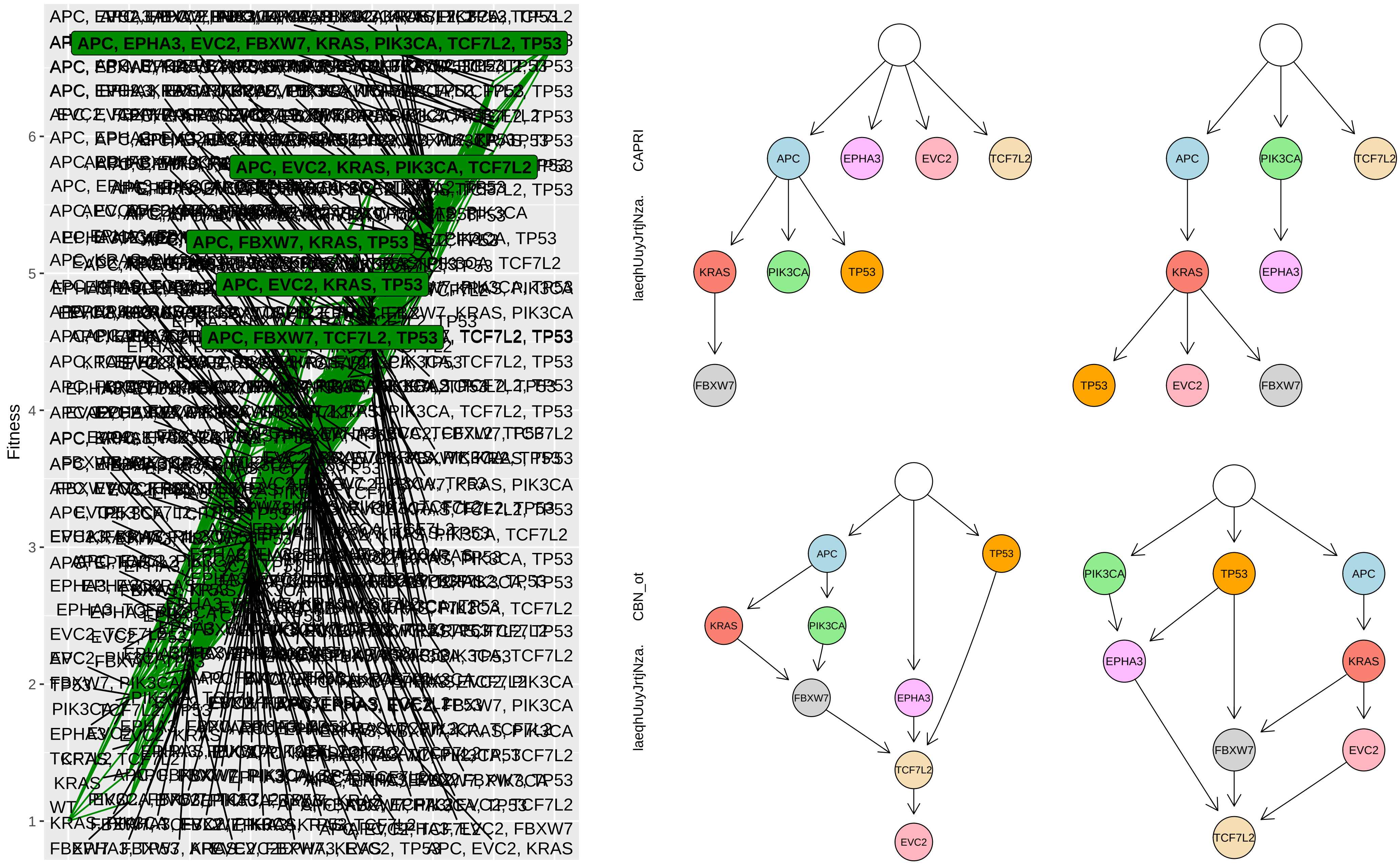

ID	p−value	Accessible Genot.
uRJrAEEUMGddFDK	0.678	102

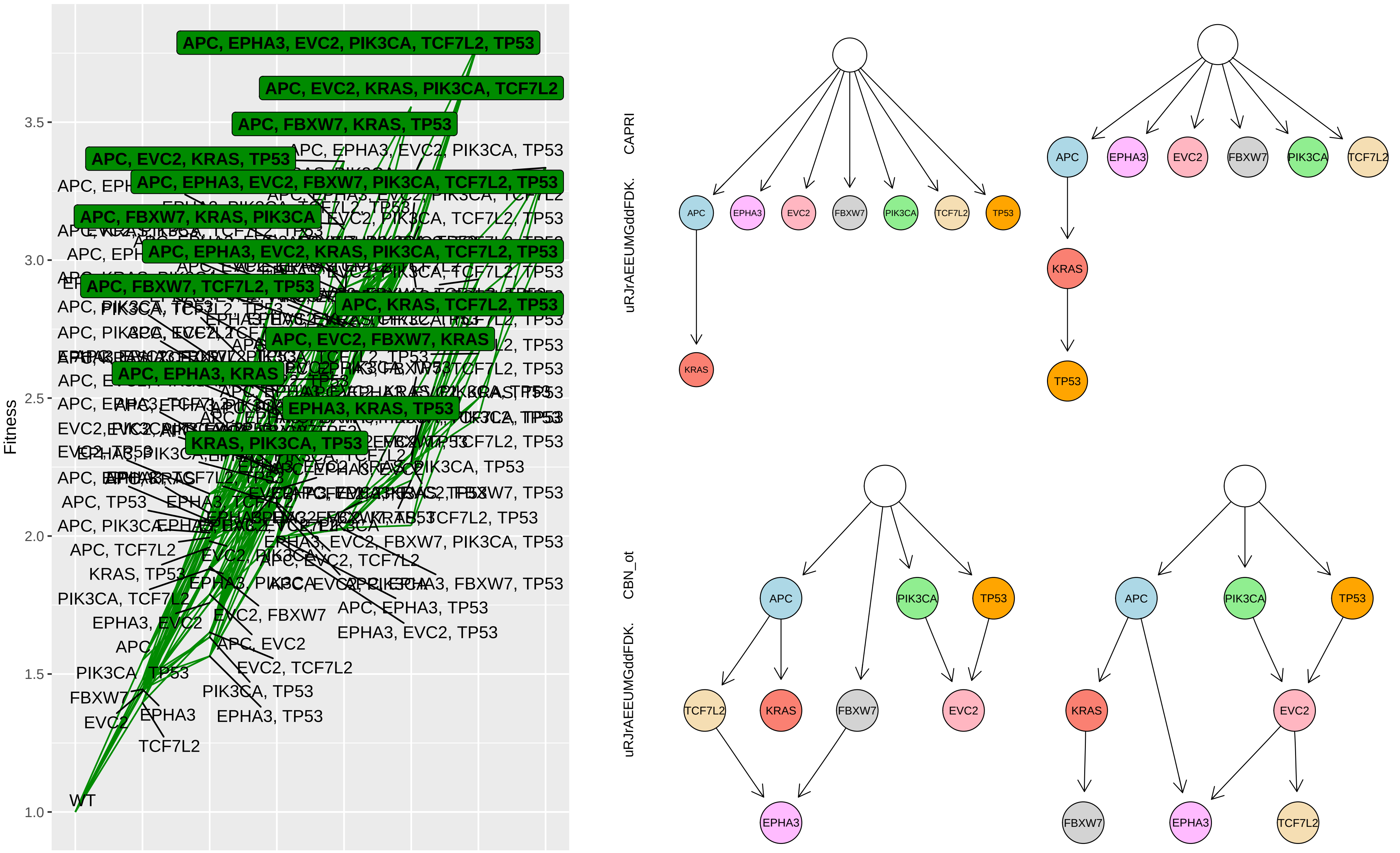

ID	p−value	Accessible Genot.
qMoyslrGIZluDGh	0.678	30

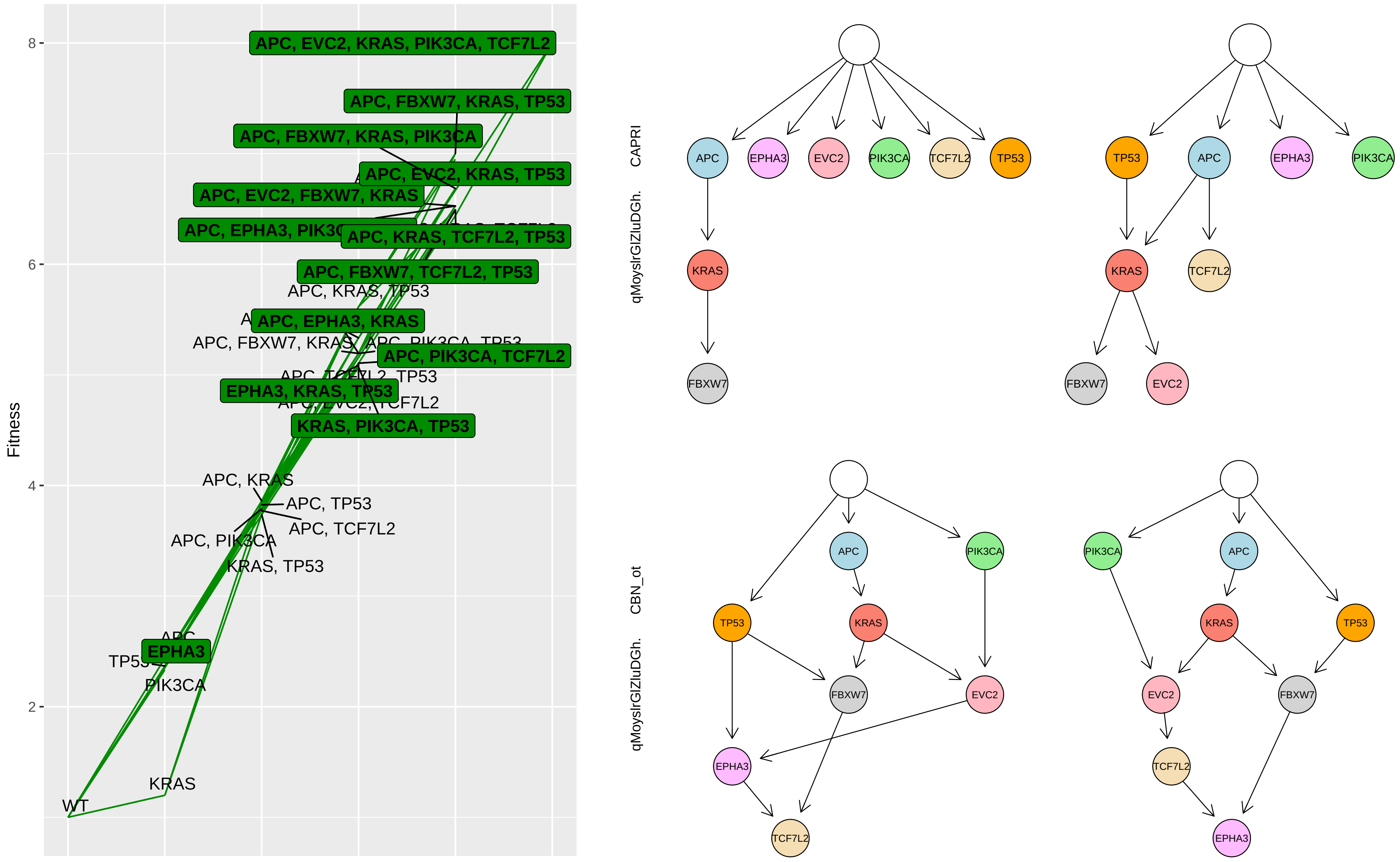

ID	p−value	Accessible Genot.
rTCQgwQnUKbsadM	0.679	67

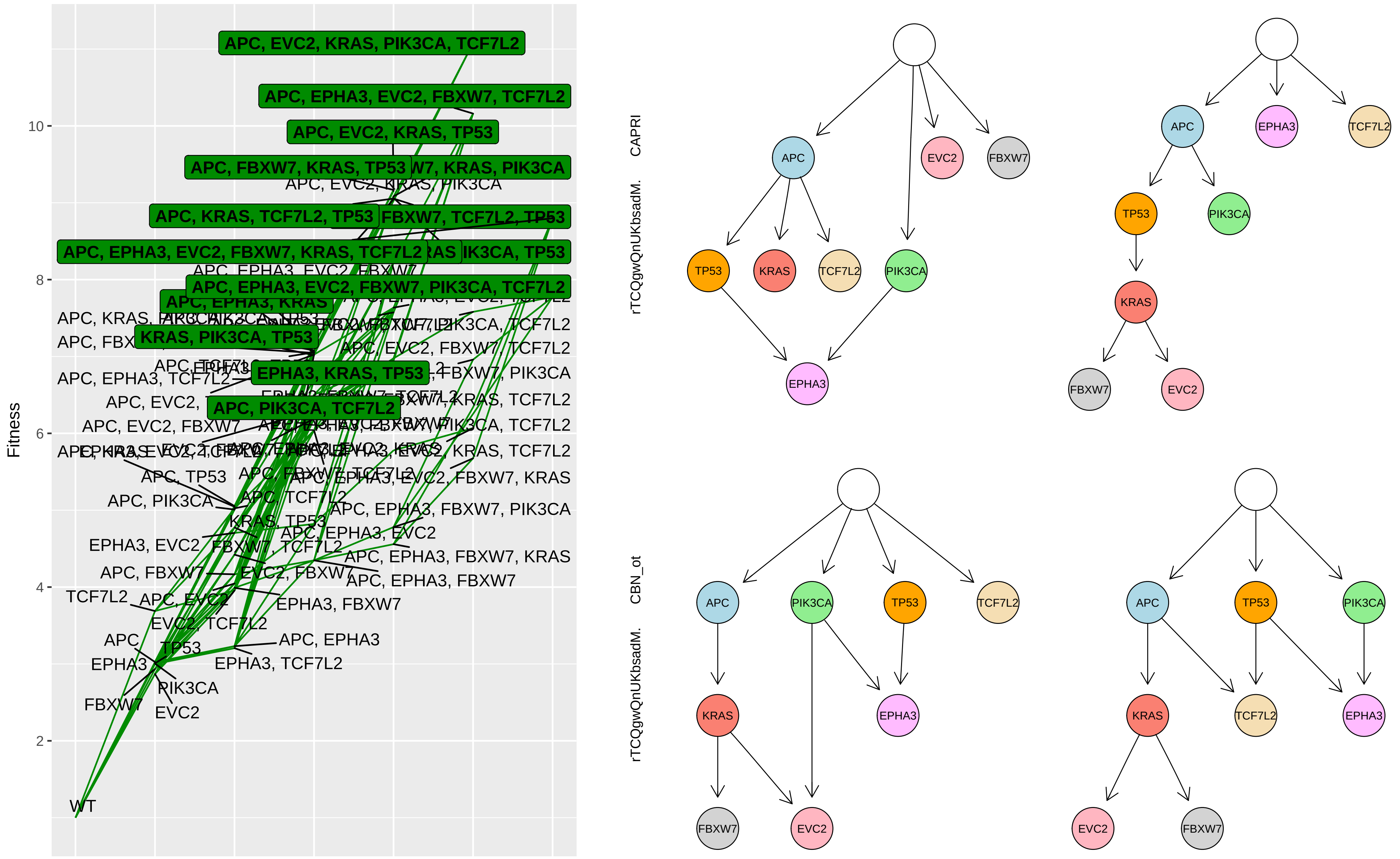

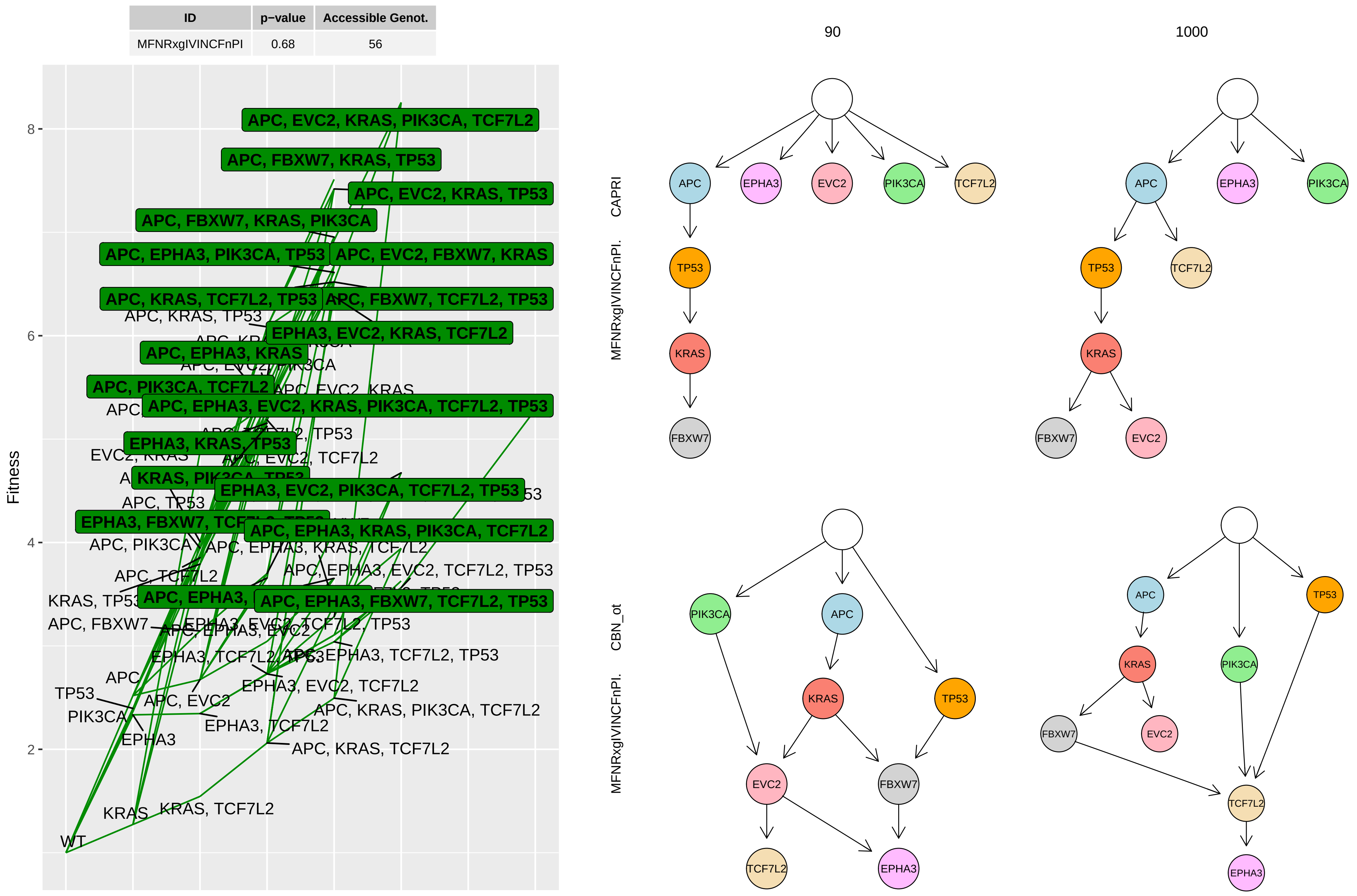

ID
p-value
Accessible Genot.
MFNRxglVINCFnPI
0.68
56
Fitness
8
6
4
2
APC, EVC2, KRAS, PIK3CA, TCF7L2
APC, FBXW7, KRAS, TP53
APC, EVC2, KRAS, TP53
APC, FBXW7, KRAS, PIK3CA
APC, EPHA3, PIK3CA, TP53
APC, EVC2, FBXW7, KRAS
APC, KRAS, TCF7L2, TP53
APC, FBXW7, TCF7L2, TP53
APC, KRAS, TP53
EPHA3, EVC2, KRAS, TCF7L2
APC, EPHA3, KRAS
APC, PIK3CA, TCF7L2
APC, EPHA3, EVC2, KRAS, PIK3CA, TCF7L2, TP53
EPHA3, KRAS, TP53
APC, EVC2, TCF7L2
EPHA3, EVC2, PIK3CA, TCF7L2, TP53
APC, TP53
APC, EPHA3, KRAS, PIK3CA, TCF7L2
APC, PIK3CA
APC, EPHA3, KRAS, TCF7L2
APC, TCF7L2
APC, EPHA3, EVC2, TCF7L2, TP53
APC, EPHA3, FBXW7, TCF7L2, TP53
APC, FBXW7
APC, EPHA3, EVC2
EPHA3, TCF7L2, TP53
EPHA3, TCF7L2, TP53
APC
EPHA3, EVC2, TCF7L2
TP53
APC, EVC2
APC, KRAS, PIK3CA, TCF7L2
PIK3CA
EPHA3, TCF7L2
EPHA3
APC, KRAS, TCF7L2
KRAS
KRAS, TCF7L2
WT
90
1000
MFNRxglVINCFnPI. CAPRI
MFNRxglVINCFnPI. CBN_ot
APC
EPHA3
EVC2
PIK3CA
TCF7L2
TP53
KRAS
FBXW7

ID	p−value	Accessible Genot.
KPNjzXOkyXcLEFR	0.68	35

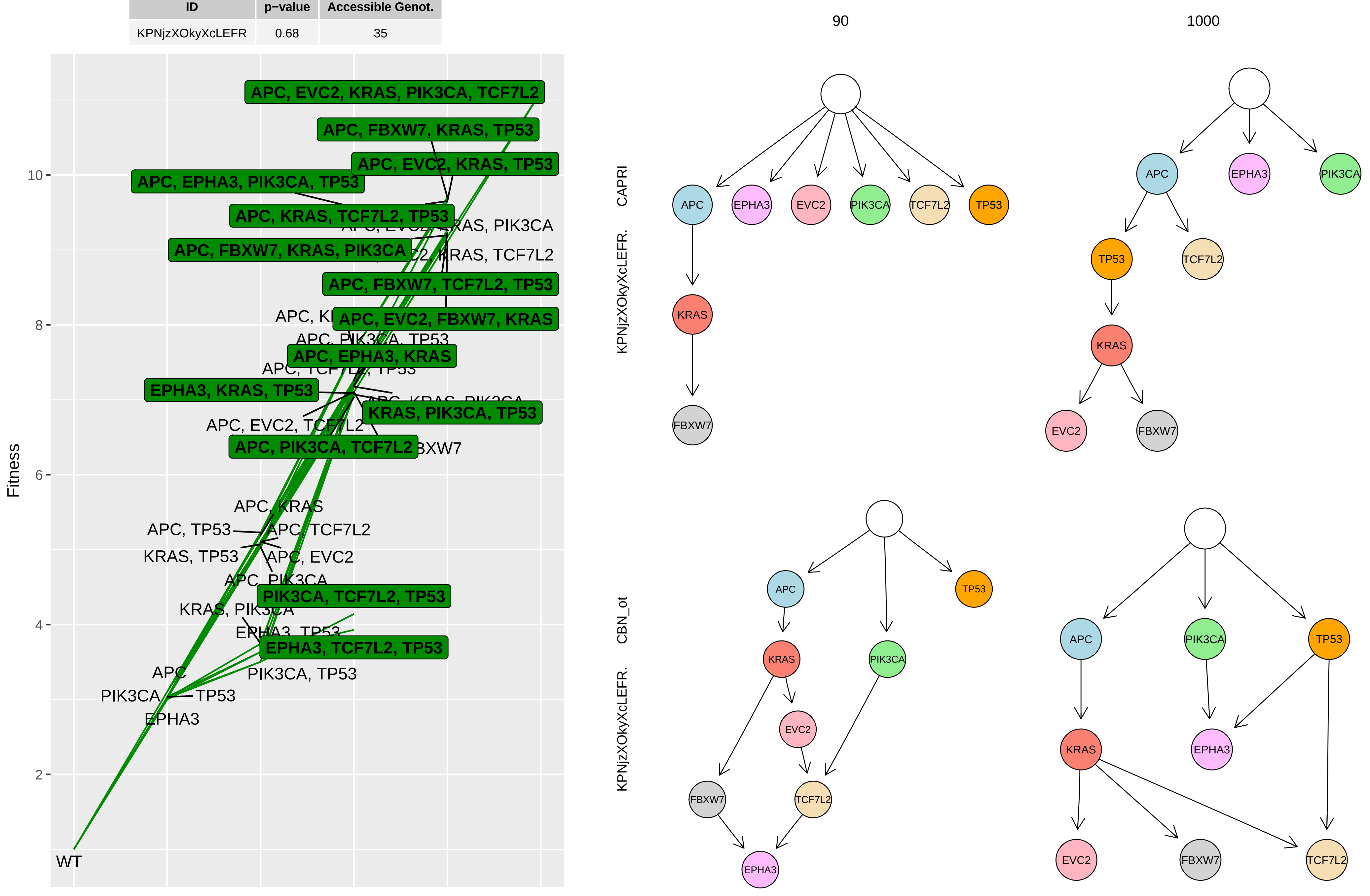

ID	p−value	Accessible Genot.
oMbQpcBZlsYLPik	0.685	215

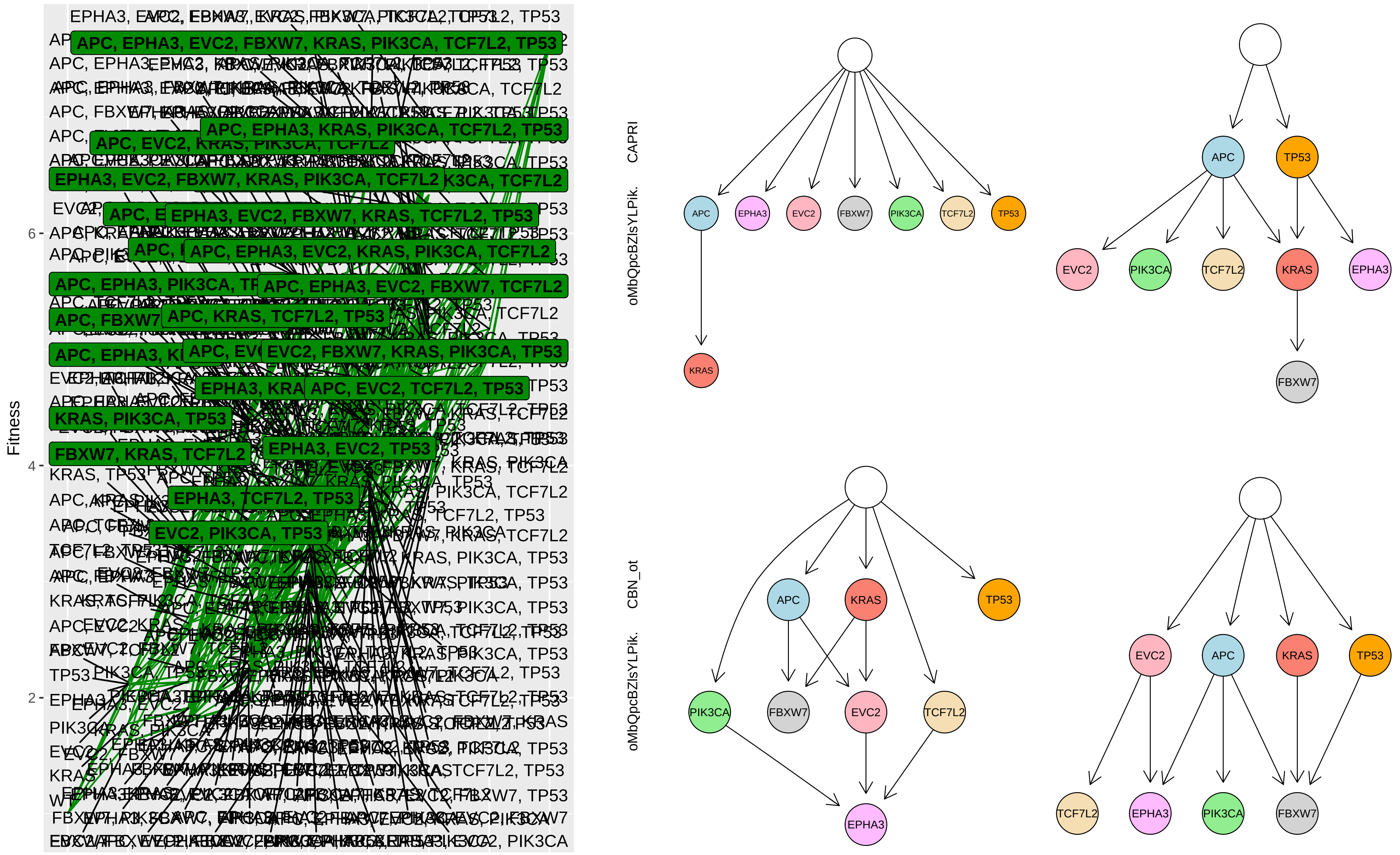

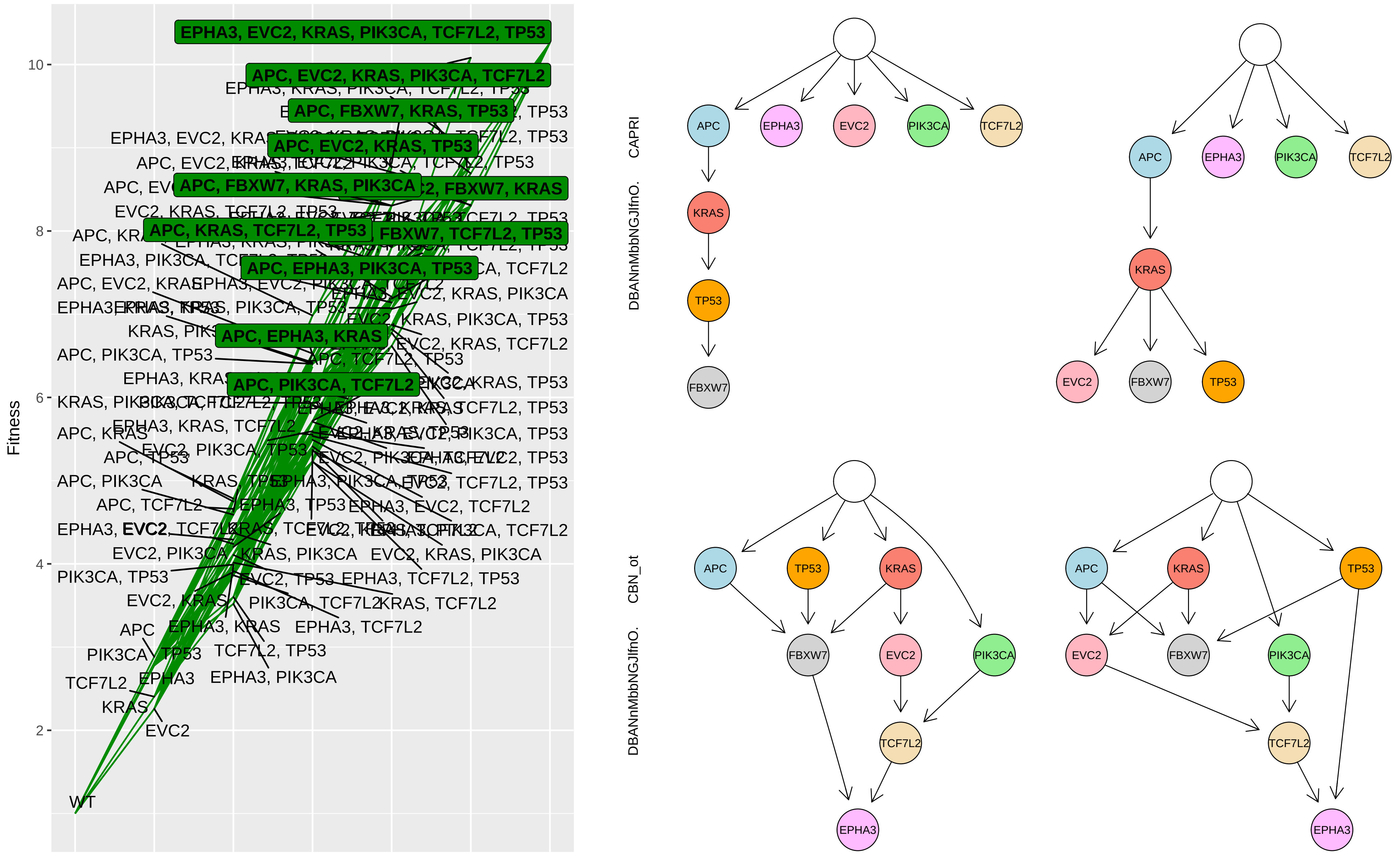

ID	p−value	Accessible Genot.
DBANnMbbNGJlfnO	0.686	85

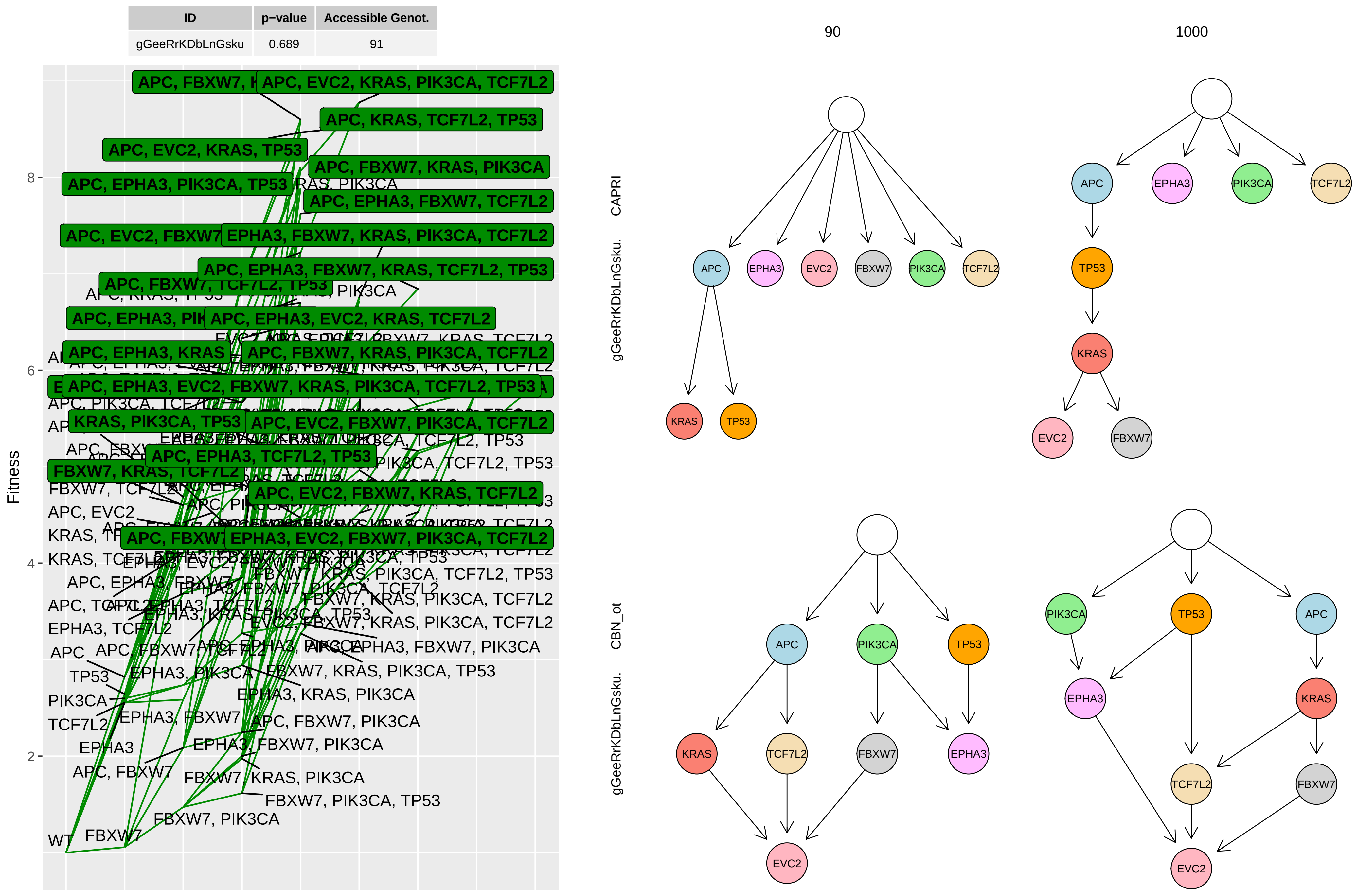

ID
p−value
Accessible Genot.
gGeeRrKDbLnGsku
0.689
91
Fitness
8
6
4
2
APC, EVC2, KRAS, PIK3CA, TCF7L2
APC, KRAS, TCF7L2, TP53
APC, EVC2, KRAS, TP53
APC, FBXW7, KRAS, PIK3CA
APC, EPHA3, PIK3CA, TP53
APC, EPHA3, FBXW7, TCF7L2
EPHA3, FBXW7, KRAS, PIK3CA, TCF7L2
APC, EPHA3, FBXW7, KRAS, TCF7L2, TP53
APC, FBXW7, TCF7L2, TP53
APC, EPHA3, EVC2, KRAS, TCF7L2
APC, EPHA3, KRAS
APC, FBXW7, KRAS, PIK3CA, TCF7L2
APC, EPHA3, EVC2, FBXW7, KRAS, PIK3CA, TCF7L2, TP53
KRAS, PIK3CA, TP53
APC, EVC2, FBXW7, PIK3CA, TCF7L2
APC, EPHA3, TCF7L2, TP53
FBXW7, KRAS, TCF7L2
APC, EVC2, FBXW7, KRAS, TCF7L2
EPHA3, EVC2, FBXW7, PIK3CA, TCF7L2
APC, PIK3CA, TCF7L2
FBXW7, TCF7L2
APC, EVC2
EPHA3, TCF7L2
APC
TP53
PIK3CA
TCF7L2
EPHA3
APC, FBXW7
WT
FBXW7
FBXW7, PIK3CA
FBXW7, PIK3CA, TP53
FBXW7, KRAS, PIK3CA
EPHA3, FBXW7, PIK3CA
APC, FBXW7, PIK3CA
EPHA3, KRAS, PIK3CA
EPHA3, FBXW7
EPHA3, PIK3CA
FBXW7, KRAS, PIK3CA, TP53
APC, EPHA3, FBXW7, PIK3CA
FBXW7, KRAS, PIK3CA, TCF7L2, TP53
90
1000
CAPRI
CBN_ot
gGeeRrKDbLnGsku.
APC
EPHA3
EVC2
FBXW7
PIK3CA
TCF7L2
KRAS
TP53

ID	p−value	Accessible Genot.
JkAVzPlUpNxoWsg	0.692	39

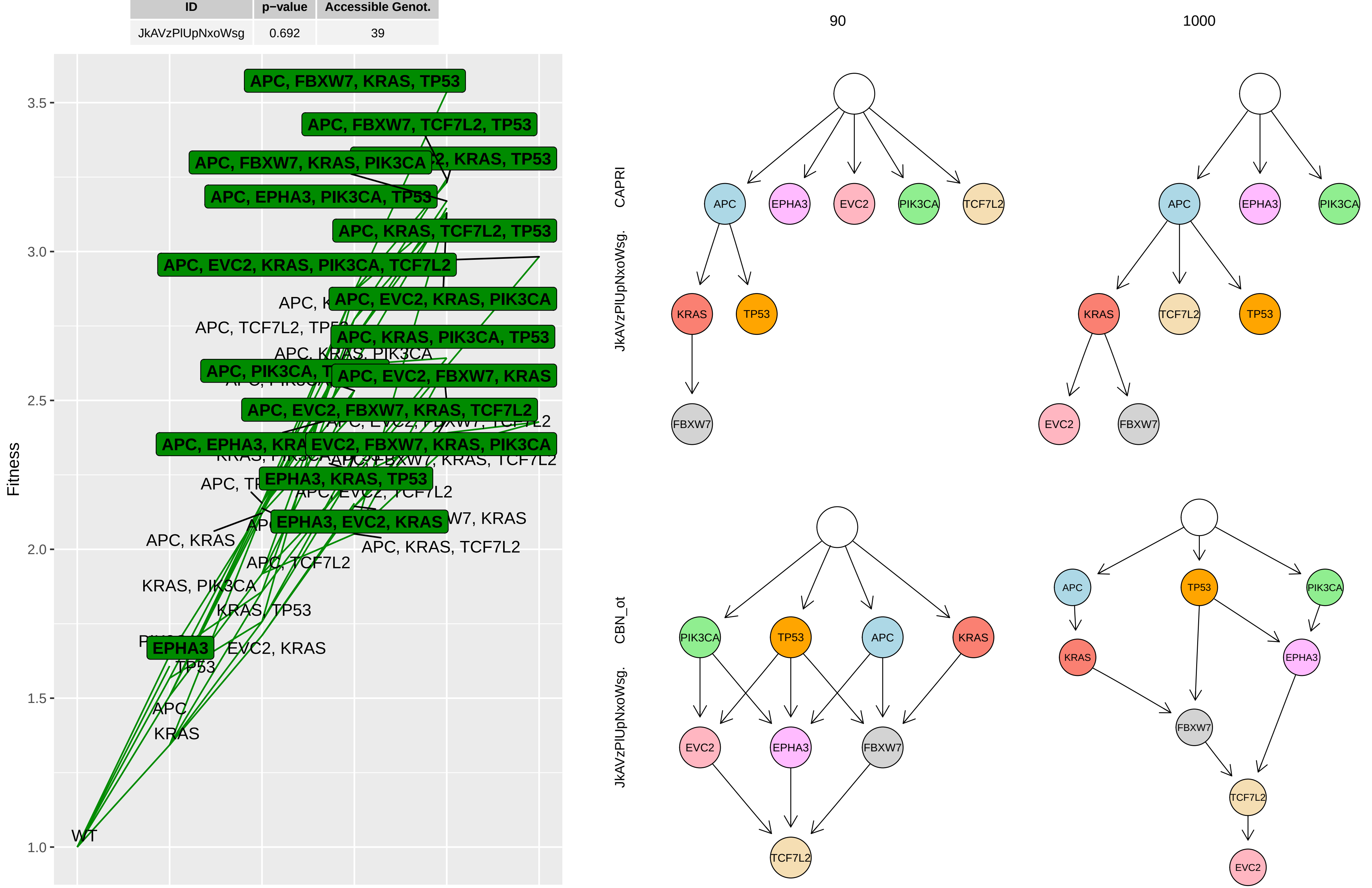

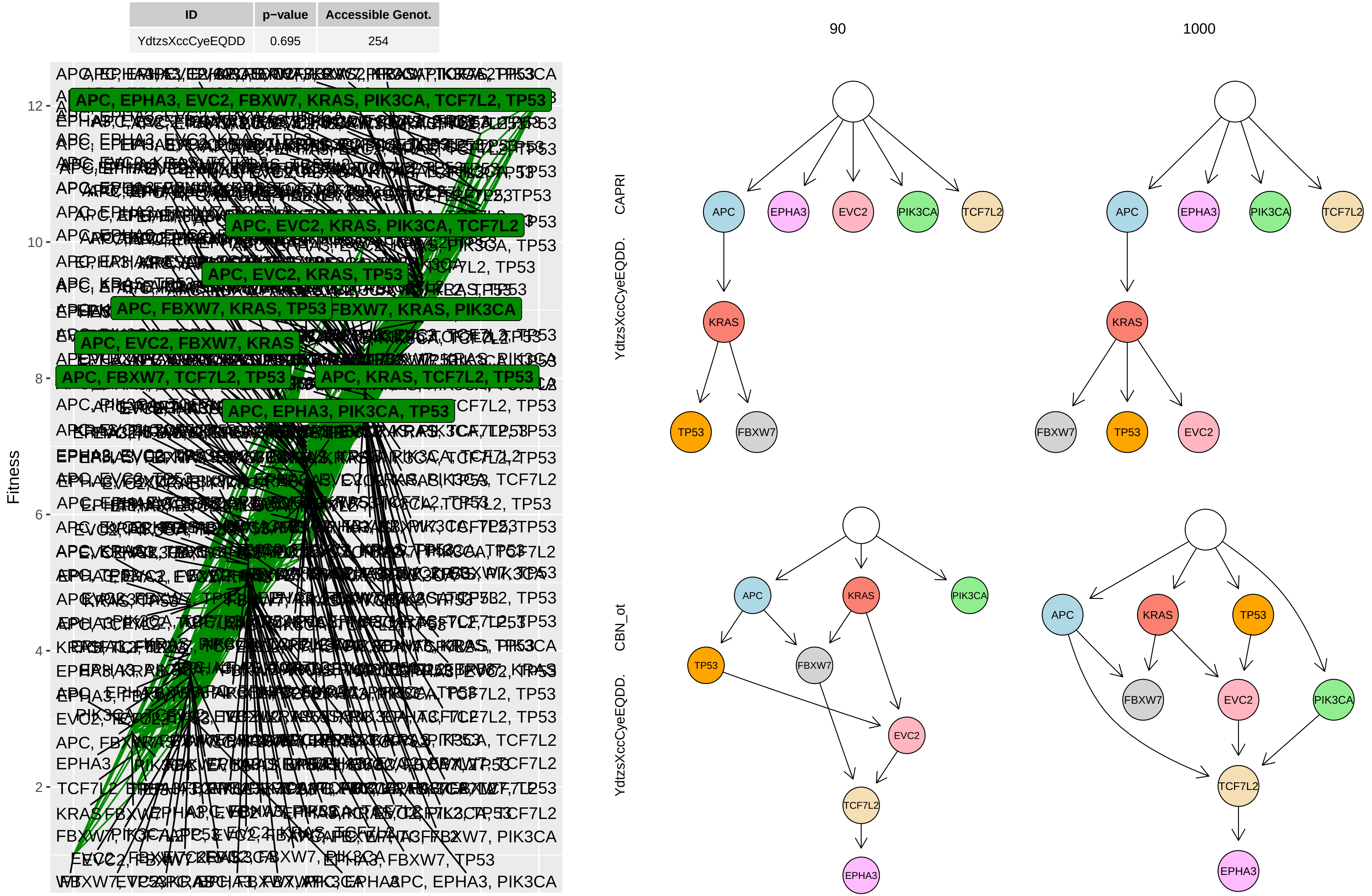

ID
p-value
Accessible Genot.
YdtzsXccCyeEQDD
0.695
254
Fitness
APC, EPHA3, EVC2, FBXW7, KRAS, PIK3CA, TCF7L2, TP53
APC, EVC2, KRAS, PIK3CA, TCF7L2
APC, EVC2, KRAS, TP53
APC, FBXW7, KRAS, TP53
APC, EVC2, FBXW7, KRAS
APC, FBXW7, TCF7L2, TP53
APC, KRAS, TCF7L2, TP53
APC, EPHA3, PIK3CA, TP53
90
1000
YdtzsXccCyeEQDD. CAPRI
YdtzsXccCyeEQDD. CBN_ot
APC
EPHA3
EVC2
PIK3CA
TCF7L2
KRAS
TP53
FBXW7

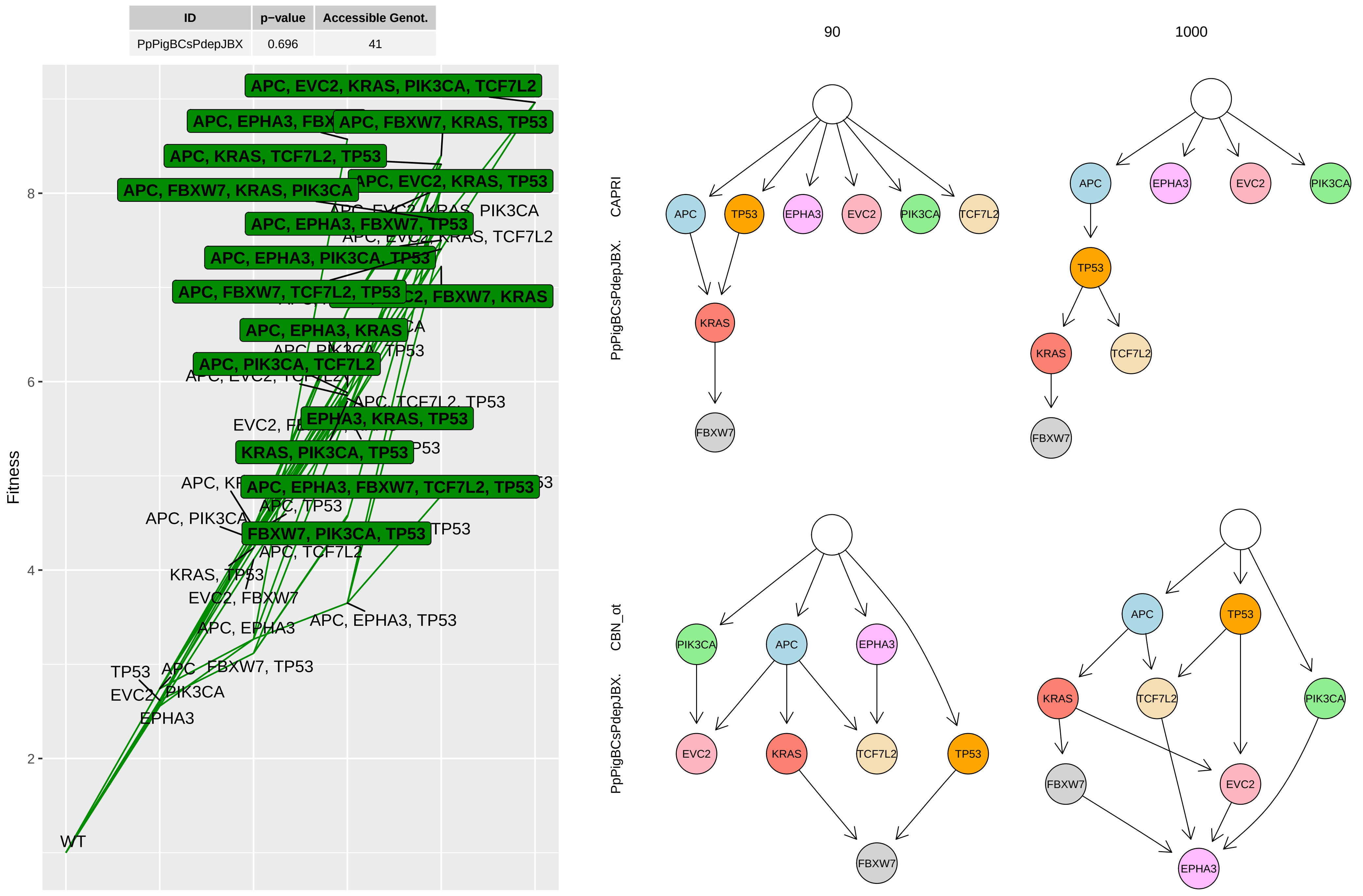

ID
p−value
Accessible Genot.
PpPigBCsPdepJBX
0.696
41
Fitness
8
6
4
2
APC, EVC2, KRAS, PIK3CA, TCF7L2
APC, FBXW7, KRAS, TP53
APC, KRAS, TCF7L2, TP53
APC, EVC2, KRAS, TP53
APC, FBXW7, KRAS, PIK3CA
APC, EPHA3, FBXW7, TP53
APC, EPHA3, PIK3CA, TP53
APC, FBXW7, TCF7L2, TP53
APC, EPHA3, KRAS
APC, PIK3CA, TCF7L2
APC, TCF7L2, TP53
EPHA3, KRAS, TP53
KRAS, PIK3CA, TP53
APC, EPHA3, FBXW7, TCF7L2, TP53
APC, TP53
APC, PIK3CA
FBXW7, PIK3CA, TP53
APC, TCF7L2
KRAS, TP53
EVC2, FBXW7
APC, EPHA3
APC, EPHA3, TP53
FBXW7, TP53
TP53
APC
EVC2
PIK3CA
EPHA3
WT
90
1000
CAPRI
PpPigBCsPdepJBX.
CBN_ot
PpPigBCsPdepJBX.
APC
TP53
EPHA3
EVC2
PIK3CA
TCF7L2
KRAS
FBXW7

ID	p−value	Accessible Genot.
ihVsgJHsyJoUOyw	0.697	166

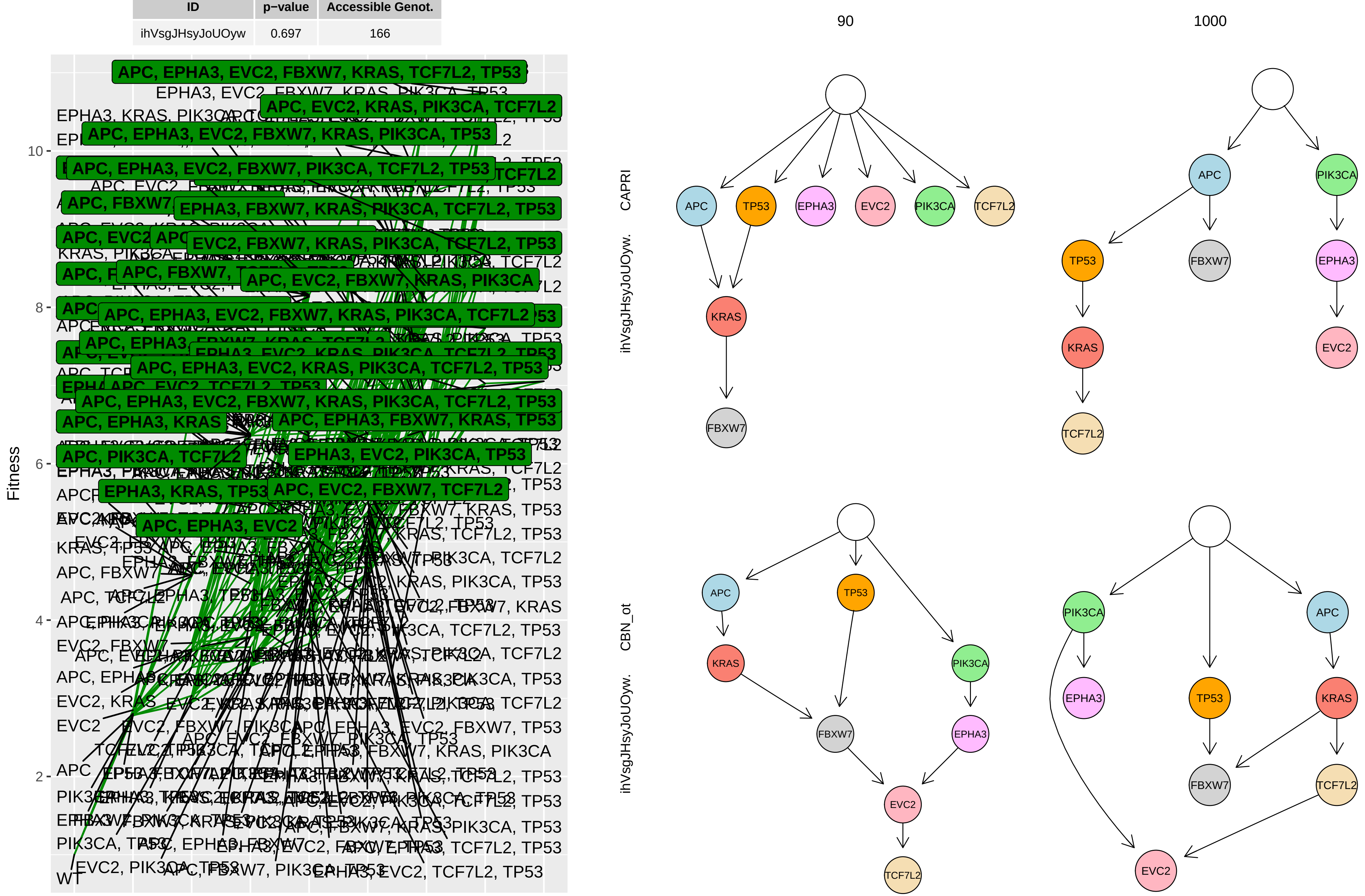

ID	p-value	Accessible Genot.
CDsggVxDjWpikjM	0.698	57

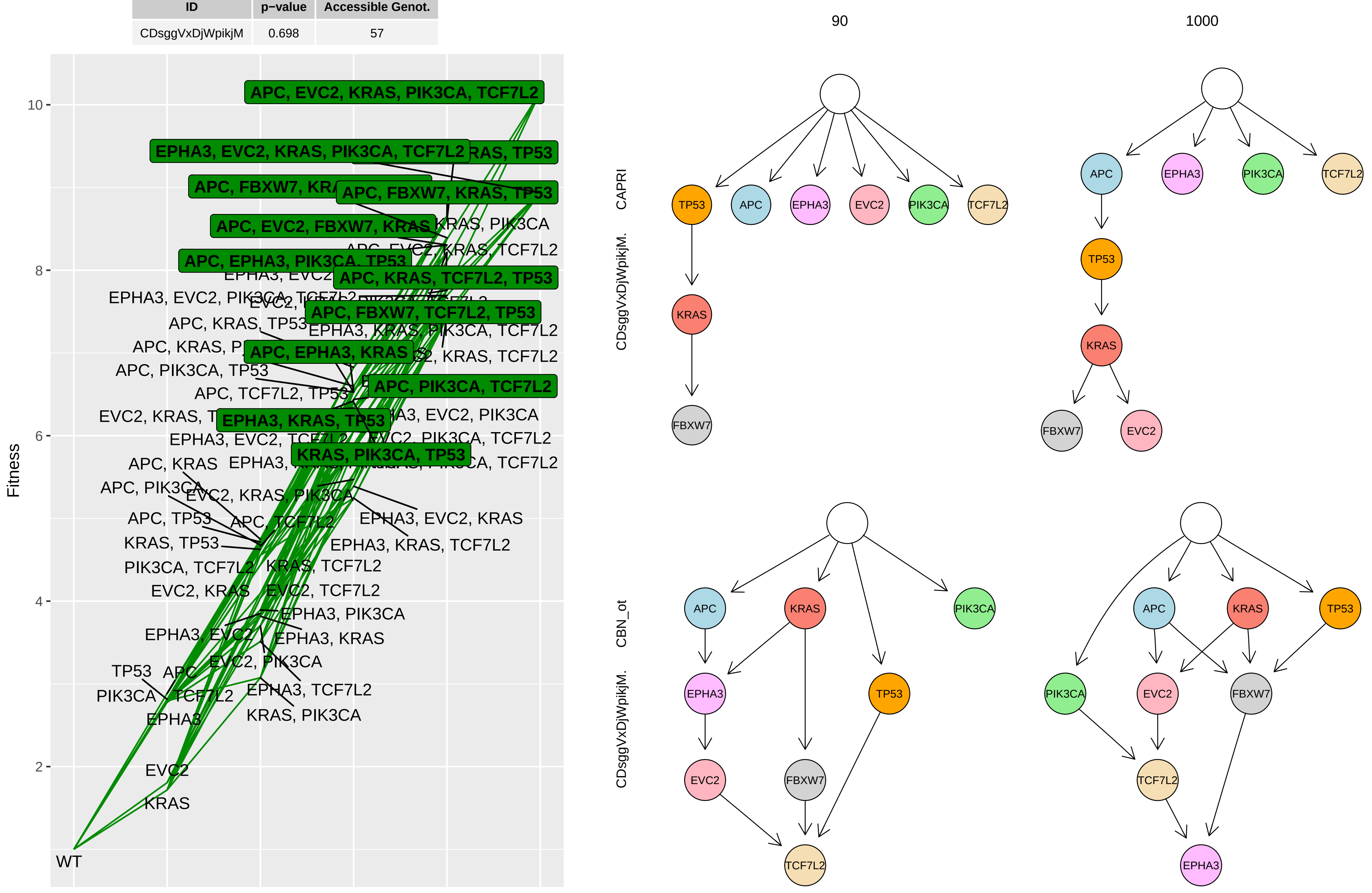

ID	p−value	Accessible Genot.
gYJnILHgYduOtoF	0.701	40

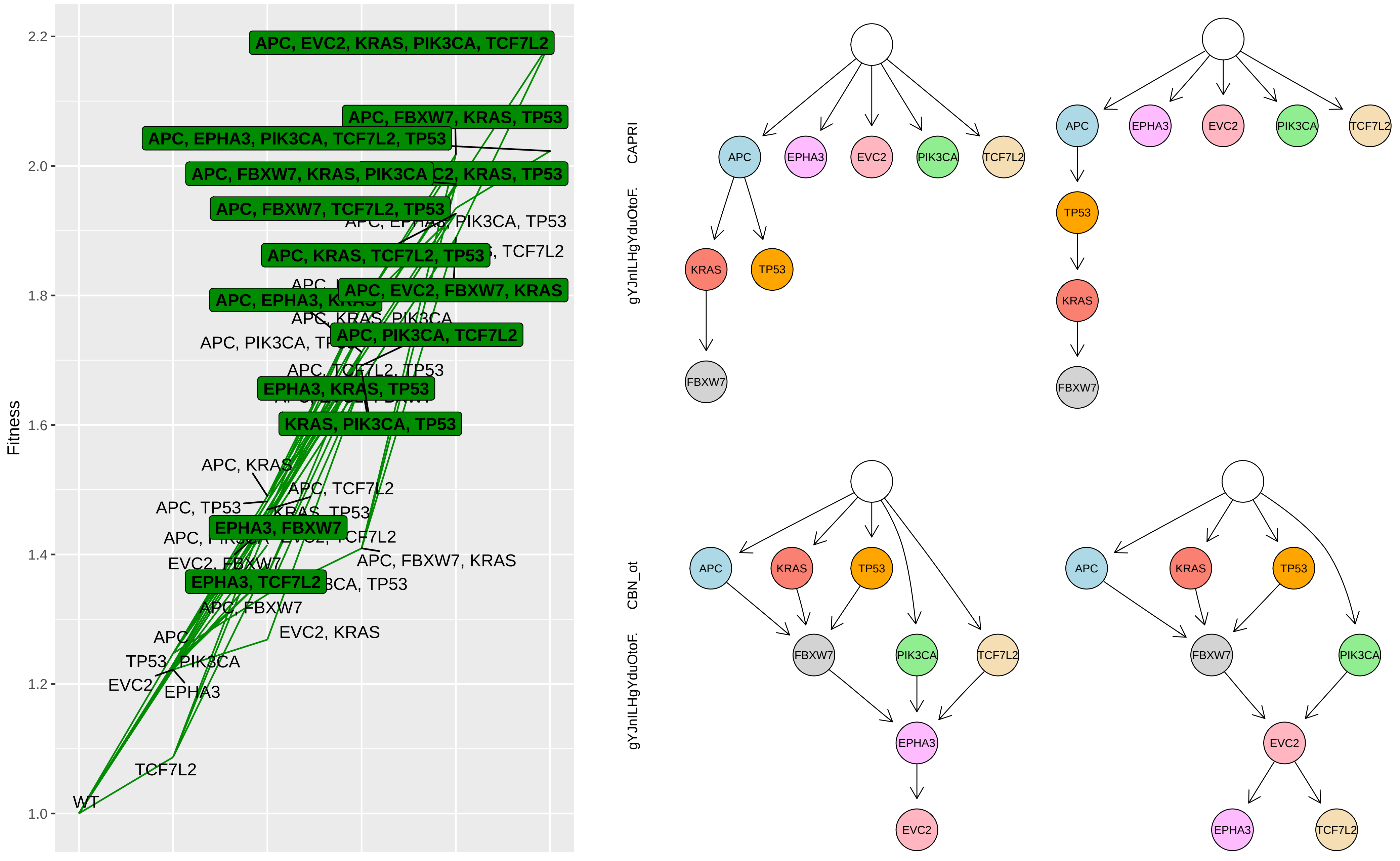

ID	p−value	Accessible Genot.
ZFUUJCJkjETBJvd	0.701	97

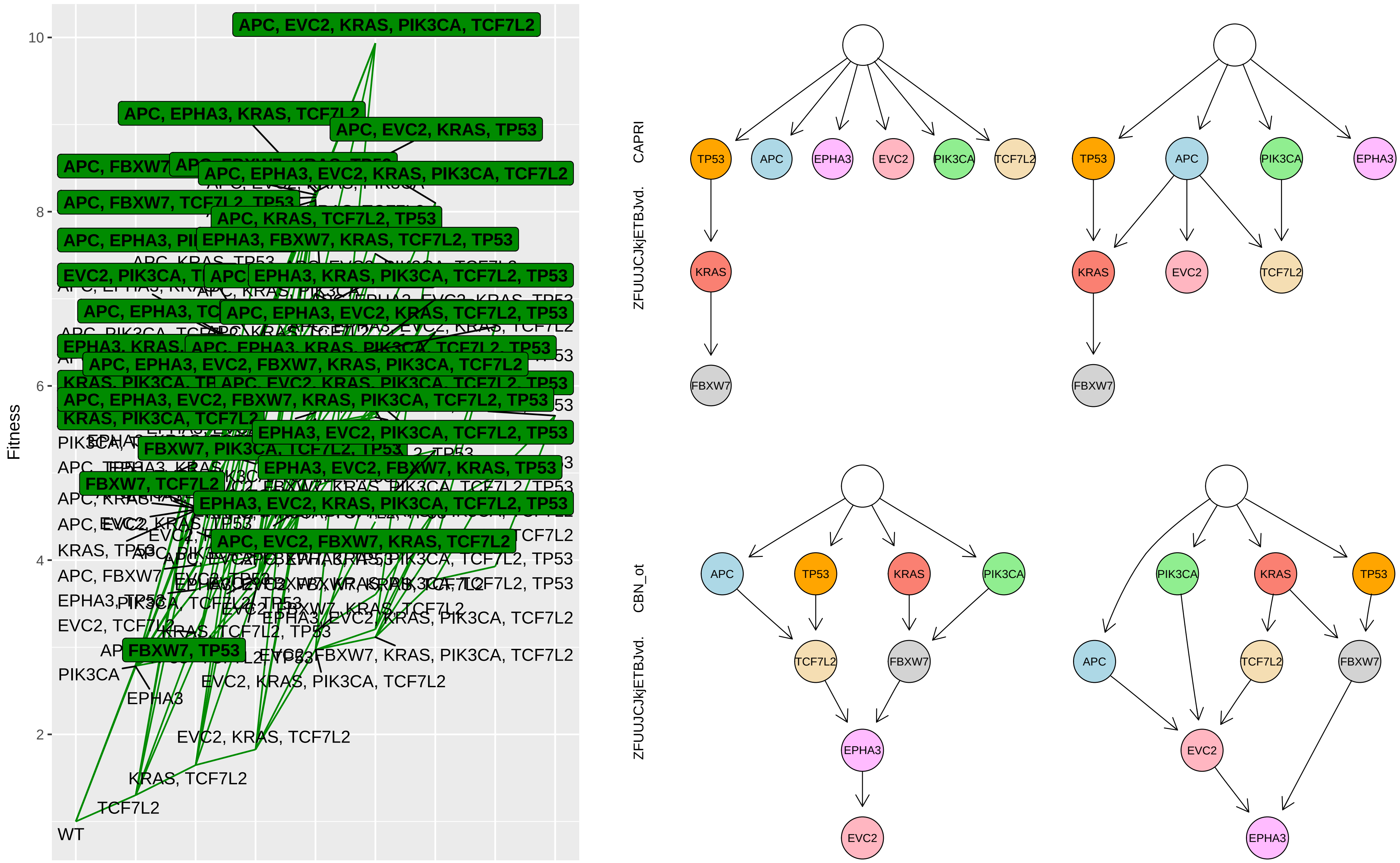

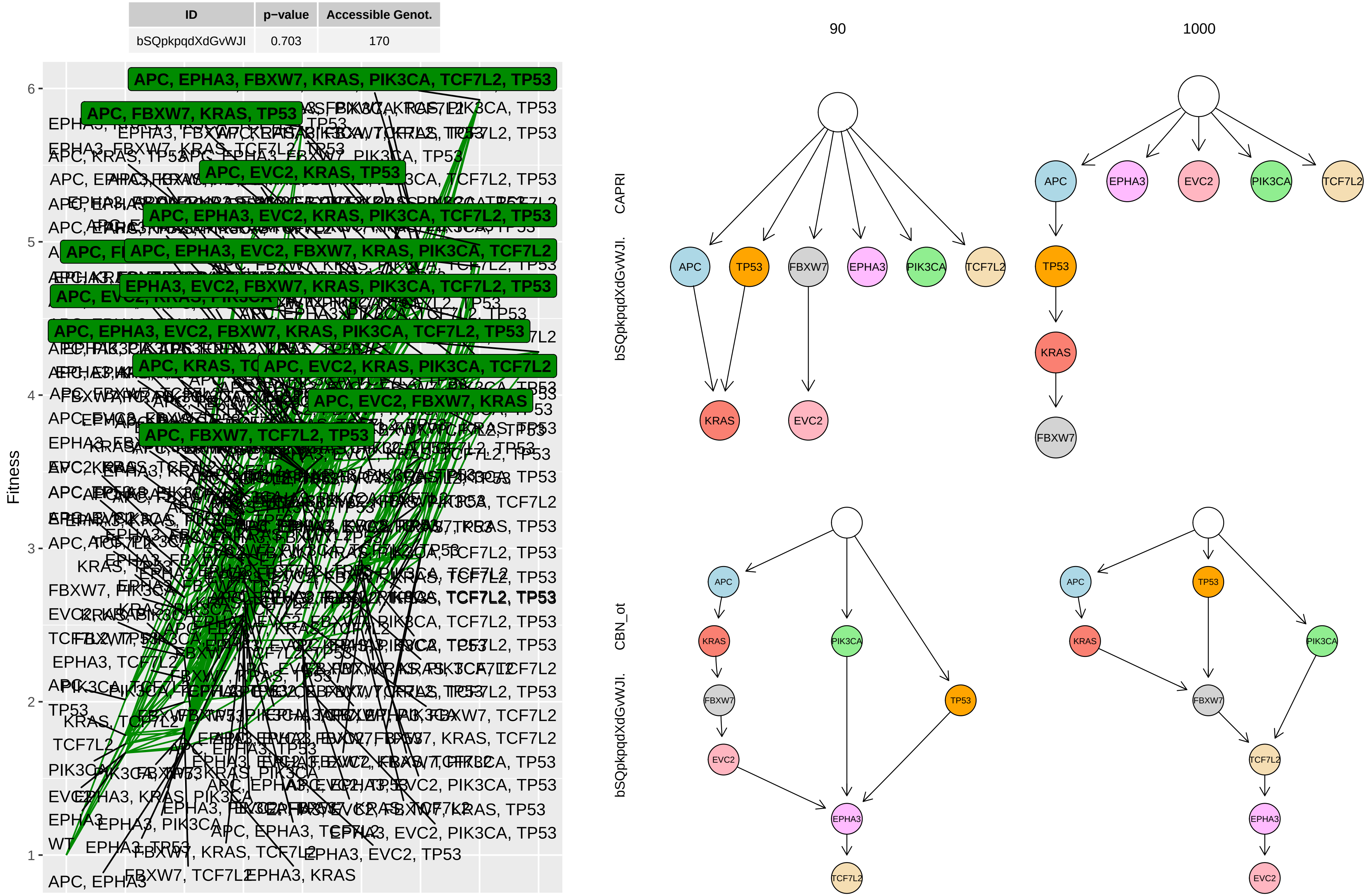

ID
p−value
Accessible Genot.
bSQpkpqdXdGvWJI
0.703
170
Fitness
APC, EPHA3, FBXW7, KRAS, PIK3CA, TCF7L2, TP53
APC, FBXW7, KRAS, TP53
APC, EVC2, KRAS, TP53
APC, EPHA3, EVC2, KRAS, PIK3CA, TCF7L2, TP53
APC, EPHA3, EVC2, FBXW7, KRAS, PIK3CA, TCF7L2
EPHA3, EVC2, FBXW7, KRAS, PIK3CA, TCF7L2, TP53
APC, EPHA3, EVC2, FBXW7, KRAS, PIK3CA, TCF7L2, TP53
APC, EVC2, KRAS, PIK3CA, TCF7L2
APC, EVC2, FBXW7, KRAS
APC, FBXW7, TCF7L2, TP53
WT
90
1000
CAPRI
bSQpkpqdXdGvWJI.
CBN_ot
bSQpkpqdXdGvWJI.
APC
TP53
FBXW7
EPHA3
PIK3CA
TCF7L2
KRAS
EVC2

ID	p−value	Accessible Genot.
XVINeUpZbuwtNDT	0.706	151

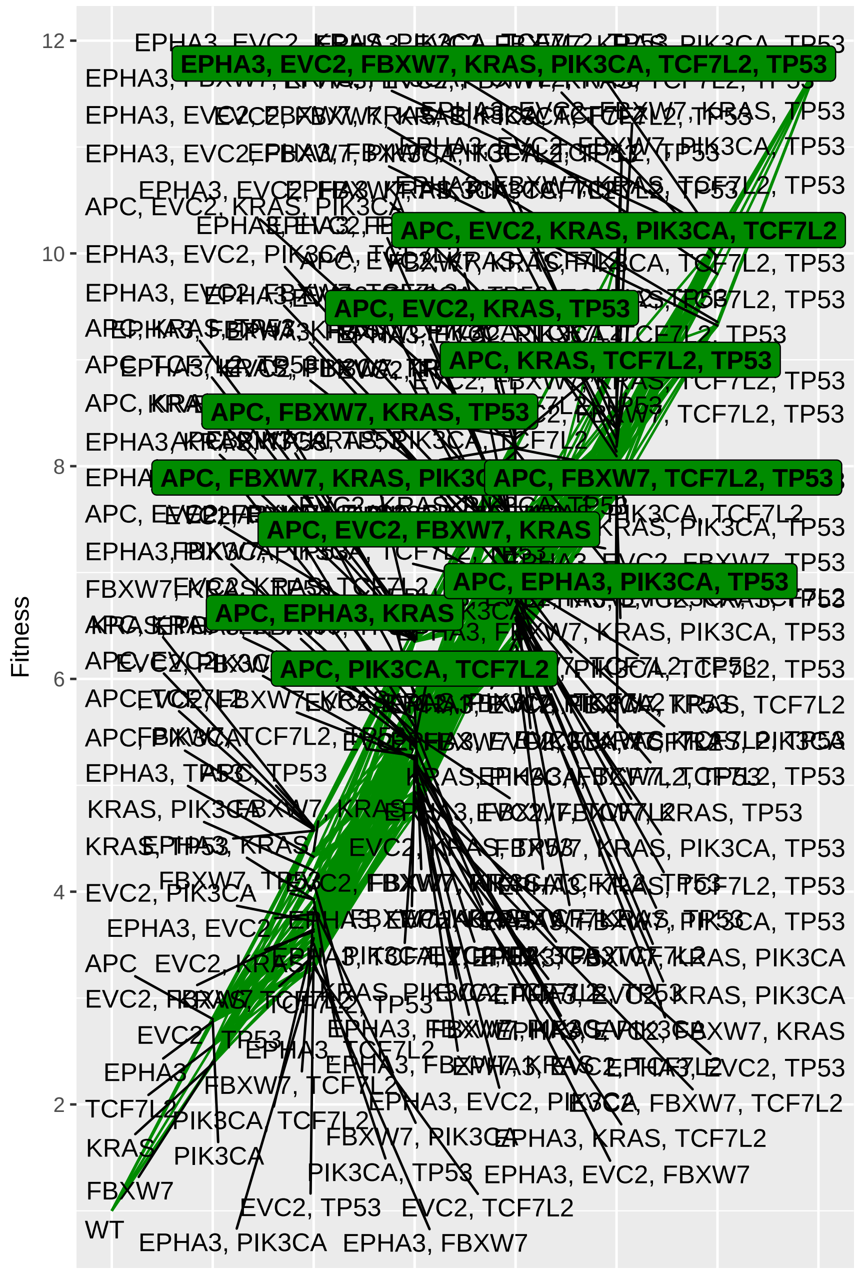

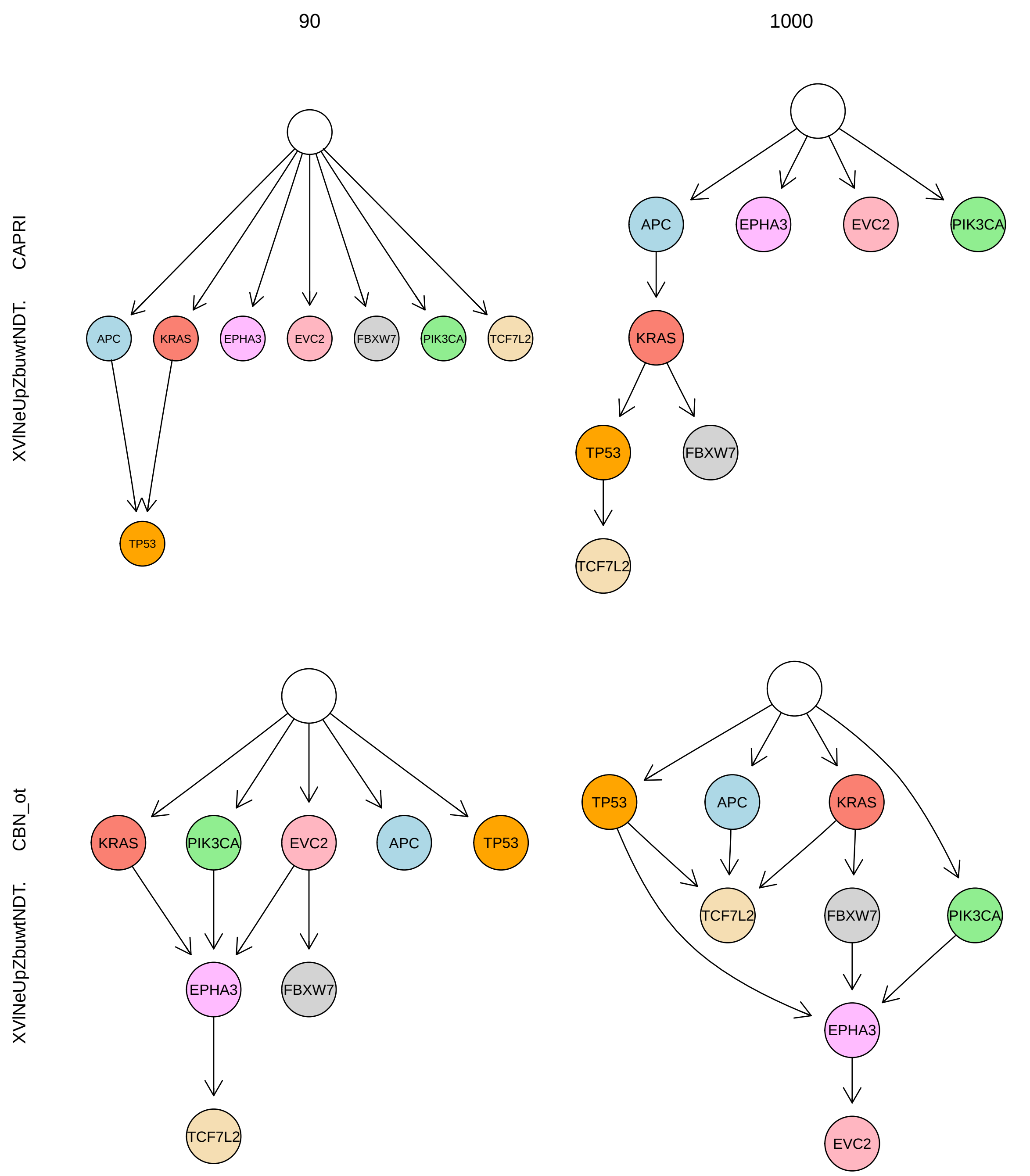

ID	p−value	Accessible Genot.
rMVtIrzWDqBvdLv	0.706	30

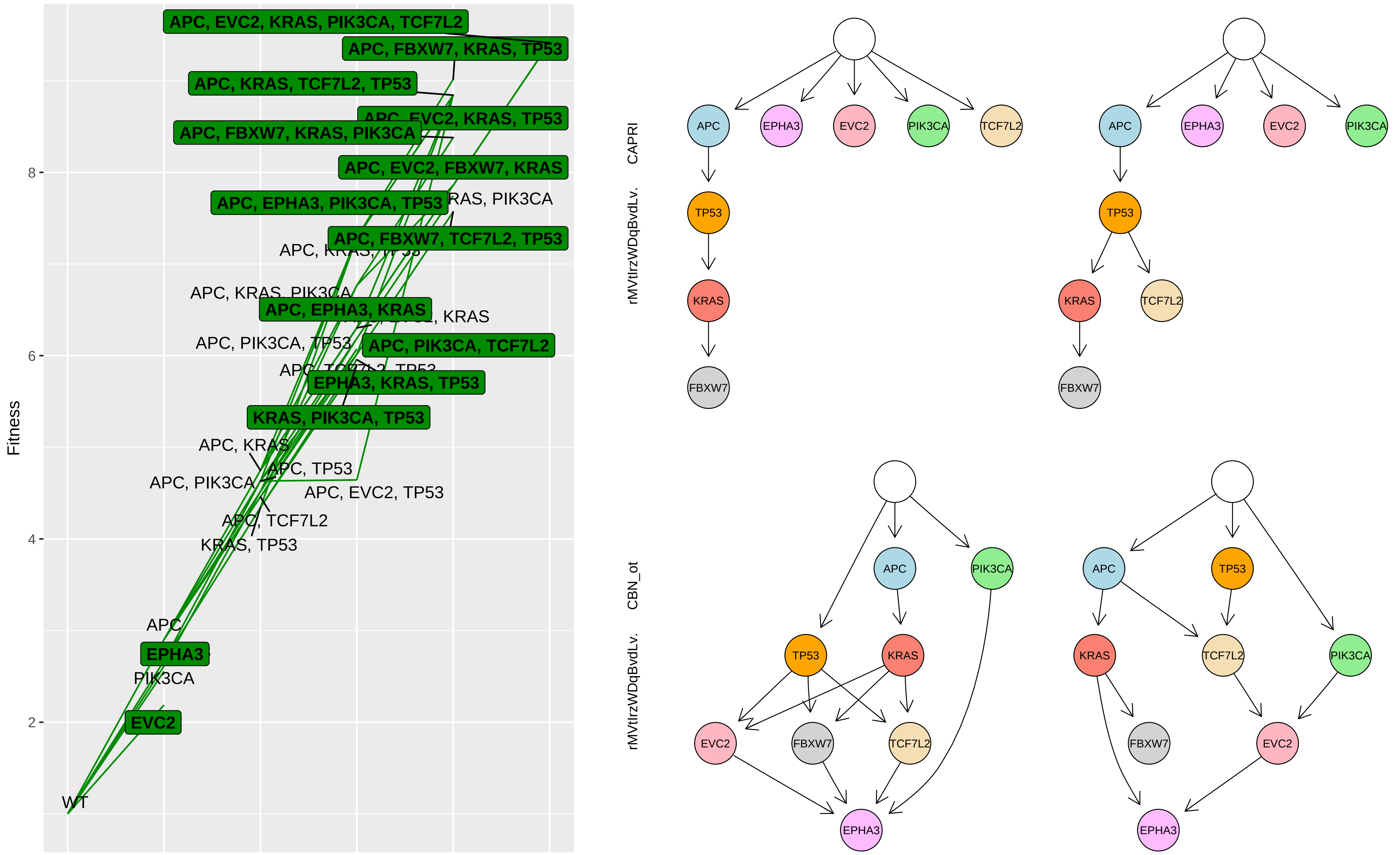

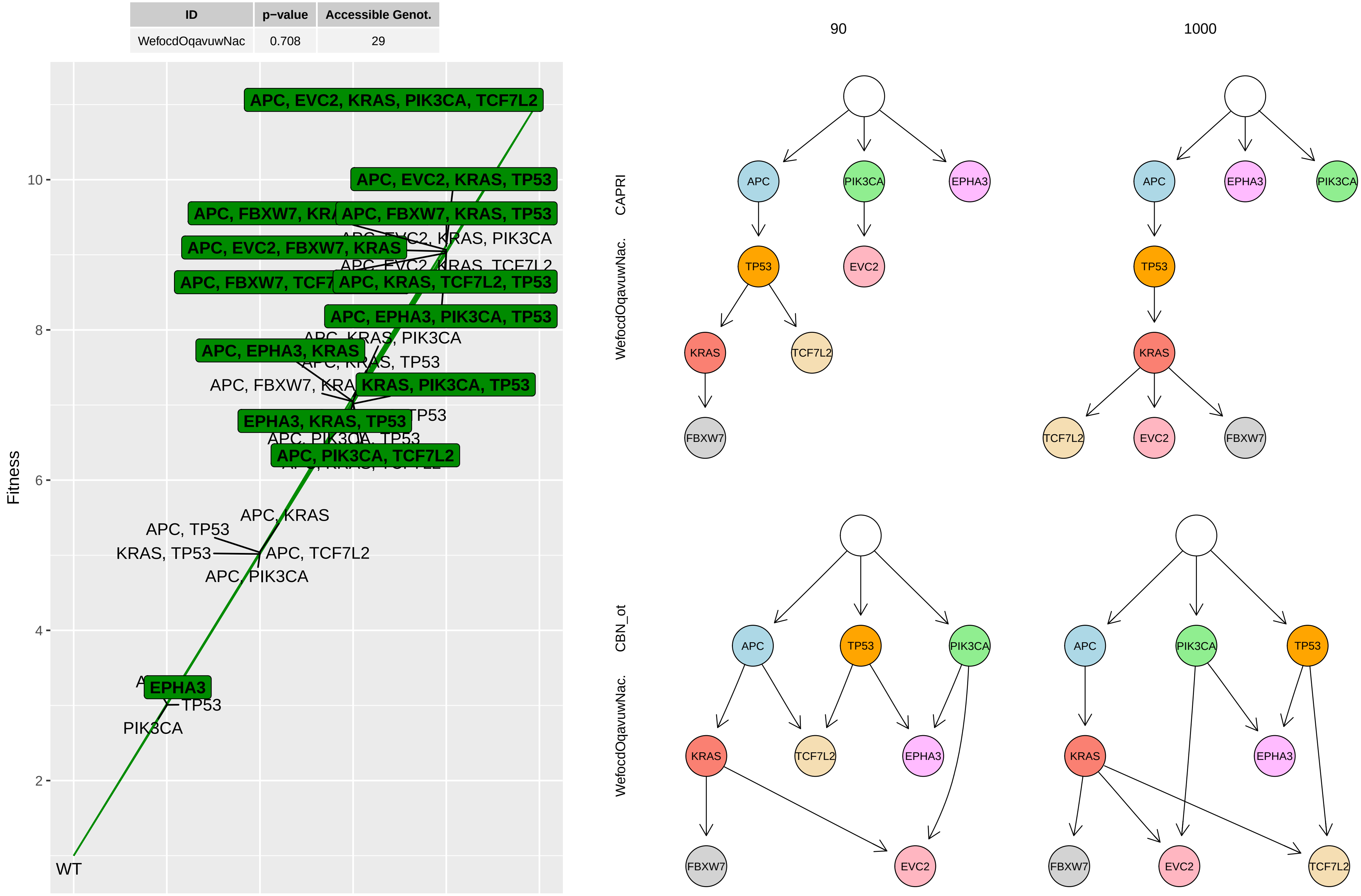

ID
p−value
Accessible Genot.
WefocdOqavuwNac
0.708
29
Fitness
APC, EVC2, KRAS, PIK3CA, TCF7L2
APC, EVC2, KRAS, TP53
APC, FBXW7, KRAS, TP53
APC, EVC2, FBXW7, KRAS
APC, KRAS, TCF7L2, TP53
APC, EPHA3, PIK3CA, TP53
APC, EPHA3, KRAS
KRAS, PIK3CA, TP53
EPHA3, KRAS, TP53
APC, PIK3CA, TCF7L2
APC, KRAS
APC, TP53
KRAS, TP53
APC, TCF7L2
APC, PIK3CA
EPHA3
TP53
PIK3CA
WT
2
4
6
8
10
90
1000
WefocdOqavuwNac. CAPRI
WefocdOqavuwNac. CBN_ot
APC
PIK3CA
EPHA3
TP53
EVC2
KRAS
TCF7L2
FBXW7

ID	p−value	Accessible Genot.
kvxxtFoBxlNgODx	0.71	29

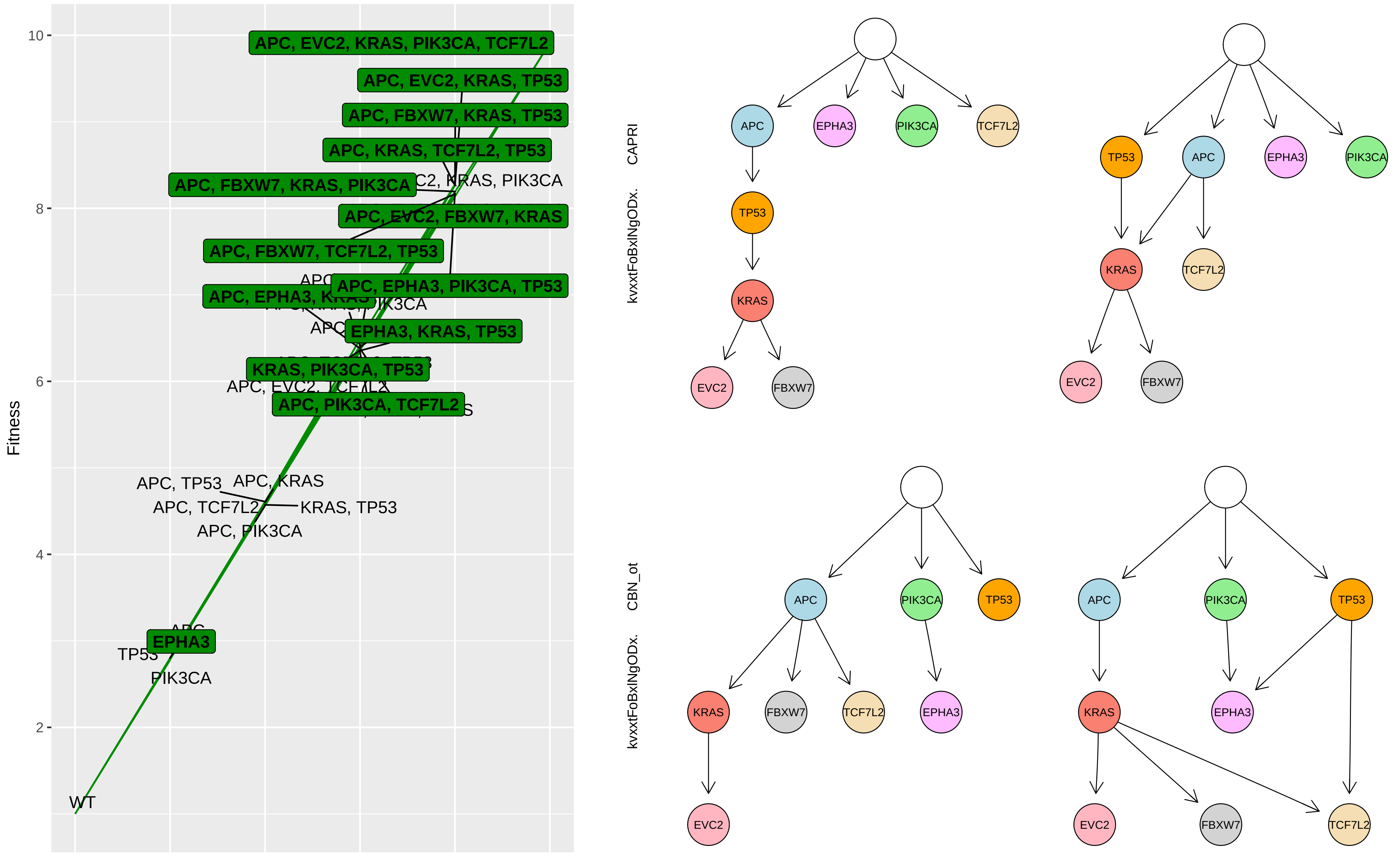

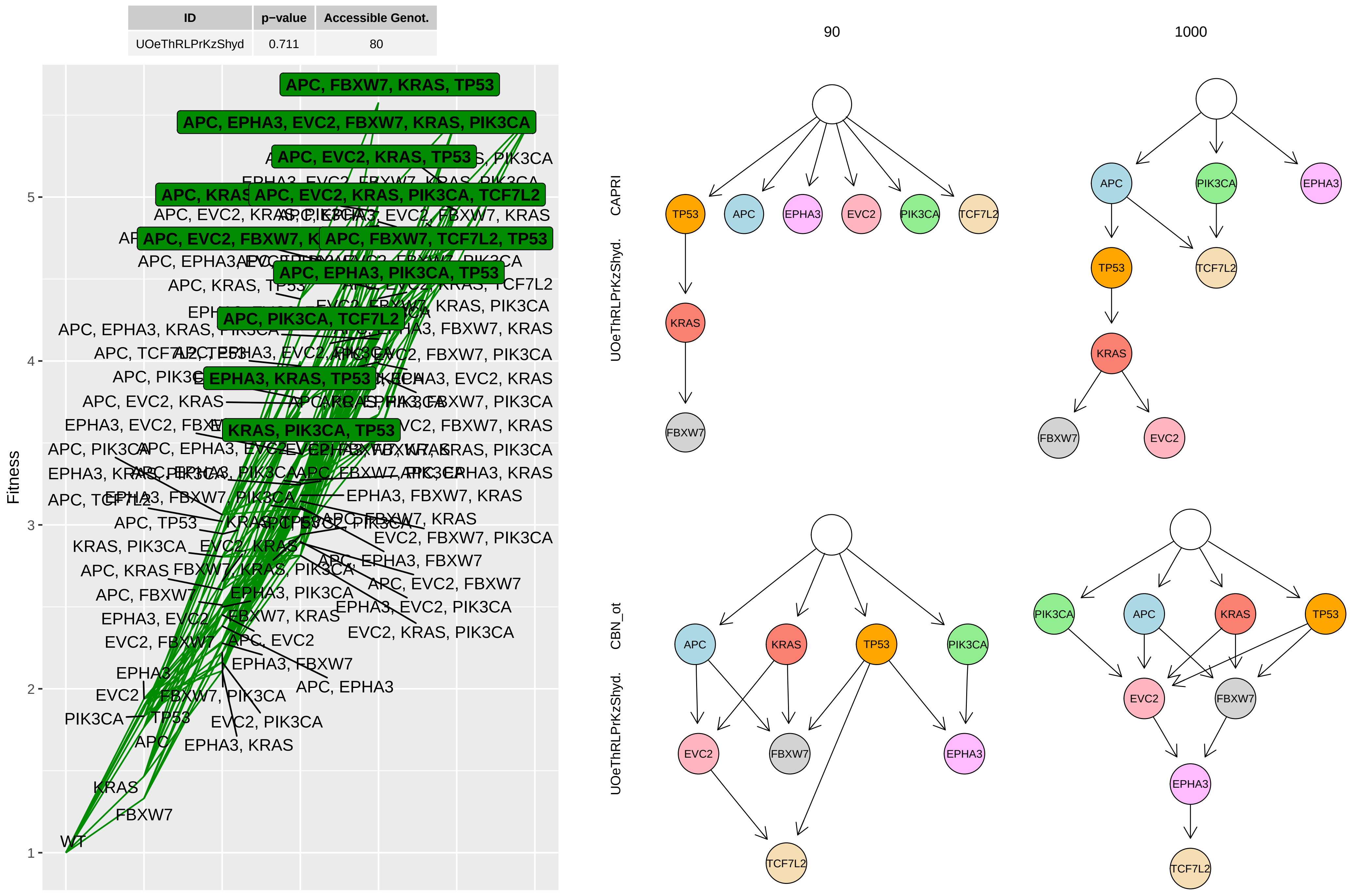
ID
p−value
Accessible Genot.
UOeThRLPrKzShyd
0.711
80
Fitness
APC, FBXW7, KRAS, TP53
APC, EPHA3, EVC2, FBXW7, KRAS, PIK3CA
APC, EVC2, KRAS, TP53
APC, EVC2, KRAS, PIK3CA, TCF7L2
APC, FBXW7, TCF7L2, TP53
APC, EPHA3, PIK3CA, TP53
APC, PIK3CA, TCF7L2
EPHA3, KRAS, TP53
KRAS, PIK3CA, TP53
APC, KRAS, TP53
APC, EPHA3, KRAS, PIK3CA
APC, EVC2, KRAS
EPHA3, FBXW7, KRAS
EVC2, FBXW7, PIK3CA
APC, TP53
KRAS, PIK3CA
APC, KRAS
APC, FBXW7
APC, EPHA3, FBXW7
APC, EVC2, FBXW7
EPHA3, EVC2, PIK3CA
EVC2, KRAS, PIK3CA
EPHA3, EVC2
EVC2, FBXW7
FBXW7, KRAS
APC, EVC2
EPHA3, PIK3CA
EPHA3, FBXW7
APC, EPHA3
EPHA3
EVC2
FBXW7, PIK3CA
PIK3CA
TP53
EVC2, PIK3CA
APC
EPHA3, KRAS
KRAS
FBXW7
WT
1
2
3
4
5
90
1000
CAPRI
UOeThRLPrKzShyd.
CBN_ot
UOeThRLPrKzShyd.
TP53
APC
EPHA3
EVC2
PIK3CA
TCF7L2
KRAS
FBXW7

ID	p−value	Accessible Genot.
kPvIKJpQkAwWLzj	0.711	28

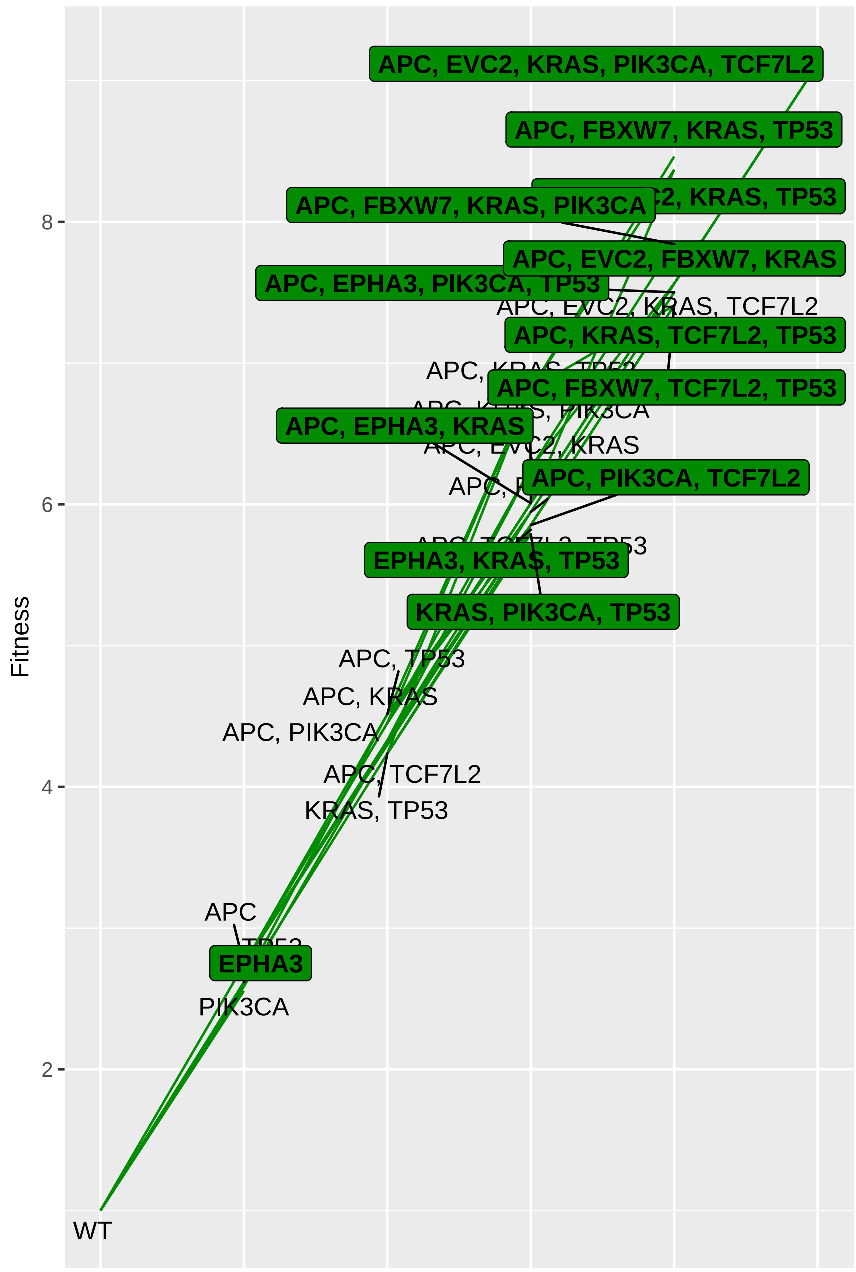

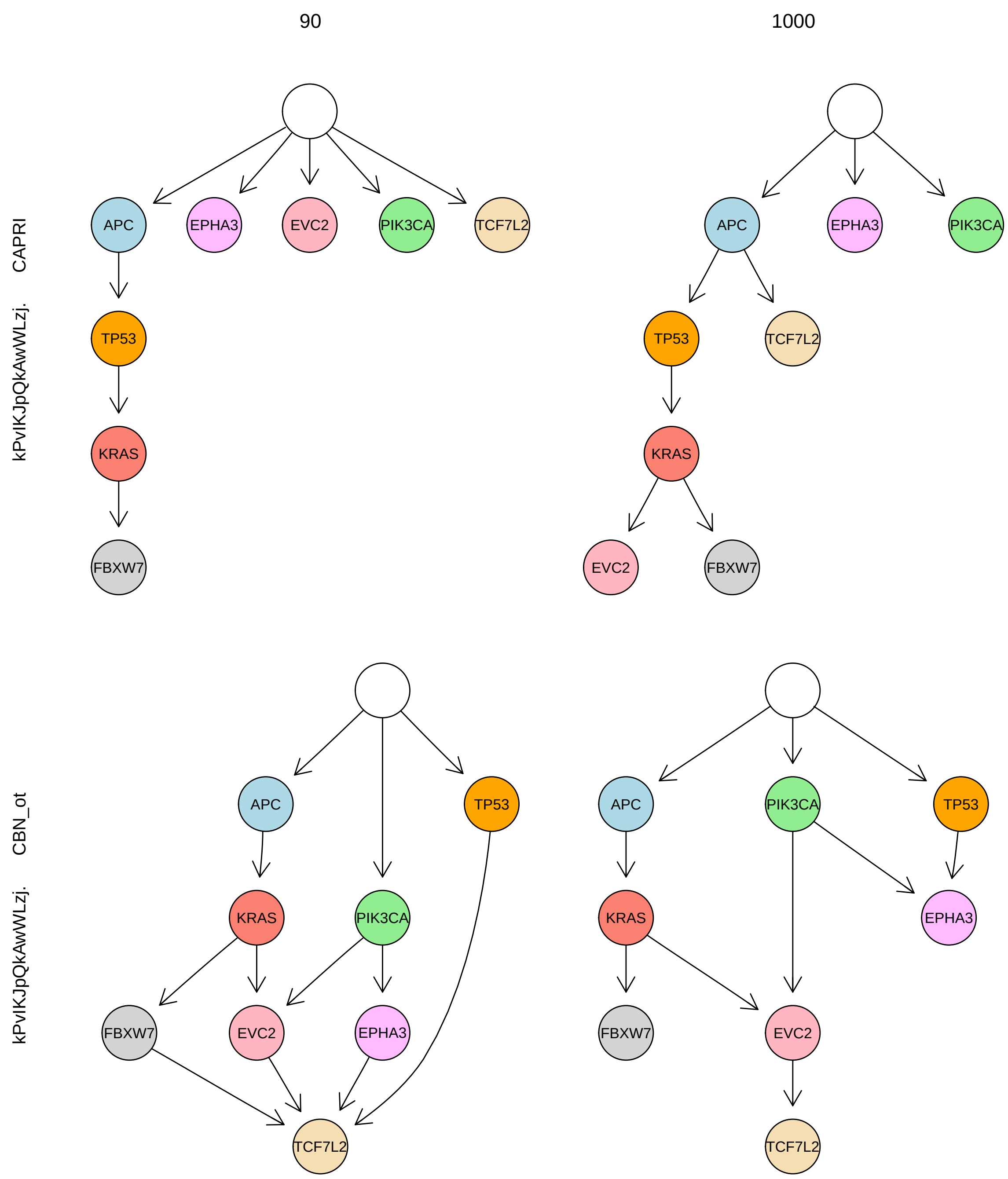

ID	p−value	Accessible Genot.
fiHFUPNpldNZIPU	0.712	255

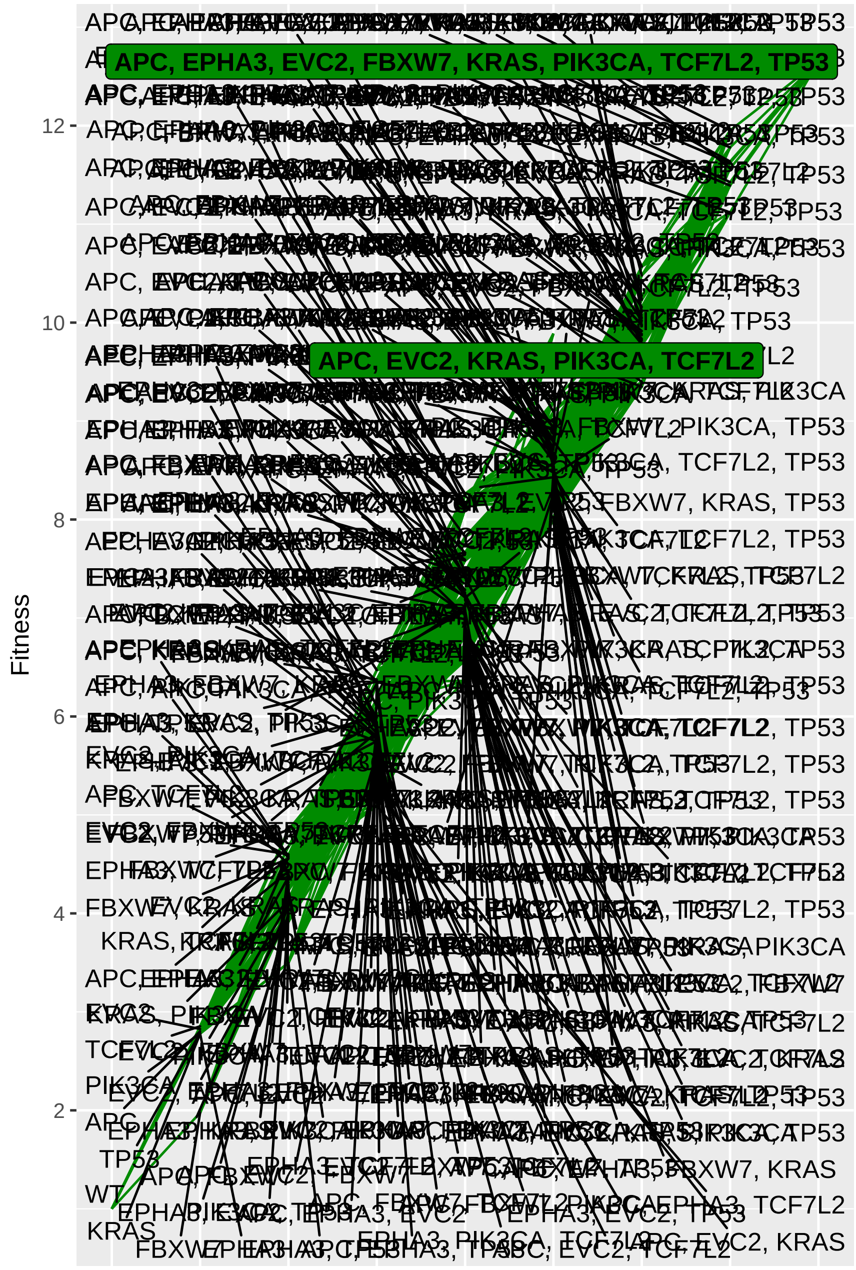

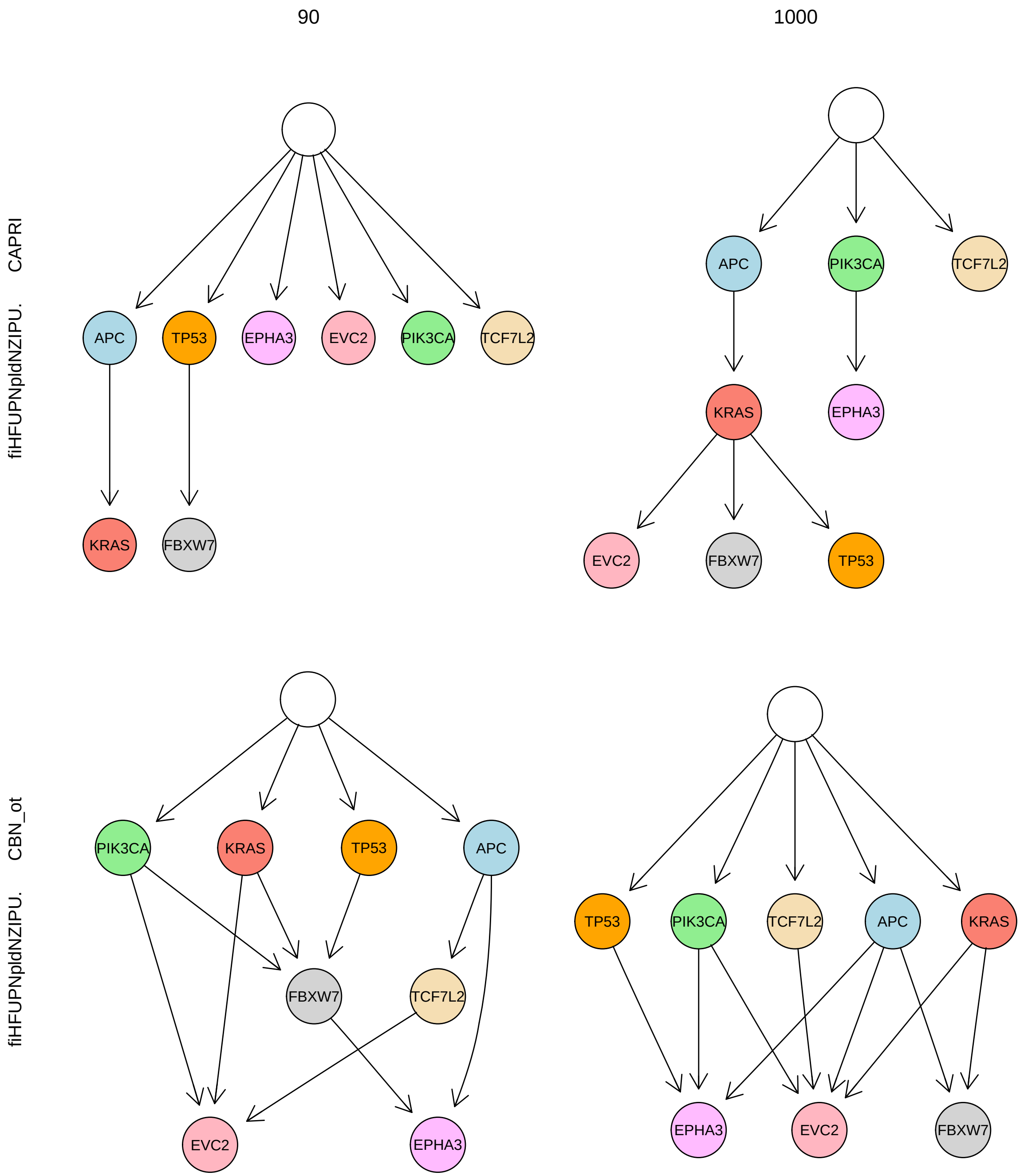

ID	p−value	Accessible Genot.
ElyeJeBTMIuUpOL	0.713	46

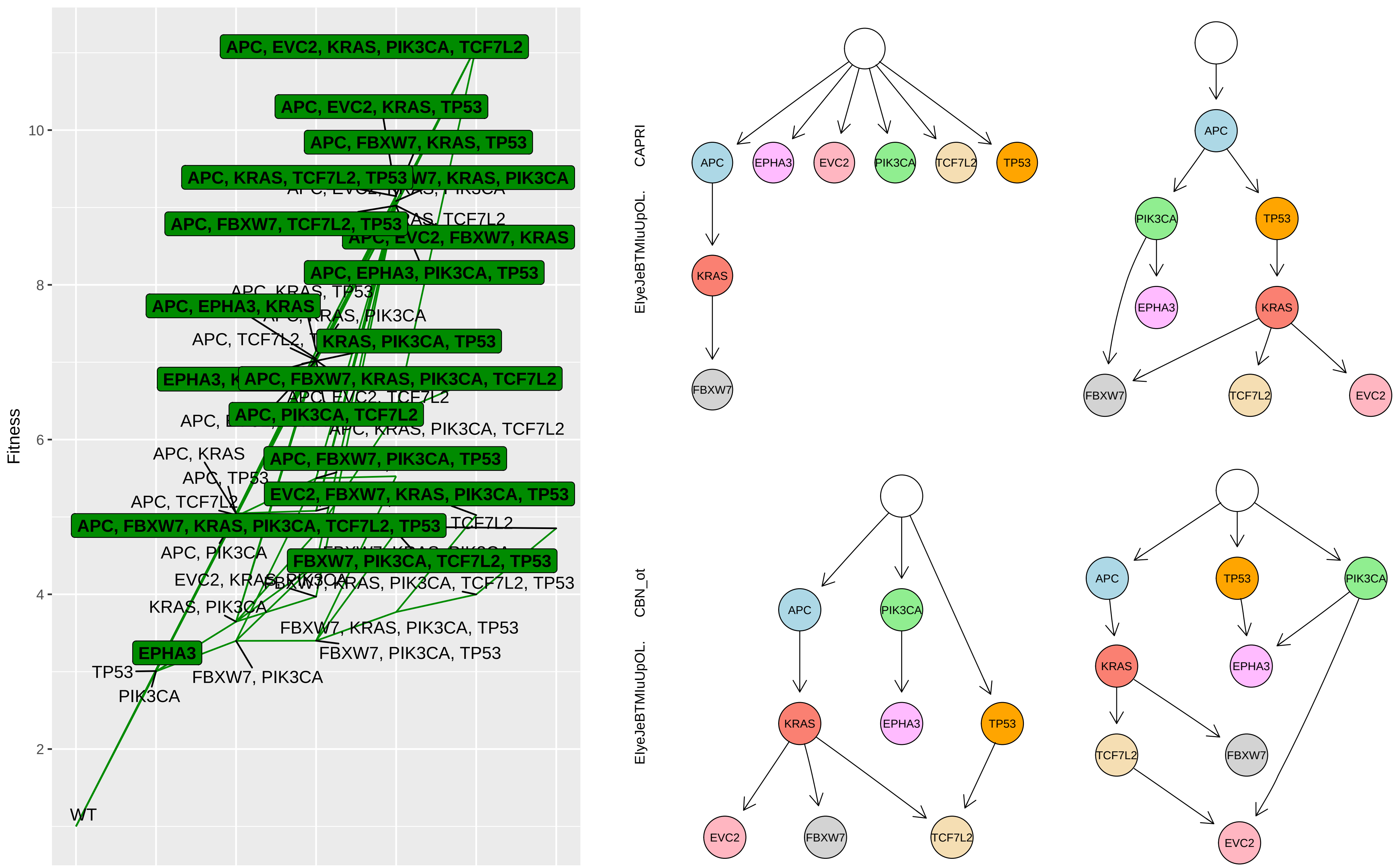

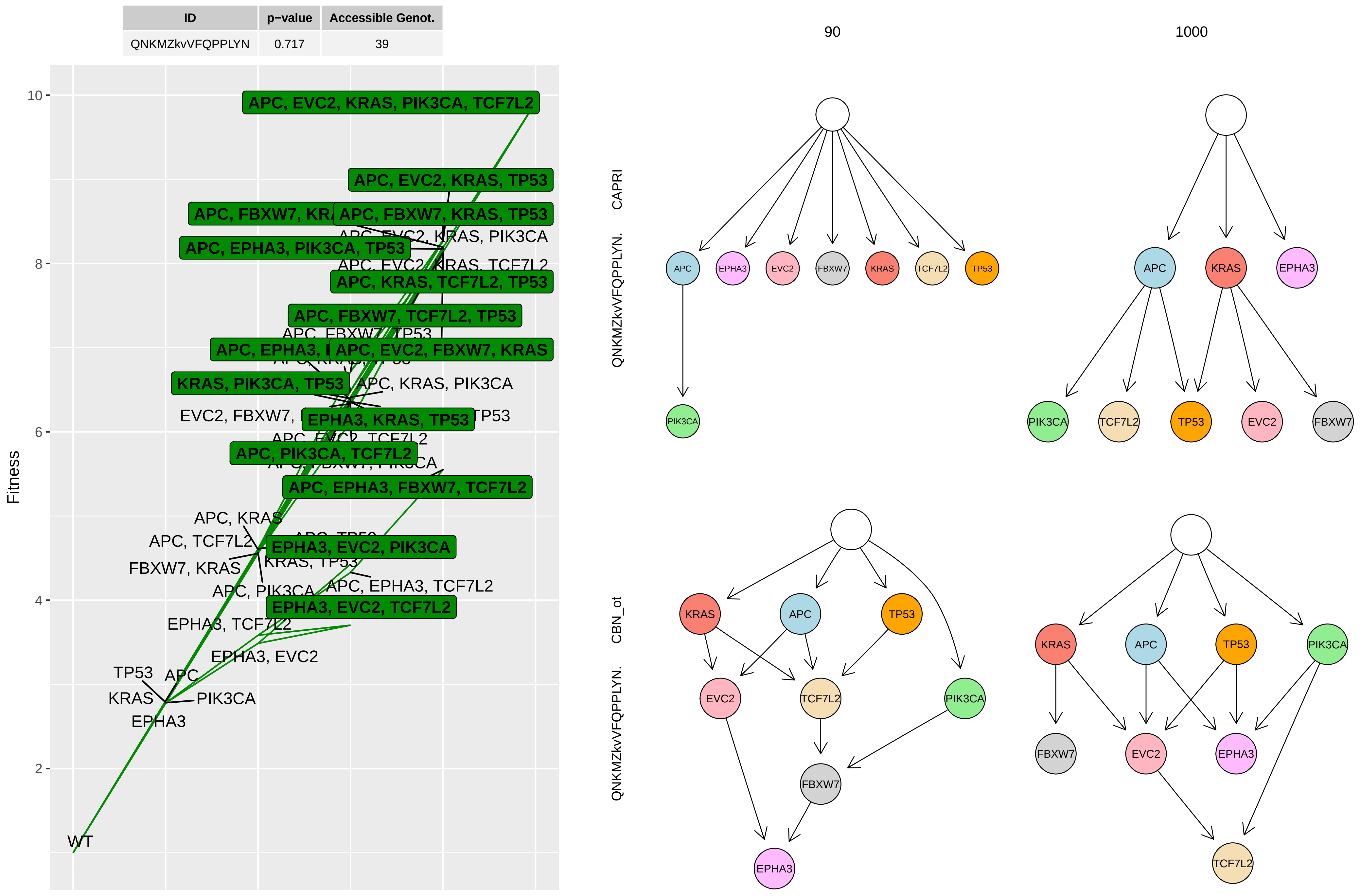

ID
p−value
Accessible Genot.
QNKMZkvVFQPPLYN
0.717
39
Fitness
10
8
6
4
2
WT
APC, EVC2, KRAS, PIK3CA, TCF7L2
APC, EVC2, KRAS, TP53
APC, FBXW7, KRAS, TP53
APC, EPHA3, PIK3CA, TP53
APC, KRAS, TCF7L2, TP53
APC, FBXW7, TCF7L2, TP53
APC, EVC2, FBXW7, KRAS
KRAS, PIK3CA, TP53
APC, KRAS, PIK3CA
EPHA3, KRAS, TP53
APC, PIK3CA, TCF7L2
APC, EPHA3, FBXW7, TCF7L2
APC, KRAS
APC, TCF7L2
FBXW7, KRAS
EPHA3, EVC2, PIK3CA
APC, PIK3CA
APC, EPHA3, TCF7L2
EPHA3, EVC2, TCF7L2
EPHA3, TCF7L2
EPHA3, EVC2
TP53
APC
KRAS
PIK3CA
EPHA3
90
1000
QNKMZkvVFQPPLYN. CAPRI
QNKMZkvVFQPPLYN. CBN_ot
APC
EPHA3
EVC2
FBXW7
KRAS
TCF7L2
TP53
PIK3CA

ID	p−value	Accessible Genot.
tMXqqtTZRzACDVt	0.721	157

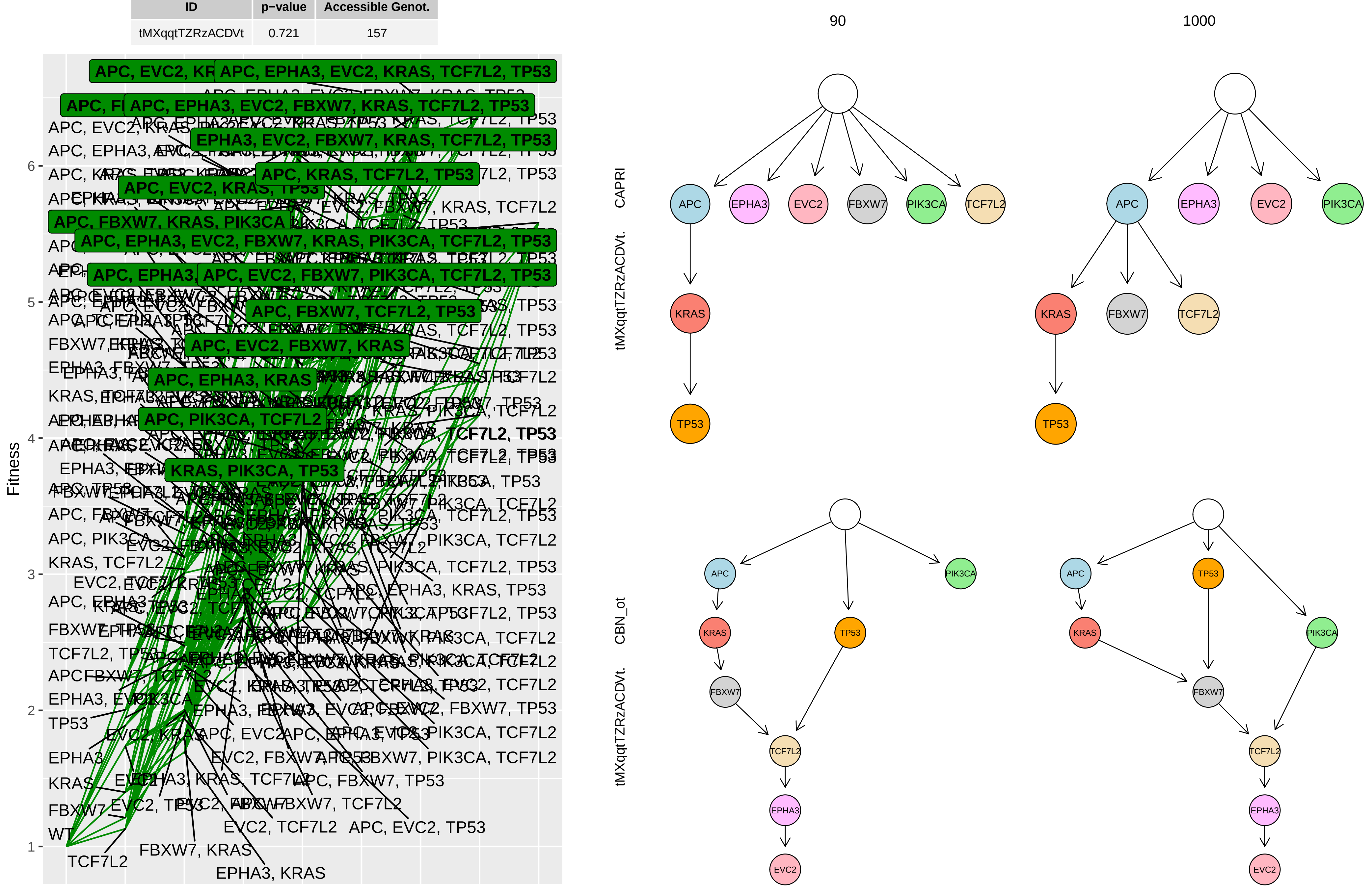

ID	p−value	Accessible Genot.
XCzCwuhzXhvuRLV	0.722	255

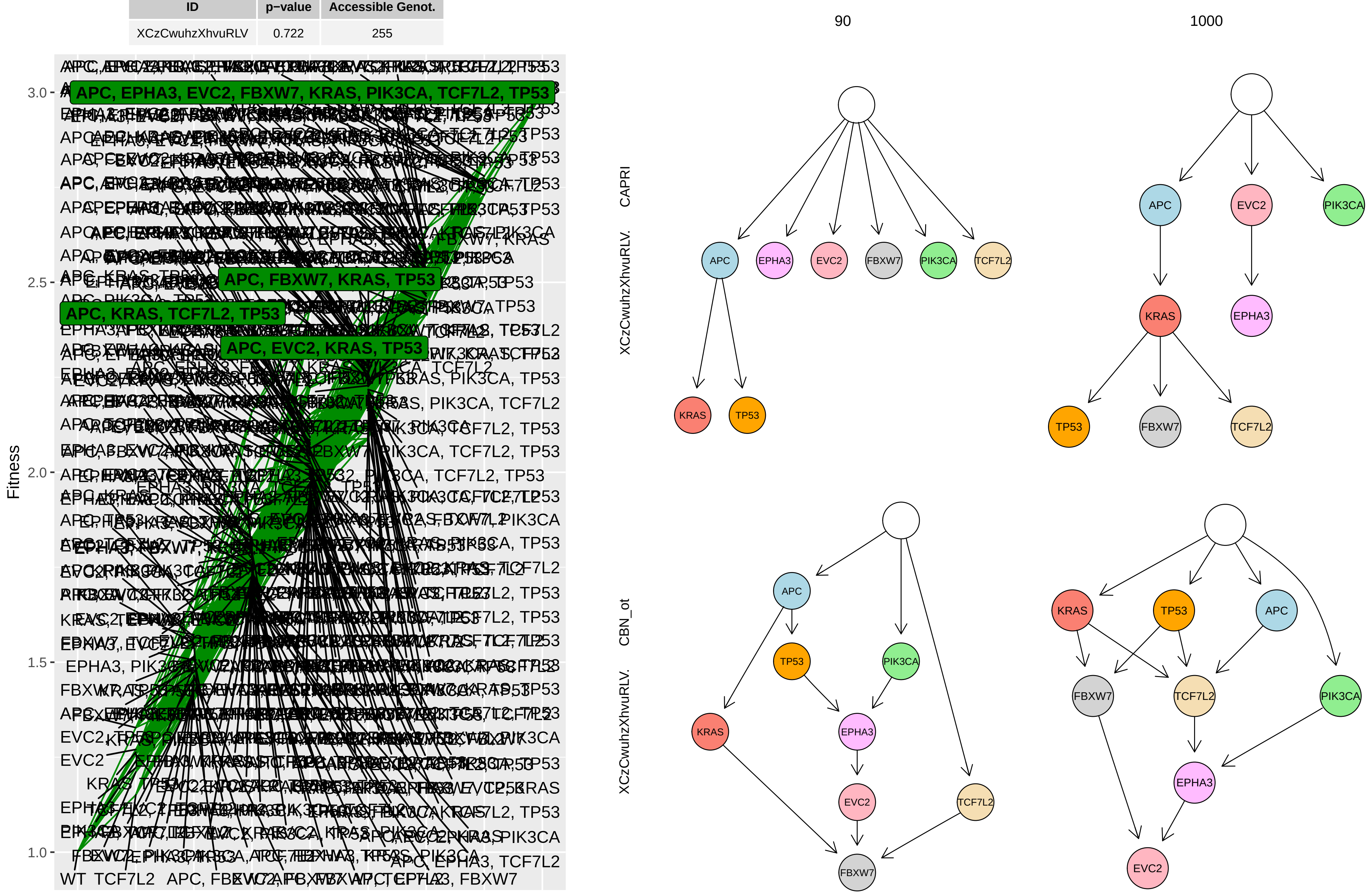

ID	p−value	Accessible Genot.
civzfMNHbwhrXjE	0.725	44

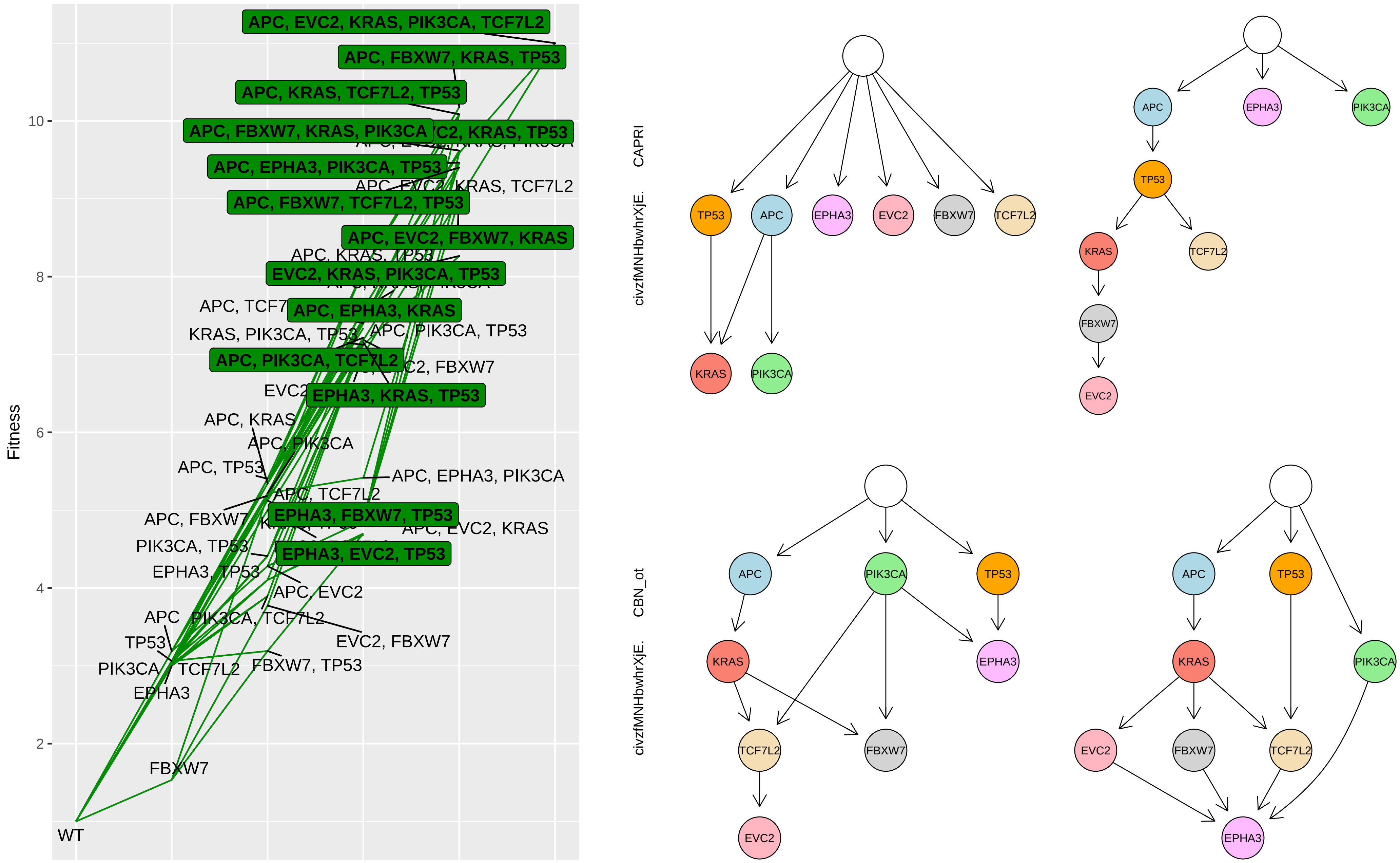

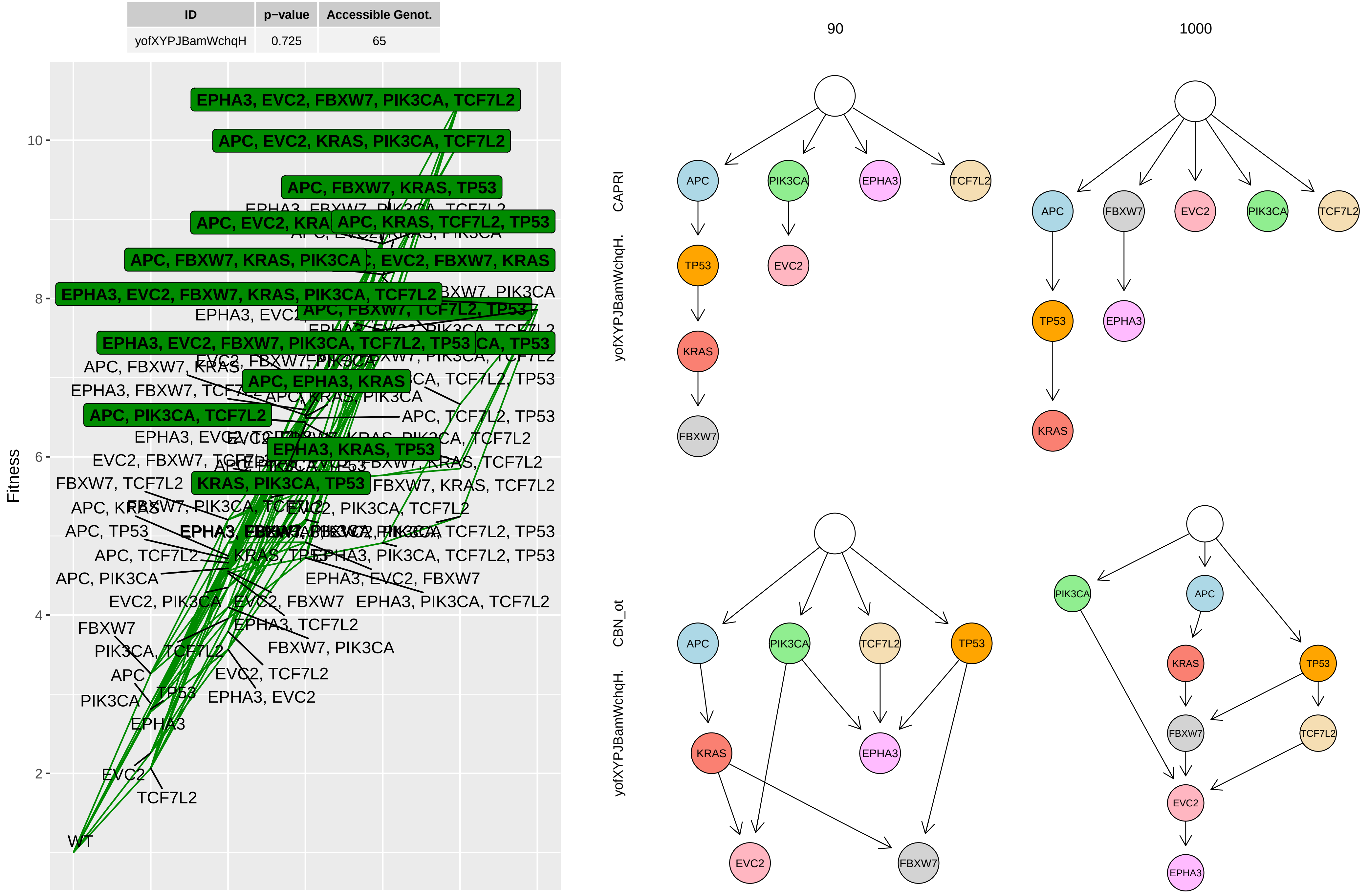

ID
p−value
Accessible Genot.
yofXYPJBamWchqH
0.725
65
Fitness
10
8
6
4
2
WT
EPHA3, EVC2, FBXW7, PIK3CA, TCF7L2
APC, EVC2, KRAS, PIK3CA, TCF7L2
APC, FBXW7, KRAS, TP53
APC, KRAS, TCF7L2, TP53
APC, FBXW7, KRAS, PIK3CA
EPHA3, EVC2, FBXW7, KRAS, PIK3CA, TCF7L2
EPHA3, EVC2, FBXW7, PIK3CA, TCF7L2, TP53
APC, EPHA3, KRAS
APC, PIK3CA, TCF7L2
EPHA3, KRAS, TP53
KRAS, PIK3CA, TP53
APC, TCF7L2, TP53
FBXW7, KRAS, TCF7L2
EPHA3, PIK3CA, TCF7L2, TP53
EPHA3, EVC2, FBXW7
EPHA3, PIK3CA, TCF7L2
FBXW7, TCF7L2
APC, TP53
APC, TCF7L2
APC, PIK3CA
EVC2, PIK3CA
EVC2, FBXW7
EPHA3, TCF7L2
FBXW7, PIK3CA
EVC2, TCF7L2
EPHA3, EVC2
FBXW7
PIK3CA, TCF7L2
APC
PIK3CA
TP53
EPHA3
EVC2
TCF7L2
90
1000
CAPRI
CBN_ot
yofXYPJBamWchqH.
APC
PIK3CA
EPHA3
TCF7L2
TP53
EVC2
KRAS
FBXW7

ID	p−value	Accessible Genot.
wAEqpmAuiEPBcnY	0.726	137

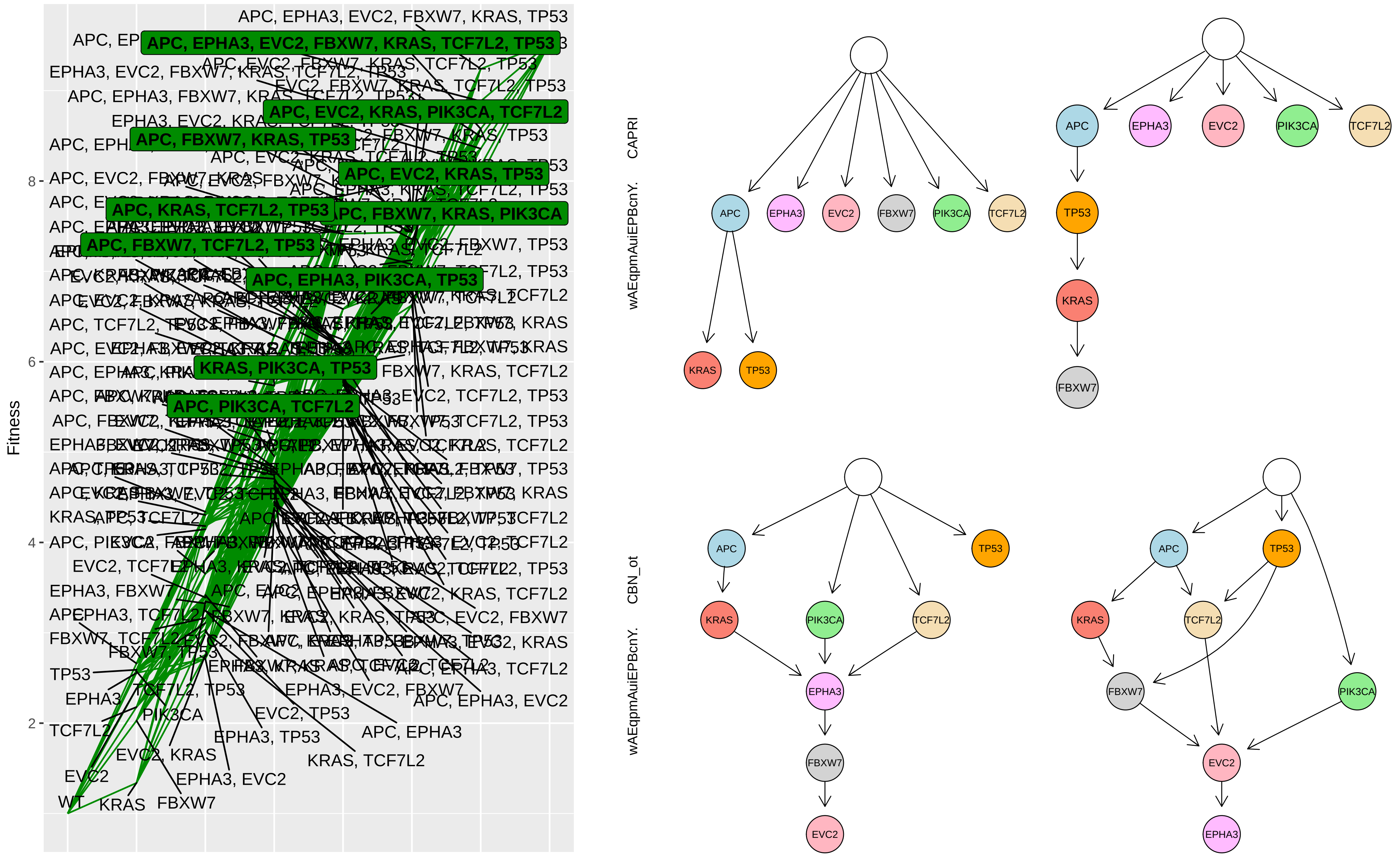

ID	p−value	Accessible Genot.
PAaoKilOJtxaaJo	0.726	40

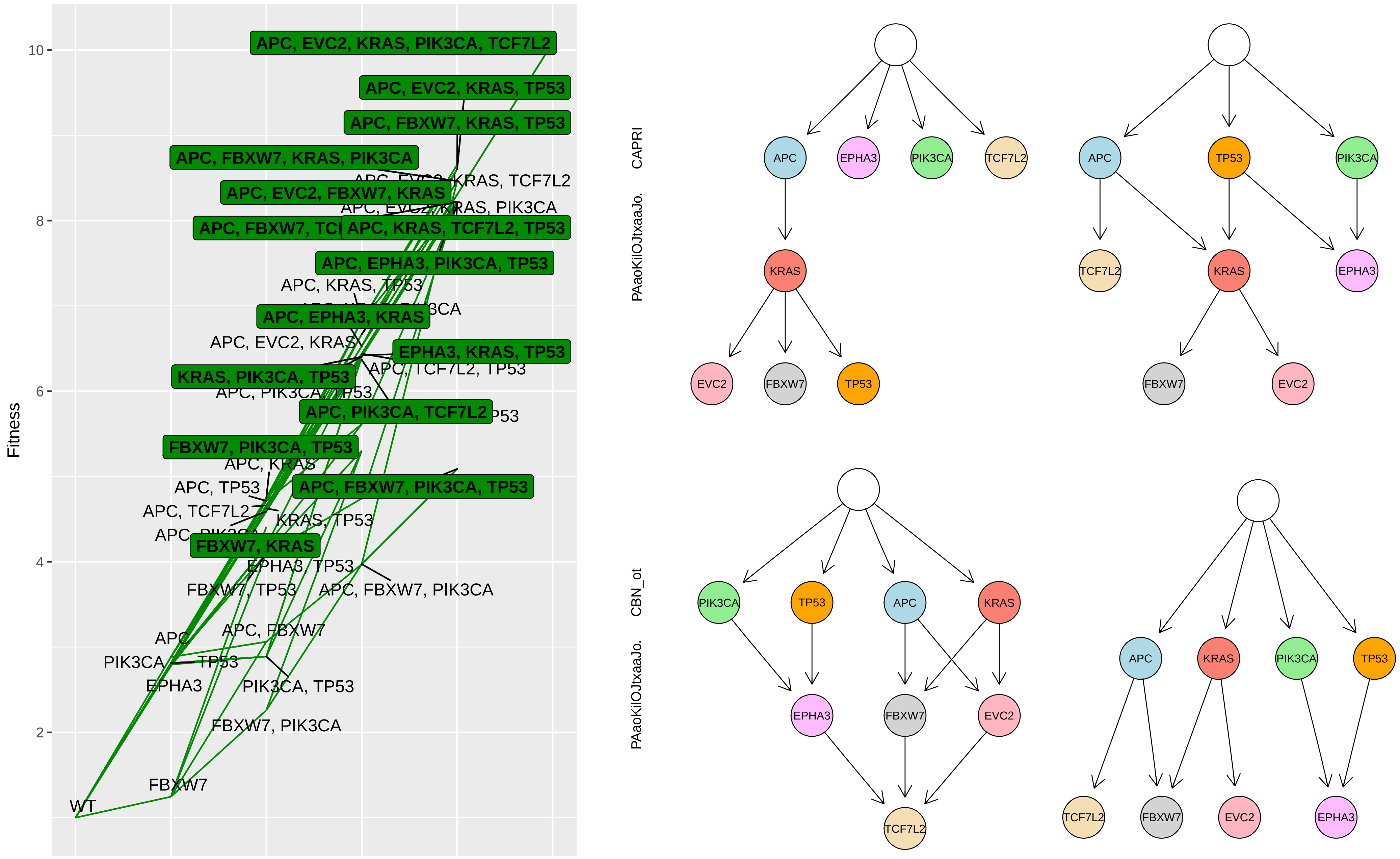

ID	p−value	Accessible Genot.
hYmrbXzqCTaahYW	0.726	156

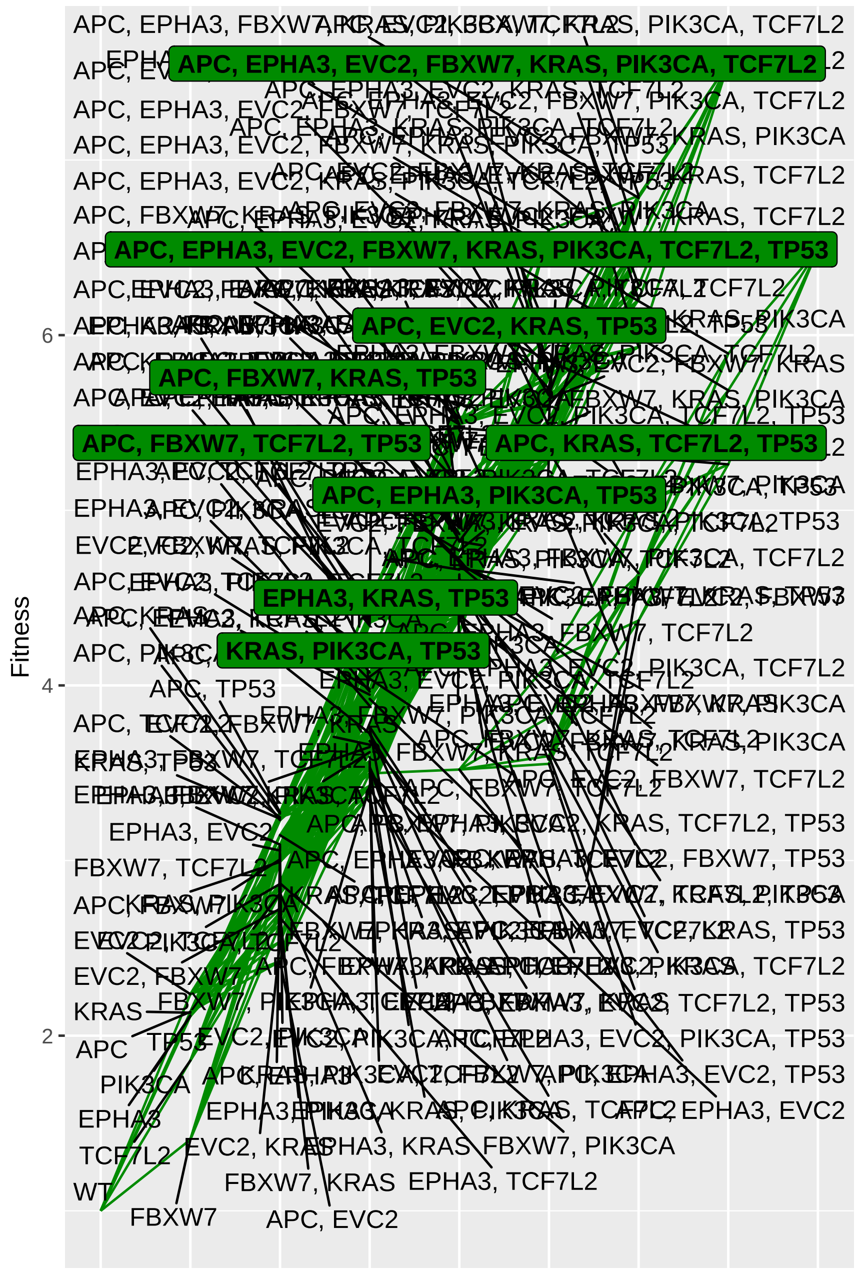

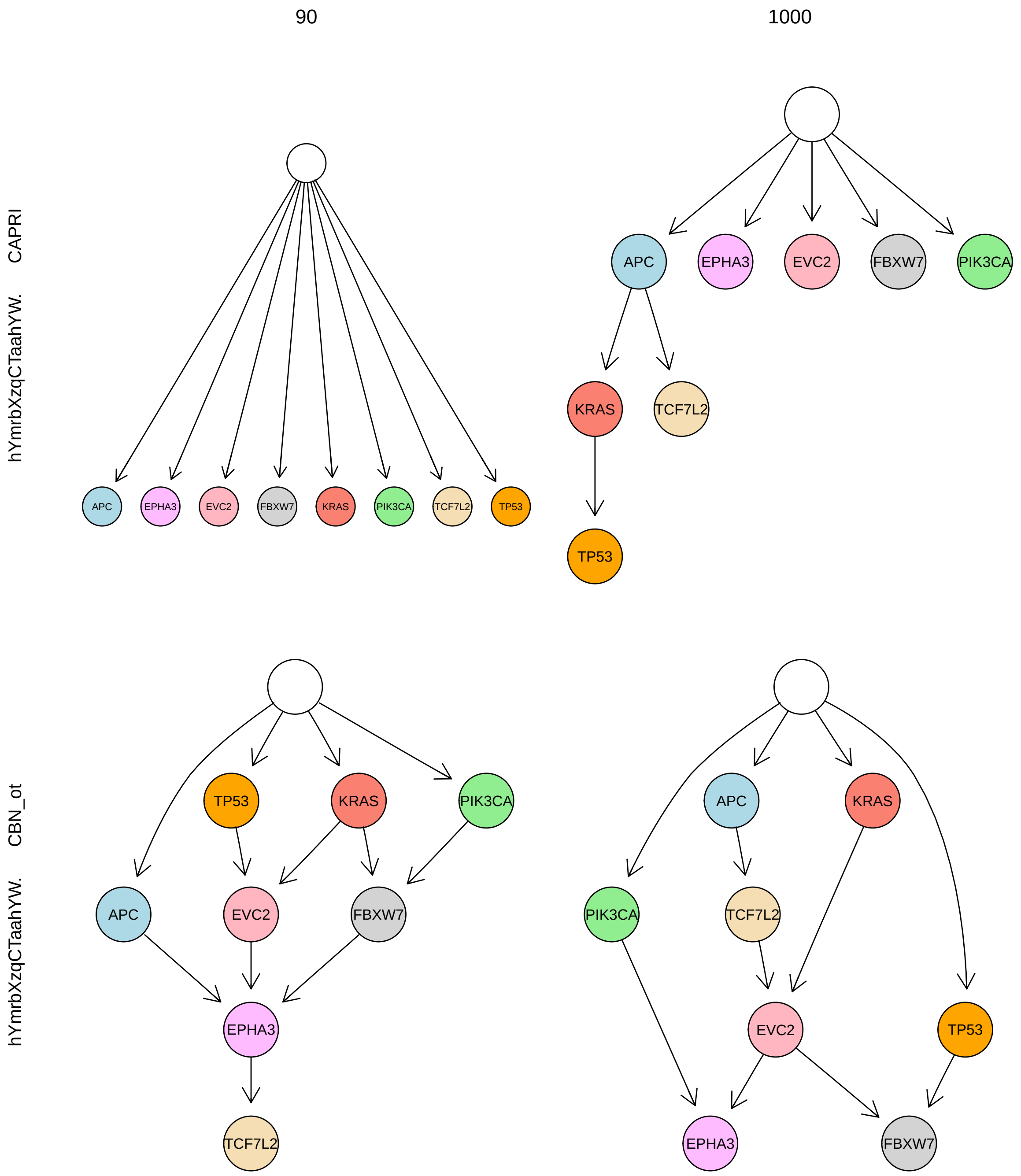

ID	p−value	Accessible Genot.
djUducjZtQAfBMK	0.726	33

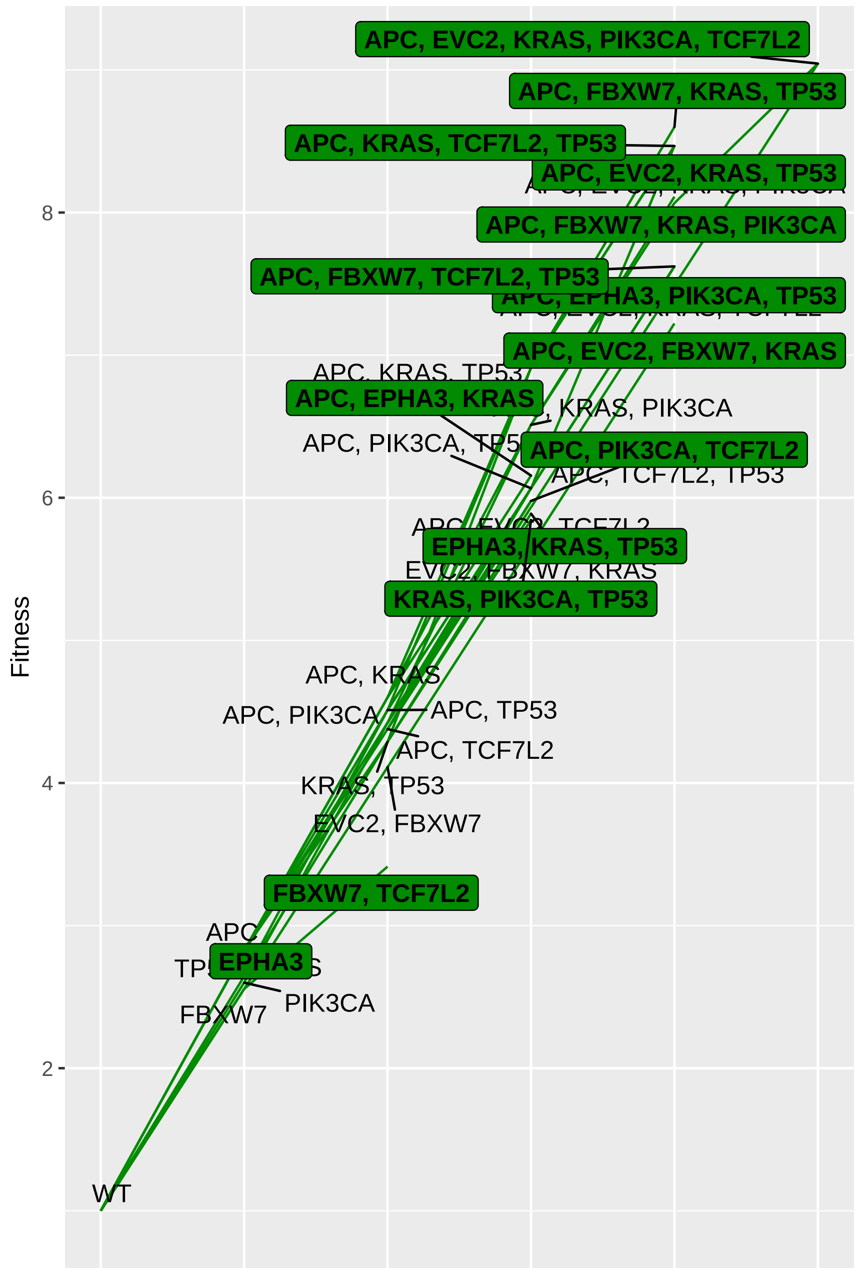

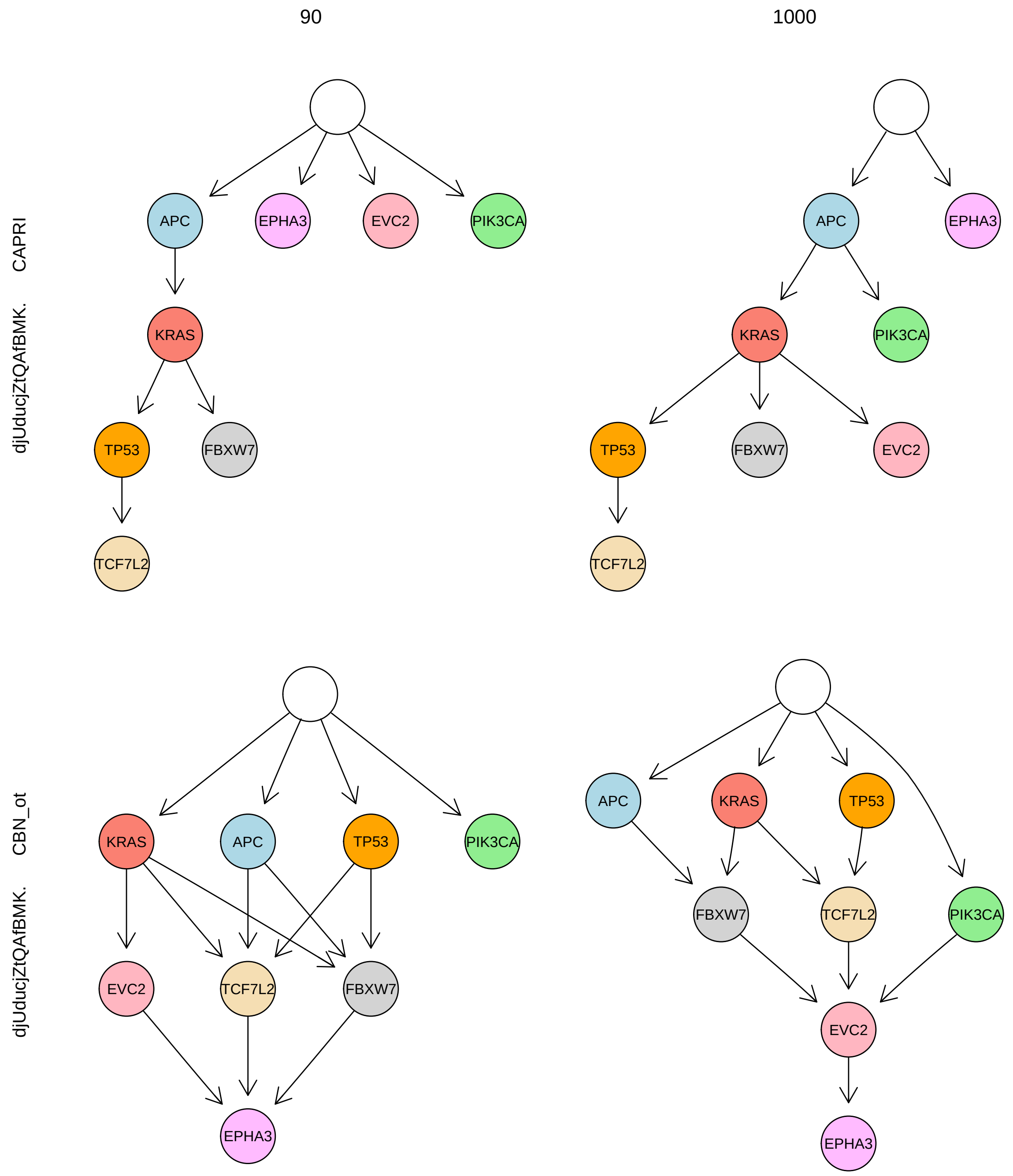

ID	p−value	Accessible Genot.
PThOprNbwwHDhEI	0.727	40

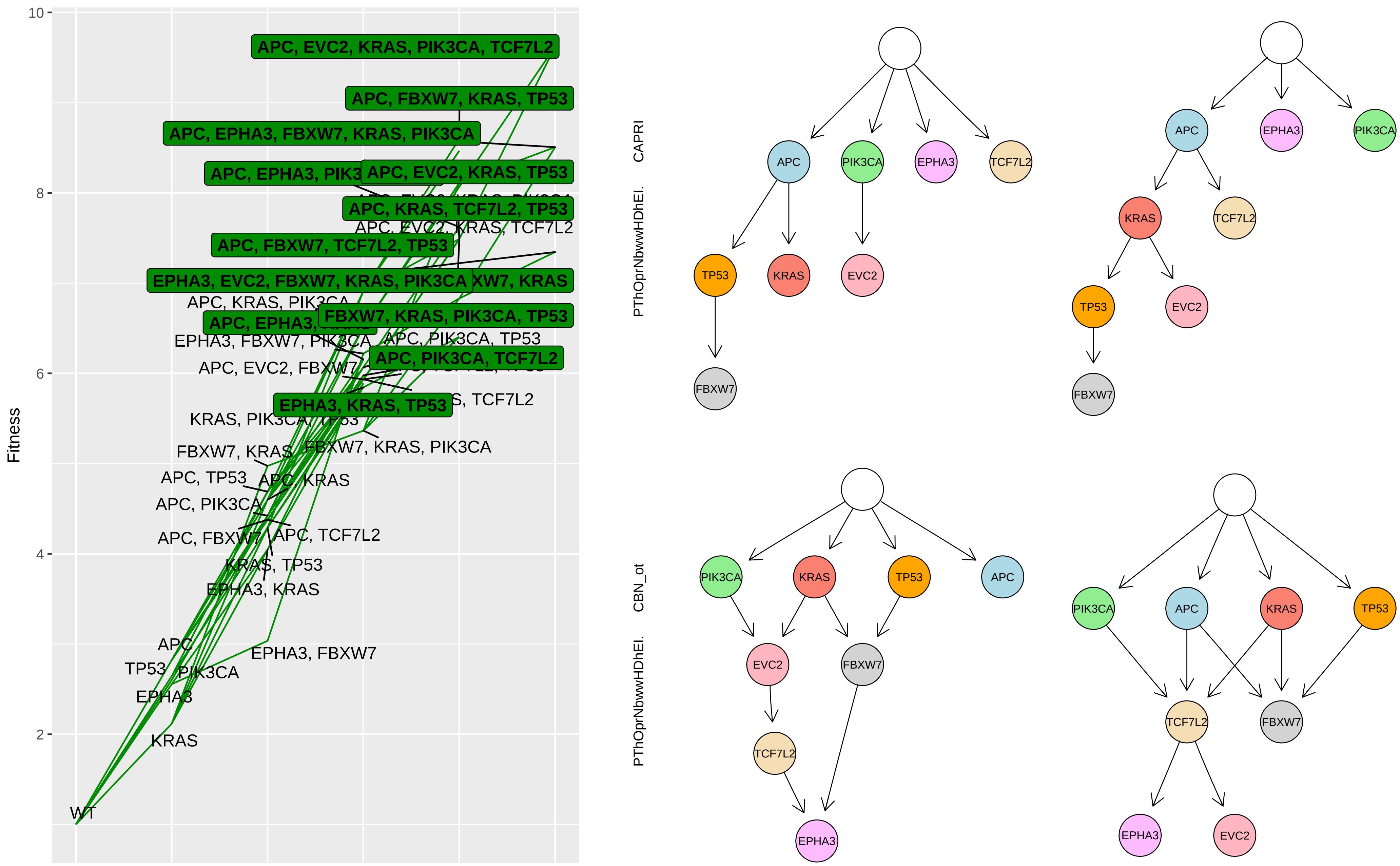

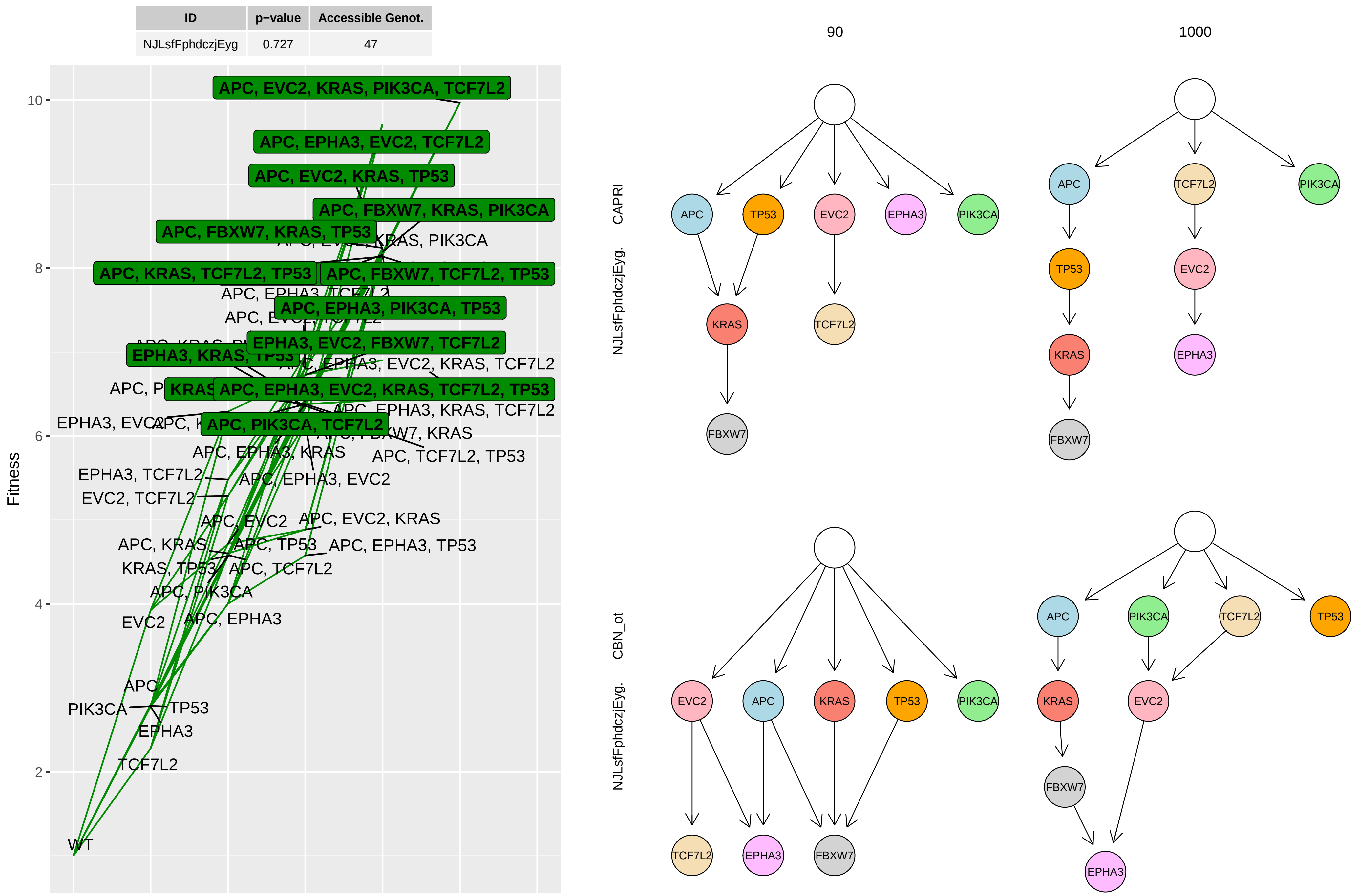

ID
p−value
Accessible Genot.
NJLsfFphdczjEyg
0.727
47
Fitness
10
8
6
4
2
APC, EVC2, KRAS, PIK3CA, TCF7L2
APC, EPHA3, EVC2, TCF7L2
APC, EVC2, KRAS, TP53
APC, FBXW7, KRAS, PIK3CA
APC, FBXW7, KRAS, TP53
APC, KRAS, TCF7L2, TP53
APC, FBXW7, TCF7L2, TP53
APC, EPHA3, PIK3CA, TP53
EPHA3, EVC2, FBXW7, TCF7L2
EPHA3, KRAS, TP53
APC, EPHA3, EVC2, KRAS, TCF7L2
APC, EPHA3, EVC2, KRAS, TCF7L2, TP53
APC, EPHA3, KRAS, TCF7L2
APC, PIK3CA, TCF7L2
APC, EPHA3, KRAS
APC, TCF7L2, TP53
EPHA3, TCF7L2
APC, EPHA3, EVC2
EVC2, TCF7L2
APC, EVC2
APC, EVC2, KRAS
APC, KRAS
APC, TP53
APC, EPHA3, TP53
KRAS, TP53
APC, TCF7L2
APC, PIK3CA
EVC2
APC, EPHA3
APC
PIK3CA
TP53
EPHA3
TCF7L2
WT
90
1000
CAPRI
CBN_ot
NJLsfFphdczjEyg.
APC
TP53
EVC2
EPHA3
PIK3CA
KRAS
TCF7L2
FBXW7

ID	p−value	Accessible Genot.
yjaNNQIHNpkVOxb	0.728	37

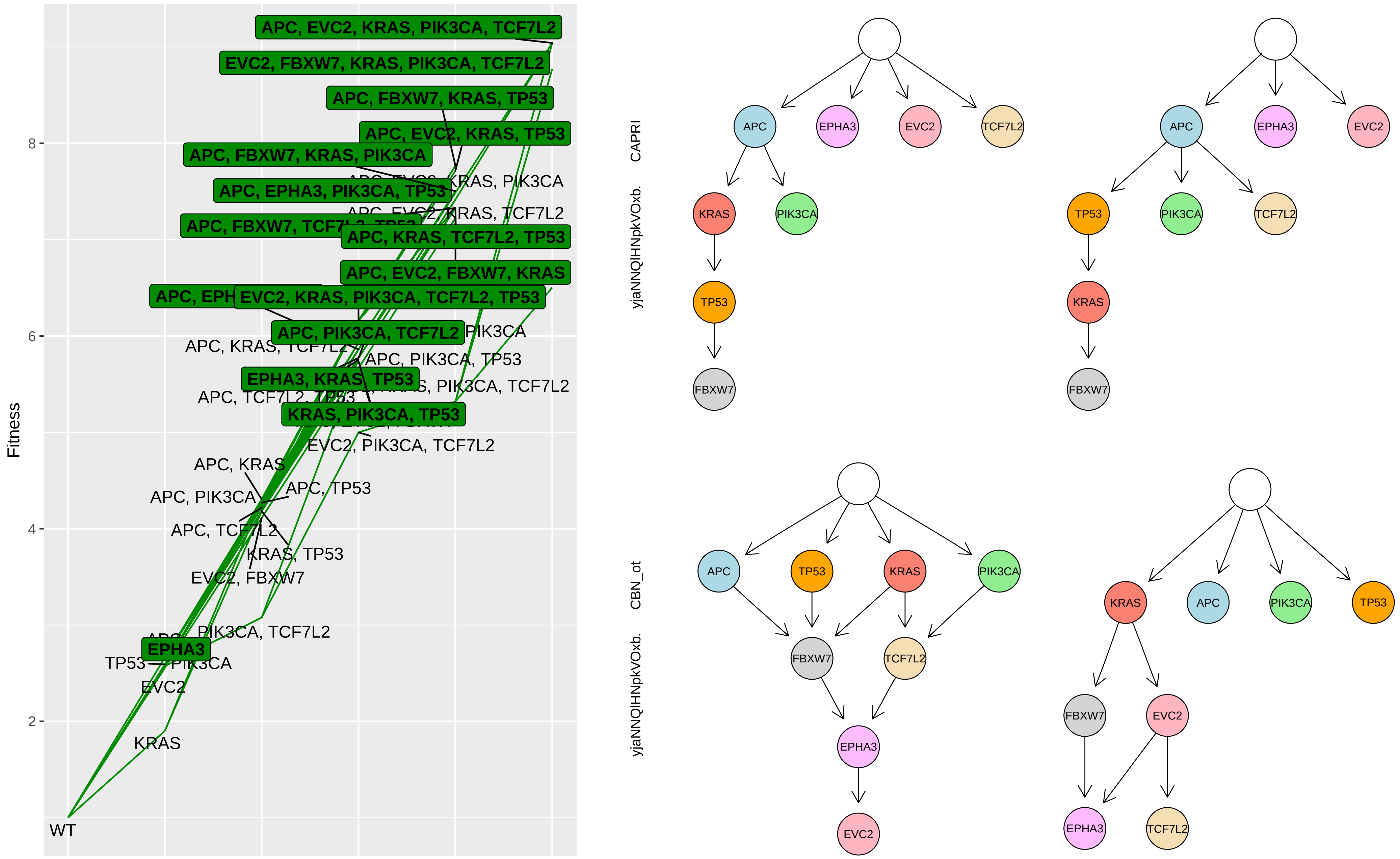

ID	p−value	Accessible Genot.
FkhPdcWYVfpoWcM	0.732	61

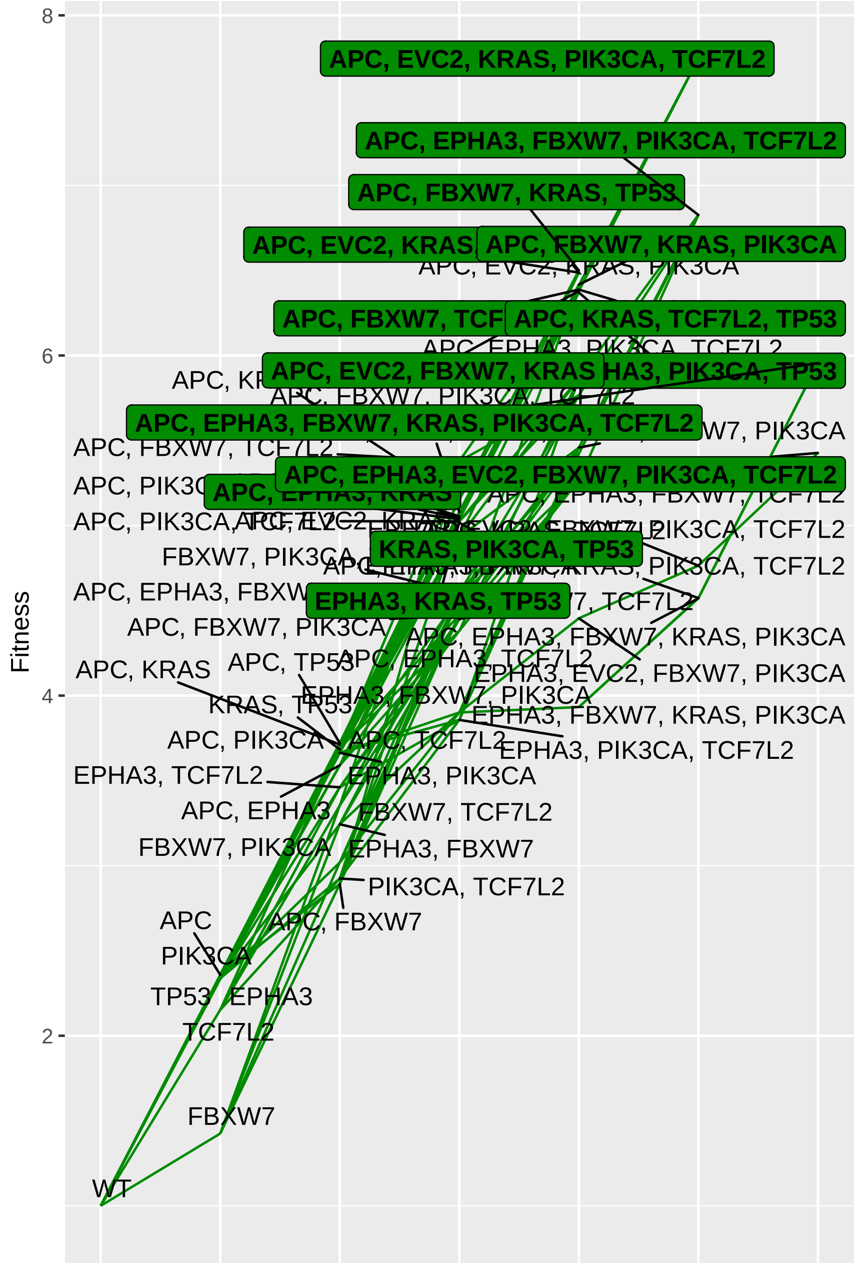

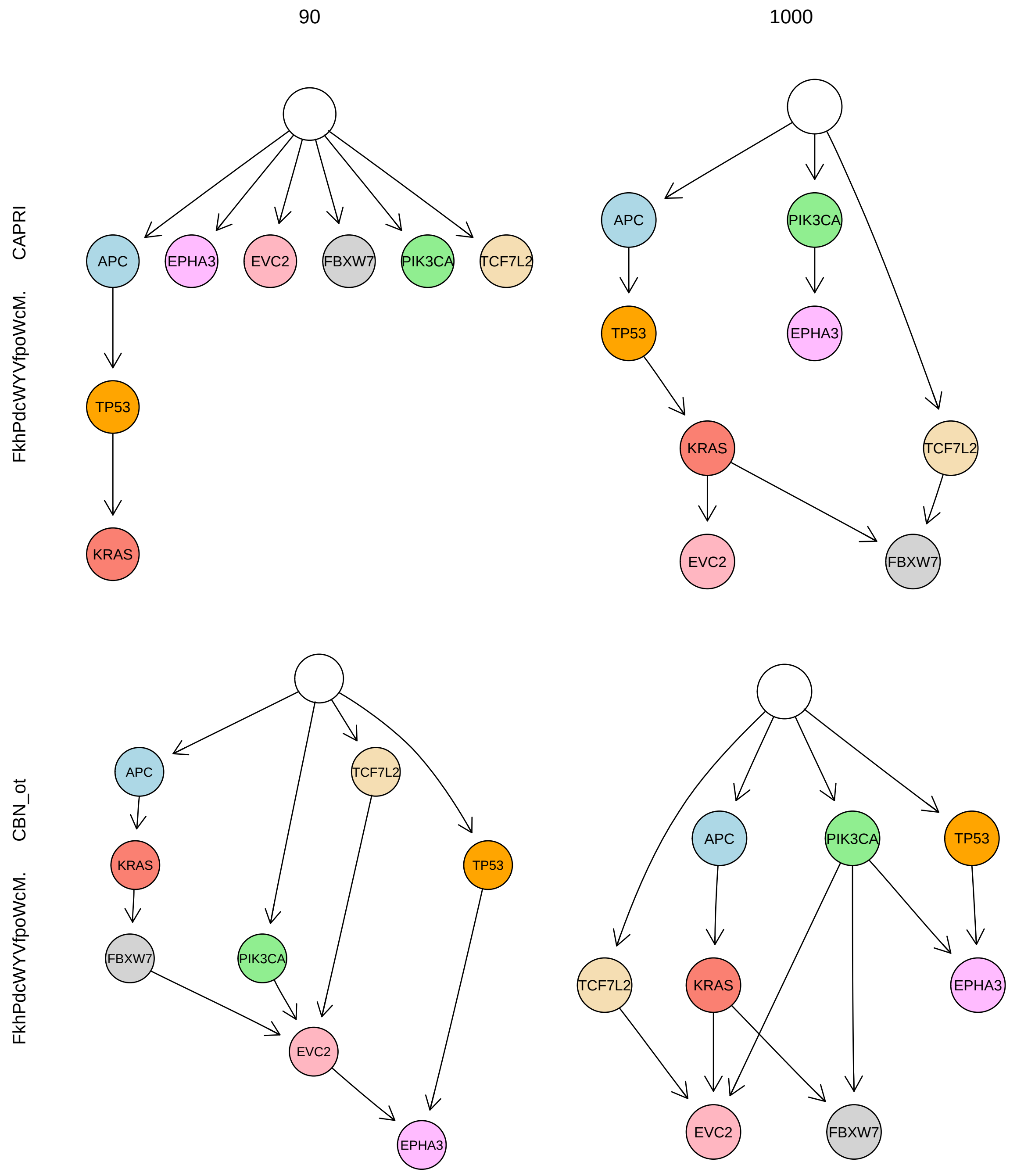

ID	p−value	Accessible Genot.
odpzCpAHfkSKeUL	0.732	35

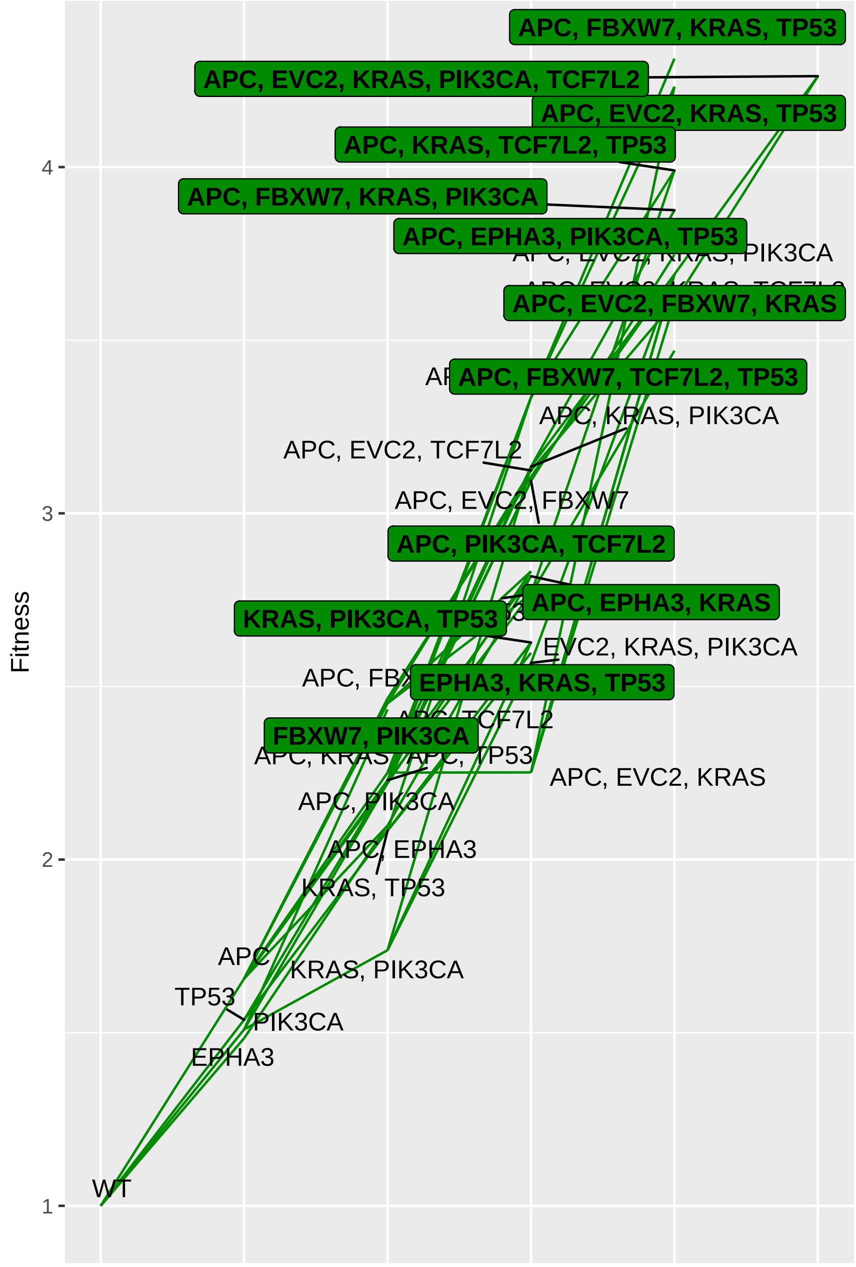

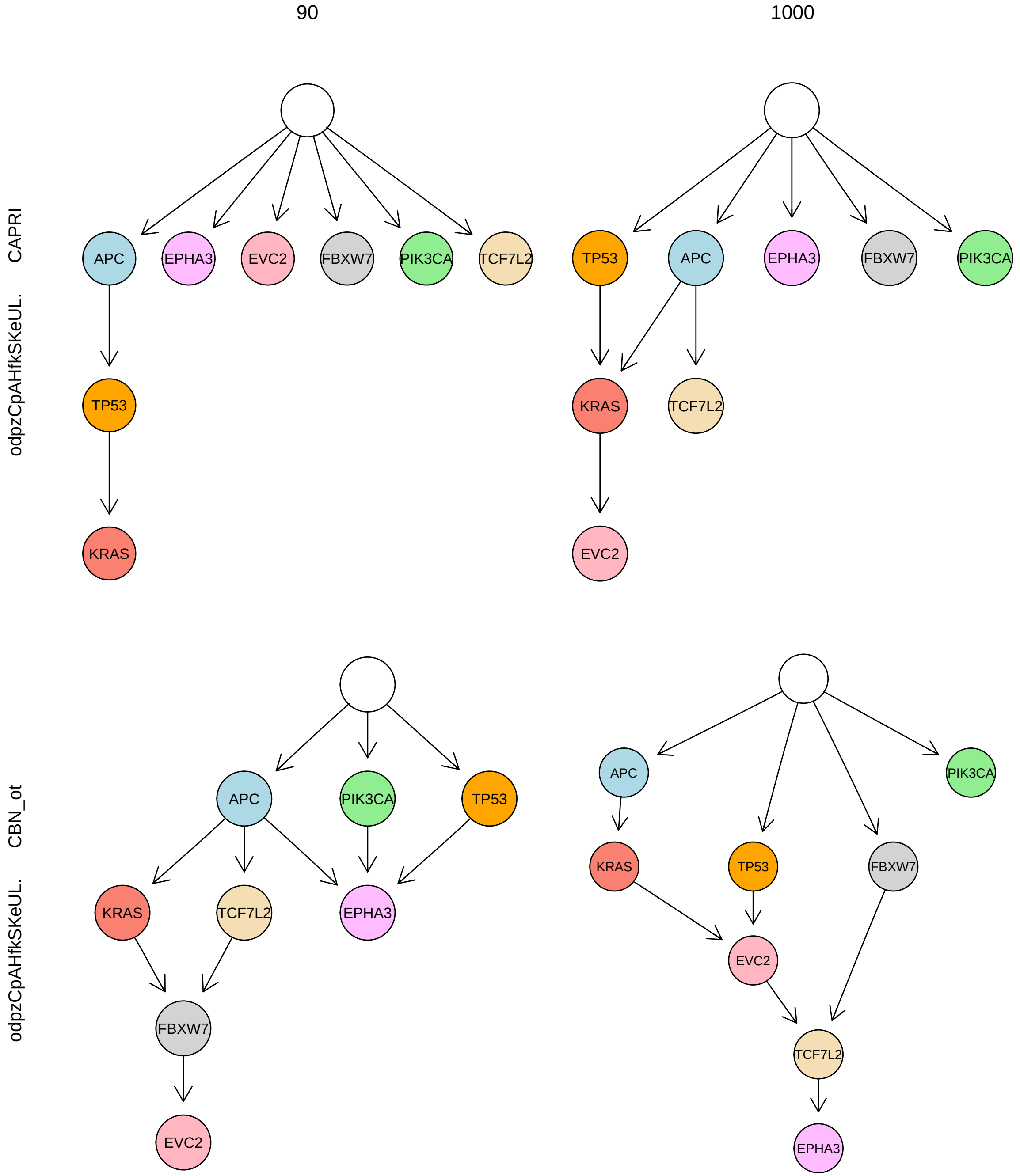

ID	p−value	Accessible Genot.
mvRIbUzJkkzVMXb	0.737	30

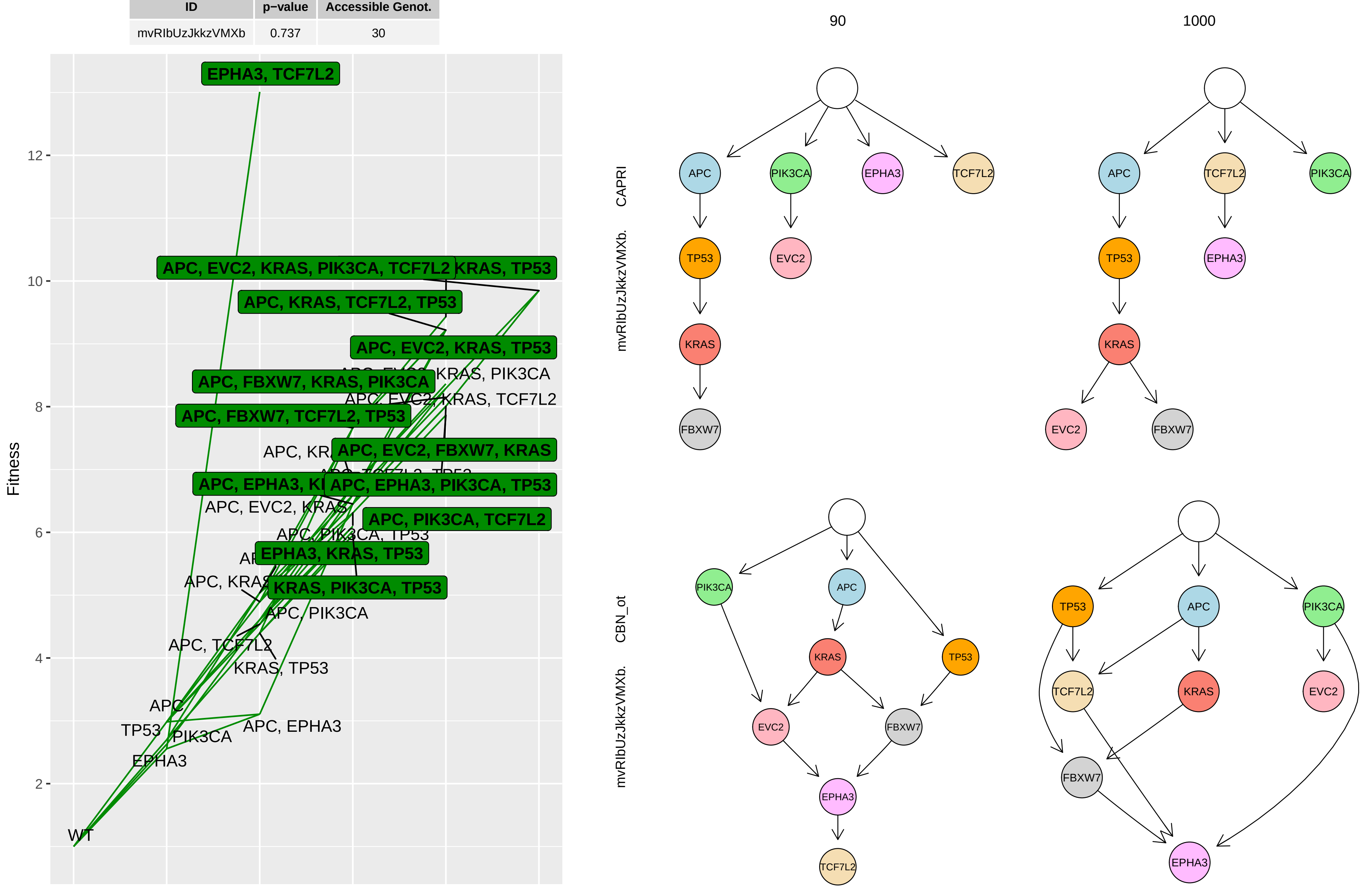

ID	p-value	Accessible Genot.
EXYAXXmGsqKrLbE	0.737	98

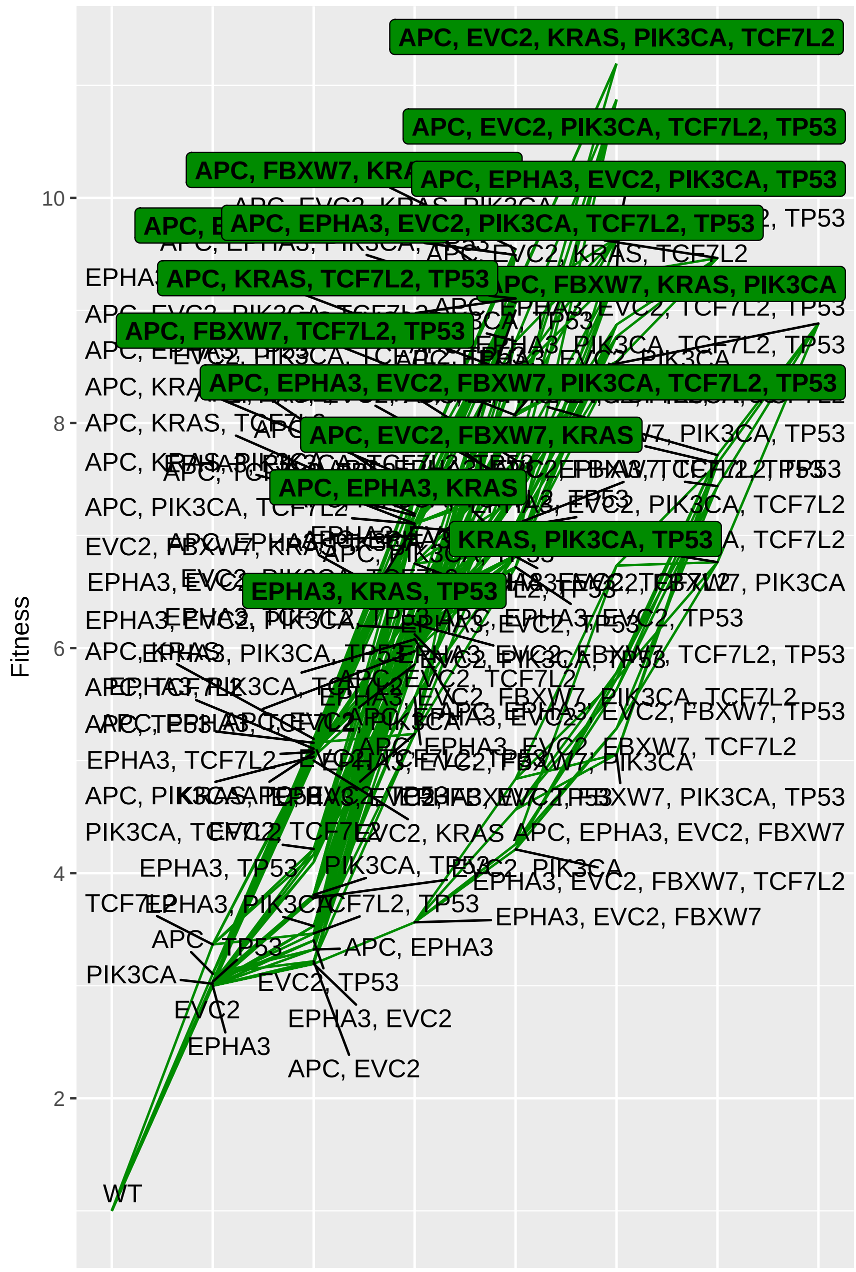

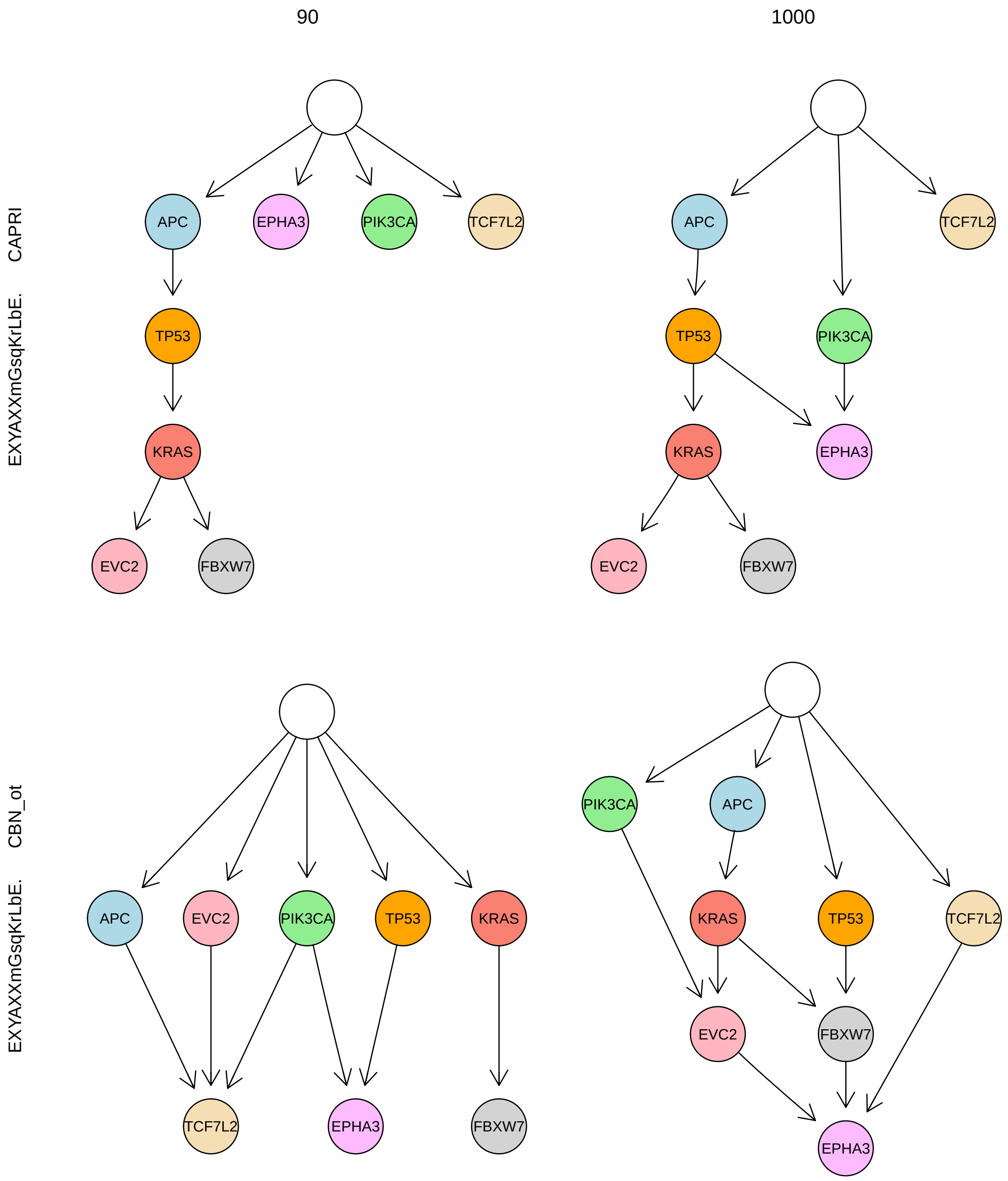

ID	p−value	Accessible Genot.
uRzHXIHtTfUutbL	0.743	38

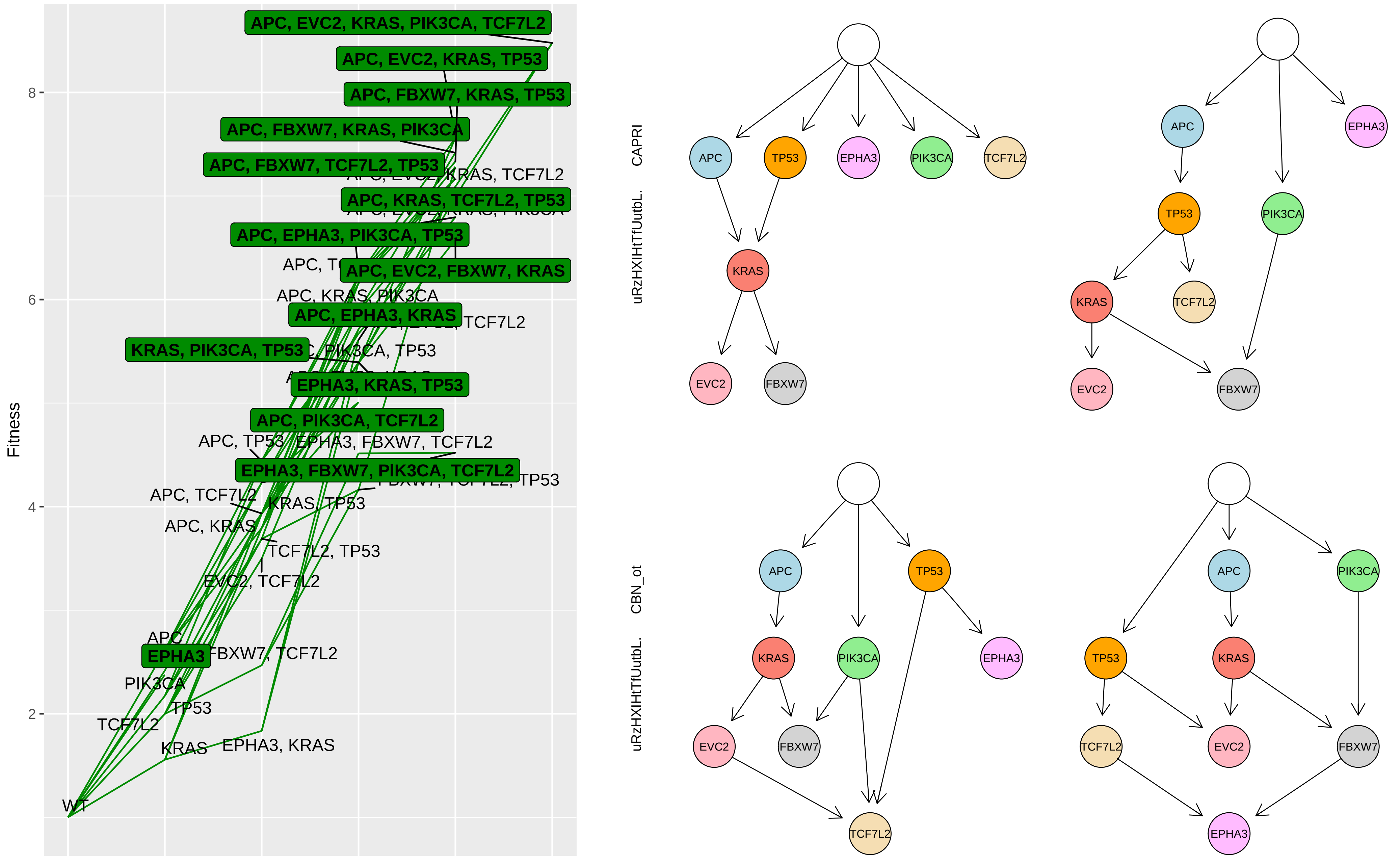

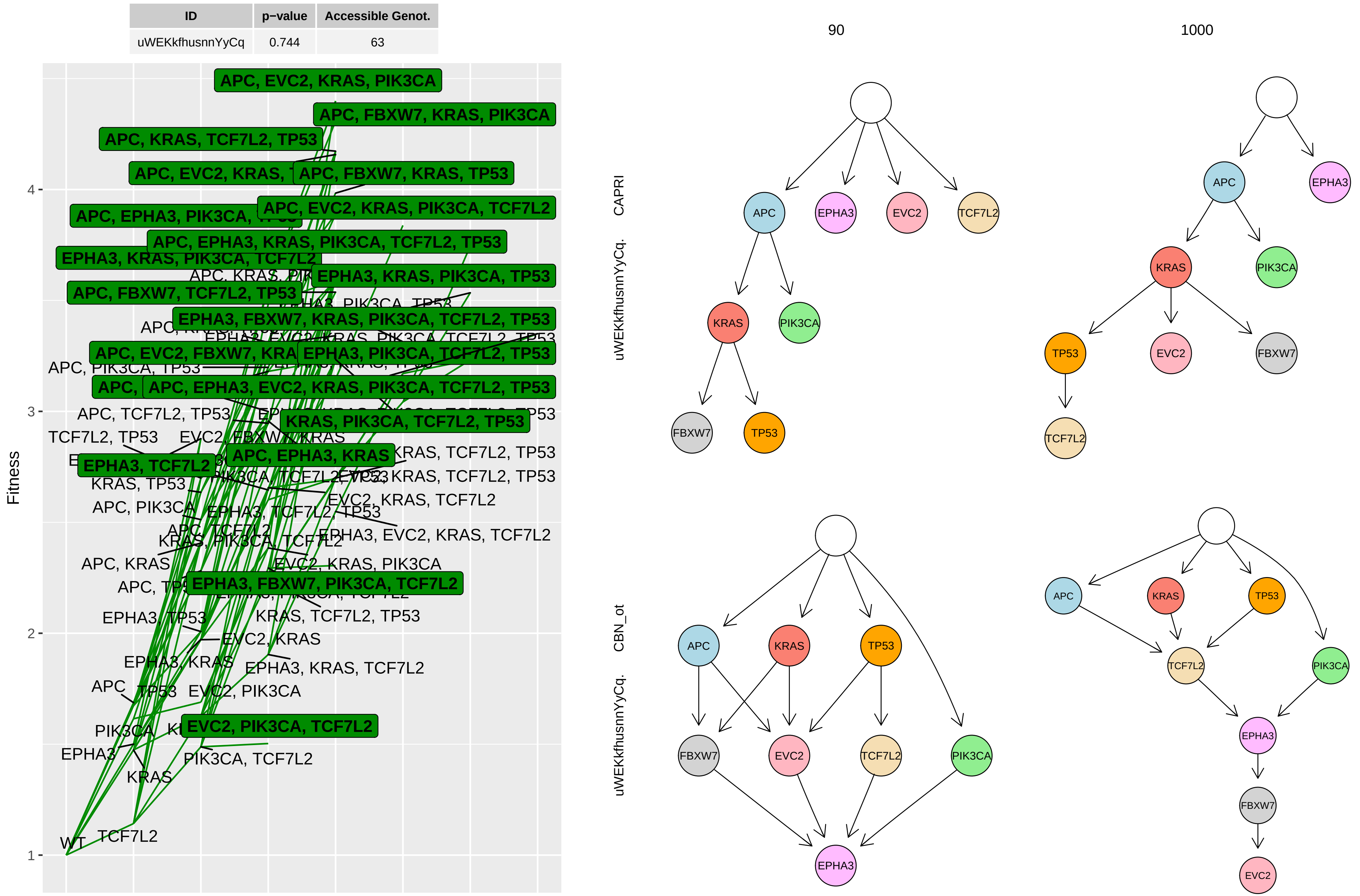

ID
p−value
Accessible Genot.
uWEKkfhusnnYyCq
0.744
63
Fitness
1
2
3
4
WT
TCF7L2
KRAS
EPHA3
PIK3CA
APC
TP53
APC, EVC2, KRAS, PIK3CA
APC, FBXW7, KRAS, PIK3CA
APC, KRAS, TCF7L2, TP53
APC, FBXW7, KRAS, TP53
APC, EVC2, KRAS, PIK3CA, TCF7L2
APC, EPHA3, KRAS, PIK3CA, TCF7L2, TP53
EPHA3, KRAS, PIK3CA, TCF7L2
EPHA3, KRAS, PIK3CA, TP53
APC, FBXW7, TCF7L2, TP53
EPHA3, FBXW7, KRAS, PIK3CA, TCF7L2, TP53
EPHA3, PIK3CA, TCF7L2, TP53
APC, EPHA3, EVC2, KRAS, PIK3CA, TCF7L2, TP53
KRAS, PIK3CA, TCF7L2, TP53
APC, EPHA3, KRAS
EPHA3, TCF7L2
EPHA3, FBXW7, PIK3CA, TCF7L2
EVC2, PIK3CA, TCF7L2
APC, TCF7L2, TP53
TCF7L2, TP53
KRAS, TP53
APC, PIK3CA
EVC2, KRAS, TCF7L2
EPHA3, TCF7L2, TP53
EPHA3, EVC2, KRAS, TCF7L2
APC, KRAS
EVC2, KRAS, PIK3CA
KRAS, TCF7L2, TP53
EPHA3, TP53
EVC2, KRAS
EPHA3, KRAS
EPHA3, KRAS, TCF7L2
EVC2, PIK3CA
PIK3CA, TCF7L2
90
1000
CAPRI
CBN_ot
uWEKkfhusnnYyCq.
APC
EPHA3
EVC2
TCF7L2
KRAS
PIK3CA
FBXW7
TP53

ID	p−value	Accessible Genot.
aaScqrCieKfLmyq	0.744	165

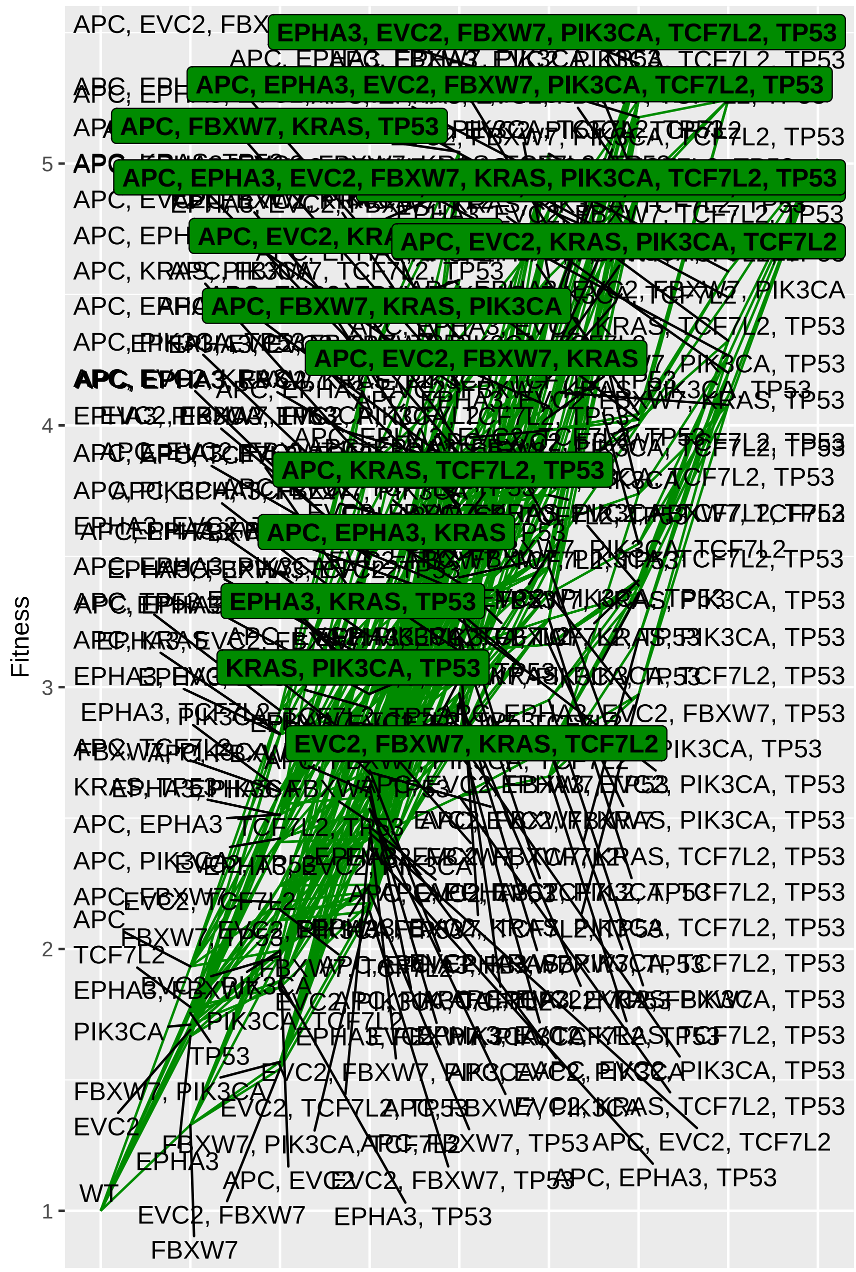

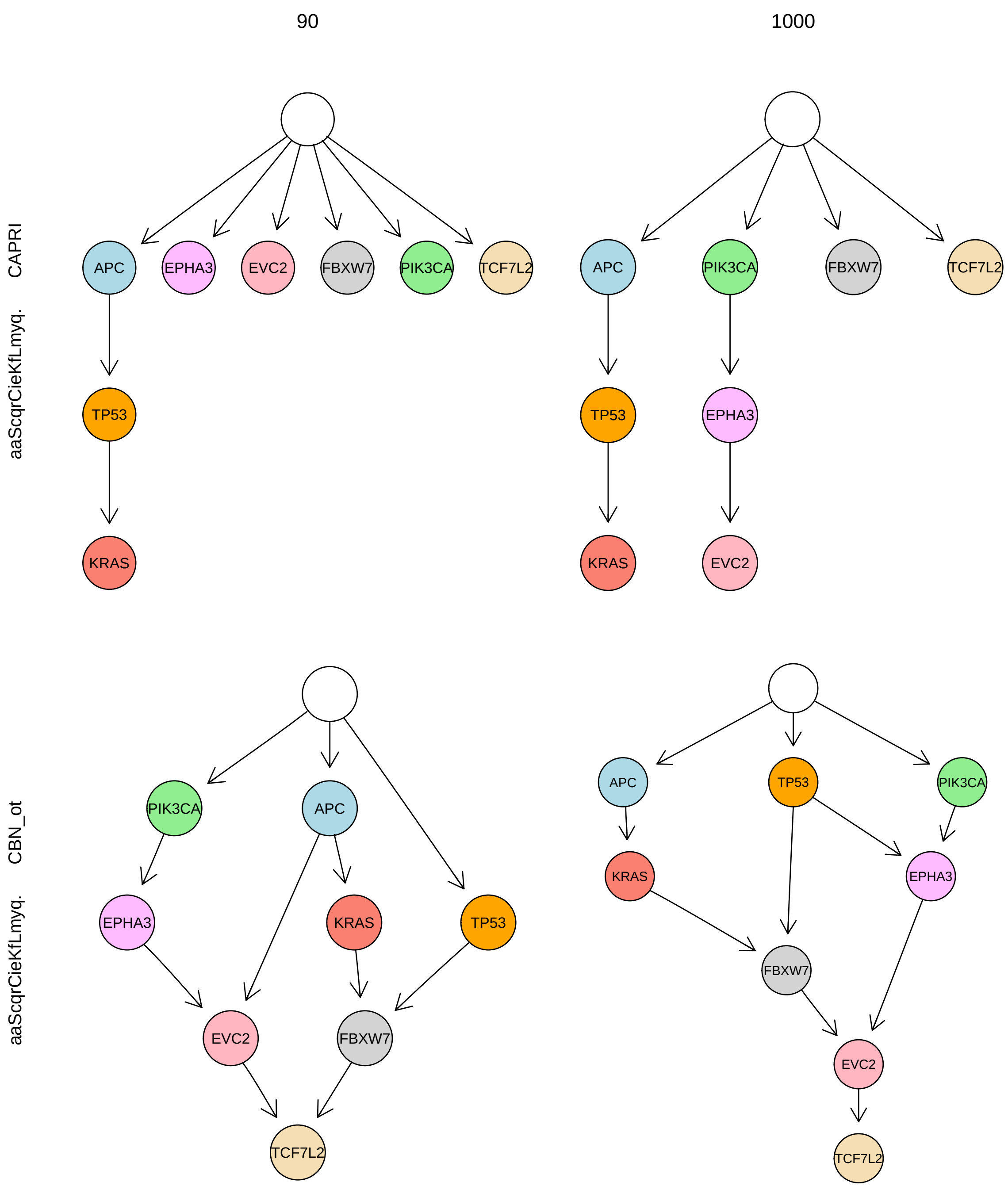

ID	p−value	Accessible Genot.
KVkICHaSwEoVfMq	0.746	42

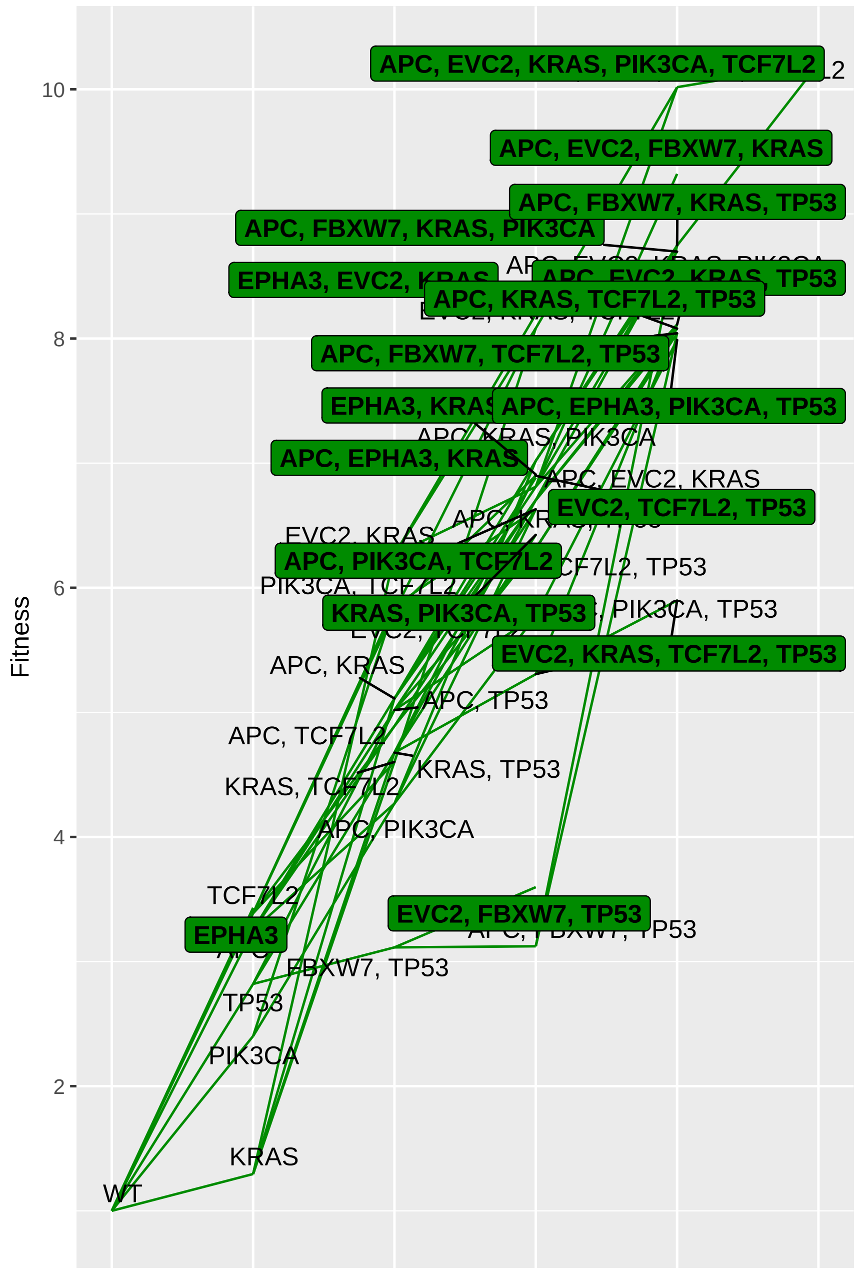

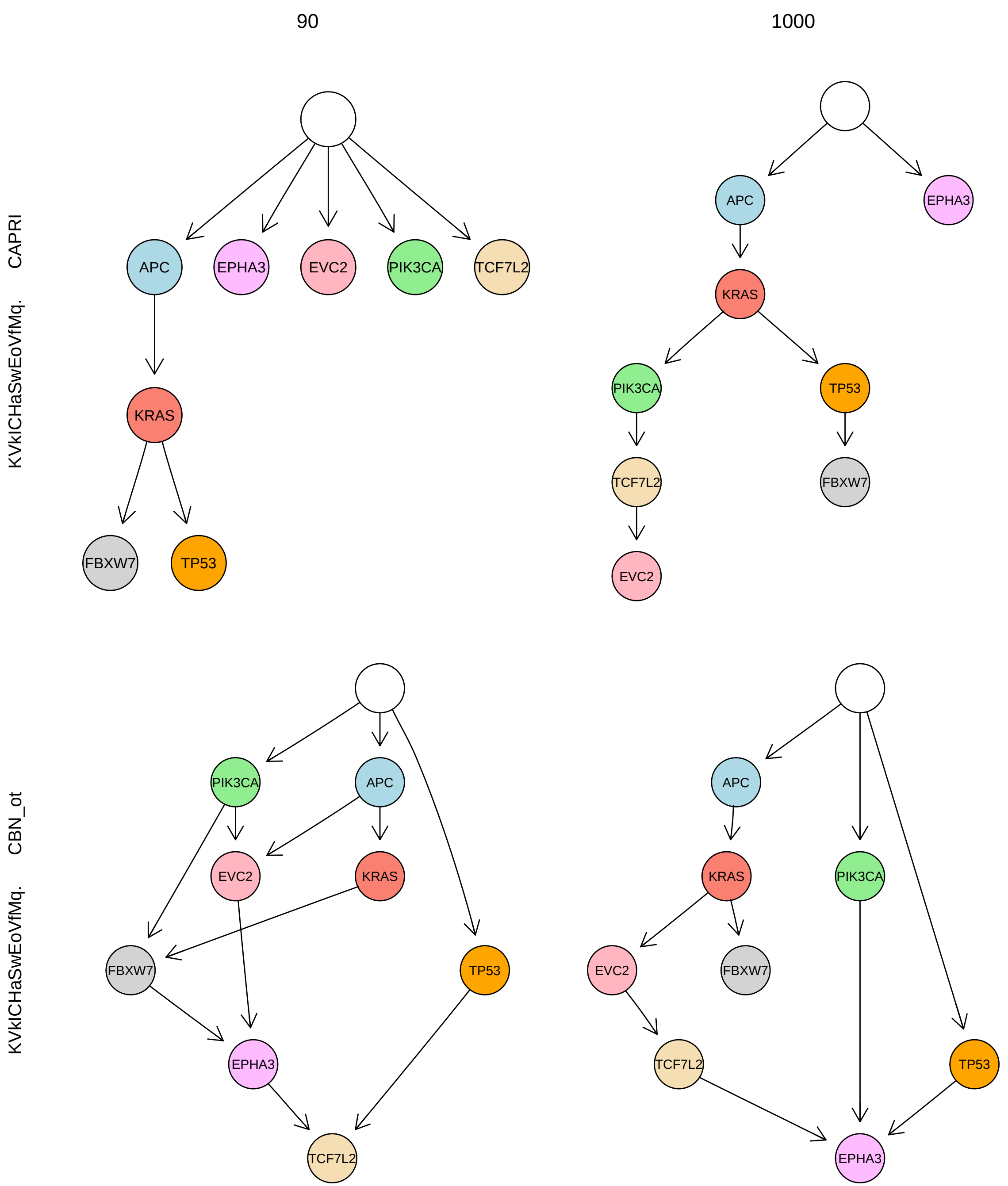

ID	p−value	Accessible Genot.
QaaGXPQtlevlzdp	0.75	36

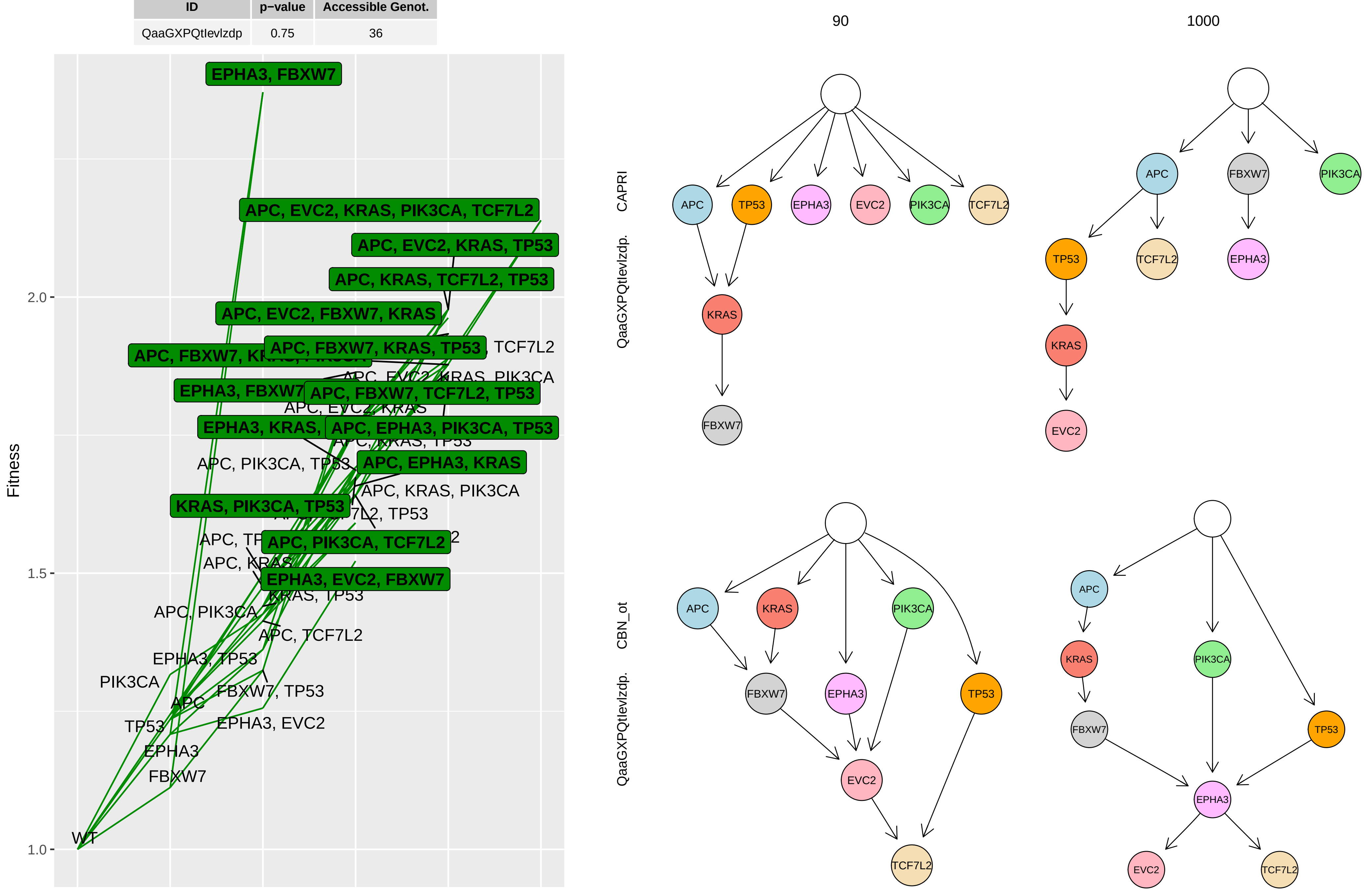

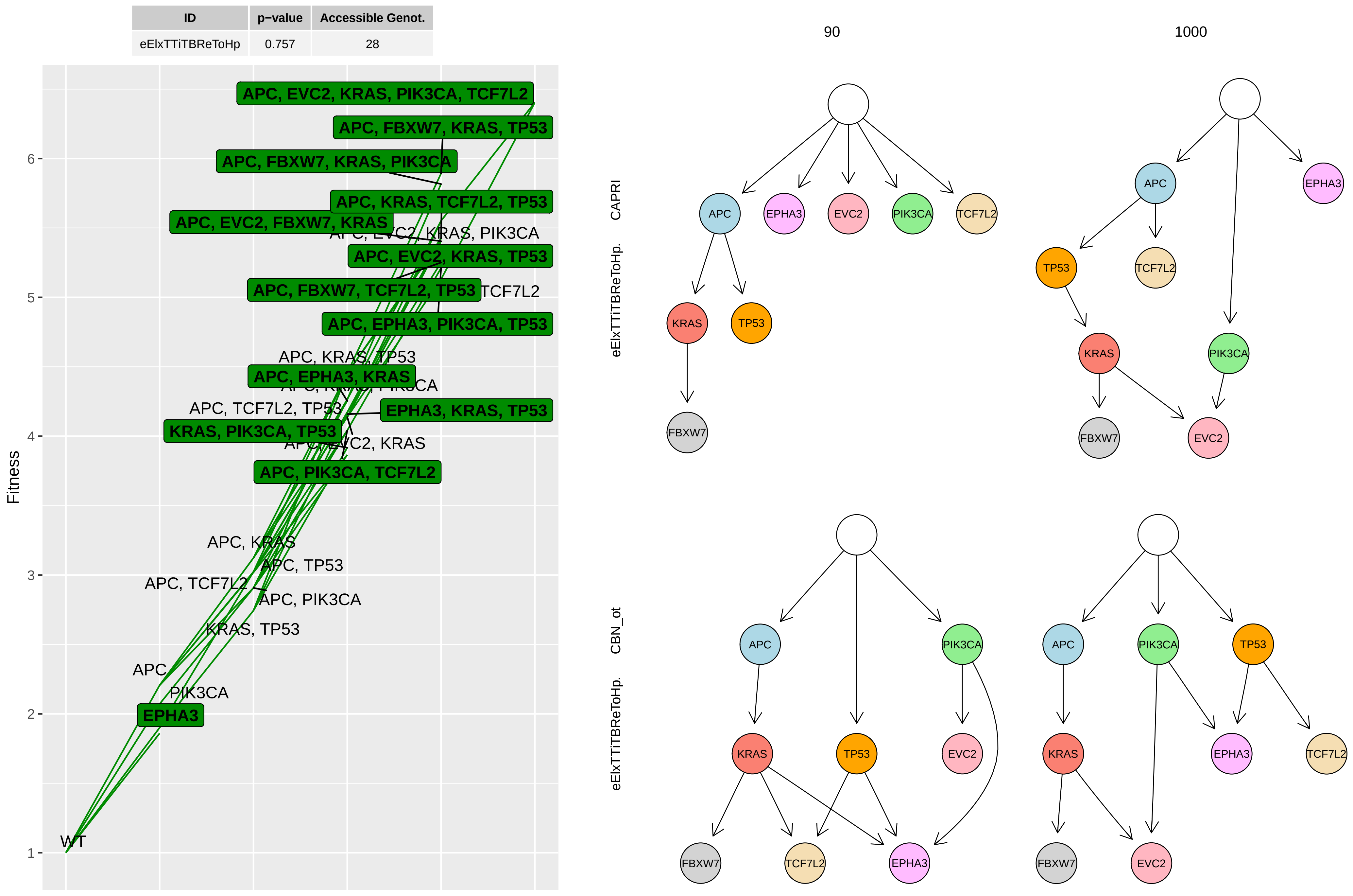

ID
p−value
Accessible Genot.
eElxTTiTBReToHp
0.757
28
Fitness
APC, EVC2, KRAS, PIK3CA, TCF7L2
APC, FBXW7, KRAS, TP53
APC, FBXW7, KRAS, PIK3CA
APC, KRAS, TCF7L2, TP53
APC, EVC2, FBXW7, KRAS
APC, EVC2, KRAS, TP53
APC, FBXW7, TCF7L2, TP53
APC, EPHA3, PIK3CA, TP53
APC, KRAS, TP53
APC, EPHA3, KRAS
APC, TCF7L2, TP53
EPHA3, KRAS, TP53
KRAS, PIK3CA, TP53
APC, PIK3CA, TCF7L2
APC, KRAS
APC, TP53
APC, TCF7L2
APC, PIK3CA
KRAS, TP53
APC
PIK3CA
EPHA3
WT
90
1000
CAPRI
eElxTTiTBReToHp.
CBN_ot
APC
EPHA3
EVC2
PIK3CA
TCF7L2
KRAS
TP53
FBXW7

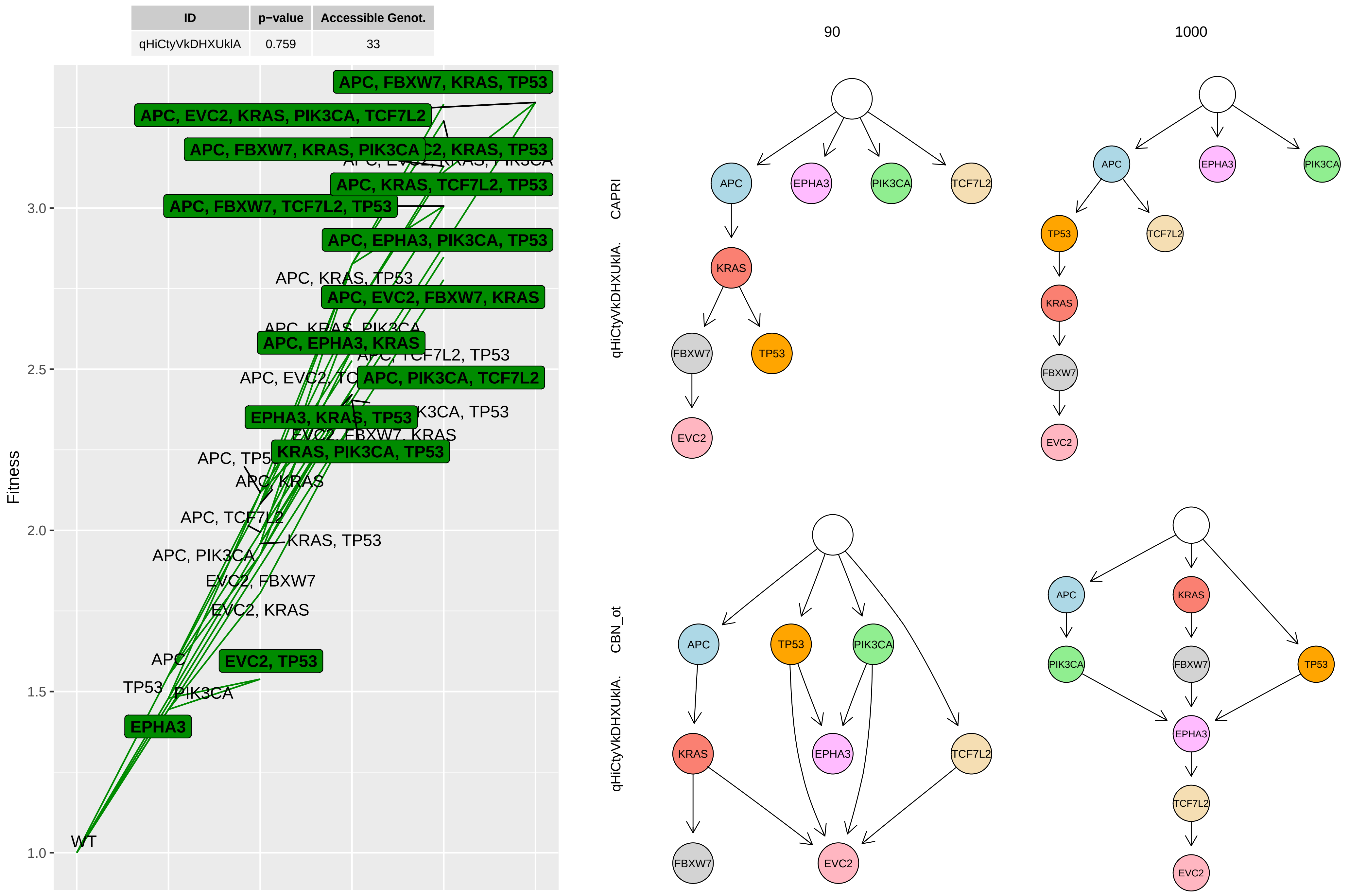

ID
p−value
Accessible Genot.
qHiCtyVkDHXUklA
0.759
33
Fitness
3.0
2.5
2.0
1.5
1.0
APC, FBXW7, KRAS, TP53
APC, EVC2, KRAS, PIK3CA, TCF7L2
APC, FBXW7, KRAS, PIK3CA
APC, KRAS, TCF7L2, TP53
APC, FBXW7, TCF7L2, TP53
APC, EPHA3, PIK3CA, TP53
APC, KRAS, TP53
APC, EVC2, FBXW7, KRAS
APC, EPHA3, KRAS
APC, PIK3CA, TCF7L2
EPHA3, KRAS, TP53
KRAS, PIK3CA, TP53
APC, KRAS
APC, TCF7L2
KRAS, TP53
APC, PIK3CA
EVC2, FBXW7
EVC2, KRAS
APC
EVC2, TP53
TP53
PIK3CA
EPHA3
WT
90
1000
qHiCtyVkDHXUklA. CAPRI
qHiCtyVkDHXUklA. CBN_ot
APC
EPHA3
PIK3CA
TCF7L2
KRAS
FBXW7
TP53
EVC2

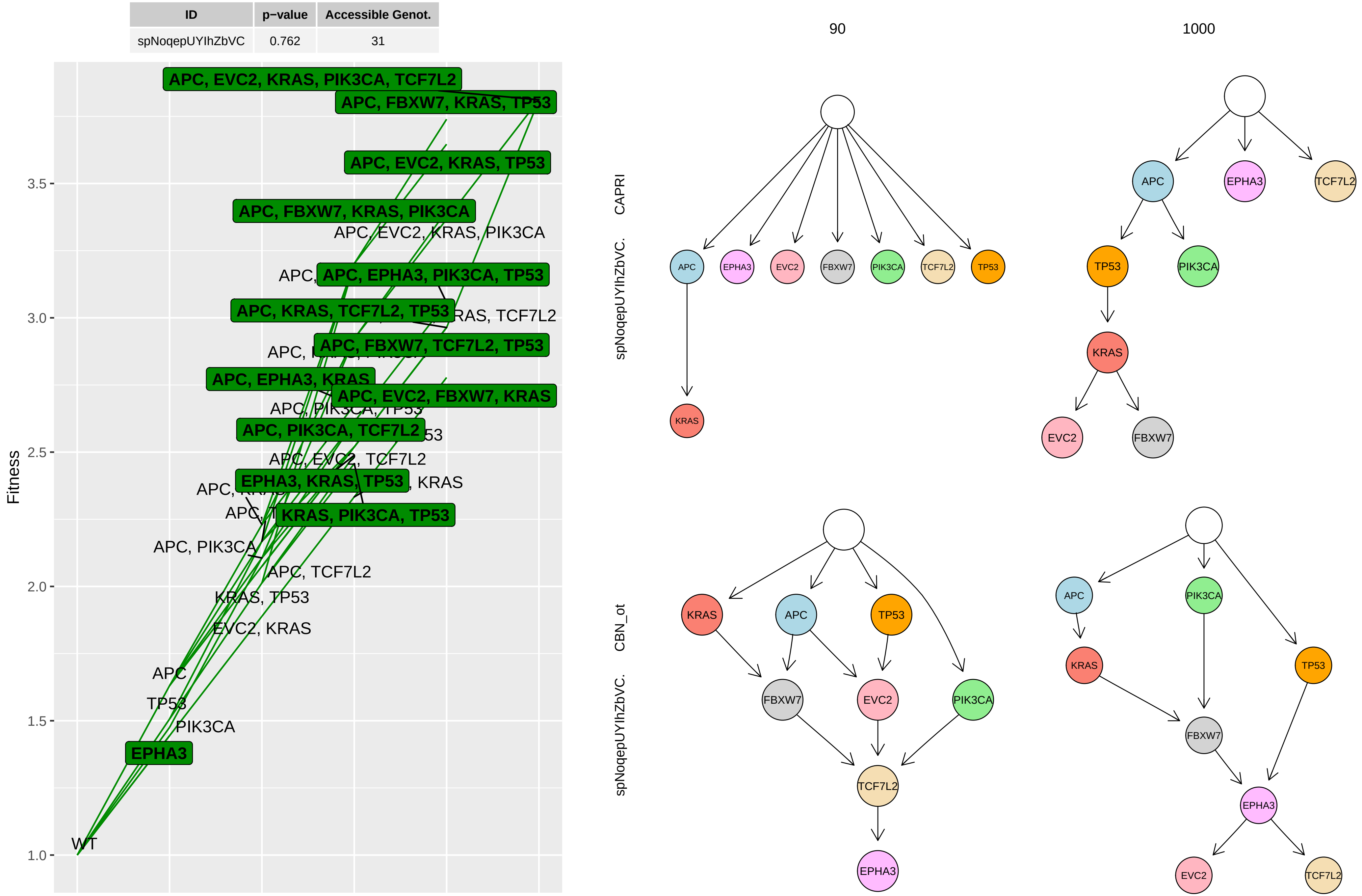

ID
p−value
Accessible Genot.
spNoqepUYIhZbVC
0.762
31
Fitness
3.5
3.0
2.5
2.0
1.5
1.0
APC, EVC2, KRAS, PIK3CA, TCF7L2
APC, FBXW7, KRAS, TP53
APC, EVC2, KRAS, TP53
APC, FBXW7, KRAS, PIK3CA
APC, EVC2, KRAS, PIK3CA
APC, EPHA3, PIK3CA, TP53
APC, KRAS, TCF7L2, TP53
APC, FBXW7, TCF7L2, TP53
APC, EPHA3, KRAS
APC, EVC2, FBXW7, KRAS
APC, PIK3CA, TCF7L2
APC, EVC2, TCF7L2
EPHA3, KRAS, TP53
KRAS, PIK3CA, TP53
APC, PIK3CA
APC, TCF7L2
KRAS, TP53
EVC2, KRAS
APC
TP53
PIK3CA
EPHA3
WT
90
1000
CAPRI
spNoqepUYIhZbVC.
CBN_ot
spNoqepUYIhZbVC.
APC
EPHA3
EVC2
FBXW7
PIK3CA
TCF7L2
TP53
KRAS

ID	p−value	Accessible Genot.
DcBFTyWKNWgEGkn	0.765	185

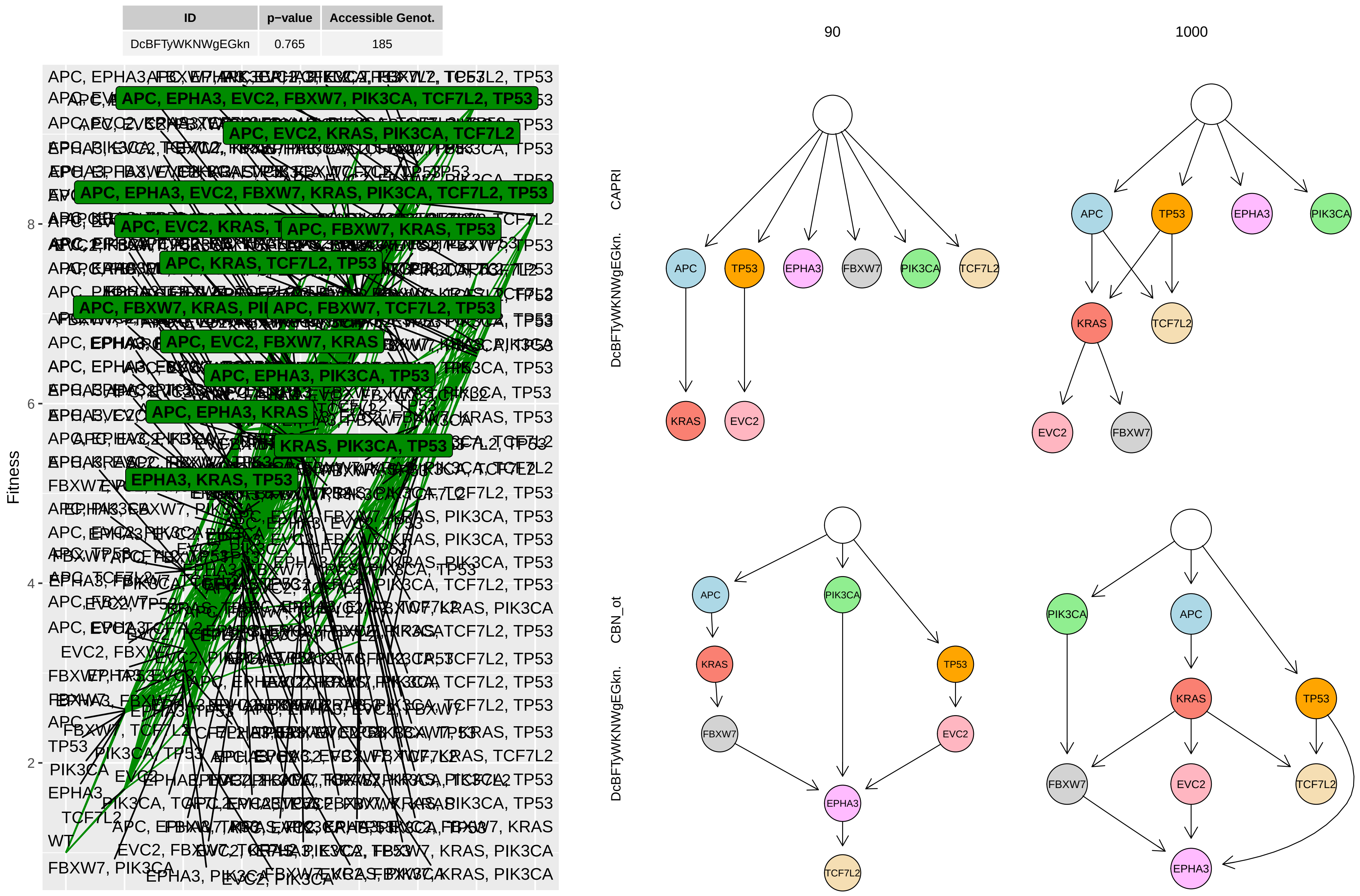

ID	p−value	Accessible Genot.
PXdBsoQNhOHMBYV	0.767	73

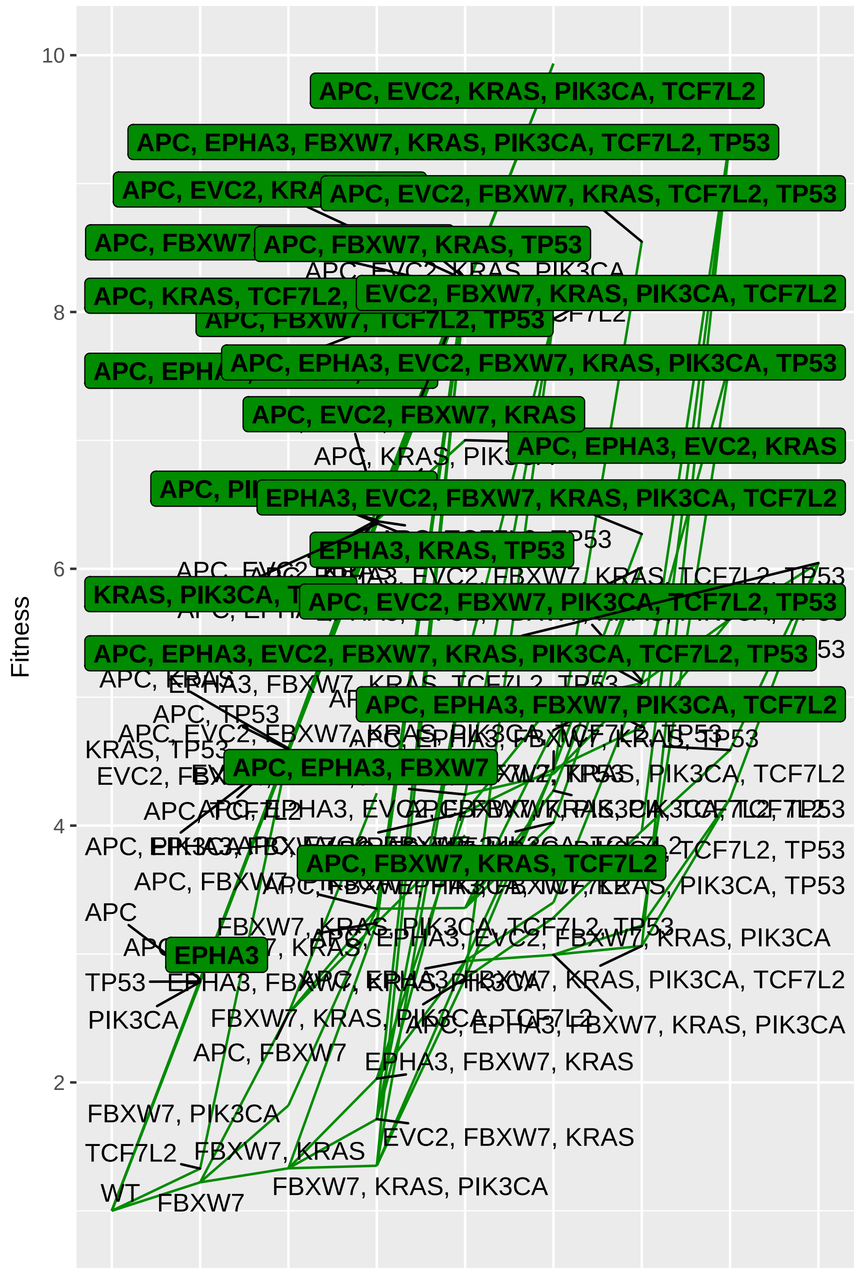

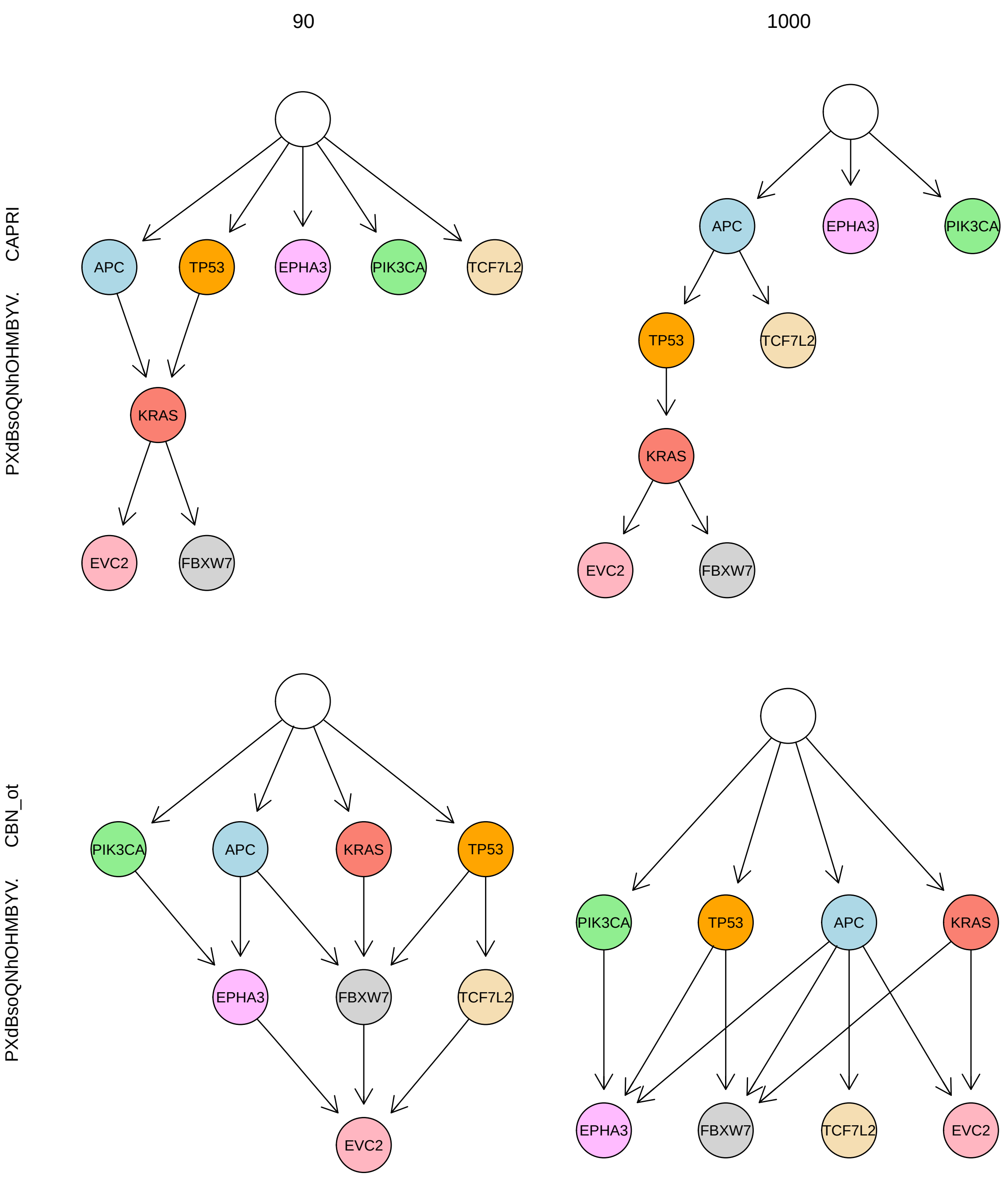

ID	p-value	Accessible Genot.
EgqTyMxCLHJXMjN	0.779	68

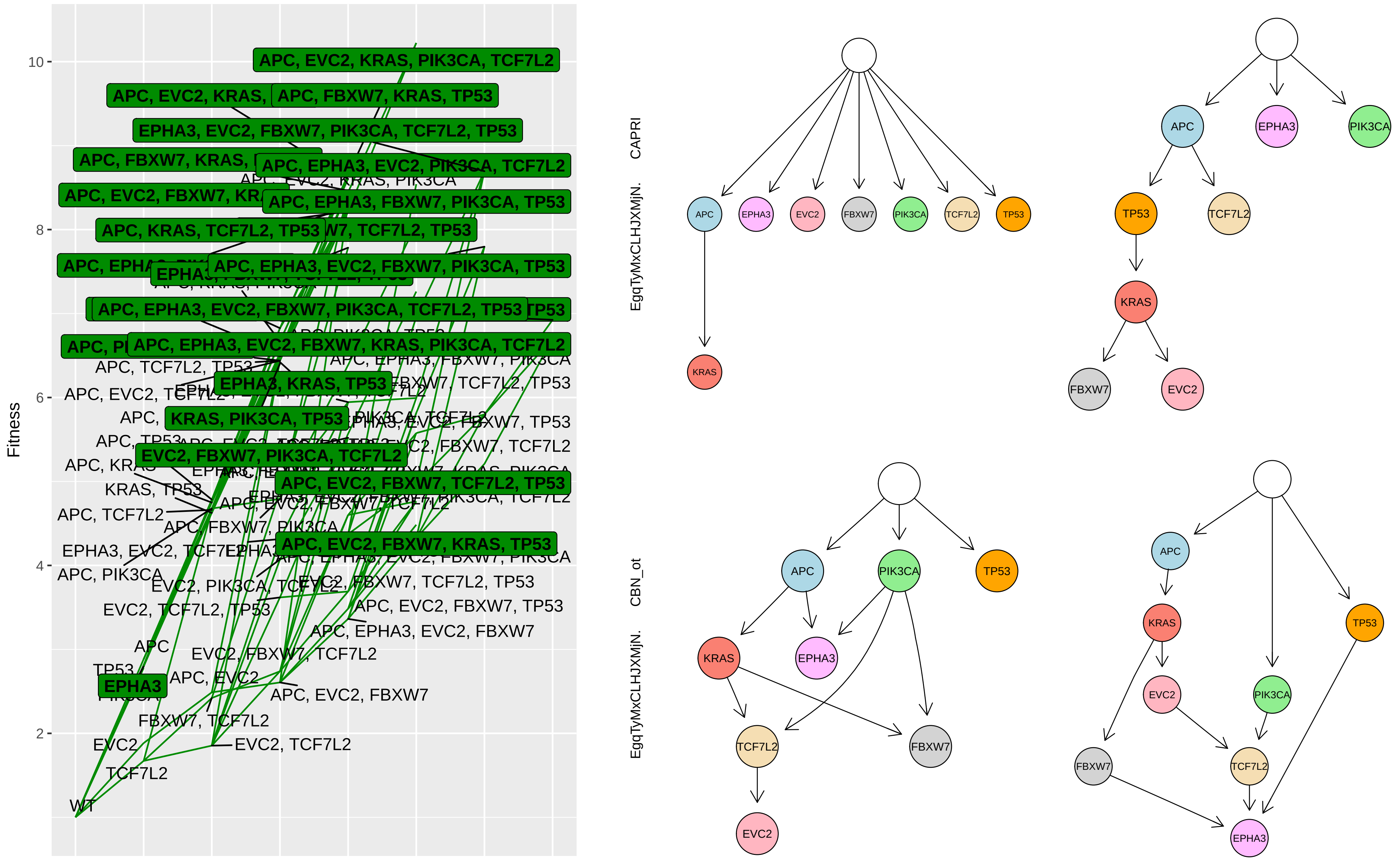

ID	p−value	Accessible Genot.
FZSlVmFgrCkzEFs	0.779	239

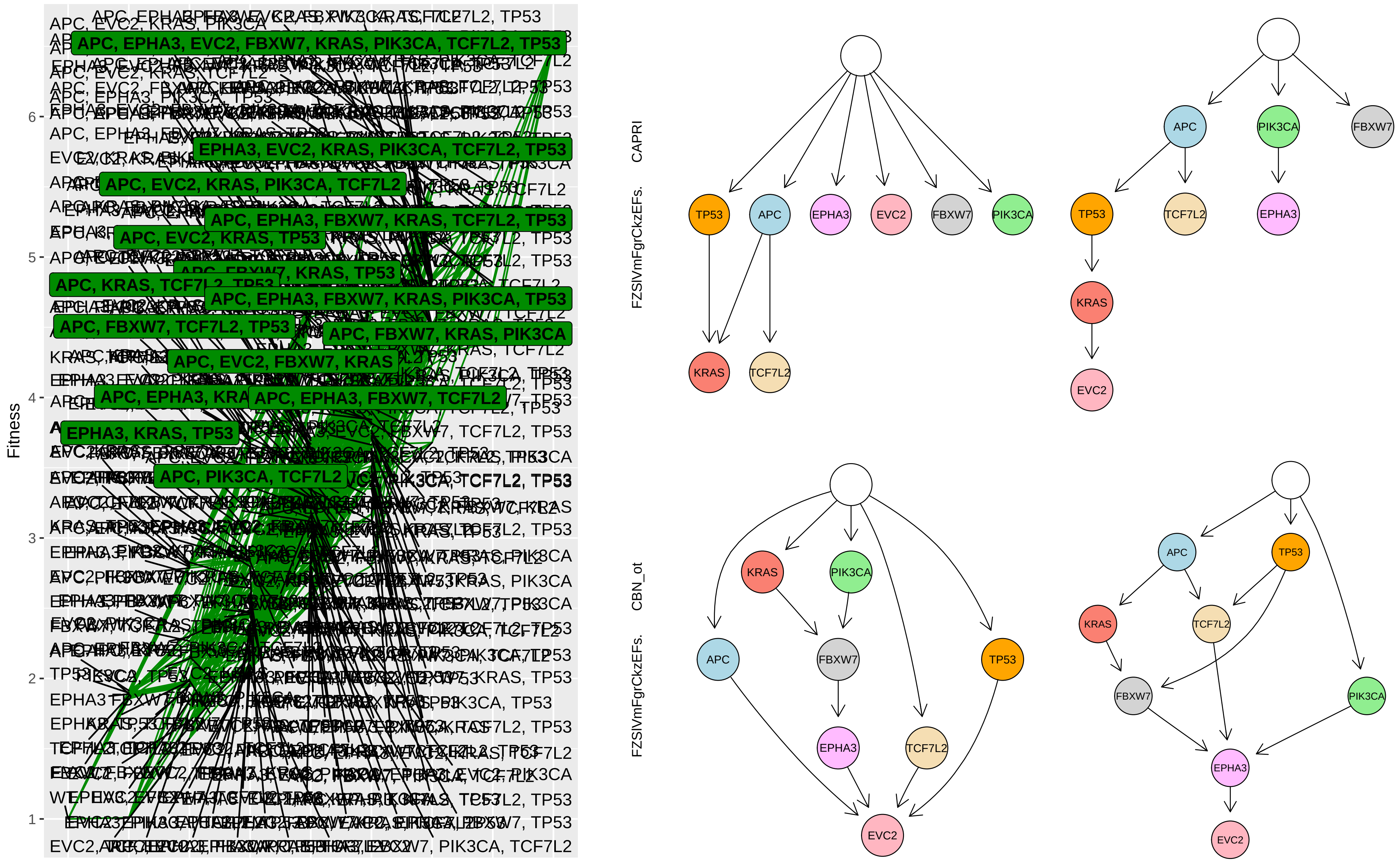

ID	p−value	Accessible Genot.
pSDswVPHQMGXCOn	0.782	31

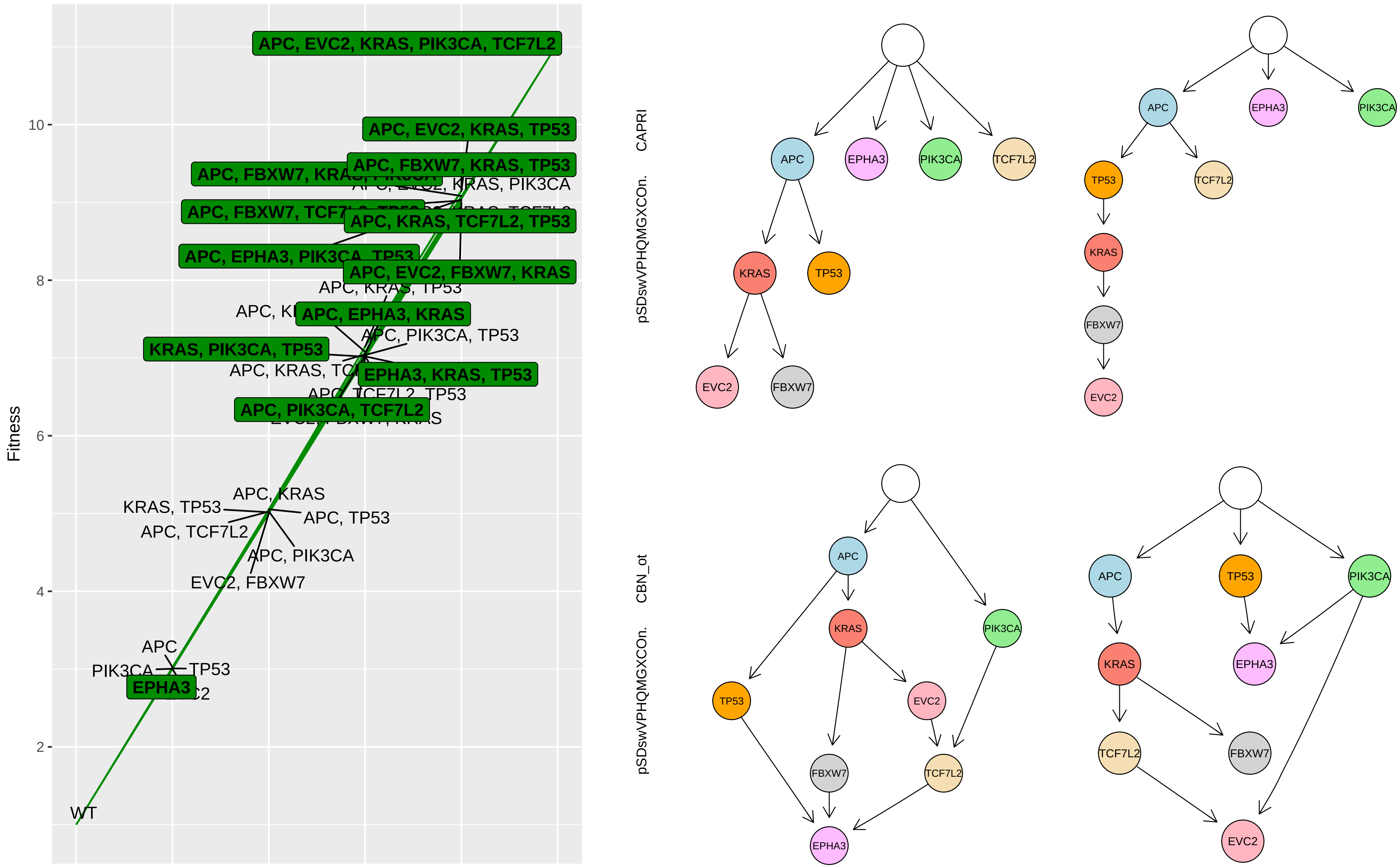

ID	p-value	Accessible Genot.
eYvlNZfAazKnSIz	0.782	39

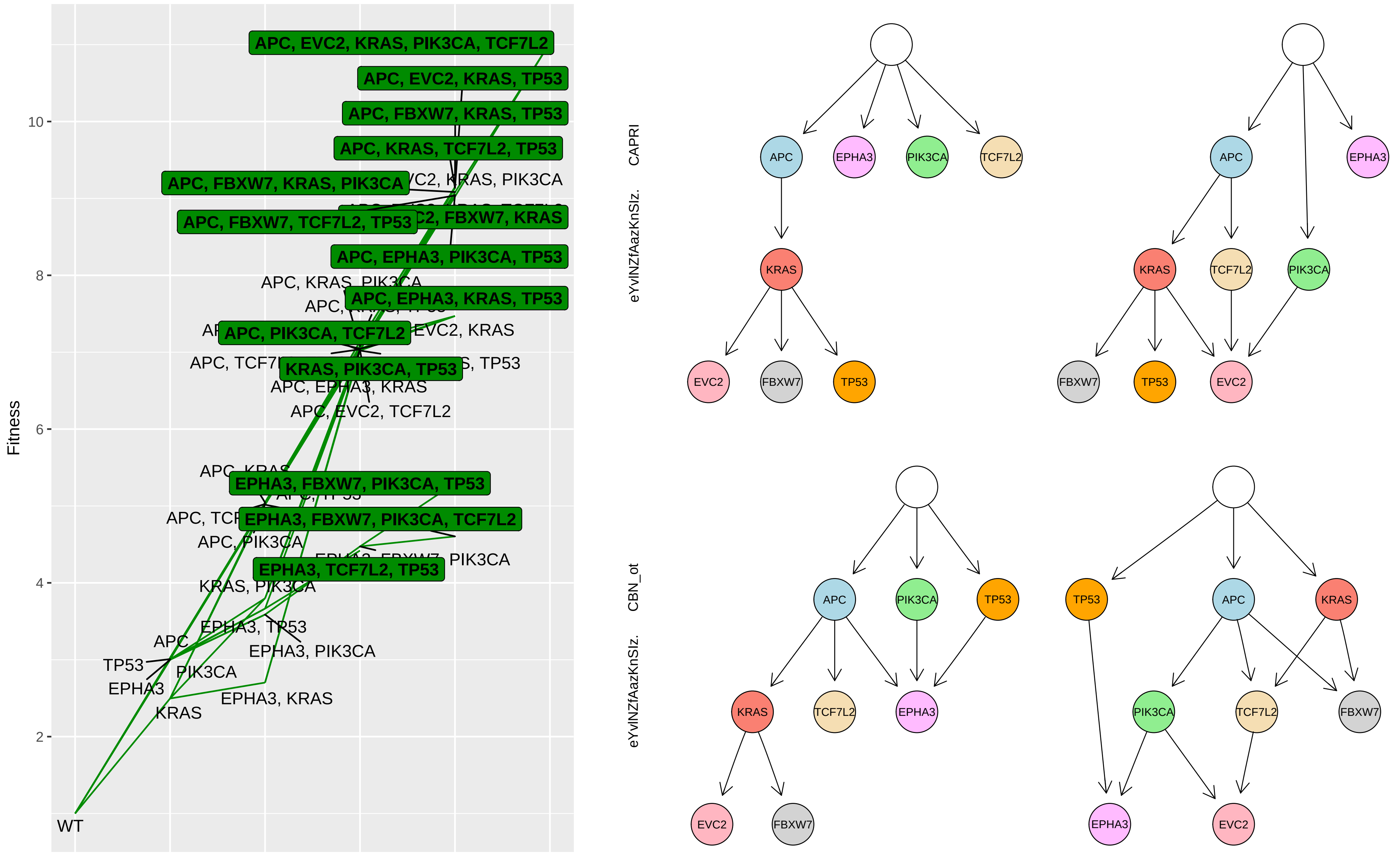

ID	p−value	Accessible Genot.
rgBThSlBMvPEVjN	0.787	126

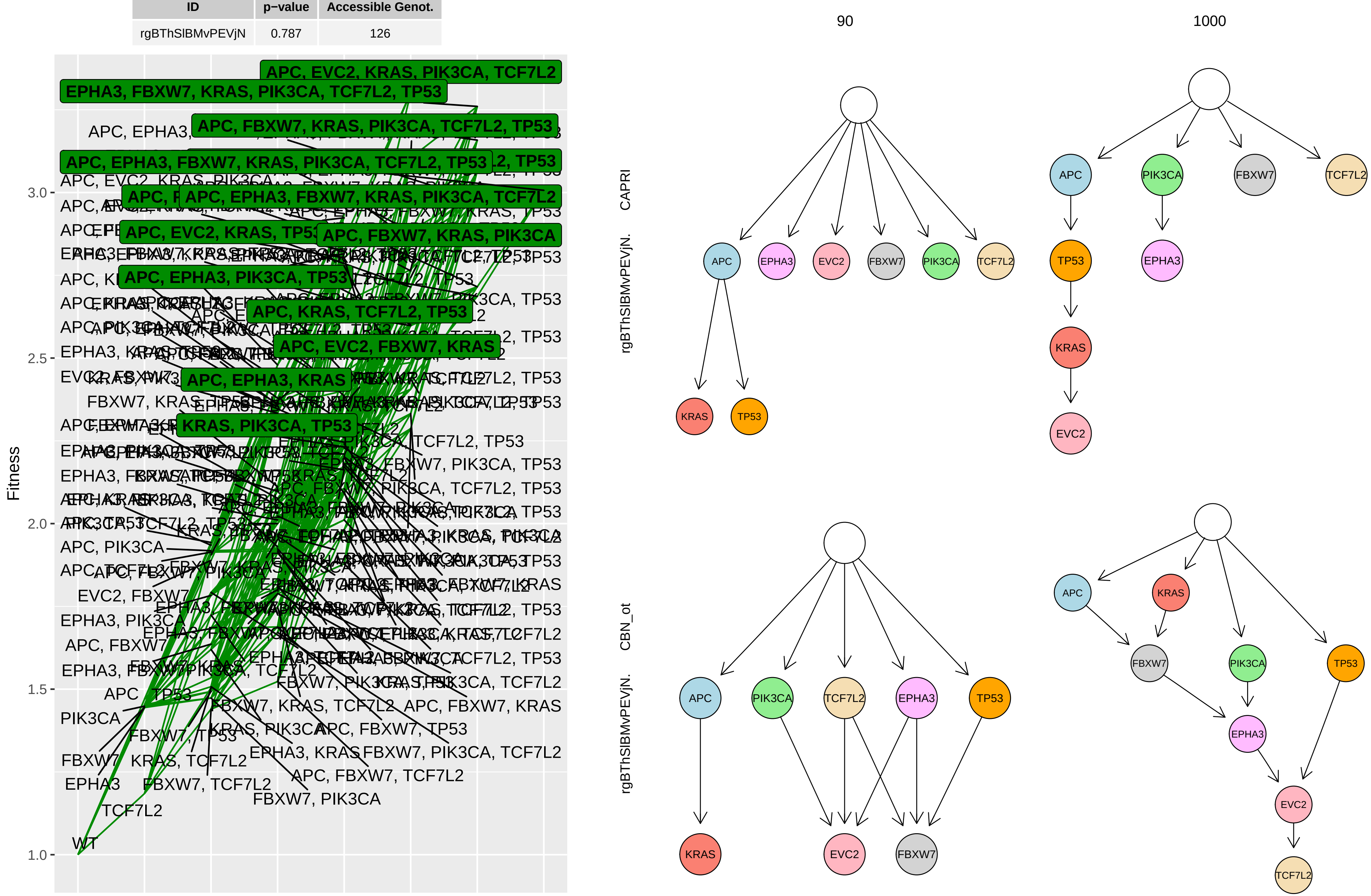

ID	p−value	Accessible Genot.
AvkobTIjBTPcKut	0.789	254

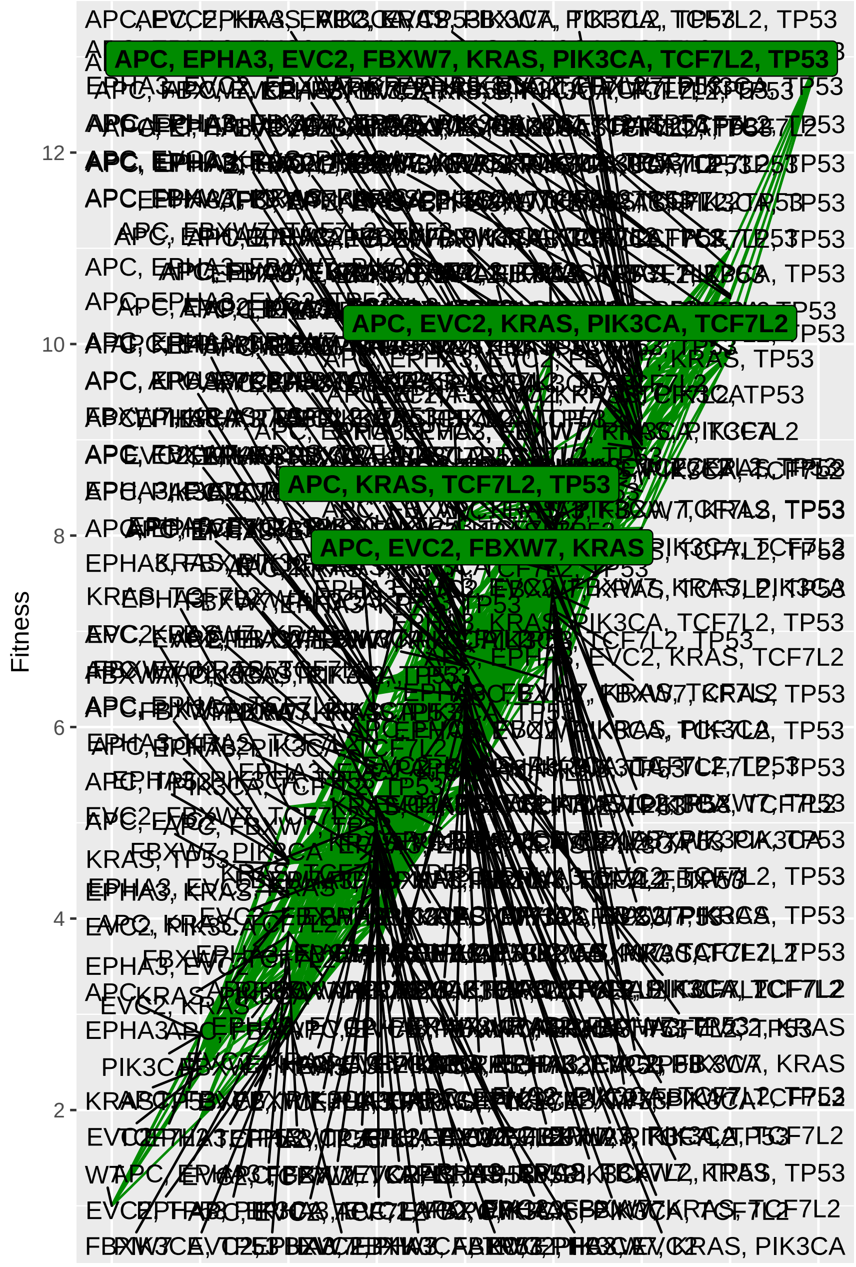

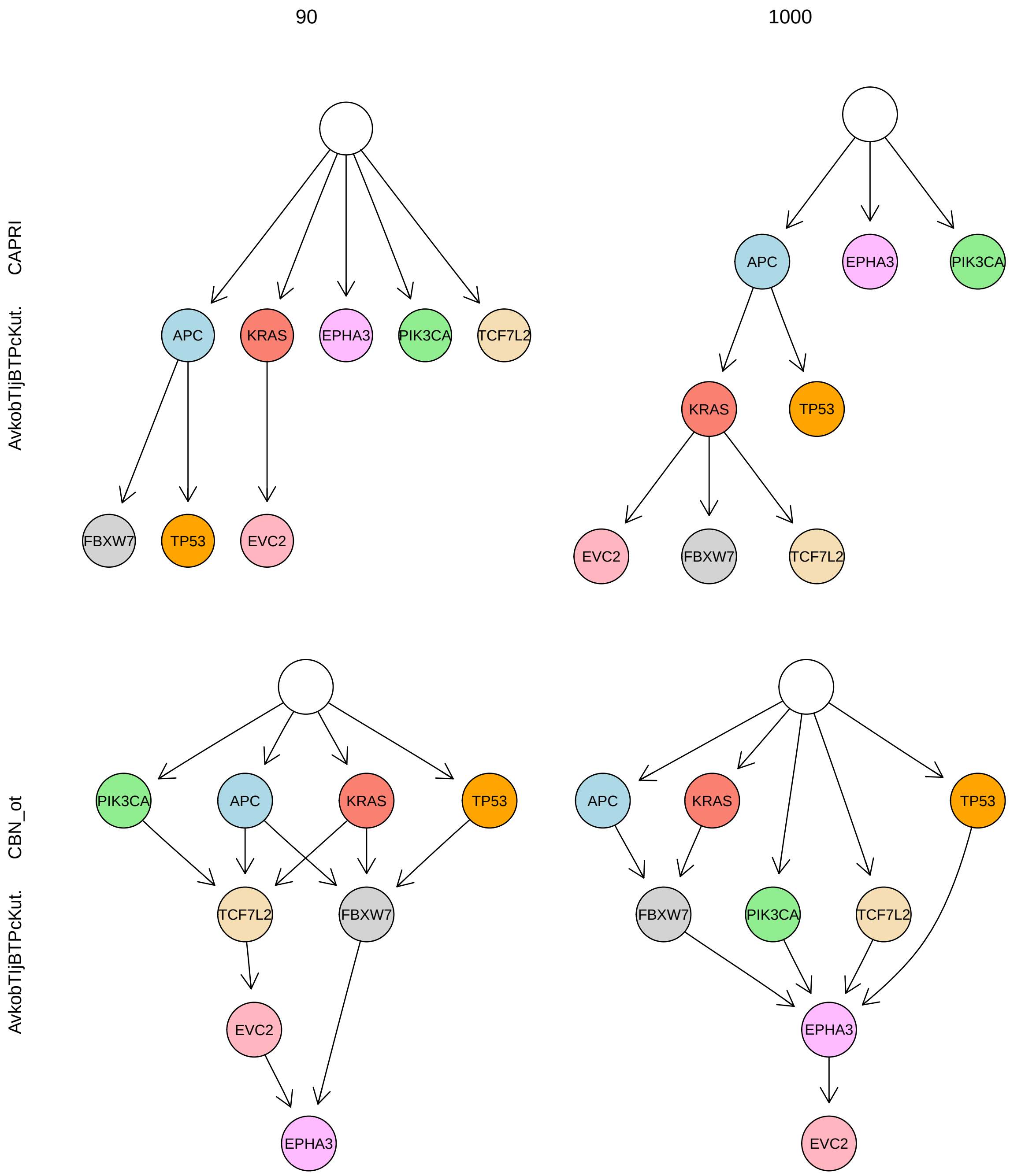

ID	p−value	Accessible Genot.
XExlqfStDqgRvmZ	0.79	42

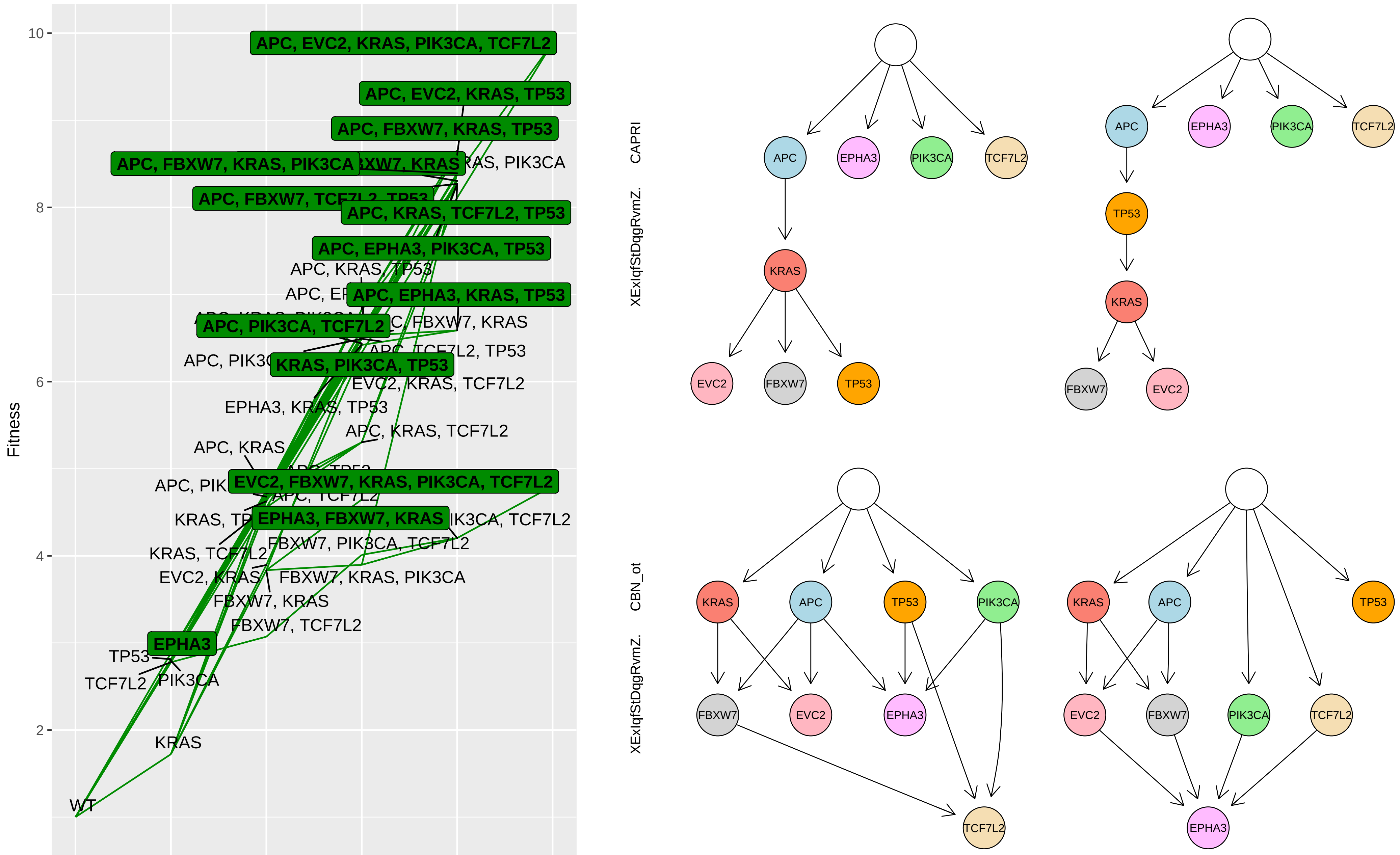

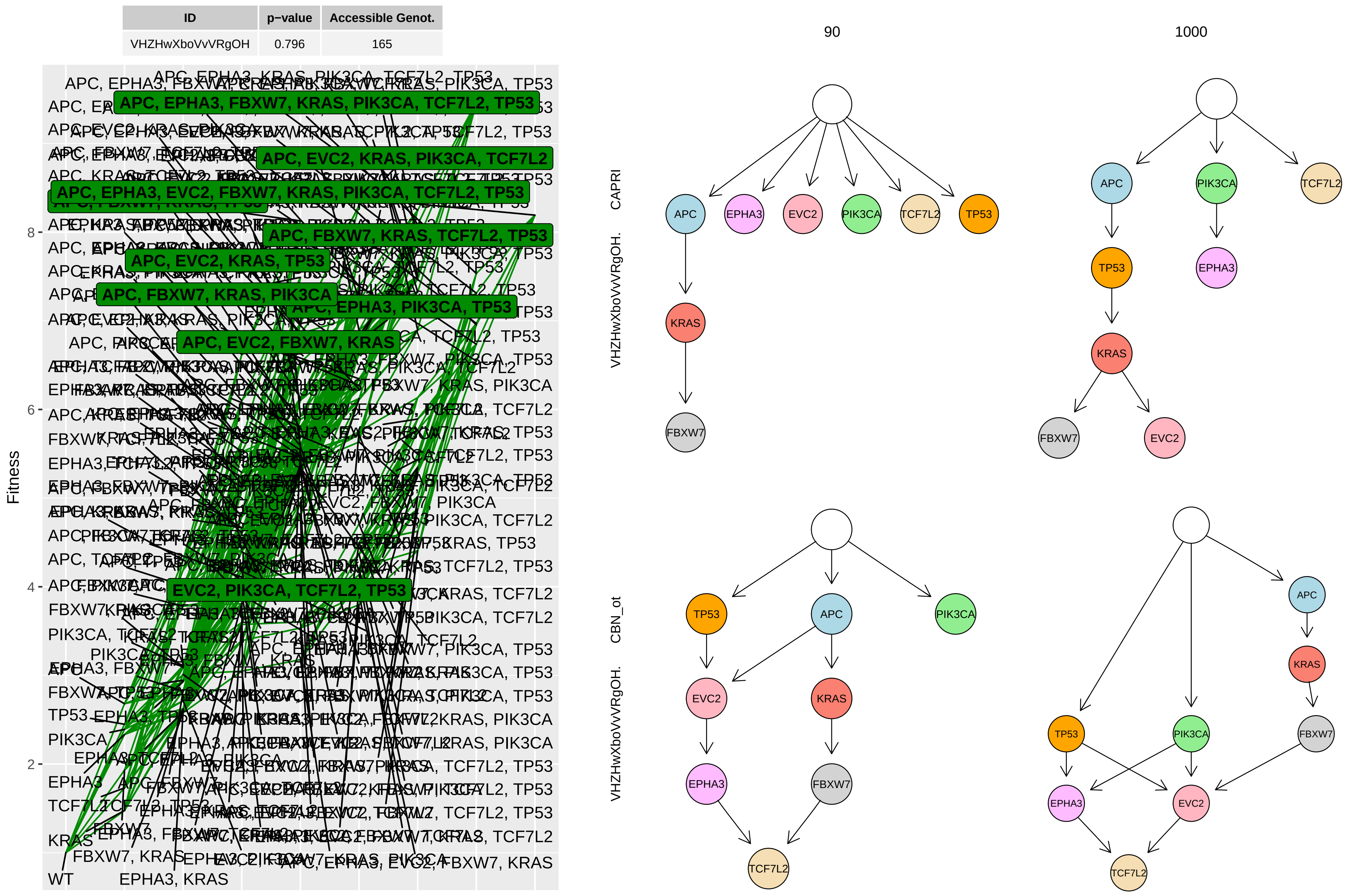

ID
p−value
Accessible Genot.
VHZHwXboVvVRgOH
0.796
165
Fitness
8
6
4
2
WT
APC, EPHA3, FBXW7, KRAS, PIK3CA, TCF7L2, TP53
APC, EVC2, KRAS, PIK3CA, TCF7L2
APC, EPHA3, EVC2, FBXW7, KRAS, PIK3CA, TCF7L2, TP53
APC, FBXW7, KRAS, TCF7L2, TP53
APC, EVC2, KRAS, TP53
APC, FBXW7, KRAS, PIK3CA
APC, EPHA3, PIK3CA, TP53
APC, EVC2, FBXW7, KRAS
EVC2, PIK3CA, TCF7L2, TP53
90
1000
CAPRI
CBN_ot
VHZHwXboVvVRgOH.
APC
EPHA3
EVC2
PIK3CA
TCF7L2
TP53
KRAS
FBXW7

ID	p-value	Accessible Genot.
hfwaiDocFTiRYyH	0.796	146

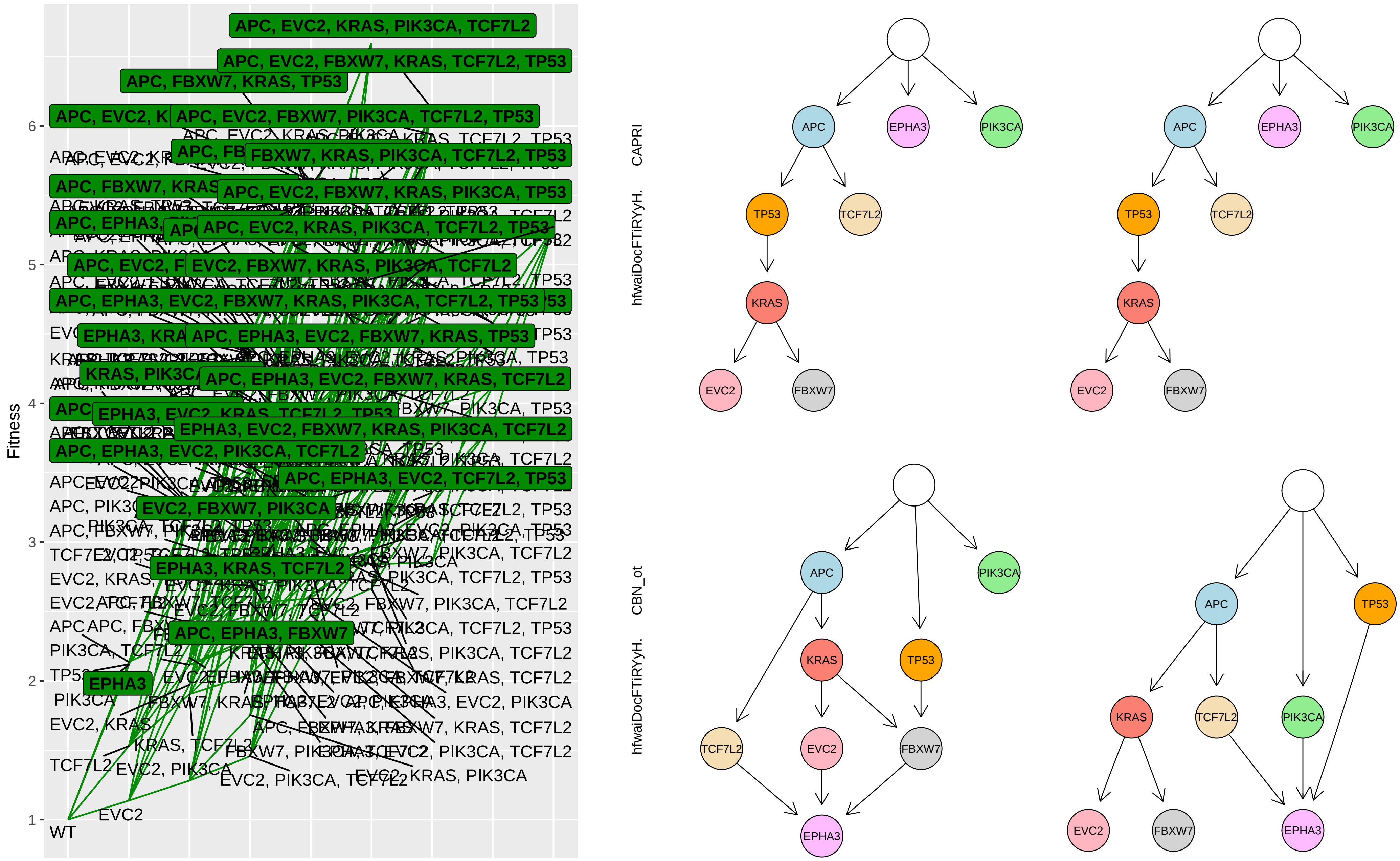

ID	p−value	Accessible Genot.
ZZSVNUuWxDqiEWO	0.799	30

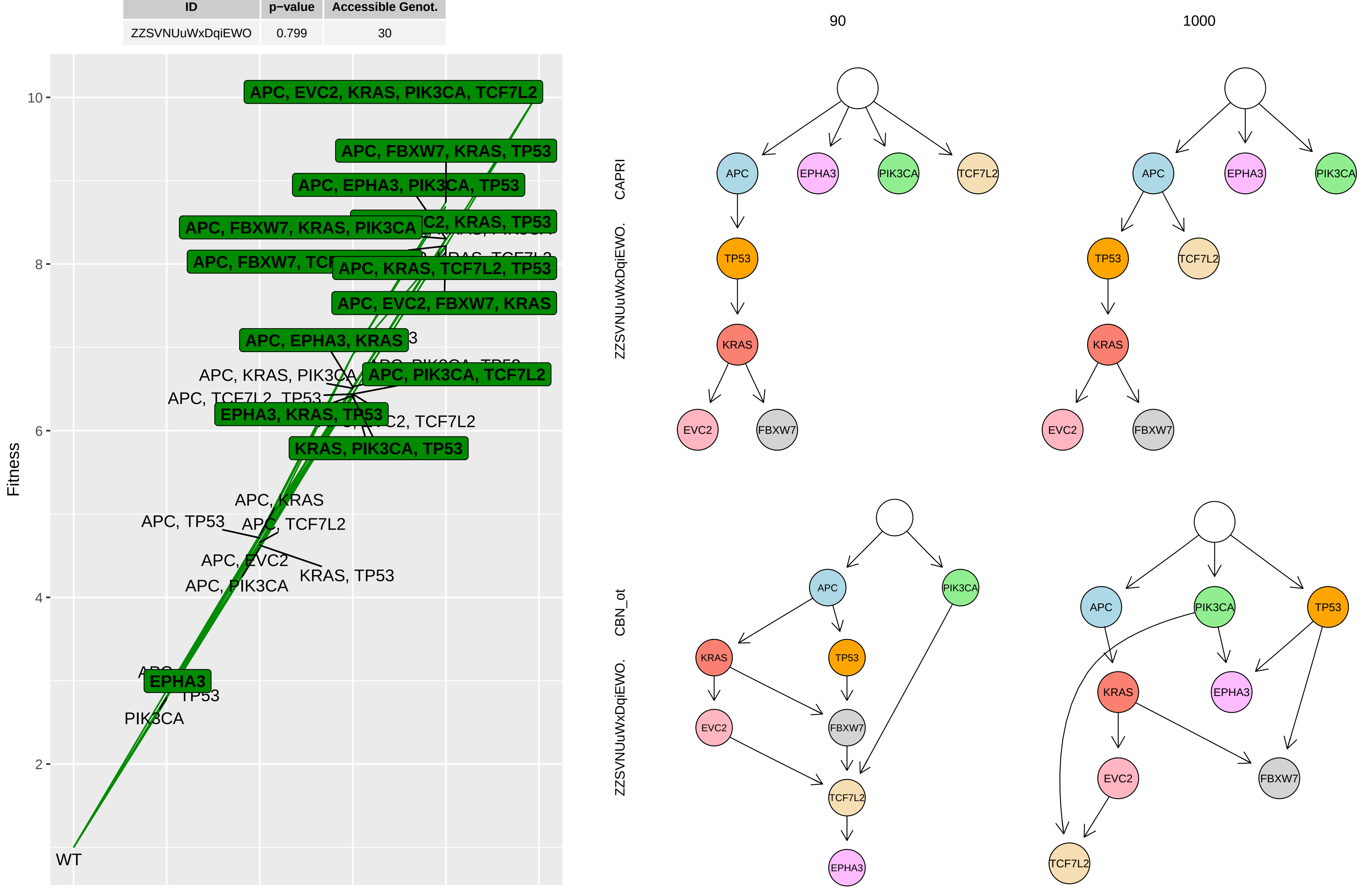

ID	p−value	Accessible Genot.
KnHozGkfcPqdZza	0.809	62

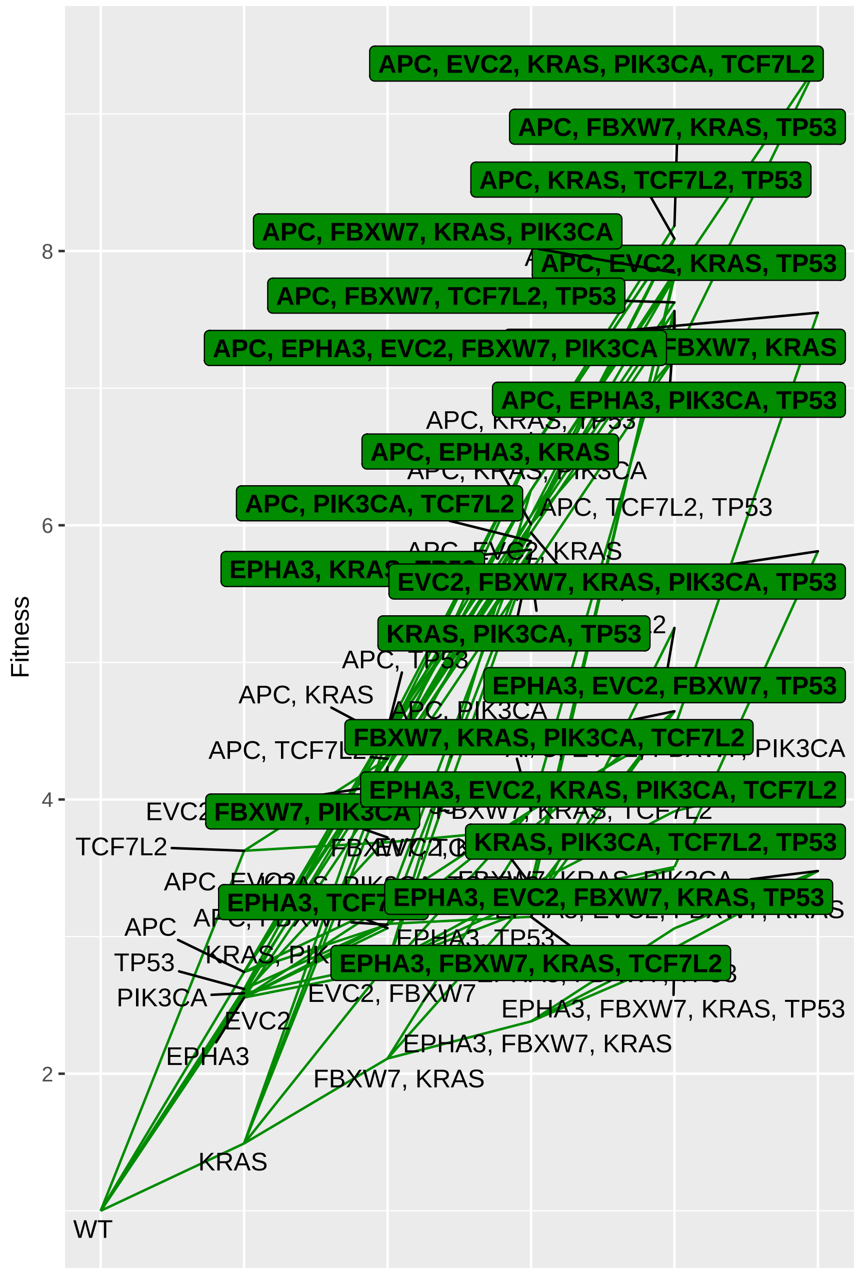

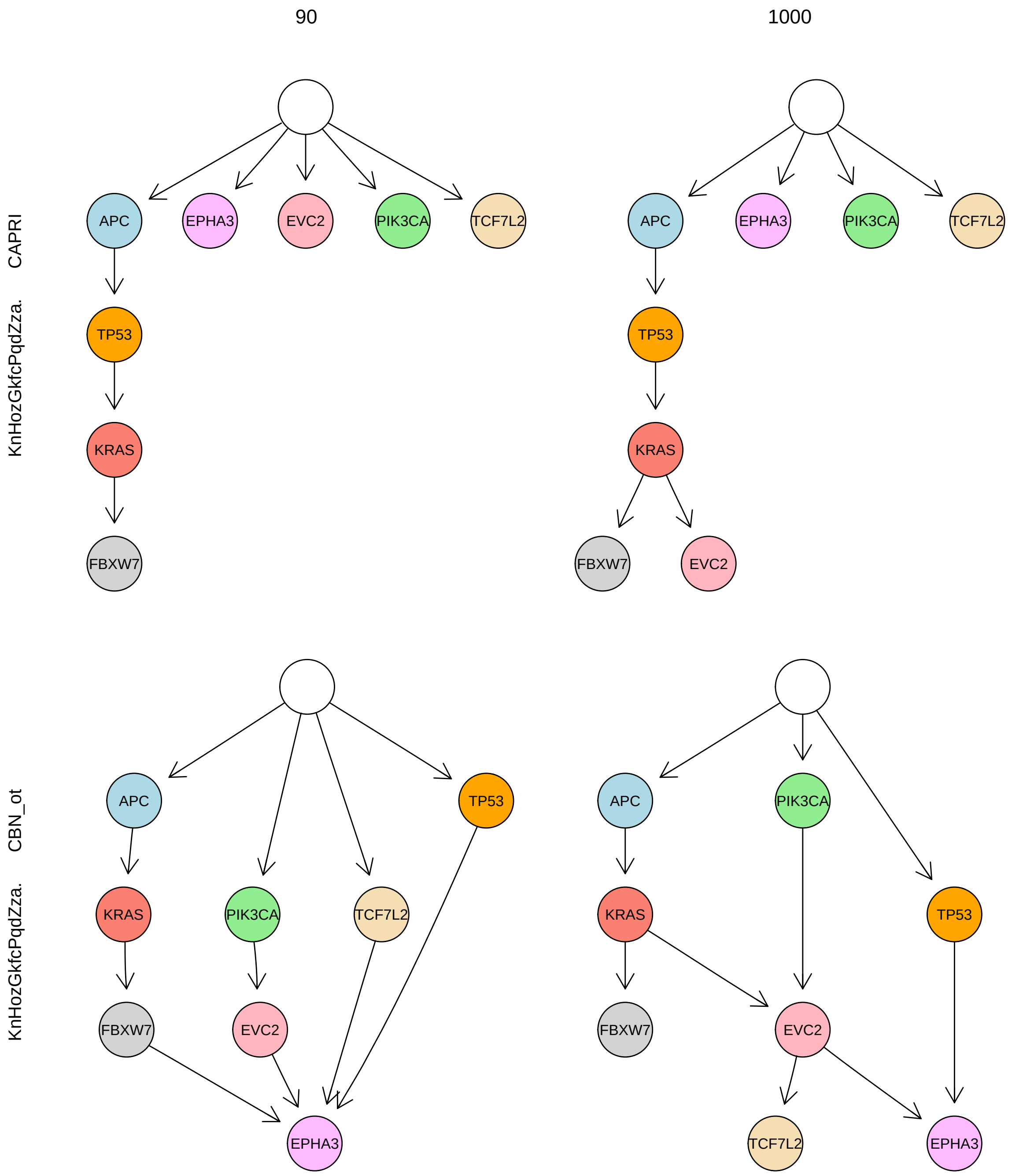

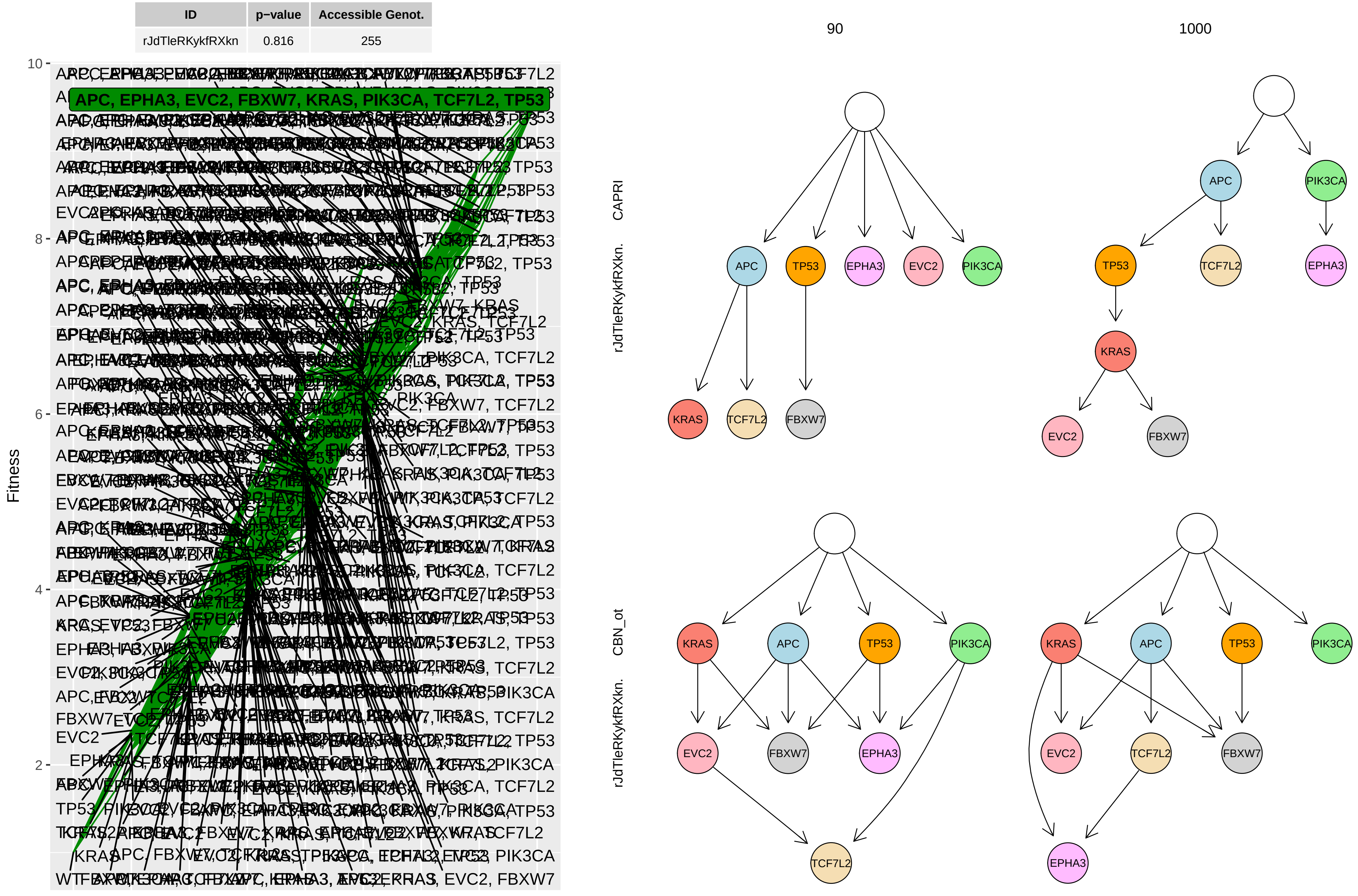

ID
p−value
Accessible Genot.
rJdTleRKykfRXkn
0.816
255
APC, EPHA3, EVC2, FBXW7, KRAS, PIK3CA, TCF7L2, TP53
Fitness
10
8
6
4
2
90
1000
CAPRI
CBN_ot
rJdTleRKykfRXkn.
APC
TP53
EPHA3
EVC2
PIK3CA
KRAS
TCF7L2
FBXW7

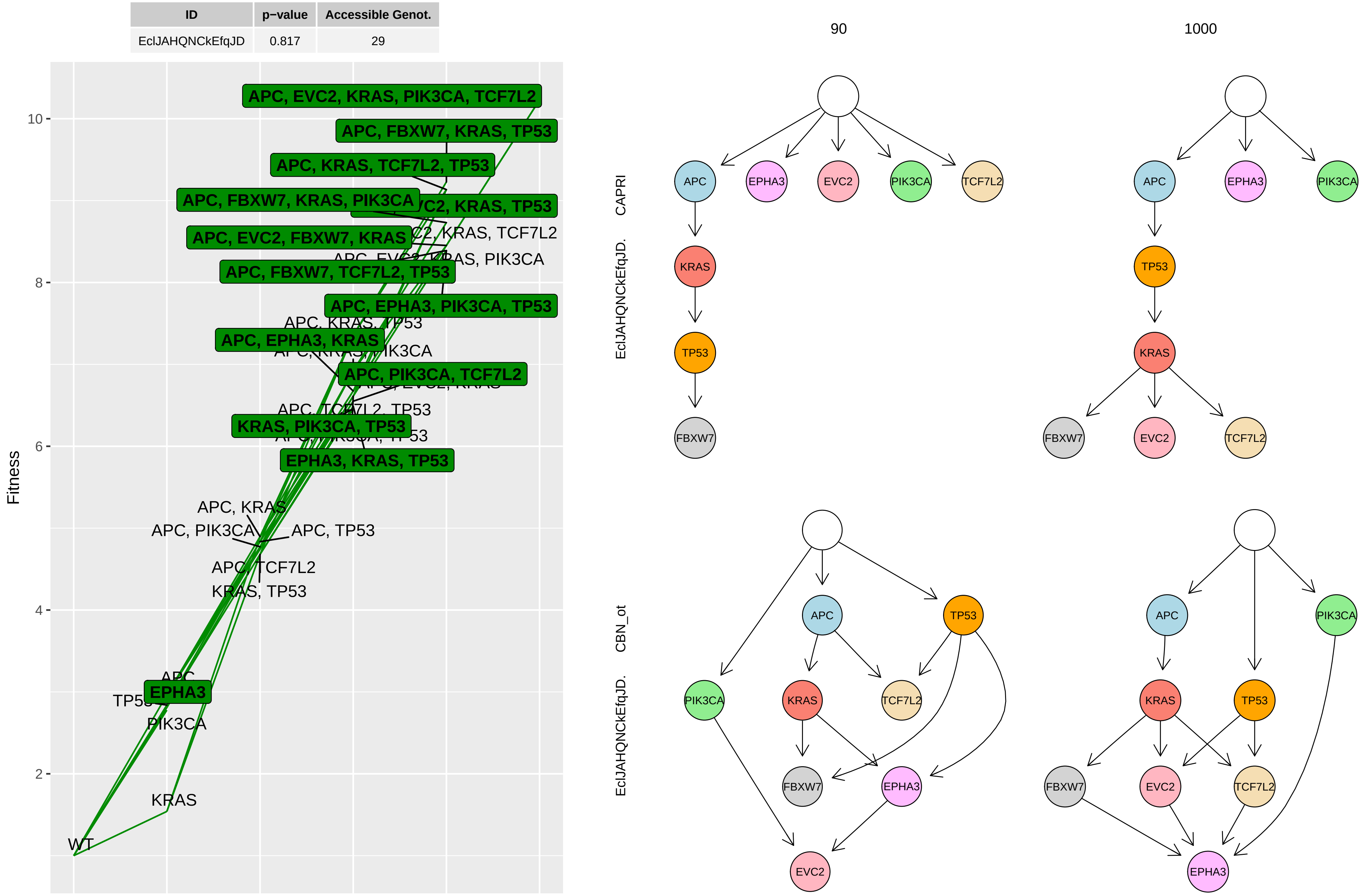

ID
p−value
Accessible Genot.
EclJAHQNCkEfqJD
0.817
29
Fitness
10
8
6
4
2
APC, EVC2, KRAS, PIK3CA, TCF7L2
APC, FBXW7, KRAS, TP53
APC, KRAS, TCF7L2, TP53
APC, FBXW7, KRAS, PIK3CA
APC, EVC2, FBXW7, KRAS
APC, FBXW7, TCF7L2, TP53
APC, EPHA3, PIK3CA, TP53
APC, KRAS, TP53
APC, EPHA3, KRAS
APC, PIK3CA, TCF7L2
APC, TCF7L2, TP53
KRAS, PIK3CA, TP53
EPHA3, KRAS, TP53
APC, KRAS
APC, PIK3CA
APC, TP53
APC, TCF7L2
KRAS, TP53
EPHA3
PIK3CA
KRAS
WT
90
1000
EclJAHQNCkEfqJD. CAPRI
EclJAHQNCkEfqJD. CBN_ot
APC
EPHA3
EVC2
PIK3CA
TCF7L2
KRAS
TP53
FBXW7

ID	p−value	Accessible Genot.
tTsybCMjXGOBVQZ	0.821	31

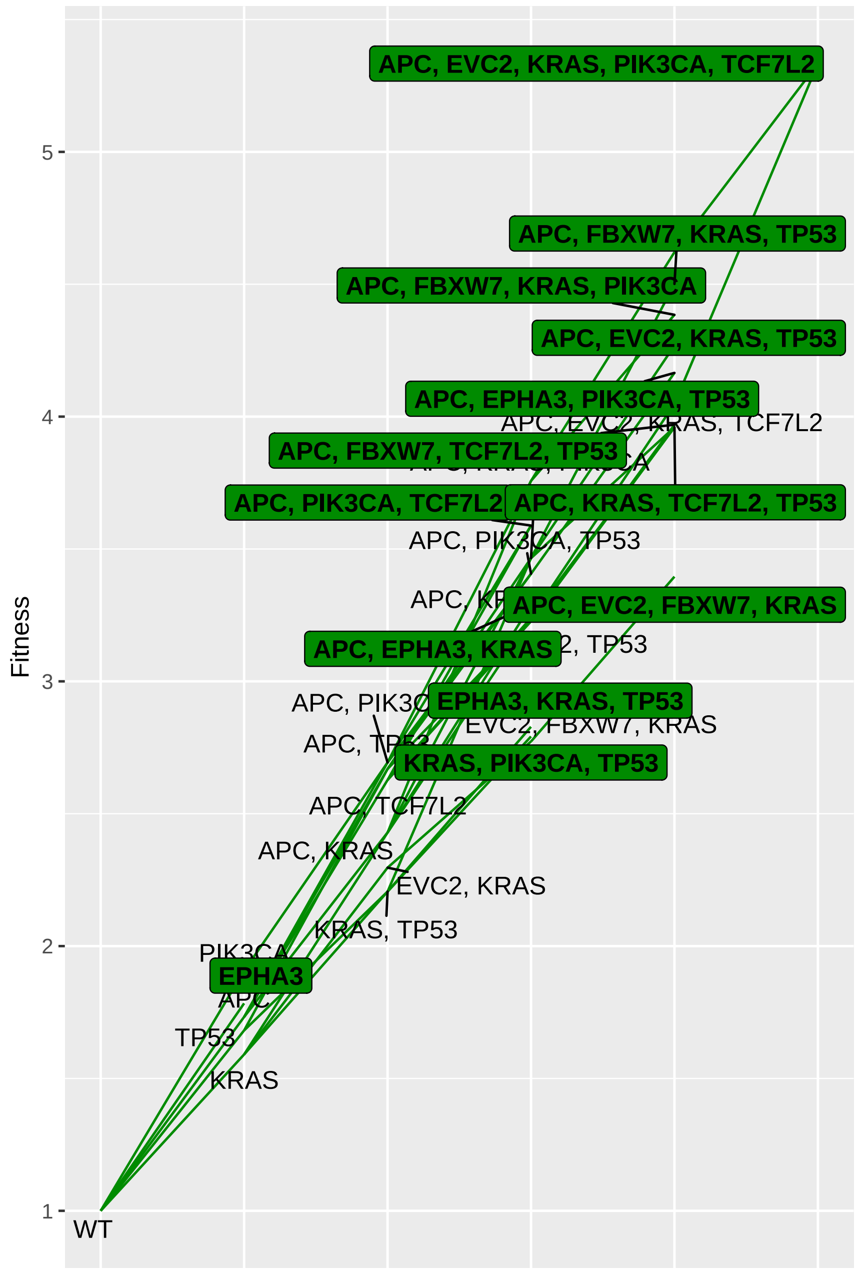

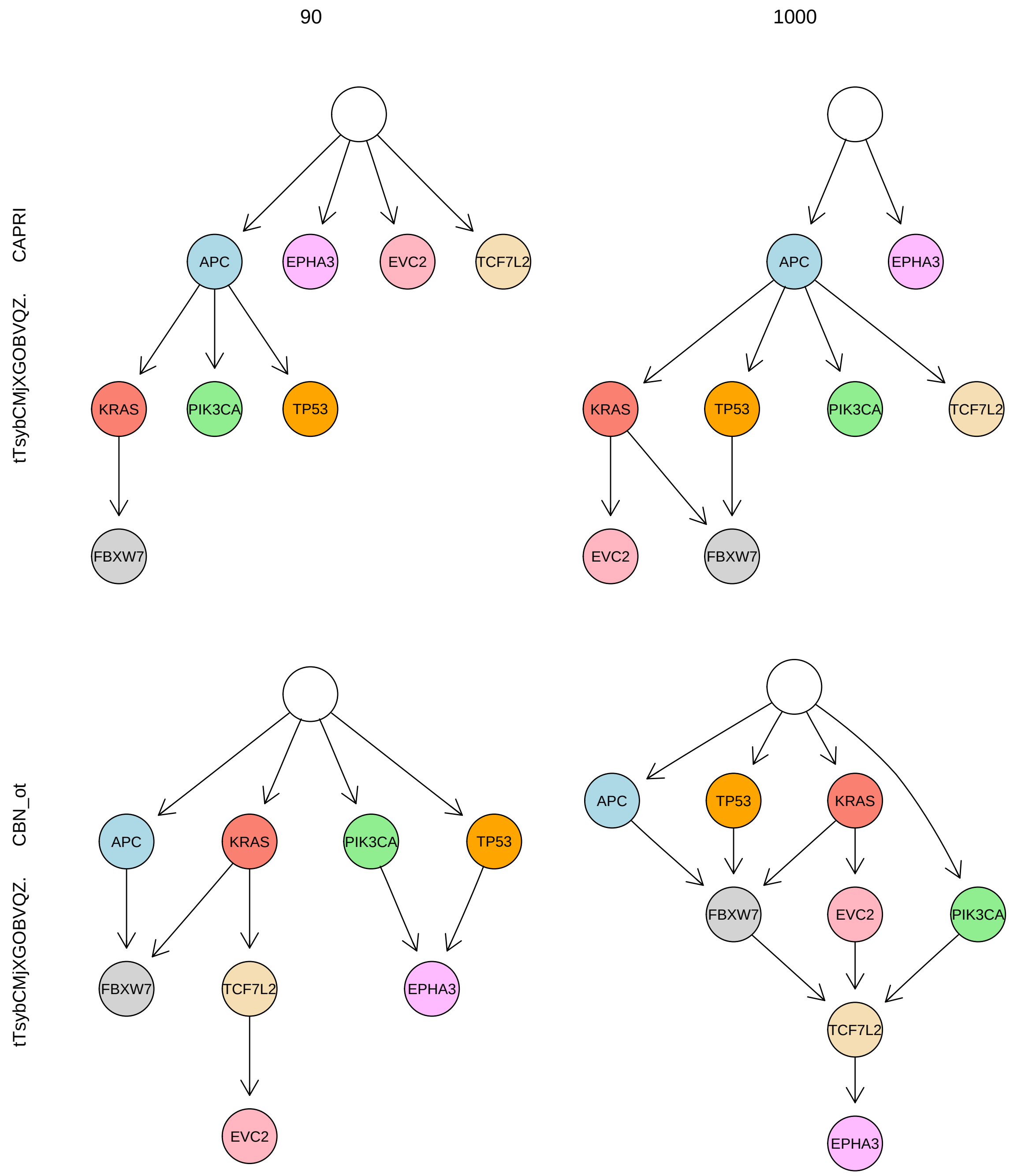

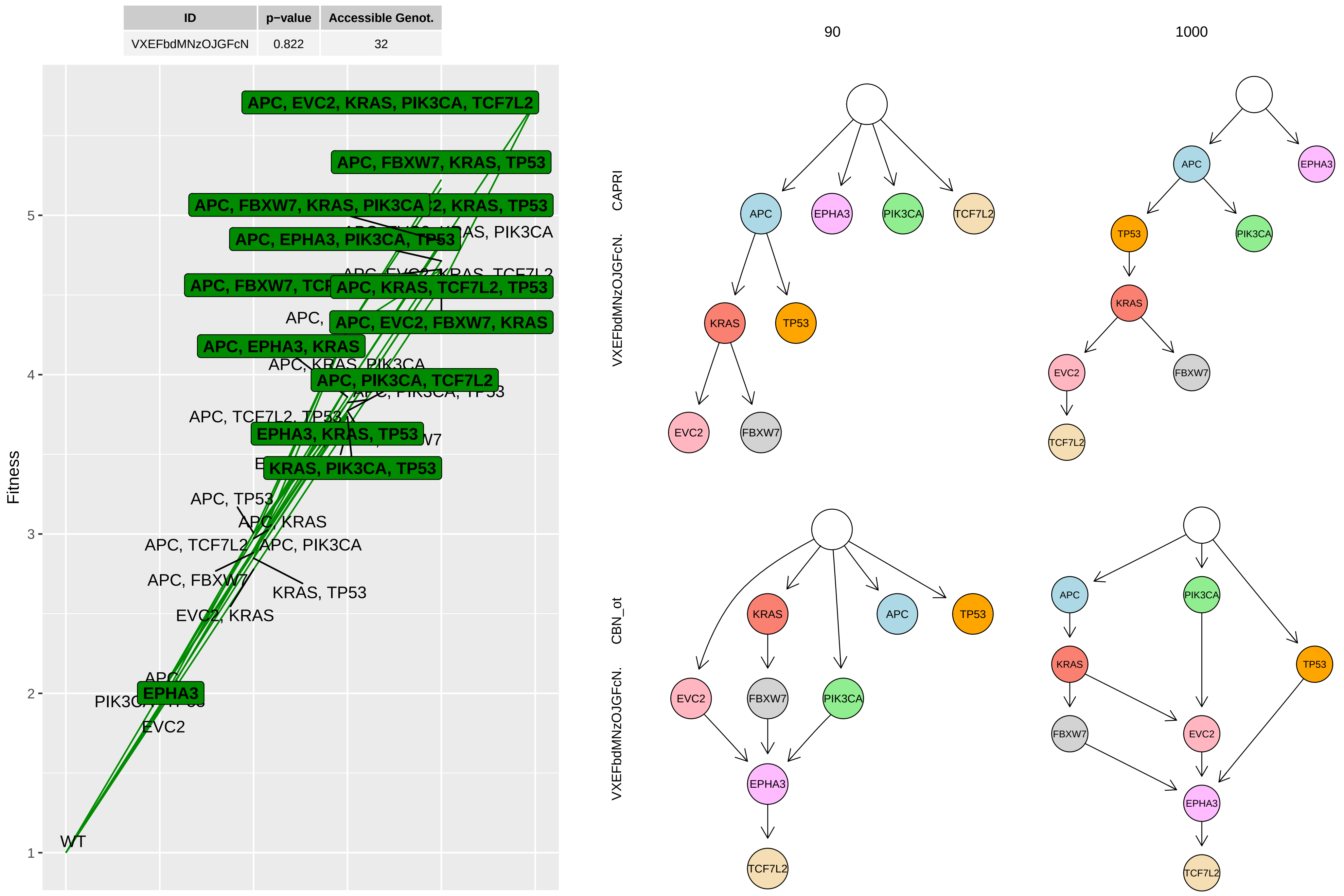

ID
p−value
Accessible Genot.
VXEFbdMNzOJGFcN
0.822
32
Fitness
1
2
3
4
5
WT
EVC2
EPHA3
APC, EVC2, KRAS, PIK3CA, TCF7L2
APC, FBXW7, KRAS, TP53
APC, FBXW7, KRAS, PIK3CA
APC, EPHA3, PIK3CA, TP53
APC, KRAS, TCF7L2, TP53
APC, EVC2, FBXW7, KRAS
APC, EPHA3, KRAS
APC, KRAS, PIK3CA
APC, PIK3CA, TCF7L2
APC, TCF7L2, TP53
EPHA3, KRAS, TP53
KRAS, PIK3CA, TP53
APC, TP53
APC, KRAS
APC, TCF7L2
APC, PIK3CA
APC, FBXW7
KRAS, TP53
EVC2, KRAS
90
1000
CAPRI
CBN_ot
VXEFbdMNzOJGFcN.
APC
EPHA3
PIK3CA
TCF7L2
KRAS
TP53
EVC2
FBXW7

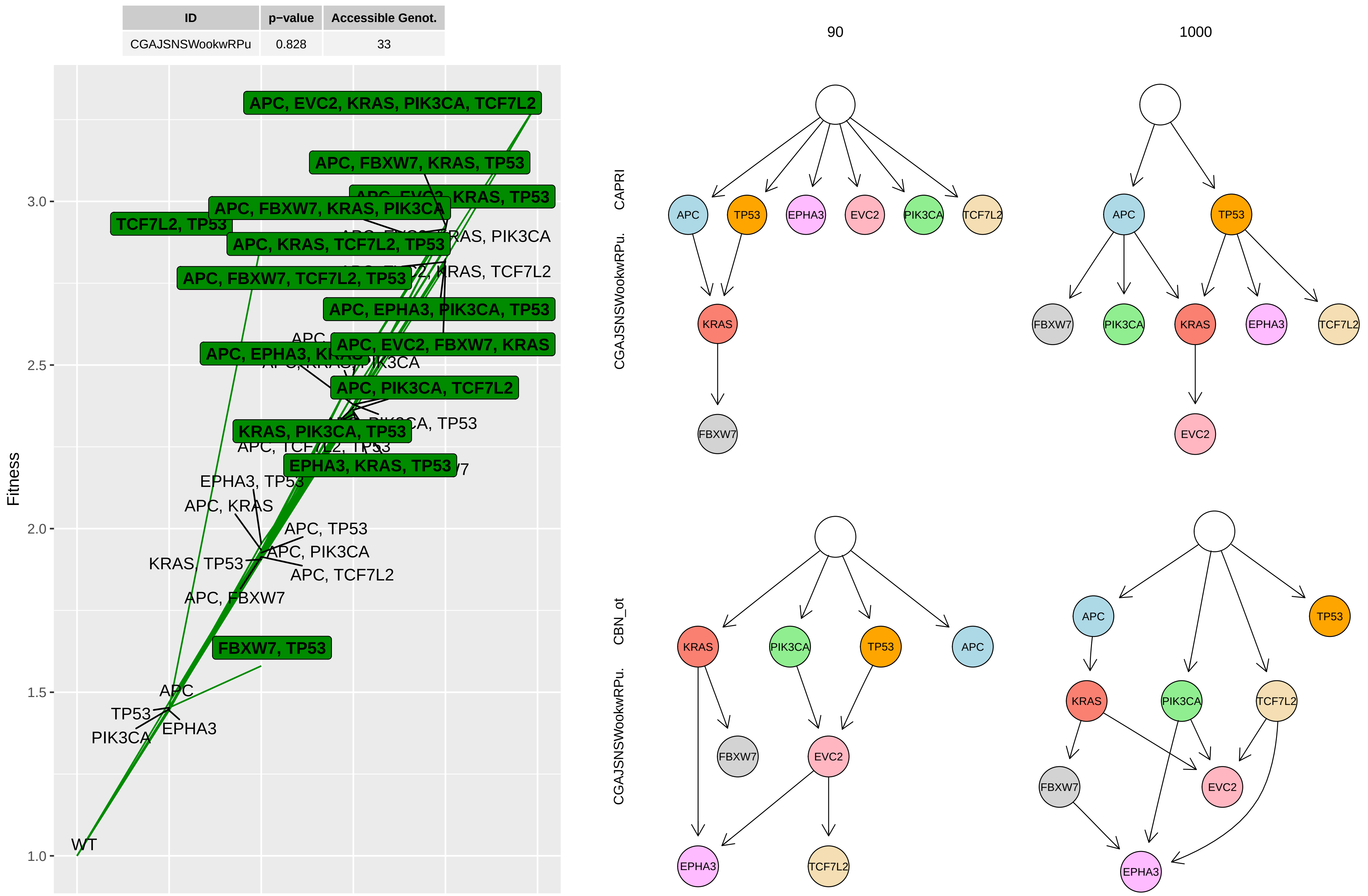

ID
p−value
Accessible Genot.
CGAJSNSWookwRPu
0.828
33
Fitness
3.0
2.5
2.0
1.5
1.0
APC, EVC2, KRAS, PIK3CA, TCF7L2
APC, FBXW7, KRAS, TP53
APC, FBXW7, KRAS, PIK3CA
TCF7L2, TP53
APC, KRAS, TCF7L2, TP53
APC, FBXW7, TCF7L2, TP53
APC, EPHA3, PIK3CA, TP53
APC, EVC2, FBXW7, KRAS
APC, PIK3CA, TCF7L2
KRAS, PIK3CA, TP53
EPHA3, KRAS, TP53
EPHA3, TP53
APC, KRAS
APC, TP53
APC, PIK3CA
KRAS, TP53
APC, TCF7L2
APC, FBXW7
FBXW7, TP53
APC
TP53
PIK3CA
EPHA3
WT
90
1000
CAPRI
CGAJSNSWookwRPu.
CBN_ot
CGAJSNSWookwRPu.
APC
TP53
EPHA3
EVC2
PIK3CA
TCF7L2
KRAS
FBXW7

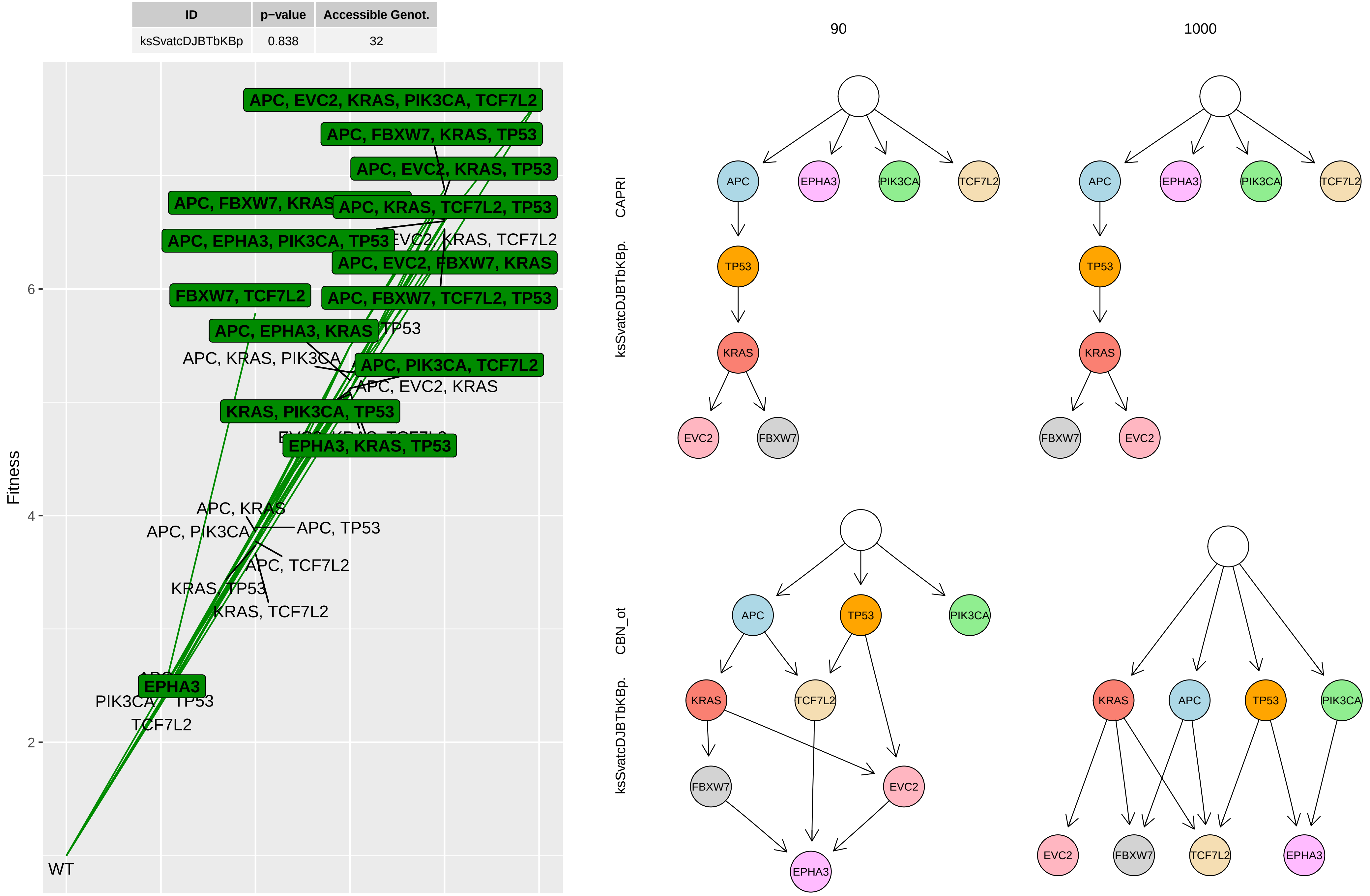
ID
p−value
Accessible Genot.
ksSvatcDJBTbKBp
0.838
32
Fitness
APC, EVC2, KRAS, PIK3CA, TCF7L2
APC, FBXW7, KRAS, TP53
APC, EVC2, KRAS, TP53
APC, FBXW7, KRAS
APC, KRAS, TCF7L2, TP53
APC, EPHA3, PIK3CA, TP53
EVC2, KRAS, TCF7L2
APC, EVC2, FBXW7, KRAS
FBXW7, TCF7L2
APC, FBXW7, TCF7L2, TP53
APC, EPHA3, KRAS
TP53
APC, KRAS, PIK3CA
APC, PIK3CA, TCF7L2
APC, EVC2, KRAS
KRAS, PIK3CA, TP53
EPHA3, KRAS, TP53
APC, KRAS
APC, PIK3CA
APC, TP53
APC, TCF7L2
KRAS, TP53
KRAS, TCF7L2
EPHA3
PIK3CA
TP53
TCF7L2
WT
6
4
2
90
1000
CAPRI
CBN_ot
ksSvatcDJBTbKBp.
APC
EPHA3
PIK3CA
TCF7L2
TP53
KRAS
EVC2
FBXW7

ID	p−value	Accessible Genot.
PKxBFVlKNHZrXjL	0.838	255

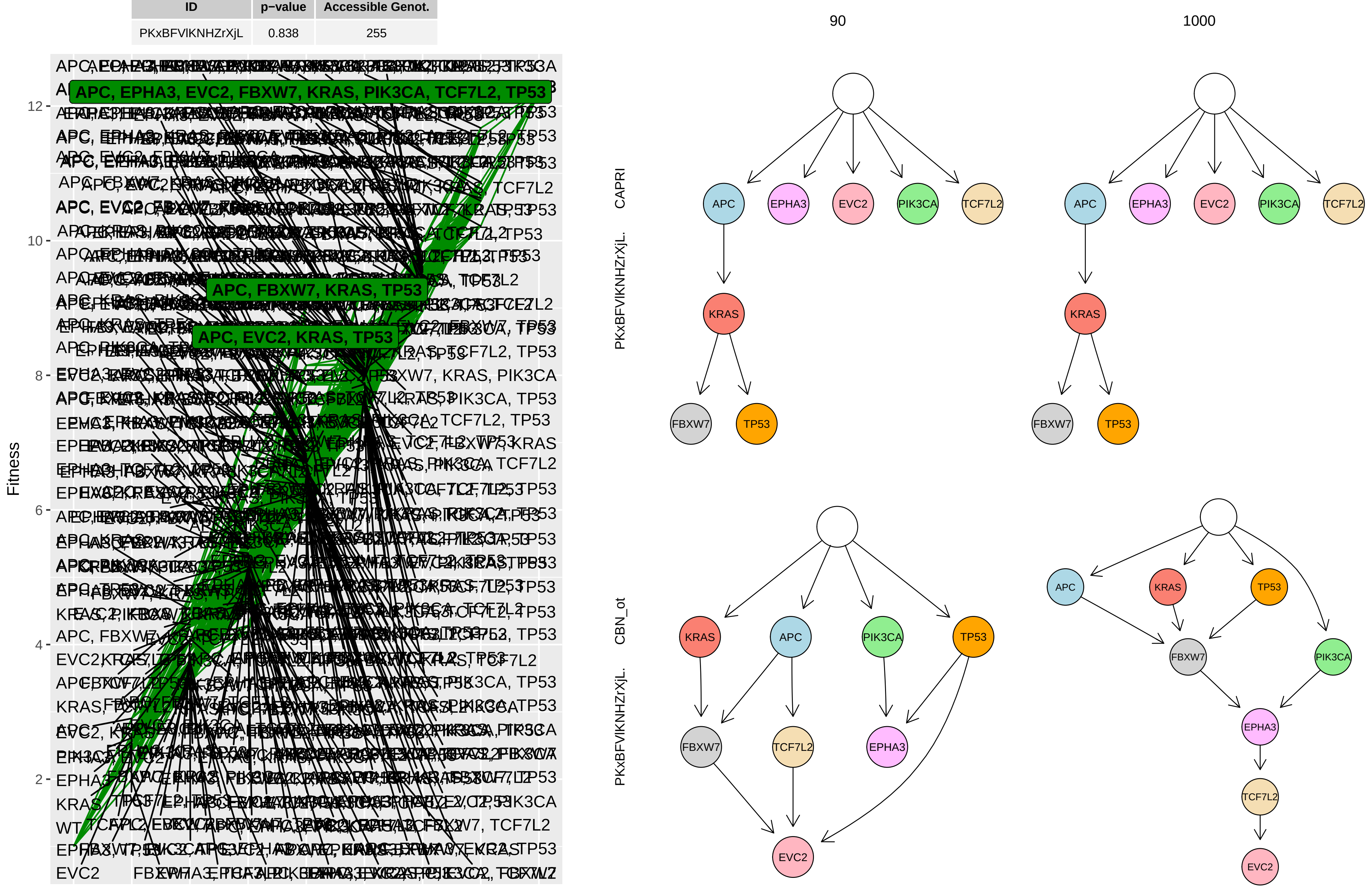

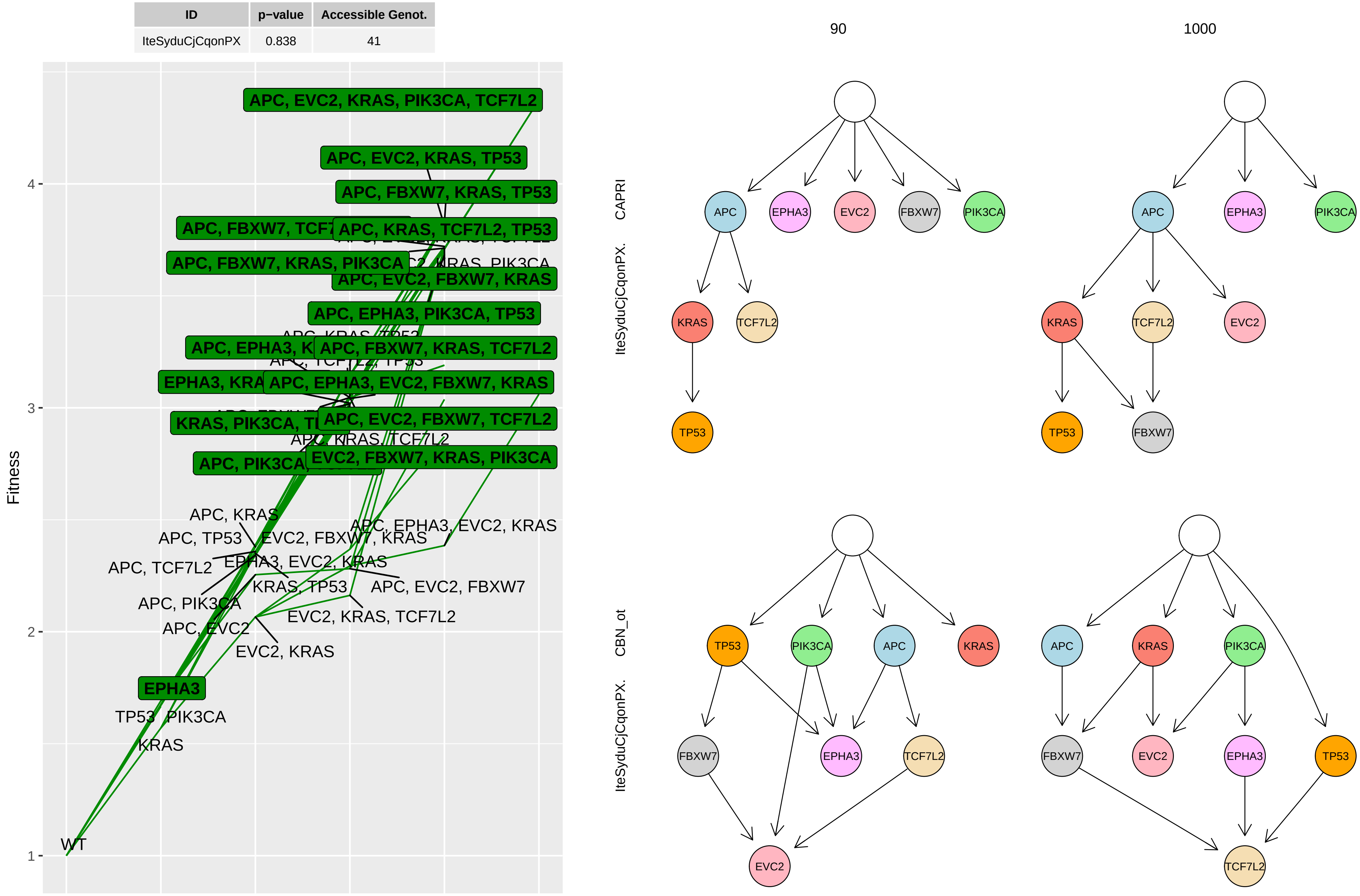

ID
p−value
Accessible Genot.
IteSyduCjCqonPX
0.838
41
Fitness
4
3
2
1
APC, EVC2, KRAS, PIK3CA, TCF7L2
APC, EVC2, KRAS, TP53
APC, FBXW7, KRAS, TP53
APC, KRAS, TCF7L2, TP53
APC, FBXW7, KRAS, PIK3CA
APC, EVC2, FBXW7, KRAS
APC, EPHA3, PIK3CA, TP53
APC, FBXW7, KRAS, TCF7L2
APC, EPHA3, EVC2, FBXW7, KRAS
APC, EVC2, FBXW7, TCF7L2
APC, KRAS, TCF7L2
EVC2, FBXW7, KRAS, PIK3CA
APC, KRAS
APC, TP53
EVC2, FBXW7, KRAS
APC, EPHA3, EVC2, KRAS
APC, TCF7L2
EPHA3, EVC2, KRAS
KRAS, TP53
APC, EVC2, FBXW7
APC, PIK3CA
APC, EVC2
EVC2, KRAS, TCF7L2
EVC2, KRAS
EPHA3
TP53
PIK3CA
KRAS
WT
90
1000
IteSyduCjCqonPX. CAPRI
IteSyduCjCqonPX. CBN_ot
APC
EPHA3
EVC2
FBXW7
PIK3CA
KRAS
TCF7L2
TP53

ID	p−value	Accessible Genot.
XOCPeJmmBdzZKaQ	0.84	34

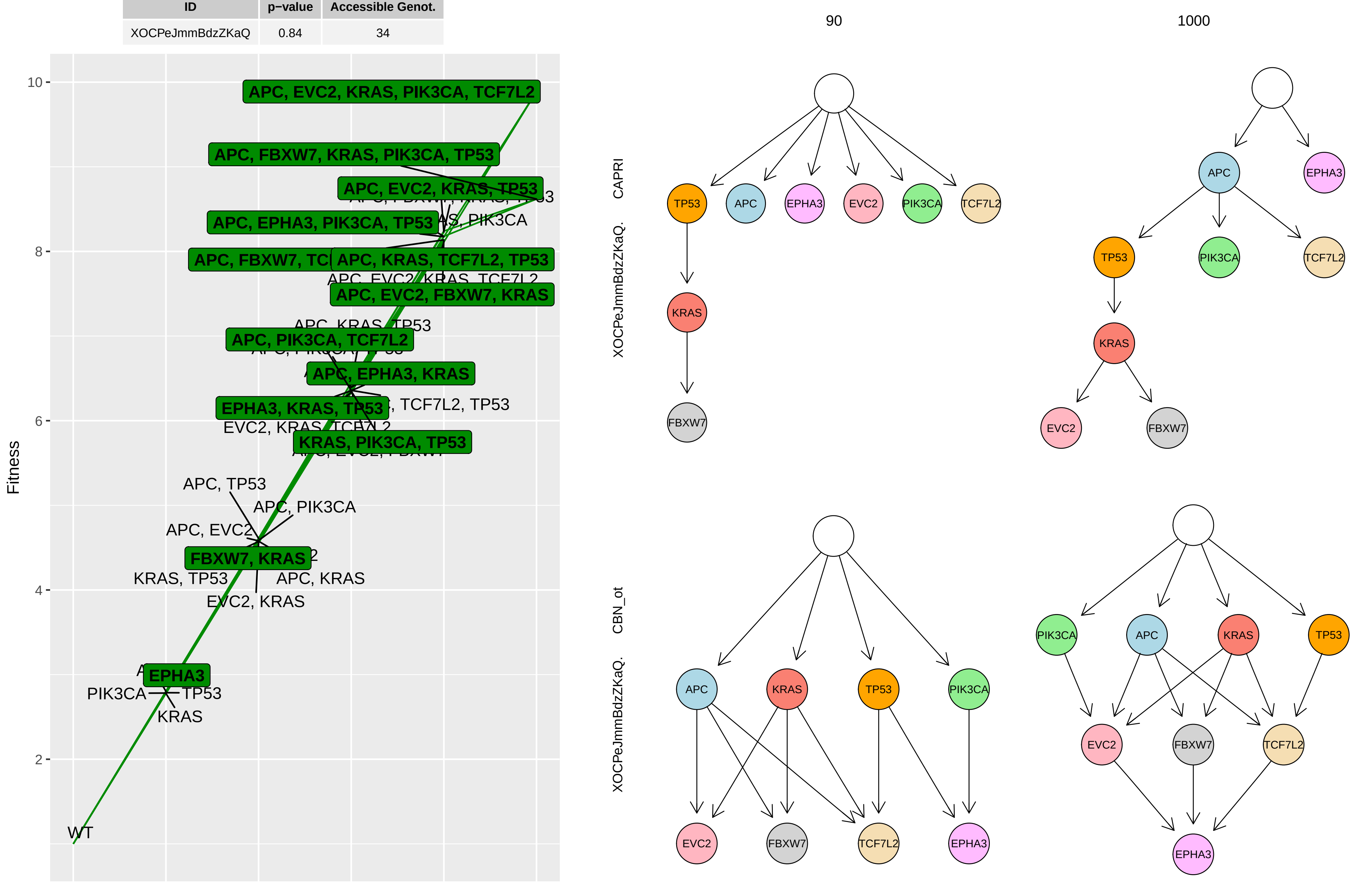

ID	p−value	Accessible Genot.
VfKtDIMdIqIgEEi	0.841	254

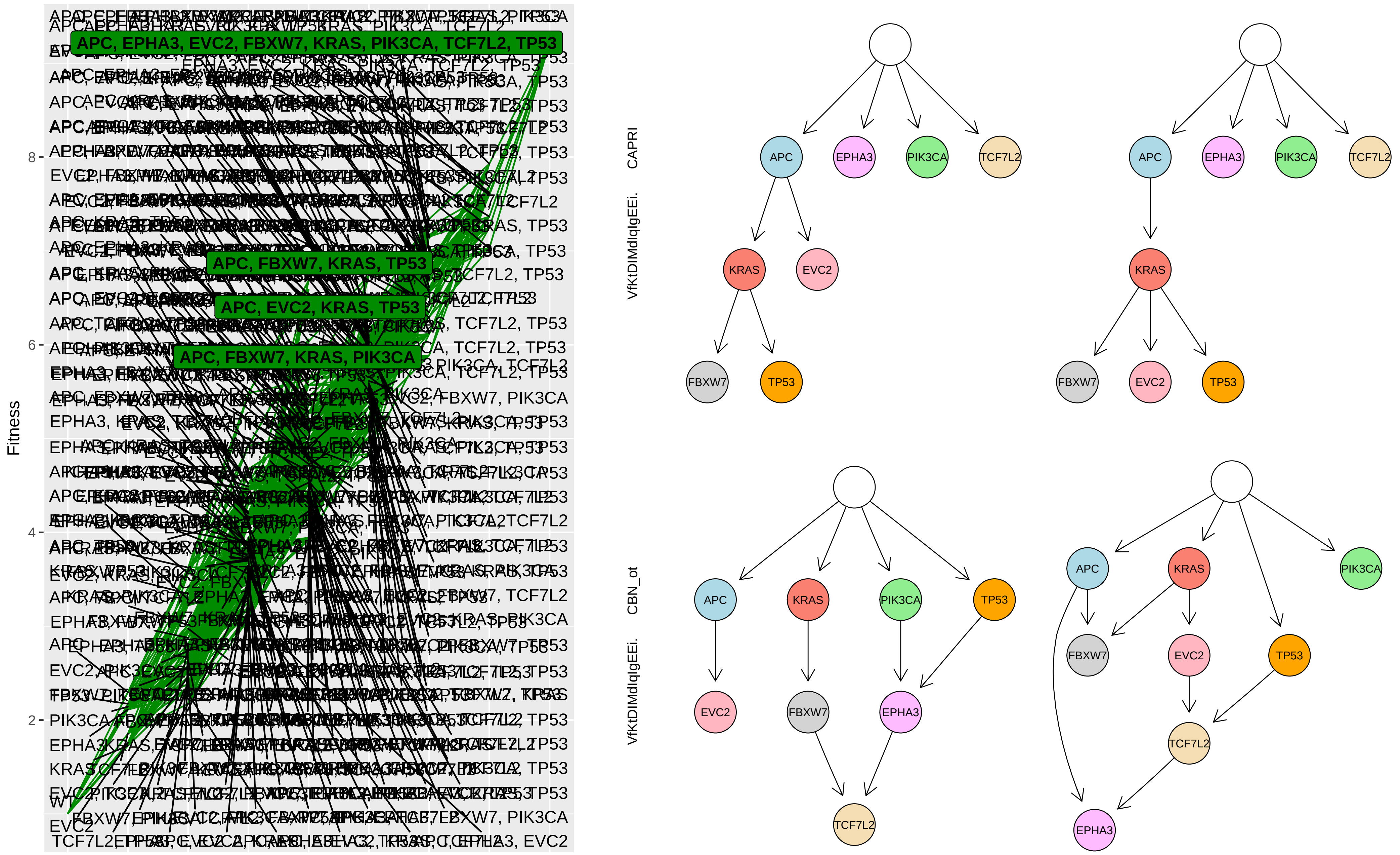

ID	p−value	Accessible Genot.
rWtkgnSJlXKLxZU	0.851	78

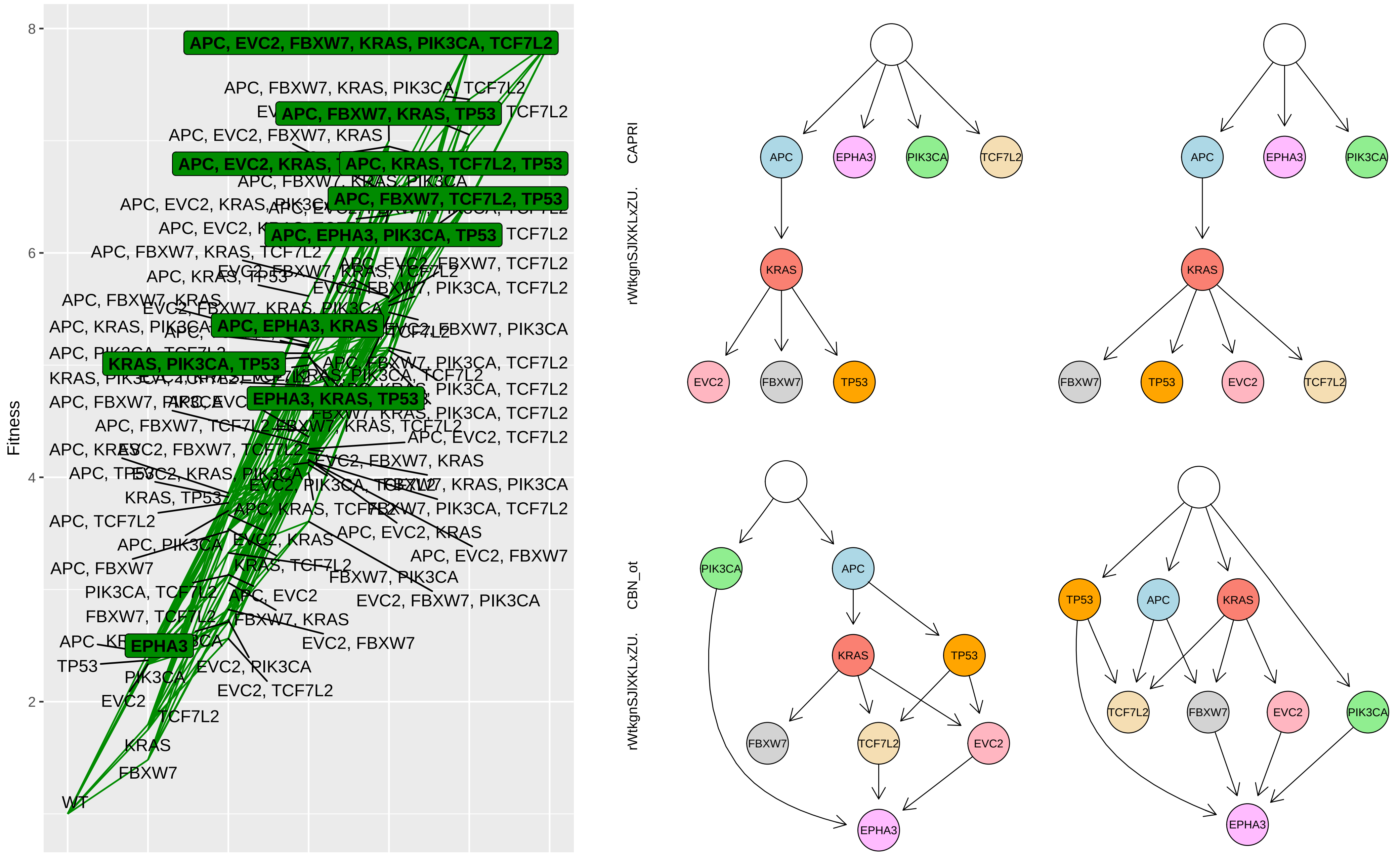

ID	p−value	Accessible Genot.
WzUPRHWrqQaZgDY	0.853	31

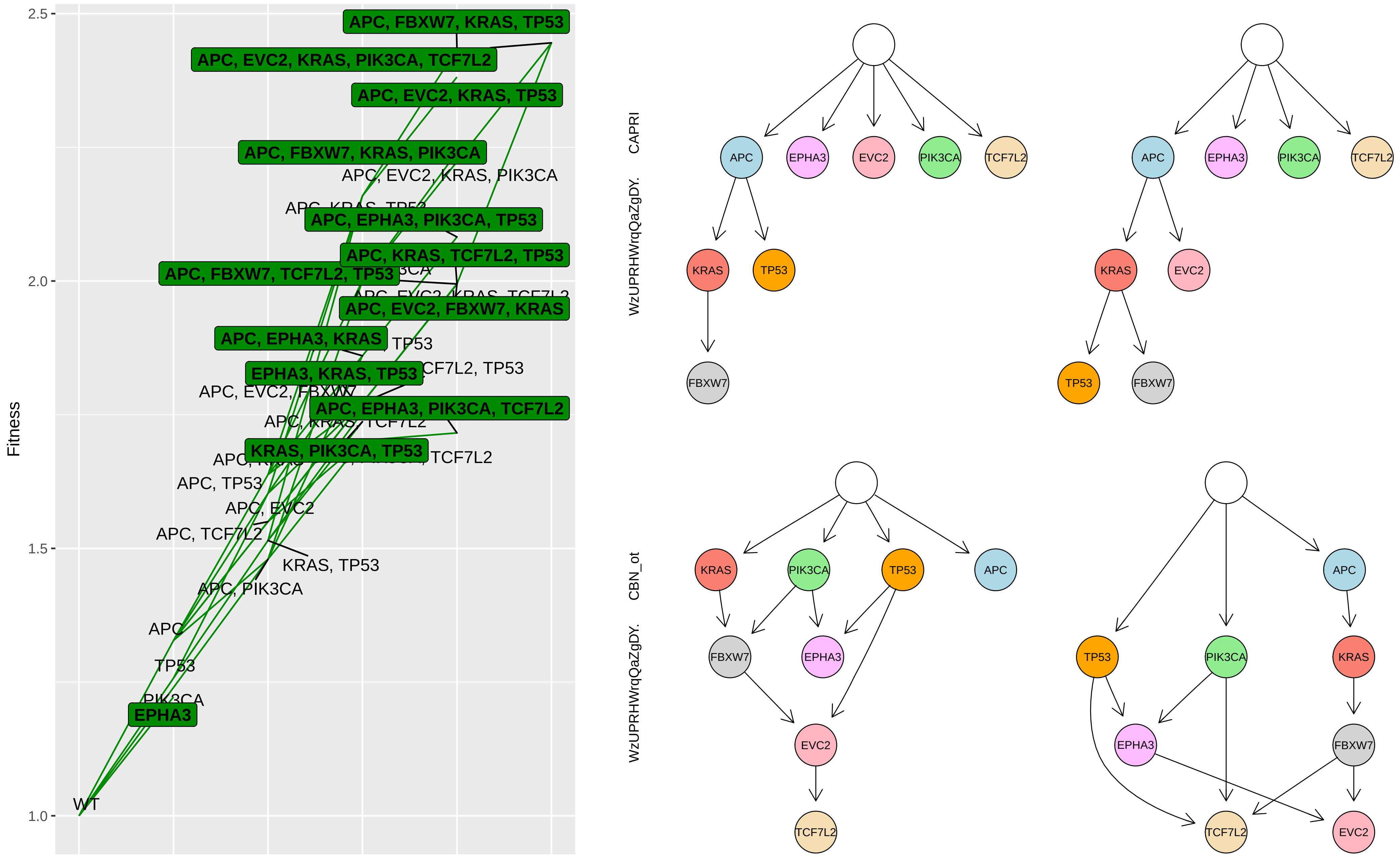

ID	p−value	Accessible Genot.
jolREUXTvFLInKd	0.859	62

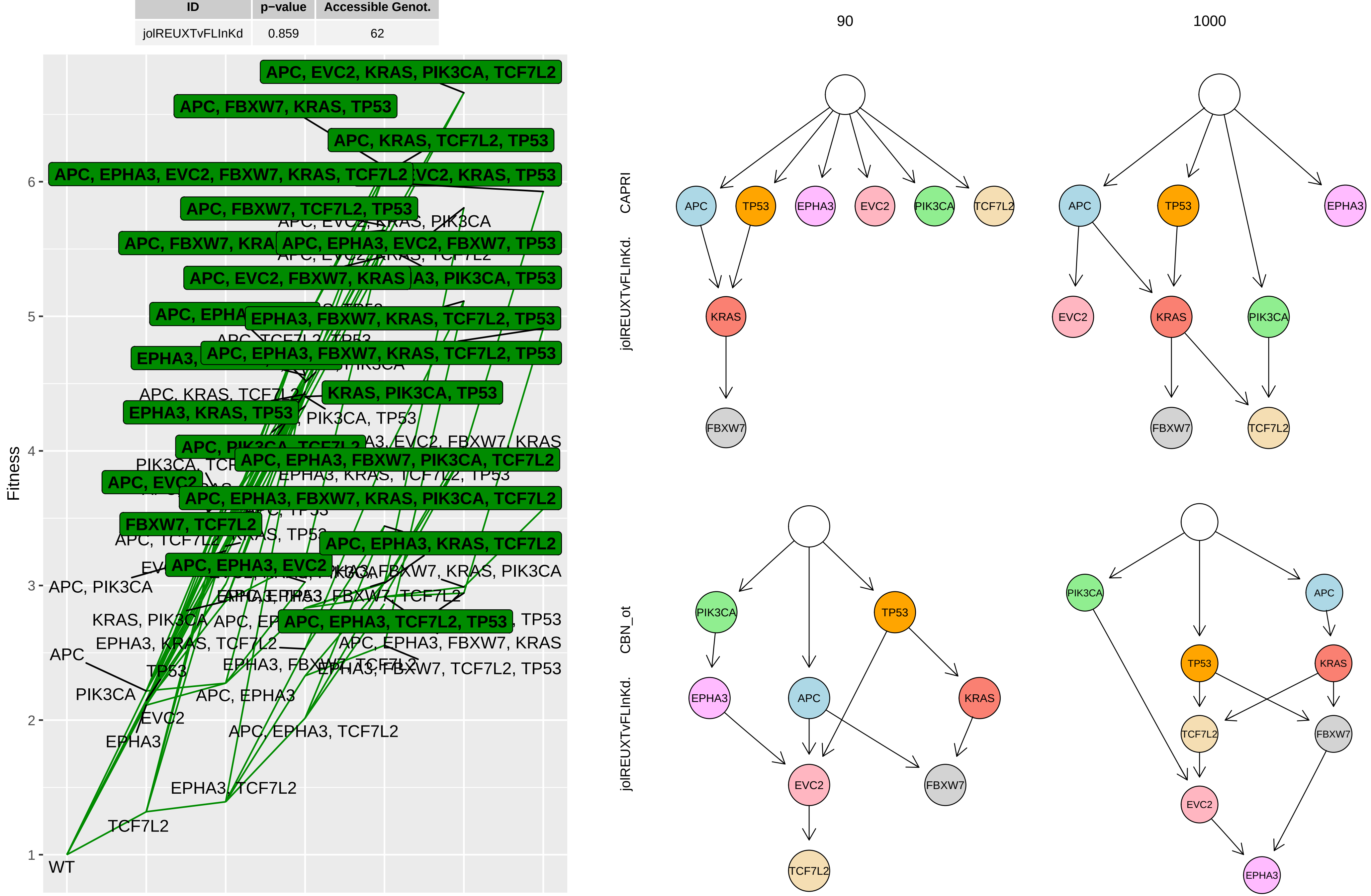

ID	p−value	Accessible Genot.
LqSYzcJxbhOoiZQ	0.86	33

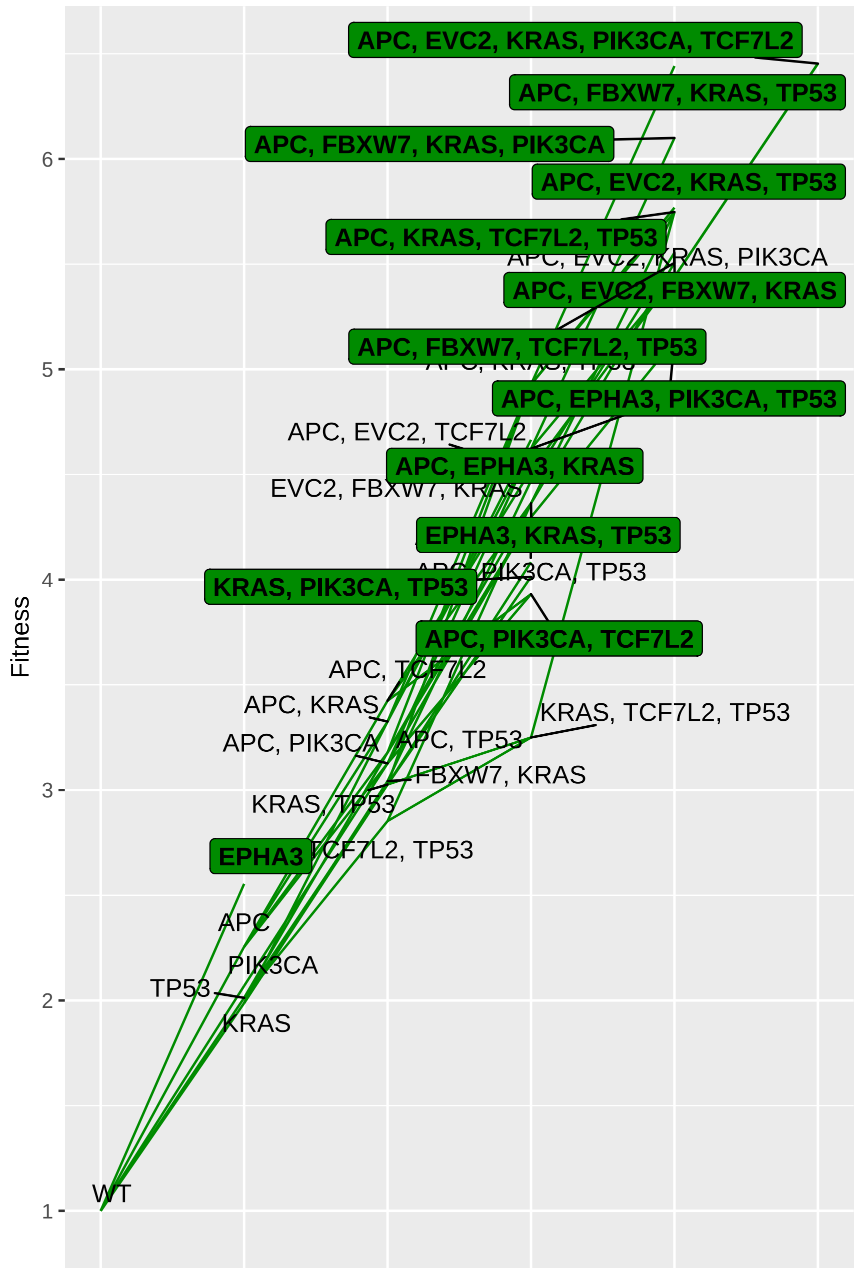

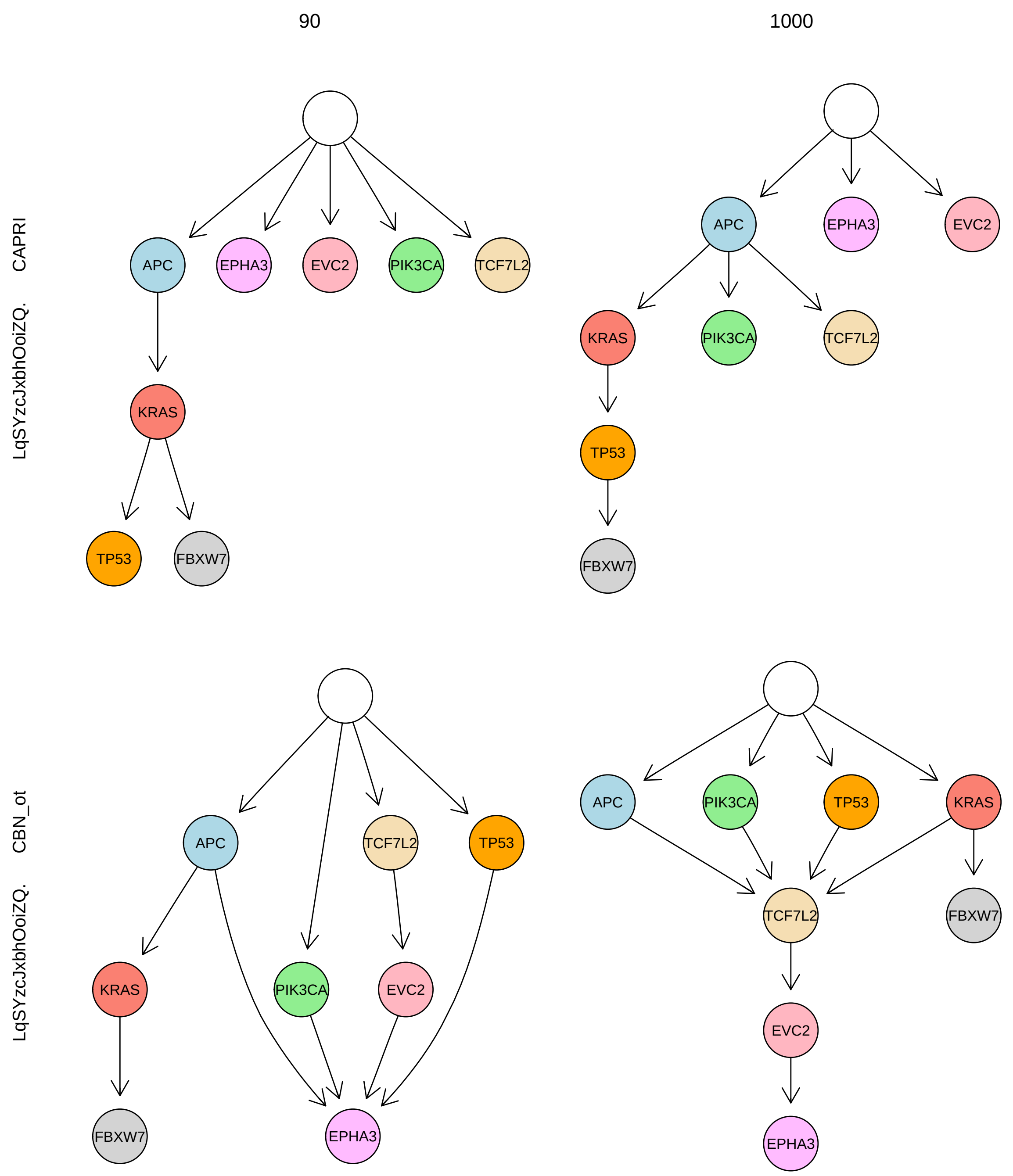

ID	p−value	Accessible Genot.
RCxKUqHAnyMgLPI	0.863	130

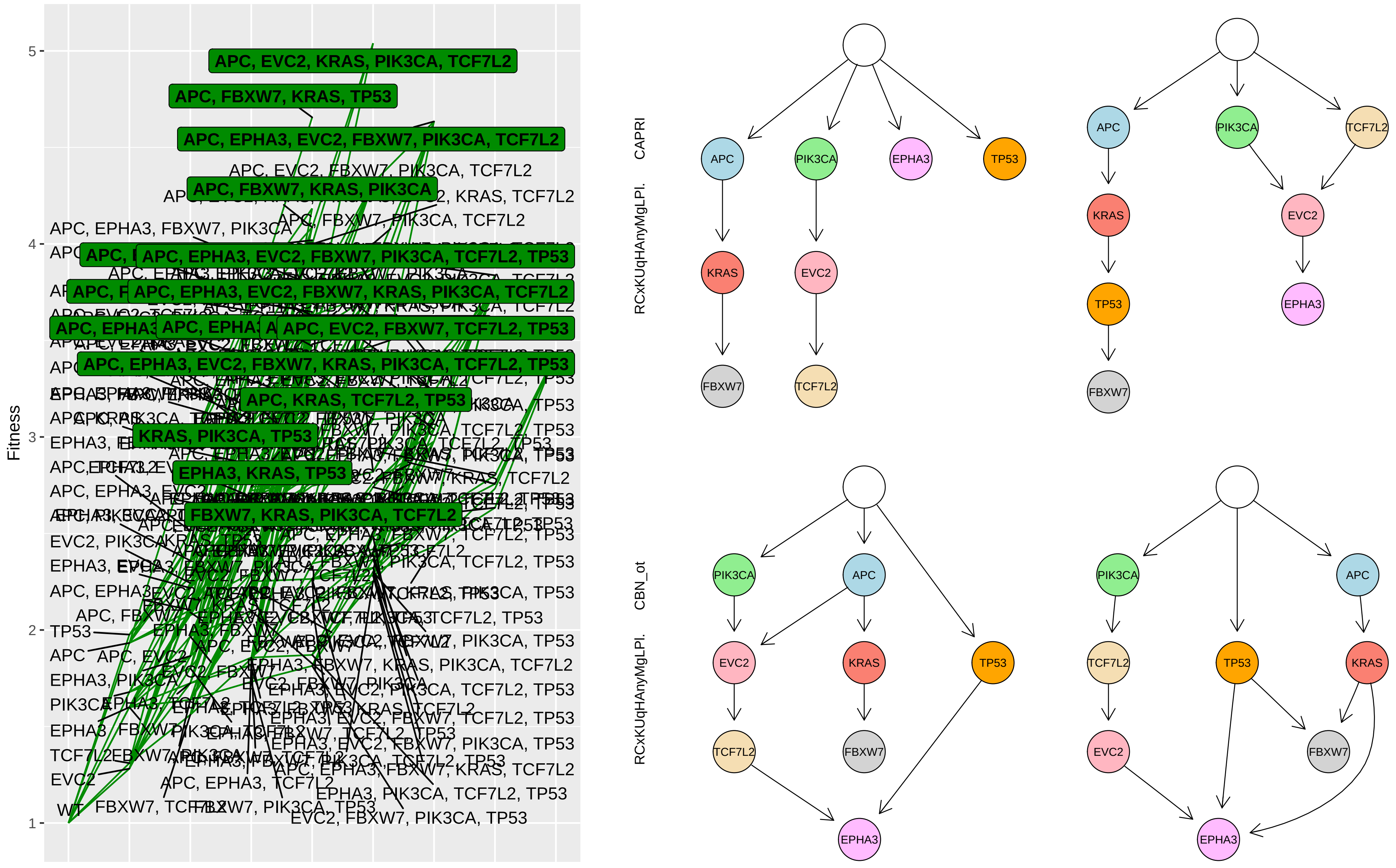

ID	p−value	Accessible Genot.
mwfMHnOaEcPoJYB	0.865	150

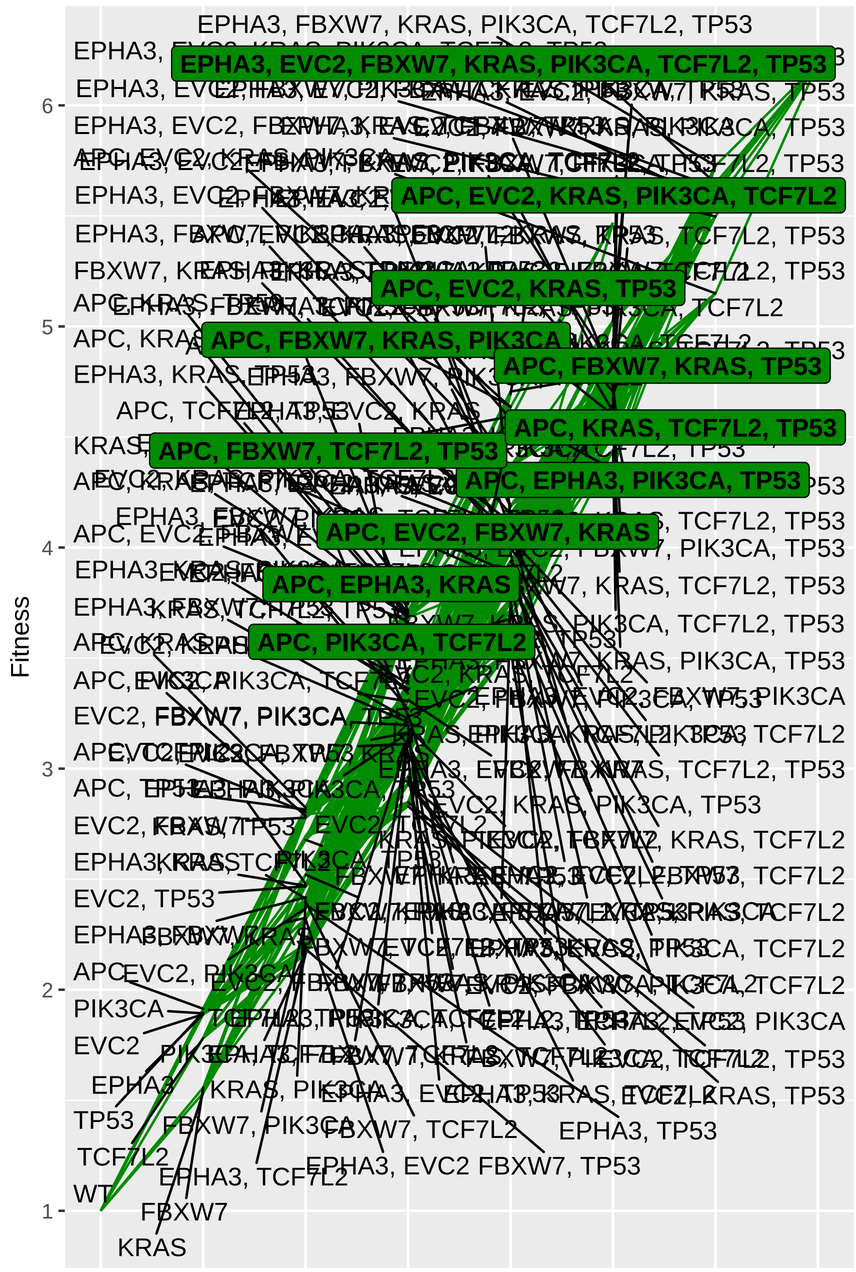

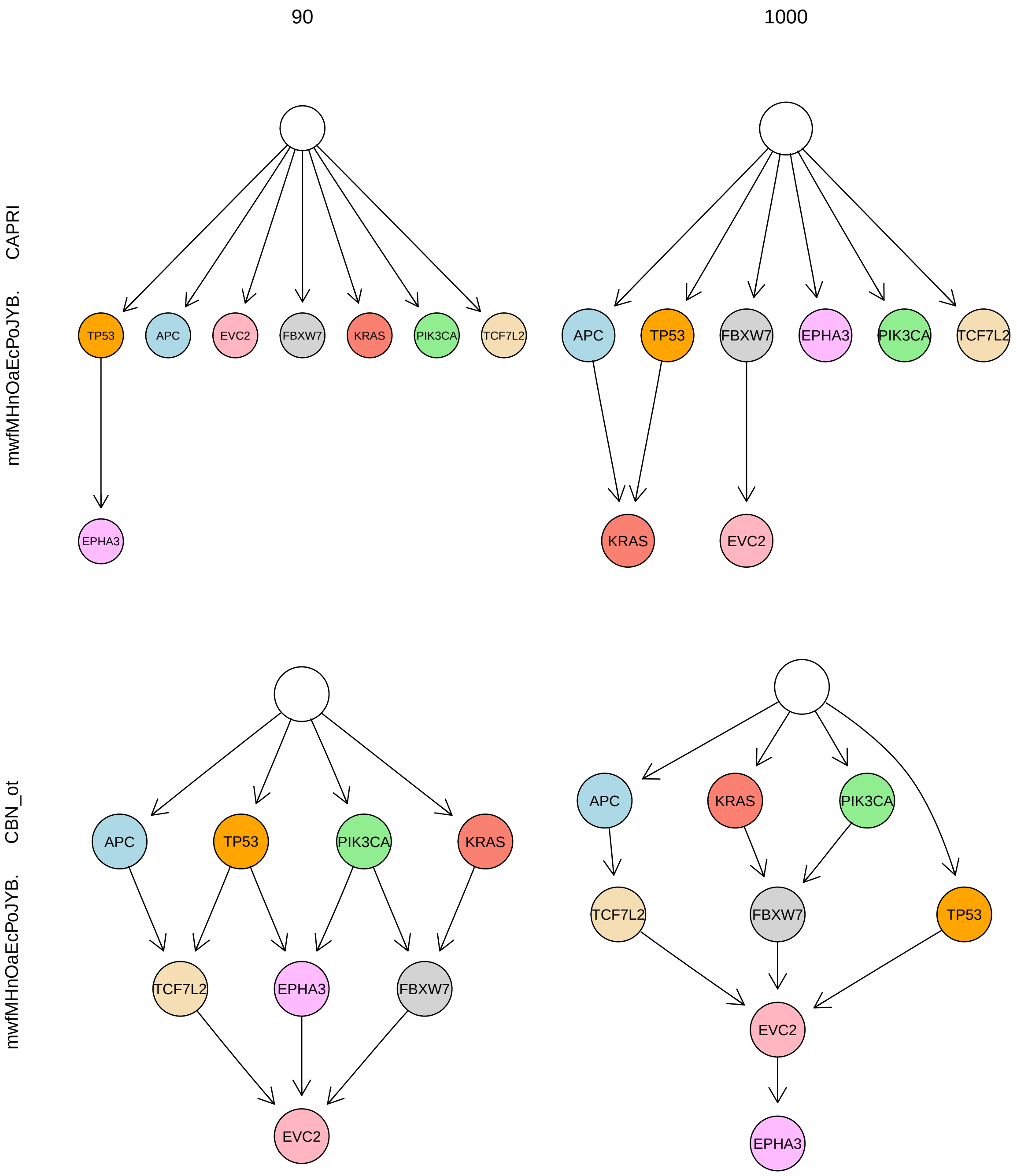

ID	p−value	Accessible Genot.
IPAVZDgfXqGSNII	0.869	198

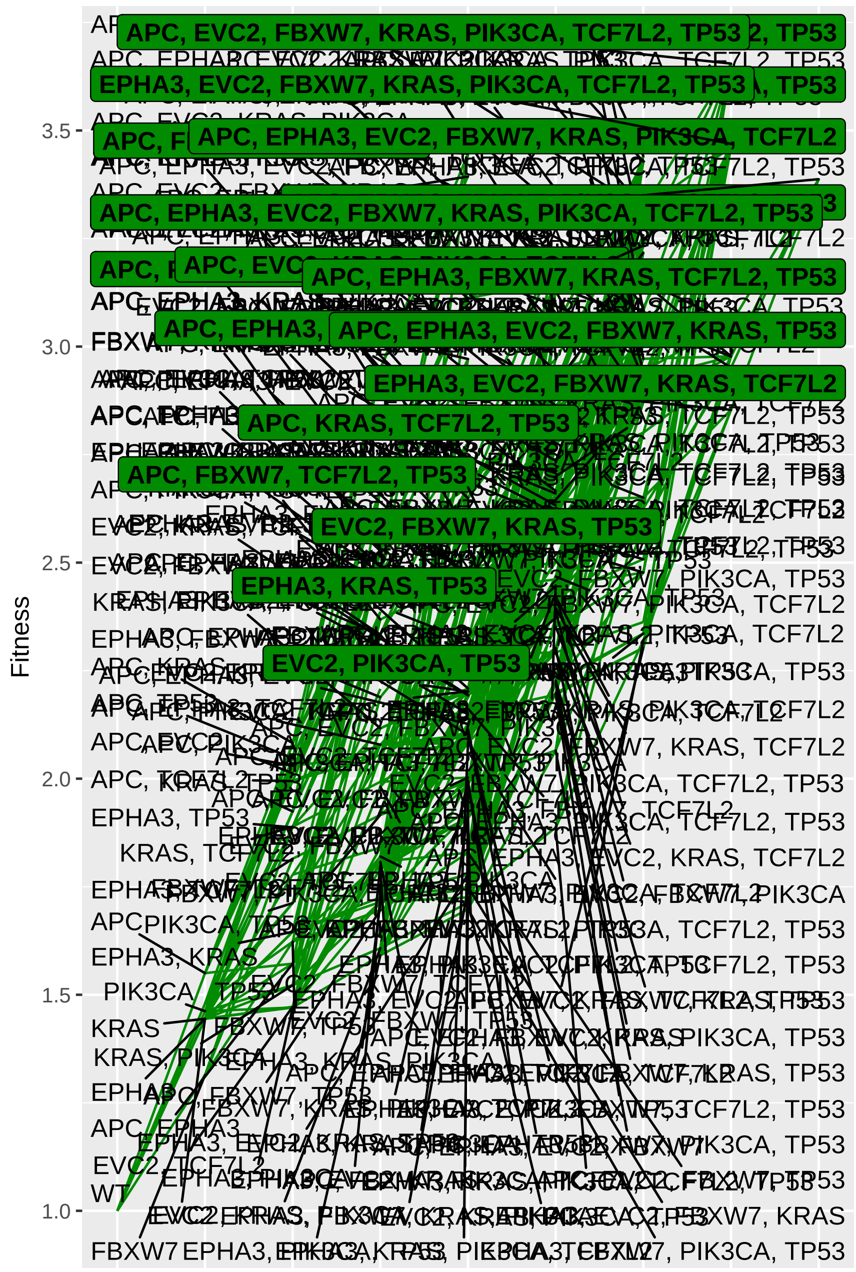

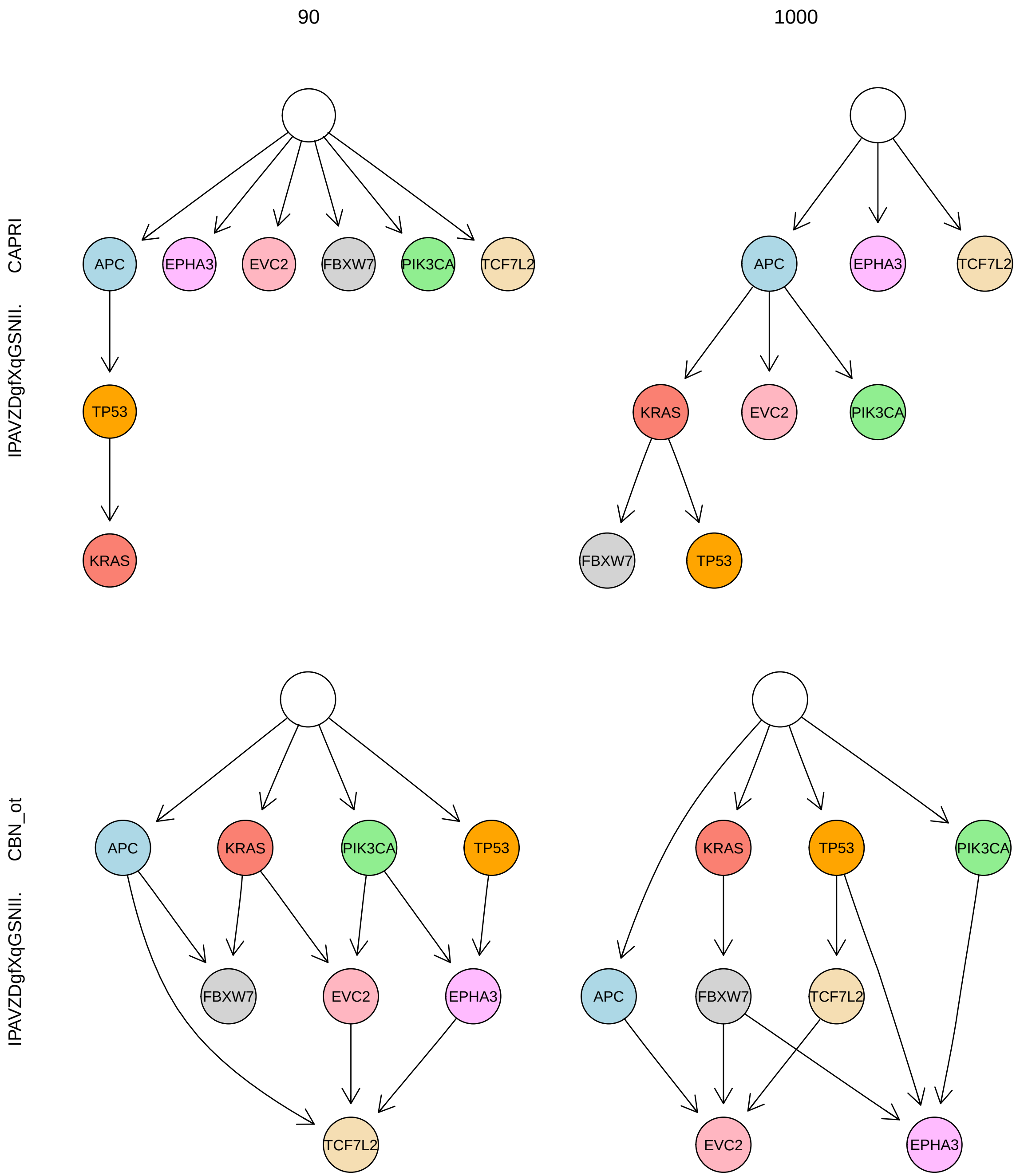

ID	p−value	Accessible Genot.
EUrCyBRZZbOaSSI	0.869	31

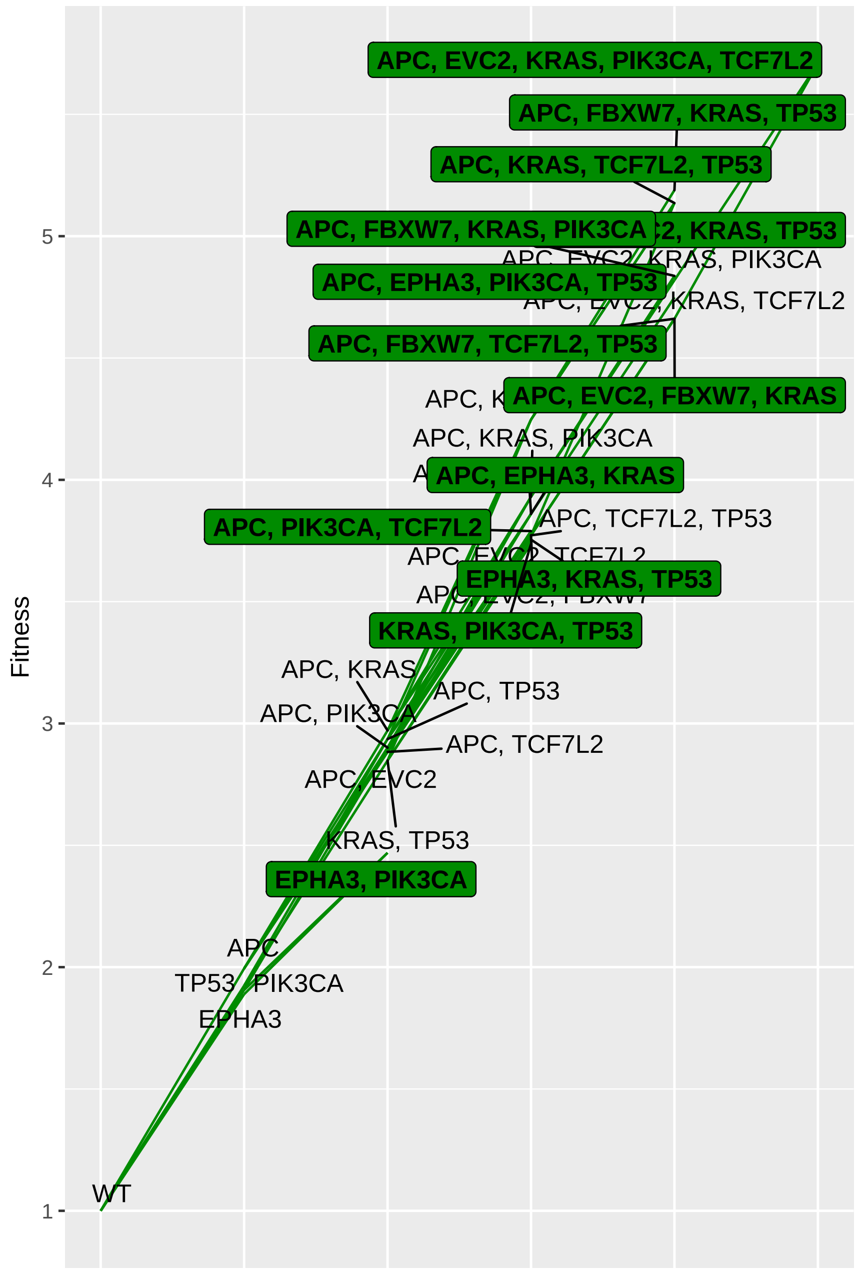

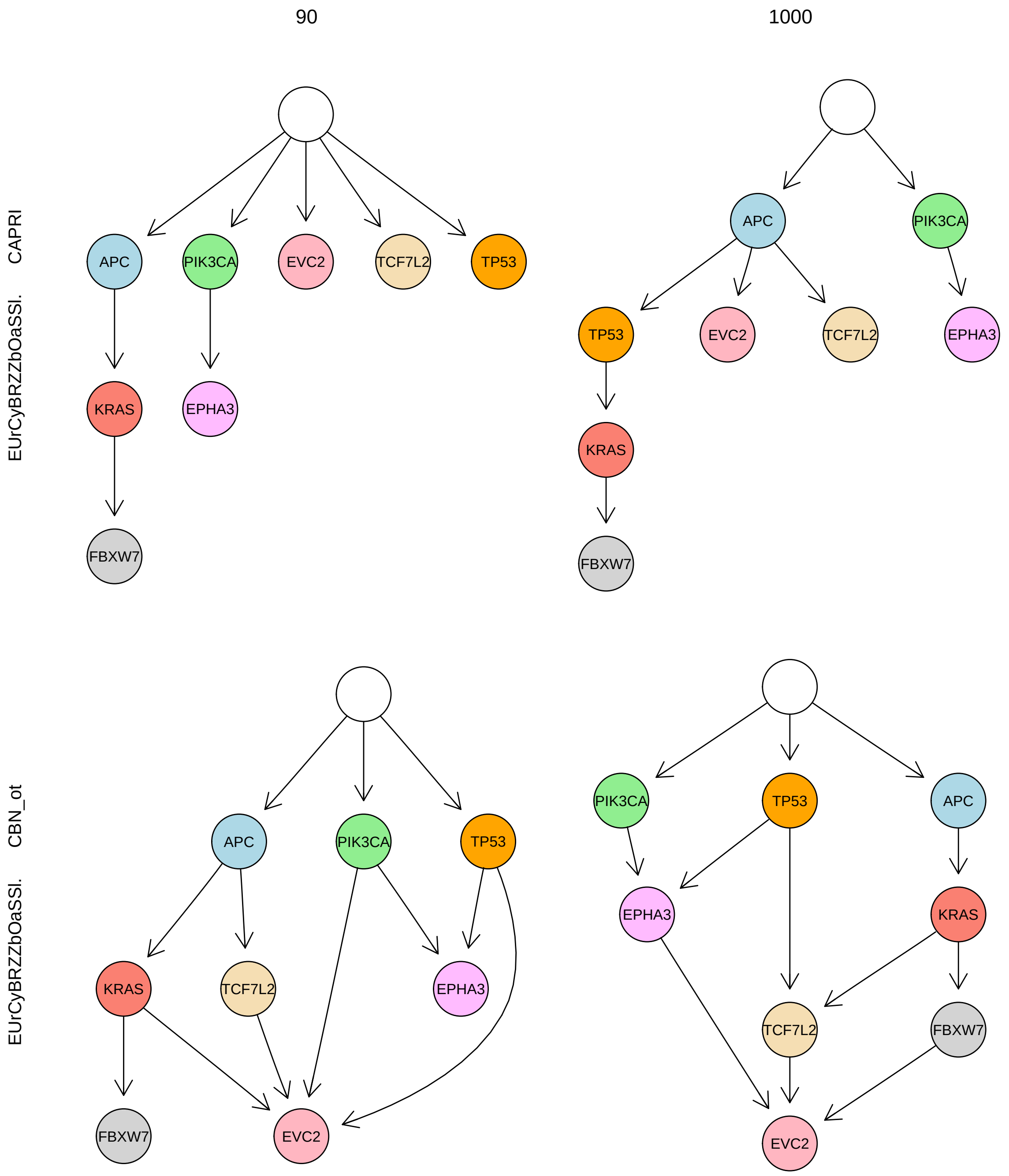

ID	p−value	Accessible Genot.
ncdNZplegjhmMpc	0.87	140

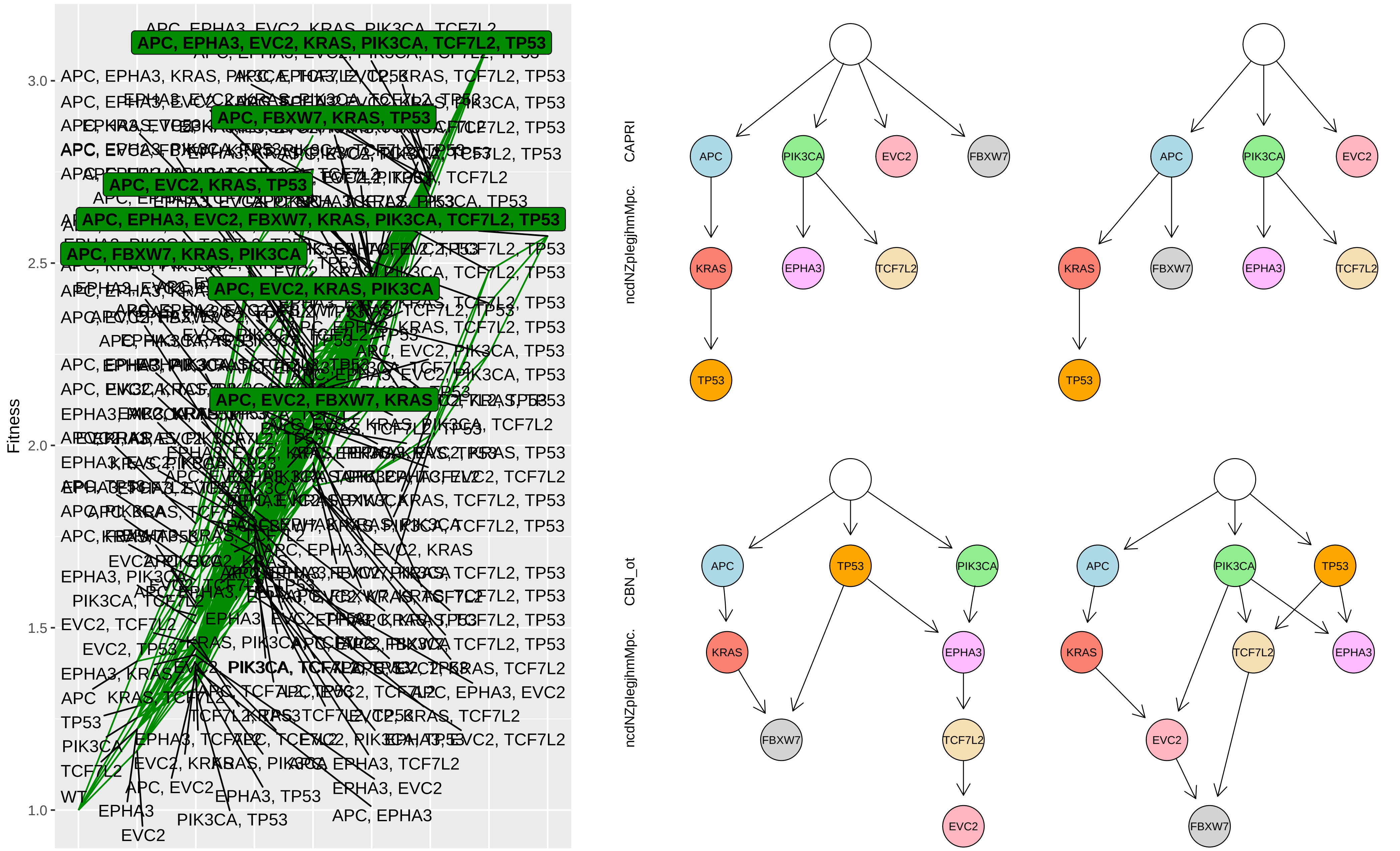

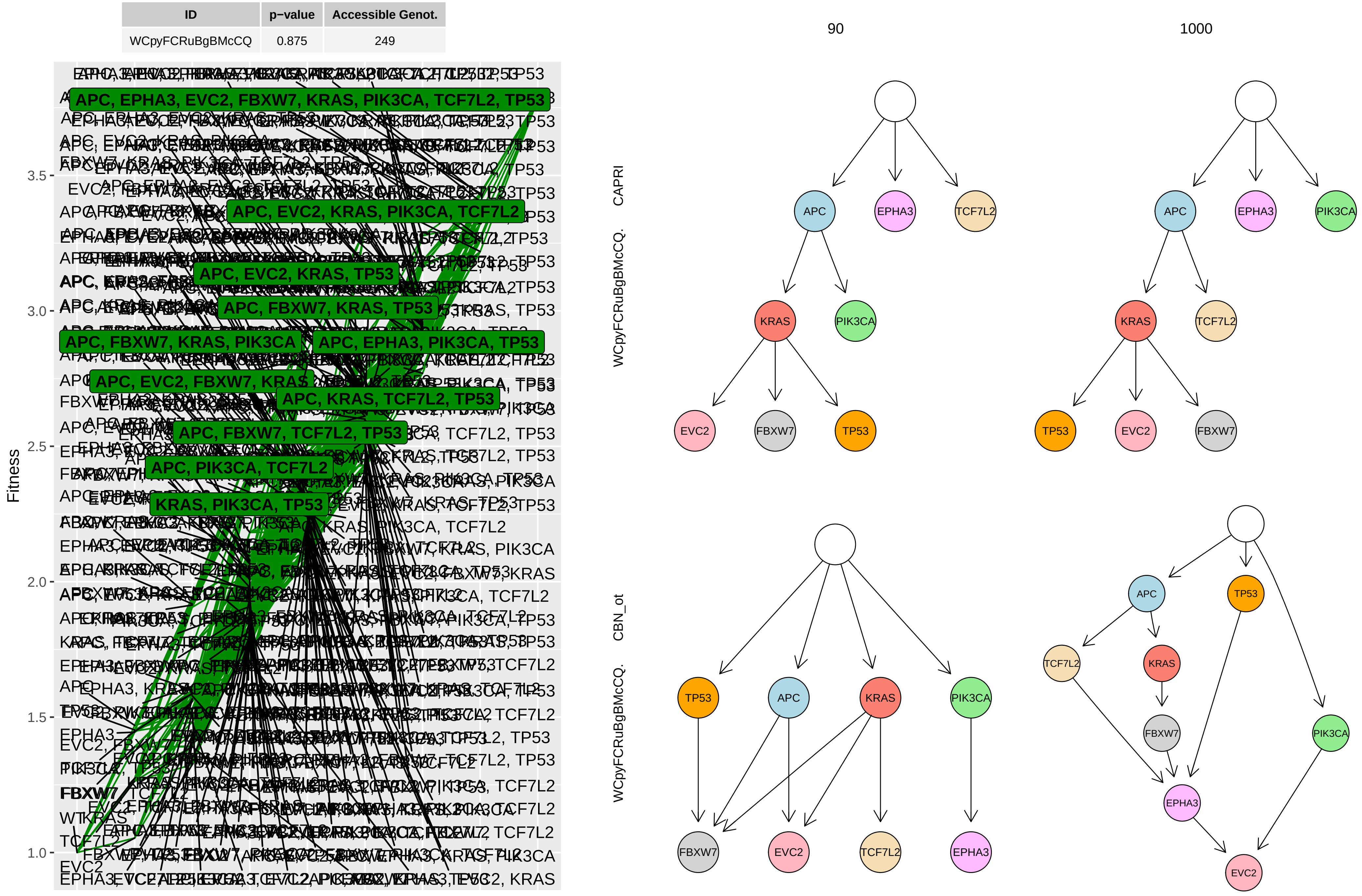

ID
p−value
Accessible Genot.
WCpyFCRuBgBMcCQ
0.875
249
Fitness
3.5
3.0
2.5
2.0
1.5
1.0
APC, EPHA3, EVC2, FBXW7, KRAS, PIK3CA, TCF7L2, TP53
APC, EVC2, KRAS, PIK3CA, TCF7L2
APC, EVC2, KRAS, TP53
APC, FBXW7, KRAS, TP53
APC, FBXW7, KRAS, PIK3CA
APC, EPHA3, PIK3CA, TP53
APC, EVC2, FBXW7, KRAS
APC, KRAS, TCF7L2, TP53
APC, FBXW7, TCF7L2, TP53
APC, PIK3CA, TCF7L2
KRAS, PIK3CA, TP53
90
1000
WCpyFCRuBgBMcCQ. CAPRI
WCpyFCRuBgBMcCQ. CBN_ot
APC
EPHA3
TCF7L2
KRAS
PIK3CA
EVC2
FBXW7
TP53

ID	p−value	Accessible Genot.
xwKpacPBkfphEGp	0.897	80

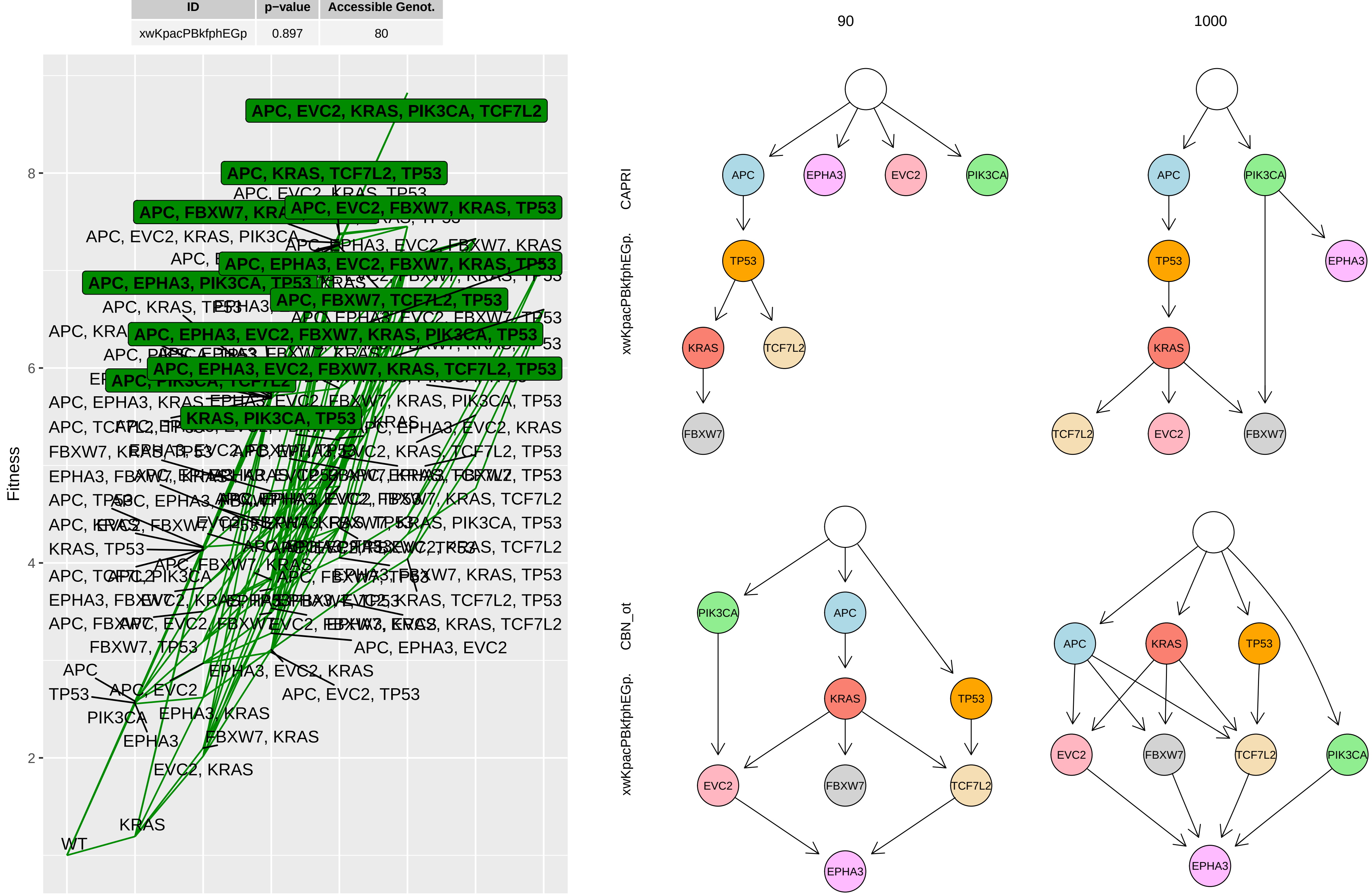

ID	p−value	Accessible Genot.
KNZGXNBOgVyJWnq	0.898	85

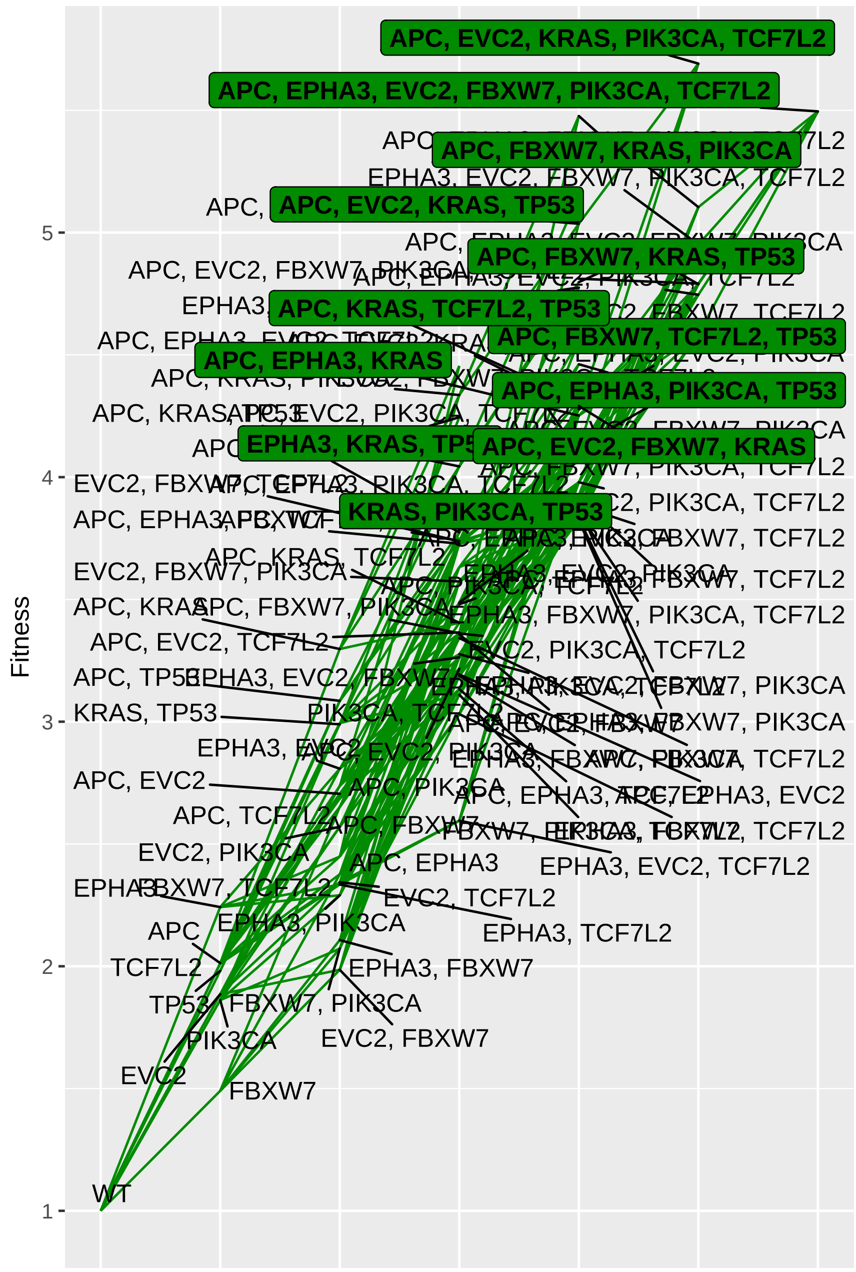

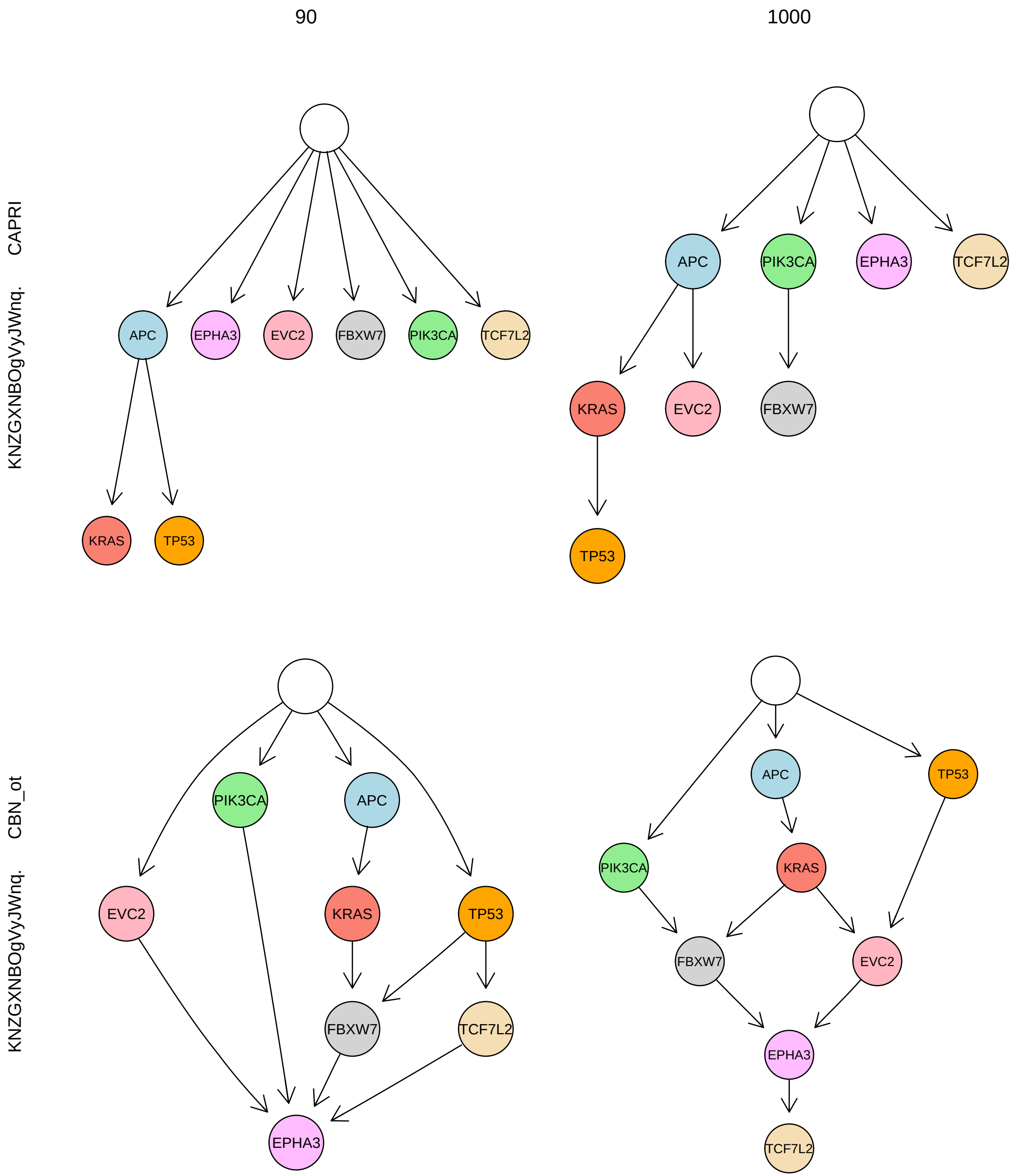

ID	p−value	Accessible Genot.
gvMAFmewheLpbAe	0.904	28

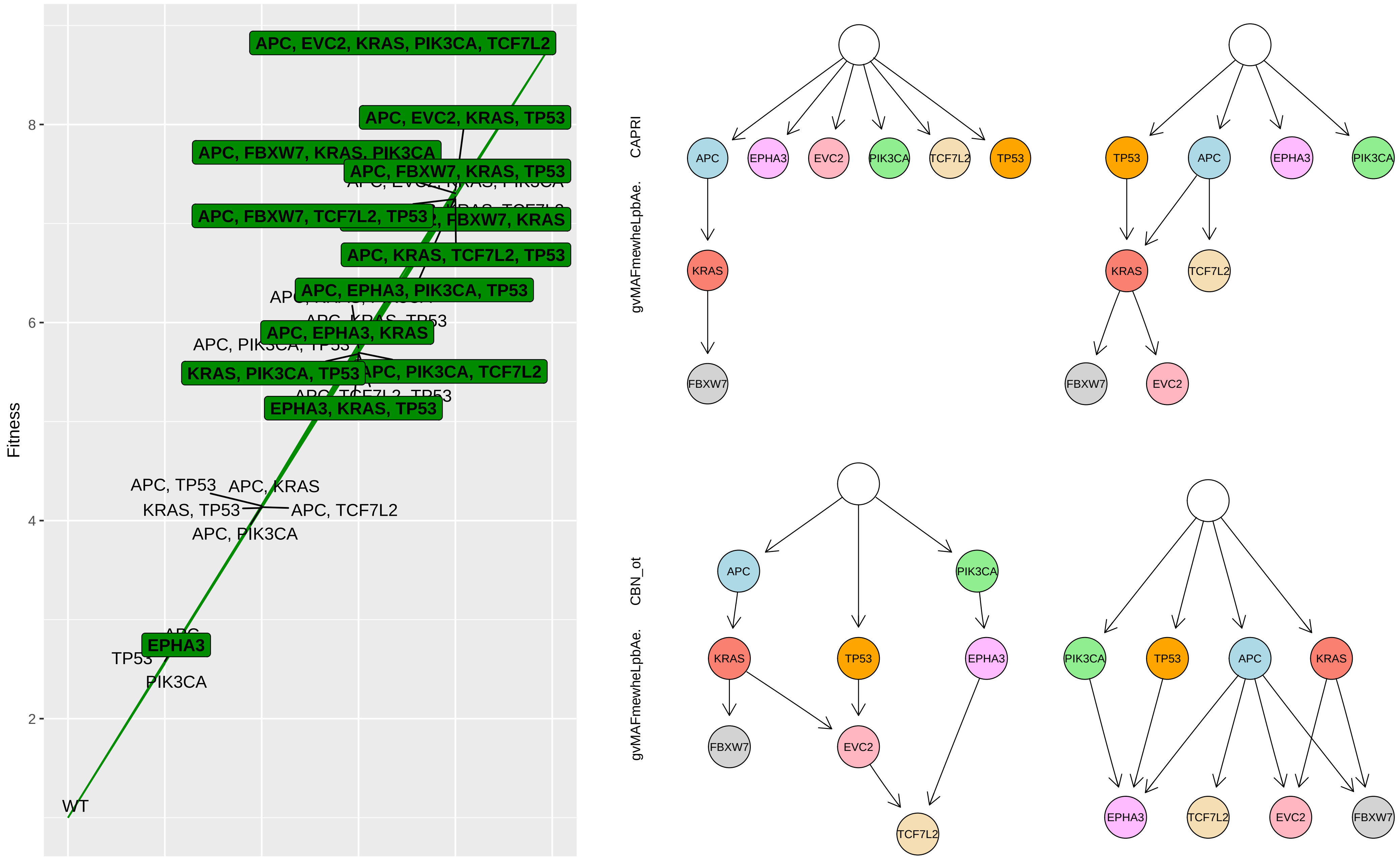

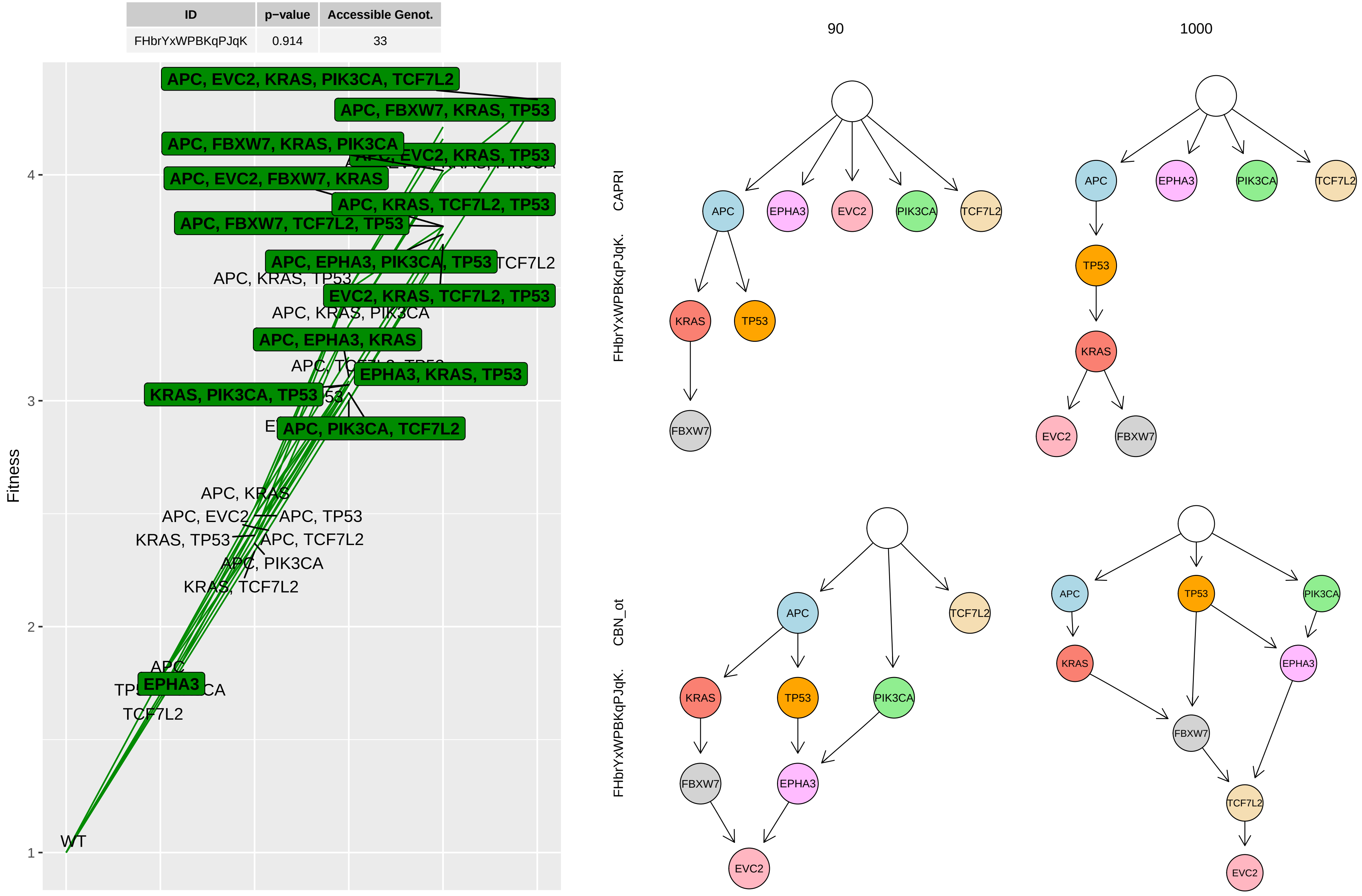

ID
p−value
Accessible Genot.
FHbrYxWPBKqPJqK
0.914
33
Fitness
APC, EVC2, KRAS, PIK3CA, TCF7L2
APC, FBXW7, KRAS, TP53
APC, FBXW7, KRAS, PIK3CA
APC, EVC2, FBXW7, KRAS
APC, KRAS, TCF7L2, TP53
APC, FBXW7, TCF7L2, TP53
APC, EPHA3, PIK3CA, TP53
APC, KRAS, TP53
EVC2, KRAS, TCF7L2, TP53
APC, KRAS, PIK3CA
APC, EPHA3, KRAS
EPHA3, KRAS, TP53
KRAS, PIK3CA, TP53
APC, PIK3CA, TCF7L2
APC, KRAS
APC, EVC2
APC, TP53
KRAS, TP53
APC, TCF7L2
APC, PIK3CA
KRAS, TCF7L2
APC
EPHA3
TCF7L2
WT
1
2
3
4
90
1000
FHbrYxWPBKqPJqK. CAPRI
FHbrYxWPBKqPJqK. CBN_ot
APC
EPHA3
EVC2
PIK3CA
TCF7L2
KRAS
TP53
FBXW7

ID	p−value	Accessible Genot.
HlgfKlwczmEthRN	0.928	77

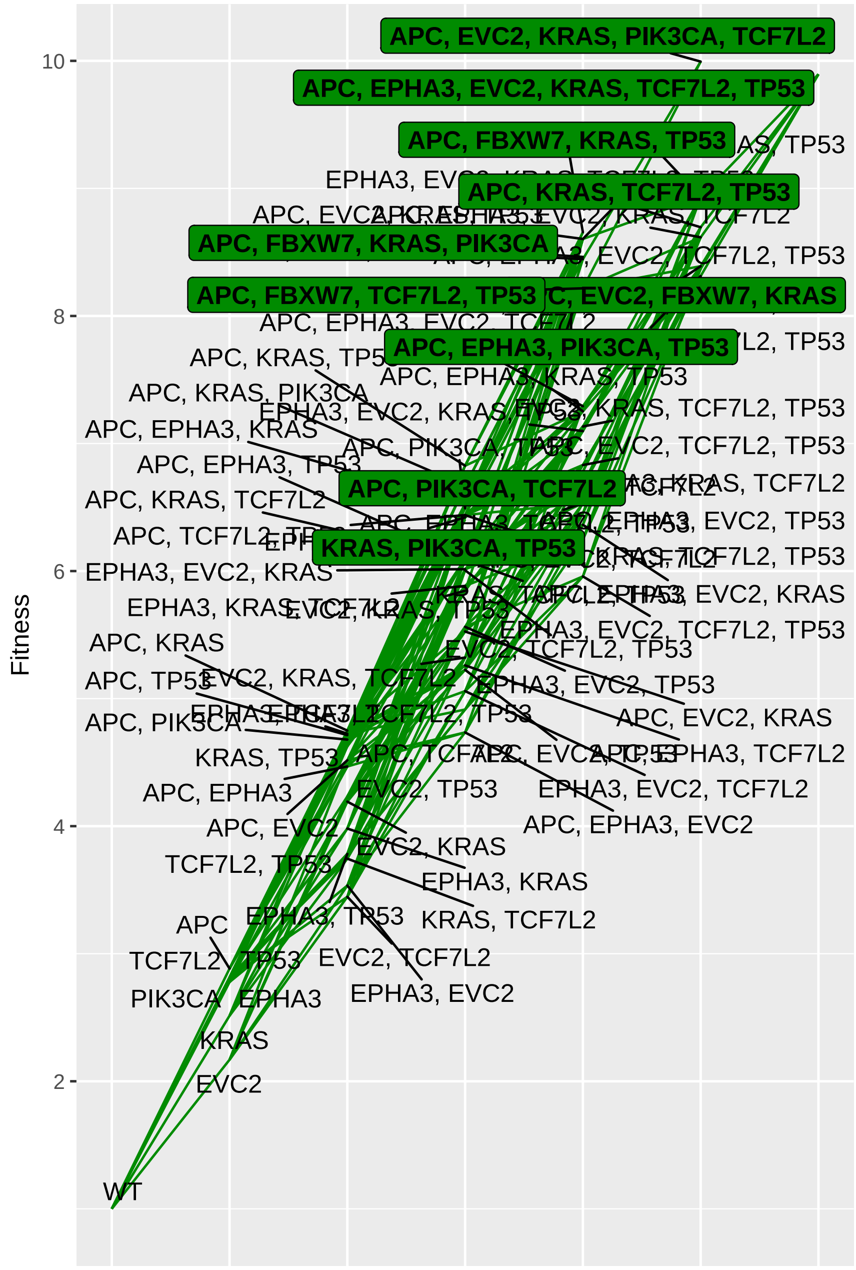

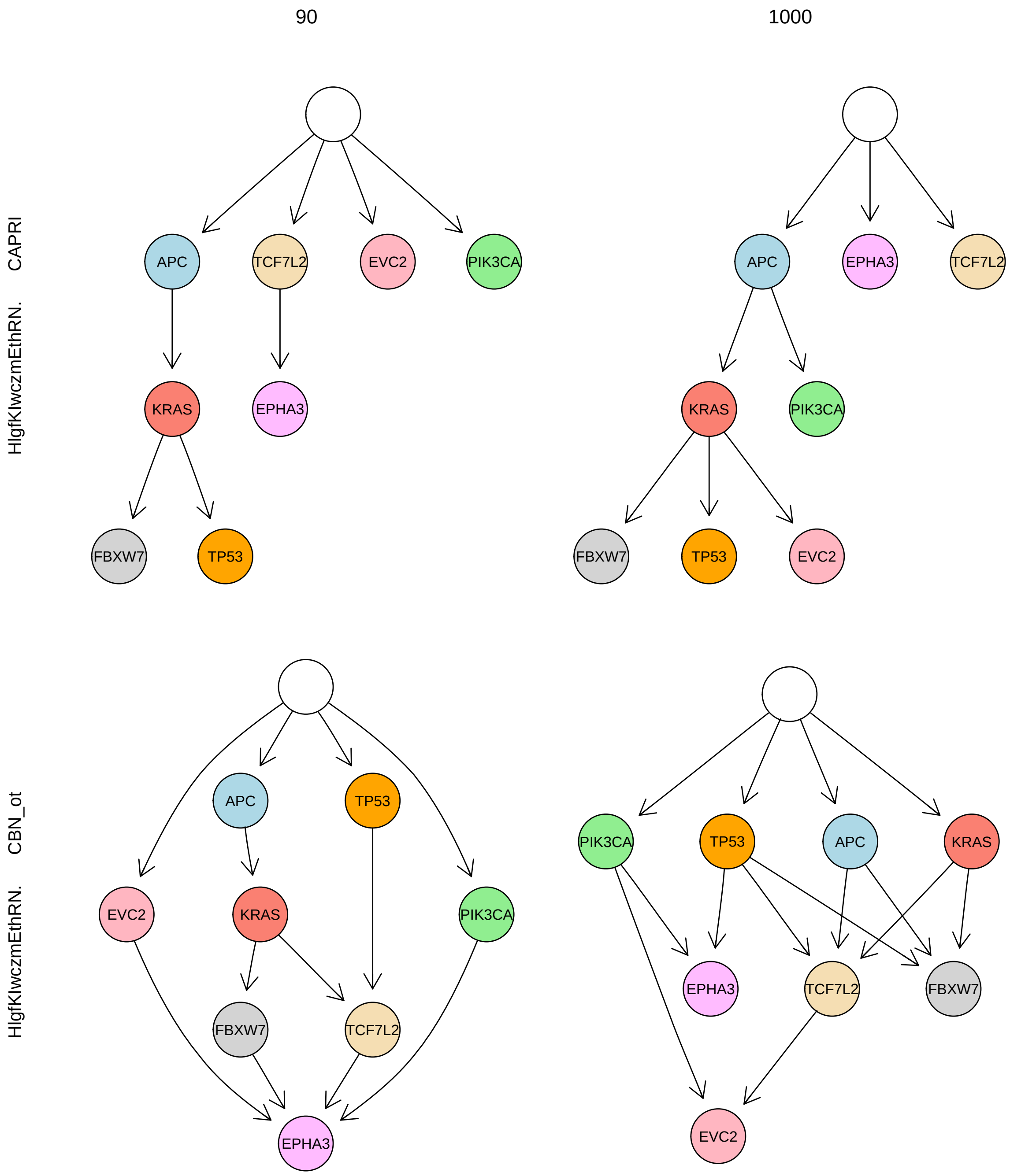

ID	p−value	Accessible Genot.
gTFOzQPmeAqwcep	0.928	56

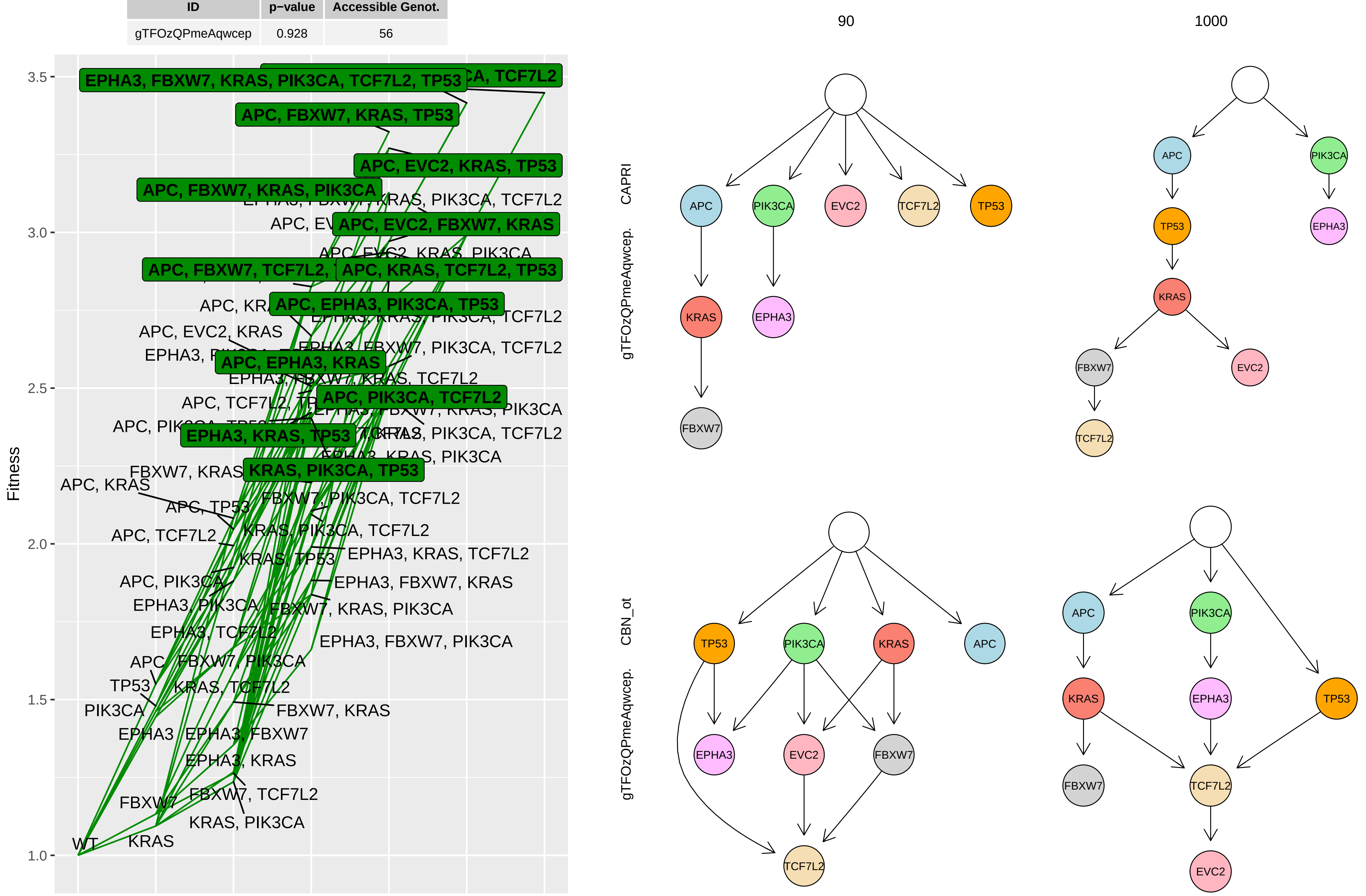

ID	p−value	Accessible Genot.
xbhtroSGGFWtvTr	0.93	30

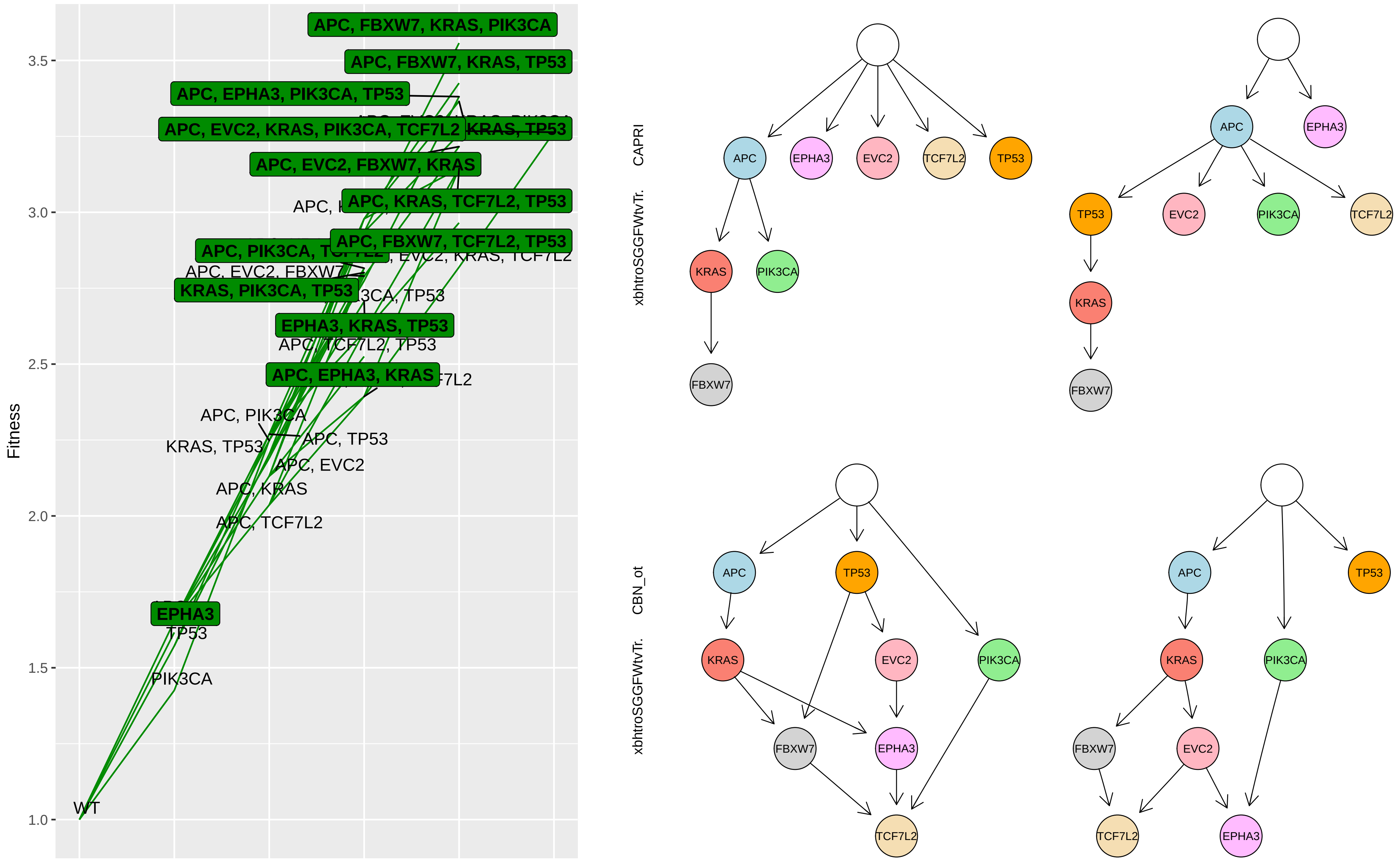

ID	p−value	Accessible Genot.
uOweDDqGdpbrDUe	0.935	93

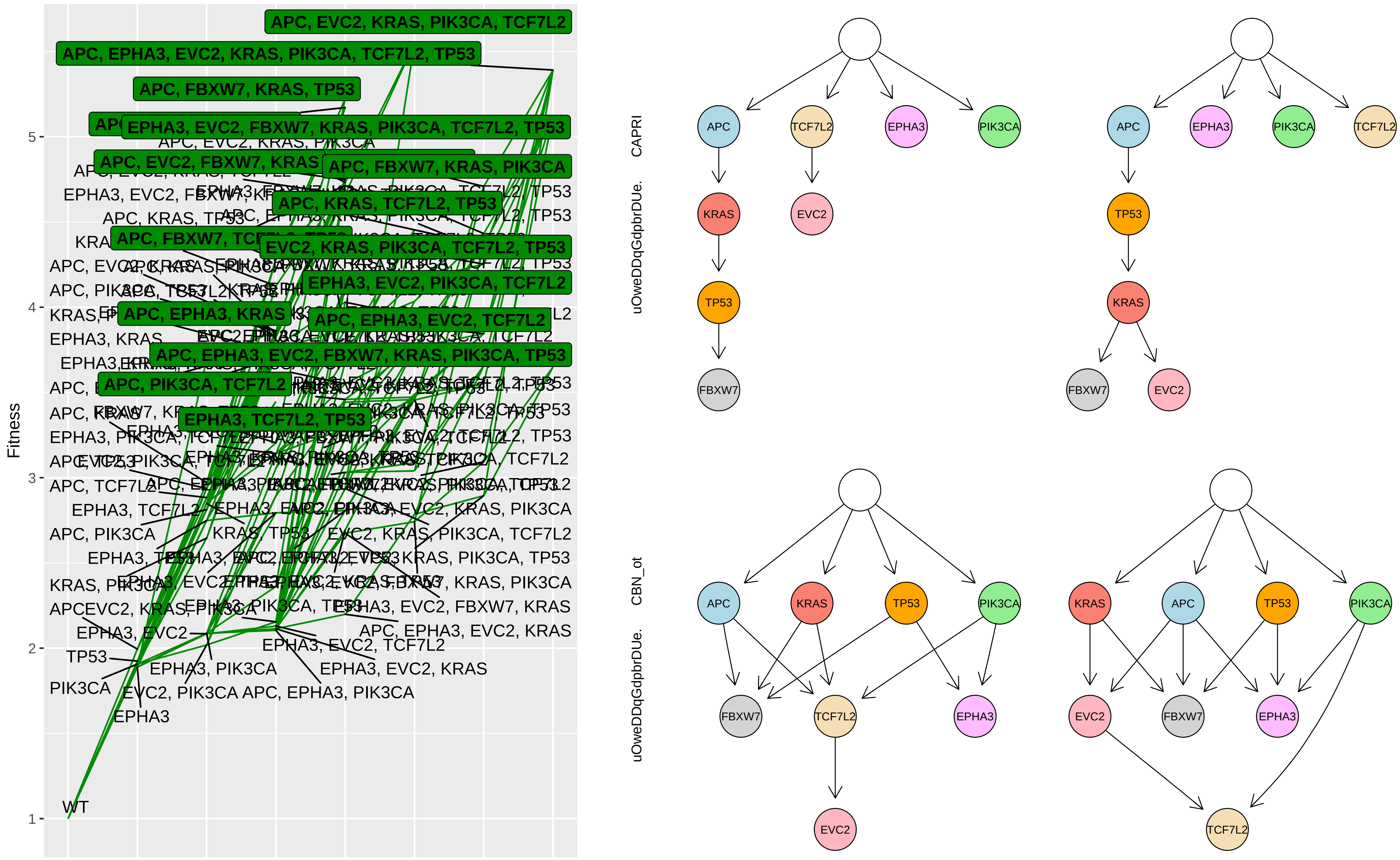

ID	p−value	Accessible Genot.
yNUePNPyKZbqKYD	0.942	30

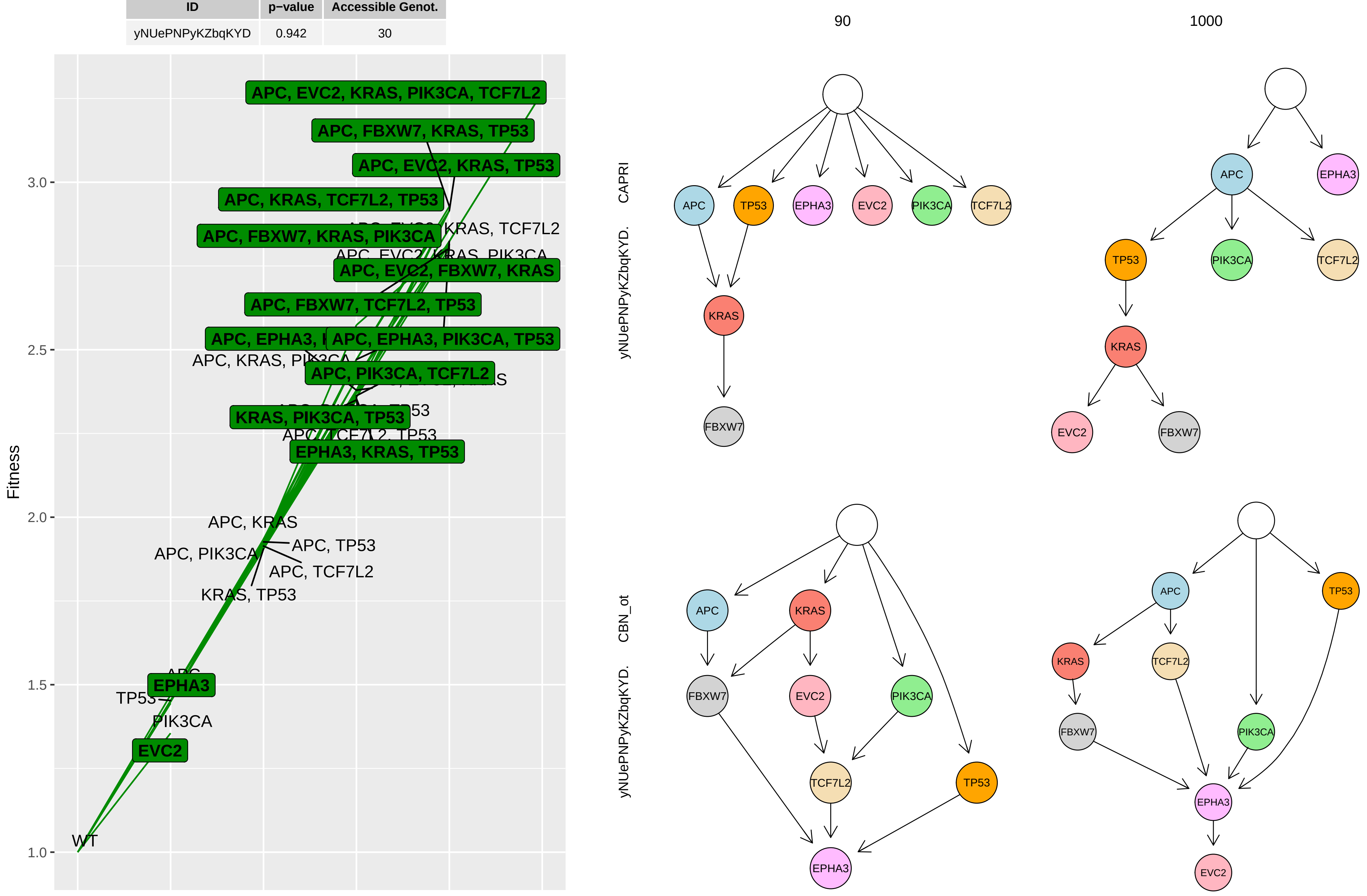

ID	p−value	Accessible Genot.
qOJTXMQOZrdNWar	0.945	41

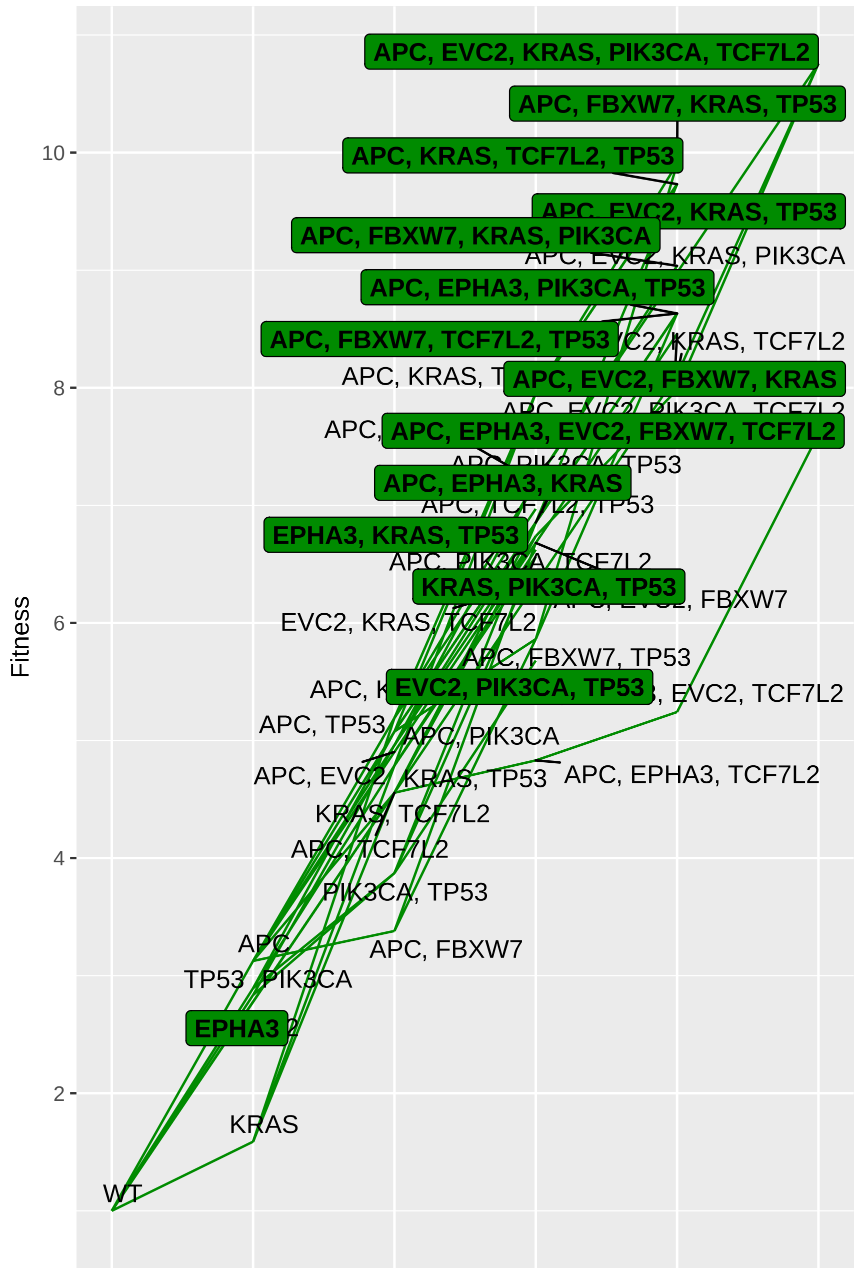

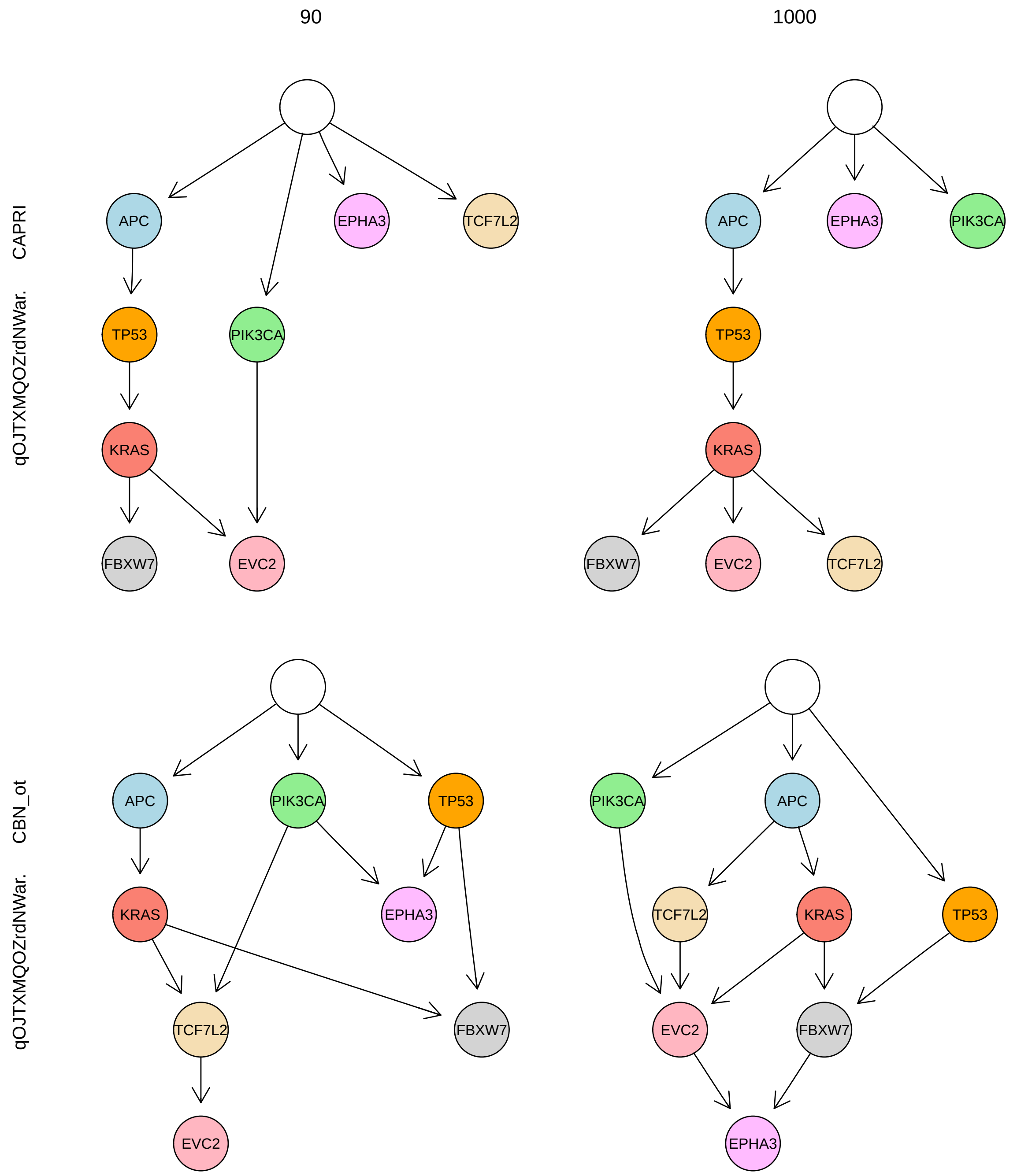

ID	p−value	Accessible Genot.
DCqTeozUJMssMFo	0.946	30

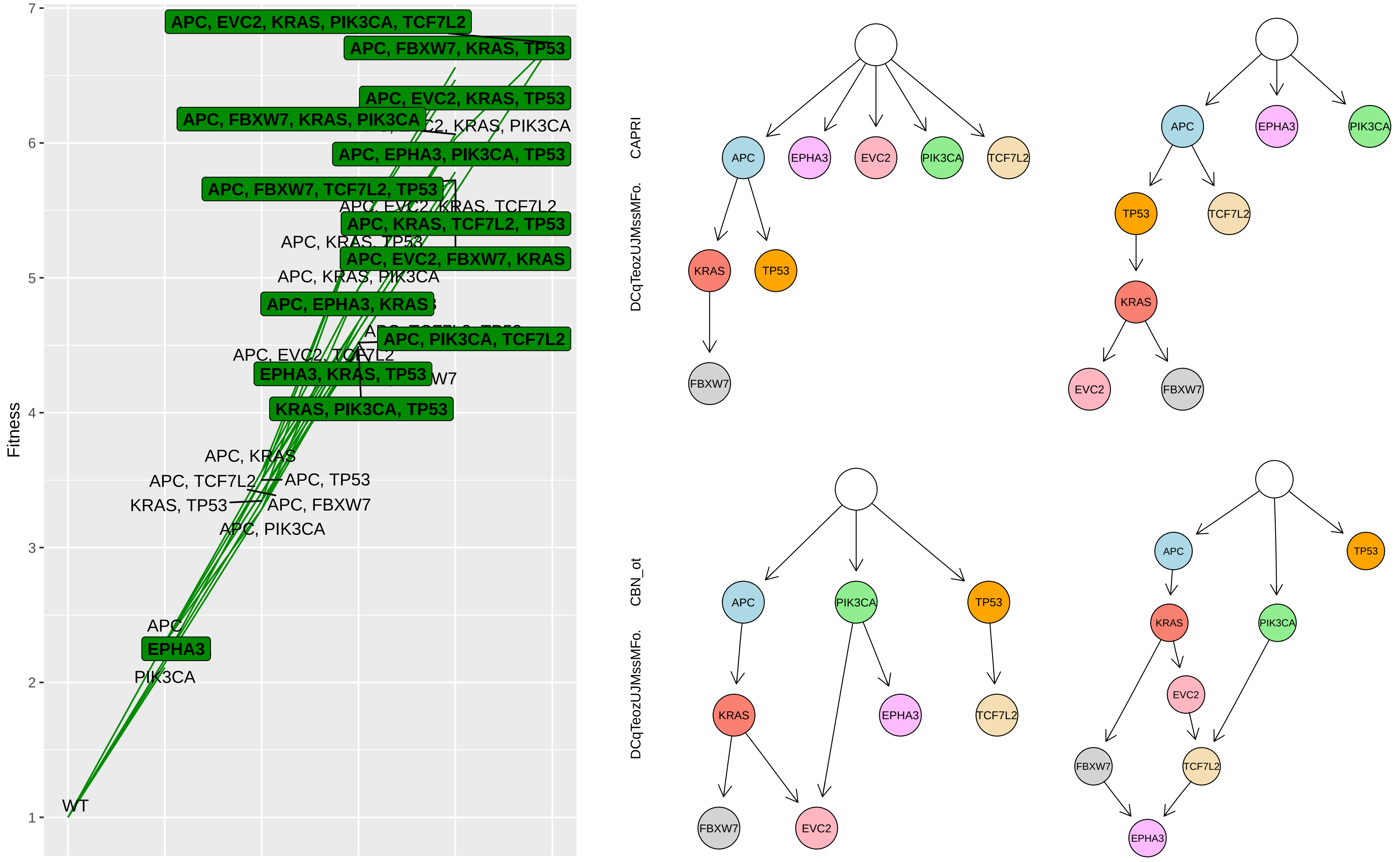

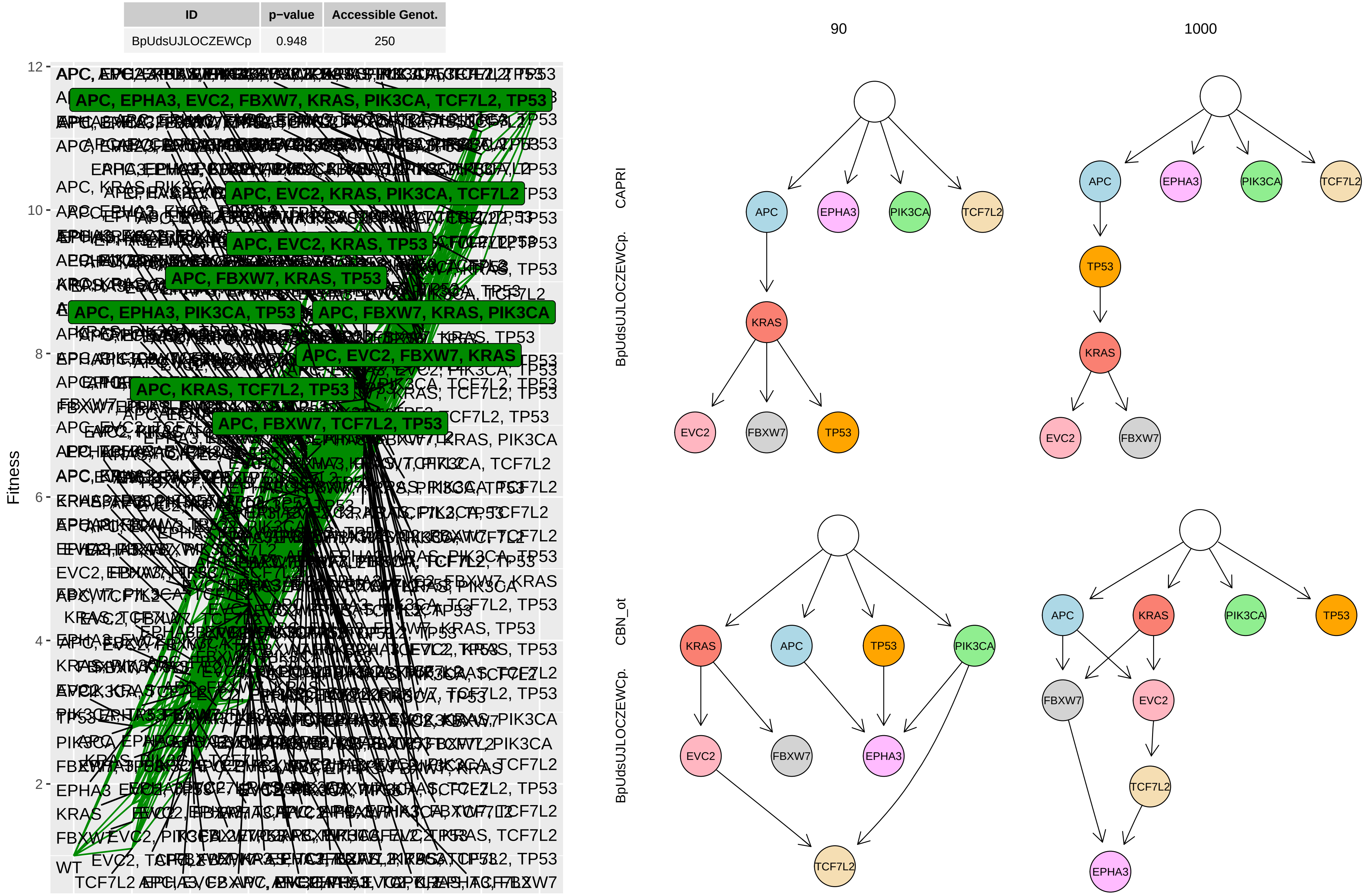

ID
p−value
Accessible Genot.
BpUdsUJLOCZEWCp
0.948
250
Fitness
12
10
8
6
4
2
WT
APC, EPHA3, EVC2, FBXW7, KRAS, PIK3CA, TCF7L2, TP53
APC, EVC2, KRAS, PIK3CA, TCF7L2
APC, EVC2, KRAS, TP53
APC, FBXW7, KRAS, TP53
APC, EPHA3, PIK3CA, TP53
APC, FBXW7, KRAS, PIK3CA
APC, EVC2, FBXW7, KRAS
APC, KRAS, TCF7L2, TP53
APC, FBXW7, TCF7L2, TP53
90
1000
BpUdsUJLOCZEWCp. CAPRI
BpUdsUJLOCZEWCp. CBN_ot
APC
EPHA3
PIK3CA
TCF7L2
KRAS
EVC2
FBXW7
TP53
