# Supplementary material for: Cancer progression models and fitness landscapes: a many-to-many relationship
Source: Bioinformatics. 2017 Oct 18;34(5):836–44. doi: 10.1093/bioinformatics/btx663 (PMC6031050; doi:10.1093/bioinformatics/btx663)
Supplement: Supplementary Data [file btx663_supp.zip › btx663-suppl_data/gliob-landscapes-dags.pdf]

ID	p−value	Accessible Genot.
cARBYlkOOZrRNYr	0.601	75

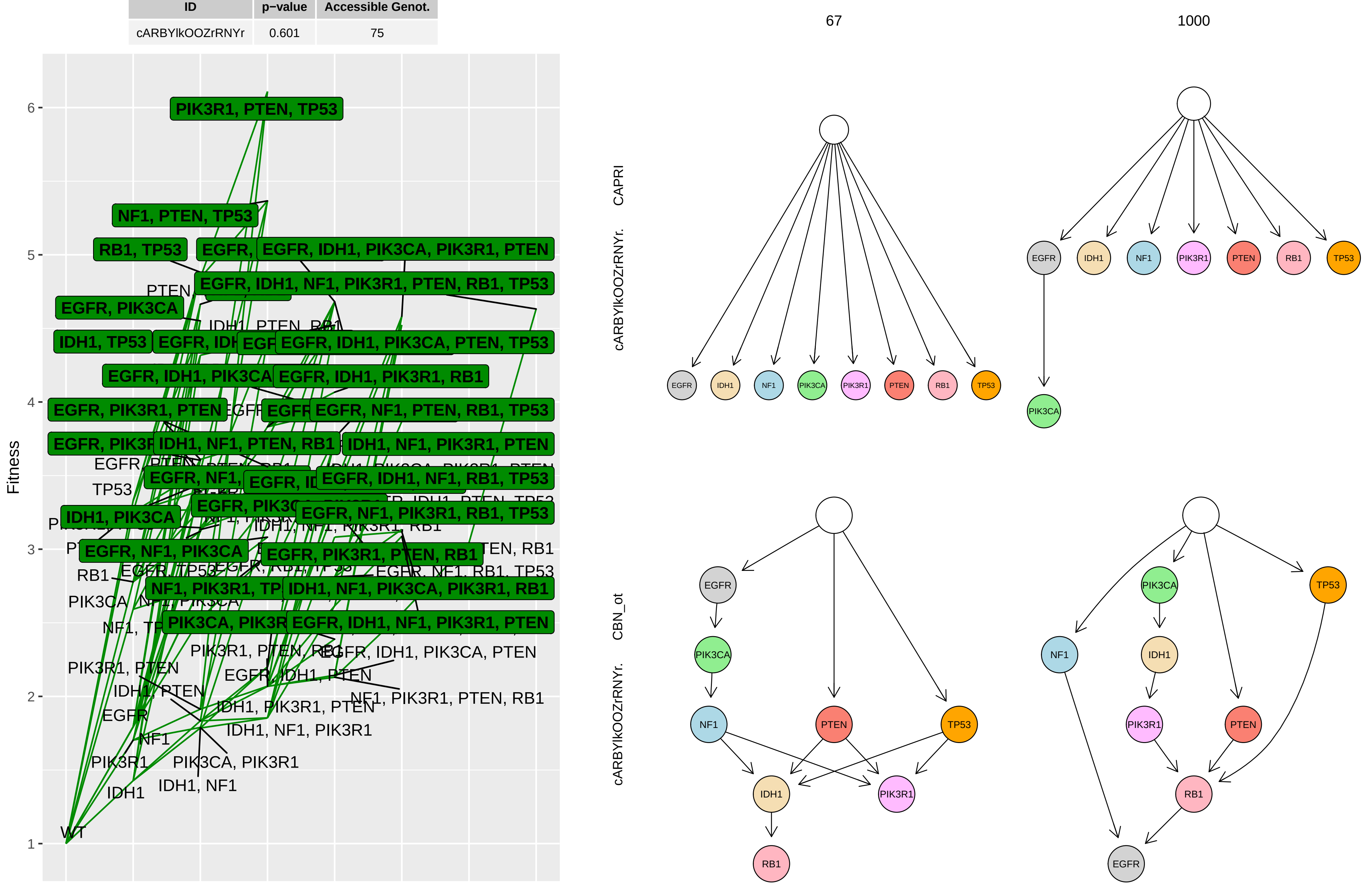

ID	p−value	Accessible Genot.
DzCrpQVHXirVDus	0.602	52

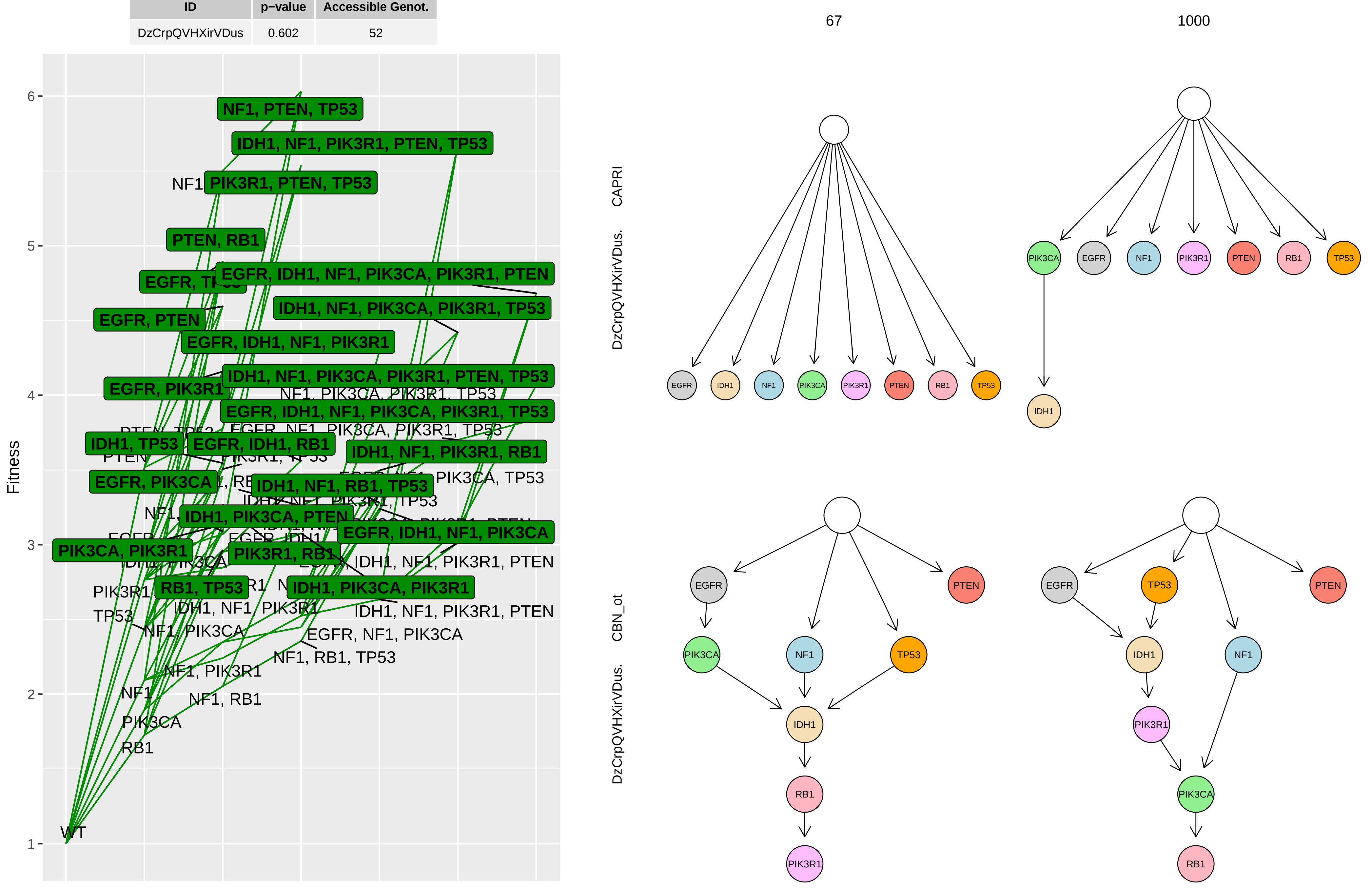

ID	p−value	Accessible Genot.
rrjKQOrRzsVJgvc	0.602	24

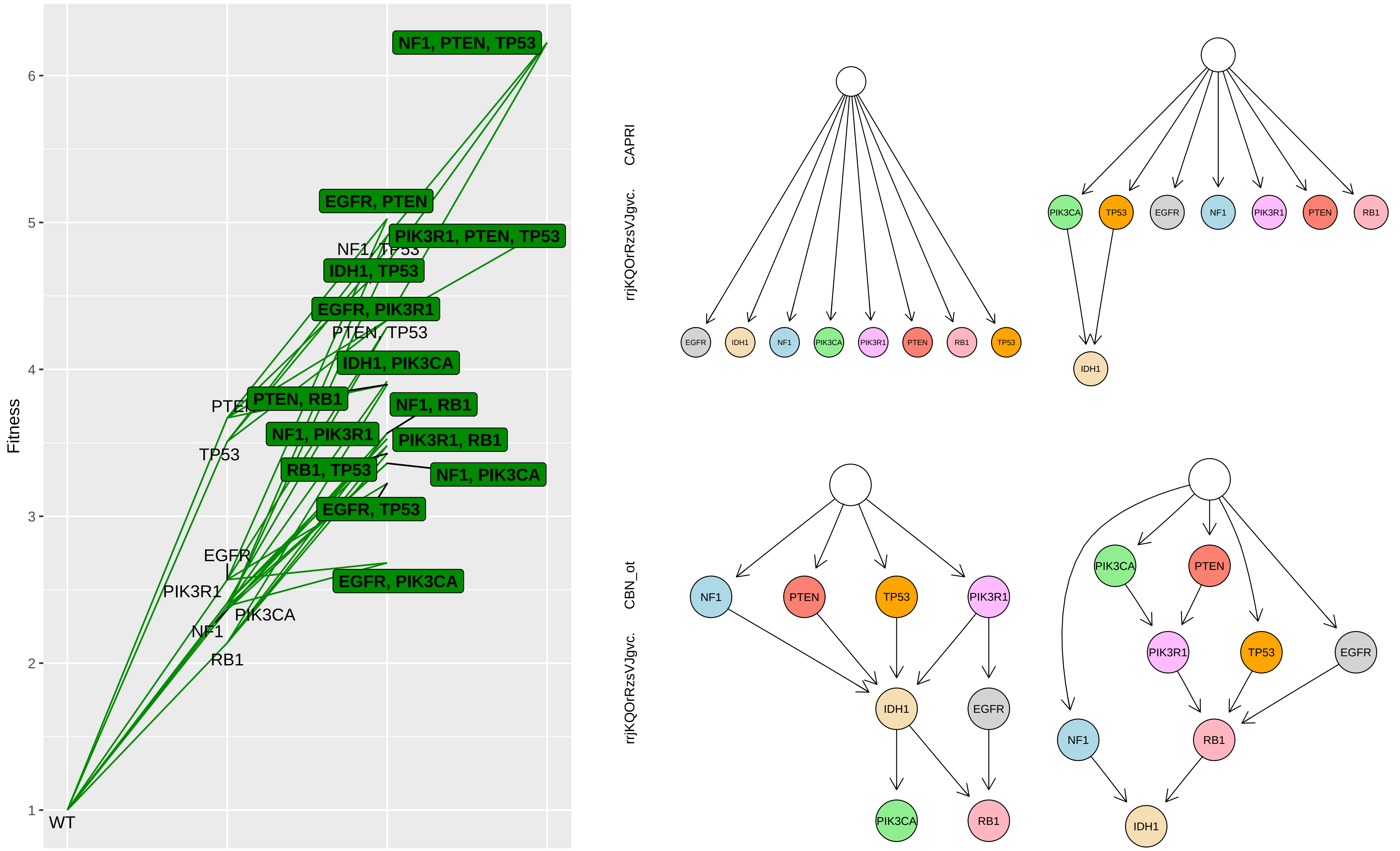

ID	p-value	Accessible Genot.
NTNExCRLdtSRIPI	0.603	245

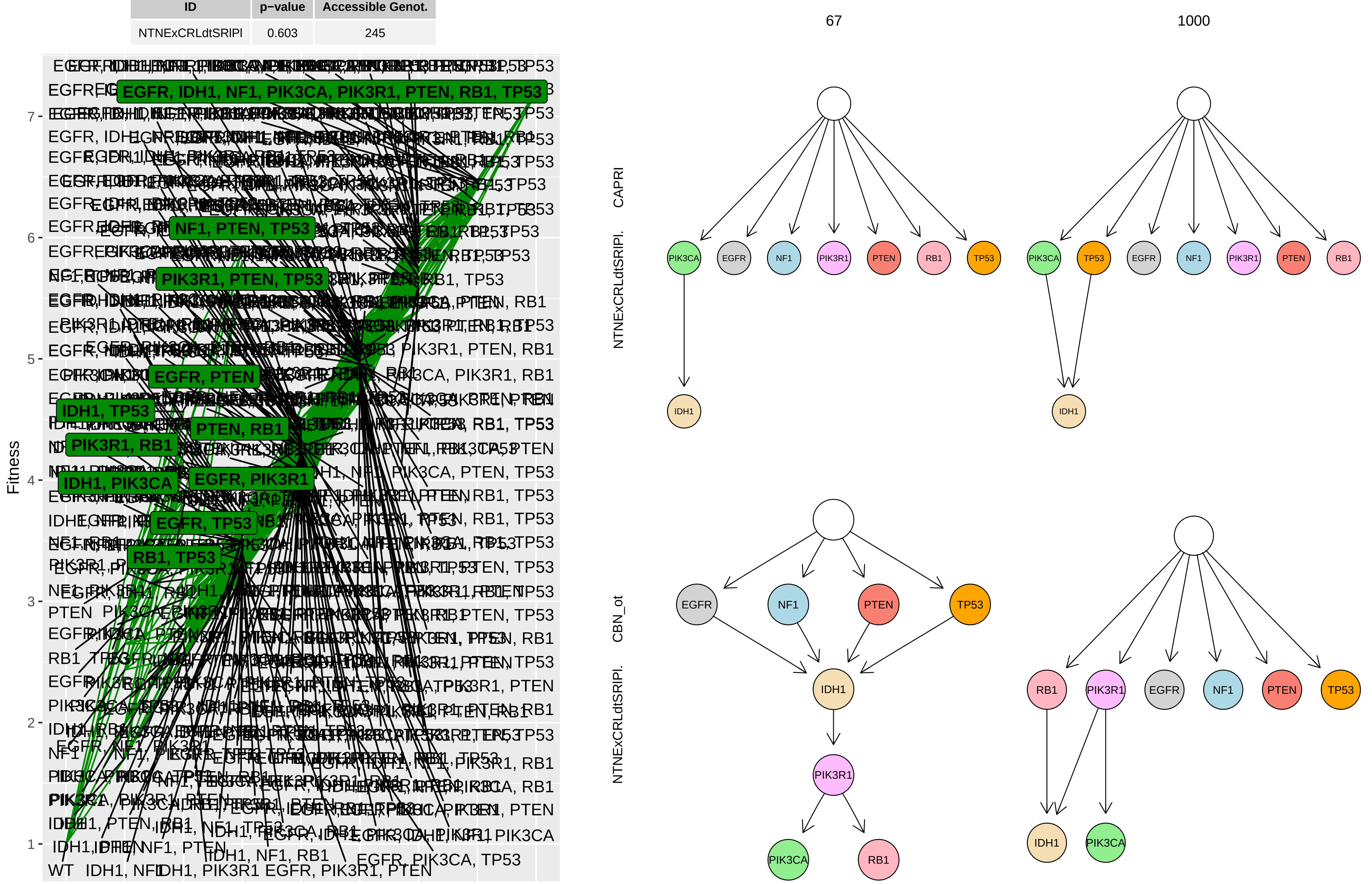

ID	p−value	Accessible Genot.
uBAkaTyRNEibOqT	0.603	28

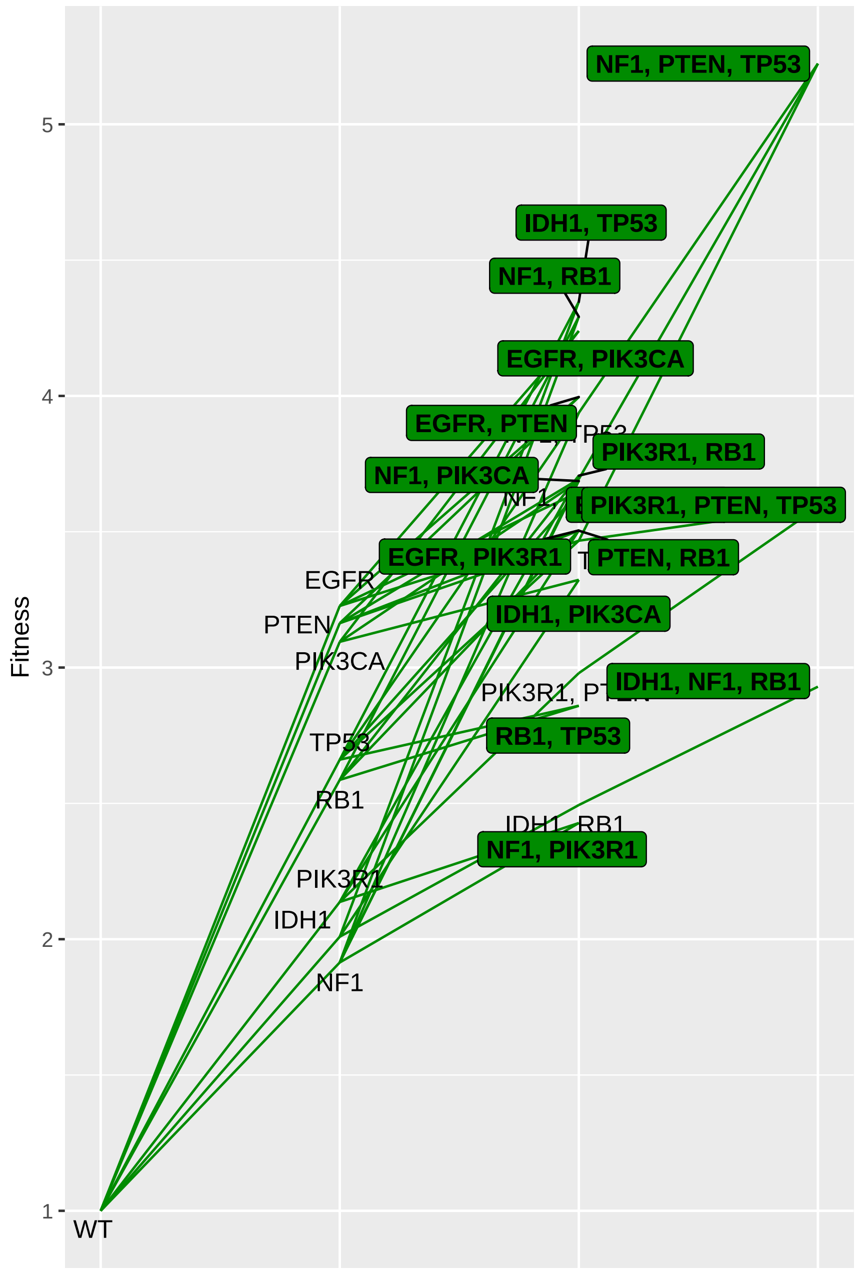

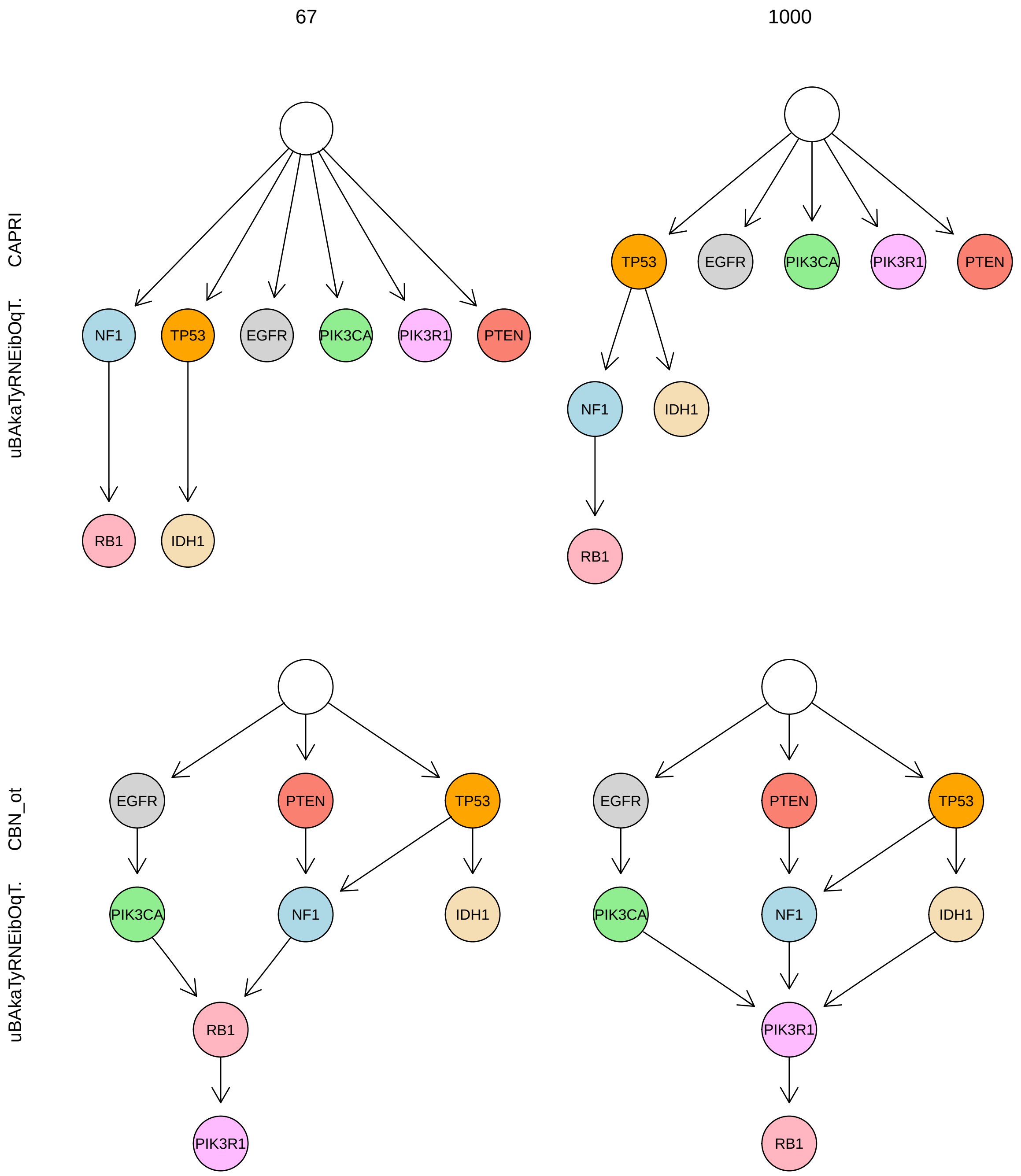

ID	p−value	Accessible Genot.
HUTPlkEwwEEBDLj	0.604	25

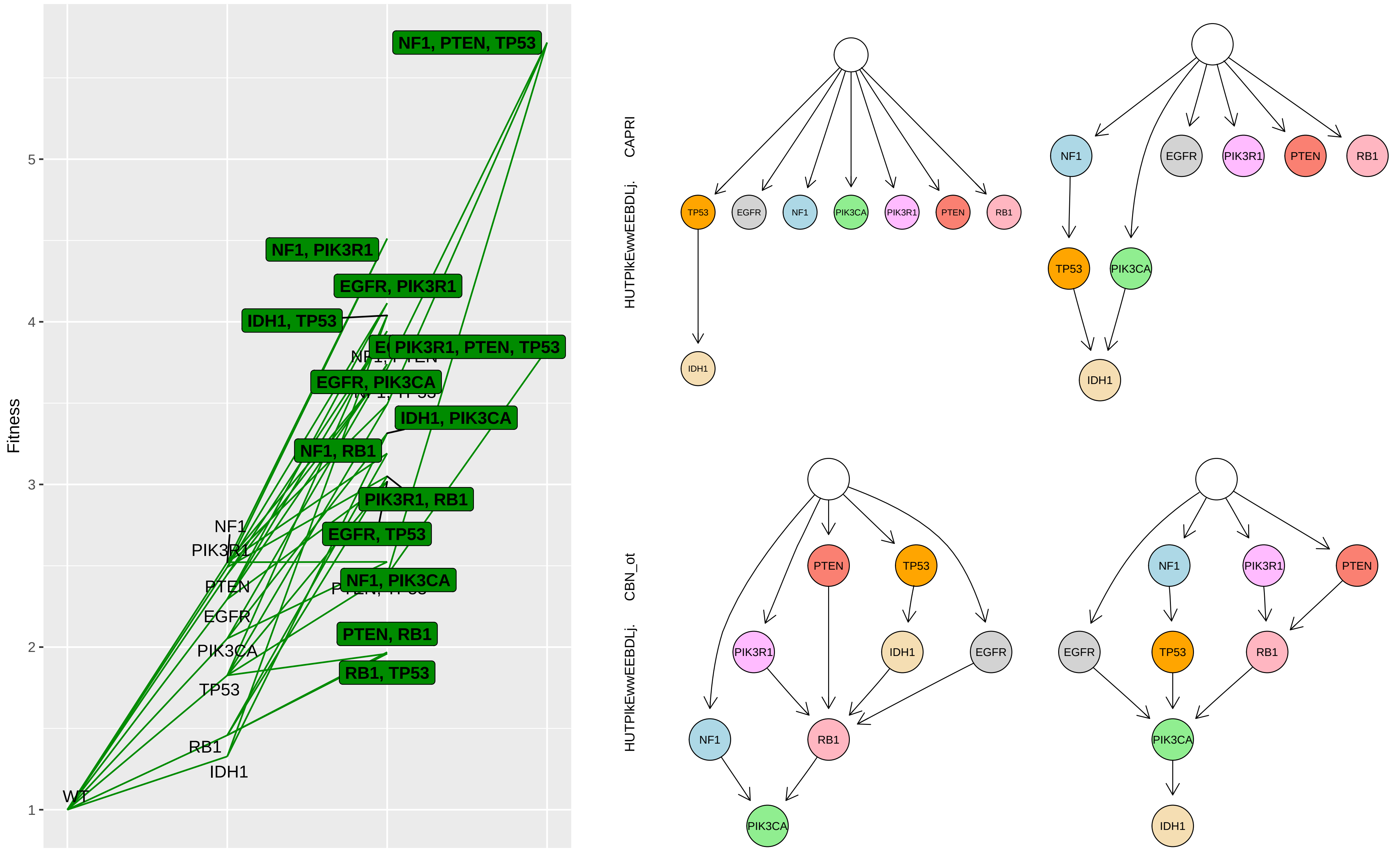

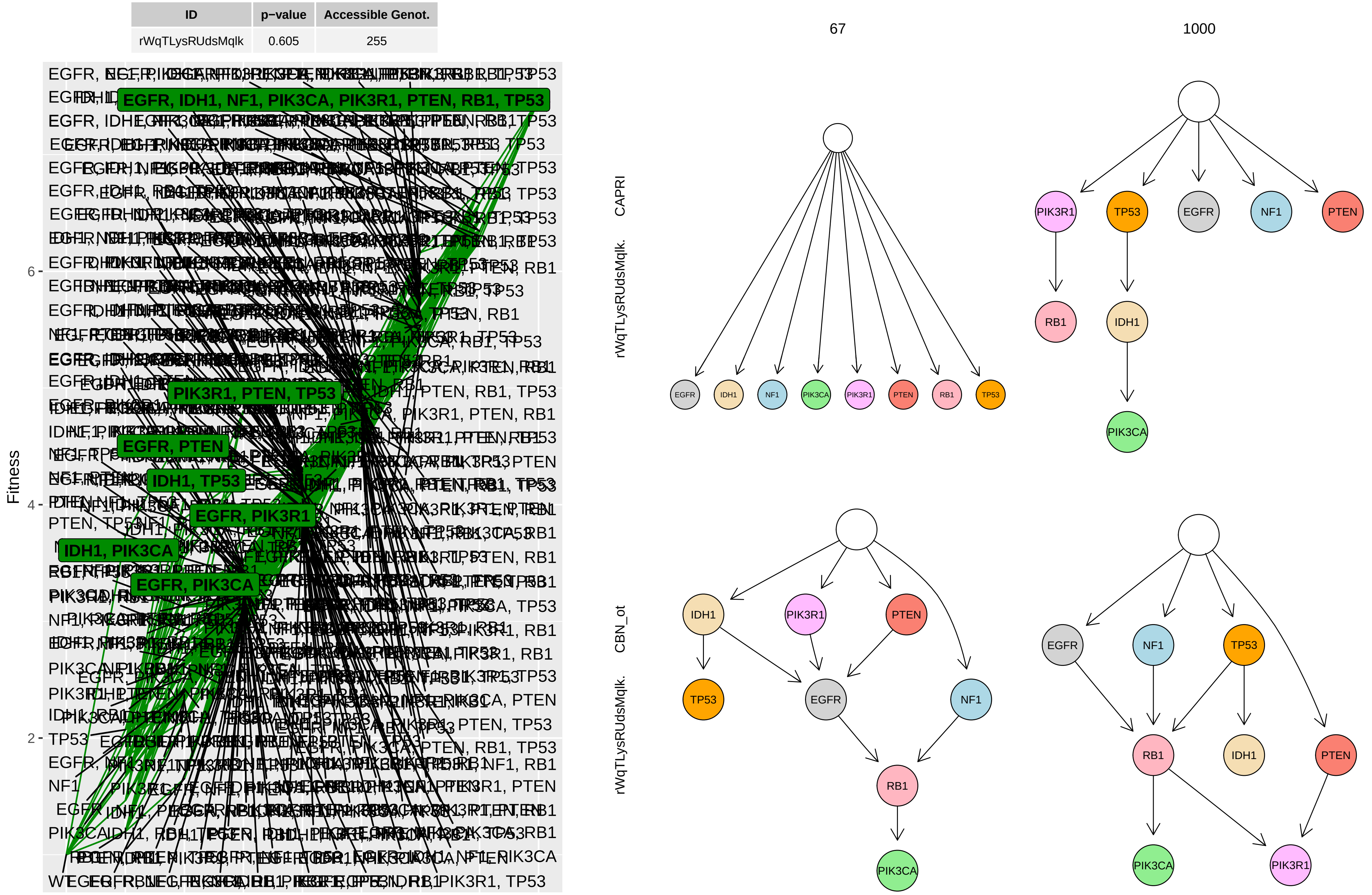
ID
p-value
Accessible Genot.
rWqTLysRUdsMqlk
0.605
255
EGFR, IDH1, NF1, PIK3CA, PIK3R1, PTEN, RB1, TP53
PIK3R1, PTEN, TP53
EGFR, PTEN
IDH1, TP53
EGFR, PIK3R1
IDH1, PIK3CA
EGFR, PIK3CA
Fitness
6
4
2
67
1000
rWqTLysRUdsMqlk.   CAPRI
rWqTLysRUdsMqlk.   CBN_ot
EGFR
IDH1
NF1
PIK3CA
PIK3R1
PTEN
RB1
TP53

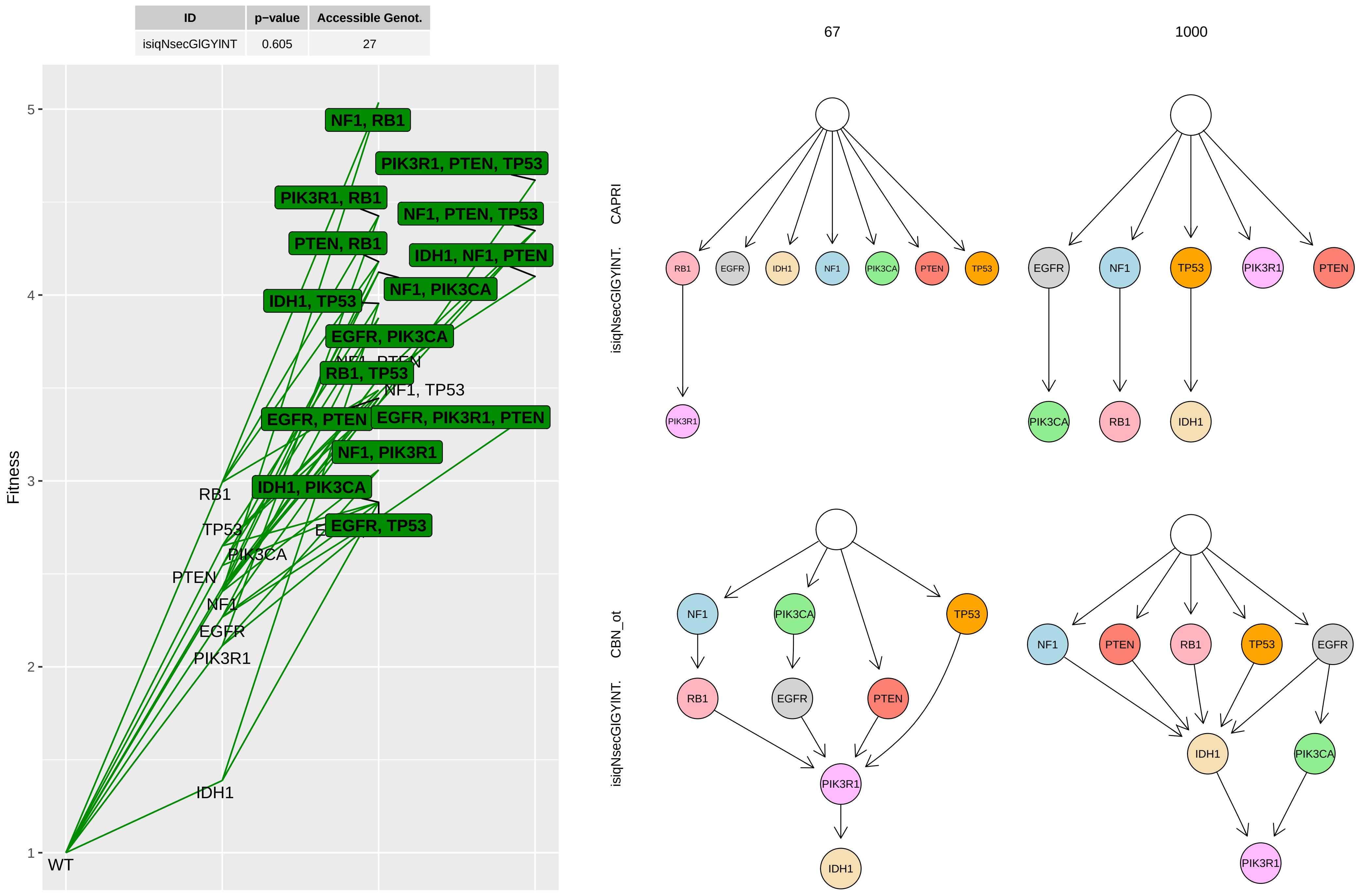
ID | p−value | Accessible Genot.
isiqNsecGIGYINT | 0.605 | 27
Fitness
1
2
3
4
5
WT
IDH1
PIK3R1
EGFR
NF1
PTEN
PIK3CA
TP53
RB1
NF1, RB1
PIK3R1, PTEN, TP53
PIK3R1, RB1
NF1, PTEN, TP53
PTEN, RB1
IDH1, NF1, PTEN
NF1, PIK3CA
IDH1, TP53
EGFR, PIK3CA
RB1, TP53
NF1, TP53
EGFR, PTEN
EGFR, PIK3R1, PTEN
NF1, PIK3R1
IDH1, PIK3CA
EGFR, TP53
67
1000
isiqNsecGIGYINT. CAPRI
isiqNsecGIGYINT. CBN_ot
RB1
EGFR
IDH1
NF1
PIK3CA
PTEN
TP53
PIK3R1

ID	p-value	Accessible Genot.
OrnWkYQWHwAMzrP	0.606	36

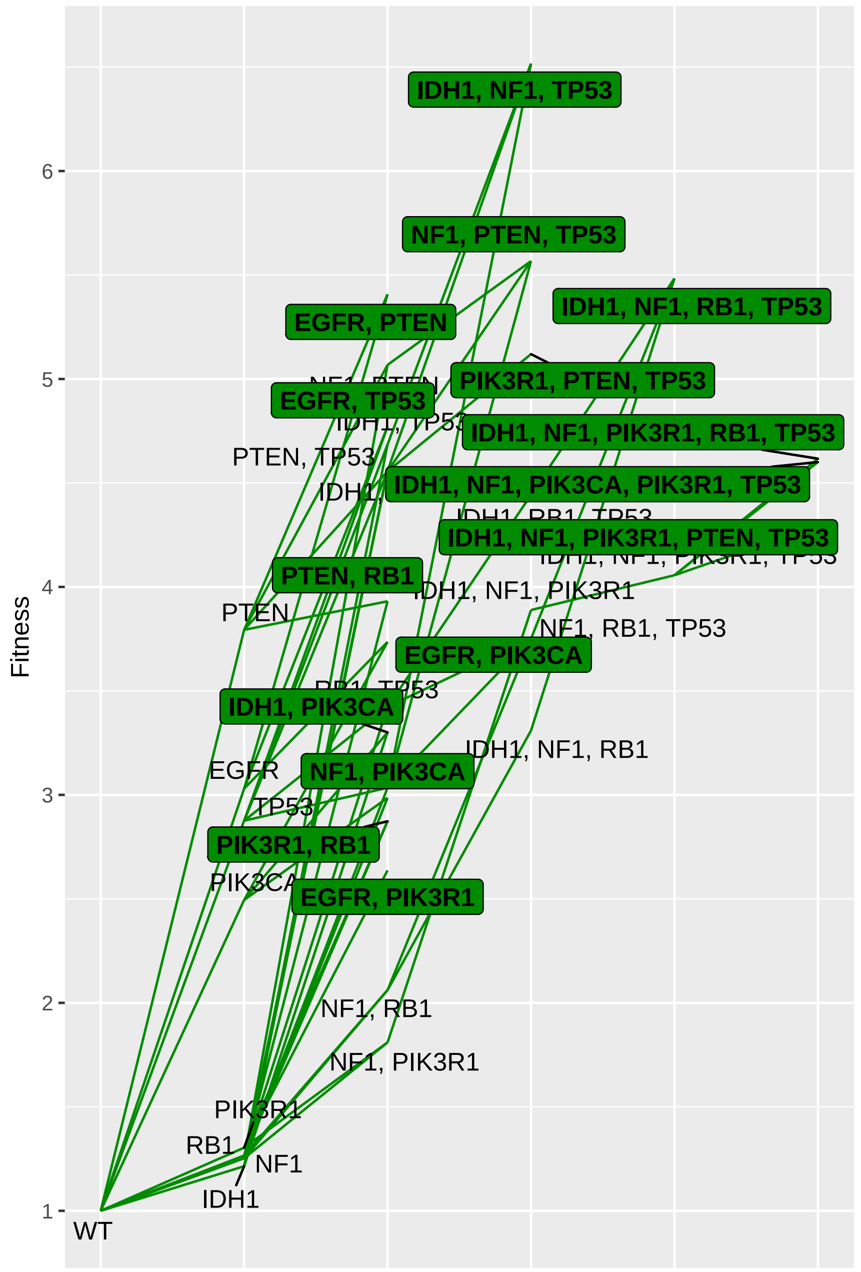

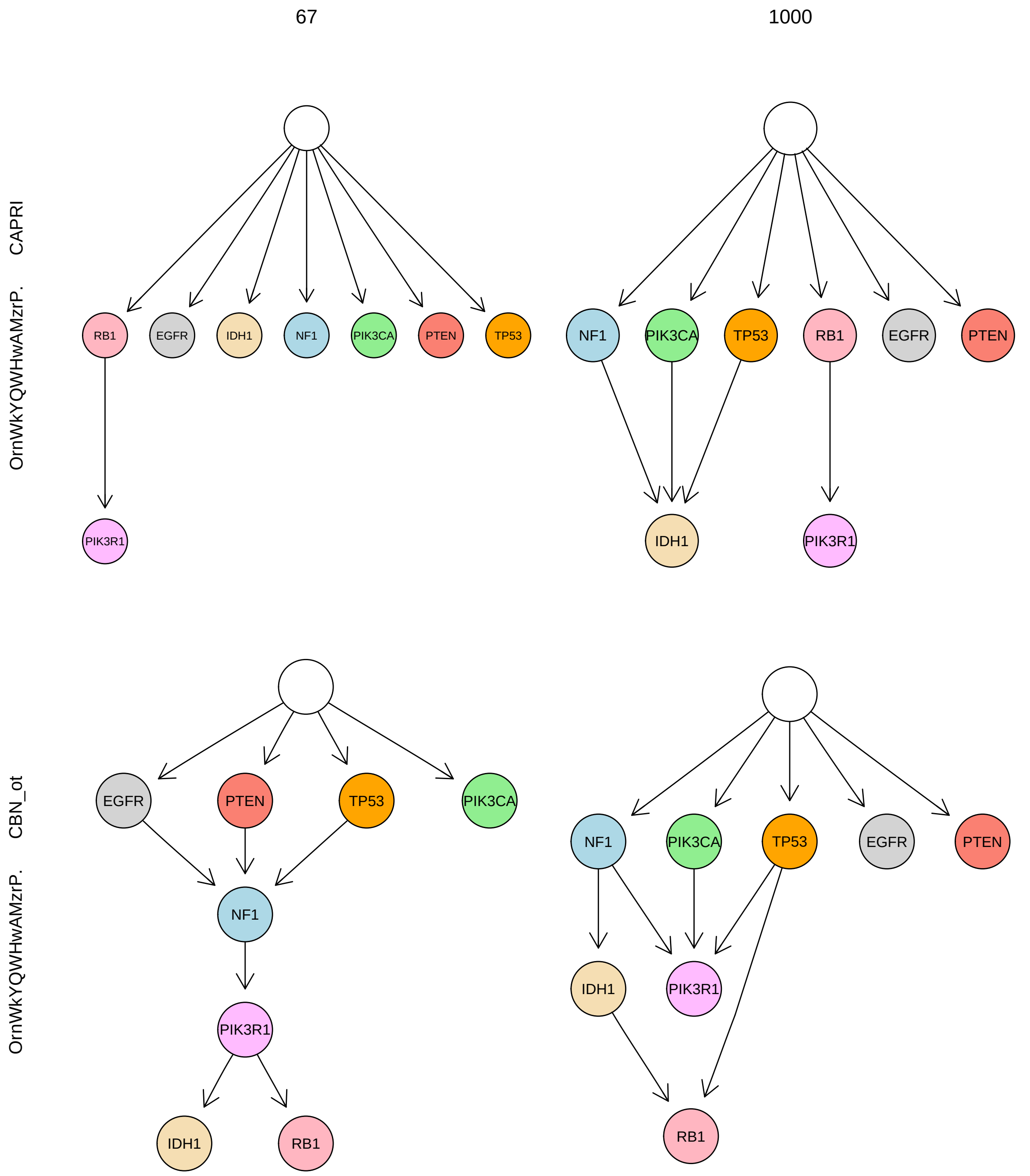

ID	p−value	Accessible Genot.
hAmGulLNhiRGzea	0.606	54

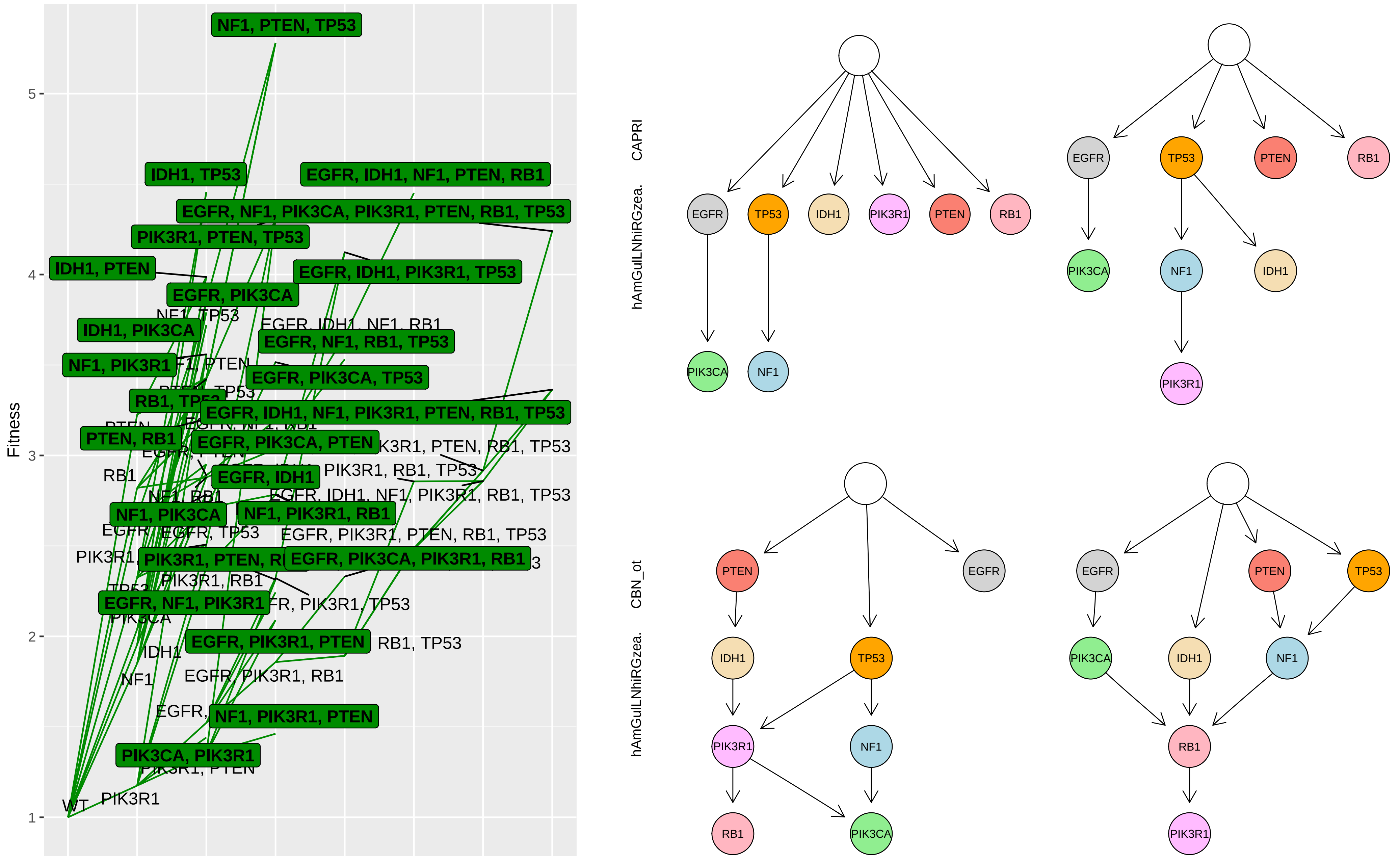

ID	p−value	Accessible Genot.
JyNmLqwppKrhpgj	0.607	67

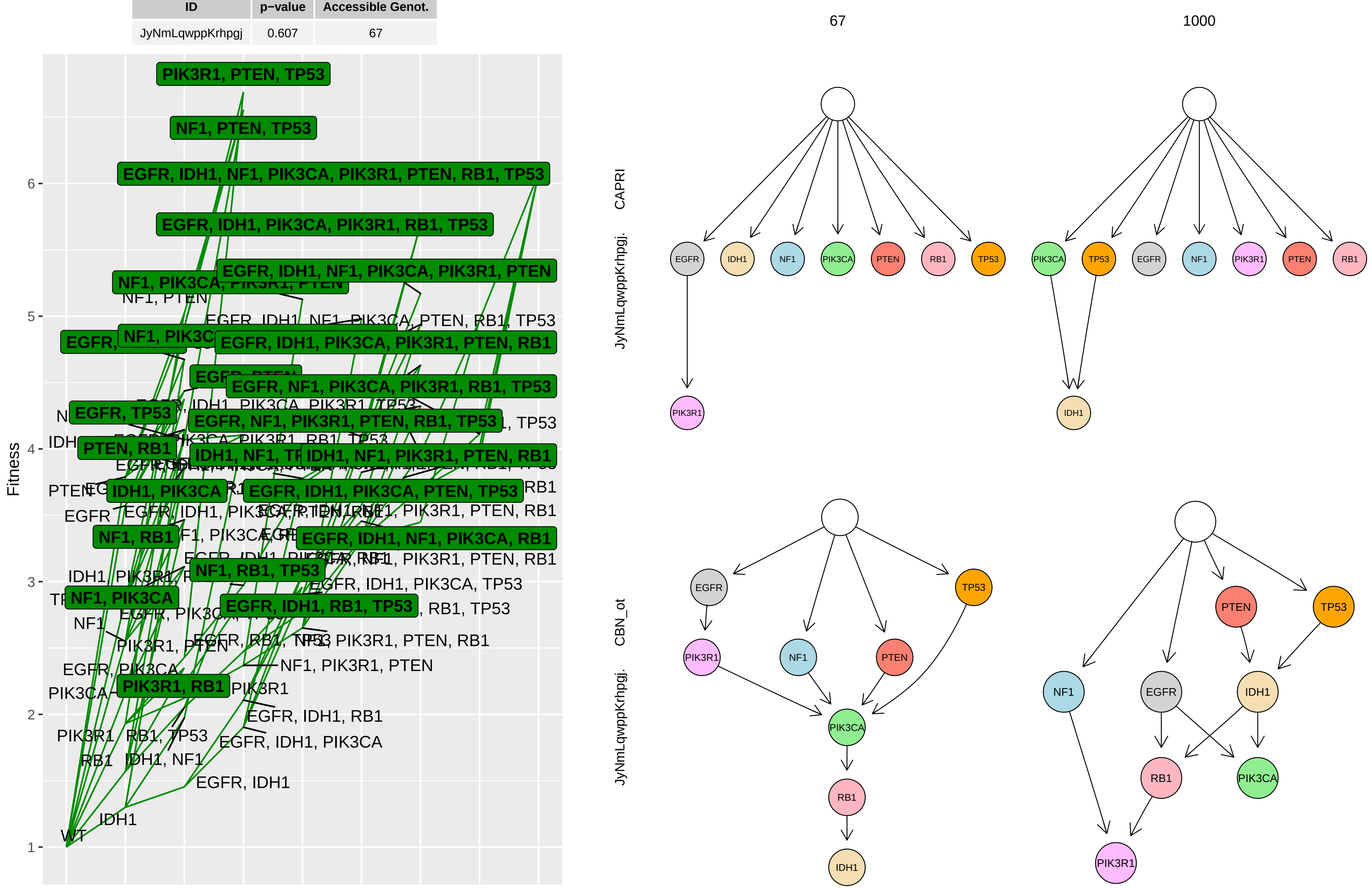

ID	p−value	Accessible Genot.
IEHyLnANpoQEWjd	0.609	30

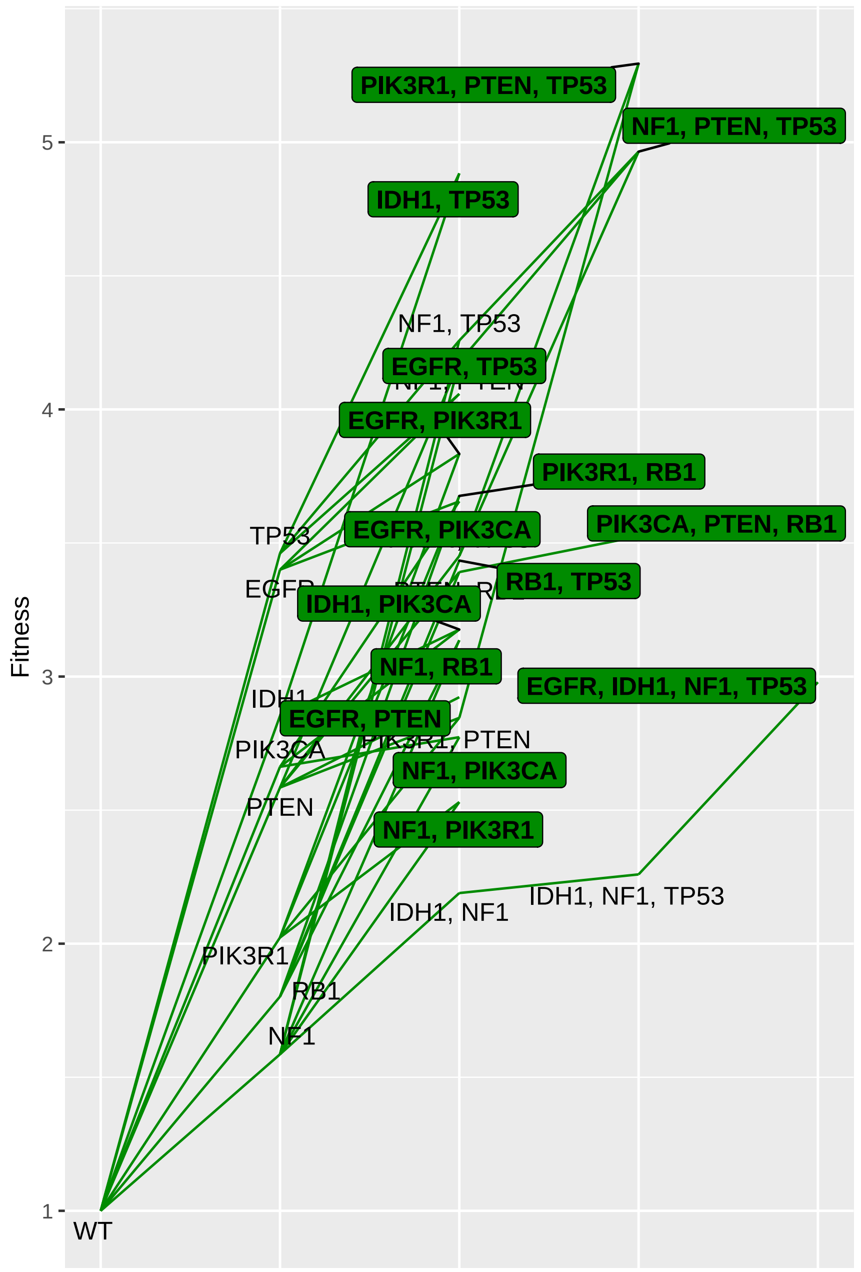

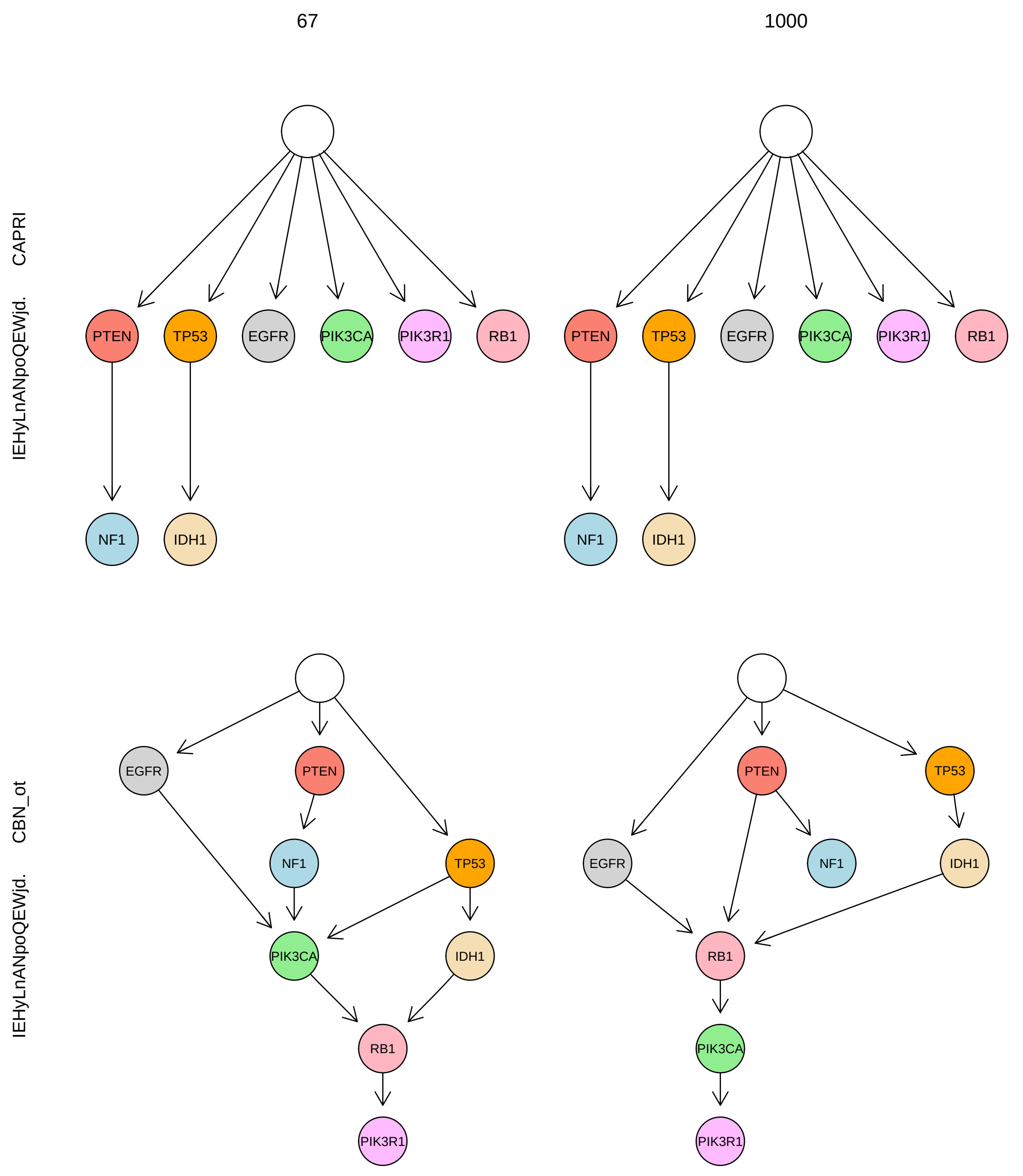

ID	p−value	Accessible Genot.
XCLcvNBXqIHhtlh	0.61	71

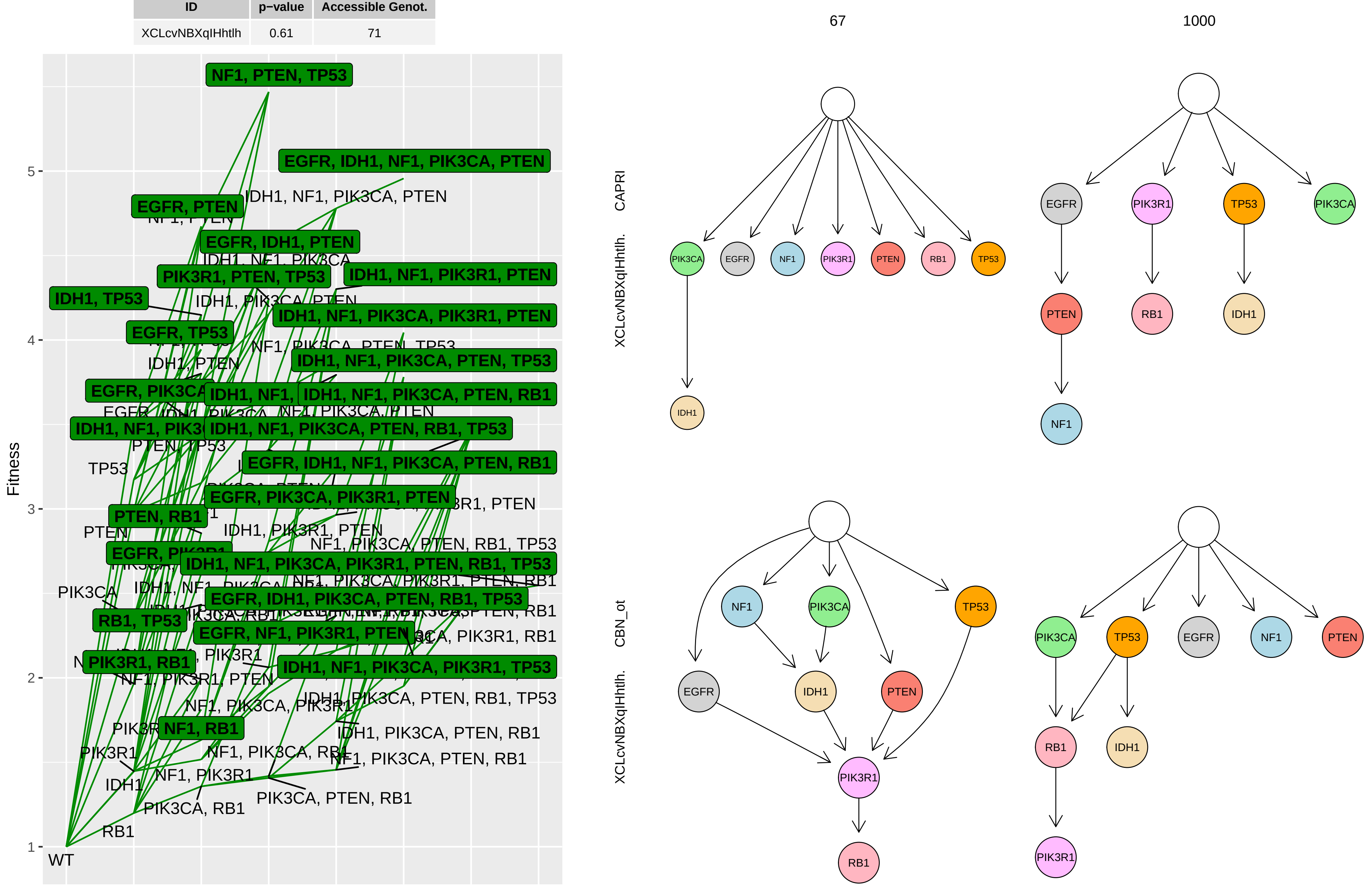

ID	p−value	Accessible Genot.
IcQizheFMAfSDbD	0.61	31

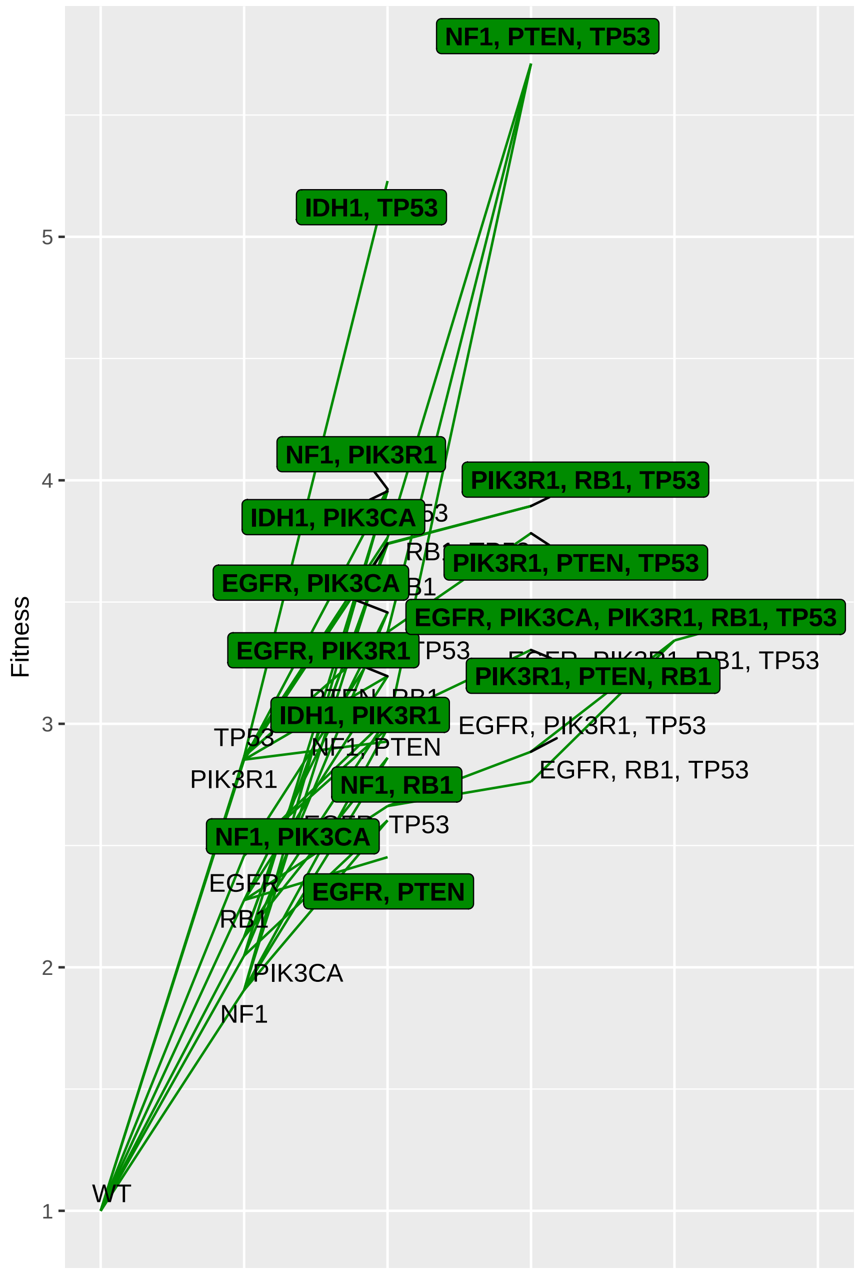

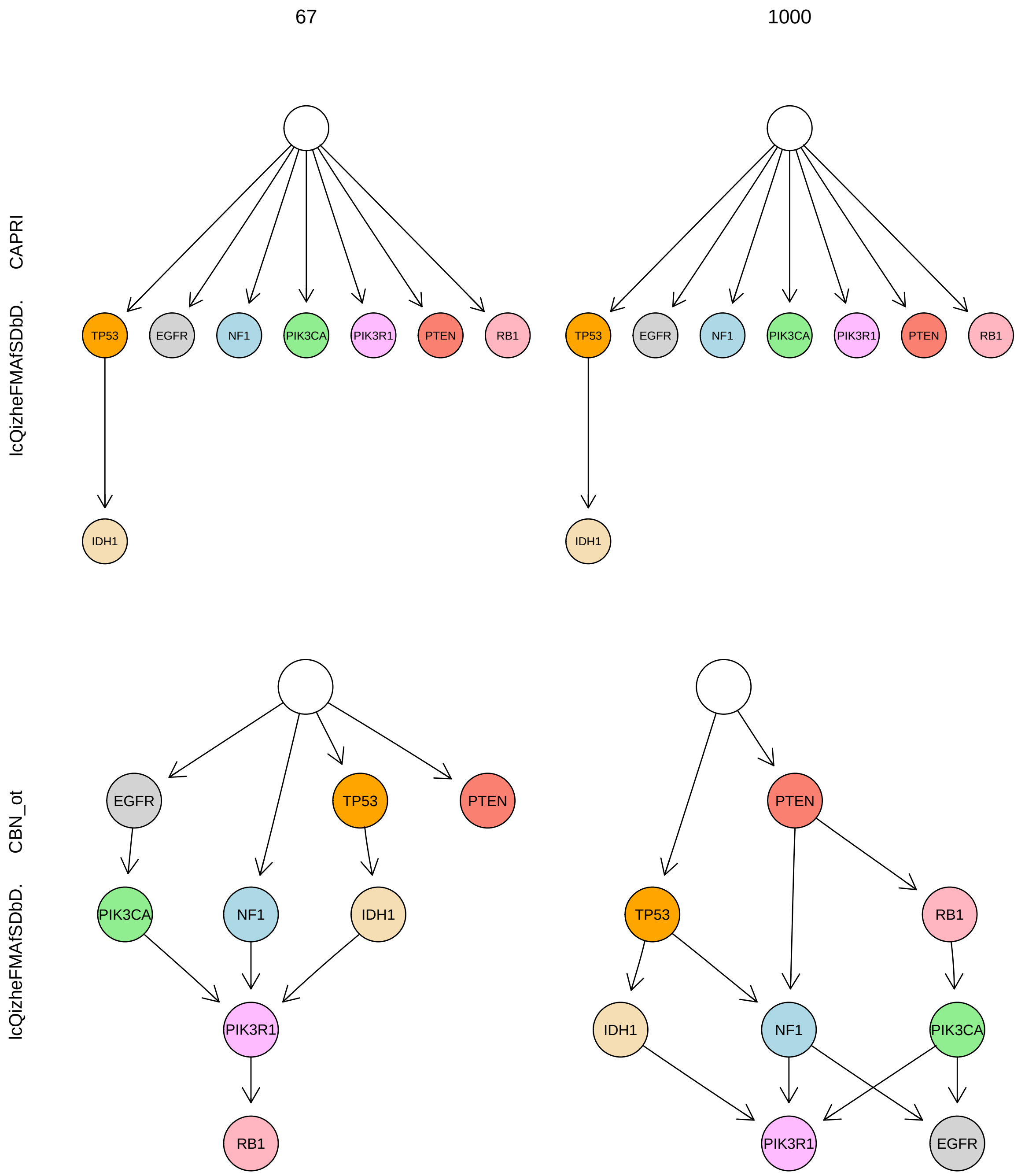

ID	p-value	Accessible Genot.
HCLfduPHzOgYtch	0.611	46

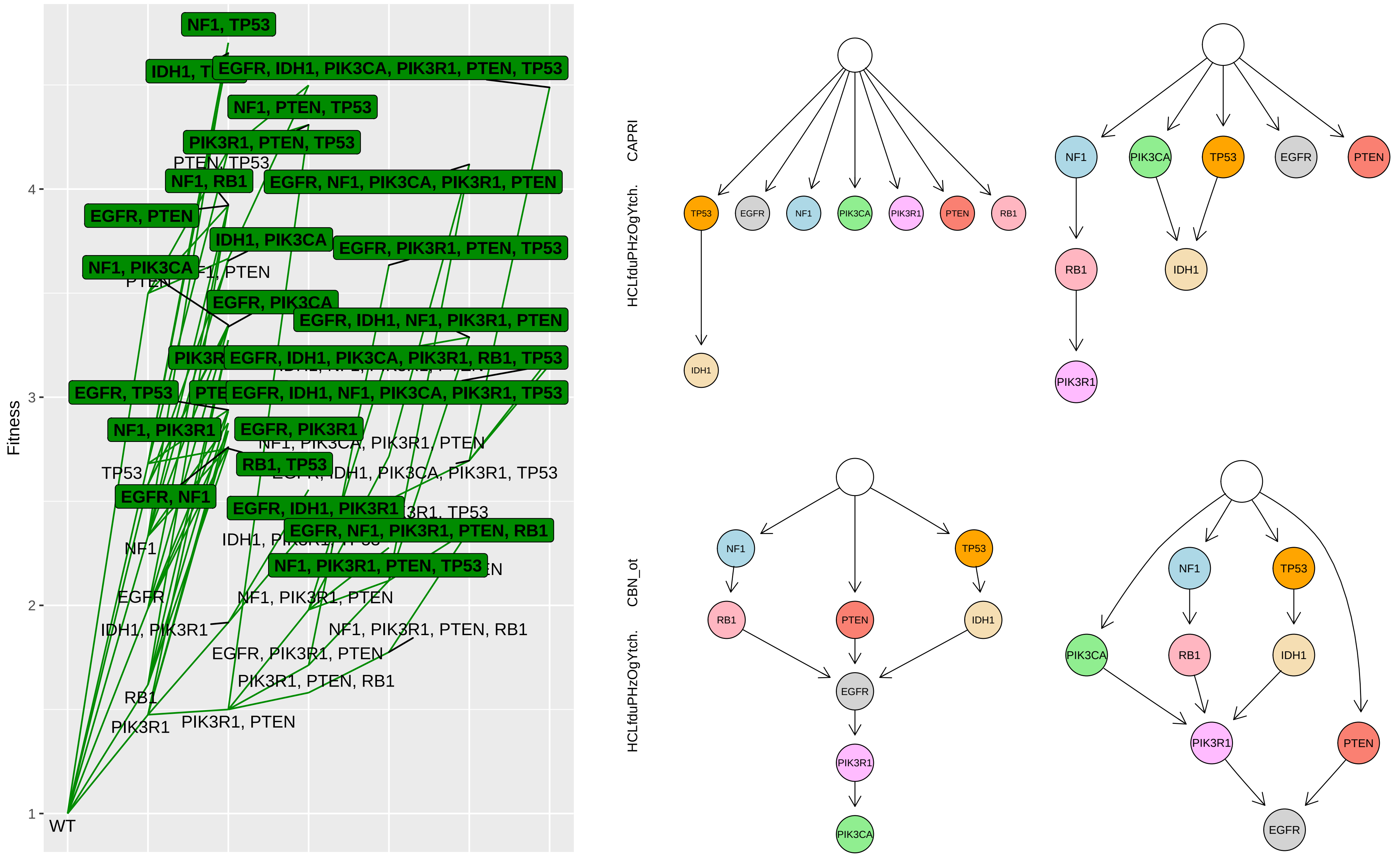

ID	p−value	Accessible Genot.
wCwZQEPLectHCnl	0.612	79

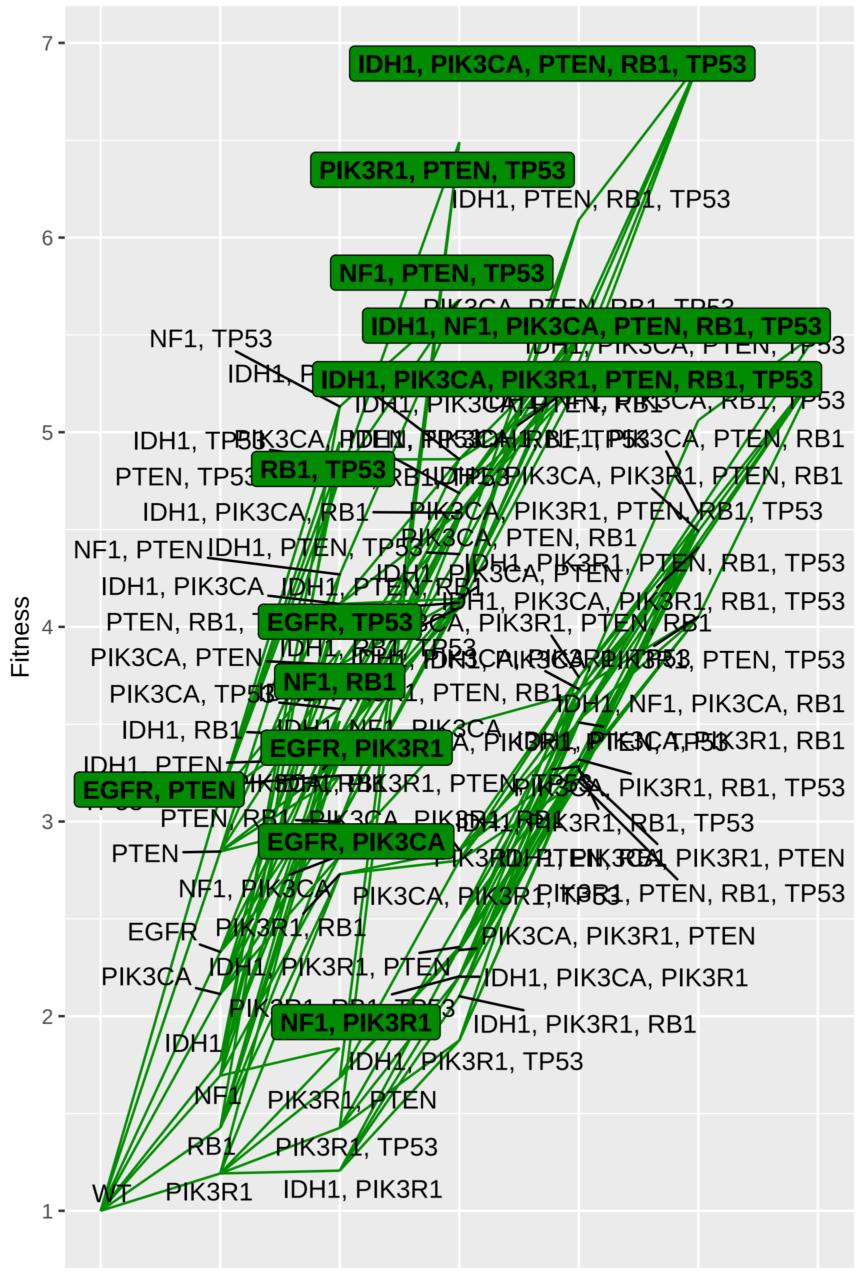

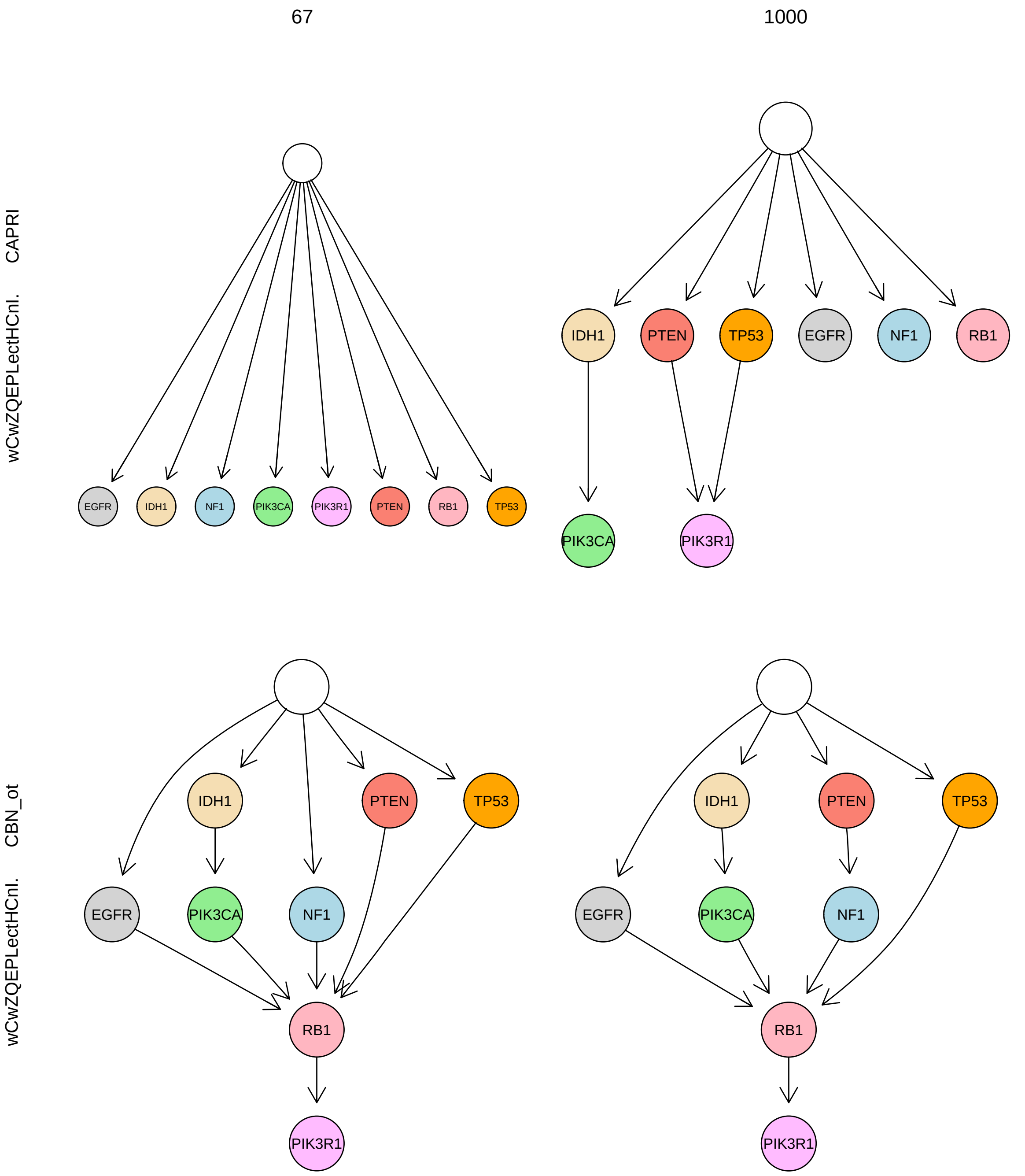

ID	p−value	Accessible Genot.
OWDqppokHzzdzDU	0.615	30

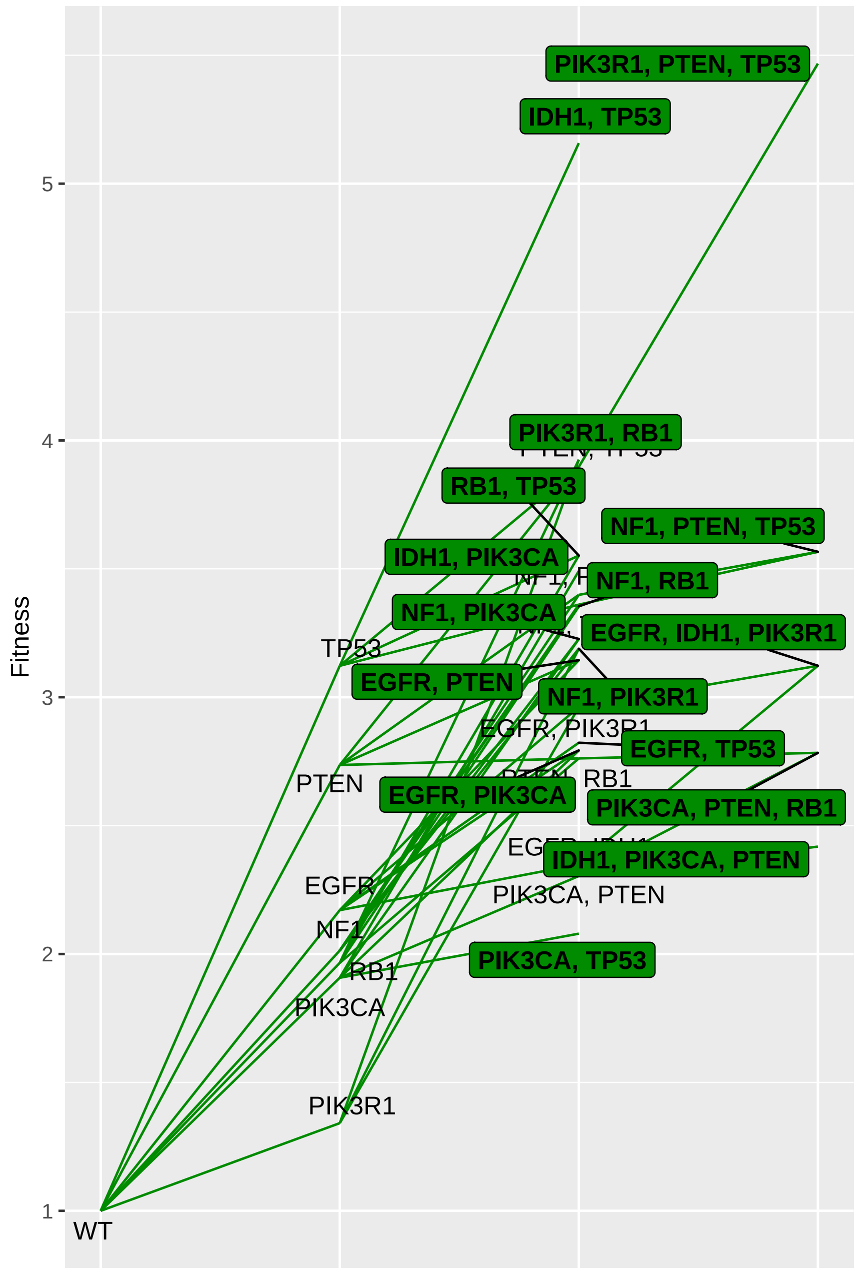

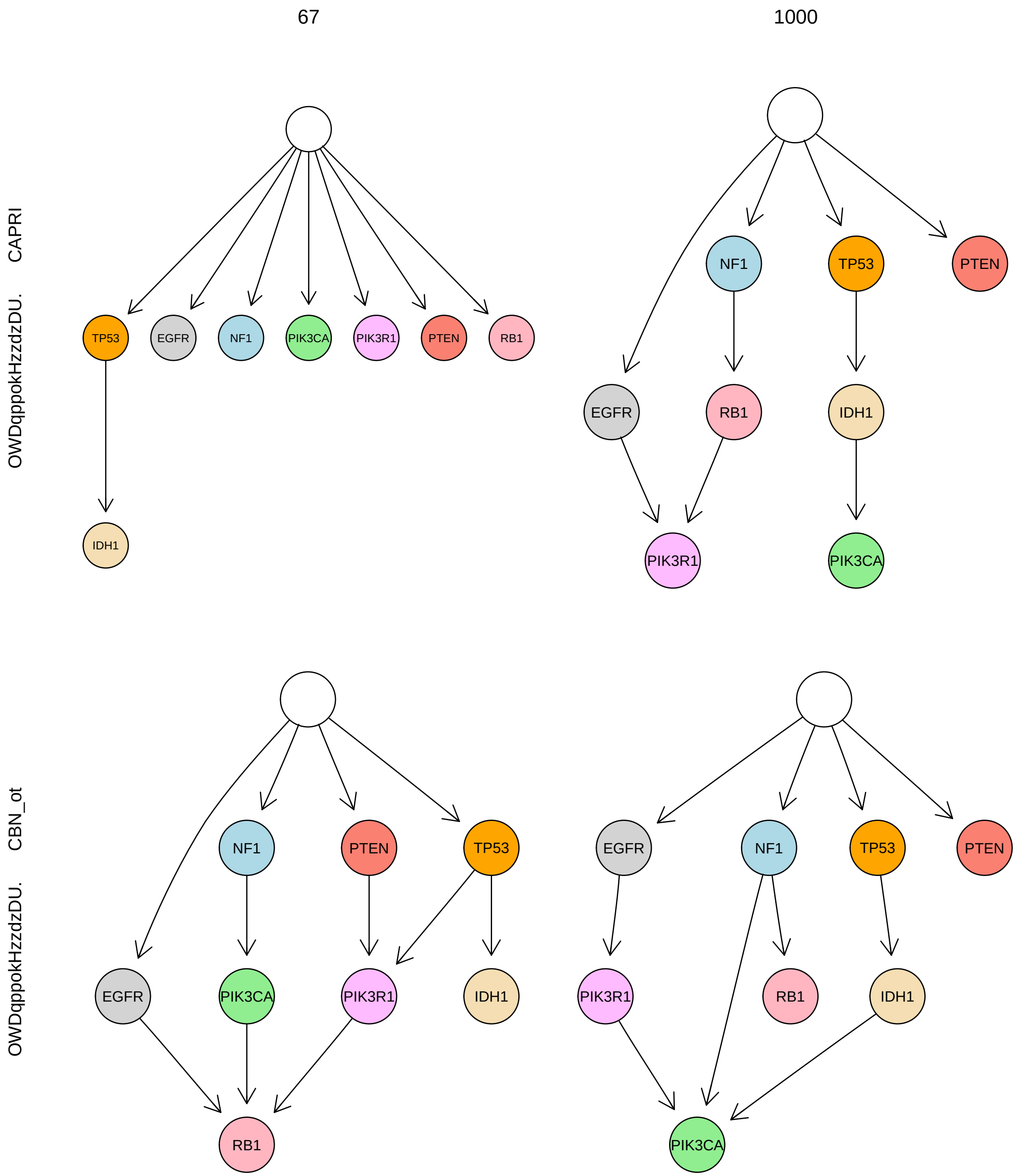

ID	p−value	Accessible Genot.
ZAEcXrulyyOWyel	0.615	84

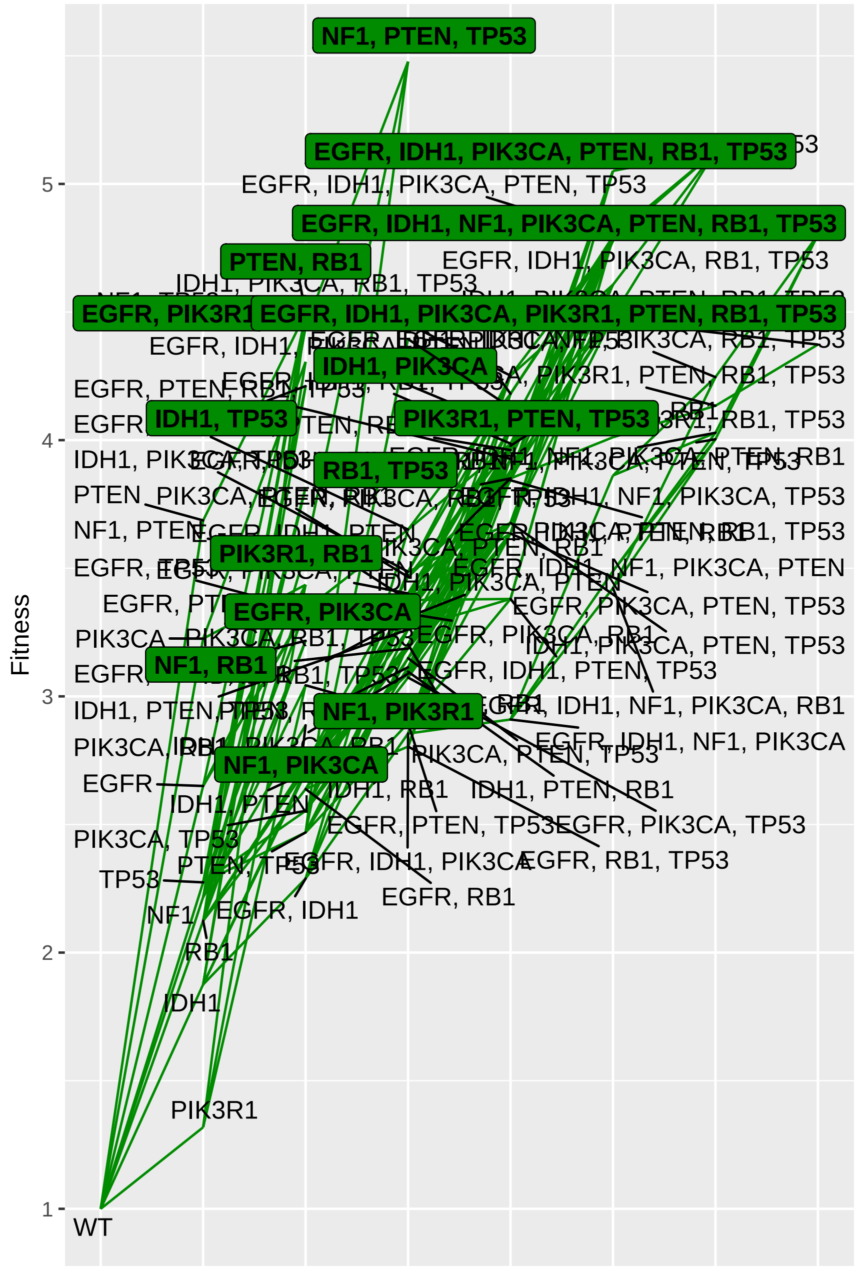

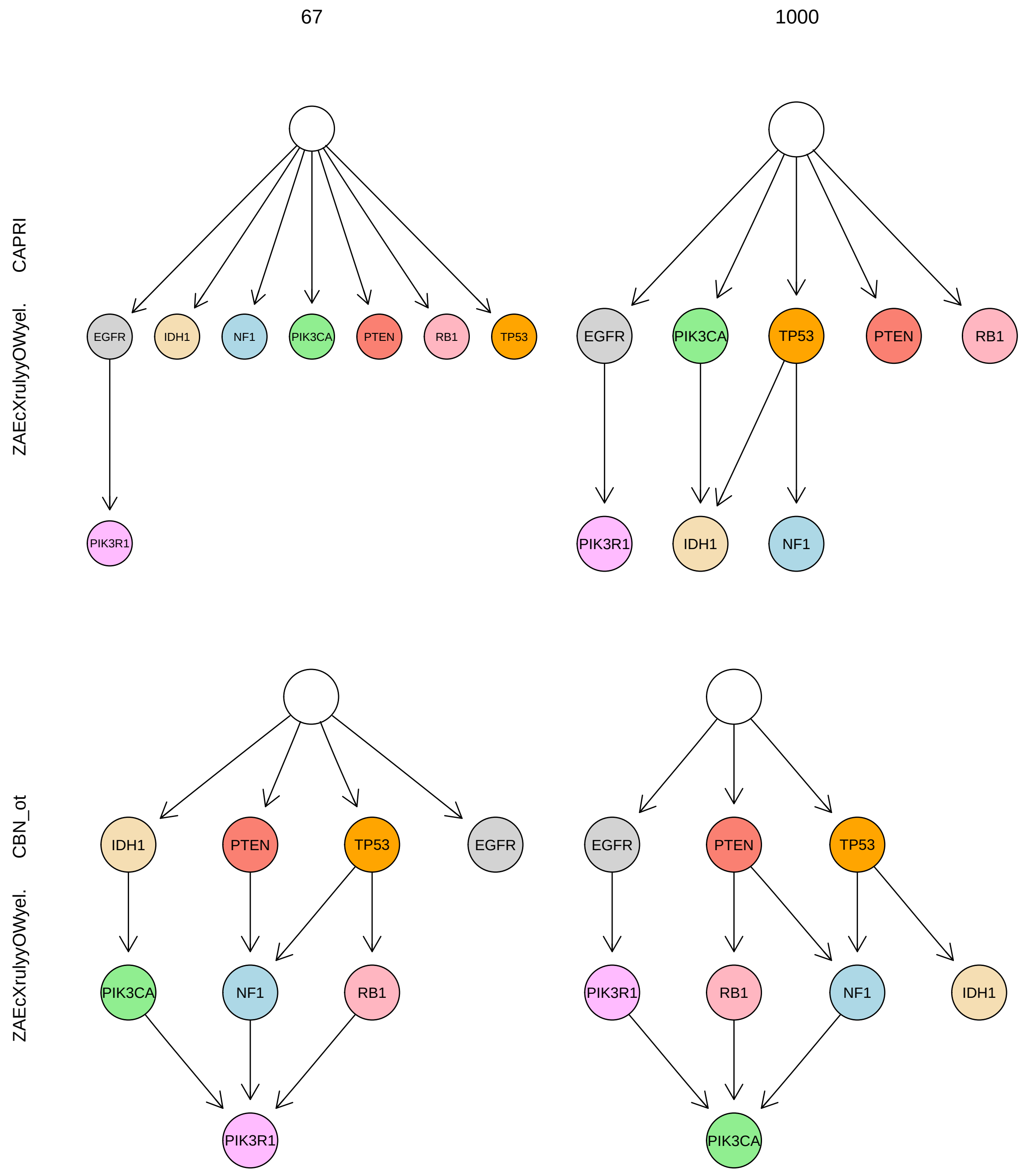

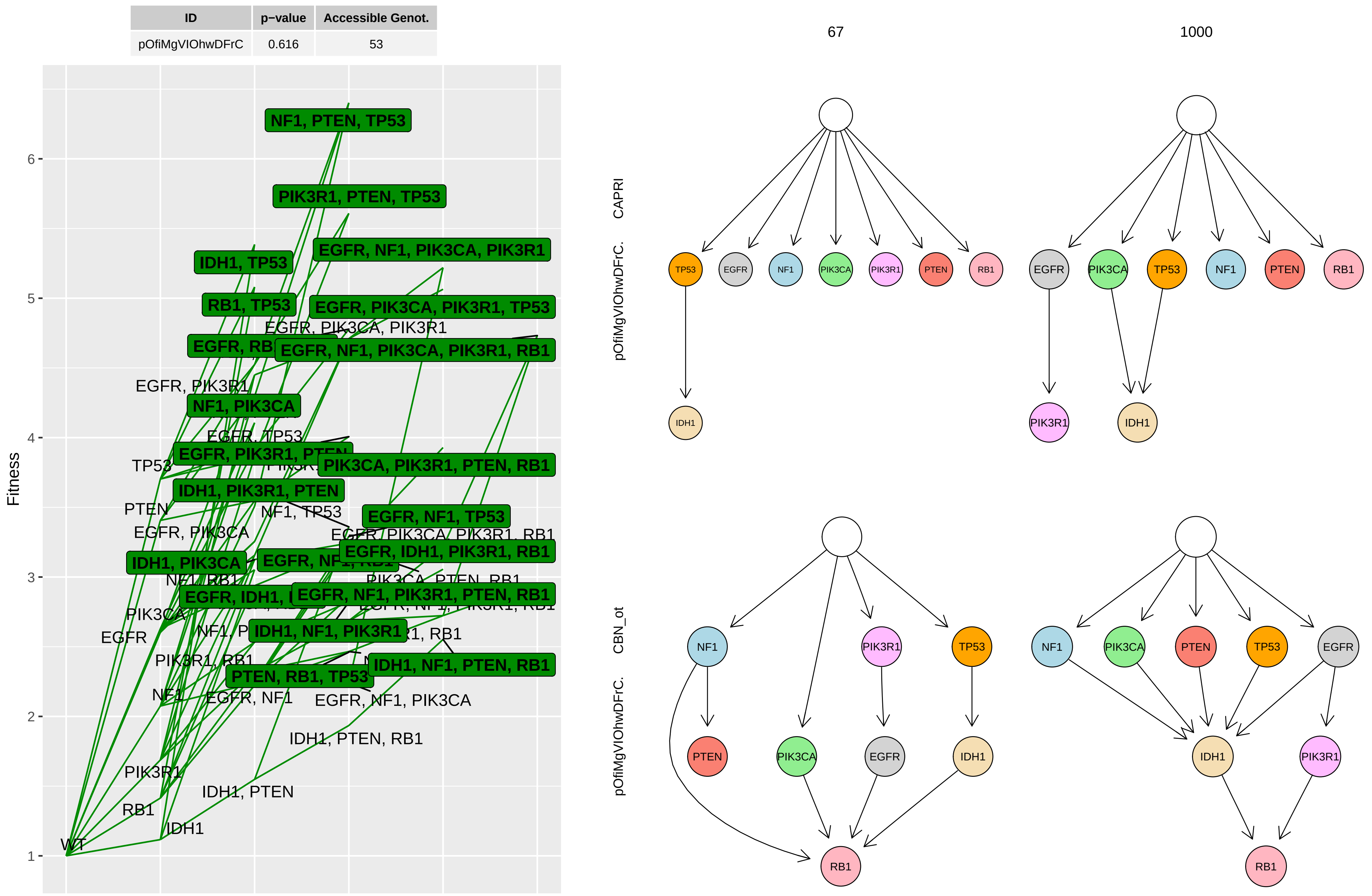
ID
p−value
Accessible Genot.
pOfiMgVIOhwDFrC
0.616
53
Fitness
1
2
3
4
5
6
NF1, PTEN, TP53
PIK3R1, PTEN, TP53
EGFR, NF1, PIK3CA, PIK3R1
IDH1, TP53
RB1, TP53
EGFR, PIK3CA, PIK3R1, TP53
EGFR, PIK3CA, PIK3R1
EGFR, NF1, PIK3CA, PIK3R1, RB1
EGFR, PIK3R1
NF1, PIK3CA
EGFR, TP53
TP53
PIK3CA, PIK3R1, PTEN, RB1
IDH1, PIK3R1, PTEN
PTEN
NF1, TP53
EGFR, NF1, TP53
EGFR, PIK3CA
EGFR, PIK3CA, PIK3R1, RB1
IDH1, PIK3CA
EGFR, IDH1, PIK3R1, RB1
NF1, RB1
PIK3CA, PTEN, RB1
EGFR, NF1, PIK3R1, PTEN, RB1
PIK3CA
EGFR
IDH1, NF1, PIK3R1
PIK3R1, RB1
IDH1, NF1, PTEN, RB1
PTEN, RB1, TP53
NF1
EGFR, NF1
EGFR, NF1, PIK3CA
IDH1, PTEN, RB1
PIK3R1
IDH1, PTEN
RB1
IDH1
WT
67
1000
pOfiMgVIOhwDFrC.
CAPRI
TP53
EGFR
NF1
PIK3CA
PIK3R1
PTEN
RB1
IDH1
CBN_ot

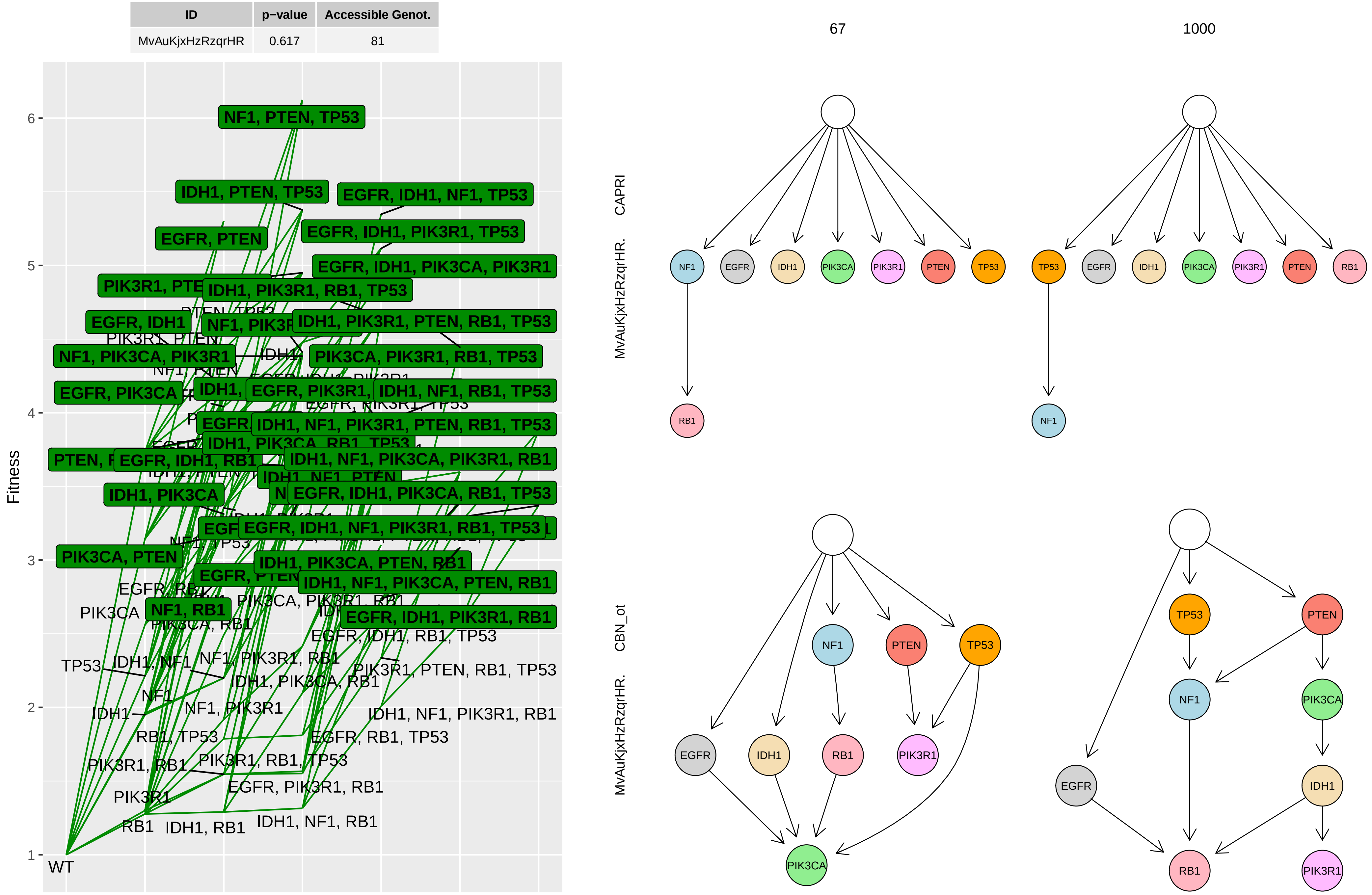
ID
p−value
Accessible Genot.
MvAuKjxHzRzqrHR
0.617
81
Fitness
WT
67
1000
CAPRI
CBN_ot
MvAuKjxHzRzqrHR.
NF1
EGFR
IDH1
PIK3CA
PIK3R1
PTEN
TP53
RB1

ID	p−value	Accessible Genot.
EgXBBOQLGOUhmYR	0.617	29

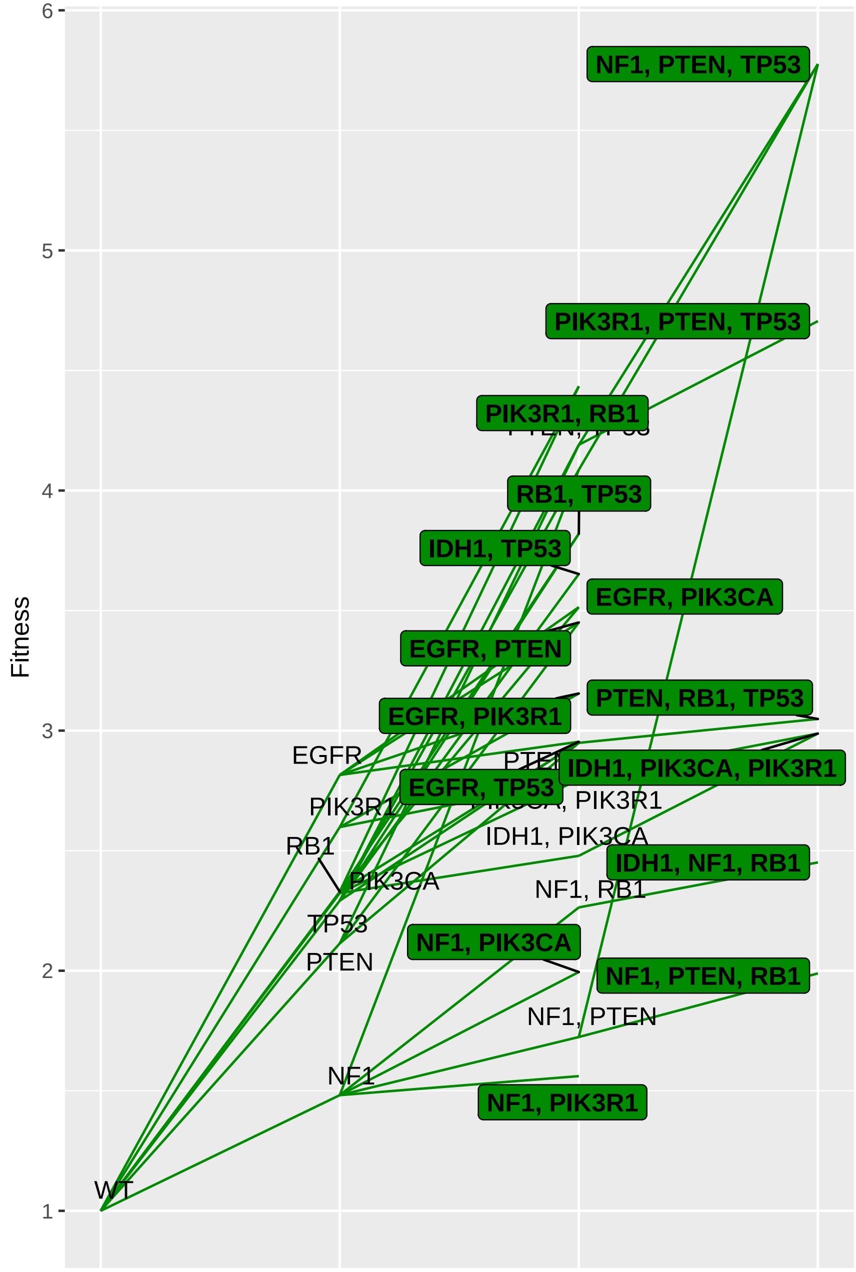

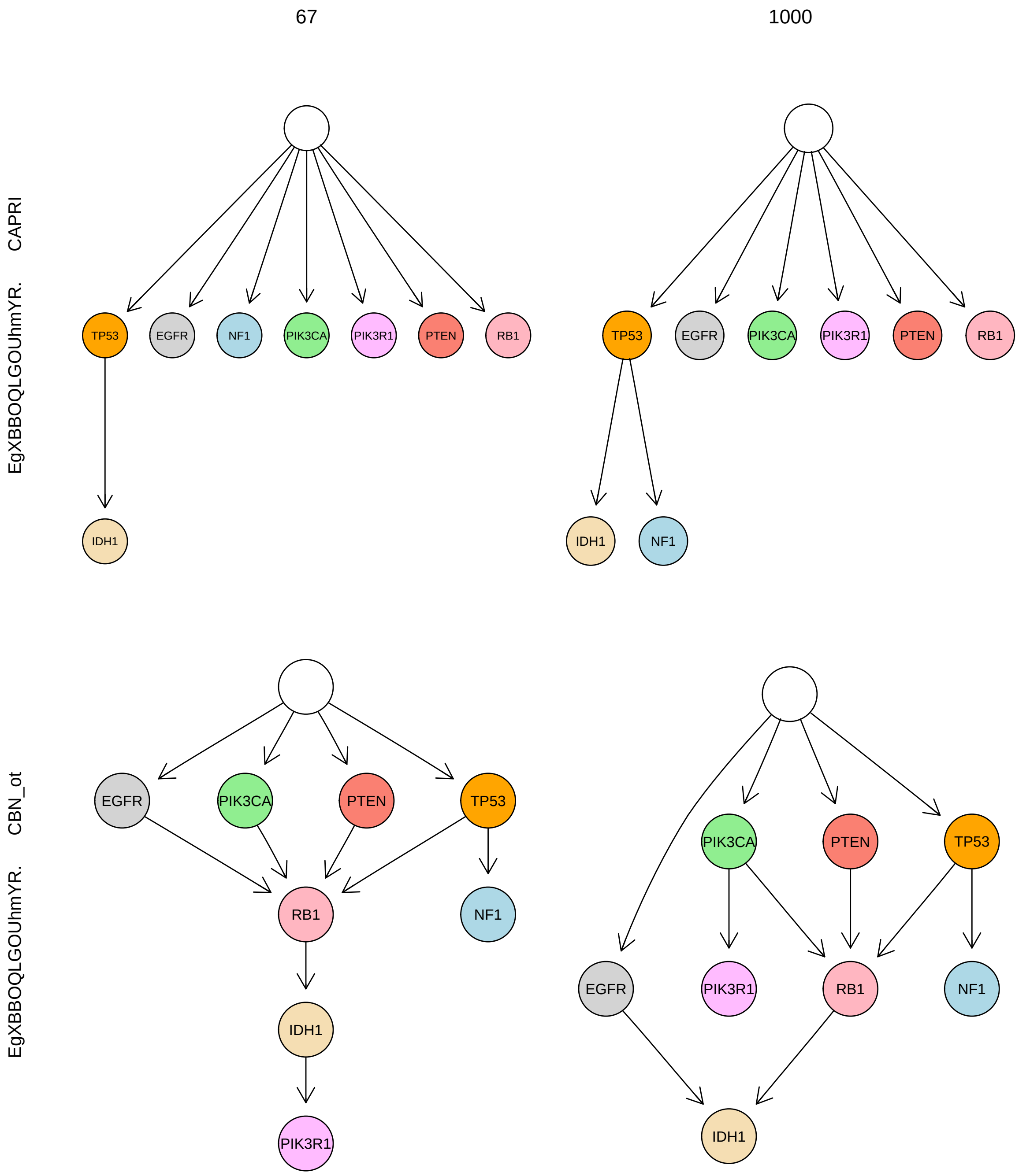

ID	p−value	Accessible Genot.
GHSuJcrTIWRgWOC	0.619	26

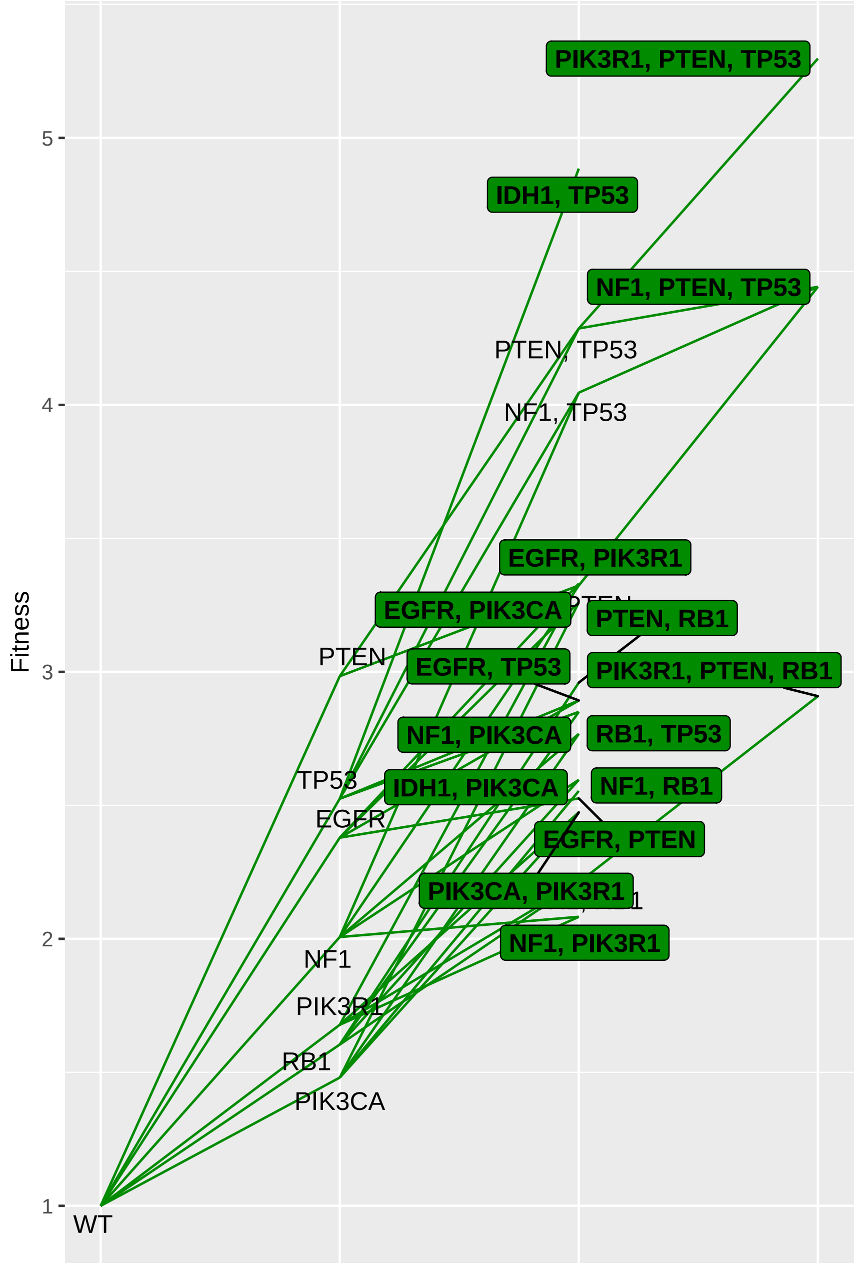

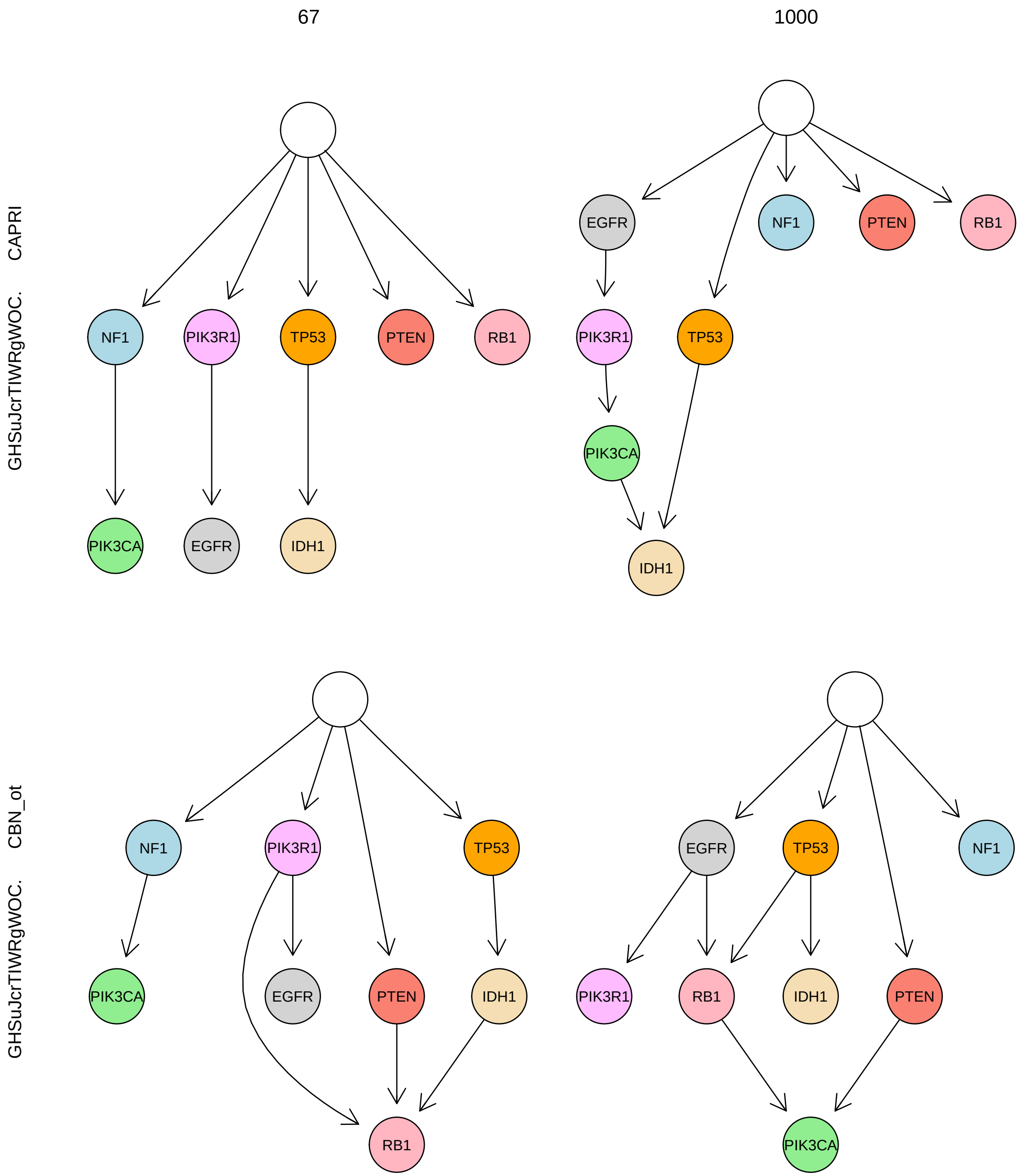

ID	p−value	Accessible Genot.
RMuwUuiFymtcMWV	0.62	37

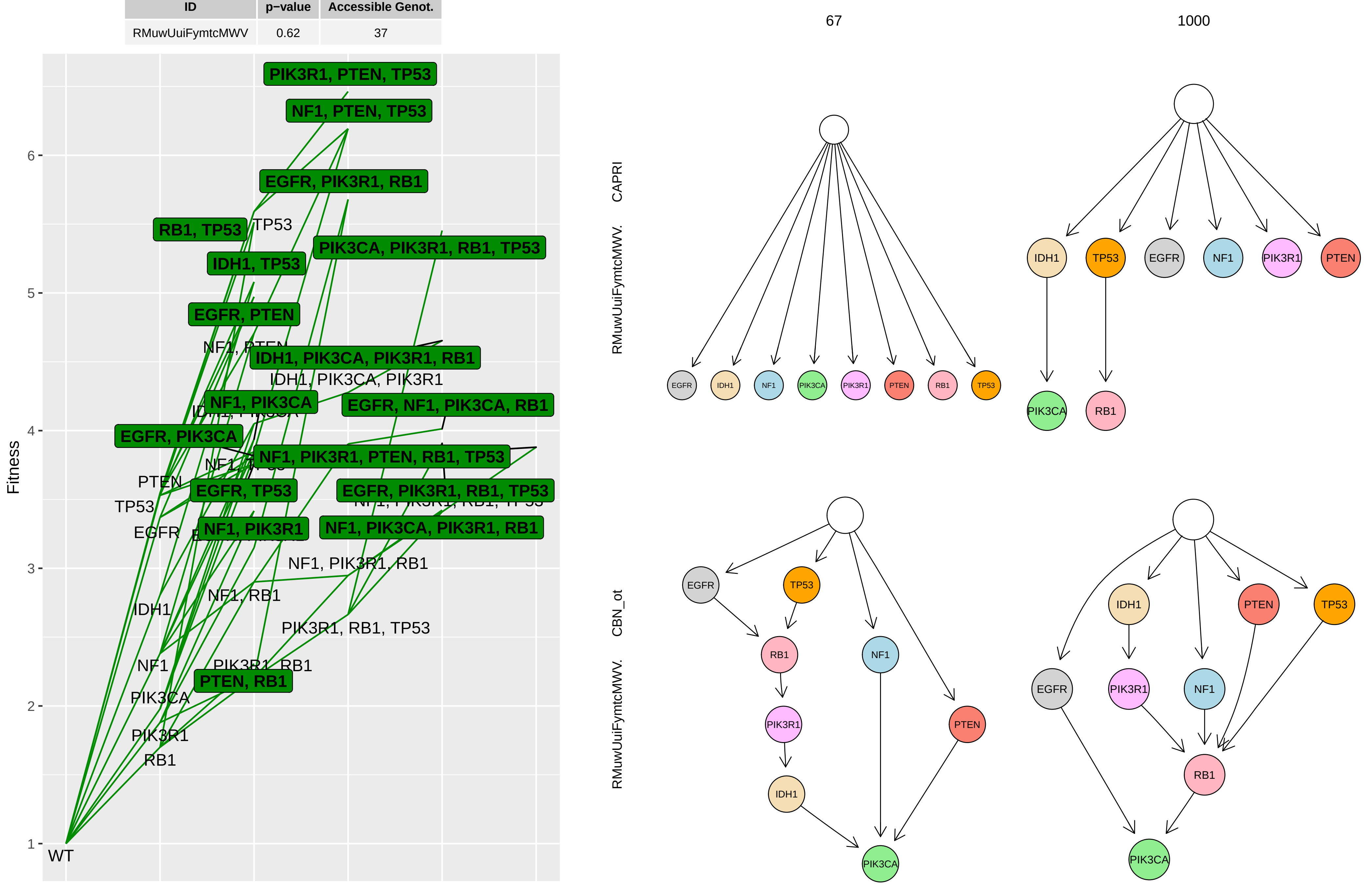

ID	p−value	Accessible Genot.
wRoBpDCGJwQTcKd	0.622	36

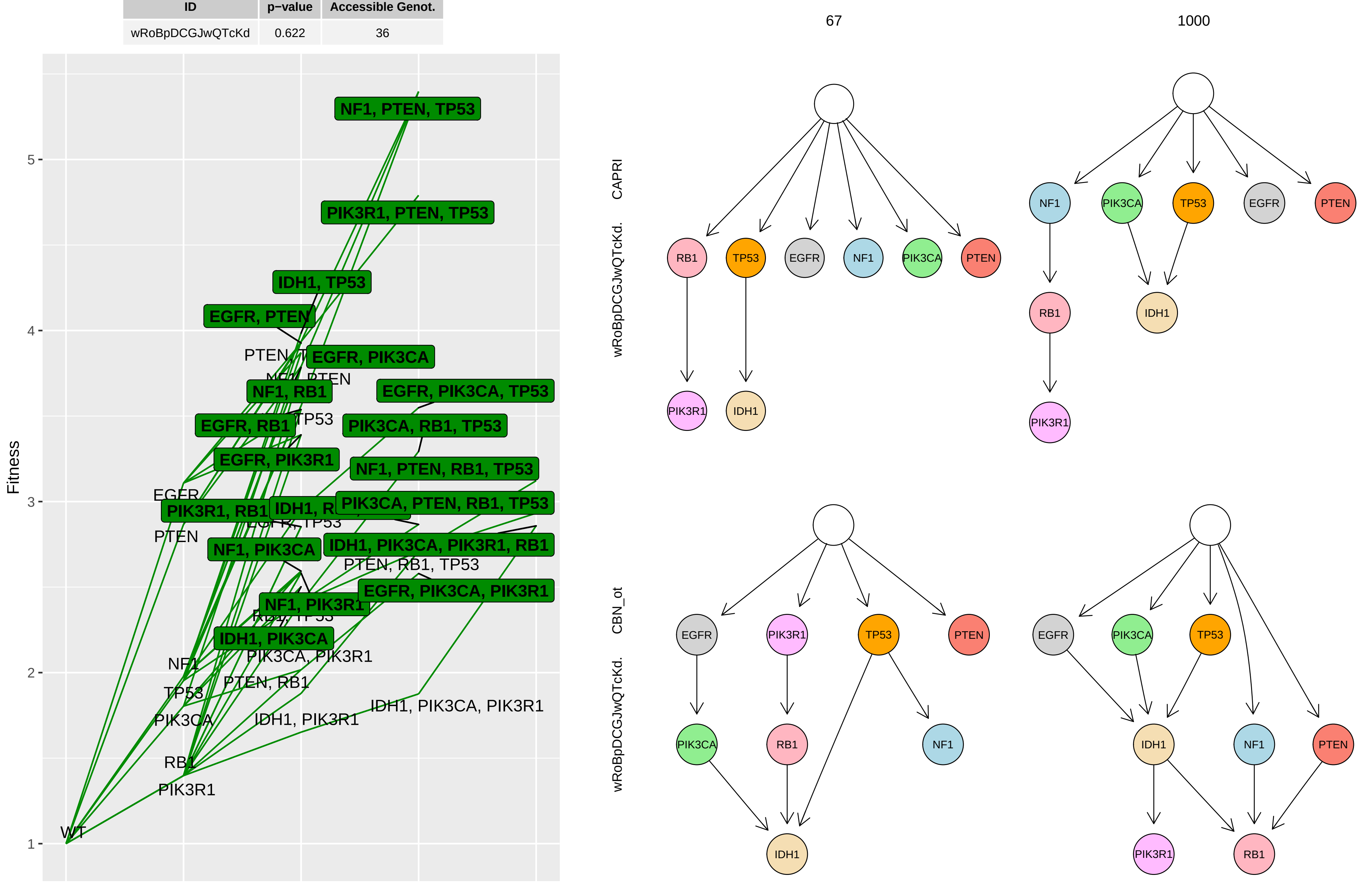

ID	p−value	Accessible Genot.
MCMKmqmllzmsGQP	0.623	61

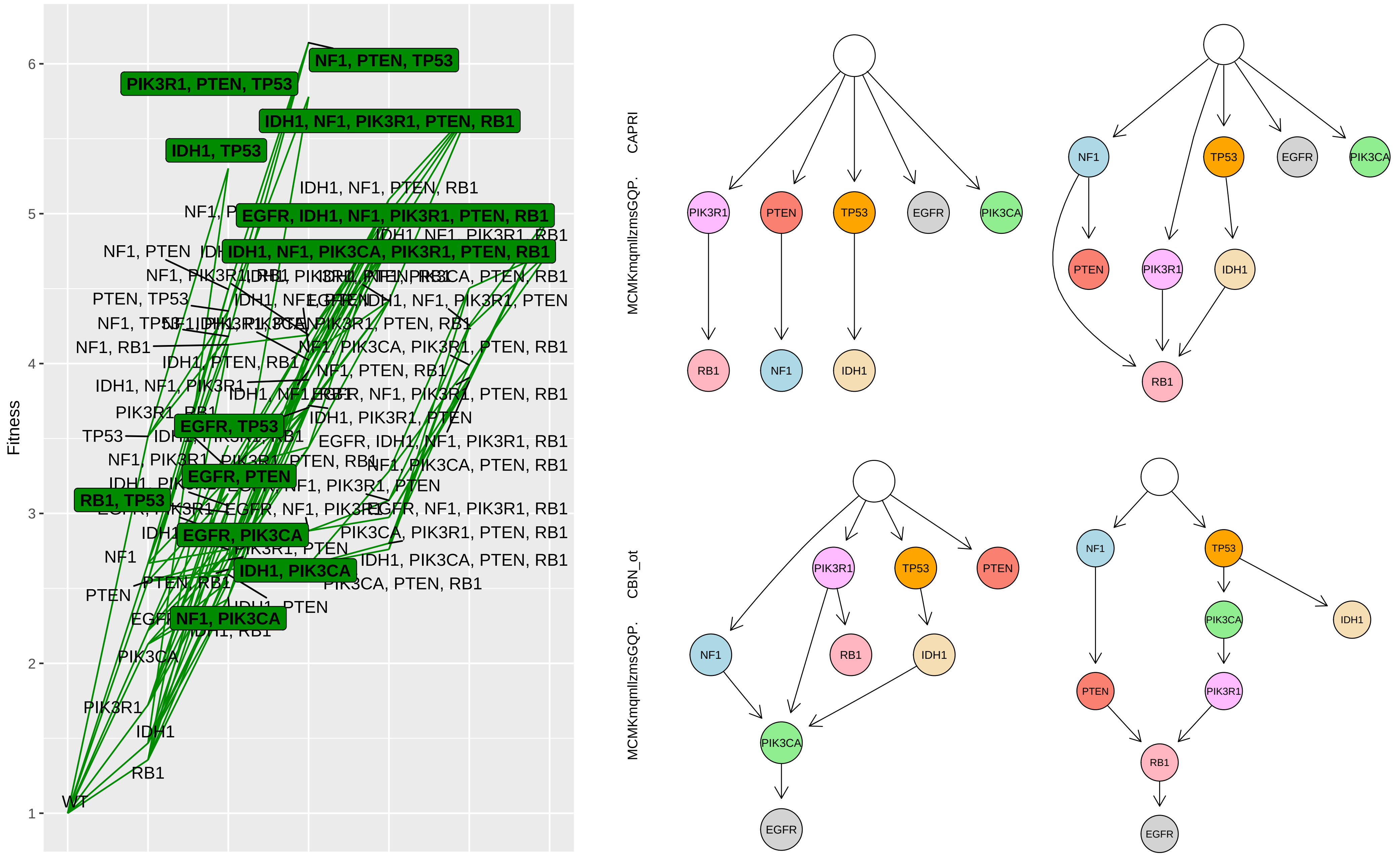

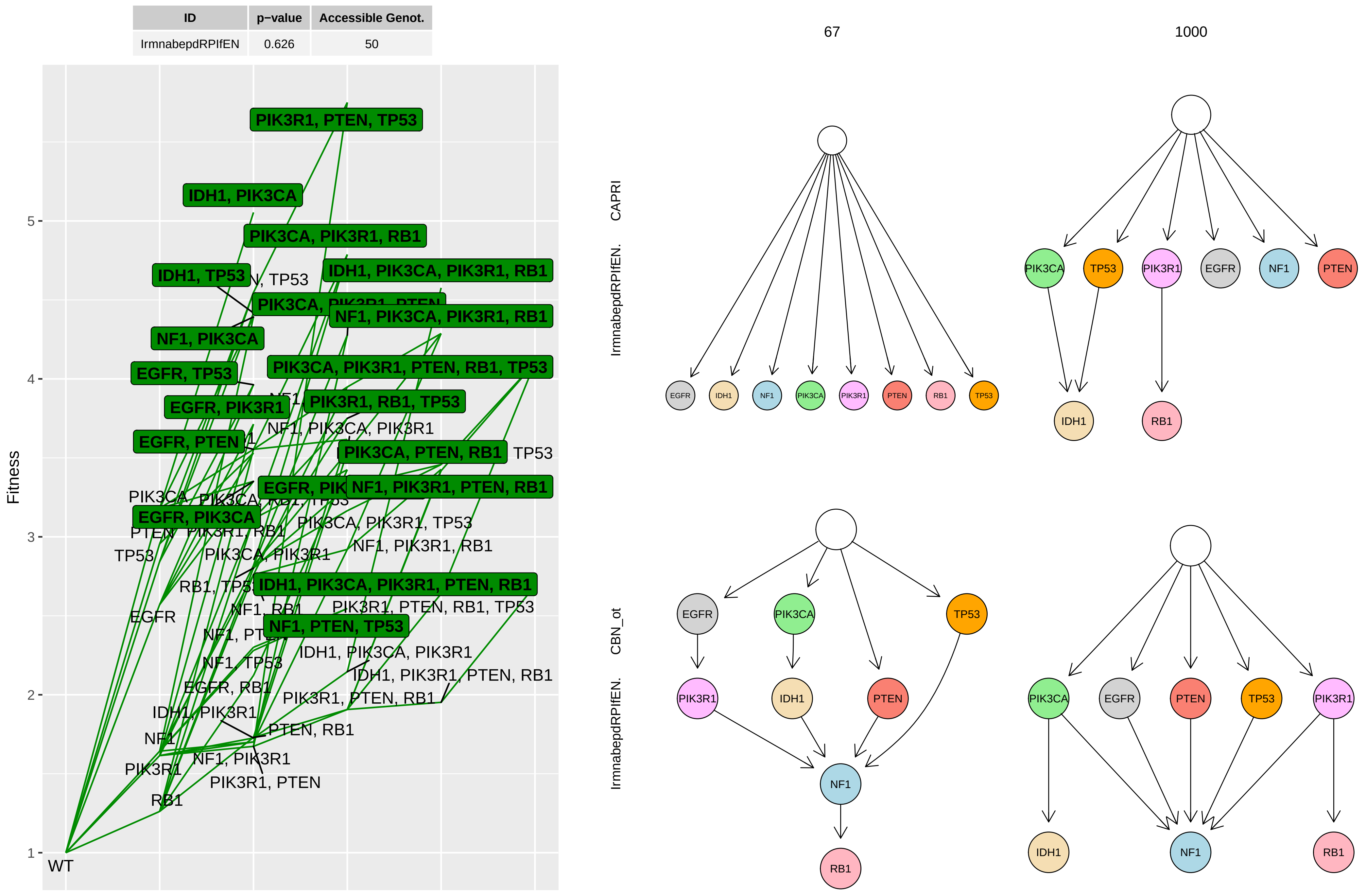

ID
p−value
Accessible Genot.
IrmnabepdRPIfEN
0.626
50
Fitness
WT
PIK3R1, PTEN, TP53
IDH1, PIK3CA
PIK3CA, PIK3R1, RB1
IDH1, TP53
IDH1, PIK3CA, PIK3R1, RB1
NF1, PIK3CA, PIK3R1, RB1
NF1, PIK3CA
EGFR, TP53
PIK3CA, PIK3R1, PTEN, RB1, TP53
EGFR, PIK3R1
PIK3R1, RB1, TP53
NF1, PIK3CA, PIK3R1
EGFR, PTEN
PIK3CA, PTEN, RB1
TP53
NF1, PIK3R1, PTEN, RB1
EGFR, PIK3CA
PIK3CA, PIK3R1, TP53
NF1, PIK3R1, RB1
PIK3CA, PIK3R1
IDH1, PIK3CA, PIK3R1, PTEN, RB1
PIK3R1, PTEN, RB1, TP53
EGFR
NF1, PTEN, TP53
IDH1, PIK3CA, PIK3R1
NF1, TP53
IDH1, PIK3R1, PTEN, RB1
EGFR, RB1
PIK3R1, PTEN, RB1
IDH1, PIK3R1
PTEN, RB1
NF1
NF1, PIK3R1
PIK3R1
PIK3R1, PTEN
RB1
67
1000
IrmnabepdRPIfEN. CAPRI
IrmnabepdRPIfEN. CBN_ot
EGFR
IDH1
NF1
PIK3CA
PIK3R1
PTEN
RB1
TP53

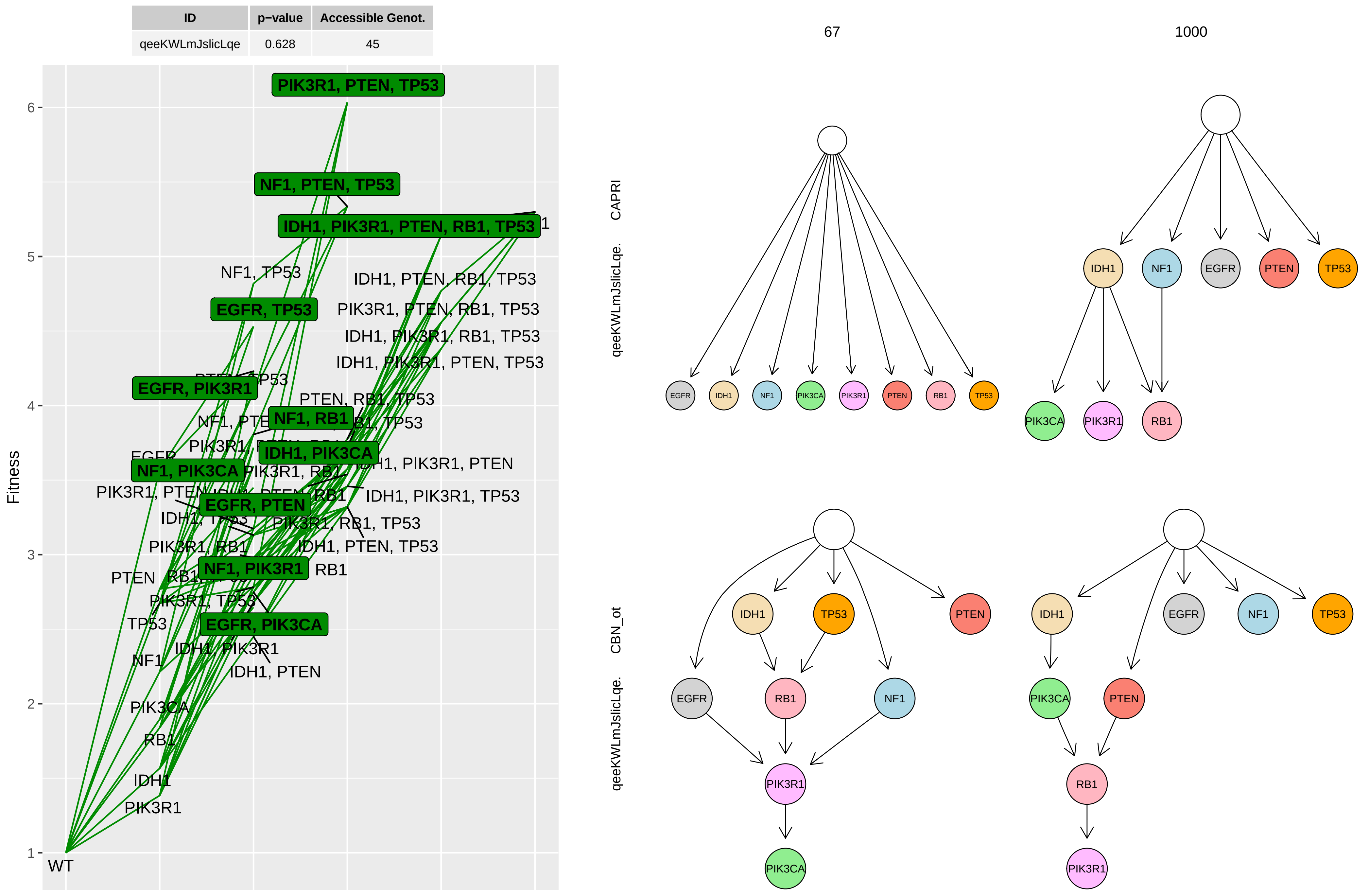
ID
p−value
Accessible Genot.
qeeKWLmJslicLqe
0.628
45
Fitness
PIK3R1, PTEN, TP53
NF1, PTEN, TP53
IDH1, PIK3R1, PTEN, RB1, TP53
NF1, TP53
IDH1, PTEN, RB1, TP53
EGFR, TP53
PIK3R1, PTEN, RB1, TP53
IDH1, PIK3R1, RB1, TP53
IDH1, PIK3R1, PTEN, TP53
EGFR, PIK3R1
PTEN, RB1, TP53
NF1, RB1
IDH1, PIK3CA
NF1, PIK3CA
PIK3R1, RB1
IDH1, PIK3R1, TP53
EGFR, PTEN
PIK3R1, RB1, TP53
IDH1, PTEN, TP53
NF1, PIK3R1
RB1
PTEN
PIK3R1, TP53
EGFR, PIK3CA
TP53
IDH1, PIK3R1
NF1
IDH1, PTEN
PIK3CA
IDH1
PIK3R1
WT
67
1000
qeeKWLmJslicLqe. CAPRI
qeeKWLmJslicLqe. CBN_ot
EGFR
IDH1
NF1
PIK3CA
PIK3R1
PTEN
RB1
TP53

ID	p−value	Accessible Genot.
gxwtRRlzybialLg	0.628	77

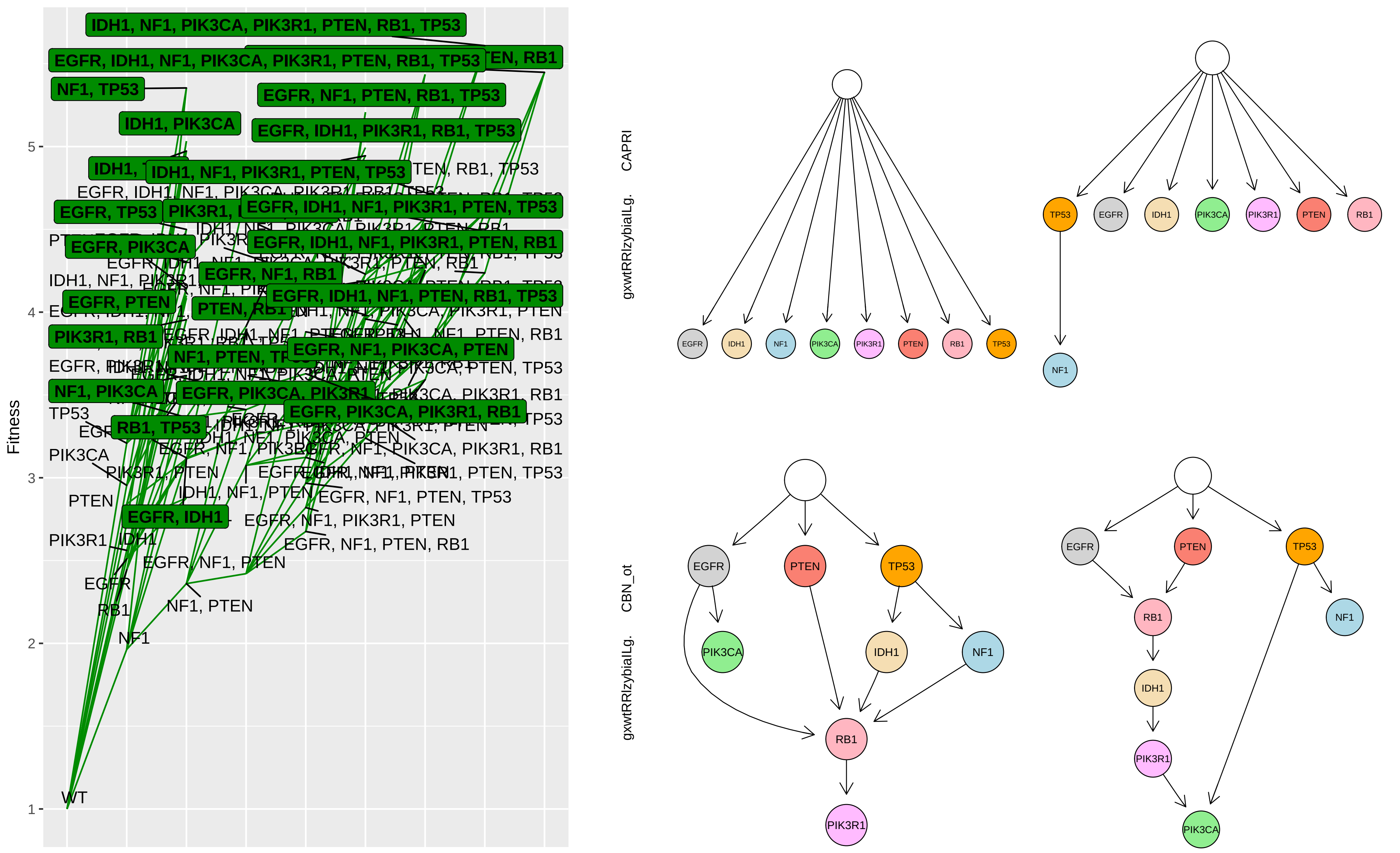

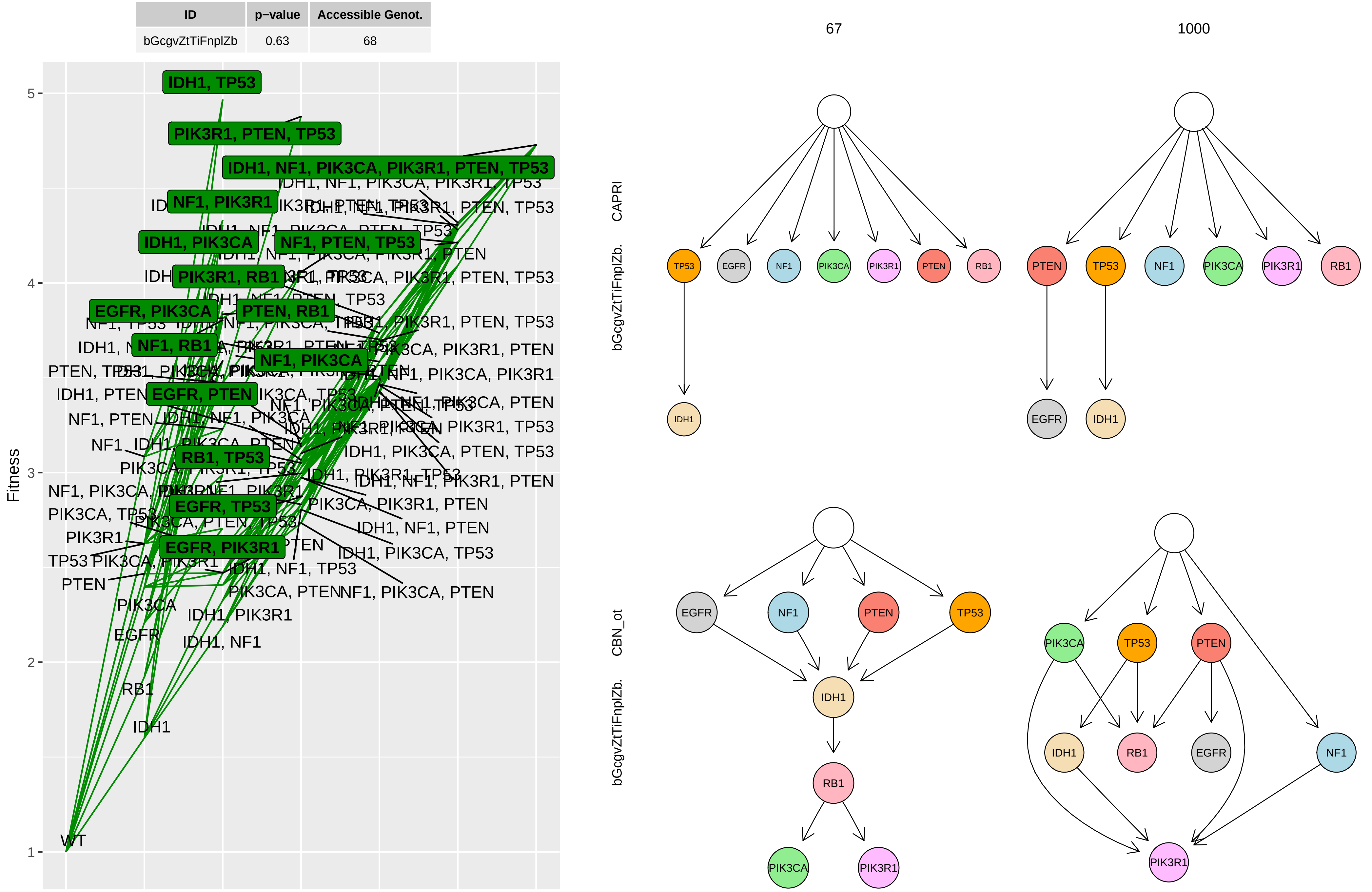

ID
p−value
Accessible Genot.
bGcgvZtTiFnplZb
0.63
68
Fitness
5
4
3
2
1
WT
IDH1
RB1
EGFR
PIK3CA
PTEN
IDH1, TP53
PIK3R1, PTEN, TP53
IDH1, NF1, PIK3CA, PIK3R1, PTEN, TP53
NF1, PIK3R1
IDH1, PIK3CA
NF1, PTEN, TP53
PIK3R1, RB1
EGFR, PIK3CA
PTEN, RB1
NF1, RB1
NF1, PIK3CA
EGFR, PTEN
RB1, TP53
EGFR, TP53
EGFR, PIK3R1
IDH1, NF1
IDH1, PIK3R1
PIK3CA, PTEN
NF1, PIK3CA, PTEN
IDH1, PIK3CA, TP53
IDH1, NF1, PTEN
PIK3CA, PIK3R1, PTEN
IDH1, NF1, PIK3R1, PTEN
IDH1, PIK3CA, PTEN, TP53
67
1000
CAPRI
CBN_ot
bGcgvZtTiFnplZb.
TP53
EGFR
NF1
PIK3CA
PIK3R1
PTEN
RB1
IDH1

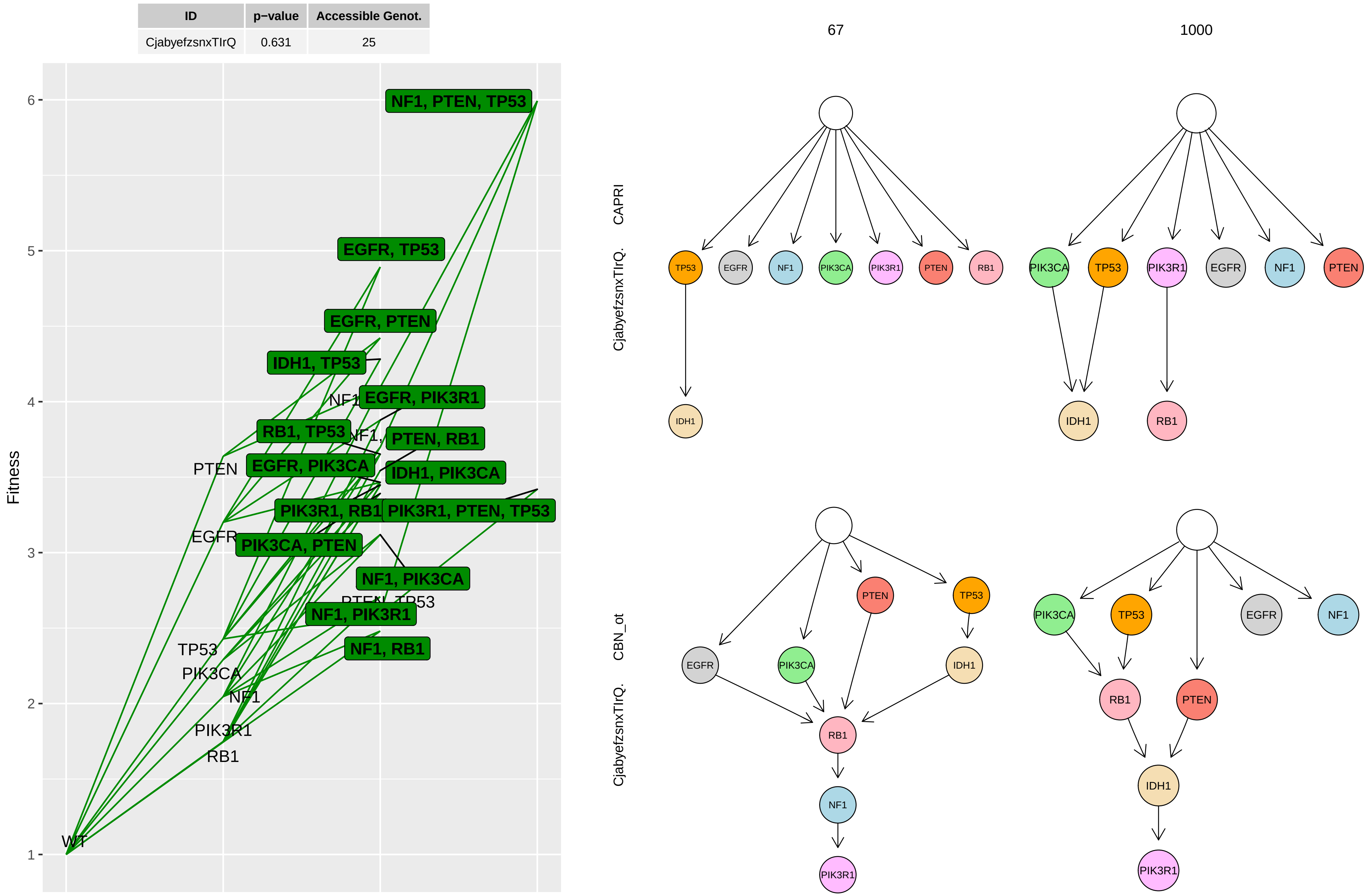
ID
p−value
Accessible Genot.
CjabyefzsnxTIrQ
0.631
25
Fitness
NF1, PTEN, TP53
EGFR, TP53
EGFR, PTEN
IDH1, TP53
EGFR, PIK3R1
RB1, TP53
PTEN, RB1
EGFR, PIK3CA
IDH1, PIK3CA
PIK3R1, RB1
PIK3R1, PTEN, TP53
PIK3CA, PTEN
NF1, PIK3CA
NF1, PIK3R1
NF1, RB1
PTEN
EGFR
TP53
PIK3CA
NF1
PIK3R1
RB1
WT
67
1000
CjabyefzsnxTIrQ. CAPRI
CjabyefzsnxTIrQ. CBN_ot
TP53
EGFR
NF1
PIK3CA
PIK3R1
PTEN
RB1
IDH1

ID	p−value	Accessible Genot.
QaeryUriHMjYqUz	0.631	141

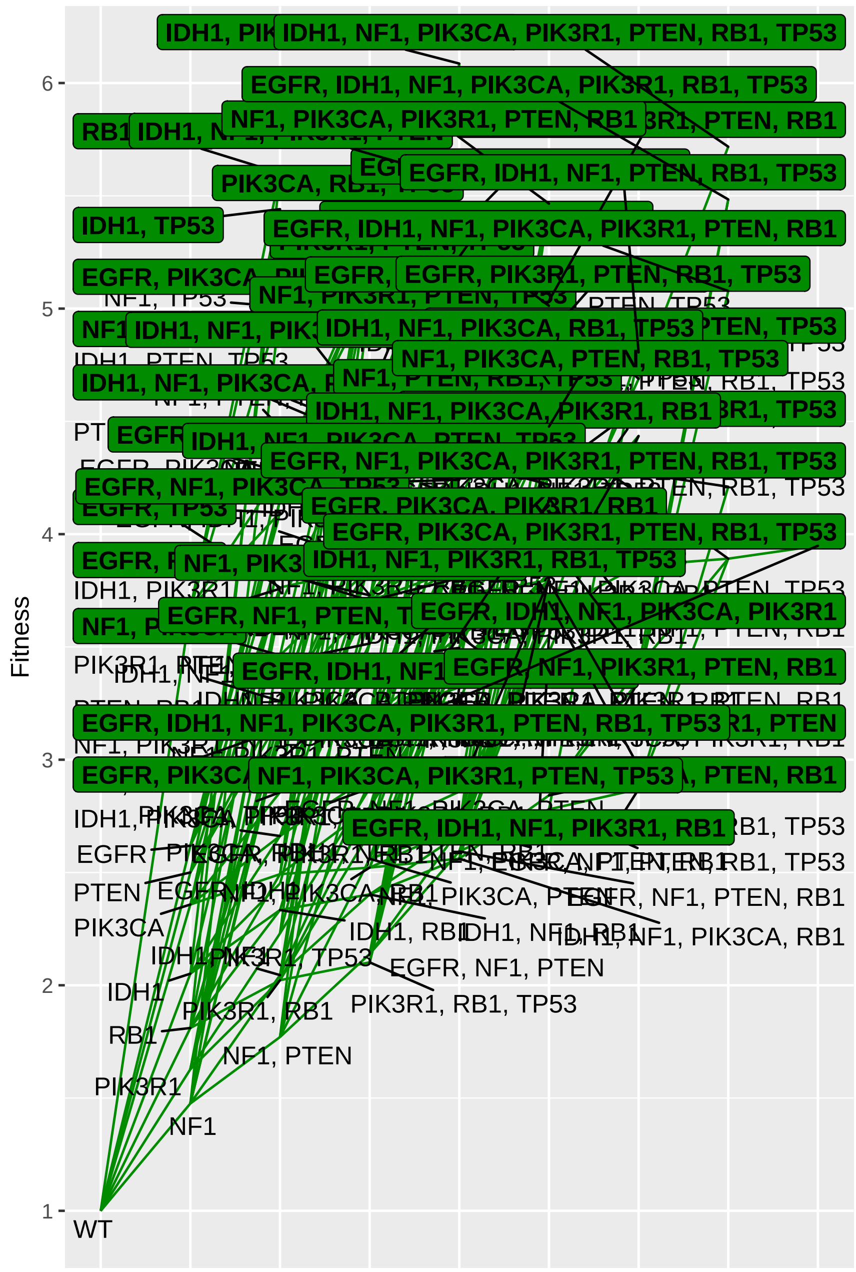

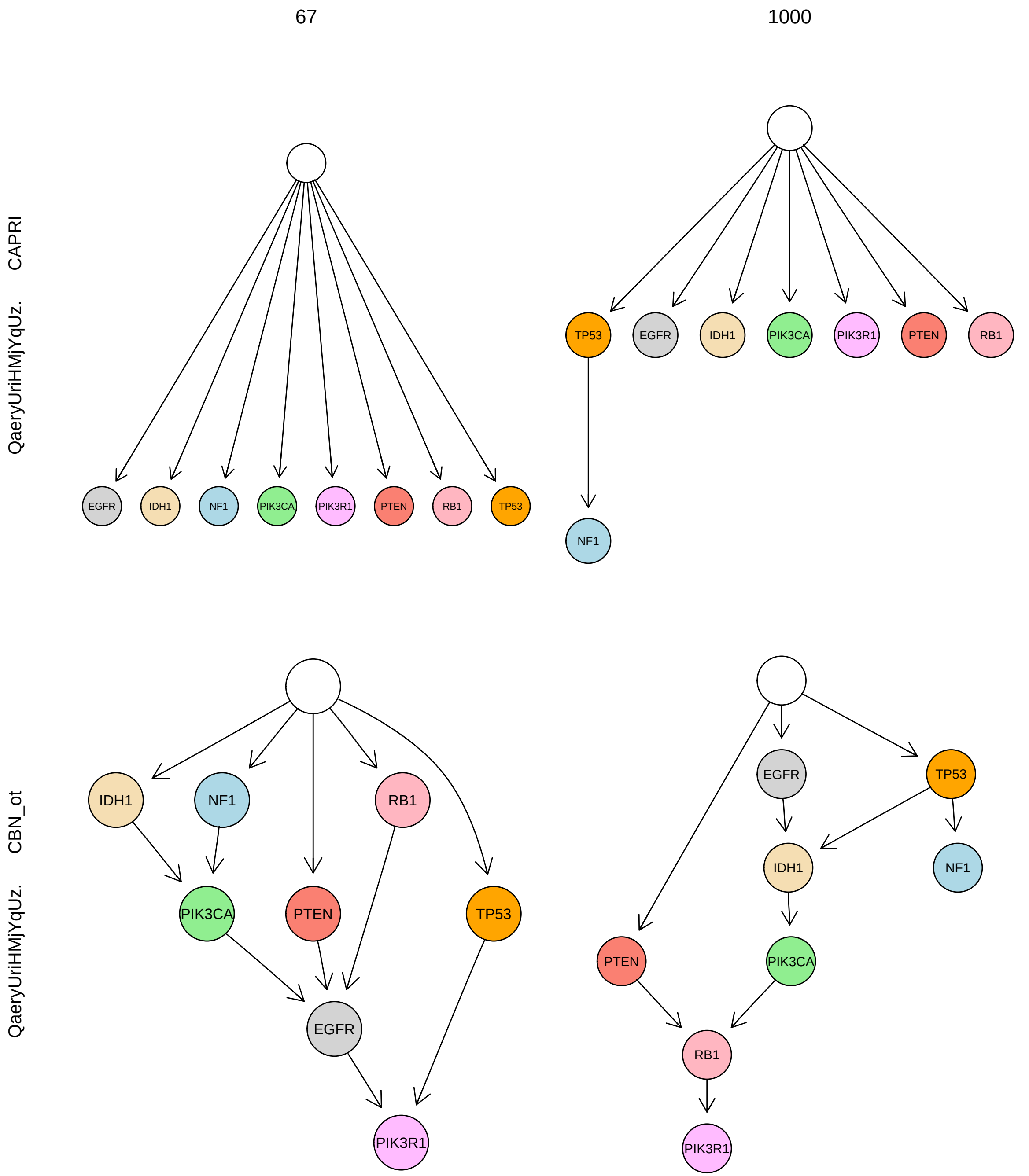

ID	p-value	Accessible Genot.
HeeOVLgjVlVVRsL	0.632	30

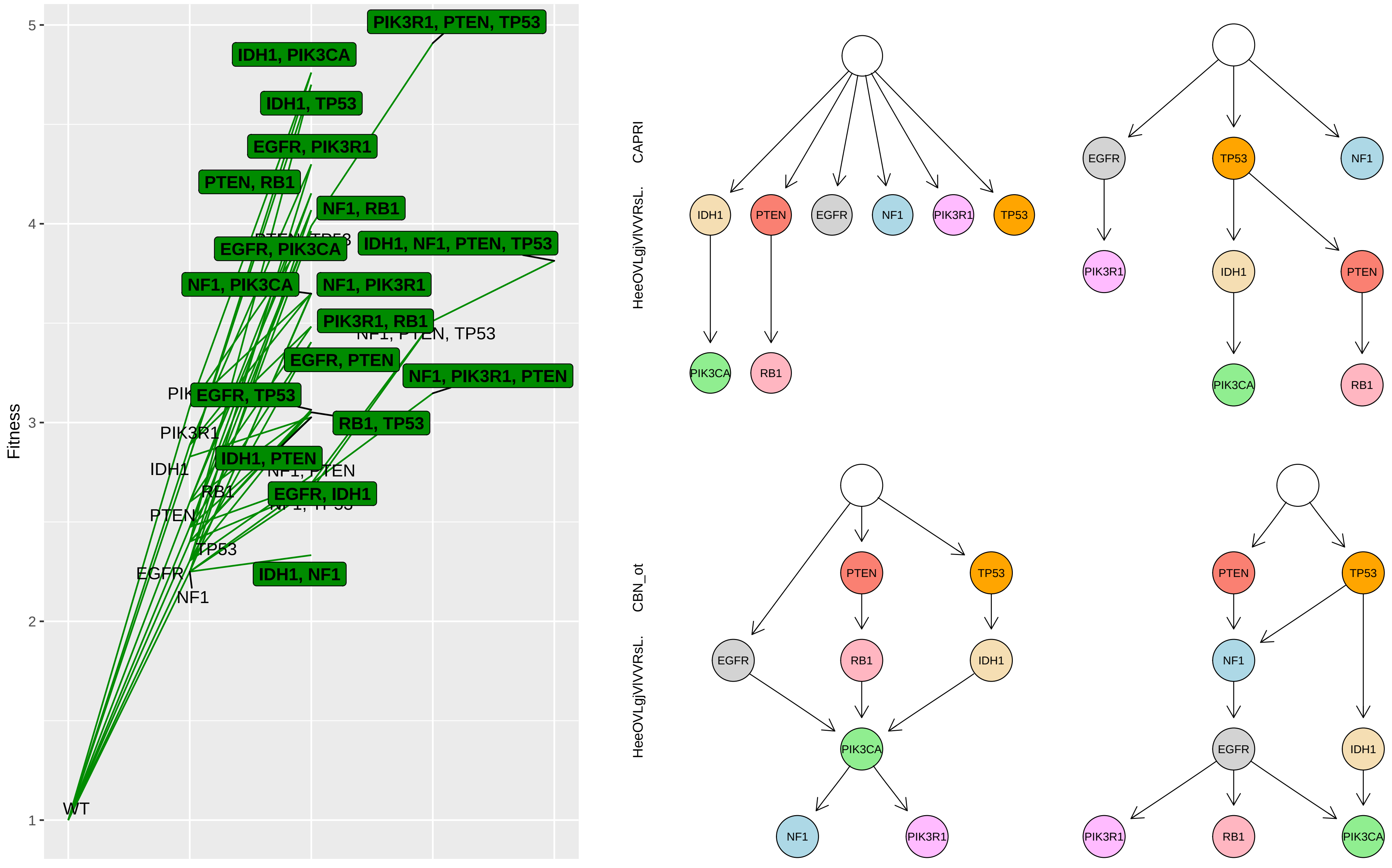

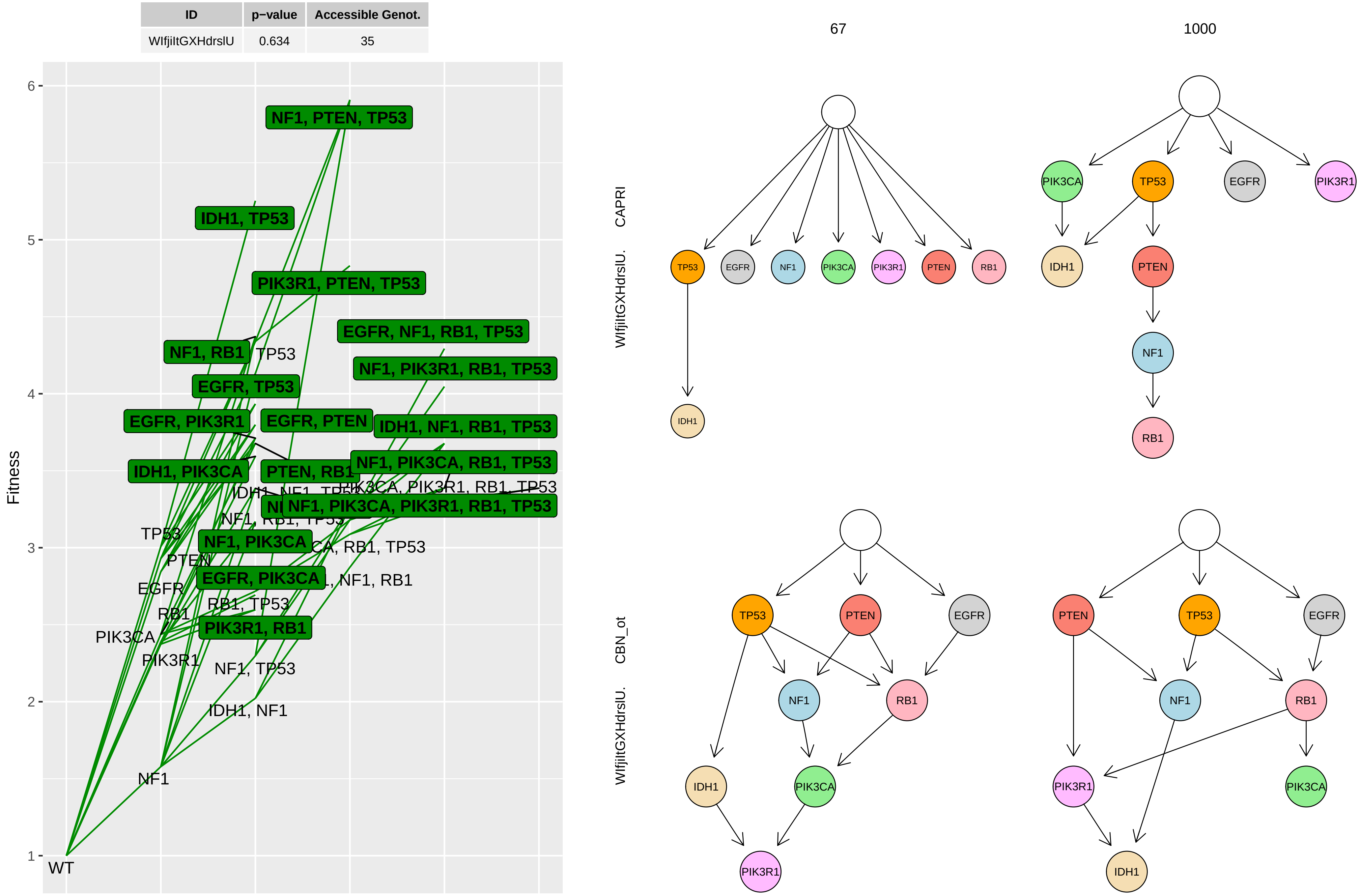

ID
p−value
Accessible Genot.
WIfjiltGXHdrslU
0.634
35
Fitness
WT
NF1
NF1, PTEN, TP53
IDH1, TP53
PIK3R1, PTEN, TP53
EGFR, NF1, RB1, TP53
NF1, RB1
NF1, PIK3R1, RB1, TP53
EGFR, TP53
EGFR, PIK3R1
EGFR, PTEN
IDH1, NF1, RB1, TP53
IDH1, PIK3CA
PTEN, RB1
NF1, PIK3CA, RB1, TP53
NF1, PIK3CA, PIK3R1, RB1, TP53
NF1, PIK3CA
EGFR, PIK3CA
PIK3R1, RB1
NF1, TP53
IDH1, NF1
TP53
PTEN
EGFR
RB1
PIK3CA
PIK3R1
67
1000
CAPRI
CBN_ot
WIfjiltGXHdrslU.
TP53
EGFR
NF1
PIK3CA
PIK3R1
PTEN
RB1
IDH1

ID	p−value	Accessible Genot.
XKiLHYXzmGWxcuh	0.634	239

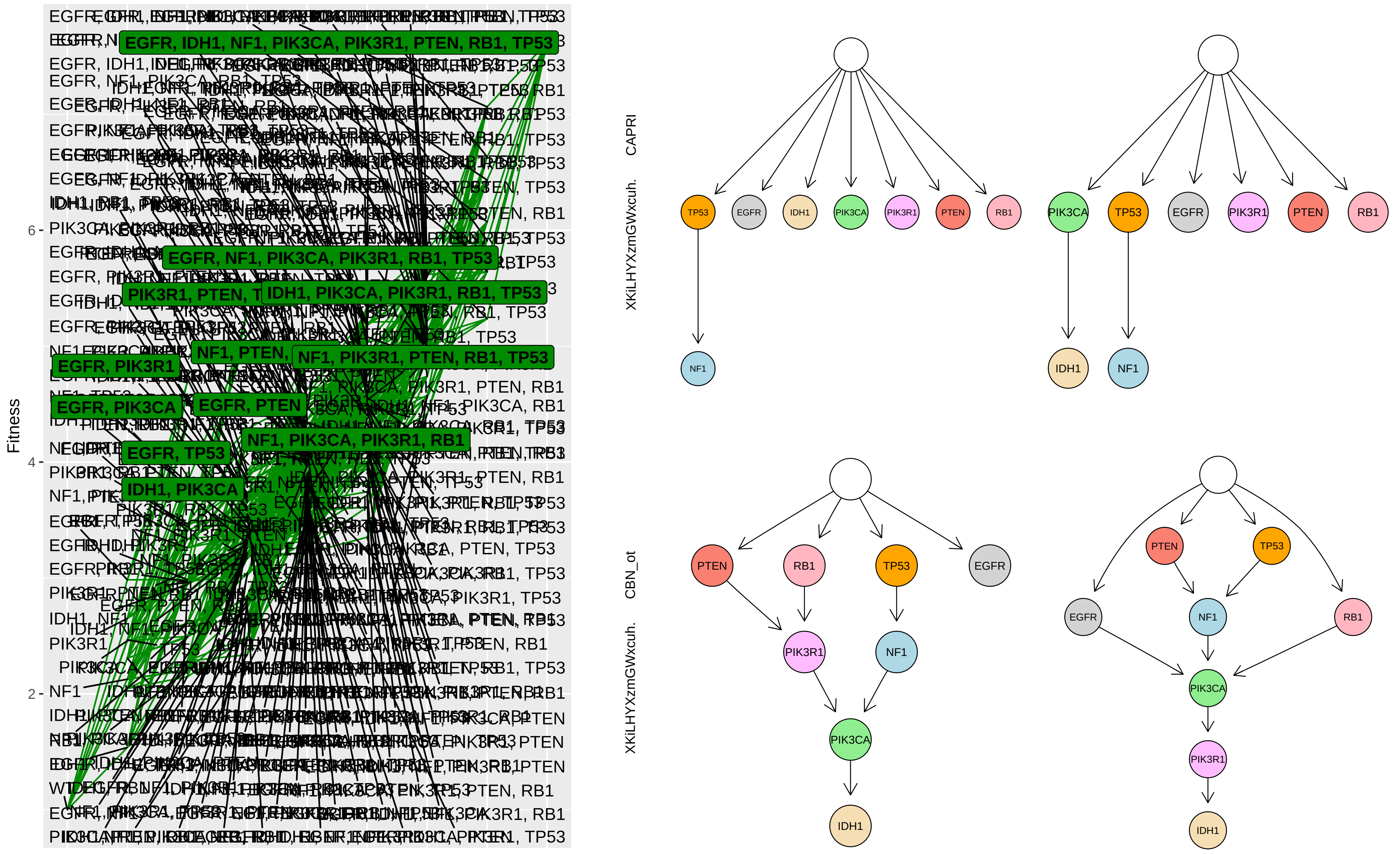

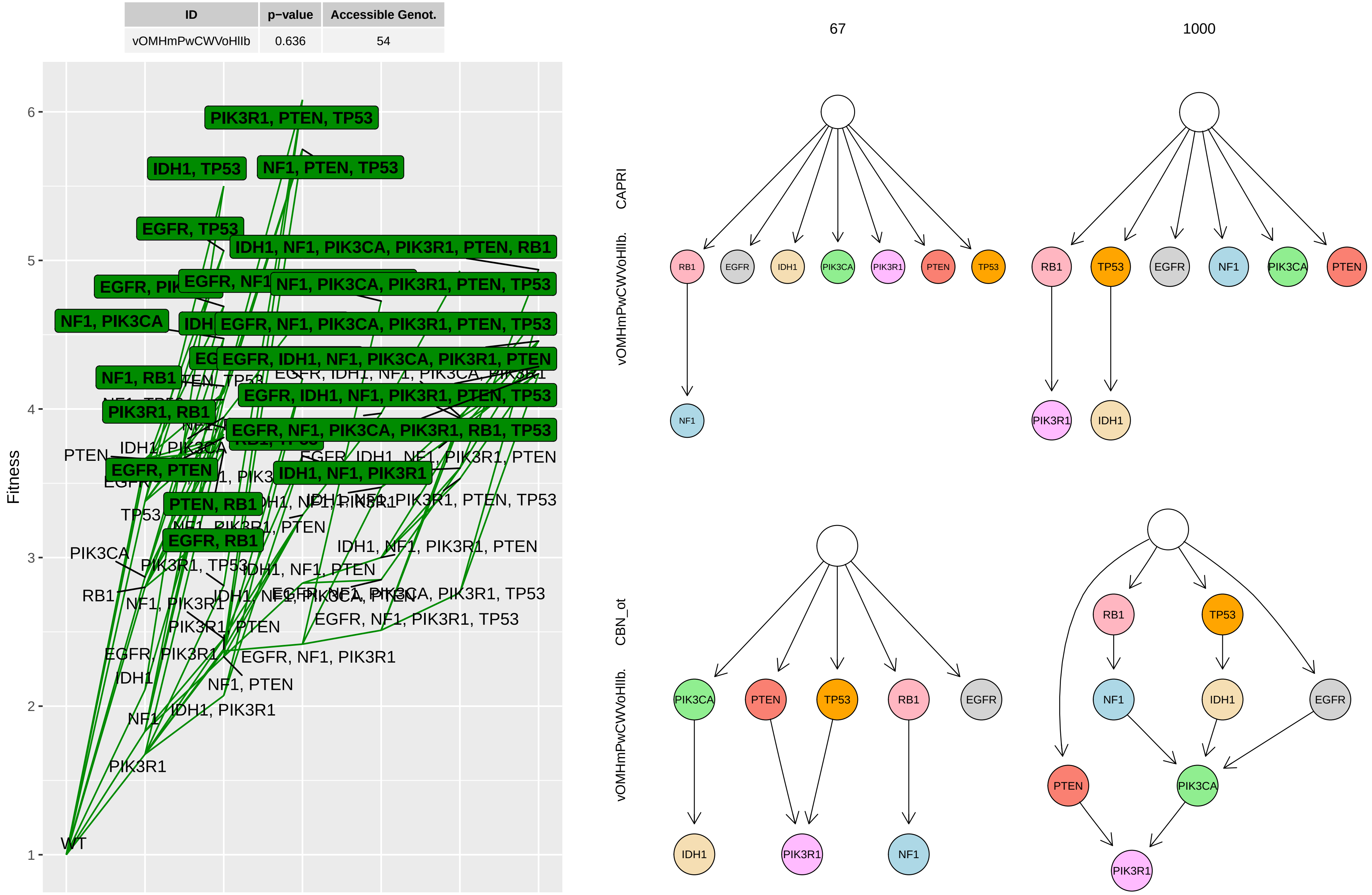
ID
p−value
Accessible Genot.
vOMHmPwCWVoHllb
0.636
54
Fitness
PIK3R1, PTEN, TP53
IDH1, TP53
NF1, PTEN, TP53
EGFR, TP53
IDH1, NF1, PIK3CA, PIK3R1, PTEN, RB1
NF1, PIK3CA, PIK3R1, PTEN, TP53
NF1, PIK3CA
EGFR, NF1, PIK3CA, PIK3R1, PTEN, TP53
EGFR, IDH1, NF1, PIK3CA, PIK3R1, PTEN
NF1, RB1
EGFR, IDH1, NF1, PIK3R1, PTEN, TP53
PIK3R1, RB1
EGFR, NF1, PIK3CA, PIK3R1, RB1, TP53
PTEN
EGFR, PTEN
EGFR, IDH1, NF1, PIK3R1, PTEN
IDH1, NF1, PIK3R1
PTEN, RB1
TP53
EGFR, RB1
IDH1, NF1, PIK3R1, PTEN
PIK3CA
IDH1, NF1, PTEN
RB1
EGFR, NF1, PIK3CA, PIK3R1, TP53
NF1, PIK3R1
PIK3R1, PTEN
EGFR, NF1, PIK3R1, TP53
EGFR, PIK3R1
EGFR, NF1, PIK3R1
IDH1
NF1, PTEN
NF1
IDH1, PIK3R1
PIK3R1
WT
67
1000
vOMHmPwCWVoHllb. CAPRI
vOMHmPwCWVoHllb. CBN_ot
RB1
EGFR
IDH1
PIK3CA
PIK3R1
PTEN
TP53
NF1

ID	p−value	Accessible Genot.
OCjkqLMcHjlwoof	0.637	38

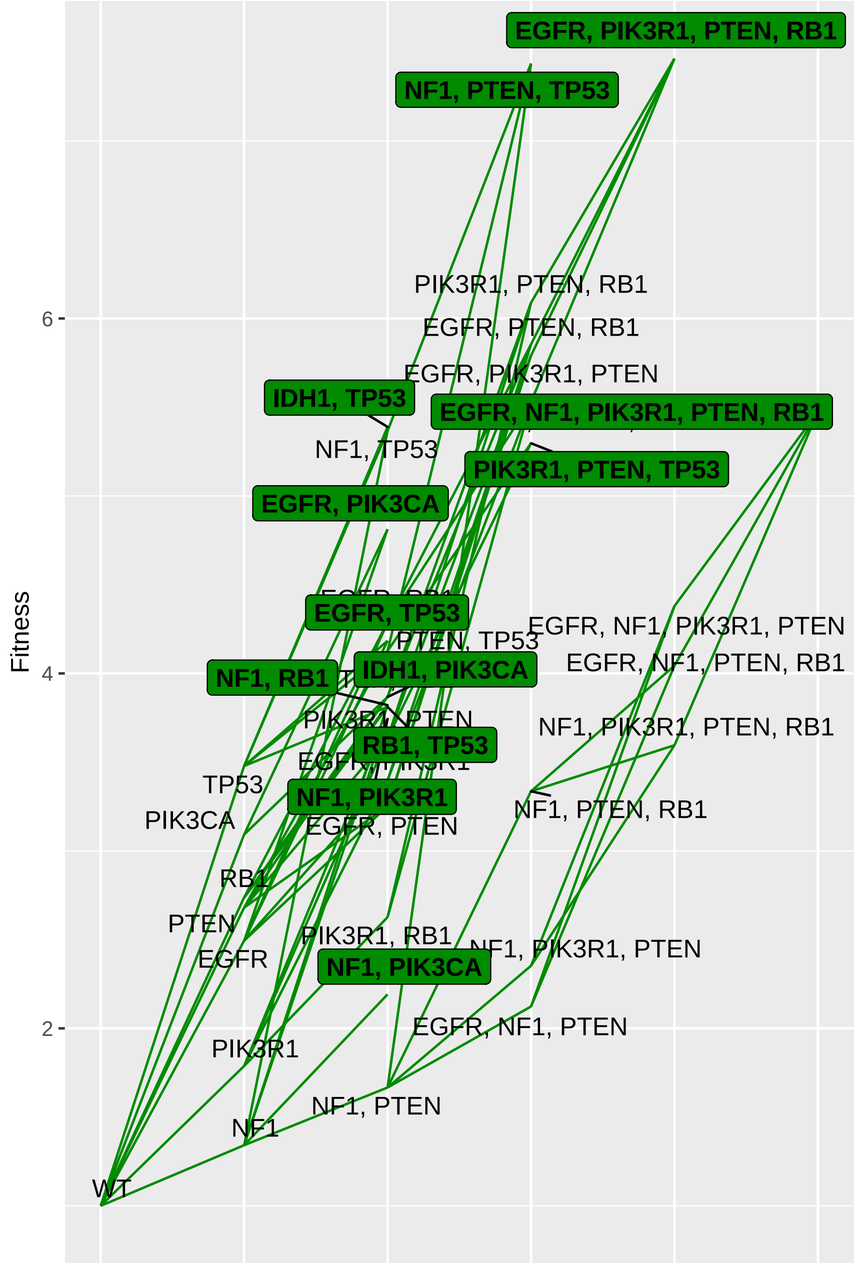

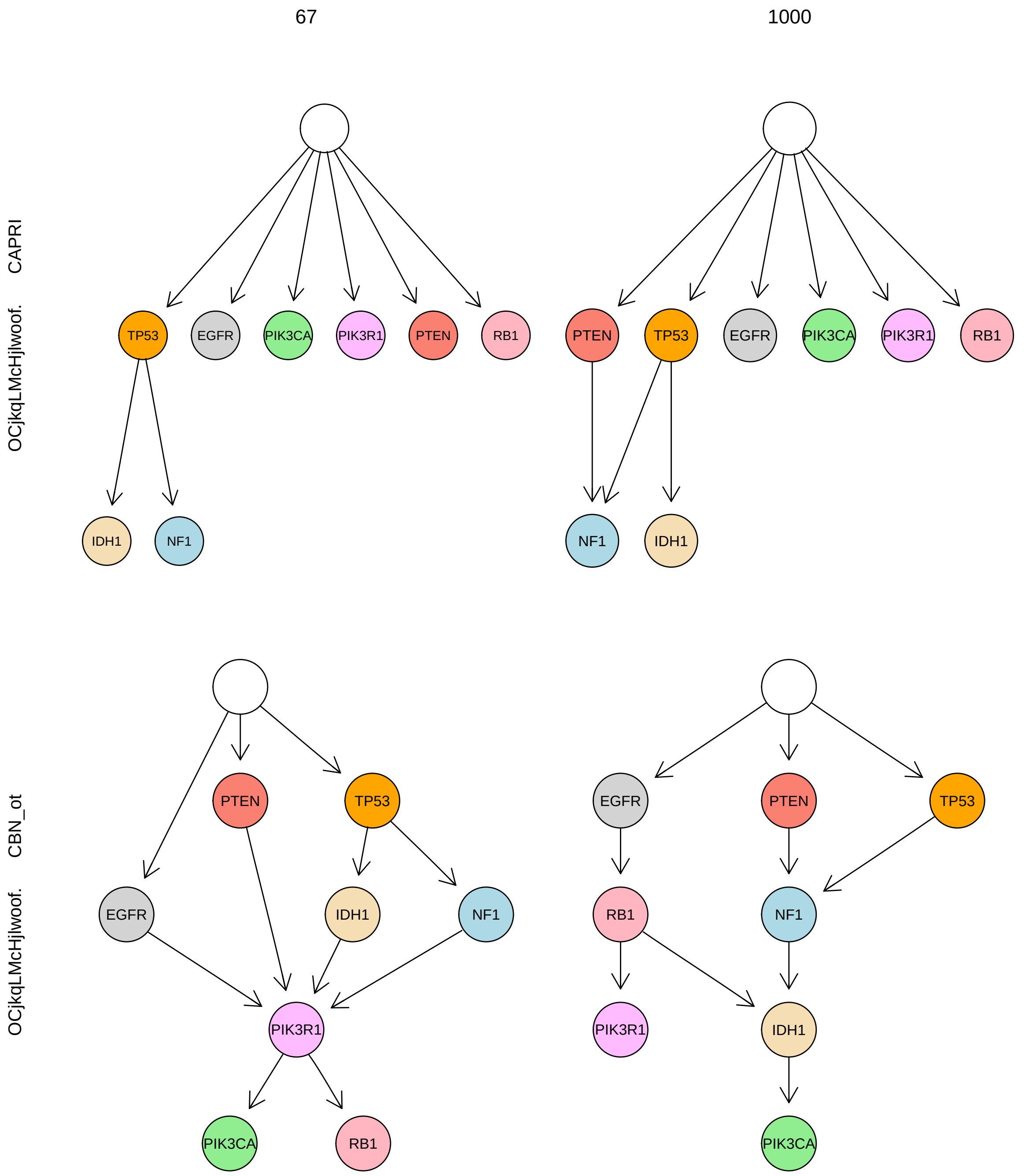

ID	p-value	Accessible Genot.
gAeWsinUwaASmzu	0.638	45

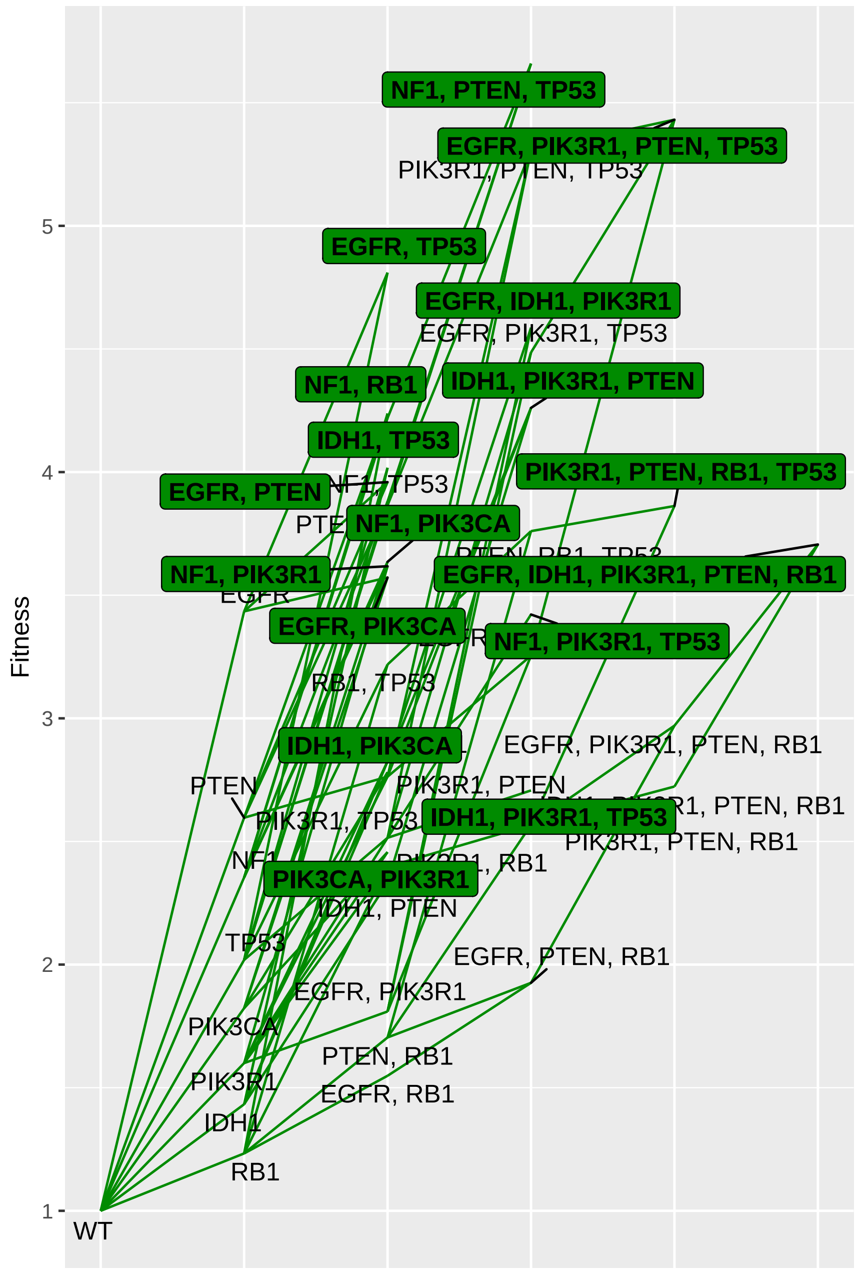

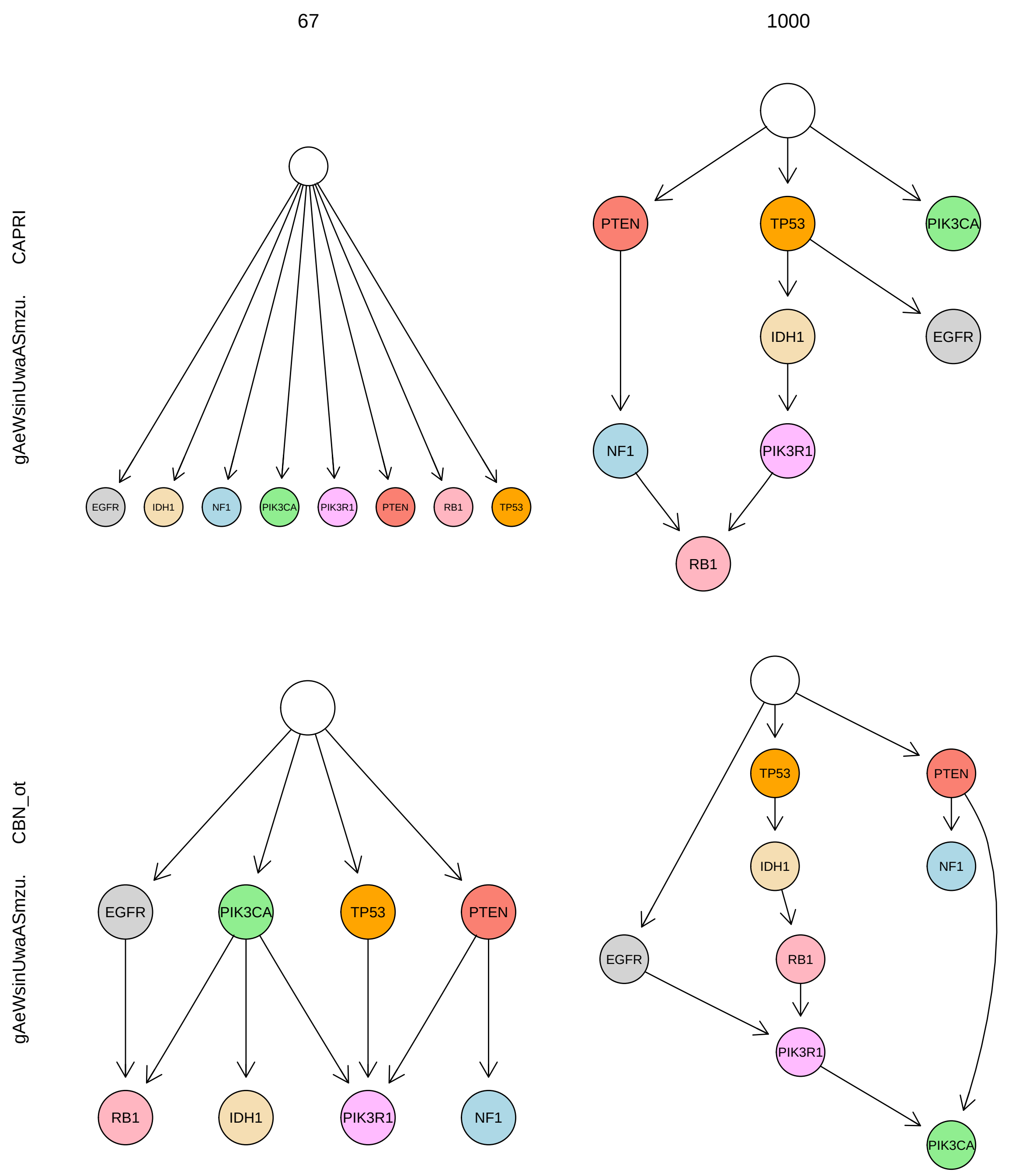

ID	p−value	Accessible Genot.
BfzxtgDRfkbPlHC	0.639	34

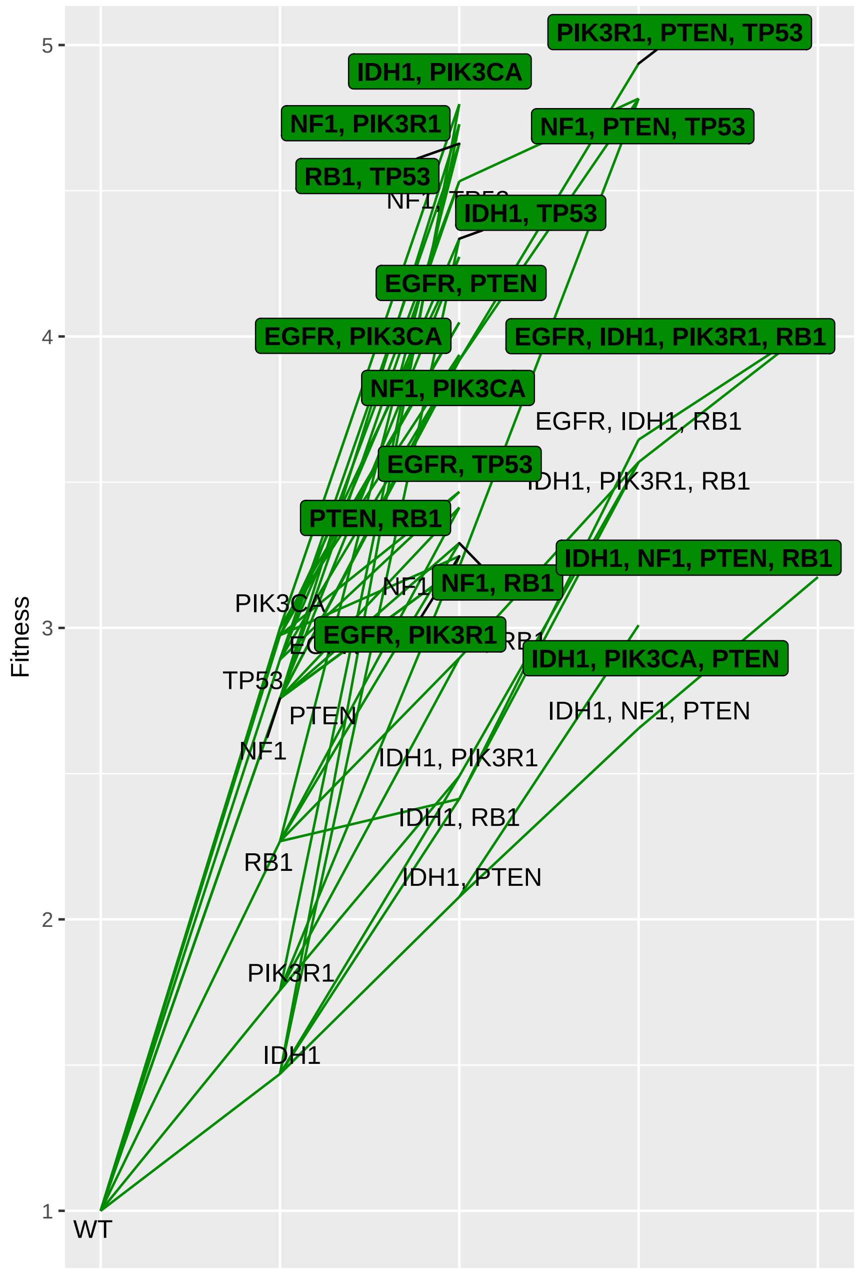

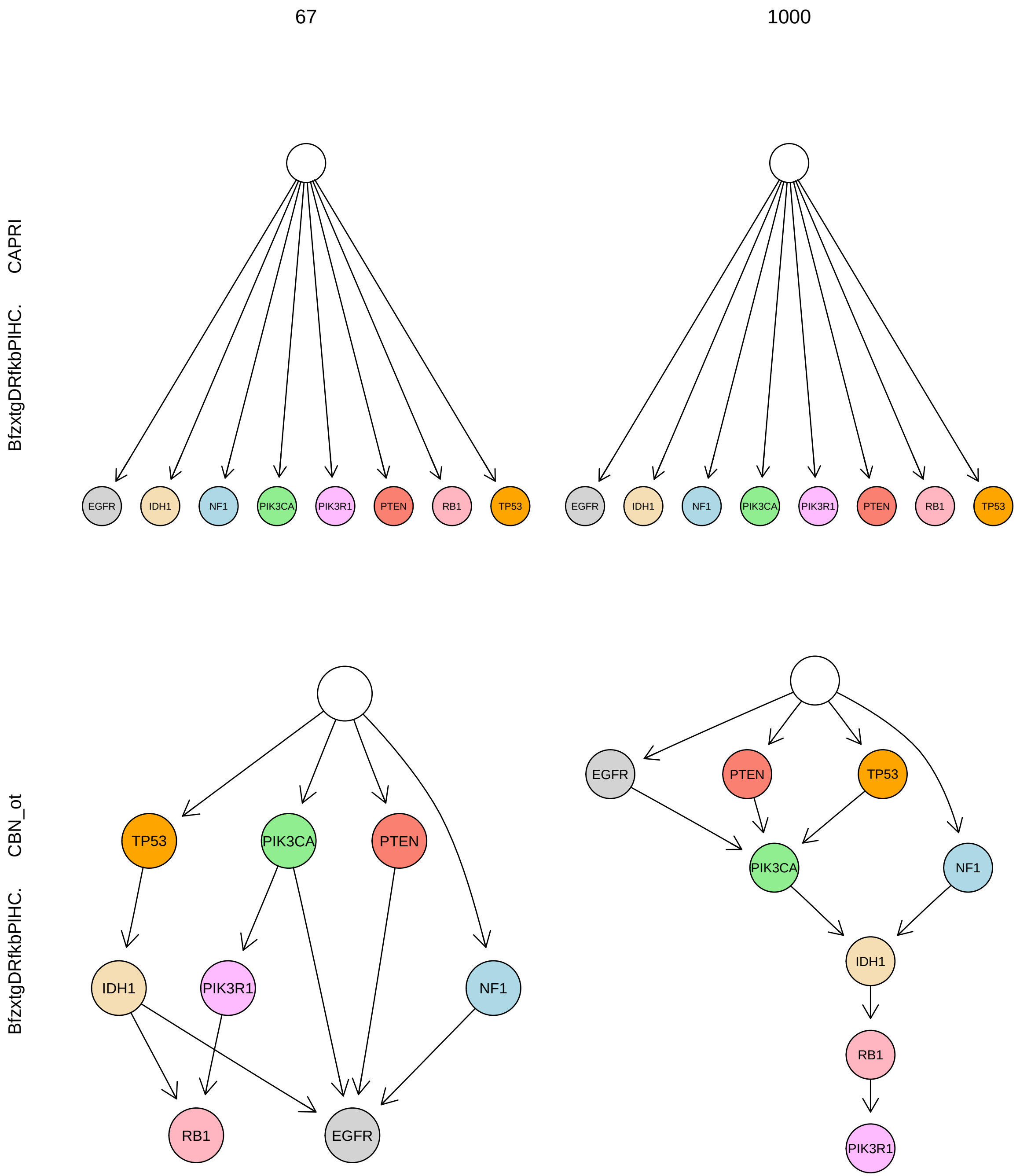

ID	p−value	Accessible Genot.
FfxgRyJhdYwcdRM	0.64	36

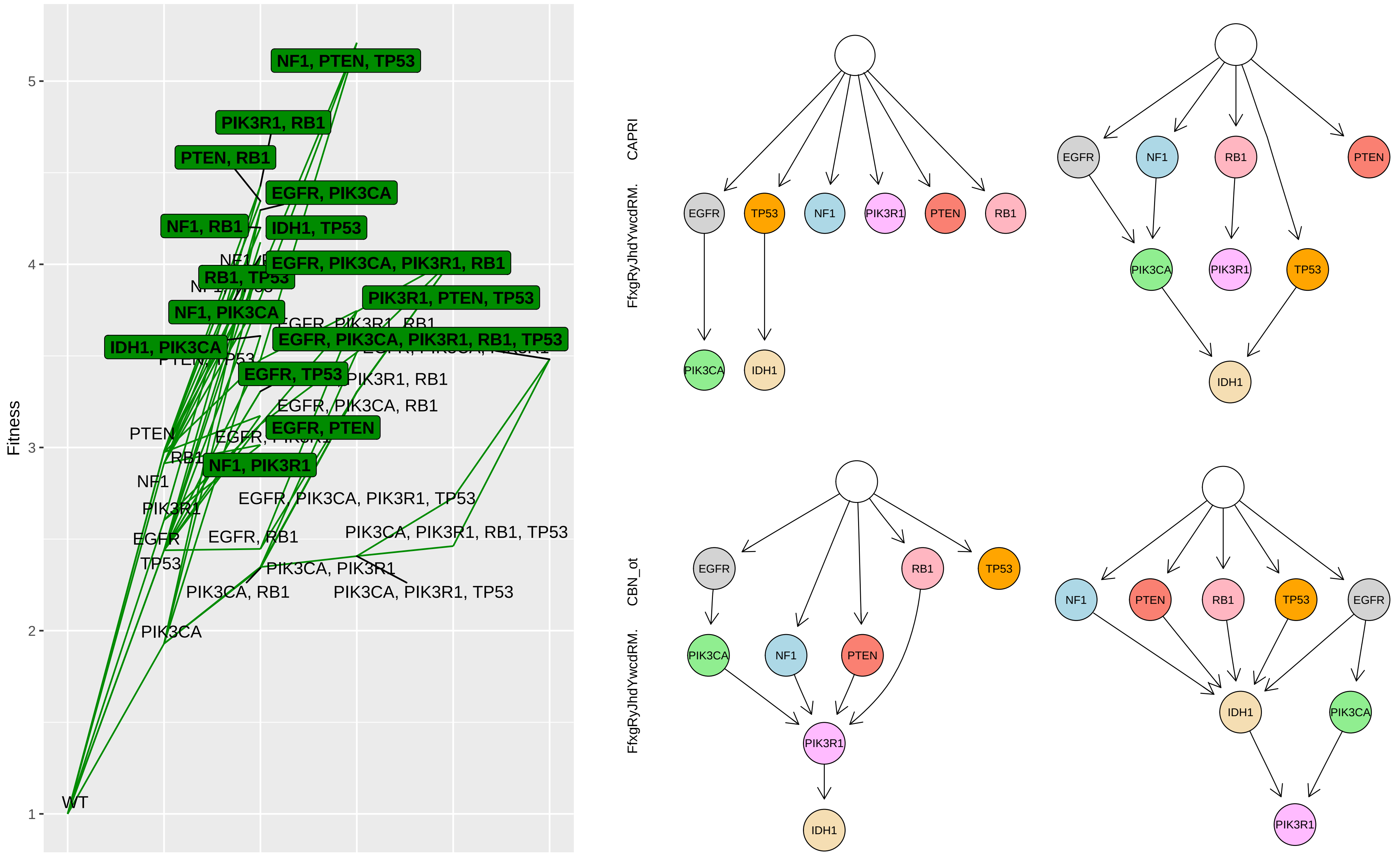

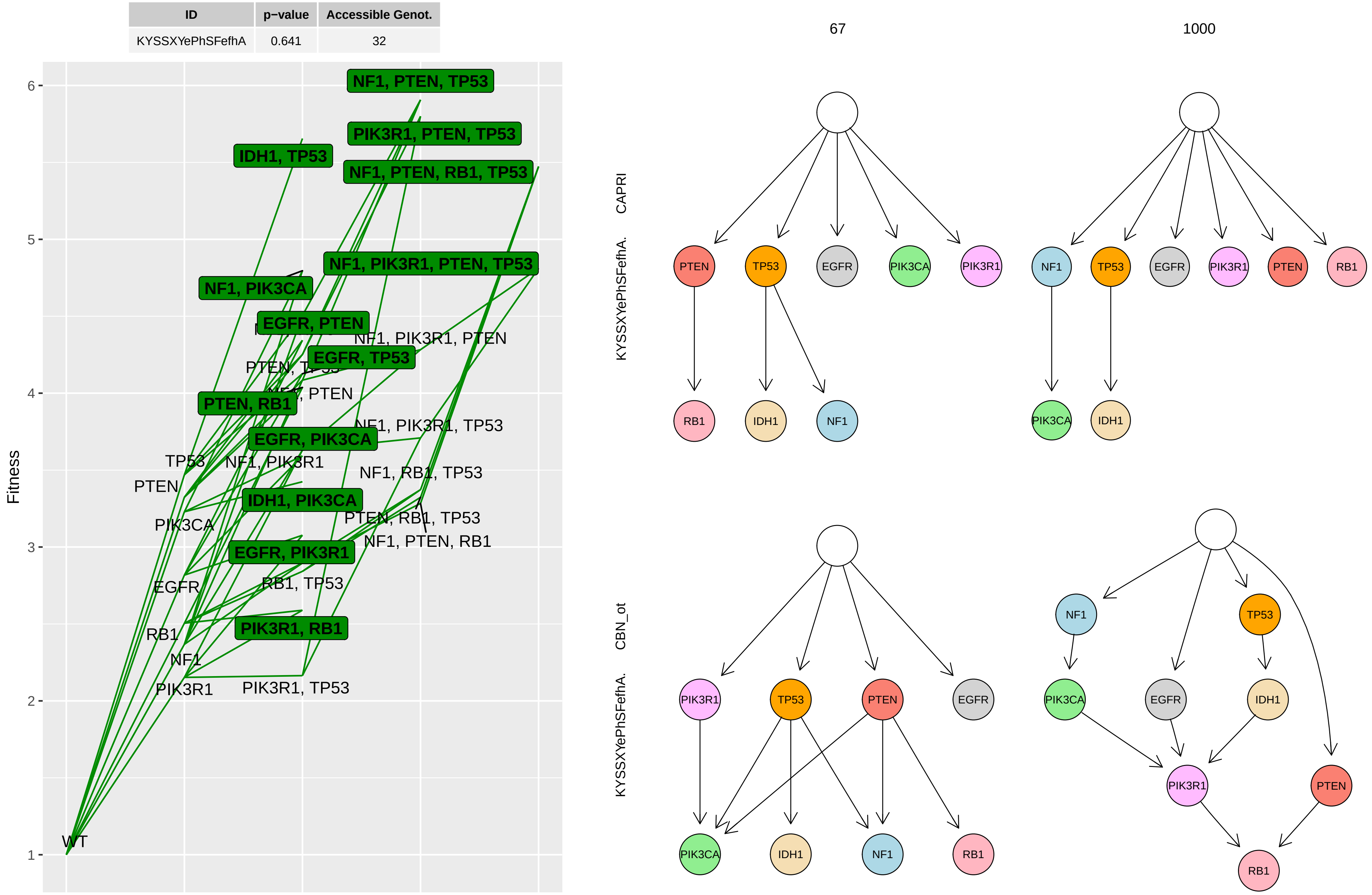
ID
p−value
Accessible Genot.
KYSSXYePhSFefhA
0.641
32
Fitness
NF1, PTEN, TP53
PIK3R1, PTEN, TP53
IDH1, TP53
NF1, PTEN, RB1, TP53
NF1, PIK3R1, PTEN, TP53
NF1, PIK3CA
EGFR, PTEN
NF1, PIK3R1, PTEN
EGFR, TP53
PTEN, RB1
NF1, PIK3R1, TP53
EGFR, PIK3CA
TP53
NF1, PIK3R1
PTEN
NF1, RB1, TP53
IDH1, PIK3CA
PTEN, RB1, TP53
PIK3CA
NF1, PTEN, RB1
EGFR, PIK3R1
RB1, TP53
EGFR
PIK3R1, RB1
RB1
NF1
PIK3R1
PIK3R1, TP53
WT
67
1000
KYSSXYePhSFefhA. CAPRI
KYSSXYePhSFefhA. CBN_ot
PTEN
TP53
EGFR
PIK3CA
PIK3R1
RB1
IDH1
NF1

ID	p−value	Accessible Genot.
MwwjpnmgsfhpPpU	0.644	118

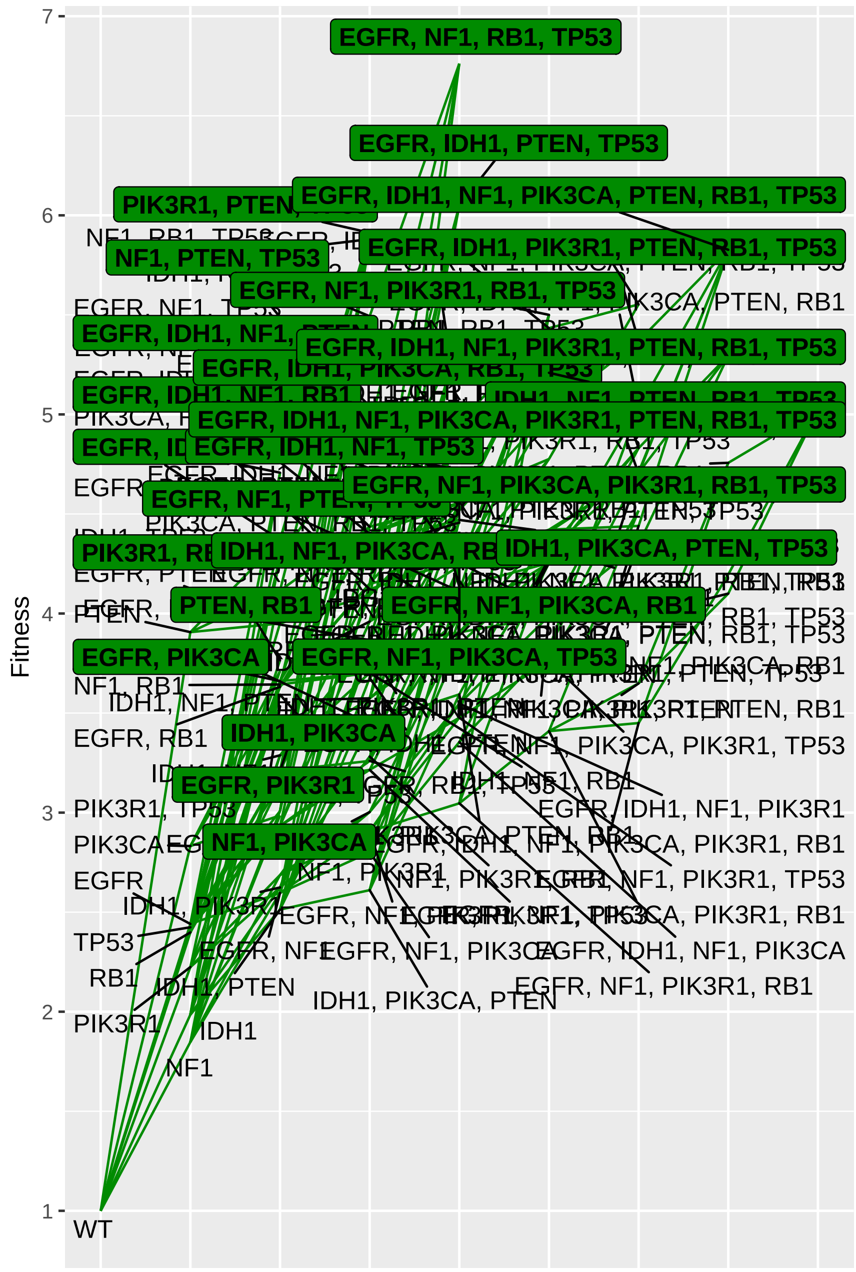

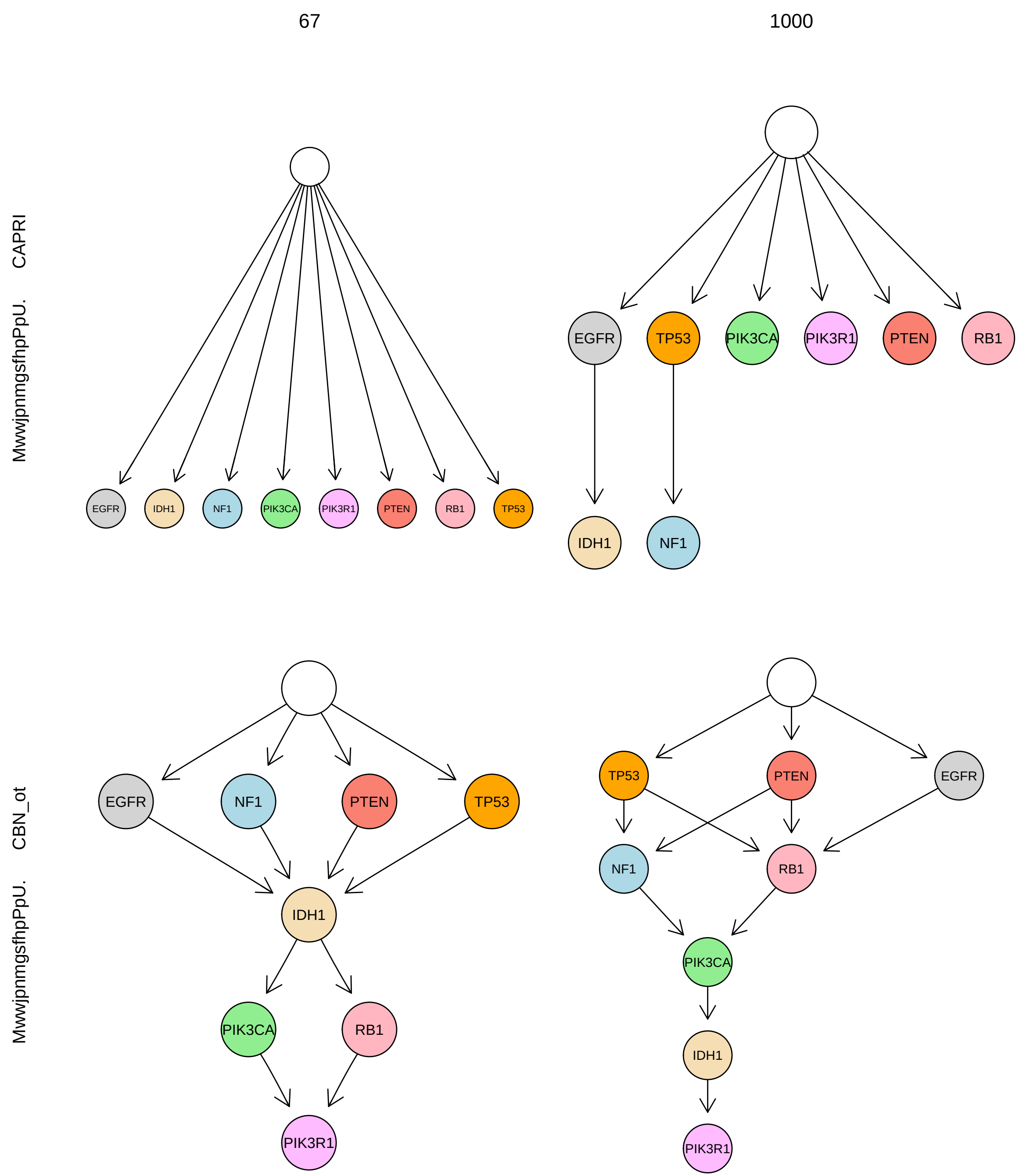

ID	p−value	Accessible Genot.
MYgNtbnqZwMtsRR	0.646	172

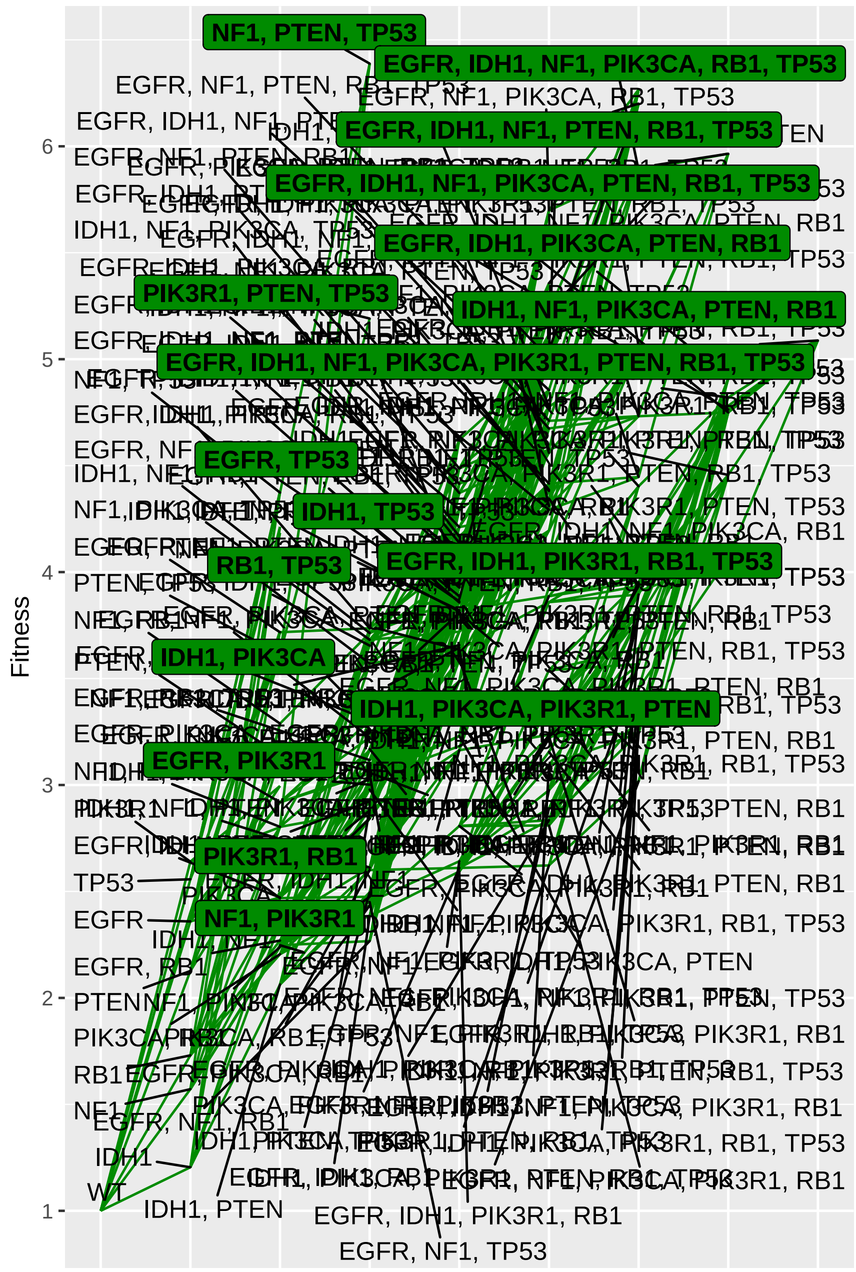

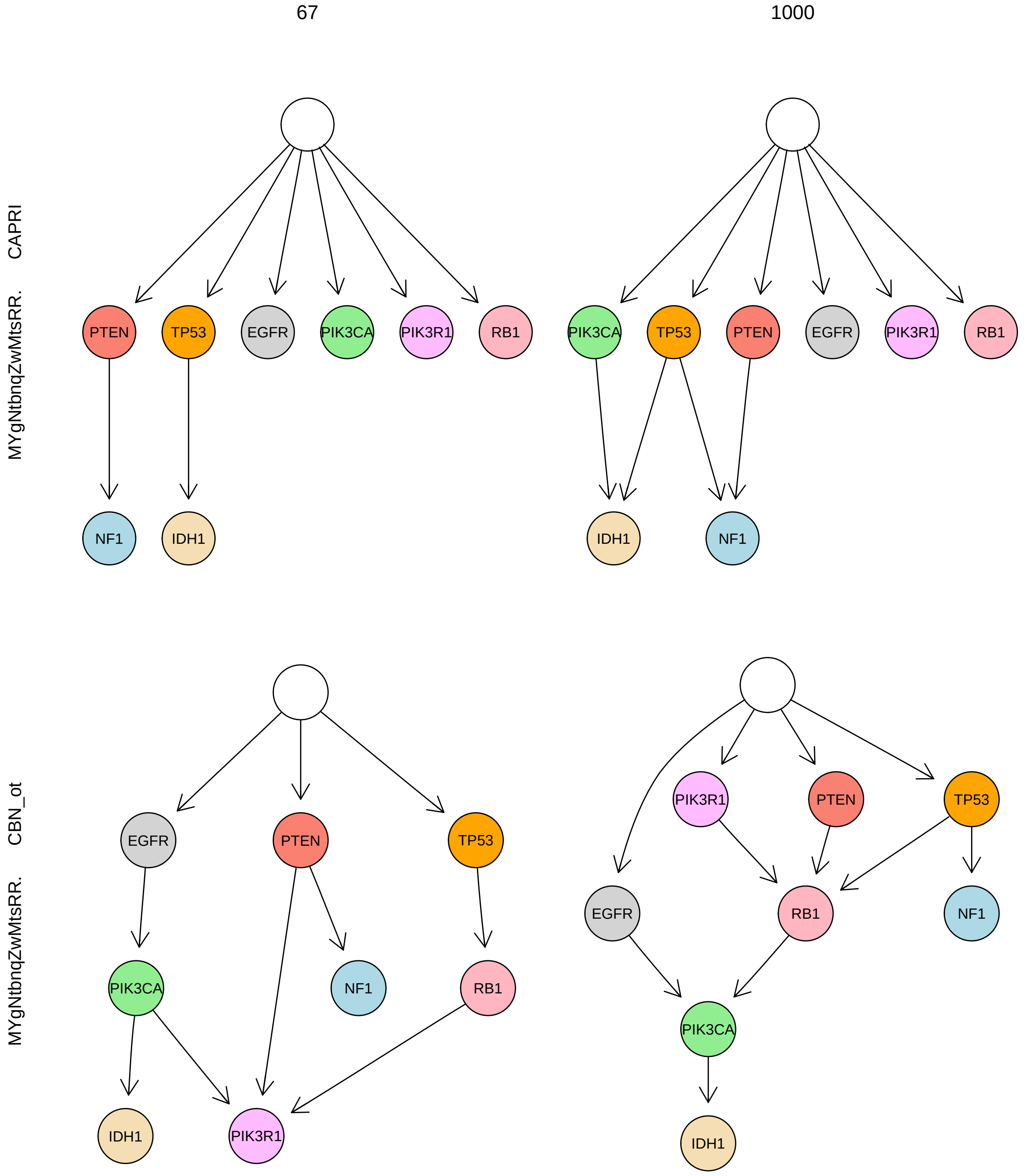

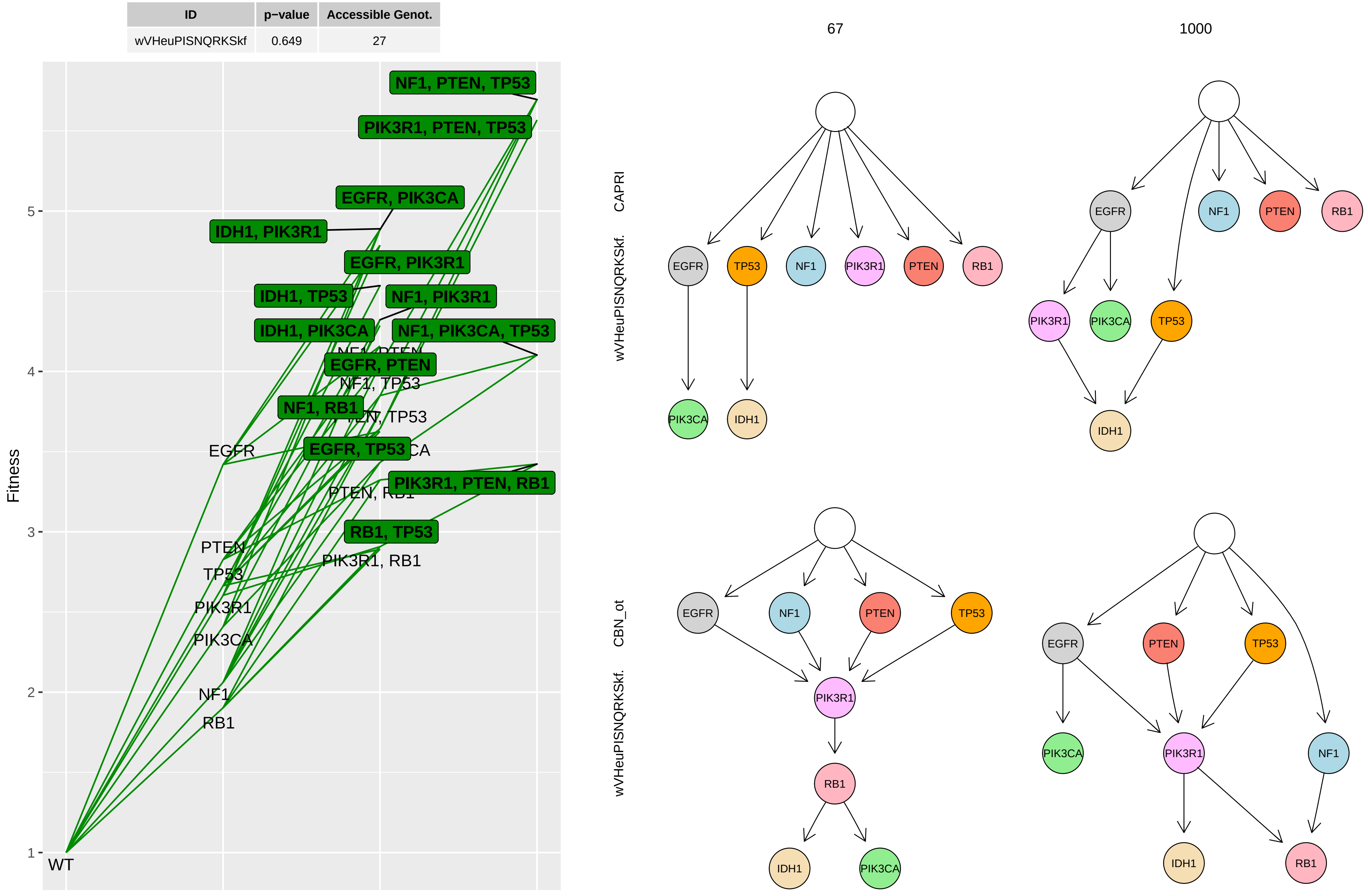

ID
p−value
Accessible Genot.
wVHeuPISNQRKSkf
0.649
27
Fitness
1
2
3
4
5
WT
EGFR
PTEN
TP53
PIK3R1
PIK3CA
NF1
RB1
NF1, PTEN, TP53
PIK3R1, PTEN, TP53
EGFR, PIK3CA
IDH1, PIK3R1
EGFR, PIK3R1
IDH1, TP53
NF1, PIK3R1
IDH1, PIK3CA
NF1, PIK3CA, TP53
EGFR, PTEN
NF1, TP53
NF1, RB1
EGFR, TP53
PIK3R1, PTEN, RB1
RB1, TP53
PIK3R1, RB1
67
1000
CAPRI
wVHeuPISNQRKSkf.
CBN_ot
wVHeuPISNQRKSkf.
EGFR
TP53
NF1
PIK3R1
PTEN
RB1
PIK3CA
IDH1

ID	p−value	Accessible Genot.
pOvfzKjDaTzoNcS	0.65	32

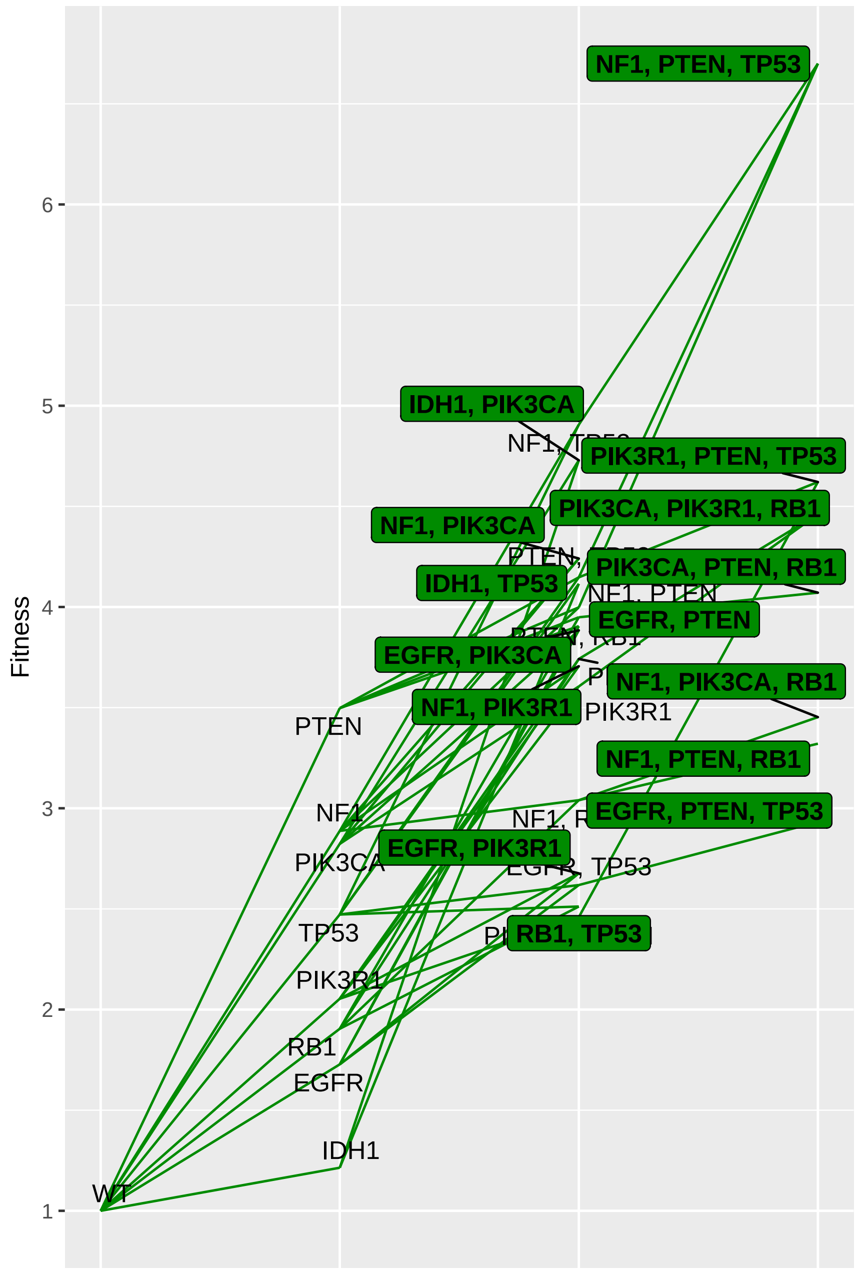

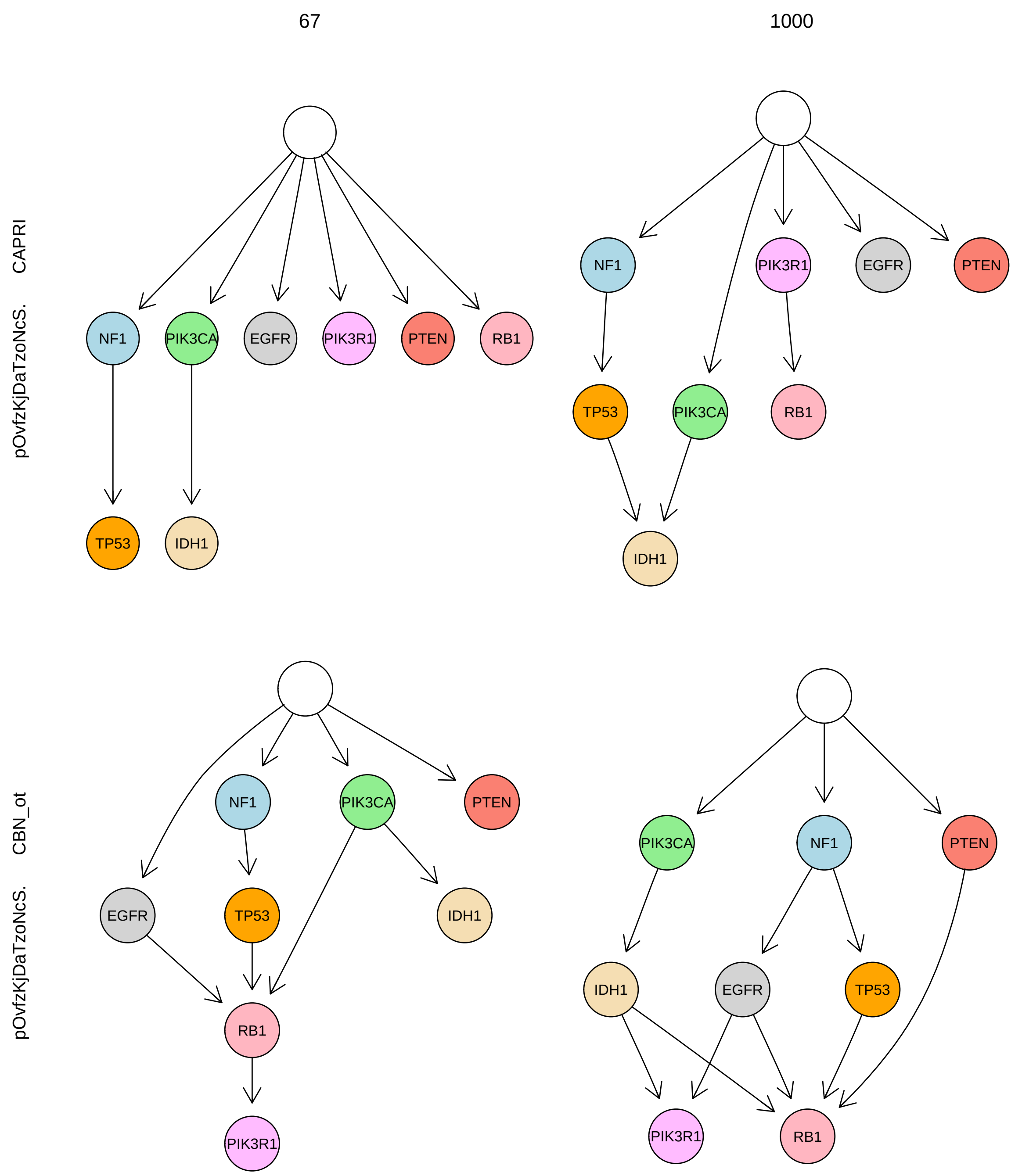

ID	p−value	Accessible Genot.
oHvGqADbRGjCNVm	0.653	36

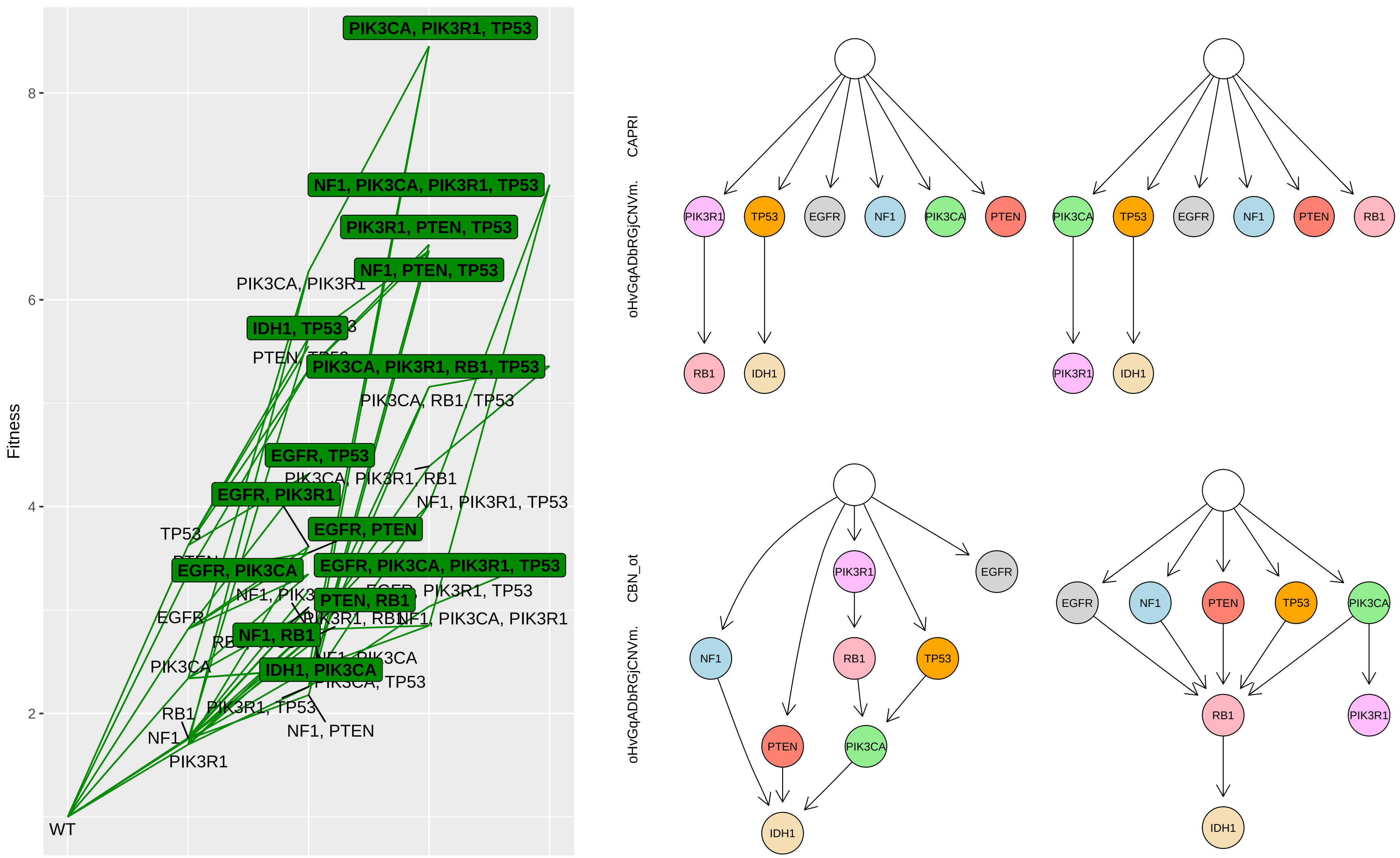

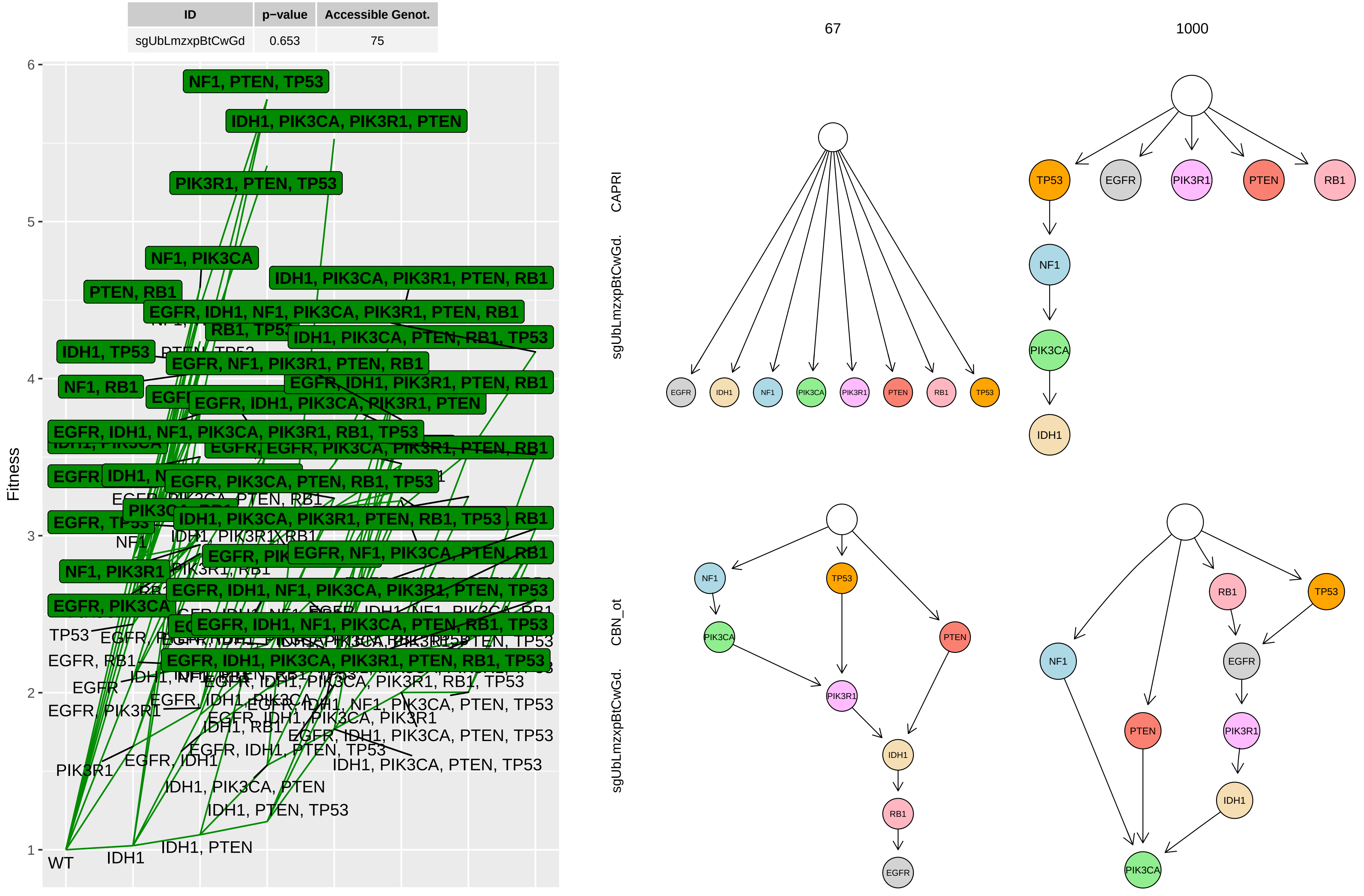

ID
p−value
Accessible Genot.
sgUbLmzxpBtCwGd
0.653
75
Fitness
6
5
4
3
2
1
WT
IDH1
IDH1, PTEN
IDH1, PTEN, TP53
IDH1, PIK3CA, PTEN
IDH1, PIK3CA, PTEN, TP53
NF1, PTEN, TP53
IDH1, PIK3CA, PIK3R1, PTEN
PIK3R1, PTEN, TP53
NF1, PIK3CA
PTEN, RB1
IDH1, PIK3CA, PIK3R1, PTEN, RB1
EGFR, IDH1, NF1, PIK3CA, PIK3R1, PTEN, RB1
IDH1, PIK3CA, PTEN, RB1, TP53
IDH1, TP53
EGFR, NF1, PIK3R1, PTEN, RB1
NF1, RB1
EGFR, IDH1, PIK3R1, PTEN, RB1
EGFR, IDH1, PIK3CA, PIK3R1, PTEN
EGFR, IDH1, NF1, PIK3CA, PIK3R1, RB1, TP53
EGFR, PIK3CA, PTEN, RB1, TP53
IDH1, PIK3CA, PIK3R1, PTEN, RB1, TP53
EGFR, TP53
EGFR, NF1, PIK3CA, PTEN, RB1
NF1, PIK3R1
EGFR, IDH1, NF1, PIK3CA, PIK3R1, PTEN, TP53
EGFR, PIK3CA
EGFR, IDH1, NF1, PIK3CA, PTEN, RB1, TP53
TP53
EGFR, RB1
EGFR, IDH1, PIK3CA, PIK3R1, PTEN, RB1, TP53
EGFR
EGFR, PIK3R1
EGFR, IDH1, PIK3CA, PIK3R1, RB1, TP53
EGFR, IDH1, NF1, PIK3CA, PTEN, TP53
EGFR, IDH1, PIK3CA, PIK3R1
EGFR, IDH1, PIK3CA, PTEN, TP53
EGFR, IDH1, PTEN, TP53
PIK3R1
EGFR, IDH1
IDH1, RB1
67
1000
sgUbLmzxpBtCwGd. CAPRI
sgUbLmzxpBtCwGd. CBN_ot
EGFR
IDH1
NF1
PIK3CA
PIK3R1
PTEN
RB1
TP53

ID	p−value	Accessible Genot.
mKaPYIjVOtoKAFC	0.653	34

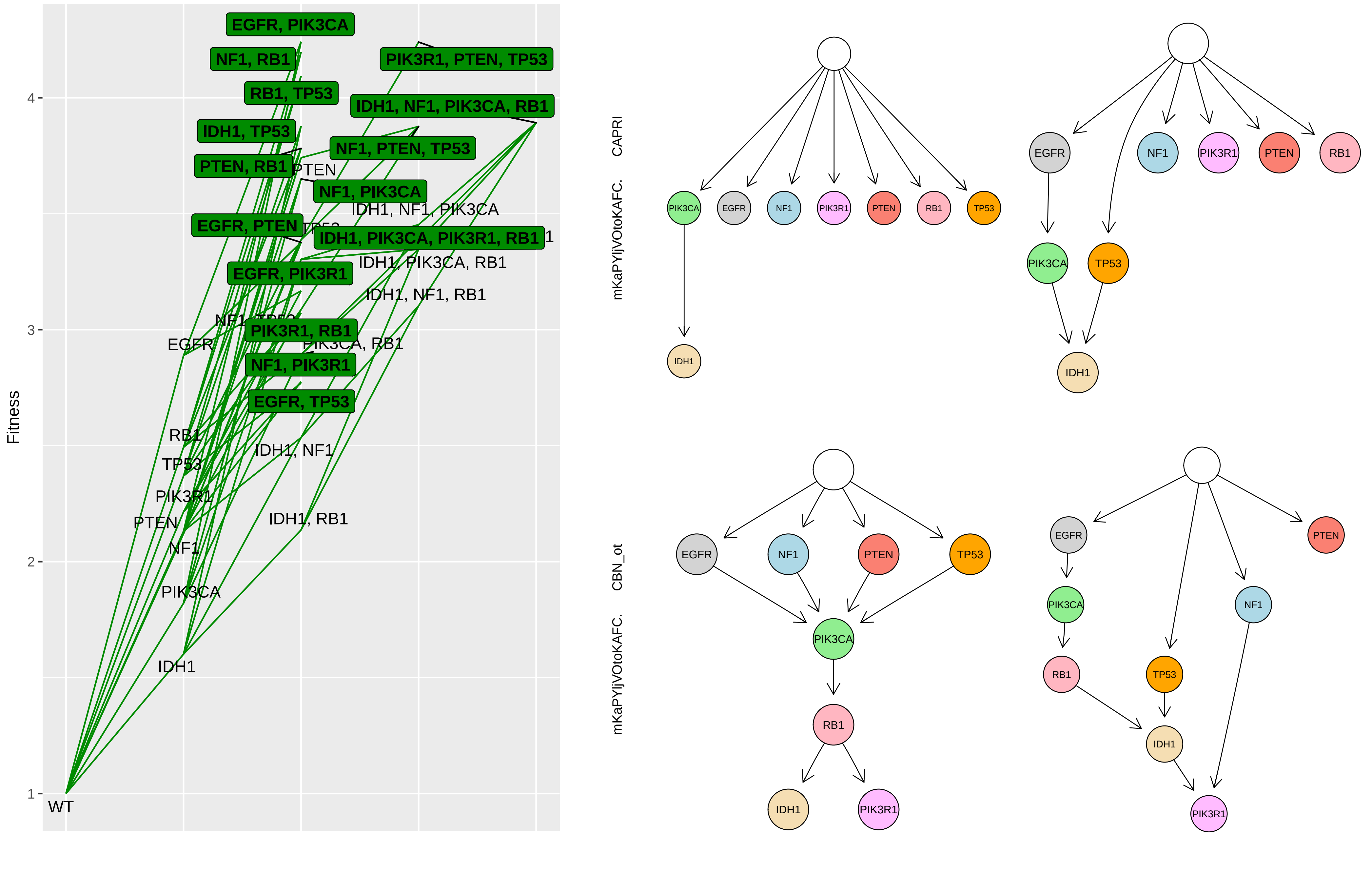

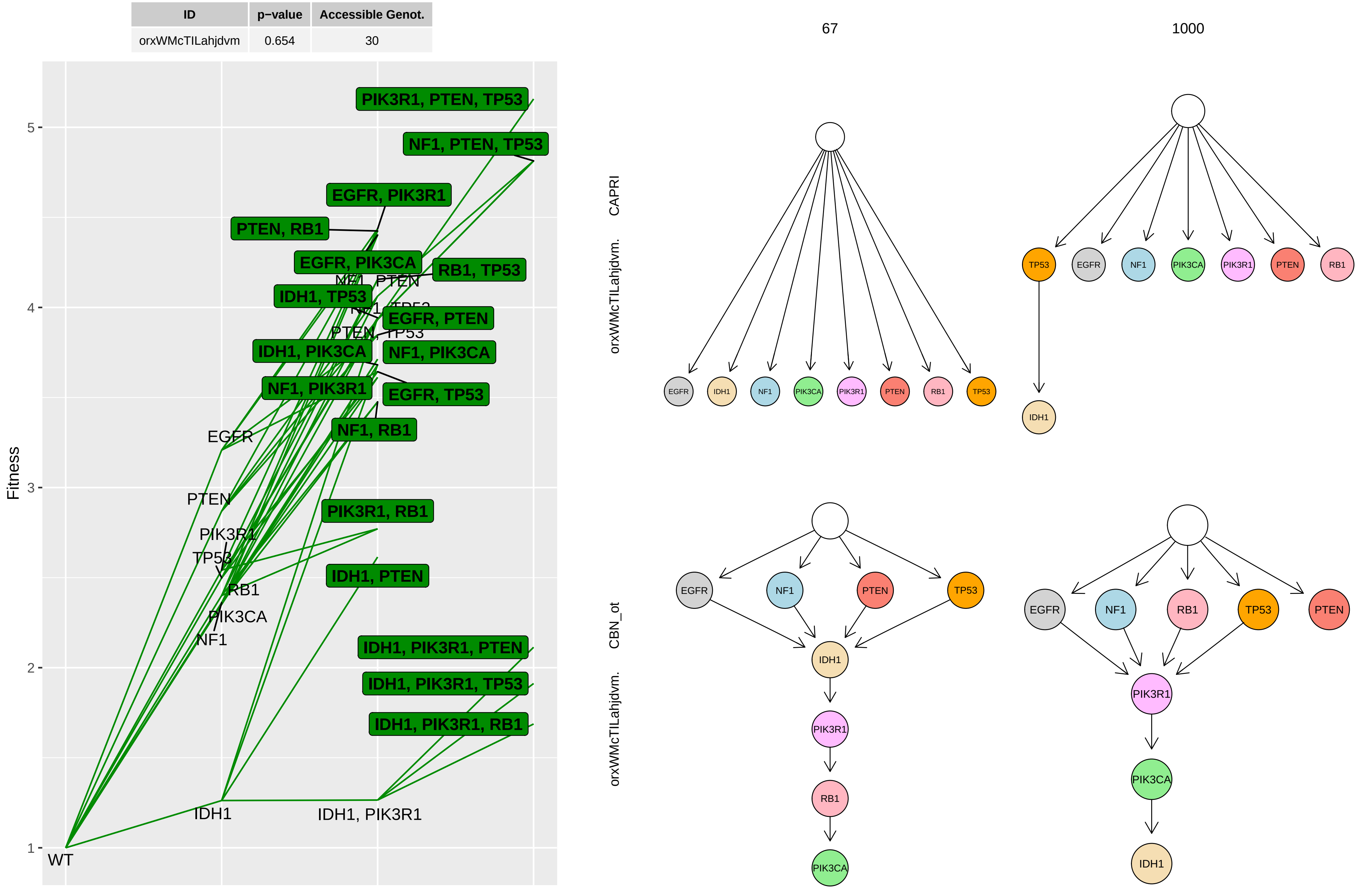

ID
p−value
Accessible Genot.
orxWMcTILahjdvm
0.654
30
Fitness
1
2
3
4
5
WT
IDH1
IDH1, PIK3R1
EGFR
PTEN
PIK3R1
TP53
RB1
PIK3CA
NF1
PIK3R1, PTEN, TP53
NF1, PTEN, TP53
EGFR, PIK3R1
PTEN, RB1
EGFR, PIK3CA
RB1, TP53
IDH1, TP53
EGFR, PTEN
PTEN, TP53
IDH1, PIK3CA
NF1, PIK3CA
NF1, PIK3R1
EGFR, TP53
NF1, RB1
PIK3R1, RB1
IDH1, PTEN
IDH1, PIK3R1, PTEN
IDH1, PIK3R1, TP53
IDH1, PIK3R1, RB1
67
1000
orxWMcTILahjdvm. CAPRI
orxWMcTILahjdvm. CBN_ot
EGFR
IDH1
NF1
PIK3CA
PIK3R1
PTEN
RB1
TP53

ID	p−value	Accessible Genot.
RQAWptSNnlaMOHH	0.654	24

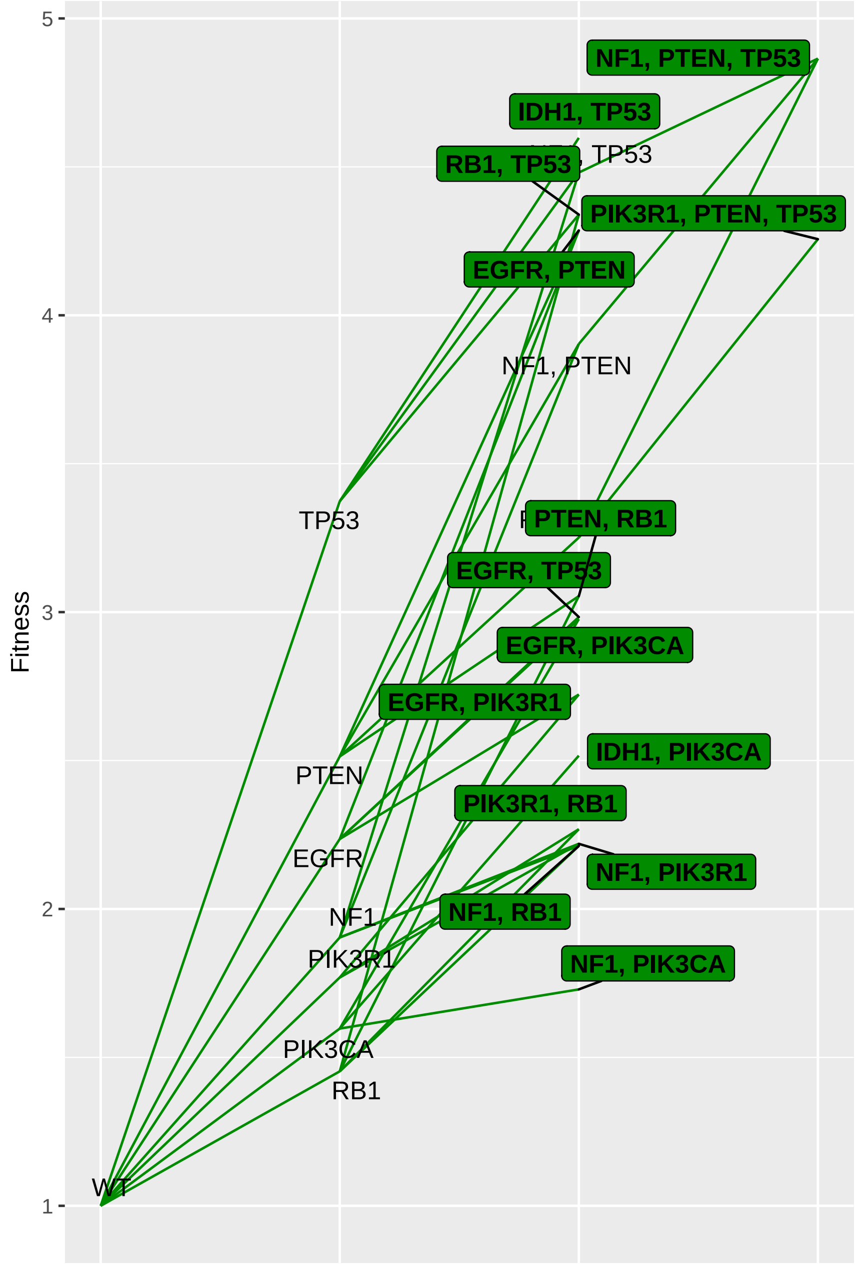

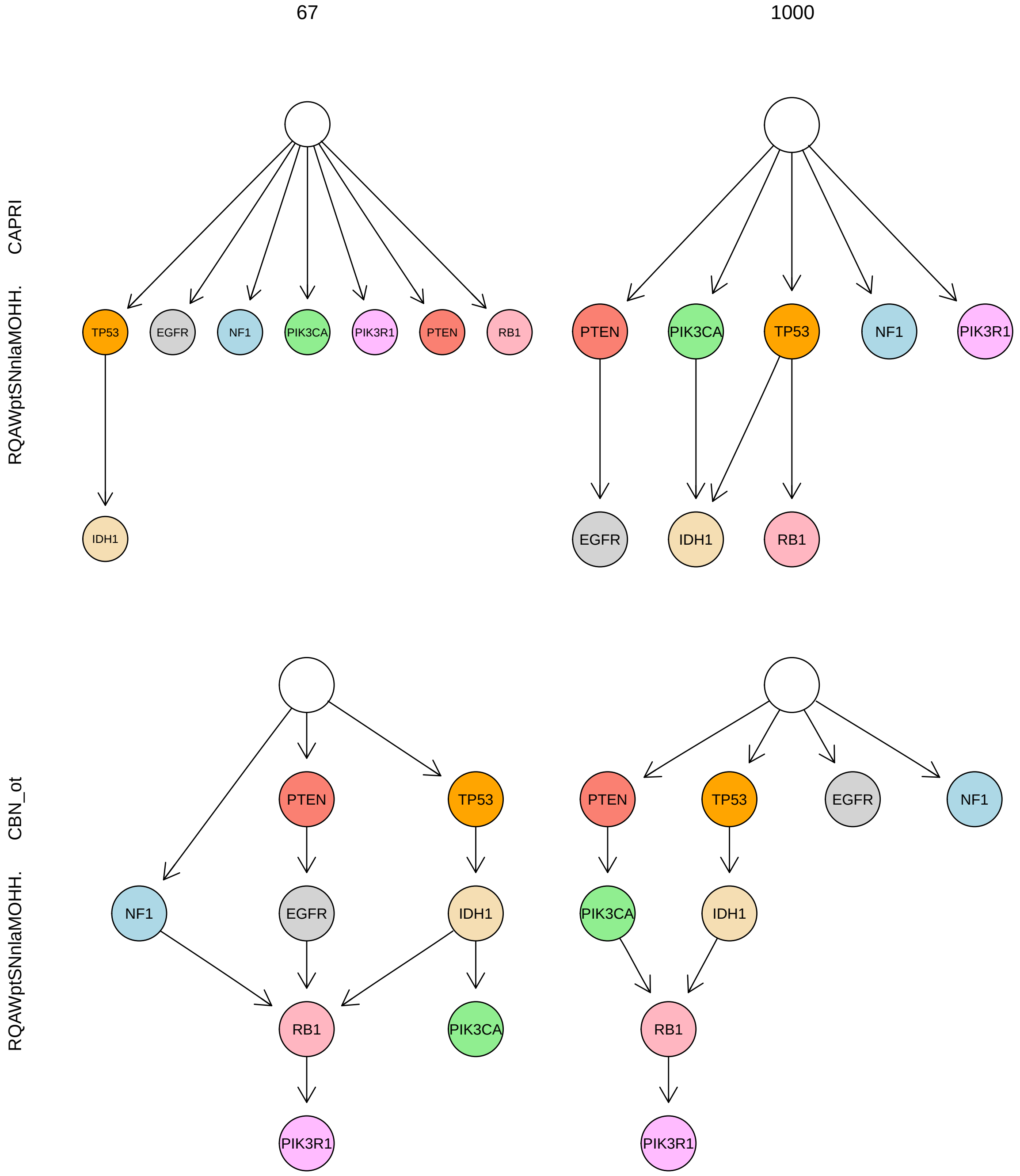

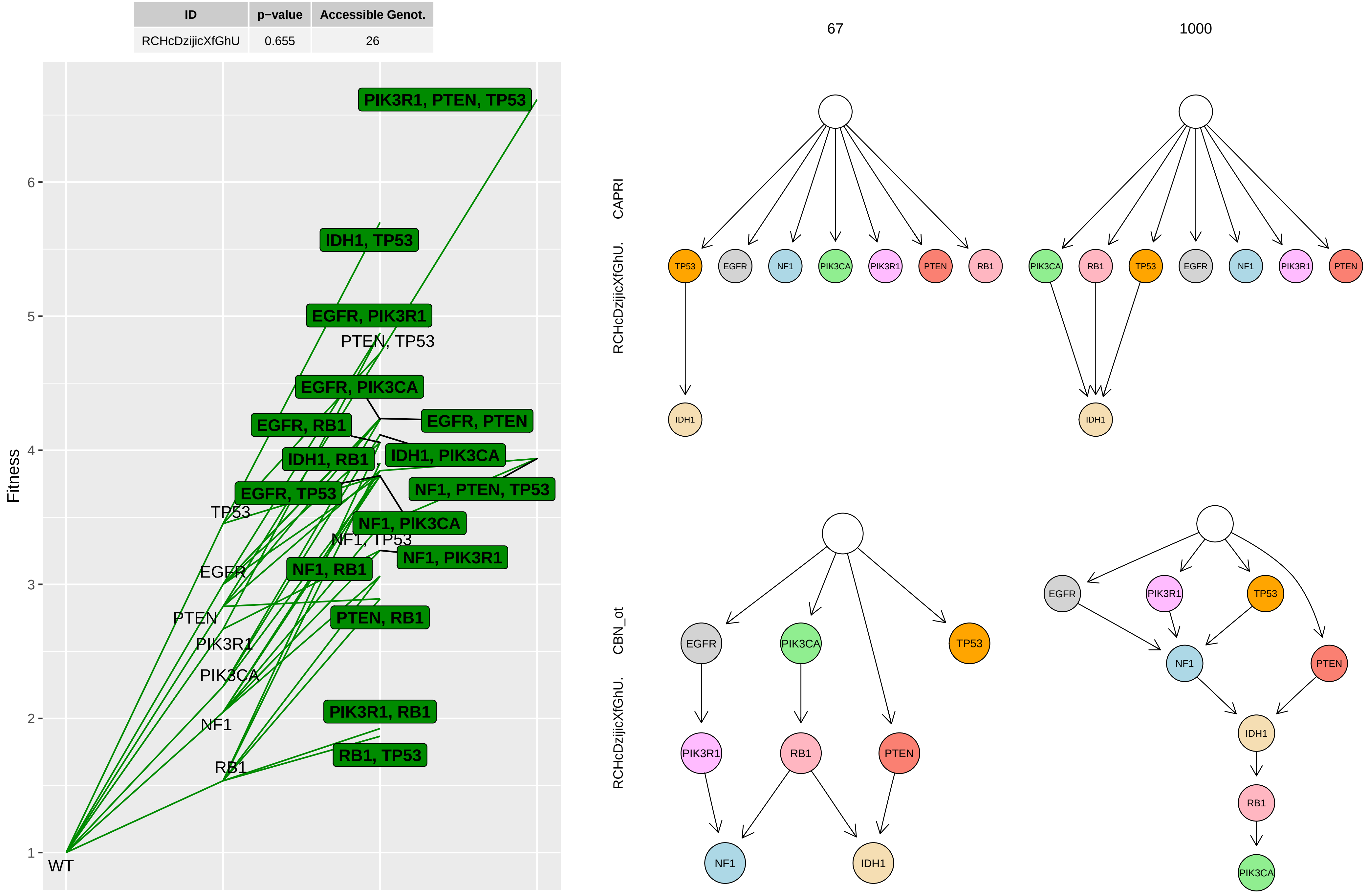
ID
p−value
Accessible Genot.
RCHcDzijicXfGhU
0.655
26
Fitness
WT
TP53
EGFR
PTEN
PIK3R1
PIK3CA
NF1
RB1
PIK3R1, PTEN, TP53
IDH1, TP53
EGFR, PIK3R1
PTEN, TP53
EGFR, PIK3CA
EGFR, PTEN
EGFR, RB1
IDH1, RB1
IDH1, PIK3CA
NF1, PTEN, TP53
EGFR, TP53
NF1, PIK3CA
NF1, TP53
NF1, PIK3R1
NF1, RB1
PTEN, RB1
PIK3R1, RB1
RB1, TP53
67
1000
RCHcDzijicXfGhU. CAPRI
RCHcDzijicXfGhU. CBN_ot
TP53
EGFR
NF1
PIK3CA
PIK3R1
PTEN
RB1
IDH1

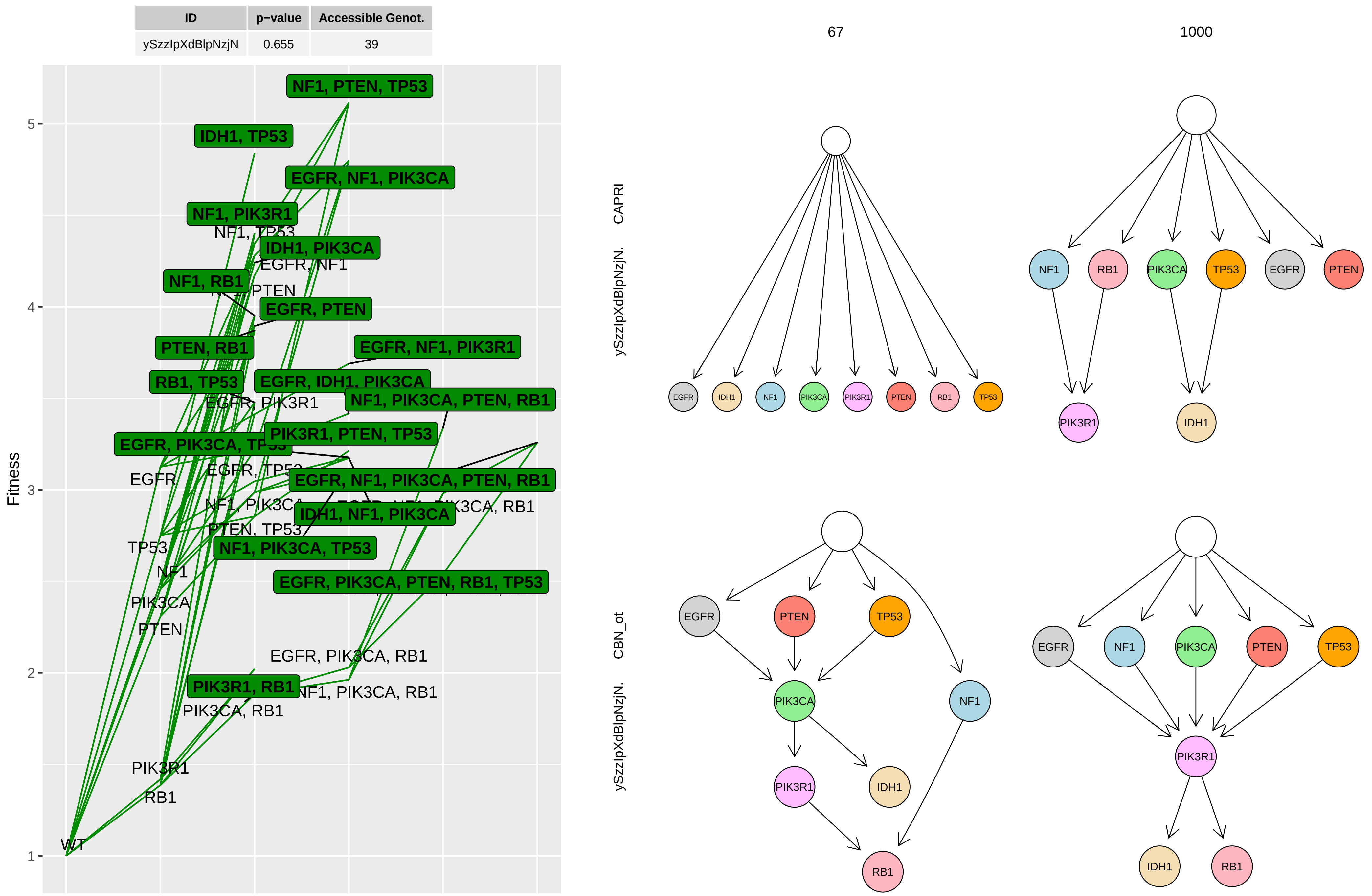
ID
p−value
Accessible Genot.
ySzzIpXdBlpNzjN
0.655
39
Fitness
NF1, PTEN, TP53
IDH1, TP53
EGFR, NF1, PIK3CA
NF1, PIK3R1
IDH1, PIK3CA
NF1, RB1
EGFR, PTEN
EGFR, NF1, PIK3R1
PTEN, RB1
RB1, TP53
EGFR, IDH1, PIK3CA
NF1, PIK3CA, PTEN, RB1
PIK3R1, PTEN, TP53
EGFR, NF1, PIK3CA, PTEN, RB1
IDH1, NF1, PIK3CA
NF1, PIK3CA, TP53
EGFR, PIK3CA, PTEN, RB1, TP53
PIK3R1, RB1
EGFR, PIK3R1
EGFR
TP53
NF1
PIK3CA
PTEN
EGFR, PIK3CA, RB1
NF1, PIK3CA, RB1
PIK3CA, RB1
PIK3R1
RB1
WT
67
1000
ySzzIpXdBlpNzjN. CAPRI
ySzzIpXdBlpNzjN. CBN_ot
EGFR
IDH1
NF1
PIK3CA
PIK3R1
PTEN
RB1
TP53

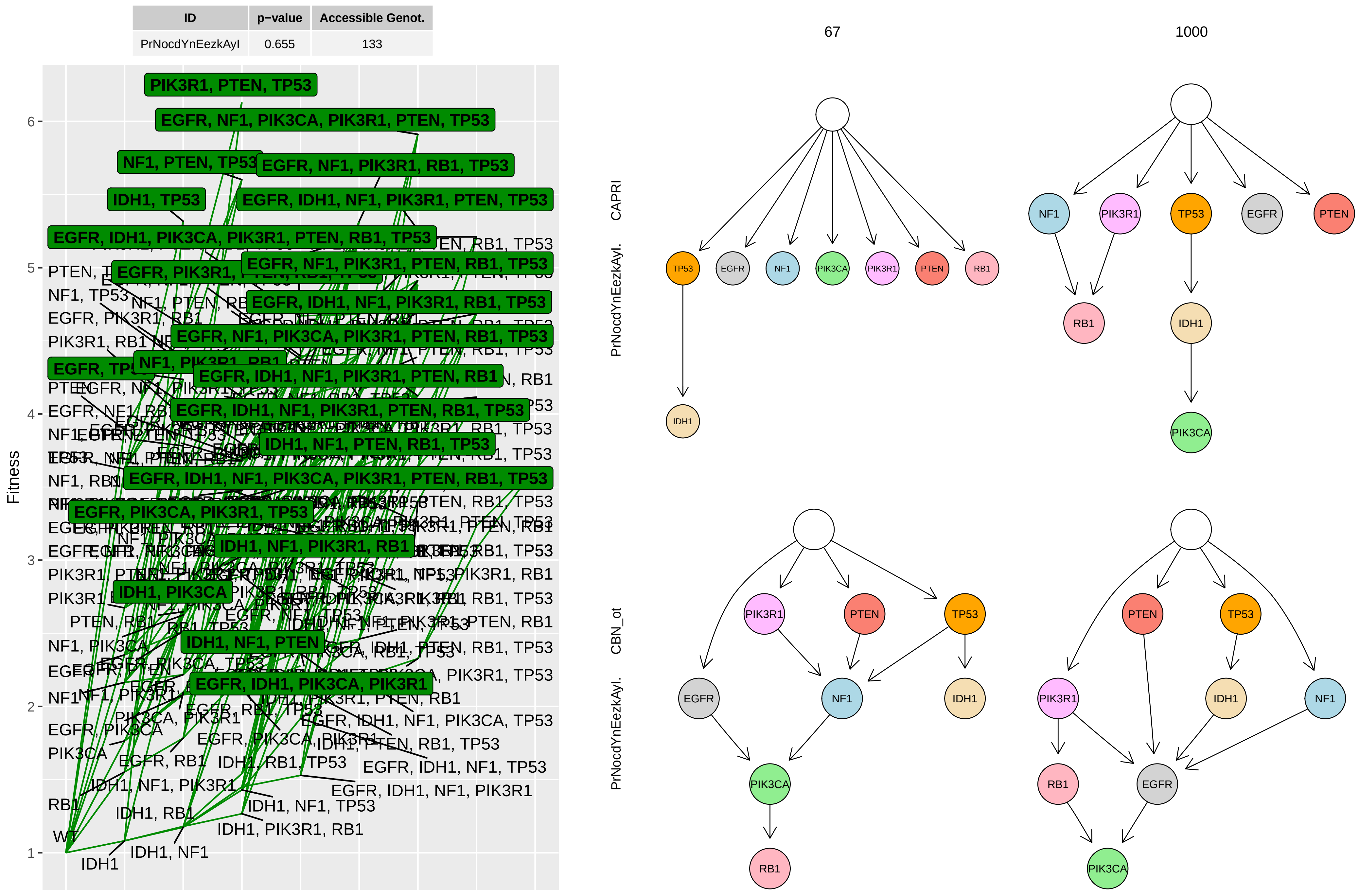

ID
p−value
Accessible Genot.
PrNocdYnEezkAyl
0.655
133
Fitness
67
1000
PrNocdYnEezkAyl.   CAPRI
PrNocdYnEezkAyl.   CBN_ot
TP53
EGFR
NF1
PIK3CA
PIK3R1
PTEN
RB1
IDH1

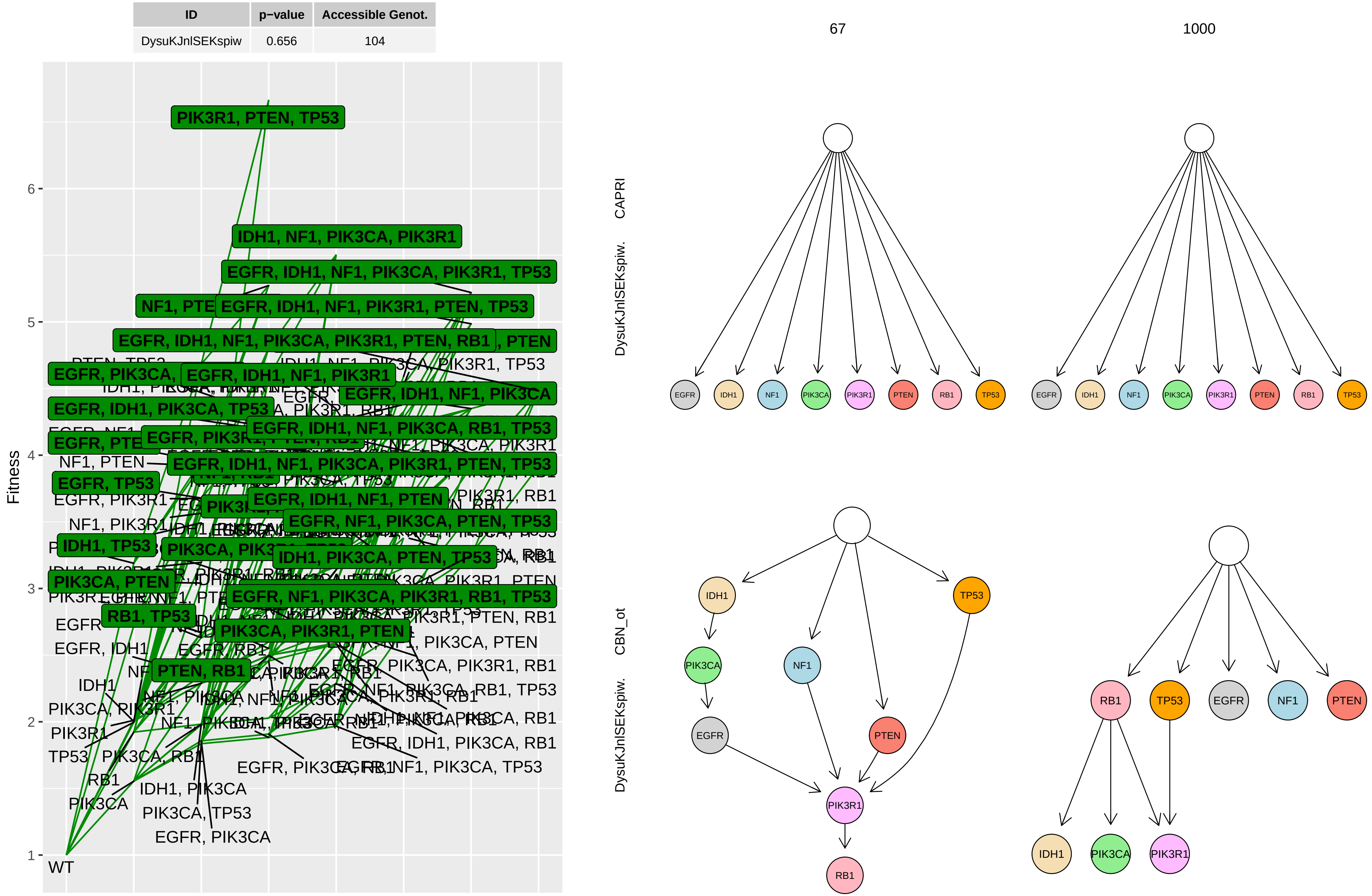

ID
p−value
Accessible Genot.
DysuKJnlSEKspiw
0.656
104
Fitness
WT
PIK3R1, PTEN, TP53
IDH1, NF1, PIK3CA, PIK3R1
EGFR, IDH1, NF1, PIK3CA, PIK3R1, TP53
EGFR, IDH1, NF1, PIK3R1, PTEN, TP53
EGFR, IDH1, NF1, PIK3CA, PIK3R1, PTEN, RB1
EGFR, IDH1, NF1, PIK3R1
EGFR, IDH1, NF1, PIK3CA
EGFR, IDH1, PIK3CA, TP53
EGFR, IDH1, NF1, PIK3CA, RB1, TP53
EGFR, IDH1, NF1, PIK3CA, PIK3R1, PTEN, TP53
EGFR, TP53
EGFR, IDH1, NF1, PTEN
EGFR, NF1, PIK3CA, PTEN, TP53
IDH1, TP53
IDH1, PIK3CA, PTEN, TP53
PIK3CA, PTEN
EGFR, NF1, PIK3CA, PIK3R1, RB1, TP53
RB1, TP53
PIK3CA, PIK3R1, PTEN
PTEN, RB1
NF1, PTEN
EGFR, PIK3R1
NF1, PIK3R1
EGFR, IDH1
IDH1
PIK3CA, PIK3R1
PIK3R1
TP53
PIK3CA, RB1
RB1
PIK3CA
IDH1, PIK3CA
PIK3CA, TP53
EGFR, PIK3CA
IDH1, NF1, PIK3CA, PTEN
EGFR, PIK3CA, PIK3R1, RB1
EGFR, NF1, PIK3CA, RB1, TP53
EGFR, IDH1, PIK3CA, RB1
67
1000
DysuKJnlSEKspiw. CAPRI
DysuKJnlSEKspiw. CBN_ot
EGFR
IDH1
NF1
PIK3CA
PIK3R1
PTEN
RB1
TP53

ID	p−value	Accessible Genot.
IRCZNOKQjGLYHuB	0.656	25

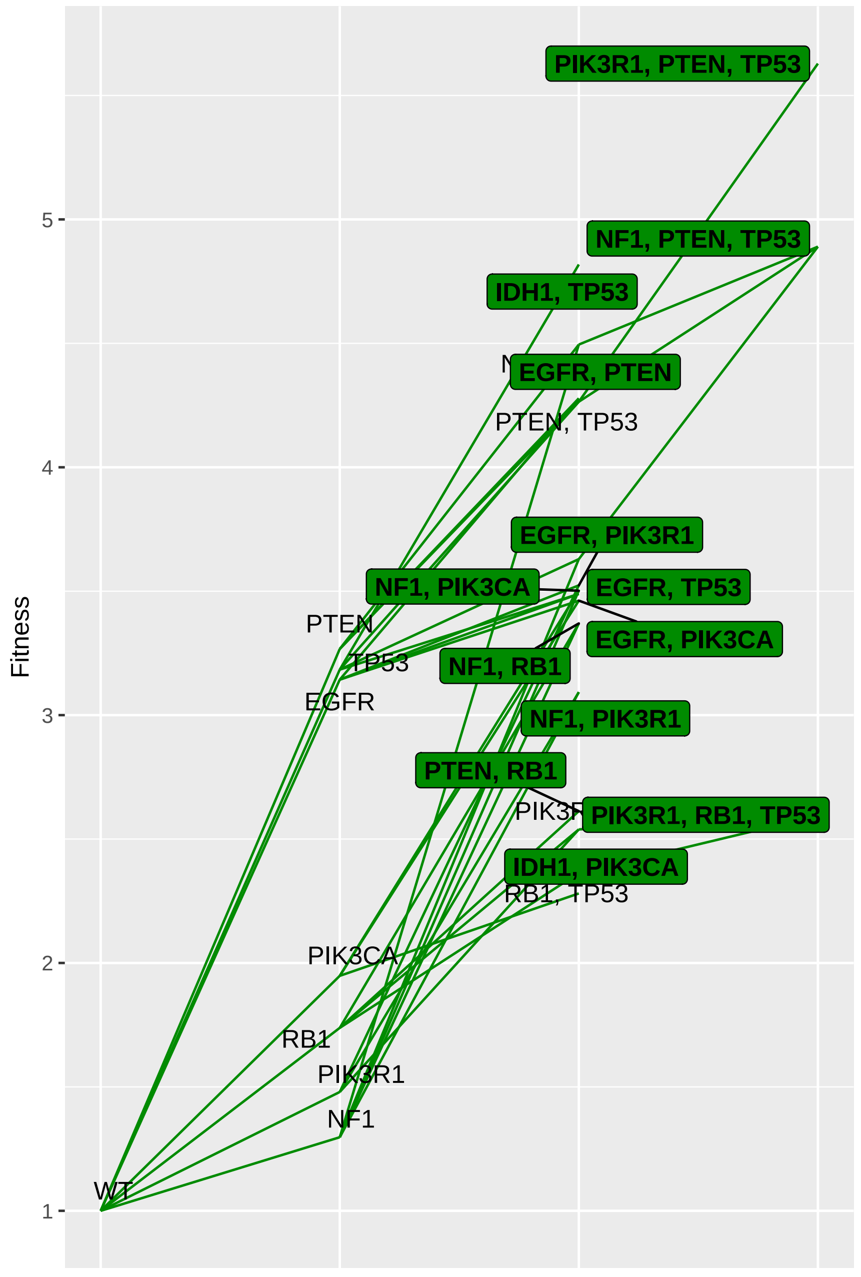

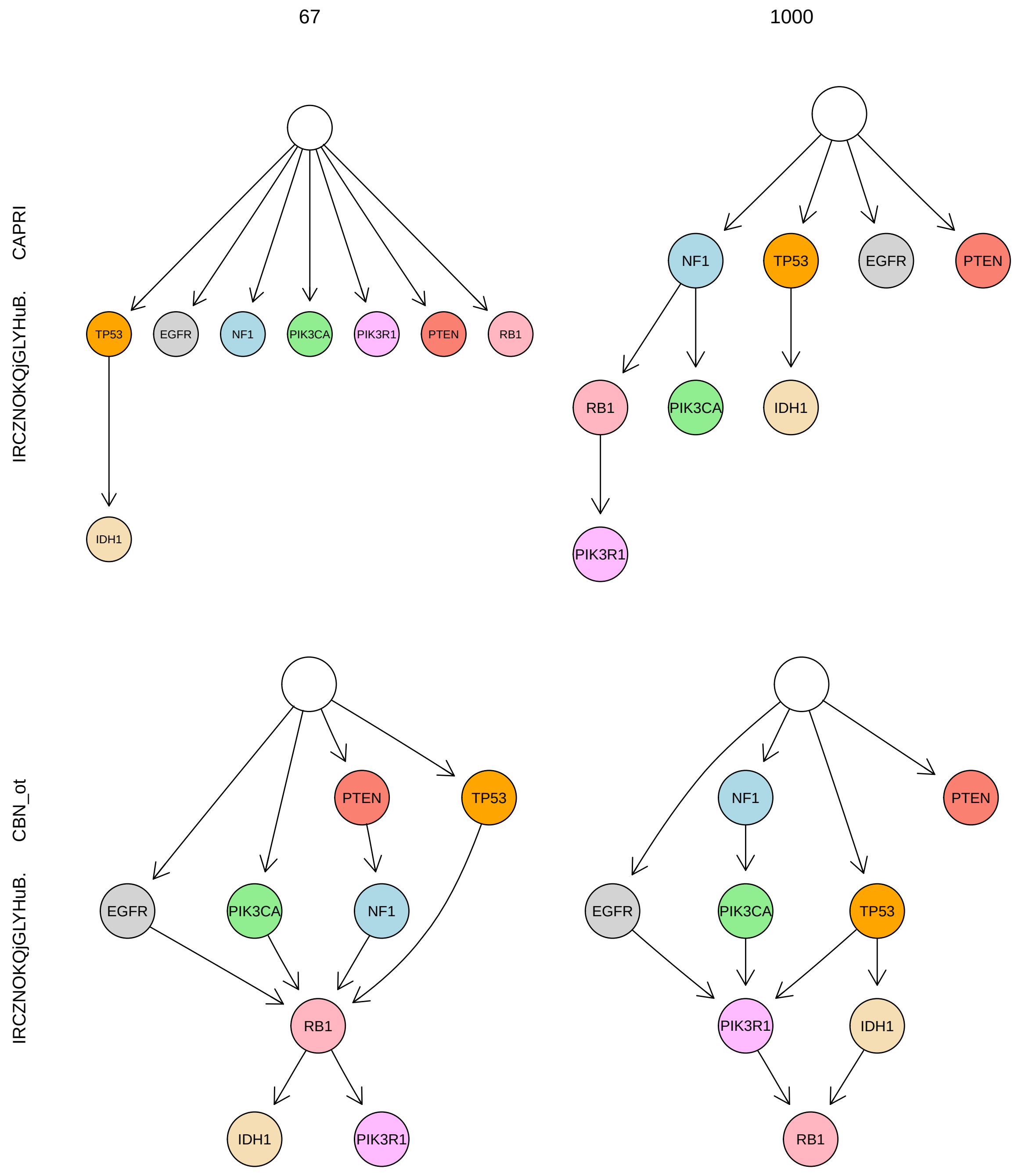

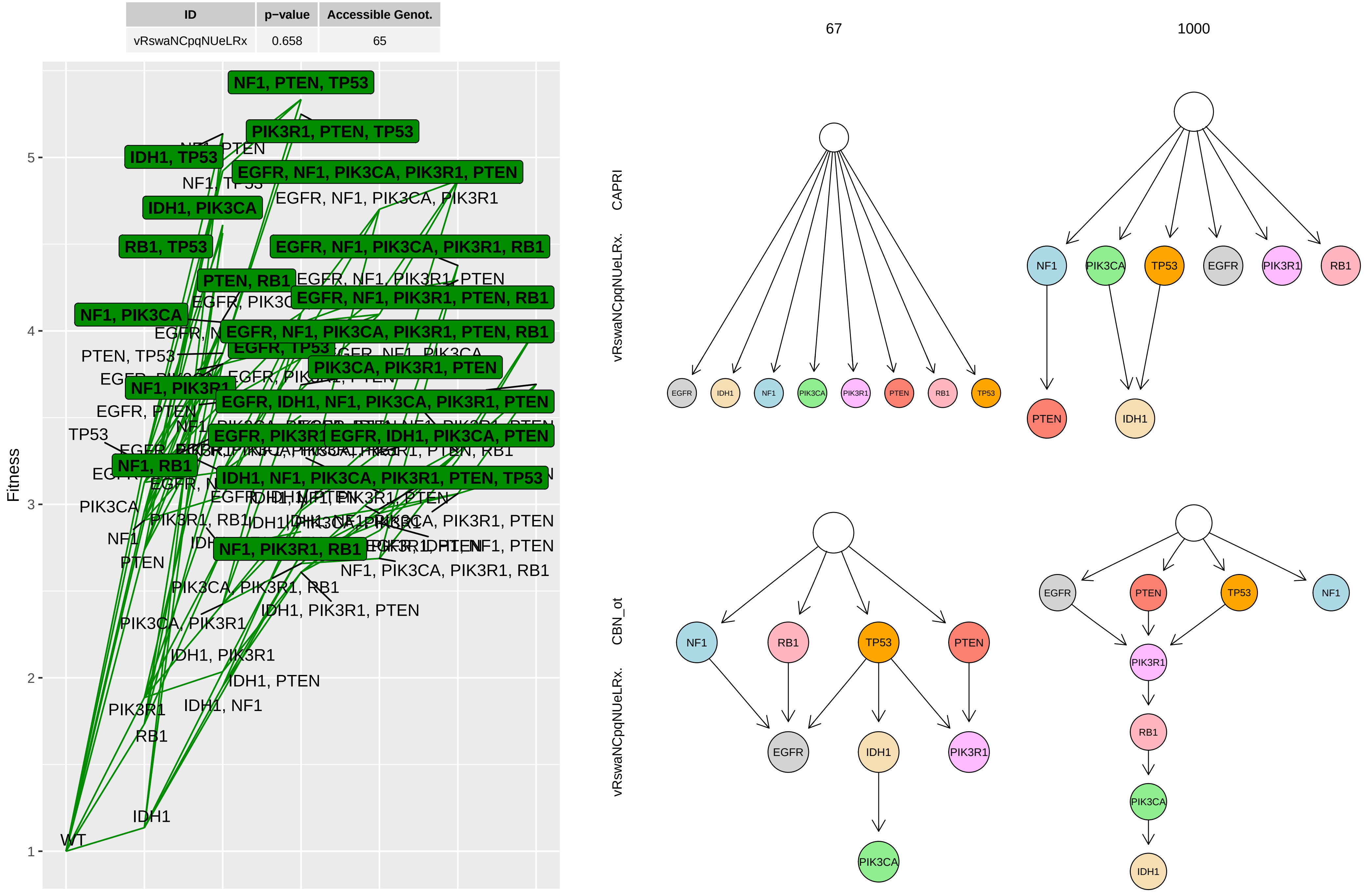
ID | p−value | Accessible Genot.
vRswaNCpqNUeLRx | 0.658 | 65
Fitness
1
2
3
4
5
WT
IDH1
RB1
PIK3R1
PTEN
NF1
PIK3CA
TP53
IDH1, NF1
IDH1, PTEN
IDH1, PIK3R1
PIK3CA, PIK3R1
IDH1, PIK3R1, PTEN
PIK3CA, PIK3R1, RB1
NF1, PIK3CA, PIK3R1, RB1
NF1, PIK3R1, RB1
NF1, RB1
NF1, PIK3R1
NF1, PIK3CA
PTEN, TP53
EGFR, PTEN
PIK3R1, RB1
IDH1, PIK3CA
IDH1, TP53
RB1, TP53
PTEN, RB1
NF1, PTEN, TP53
PIK3R1, PTEN, TP53
EGFR, NF1, PIK3CA, PIK3R1, PTEN
EGFR, NF1, PIK3CA, PIK3R1
EGFR, NF1, PIK3CA, PIK3R1, RB1
EGFR, NF1, PIK3R1, PTEN
EGFR, NF1, PIK3R1, PTEN, RB1
EGFR, NF1, PIK3CA, PIK3R1, PTEN, RB1
EGFR, TP53
PIK3CA, PIK3R1, PTEN
EGFR, IDH1, NF1, PIK3CA, PIK3R1, PTEN
EGFR, IDH1, PIK3CA, PTEN
IDH1, NF1, PIK3CA, PIK3R1, PTEN, TP53
67
1000
vRswaNCpqNUeLRx. CAPRI
vRswaNCpqNUeLRx. CBN_ot
EGFR
IDH1
NF1
PIK3CA
PIK3R1
PTEN
RB1
TP53

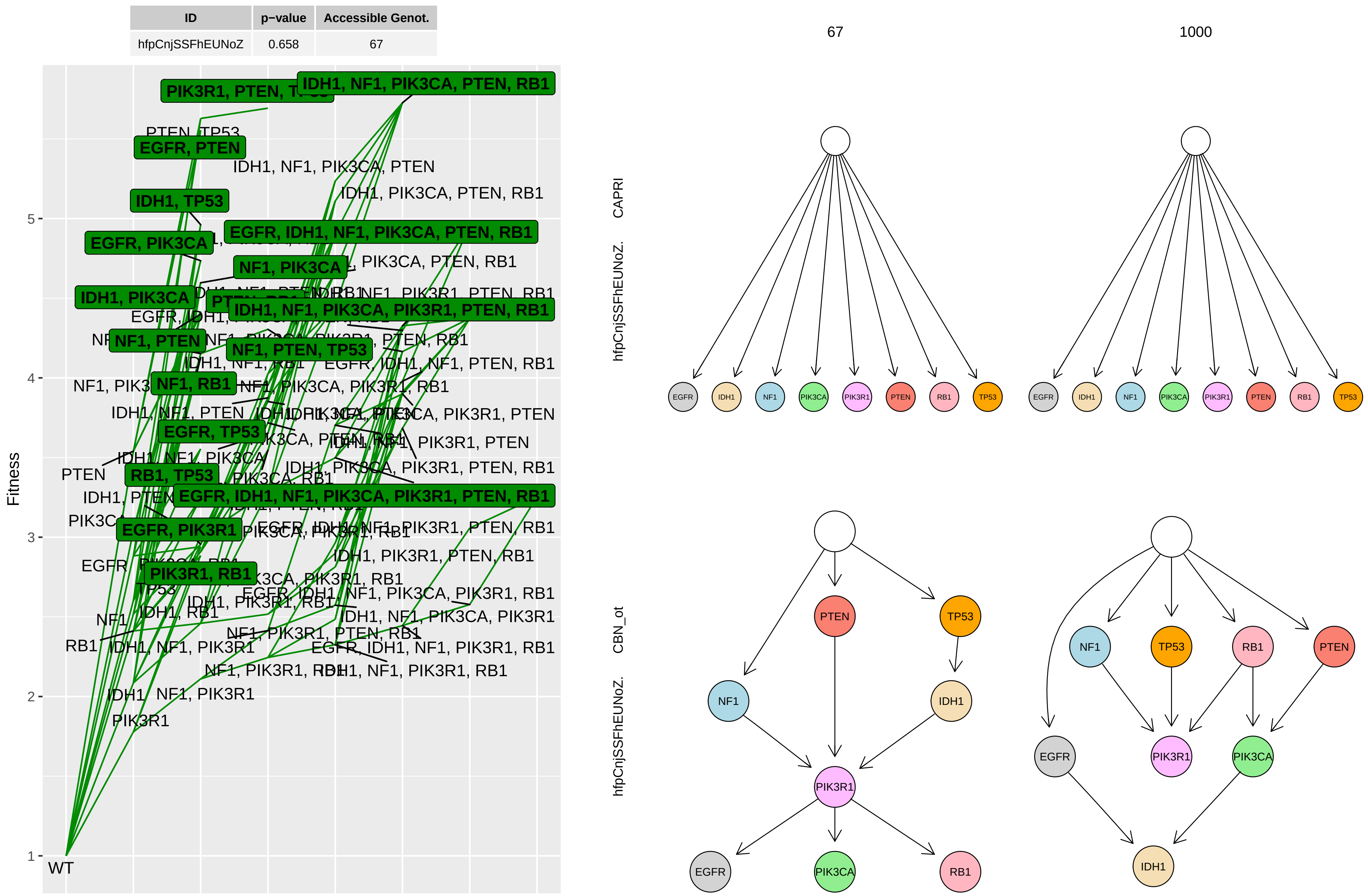

ID
p−value
Accessible Genot.
hfpCnjSSFhEUNoZ
0.658
67
Fitness
1
2
3
4
5
WT
PIK3R1
IDH1, NF1, PIK3R1
IDH1, NF1, PIK3CA, PTEN, RB1
EGFR, IDH1, NF1, PIK3CA, PTEN, RB1
IDH1, NF1, PIK3CA, PIK3R1, PTEN, RB1
EGFR, IDH1, NF1, PIK3CA, PIK3R1, PTEN, RB1
EGFR, PTEN
IDH1, TP53
EGFR, PIK3CA
NF1, PIK3CA
IDH1, PIK3CA
NF1, PTEN
NF1, PTEN, TP53
NF1, RB1
EGFR, TP53
RB1, TP53
EGFR, PIK3R1
PIK3R1, RB1
IDH1, NF1, PIK3CA, PTEN
IDH1, PIK3CA, PTEN, RB1
EGFR, IDH1, NF1, PTEN, RB1
IDH1, NF1, PTEN
IDH1, PIK3CA, PIK3R1, PTEN, RB1
EGFR, IDH1, NF1, PIK3R1, PTEN, RB1
IDH1, PIK3R1, PTEN, RB1
EGFR, IDH1, NF1, PIK3CA, PIK3R1, RB1
IDH1, NF1, PIK3CA, PIK3R1
EGFR, IDH1, NF1, PIK3R1, RB1
IDH1, NF1, PIK3R1, RB1
67
1000
hfpCnjSSFhEUNoZ.
CAPRI
CBN_ot
EGFR
IDH1
NF1
PIK3CA
PIK3R1
PTEN
RB1
TP53

ID	p−value	Accessible Genot.
LVNsdgkaXhhEhCG	0.659	24

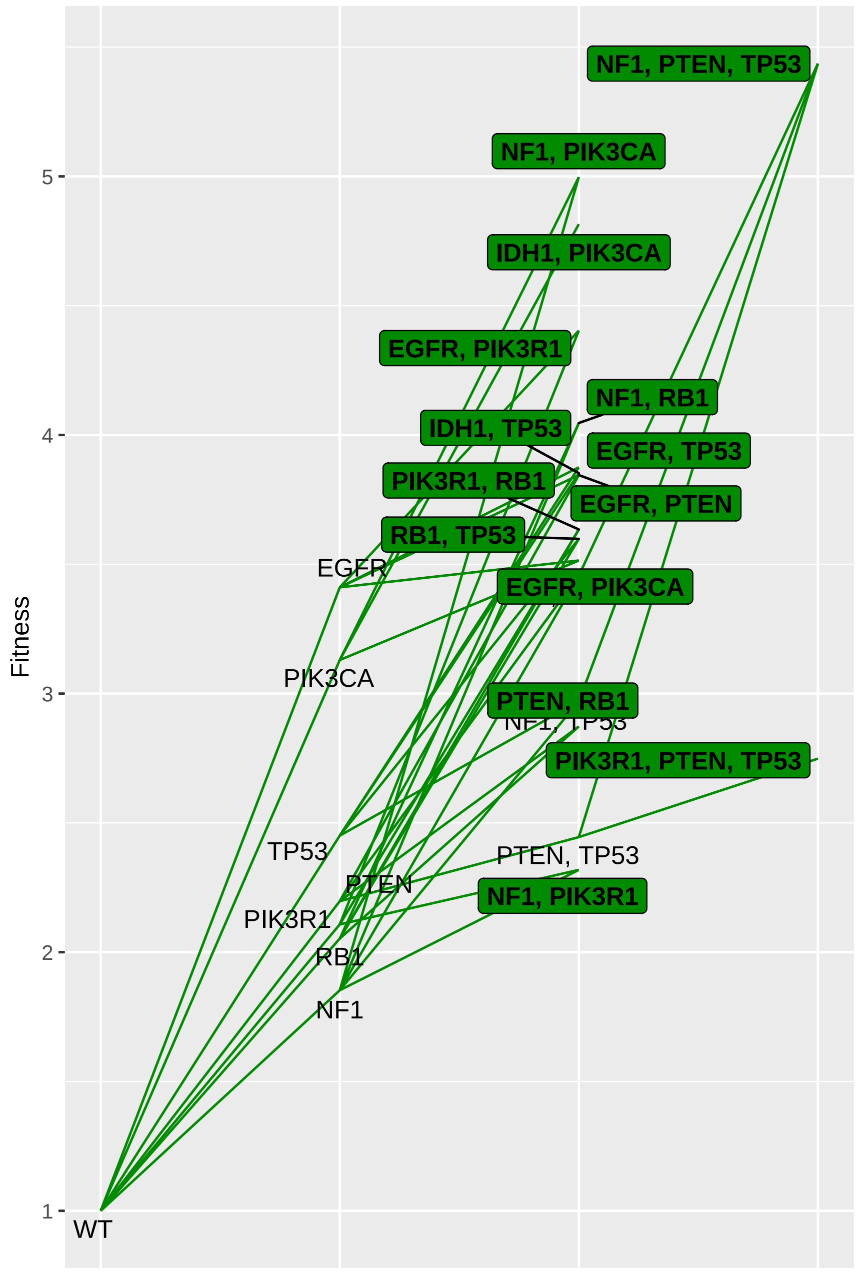

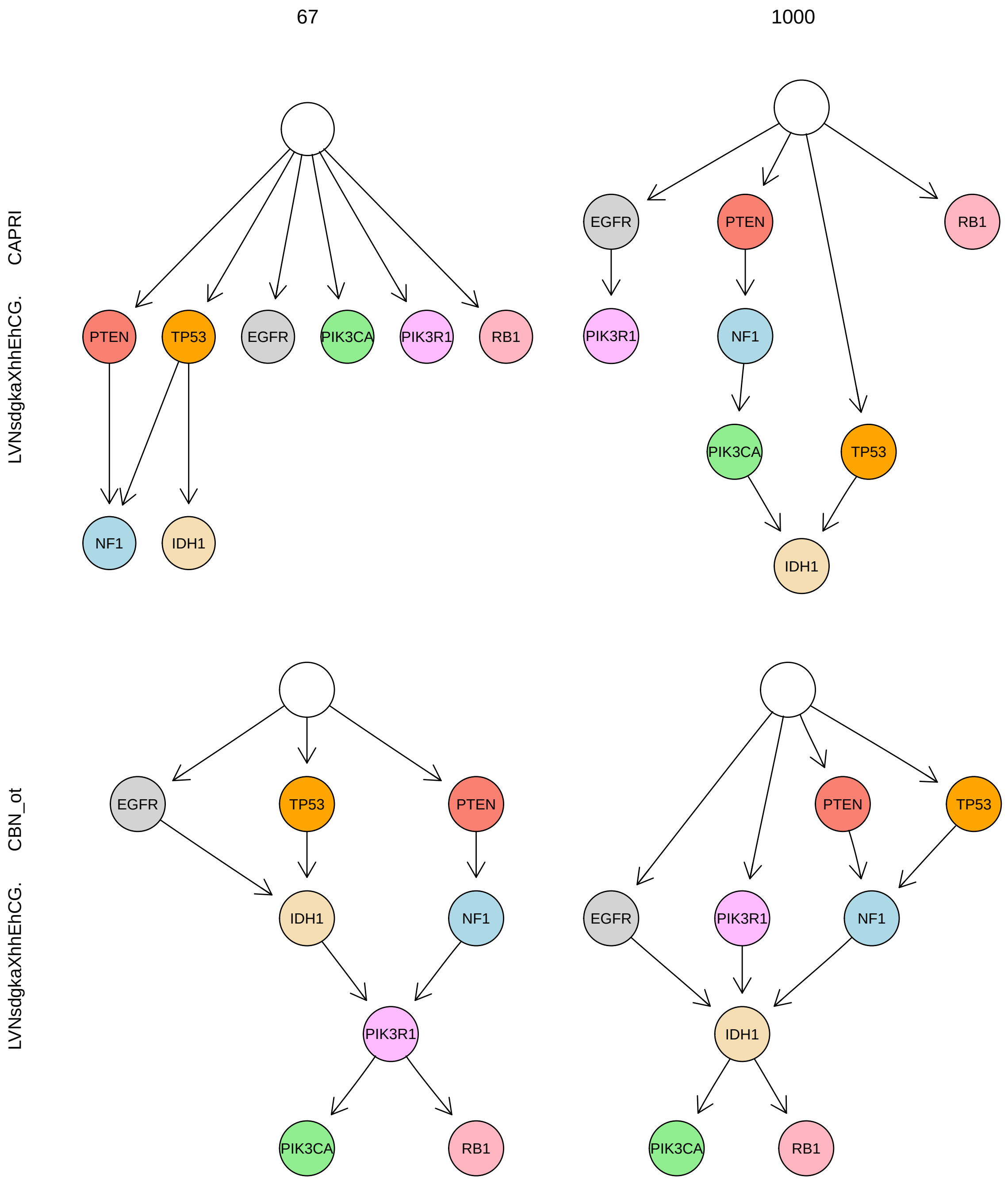

ID	p−value	Accessible Genot.
pKFzyBFYLxBdtiS	0.659	28

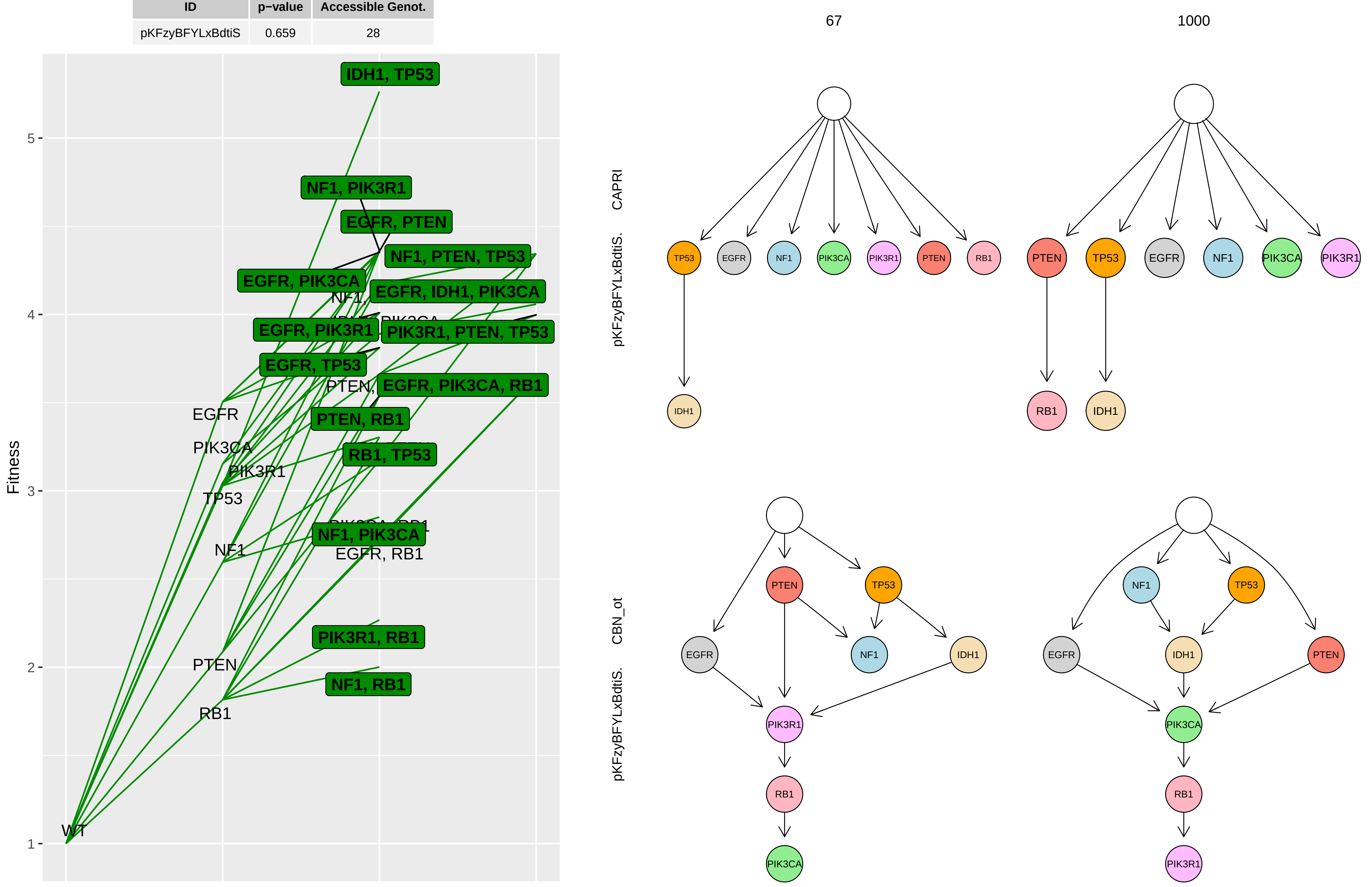

ID	p−value	Accessible Genot.
tpGyhyuOnoqNFUq	0.662	32

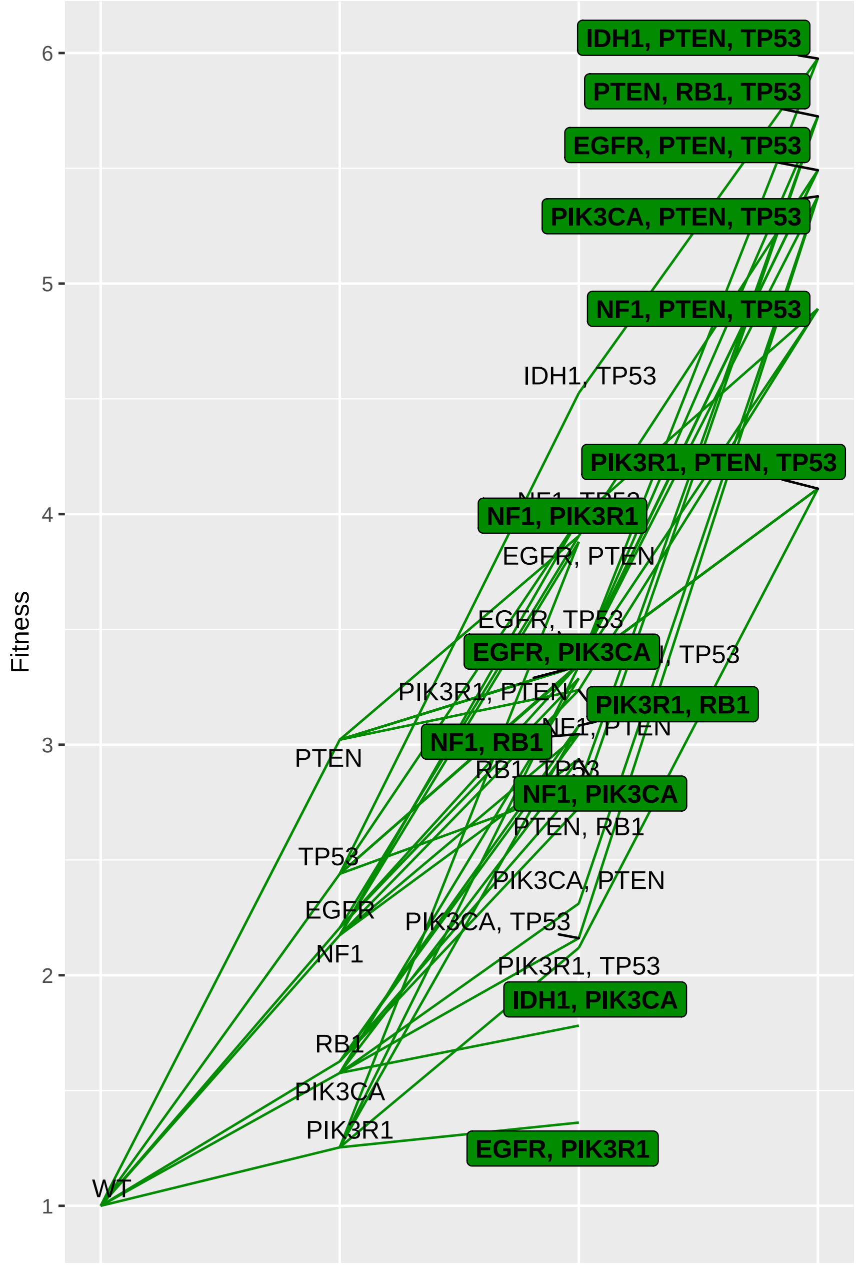

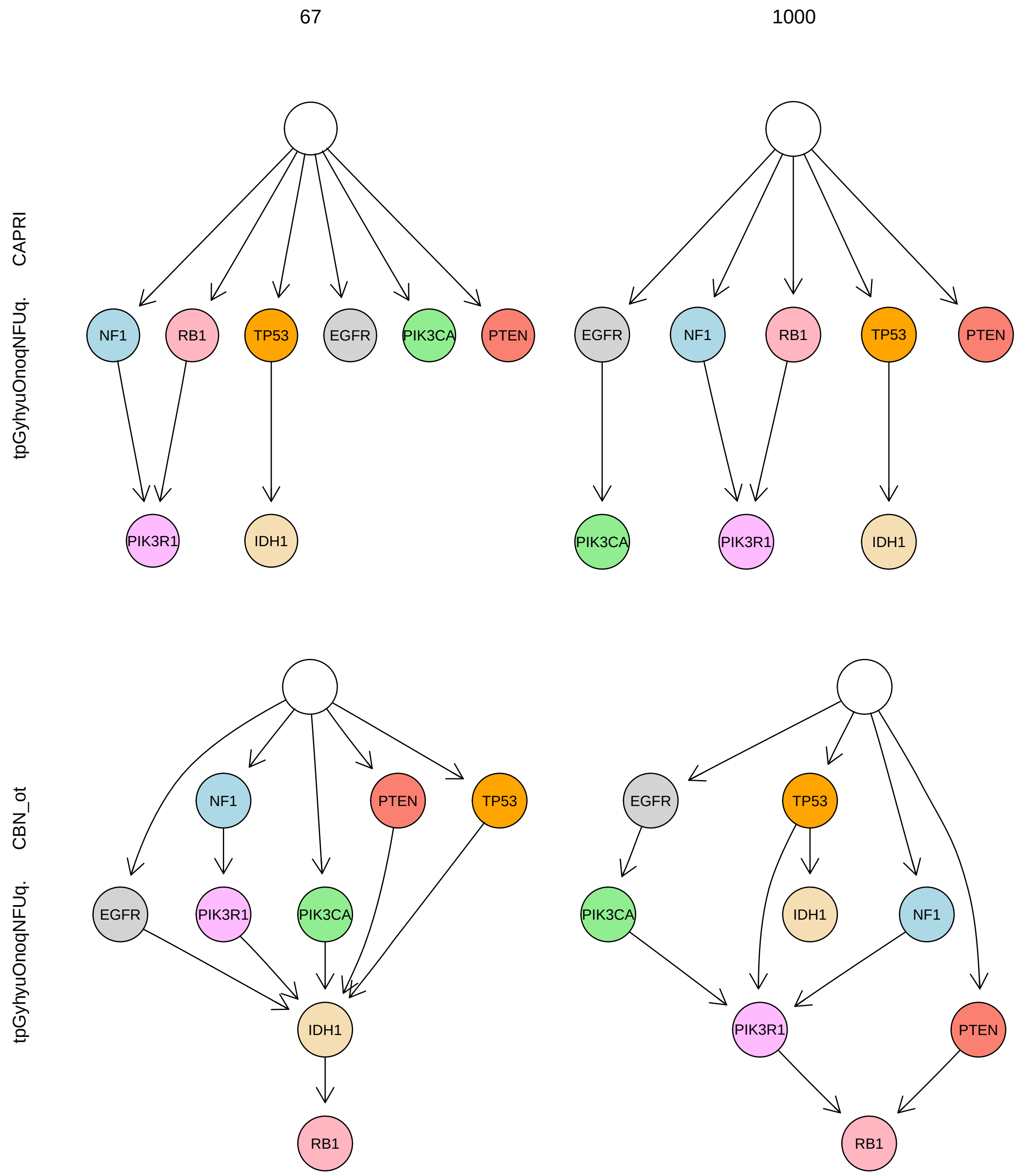

ID	p−value	Accessible Genot.
PFvXGwZKrALCihg	0.664	244

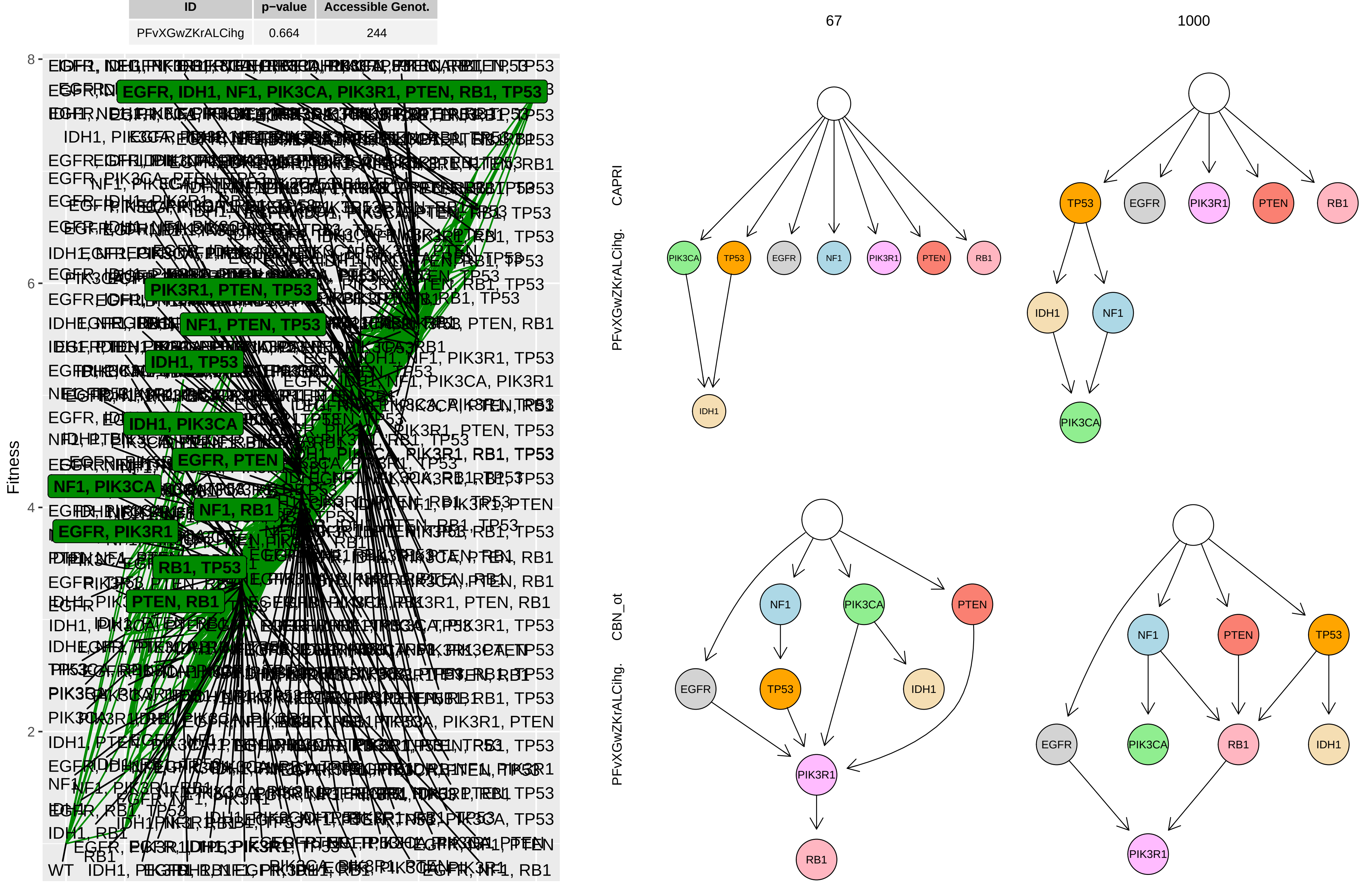

ID	p−value	Accessible Genot.
OHibERwgEsKAeRR	0.667	24

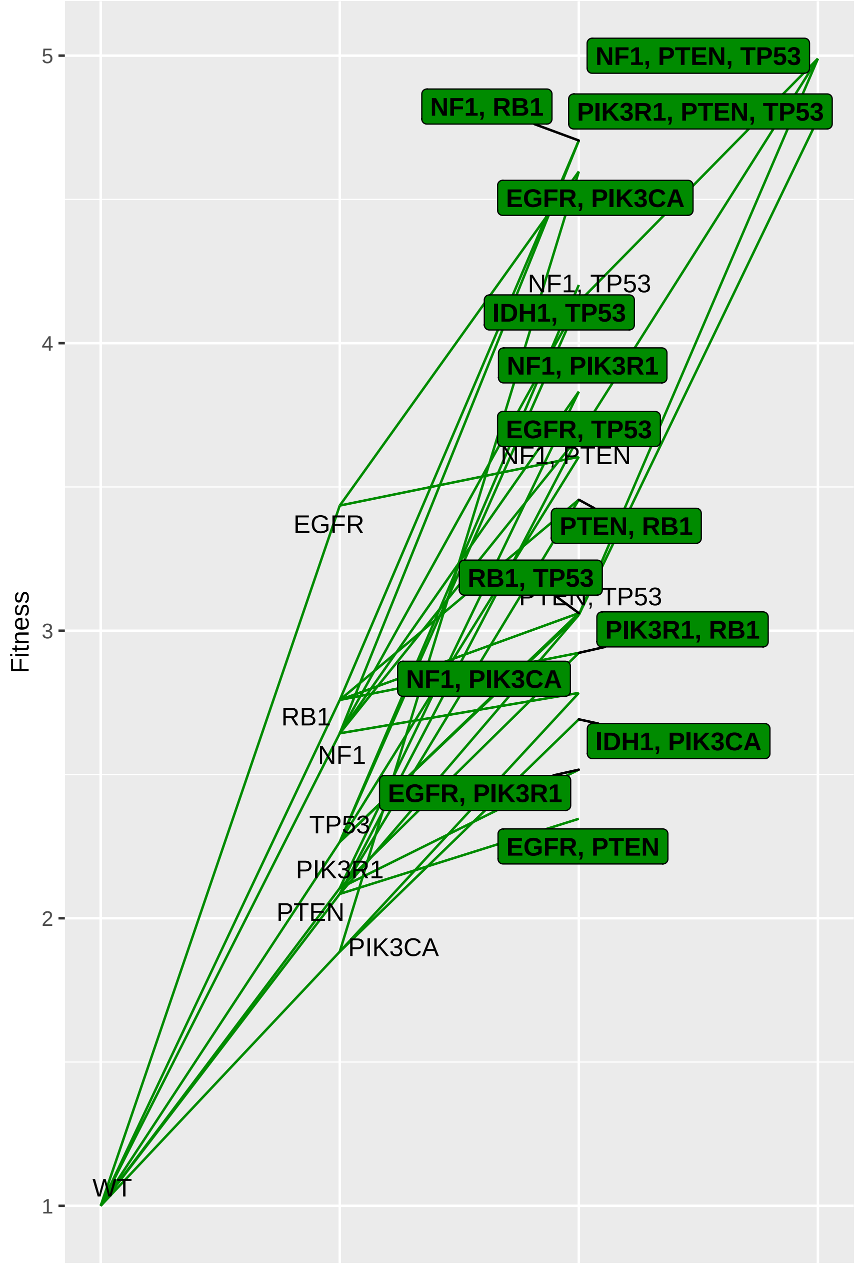

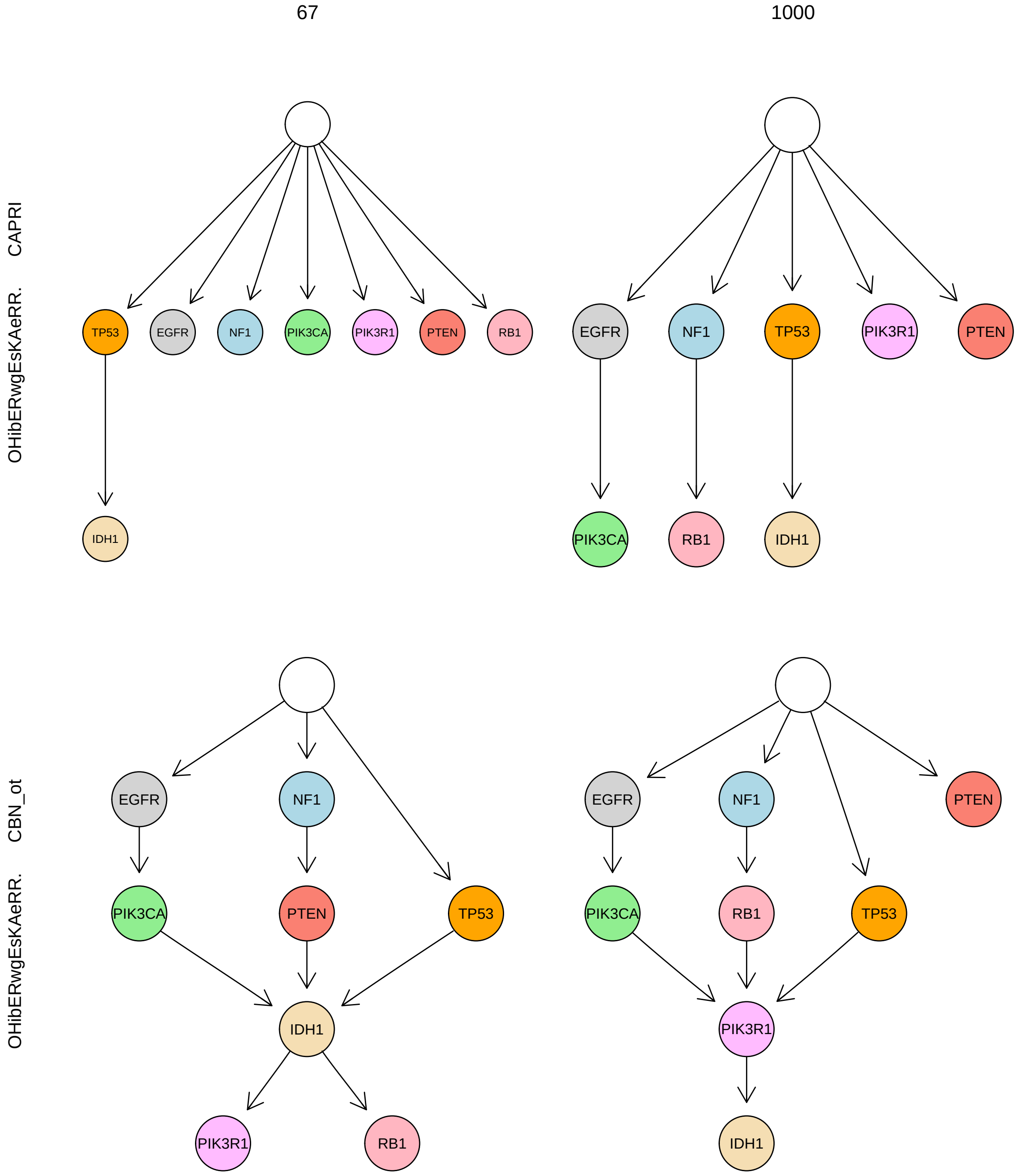

ID	p−value	Accessible Genot.
GrCwiuKvovcIIls	0.668	72

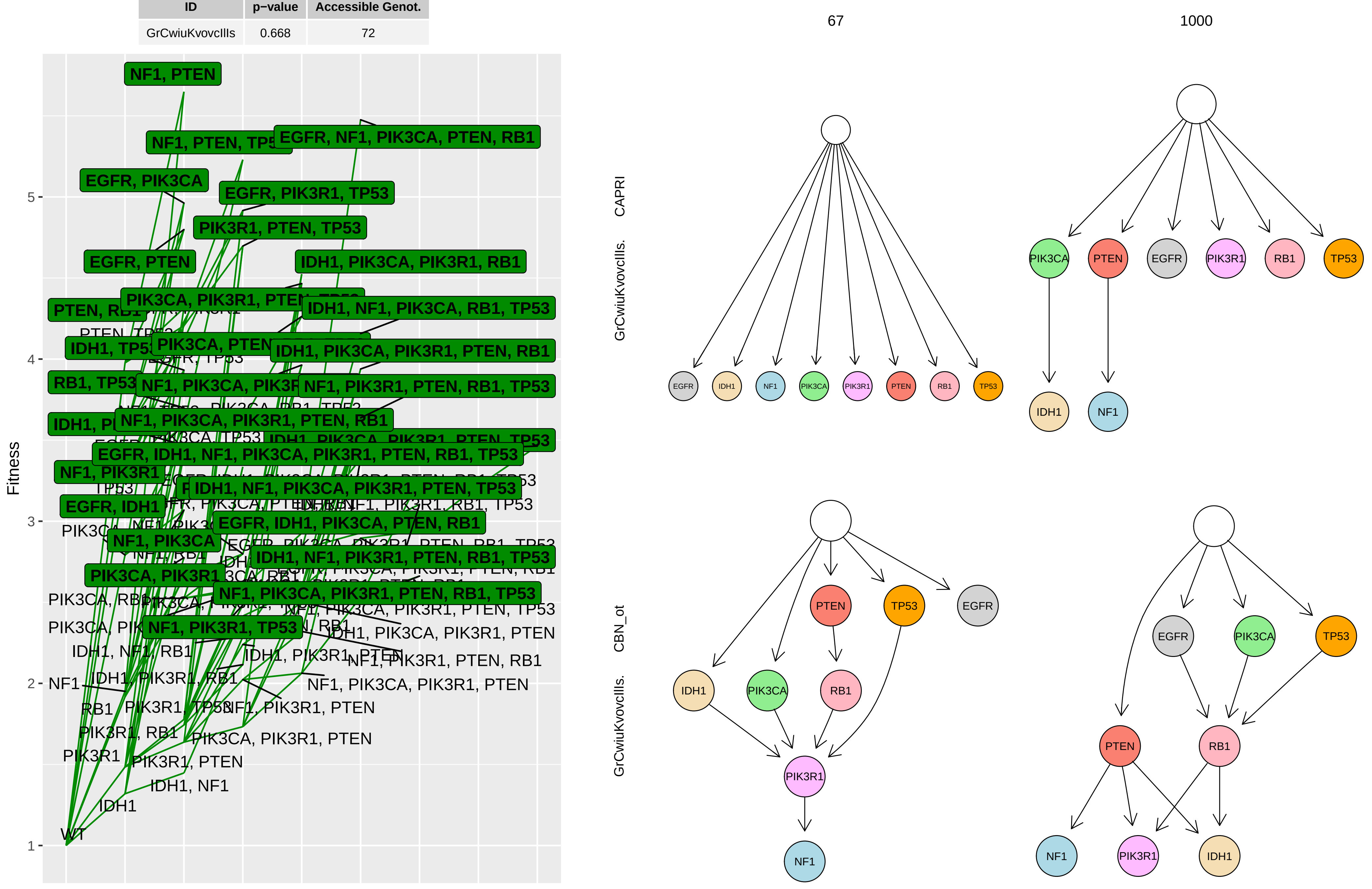

ID	p−value	Accessible Genot.
rbAGUiOlGLrFRNs	0.671	30

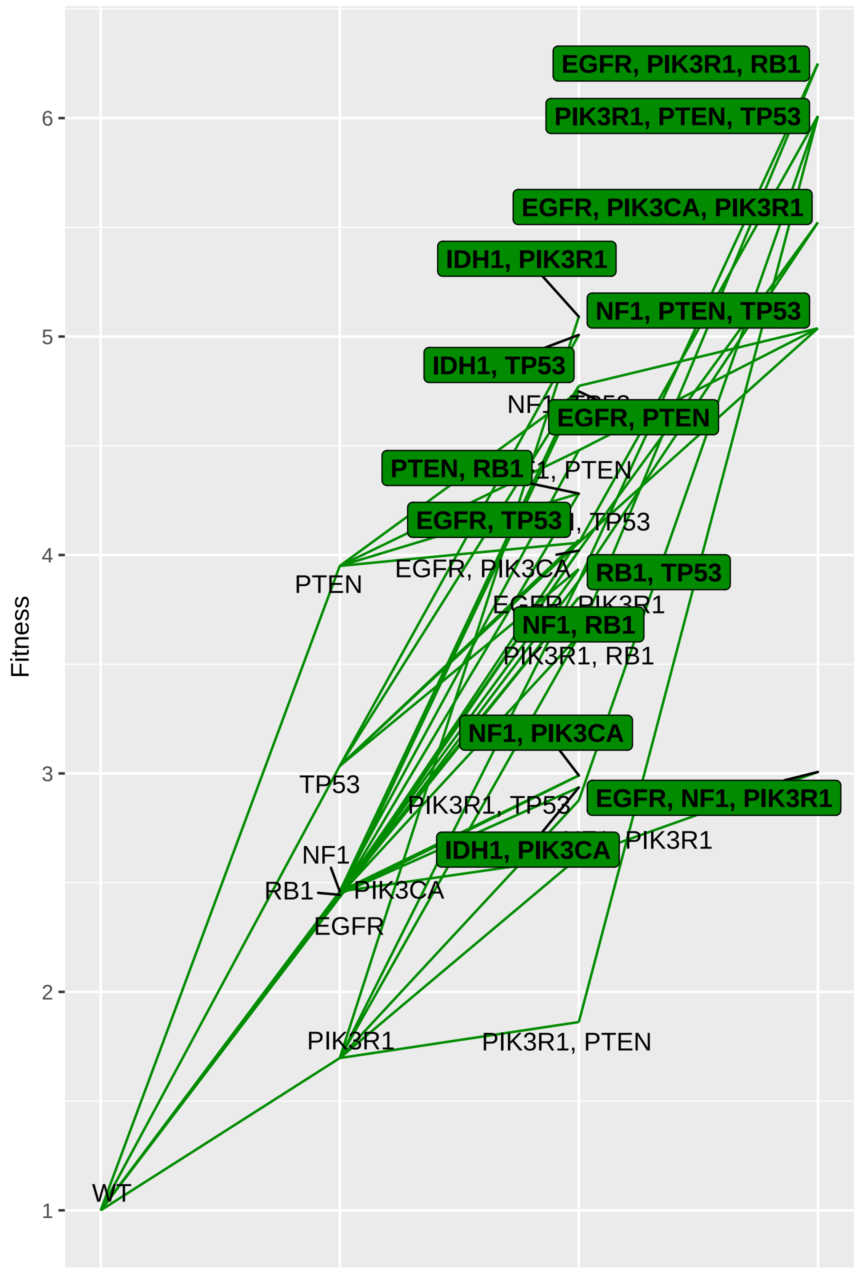

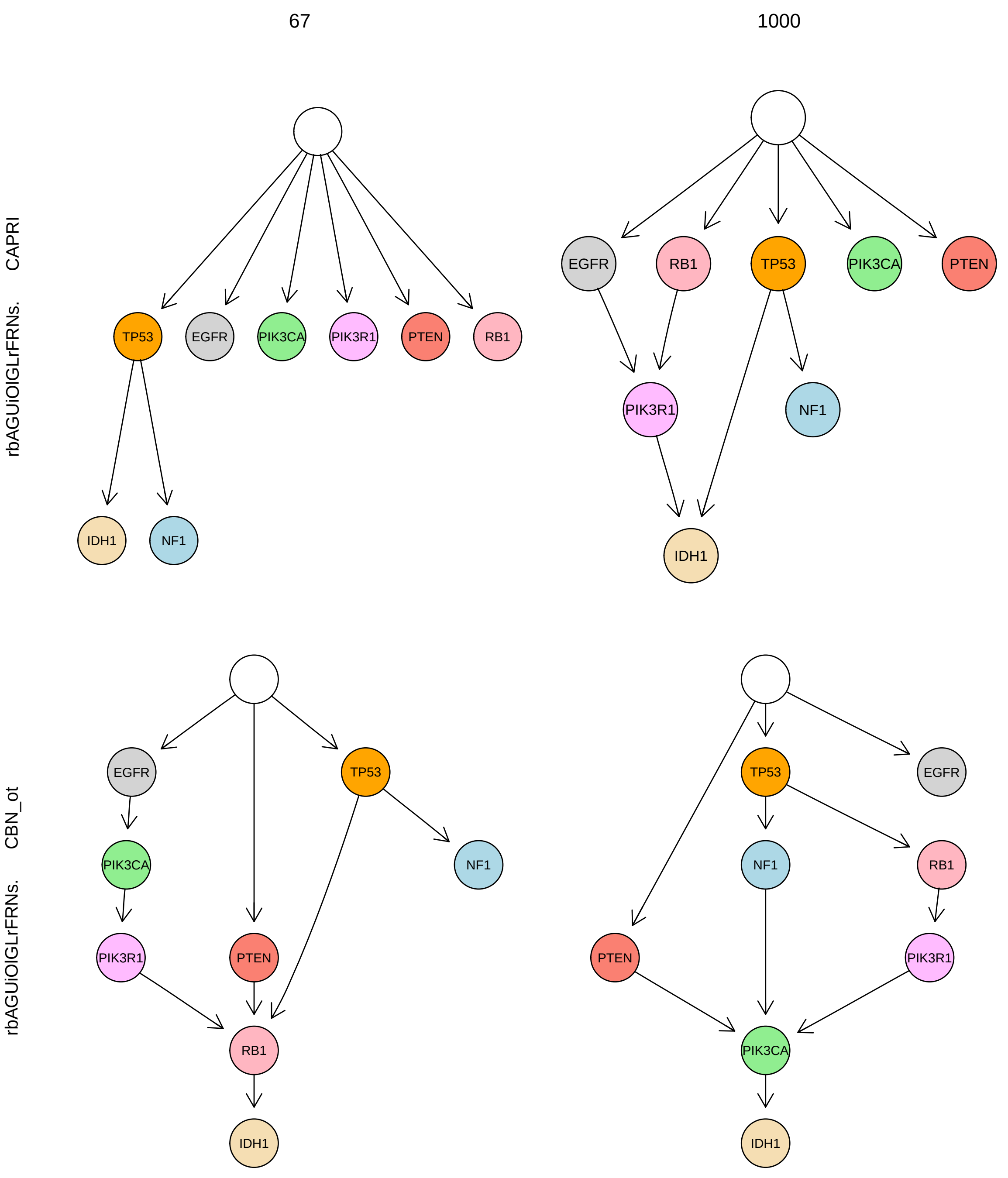

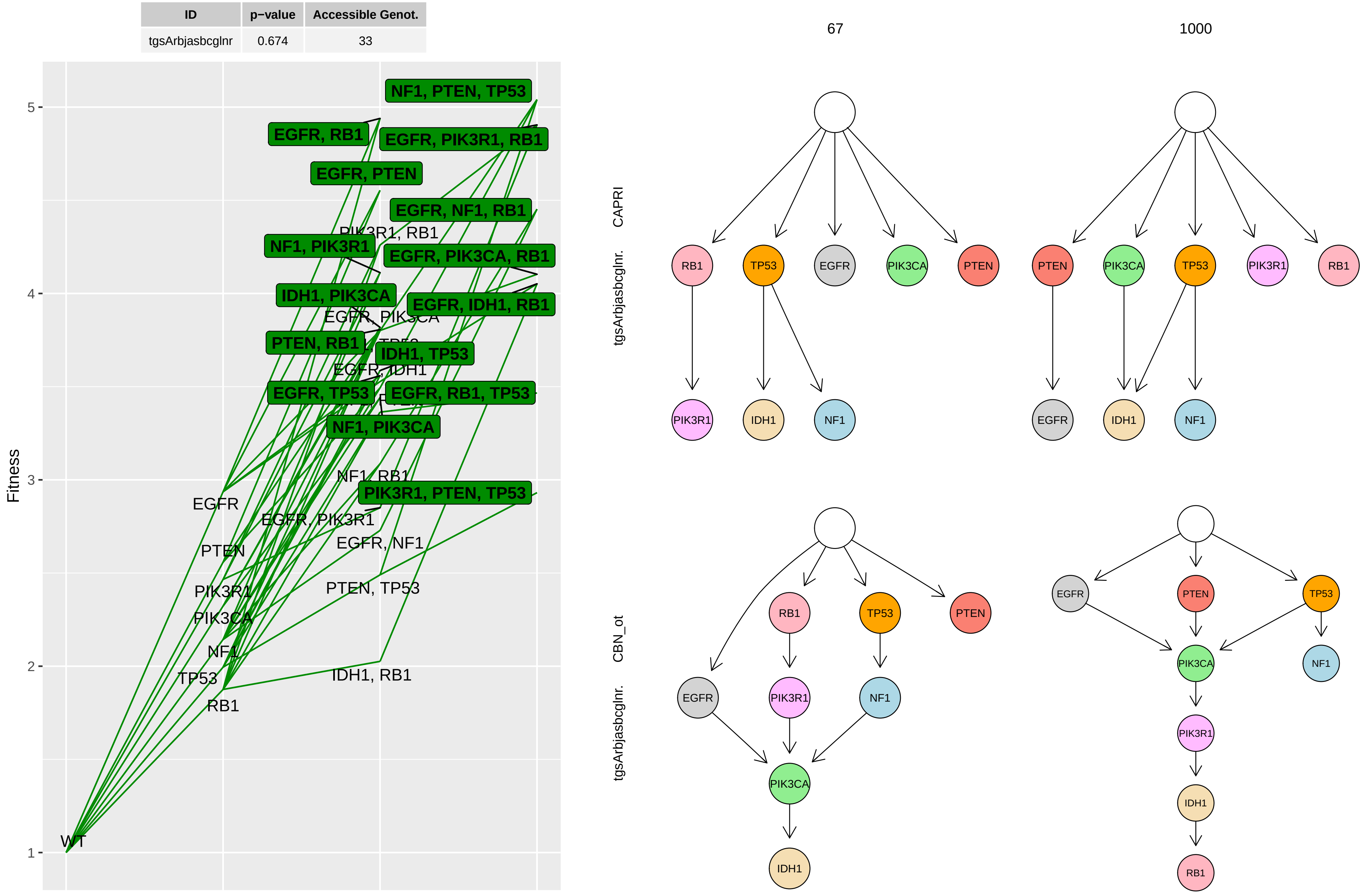
ID
p−value
Accessible Genot.
tgsArbjasbcglnr
0.674
33
Fitness
5
4
3
2
1
WT
EGFR
PTEN
PIK3R1
PIK3CA
NF1
TP53
RB1
NF1, PTEN, TP53
EGFR, RB1
EGFR, PIK3R1, RB1
EGFR, PTEN
EGFR, NF1, RB1
NF1, PIK3R1
EGFR, PIK3CA, RB1
IDH1, PIK3CA
EGFR, IDH1, RB1
EGFR, PIK3CA
PTEN, RB1
IDH1, TP53
EGFR, IDH1
EGFR, TP53
EGFR, RB1, TP53
NF1, PIK3CA
NF1, RB1
PIK3R1, PTEN, TP53
EGFR, PIK3R1
EGFR, NF1
PTEN, TP53
IDH1, RB1
67
1000
CAPRI
tgsArbjasbcglnr.
CBN_ot
tgsArbjasbcglnr.
RB1
TP53
EGFR
PIK3CA
PTEN
PIK3R1
IDH1
NF1

ID	p−value	Accessible Genot.
mBPfKuGyVTbsdio	0.674	38

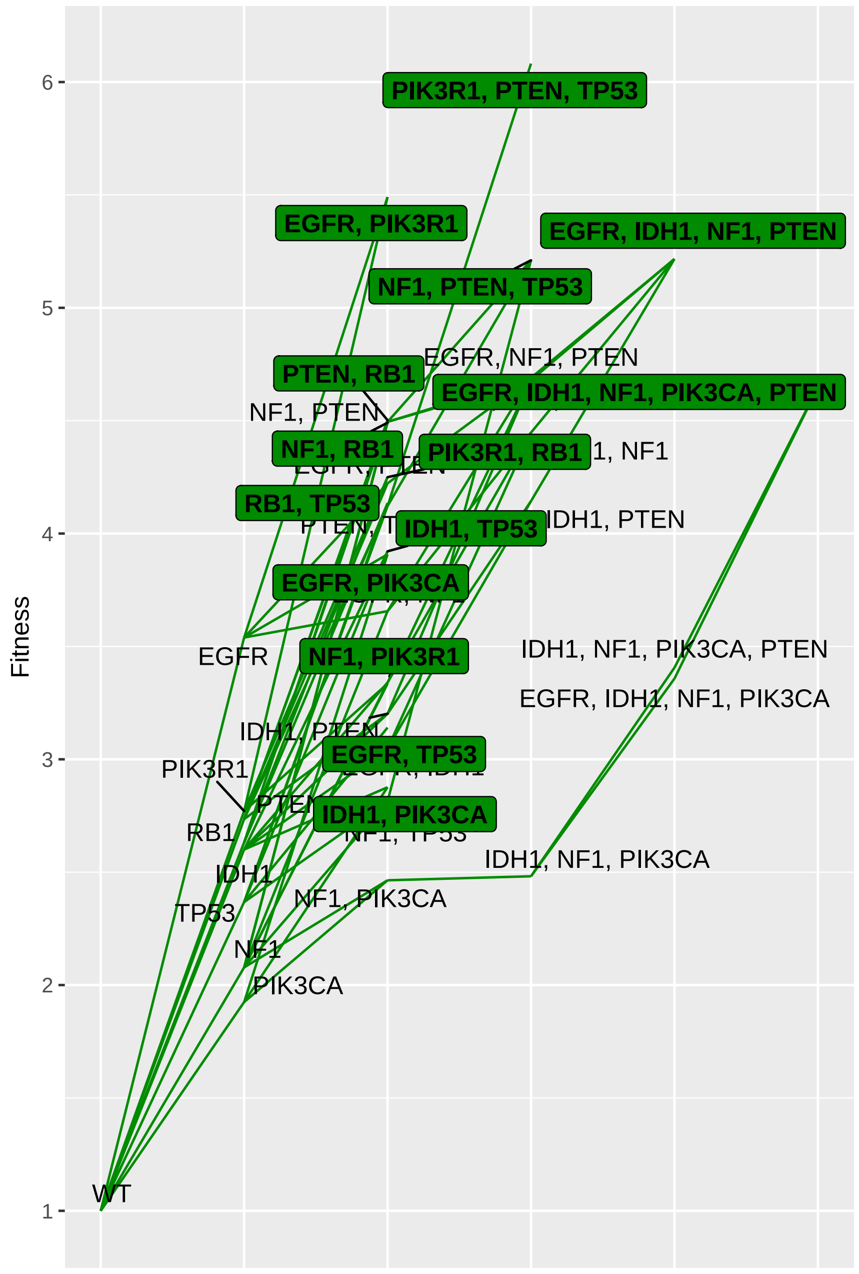

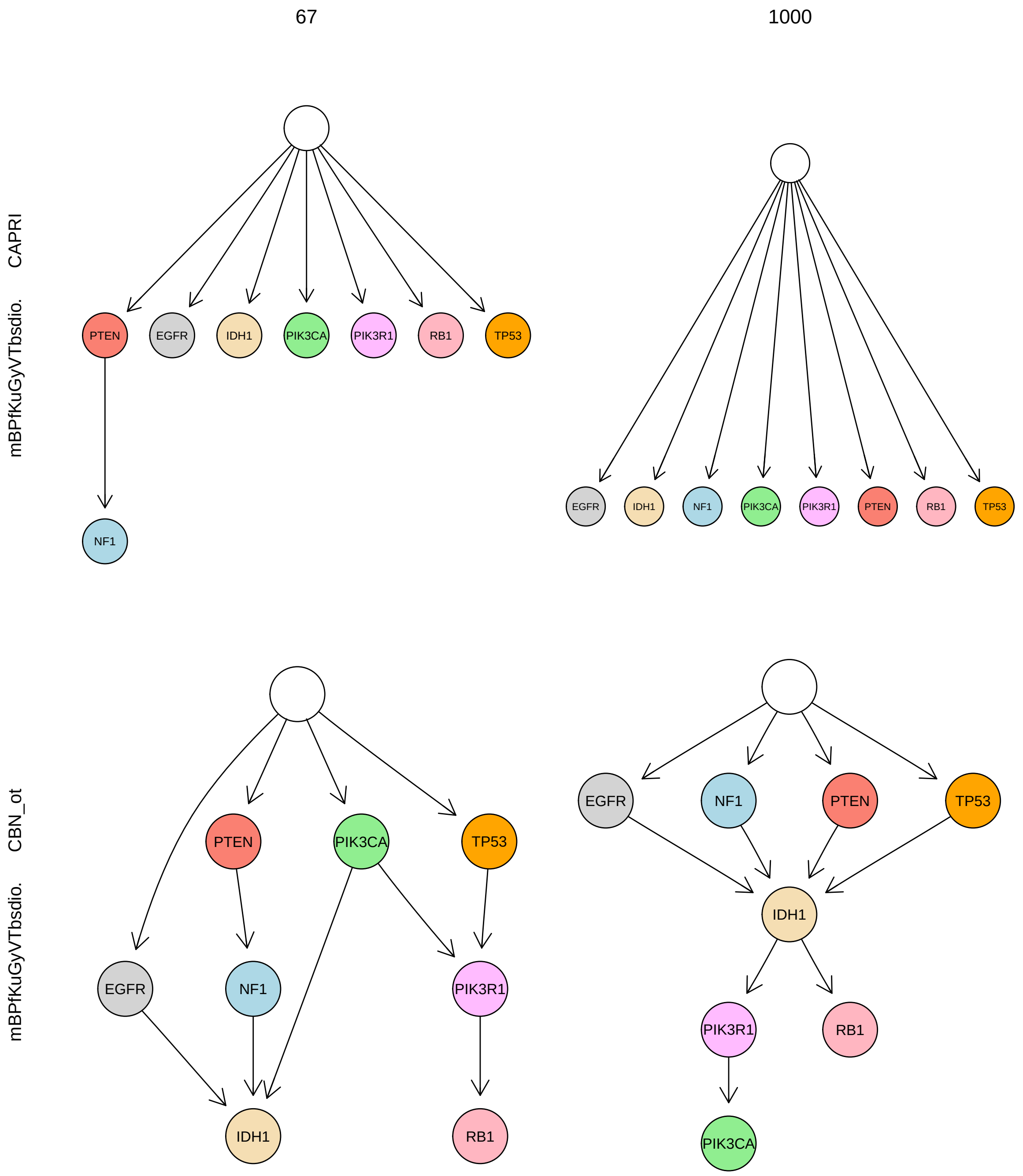

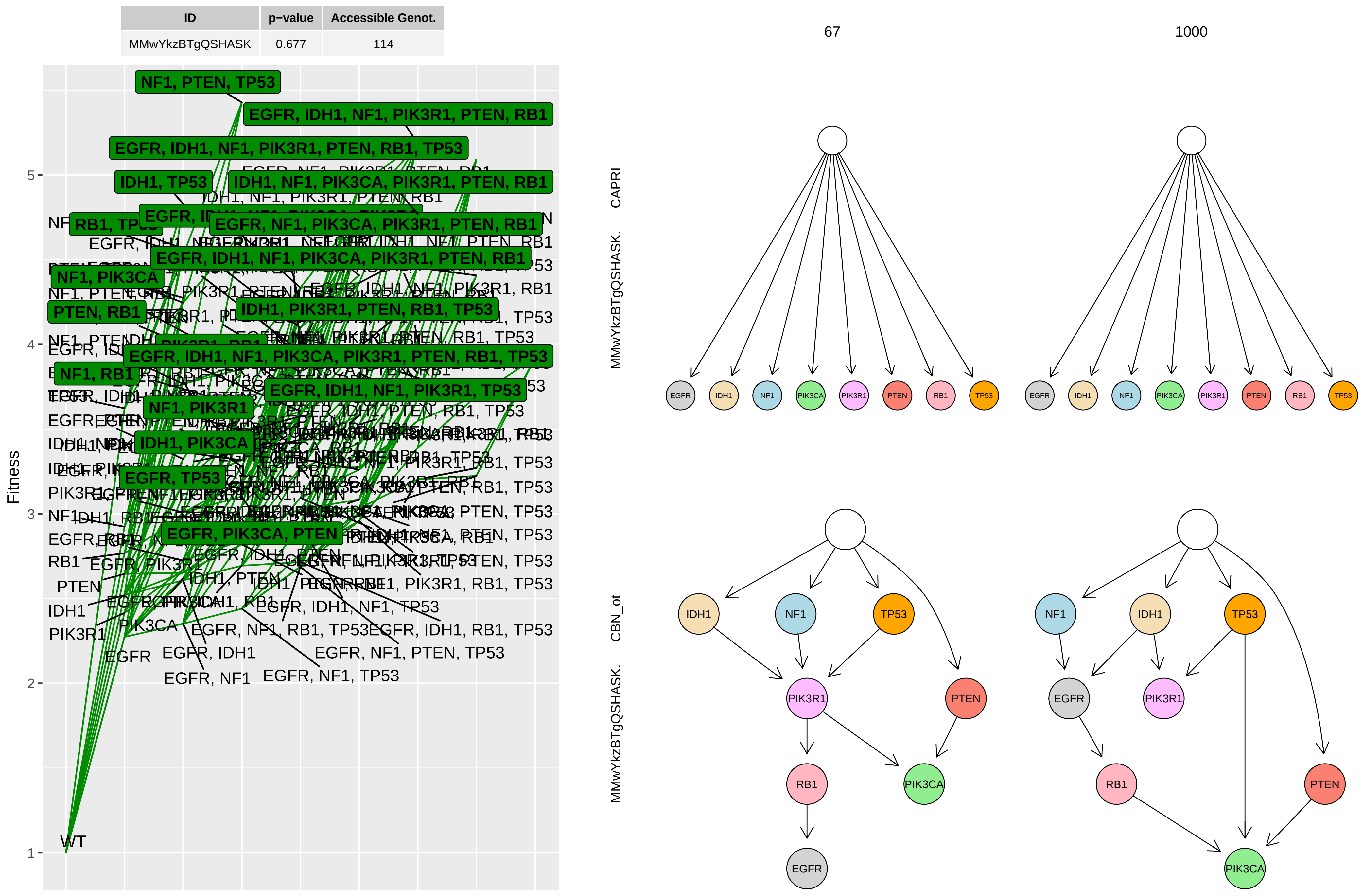
ID
p−value
Accessible Genot.
MMwYkzBTgQSHASK
0.677
114
Fitness
WT
NF1, PTEN, TP53
EGFR, IDH1, NF1, PIK3R1, PTEN, RB1
EGFR, IDH1, NF1, PIK3R1, PTEN, RB1, TP53
IDH1, TP53
IDH1, NF1, PIK3CA, PIK3R1, PTEN, RB1
EGFR, NF1, PIK3CA, PIK3R1, PTEN, RB1
EGFR, IDH1, NF1, PIK3CA, PIK3R1, PTEN, RB1
NF1, PIK3CA
PTEN, RB1
IDH1, PIK3R1, PTEN, RB1, TP53
EGFR, IDH1, NF1, PIK3CA, PIK3R1, PTEN, RB1, TP53
NF1, RB1
EGFR, IDH1, NF1, PIK3R1, TP53
NF1, PIK3R1
IDH1, PIK3CA
EGFR, TP53
EGFR, PIK3CA, PTEN
EGFR, IDH1, NF1, TP53
EGFR, NF1, RB1, TP53
EGFR, IDH1, RB1, TP53
EGFR, IDH1
EGFR, NF1, PTEN, TP53
EGFR, NF1
EGFR, NF1, TP53
EGFR
PIK3CA
PIK3R1
IDH1
PTEN
RB1
67
1000
MMwYkzBTgQSHASK. CAPRI
MMwYkzBTgQSHASK. CBN_ot
EGFR
IDH1
NF1
PIK3CA
PIK3R1
PTEN
RB1
TP53

ID	p−value	Accessible Genot.
FxZGSvwNJZjiylW	0.679	24

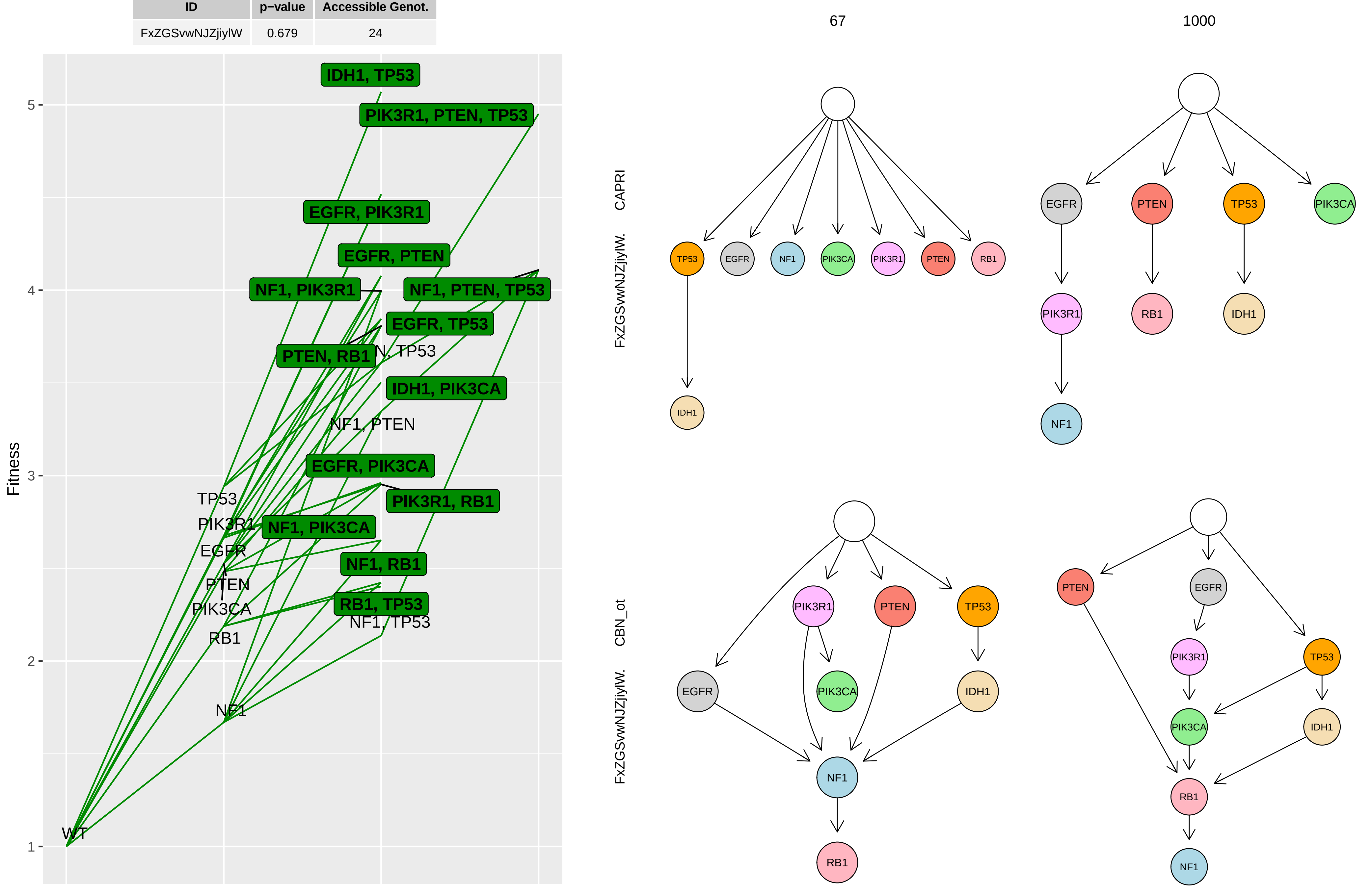

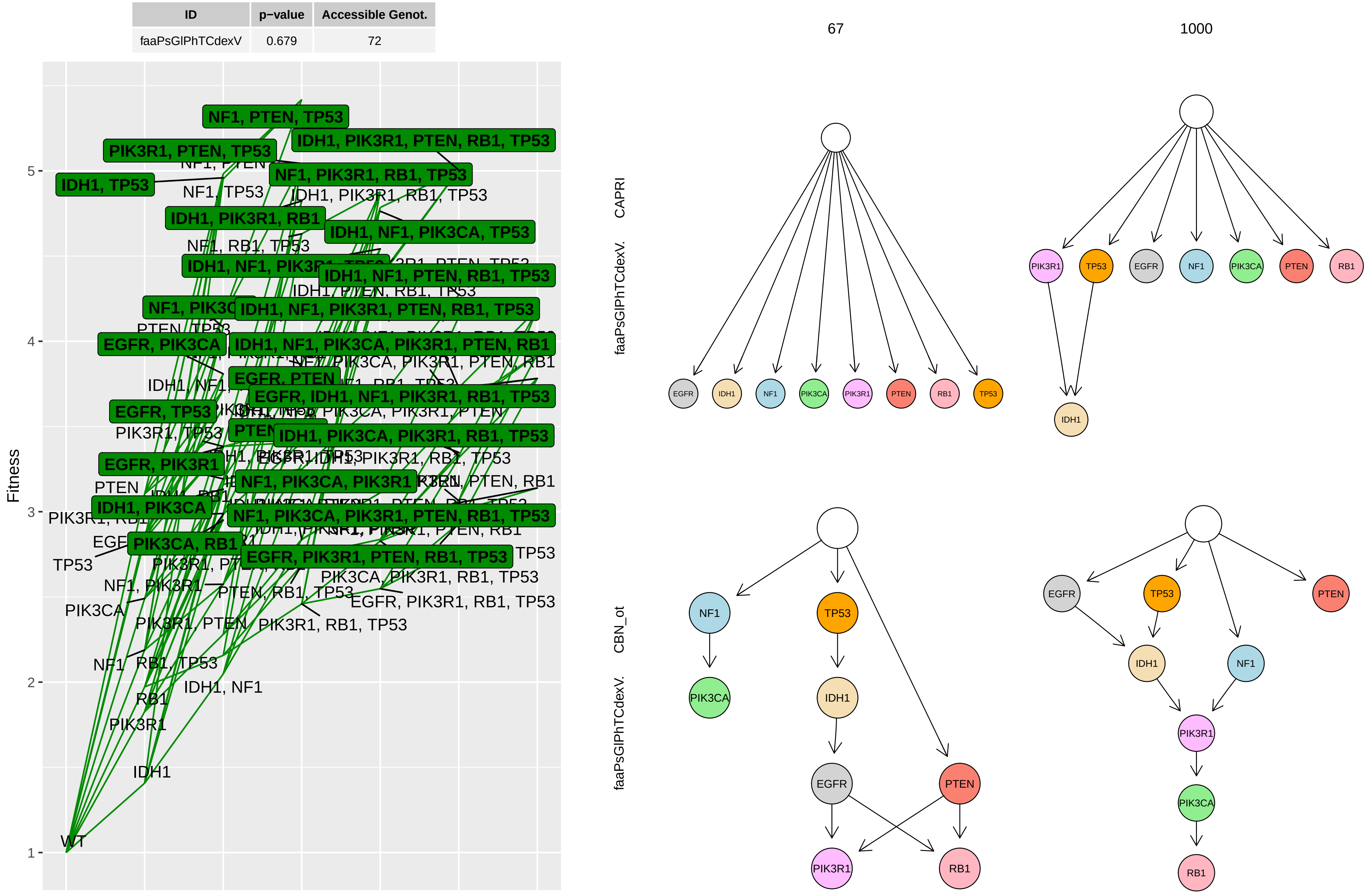
ID
p−value
Accessible Genot.
faaPsGlPhTCdexV
0.679
72
Fitness
NF1, PTEN, TP53
PIK3R1, PTEN, TP53
IDH1, PIK3R1, PTEN, RB1, TP53
NF1, PIK3R1, RB1, TP53
IDH1, TP53
NF1, TP53
IDH1, PIK3R1, RB1, TP53
IDH1, PIK3R1, RB1
IDH1, NF1, PIK3CA, TP53
NF1, RB1, TP53
IDH1, NF1, PTEN, RB1, TP53
IDH1, NF1, PIK3R1, PTEN, RB1, TP53
EGFR, PIK3CA
IDH1, NF1, PIK3CA, PIK3R1, PTEN, RB1
NF1, PIK3CA, PIK3R1, PTEN, RB1
EGFR, PTEN
EGFR, IDH1, NF1, PIK3R1, RB1, TP53
EGFR, TP53
PIK3R1, TP53
IDH1, PIK3CA, PIK3R1, RB1, TP53
EGFR, PIK3R1
NF1, PIK3CA, PIK3R1
PTEN
IDH1, PIK3CA
NF1, PIK3CA, PIK3R1, PTEN, RB1, TP53
PIK3CA, RB1
EGFR, PIK3R1, PTEN, RB1, TP53
TP53
NF1, PIK3R1
PTEN, RB1, TP53
PIK3CA, PIK3R1, RB1, TP53
EGFR, PIK3R1, RB1, TP53
PIK3CA
PIK3R1, PTEN
PIK3R1, RB1, TP53
NF1
RB1, TP53
IDH1, NF1
RB1
PIK3R1
IDH1
WT
1
2
3
4
5
67
1000
faaPsGlPhTCdexV. CAPRI
faaPsGlPhTCdexV. CBN_ot
EGFR
IDH1
NF1
PIK3CA
PIK3R1
PTEN
RB1
TP53

ID	p−value	Accessible Genot.
teyfmduQNuGCgrb	0.68	65

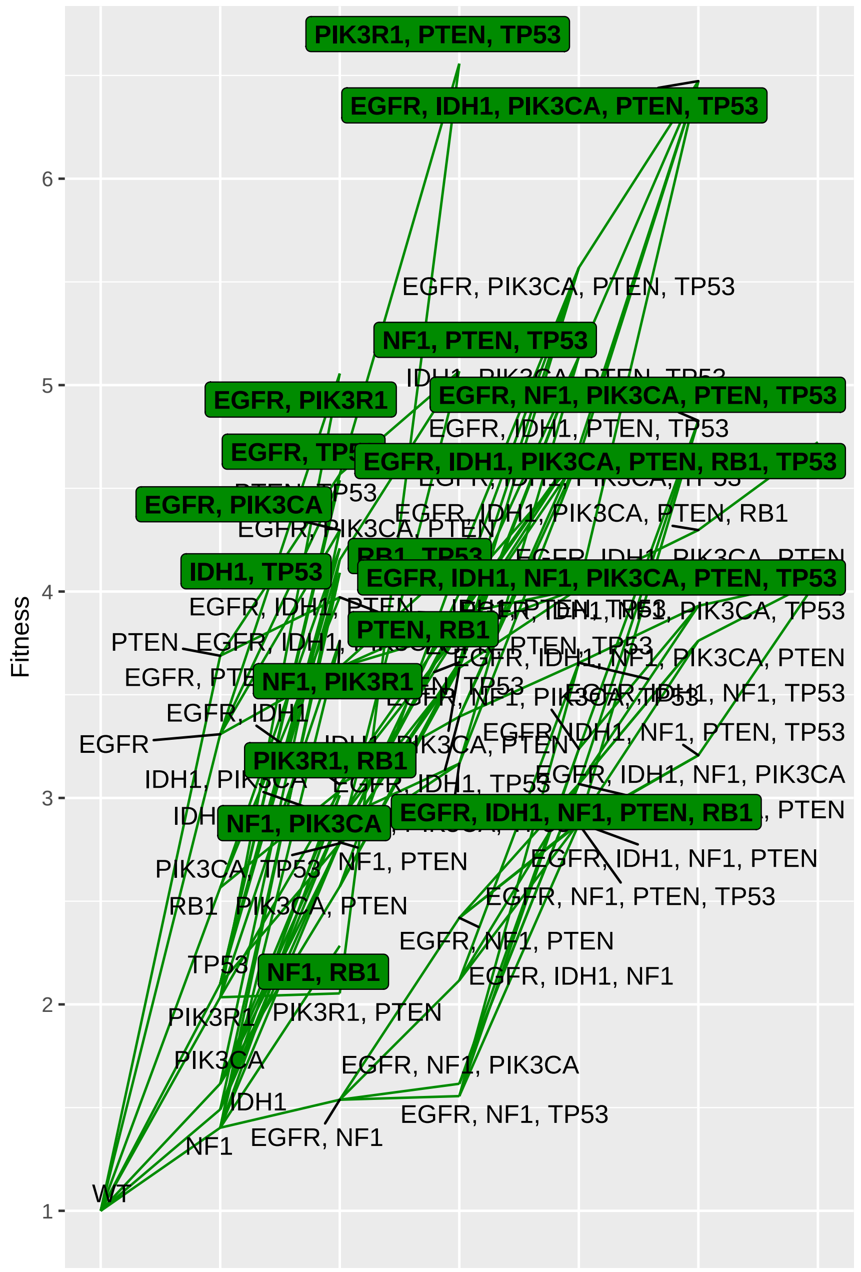

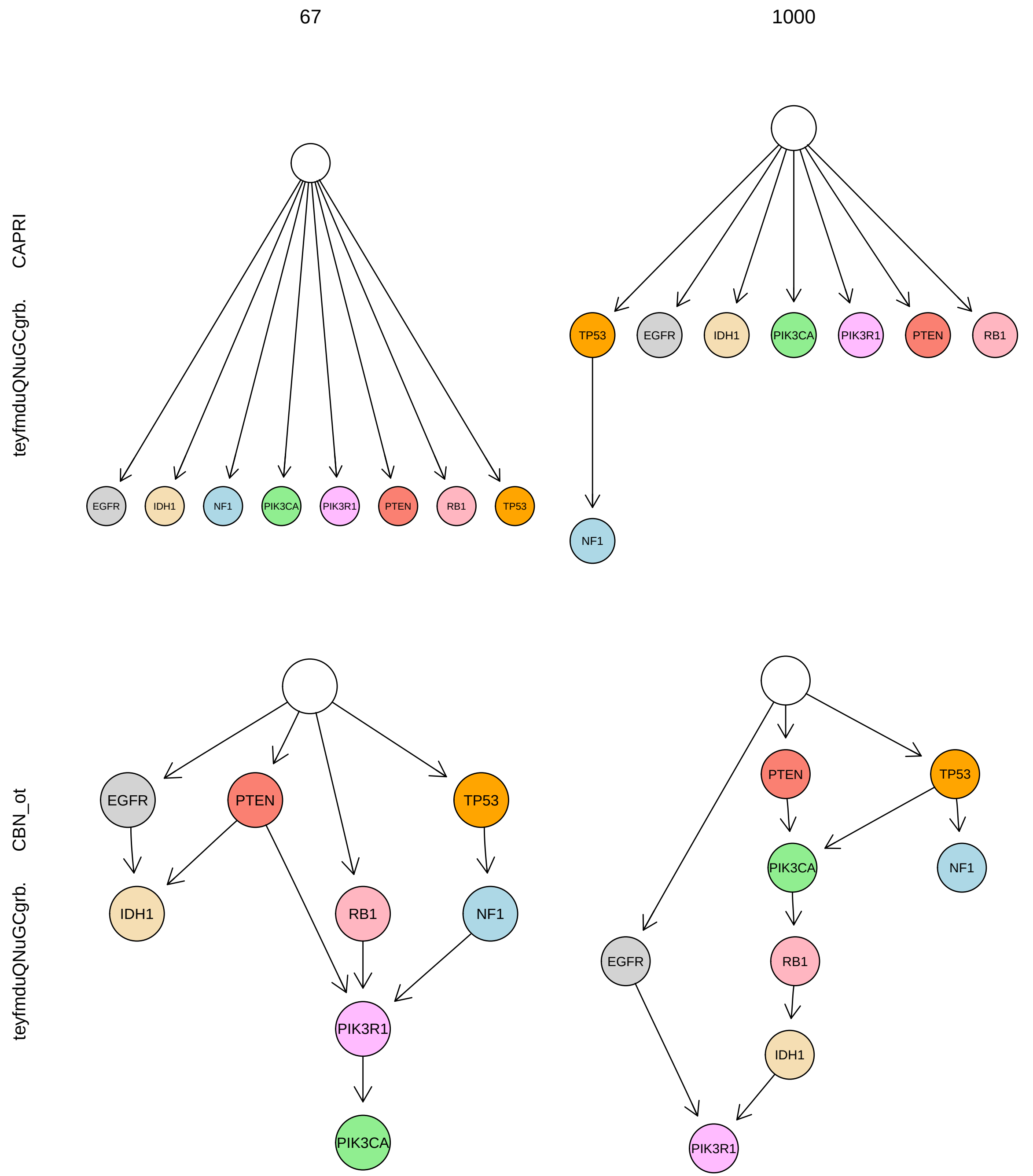

ID	p−value	Accessible Genot.
tHjbNvWpALAHWXA	0.681	24

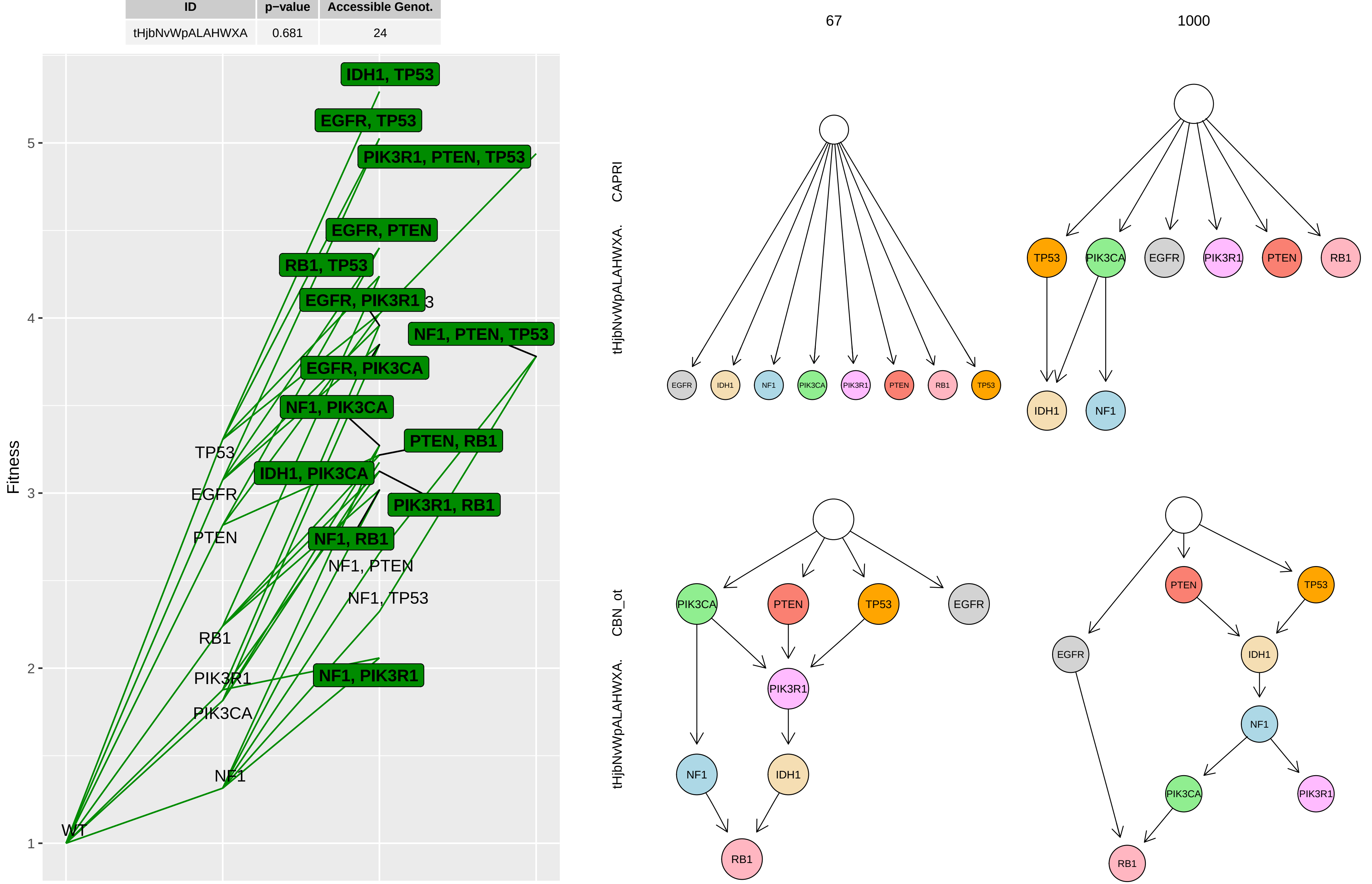

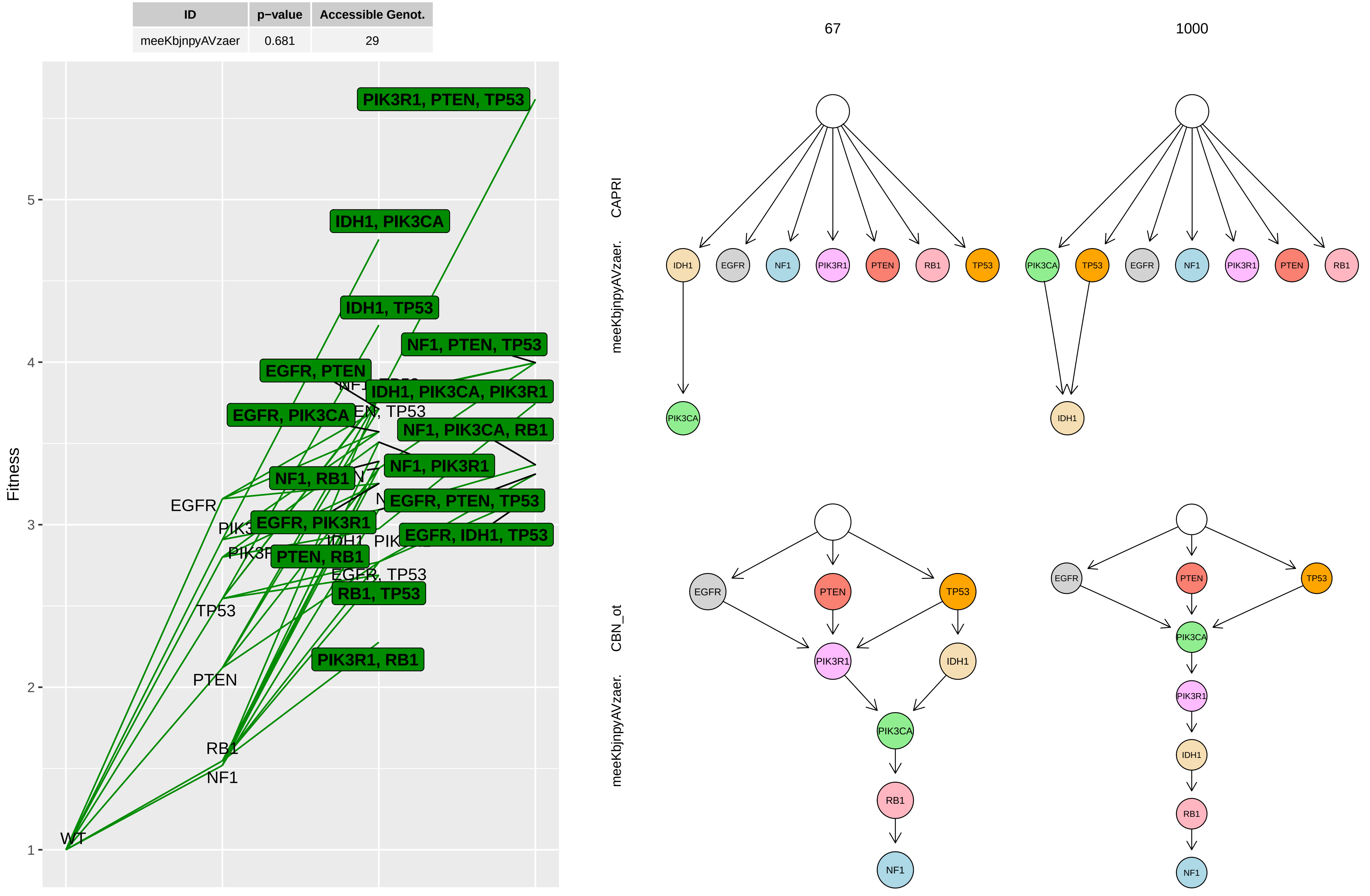

ID
p−value
Accessible Genot.
meeKbjnpyAVzaer
0.681
29
Fitness
5
4
3
2
1
WT
EGFR
TP53
PTEN
RB1
NF1
PIK3R1, PTEN, TP53
IDH1, PIK3CA
IDH1, TP53
NF1, PTEN, TP53
EGFR, PTEN
IDH1, PIK3CA, PIK3R1
EGFR, PIK3CA
NF1, PIK3CA, RB1
NF1, PIK3R1
NF1, RB1
EGFR, PTEN, TP53
EGFR, PIK3R1
EGFR, IDH1, TP53
PTEN, RB1
EGFR, TP53
RB1, TP53
PIK3R1, RB1
67
1000
meeKbjnpyAVzaer. CAPRI
meeKbjnpyAVzaer. CBN_ot
IDH1
EGFR
NF1
PIK3R1
PTEN
RB1
TP53
PIK3CA

ID	p−value	Accessible Genot.
oxYDQJcerqcoJFF	0.682	24

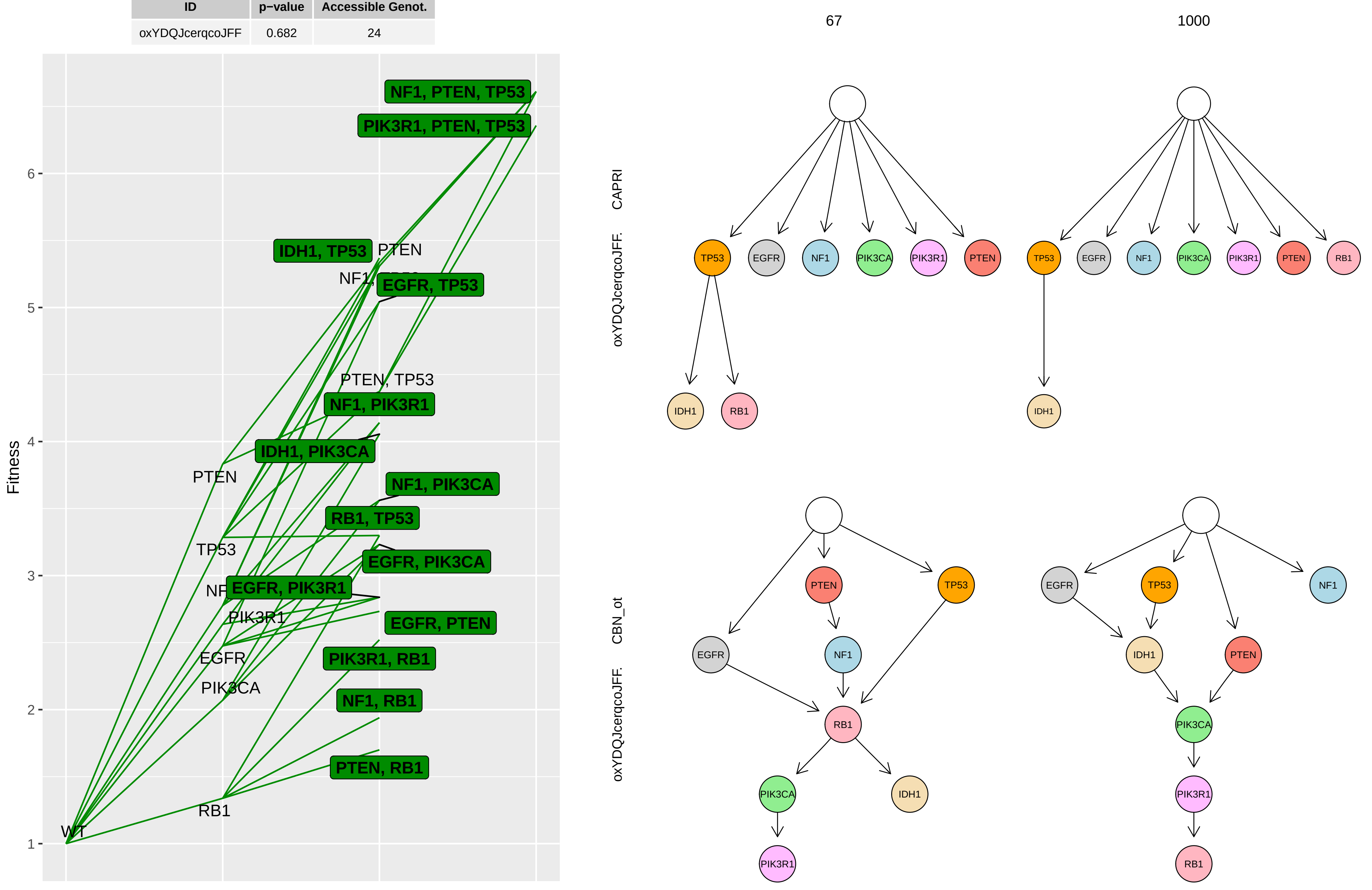

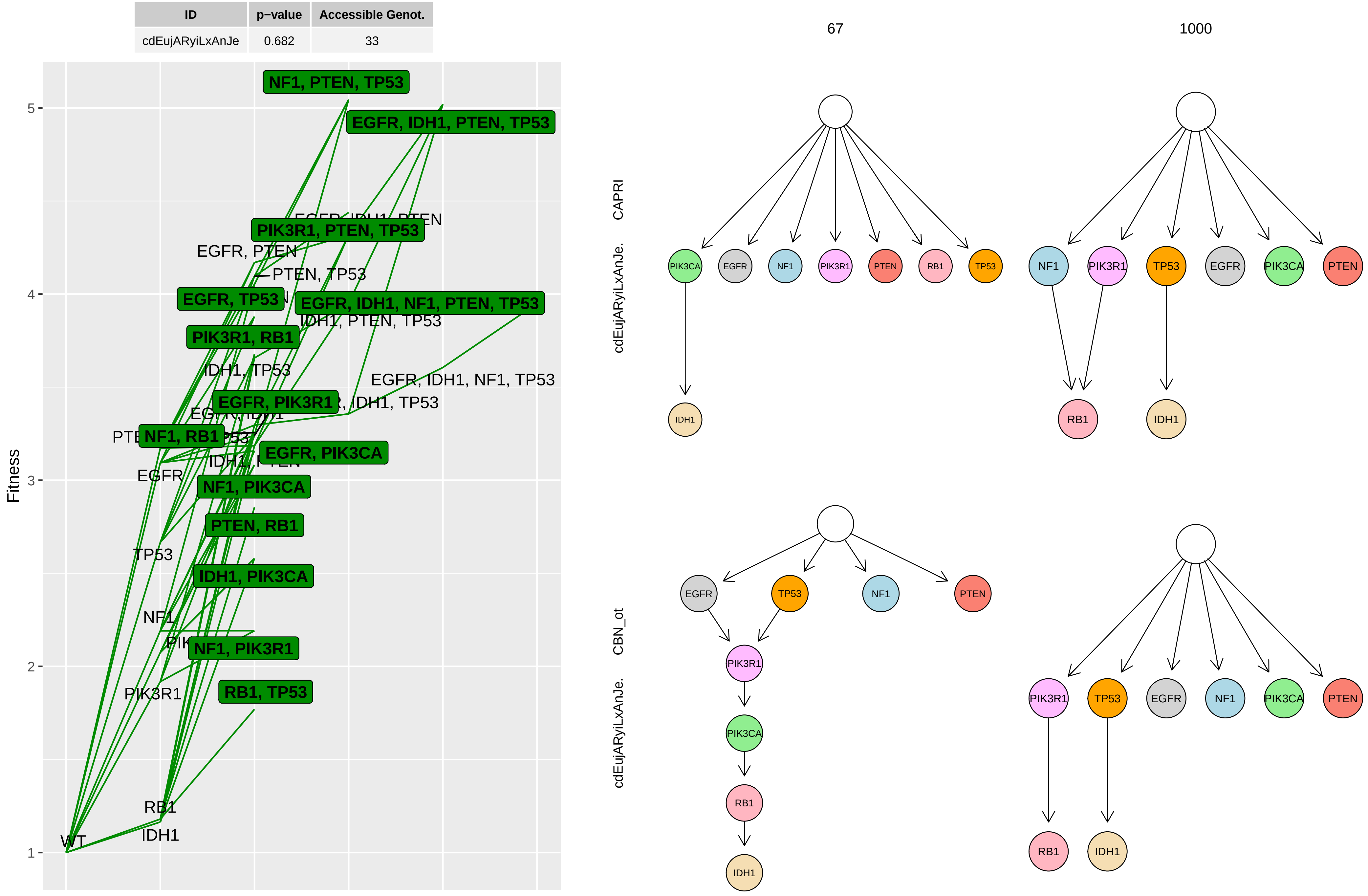

ID
p−value
Accessible Genot.
cdEujARyiLxAnJe
0.682
33
Fitness
NF1, PTEN, TP53
EGFR, IDH1, PTEN, TP53
PIK3R1, PTEN, TP53
EGFR, PTEN
PTEN, TP53
EGFR, TP53
EGFR, IDH1, NF1, PTEN, TP53
IDH1, PTEN, TP53
PIK3R1, RB1
IDH1, TP53
EGFR, IDH1, NF1, TP53
EGFR, PIK3R1
NF1, RB1
EGFR, PIK3CA
EGFR
NF1, PIK3CA
PTEN, RB1
TP53
IDH1, PIK3CA
NF1
NF1, PIK3R1
PIK3R1
RB1, TP53
RB1
WT
IDH1
67
1000
CAPRI
cdEujARyiLxAnJe.
CBN_ot
cdEujARyiLxAnJe.
PIK3CA
EGFR
NF1
PIK3R1
PTEN
RB1
TP53
IDH1

ID	p−value	Accessible Genot.
NnBFwTmnjbjUNYK	0.683	48

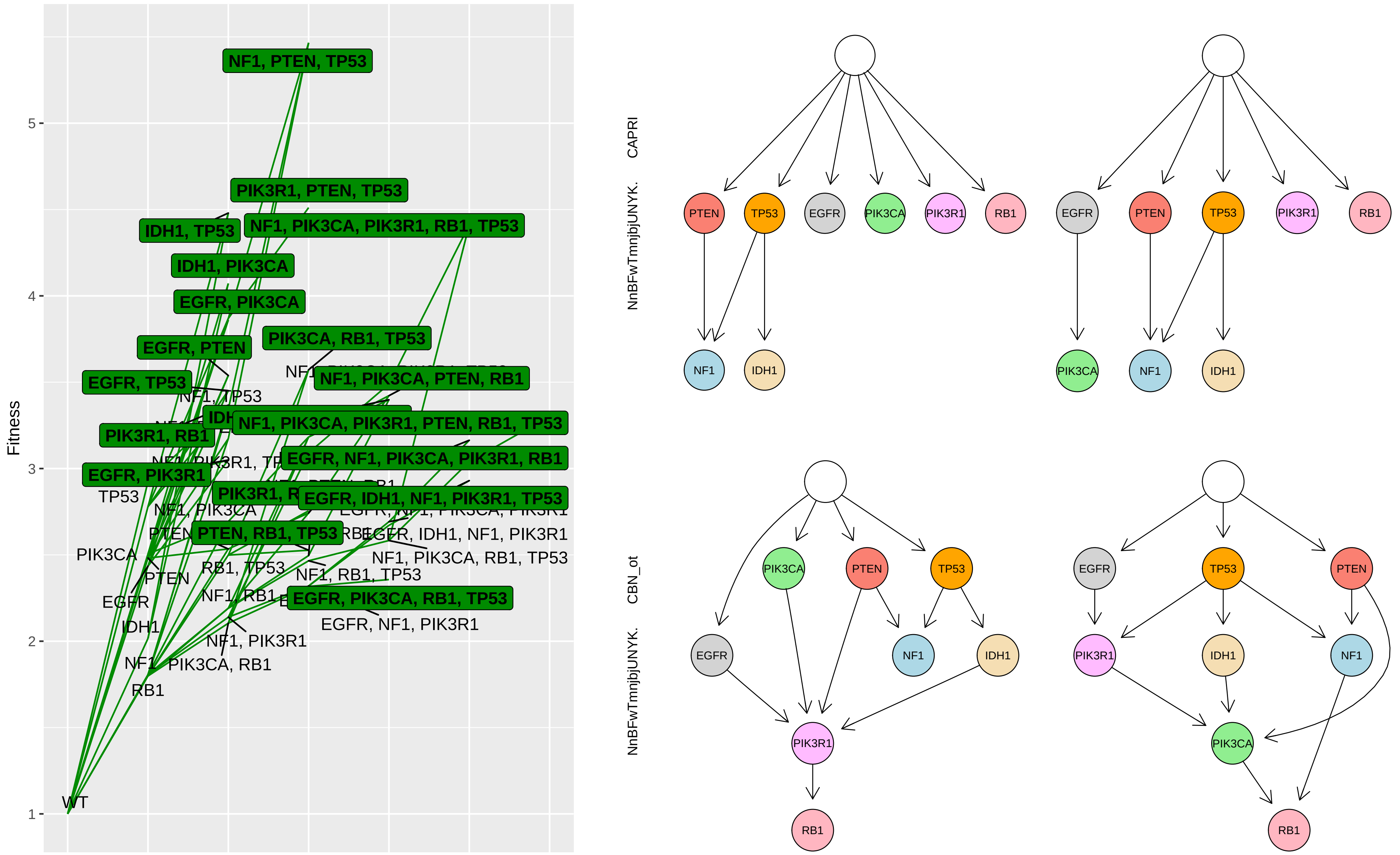

ID	p−value	Accessible Genot.
pLhxazACsClDbEF	0.689	126

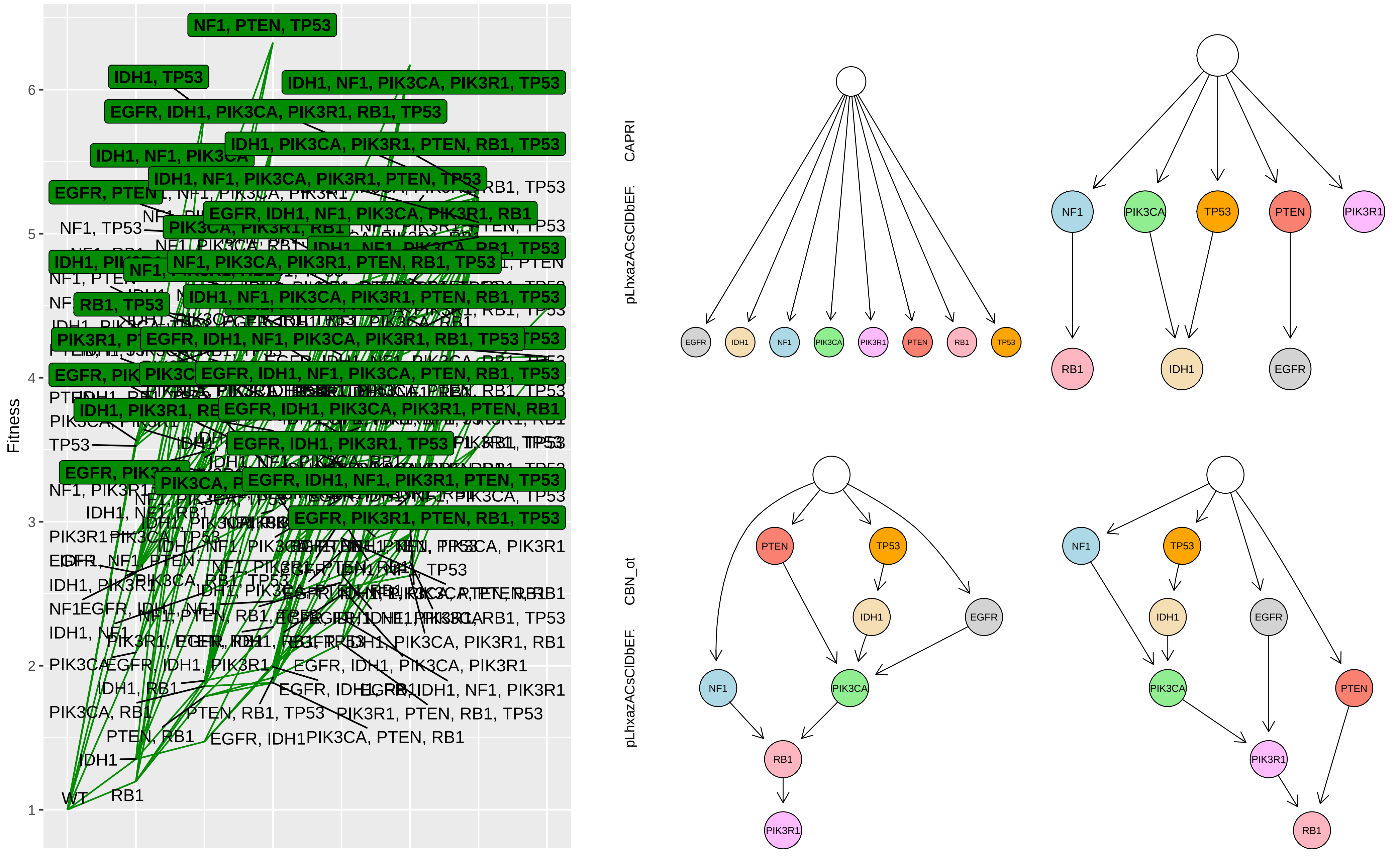

ID	p−value	Accessible Genot.
UFeBWMktsvtvrlO	0.69	52

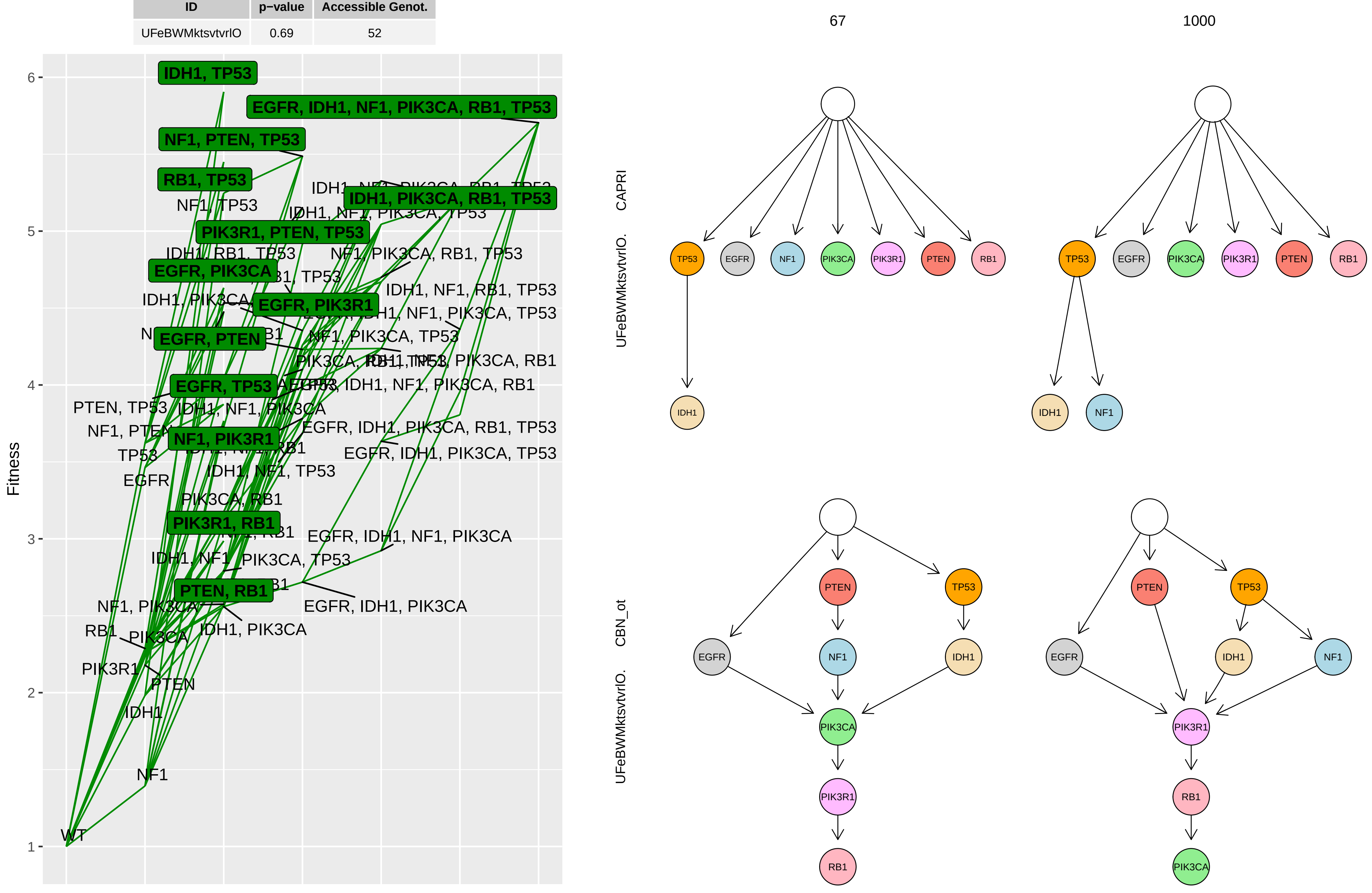

ID	p−value	Accessible Genot.
jHXZAoSvMhIvpUj	0.69	32

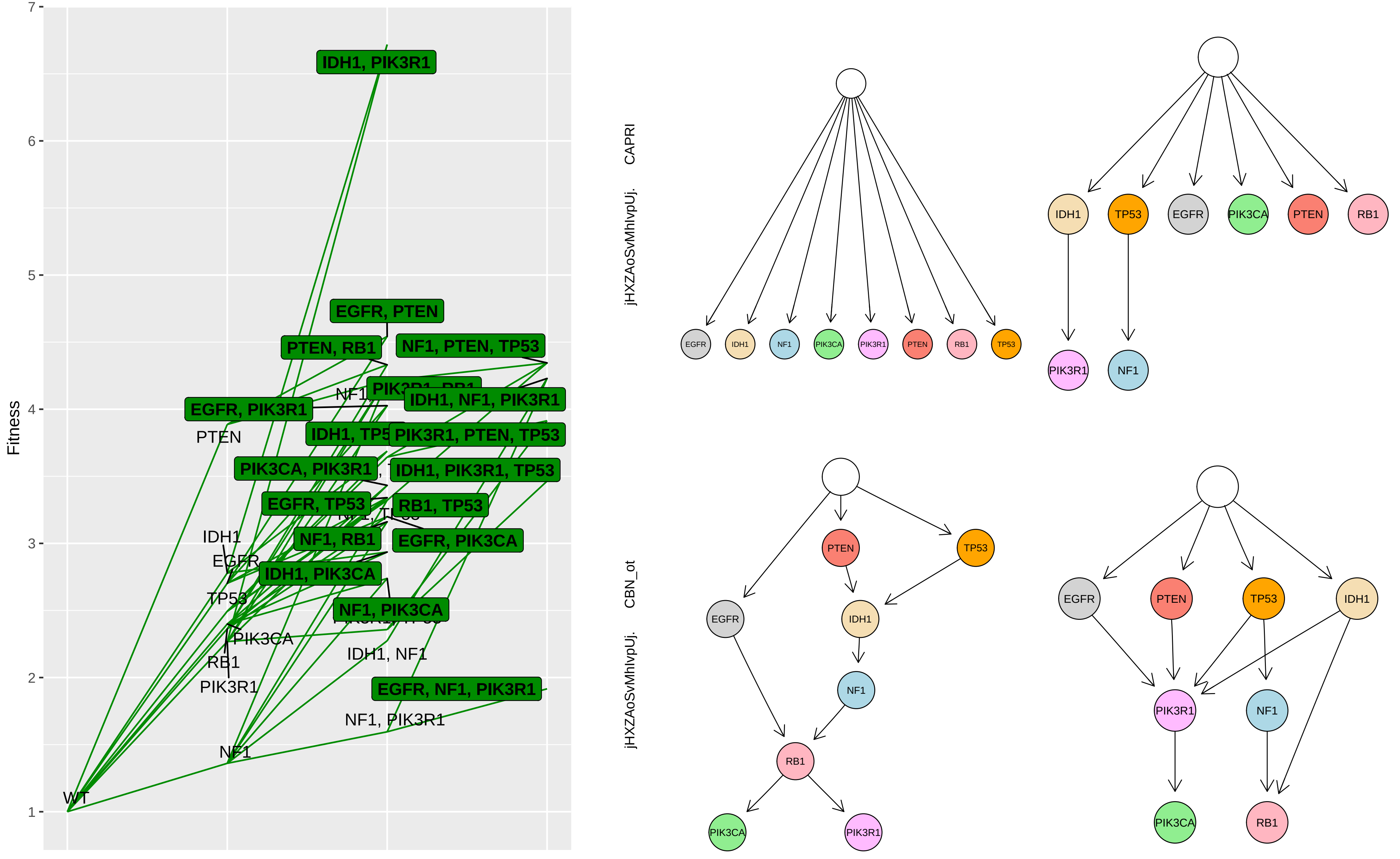

ID	p−value	Accessible Genot.
aTYBLzskRoypDoe	0.693	70

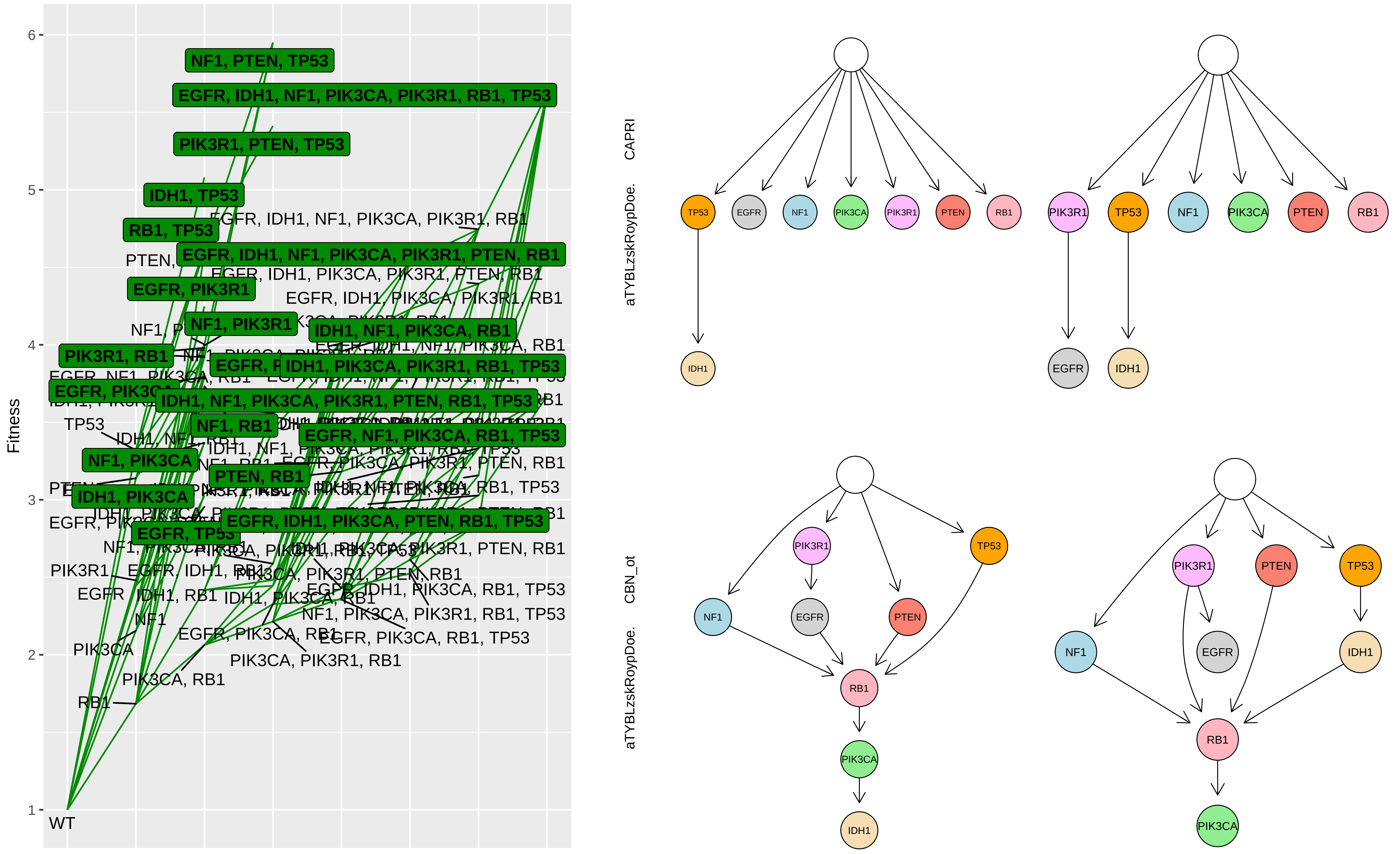

ID	p−value	Accessible Genot.
GchpCabsfwceMxr	0.693	81

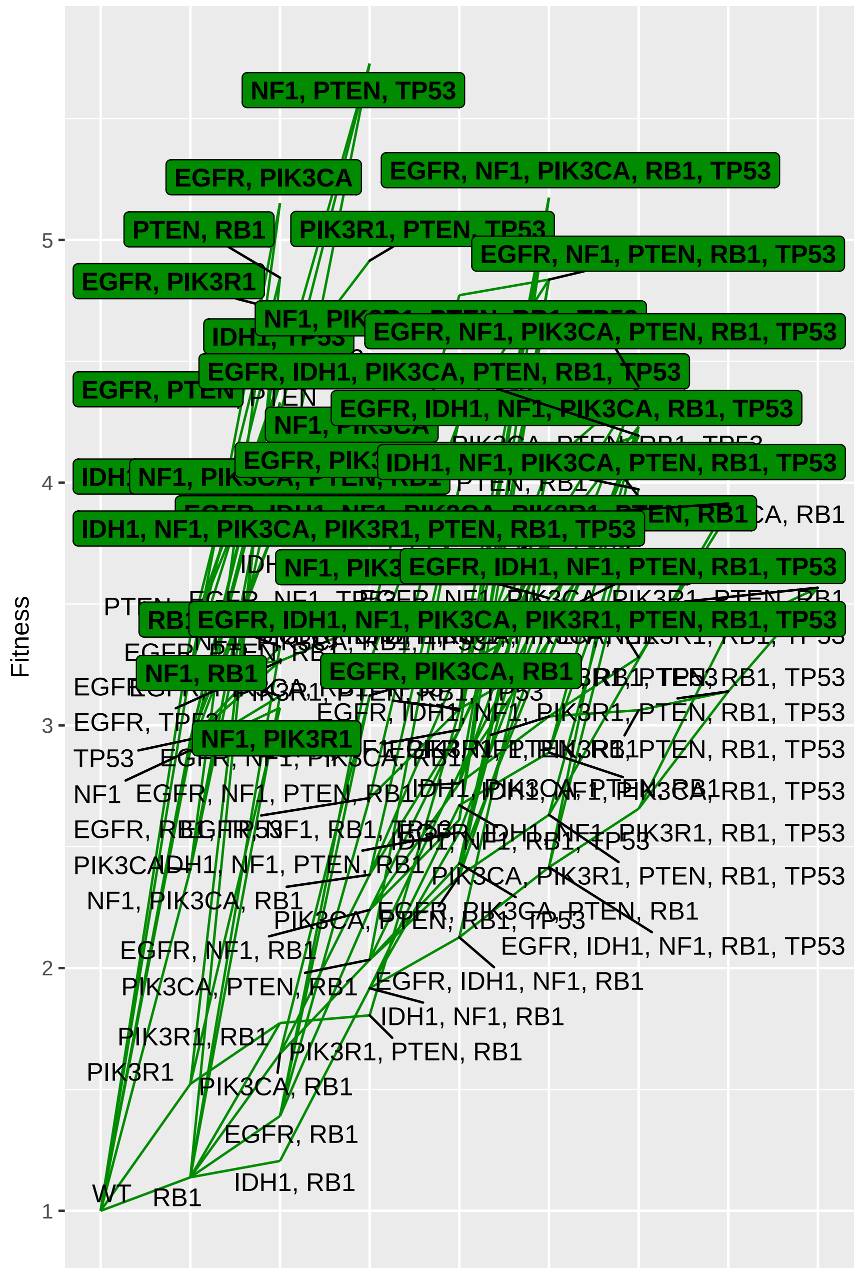

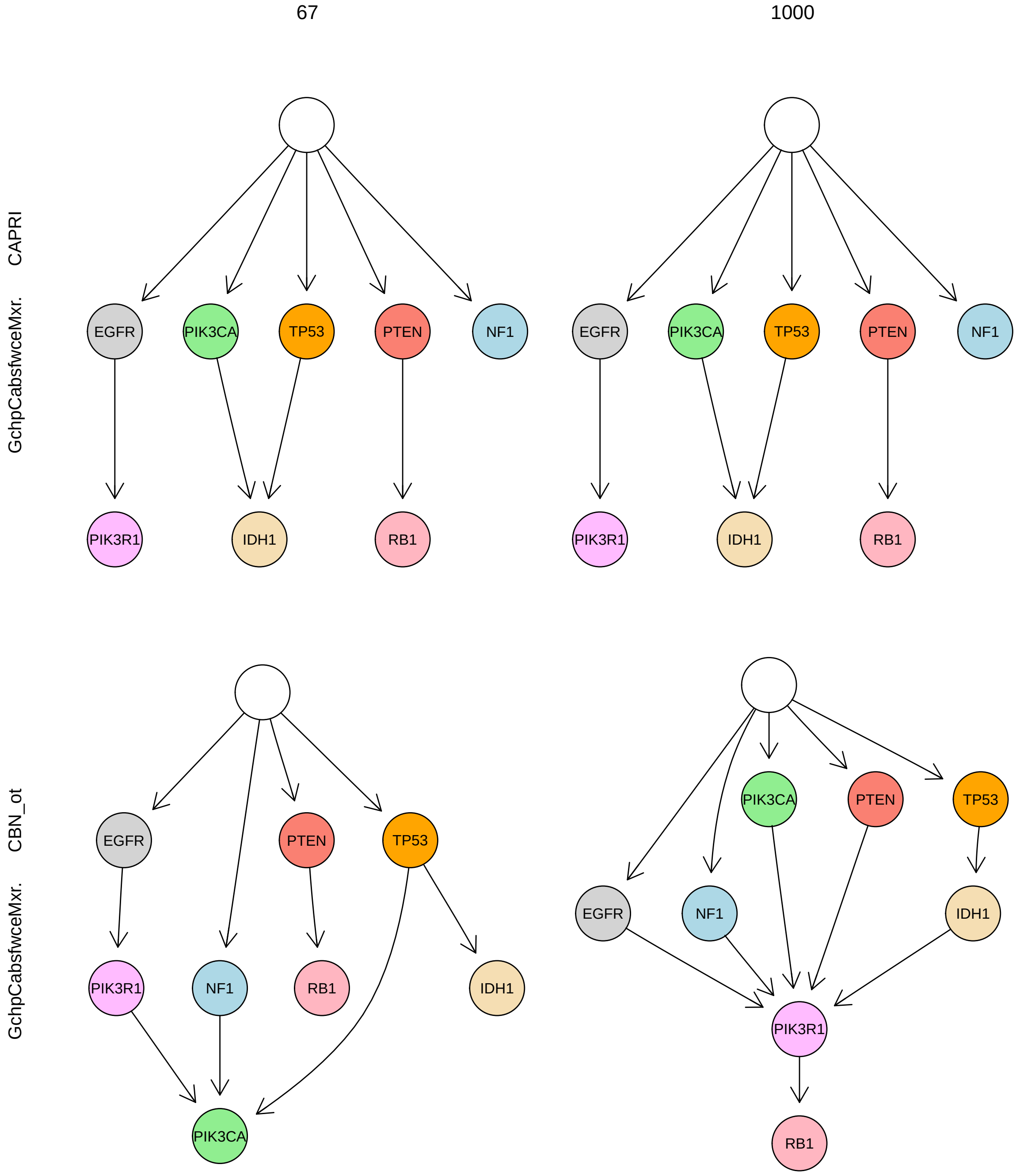

ID	p−value	Accessible Genot.
elyqExgczQSjncf	0.694	28

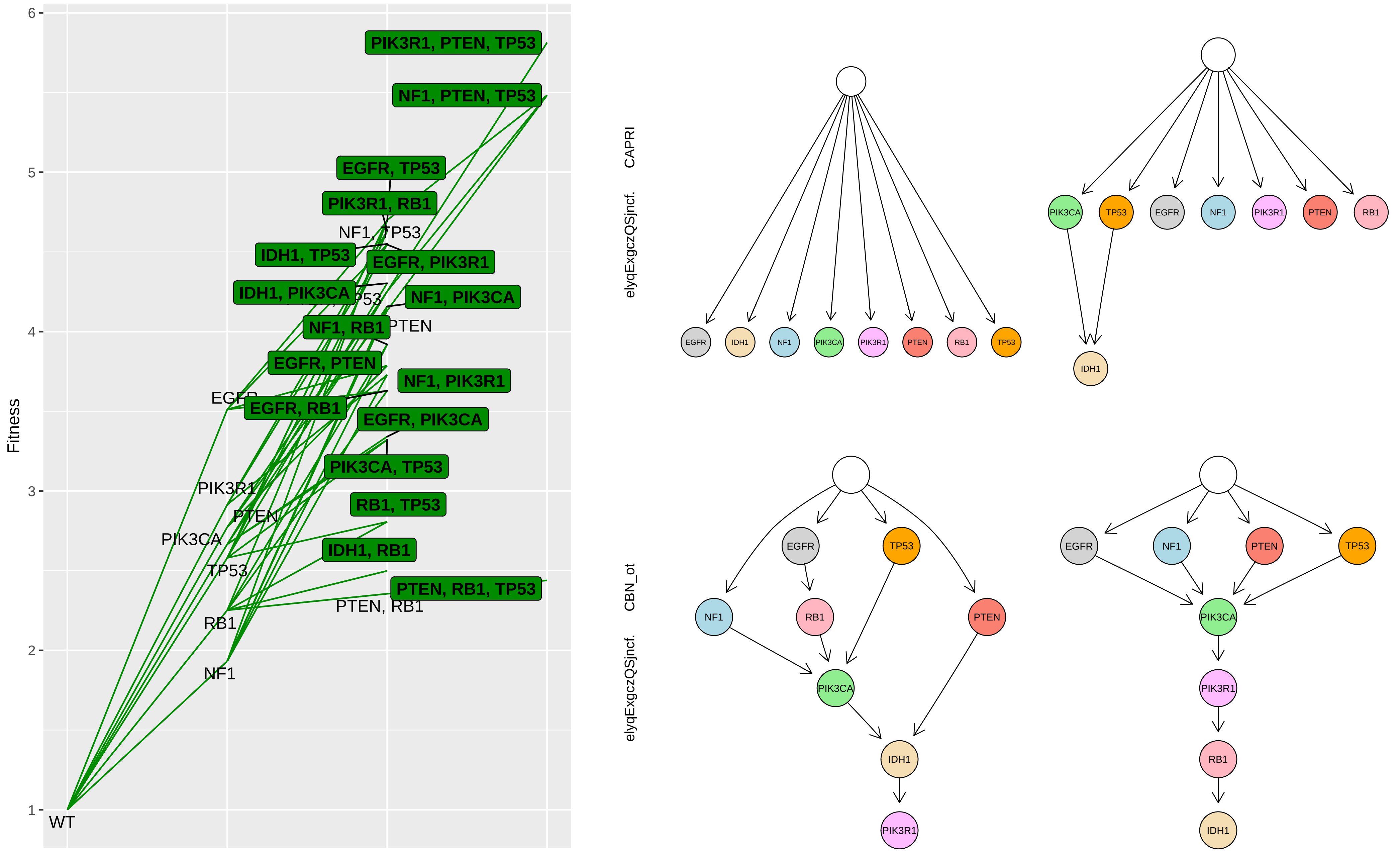

ID	p−value	Accessible Genot.
aHcUoDWoYDdyrAt	0.697	121

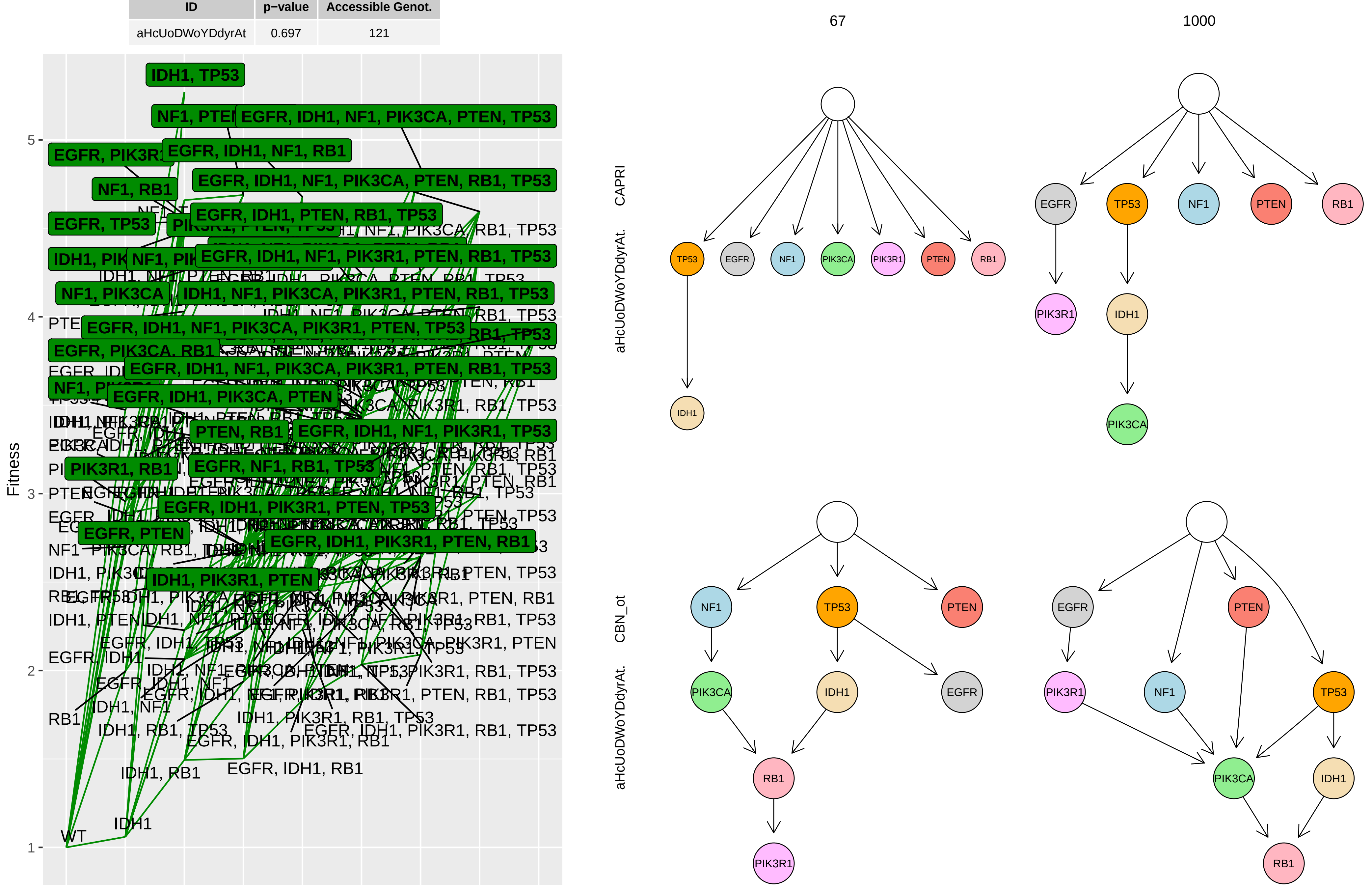

ID	p−value	Accessible Genot.
vNJhDrGqbXGOGZy	0.701	118

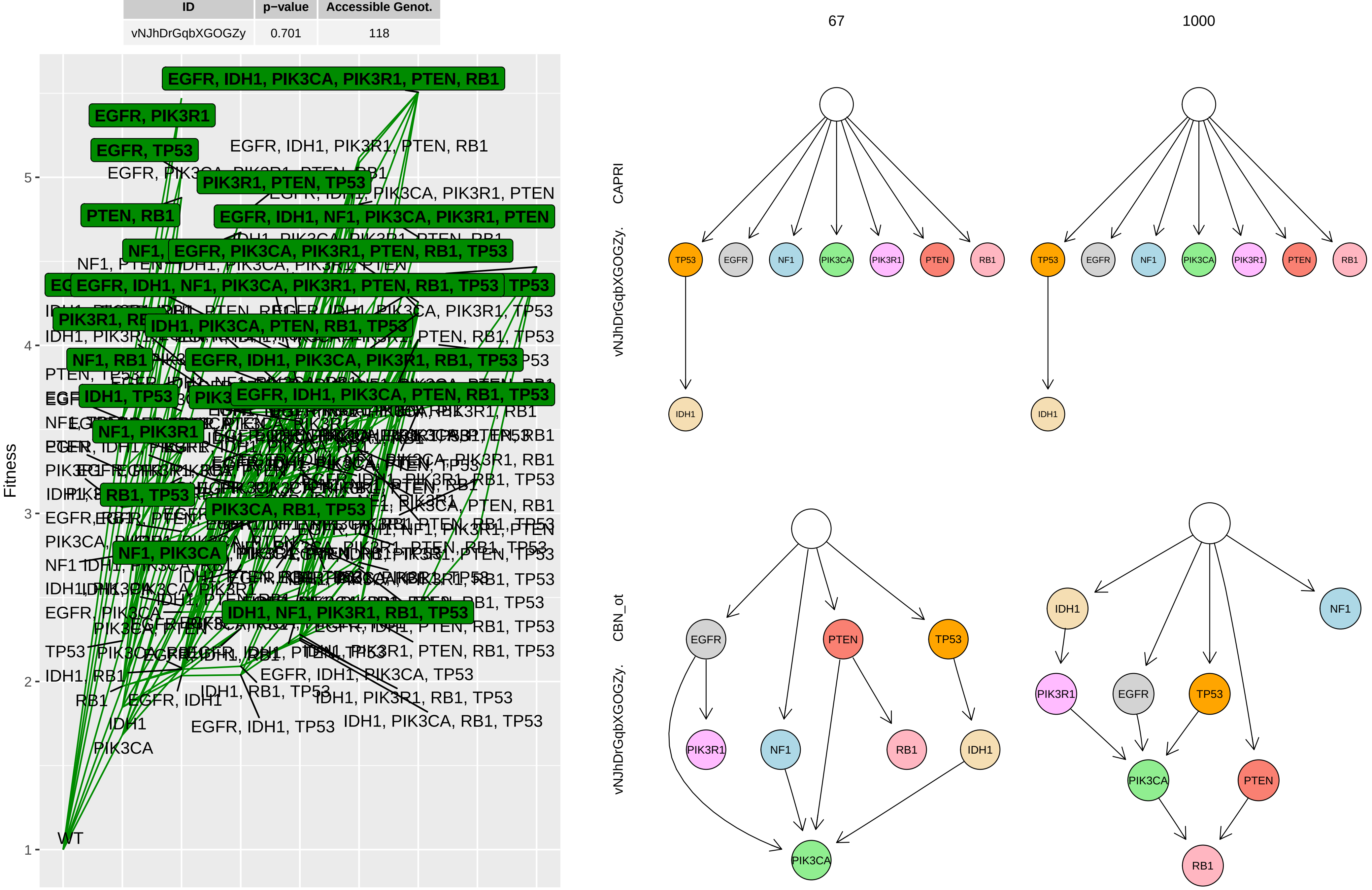

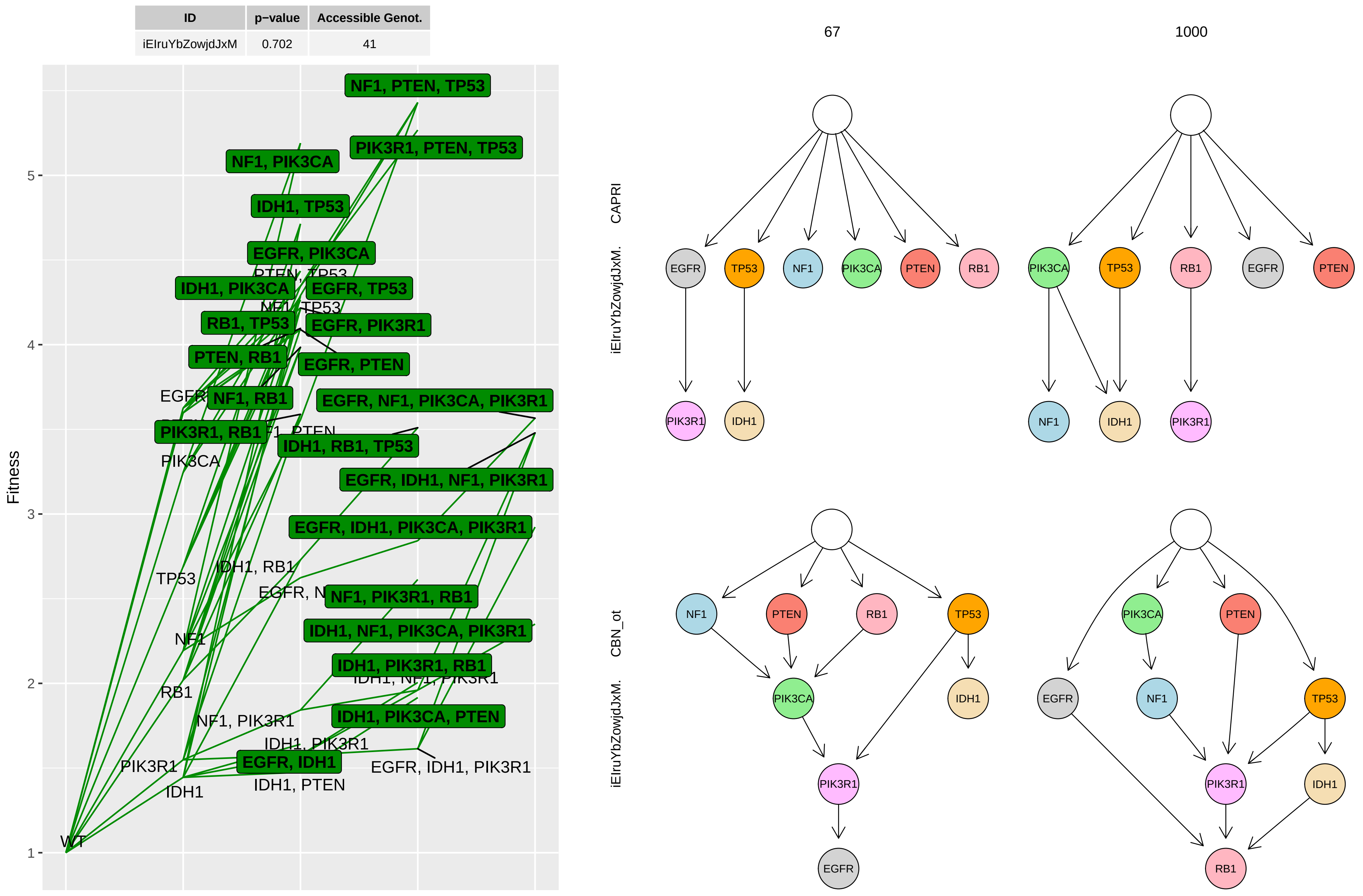

ID
p−value
Accessible Genot.
iElruYbZowjdJxM
0.702
41
Fitness
WT
EGFR
PIK3CA
TP53
NF1
RB1
PIK3R1
IDH1
IDH1, RB1
NF1, PIK3R1
IDH1, PIK3R1
IDH1, PTEN
EGFR, IDH1, PIK3R1
NF1, PTEN, TP53
PIK3R1, PTEN, TP53
NF1, PIK3CA
IDH1, TP53
EGFR, PIK3CA
IDH1, PIK3CA
EGFR, TP53
RB1, TP53
EGFR, PIK3R1
PTEN, RB1
EGFR, PTEN
NF1, RB1
EGFR, NF1, PIK3CA, PIK3R1
PIK3R1, RB1
IDH1, RB1, TP53
EGFR, IDH1, NF1, PIK3R1
EGFR, IDH1, PIK3CA, PIK3R1
NF1, PIK3R1, RB1
IDH1, NF1, PIK3CA, PIK3R1
IDH1, PIK3R1, RB1
IDH1, PIK3CA, PTEN
EGFR, IDH1
67
1000
iElruYbZowjdJxM. CAPRI
iElruYbZowjdJxM. CBN_ot
EGFR
TP53
NF1
PIK3CA
PTEN
RB1
PIK3R1
IDH1

ID	p−value	Accessible Genot.
iXLBNfIOkLmHZcQ	0.702	30

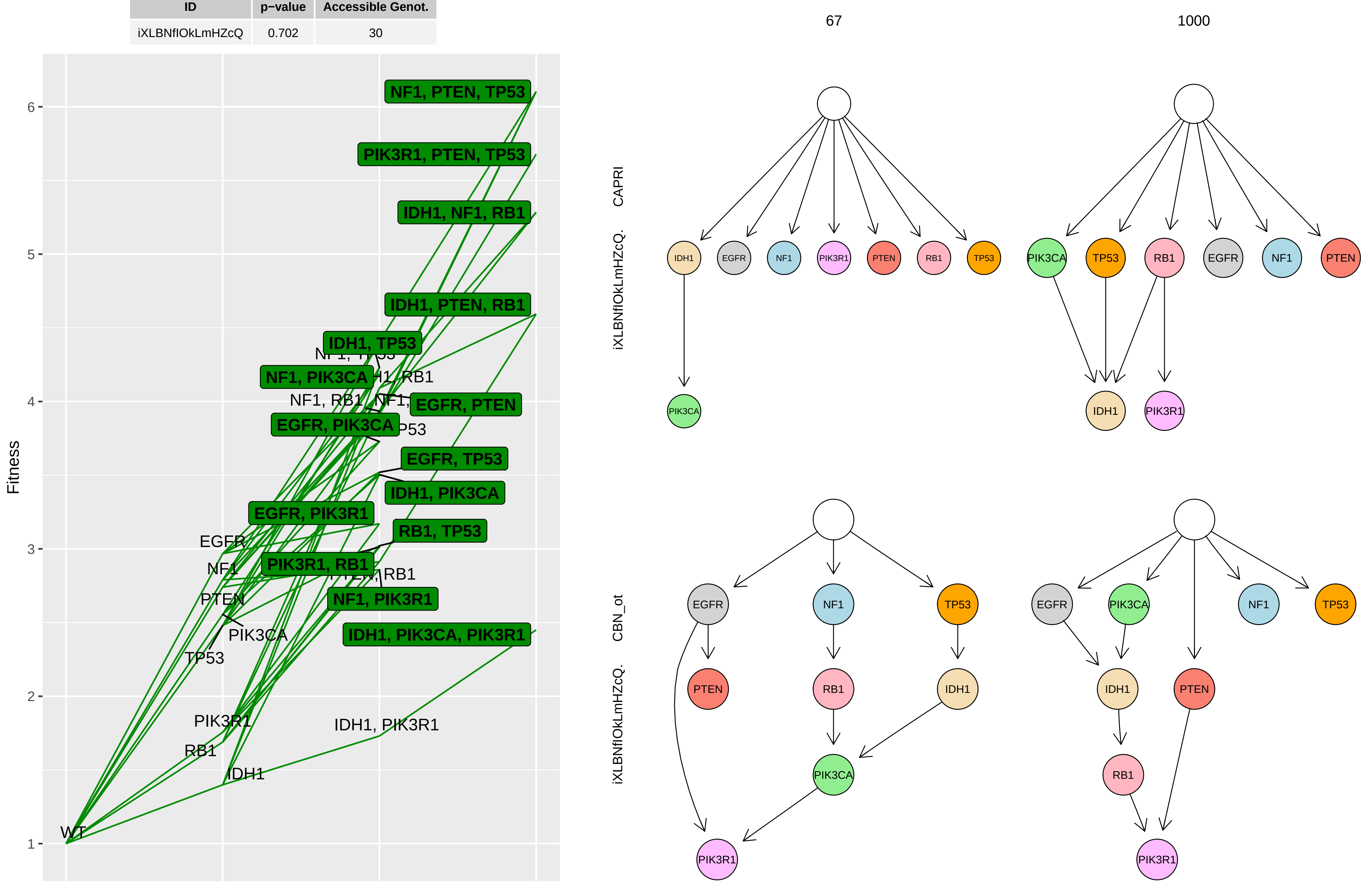

ID	p−value	Accessible Genot.
mJhpJmSTKXPXONE	0.702	28

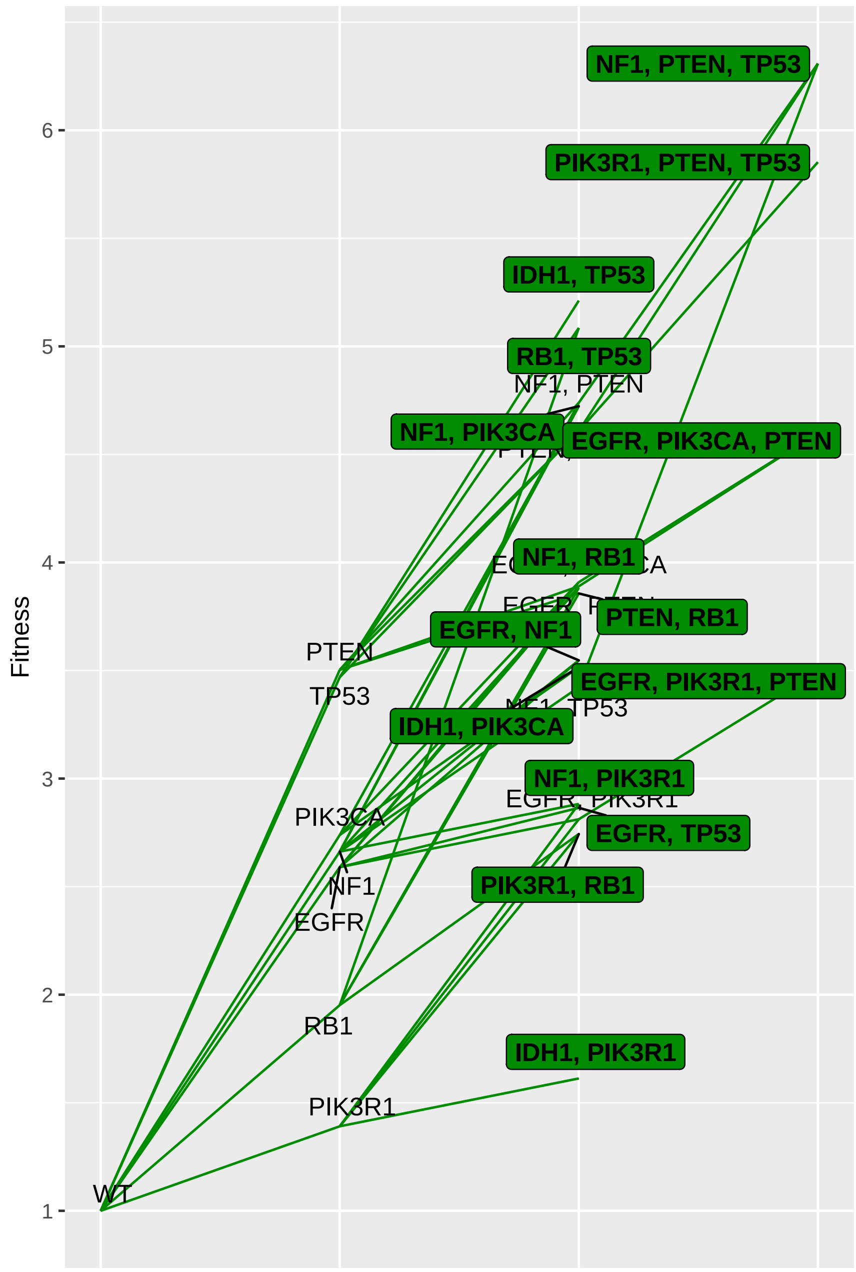

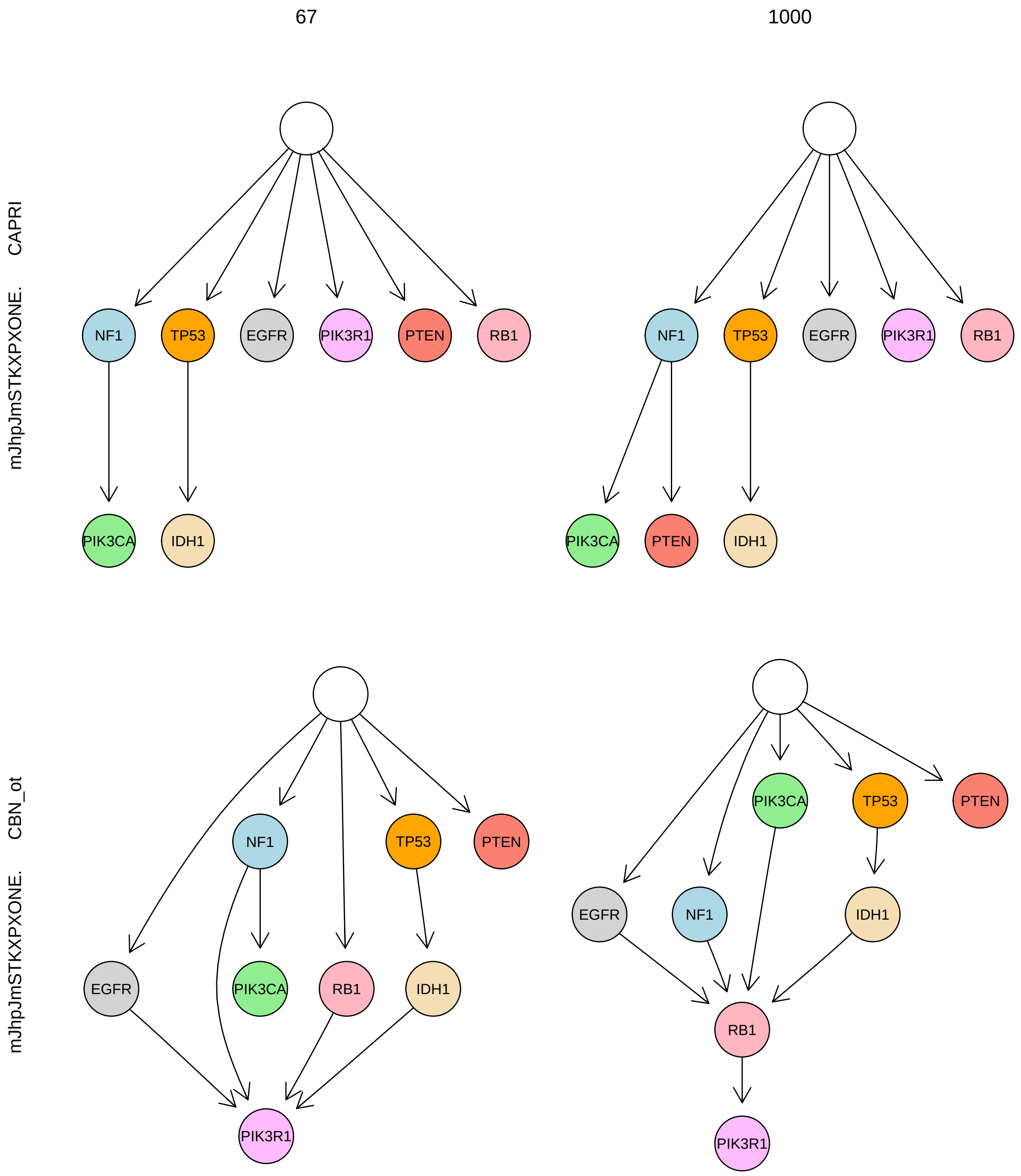

ID	p−value	Accessible Genot.
fAFNnTBFdHQlhxm	0.703	31

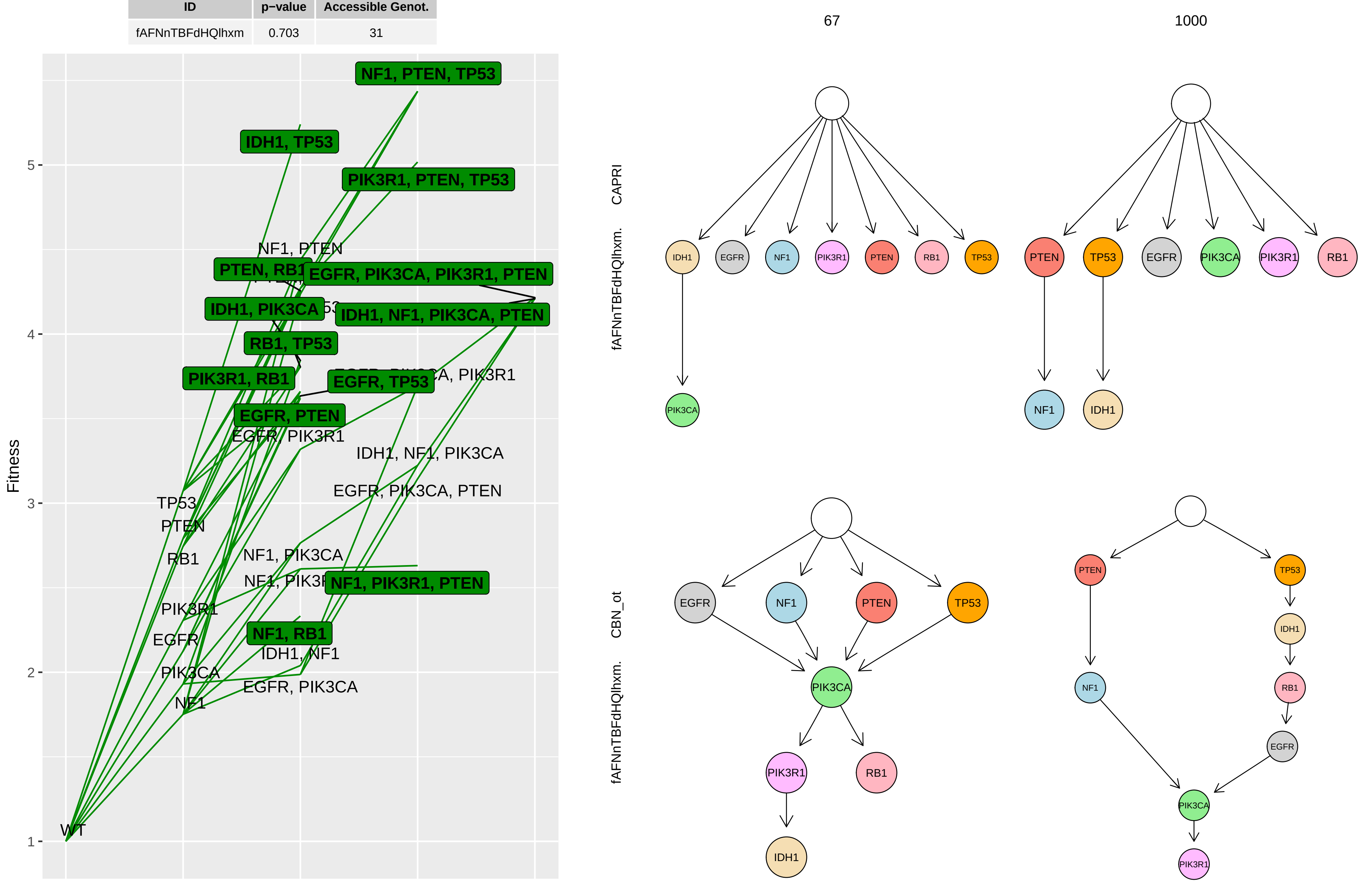

ID	p−value	Accessible Genot.
jnvhViVXoUAxSDq	0.708	221

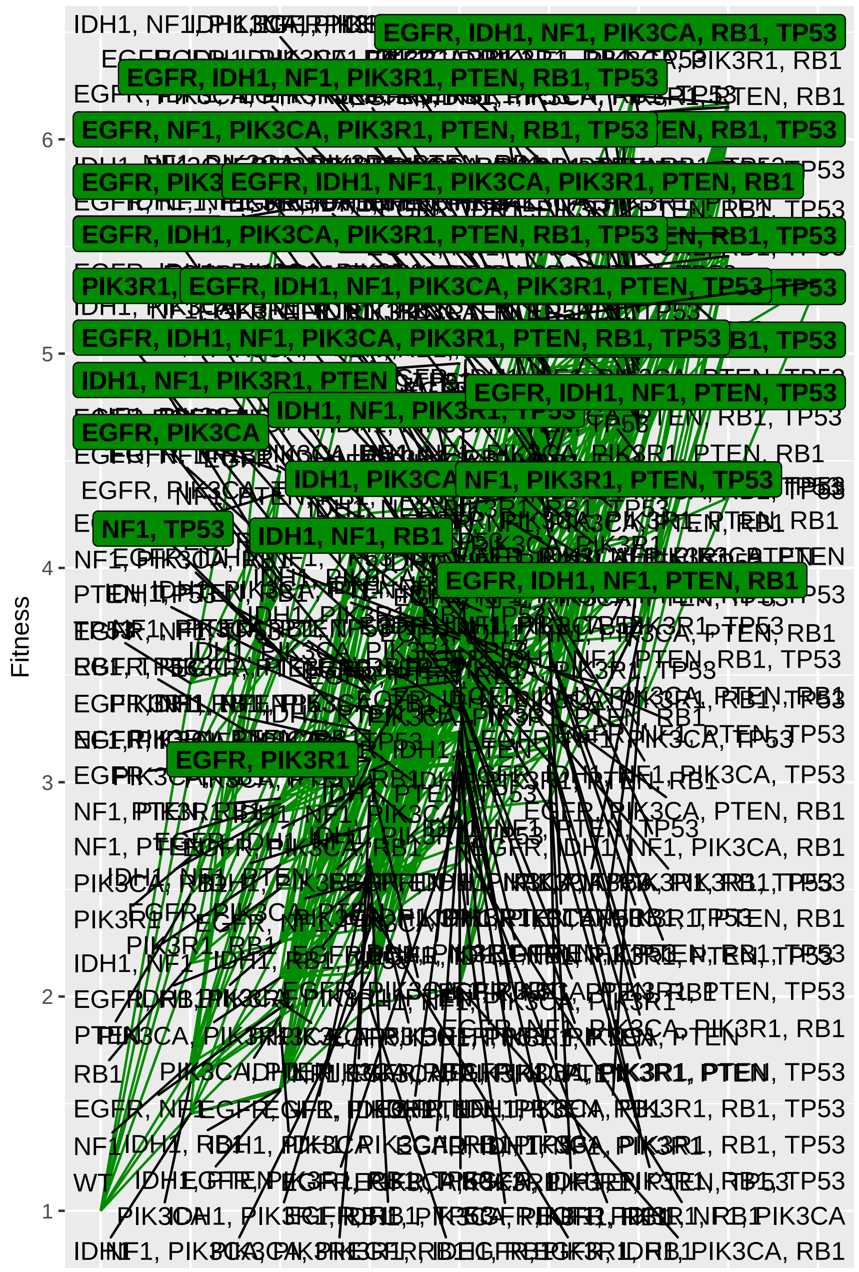

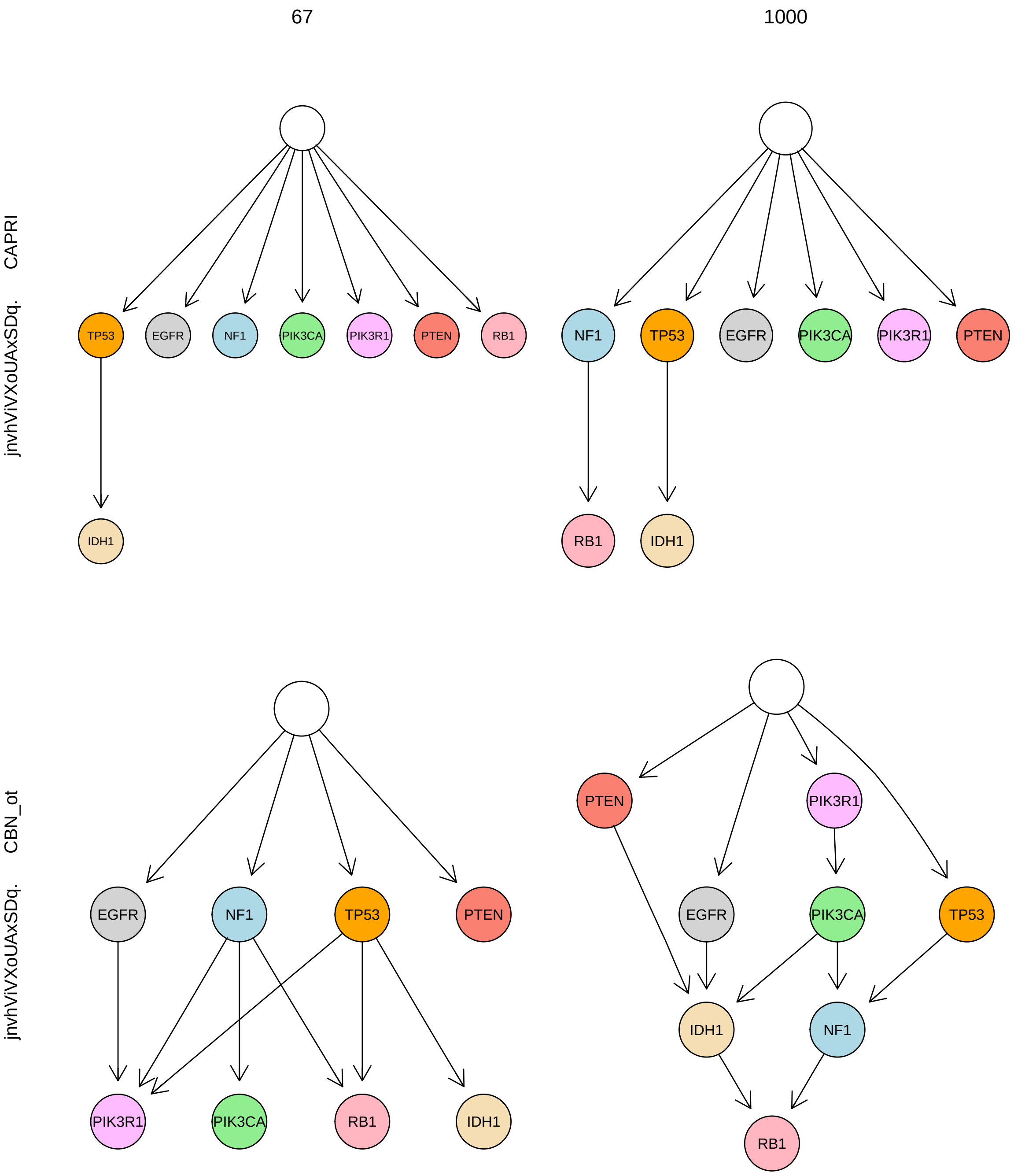

ID	p−value	Accessible Genot.
CuapFjyulbyMezX	0.711	24

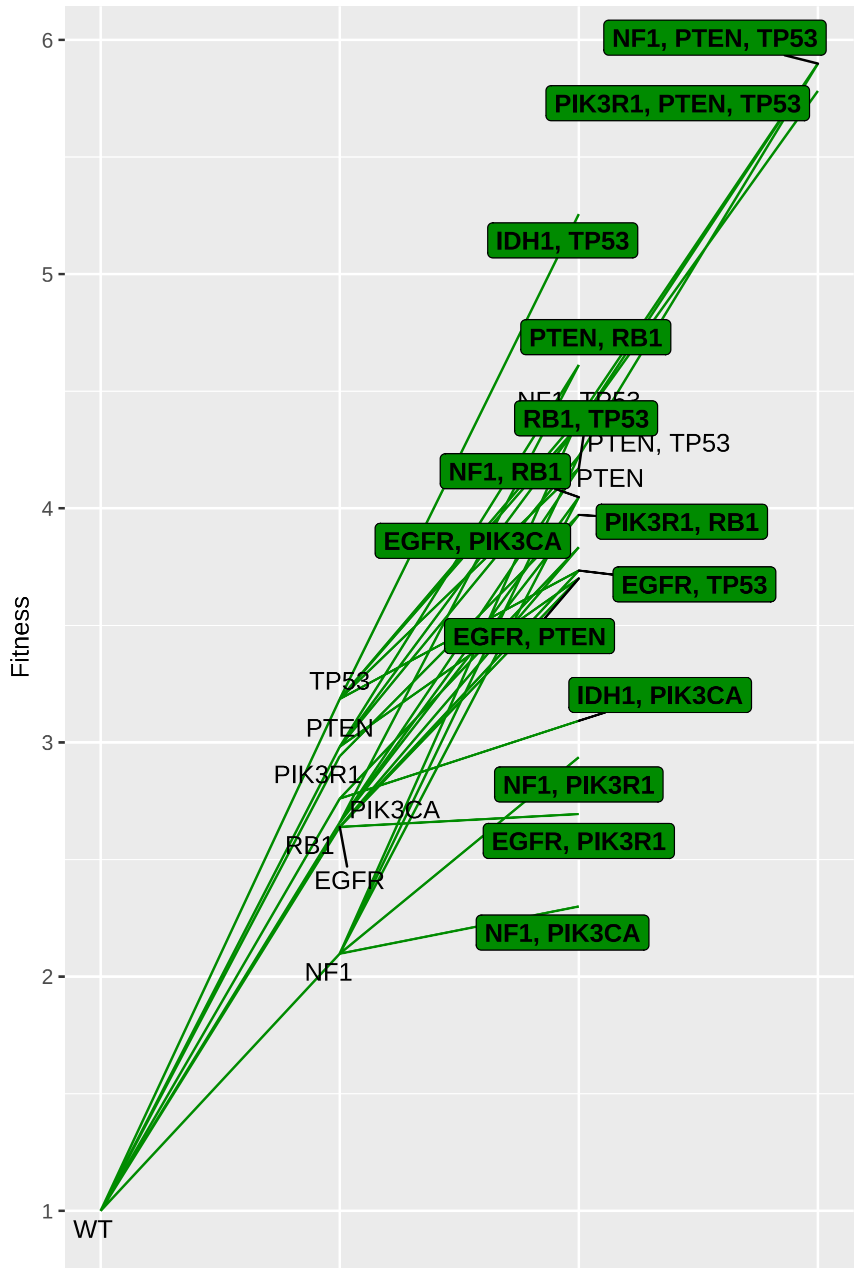

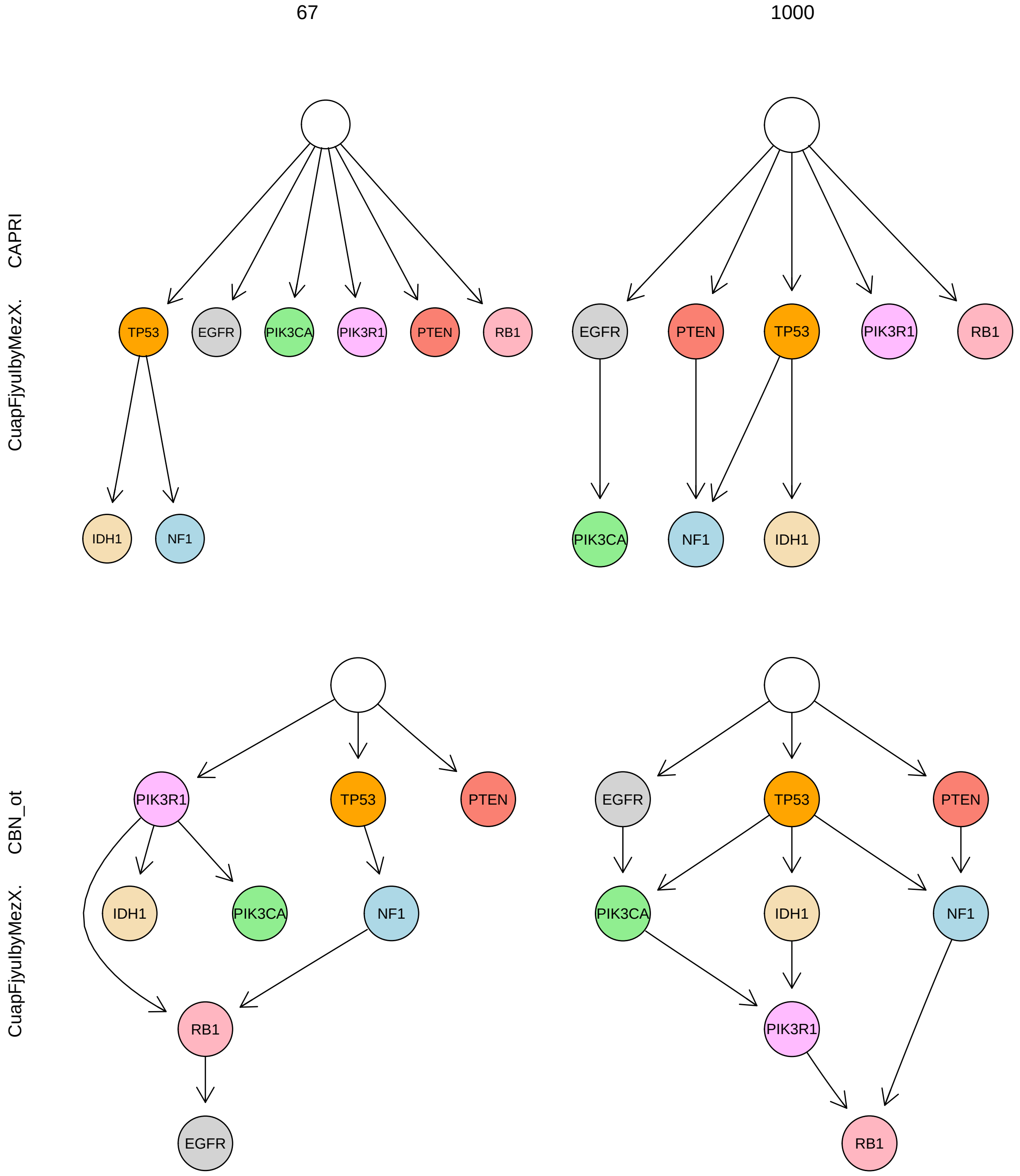

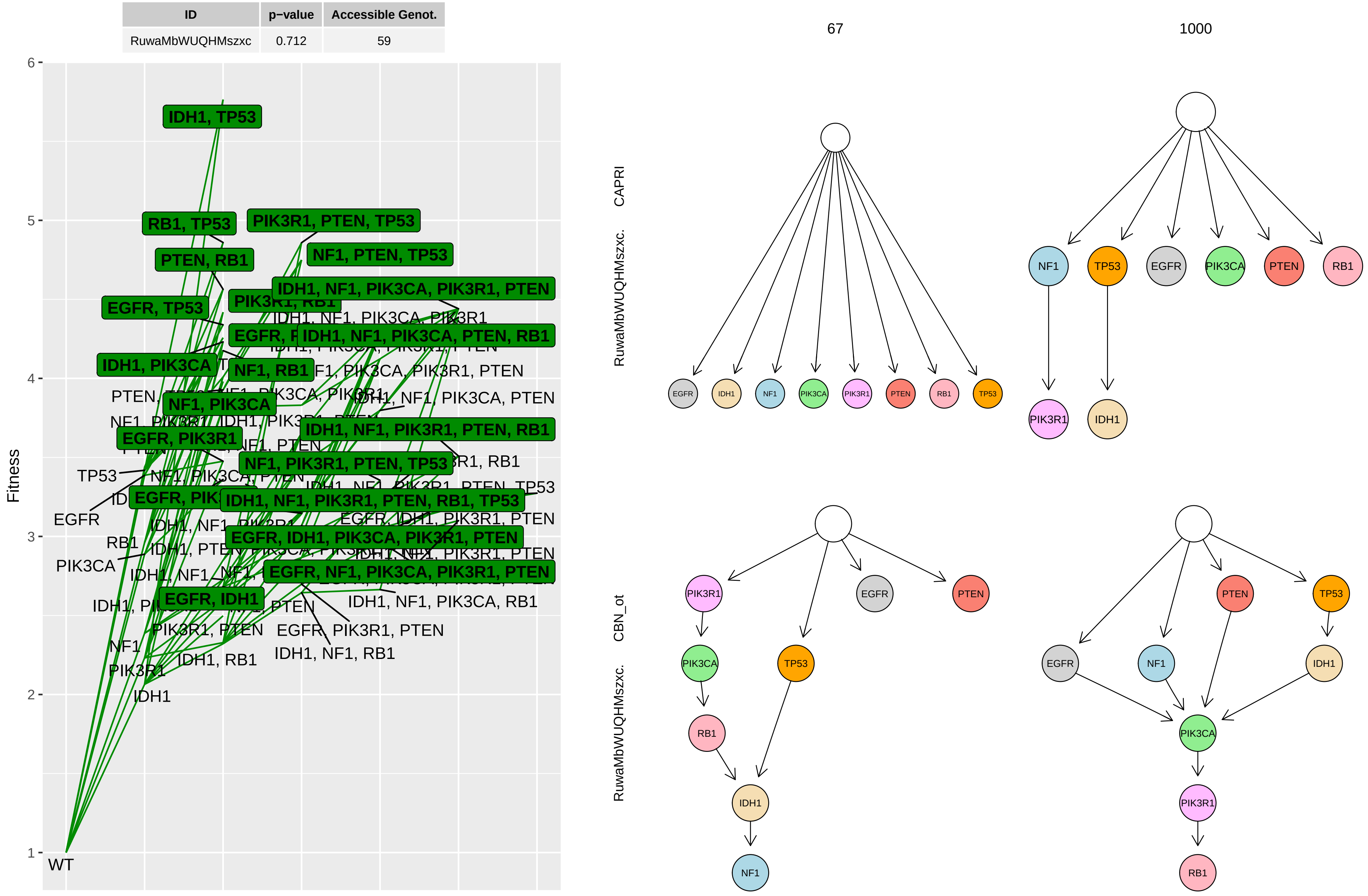

ID
p−value
Accessible Genot.
RuwaMbWUQHMszxc
0.712
59
Fitness
WT
IDH1, TP53
RB1, TP53
PIK3R1, PTEN, TP53
PTEN, RB1
NF1, PTEN, TP53
IDH1, NF1, PIK3CA, PIK3R1, PTEN
EGFR, TP53
IDH1, NF1, PIK3CA, PTEN, RB1
IDH1, PIK3CA
NF1, RB1
NF1, PIK3CA
IDH1, NF1, PIK3CA, PTEN
IDH1, NF1, PIK3R1, PTEN, RB1
EGFR, PIK3R1
NF1, PIK3R1, PTEN, TP53
TP53
IDH1, NF1, PIK3R1, PTEN, RB1, TP53
EGFR
EGFR, IDH1, PIK3R1, PTEN
EGFR, IDH1, PIK3CA, PIK3R1, PTEN
RB1
PIK3CA
EGFR, NF1, PIK3CA, PIK3R1, PTEN
EGFR, IDH1
IDH1, NF1, PIK3CA, RB1
PIK3R1, PTEN
EGFR, PIK3R1, PTEN
NF1
IDH1, RB1
IDH1, NF1, RB1
PIK3R1
IDH1
67
1000
CAPRI
RuwaMbWUQHMszxc.
CBN_ot
EGFR
IDH1
NF1
PIK3CA
PIK3R1
PTEN
RB1
TP53

ID	p−value	Accessible Genot.
DFcWXTbWWoyMfJS	0.713	26

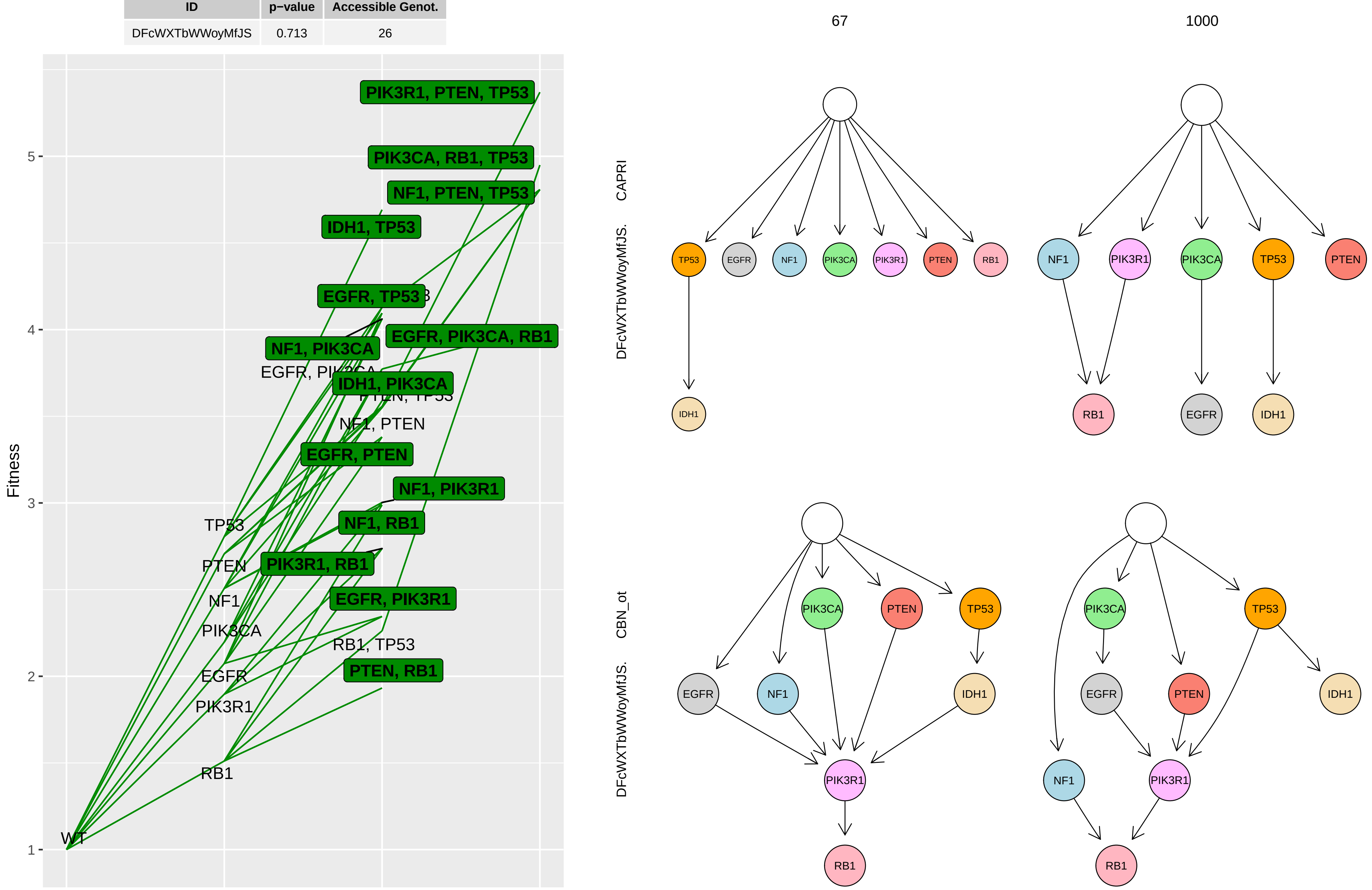

ID	p−value	Accessible Genot.
aJTpbrkMMPRmODF	0.713	246

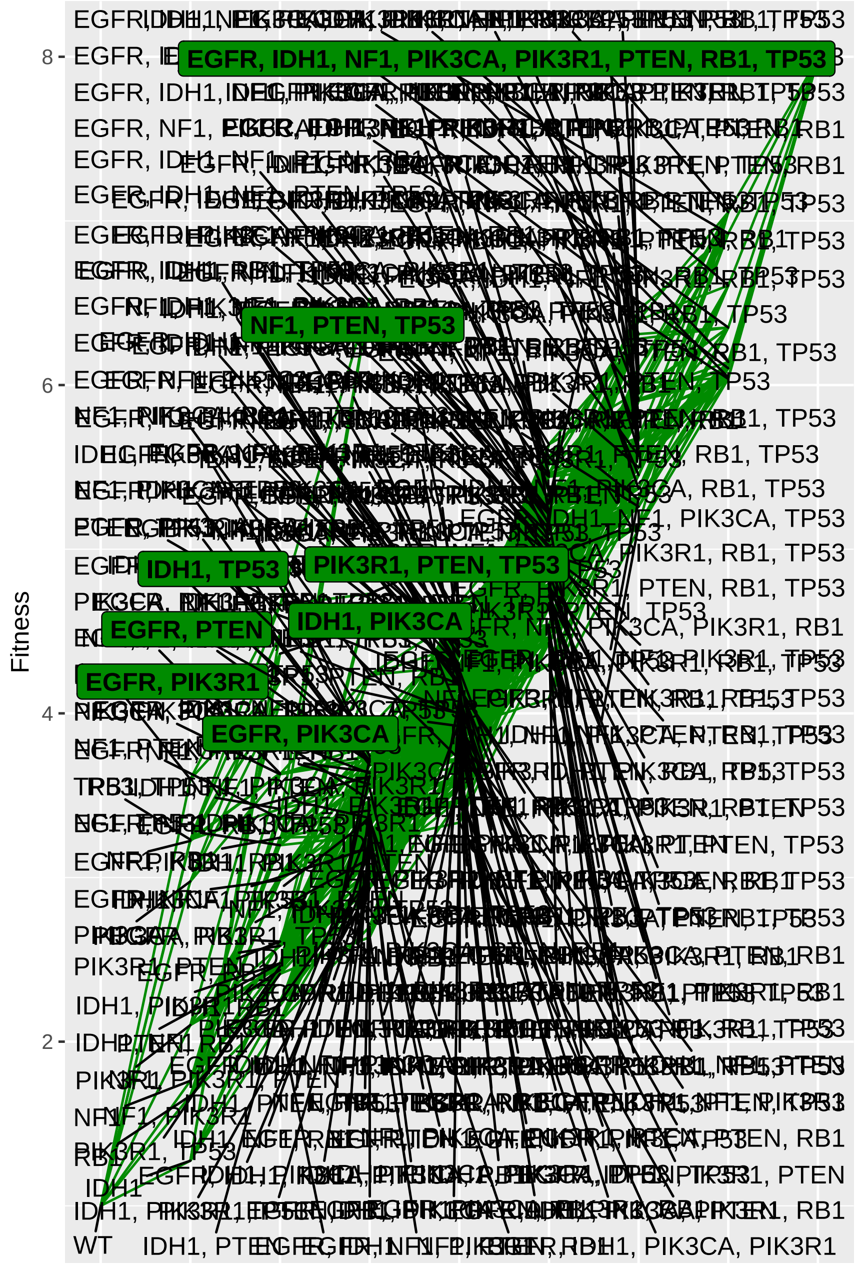

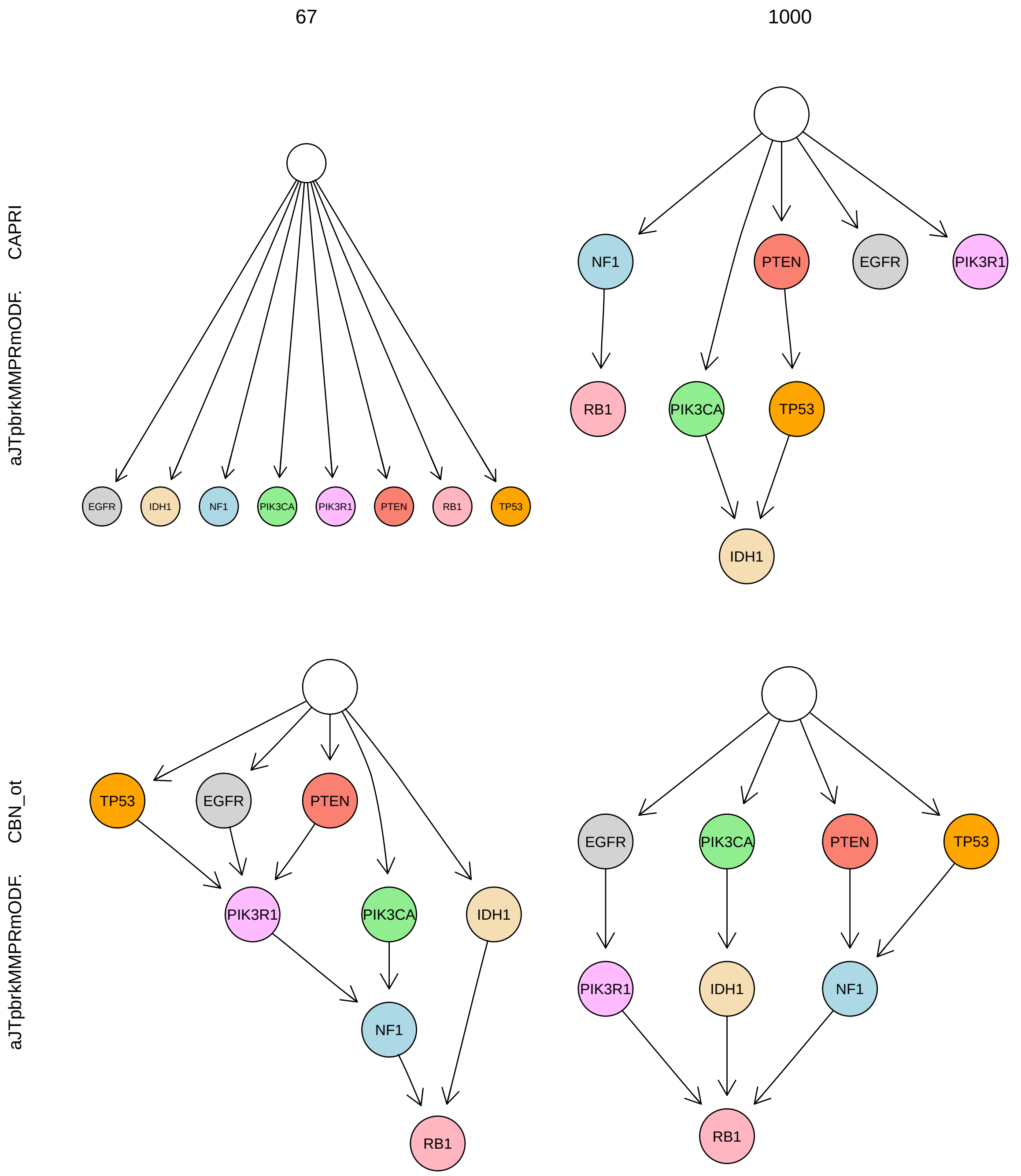

ID	p−value	Accessible Genot.
lxgZISmfXYgXcop	0.714	25

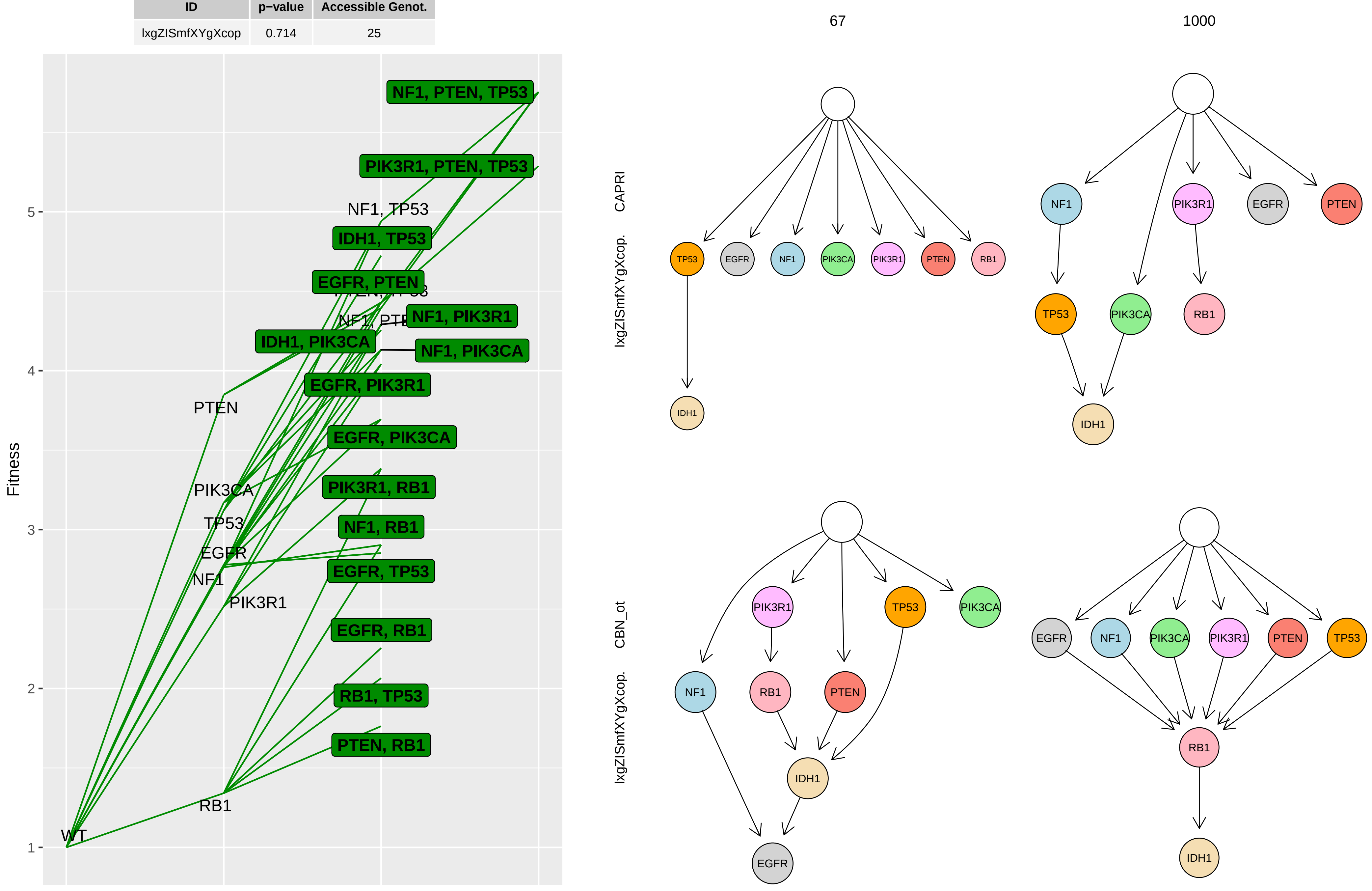

ID	p−value	Accessible Genot.
BytxYGMMDZWFYnY	0.715	102

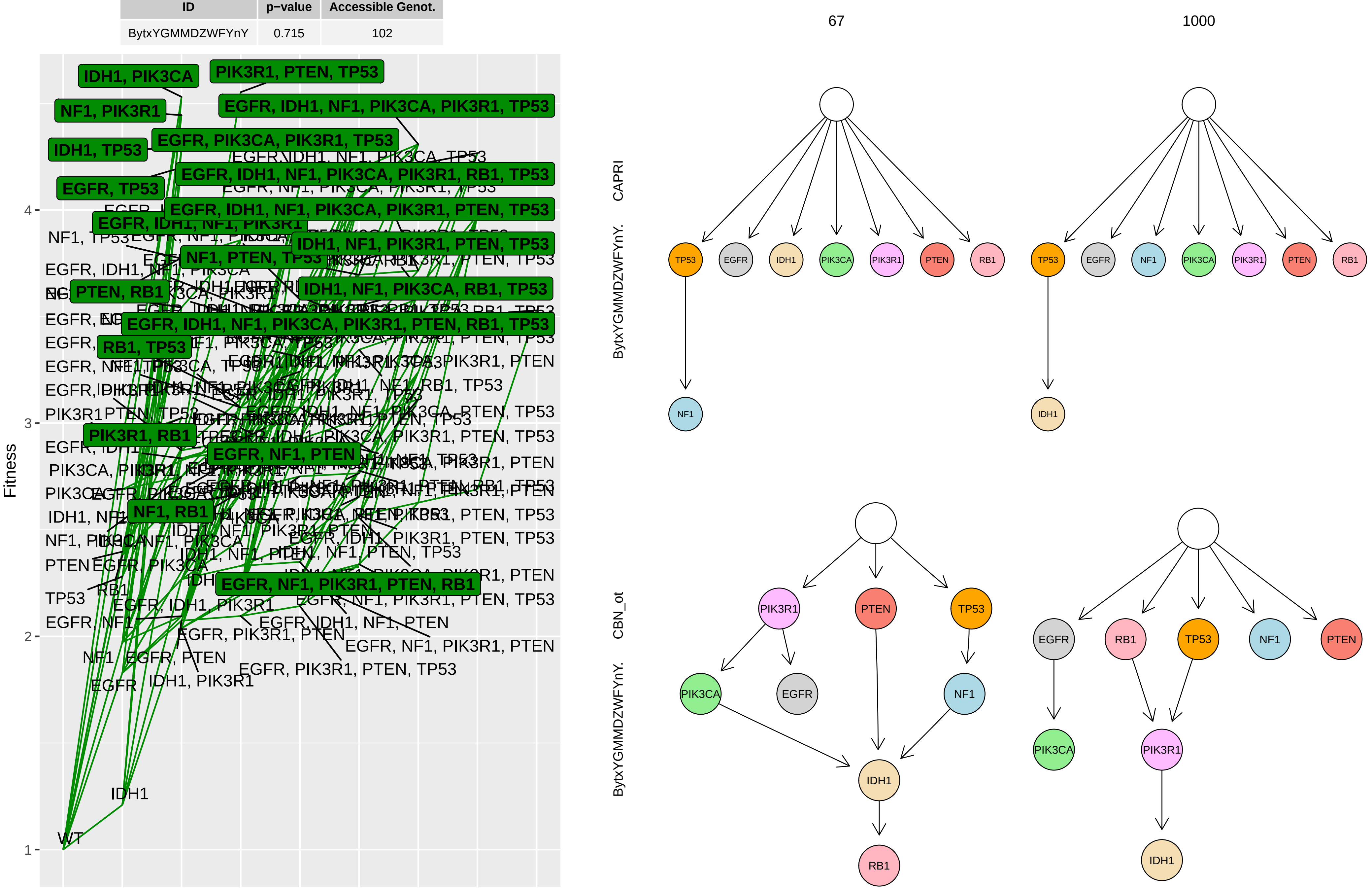

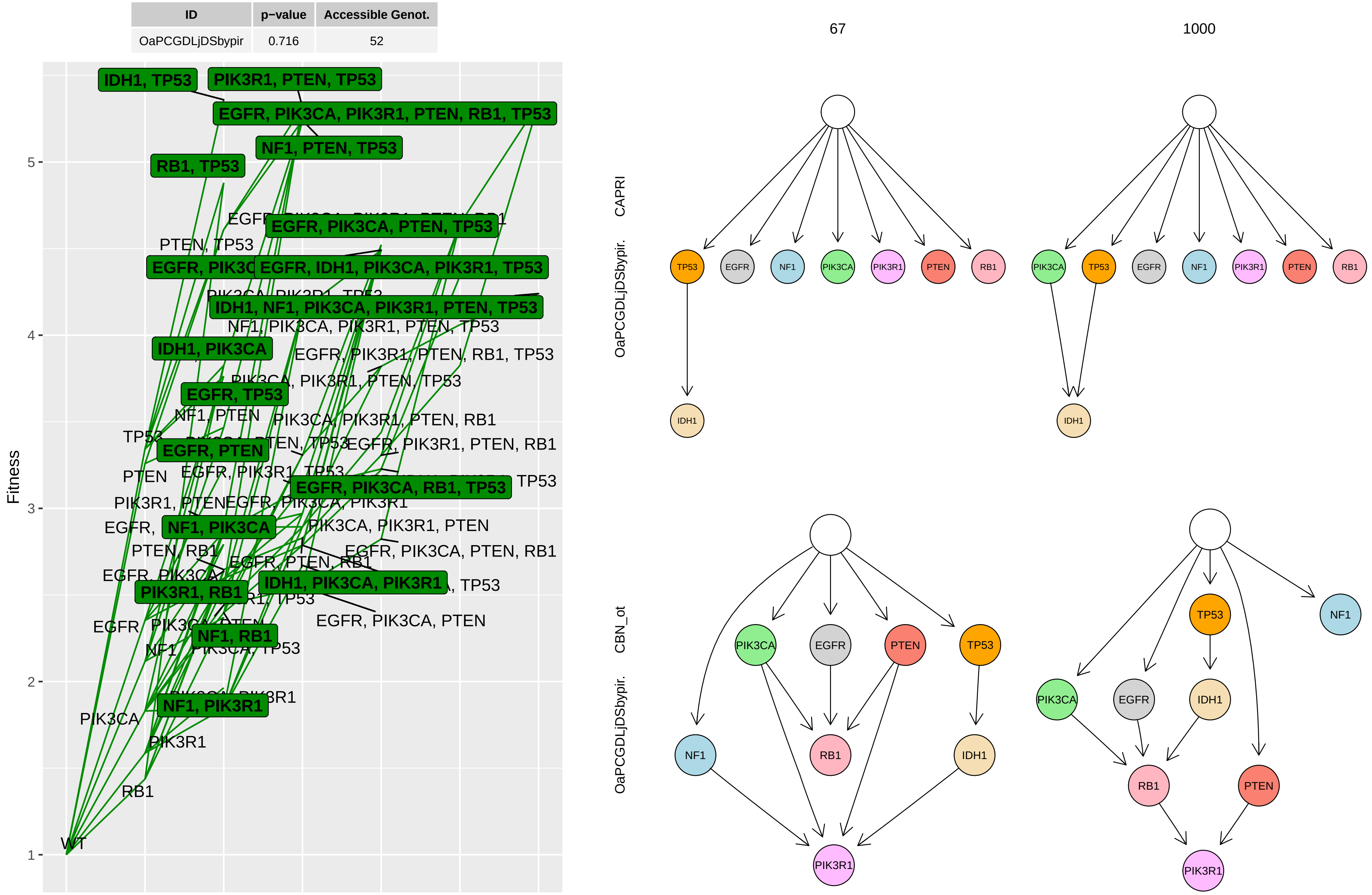

ID
p−value
Accessible Genot.
OaPCGDLjDSbypir
0.716
52
Fitness
1
2
3
4
5
WT
RB1
PIK3R1
PIK3CA
PTEN
TP53
IDH1, TP53
PIK3R1, PTEN, TP53
EGFR, PIK3CA, PIK3R1, PTEN, RB1, TP53
NF1, PTEN, TP53
RB1, TP53
EGFR, PIK3CA, PTEN, TP53
PTEN, TP53
EGFR, IDH1, PIK3CA, PIK3R1, TP53
IDH1, NF1, PIK3CA, PIK3R1, PTEN, TP53
NF1, PIK3CA, PIK3R1, PTEN, TP53
IDH1, PIK3CA
EGFR, PIK3R1, PTEN, RB1, TP53
EGFR, TP53
NF1, PTEN
PIK3CA, PIK3R1, PTEN, RB1
EGFR, PTEN
EGFR, PIK3R1, PTEN, RB1
EGFR, PIK3CA, RB1, TP53
PIK3R1, PTEN
NF1, PIK3CA
PIK3CA, PIK3R1, PTEN
PTEN, RB1
EGFR, PIK3CA, PTEN, RB1
EGFR, PTEN, RB1
IDH1, PIK3CA, PIK3R1
PIK3R1, RB1
EGFR, PIK3CA, PTEN
NF1, RB1
NF1, PIK3R1
67
1000
CAPRI
CBN_ot
OaPCGDLjDSbypir.
TP53
EGFR
NF1
PIK3CA
PIK3R1
PTEN
RB1
IDH1

ID	p-value	Accessible Genot.
qEjdFGGAYftclNv	0.716	24

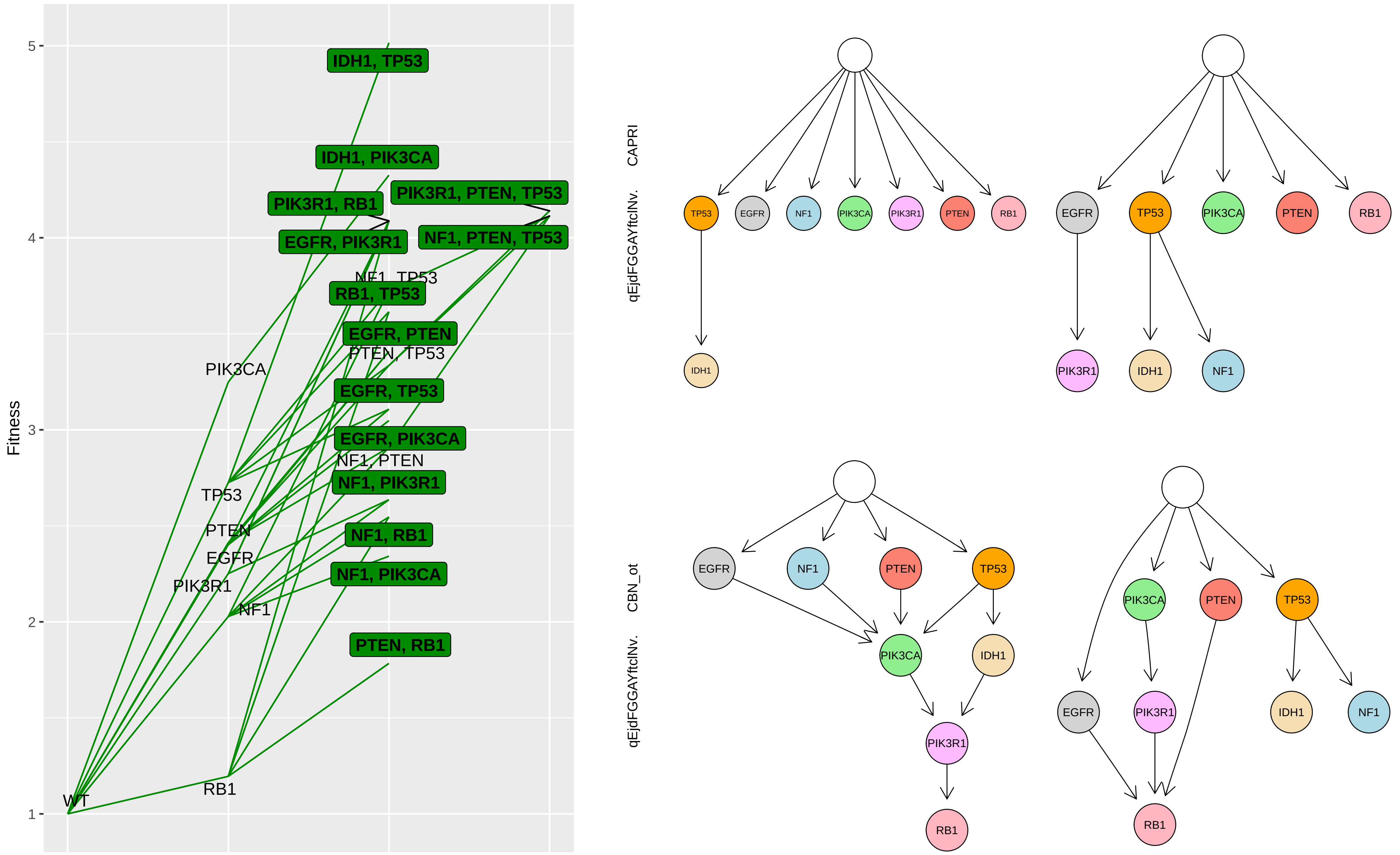

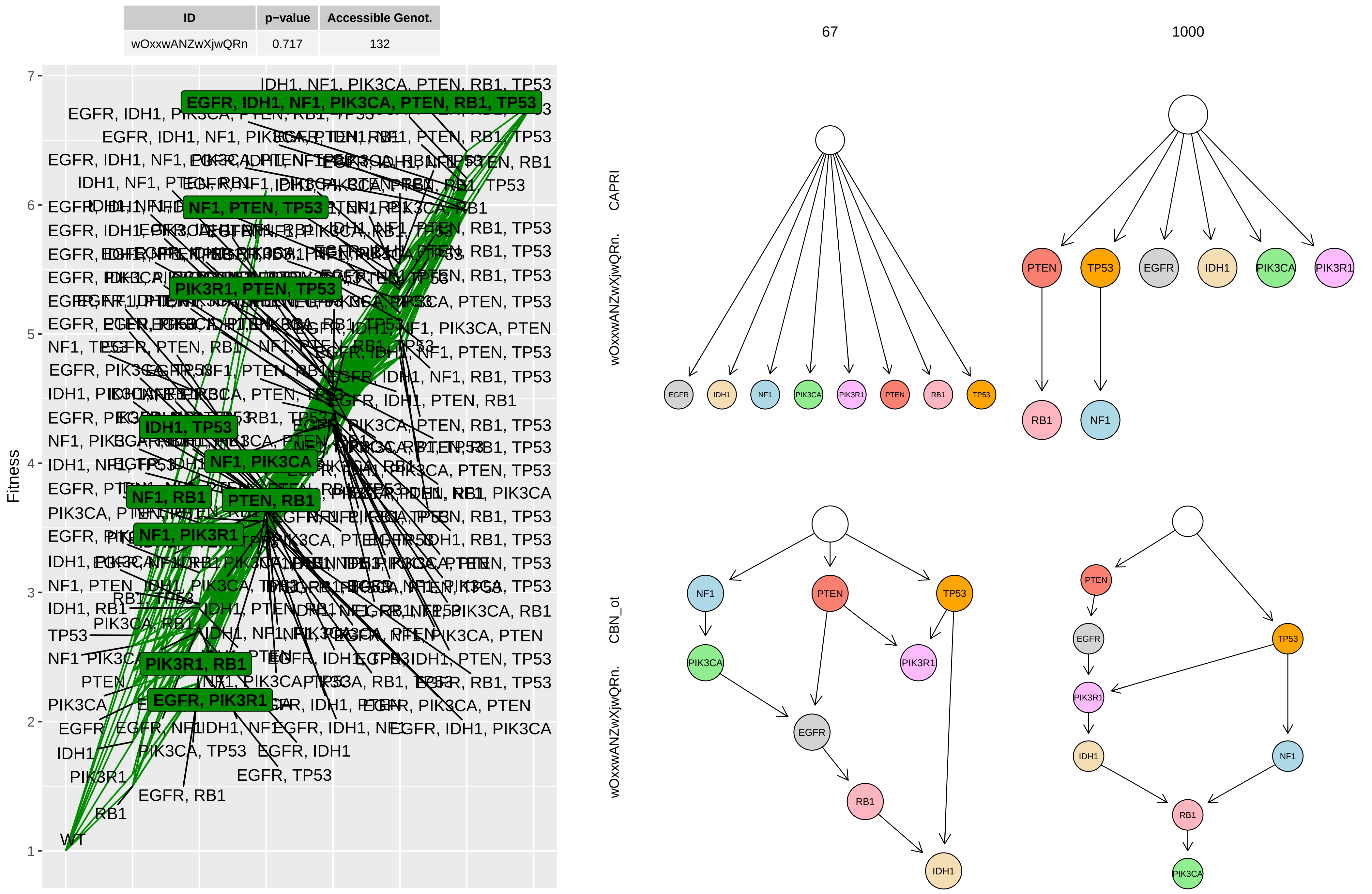
ID
p−value
Accessible Genot.
wOxxwANZwXjwQRn
0.717
132
Fitness
EGFR, IDH1, NF1, PIK3CA, PTEN, RB1, TP53
NF1, PTEN, TP53
PIK3R1, PTEN, TP53
IDH1, TP53
NF1, PIK3CA
NF1, RB1
PTEN, RB1
NF1, PIK3R1
PIK3R1, RB1
EGFR, PIK3R1
WT
67
1000
CAPRI
CBN_ot
wOxxwANZwXjwQRn.
EGFR
IDH1
NF1
PIK3CA
PIK3R1
PTEN
RB1
TP53

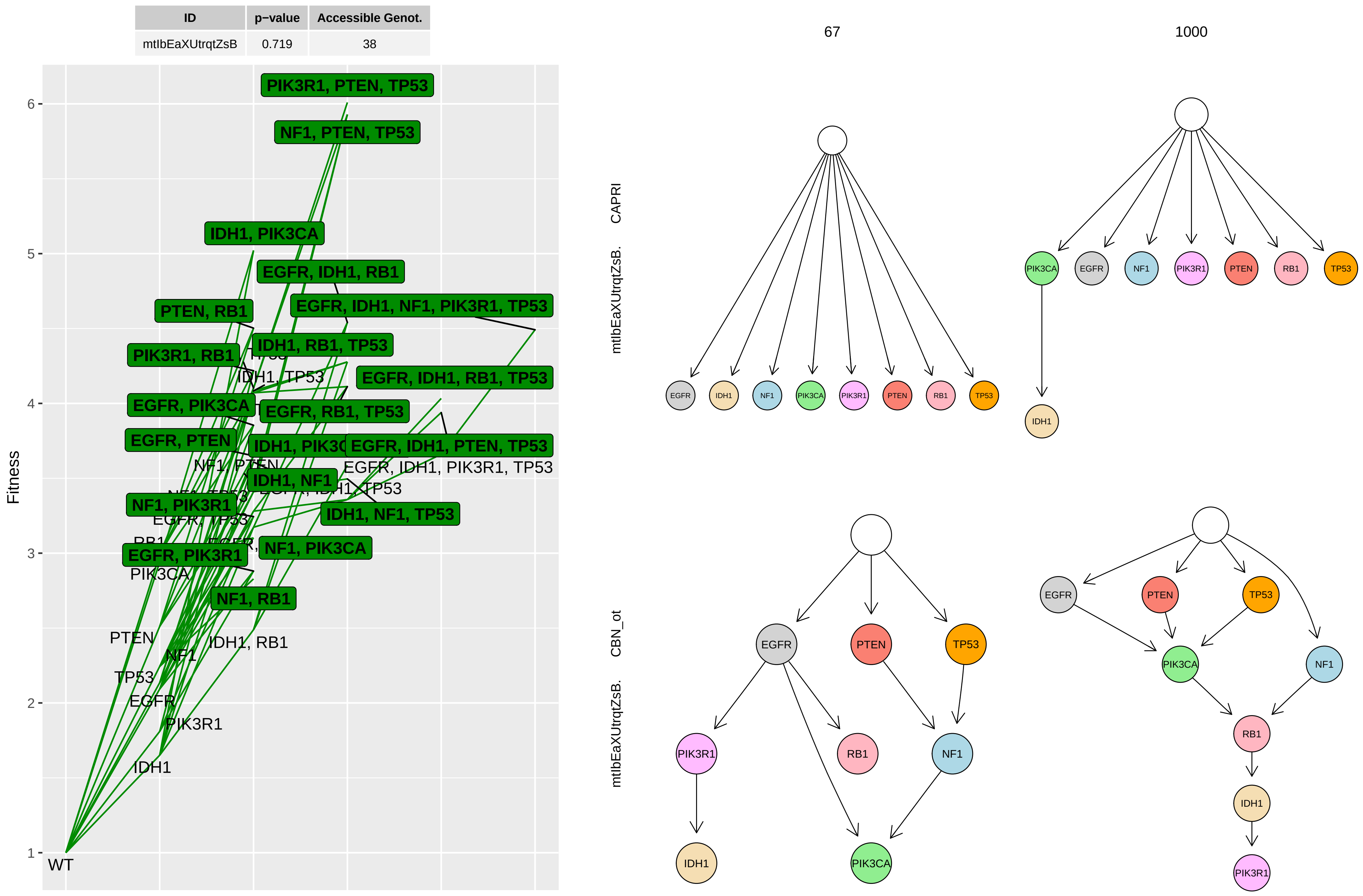
ID
p−value
Accessible Genot.
mtIbEaXUtrqtZsB
0.719
38
Fitness
PIK3R1, PTEN, TP53
NF1, PTEN, TP53
IDH1, PIK3CA
EGFR, IDH1, RB1
PTEN, RB1
EGFR, IDH1, NF1, PIK3R1, TP53
IDH1, RB1, TP53
PIK3R1, RB1
IDH1, TP53
EGFR, IDH1, RB1, TP53
EGFR, PIK3CA
EGFR, RB1, TP53
EGFR, PTEN
EGFR, IDH1, PTEN, TP53
EGFR, IDH1, PIK3R1, TP53
IDH1, NF1
NF1, PIK3R1
IDH1, NF1, TP53
EGFR, PIK3R1
NF1, PIK3CA
PIK3CA
NF1, RB1
PTEN
IDH1, RB1
NF1
TP53
EGFR
PIK3R1
IDH1
WT
67
1000
mtIbEaXUtrqtZsB. CAPRI
mtIbEaXUtrqtZsB. CBN_ot
EGFR
IDH1
NF1
PIK3CA
PIK3R1
PTEN
RB1
TP53

ID	p−value	Accessible Genot.
vefUaQZUyczGcgz	0.72	30

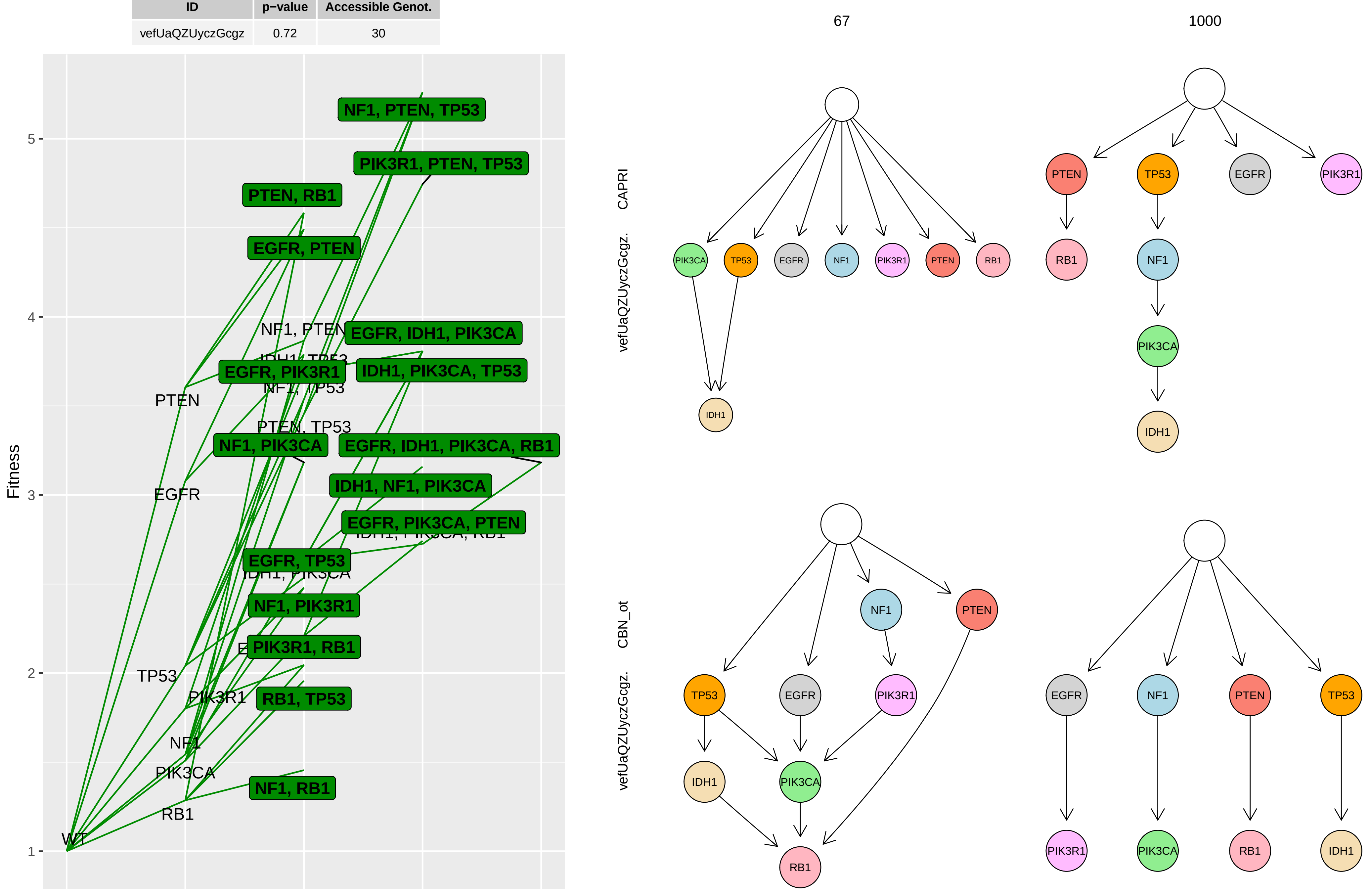

ID	p−value	Accessible Genot.
xGrYtsTTcoKyvsg	0.72	58

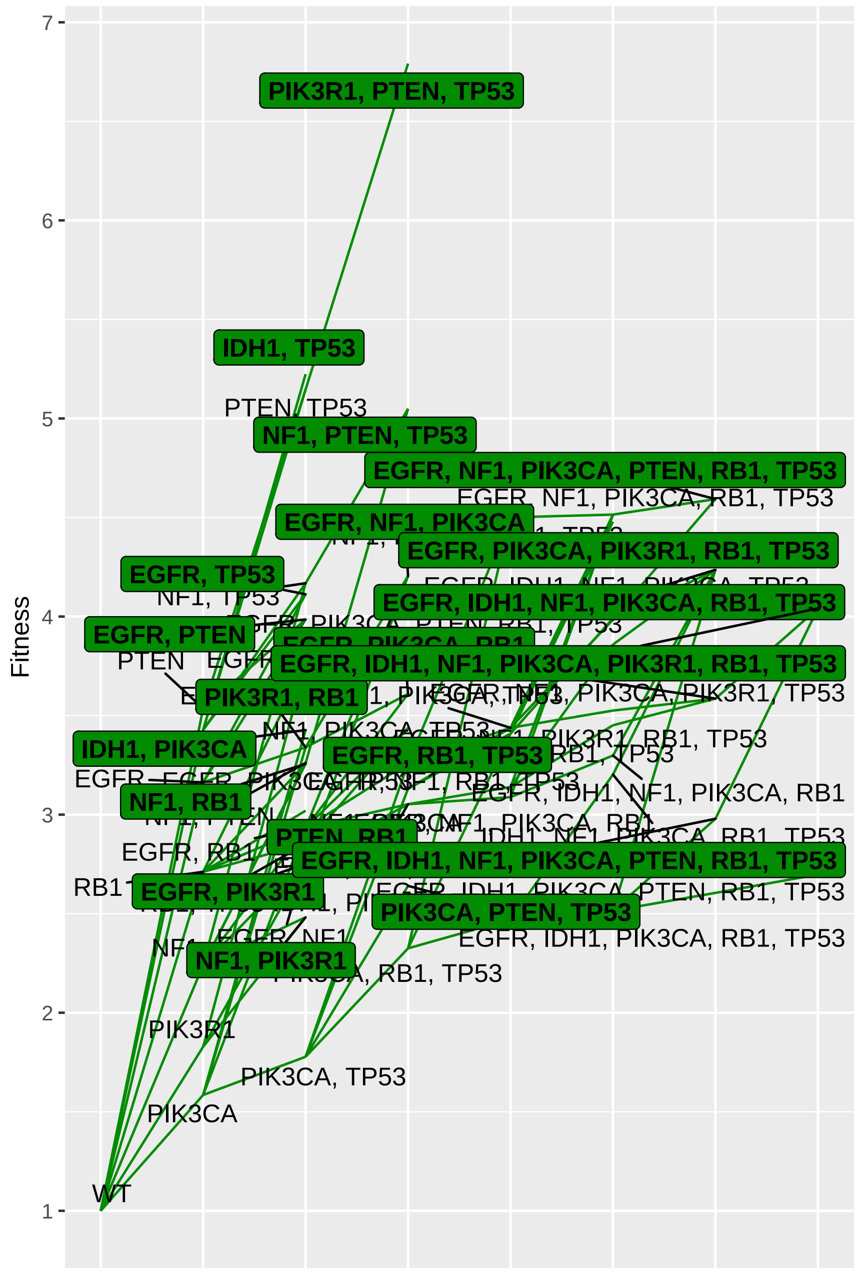

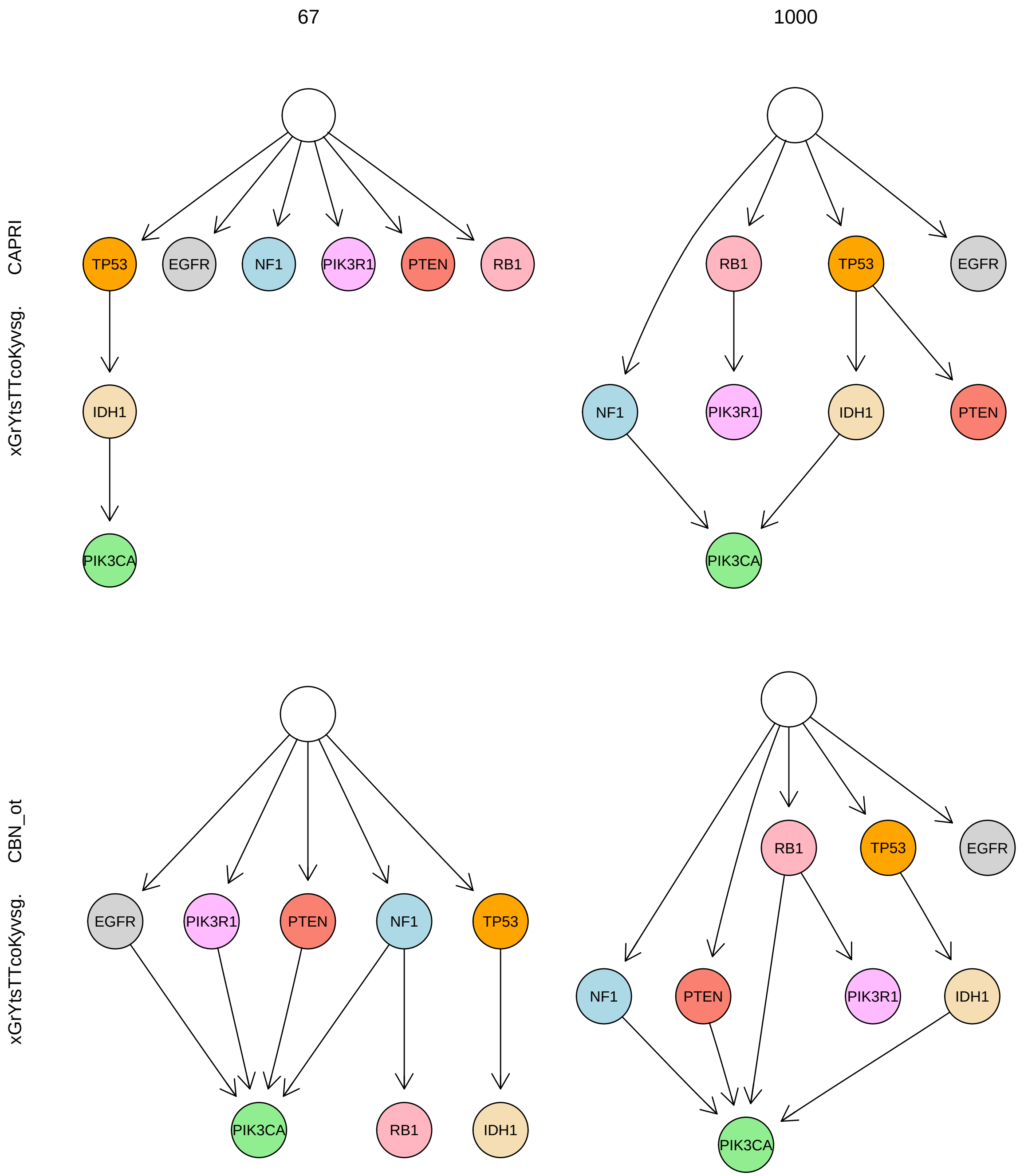

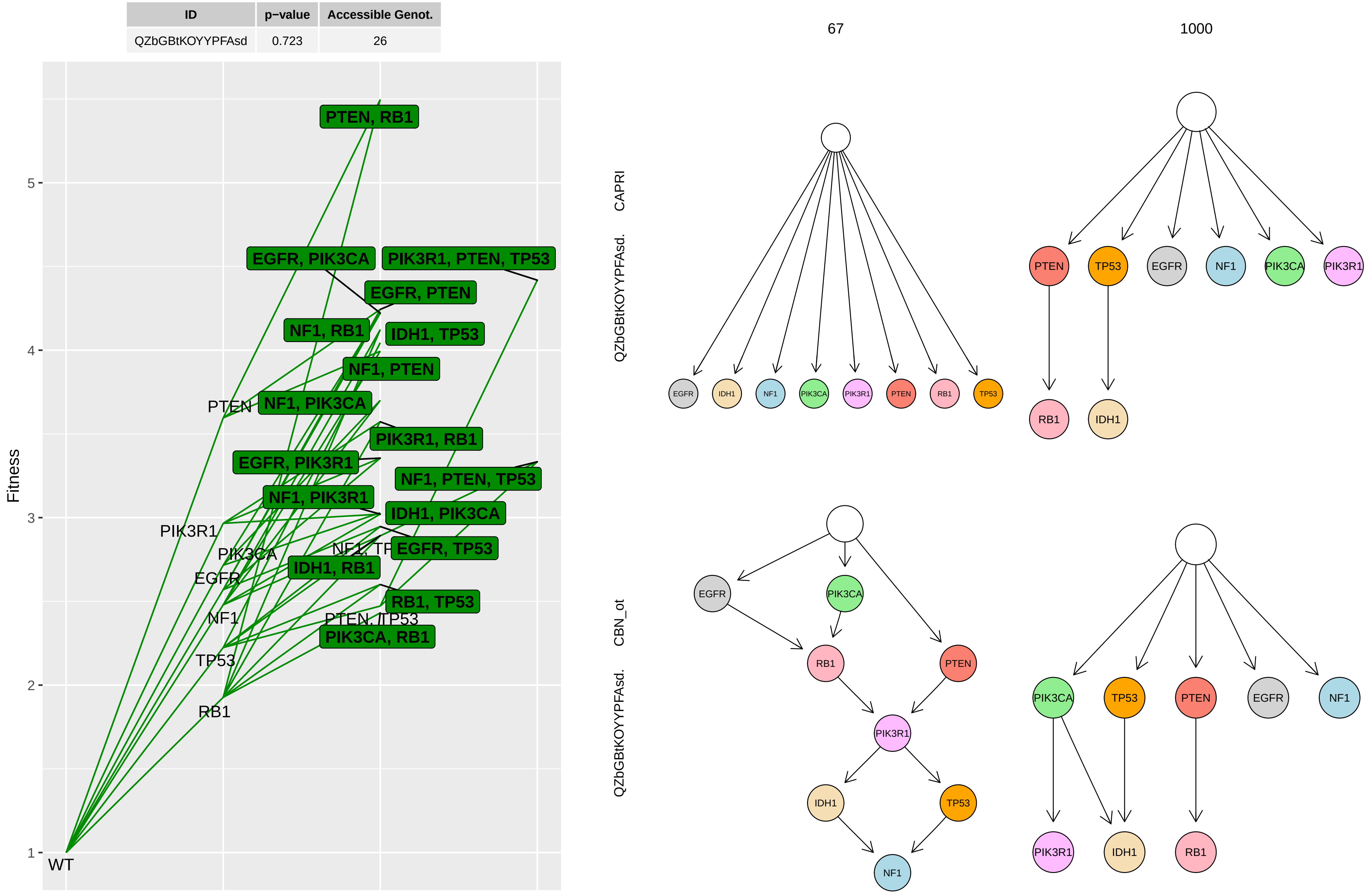

ID
p−value
Accessible Genot.
QZbGBtKOYYPFAsd
0.723
26
Fitness
WT
RB1
TP53
NF1
EGFR
PIK3CA
PIK3R1
PTEN
PTEN, RB1
EGFR, PIK3CA
PIK3R1, PTEN, TP53
EGFR, PTEN
NF1, RB1
IDH1, TP53
NF1, PTEN
NF1, PIK3CA
PIK3R1, RB1
EGFR, PIK3R1
NF1, PTEN, TP53
NF1, PIK3R1
IDH1, PIK3CA
EGFR, TP53
IDH1, RB1
RB1, TP53
PTEN, TP53
PIK3CA, RB1
67
1000
QZbGBtKOYYPFAsd. CAPRI
QZbGBtKOYYPFAsd. CBN_ot
EGFR
IDH1
NF1
PIK3CA
PIK3R1
PTEN
RB1
TP53

ID	p−value	Accessible Genot.
OohlWXgXWEdVOzb	0.727	26

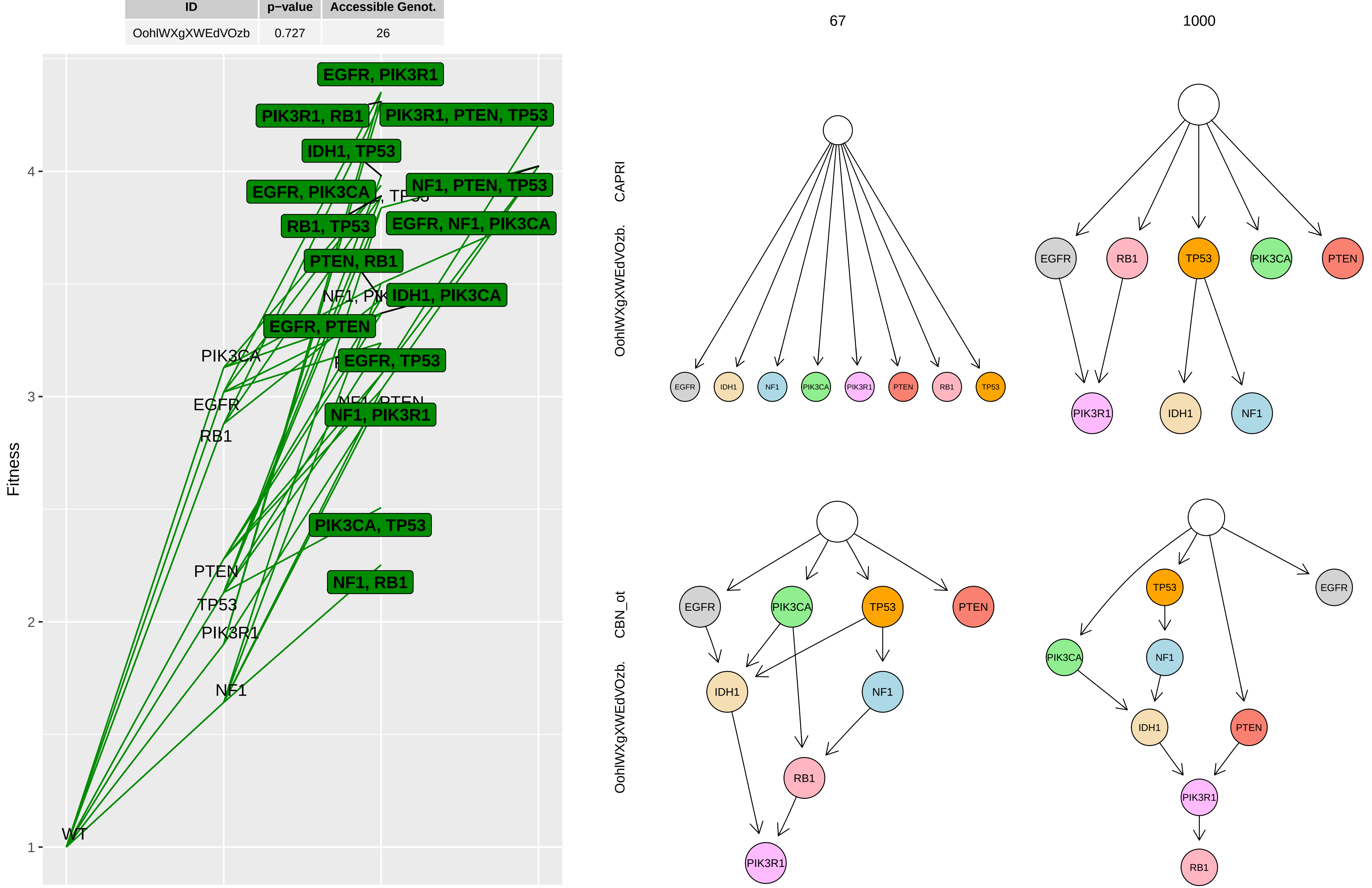

ID	p−value	Accessible Genot.
uzwZmtQDEVBoEUb	0.728	58

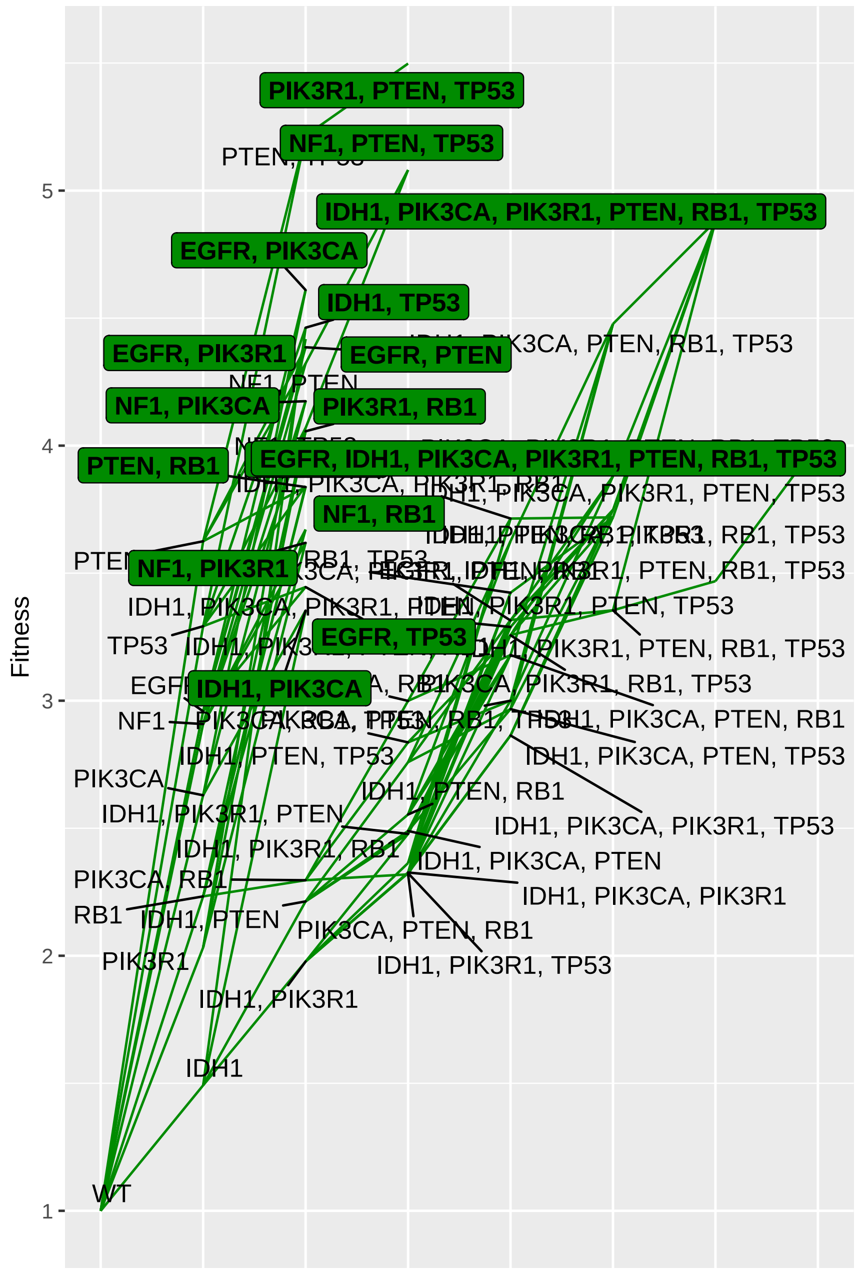

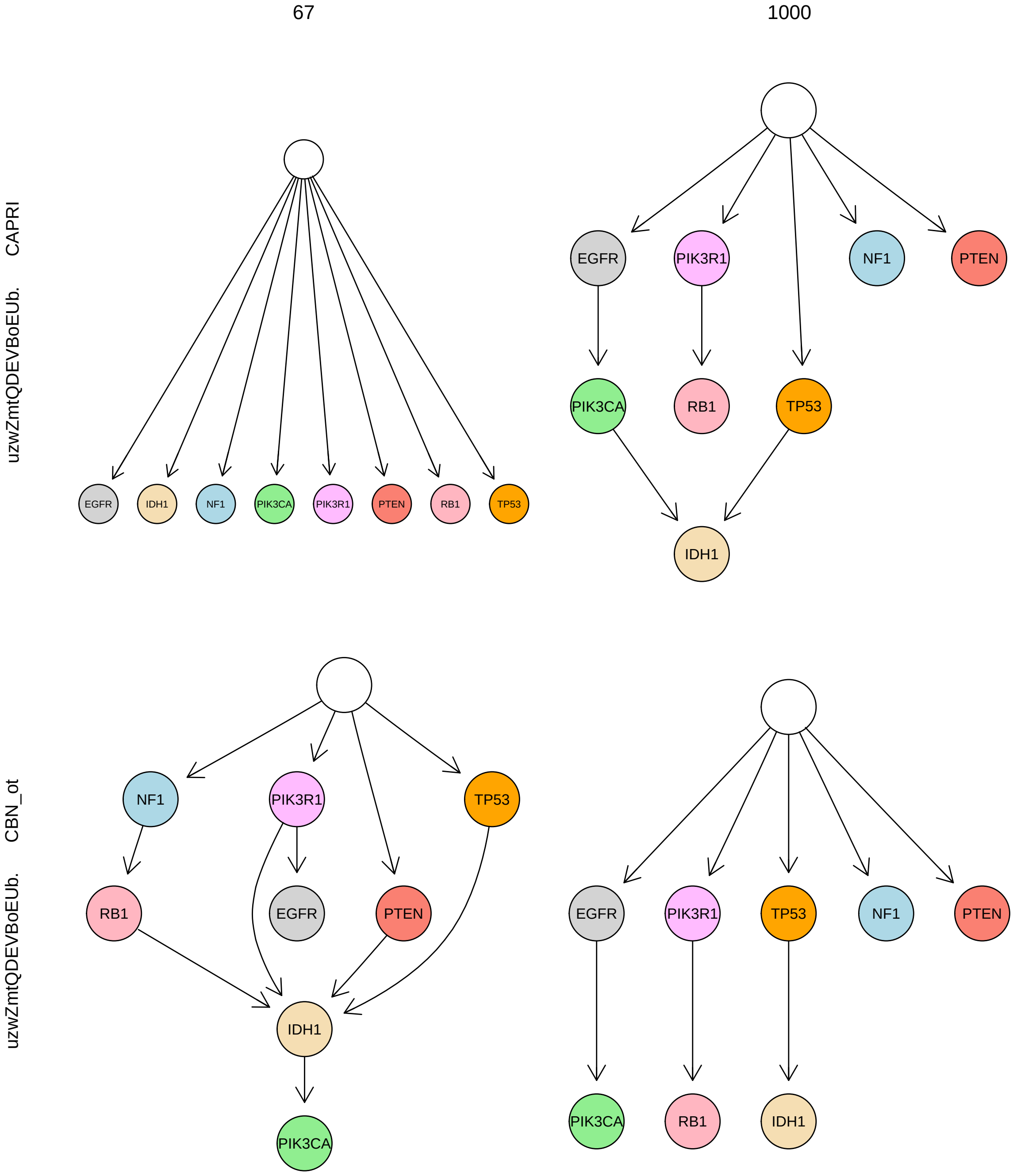

ID	p−value	Accessible Genot.
WxejMcFroHpyWfd	0.731	247

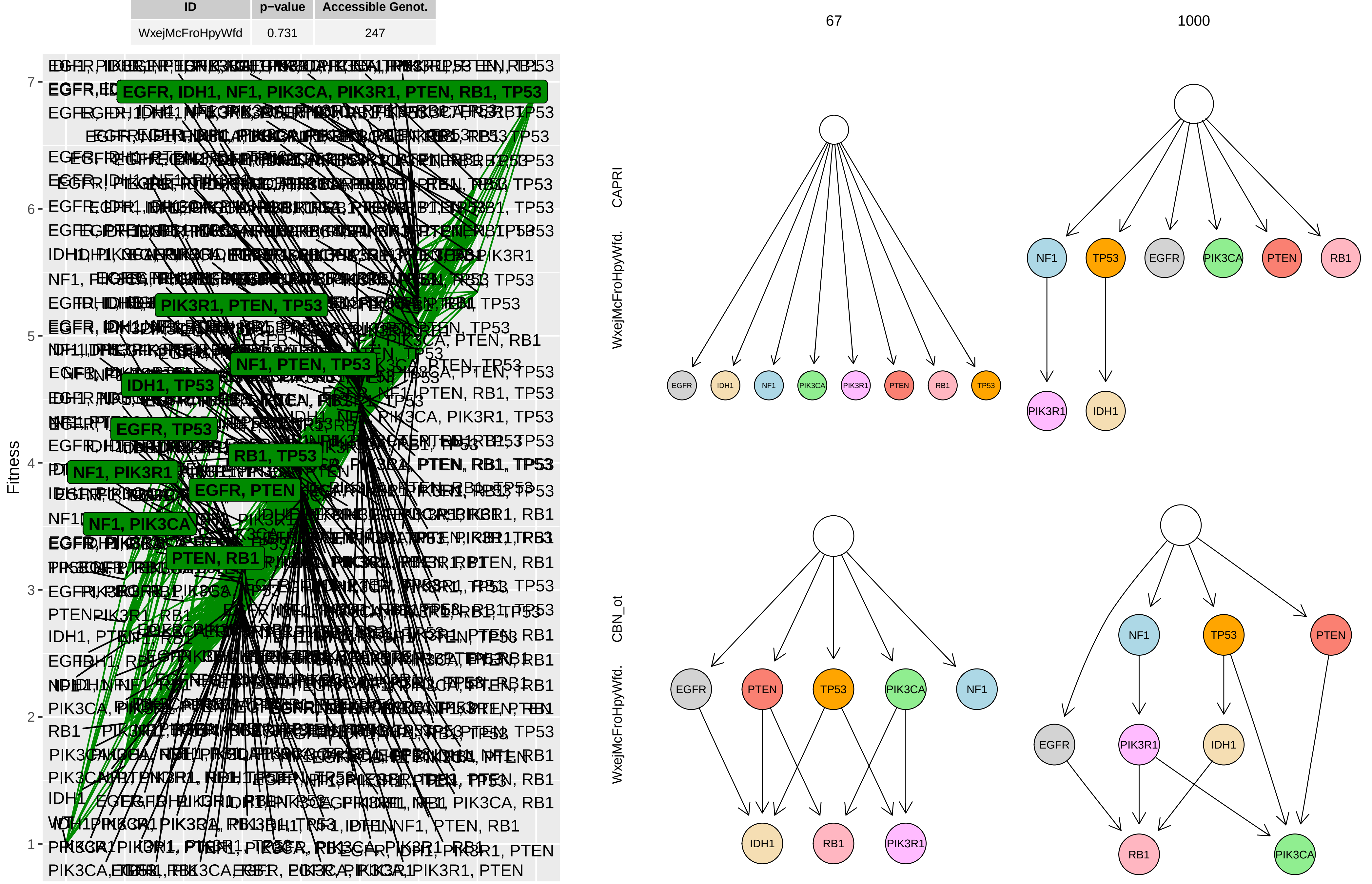

ID	p-value	Accessible Genot.
GxAcMcNEYaqBsxM	0.732	26

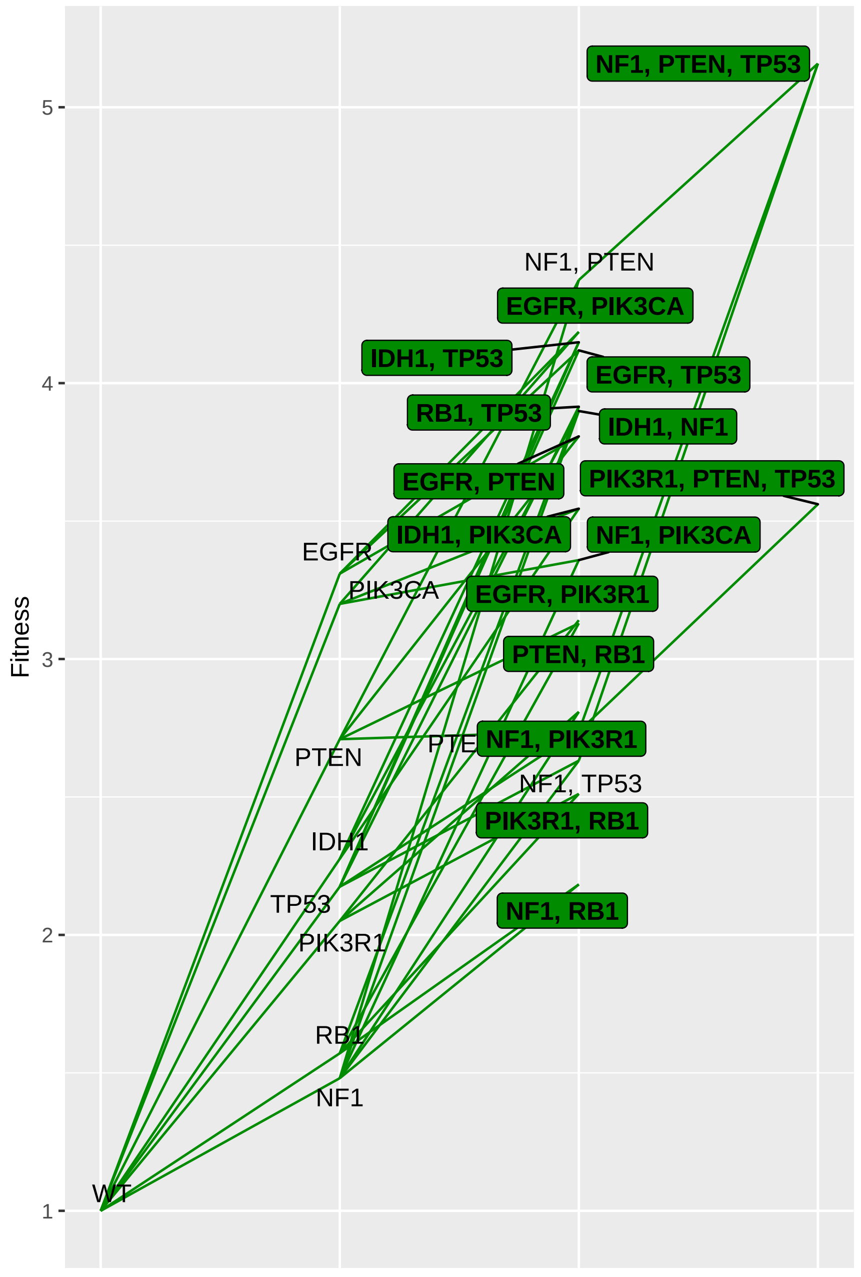

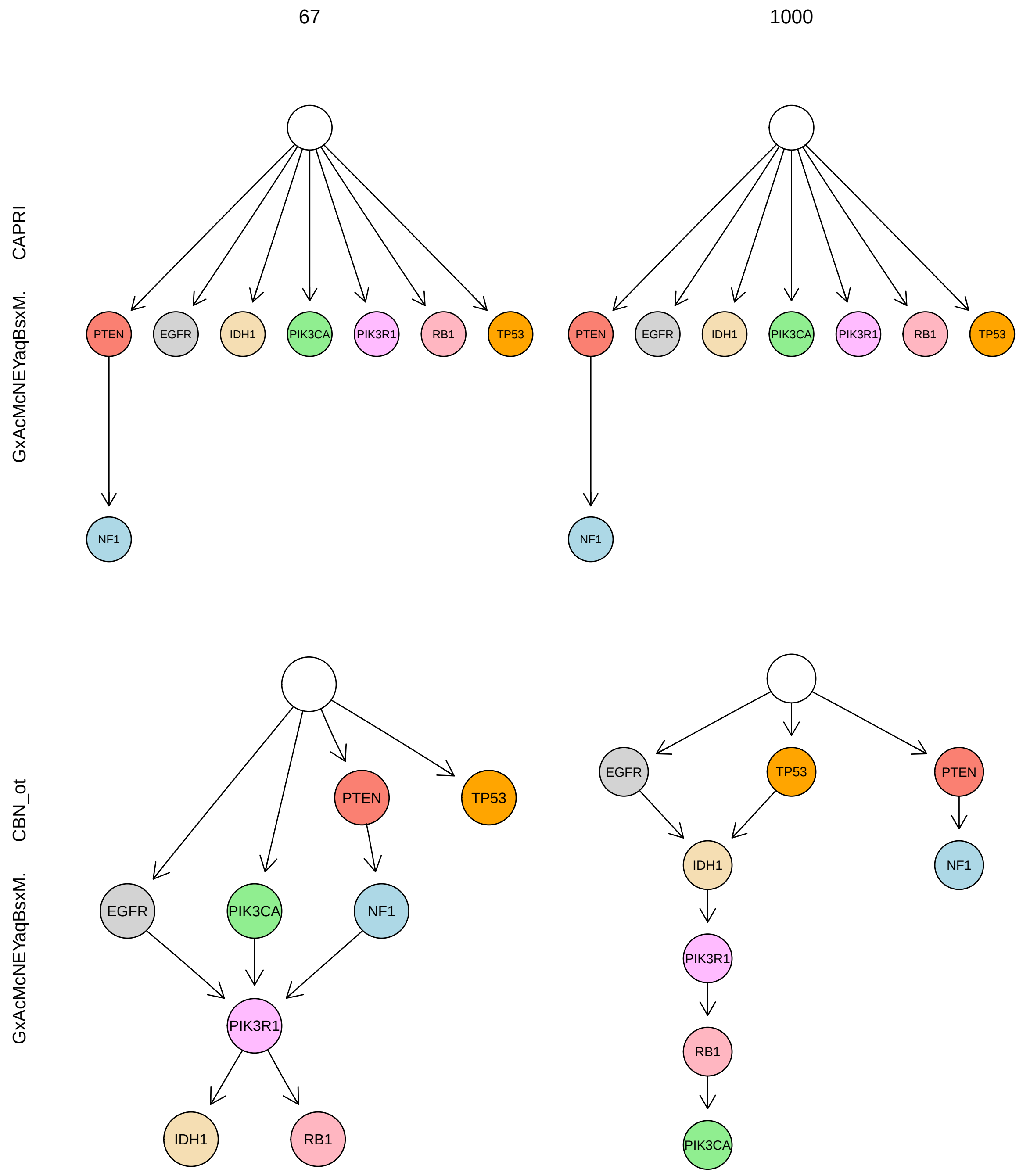

ID	p−value	Accessible Genot.
tiFTzXamOJTKnHG	0.735	26

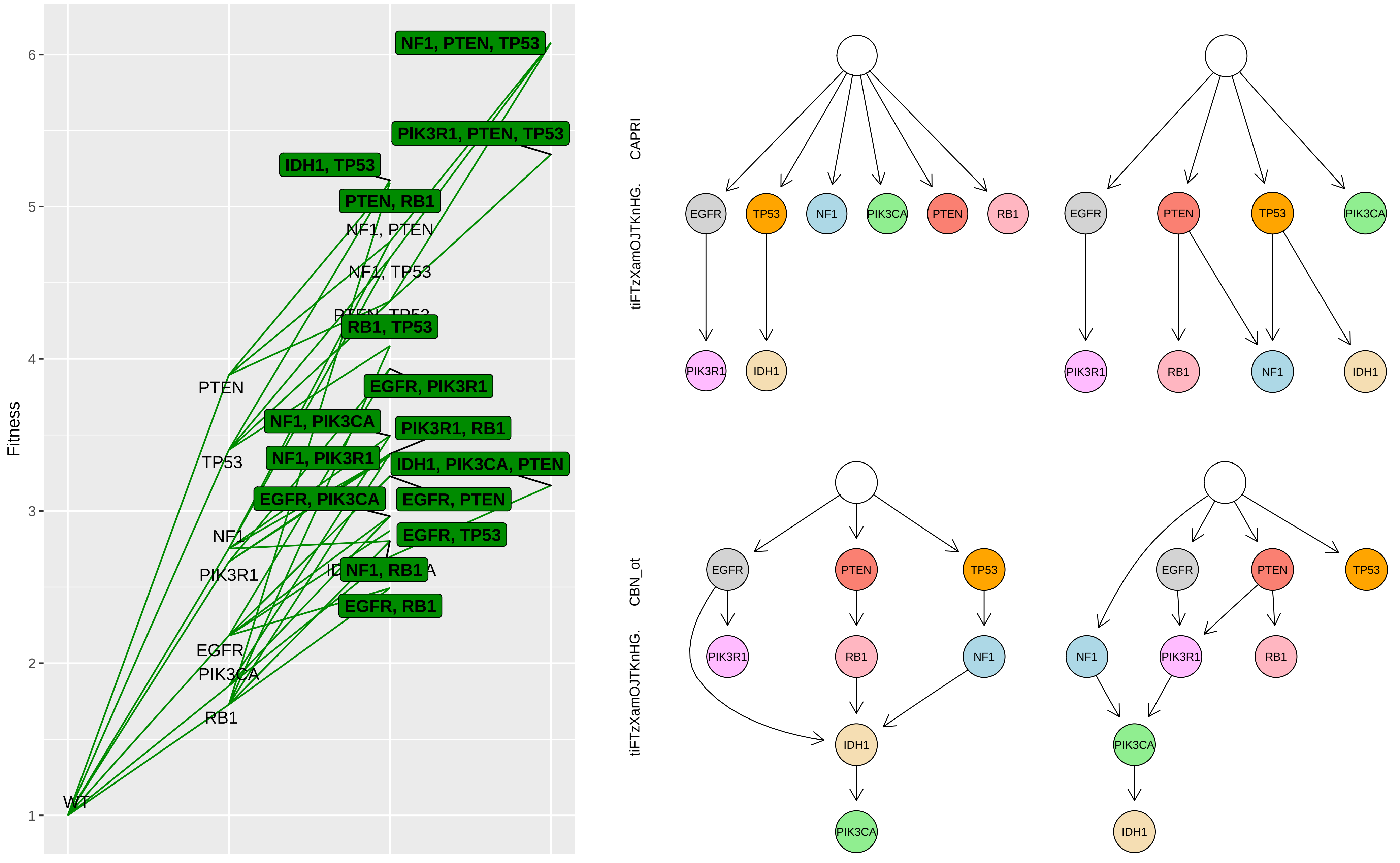

ID	p−value	Accessible Genot.
qdlyHYQCNrPmTQi	0.741	248

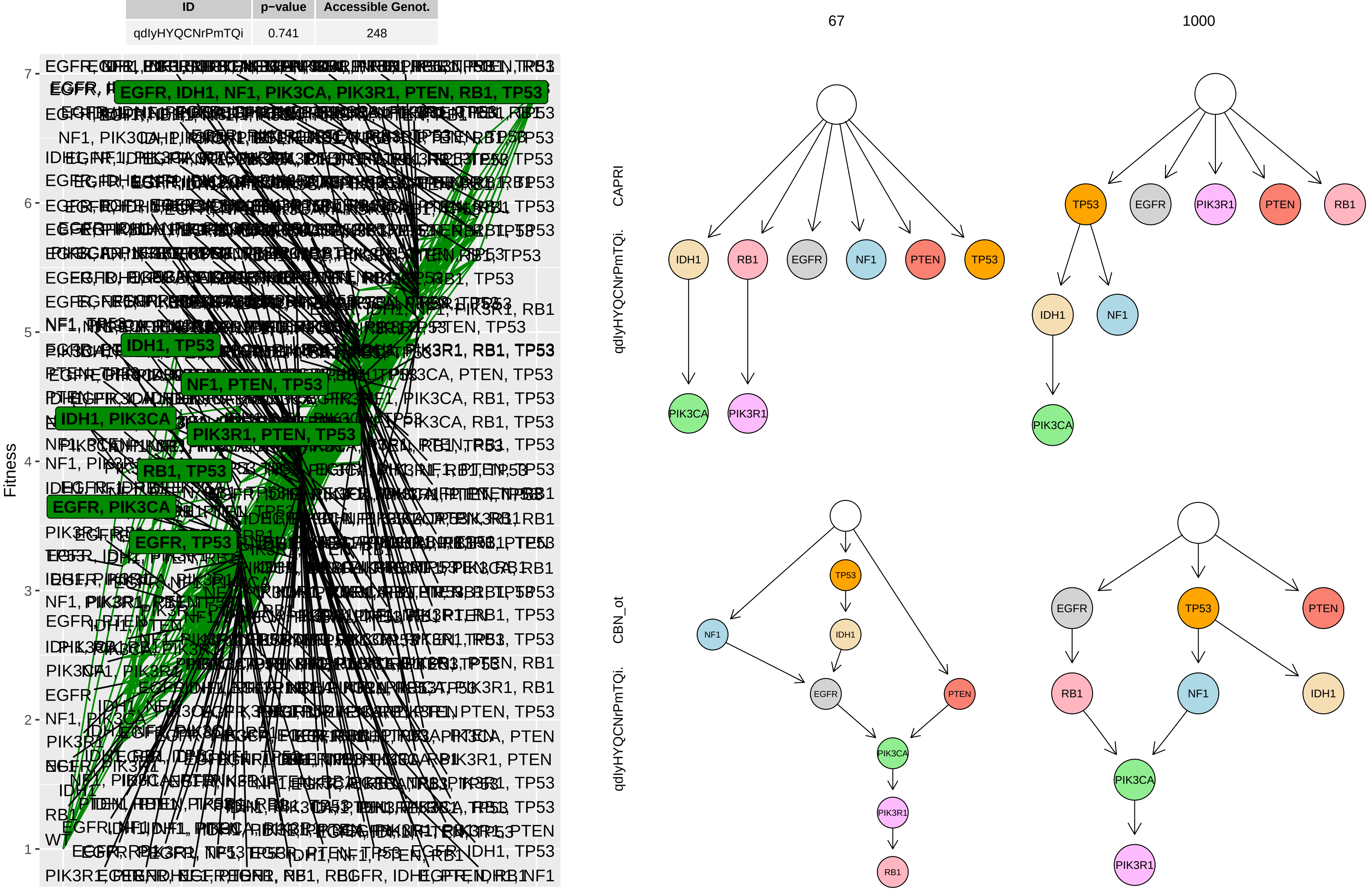

ID	p-value	Accessible Genot.
wgKJeKBXJPlFgNp	0.744	51

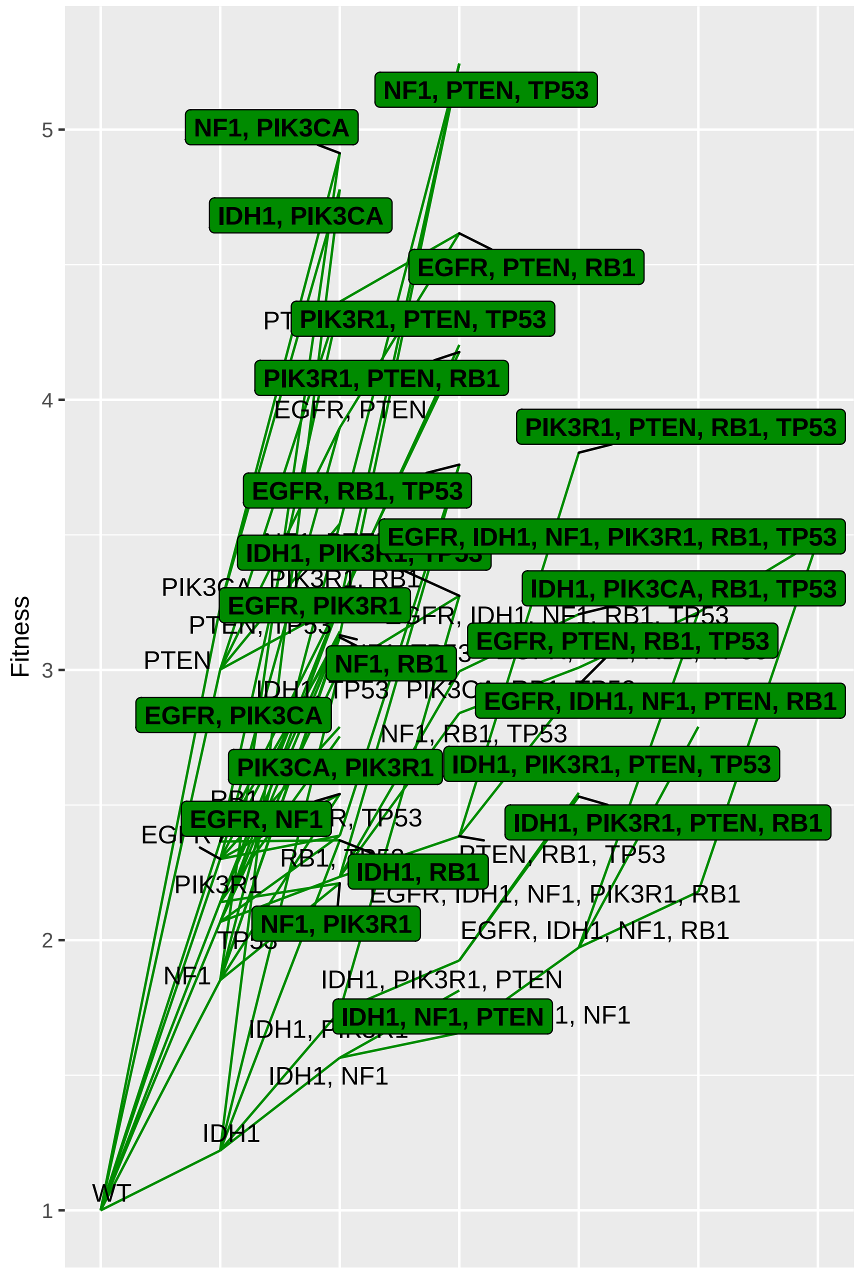

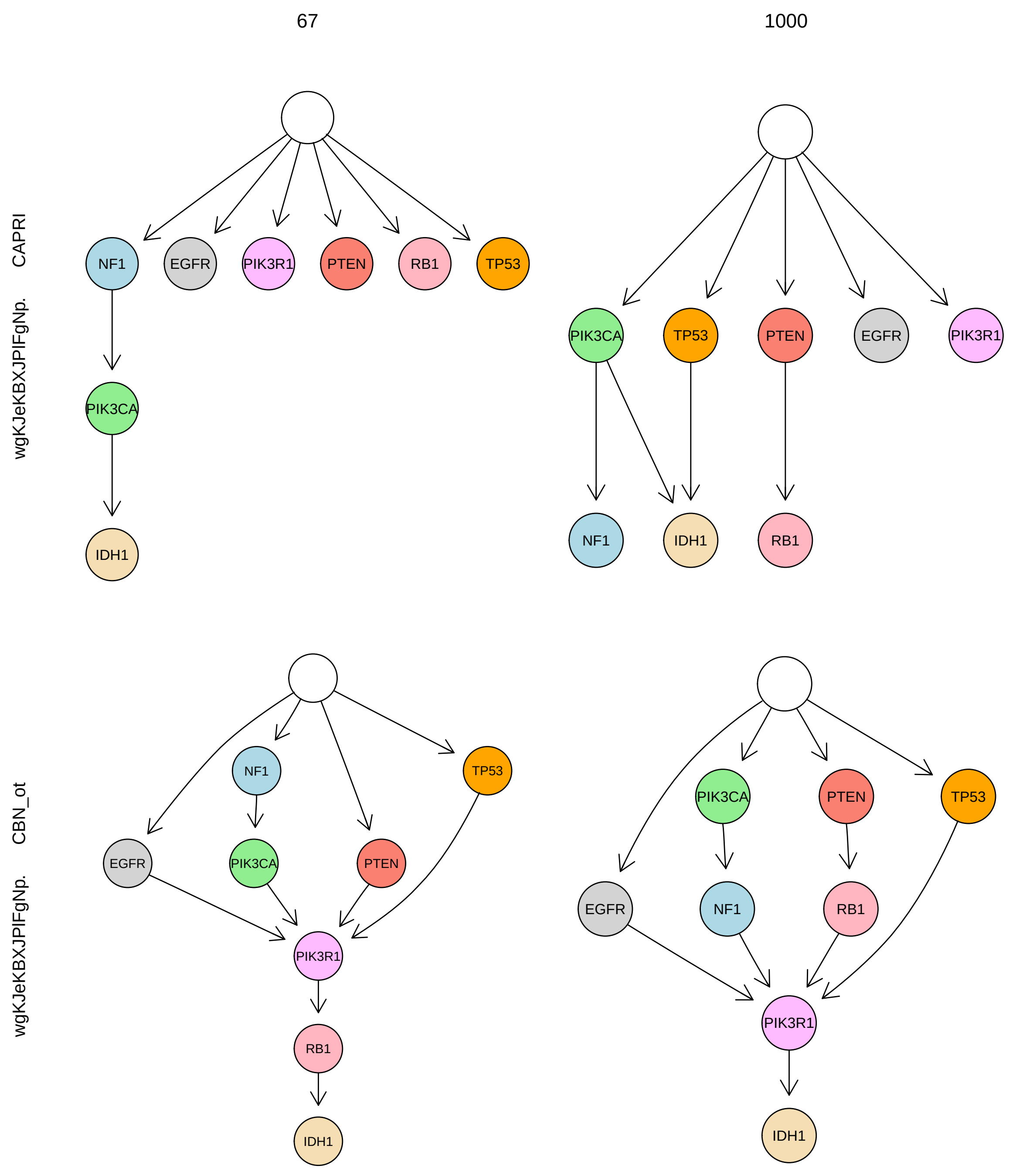

ID	p−value	Accessible Genot.
ffupZopzKWnjxWJ	0.745	113

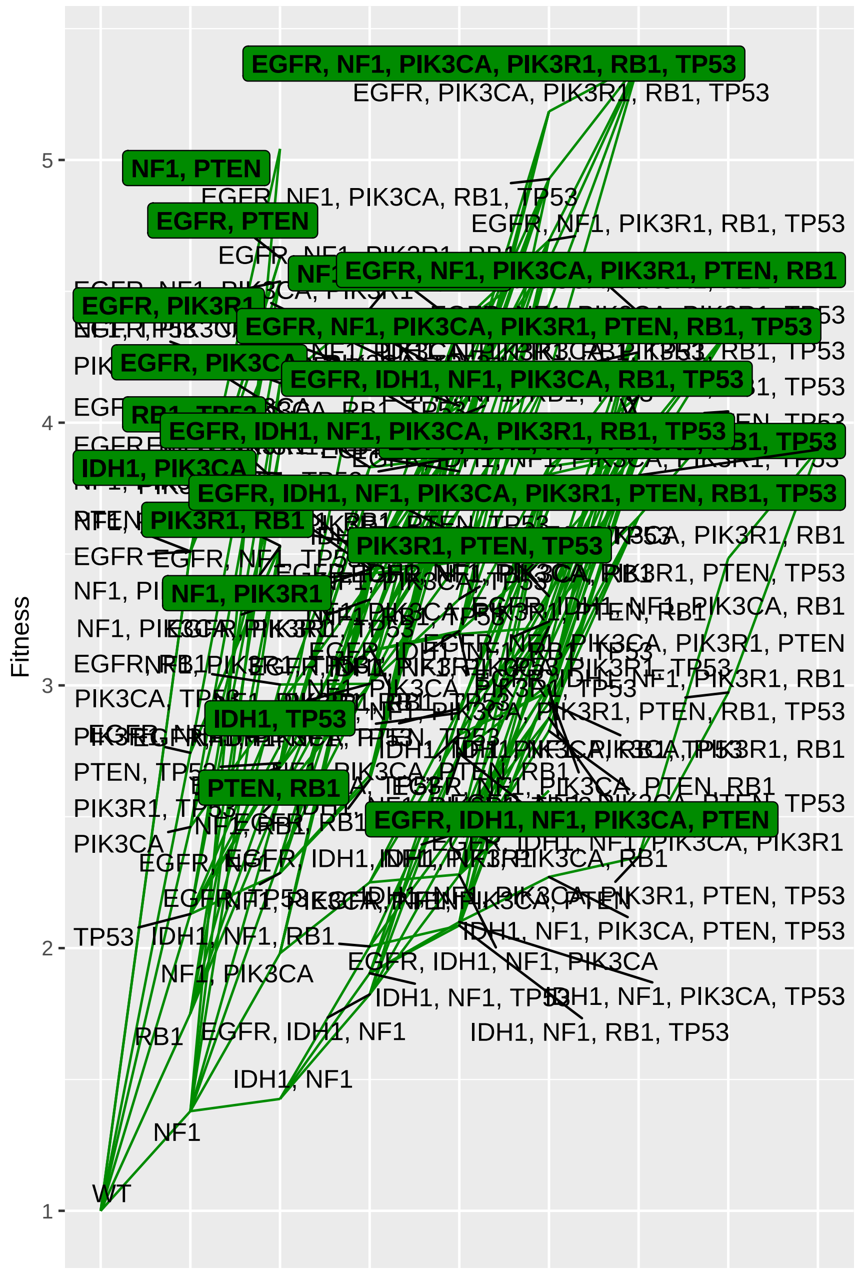

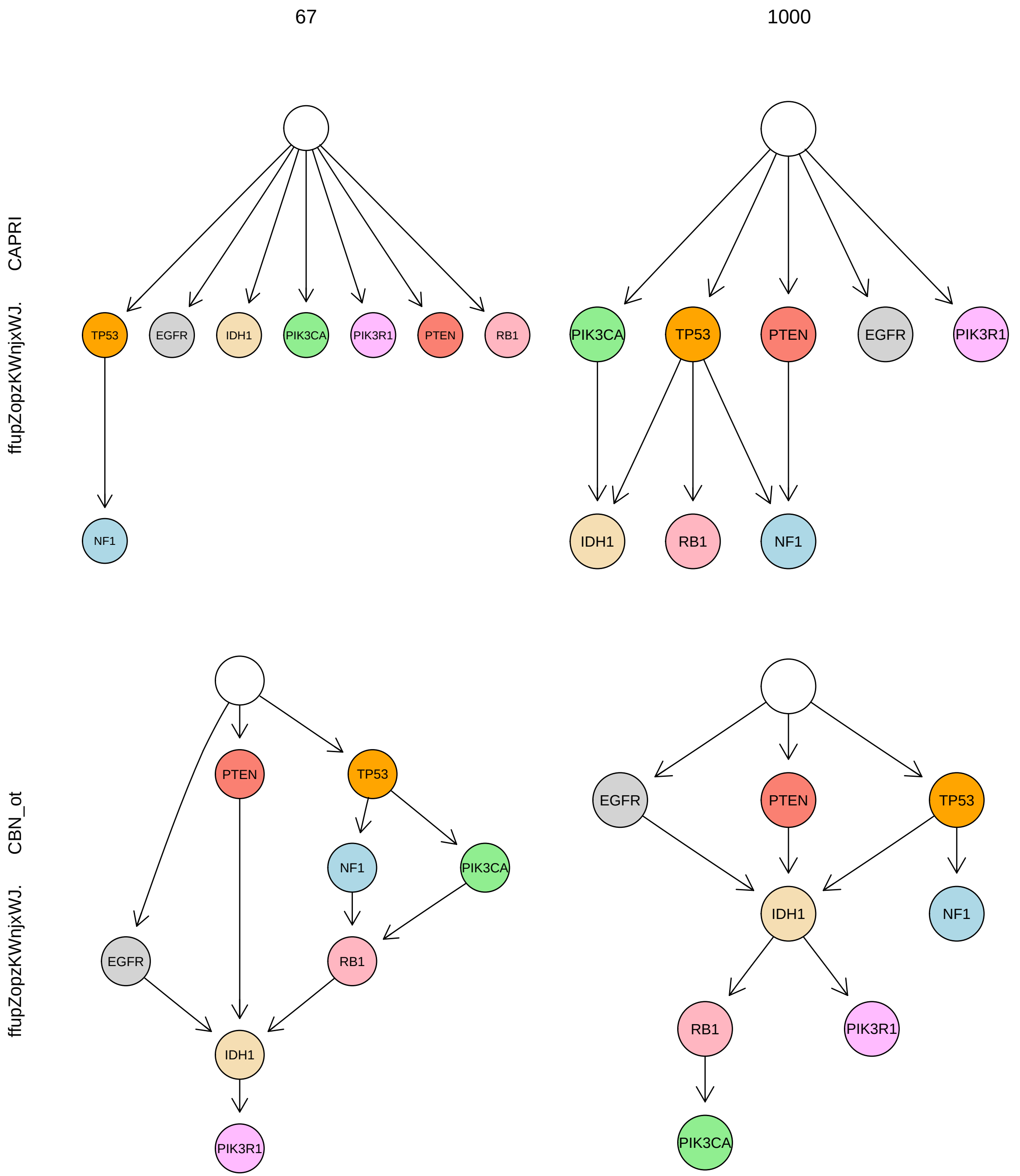

ID	p−value	Accessible Genot.
evnblqklaVgQllR	0.747	31

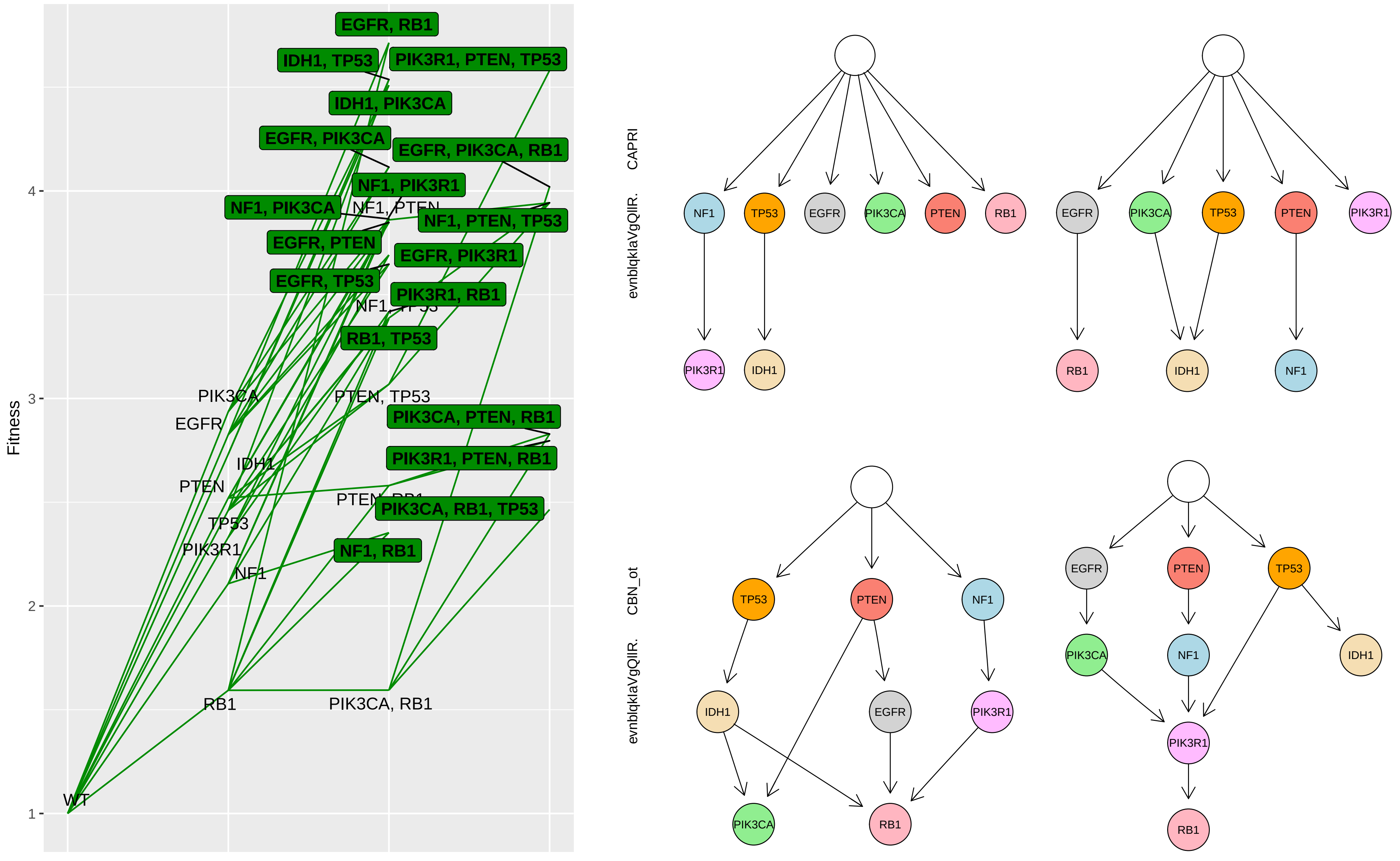

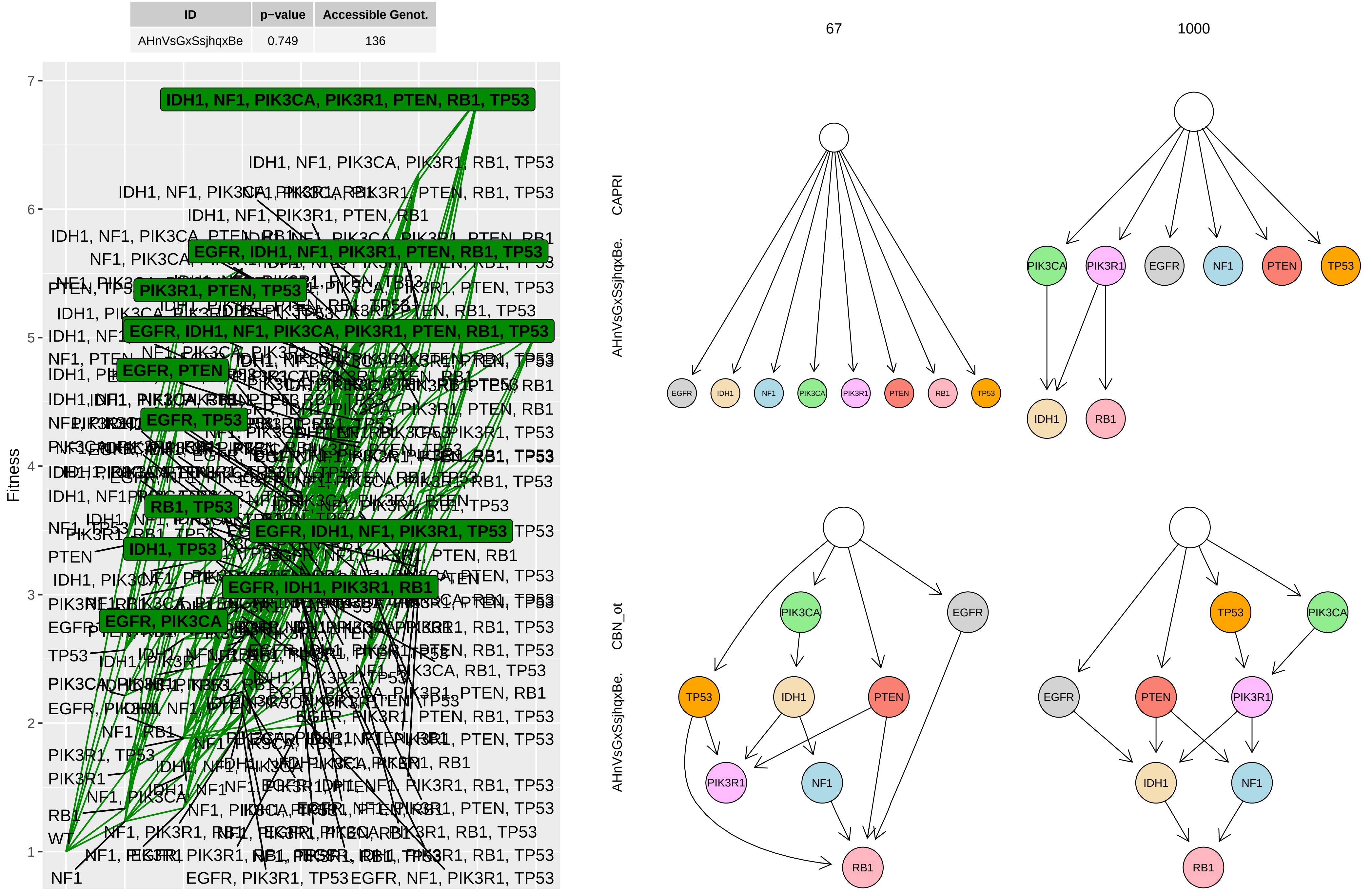

ID
p−value
Accessible Genot.
AHnVsGxSsjhqxBe
0.749
136
Fitness
IDH1, NF1, PIK3CA, PIK3R1, PTEN, RB1, TP53
IDH1, NF1, PIK3CA, PIK3R1, RB1, TP53
IDH1, NF1, PIK3R1, PTEN, RB1
EGFR, IDH1, NF1, PIK3R1, PTEN, RB1, TP53
PIK3R1, PTEN, TP53
EGFR, IDH1, NF1, PIK3CA, PIK3R1, PTEN, RB1, TP53
EGFR, PTEN
EGFR, TP53
RB1, TP53
EGFR, IDH1, NF1, PIK3R1, TP53
IDH1, TP53
EGFR, IDH1, PIK3R1, RB1
EGFR, PIK3CA
PTEN
TP53
PIK3R1
RB1
WT
NF1
EGFR, PIK3R1, TP53
EGFR, NF1, PIK3R1, TP53
67
1000
CAPRI
CBN_ot
AHnVsGxSsjhqxBe.
EGFR
IDH1
NF1
PIK3CA
PIK3R1
PTEN
RB1
TP53

ID	p−value	Accessible Genot.
FxOJhxDsNyKtstL	0.755	27

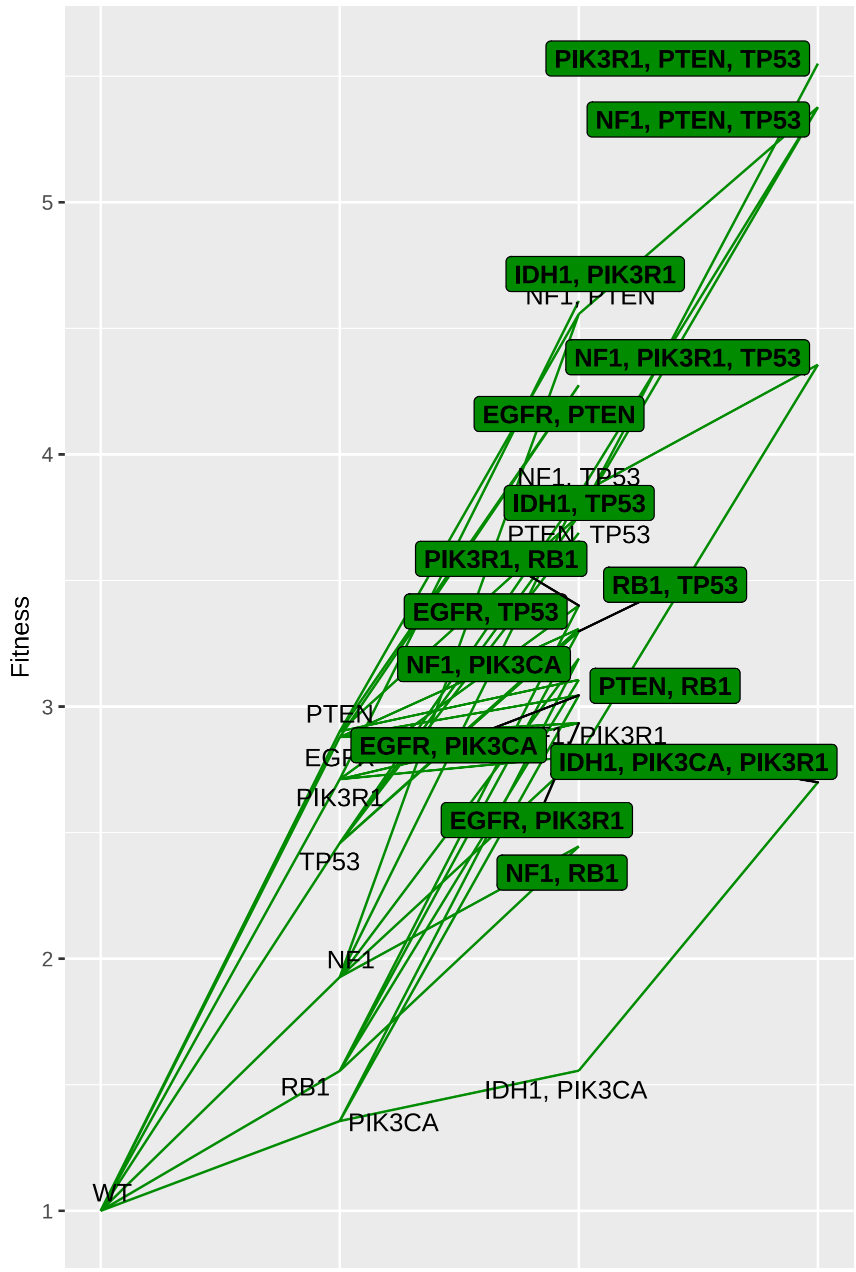

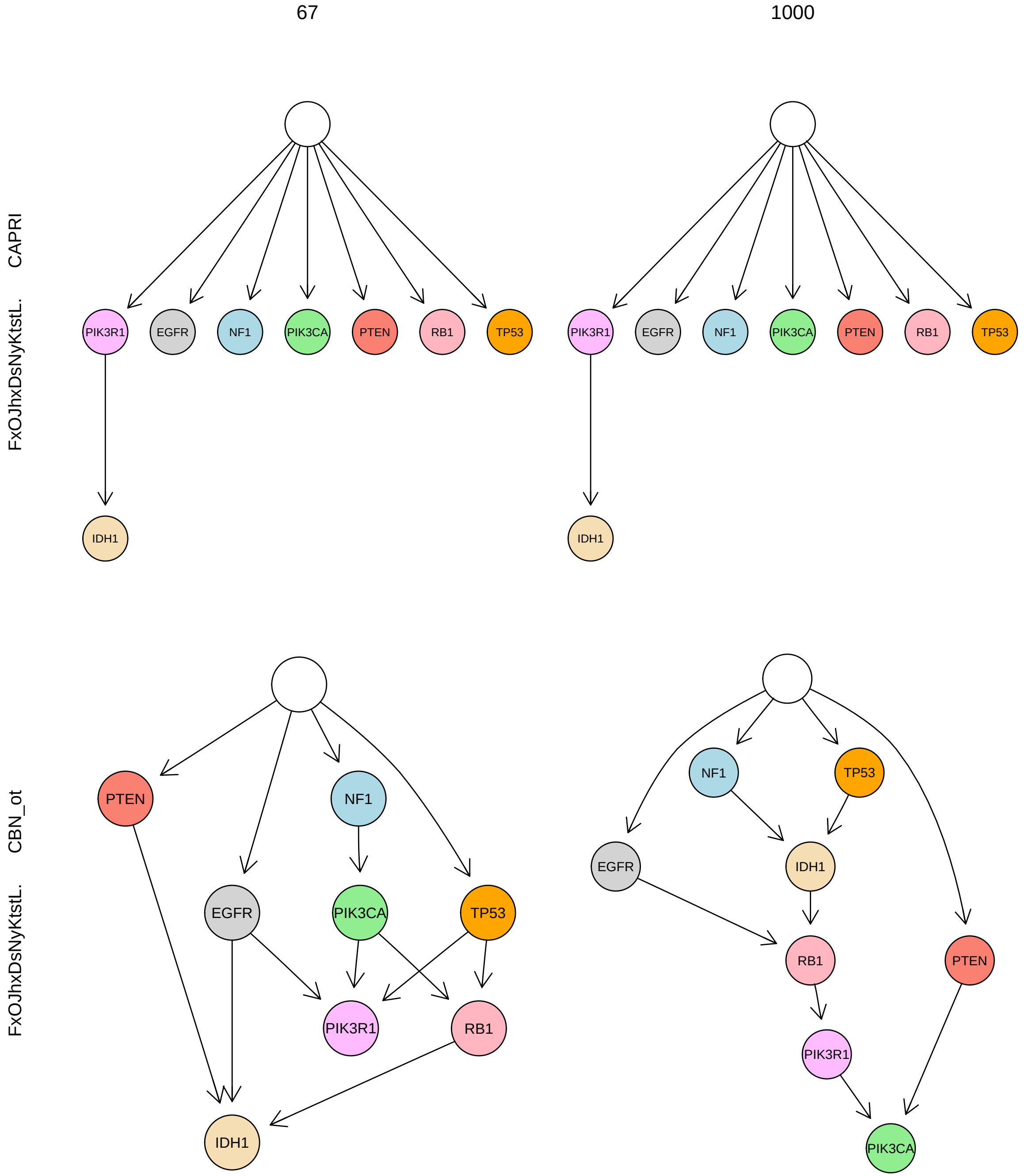

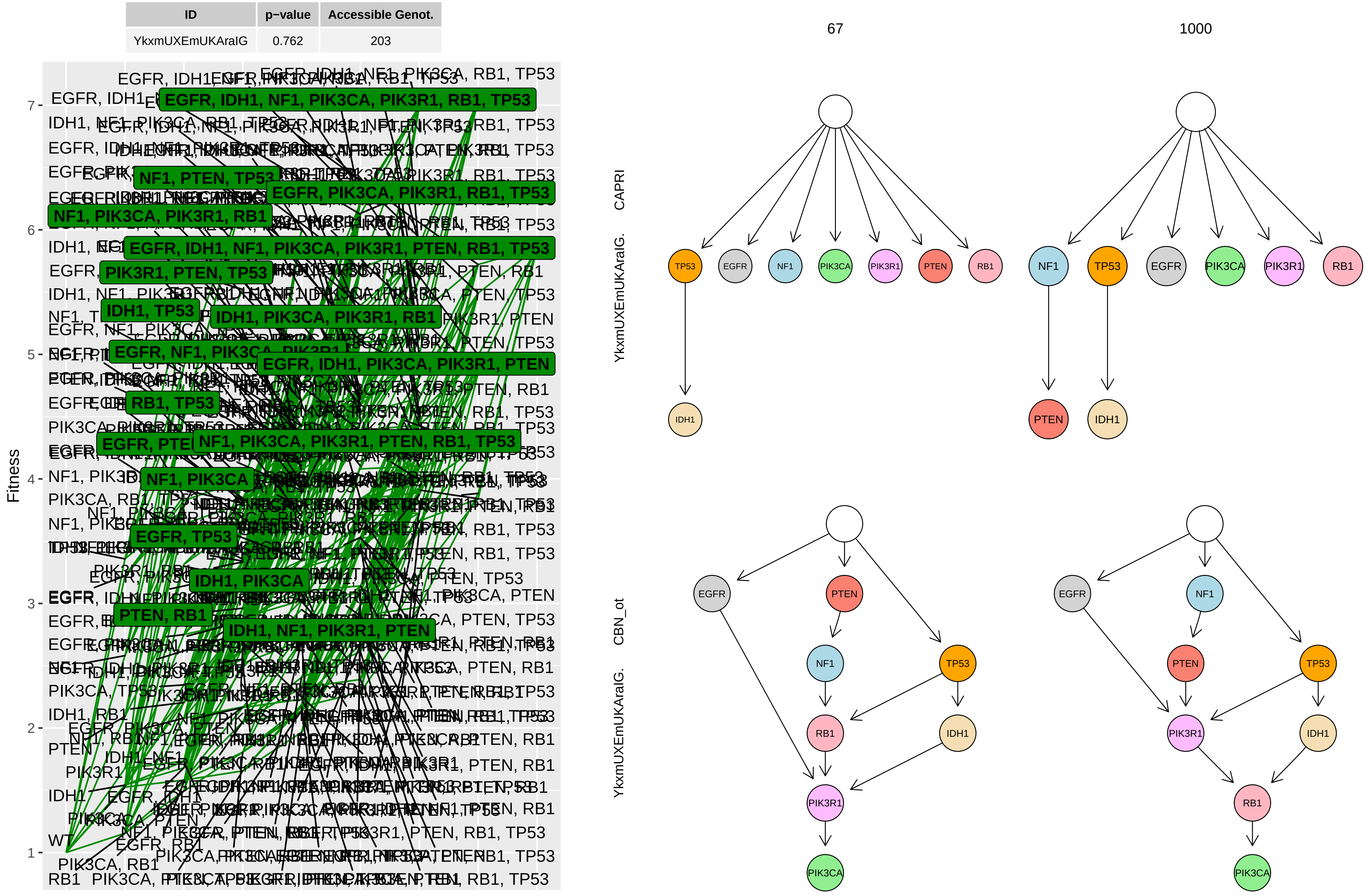

ID	p−value	Accessible Genot.
YkxmUXEmUKAraIG	0.762	203

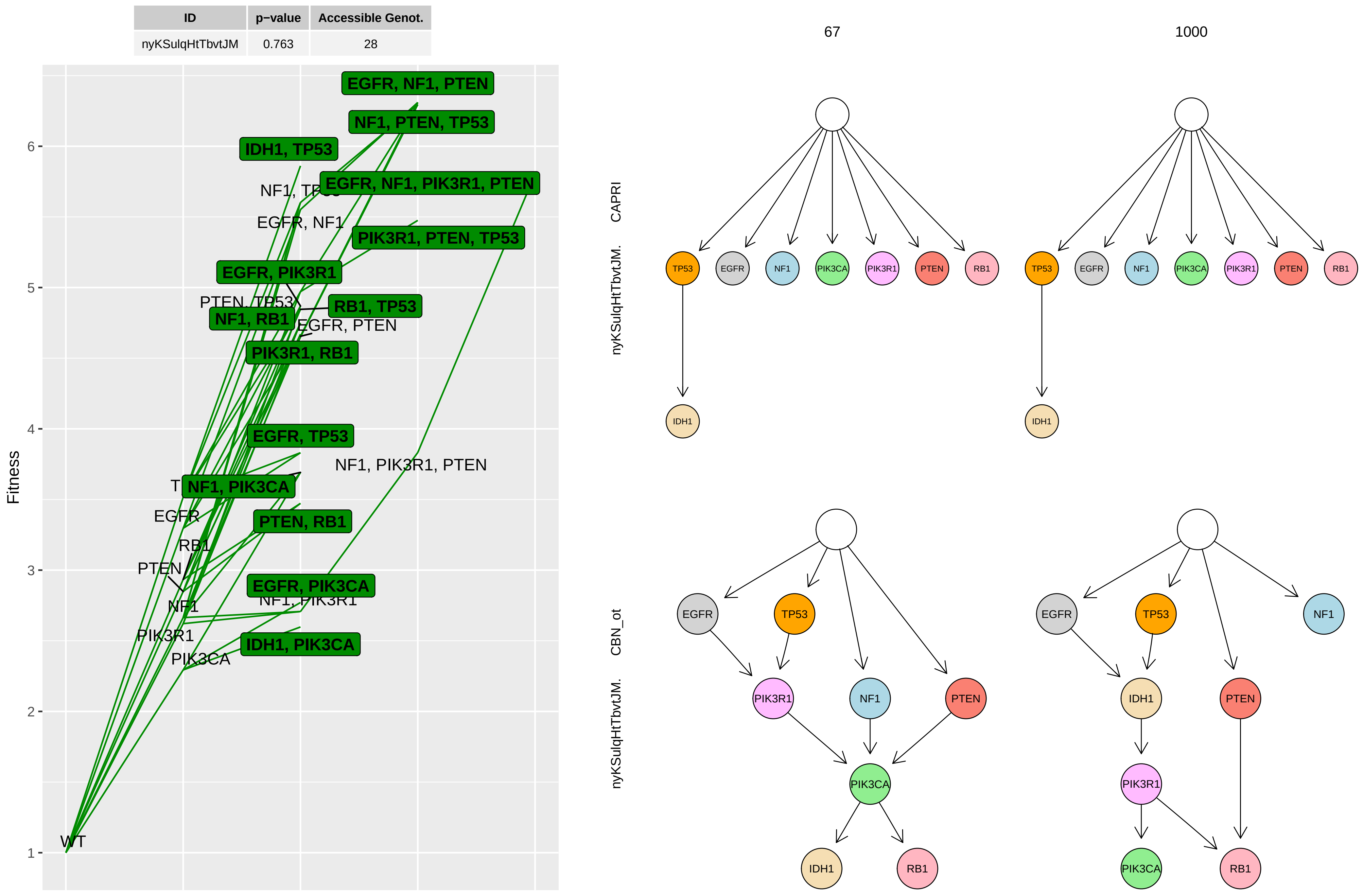

ID
p−value
Accessible Genot.
nyKSulqHtTbvtJM
0.763
28
Fitness
WT
PIK3CA
PIK3R1
NF1
PTEN
RB1
EGFR
IDH1, PIK3CA
EGFR, PIK3CA
NF1, PIK3R1
PTEN, RB1
NF1, PIK3CA
NF1, PIK3R1, PTEN
EGFR, TP53
PIK3R1, RB1
NF1, RB1
EGFR, PTEN
RB1, TP53
PTEN, TP53
EGFR, PIK3R1
PIK3R1, PTEN, TP53
EGFR, NF1
EGFR, NF1, PIK3R1, PTEN
IDH1, TP53
NF1, PTEN, TP53
EGFR, NF1, PTEN
67
1000
CAPRI
nyKSulqHtTbvtJM.
CBN_ot
nyKSulqHtTbvtJM.
TP53
EGFR
NF1
PIK3CA
PIK3R1
PTEN
RB1
IDH1

ID	p−value	Accessible Genot.
dTPLlaGqCIzandp	0.764	24

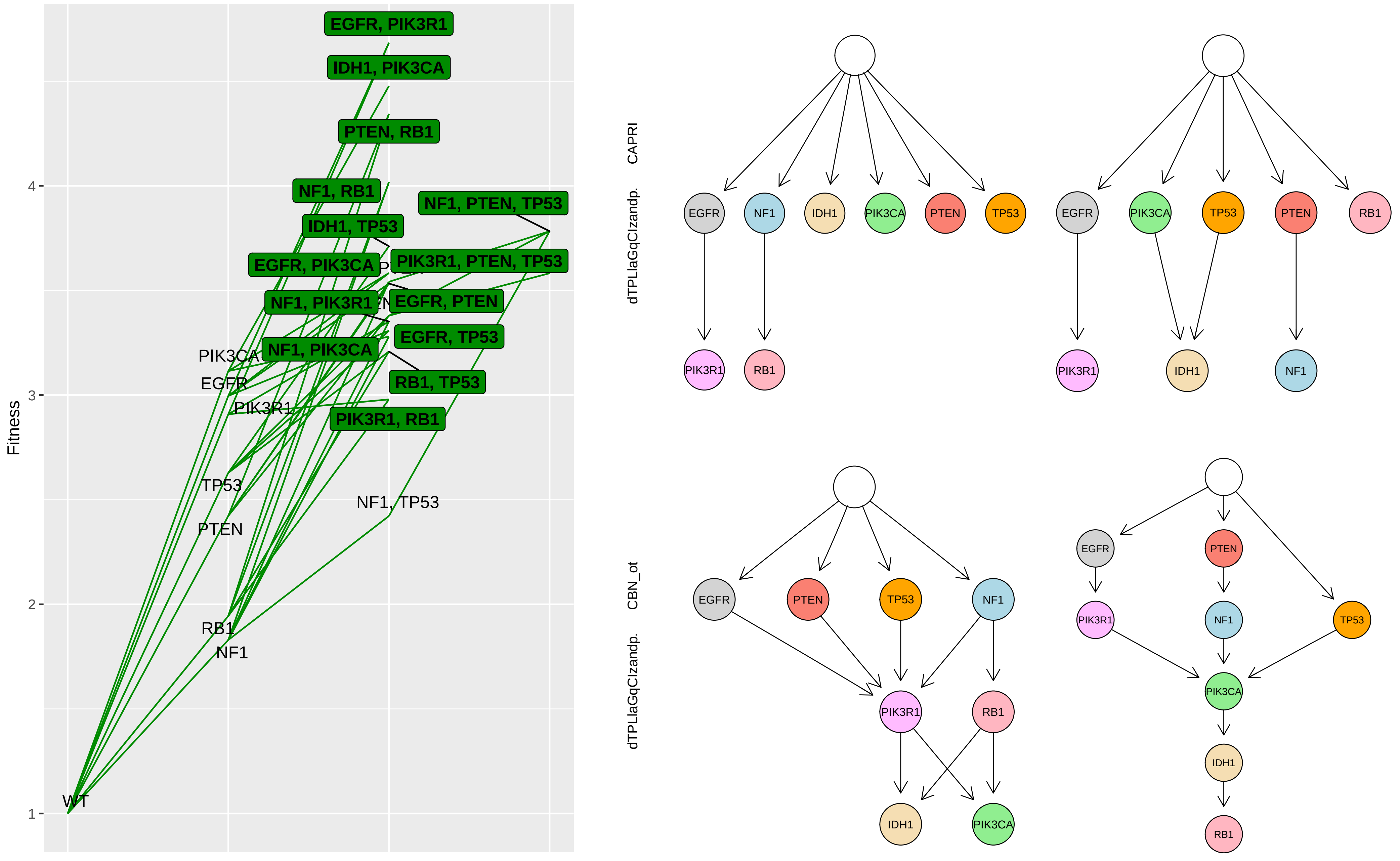

ID	p−value	Accessible Genot.
aSwpleAdiGDFMly	0.766	40

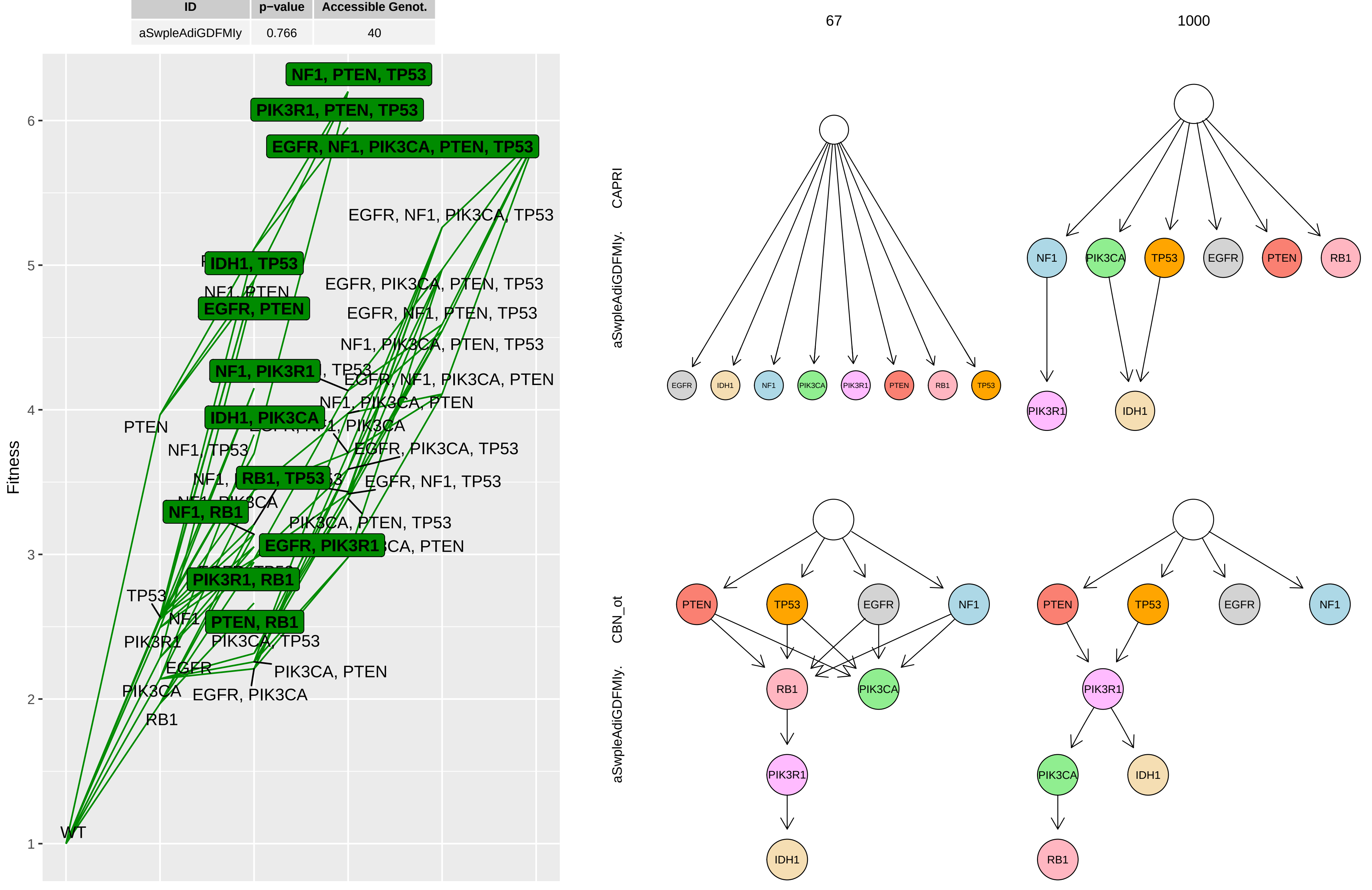

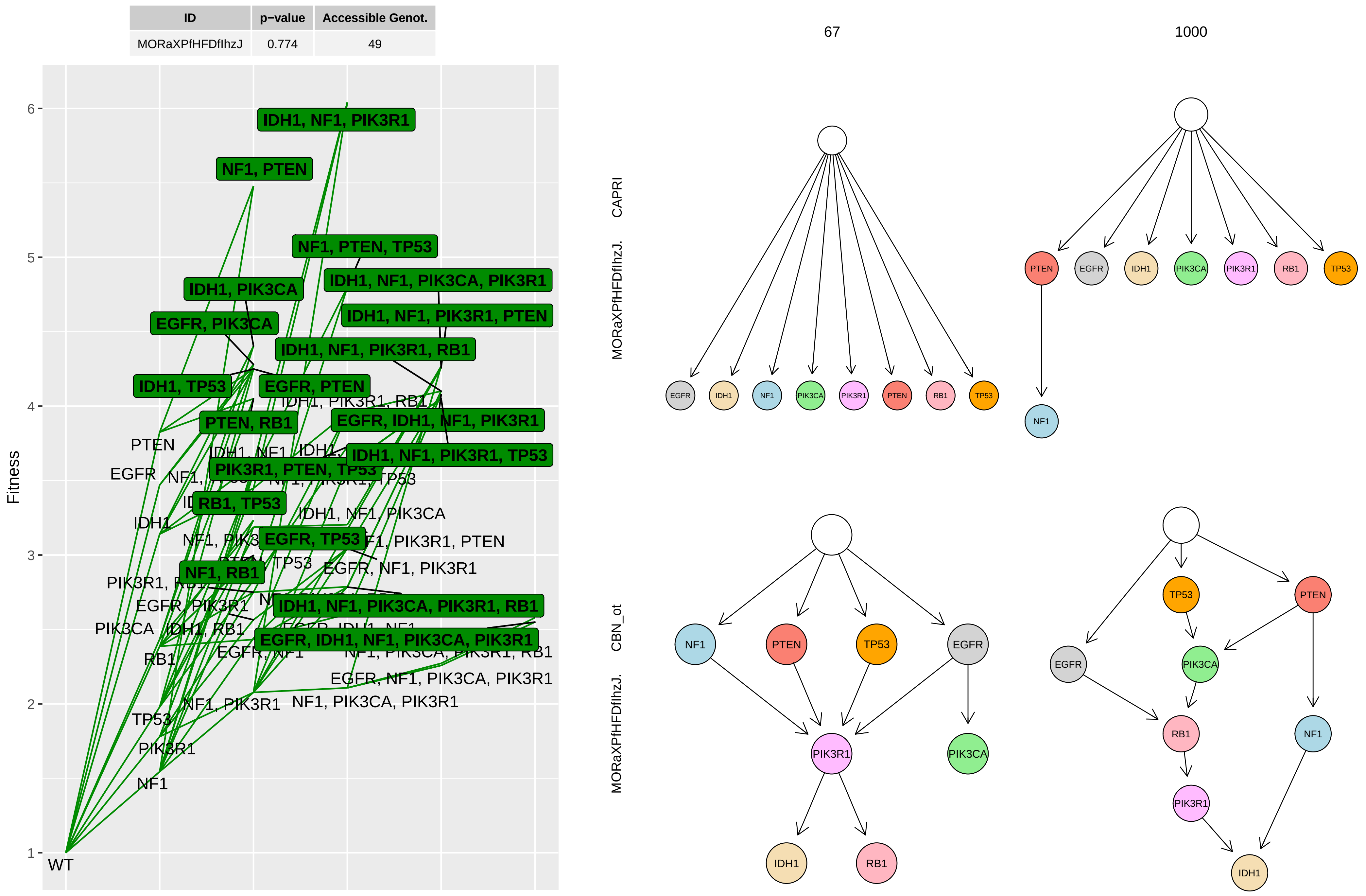

ID
p−value
Accessible Genot.
MORaXPfHFDflhzJ
0.774
49
Fitness
WT
IDH1, NF1, PIK3R1
NF1, PTEN
NF1, PTEN, TP53
IDH1, PIK3CA
IDH1, NF1, PIK3CA, PIK3R1
EGFR, PIK3CA
IDH1, NF1, PIK3R1, PTEN
IDH1, NF1, PIK3R1, RB1
IDH1, TP53
EGFR, PTEN
EGFR, IDH1, NF1, PIK3R1
PTEN, RB1
IDH1, NF1, PIK3R1, TP53
PIK3R1, PTEN, TP53
RB1, TP53
EGFR, TP53
NF1, RB1
IDH1, NF1, PIK3CA, PIK3R1, RB1
EGFR, IDH1, NF1, PIK3CA, PIK3R1
PTEN
EGFR
IDH1
PIK3CA
RB1
TP53
PIK3R1
NF1
IDH1, NF1, PIK3CA
EGFR, NF1, PIK3R1
EGFR, PIK3R1
IDH1, RB1
EGFR, NF1
EGFR, NF1, PIK3CA, PIK3R1
NF1, PIK3R1
NF1, PIK3CA, PIK3R1
67
1000
CAPRI
CBN_ot
MORaXPfHFDflhzJ.
EGFR
IDH1
NF1
PIK3CA
PIK3R1
PTEN
RB1
TP53

ID	p−value	Accessible Genot.
TSXdyDhabLfoTmp	0.776	43

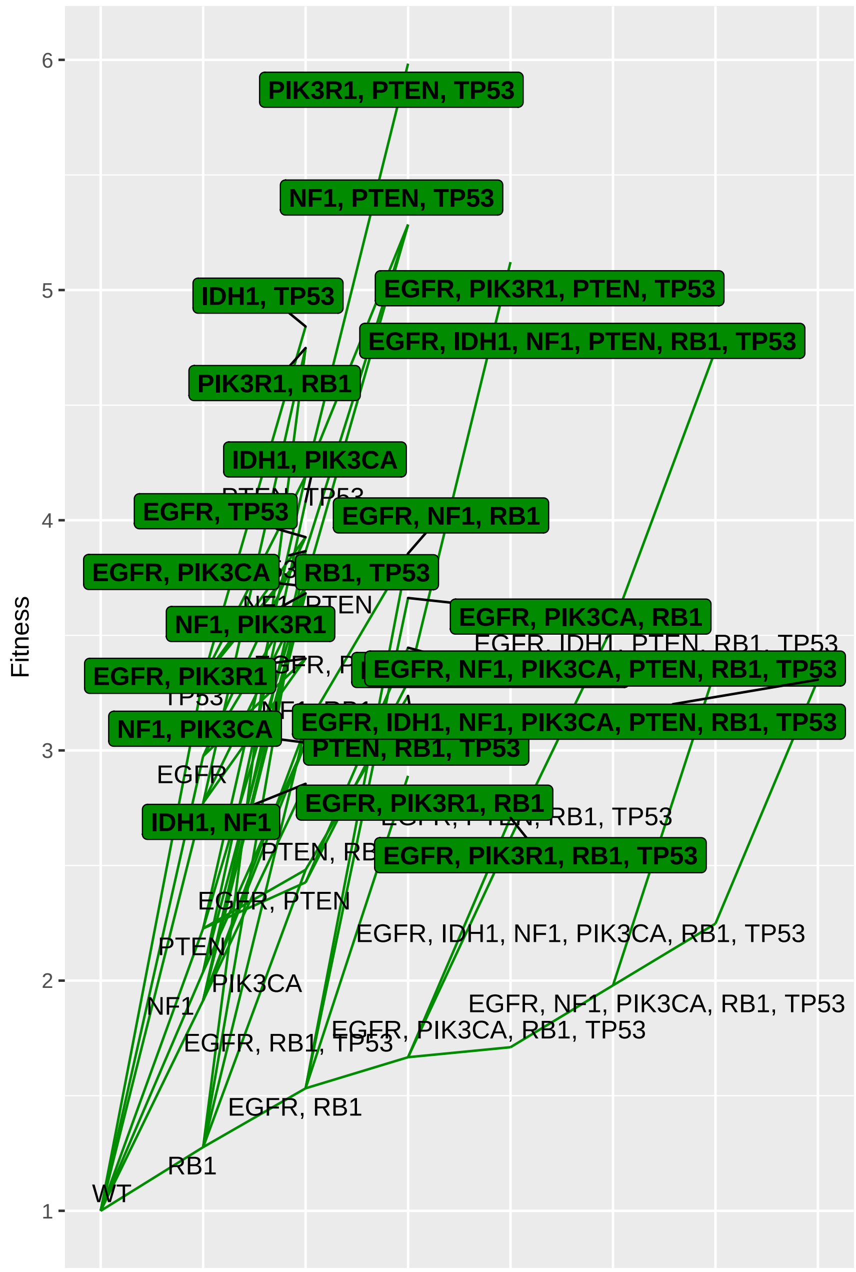

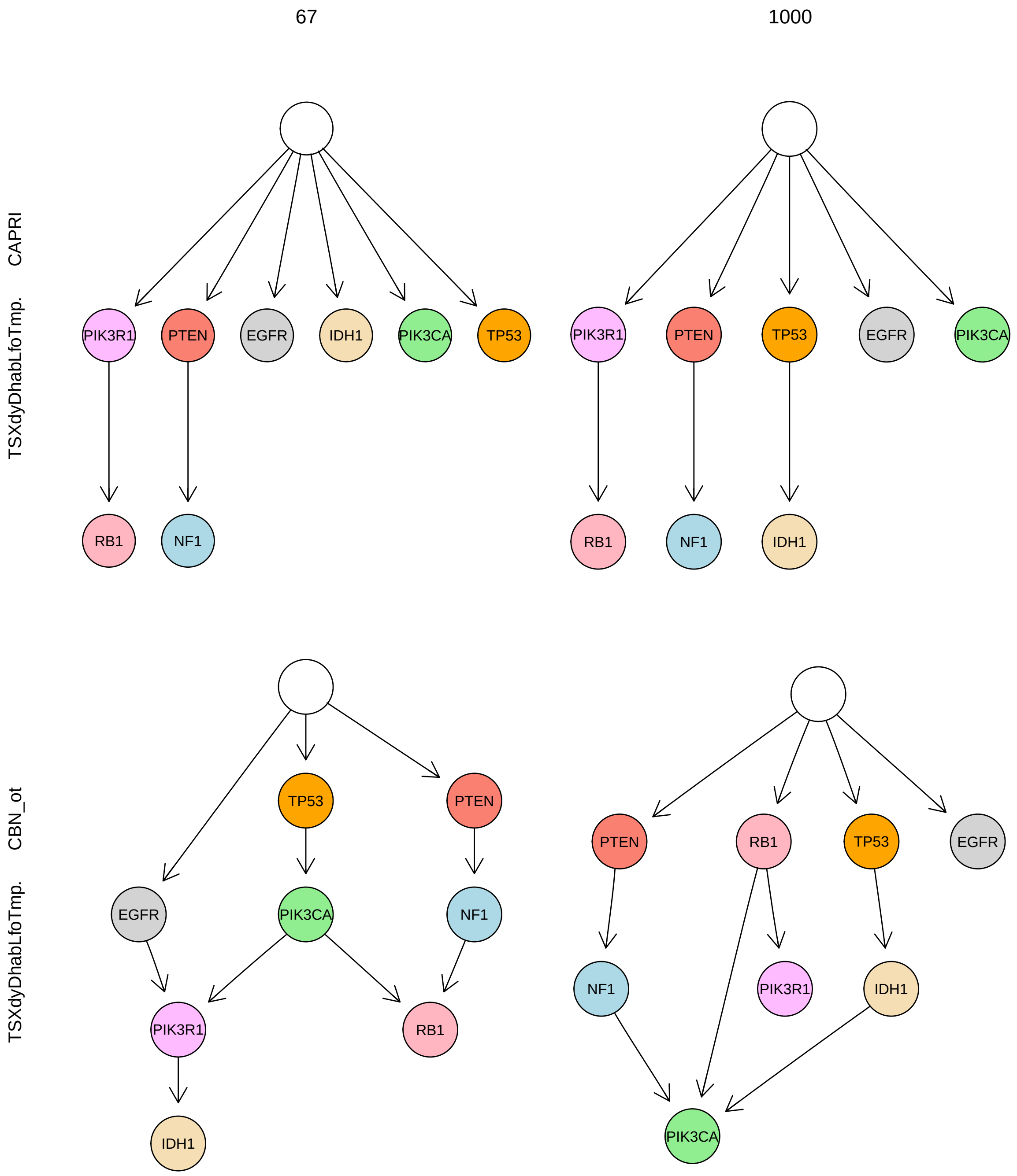

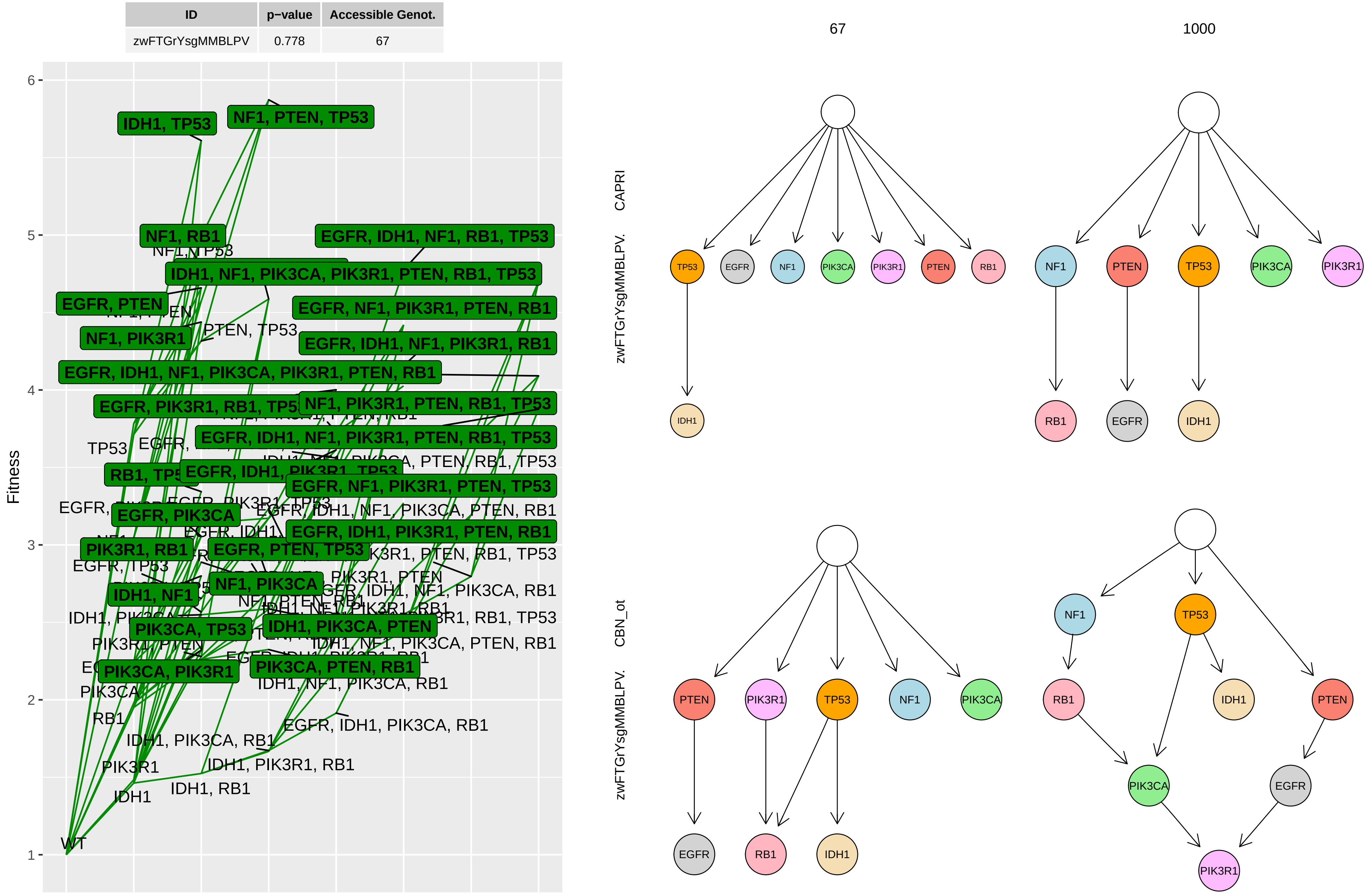
ID
p−value
Accessible Genot.
zwFTGrYsgMMBLPV
0.778
67
Fitness
WT
67
1000
CAPRI
CBN_ot
zwFTGrYsgMMBLPV.
TP53
EGFR
NF1
PIK3CA
PIK3R1
PTEN
RB1
IDH1

ID	p−value	Accessible Genot.
cFyhDLSGLOmZpll	0.781	26

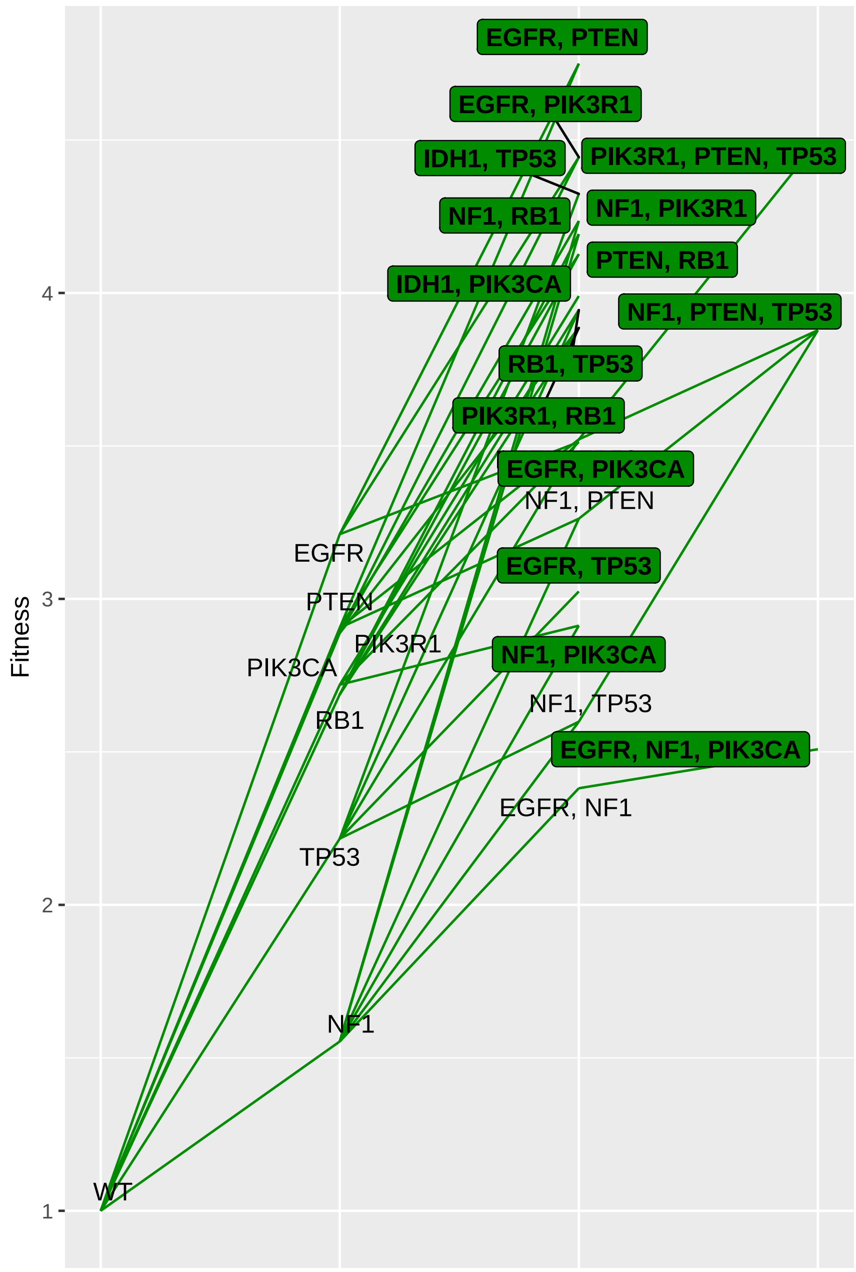

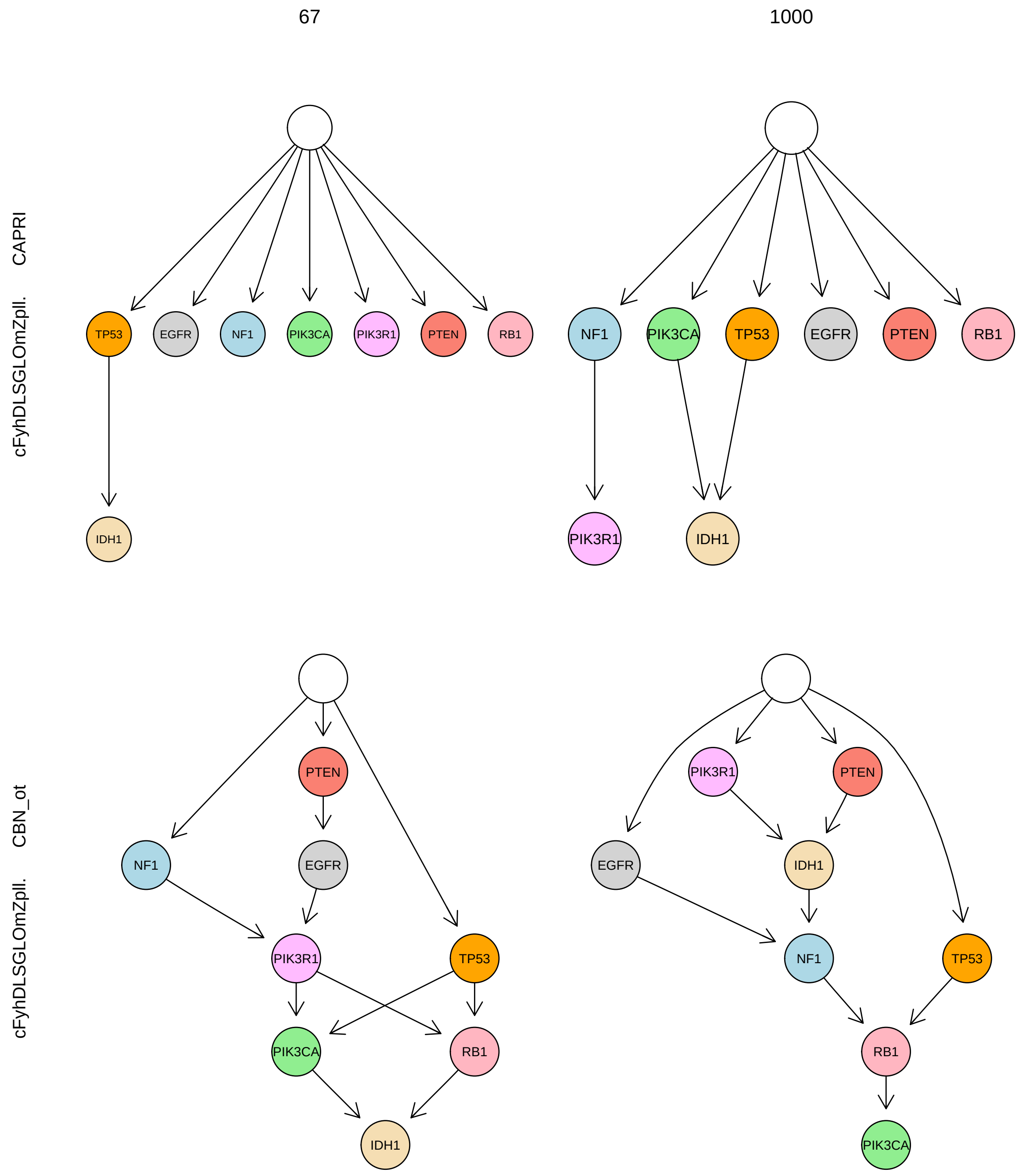

ID	p−value	Accessible Genot.
wjMddqoATULUujh	0.784	44

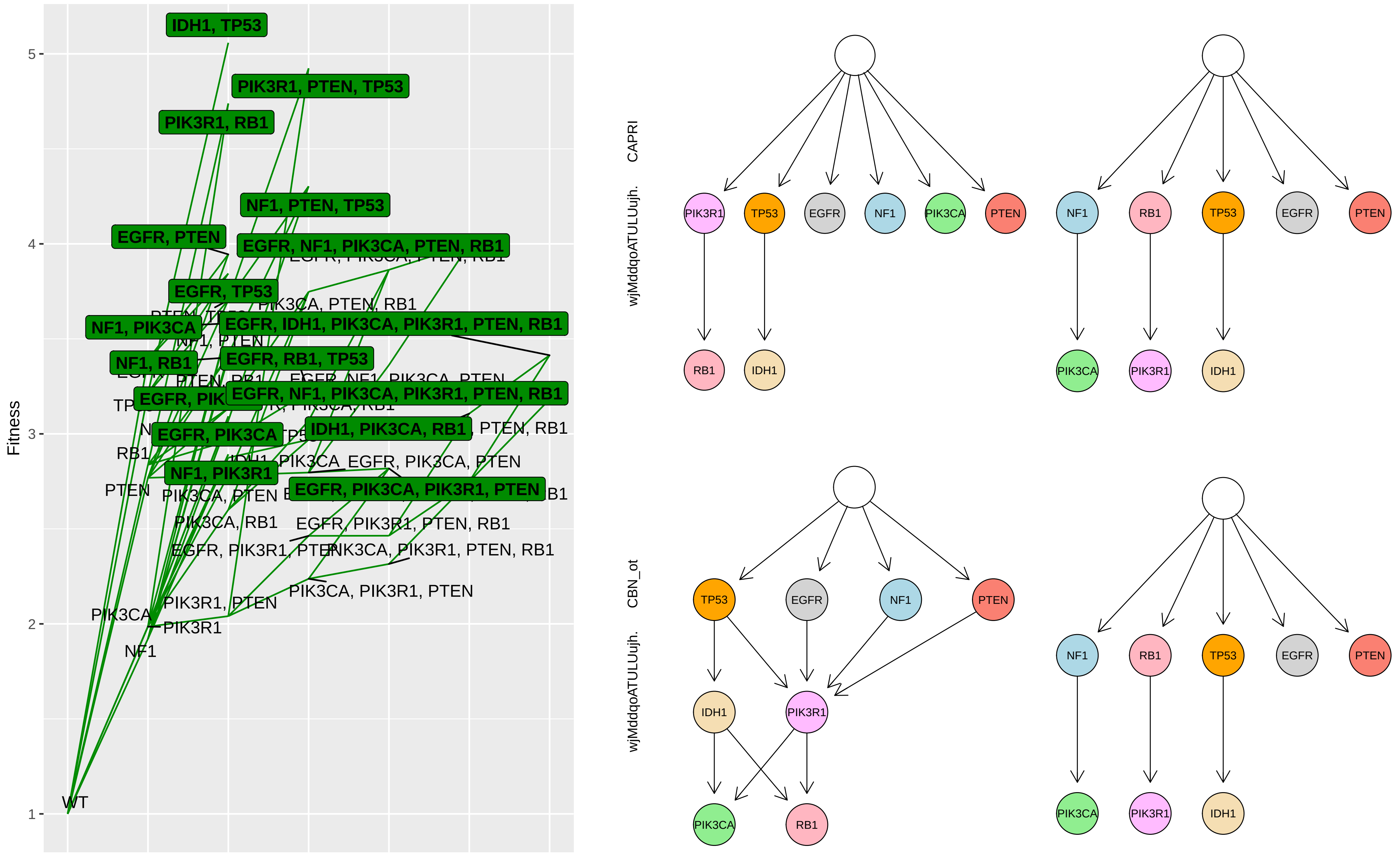

ID	p−value	Accessible Genot.
ADObYQOYvKnzGZT	0.794	112

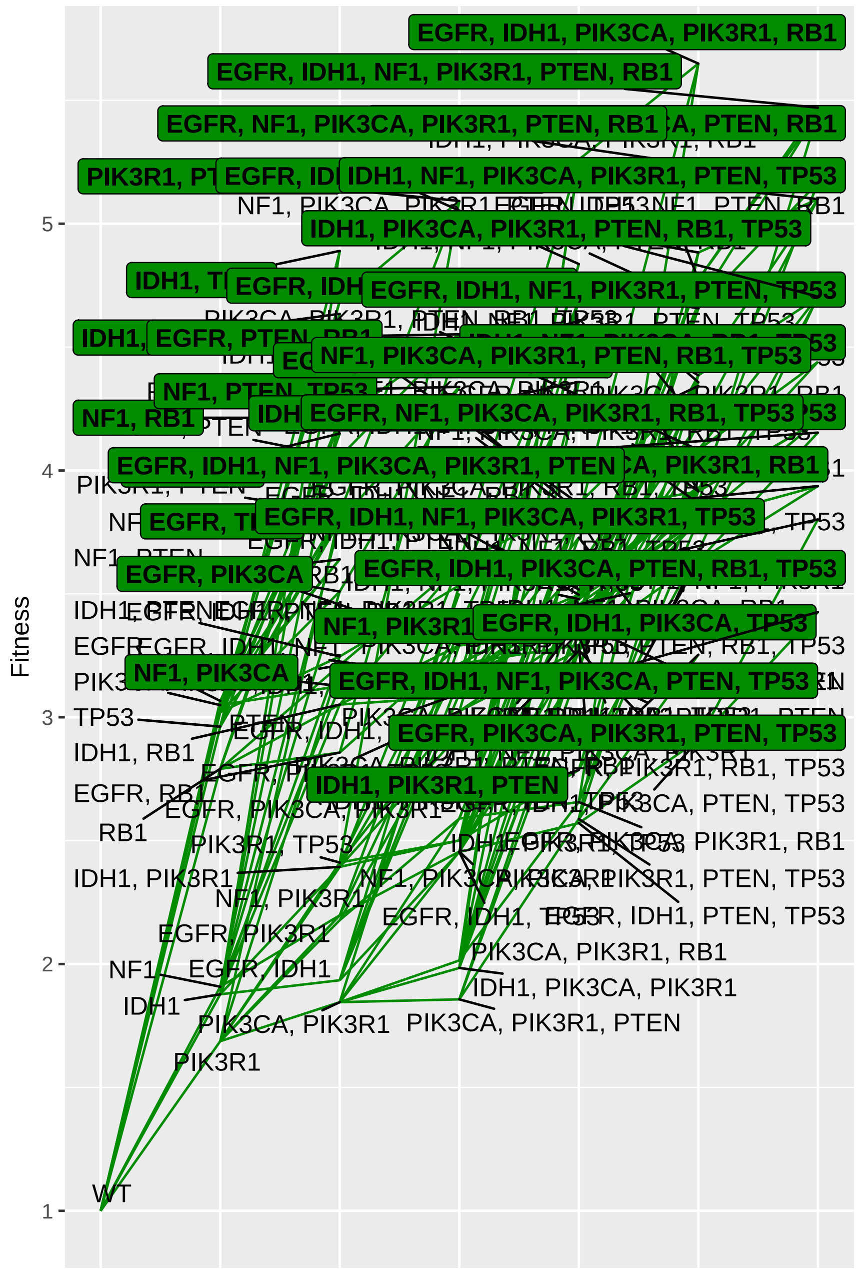

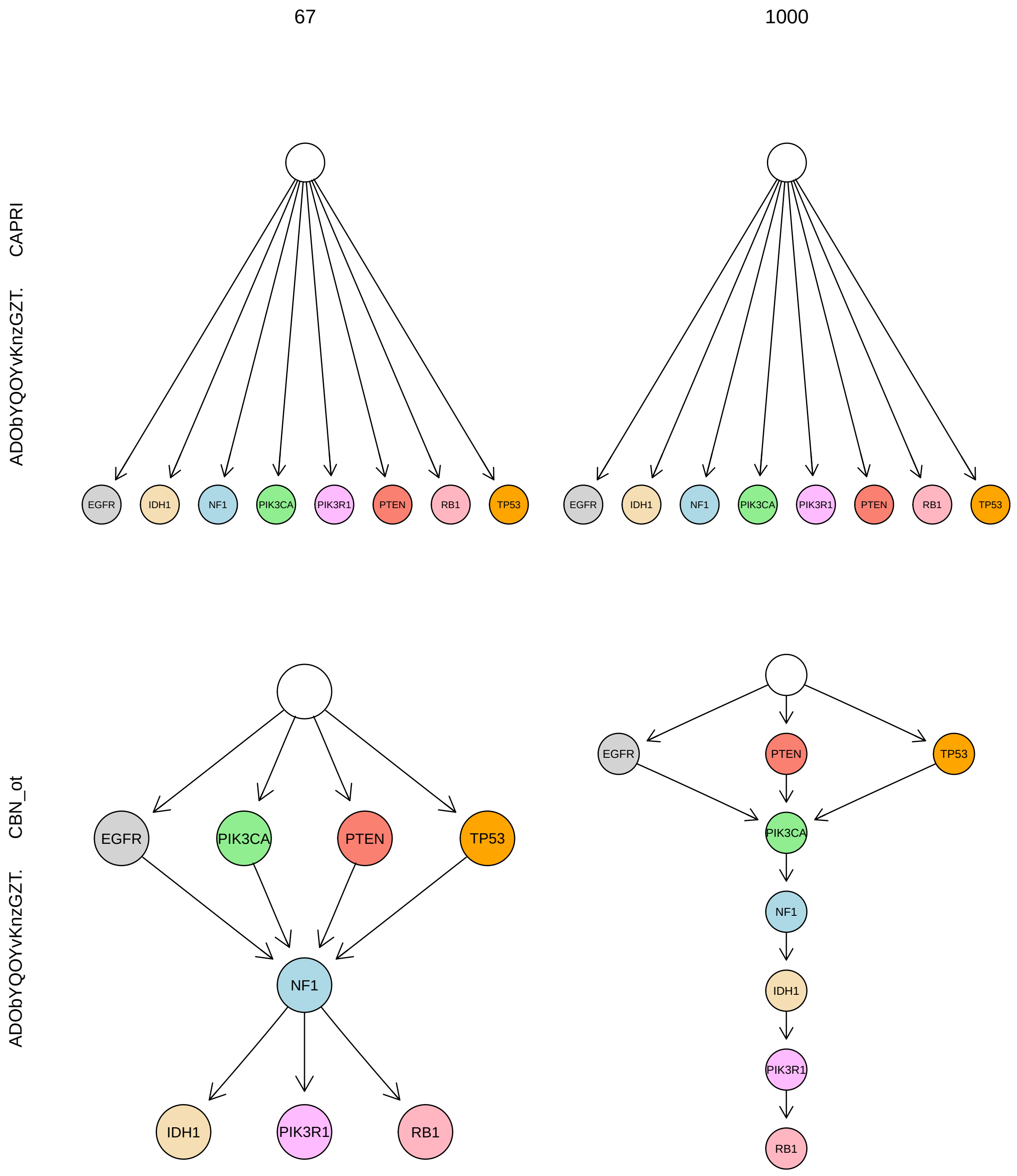

ID	p−value	Accessible Genot.
ZKgcaJQnwVUmttB	0.794	30

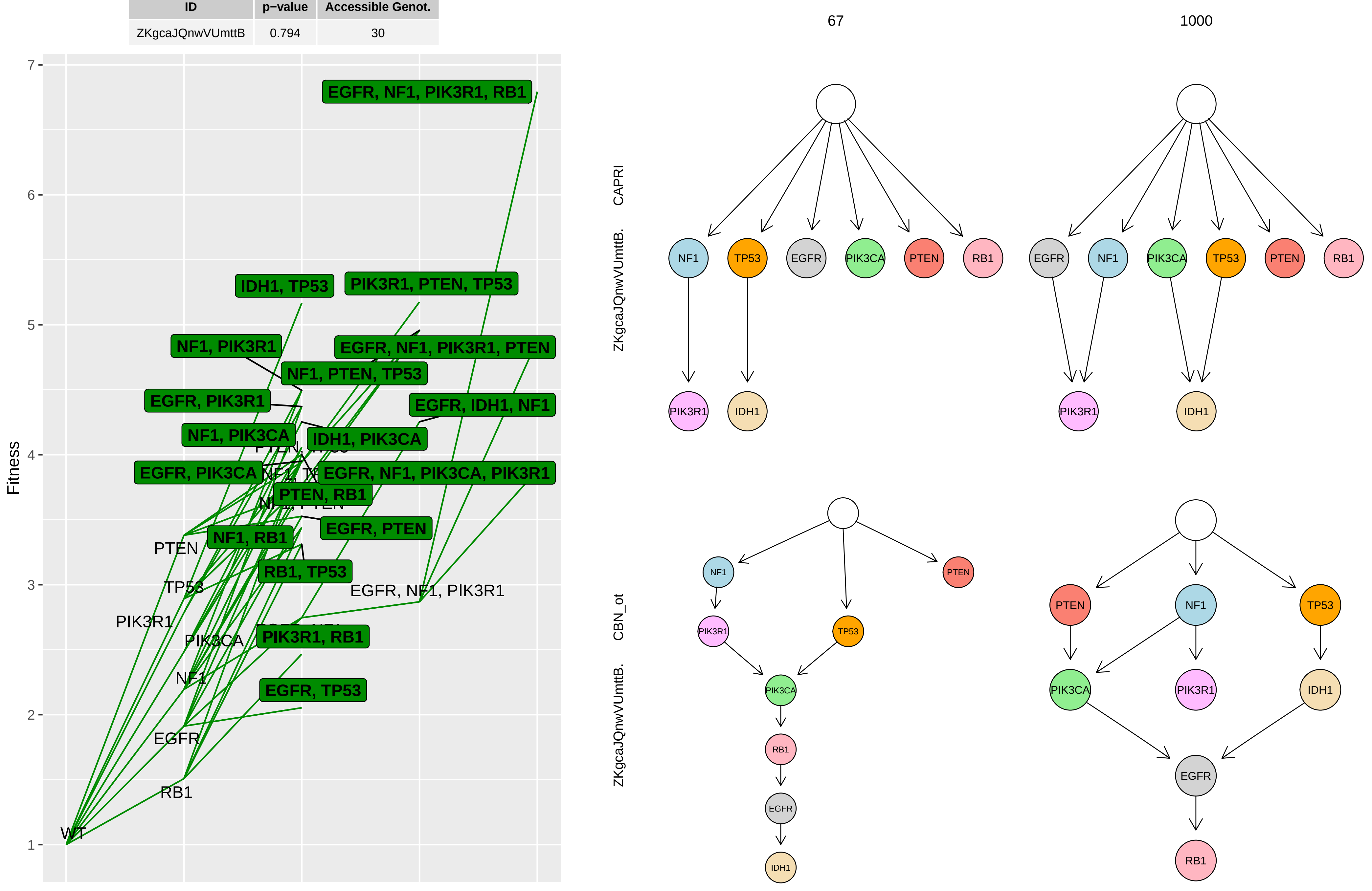

ID	p−value	Accessible Genot.
pdwfTBmqsZpomPb	0.8	238

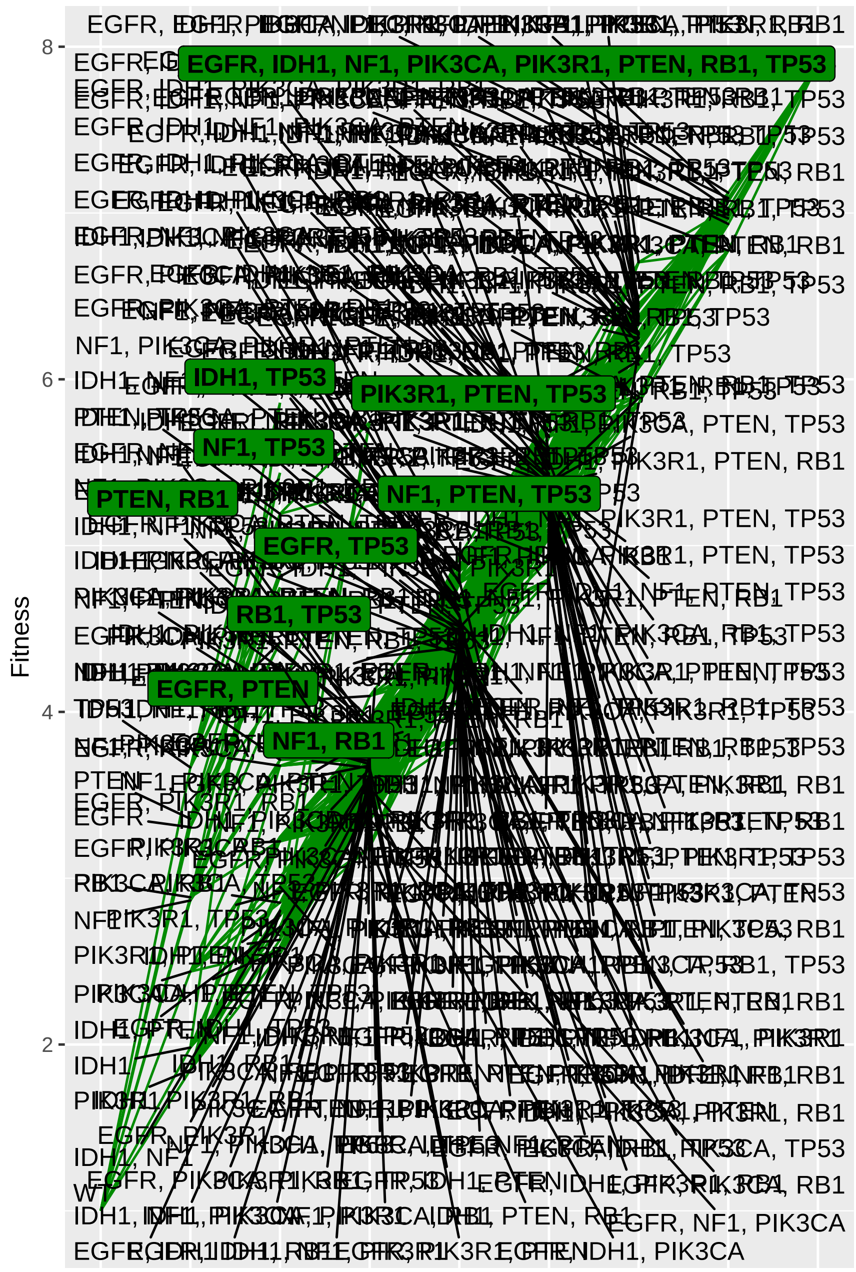

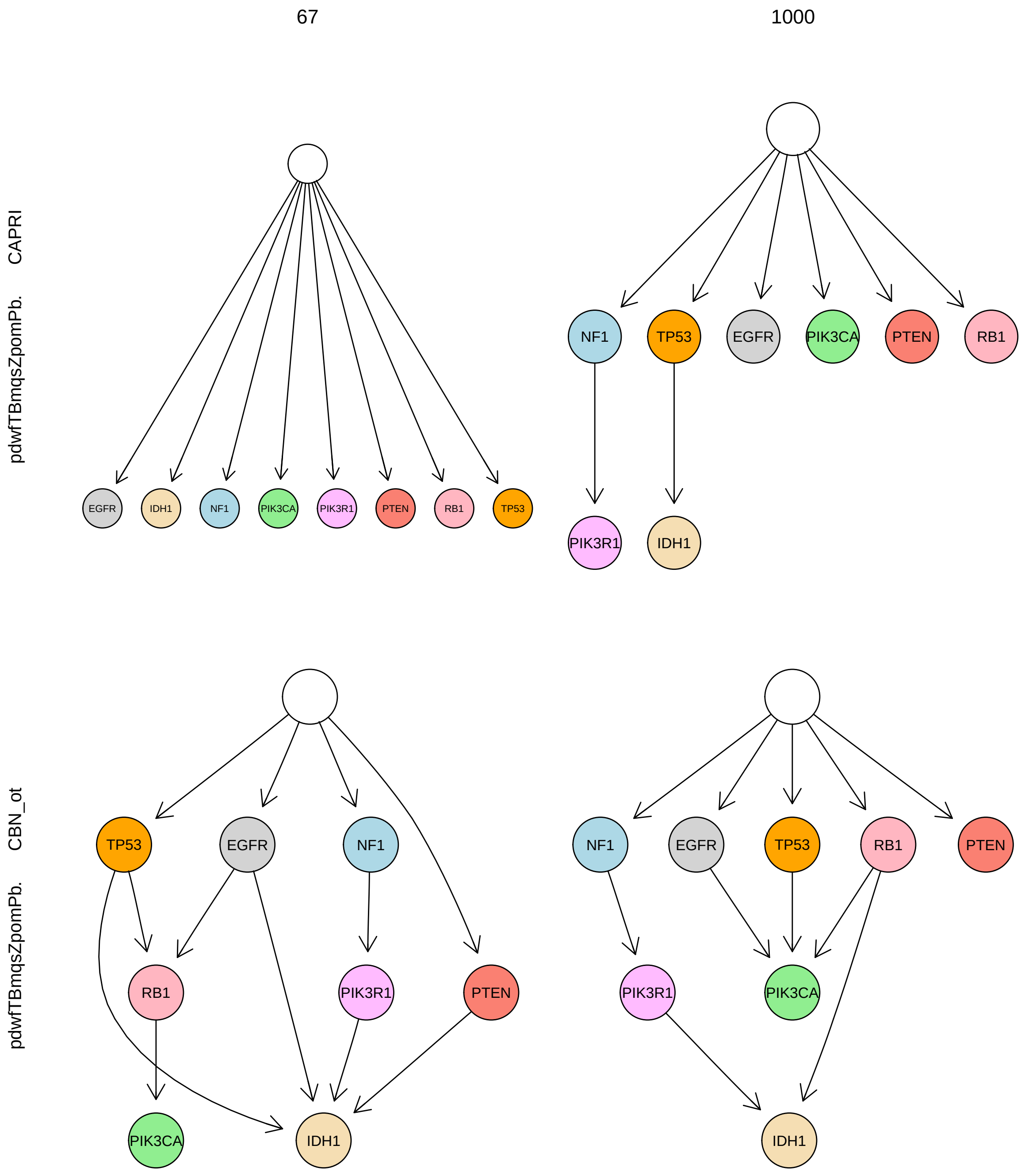

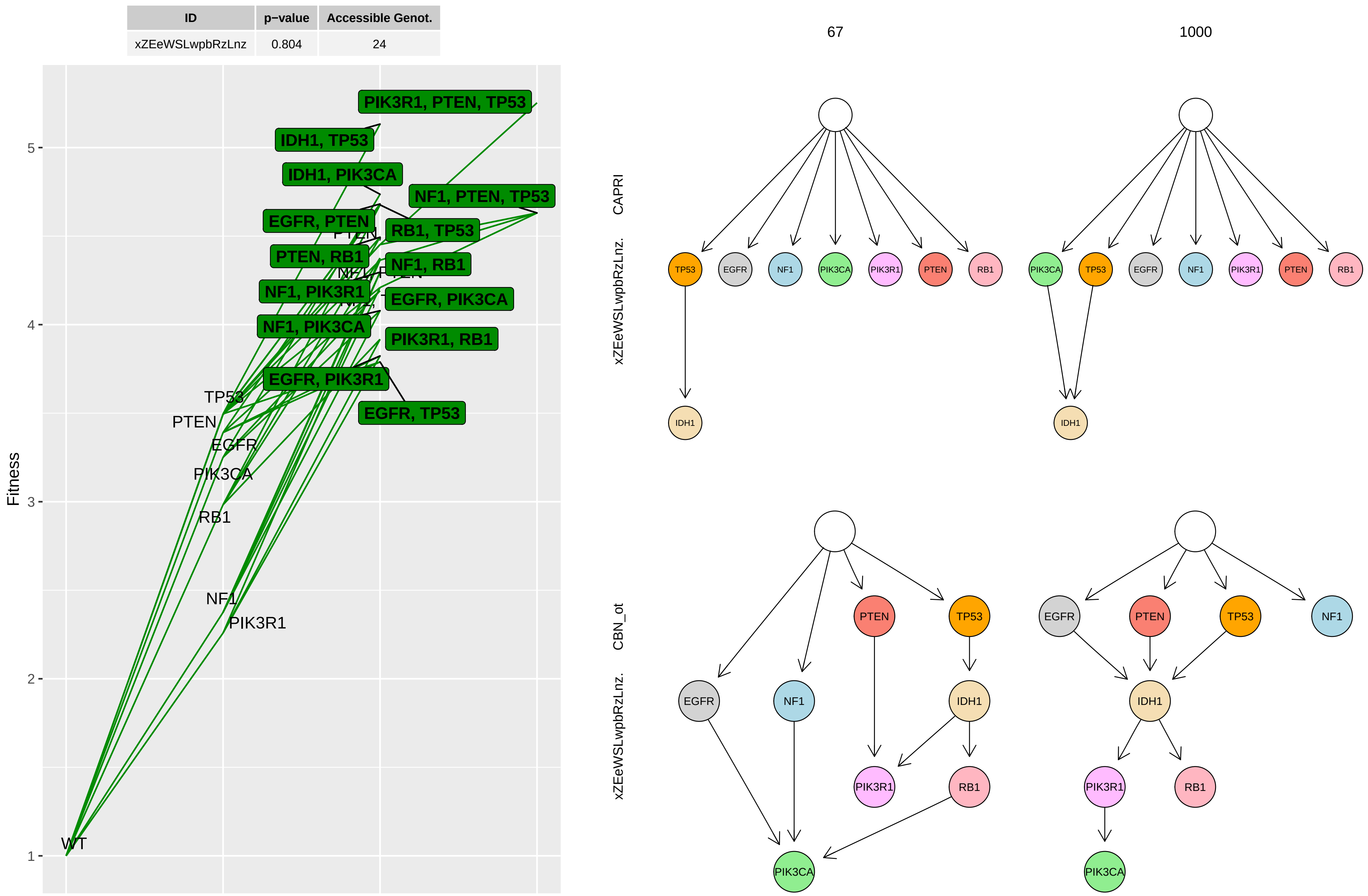
ID
p−value
Accessible Genot.
xZEeWSLwpbRzLnz
0.804
24
Fitness
PIK3R1, PTEN, TP53
IDH1, TP53
IDH1, PIK3CA
NF1, PTEN, TP53
EGFR, PTEN
RB1, TP53
PTEN, RB1
NF1, RB1
NF1, PIK3R1
EGFR, PIK3CA
NF1, PIK3CA
PIK3R1, RB1
EGFR, PIK3R1
EGFR, TP53
TP53
PTEN
EGFR
PIK3CA
RB1
NF1
PIK3R1
WT
67
1000
CAPRI
CBN_ot
xZEeWSLwpbRzLnz.
TP53
EGFR
NF1
PIK3CA
PIK3R1
PTEN
RB1
IDH1

ID	p−value	Accessible Genot.
SSDCaovHSzmtTdZ	0.805	34

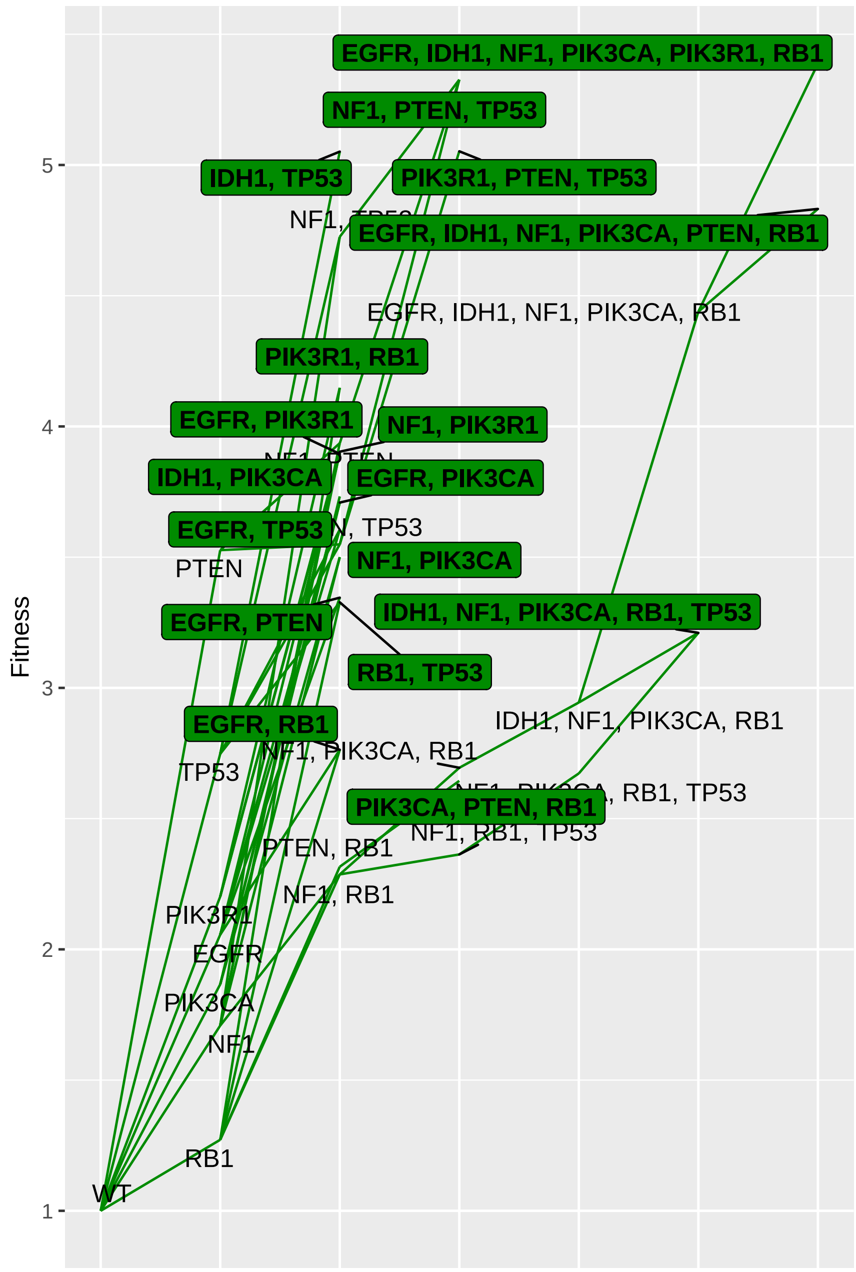

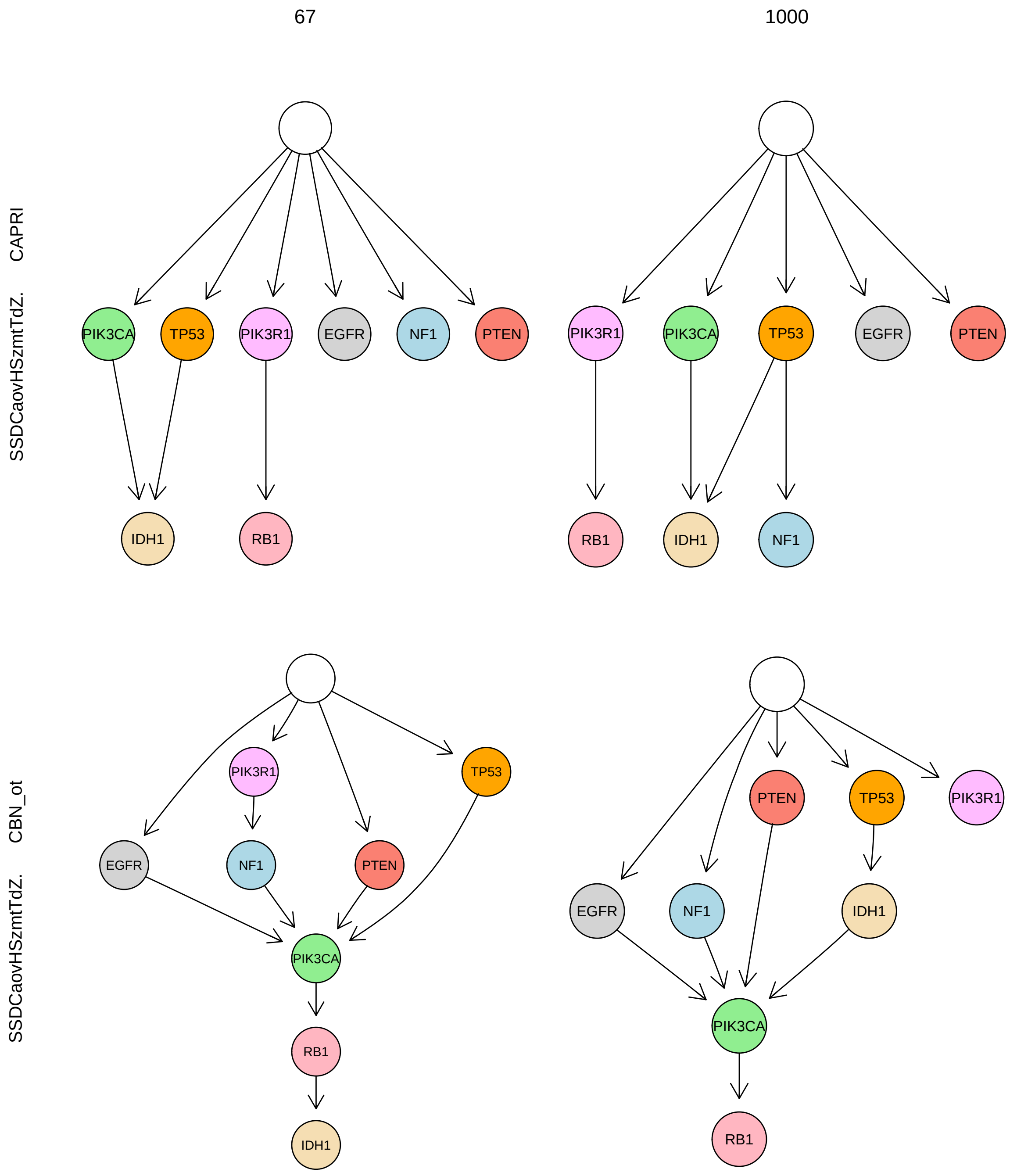

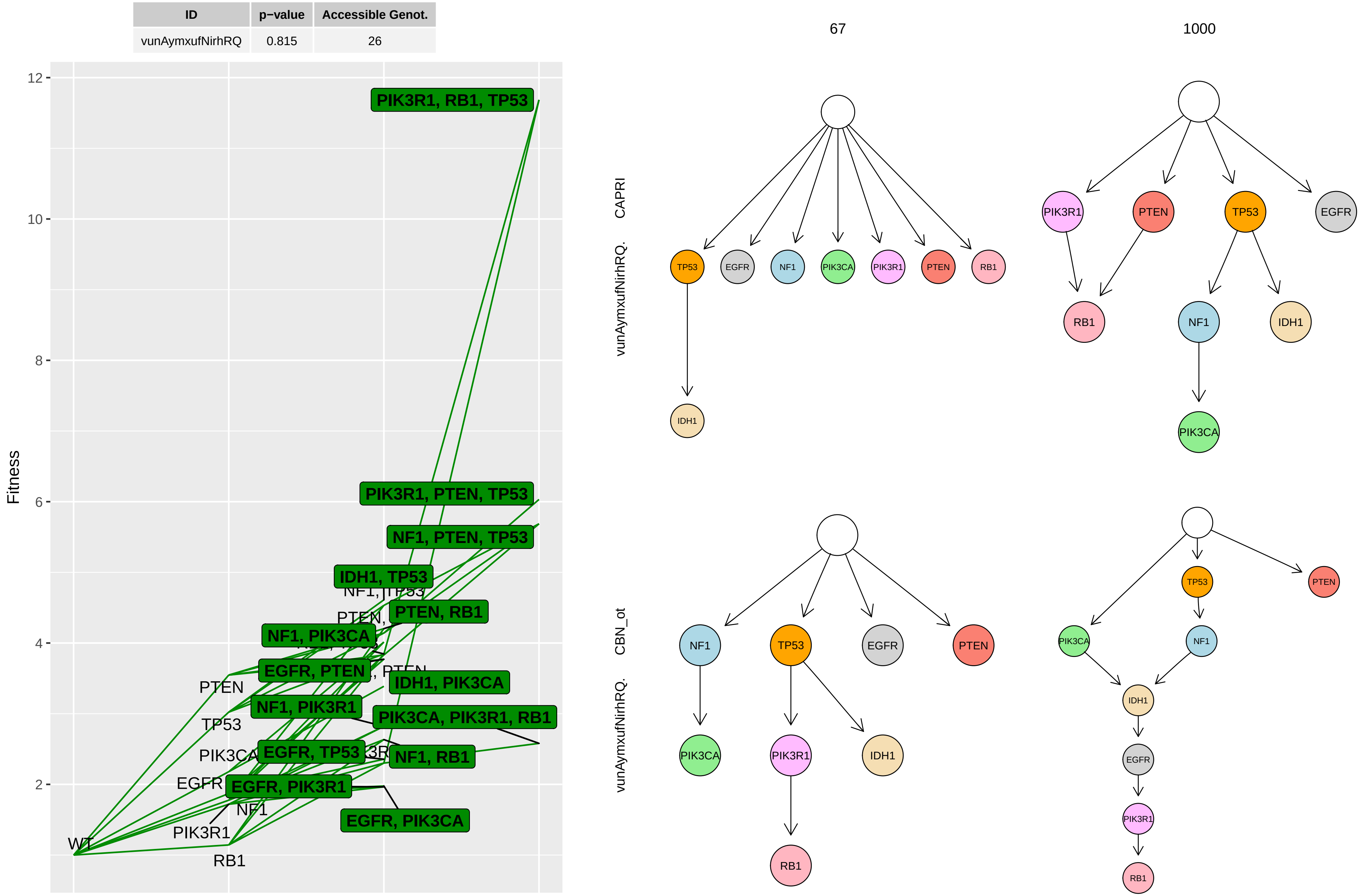
ID
p−value
Accessible Genot.
vunAymxufNirhRQ
0.815
26
Fitness
12
10
8
6
4
2
PIK3R1, RB1, TP53
PIK3R1, PTEN, TP53
NF1, PTEN, TP53
IDH1, TP53
PTEN, RB1
NF1, PIK3CA
EGFR, PTEN
IDH1, PIK3CA
NF1, PIK3R1
PIK3CA, PIK3R1, RB1
EGFR, TP53
NF1, RB1
EGFR, PIK3R1
EGFR, PIK3CA
PTEN
TP53
PIK3CA
EGFR
NF1
PIK3R1
RB1
WT
67
1000
vunAymxufNirhRQ. CAPRI
vunAymxufNirhRQ. CBN_ot
TP53
EGFR
NF1
PIK3CA
PIK3R1
PTEN
RB1
IDH1
IDH1

ID	p−value	Accessible Genot.
WxZnWLRYpbnowCD	0.818	24

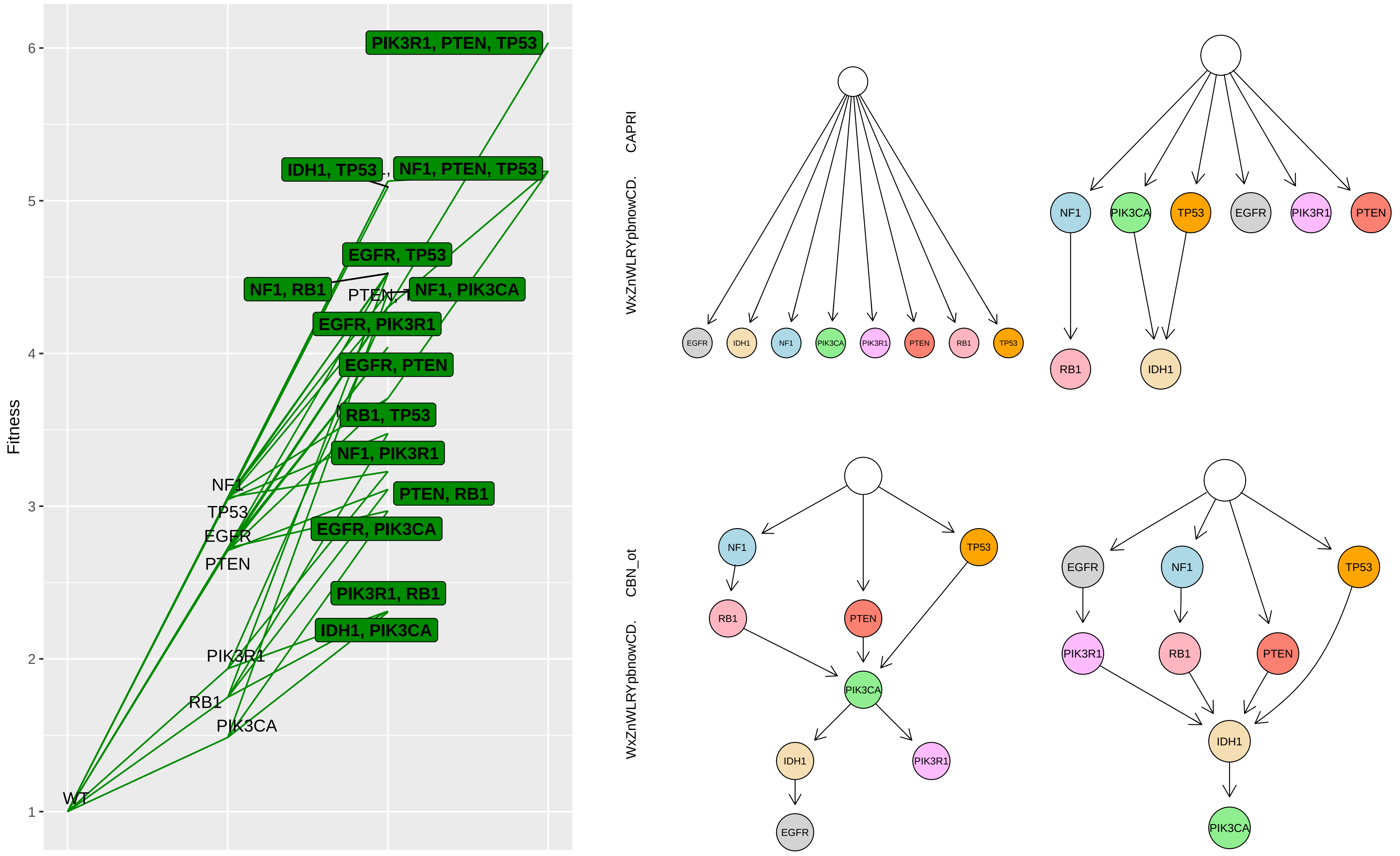

ID	p−value	Accessible Genot.
xSCPnyYWByXytqD	0.82	78

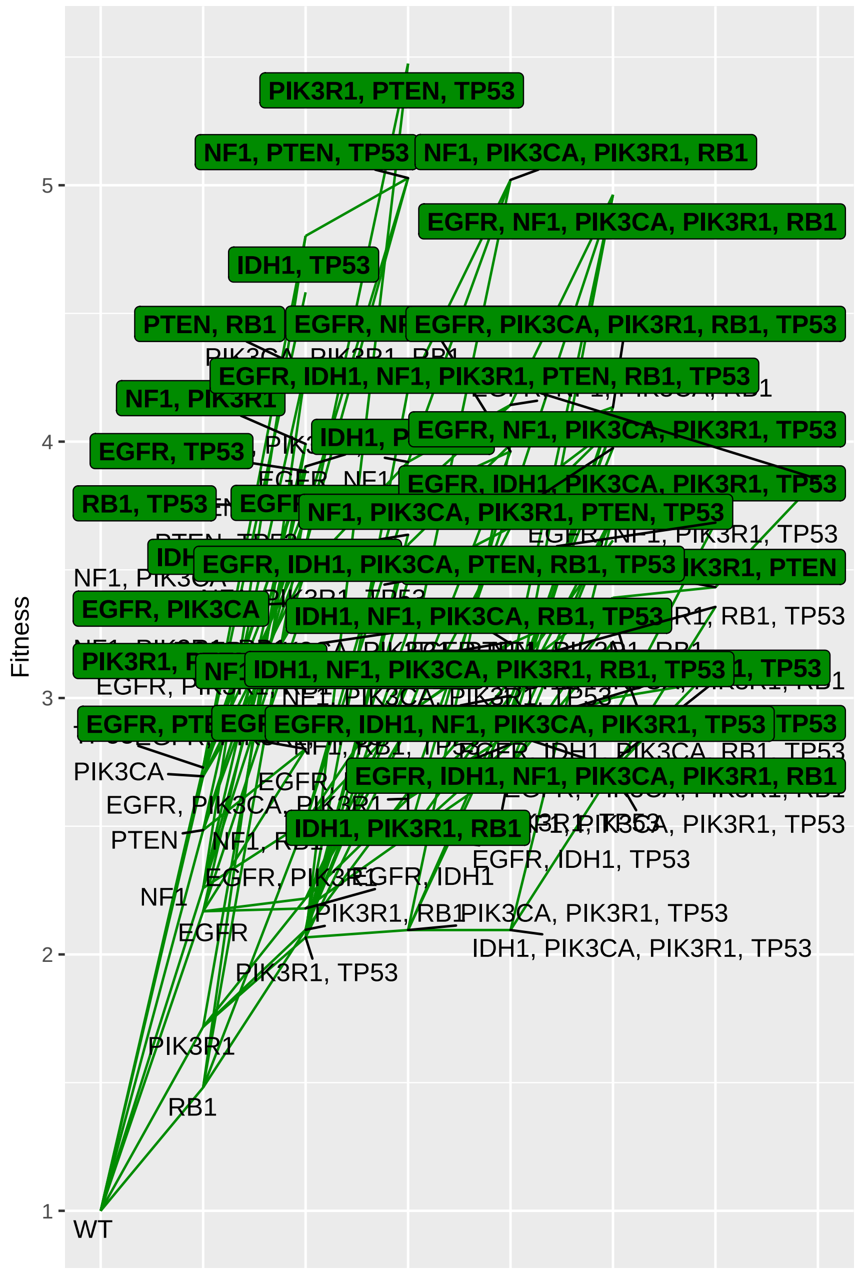

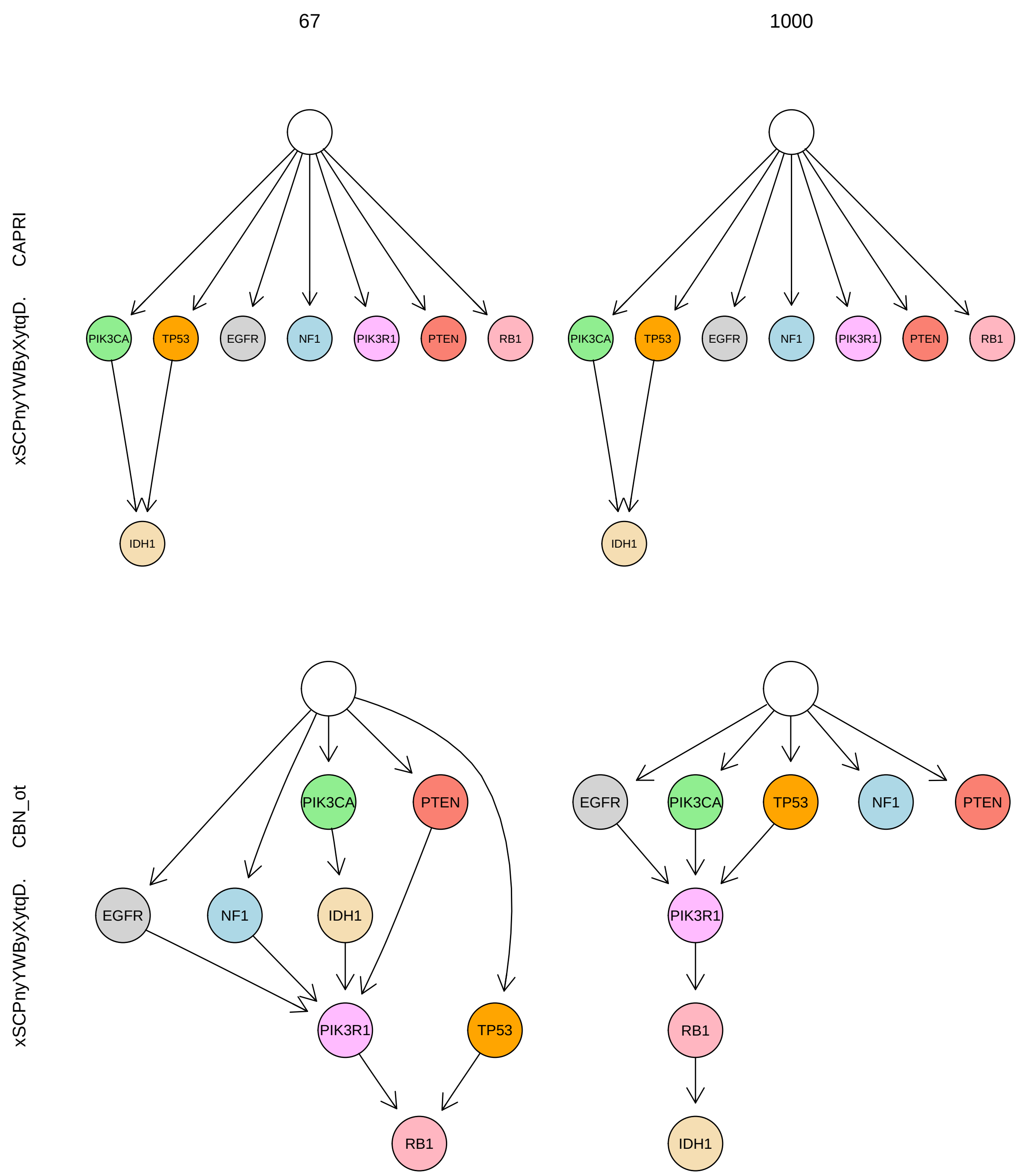

ID	p−value	Accessible Genot.
zbQtockjhtqCcWP	0.822	109

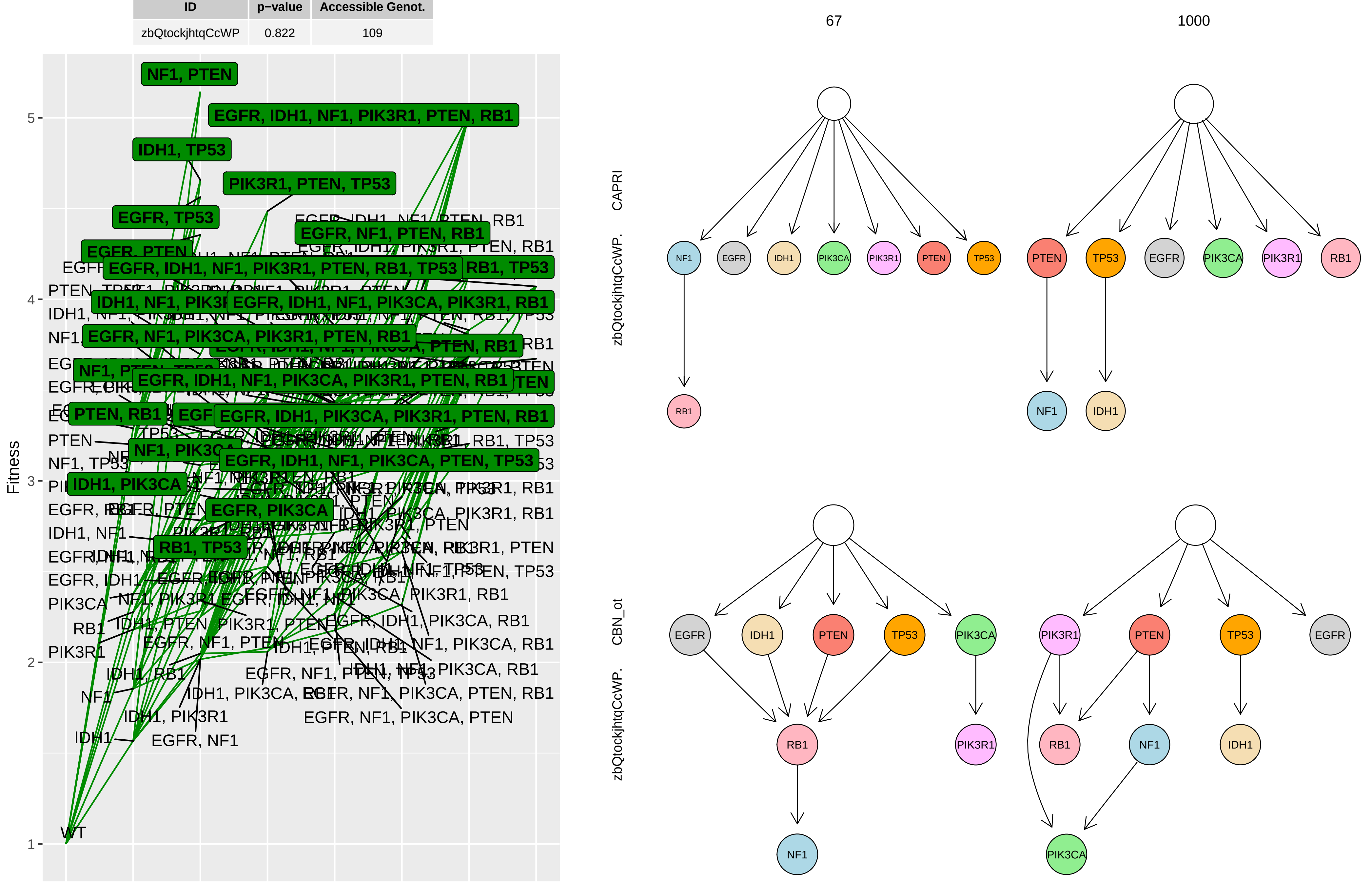

ID	p−value	Accessible Genot.
AIArfaCwHjiLFEP	0.824	31

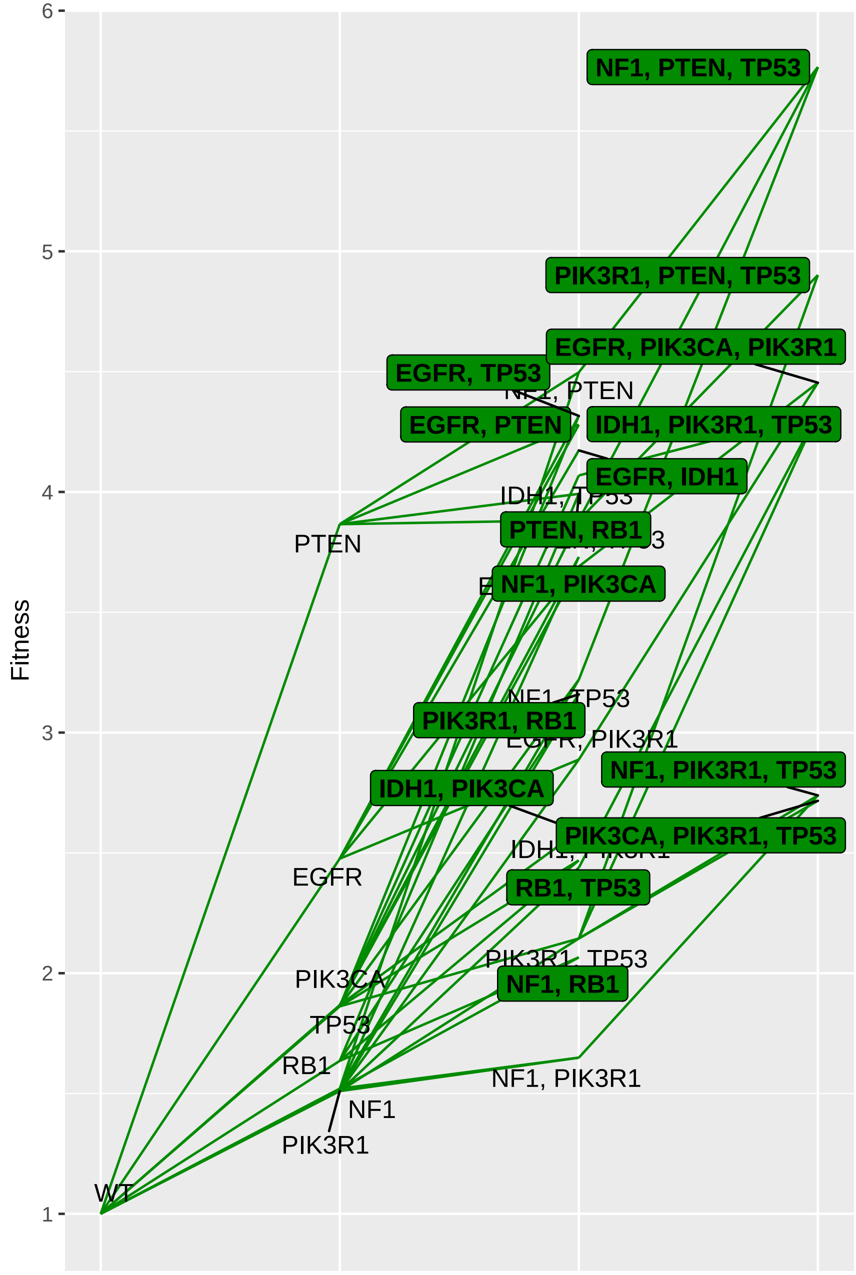

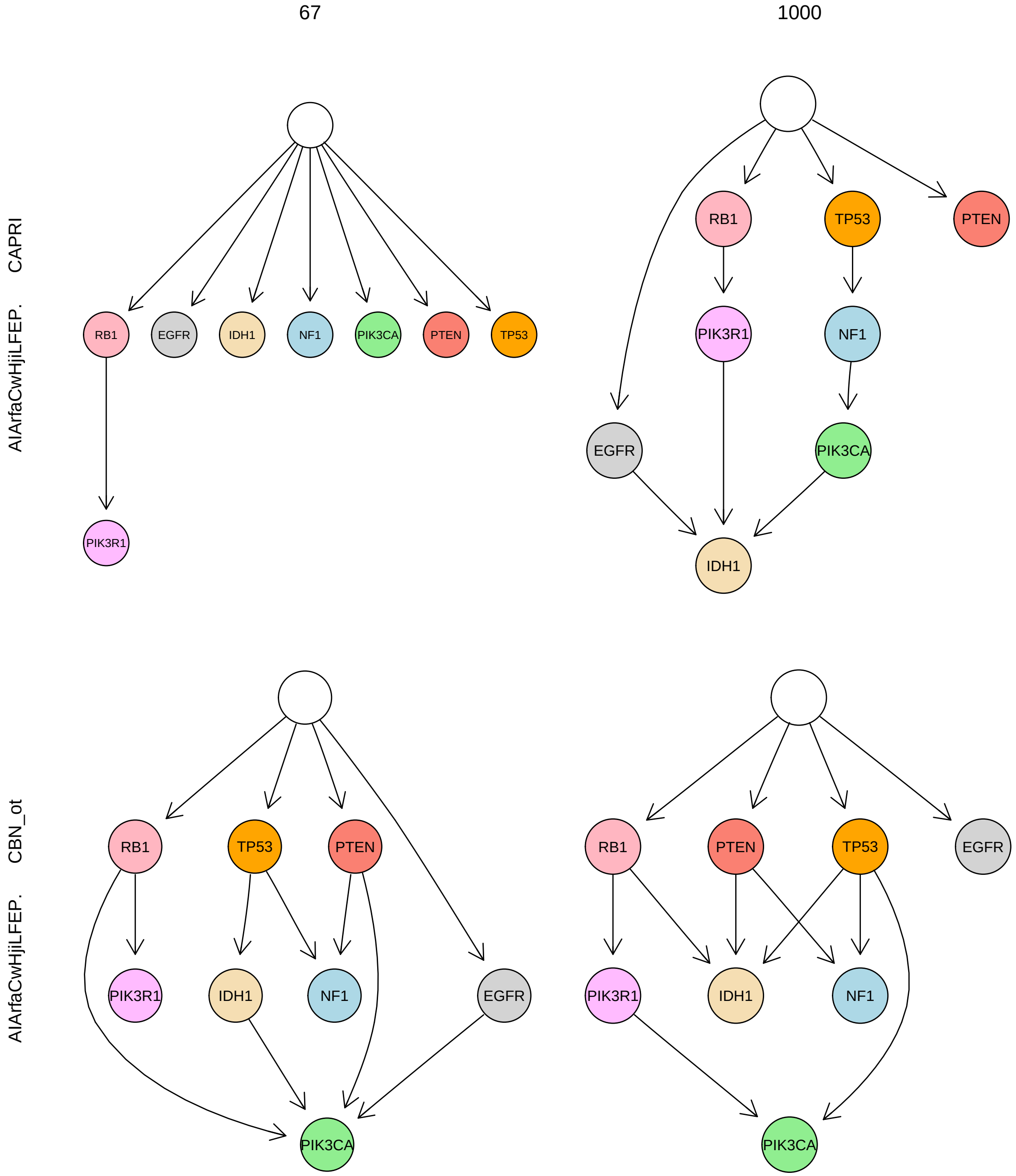

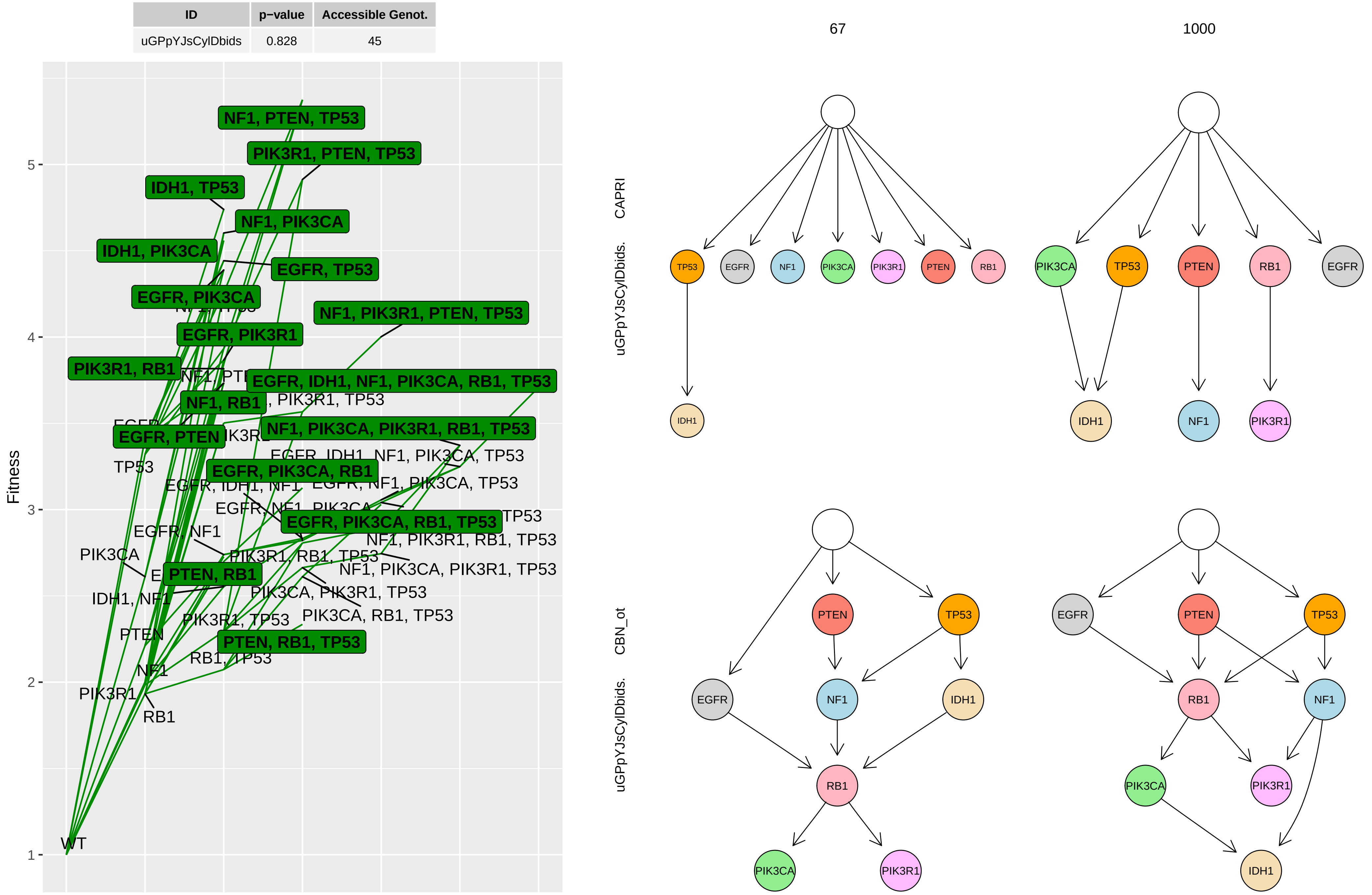

ID
p−value
Accessible Genot.
uGPpYJsCylDbids
0.828
45
Fitness
NF1, PTEN, TP53
PIK3R1, PTEN, TP53
IDH1, TP53
NF1, PIK3CA
IDH1, PIK3CA
EGFR, TP53
EGFR, PIK3CA
NF1, PIK3R1, PTEN, TP53
EGFR, PIK3R1
PIK3R1, RB1
EGFR, IDH1, NF1, PIK3CA, RB1, TP53
NF1, RB1
NF1, PIK3CA, PIK3R1, RB1, TP53
EGFR, PTEN
TP53
EGFR, IDH1, NF1, PIK3CA, TP53
EGFR, PIK3CA, RB1
EGFR, NF1, PIK3CA, TP53
EGFR, PIK3CA, RB1, TP53
EGFR, NF1
NF1, PIK3R1, RB1, TP53
PIK3CA
PIK3R1, RB1, TP53
PTEN, RB1
NF1, PIK3CA, PIK3R1, TP53
IDH1, NF1
PIK3CA, PIK3R1, TP53
PIK3R1, TP53
PIK3CA, RB1, TP53
PTEN
PTEN, RB1, TP53
RB1, TP53
NF1
PIK3R1
RB1
WT
1
2
3
4
5
67
1000
CAPRI
uGPpYJsCylDbids.
CBN_ot
uGPpYJsCylDbids.
TP53
EGFR
NF1
PIK3CA
PIK3R1
PTEN
RB1
IDH1
PIK3CA
TP53
PTEN
RB1
EGFR
IDH1
NF1
PIK3R1
PTEN
TP53
EGFR
NF1
IDH1
RB1
PIK3CA
PIK3R1
EGFR
PTEN
TP53
RB1
NF1
PIK3CA
PIK3R1
IDH1

ID	p-value	Accessible Genot.
JnVYJxasVwQRFxe	0.83	111

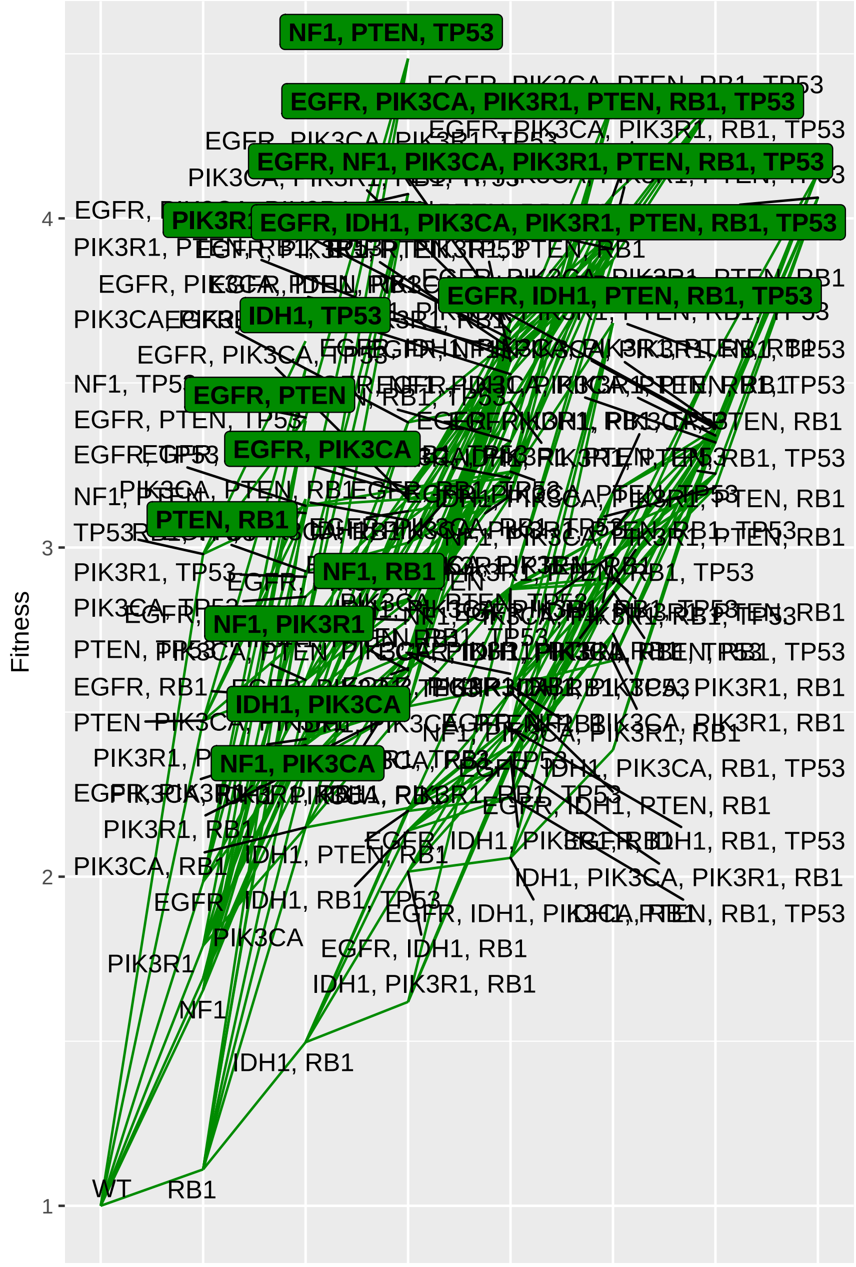

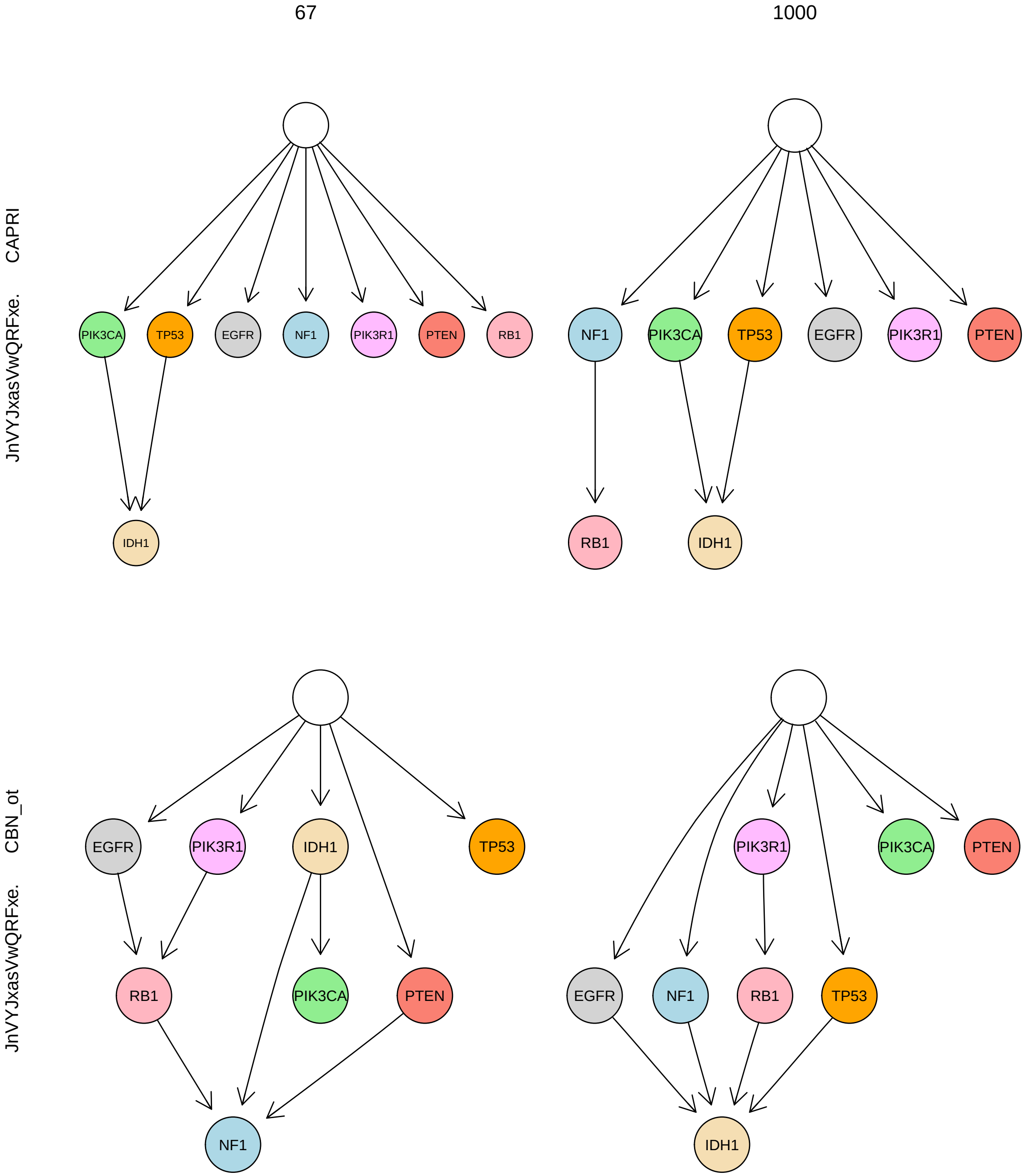

ID	p−value	Accessible Genot.
OfpbpfuETZmlPUU	0.833	129

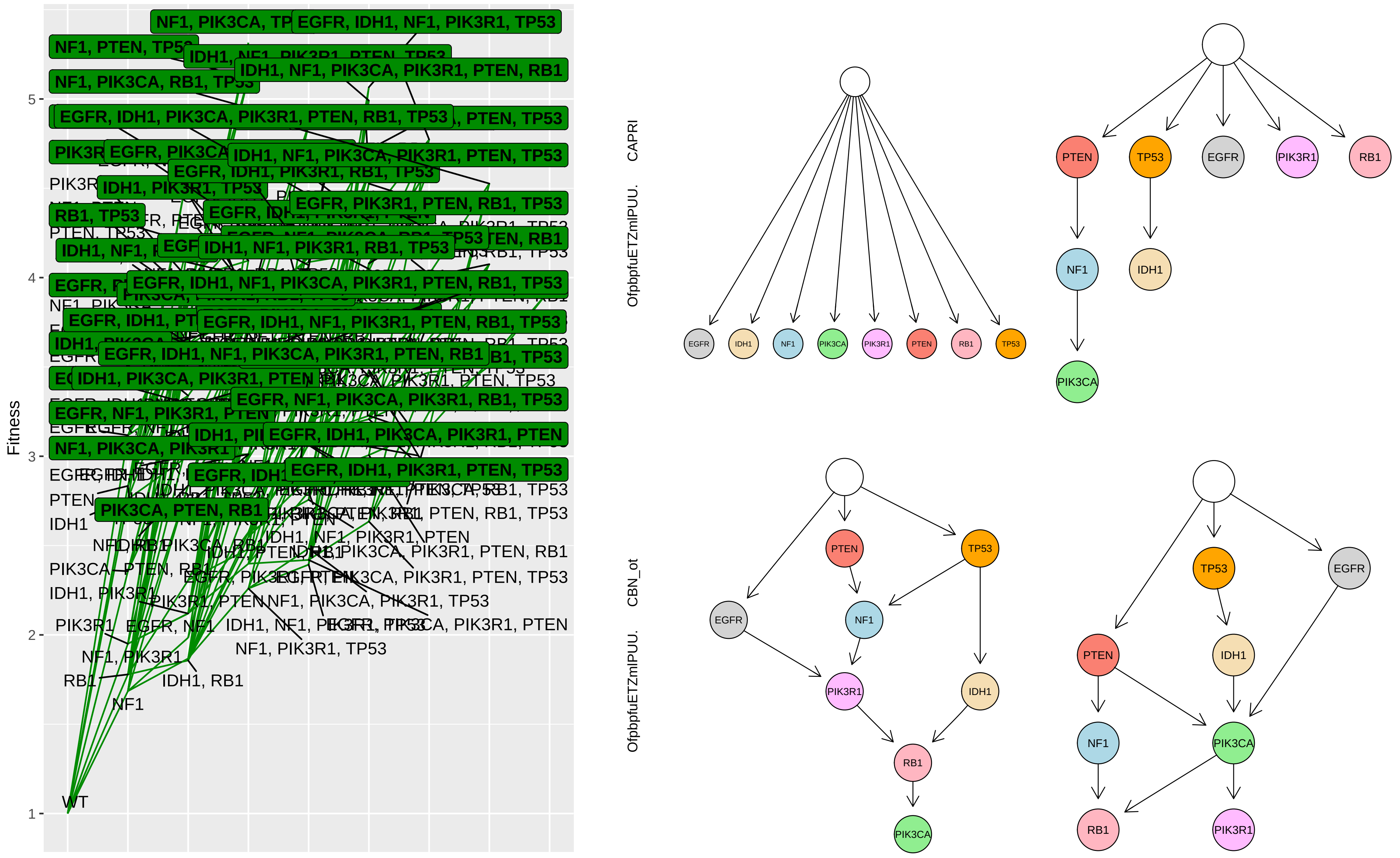

ID	p−value	Accessible Genot.
TxttAuYPzVLMHzX	0.839	32

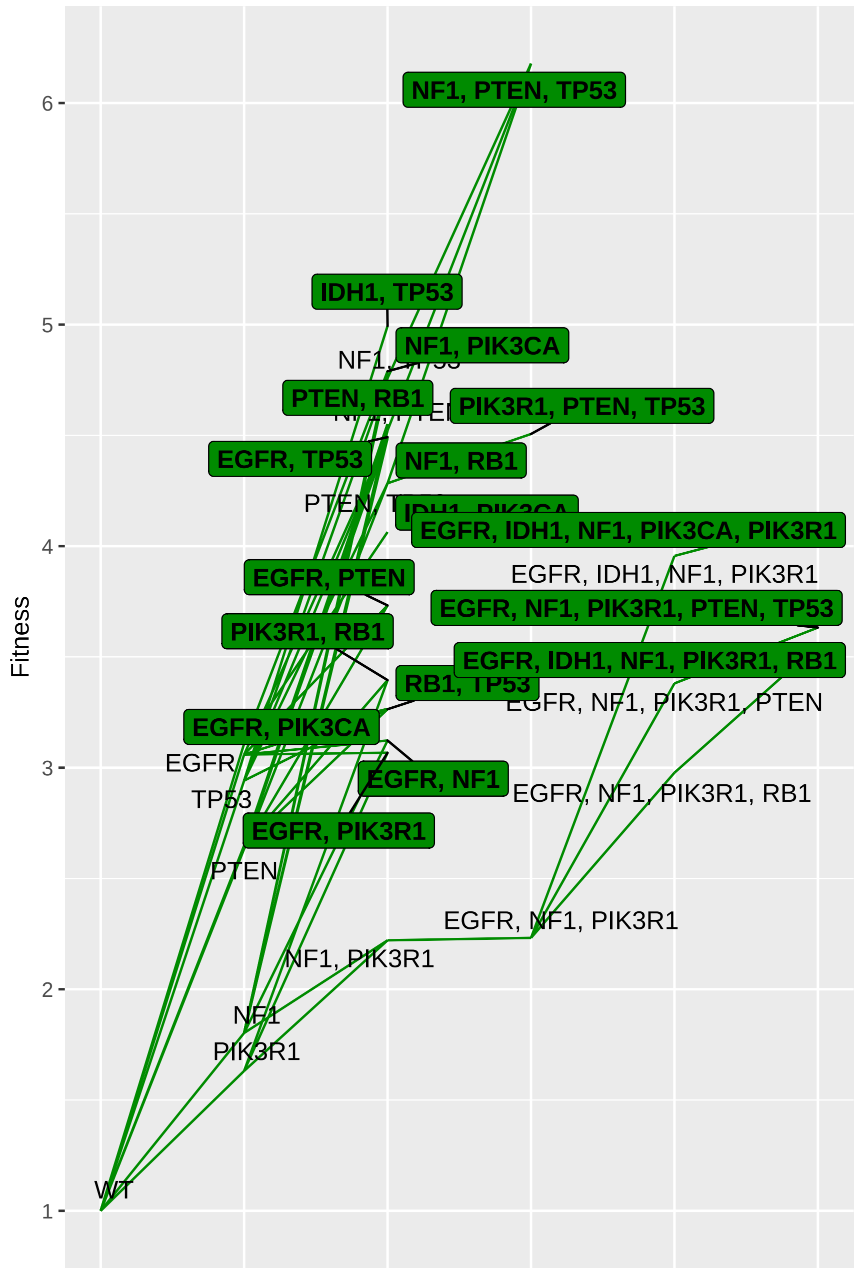

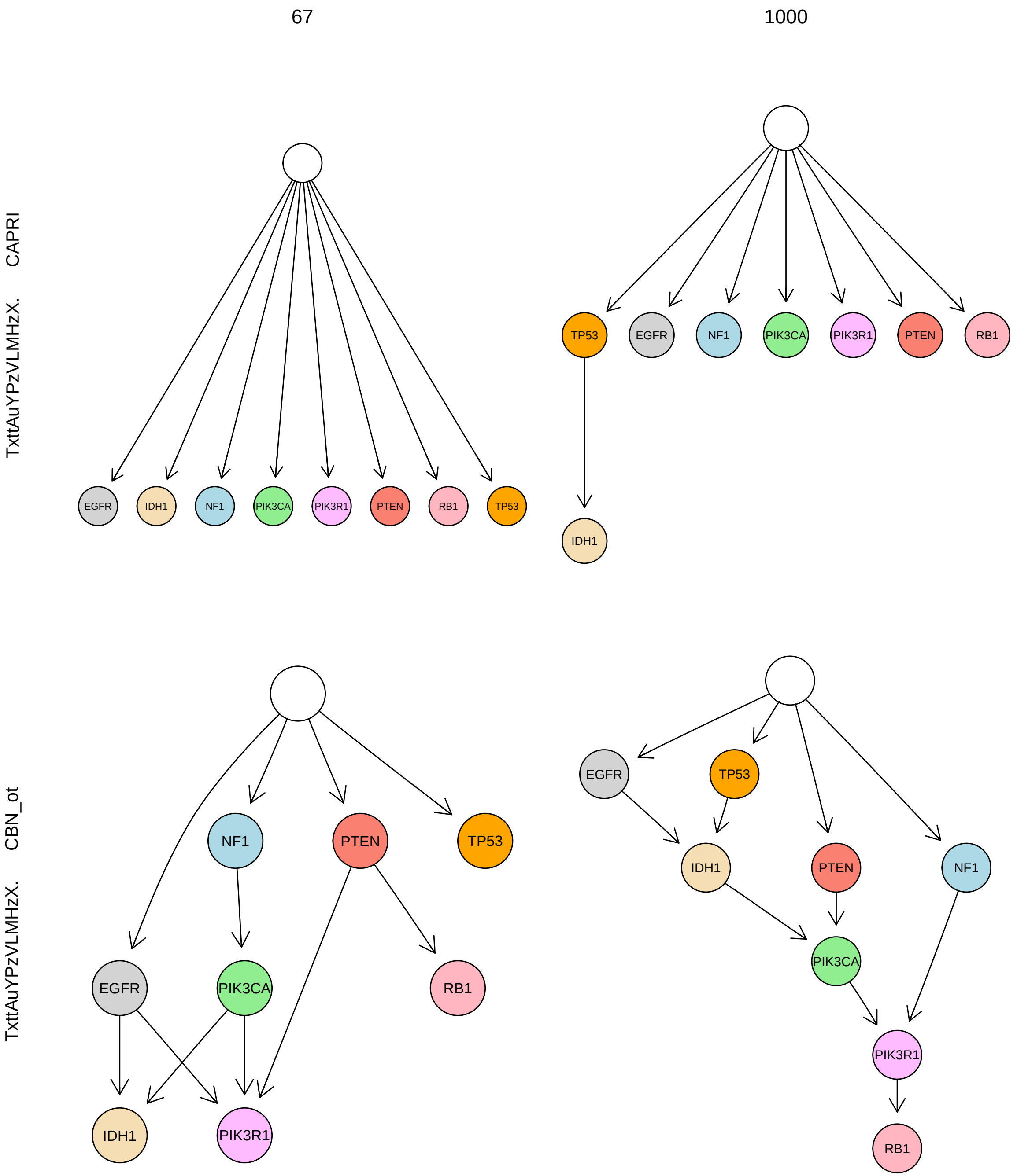

ID	p-value	Accessible Genot.
jKeUyaNiYRvOiHr	0.849	24

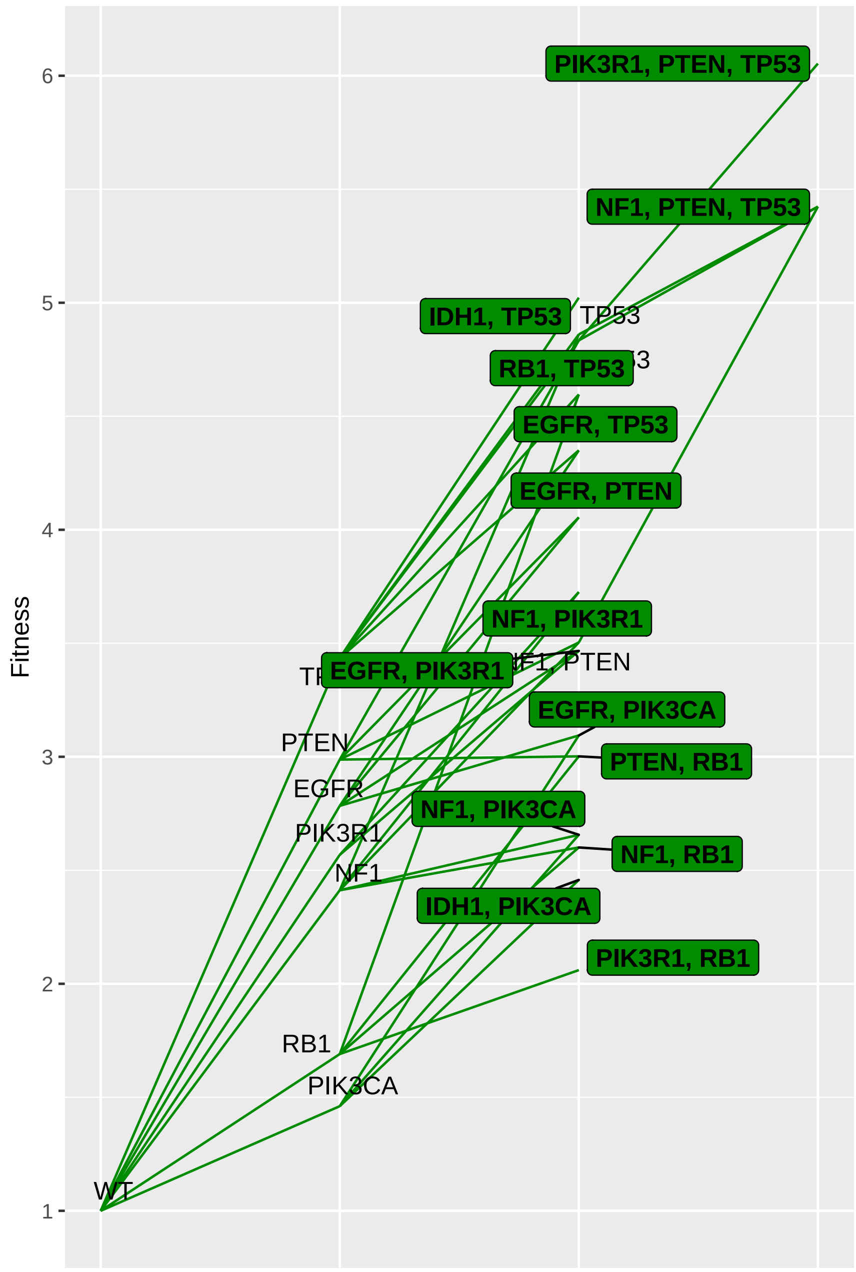

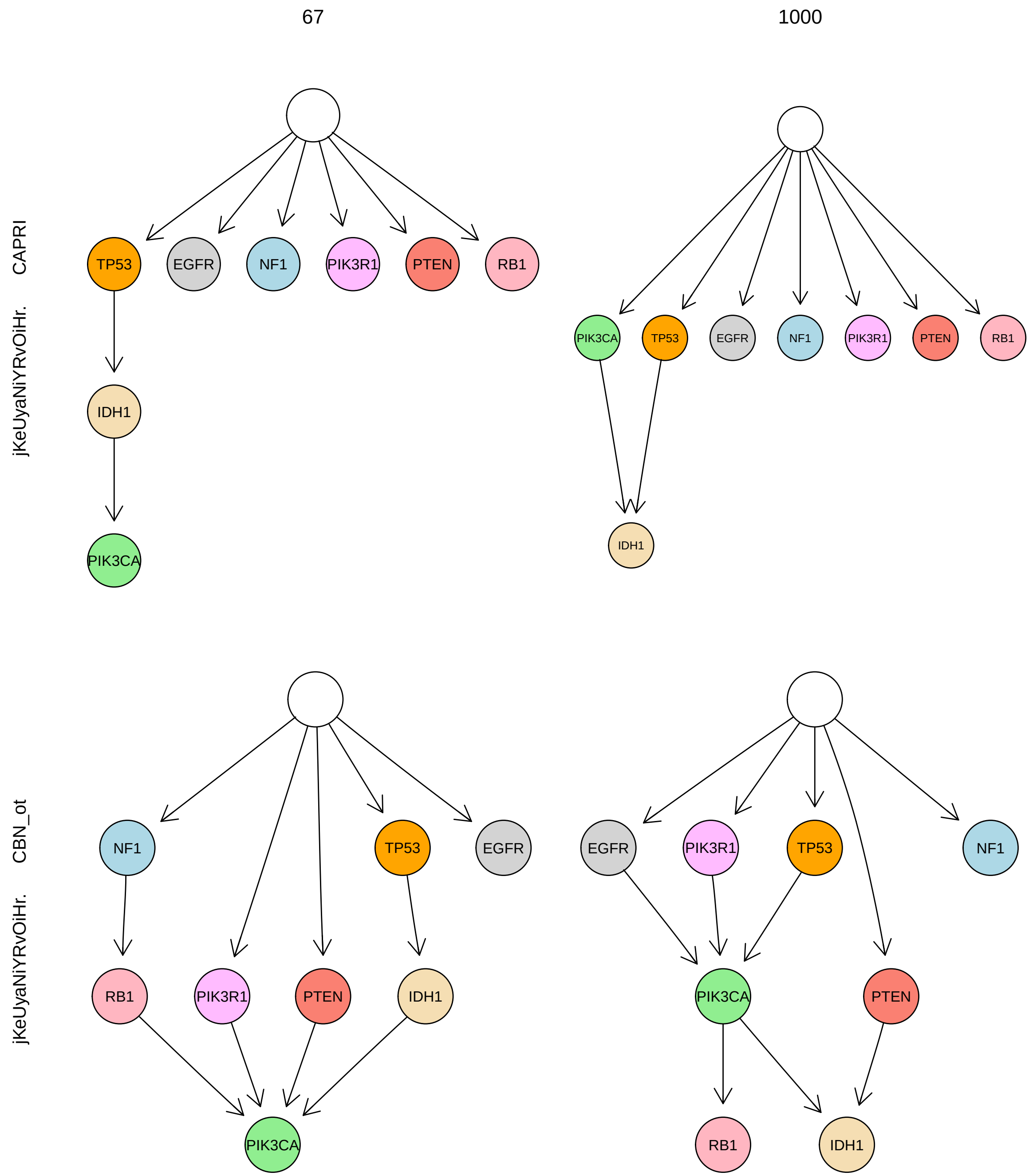

ID	p−value	Accessible Genot.
NLnJwEHXBlgowlu	0.85	29

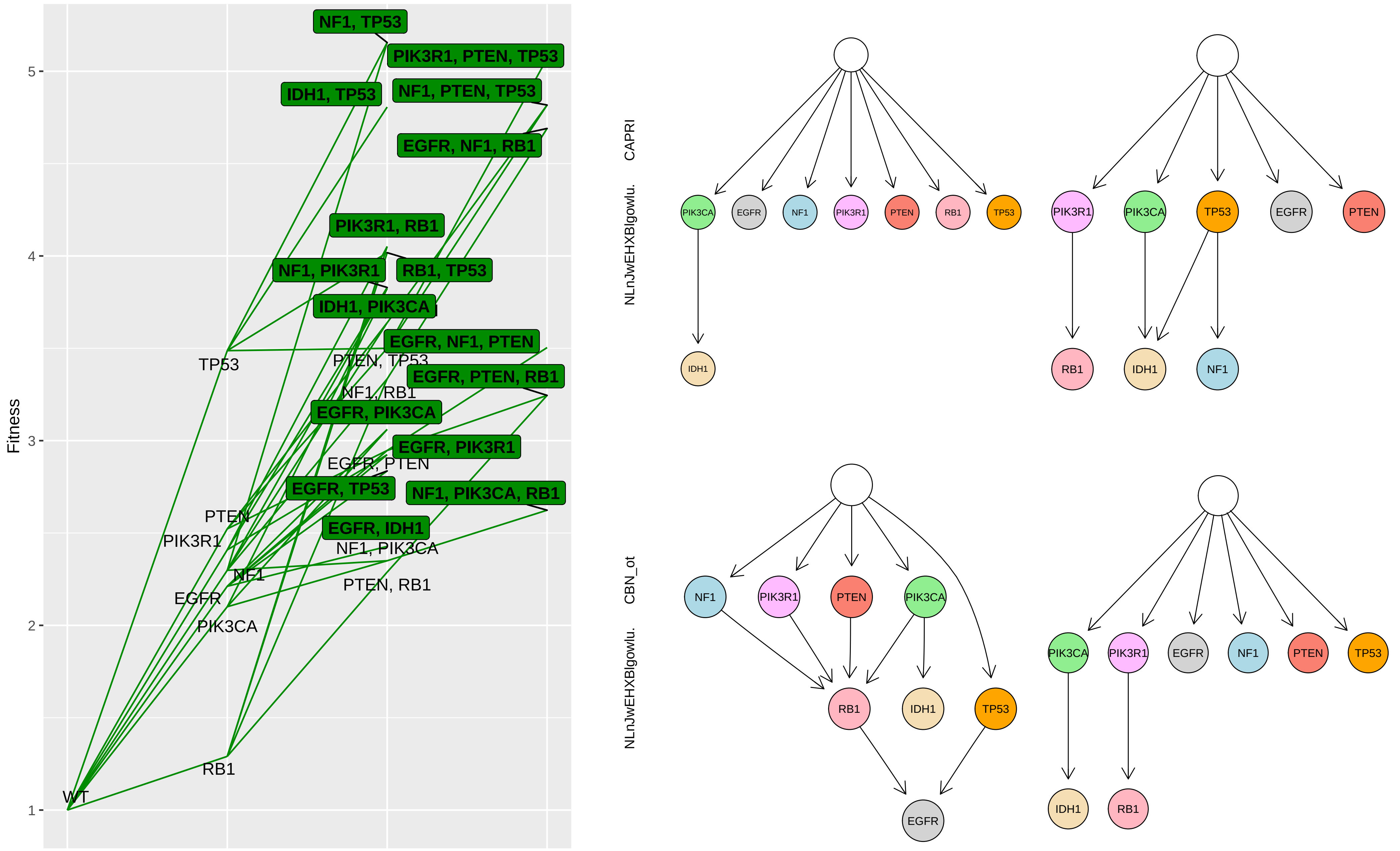

ID	p−value	Accessible Genot.
CWVyQzmujUIKLsL	0.859	26

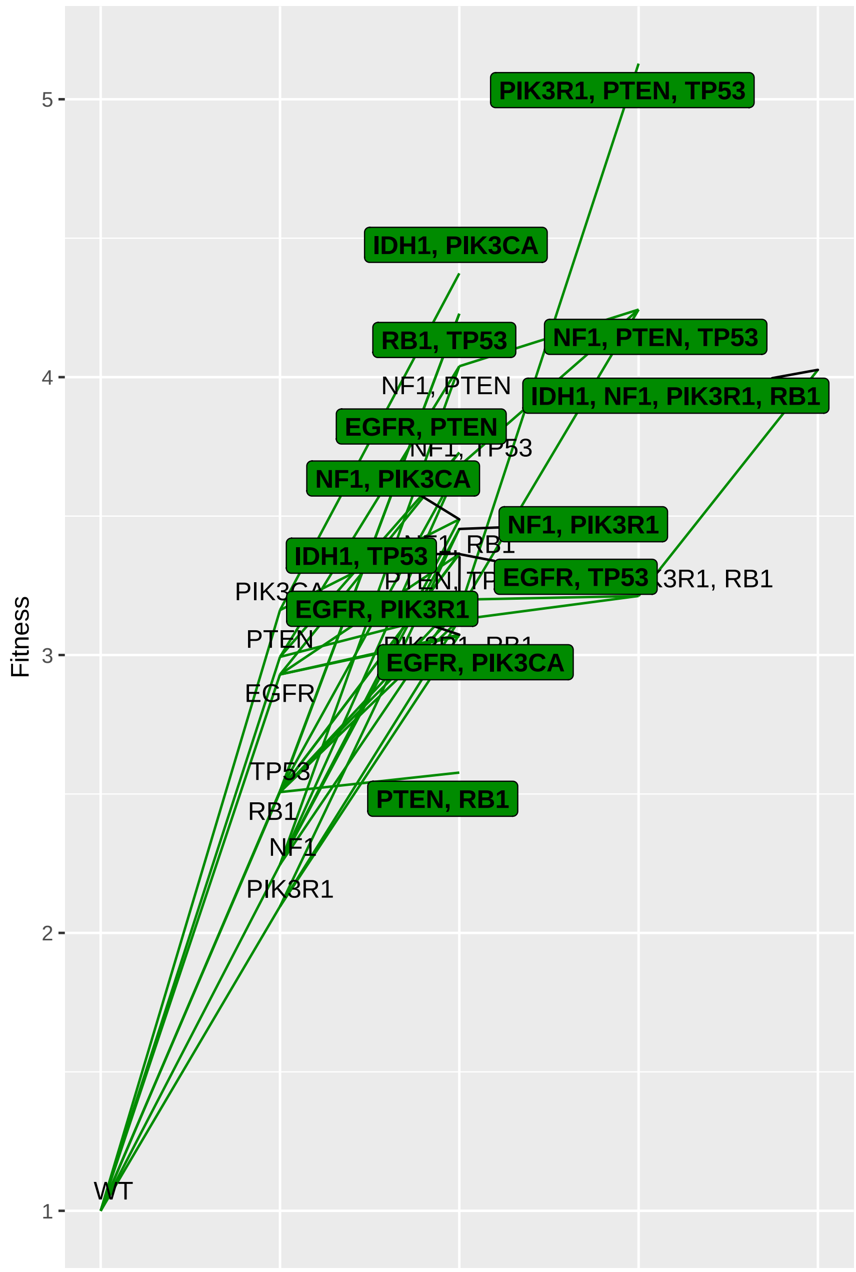

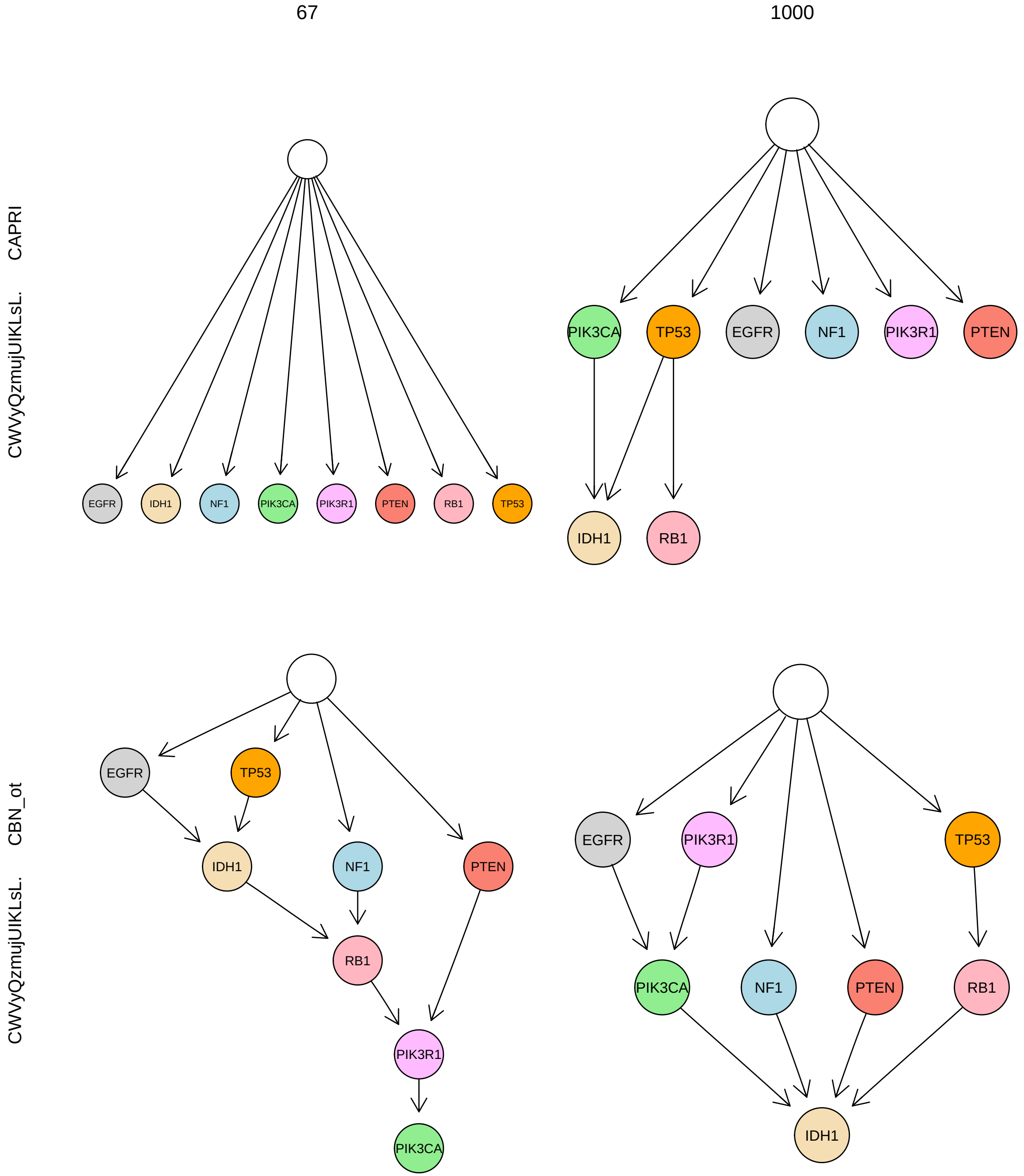

ID	p-value	Accessible Genot.
qJvVuWrAGDuojjP	0.861	24

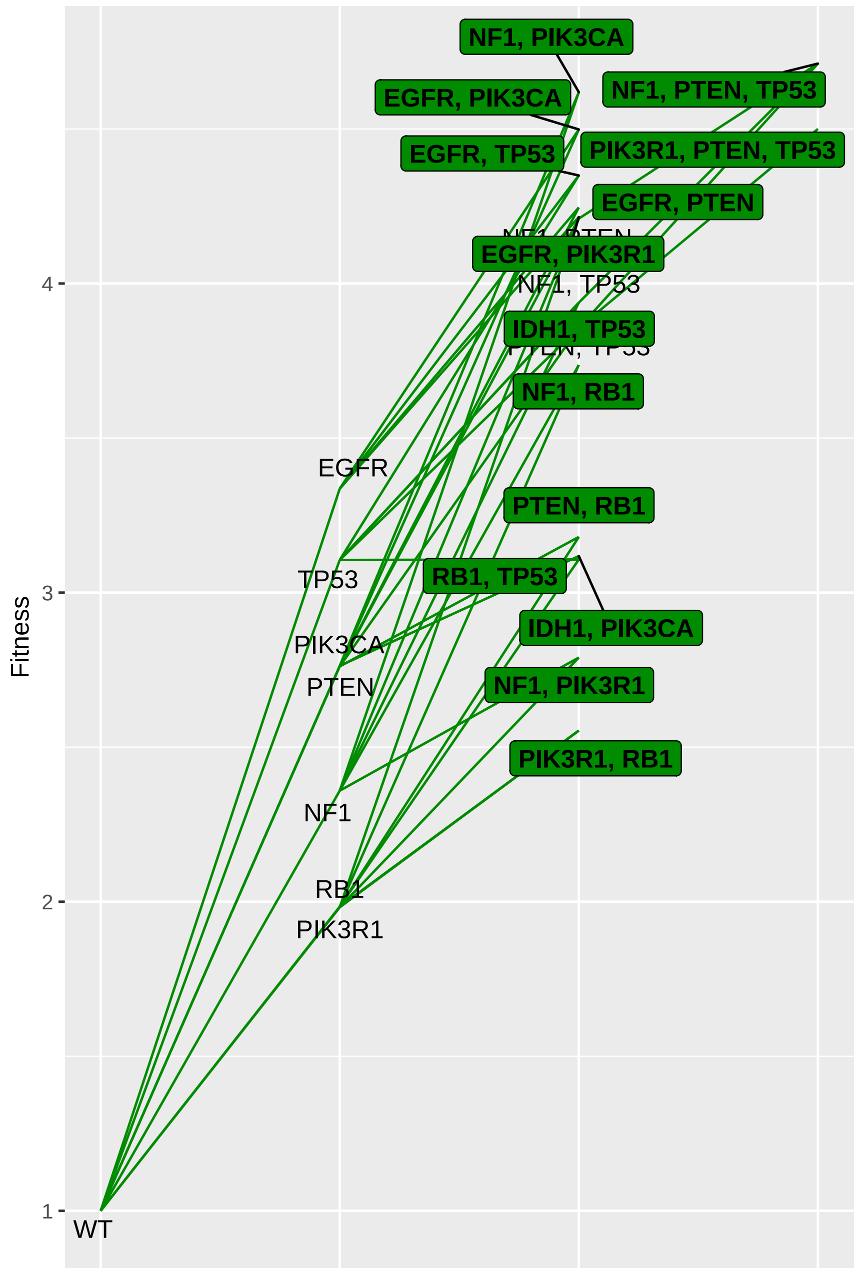

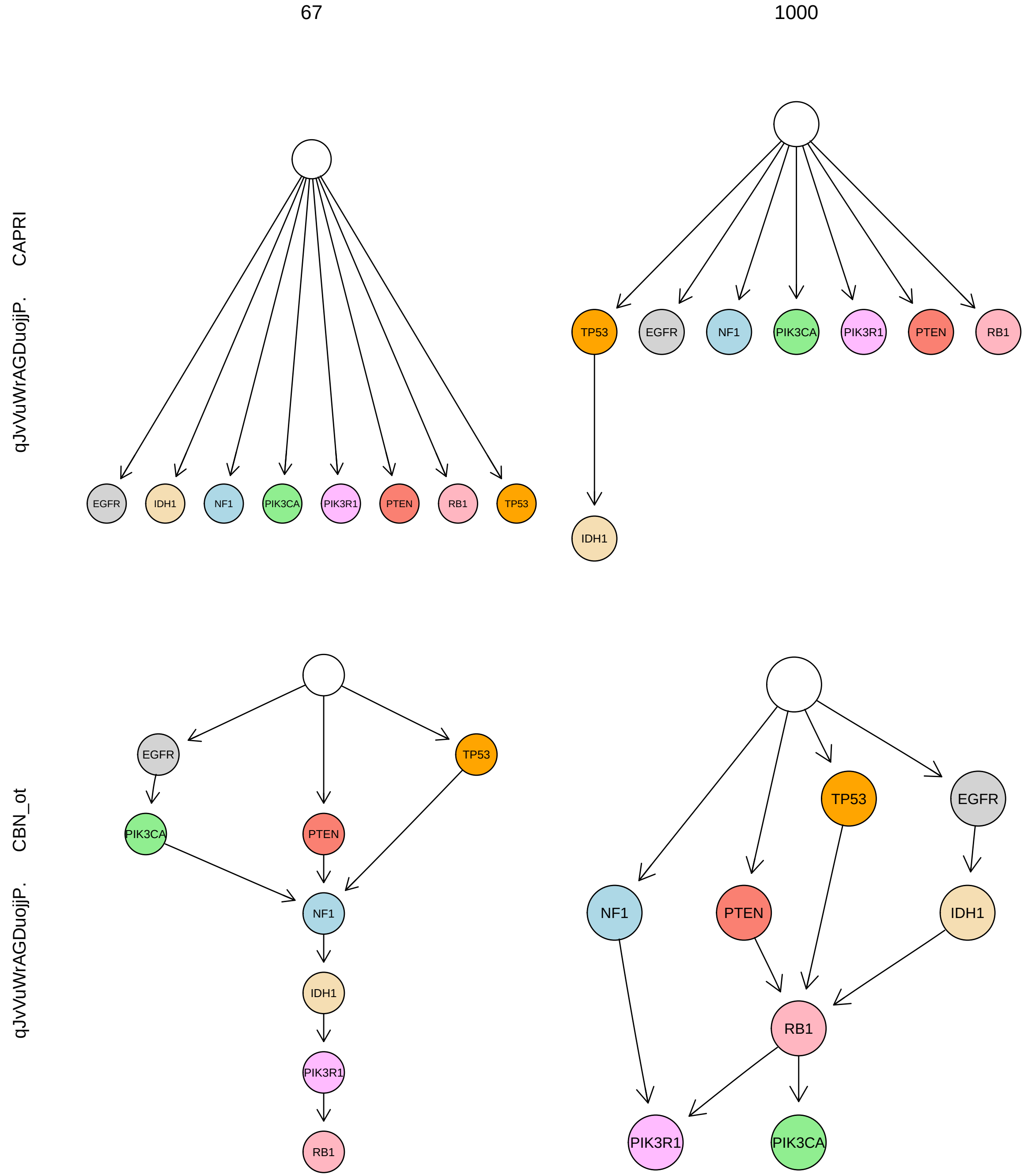

ID	p−value	Accessible Genot.
ENYdKZxIuUGoicz	0.866	28

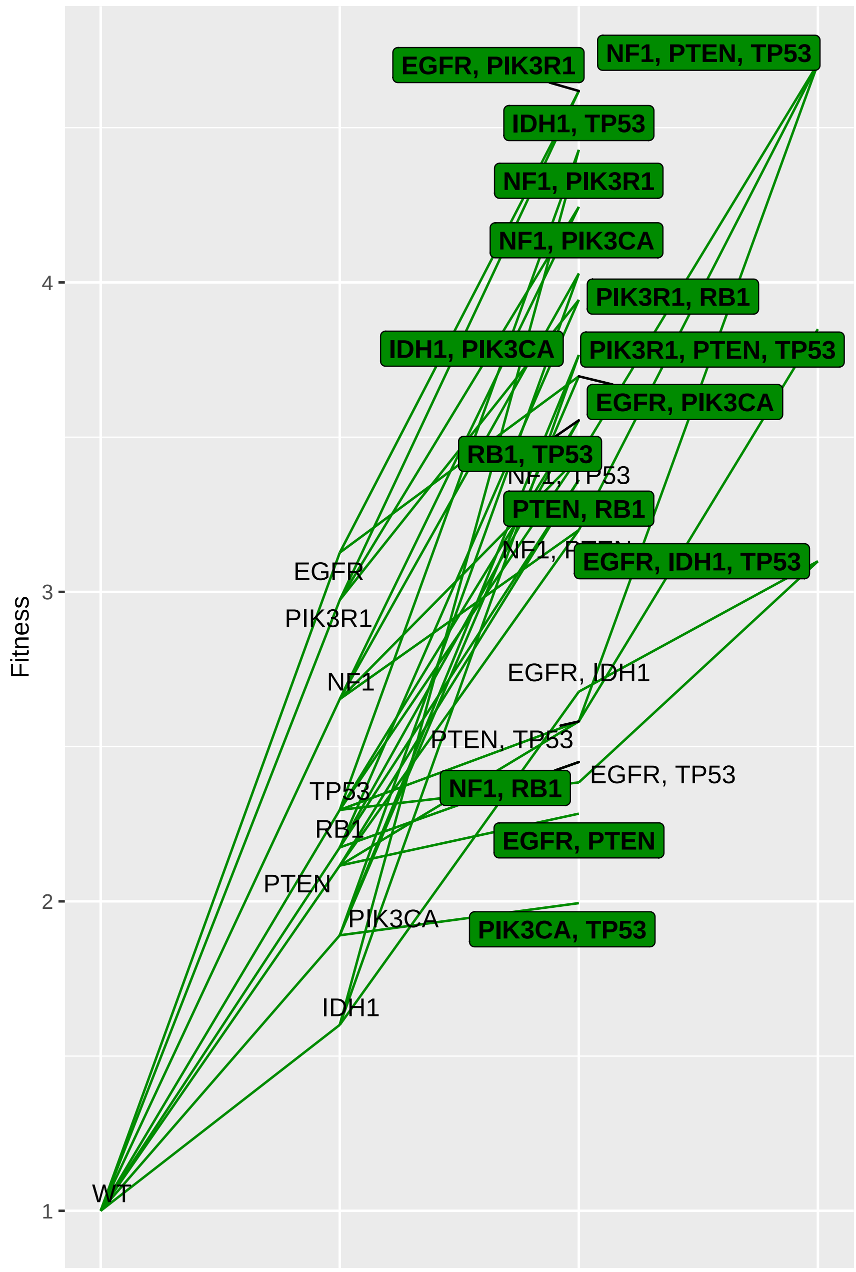

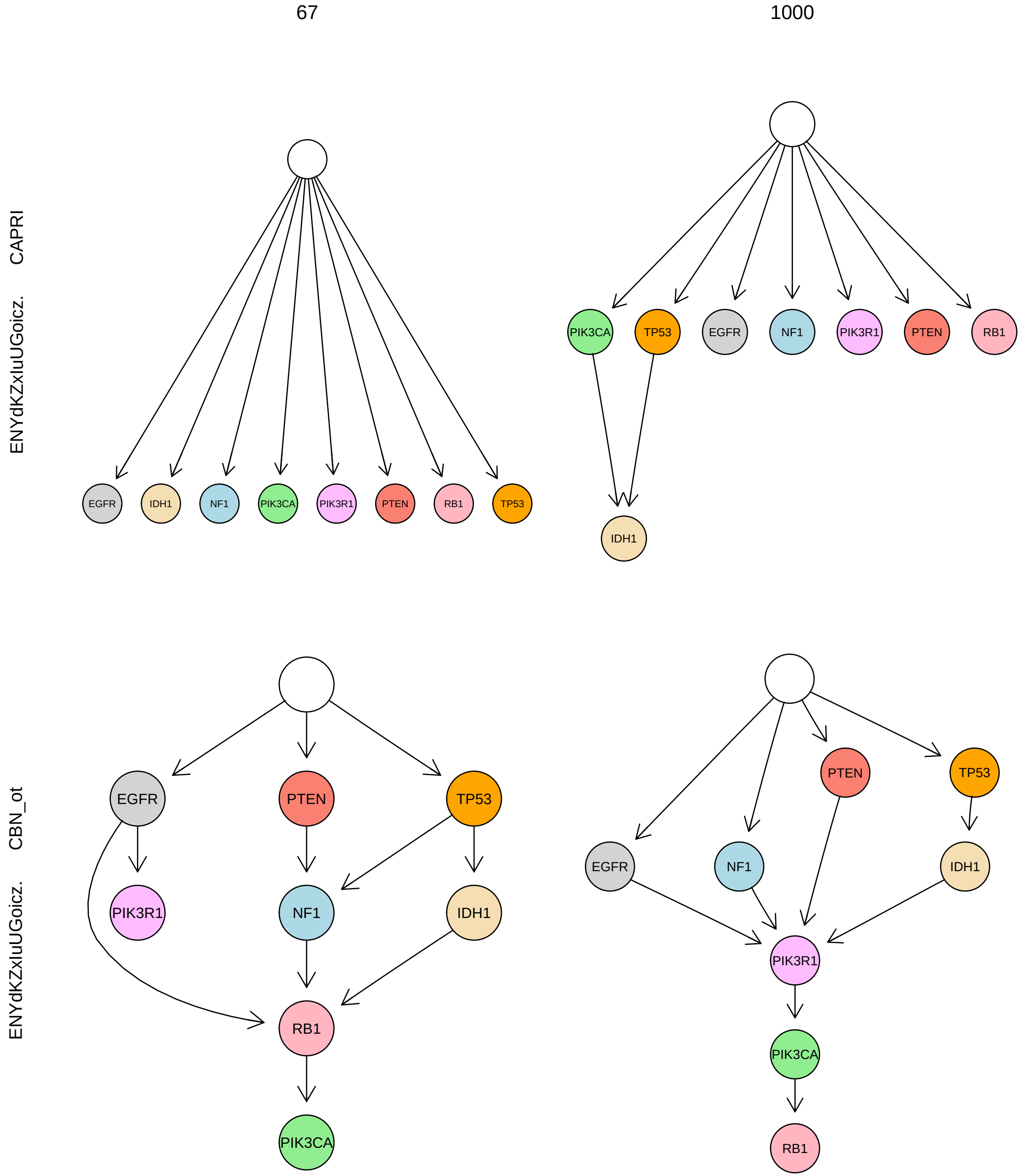

ID	p−value	Accessible Genot.
LvMbITOAfbxtffQ	0.869	25

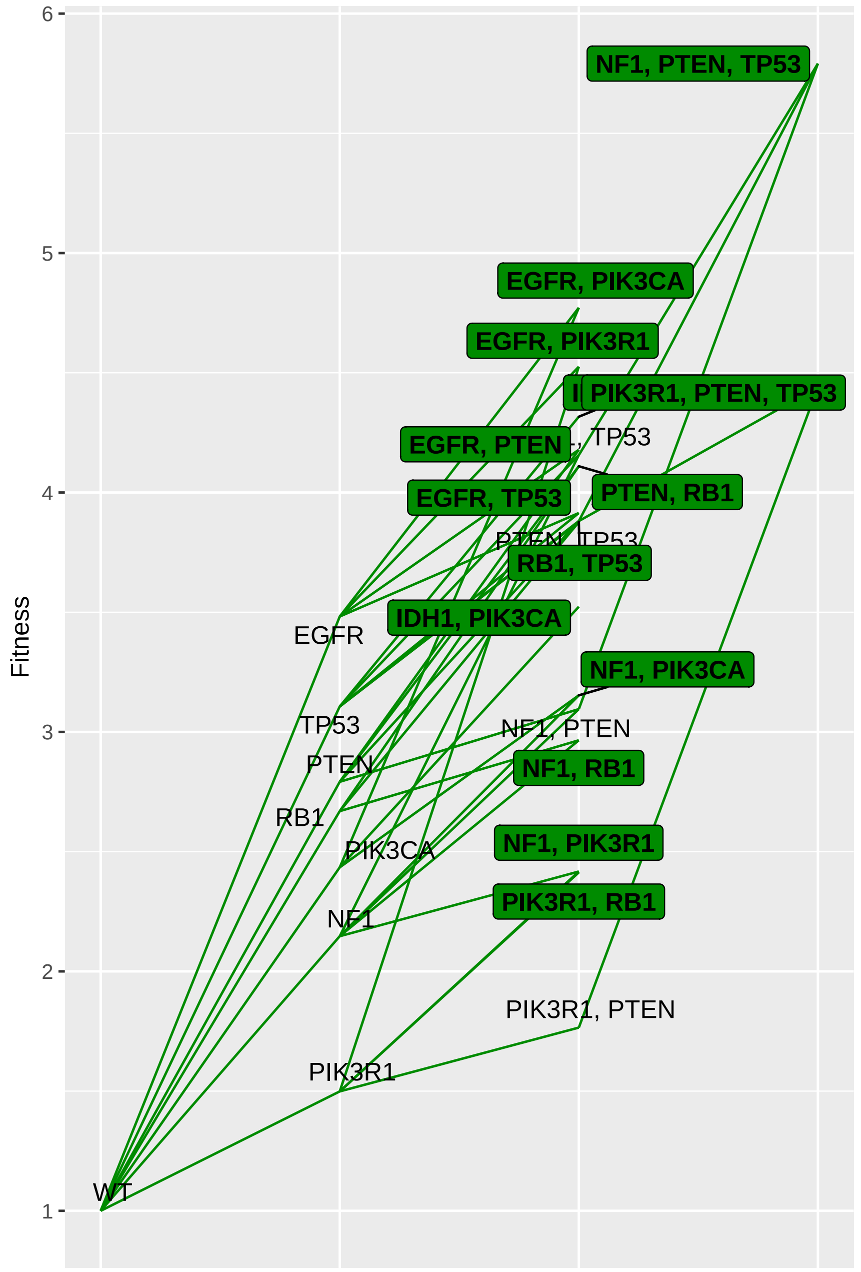

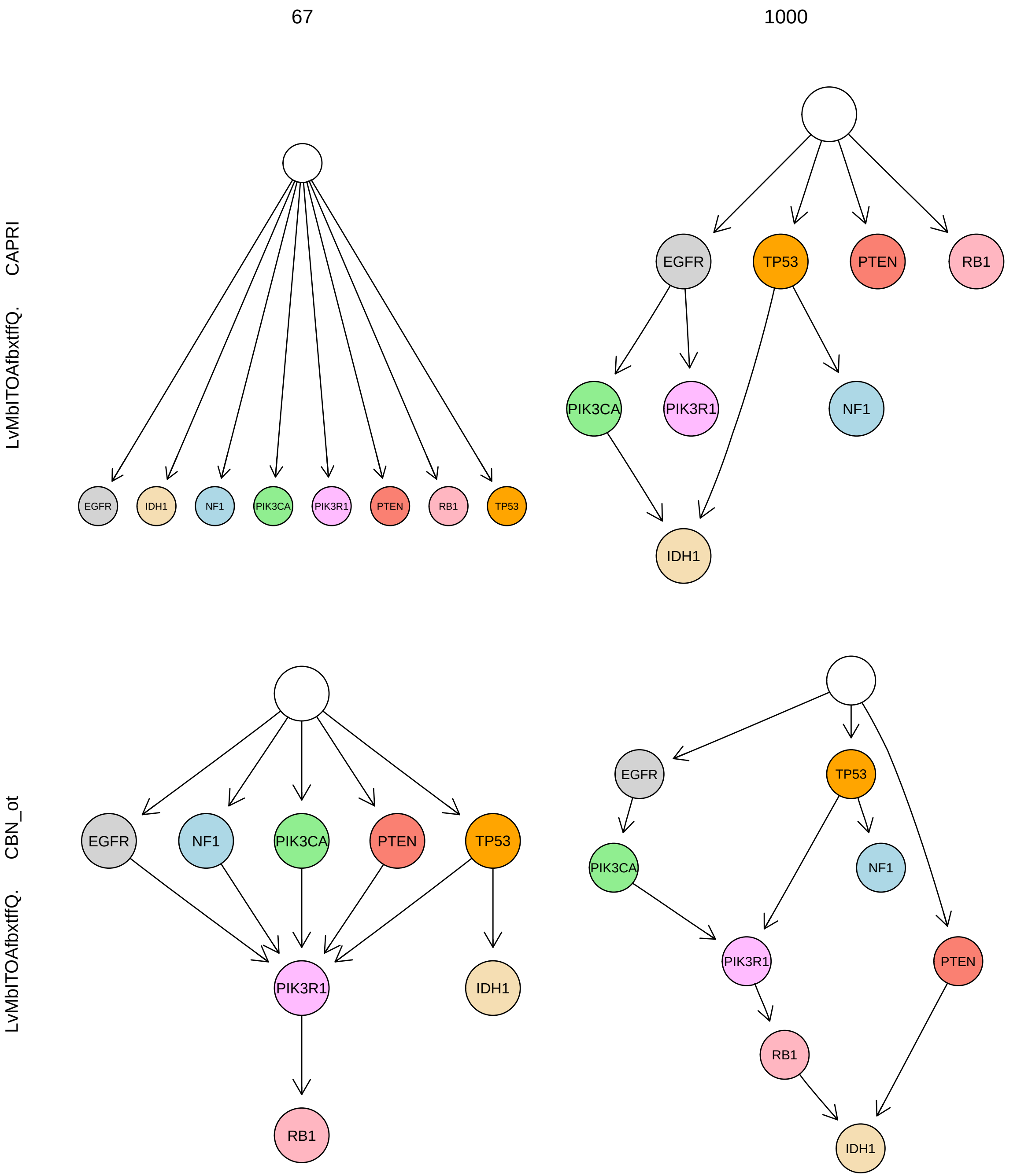

ID	p−value	Accessible Genot.
oUPIxXGiZaybsGU	0.869	61

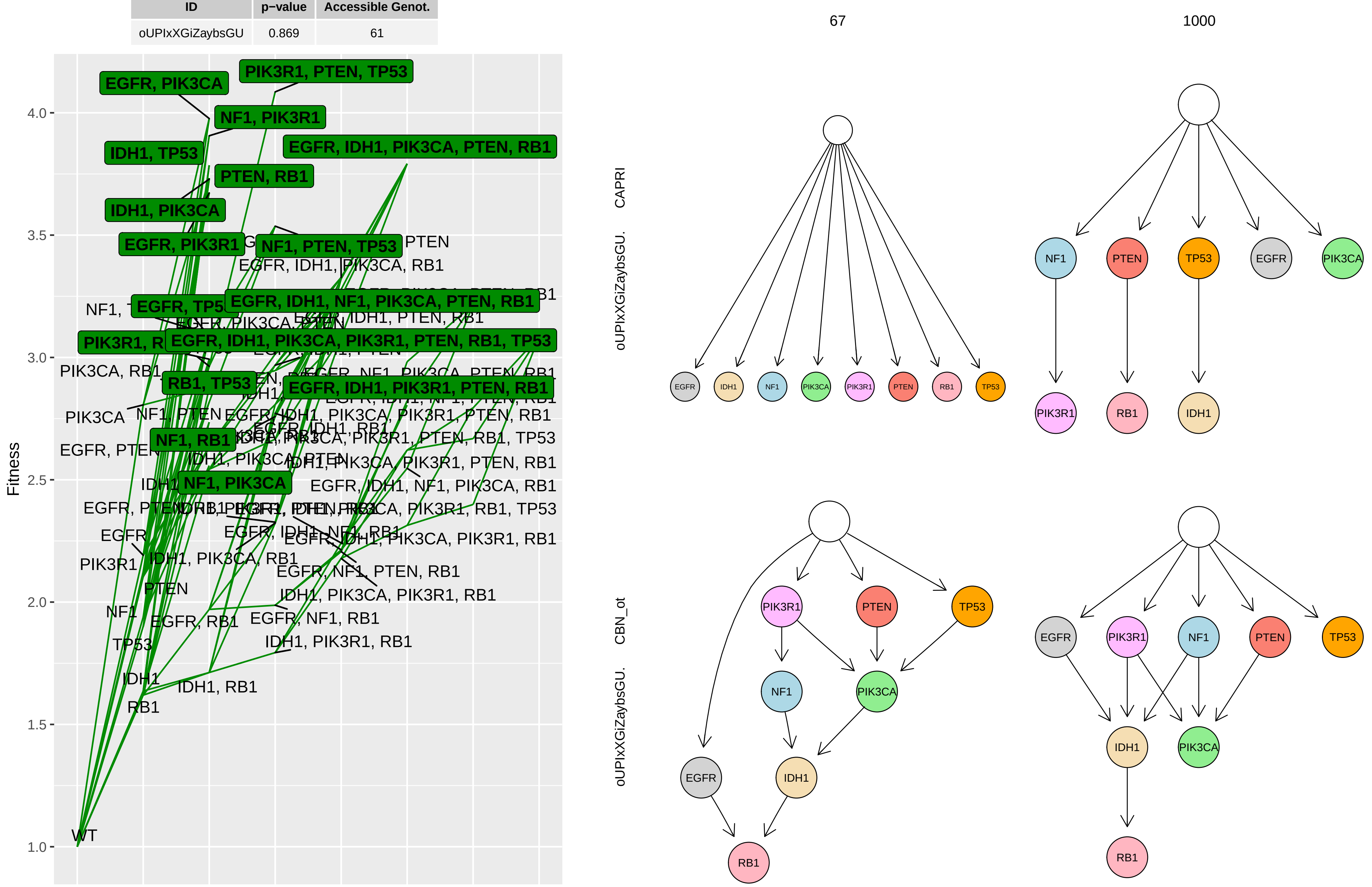

ID	p−value	Accessible Genot.
HUQrRCFYlUkXfgA	0.877	41

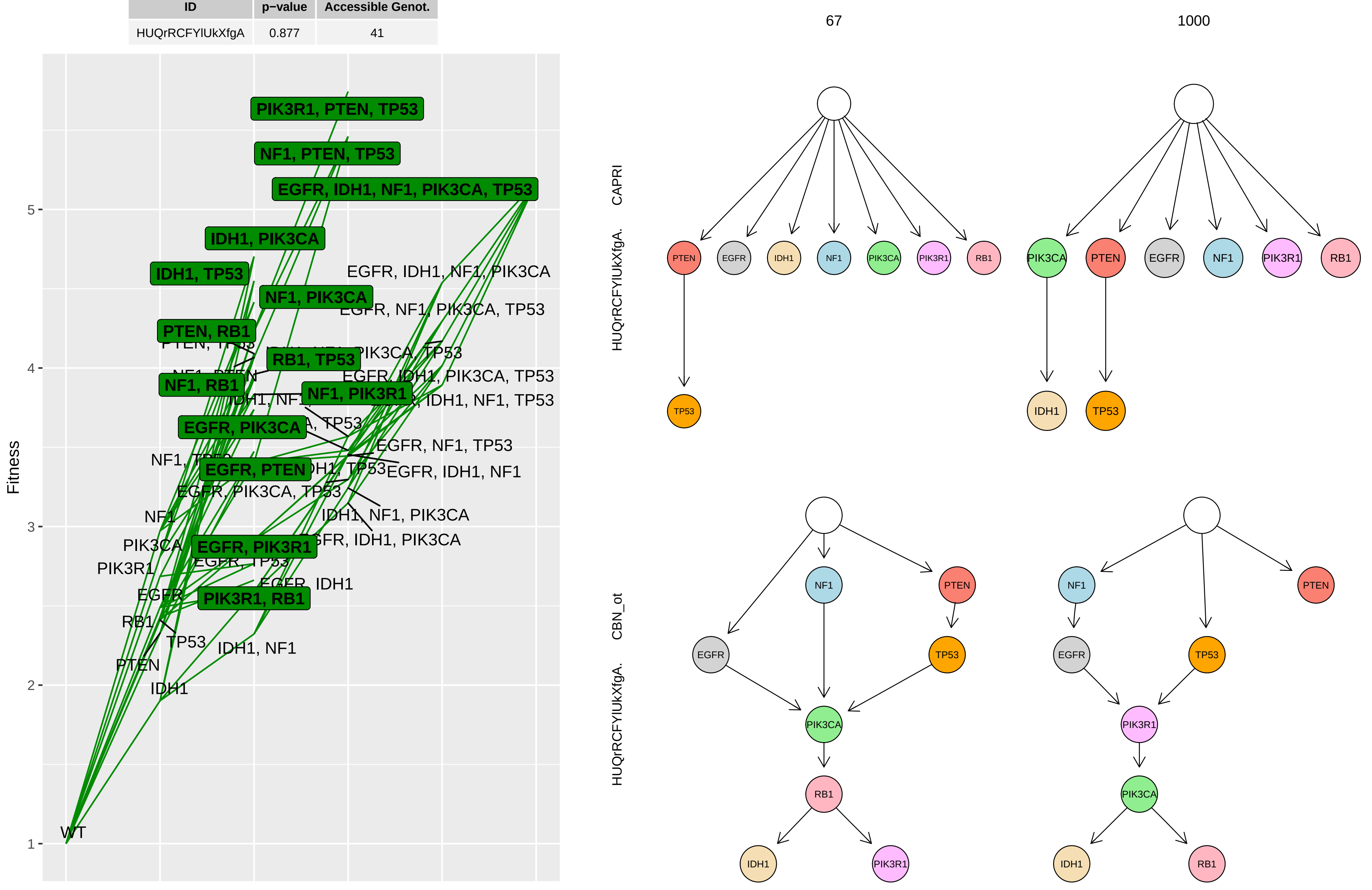

ID	p−value	Accessible Genot.
vpgGgMyXKjInvgV	0.887	25

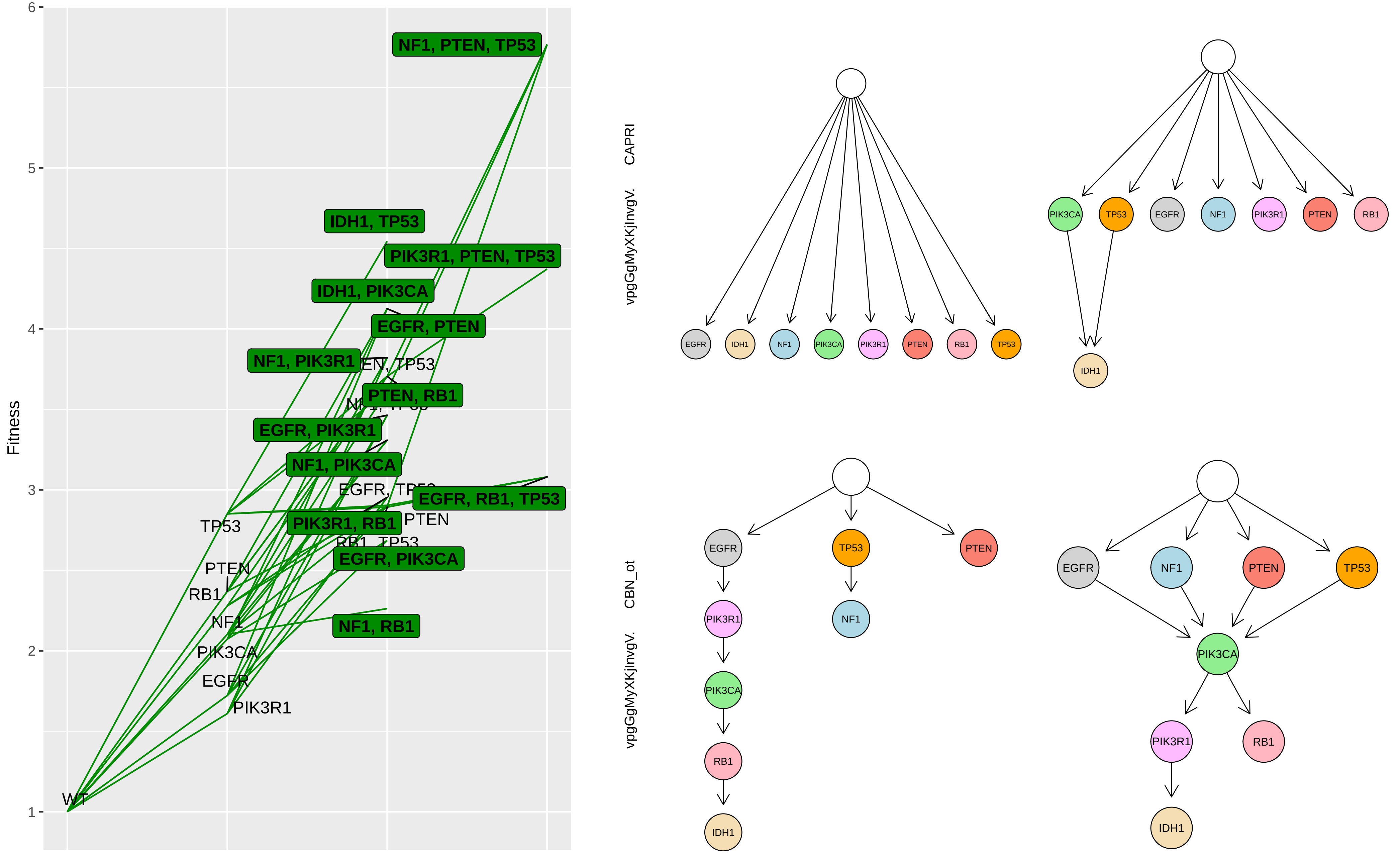

ID	p−value	Accessible Genot.
yEcaDboCIYFAQZB	0.892	26

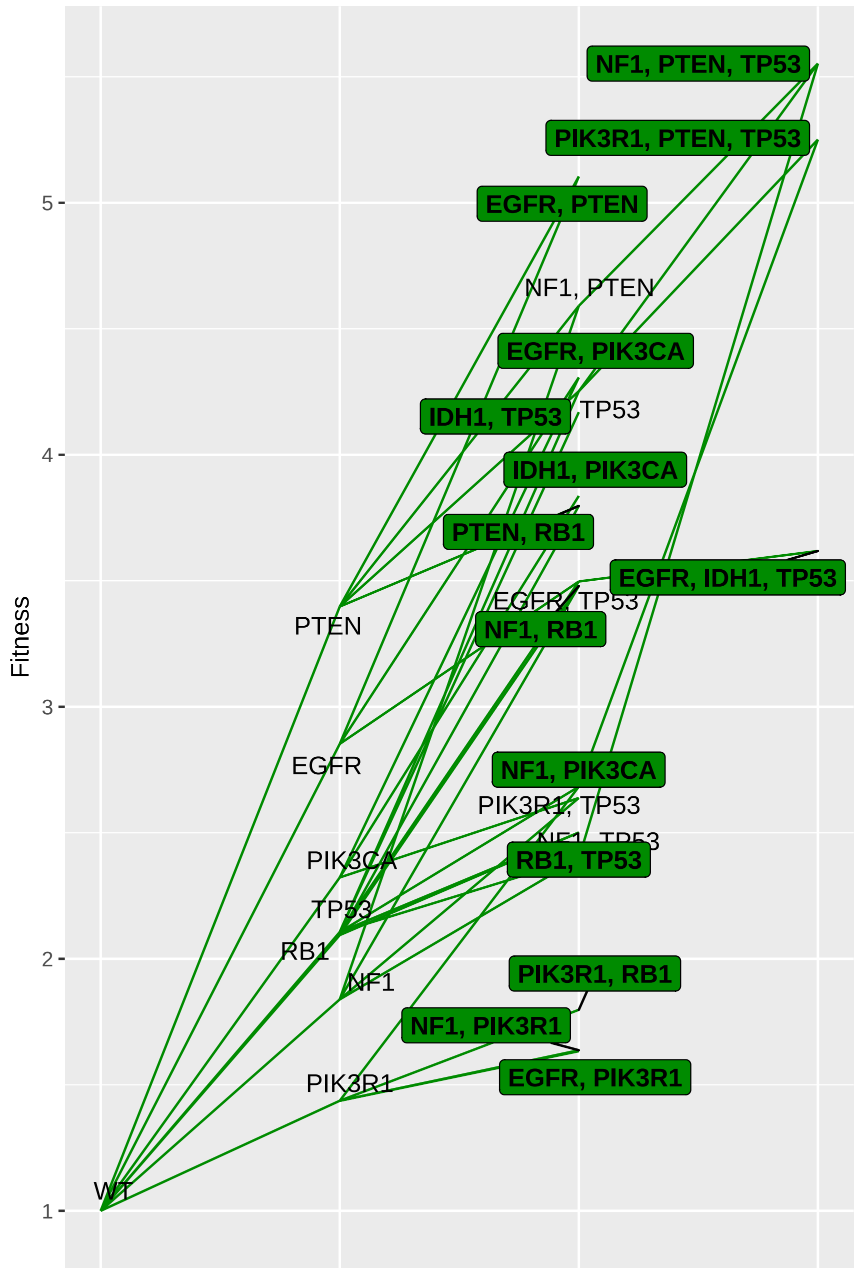

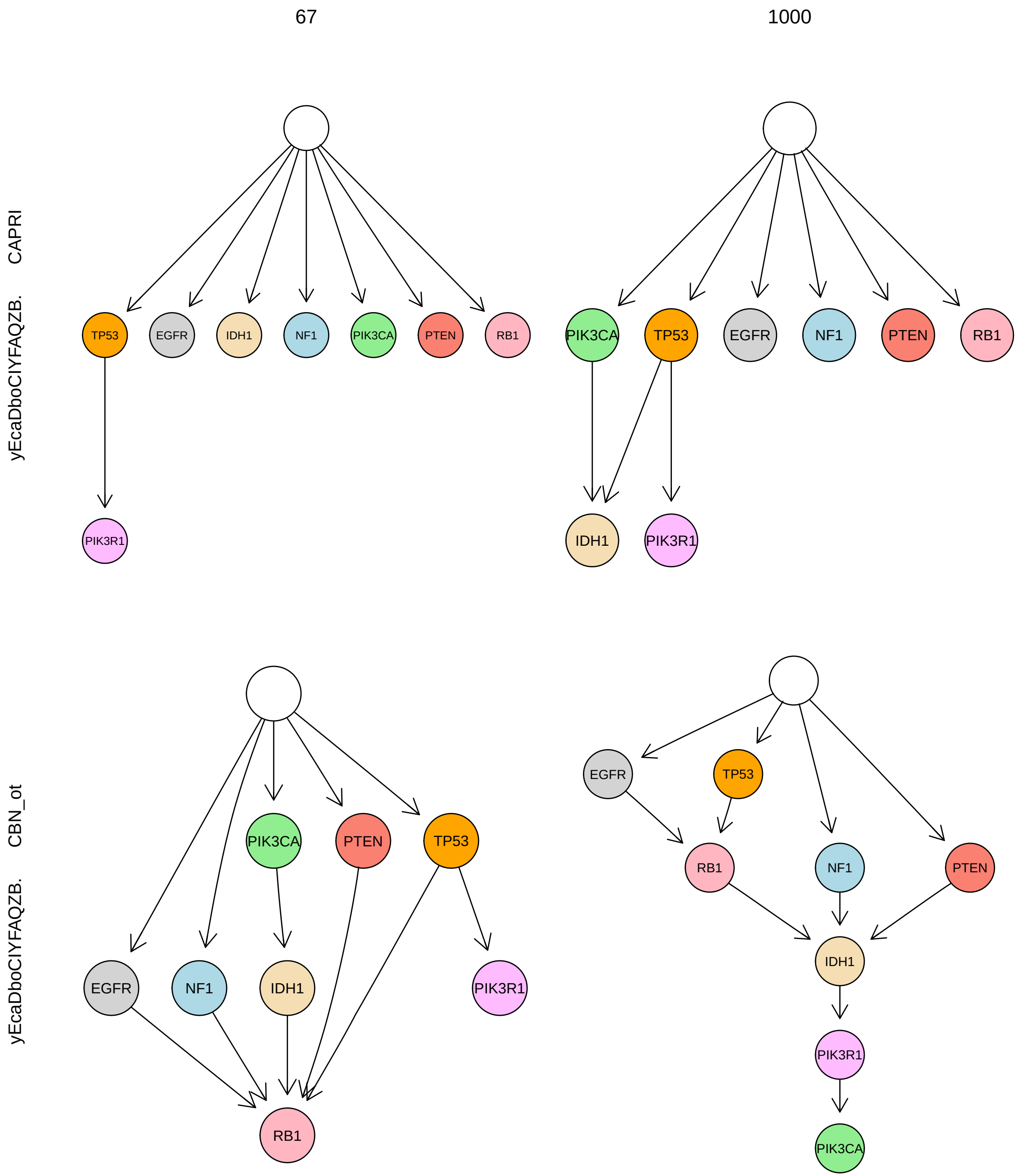

ID	p−value	Accessible Genot.
MACHKsWnyFLCPzl	0.901	65

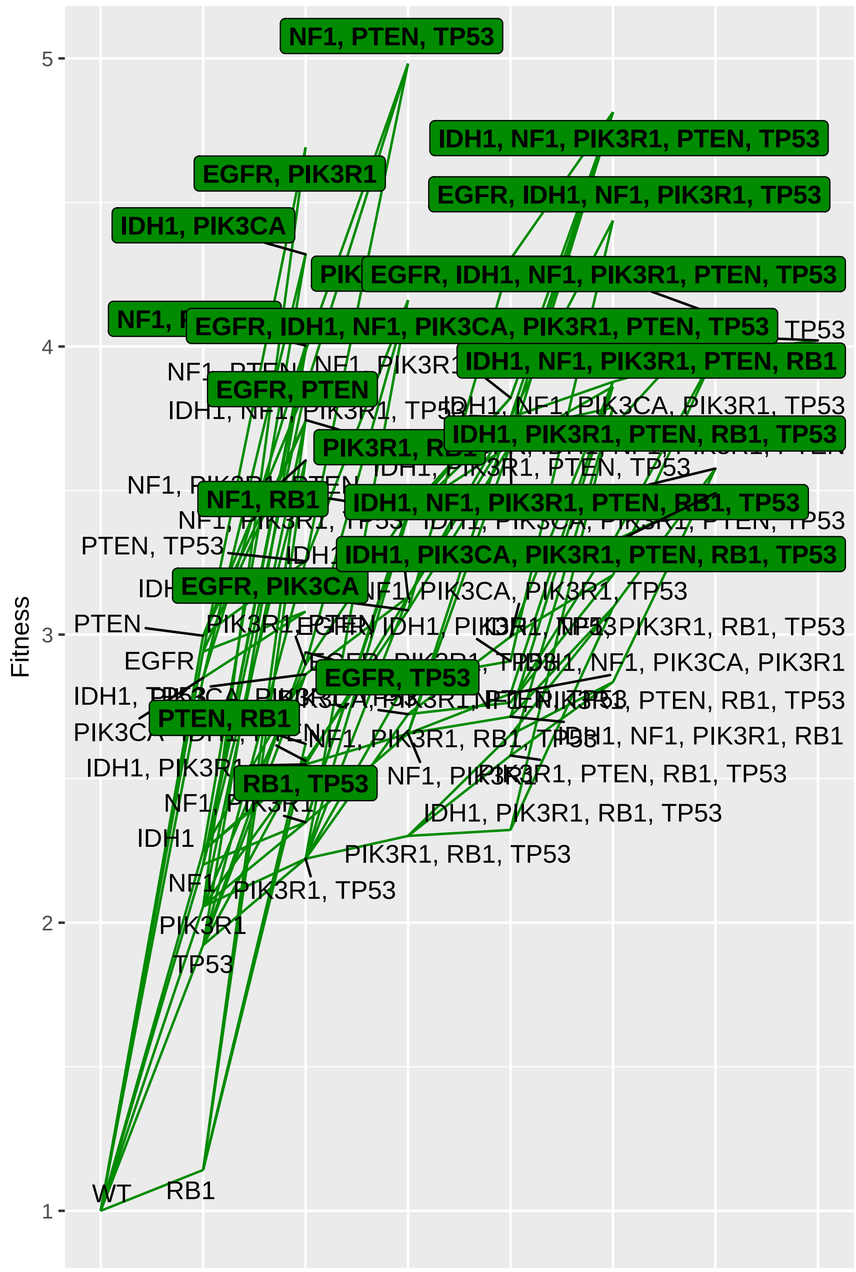

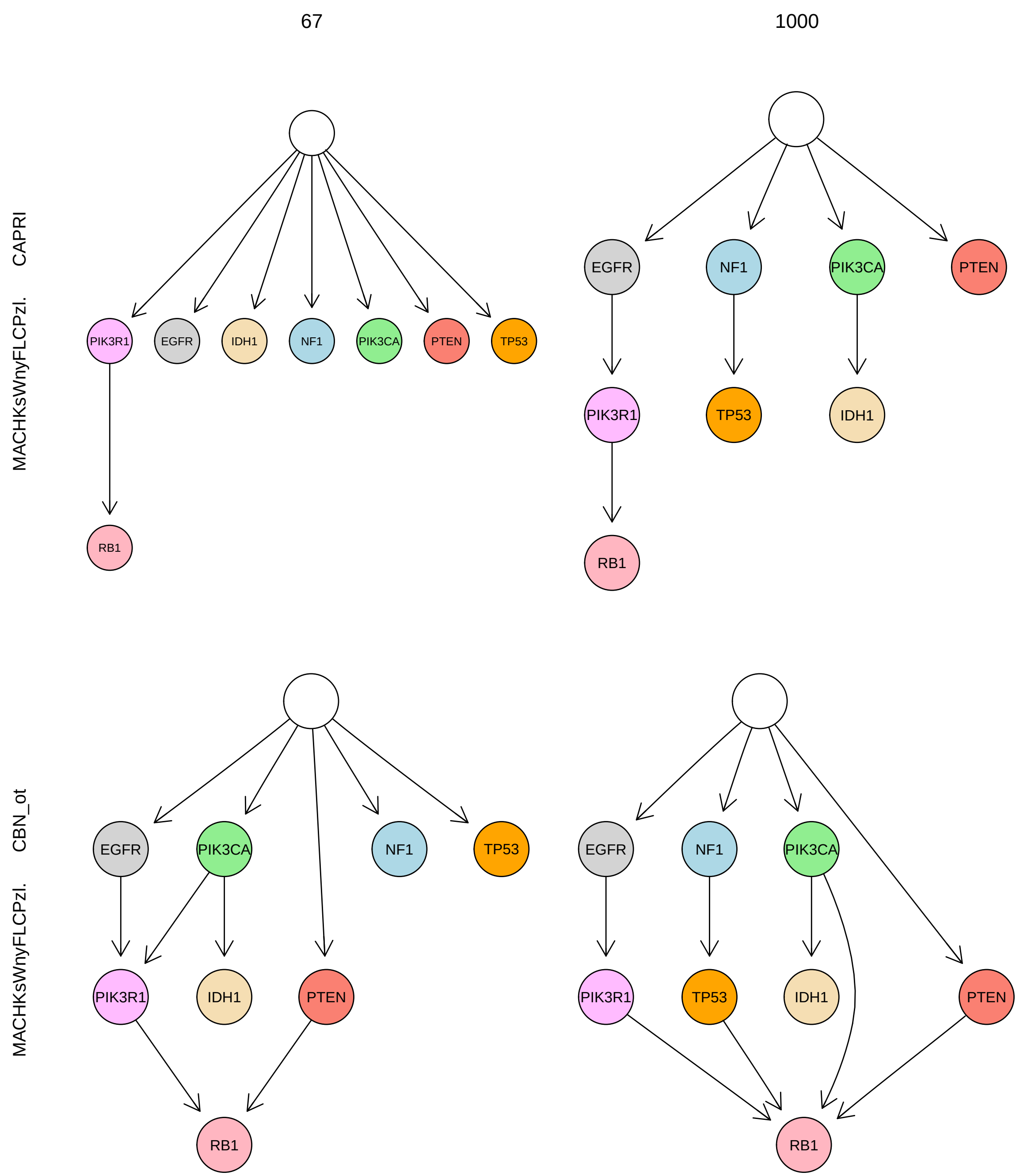

ID	p−value	Accessible Genot.
XbiTCWnybelrRyO	0.905	254

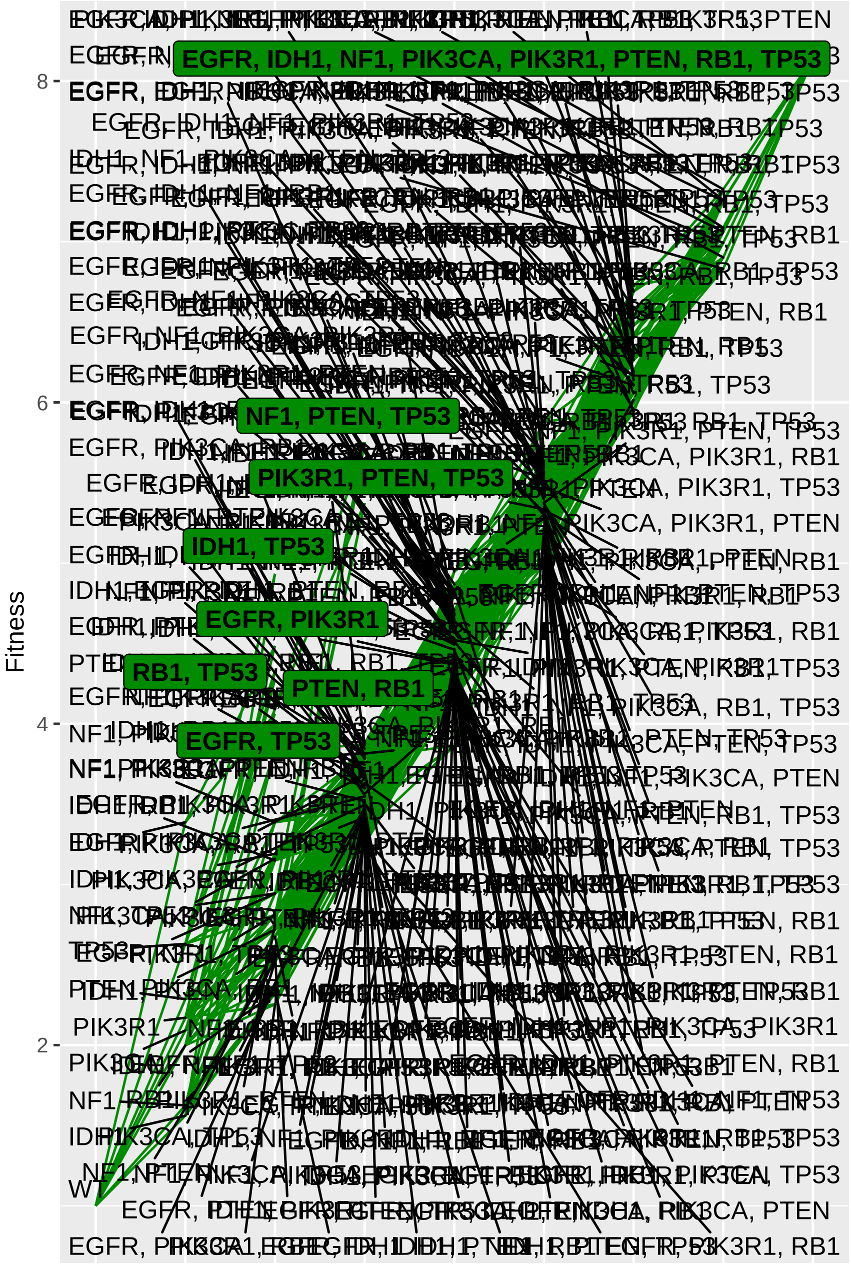

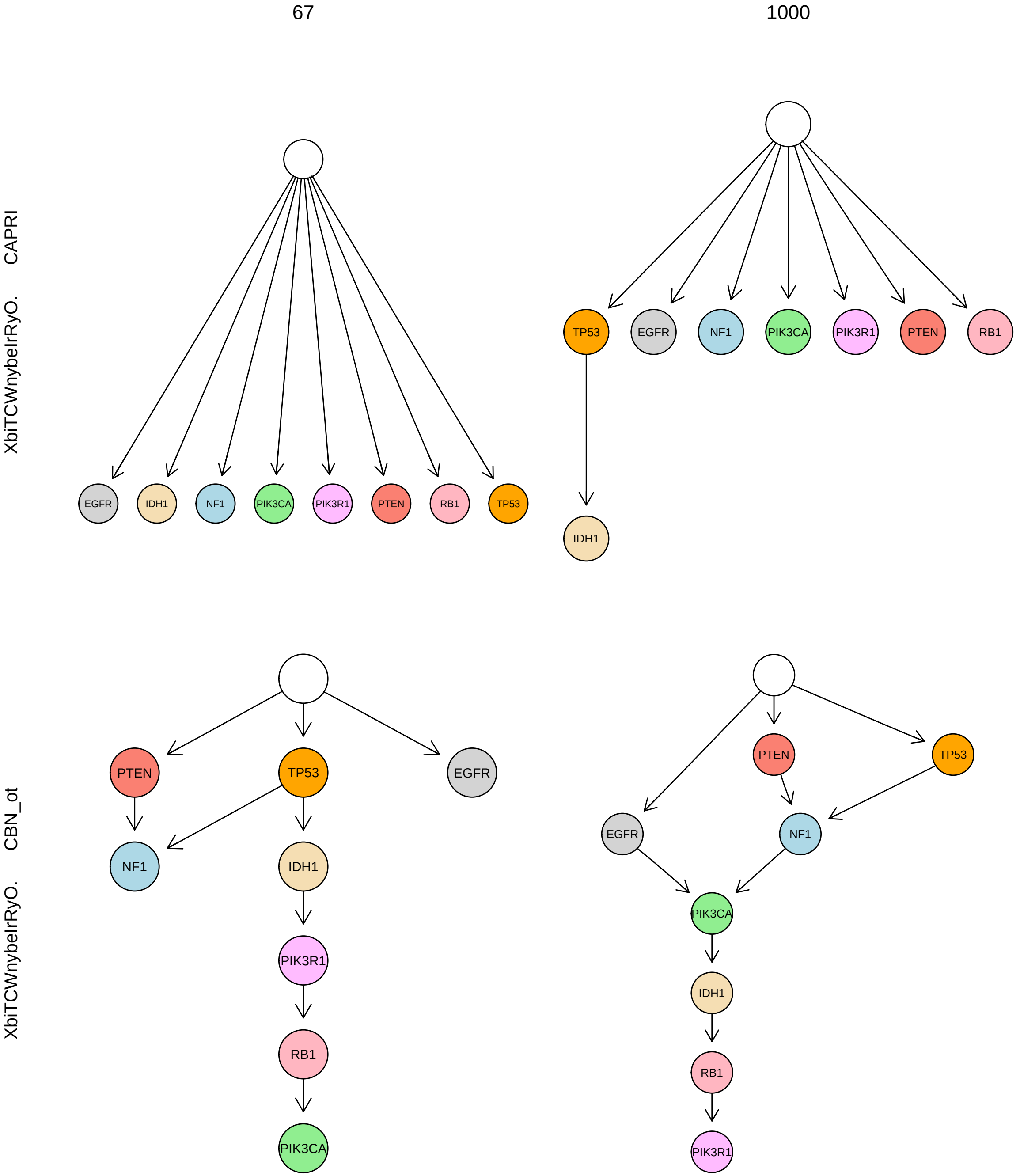

ID	p-value	Accessible Genot.
xPfwgeSvlKvvKJq	0.931	46

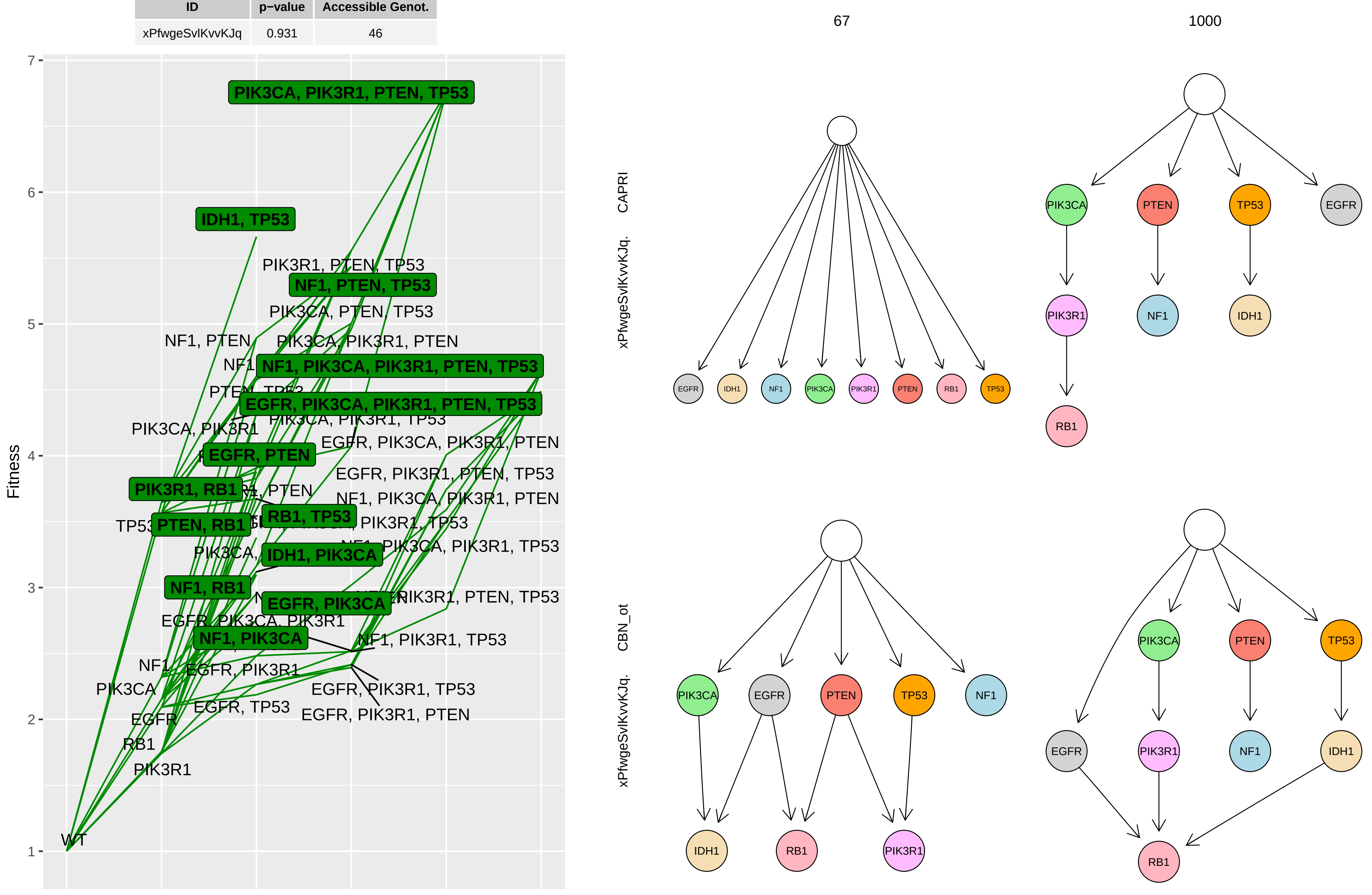

ID	p−value	Accessible Genot.
vemXhGilbRJWpKr	0.936	35

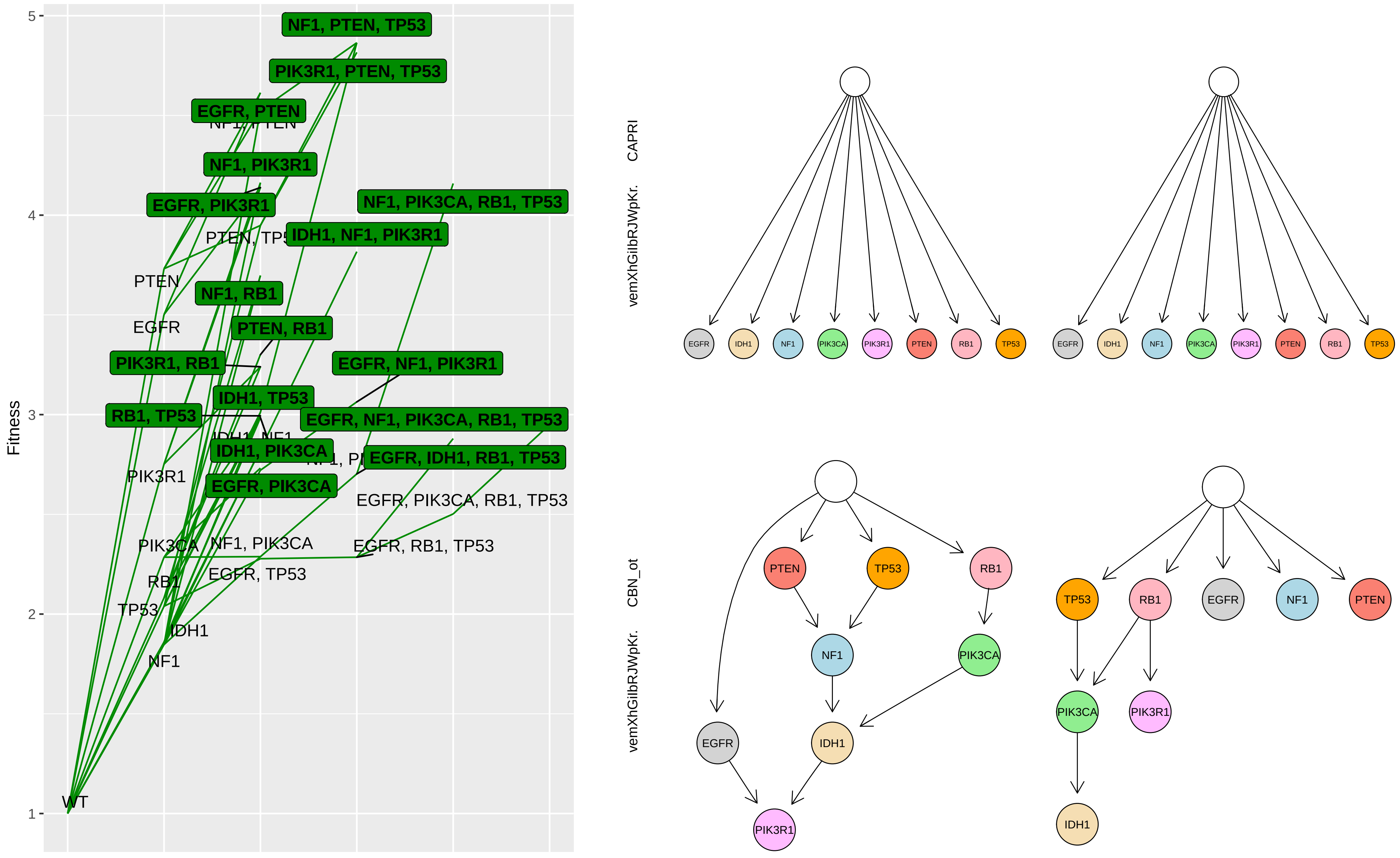

ID	p−value	Accessible Genot.
WKFYqalyAXBXpBm	0.947	47

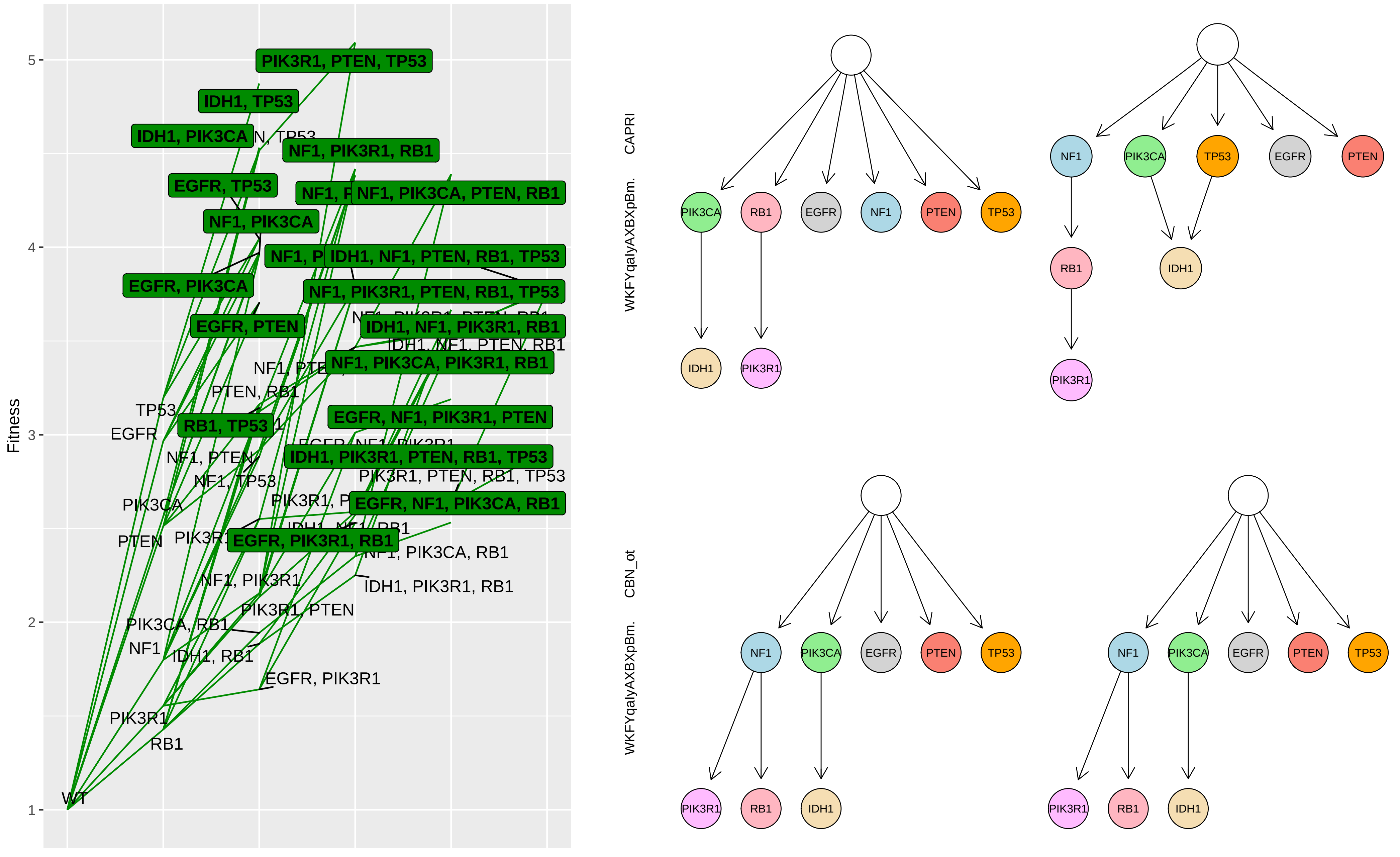

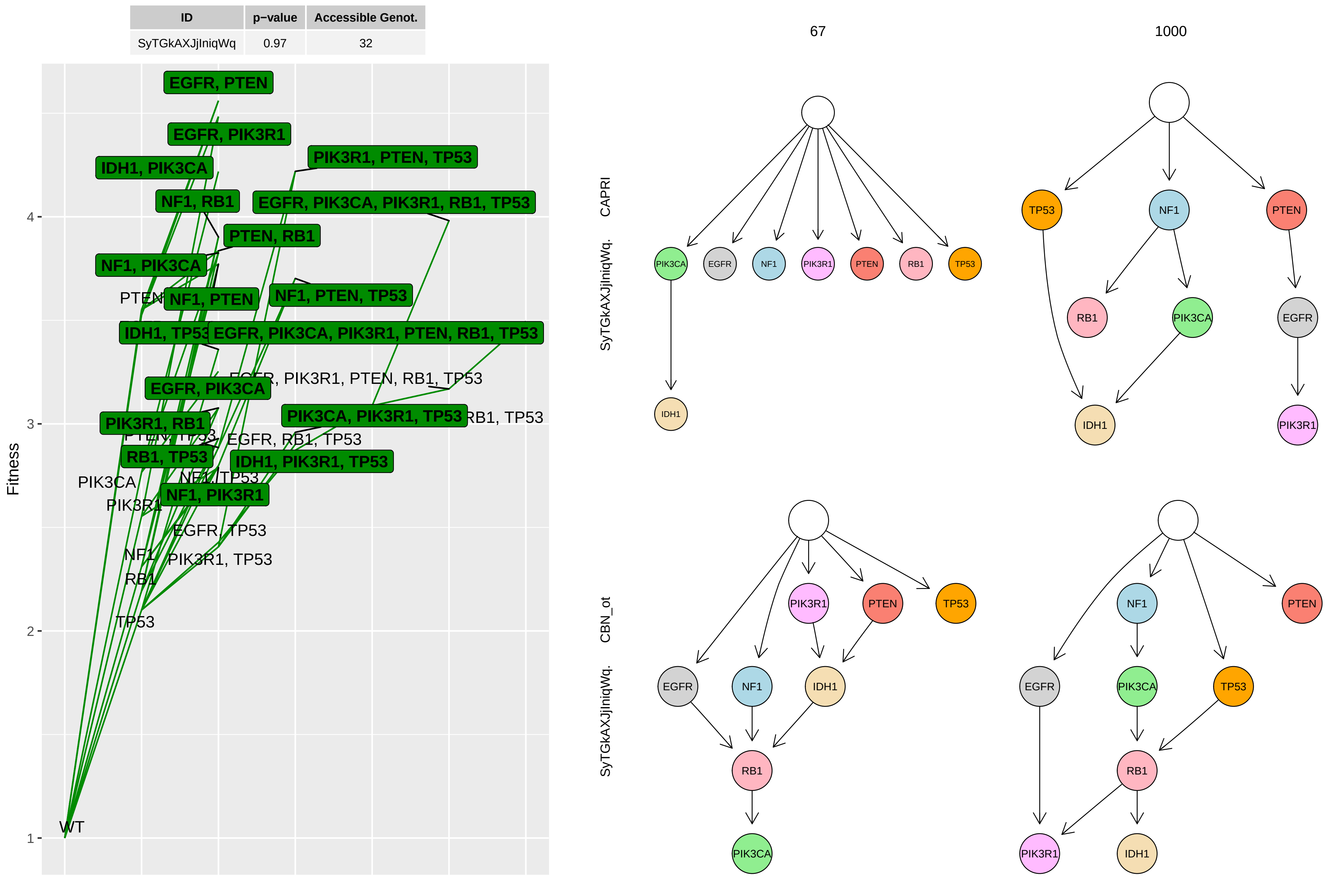

ID
p−value
Accessible Genot.
SyTGkAXJjIniqWq
0.97
32
Fitness
EGFR, PTEN
EGFR, PIK3R1
IDH1, PIK3CA
PIK3R1, PTEN, TP53
NF1, RB1
EGFR, PIK3CA, PIK3R1, RB1, TP53
PTEN, RB1
NF1, PIK3CA
NF1, PTEN
NF1, PTEN, TP53
PTEN
IDH1, TP53
EGFR, PIK3CA, PIK3R1, PTEN, RB1, TP53
EGFR, PIK3CA
PIK3R1, RB1
PIK3CA, PIK3R1, TP53
RB1, TP53
EGFR, RB1, TP53
IDH1, PIK3R1, TP53
PIK3CA
NF1, PIK3R1
PIK3R1
EGFR, TP53
NF1
PIK3R1, TP53
RB1
TP53
WT
1
2
3
4
67
1000
CAPRI
CBN_ot
SyTGkAXJjIniqWq.
PIK3CA
EGFR
NF1
PIK3R1
PTEN
RB1
TP53
IDH1
