# Supplementary material for: Cancer progression models and fitness landscapes: a many-to-many relationship
Source: Bioinformatics. 2017 Oct 18;34(5):836–44. doi: 10.1093/bioinformatics/btx663 (PMC6031050; doi:10.1093/bioinformatics/btx663)

ID	p−value	Accessible Genot.
PWlvCDOwmMzejBo	0.602	20

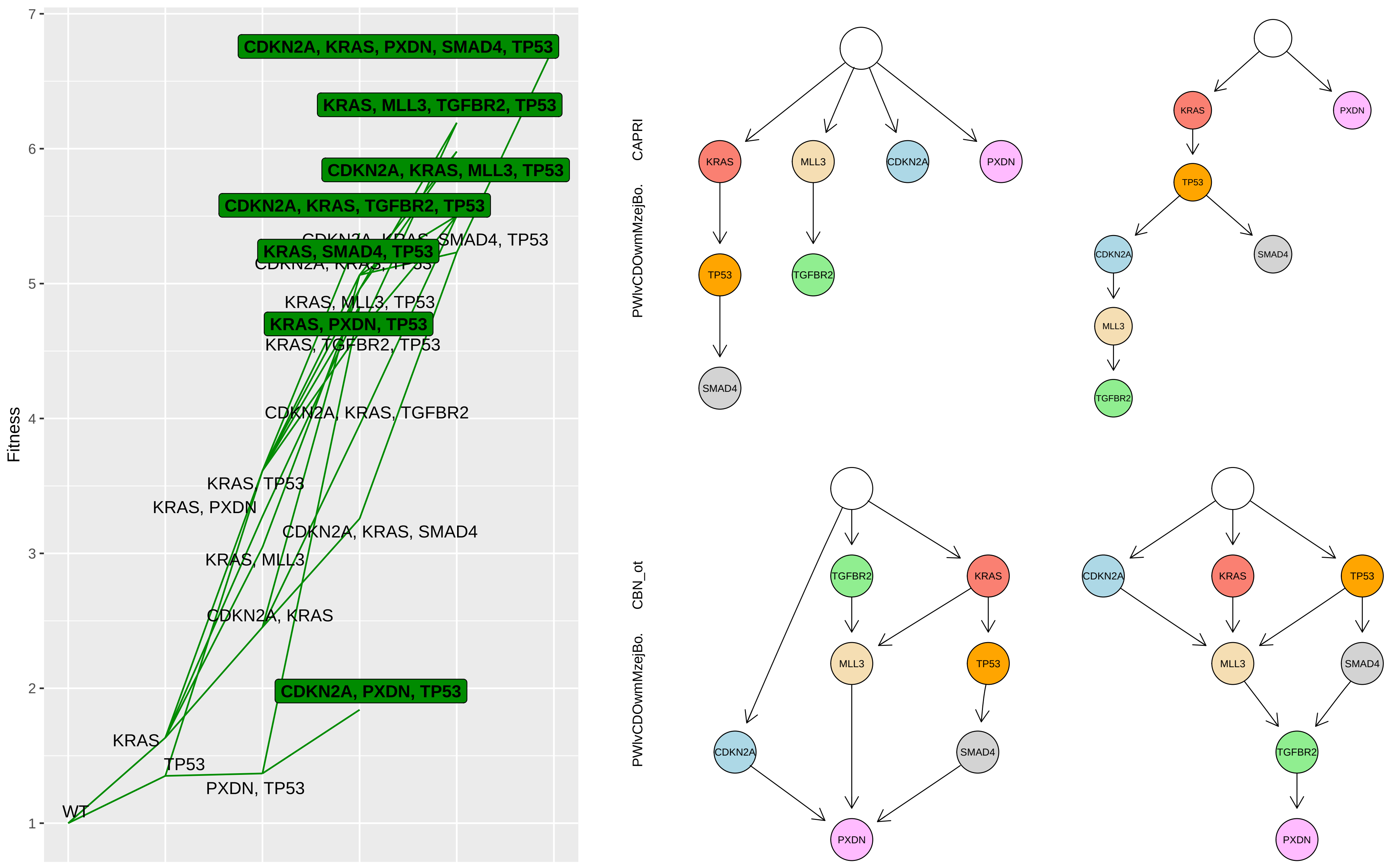

ID	p−value	Accessible Genot.
EzvzeSeelQdZGzY	0.602	18

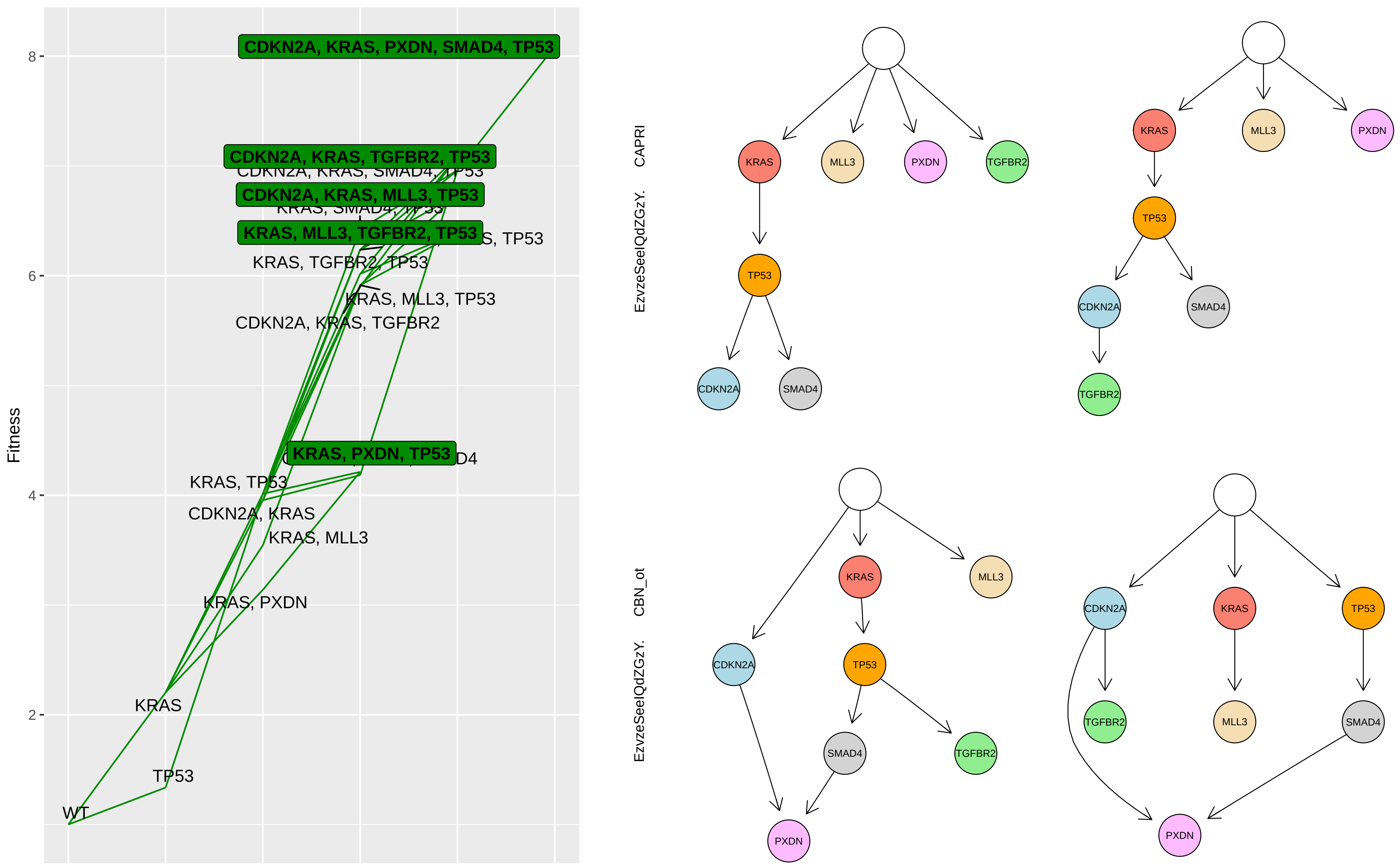

ID	p−value	Accessible Genot.
AZhpnTfAXIHWfzJ	0.603	19

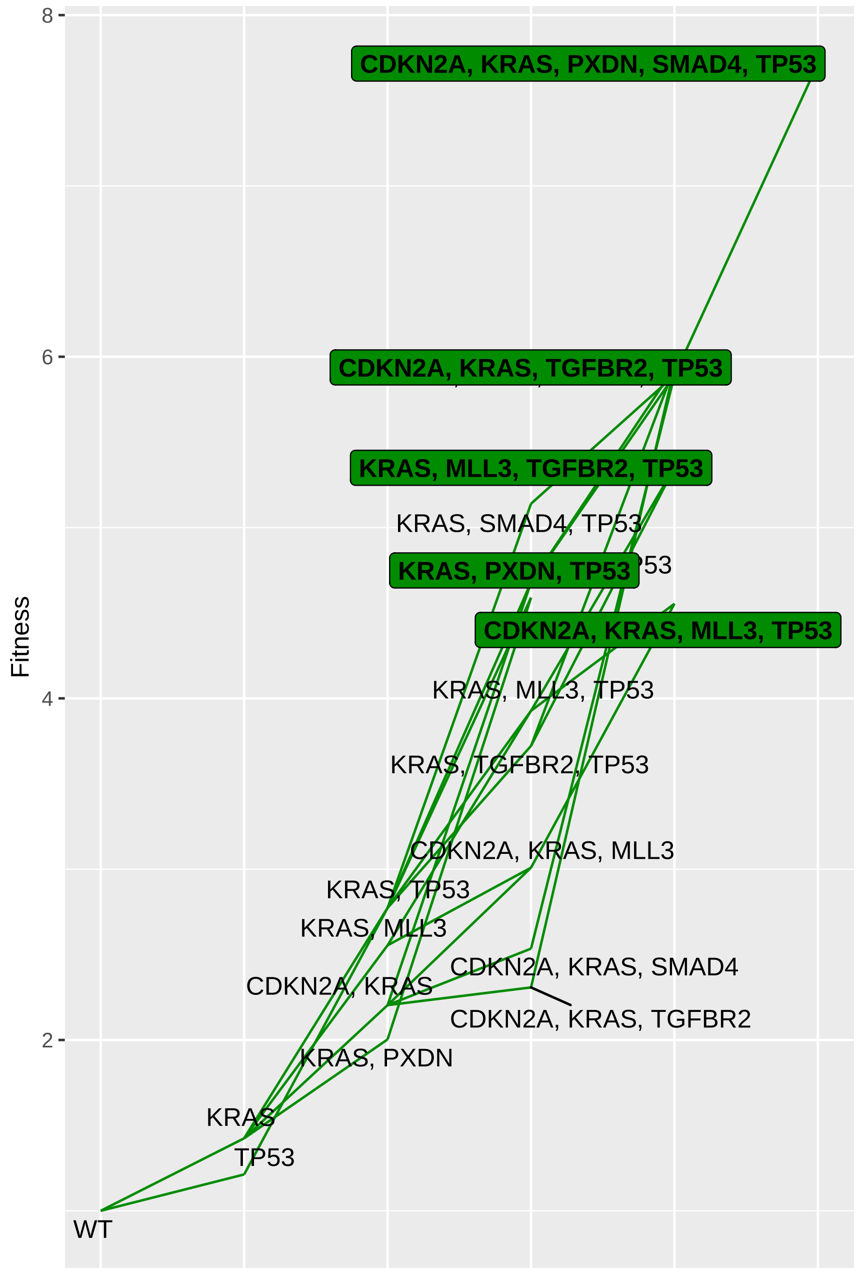

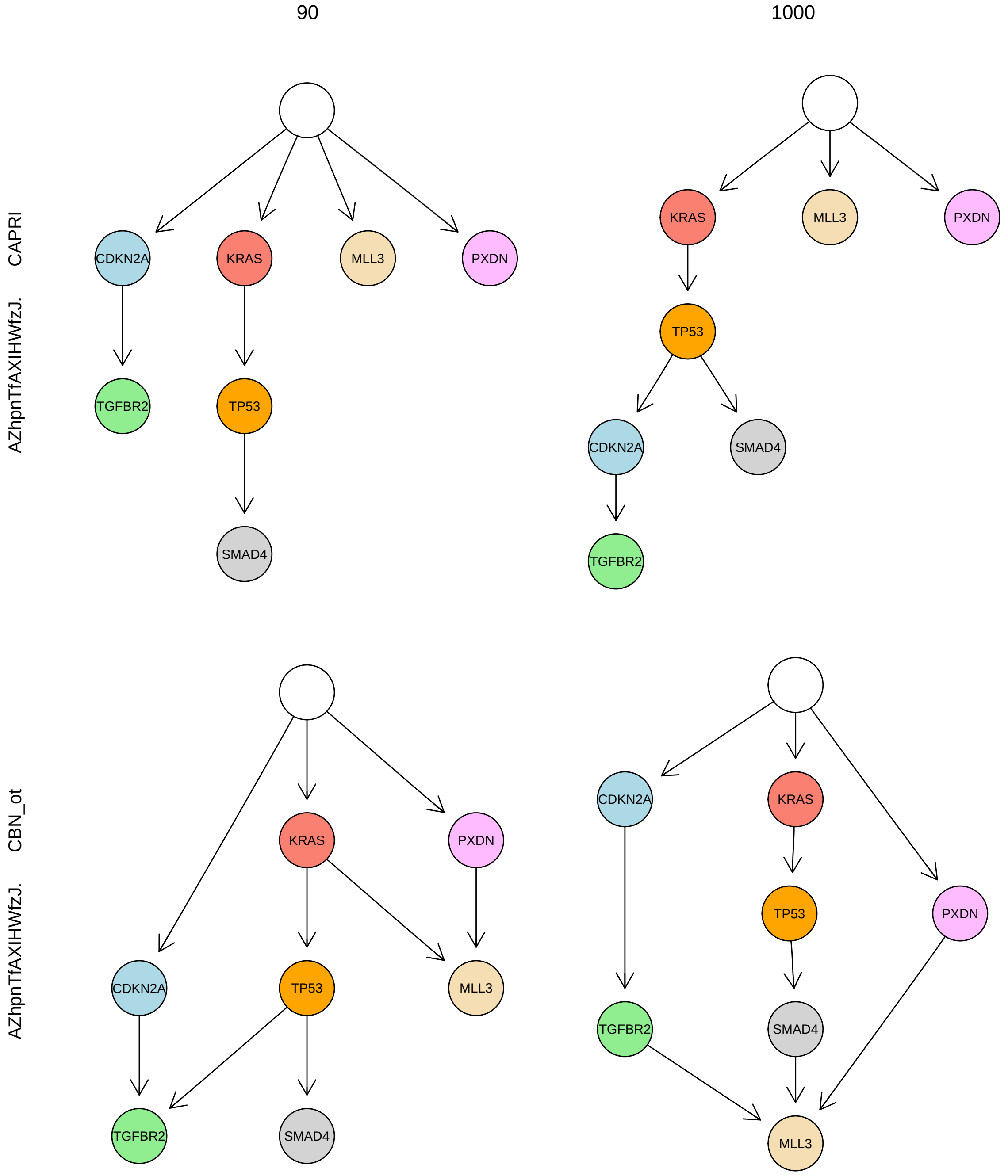

ID	p−value	Accessible Genot.
VmZgmYqgyrxtYGa	0.604	18

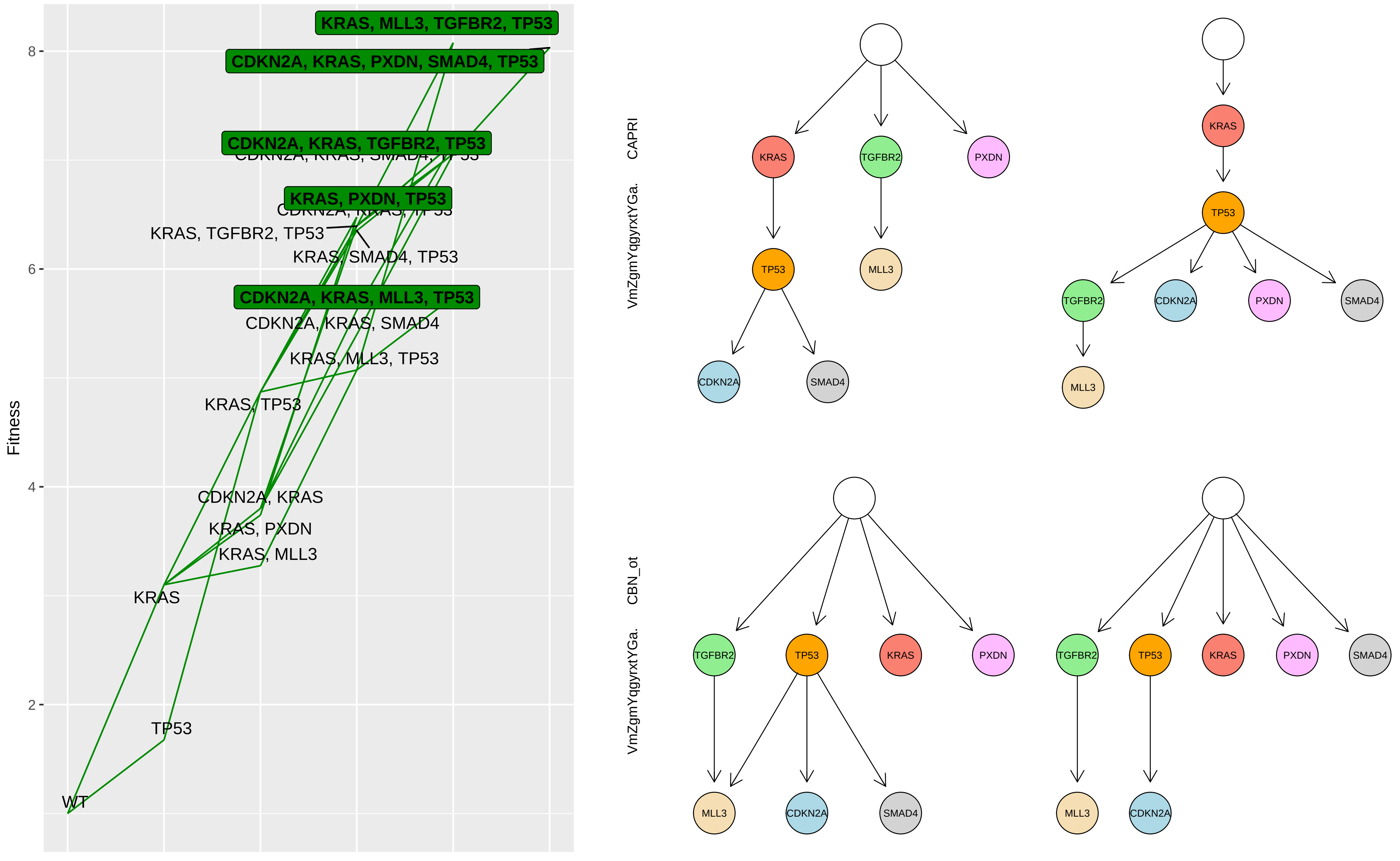

ID	p−value	Accessible Genot.
MXxfpbHTnDvQqWu	0.605	127

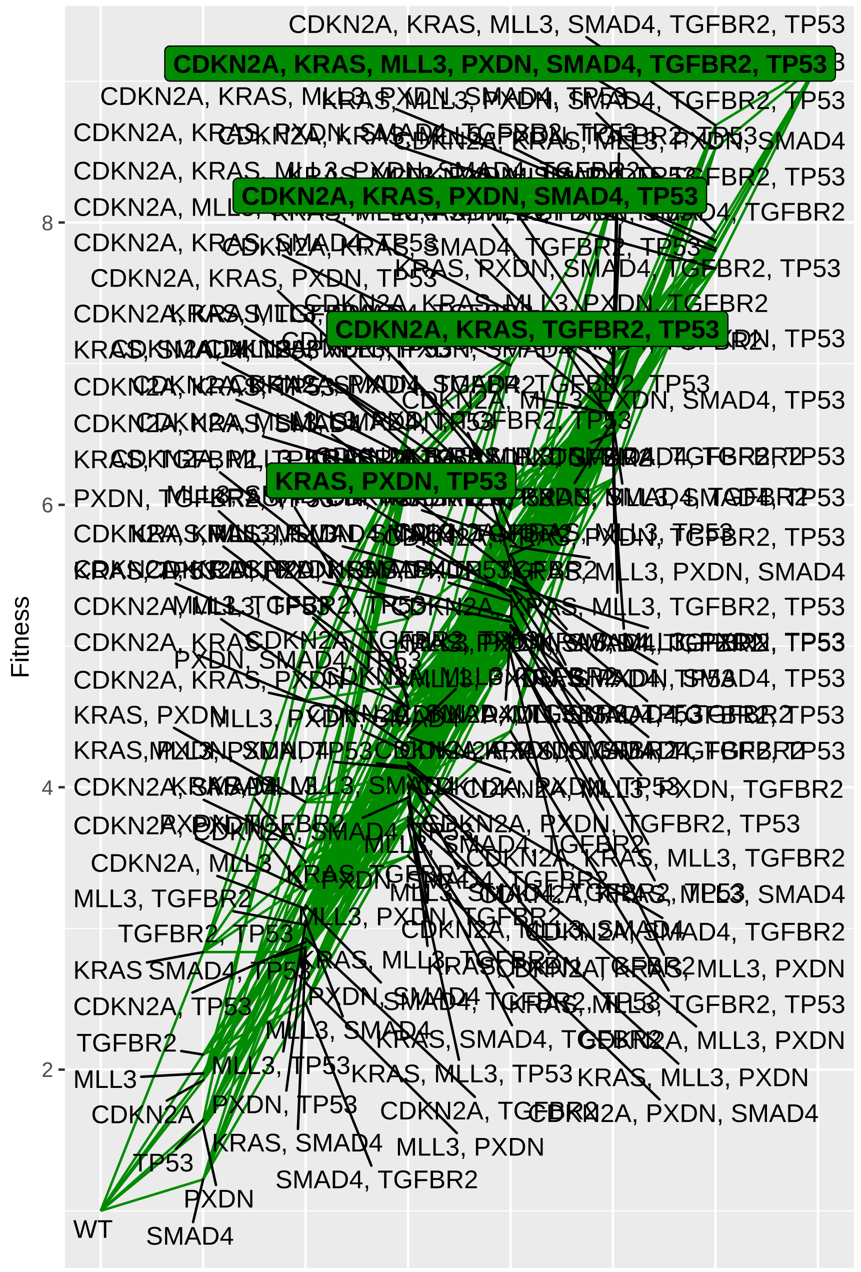

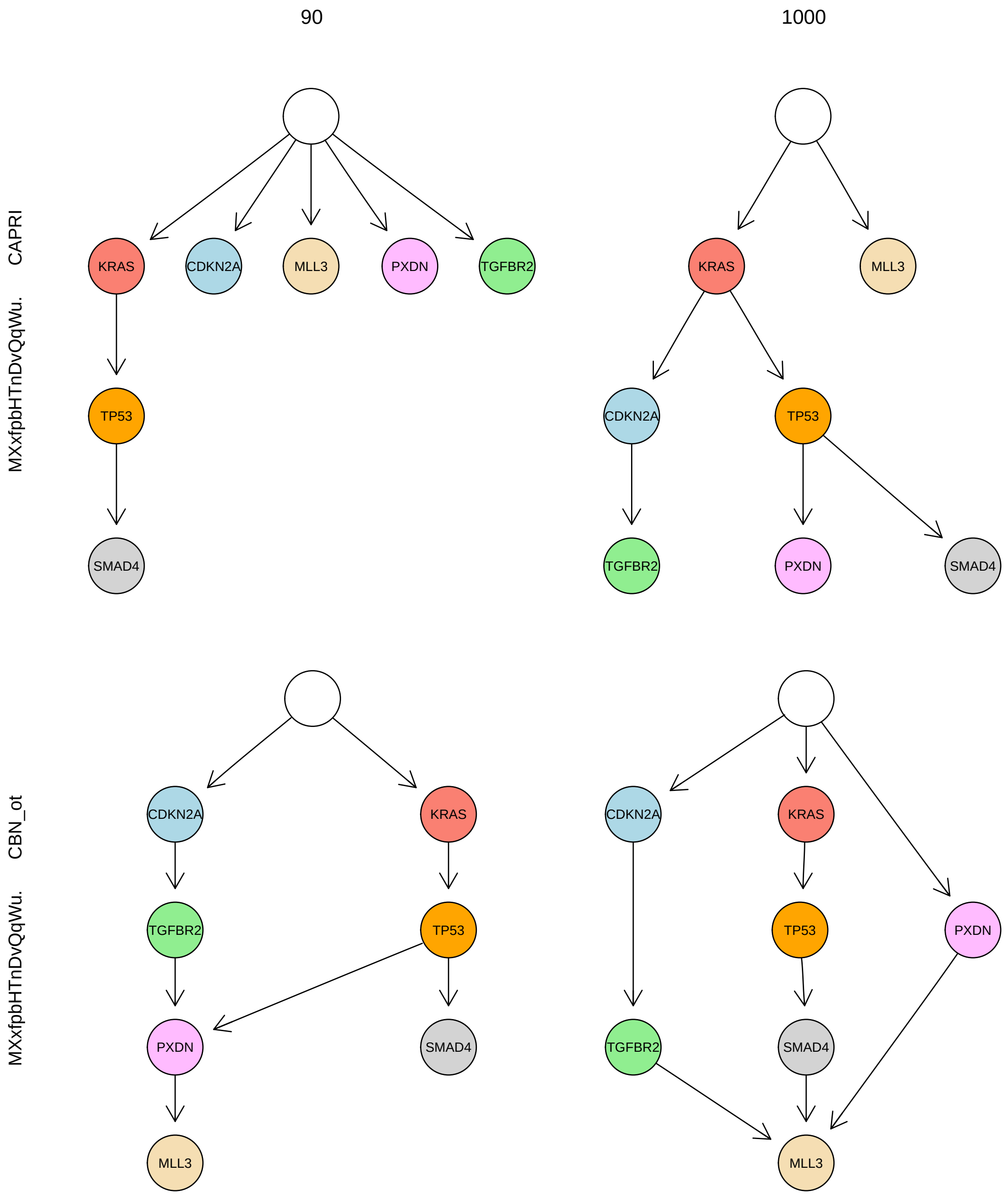

ID	p−value	Accessible Genot.
WOopmkQOnlnpXPM	0.606	67

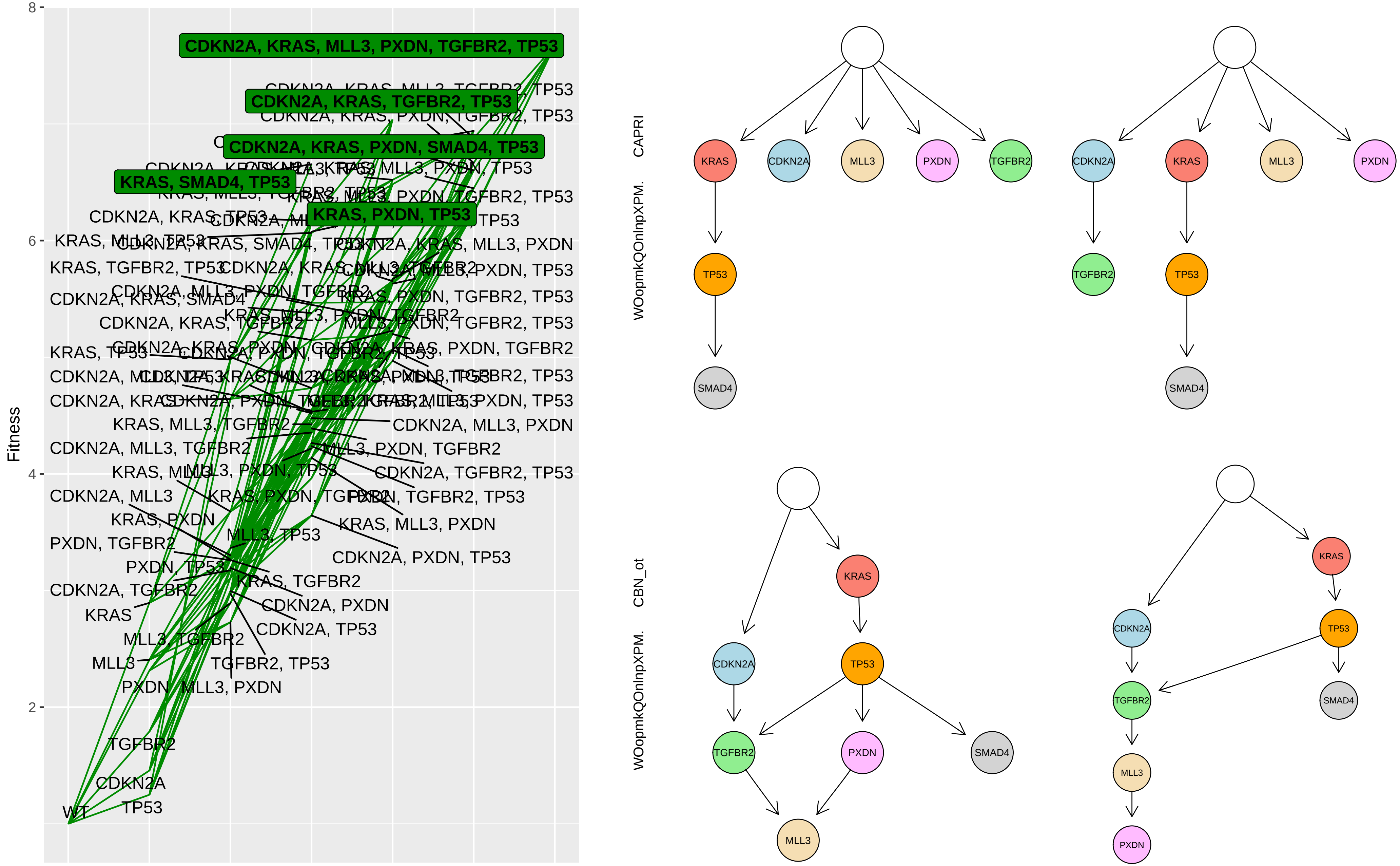

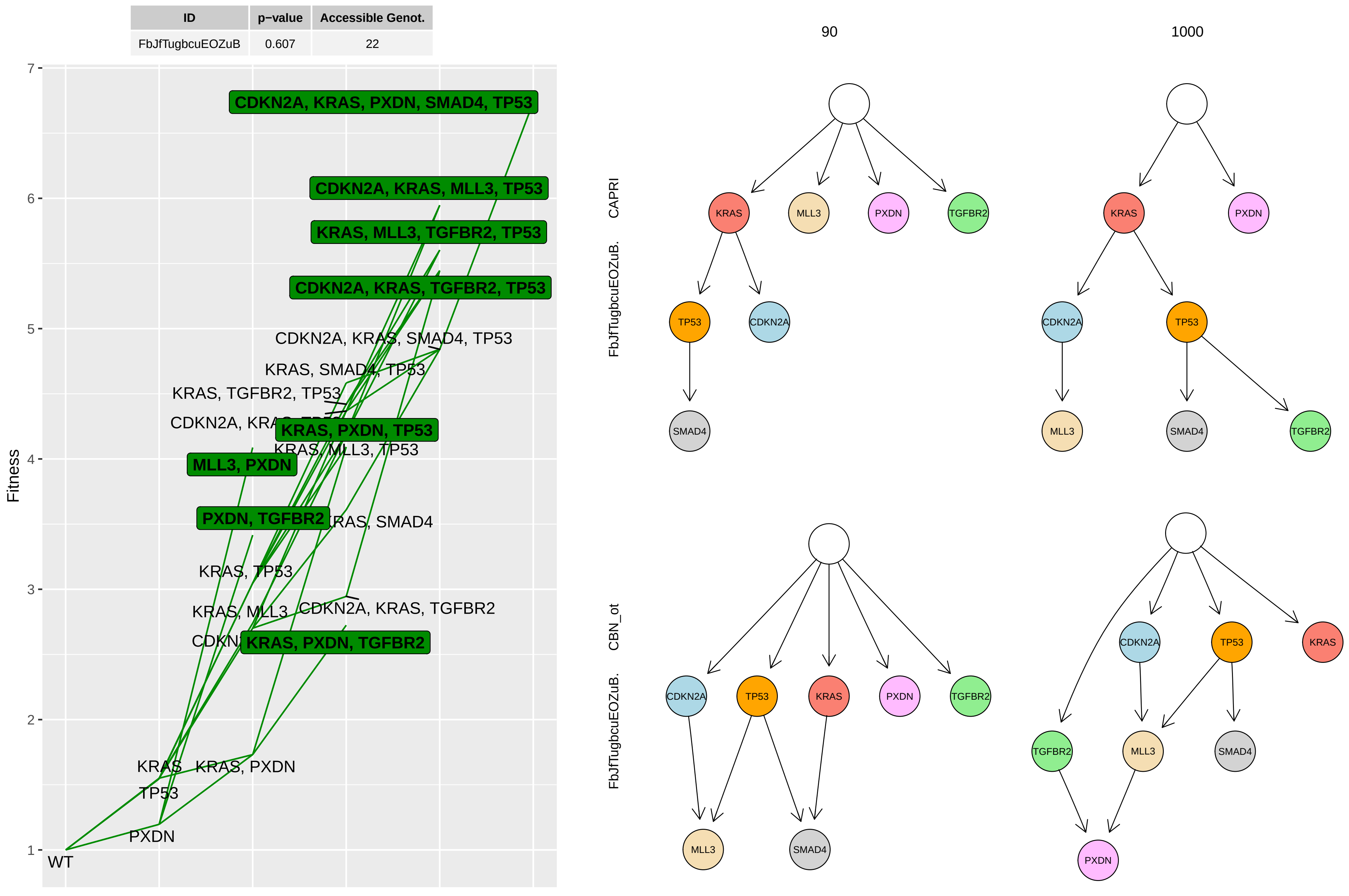

ID
p−value
Accessible Genot.
FbJfTugbcuEOZuB
0.607
22
Fitness
7
6
5
4
3
2
1
CDKN2A, KRAS, PXDN, SMAD4, TP53
CDKN2A, KRAS, MLL3, TP53
KRAS, MLL3, TGFBR2, TP53
CDKN2A, KRAS, TGFBR2, TP53
CDKN2A, KRAS, SMAD4, TP53
KRAS, SMAD4, TP53
KRAS, TGFBR2, TP53
KRAS, PXDN, TP53
KRAS, MLL3, TP53
MLL3, PXDN
PXDN, TGFBR2
KRAS, SMAD4
KRAS, TP53
KRAS, MLL3
CDKN2A, KRAS, TGFBR2
KRAS, PXDN, TGFBR2
KRAS
KRAS, PXDN
TP53
PXDN
WT
90
1000
FbJfTugbcuEOZuB. CAPRI
FbJfTugbcuEOZuB. CBN_ot
KRAS
MLL3
PXDN
TGFBR2
TP53
CDKN2A
SMAD4

ID	p−value	Accessible Genot.
gOYmbYfMonpWkTz	0.608	20

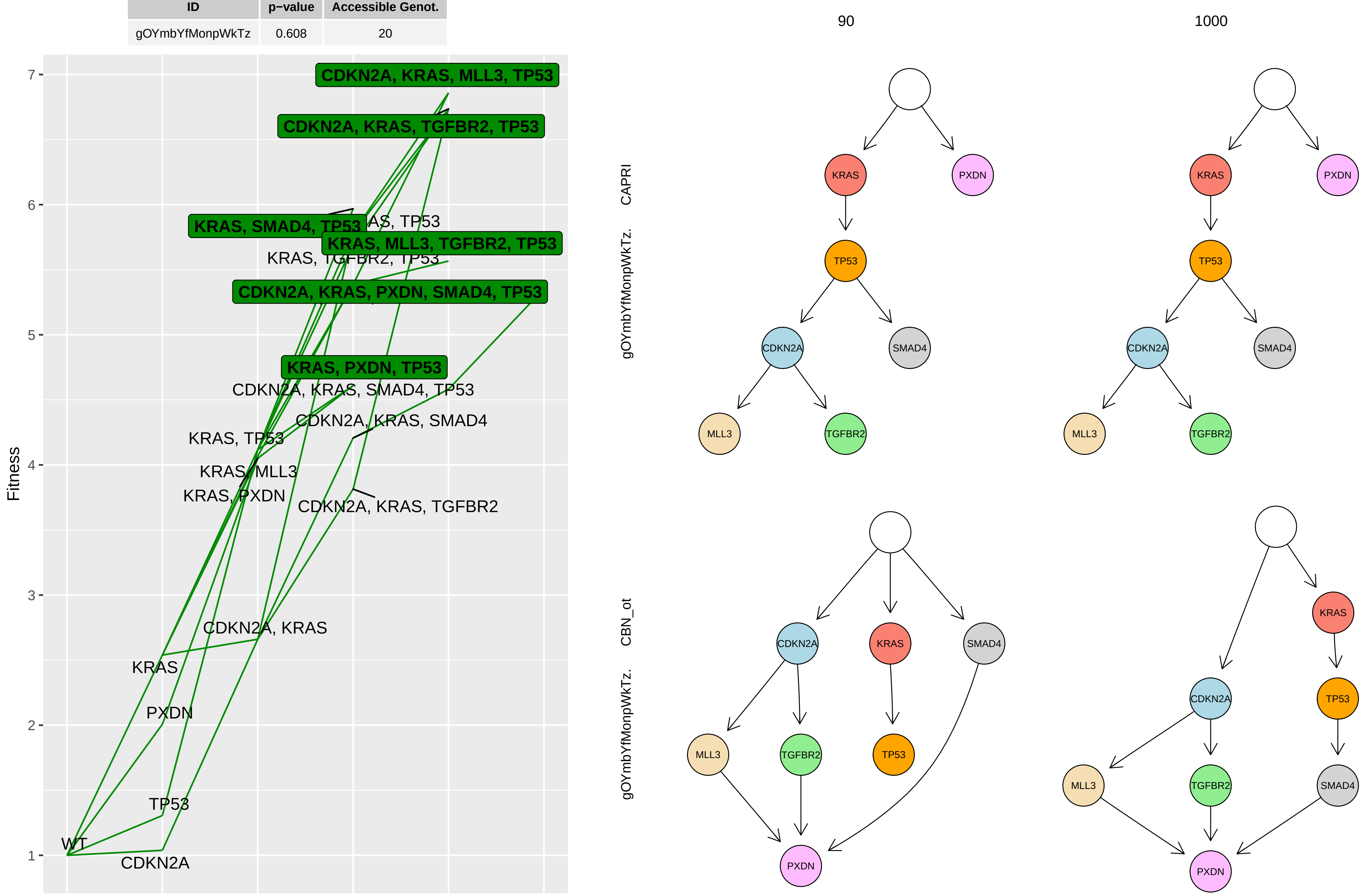

ID	p−value	Accessible Genot.
dnhMiexfVzaNvyx	0.61	27

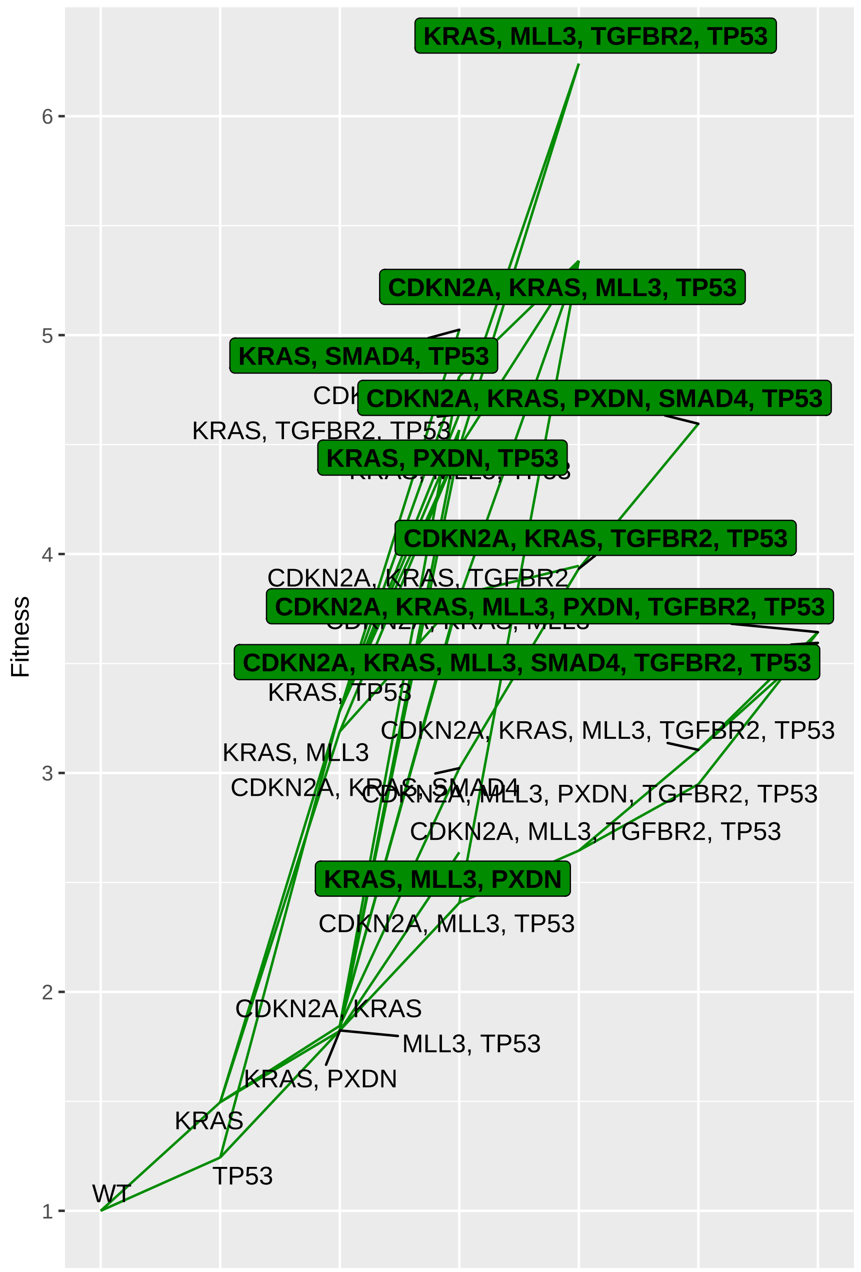

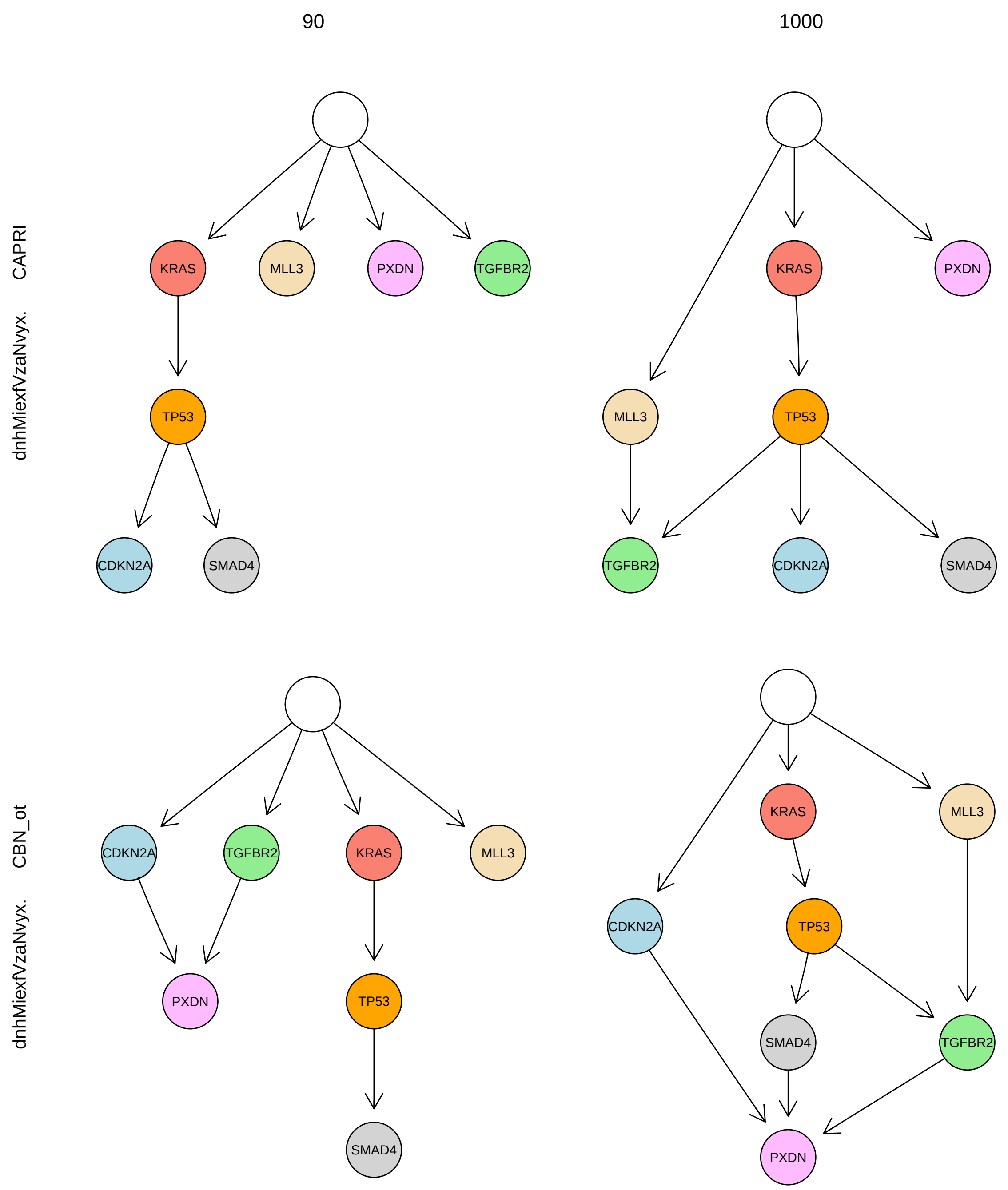

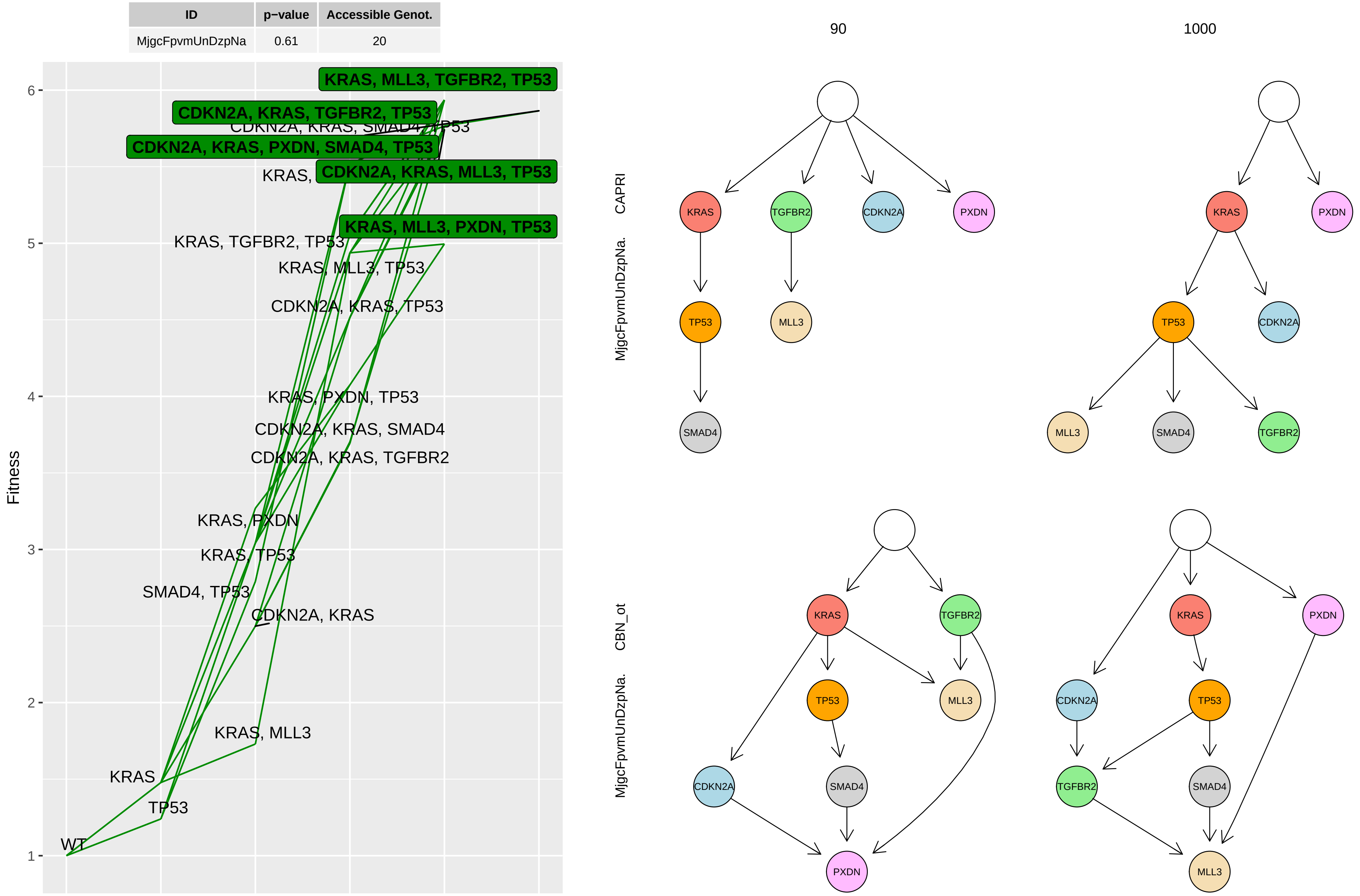

ID
p−value
Accessible Genot.
MjgcFpvmUnDzpNa
0.61
20
Fitness
6
5
4
3
2
1
KRAS, MLL3, TGFBR2, TP53
CDKN2A, KRAS, TGFBR2, TP53
CDKN2A, KRAS, PXDN, SMAD4, TP53
CDKN2A, KRAS, MLL3, TP53
KRAS, MLL3, PXDN, TP53
KRAS, TGFBR2, TP53
KRAS, MLL3, TP53
CDKN2A, KRAS, TP53
KRAS, PXDN, TP53
CDKN2A, KRAS, SMAD4
CDKN2A, KRAS, TGFBR2
KRAS, PXDN
KRAS, TP53
SMAD4, TP53
CDKN2A, KRAS
KRAS, MLL3
KRAS
TP53
WT
90
1000
MjgcFpvmUnDzpNa. CAPRI
MjgcFpvmUnDzpNa. CBN_ot
KRAS
TGFBR2
CDKN2A
PXDN
TP53
MLL3
SMAD4

ID	p−value	Accessible Genot.
DAYEsxQIJhTjQdf	0.612	23

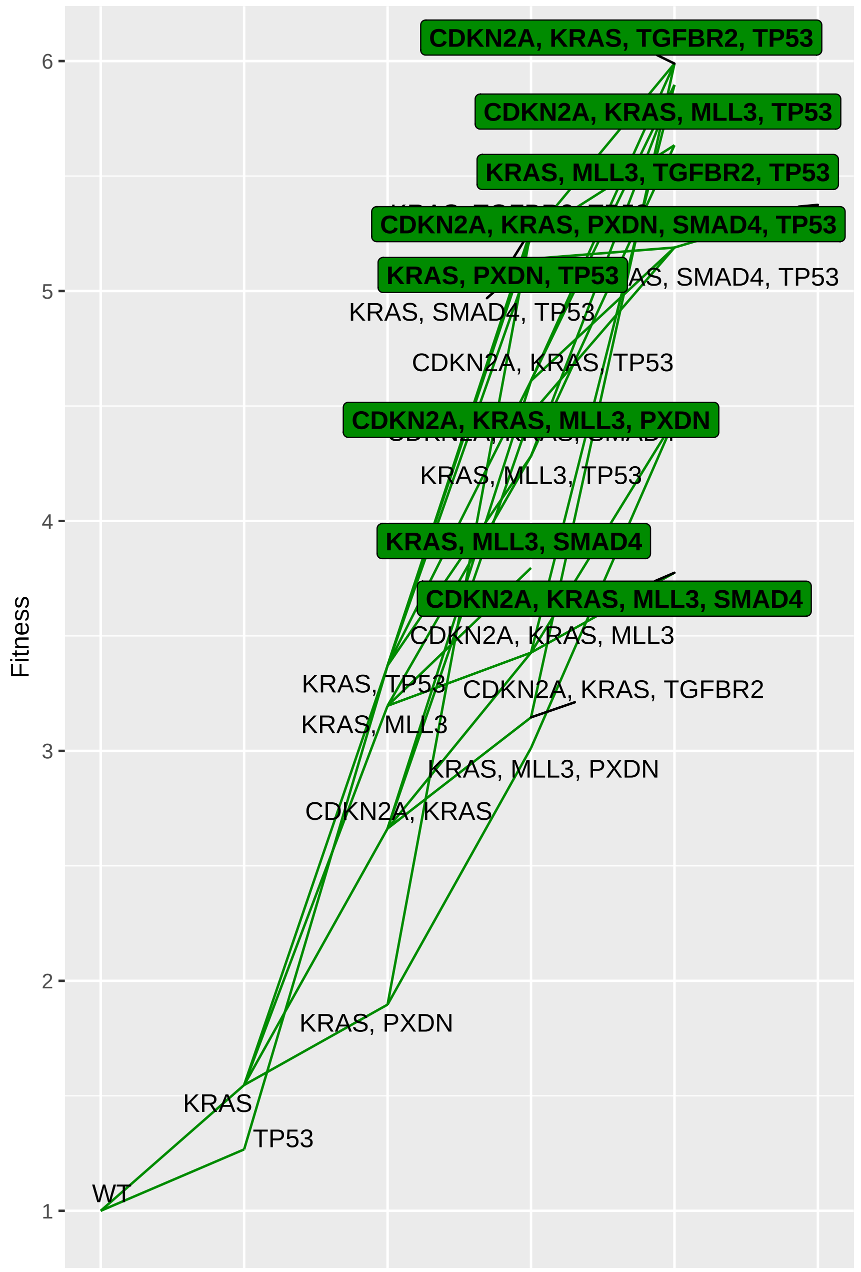

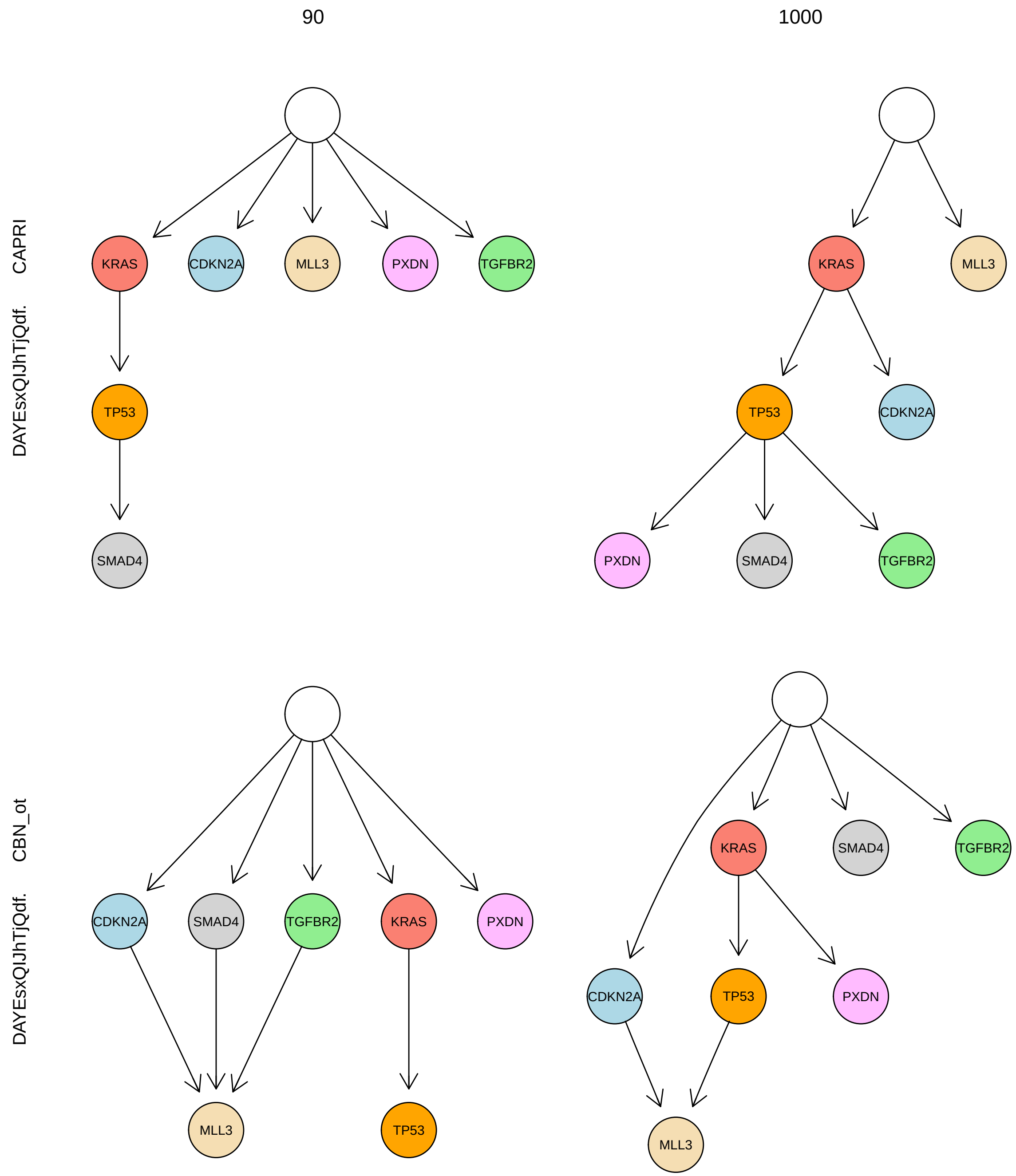

ID	p−value	Accessible Genot.
YBPmQpWwVarTKmS	0.613	127

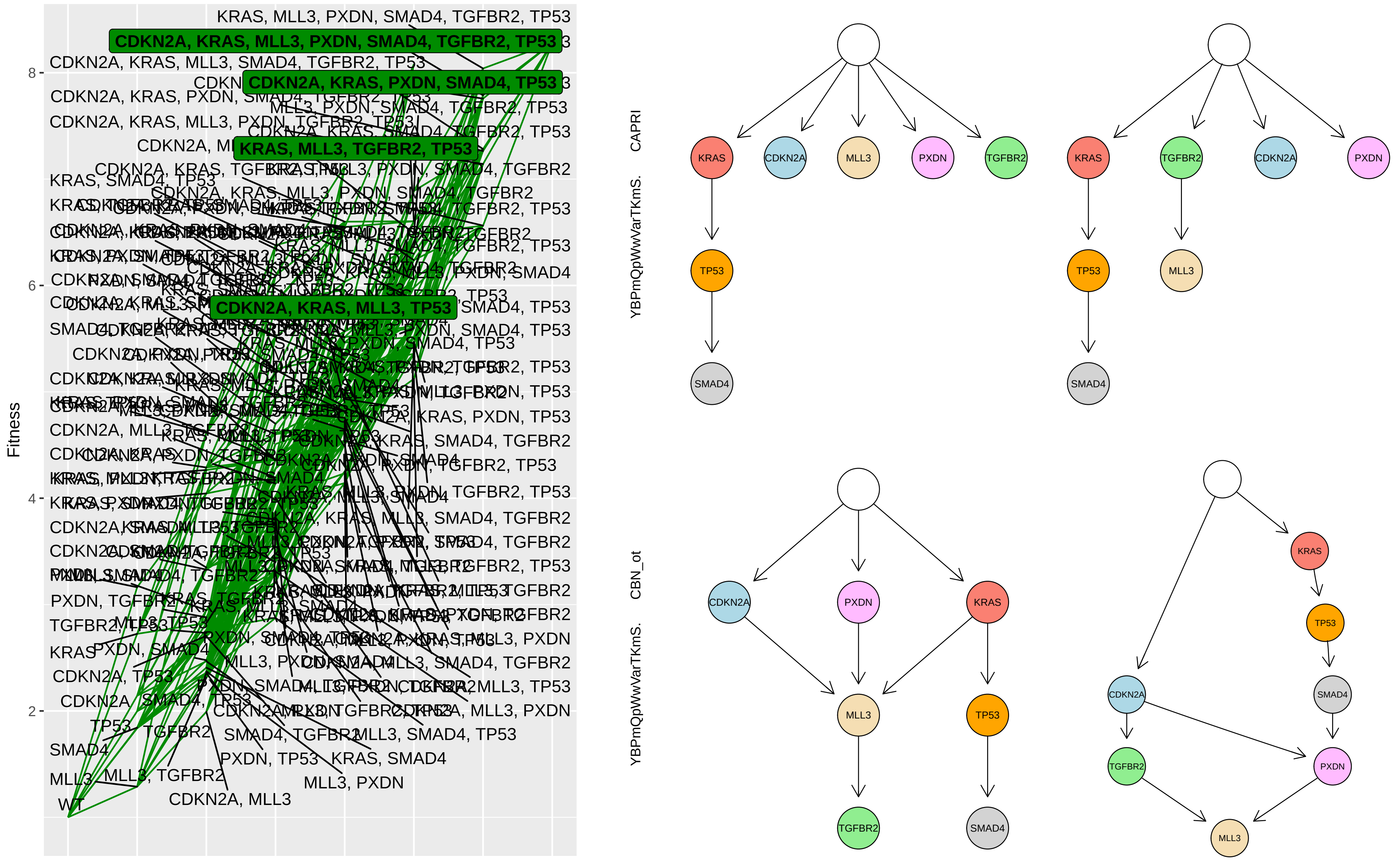

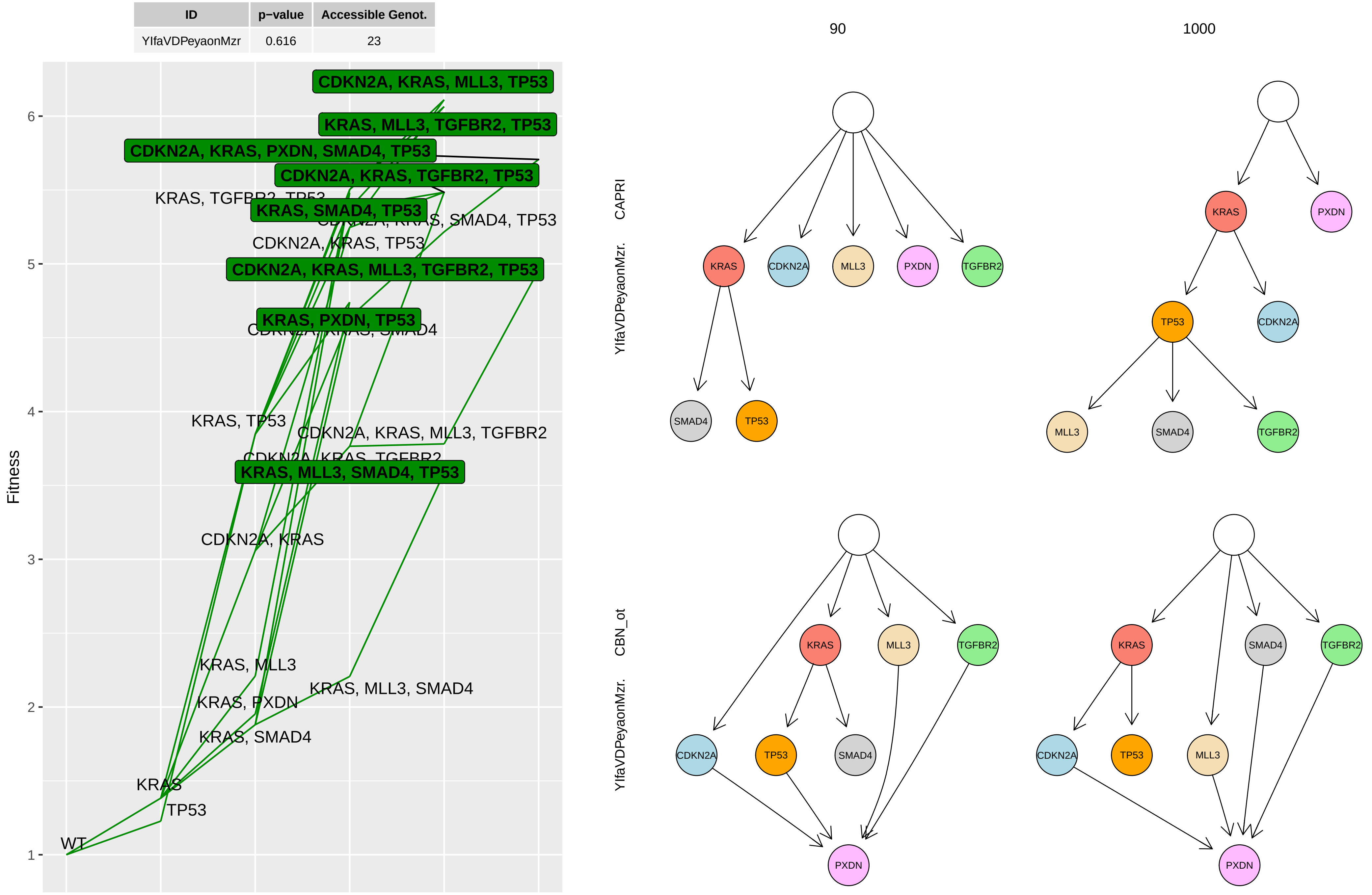

ID
p−value
Accessible Genot.
YIfaVDPeyaonMzr
0.616
23
Fitness
6
5
4
3
2
1
CDKN2A, KRAS, MLL3, TP53
KRAS, MLL3, TGFBR2, TP53
CDKN2A, KRAS, PXDN, SMAD4, TP53
CDKN2A, KRAS, TGFBR2, TP53
KRAS, SMAD4, TP53
CDKN2A, KRAS, TP53
CDKN2A, KRAS, MLL3, TGFBR2, TP53
KRAS, PXDN, TP53
KRAS, TP53
CDKN2A, KRAS, MLL3, TGFBR2
KRAS, MLL3, SMAD4, TP53
CDKN2A, KRAS
KRAS, MLL3
KRAS, MLL3, SMAD4
KRAS, PXDN
KRAS, SMAD4
KRAS
TP53
WT
90
1000
YIfaVDPeyaonMzr. CAPRI
YIfaVDPeyaonMzr. CBN_ot
KRAS
CDKN2A
MLL3
PXDN
TGFBR2
SMAD4
TP53

ID	p−value	Accessible Genot.
DXbDIoYMcTpSVmN	0.616	20

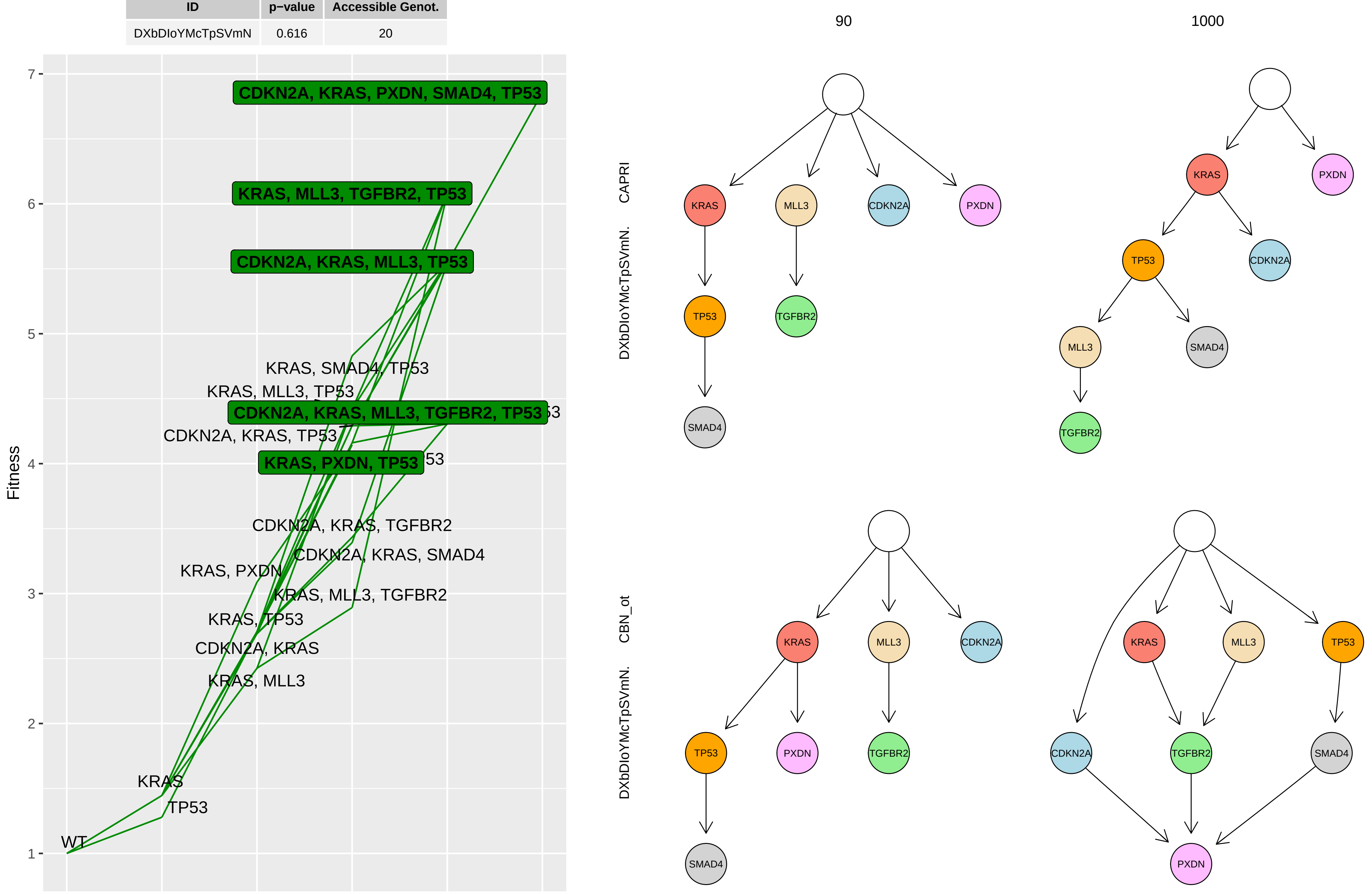

ID	p−value	Accessible Genot.
BZIGaZPWnvSQrpB	0.618	125

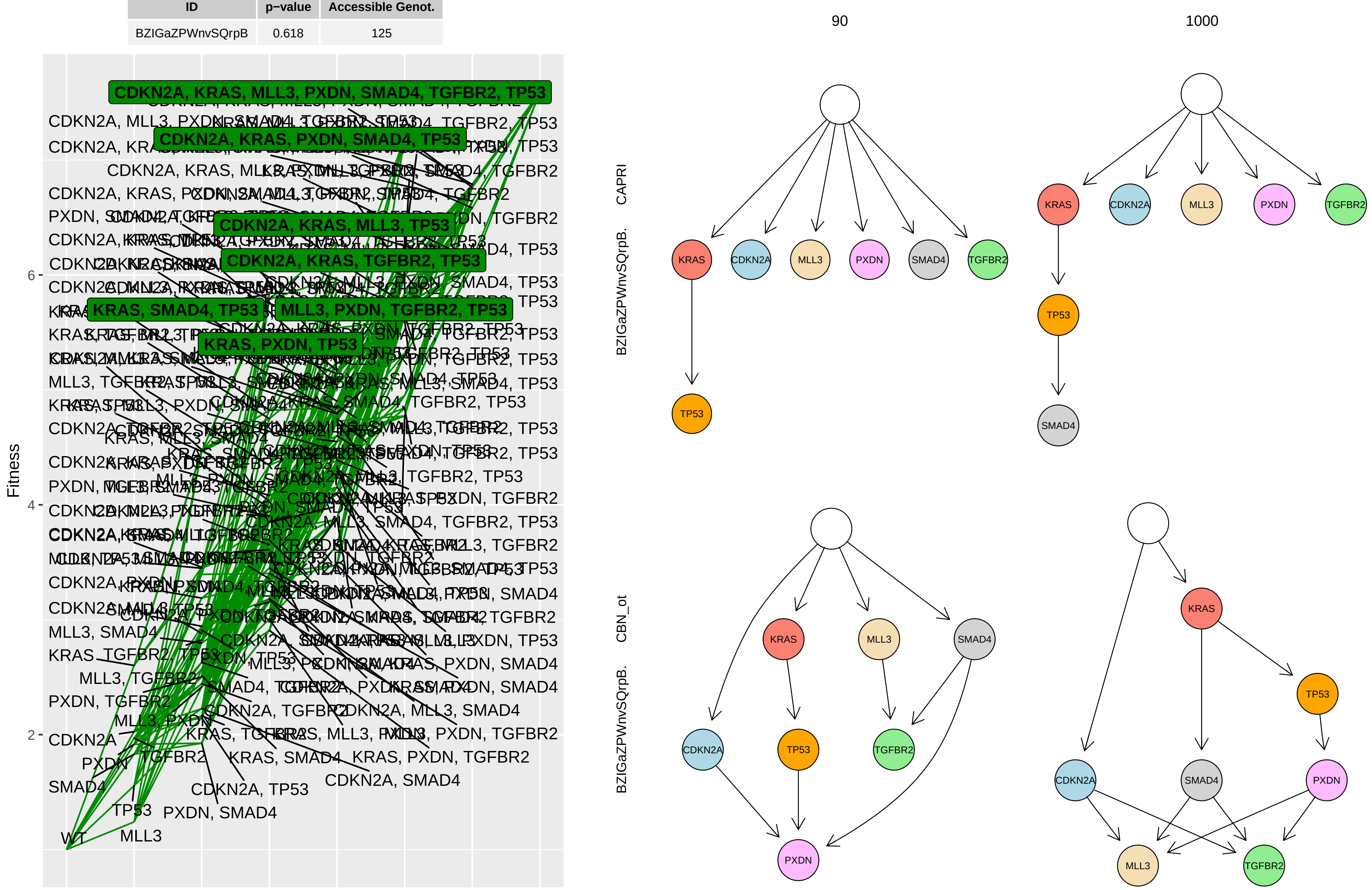

ID	p−value	Accessible Genot.
mOnWeWGFnVnbgPf	0.618	70

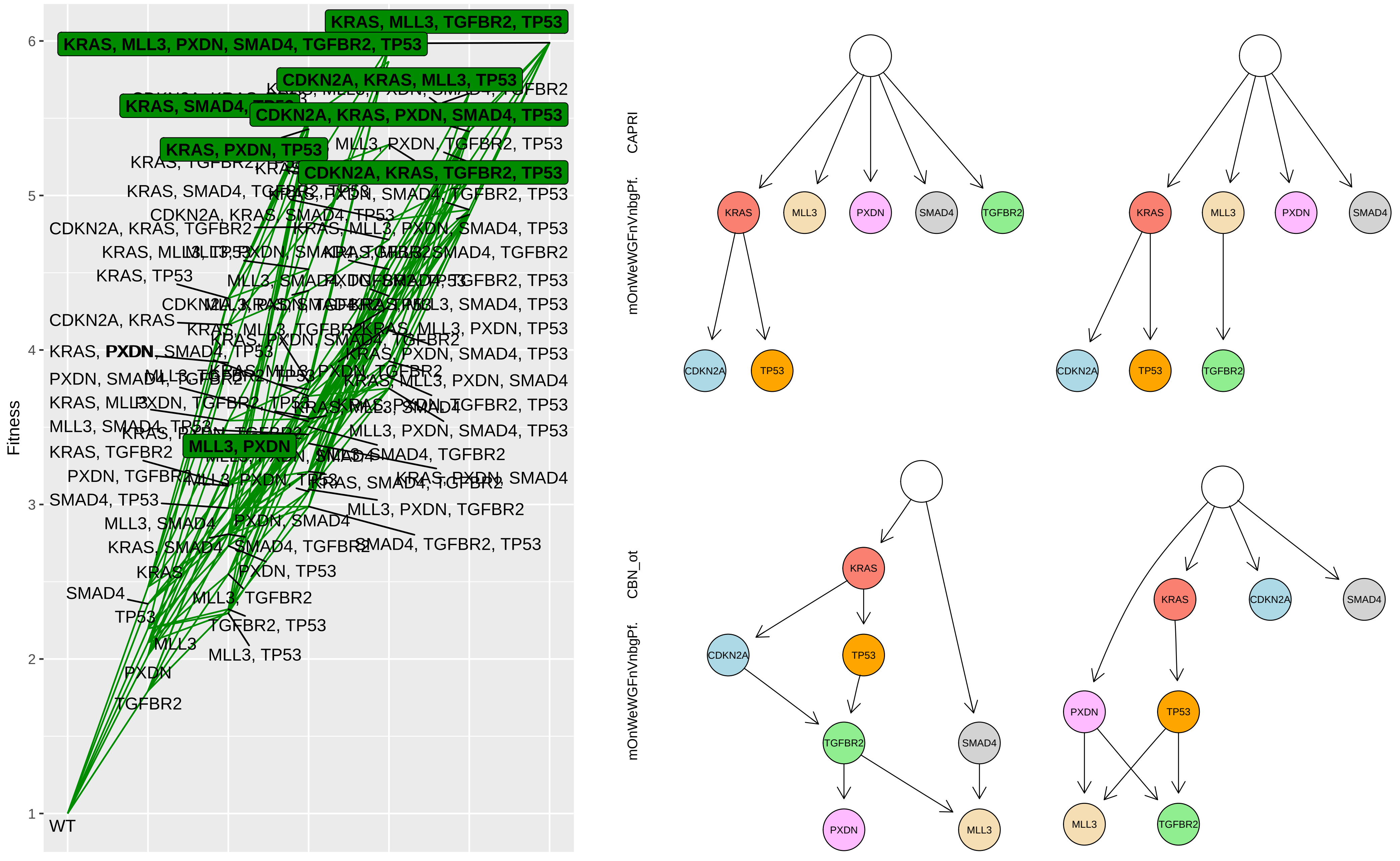

ID	p−value	Accessible Genot.
GrVMbbYJeRFTmFE	0.618	109

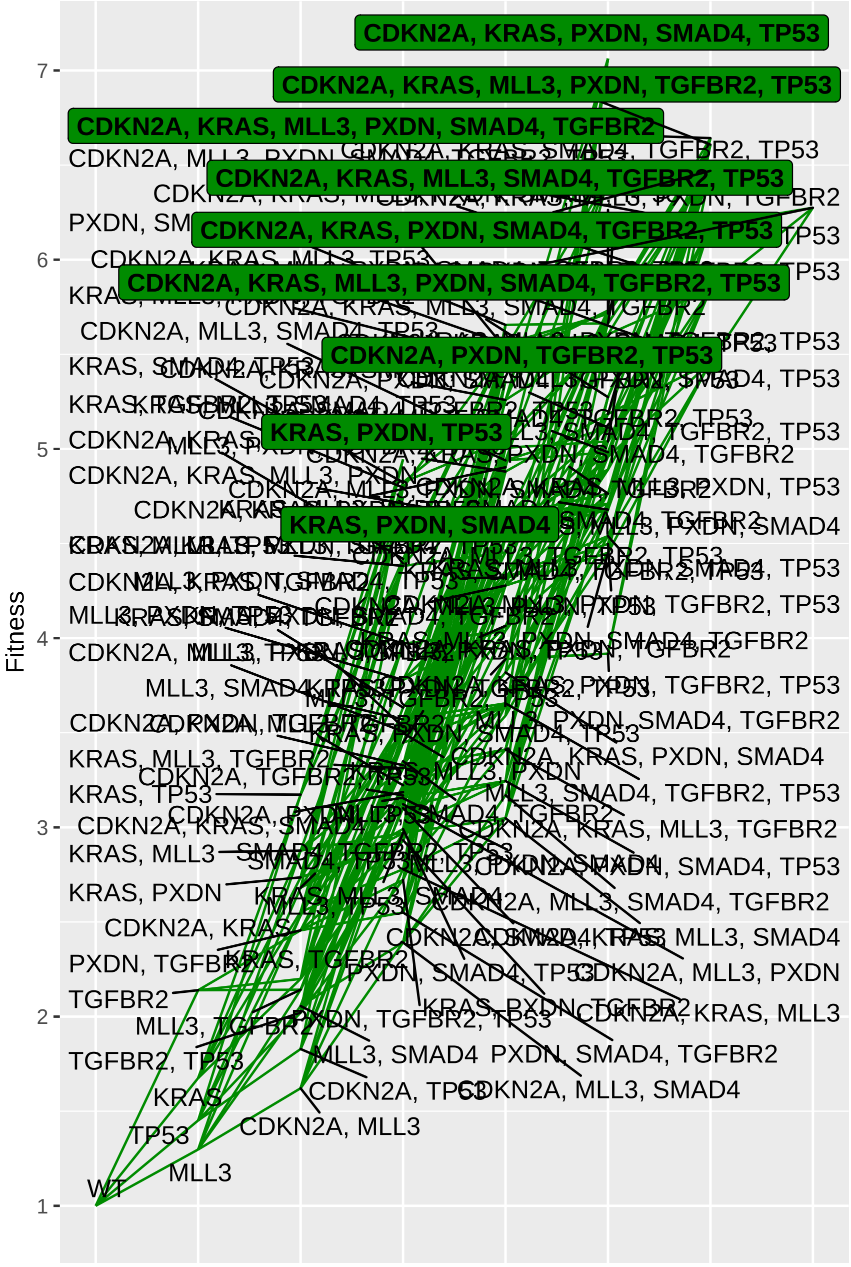

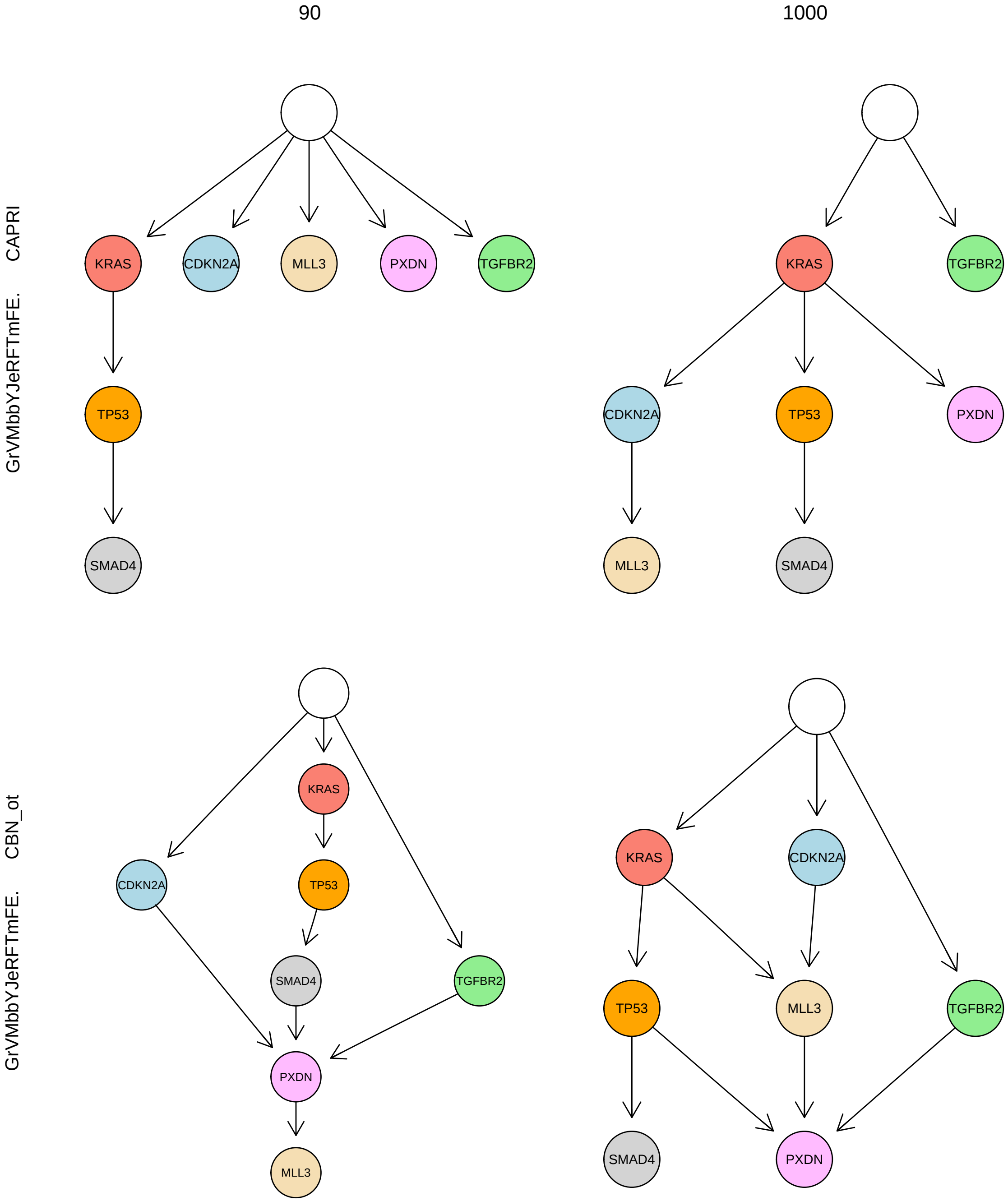

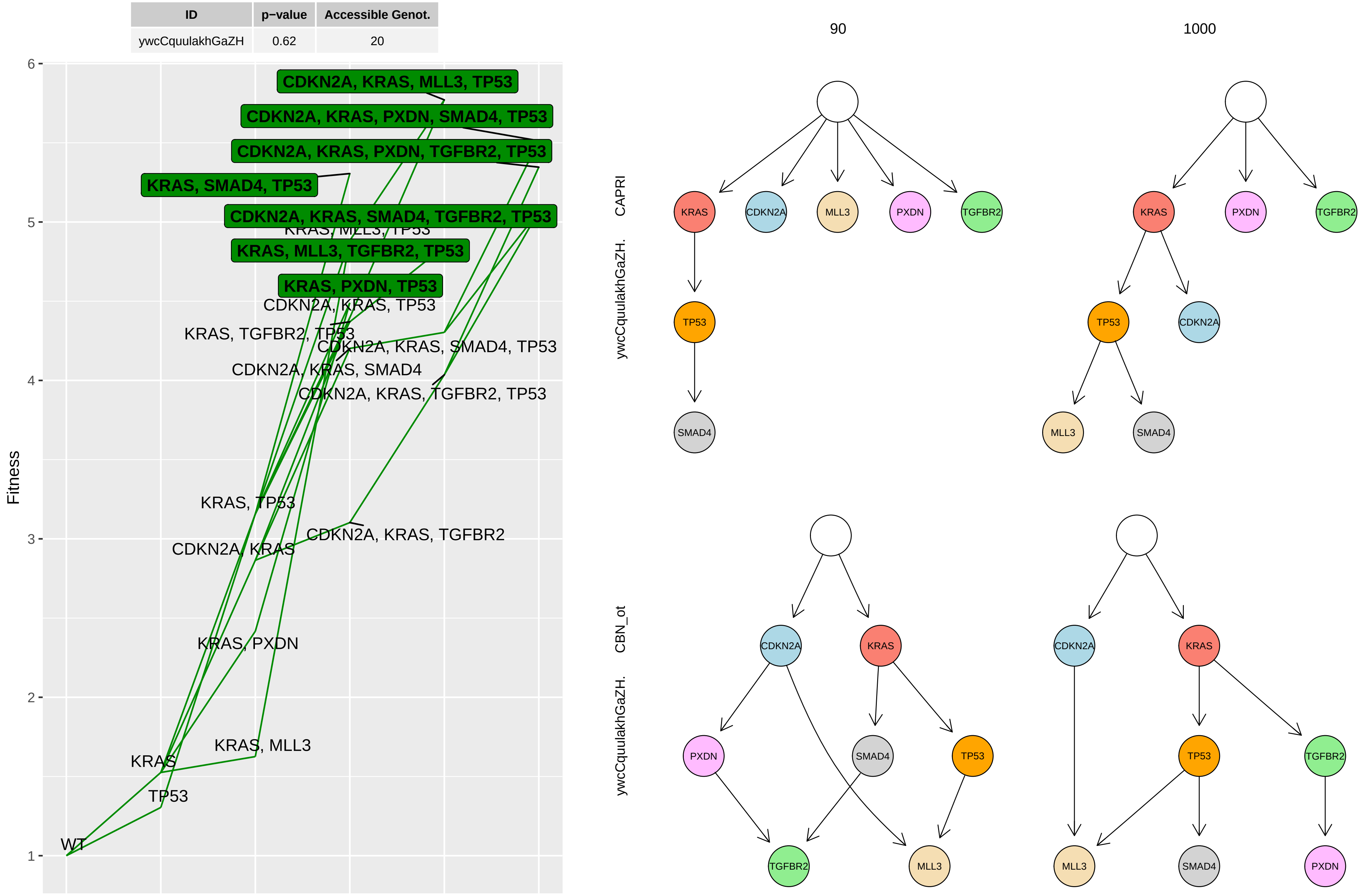
ID
p−value
Accessible Genot.
ywcCquulakhGaZH
0.62
20
Fitness
CDKN2A, KRAS, MLL3, TP53
CDKN2A, KRAS, PXDN, SMAD4, TP53
CDKN2A, KRAS, PXDN, TGFBR2, TP53
KRAS, SMAD4, TP53
CDKN2A, KRAS, SMAD4, TGFBR2, TP53
KRAS, MLL3, TGFBR2, TP53
KRAS, PXDN, TP53
CDKN2A, KRAS, TP53
KRAS, TGFBR2, TP53
CDKN2A, KRAS, SMAD4, TP53
CDKN2A, KRAS, SMAD4
CDKN2A, KRAS, TGFBR2, TP53
KRAS, TP53
CDKN2A, KRAS, TGFBR2
CDKN2A, KRAS
KRAS, PXDN
KRAS, MLL3
KRAS
TP53
WT
90
1000
ywcCquulakhGaZH. CAPRI
ywcCquulakhGaZH. CBN_ot
KRAS
CDKN2A
MLL3
PXDN
TGFBR2
TP53
SMAD4

ID	p−value	Accessible Genot.
whjBUefgvZexmAZ	0.621	124

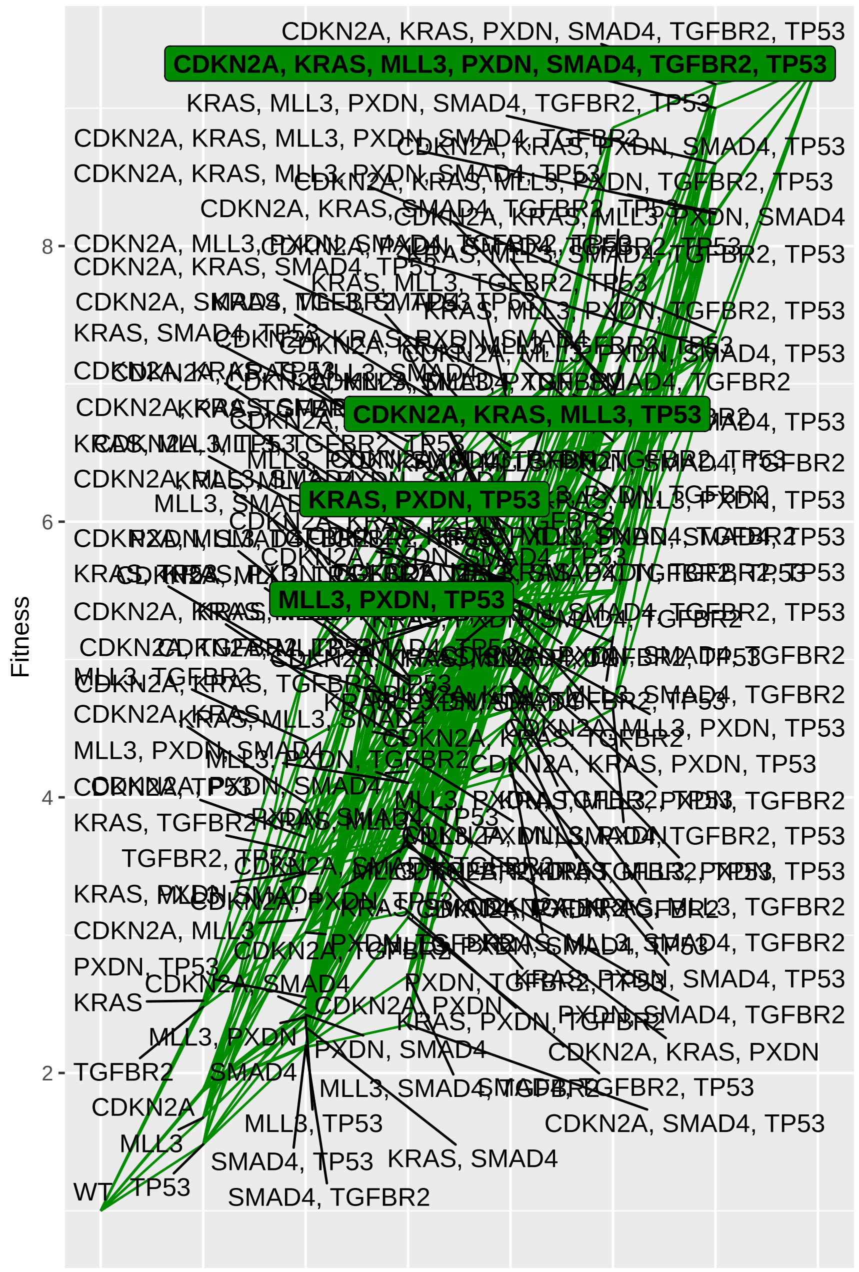

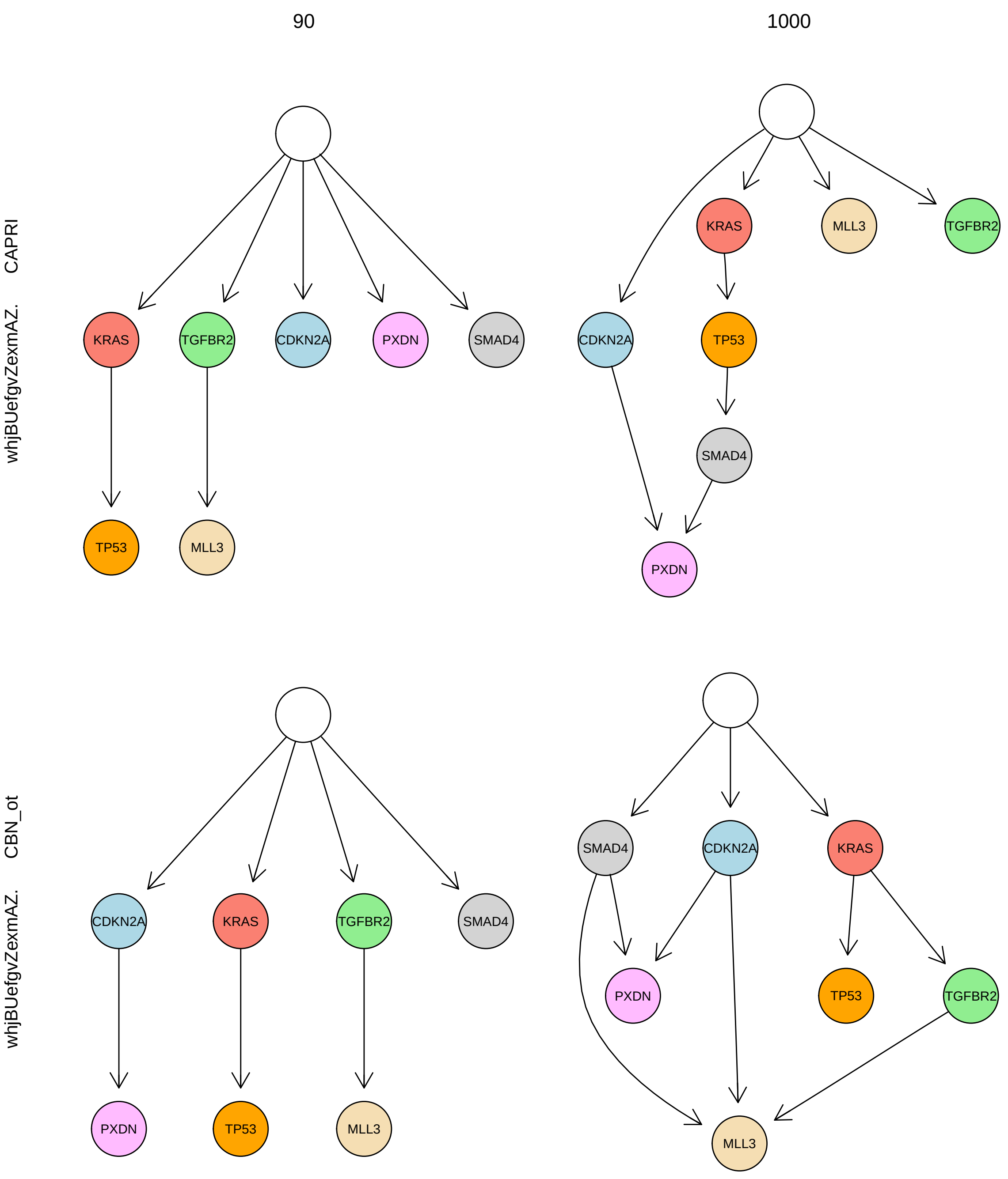

ID	p−value	Accessible Genot.
NWjlTOHMdcuqSqF	0.621	32

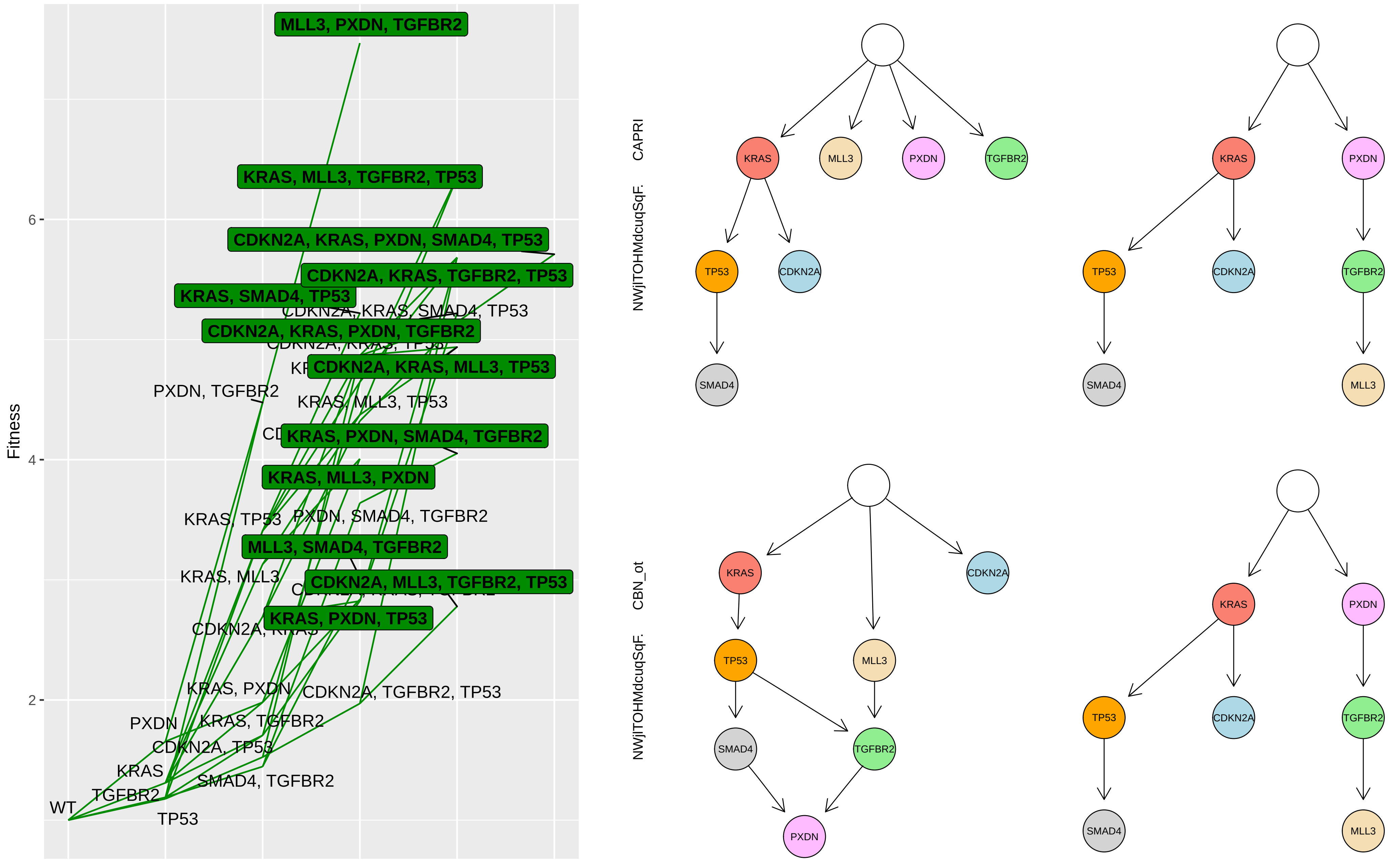

ID	p−value	Accessible Genot.
RxPJdHYyHkSlxEH	0.622	105

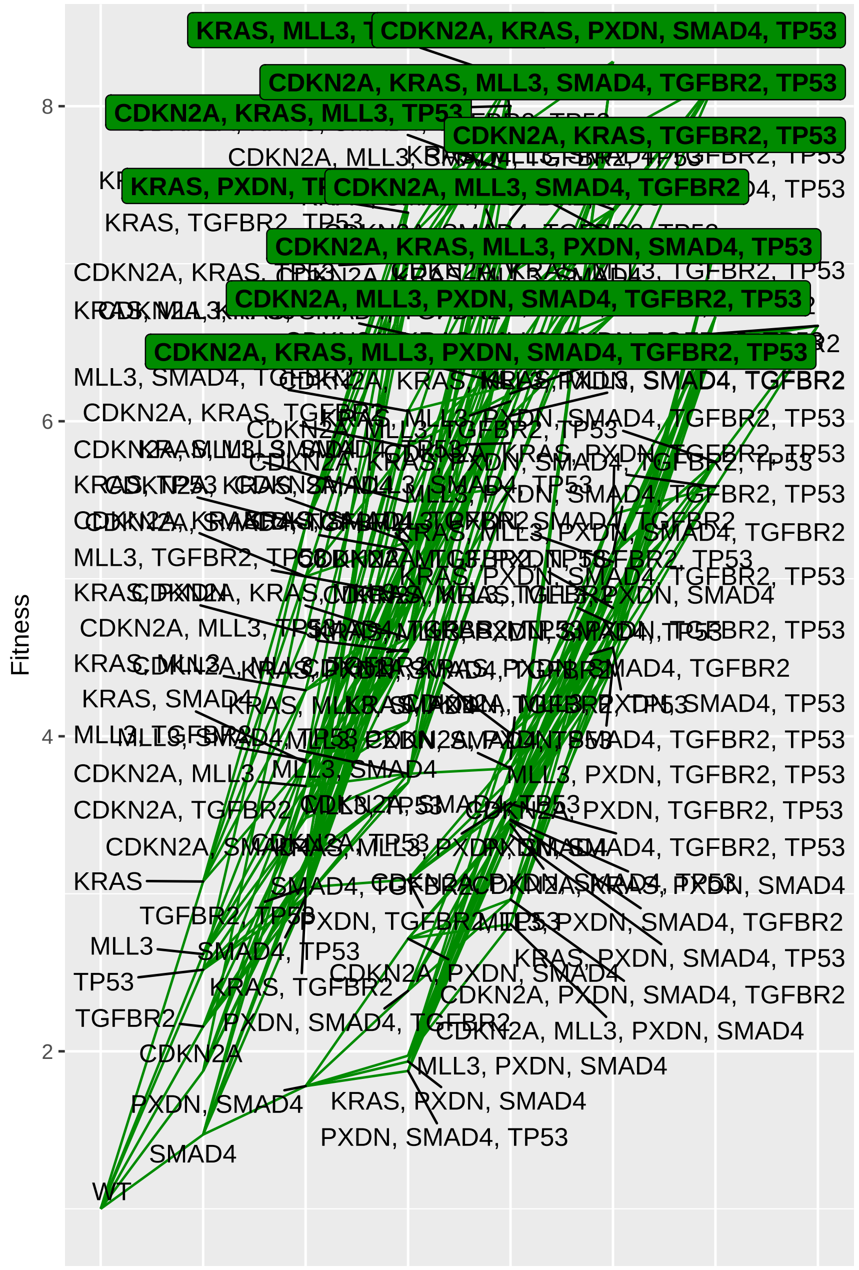

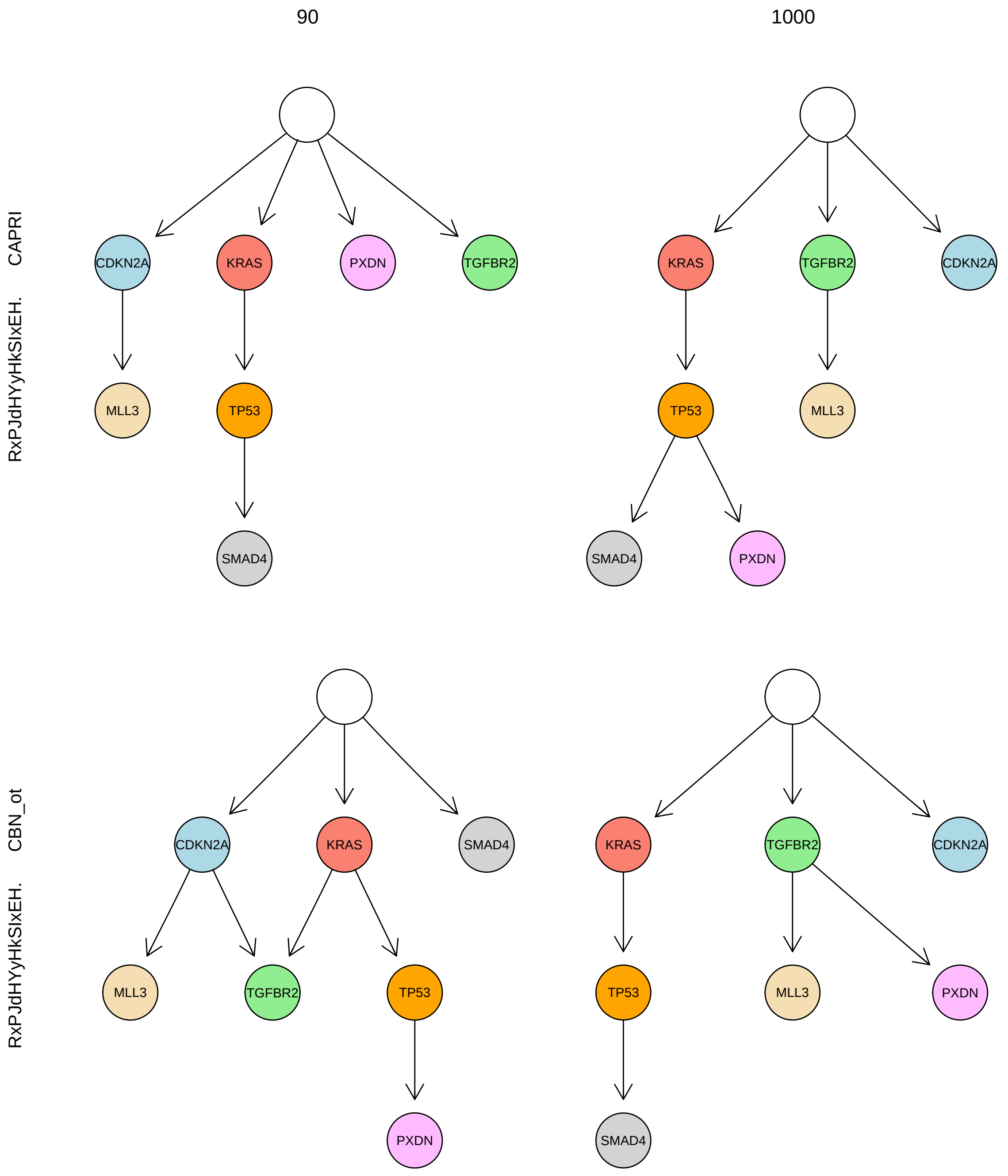

ID	p−value	Accessible Genot.
ahFnkuWgQuBdQID	0.625	19

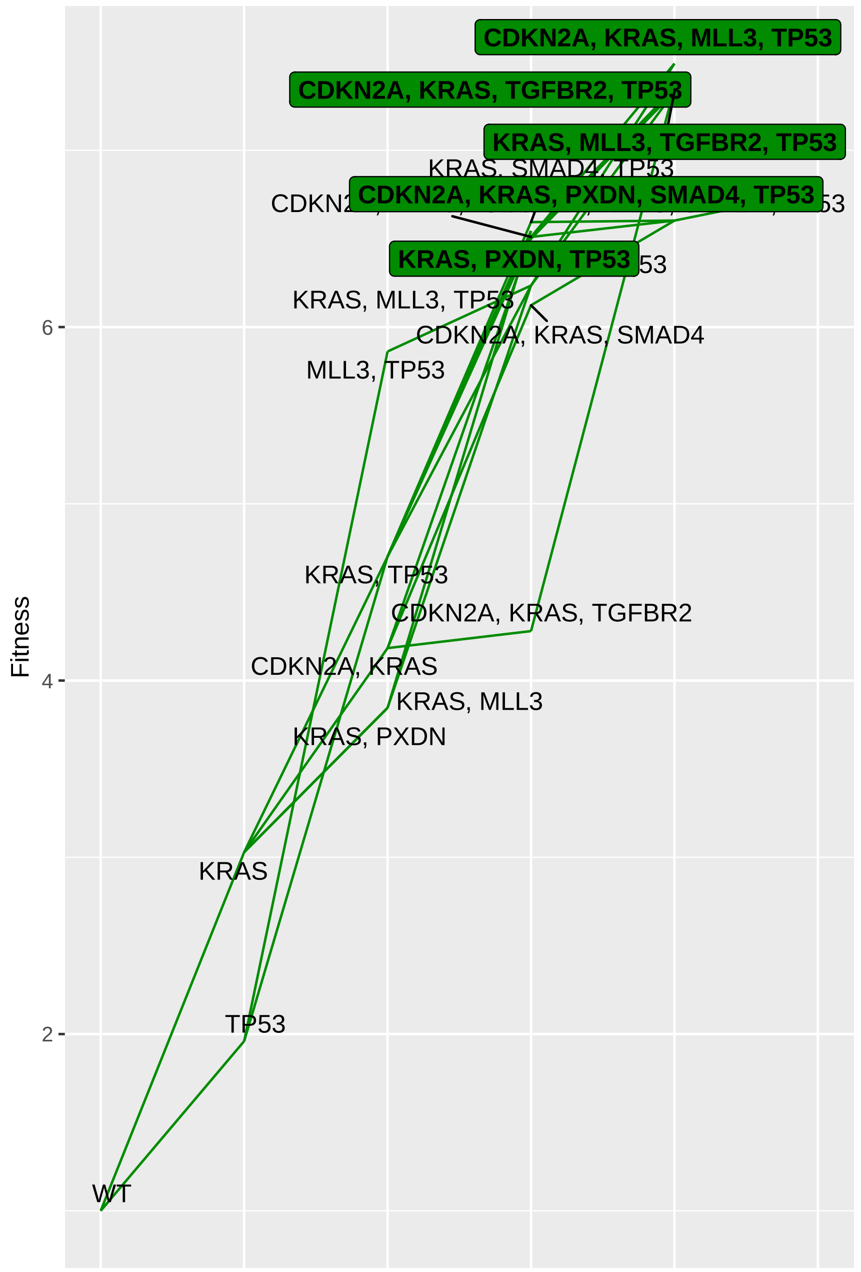

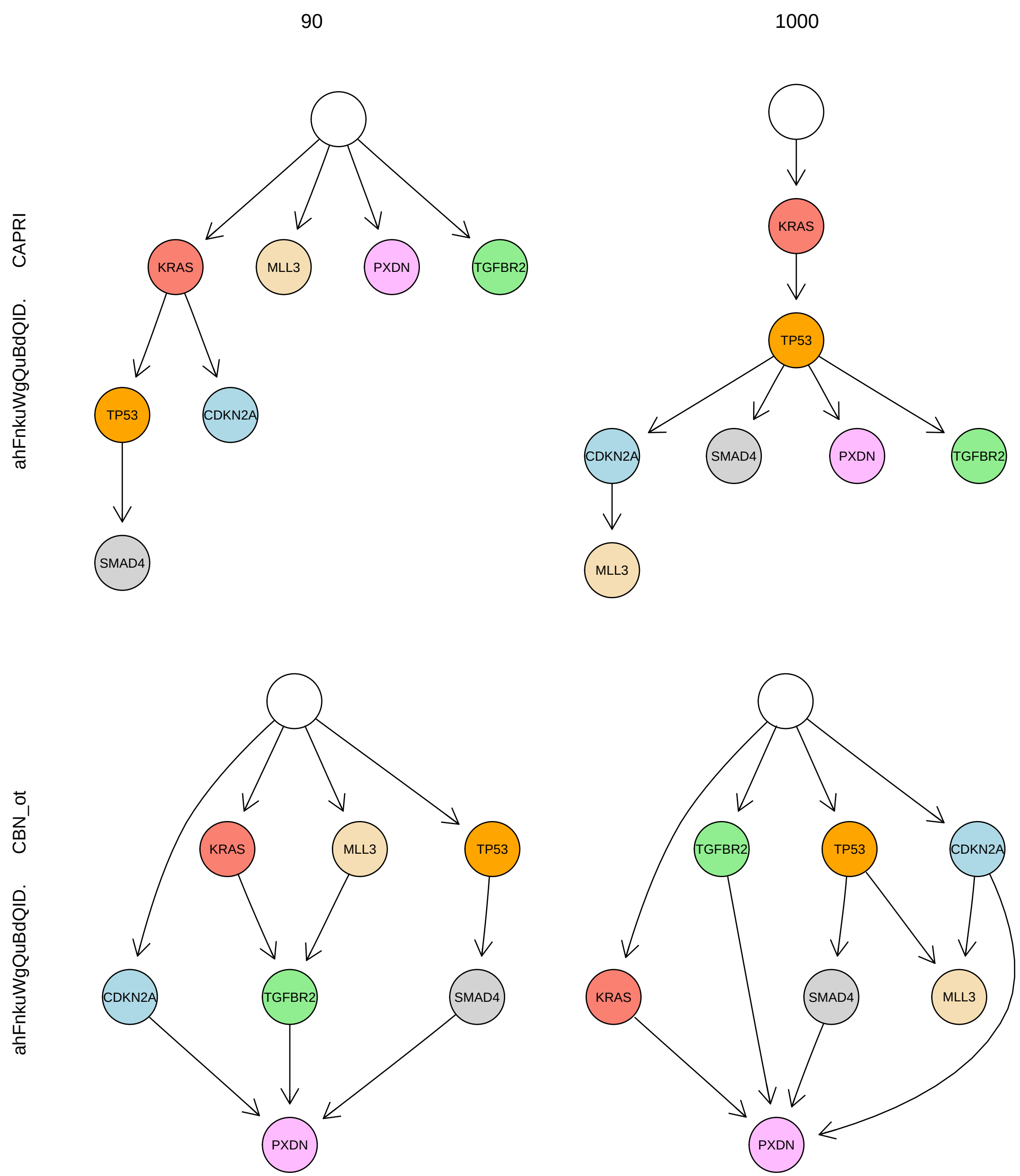

ID	p−value	Accessible Genot.
QCdDoSFysfACREI	0.626	41

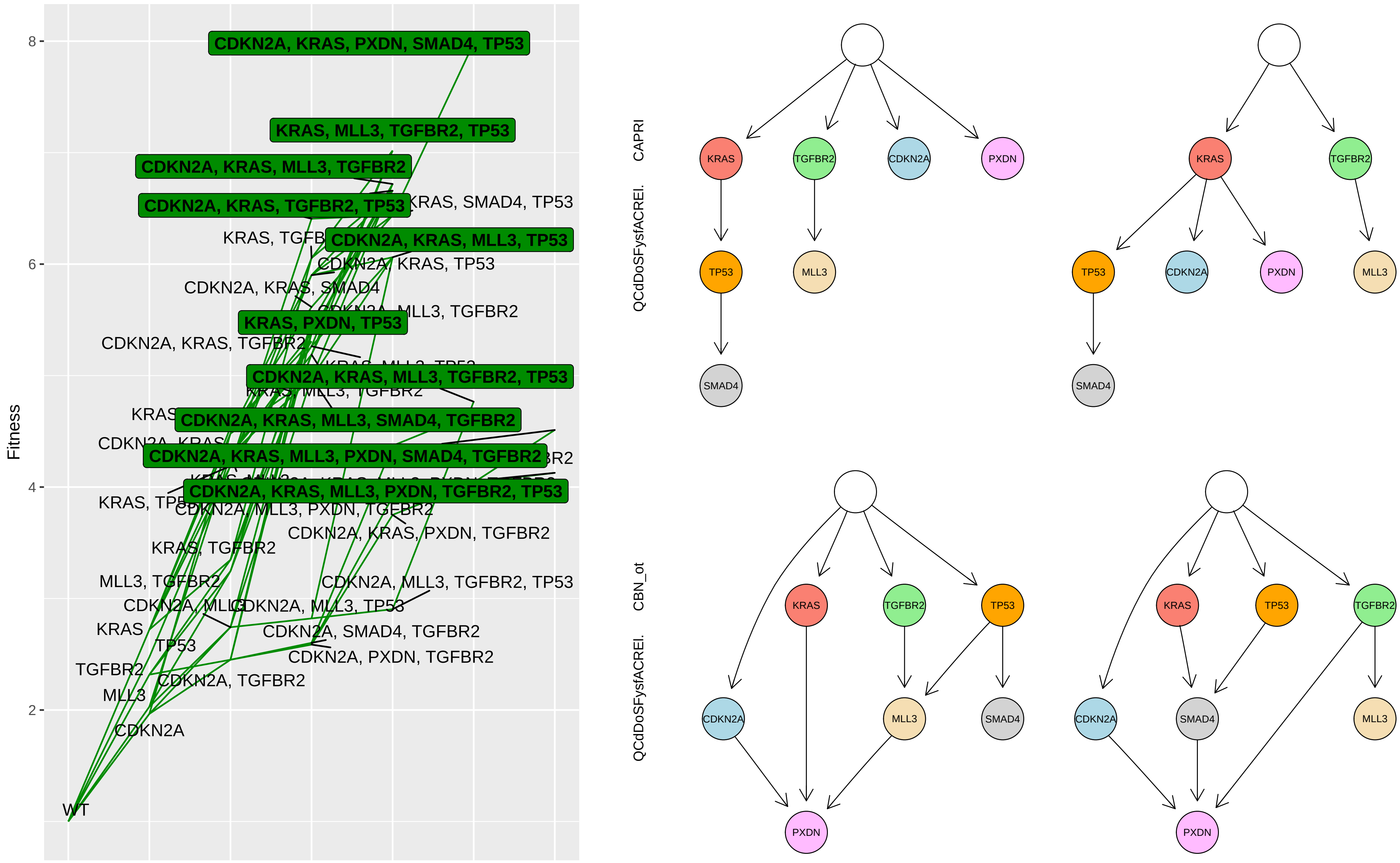

ID	p−value	Accessible Genot.
bPuzdkfXxirCktl	0.628	25

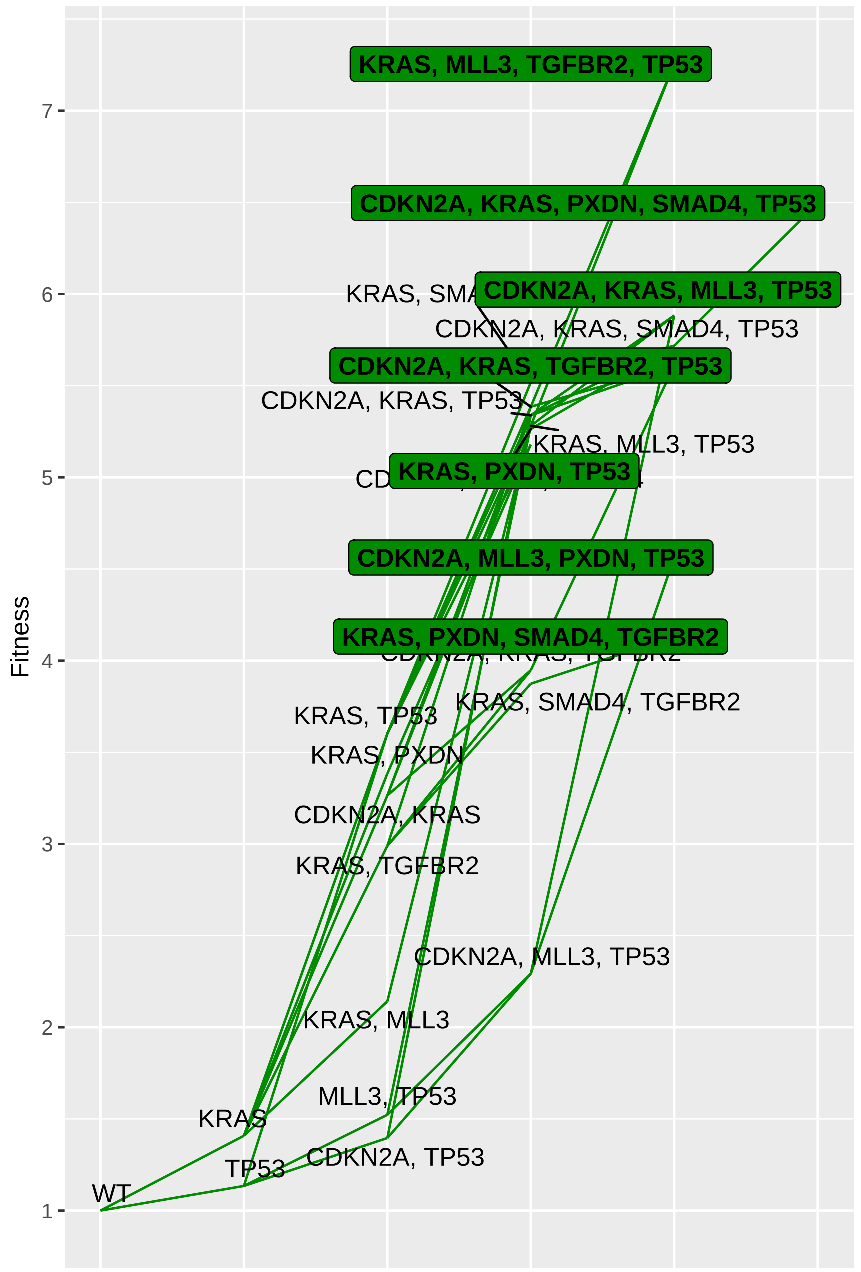

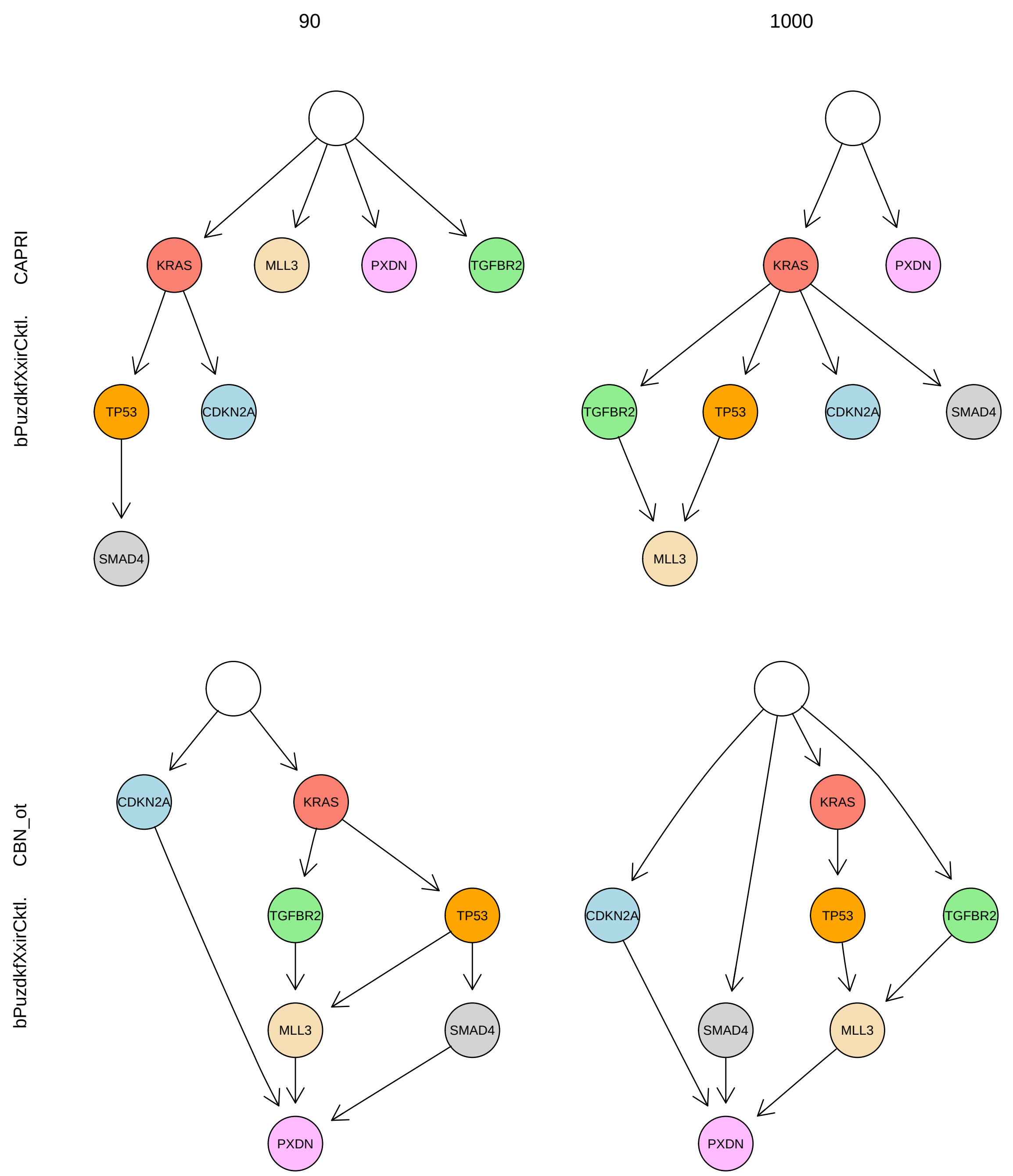

ID	p−value	Accessible Genot.
vHVhpsfORVPlmat	0.628	20

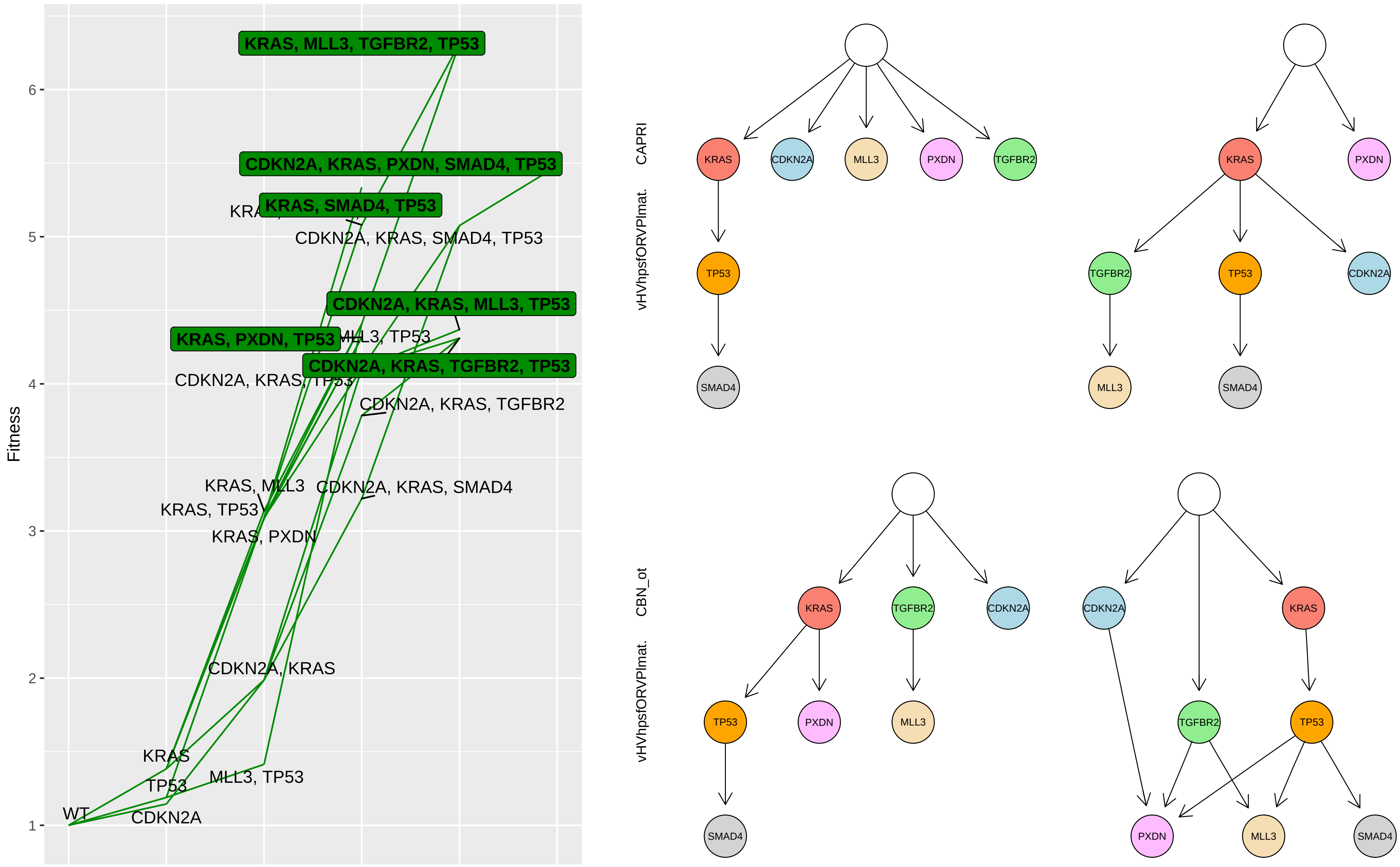

ID	p−value	Accessible Genot.
CswjtlEATgEKBdd	0.628	83

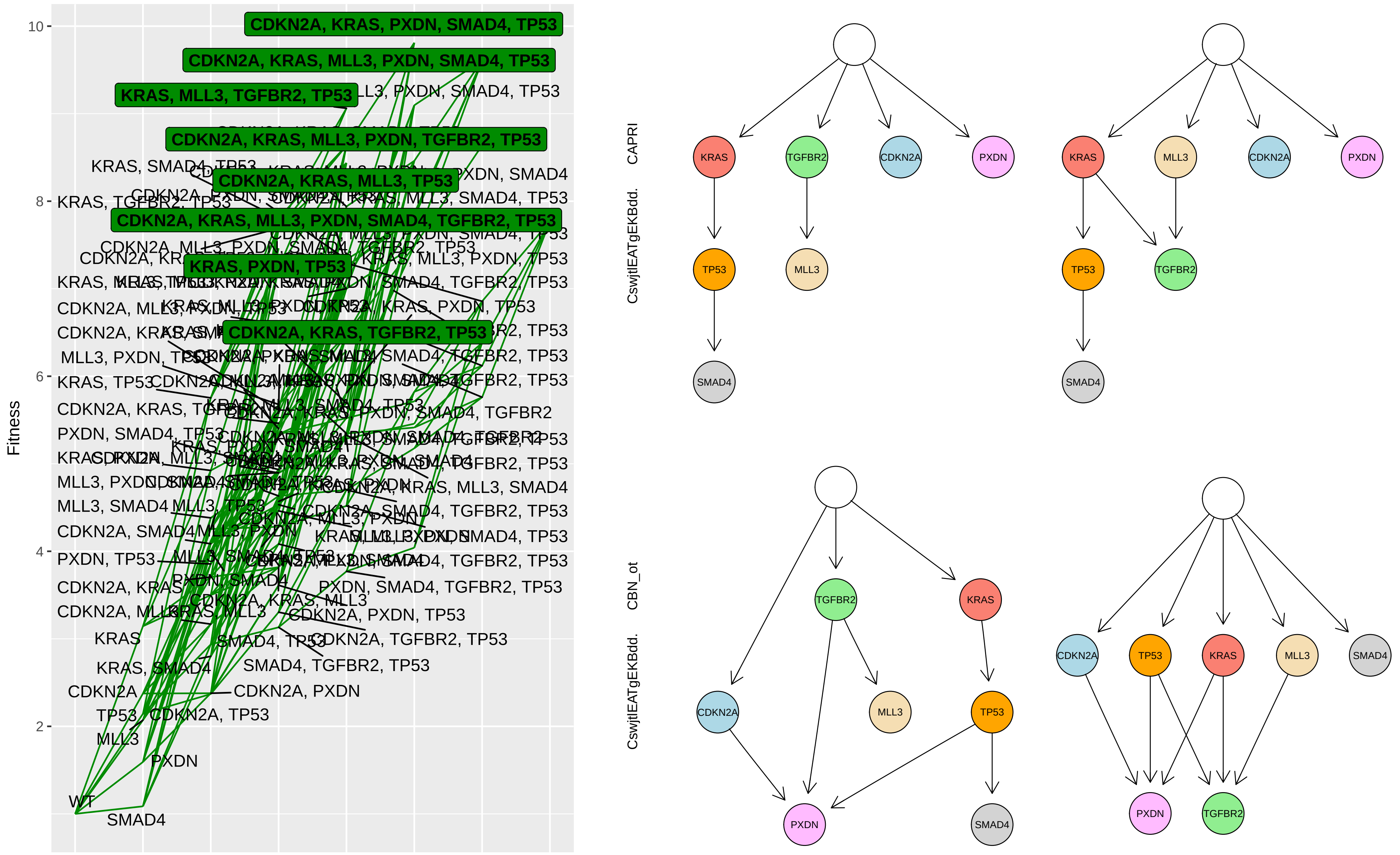

ID	p−value	Accessible Genot.
YYnDklUnLGsdLEe	0.629	20

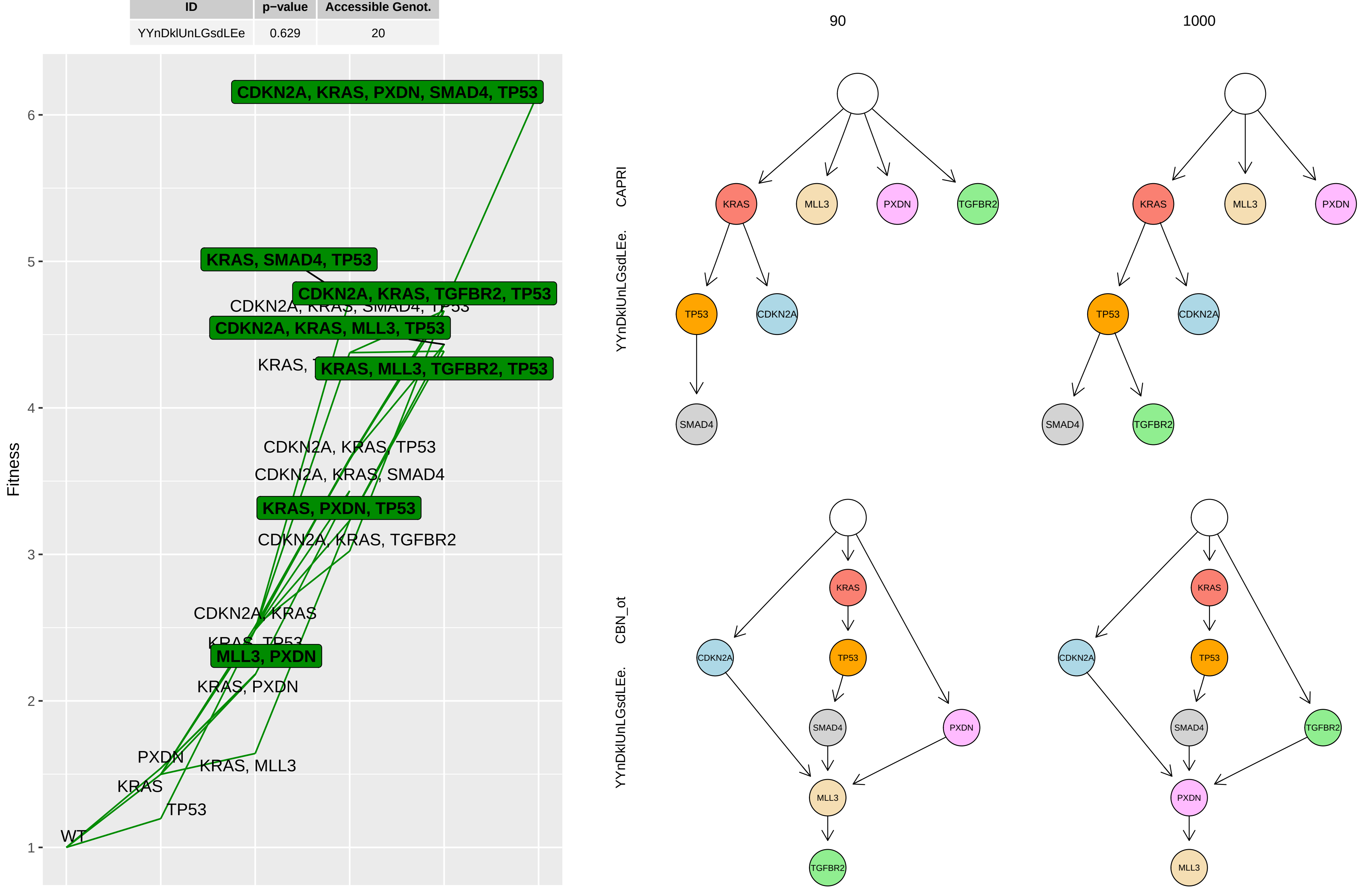

ID	p−value	Accessible Genot.
wUIByJeOHzIUKaU	0.629	52

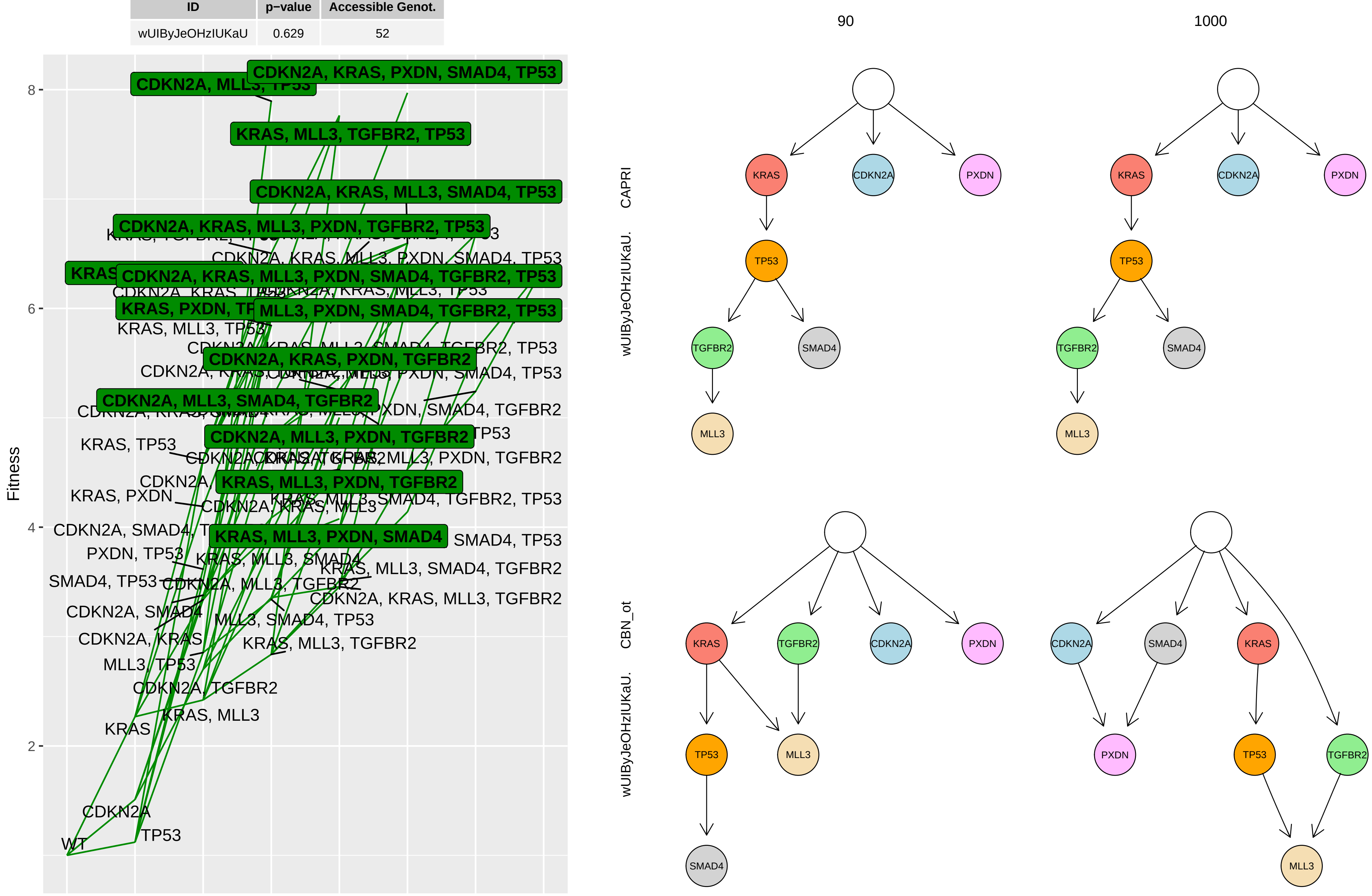

ID	p−value	Accessible Genot.
WDBkcHNUJEiUipB	0.639	33

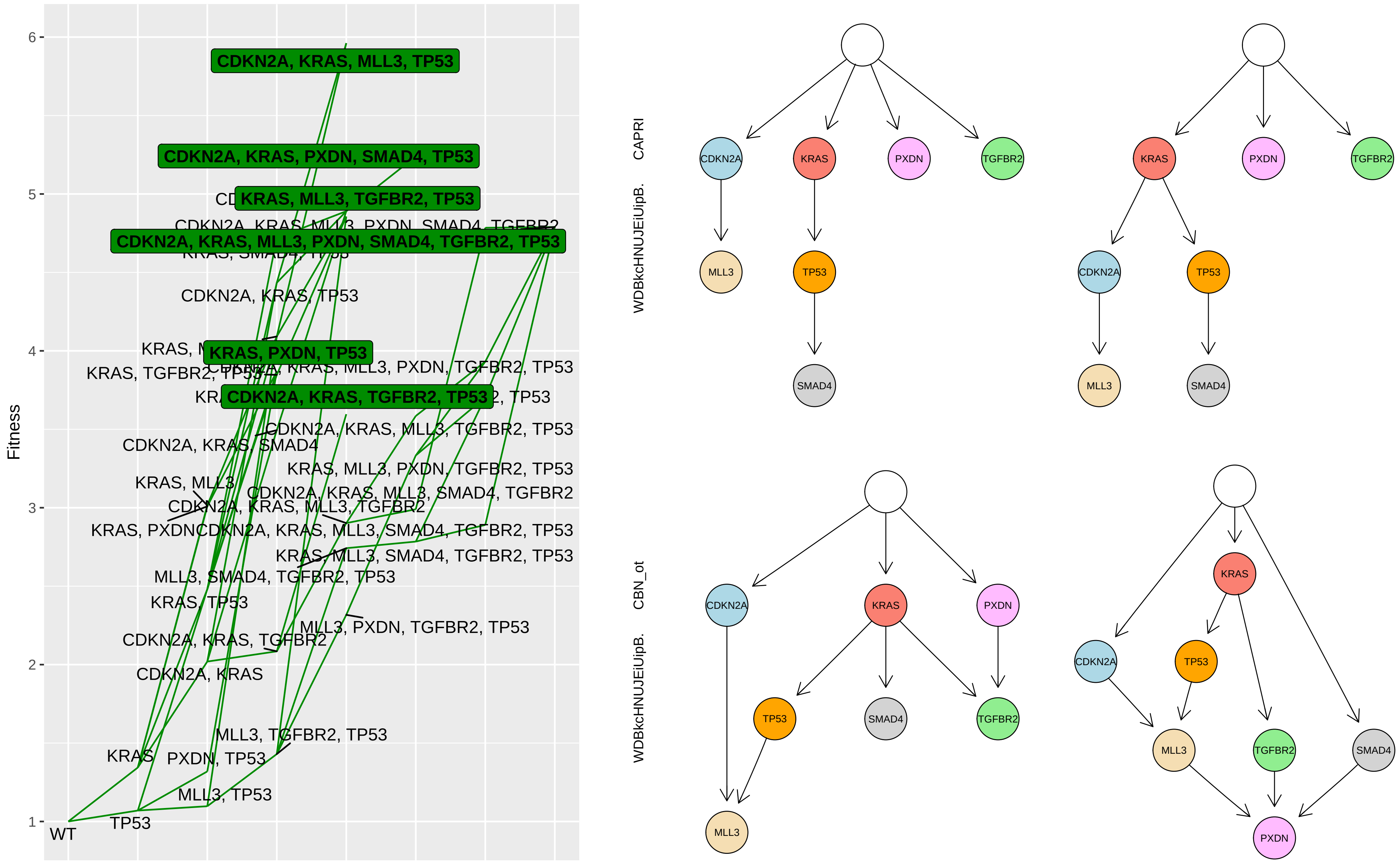

ID	p−value	Accessible Genot.
QekmkcmaGTEndpG	0.639	72

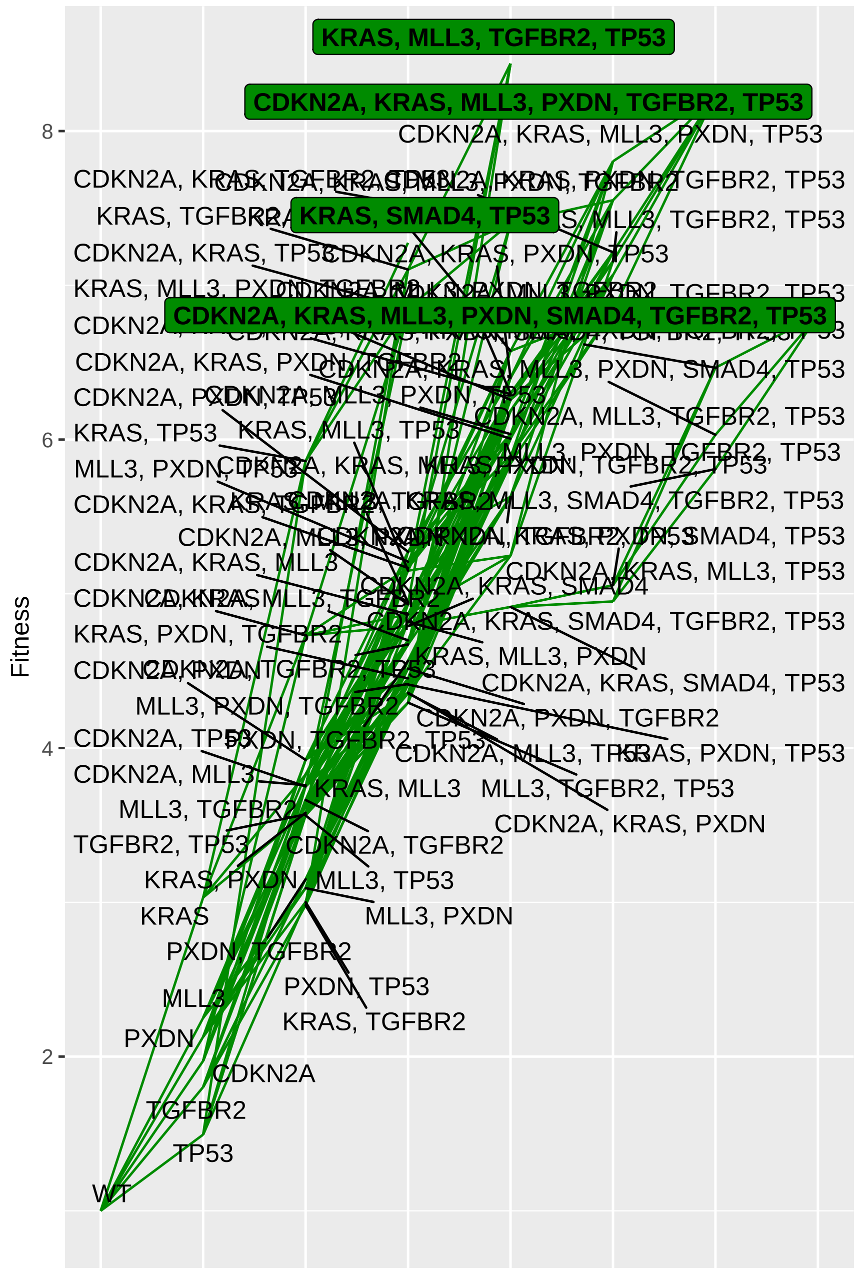

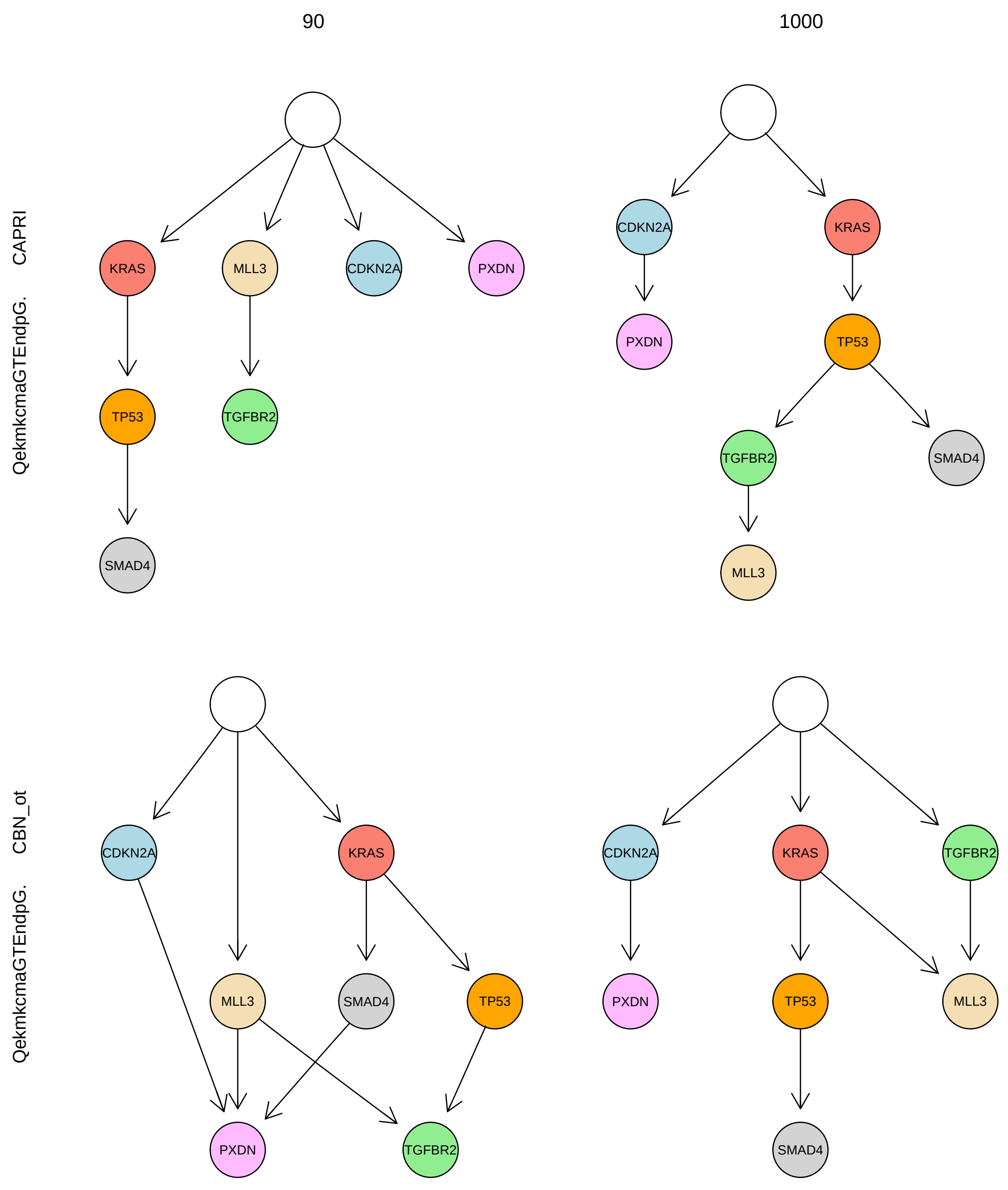

ID	p−value	Accessible Genot.
jJpOmuAQzqlhISo	0.641	18

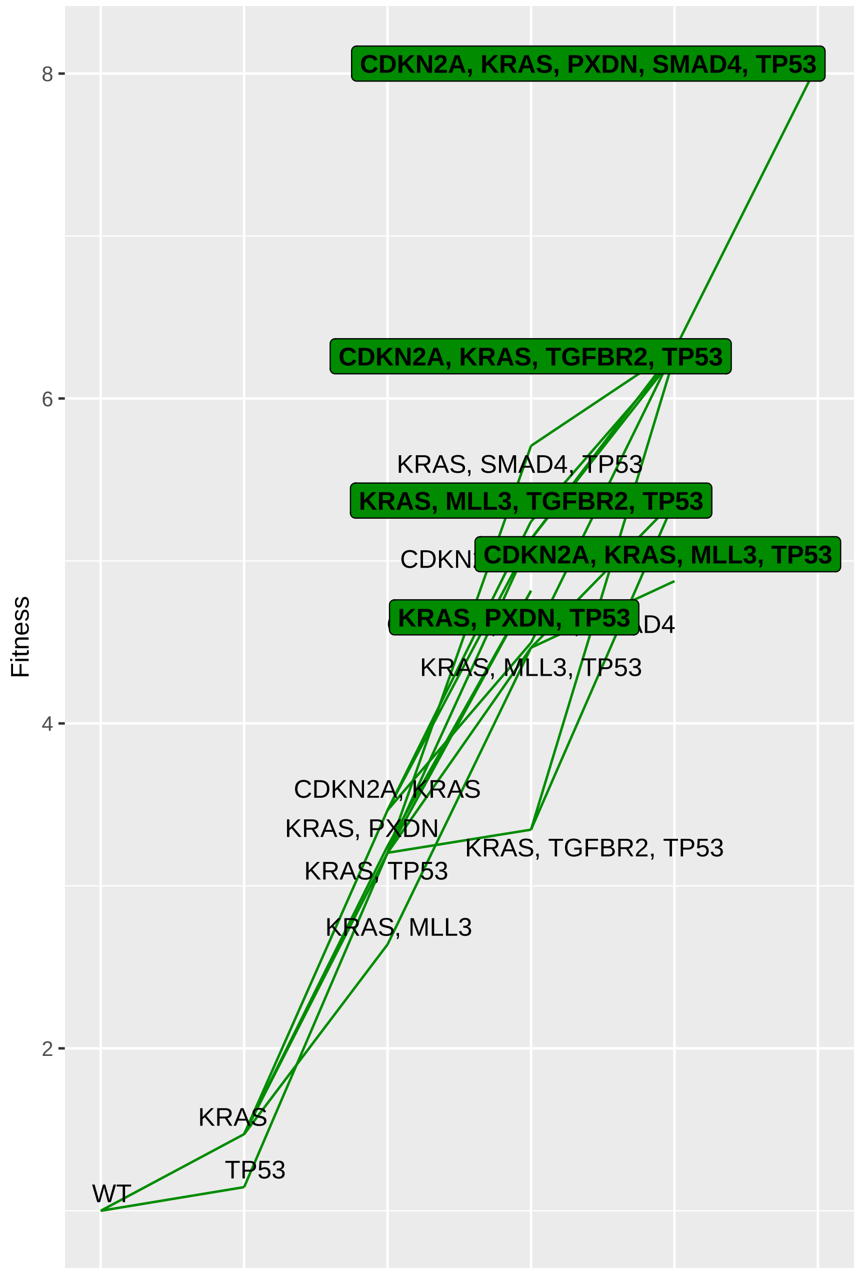

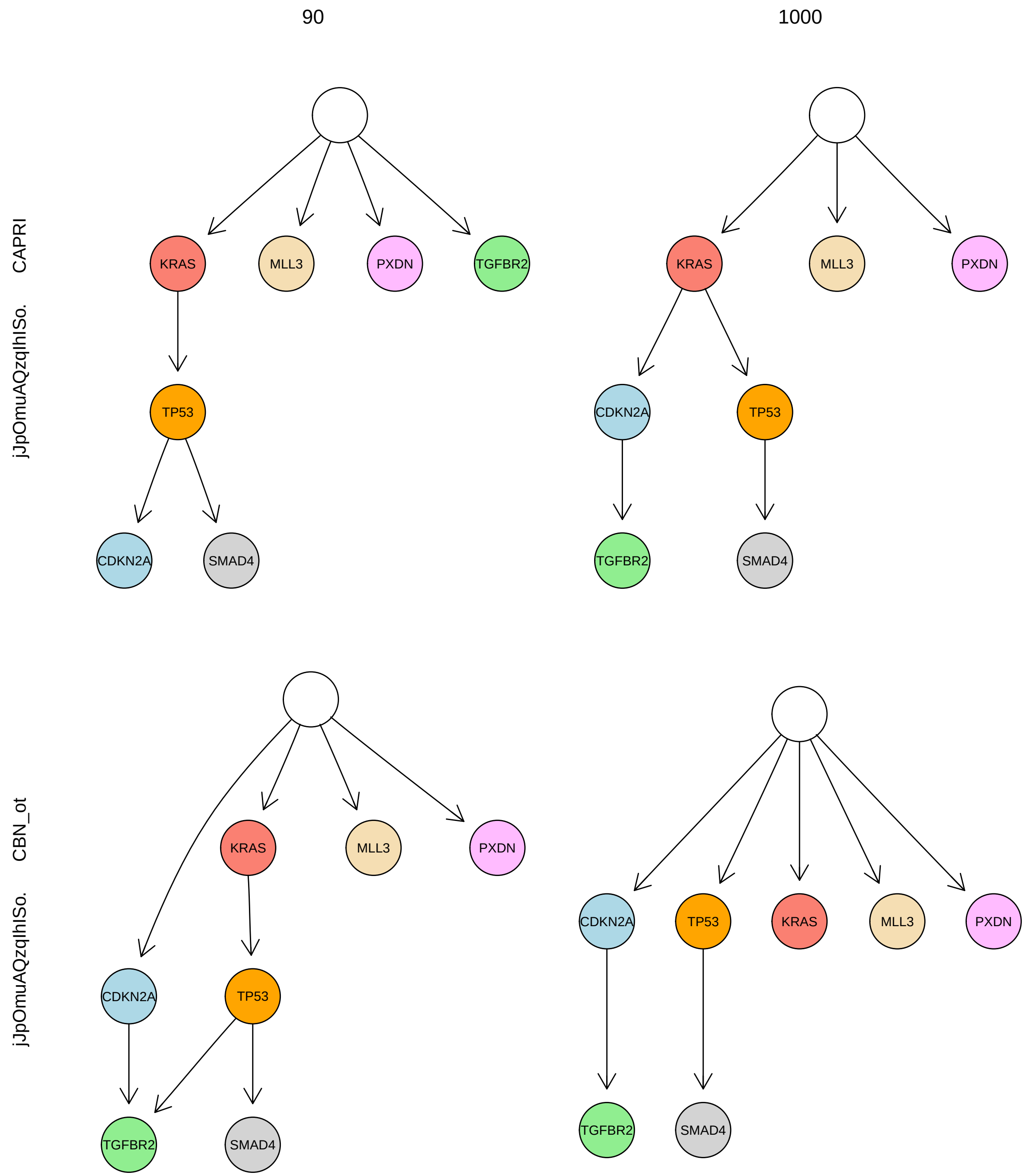

ID	p-value	Accessible Genot.
EvjtKZOaPqpancl	0.641	21

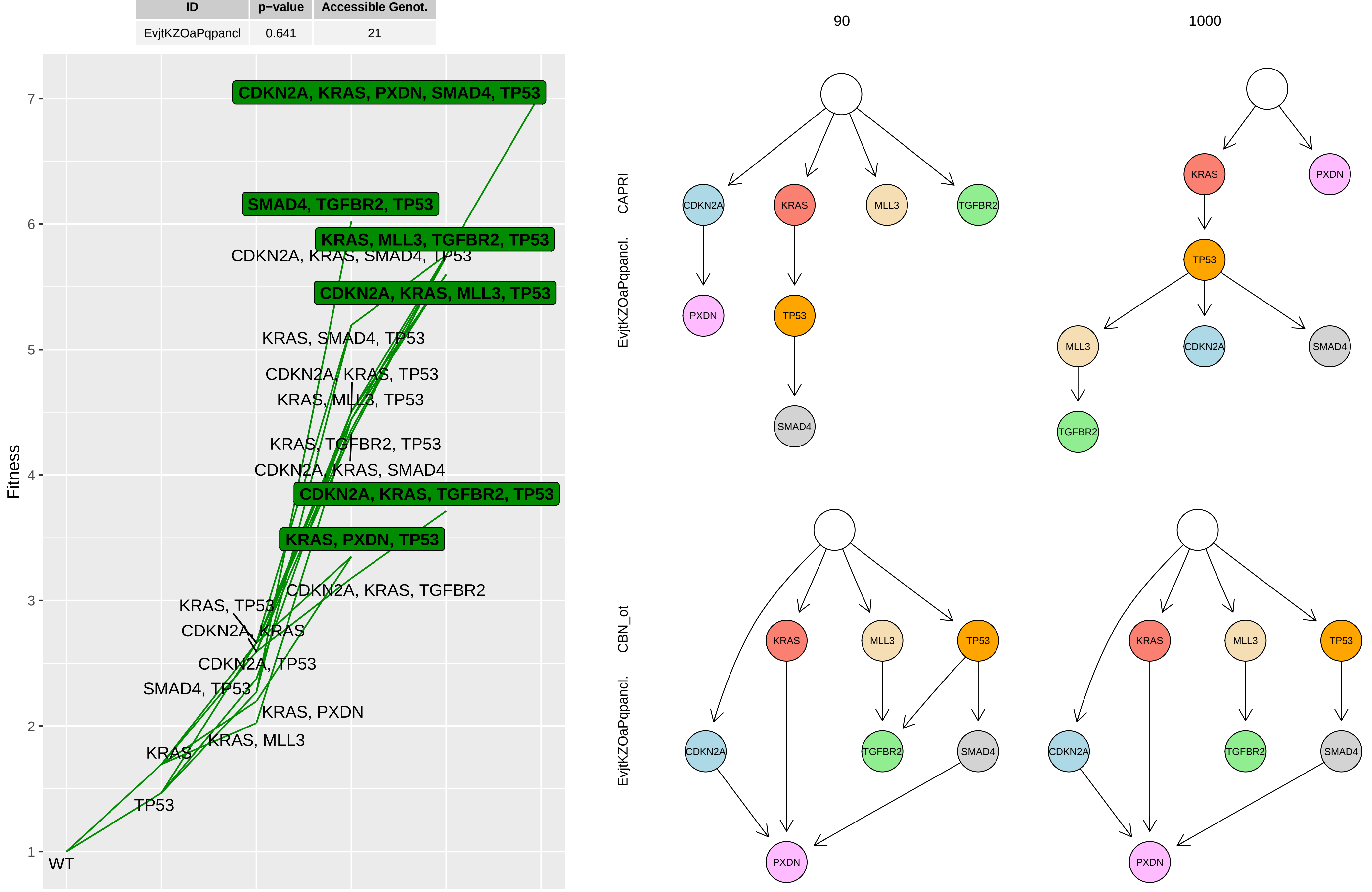

ID	p−value	Accessible Genot.
EoeHkDDzNHArHLk	0.643	24

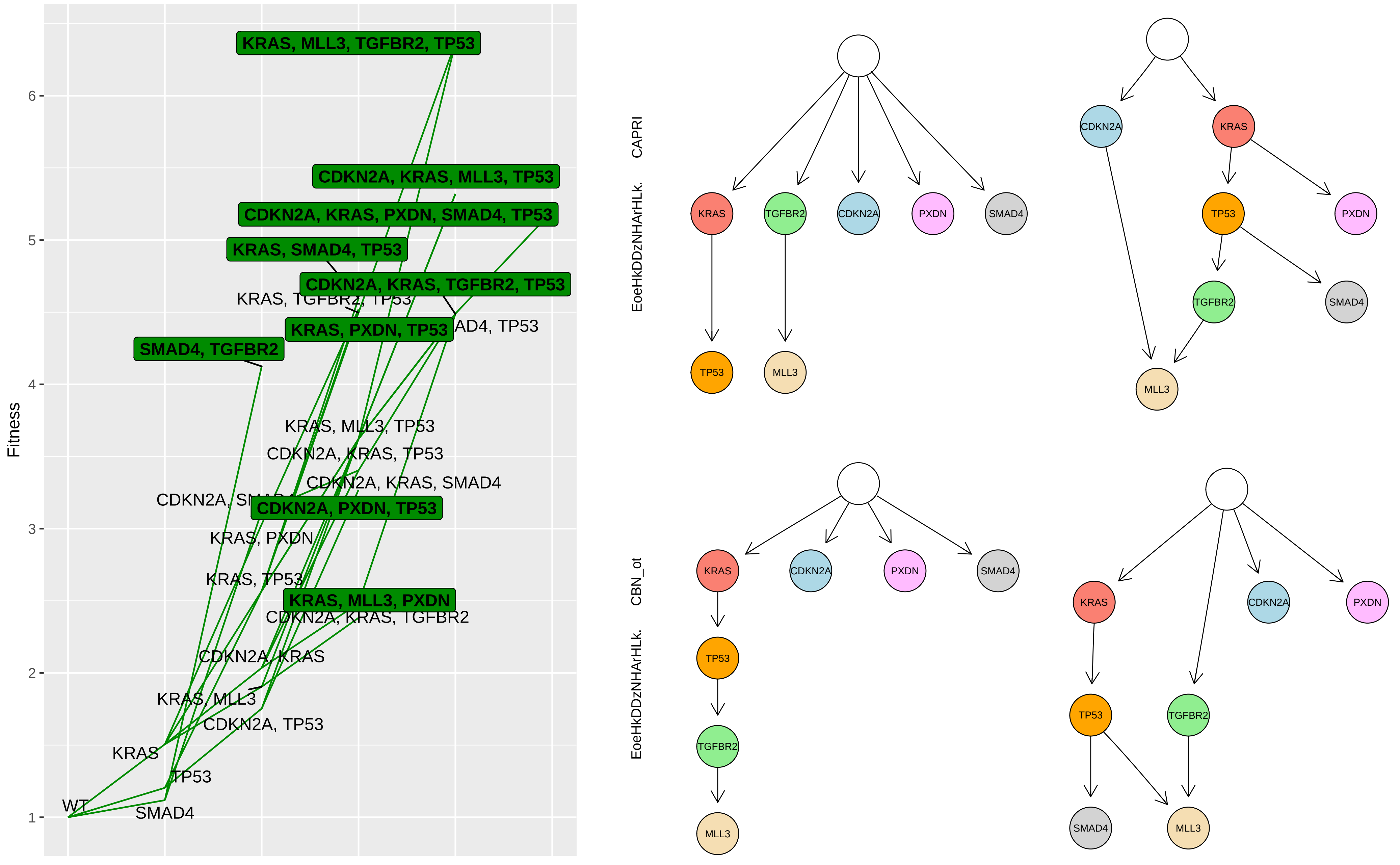

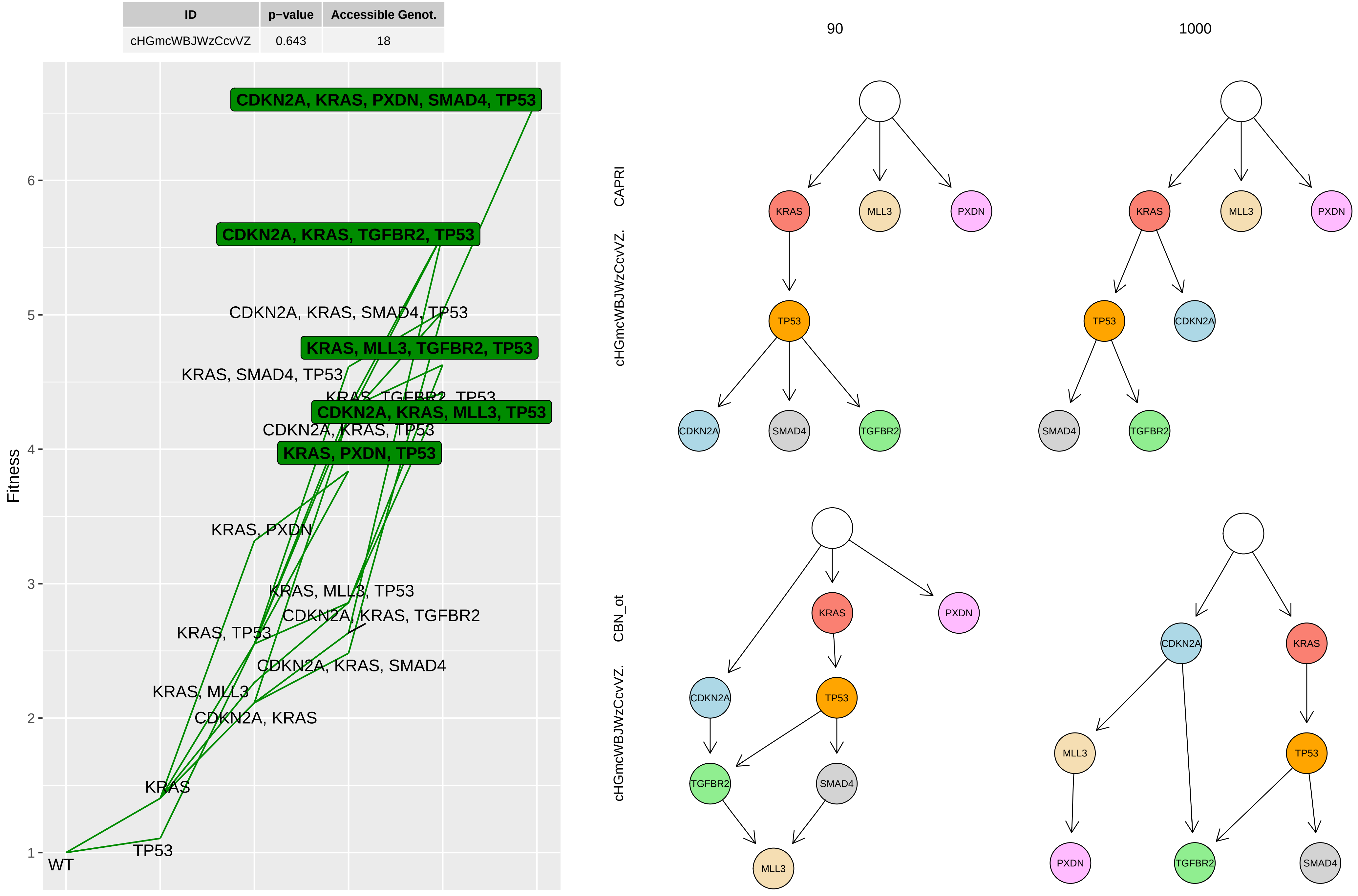

ID
p−value
Accessible Genot.
cHGmcWBJWzCcvVZ
0.643
18
Fitness
CDKN2A, KRAS, PXDN, SMAD4, TP53
CDKN2A, KRAS, TGFBR2, TP53
CDKN2A, KRAS, SMAD4, TP53
KRAS, MLL3, TGFBR2, TP53
KRAS, SMAD4, TP53
KRAS, TGFBR2, TP53
CDKN2A, KRAS, MLL3, TP53
CDKN2A, KRAS, TP53
KRAS, PXDN, TP53
KRAS, PXDN
KRAS, MLL3, TP53
CDKN2A, KRAS, TGFBR2
KRAS, TP53
CDKN2A, KRAS, SMAD4
KRAS, MLL3
CDKN2A, KRAS
KRAS
TP53
WT
90
1000
CAPRI
cHGmcWBJWzCcvVZ.
CBN_ot
cHGmcWBJWzCcvVZ.
KRAS
MLL3
PXDN
TP53
CDKN2A
SMAD4
TGFBR2

ID	p−value	Accessible Genot.
DDXJczHRWZStLGK	0.644	18

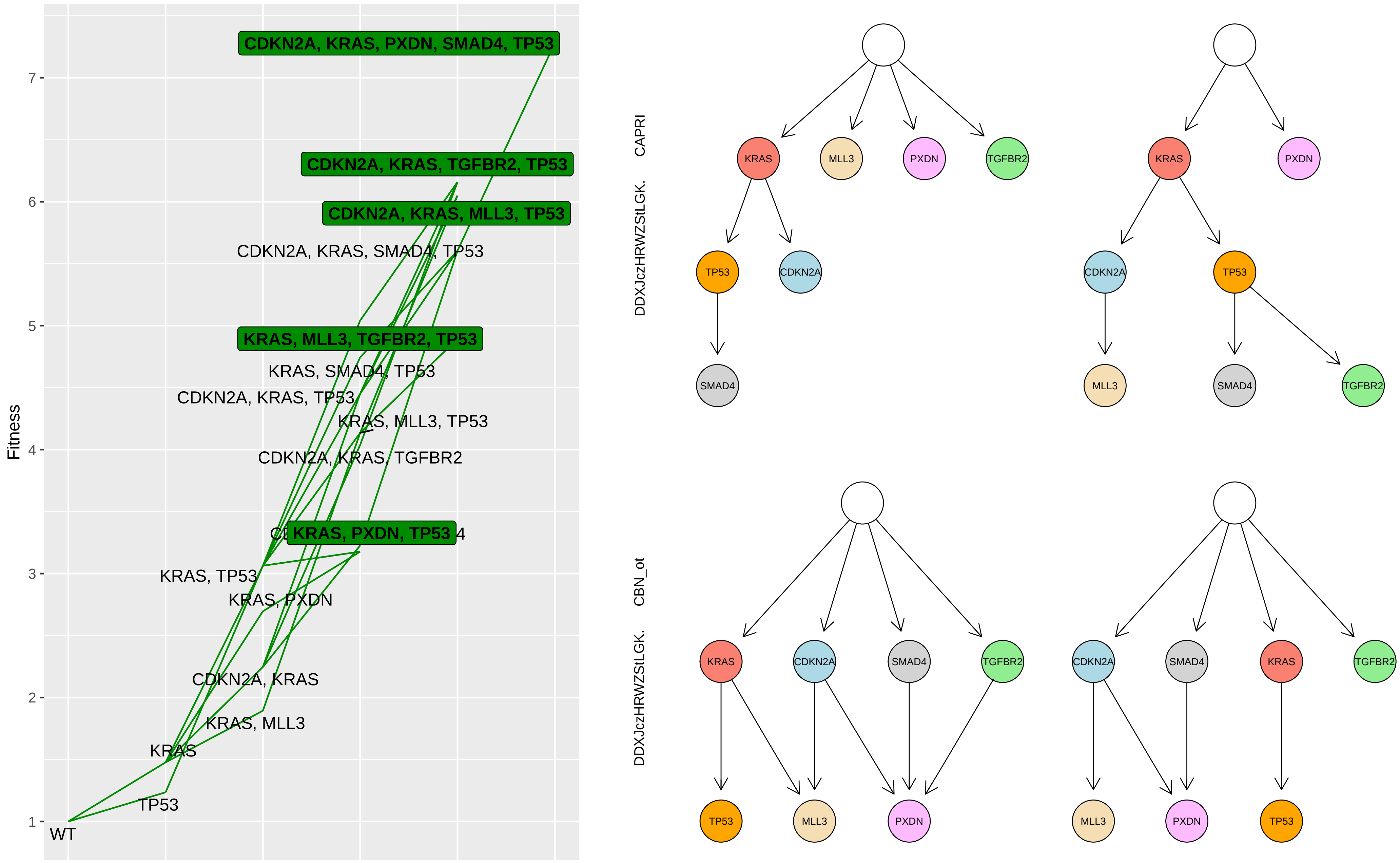

ID	p−value	Accessible Genot.
RErpJLoELJIUmLg	0.644	126

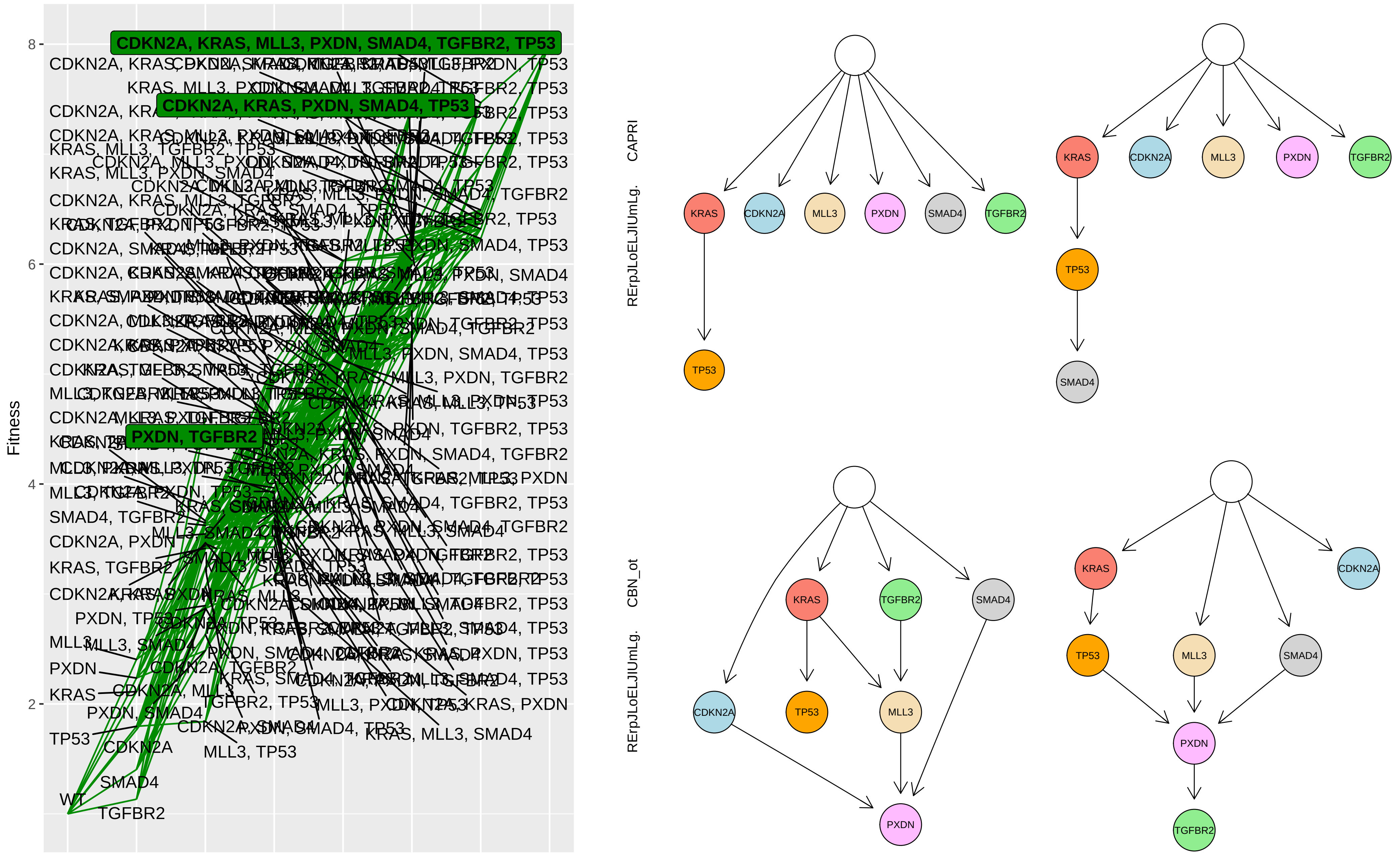

ID	p−value	Accessible Genot.
gZRzsdiuOqZMXrt	0.647	26

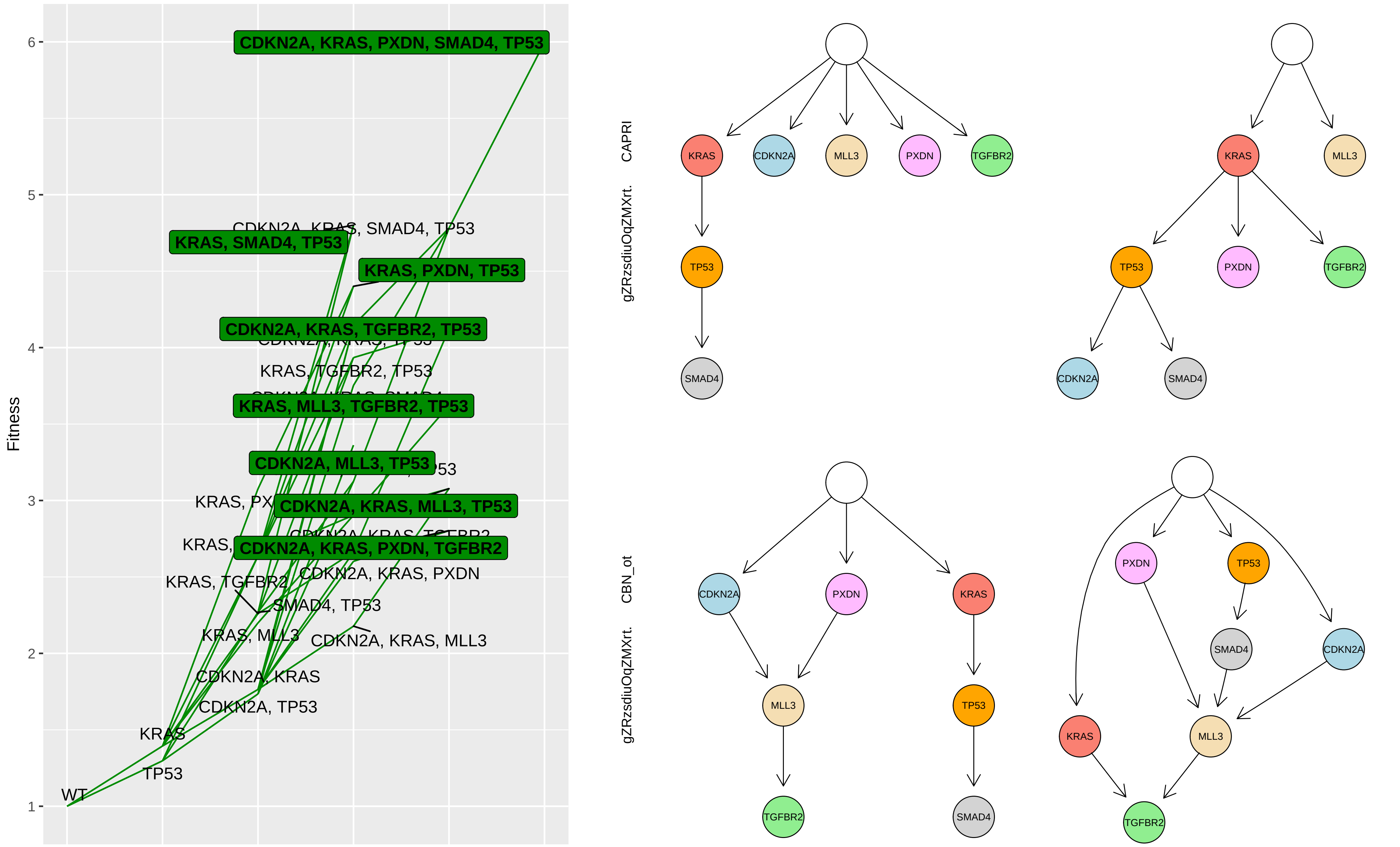

ID	p−value	Accessible Genot.
gjTsMGXOxFCuDbP	0.65	28

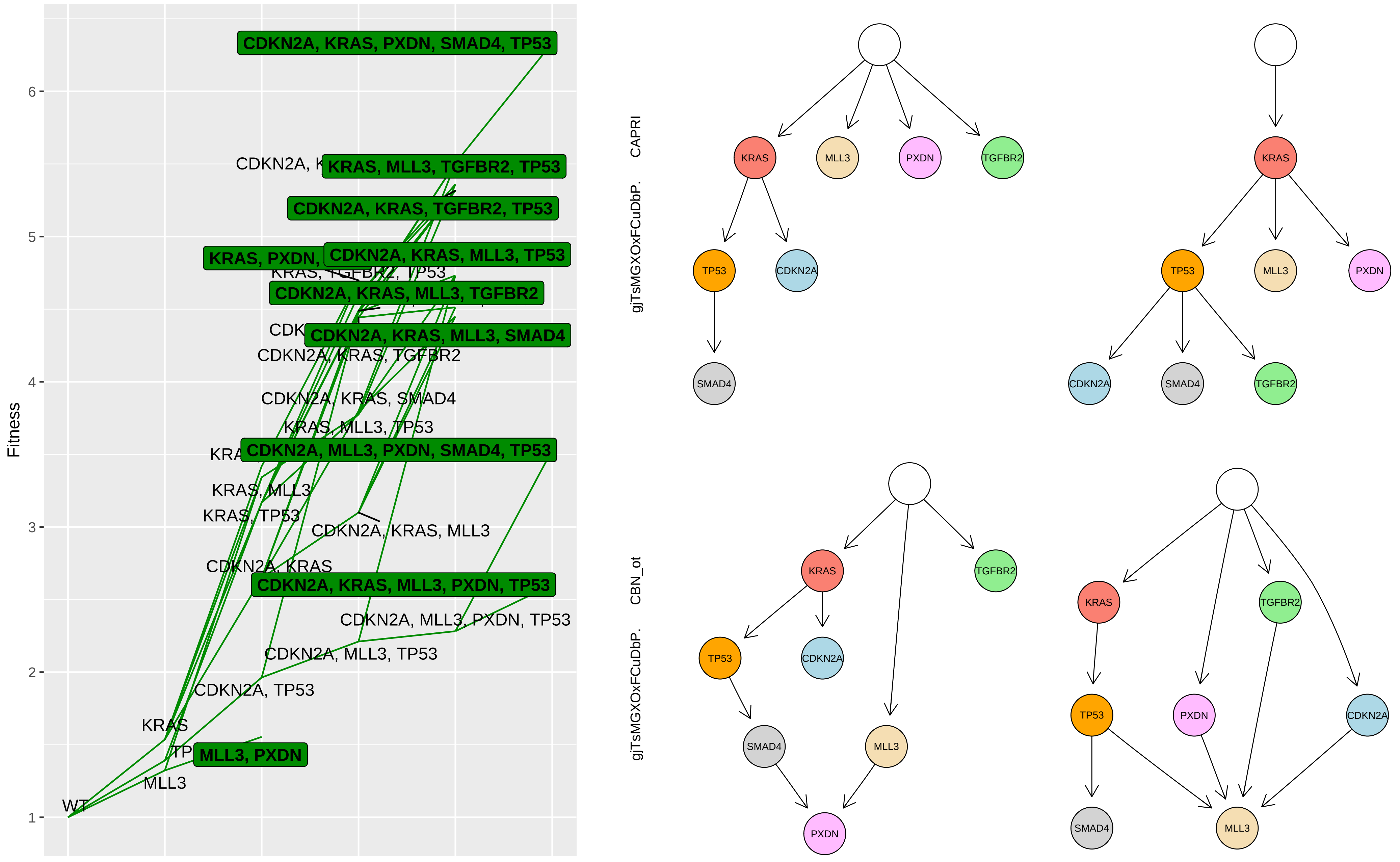

ID	p−value	Accessible Genot.
IMdYyqtgrBQvdsA	0.651	18

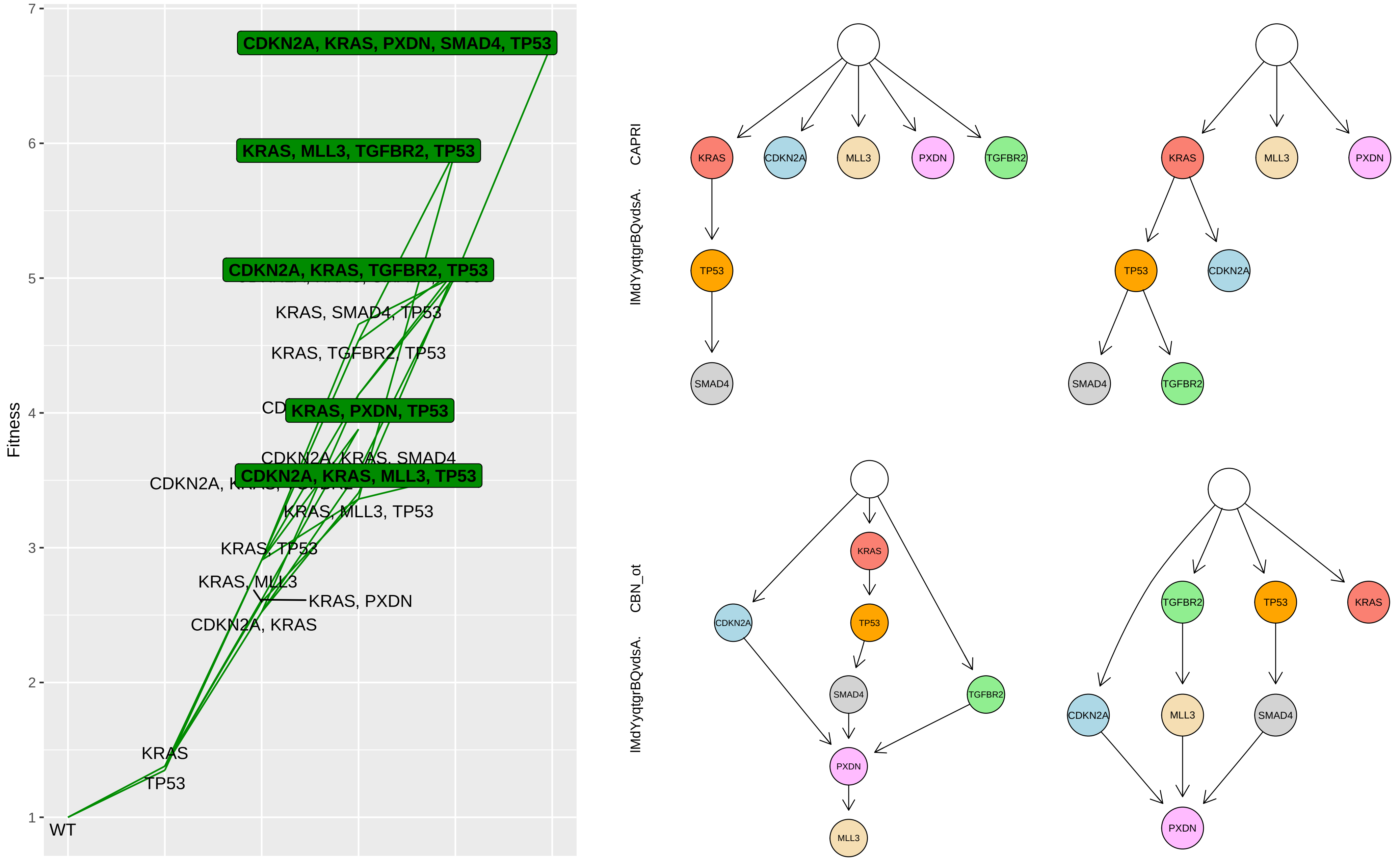

ID	p−value	Accessible Genot.
oOFZMvCrMajuFFV	0.652	30

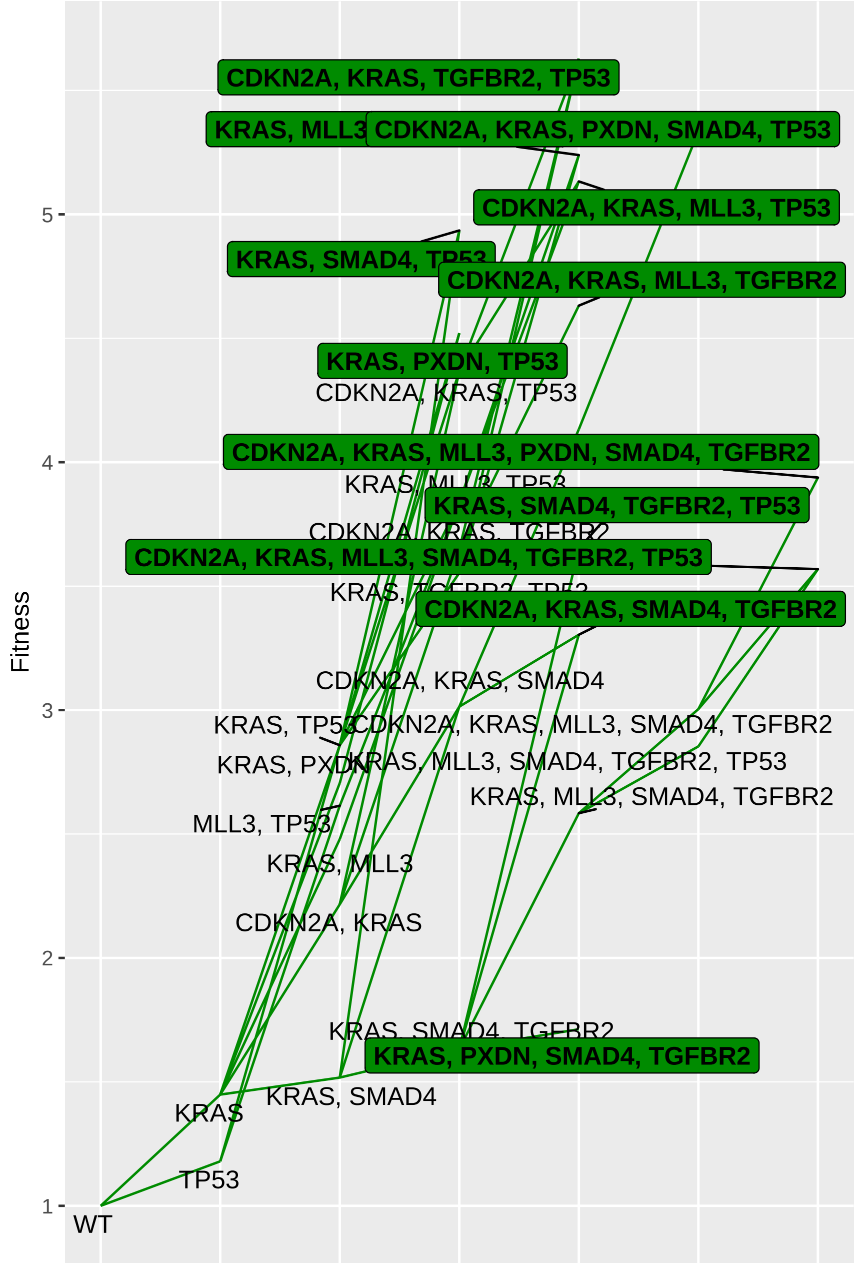

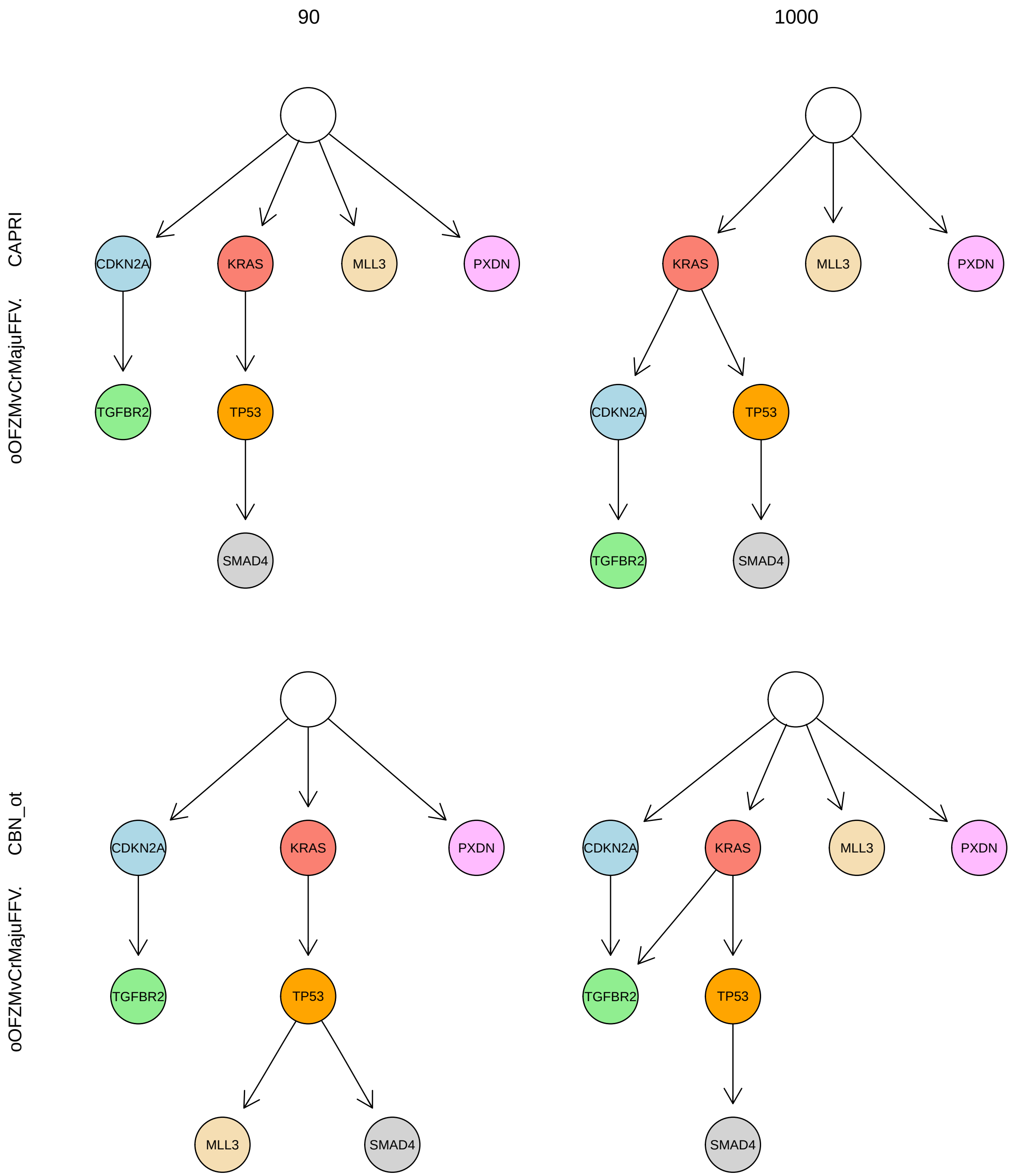

ID	p−value	Accessible Genot.
joDdwOeKmmHZxco	0.653	20

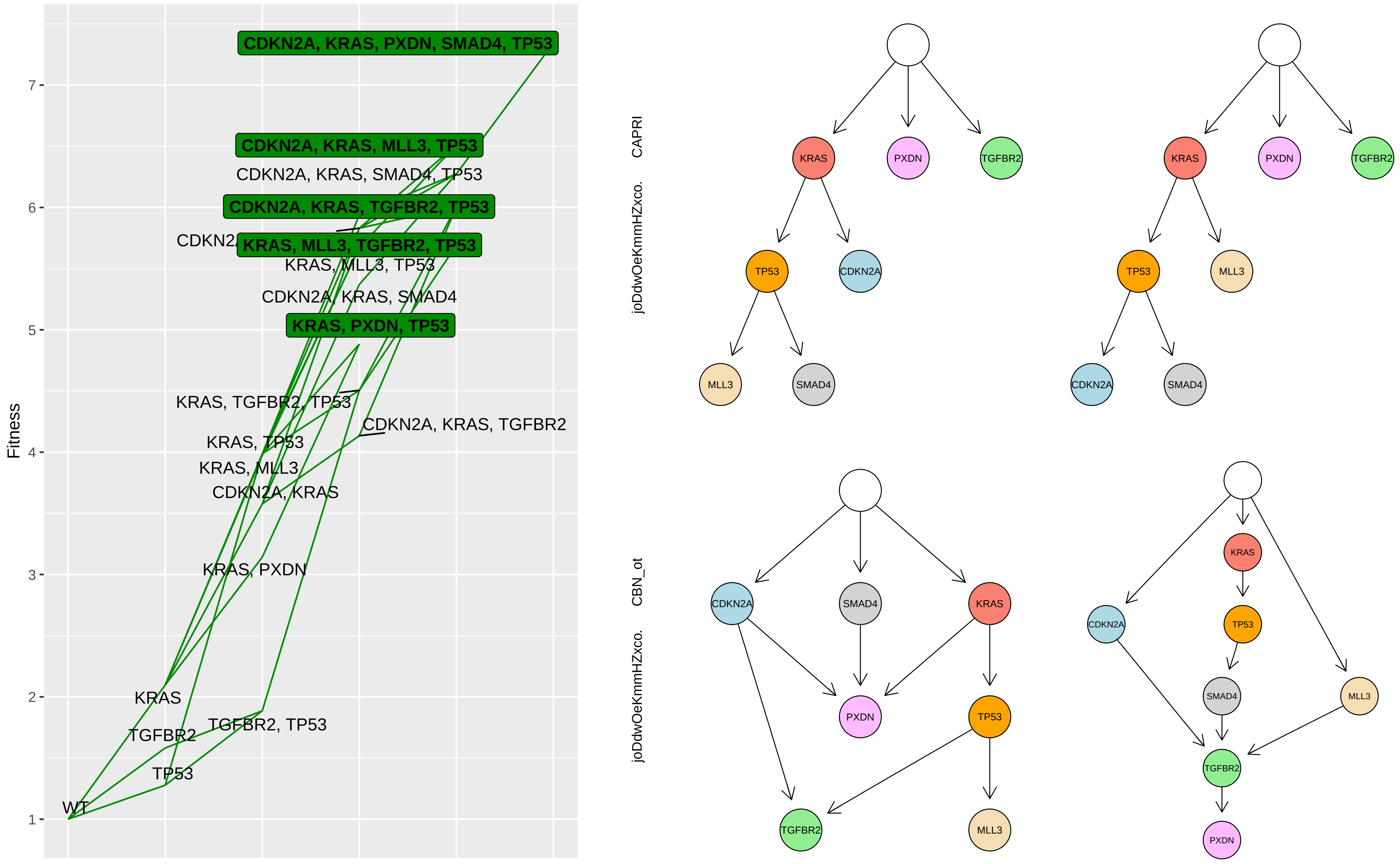

ID	p−value	Accessible Genot.
CjROlWyTvkfpQbd	0.654	19

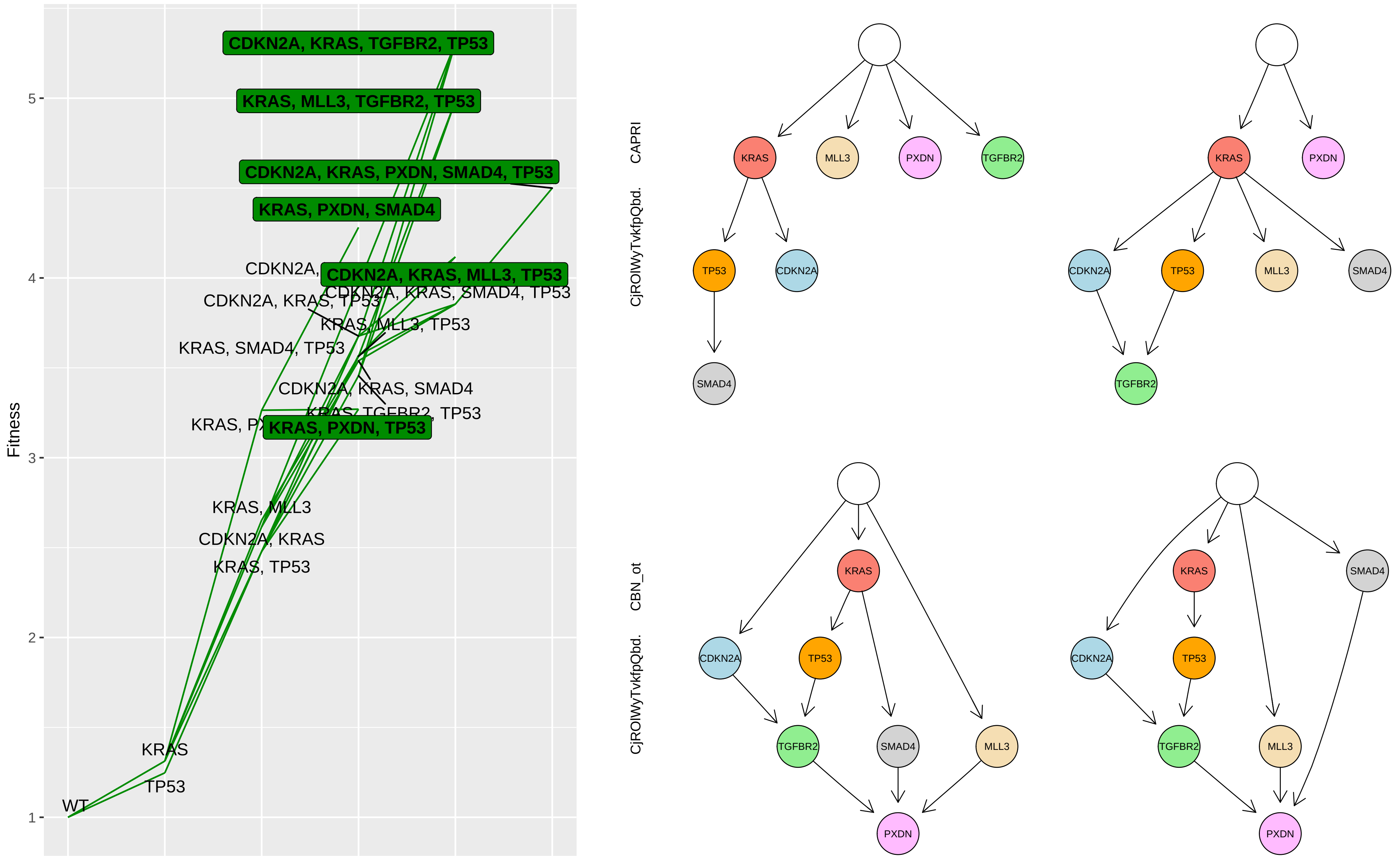

ID	p−value	Accessible Genot.
MznmMETnchPqBjz	0.656	71

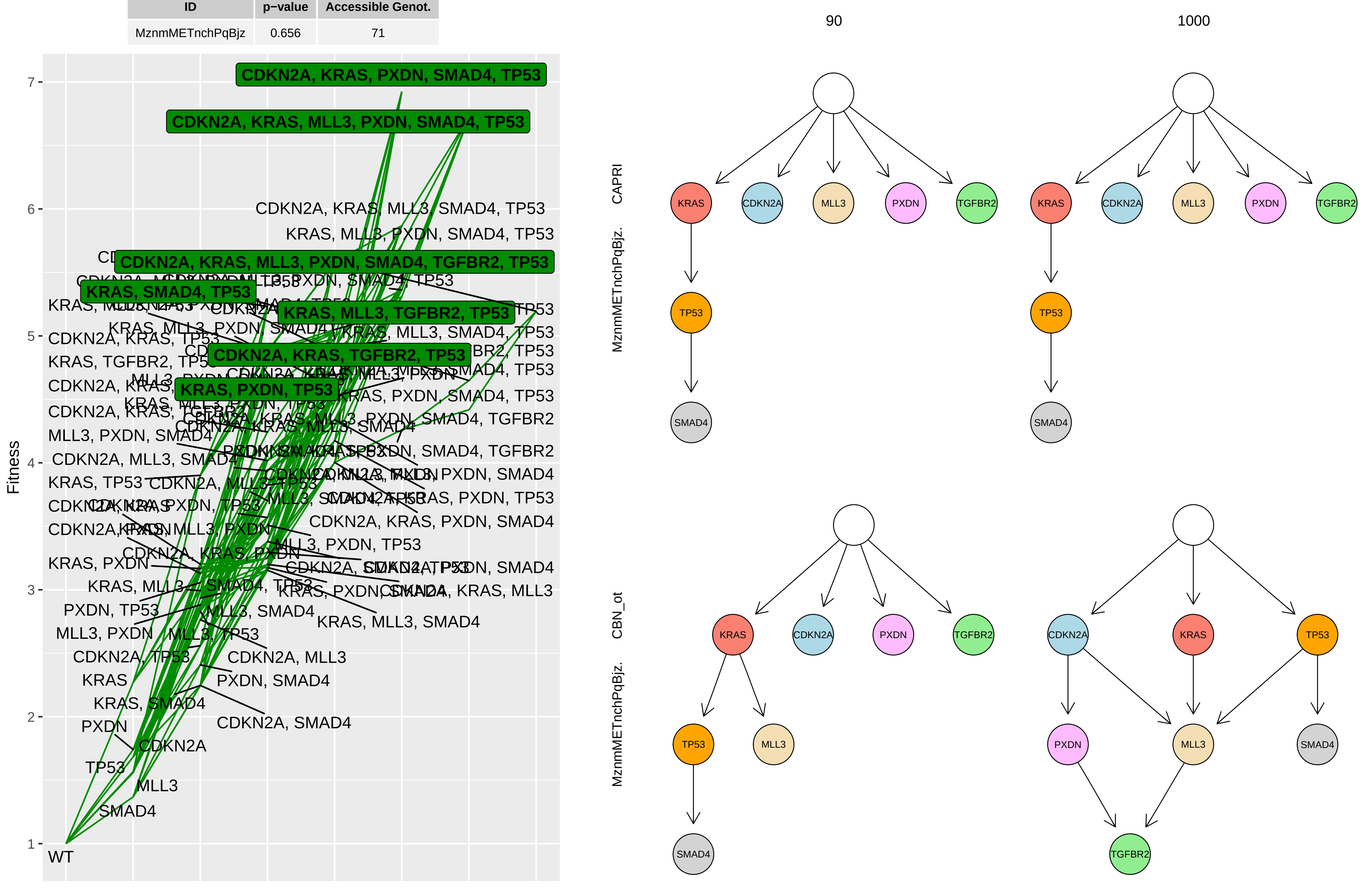

ID	p−value	Accessible Genot.
gQUclGVzfElkyJR	0.658	20

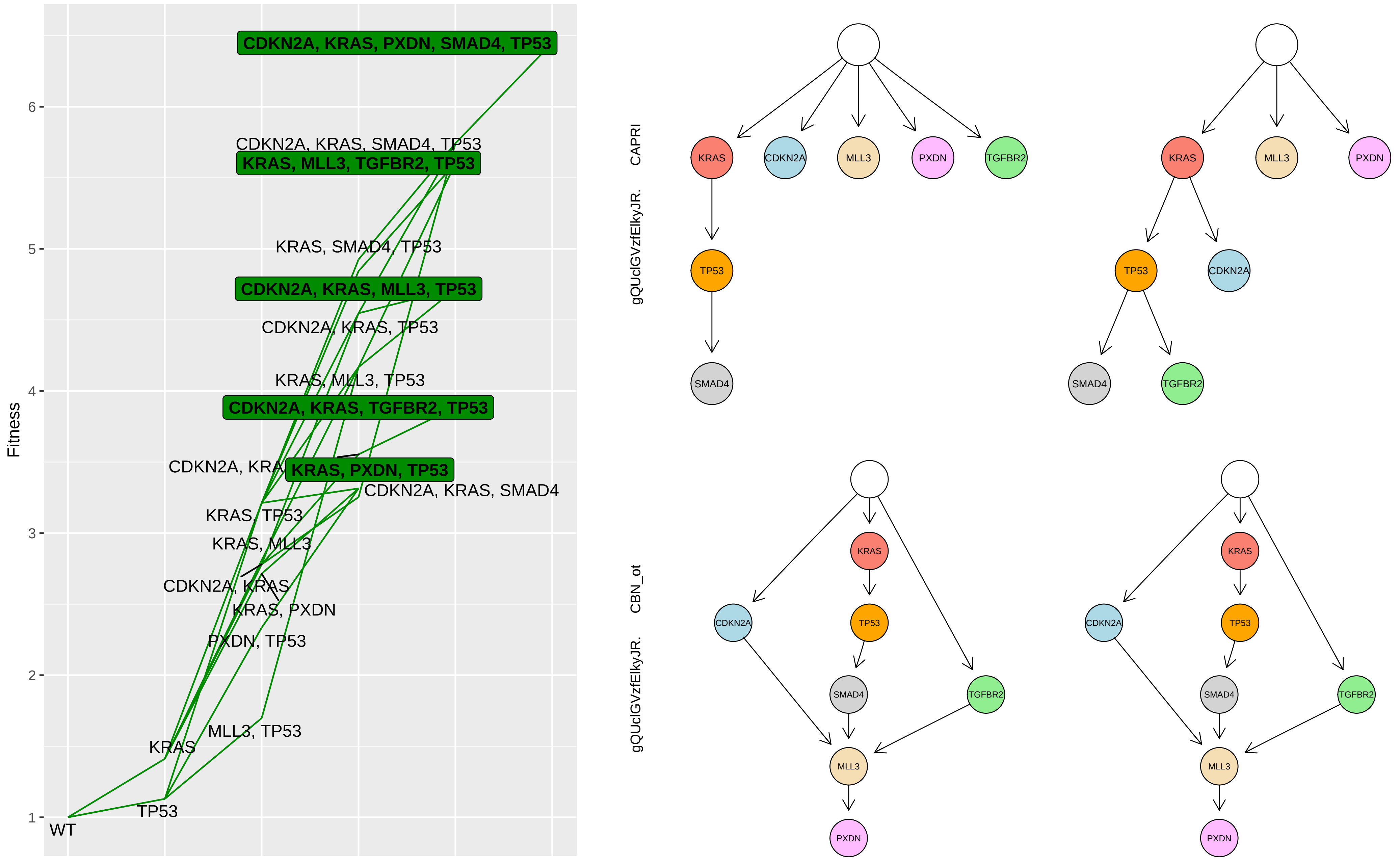

ID	p−value	Accessible Genot.
NdmQbfFarUgMhEj	0.662	75

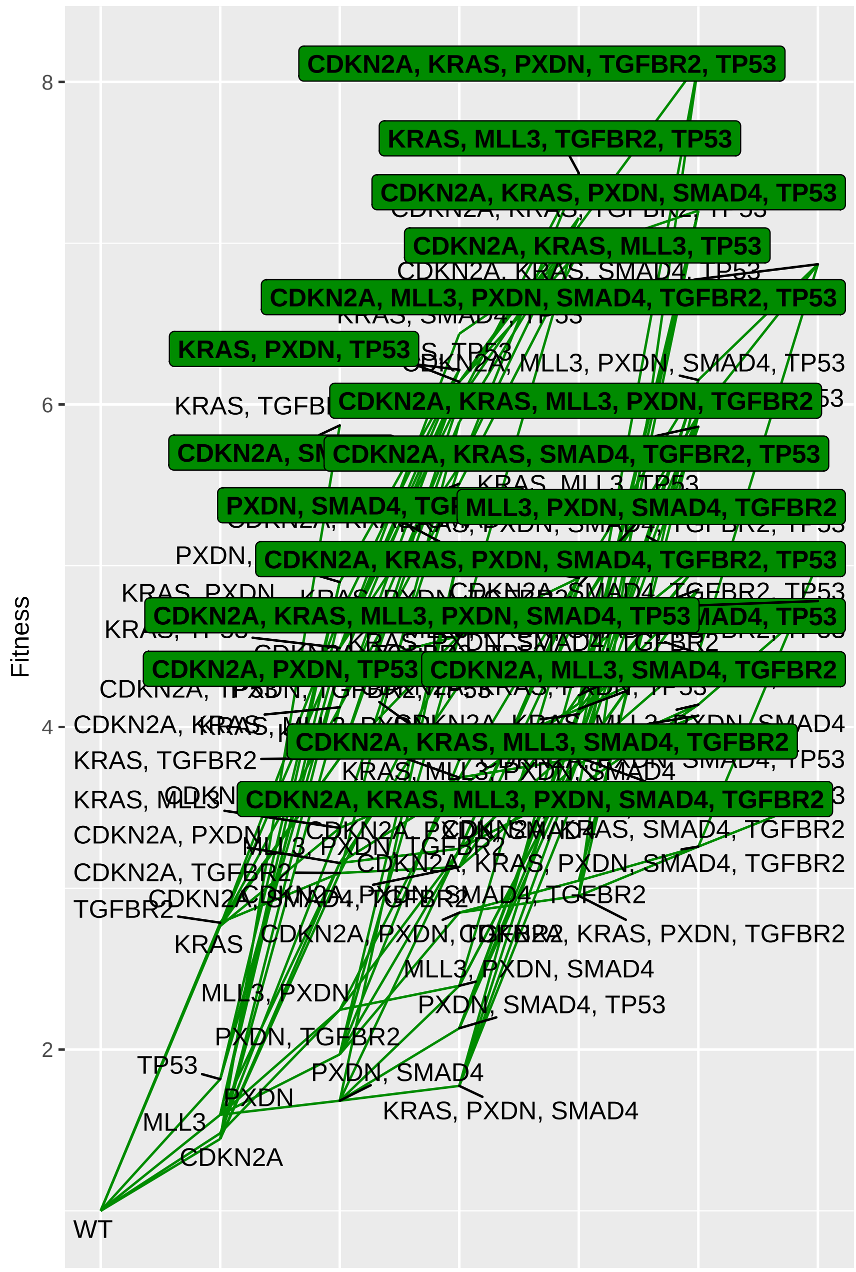

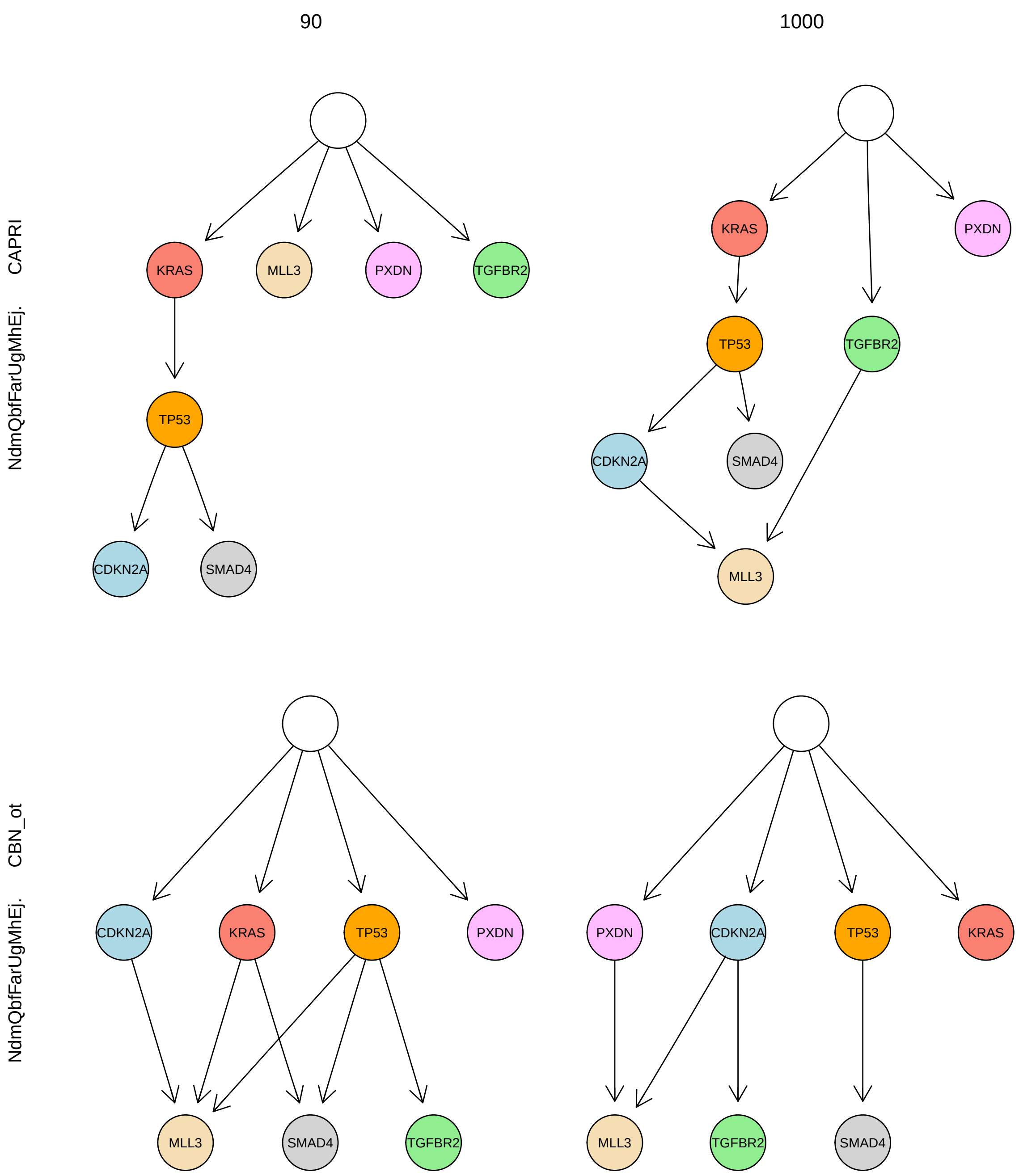

ID	p−value	Accessible Genot.
DXdEJQdsRtZyfKb	0.667	20

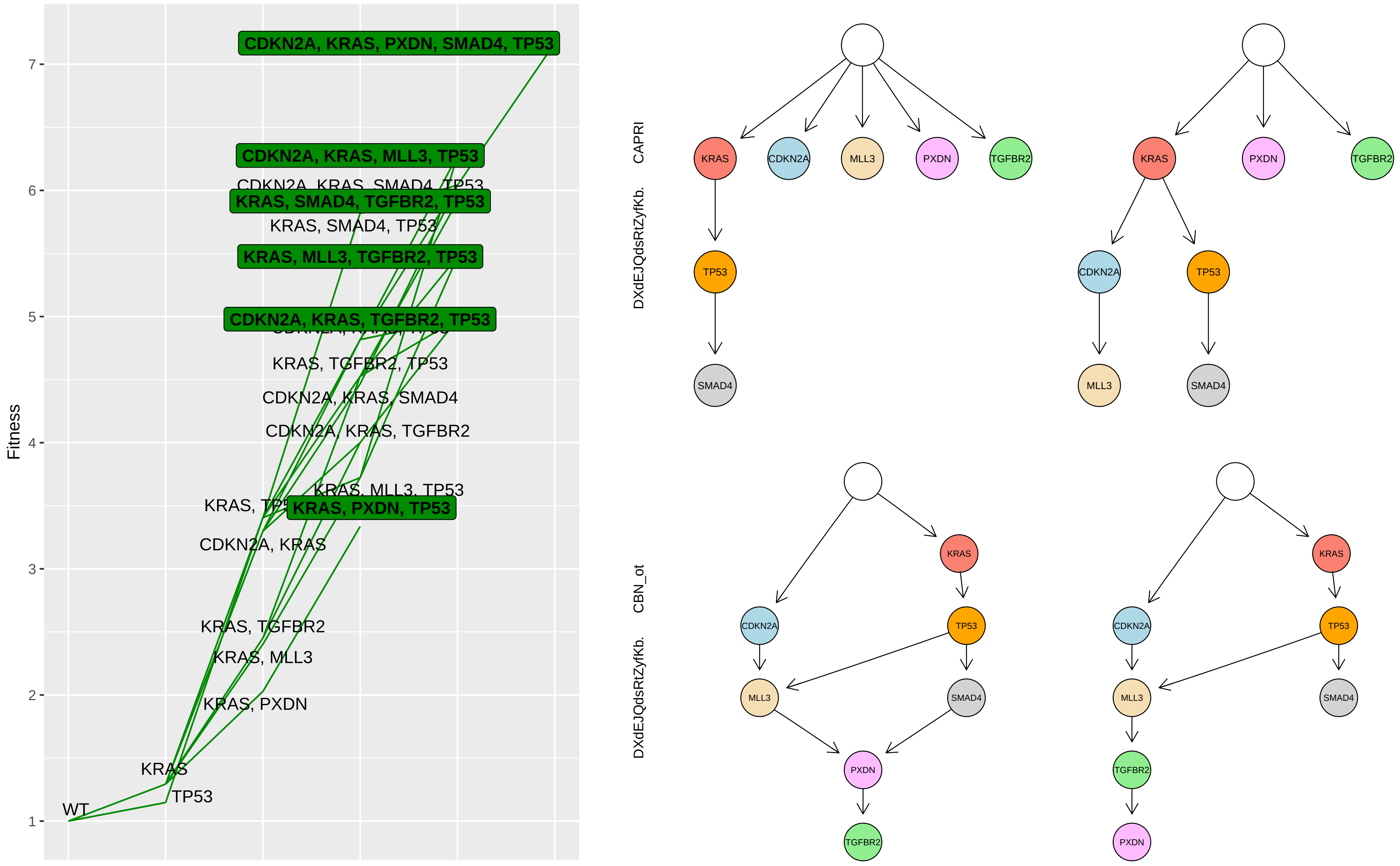

ID	p−value	Accessible Genot.
izsLFOsDhEiOYTq	0.67	18

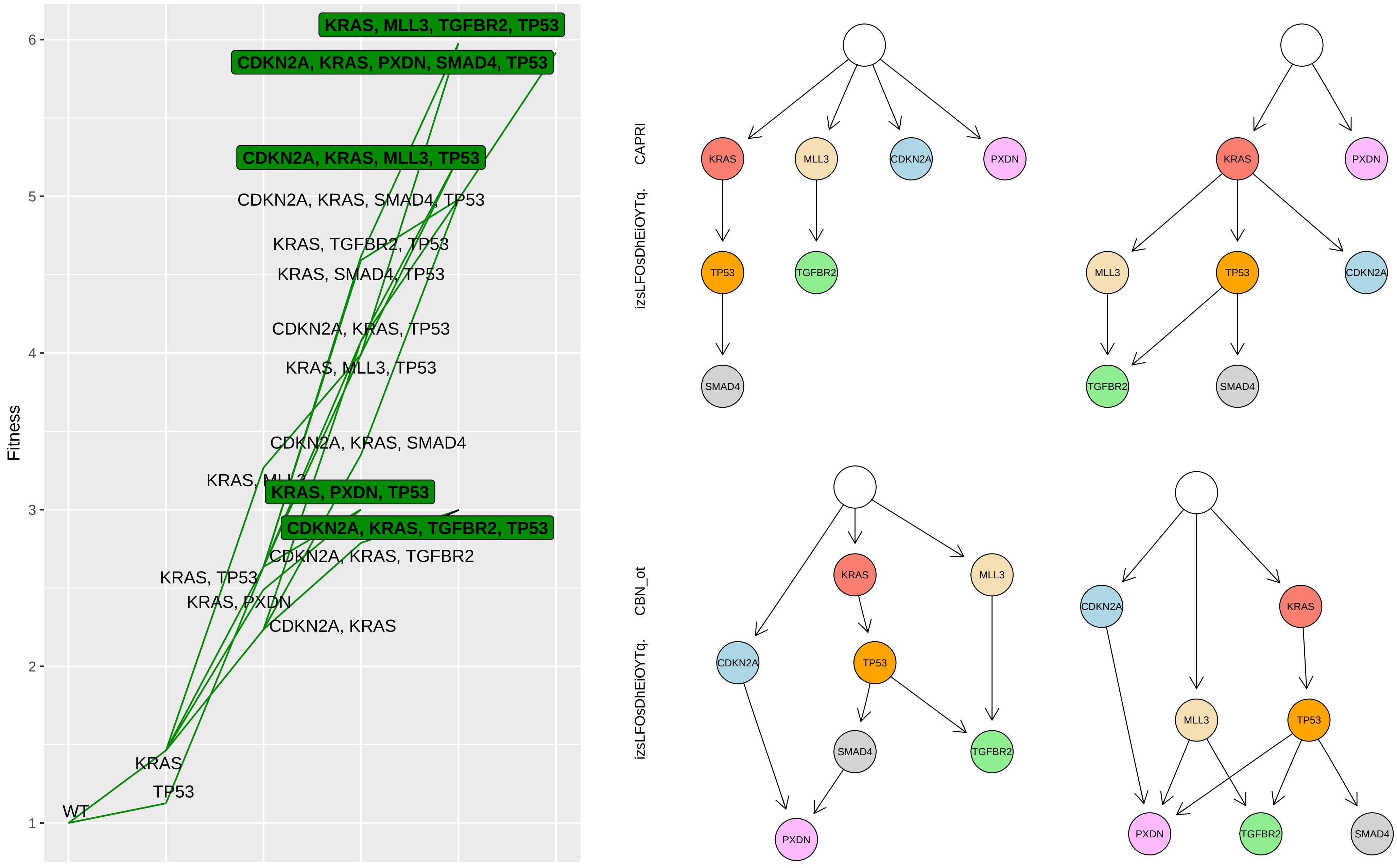

ID	p−value	Accessible Genot.
dzwPVybWdOBwVtG	0.672	18

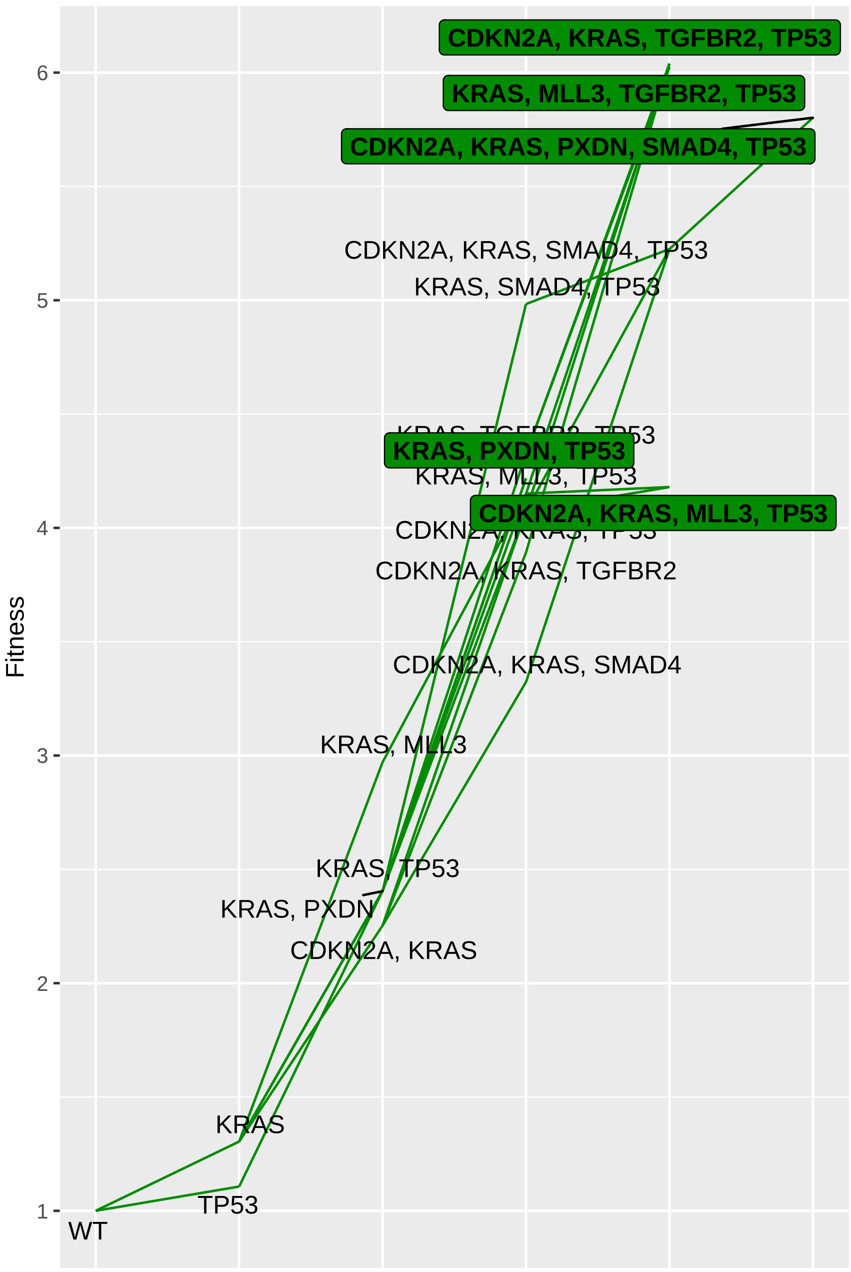

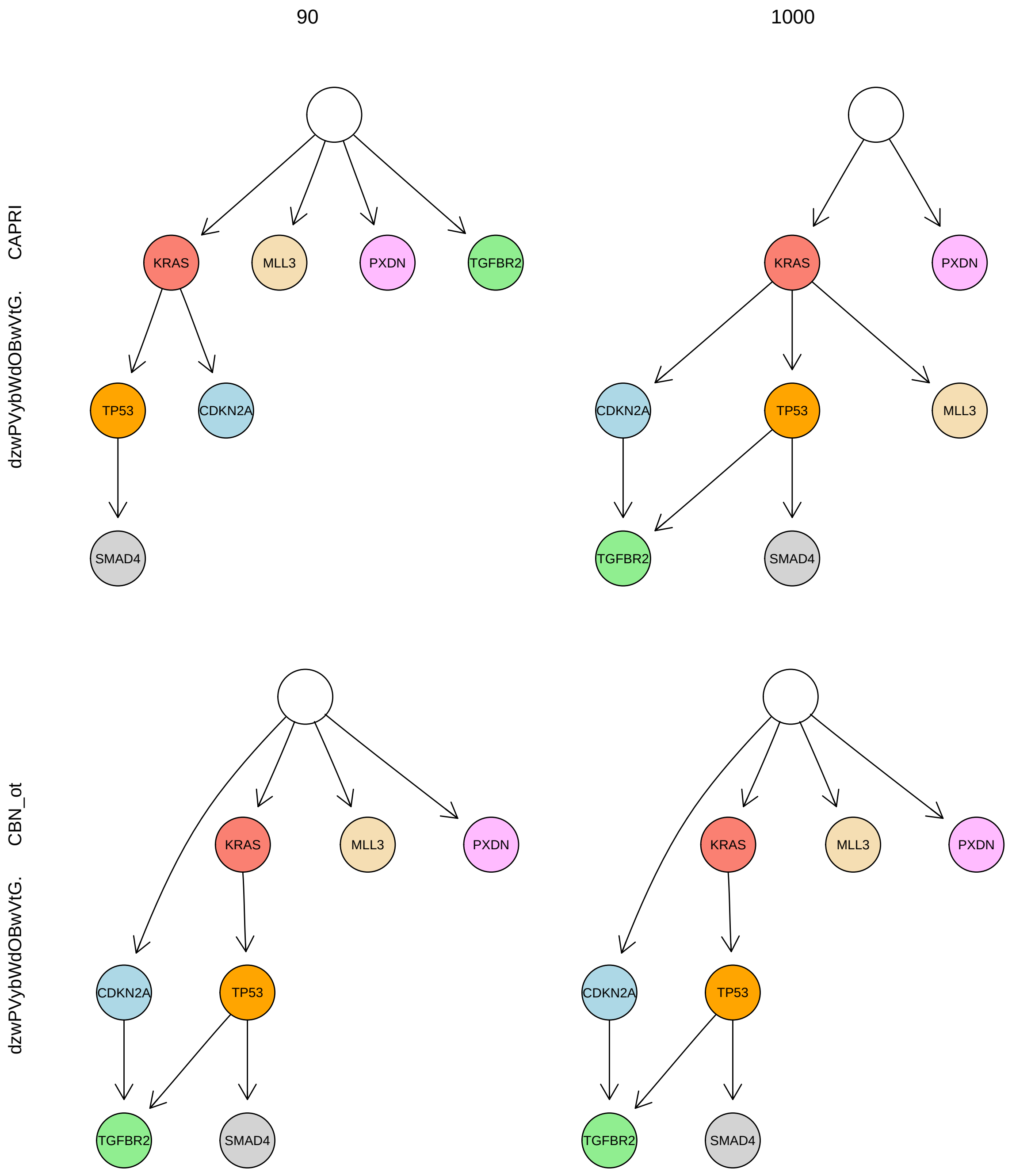

ID	p−value	Accessible Genot.
hrCeaxnmAqDrLLa	0.675	18

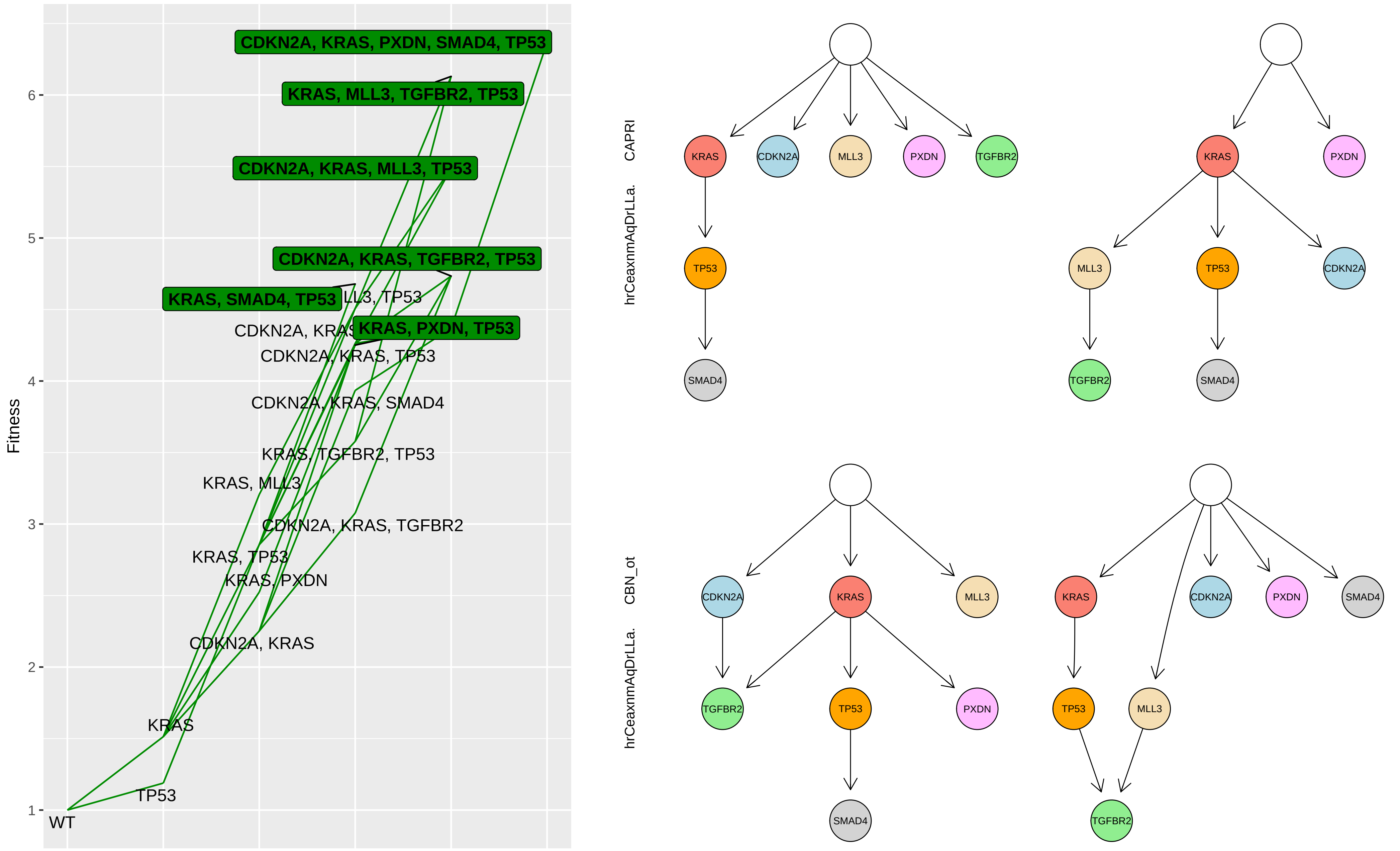

ID	p-value	Accessible Genot.
vCjjmAQzEuCjCja	0.677	18

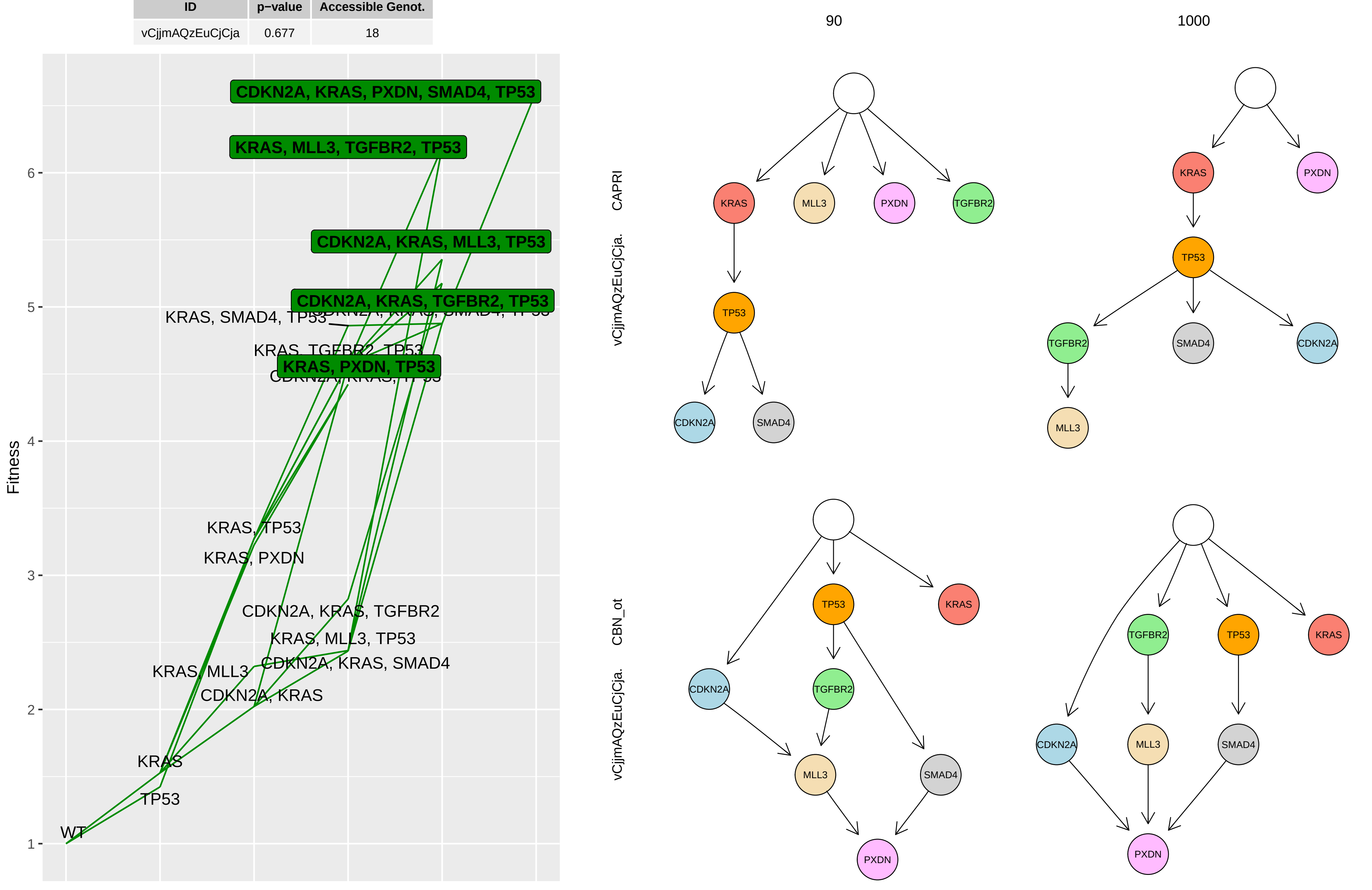

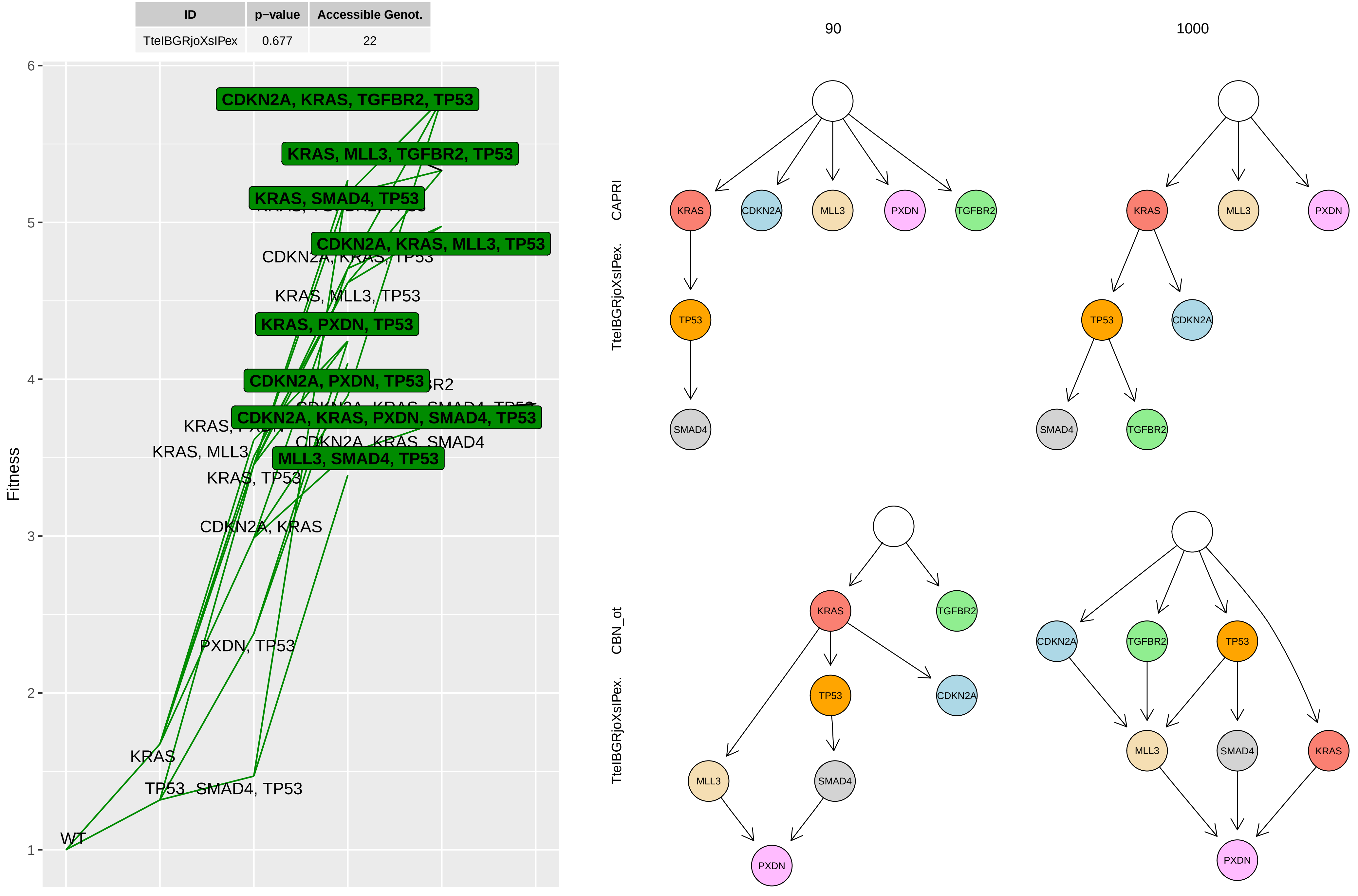

ID
p-value
Accessible Genot.
TteIBGRjoXsIPex
0.677
22
Fitness
CDKN2A, KRAS, TGFBR2, TP53
KRAS, MLL3, TGFBR2, TP53
KRAS, SMAD4, TP53
CDKN2A, KRAS, MLL3, TP53
CDKN2A, KRAS, TP53
KRAS, MLL3, TP53
KRAS, PXDN, TP53
CDKN2A, PXDN, TP53
CDKN2A, KRAS, PXDN, SMAD4, TP53
CDKN2A, KRAS, SMAD4
MLL3, SMAD4, TP53
KRAS, MLL3
KRAS, TP53
CDKN2A, KRAS
PXDN, TP53
KRAS
TP53
SMAD4, TP53
WT
90
1000
TteIBGRjoXsIPex. CAPRI
TteIBGRjoXsIPex. CBN_ot
KRAS
CDKN2A
MLL3
PXDN
TGFBR2
TP53
SMAD4

ID	p−value	Accessible Genot.
APJvlgBbEikzFFc	0.678	30

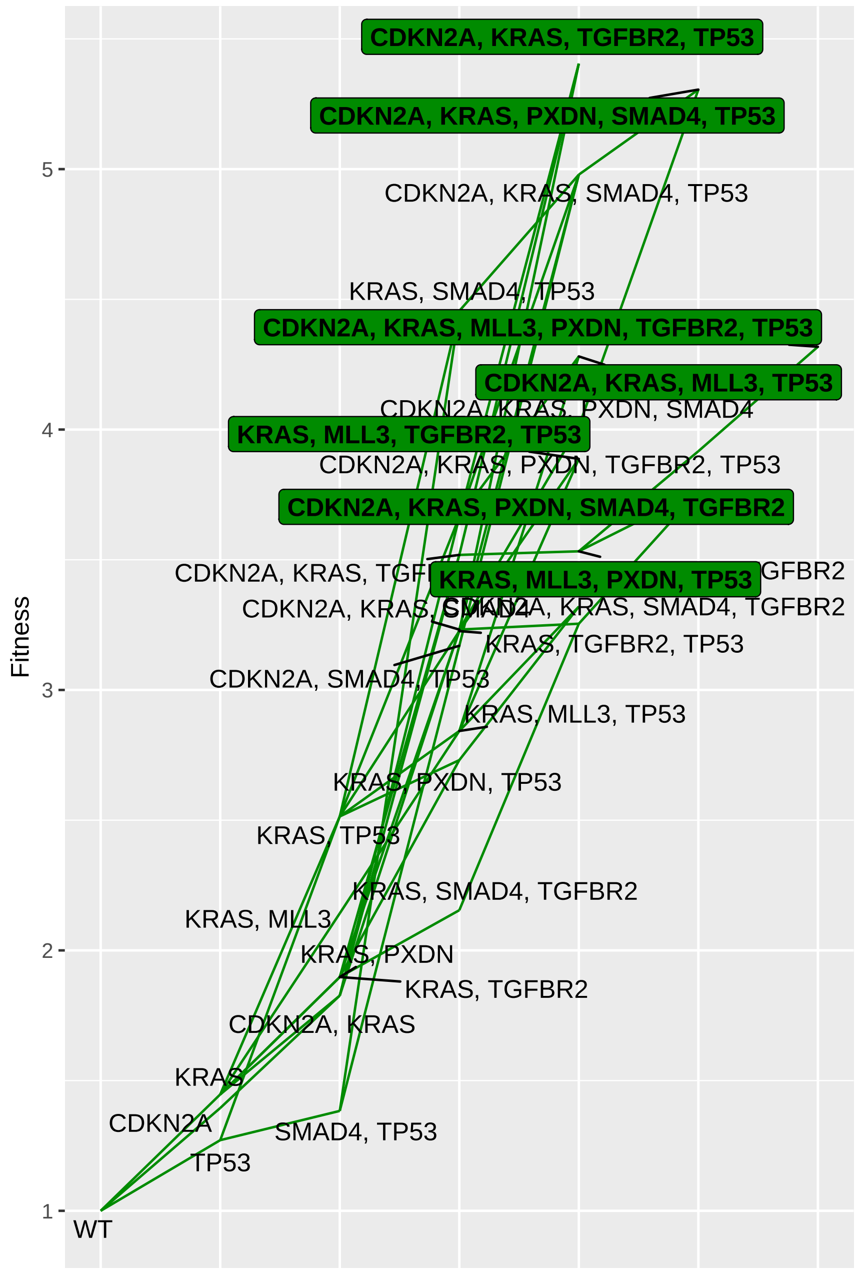

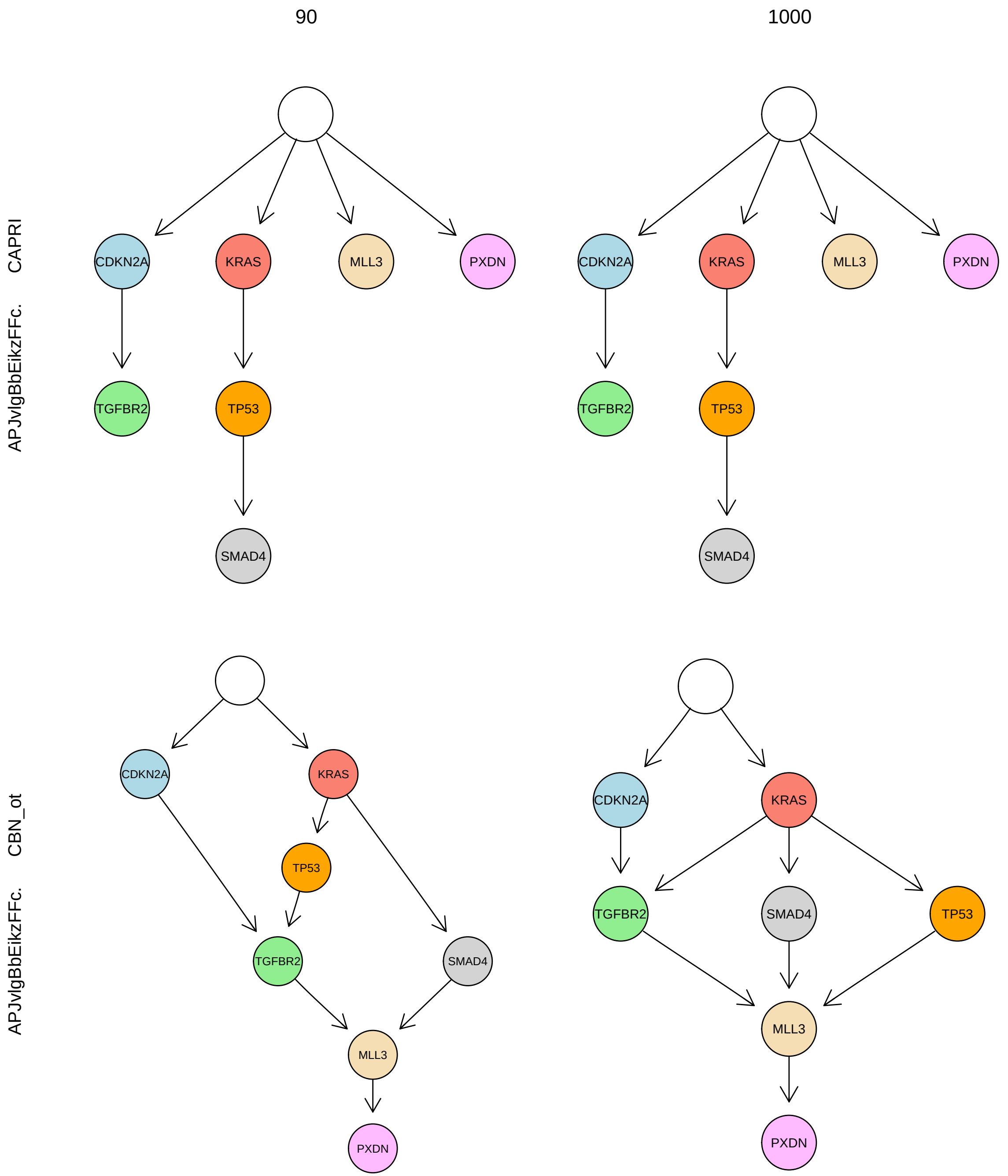

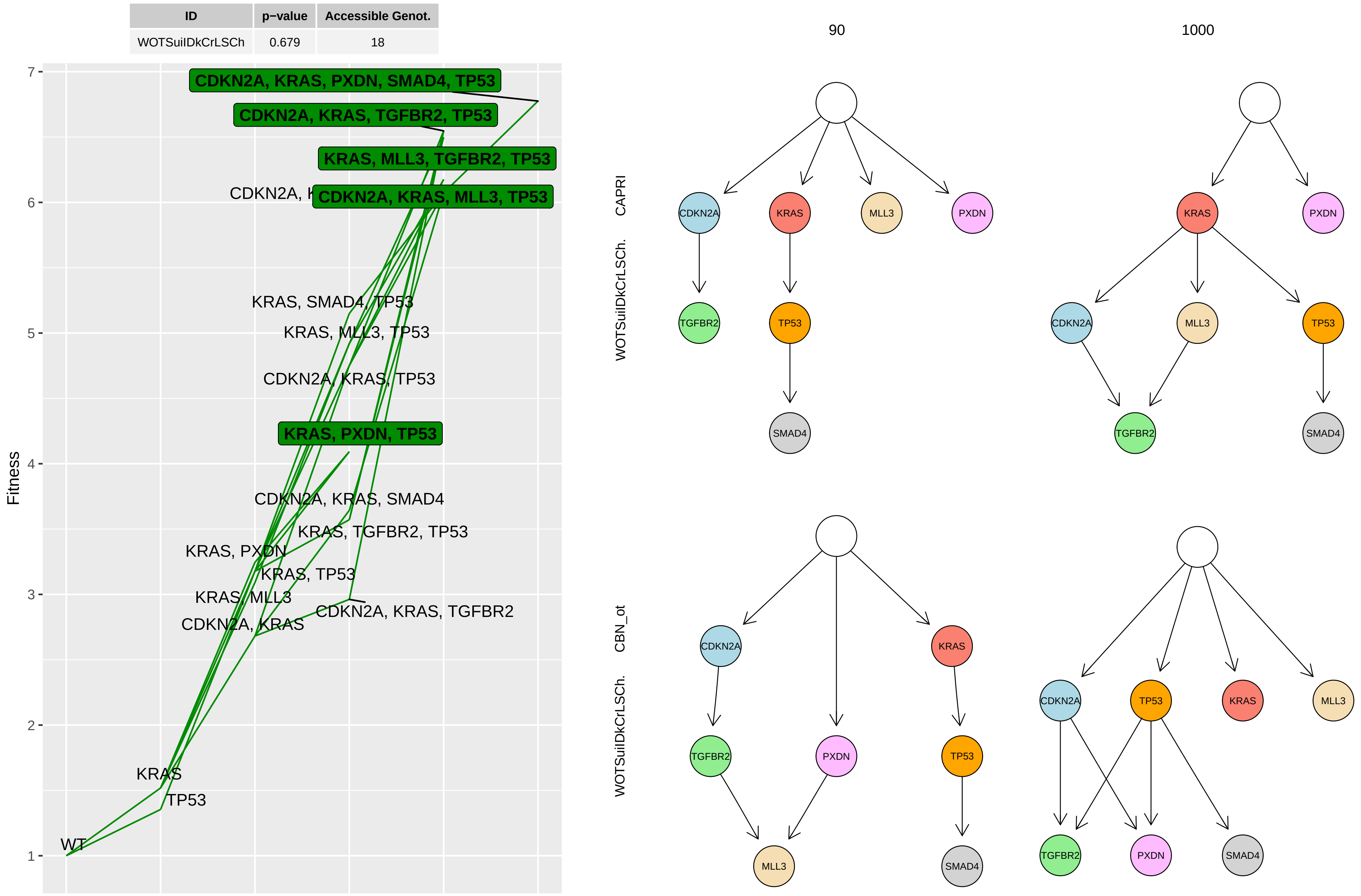

ID
p−value
Accessible Genot.
WOTSuilDkCrLSCh
0.679
18
Fitness
CDKN2A, KRAS, PXDN, SMAD4, TP53
CDKN2A, KRAS, TGFBR2, TP53
KRAS, MLL3, TGFBR2, TP53
CDKN2A, KRAS, MLL3, TP53
KRAS, SMAD4, TP53
KRAS, MLL3, TP53
CDKN2A, KRAS, TP53
KRAS, PXDN, TP53
CDKN2A, KRAS, SMAD4
KRAS, TGFBR2, TP53
KRAS, PXDN
KRAS, TP53
KRAS, MLL3
CDKN2A, KRAS, TGFBR2
CDKN2A, KRAS
KRAS
TP53
WT
90
1000
WOTSuilDkCrLSCh. CAPRI
WOTSuilDkCrLSCh. CBN_ot
CDKN2A
KRAS
MLL3
PXDN
TGFBR2
TP53
SMAD4

ID	p−value	Accessible Genot.
caxfyLnWfZWIYZT	0.68	18

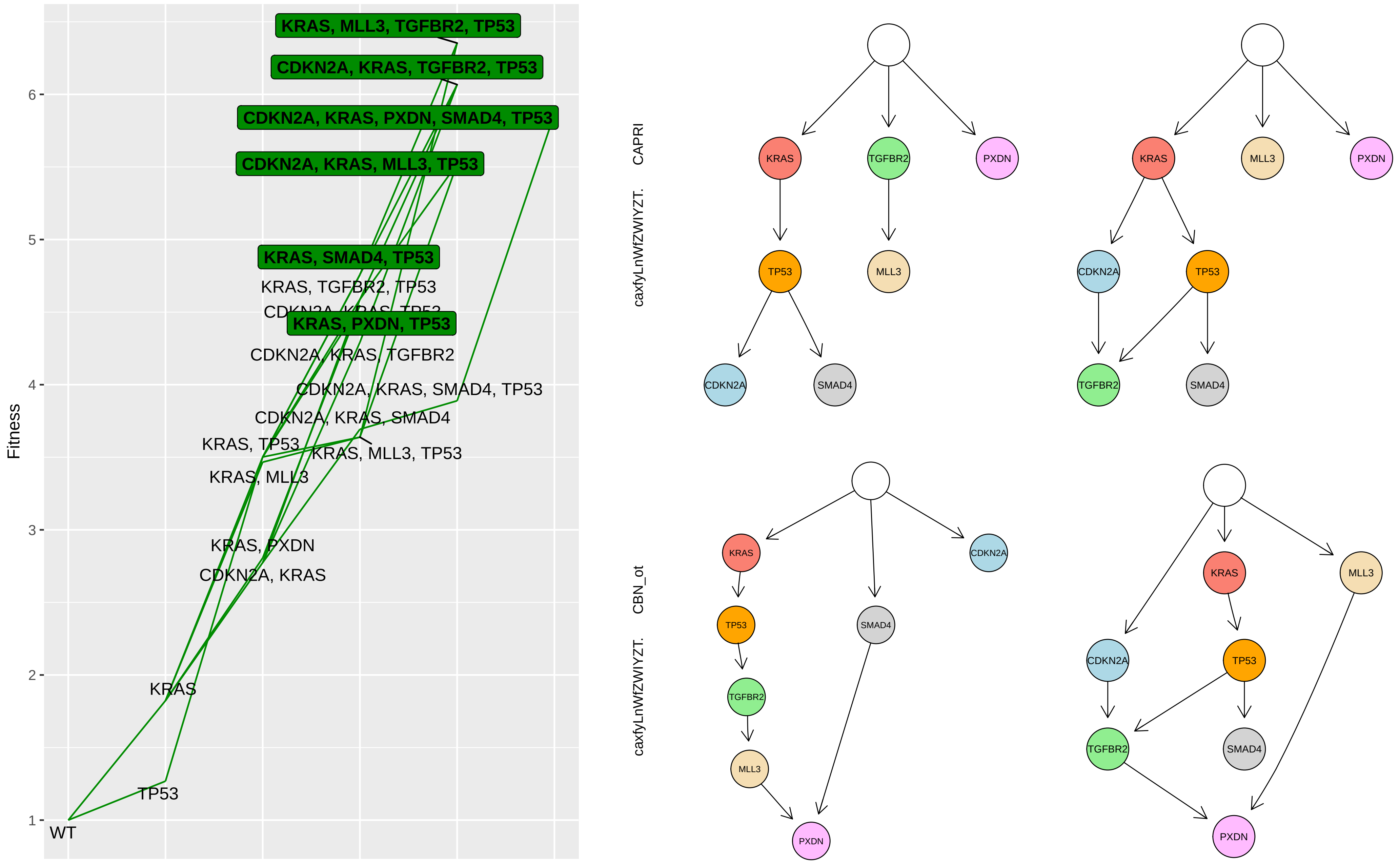

ID	p−value	Accessible Genot.
qomqrhKhMCUrsWd	0.681	21

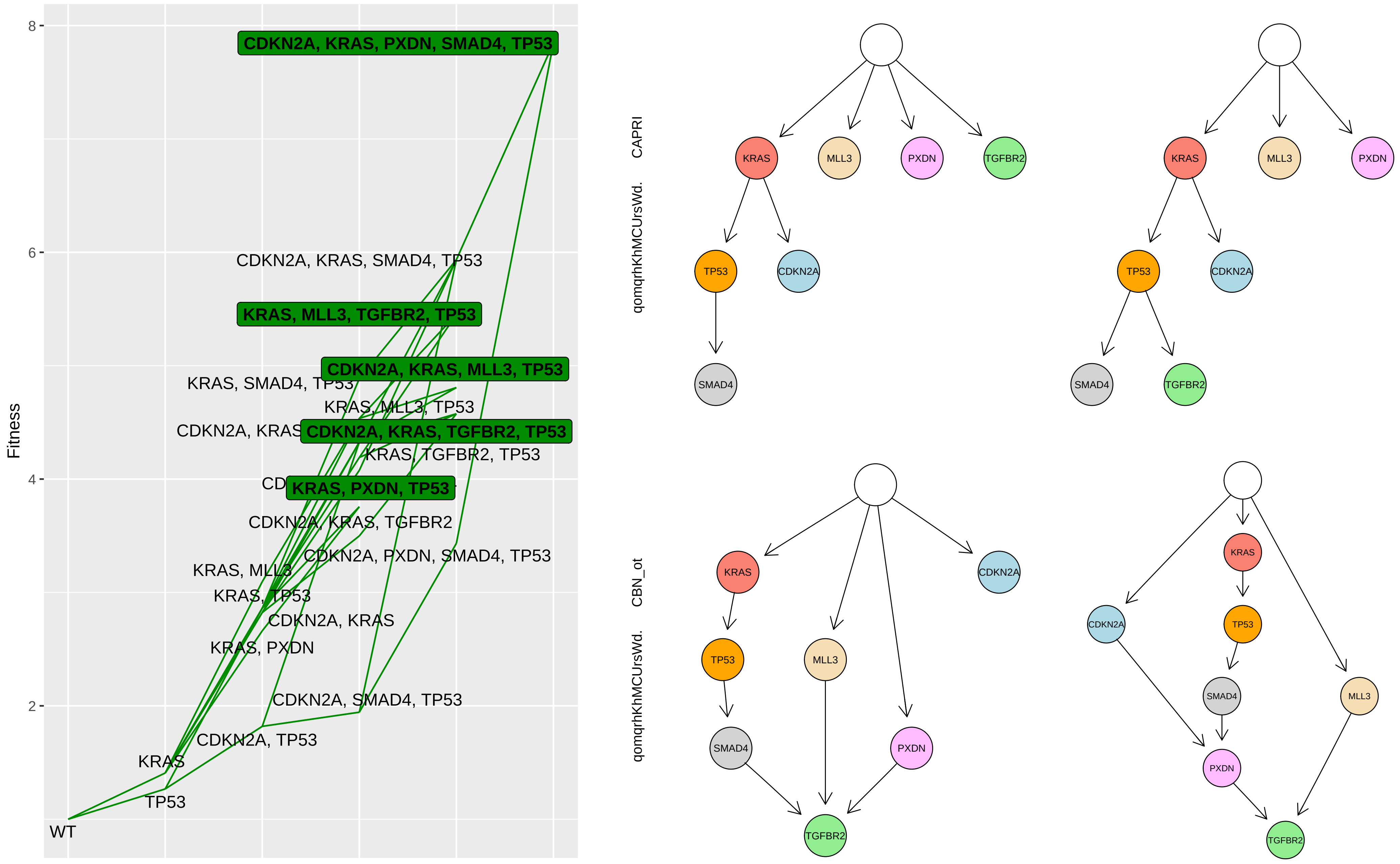

ID	p−value	Accessible Genot.
kXUiPyVjpeTWIca	0.681	88

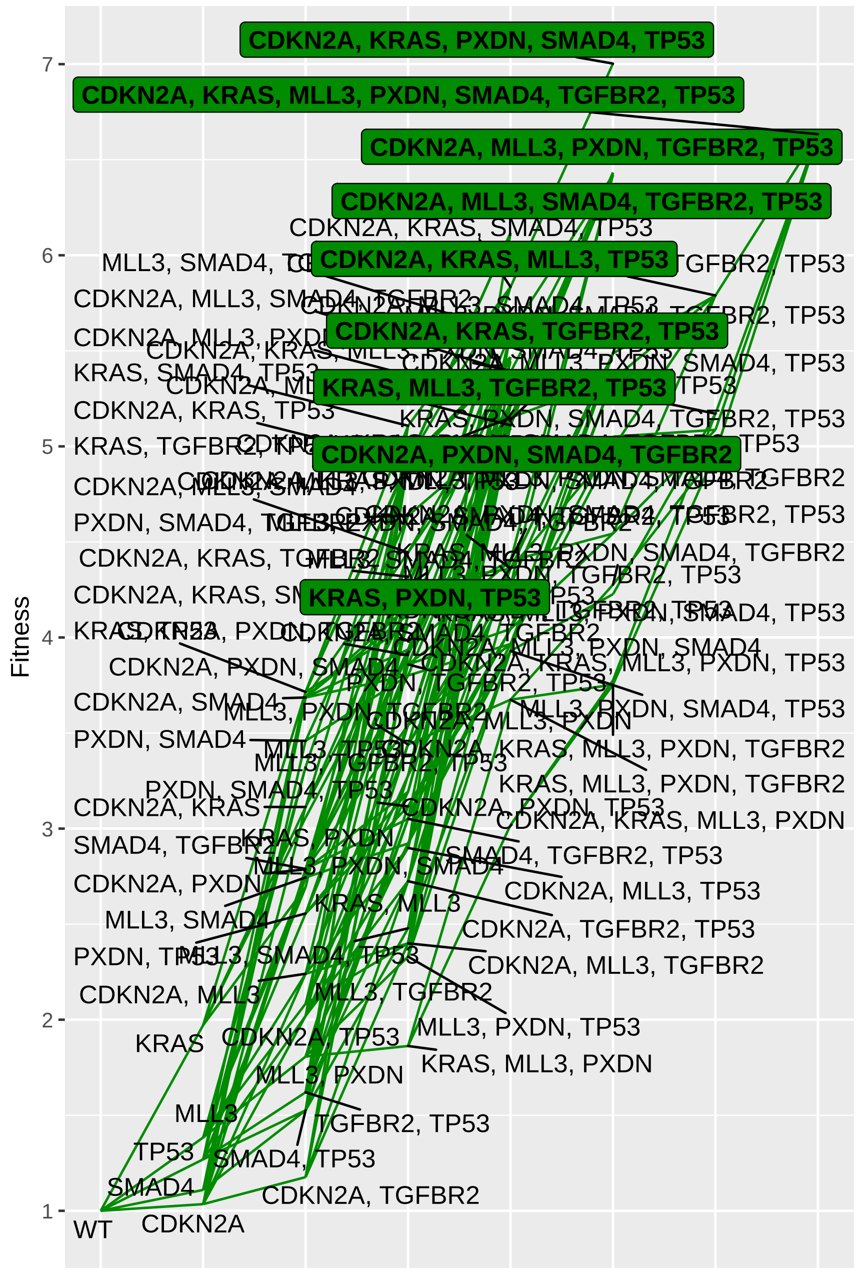

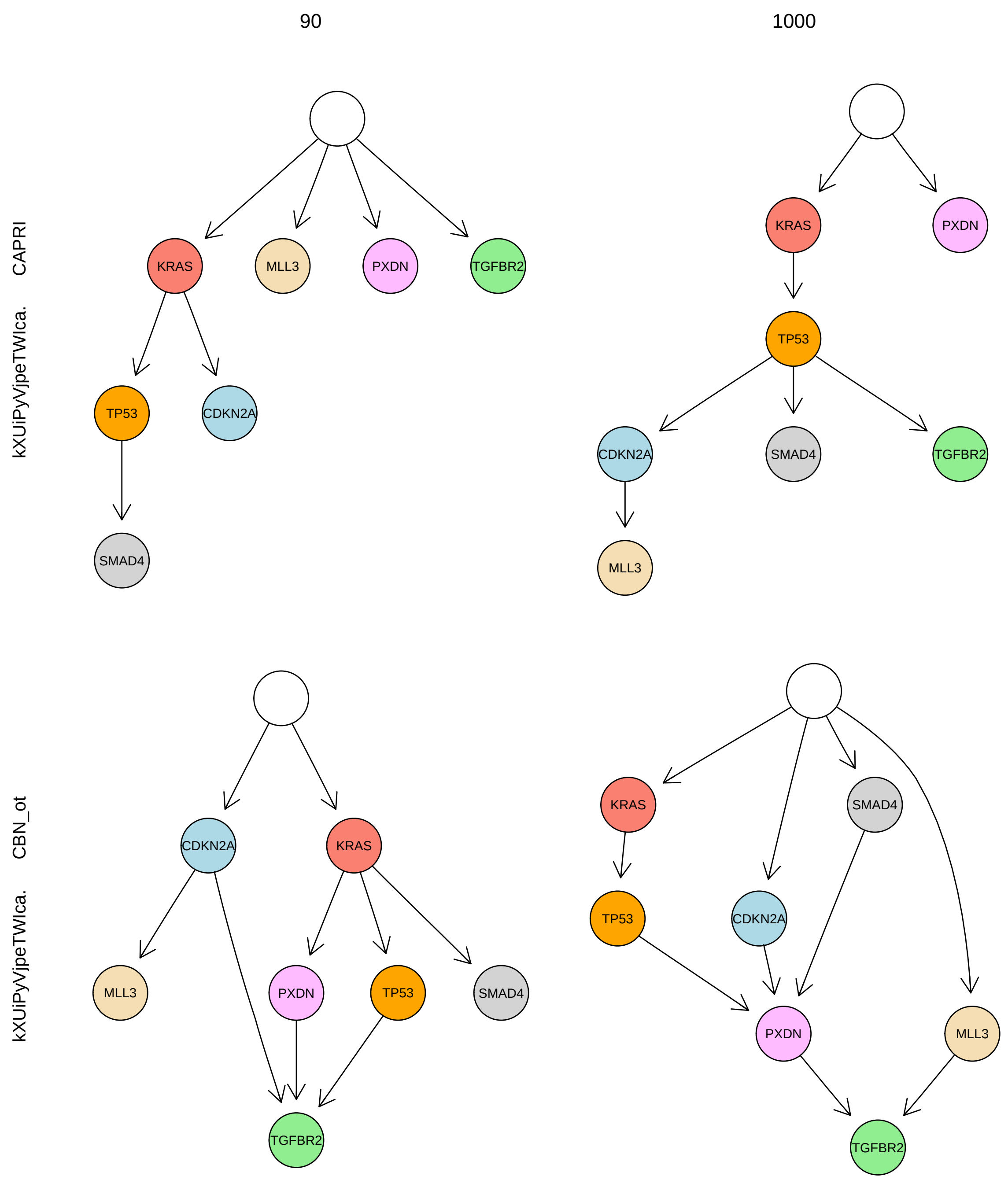

ID	p−value	Accessible Genot.
MiHznKEYZIEVMqm	0.681	38

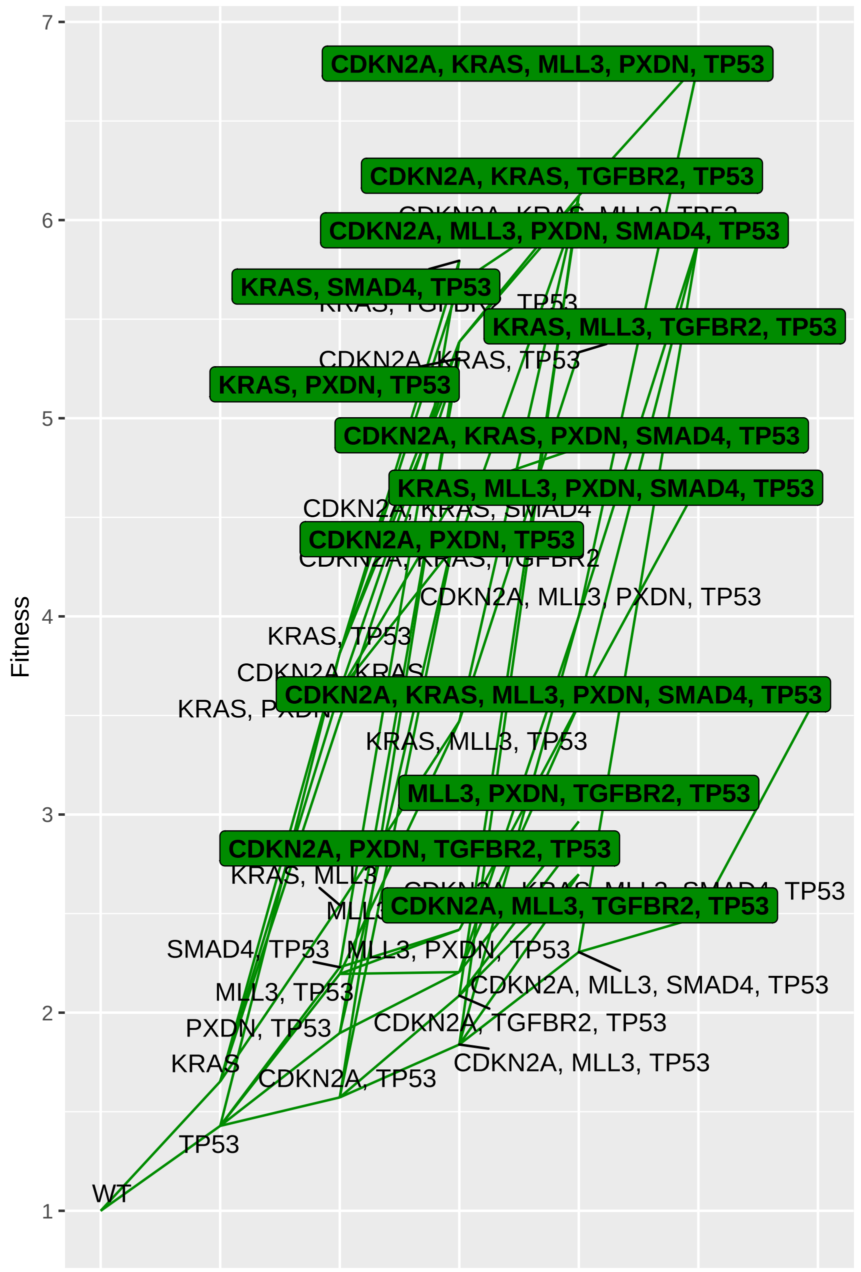

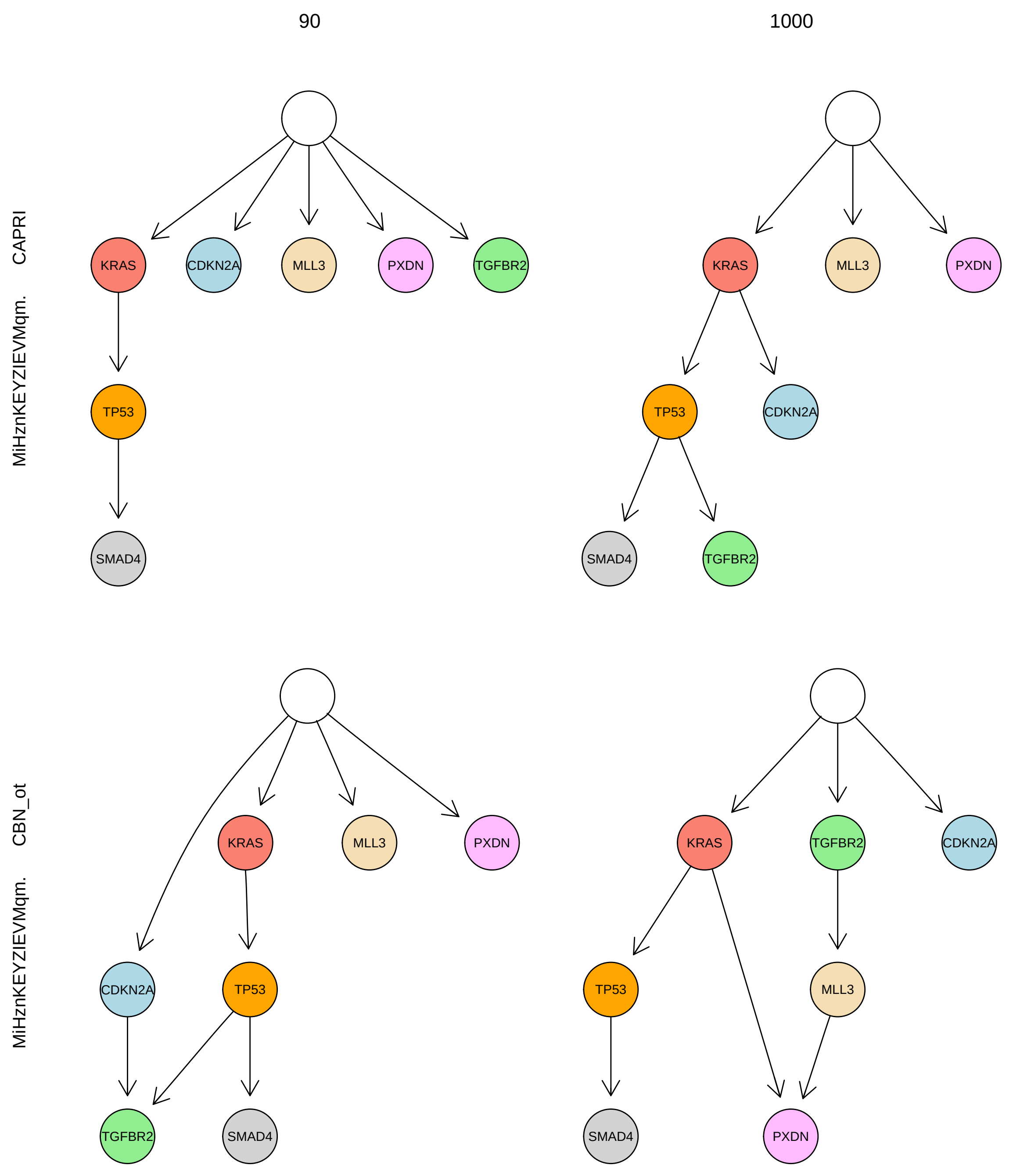

ID	p−value	Accessible Genot.
iQCQJbxfSOFKrcW	0.686	20

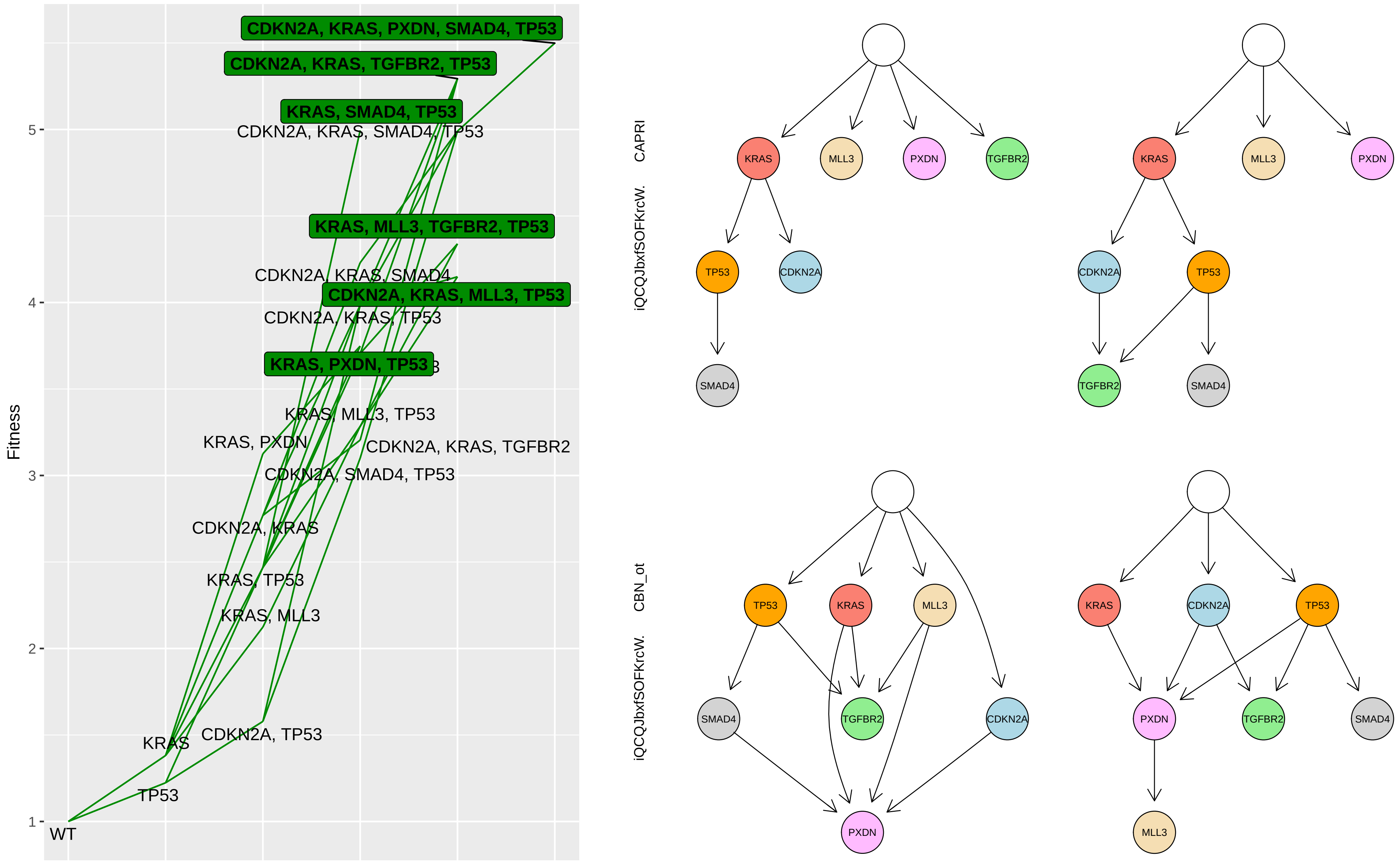

ID	p−value	Accessible Genot.
zwmQsdWCgvLUcVz	0.687	18

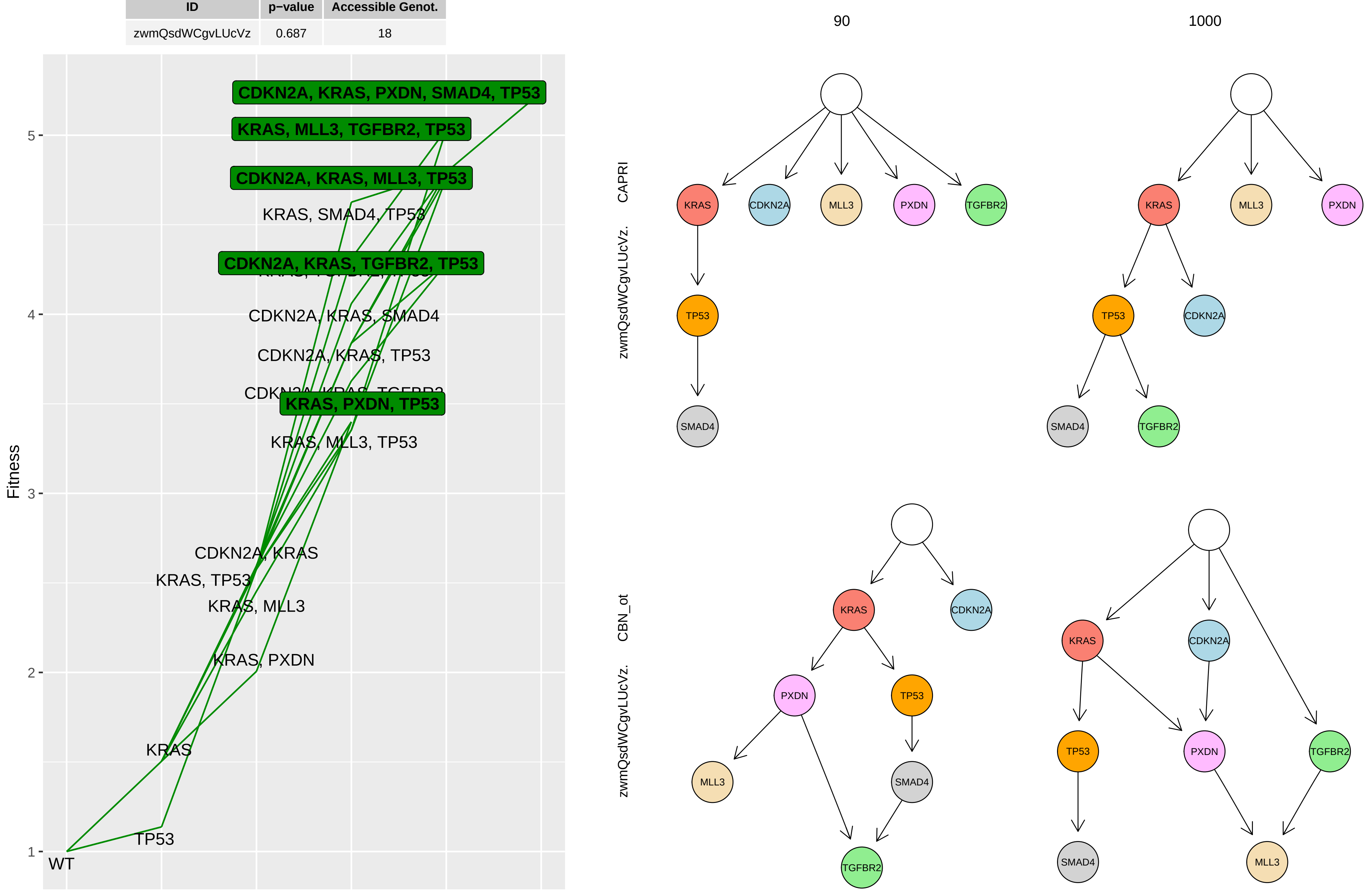

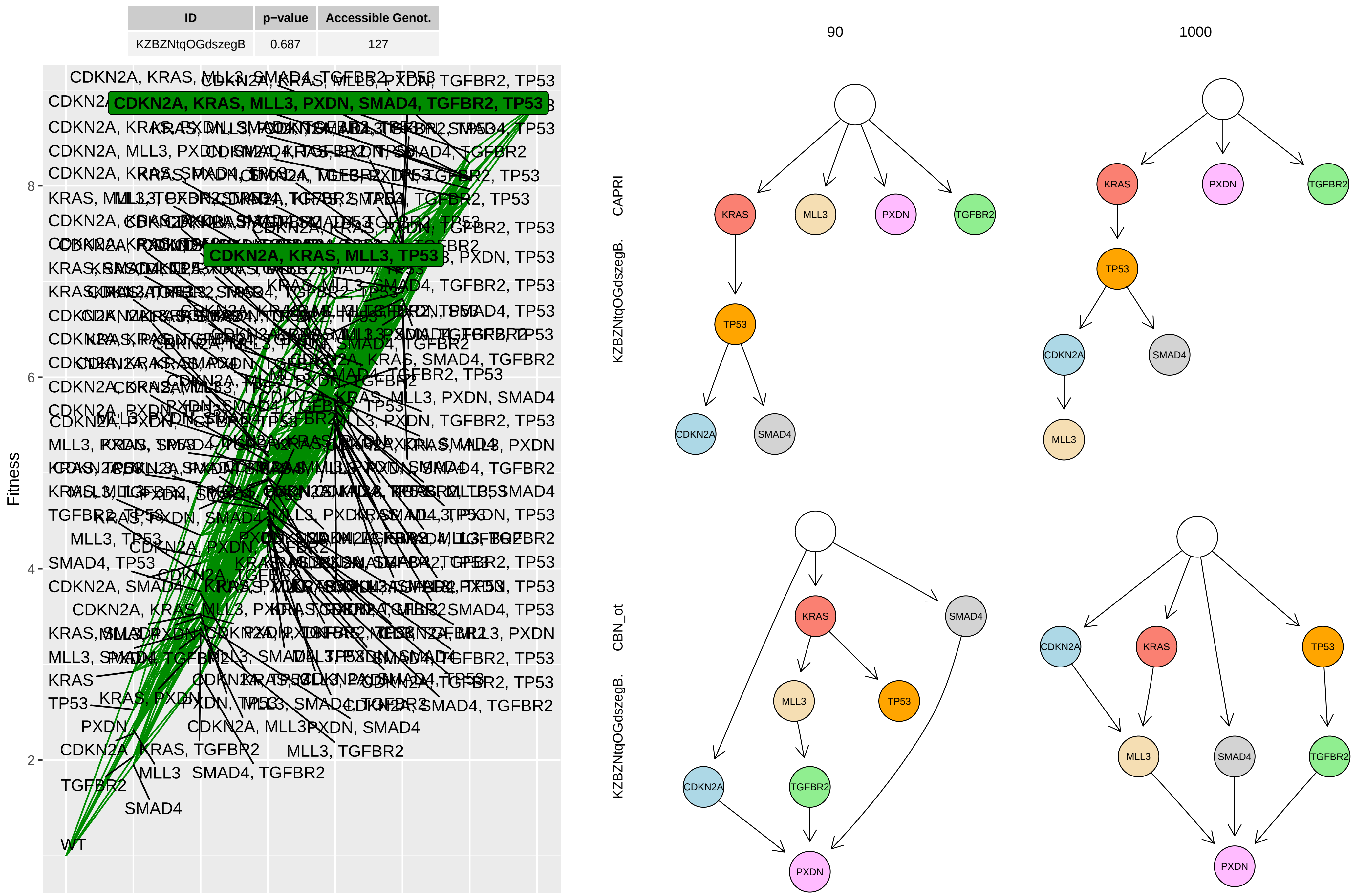
ID
p−value
Accessible Genot.
KZBZNtqOGdszegB
0.687
127
CDKN2A, KRAS, MLL3, PXDN, SMAD4, TGFBR2, TP53
CDKN2A, KRAS, MLL3, TP53
Fitness
8
6
4
2
WT
KRAS
TP53
PXDN
CDKN2A
TGFBR2
MLL3
SMAD4
KRAS, TGFBR2
SMAD4, TGFBR2
MLL3, TGFBR2
CDKN2A, MLL3
PXDN, SMAD4
SMAD4, TP53
MLL3, TP53
90
1000
CAPRI
CBN_ot
KZBZNtqOGdszegB.
KRAS
MLL3
PXDN
TGFBR2
TP53
CDKN2A
SMAD4

ID	p−value	Accessible Genot.
KRWHmWNphsHKidK	0.688	36

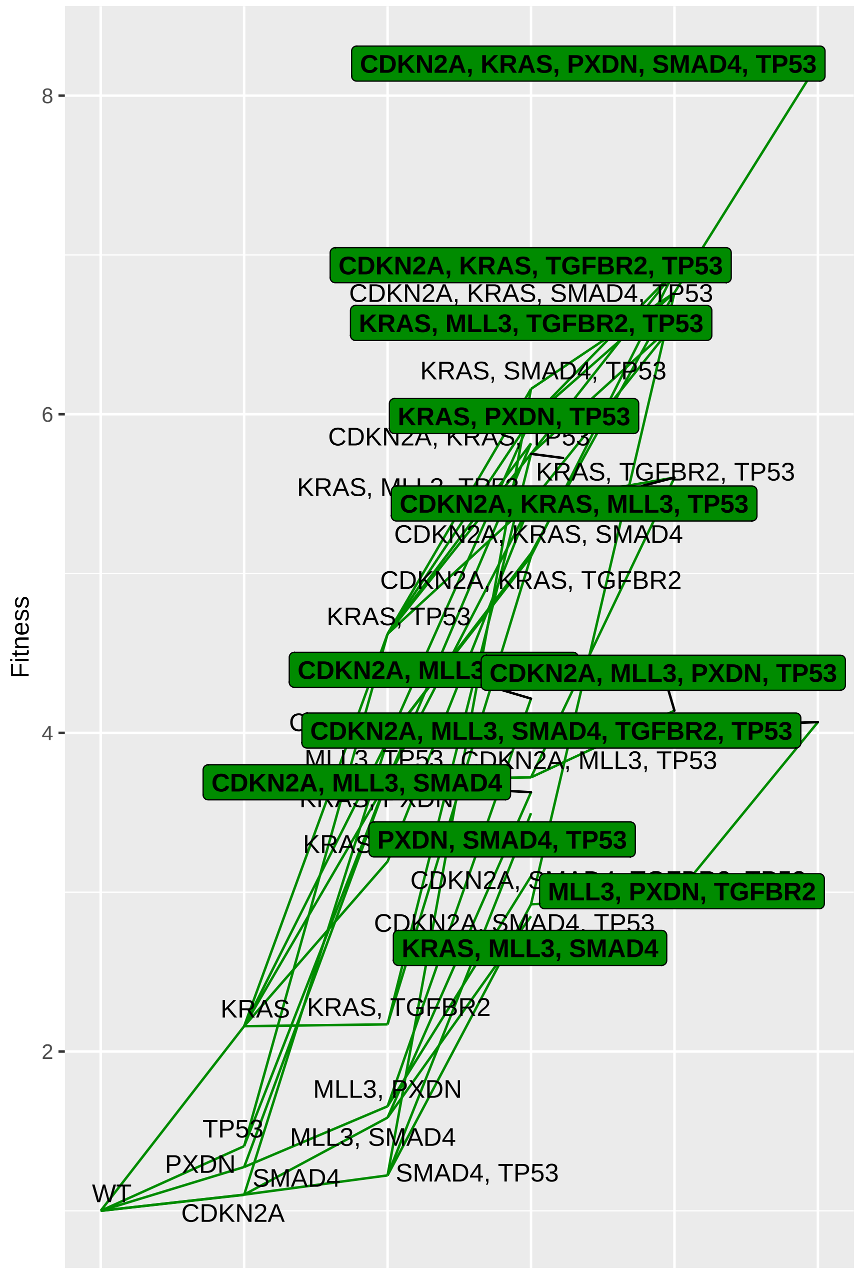

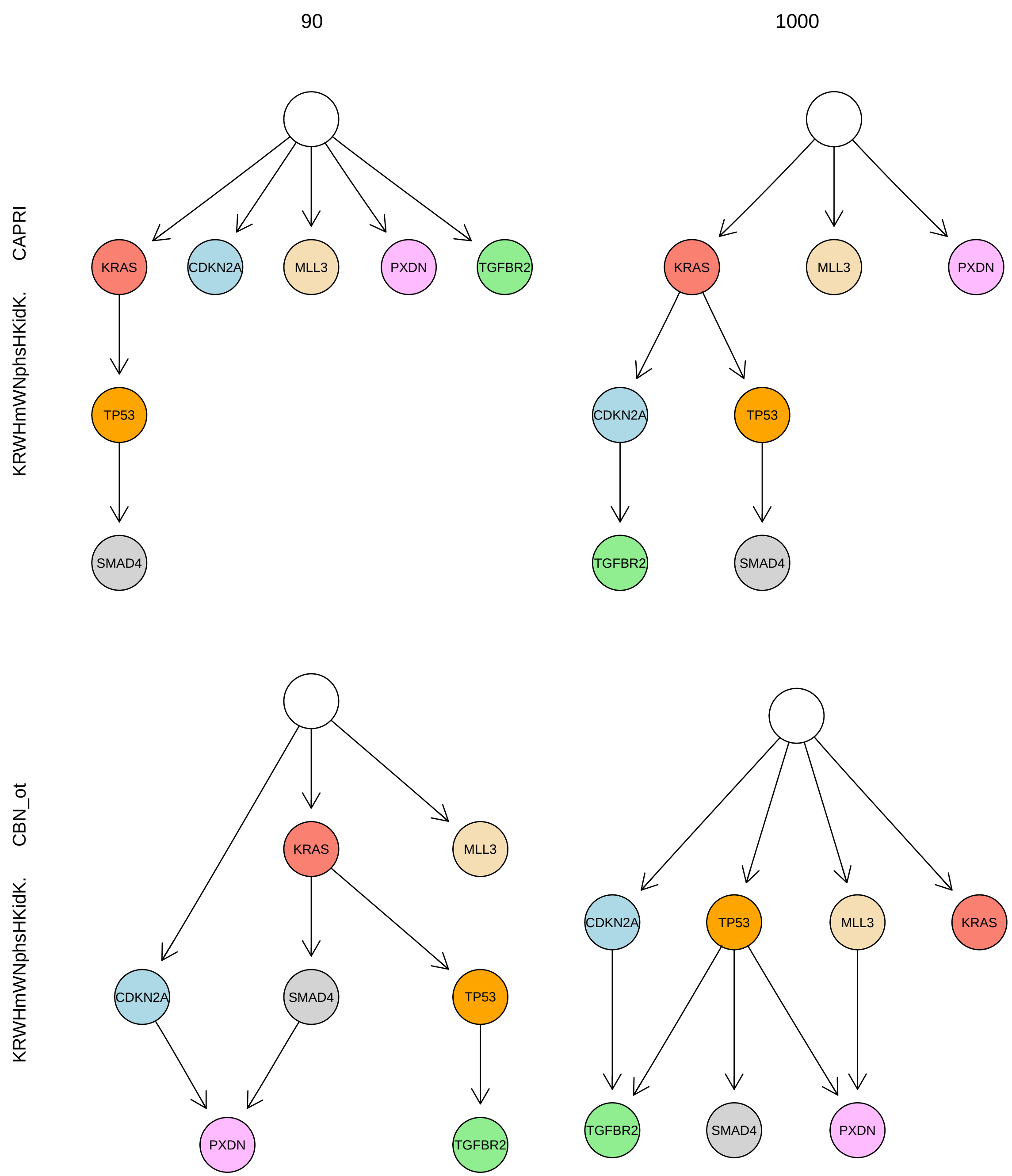

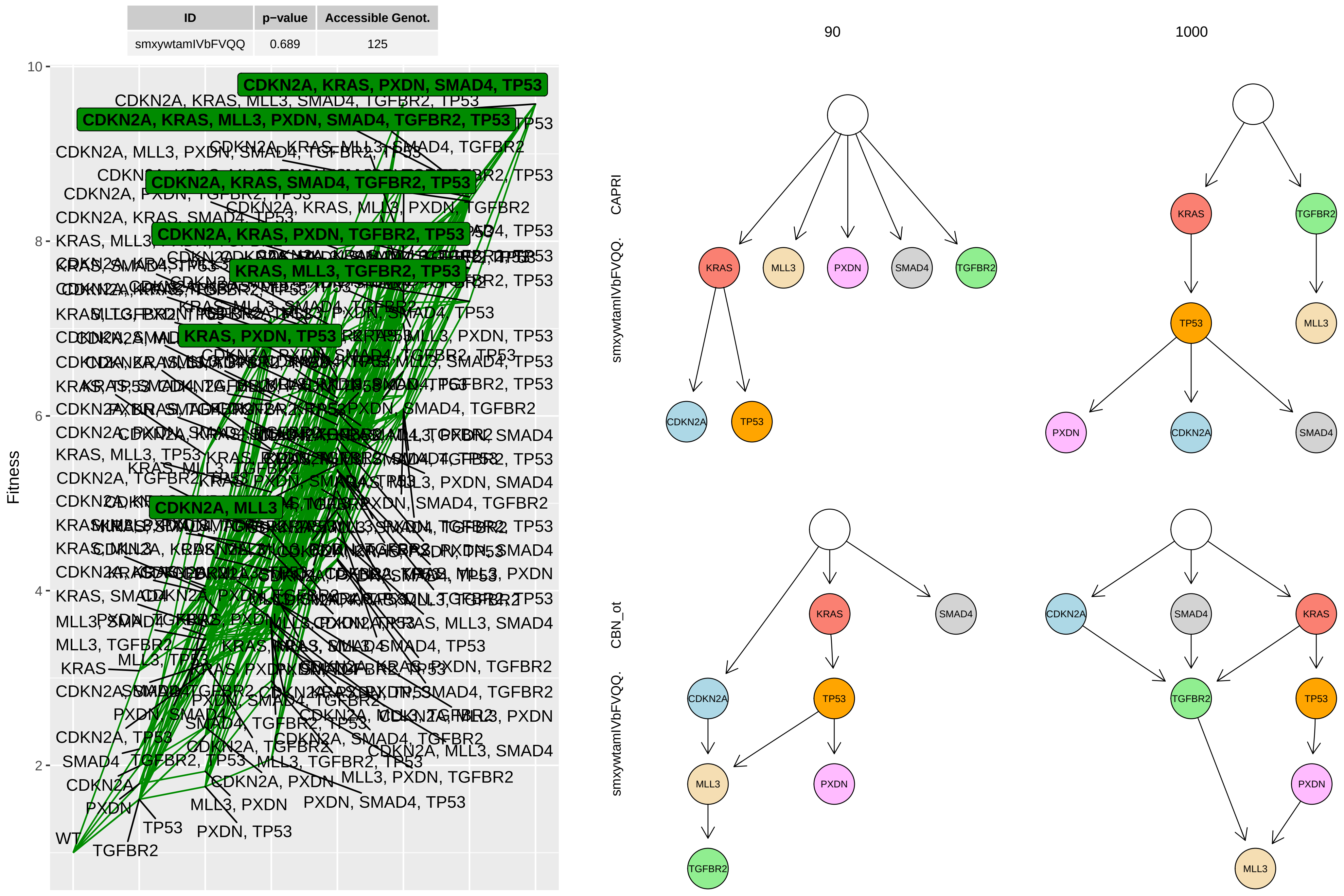
ID
p−value
Accessible Genot.
smxywtamIVbFVQQ
0.689
125
Fitness
CDKN2A, KRAS, PXDN, SMAD4, TP53
CDKN2A, KRAS, MLL3, PXDN, SMAD4, TGFBR2, TP53
CDKN2A, KRAS, SMAD4, TGFBR2, TP53
CDKN2A, KRAS, PXDN, TGFBR2, TP53
KRAS, MLL3, TGFBR2, TP53
KRAS, PXDN, TP53
CDKN2A, MLL3
WT
90
1000
CAPRI
CBN_ot
smxywtamIVbFVQQ.
KRAS
MLL3
PXDN
SMAD4
TGFBR2
CDKN2A
TP53

ID	p−value	Accessible Genot.
yoEMiKgeDFpPywX	0.689	19

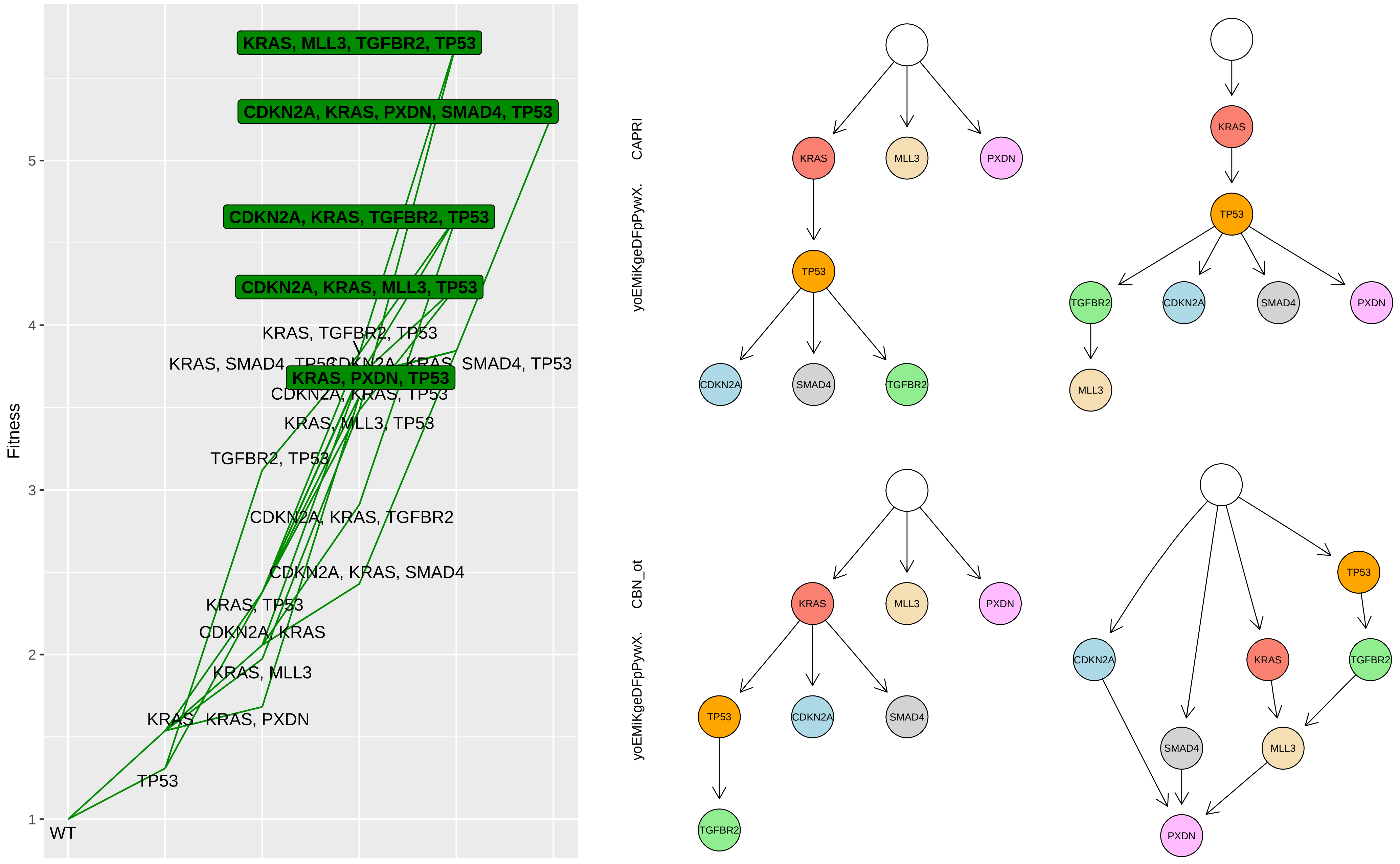

ID	p−value	Accessible Genot.
zdielxGzWGHNeOt	0.69	25

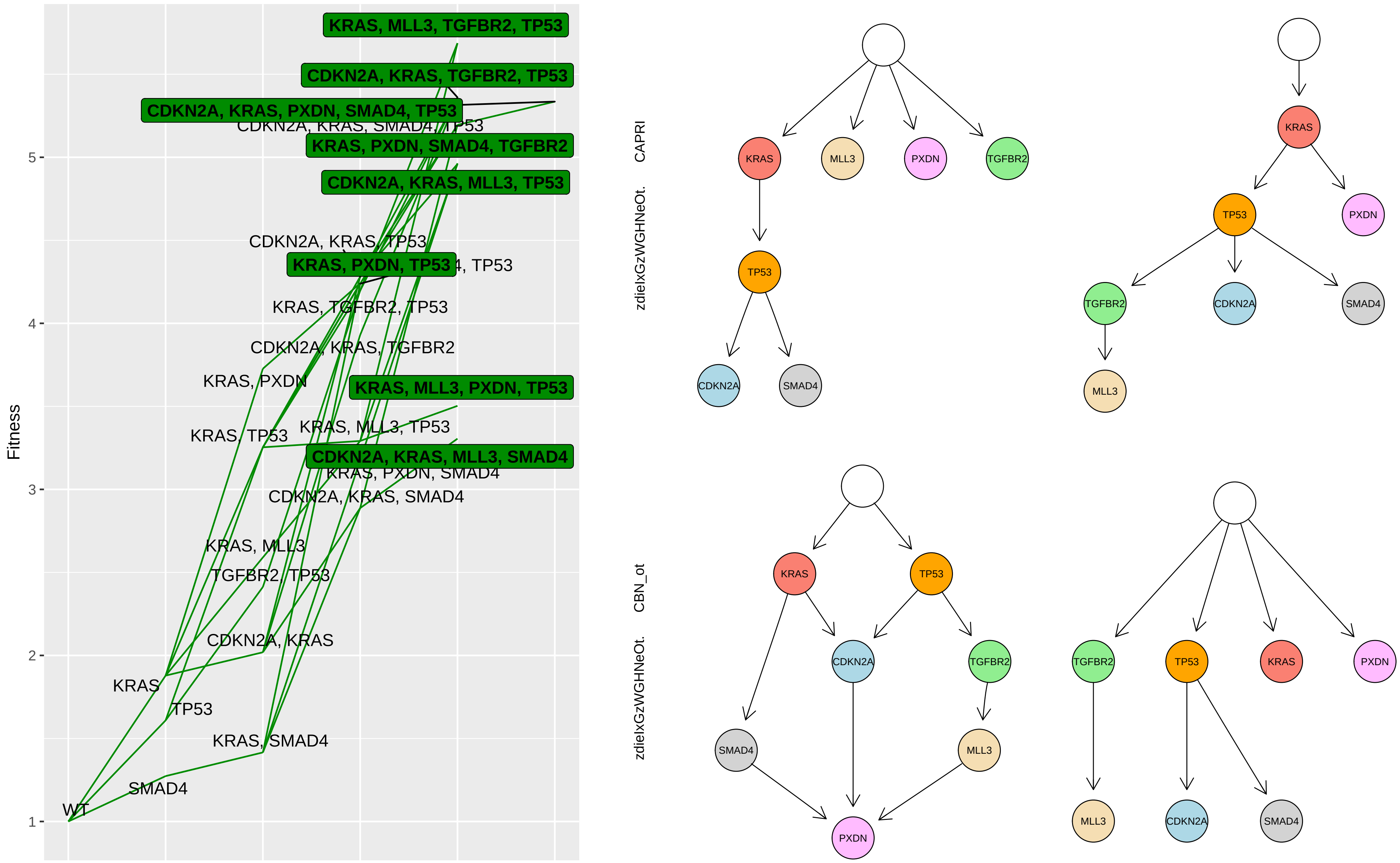

ID	p−value	Accessible Genot.
pSdGXhEpMUNKXCi	0.69	18

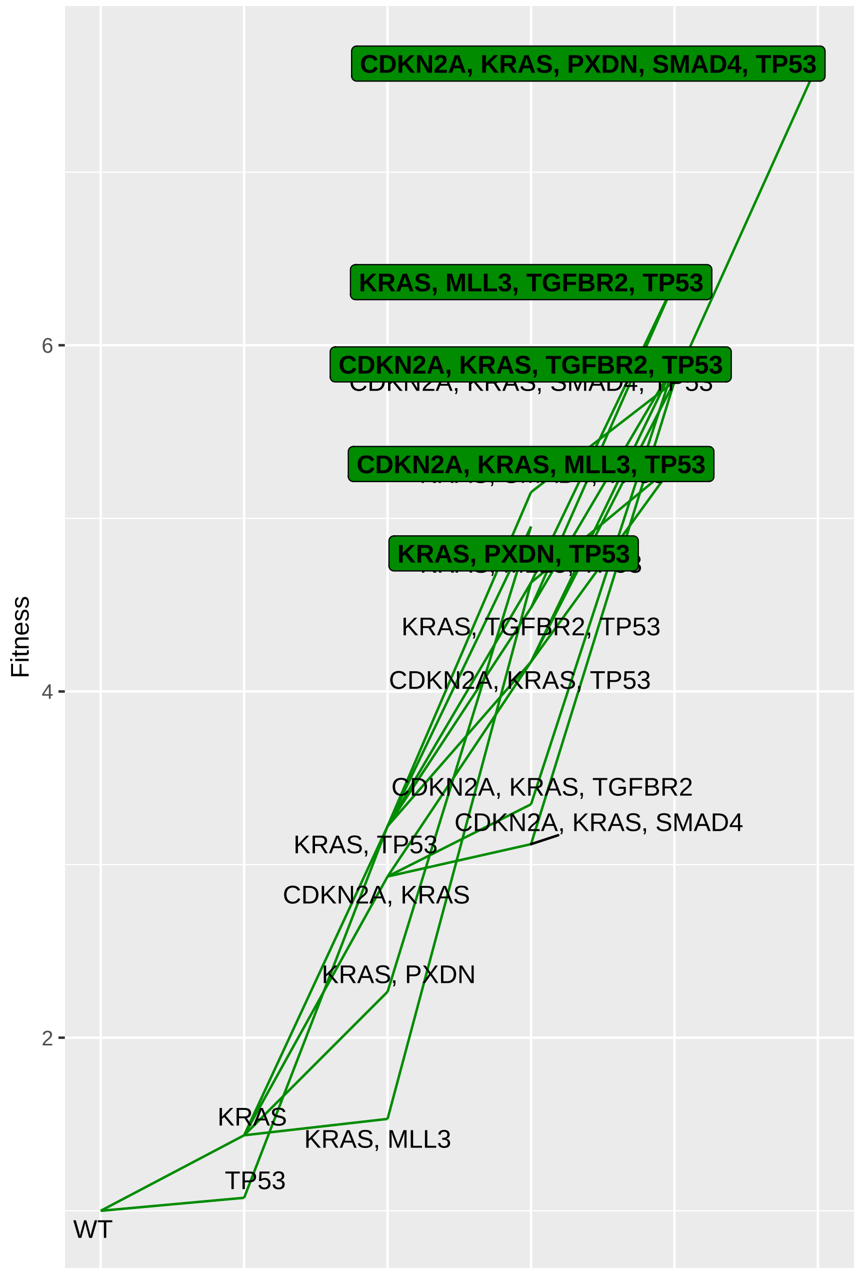

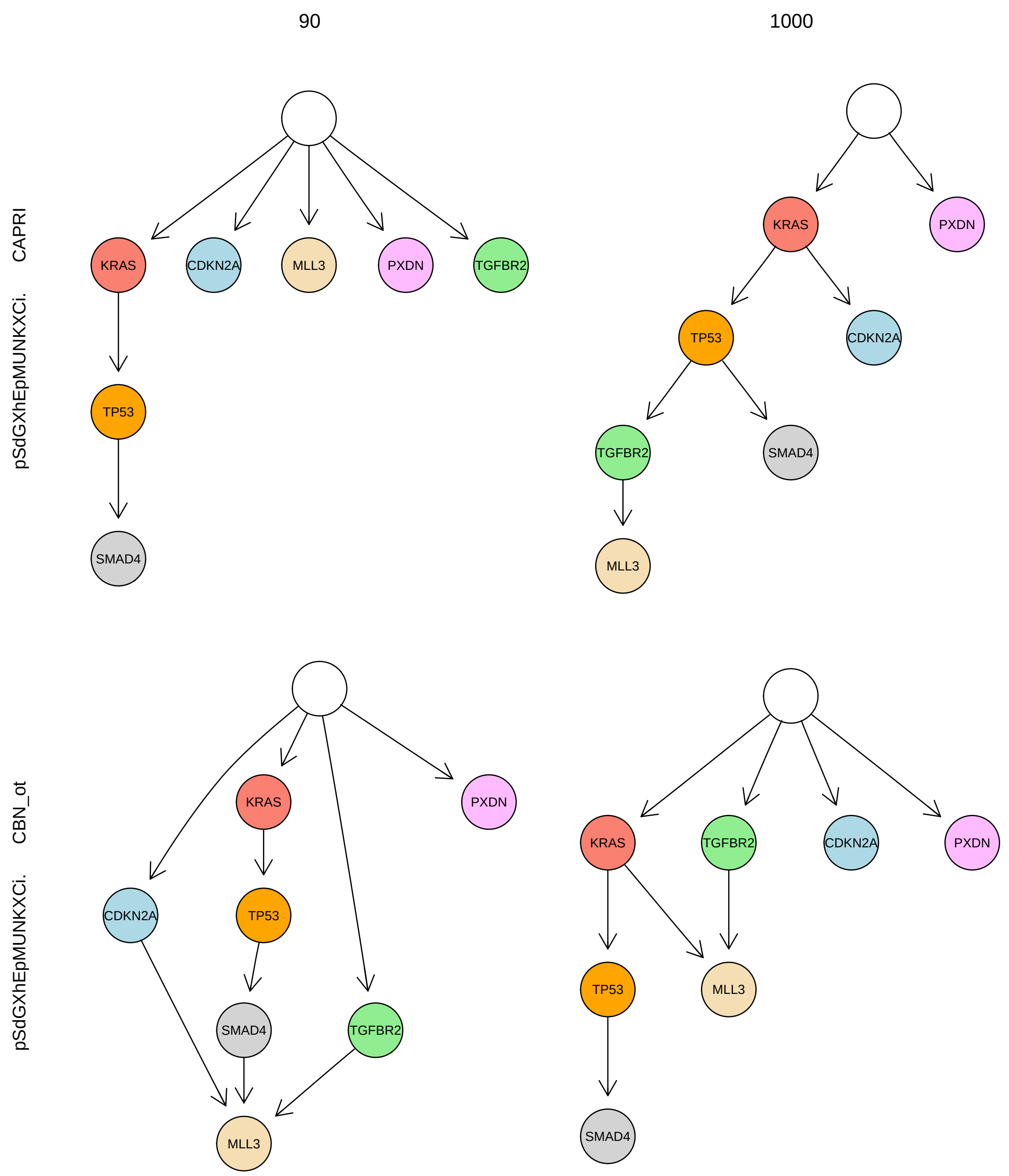

ID	p−value	Accessible Genot.
BJBWBFZzPEoYRLB	0.69	20

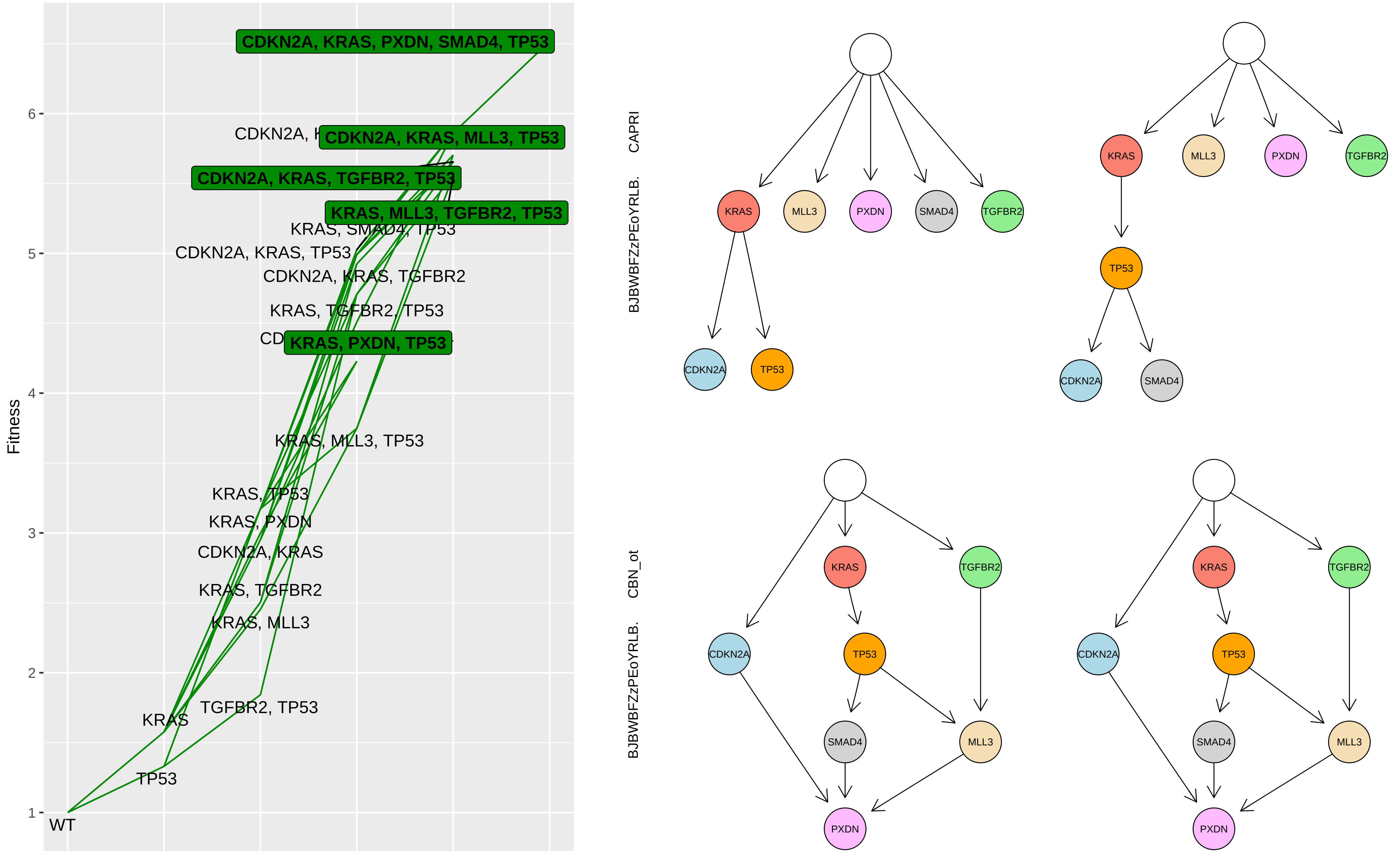

ID	p−value	Accessible Genot.
KqHVfezTiNNJfeQ	0.694	18

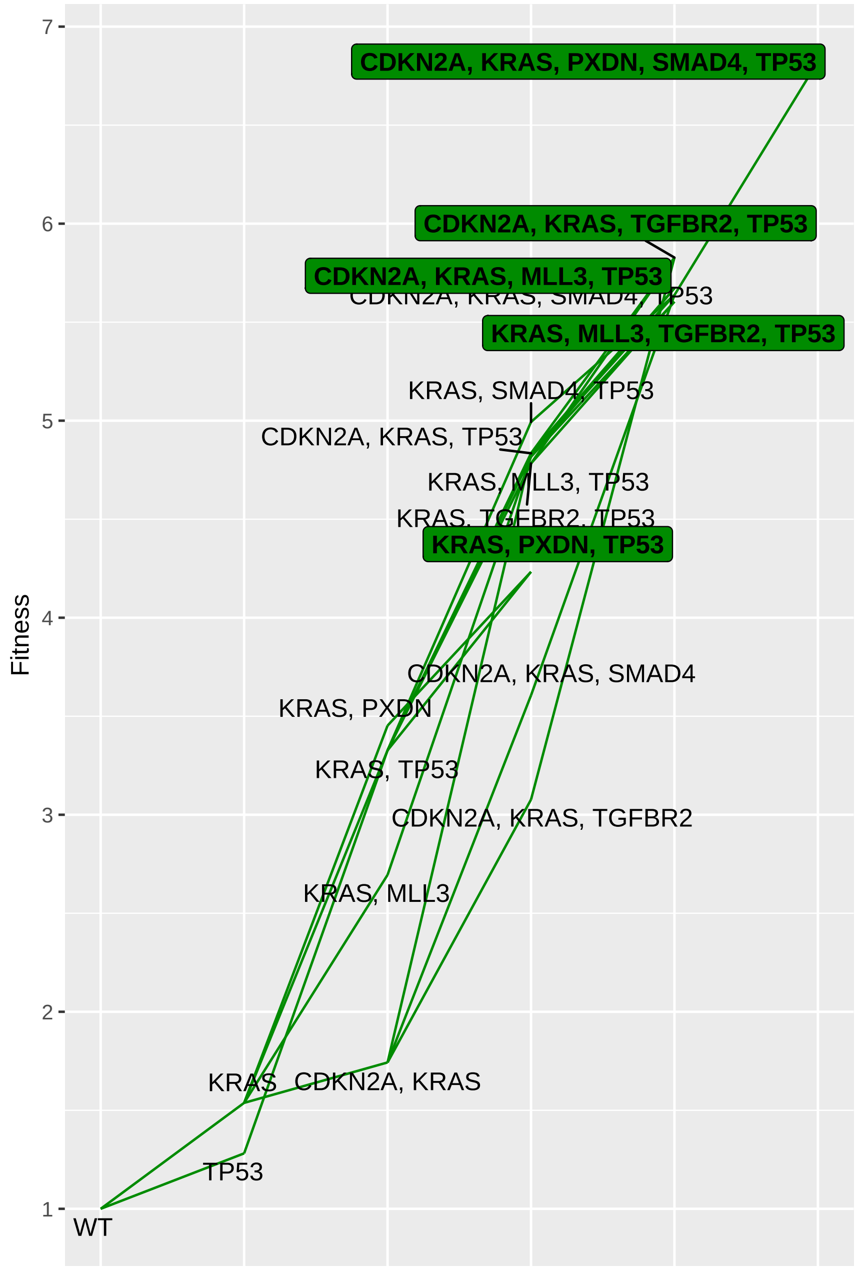

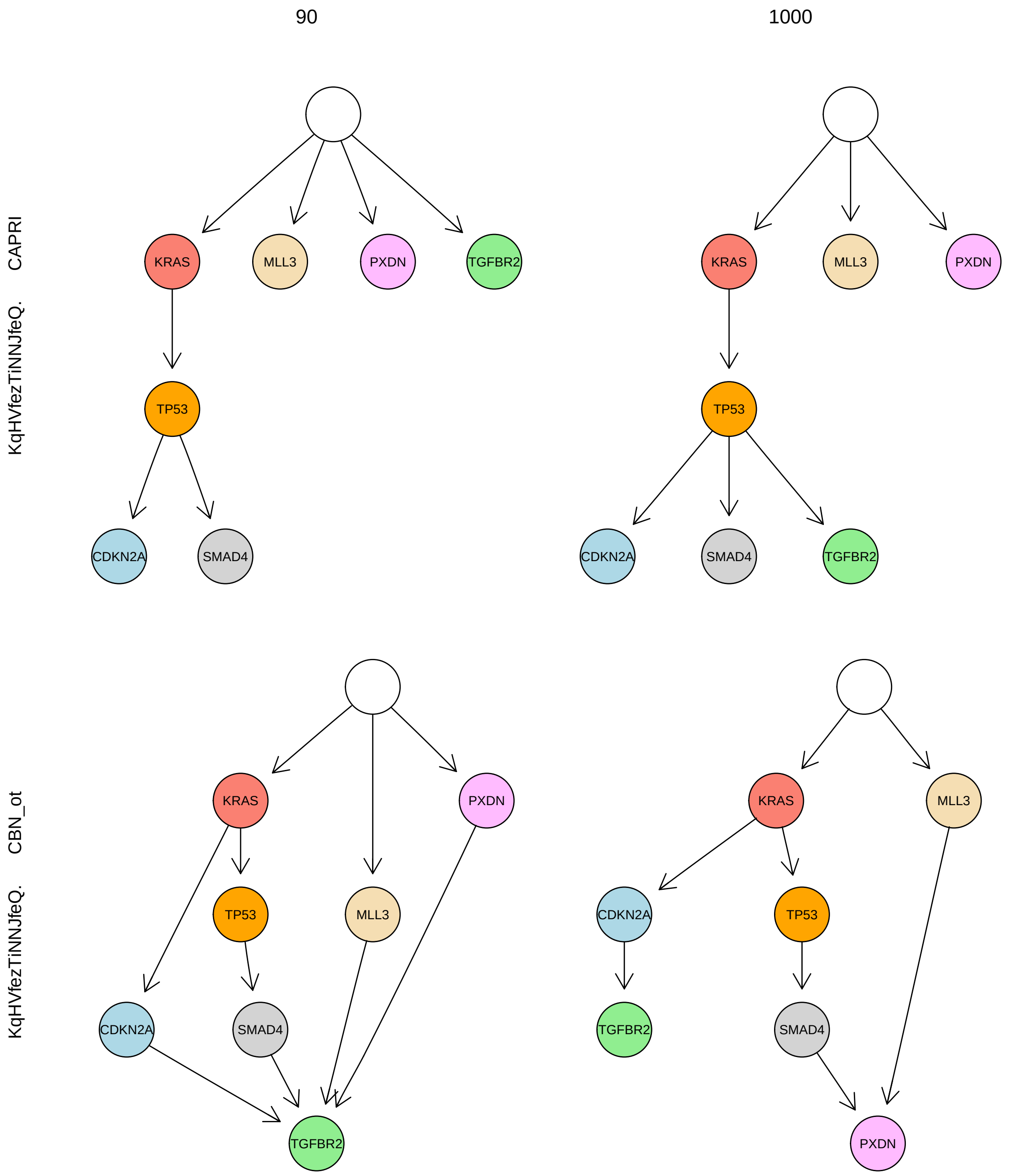

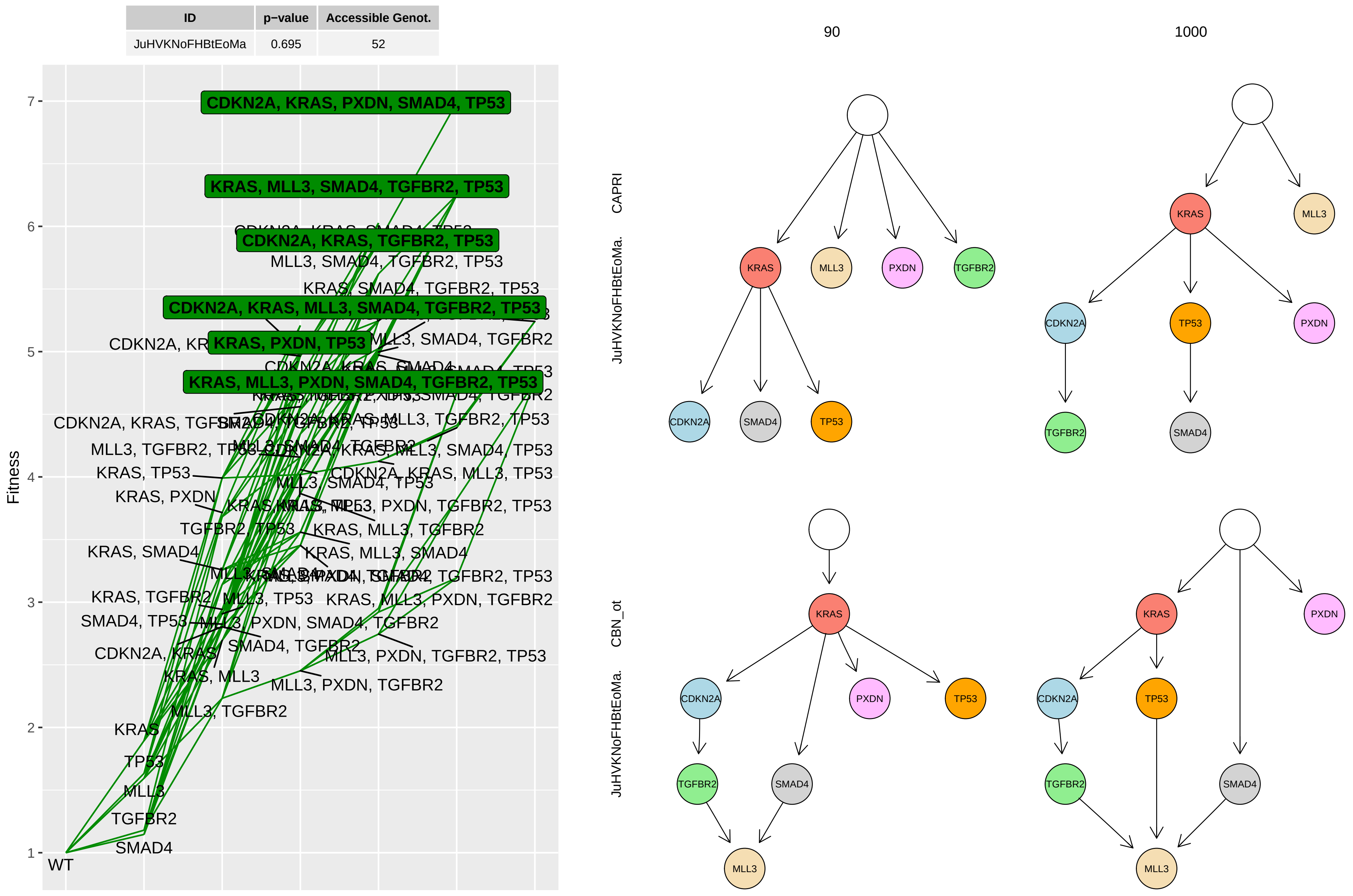

ID
p−value
Accessible Genot.
JuHVKNoFHBtEoMa
0.695
52
Fitness
7
6
5
4
3
2
1
WT
CDKN2A, KRAS, PXDN, SMAD4, TP53
KRAS, MLL3, SMAD4, TGFBR2, TP53
CDKN2A, KRAS, TGFBR2, TP53
MLL3, SMAD4, TGFBR2, TP53
KRAS, SMAD4, TGFBR2, TP53
CDKN2A, KRAS, MLL3, SMAD4, TGFBR2, TP53
KRAS, PXDN, TP53
KRAS, MLL3, PXDN, SMAD4, TGFBR2, TP53
KRAS, TP53
KRAS, PXDN
TGFBR2, TP53
KRAS, SMAD4
KRAS, TGFBR2
SMAD4, TP53
CDKN2A, KRAS
KRAS, MLL3
KRAS
TP53
MLL3
TGFBR2
SMAD4
CDKN2A, KRAS, MLL3, TP53
MLL3, SMAD4, TP53
KRAS, MLL3, TGFBR2
KRAS, MLL3, SMAD4
KRAS, MLL3, PXDN, TGFBR2
MLL3, PXDN, SMAD4, TGFBR2
SMAD4, TGFBR2
MLL3, PXDN, TGFBR2, TP53
MLL3, PXDN, TGFBR2
MLL3, TGFBR2
MLL3, TP53
CDKN2A, KRAS, MLL3, TGFBR2, TP53
90
1000
CAPRI
CBN_ot
JuHVKNoFHBtEoMa.
KRAS
MLL3
PXDN
TGFBR2
CDKN2A
SMAD4
TP53

ID	p−value	Accessible Genot.
ylJOPwxOeHfMJXk	0.696	20

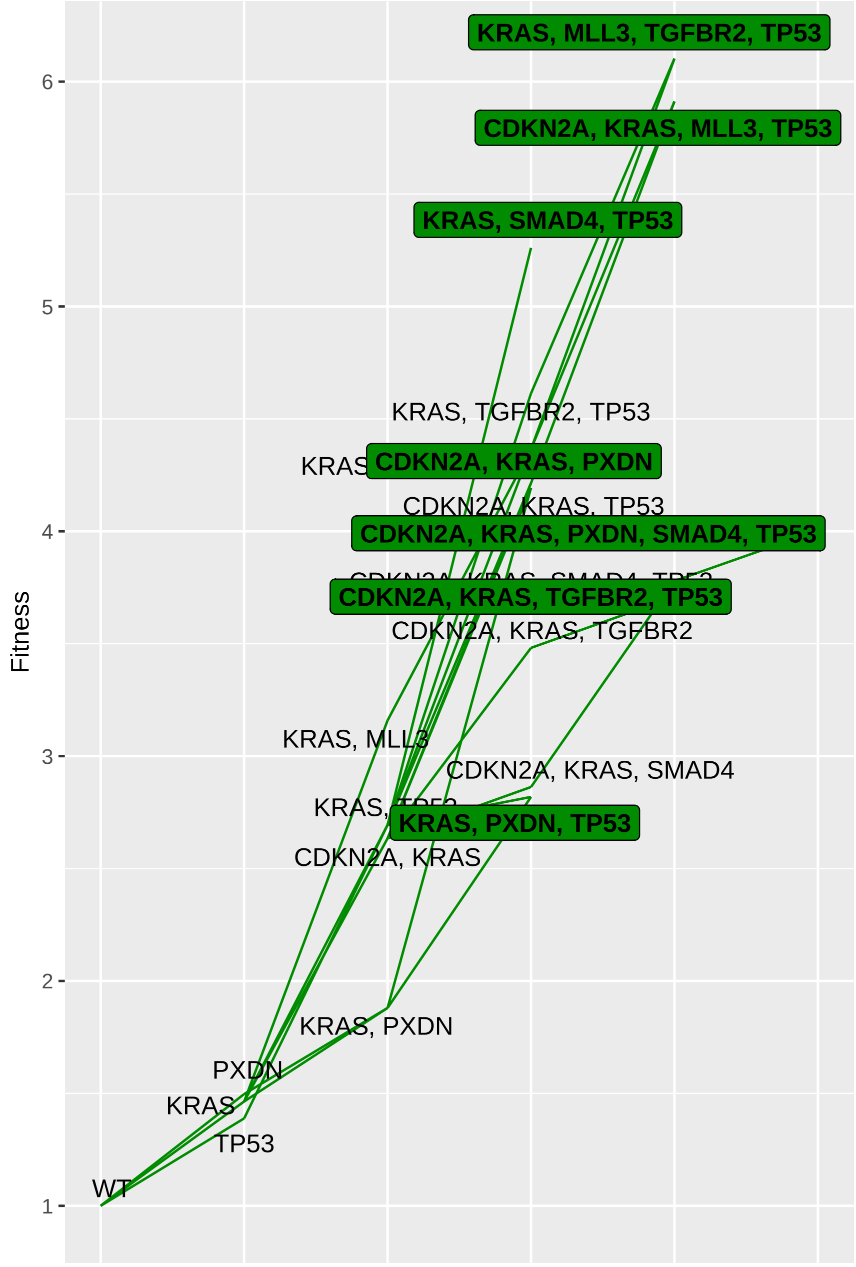

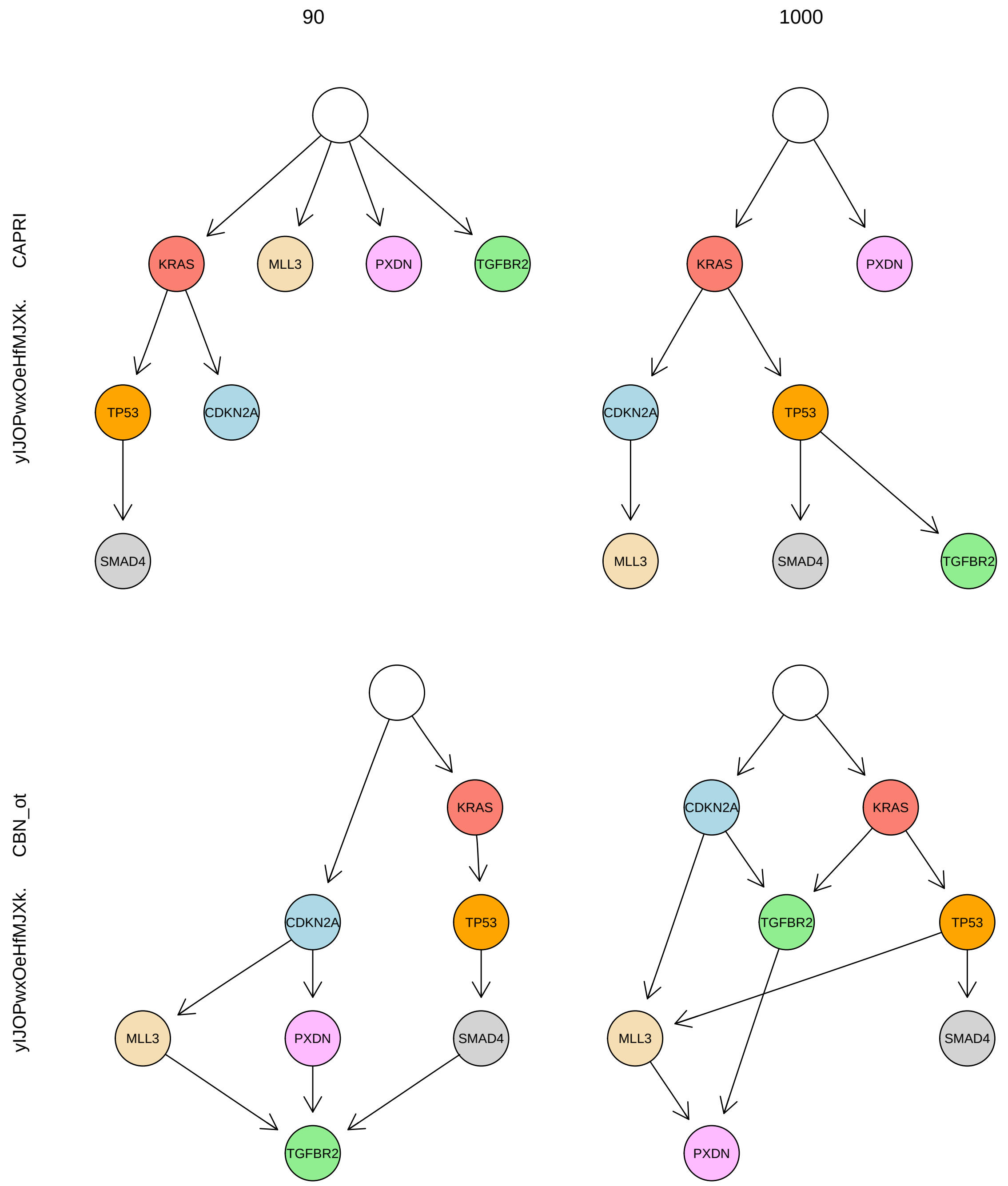

ID	p−value	Accessible Genot.
RIllPgpaPVnHViK	0.697	18

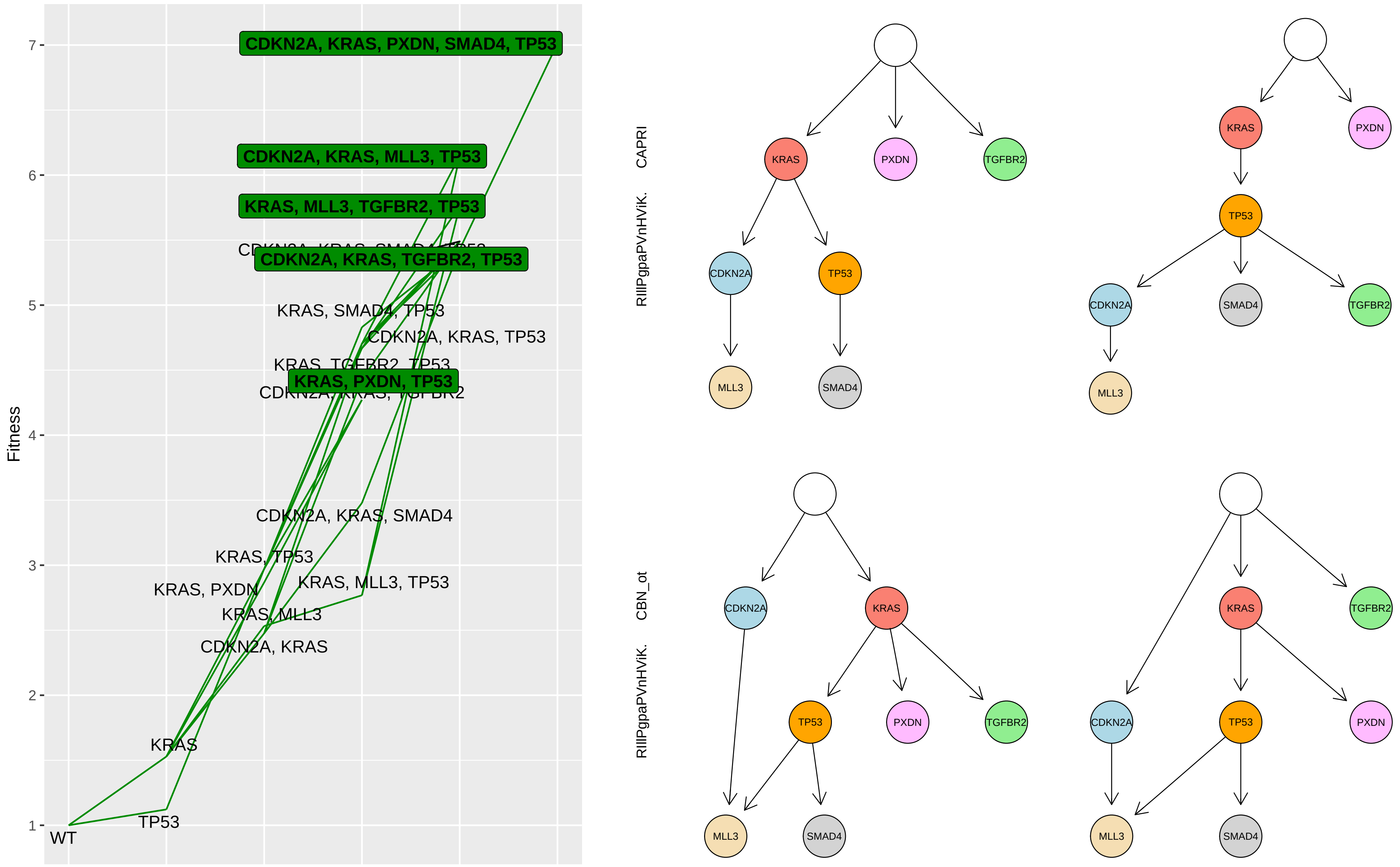

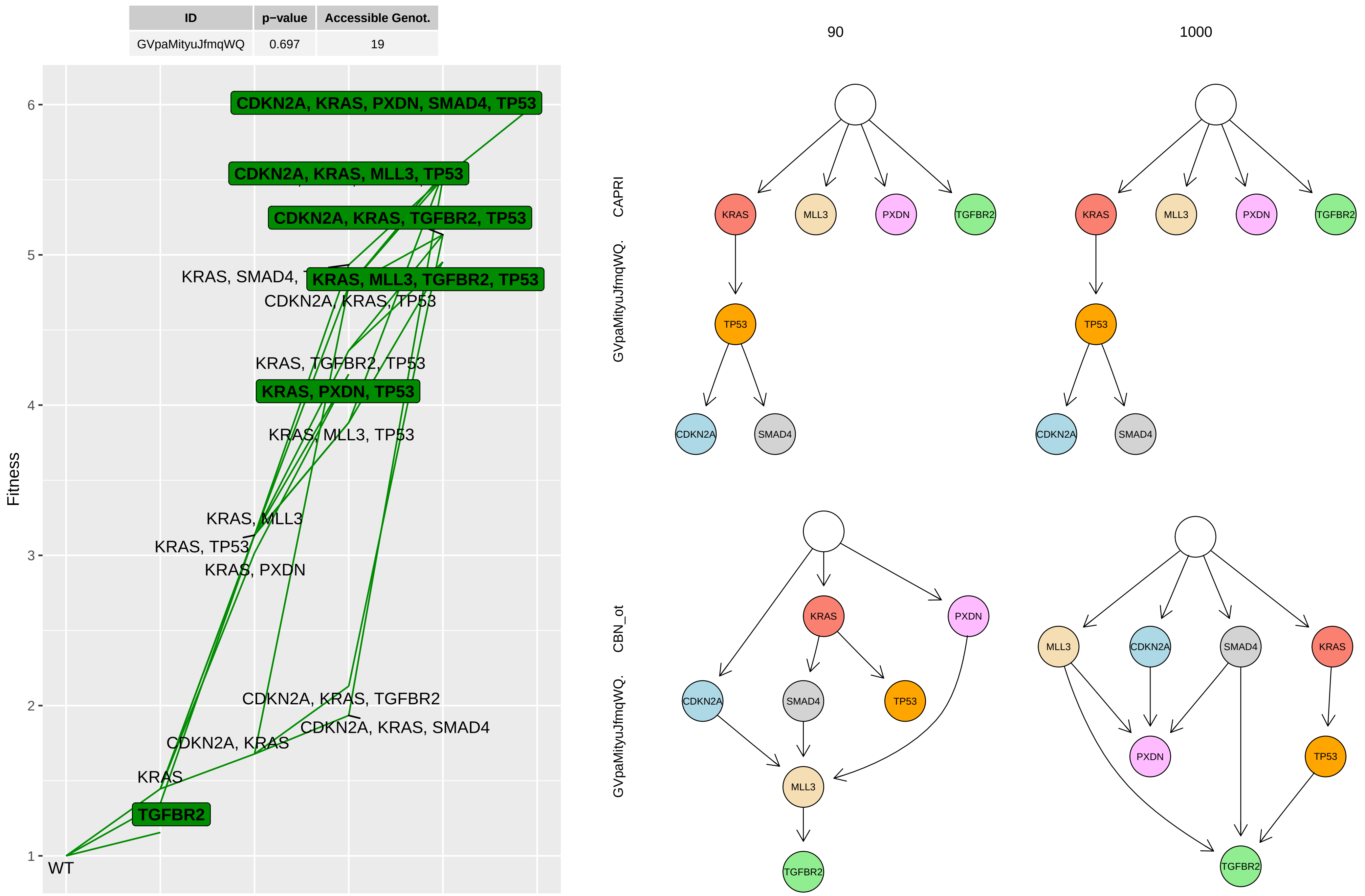

ID
p−value
Accessible Genot.
GVpaMityuJfmqWQ
0.697
19
Fitness
CDKN2A, KRAS, PXDN, SMAD4, TP53
CDKN2A, KRAS, MLL3, TP53
CDKN2A, KRAS, TGFBR2, TP53
KRAS, MLL3, TGFBR2, TP53
CDKN2A, KRAS, TP53
KRAS, TGFBR2, TP53
KRAS, PXDN, TP53
KRAS, MLL3, TP53
KRAS, MLL3
KRAS, TP53
KRAS, PXDN
CDKN2A, KRAS, TGFBR2
CDKN2A, KRAS, SMAD4
CDKN2A, KRAS
KRAS
TGFBR2
WT
90
1000
GVpaMityuJfmqWQ. CAPRI
GVpaMityuJfmqWQ. CBN_ot
KRAS
MLL3
PXDN
TGFBR2
TP53
CDKN2A
SMAD4

ID	p−value	Accessible Genot.
iRuBLdQGbxPBRZQ	0.7	23

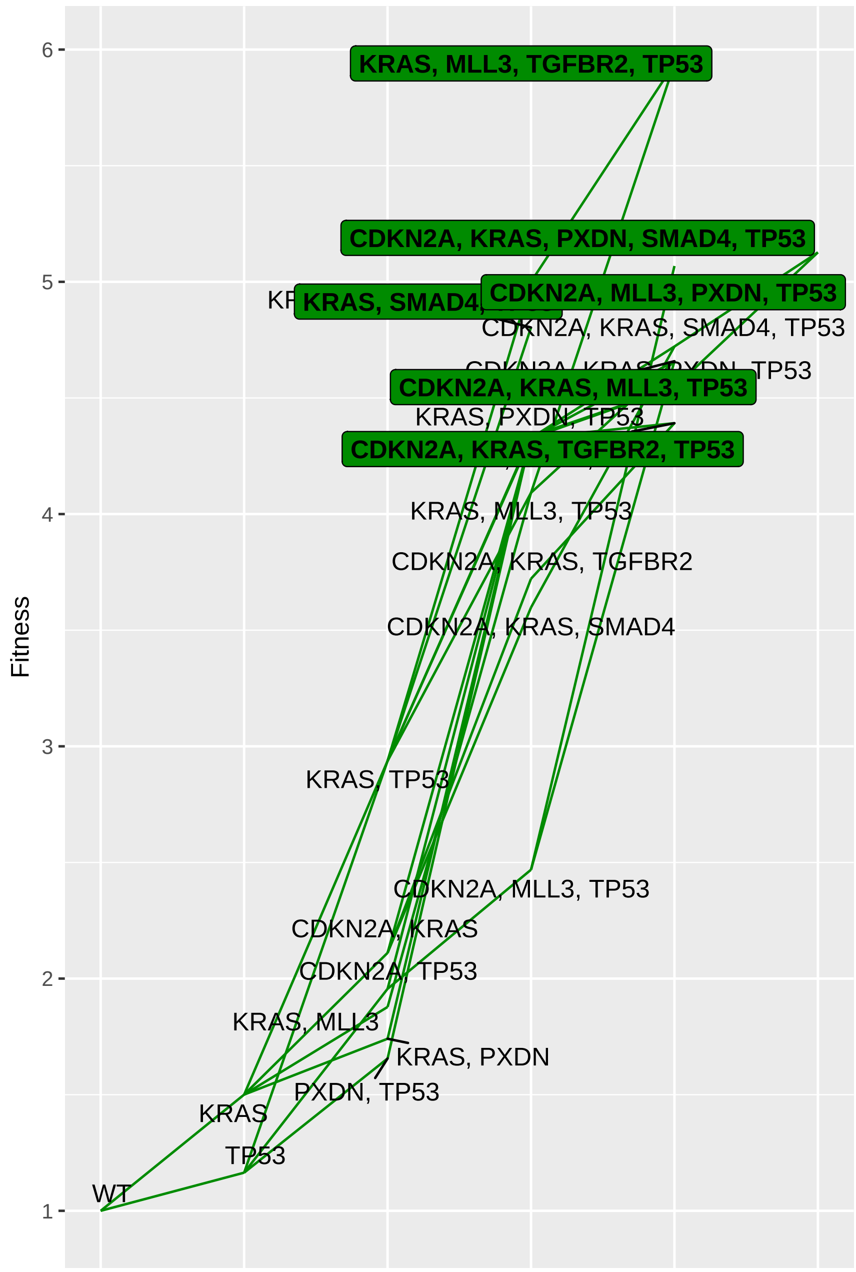

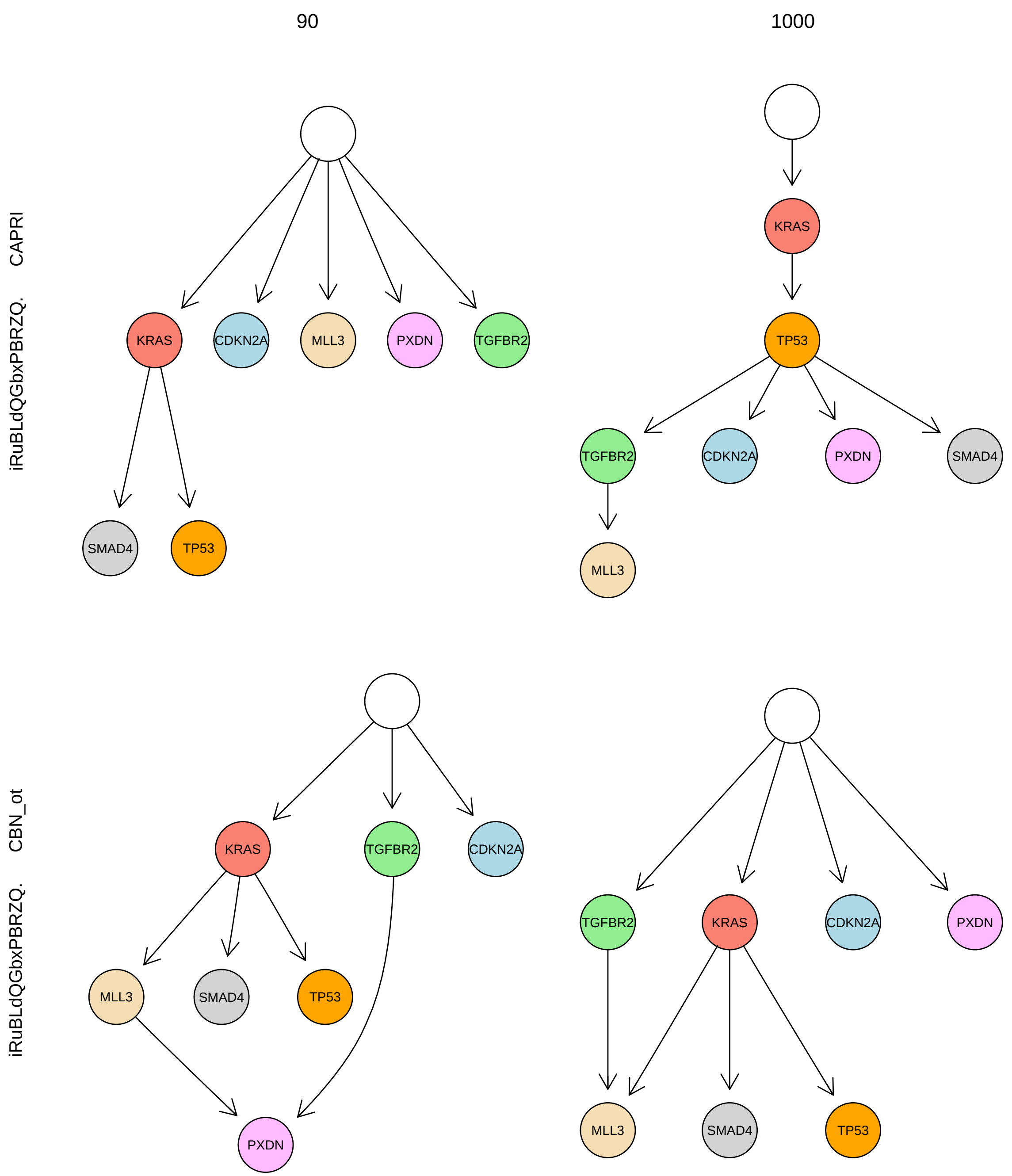

ID	p−value	Accessible Genot.
UMyHChRovEzVAqD	0.701	25

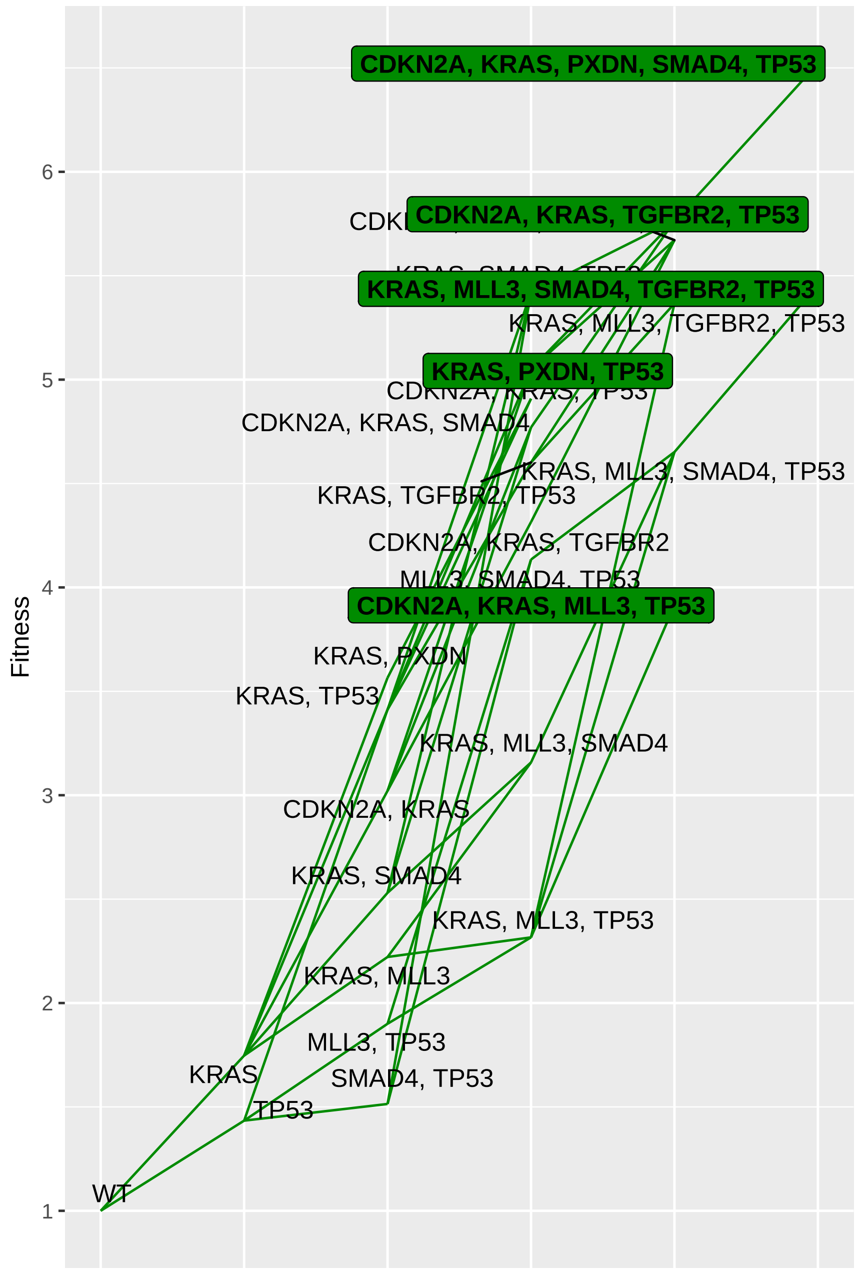

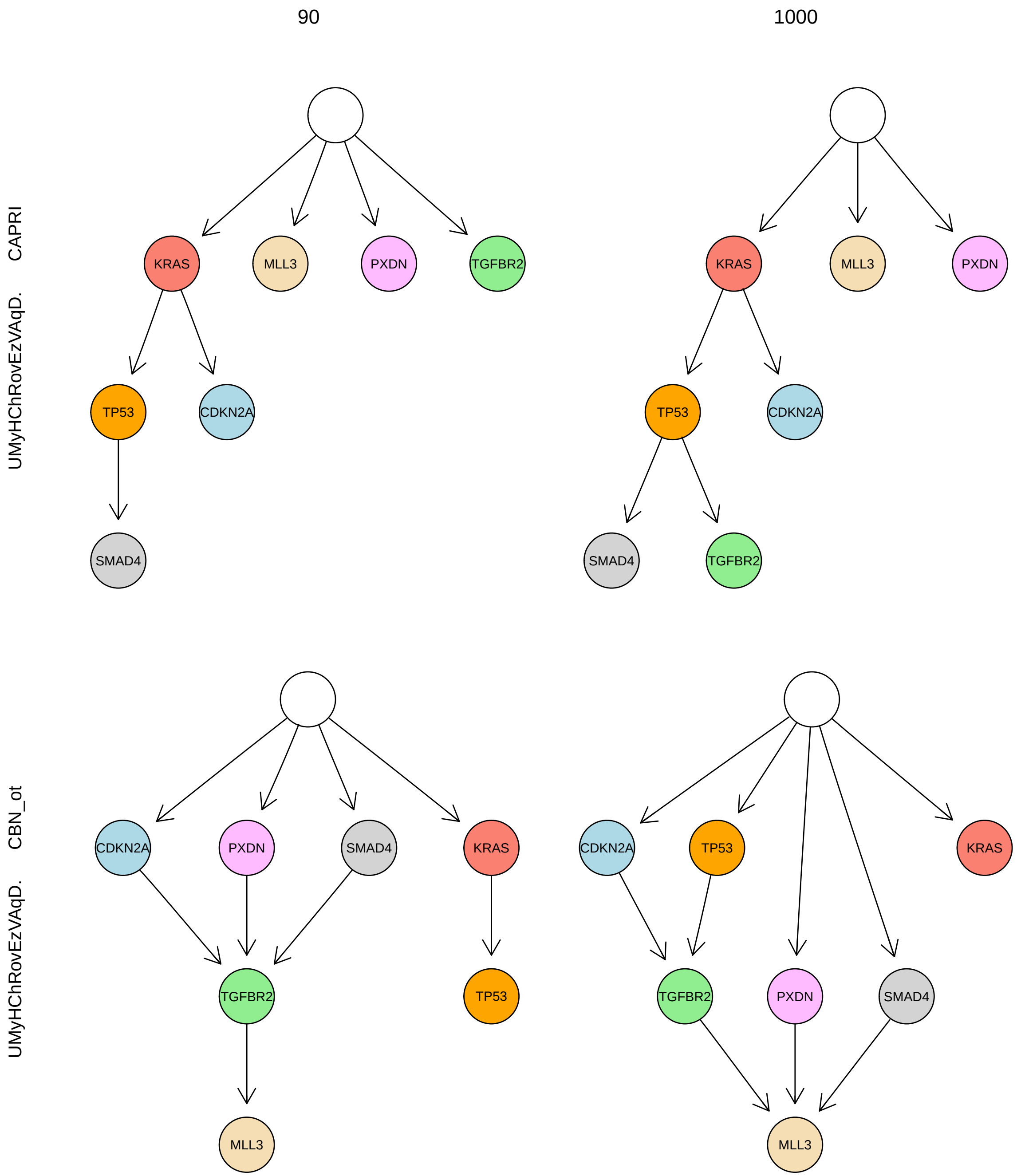

ID	p−value	Accessible Genot.
xqVkXLTPwKAQkeT	0.701	19

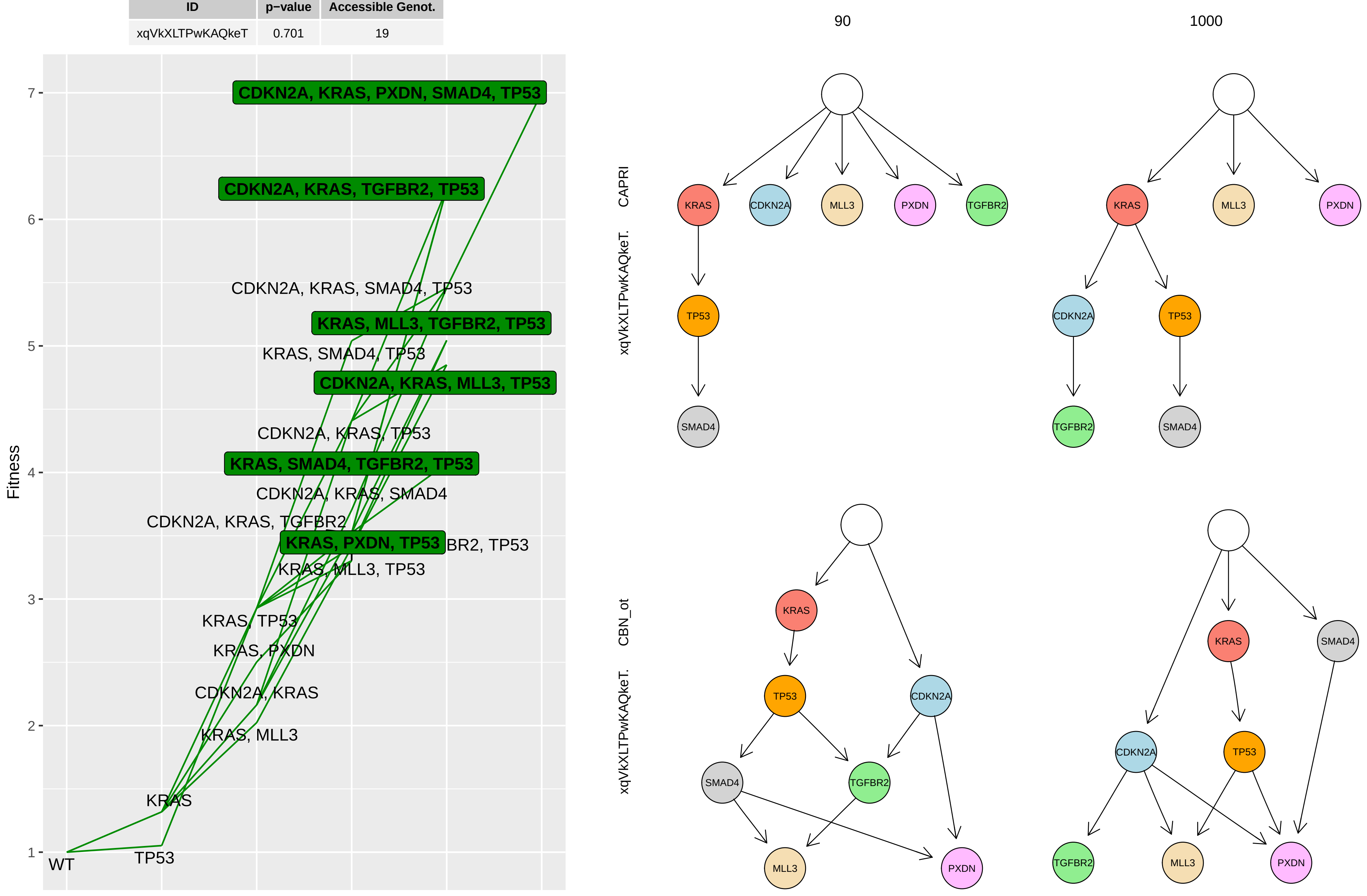

ID	p−value	Accessible Genot.
WcBWNhouhaGVDME	0.703	18

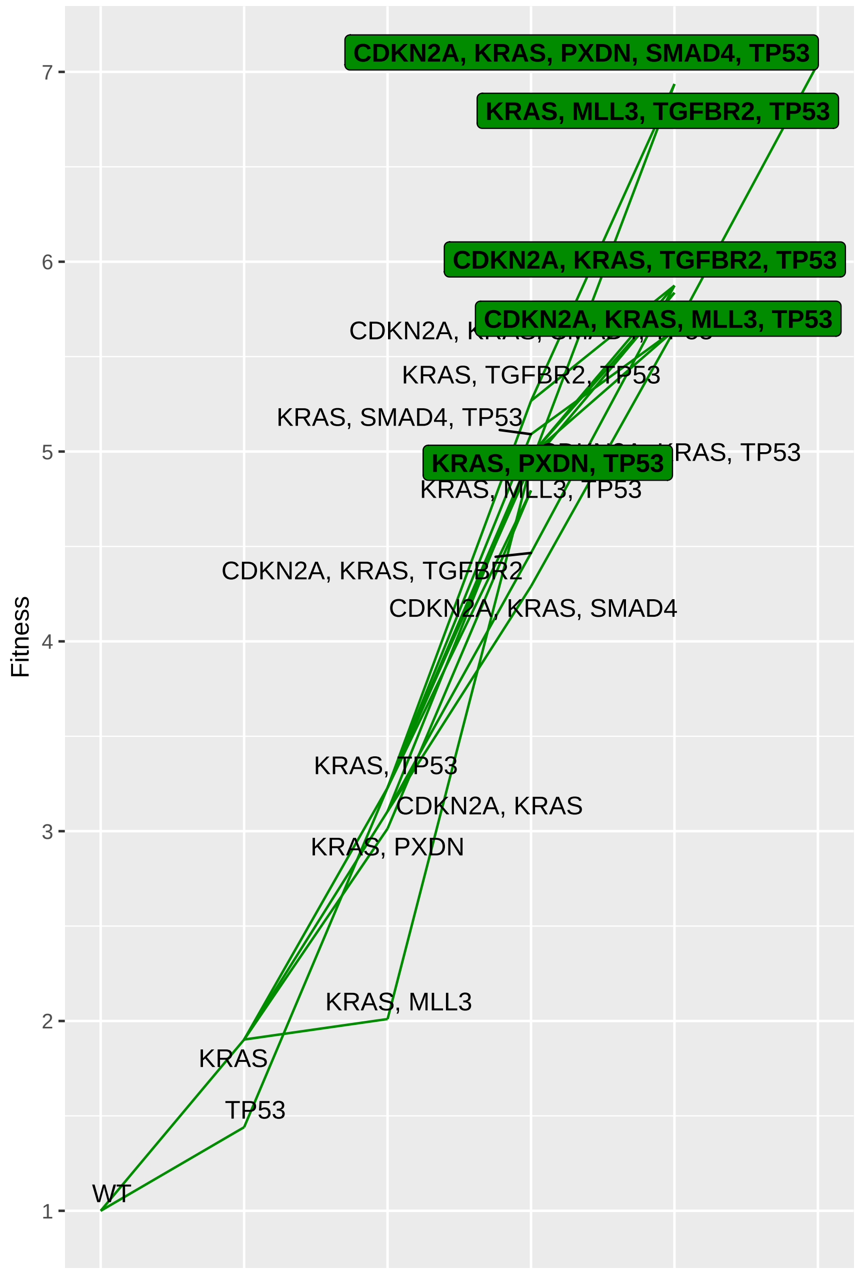

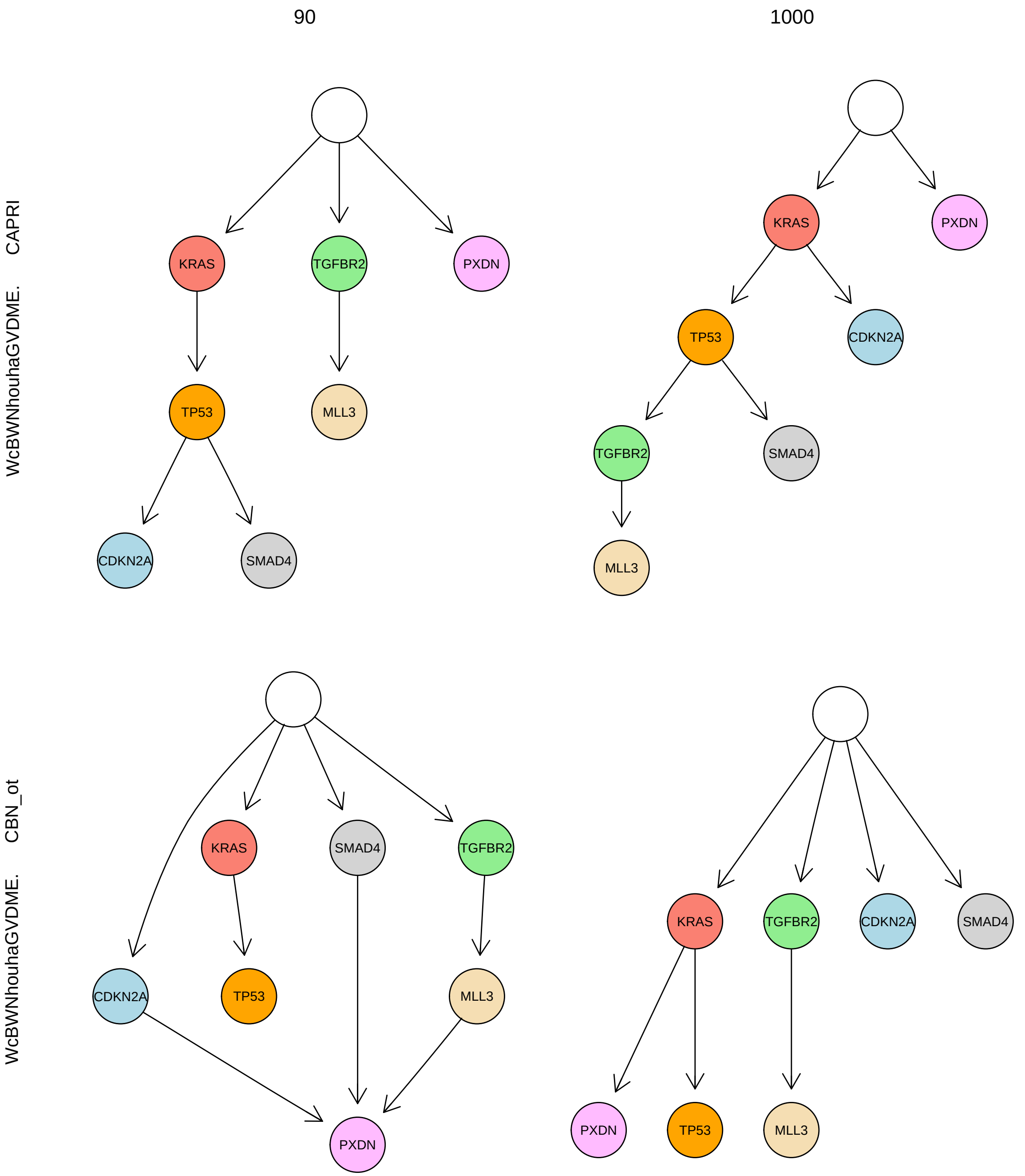

ID	p−value	Accessible Genot.
ubTvuceBuGIhReG	0.703	27

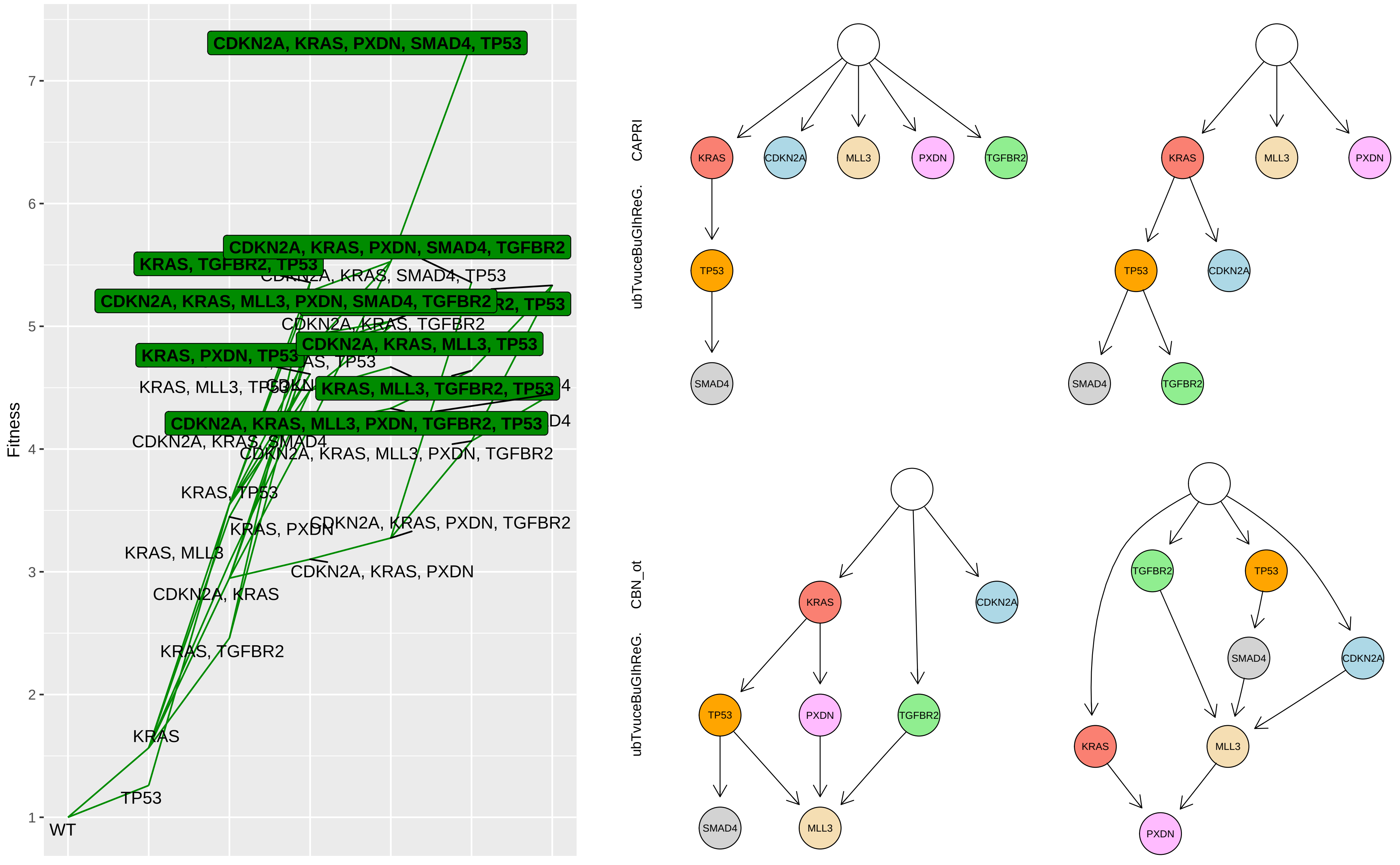

ID	p−value	Accessible Genot.
mJkdgAXfjvsdbkc	0.703	19

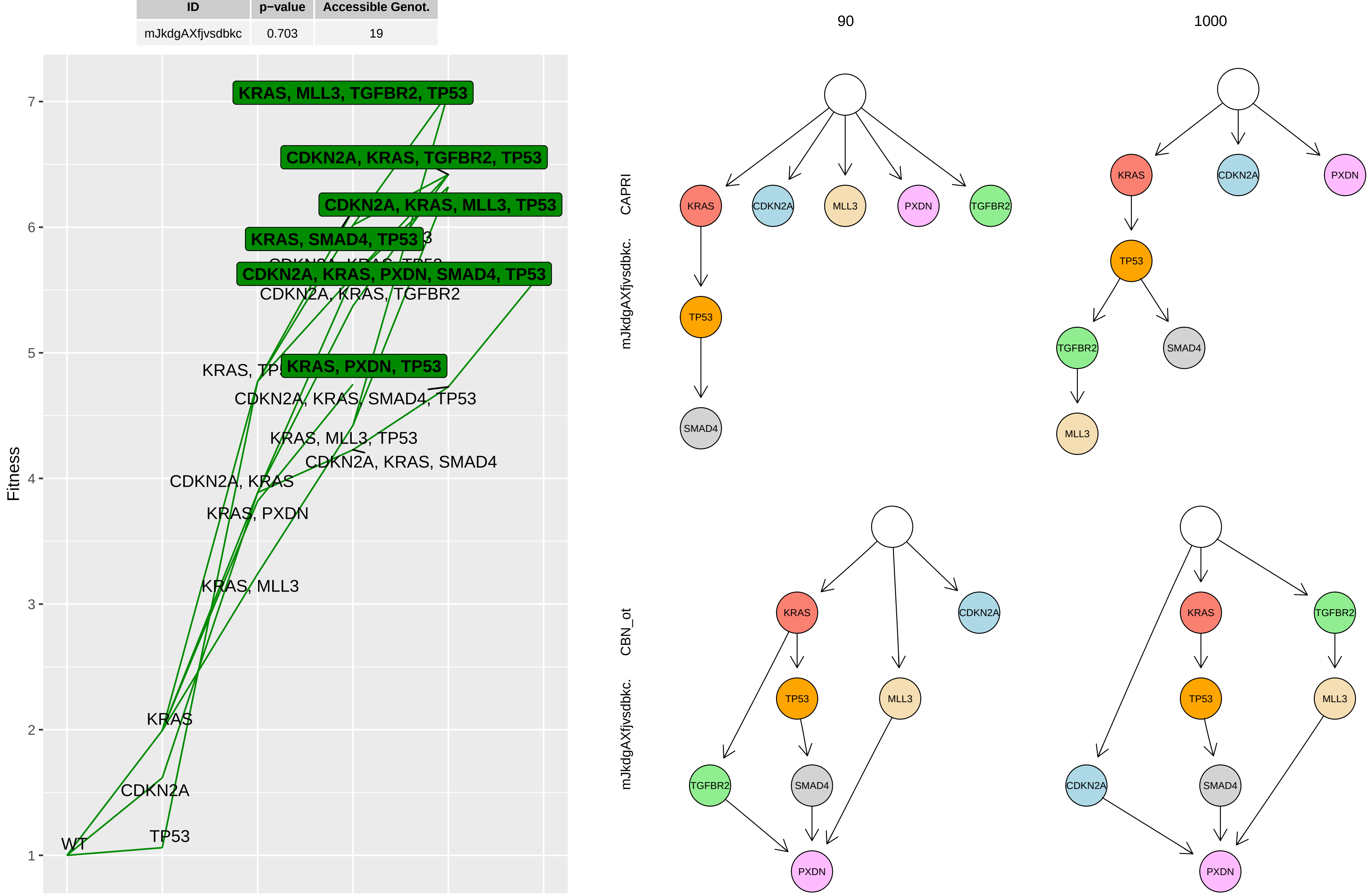

ID	p−value	Accessible Genot.
hKfYYnINcUGDuNK	0.704	48

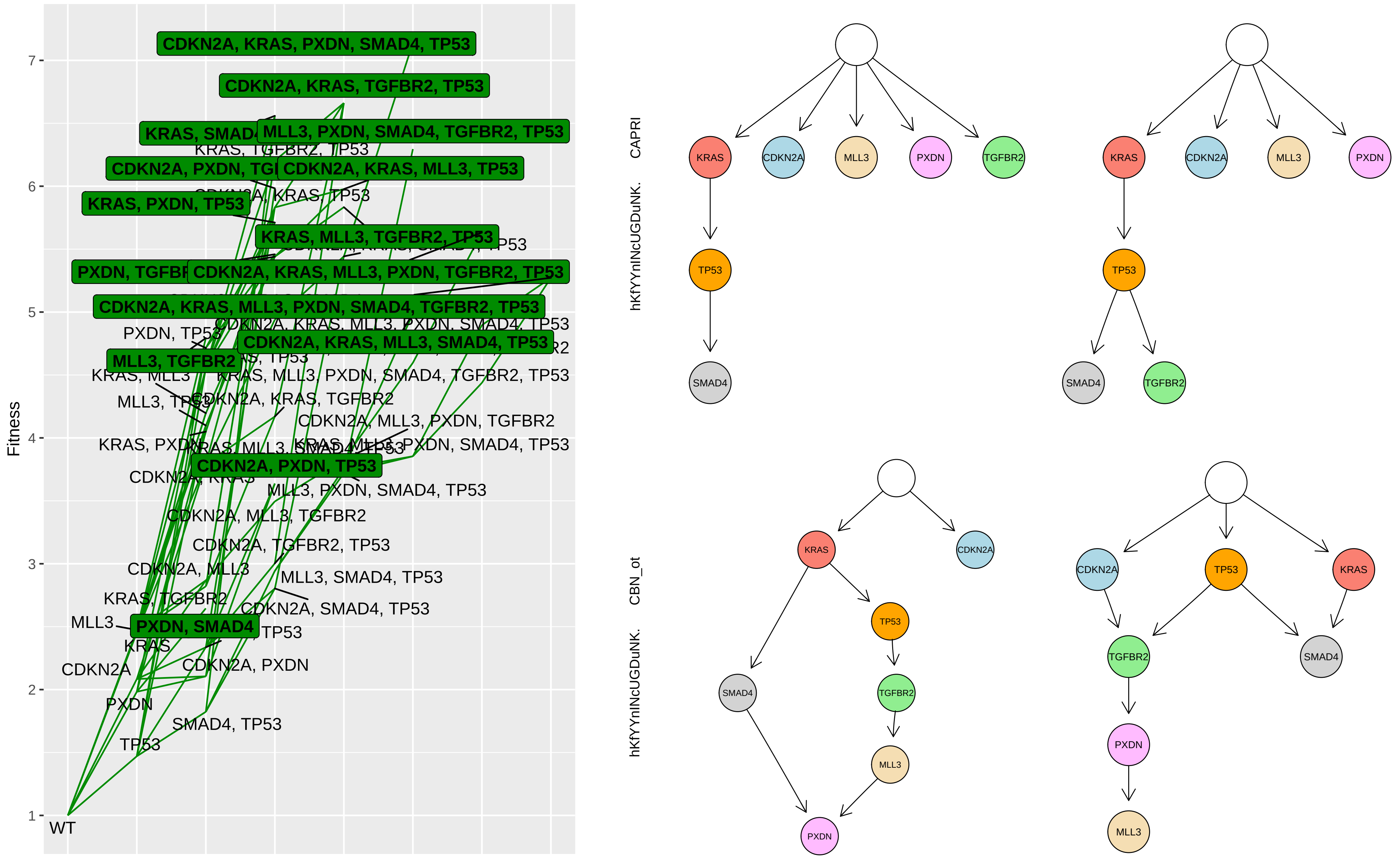

ID	p−value	Accessible Genot.
xBLZwlvhPrGxmLy	0.704	20

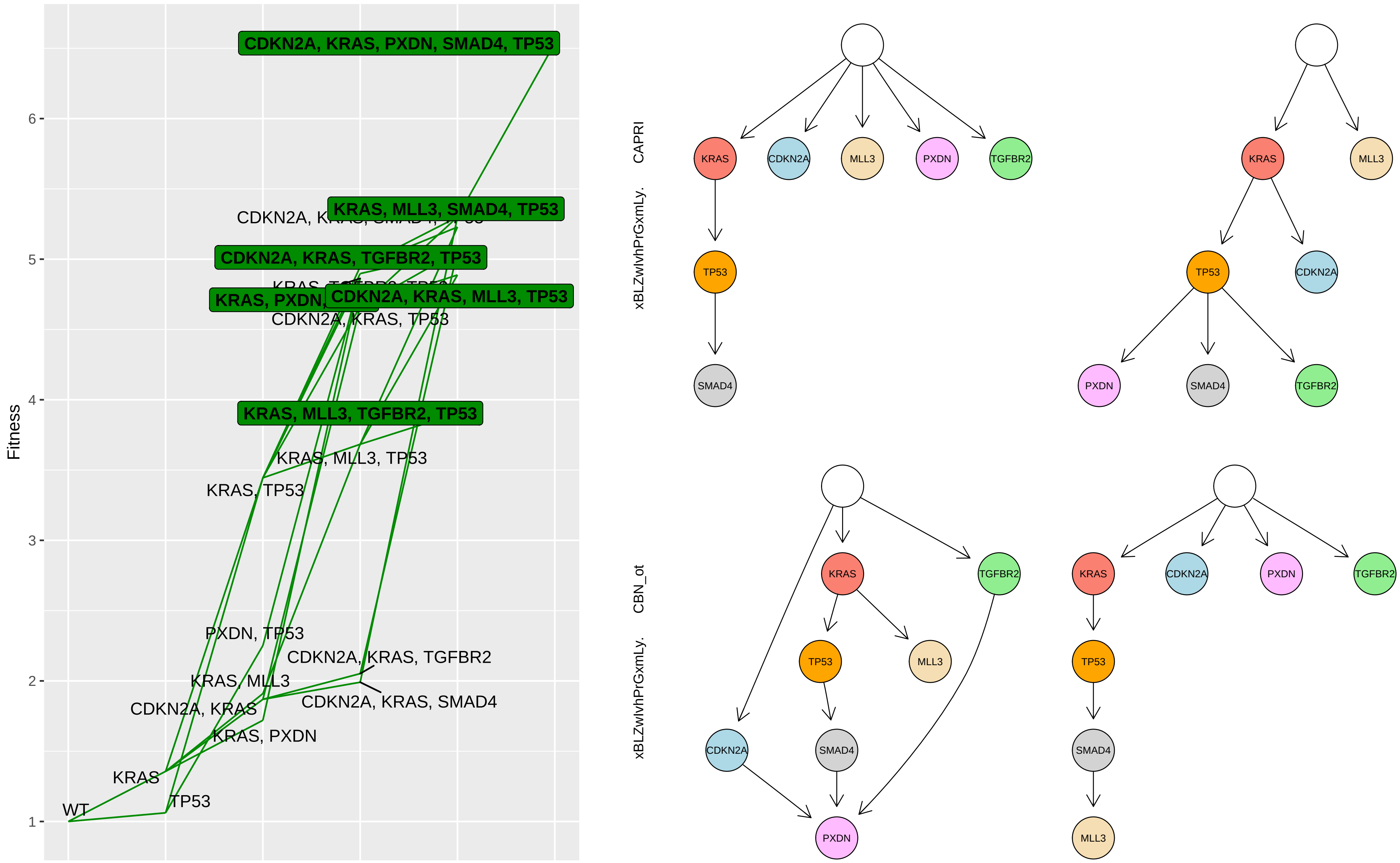

ID	p−value	Accessible Genot.
jIDkQpJlsUBRYBx	0.705	18

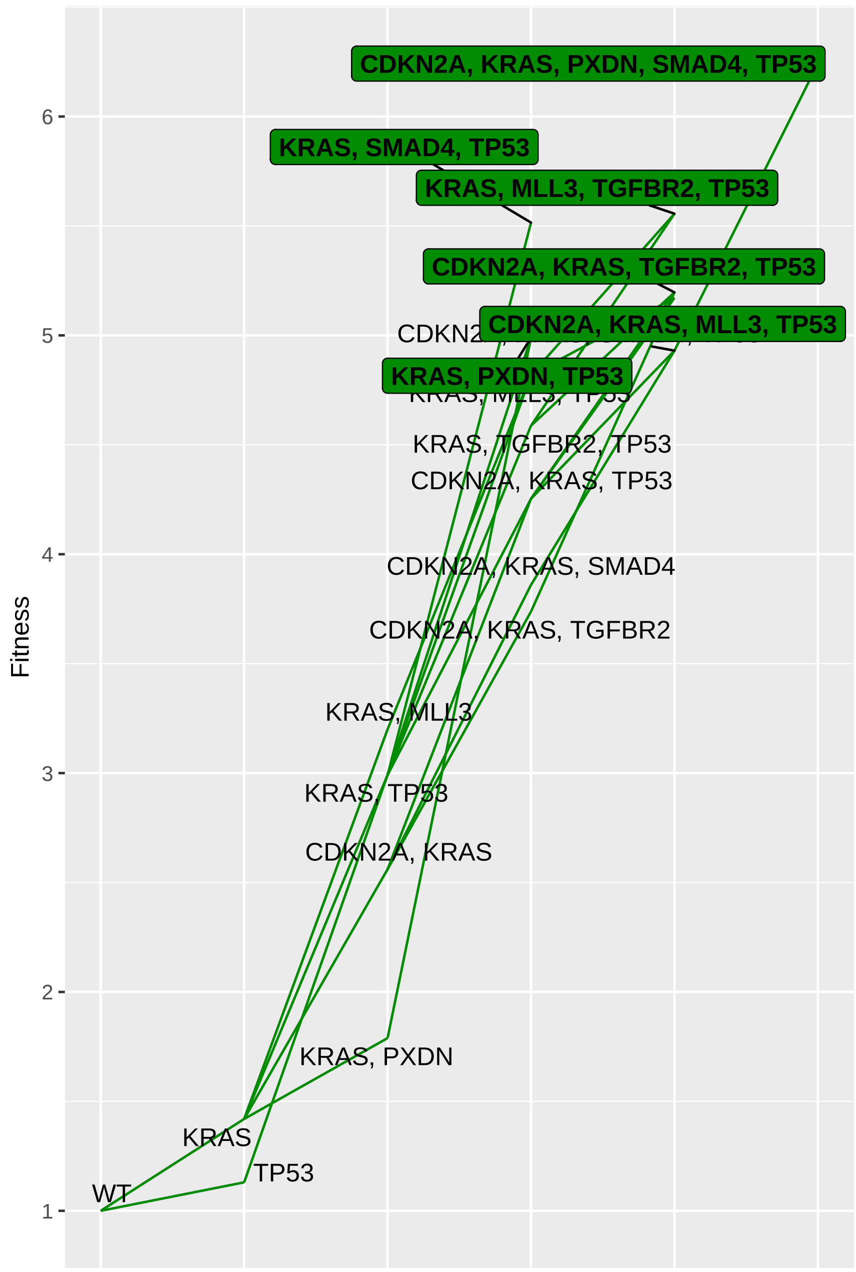

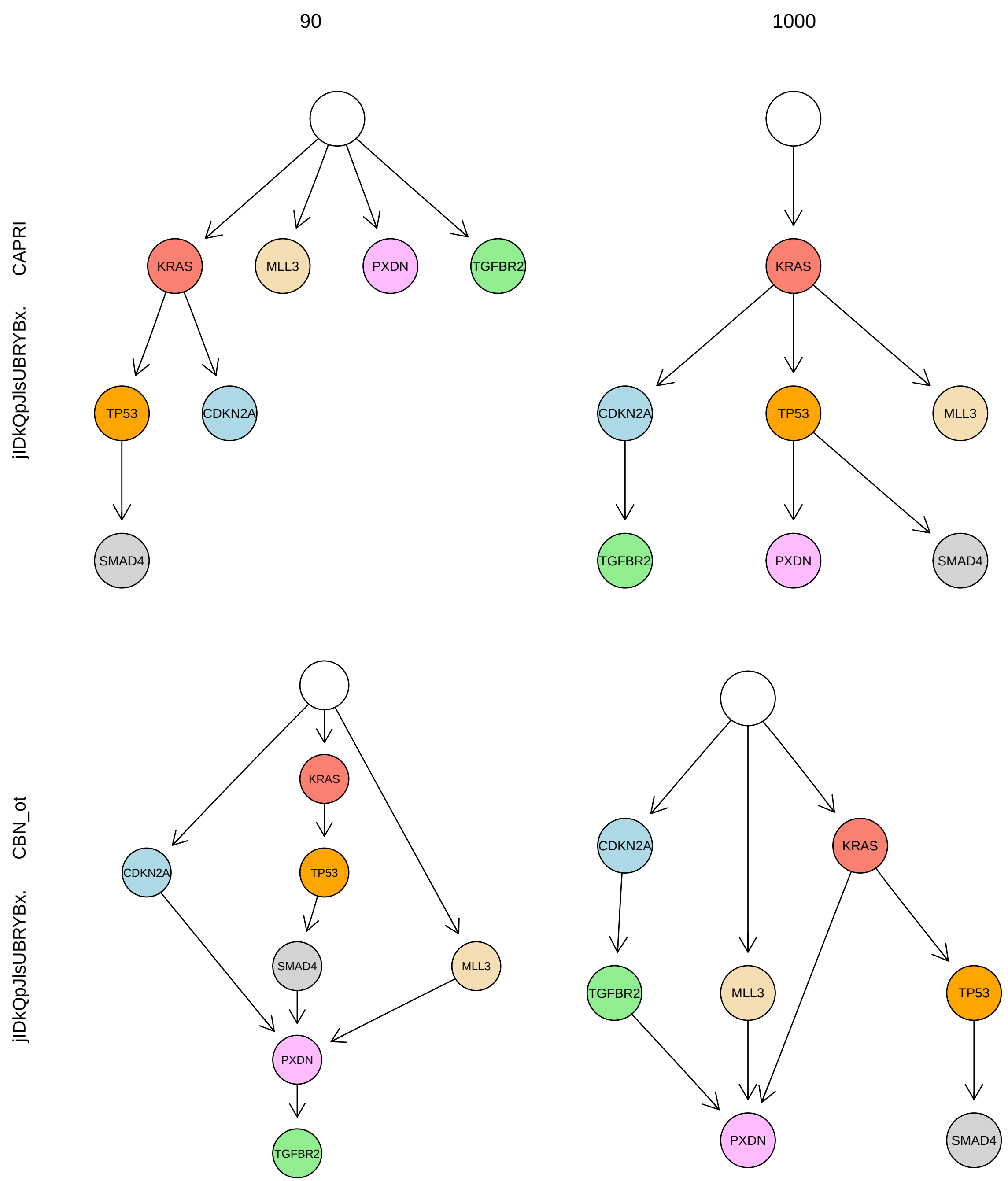

ID	p−value	Accessible Genot.
zngoGlCfFUIxqkF	0.706	124

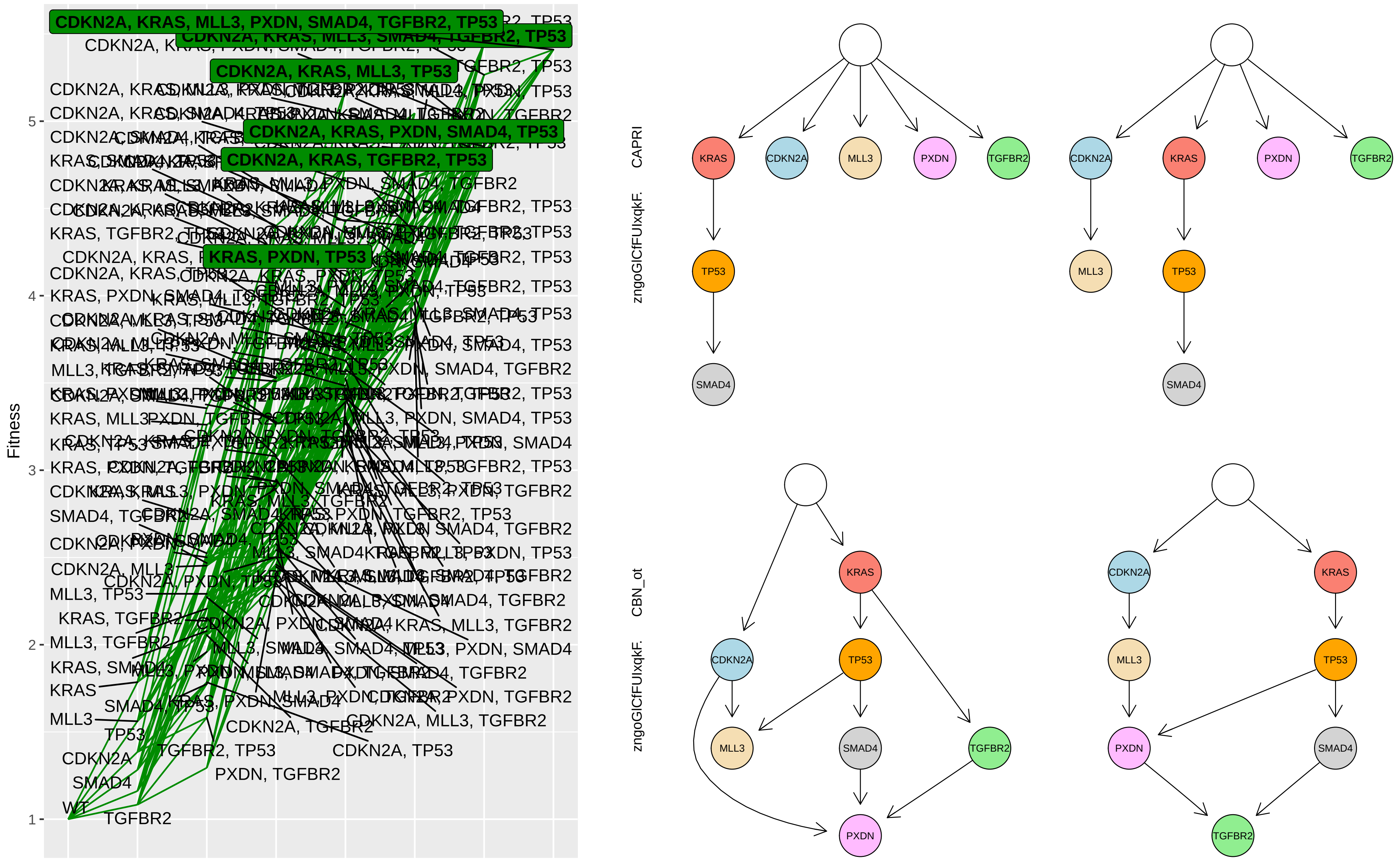

ID	p−value	Accessible Genot.
smNBrYbbnpNiMcR	0.707	18

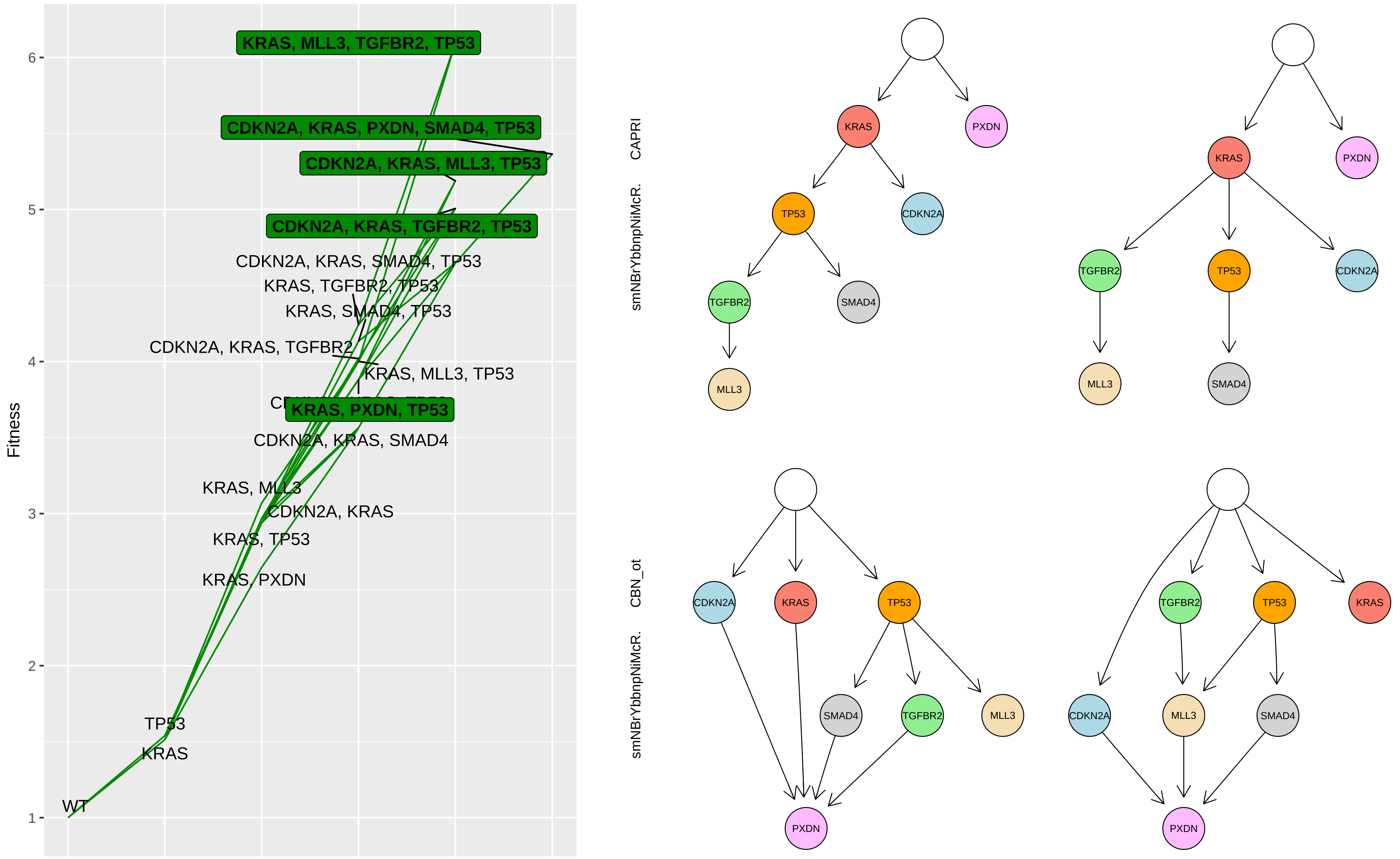

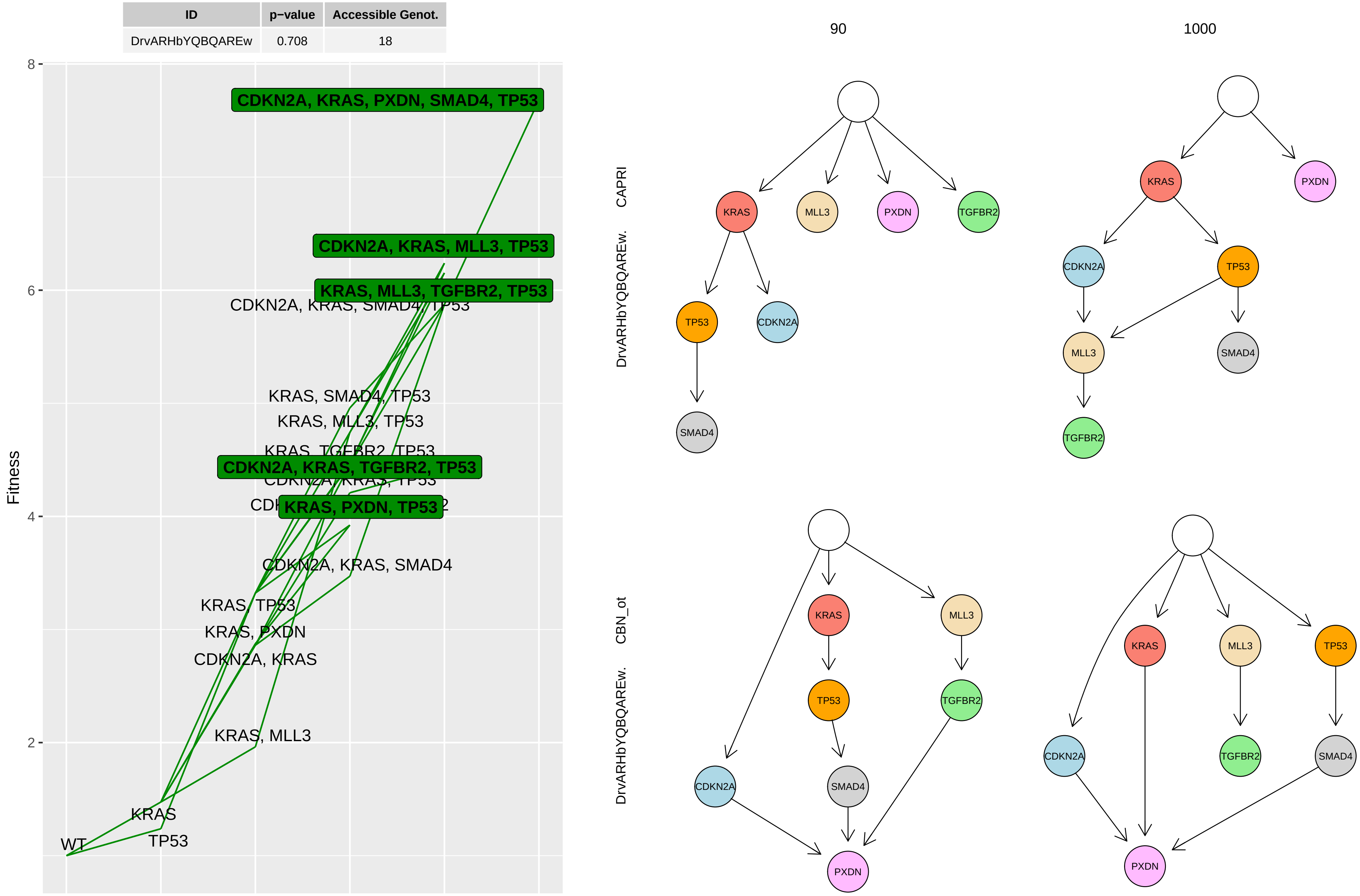

ID
p−value
Accessible Genot.
DrvARHbYQBQAREw
0.708
18
Fitness
8
6
4
2
CDKN2A, KRAS, PXDN, SMAD4, TP53
CDKN2A, KRAS, MLL3, TP53
KRAS, MLL3, TGFBR2, TP53
CDKN2A, KRAS, SMAD4, TP53
KRAS, SMAD4, TP53
KRAS, MLL3, TP53
KRAS, TGFBR2, TP53
CDKN2A, KRAS, TGFBR2, TP53
KRAS, PXDN, TP53
CDKN2A, KRAS, SMAD4
KRAS, TP53
KRAS, PXDN
CDKN2A, KRAS
KRAS, MLL3
KRAS
TP53
WT
90
1000
DrvARHbYQBQAREw. CAPRI
DrvARHbYQBQAREw. CBN_ot
KRAS
MLL3
PXDN
TGFBR2
TP53
CDKN2A
SMAD4

ID	p−value	Accessible Genot.
iUJKdQmmZrvWUhp	0.71	89

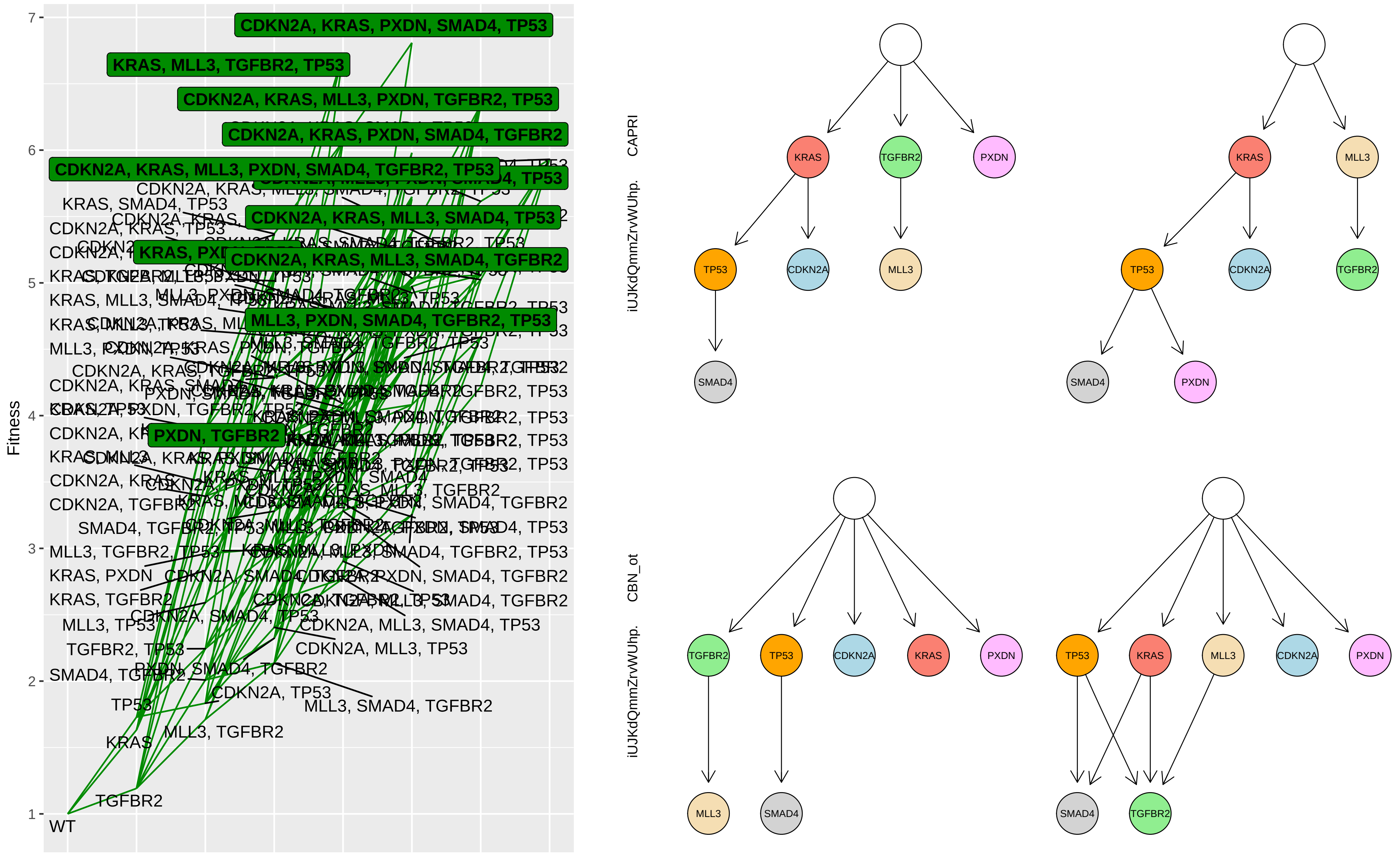

ID	p−value	Accessible Genot.
gKiXyvsprUUhfdF	0.716	18

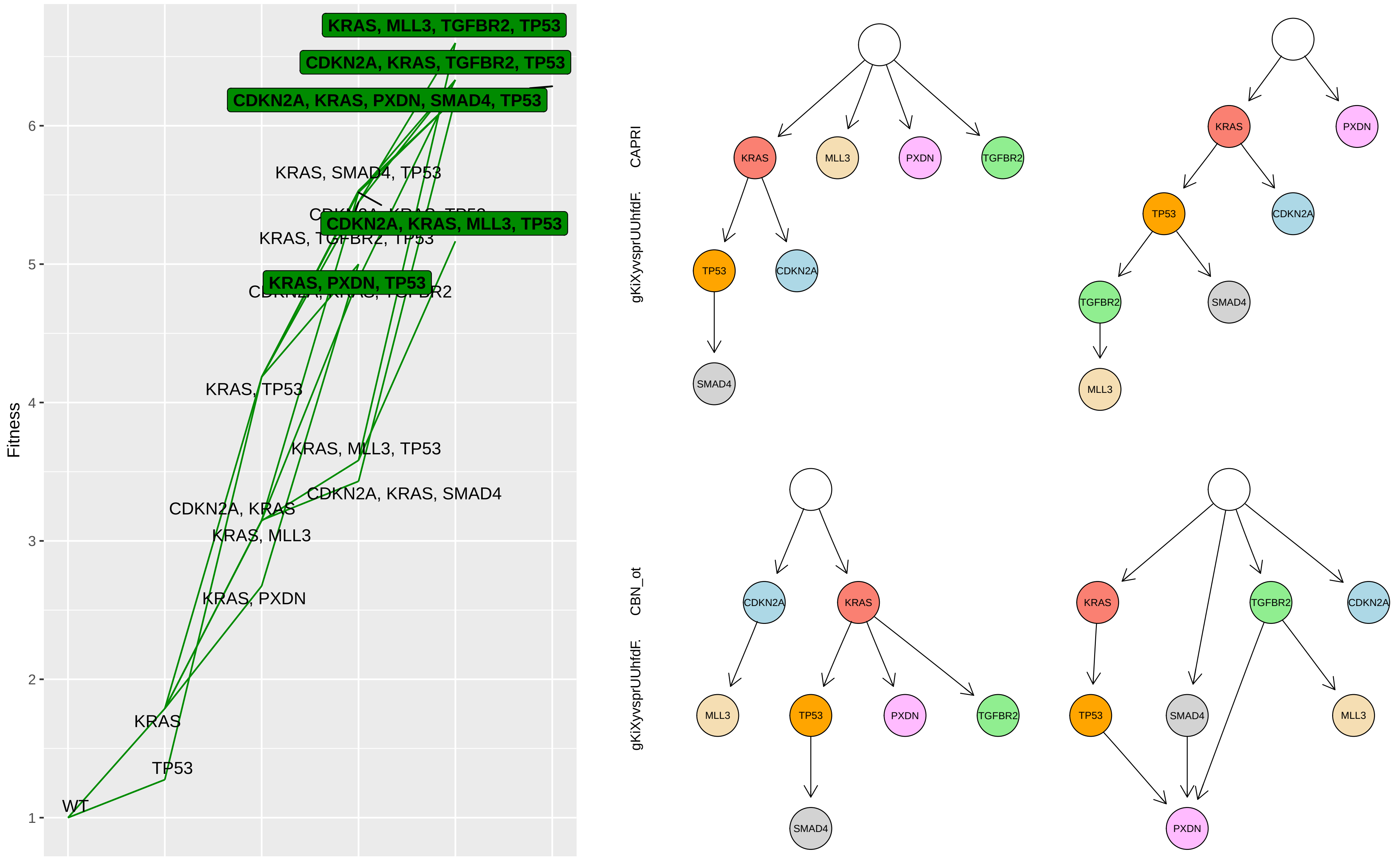

ID	p−value	Accessible Genot.
IZyzqRkfRyteDEw	0.716	19

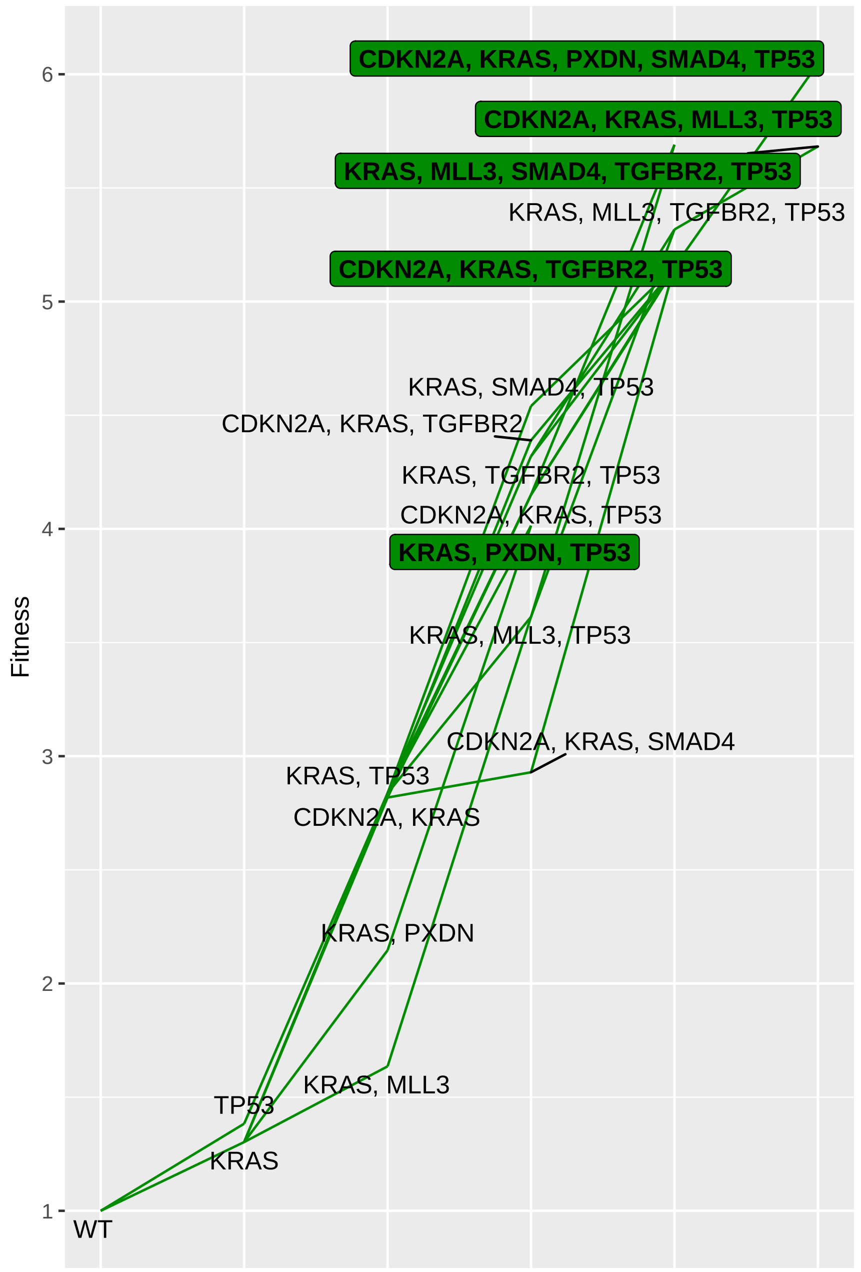

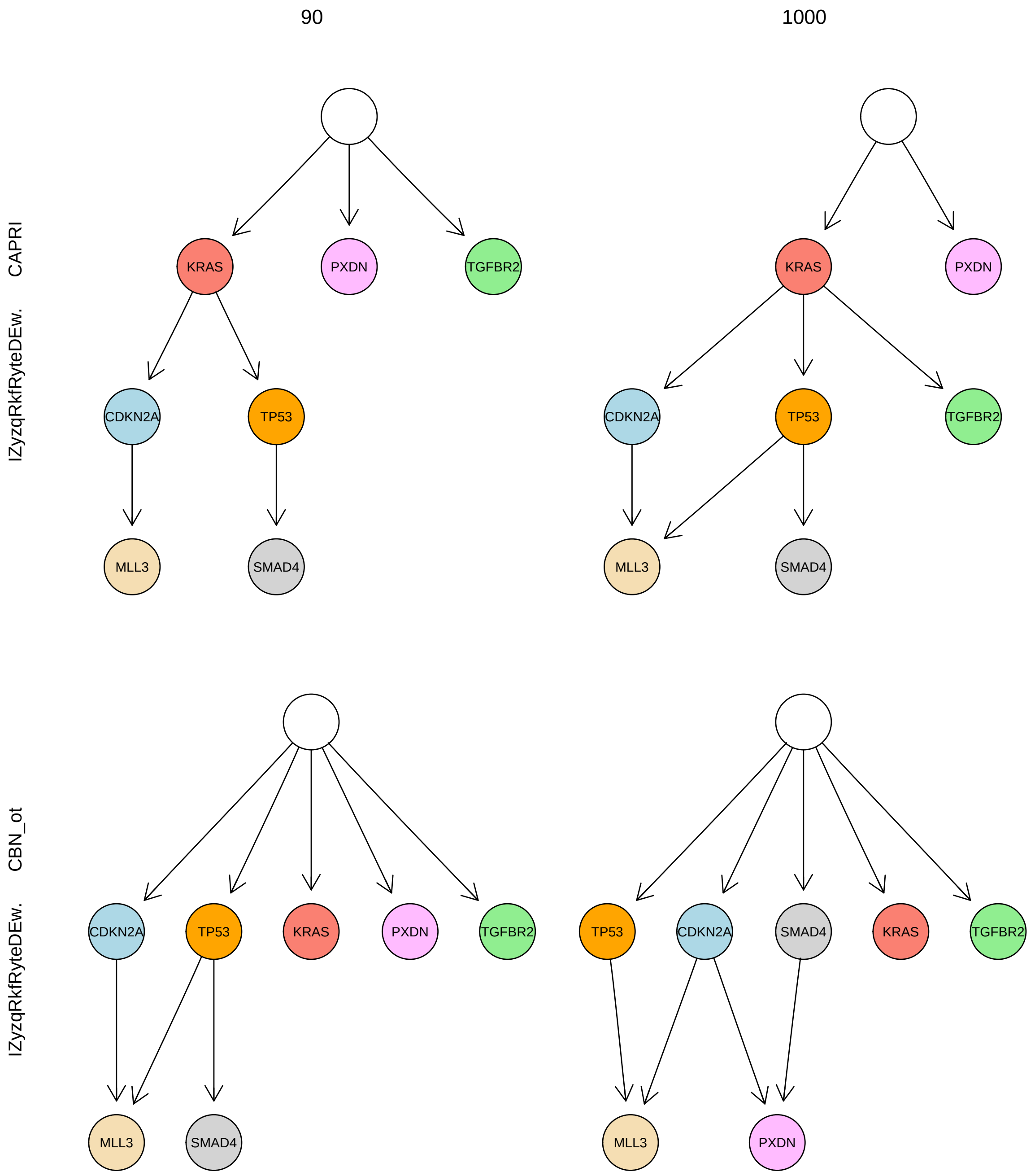

ID	p−value	Accessible Genot.
SluKGOLVrwNQtbg	0.718	18

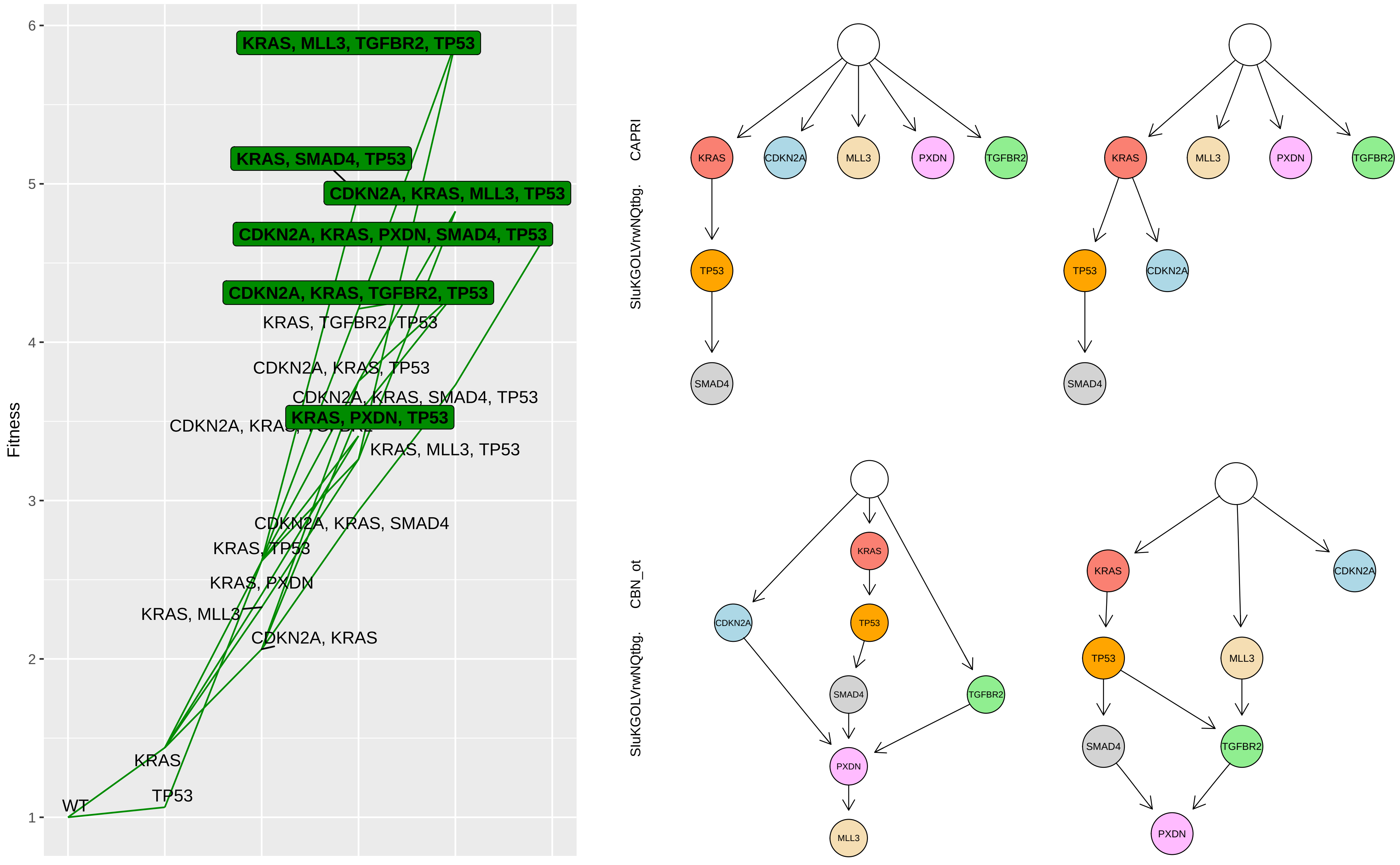

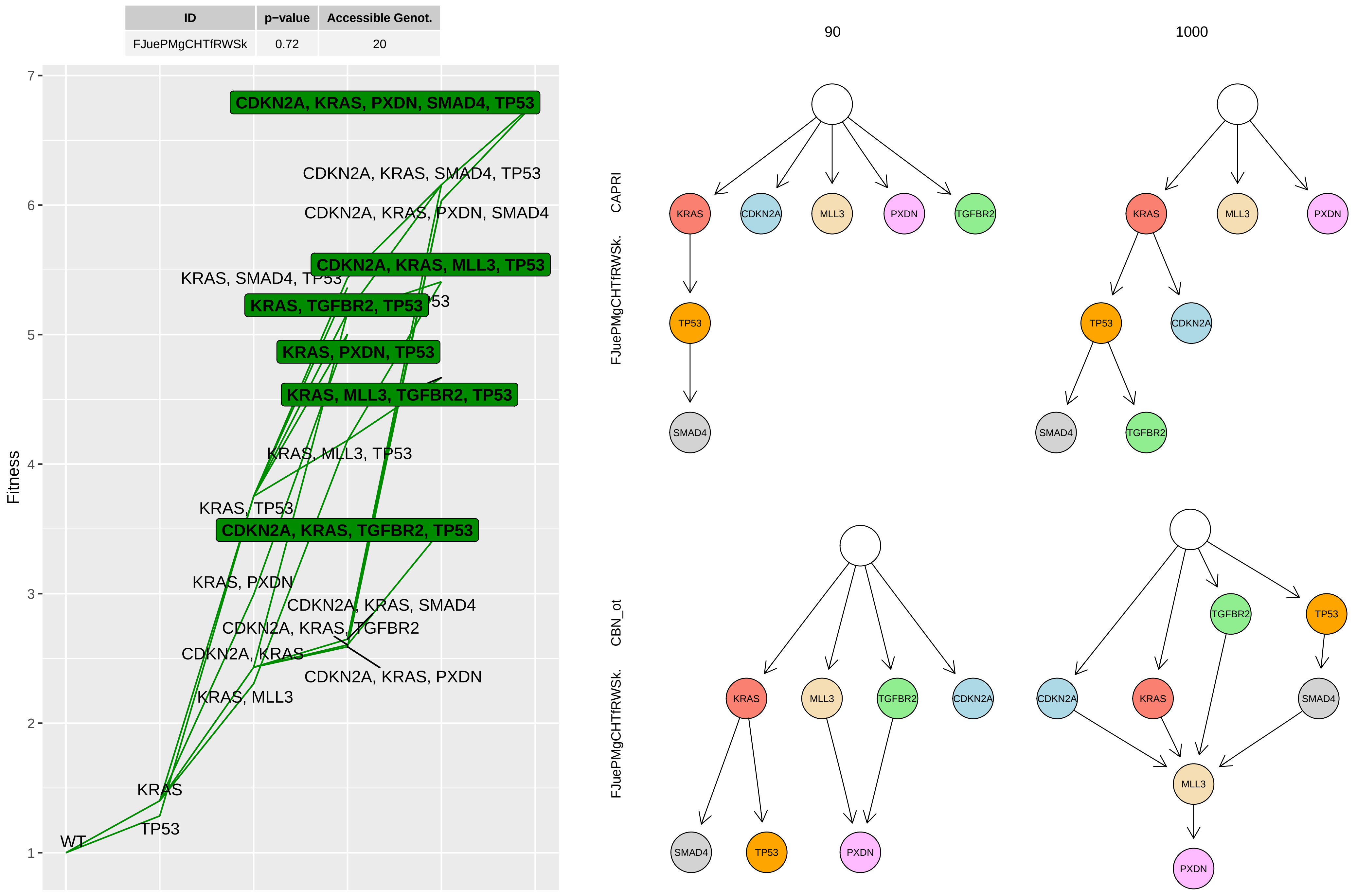

ID
p−value
Accessible Genot.
FJuePMgCHTfRWSk
0.72
20
Fitness
CDKN2A, KRAS, PXDN, SMAD4, TP53
CDKN2A, KRAS, SMAD4, TP53
CDKN2A, KRAS, PXDN, SMAD4
CDKN2A, KRAS, MLL3, TP53
KRAS, SMAD4, TP53
KRAS, TGFBR2, TP53
KRAS, PXDN, TP53
KRAS, MLL3, TGFBR2, TP53
KRAS, MLL3, TP53
KRAS, TP53
CDKN2A, KRAS, TGFBR2, TP53
KRAS, PXDN
CDKN2A, KRAS, SMAD4
CDKN2A, KRAS, TGFBR2
CDKN2A, KRAS
CDKN2A, KRAS, PXDN
KRAS, MLL3
KRAS
TP53
WT
90
1000
FJuePMgCHTfRWSk. CAPRI
FJuePMgCHTfRWSk. CBN_ot
KRAS
CDKN2A
MLL3
PXDN
TGFBR2
TP53
SMAD4

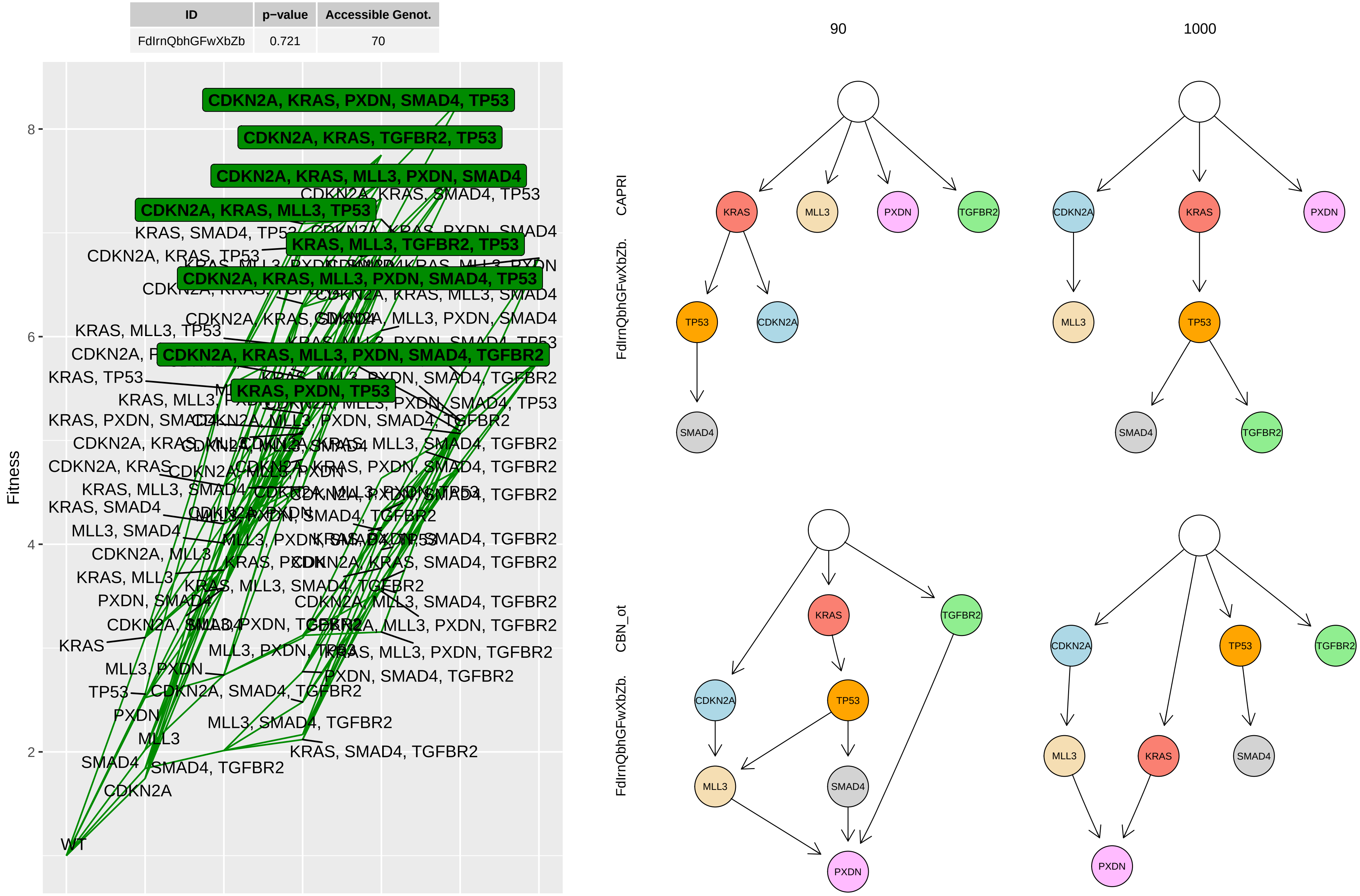

ID
p−value
Accessible Genot.
FdIrnQbhGFwXbZb
0.721
70
Fitness
CDKN2A, KRAS, PXDN, SMAD4, TP53
CDKN2A, KRAS, TGFBR2, TP53
CDKN2A, KRAS, MLL3, PXDN, SMAD4
CDKN2A, KRAS, MLL3, TP53
KRAS, MLL3, TGFBR2, TP53
CDKN2A, KRAS, MLL3, PXDN, SMAD4, TP53
CDKN2A, KRAS, MLL3, PXDN, SMAD4, TGFBR2
KRAS, PXDN, TP53
WT
90
1000
CAPRI
CBN_ot
FdIrnQbhGFwXbZb.
KRAS
MLL3
PXDN
TGFBR2
TP53
CDKN2A
SMAD4

ID	p−value	Accessible Genot.
BYWKTlnRfvlxUTA	0.721	70

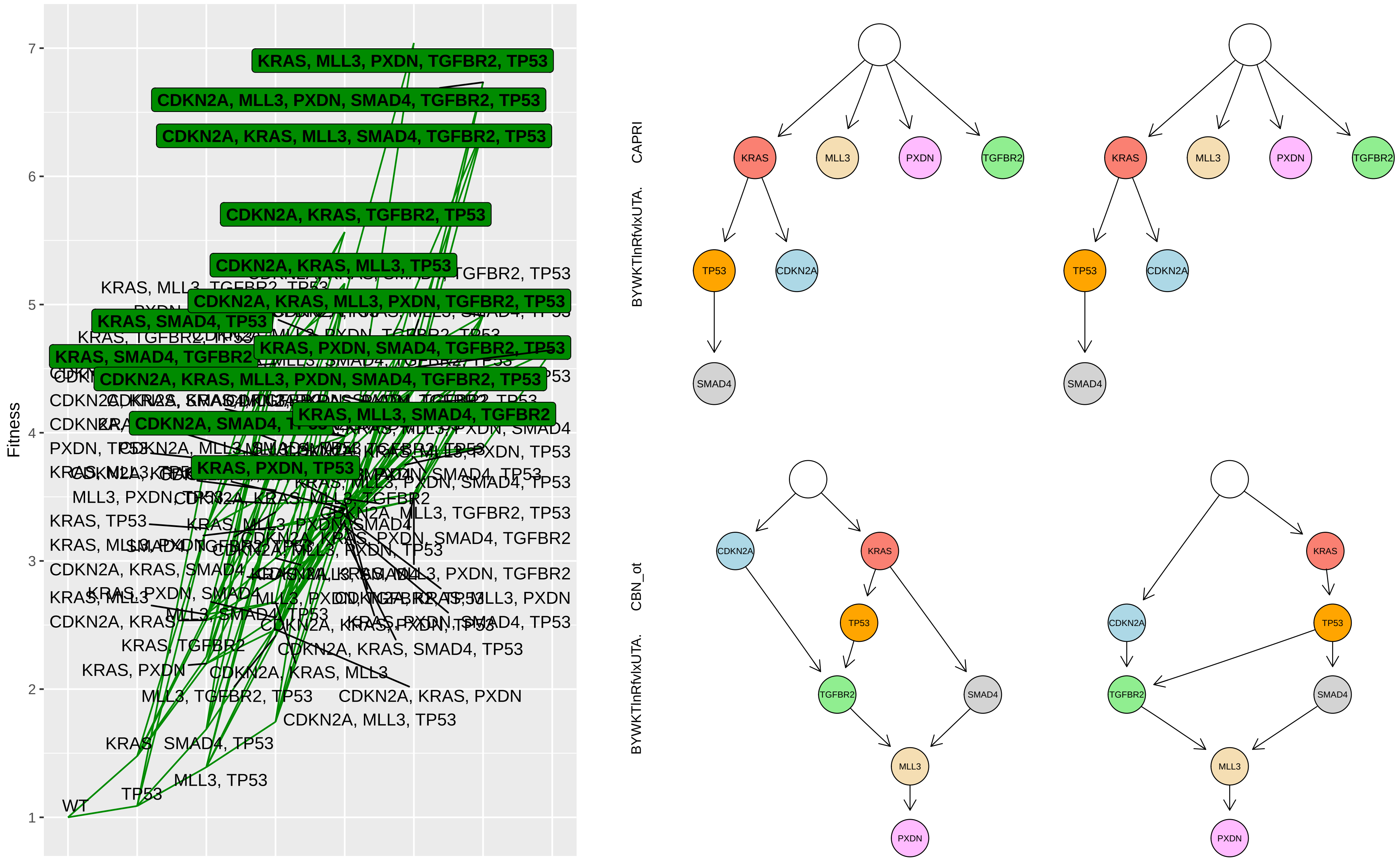

ID	p−value	Accessible Genot.
pEUpEntHIMbebXS	0.724	23

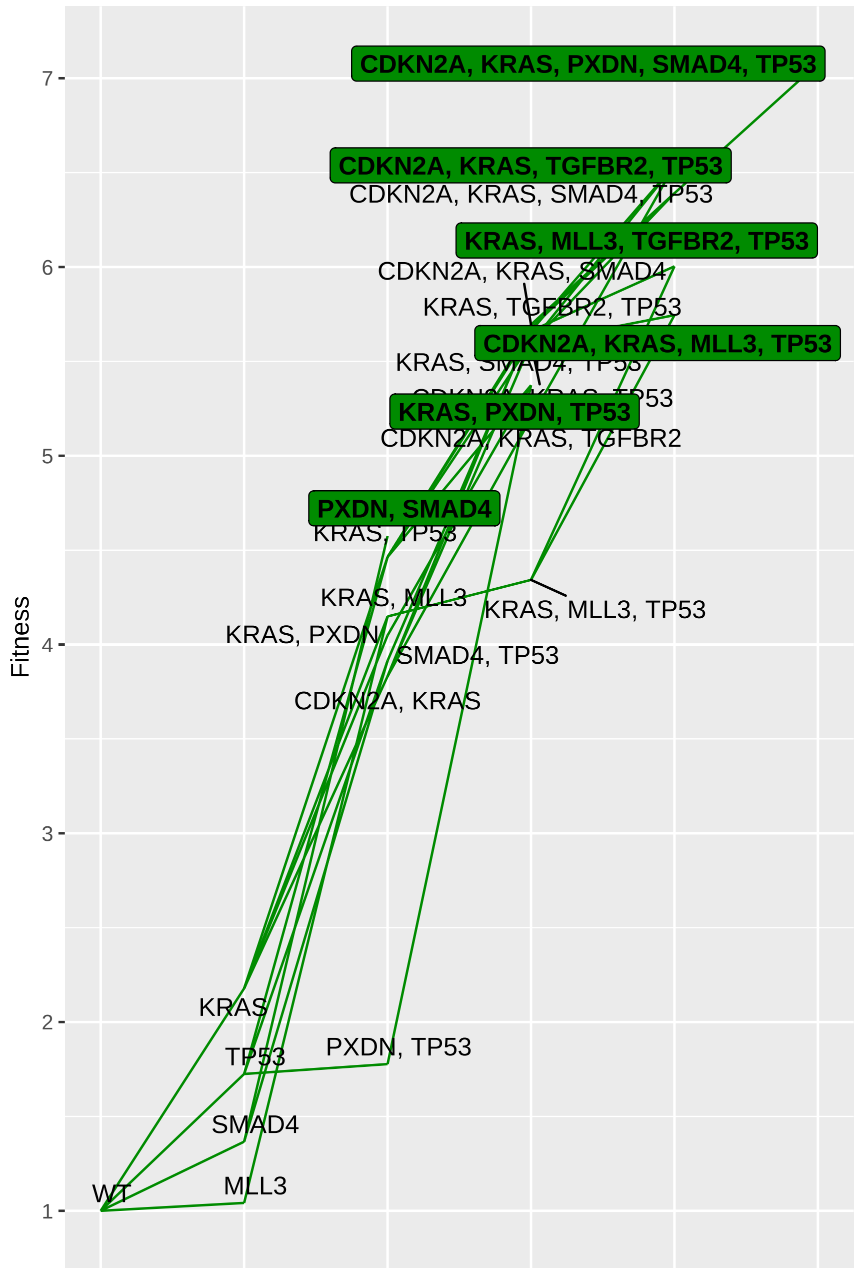

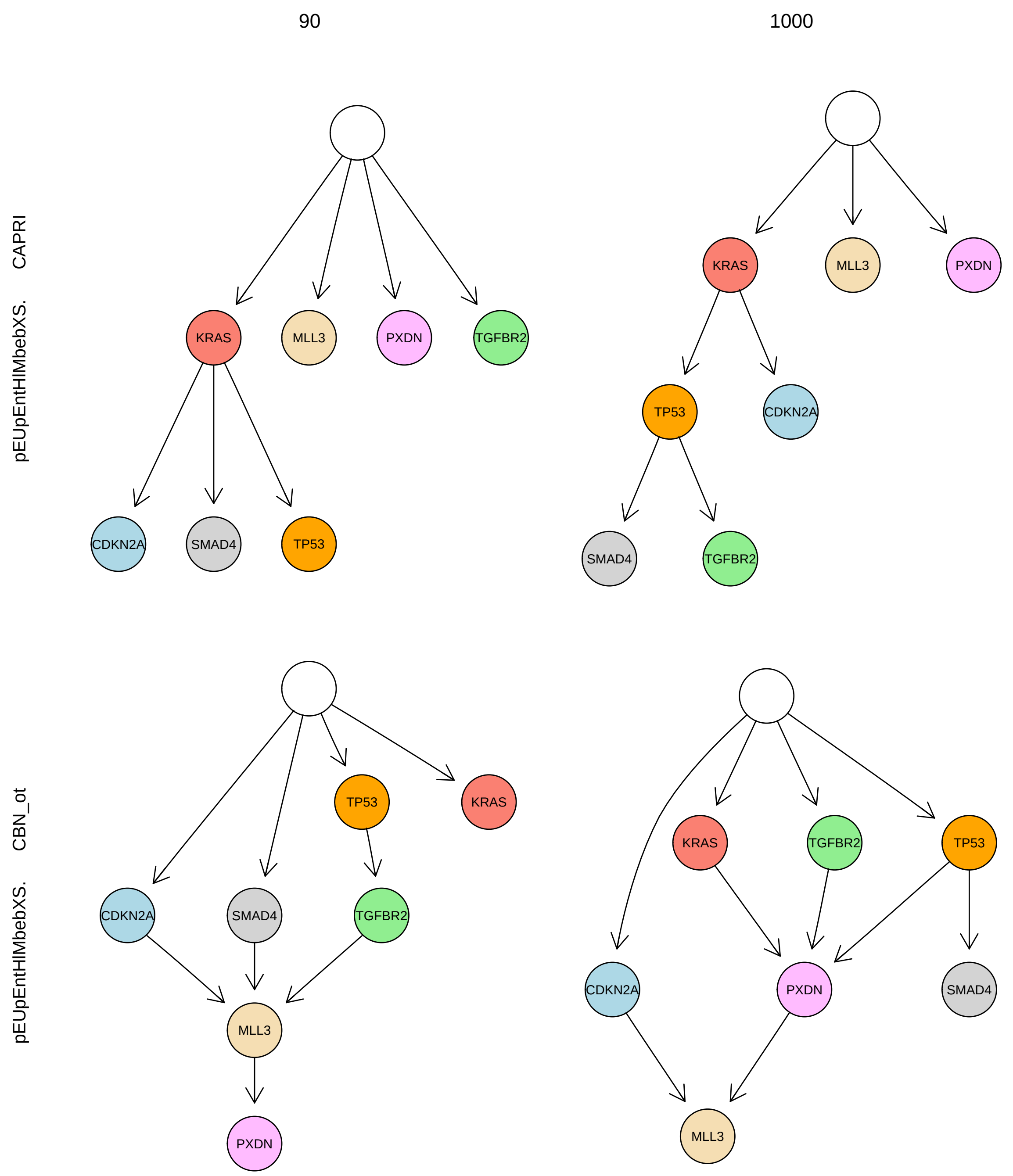

ID	p−value	Accessible Genot.
UDVgLTfODYJadCu	0.726	30

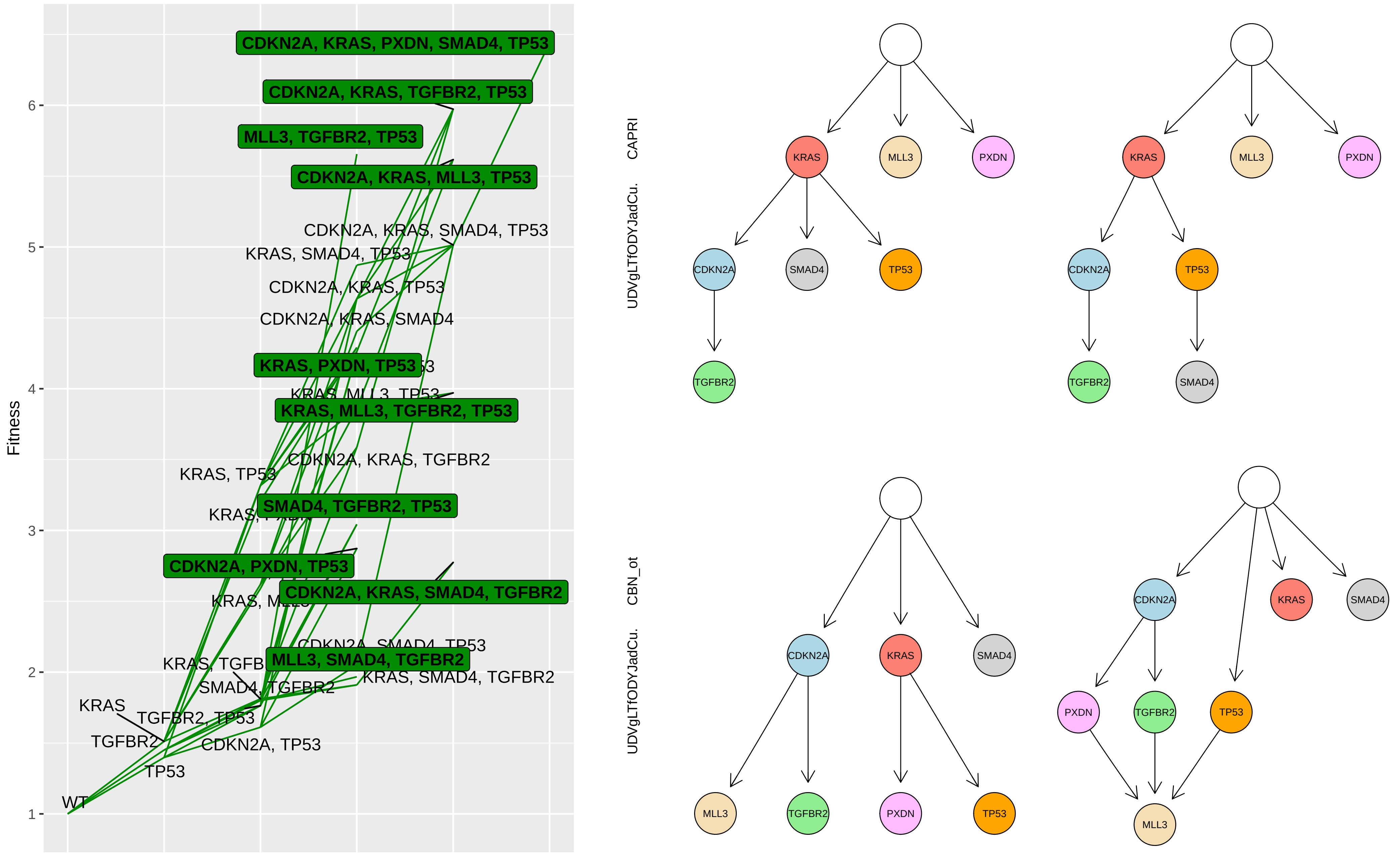

ID	p−value	Accessible Genot.
HPFYPawEYovipZz	0.729	66

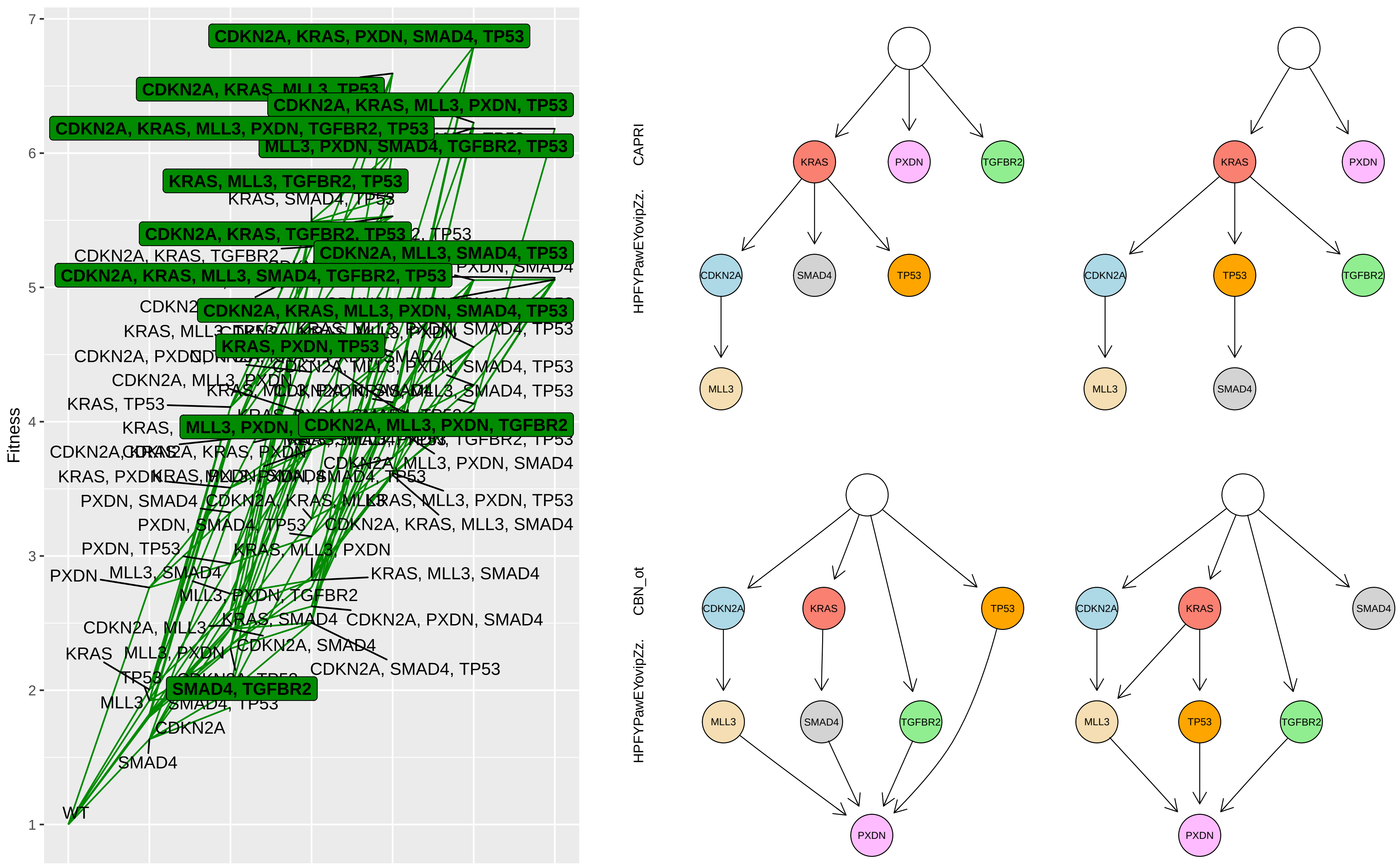

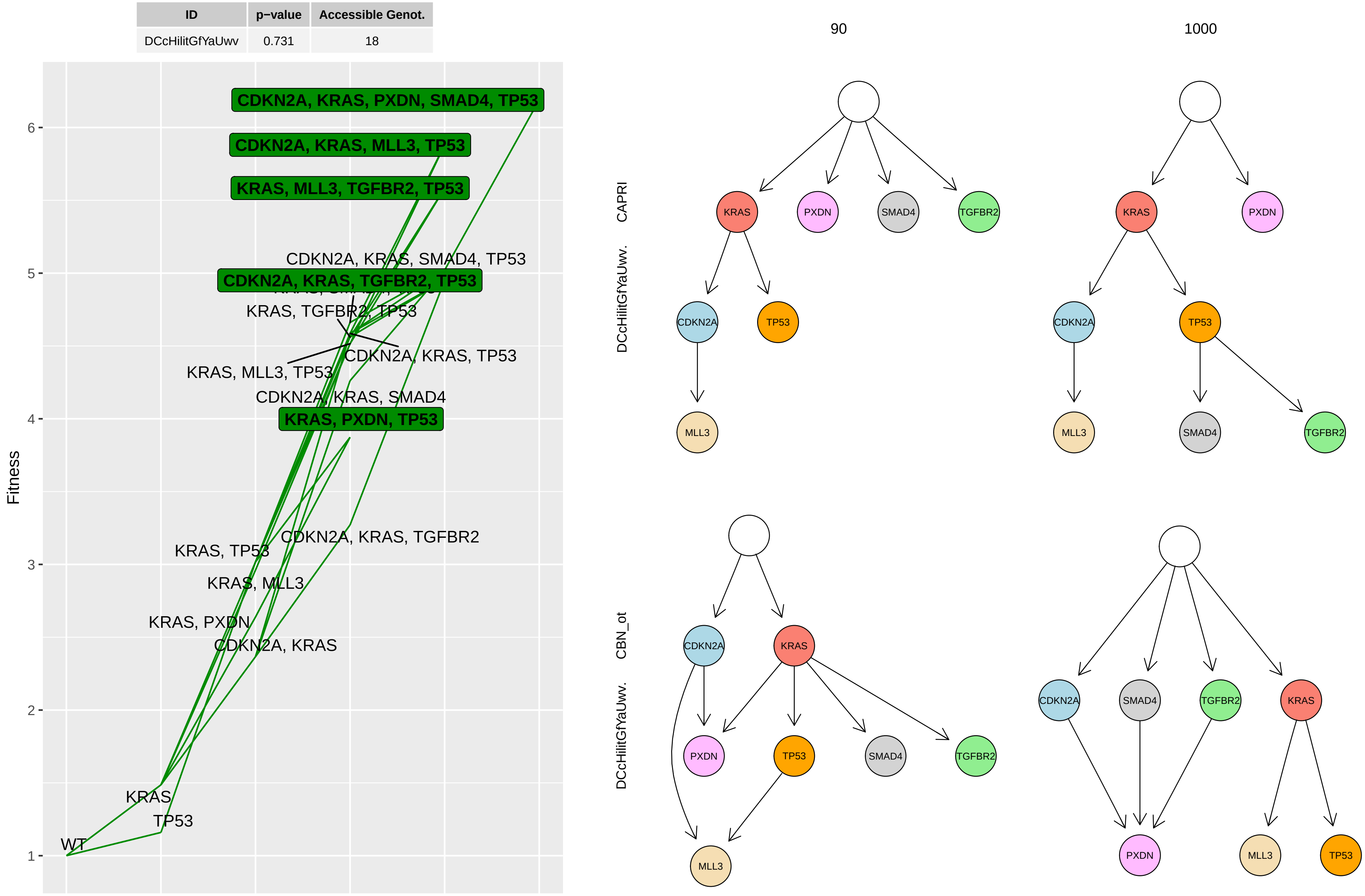

ID
p−value
Accessible Genot.
DCcHilitGfYaUwv
0.731
18
Fitness
CDKN2A, KRAS, PXDN, SMAD4, TP53
CDKN2A, KRAS, MLL3, TP53
KRAS, MLL3, TGFBR2, TP53
CDKN2A, KRAS, SMAD4, TP53
CDKN2A, KRAS, TGFBR2, TP53
KRAS, TGFBR2, TP53
CDKN2A, KRAS, TP53
KRAS, MLL3, TP53
CDKN2A, KRAS, SMAD4
KRAS, PXDN, TP53
CDKN2A, KRAS, TGFBR2
KRAS, TP53
KRAS, MLL3
KRAS, PXDN
CDKN2A, KRAS
KRAS
TP53
WT
90
1000
DCcHilitGfYaUwv. CAPRI
DCcHilitGfYaUwv. CBN_ot
KRAS
PXDN
SMAD4
TGFBR2
CDKN2A
TP53
MLL3

ID	p−value	Accessible Genot.
fsiECeCUVxEAlRu	0.732	19

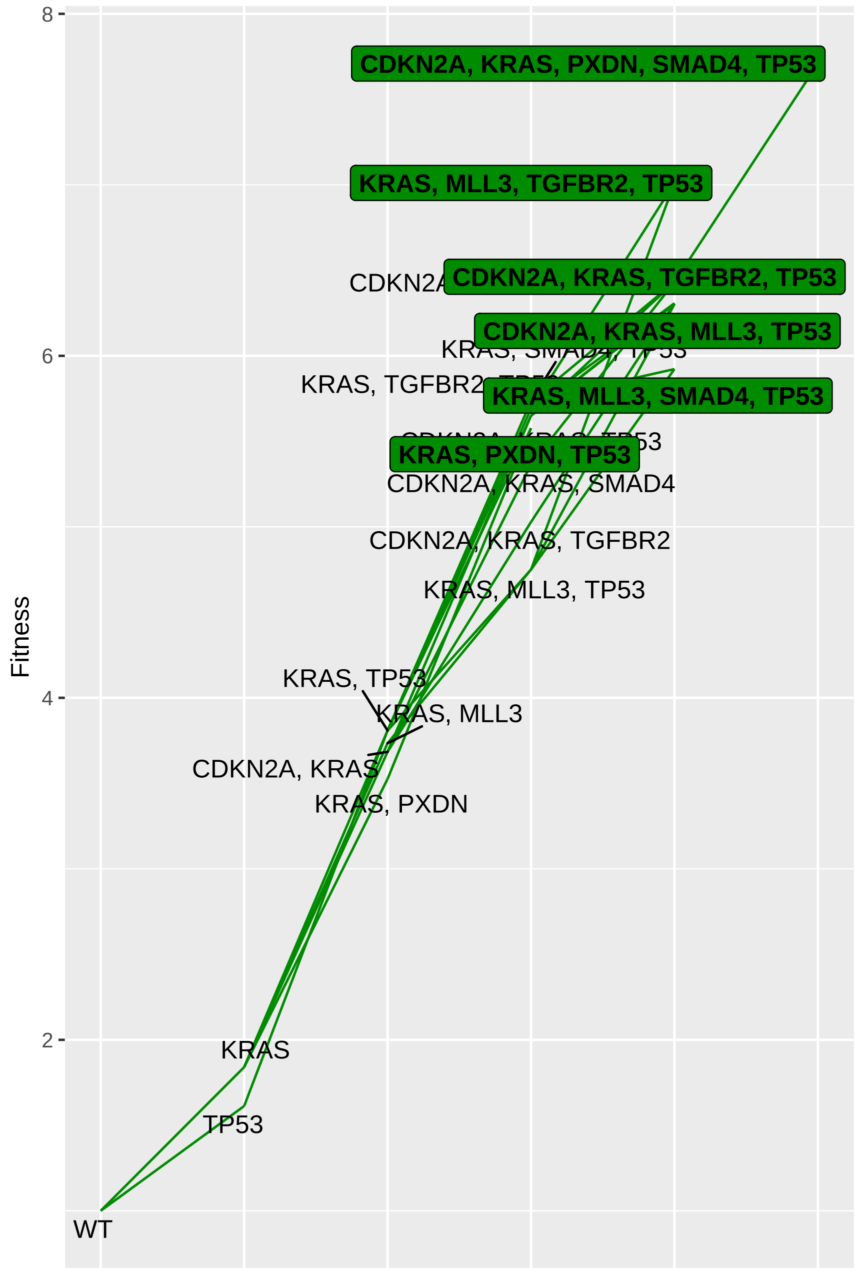

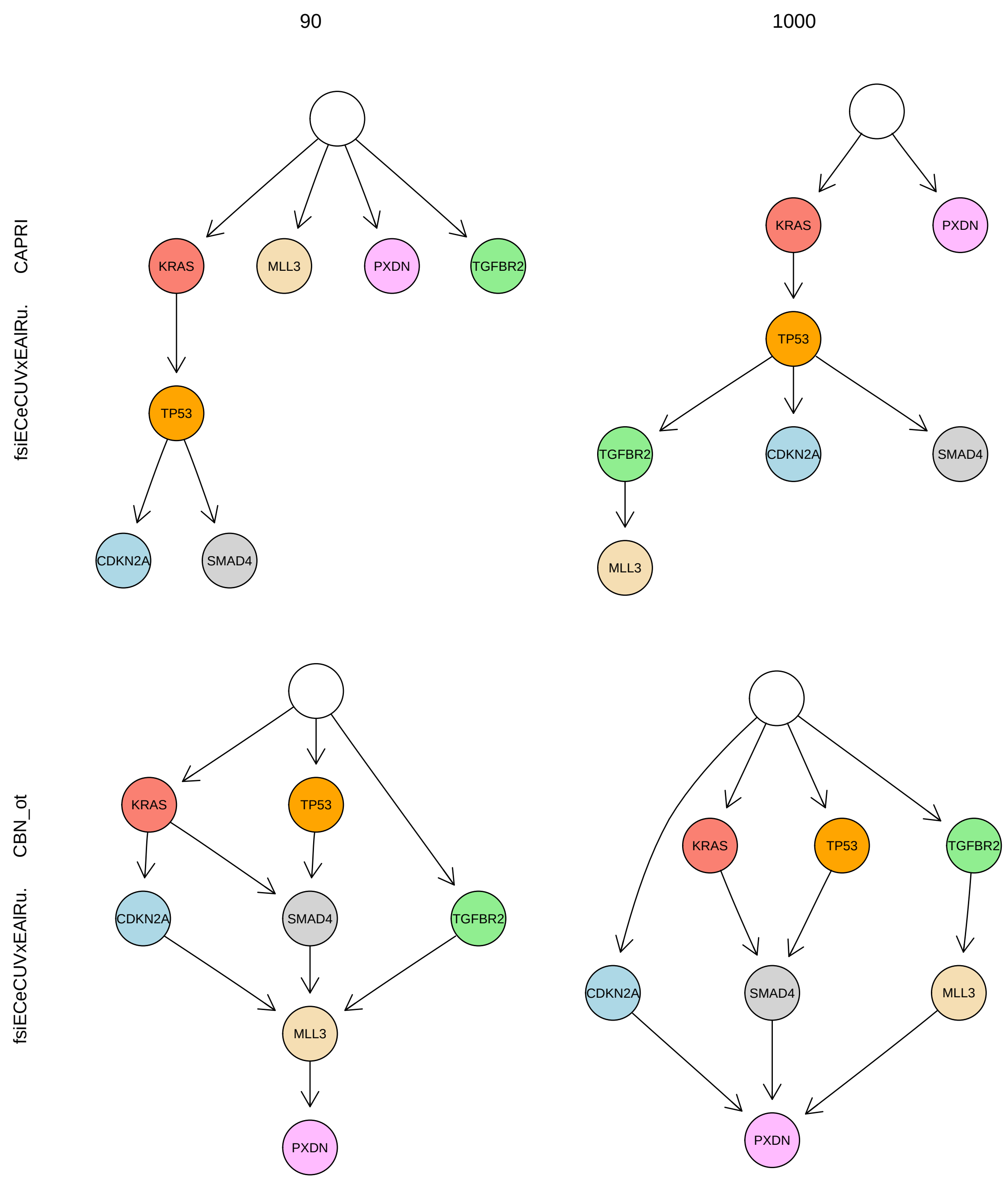

ID	p−value	Accessible Genot.
oRMoDVzrYrZkgTo	0.738	24

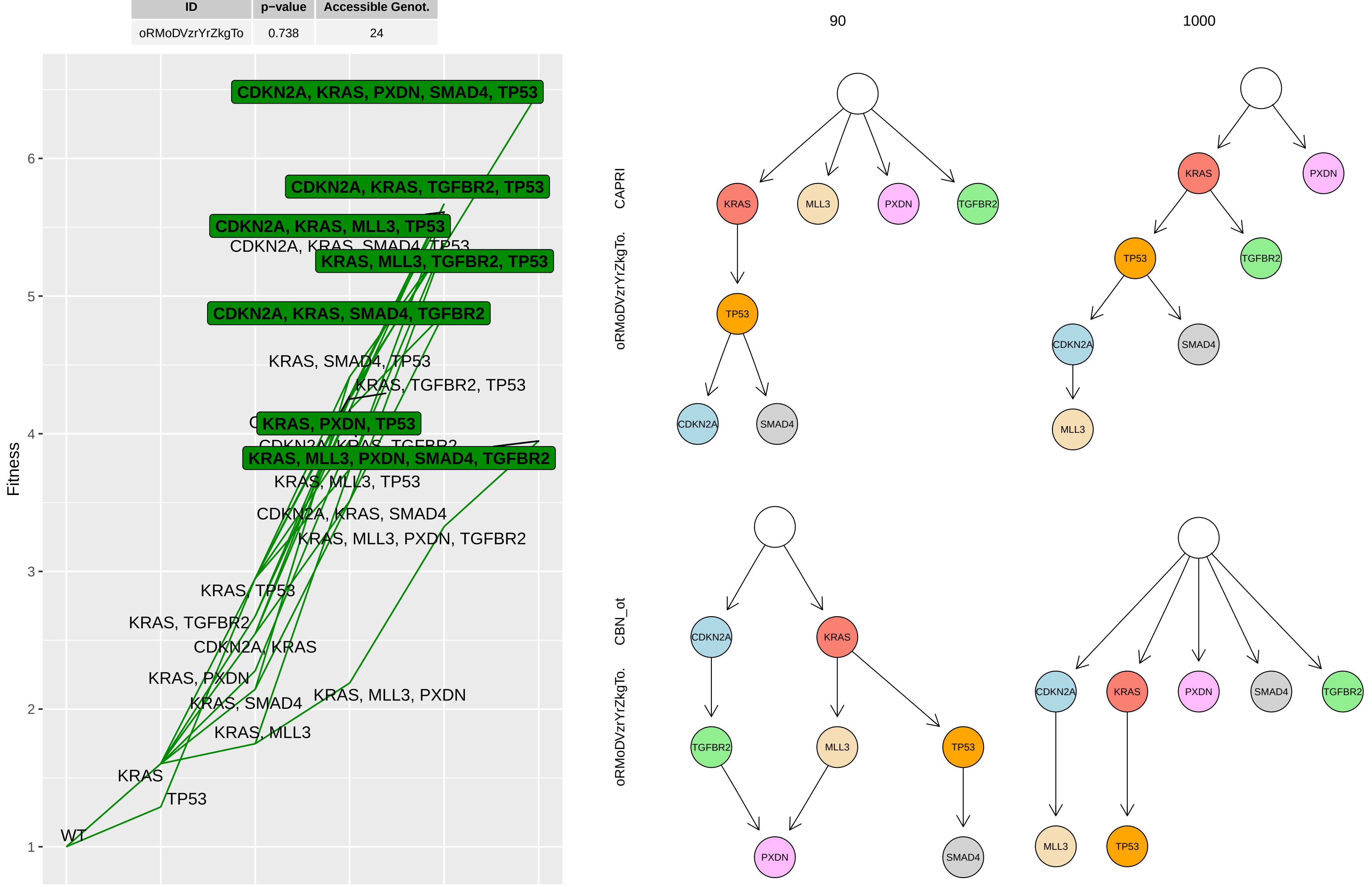

ID	p−value	Accessible Genot.
nyzVmOoHQVkGFkZ	0.739	35

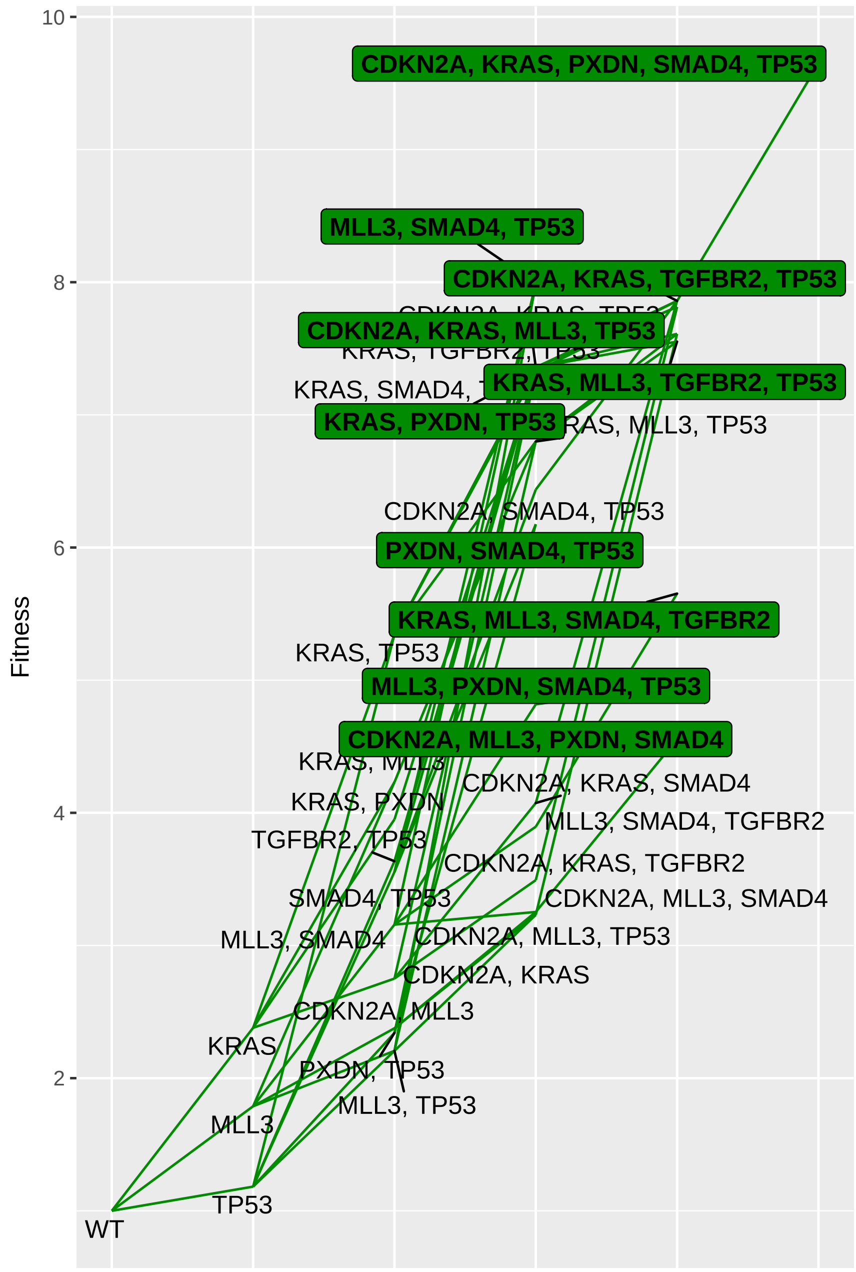

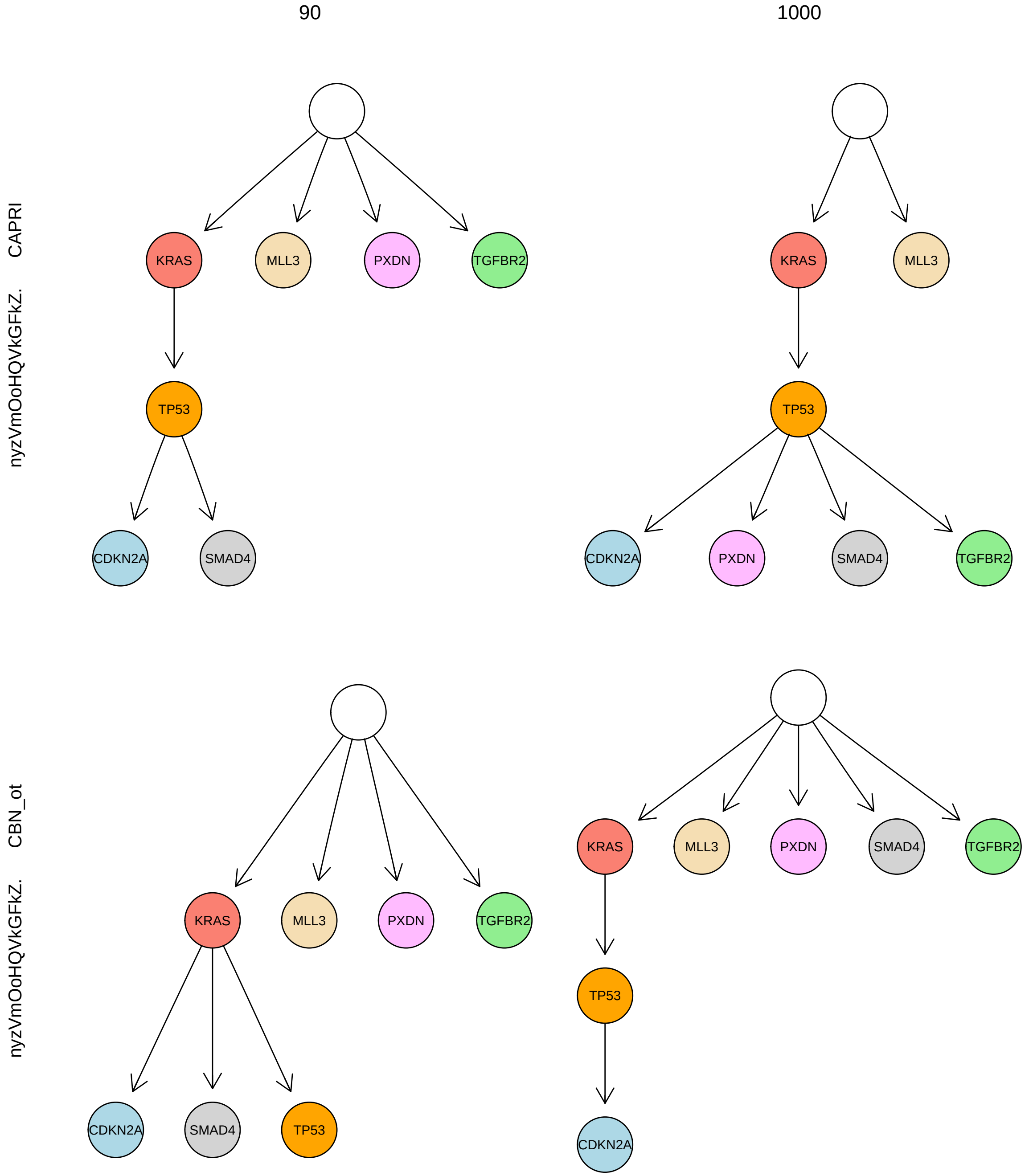

ID	p−value	Accessible Genot.
YZptEKkxltDedzo	0.741	61

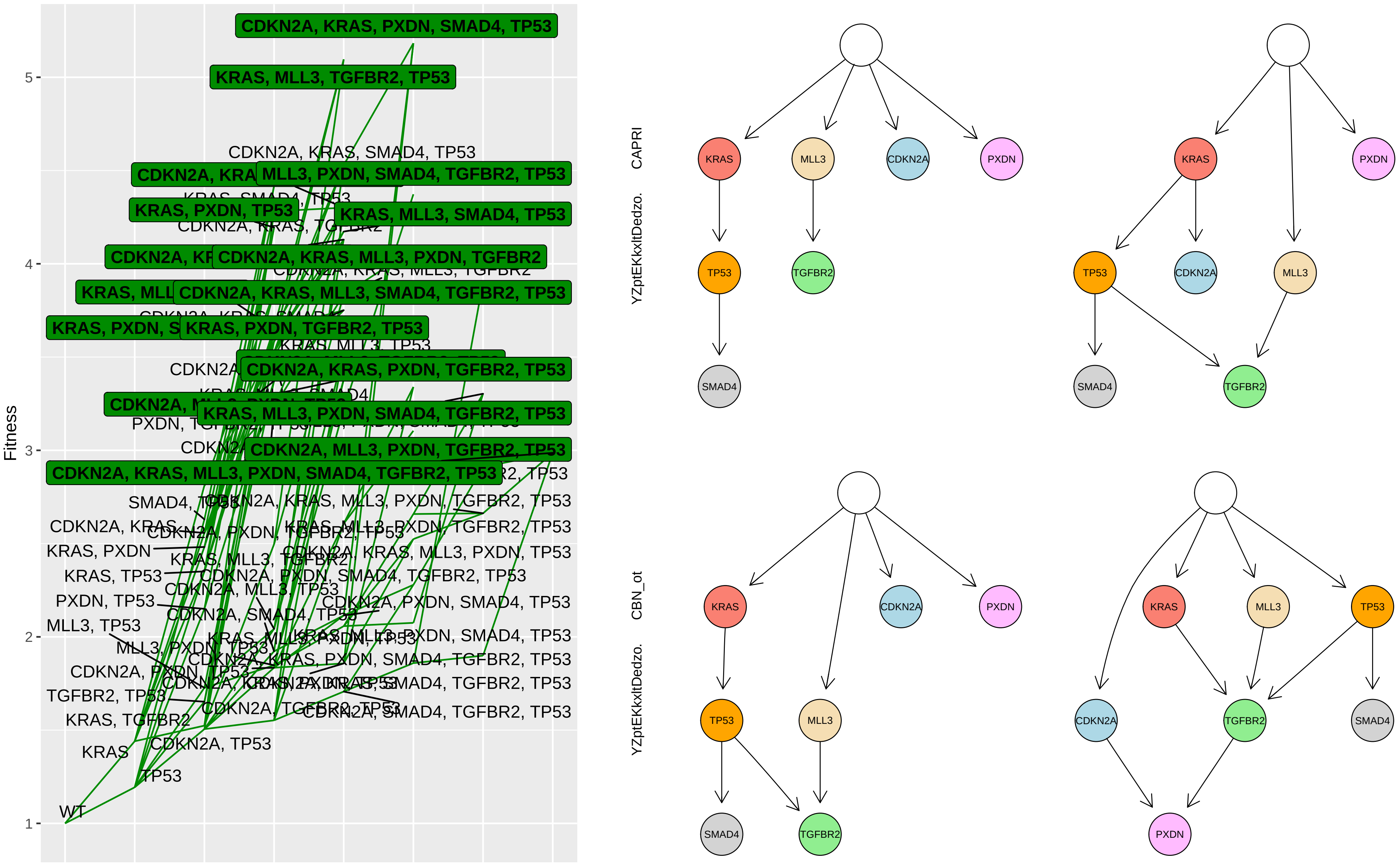

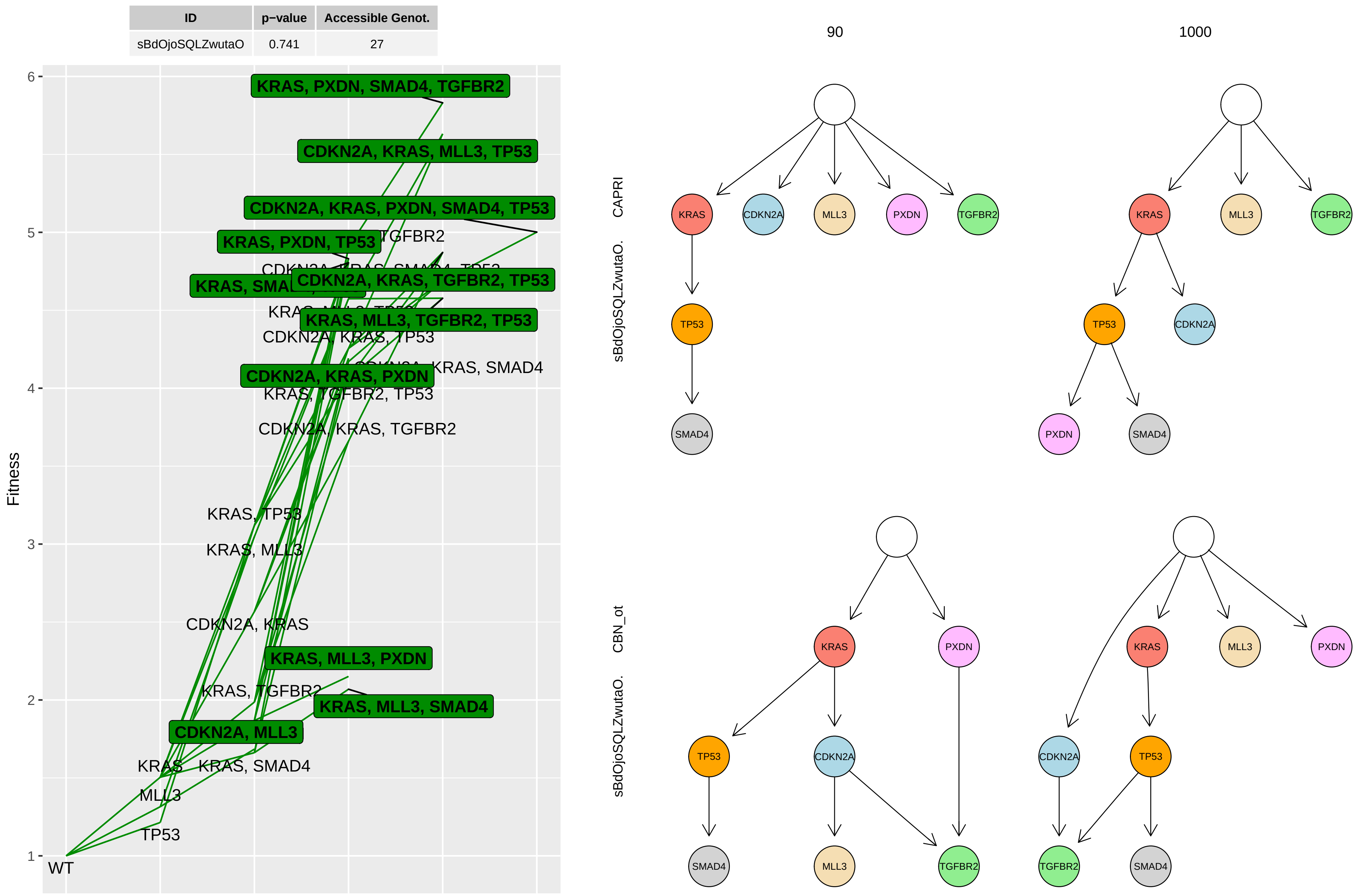

ID
p−value
Accessible Genot.
sBdOjoSQLZwutaO
0.741
27
Fitness
KRAS, PXDN, SMAD4, TGFBR2
CDKN2A, KRAS, MLL3, TP53
CDKN2A, KRAS, PXDN, SMAD4, TP53
KRAS, PXDN, TP53
CDKN2A, KRAS, TGFBR2, TP53
KRAS, MLL3, TGFBR2, TP53
CDKN2A, KRAS, TP53
CDKN2A, KRAS, PXDN
KRAS, TGFBR2, TP53
CDKN2A, KRAS, TGFBR2
KRAS, TP53
KRAS, MLL3
CDKN2A, KRAS
KRAS, MLL3, PXDN
KRAS, TGFBR2
KRAS, MLL3, SMAD4
CDKN2A, MLL3
KRAS
KRAS, SMAD4
MLL3
TP53
WT
90
1000
CAPRI
CBN_ot
sBdOjoSQLZwutaO.
KRAS
CDKN2A
MLL3
PXDN
TGFBR2
TP53
SMAD4

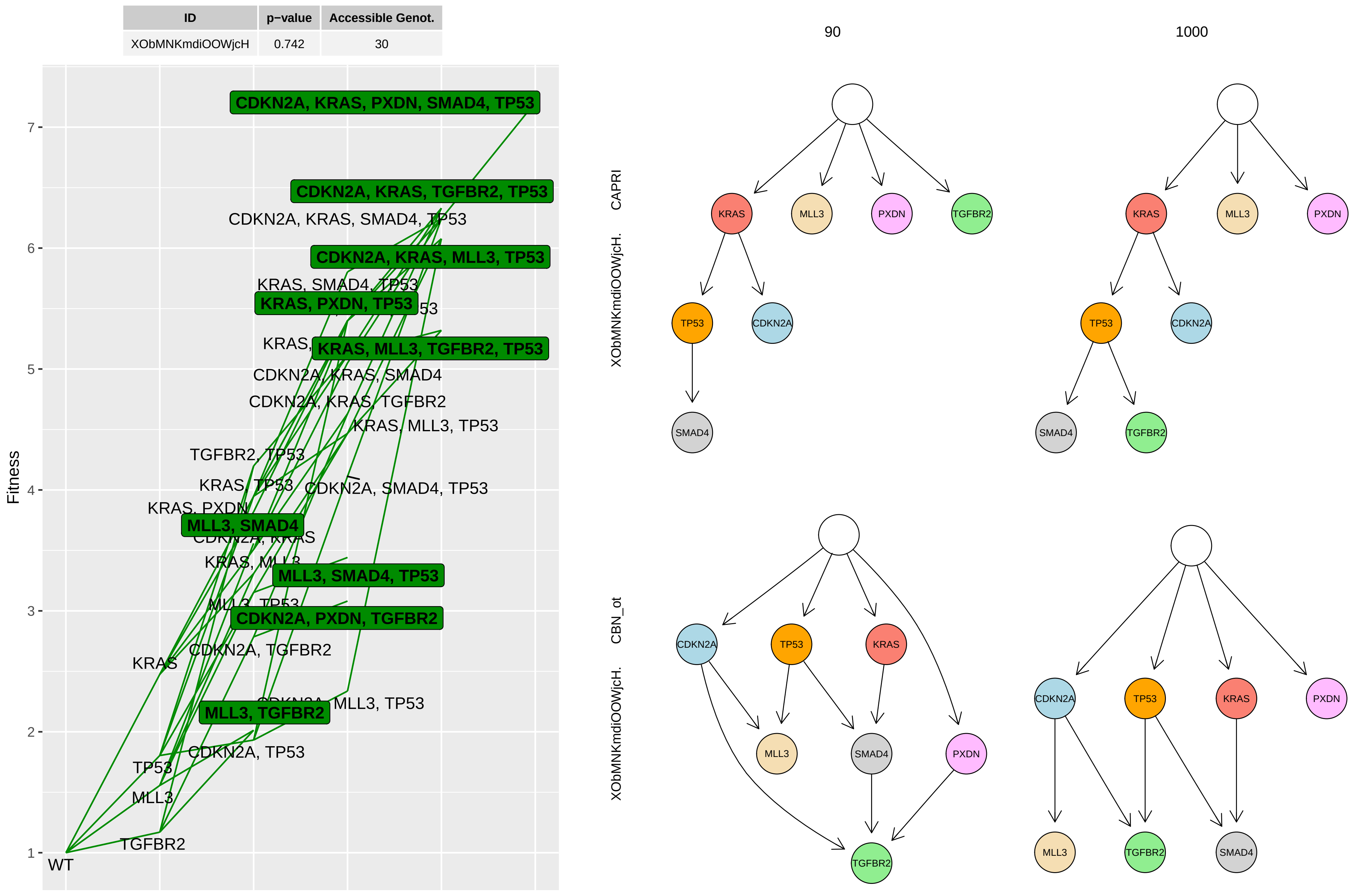
ID
p−value
Accessible Genot.
XObMNKmdiOOWjcH
0.742
30
Fitness
CDKN2A, KRAS, PXDN, SMAD4, TP53
CDKN2A, KRAS, TGFBR2, TP53
CDKN2A, KRAS, SMAD4, TP53
CDKN2A, KRAS, MLL3, TP53
KRAS, SMAD4, TP53
KRAS, PXDN, TP53
KRAS, MLL3, TGFBR2, TP53
CDKN2A, KRAS, SMAD4
CDKN2A, KRAS, TGFBR2
KRAS, MLL3, TP53
TGFBR2, TP53
KRAS, TP53
CDKN2A, SMAD4, TP53
KRAS, PXDN
MLL3, SMAD4
MLL3, SMAD4, TP53
CDKN2A, PXDN, TGFBR2
KRAS
CDKN2A, TGFBR2
MLL3, TP53
MLL3, TGFBR2
CDKN2A, TP53
TP53
MLL3
TGFBR2
WT
90
1000
CAPRI
CBN_ot
XObMNKmdiOOWjcH.
KRAS
MLL3
PXDN
TGFBR2
TP53
CDKN2A
SMAD4

ID	p−value	Accessible Genot.
LBQAbxorwpWUvDN	0.742	122

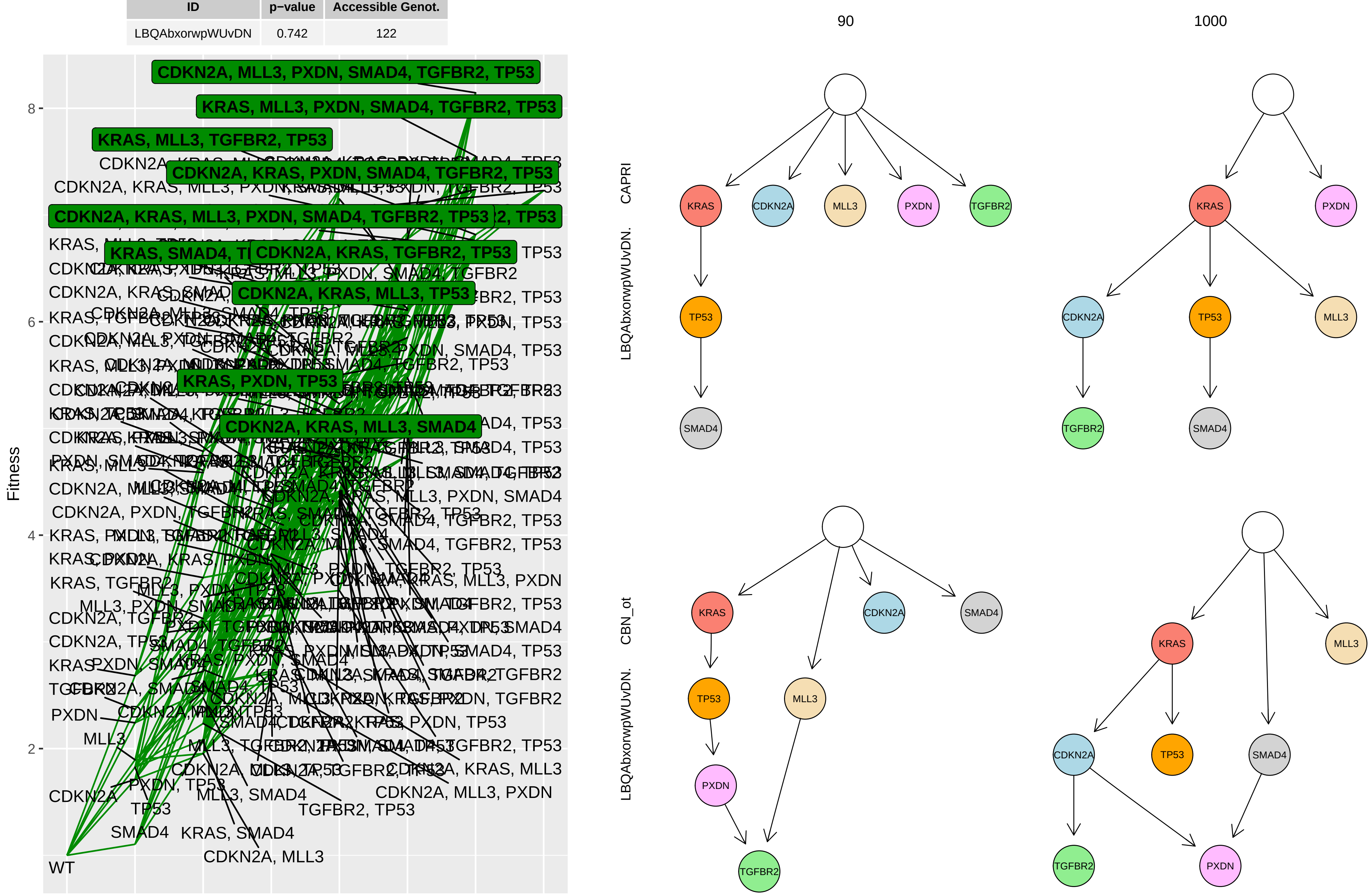

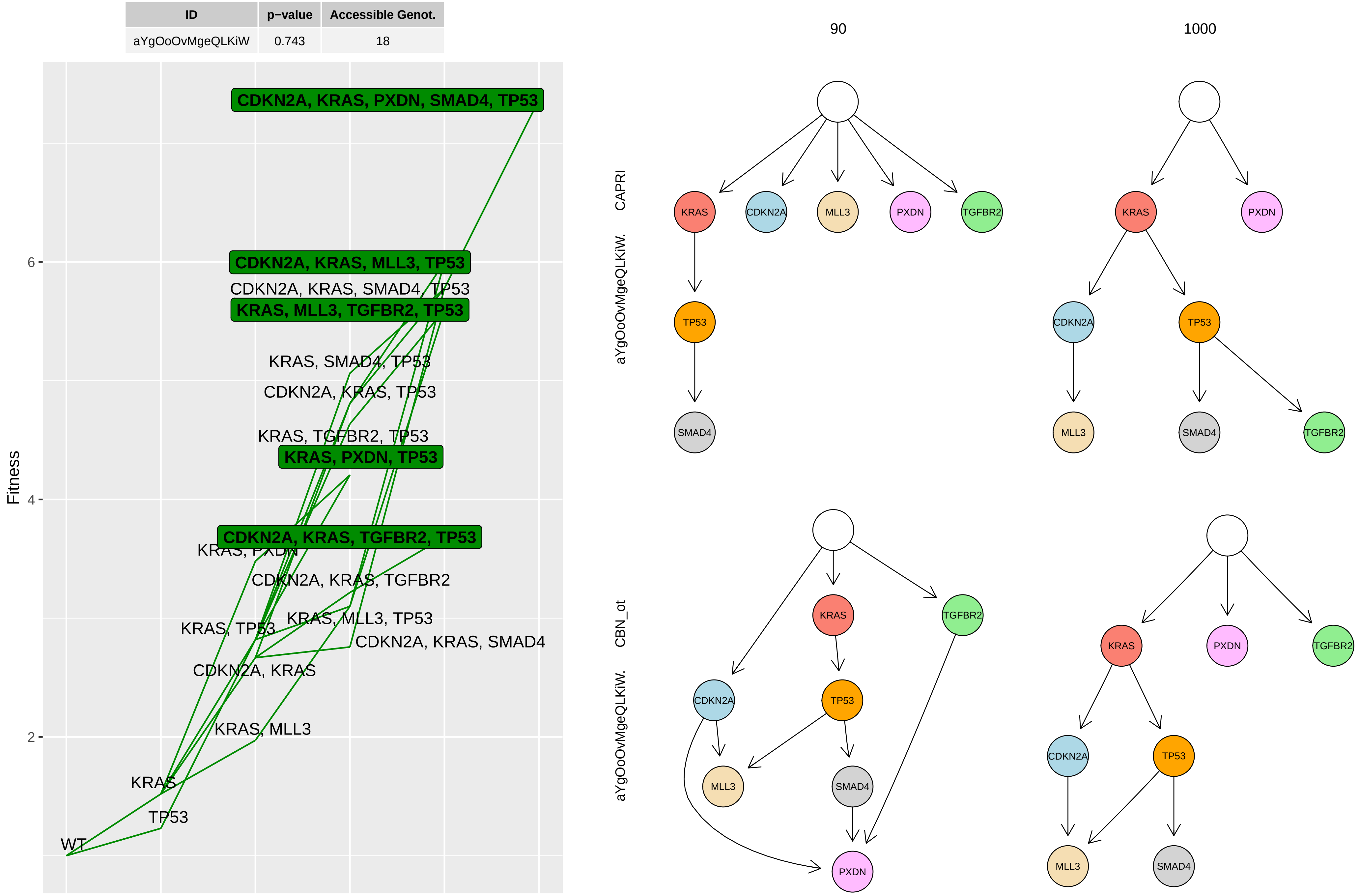
ID
p−value
Accessible Genot.
aYgOoOvMgeQLKiW
0.743
18
Fitness
6
4
2
CDKN2A, KRAS, PXDN, SMAD4, TP53
CDKN2A, KRAS, MLL3, TP53
CDKN2A, KRAS, SMAD4, TP53
KRAS, MLL3, TGFBR2, TP53
KRAS, SMAD4, TP53
CDKN2A, KRAS, TP53
KRAS, TGFBR2, TP53
KRAS, PXDN, TP53
CDKN2A, KRAS, TGFBR2, TP53
KRAS, PXDN
CDKN2A, KRAS, TGFBR2
KRAS, MLL3, TP53
KRAS, TP53
CDKN2A, KRAS, SMAD4
CDKN2A, KRAS
KRAS, MLL3
KRAS
TP53
WT
90
1000
aYgOoOvMgeQLKiW. CAPRI
aYgOoOvMgeQLKiW. CBN_ot
KRAS
CDKN2A
MLL3
PXDN
TGFBR2
TP53
SMAD4

ID	p−value	Accessible Genot.
AnVoQXZmJJszPVH	0.744	25

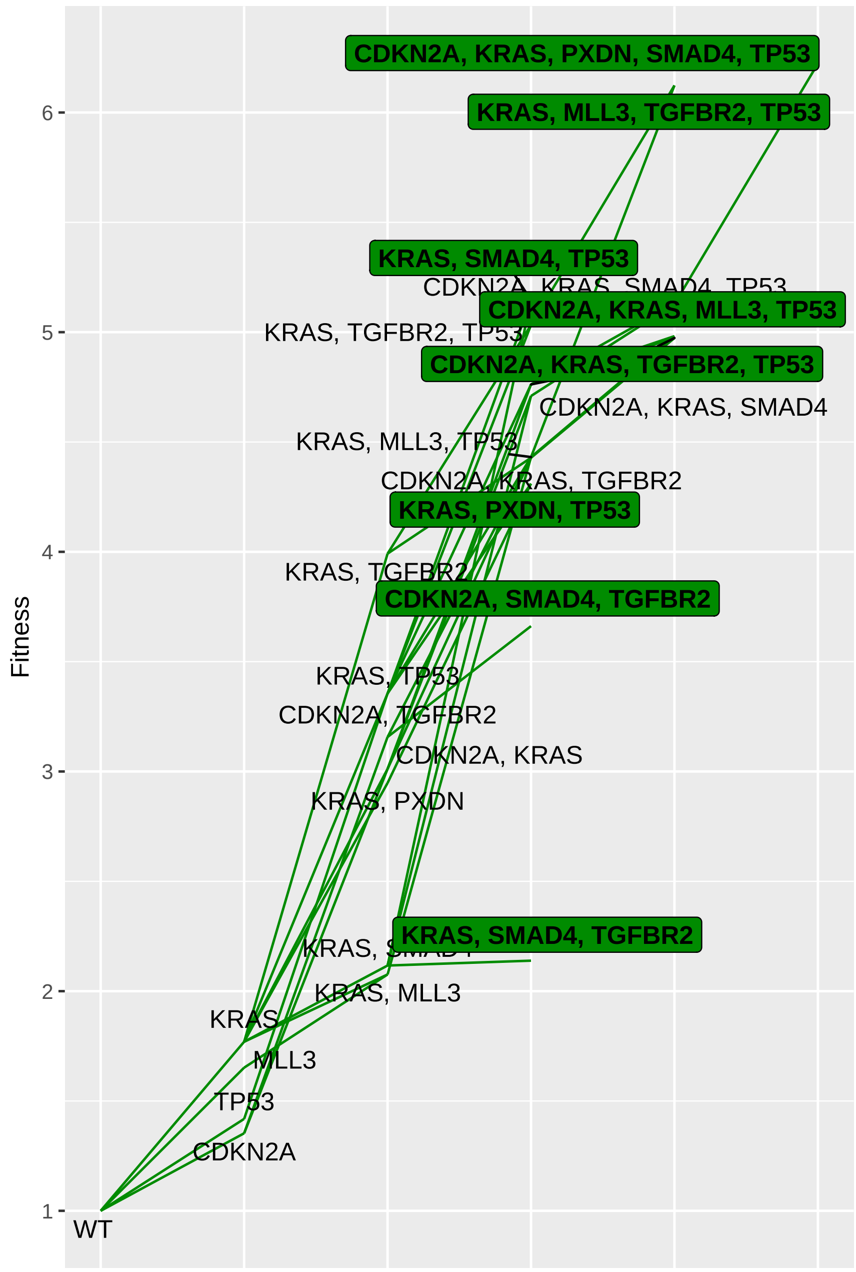

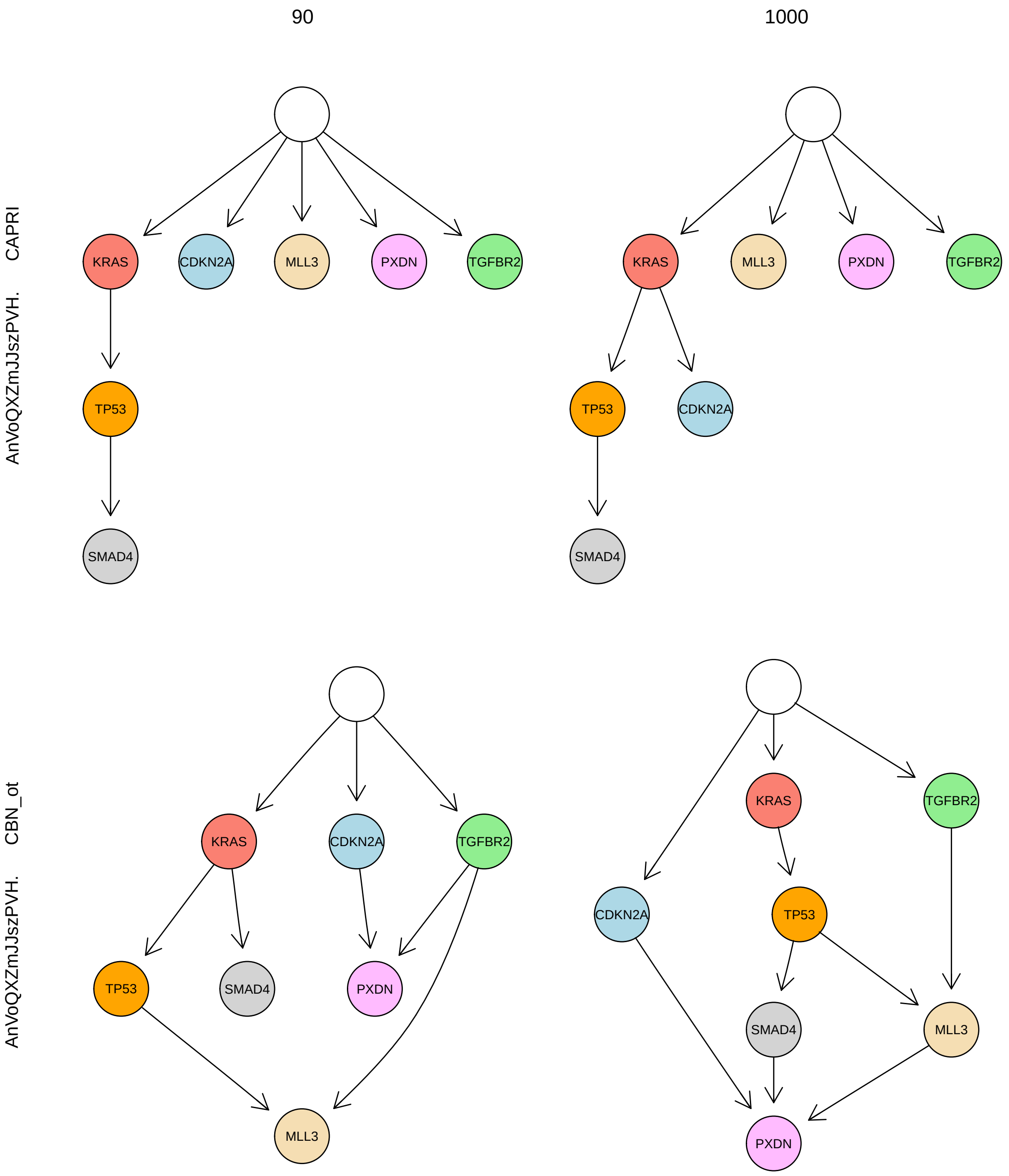

ID	p-value	Accessible Genot.
UlEOholloMpzsbs	0.746	19

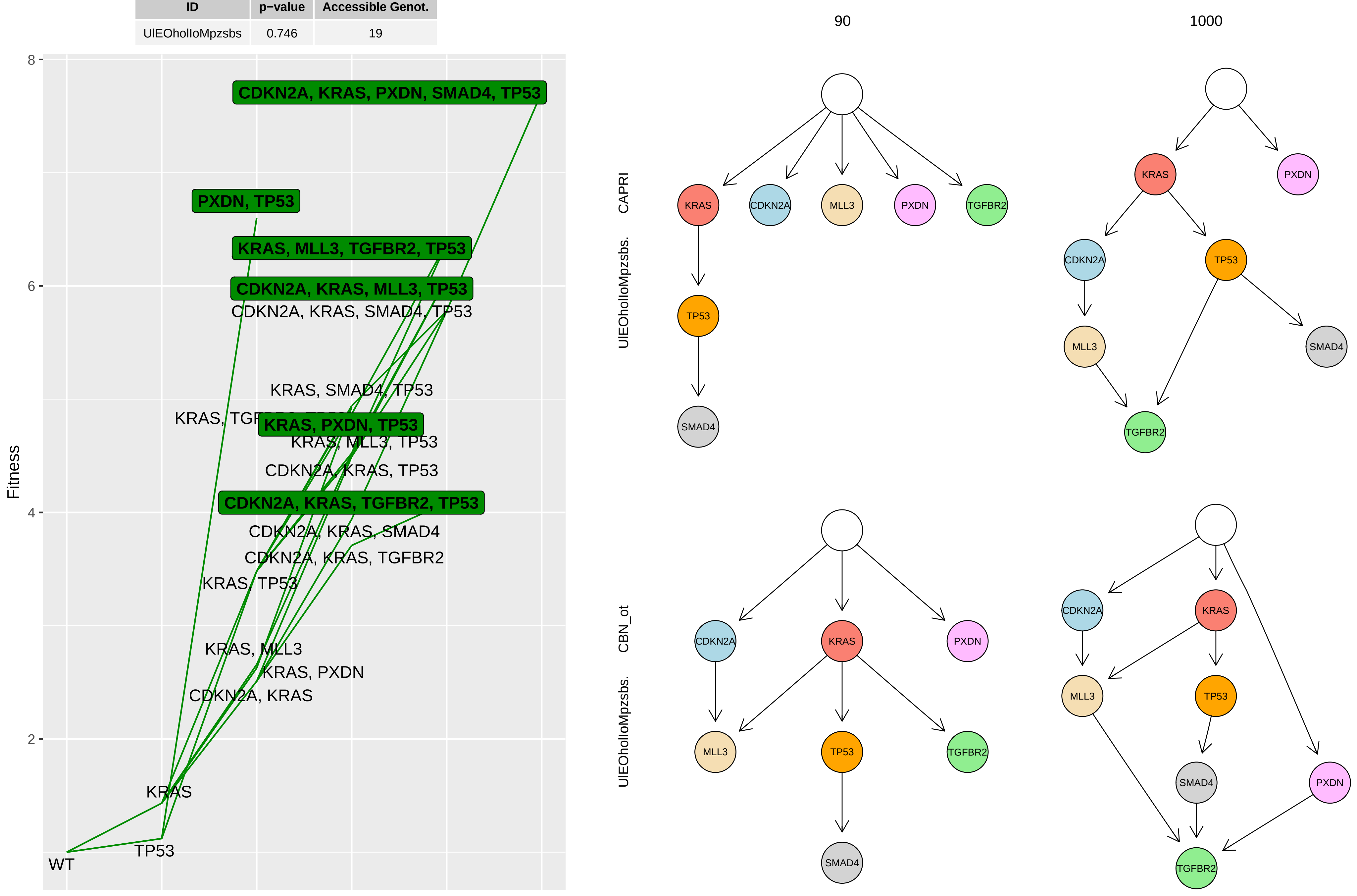

ID	p−value	Accessible Genot.
qLApCxSAjYTHSVA	0.747	71

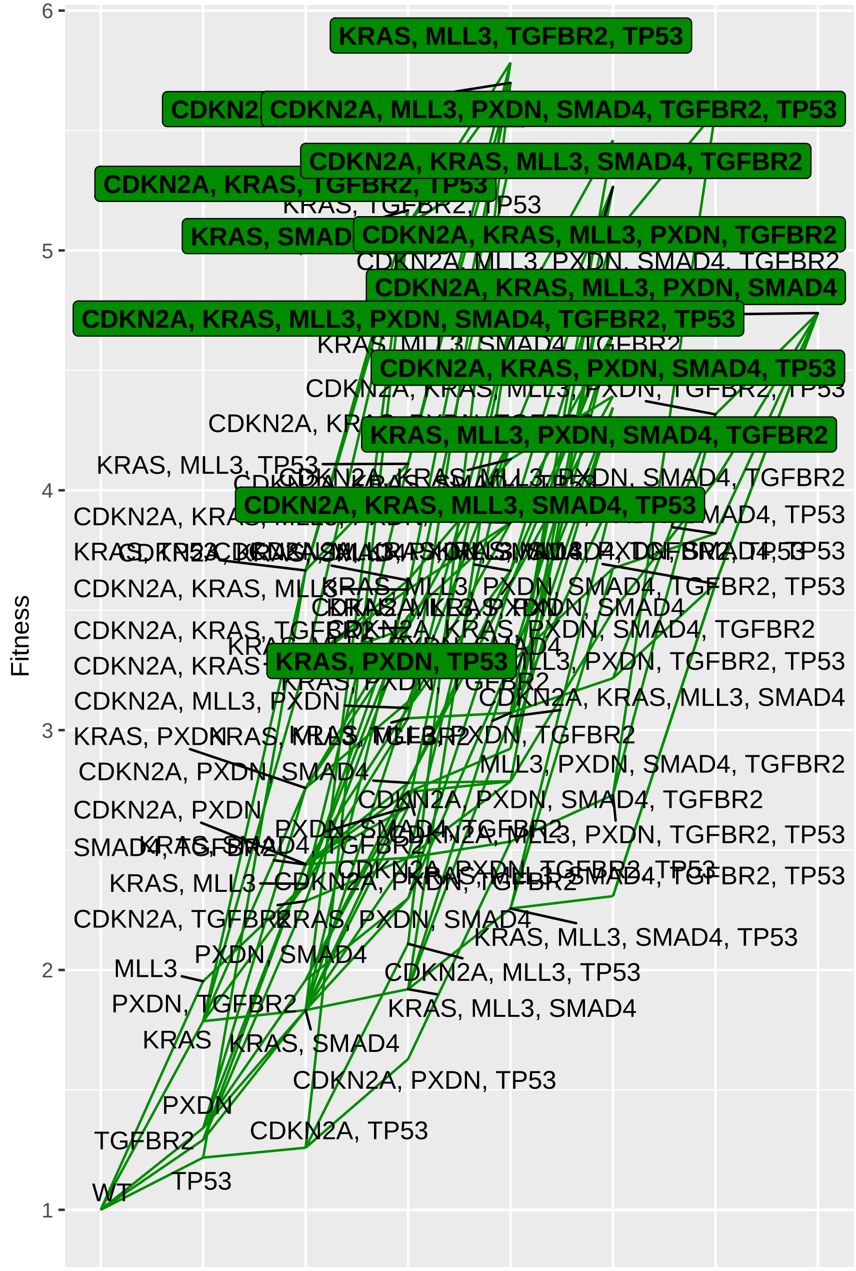

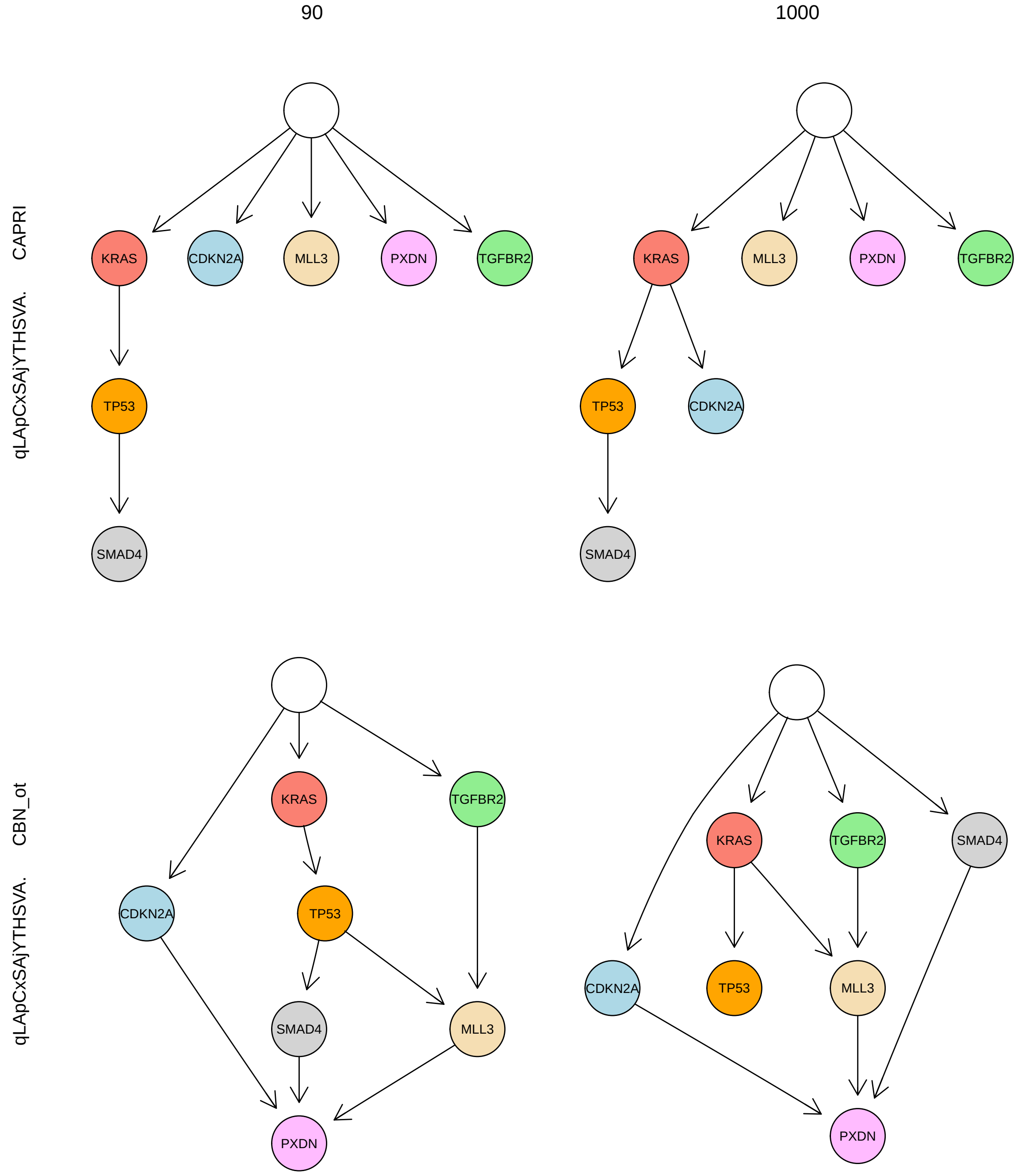

ID	p−value	Accessible Genot.
cMFFnxwXORFvfSX	0.747	18

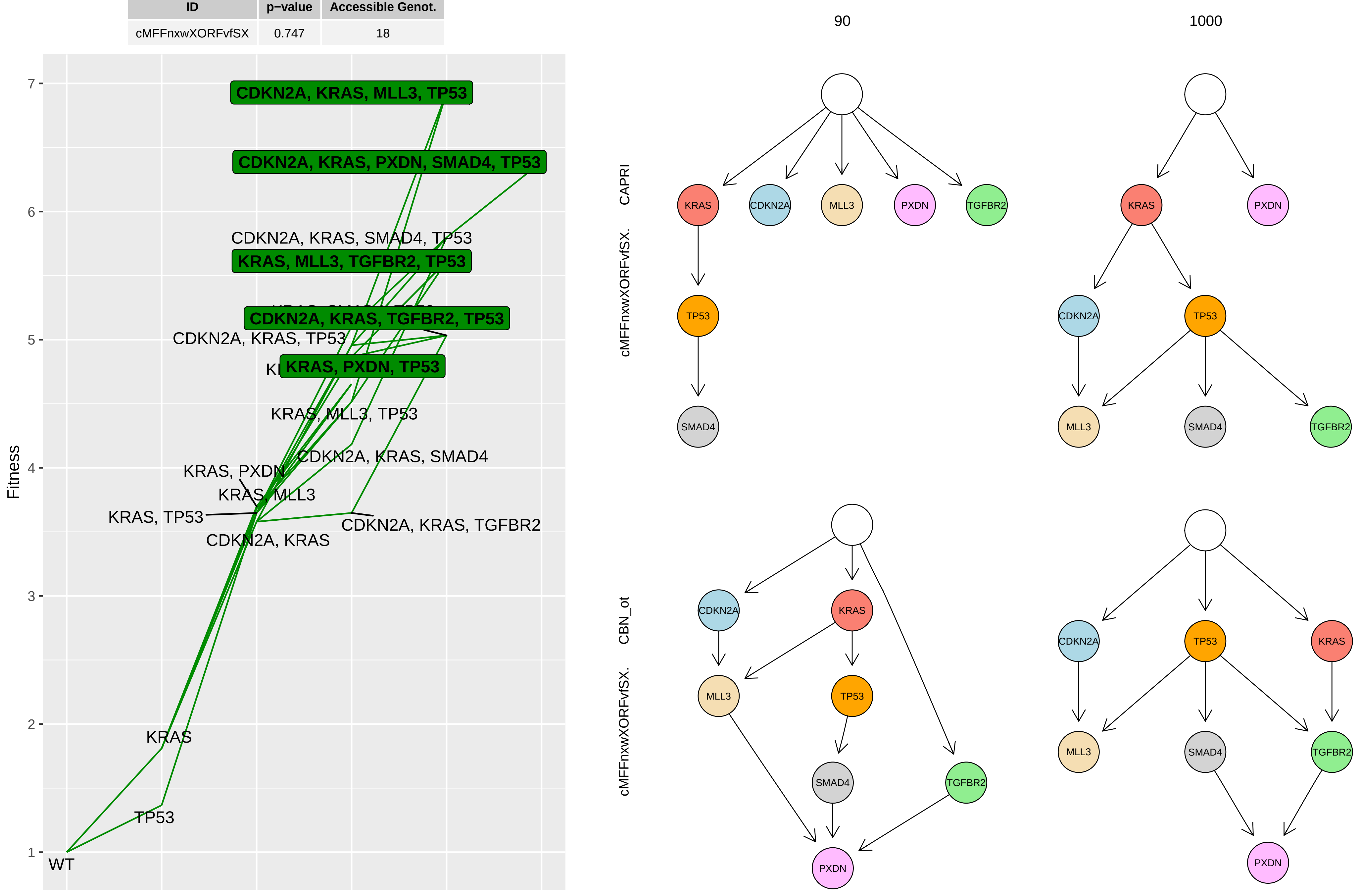

ID	p-value	Accessible Genot.
XhOFvlXUNEZBJrH	0.748	19

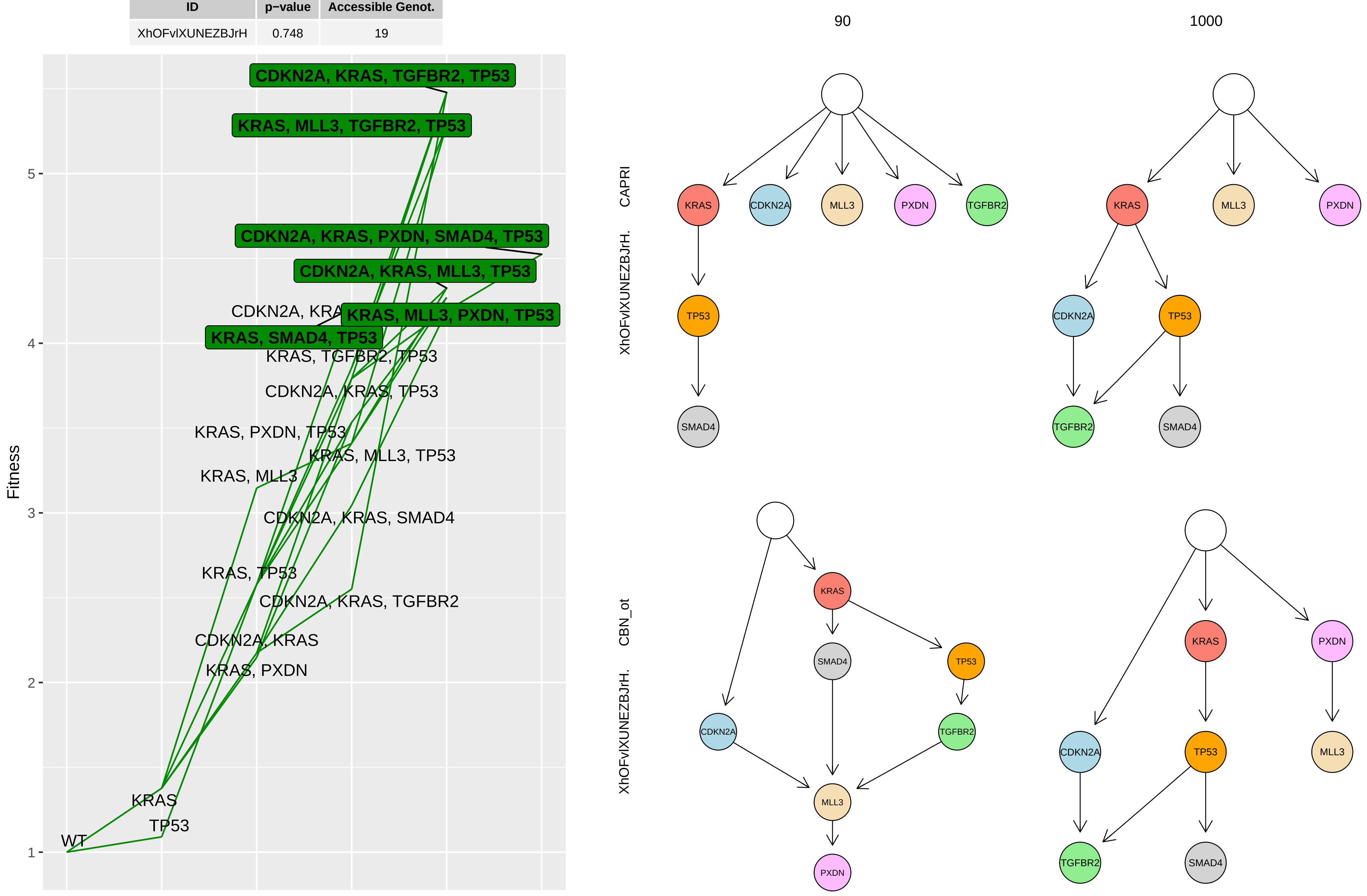

ID	p−value	Accessible Genot.
emPgHRKXoXDLzWQ	0.749	18

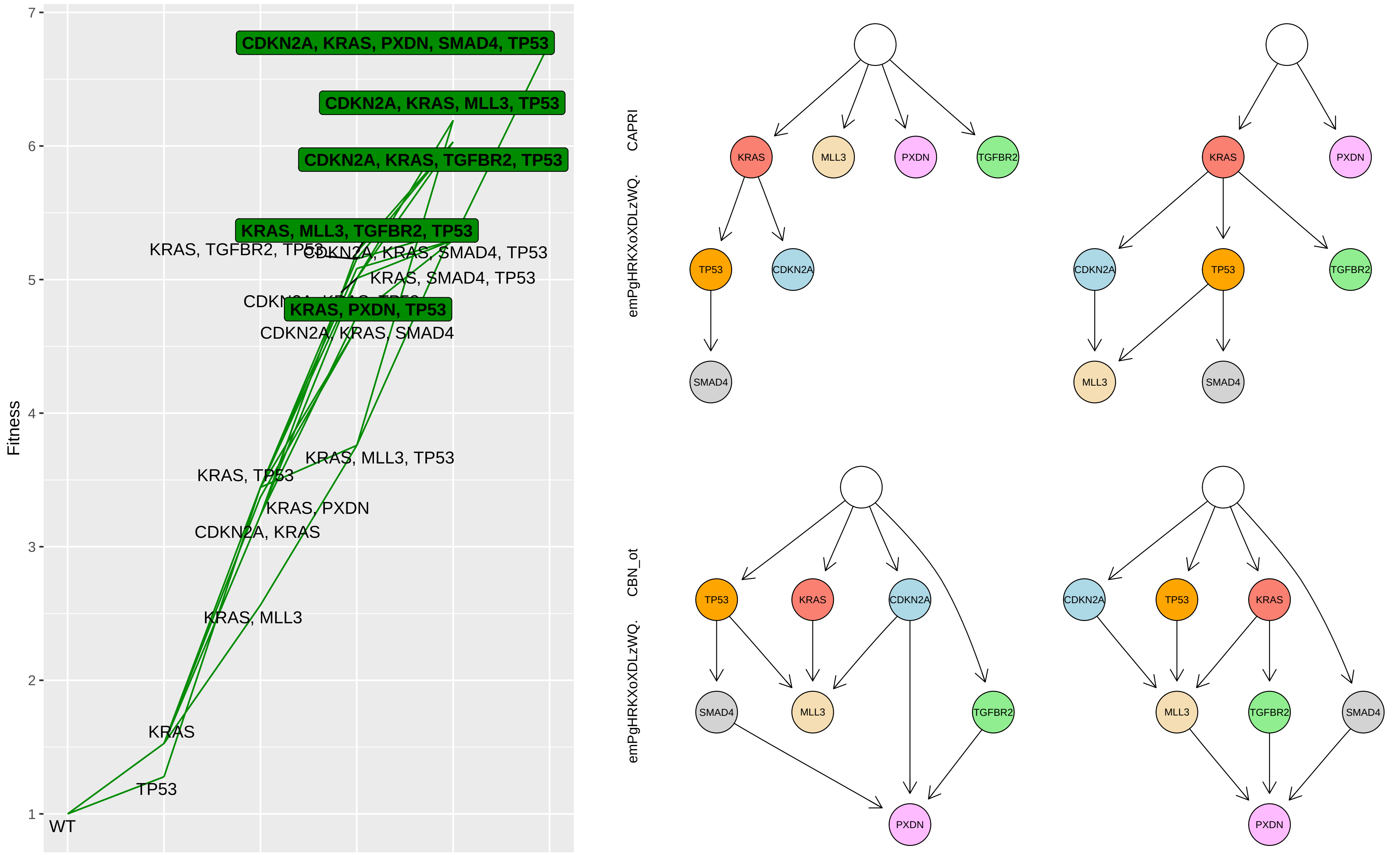

ID	p−value	Accessible Genot.
tQaXHQWHDwXQGGk	0.749	34

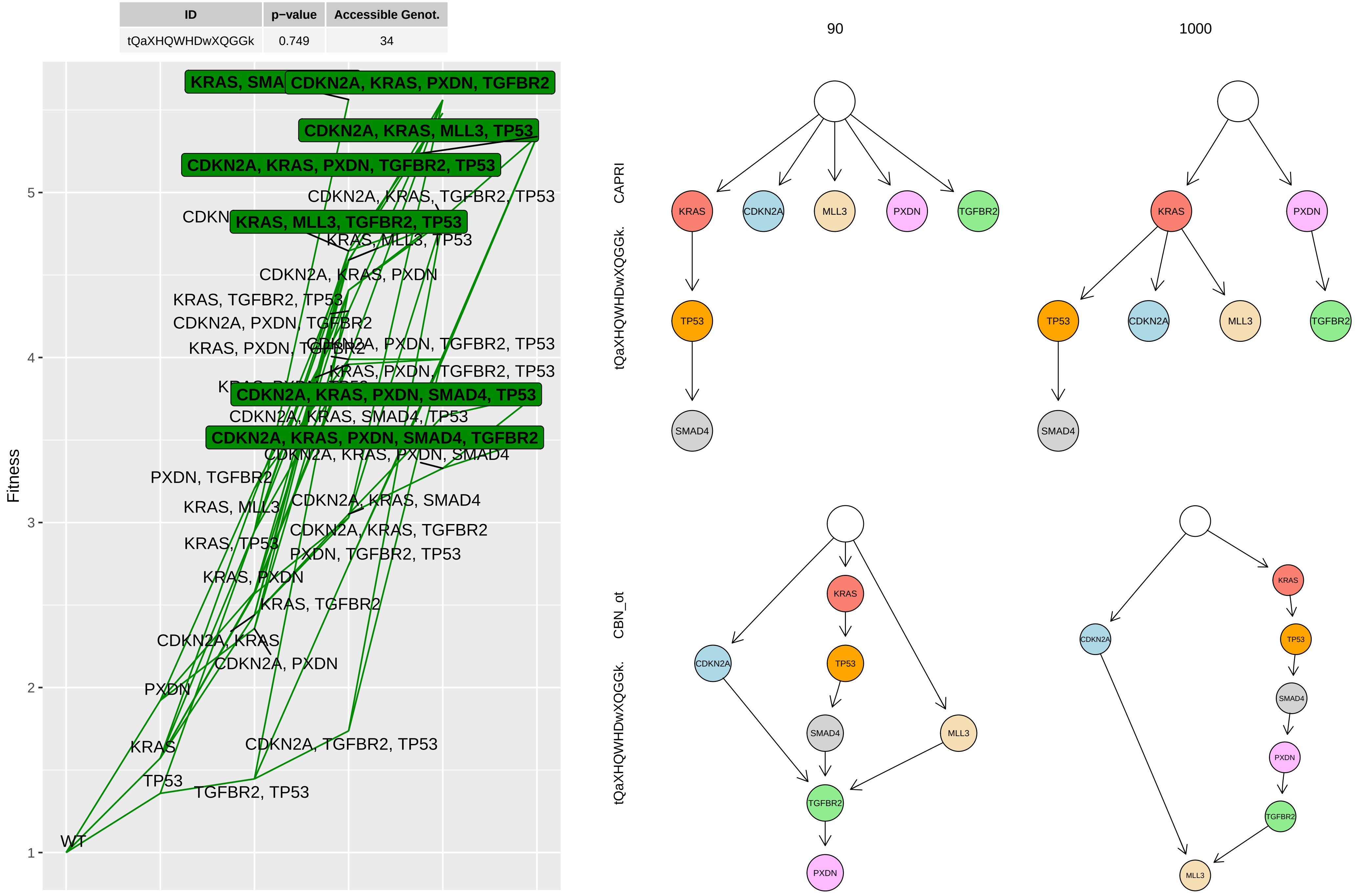

ID	p−value	Accessible Genot.
tQcurSjqUnqcmiq	0.751	28

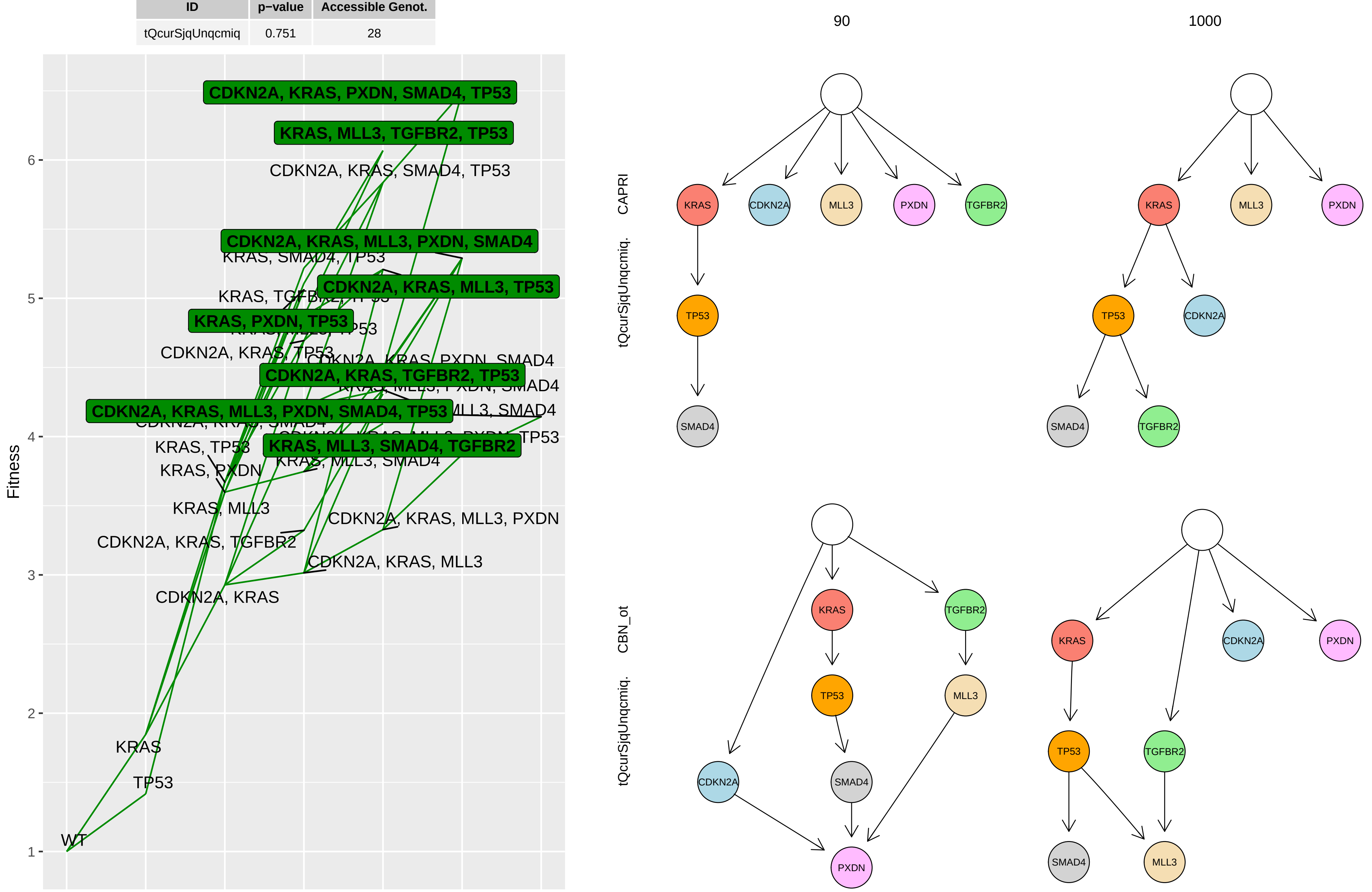

ID	p−value	Accessible Genot.
AyWcqiQpXCFFQBi	0.753	111

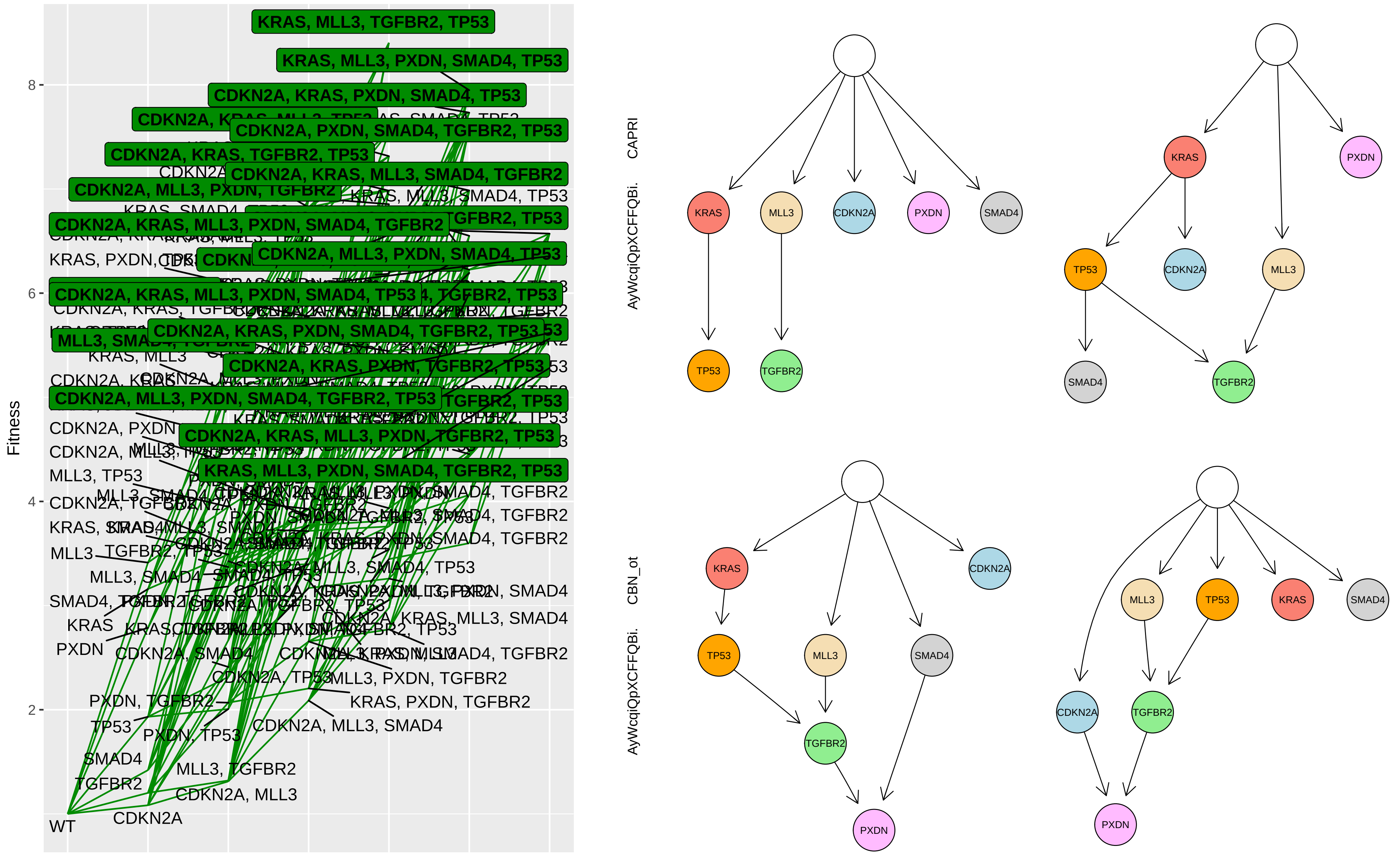

ID	p−value	Accessible Genot.
bZtDCeJMUhixeyn	0.755	25

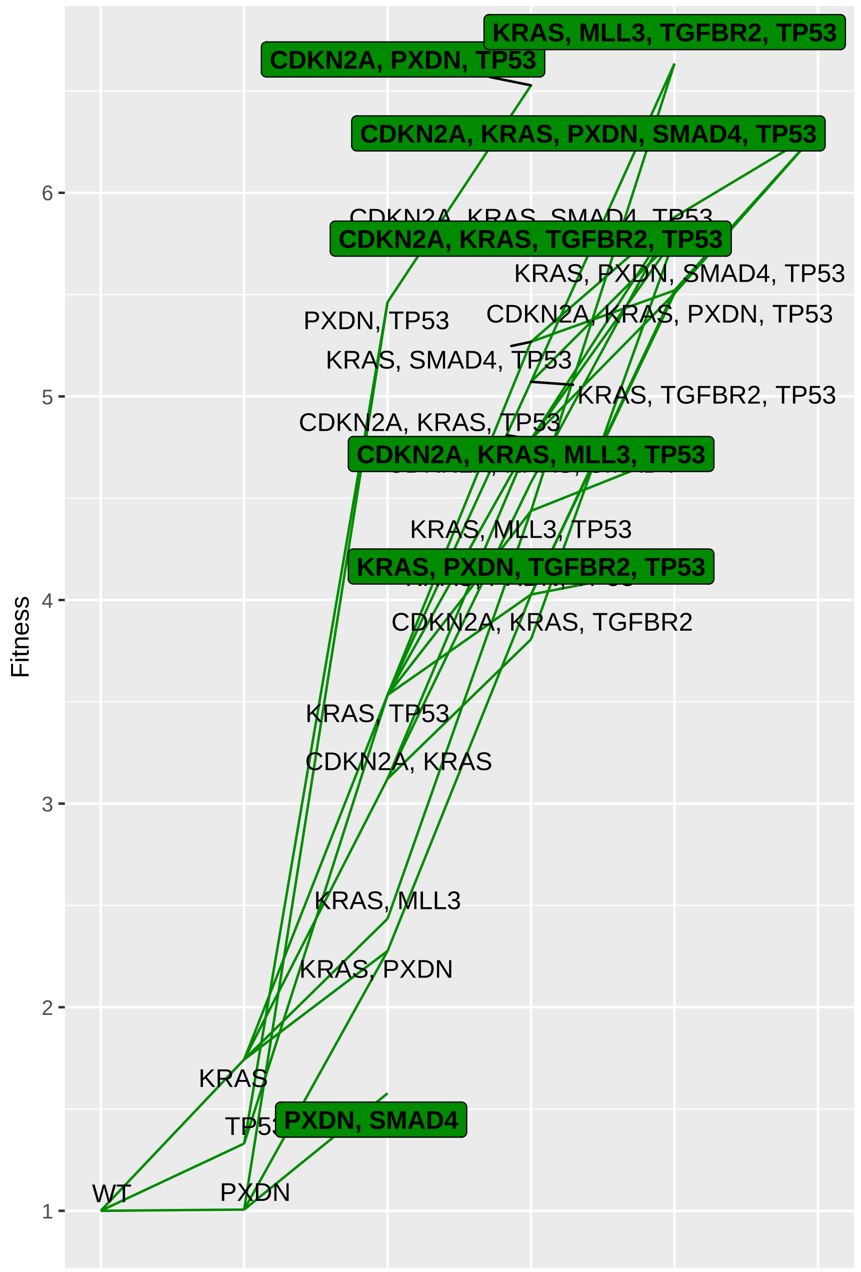

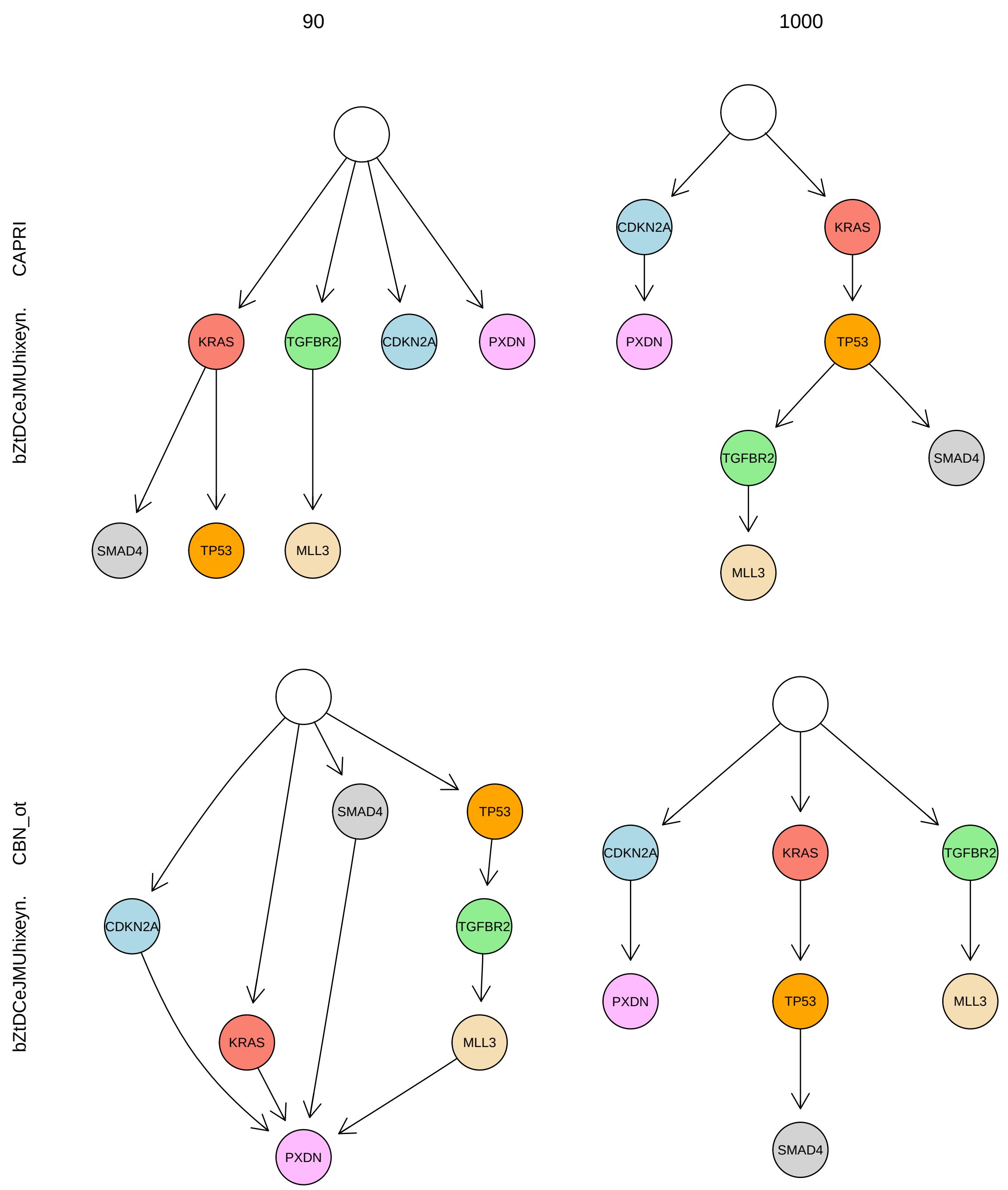

ID	p−value	Accessible Genot.
dvGfwRozBhylITQ	0.756	18

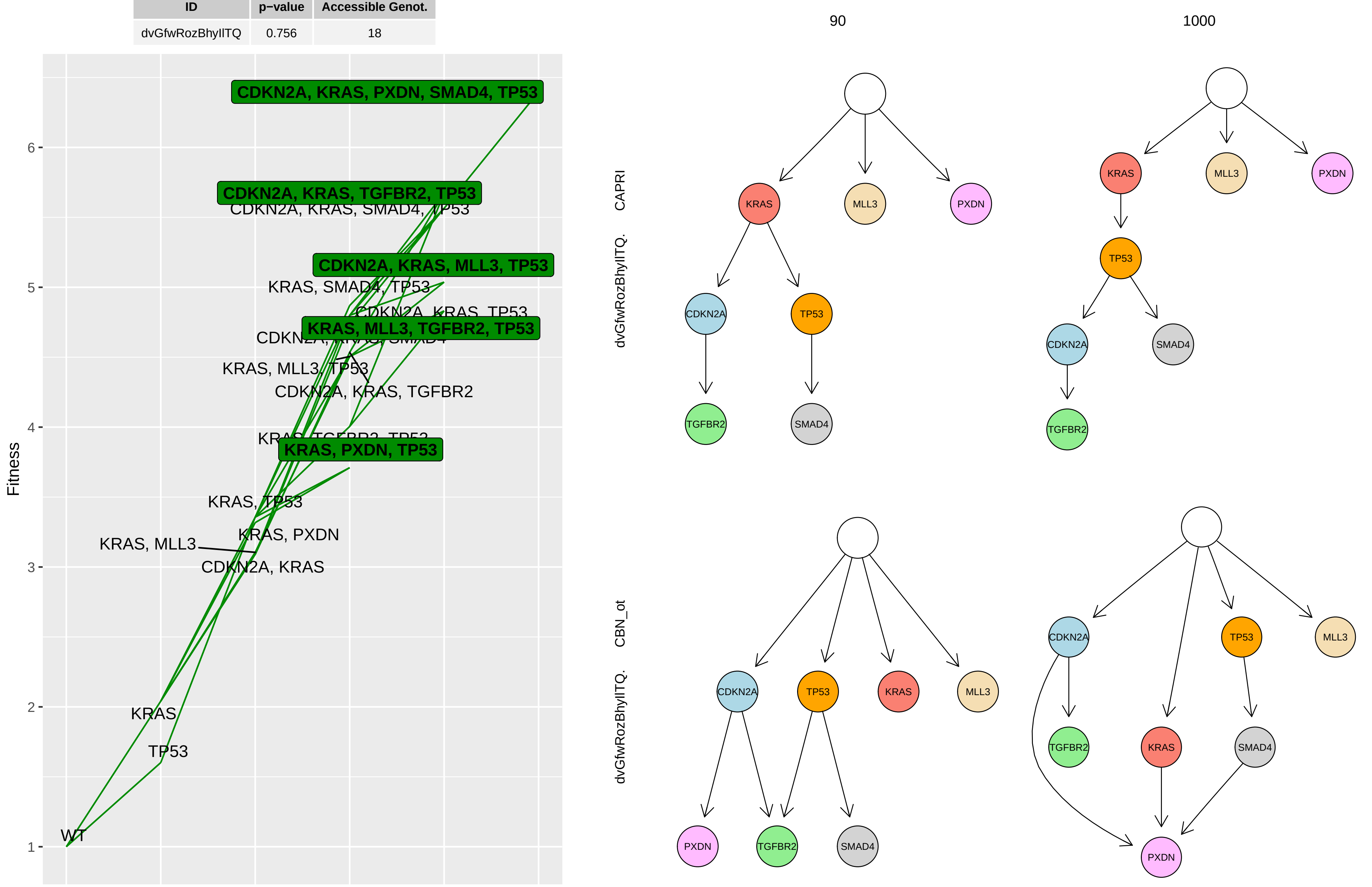

ID	p−value	Accessible Genot.
gujCoVxhFSuNUcm	0.761	31

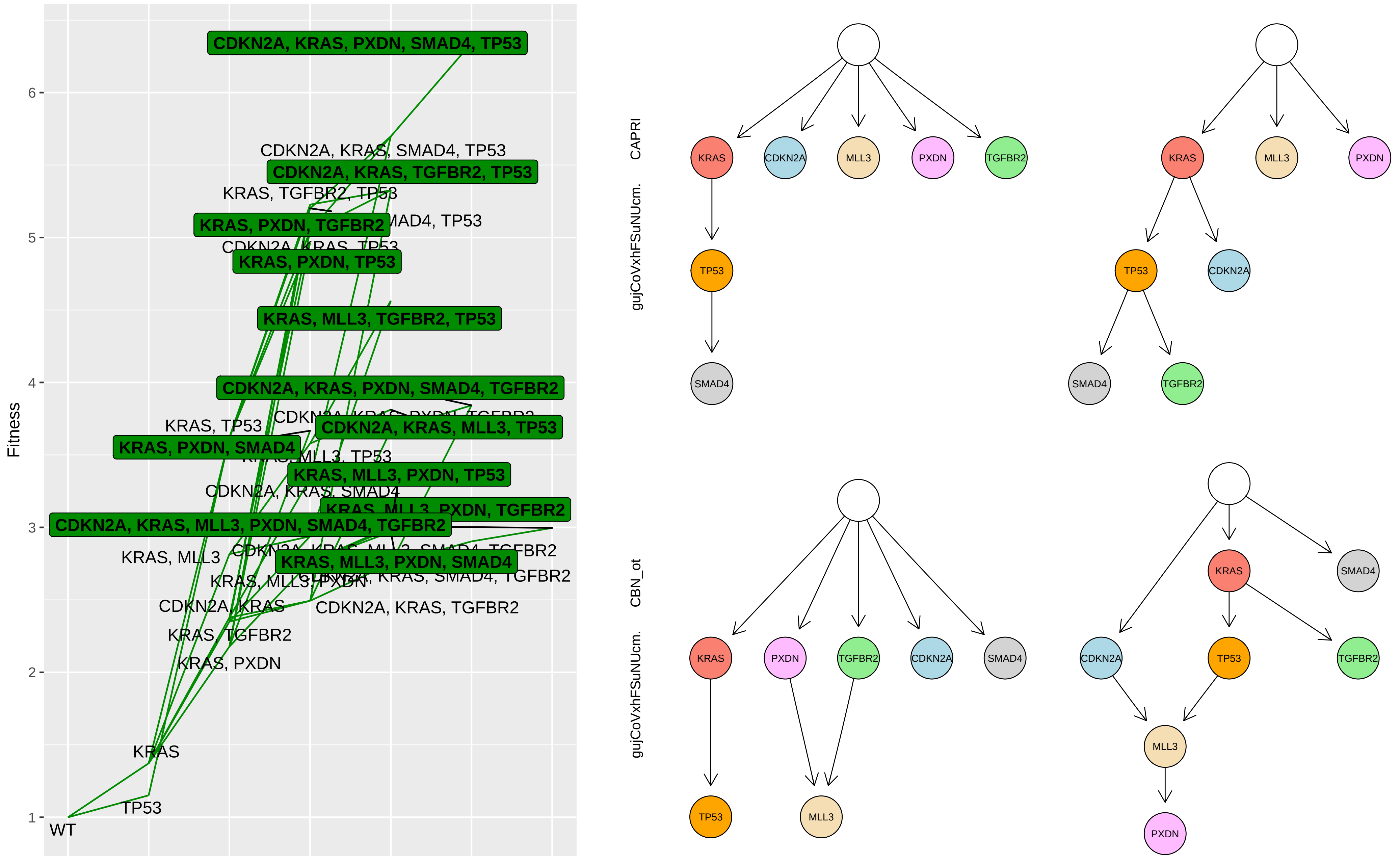

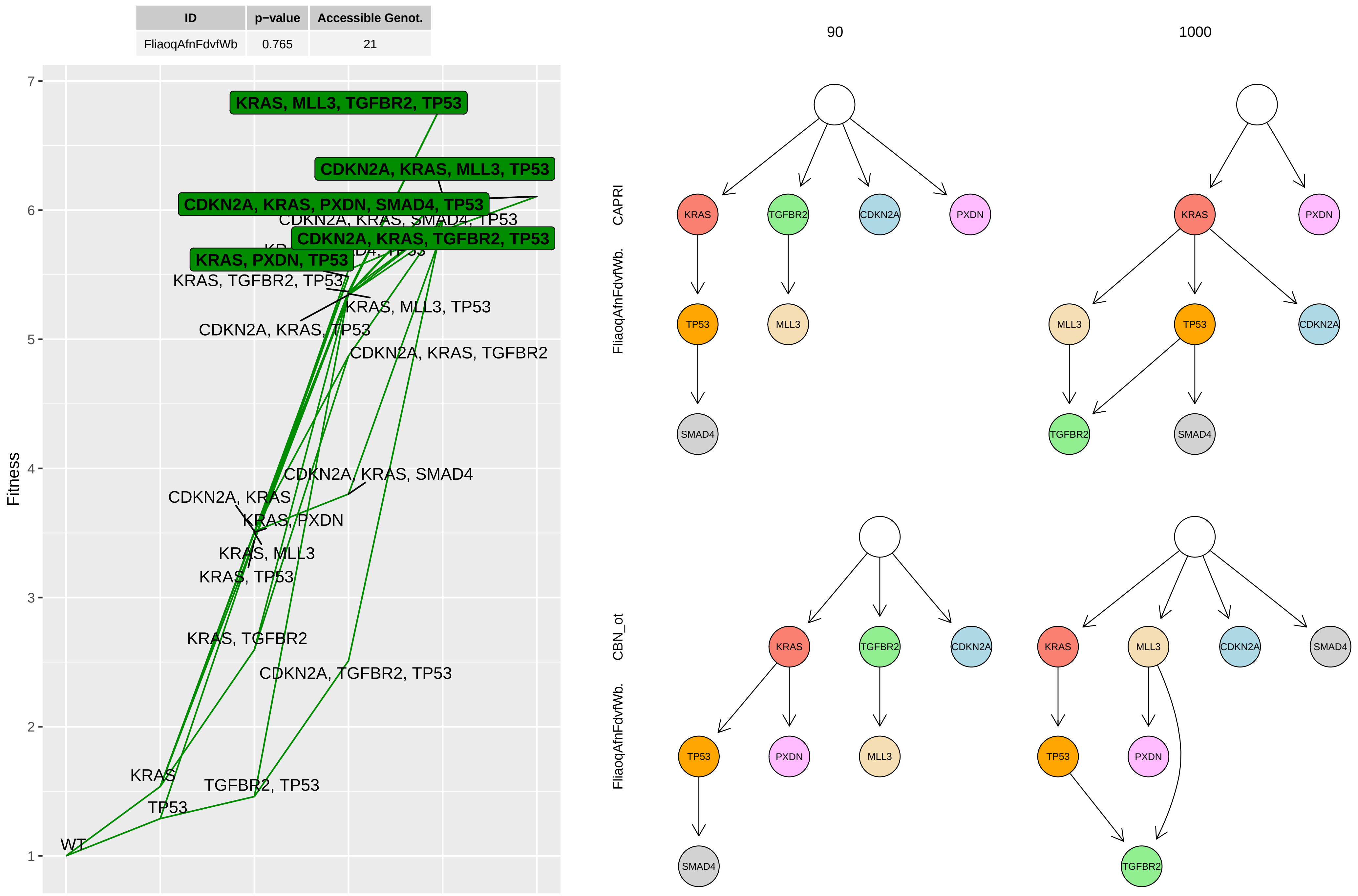

ID
p−value
Accessible Genot.
FliaoqAfnFdvfWb
0.765
21
Fitness
KRAS, MLL3, TGFBR2, TP53
CDKN2A, KRAS, MLL3, TP53
CDKN2A, KRAS, PXDN, SMAD4, TP53
CDKN2A, KRAS, SMAD4, TP53
CDKN2A, KRAS, TGFBR2, TP53
KRAS, PXDN, TP53
KRAS, TGFBR2, TP53
KRAS, MLL3, TP53
CDKN2A, KRAS, TP53
CDKN2A, KRAS, TGFBR2
CDKN2A, KRAS, SMAD4
CDKN2A, KRAS
KRAS, PXDN
KRAS, MLL3
KRAS, TP53
KRAS, TGFBR2
CDKN2A, TGFBR2, TP53
KRAS
TGFBR2, TP53
TP53
WT
90
1000
FliaoqAfnFdvfWb. CAPRI
FliaoqAfnFdvfWb. CBN_ot
KRAS
TGFBR2
CDKN2A
PXDN
TP53
MLL3
SMAD4

ID	p−value	Accessible Genot.
RmctJoZXVDyoHPV	0.768	66

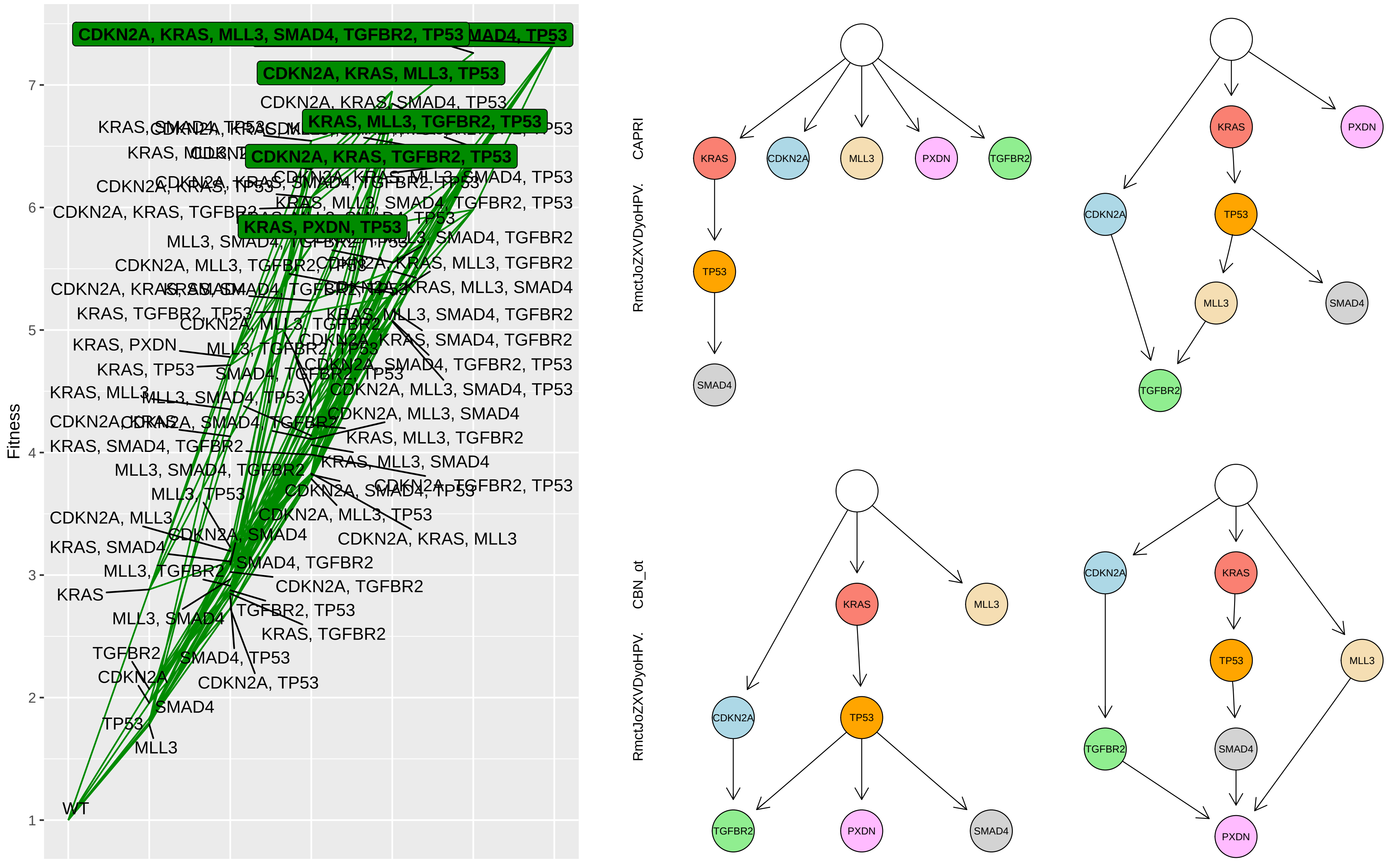

ID	p−value	Accessible Genot.
LGdIicnkgVVvmvK	0.771	77

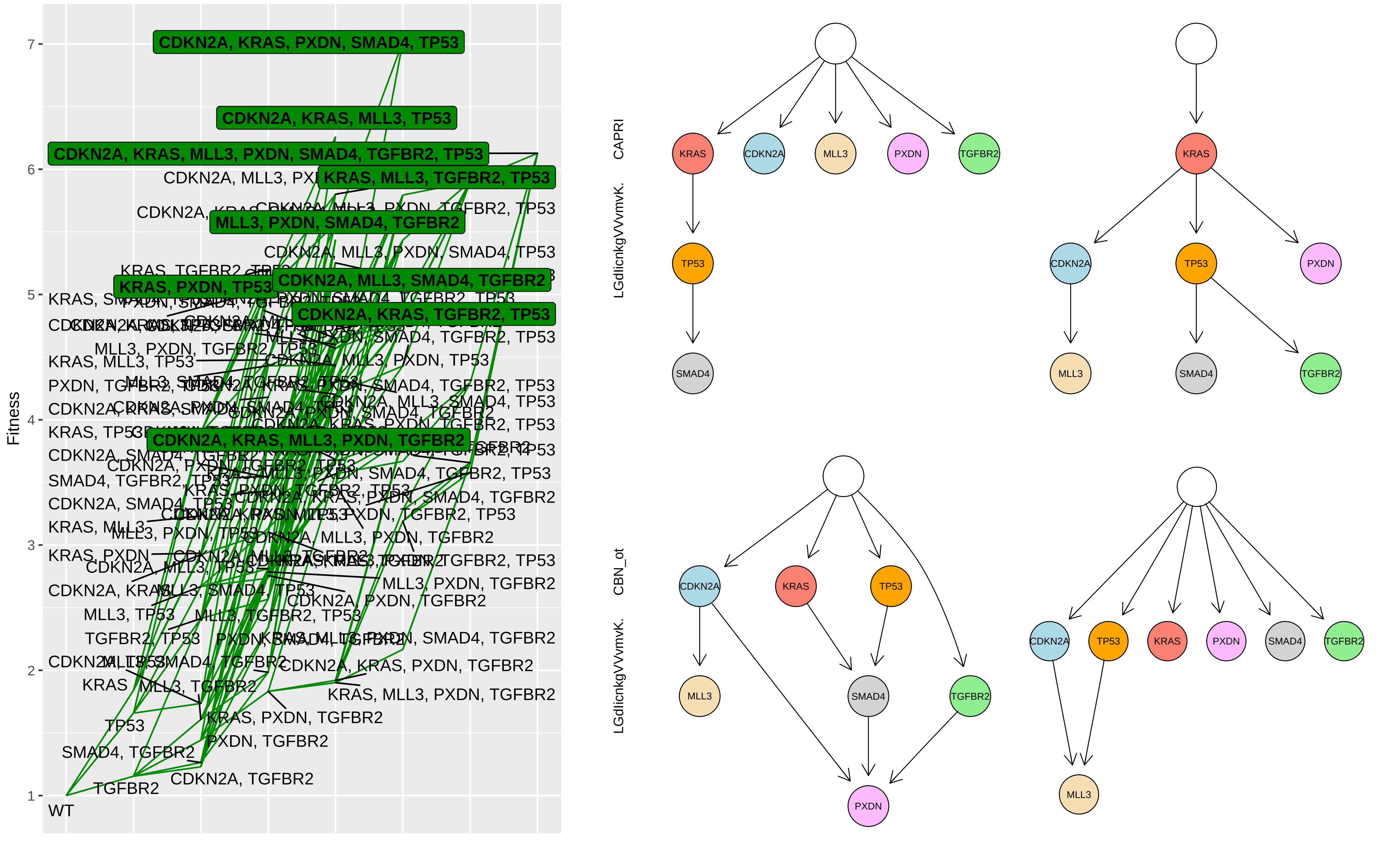

ID	p−value	Accessible Genot.
IYJCvGmCeOoOTOO	0.775	21

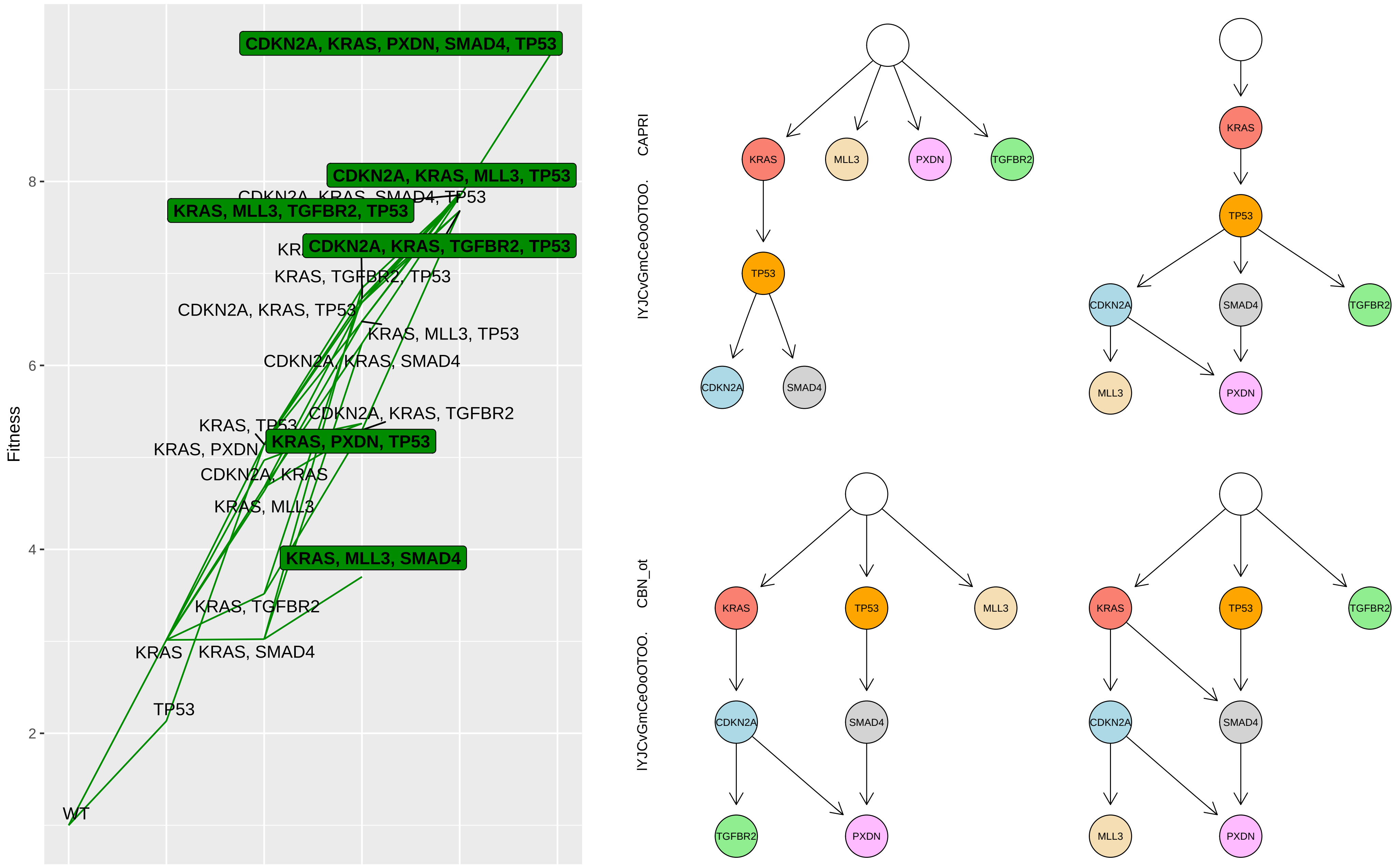

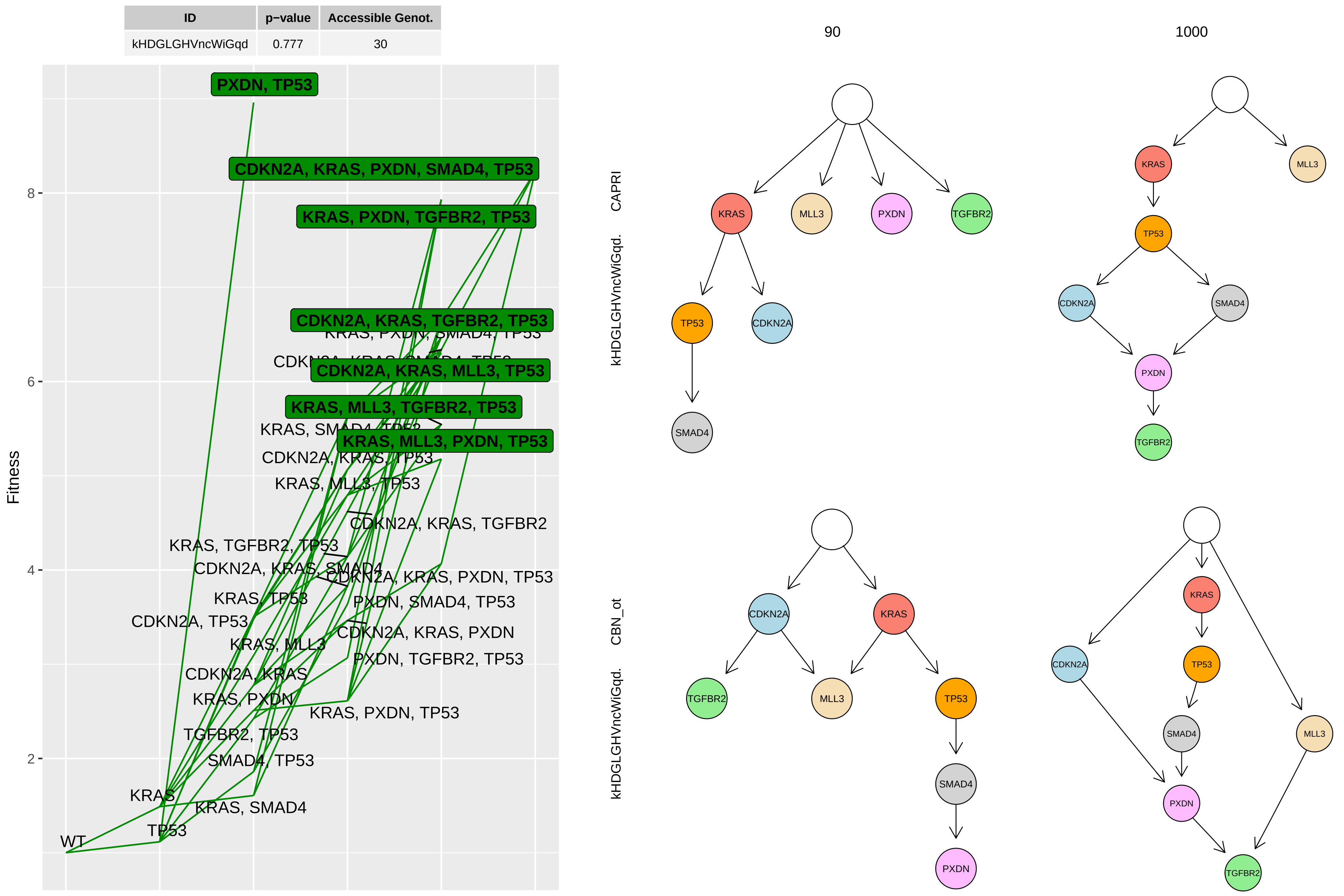
ID
p-value
Accessible Genot.
kHDGLGHVncWiGqd
0.777
30
Fitness
PXDN, TP53
CDKN2A, KRAS, PXDN, SMAD4, TP53
KRAS, PXDN, TGFBR2, TP53
CDKN2A, KRAS, TGFBR2, TP53
CDKN2A, KRAS, MLL3, TP53
KRAS, MLL3, TGFBR2, TP53
KRAS, MLL3, PXDN, TP53
CDKN2A, KRAS, TP53
KRAS, MLL3, TP53
CDKN2A, KRAS, TGFBR2
KRAS, TGFBR2, TP53
CDKN2A, KRAS, SMAD4
CDKN2A, KRAS, PXDN, TP53
KRAS, TP53
PXDN, SMAD4, TP53
CDKN2A, TP53
CDKN2A, KRAS, PXDN
KRAS, MLL3
PXDN, TGFBR2, TP53
CDKN2A, KRAS
KRAS, PXDN
KRAS, PXDN, TP53
TGFBR2, TP53
SMAD4, TP53
KRAS
KRAS, SMAD4
TP53
WT
90
1000
kHDGLGHVncWiGqd. CAPRI
kHDGLGHVncWiGqd. CBN_ot
KRAS
MLL3
PXDN
TGFBR2
TP53
CDKN2A
SMAD4

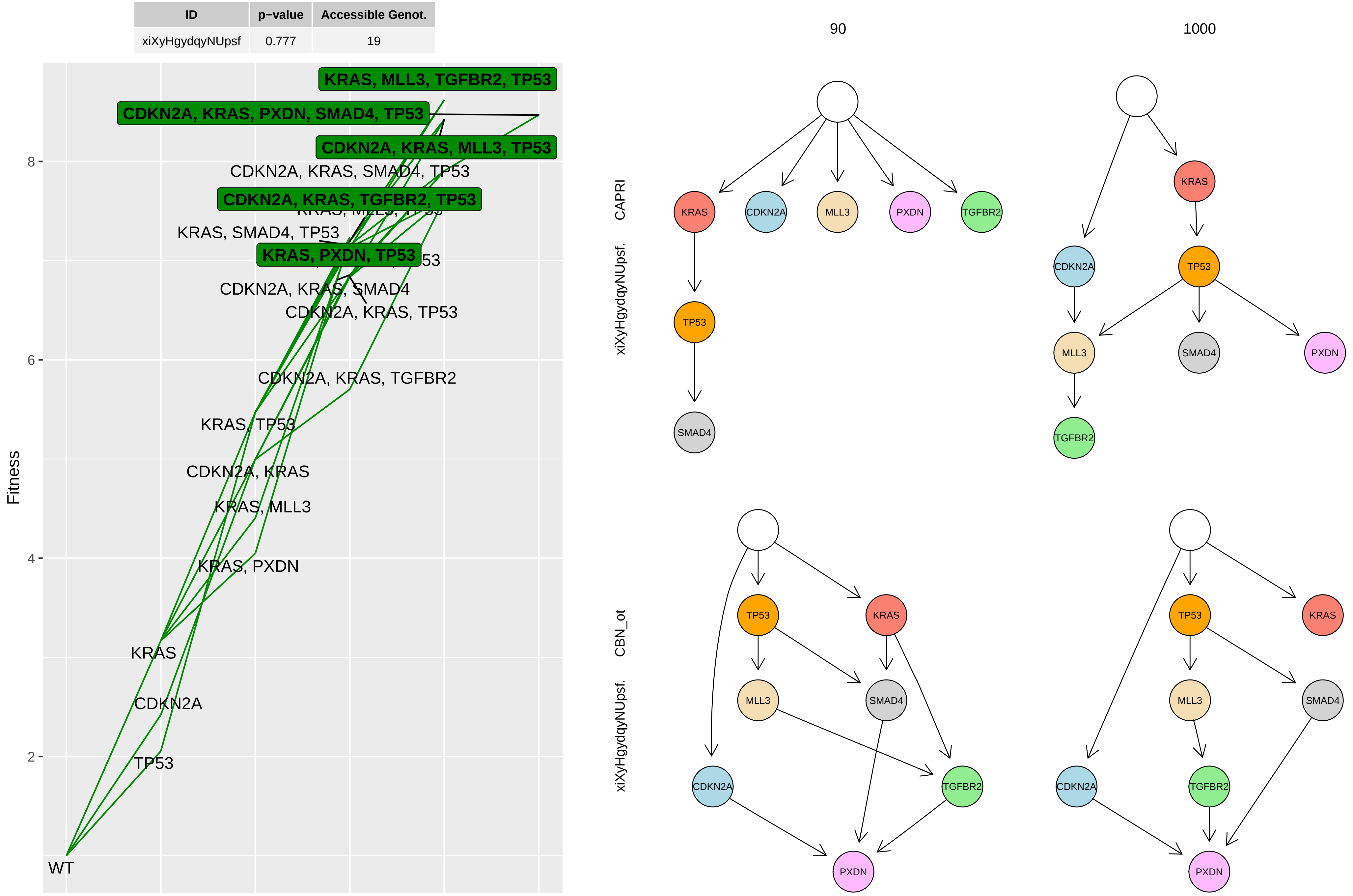

ID
p−value
Accessible Genot.
xiXyHgydqyNUpsf
0.777
19
KRAS, MLL3, TGFBR2, TP53
CDKN2A, KRAS, PXDN, SMAD4, TP53
CDKN2A, KRAS, MLL3, TP53
CDKN2A, KRAS, SMAD4, TP53
CDKN2A, KRAS, TGFBR2, TP53
KRAS, SMAD4, TP53
KRAS, PXDN, TP53
CDKN2A, KRAS, SMAD4
CDKN2A, KRAS, TP53
CDKN2A, KRAS, TGFBR2
KRAS, TP53
CDKN2A, KRAS
KRAS, MLL3
KRAS, PXDN
KRAS
CDKN2A
TP53
WT
Fitness
8
6
4
2
90
1000
CAPRI
CBN_ot
xiXyHgydqyNUpsf.
KRAS
CDKN2A
MLL3
PXDN
TGFBR2
TP53
SMAD4

ID	p−value	Accessible Genot.
nPnZmqawbckpXfk	0.786	46

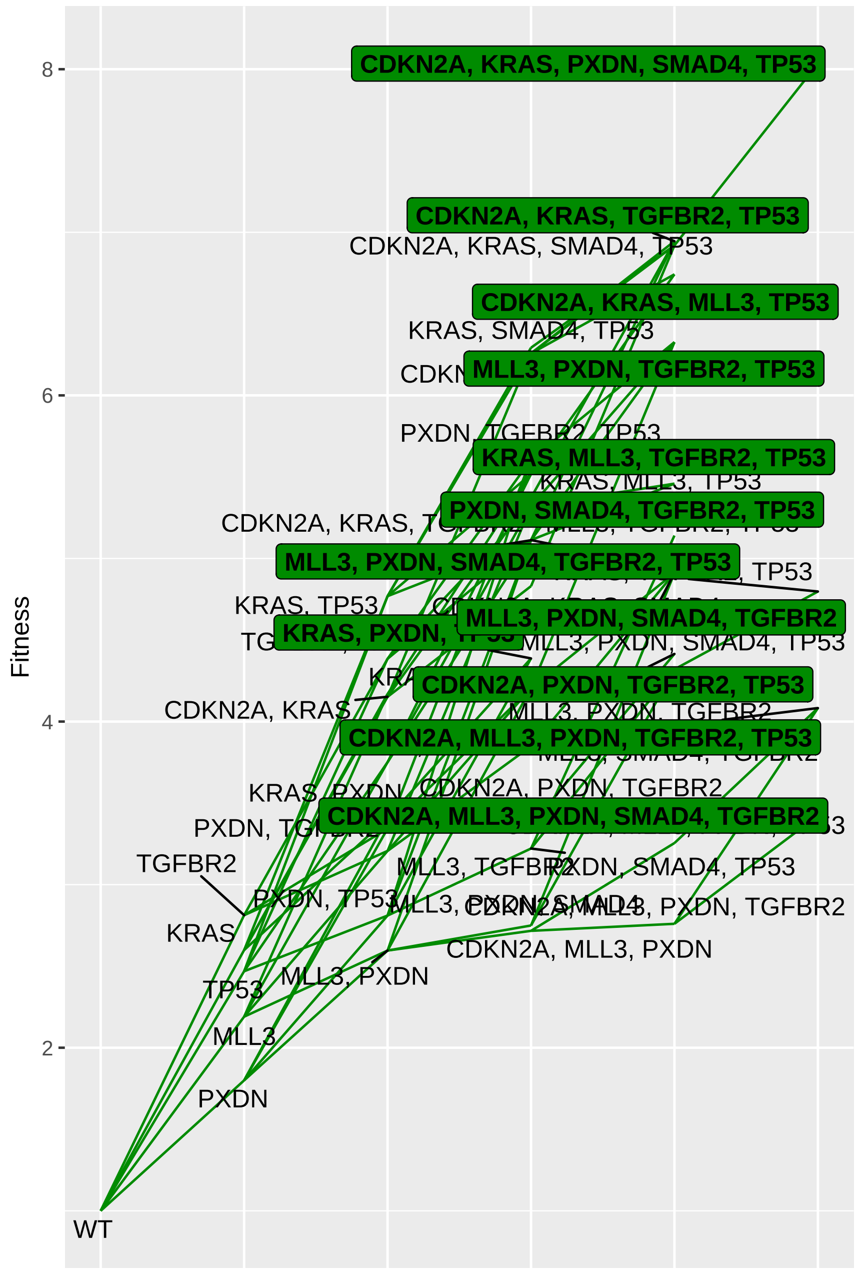

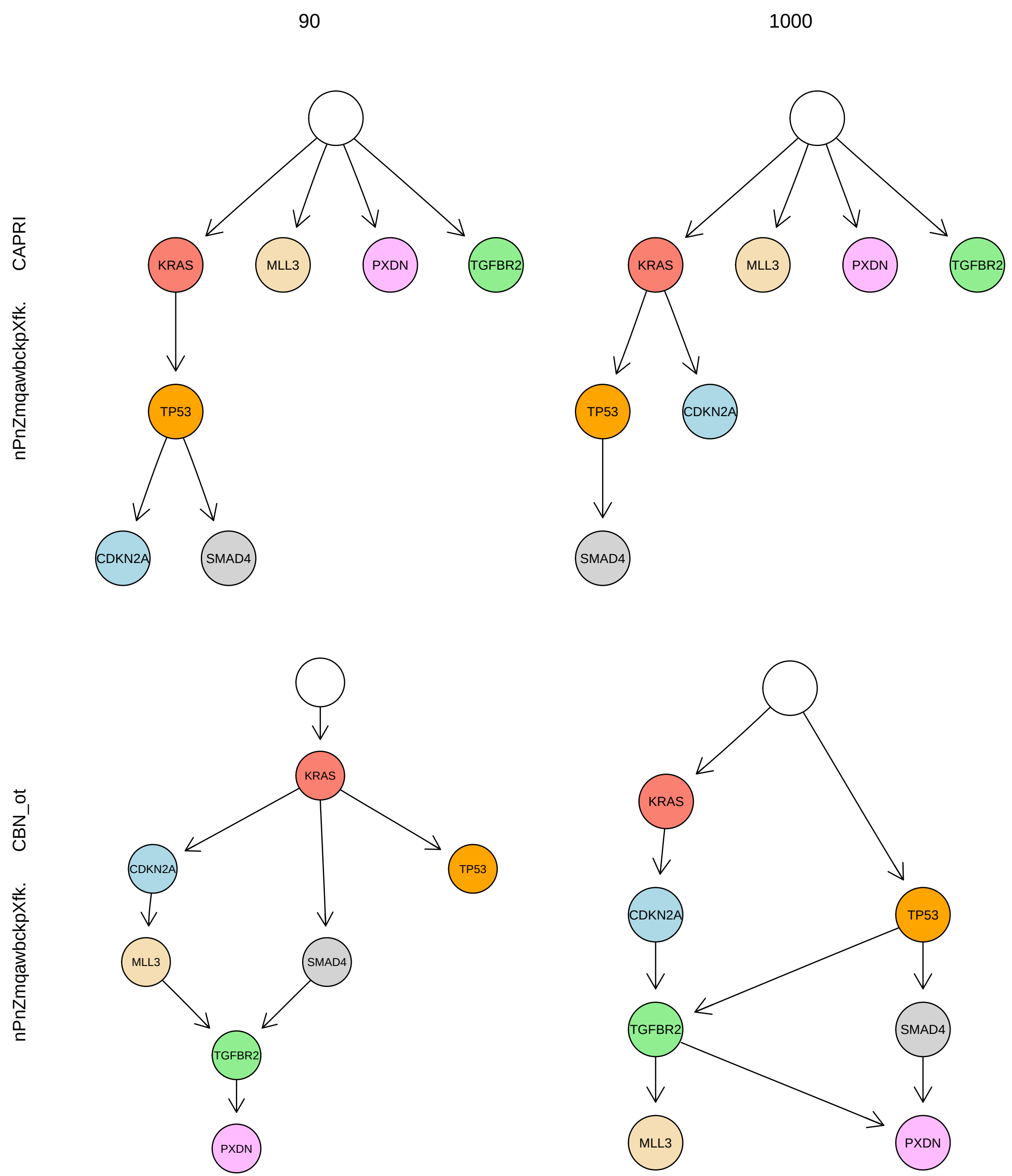

ID	p−value	Accessible Genot.
OshiYpNZjRiNOAf	0.787	77

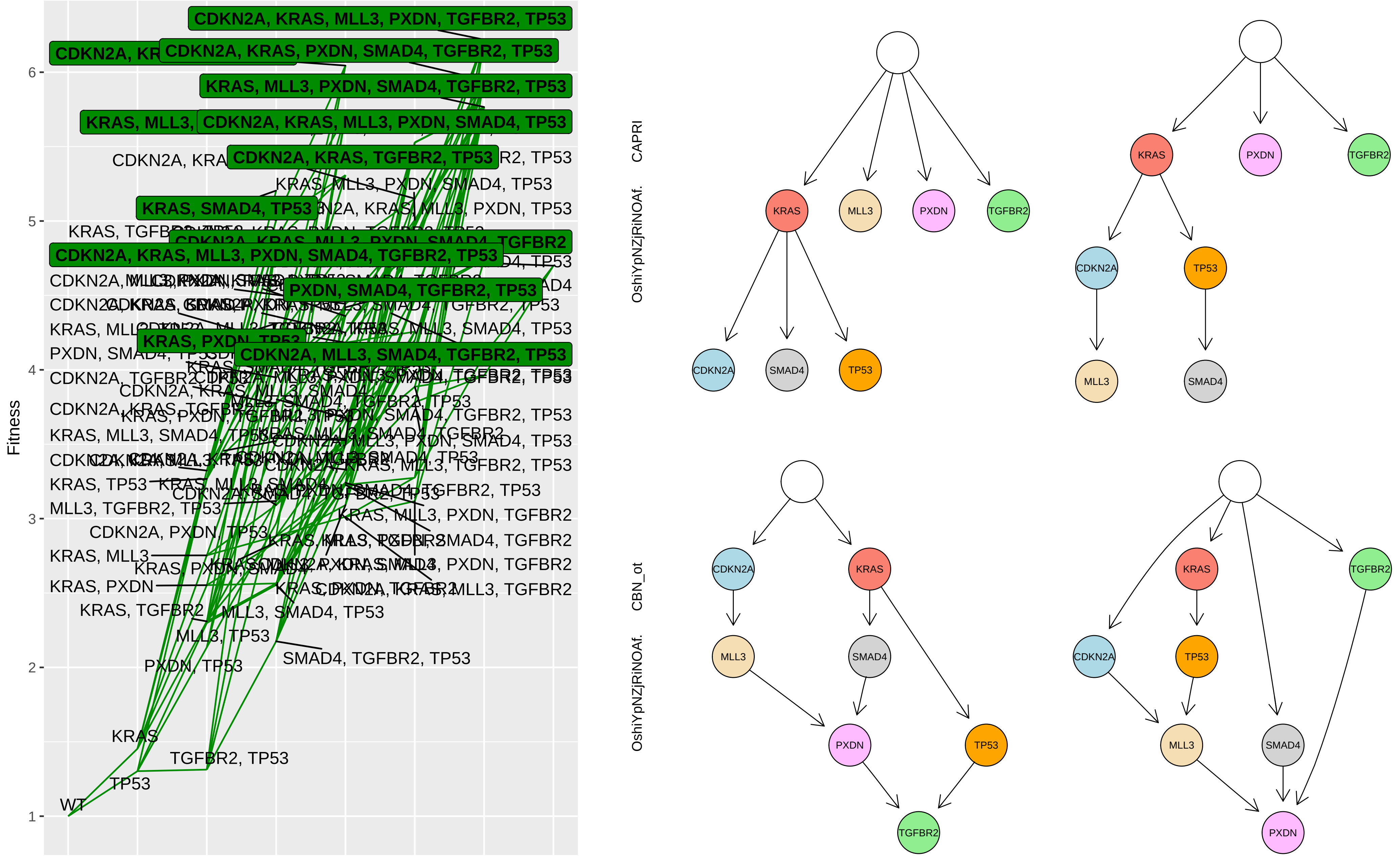

ID	p−value	Accessible Genot.
salMshsHhwCDjjG	0.788	55

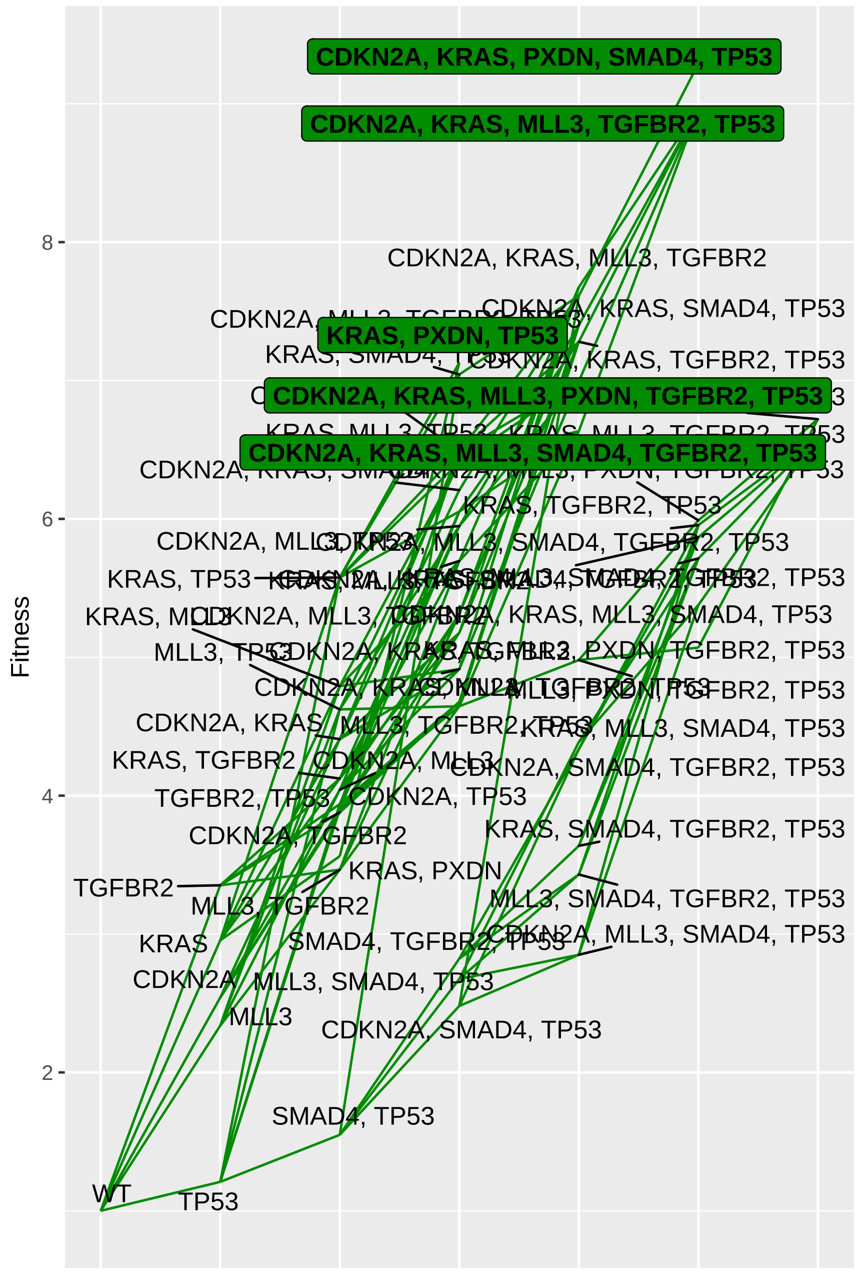

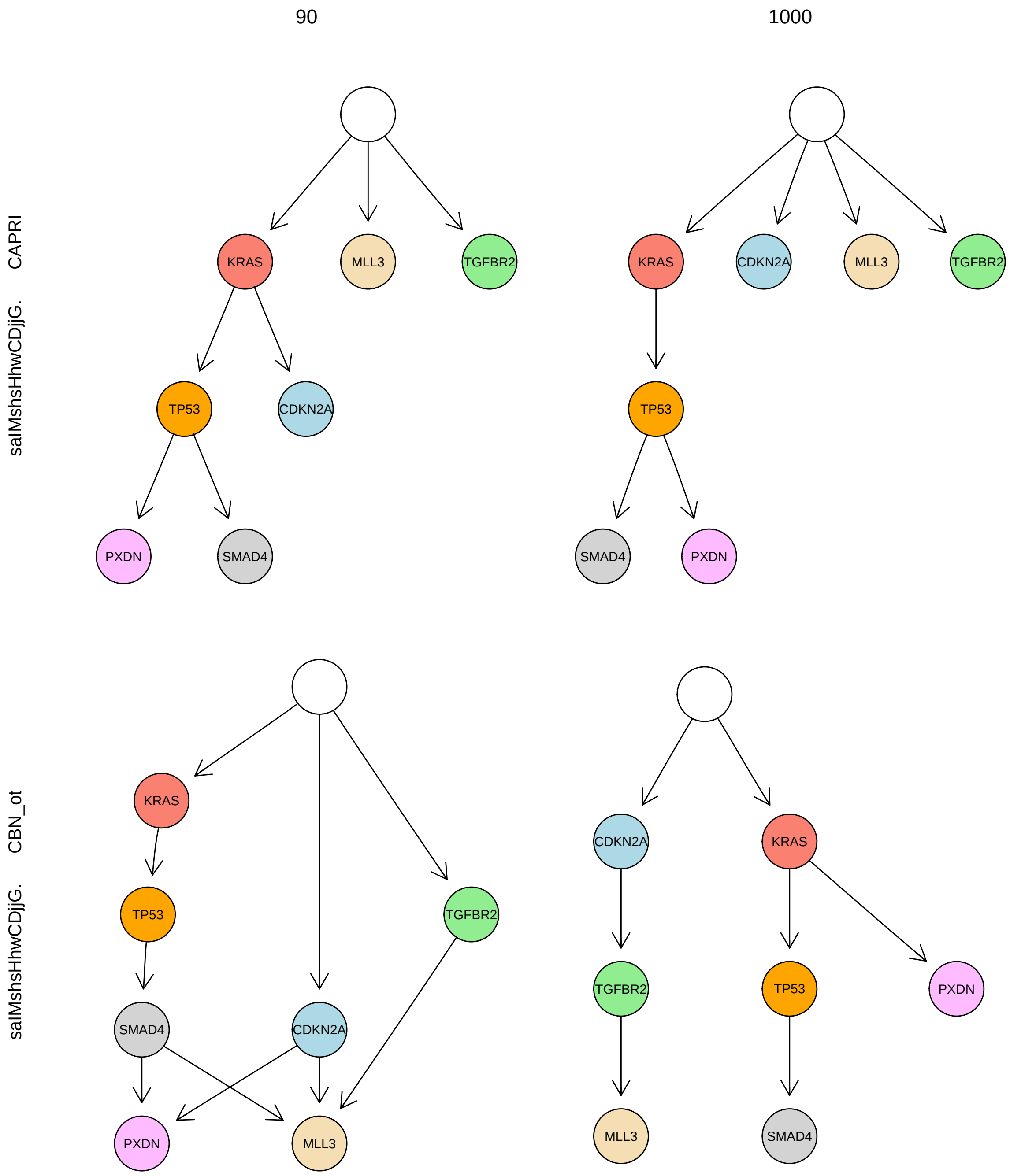

ID	p−value	Accessible Genot.
rasquFMgHkPCKgo	0.798	20

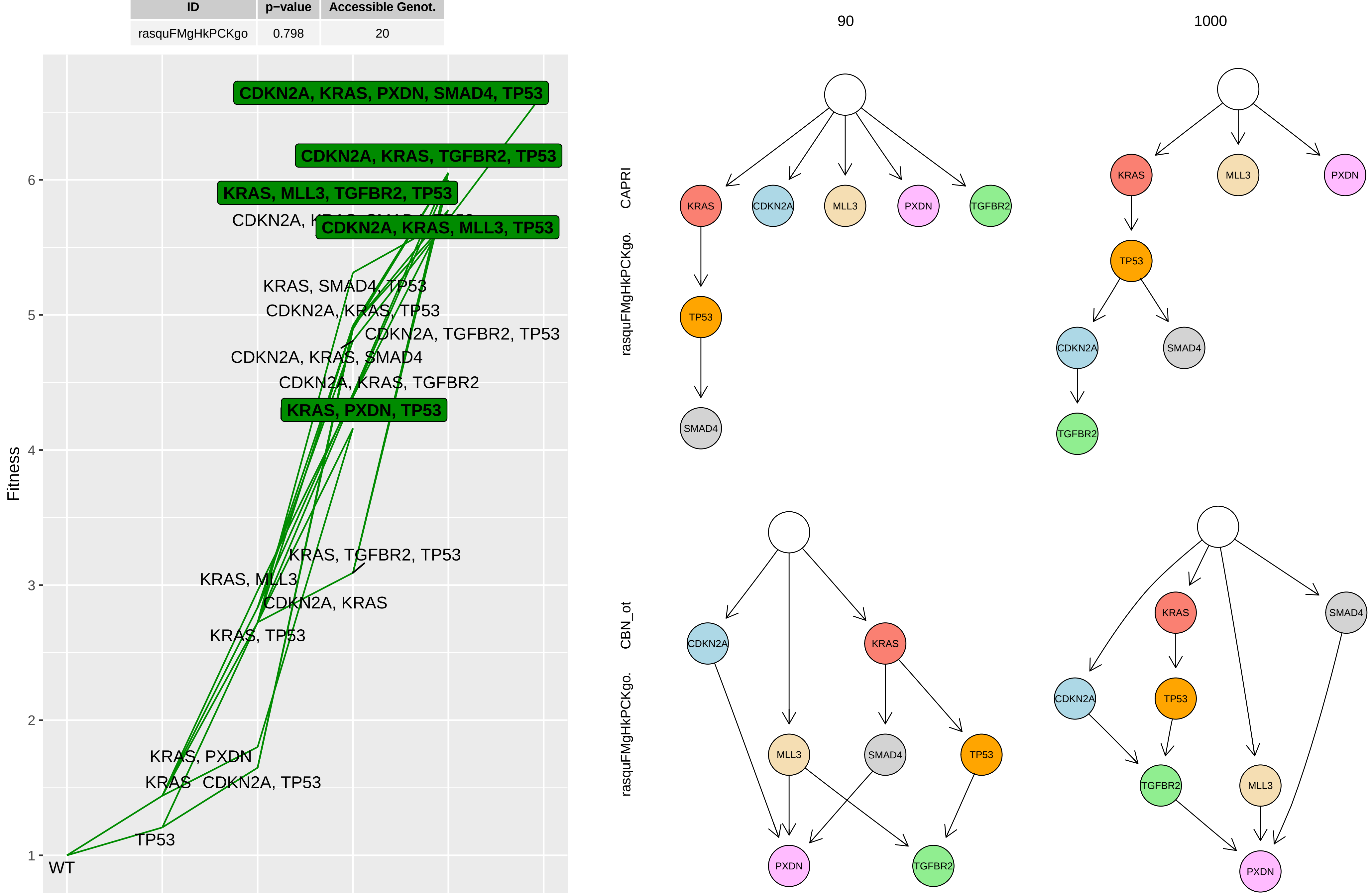

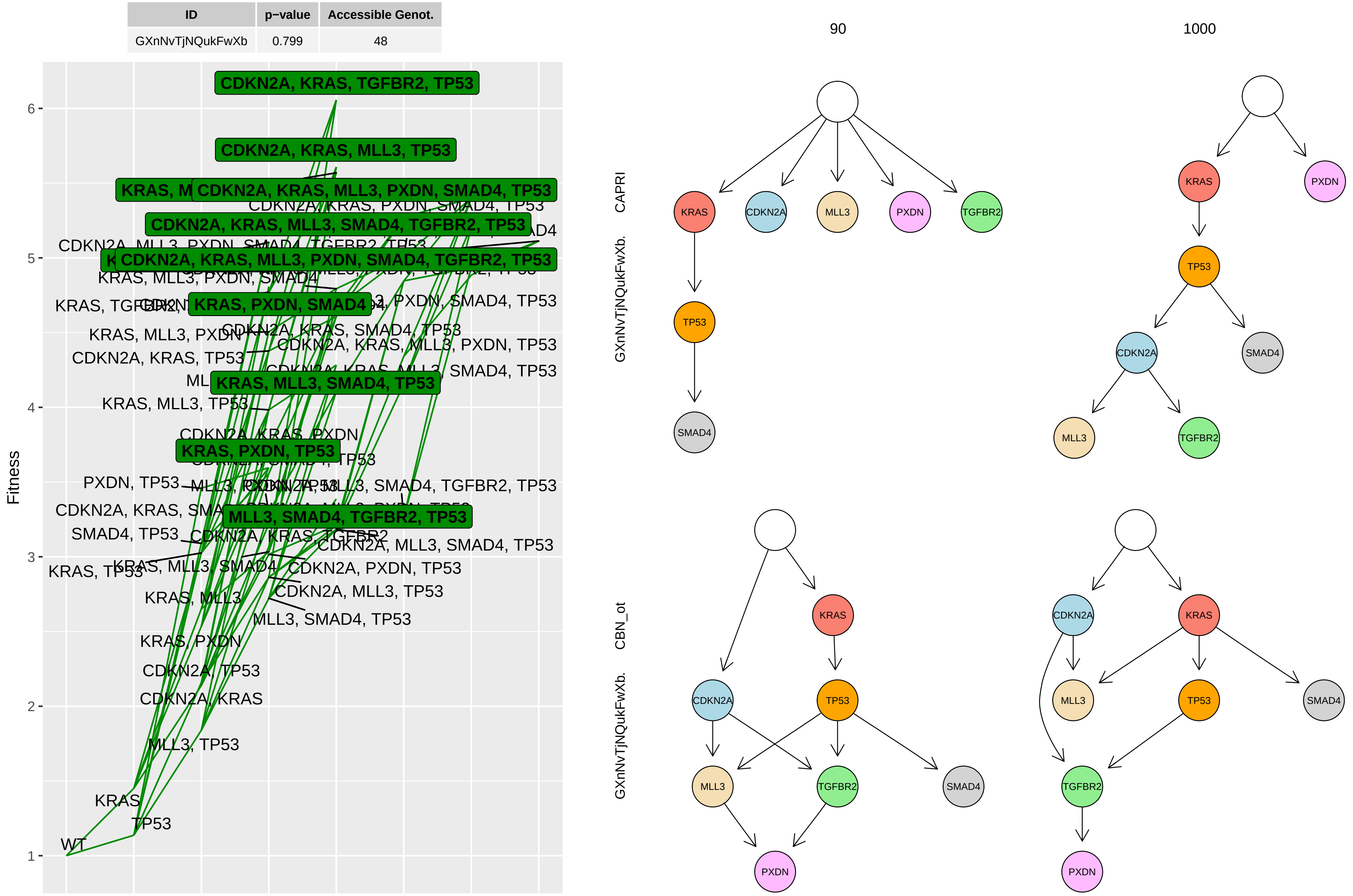

ID	p−value	Accessible Genot.
GXnNvTjNQukFwXb	0.799	48

ID	p−value	Accessible Genot.
bXdYUPwDkPHGEyD	0.799	20

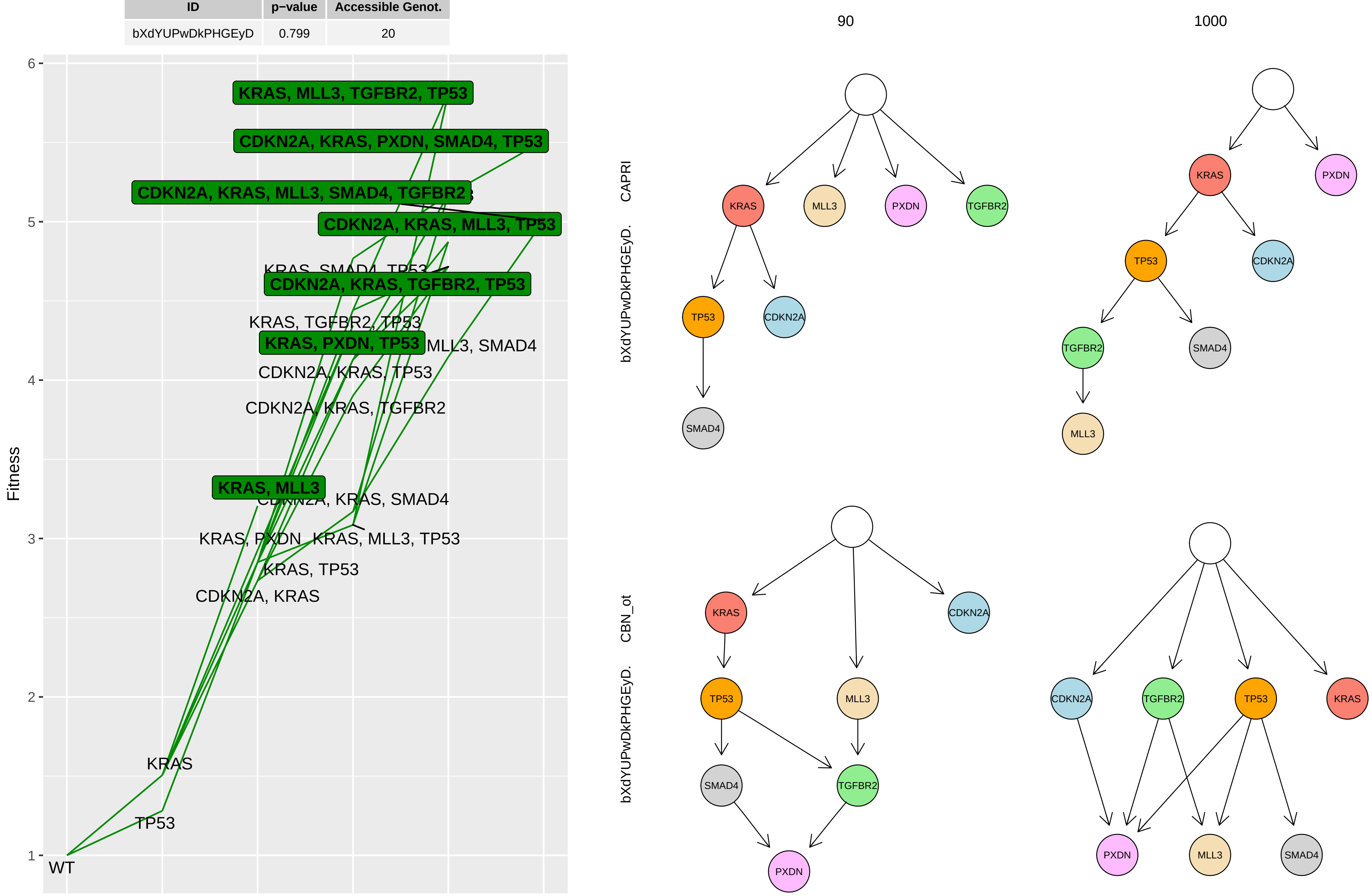

ID	p−value	Accessible Genot.
JbMDfOXigxyuWUY	0.801	32

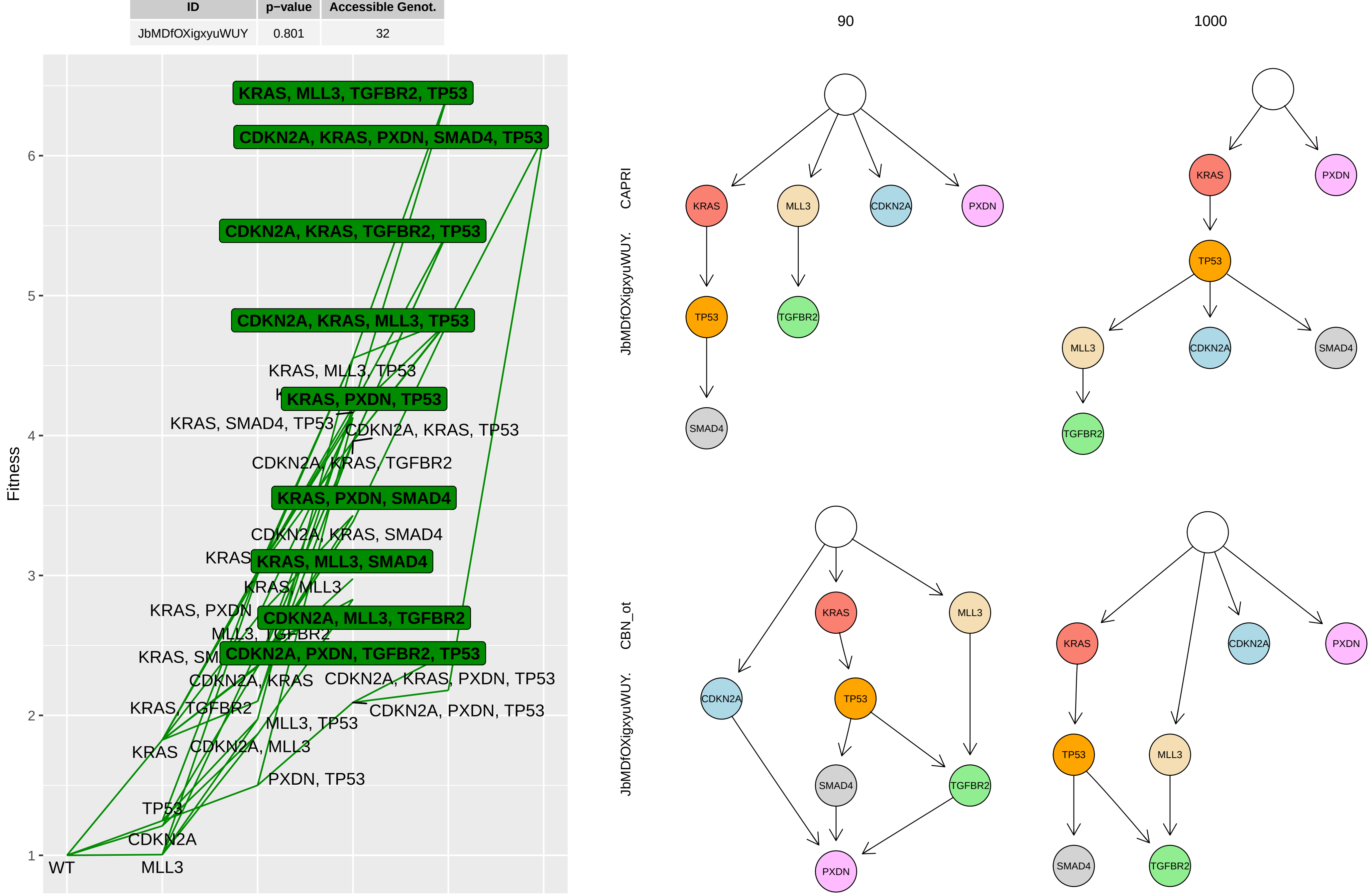

ID	p−value	Accessible Genot.
bngjYcXfsHXmPhU	0.808	18

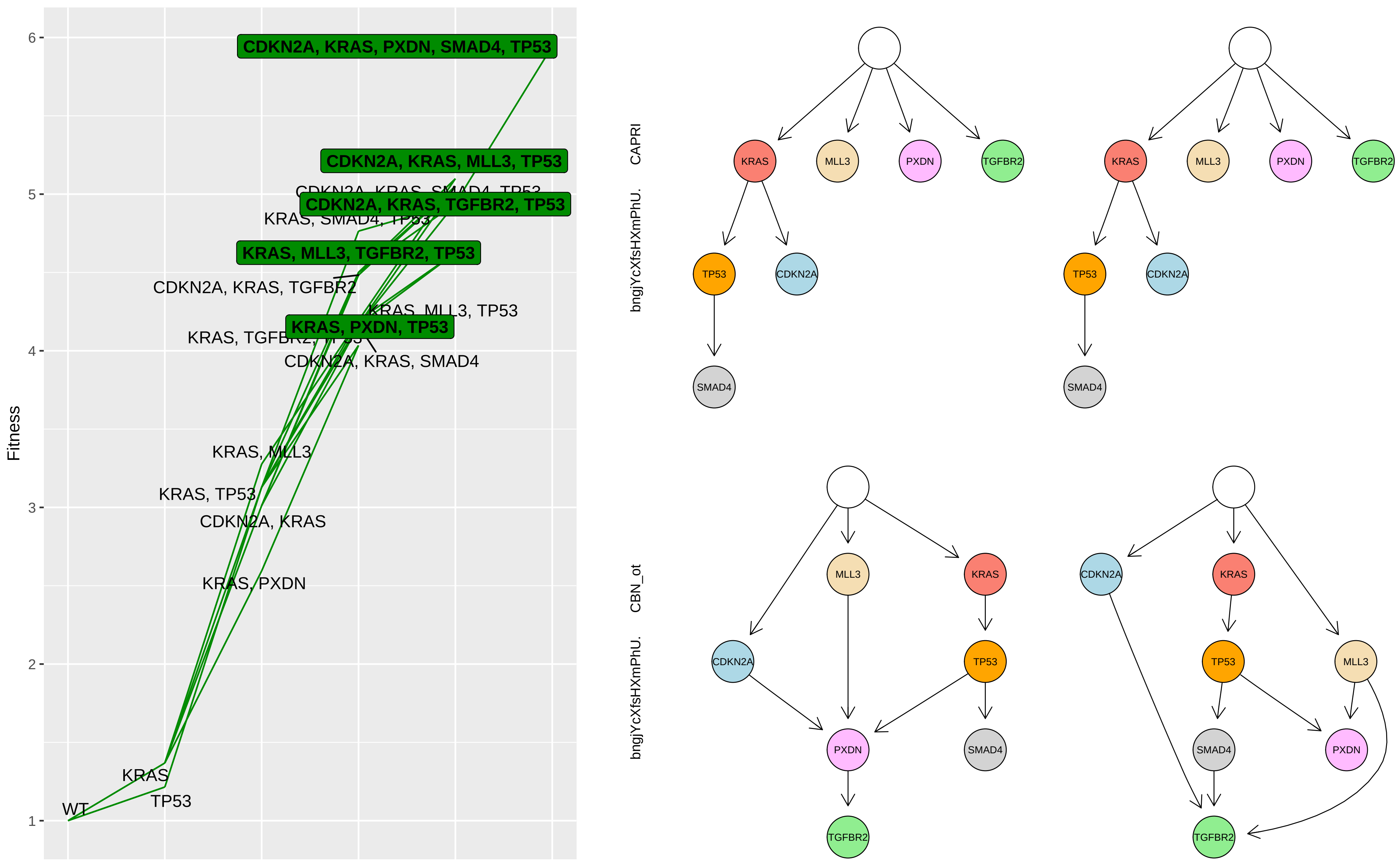

ID	p−value	Accessible Genot.
GePbvsCBWXUCyMo	0.811	45

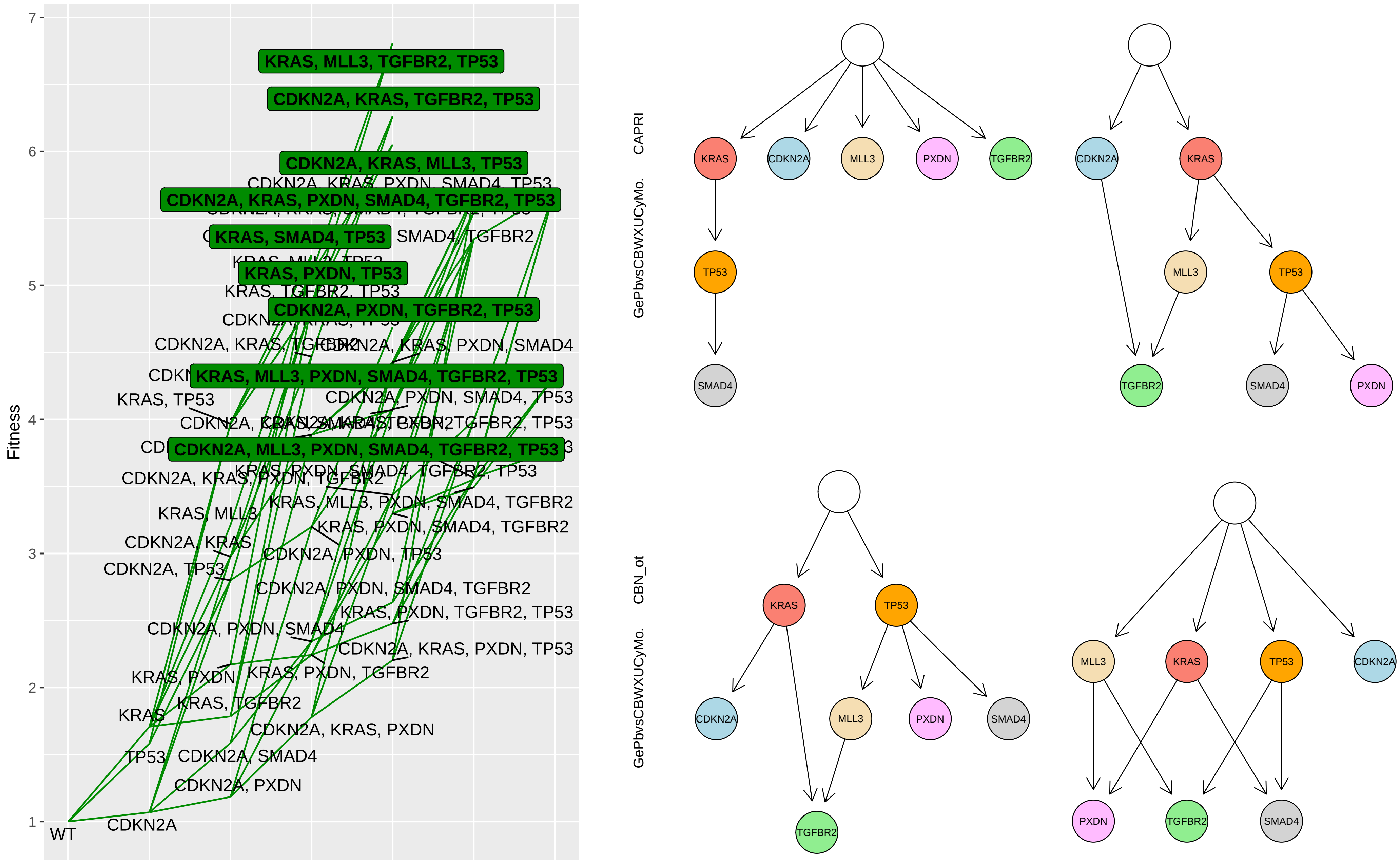

ID	p−value	Accessible Genot.
OFMGCWctuGAXJzJ	0.822	18

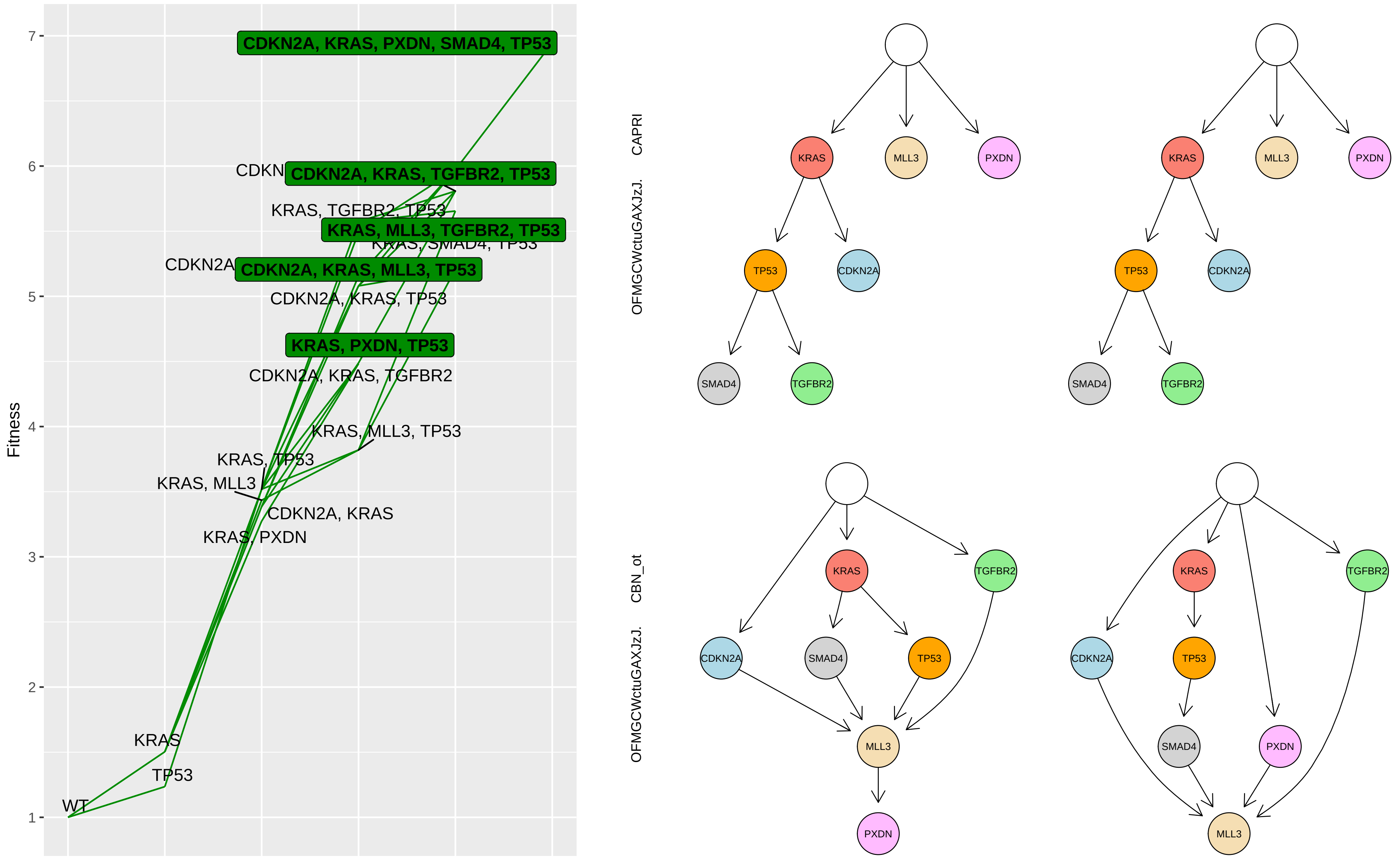

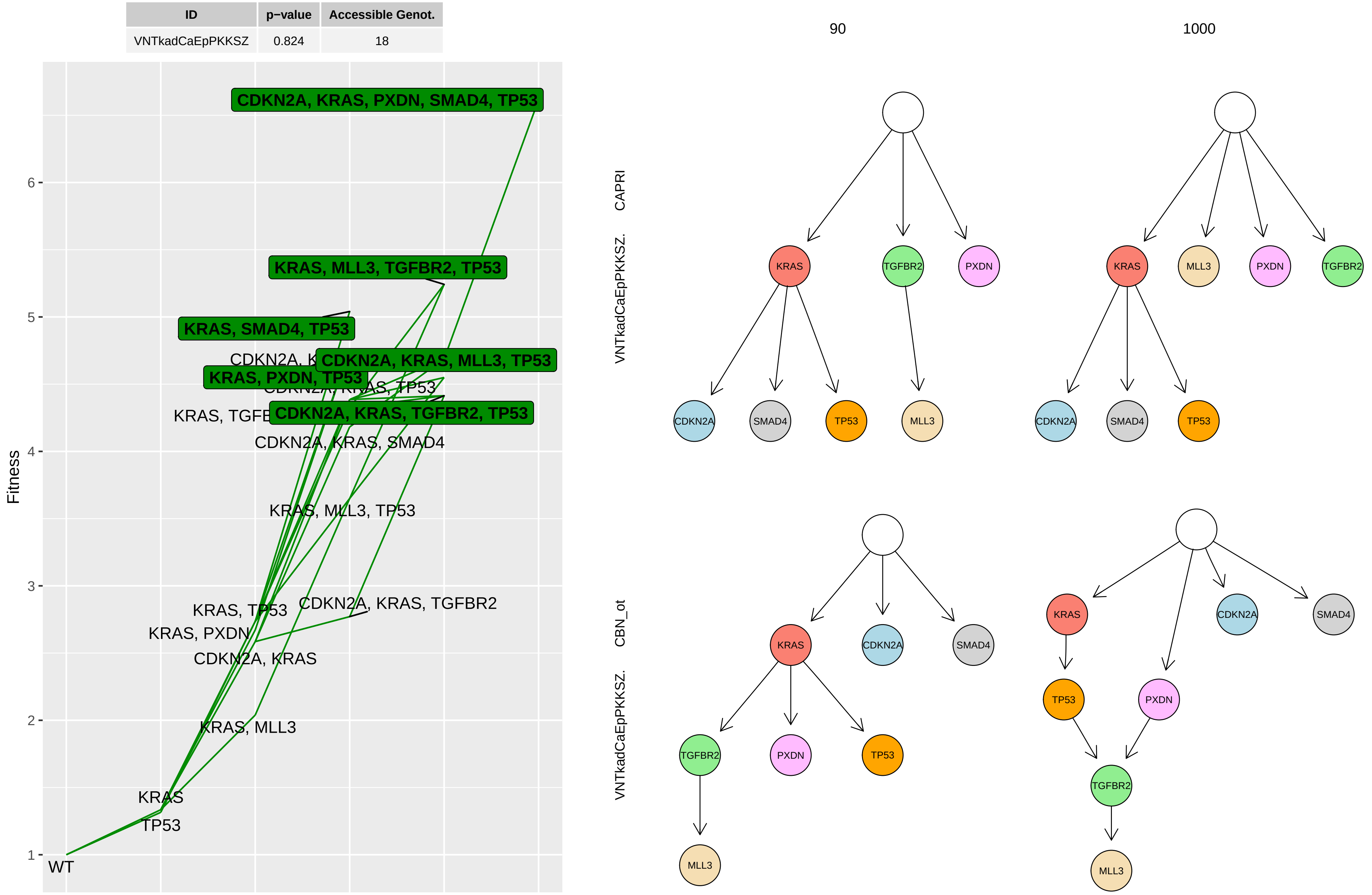

ID	p−value	Accessible Genot.
VNTkadCaEpPKKSZ	0.824	18

ID	p−value	Accessible Genot.
cRawDaWmjyvPpRk	0.834	85

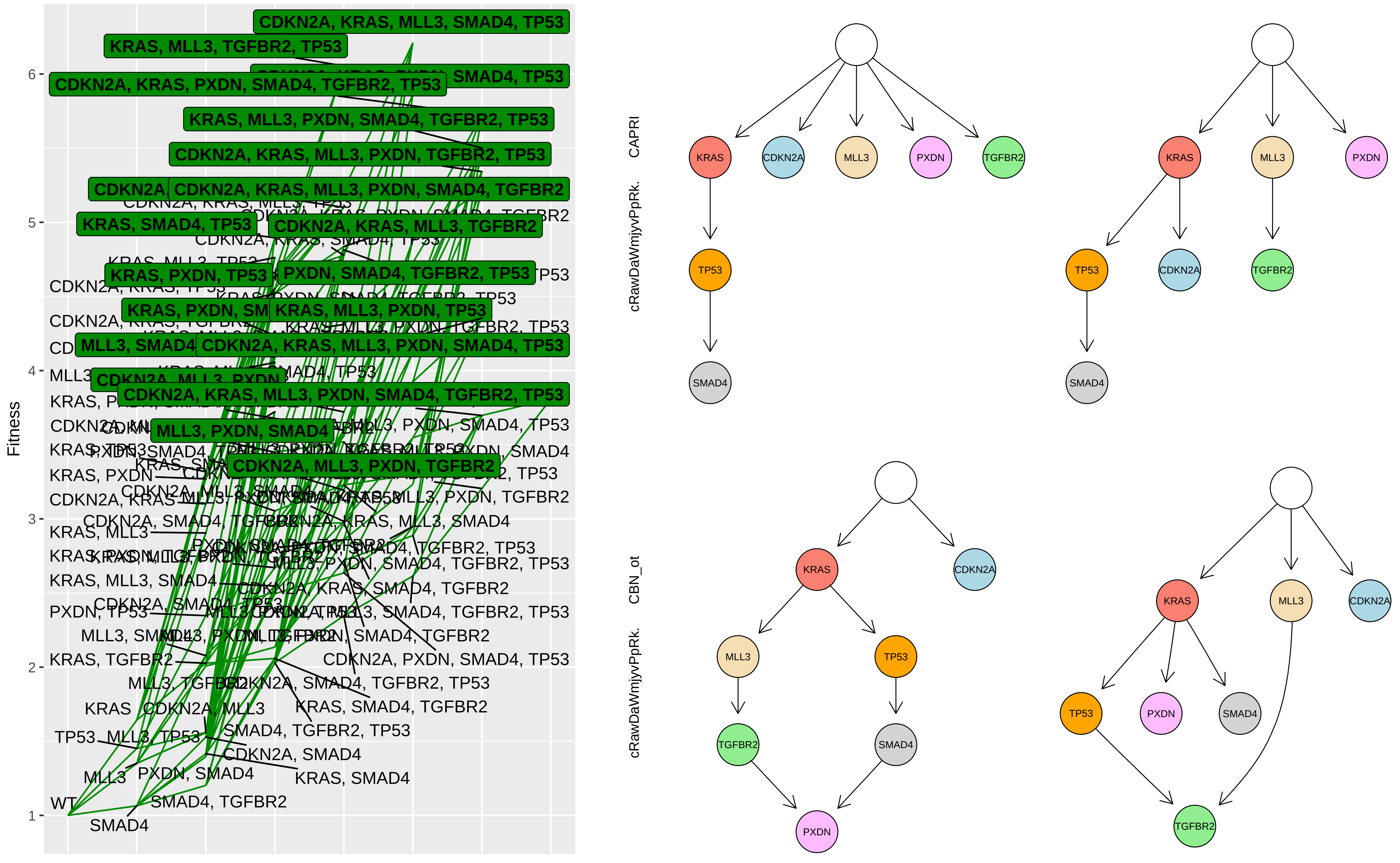

ID	p−value	Accessible Genot.
AGGxgbQHTcCuBSU	0.84	19

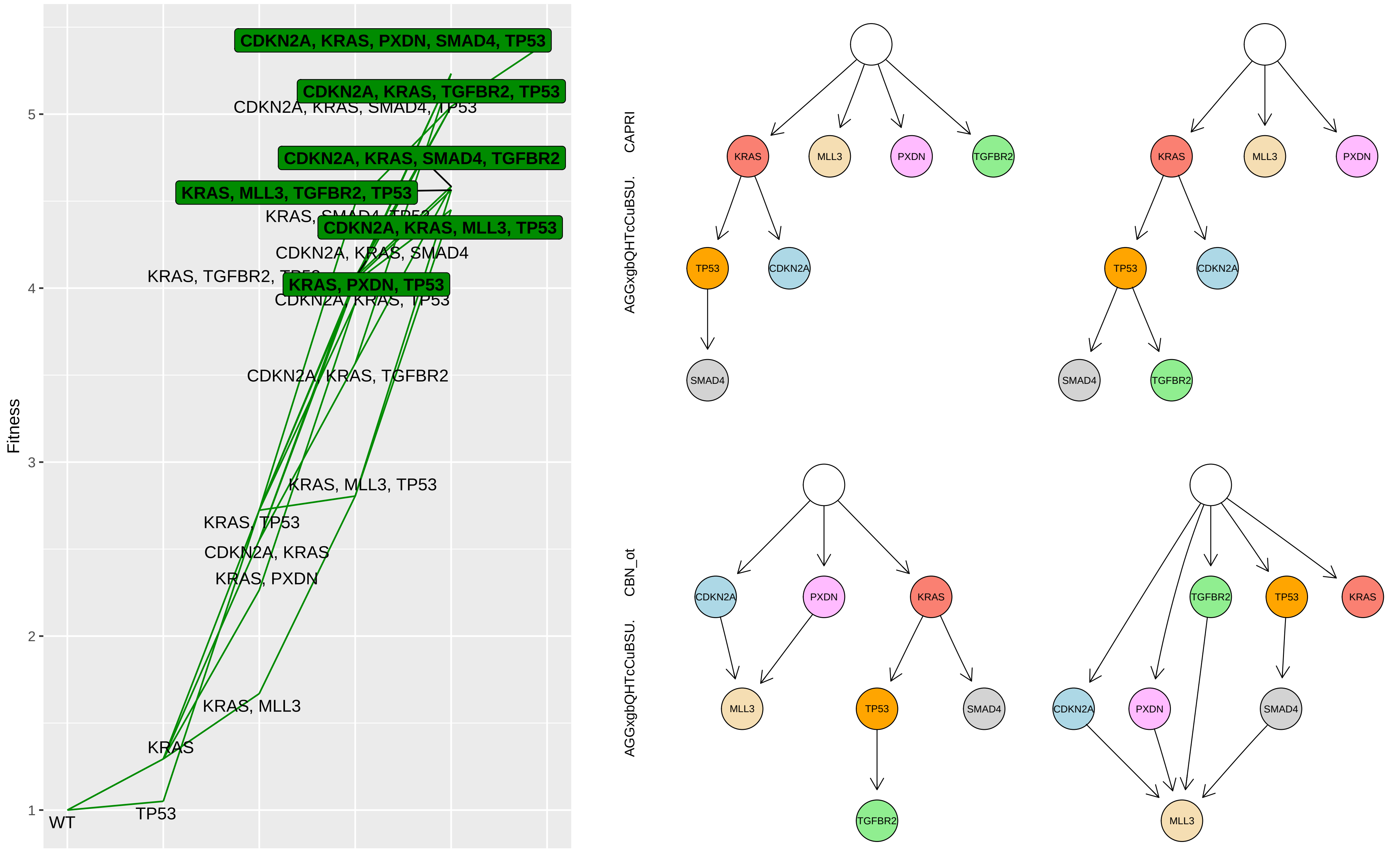

ID	p−value	Accessible Genot.
UrfLznlzgvMXaCA	0.84	18

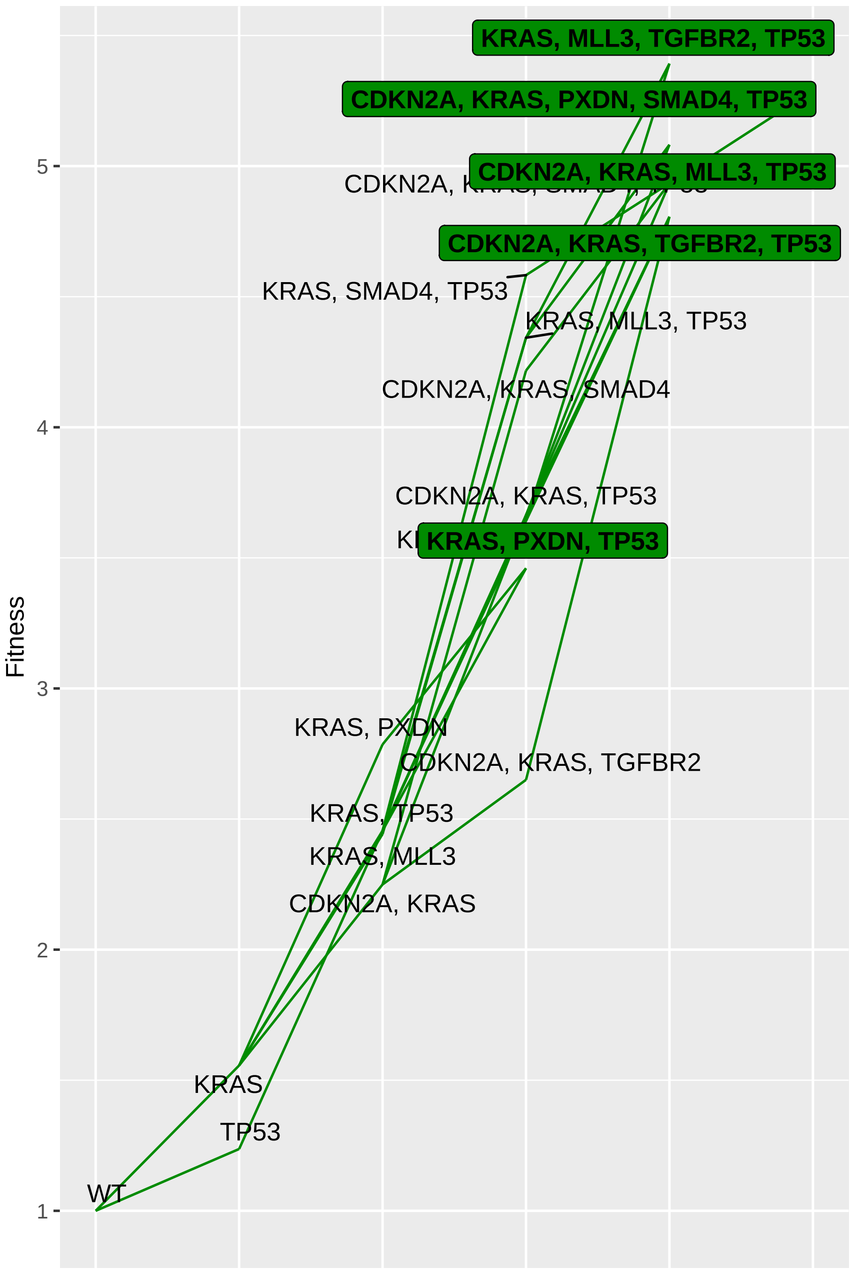

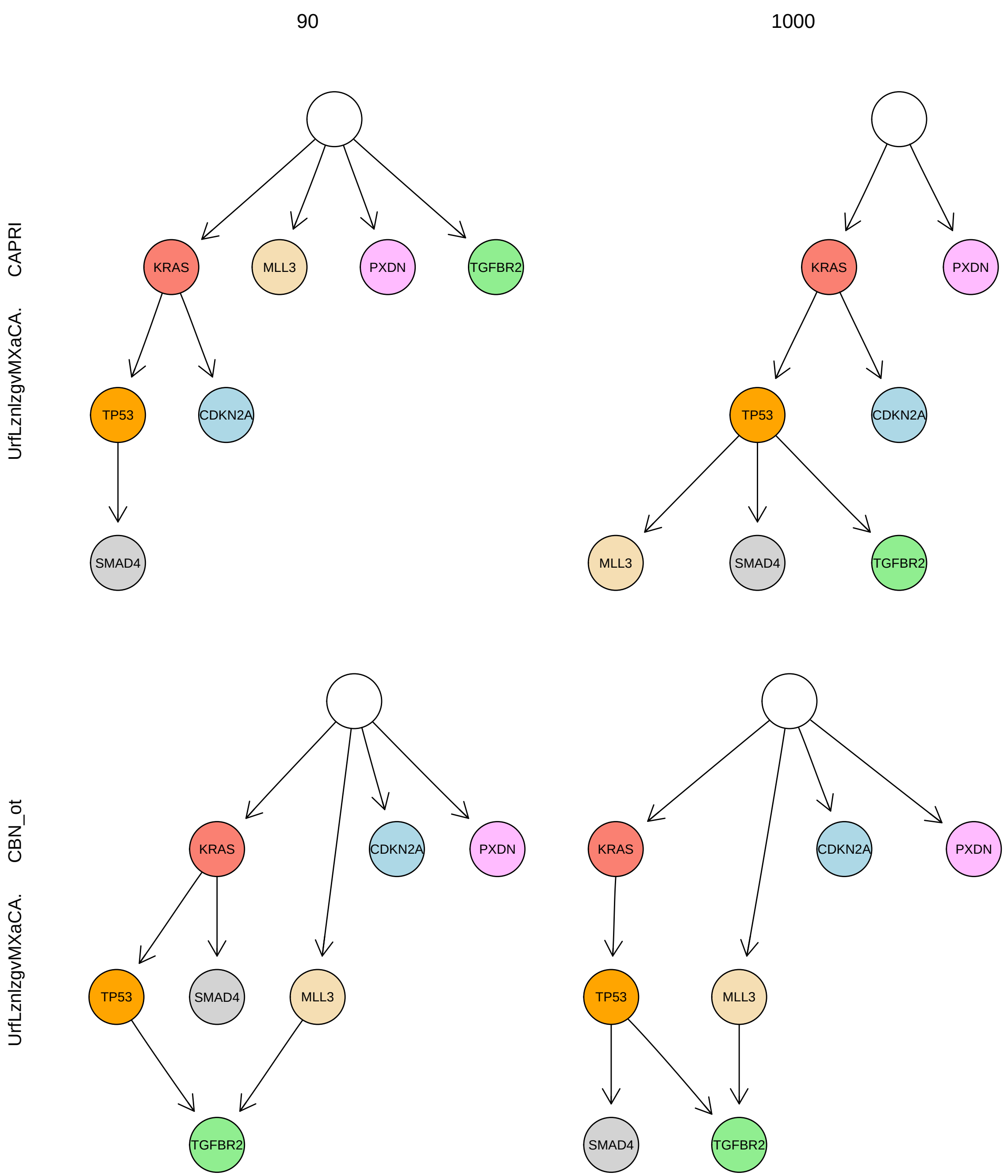

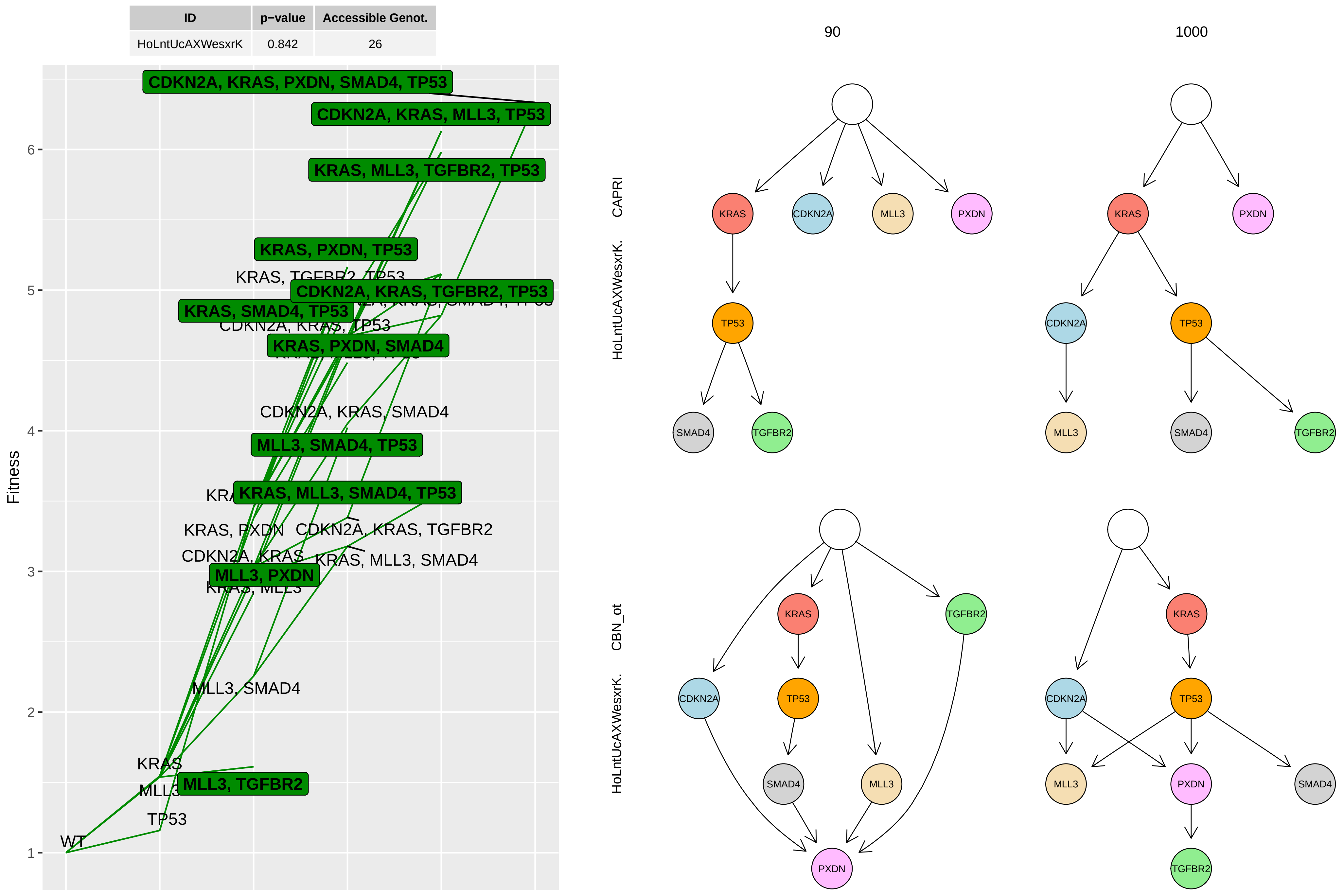
ID
p−value
Accessible Genot.
HoLntUcAXWesxrK
0.842
26
Fitness
CDKN2A, KRAS, PXDN, SMAD4, TP53
CDKN2A, KRAS, MLL3, TP53
KRAS, MLL3, TGFBR2, TP53
KRAS, PXDN, TP53
CDKN2A, KRAS, TGFBR2, TP53
KRAS, SMAD4, TP53
KRAS, PXDN, SMAD4
CDKN2A, KRAS, SMAD4
MLL3, SMAD4, TP53
KRAS, MLL3, SMAD4, TP53
KRAS, PXDN
CDKN2A, KRAS, TGFBR2
CDKN2A, KRAS
KRAS, MLL3, SMAD4
MLL3, PXDN
MLL3, SMAD4
KRAS
MLL3, TGFBR2
TP53
WT
90
1000
HoLntUcAXWesxrK. CAPRI
HoLntUcAXWesxrK. CBN_ot
KRAS
CDKN2A
MLL3
PXDN
TP53
SMAD4
TGFBR2

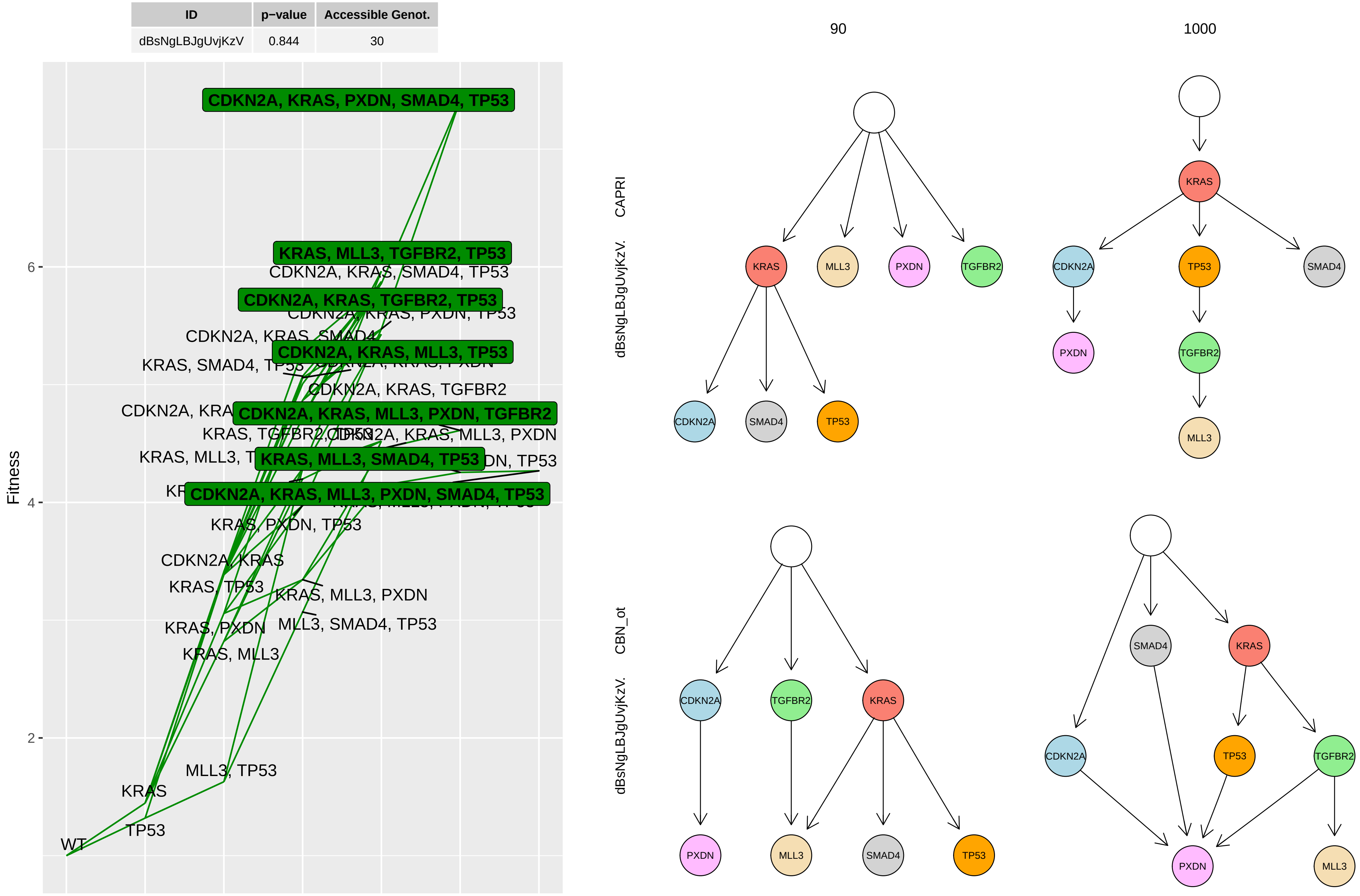
ID
p−value
Accessible Genot.
dBsNgLBJgUvjKzV
0.844
30
Fitness
CDKN2A, KRAS, PXDN, SMAD4, TP53
KRAS, MLL3, TGFBR2, TP53
CDKN2A, KRAS, SMAD4, TP53
CDKN2A, KRAS, TGFBR2, TP53
CDKN2A, KRAS, MLL3, TP53
CDKN2A, KRAS, TGFBR2
CDKN2A, KRAS, MLL3, PXDN, TGFBR2
CDKN2A, KRAS, MLL3, PXDN
KRAS, MLL3, SMAD4, TP53
CDKN2A, KRAS, MLL3, PXDN, SMAD4, TP53
KRAS, PXDN, TP53
CDKN2A, KRAS
KRAS, TP53
KRAS, MLL3, PXDN
MLL3, SMAD4, TP53
KRAS, PXDN
KRAS, MLL3
MLL3, TP53
KRAS
TP53
WT
90
1000
dBsNgLBJgUvjKzV. CAPRI
dBsNgLBJgUvjKzV. CBN_ot
KRAS
MLL3
PXDN
TGFBR2
CDKN2A
SMAD4
TP53

ID	p−value	Accessible Genot.
SlkVSzVzhkpJrXl	0.849	18

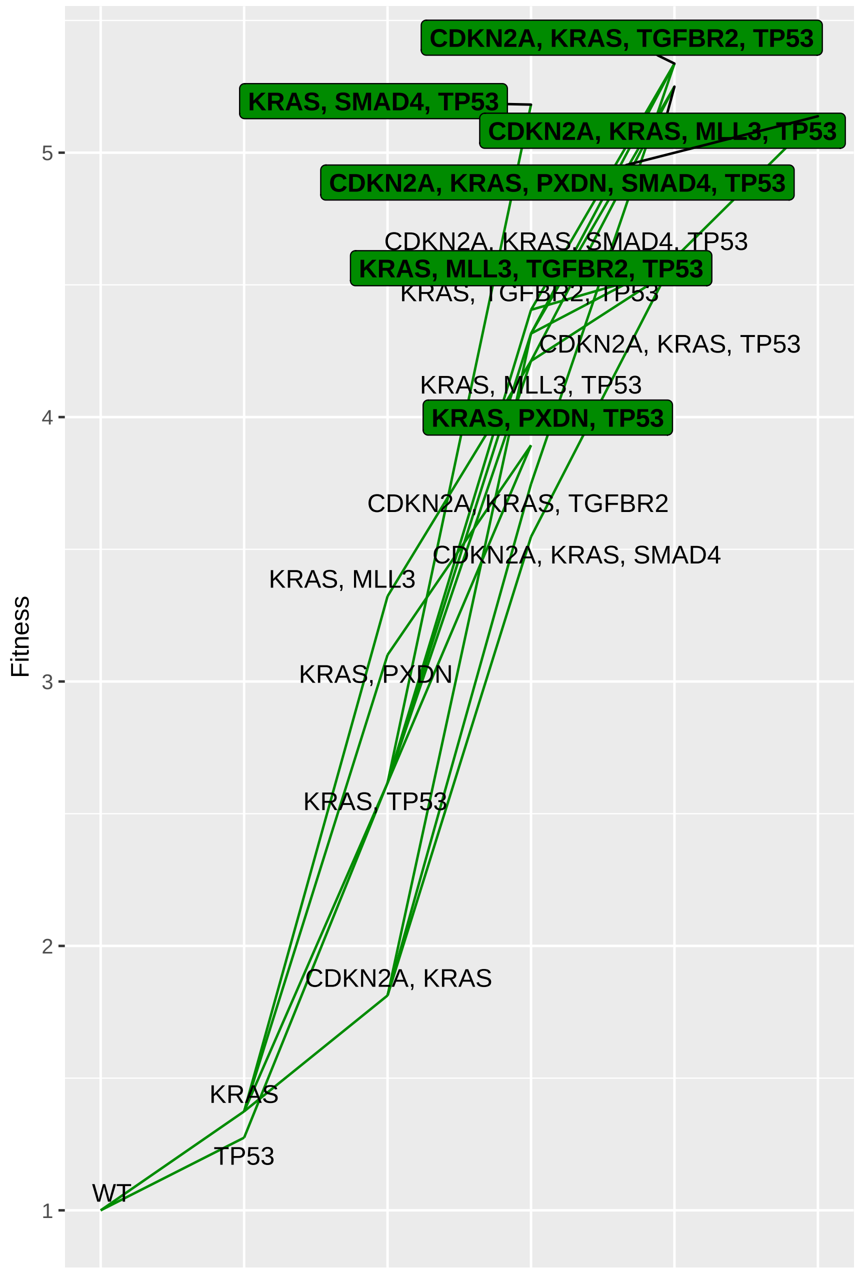

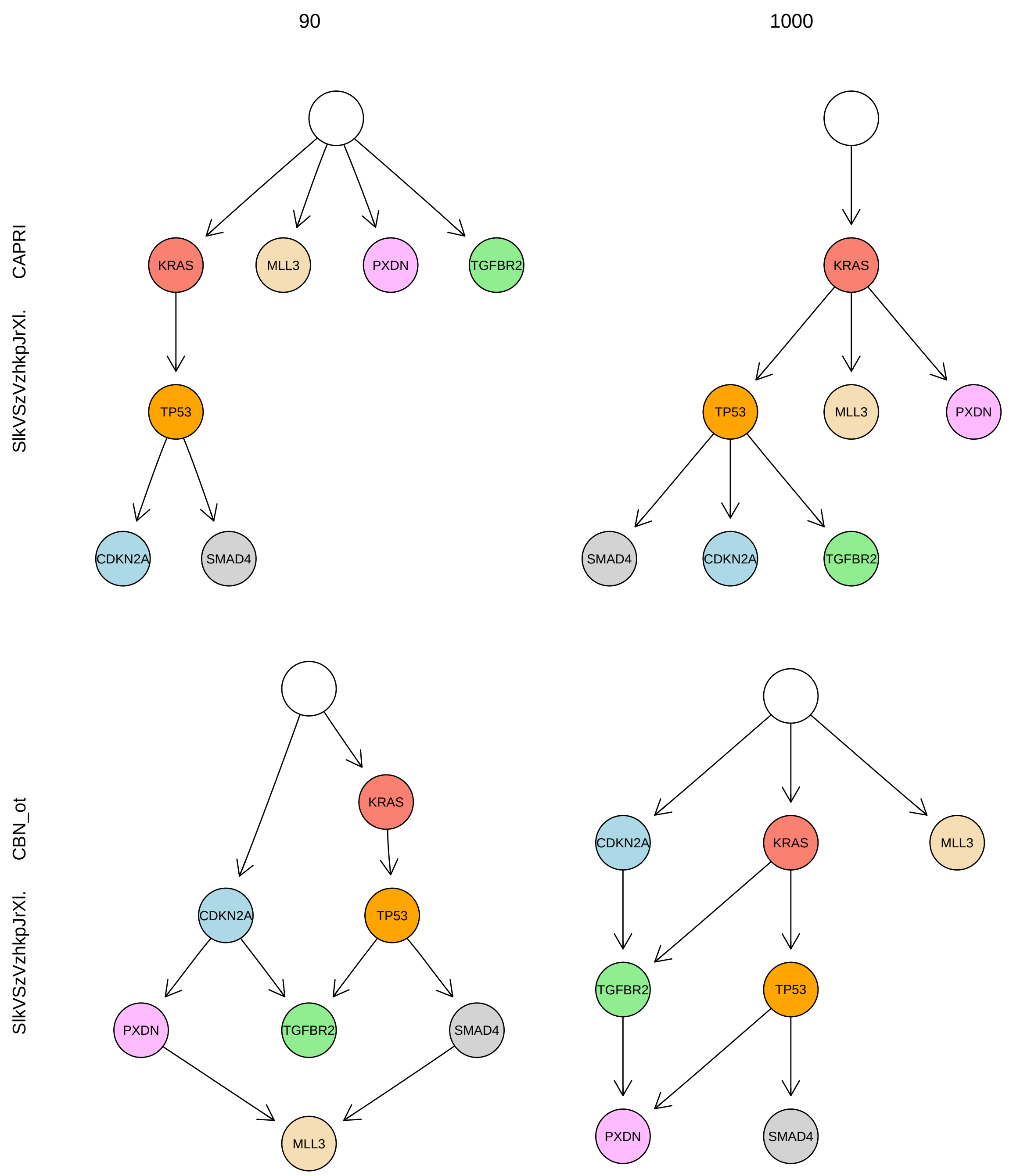

ID	p−value	Accessible Genot.
mxllWAxzViosuJQ	0.85	19

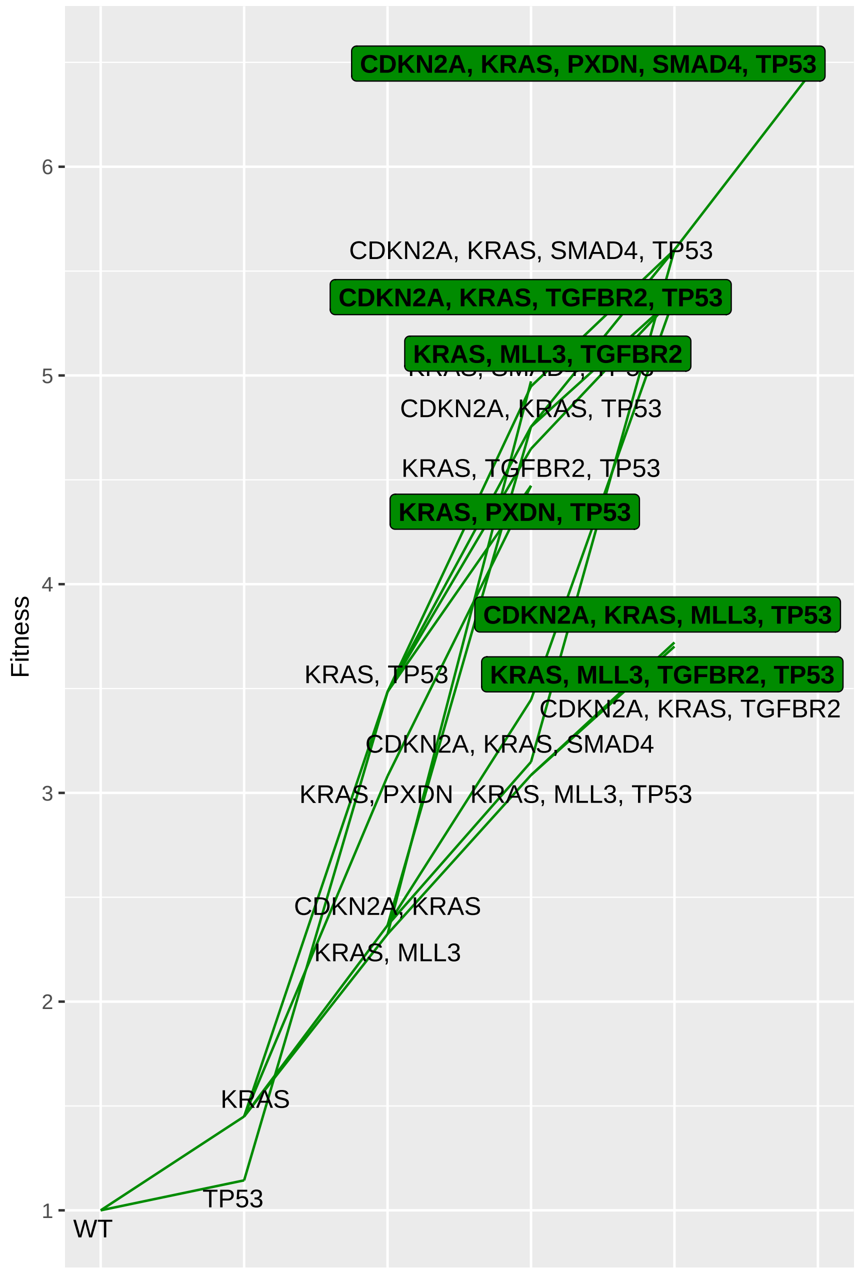

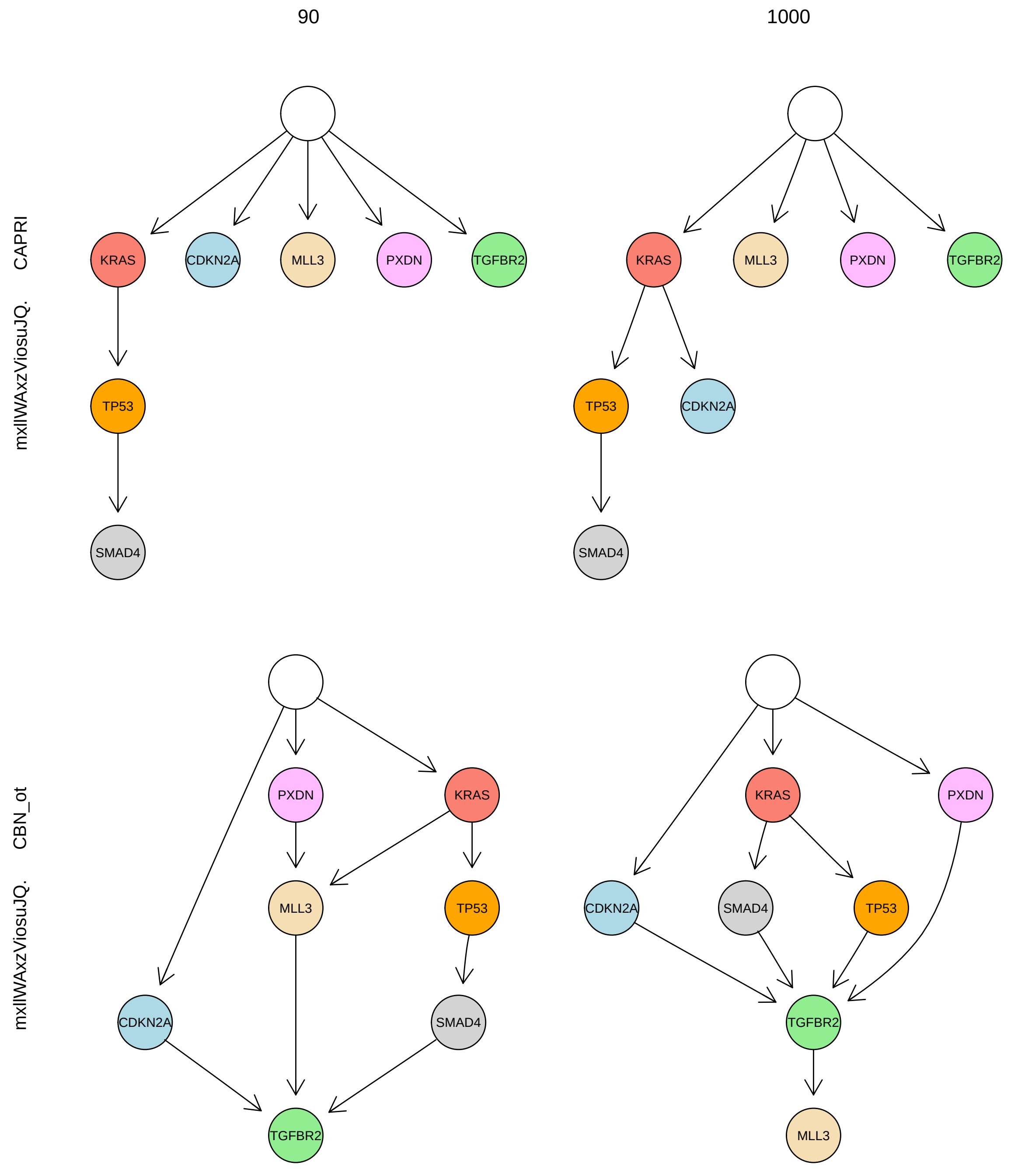

ID	p−value	Accessible Genot.
HogFyEjKyqwESaA	0.861	18

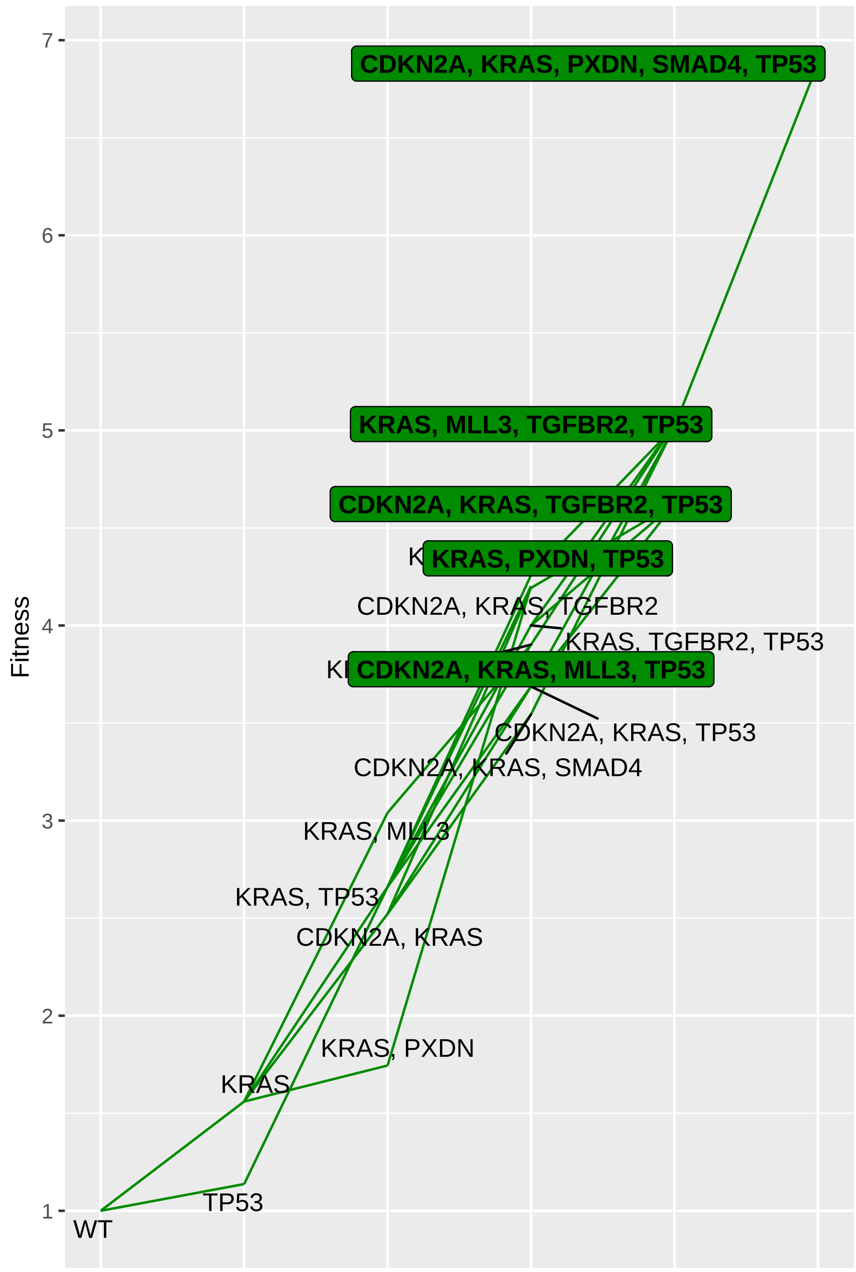

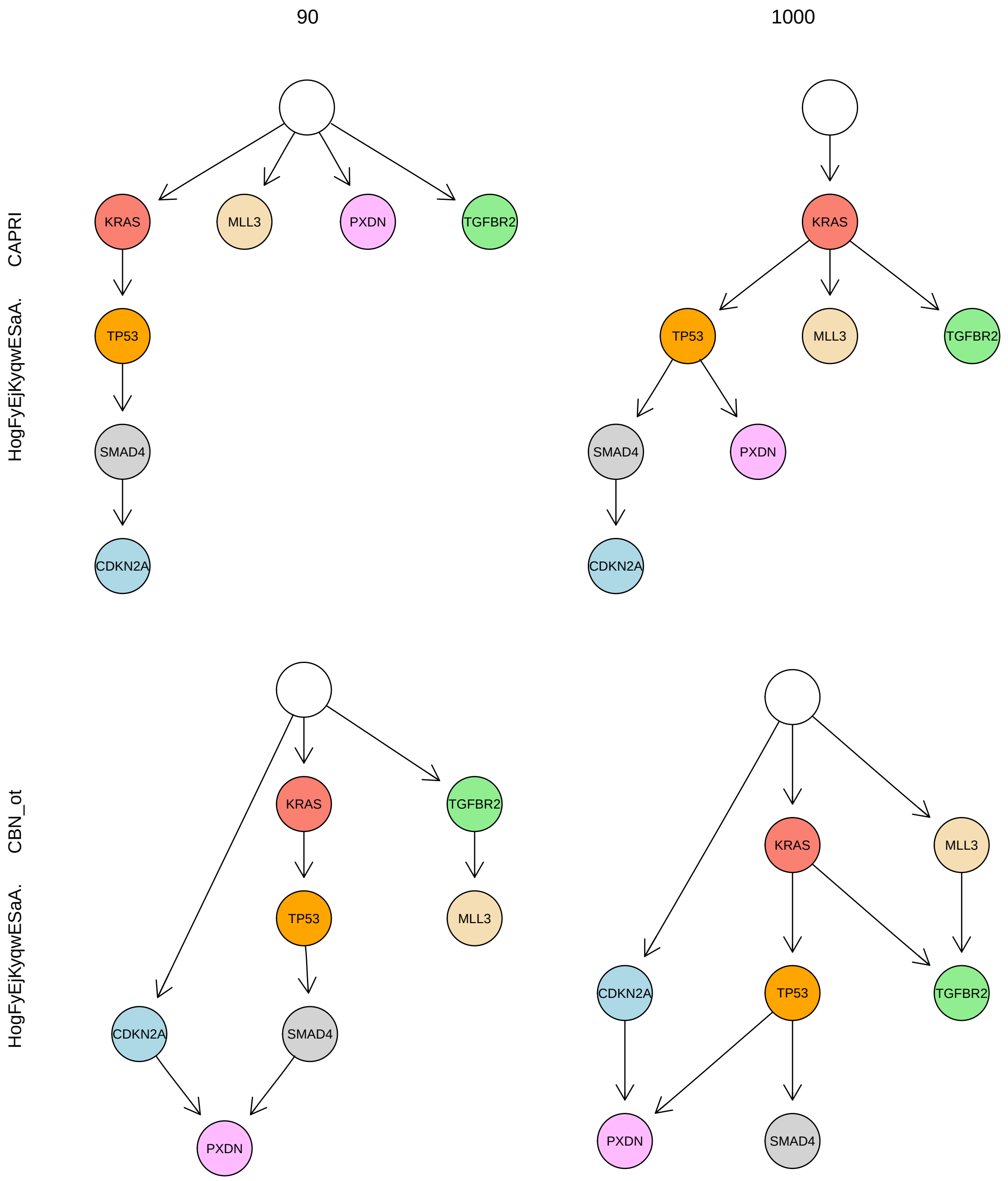

ID	p−value	Accessible Genot.
FSmOYexBYoytitx	0.864	18

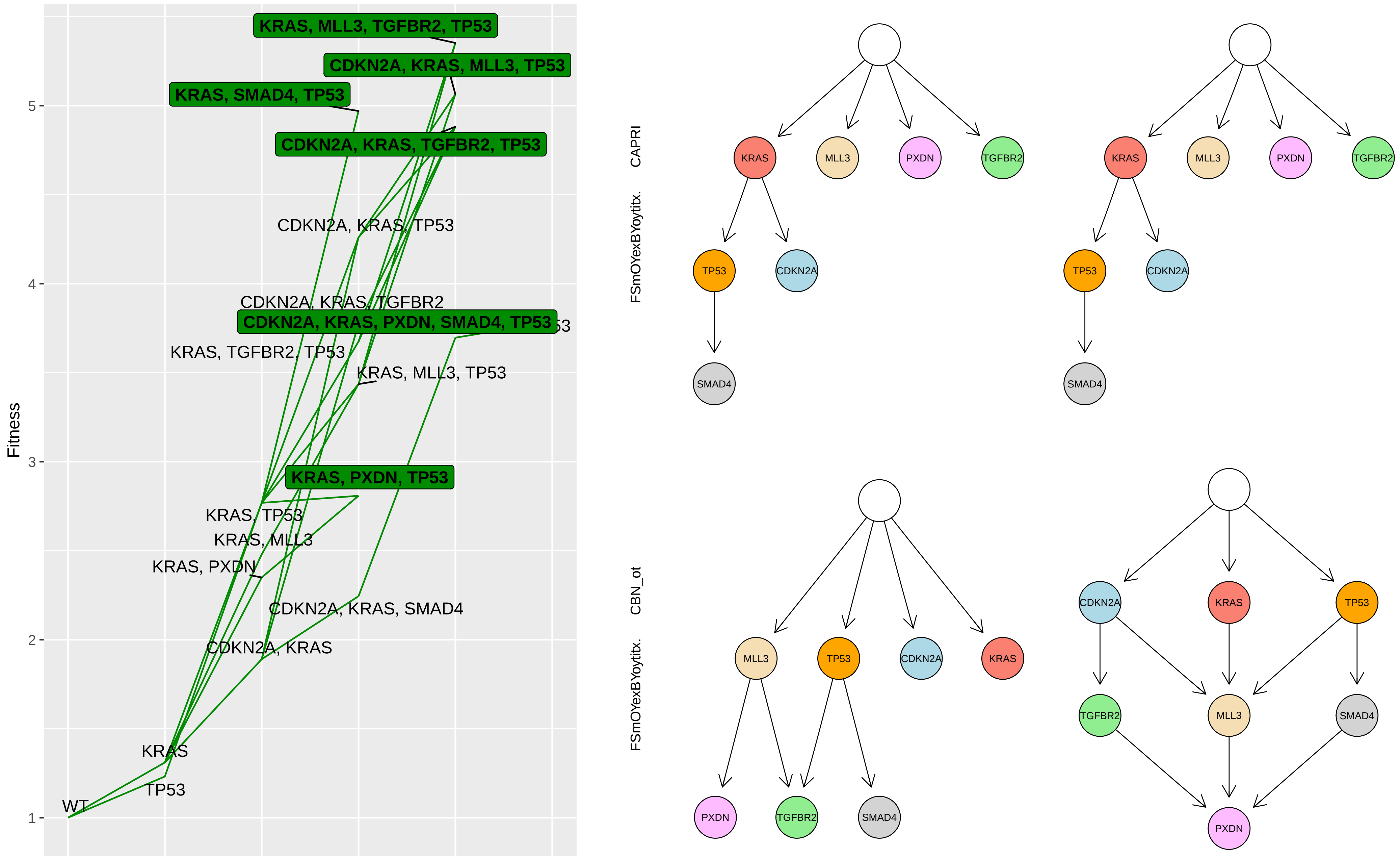

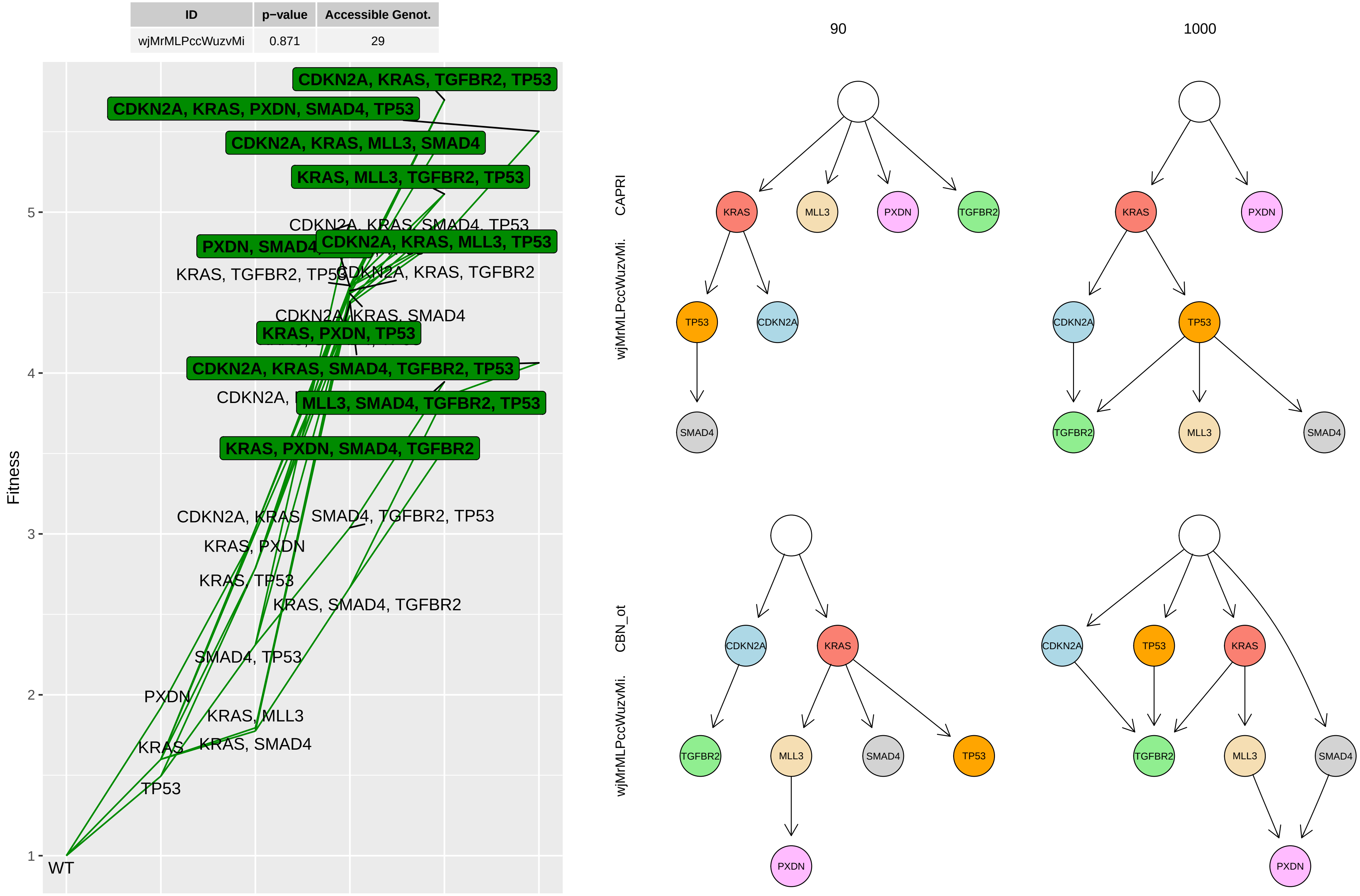

ID
p−value
Accessible Genot.
wjMrMLPccWuzvMi
0.871
29
Fitness
1
2
3
4
5
WT
CDKN2A, KRAS, TGFBR2, TP53
CDKN2A, KRAS, PXDN, SMAD4, TP53
CDKN2A, KRAS, MLL3, SMAD4
KRAS, MLL3, TGFBR2, TP53
CDKN2A, KRAS, MLL3, TP53
KRAS, TGFBR2, TP53
CDKN2A, KRAS, TGFBR2
KRAS, PXDN, TP53
CDKN2A, KRAS, SMAD4, TGFBR2, TP53
MLL3, SMAD4, TGFBR2, TP53
KRAS, PXDN, SMAD4, TGFBR2
CDKN2A, KRAS, SMAD4, TGFBR2, TP53
KRAS, PXDN
KRAS, TP53
KRAS, SMAD4, TGFBR2
SMAD4, TP53
PXDN
KRAS, MLL3
KRAS
KRAS, SMAD4
TP53
90
1000
wjMrMLPccWuzvMi. CAPRI
wjMrMLPccWuzvMi. CBN_ot
KRAS
MLL3
PXDN
TGFBR2
TP53
CDKN2A
SMAD4

ID	p−value	Accessible Genot.
FZxXijBdqDLUOAM	0.875	118

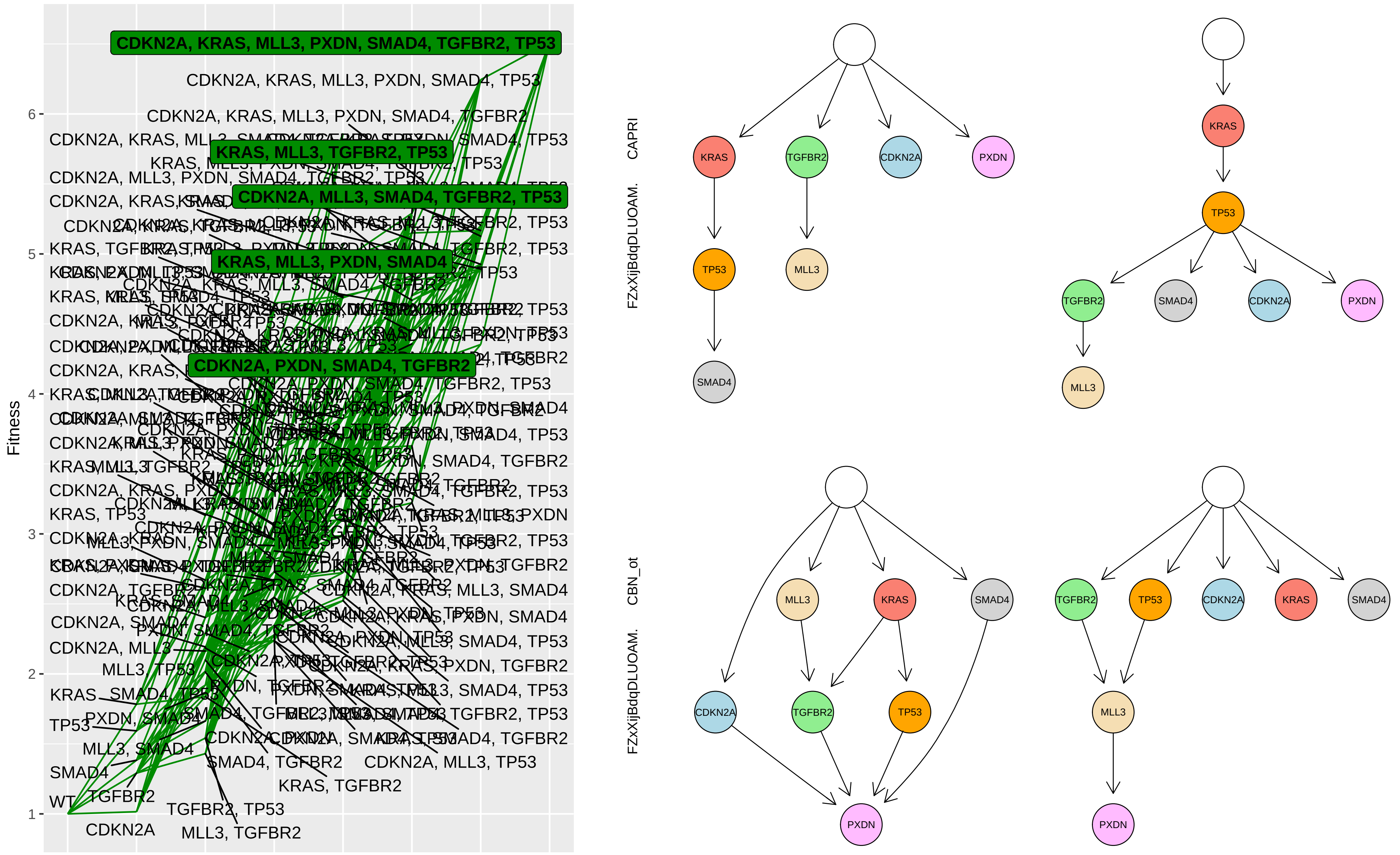

ID	p−value	Accessible Genot.
EOSJsxwultmsYPt	0.884	19

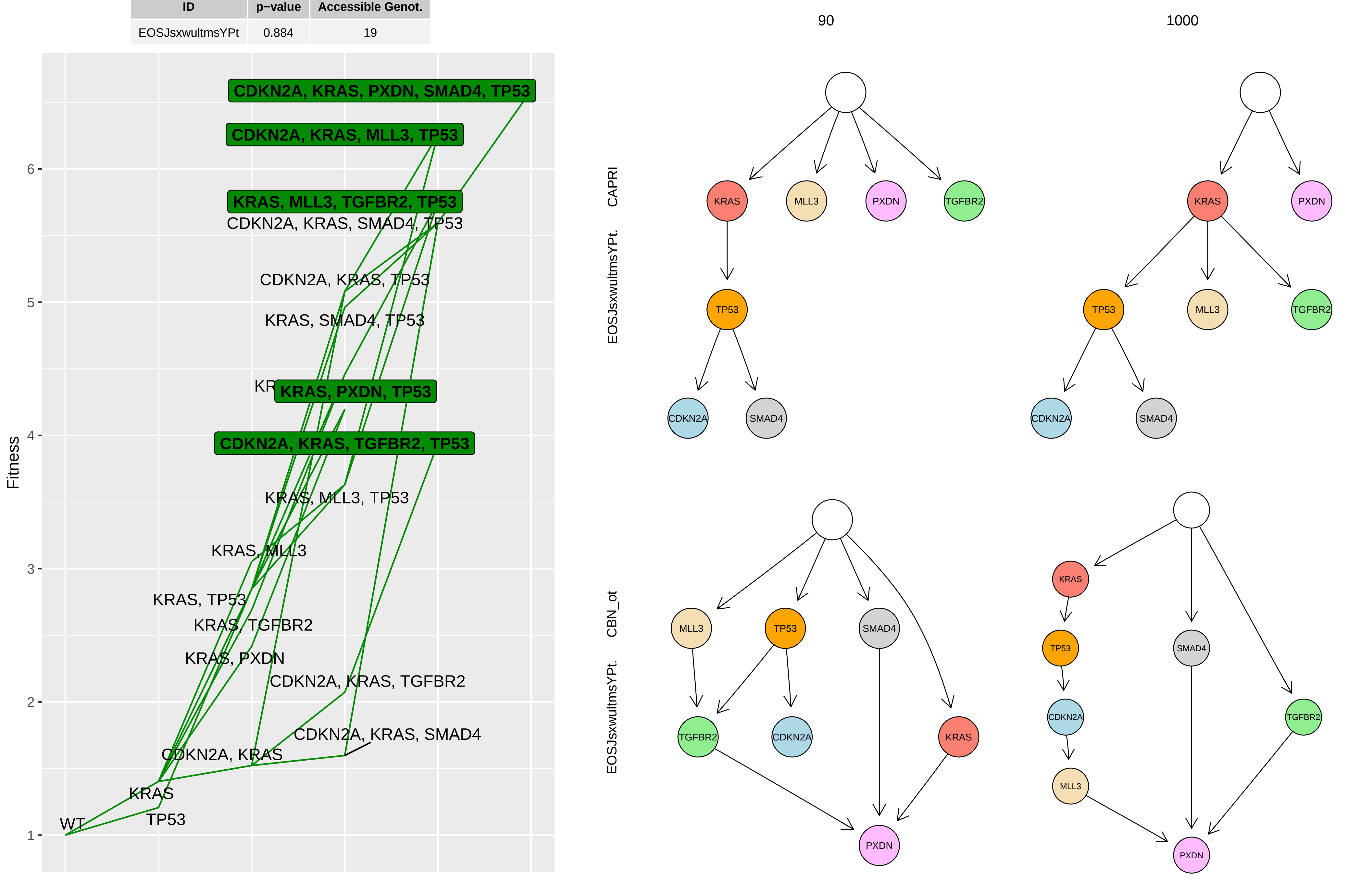

ID	p−value	Accessible Genot.
jLqUgFkiLHXoIYQ	0.887	67

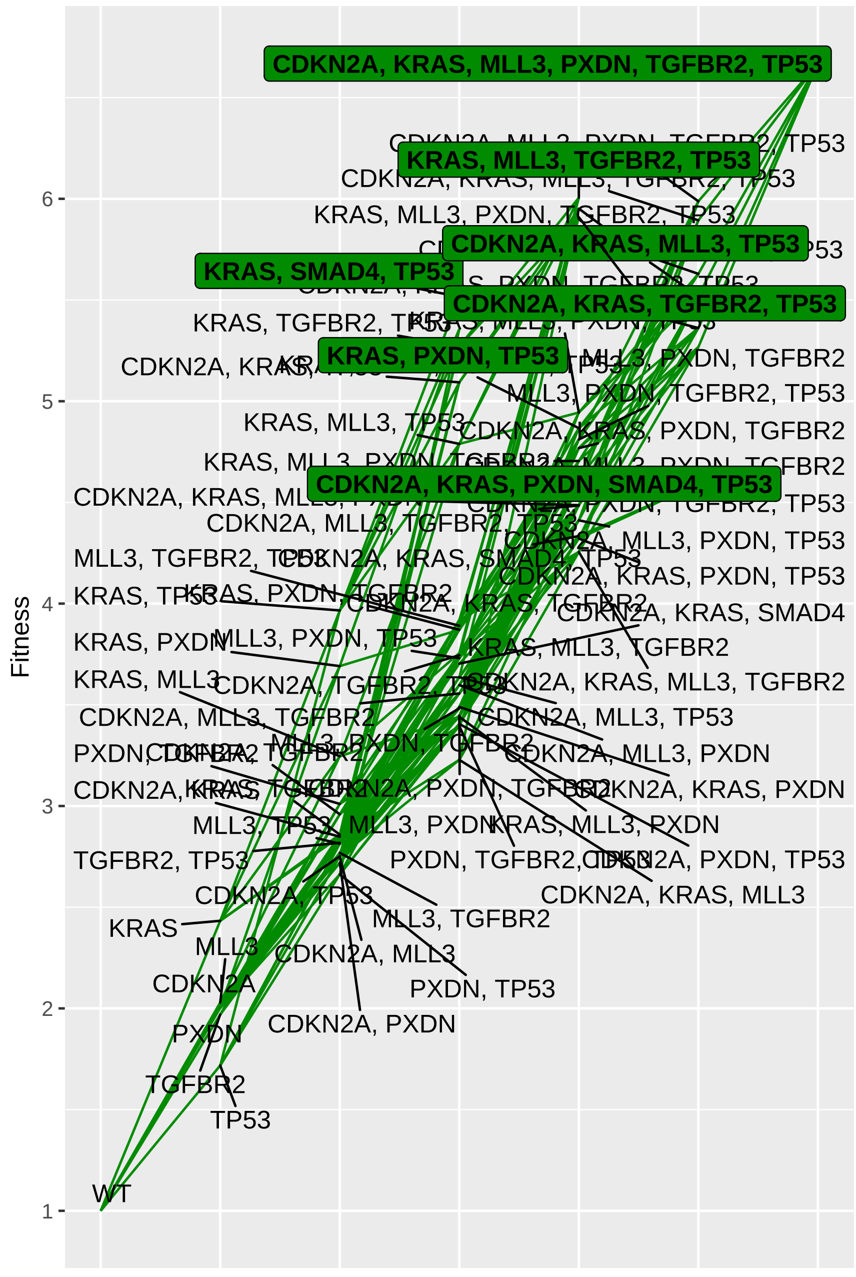

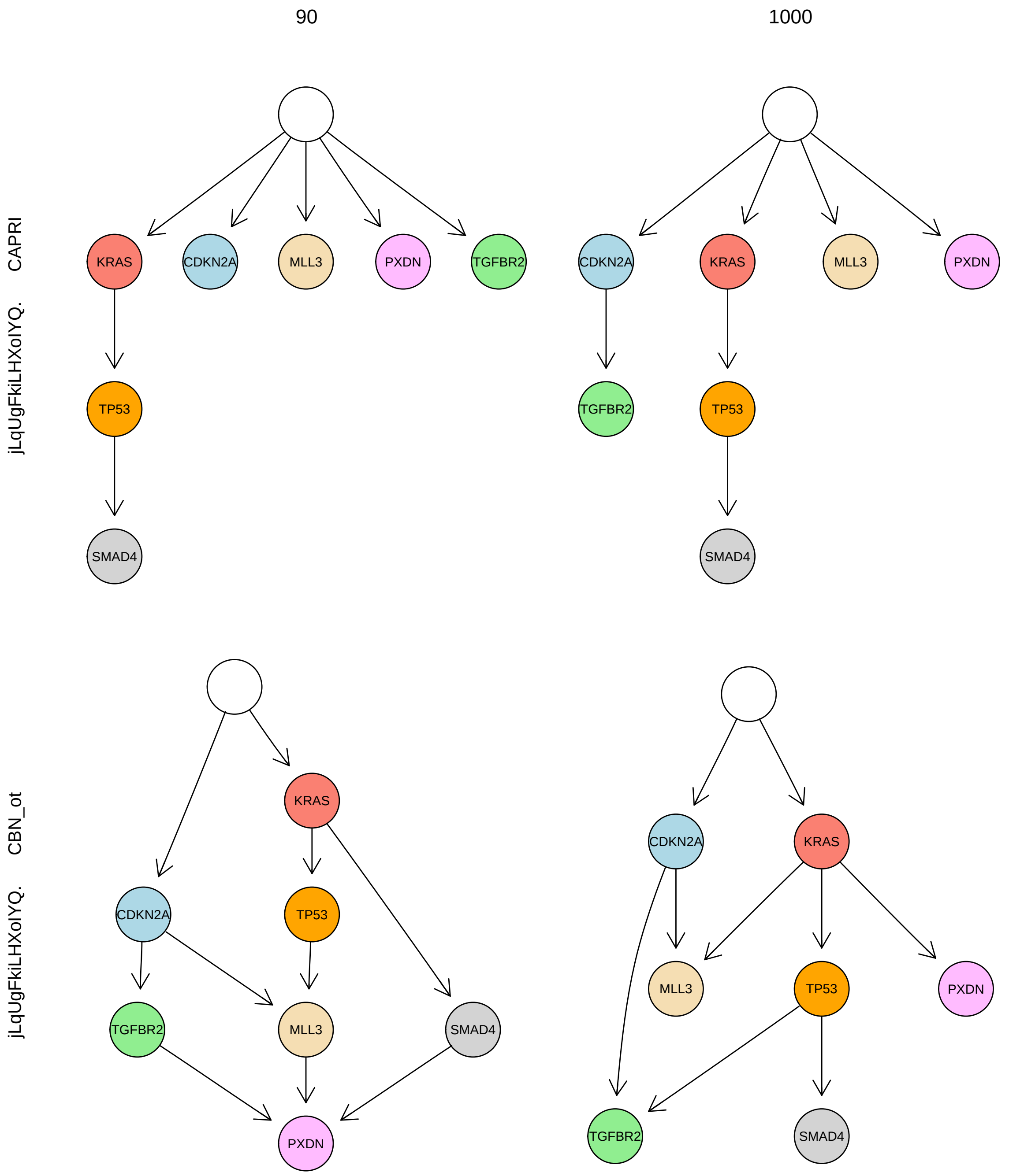

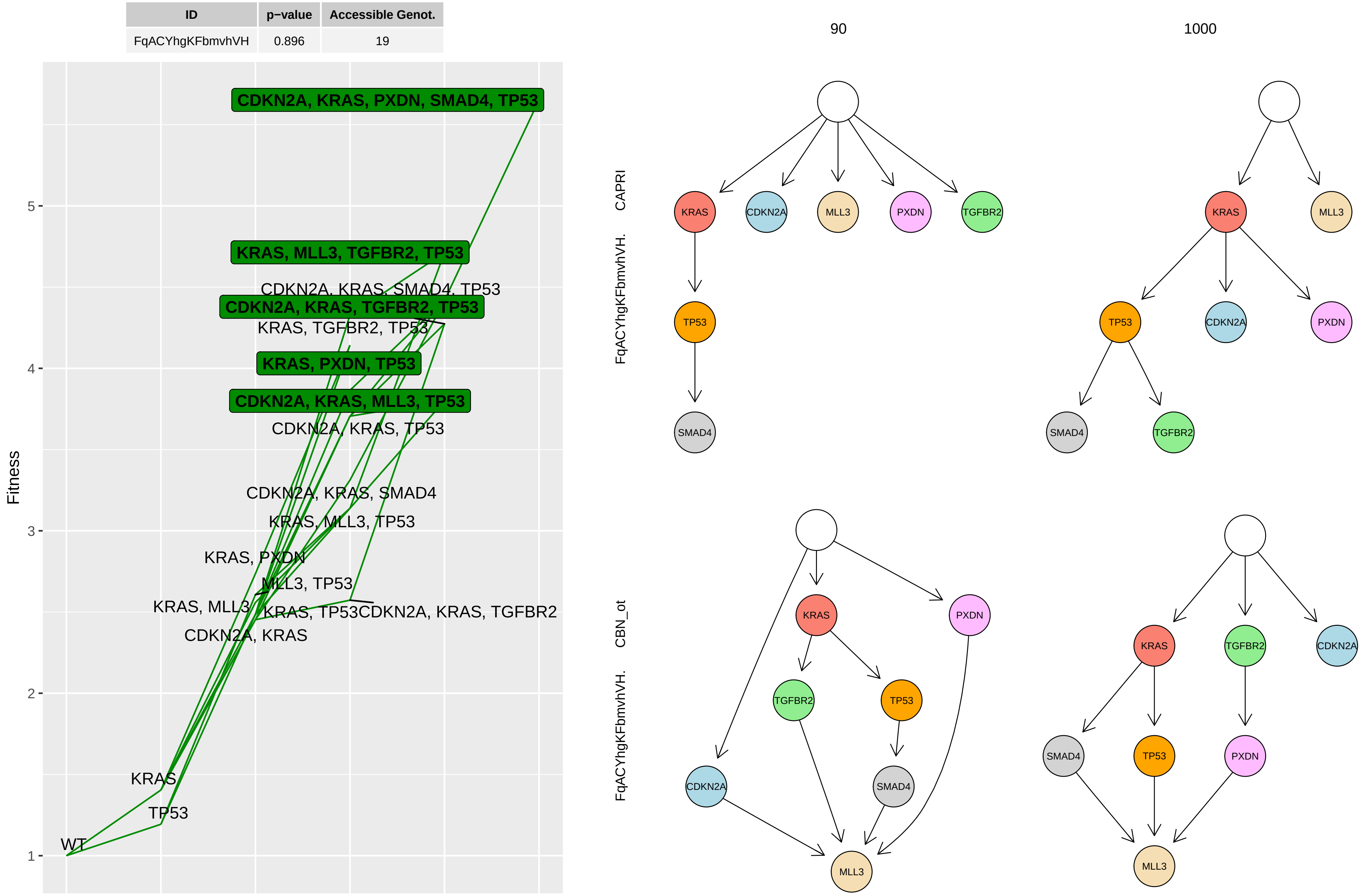

ID
p−value
Accessible Genot.
FqACYhgKFbmvhVH
0.896
19
Fitness
CDKN2A, KRAS, PXDN, SMAD4, TP53
KRAS, MLL3, TGFBR2, TP53
CDKN2A, KRAS, SMAD4, TP53
CDKN2A, KRAS, TGFBR2, TP53
KRAS, TGFBR2, TP53
KRAS, PXDN, TP53
CDKN2A, KRAS, MLL3, TP53
CDKN2A, KRAS, TP53
CDKN2A, KRAS, SMAD4
KRAS, MLL3, TP53
KRAS, PXDN
MLL3, TP53
KRAS, MLL3
KRAS, TP53
CDKN2A, KRAS, TGFBR2
CDKN2A, KRAS
KRAS
TP53
WT
90
1000
FqACYhgKFbmvhVH. CAPRI
FqACYhgKFbmvhVH. CBN_ot
KRAS
CDKN2A
MLL3
PXDN
TGFBR2
TP53
SMAD4

ID	p−value	Accessible Genot.
bWpfWzJAjyjZJKp	0.908	18

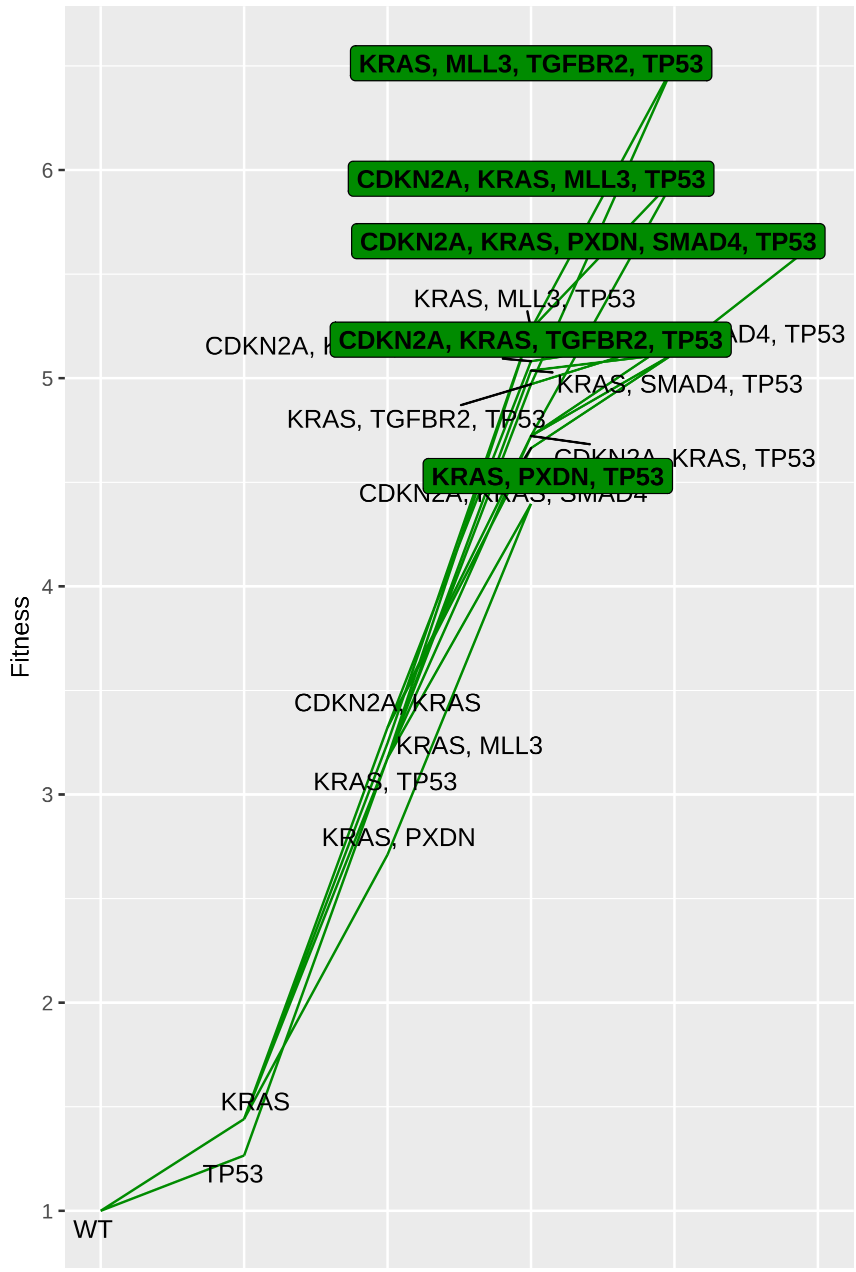

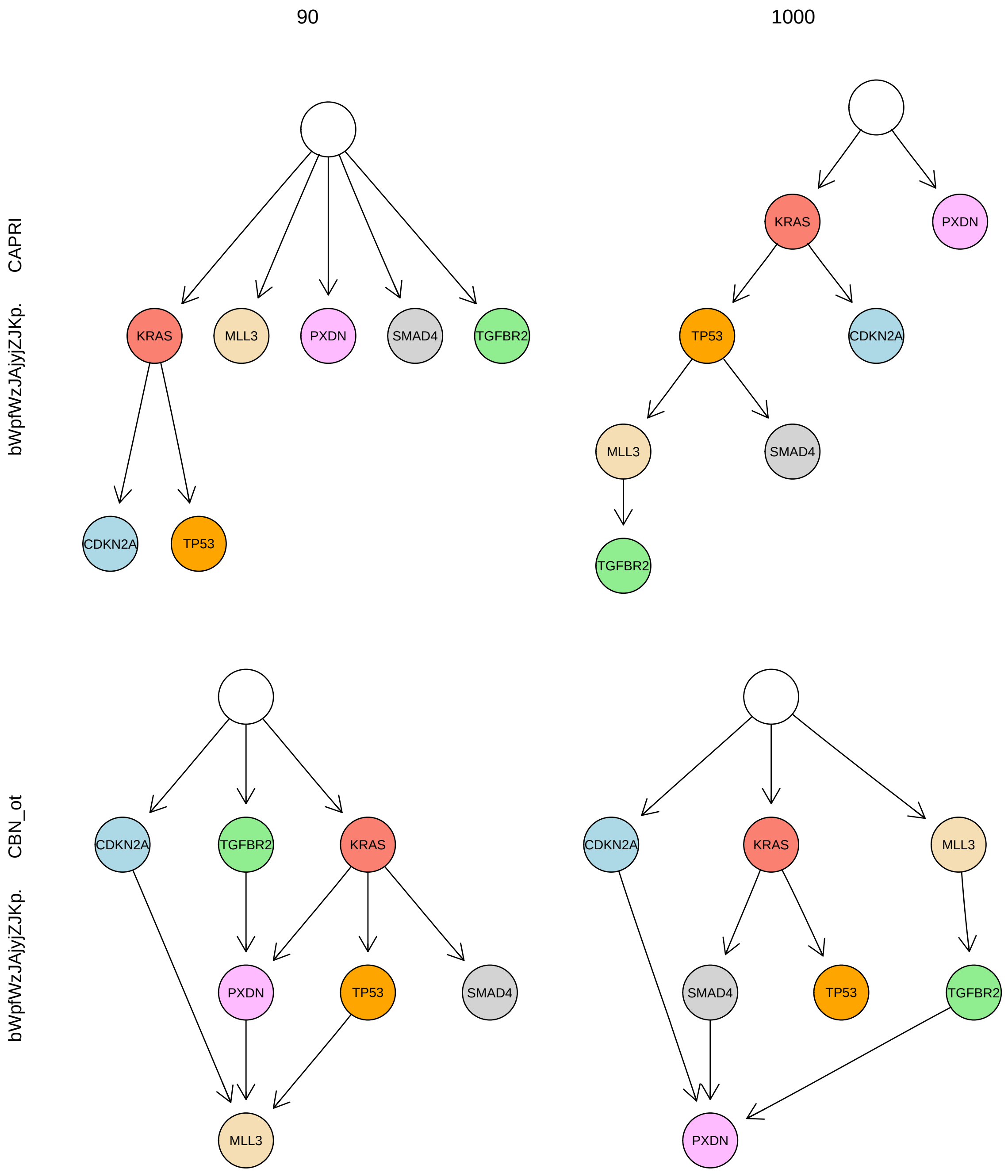

ID	p−value	Accessible Genot.
tjeWiRJbBVyqRNU	0.911	127

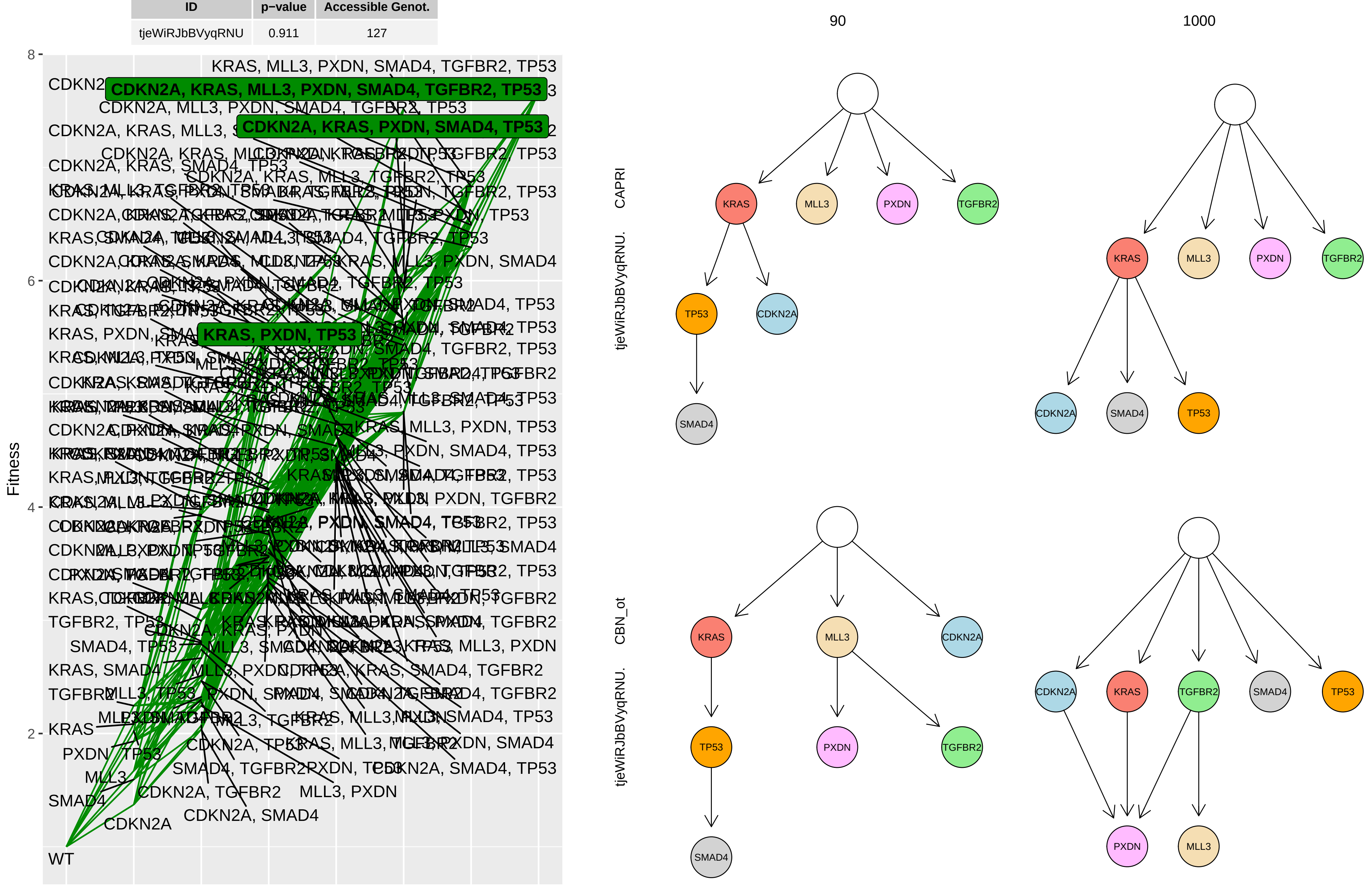

ID	p−value	Accessible Genot.
hsqWYerNoNODQwP	0.926	18

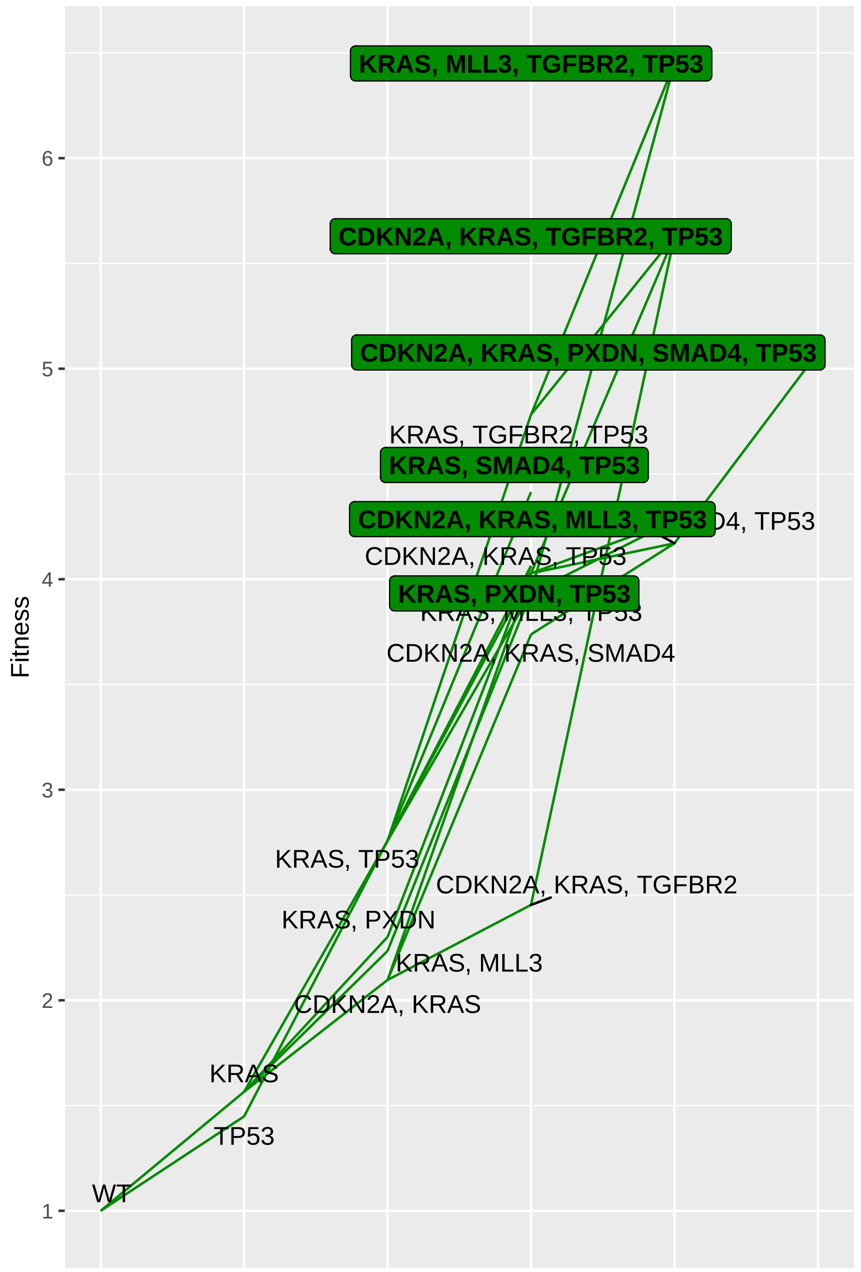

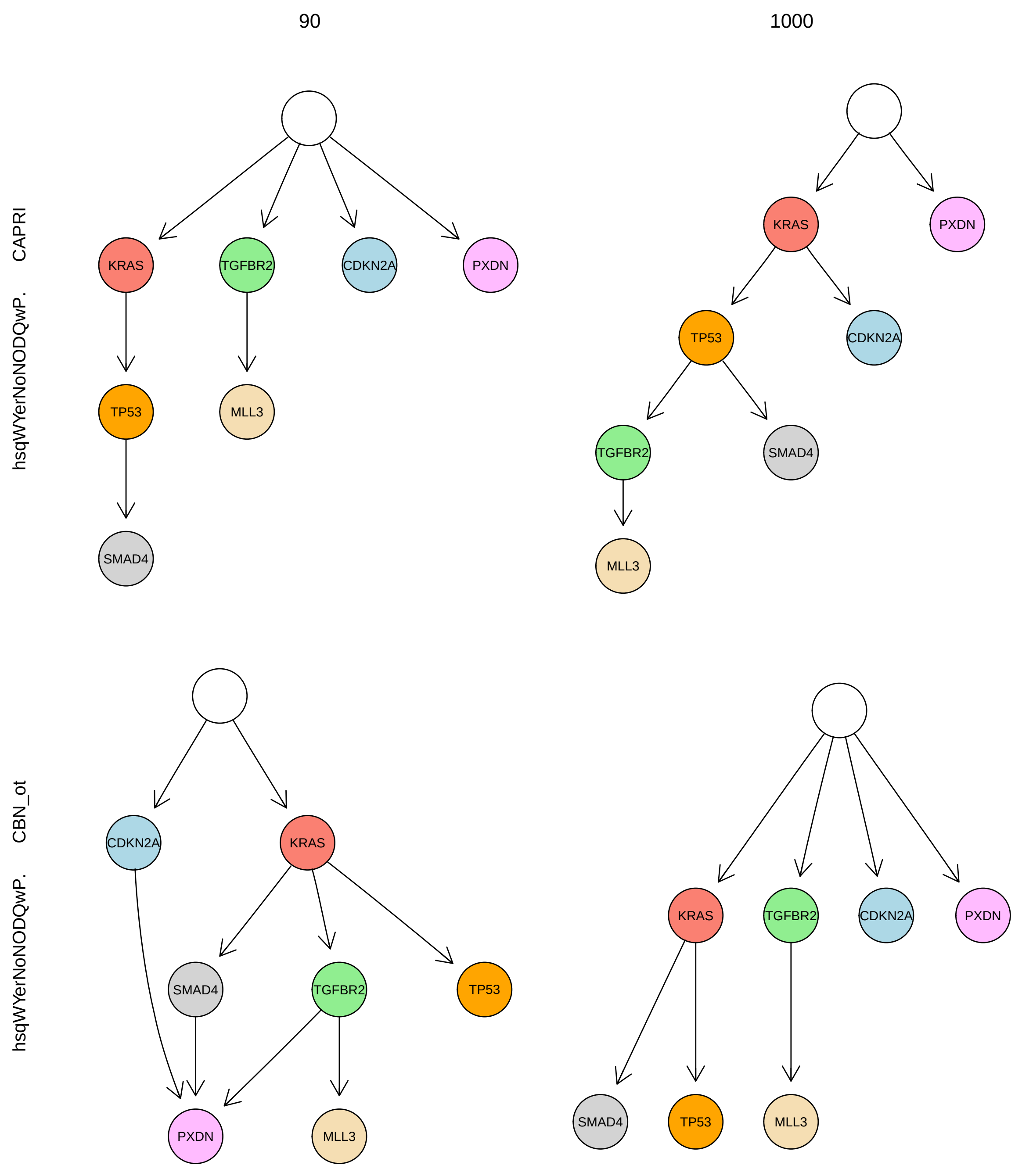

ID	p−value	Accessible Genot.
IZOZBjekwlFZAlm	0.935	66

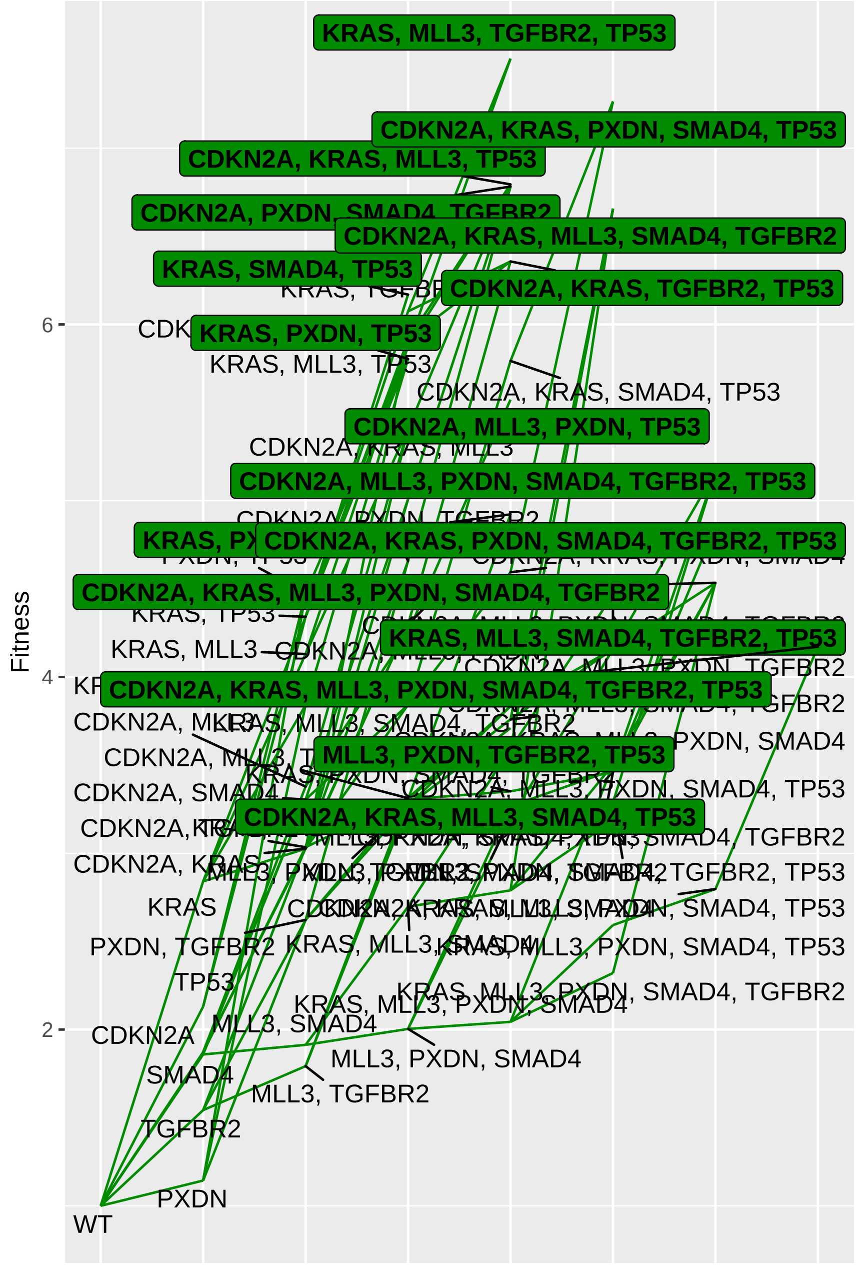

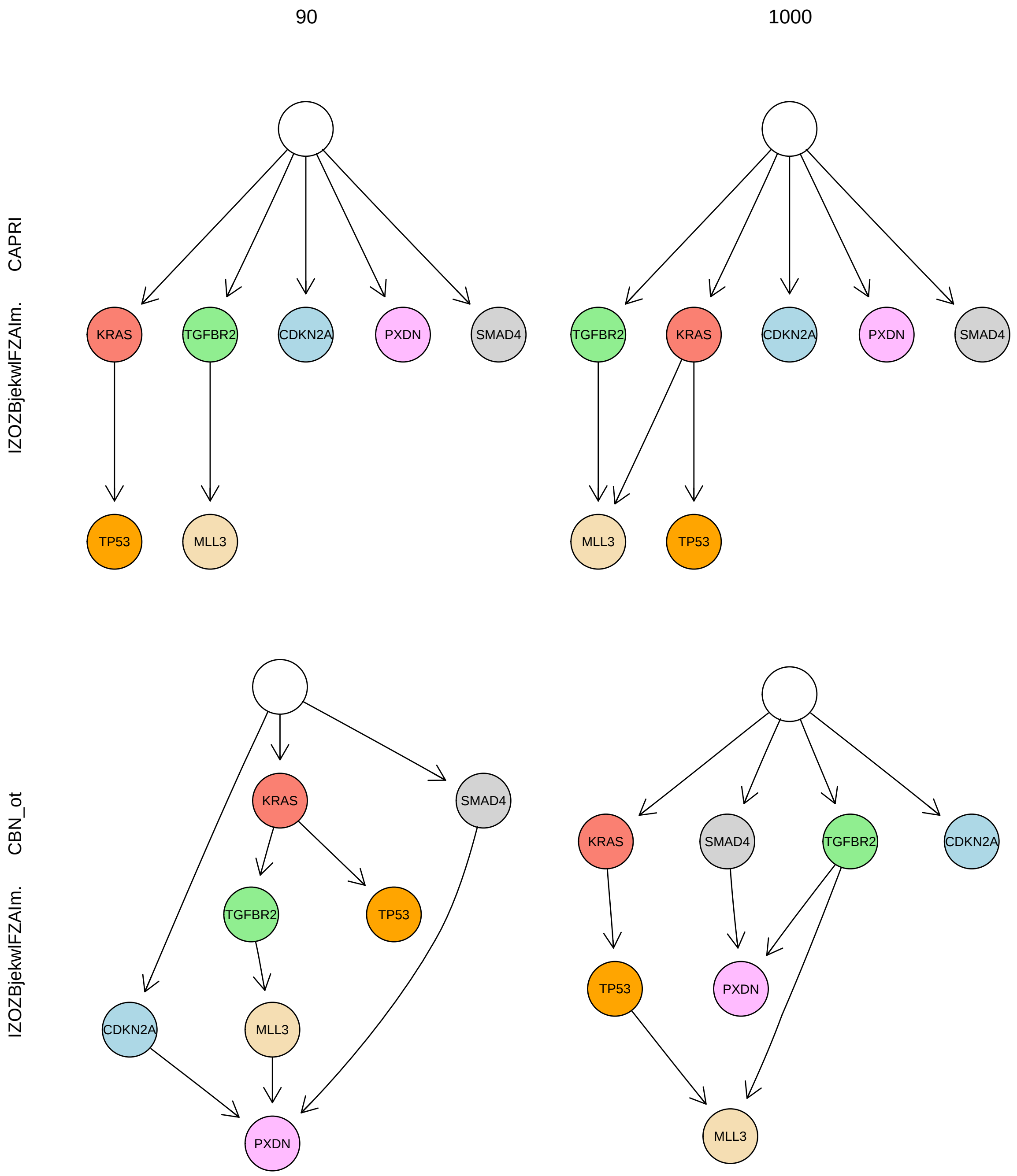

ID	p−value	Accessible Genot.
DbTYxBBsAytqwzO	0.943	34

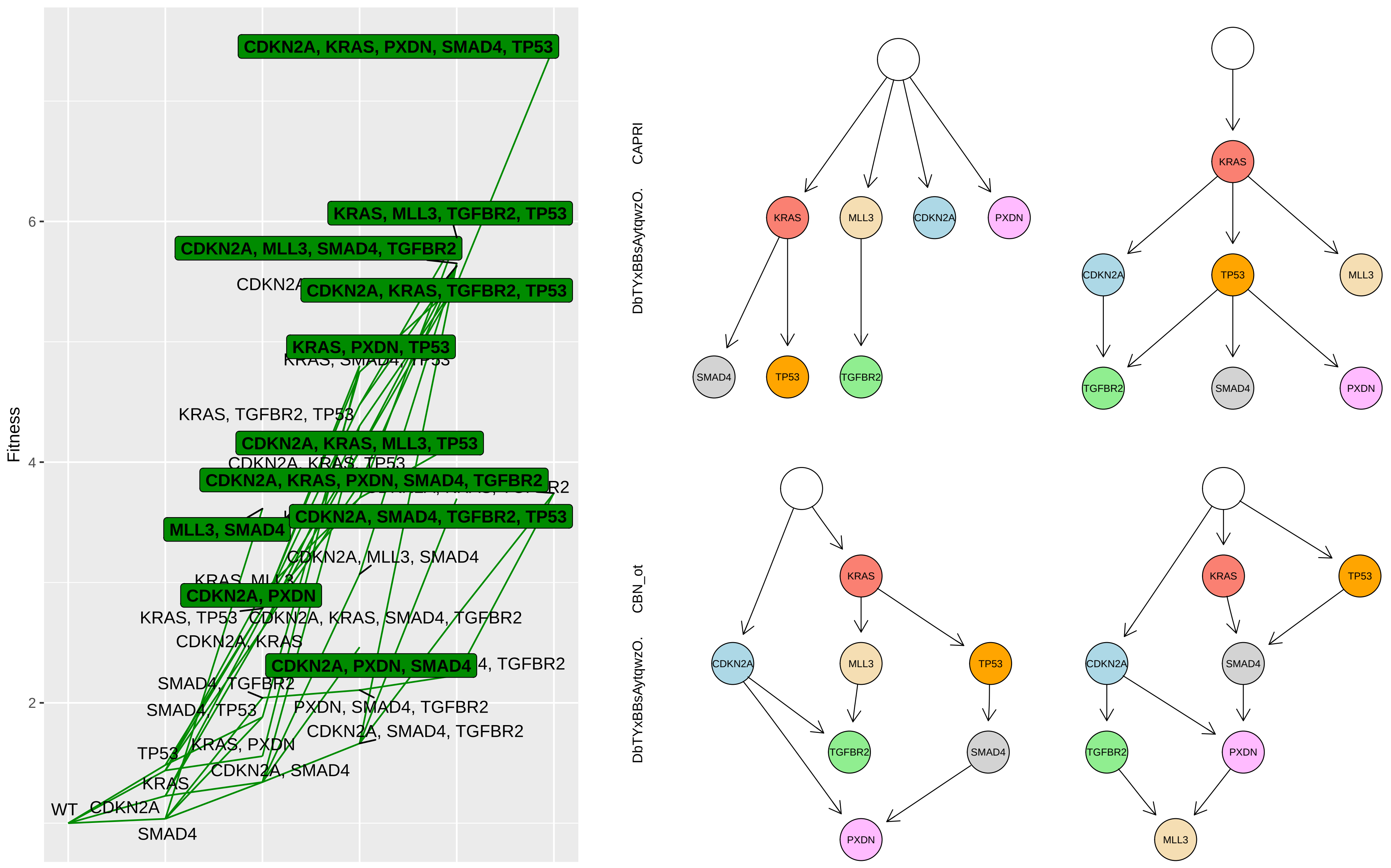

Supplement: Supplementary Data [file btx663_supp.zip › btx663-suppl_data/pancreas-landscapes-dags.pdf]
