# Supplementary material for: Cancer progression models and fitness landscapes: a many-to-many relationship
Source: Bioinformatics. 2017 Oct 18;34(5):836–44. doi: 10.1093/bioinformatics/btx663 (PMC6031050; doi:10.1093/bioinformatics/btx663)
Supplement: Supplementary Data [file btx663_supp.zip › btx663-suppl_data/SupplementaryMaterial-methods-results.pdf]

Supplementary Information for " Cancer progression models and fitness landscapes: a many-to-many relationship"

Ramon Diaz-Uriarte
Dept. Biochemistry, Universidad Autónoma de Madrid
Instituto de Investigaciones Biomédicas "Alberto Sols" (UAM-CSIC)
Madrid, Spain*
http://ligarto.org/rdiaz

Contents

1 Representable landscapes with reciprocal sign epistasis? 3

2 Random fitness landscapes 5
2.1 DAG-derived, representable fitness landscapes 5
2.2 DAG-derived, non-representable fitness landscapes 5
2.3 Rough Mount Fuji 5
2.4 Random fitness landscapes for the cancer data sets 6

3 Fitness landscapes characteristics 7

4 Selecting simulations for the cancer data sets 7

5 Simulations: parameters and detection 9
5.1 Stopping the simulations: detection 9
5.2 Other parameters of the simulations 9

6 Details on measures of DAG performance and variability, landscape variability, and reciprocal sign epistasis 9

7 Linear mixed-effects models 10
7.1 Coefficients of linear models 11

8 Paths through non-accessible genotypes 16
8.1 Lines of Descent and Path of the Maximum 16

9 Plots of fitness landscapes and inferred DAGs 17

10 Inferring the same DAG from different fitness landscapes 17
10.1 Circos plots of landscape connections 19

11 Cancer data sets: landscapes and inferred DAGs 20
11.1 Three cancer data sets: scheme 20
11.2 Three cancer data sets: plots of landscapes and DAGs 20
11.3 Three cancer data sets: DAG variability, reciprocal sign epistasis and number of peaks in the fitness landscape, and number of accessible genotypes 21

*ramon.diaz@iib.uam.es, rdiaz02@gmail.com

12 Canonical DAG 22

13 Cancer progression models and other software 22
13.1 CBN and CAPRI software . . . 23
13.2 Preprocessing of data for CPMs . . . 23
13.3 Other software . . . 23
13.4 Code and data for generating fitness landscapes and simulating from them . . . 23

1 Representable landscapes with reciprocal sign epistasis?

Figure 1 shows three examples of fitness landscapes with reciprocal sign epistasis that are representable by the DAGs of restrictions on the right. They are representable in the sense that all the genotypes predicted to exist/not exist by the DAG are matched in the fitness landscape. However, the fitness landscape is missing some paths to accessible genotypes that are implied by the DAGs. For example, in (a), the paths $A \rightarrow AB \rightarrow ABC$, $A \rightarrow AC \rightarrow ABC$, and $B \rightarrow BC \rightarrow ABC$, are not possible. In this case there is reciprocal sign epistasis between A and C in the B background (we can go from A to AB and BC, but ABC has smaller fitness than AB and BC). (Note: see Figure 2 in [14] or Figure 1 in [7] for a simple graphical way to quickly determine if there is reciprocal sign epistasis).

So even if these landscapes are representable in the sense of the genotypes that exist or not, they cannot be appropriately represented by the DAGs in terms of paths: the DAG cannot capture that certain paths are not available. The criterion used in the paper is, thus, a lot more permissive that examining paths.

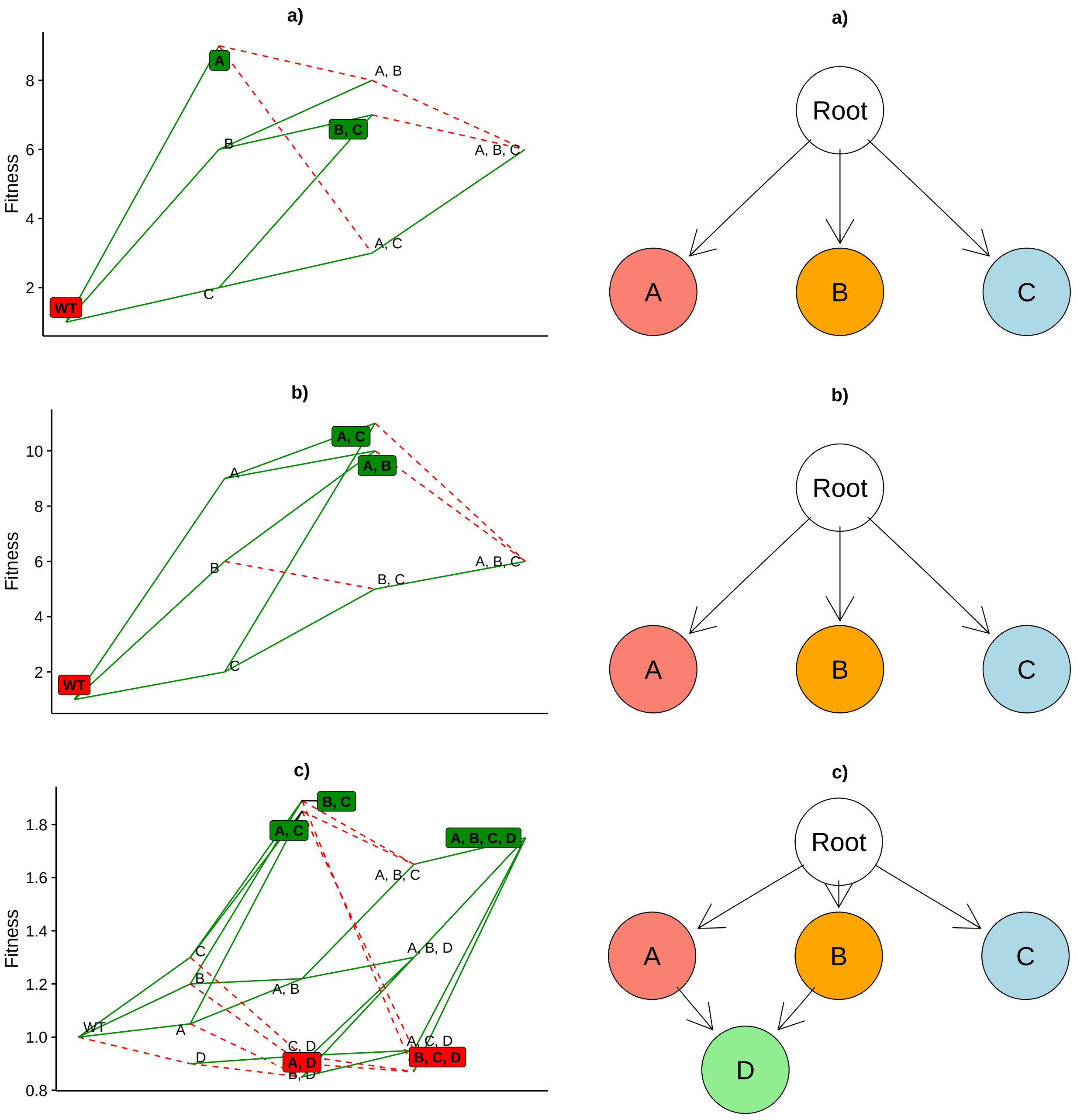

Figure 1: Examples of representable fitness landscapes (in the sense that all the genotypes predicted to exist/not exist by the DAG of restrictions are matched in the fitness landscape) with reciprocal sign epistasis. Fractions of reciprocal sign epistasis are 1/6, 1/6 , and 1/4 for (a), (b), and (c), respectively.

2 Random fitness landscapes

2.1 DAG-derived, representable fitness landscapes

Further details on the the generation of the representable fitness landscapes are provided here. This process had two steps: a) generating a random DAG of restrictions; b) assigning fitness values to mutations encoded in the DAG. To generate random DAGs, the genes were first randomly split in a number of levels, where the number of levels used was a randomly chosen integer between 2 and 5, both included. Then, each gene from each level i was randomly connected (as descendant) to randomly chosen genes (the ancestors) from levels j, where $j < i$; the number of incoming connections of each gene is a randomly chosen integer between 1 and $maxp$ (both included), where $maxp$ is a randomly chosen integer between 2 and 5 ($maxp$ is common for all genes in a DAG, but can vary between DAGs). The final DAG is the transitive reduction of the above generated DAG. (Note that this procedure can occasionally result in star DAGs, i.e., DAGs without any dependencies; in such a case, the DAG was discarded and a new one obtained). To generate random DAGs the function `simOGraph` from the OncoSimulR package [10] was used. From this DAG we now produce a fitness landscape by: b.1) setting to 10^{-9} the fitness of any genotype that is not possible under the DAG (a value this low makes it almost impossible to ever see a genotype of that kind —see section 8); b.2) assigning to every mutation with its dependencies satisfied (as specified by DAG) a random fitness effect uniformly distributed between 0.1 and 0.7 (these are values within those previously used in the literature [26]). To give an example, suppose the DAG specifies that mutating gene C requires having genes A and B mutated, and genes A and B require no previous mutation; suppose fitness effects of mutations (with restrictions satisfied) are 0.1, 0.2, 0.3, respectively. The fitness of genotypes will be: A: 1.1 (=$1 + 0.1$); B: 1.2; AB: $1.1 * 1.2$; C, AC, BC: $1e - 9$ (since dependencies are not satisfied); ABC: $1.1 * 1.2 * 1.3$, with wild-type always having fitness of 1. None of these fitness landscapes had any reciprocal sign epistasis (see also Figure 2).

2.2 DAG-derived, non-representable fitness landscapes

Generation of DAG-derived but not representable fitness landscapes started by generating a representable DAG-derived fitness landscape as just described. Then, the fitness of a randomly chosen subset of genotypes with two or more mutations and accessible under the DAG was set to 0.2; this value makes it very unlikely, though not impossible, to go through these genotypes (see section 8). Simply put, we create holes, or synthetic lethals, in a DAG-representable landscape. The number of genotypes that were set to zero (i.e., the number of holes or synthetic lethals) was a randomly chosen integer between 3 and the total number of accessible genotypes with two or more mutations. This fitness landscape was then checked to ensure that all seven genes were present in at least one accessible genotype; if they were not, the process of setting fitness to zero was repeated. If this condition was not fulfilled after 20 attempts, a new representable DAG-derived landscape was generated, and the process started again. All fitness landscapes were checked for being non-representable (see also section 12). The process of removing accessible genotypes lead to variable amounts of reciprocal sign epistasis, from 0 (in three cases) to 0.13 (see Figure 2).

See section 13.4 for code for generating these fitness landscapes.

2.3 Rough Mount Fuji

In the Rough Mount Fuji models for the 200 random fitness landscapes the reference genotype (i.e., the genotype with maximum fitness) was randomly chosen (so that the probability that the reference has m mutations, for $1 \geq m \leq 7$, is 1/7 and all genotypes with the same number of mutations are equiprobable). The standard deviation, sd, of the random normal variate was set to 1.5 and the decrease in fitness of a genotype per each unit increase in Hamming distance

from the reference genotype, c, was chosen from a uniform $U(0, 2)$ distribution. This gives a wide variety of fitness landscapes that encompass from close to additive (large values of c) to House of Cards (c close to 0). This fitness landscape was checked to ensure that all seven genes were present in at least one accessible genotype; if they were not, a new fitness landscape was generated (with, possibly, different values of c and reference genotype). Function `rfitness` from the OncoSimulR package [10] was used.

In all three cases, and to allow for comparability of fitness landscapes and DAGs under permutations of gene labels, genes were labelled so that the fitness of the accessible single mutants was in decreasing alphabetical order (i.e., for accessible single mutants, fitness is largest for genotype "A", then genotype "B", etc.)

2.4 Random fitness landscapes for the cancer data sets

As explained in the paper, I repeatedly simulated data using a modified Rough Mount Fuji random fitness landscape model where the observed genotype combinations in the empirical data sets were guaranteed to be accessible. The scheme is the same for all three data sets.

The procedure to create fitness landscapes involved two steps. First, fitness values were randomly assigned to the observed genotypes in the empirical data so that the observed genotypes were guaranteed to be accessible; this resulted in fitness values for the observed genotypes (18 for pancreatic cancer, 24 for glioblastoma, and 27 for colorectal cancer — I am not counting the five cases without any mutations in colorectal cancer). Second, and independently of the first step, a Rough Mount Fuji fitness landscape was generated (see 2.3) but the fitnesses of the observed genotypes were set to those obtained in the first step. This guarantees that, regardless of the fitness of the rest of the genotypes in the landscape, the genotypes in the reference data set remain accessible.

Generating fitness values so that the observed genotypes in the empirical data are accessible was done by creating a modified fitness graph [7] where each observed genotype with m mutations was made a descendant of exactly another observed genotype with $m - 1$ mutations (or the wild-type, if $m = 1$) from which it differs in exactly one mutated gene; when multiple candidate ancestors were available, one was chosen randomly. Next, a random fitness value was assigned to genotypes with $m = 1$; finally, iterating over genotypes with $m = 2 \ldots maxmut$ (where $maxmut$ is the largest number of mutations in the data) the fitness of each genotype was set to the sum of the genotype of its immediate ancestor plus a random deviate (larger than 0). In initial runs, I found that finding compatible landscapes worked much faster if the fitness of each observed genotype was increased by an amount proportional to its actual frequency in the observed data (this addition was performed **before** the operation of setting the fitness of a child equal to that of the parent plus a random deviate). Likewise, mutation rates were made proportional to the marginal frequencies of the individual genes. Values for the detection process, mutation rates, and random variates used in fitness were adapted to the data sets based on preliminary runs (all values used are available from the code; see section 13.4). Fitness values of all genotypes (observed and non-observed) were scaled to a common standard deviation.

The Rough Mount Fuji models for the cancer data sets were similar to the ones above (section 2.3), but with a wider range of value for the parameters, and avoiding the purely House of Cards models (since initial runs suggested those were unlikely to result in acceptable runs). The reference genotype was set as above. The c parameter was drawn so that its $\log_{10}$ was uniformly distributed between 0.2 and 5, with the standard deviation, sd, fixed at 1. The c values of the finally accepted fitness landscapes had median, first, and third quartiles of 1.3, 0.5 and 2.3, respectively (note that the value of 2.3 is actually close to the 2/1.5 value for the c/sd ratio used above). The value of sd was fixed to 1 as it simplifies the procedure. See also section 3.

See section 13.4 for the code used.

3 Fitness landscapes characteristics

Here we summarize the main characteristics of the 500 fitness landscapes in terms of accessible genotypes, reciprocal sign epistasis, and number of peaks.

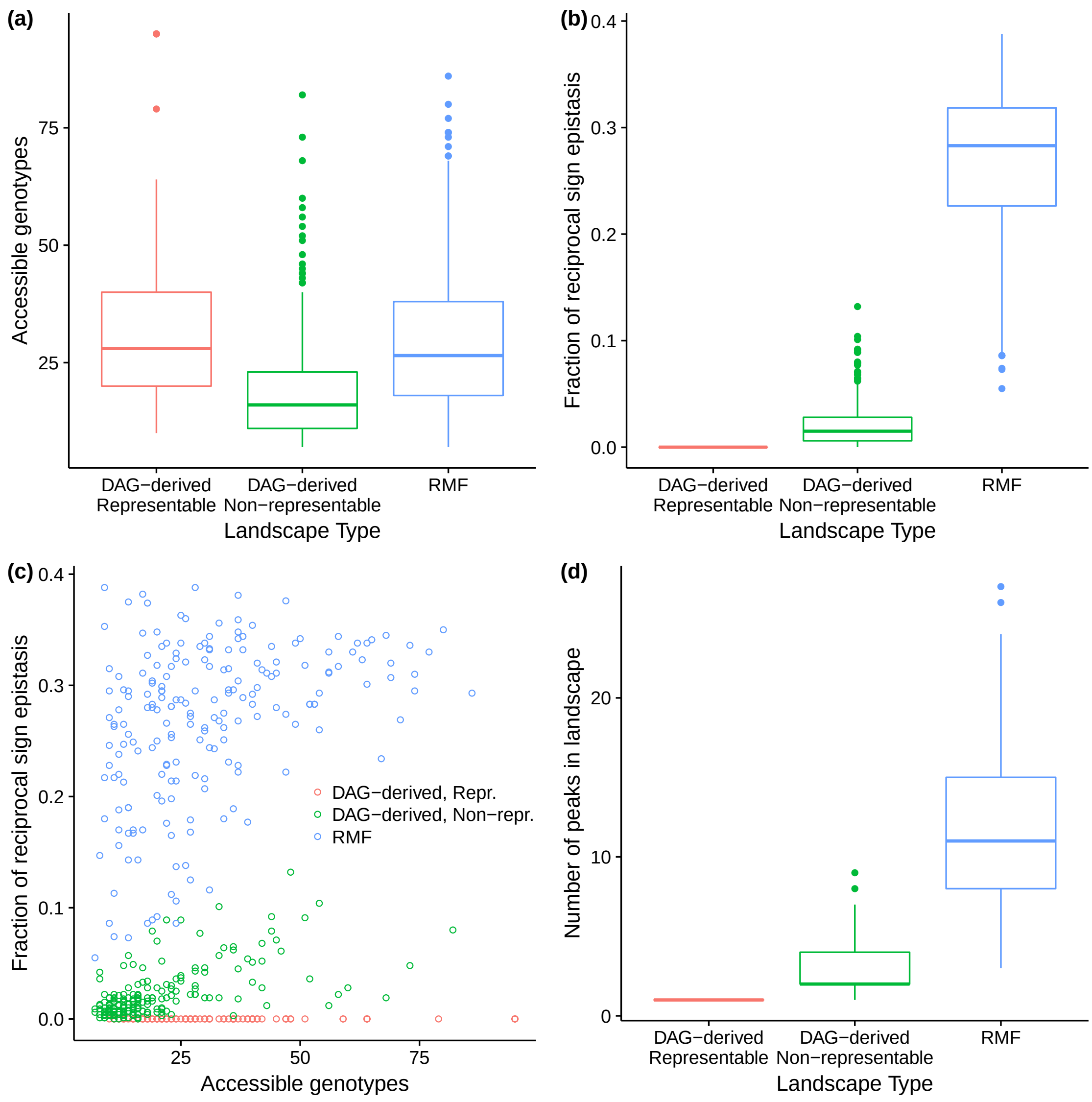

Figure 2: Simulated landscape characteristics. Number of accessible genotypes, reciprocal sign epistasis, and number of isolated peaks by type of landscape.

4 Selecting simulations for the cancer data sets

Once the fitness landscape had been generated, I repeated ten times the process of simulating 90 genotypes (pancreas and colon) or 67 genotypes (glioblastoma) from it (see section 5) using

randomly selected parameters for mutation rates and detection regime. Those are the numbers of genotypes with at least one mutation in the original data set.

I kept those simulations that fulfilled that, in at least three of the ten iterations, the simulated data set fulfilled that the p-value was > 0.6 (for both the reference χ^2 distribution and a permutation test, because of possible cells of low counts) and all genes had been observed. A p-value > 0.6 has been used because it is the next real number rounded to one significant figure that is larger than 0.5; it is large enough that no user would say that the data sets are different (the probability, under the H_0 that both data are coming from the same distribution of frequencies, that the data differ by this much is more than 1/2). The requirement of the minimal of three cases is used to prevent occasionally achieving a large p-value from a fitness landscape that rarely produces data comparable to the observed one.

This procedure is similar to some of the steps in a rejection ABC (Approximate Bayesian Computation) algorithm [23, 30] using the χ^2 $p - value$ as the distance function, with $\epsilon = 0.6$ (with the additional restriction of positive frequency for all seven genes and having observed this to happen in at least three of ten times). But this is not ABC: we are not trying to infer the distribution of the parameters in any model, only to find fitness landscapes that produce data sets that have genotype frequency distributions indistinguishable from the original ones using standard approaches (a χ^2 test).

See section 13.4 for the code used to simulate from the random fitness landscapes.

5 Simulations: parameters and detection

5.1 Stopping the simulations: detection

The probability of tumor detection increased with total tumor size according to the model $P(N) = 1 - e^{-\delta(N-B)}$ if $N > B$ and 0 otherwise, where $P(N)$ is the probability that a tumor with a population size N will be detected when checked, δ controls the increase in $P(N)$ with population size and B is the minimal population size at which a tumor could be detected. $P(N)$ refers to the probability of detection at each one of the occasions when it is checked: the interval between checks was fixed at 20 units. Two values of δ were used, $7.526 * 10^{-5}$ and $7.179 * 10^{-6}$ that correspond to probabilities of detection of 0.1 and 0.01, respectively, when the population size has doubled; the first corresponds to the "fast" detection regime and the second to the "slow" detection regime. (We could suitably increase δ and decrease the time between checks and obtain similar behavior.) Simulations used the OncoSimulR package [10]. The final time of the simulations was variable; in original simulation time units, the mean, median, 1st and 3rd quantiles of final simulation times were 760, 240, 80, and 660, respectively. Since we are using a continuous time model where initial birth and death rates are equal to 1, those values could roughly correspond to 3000, 960, 320, and 2640 days, respectively, if the original cells divide about once every four days (e.g., [4]). These numbers might seem small, but remember that in McFarland's model [26] we start from hyperplasias (so we are not modelling time to initiation), and we are only modelling time until detection; this is consistent with the assumption stated in the manuscript that "Because we want to simulate data consistent with cross-sectional sampling, we absorb all the cancer initiation process in the root node; as in [2] all tumors start cancer progression without any of the mutations shown in the DAGs (though other mutations could already be present that caused the initial tumor growth)."

When a tumor was detected, a whole-tumor sample was taken, as in [9], with a detection threshold of 50%, meaning that a gene was considered mutated if it was mutated in 50% of the cells, as in [31].

5.2 Other parameters of the simulations

Simulations used the implementation of the McFarland model in the OncoSimulR package [10]. In addition to the parameters specified in the main text, other parameters for the simulations on the 500 fitness landscapes were (see specific meaning in documentation of OncoSimulR [10]): $finalTime = 25000$, $keepEvery = 1$ (this value is set to a small value to obtain good estimates of POM diversity), $sampleEvery = 0.03$.

The actual mutation rates in the variable, or gene-specific condition, were 5.848×10^{-6}, 5×10^{-5}, 3.42×10^{-6}, 2.924×10^{-5}, 1.71×10^{-5}, 2×10^{-6} and 1×10^{-5} for genes A to G, respectively; these mutation rates have a geometric mean of 1×10^{-5}, with maximum spread between successive values, within the maximum and minimum of 5×10^{-5} and 2×10^{-6}, respectively.

6 Details on measures of DAG performance and variability, landscape variability, and reciprocal sign epistasis

Reciprocal sign epistasis is the fraction of all pairs of mutations that had reciprocal sign epistasis (i.e., both mutations have an opposite effects on the other background) [5, 14] and was computed using MAGELLAN [5].

Distance between two DAGs is the number of the edges that differ between the transitive reduction of the two DAGs. This is the same as the sum of the absolute values of the entries in the matrix difference of the adjacency matrices of two DAGs or, equivalently, the cardinality of

the symmetric difference of the sets of edges of the DAGs. Or, if you prefer, the square of the Frobenius norm [18] of the matrix difference of the adjacency matrices of the DAGs.

CAPRI can return DAGs that contain both direct and indirect edges between nodes (i.e., DAGs that contain more edges than a similar smaller DAG with the same reachability conditions): all comparisons involved the transitive reduction of the DAGs.

Relative distance between DAGs, used to assess DAG-to-DAG variability, is the distance between two DAGs divided by the total number of distinct edges in the two DAGs. Thus, this is the cardinality of the union of the sets of edges of the two DAGs. This provides an easy to interpret measure in the range $[0, 1]$ (where 0 means identical DAGs and 1 means the two DAGs have no edges in common).

PND genotype mispredictions is the ratio of false negative genotype mispredictions over the total number of genotypes that are accessible in a landscape; this is a measure of really accessible genotypes that a DAG fails to predict relative to the total number of accessible genotypes . A genotype, even if accessible under a given landscape, might actually never be observable under small mutation rates and fast detection regimes. To avoid penalizing inferences for non-observable genotypes, I corrected the count of false negatives using, as reference, not the full set of accessible genotypes under a fitness landscape, but only the subset of accessible genotypes that had been observed with a frequency larger than 5 in 1,000 (measured on the 20,000 simulations) so that the probability of not observing the minimal frequency genotype in a sample of 1,000 genotypes is less than 1%. PND is equivalent to $1 -$ recall or $1 -$ sensitivity.

PFD genotype mispredictions is the ratio of false positive genotype mispredictions over the total number of genotypes that can exist according to the DAG; this is a measure of how many genotypes that are not really accessible are predicted relative to the number of genotypes predicted by a DAG; this is equivalent to $1 -$ precision or $1 -$ positive predictive value.

The **pairwise difference of accessible genotypes** between two landscapes is the sum of the number of genotypes accessible under one landscape and not accessible under the other (i.e., the cardinality of the symmetric difference of the sets of accessible genotypes of each landscape). The **relative pairwise difference of accessible genotypes** divides the pairwise difference by the total number of distinct accessible genotypes in the two landscapes (i.e., the cardinality of the of the union of the sets of accessible landscapes of the two landscapes).

7 Linear mixed-effects models

The response variables were the average of 20 replicates for all variables, except for the relative pairwise distances (average of 190 $[= 20 * 19/2]$ and 7140 $[= (20 * 6) * ((20 * 6) - 1)/2]$ for "Same mutation*detection" and "Over mutation*detection", respectively —Figure 3 in the paper). Number of accessible genotypes was not a regressor in models for PND as it is the denominator of the dependent variable. As suggested by residual and partial residual plots of the fitted models, regressor "number of accessible genotypes" was log transformed.

Replicates are, except for the random data partition, identical replicates and there are no replicate-specific covariates, which suggests using the average as the response: it is more relevant for users and simplifies statistical modeling. Models used fitness landscape as random effect except for relative pairwise DAG distance over mutation and detection as here, since I averaged over all mutation and detection regimes, only a single value per landscape is used. Type of landscape, number of accessible genotypes, and amount of reciprocal sign epistasis were always the same for a landscape.

PND and PFD used a linear mixed effect model of those ratios. Because of the number of cases that were exactly zero (18% in PFD, 2.4% in PND) I reanalyzed the data using Tweedie compound Poisson linear mixed effects models [35], which lead to similar qualitative conclusions.

Model adequacy and transformations were assessed using standard residual and partial residual plots, including randomized quantile residuals [11] for Tweedie regression. All model fitting

was done in R, using packages lme4 [3], cpglm [35], effects [15], and car [17] with additional, custom code, for diagnostics of mixed effects and Tweedie regressions (see section 13.4).

Since the purpose of these models is interpretation, not prediction, I concentrated on factors with relevant and interpretable effects [1] and started model fitting from models that included all possible two-way interactions, even when for most variables there were evidences of three-, four-, and five-way interactions, generally of much smaller magnitude. Also for ease of interpretation and representation and to avoid showing two- and three-way interactions with method, models were fitted separately to CBN and CAPRI (which amounts to fitting models with all interactions between Method and the remaining variables). To assess significance of terms from linear mixed effects models I have used $F-$tests with Kenward-Roger adjustment for degrees of freedom [13, 17]. All tests are Type II tests [16, 17].

In all cases, models were fitted using sum-to-zero contrasts: each main effect parameter is to be interpreted as the (marginal) deviation of that level from the overall mean, and the interaction parameter as the deviation of the linear predictor of the cell mean (for that combination of levels) from the addition of the corresponding main effect parameters (see [25]). Continuous regressors (reciprocal sign epistasis and number of accessible genotypes) were scaled (mean 0, variance 1) to allow for a simpler interpretation of the magnitude of their coefficients and so that the intercept term is interpreted as the predicted response at the average value of the regressors.

7.1 Coefficients of linear models

All coefficients from the fitted models are shown in Figure 3. The figures show coefficients from overparameterized models, and those are obviously not the models fitted. What I have done is fit the models several times, always with sum to zero contrasts, but changing the level of the factor set to $-\Sigma$ rest of levels so as to explicitly obtain the coefficients and standard errors for all levels of all terms (e.g., the coefficients that correspond to “Detection, Fast” and “Detection, Slow”). This allows not only to see the coefficient for each term, but also to obtain the predicted value under any combination of the regressors (by adding the corresponding terms).

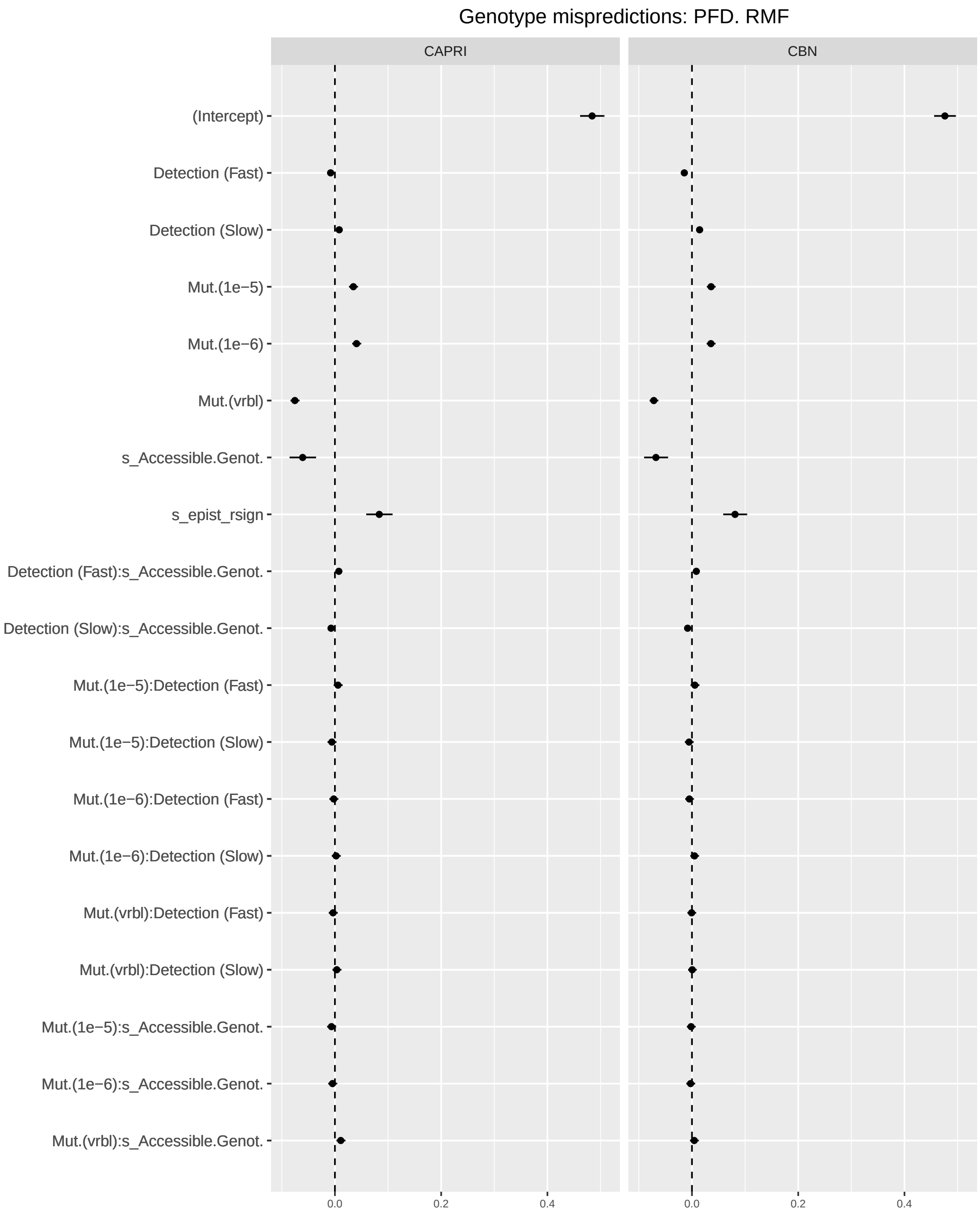

Genotype mispredictions: PFD. RMF
CAPRI
CBN
(Intercept)
Detection (Fast)
Detection (Slow)
Mut.(1e−5)
Mut.(1e−6)
Mut.(vrbl)
s_Accessible.Genot.
s_epist_rsign
Detection (Fast):s_Accessible.Genot.
Detection (Slow):s_Accessible.Genot.
Mut.(1e−5):Detection (Fast)
Mut.(1e−5):Detection (Slow)
Mut.(1e−6):Detection (Fast)
Mut.(1e−6):Detection (Slow)
Mut.(vrbl):Detection (Fast)
Mut.(vrbl):Detection (Slow)
Mut.(1e−5):s_Accessible.Genot.
Mut.(1e−6):s_Accessible.Genot.
Mut.(vrbl):s_Accessible.Genot.
0.0
0.2
0.4

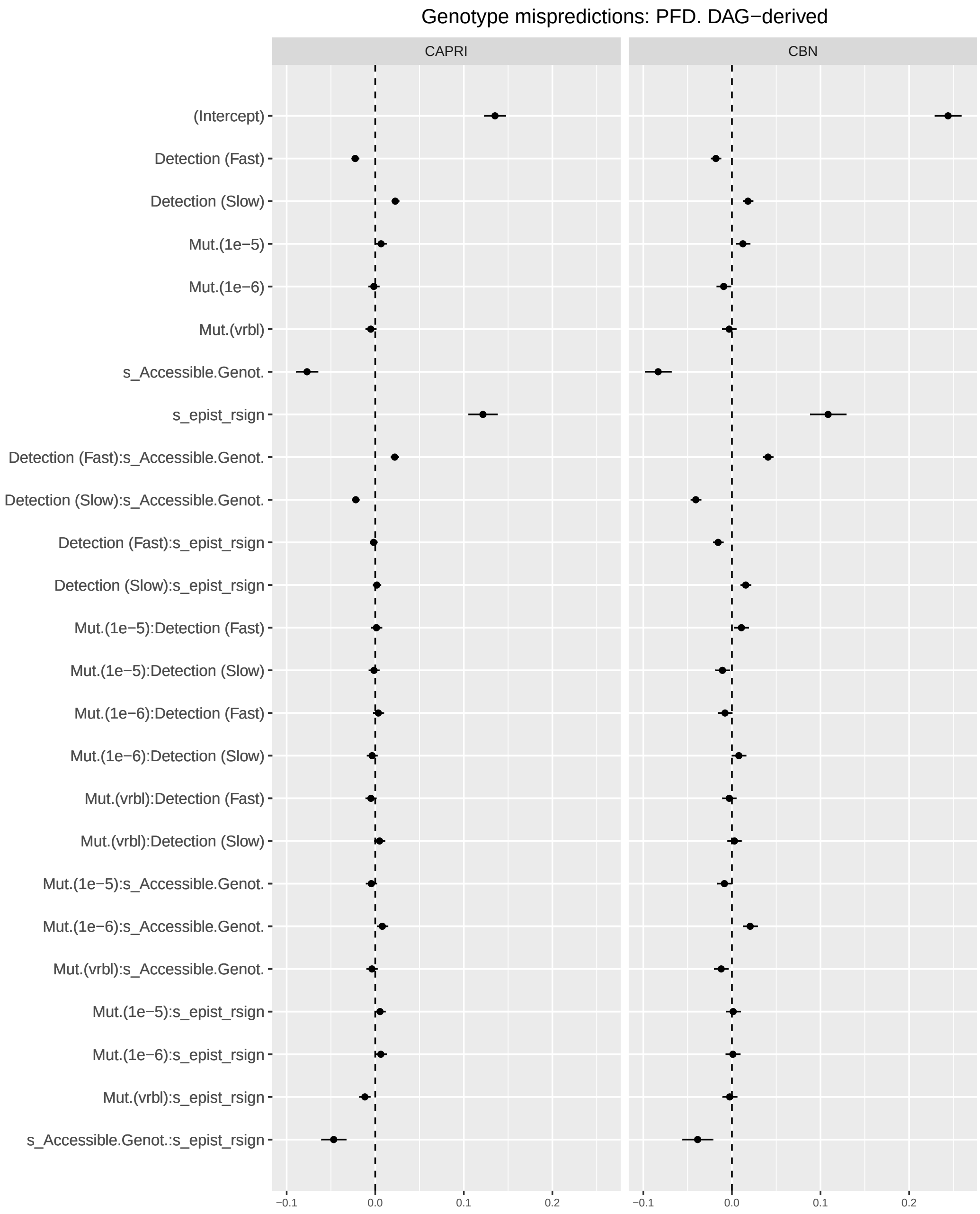

Genotype mispredictions: PFD. DAG−derived
CAPRI
CBN
(Intercept)
Detection (Fast)
Detection (Slow)
Mut.(1e−5)
Mut.(1e−6)
Mut.(vrbl)
s_Accessible.Genot.
s_epist_rsign
Detection (Fast):s_Accessible.Genot.
Detection (Slow):s_Accessible.Genot.
Detection (Fast):s_epist_rsign
Detection (Slow):s_epist_rsign
Mut.(1e−5):Detection (Fast)
Mut.(1e−5):Detection (Slow)
Mut.(1e−6):Detection (Fast)
Mut.(1e−6):Detection (Slow)
Mut.(vrbl):Detection (Fast)
Mut.(vrbl):Detection (Slow)
Mut.(1e−5):s_Accessible.Genot.
Mut.(1e−6):s_Accessible.Genot.
Mut.(vrbl):s_Accessible.Genot.
Mut.(1e−5):s_epist_rsign
Mut.(1e−6):s_epist_rsign
Mut.(vrbl):s_epist_rsign
s_Accessible.Genot.:s_epist_rsign
−0.1
0.0
0.1
0.2

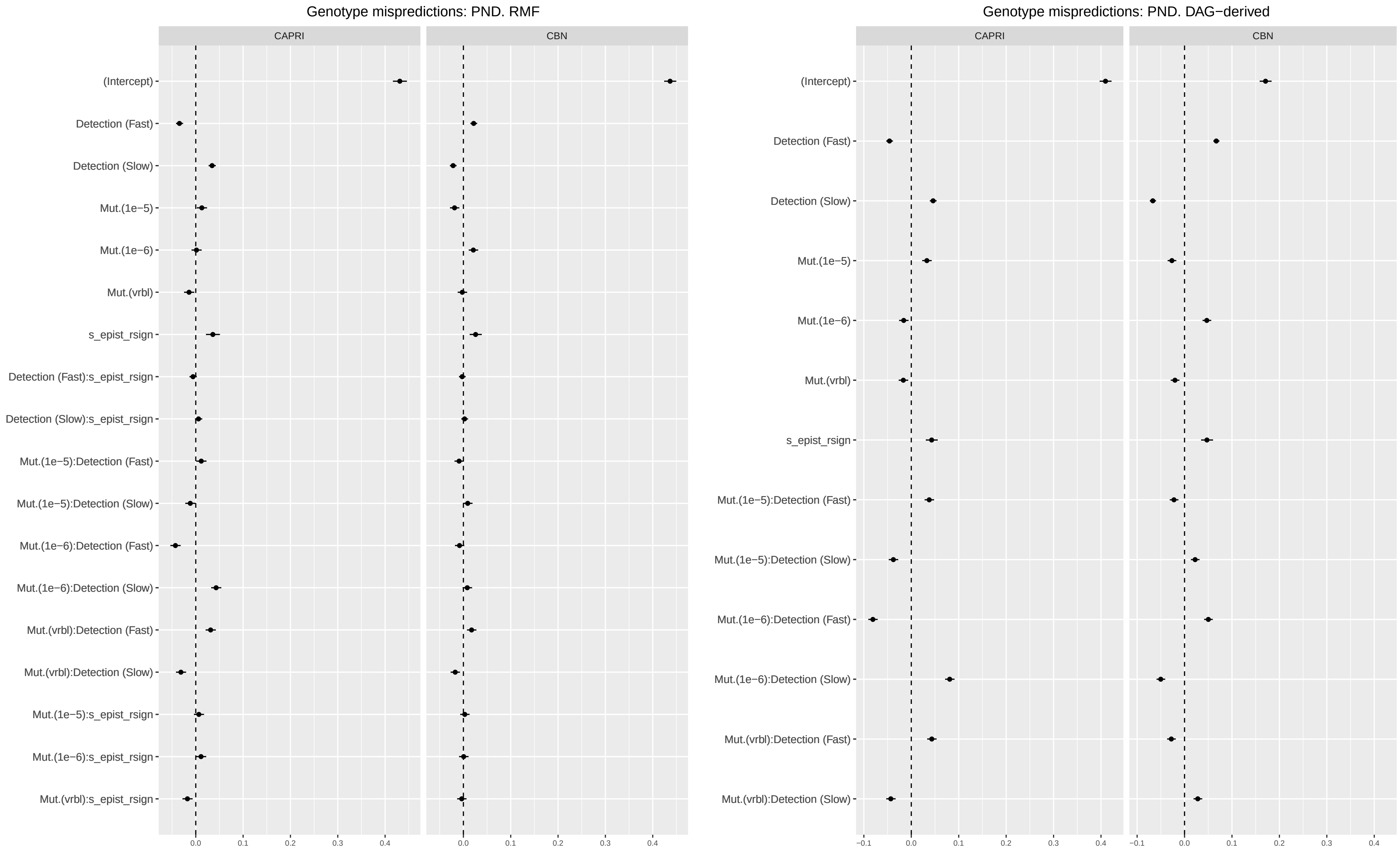
Genotype mispredictions: PND. RMF
CAPRI
CBN
(Intercept)
Detection (Fast)
Detection (Slow)
Mut.(1e−5)
Mut.(1e−6)
Mut.(vrbl)
s_epist_rsign
Detection (Fast):s_epist_rsign
Detection (Slow):s_epist_rsign
Mut.(1e−5):Detection (Fast)
Mut.(1e−5):Detection (Slow)
Mut.(1e−6):Detection (Fast)
Mut.(1e−6):Detection (Slow)
Mut.(vrbl):Detection (Fast)
Mut.(vrbl):Detection (Slow)
Mut.(1e−5):s_epist_rsign
Mut.(1e−6):s_epist_rsign
Mut.(vrbl):s_epist_rsign
0.0 0.1 0.2 0.3 0.4
Genotype mispredictions: PND. DAG−derived
CAPRI
CBN
(Intercept)
Detection (Fast)
Detection (Slow)
Mut.(1e−5)
Mut.(1e−6)
Mut.(vrbl)
s_epist_rsign
Mut.(1e−5):Detection (Fast)
Mut.(1e−5):Detection (Slow)
Mut.(1e−6):Detection (Fast)
Mut.(1e−6):Detection (Slow)
Mut.(vrbl):Detection (Fast)
Mut.(vrbl):Detection (Slow)
−0.1 0.0 0.1 0.2 0.3 0.4

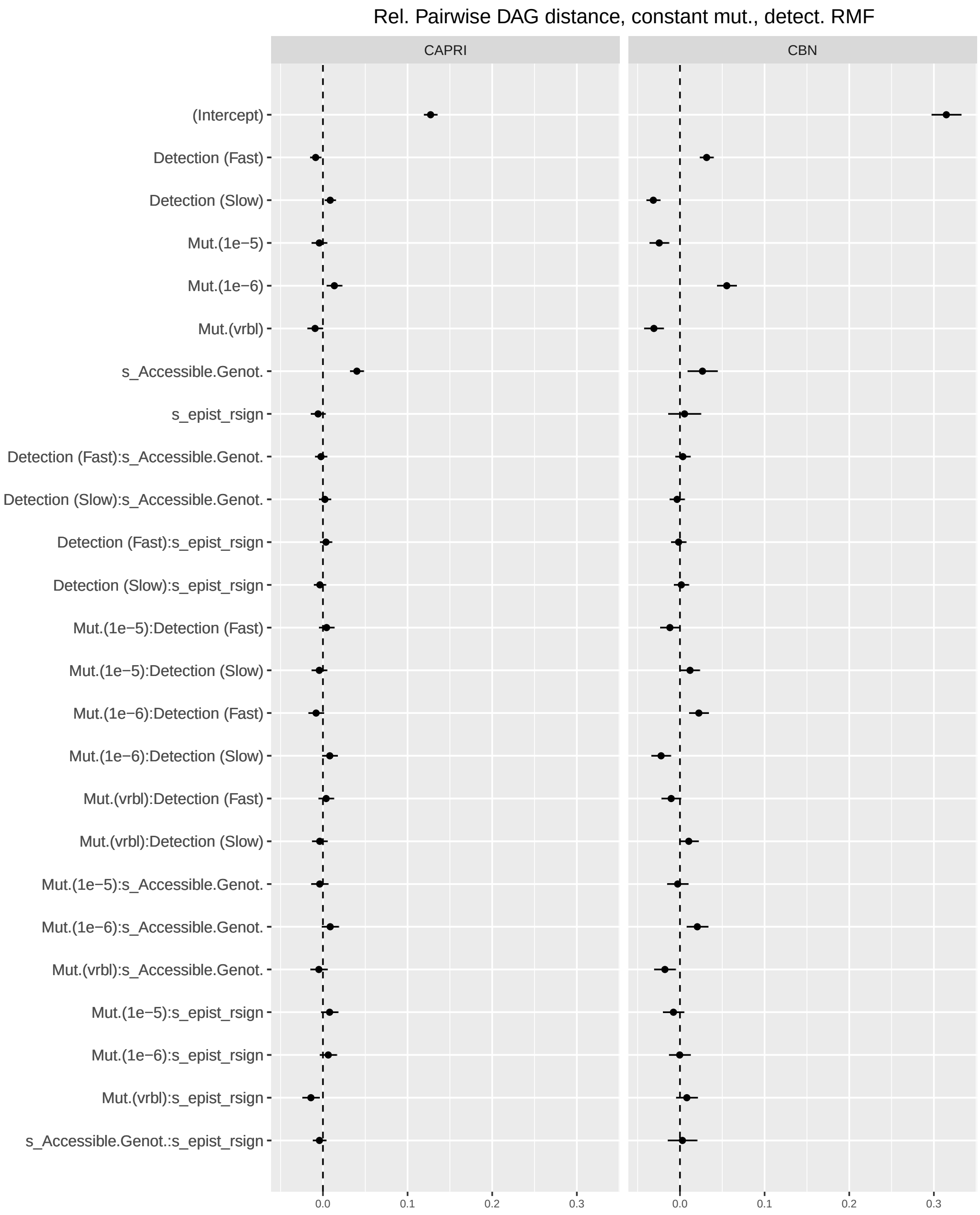
Rel. Pairwise DAG distance, constant mut., detect. RMF
CAPRI
CBN
(Intercept)
Detection (Fast)
Detection (Slow)
Mut.(1e−5)
Mut.(1e−6)
Mut.(vrbl)
s_Accessible.Genot.
s_epist_rsign
Detection (Fast):s_Accessible.Genot.
Detection (Slow):s_Accessible.Genot.
Detection (Fast):s_epist_rsign
Detection (Slow):s_epist_rsign
Mut.(1e−5):Detection (Fast)
Mut.(1e−5):Detection (Slow)
Mut.(1e−6):Detection (Fast)
Mut.(1e−6):Detection (Slow)
Mut.(vrbl):Detection (Fast)
Mut.(vrbl):Detection (Slow)
Mut.(1e−5):s_Accessible.Genot.
Mut.(1e−6):s_Accessible.Genot.
Mut.(vrbl):s_Accessible.Genot.
Mut.(1e−5):s_epist_rsign
Mut.(1e−6):s_epist_rsign
Mut.(vrbl):s_epist_rsign
s_Accessible.Genot.:s_epist_rsign
0.0
0.1
0.2
0.3
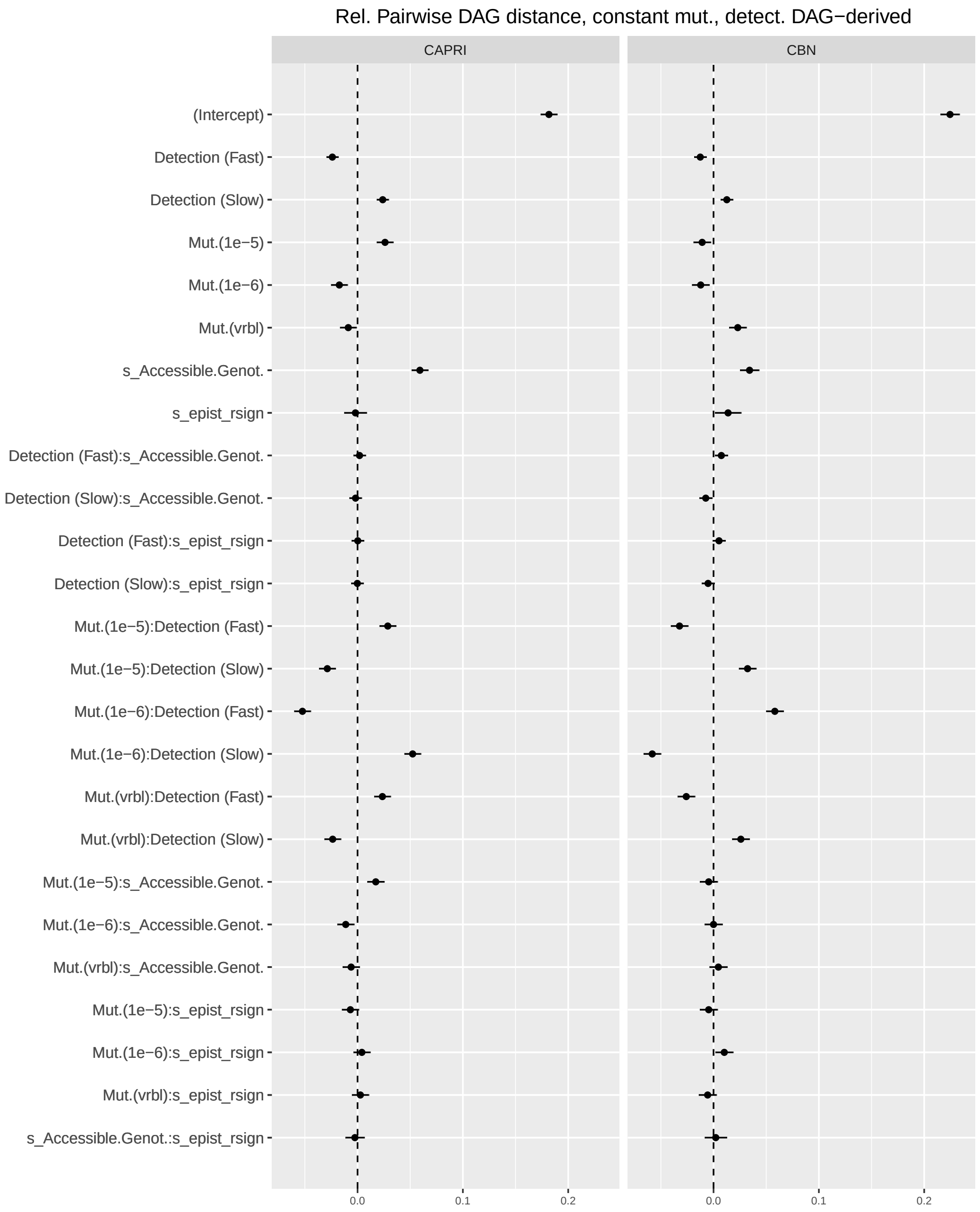
Rel. Pairwise DAG distance, constant mut., detect. DAG−derived
CAPRI
CBN
(Intercept)
Detection (Fast)
Detection (Slow)
Mut.(1e−5)
Mut.(1e−6)
Mut.(vrbl)
s_Accessible.Genot.
s_epist_rsign
Detection (Fast):s_Accessible.Genot.
Detection (Slow):s_Accessible.Genot.
Detection (Fast):s_epist_rsign
Detection (Slow):s_epist_rsign
Mut.(1e−5):Detection (Fast)
Mut.(1e−5):Detection (Slow)
Mut.(1e−6):Detection (Fast)
Mut.(1e−6):Detection (Slow)
Mut.(vrbl):Detection (Fast)
Mut.(vrbl):Detection (Slow)
Mut.(1e−5):s_Accessible.Genot.
Mut.(1e−6):s_Accessible.Genot.
Mut.(vrbl):s_Accessible.Genot.
Mut.(1e−5):s_epist_rsign
Mut.(1e−6):s_epist_rsign
Mut.(vrbl):s_epist_rsign
s_Accessible.Genot.:s_epist_rsign
0.0
0.1
0.2

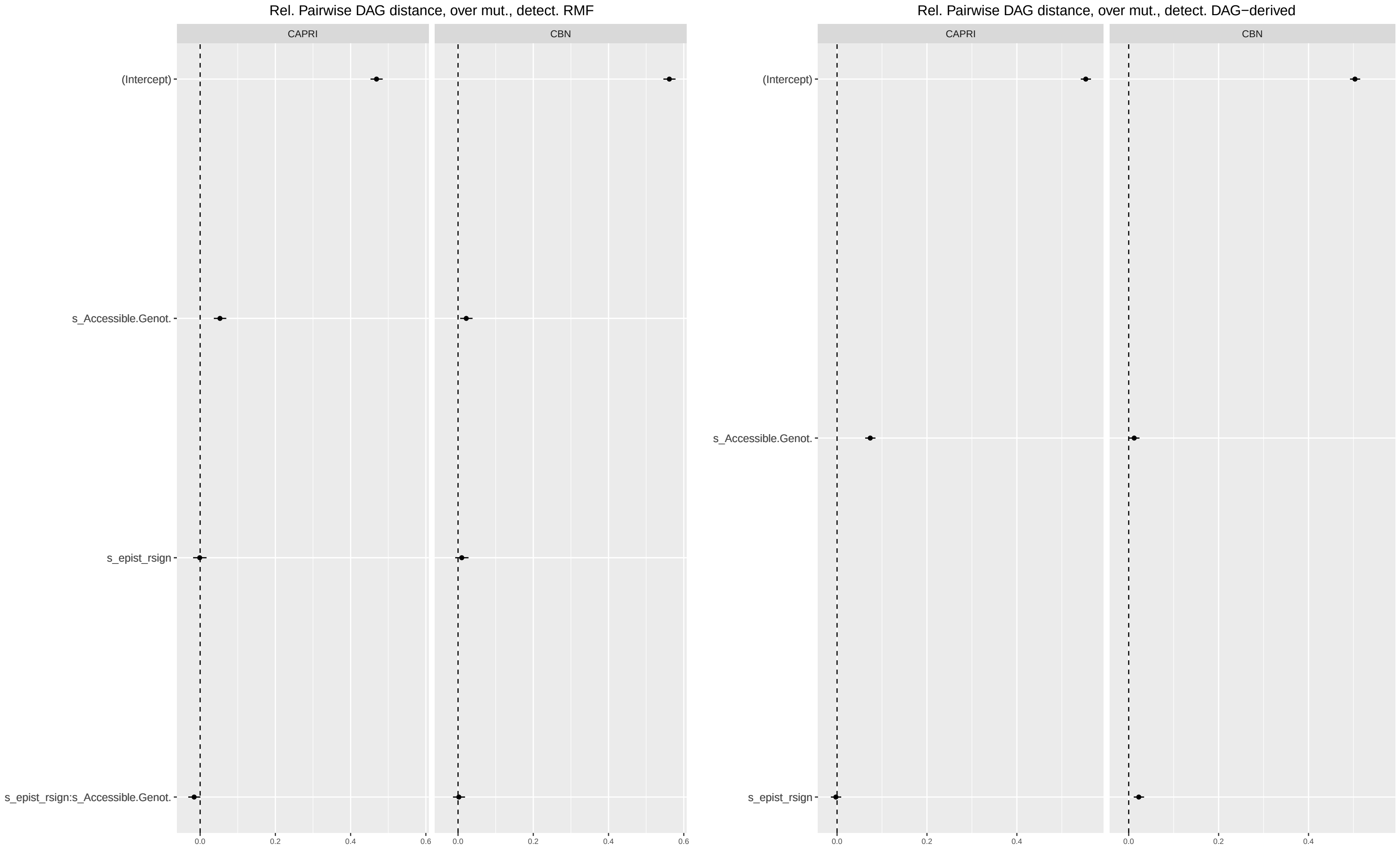

Figure 3: Coefficients from linear mixed-effects models. See text for a description of parameterization. Each page shows a different response variable; values shown are coefficients $\pm 2 * \text{standard error}$.

8 Paths through non-accessible genotypes

Do any of the paths to the sampled genotypes ever go through non-accessible genotypes? I have checked if that is the case in the simulations for the 500 fitness examining if:

- any of the sampled genotypes (i.e., any of the genotypes used for inferring DAGs) is a non-accessible genotype;
- any Path of the Maximum [34] (see section 8.1) ever visits a non-accessible genotype;
- any Line of Descent [34] (see section 8.1) ever visits a non-accessible genotype.

The results are:

- In the representable landscapes, we never have a sampled genotype among the non-accessible genotypes, nor do LOD or POM ever visit a non-accessible genotypes in any of the $1.2 * 10^7 = 20000 * 100 * 6$ simulated evolutionary trajectories. Remember that in the representable landscapes the fitness of the non-accessible genotypes was set to 10^{-9}.
- Among the simulations in DAG-derived, but non-representable, landscapes, over the $20000 * 200 * 6$ evolutionary runs, the probabilities of ever ending in a non-accessible genotype or having a LOD or POM go through a non-accessible genotype were 1.679×10^{-5}, 6.483×10^{-5}, 2.583×10^{-5}, respectively. We can go through non-accessible genotypes as the fitness of the non-accessible genotypes was set to 0.02, but the events are extremely uncommon. (The counts for POM can be larger than those for final states: we can visit or go through a non-accessible genotype but not end in one. Similar for LOD. Moreover, we can have a sampled genotype that shows a given genotype without that genotype actually ever existing in the population, since we set the threshold for detection at 0.5; suppose there are three genotypes, each with a frequency of 1/3, AB, AC, BC. Then, the sampled genotype will be reported as ABC, because the frequencies of all of A, B, C are > 0.5; see section 5.1).
- Among the simulations in RMF landscapes, the probabilities of ever ending in a non-accessible genotype or having a LOD or POM go through a non-accessible genotype were 2.032×10^{-4}, 7.928×10^{-4}, 3.046×10^{-4}. These numbers are larger than above since some of the non-accessible genotypes might only differ slightly in fitness from their accessible parent genotype thus making it possible to go through non-accessible but "almost accessible" genotypes. That many non-accessible genotypes be extremely close to being accessible is just a feature of RMF landscapes. Regardless, those frequencies are sufficiently low so as not to alter the assumptions used in the paper: the probability of sampling a genotype not among the accessible ones is about 2 in 10,000.

8.1 Lines of Descent and Path of the Maximum

I have used Lines of Descent and Path of the Maximum above, two measures of evolutionary predictability defined in [34]. In [34] "(...) paths defined as the time ordered sets of genotypes that at some time contain the largest subpopulation" are called "Path of the Maximum" (POM) (see their p. 572). In our case, using OncoSimulR [10], which does continuous time simulations, POM are obtained from the genotypes at each of the sampling times and, thus, the POMs will be affected by how often we sample and keep samples; we sampled once every time unit.

Szendro et al. [34] also define Lines of Descent (LODs) which "(...) represent the lineages that arrive at the most populated genotype at the final time". In that same page (p. 572) they provide the details on how the LODs are obtained. My implementation is not exactly identical to the definition given in p. 572 of Szendro et al. When returning a unique LOD, the LOD

returned is the first path to arrive at the genotype that eventually becomes the most populated genotype at the final time (and, in this sense, agrees with the LOD of Szendro et al.). However, in contrast to what is apparently done in Szendro ("A given genotype may undergo several episodes of colonization and extinction that are stored by the algorithm, and the last episode before the colonization of the final state is used to construct the step."), I do not check that this genotype (which is the one that will become the most populated at final time) does not become extinct before the final colonization. So there could be multiple paths that are actually the one(s) that are colonizers of the most populated genotype (with no extinction before the final colonization). All of these possible paths are kept and stored as the LODs and the statistics above about number of genotypes in LODs not among the accessible genotypes used all the recorded LODs.

9 Plots of fitness landscapes and inferred DAGs

File `plots-landscapes-dags-trans-red.pdf` shows the 500 fitness landscapes and the modal inferred DAG under each combination of mutation and detection regimes (it shows the transitive reduction of the DAGs as CAPRI sometimes returns DAGs contain both direct and indirect edges between nodes). Nodes with a name such as `D_E` denote nodes where genes D and E had identical status for all individuals, and thus cannot be distinguished (this was detected and dealt with during data preprocessing; see section 13.2). In a few cases no DAG could be inferred (e.g., not enough data that fulfilled the minimal requirements was availablbe); these are shown with "none" instead of a DAG.

To ease the visual inspection of the landscapes and DAGs, I have added, on top of each landscape, the canonical DAGs (see section 12) computed with two procedures. With representable fitness landscapes, the two canonical DAGs are identical and correspond to the true DAG that generated the landscape.

The first 100 landscapes are the representable landscapes (note that the first 100 DAGs show discrepancy measures of 0), the next 200 (101 to 300) are the DAG-derived and not-representable, and the final 200 landscapes (301 to 500) the Rough Mount Fuji (RMF) landscapes. Incidentally, note how the addition of reciprocal sign epistasis, in the DAG-derived, often, but no always, leads to multiple peaks (and this is to be expected: reciprocal sign epistasis often, but not necessarily, leads to multiple peaks [7, 27]).

10 Inferring the same DAG from different fitness landscapes

I examined whether the same DAG was inferred from distinct fitness landscapes. I regarded as distinct landscapes those fitness landscapes that differed in $> 50\%$ of their accessible genotypes (i.e., landscapes with a relative pairwise difference of accesible genotypes > 0.5 —see Methods). I first counted for each DAG in how many pairs of distinct landscapes it had been inferred. Of the inferred DAGs, 8% and 11% for CBN and CAPRI, respectively, were present in one or more pairs of distinct landscapes and some DAGs were present in hundreds of different pairs of distinct landscapes (Figure 4a).

I also counted, for each fitness landscape, with how many distinct landscapes it was connected, where two landscapes were connected if the same DAG had been inferred in both landscapes in at least one occasion. The vast majority of landscapes (96% and 90% for CBN and CAPRI, respectively) were connected with at least one distinct landscape (Figure 4b). This lead to a complex pattern of relationships between fitness landscapes via shared DAGs (Figure 4c; see also section 10.1). These results were not the consequence of the same DAG appearing in different landscapes under different mutation and detection regimes. Restricting the analysis to the same DAG appearing under identical mutation and detection conditions, there were 426 and 451 landscapes connected to a distinct landscape in CAPRI and CBN, respectively. These

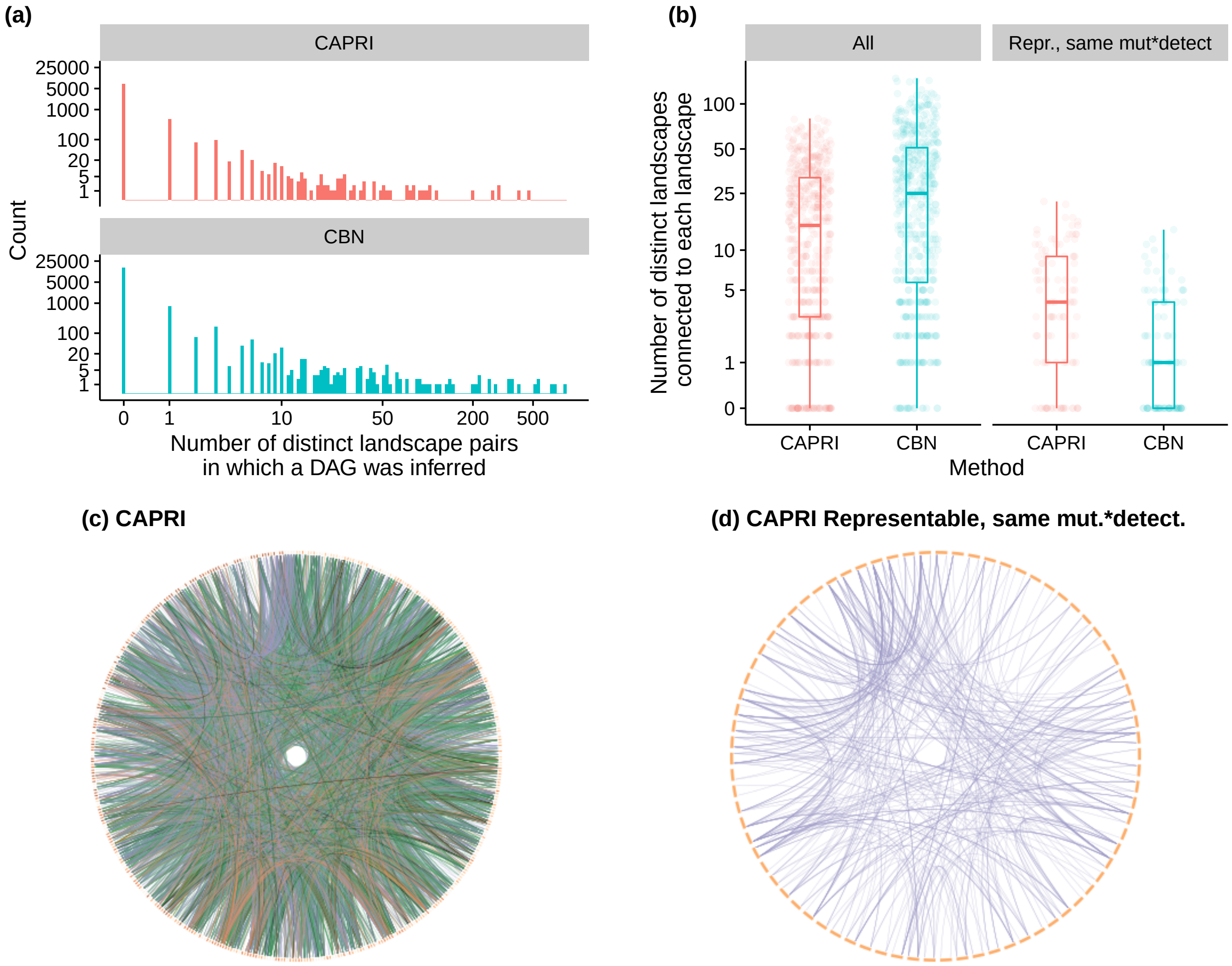

Figure 4: Distinct fitness landscapes with the same inferred DAG. (a) Histograms of the number of distinct landscapes in which DAGs were found, with both y- and x-axes in log-scale; for example, for both CAPRI and CBN about 1,000 different DAGs were found in one pair of distinct landscapes, and for CBN 50 DAGs were found in 10 distinct lanscape pairs. (b) Box-plots with overimposed, horizontally-jittered, data of number of distinct landscapes connected to each landscape for all landscapes under all conditions (left) and only representable landscapes connected under the same mutation and detection conditions (right). (c) Circos [22] plot of connections between distinct landscapes for CAPRI; 448 landscapes (ideograms) are shown, with landscapes ordered by increasing number of accessible genotypes starting from the 12 o'clock position; green links are connections where the same DAG was obtained under the same detection regime but different mutation rates; orange links for different detection regime and same mutation rates; black links for different detection and mutation; purple links for identical detection and mutation. (d) Like (c), but only for representable landscapes and same mutation and detection conditions; 78 landscapes shown.

patterns were also observed when the analysis was restricted to DAG-representable landscapes under the same mutation and detection conditions (Figure 4b, right subpanel; Figure 4d): 78 and 55, for CAPRI and CBN, respectively, of the 100 representable landscapes were connected to at least one other distinct representable landscape under the same mutation and detection conditions. Hence, the data show a "one DAG, many landscapes" phenomenon.

This complex pattern is also seen even if we only focus on DAG-representable landscapes (Figure 4 d): 84 and 63, for CAPRI and CBN, respectively, of the 100 representable landscapes

are connected to at least one other distinct representable landscape. Among the representable landscapes these numbers were 78 and 55.

10.1 Circos plots of landscape connections

In figure 5 I show the Circos plots [22] for both CBN and CAPRI.

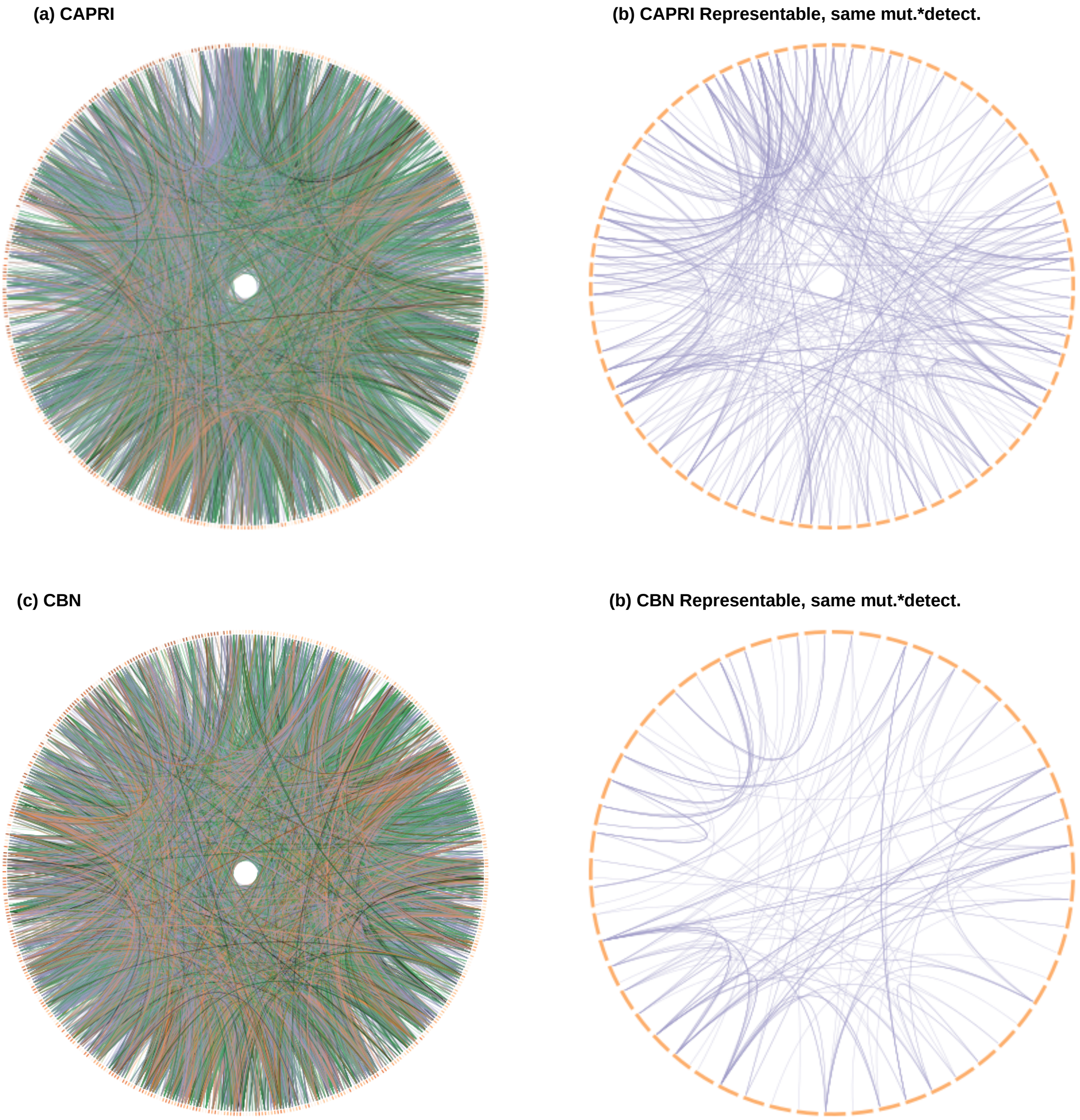

Figure 5: Circos plots [22] plot of connections between distinct landscapes. (a) and (c) for all landscapes, with CAPRI and CBN, showing 448 and 481 landscapes, respectively; (b) and (d) restricted to representable landscapes under the same mutation and detection conditions, showing 78 and 55 landscapes, respectively. In all cases, landscapes are ordered by increasing number of accessible genotypes starting from the 12 o'clock position; green links are connections where the same DAG was obtained under the same detection regime but different mutation rates; orange links for different detection regime and same mutation rates; black links for different detection and mutation; purple links for identical detection and mutation.

11 Cancer data sets: landscapes and inferred DAGs

11.1 Three cancer data sets: scheme

Figure 6 shows a diagram of the design.

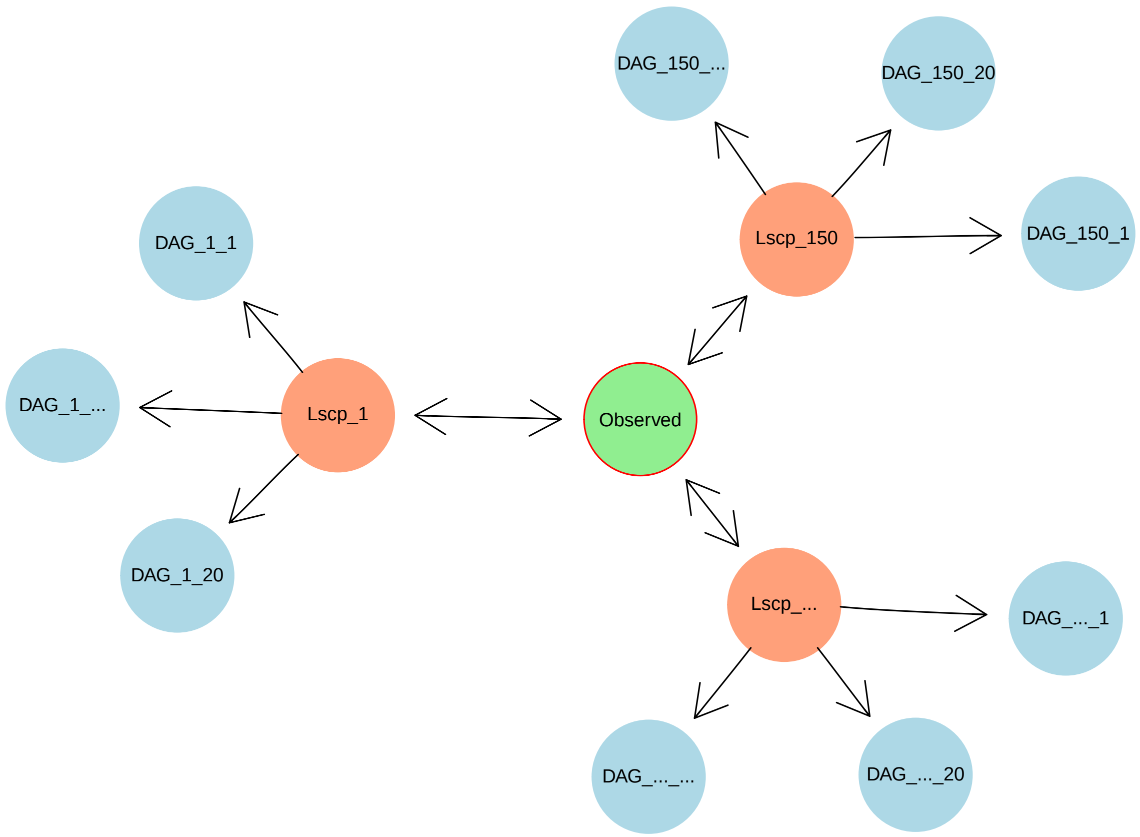

Figure 6: Schematic of the procedure and design for the cancer data set analysis and simulations. A set of 150 fitness landscapes (Lscp in the figure), together with mutation rates and detection regimes, were obtained that produced a set of 90/67 genotypes with a frequency distribution such that a χ^2 test of the null hypothesis that both data sets had the same genotype frequencies had a p-value larger than 0.6 (see details in Methods). For each of those fitness landscapes I simulated a additional 20,000 genotypes. From those 20,000 I took 20 subsets of 90/67 genotypes, and obtained 20 cancer progression DAGs for CPM and 20 for CAPRI; I repeated this procedure with 20 subsets of 1,000 genotypes. Comparisons of similarity between the landscapes are, thus, comparisons of the "Lscp" in this figure; comparisons of similarity between DAGs are comparisons of either the pairwise similarity of DAGs within "Lscp" or the average pairwise similarity of DAGs over "Lscp".

11.2 Three cancer data sets: plots of landscapes and DAGs

Files `pancreas-landscapes-dags.pdf`, `gliob-landscapes-dags.pdf`, and `colon-landscapes-dags.pdf` show, for each data set, the 150 landscapes, together with the modal inferred DAGs (their transitive reduction) with N=90 and N=1,000.

11.3 Three cancer data sets: DAG variability, reciprocal sign epistasis and number of peaks in the fitness landscape, and number of accessible genotypes

Figure 7 shows reciprocal sign epistasis, number of peaks, number of accessible genotypes, and within-landscape DAG variability for the three cancer data sets.

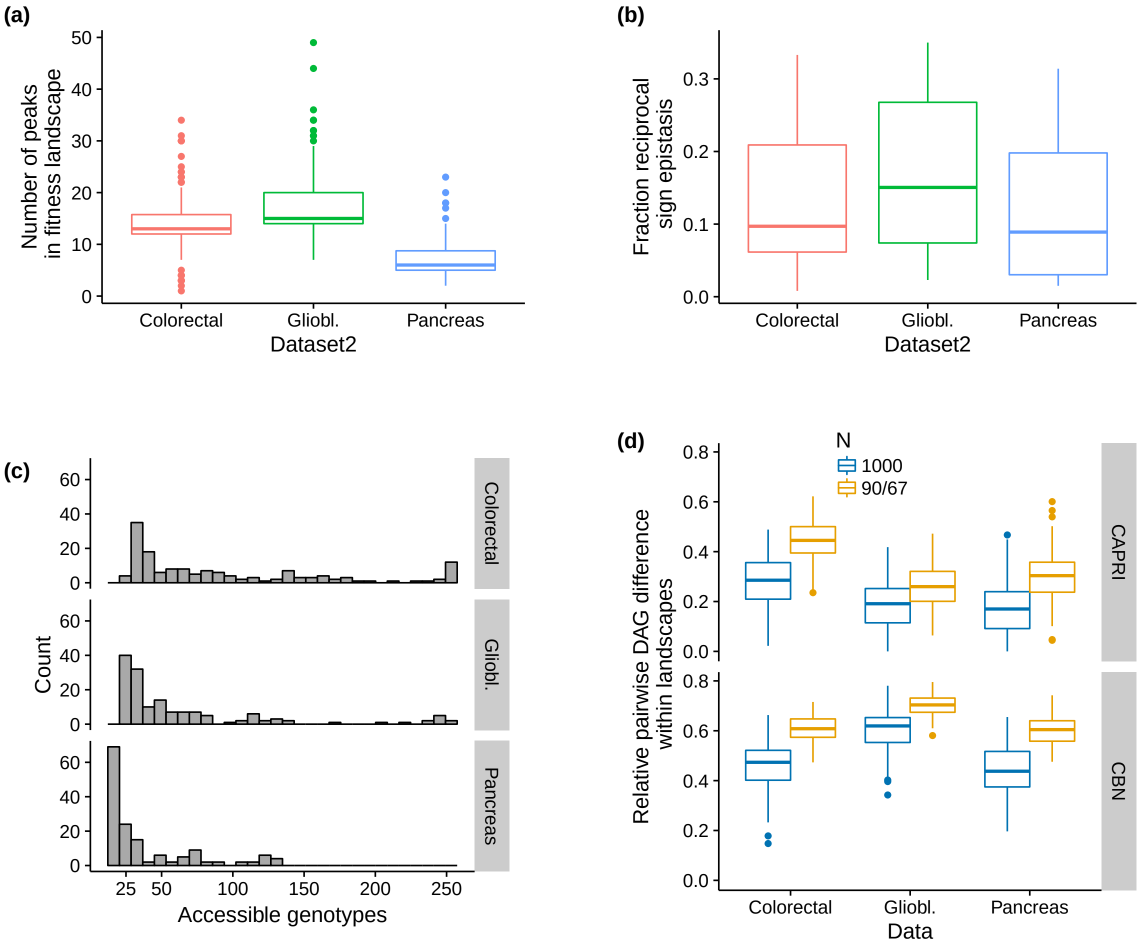

Figure 7: Fitness landscape characteristics and within-landscape DAG differences for the three cancer data sets. (a) and (b) Boxplots of number of peaks and fraction of reciprocal sign epistasis in the 150 fitness landscapes for each data set. (c) Histogram of number of accessible genotypes for each landscape. (d) Box-plots of relative pairwise differences over all pairs of the 20 DAGs inferred from each landscape with sample sizes of 90/67 and 1000 genotypes; thus, 150 points are shown, each the average of $20 * 19/2$ pairwise differences.

12 Canonical DAG

As both cancer progression DAGs of restrictions and fitness landscapes specify what genotypes can and cannot be observed, we can try to define a canonical DAG that provides a faithful representation of the accessible/inaccessible status of the genotypes in a fitness landscape. If such faithful representation exists, this is the reference DAG against which the output from any method should be compared. Even if a faithful DAG does not exist (for instance, when there is reciprocal sign epistasis), this canonical DAG can help us understand the relationships in the fitness landscape.

The procedure used here, DAG_a, finds the DAG of restrictions with the fewest edges such that all accessible genotypes under the landscape can be observed. This is equivalent to finding the DAG of restrictions so that all accessible genotypes can be "explained" or accounted for by the DAG. Alternatively, following the terminology in [20] one can think of finding, for the lattice of genotypes, the corresponding Hasse diagram of mutational events with the smallest number of edges or, equivalently, finding the smallest poset. We will refer to this procedure as DAG_a from accounting for genotypes by adding edges to the DAG.

To understand this procedure, we can refer to the fitness graph of [7], where genotypes are vertices and there is a directed edge between genotypes i and j if genotype j has larger fitness and exactly one more mutated gene than genotype i or, simply, genotype j is accessible from genotype i (in our fitness graphs no backmutations are possible: there can be no transitions from a genotype with k mutations to a genotype with $k-1$ mutations). From this fitness graph of genotypes we want an algorithm to construct another graph, a DAG with genes as vertices, where gene y is a descendant of gene(s) x if gene y can only be observed mutated if (all the) gene(s) in x have been observed mutated already. The algorithm to obtain the DAG of restrictions starts from the set of genotypes directly accessible from the Root. These are genotype(s) with only one mutated gene; these gene(s) thus depend on no other gene (only the "Root" node) and are placed in the DAG of restrictions with Root as the single parent. Then, the algorithm goes through genotypes of increasing number of mutated genes (thus, using breadth first traversal in the graph of genotypes). For each mutated gene that is not yet in the DAG of restrictions, it finds the gene(s) that are already in the DAG of restrictions on which it must establish dependencies to explain the observed genotype. Therefore, the algorithm adds the minimal set of arrows to capture the very first appearance of a mutated gene. This algorithm will add conjunctions (convergent arrows) from genes x_1 and x_2 to gene y if, and only if, we never observe y except if we also observe both of x_1 and x_2 (and this means that a genotype with x_1 and x_2 mutated must be accessible). If there are OR relationships of dependency in the fitness landscape, so that we can observe y in combination with x_1 (but without x_2), and viceversa, there will only be one edge to y (from either x_1 or x_2, depending on the order of the names of the genes).

When the fitness landscape is such that the accessible/inaccessible genotypes can be perfectly predicted by a DAG DAG_a returns a genotype misprediction, or non-representability, of 0.

Obtaining DAG_a was done with code written in R (see `canonical-DAG.R` in section 13.4).

13 Cancer progression models and other software

I have used CBN [19, 21] and CAPRI [28], the two widely available state-of-the-art methods that accommodate multiple parents (convergent arrows or conjunctions) in DAGs. Other methods for cancer progression models have been described but either they cannot represent conjunctions, such as OT [8, 32] or CAPRESE [24], or are too slow for routine use such as [29], or have dependencies on external libraries that are not open source such as DiP [12], or have no software available. The methods in [2, 6] try to infer the order of mutations within a explicit evolutionary model of tumor progression; unfortunately, working software is not really available, and thus

comparisons are not possible. See further details in [9].

13.1 CBN and CAPRI software

For CBN version 0.1.04b from March 2016, and still current as of April 2017, was downloaded from https://www.bsse.ethz.ch/cbg/software/ct-cbn.html. Defaults for CBN were used. I wrote a wrapper to call their code from R, and I used the default settings for temp ($-T = 1$) and steps ($-N =$ number of nodes^2); I started the simulated annealing search for the best poset from an initial poset built using Oncogenetic Tress [33], as preliminary runs suggested this initial poset is as good as, or better than, the default linear poset in [21].

For CAPRI, version 2.5.4, current as of October 2016, was used, downloaded from the official BioConductor site. This version would become version 2.6.1 of TRONCO when BioConductor 3.4 was released as stable version. TRONCO was run using R-3.3.1 (patched: svn release 70828). All options were left at the recommended defaults (BIC regularization, 100 bootstrap samples for the estimation of the selective advantage scores with p-value of 0.05, and heuristic search using Hill Climbing).

13.2 Preprocessing of data for CPMs

Before analyzing data with CBN and CAPRI, data were preprocessed so that:

- All columns that had all 0s (i.e., genes that were absent in all samples) were removed.
- Columns that were identical over all individuals were fused (those are, for instance, the genes with names such as "D_E" in some figures: those correspond to genes that, for a particular set of individuals, had identical values over all individuals).
- Genes that were present in less than 5% of the individuals were removed (this is a common practice in this field; see examples in [9]). Note that this does not apply to the cancer data sets, where one of the genes had, in the original pancreas data set, a frequency of 4.4%. For these cases, no genes were removed if they were present in at least one individual.
- To prevent removal of genes present in all cases, 10% of cases with no mutation whatsoever were always added to all data sets (see [9] for details).

13.3 Other software

Measures of fraction of pairs of loci with reciprocal sign epistasis (e.g., [14]) were obtained using MAGELLAN [5], with source code available from it's web page (http://wwwabi.snv.jussieu.fr/public/magellan/). Simulations and generation of random fitness landscapes and random DAGs were done using OncoSimulR [10]; see section 13.4.

13.4 Code and data for generating fitness landscapes and simulating from them

The 500 random fitness landscapes (representable, non-representable DAG-derived, and RMF) were created using file `generate-landscapes.R` (this file sources `canonical-DAG.R`). Simulations on landscapes were run using file `simuls-landscapes.R` (and this file sources `common-simul-landscapes.R`), and use the OncoSimulR package [10].

For the cancer data sets, searching for fitness landscapes was done with `find-compatible-landscape.R` (and this file sources `common-reverse.R` and reads the data file `Gerstung_PLoS_ONE_pancreas_gliob_colon_genes.RData`). The RData file was obtained using file `modified_example.py`, modified from the original `example.py` in [21], to obtain files `pancreas_genes.pat`, `colon_genes.pat`, and `gliob_genes.pat`, and I then read, added column

names, and verified the frequencies of genotypes using the code in `create-pancreas-data.R`. It is of course possible to read the data directly into R from the txt files "WoodS2007.txt", "ParsonsS2008.txt" and "JonesS2008.txt"; this is shown in `reading_txt_files_directly_from_R.R` (which serves as a third check of the procedure). `find-compatible-landscape.R` was run with low priority during idle times on a 128-cores cluster during two weeks. The file `generic-launcher.sh` provides a basic shell script to launch many of those searches in a cluster. Once a large number of hits have been found, you can select those that match the criteria of p-values and having all genes present using `select_reverse_hits.R`. Once a set of candidate landscapes were found, simulations were launched on those fitness landscapes with `simul_from_reverse_landscape.R` (which also sources `common-reverse.R` and loads `Gerstung_PLoS_ONE_pancreas_gliob_colon_genes.RData`).

File `diagnostics-utils-cpm-lmer.R` includes the custom code for statistical model checking of Tweedie and linear mixed effects models (see section 7).

All of the above files are included in file `code.zip`.

References

[1] Agresti, A., 2002. *Categorical Data Analysis, 2nd Ed.* Wiley, Hoboken, New Jersey, 2nd edition.

[2] Attolini, C., Cheng, Y., Beroukhim, R., Getz, G., Abdel-Wahab, O., Levine, R. L., Mellinghoff, I. K., Michor, F., 2010. A mathematical framework to determine the temporal sequence of somatic genetic events in cancer. *Proceedings of the National Academy of Sciences*, **107(41)**:17604–17609. doi:10.1073/pnas.1009117107/-/DCSupplemental.www.pnas.org/cgi/doi/10.1073/pnas.1009117107. URL http://www.pnas.org/content/107/41/17604.short.

[3] Bates, D., Mächler, M., Bolker, B., Walker, S., 2015. Fitting Linear Mixed-Effects Models Using lme4. *Journal of Statistical Software*, **67(1)**:1–48. doi:10.18637/jss.v067.i01. URL https://www.jstatsoft.org/article/view/v067i01/0.

[4] Bozic, I., Antal, T., Ohtsuki, H., Carter, H., Kim, D., Chen, S., Karchin, R., Kinzler, K. W., Vogelstein, B., Nowak, M. A., 2010. Accumulation of driver and passenger mutations during tumor progression. *Proceedings of the National Academy of Sciences of the United States of America*, **107**:18545–18550. doi:10.1073/pnas.1010978107. URL http://www.ncbi.nlm.nih.gov/pubmed/20876136.

[5] Brouillet, S., Annoni, H., Ferretti, L., Achaz, G., 2015. MAGELLAN: A tool to explore small fitness landscapes. *bioRxiv*, p. 031583. doi:10.1101/031583. URL http://biorxiv.org/content/early/2015/11/13/031583.

[6] Cheng, Y.-K., Beroukhim, R., Levine, R. L., Mellinghoff, I. K., Holland, E. C., Michor, F., 2012. A mathematical methodology for determining the temporal order of pathway alterations arising during gliomagenesis. *PLoS computational biology*, **8(1)**:e1002337. doi:10.1371/journal.pcbi.1002337. URL http://www.pubmedcentral.nih.gov/articlerender.fcgi?artid=3252265&tool=pmcentrez&rendertype=abstract.

[7] Crona, K., Greene, D., Barlow, M., 2013. The peaks and geometry of fitness landscapes. *Journal of Theoretical Biology*, **317**:1–10. doi:10.1016/j.jtbi.2012.09.028. URL http://www.sciencedirect.com/science/article/pii/S0022519312005061.

[8] Desper, R., Jiang, F., Kallioniemi, O. P., Moch, H., Papadimitriou, C. H., Schäffer, A. A., 1999. Inferring tree models for oncogenesis from comparative genome hybridization data. *J Comput Biol*, **6(1)**:37–51. URL http://view.ncbi.nlm.nih.gov/pubmed/10223663.

[9] Diaz-Uriarte, R., 2015. Identifying restrictions in the order of accumulation of mutations during tumor progression: Effects of passengers, evolutionary models, and sampling. *BMC Bioinformatics*, **16(41)**:0–36. doi:doi:10.1186/s12859-015-0466-7. URL http://www.biomedcentral.com/1471-2105/16/41/abstract.

[10] Diaz-Uriarte, R., 2017. OncoSimulR: Genetic simulation with arbitrary epistasis and mutator genes in asexual populations. *Bioinformatics*, **33(12)**:1898–1899. doi:10.1093/bioinformatics/btx077. URL https://academic.oup.com/bioinformatics/article/33/12/1898/2982052/OncoSimulR-genetic-simulation-with-arbitrary.

[11] Dunn, P. K., Smyth, G. K., 1996. Randomized Quantile Residuals. *Journal of Computational and Graphical Statistics*, **5(3)**:236. doi:10.2307/1390802. URL http://www.jstor.org/stable/1390802?origin=crossref.

[12] Farahani, H. S., Lagergren, J., 2013. Learning oncogenetic networks by reducing to mixed integer linear programming. *PloS ONE*, **8(6)**:e65773. doi:10.1371/journal.pone.0065773. URL http://www.pubmedcentral.nih.gov/articlerender.fcgi?artid=3683041&tool=pmcentrez&rendertype=abstract.

[13] Faraway, J. J., 2016. *Extending the Linear Model with R: Generalized Linear, Mixed Effects and Nonparametric Regression Models, Second Edition*. Chapman and Hall/CRC, Boca Raton, 2 edition edition. ISBN 978-1-4987-2096-0.

[14] Ferretti, L., Schmiegelt, B., Weinreich, D., Yamauchi, A., Kobayashi, Y., Tajima, F., Achaz, G., 2016. Measuring epistasis in fitness landscapes: The correlation of fitness effects of mutations. *Journal of Theoretical Biology*, **396**:132–143. doi:10.1016/j.jtbi.2016.01.037. URL http://www.sciencedirect.com/science/article/pii/S0022519316000771.

[15] Fox, J., 2003. Effect Displays in R for Generalised Linear Models. *Journal of Statistical Software*, **8(15)**:1–27. doi:10.18637/jss.v008.i15. URL http://www.jstatsoft.org/v08/i15/.

[16] Fox, J., 2016. *Applied Regression Analysis and Generalized Linear Models*. SAGE, Los Angeles. ISBN 978-1-4522-0566-3.

[17] Fox, J., Weisberg, S., 2011. *An R Companion to Applied Regression*. Sage, Los Angeles, Calif. ISBN 978-1-4129-7514-8.

[18] Gentle, J. E., 2007. *Matrix Algebra*. Springer, New York.

[19] Gerstung, M., Baudis, M., Moch, H., Beerenwinkel, N., 2009. Quantifying cancer progression with conjunctive Bayesian networks. *Bioinformatics (Oxford, England)*, **25(21)**:2809–2815. doi:10.1093/bioinformatics/btp505. URL http://dx.doi.org/10.1093/bioinformatics/btp505%0020http://www.bsse.ethz.ch/cbg/software/ct-cbn.

[20] Gerstung, M., Beerenwinkel, N., 2010. Waiting time models of cancer progression. *Mathematical Population Studies*, **17**:115–135. URL http://arxiv.org/abs/0807.3638.

[21] Gerstung, M., Eriksson, N., Lin, J., Vogelstein, B., Beerenwinkel, N., 2011. The Temporal Order of Genetic and Pathway Alterations in Tumorigenesis. *PLoS ONE*, **6(11)**:e27136. doi:10.1371/journal.pone.0027136. URL http://dx.plos.org/10.1371/journal.pone.0027136%0020http://www.bsse.ethz.ch/cbg/software/ct-cbn.

[22] Krzywinski, M., Schein, J., Birol, b., Connors, J., Gascoyne, R., Horsman, D., Jones, S. J., Marra, M. A., 2009. Circos: An information aesthetic for comparative genomics. *Genome Research*, **19(9)**:1639–1645.

doi:10.1101/gr.092759.109. URL http://dx.doi.org/10.1101/gr.092759.109.

[23] Lintusaari, J., Gutmann, M. U., Dutta, R., Kaski, S., Corander, J., 2017. Fundamentals and Recent Developments in Approximate Bayesian Computation. *Systematic Biology*, pp. e66–e82. doi:10.1093/sysbio/syw077. URL https://academic.oup.com/sysbio/article-lookup/doi/10.1093/sysbio/syw077.

[24] Loohuis, L. O., Caravagna, G., Graudenzi, A., Ramazzotti, D., Mauri, G., Antoniotti, M., Mishra, B., 2014. Inferring Tree Causal Models of Cancer Progression with Probability Raising. *PLoS ONE*, **9(10)**:e108358. doi:10.1371/journal.pone.0108358. URL http://dx.plos.org/10.1371/journal.pone.0108358%0020http://bimib.disco.unimib.it/index.php/Tronco%0020http://journals.plos.org/plosone/article?id=10.1371/journal.pone.0108358.

[25] McCullagh, P., Nelder, J., 1989. *Generalized Linear Models, 2nd Ed.* Chapman and Hall/CRC, London.

[26] McFarland, C. D., Korolev, K. S., Kryukov, G. V., Sunyaev, S. R., Mirny, L. A., 2013. Impact of deleterious passenger mutations on cancer progression. *Proceedings of the National Academy of Sciences of the United States of America*, **110(8)**:2910–5. doi:10.1073/pnas.1213968110. URL http://www.ncbi.nlm.nih.gov/pubmed/23388632.

[27] Poelwijk, F. J., Kiviet, D. J., Weinreich, D. M., Tans, S. J., 2007. Empirical fitness landscapes reveal accessible evolutionary paths. *Nature*, **445(7126)**:383–6. doi:http://dx.doi.org/10.1038/nature05451. URL http://search.proquest.com/docview/204523563/abstract/CED5FE881C9942BCPQ/1.

[28] Ramazzotti, D., Caravagna, G., Olde Loohuis, L., Graudenzi, A., Korsunsky, I., Mauri, G., Antoniotti, M., Mishra, B., 2015. CAPRI: Efficient inference of cancer progression models from cross-sectional data. *Bioinformatics*, **31(18)**:3016–3026. doi:10.1093/bioinformatics/btv296. URL https://academic.oup.com/bioinformatics/article-lookup/doi/10.1093/bioinformatics/btv296.

[29] Sakoparnig, T., Beerenwinkel, N., 2012. Efficient sampling for Bayesian inference of conjunctive Bayesian networks. *Bioinformatics (Oxford, England)*, **28(18)**:2318–24. doi:10.1093/bioinformatics/bts433. URL http://www.ncbi.nlm.nih.gov/pubmed/22782551%0020http://www.bsse.ethz.ch/cbg/software/bayes-cbn.

[30] Sousa, V. C., Fritz, M., Beaumont, M. A., Chikhi, L., 2009. Approximate Bayesian Computation Without Summary Statistics: The Case of Admixture. *Genetics*, **181(4)**:1507–1519. doi:10.1534/genetics.108.098129. URL http://www.genetics.org/content/181/4/1507.

[31] Sprouffske, K., Pepper, J. W., Maley, C. C., 2011. Accurate reconstruction of the temporal order of mutations in neoplastic progression. *Cancer prevention research (Philadelphia, Pa.)*, **4(7)**:1135–44. doi:10.1158/1940-6207.CAPR-10-0374. URL http://www.pubmedcentral.nih.gov/articlerender.fcgi?artid=3131446&tool=pmcentrez&rendertype=abstract.

[32] Szabo, A., Boucher, K. M., 2008. Oncogenetic trees. In Tan, W.-Y., Hanin, L., editors, *Handbook of Cancer Models with Applications*, pp. 1–24. World Scientific. URL http://www.worldscibooks.com/lifesci/6677.html.

[33] Szabo, A., Pappas, L., 2013. Oncotree: Estimating oncogenetic trees. R package version 0.3.3. URL http://cran.r-project.org/package=Oncotree.

[34] Szendro, I. G., Franke, J., de Visser, J. A. G. M., Krug, J., 2013. Predictability of evolution depends nonmonotonically on population size. *PNAS*, **110(2)**:571–576. doi:10.1073/pnas.1213613110. URL http://www.pnas.org/content/110/2/571.

[35] Zhang, Y., 2013. Likelihood-based and Bayesian methods for Tweedie compound Poisson linear mixed models. *Statistics and Computing*, **23(6)**:743–757. doi:10.1007/s11222-012-9343-7. URL http://link.springer.com/10.1007/s11222-012-9343-7.
