# Supplementary material for: Cancer progression models and fitness landscapes: a many-to-many relationship
Source: Bioinformatics. 2017 Oct 18;34(5):836–44. doi: 10.1093/bioinformatics/btx663 (PMC6031050; doi:10.1093/bioinformatics/btx663)

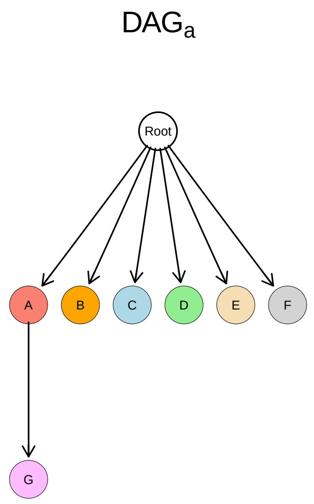

ID	Accessible Genot.	Discrep. DAG_a
S7_1	95	0

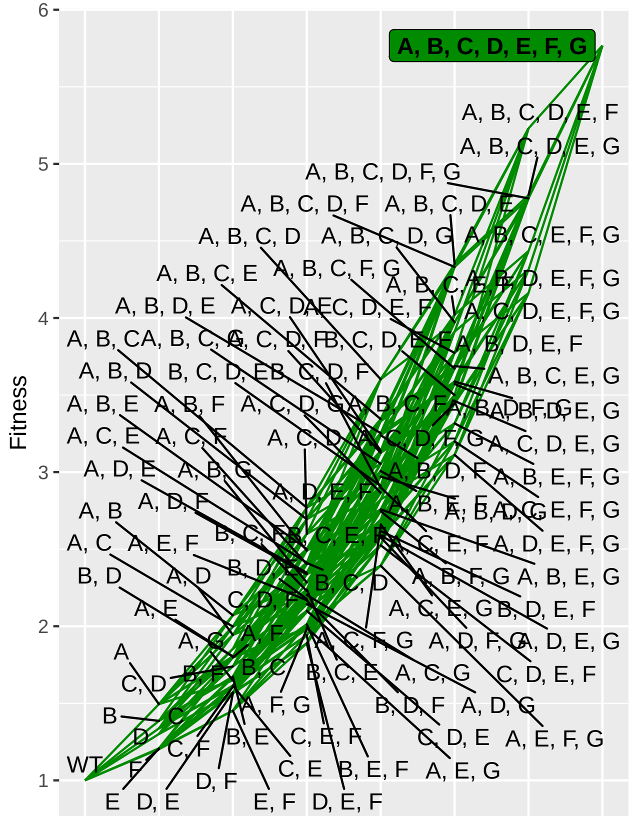

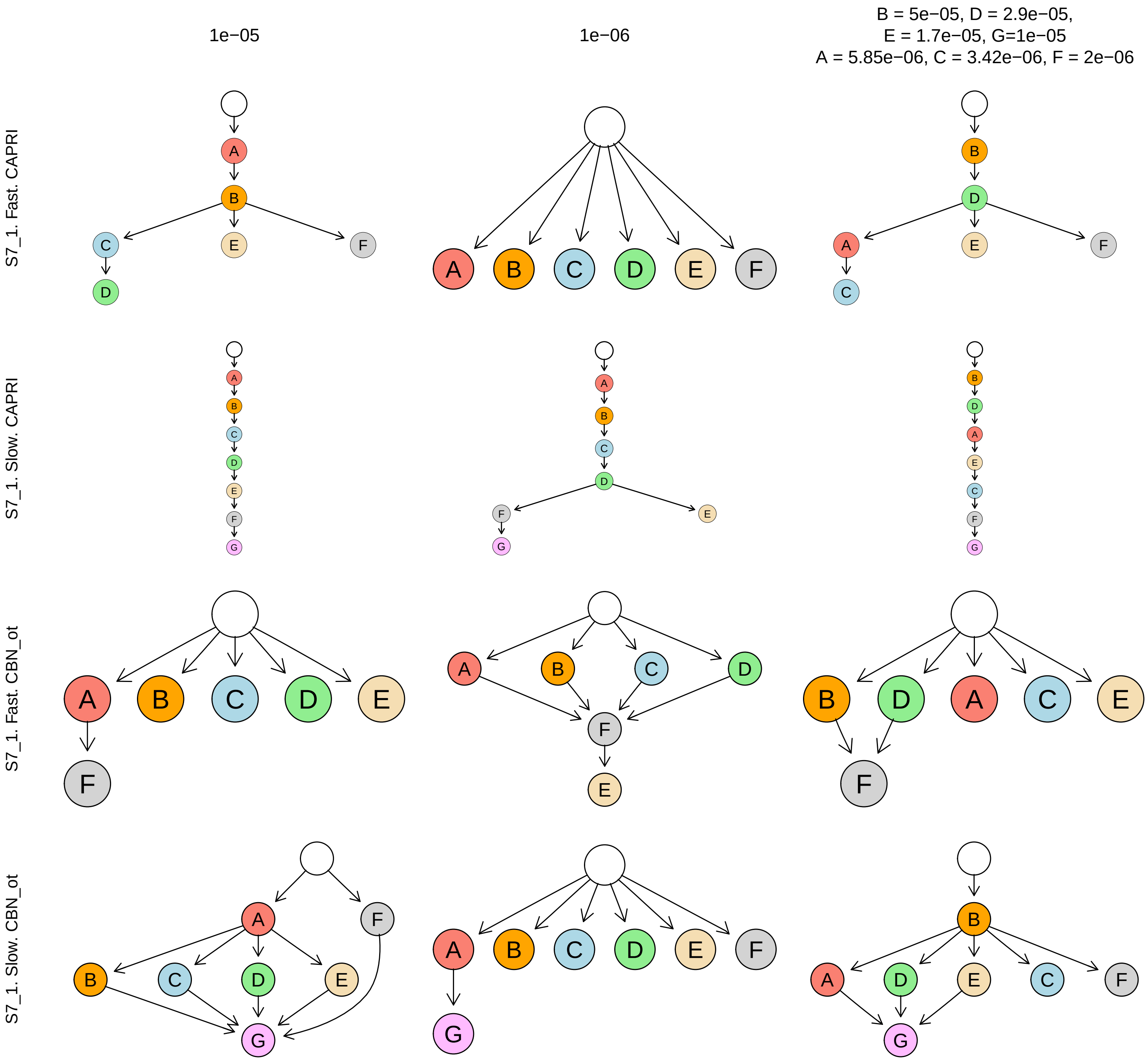

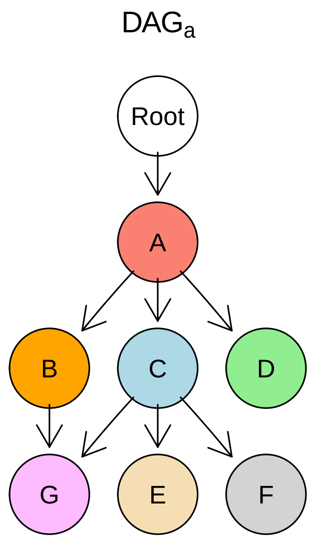

ID	Accessible Genot.	Discrep. DAG_a
S7_2	28	0

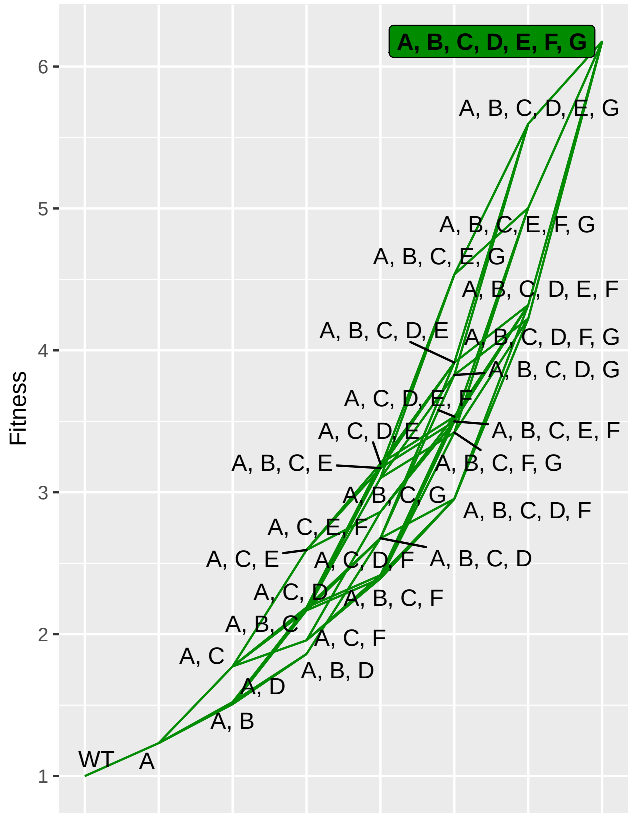

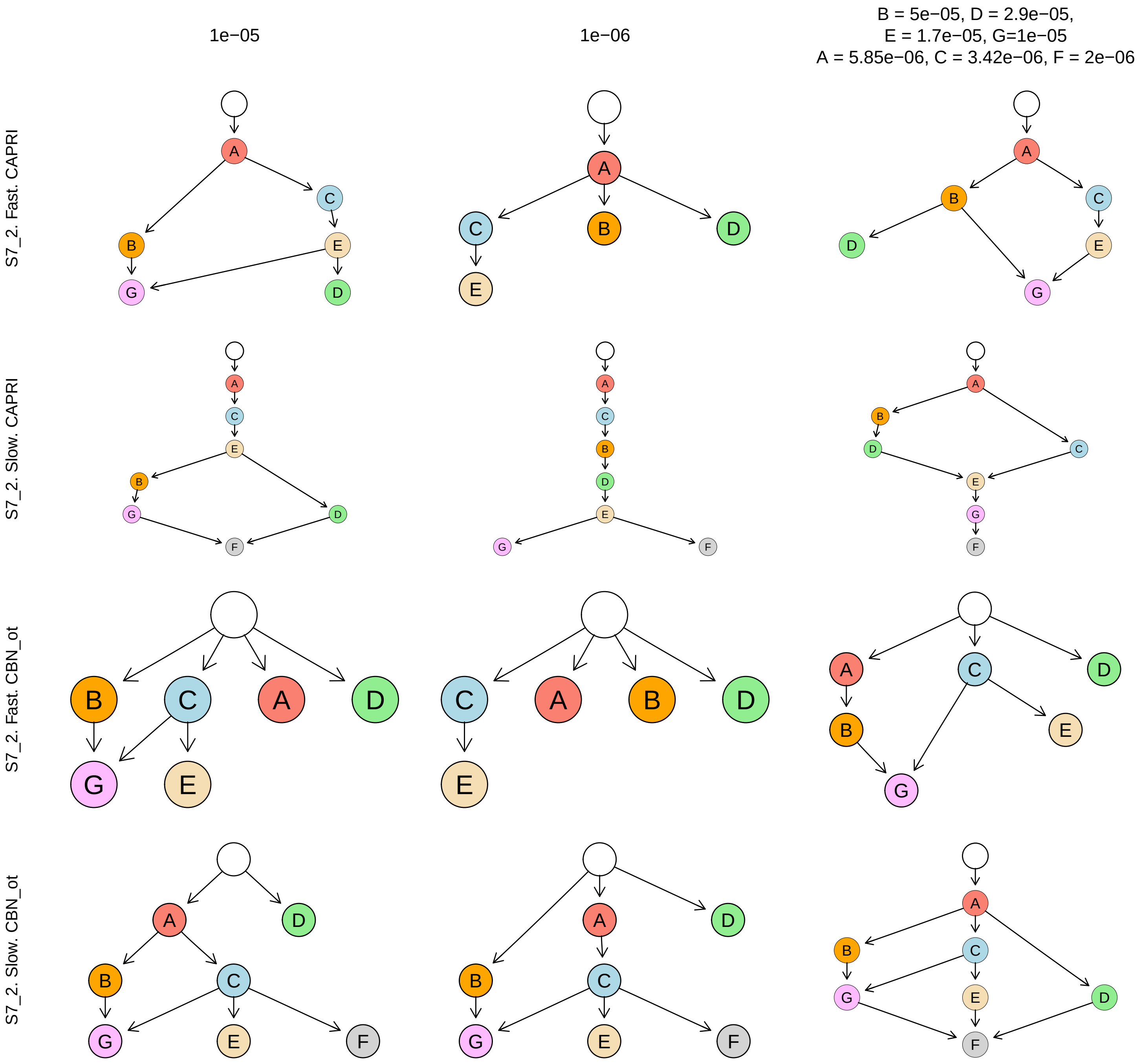

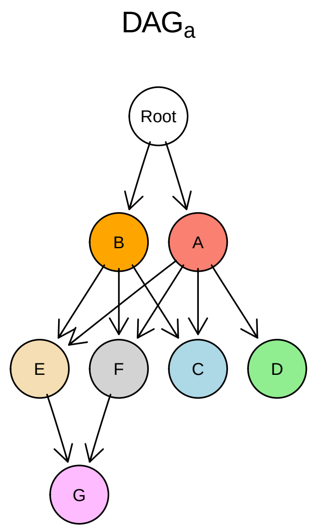

ID	Accessible Genot.	Discrep. DAG_a
S7_3	23	0

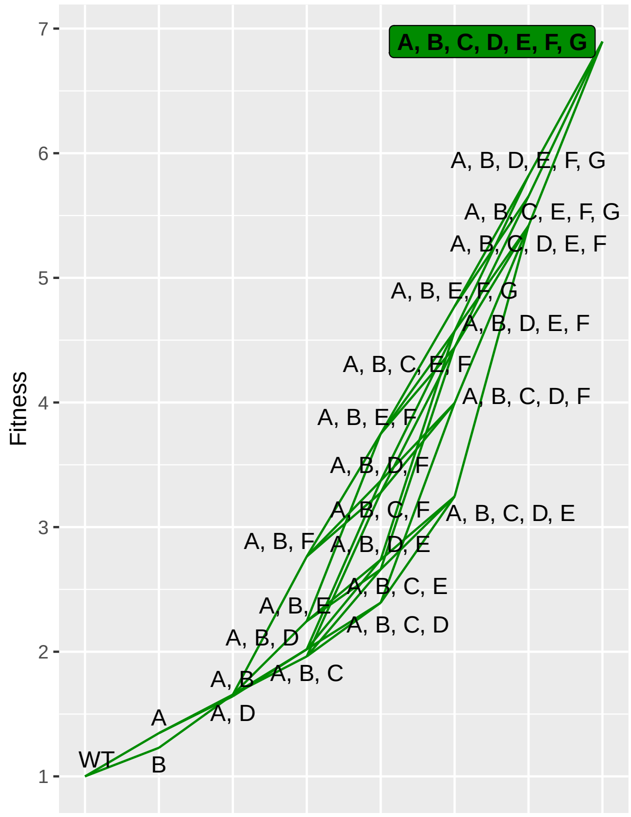

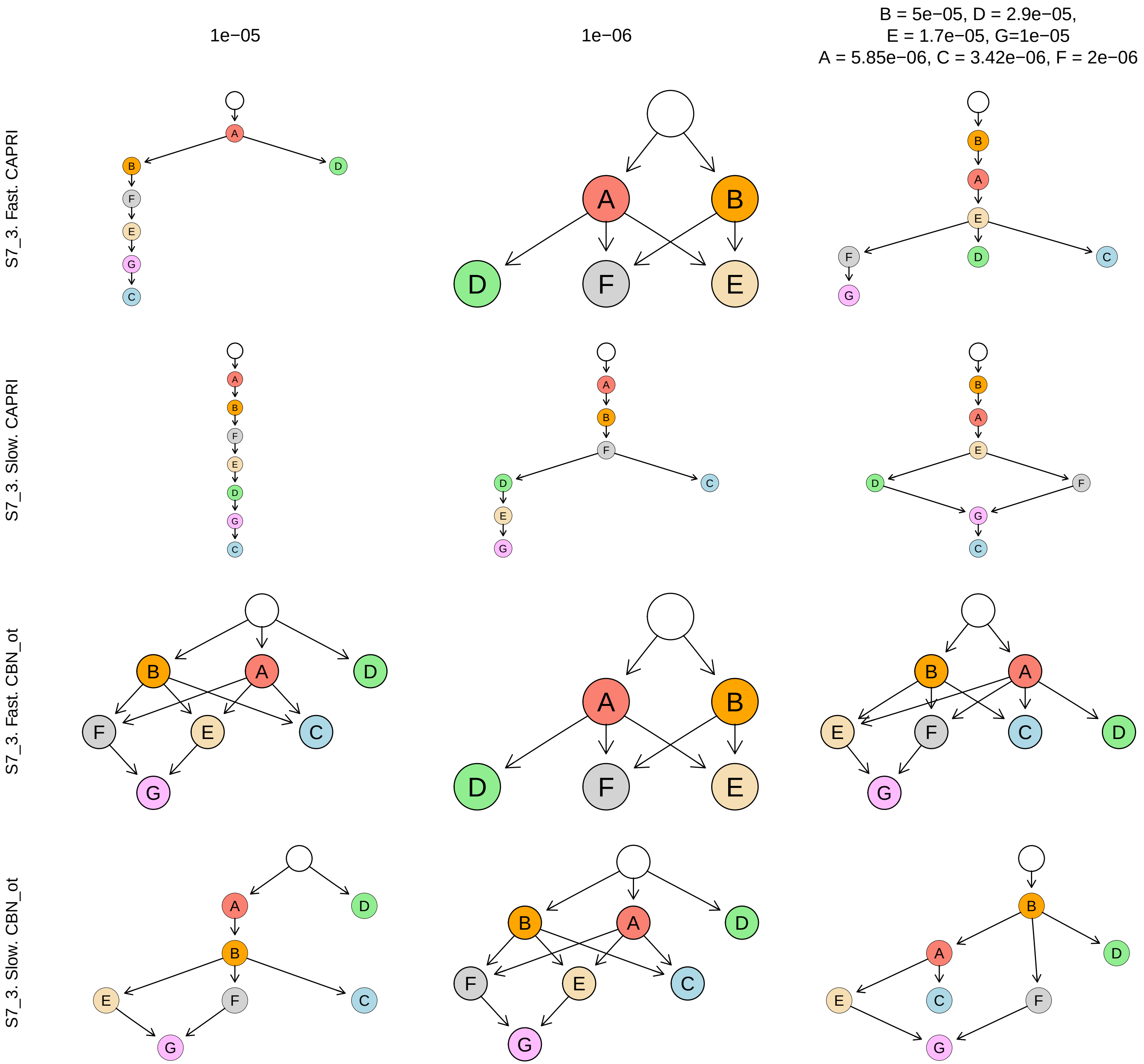

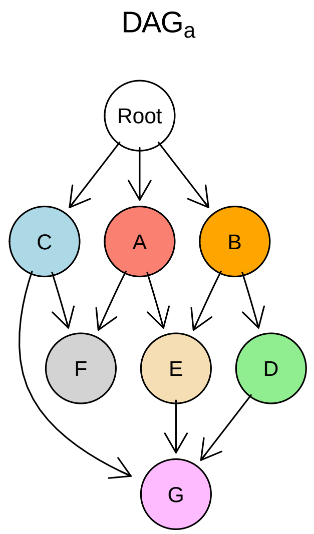

ID	Accessible Genot.	Discrep. DAG_a
S7_4	22	0

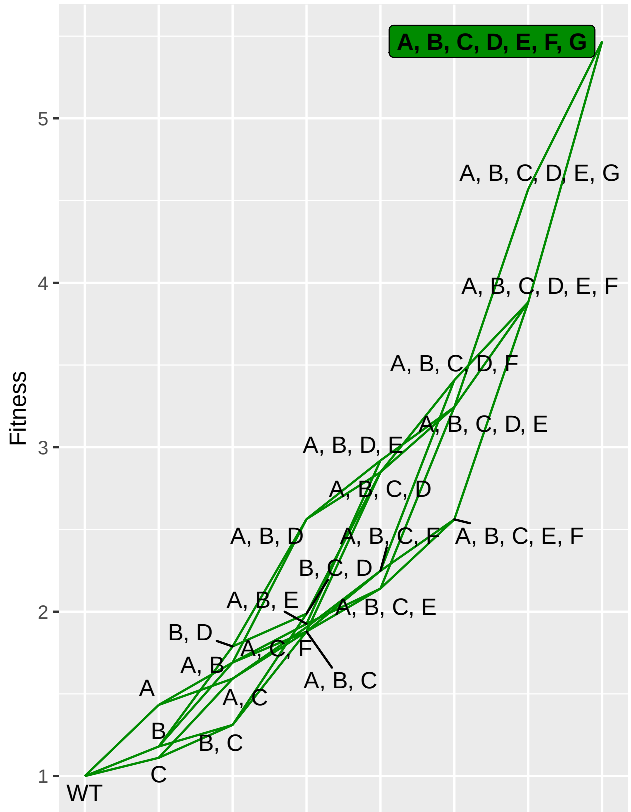

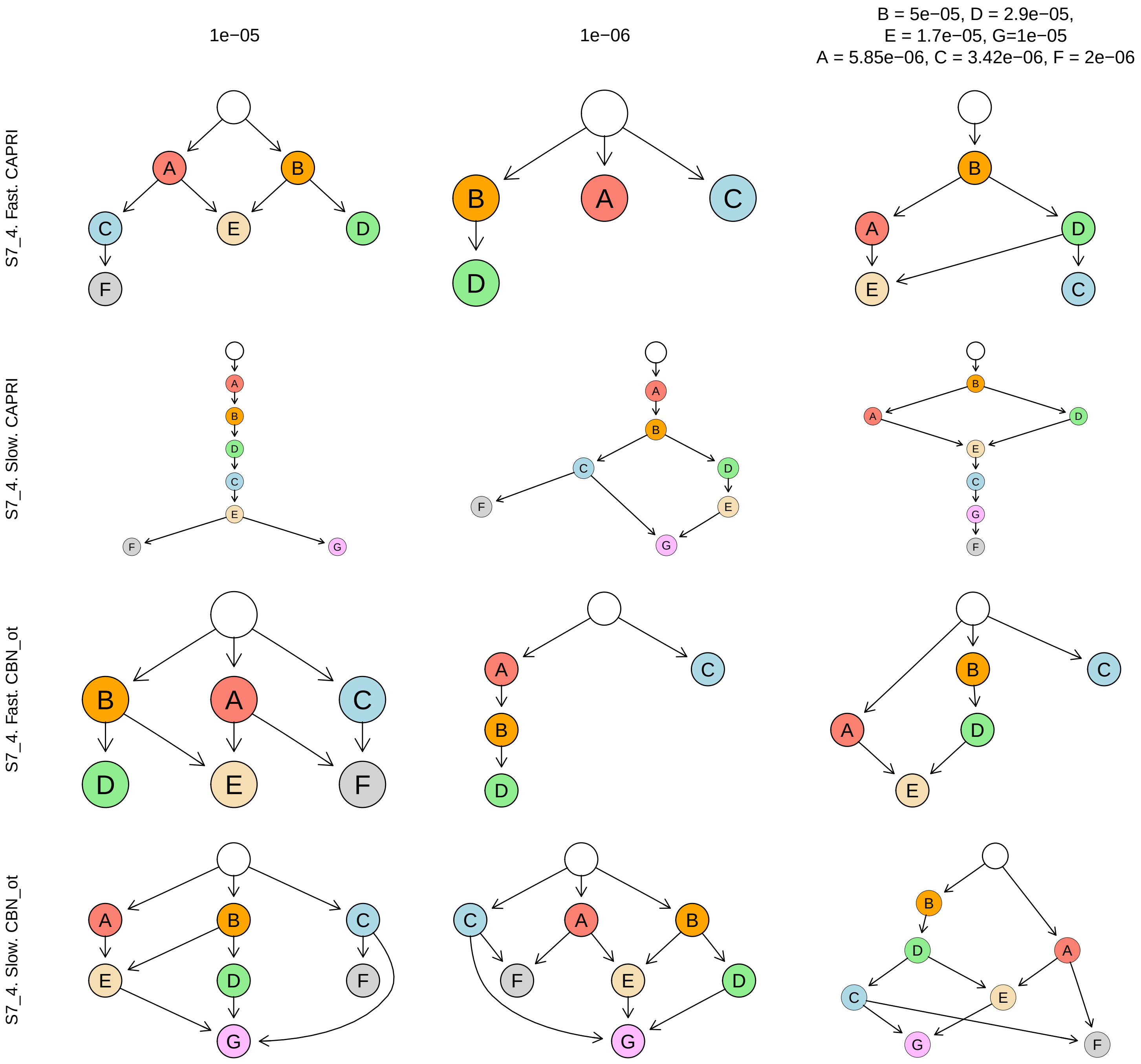

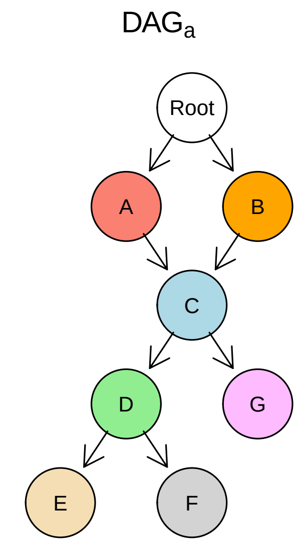

ID	Accessible Genot.	Discrep. DAG_a
S7_5	13	0

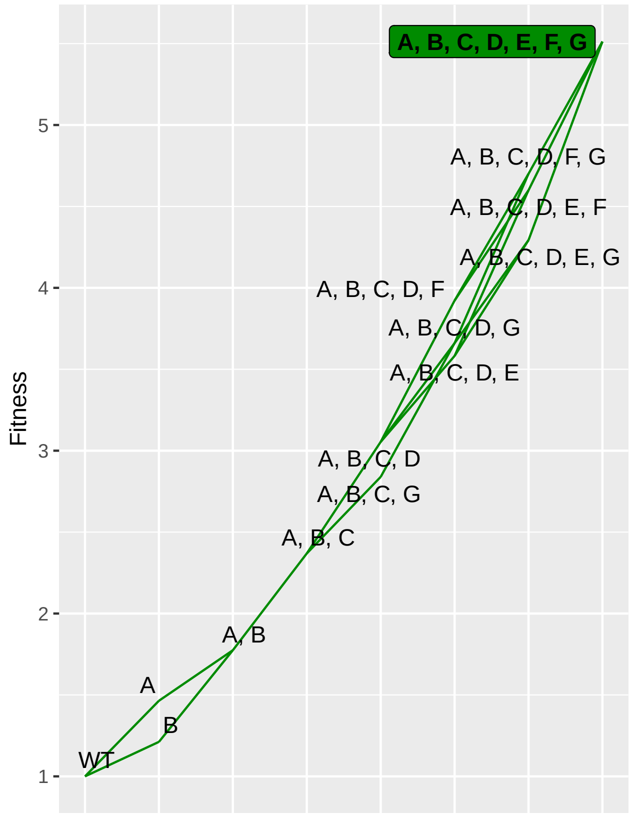

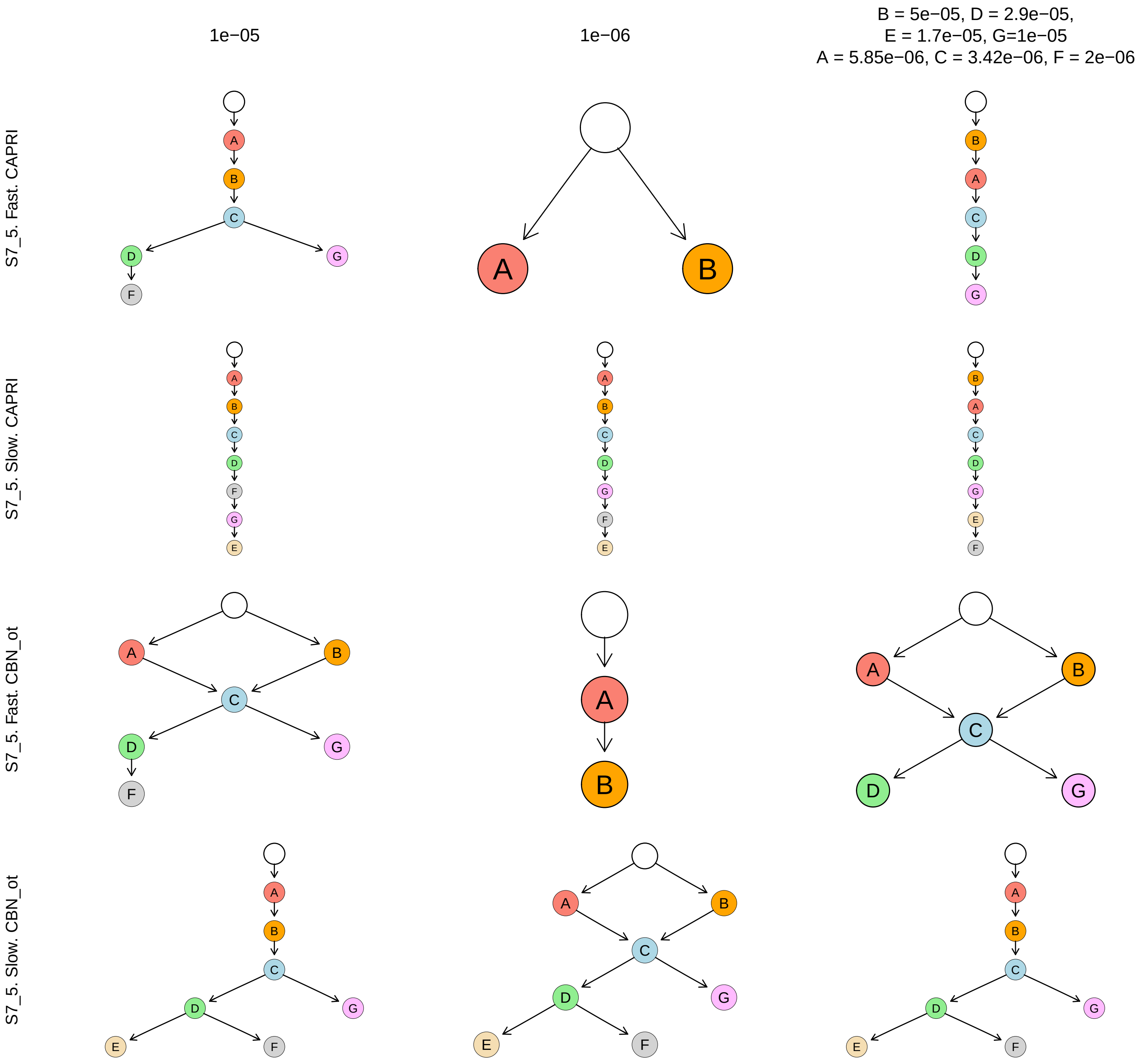

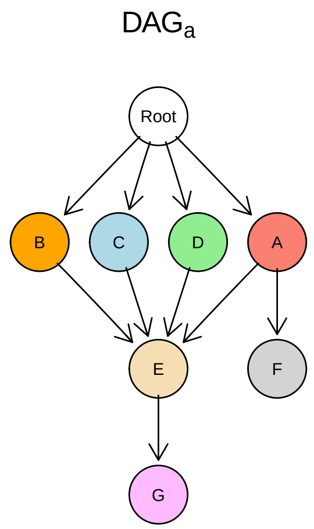

ID	Accessible Genot.	Discrep. DAG_a
S7_6	27	0

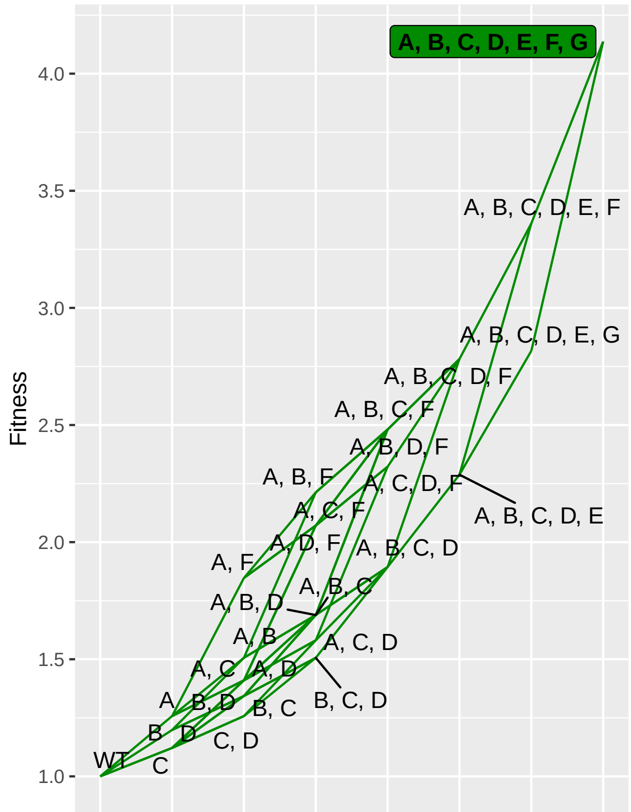

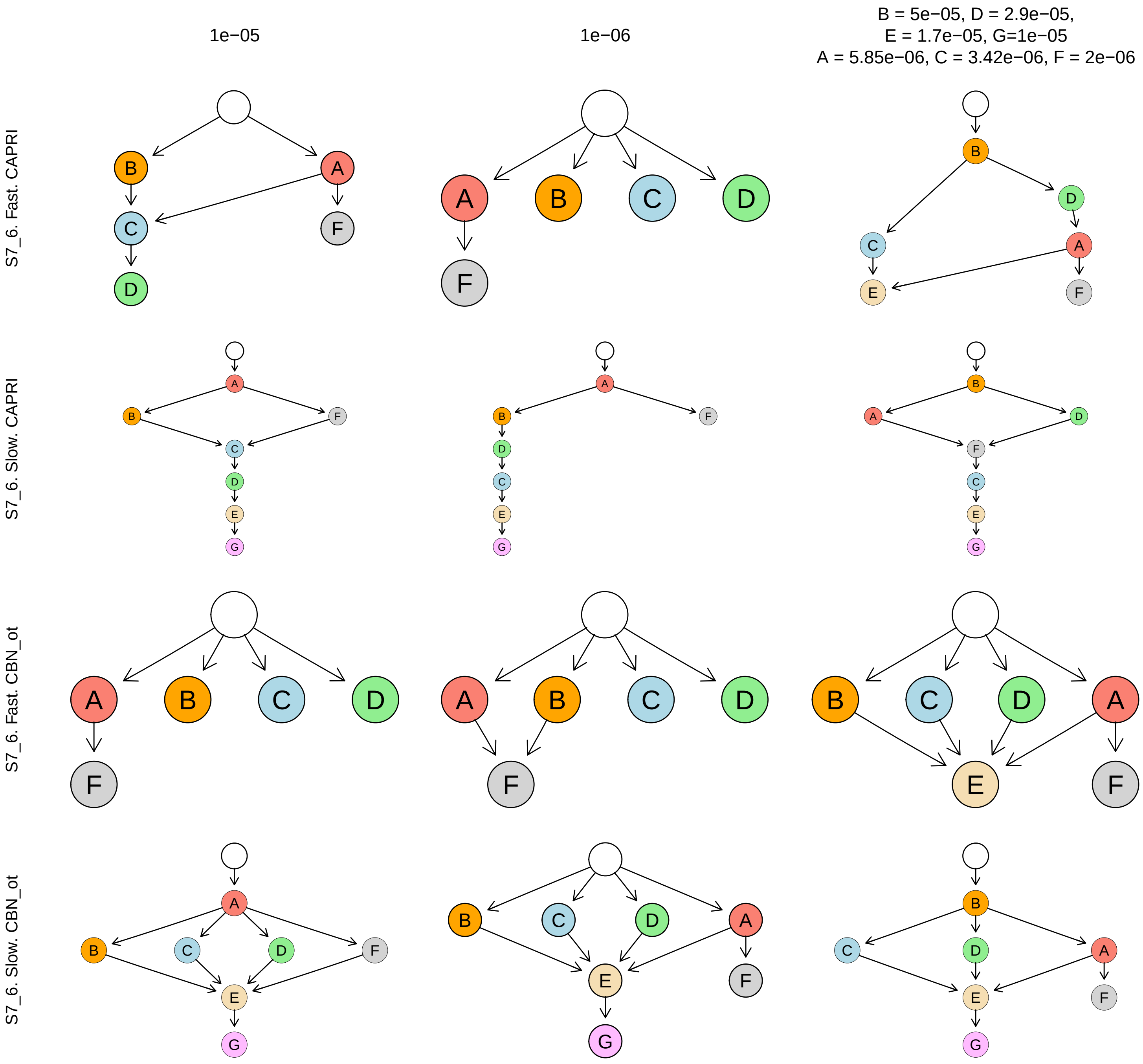

DAG_a

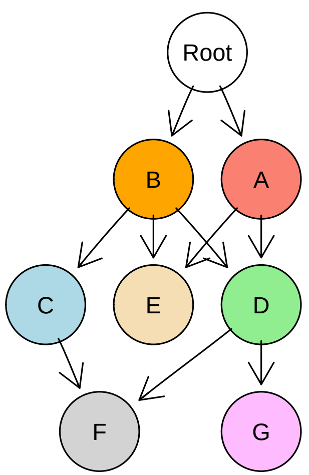

ID	Accessible Genot.	Discrep. DAG_a
S7_7	19	0

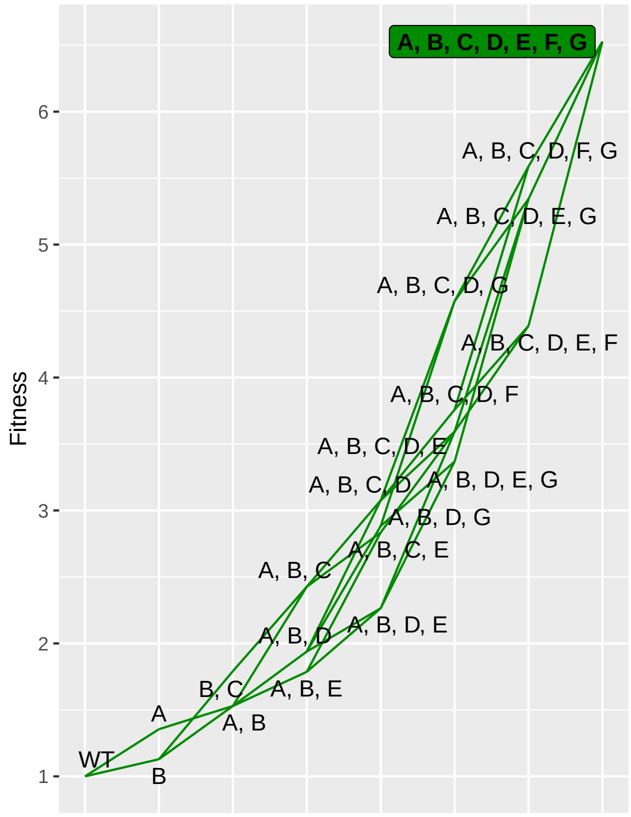

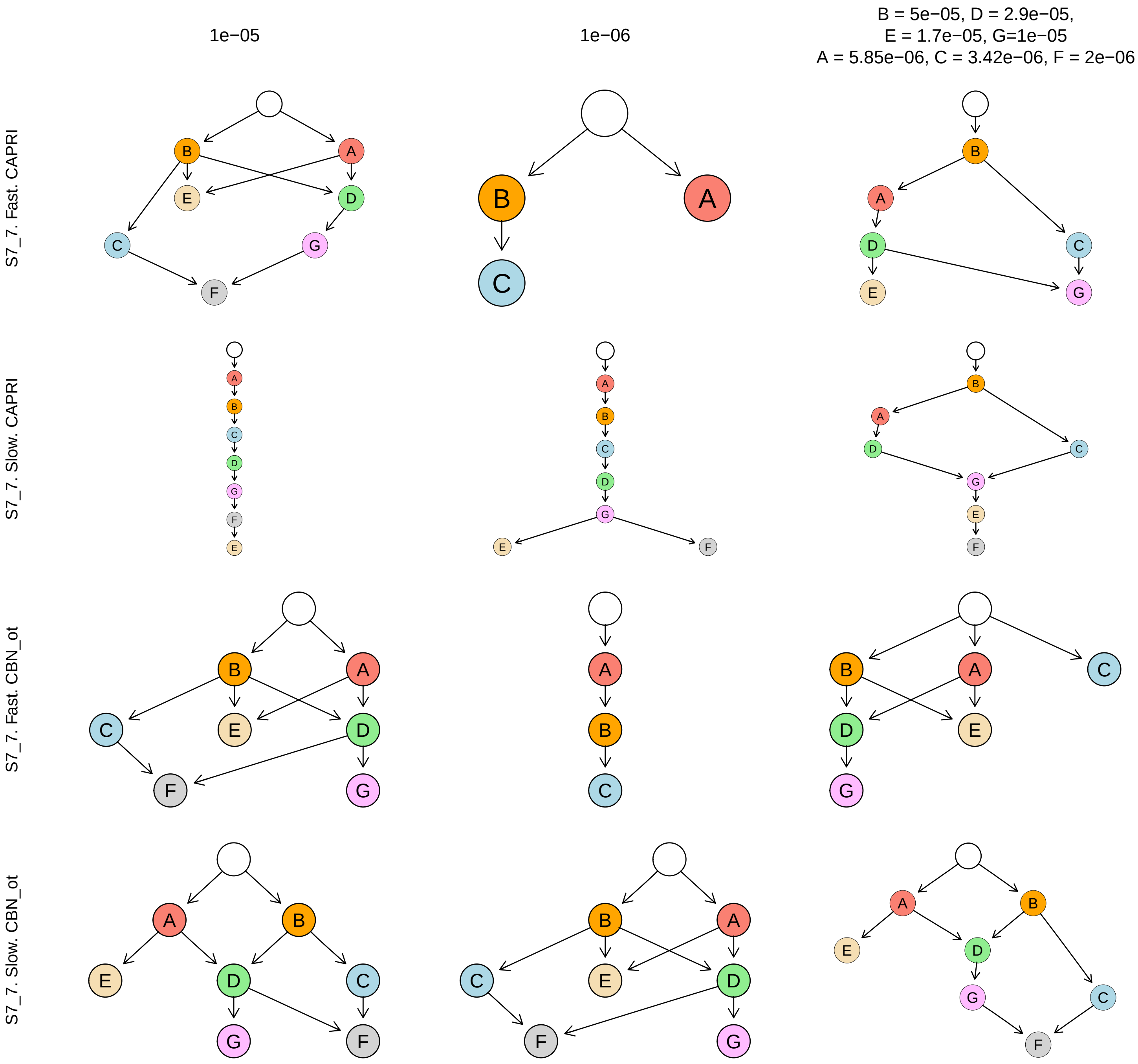

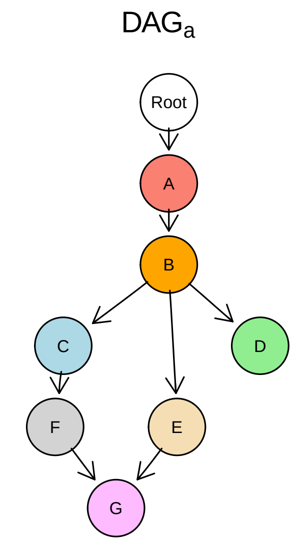

ID	Accessible Genot.	Discrep. DAG_a
S7_8	15	0

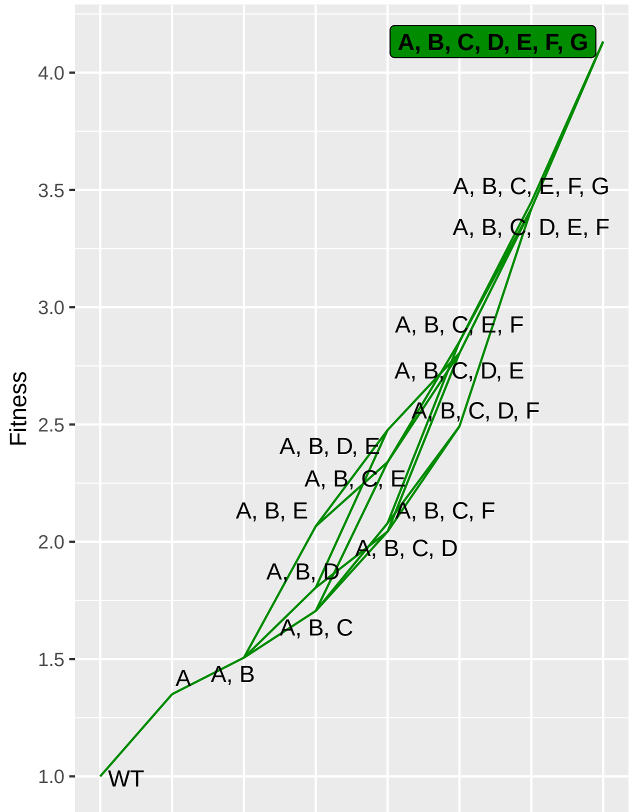

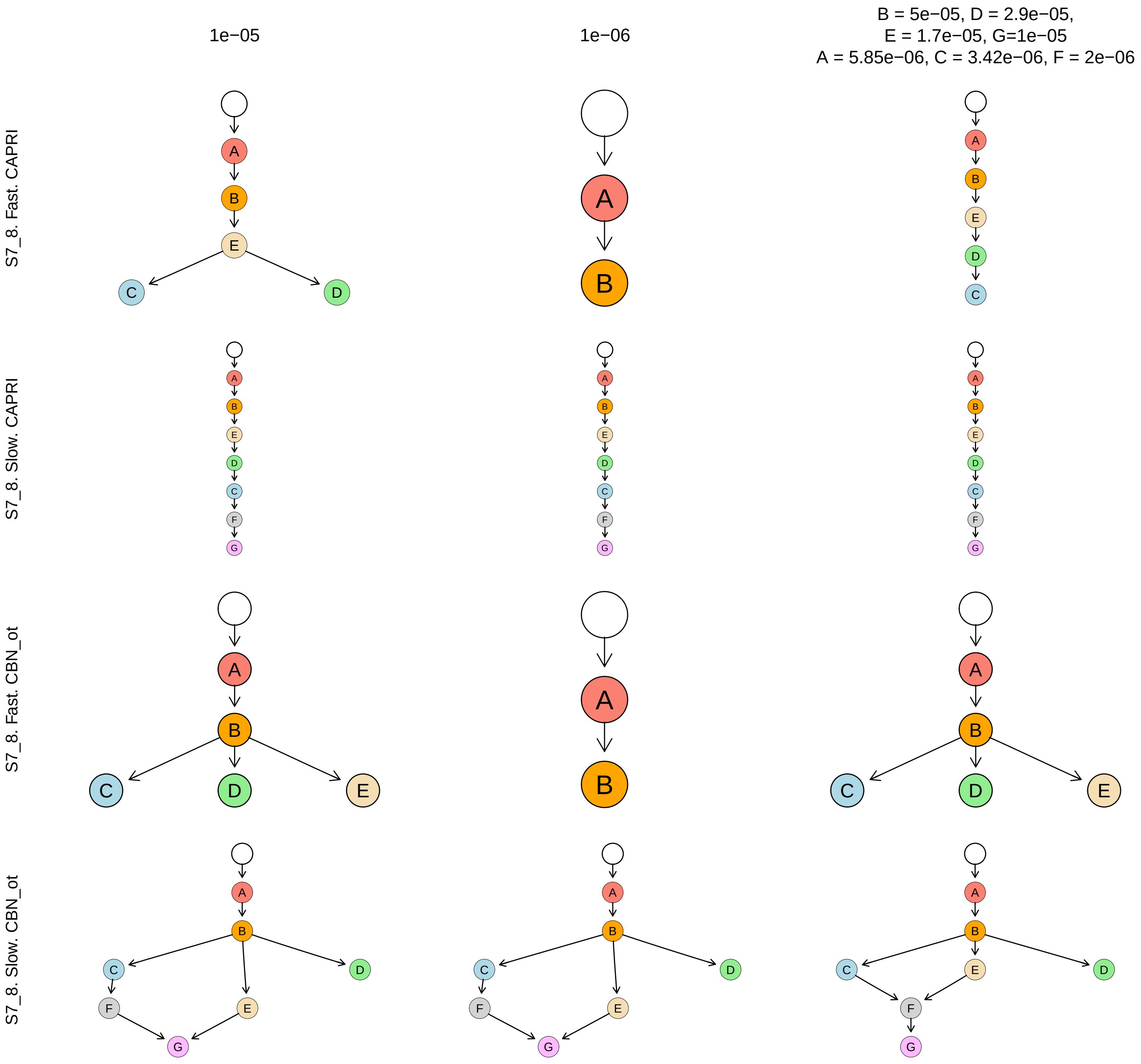

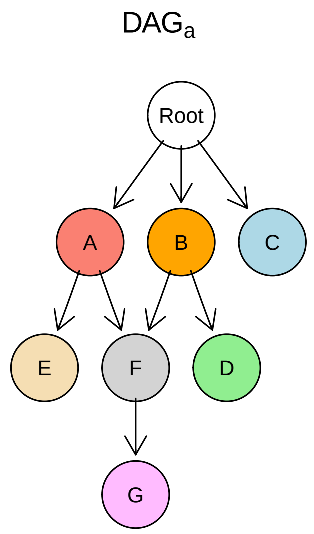

ID	Accessible Genot.	Discrep. DAG_a
S7_9	33	0

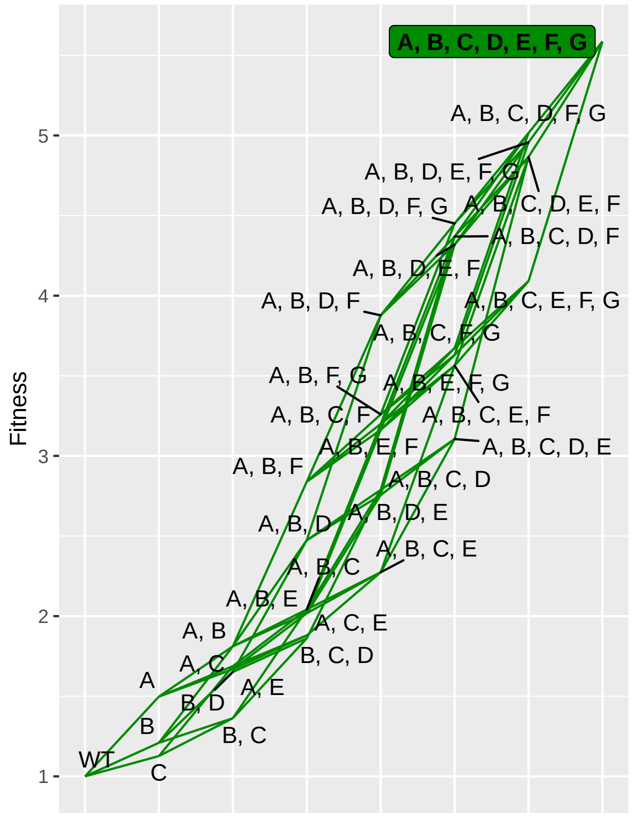

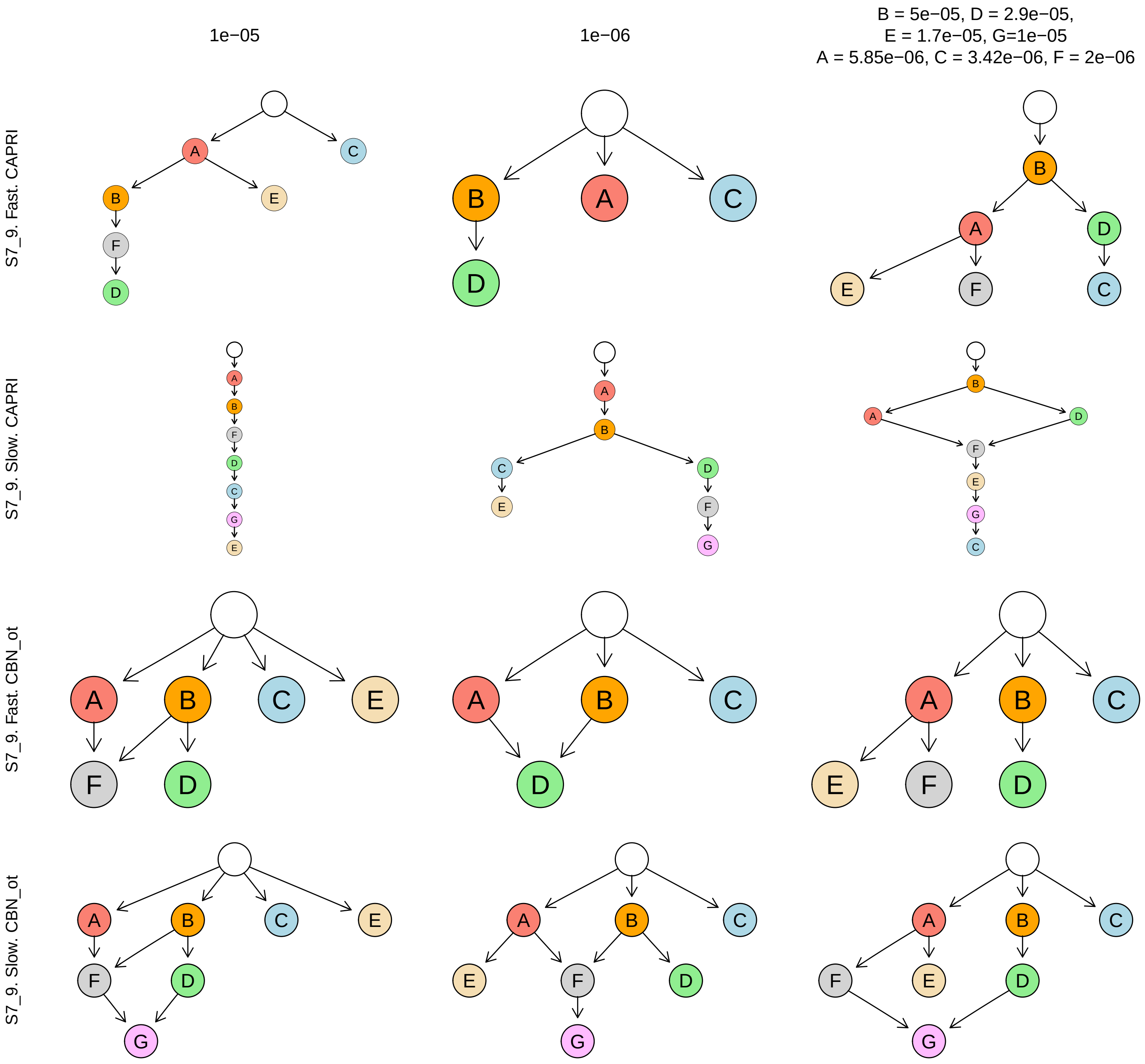

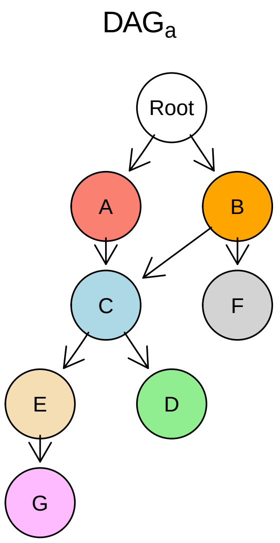

ID	Accessible Genot.	Discrep. DAG_a
S7_10	17	0

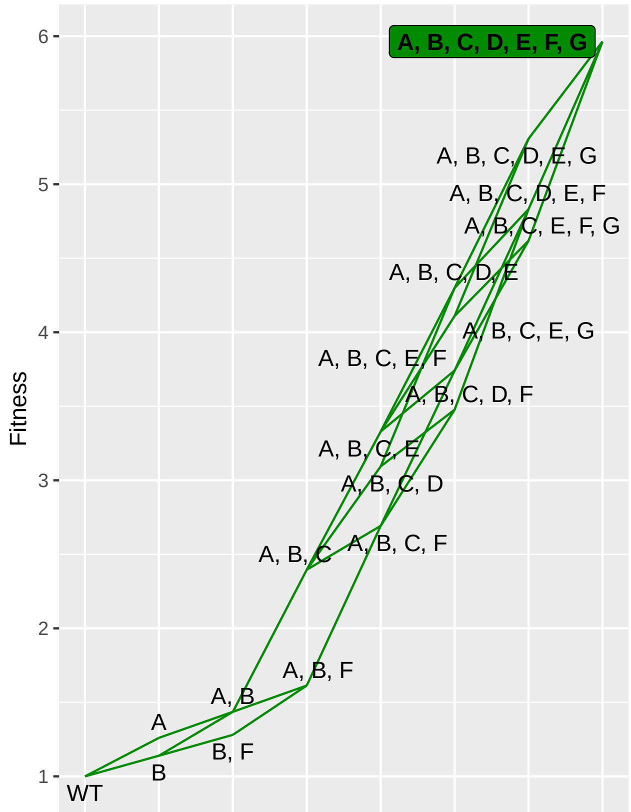

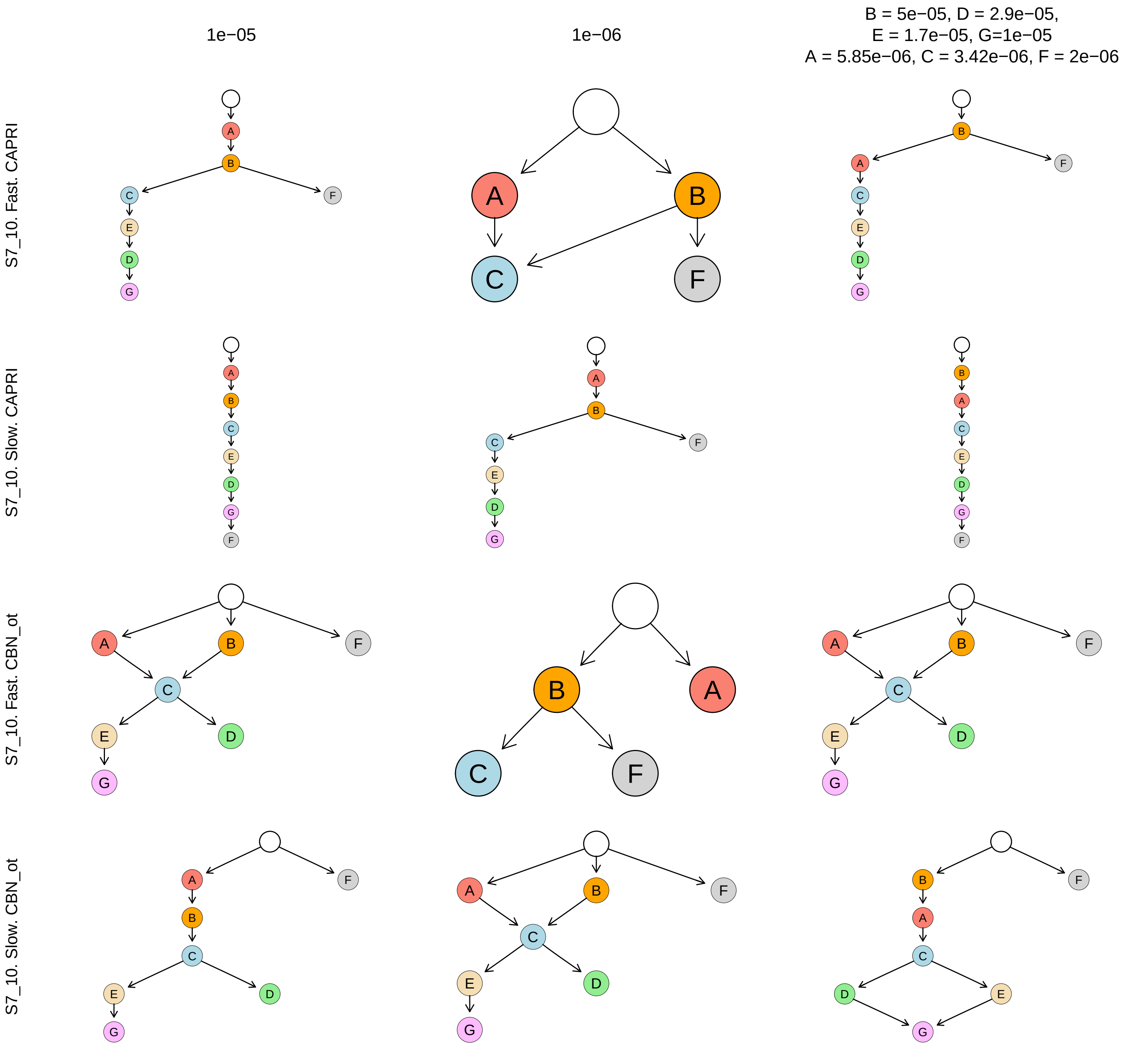

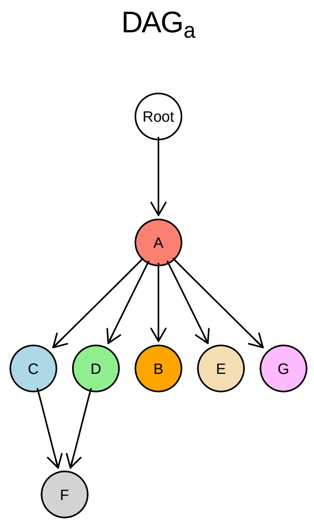

ID	Accessible Genot.	Discrep. DAG_a
S7_11	40	0

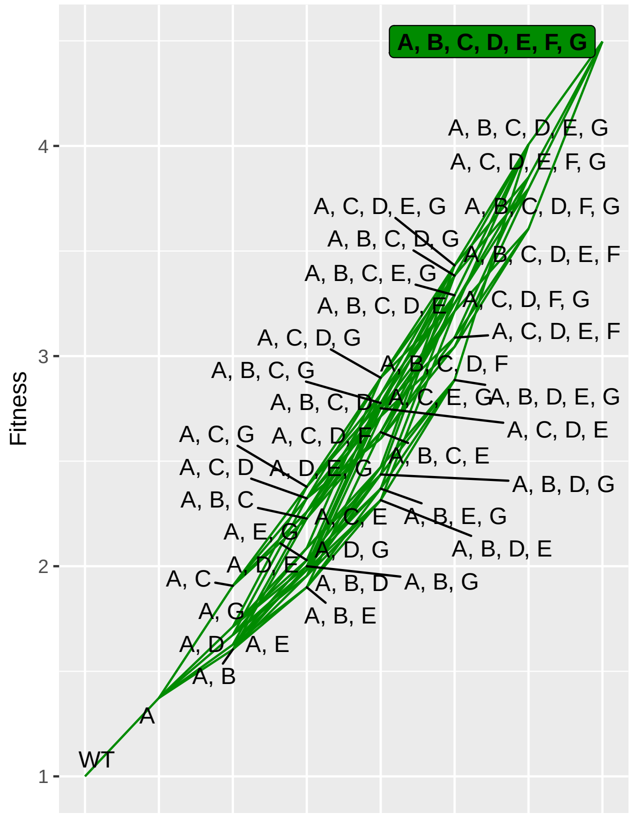

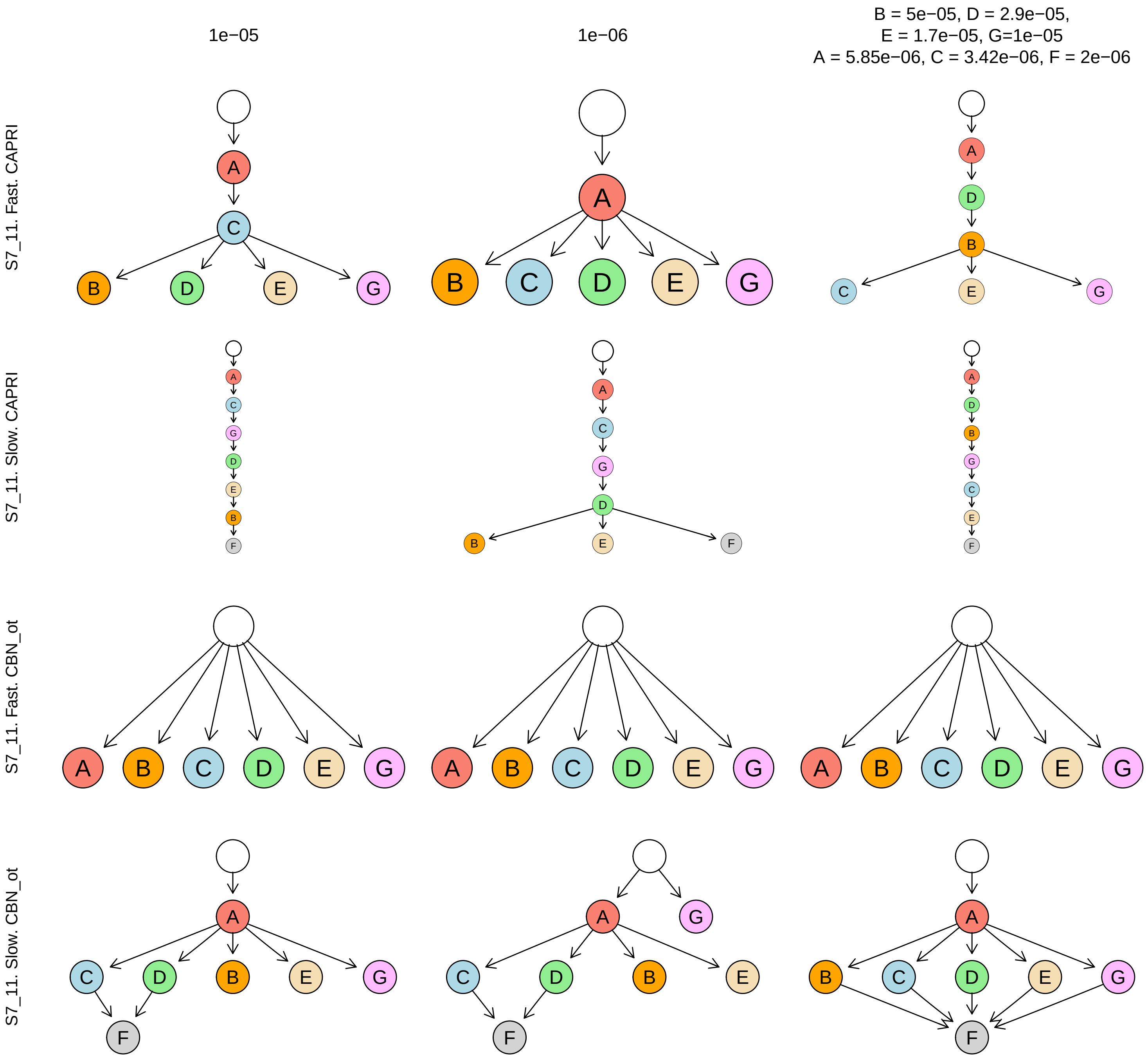

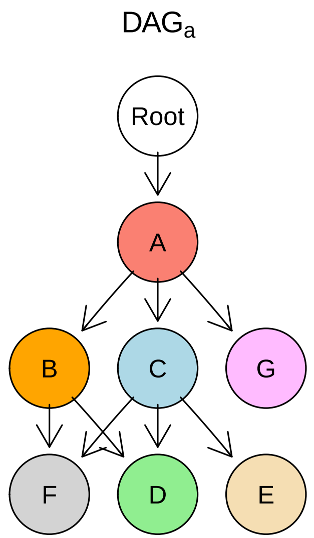

ID	Accessible Genot.	Discrep. DAG_a
S7_12	24	0

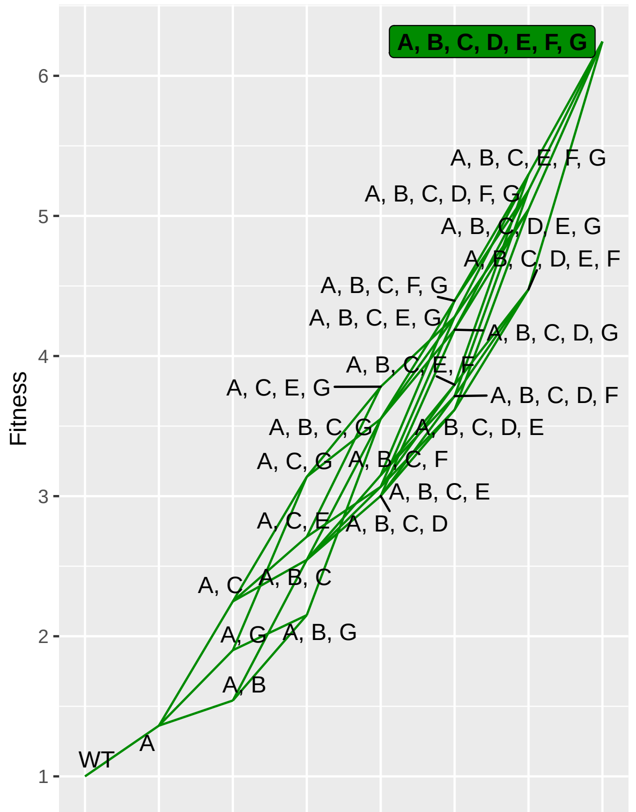

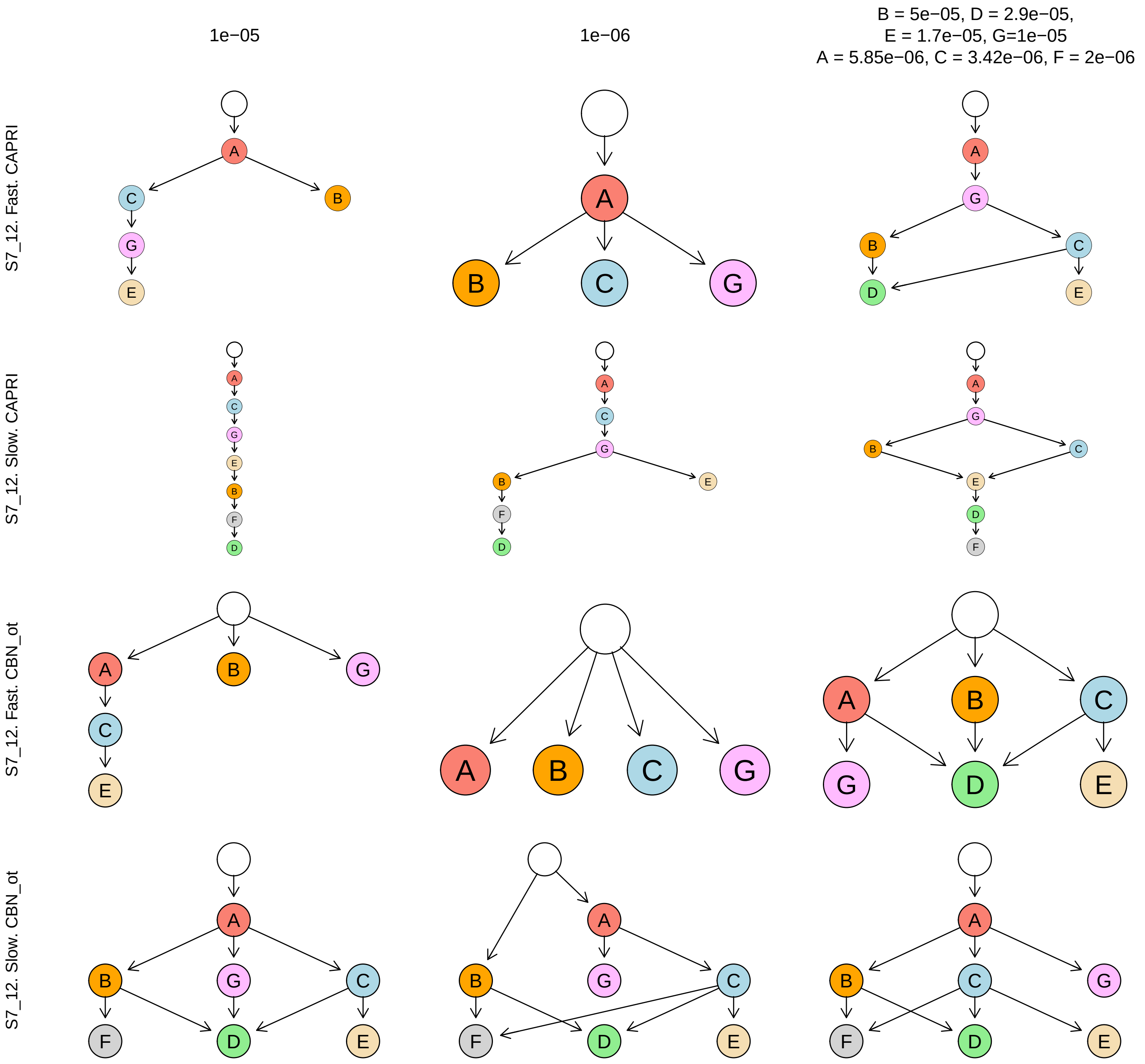

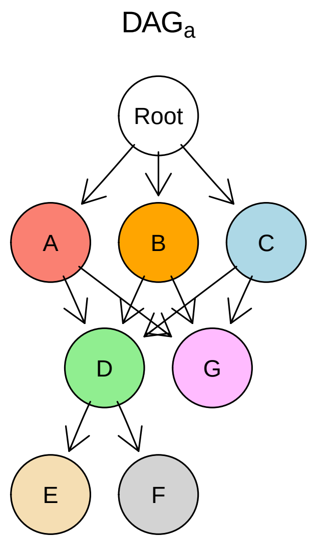

ID	Accessible Genot.	Discrep. DAG_a
S7_13	16	0

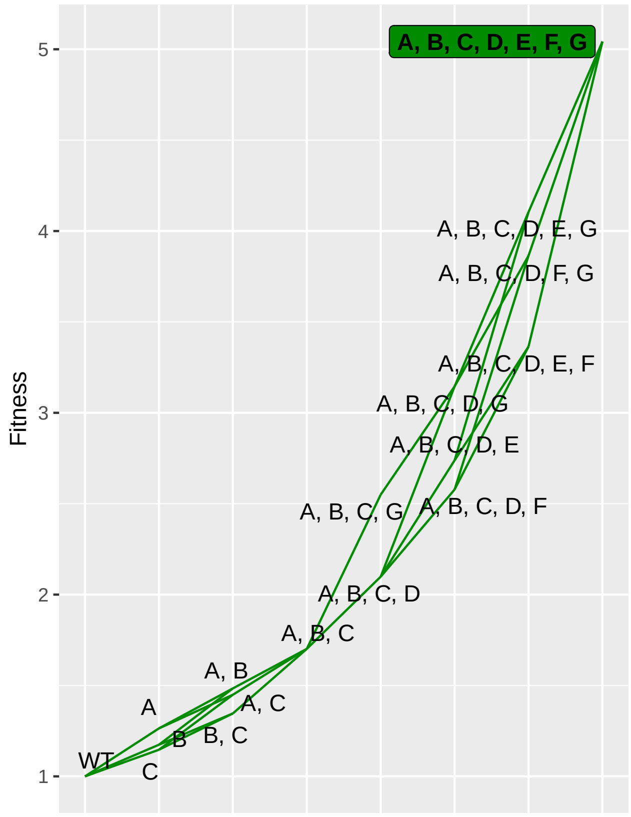

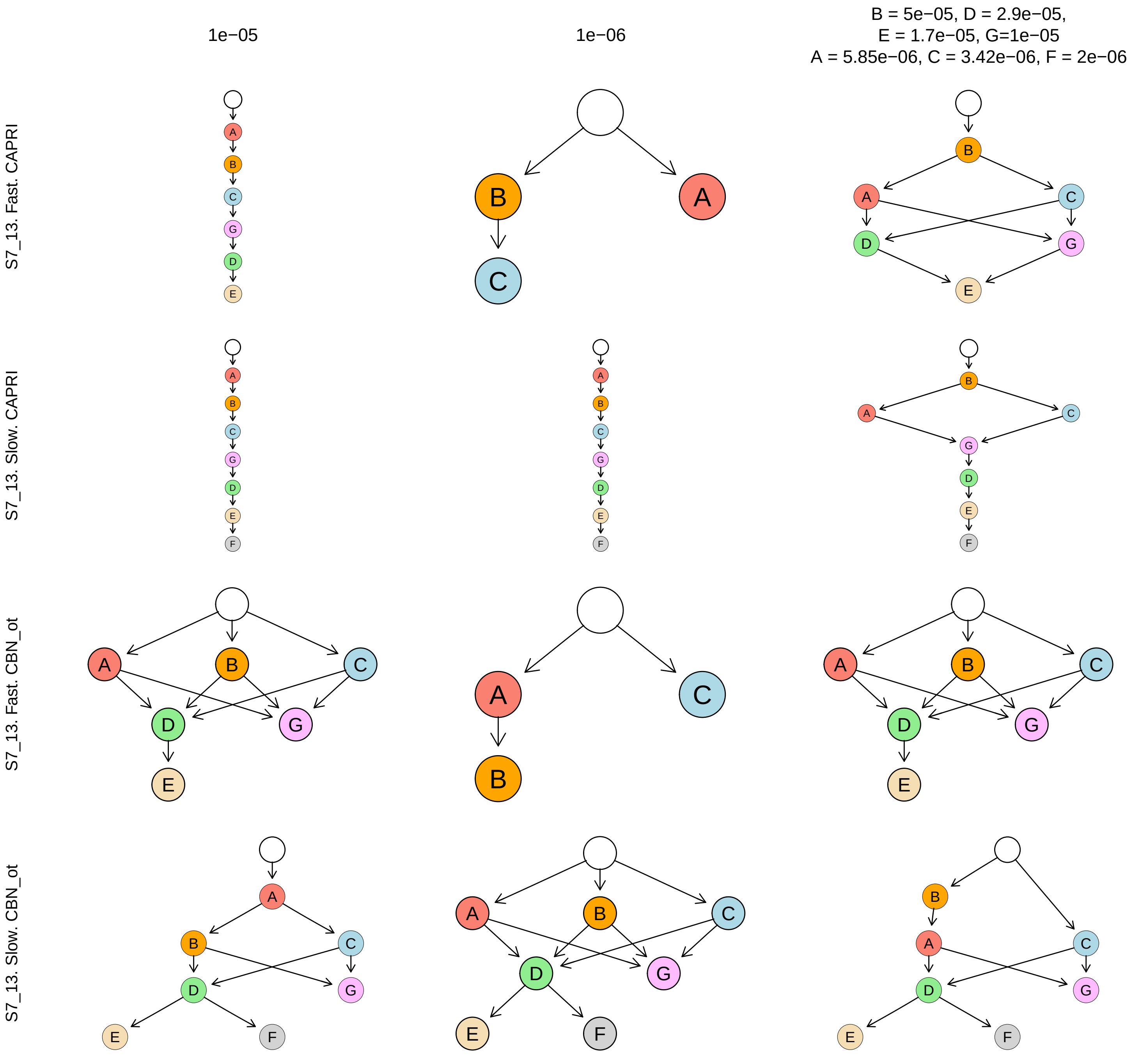

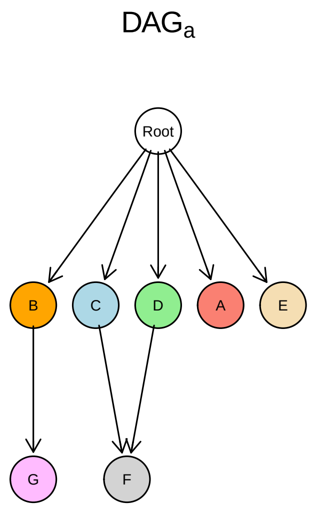

ID	Accessible Genot.	Discrep. DAG_a
S7_14	59	0

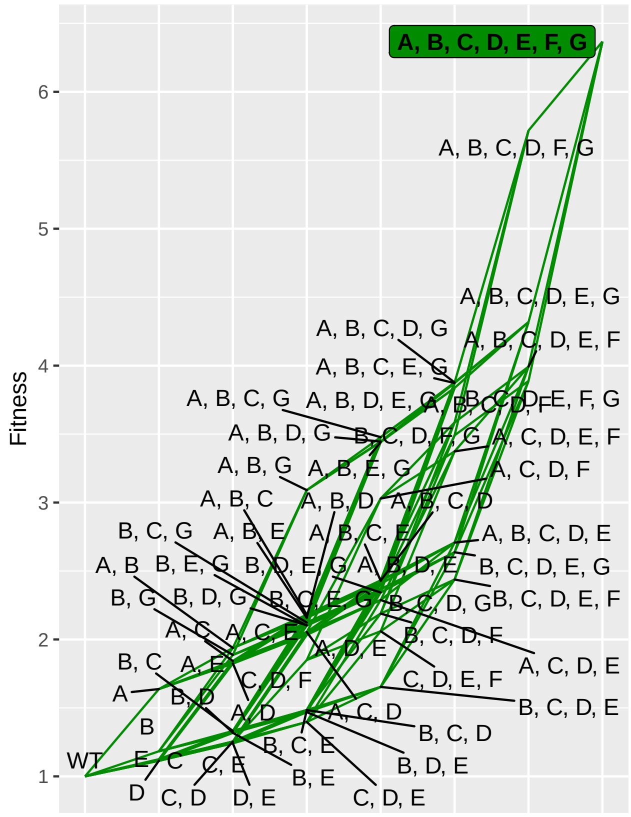

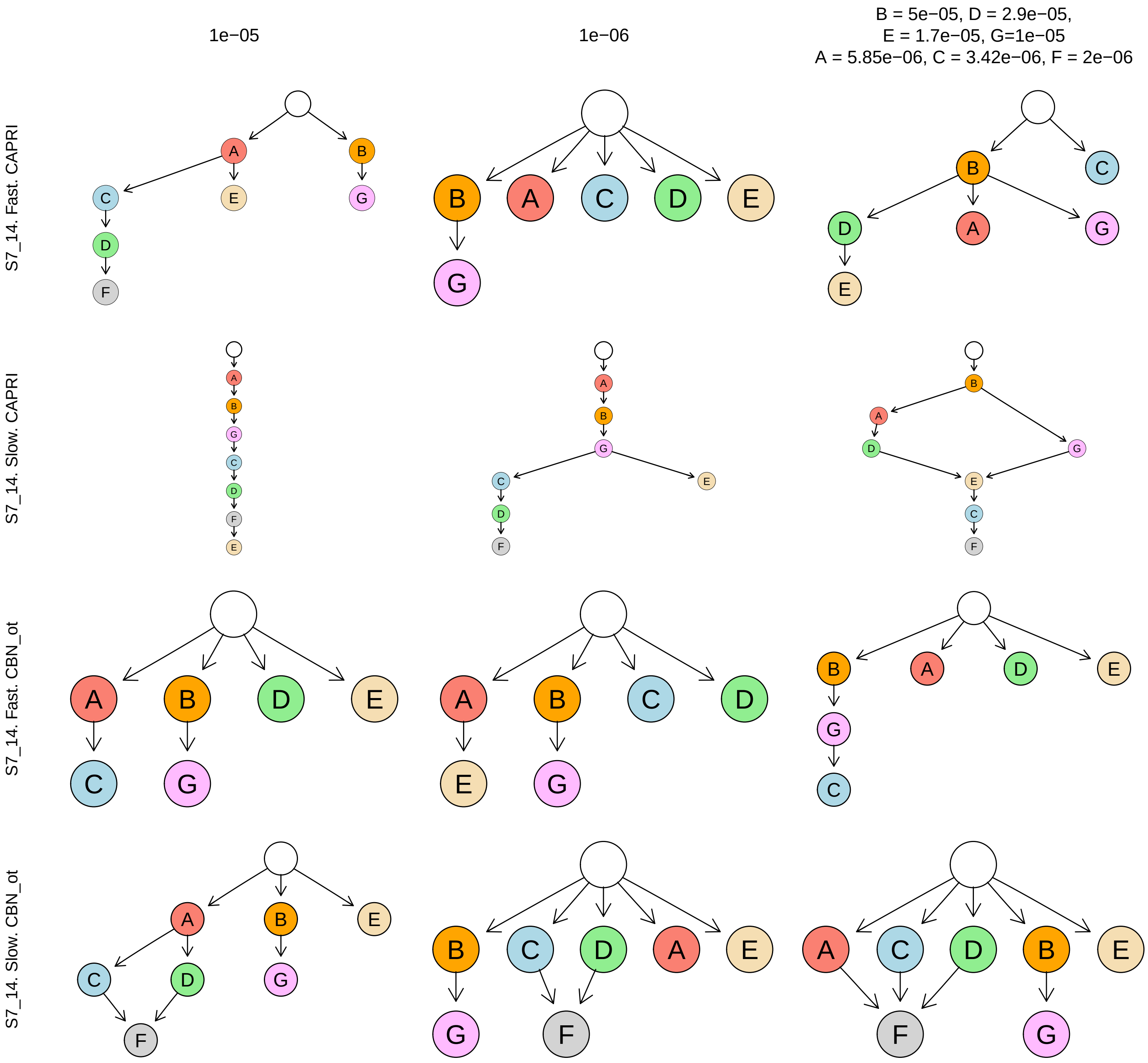

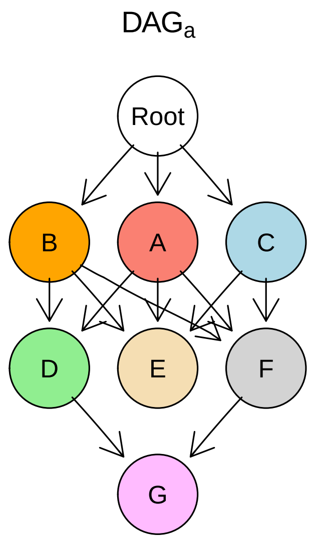

ID	Accessible Genot.	Discrep. DAG_a
S7_15	17	0

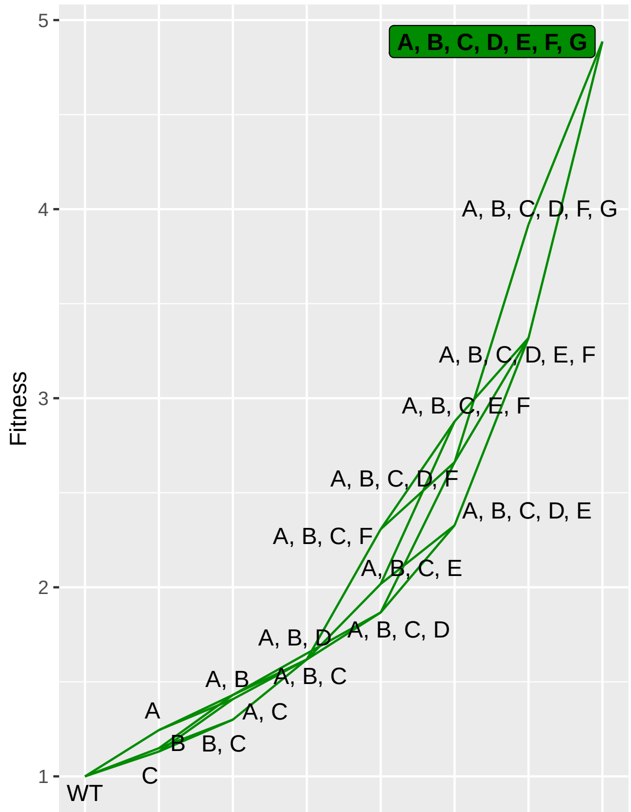

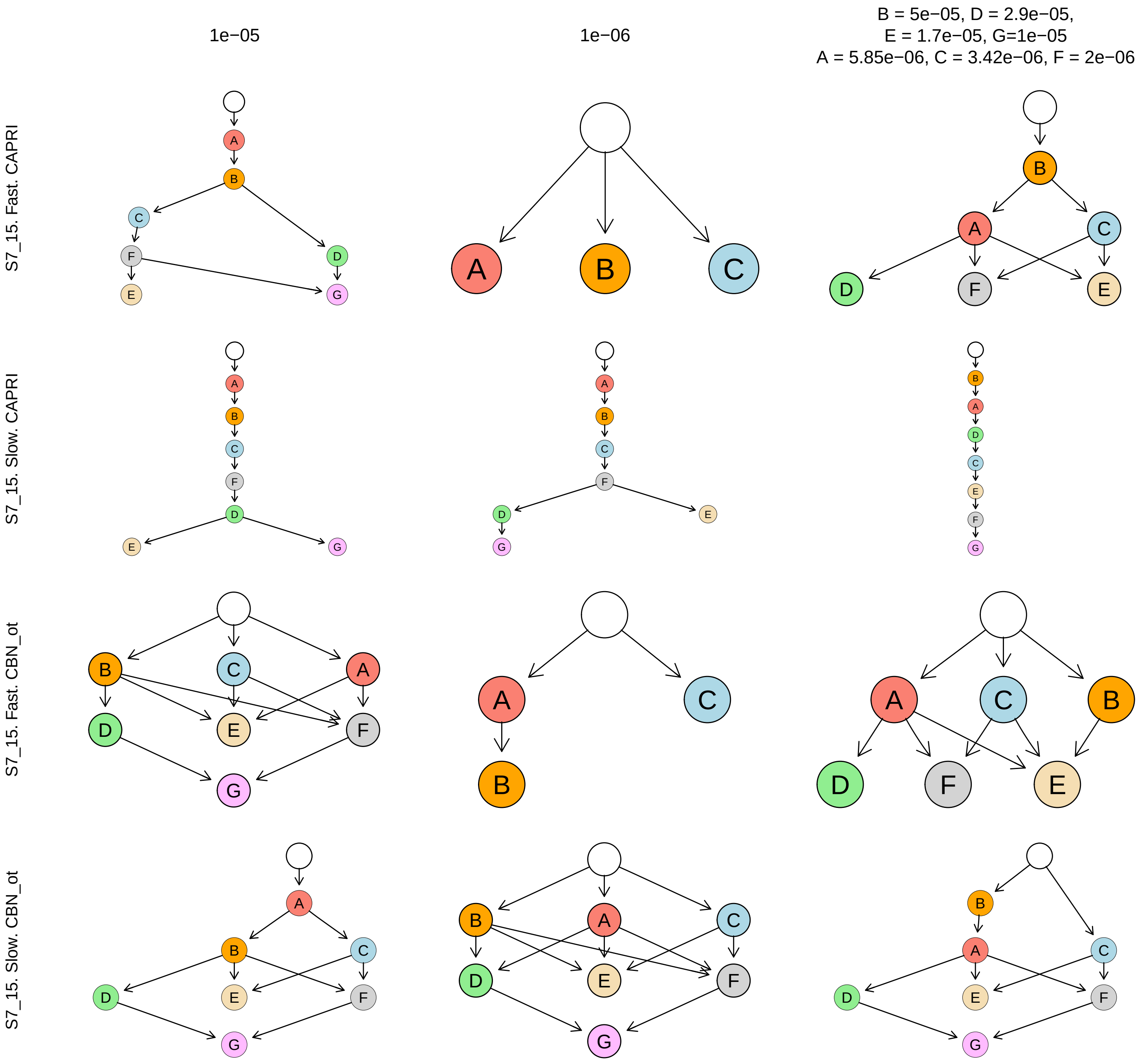

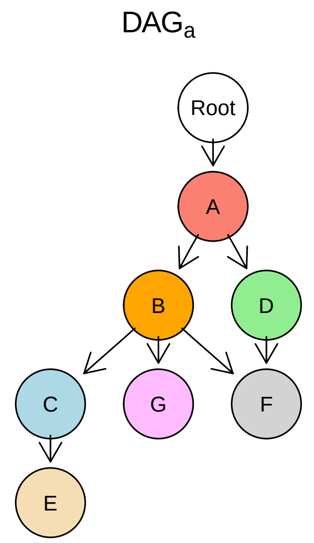

ID	Accessible Genot.	Discrep. DAG_a
S7_16	20	0

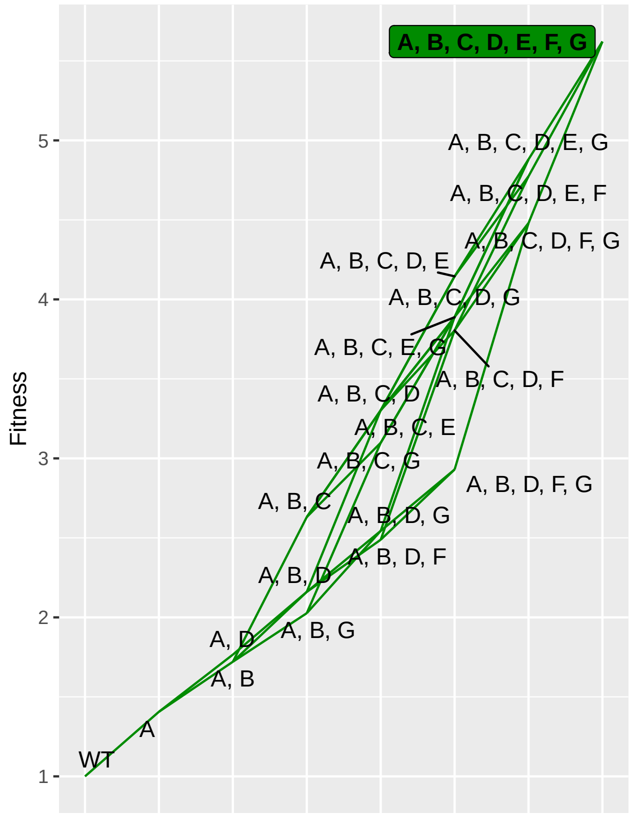

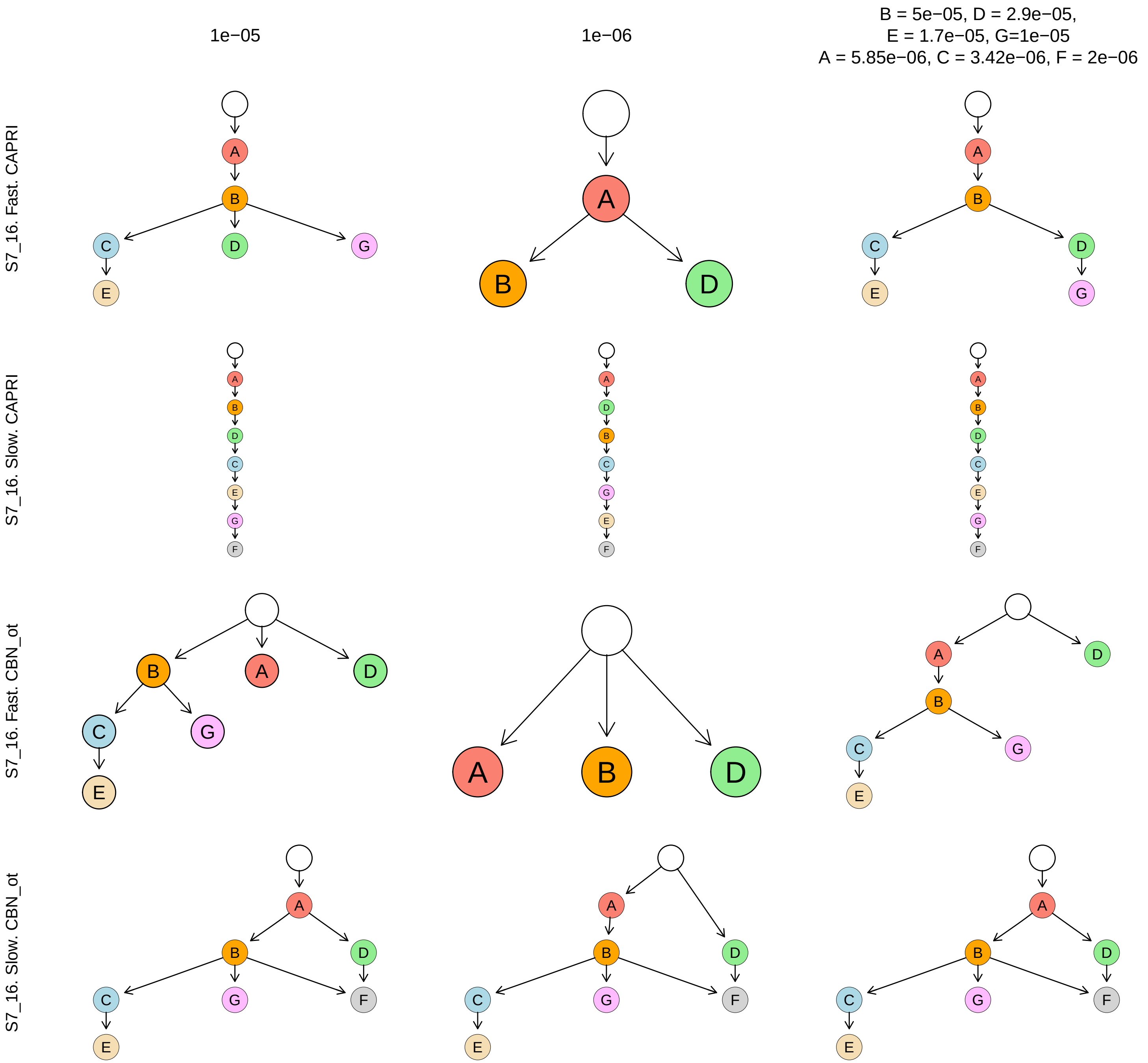

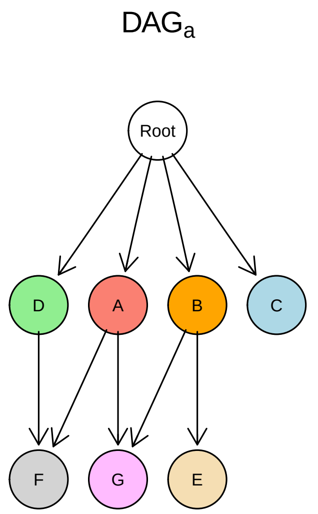

ID	Accessible Genot.	Discrep. DAG_a
S7_17	41	0

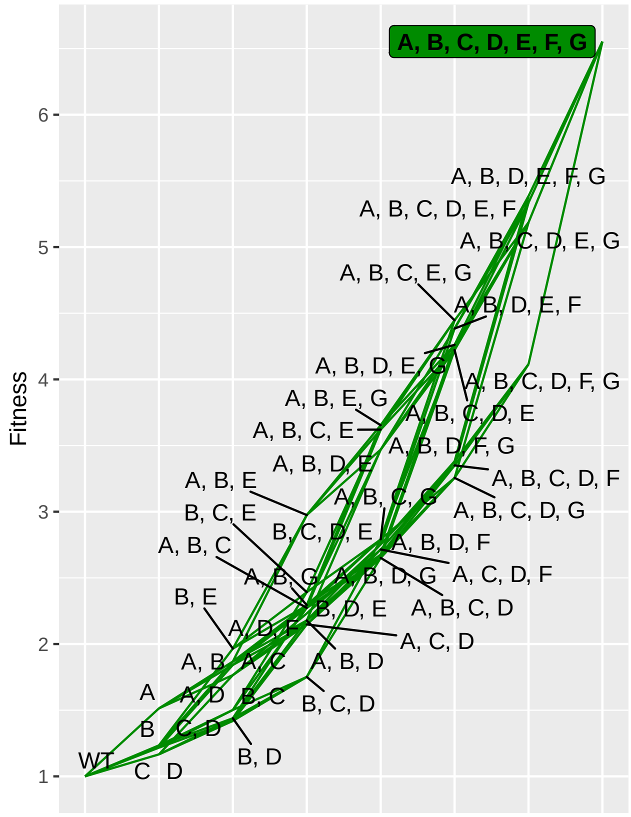

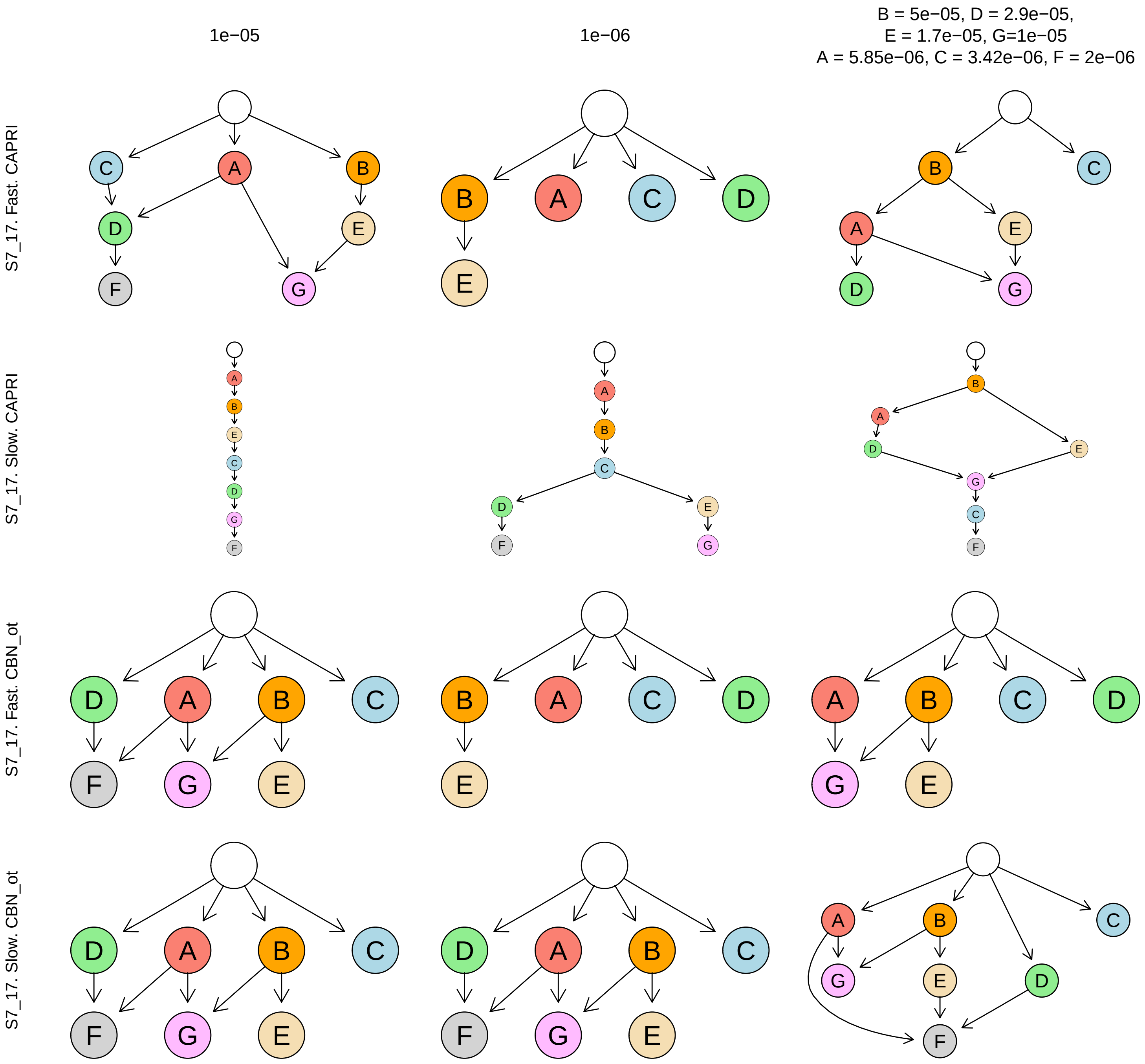

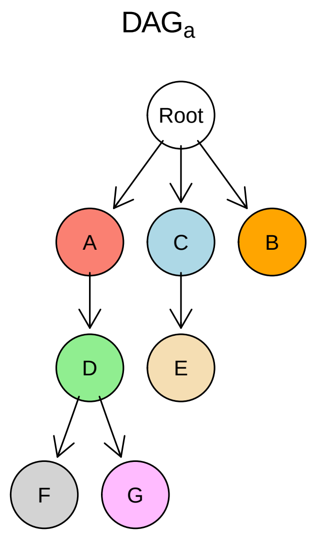

ID	Accessible Genot.	Discrep. DAG_a
S7_18	35	0

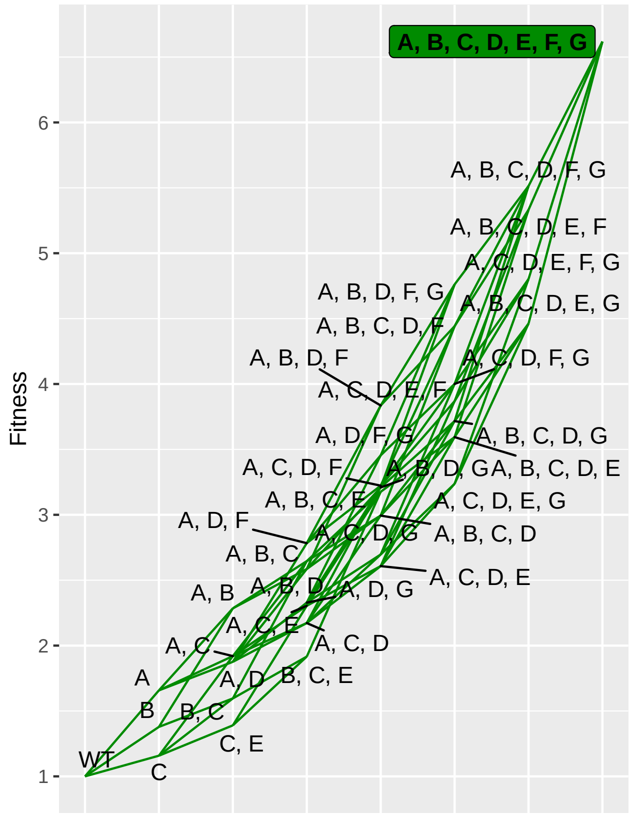

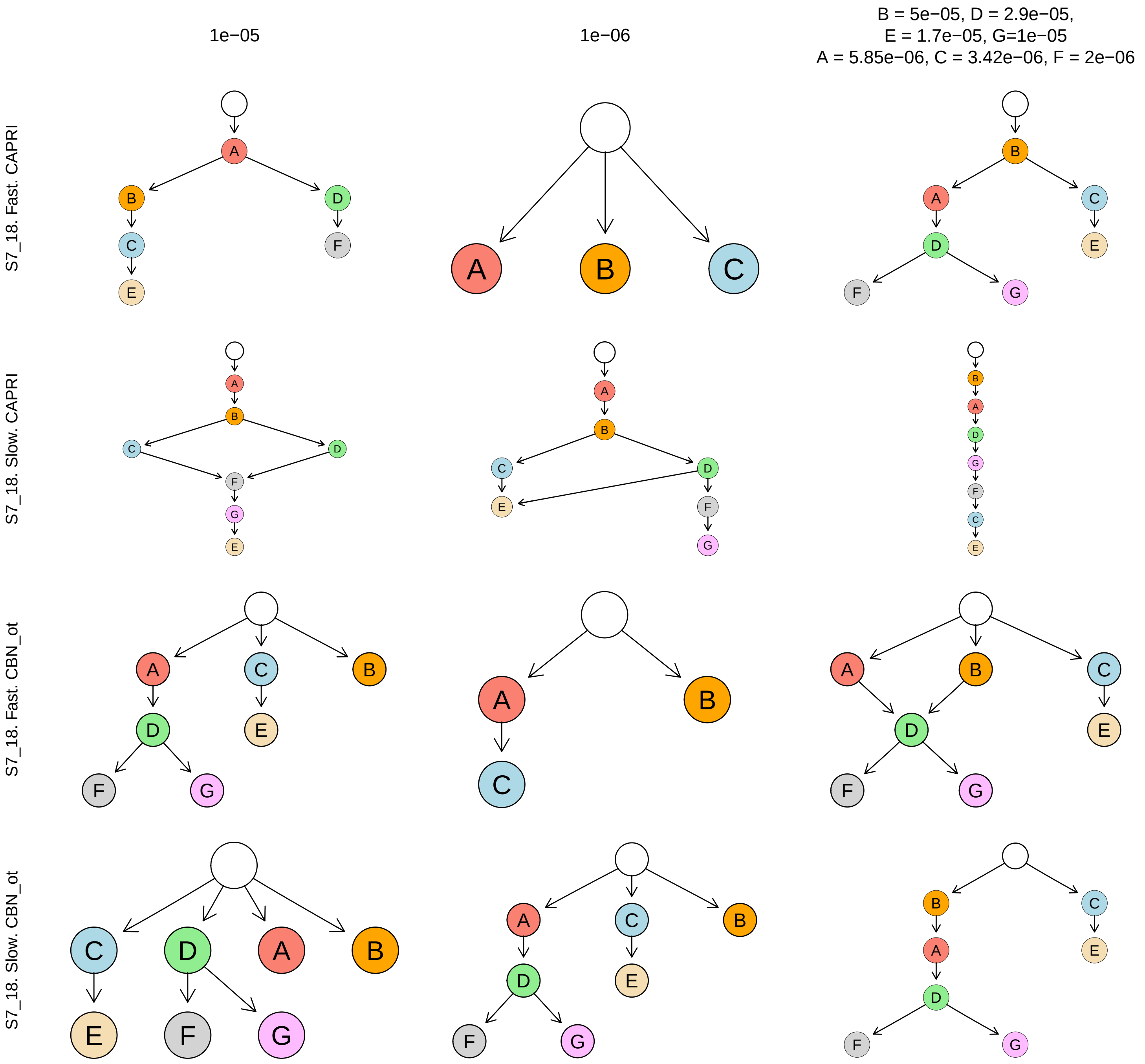

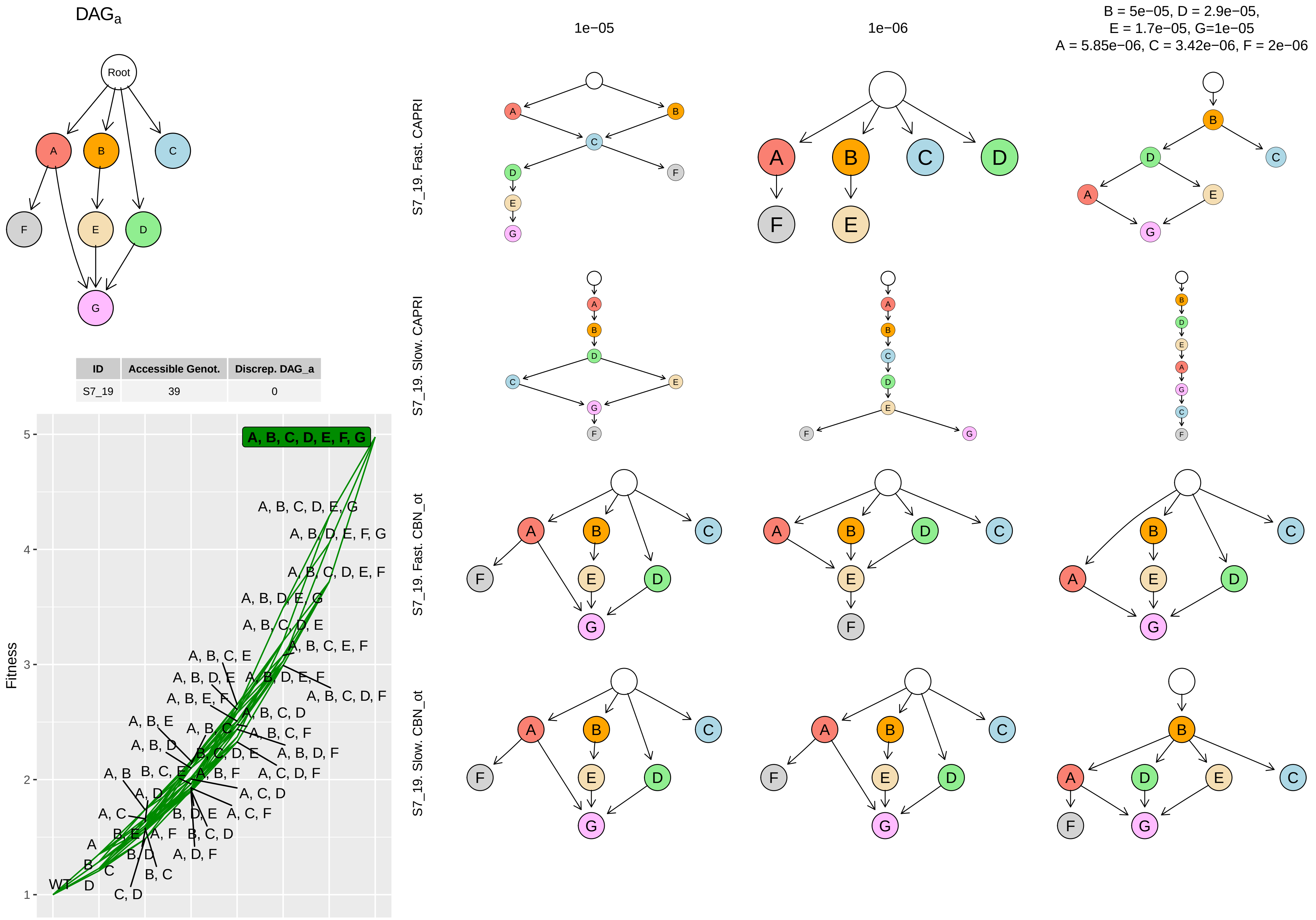

ID	Accessible Genot.	Discrep. DAG_a
S7_19	39	0

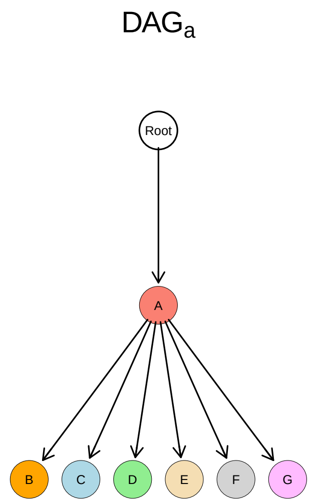

ID	Accessible Genot.	Discrep. DAG_a
S7_20	64	0

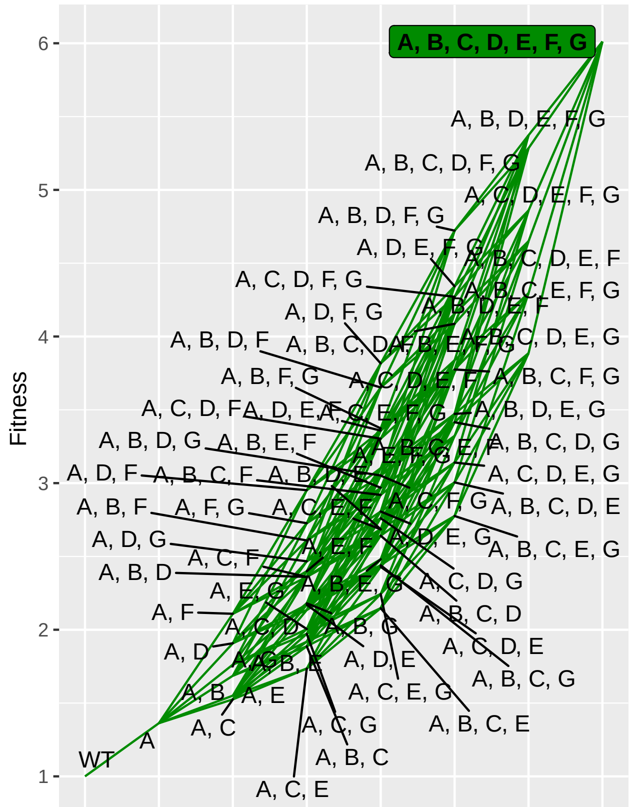

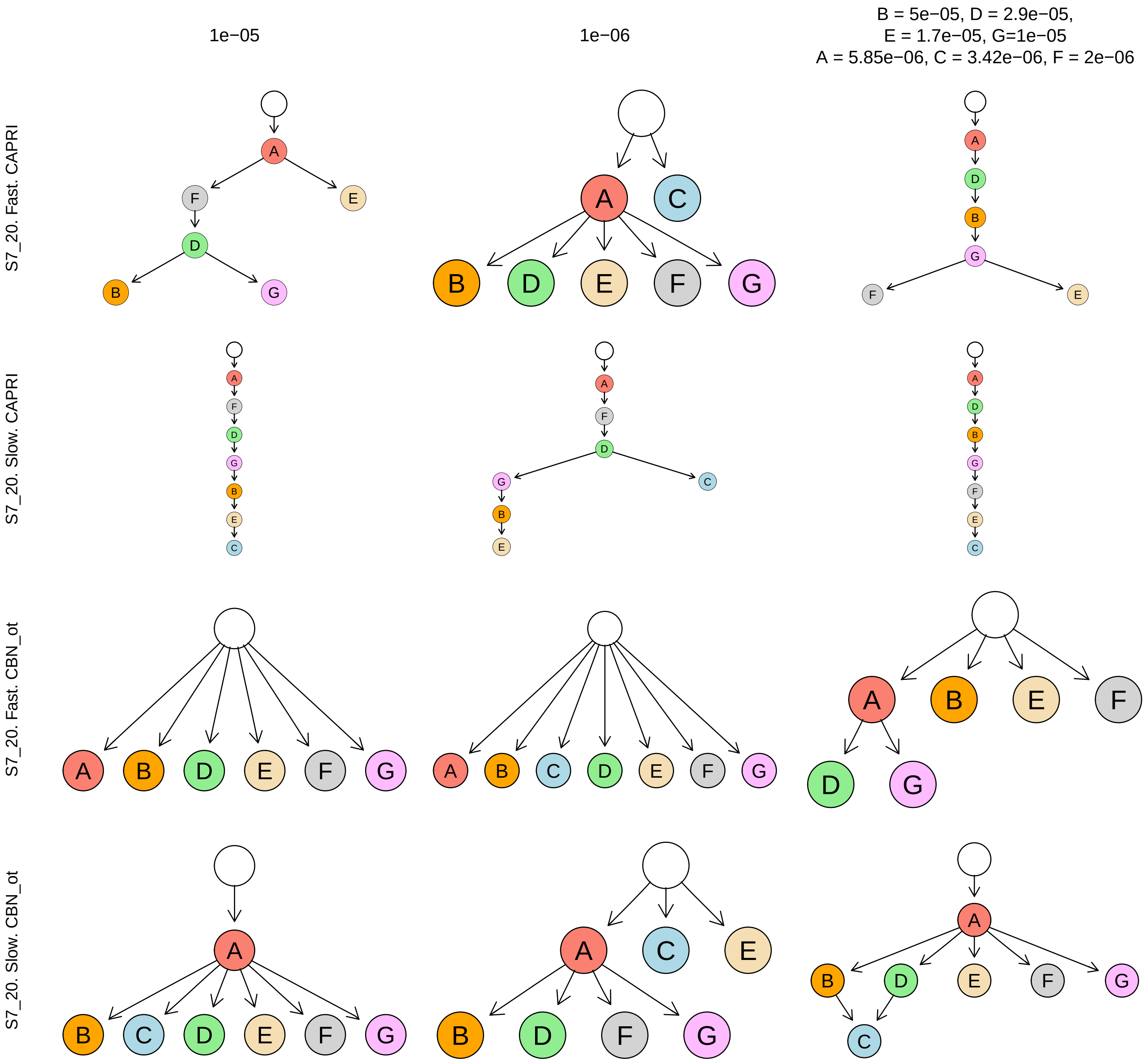

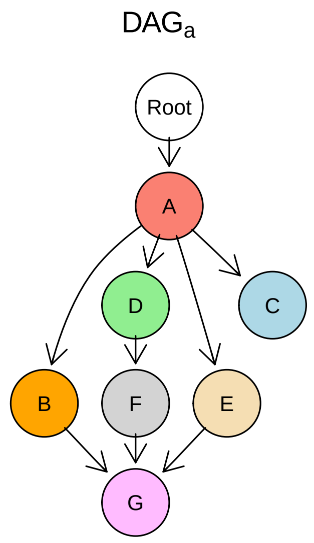

ID	Accessible Genot.	Discrep. DAG_a
S7_21	26	0

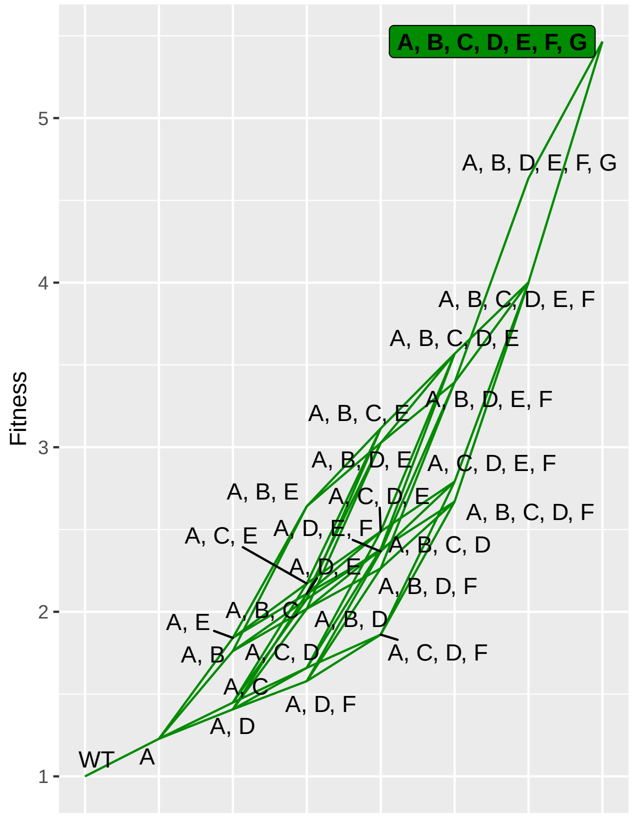

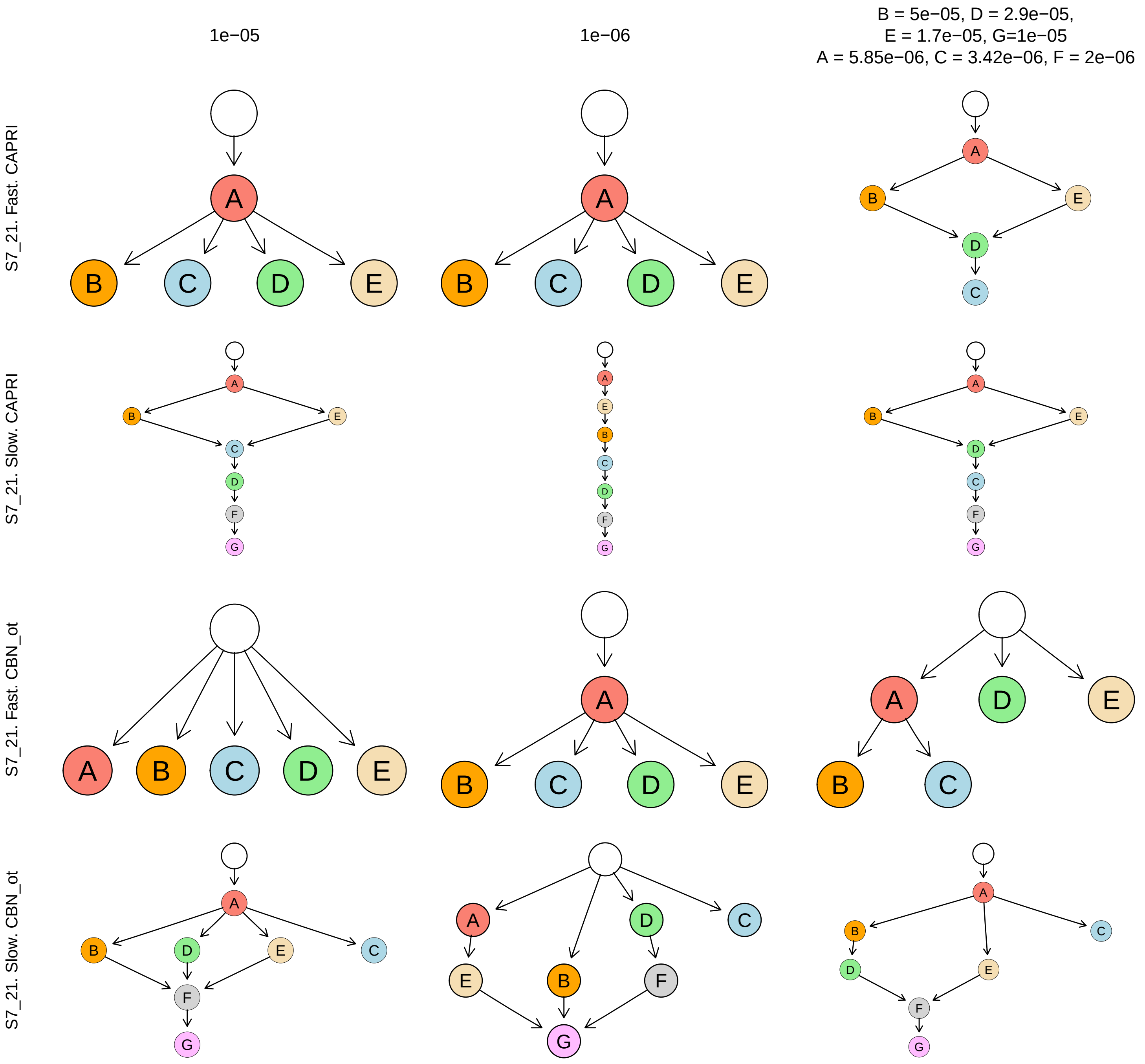

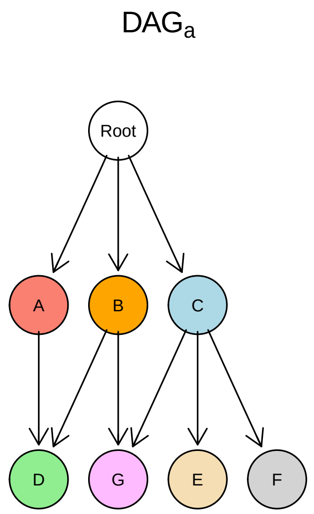

ID	Accessible Genot.	Discrep. DAG_a
S7_22	36	0

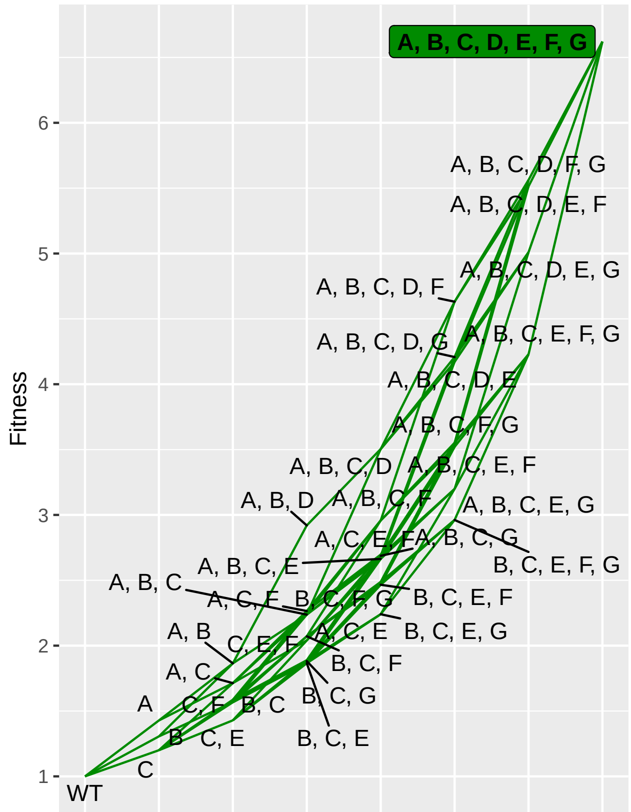

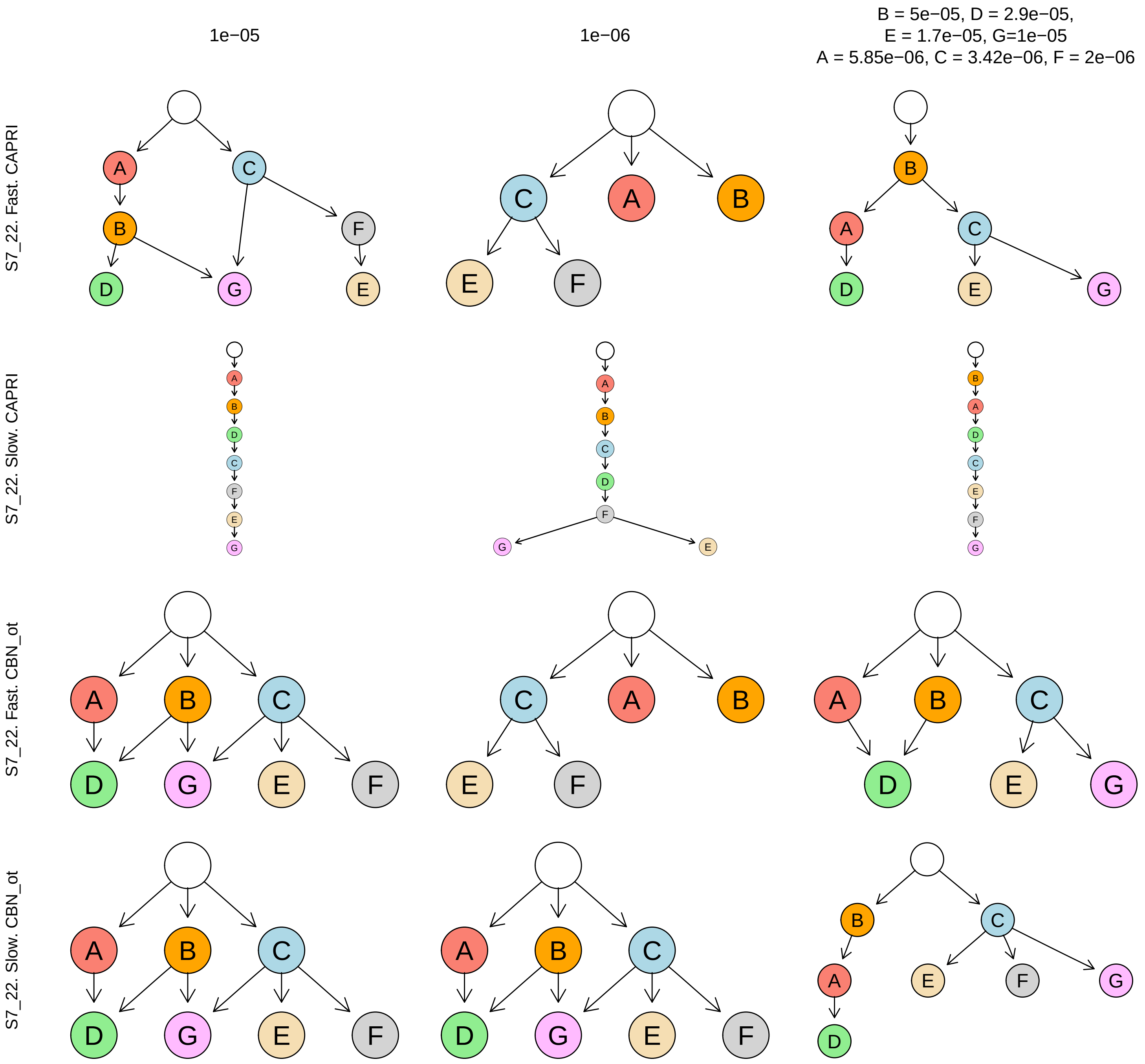

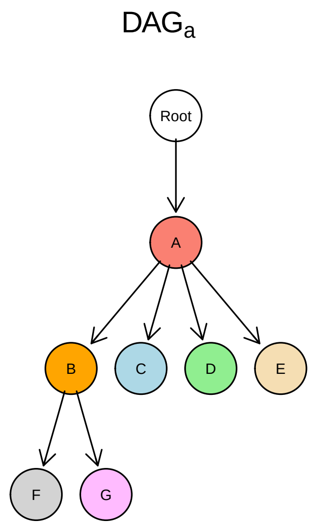

ID	Accessible Genot.	Discrep. DAG_a
S7_23	40	0

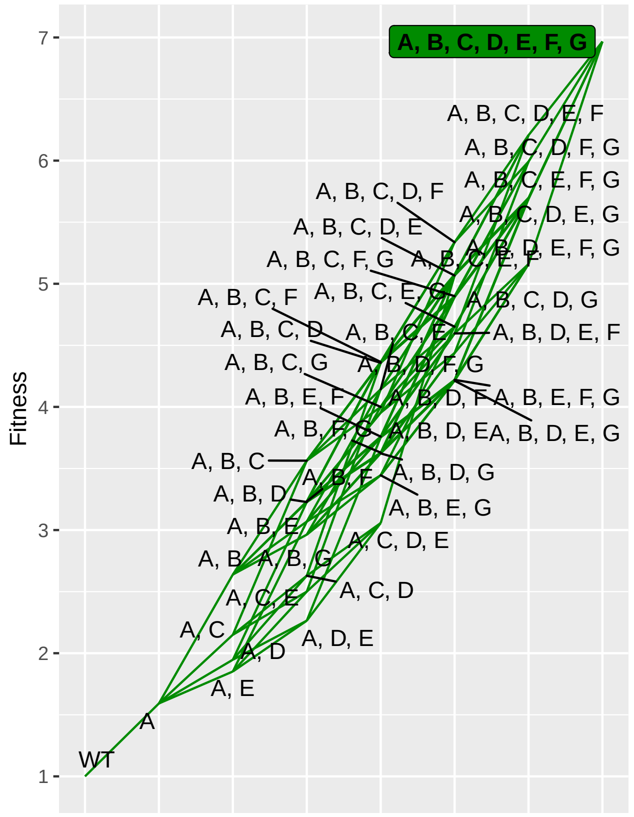

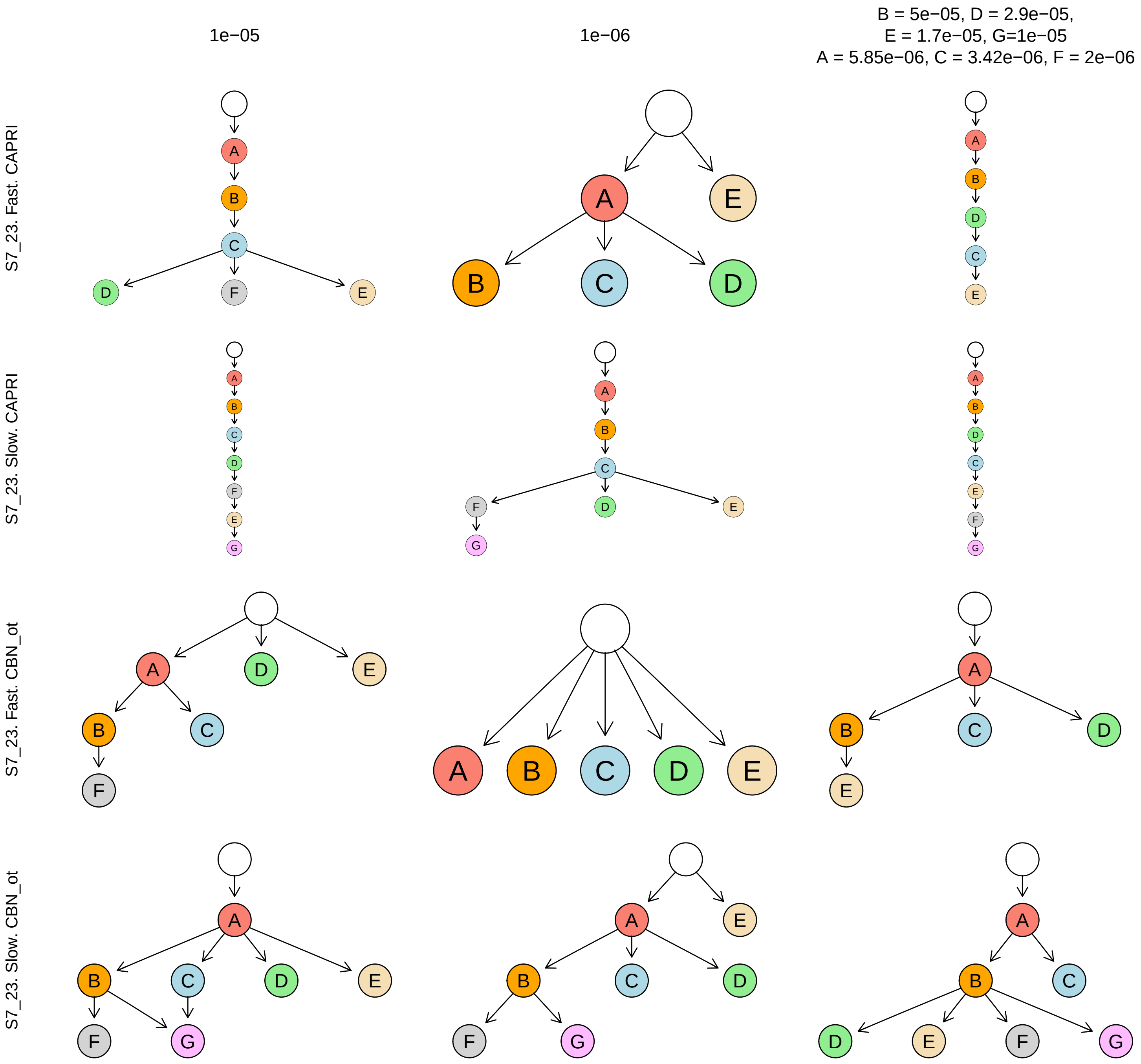

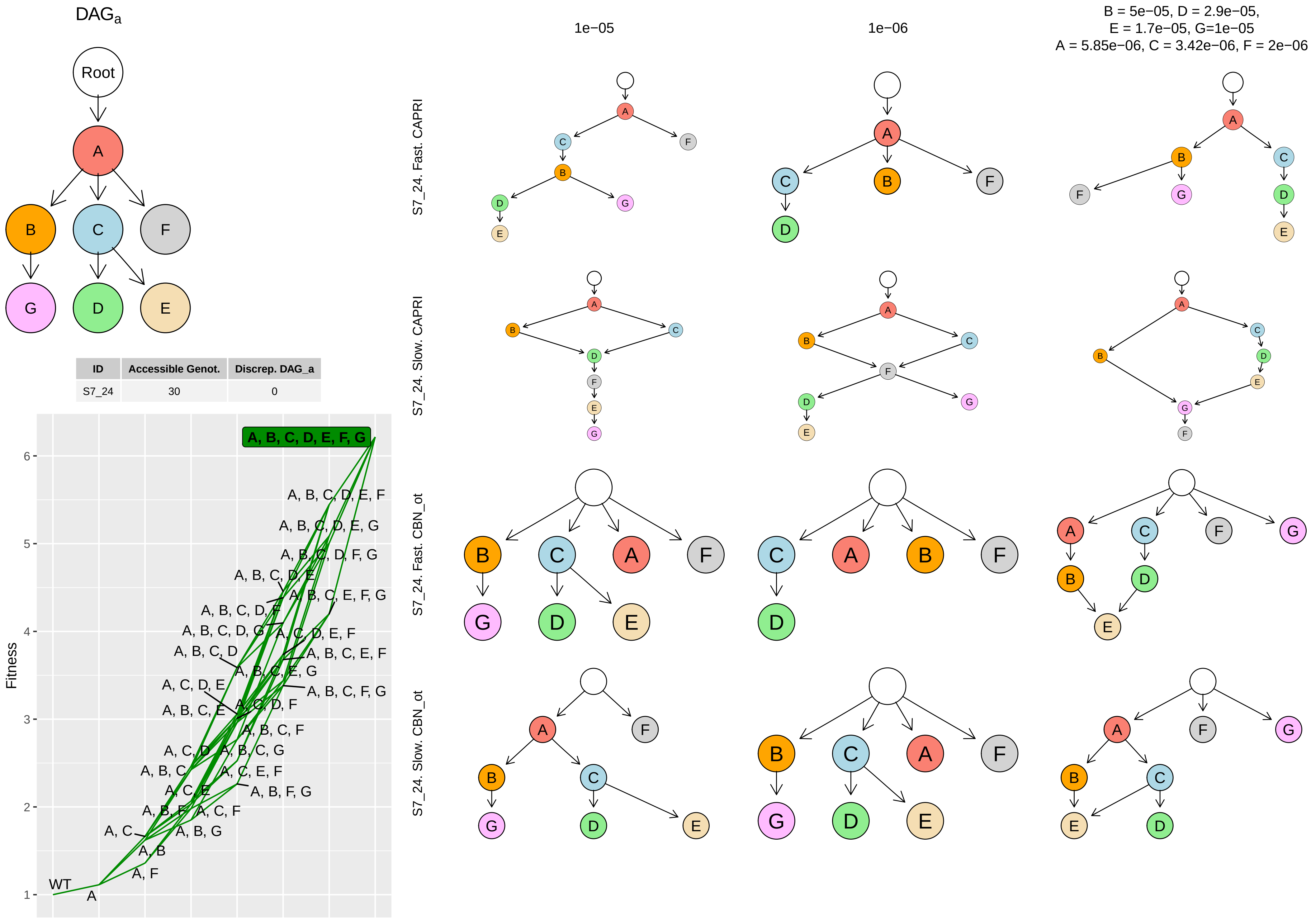

ID	Accessible Genot.	Discrep. DAG_a
S7_24	30	0

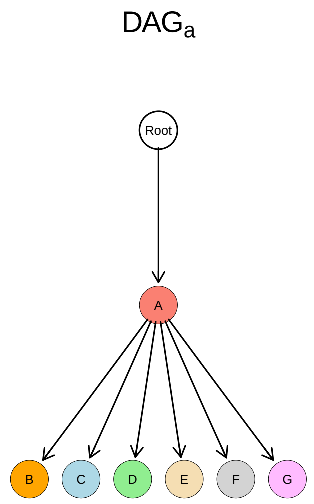

ID	Accessible Genot.	Discrep. DAG_a
S7_25	64	0

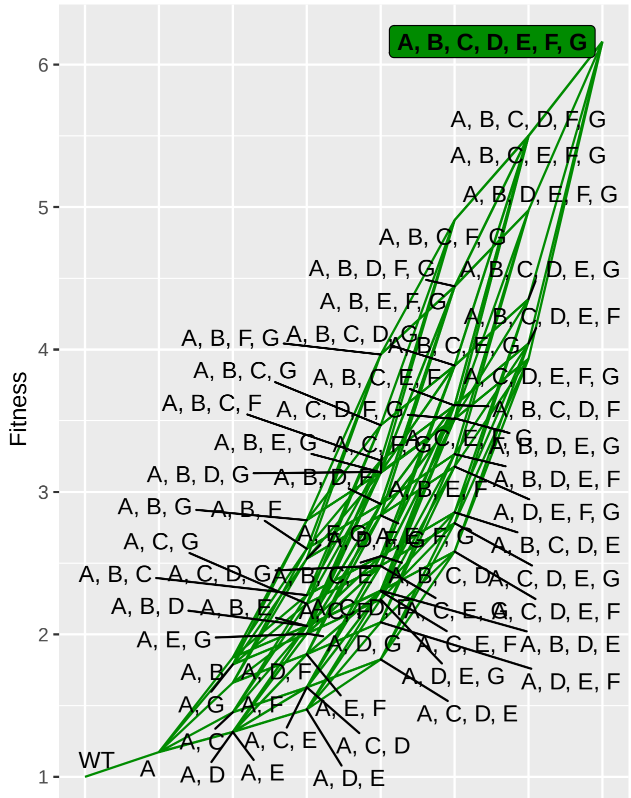

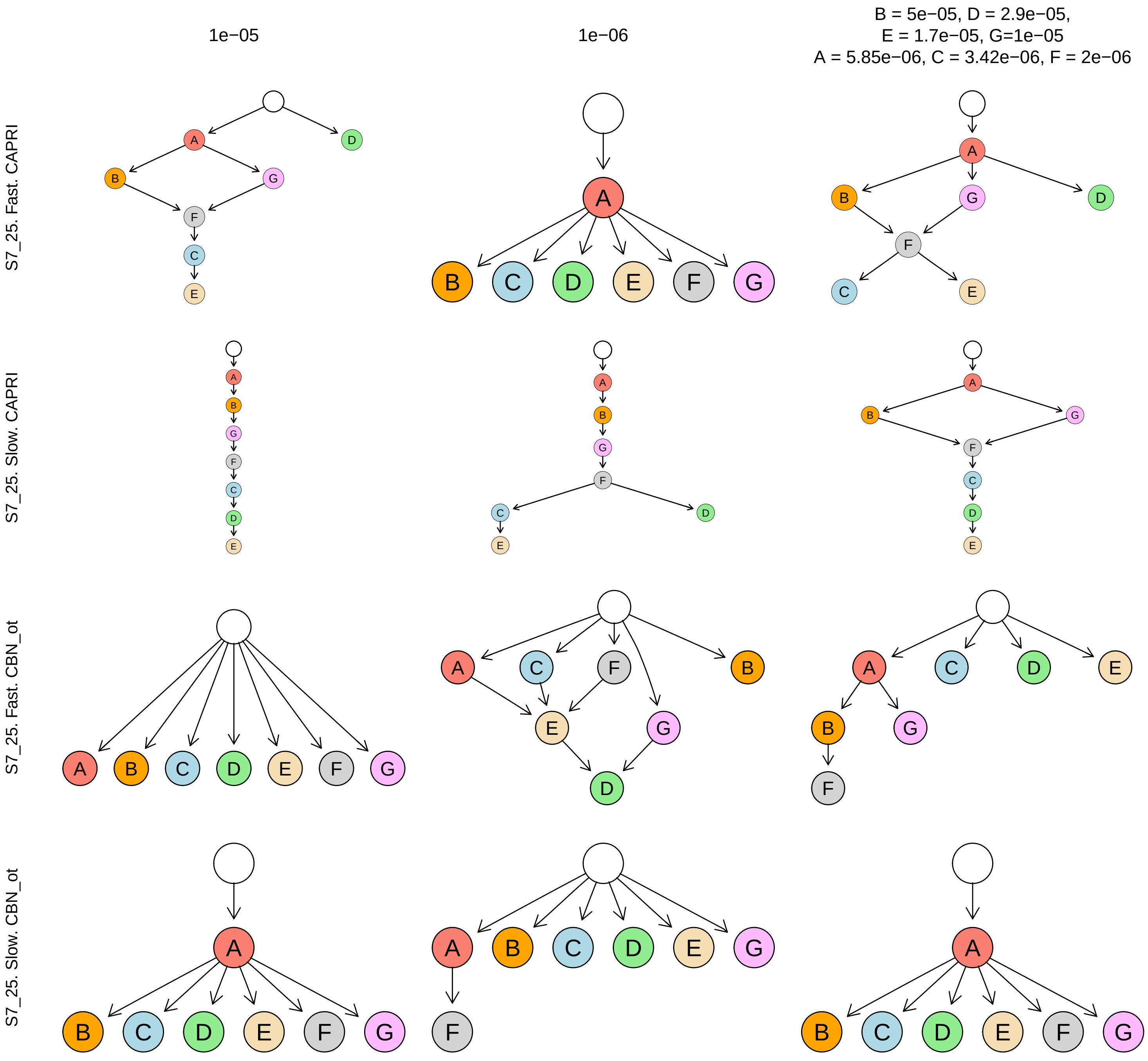

DAG$_a$

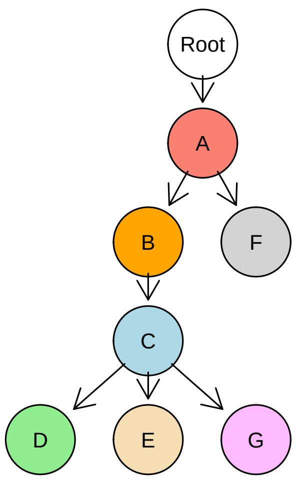

ID	Accessible Genot.	Discrep. DAG_a
S7_26	20	0

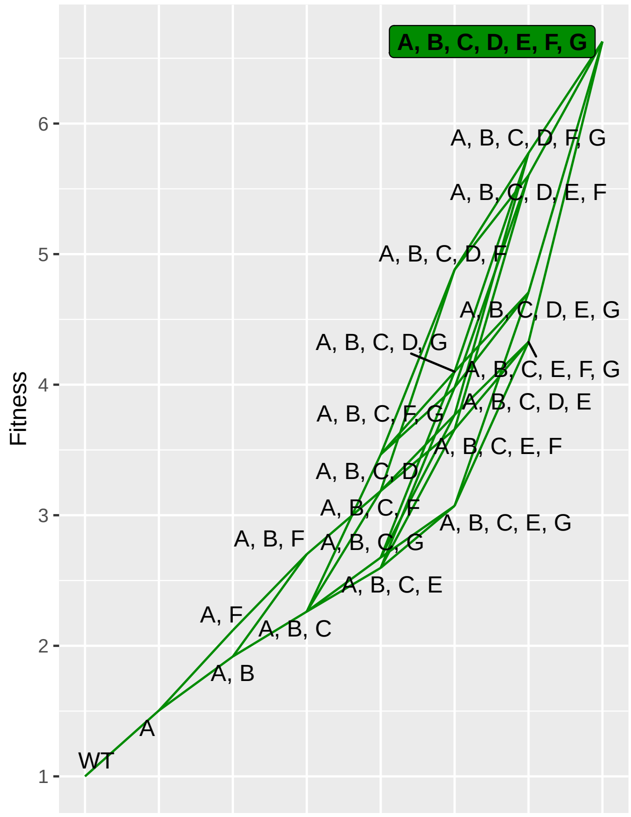

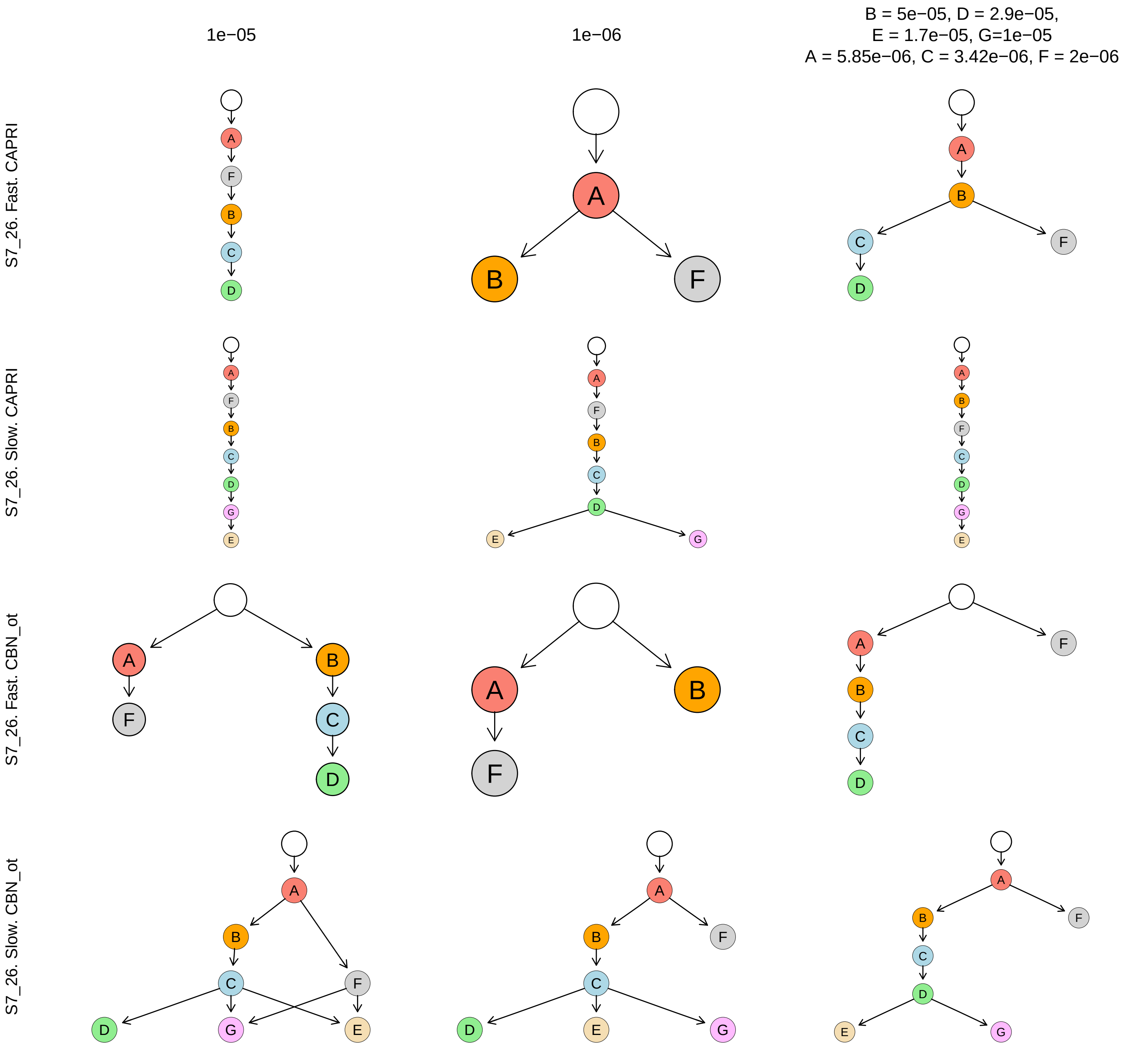

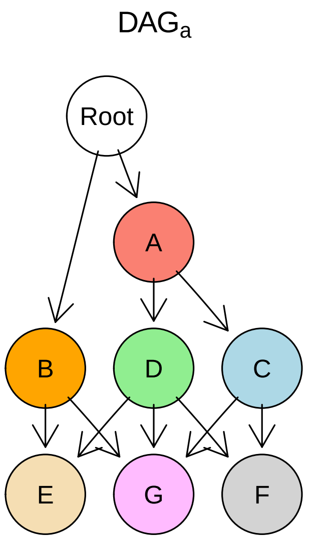

ID	Accessible Genot.	Discrep. DAG_a
S7_27	18	0

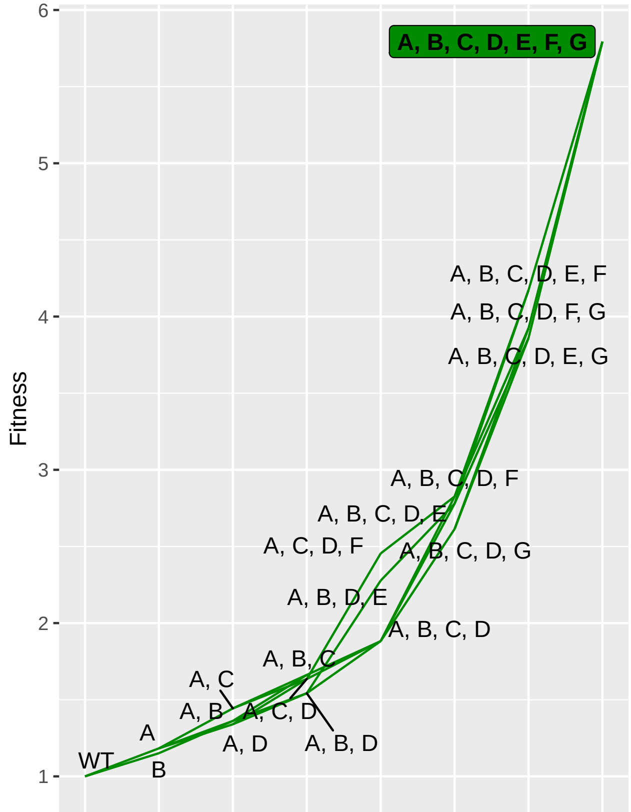

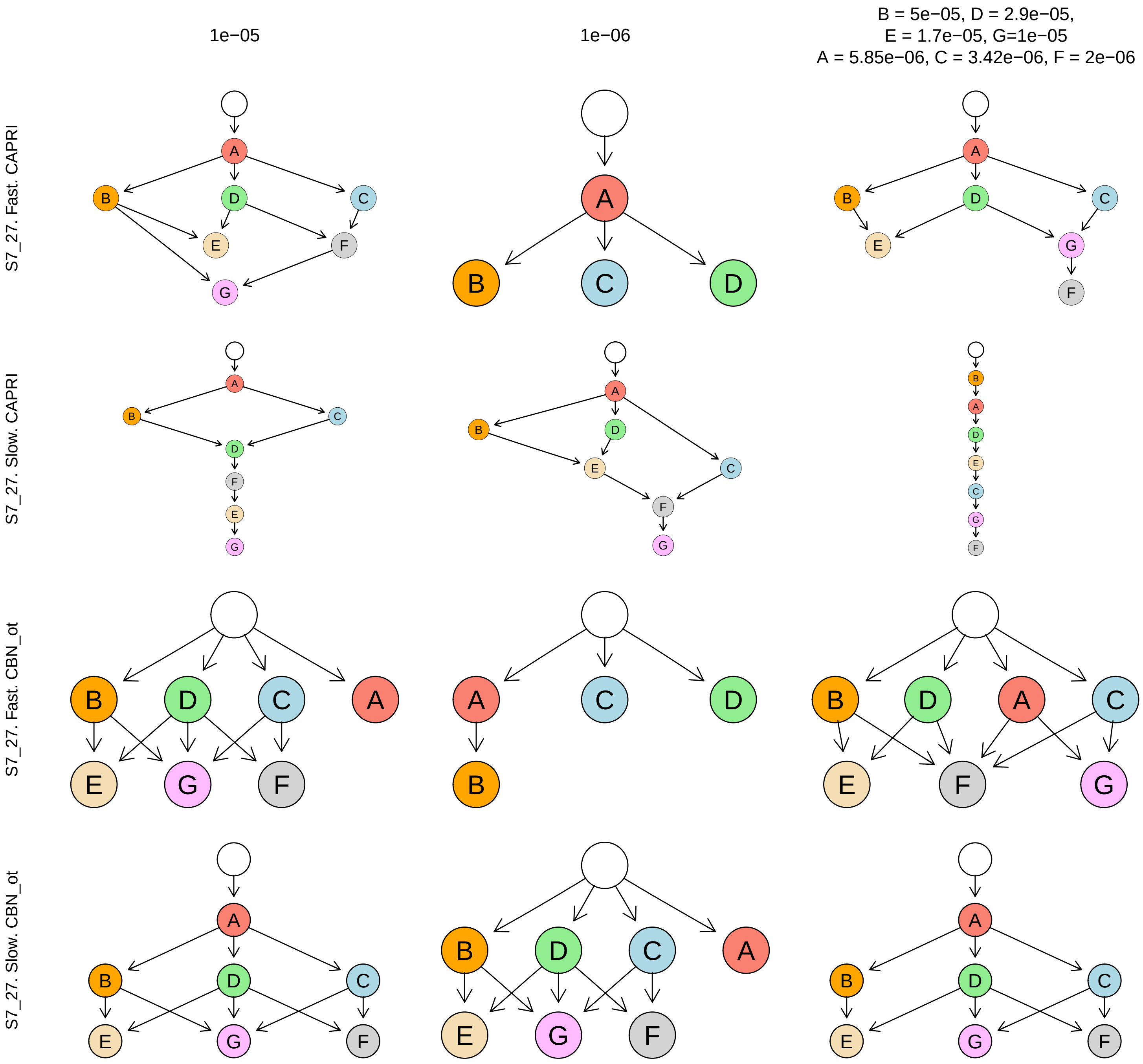

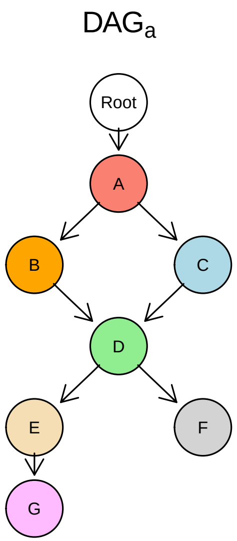

ID	Accessible Genot.	Discrep. DAG_a
S7_28	10	0

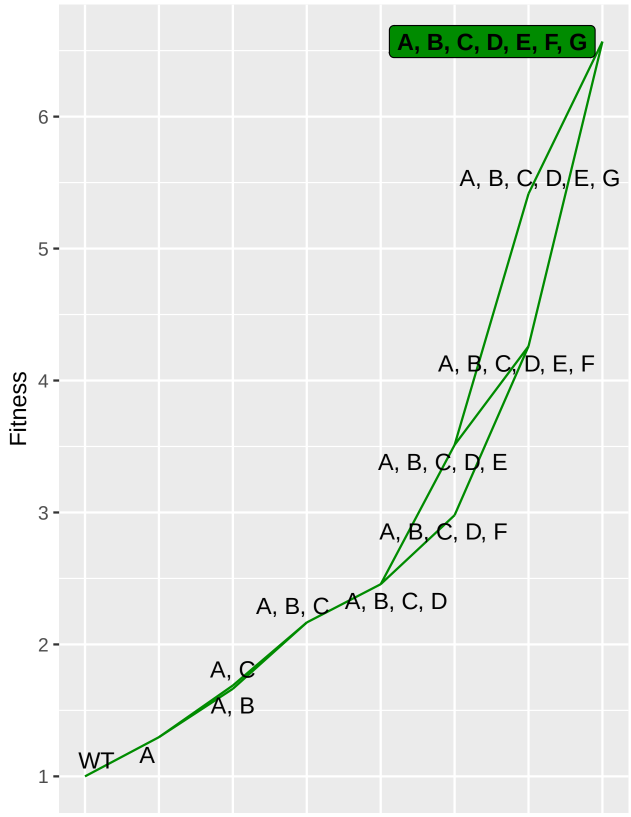

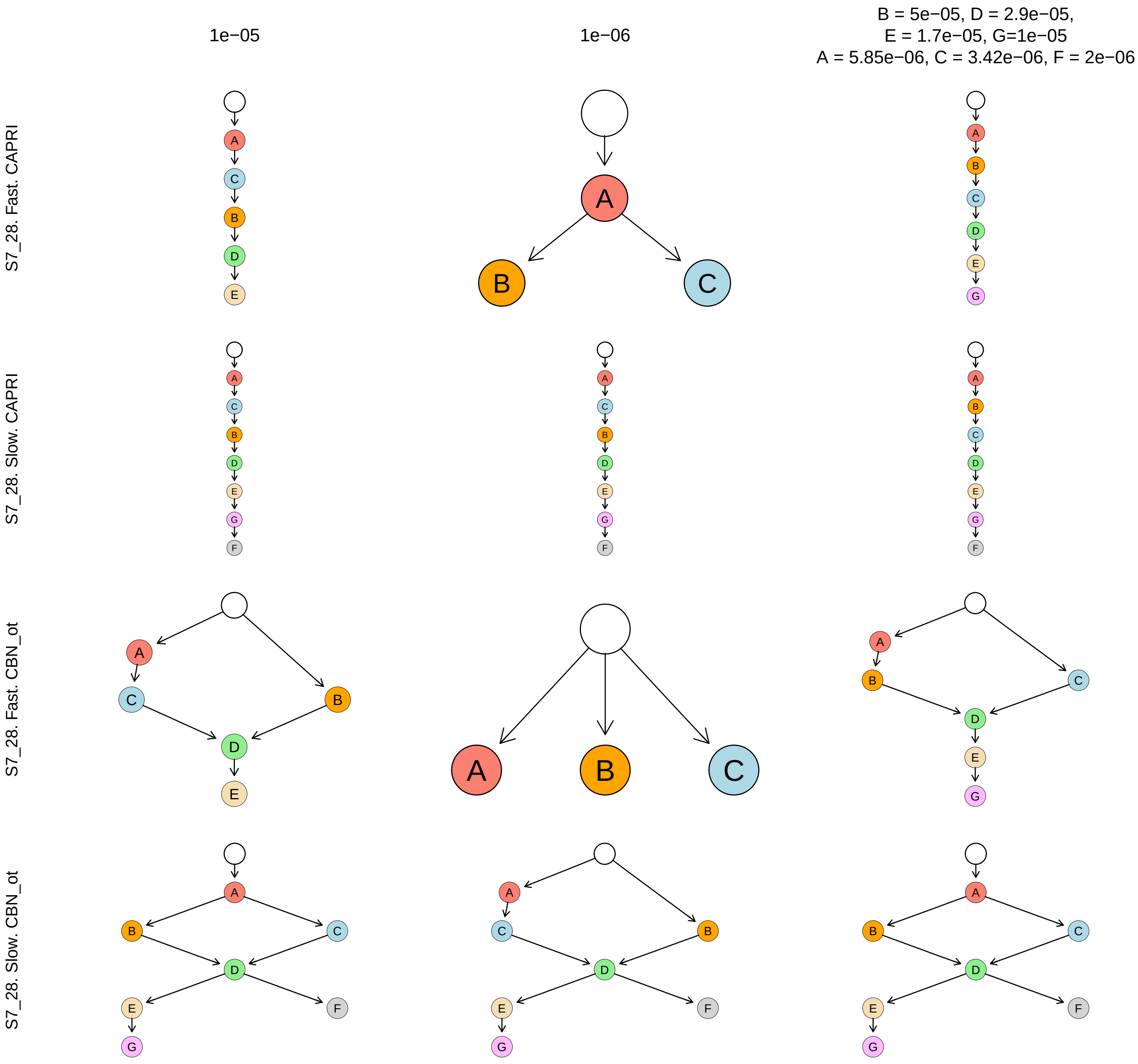

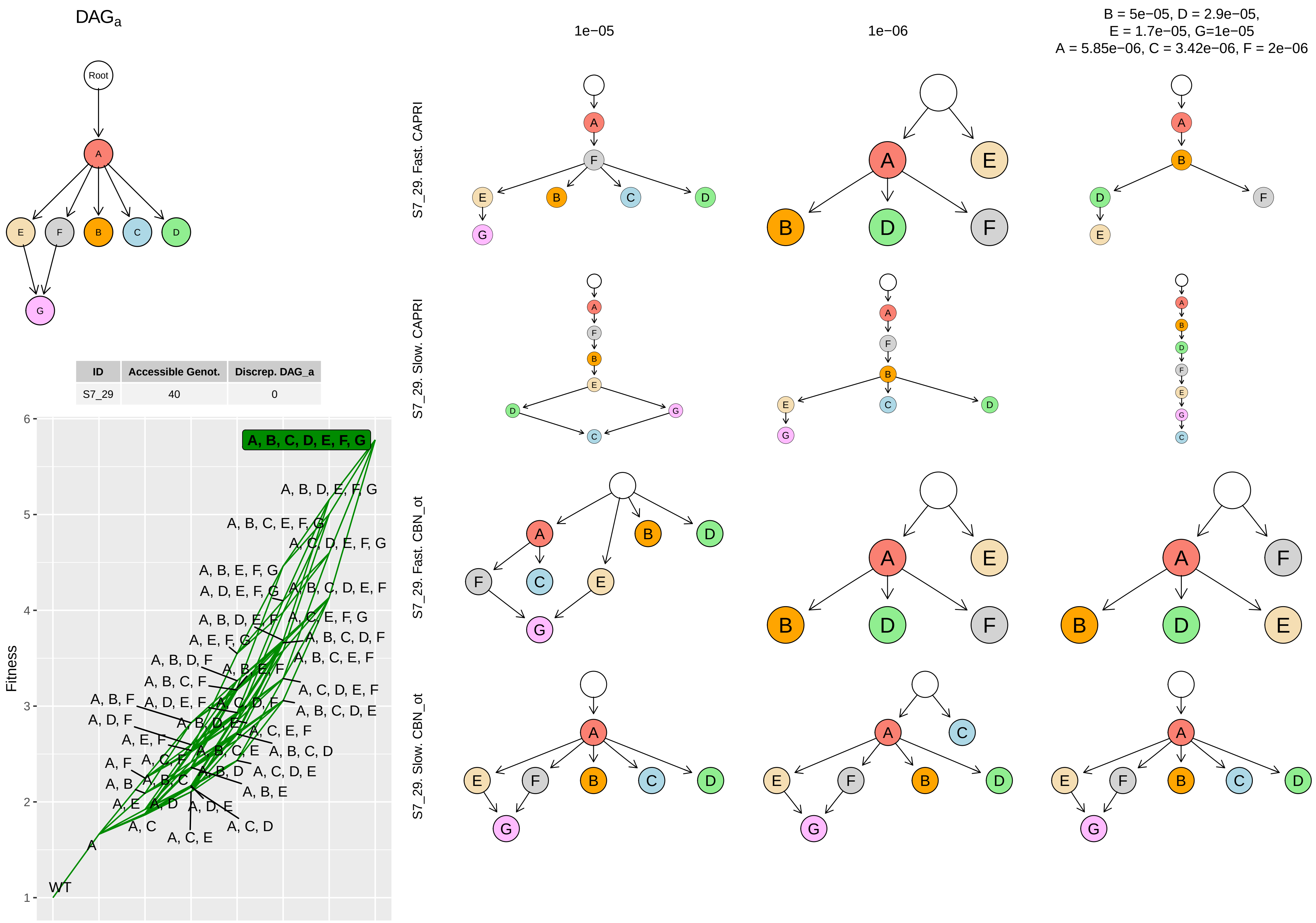

ID	Accessible Genot.	Discrep. DAG_a
S7_29	40	0

DAG$_a$

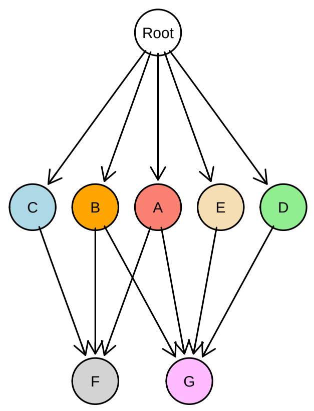

ID	Accessible Genot.	Discrep. DAG_a
S7_30	38	0

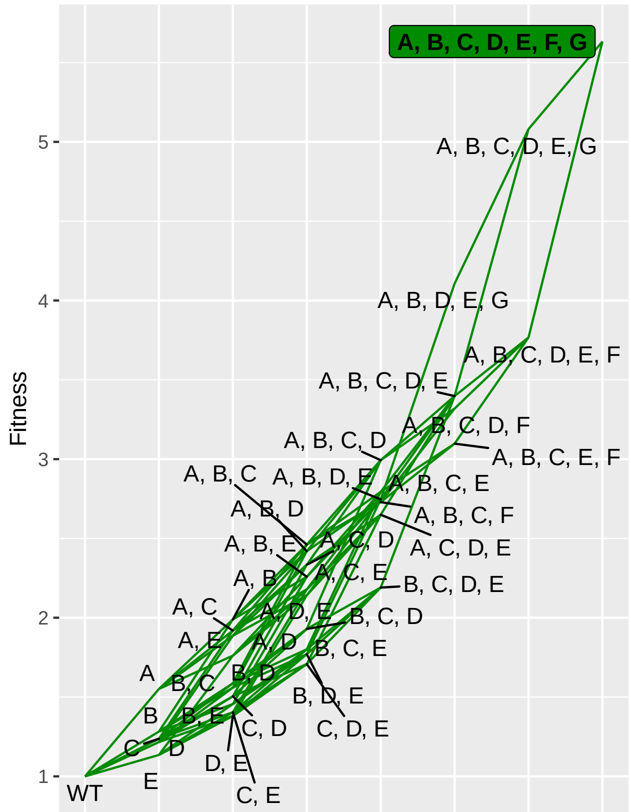

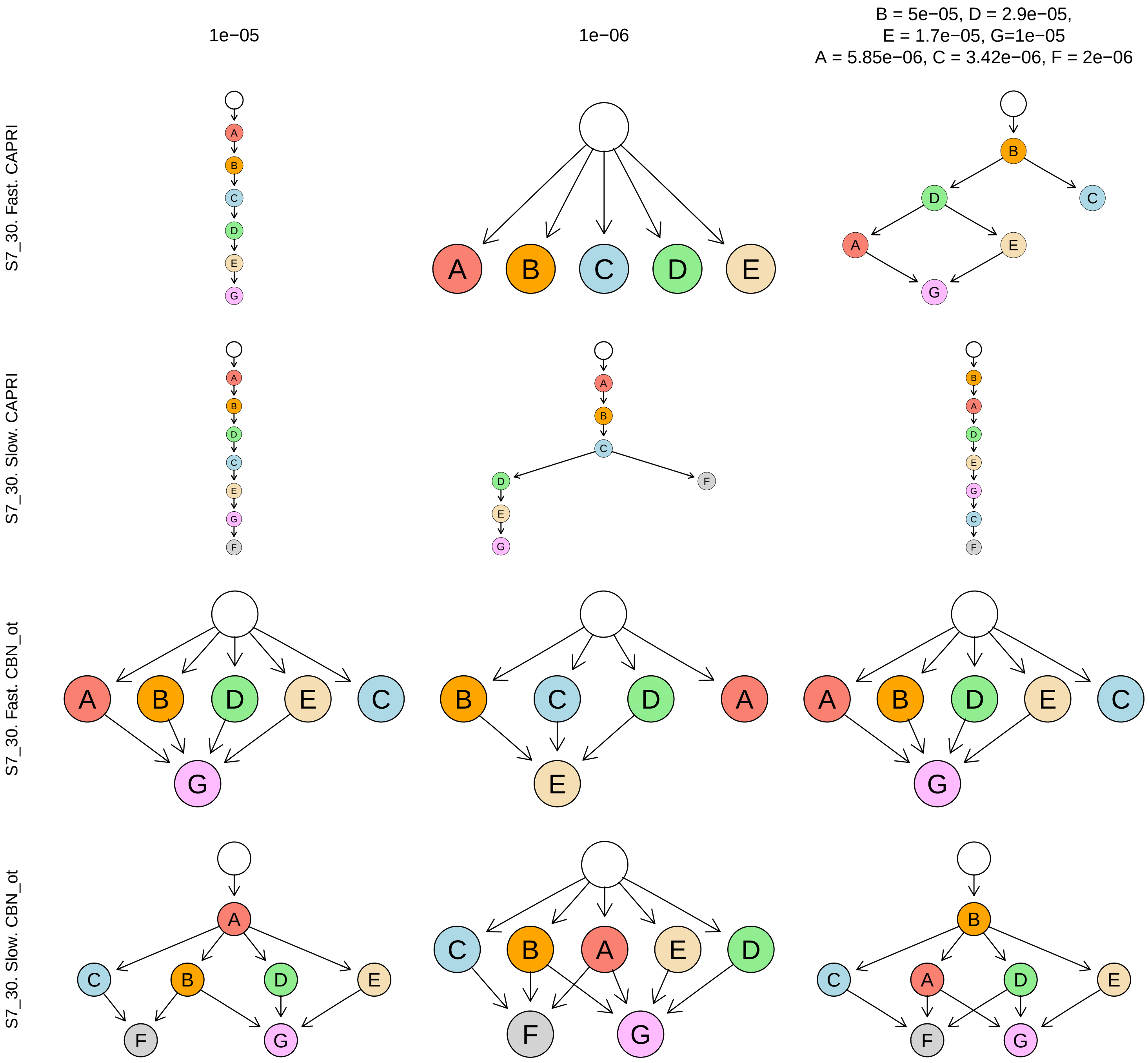

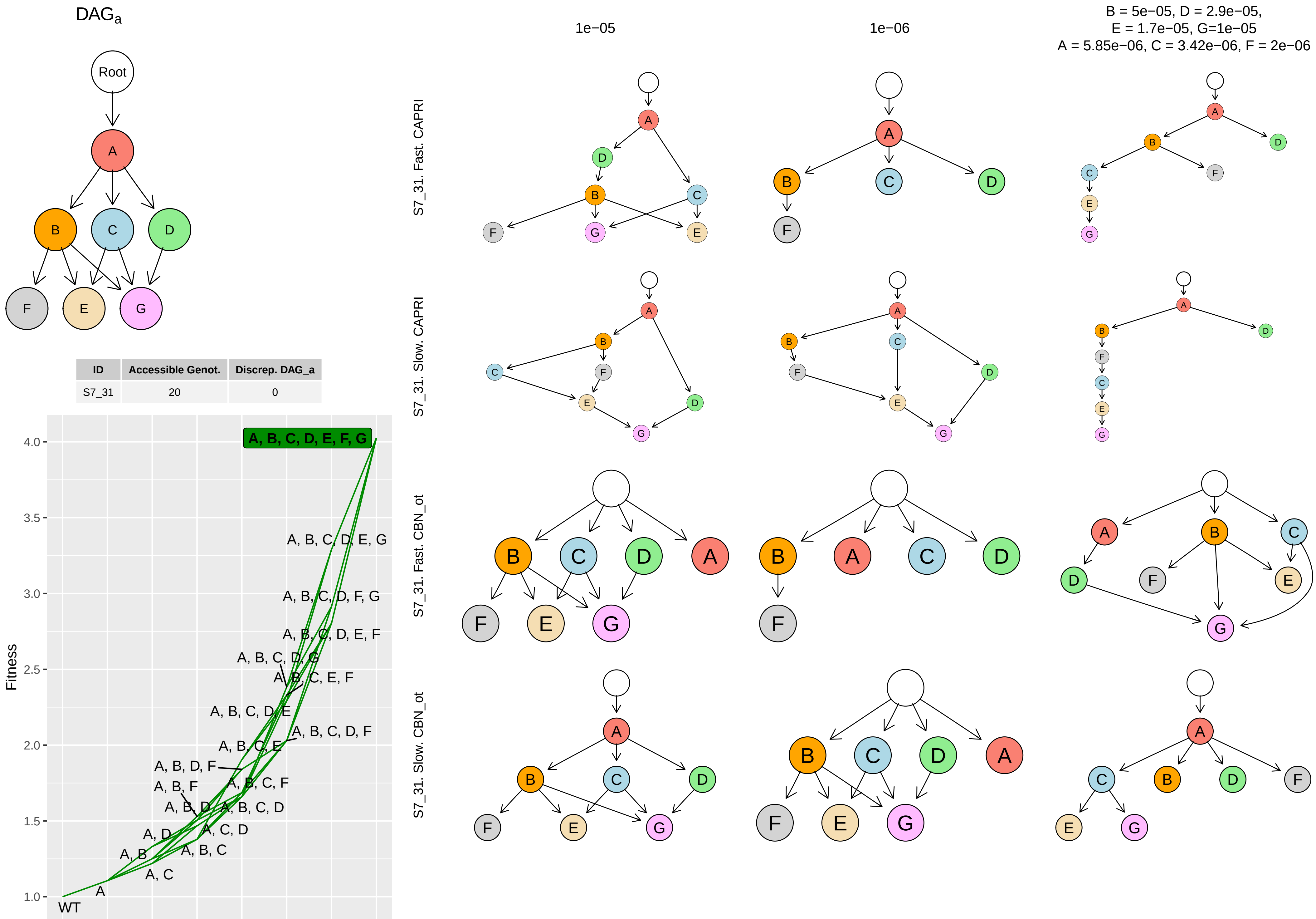

DAG_a
Root
A
B
C
D
F
E
G
ID
Accessible Genot.
Discrep. DAG_a
S7_31
20
0
Fitness
4.0
3.5
3.0
2.5
2.0
1.5
1.0
A, B, C, D, E, F, G
A, B, C, D, E, G
A, B, C, D, F, G
A, B, C, D, E, F
A, B, C, D, G
A, B, C, E, F
A, B, C, D, E
A, B, C, D, F
A, B, C, E
A, B, D, F
A, B, C, F
A, B, F
A, B, D
A, B, C, D
A, D
A, C, D
A, B
A, B, C
A, C
A
WT
1e−05
1e−06
B = 5e−05, D = 2.9e−05,
E = 1.7e−05, G=1e−05
A = 5.85e−06, C = 3.42e−06, F = 2e−06
S7_31. Fast. CAPRI
S7_31. Slow. CAPRI
S7_31. Fast. CBN_ot
S7_31. Slow. CBN_ot

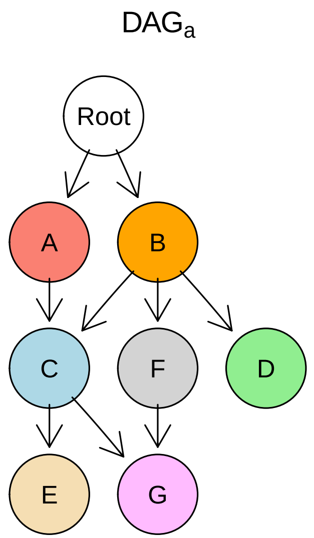

ID	Accessible Genot.	Discrep. DAG_a
S7_32	21	0

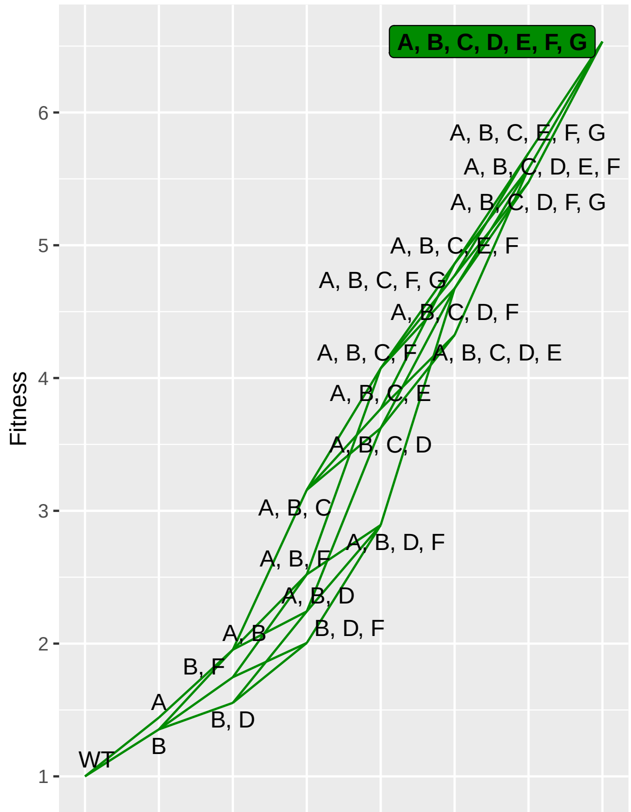

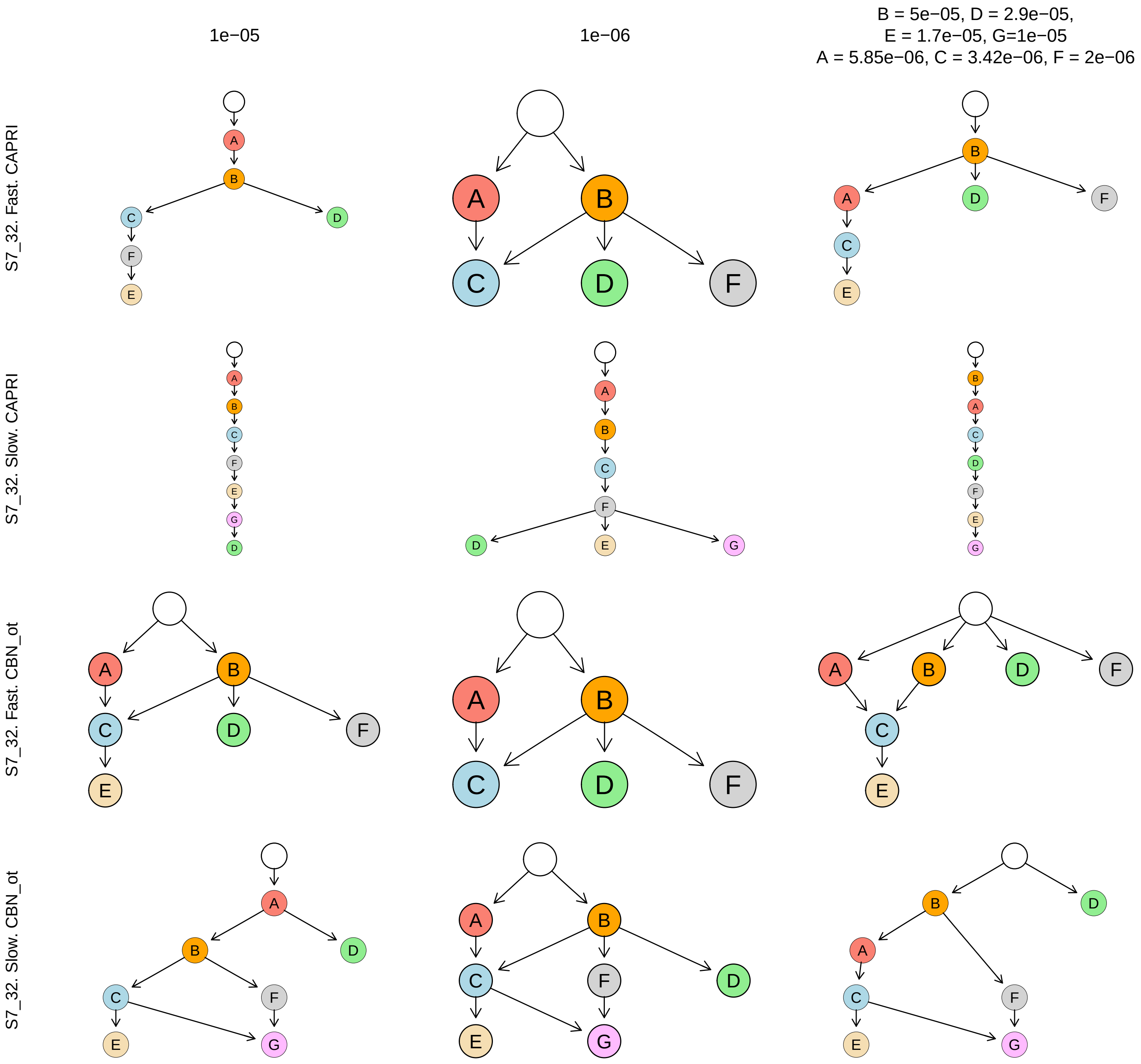

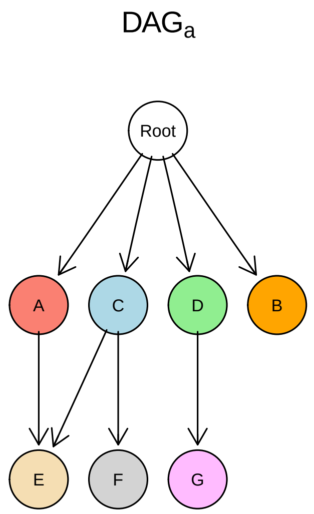

ID	Accessible Genot.	Discrep. DAG_a
S7_33	47	0

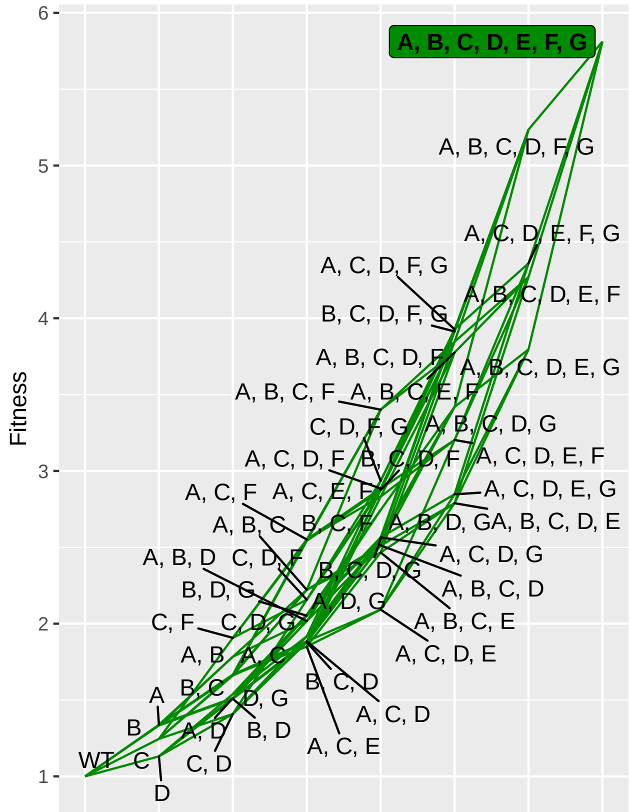

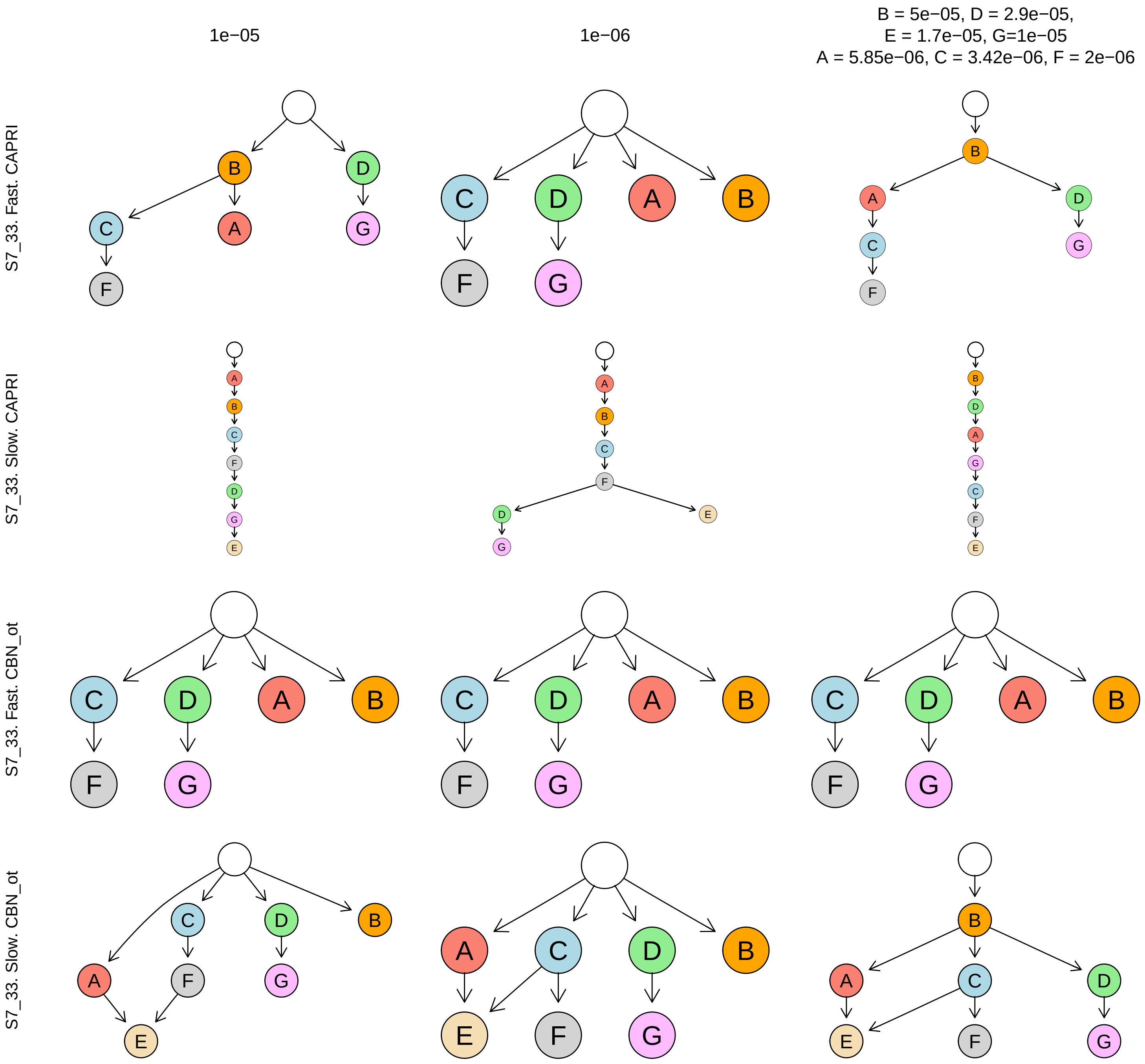

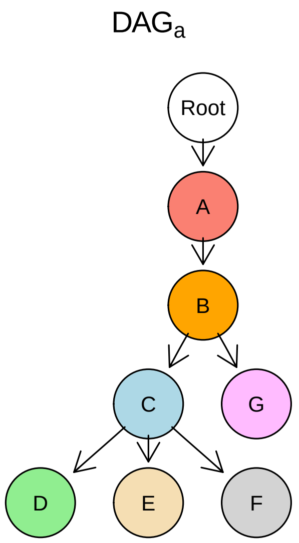

ID	Accessible Genot.	Discrep. DAG_a
S7_34	19	0

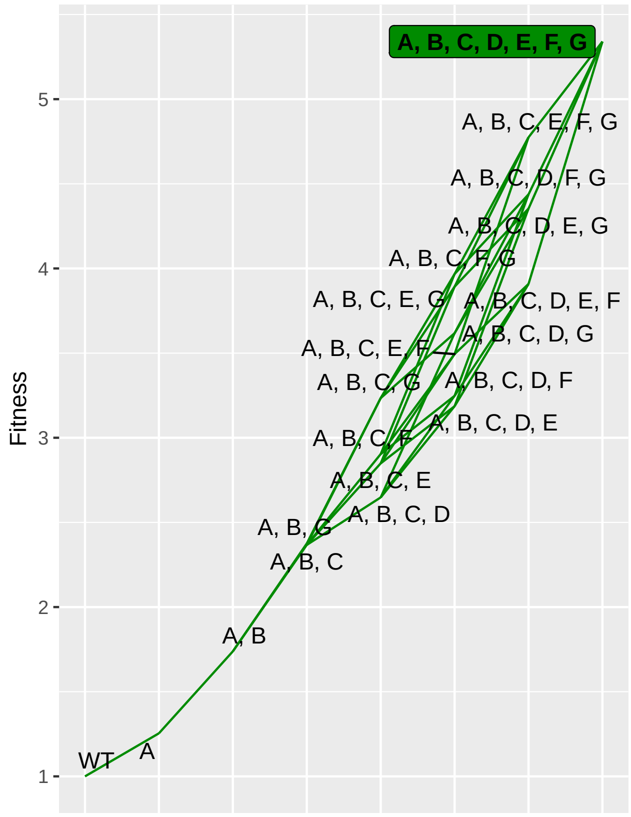

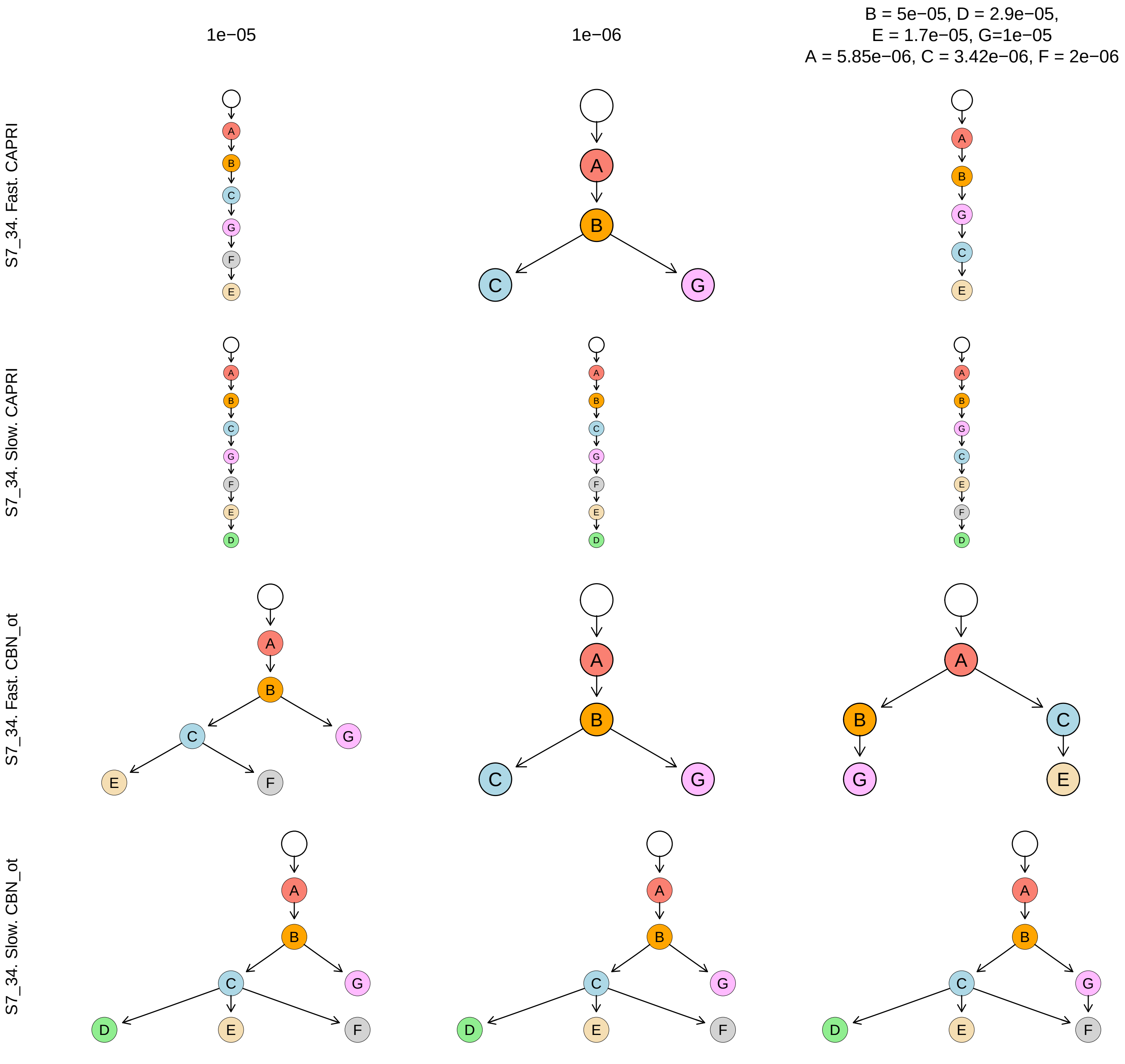

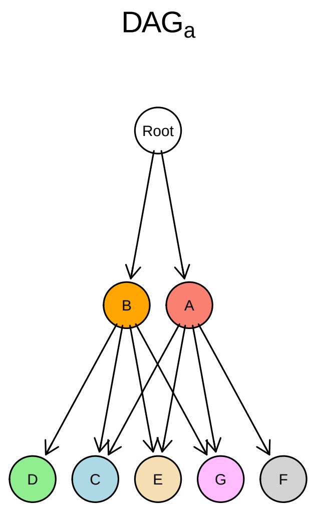

ID	Accessible Genot.	Discrep. DAG_a
S7_35	36	0

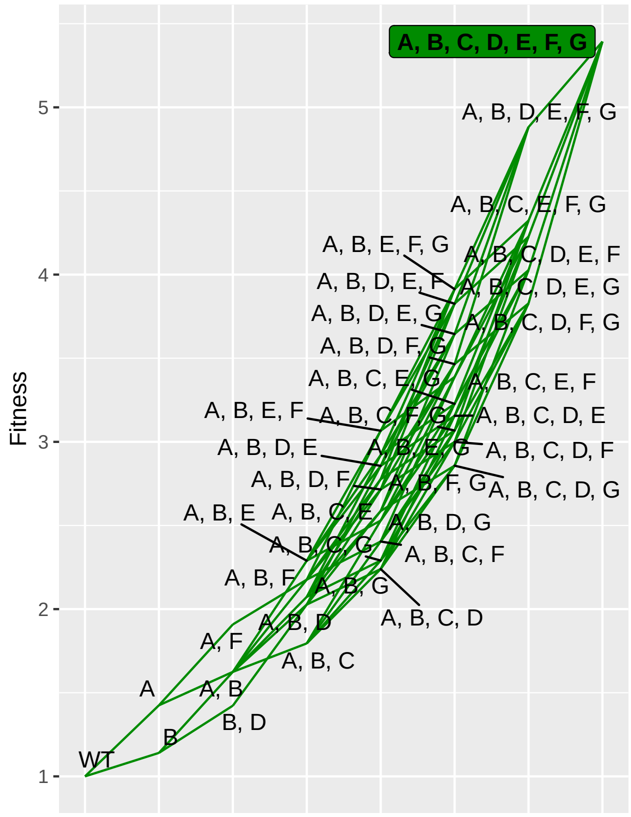

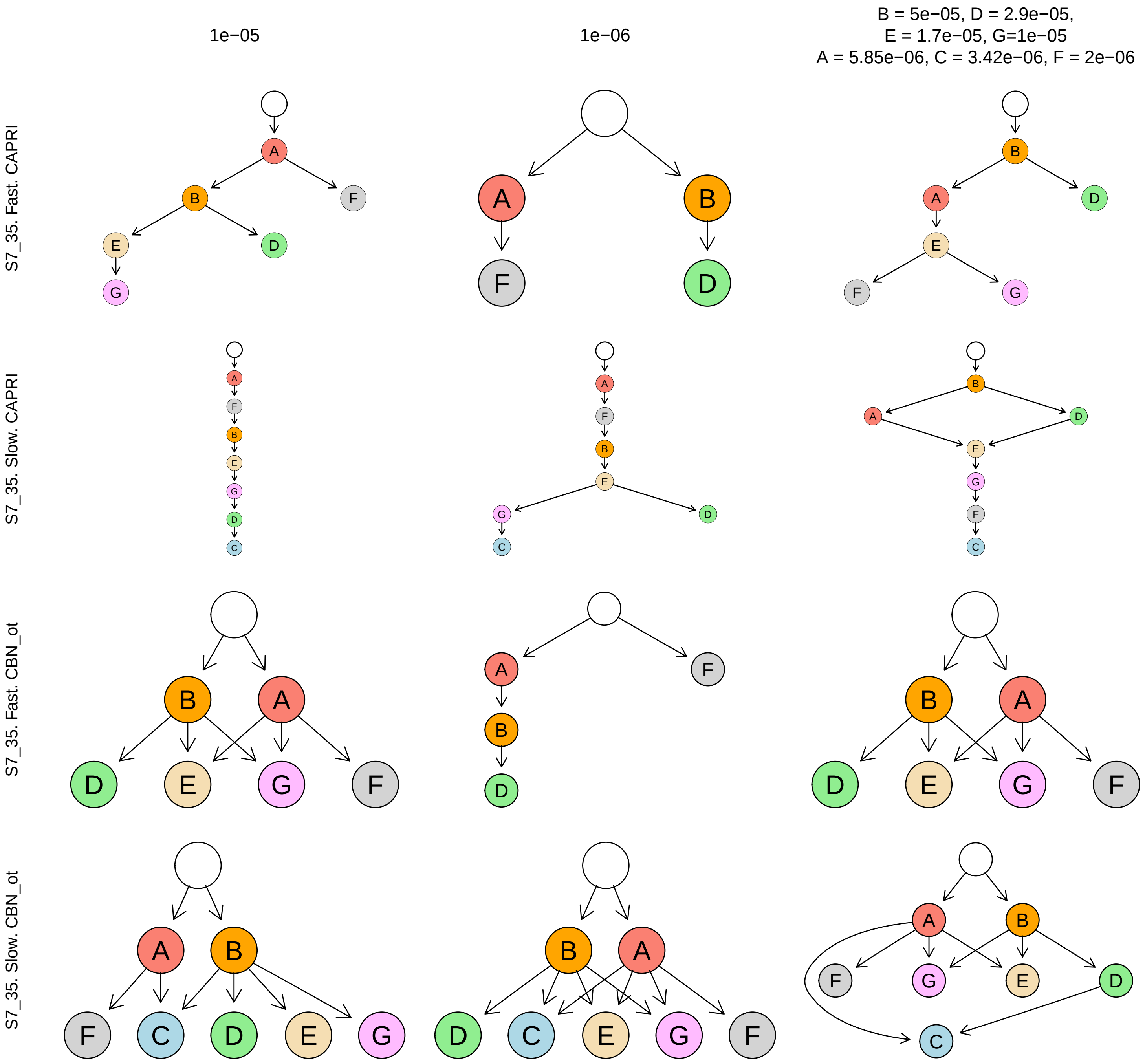

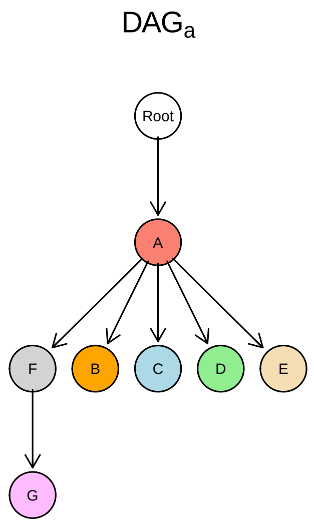

ID	Accessible Genot.	Discrep. DAG_a
S7_36	48	0

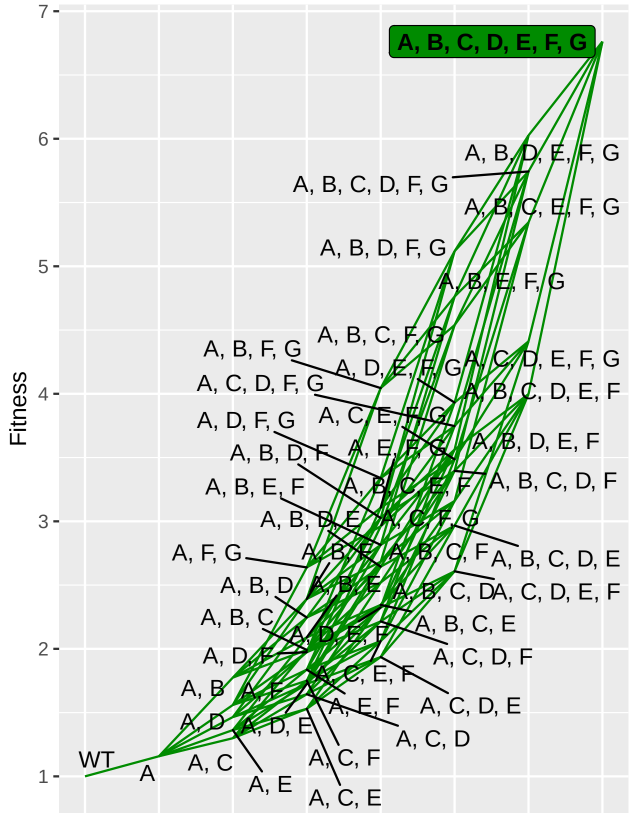

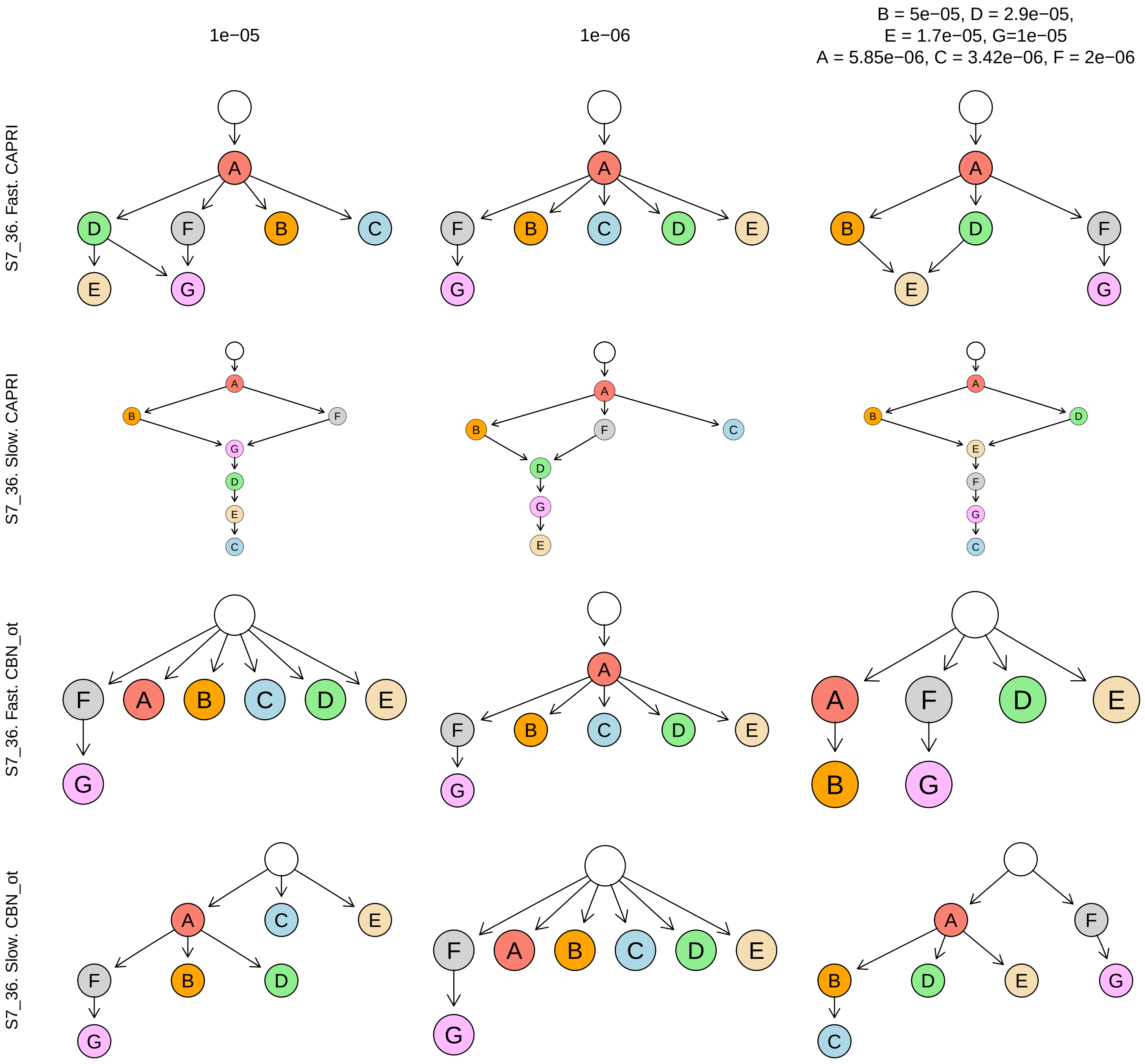

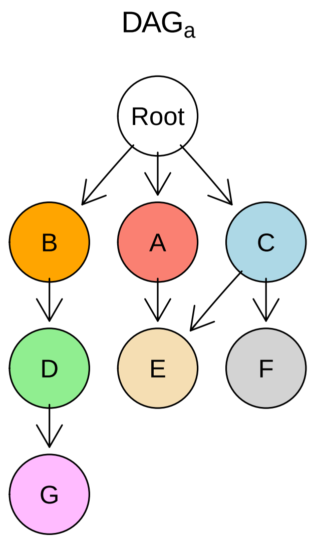

ID	Accessible Genot.	Discrep. DAG_a
S7_37	31	0

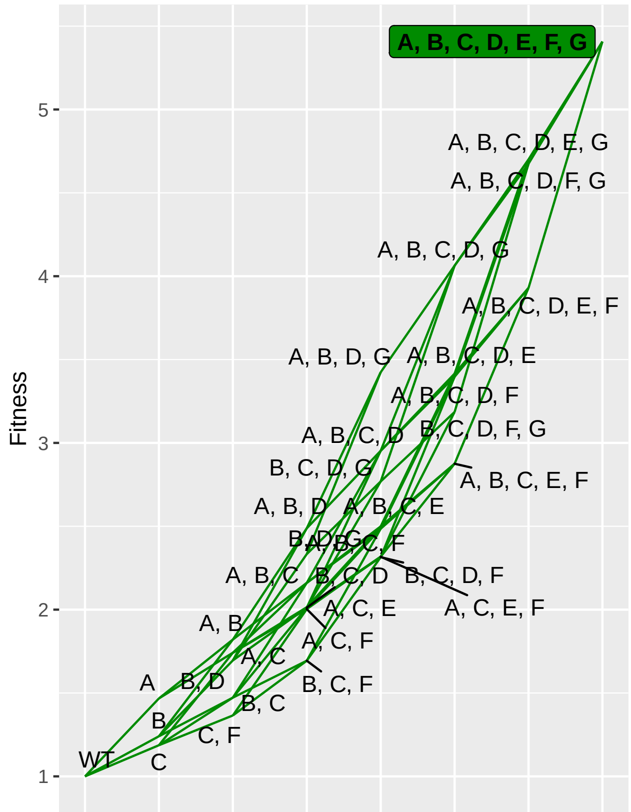

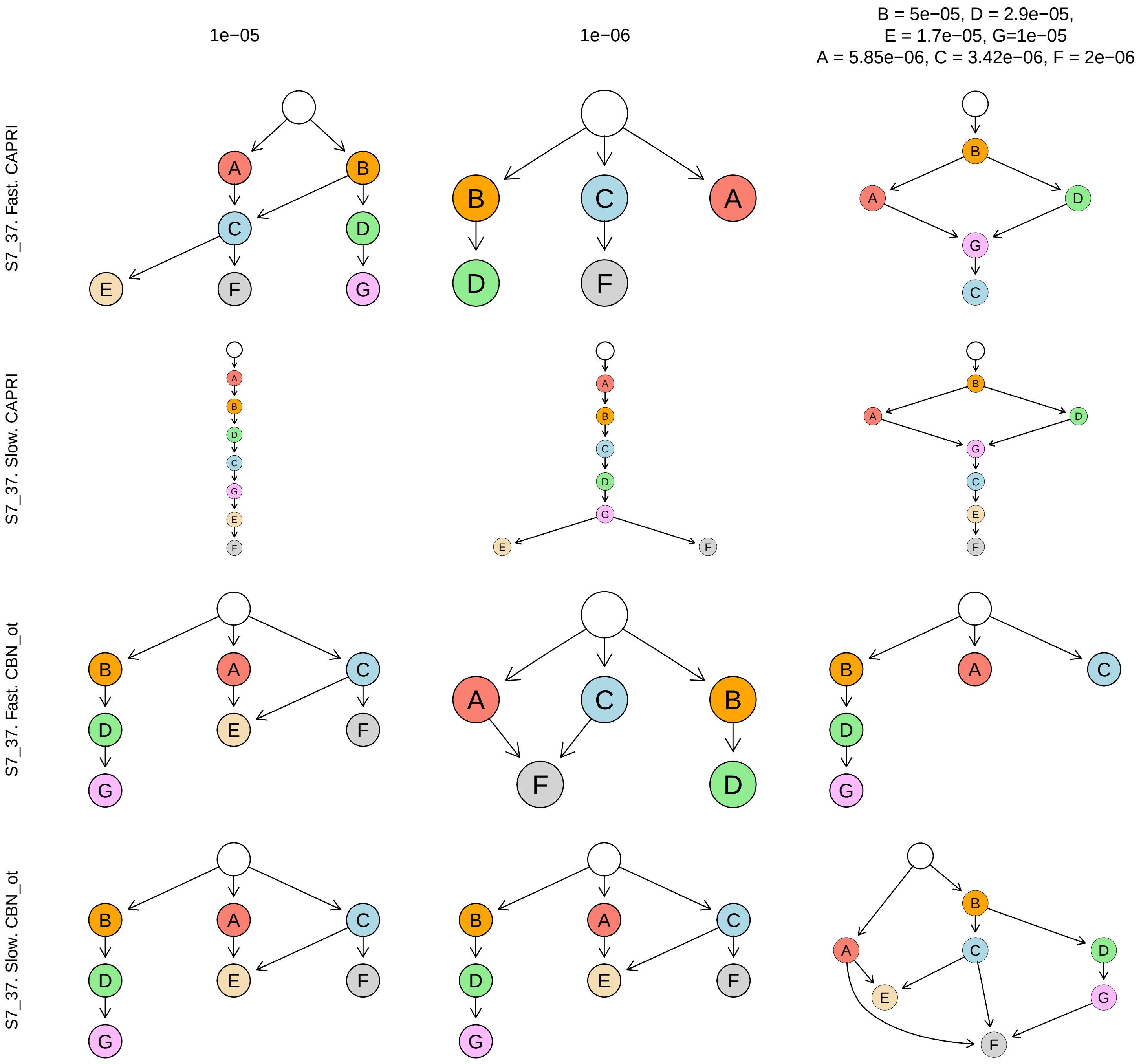

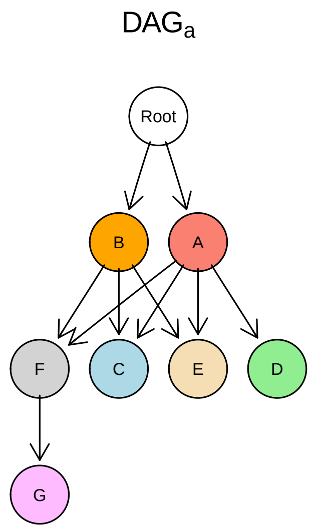

ID	Accessible Genot.	Discrep. DAG_a
S7_38	27	0

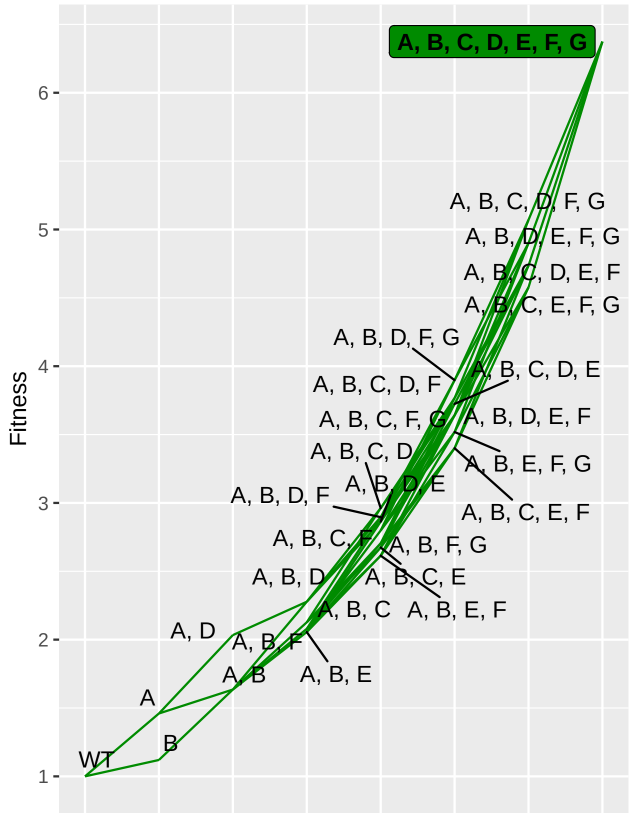

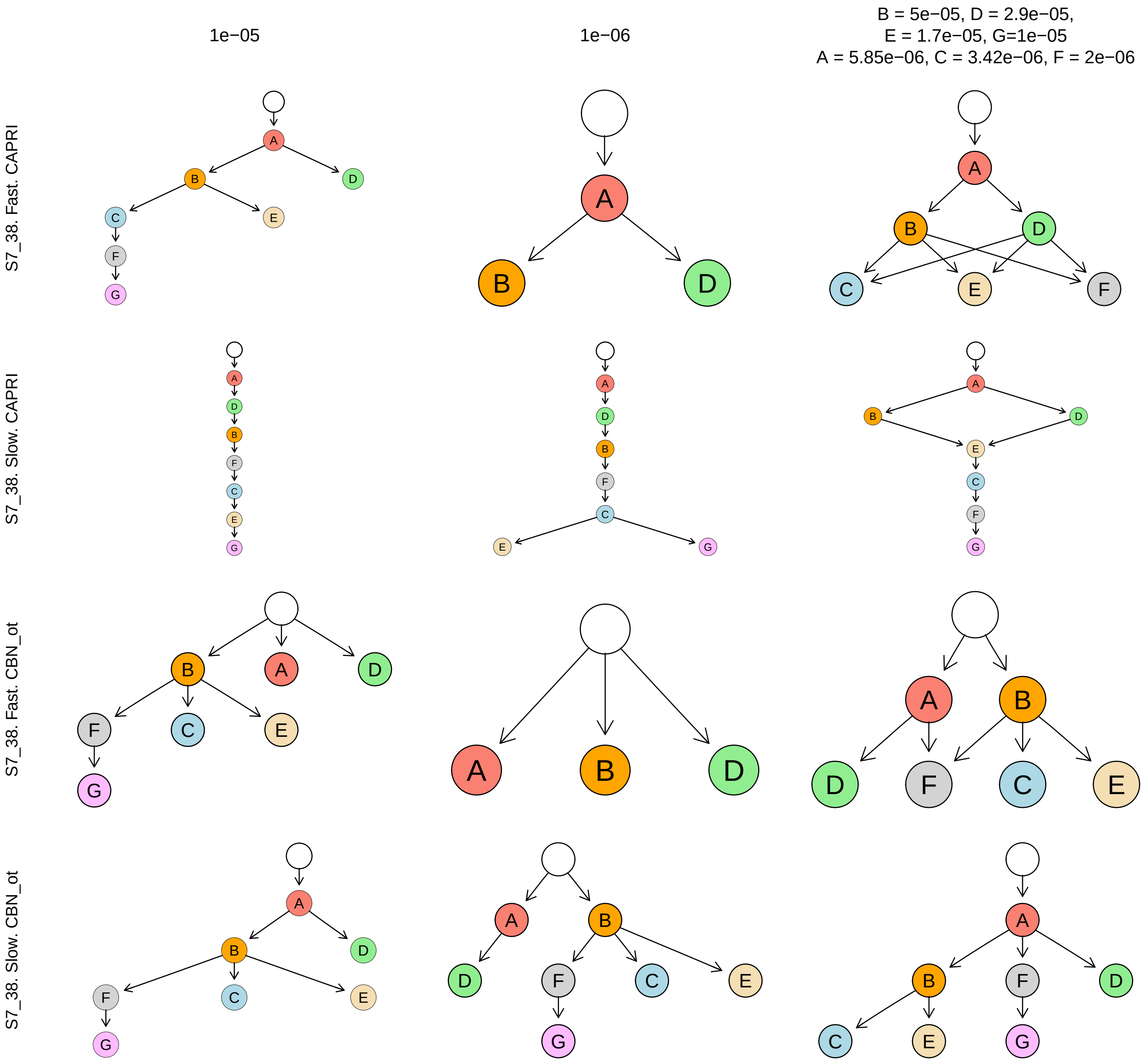

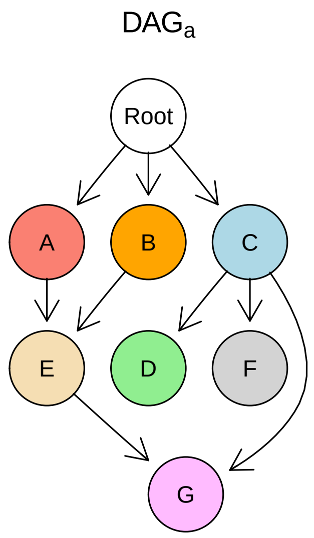

ID	Accessible Genot.	Discrep. DAG_a
S7_39	28	0

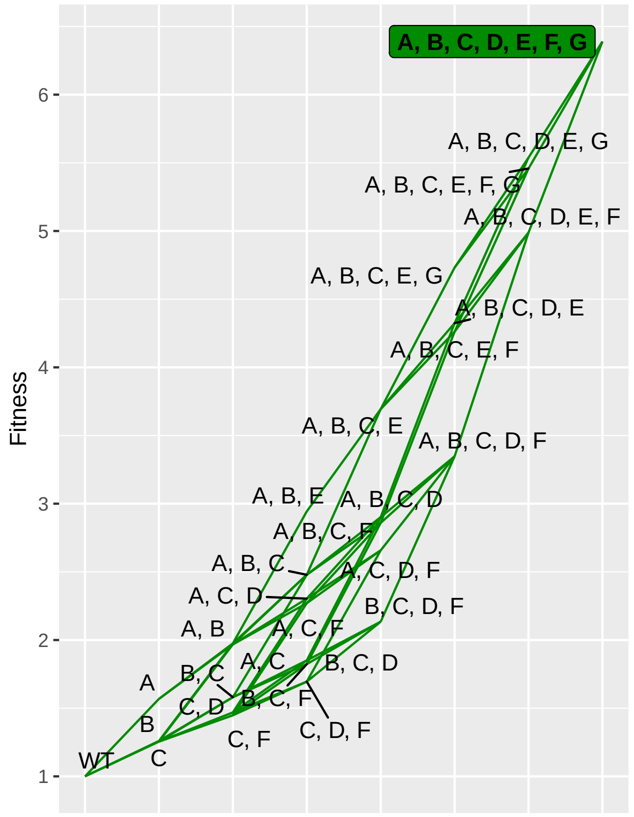

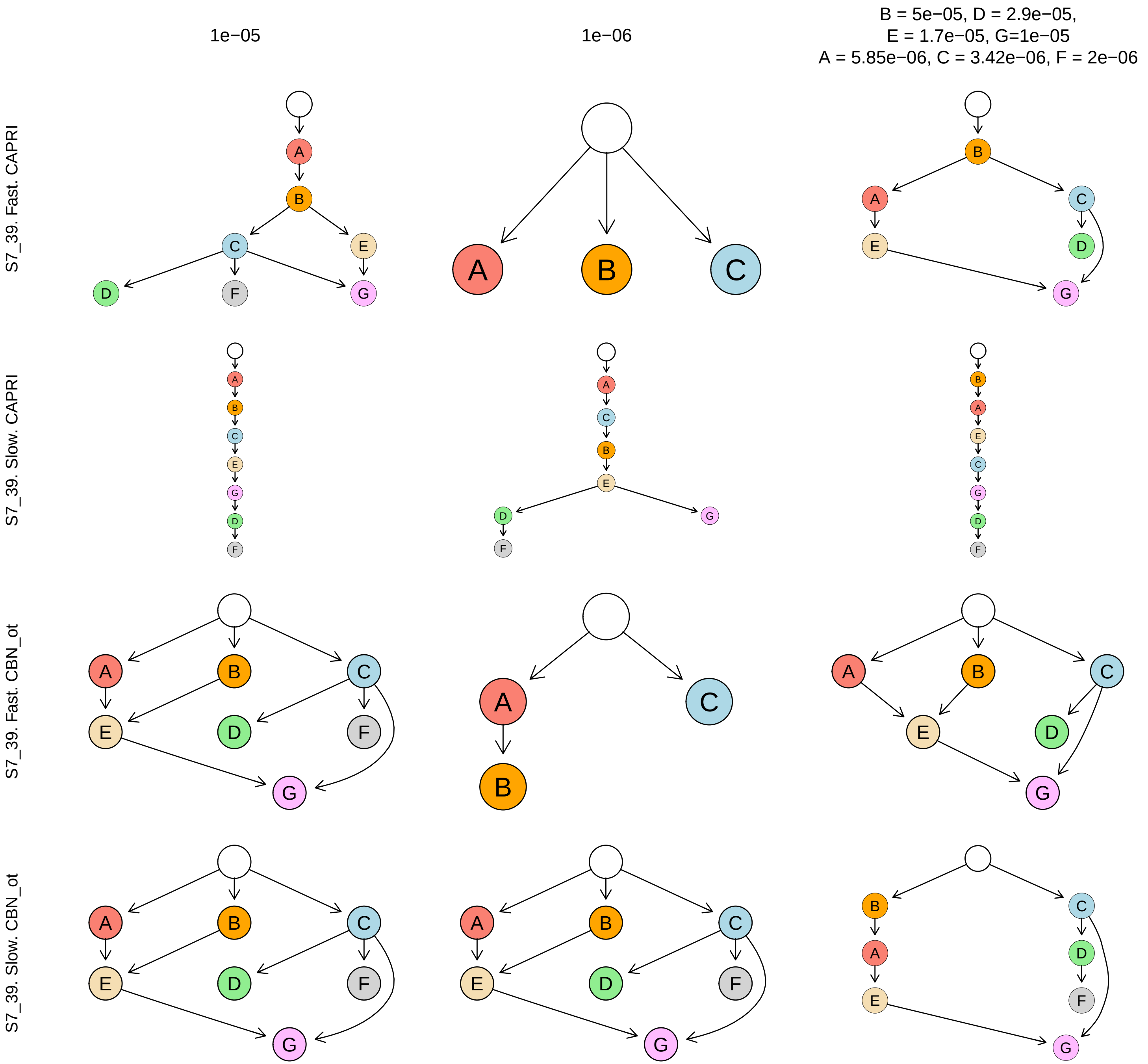

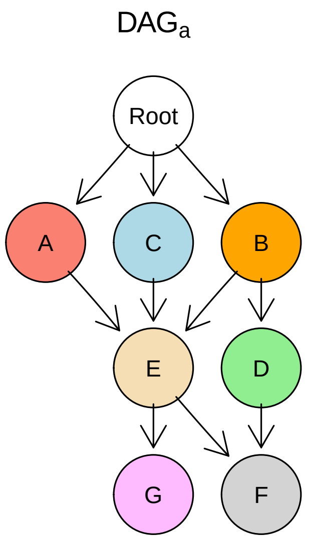

ID	Accessible Genot.	Discrep. DAG_a
S7_40	17	0

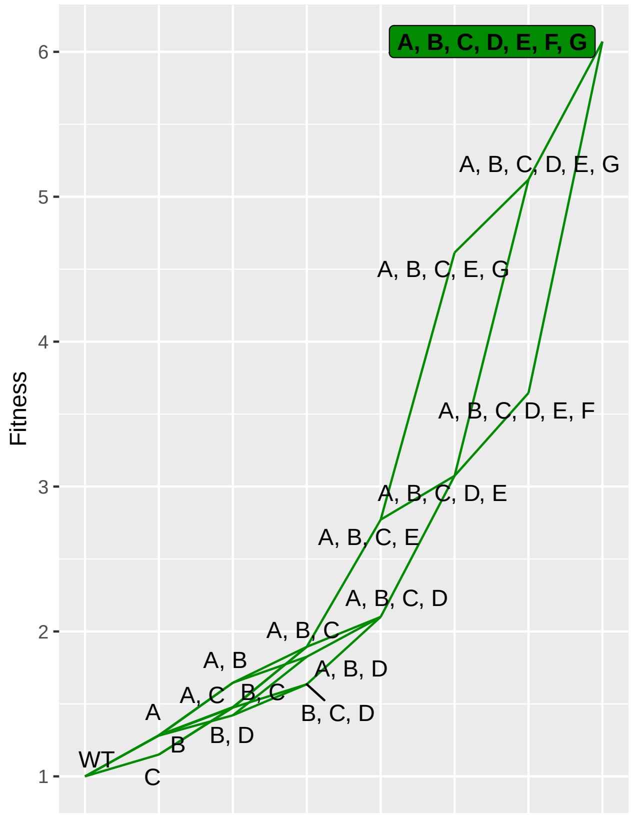

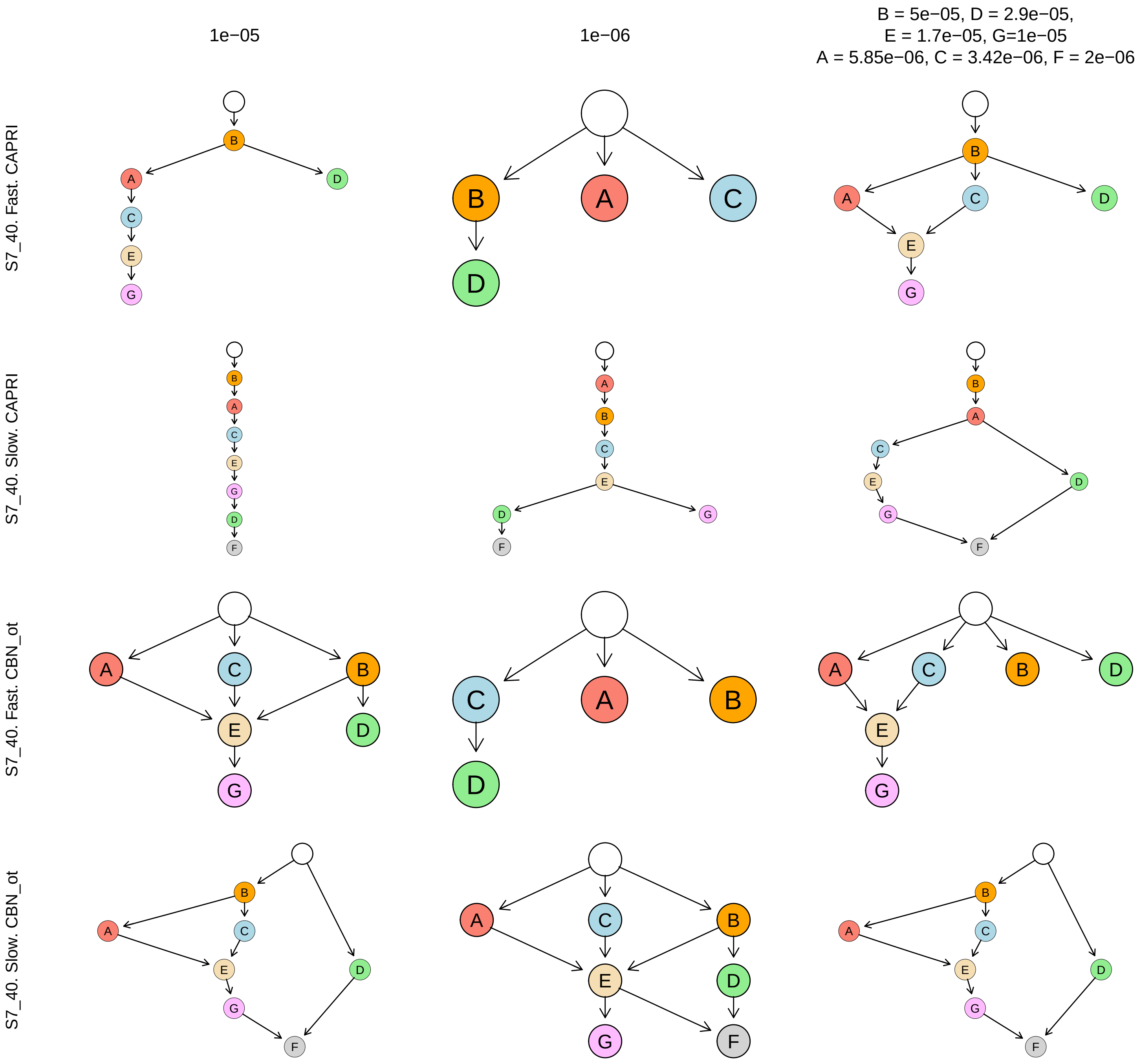

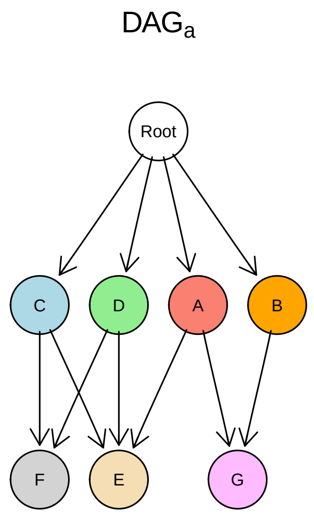

ID	Accessible Genot.	Discrep. DAG_a
S7_41	30	0

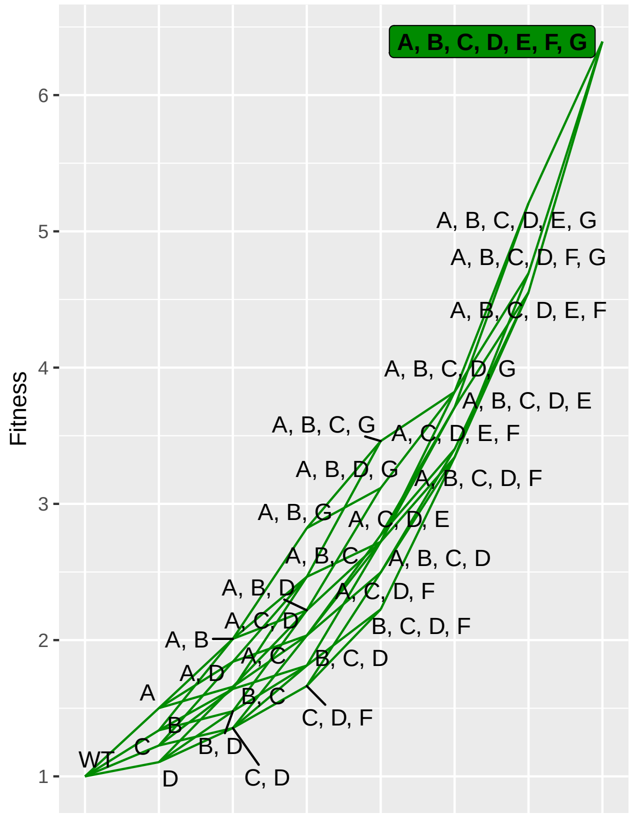

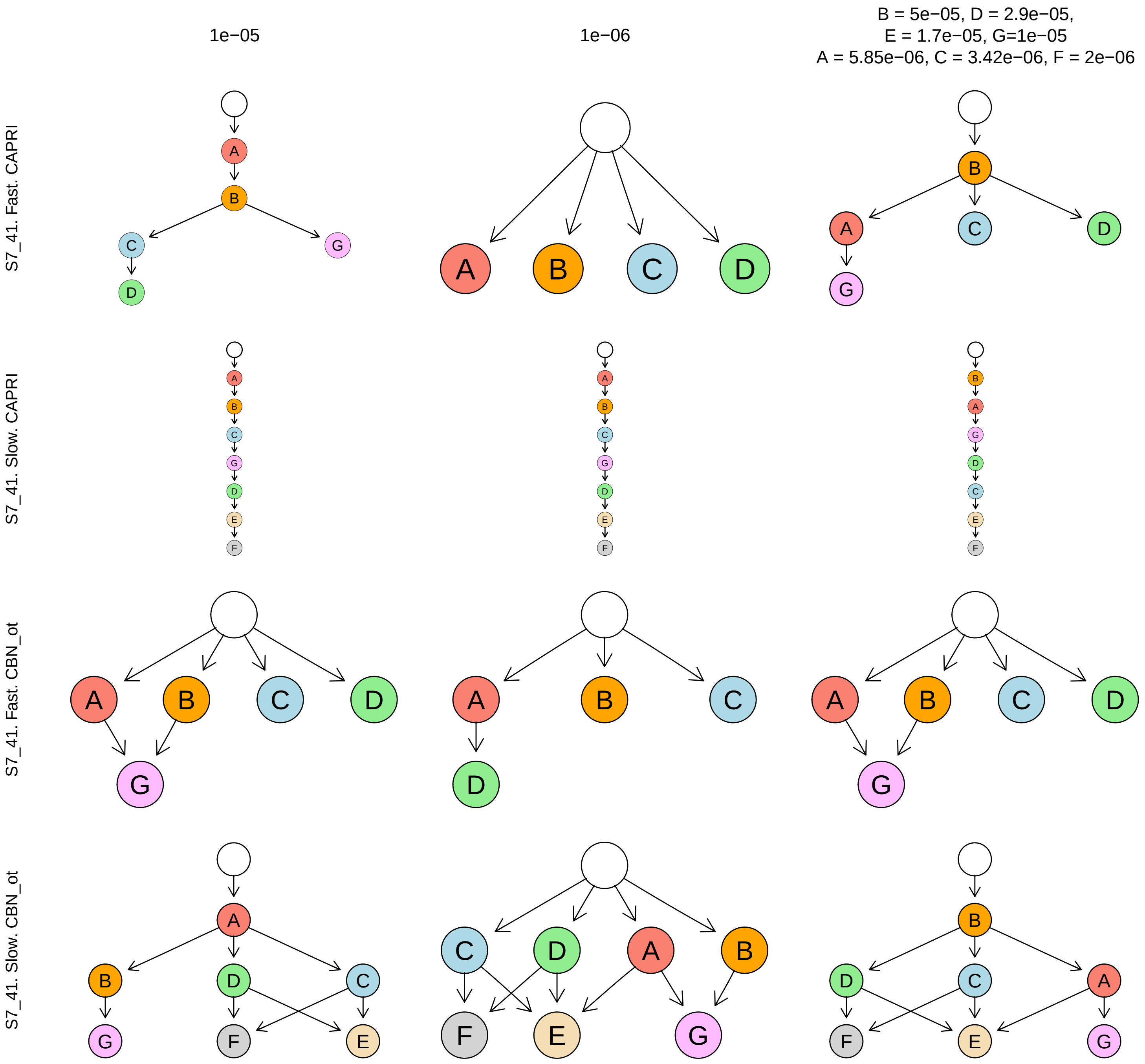

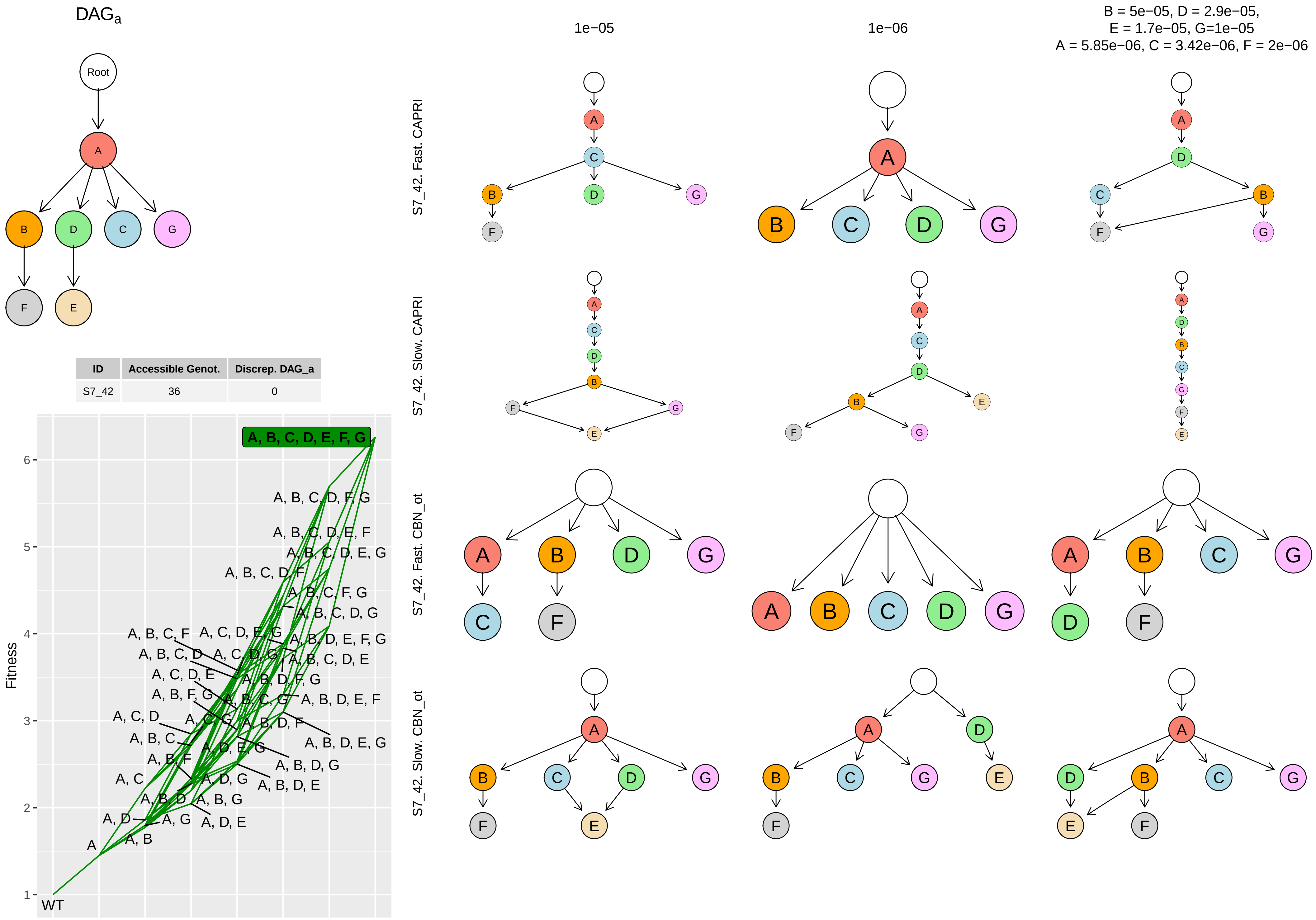

ID	Accessible Genot.	Discrep. DAG_a
S7_42	36	0

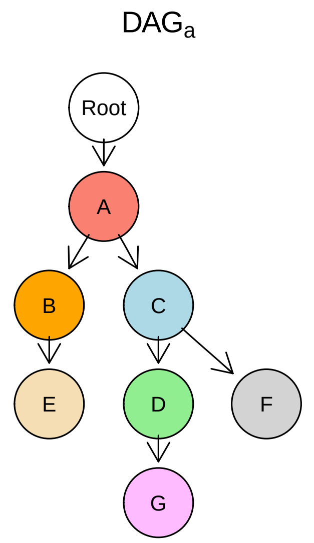

ID	Accessible Genot.	Discrep. DAG_a
S7_43	21	0

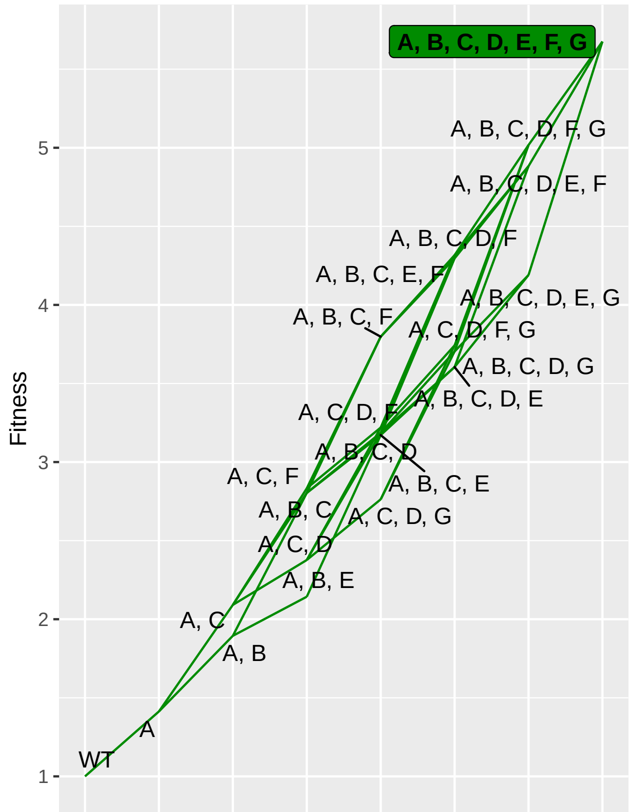

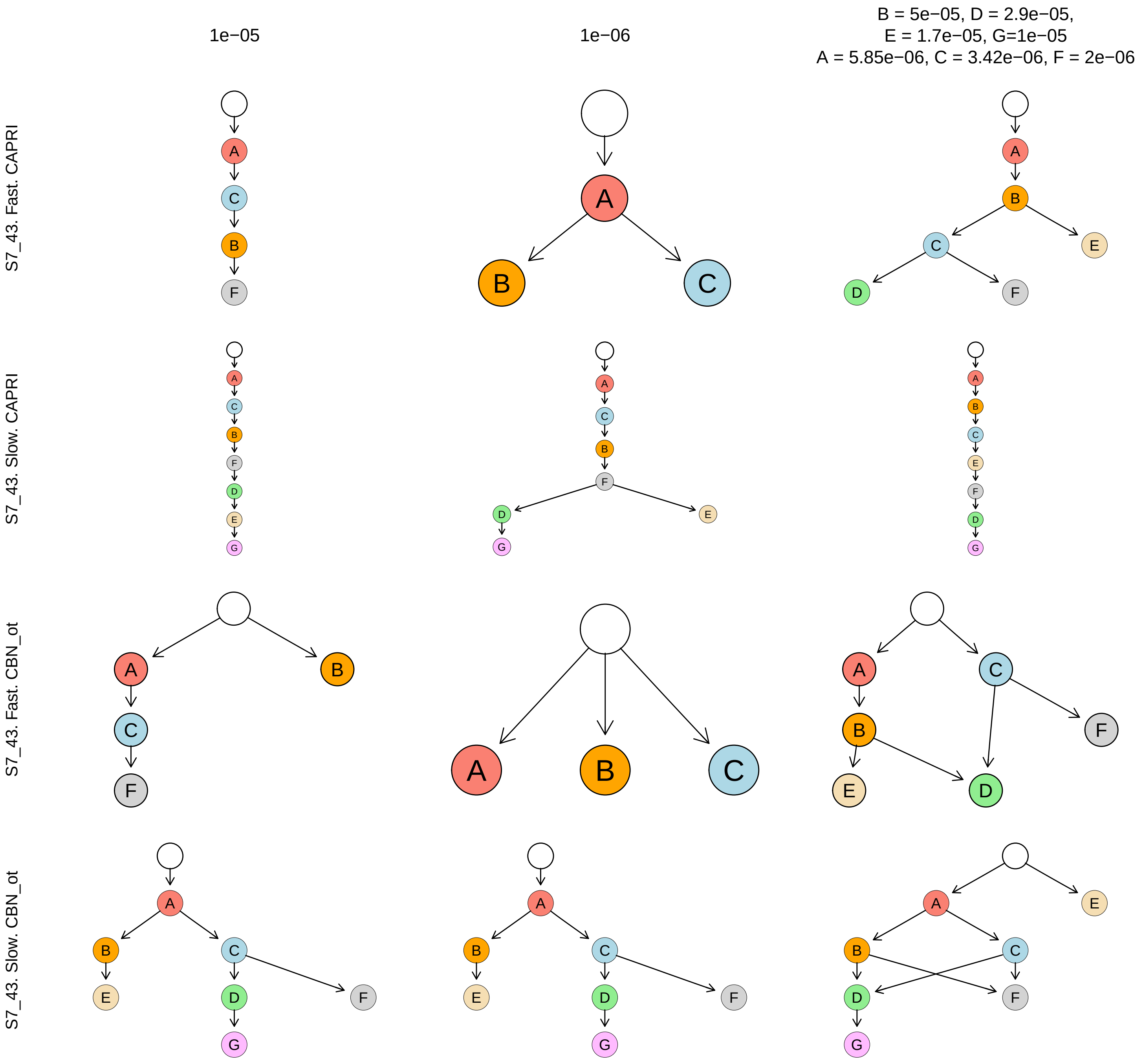

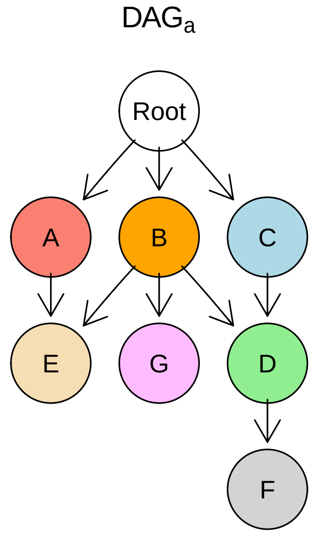

ID	Accessible Genot.	Discrep. DAG_a
S7_44	27	0

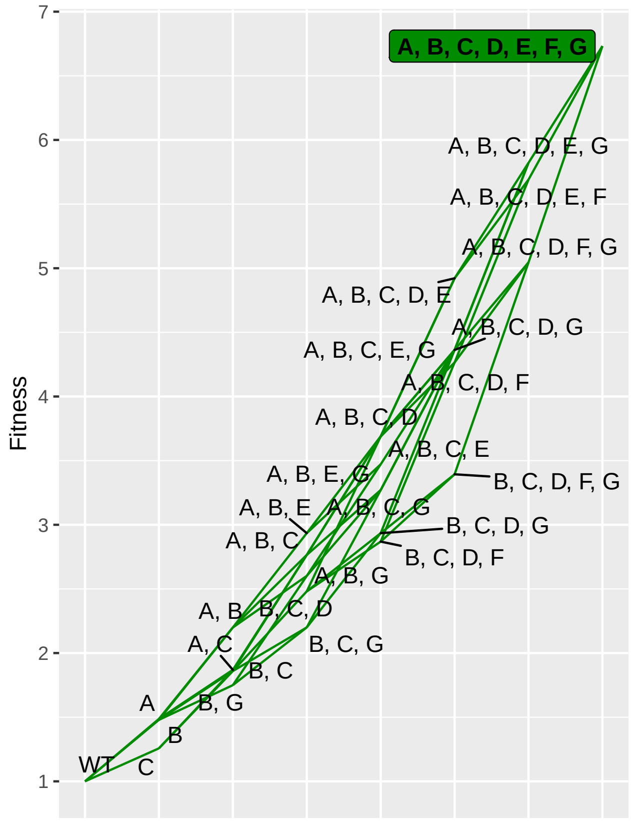

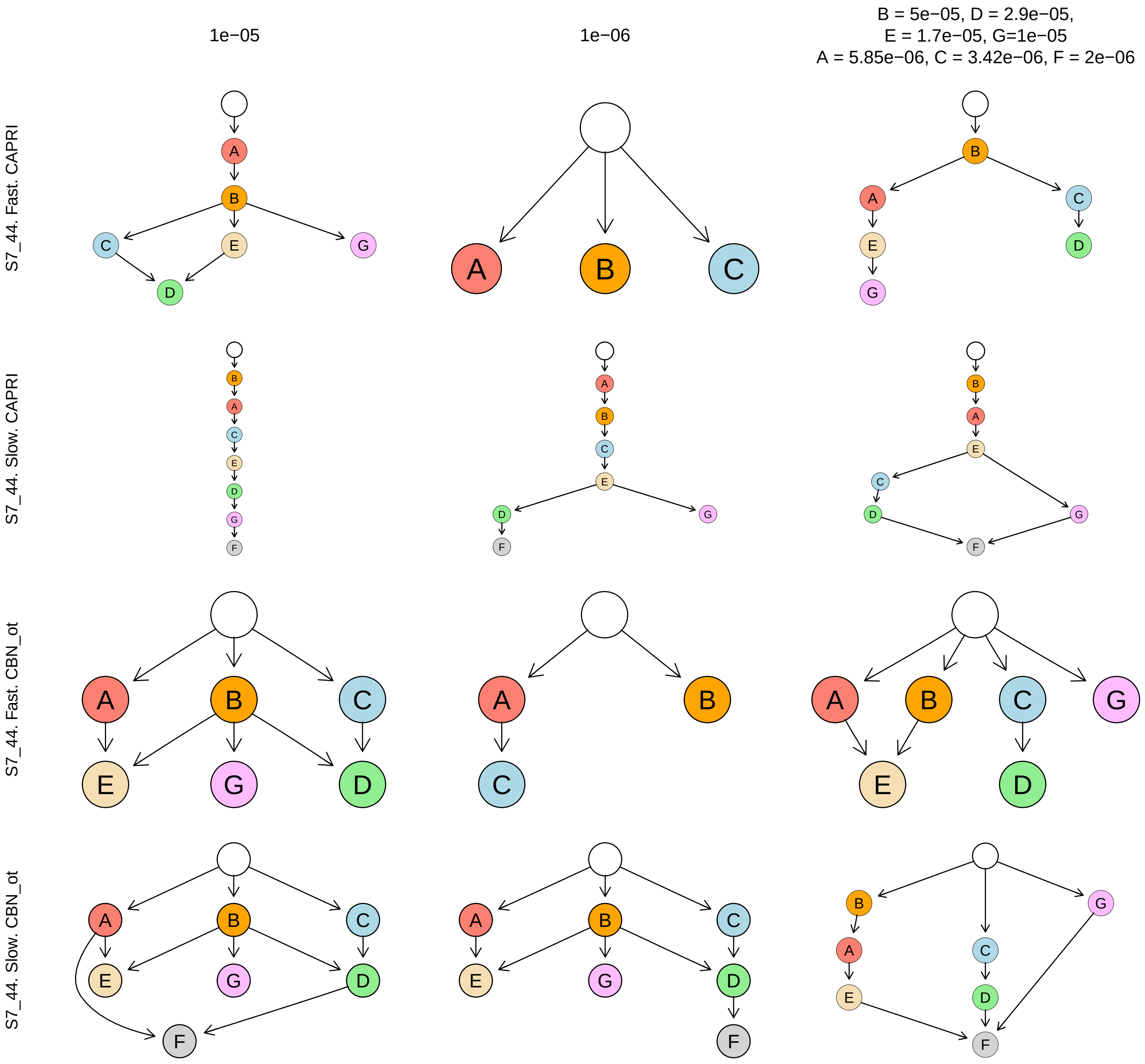

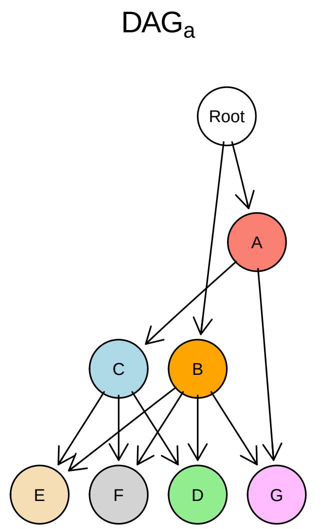

ID	Accessible Genot.	Discrep. DAG_a
S7_45	21	0

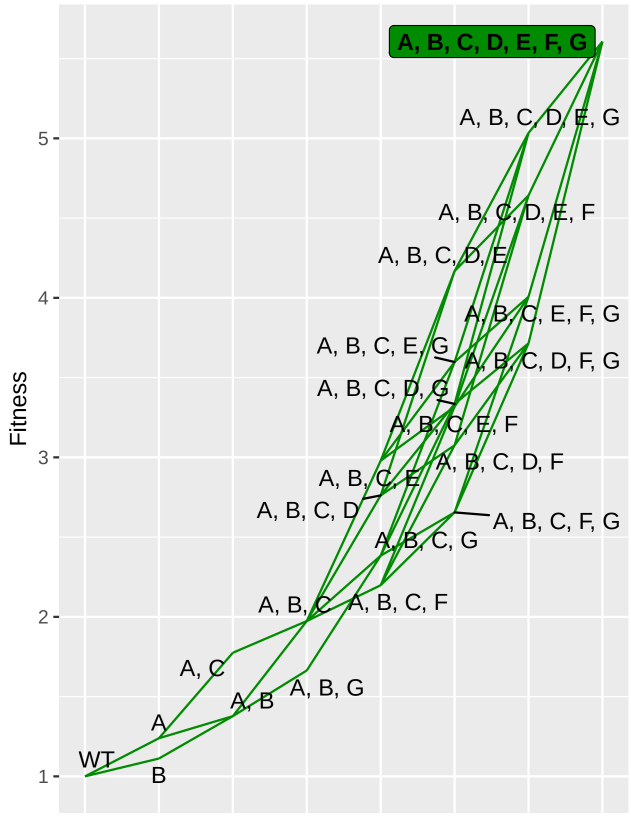

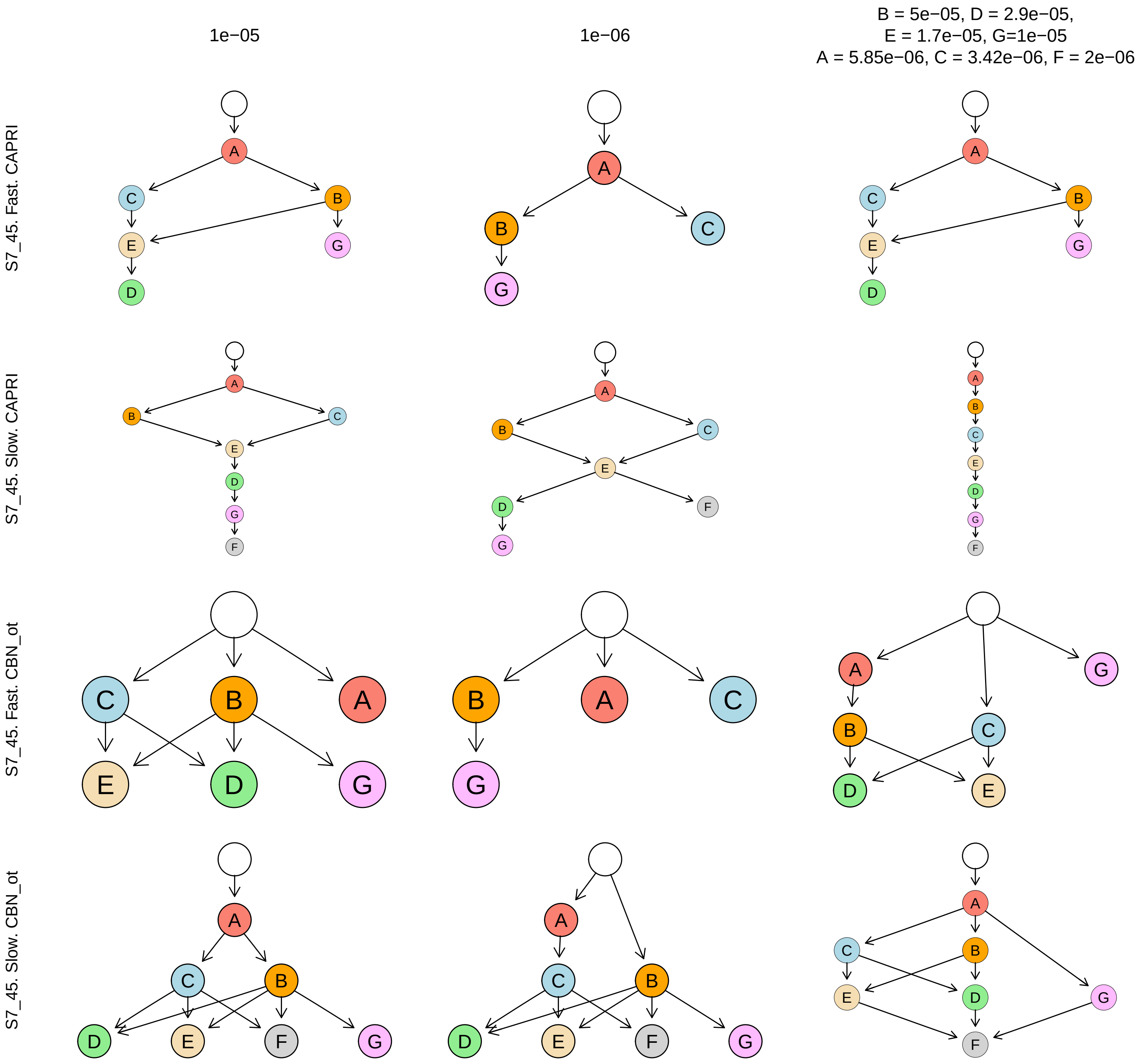

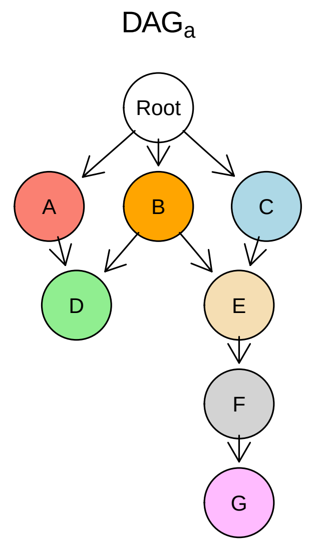

ID	Accessible Genot.	Discrep. DAG_a
S7_46	18	0

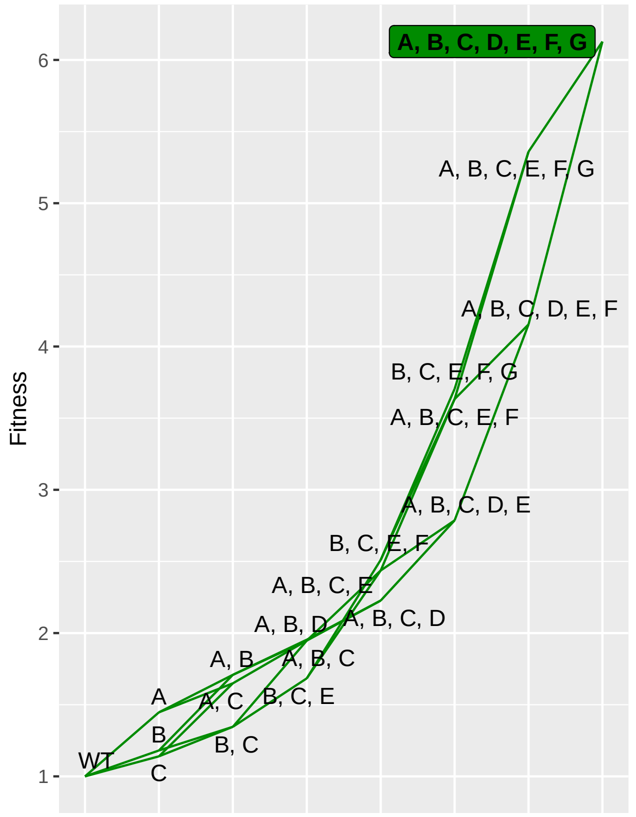

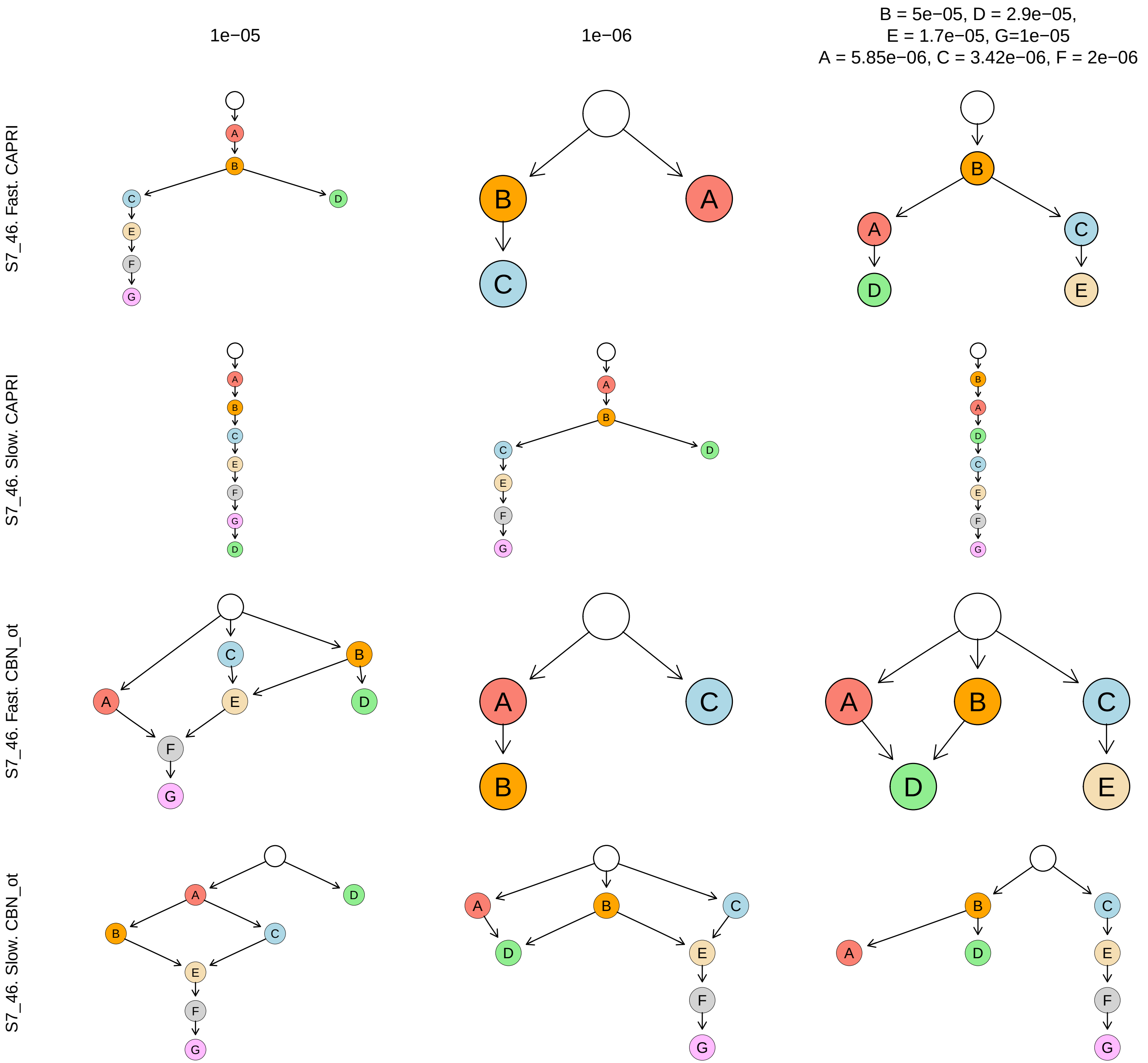

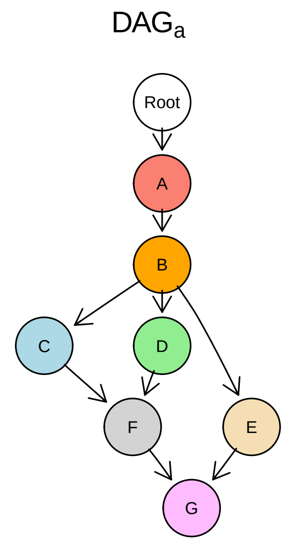

ID	Accessible Genot.	Discrep. DAG_a
S7_47	12	0

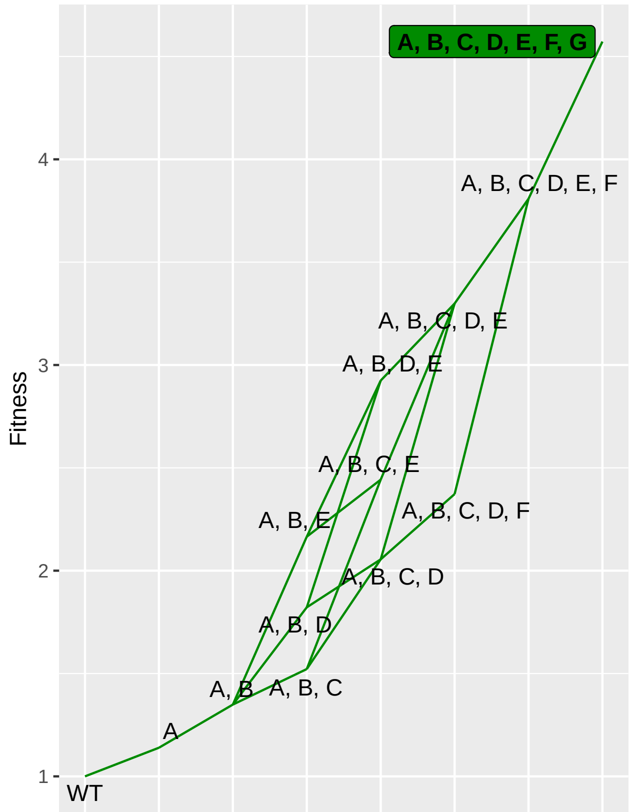

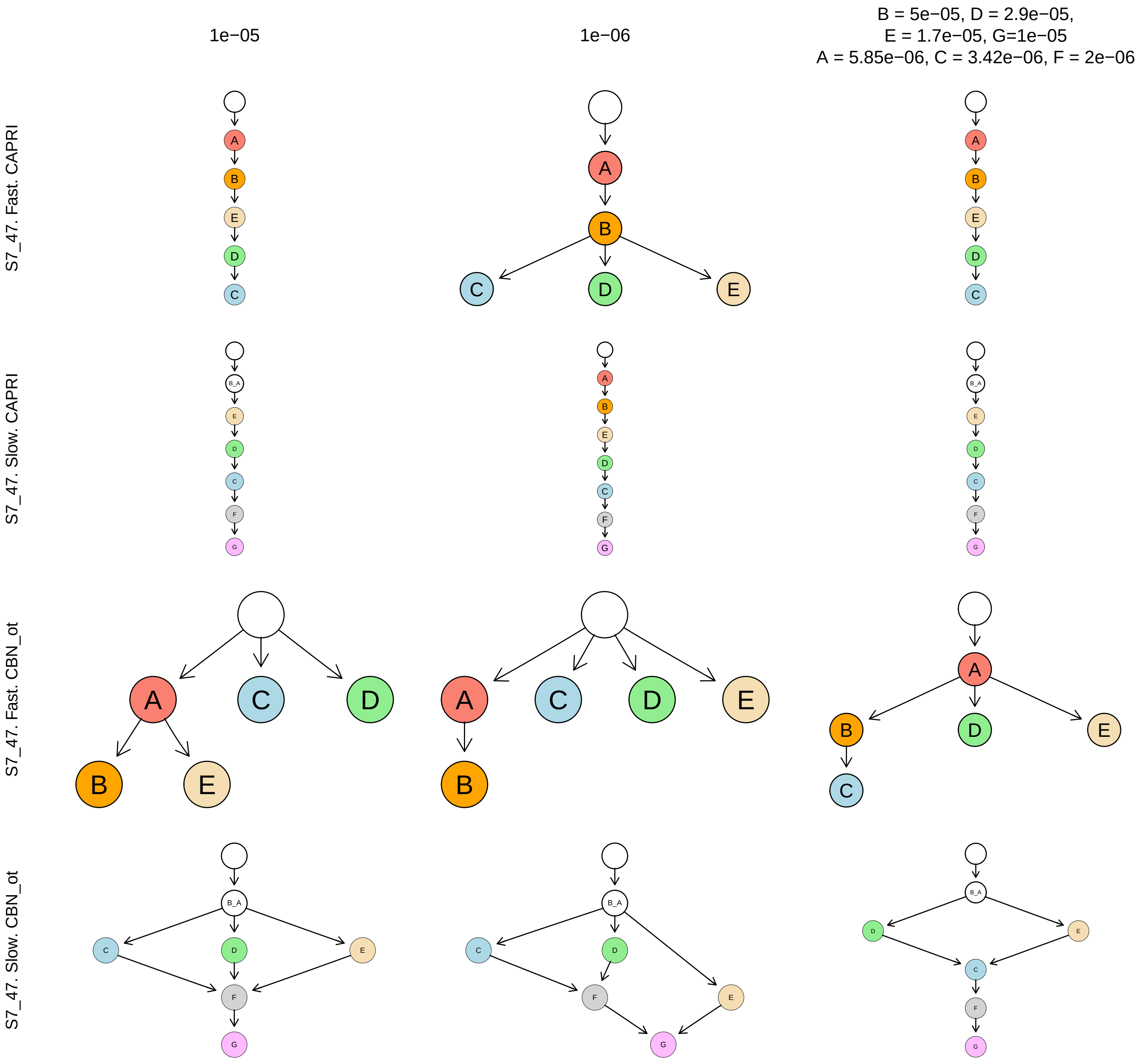

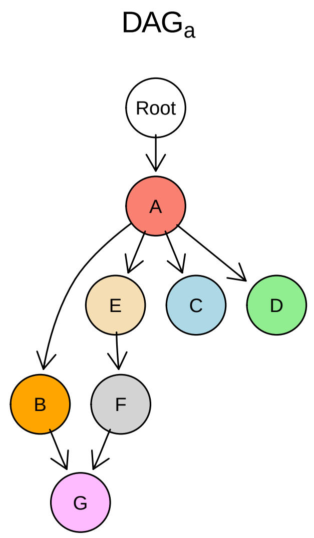

ID	Accessible Genot.	Discrep. DAG_a
S7_48	28	0

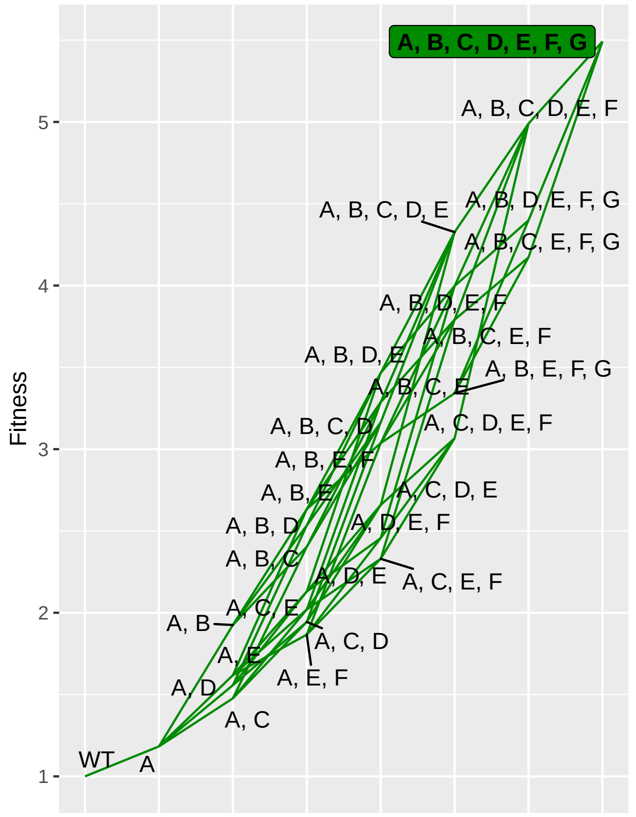

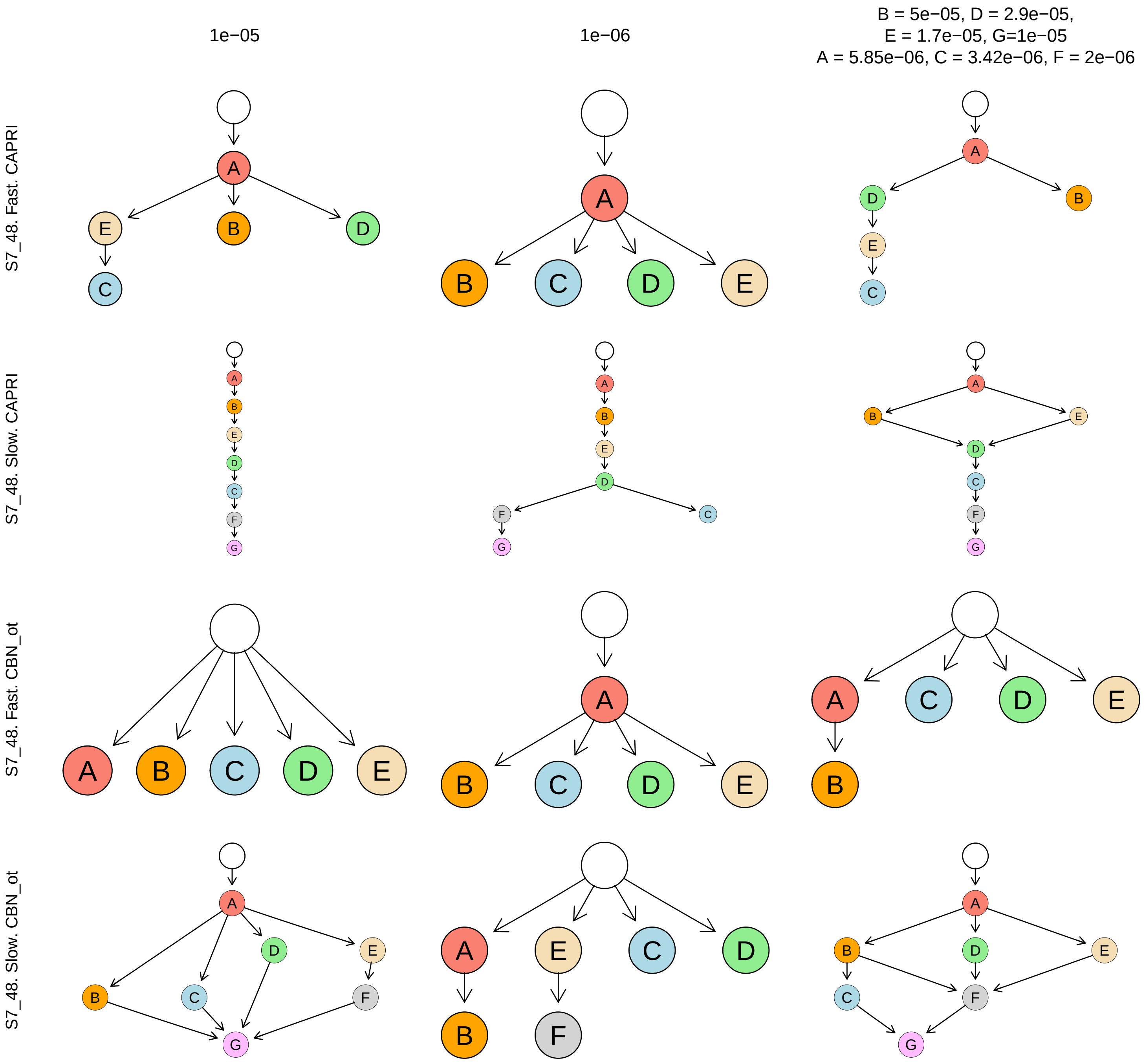

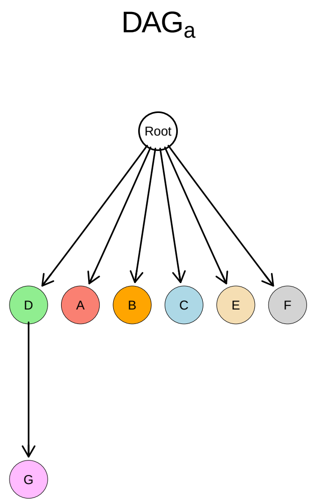

ID	Accessible Genot.	Discrep. DAG_a
S7_49	95	0

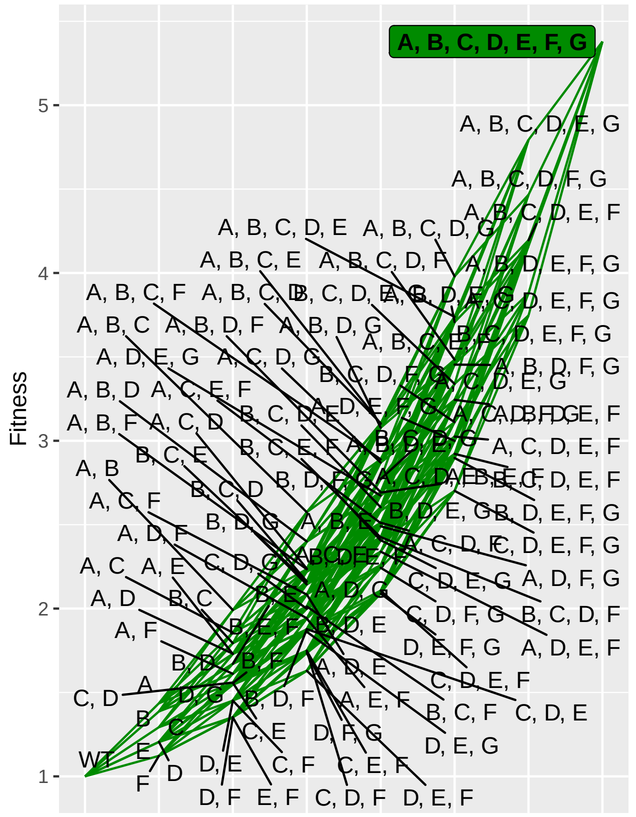

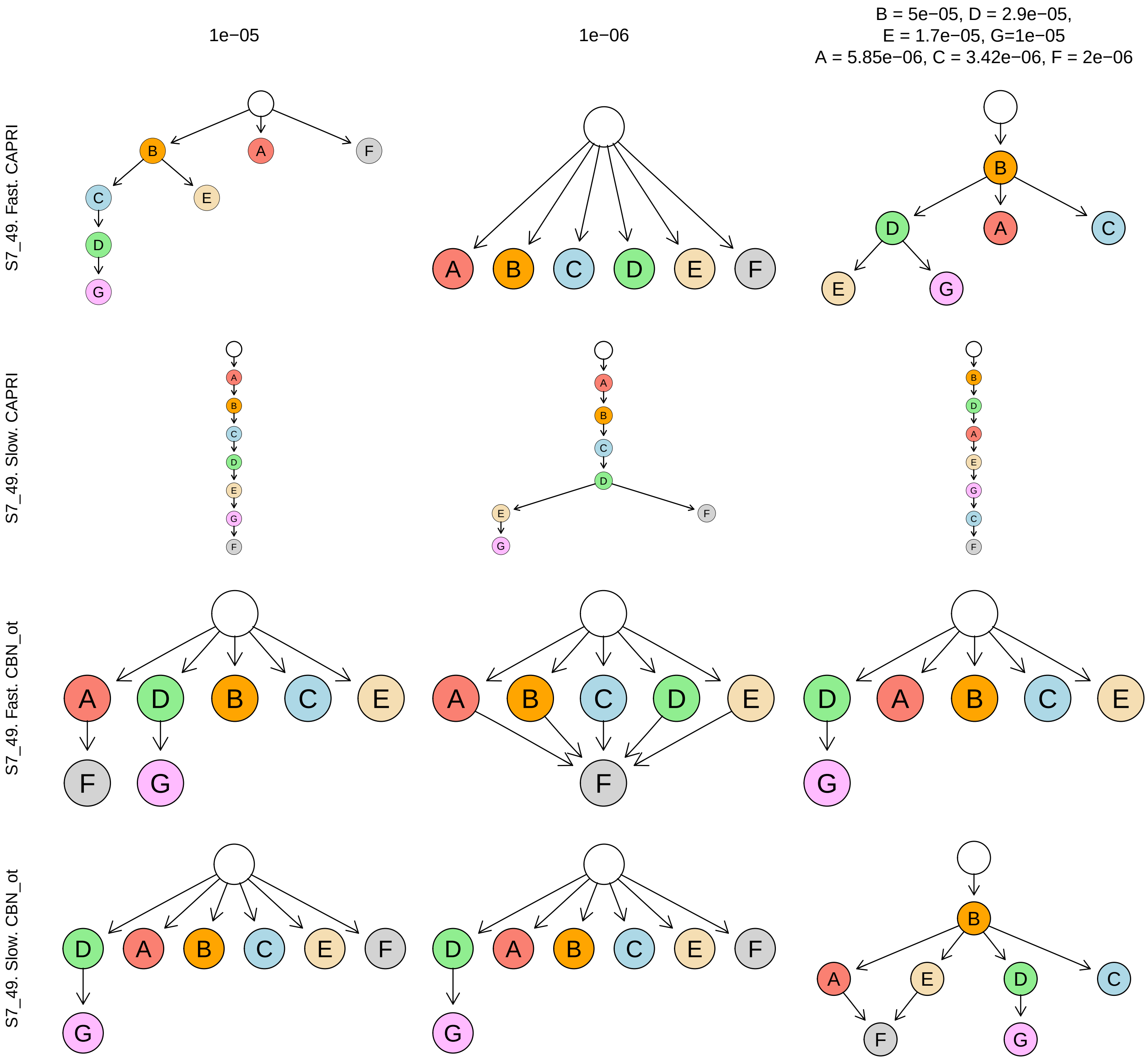

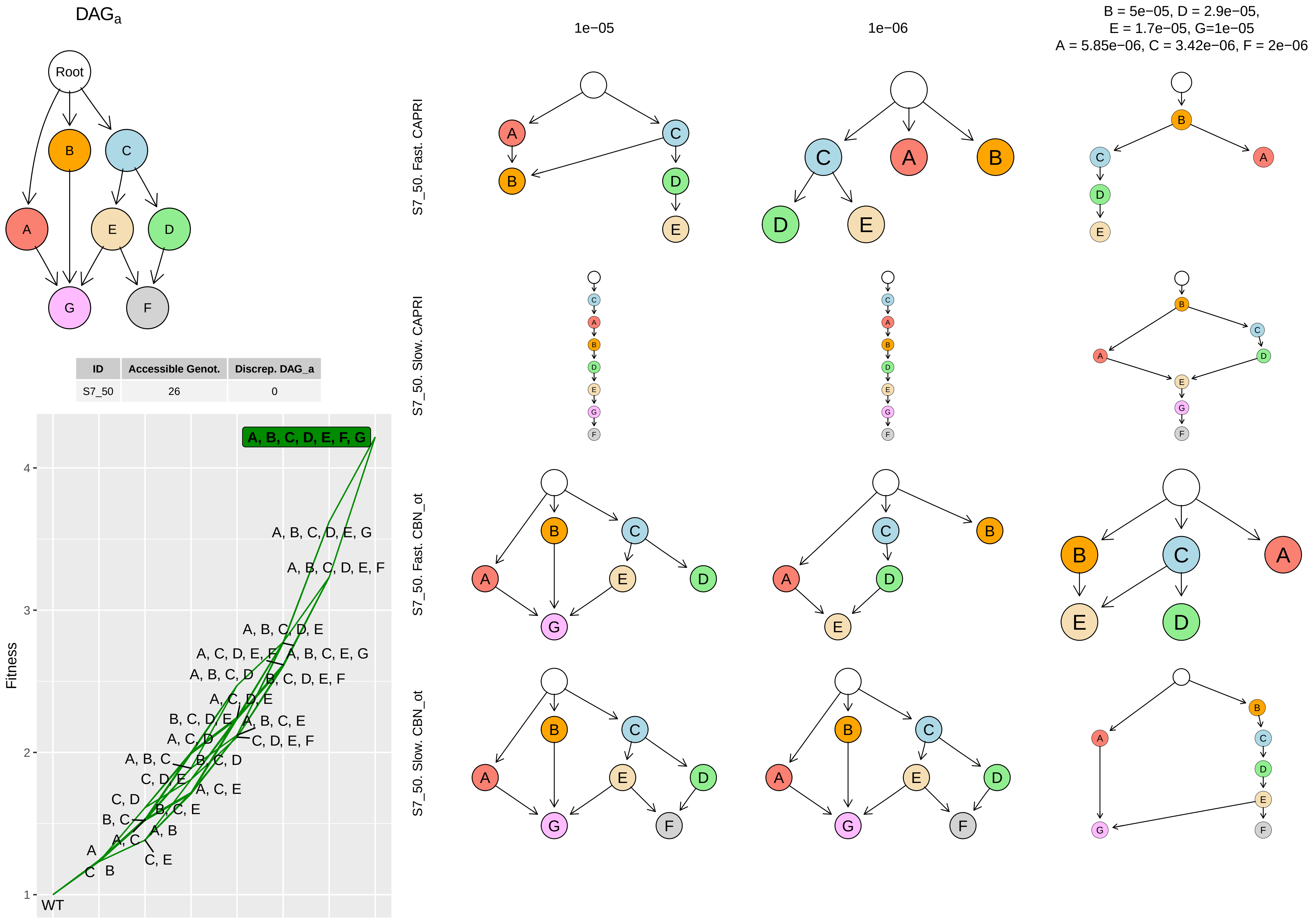

ID	Accessible Genot.	Discrep. DAG_a
S7_50	26	0

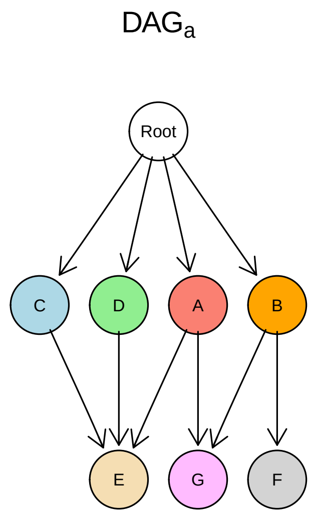

ID	Accessible Genot.	Discrep. DAG_a
S7_51	36	0

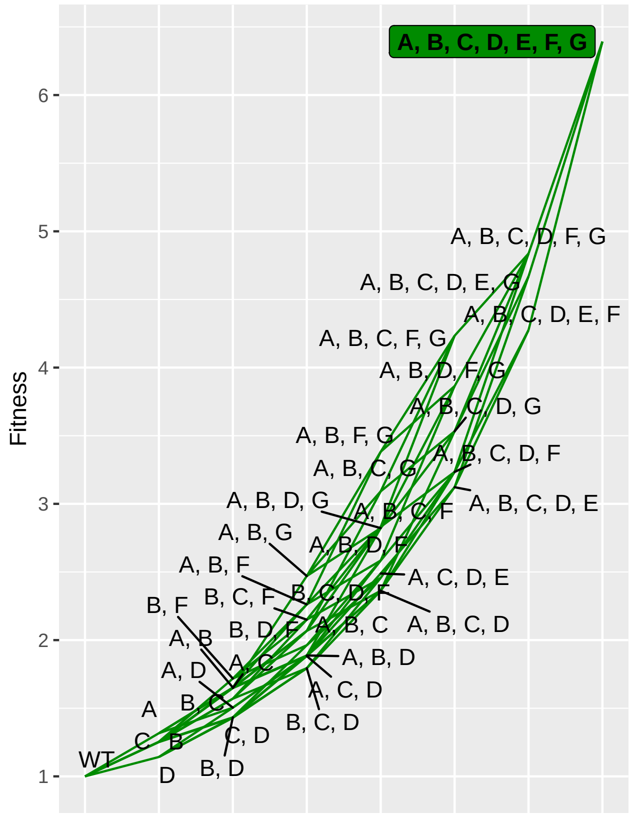

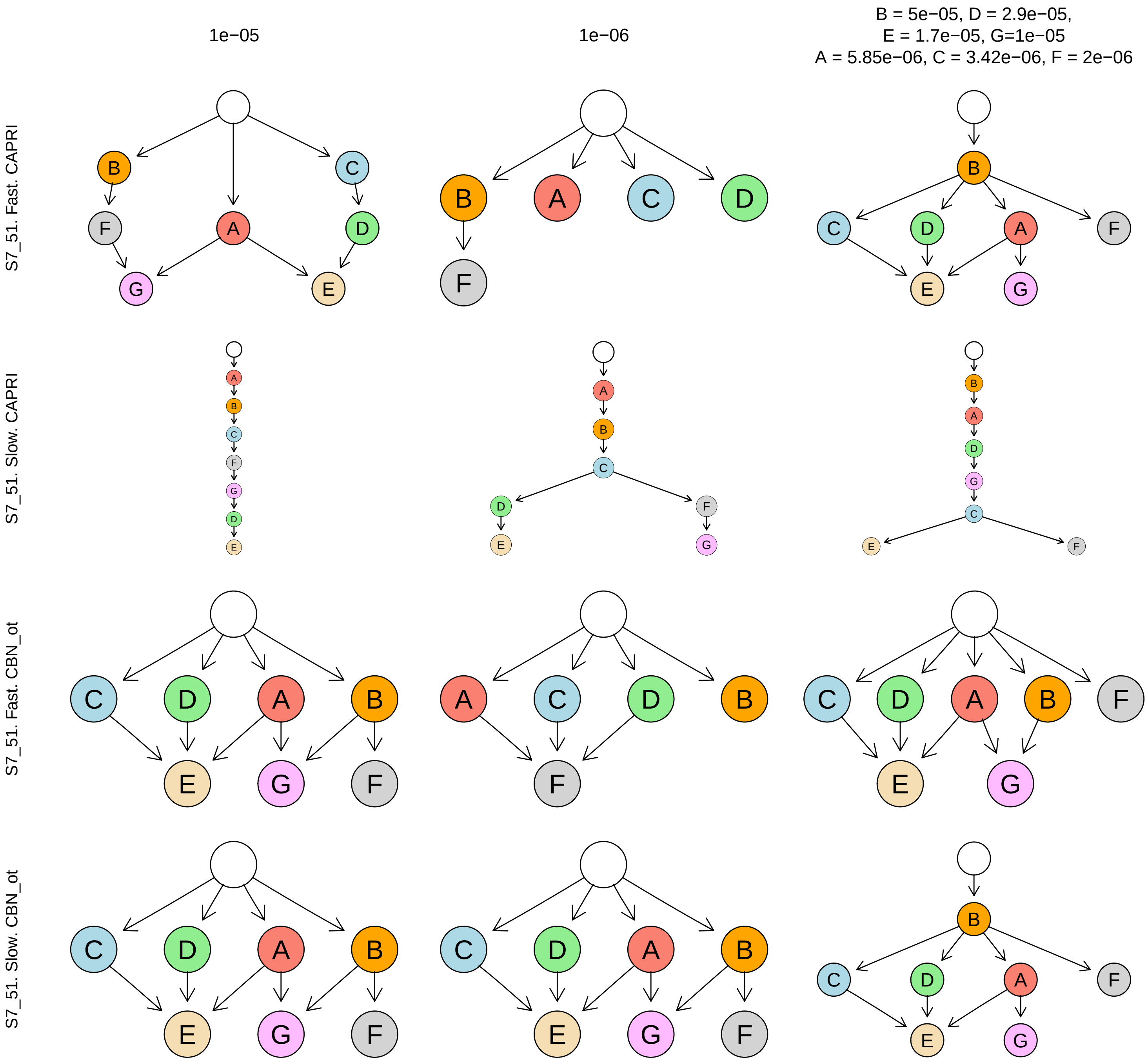

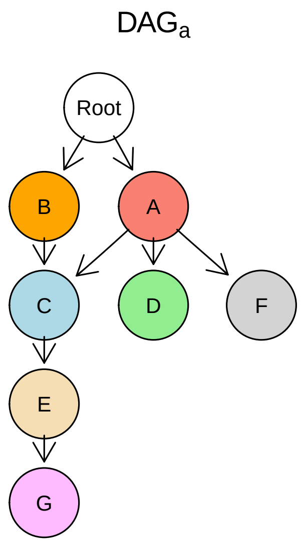

ID	Accessible Genot.	Discrep. DAG_a
S7_52	21	0

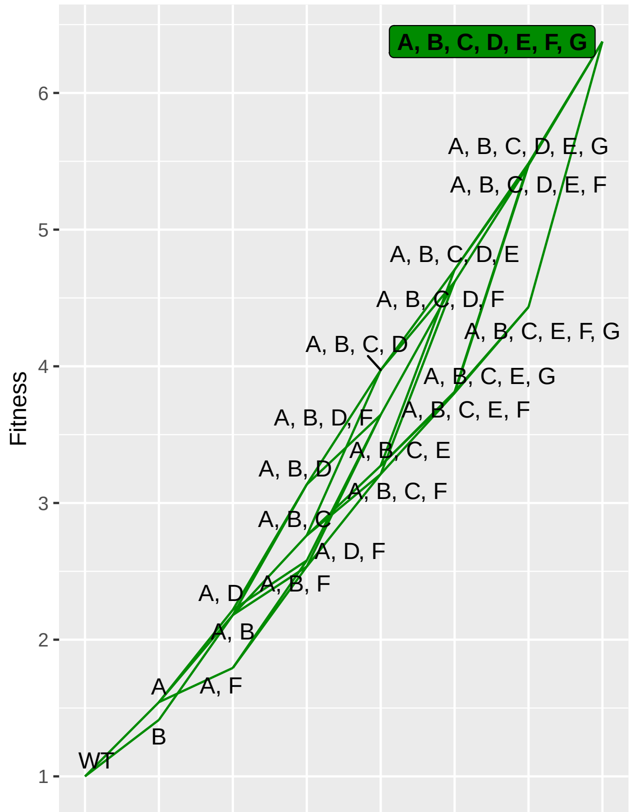

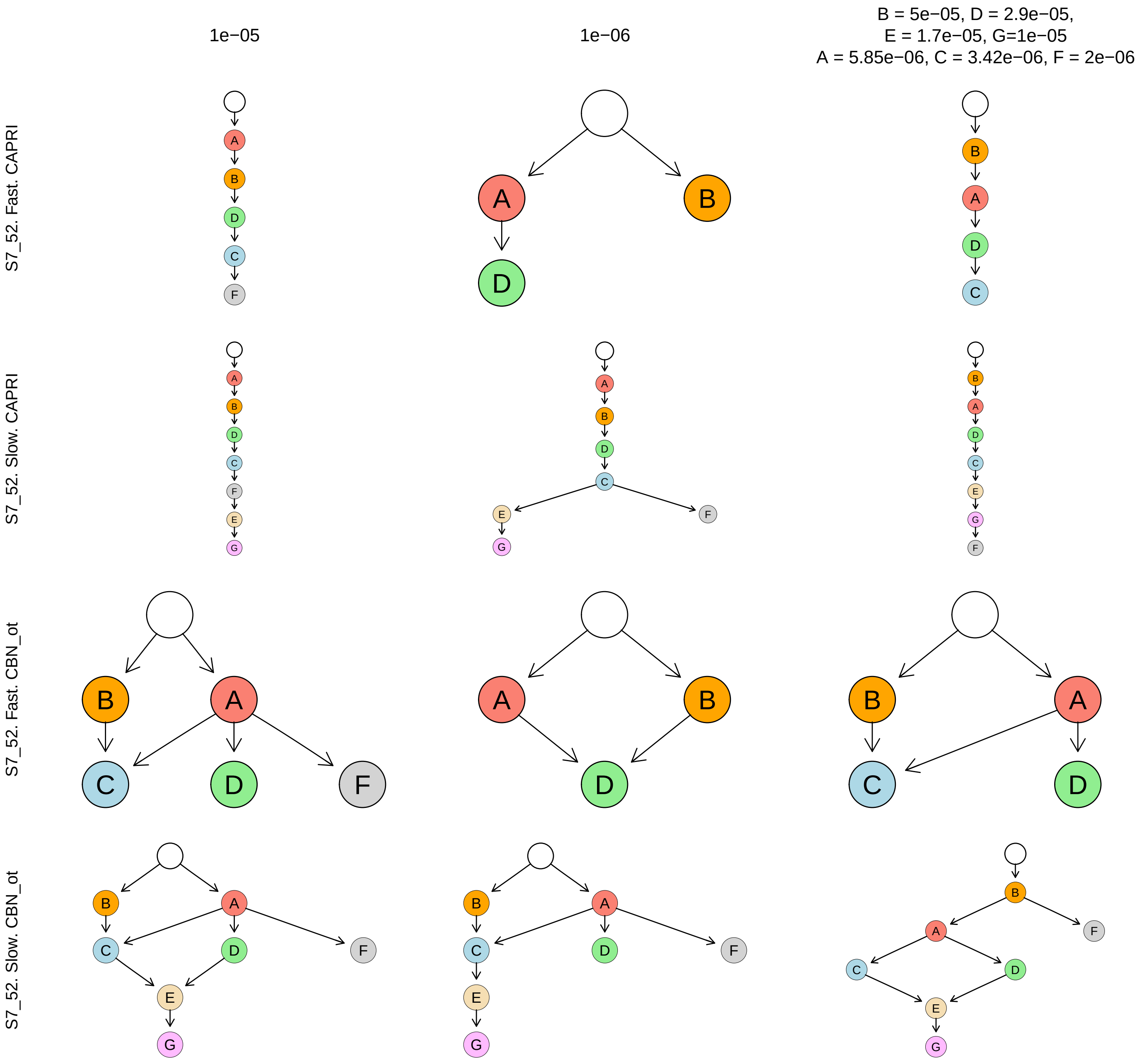

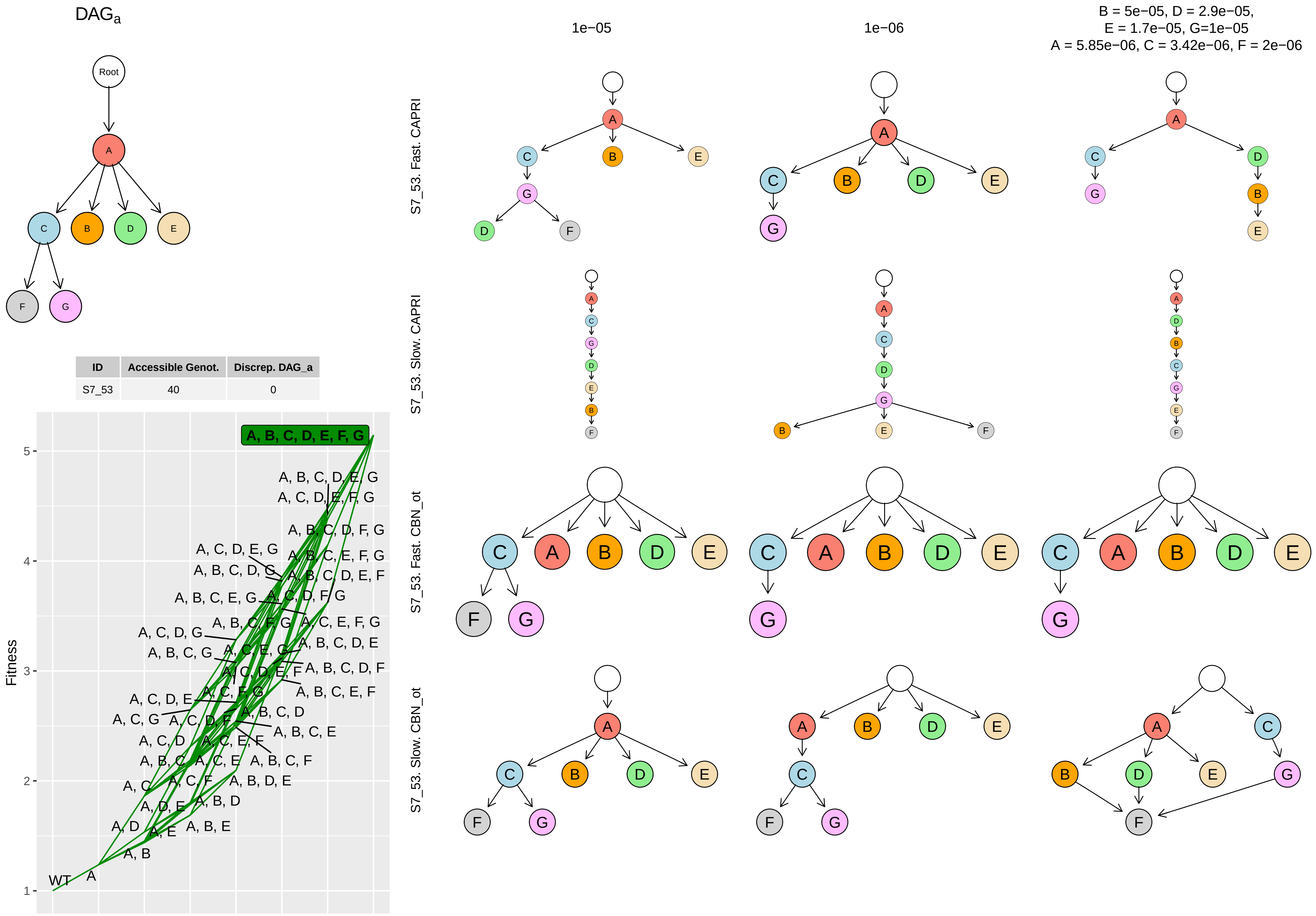

DAGa
Root
1e−05
1e−06
B = 5e−05, D = 2.9e−05,
E = 1.7e−05, G=1e−05
A = 5.85e−06, C = 3.42e−06, F = 2e−06
S7_53. Fast. CAPRI
S7_53. Slow. CAPRI
S7_53. Fast. CBN_ot
S7_53. Slow. CBN_ot
ID
Accessible Genot.
Discrep. DAG_a
S7_53
40
0
Fitness
A, B, C, D, E, F, G
WT

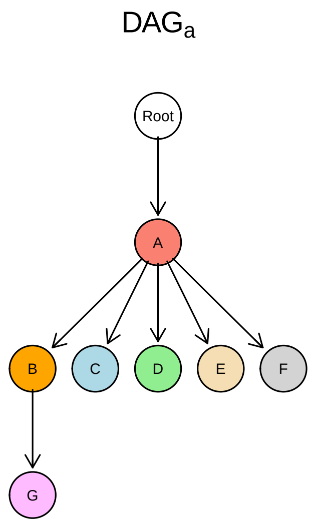

ID	Accessible Genot.	Discrep. DAG_a
S7_54	48	0

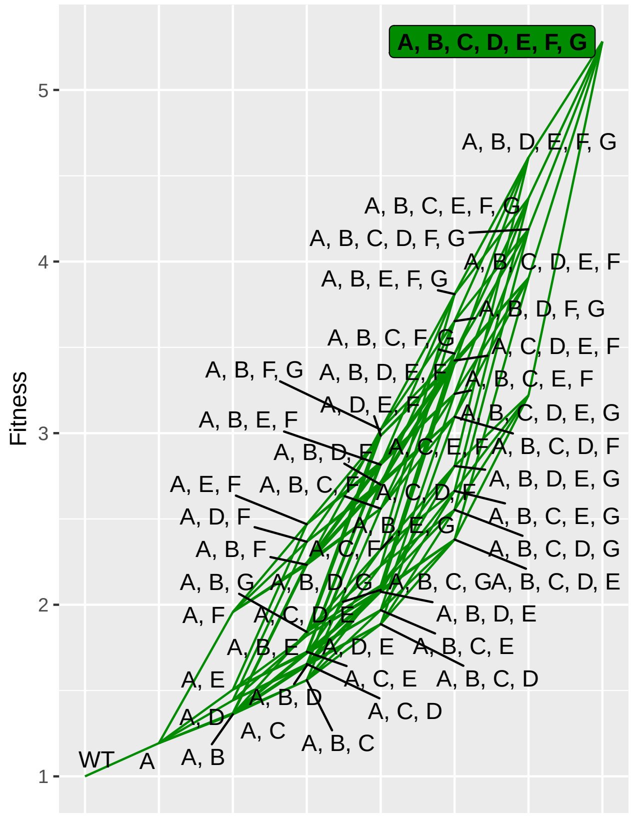

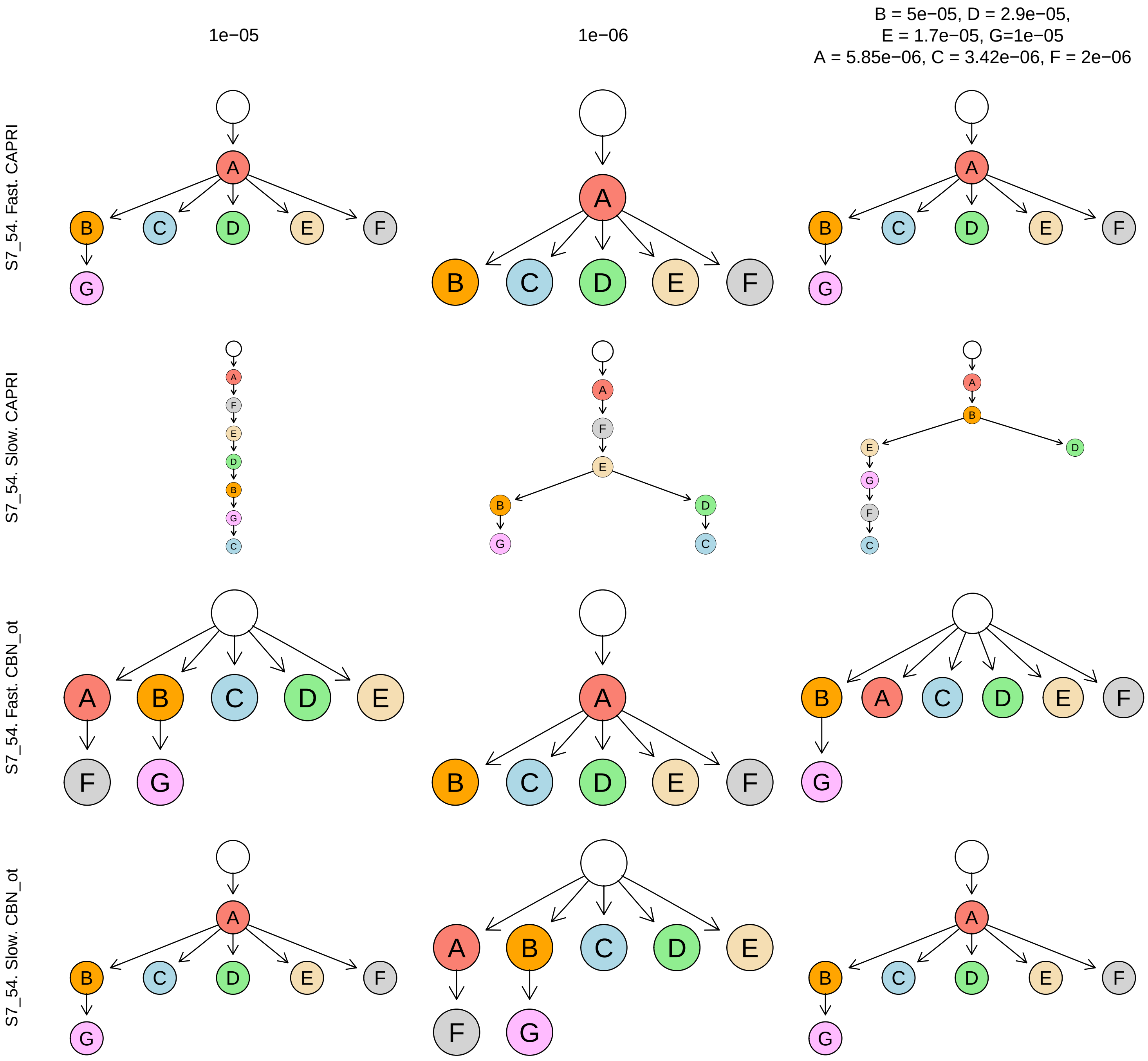

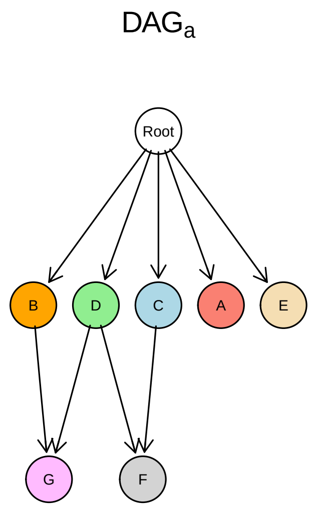

ID	Accessible Genot.	Discrep. DAG_a
S7_55	51	0

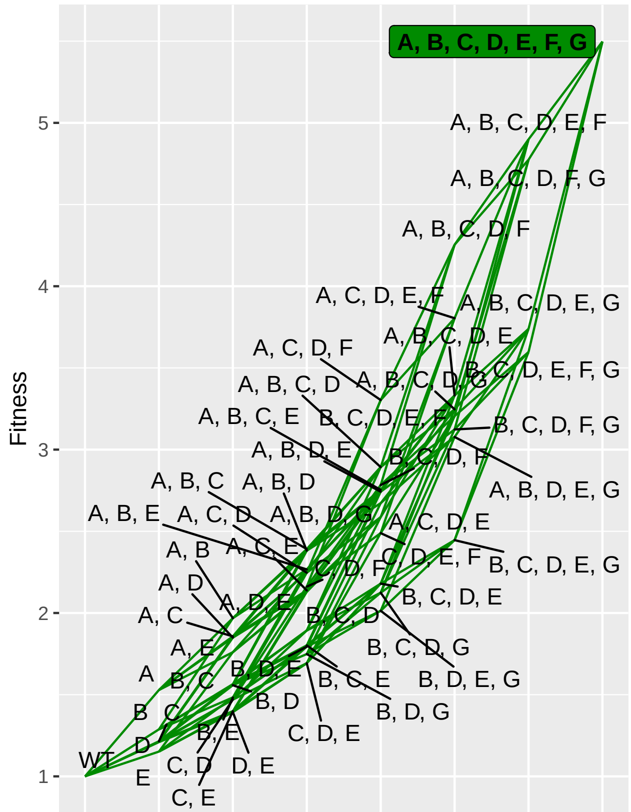

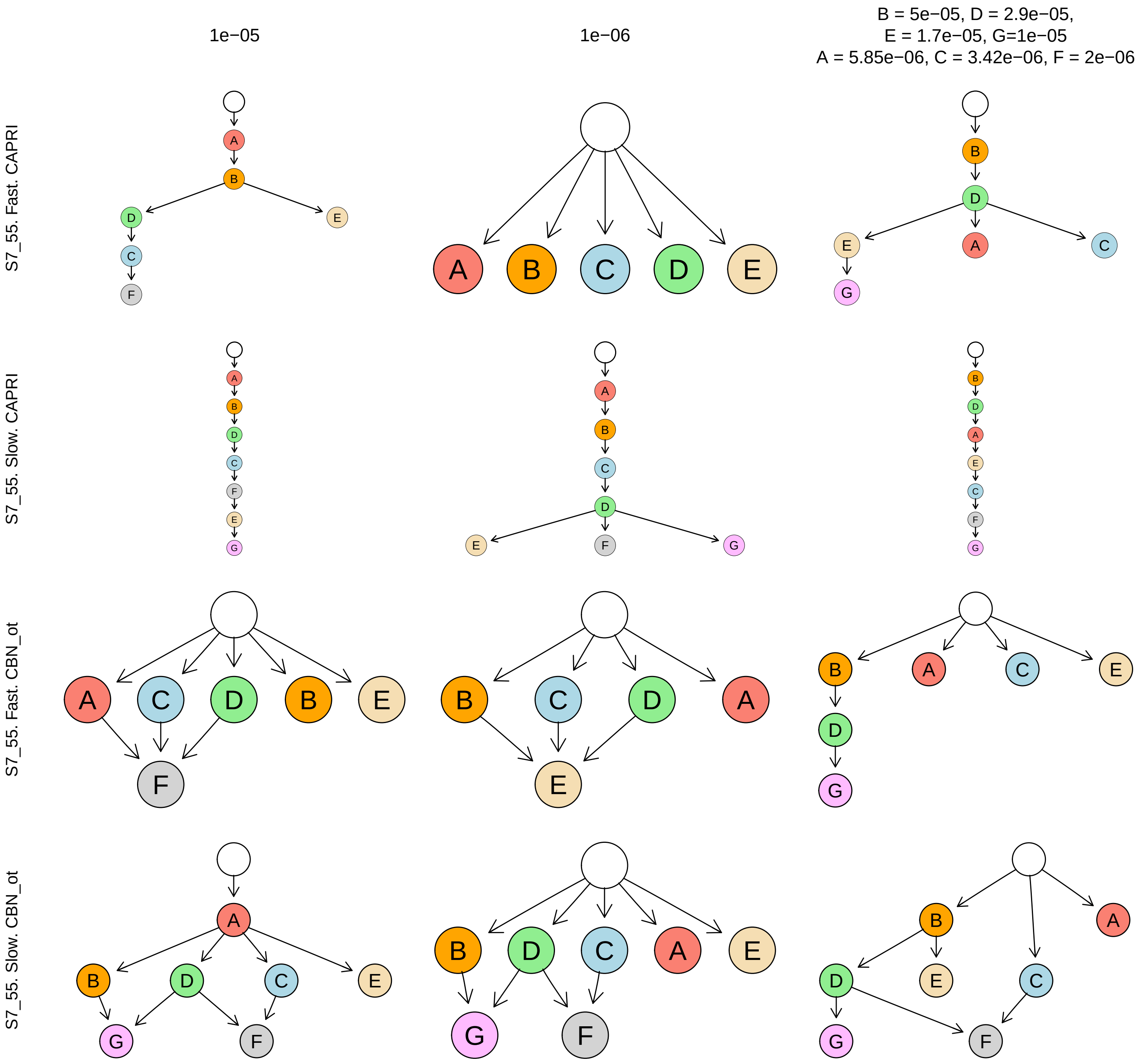

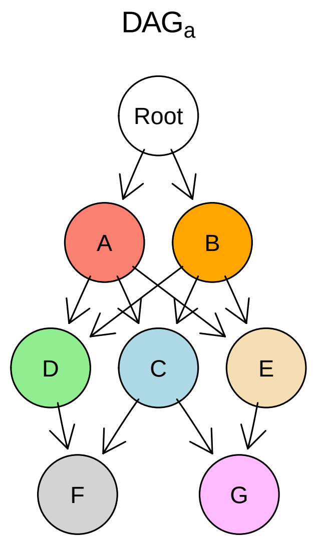

ID	Accessible Genot.	Discrep. DAG_a
S7_56	15	0

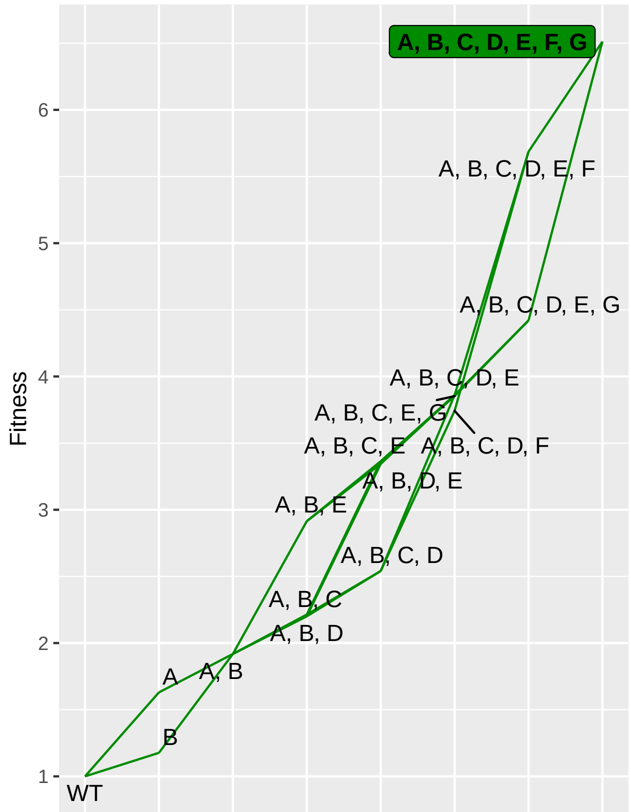

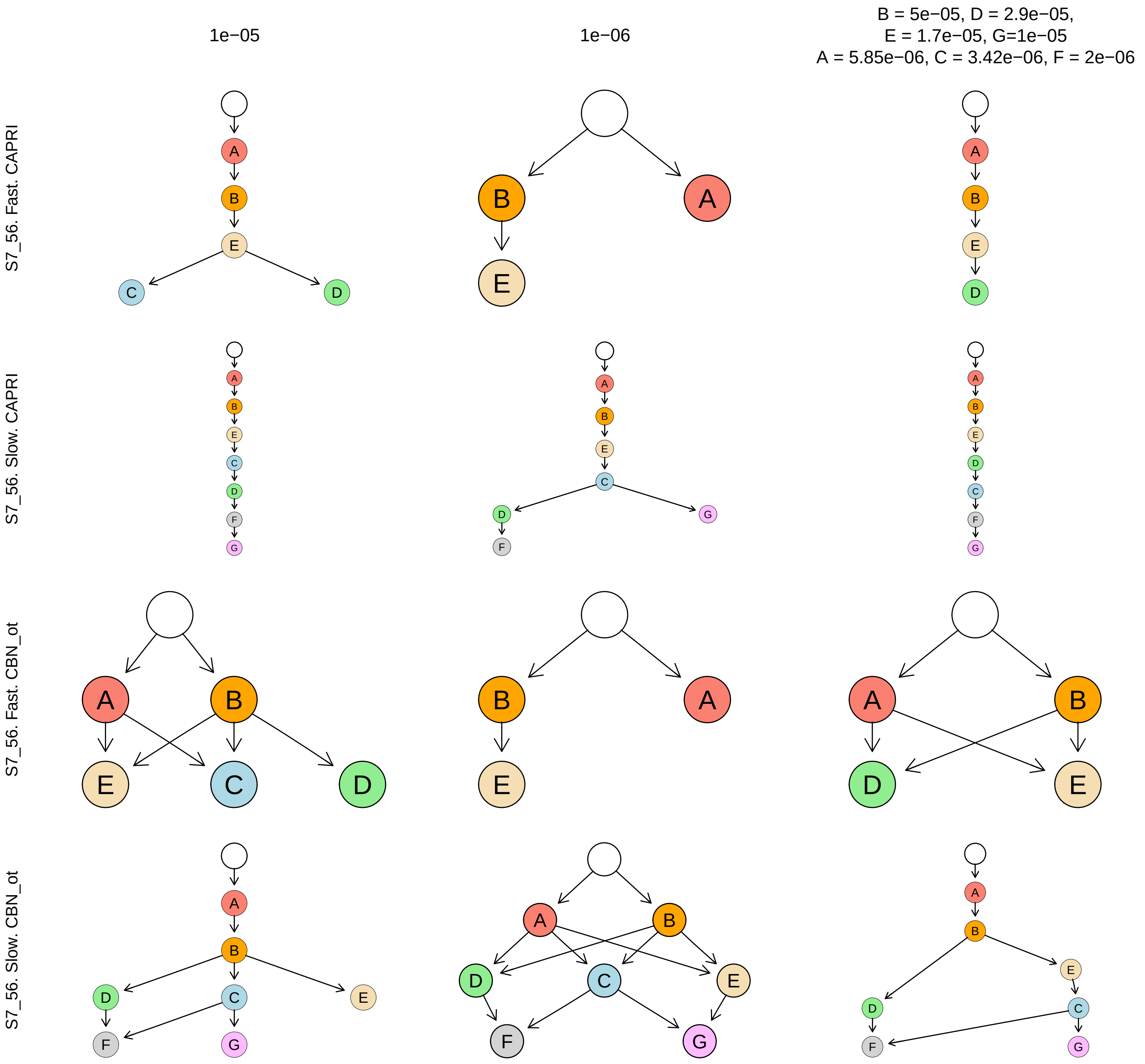

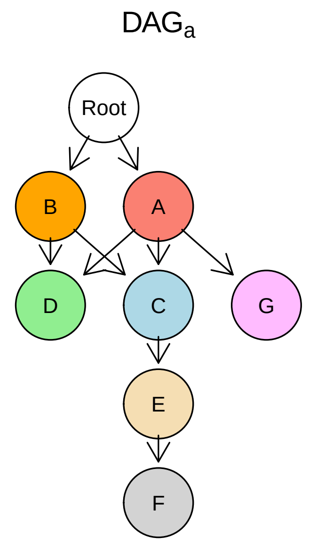

ID	Accessible Genot.	Discrep. DAG_a
S7_57	19	0

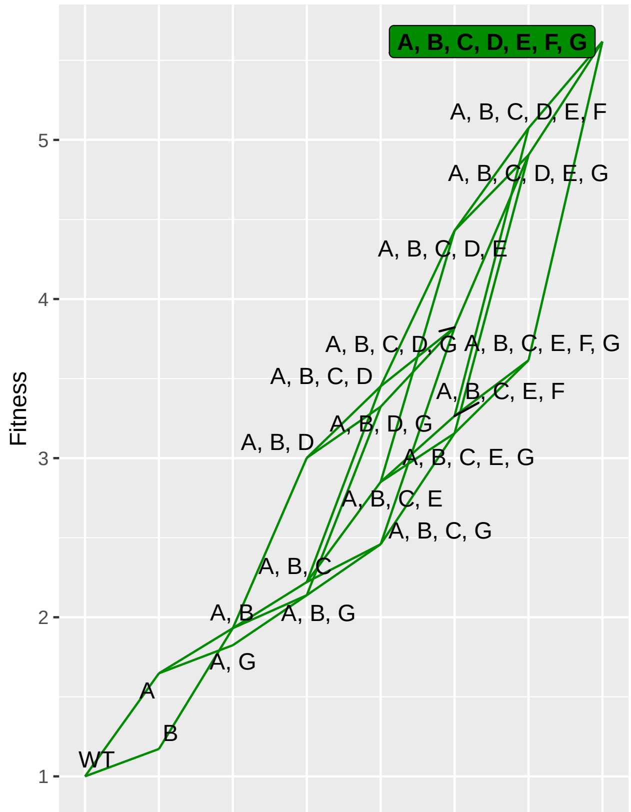

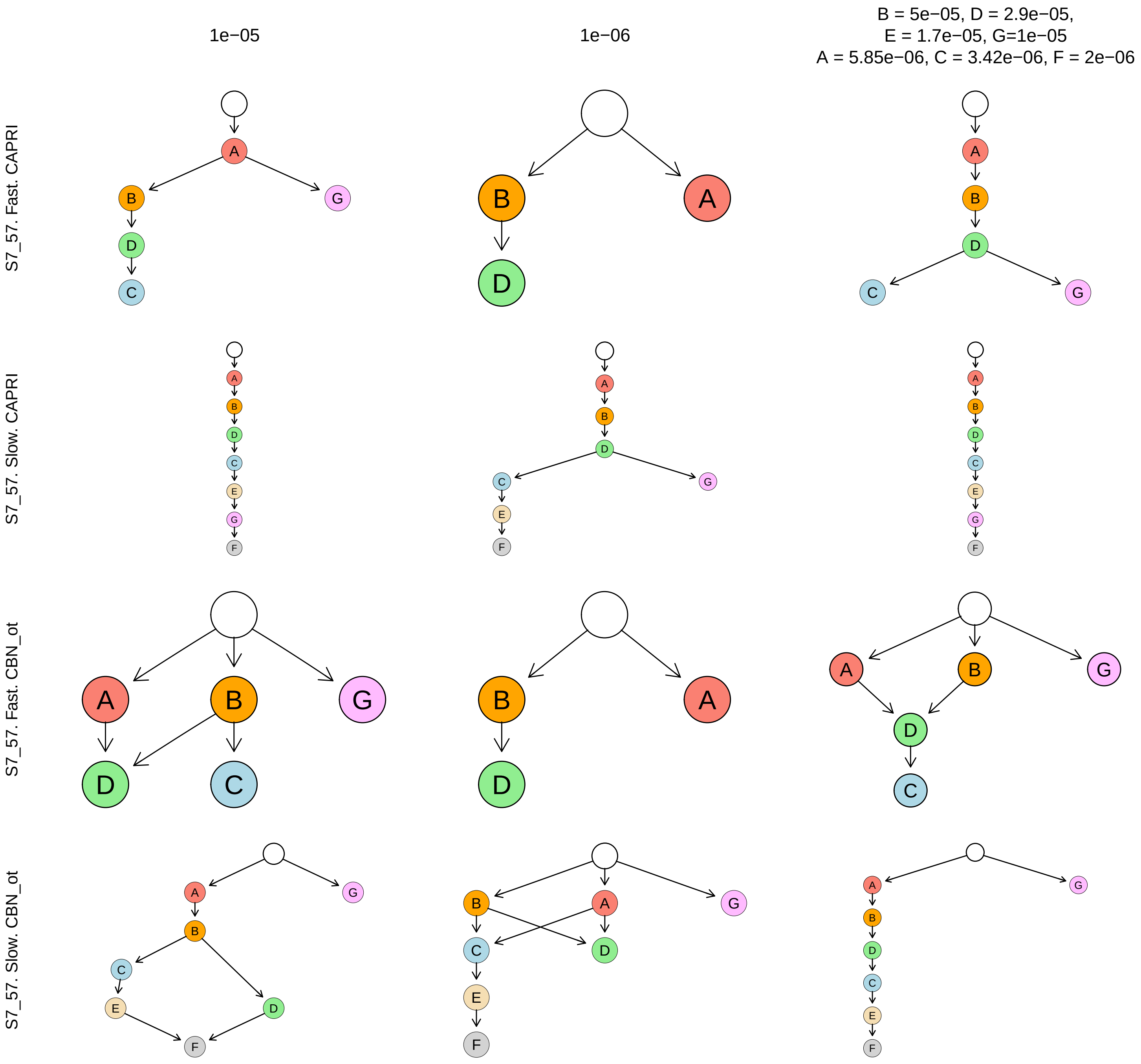

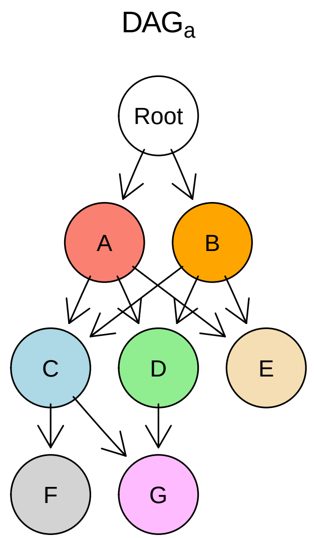

ID	Accessible Genot.	Discrep. DAG_a
S7_58	18	0

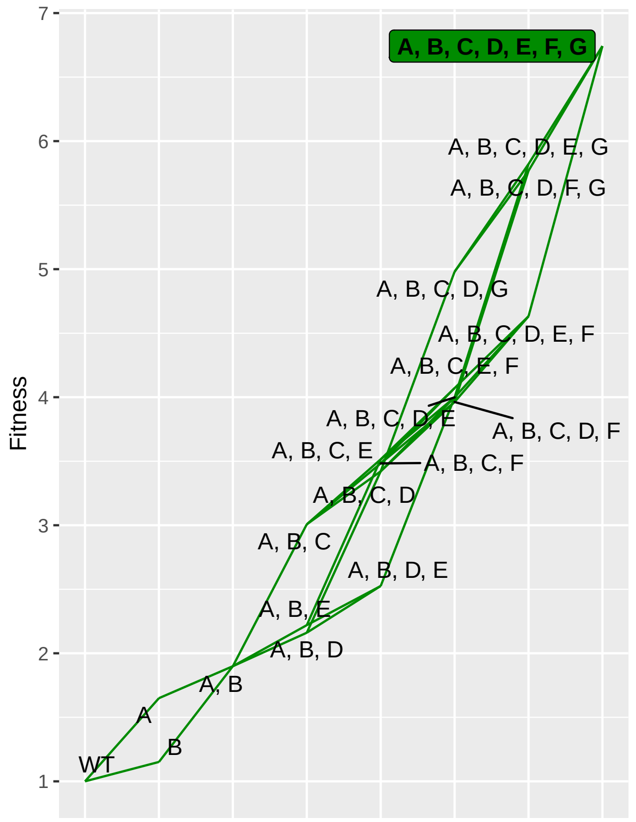

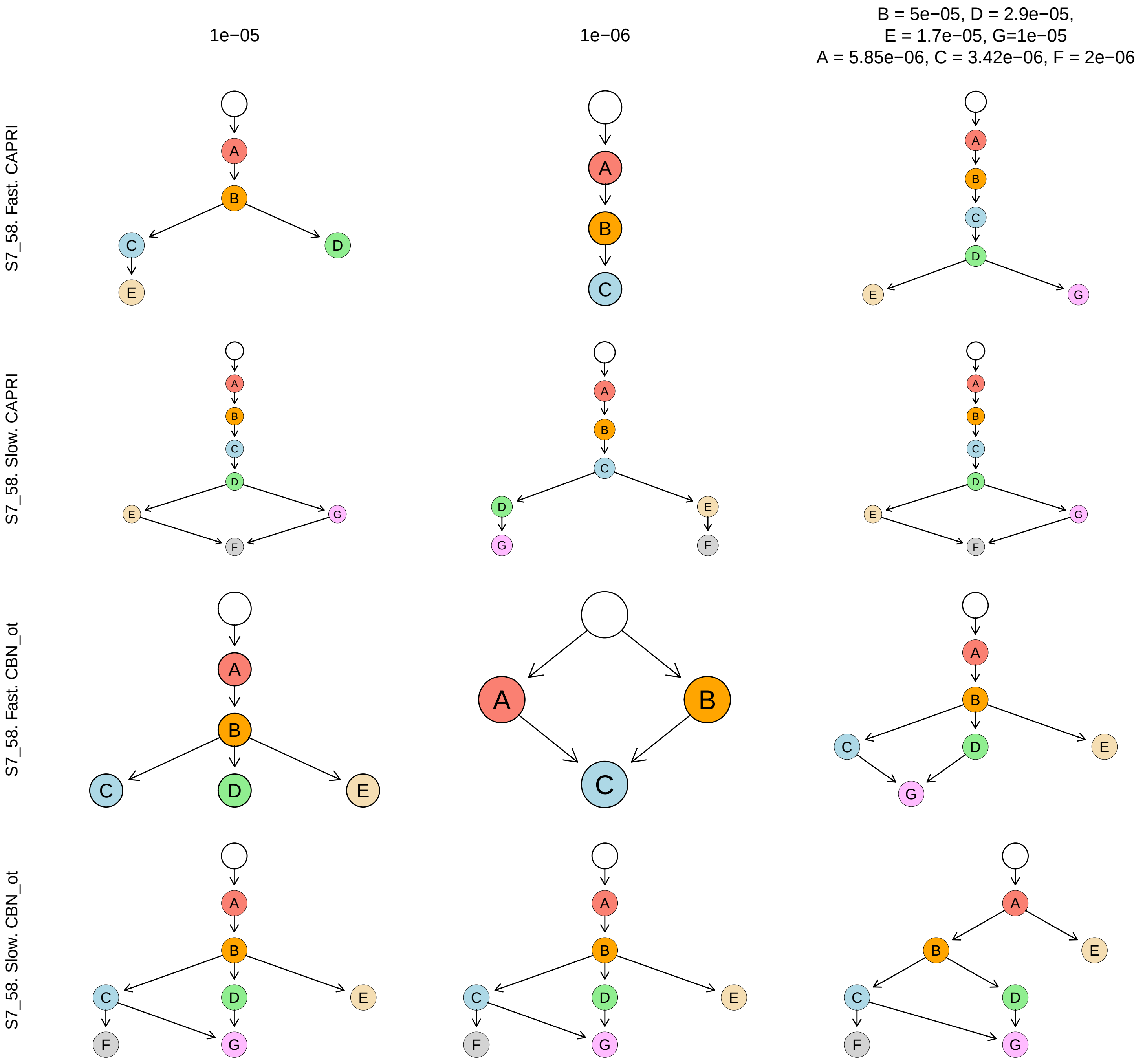

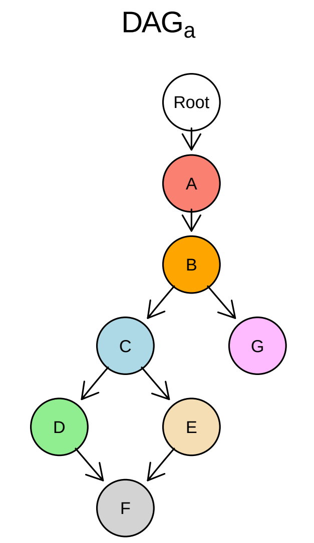

ID	Accessible Genot.	Discrep. DAG_a
S7_59	13	0

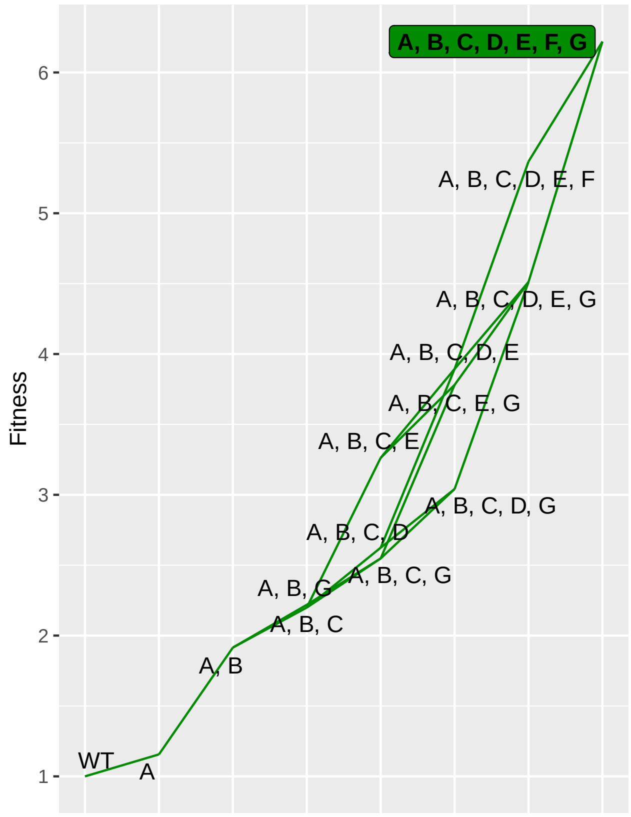

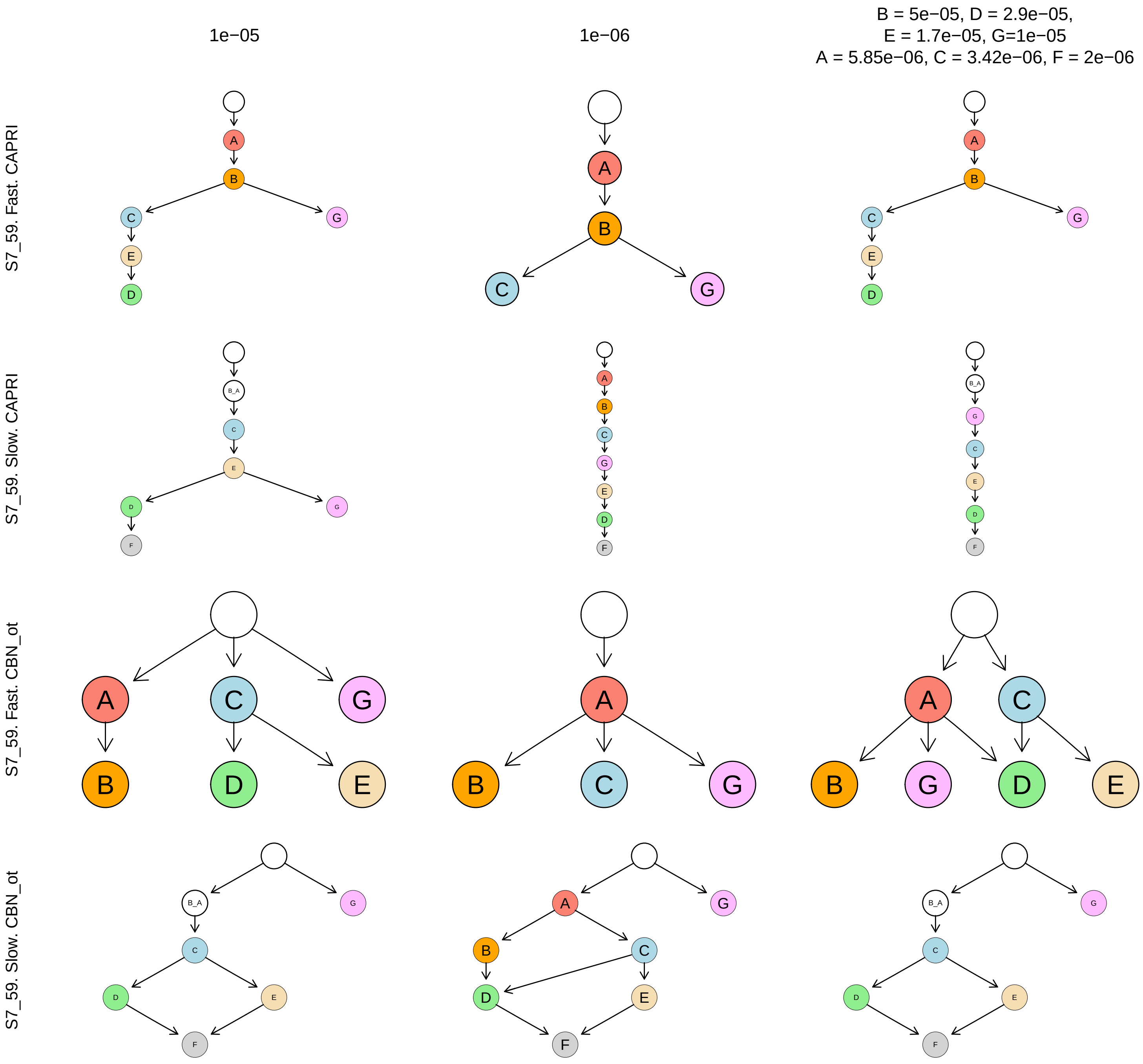

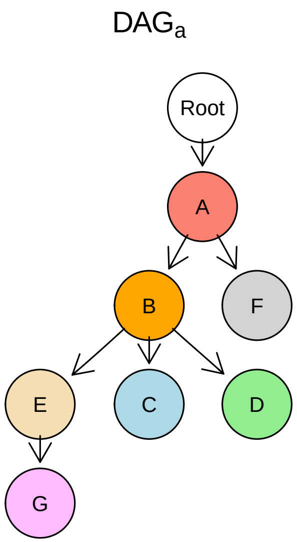

ID	Accessible Genot.	Discrep. DAG_a
S7_60	26	0

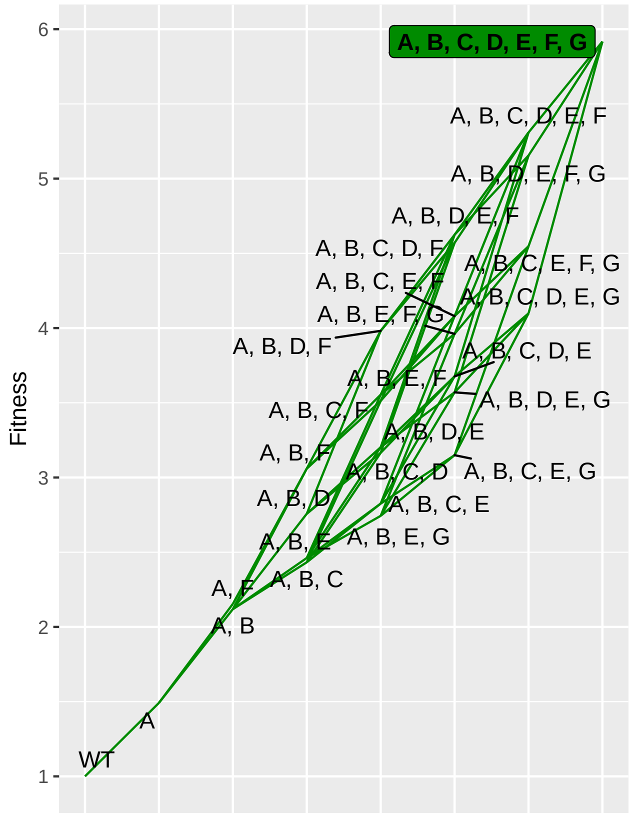

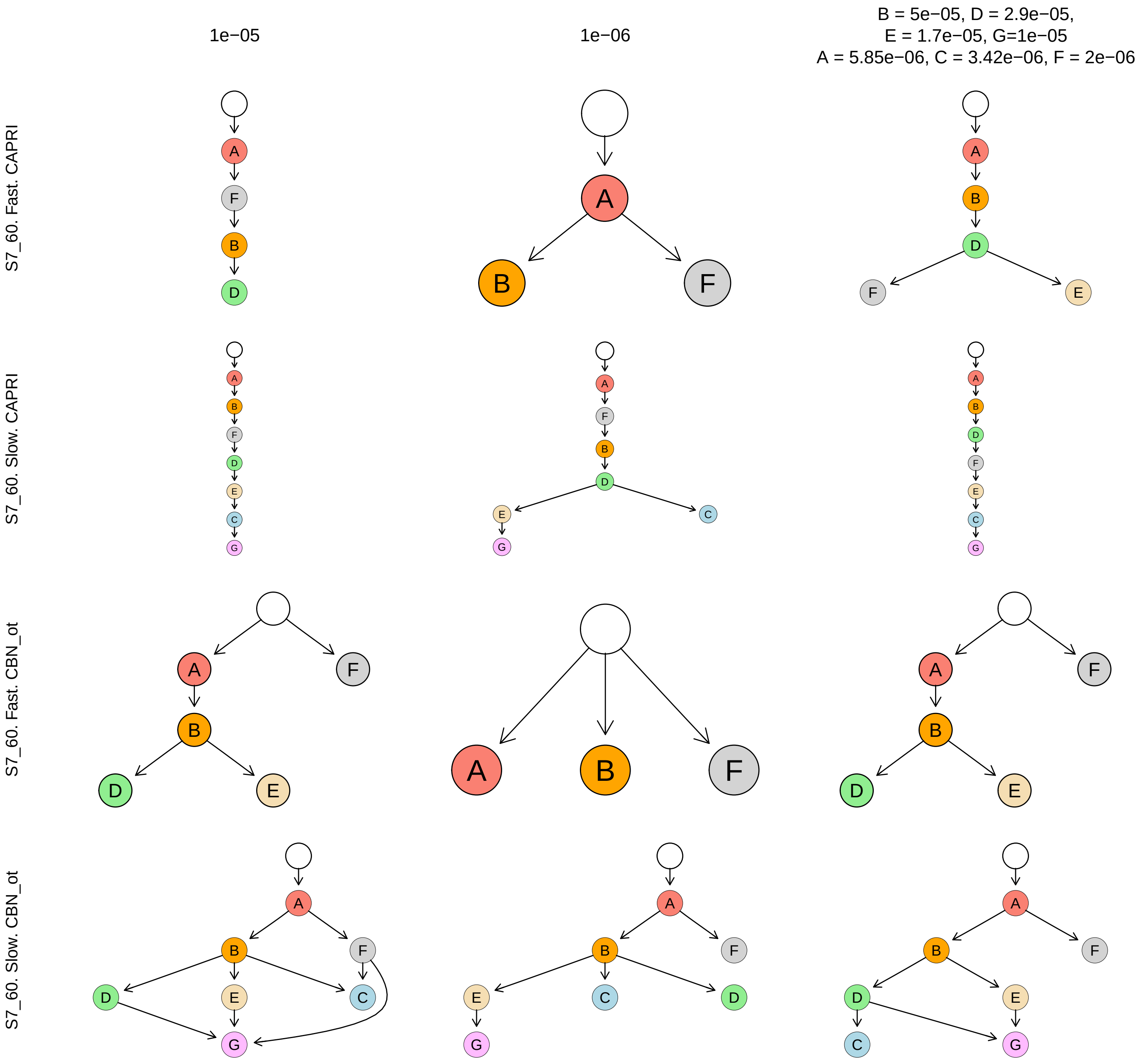

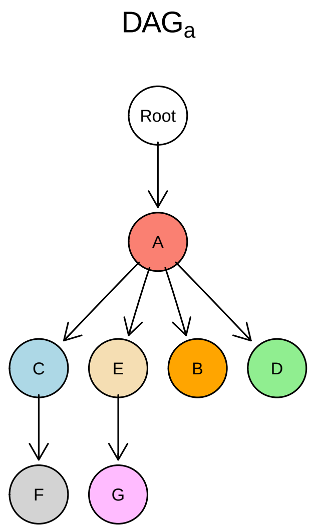

ID	Accessible Genot.	Discrep. DAG_a
S7_61	36	0

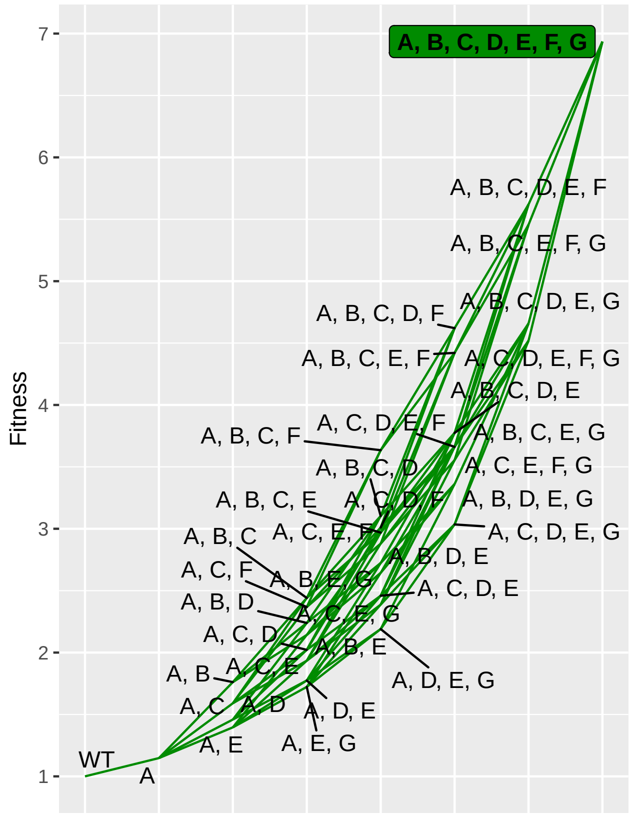

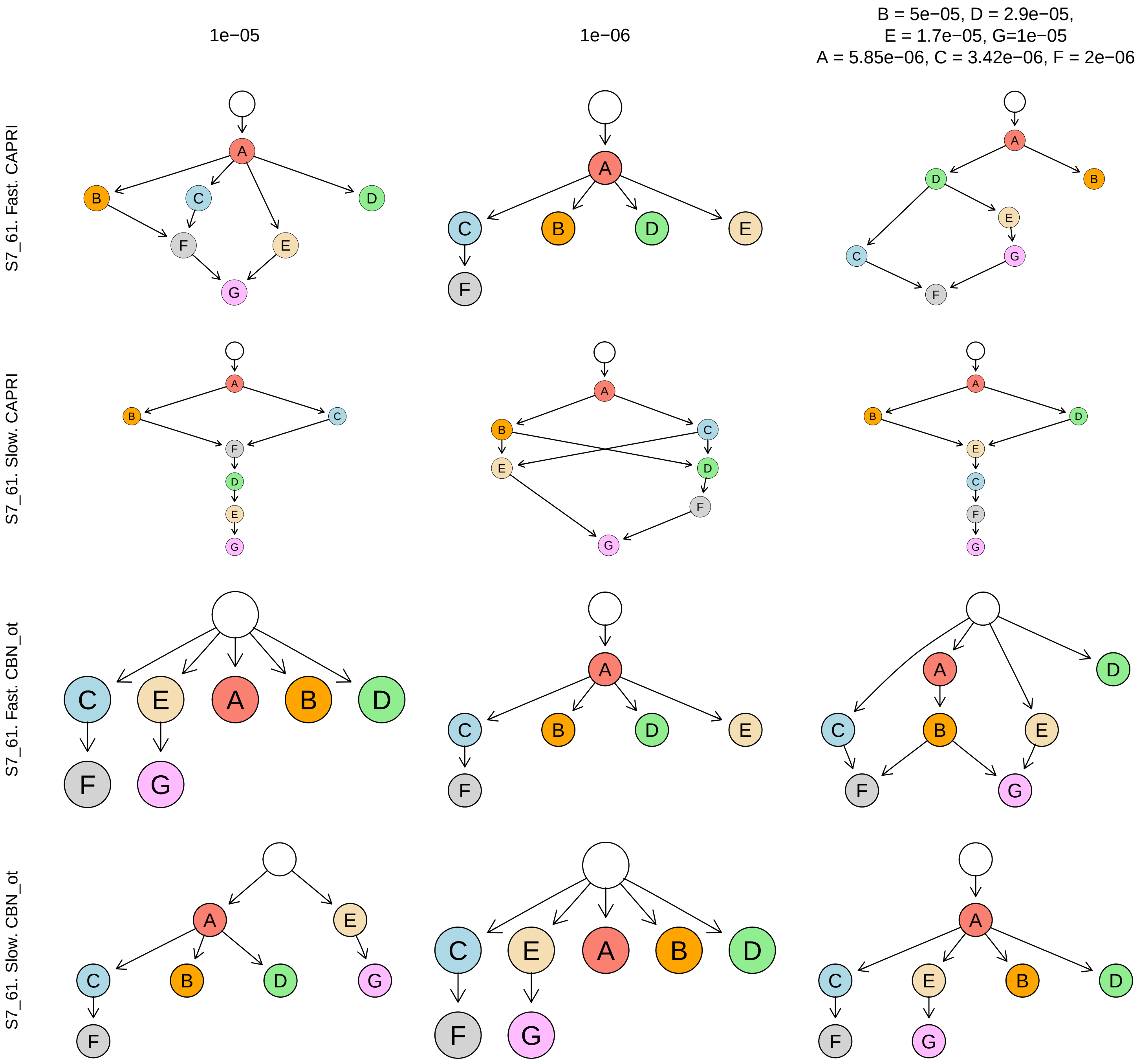

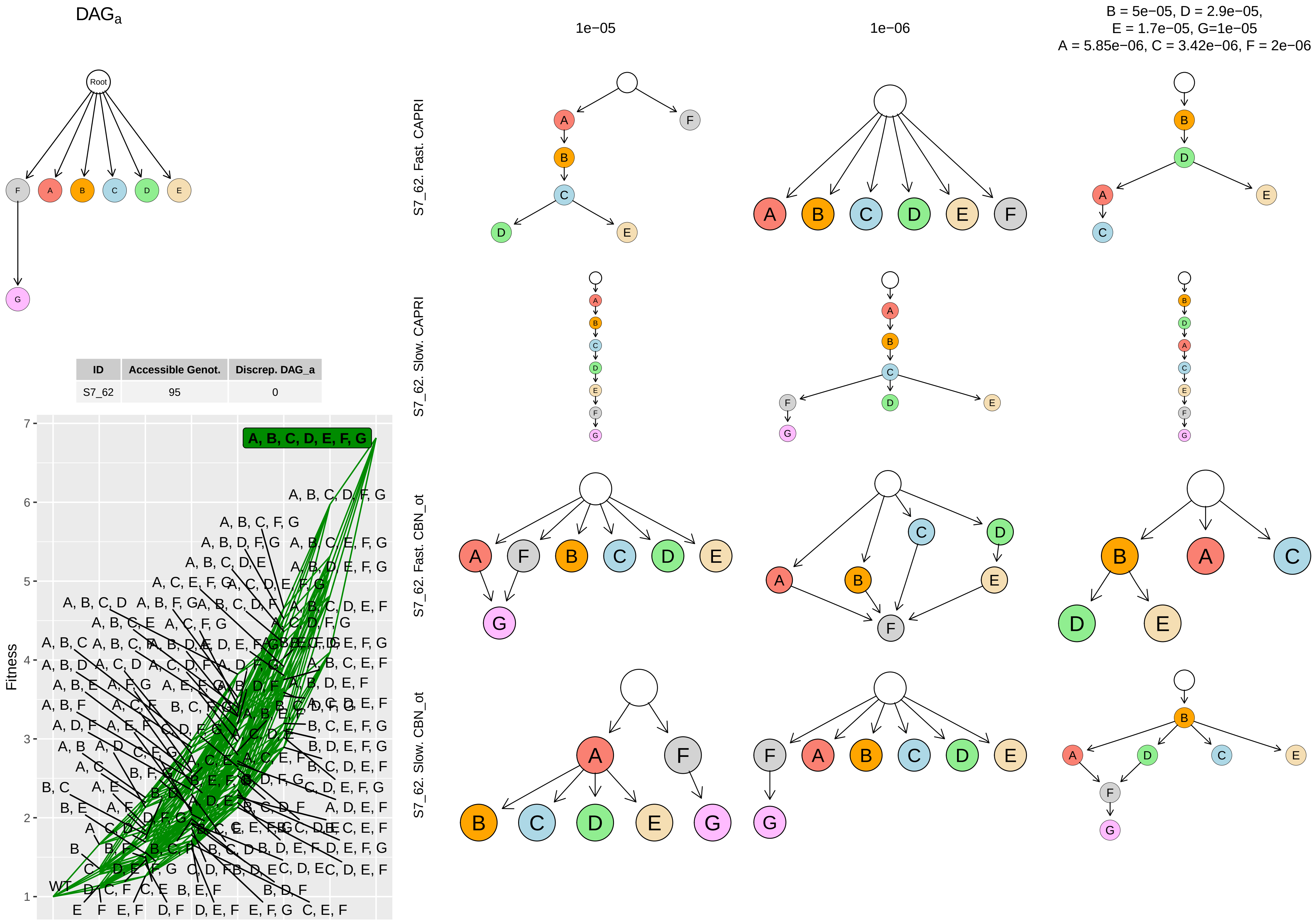

ID	Accessible Genot.	Discrep. DAG_a
S7_62	95	0

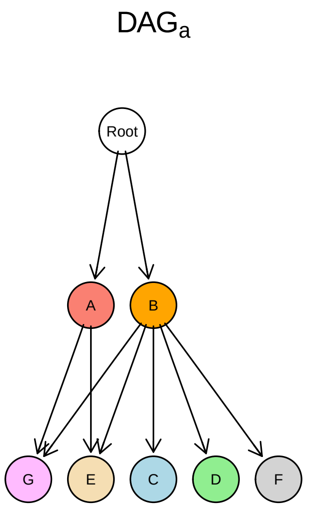

ID	Accessible Genot.	Discrep. DAG_a
S7_63	41	0

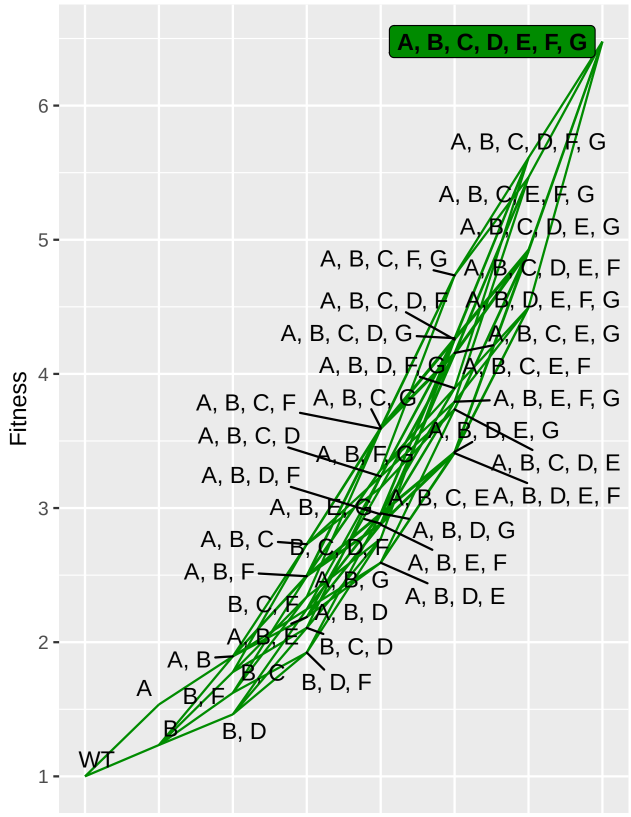

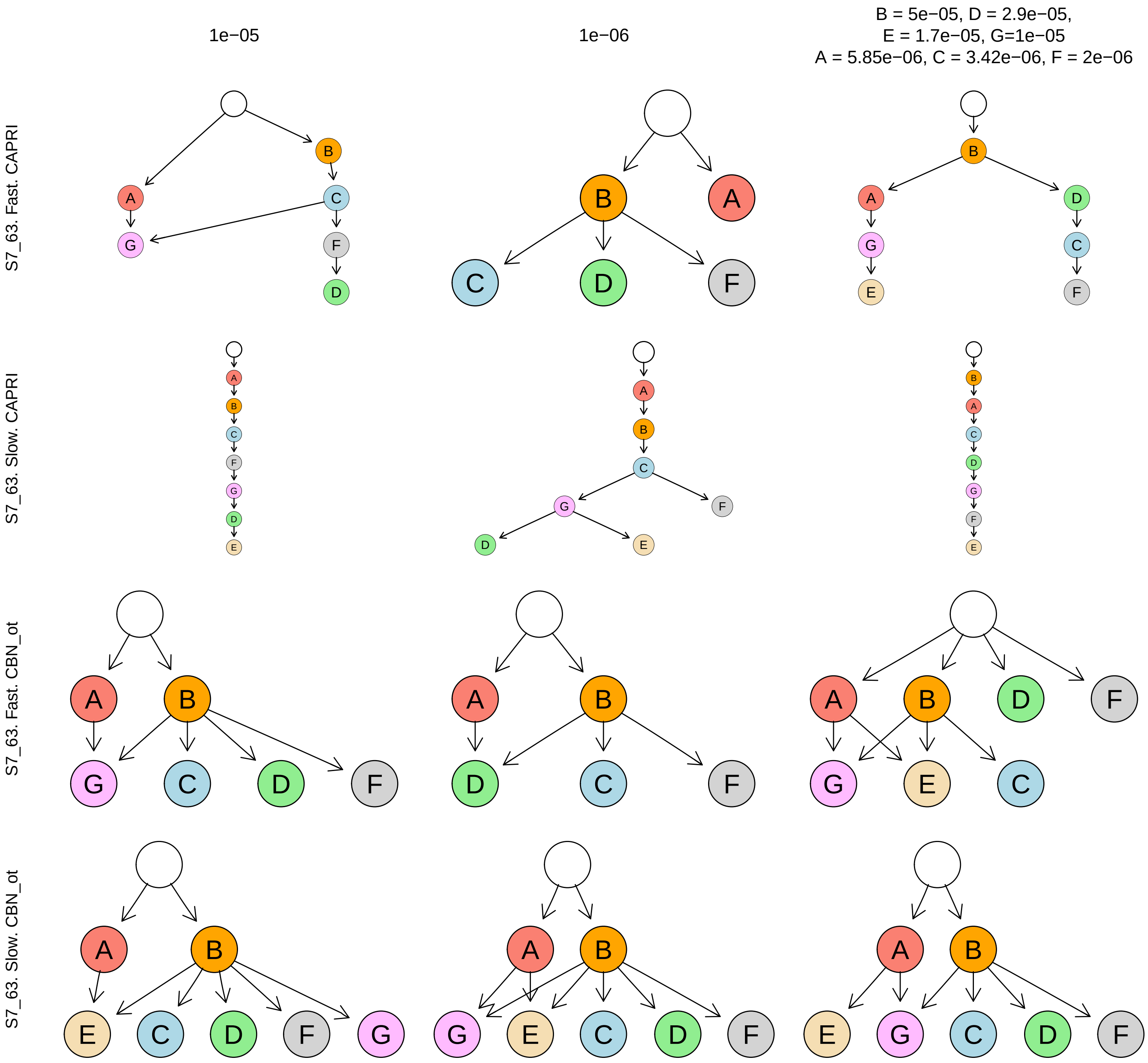

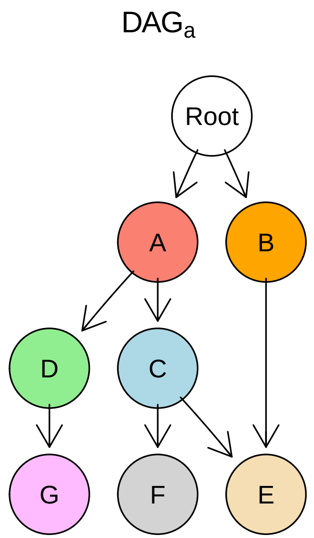

ID	Accessible Genot.	Discrep. DAG_a
S7_64	25	0

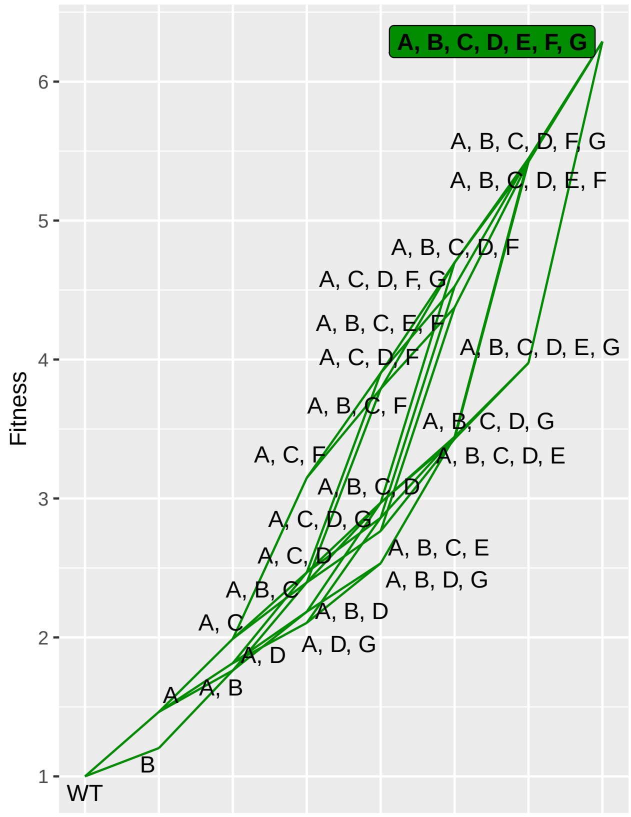

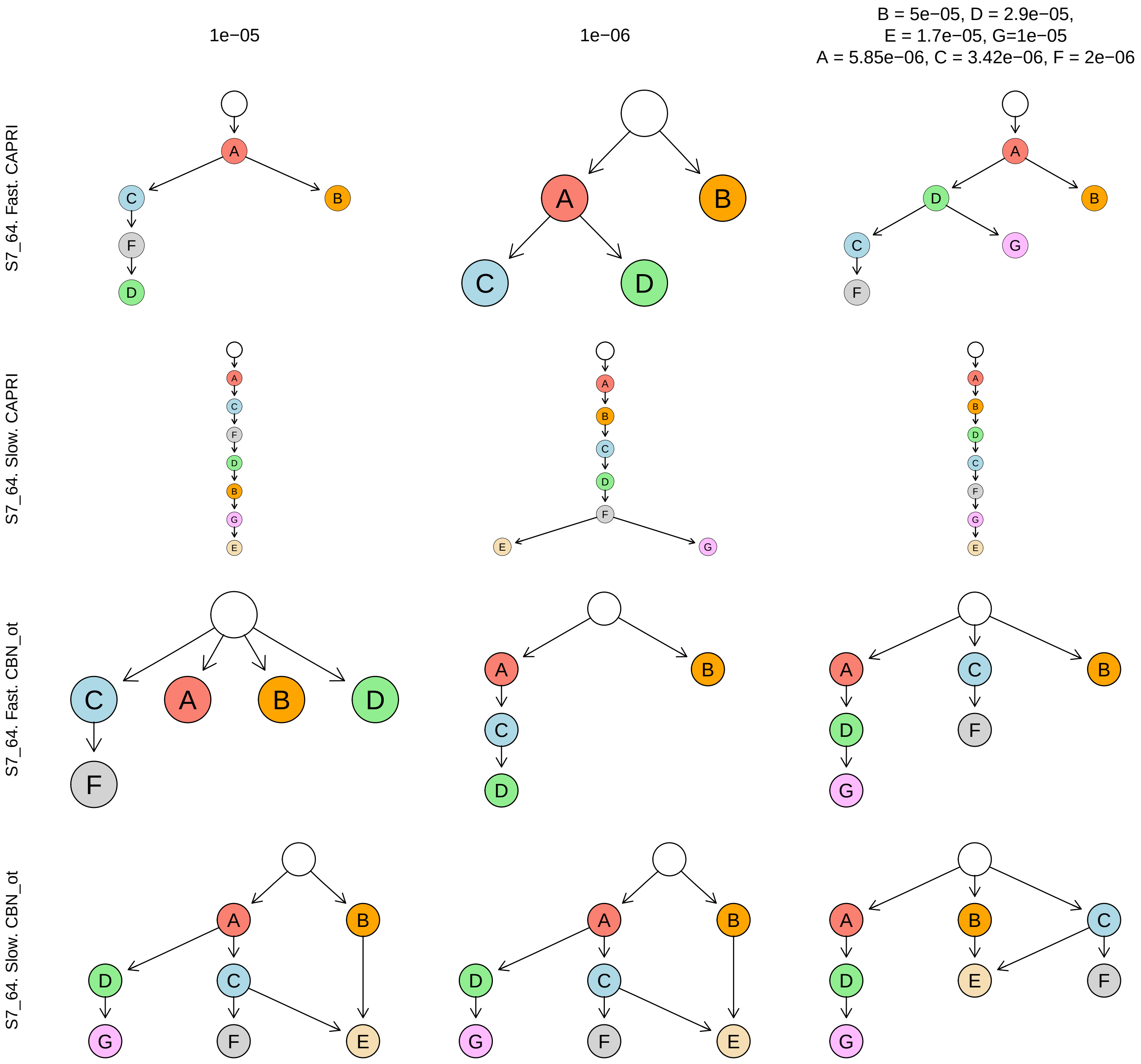

DAG$_a$

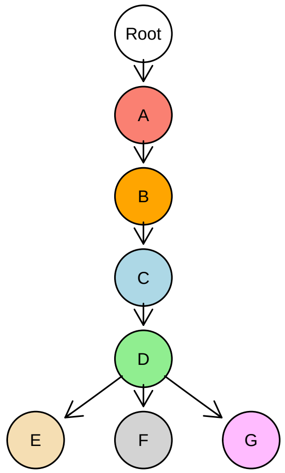

ID	Accessible Genot.	Discrep. DAG_a
S7_65	11	0

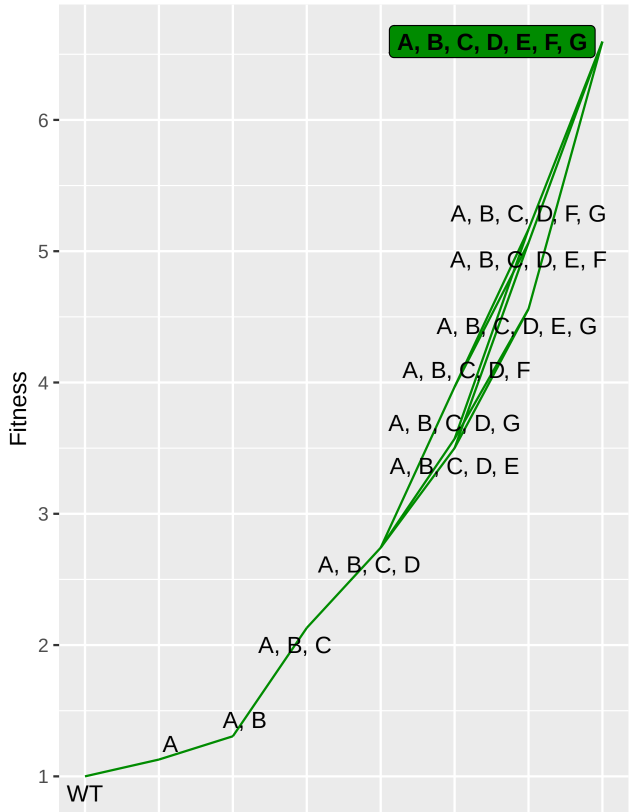

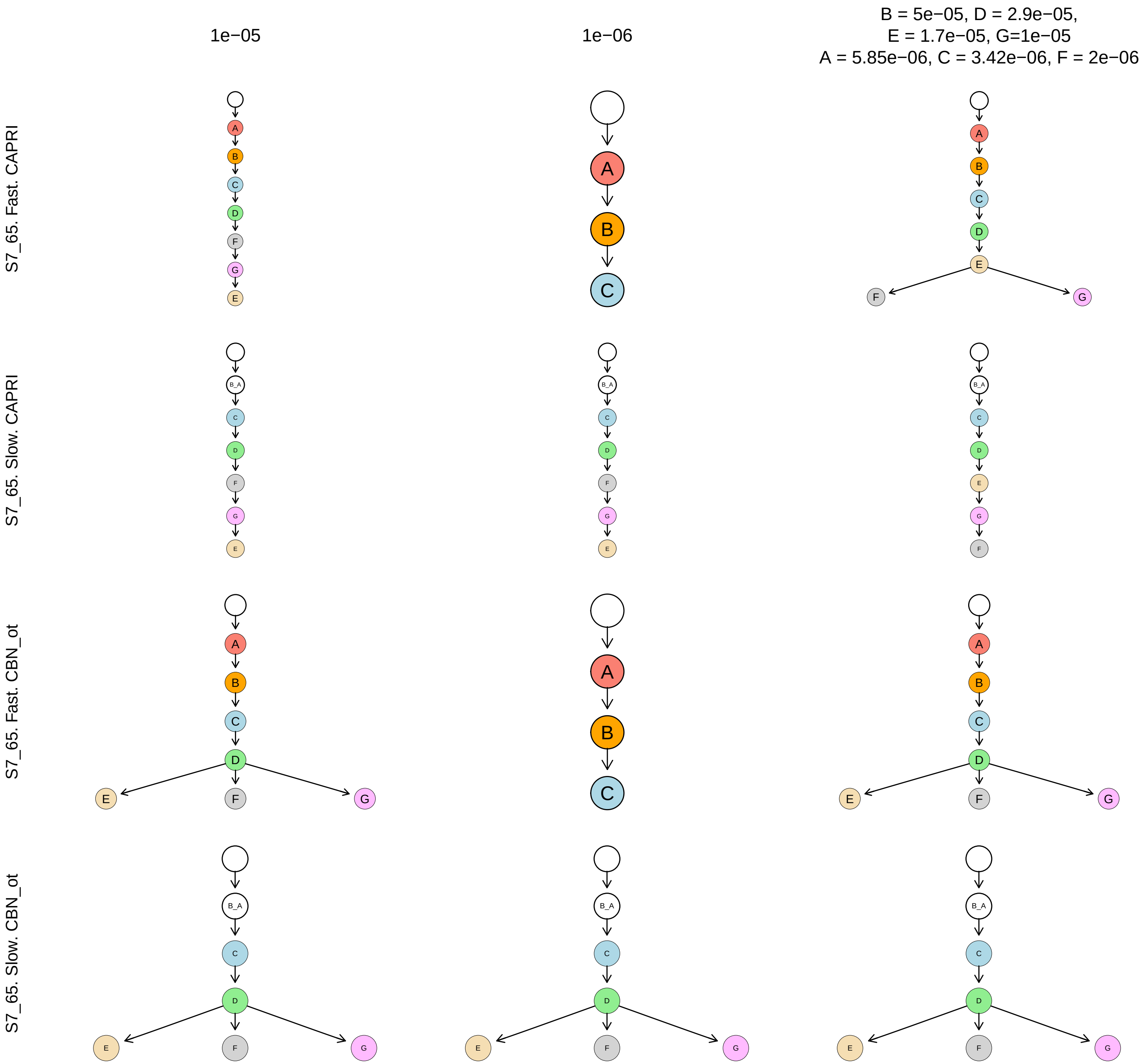

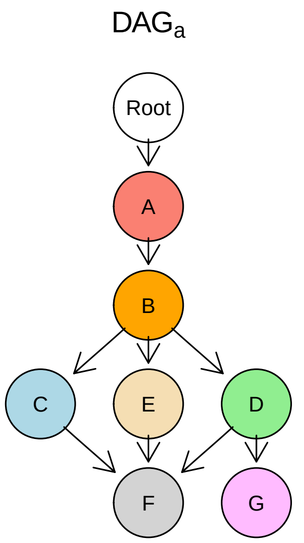

ID	Accessible Genot.	Discrep. DAG_a
S7_66	15	0

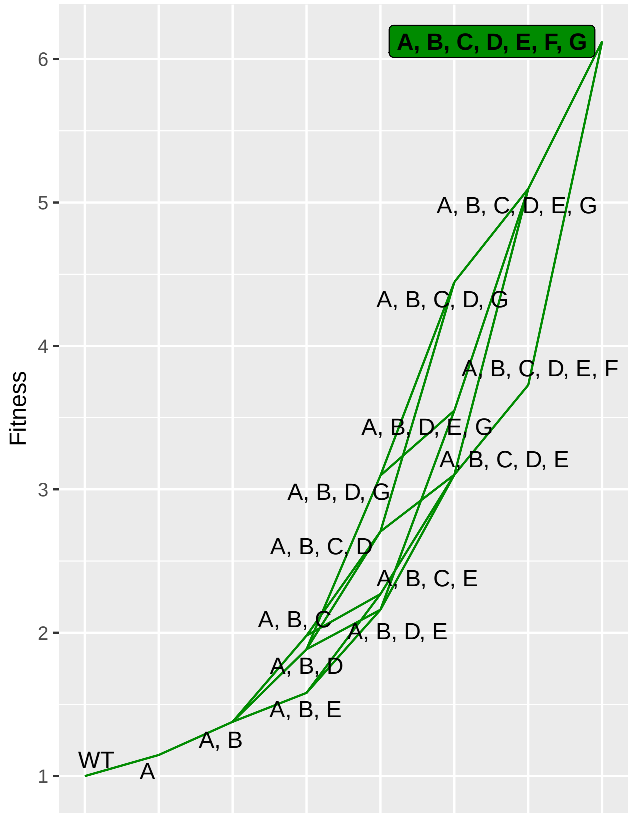

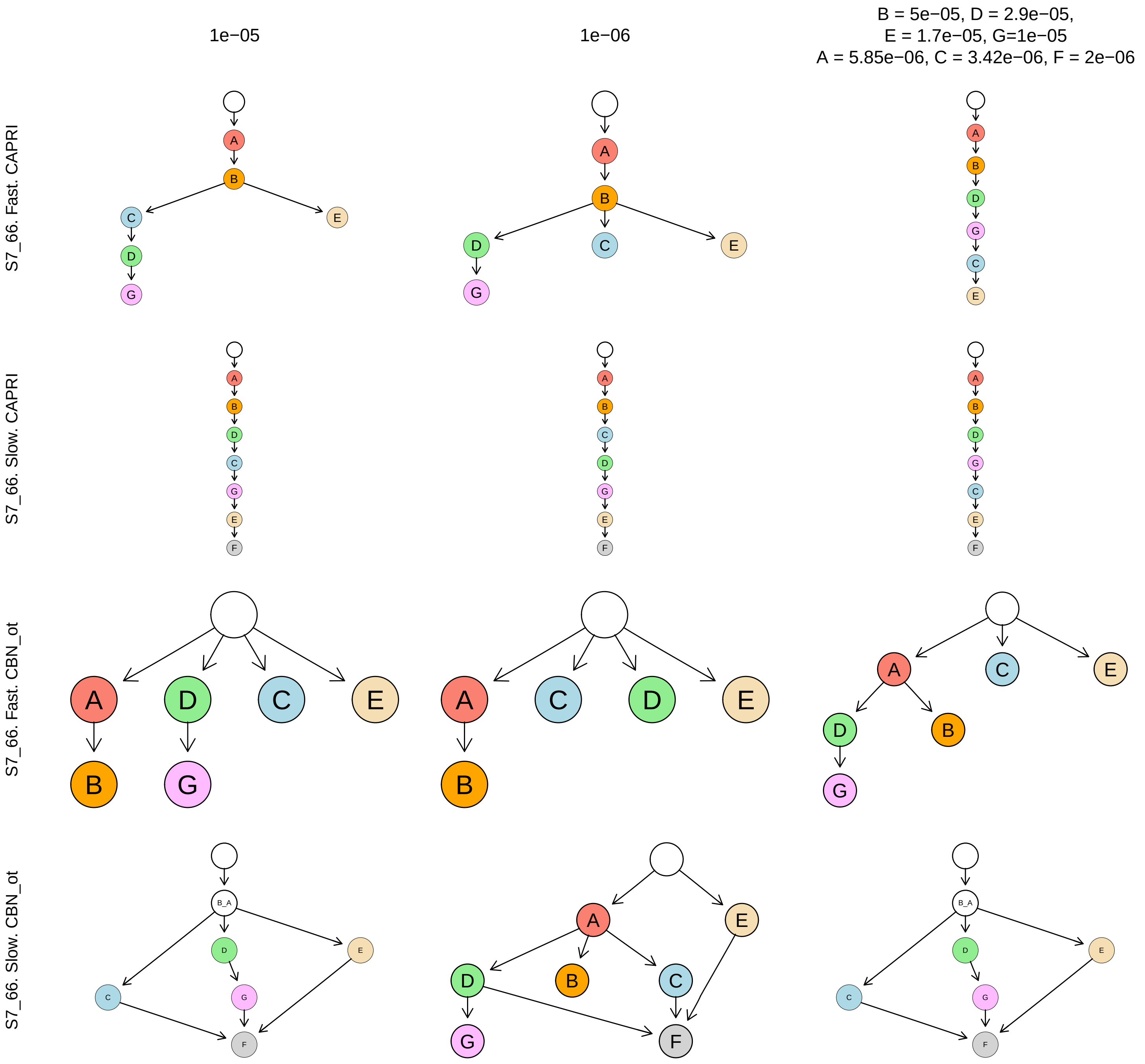

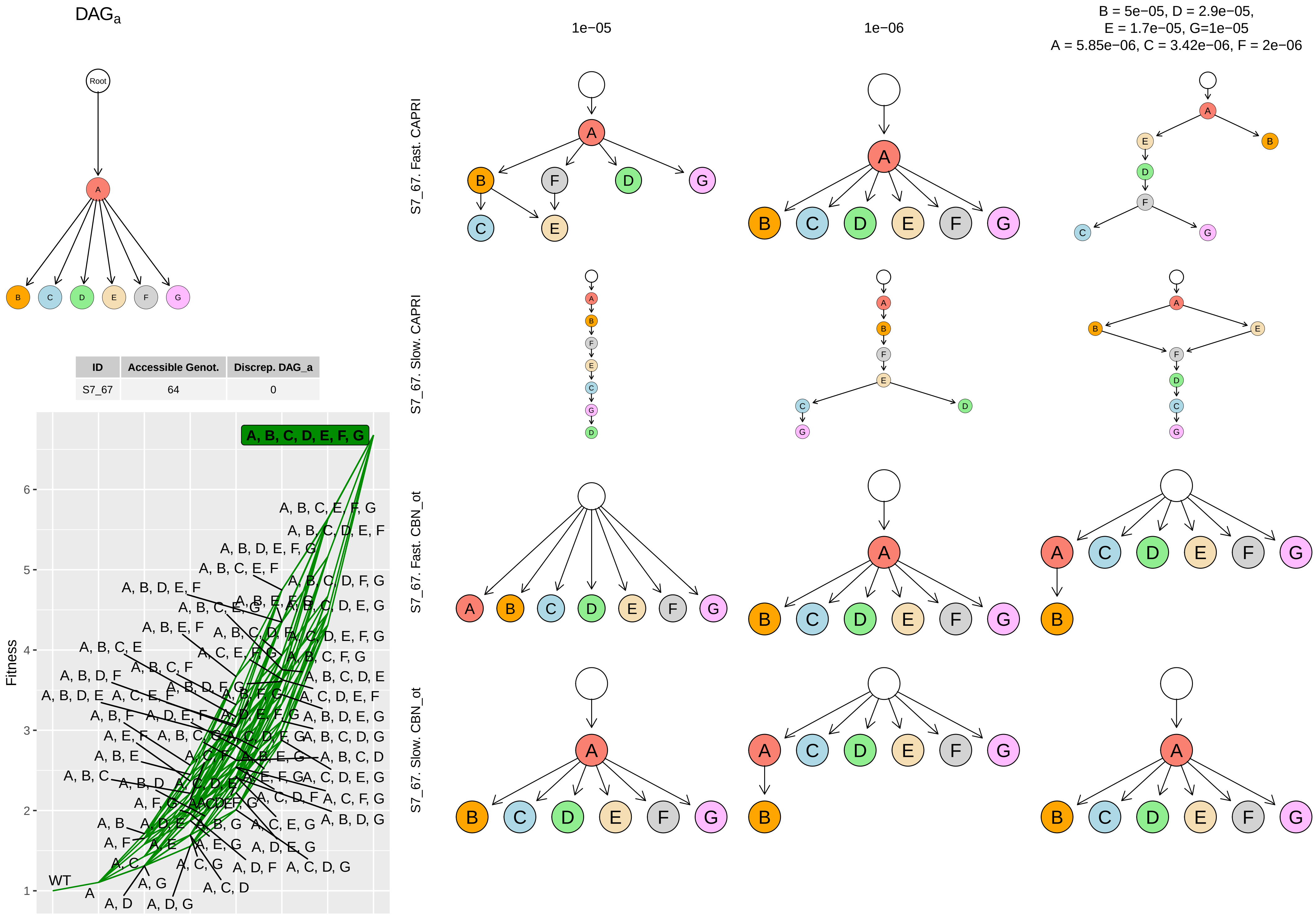

ID	Accessible Genot.	Discrep. DAG_a
S7_67	64	0

DAG_a

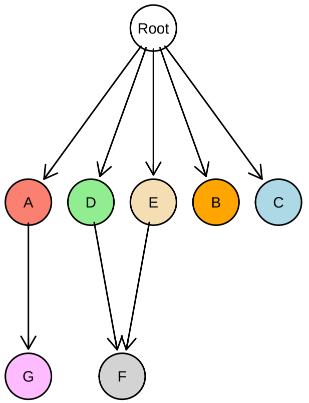

ID	Accessible Genot.	Discrep. DAG_a
S7_68	59	0

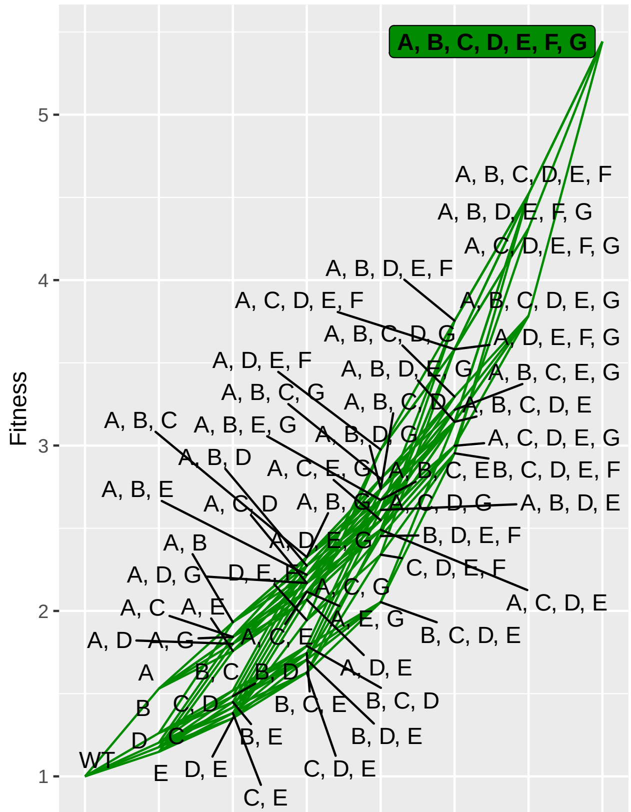

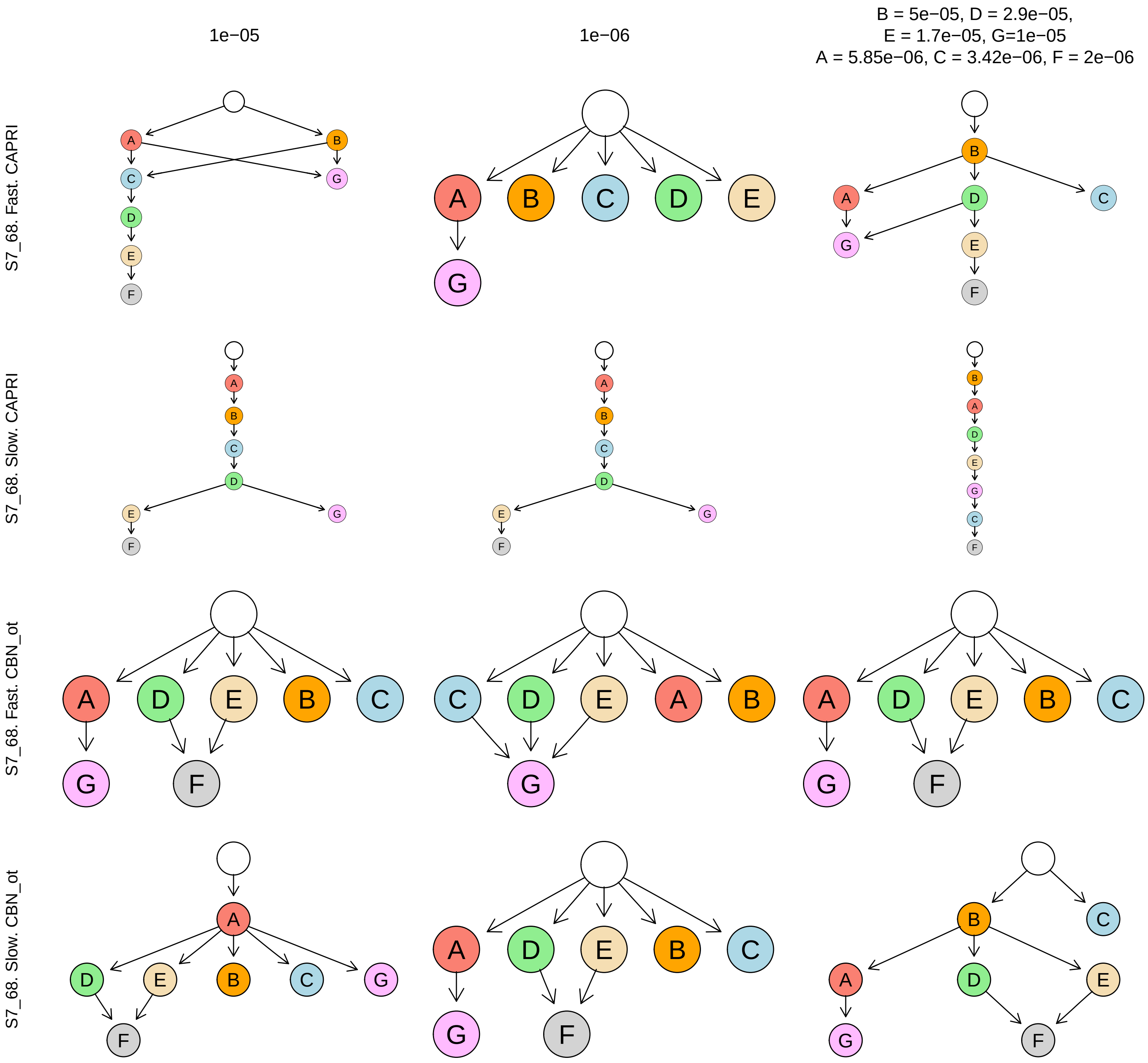

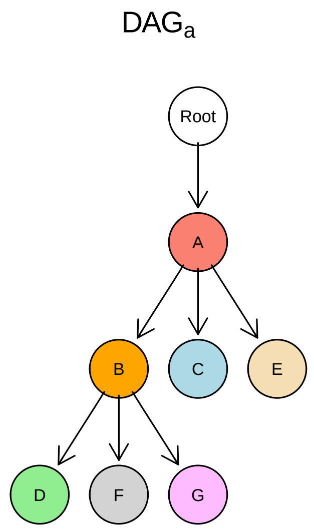

ID	Accessible Genot.	Discrep. DAG_a
S7_69	36	0

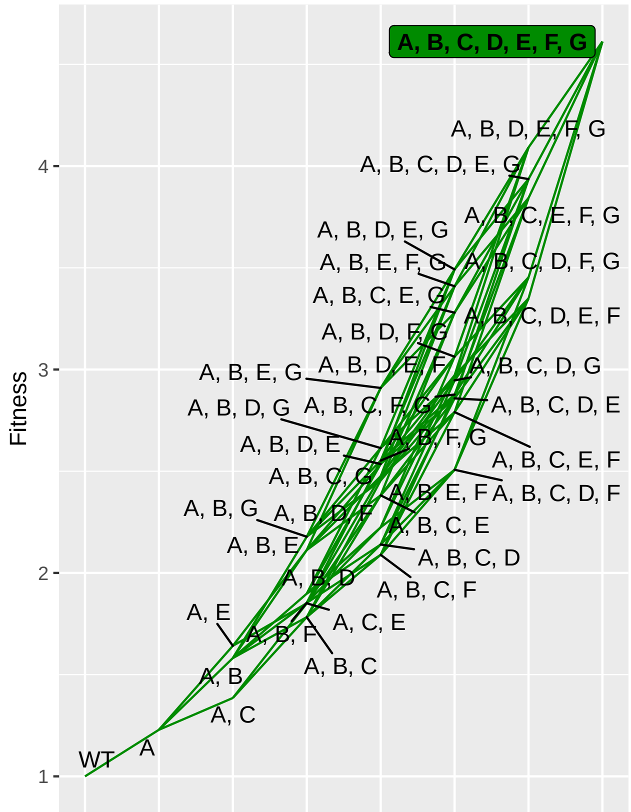

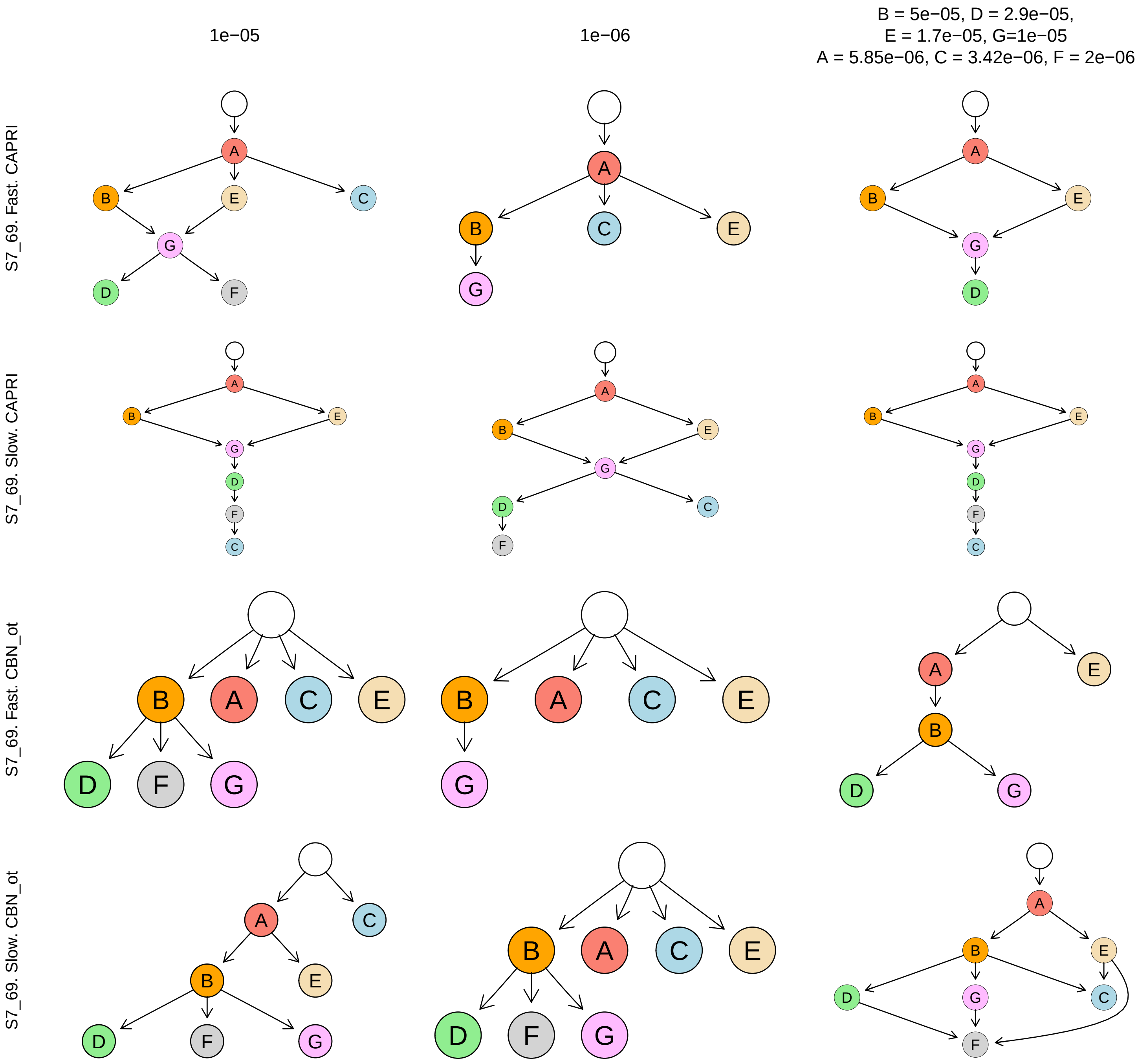

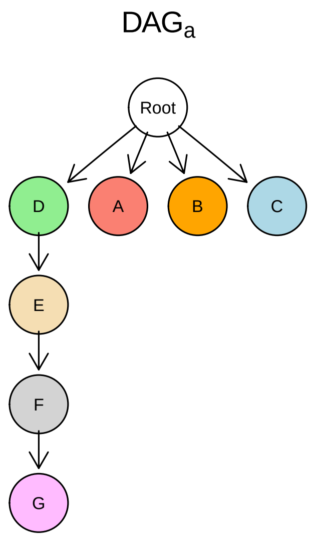

ID	Accessible Genot.	Discrep. DAG_a
S7_70	39	0

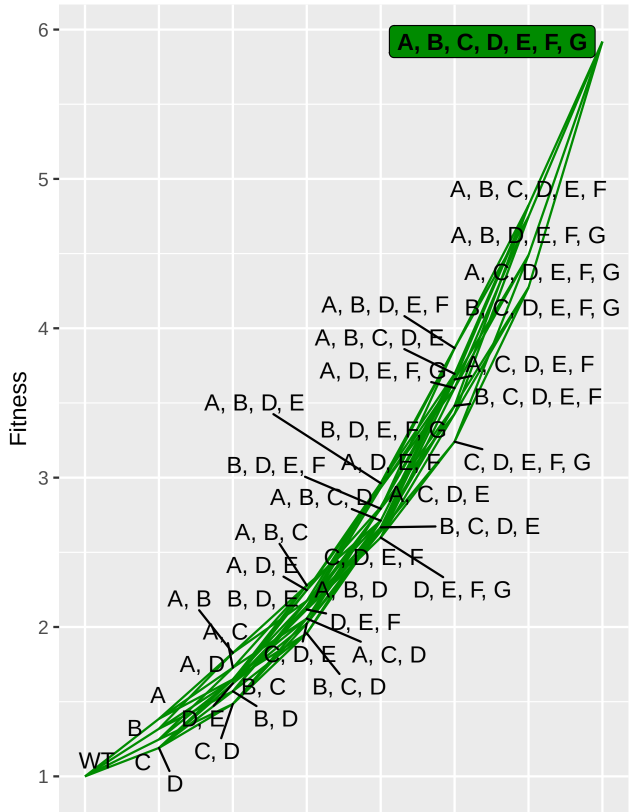

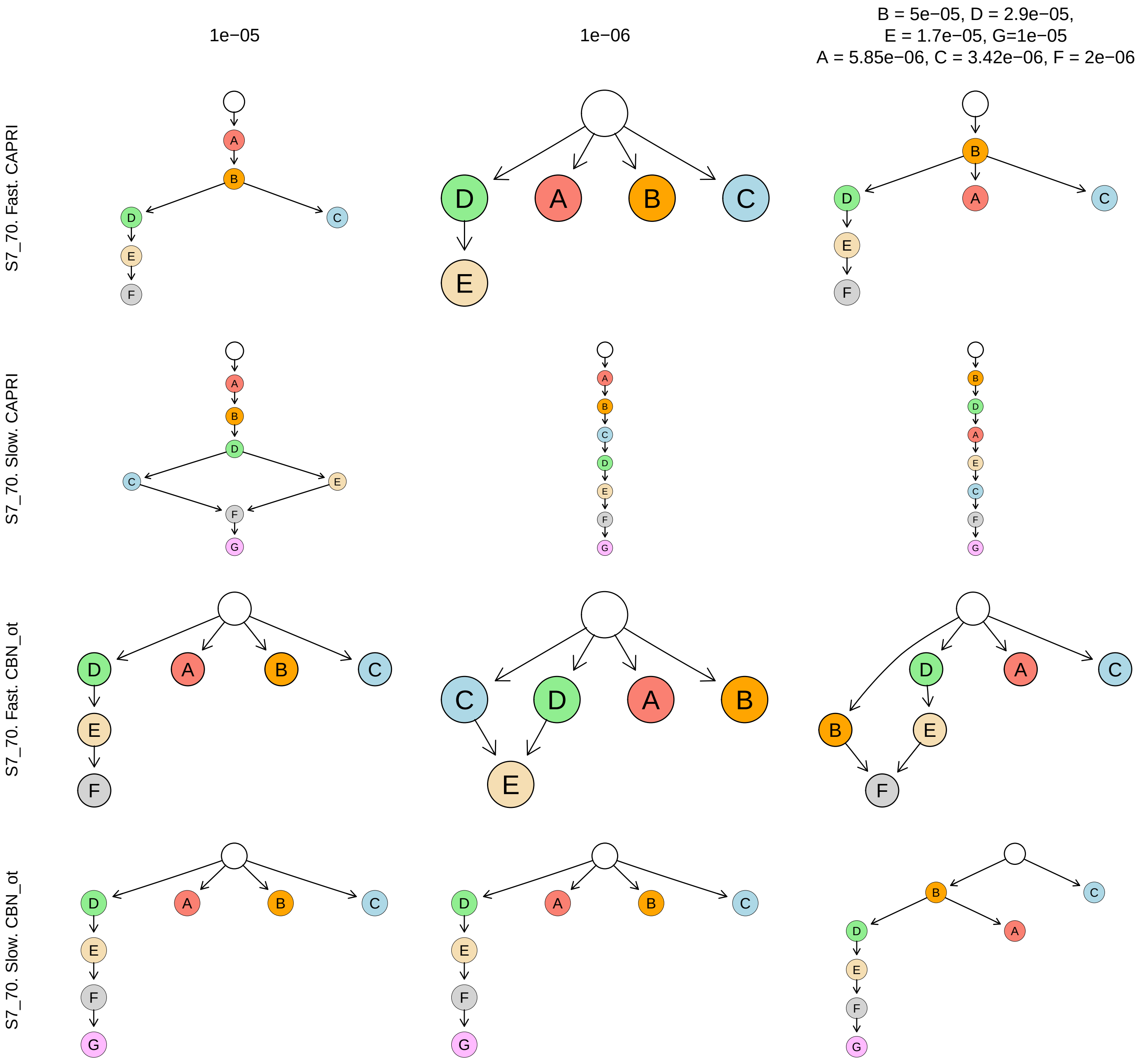

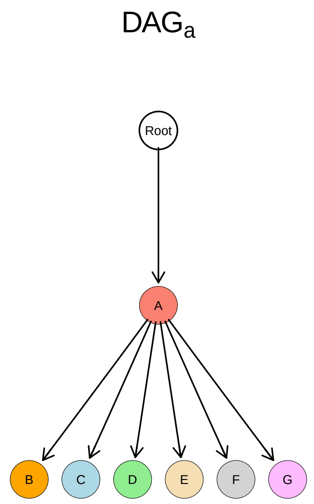

ID	Accessible Genot.	Discrep. DAG_a
S7_71	64	0

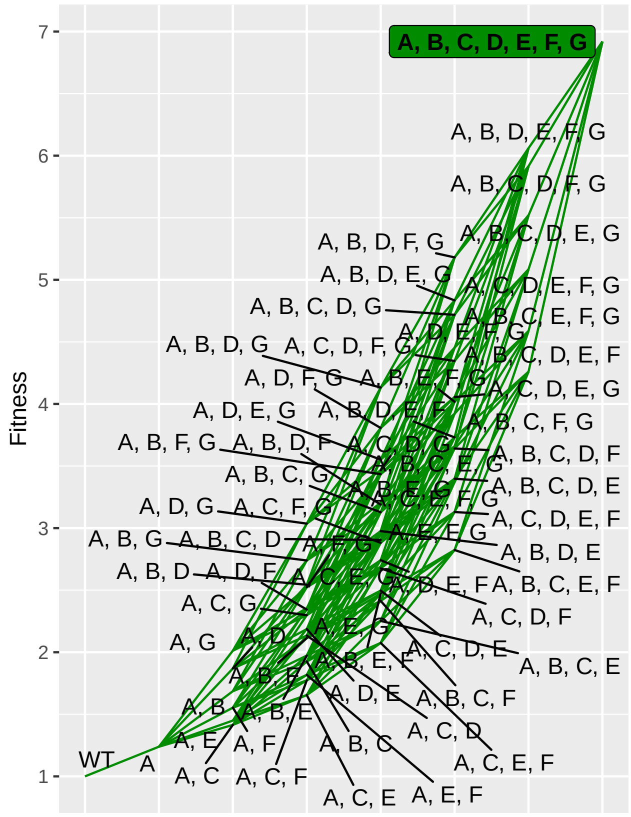

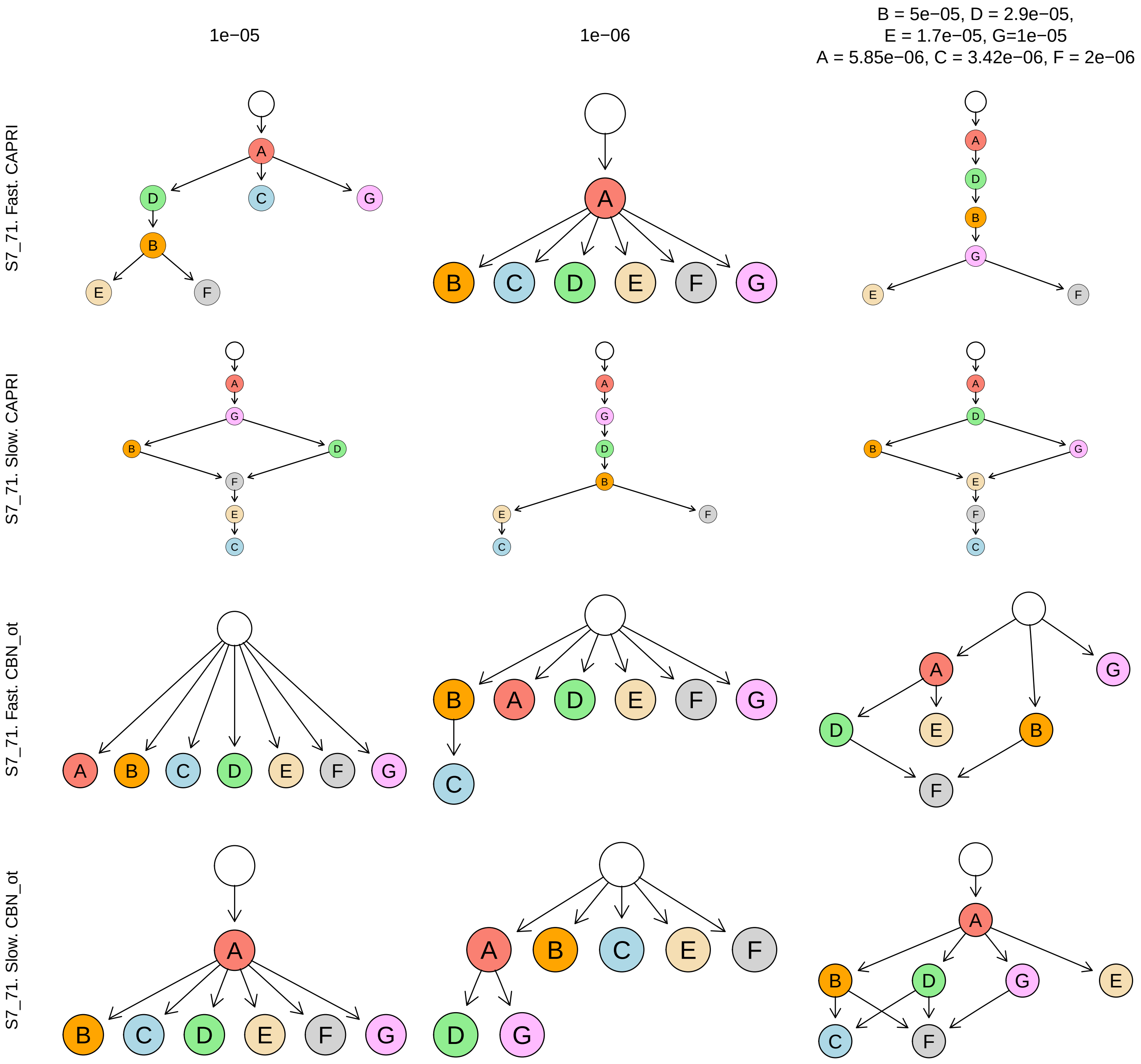

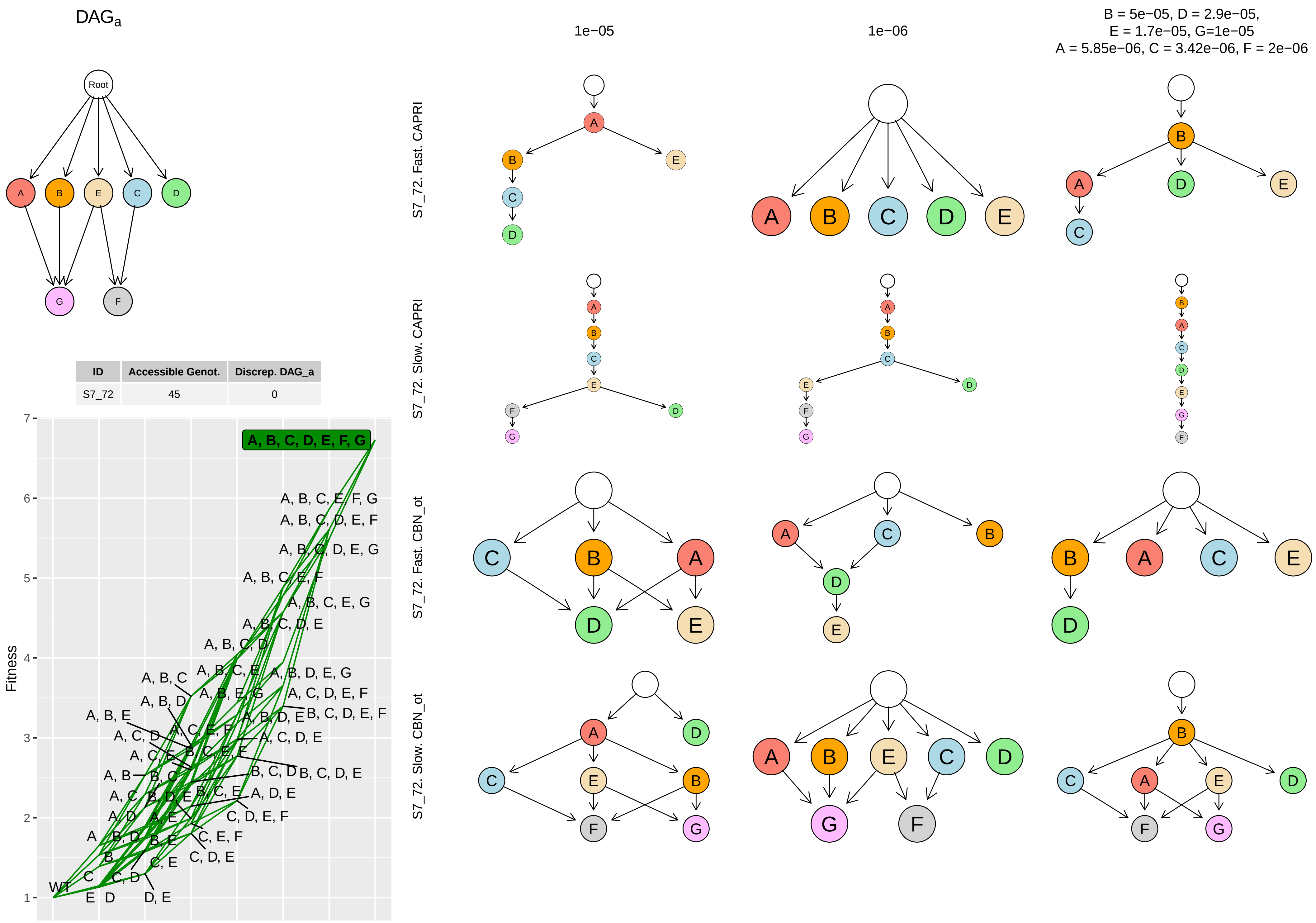

DAGa
Root
1e−05
1e−06
B = 5e−05, D = 2.9e−05,
E = 1.7e−05, G=1e−05
A = 5.85e−06, C = 3.42e−06, F = 2e−06
S7_72. Fast. CAPRI
S7_72. Slow. CAPRI
S7_72. Fast. CBN_ot
S7_72. Slow. CBN_ot
ID | Accessible Genot. | Discrep. DAG_a
S7_72 | 45 | 0
Fitness
A, B, C, D, E, F, G
A, B, C, E, F, G
A, B, C, D, E, F
A, B, C, D, E, G
WT

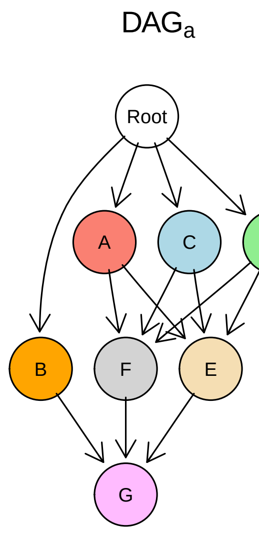

ID	Accessible Genot.	Discrep. DAG_a
S7_73	22	0

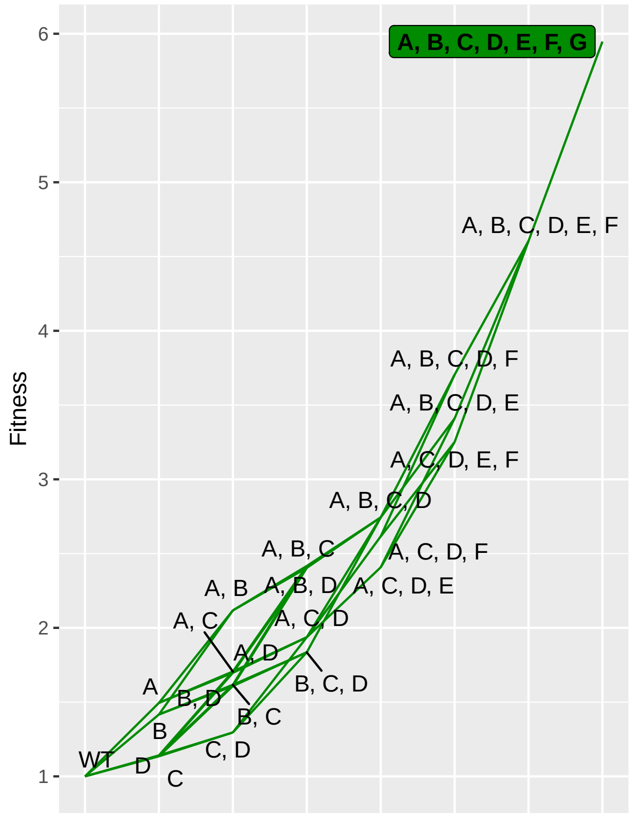

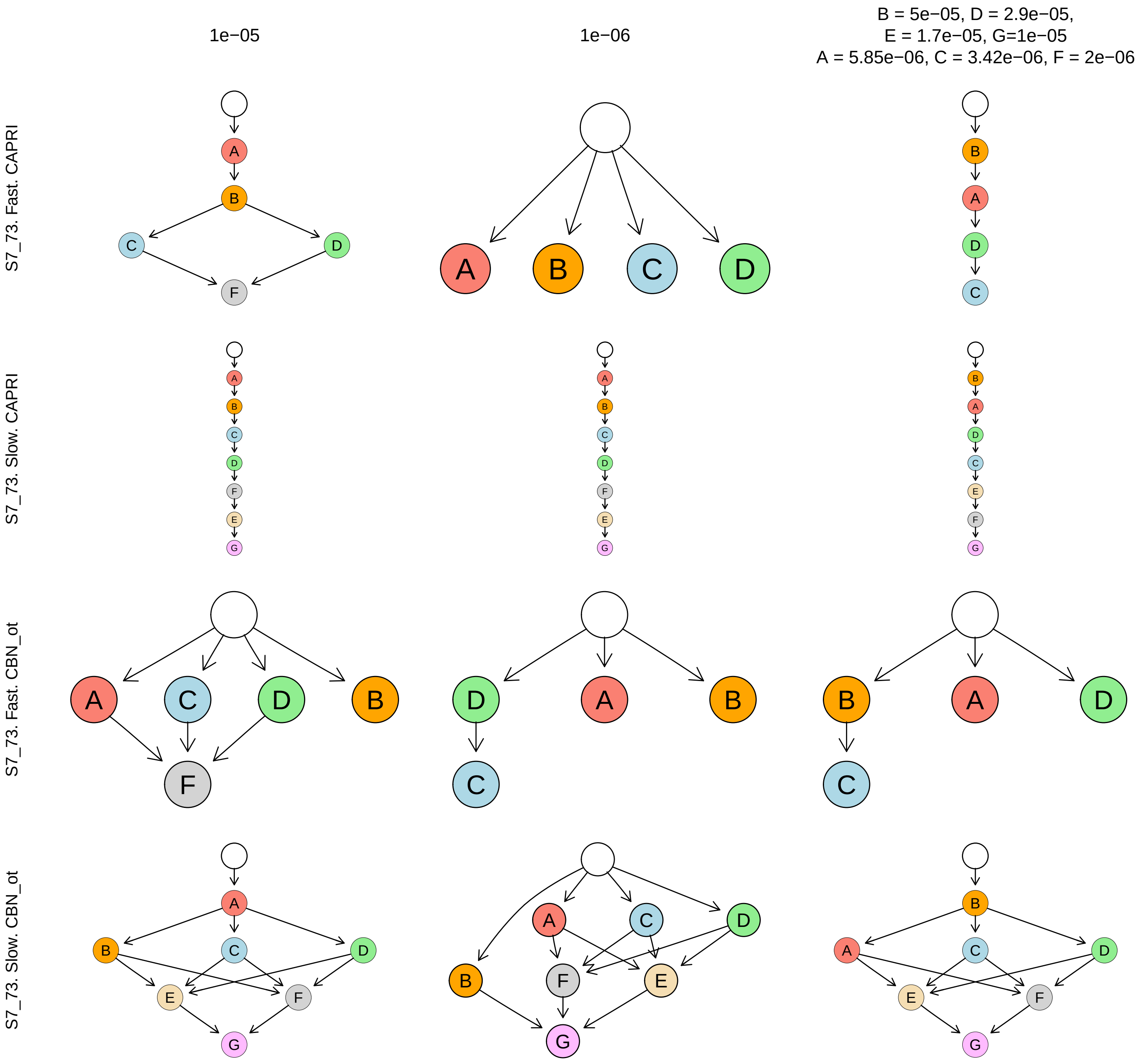

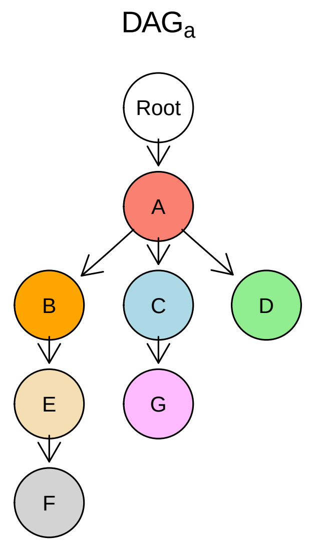

ID	Accessible Genot.	Discrep. DAG_a
S7_74	24	0

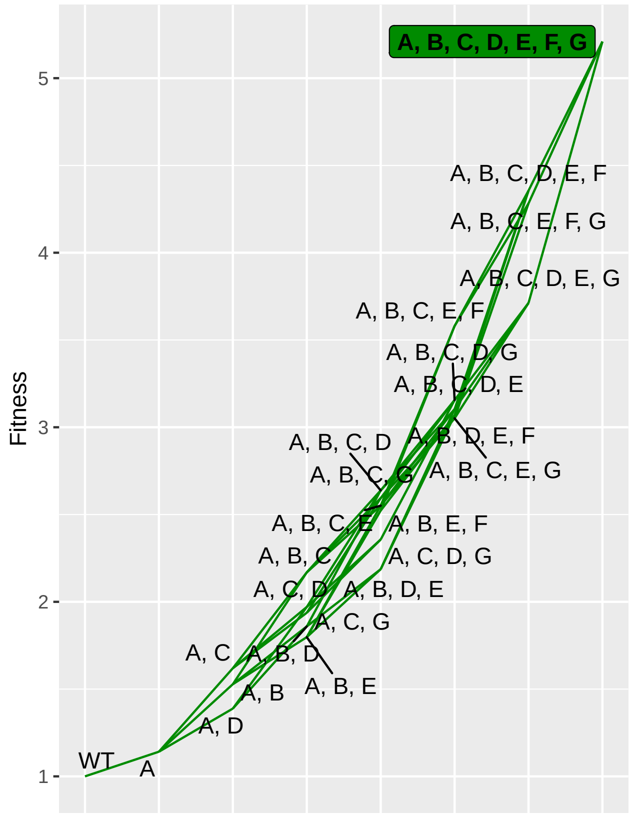

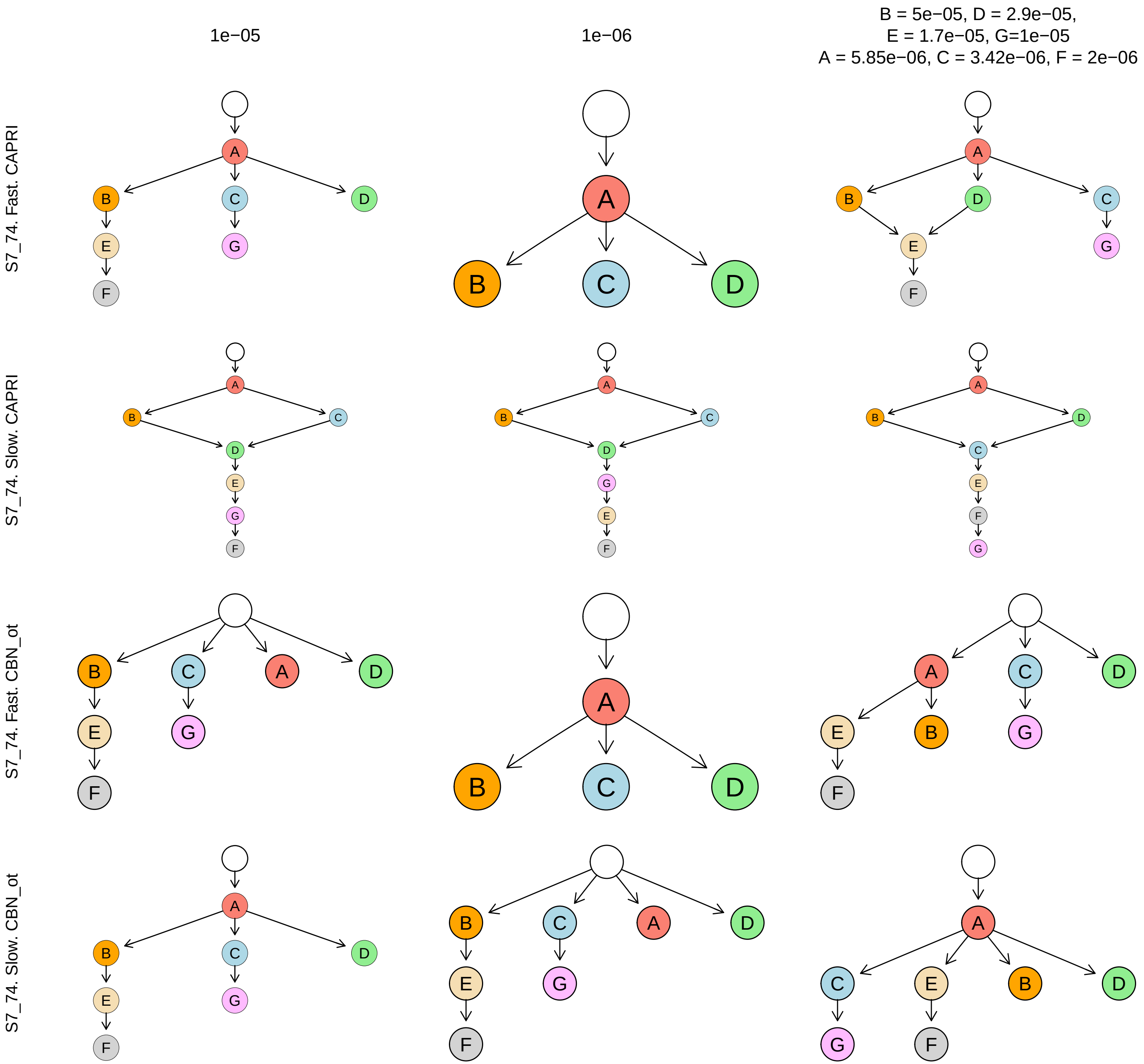

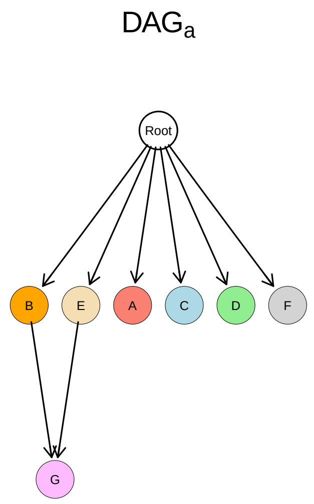

ID	Accessible Genot.	Discrep. DAG_a
S7_75	79	0

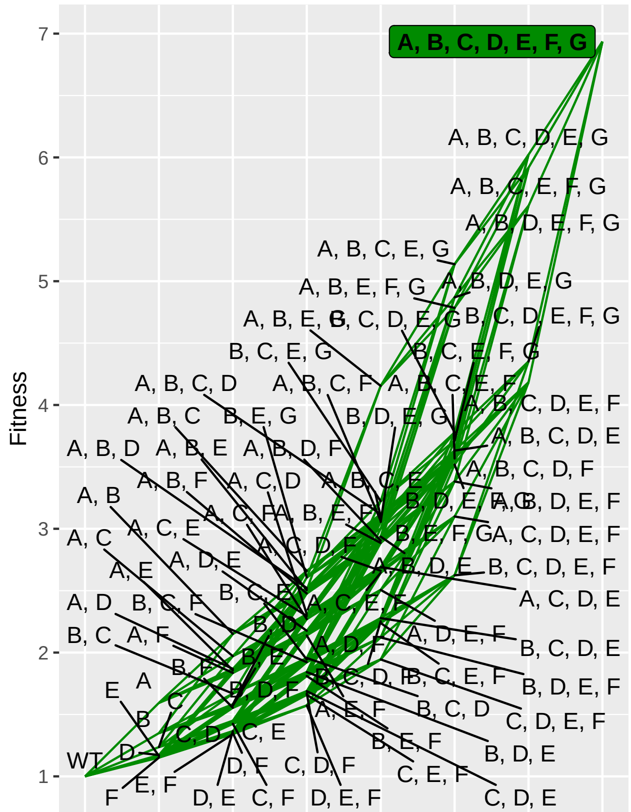

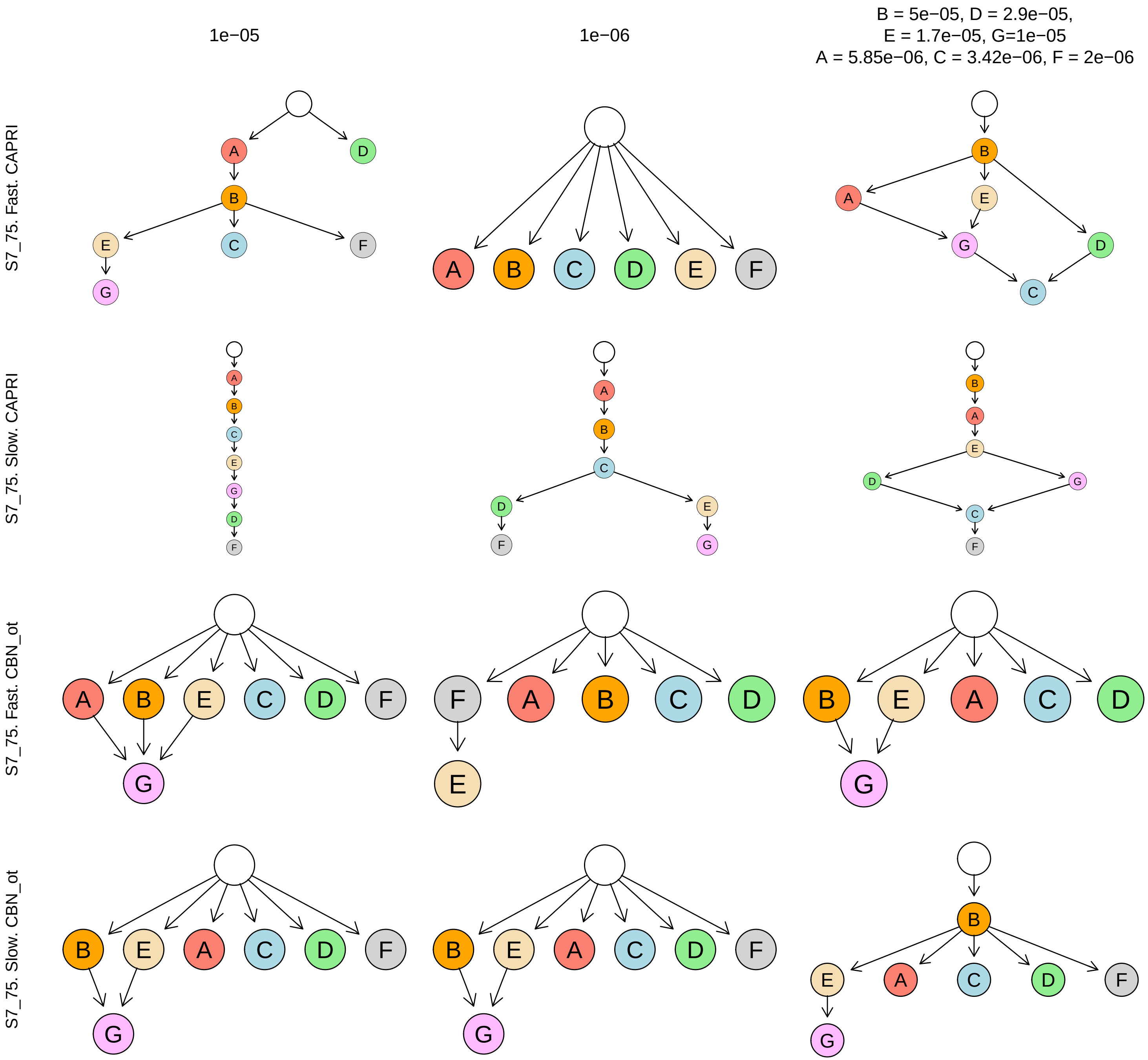

DAG$_a$

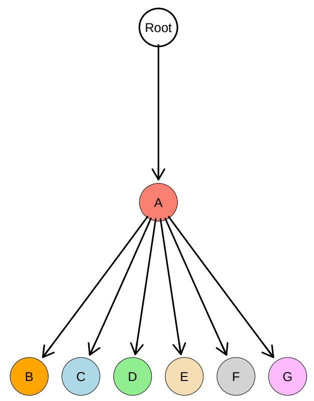

ID	Accessible Genot.	Discrep. DAG_a
S7_76	64	0

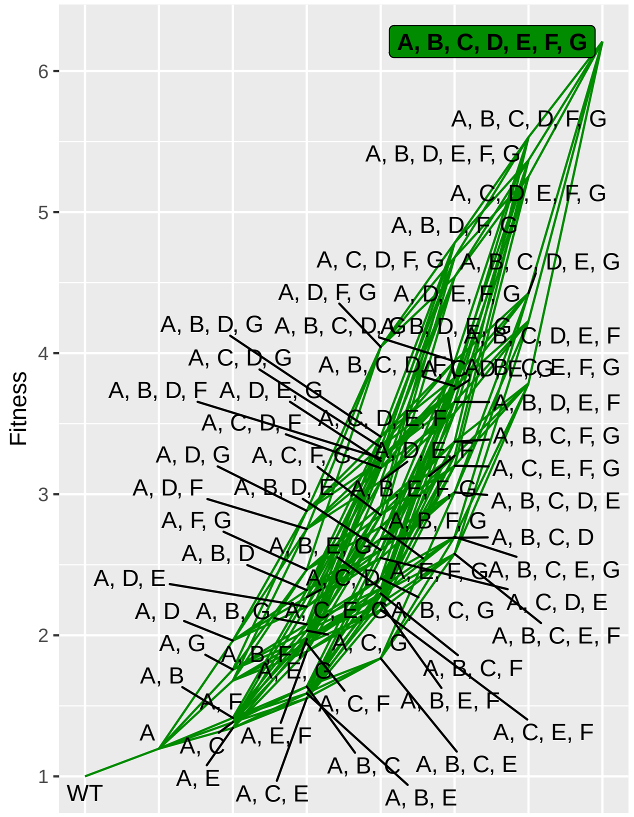

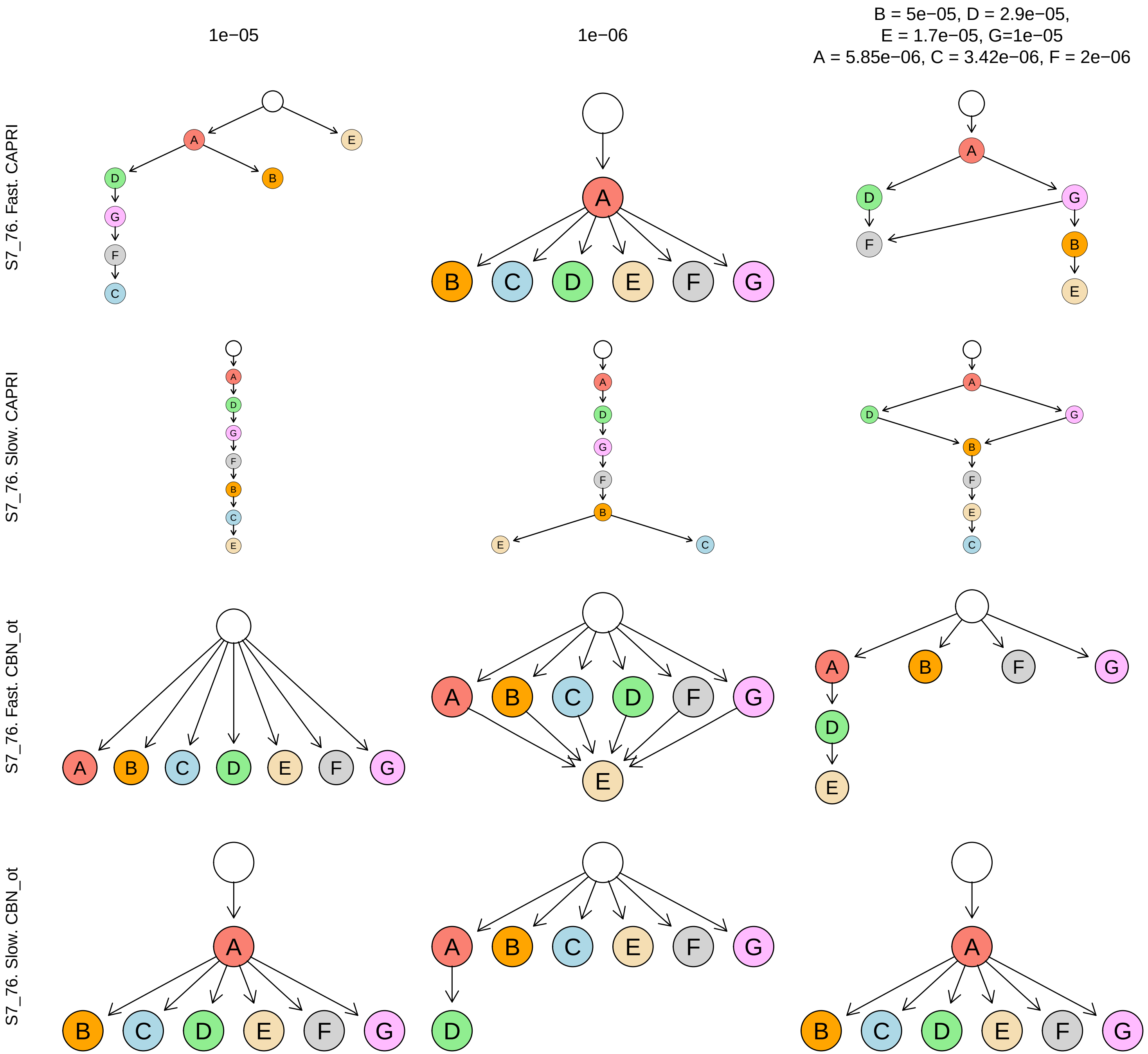

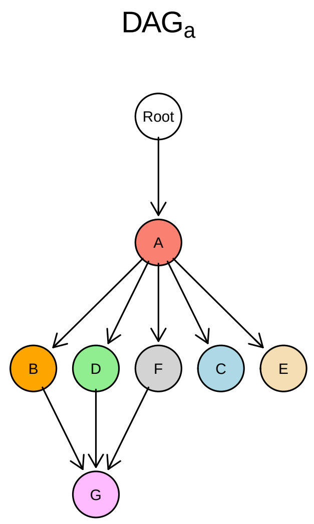

ID	Accessible Genot.	Discrep. DAG_a
S7_77	36	0

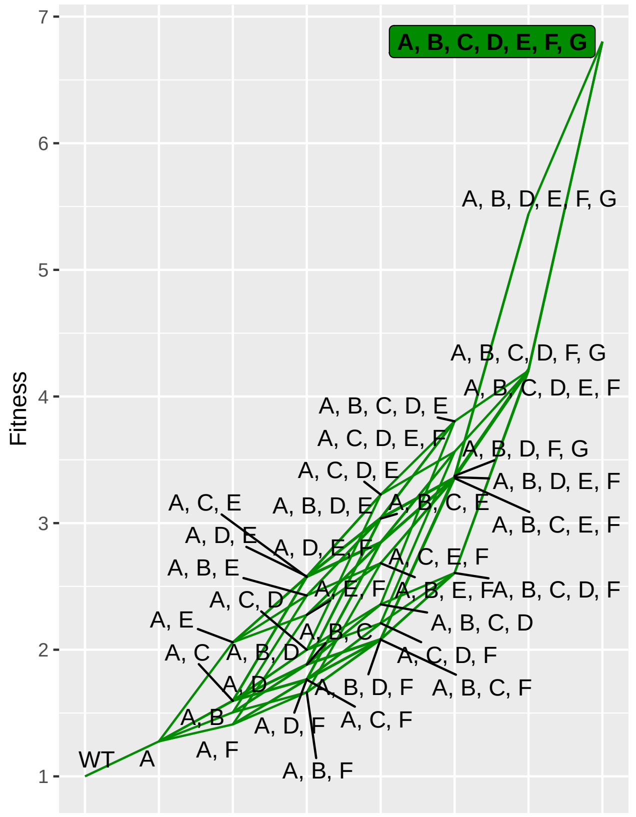

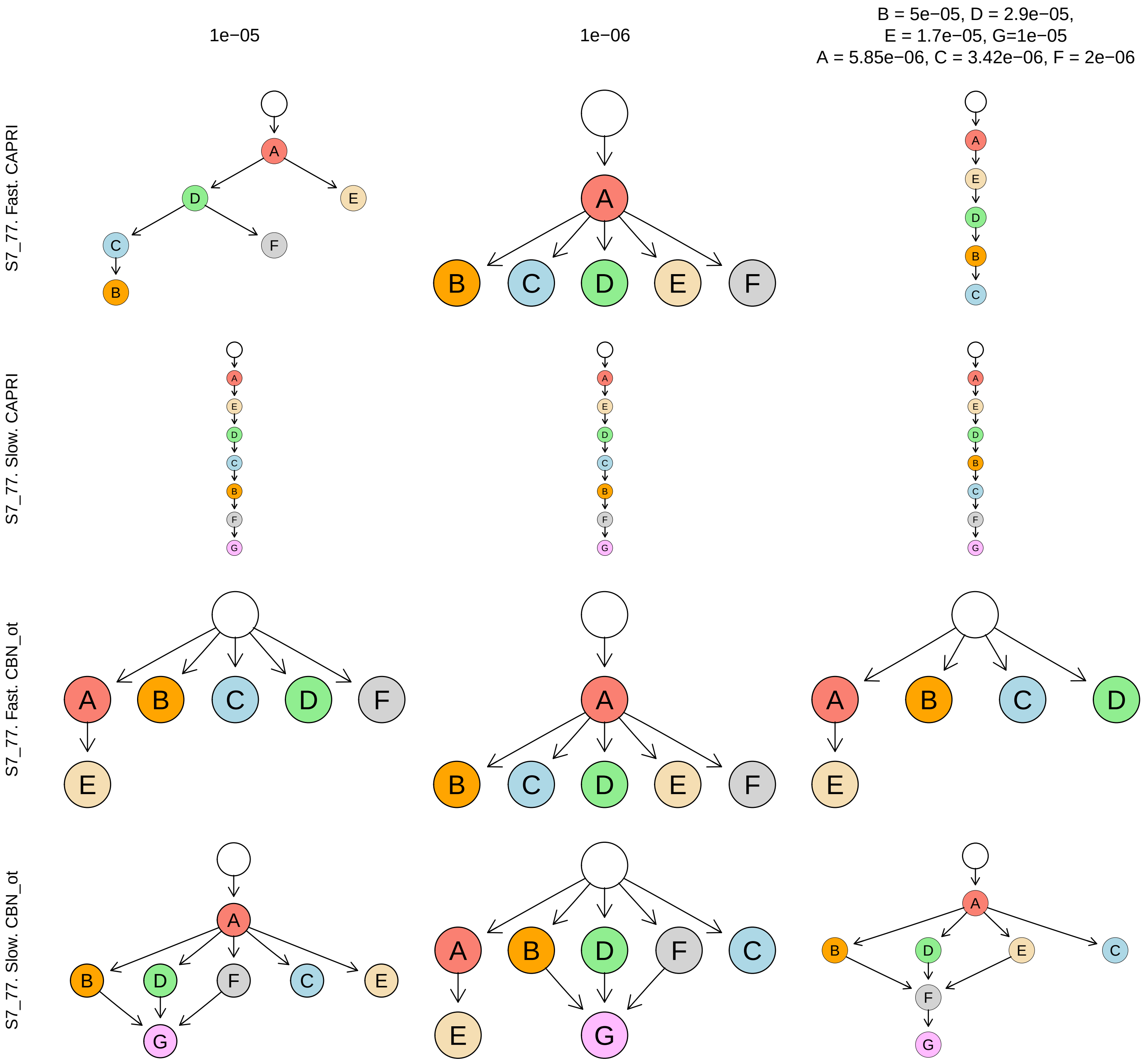

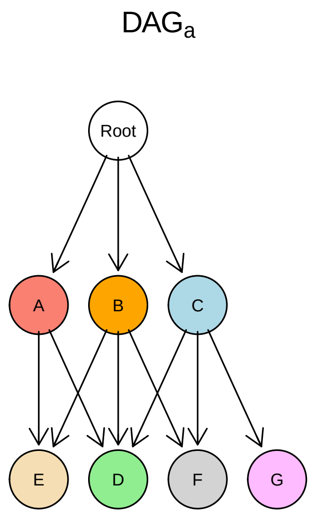

ID	Accessible Genot.	Discrep. DAG_a
S7_78	28	0

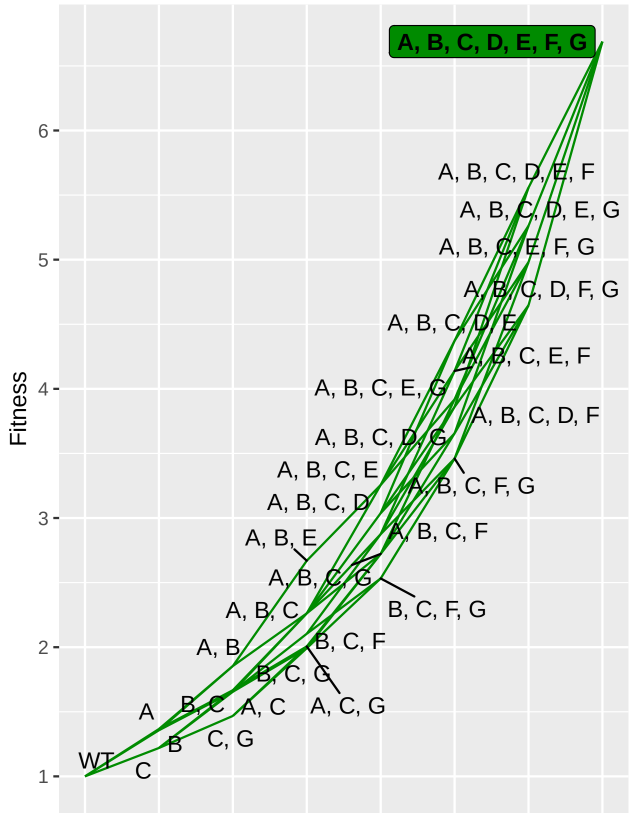

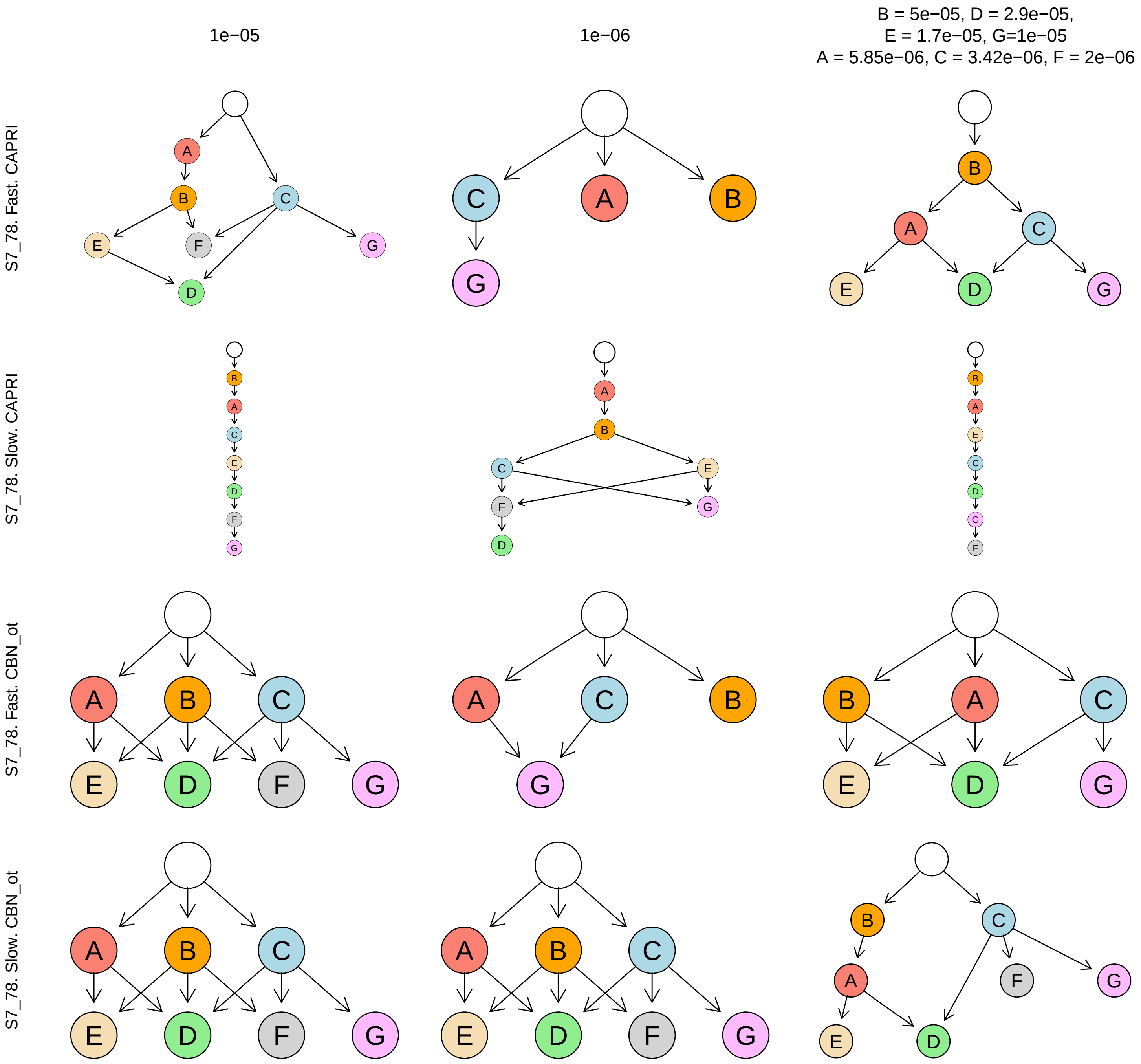

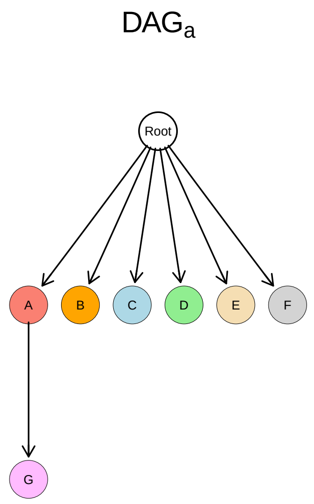

ID	Accessible Genot.	Discrep. DAG_a
S7_79	95	0

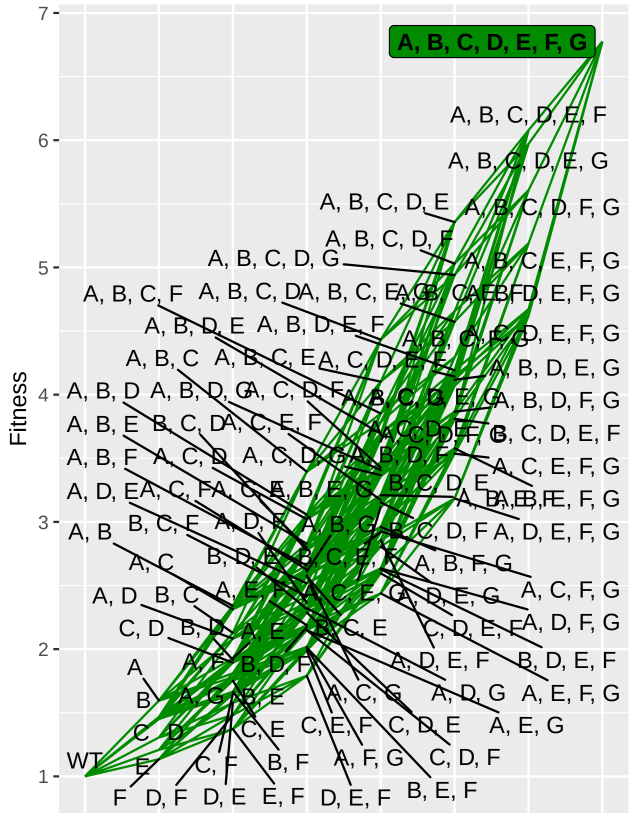

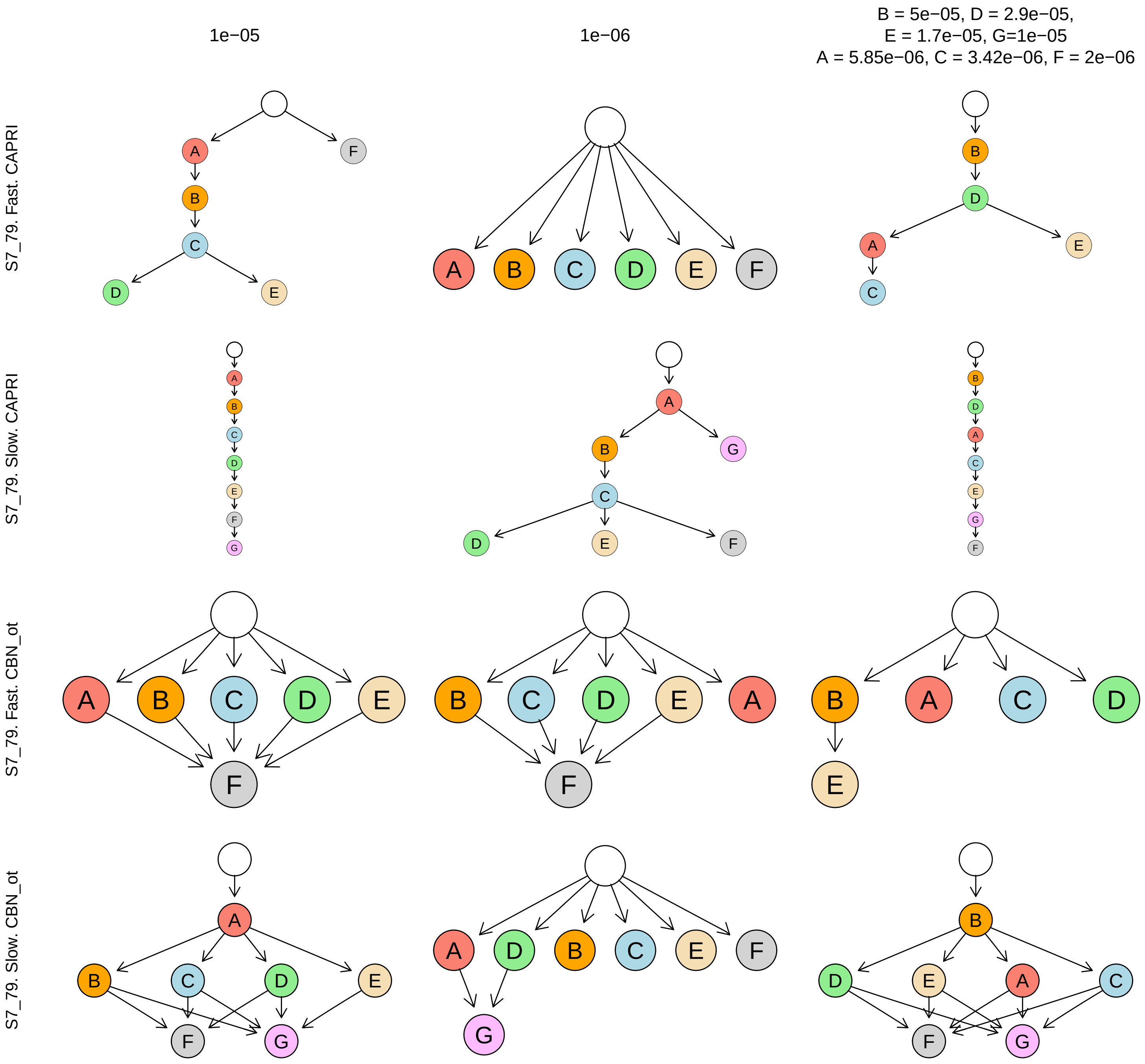

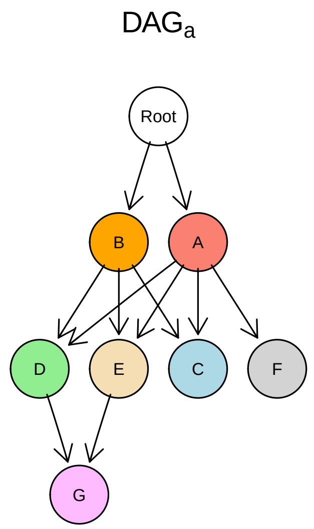

ID	Accessible Genot.	Discrep. DAG_a
S7_80	23	0

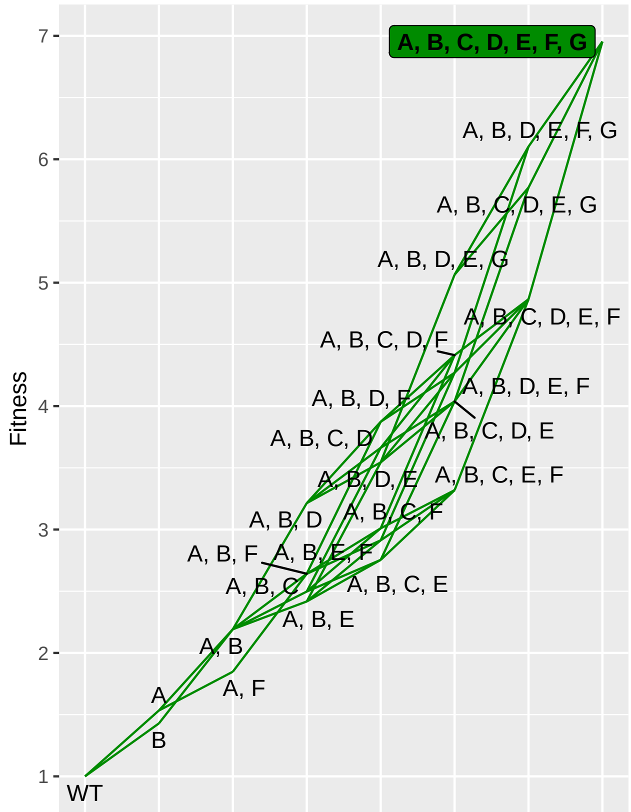

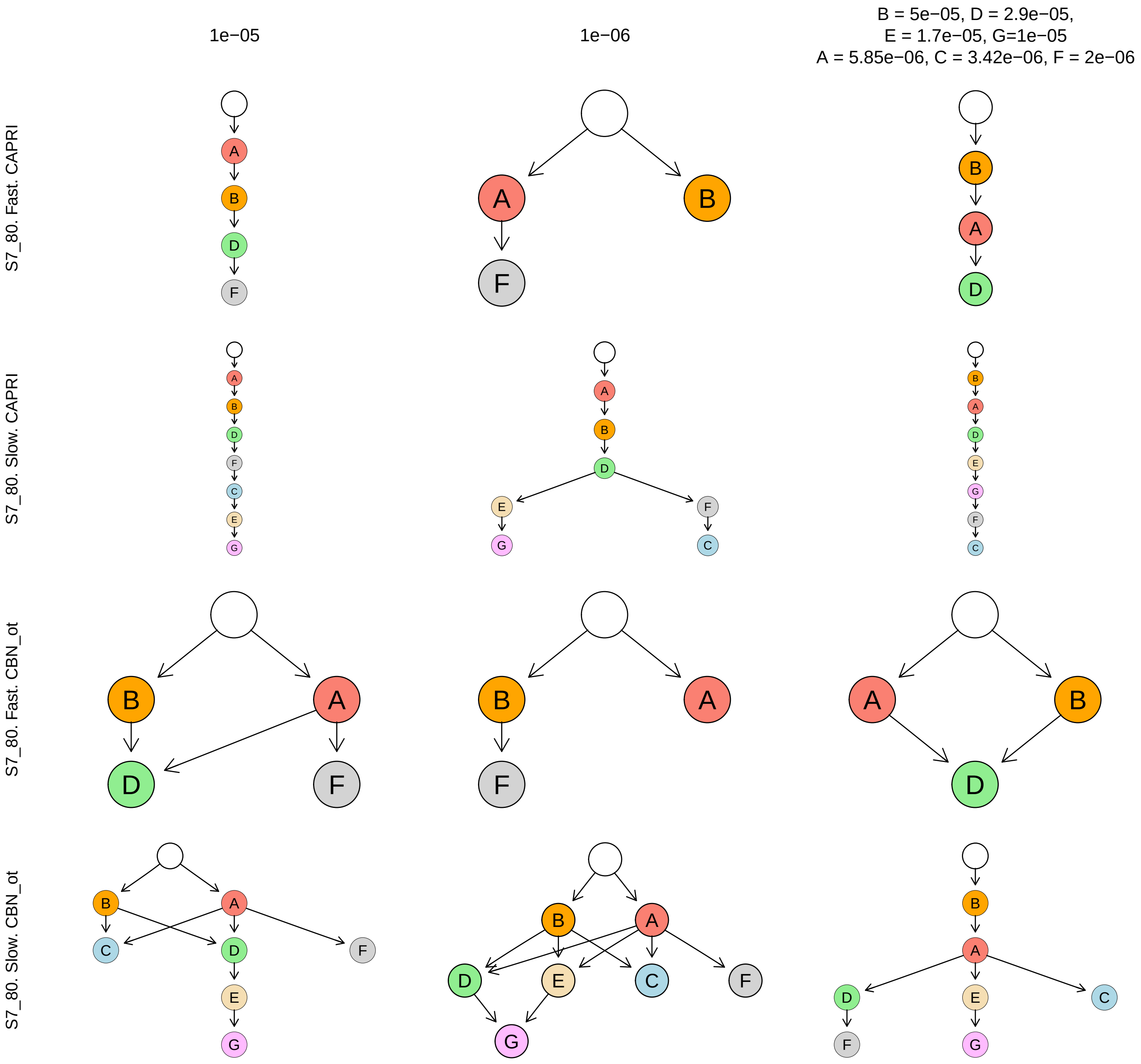

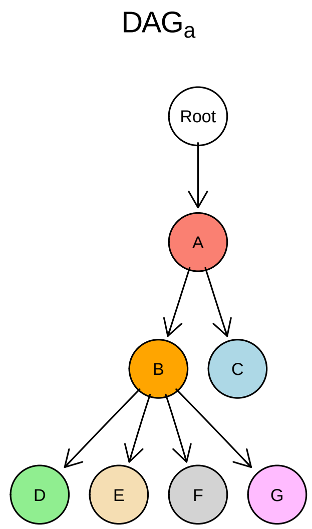

ID	Accessible Genot.	Discrep. DAG_a
S7_81	34	0

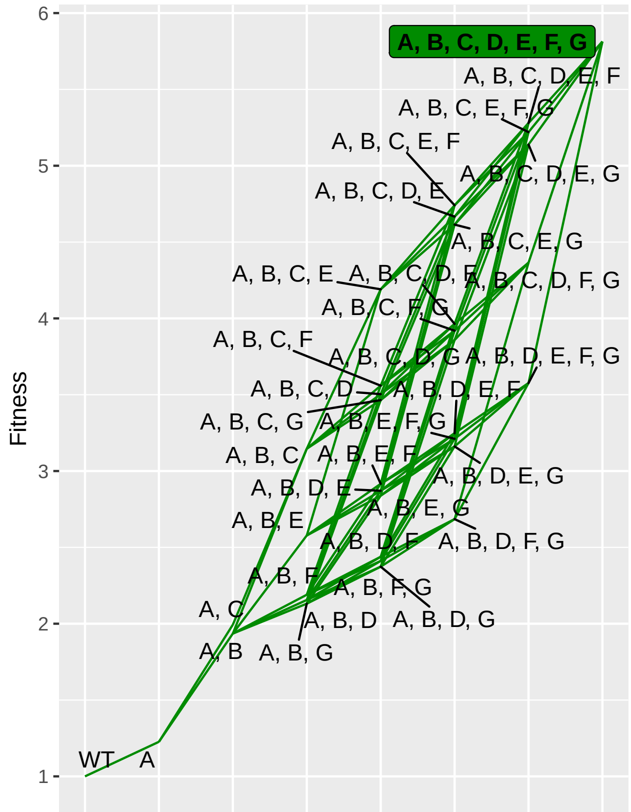

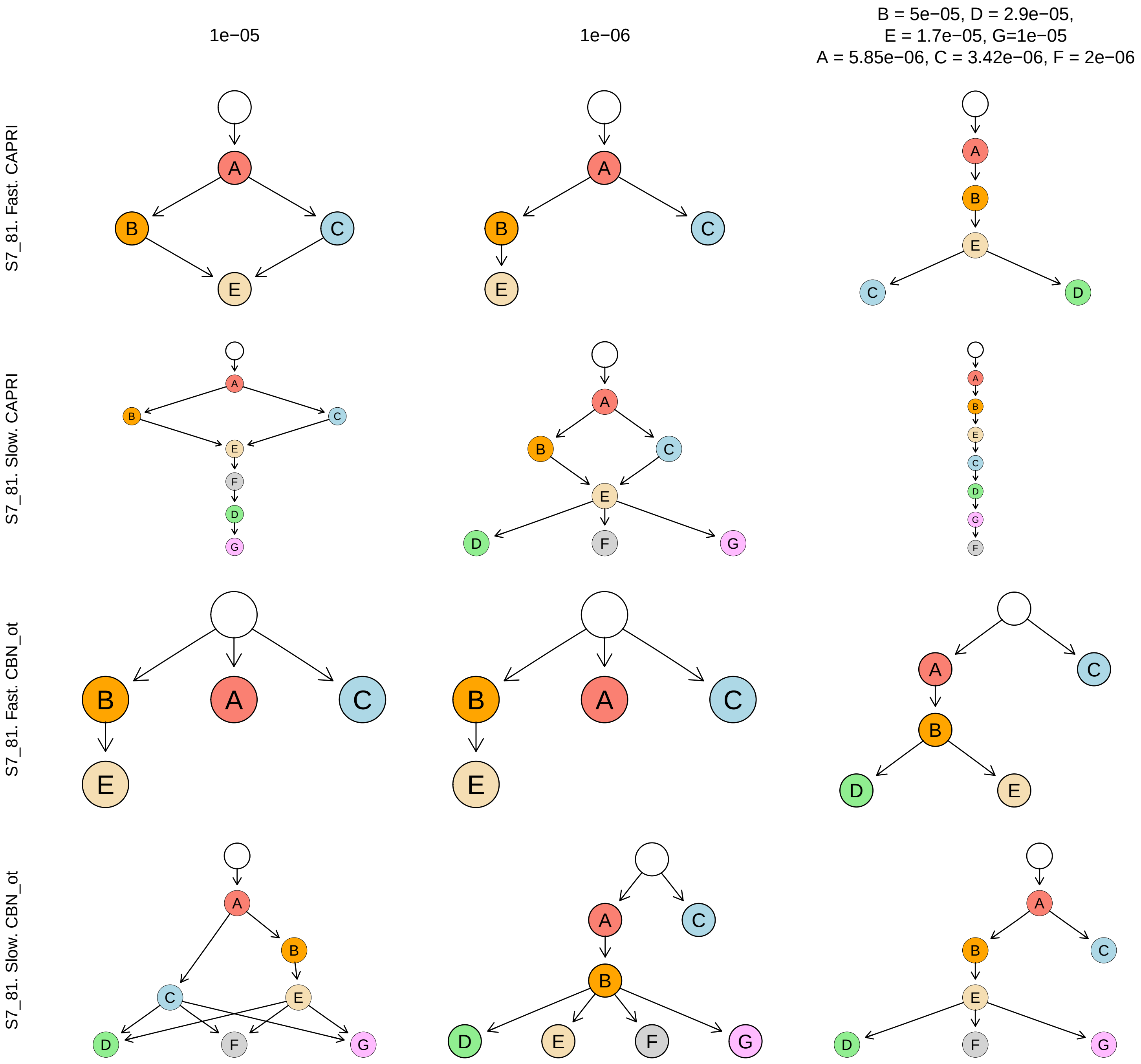

DAG_a

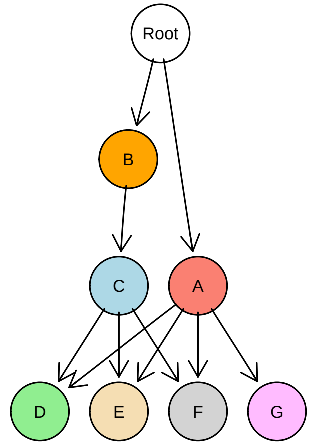

ID	Accessible Genot.	Discrep. DAG_a
S7_82	22	0

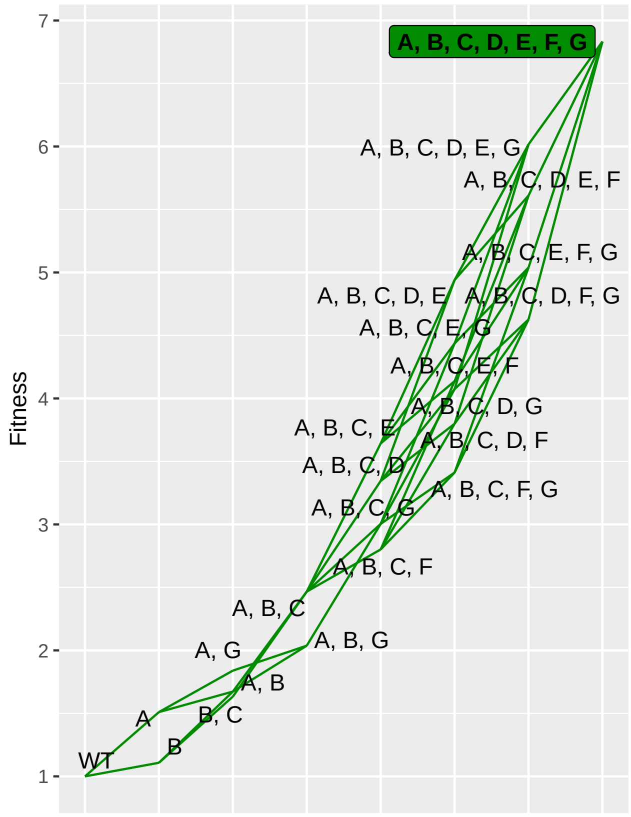

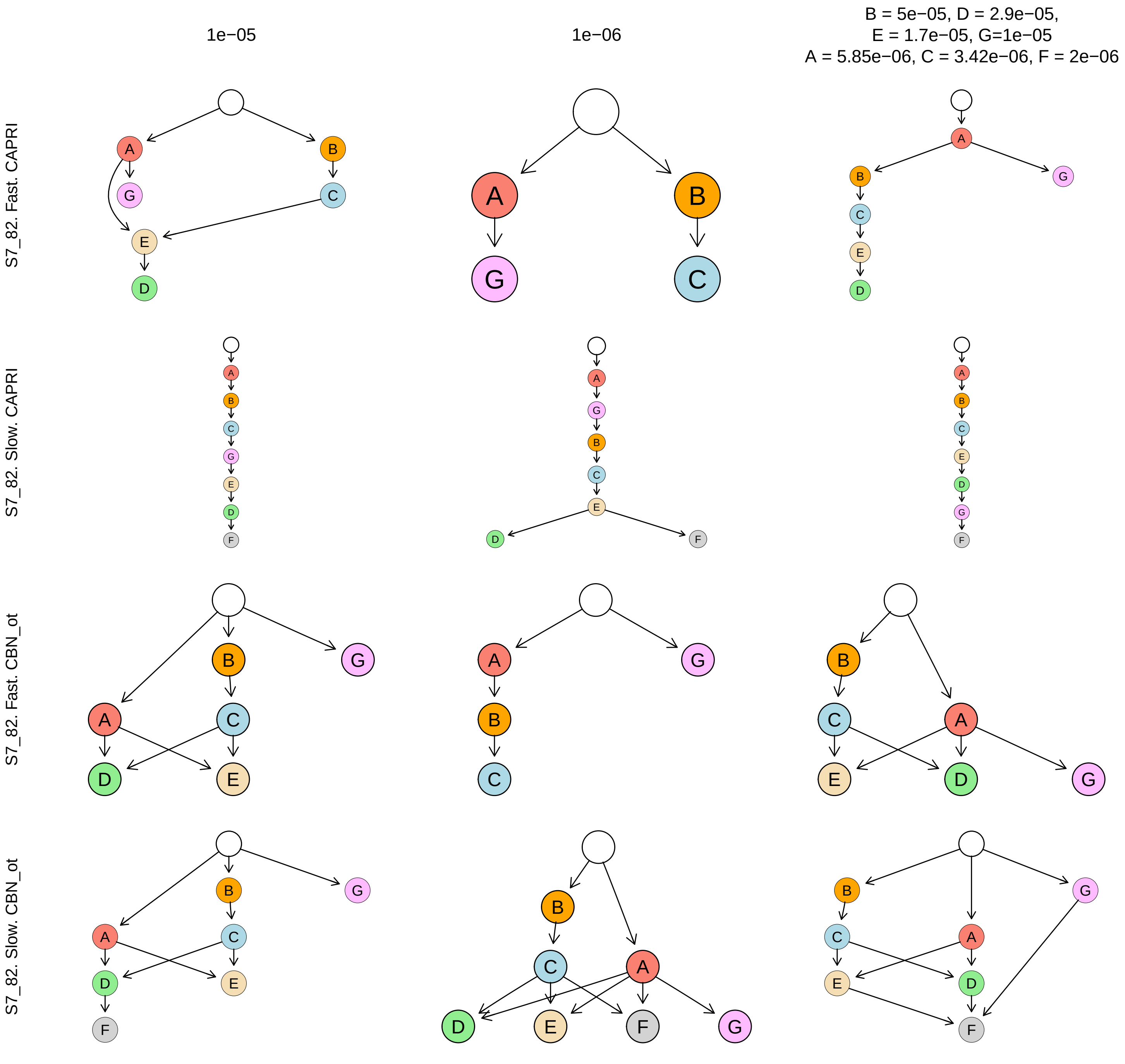

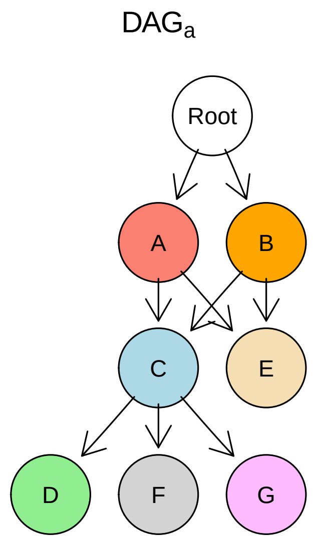

ID	Accessible Genot.	Discrep. DAG_a
S7_83	20	0

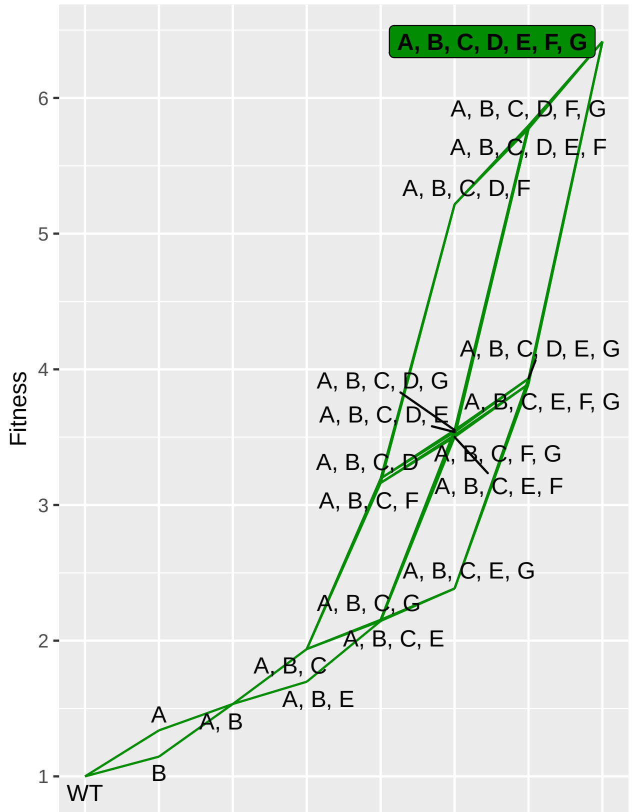

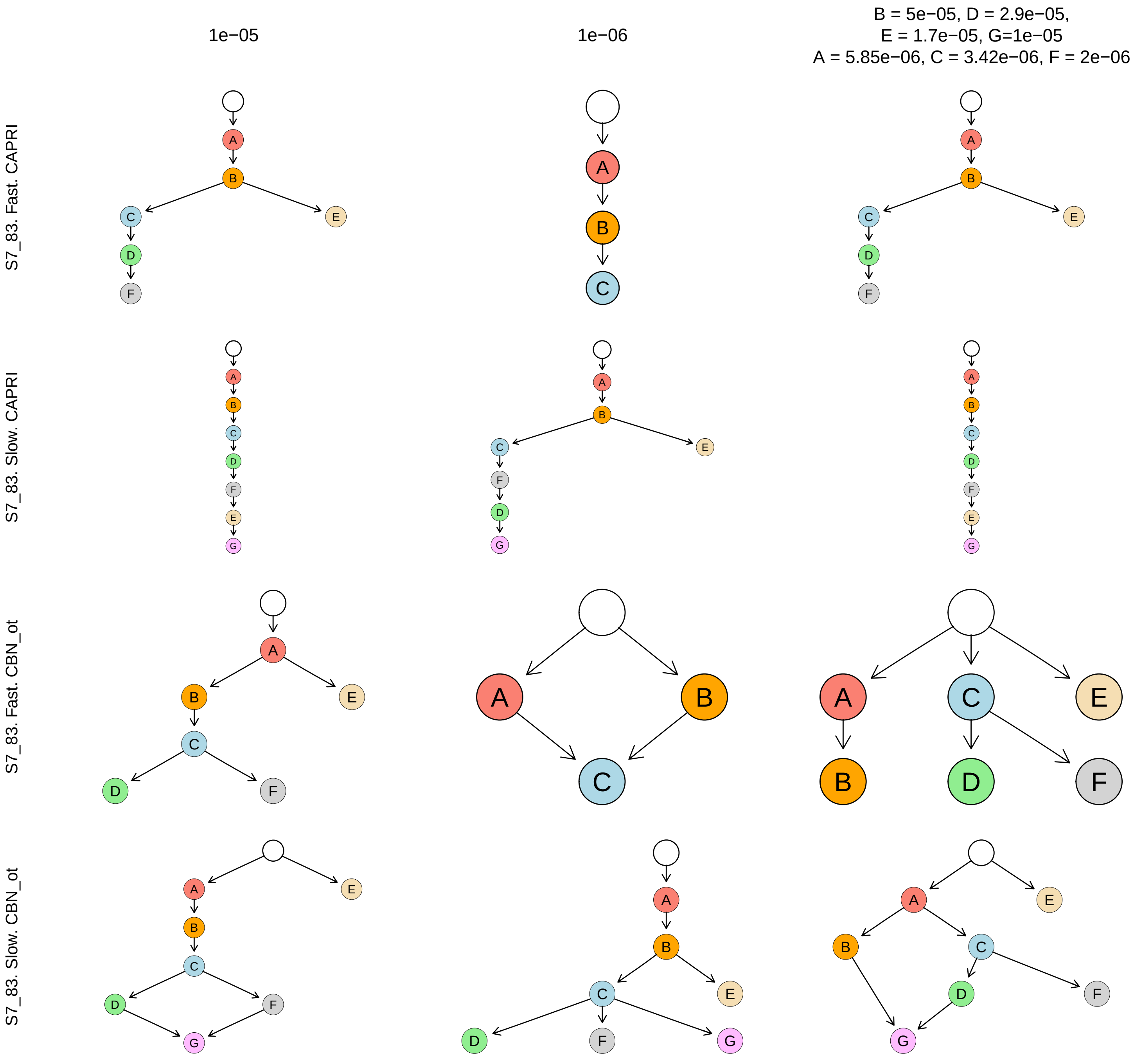

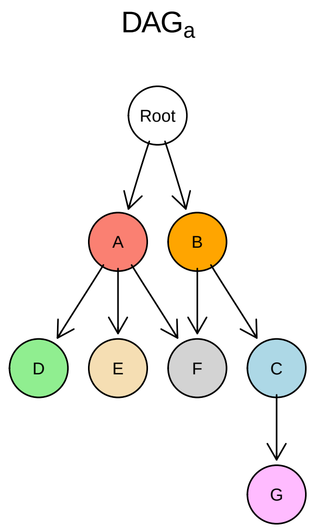

ID	Accessible Genot.	Discrep. DAG_a
S7_84	31	0

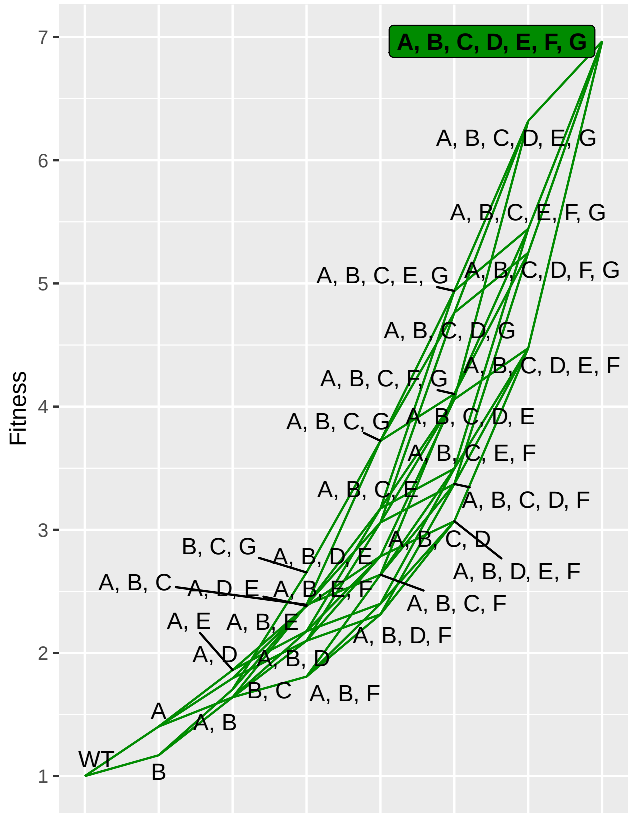

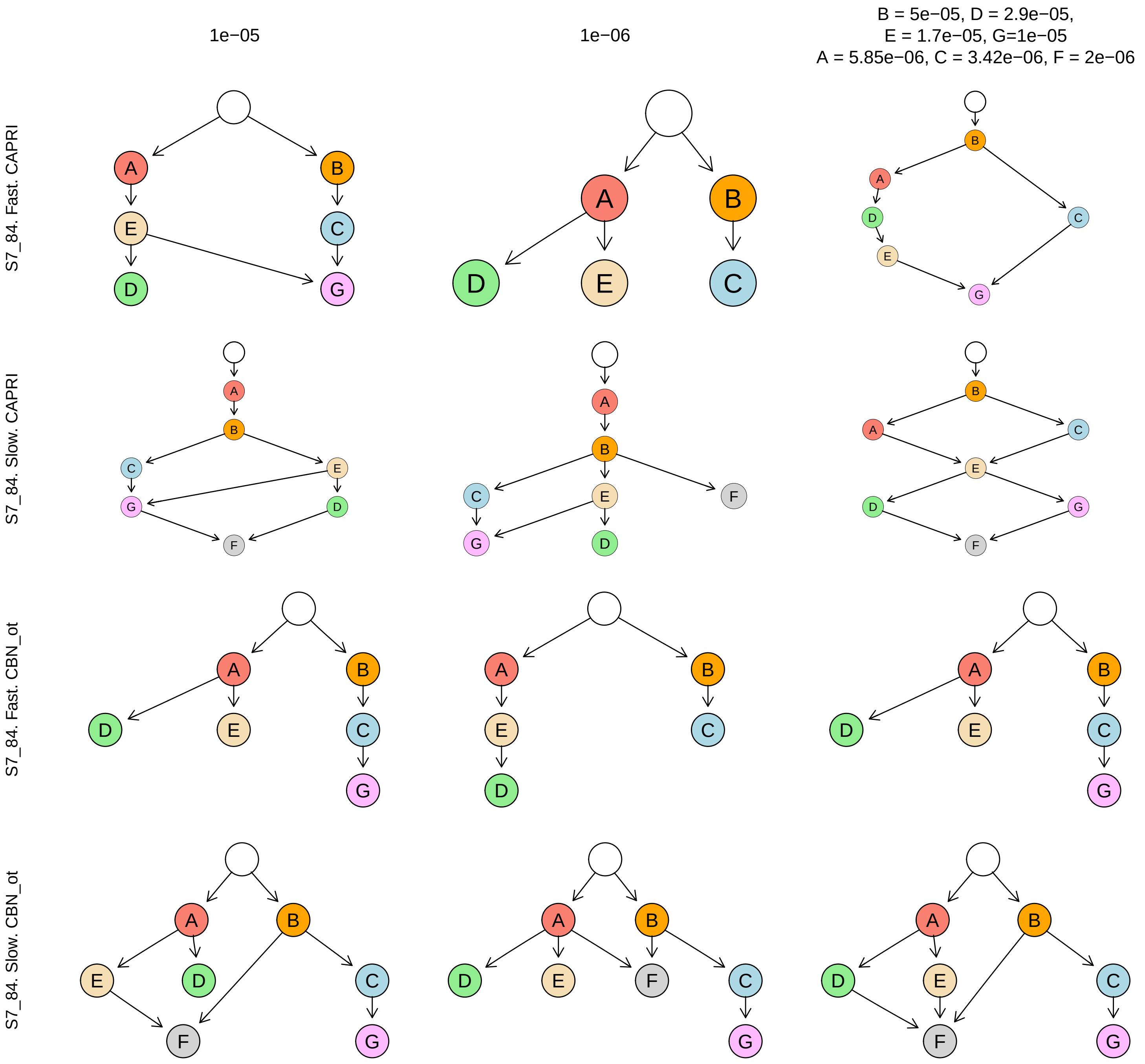

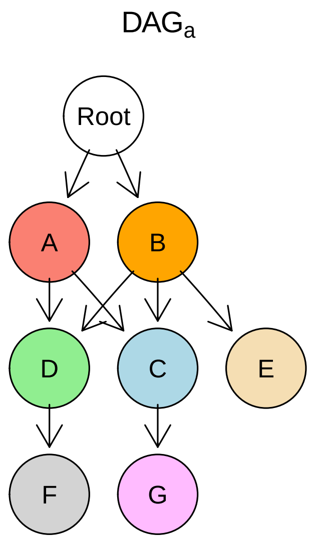

ID	Accessible Genot.	Discrep. DAG_a
S7_85	21	0

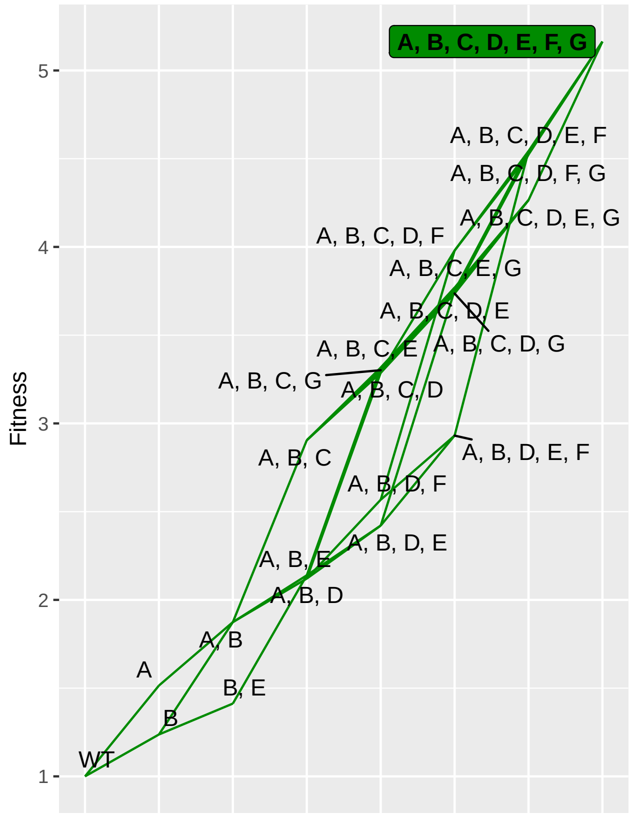

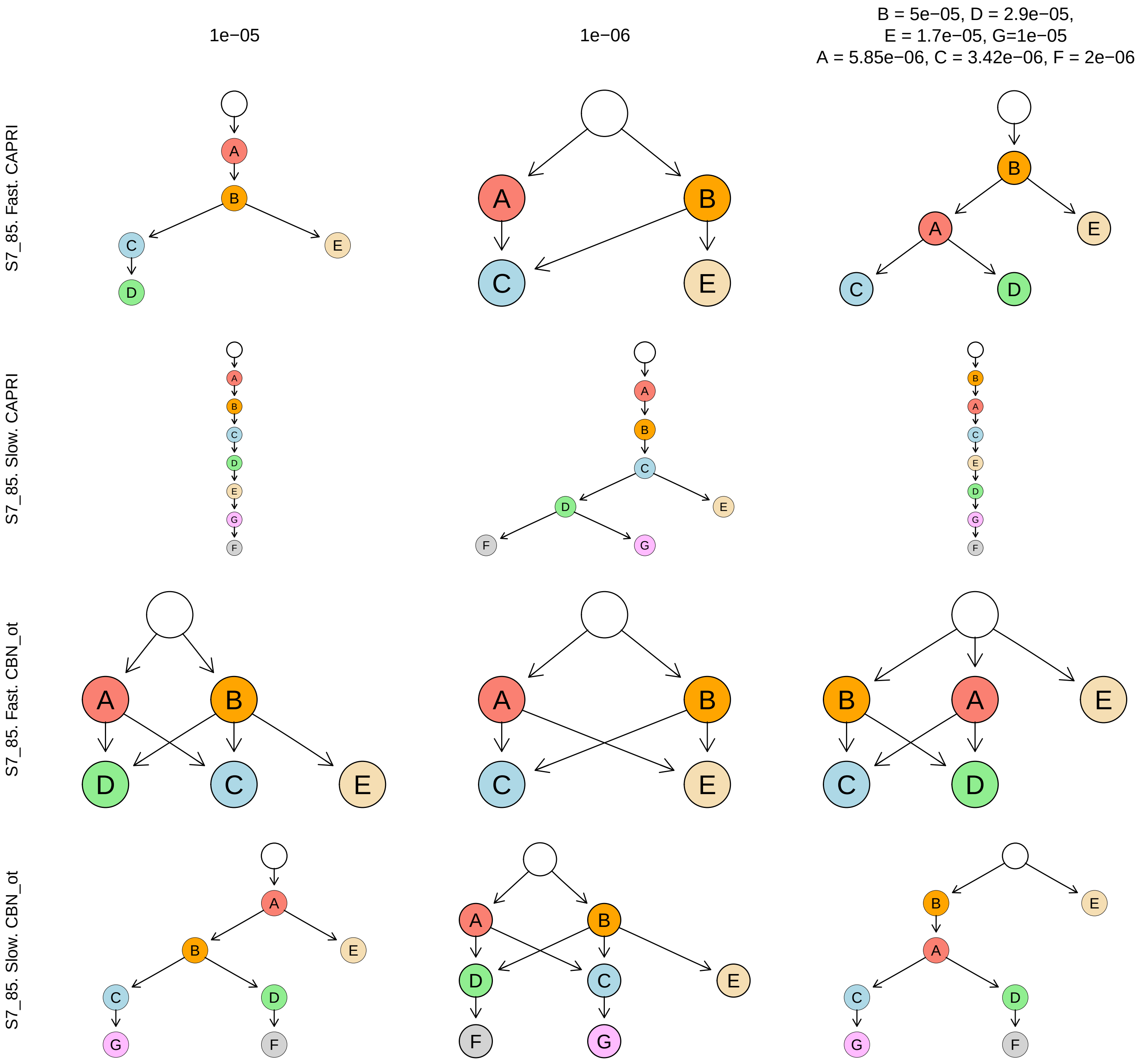

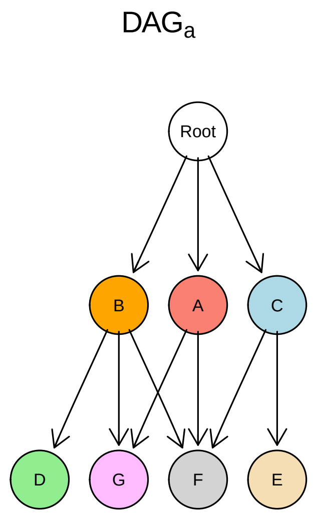

ID	Accessible Genot.	Discrep. DAG_a
S7_86	31	0

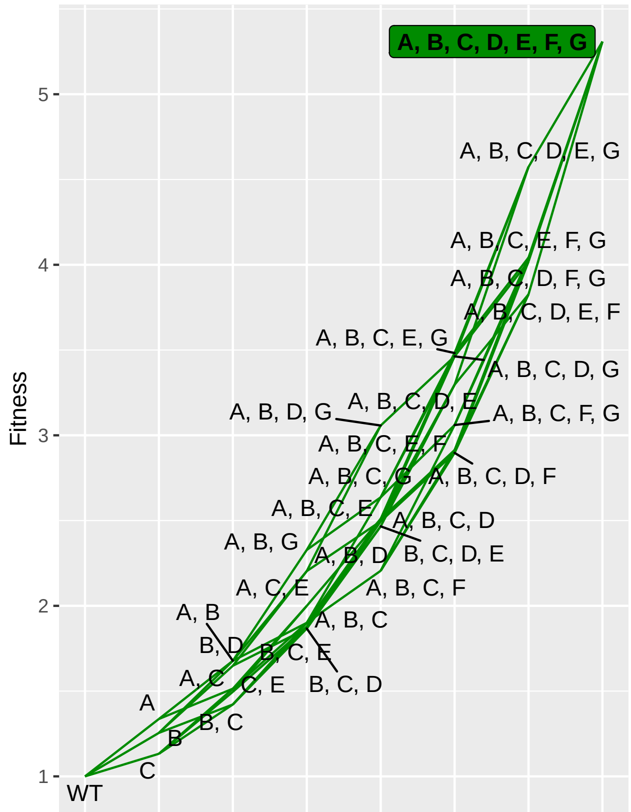

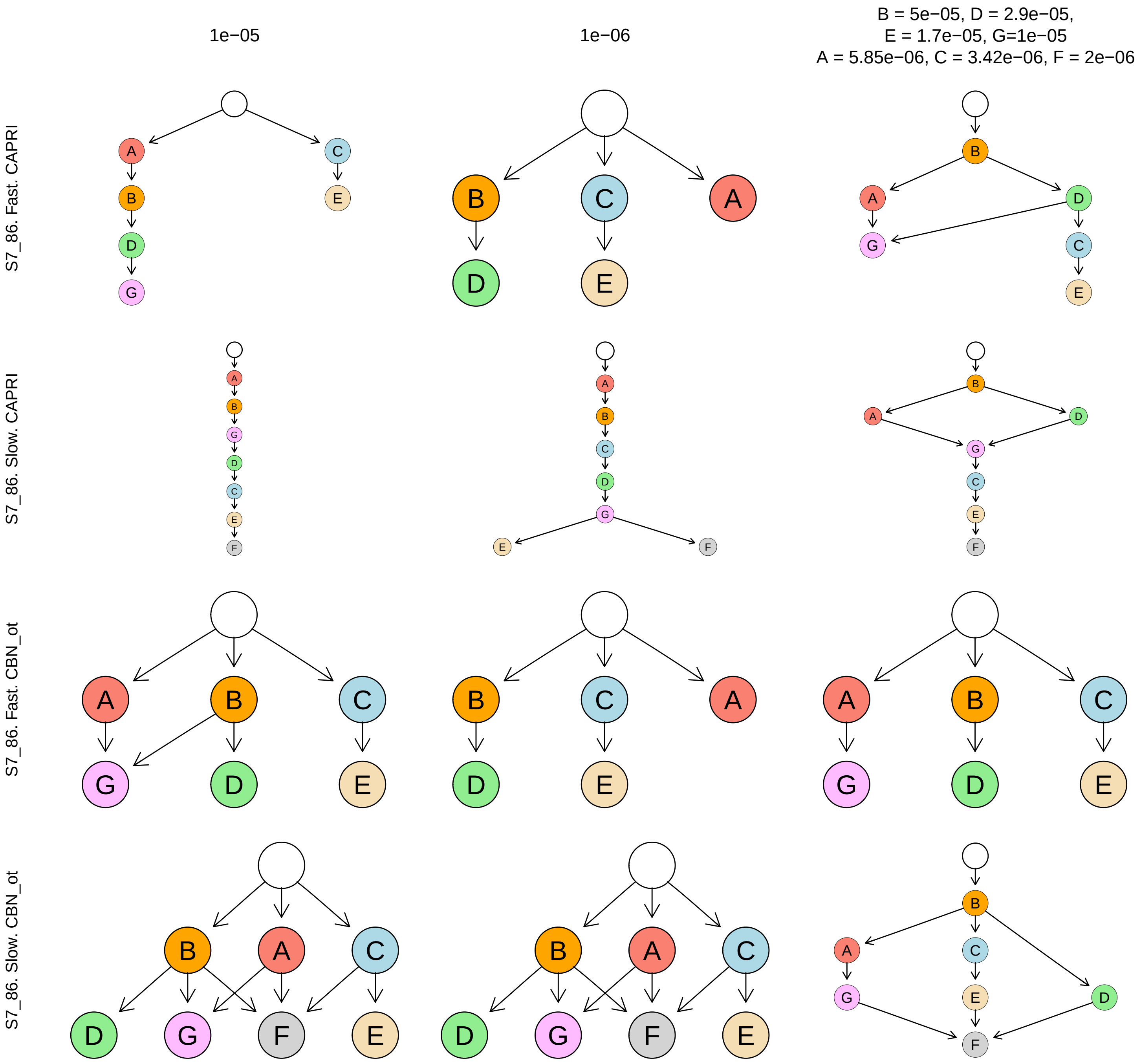

DAG$_a$

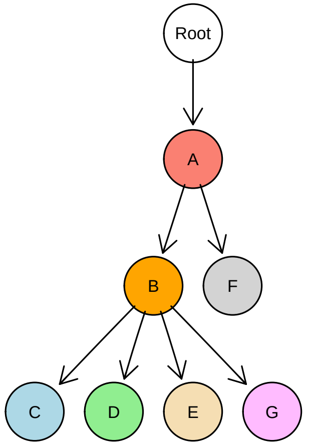

ID	Accessible Genot.	Discrep. DAG_a
S7_87	34	0

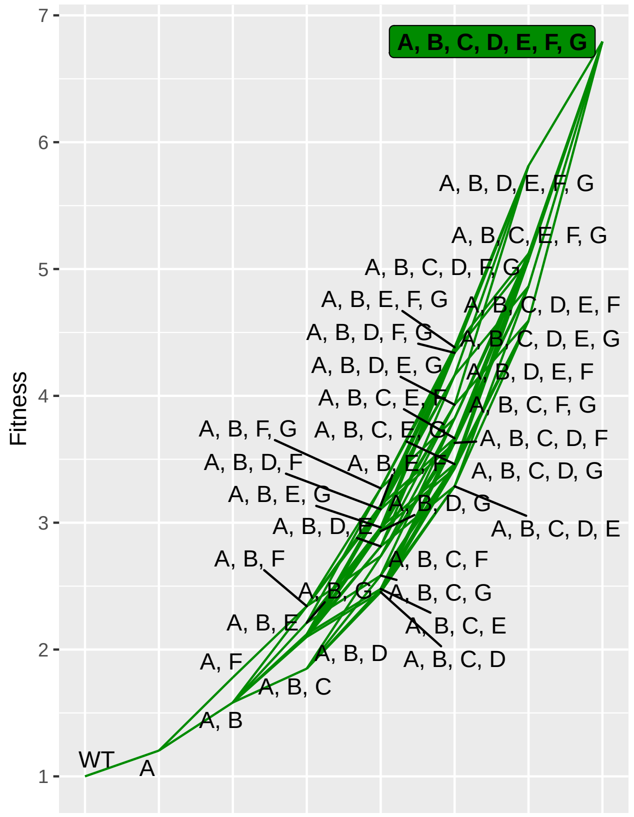

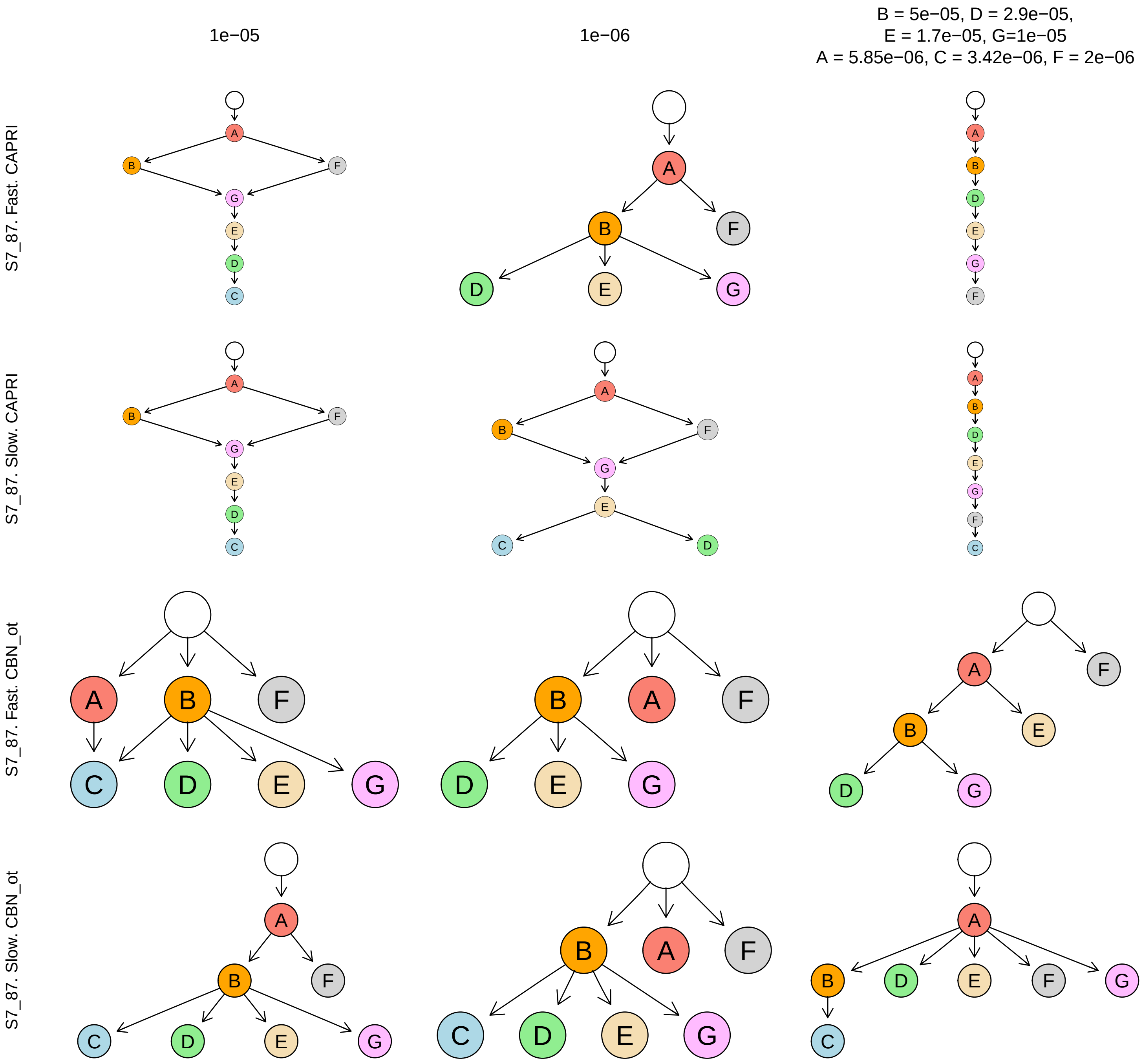

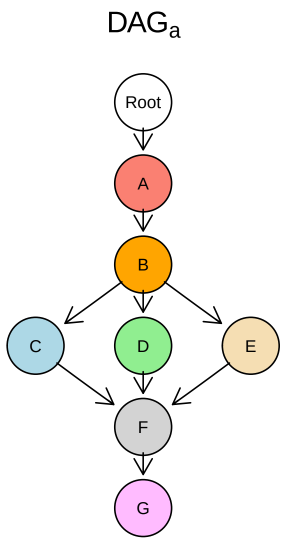

ID	Accessible Genot.	Discrep. DAG_a
S7_88	11	0

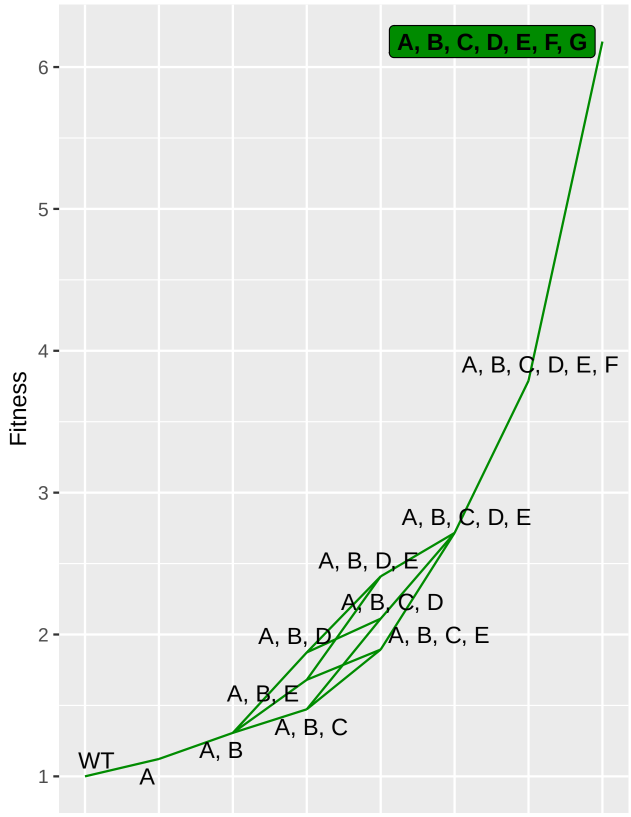

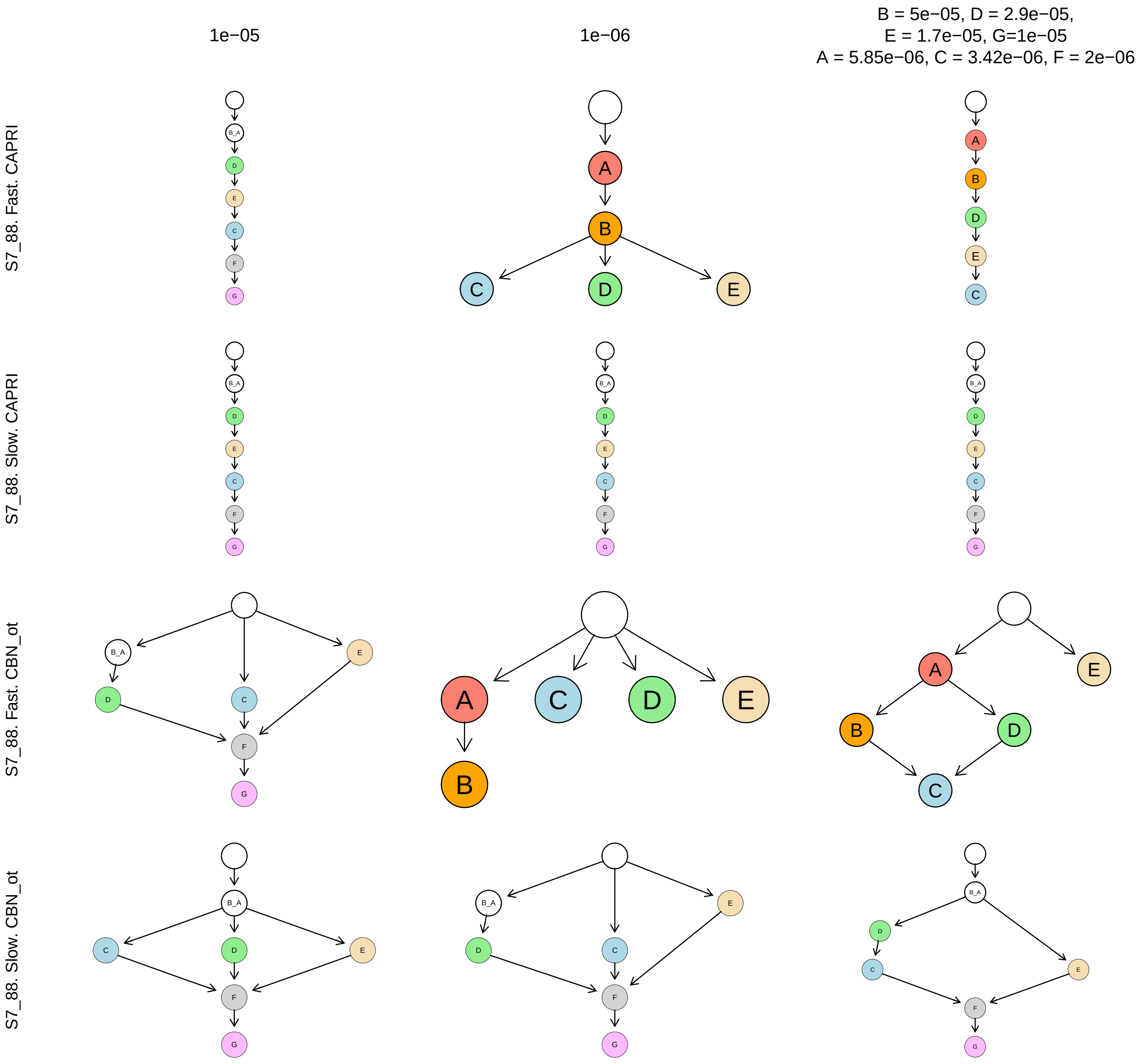

DAG$_a$

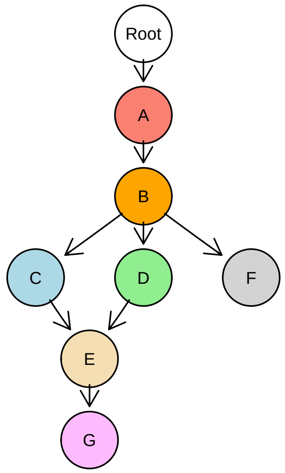

ID	Accessible Genot.	Discrep. DAG_a
S7_89	13	0

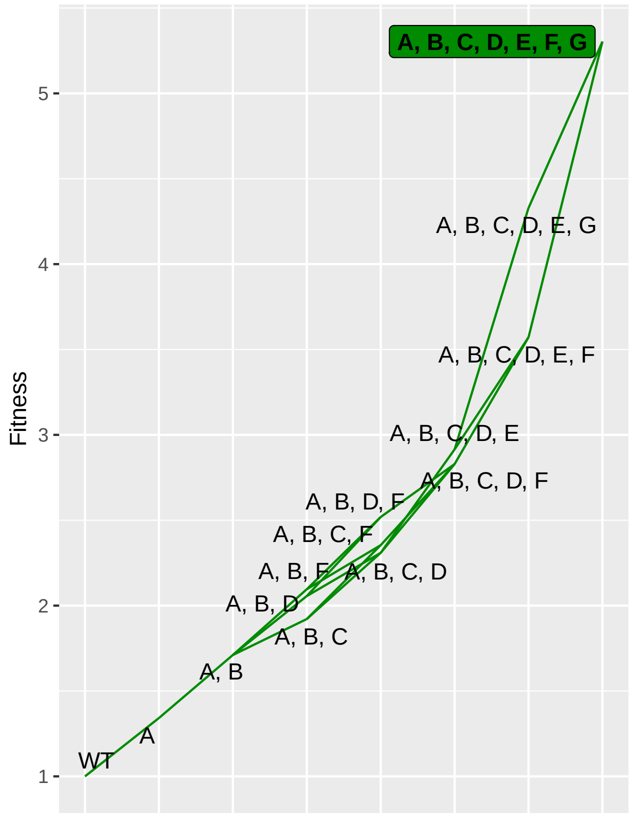

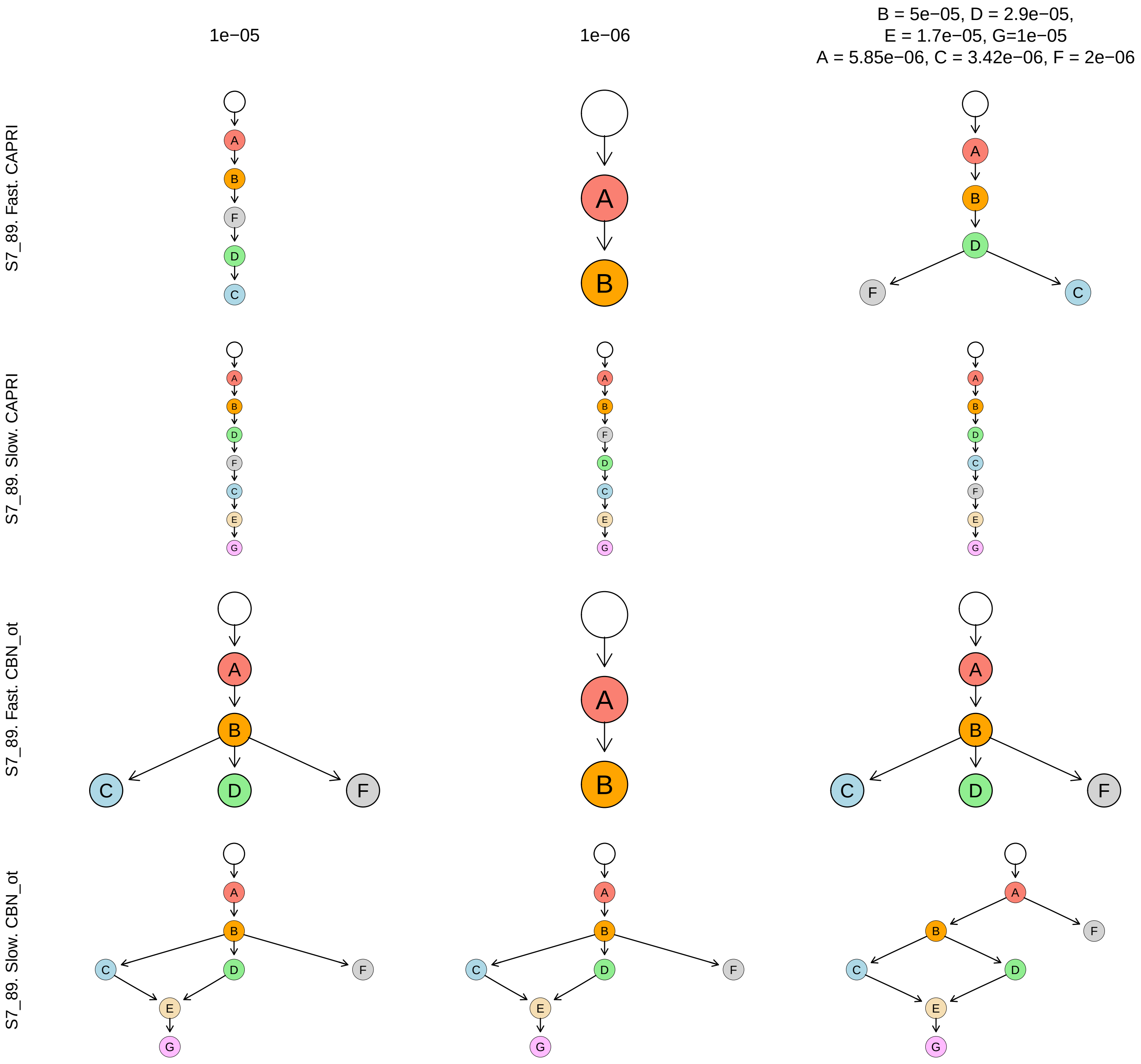

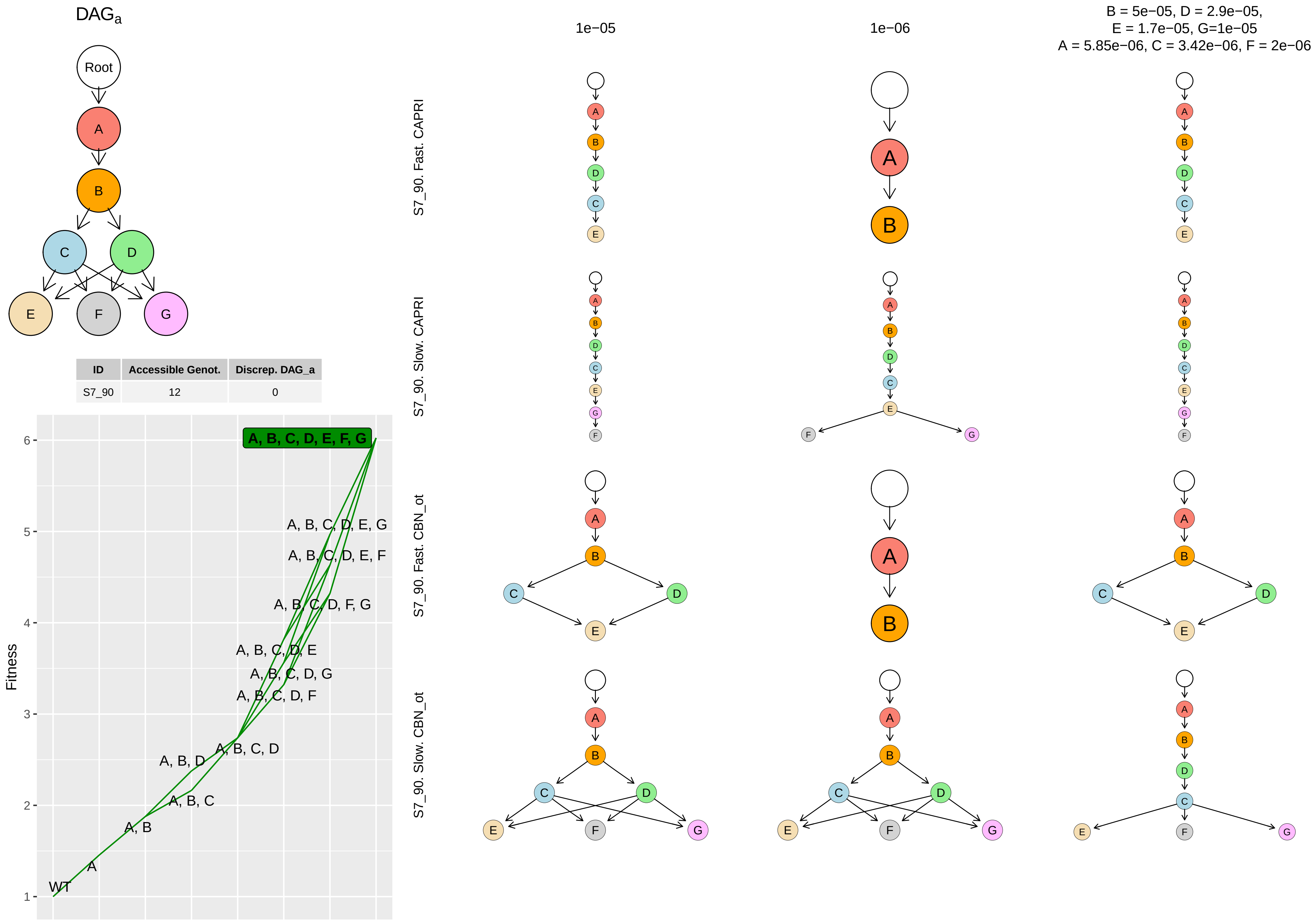

ID	Accessible Genot.	Discrep. DAG_a
S7_90	12	0

DAG$_a$

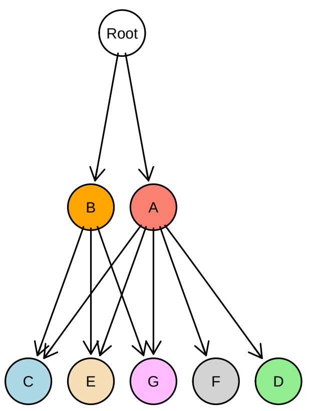

ID	Accessible Genot.	Discrep. DAG_a
S7_91	37	0

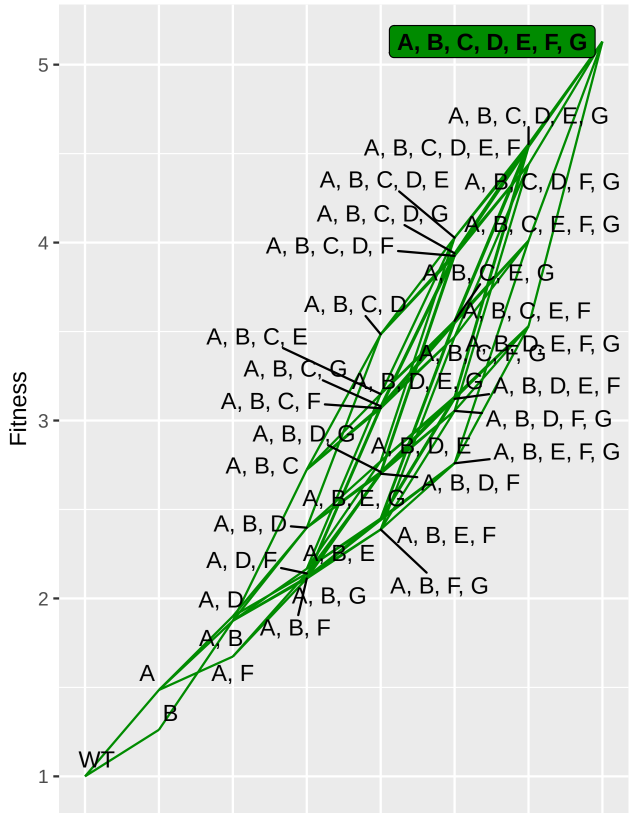

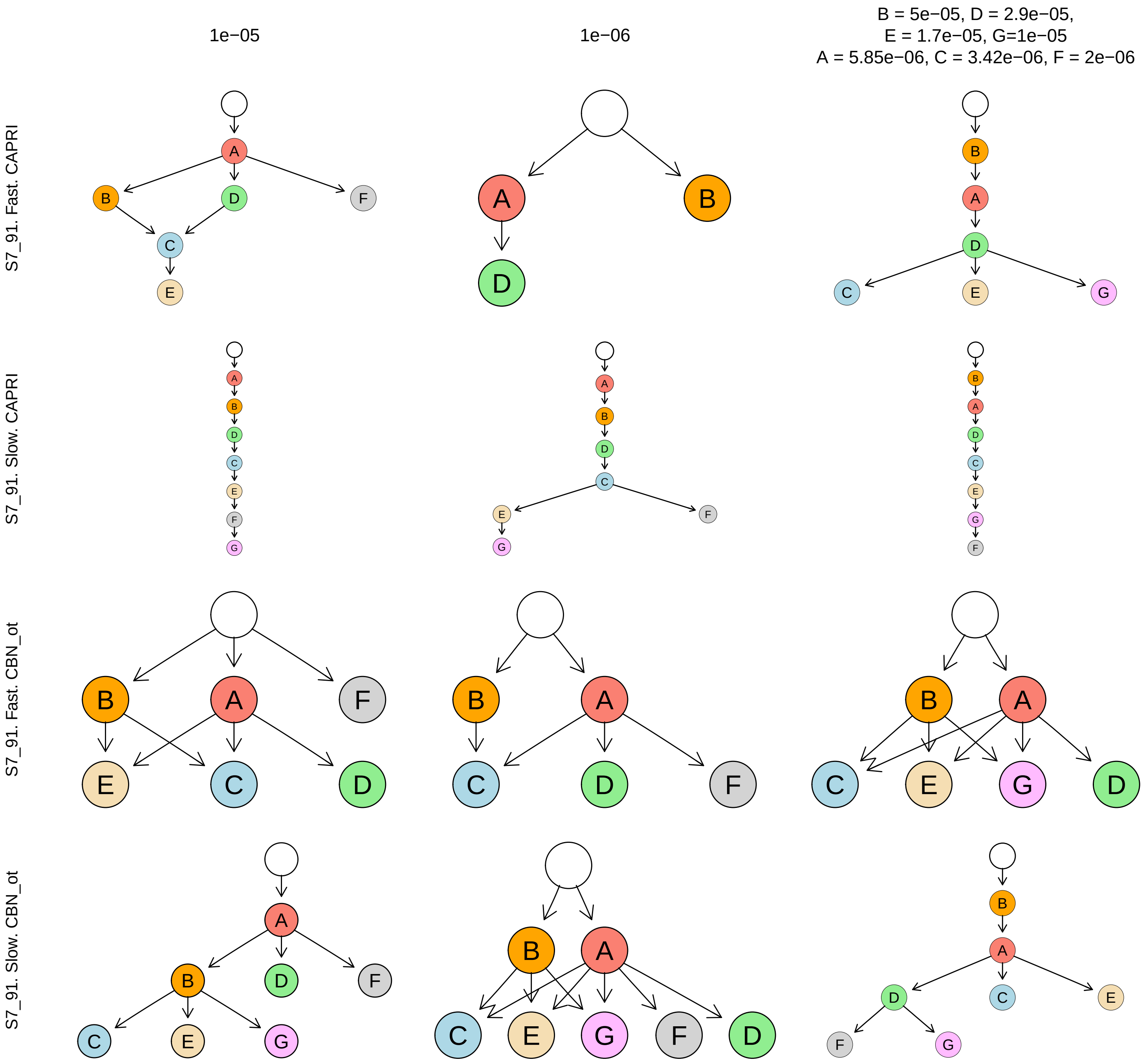

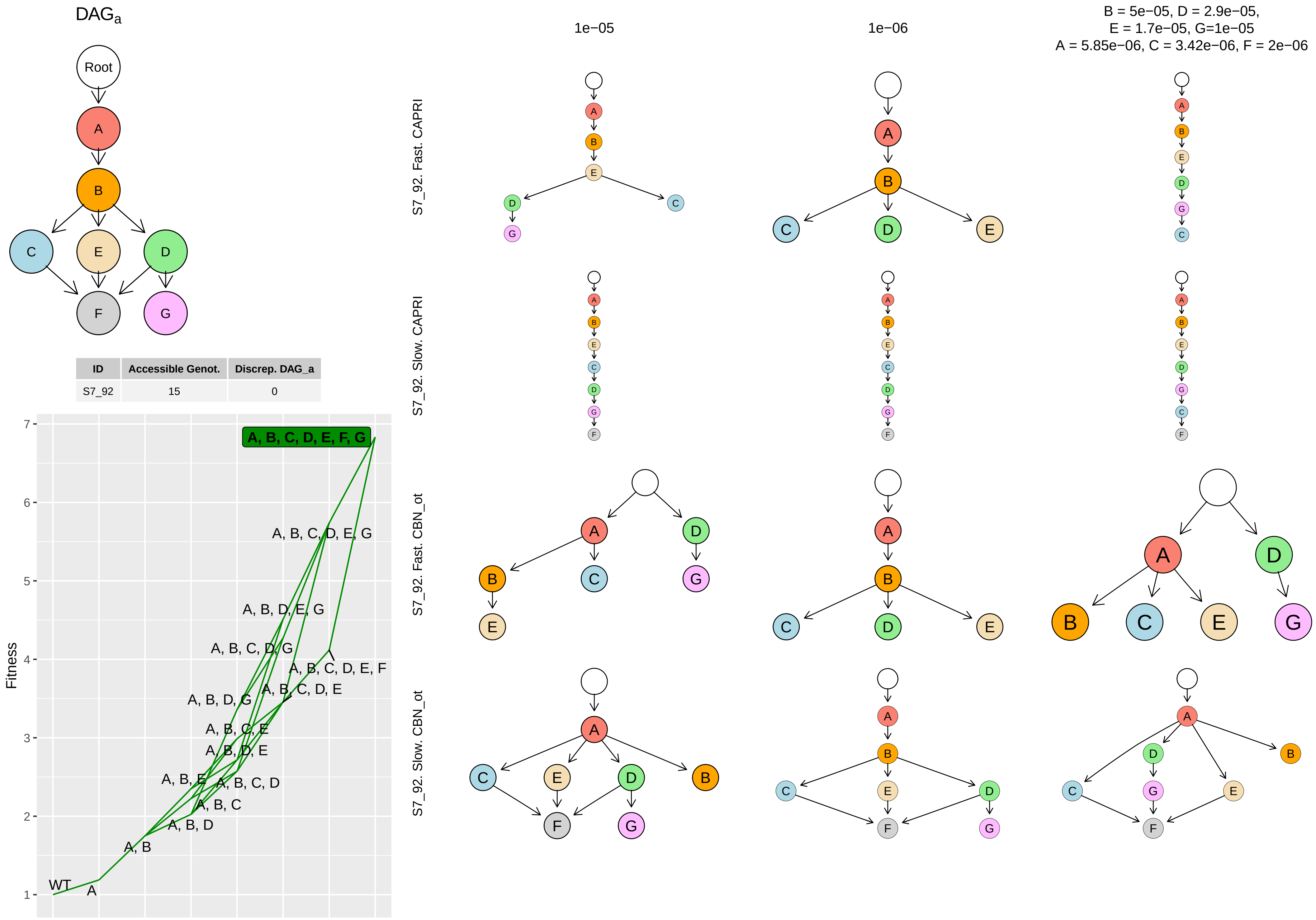

ID	Accessible Genot.	Discrep. DAG_a
S7_92	15	0

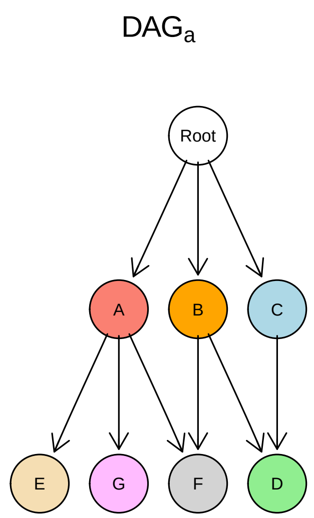

ID	Accessible Genot.	Discrep. DAG_a
S7_93	36	0

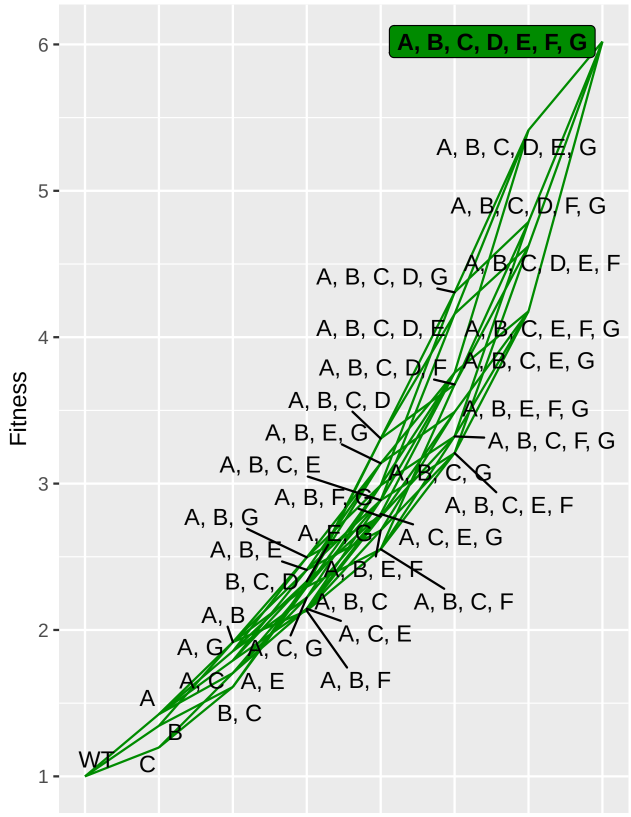

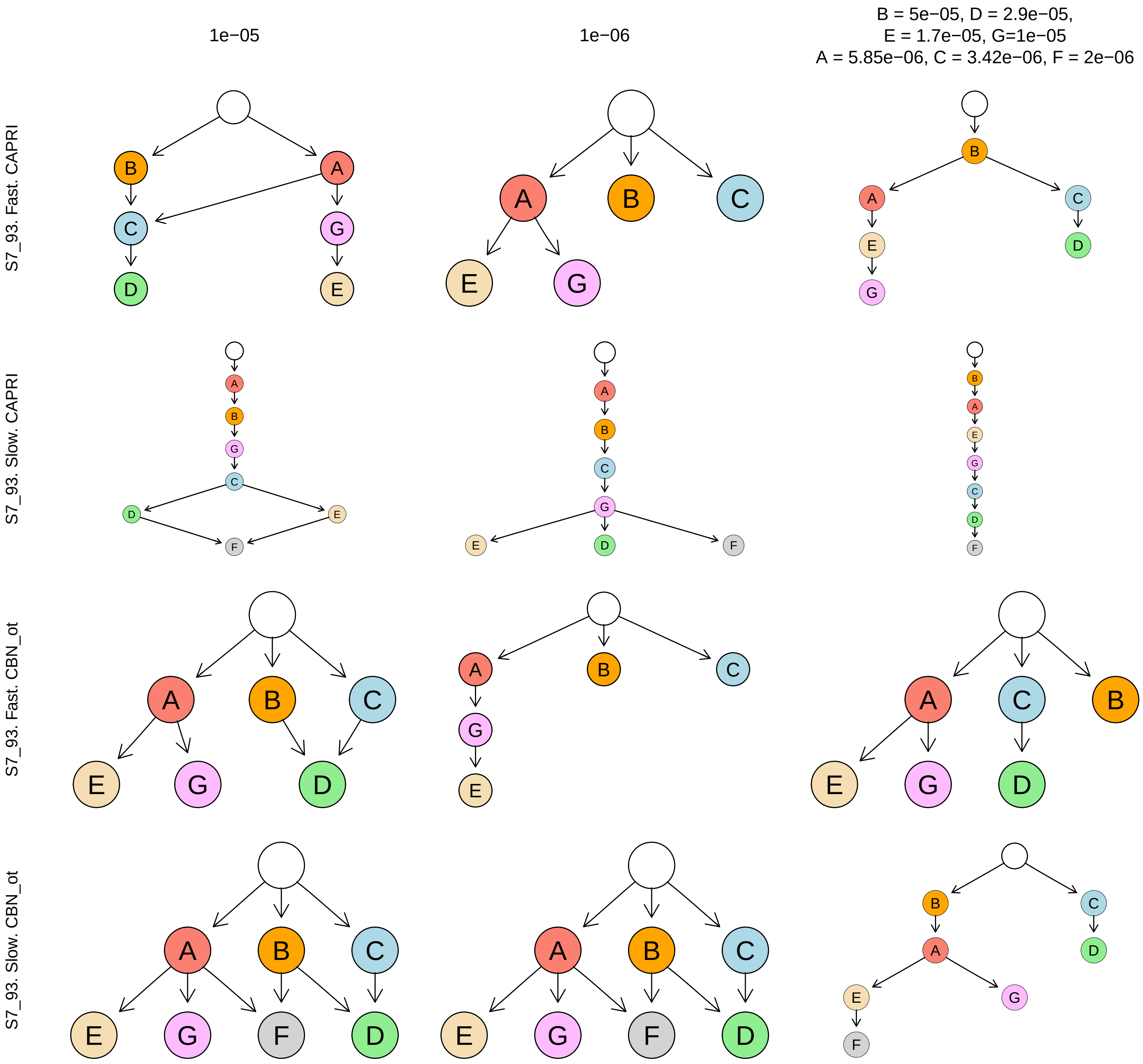

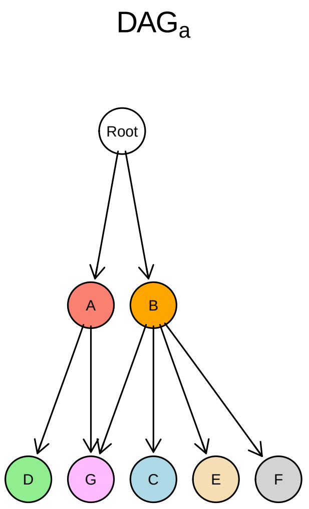

ID	Accessible Genot.	Discrep. DAG_a
S7_94	42	0

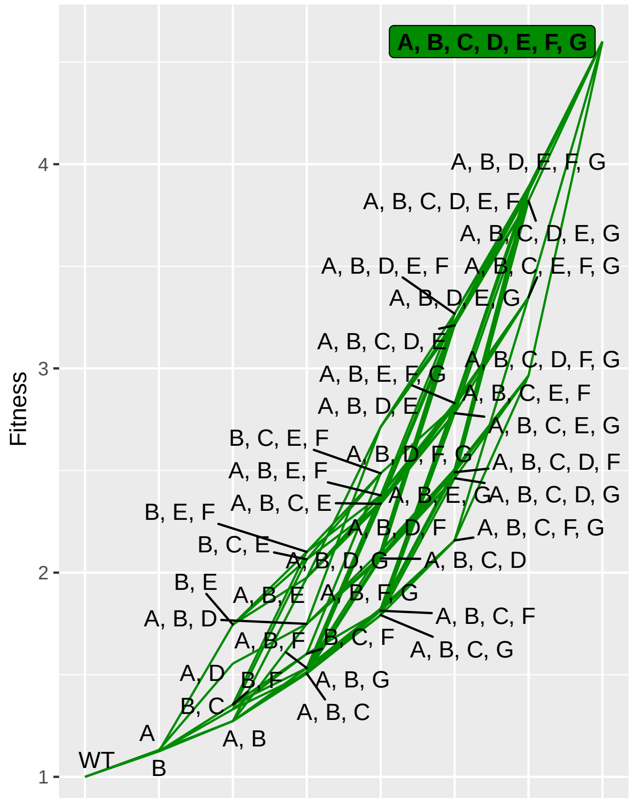

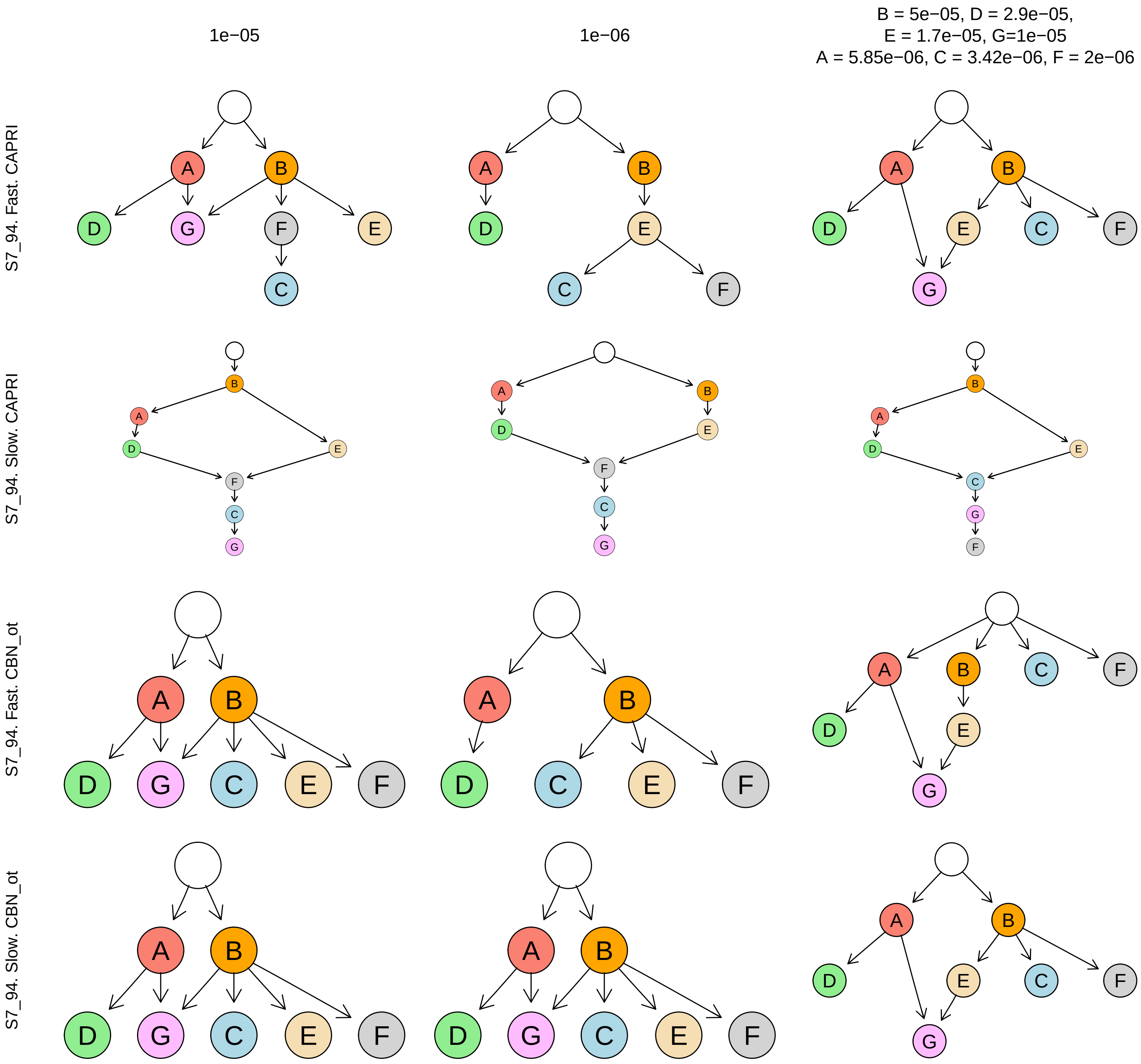

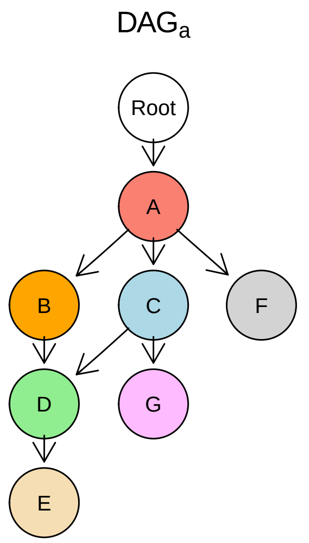

ID	Accessible Genot.	Discrep. DAG_a
S7_95	20	0

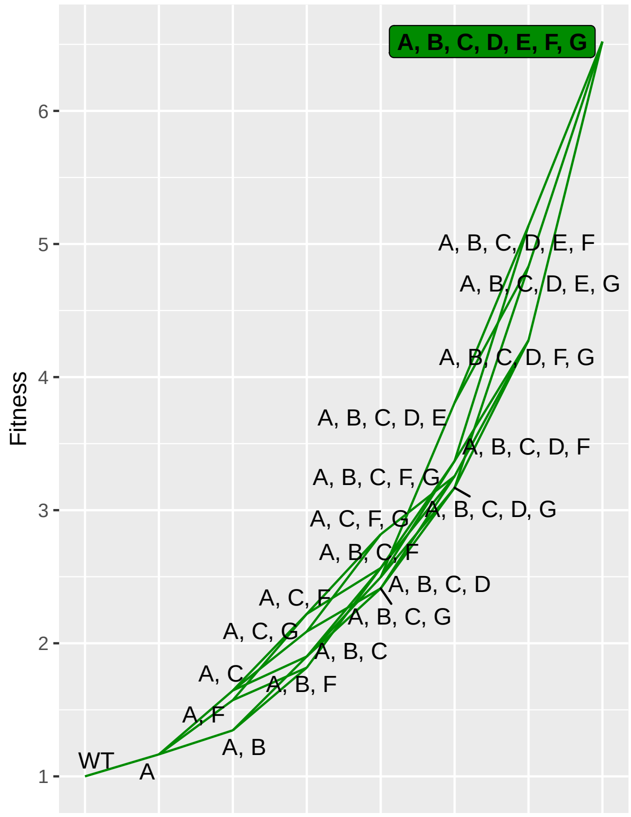

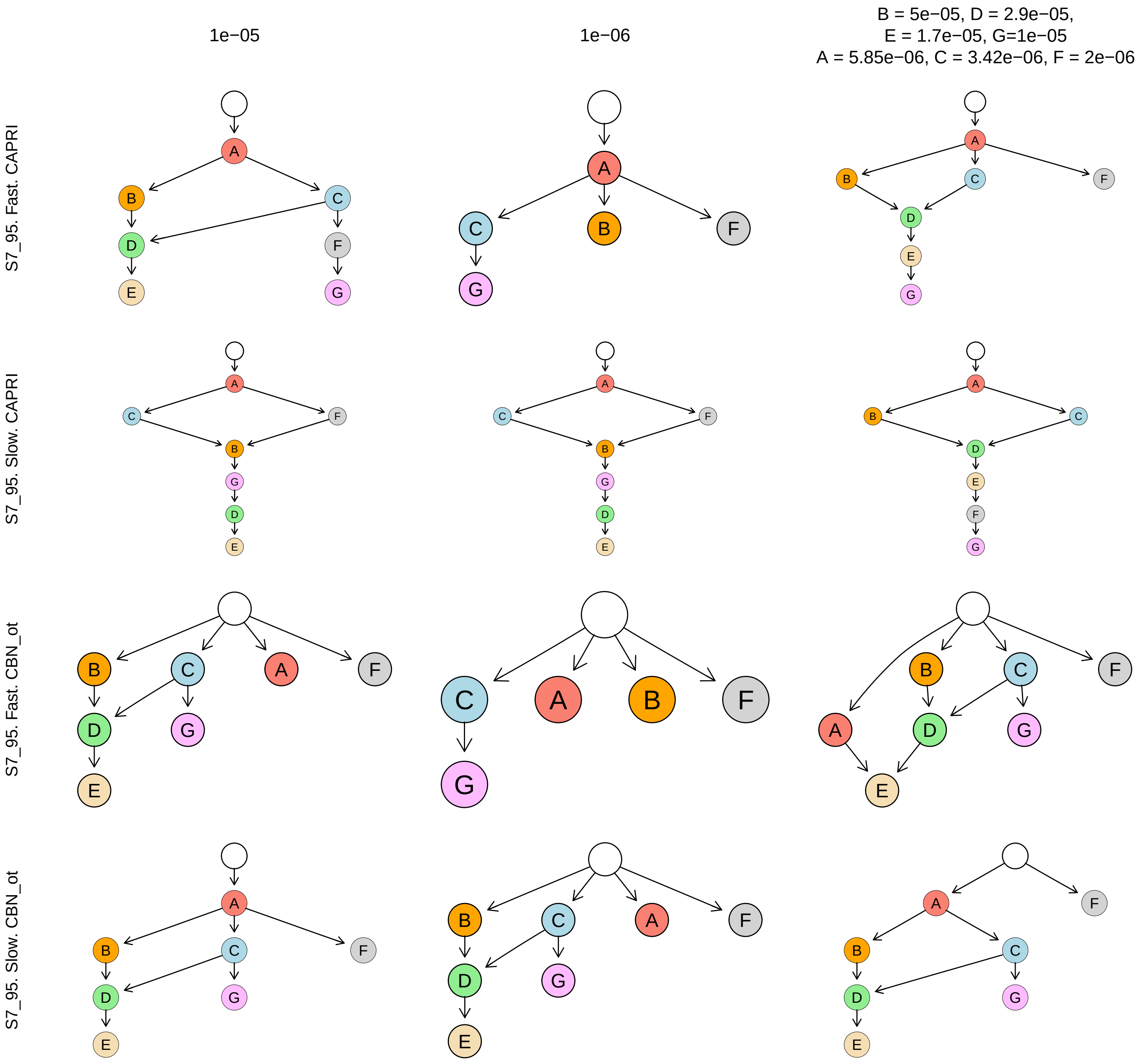

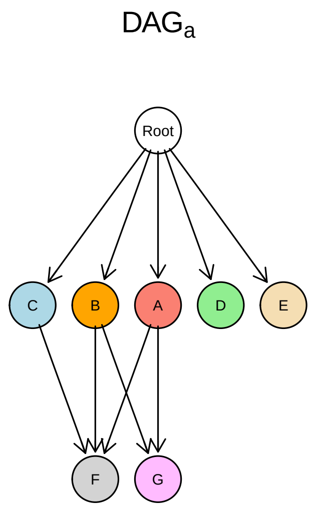

ID	Accessible Genot.	Discrep. DAG_a
S7_96	47	0

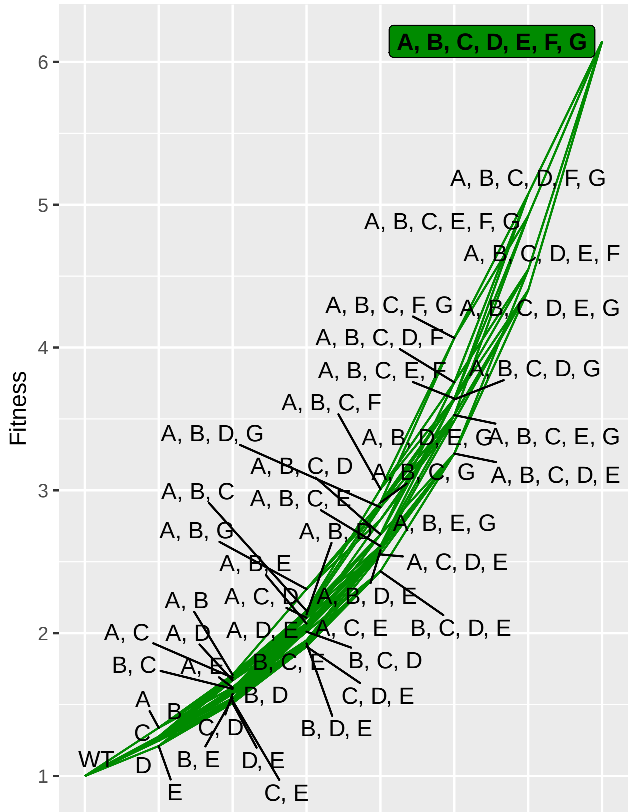

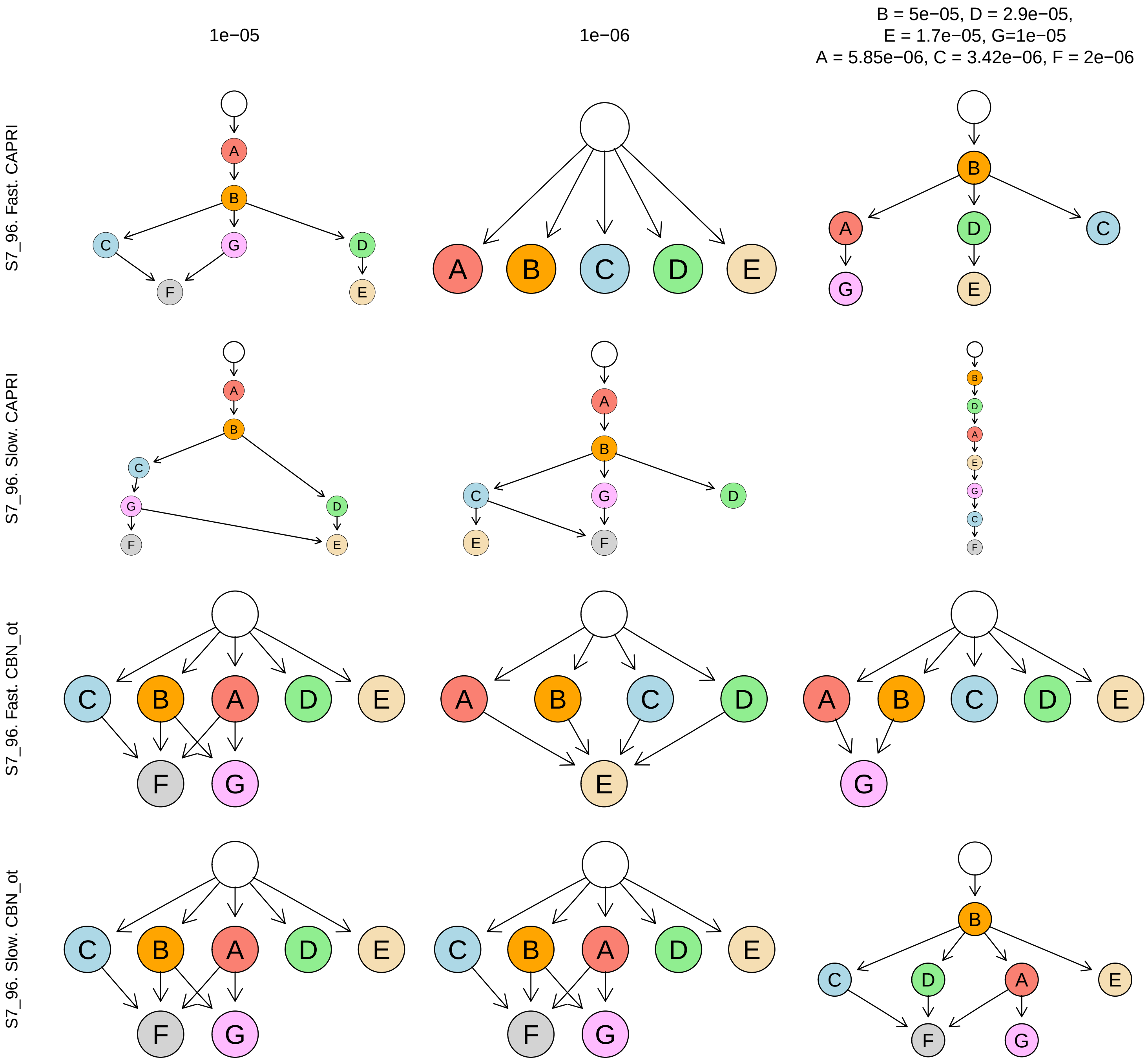

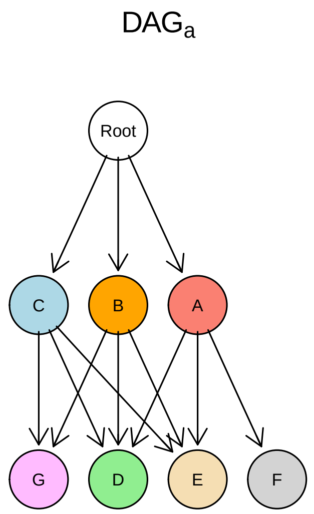

ID	Accessible Genot.	Discrep. DAG_a
S7_97	26	0

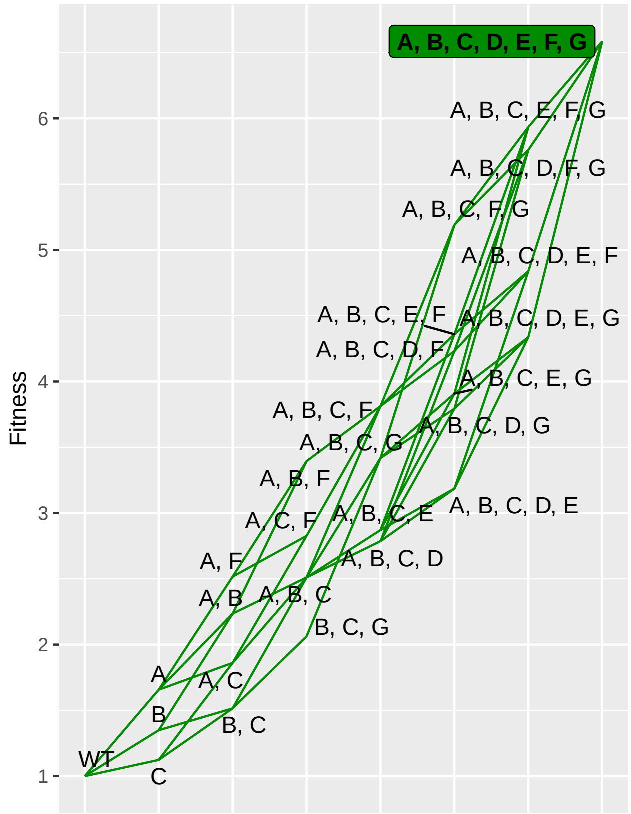

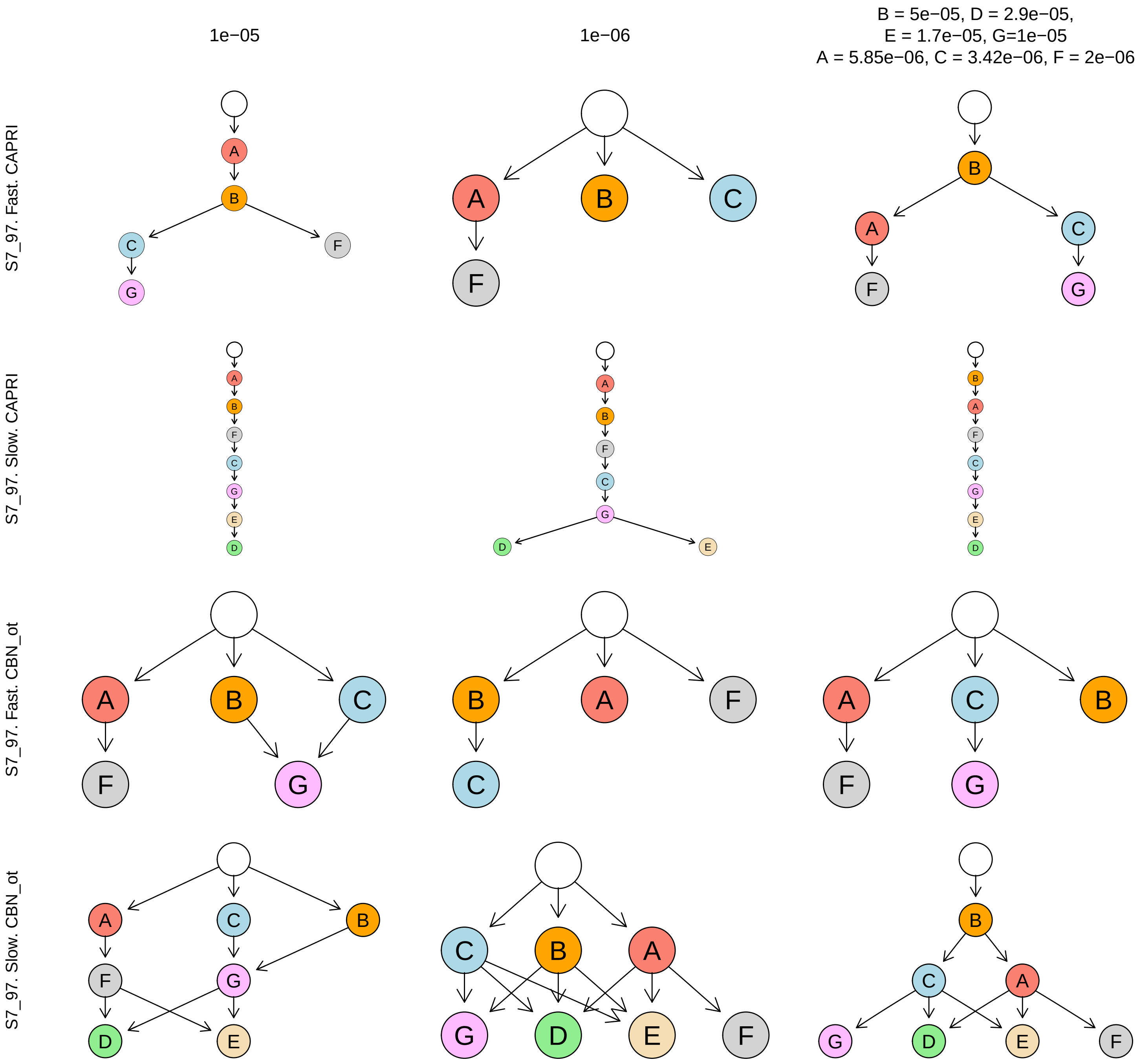

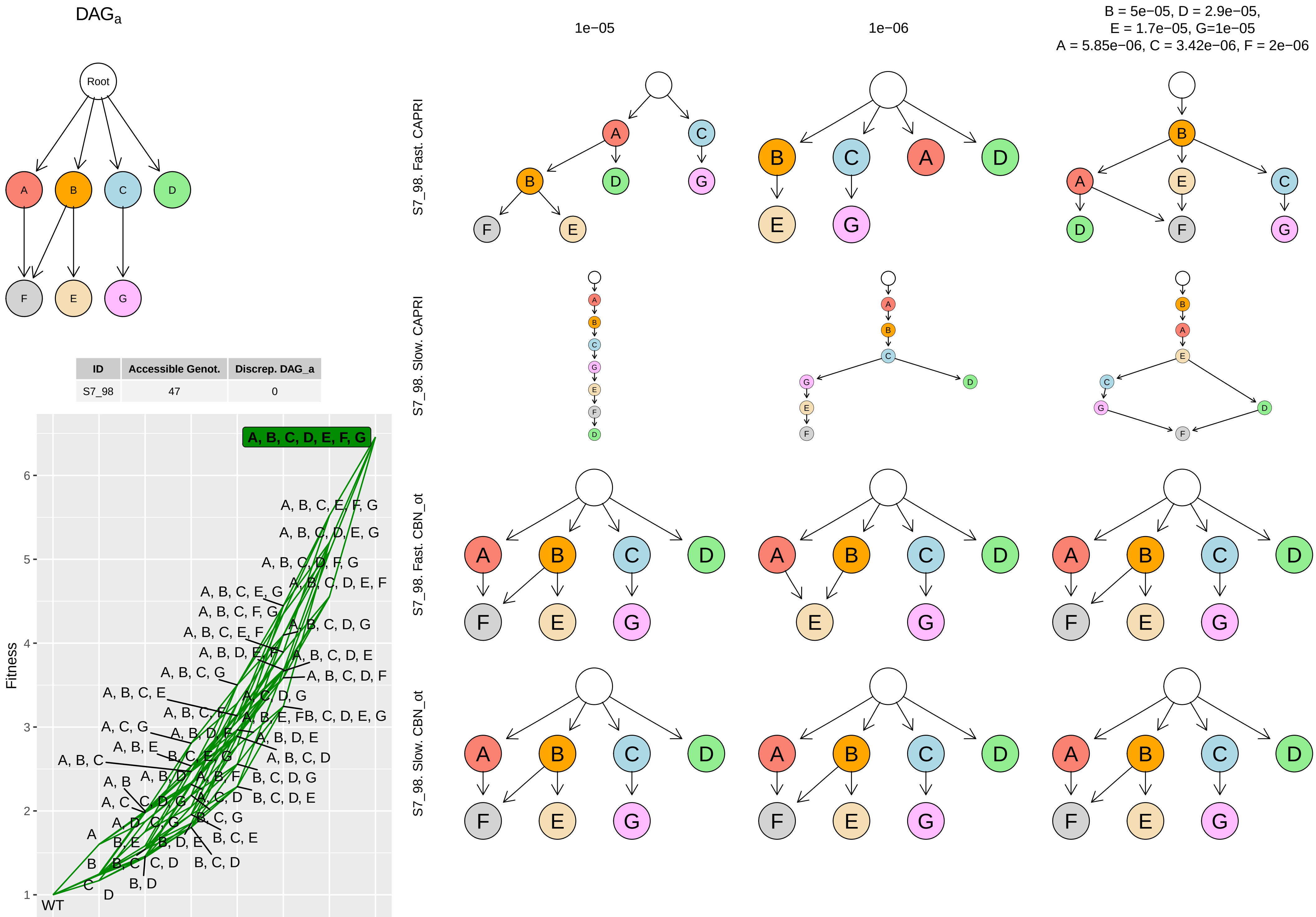

DAG_a
Root
A
B
C
D
F
E
G
ID | Accessible Genot. | Discrep. DAG_a
S7_98 | 47 | 0
A, B, C, D, E, F, G
Fitness
WT
1e−05
1e−06
B = 5e−05, D = 2.9e−05,
E = 1.7e−05, G=1e−05
A = 5.85e−06, C = 3.42e−06, F = 2e−06
S7_98. Fast. CAPRI
S7_98. Slow. CAPRI
S7_98. Fast. CBN_ot
S7_98. Slow. CBN_ot

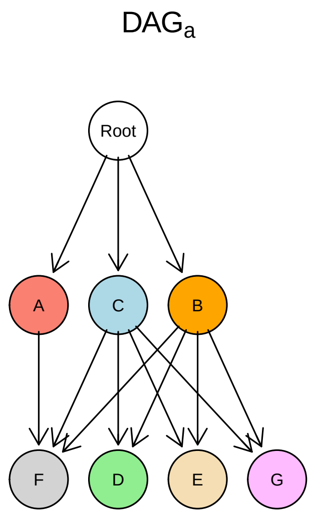

ID	Accessible Genot.	Discrep. DAG_a
S7_99	29	0

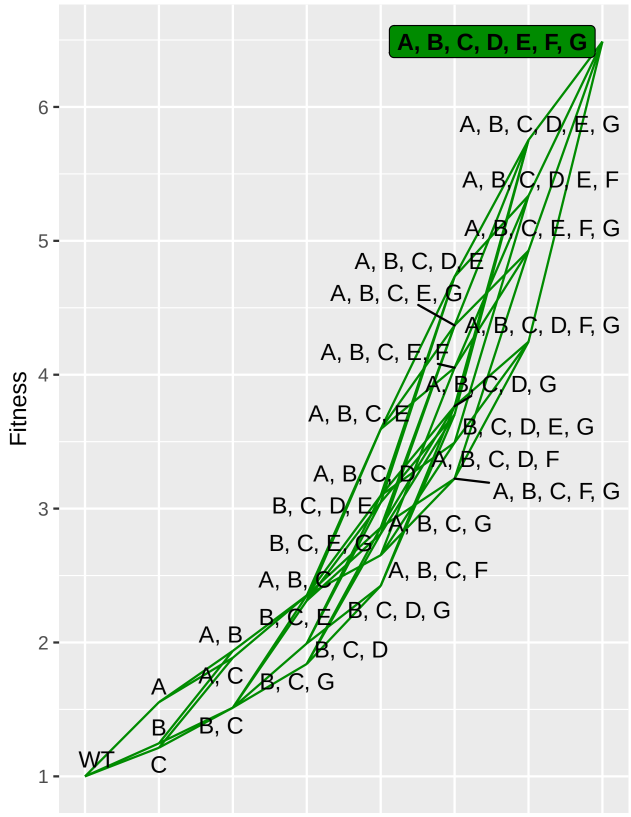

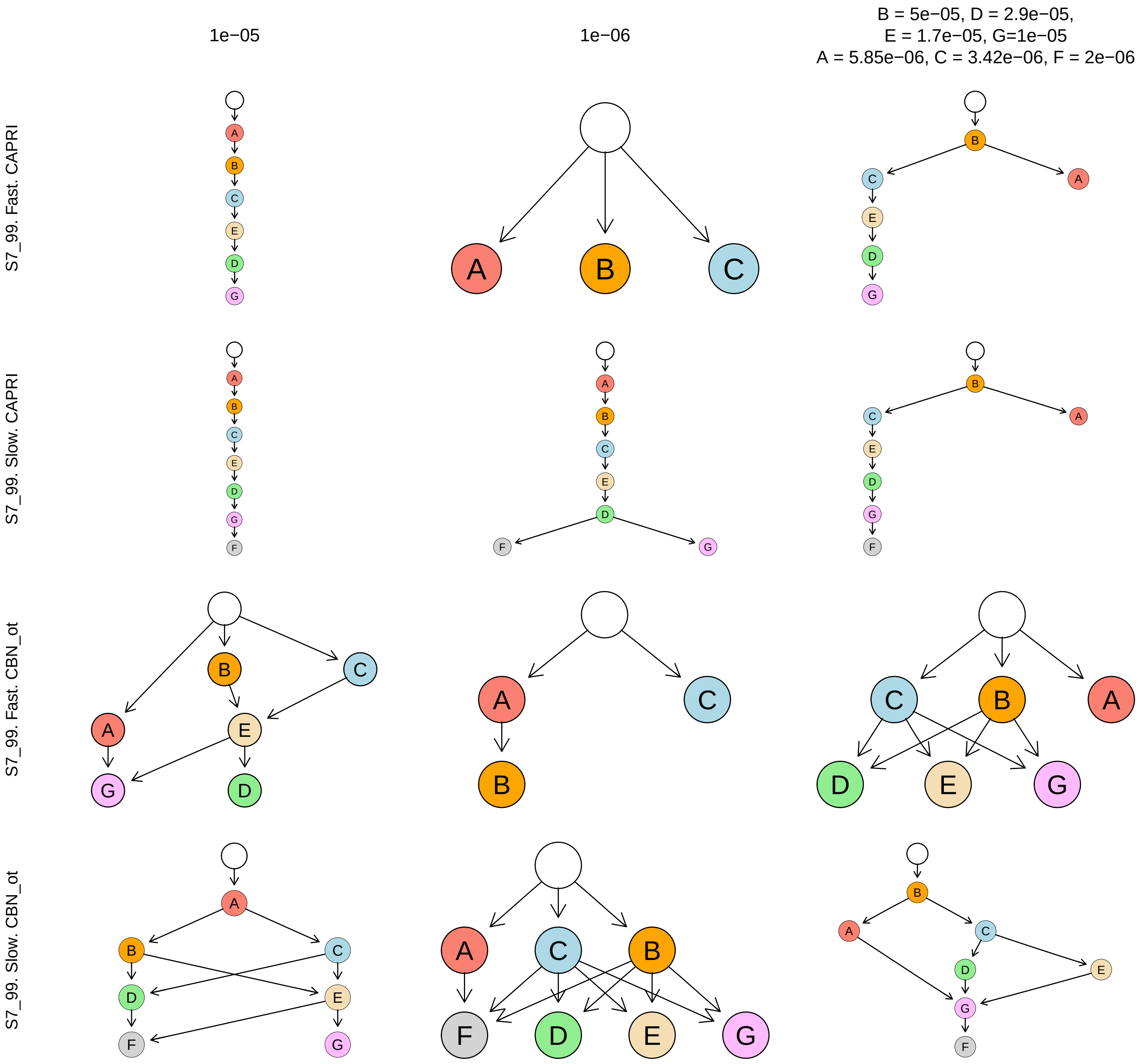

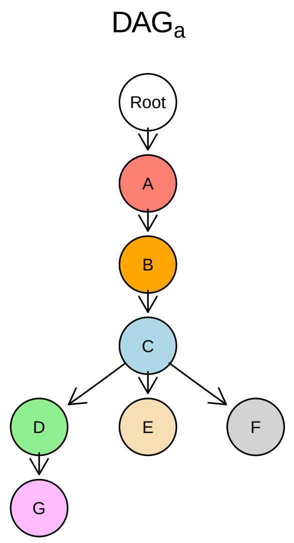

ID	Accessible Genot.	Discrep. DAG_a
S7_100	14	0

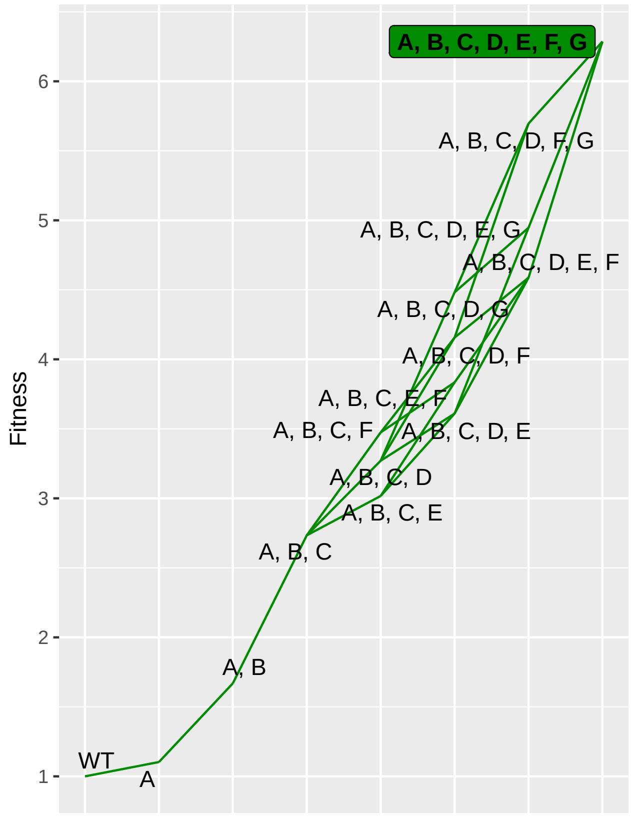

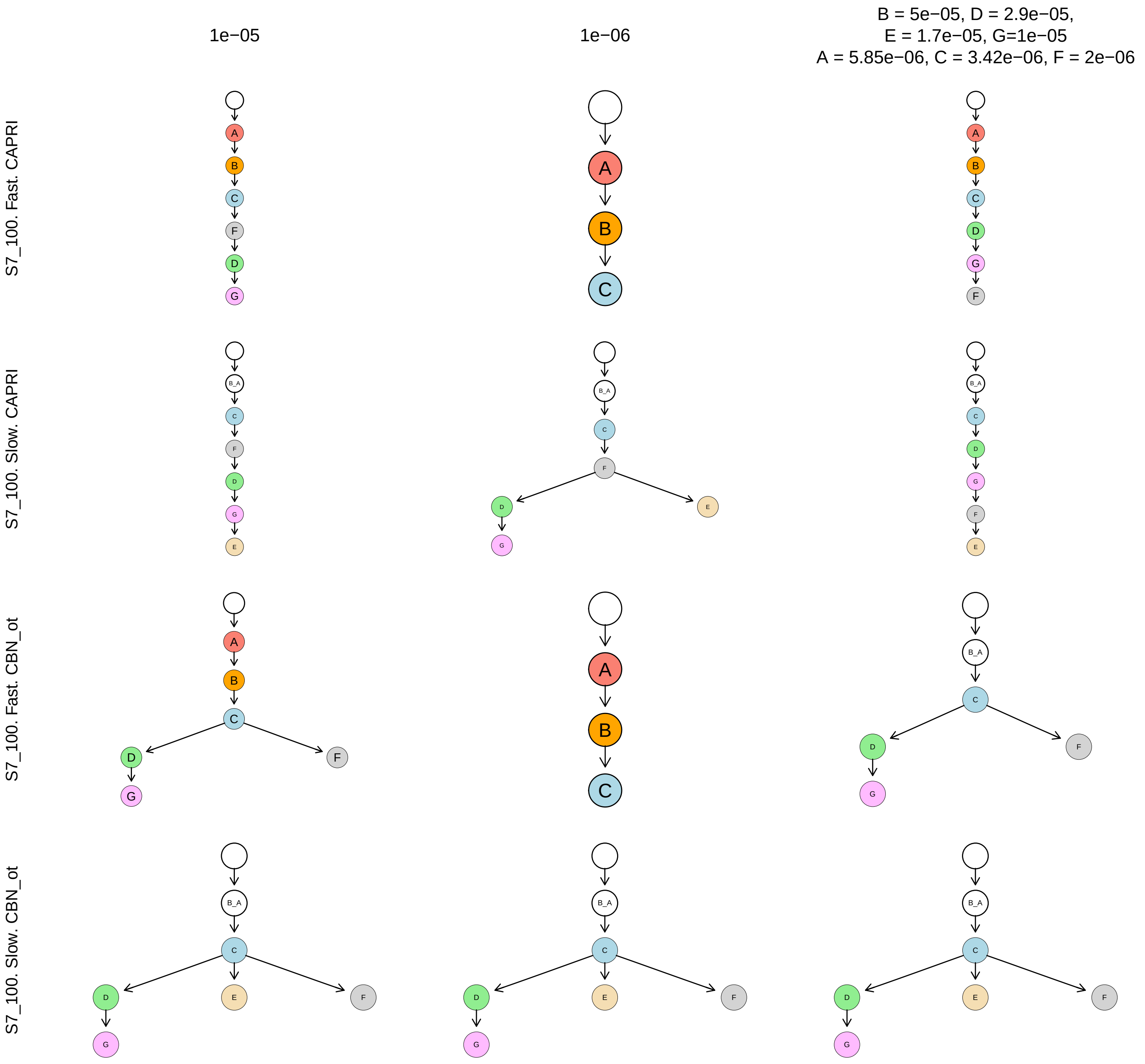

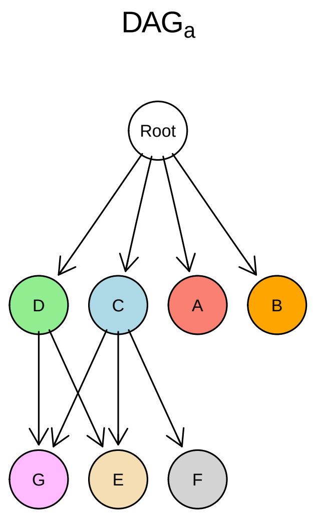

ID	Accessible Genot.	Discrep. DAG_a
S7_101	40	7

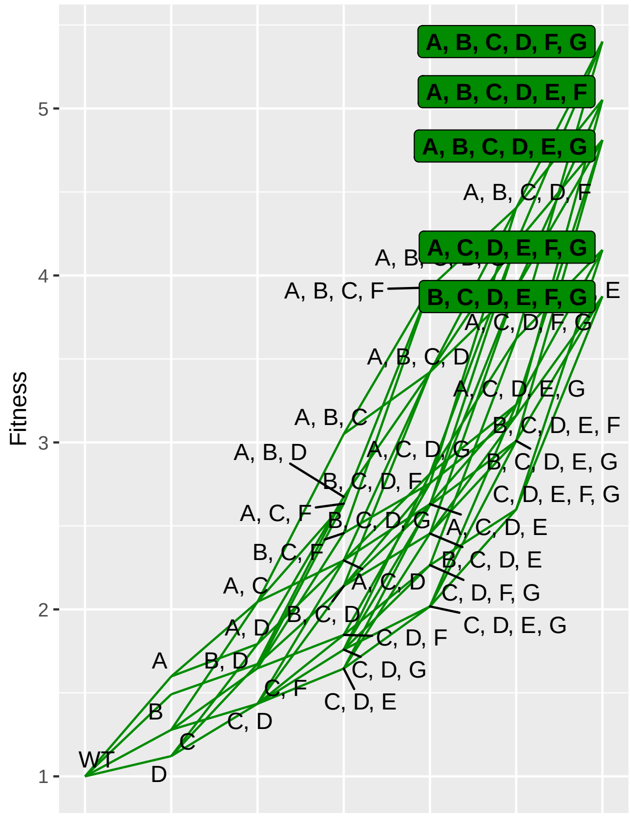

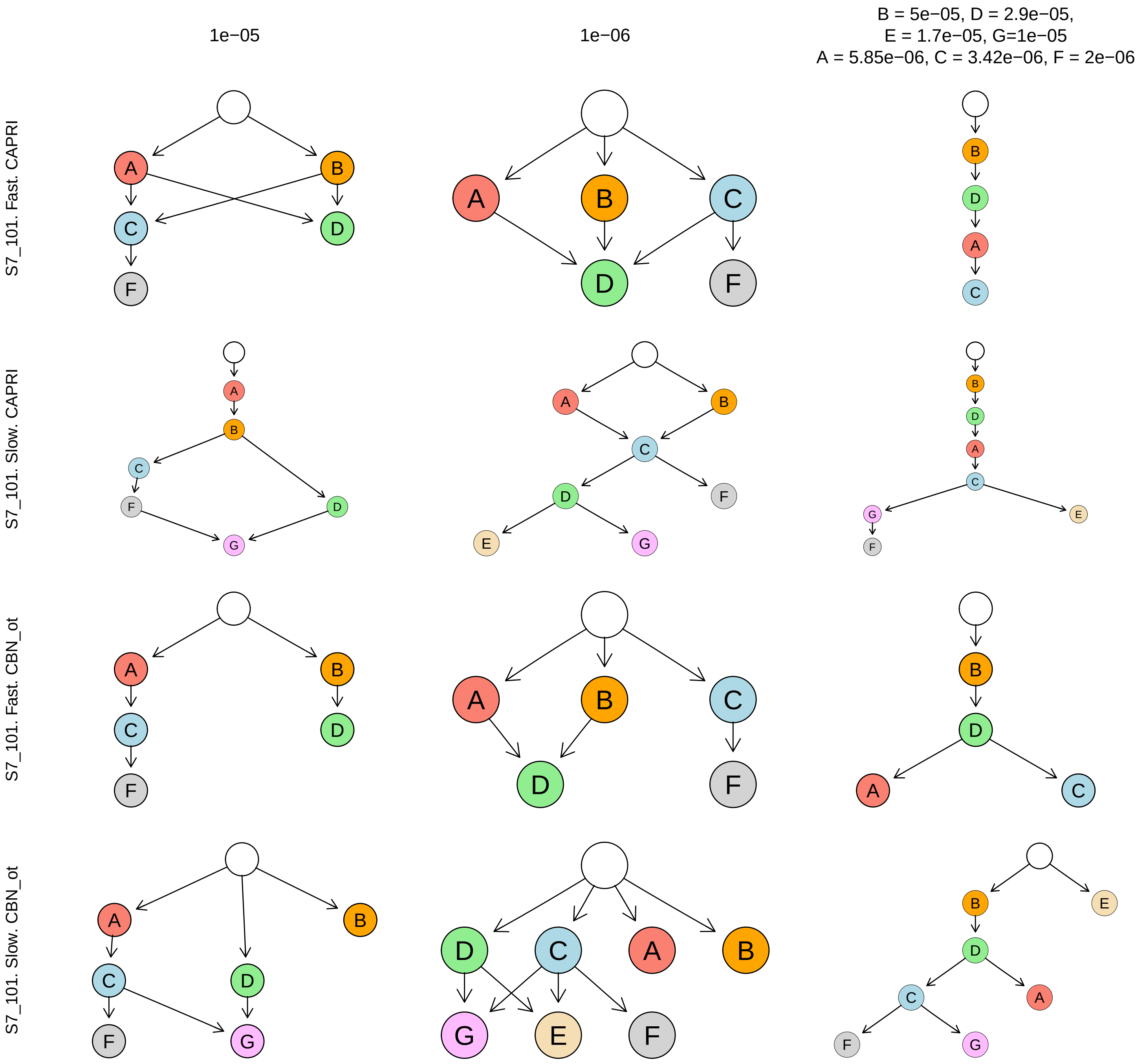

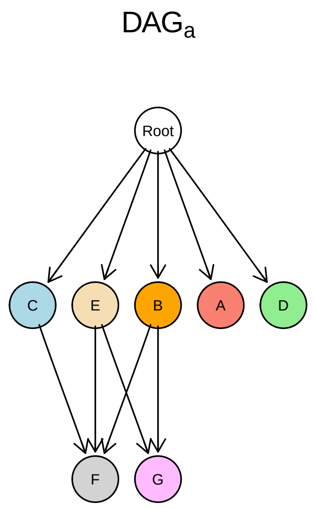

ID	Accessible Genot.	Discrep. DAG_a
S7_102	25	30

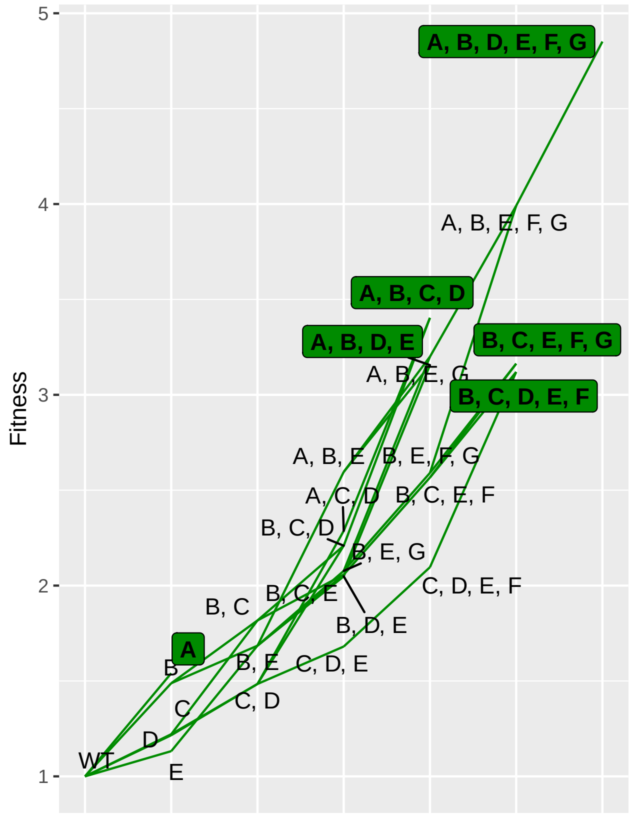

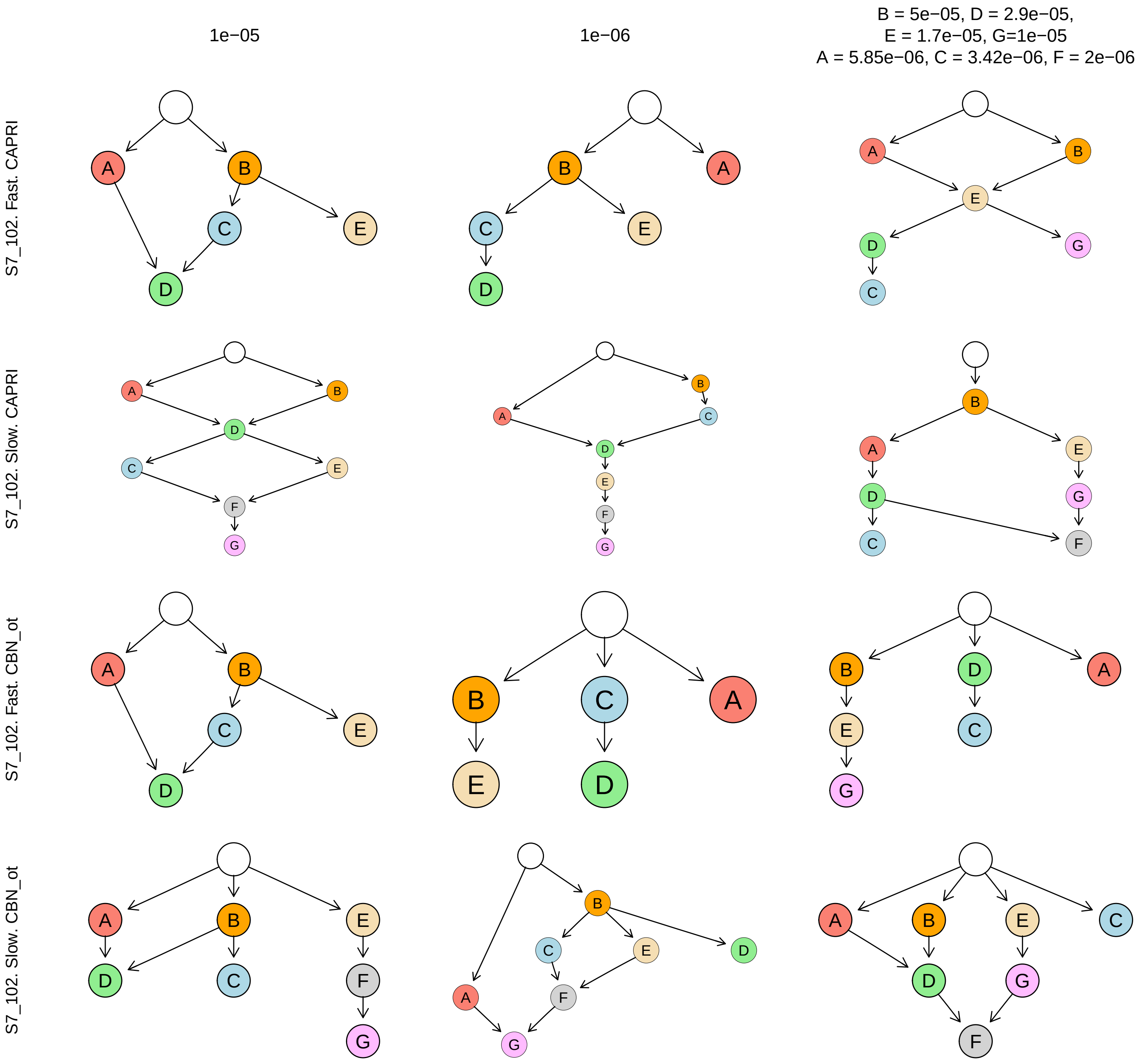

DAG_a

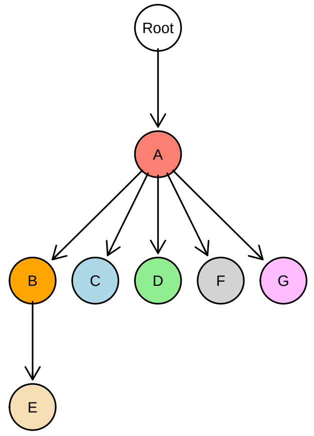

ID	Accessible Genot.	Discrep. DAG_a
S7_103	52	22

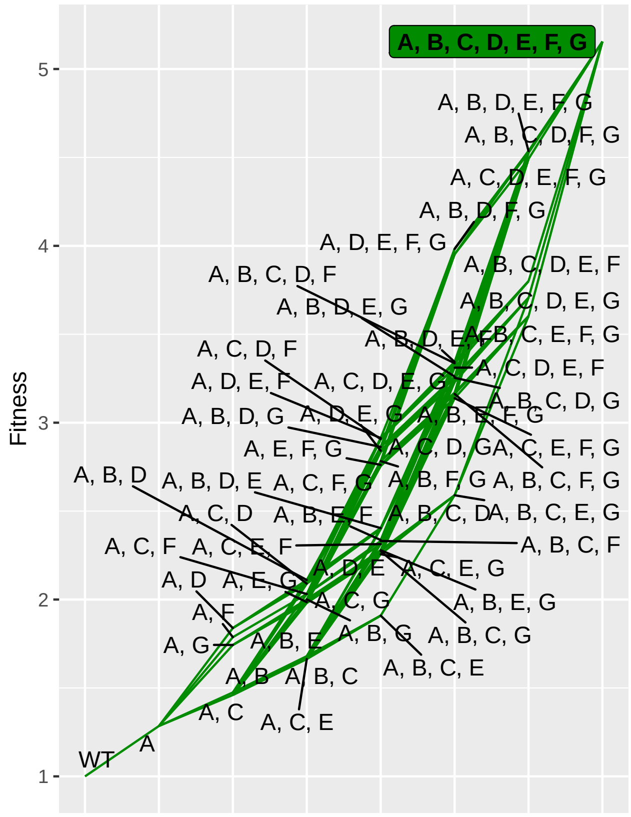

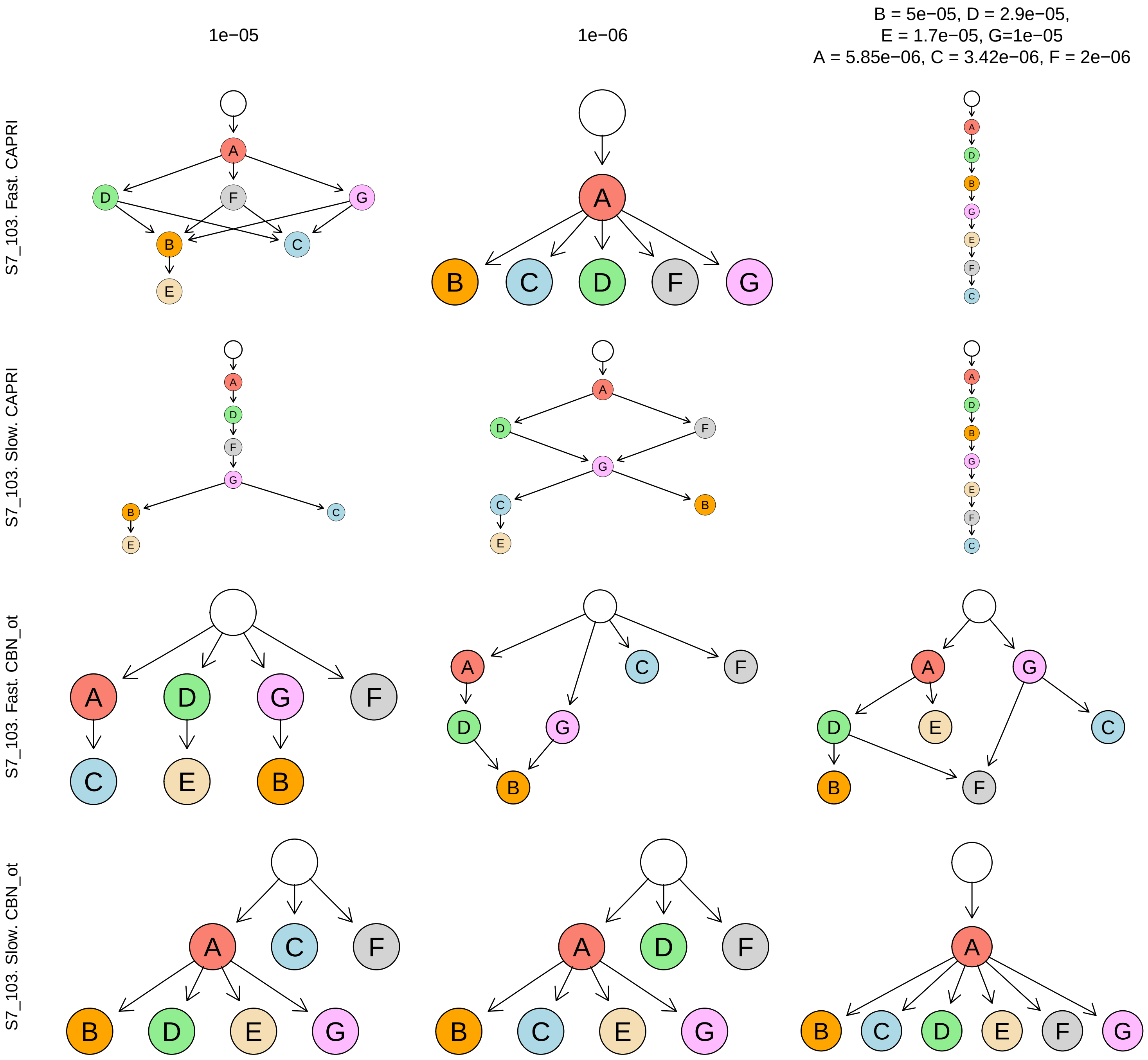

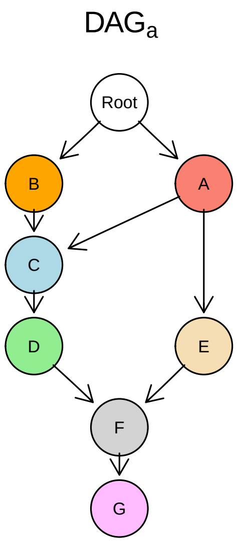

ID	Accessible Genot.	Discrep. DAG_a
S7_104	11	2

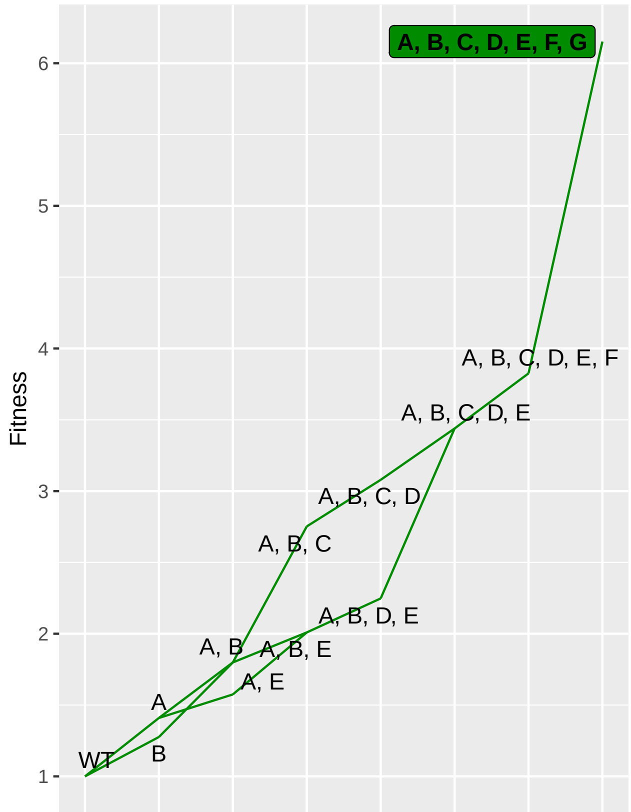

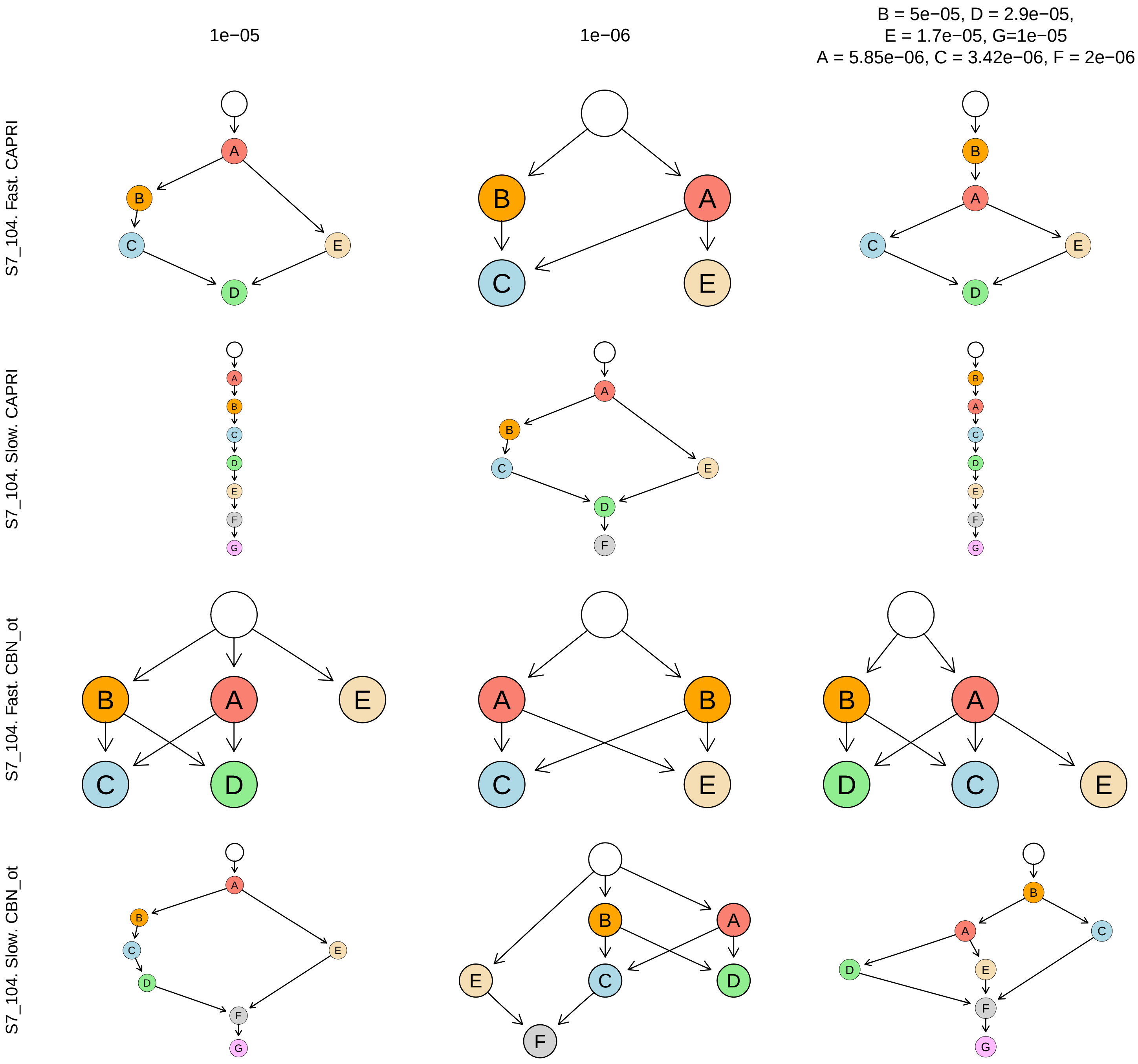

DAG$_a$

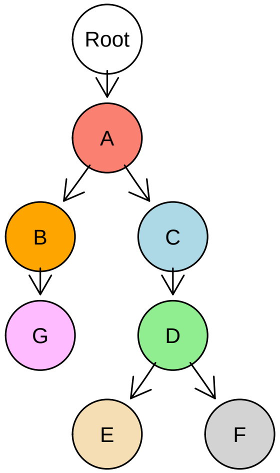

ID	Accessible Genot.	Discrep. DAG_a
S7_105	13	5

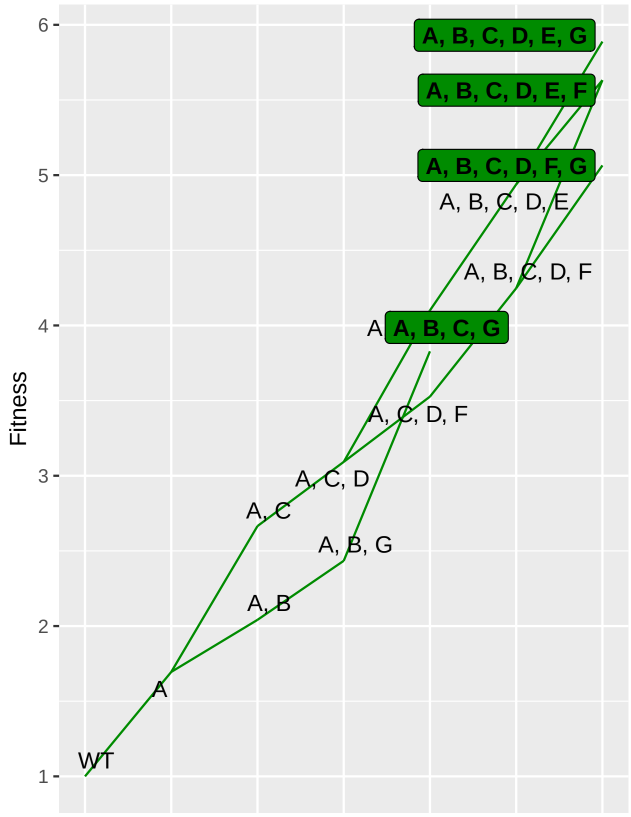

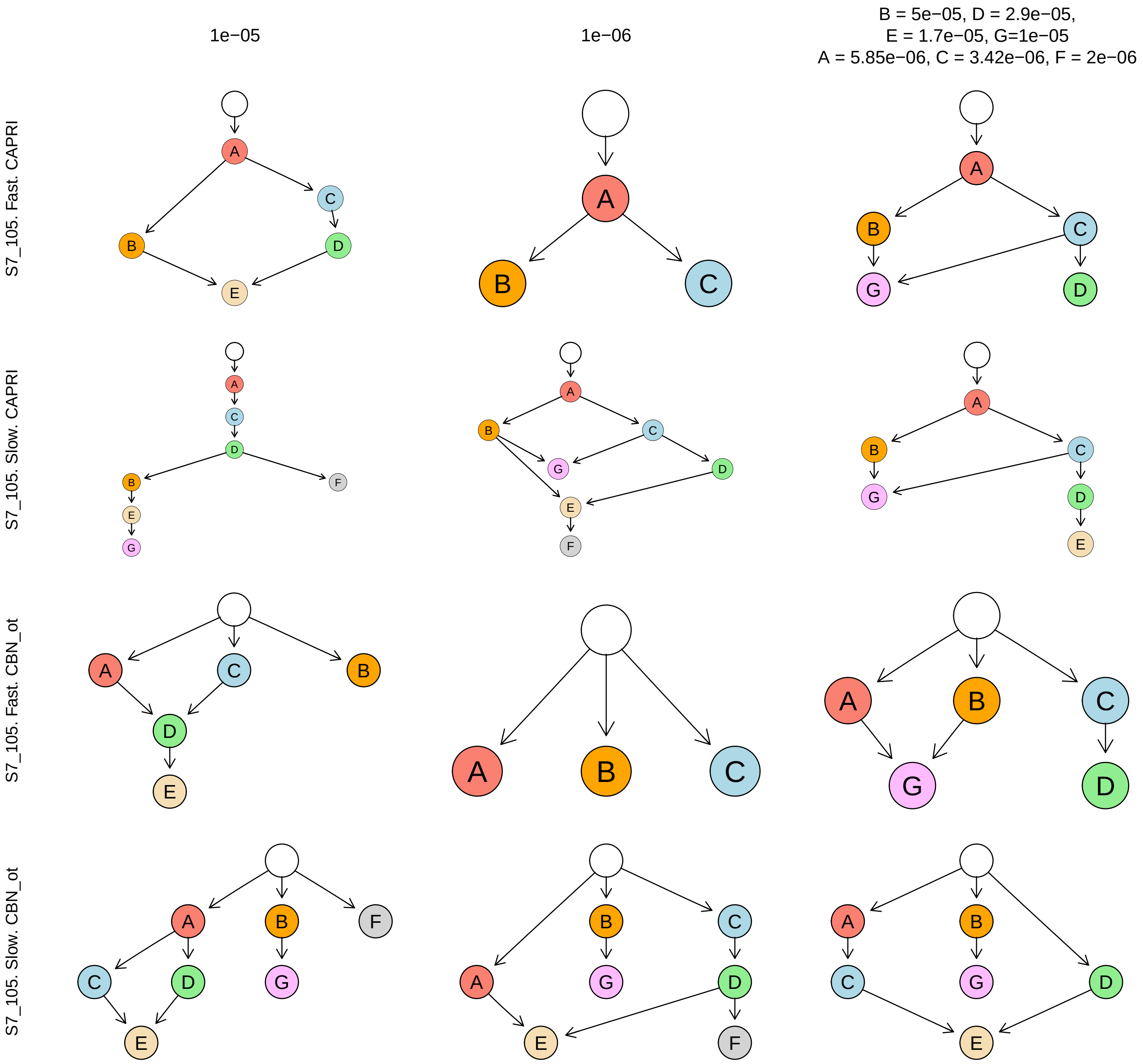

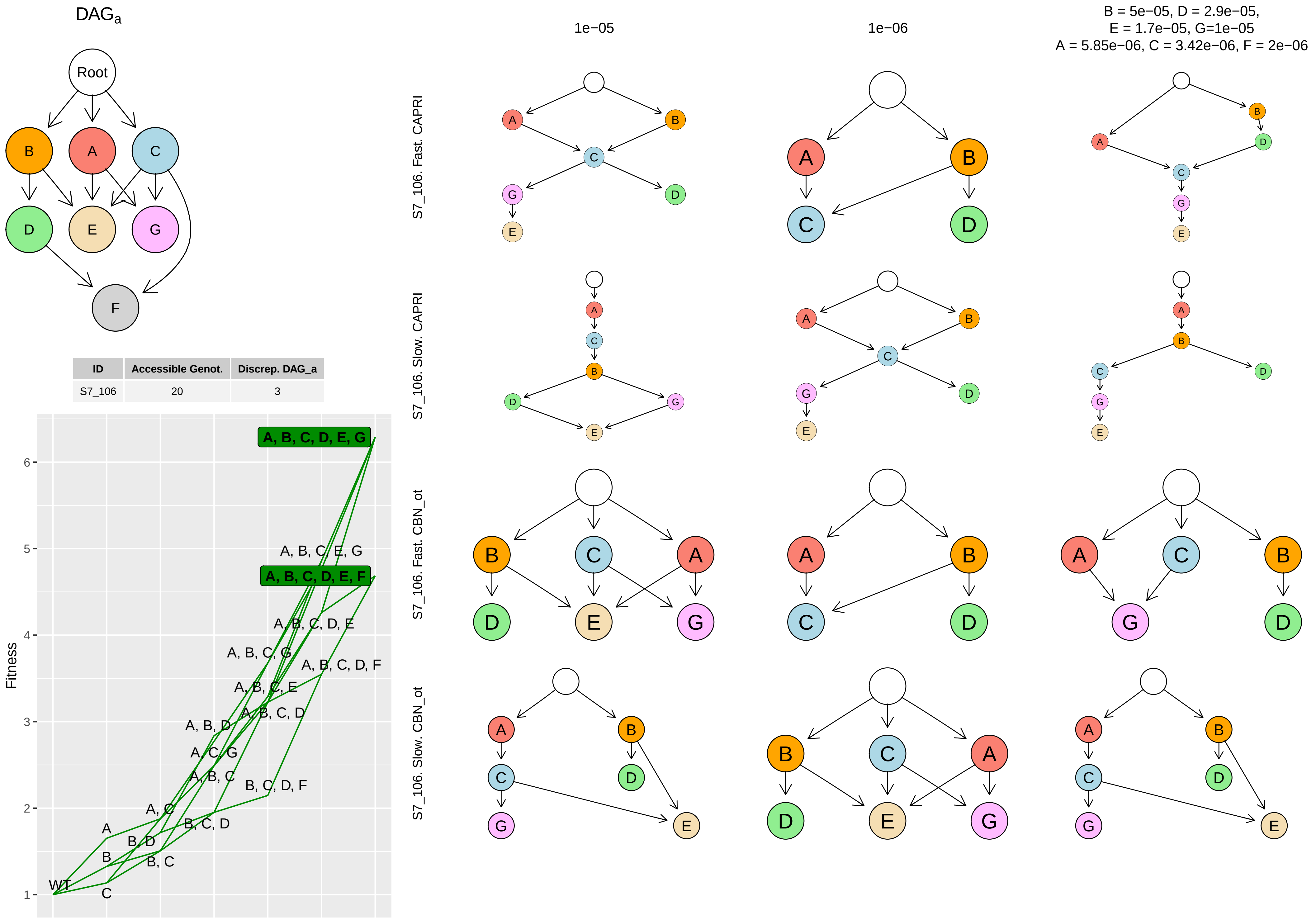

ID	Accessible Genot.	Discrep. DAG_a
S7_106	20	3

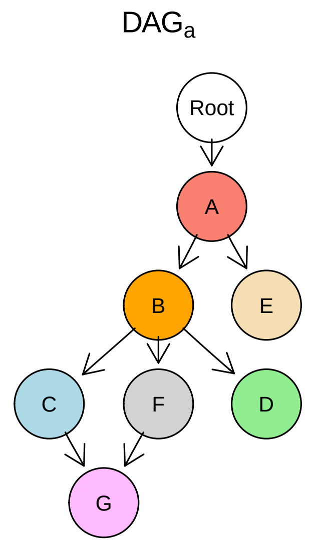

ID	Accessible Genot.	Discrep. DAG_a
S7_107	18	6

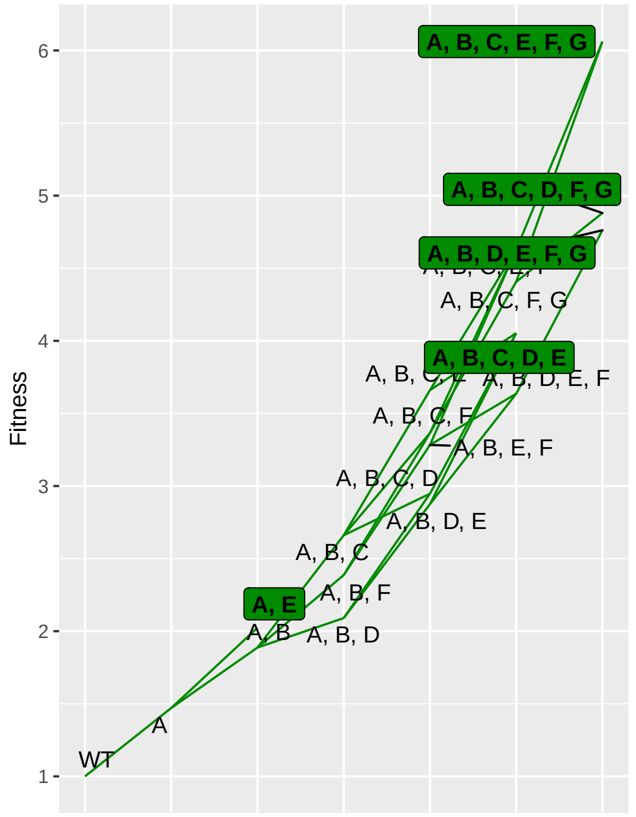

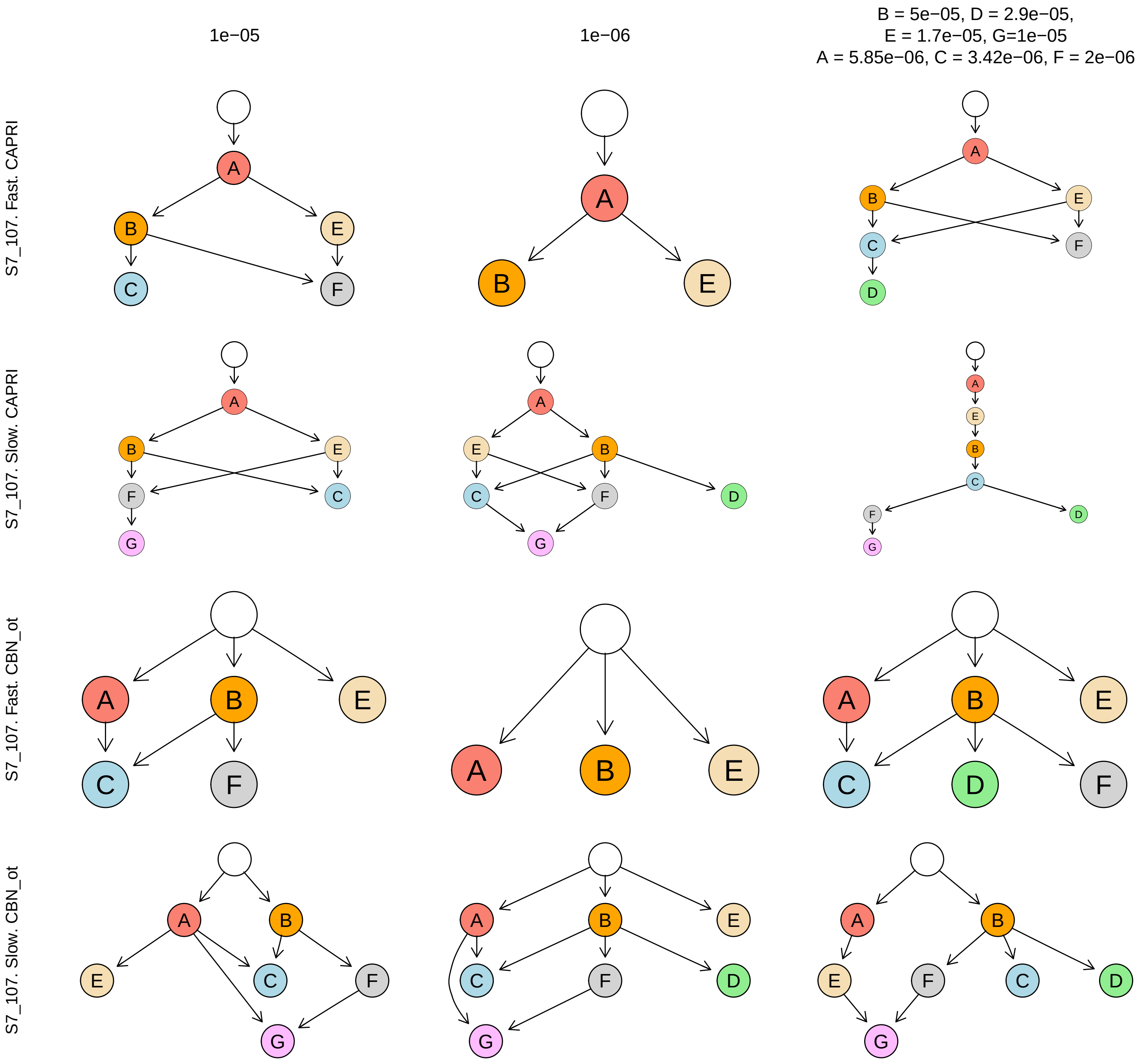

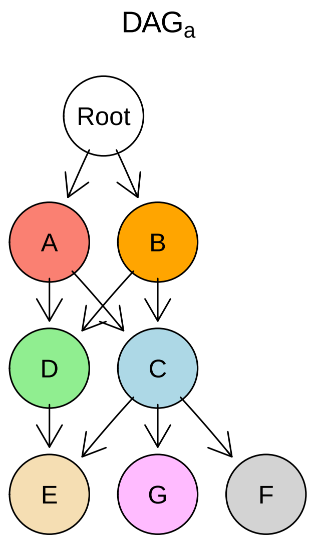

ID	Accessible Genot.	Discrep. DAG_a
S7_108	11	7

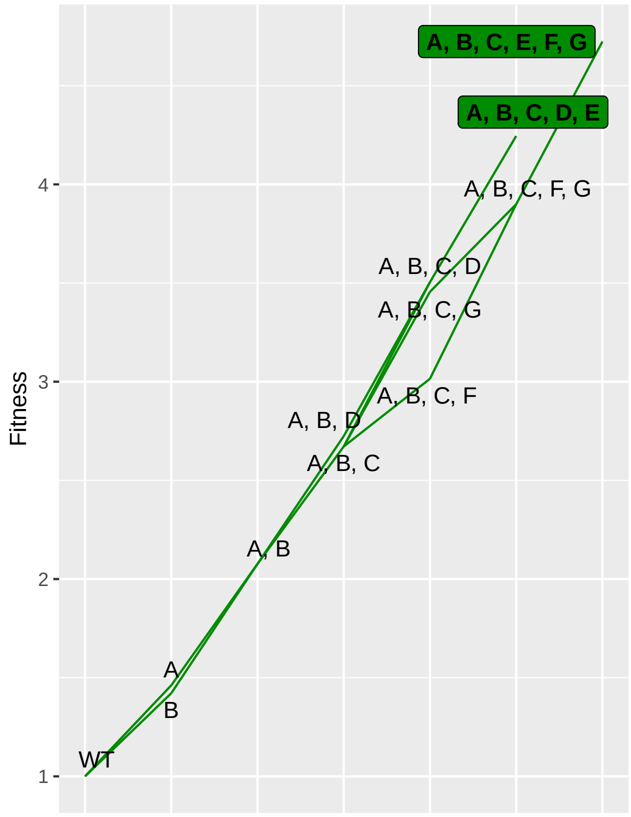

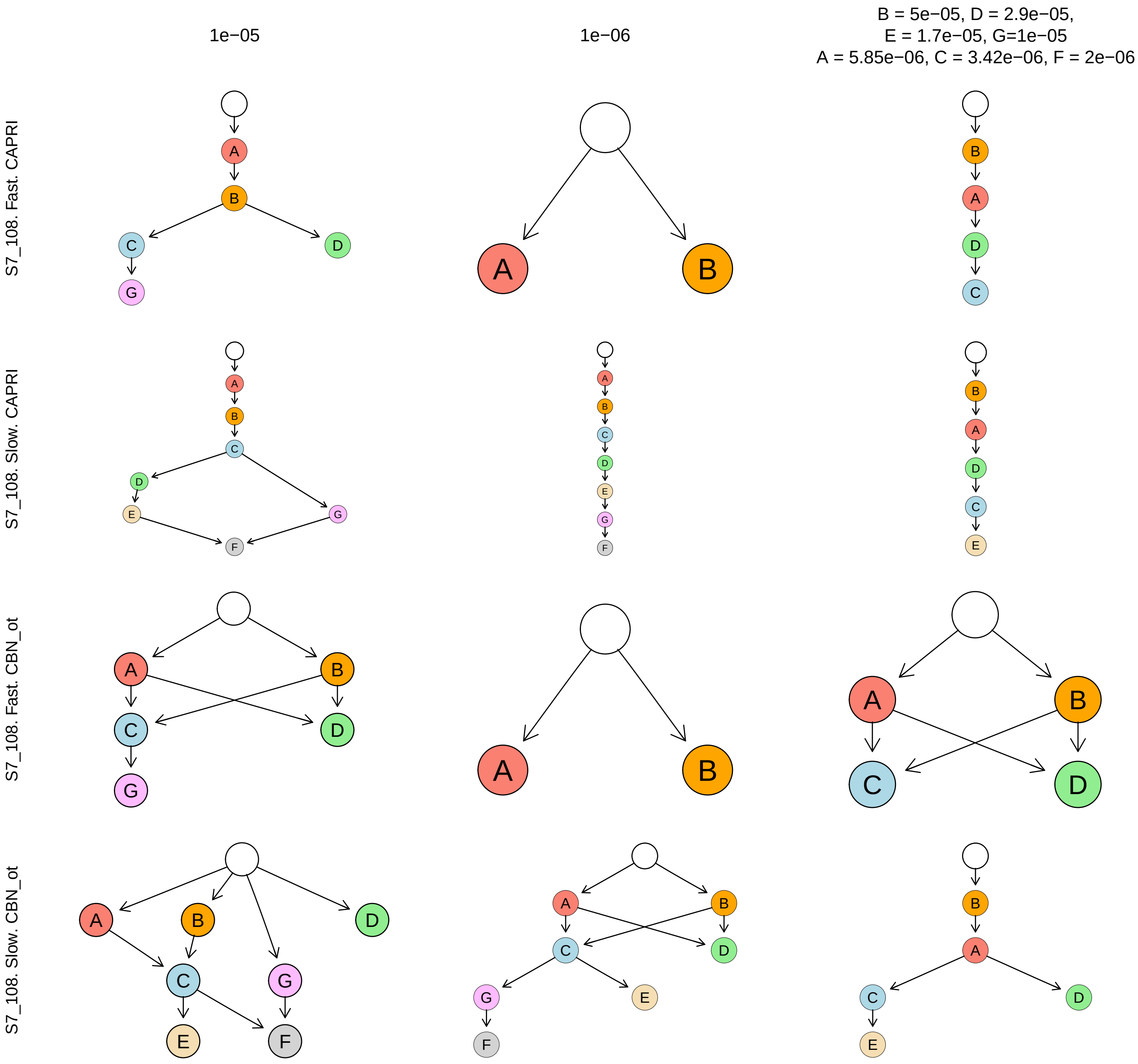

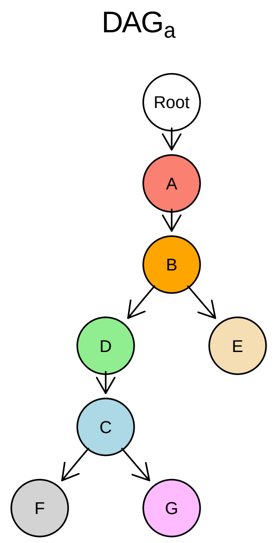

ID	Accessible Genot.	Discrep. DAG_a
S7_109	13	2

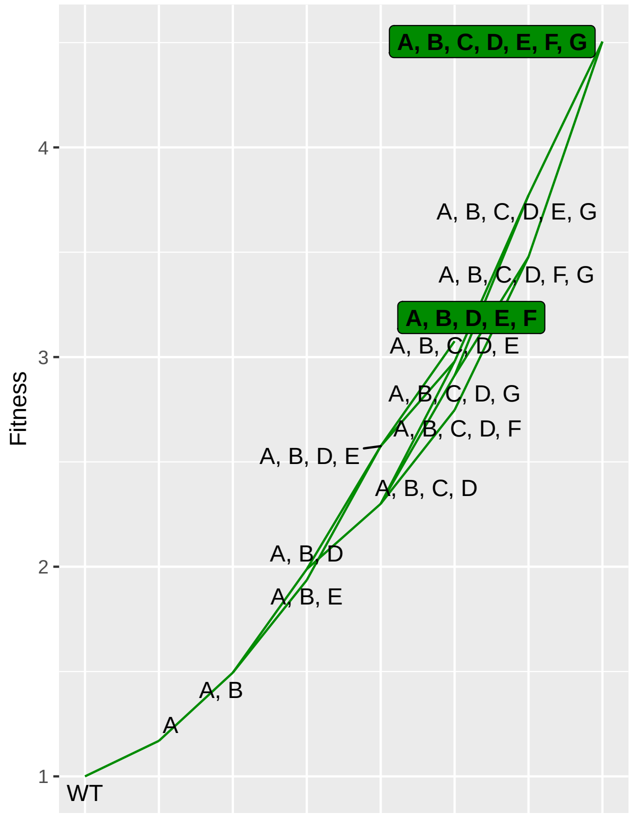

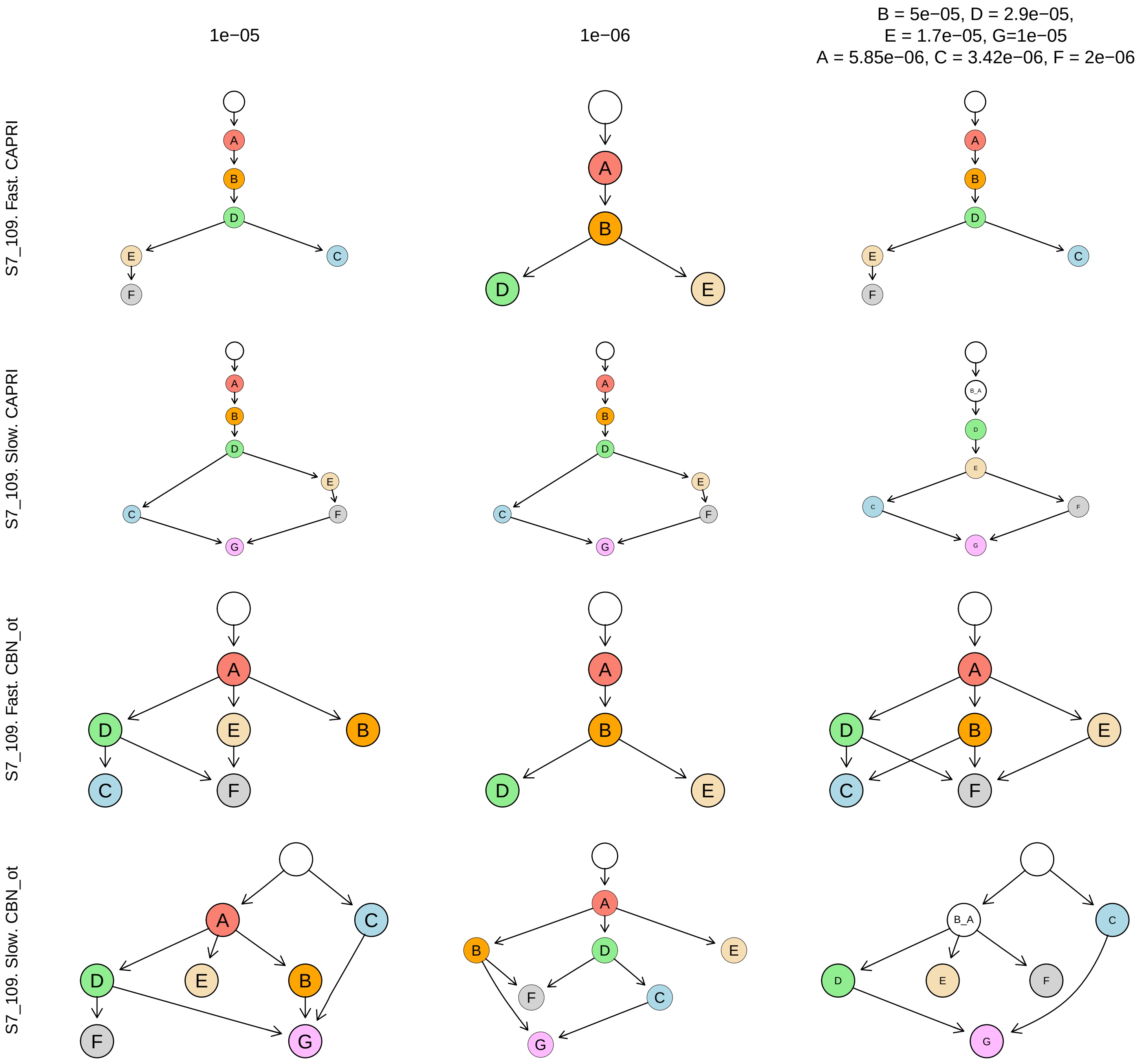

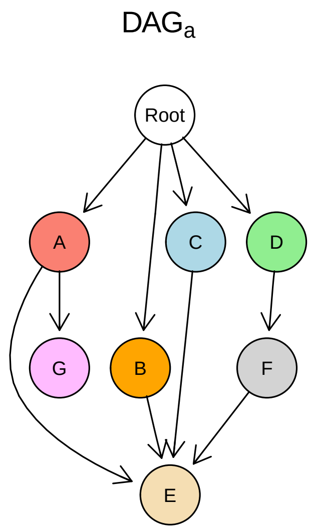

ID	Accessible Genot.	Discrep. DAG_a
S7_110	28	9

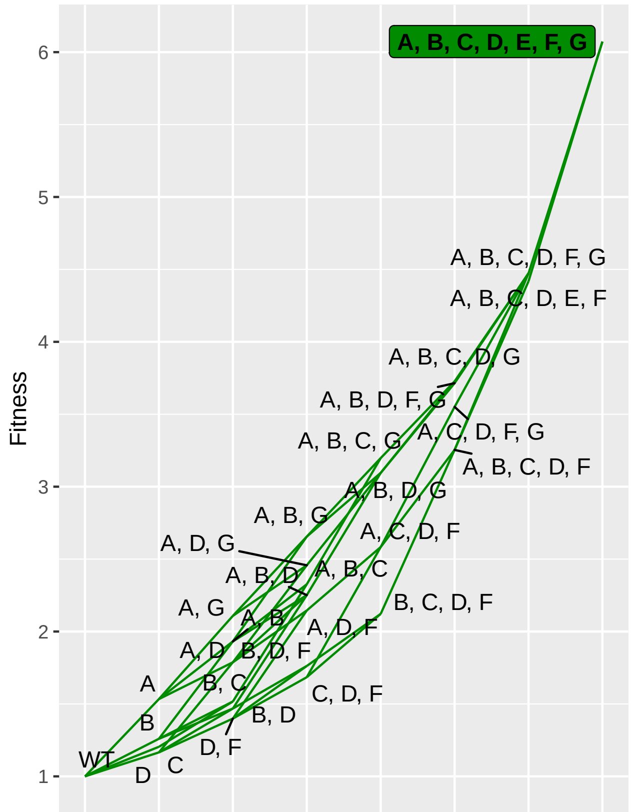

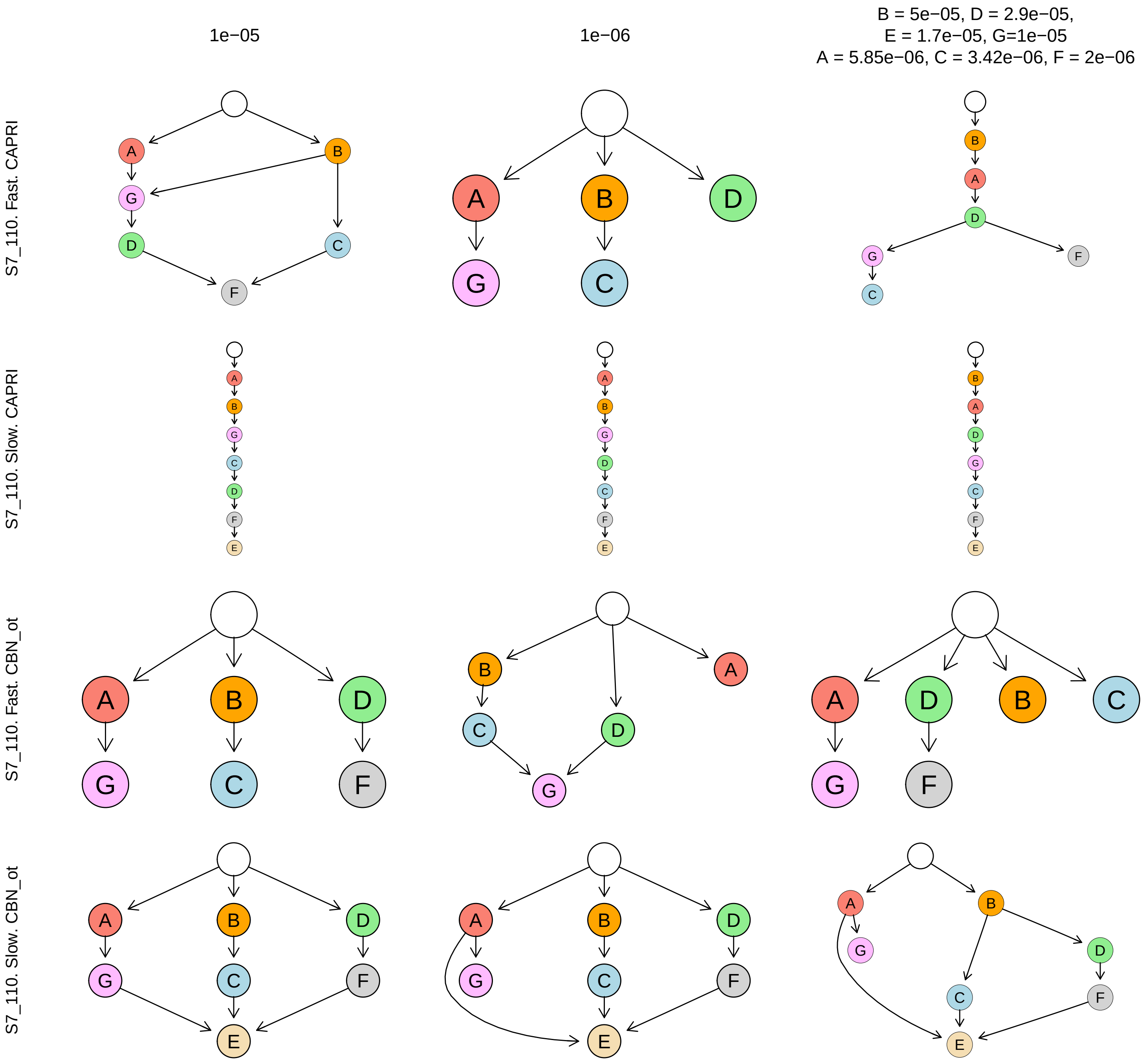

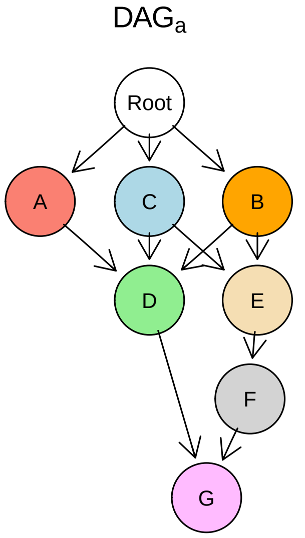

ID	Accessible Genot.	Discrep. DAG_a
S7_111	14	1

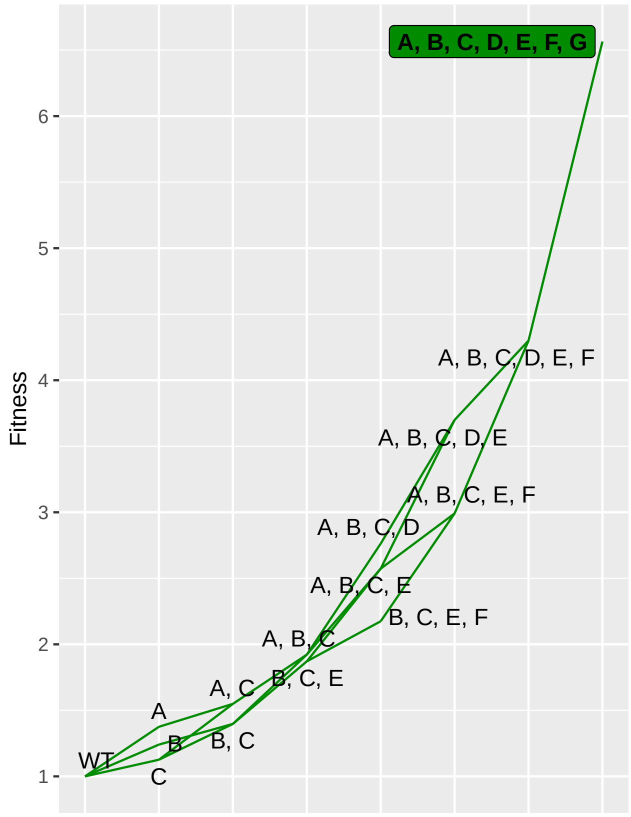

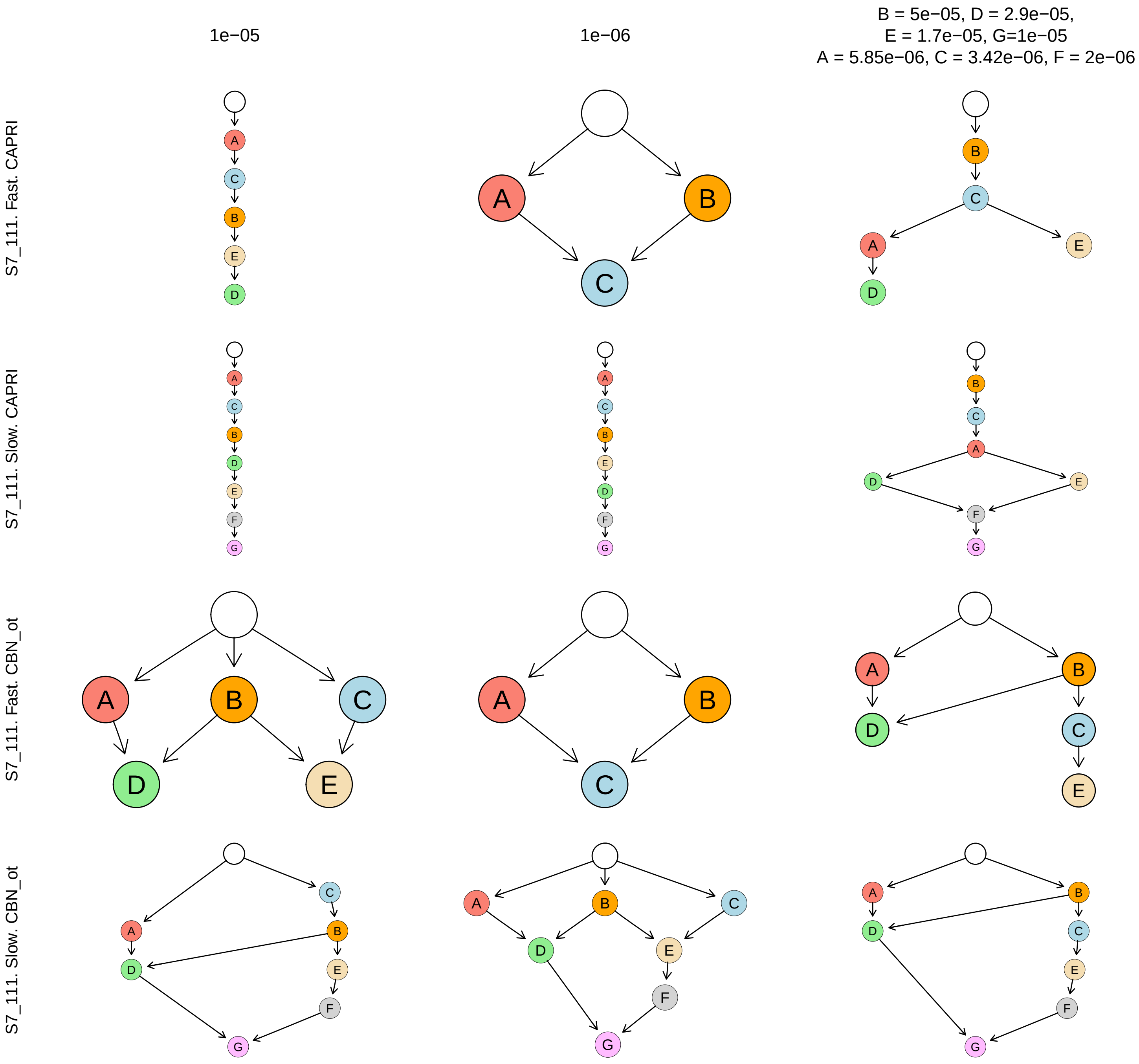

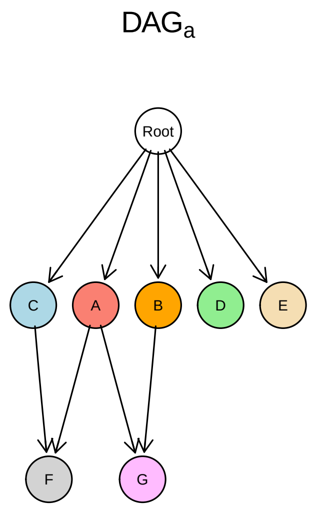

ID	Accessible Genot.	Discrep. DAG_a
S7_112	42	15

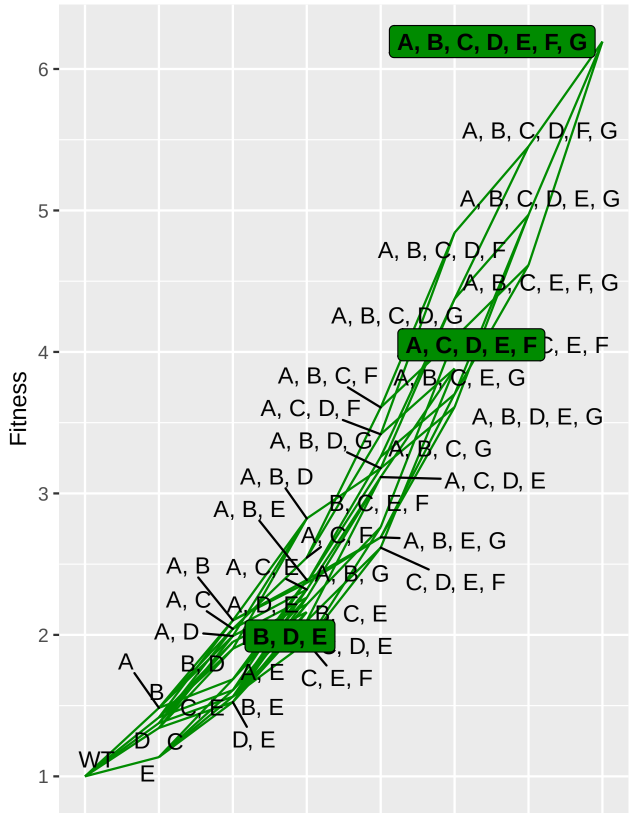

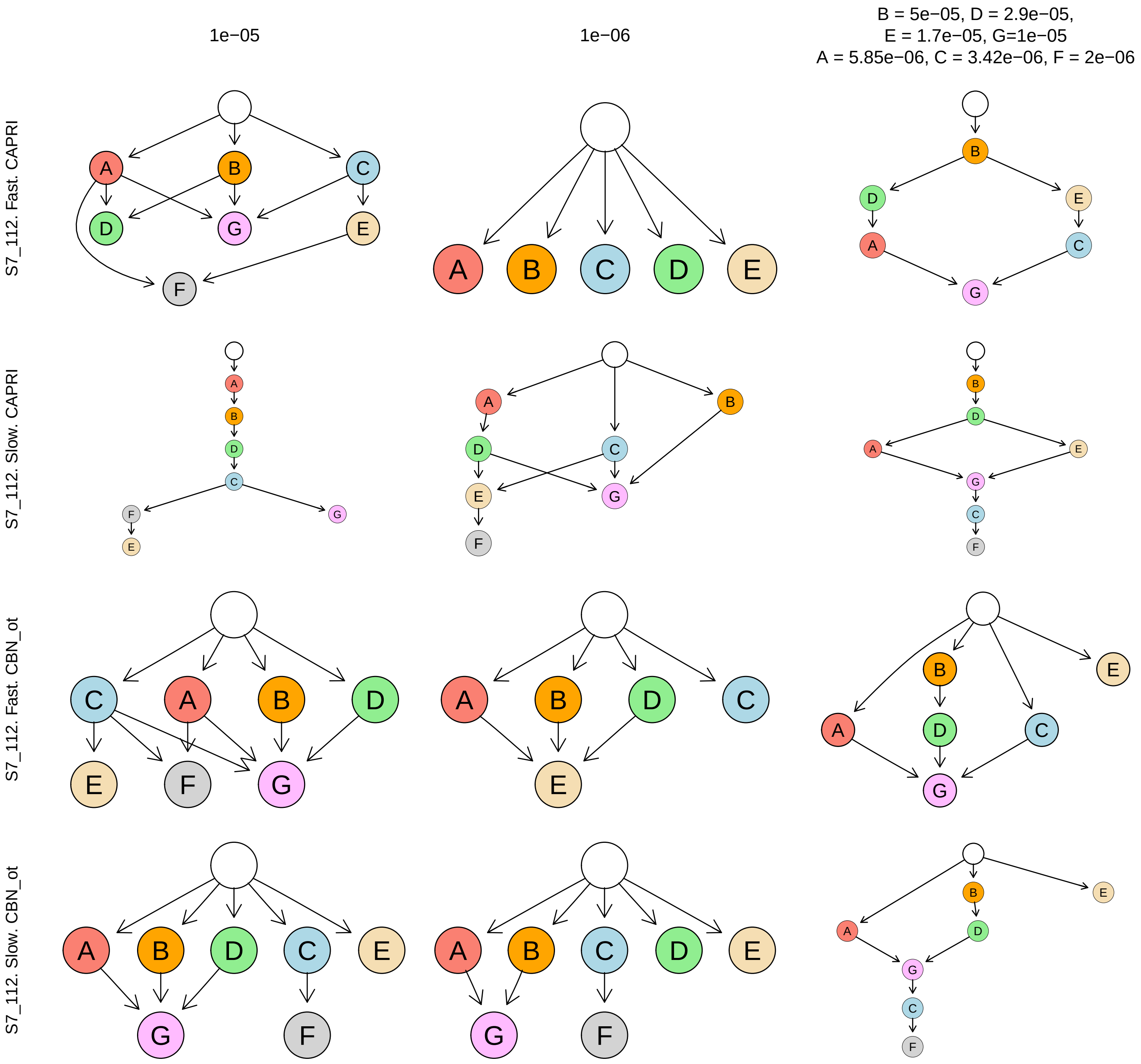

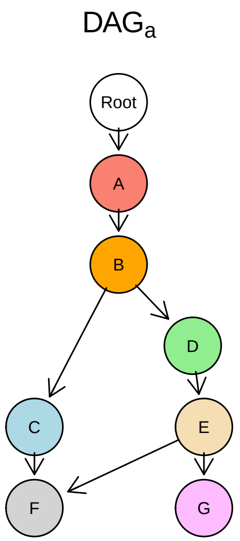

ID	Accessible Genot.	Discrep. DAG_a
S7_113	10	1

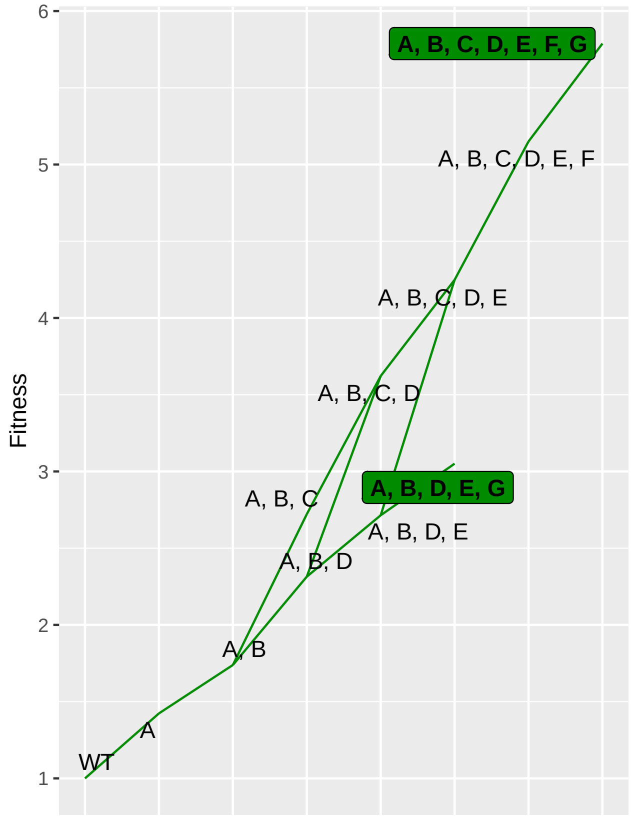

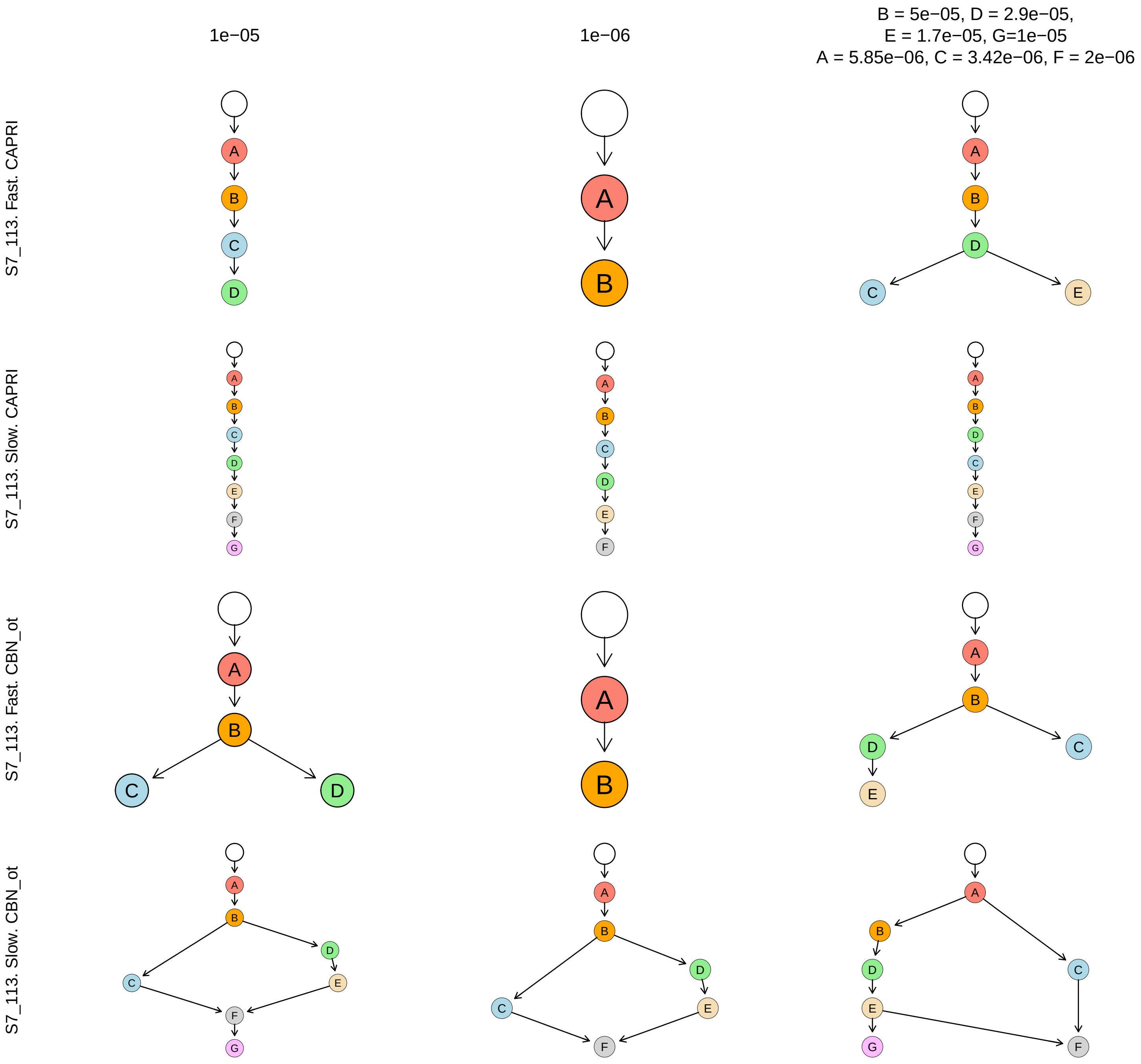

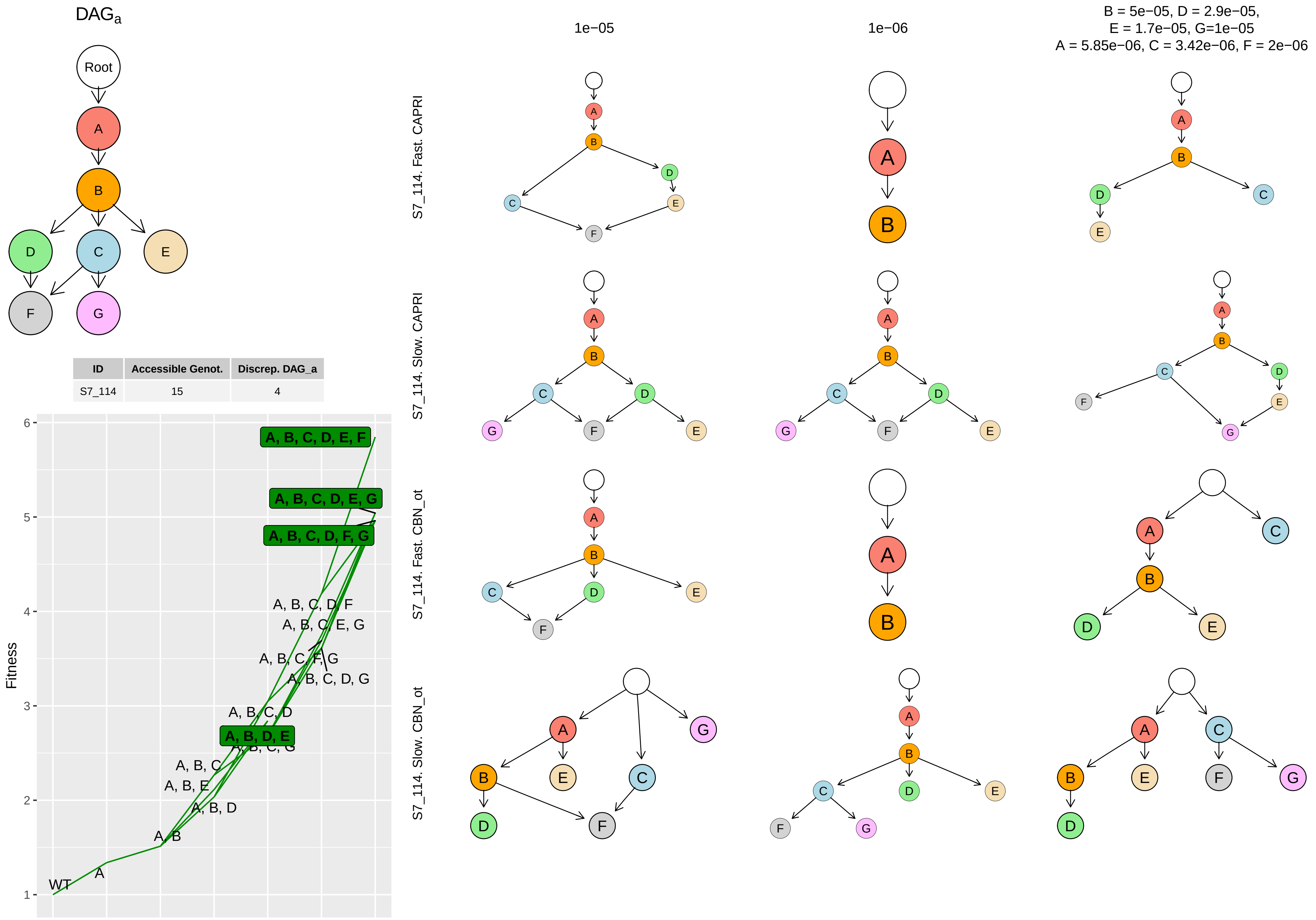

ID	Accessible Genot.	Discrep. DAG_a
S7_114	15	4

DAG_a

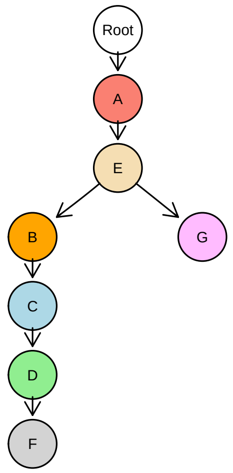

ID	Accessible Genot.	Discrep. DAG_a
S7_115	10	3

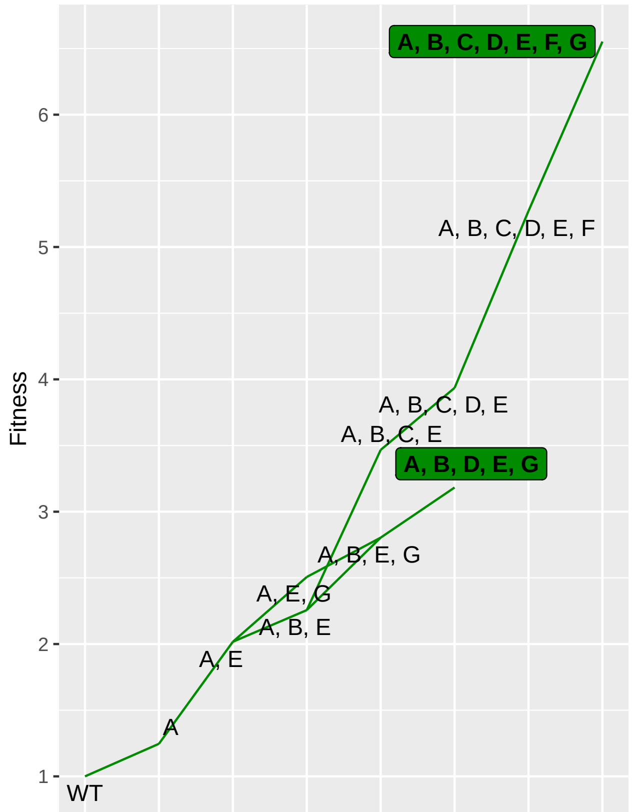

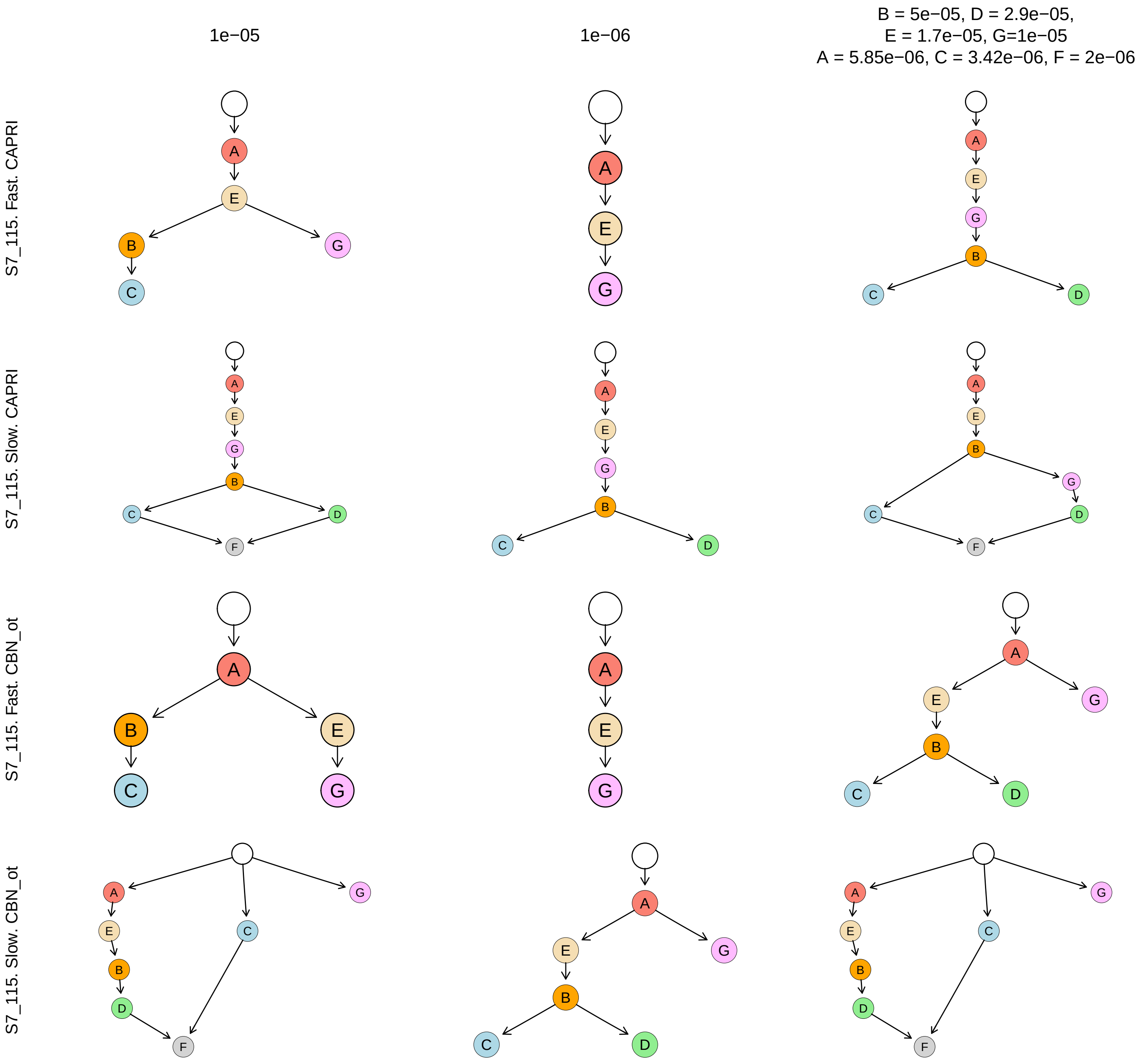

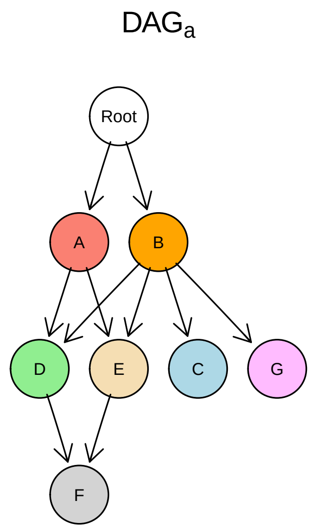

ID	Accessible Genot.	Discrep. DAG_a
S7_116	21	4

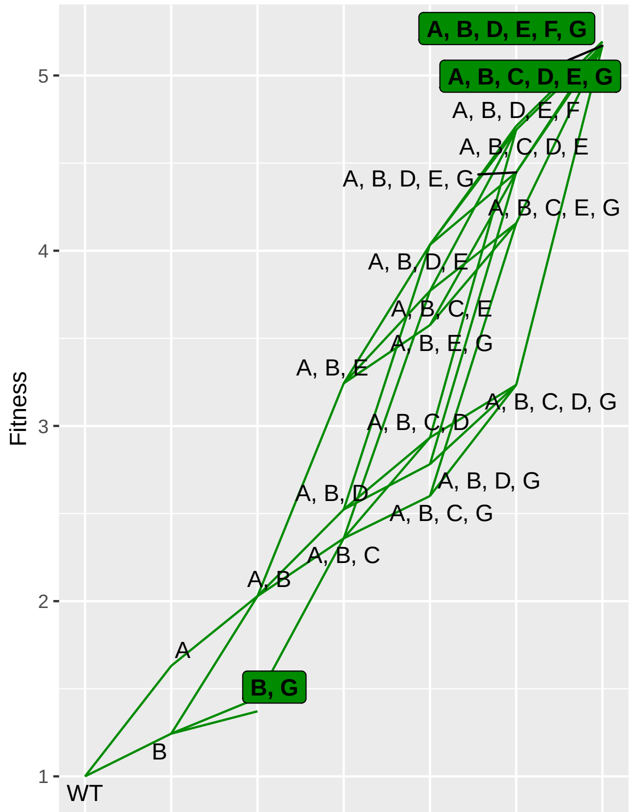

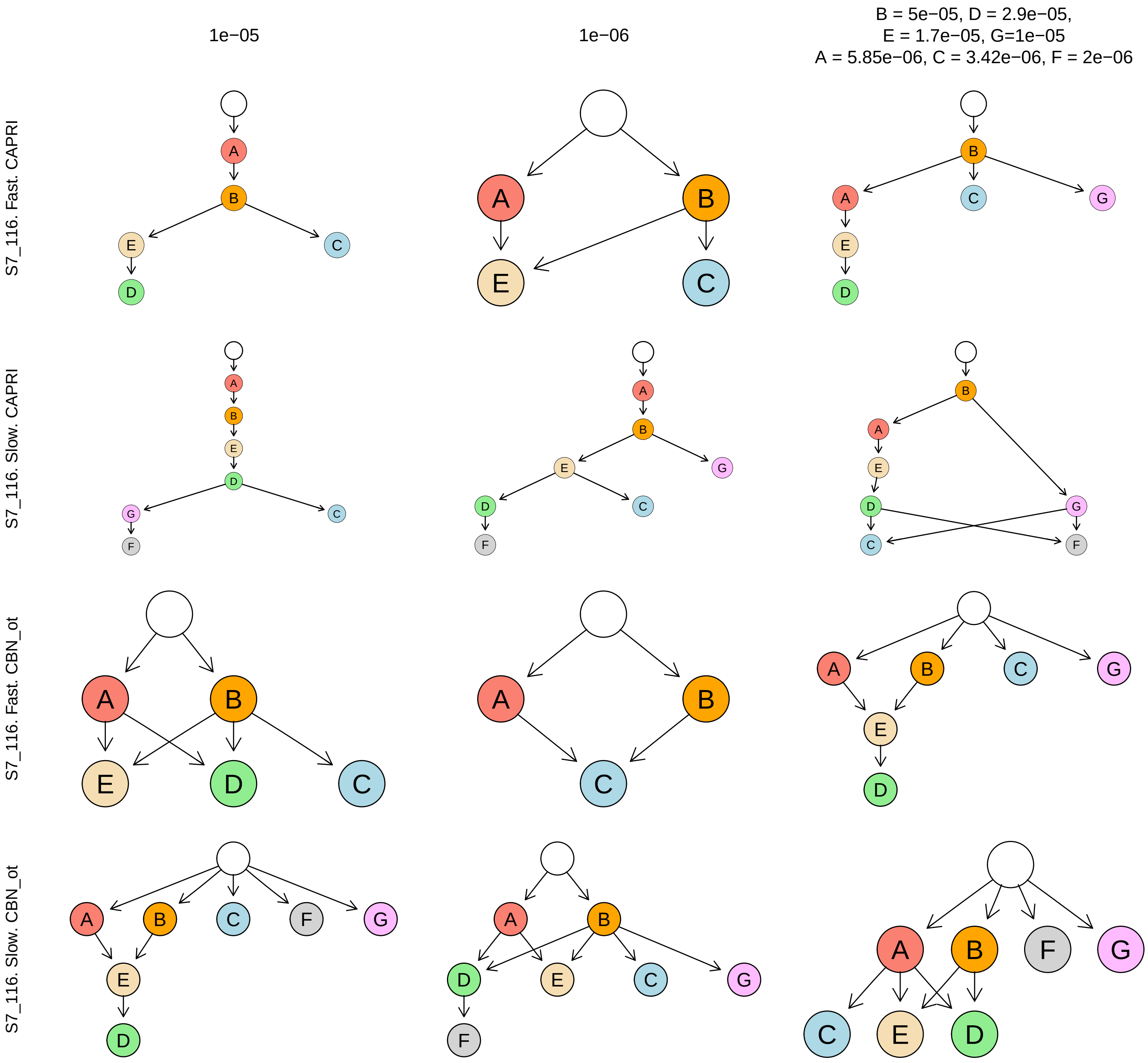

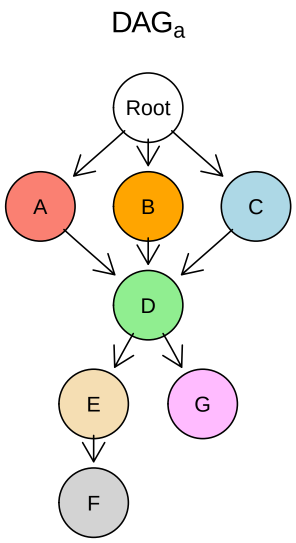

ID	Accessible Genot.	Discrep. DAG_a
S7_117	11	4

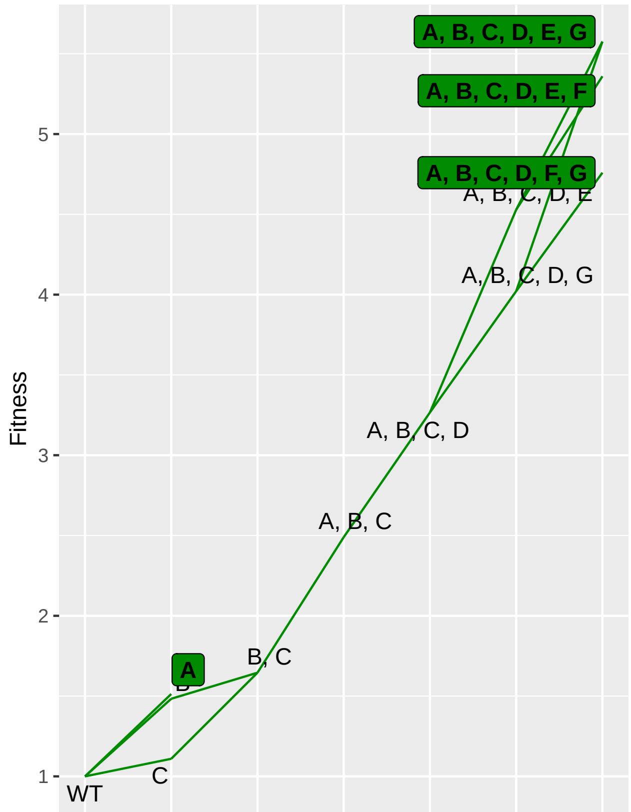

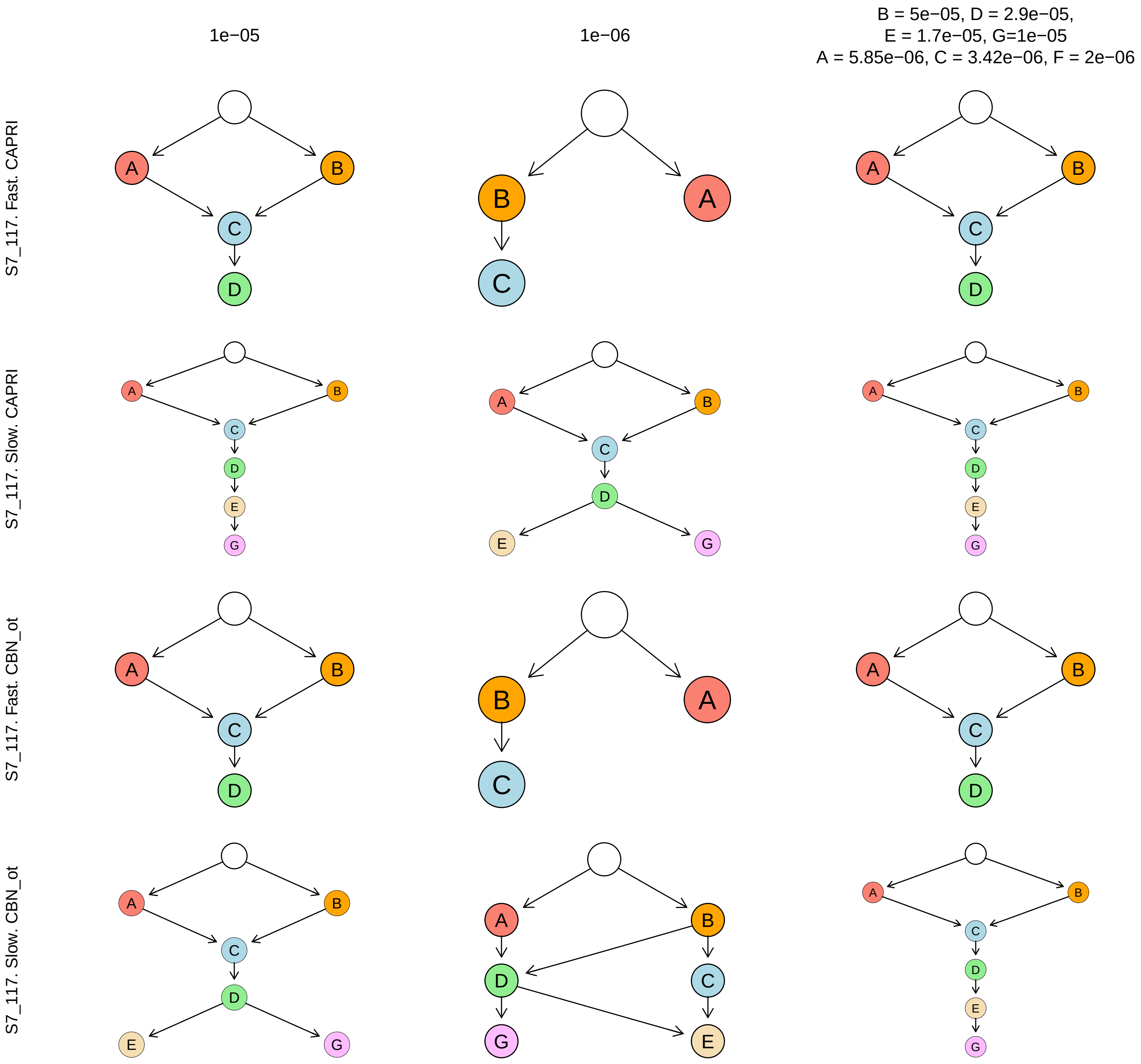

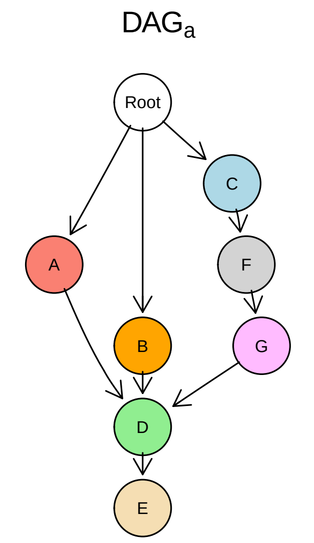

ID	Accessible Genot.	Discrep. DAG_a
S7_118	15	2

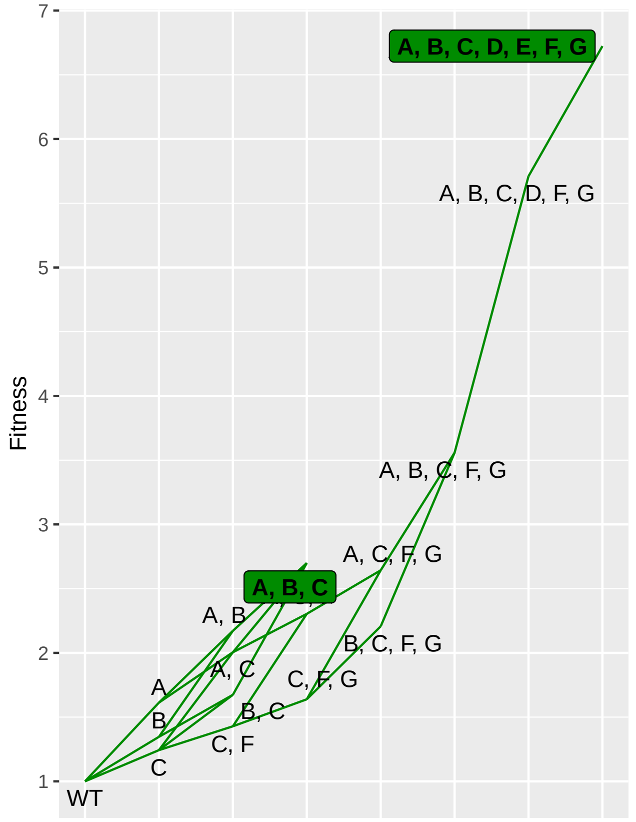

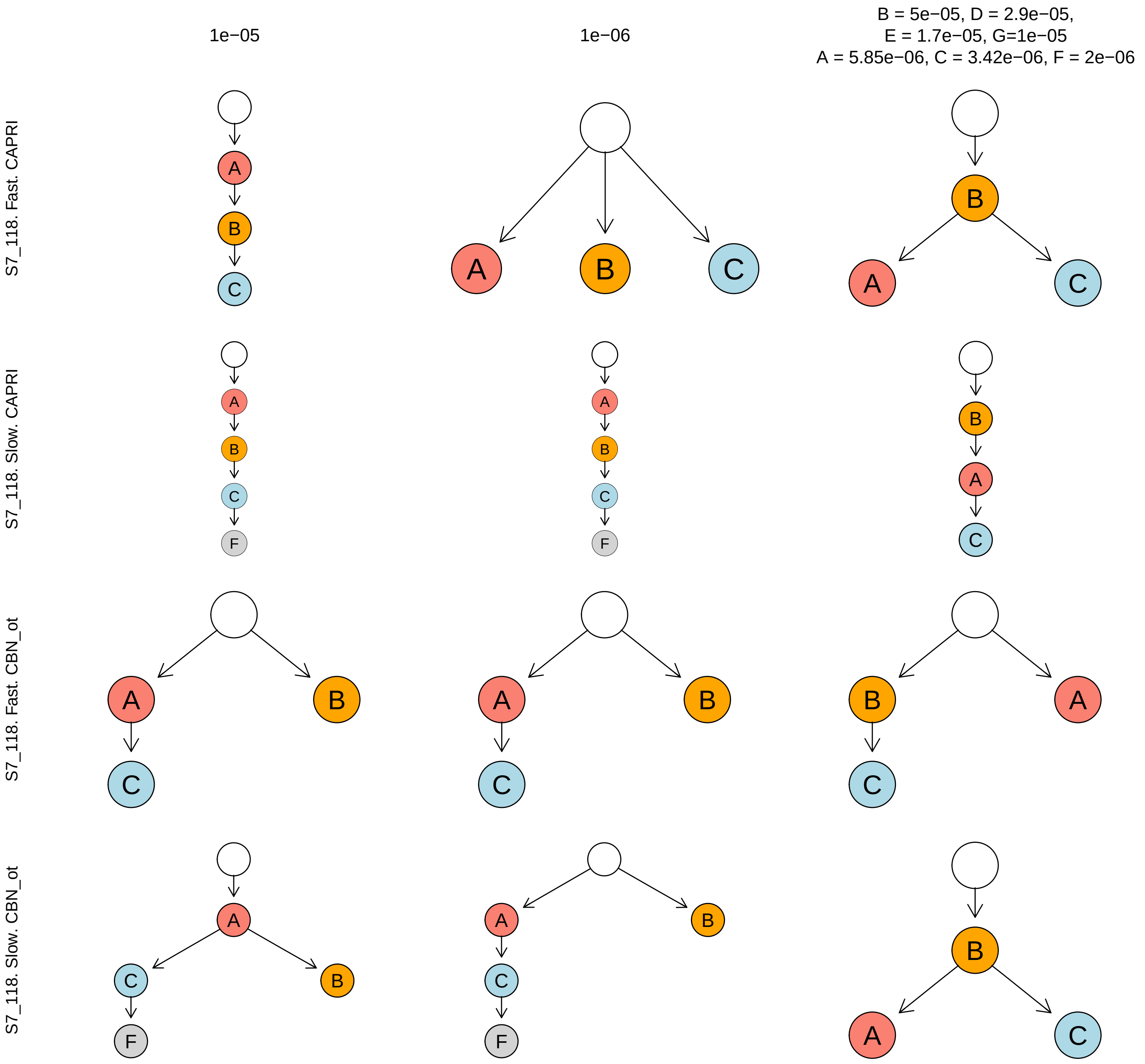

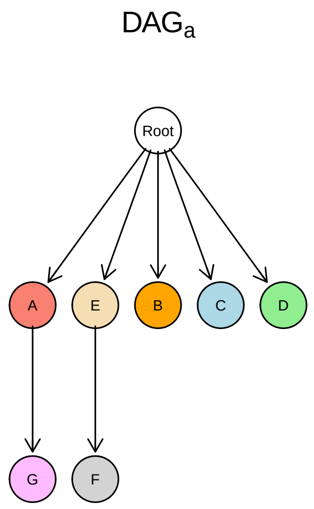

ID	Accessible Genot.	Discrep. DAG_a
S7_119	68	3

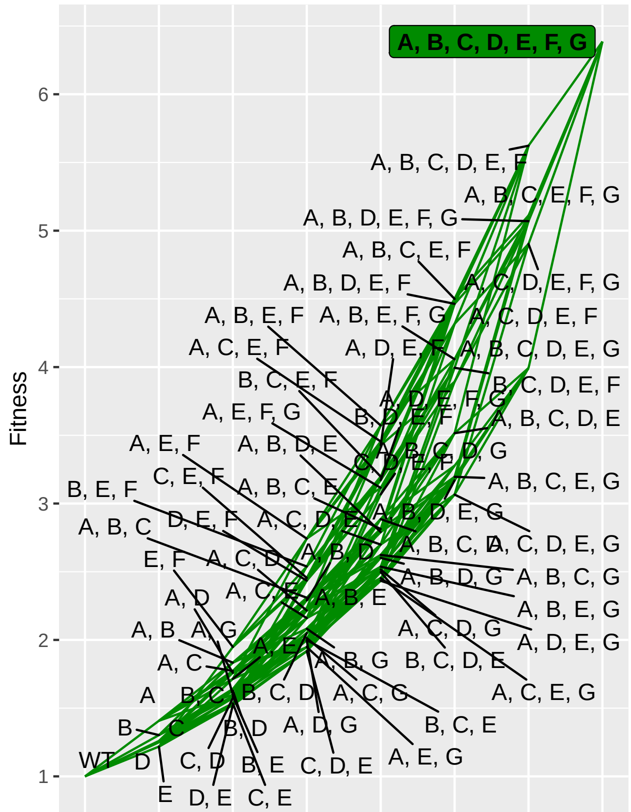

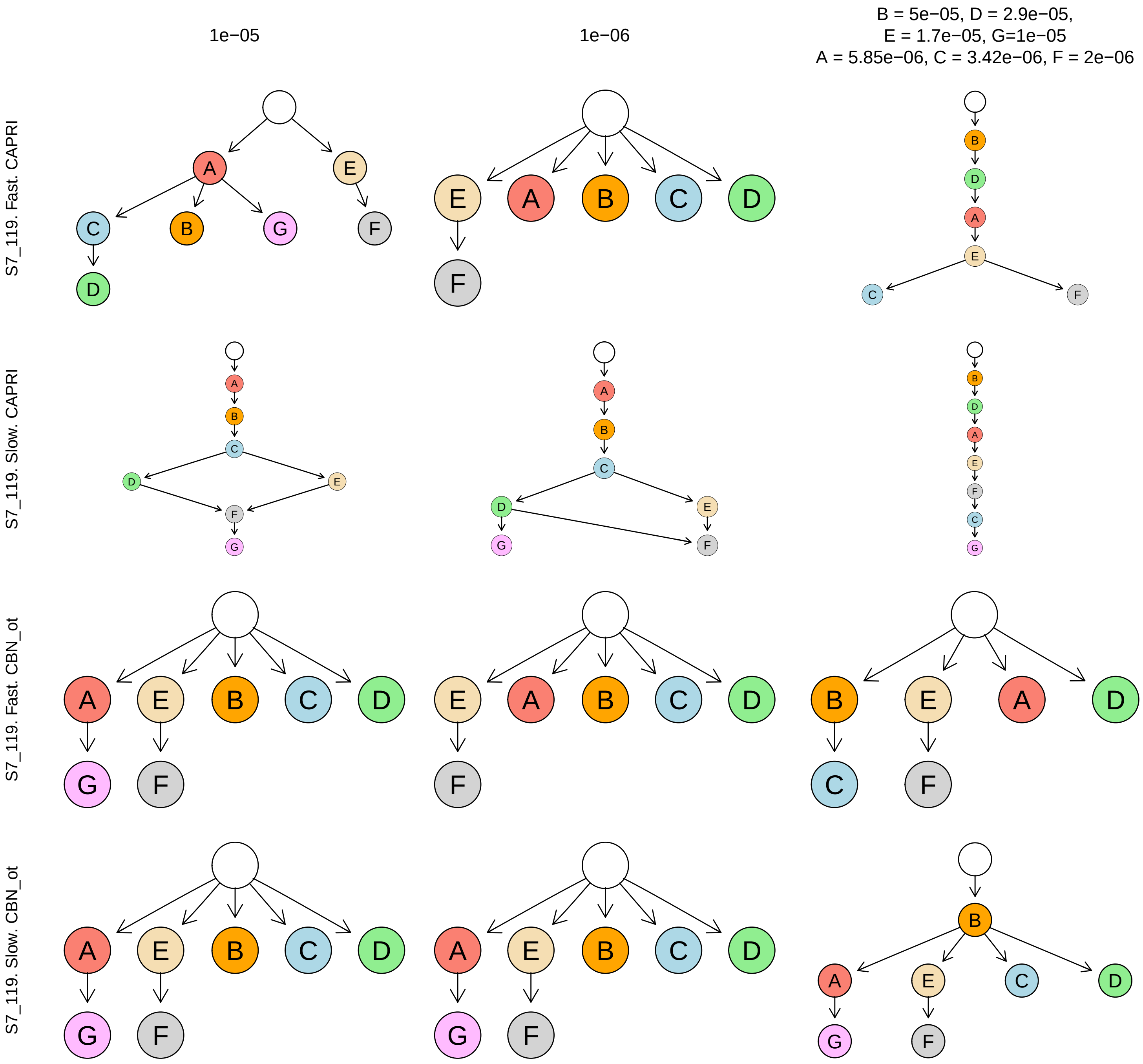

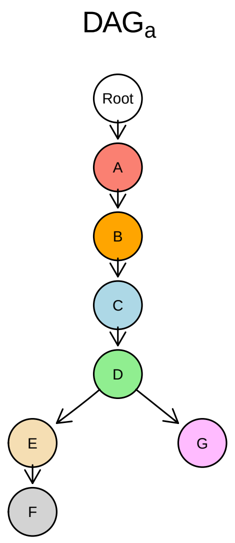

ID	Accessible Genot.	Discrep. DAG_a
S7_120	7	2

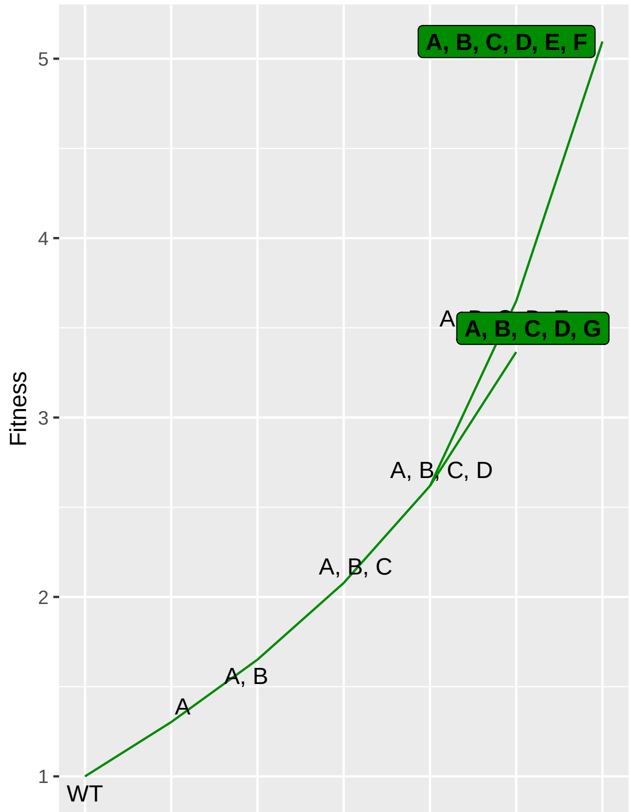

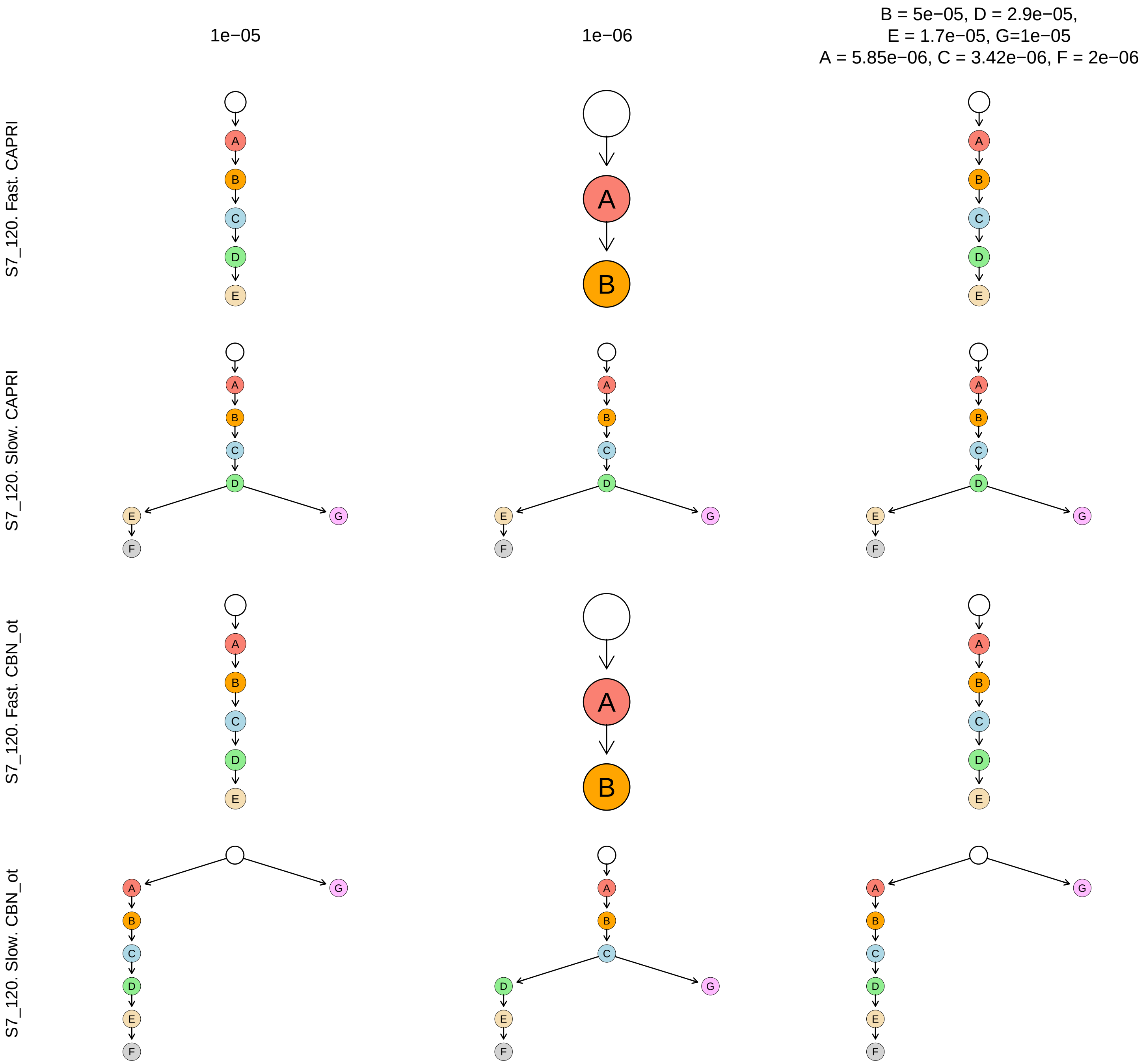

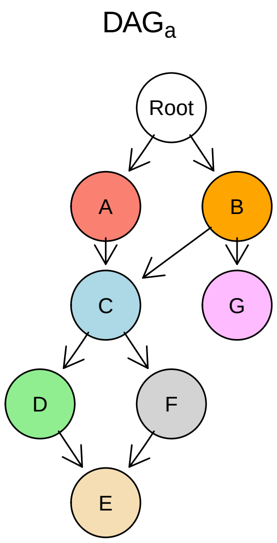

ID	Accessible Genot.	Discrep. DAG_a
S7_121	16	1

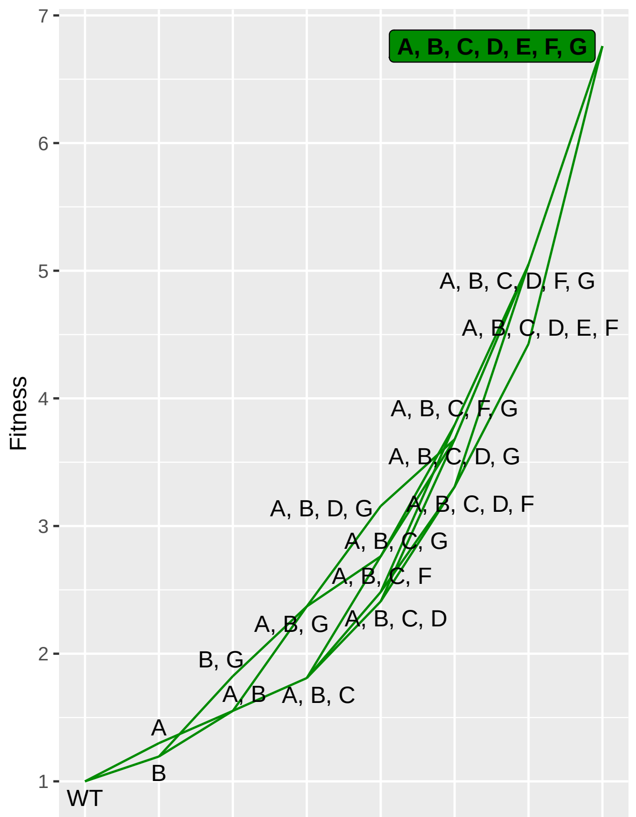

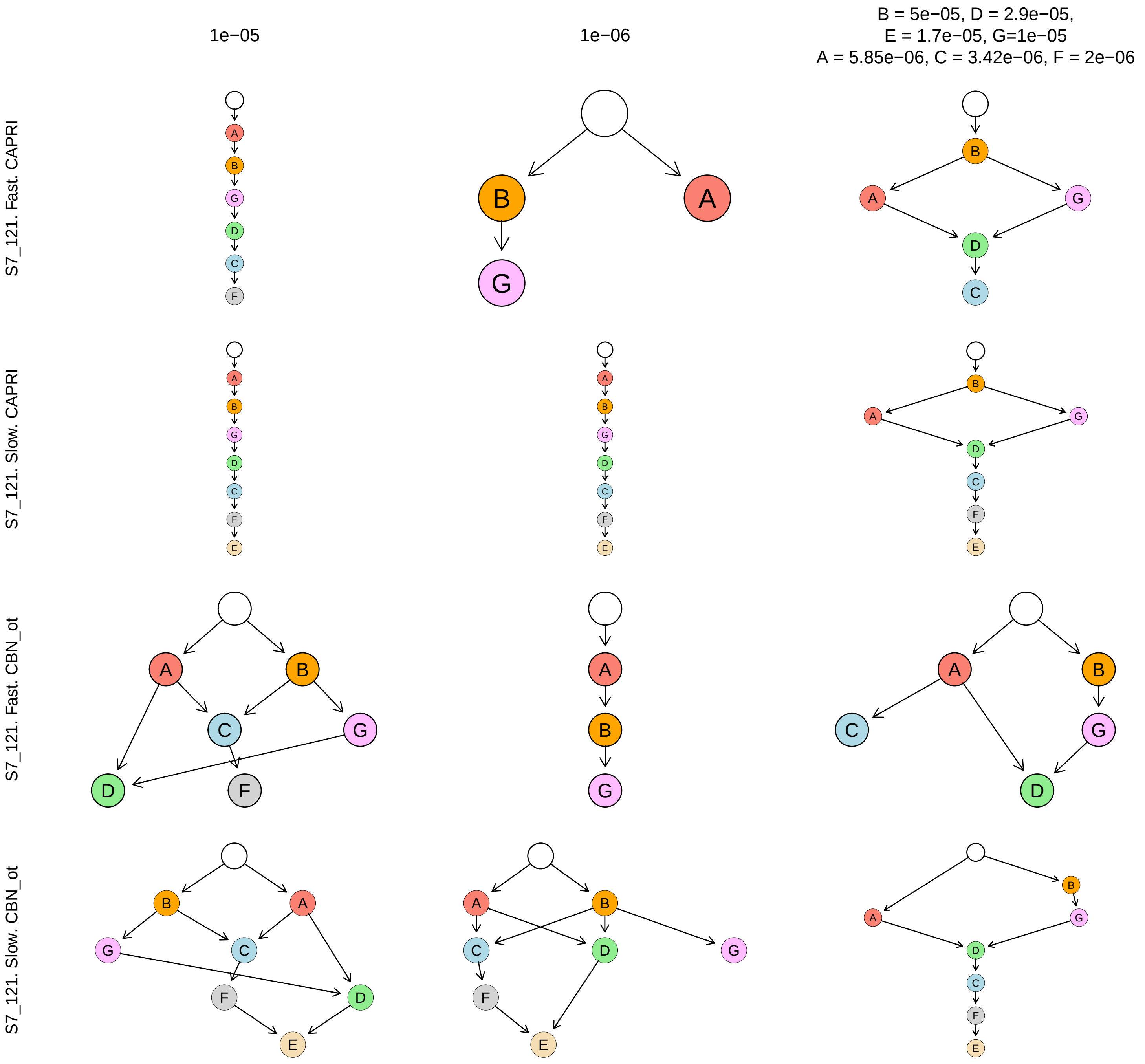

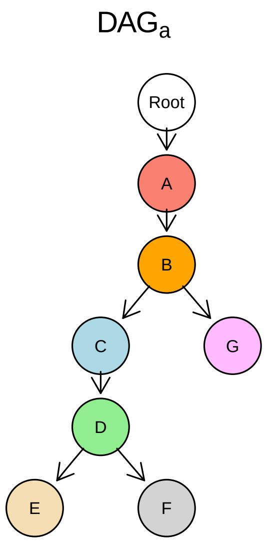

ID	Accessible Genot.	Discrep. DAG_a
S7_122	10	3

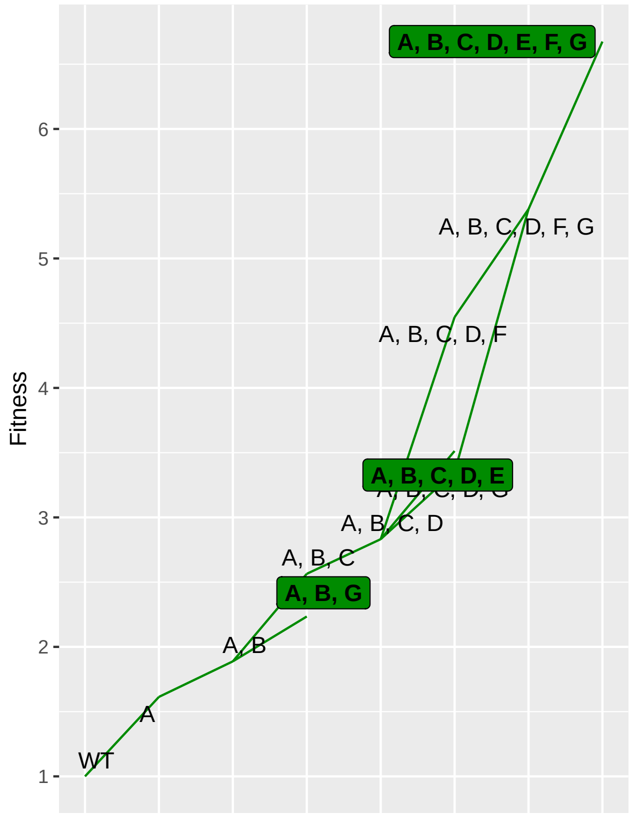

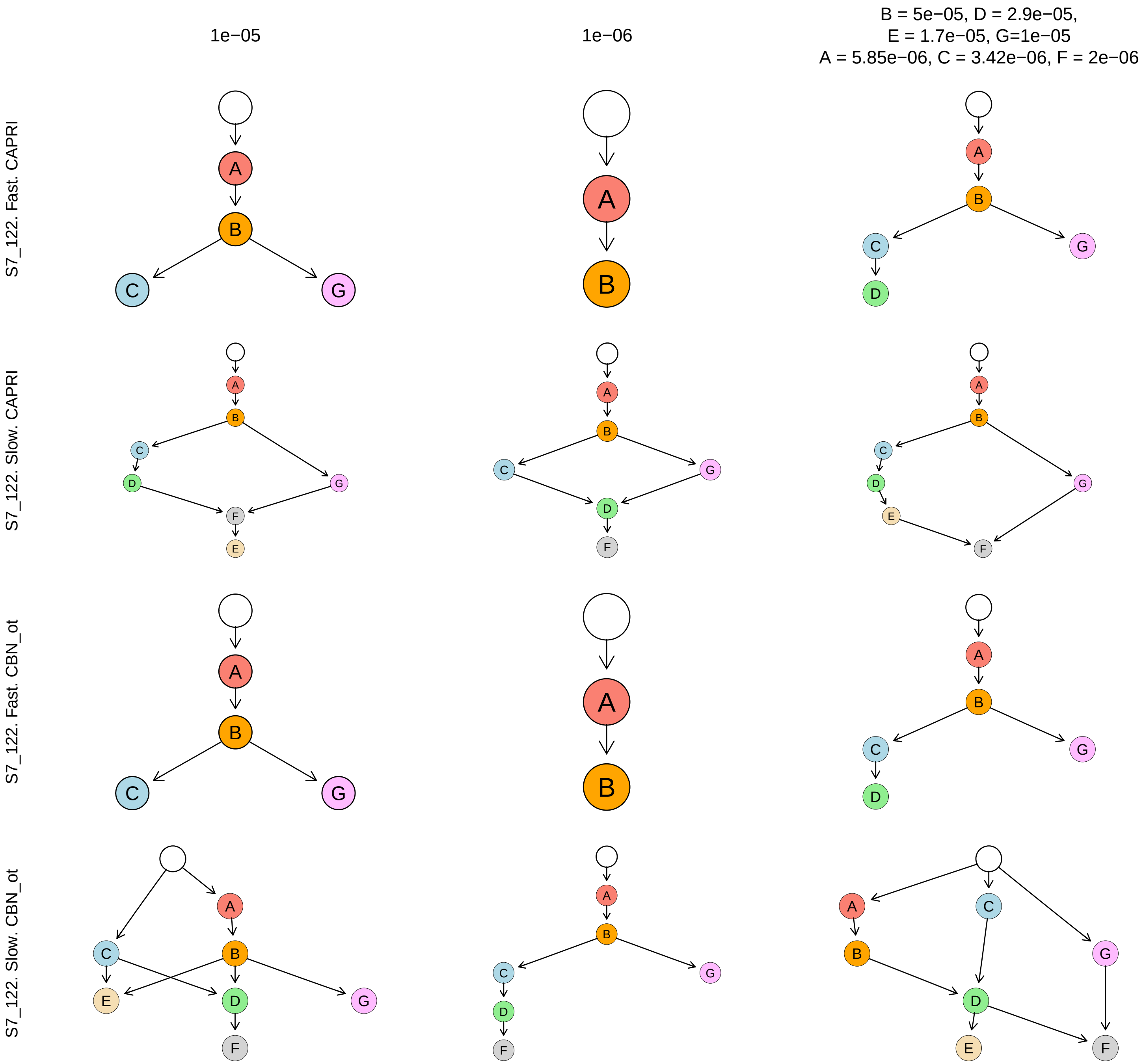

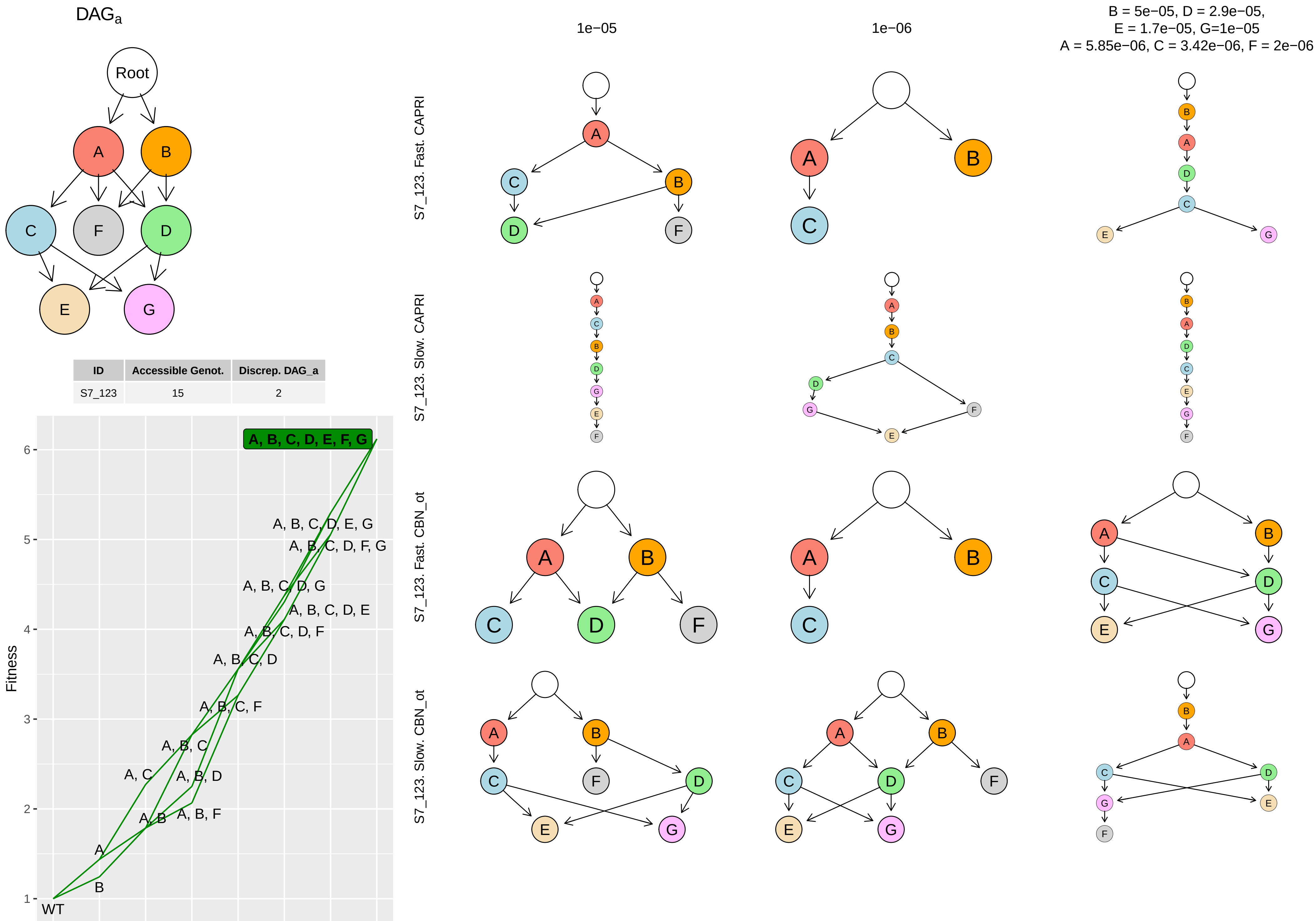

ID	Accessible Genot.	Discrep. DAG_a
S7_123	15	2

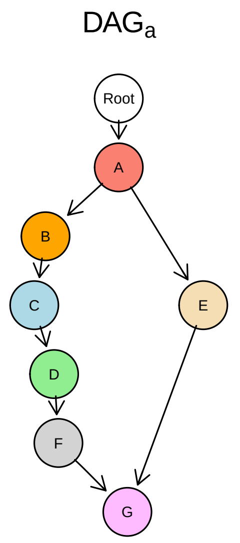

ID	Accessible Genot.	Discrep. DAG_a
S7_124	10	1

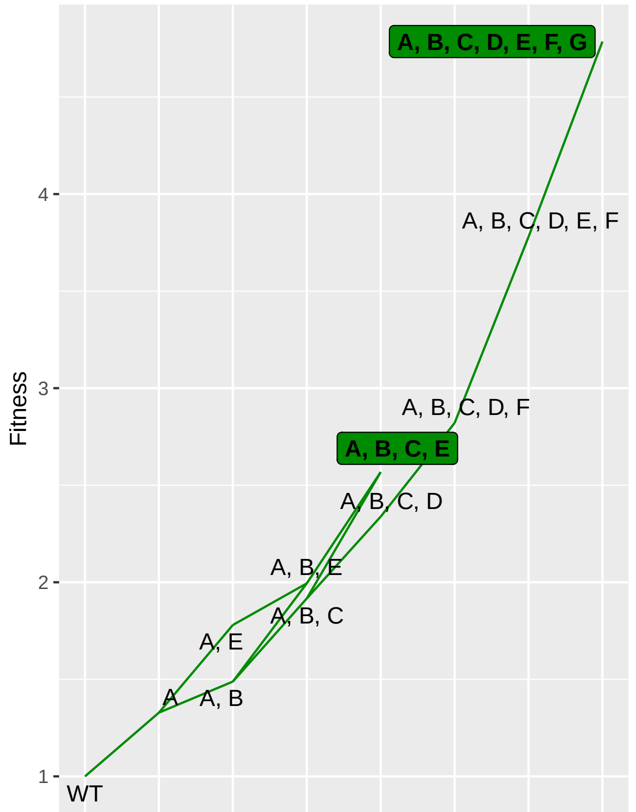

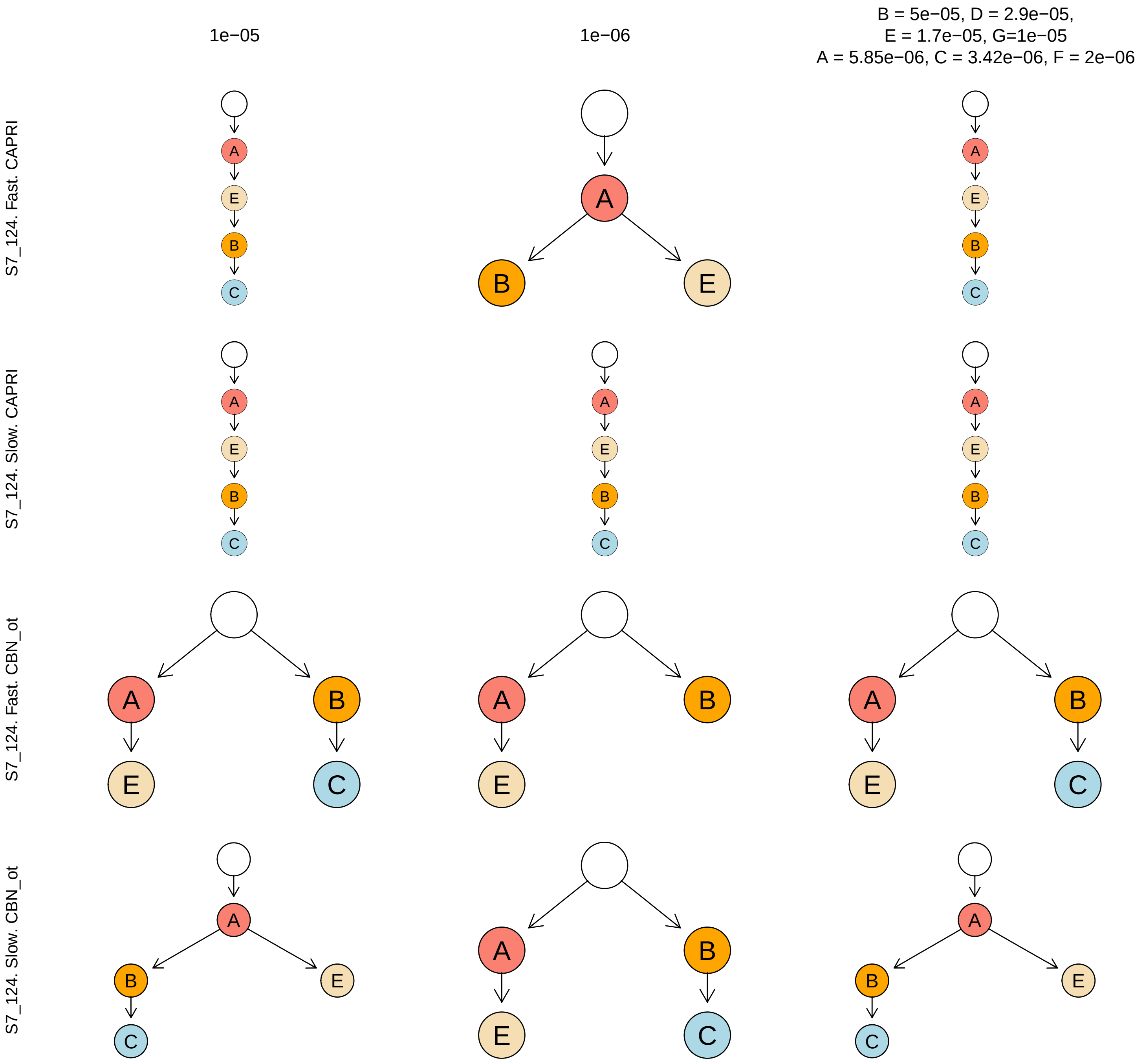

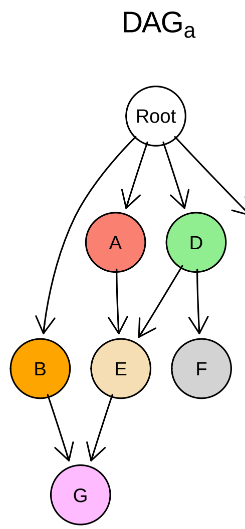

ID	Accessible Genot.	Discrep. DAG_a
S7_125	28	17

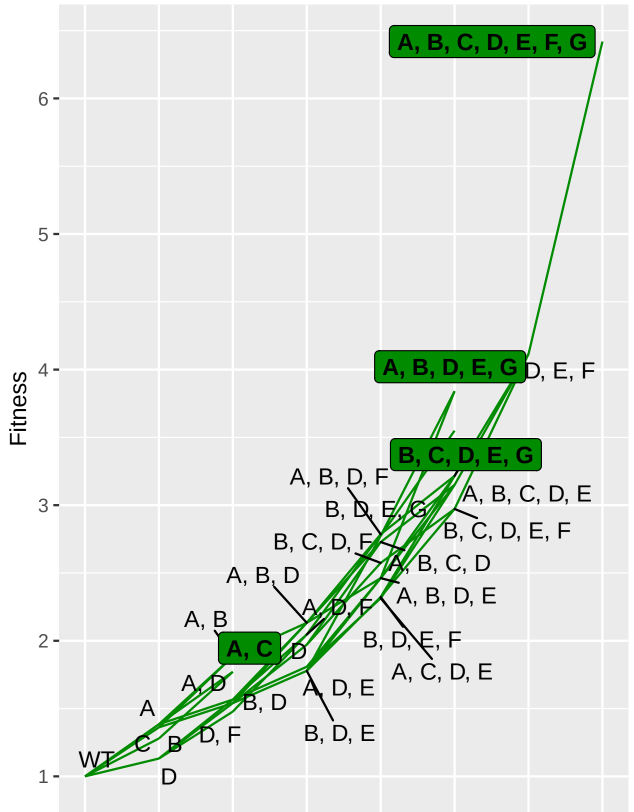

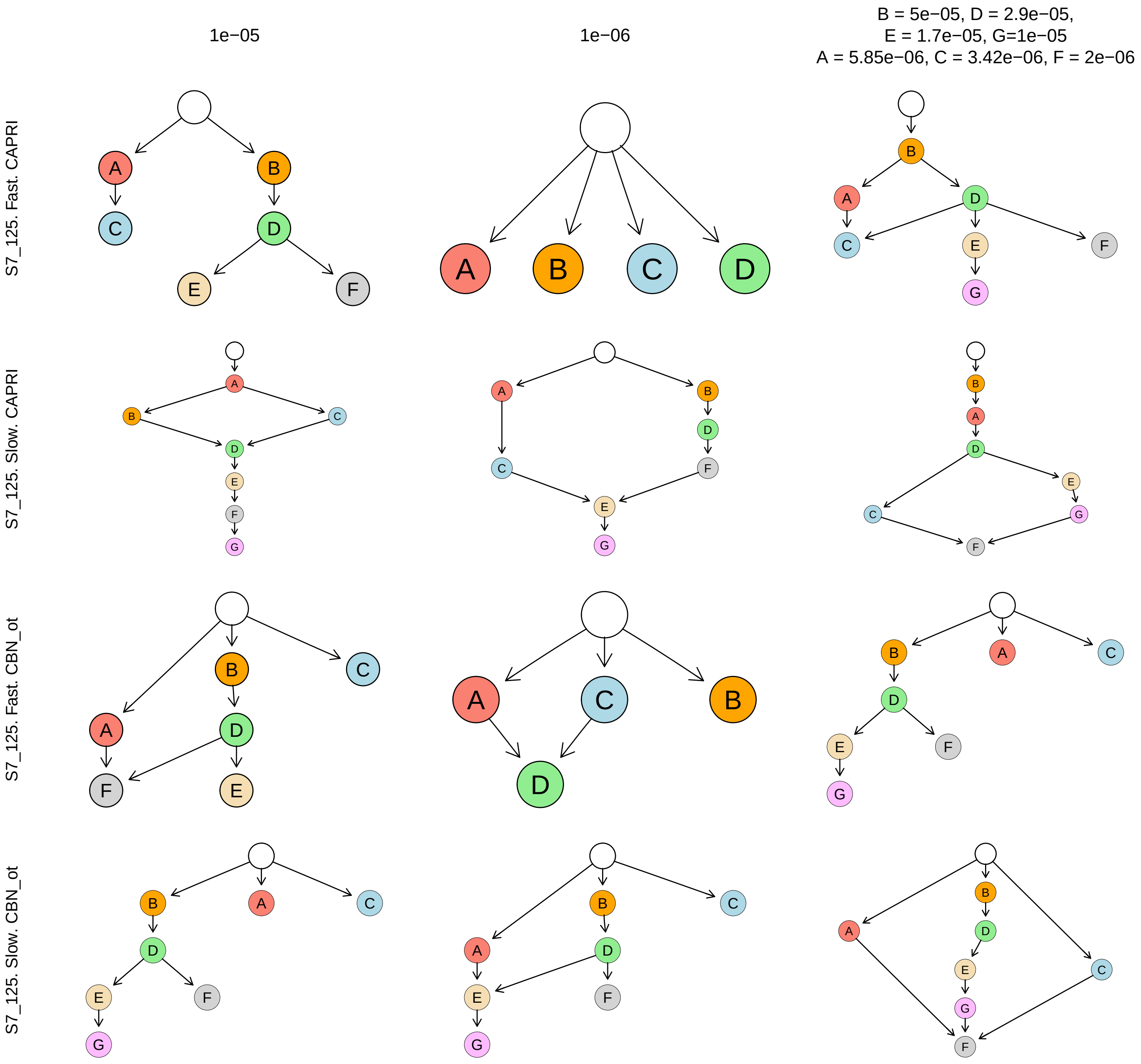

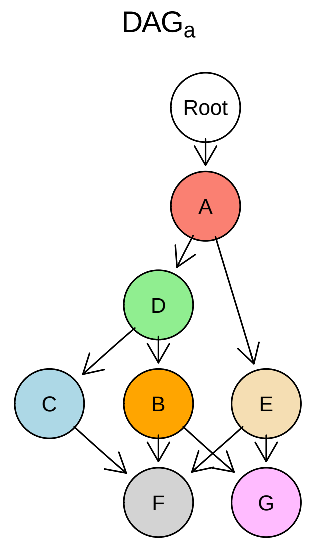

ID	Accessible Genot.	Discrep. DAG_a
S7_126	18	8

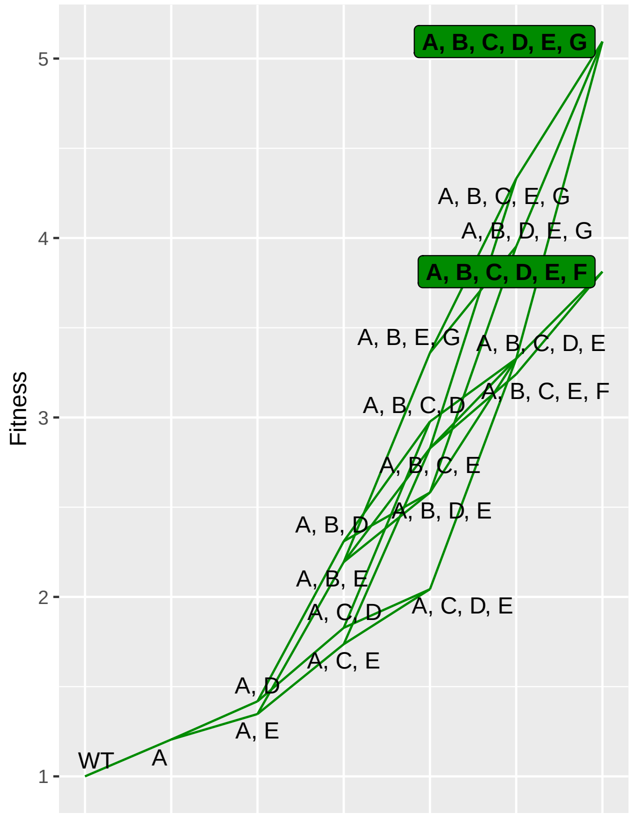

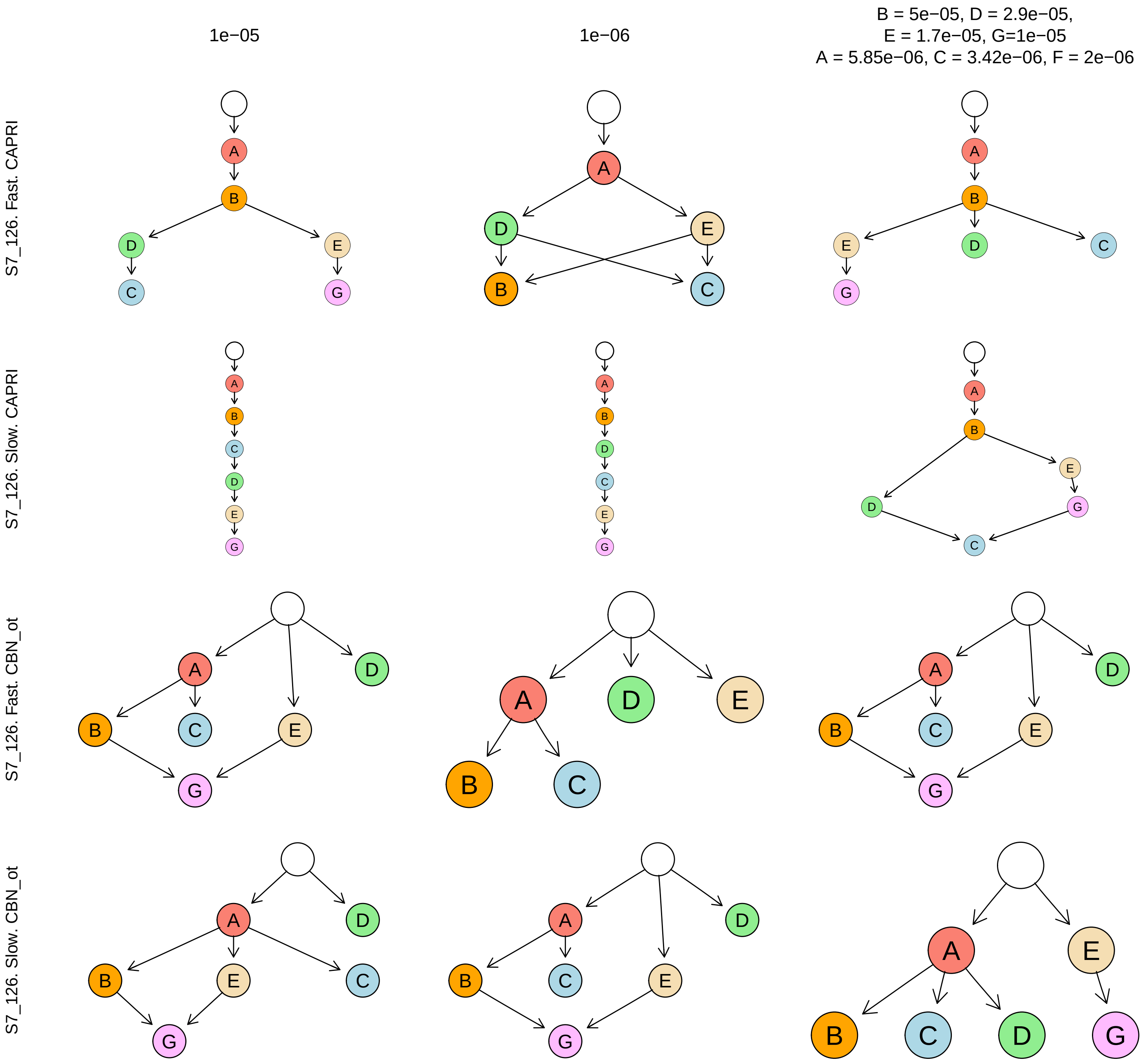

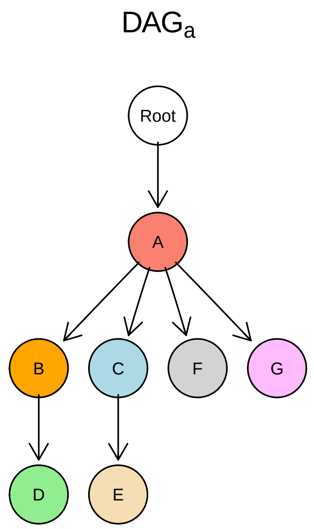

ID	Accessible Genot.	Discrep. DAG_a
S7_127	36	28

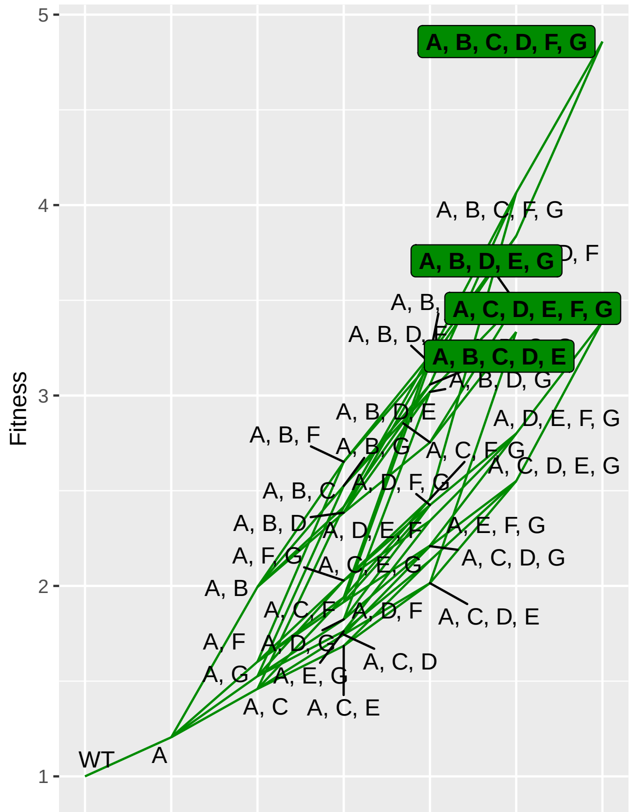

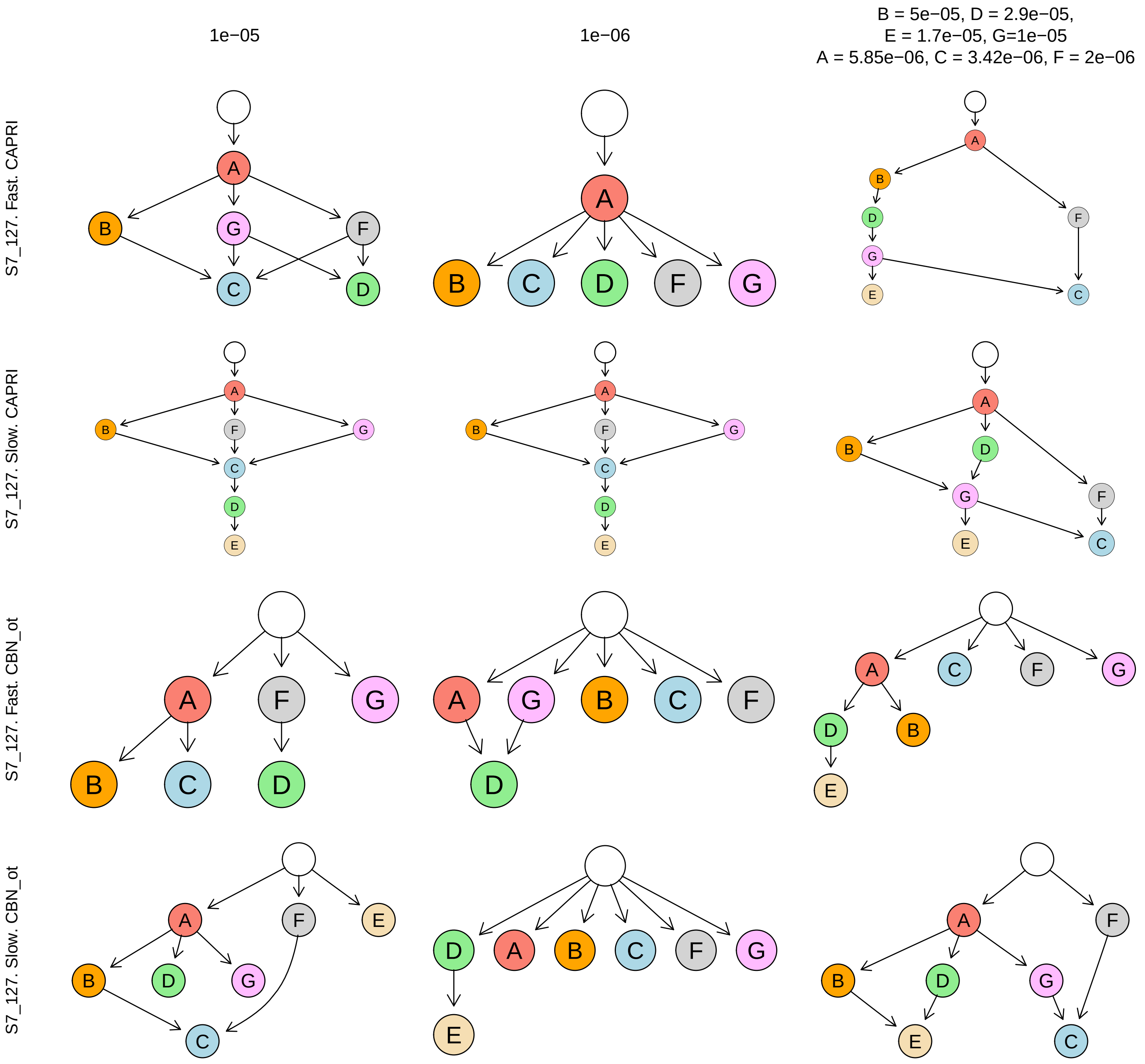

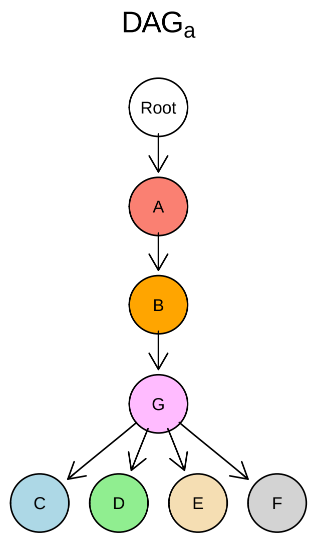

ID	Accessible Genot.	Discrep. DAG_a
S7_128	16	2

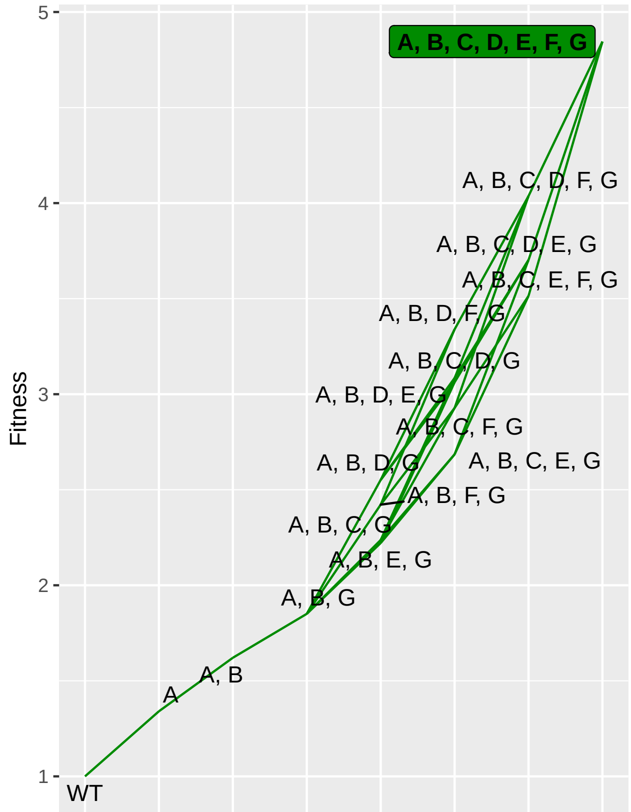

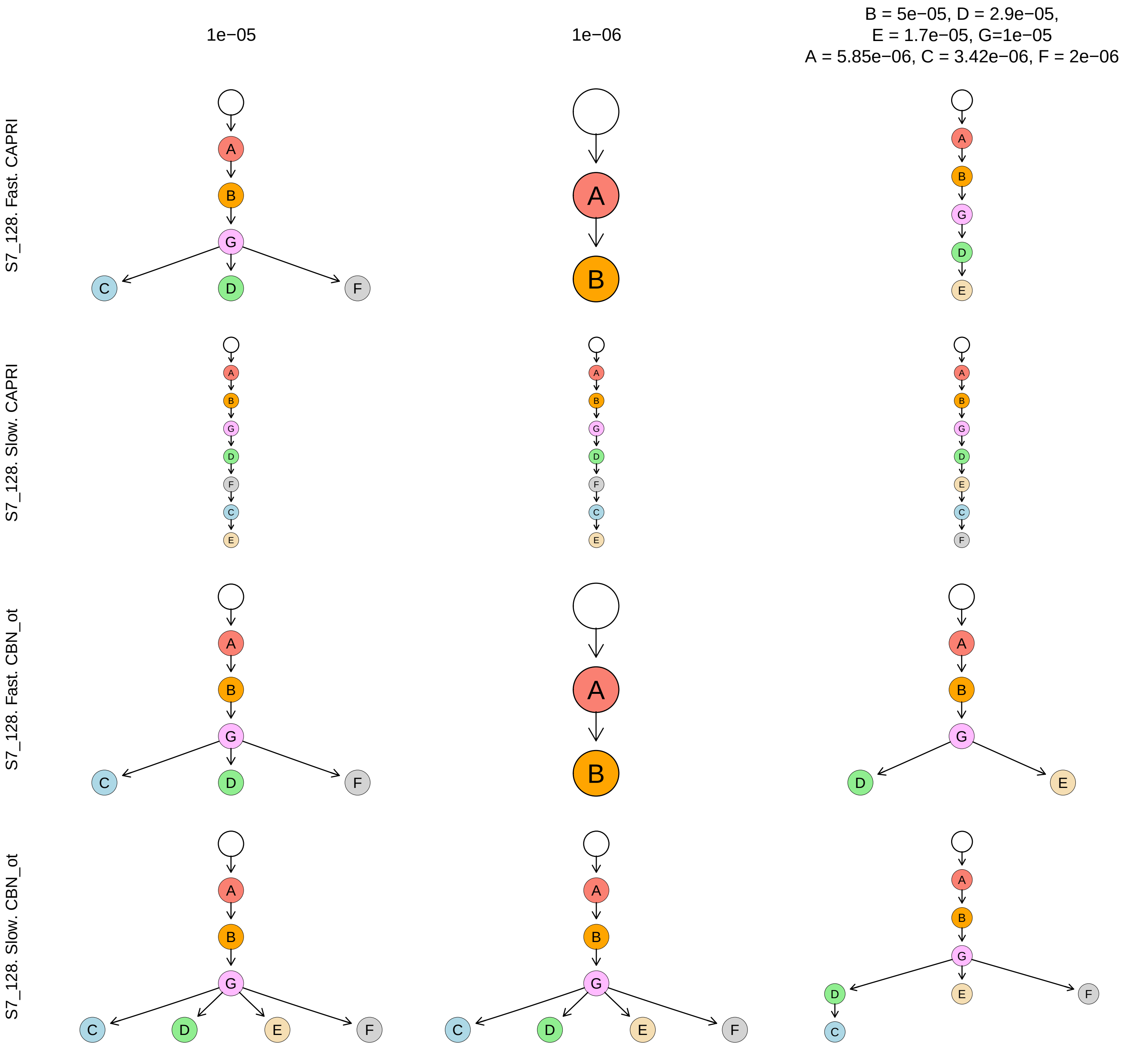

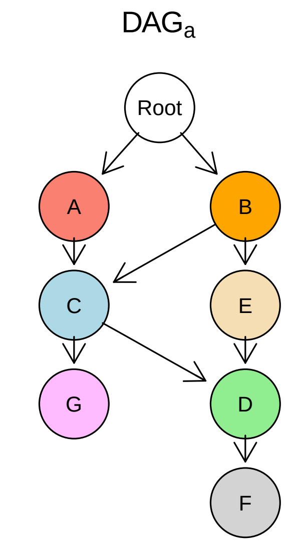

ID	Accessible Genot.	Discrep. DAG_a
S7_129	13	4

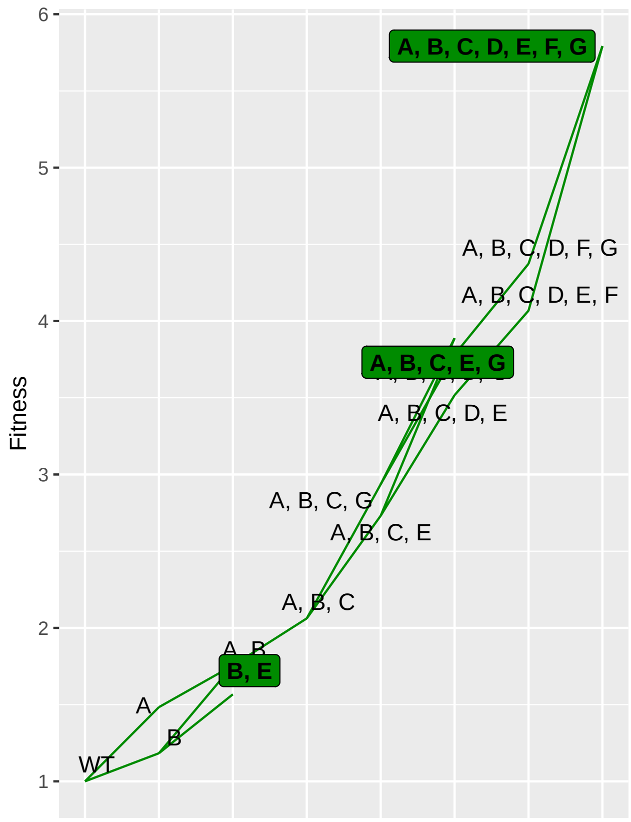

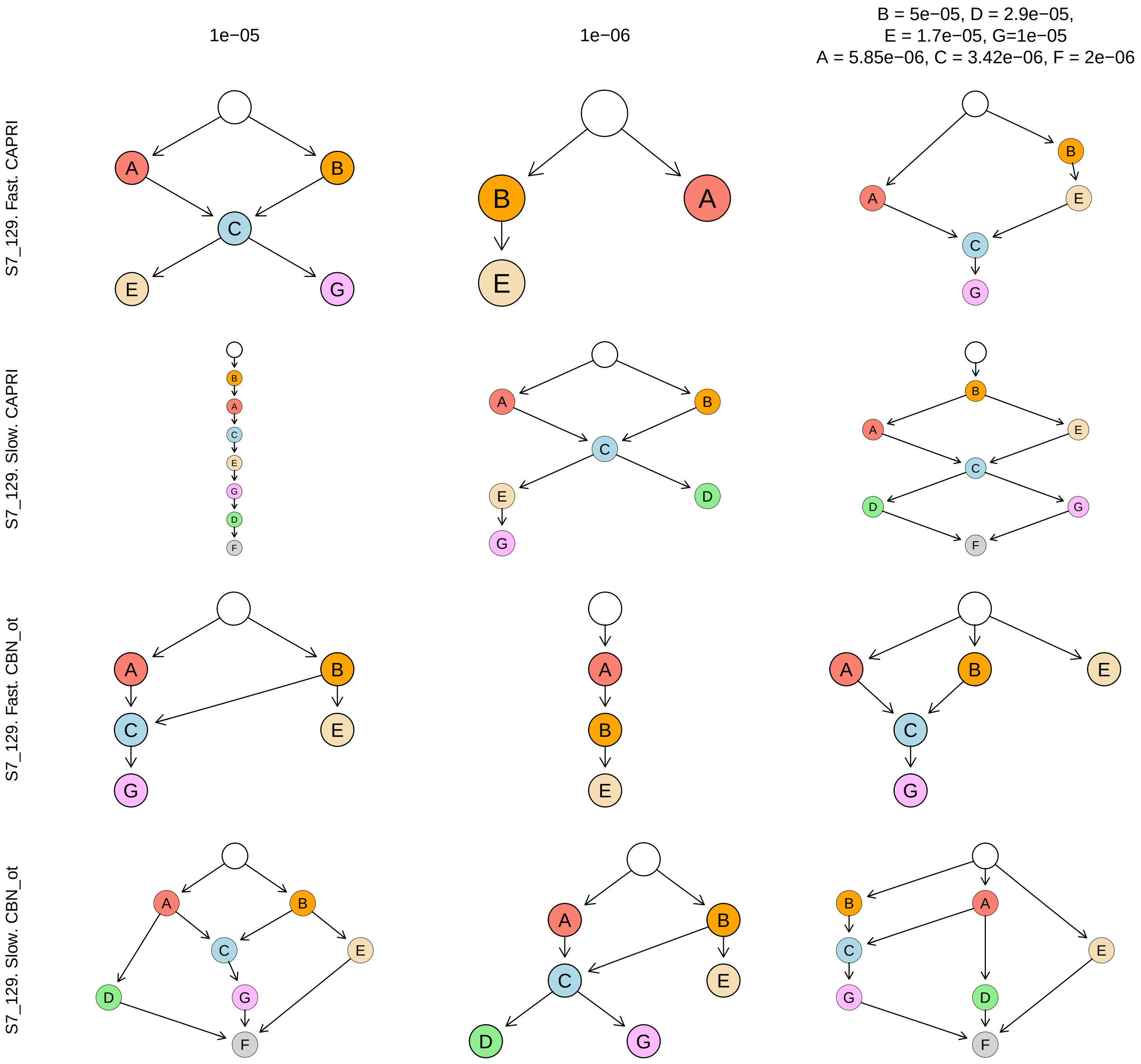

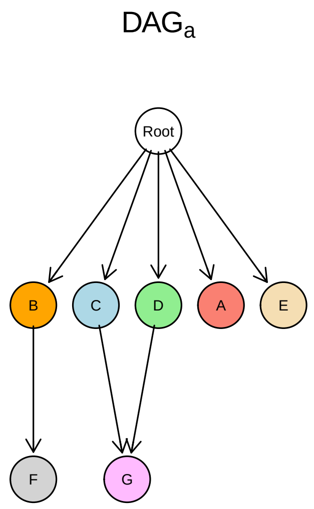

ID	Accessible Genot.	Discrep. DAG_a
S7_130	56	3

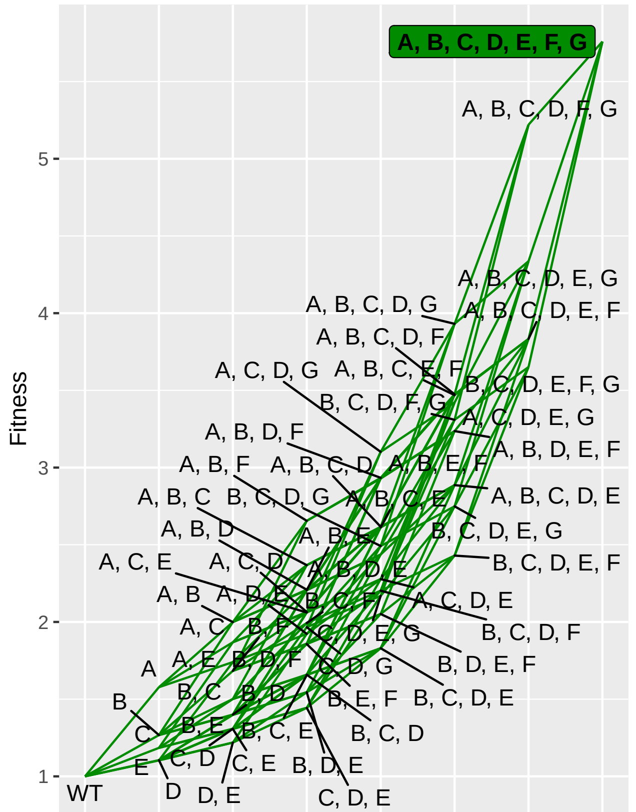

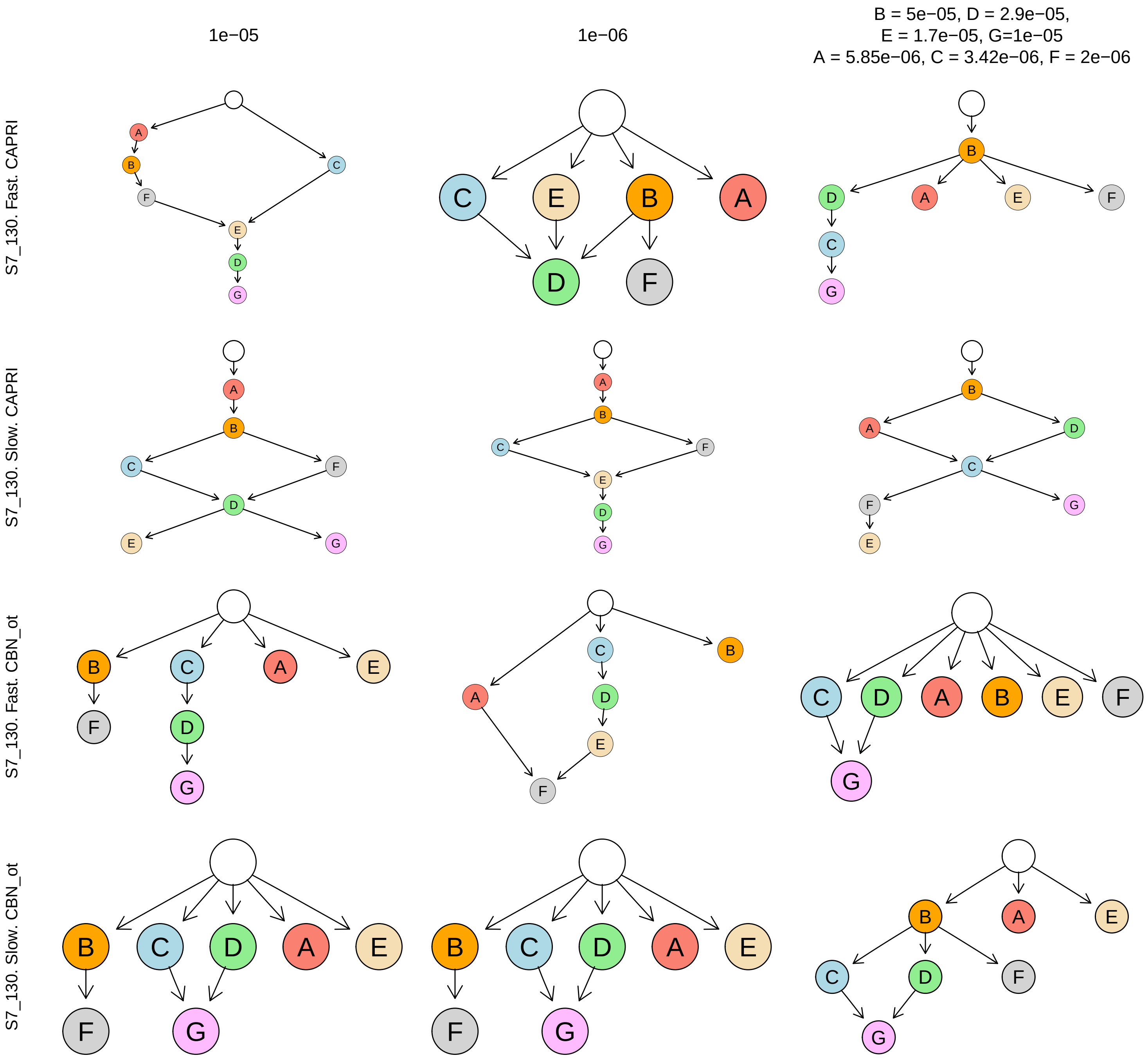

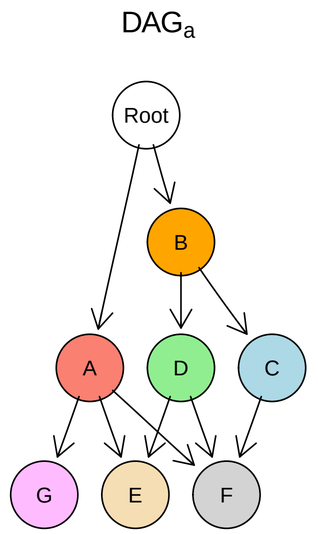

ID	Accessible Genot.	Discrep. DAG_a
S7_131	20	2

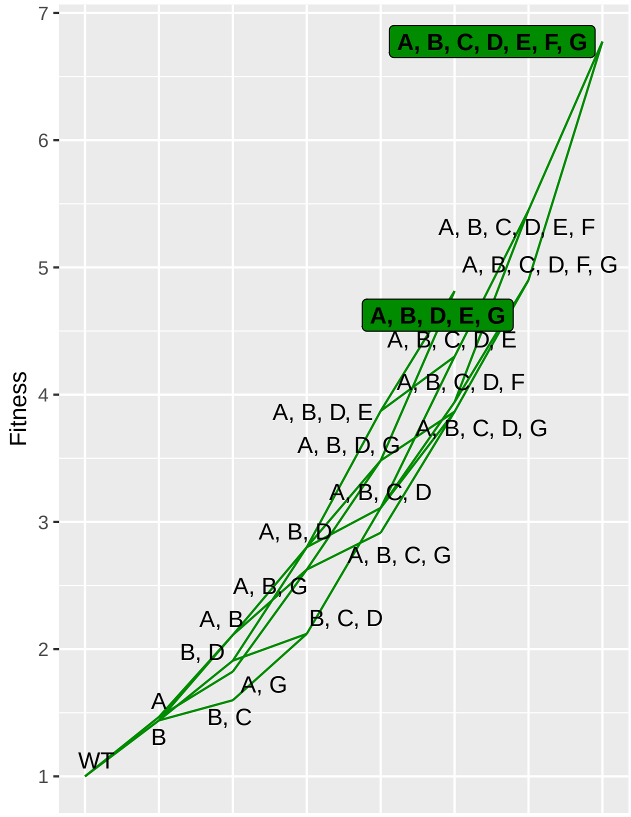

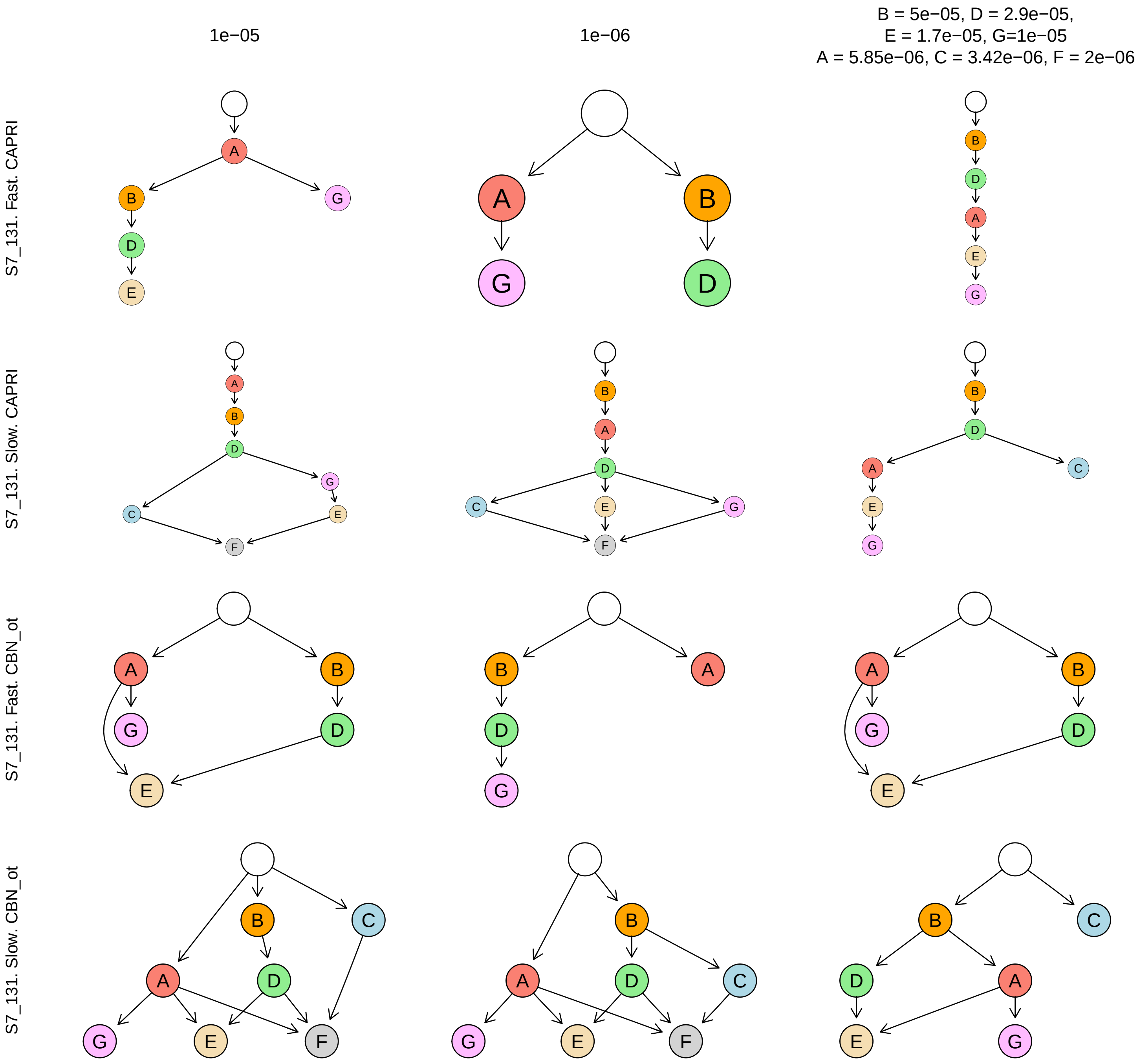

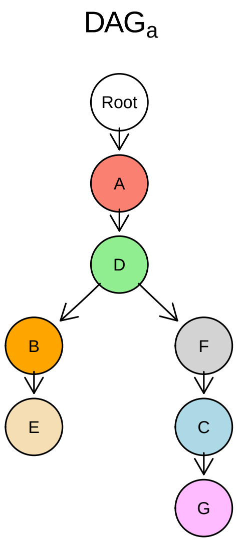

ID	Accessible Genot.	Discrep. DAG_a
S7_132	13	2

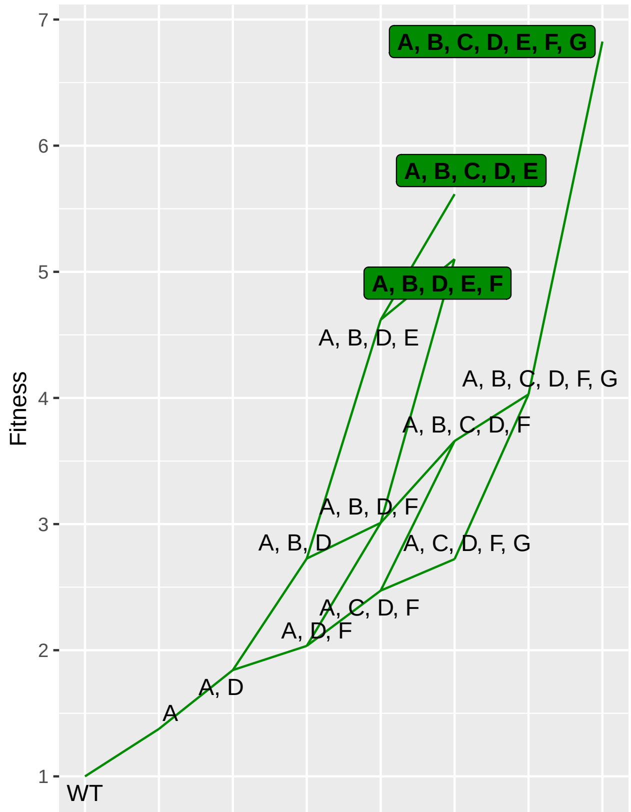

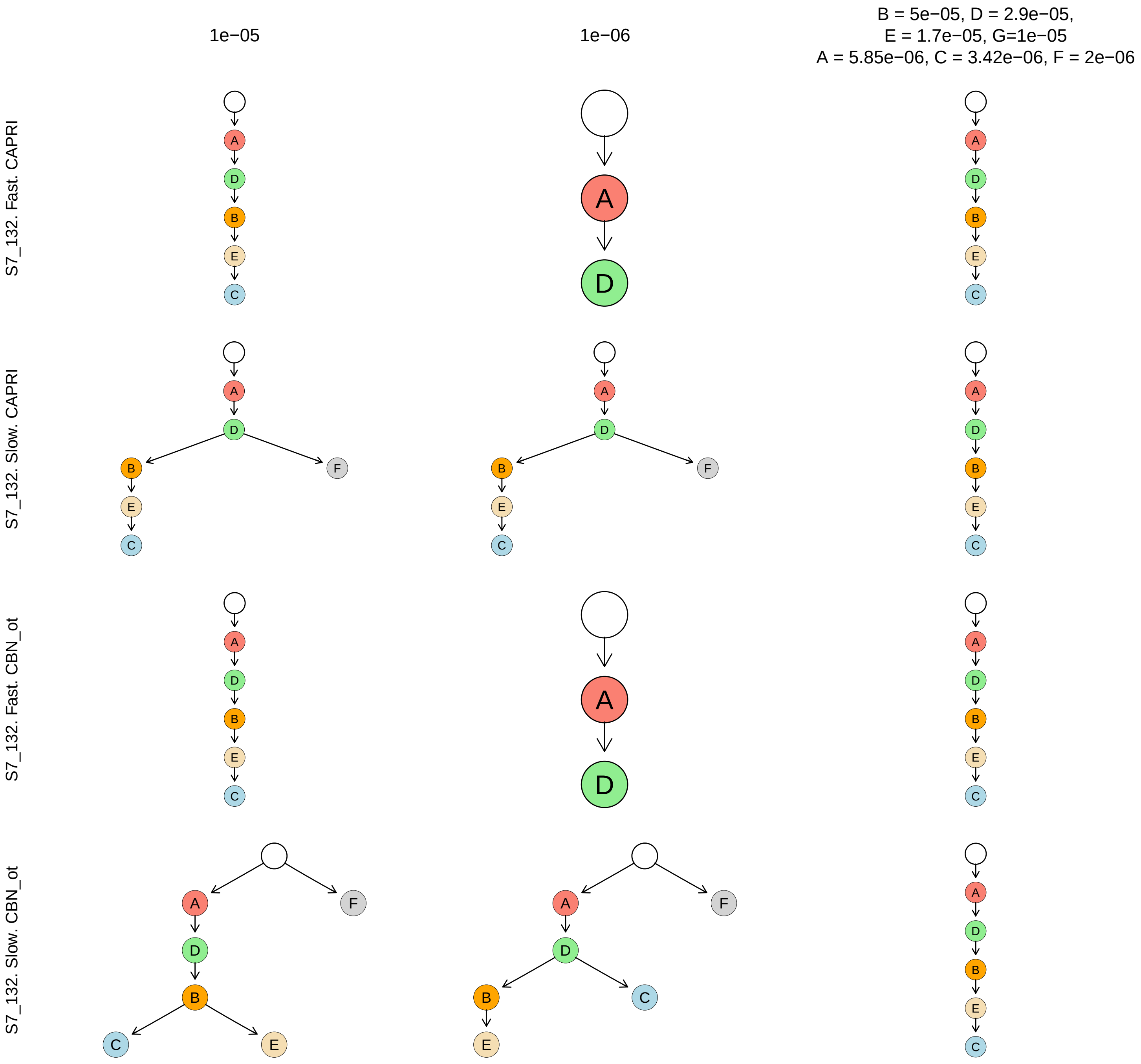

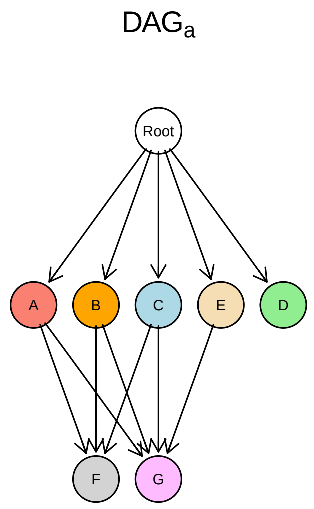

ID	Accessible Genot.	Discrep. DAG_a
S7_133	21	18

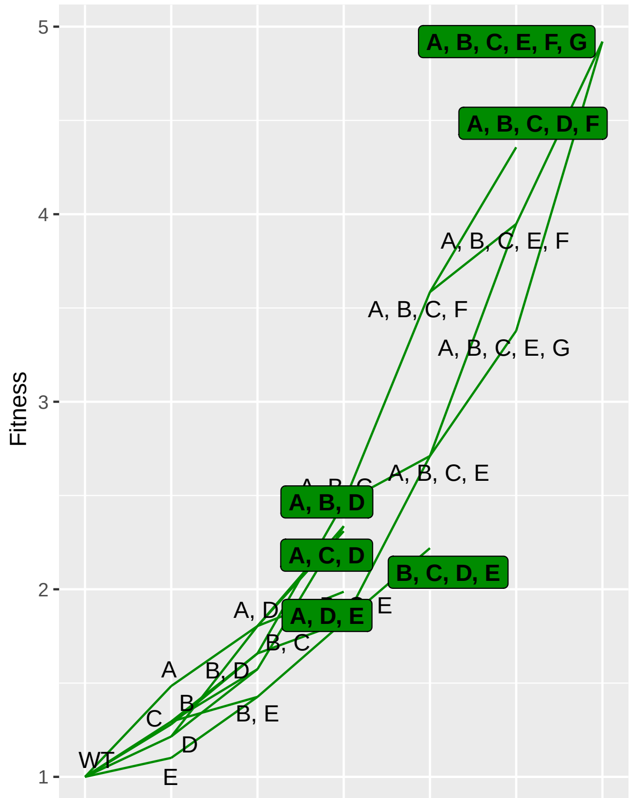

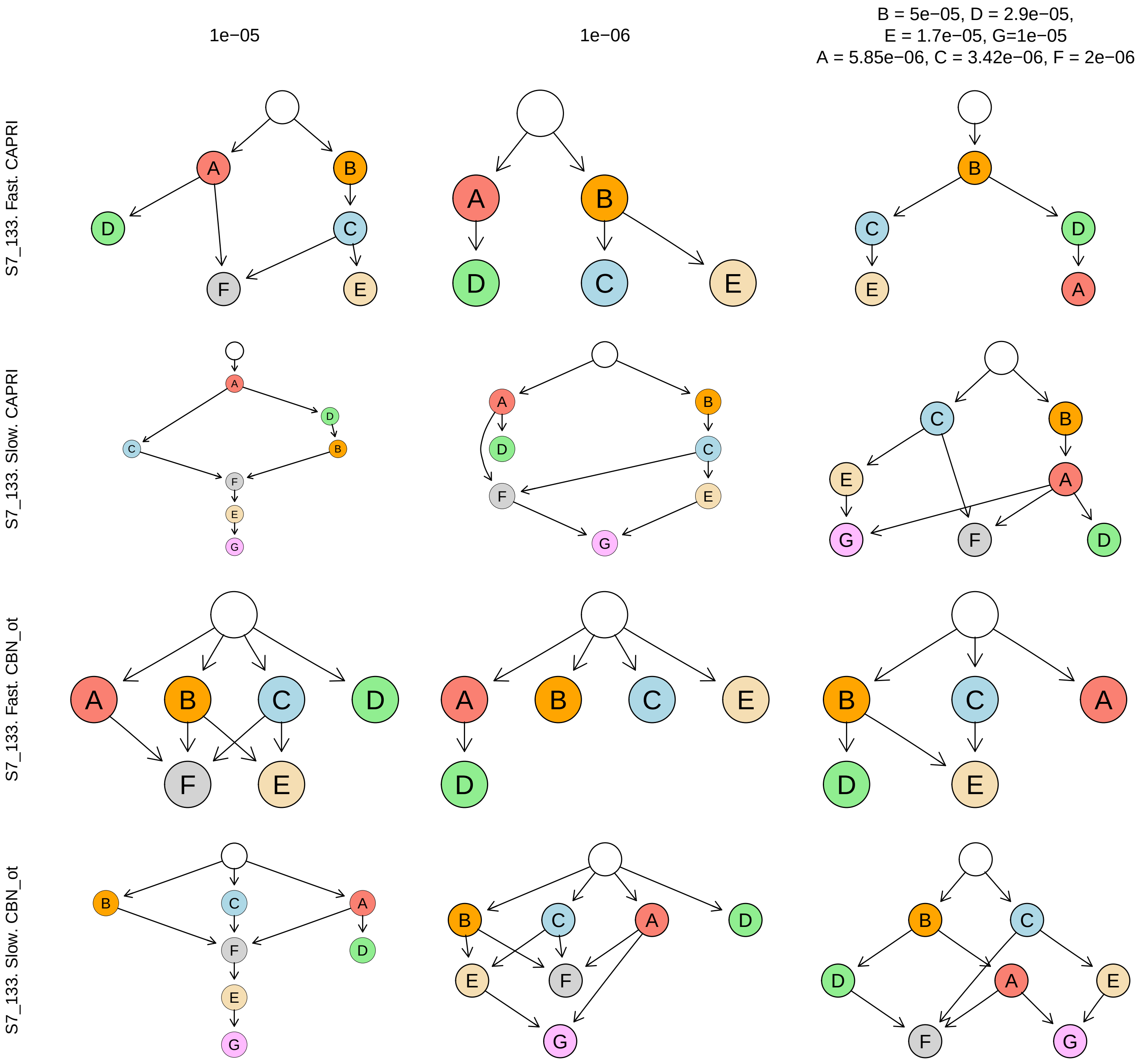

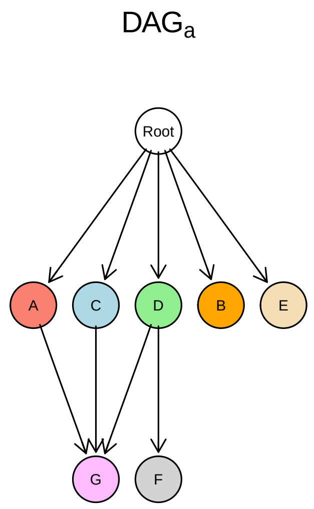

ID	Accessible Genot.	Discrep. DAG_a
S7_134	29	26

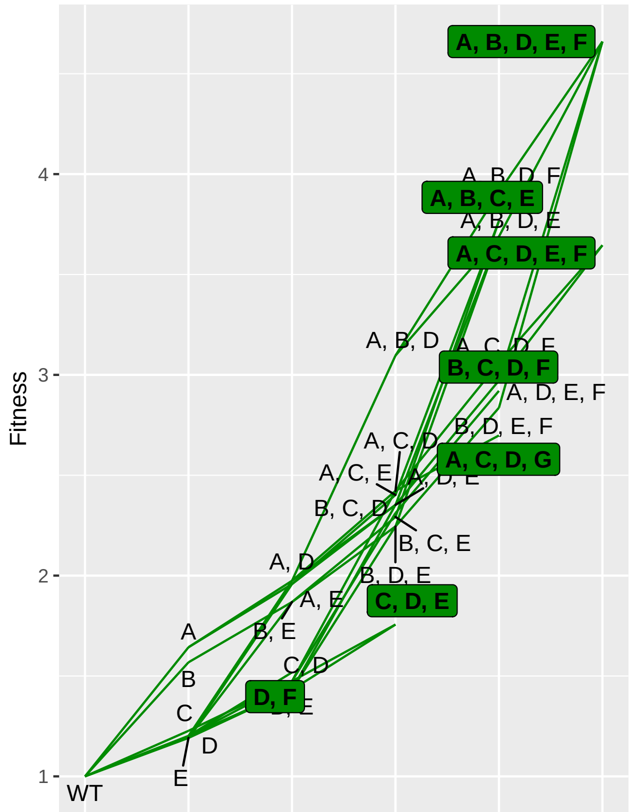

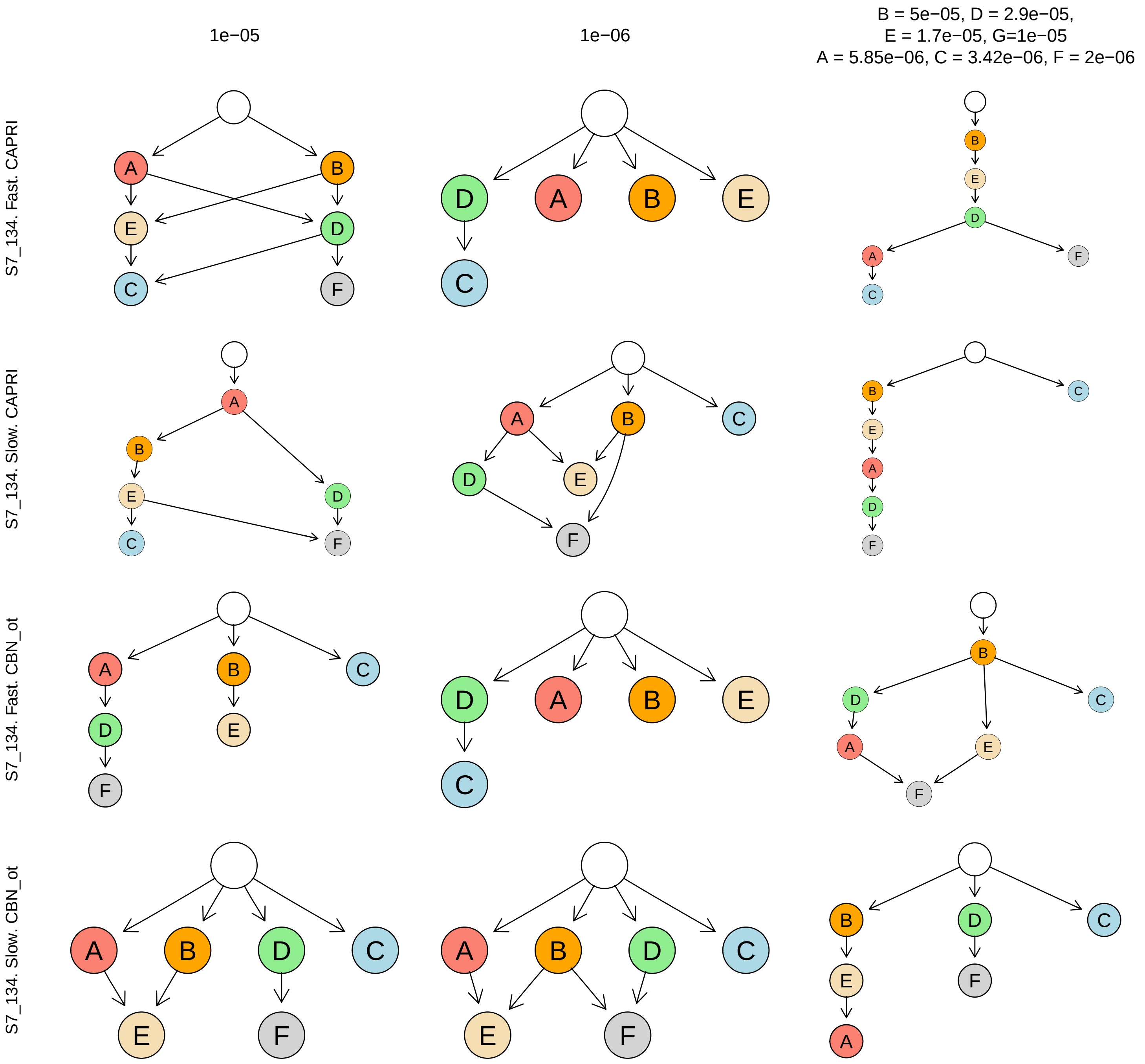

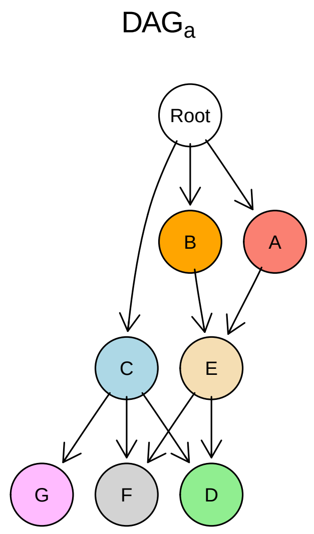

ID	Accessible Genot.	Discrep. DAG_a
S7_135	16	4

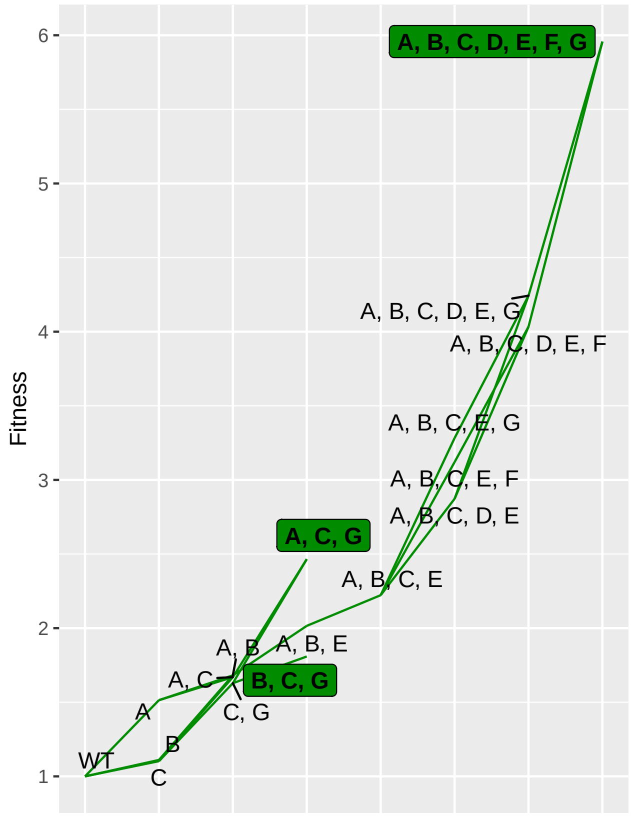

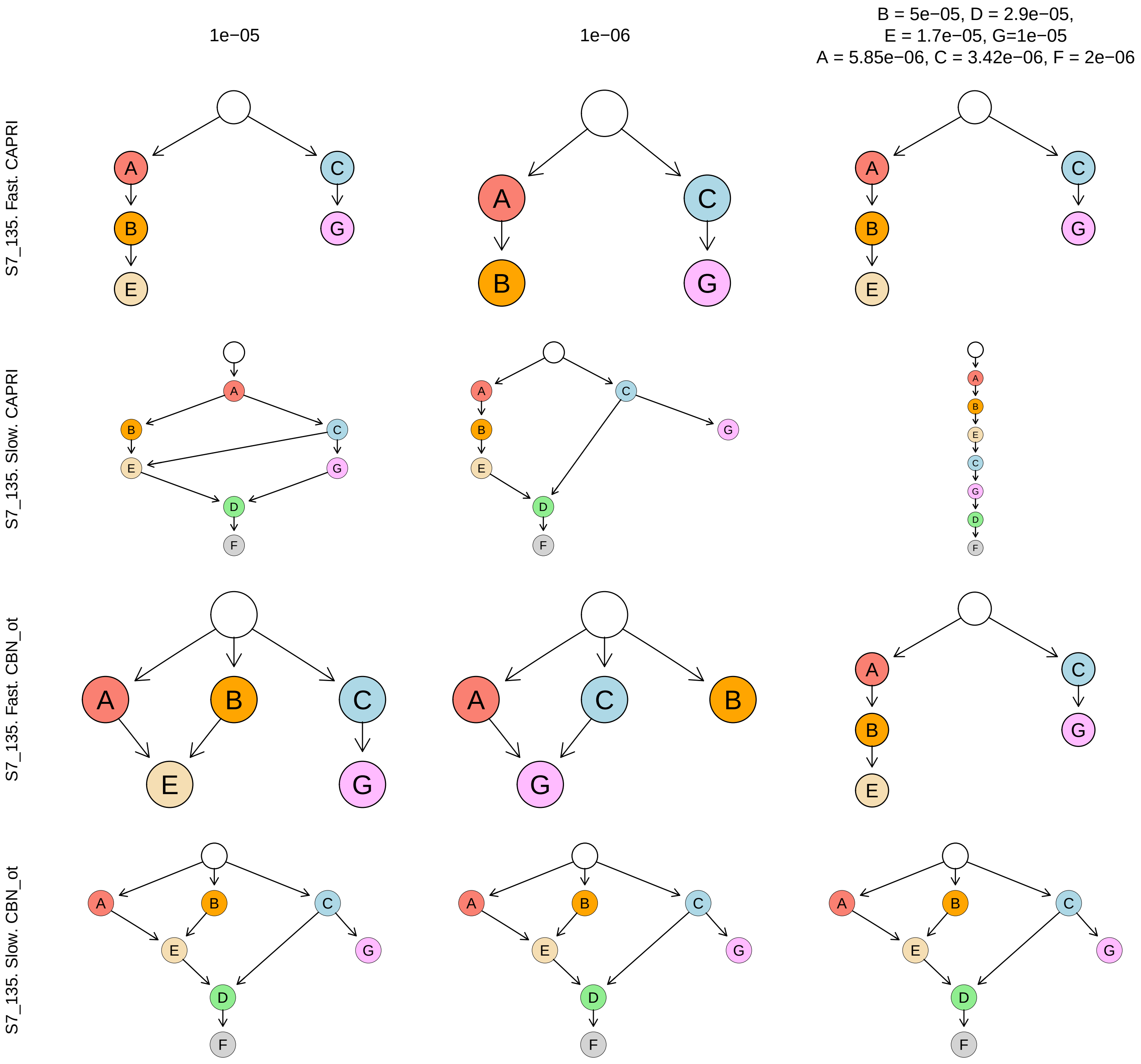

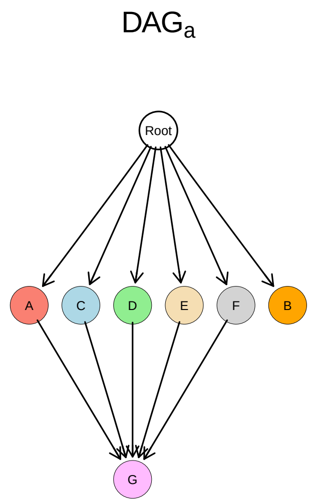

ID	Accessible Genot.	Discrep. DAG_a
S7_136	33	32

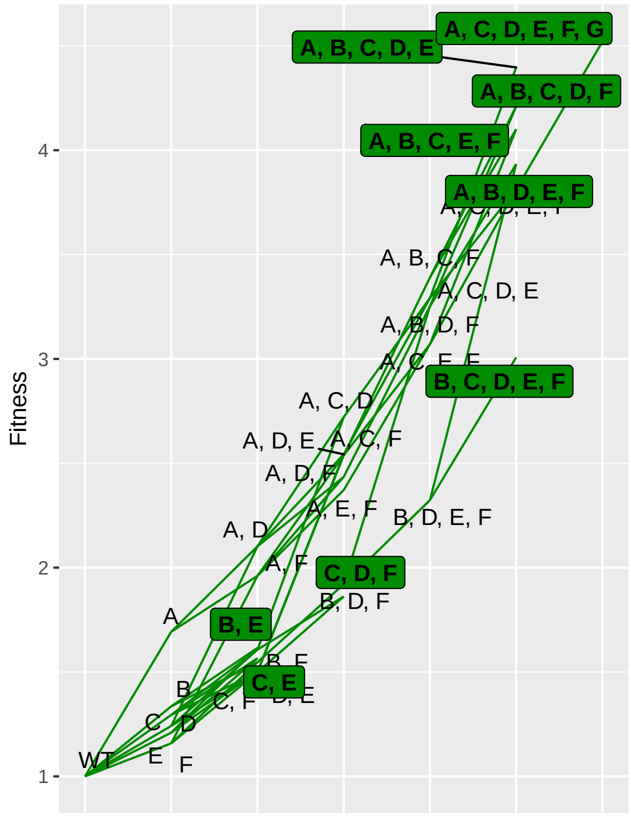

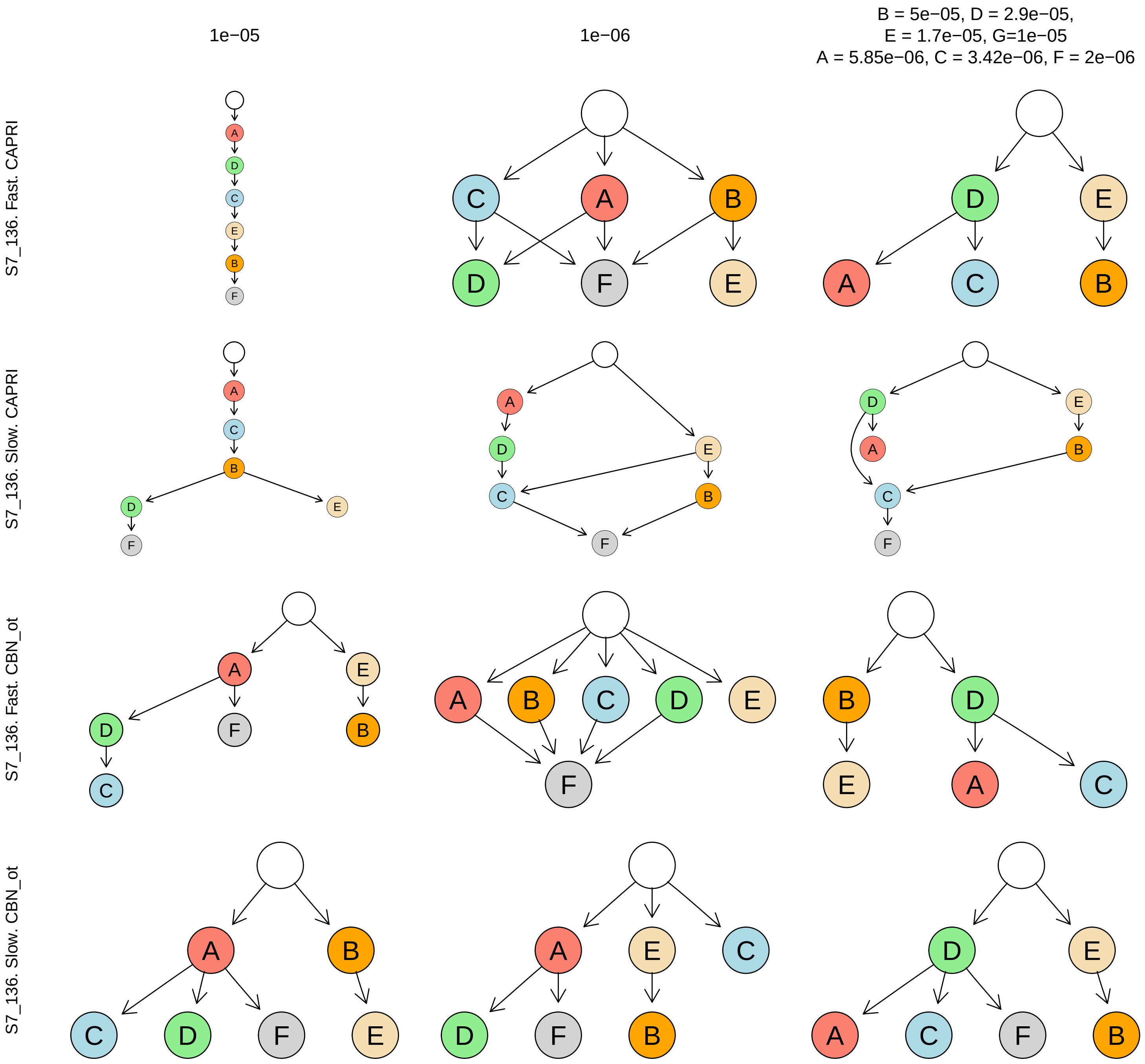

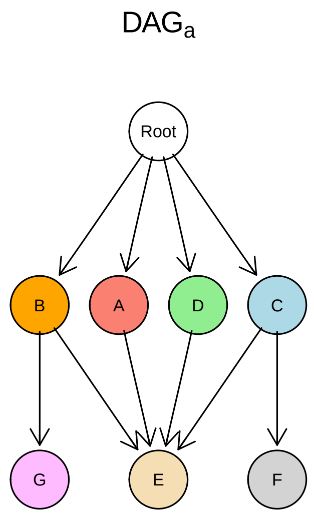

ID	Accessible Genot.	Discrep. DAG_a
S7_137	36	3

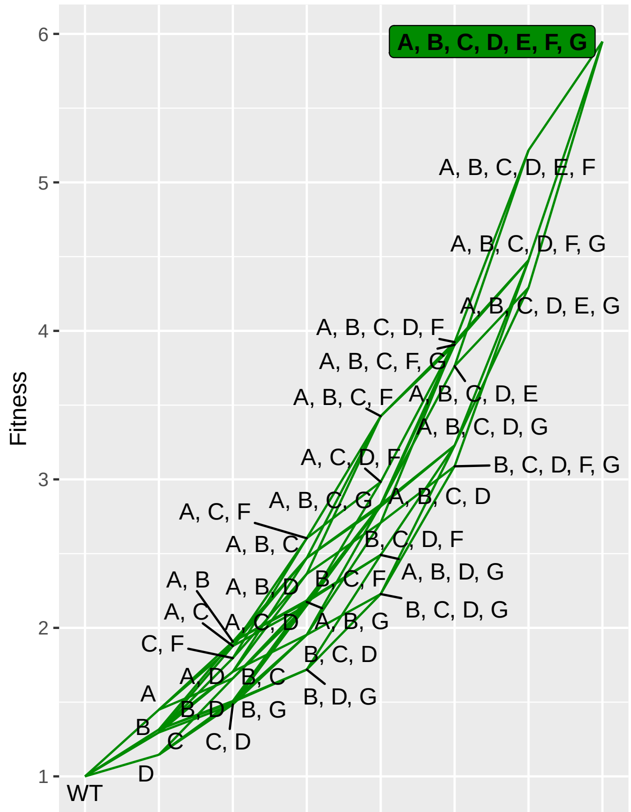

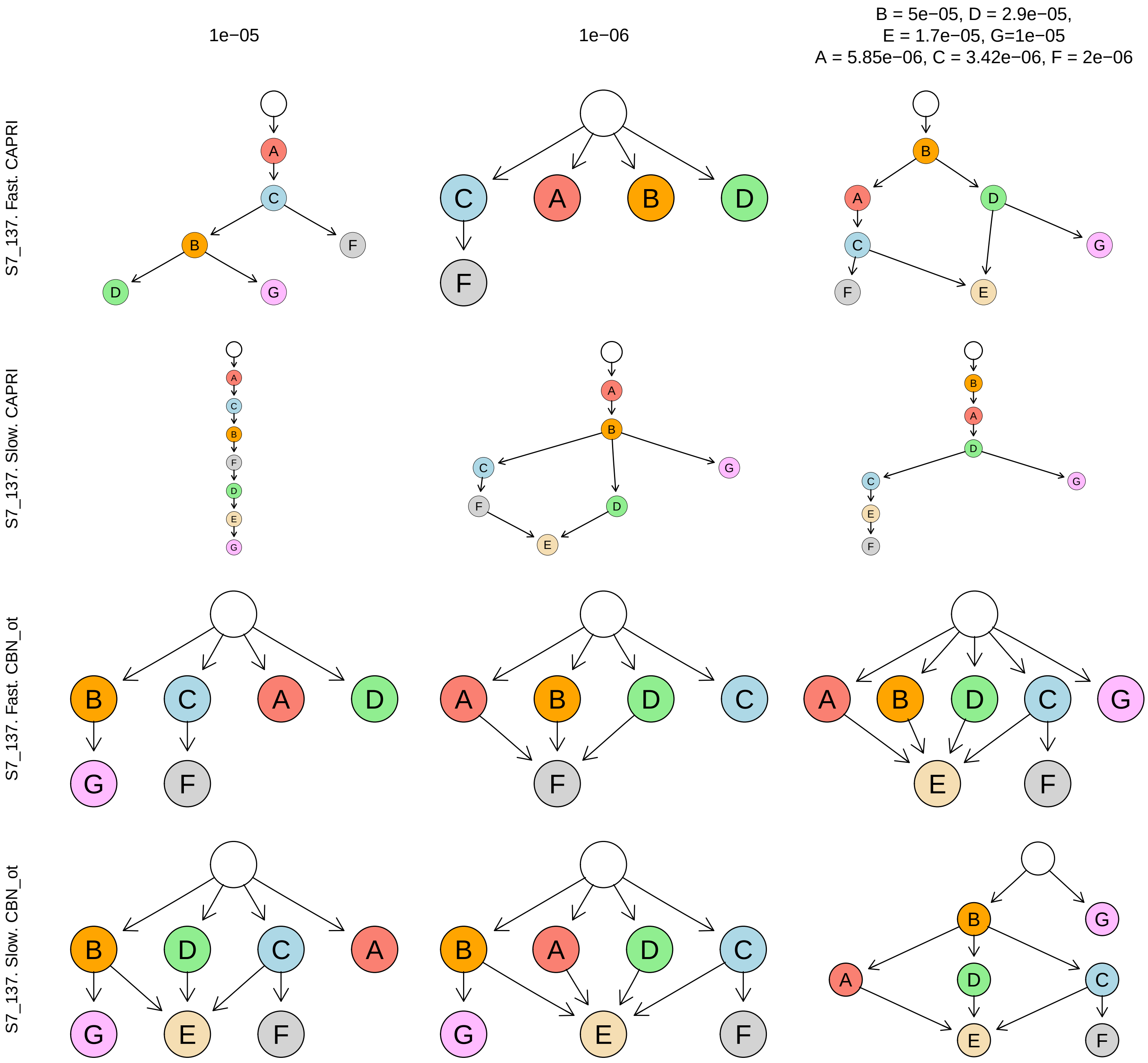

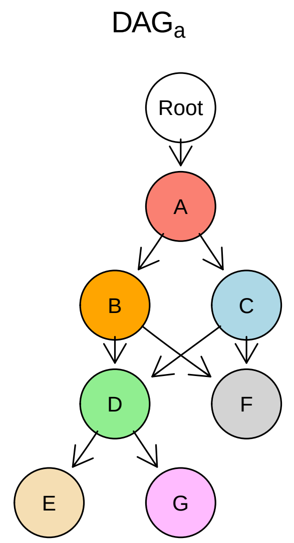

ID	Accessible Genot.	Discrep. DAG_a
S7_138	15	4

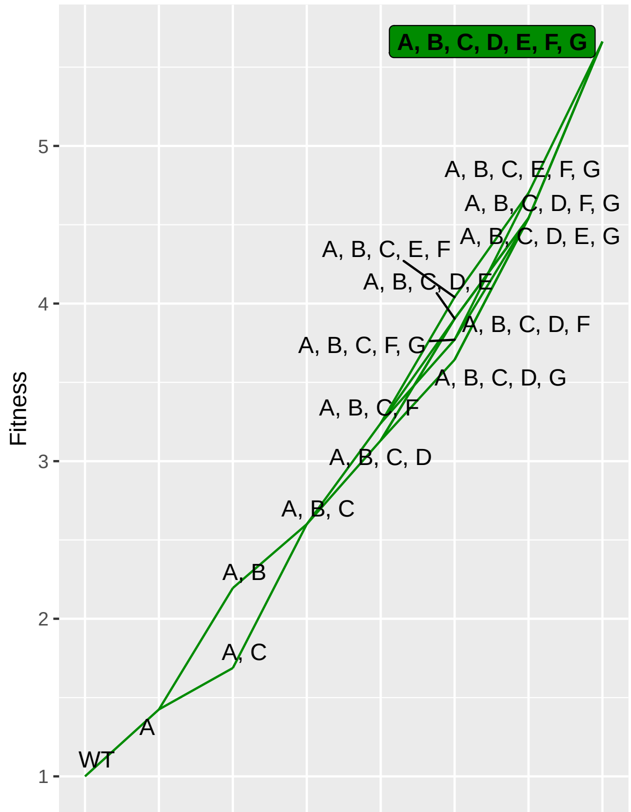

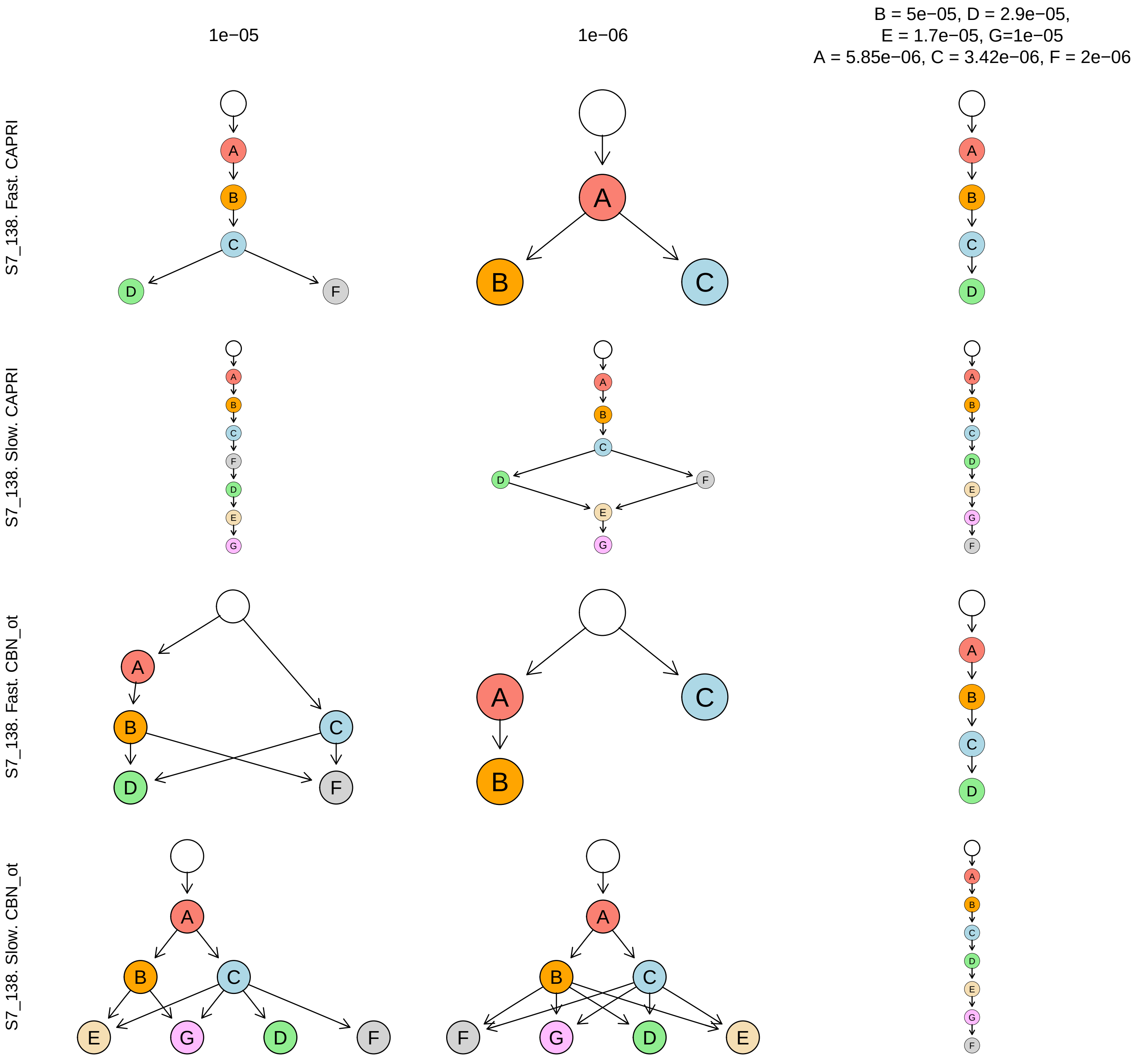

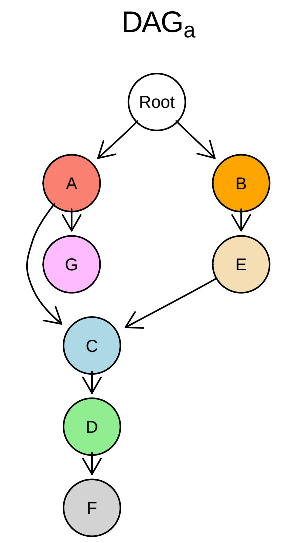

ID	Accessible Genot.	Discrep. DAG_a
S7_139	8	6

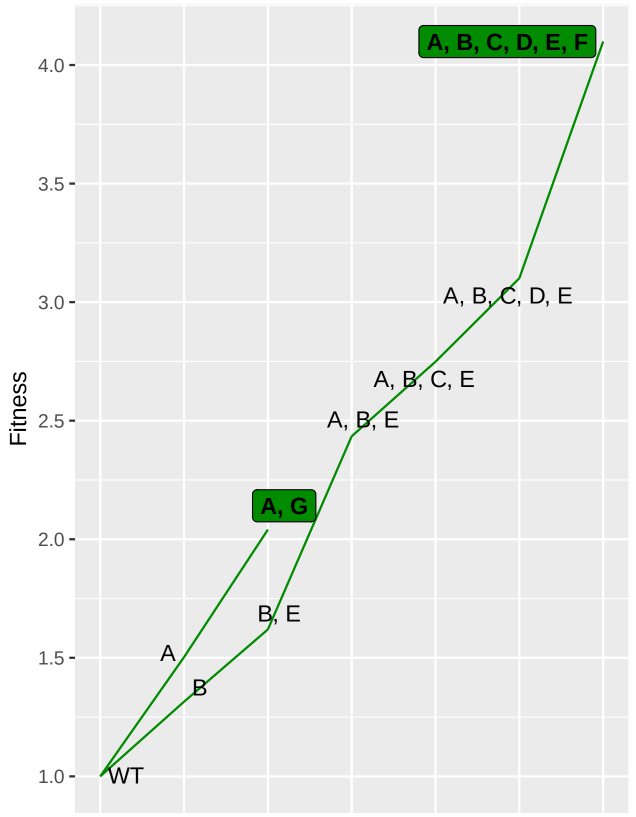

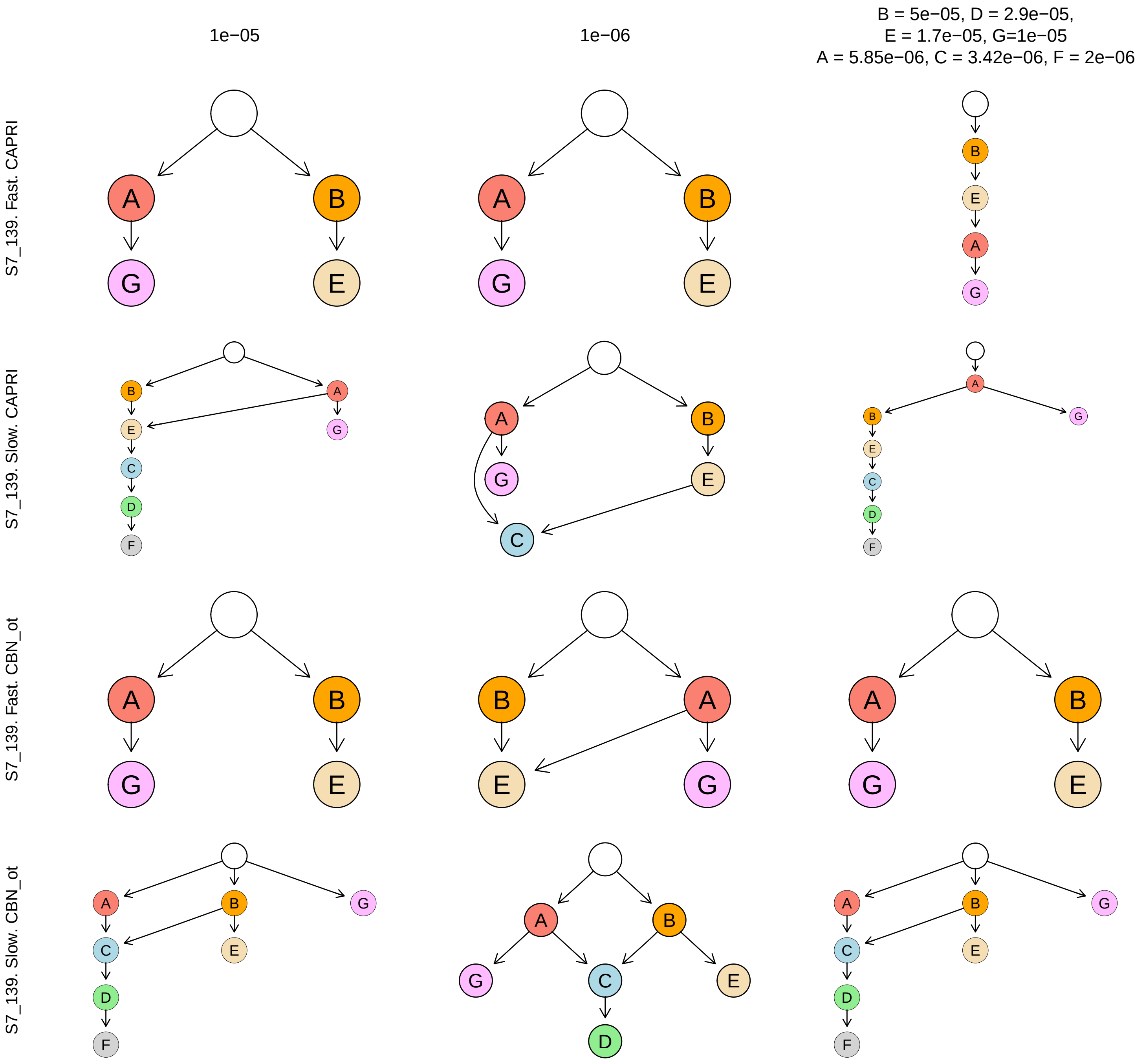

DAG$_a$

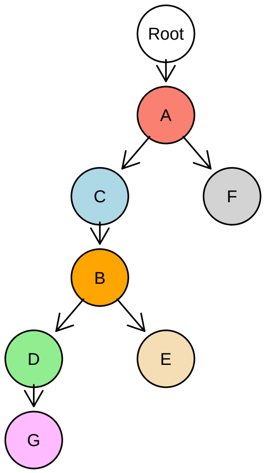

ID	Accessible Genot.	Discrep. DAG_a
S7_140	18	2

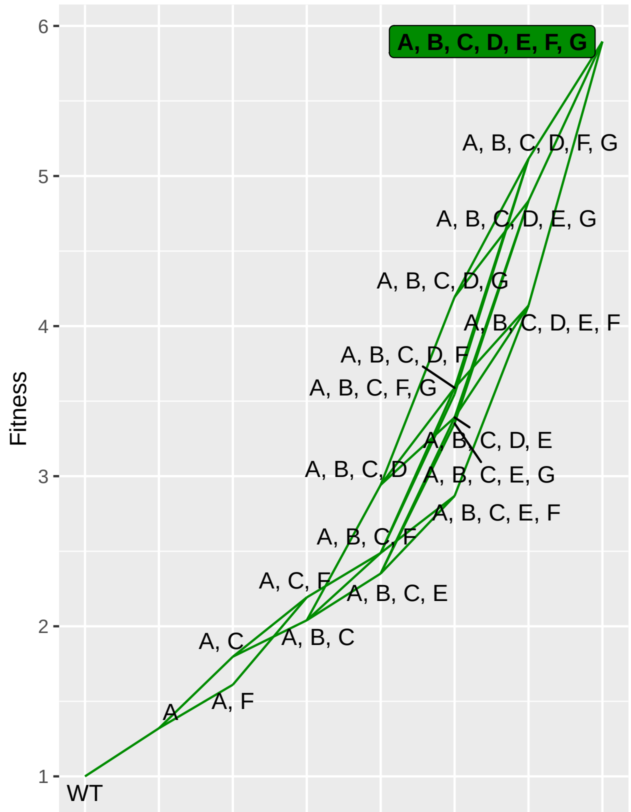

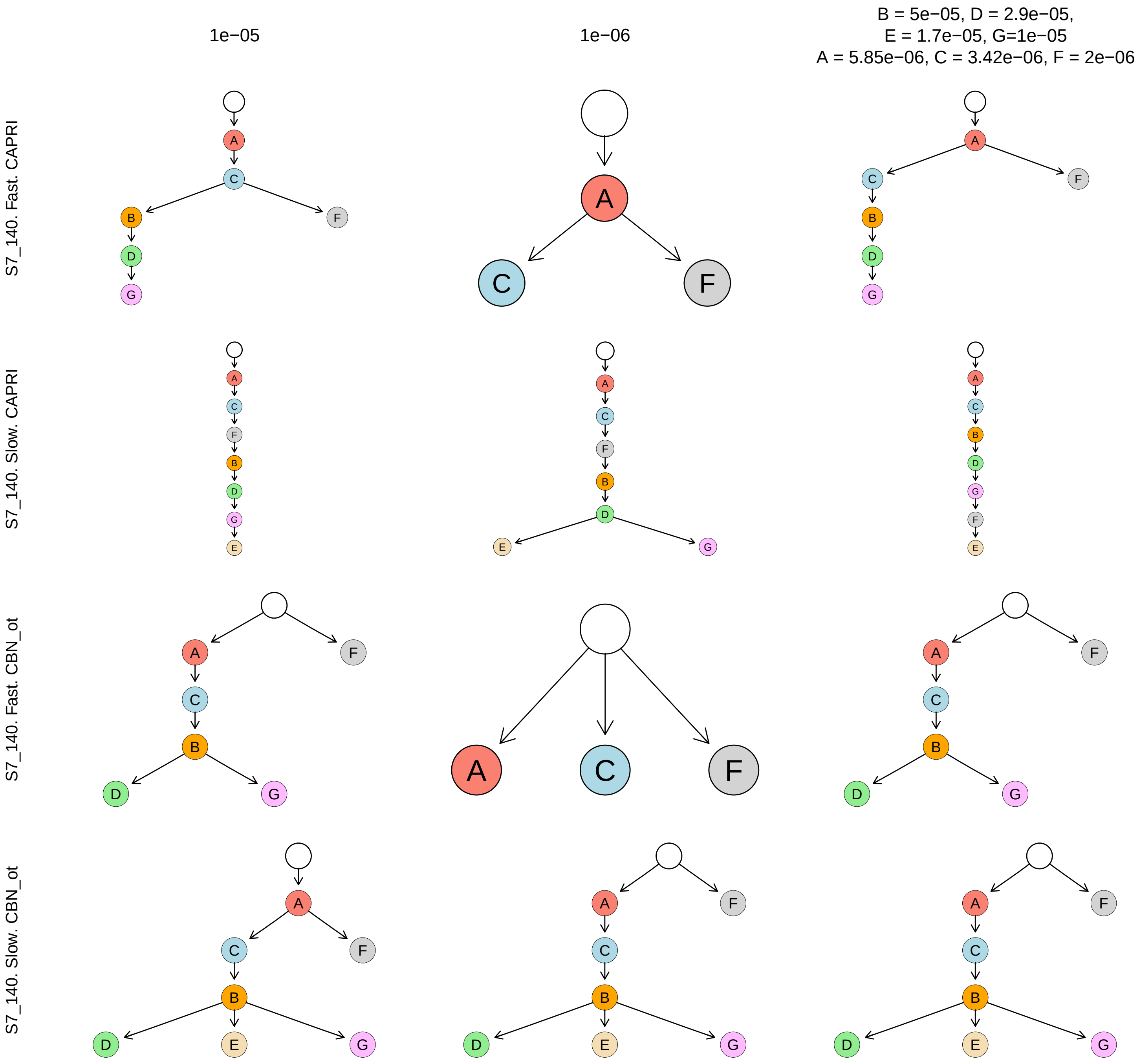

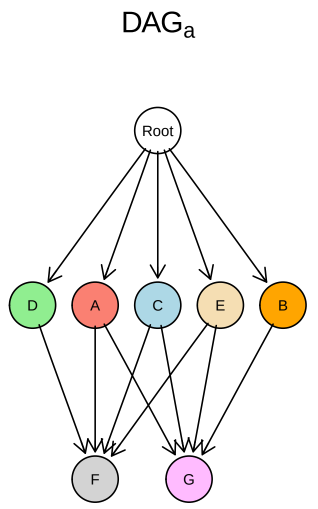

ID	Accessible Genot.	Discrep. DAG_a
S7_141	28	10

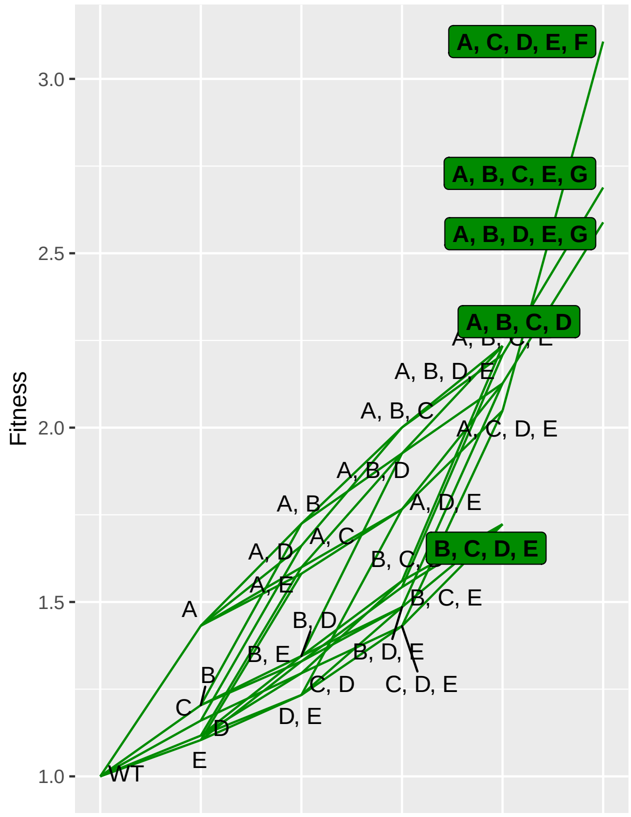

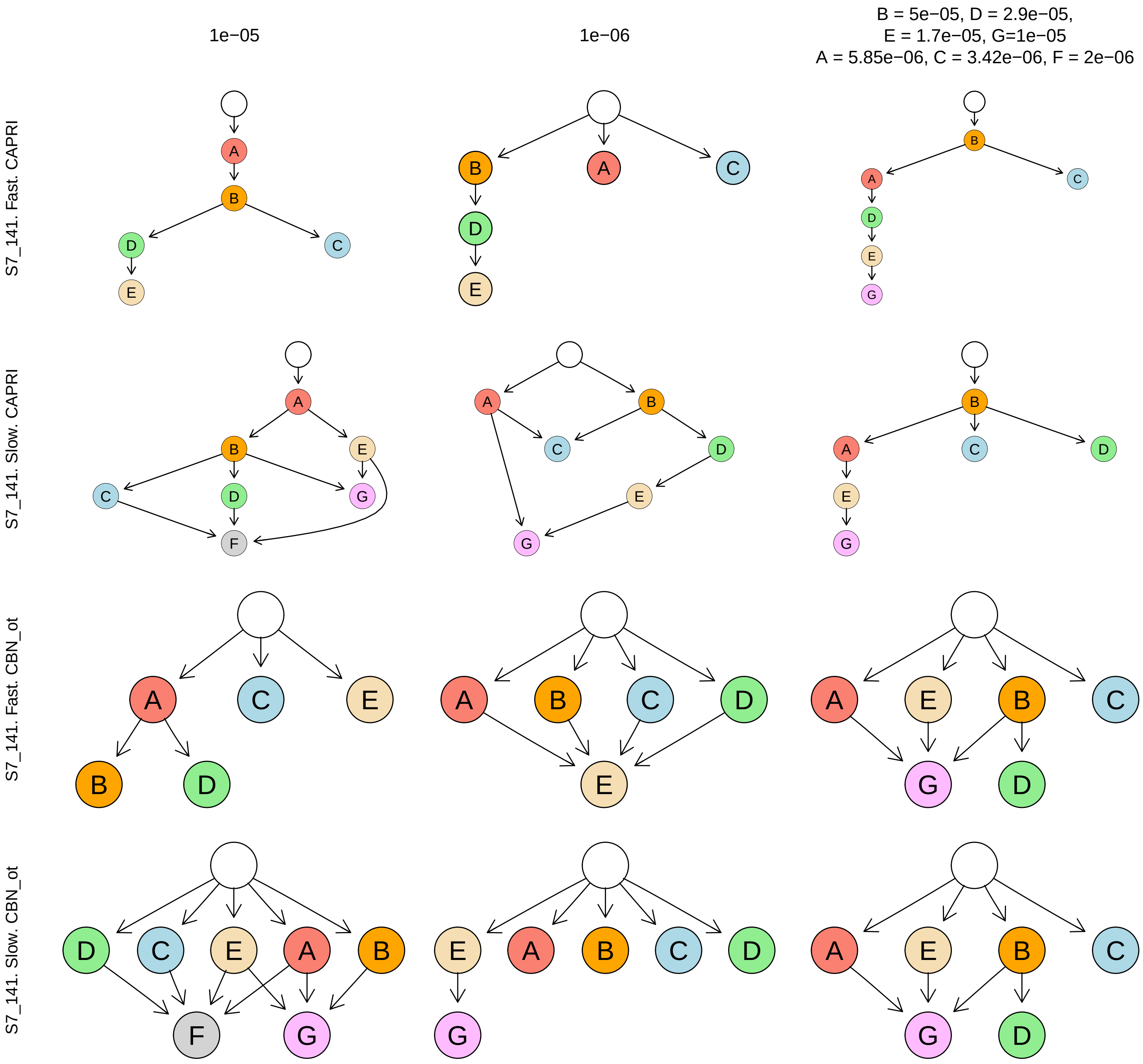

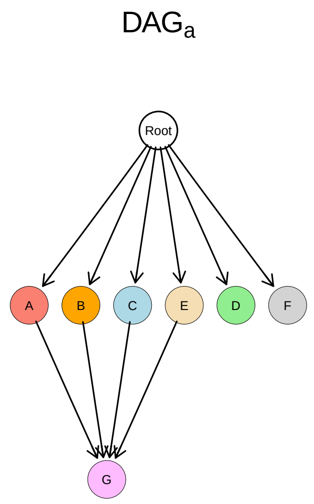

ID	Accessible Genot.	Discrep. DAG_a
S7_142	44	23

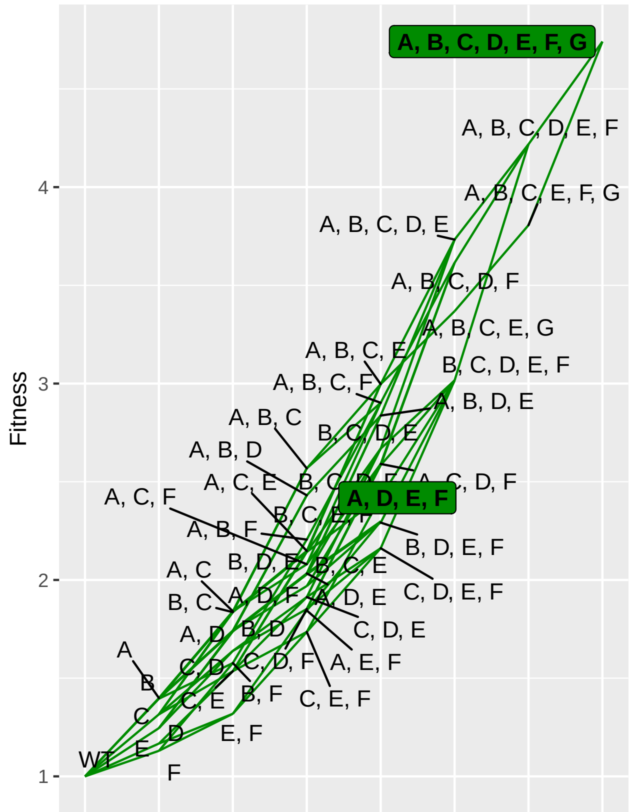

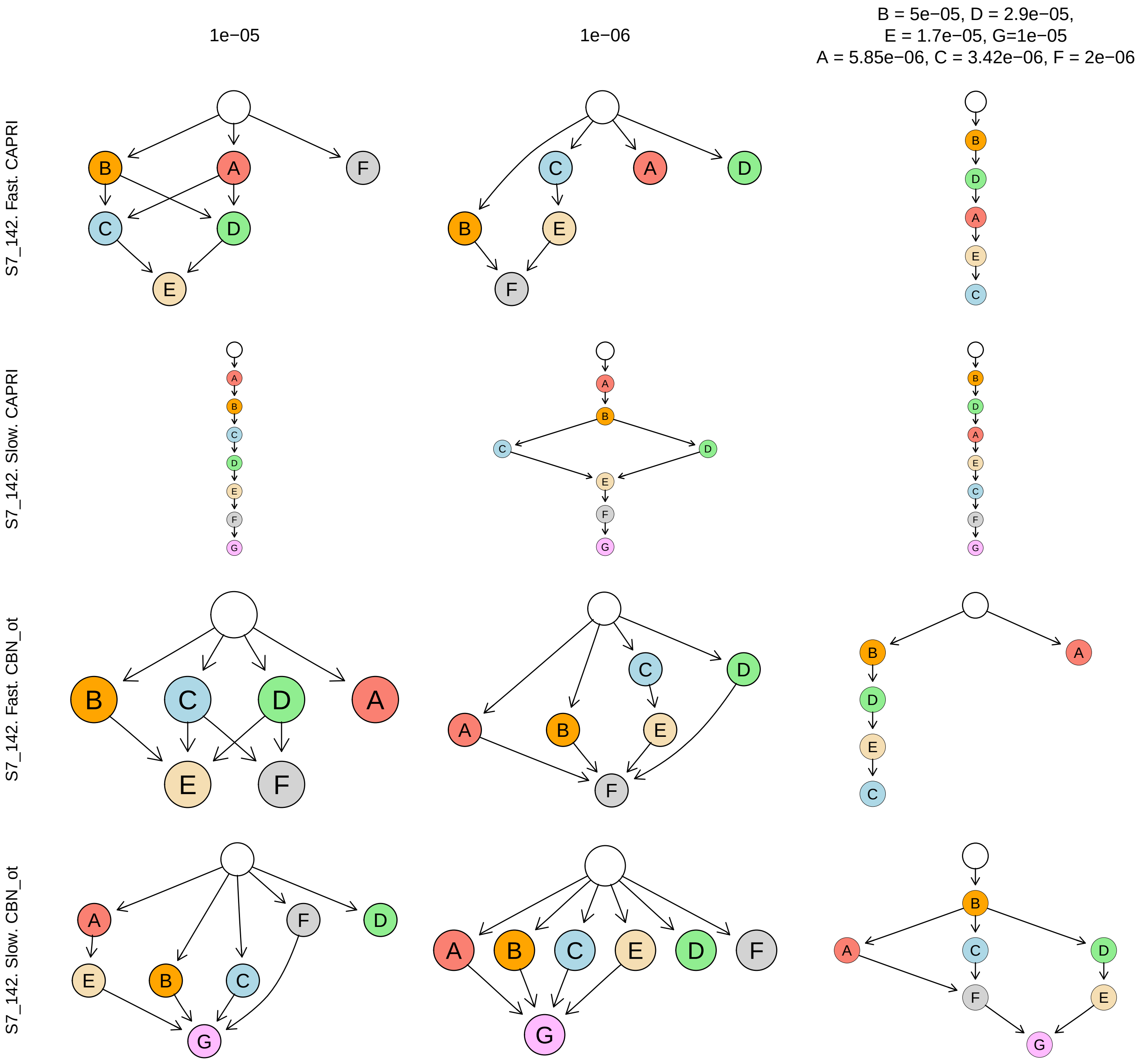

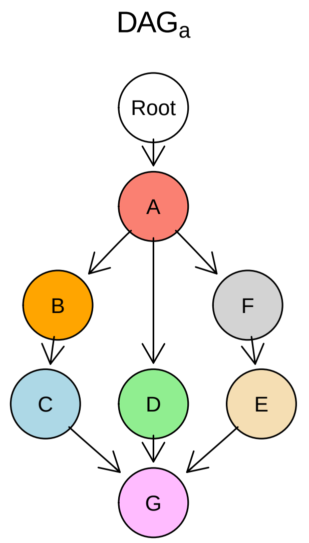

ID	Accessible Genot.	Discrep. DAG_a
S7_143	21	12

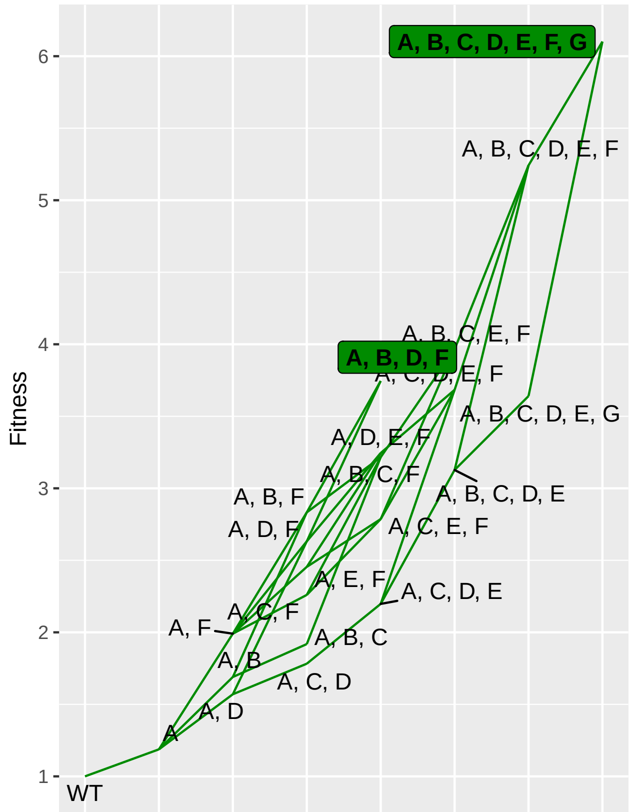

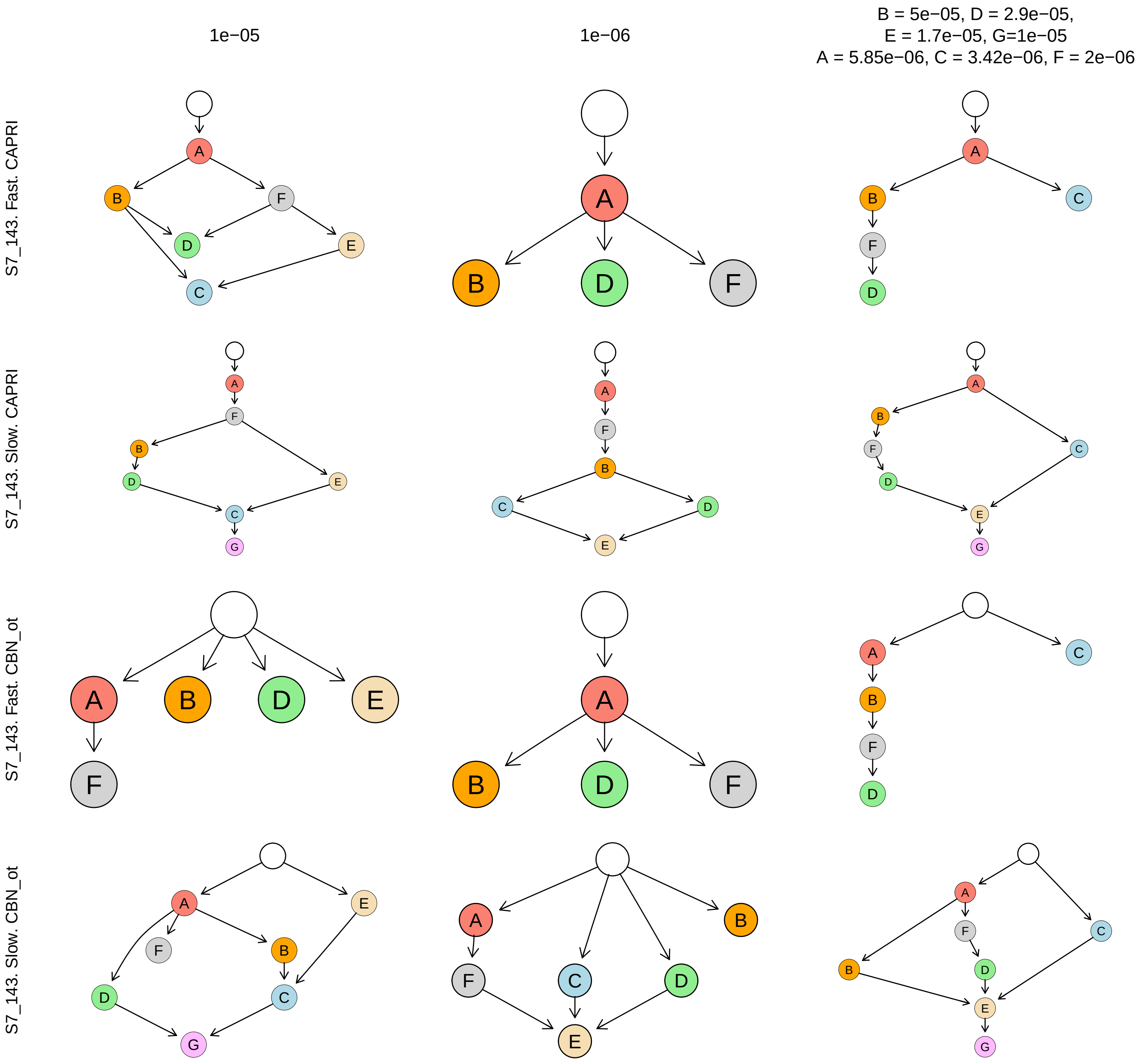

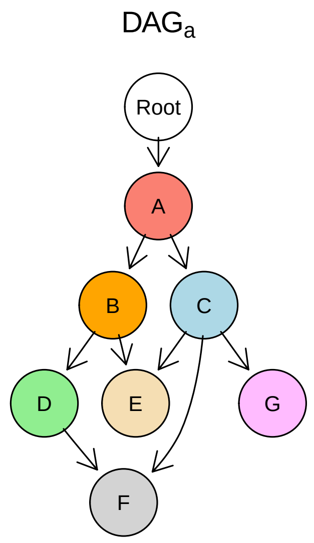

ID	Accessible Genot.	Discrep. DAG_a
S7_144	18	7

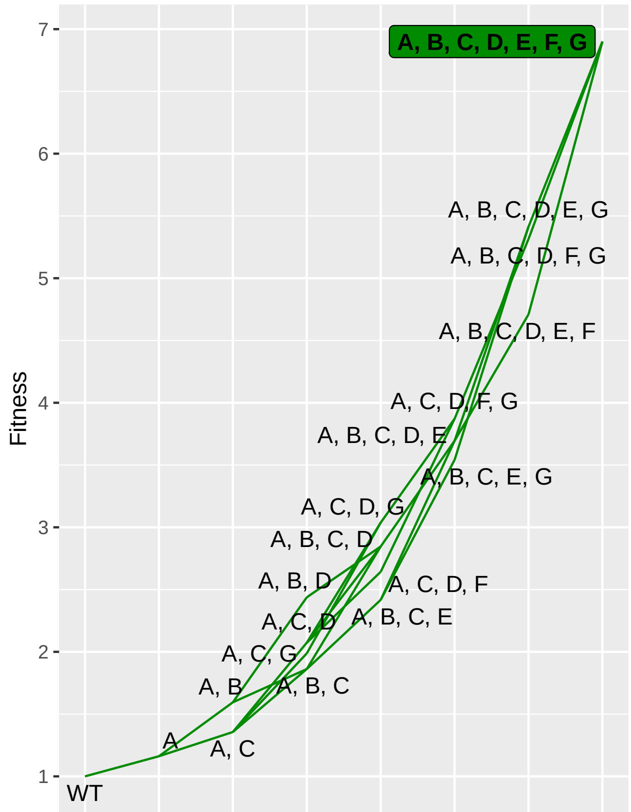

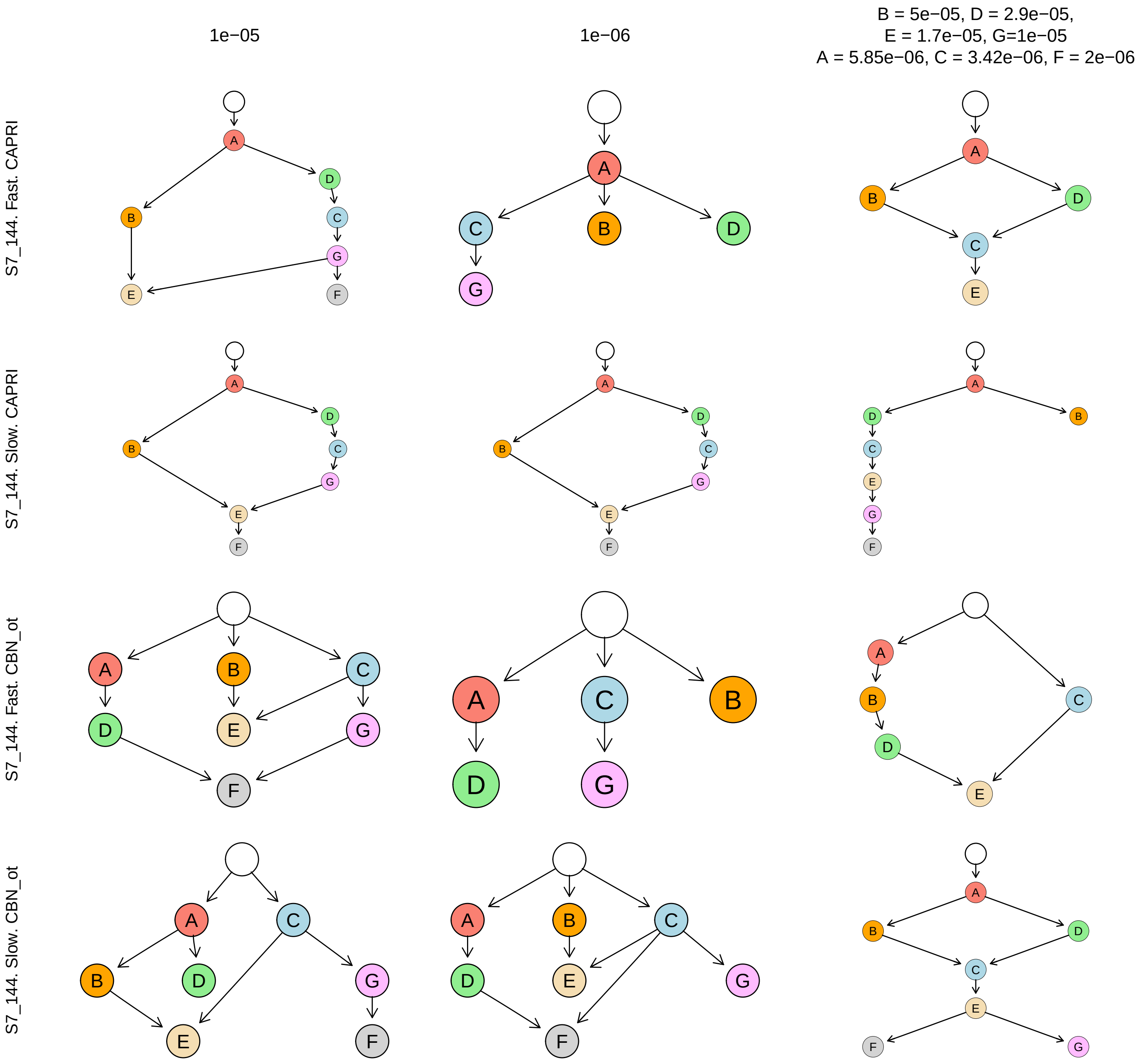

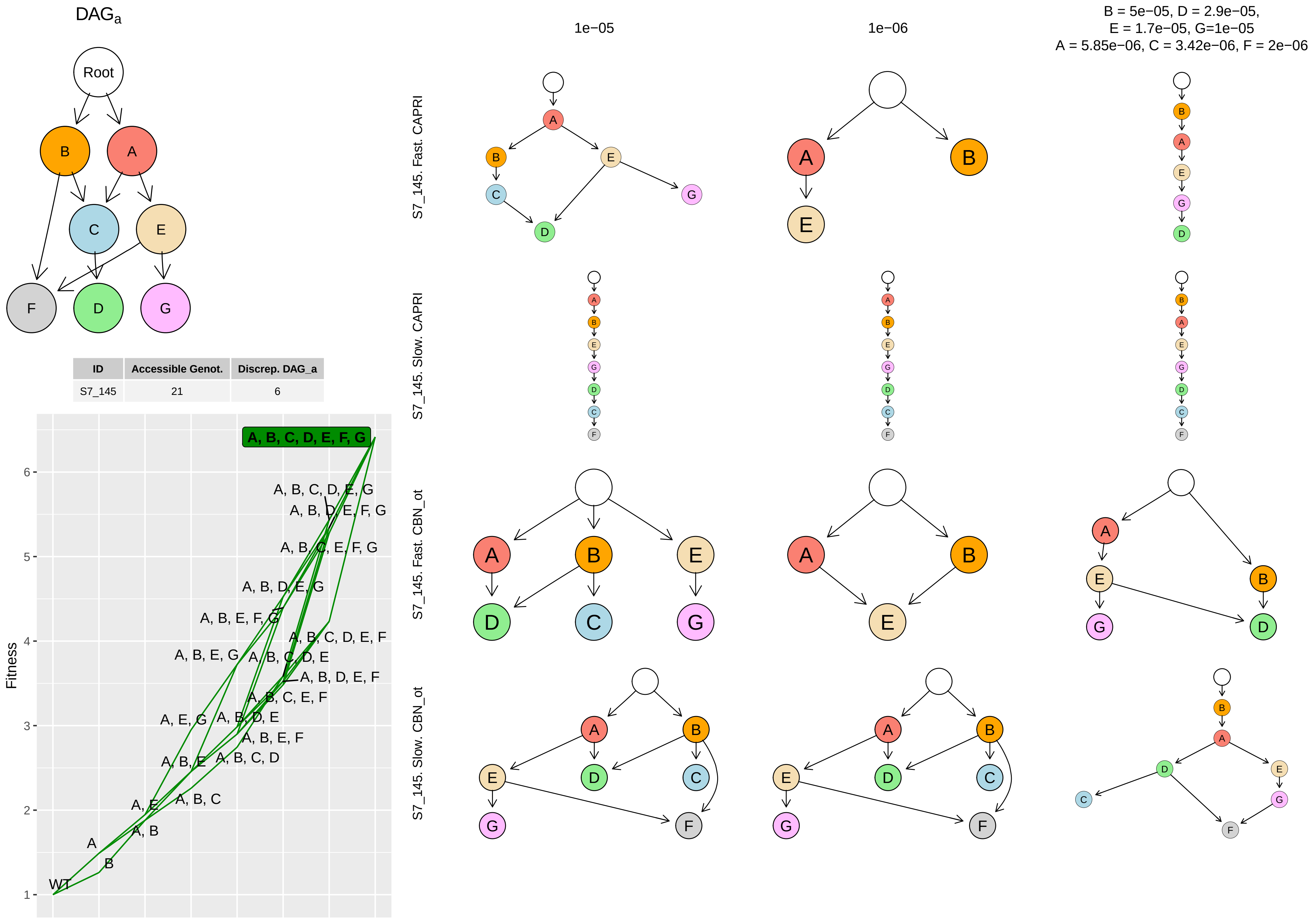

ID	Accessible Genot.	Discrep. DAG_a
S7_145	21	6

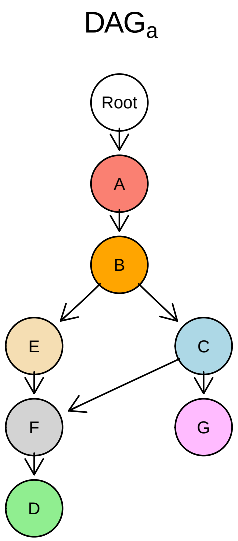

ID	Accessible Genot.	Discrep. DAG_a
S7_146	11	2

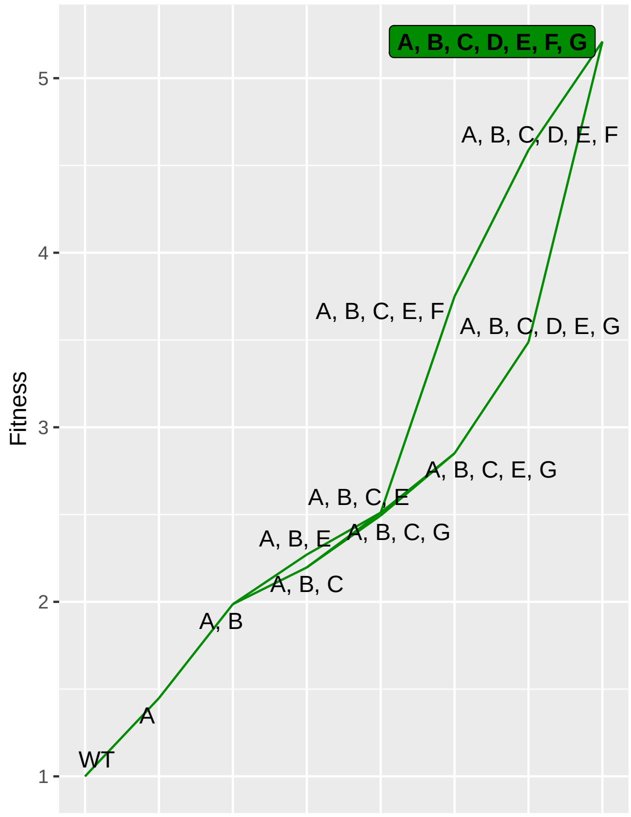

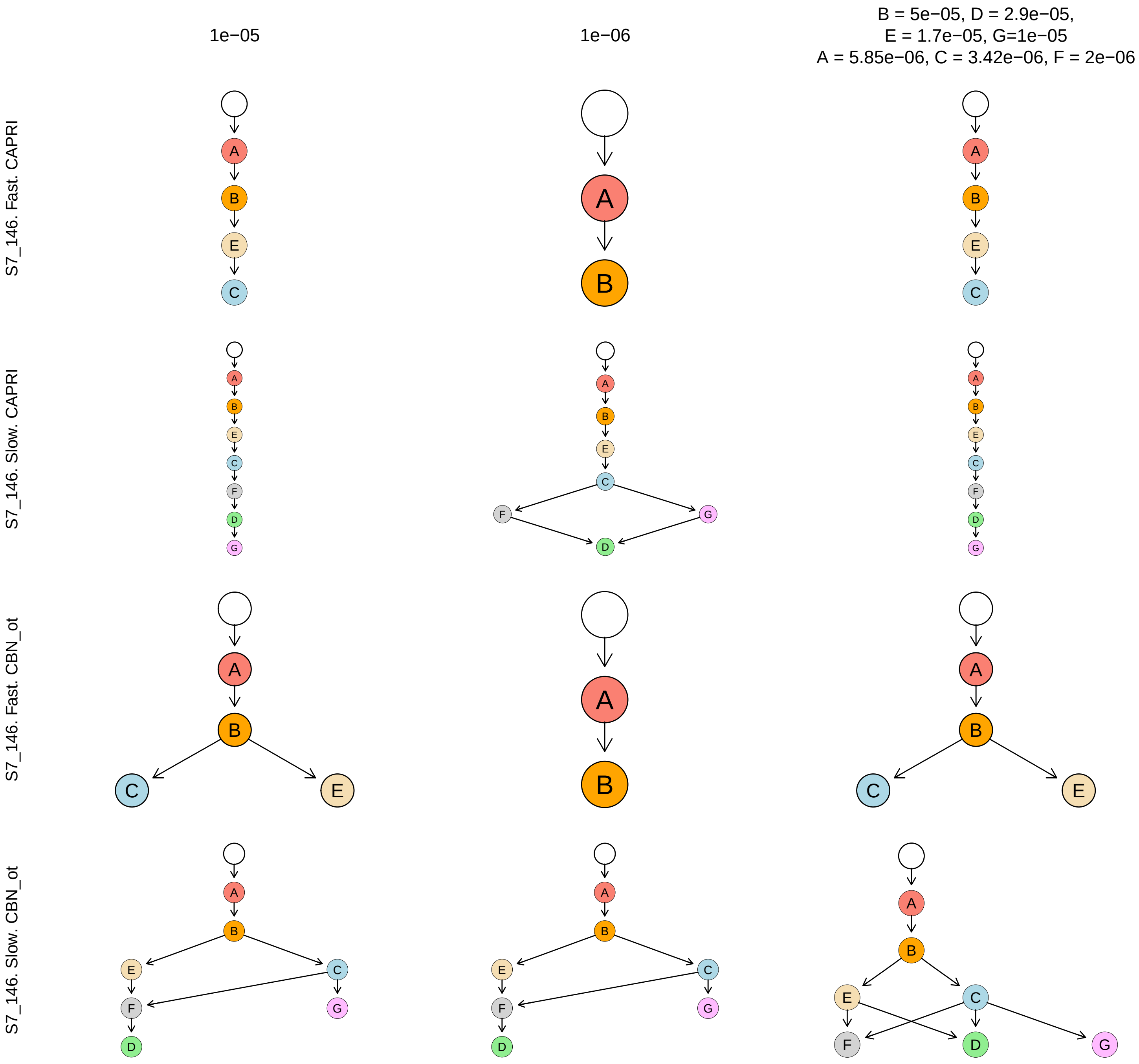

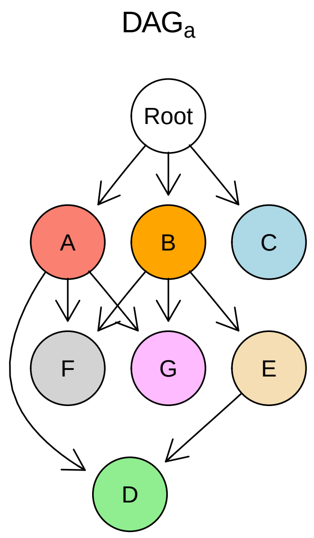

ID	Accessible Genot.	Discrep. DAG_a
S7_147	30	9

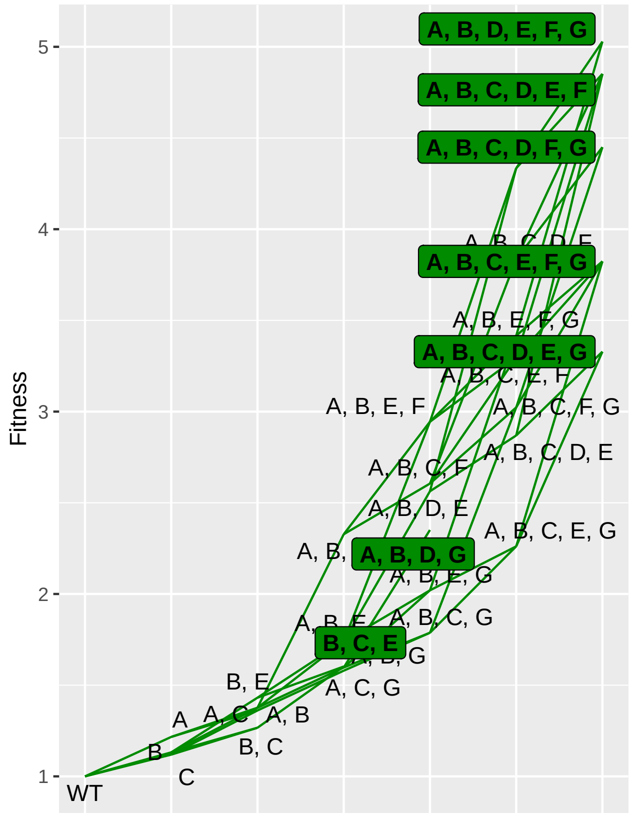

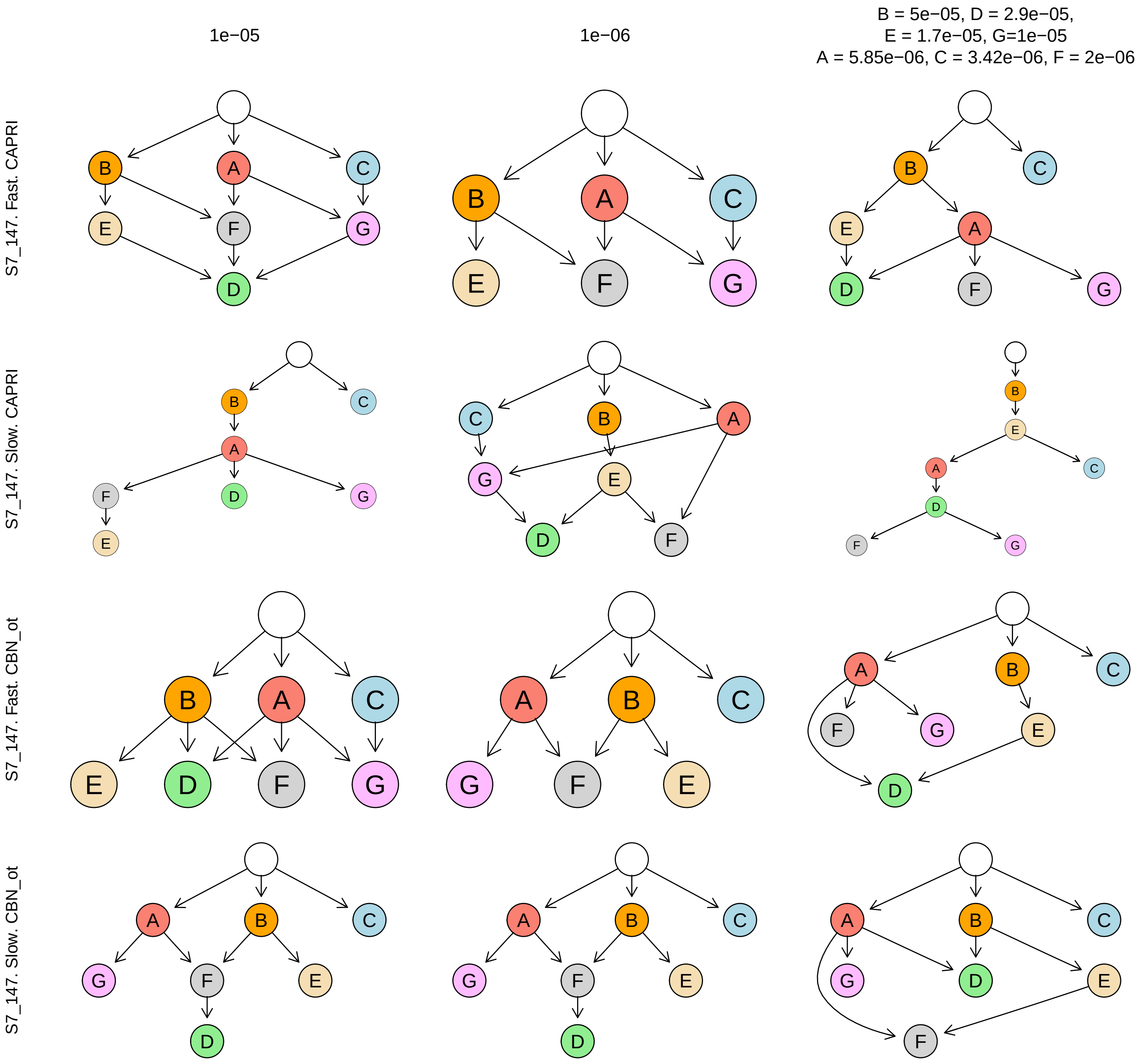

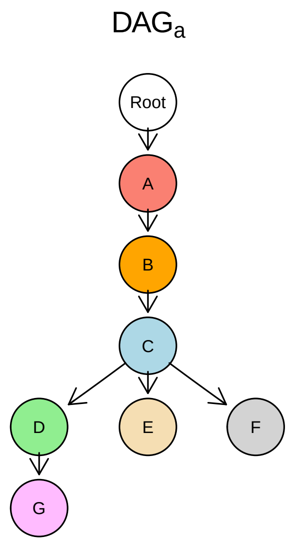

ID	Accessible Genot.	Discrep. DAG_a
S7_148	10	4

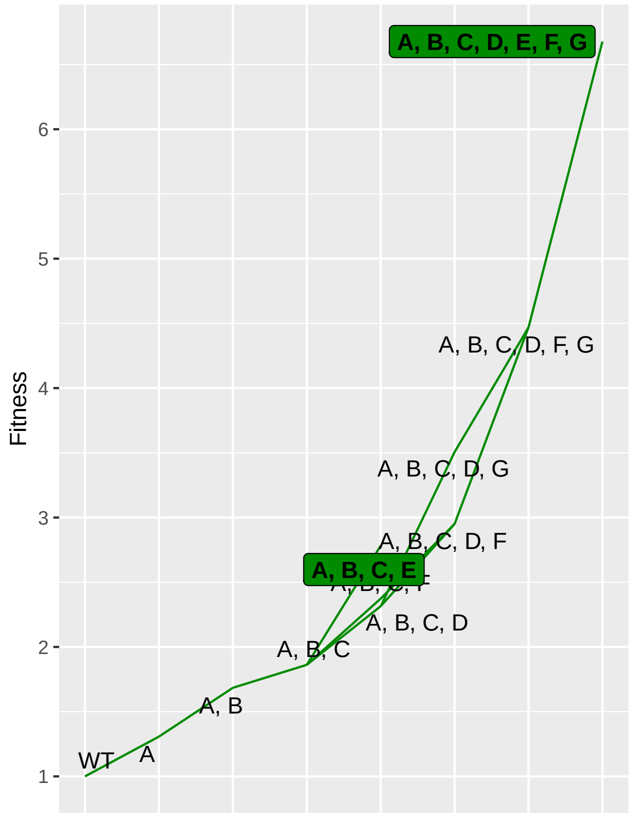

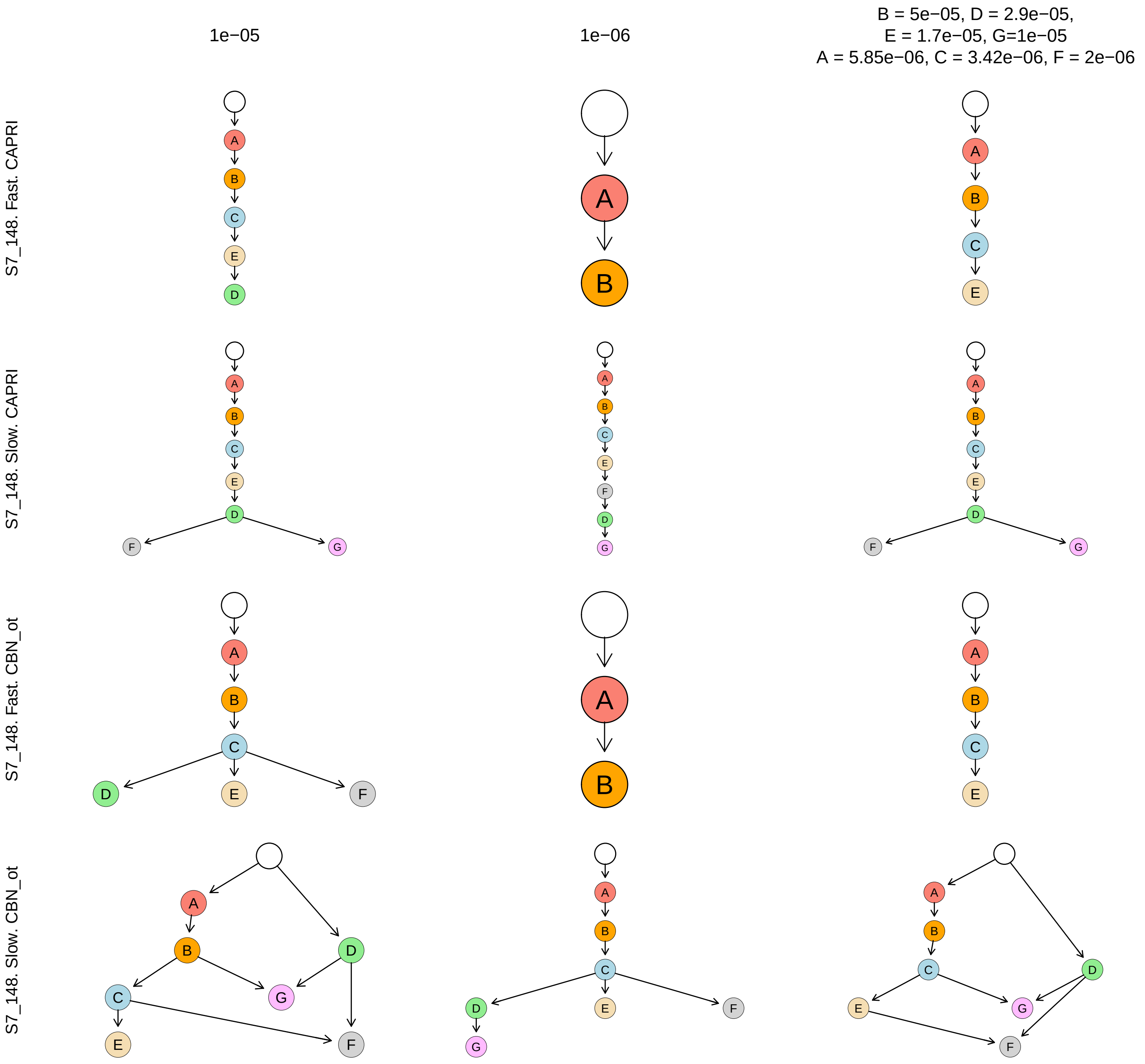

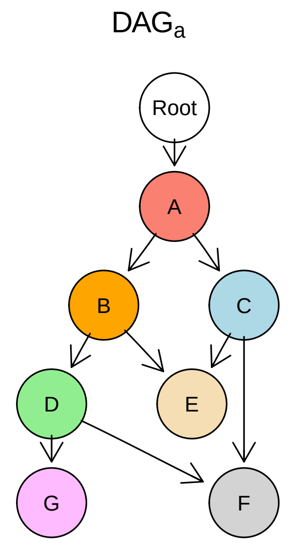

ID	Accessible Genot.	Discrep. DAG_a
S7_149	13	2

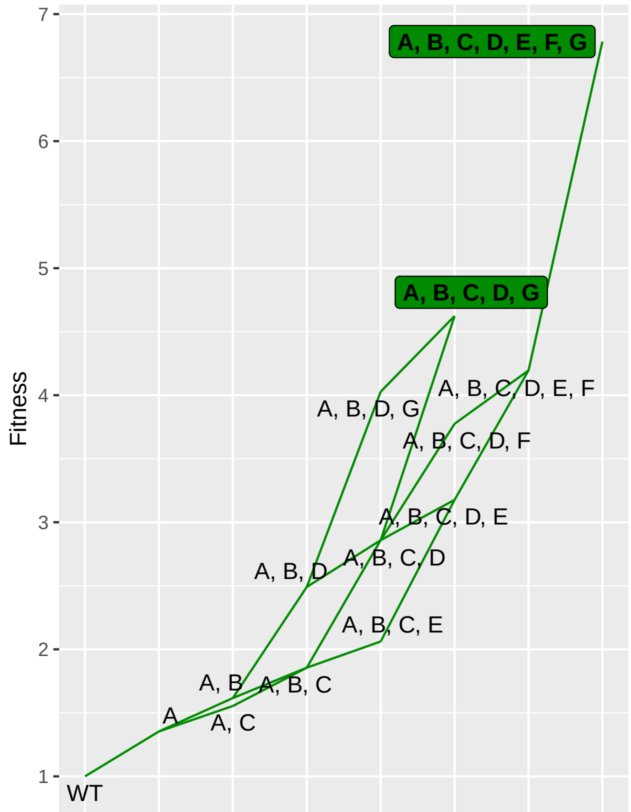

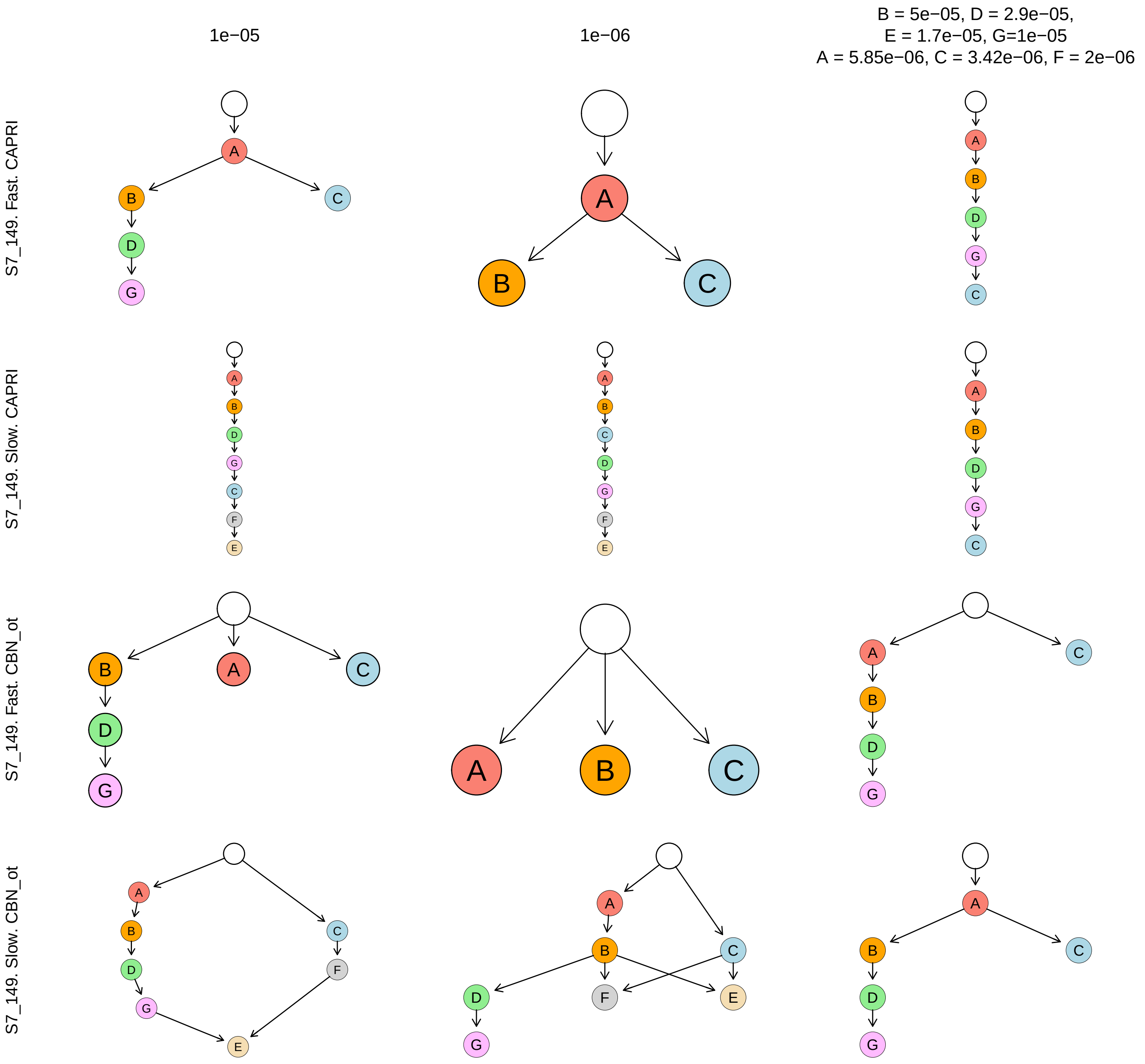

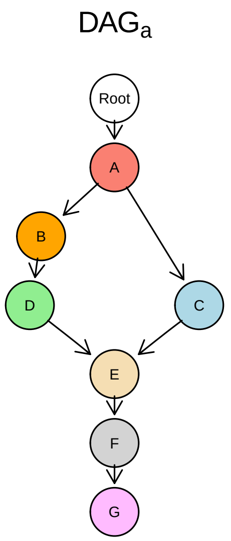

ID	Accessible Genot.	Discrep. DAG_a
S7_150	9	2

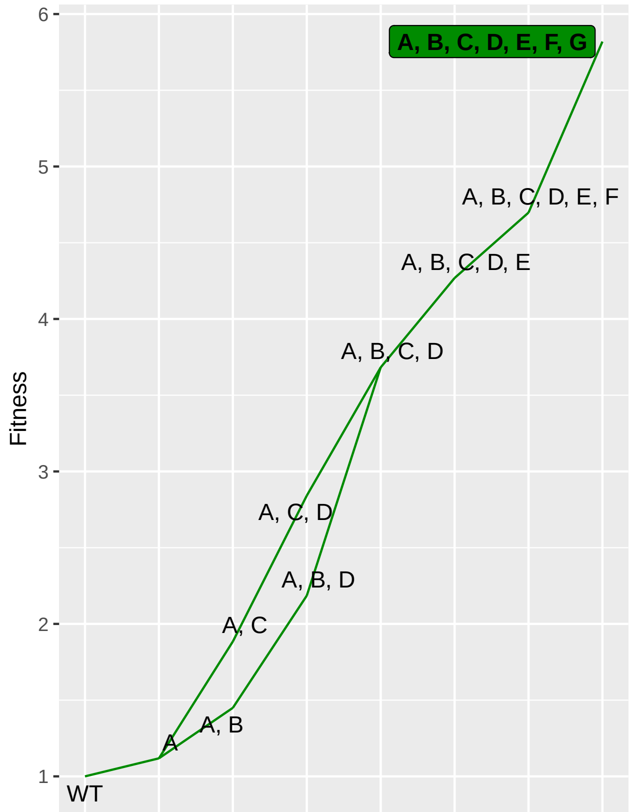

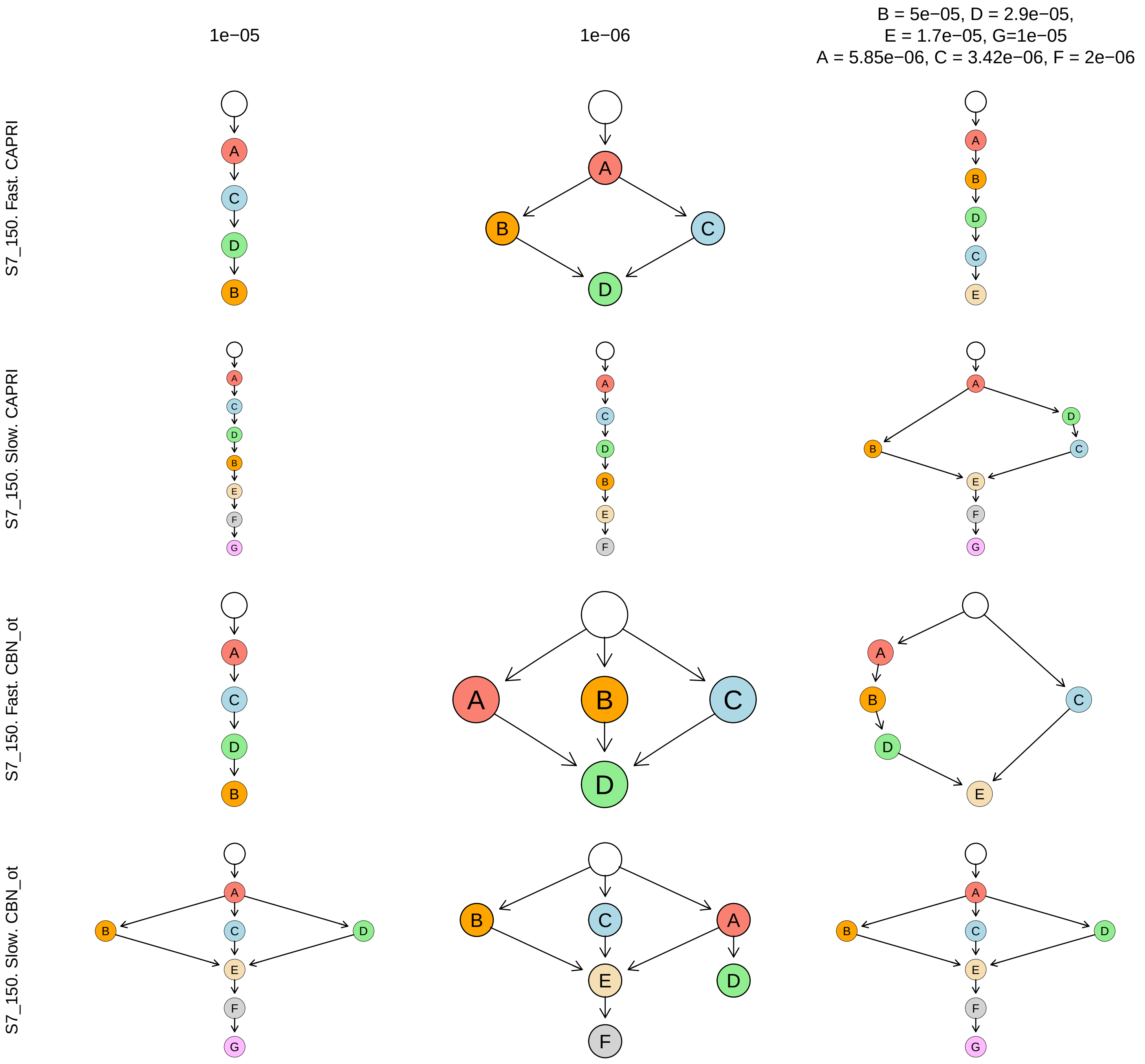

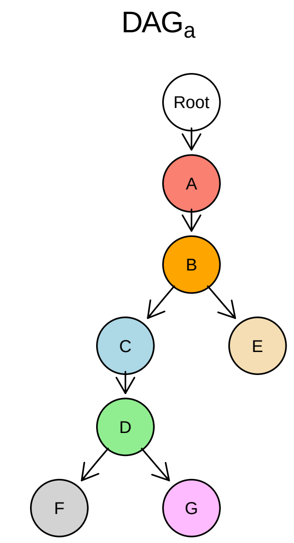

ID	Accessible Genot.	Discrep. DAG_a
S7_151	9	4

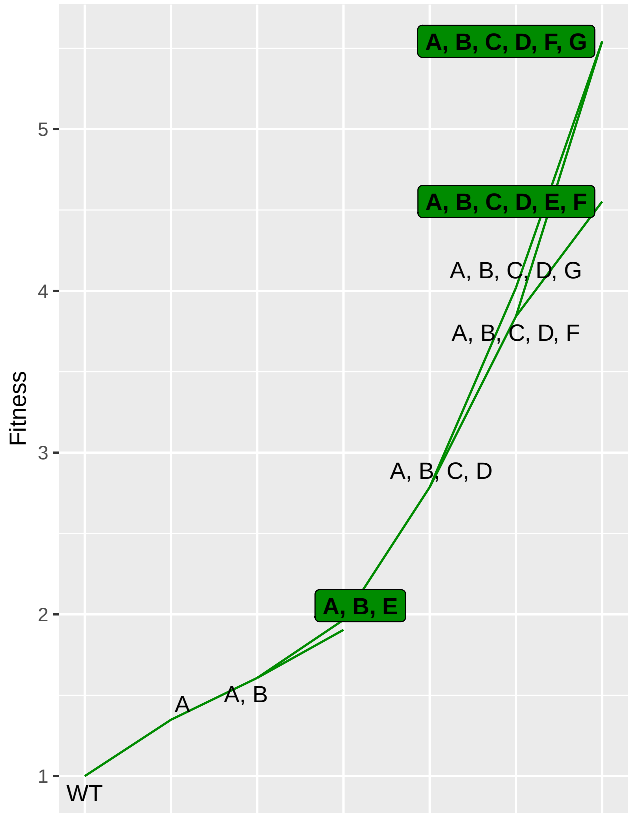

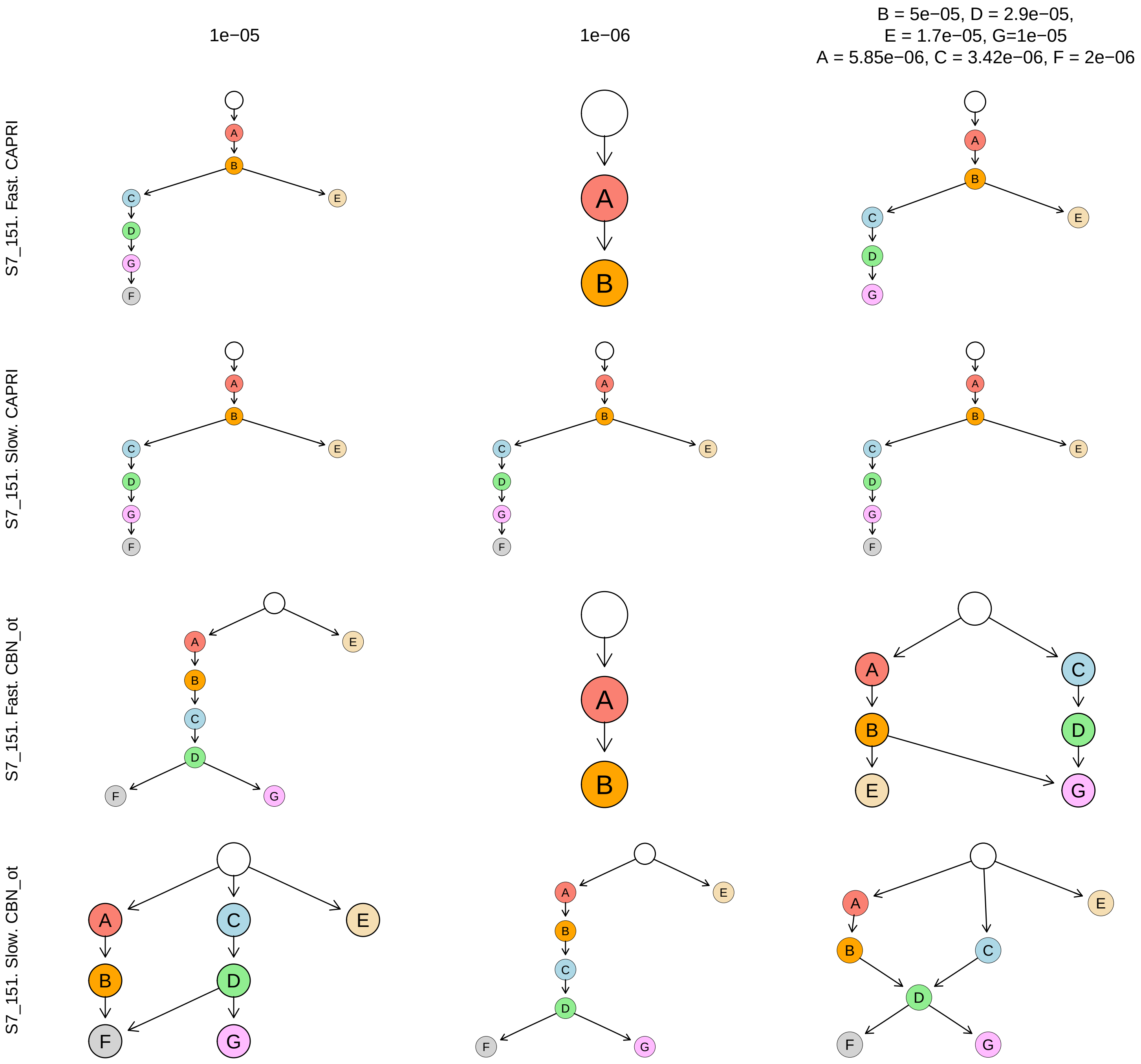

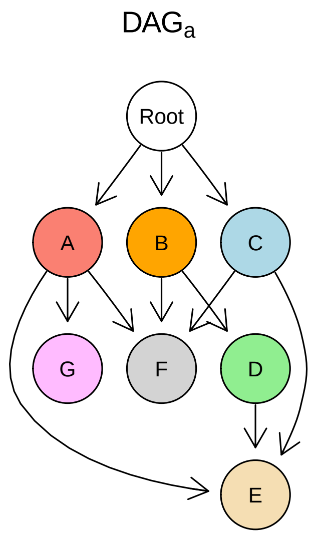

ID	Accessible Genot.	Discrep. DAG_a
S7_152	22	9

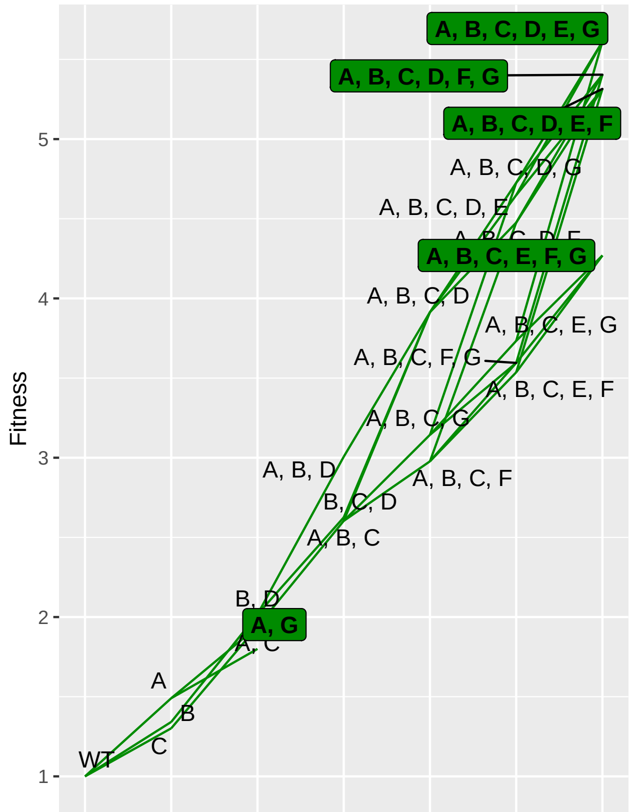

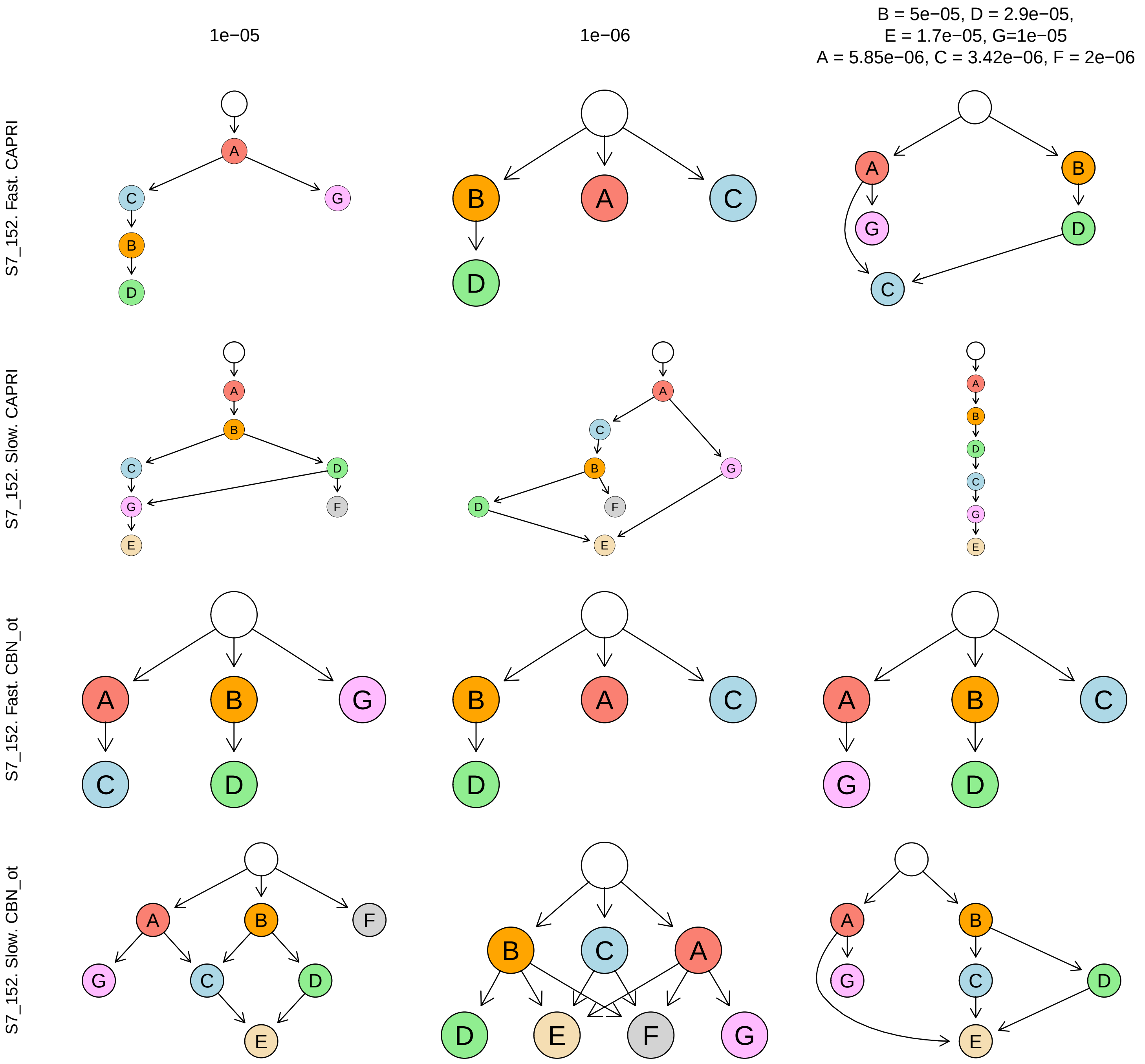

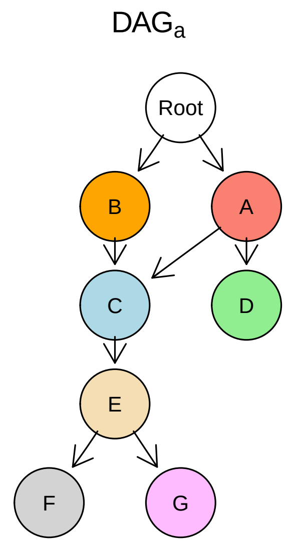

ID	Accessible Genot.	Discrep. DAG_a
S7_153	12	3

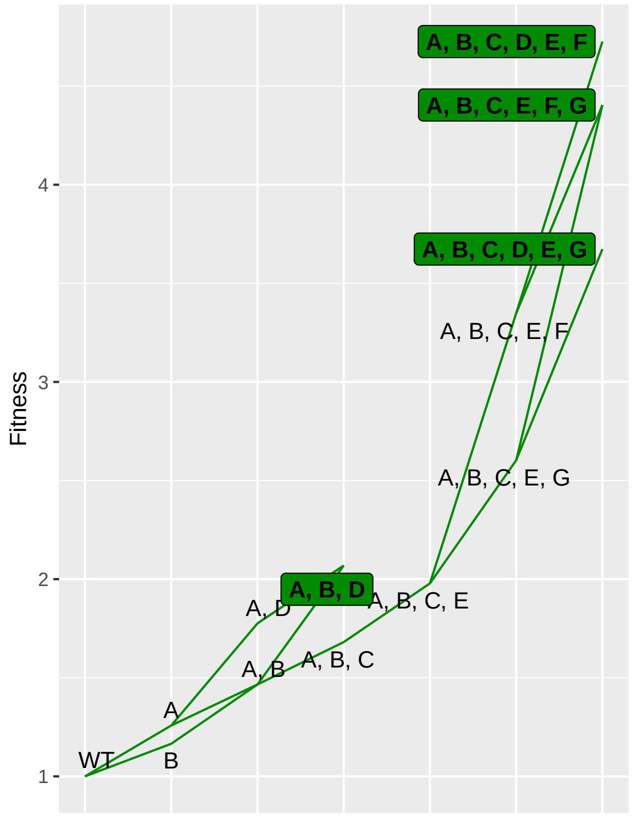

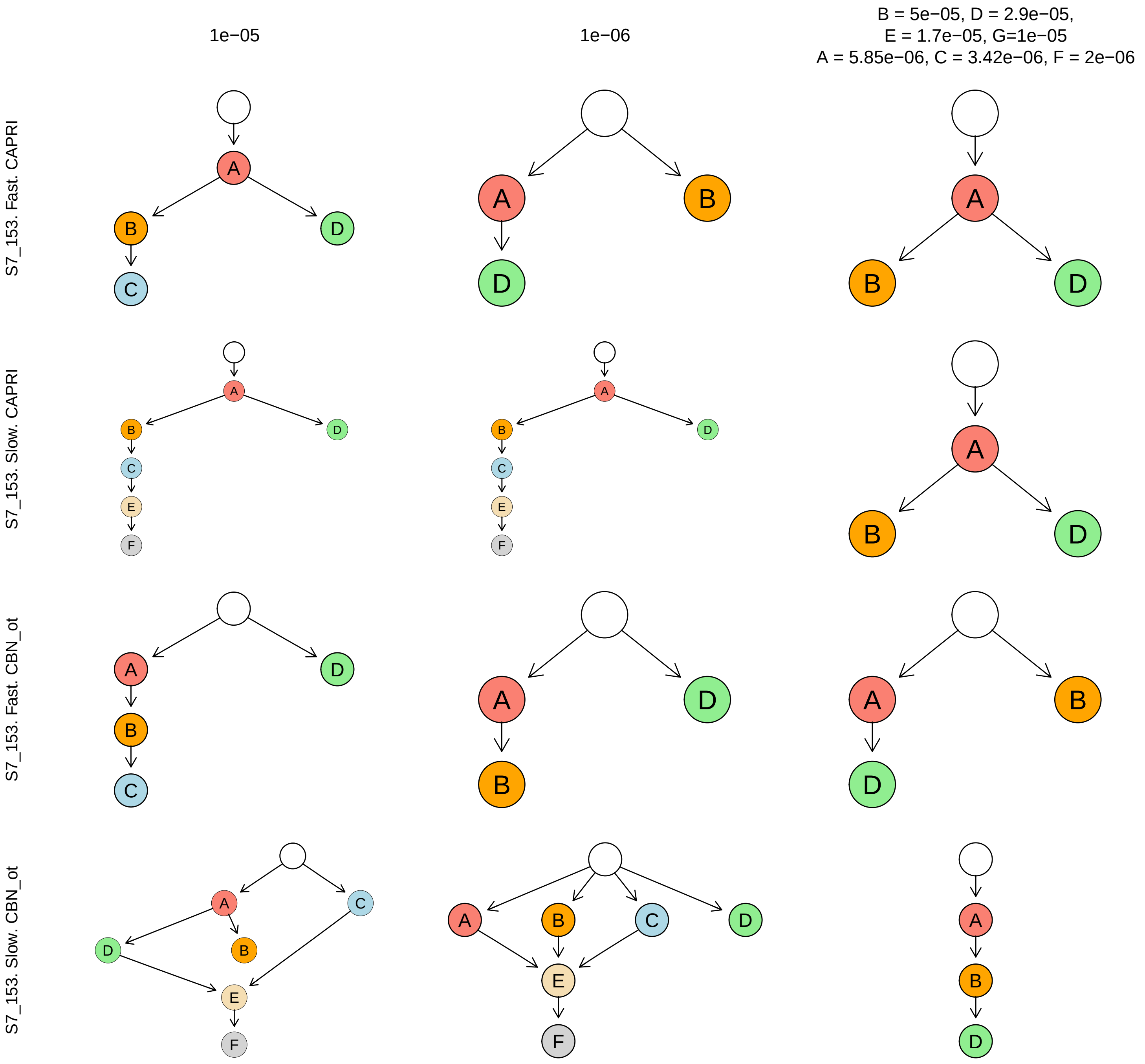

DAG_a

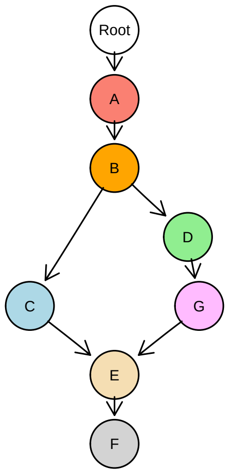

ID	Accessible Genot.	Discrep. DAG_a
S7_154	8	1

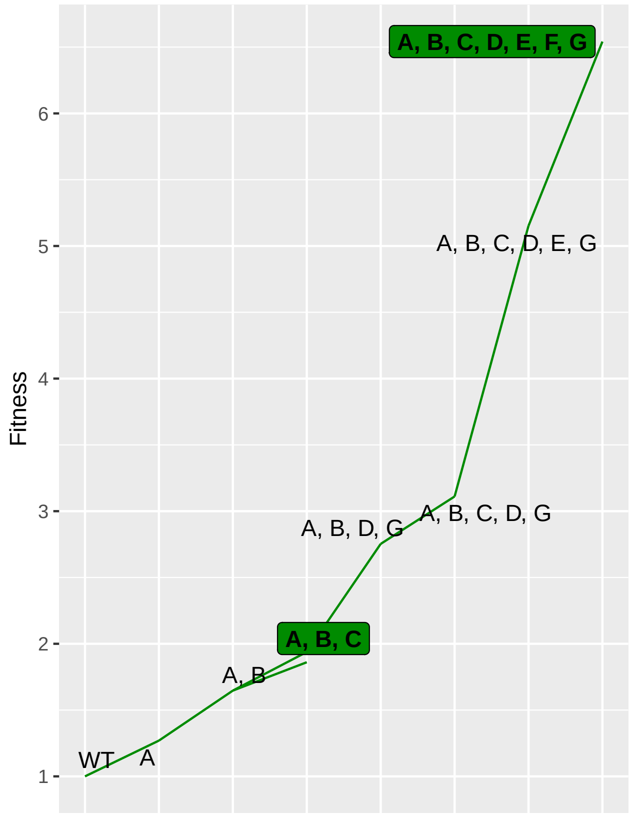

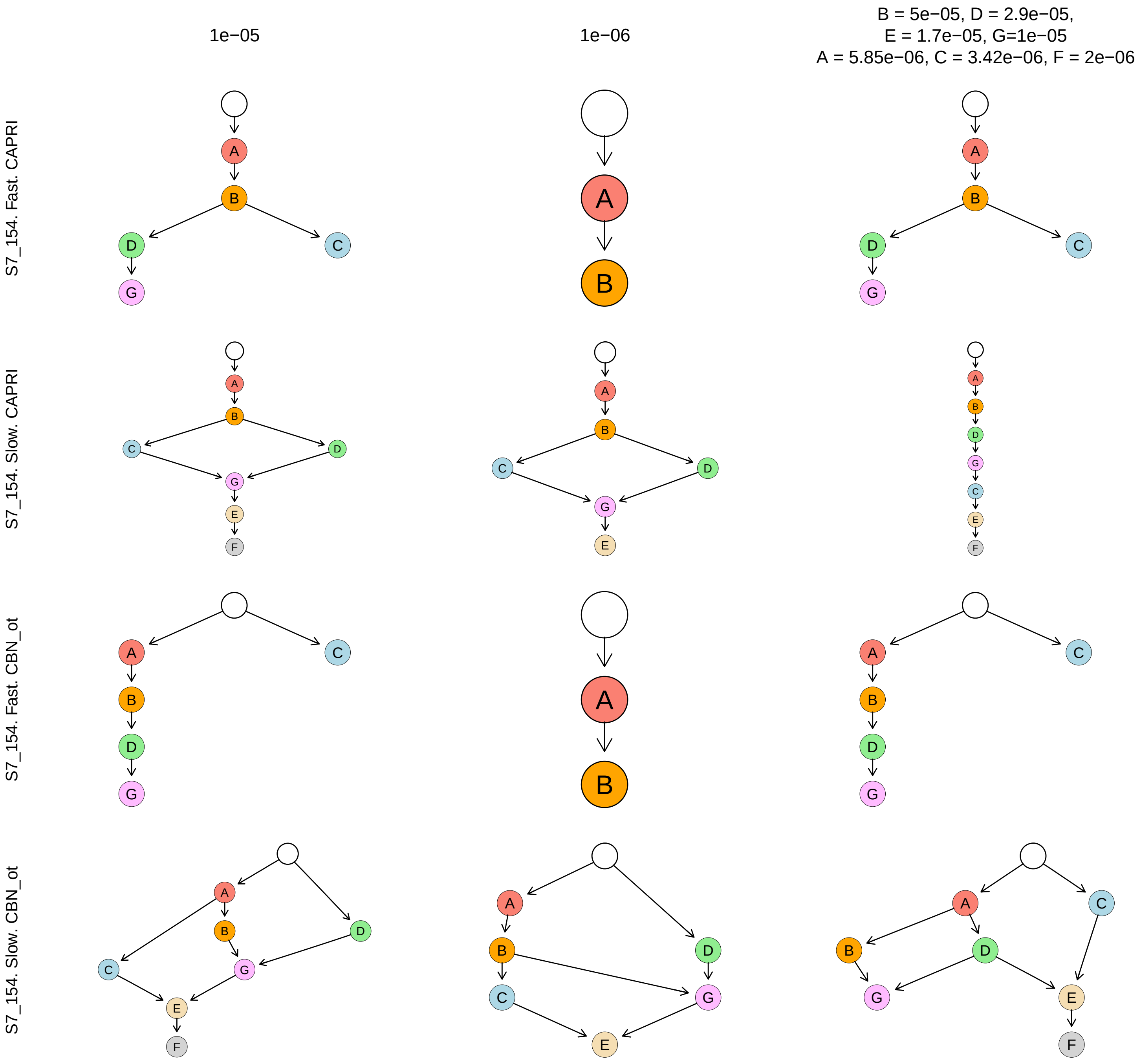

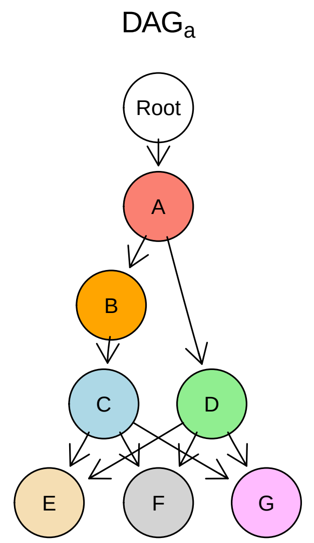

ID	Accessible Genot.	Discrep. DAG_a
S7_155	11	2

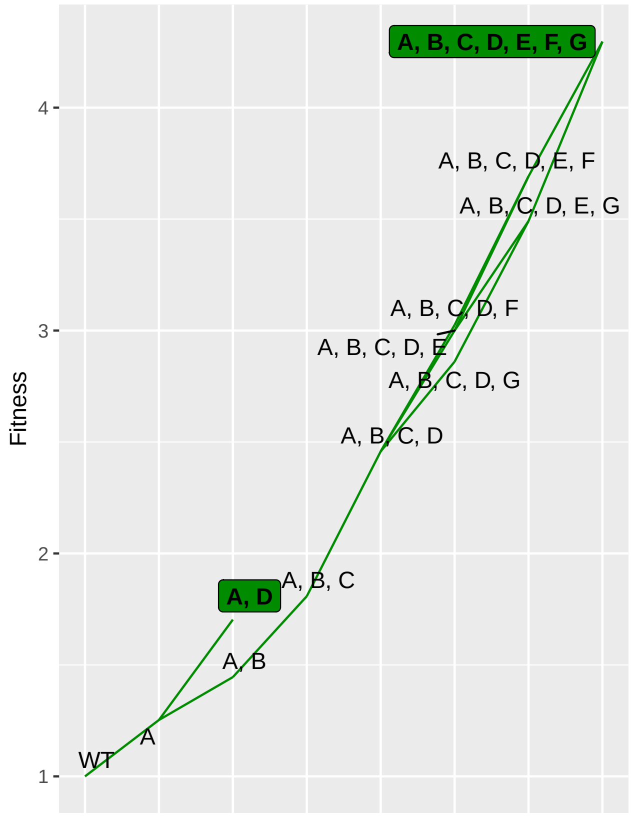

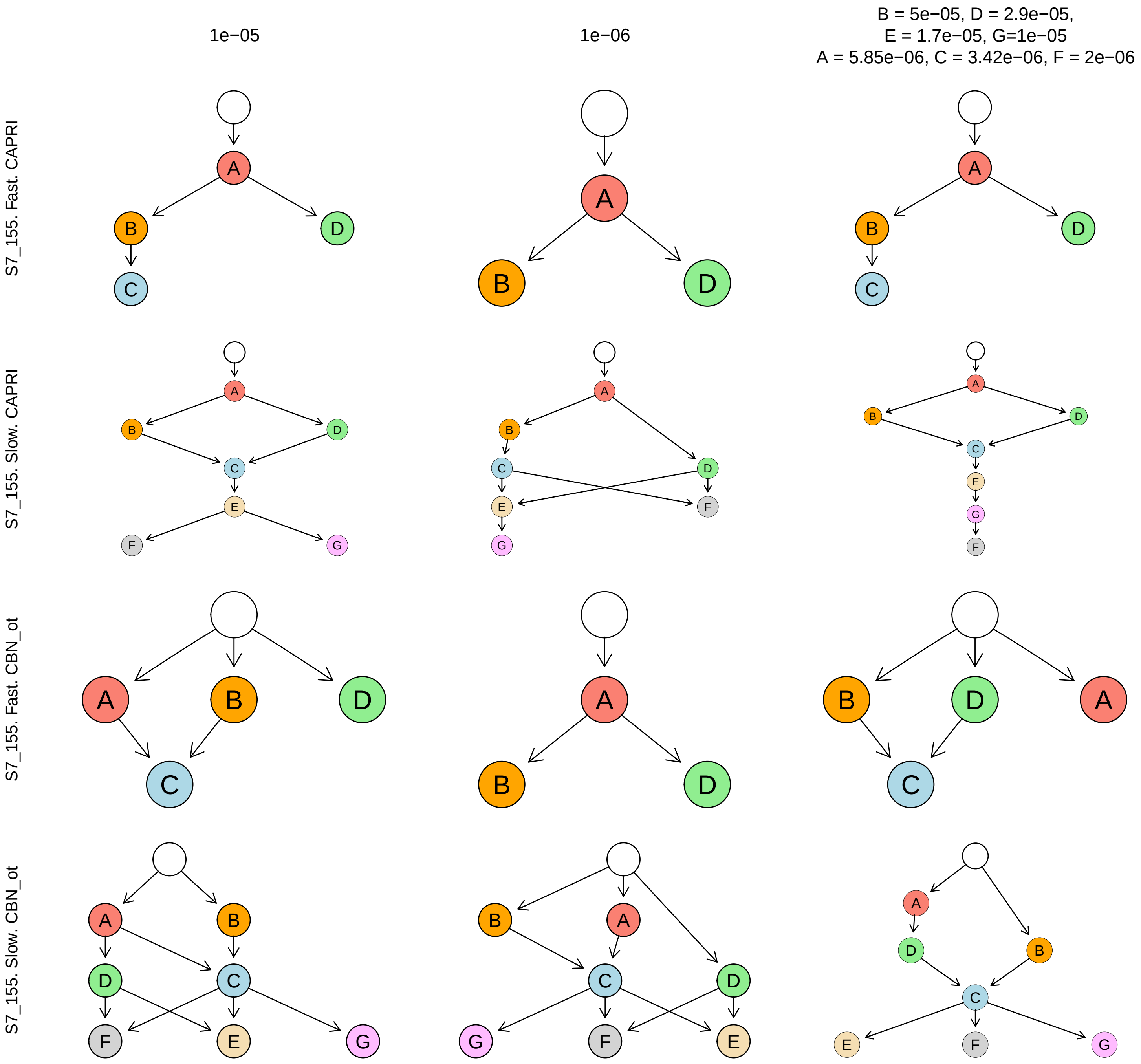

DAG_a

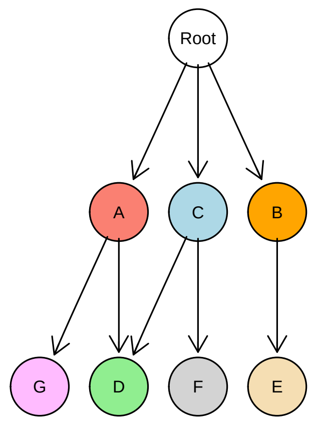

ID	Accessible Genot.	Discrep. DAG_a
S7_156	25	13

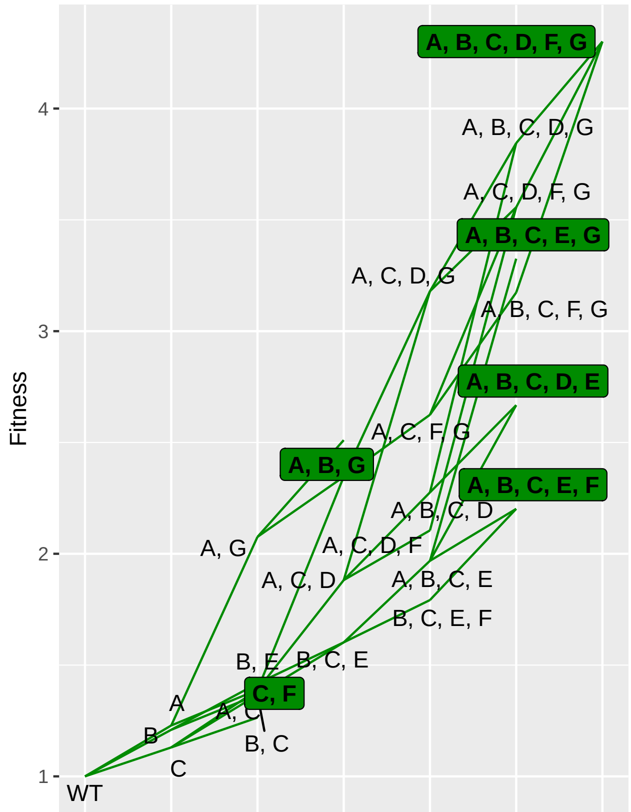

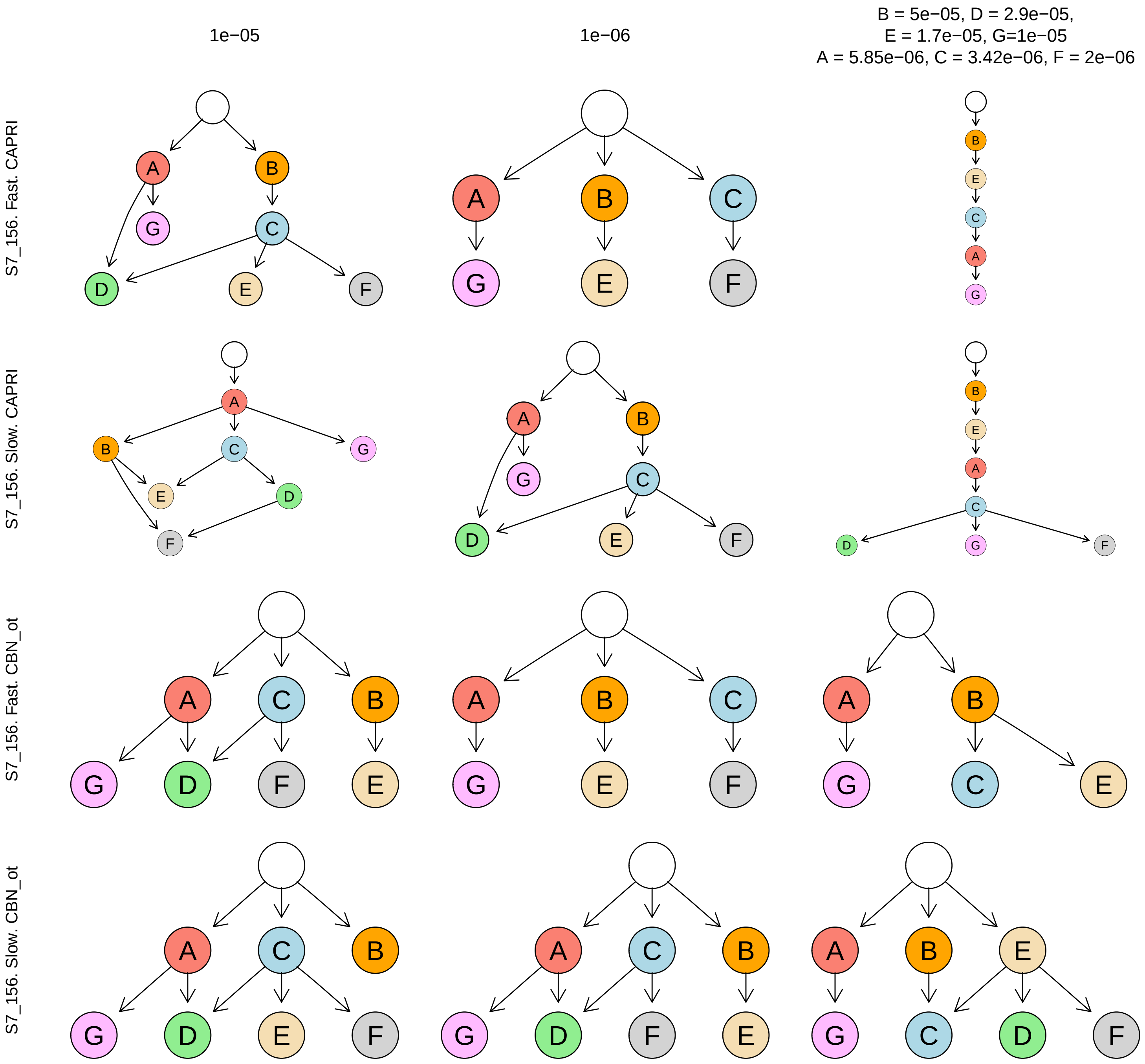

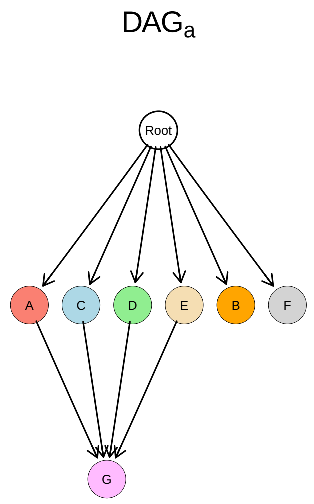

ID	Accessible Genot.	Discrep. DAG_a
S7_157	15	52

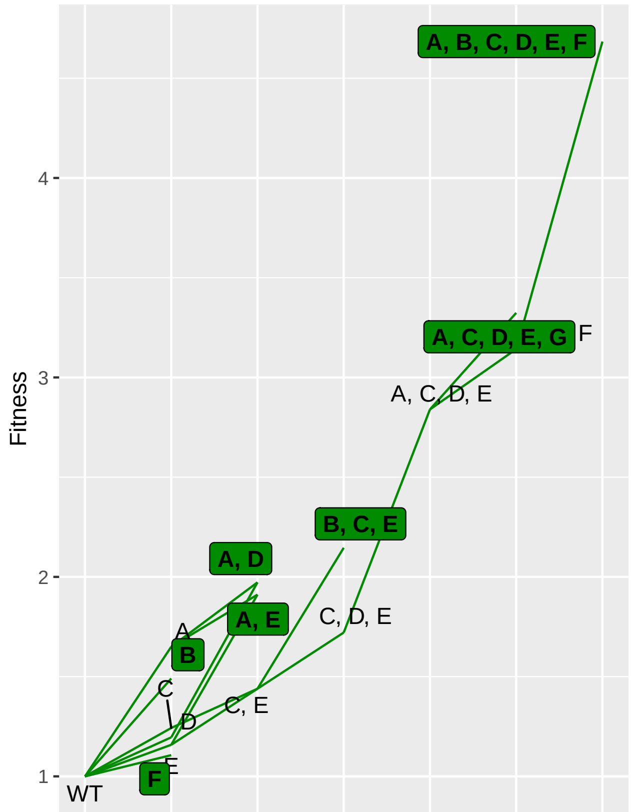

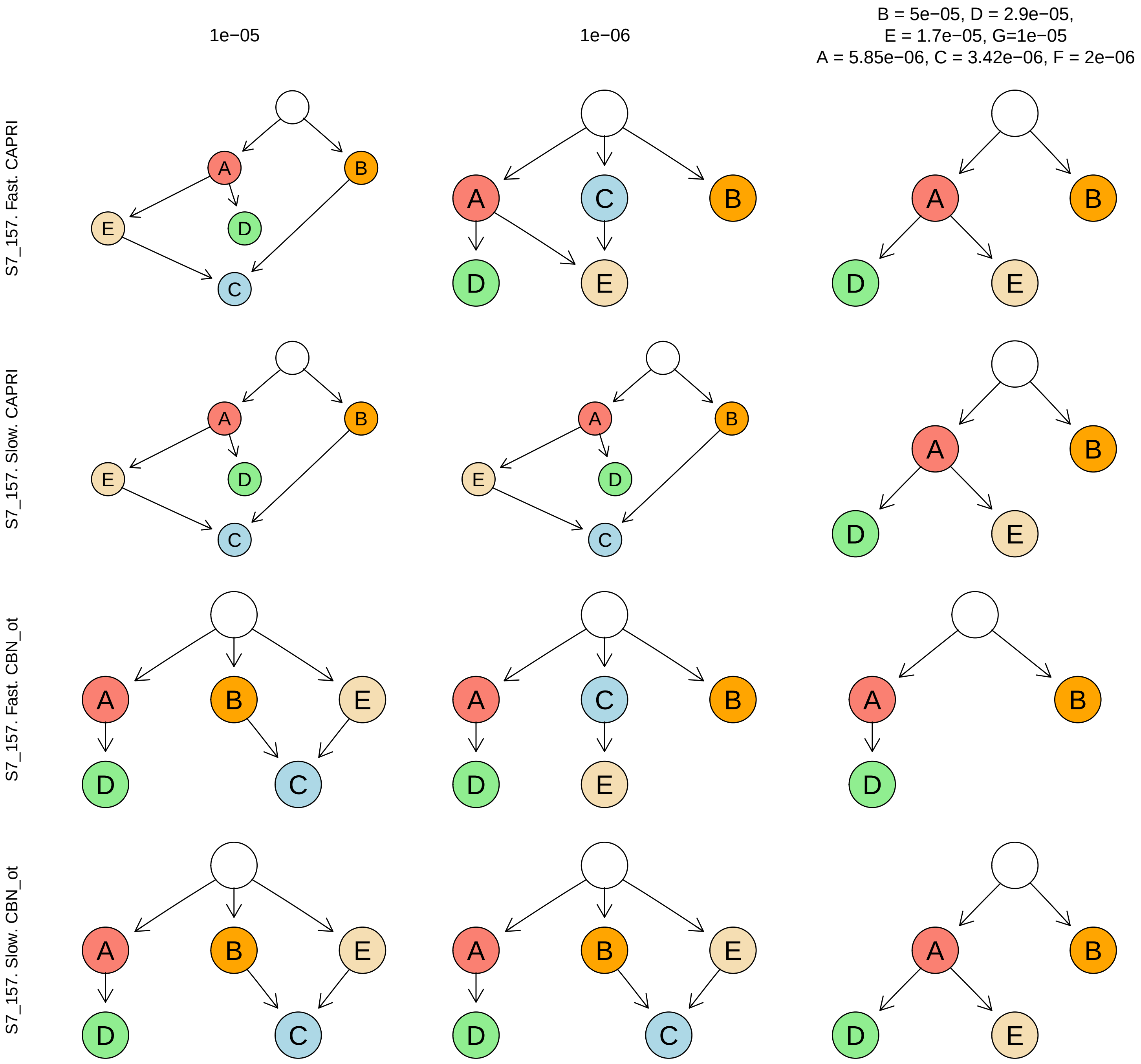

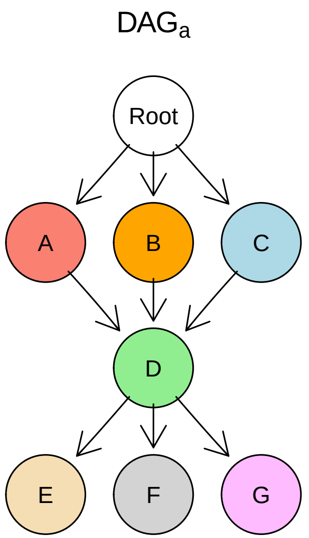

ID	Accessible Genot.	Discrep. DAG_a
S7_158	13	2

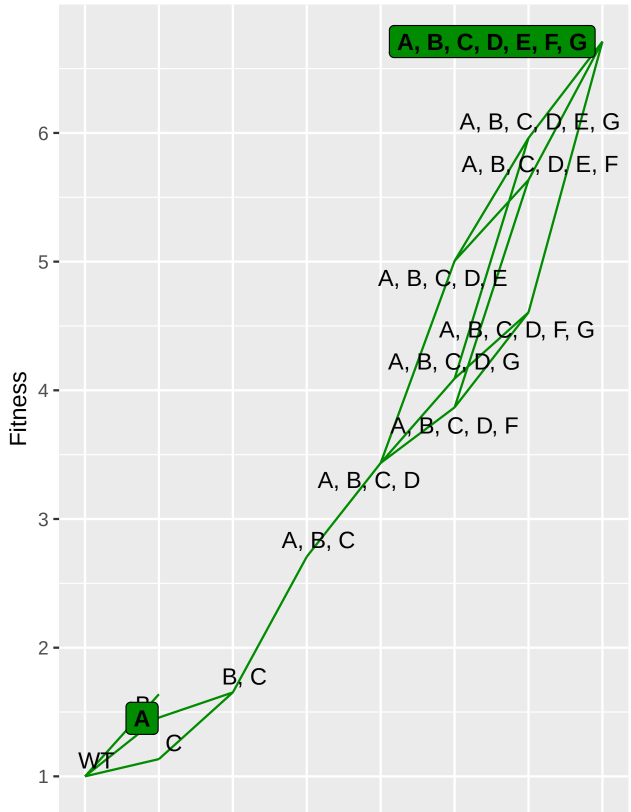

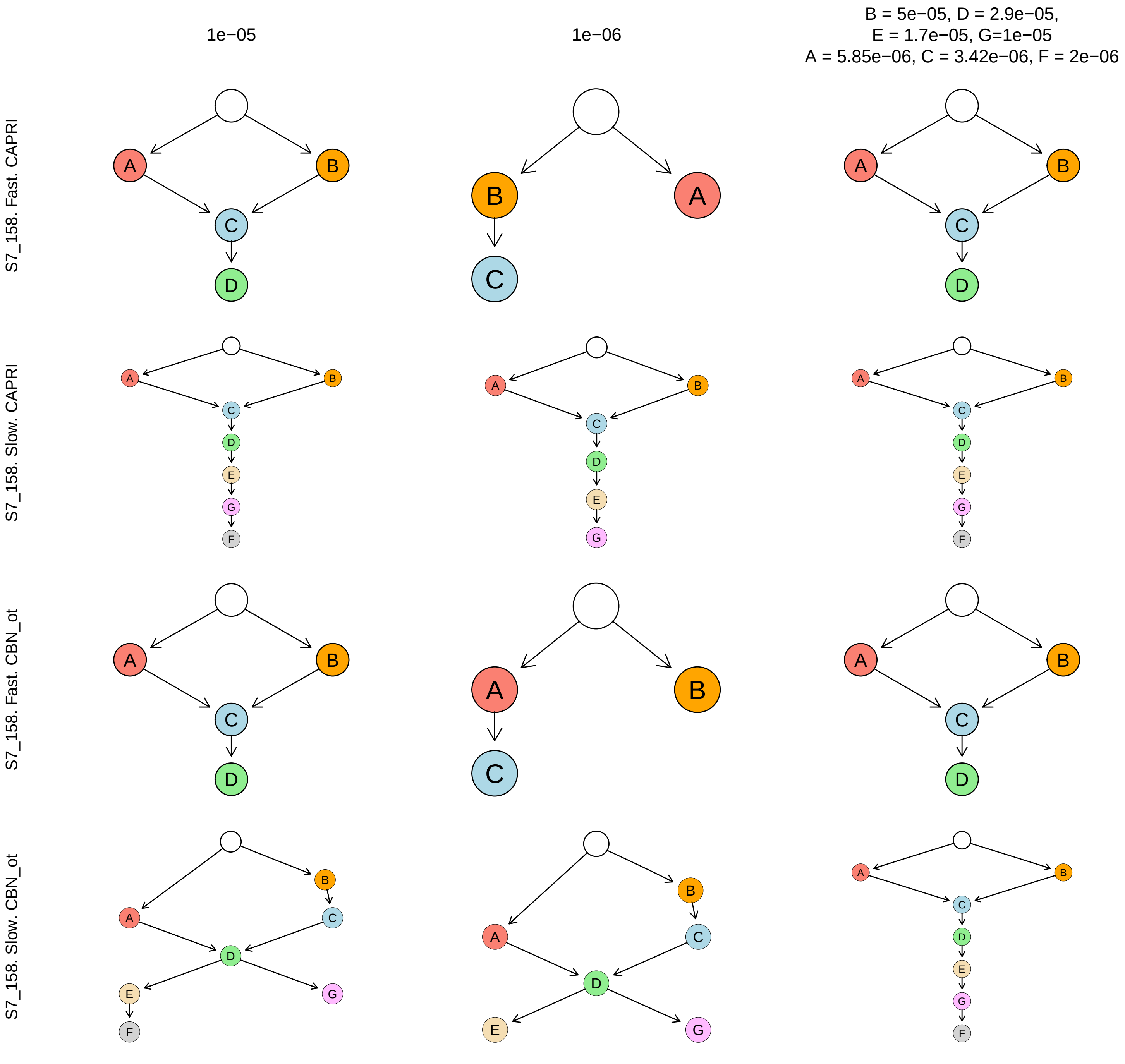

DAG$_a$

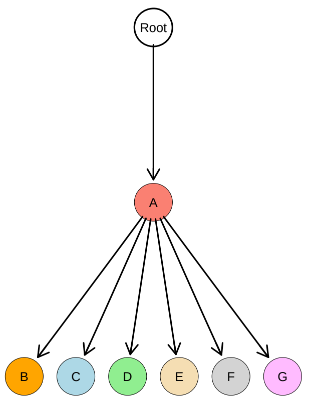

ID	Accessible Genot.	Discrep. DAG_a
S7_159	60	4

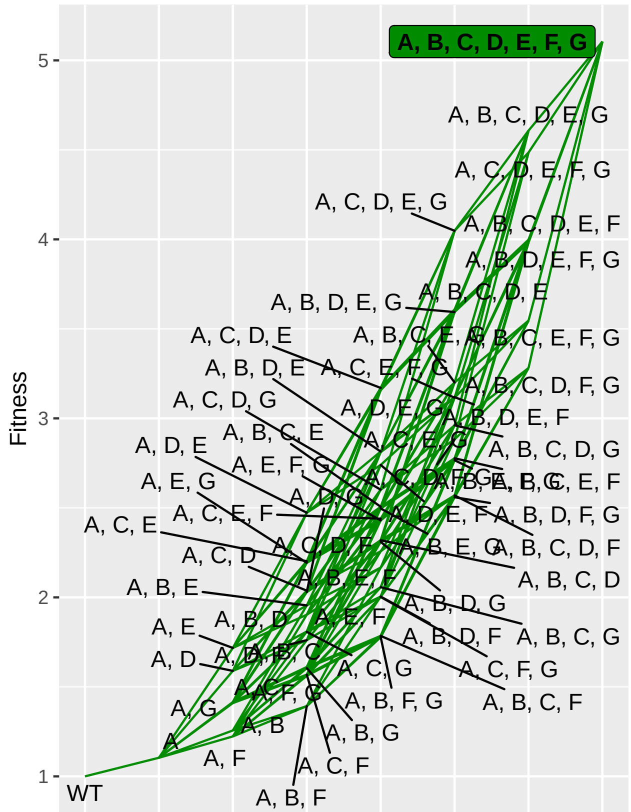

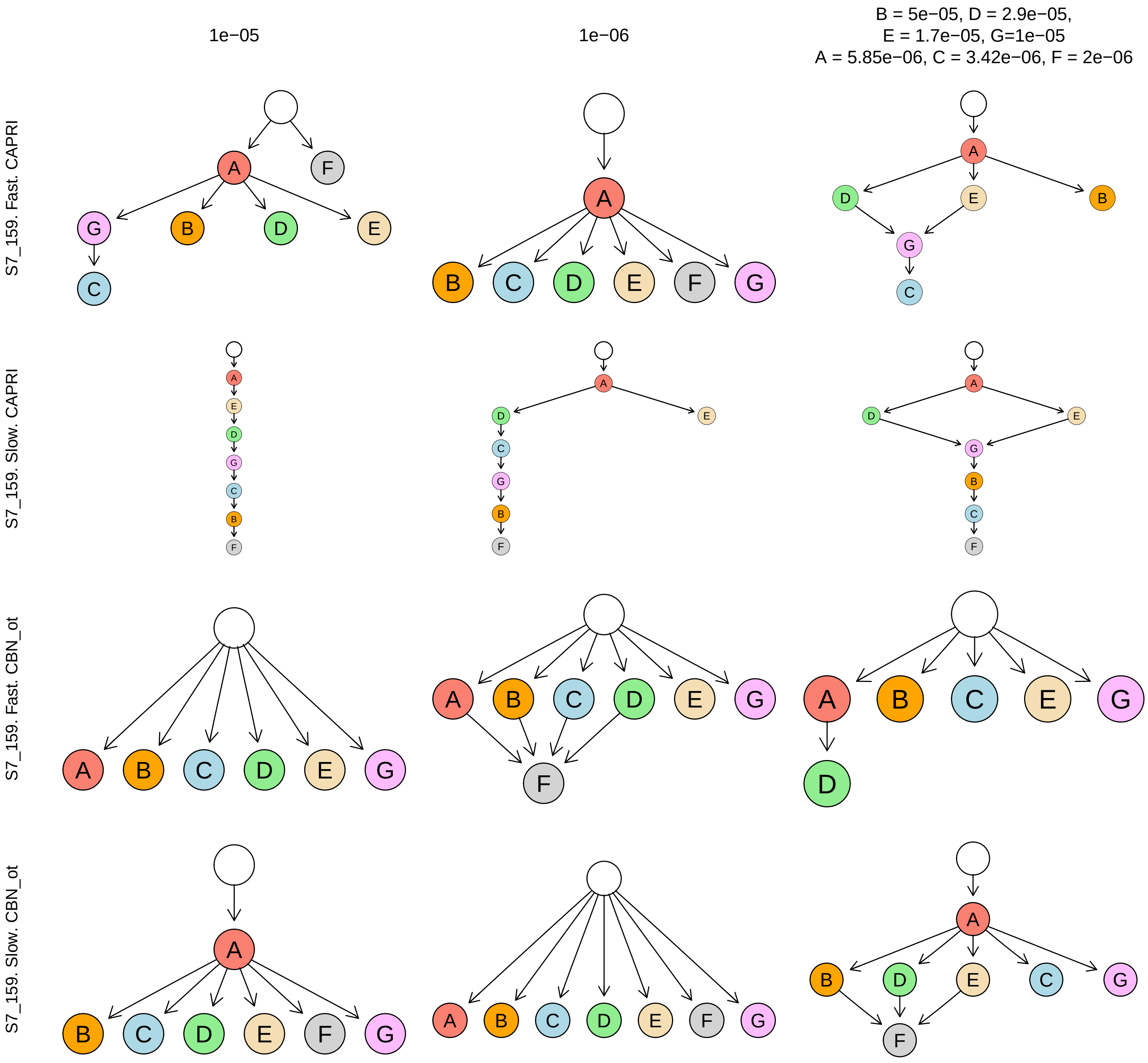

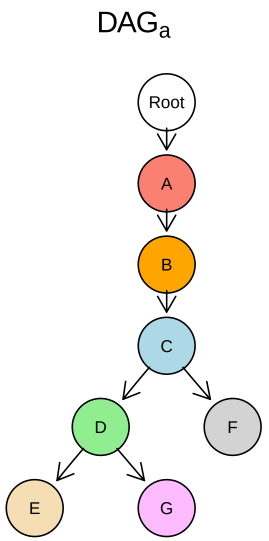

ID	Accessible Genot.	Discrep. DAG_a
S7_160	9	3

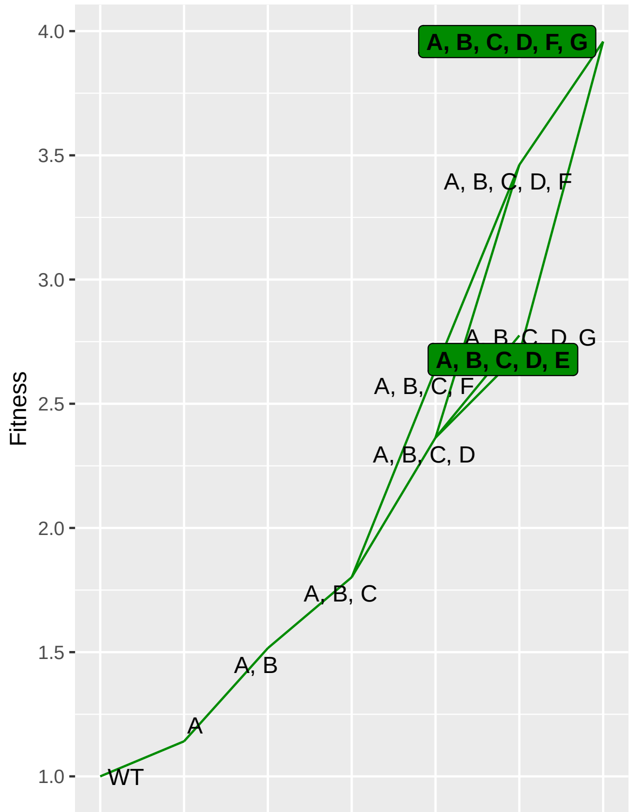

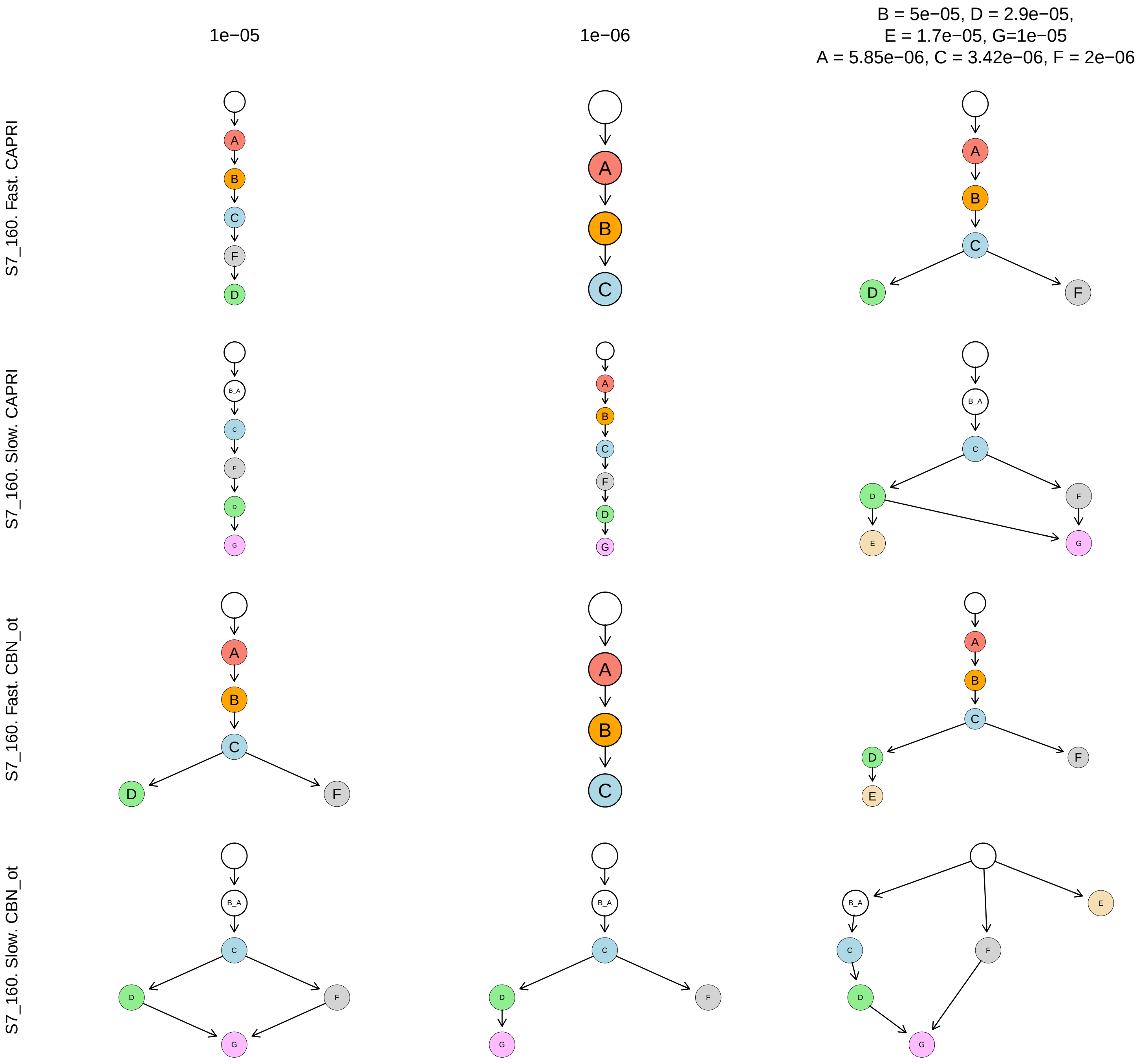

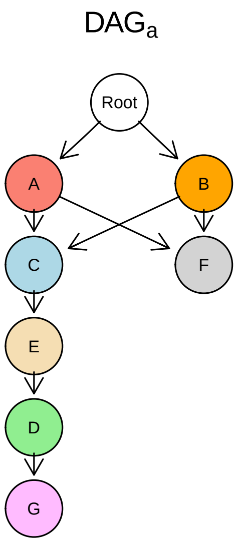

ID	Accessible Genot.	Discrep. DAG_a
S7_161	11	3

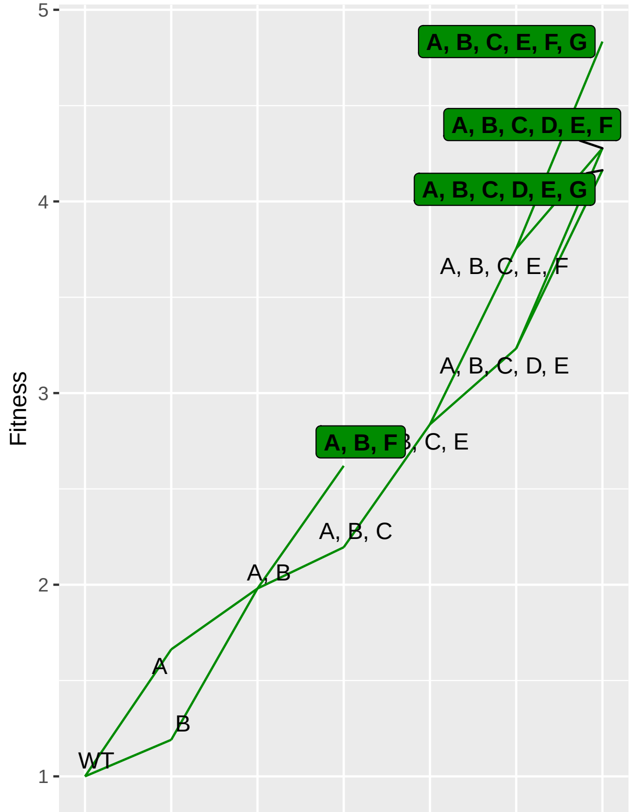

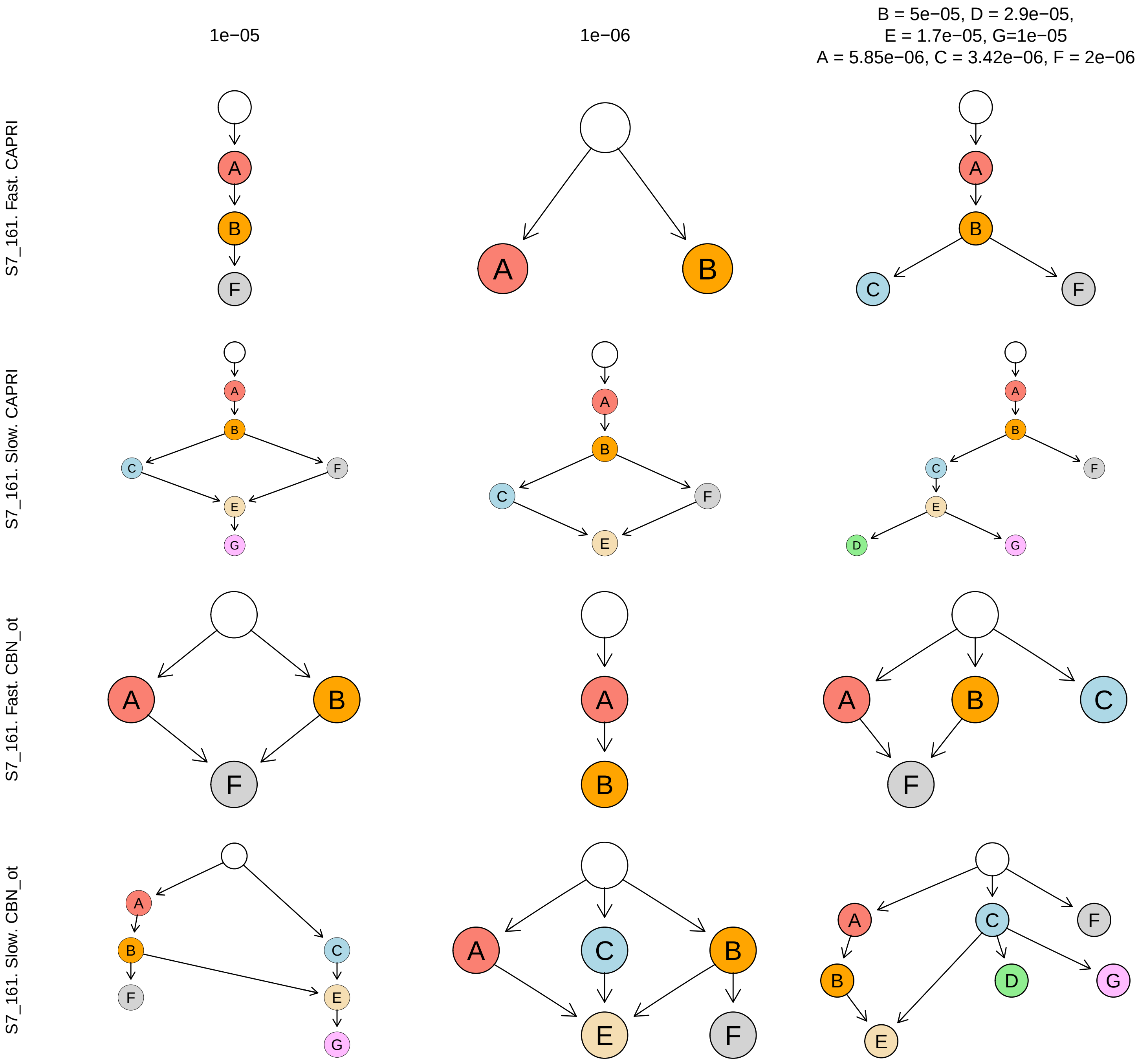

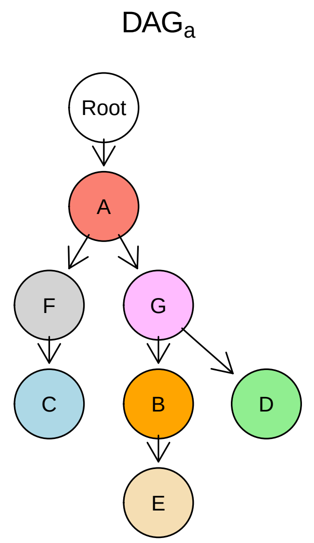

ID	Accessible Genot.	Discrep. DAG_a
S7_162	33	16

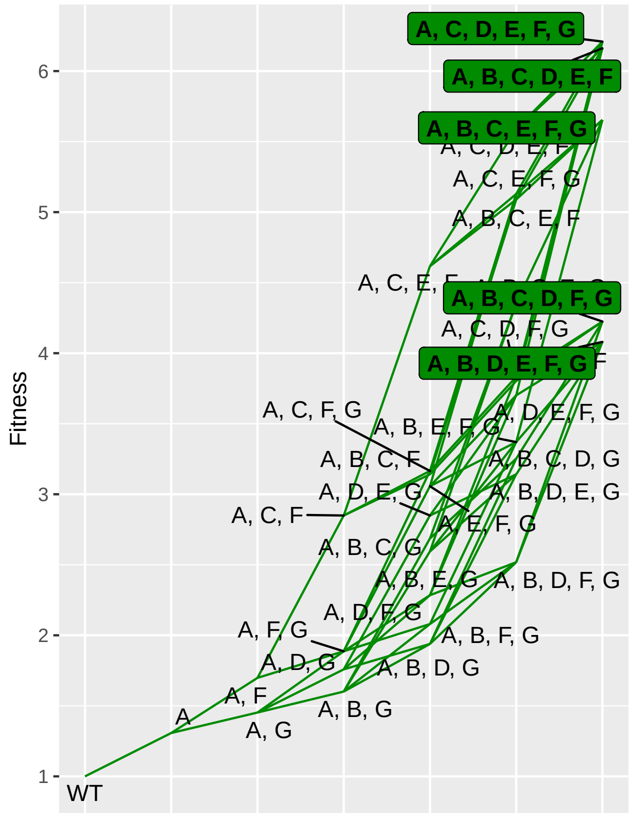

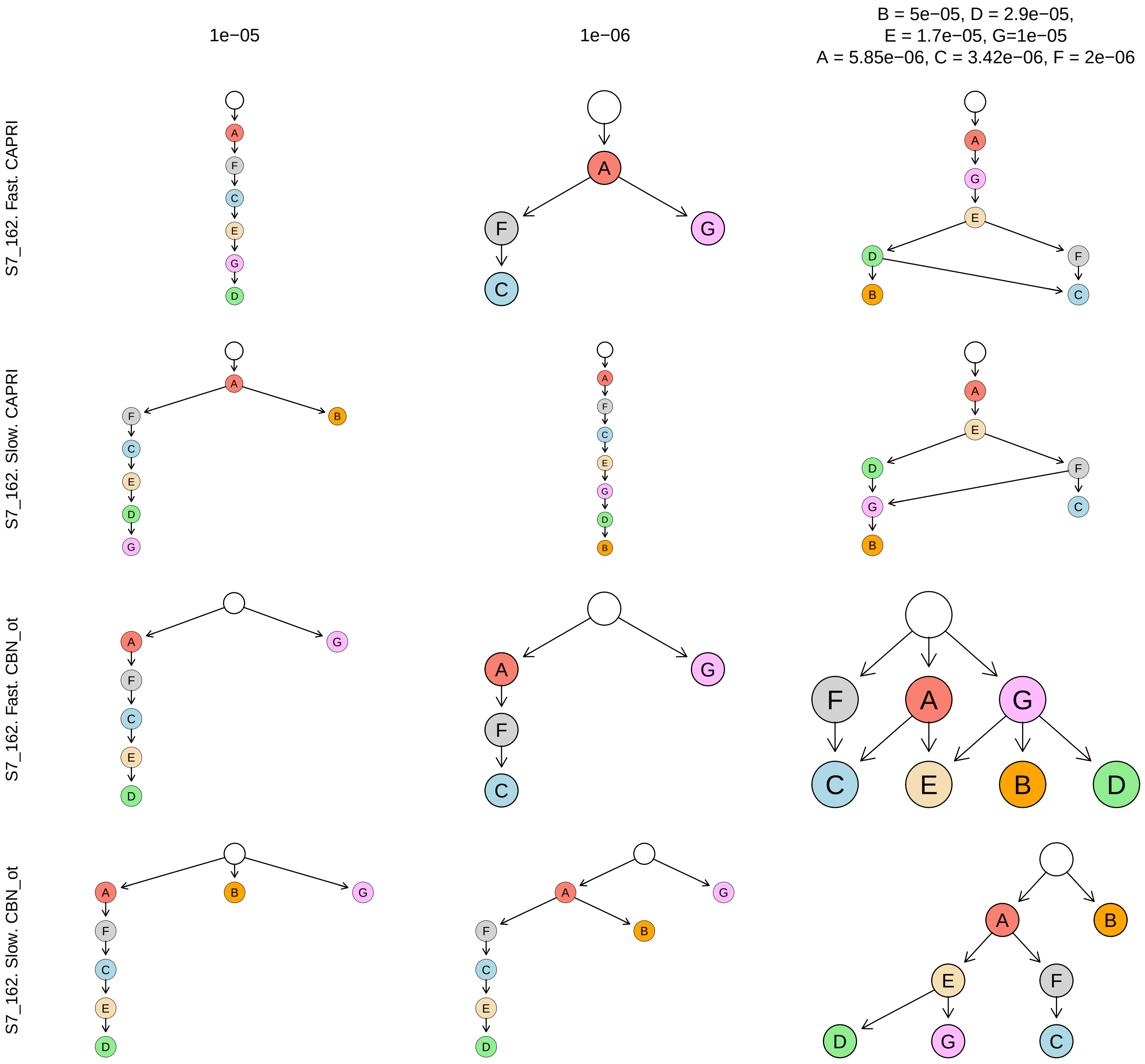

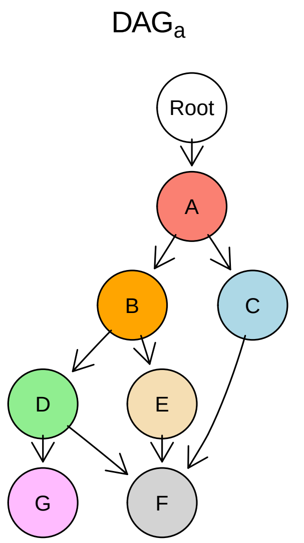

ID	Accessible Genot.	Discrep. DAG_a
S7_163	16	4

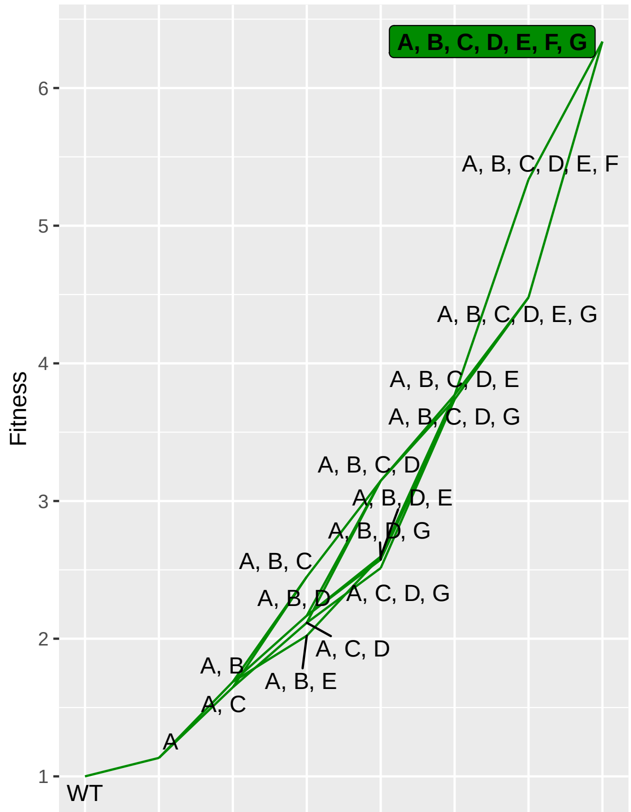

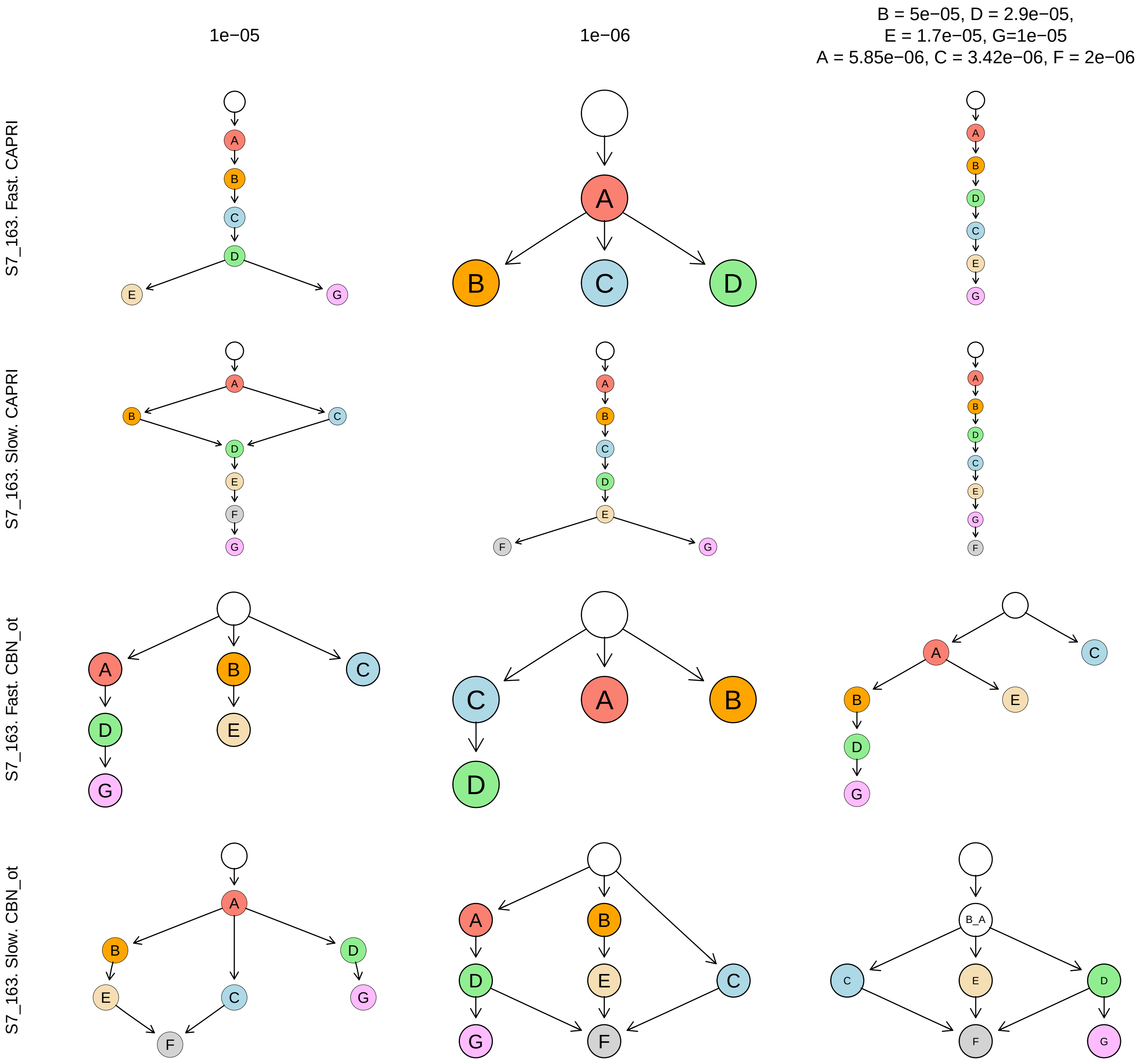

DAG_a

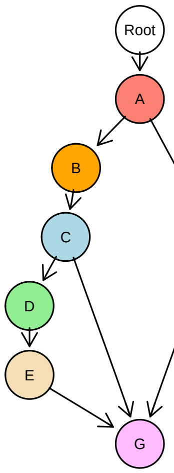

ID	Accessible Genot.	Discrep. DAG_a
S7_164	11	4

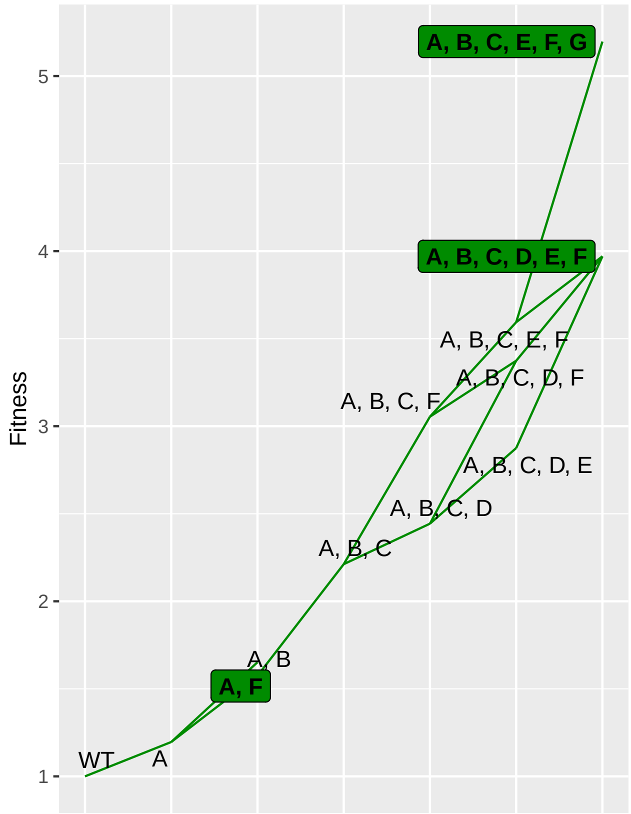

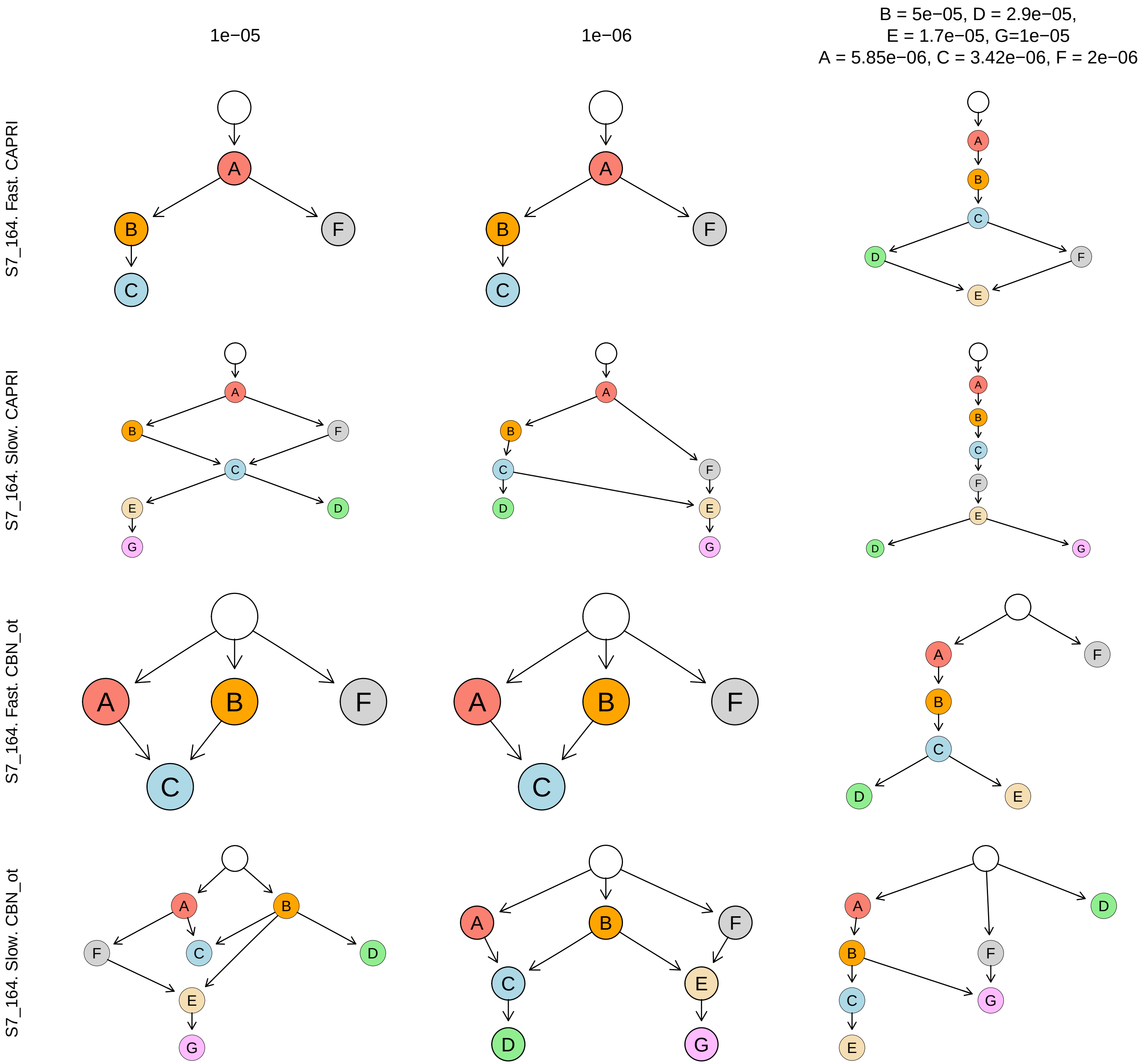

DAG$_a$

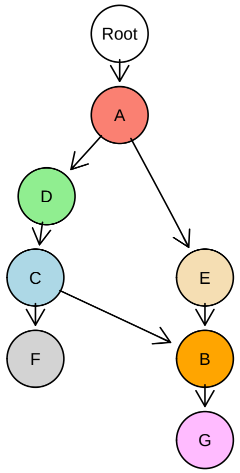

ID	Accessible Genot.	Discrep. DAG_a
S7_165	19	9

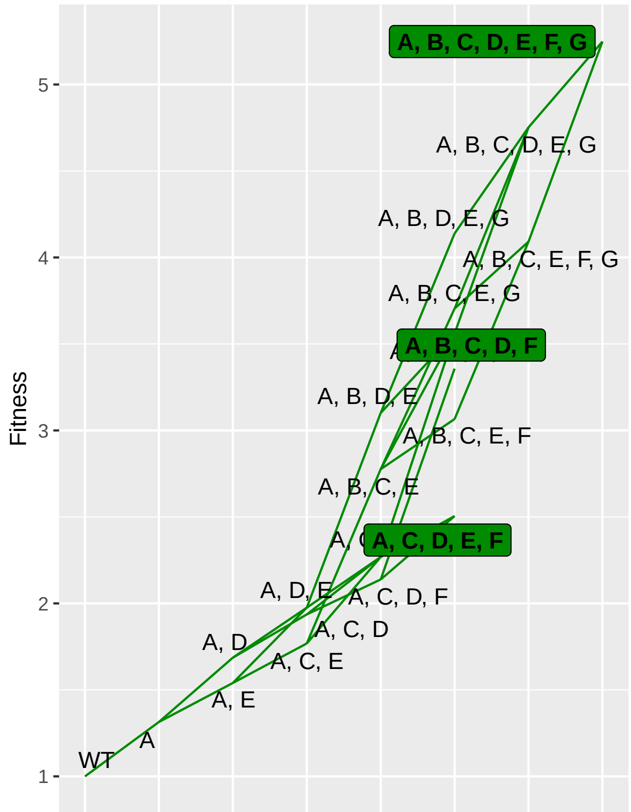

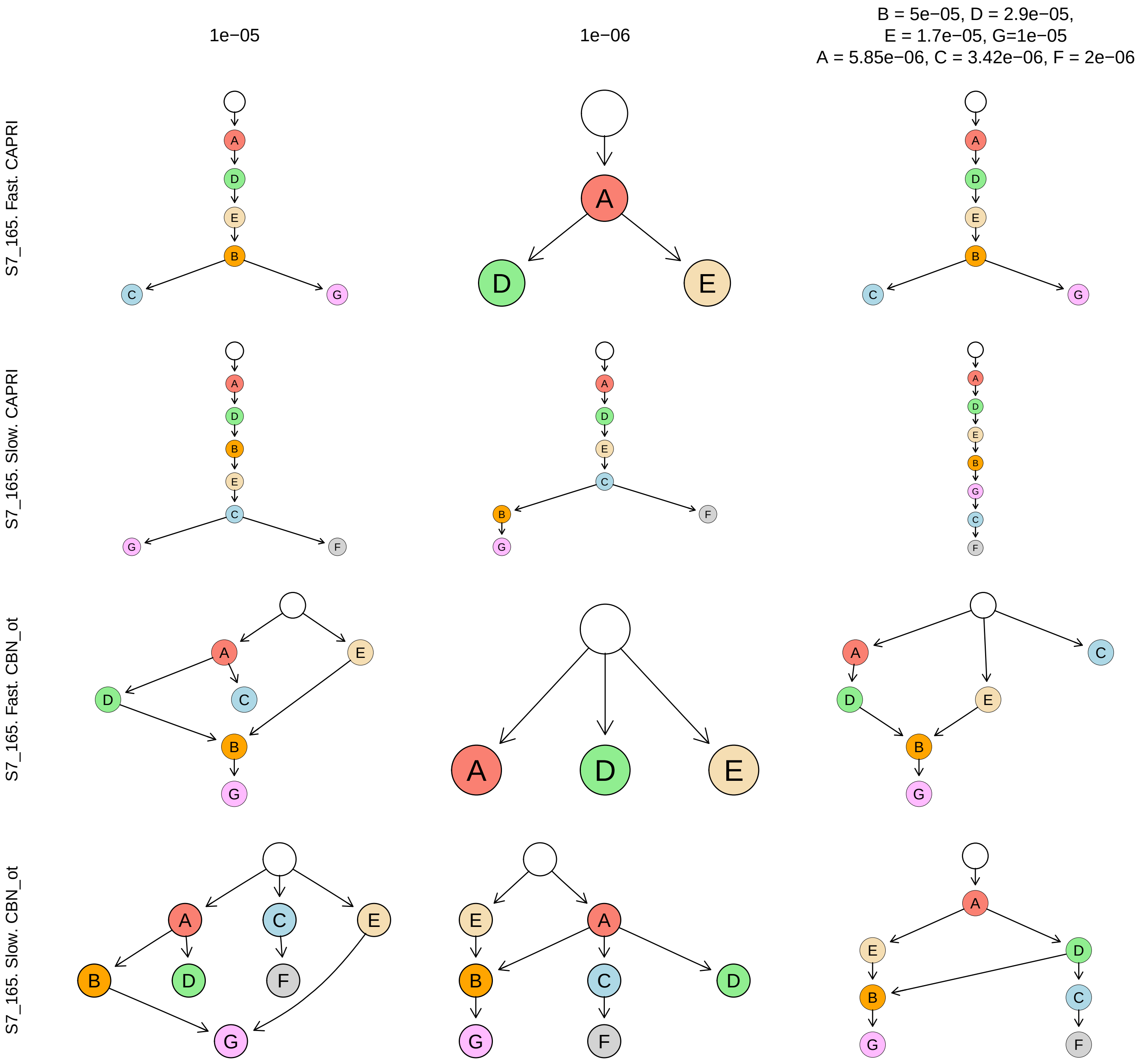

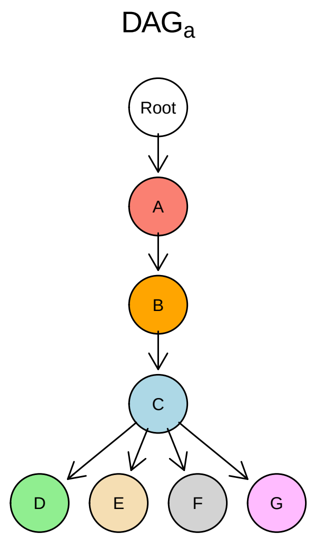

ID	Accessible Genot.	Discrep. DAG_a
S7_166	15	3

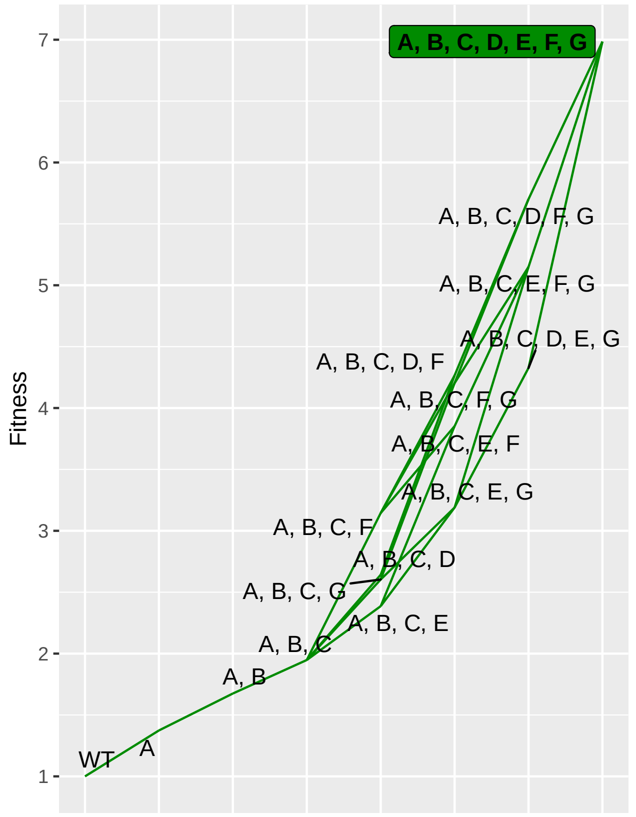

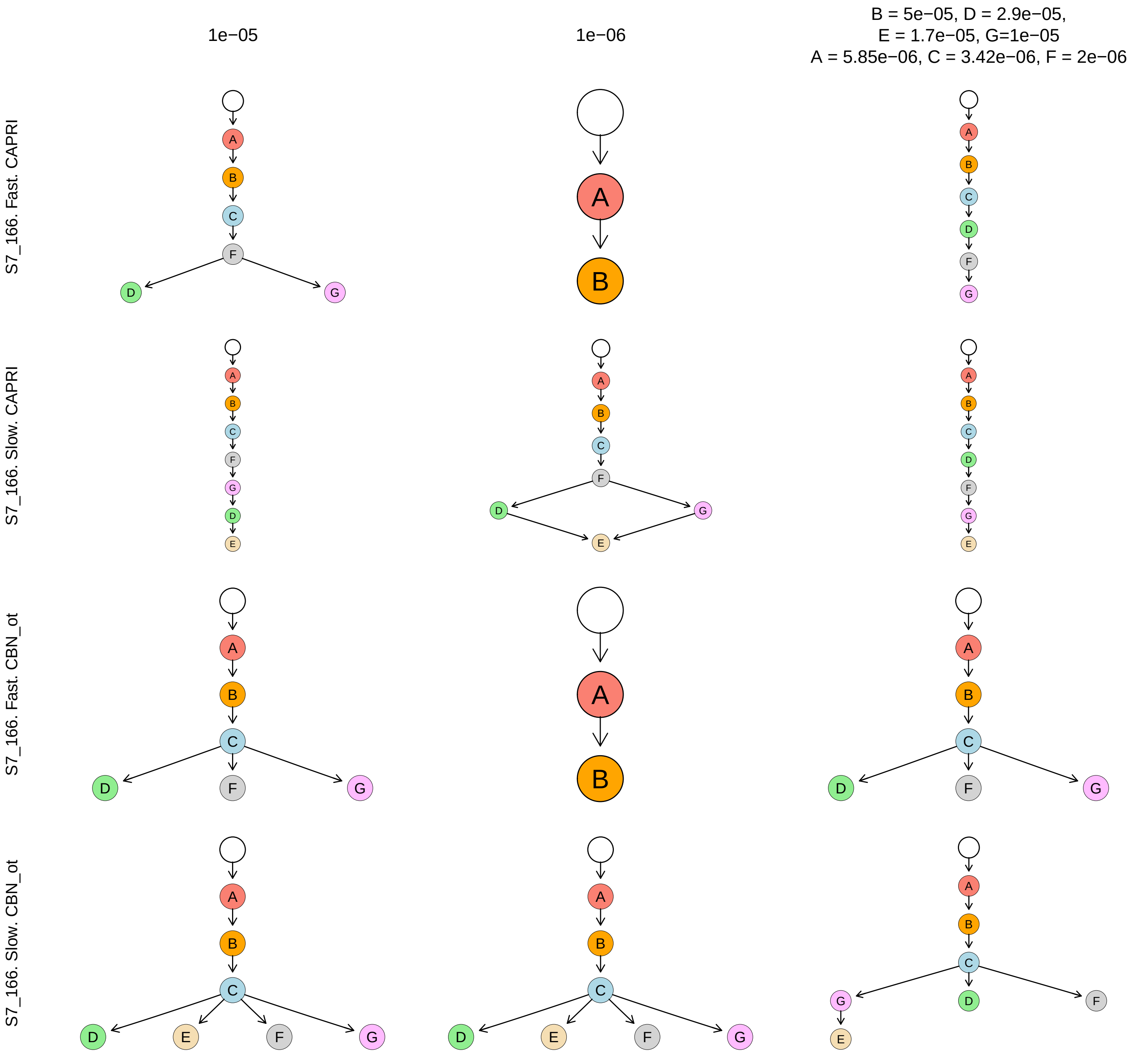

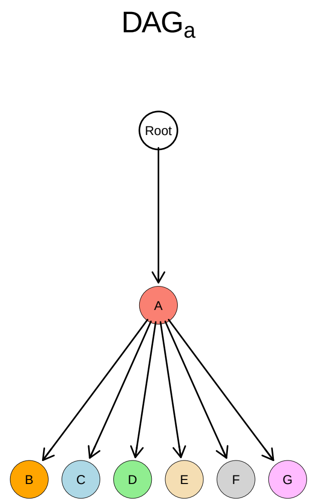

ID	Accessible Genot.	Discrep. DAG_a
S7_167	44	20

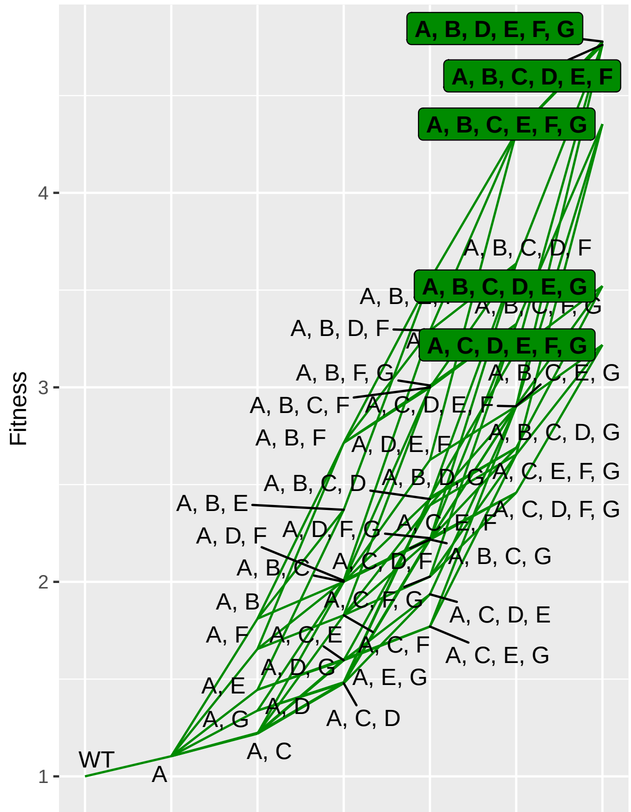

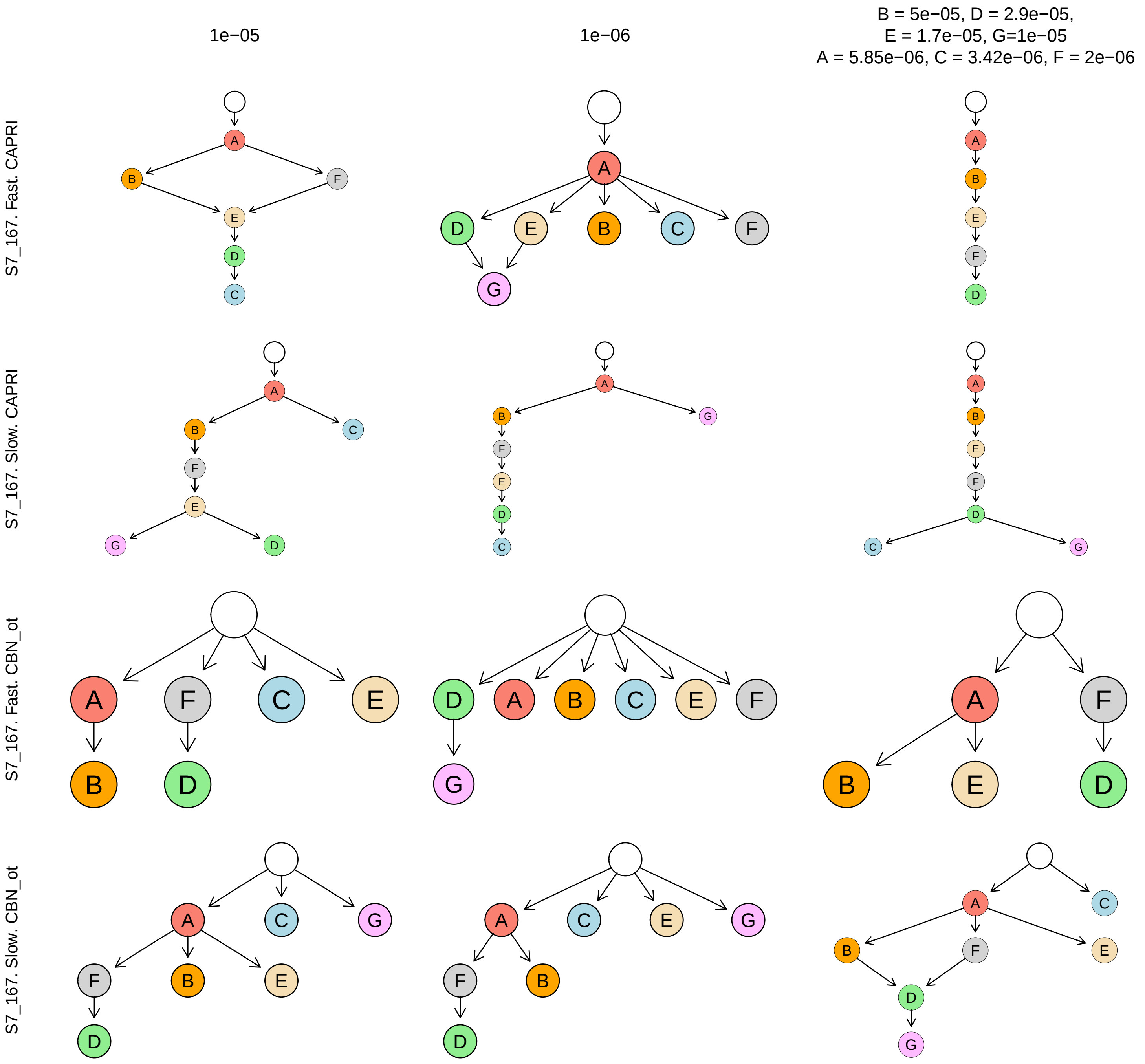

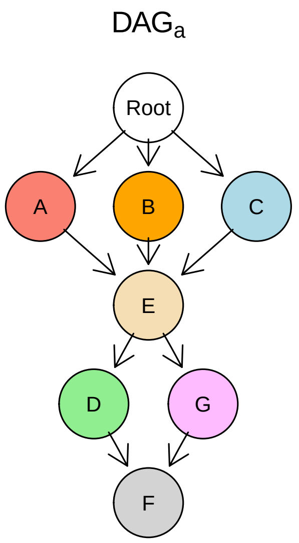

ID	Accessible Genot.	Discrep. DAG_a
S7_168	11	1

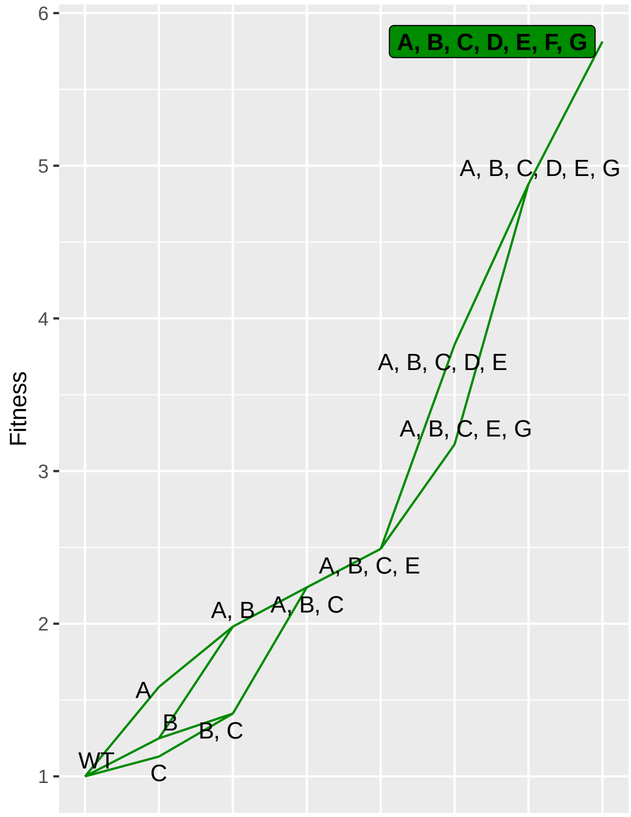

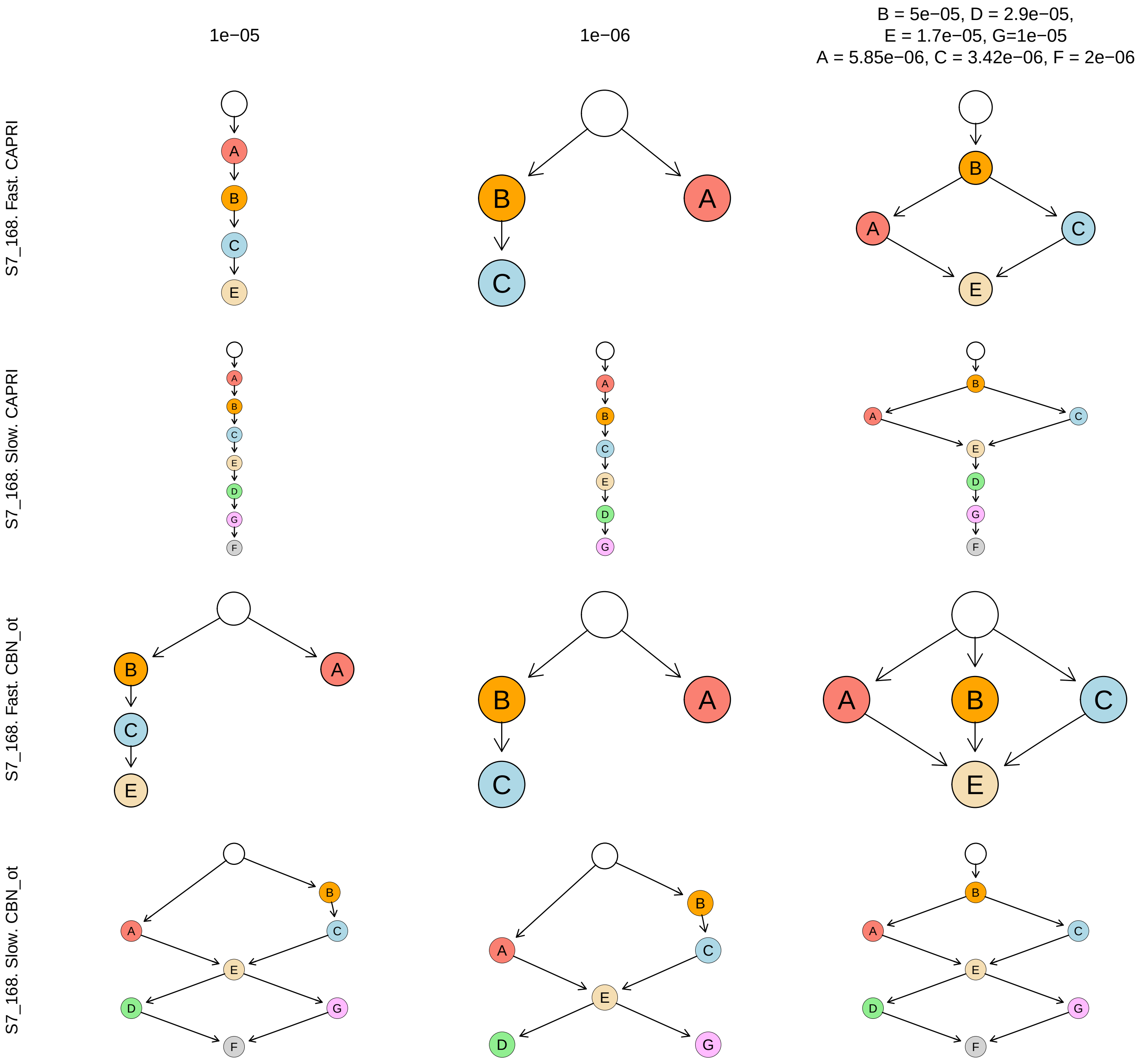

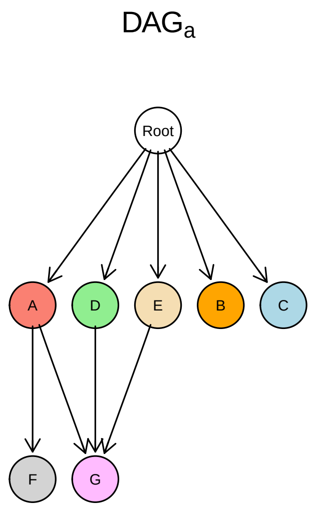

ID	Accessible Genot.	Discrep. DAG_a
S7_169	36	19

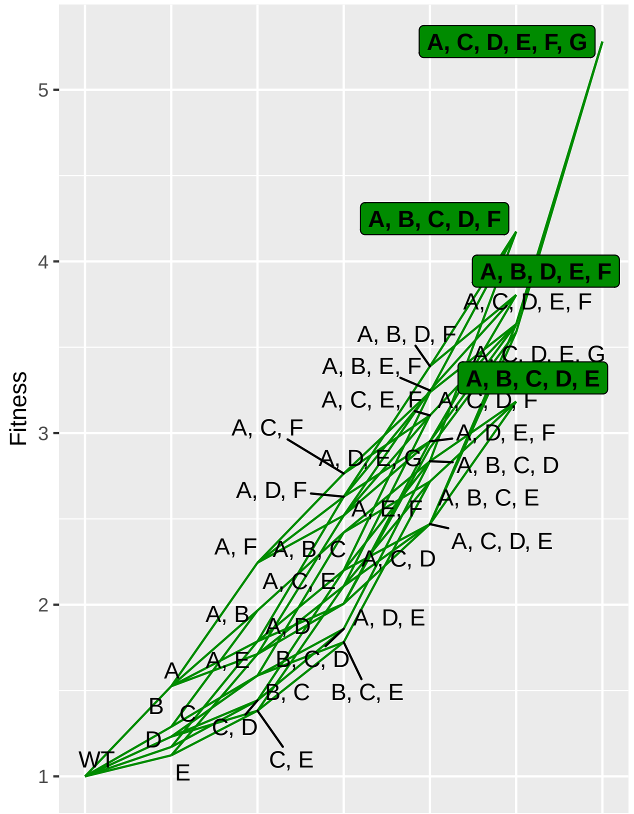

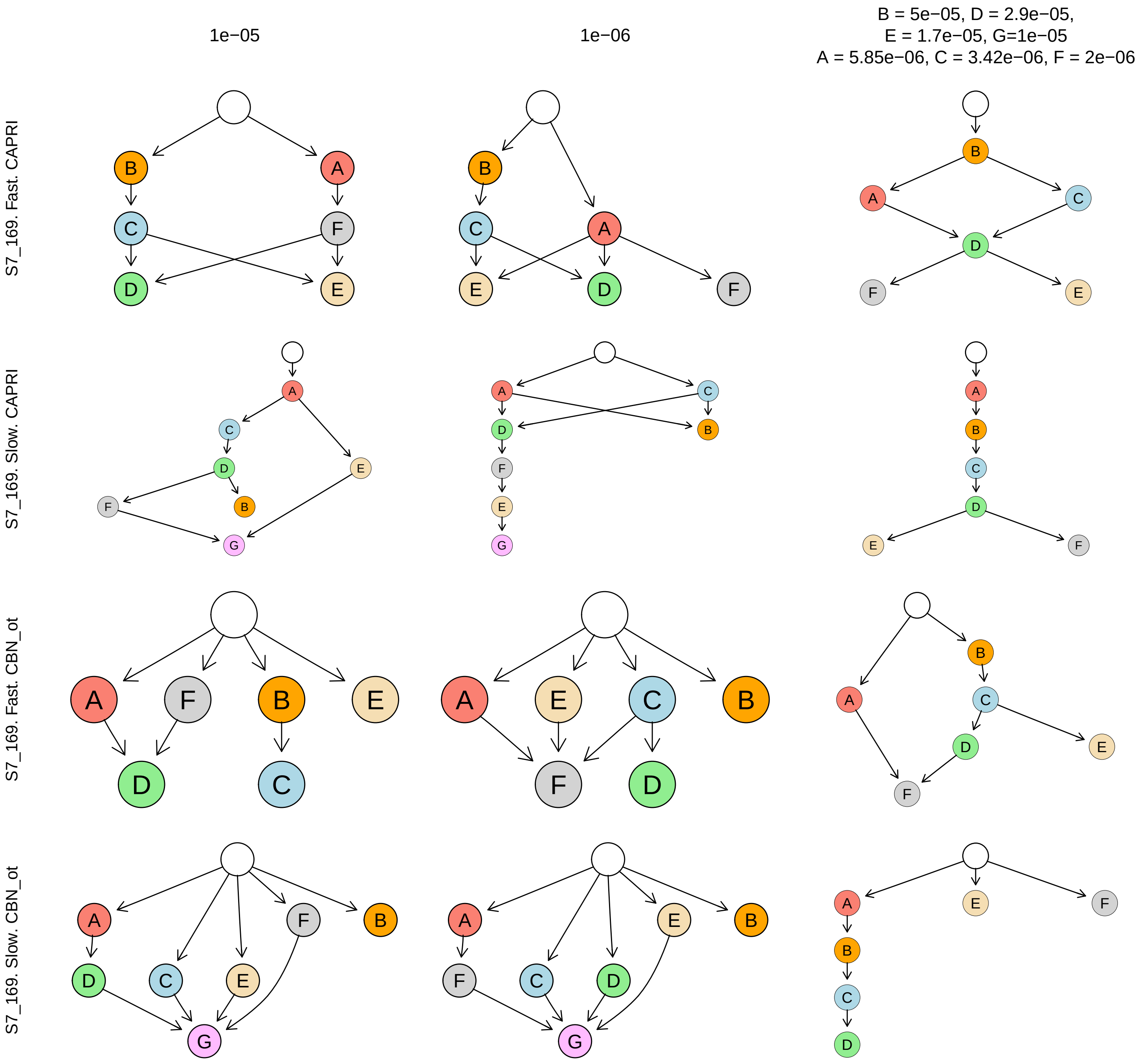

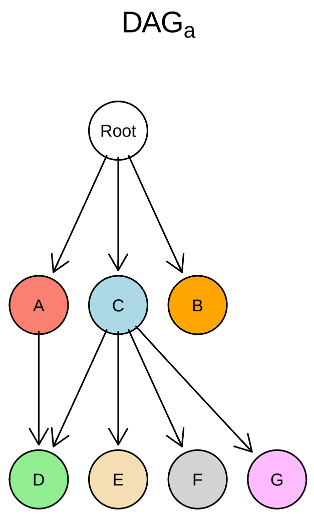

ID	Accessible Genot.	Discrep. DAG_a
S7_170	37	14

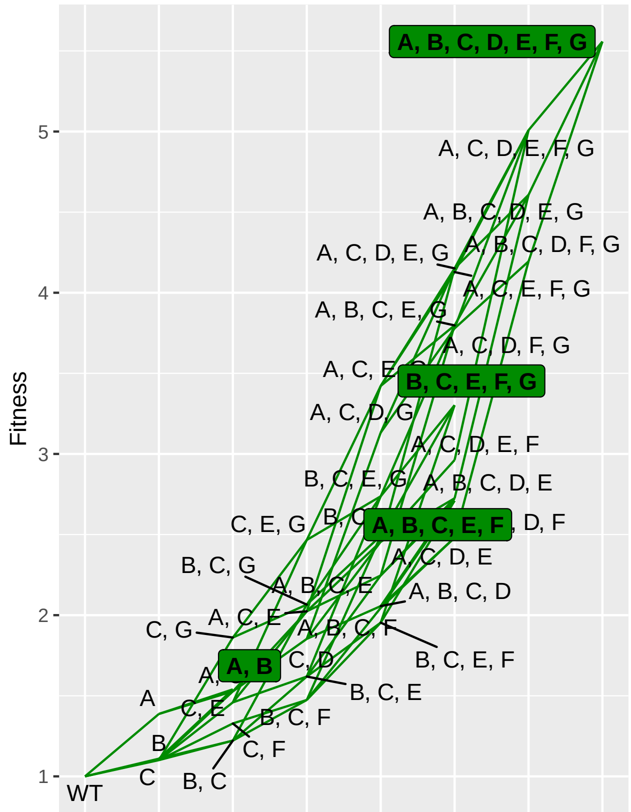

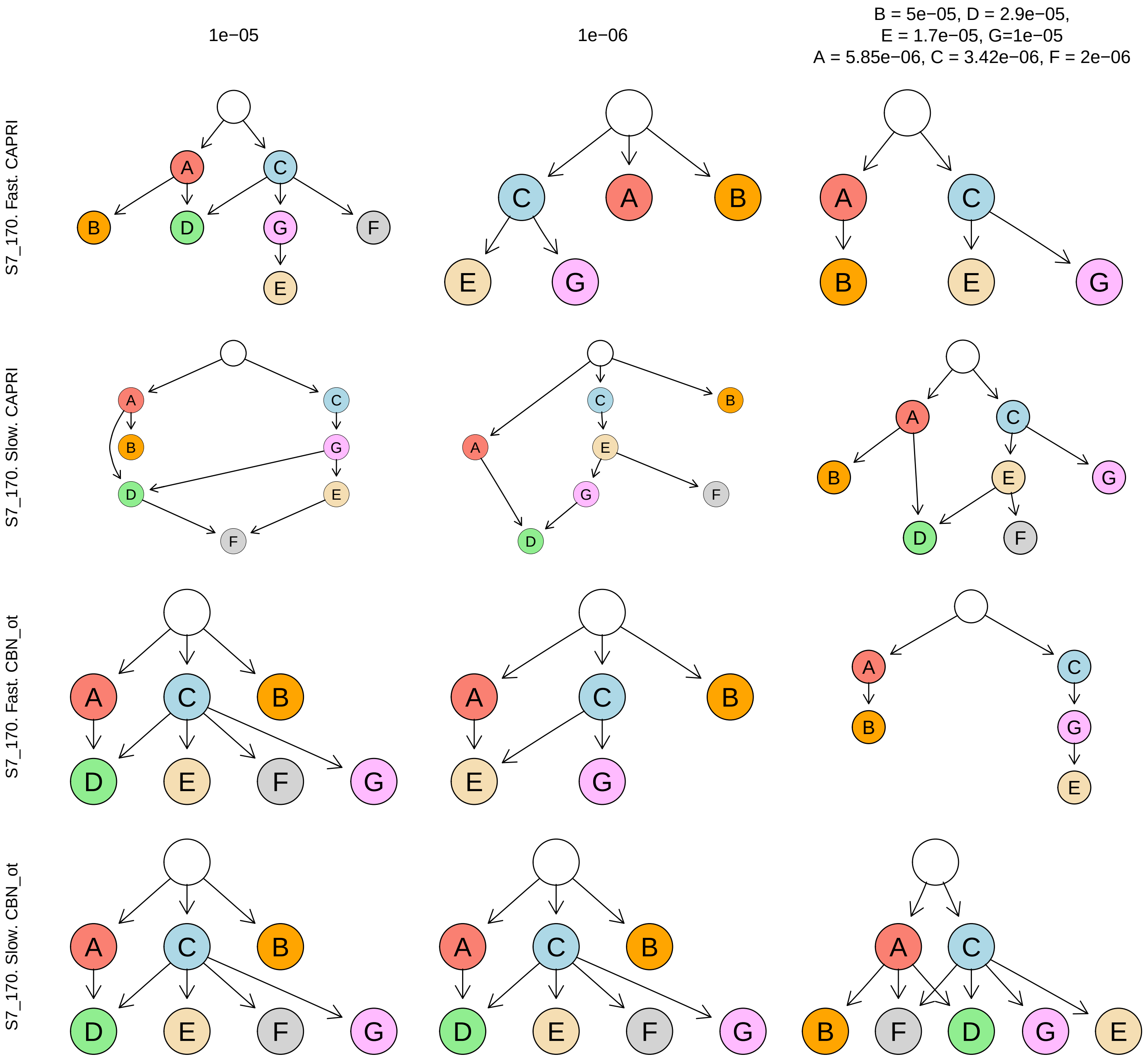

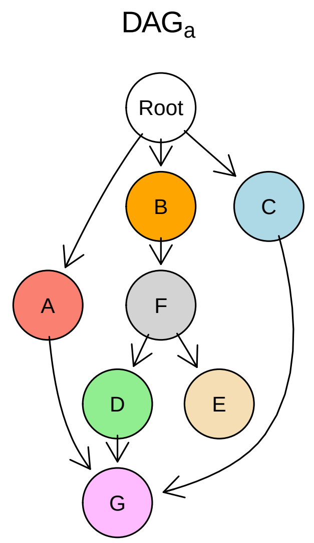

ID	Accessible Genot.	Discrep. DAG_a
S7_171	16	9

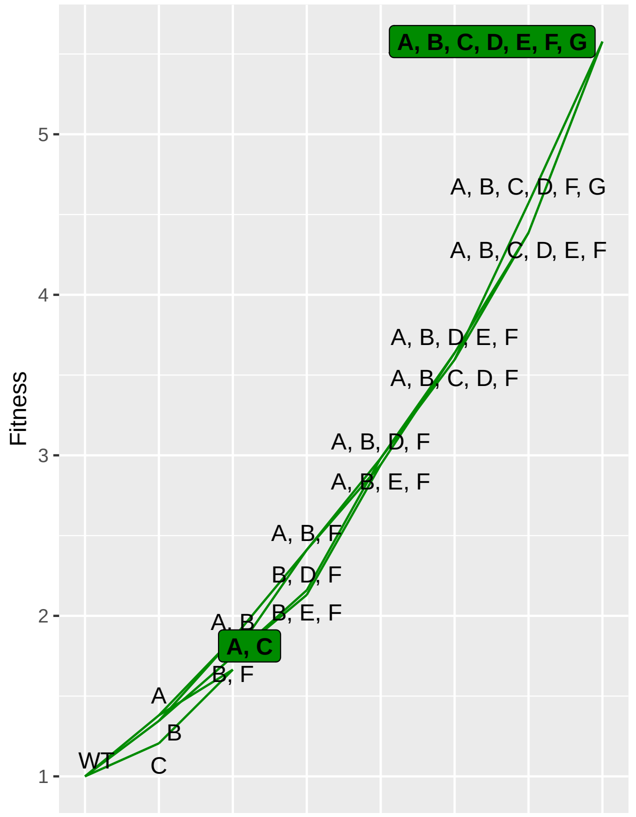

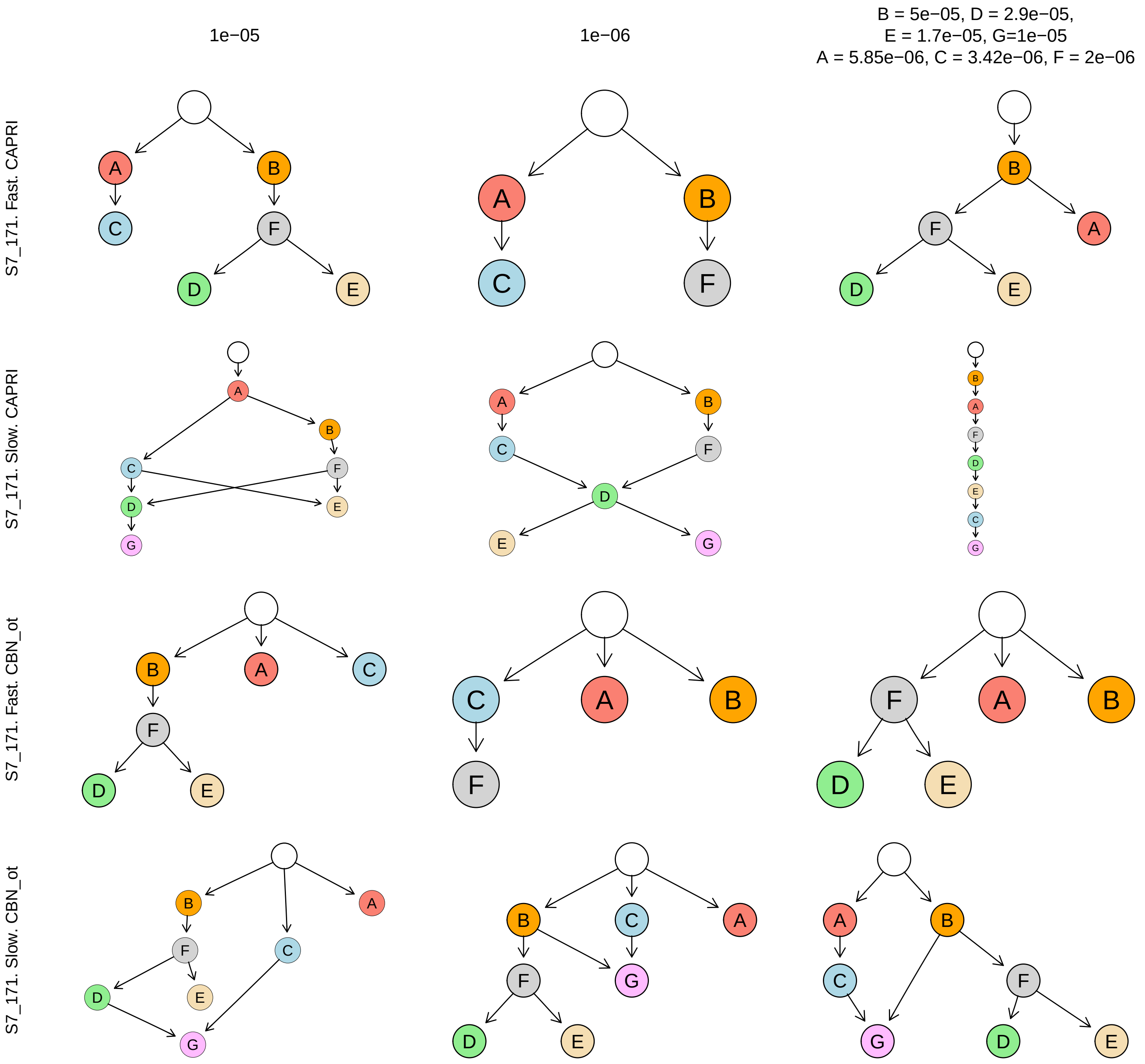

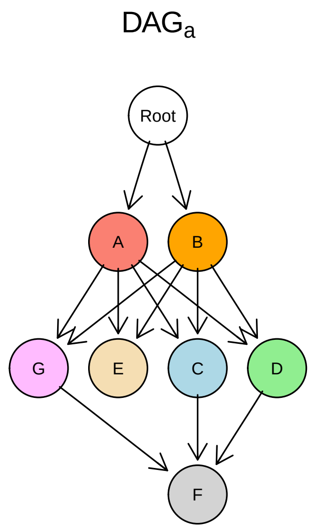

ID	Accessible Genot.	Discrep. DAG_a
S7_172	13	7

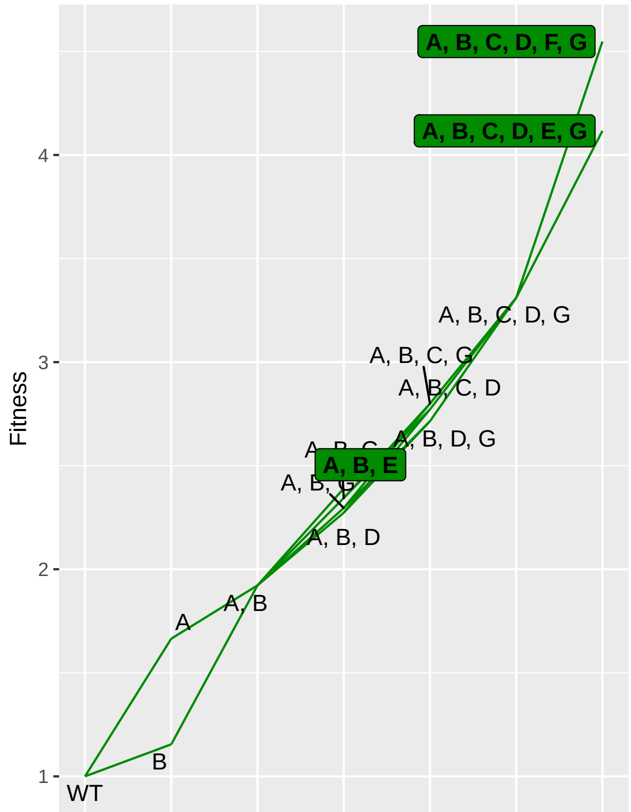

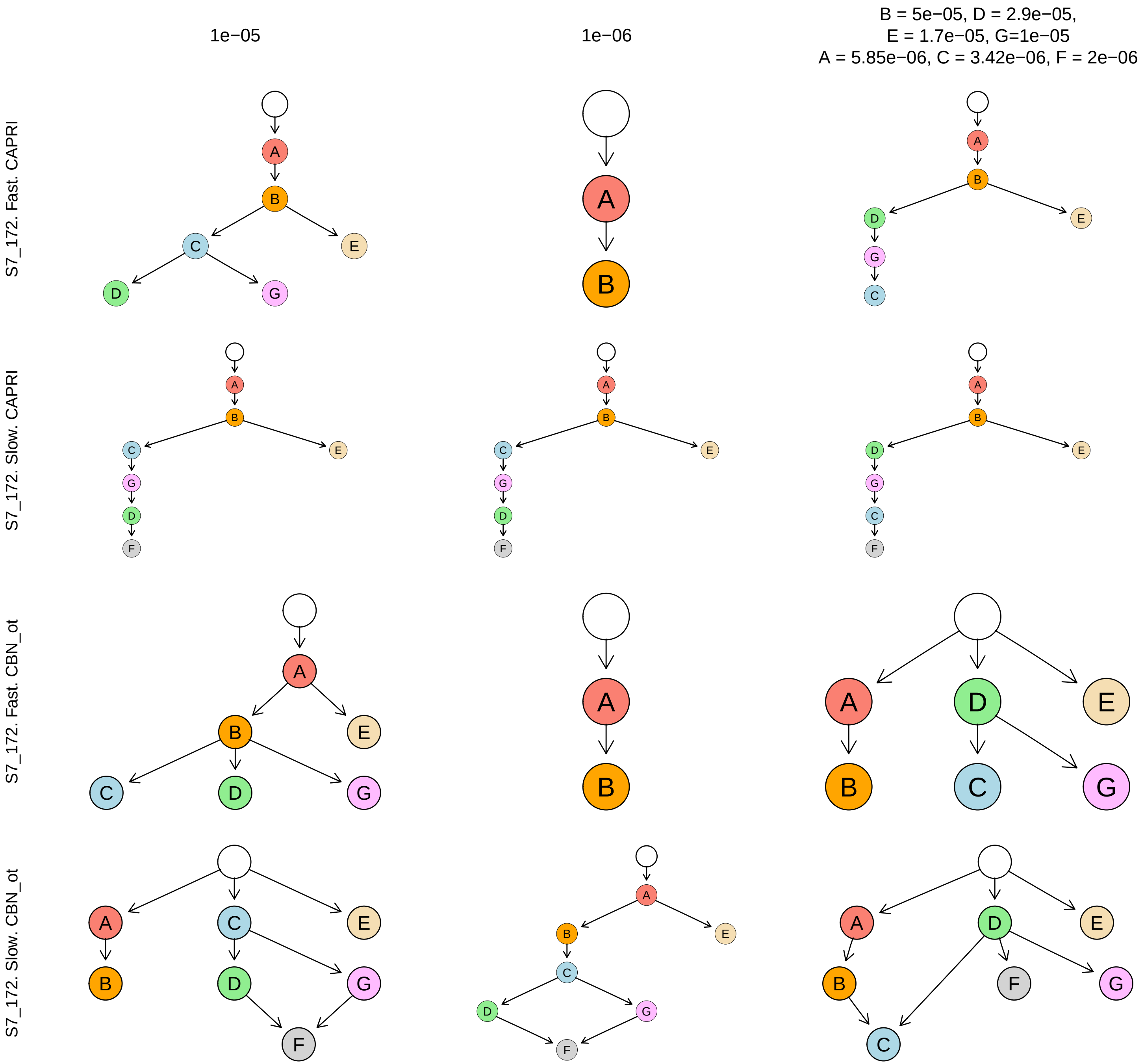

DAG$_a$

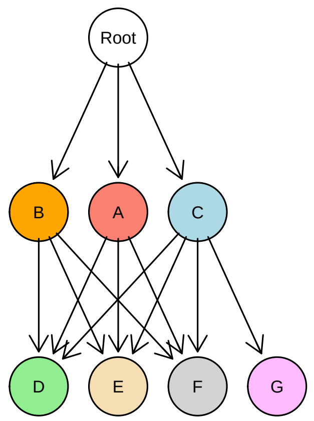

ID	Accessible Genot.	Discrep. DAG_a
S7_173	22	3

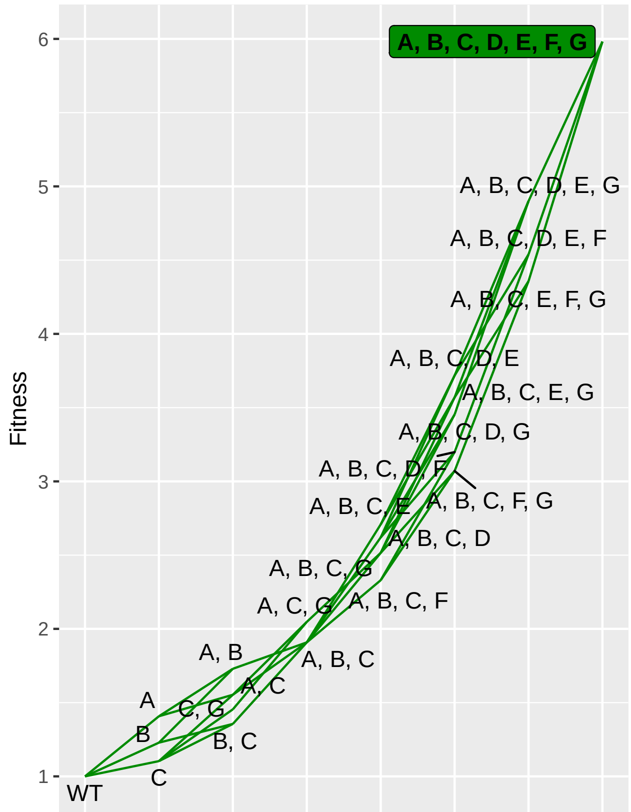

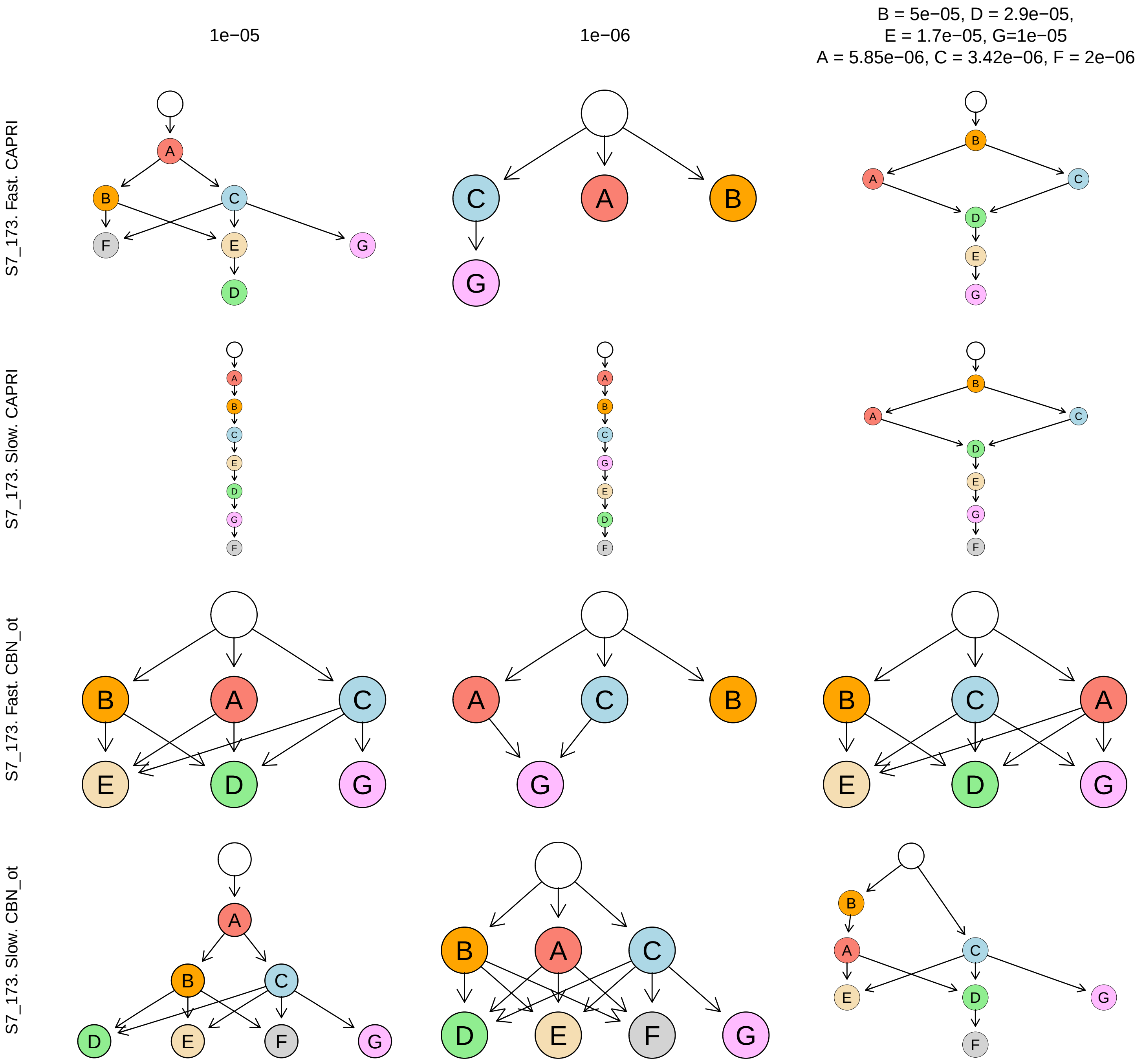

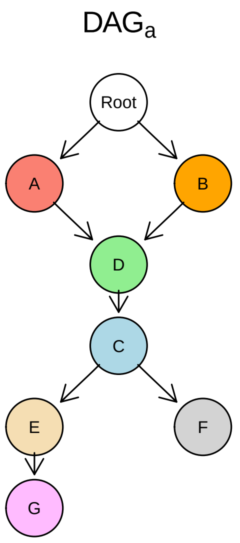

ID	Accessible Genot.	Discrep. DAG_a
S7_174	9	1

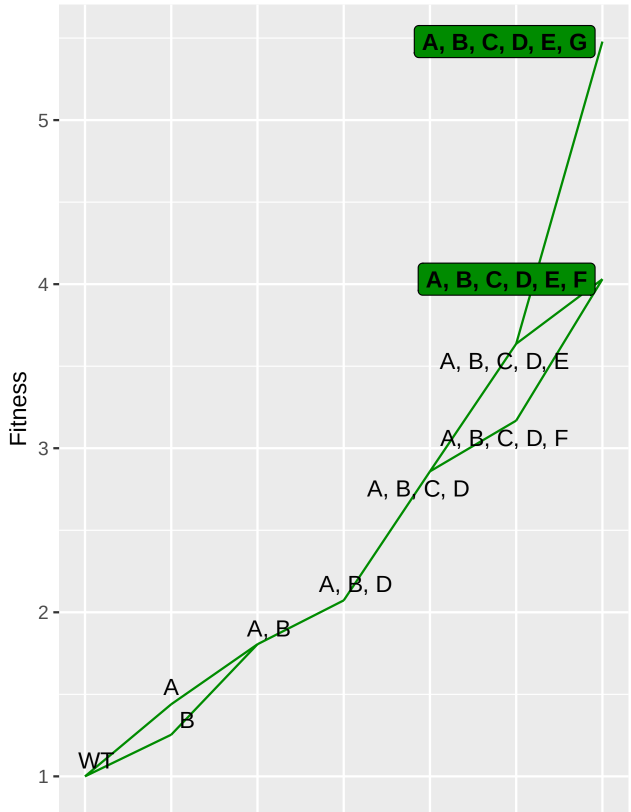

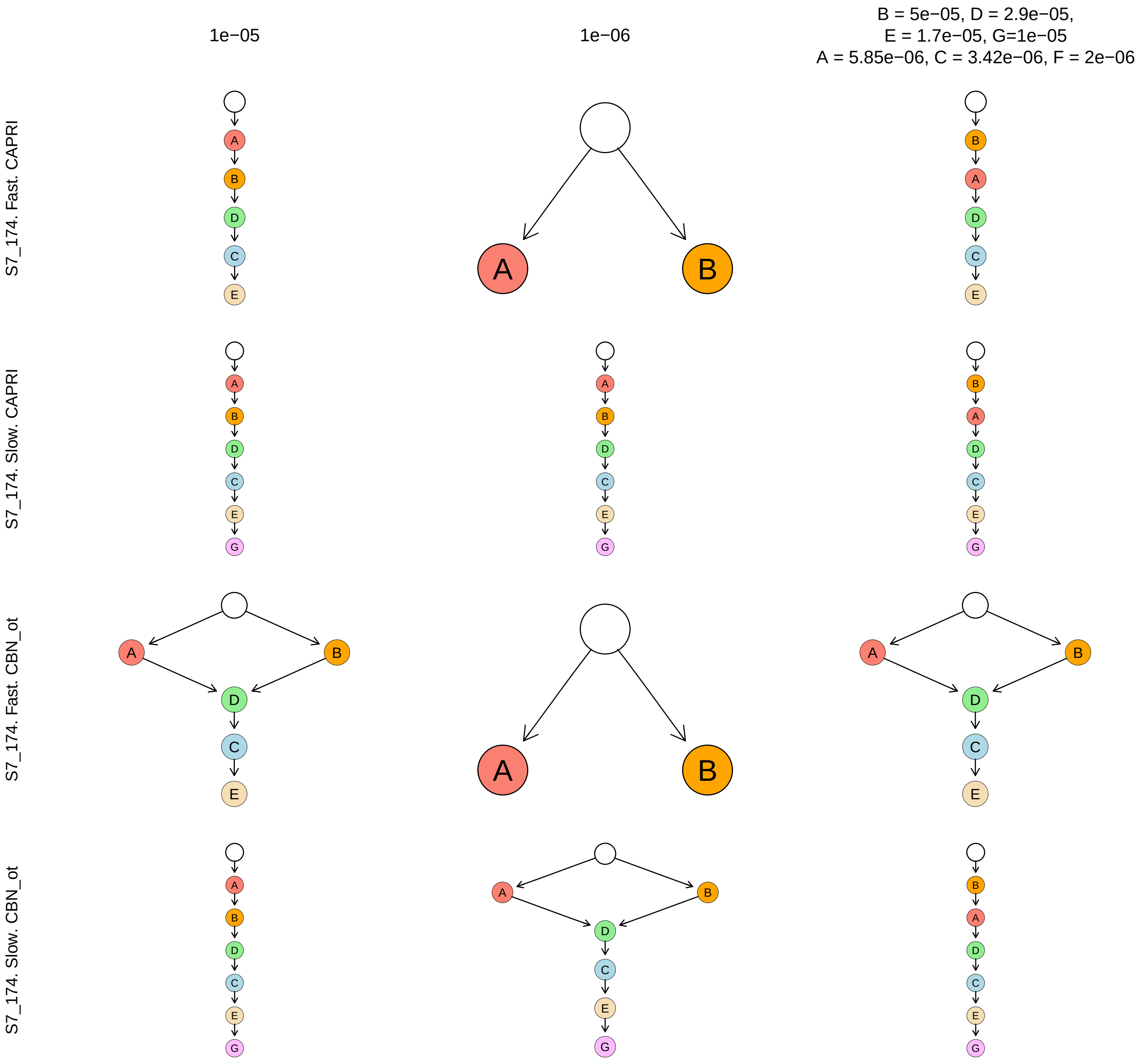

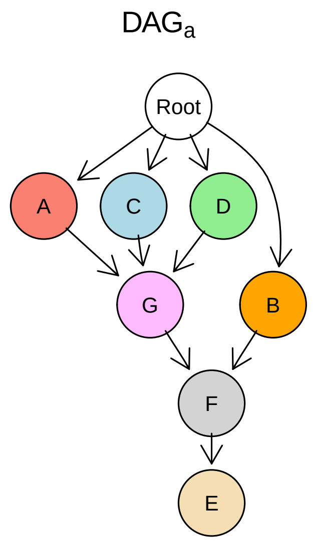

ID	Accessible Genot.	Discrep. DAG_a
S7_175	11	8

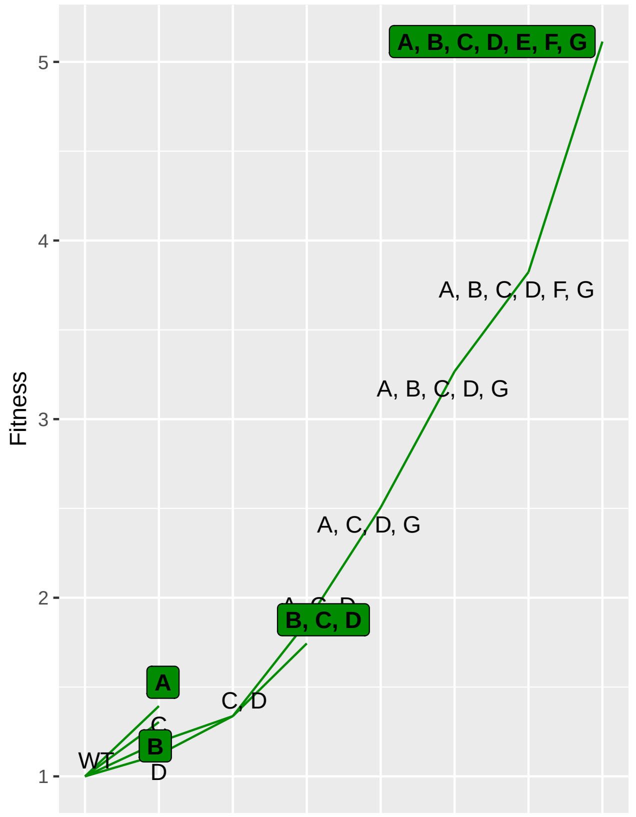

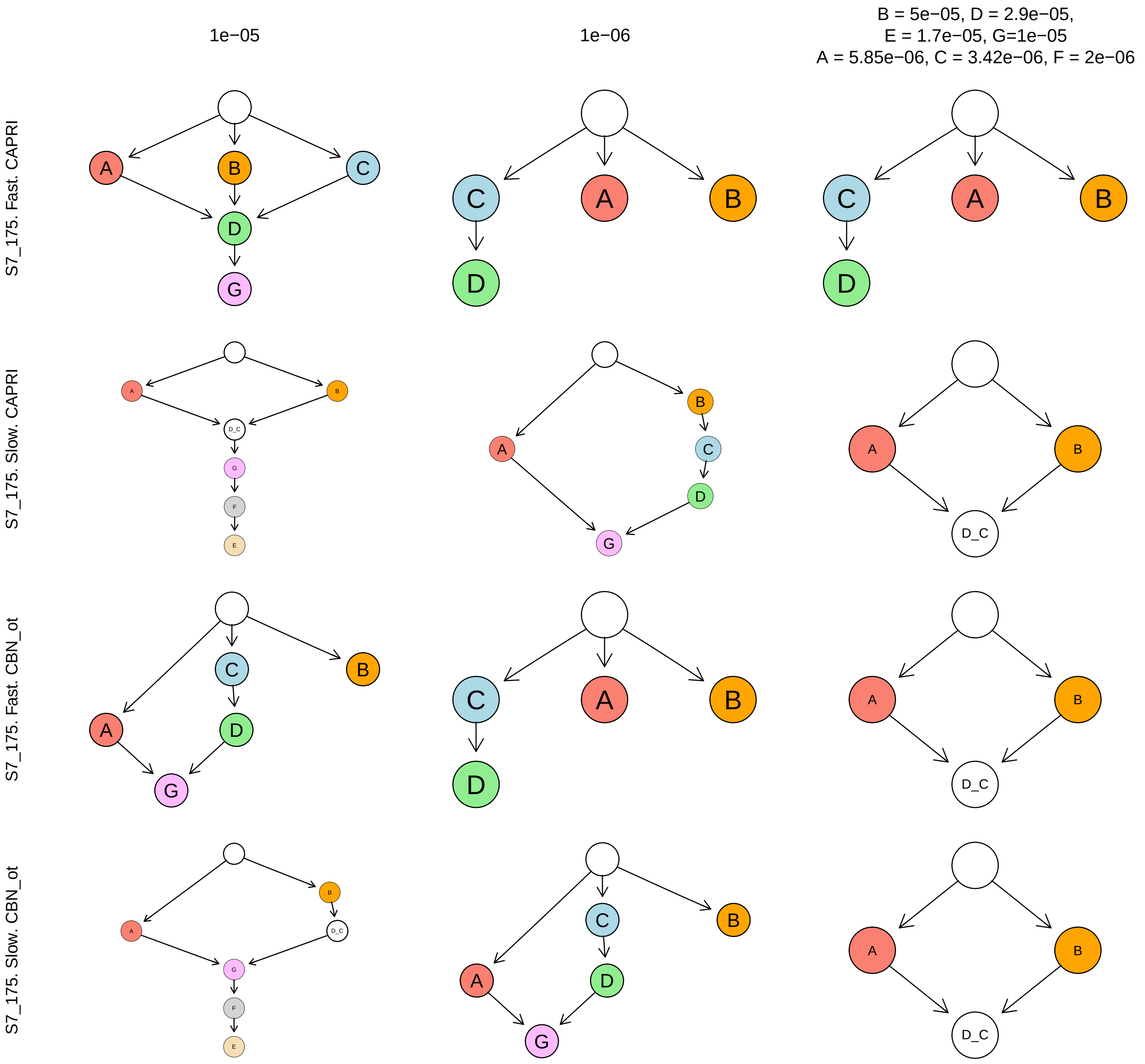

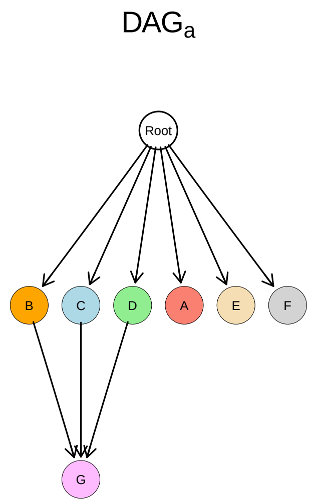

ID	Accessible Genot.	Discrep. DAG_a
S7_176	51	24

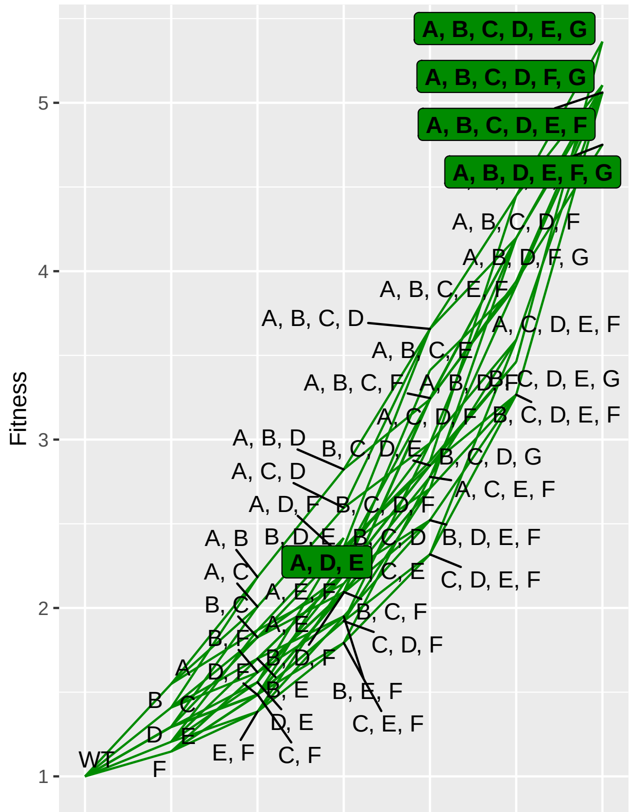

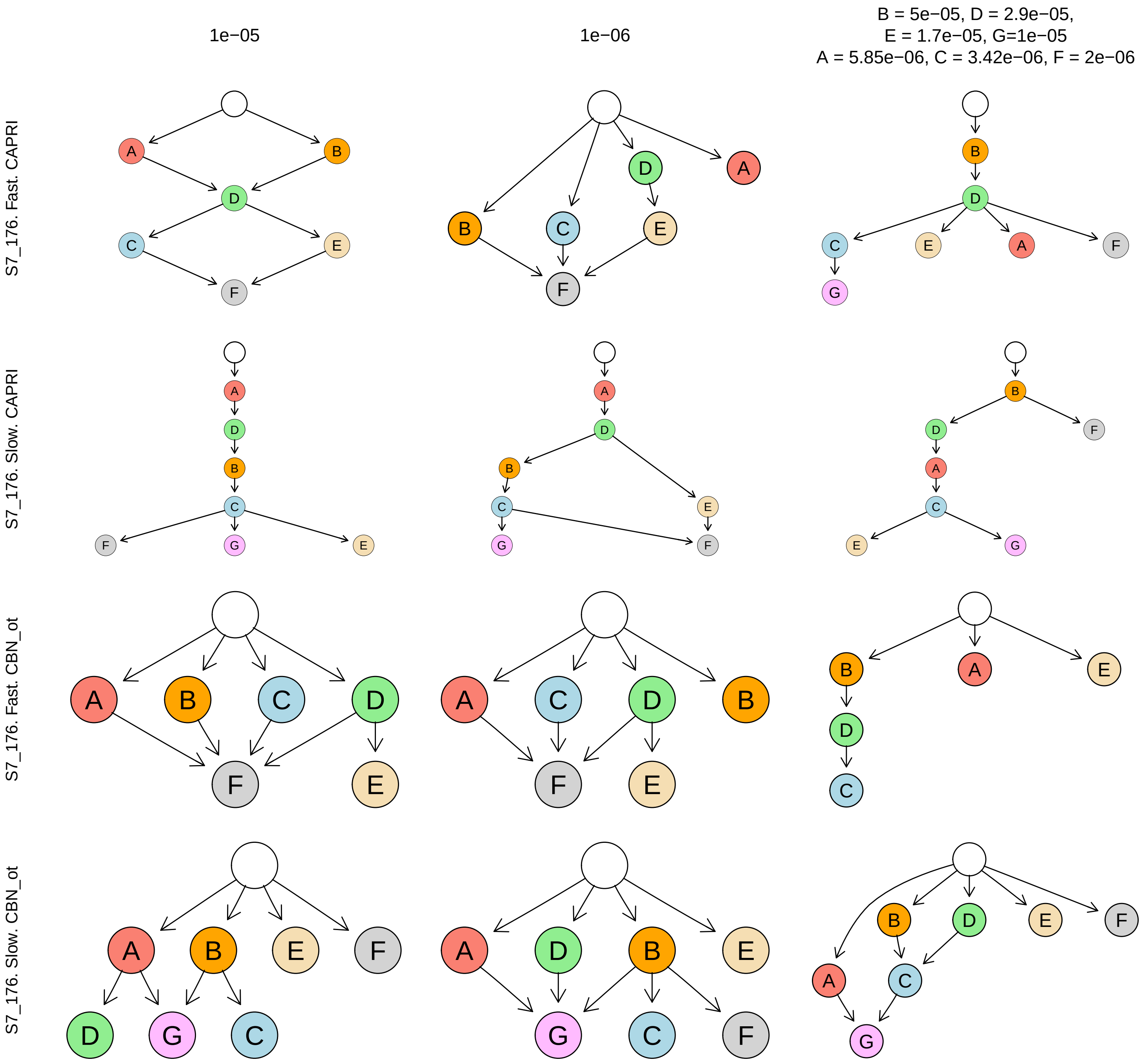

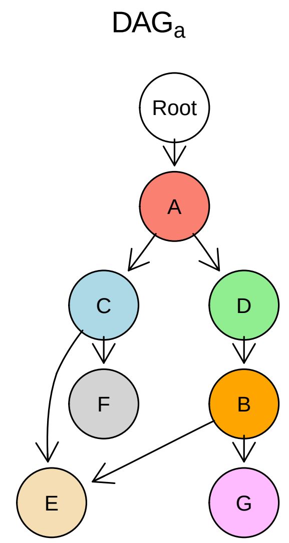

ID	Accessible Genot.	Discrep. DAG_a
S7_177	16	2

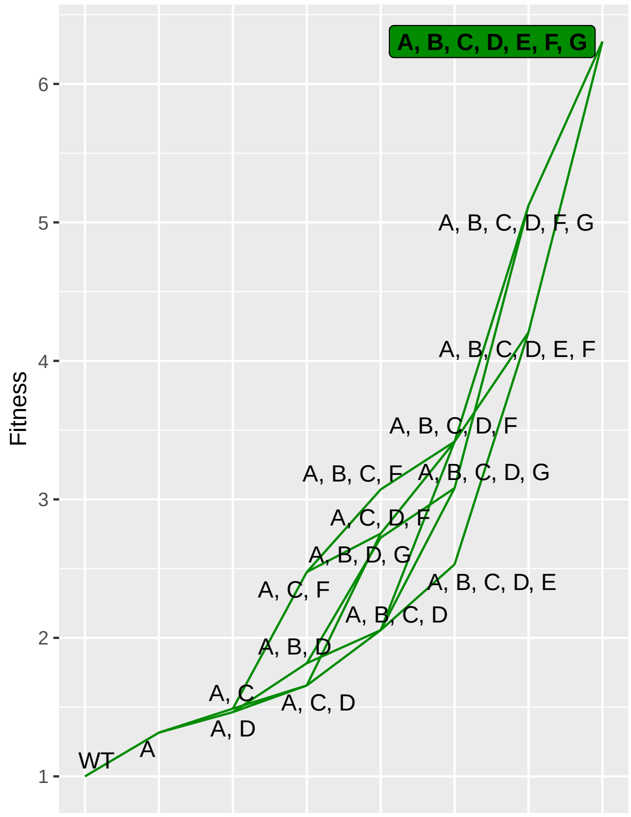

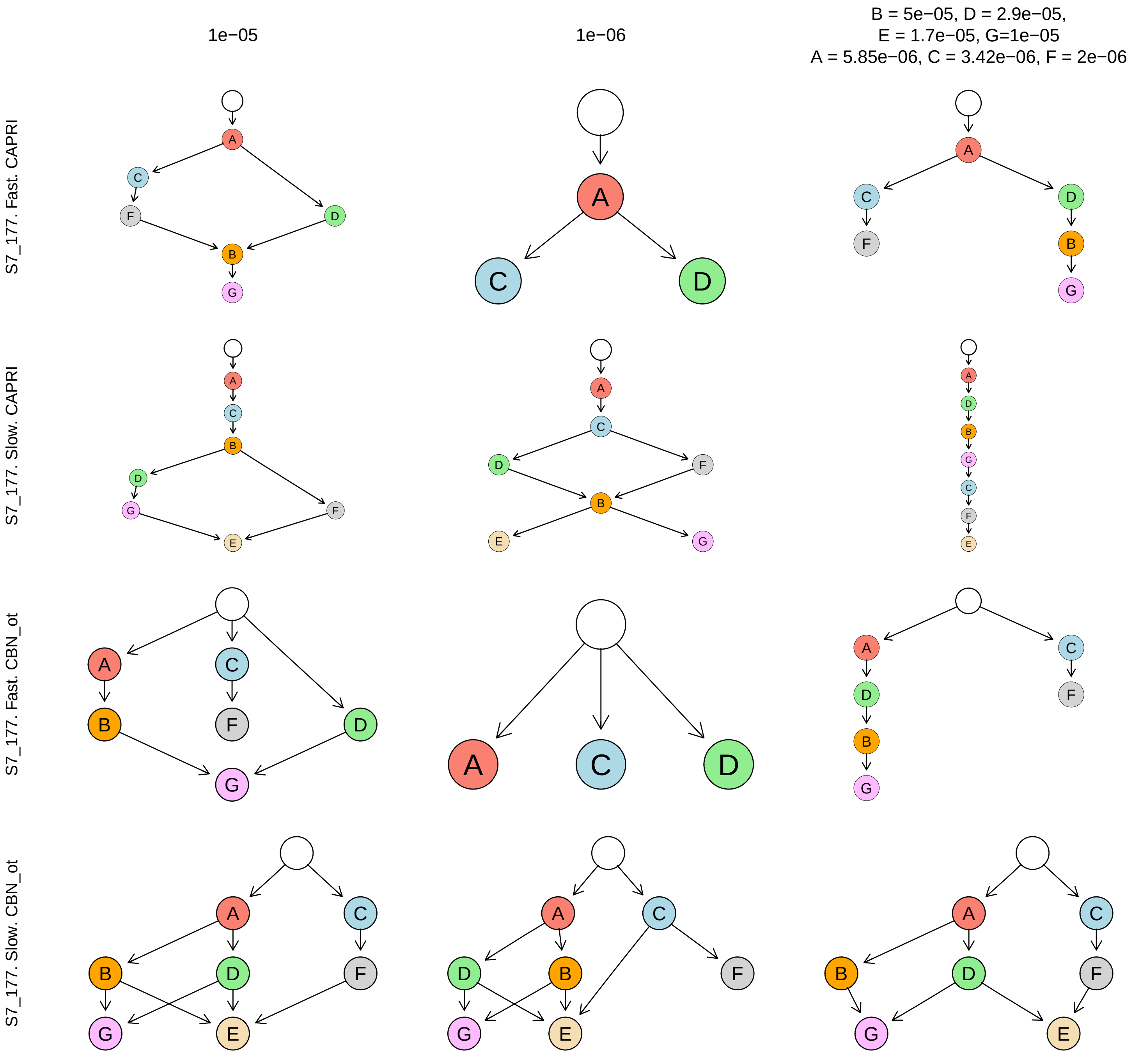

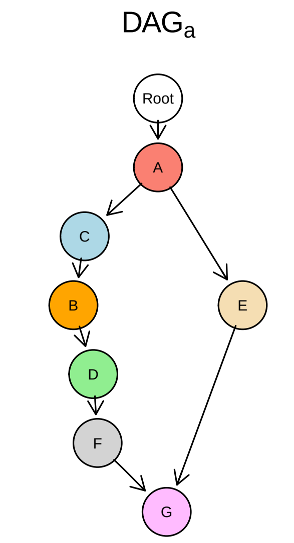

ID	Accessible Genot.	Discrep. DAG_a
S7_178	11	4

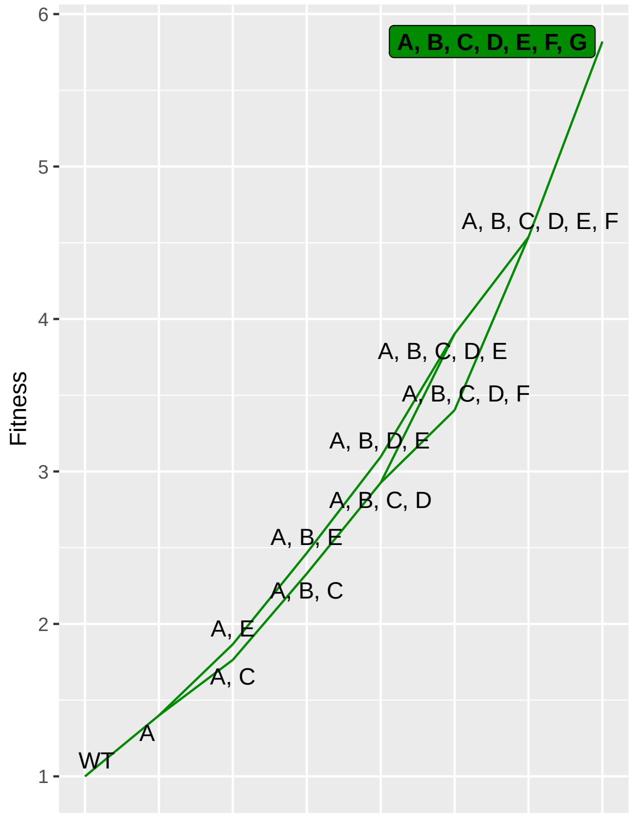

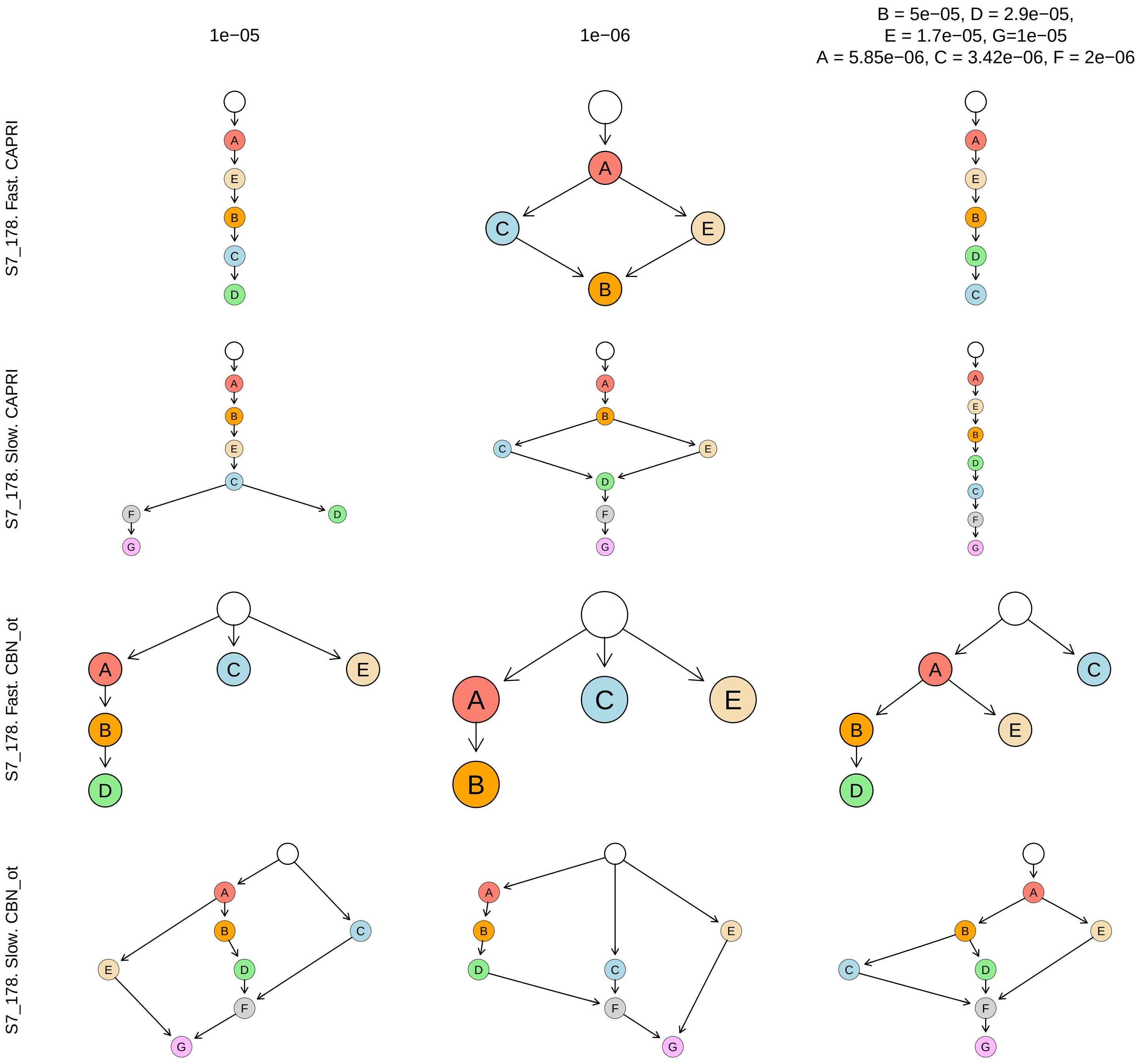

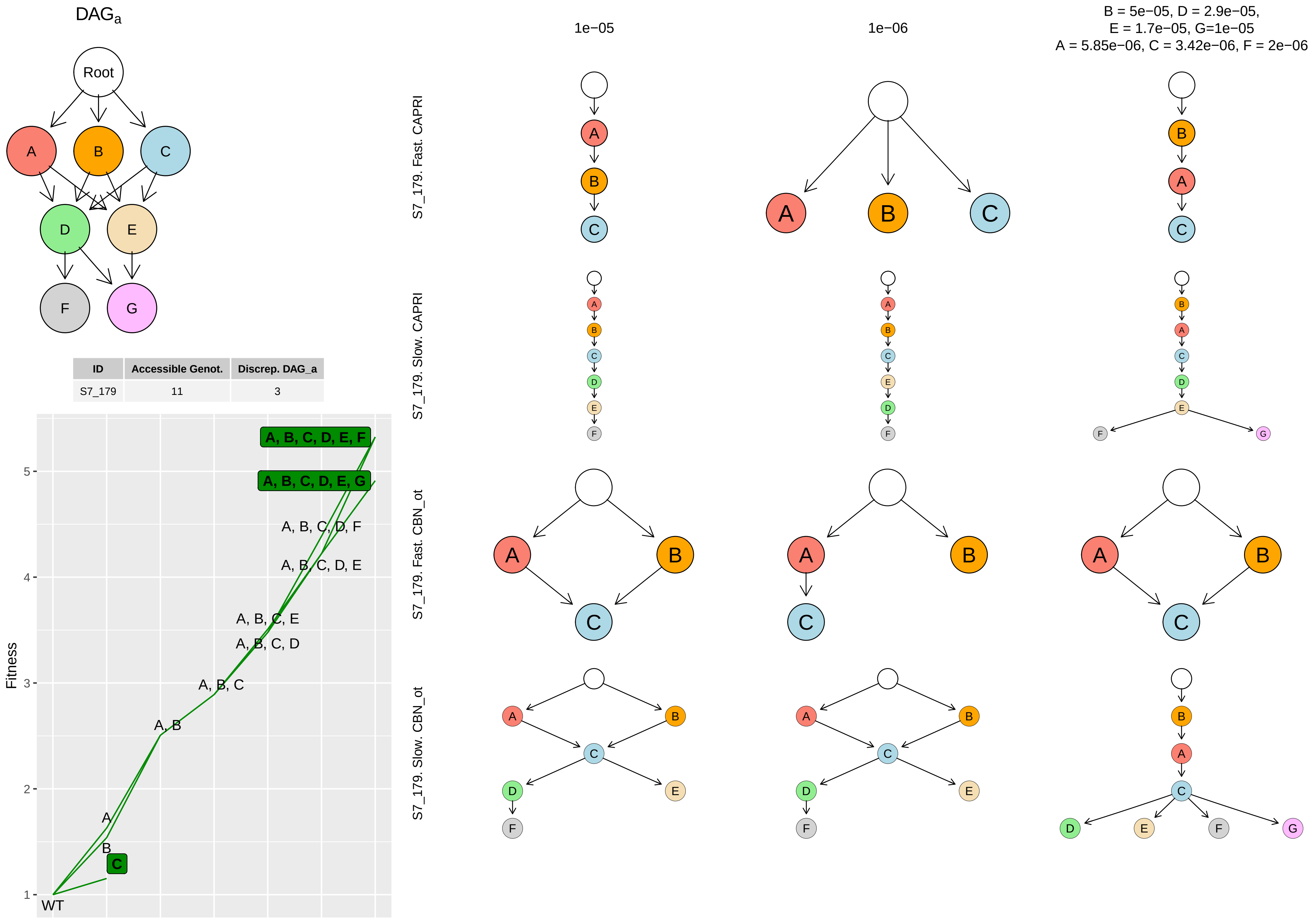

DAGa
Root
A
B
C
D
E
F
G
ID
Accessible Genot.
Discrep. DAG_a
S7_179
11
3
Fitness
WT
C
A
B
A, B
A, B, C
A, B, C, D
A, B, C, E
A, B, C, D, E
A, B, C, D, F
A, B, C, D, E, G
A, B, C, D, E, F
1e−05
1e−06
B = 5e−05, D = 2.9e−05,
E = 1.7e−05, G=1e−05
A = 5.85e−06, C = 3.42e−06, F = 2e−06
S7_179. Fast. CAPRI
S7_179. Slow. CAPRI
S7_179. Fast. CBN_ot
S7_179. Slow. CBN_ot

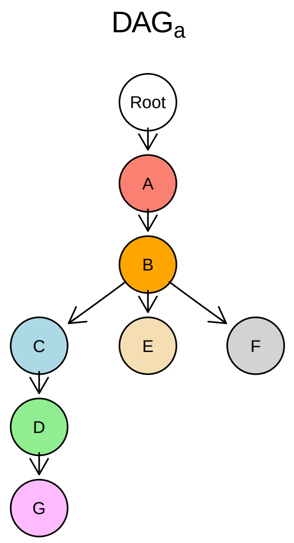

ID	Accessible Genot.	Discrep. DAG_a
S7_180	21	8

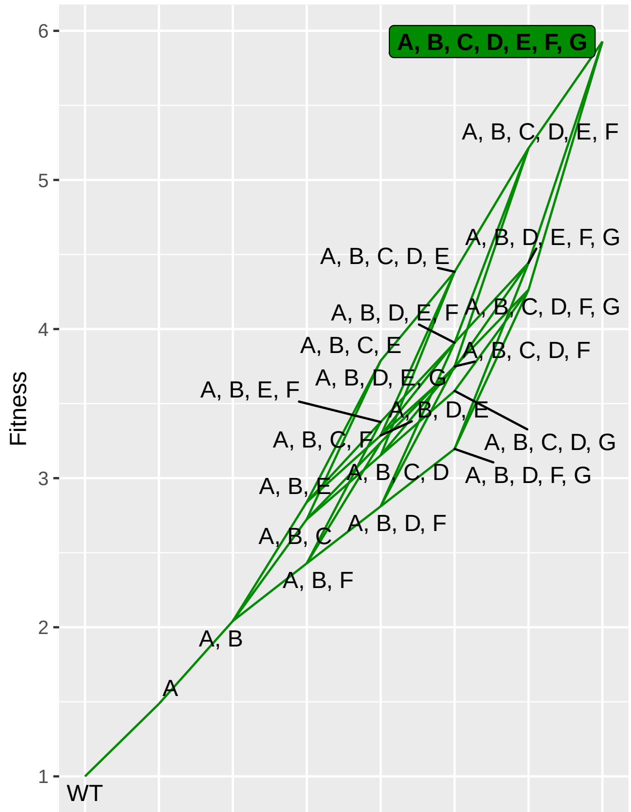

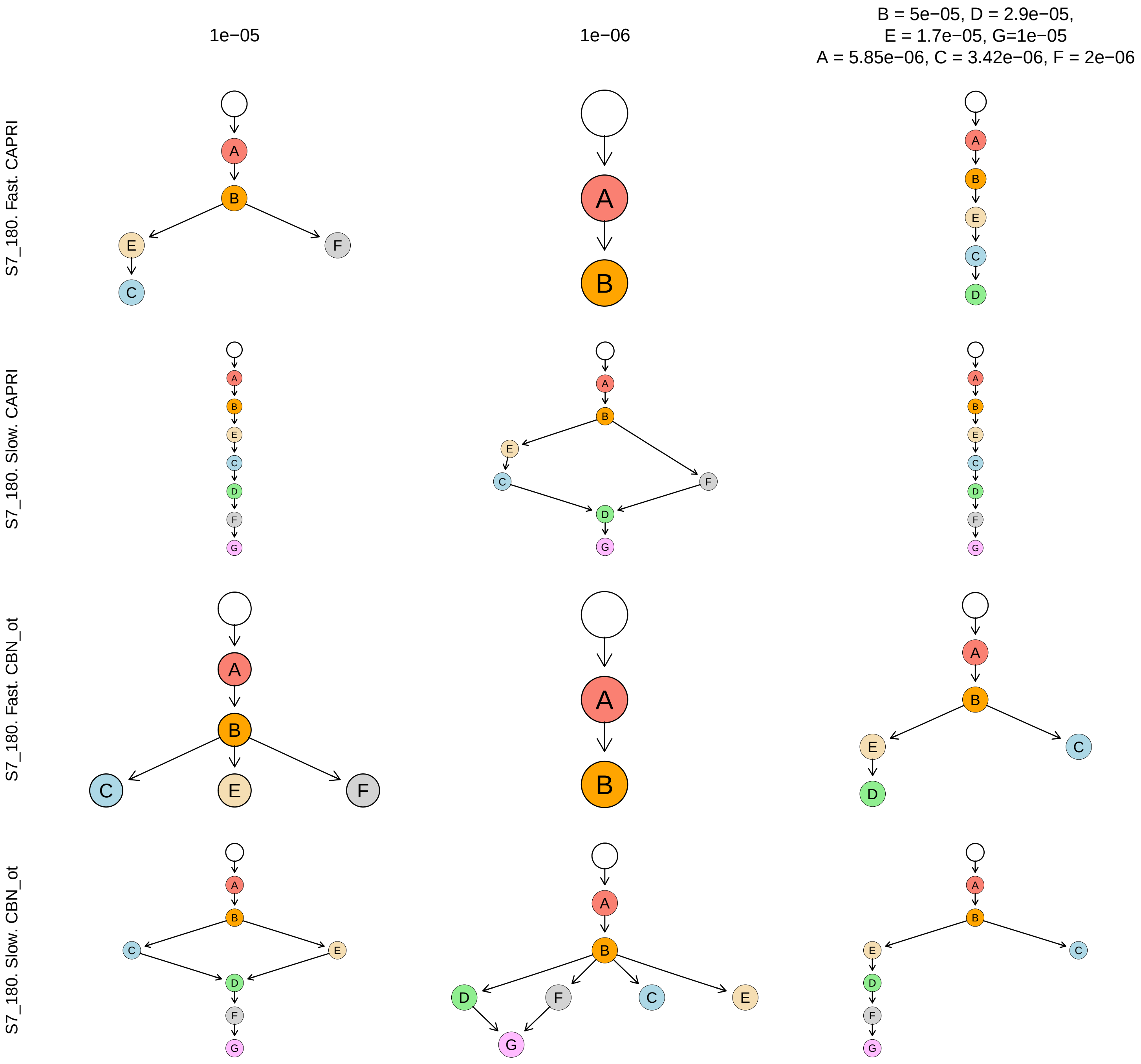

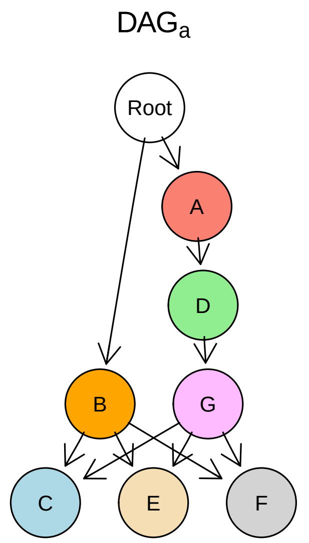

ID	Accessible Genot.	Discrep. DAG_a
S7_181	8	6

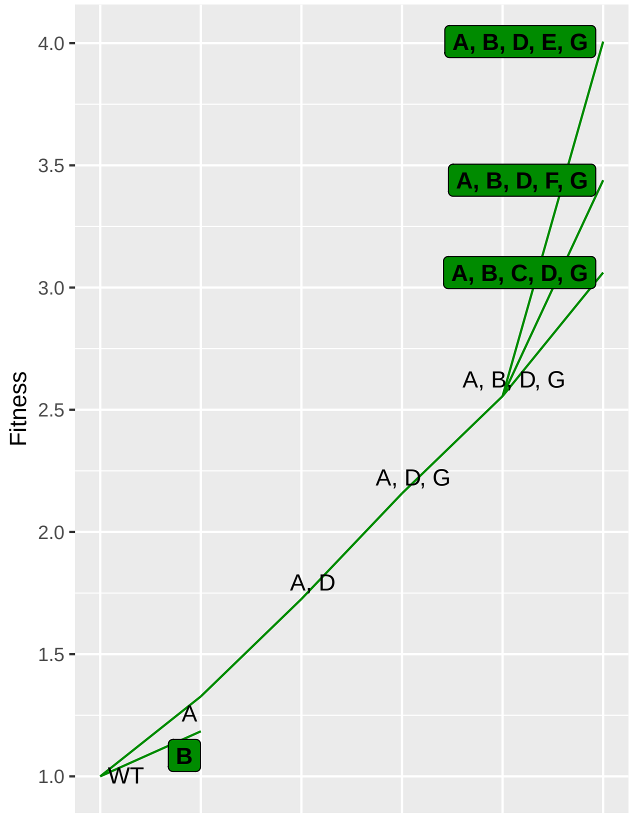

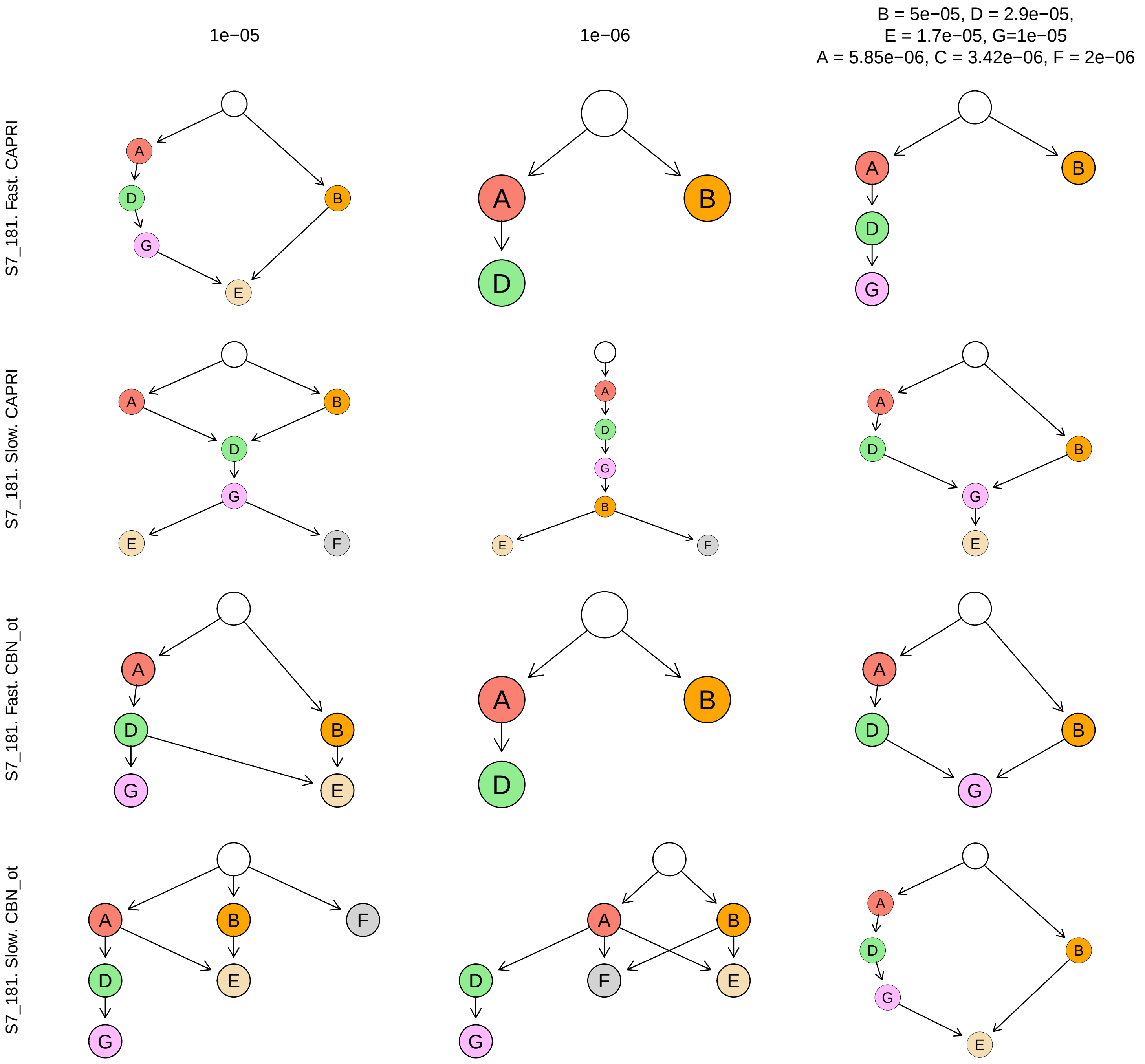

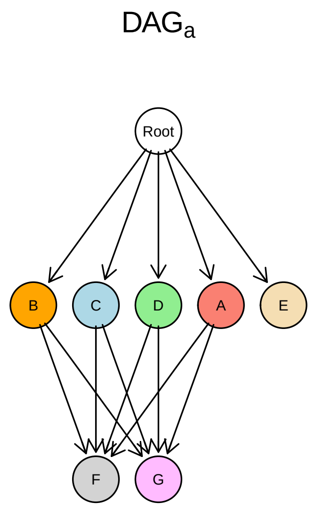

ID	Accessible Genot.	Discrep. DAG_a
S7_182	19	18

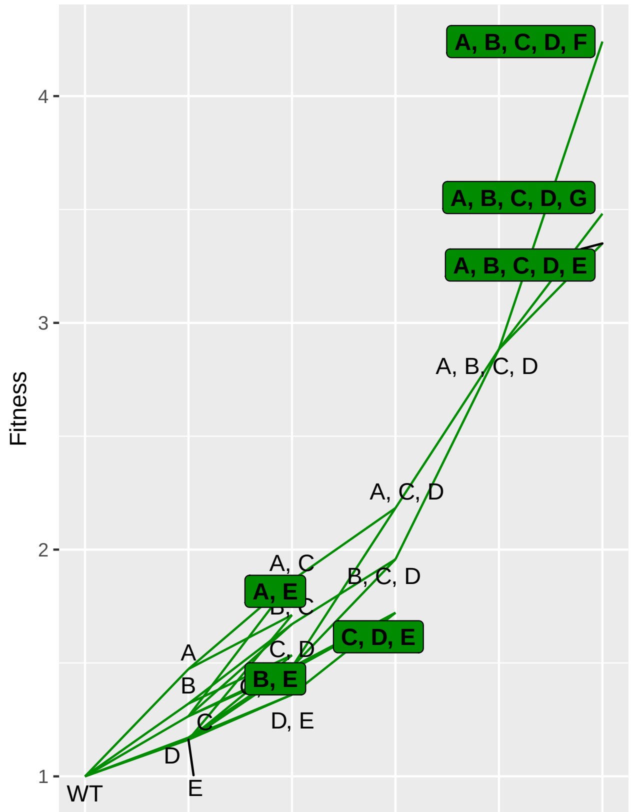

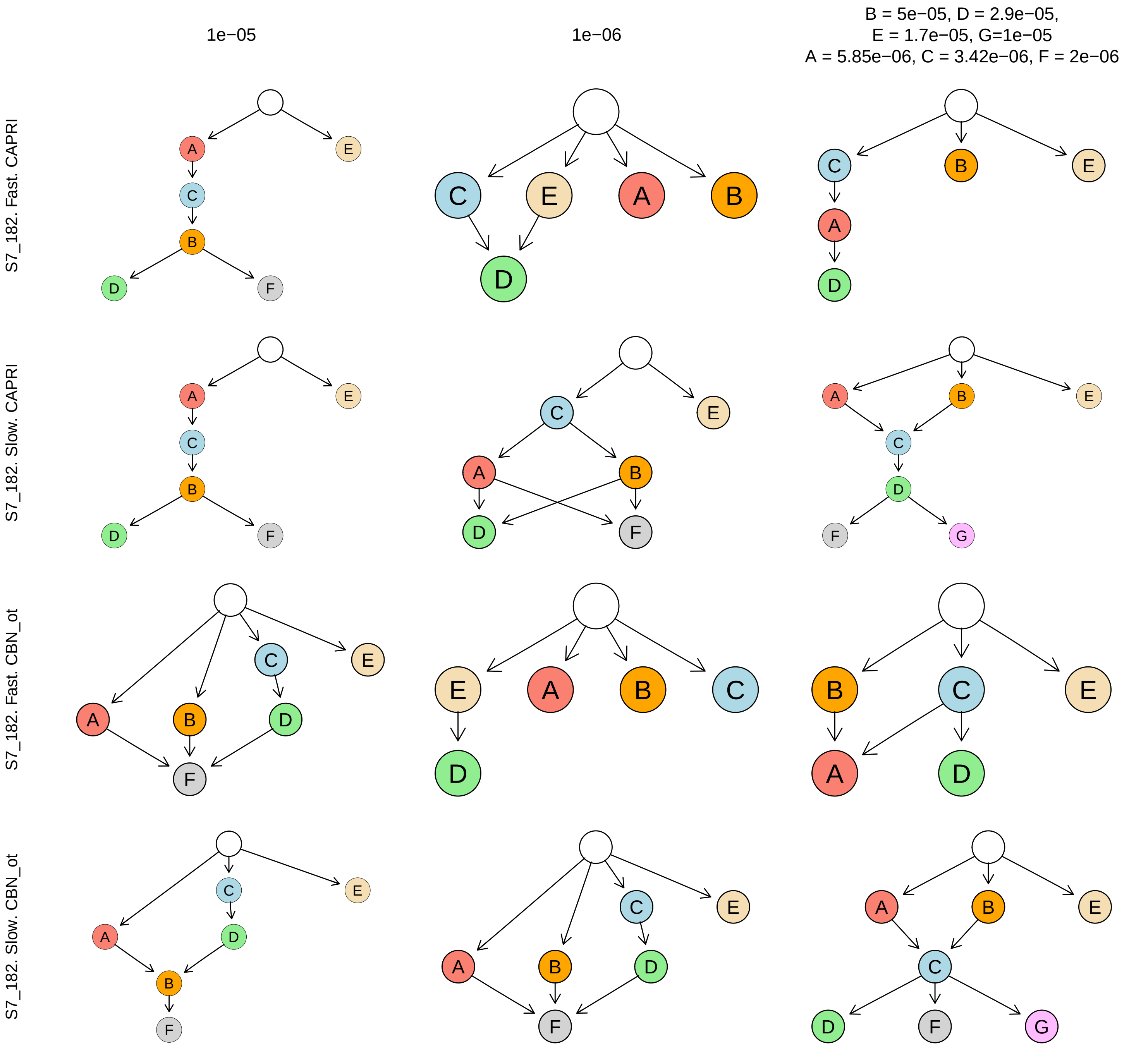

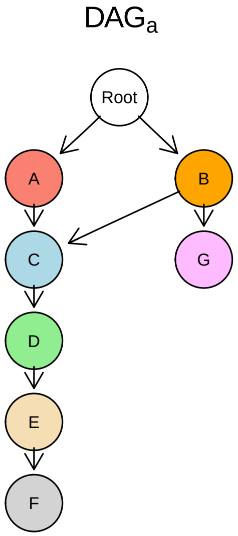

ID	Accessible Genot.	Discrep. DAG_a
S7_183	8	5

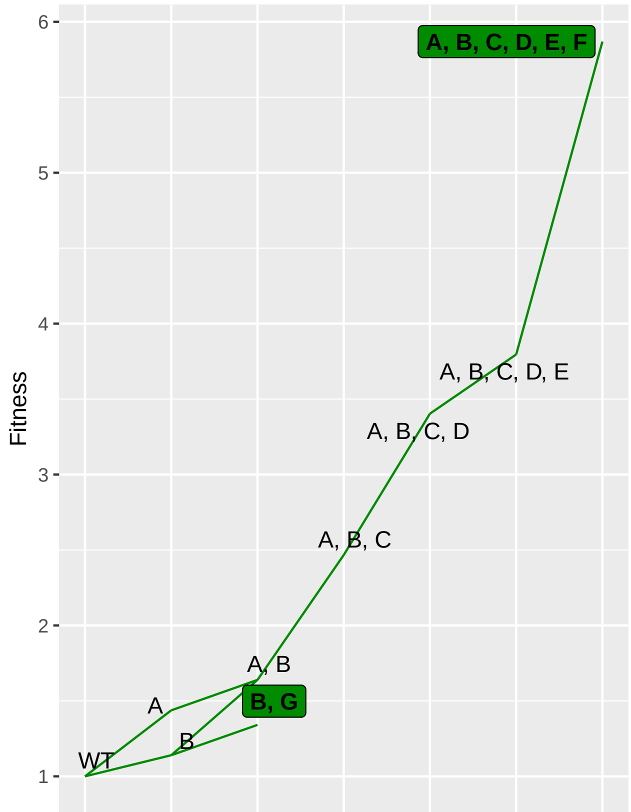

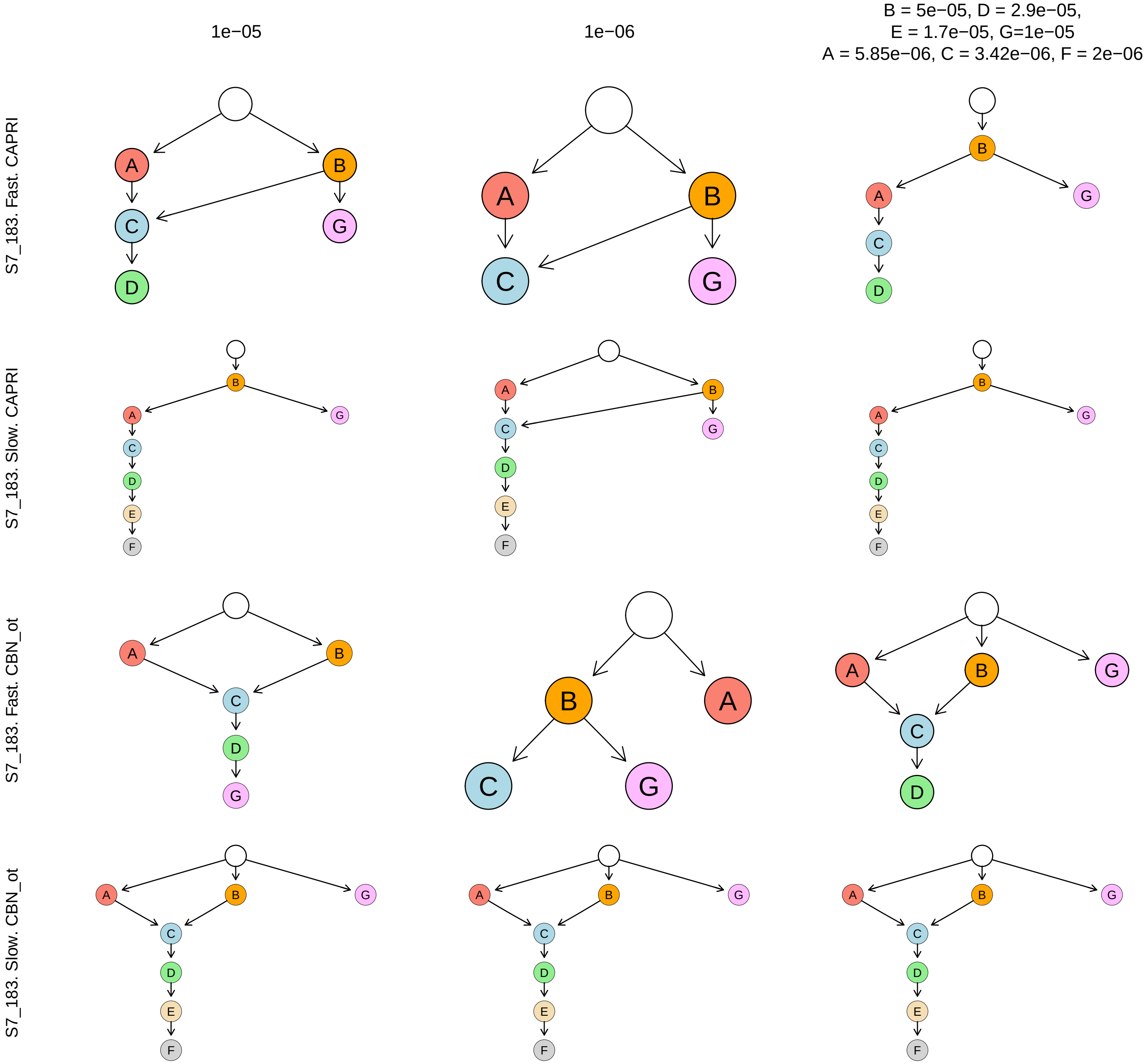

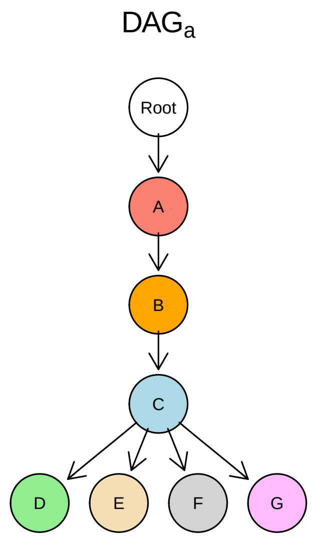

ID	Accessible Genot.	Discrep. DAG_a
S7_184	15	3

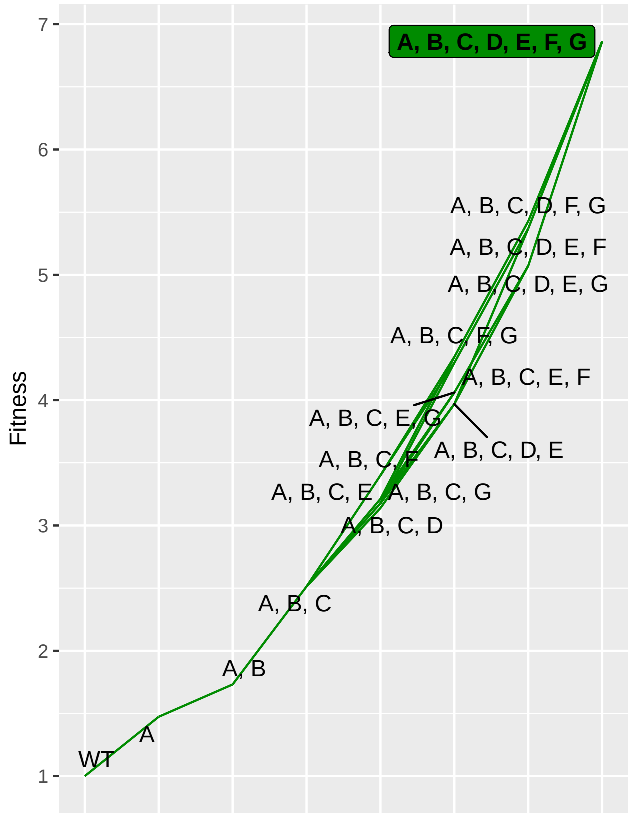

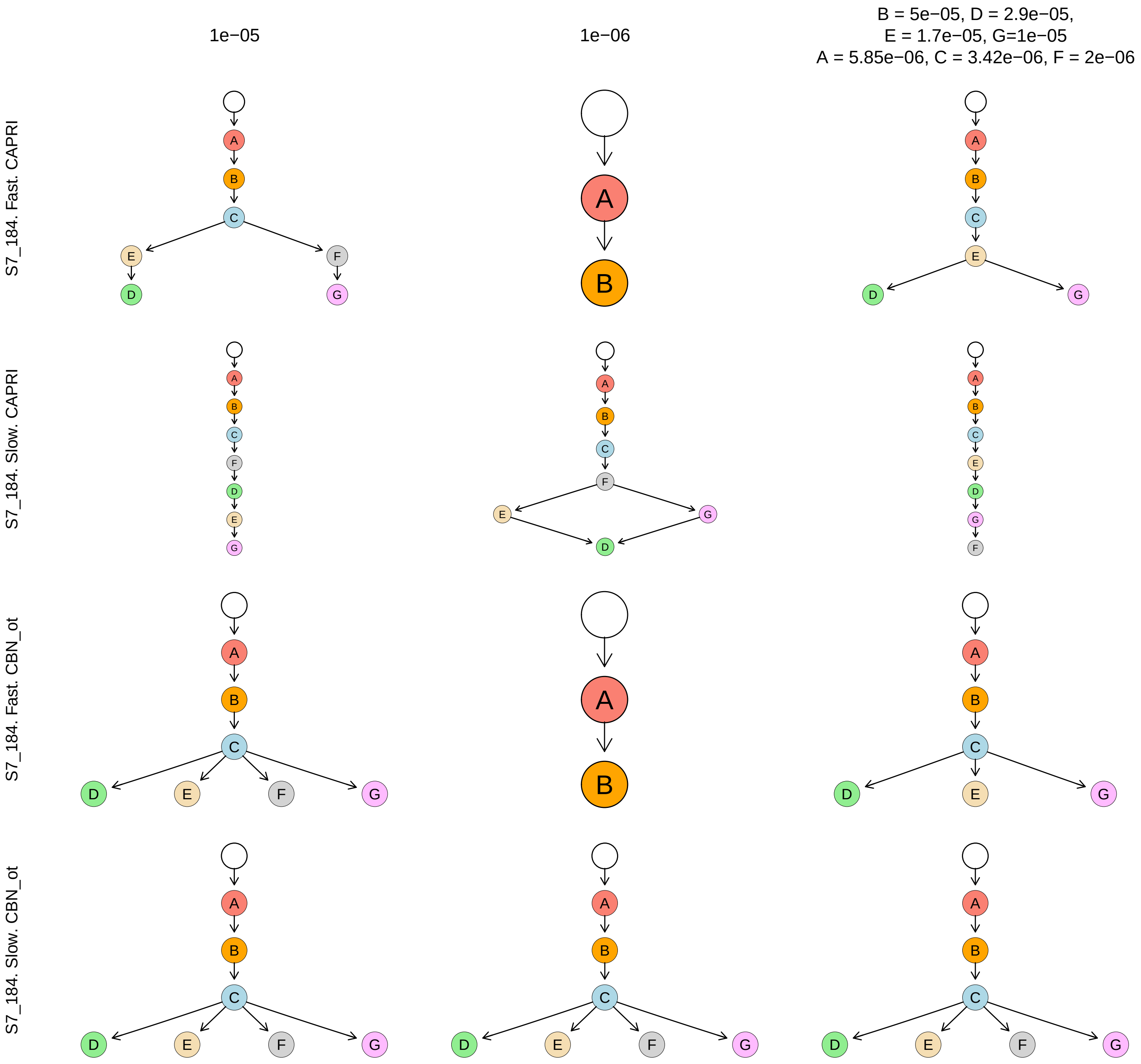

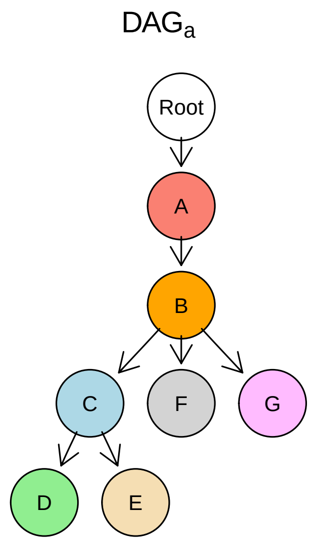

ID	Accessible Genot.	Discrep. DAG_a
S7_185	17	4

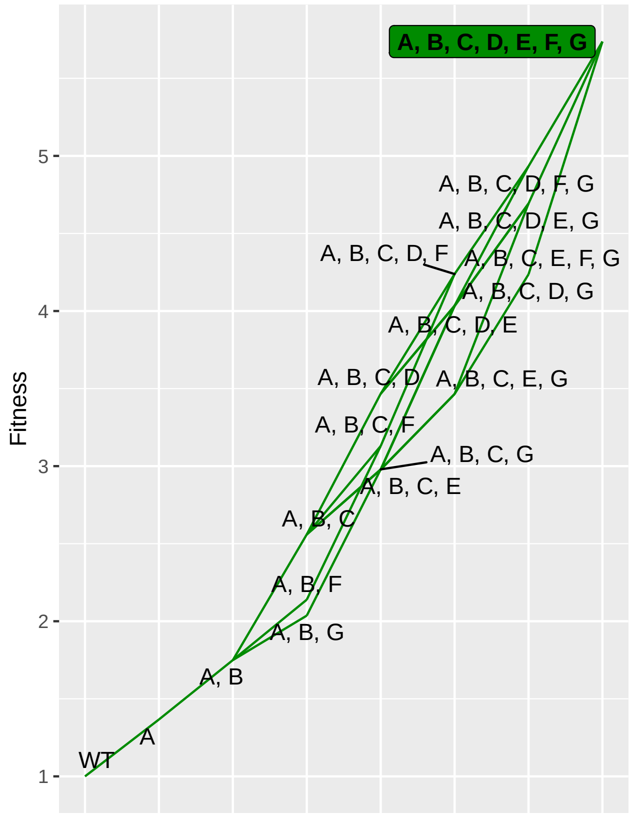

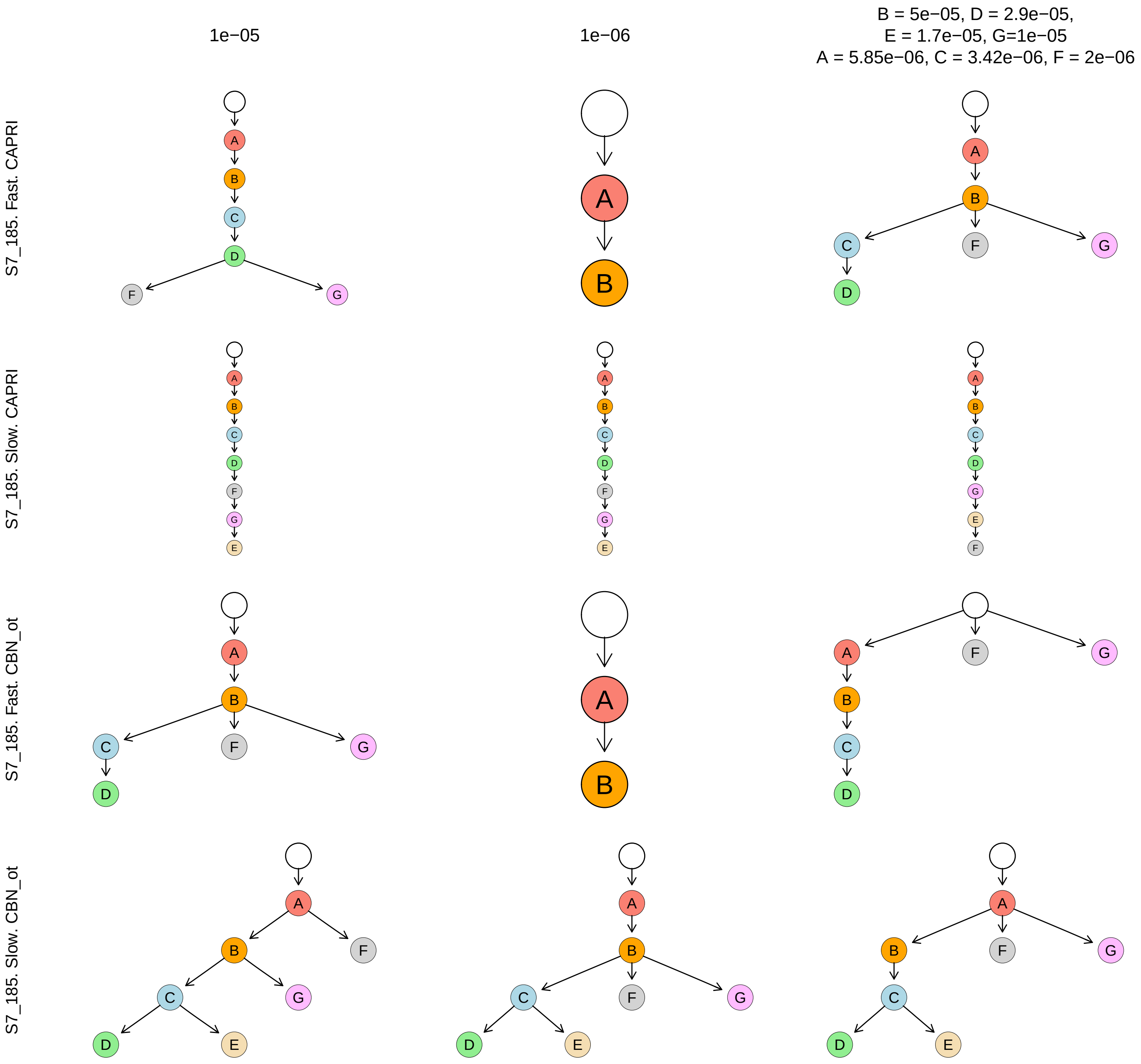

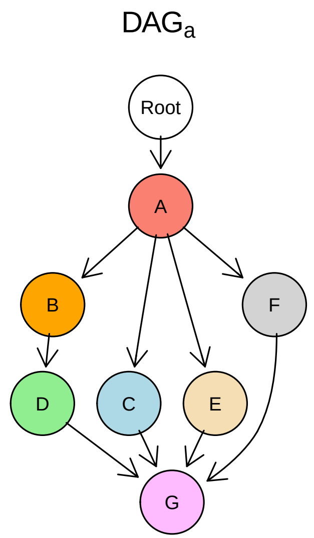

ID	Accessible Genot.	Discrep. DAG_a
S7_186	17	12

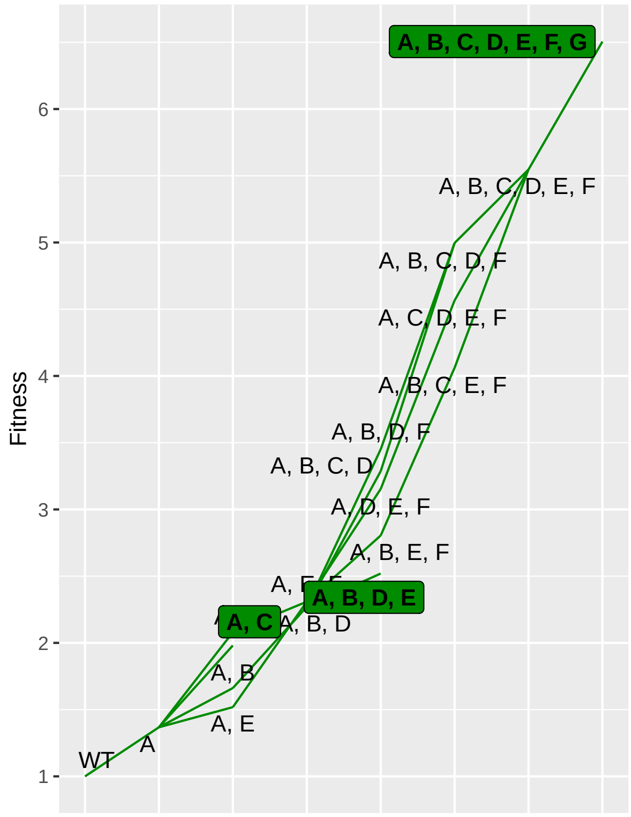

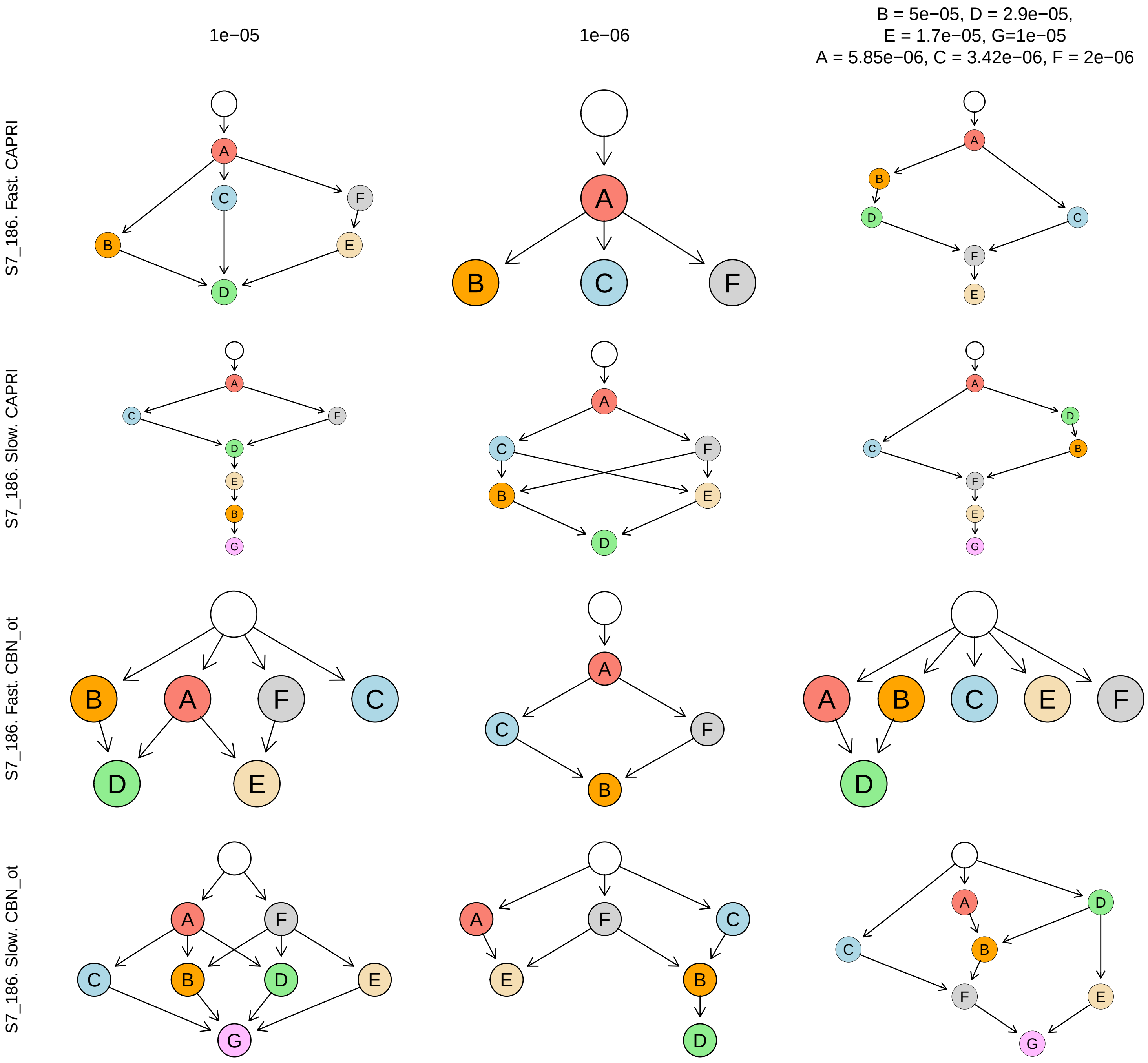

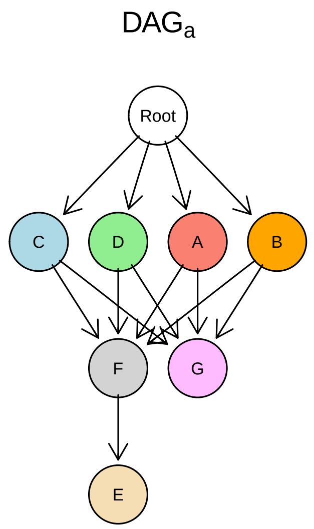

ID	Accessible Genot.	Discrep. DAG_a
S7_187	13	9

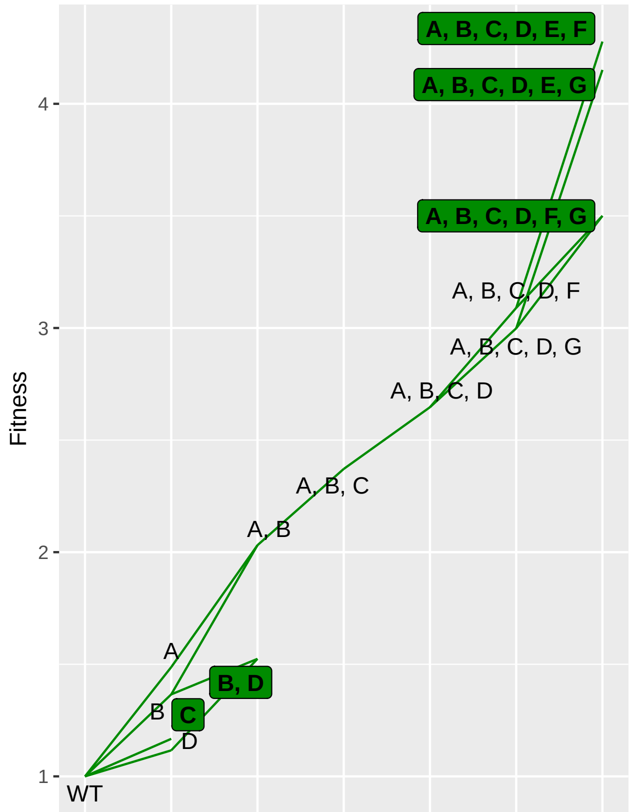

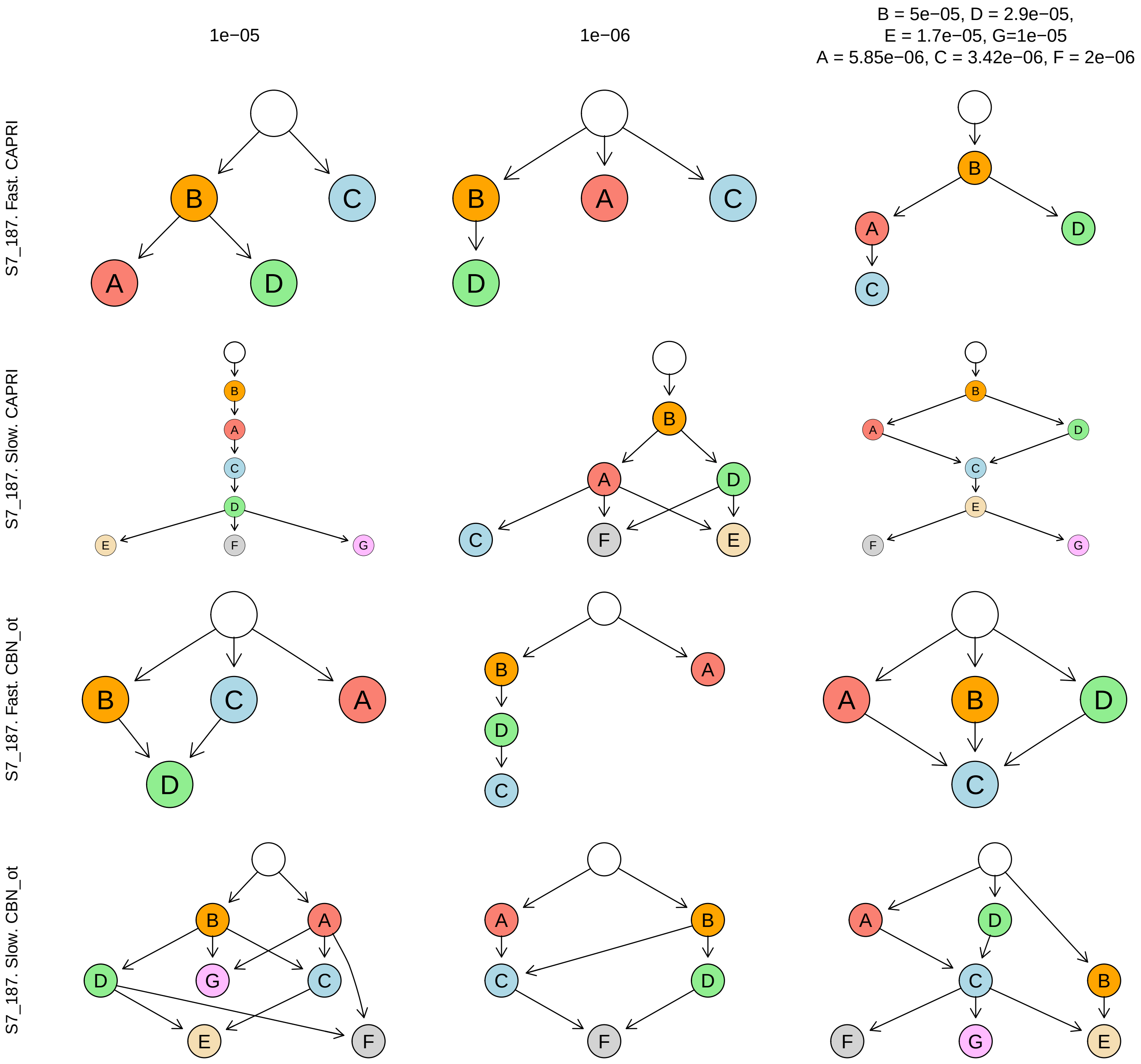

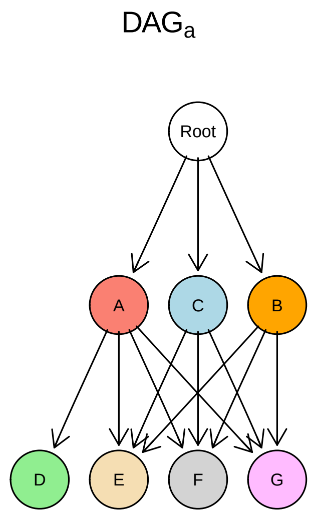

ID	Accessible Genot.	Discrep. DAG_a
S7_188	21	8

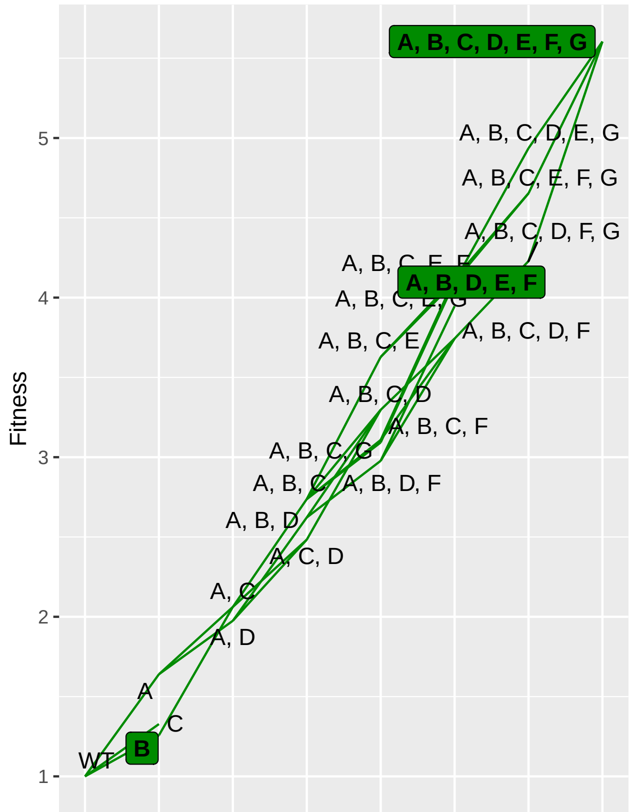

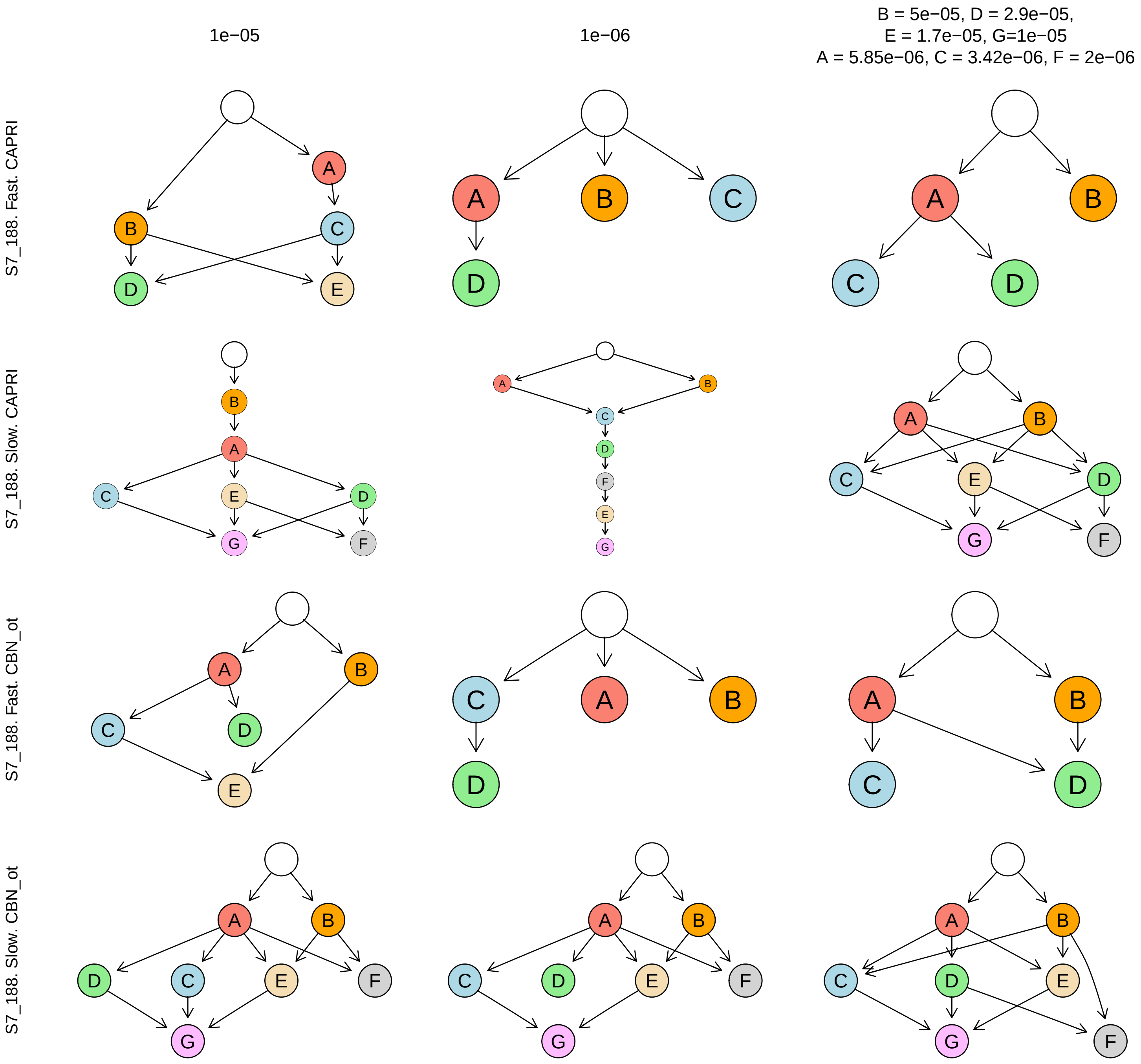

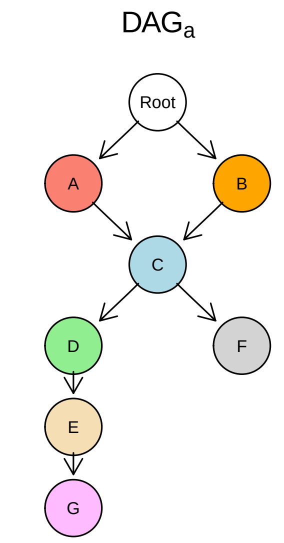

ID	Accessible Genot.	Discrep. DAG_a
S7_189	12	1

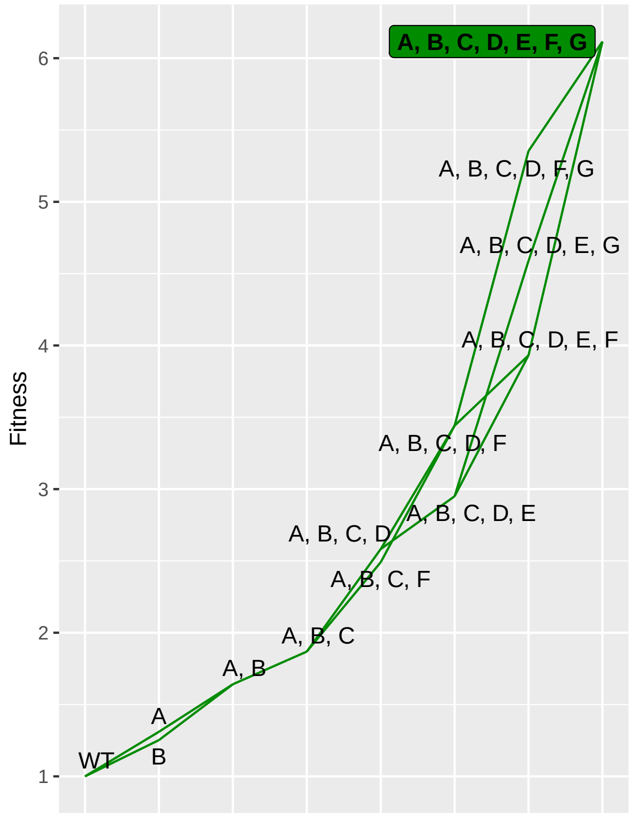

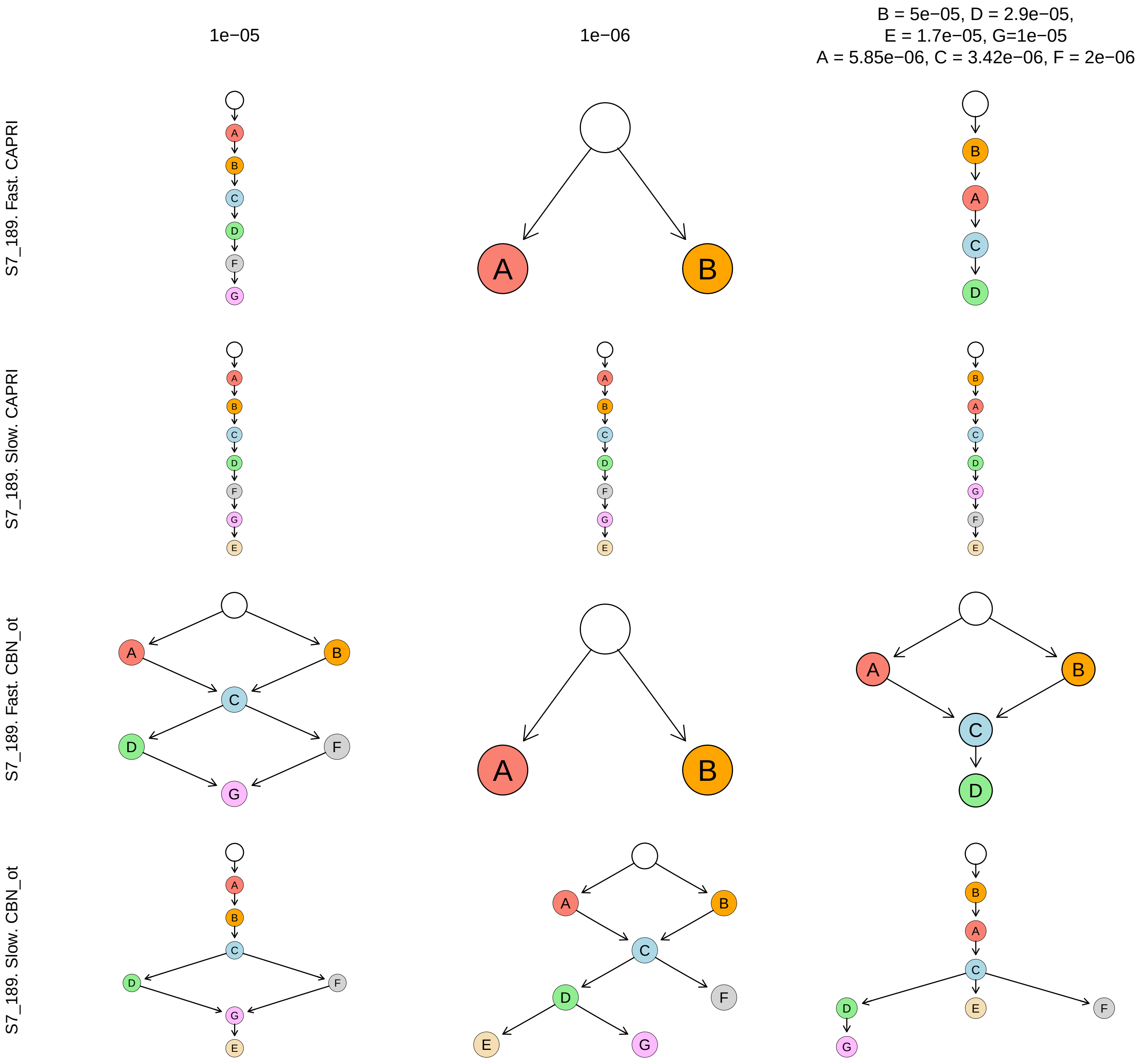

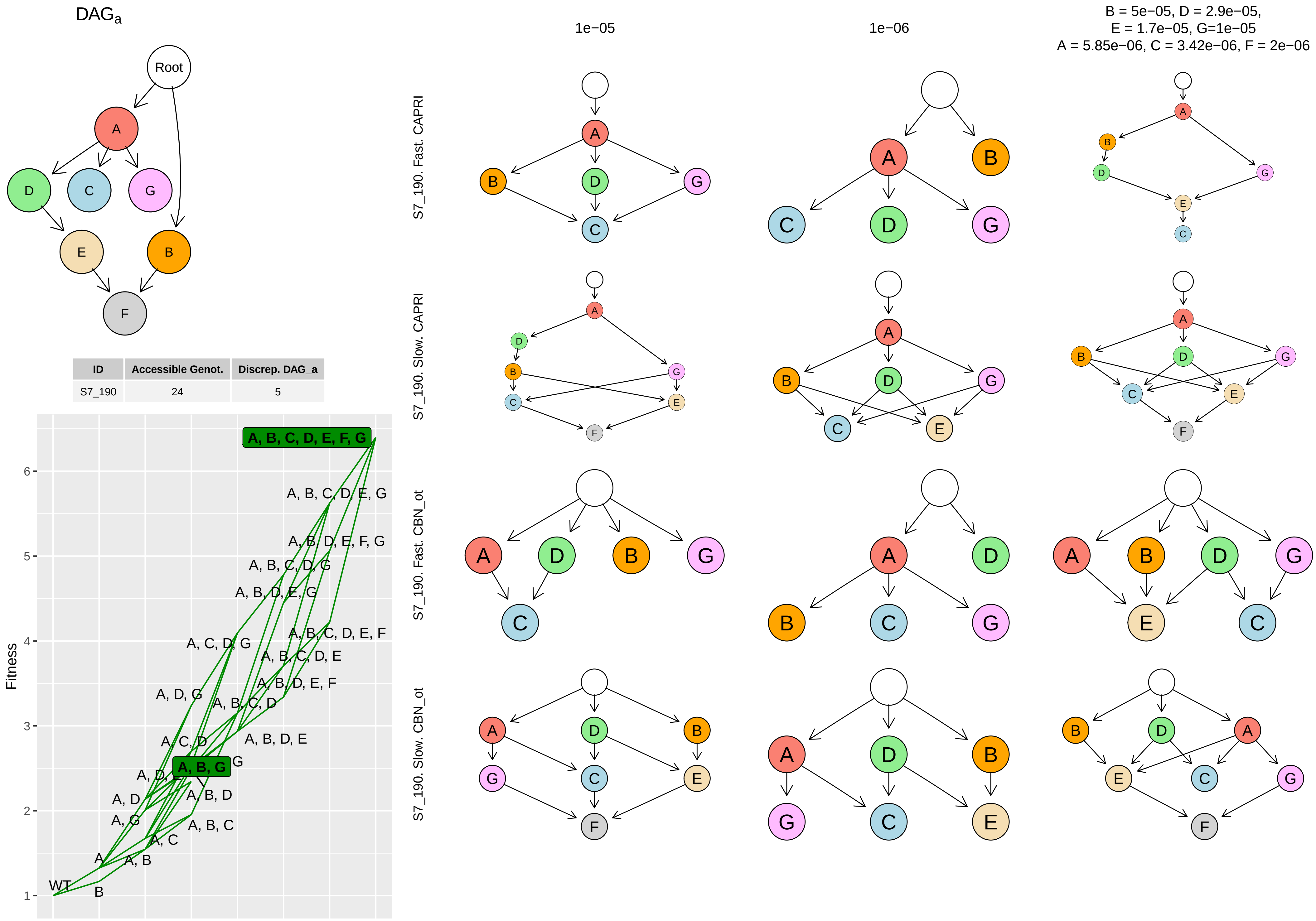

DAG_a
Root
A
D
C
G
E
B
F
ID | Accessible Genot. | Discrep. DAG_a
S7_190 | 24 | 5
Fitness
1
2
3
4
5
6
WT
B
A
A, B
A, C
A, G
A, B, C
A, D
A, B, D
A, B, G
A, C, D
A, B, D, E
A, D, G
A, B, C, D
A, B, D, E, F
A, B, C, D, E
A, C, D, G
A, B, C, D, E, F
A, B, D, E, G
A, B, C, D, G
A, B, D, E, F, G
A, B, C, D, E, G
A, B, C, D, E, F, G
1e−05
1e−06
B = 5e−05, D = 2.9e−05,
E = 1.7e−05, G=1e−05
A = 5.85e−06, C = 3.42e−06, F = 2e−06
S7_190. Fast. CAPRI
S7_190. Slow. CAPRI
S7_190. Fast. CBN_ot
S7_190. Slow. CBN_ot

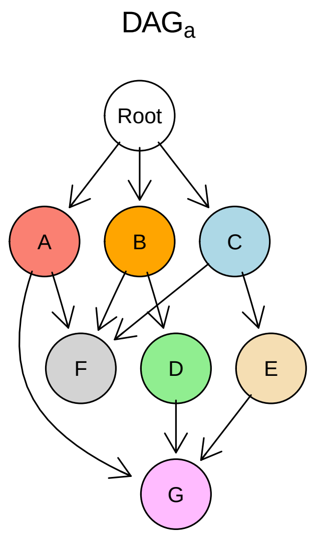

ID	Accessible Genot.	Discrep. DAG_a
S7_191	16	9

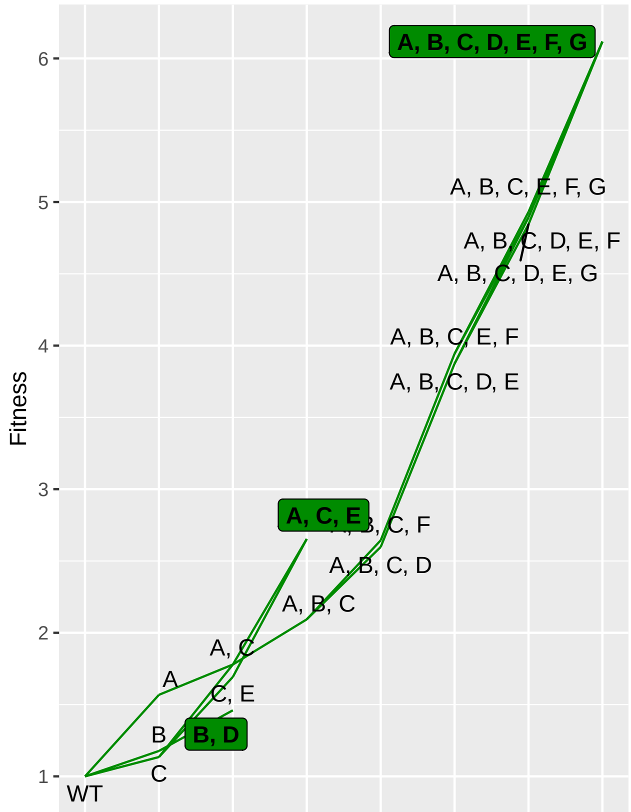

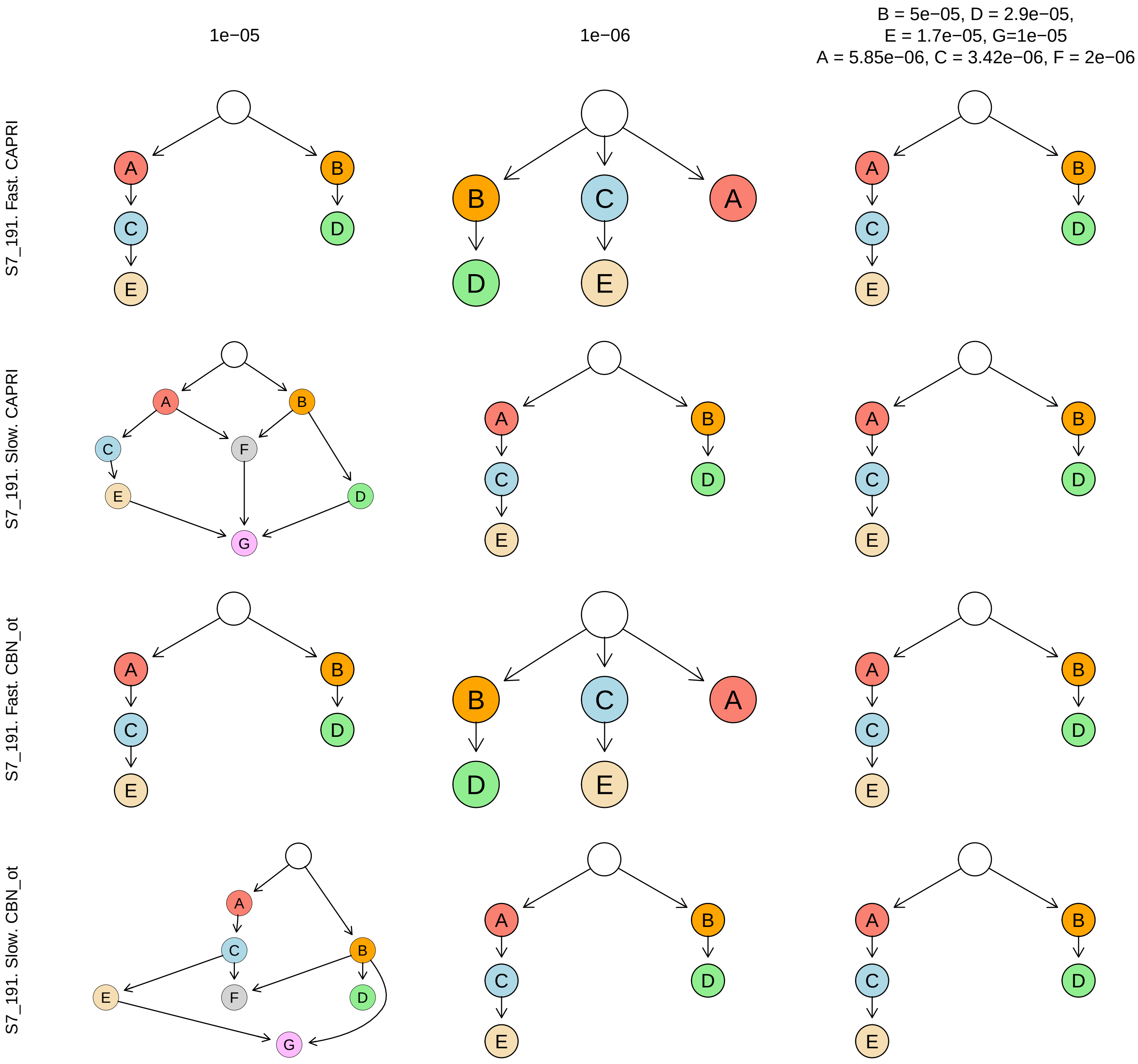

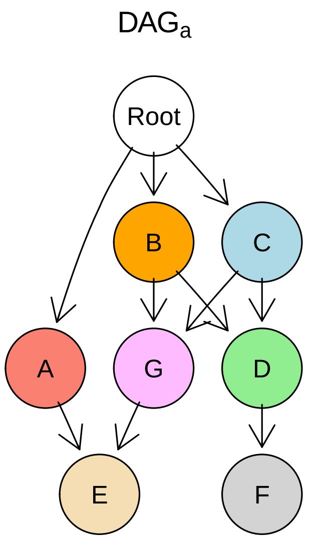

ID	Accessible Genot.	Discrep. DAG_a
S7_192	15	5

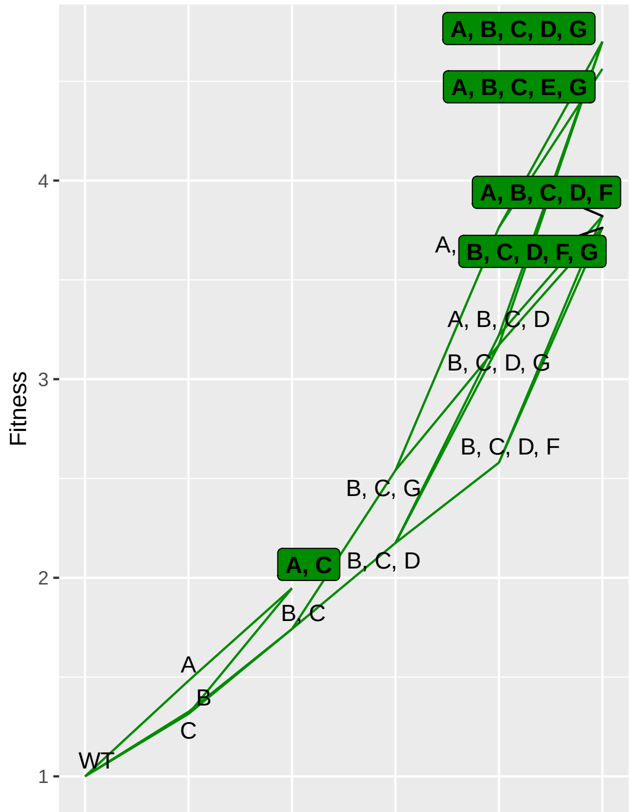

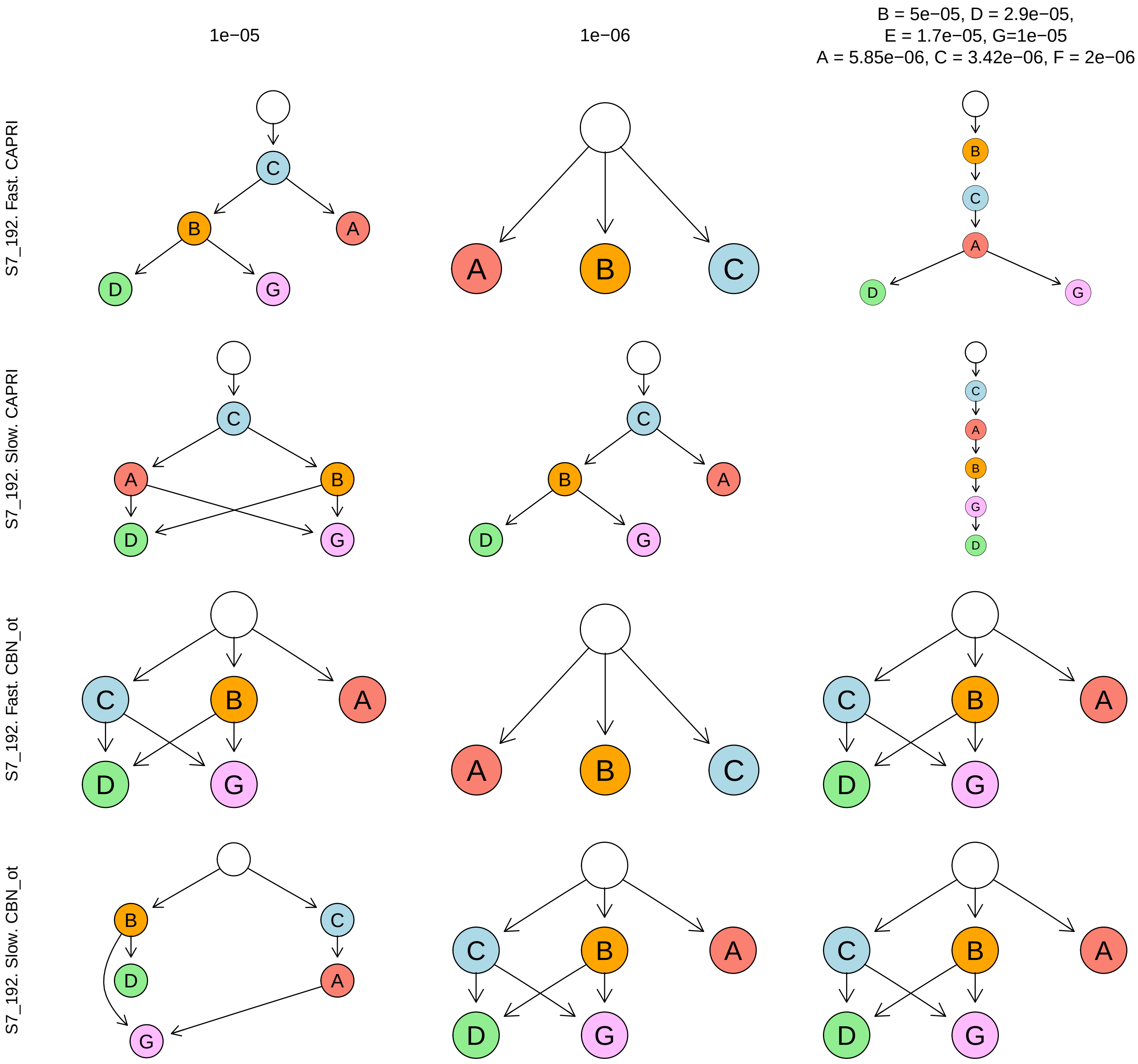

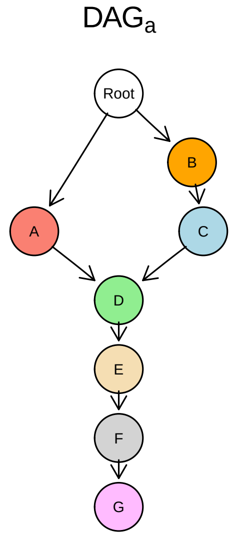

ID	Accessible Genot.	Discrep. DAG_a
S7_193	8	1

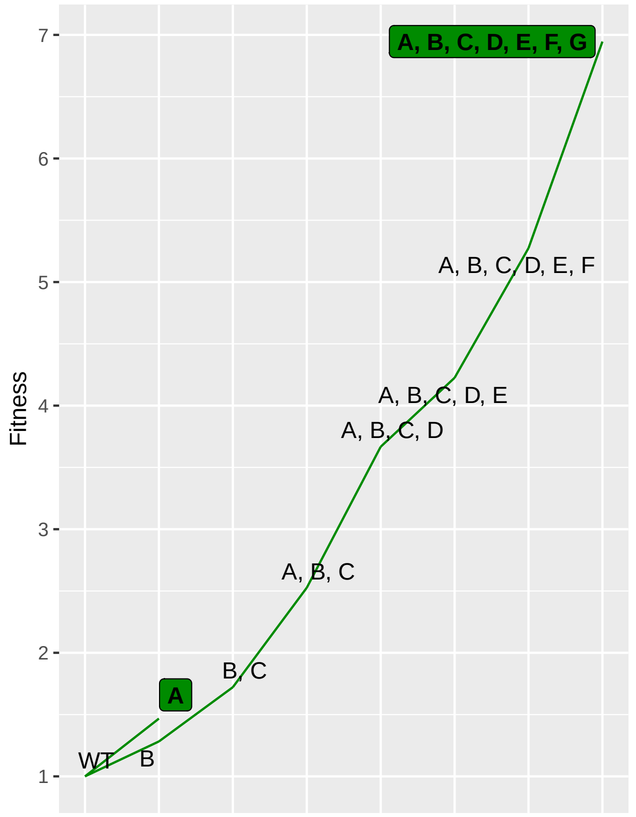

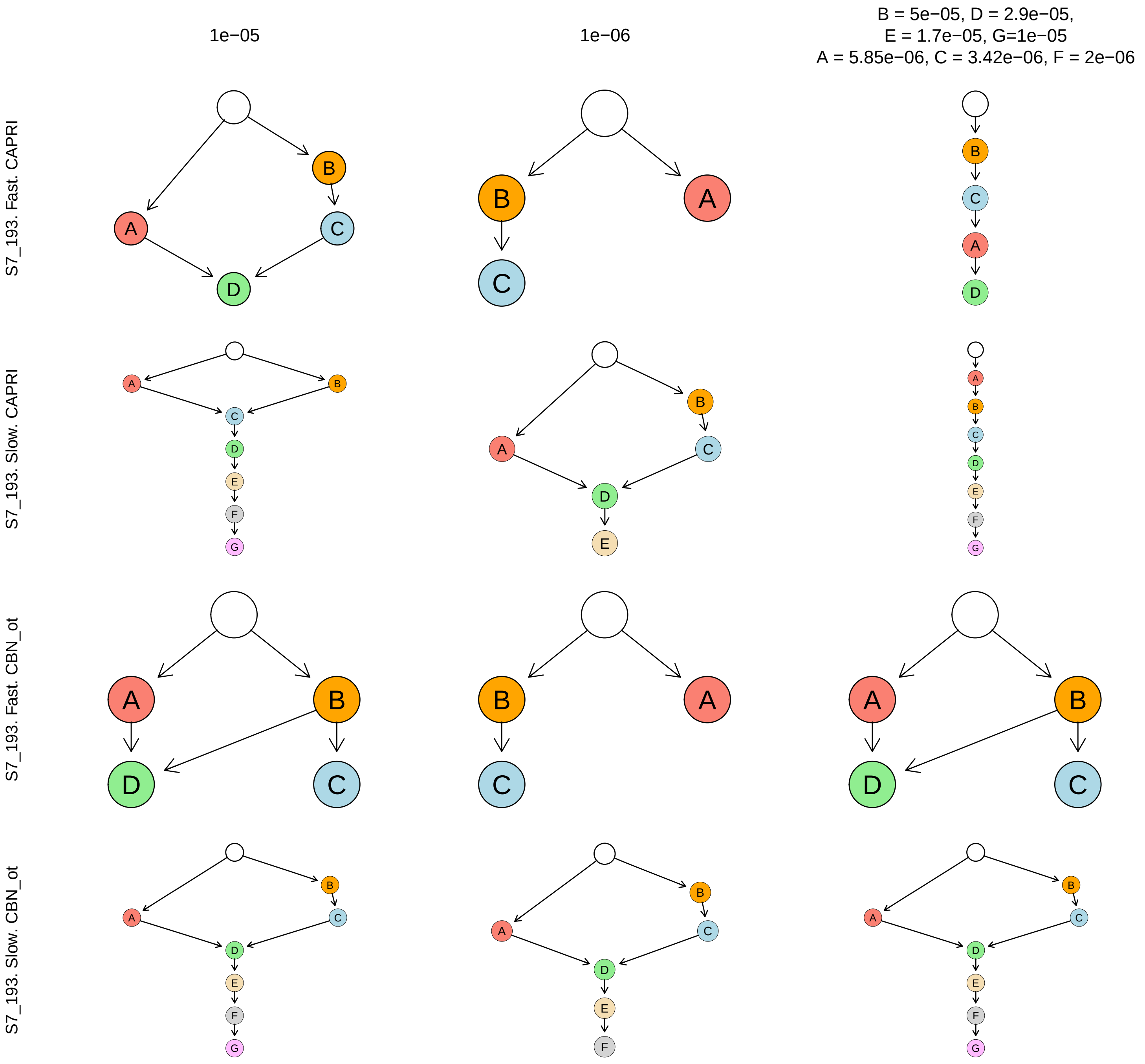

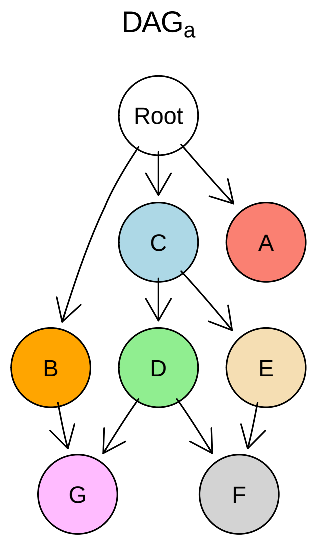

ID	Accessible Genot.	Discrep. DAG_a
S7_194	23	12

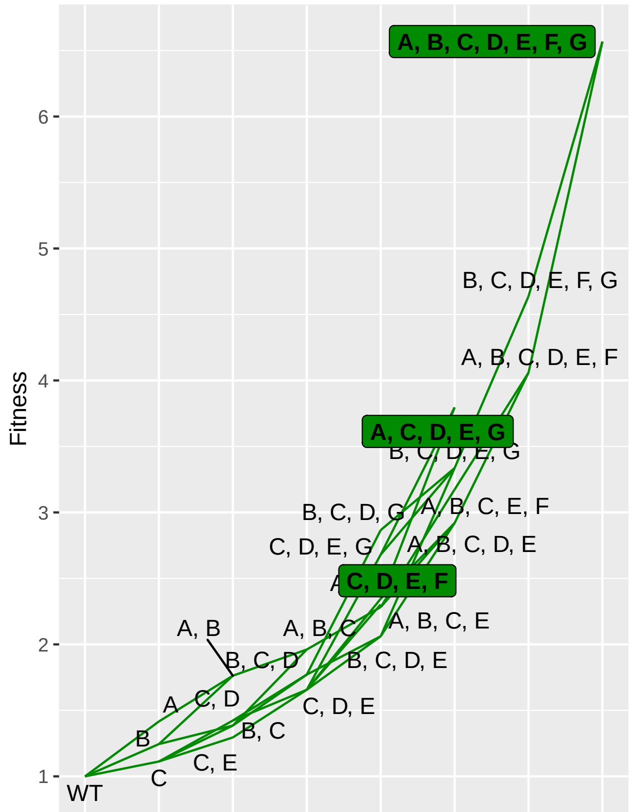

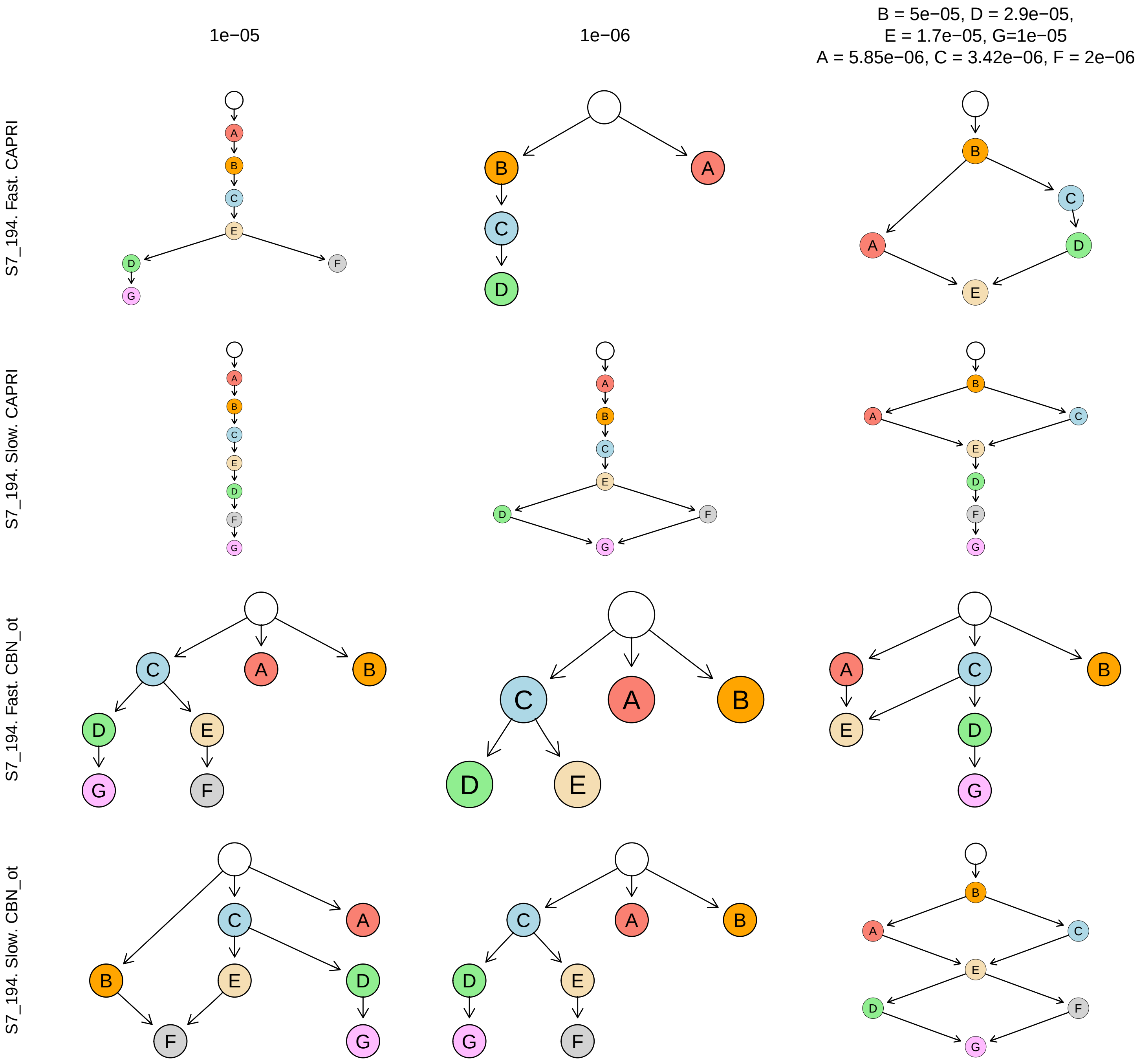

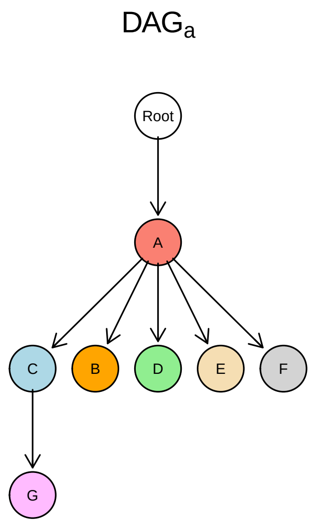

ID	Accessible Genot.	Discrep. DAG_a
S7_195	39	29

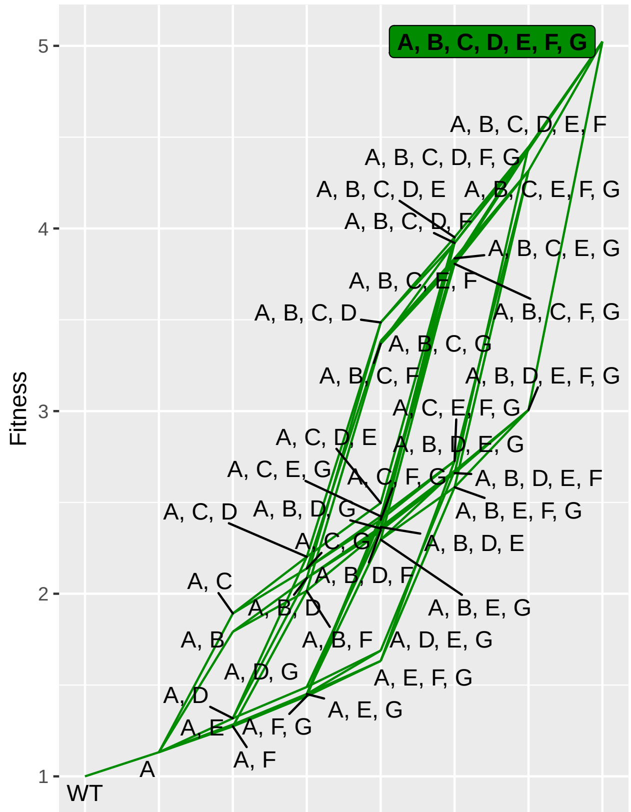

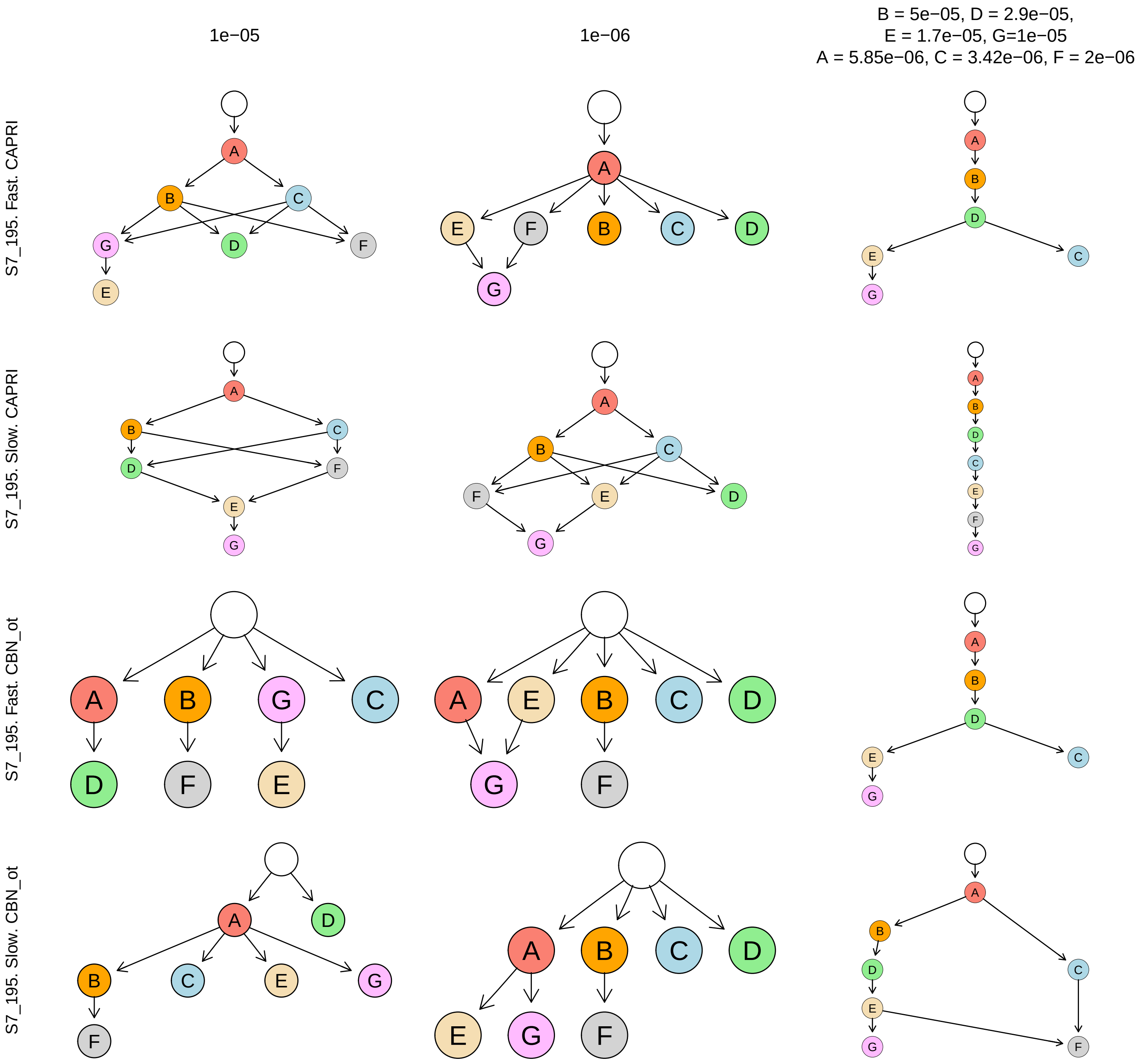

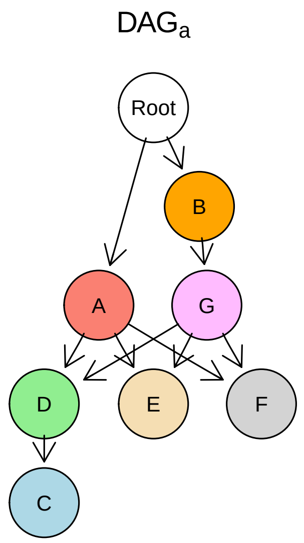

ID	Accessible Genot.	Discrep. DAG_a
S7_196	16	4

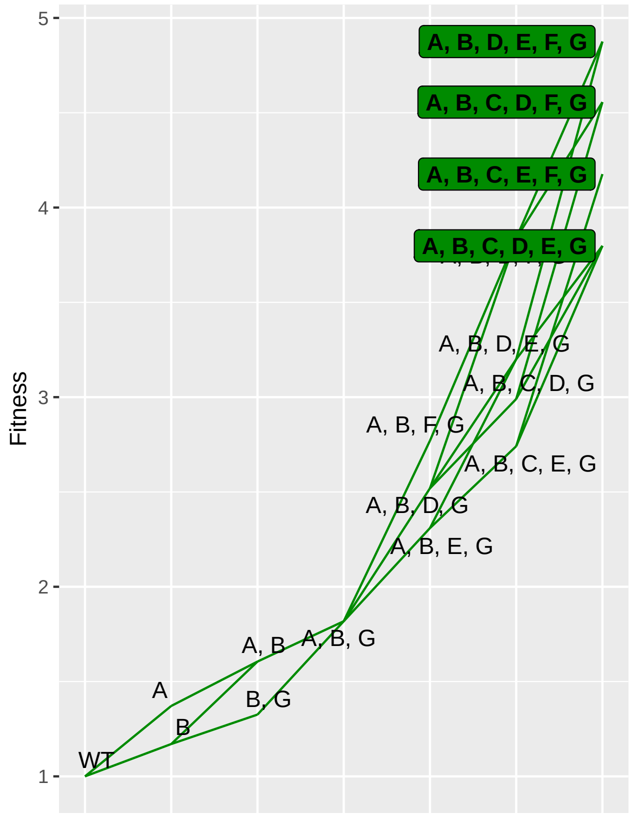

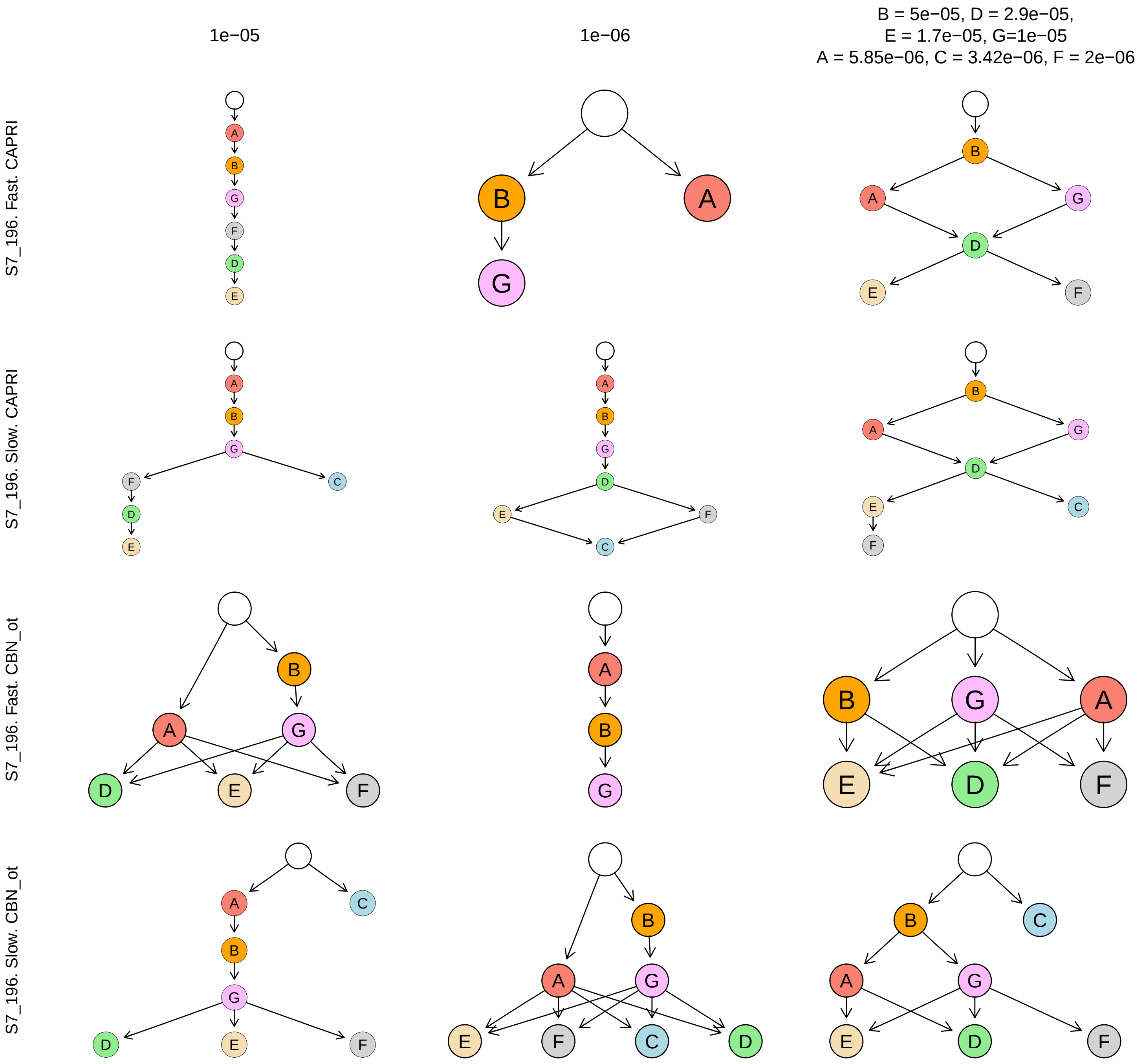

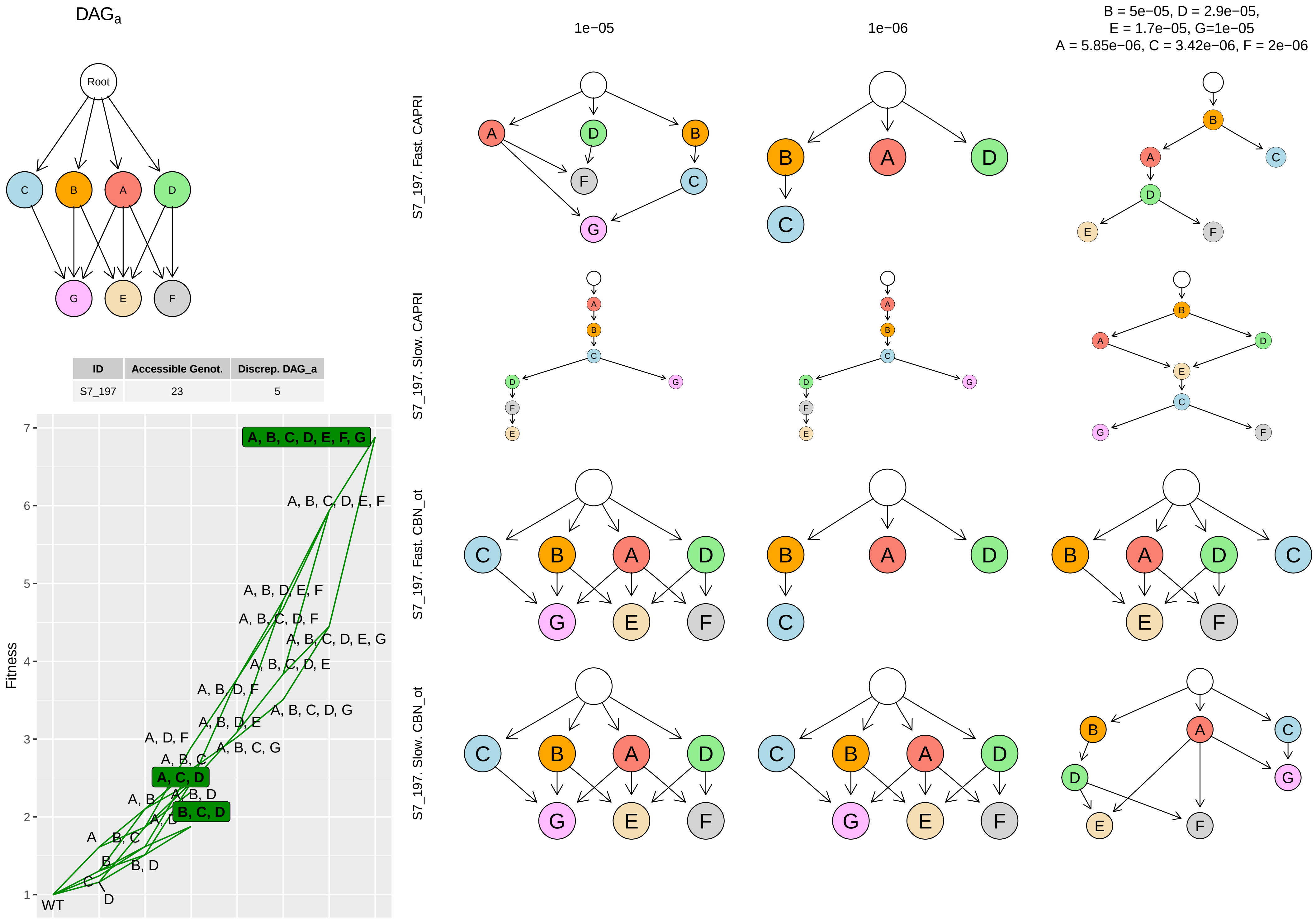

ID	Accessible Genot.	Discrep. DAG_a
S7_197	23	5

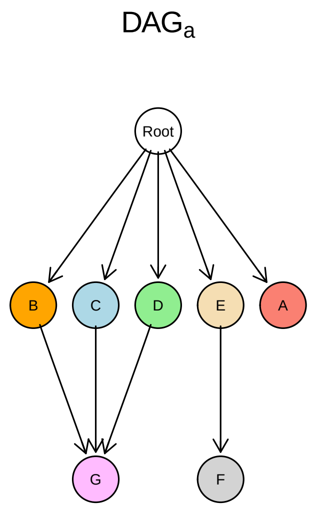

ID	Accessible Genot.	Discrep. DAG_a
S7_198	40	17

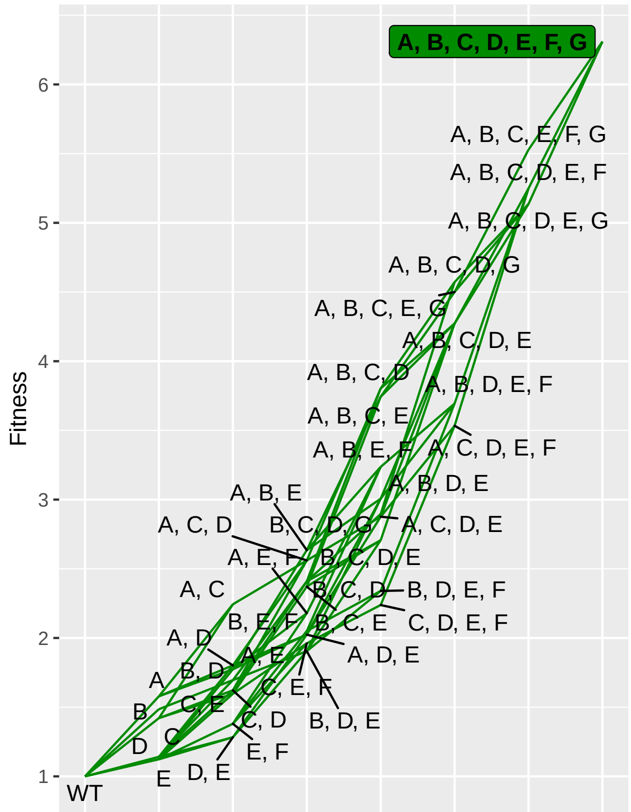

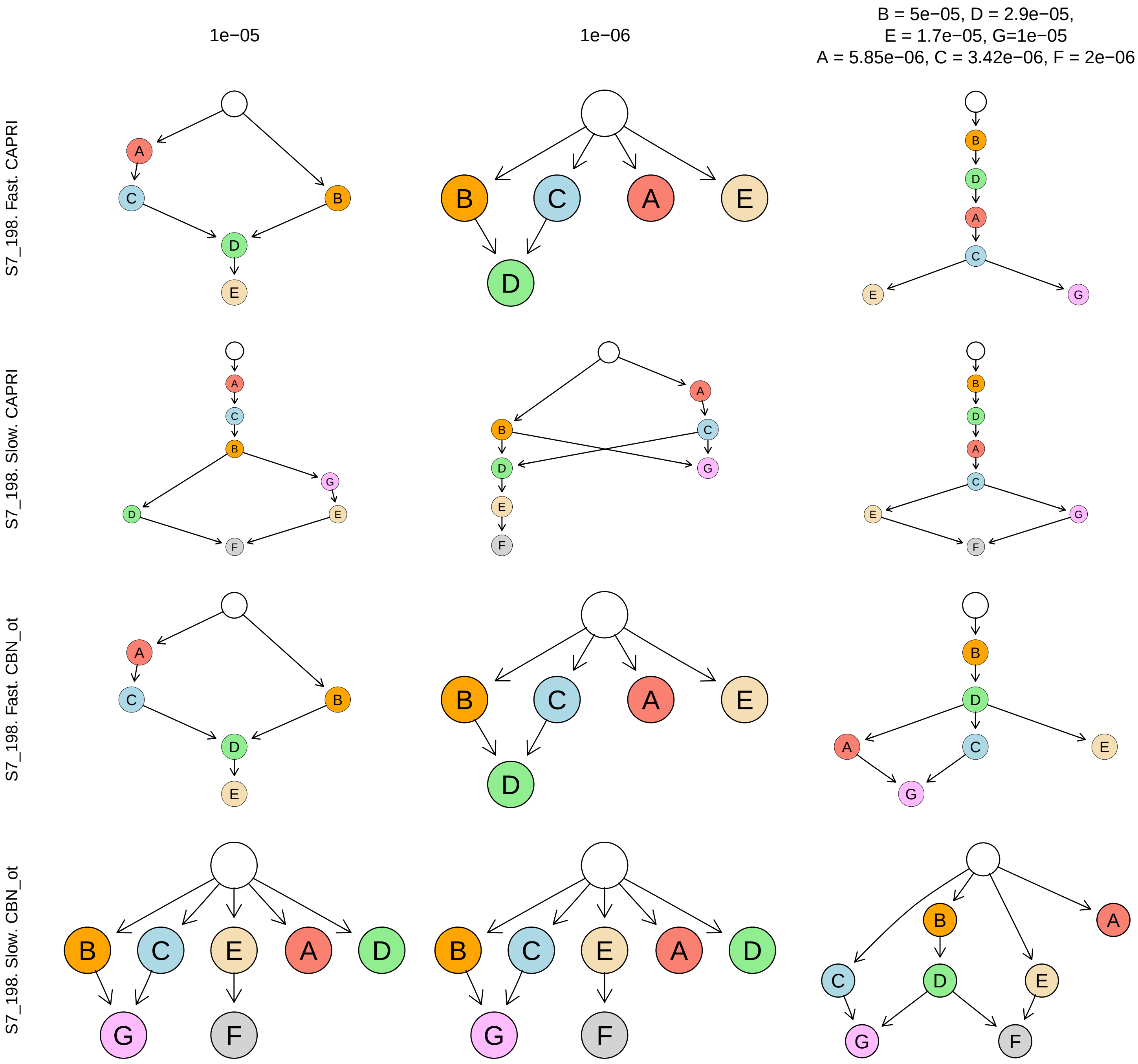

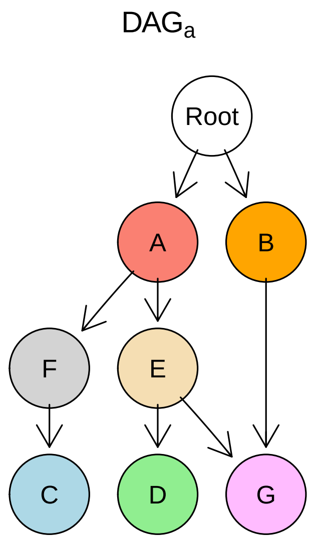

ID	Accessible Genot.	Discrep. DAG_a
S7_199	22	19

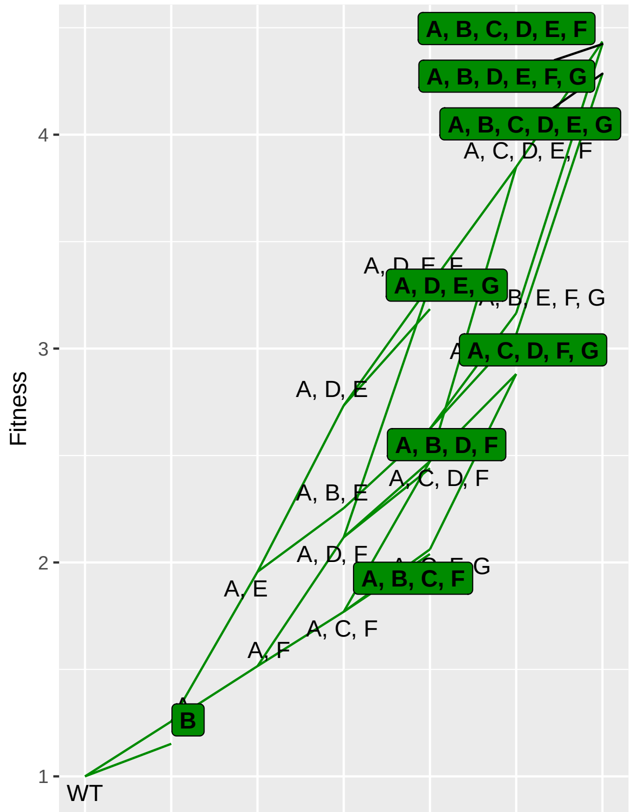

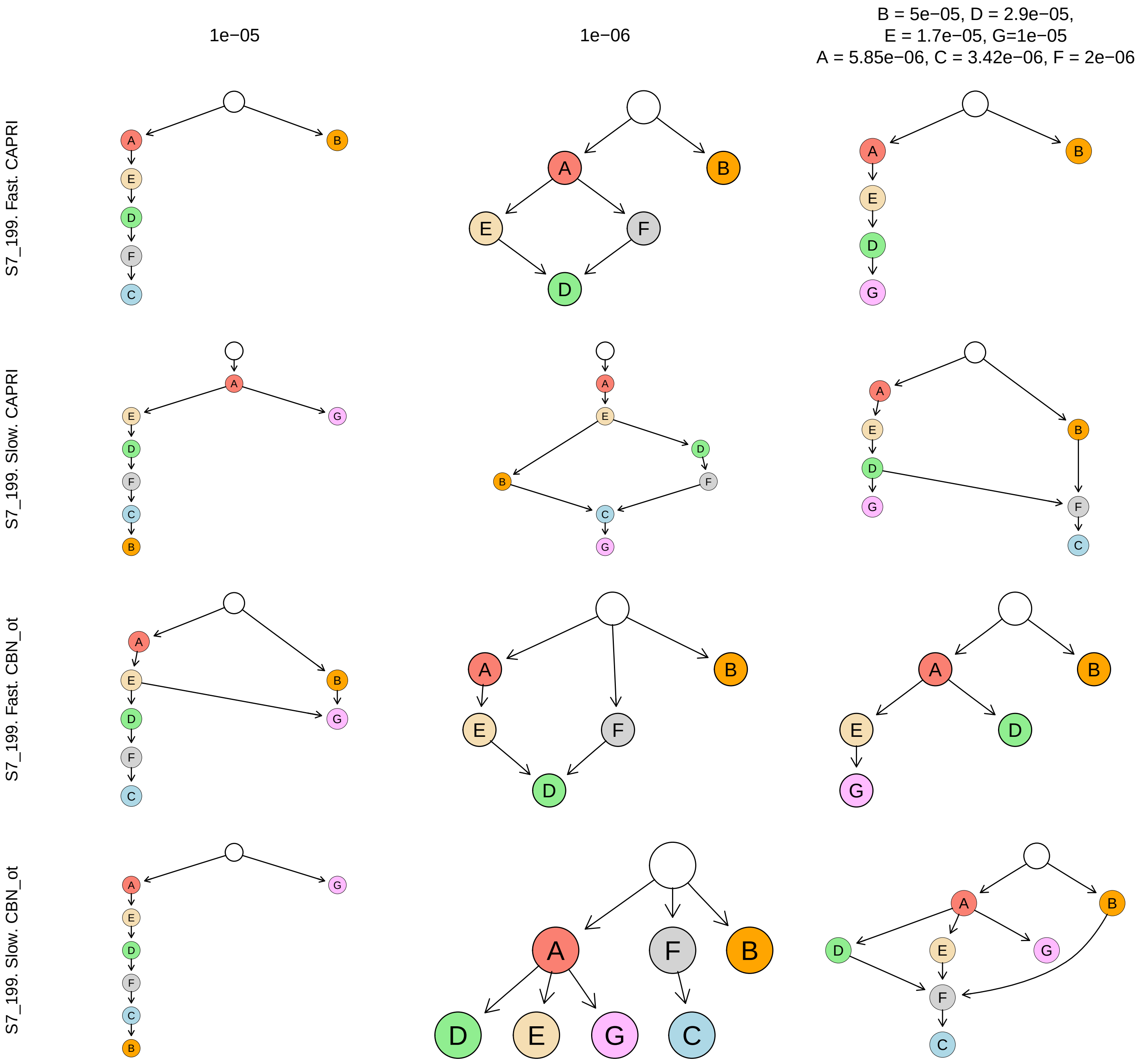

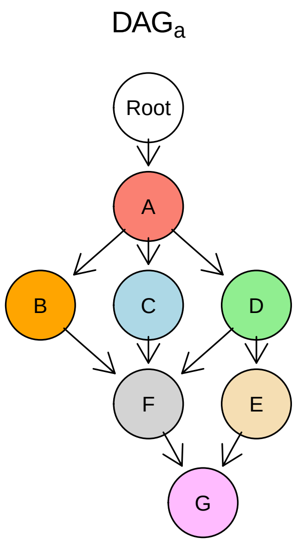

ID	Accessible Genot.	Discrep. DAG_a
S7_200	14	3

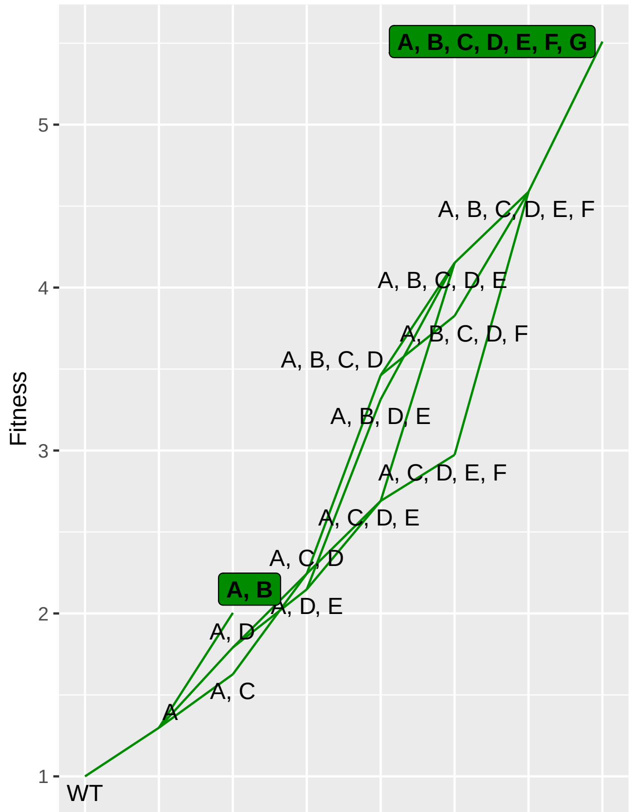

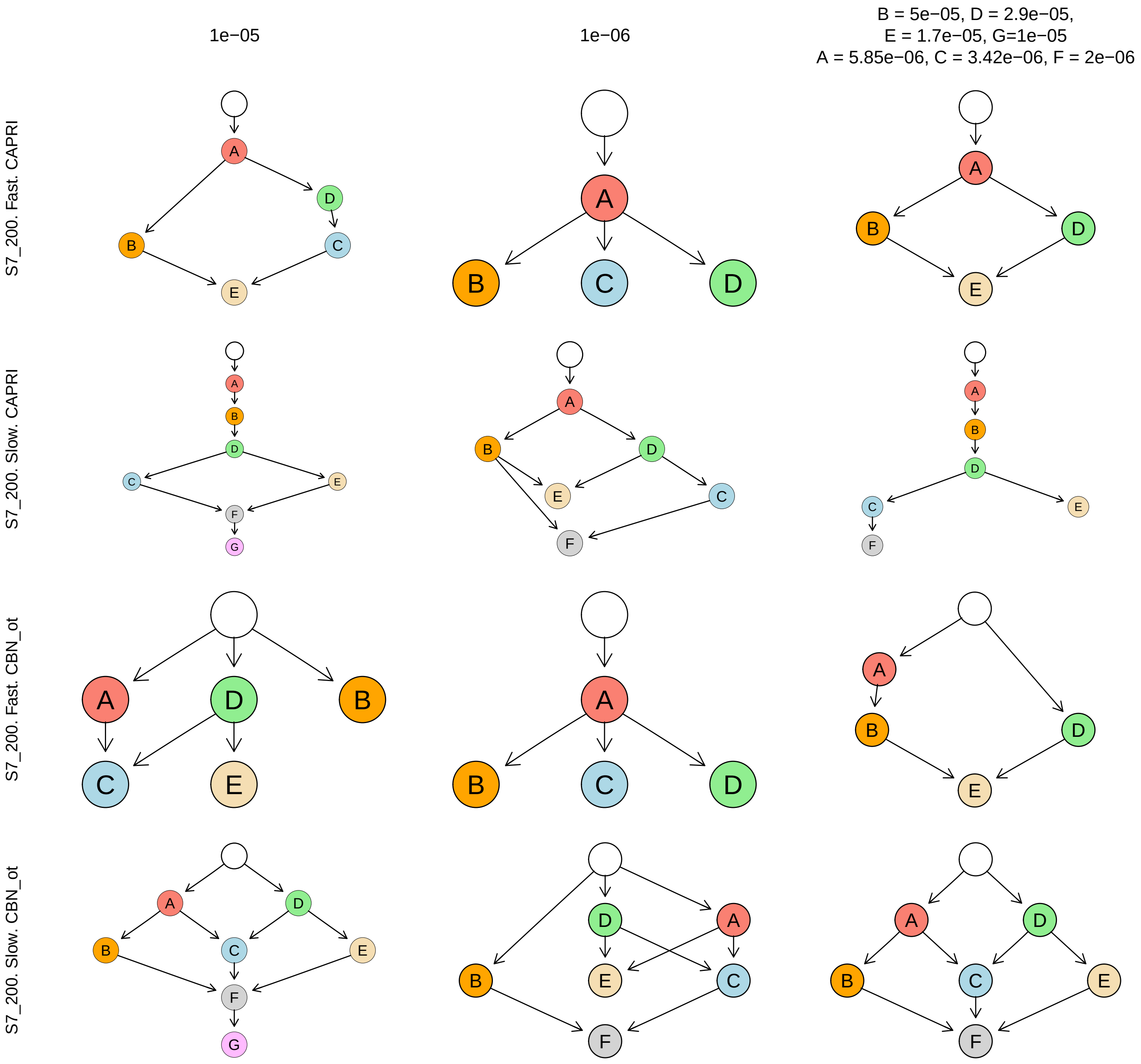

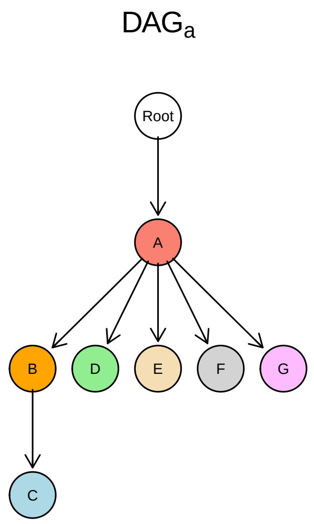

ID	Accessible Genot.	Discrep. DAG_a
S7_201	46	24

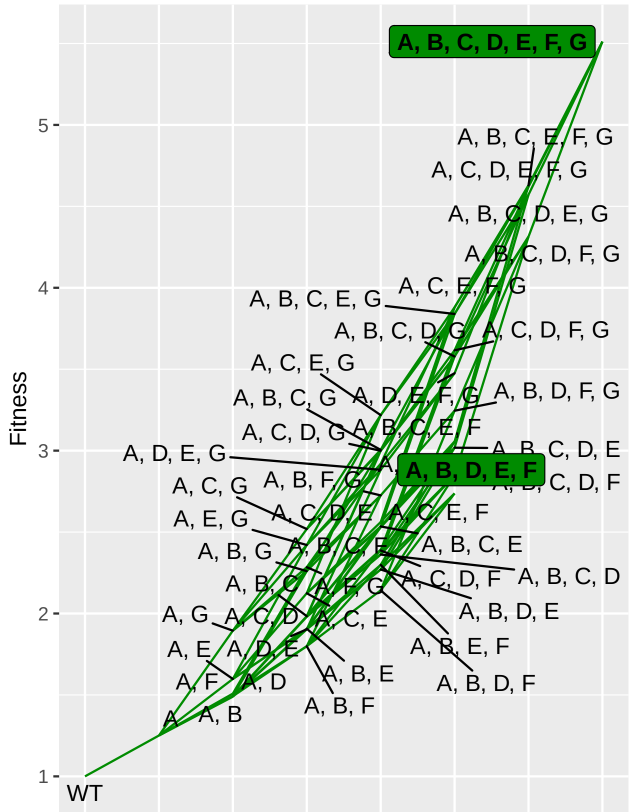

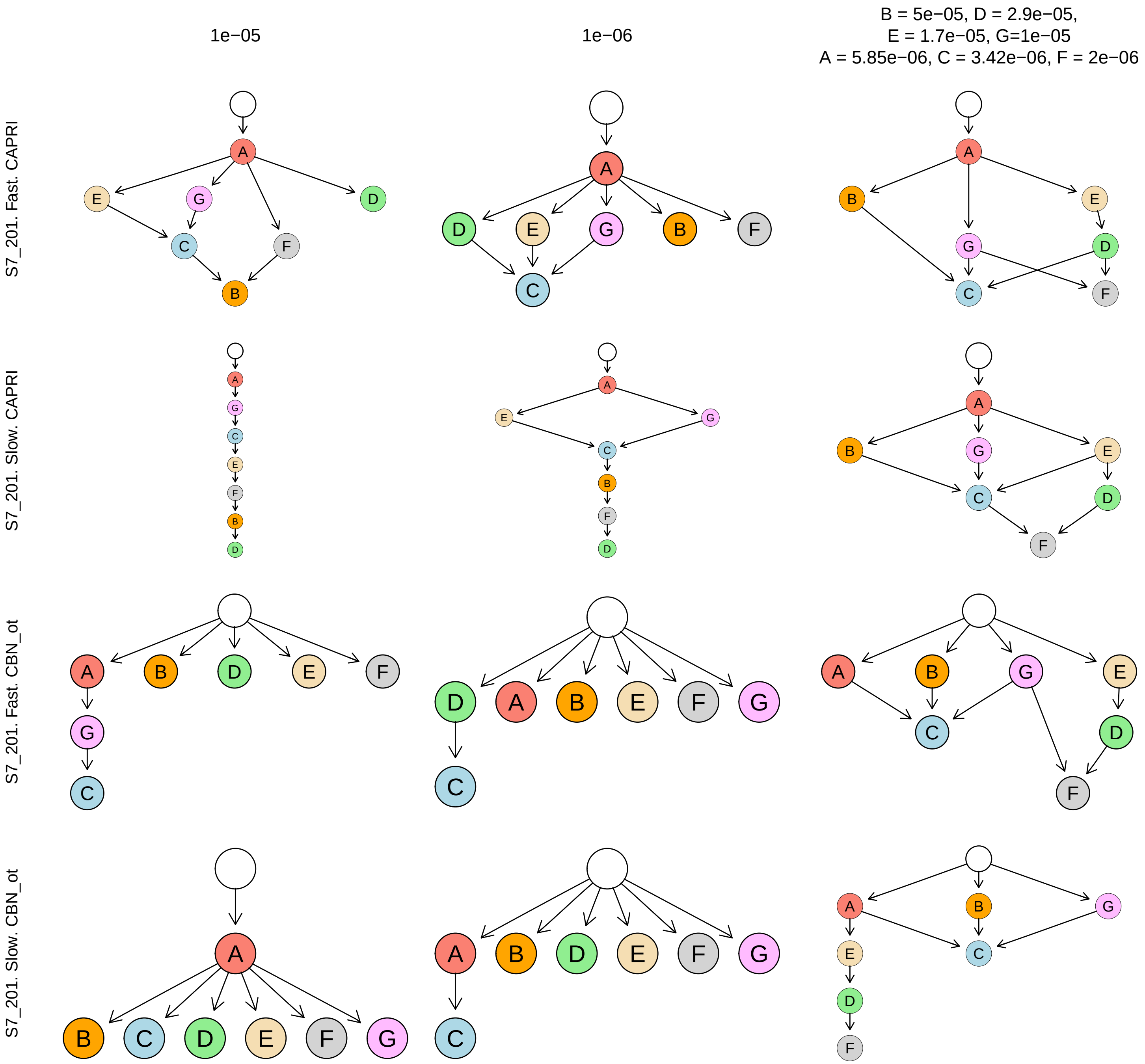

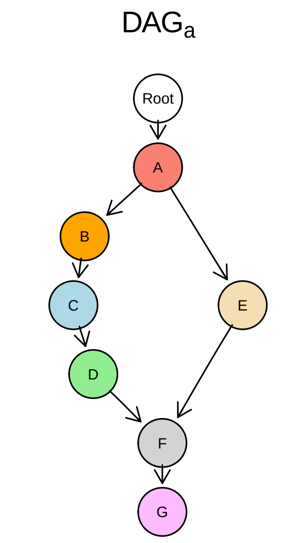

ID	Accessible Genot.	Discrep. DAG_a
S7_202	11	1

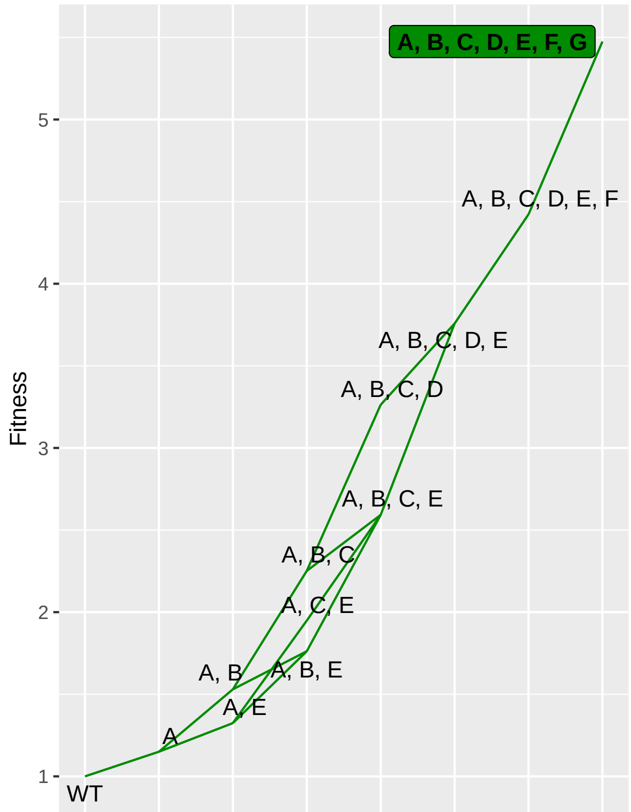

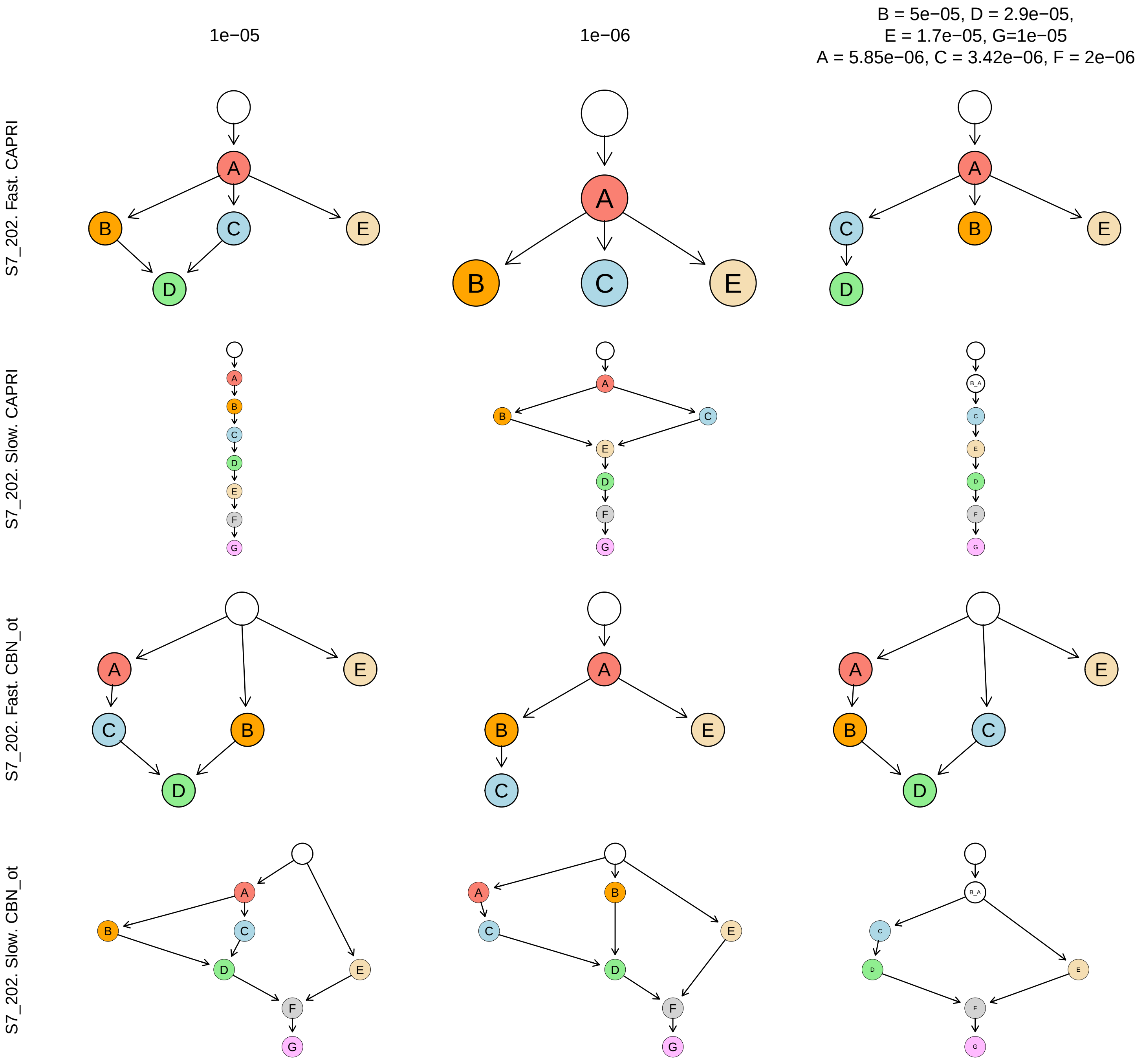

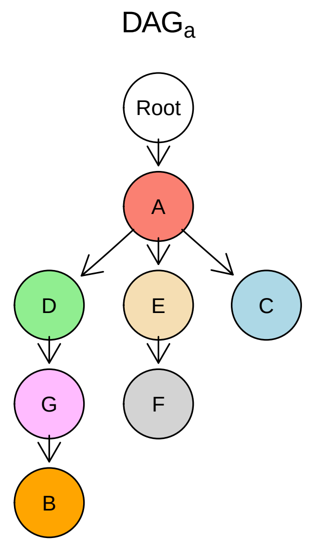

ID	Accessible Genot.	Discrep. DAG_a
S7_203	16	14

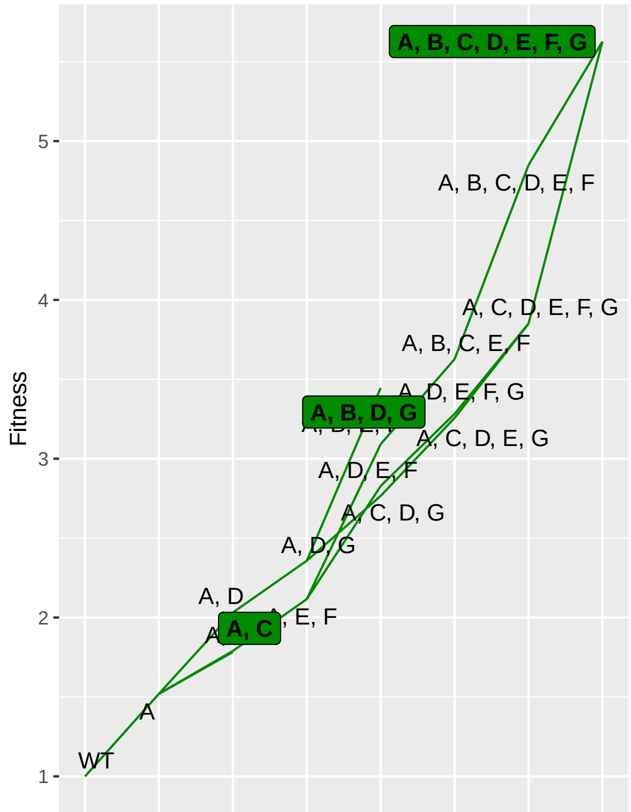

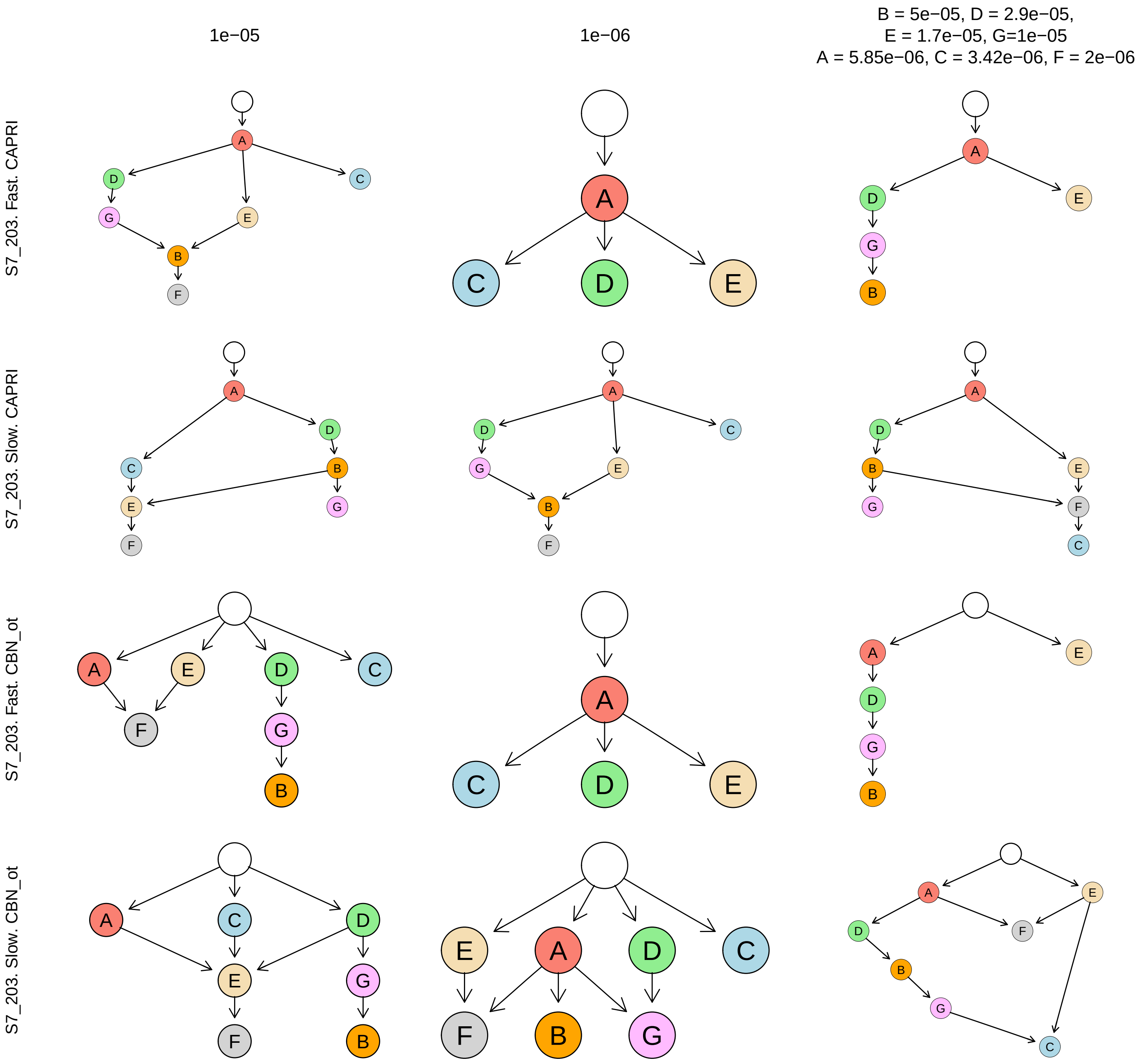

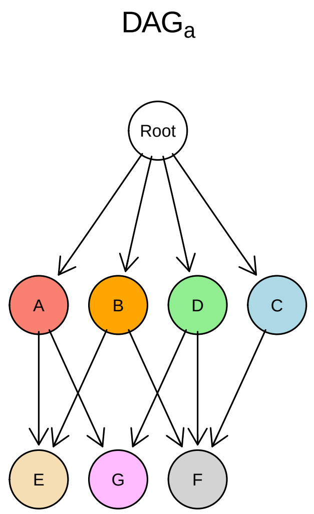

ID	Accessible Genot.	Discrep. DAG_a
S7_204	23	7

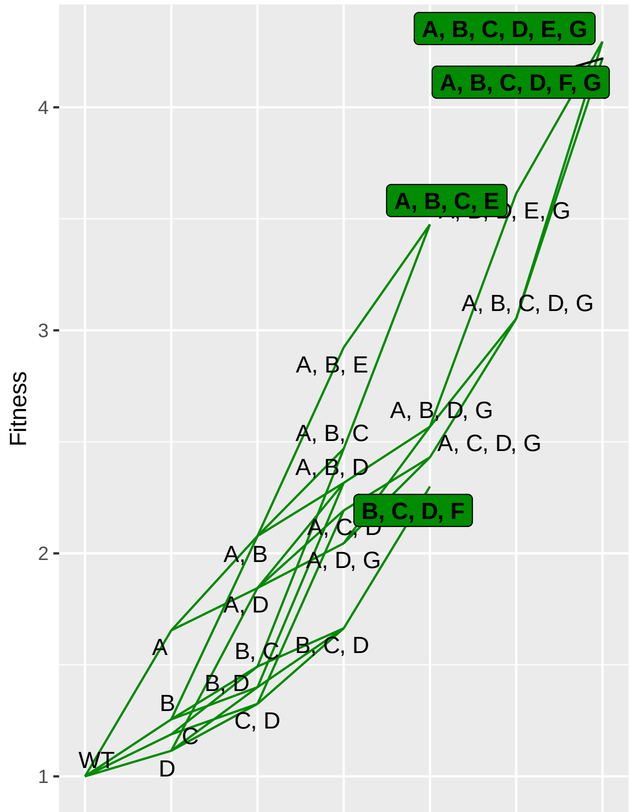

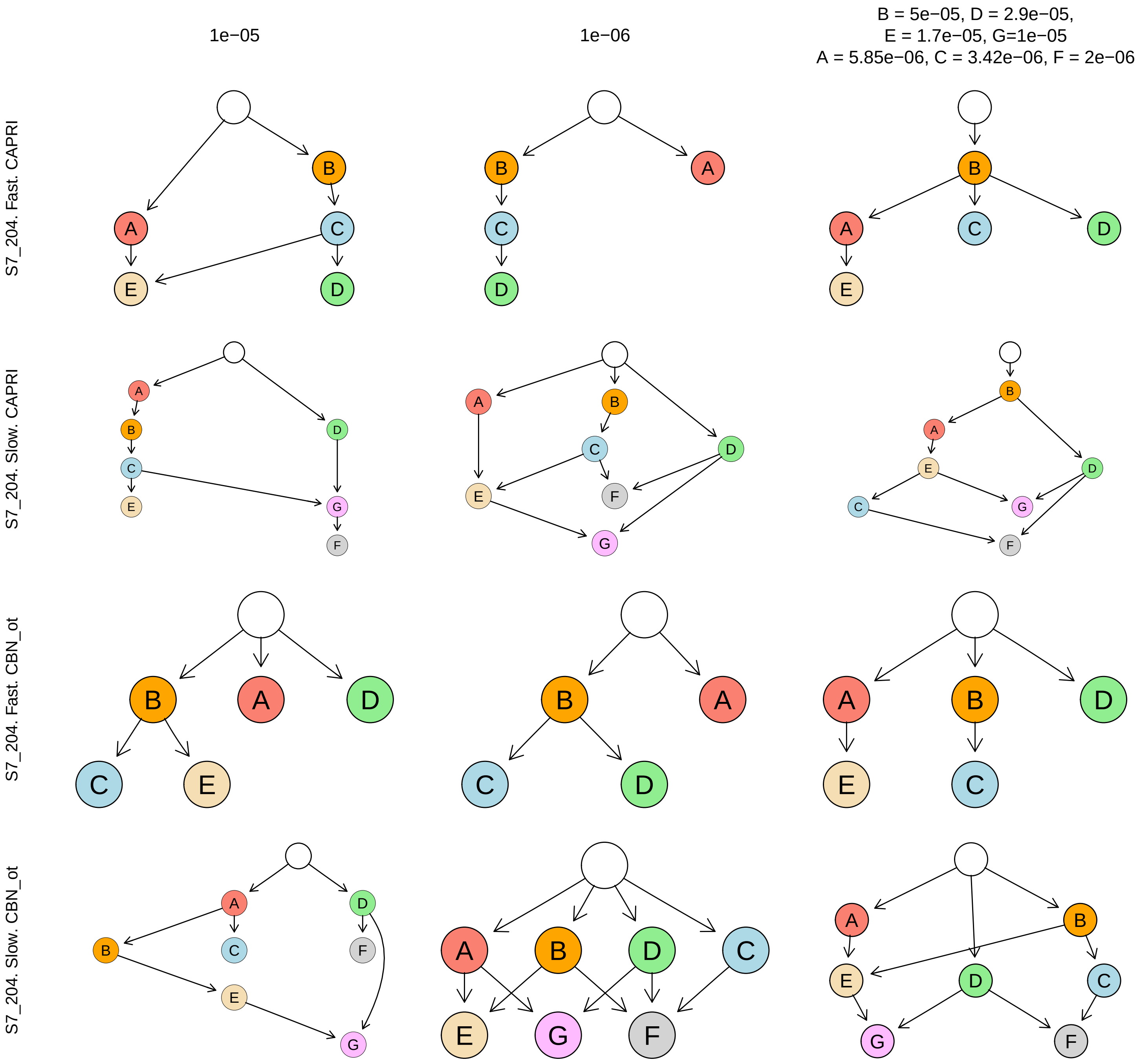

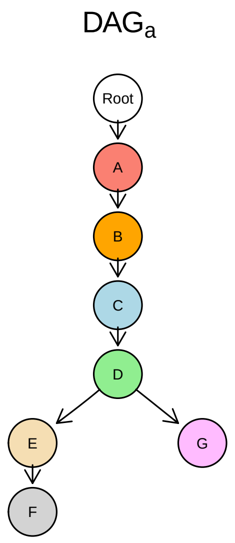

ID	Accessible Genot.	Discrep. DAG_a
S7_205	7	2

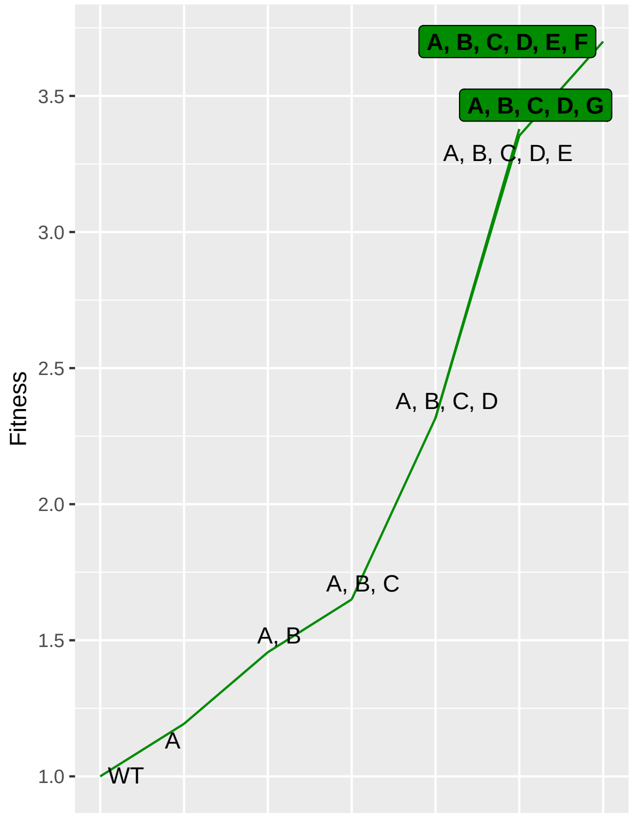

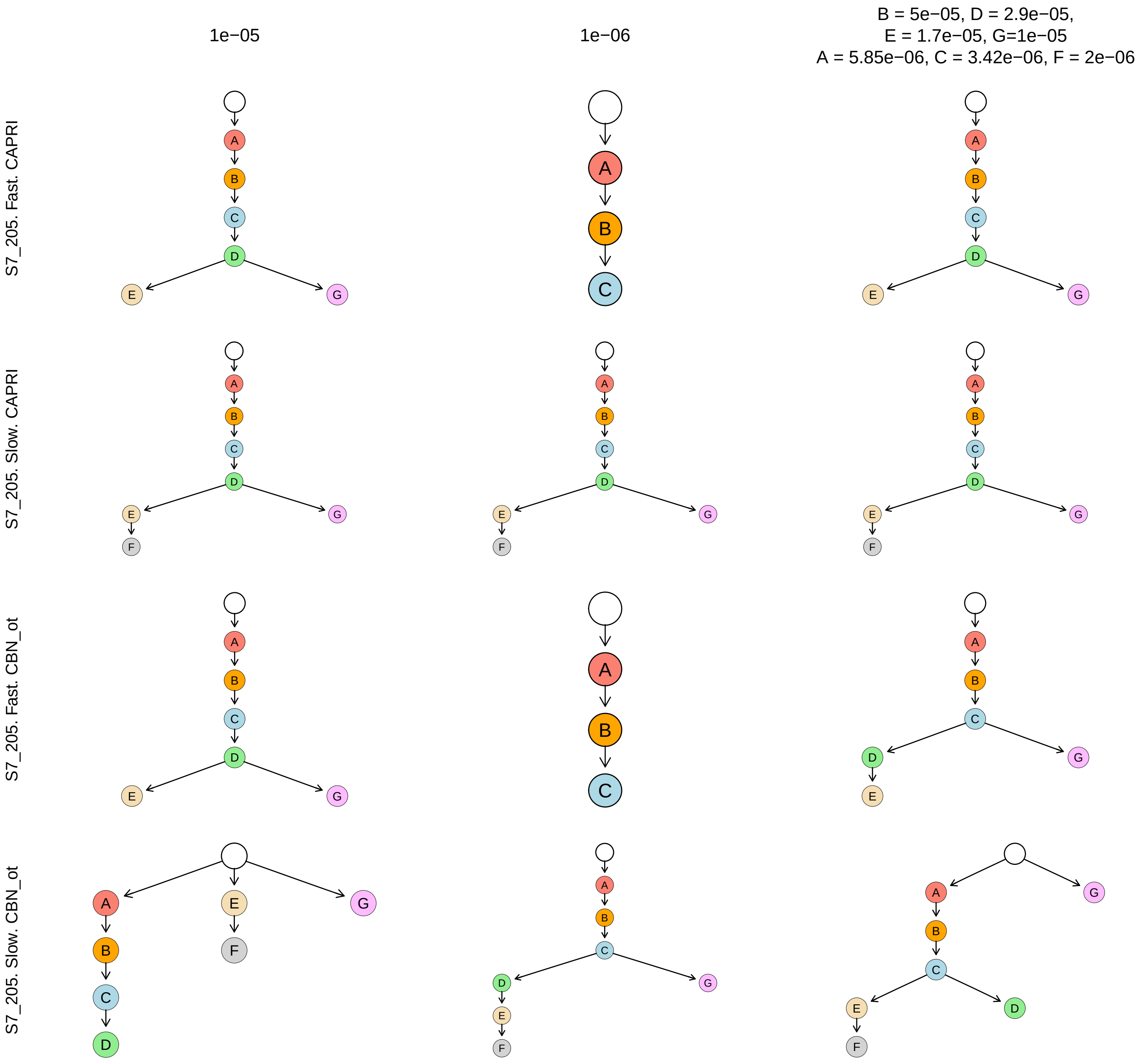

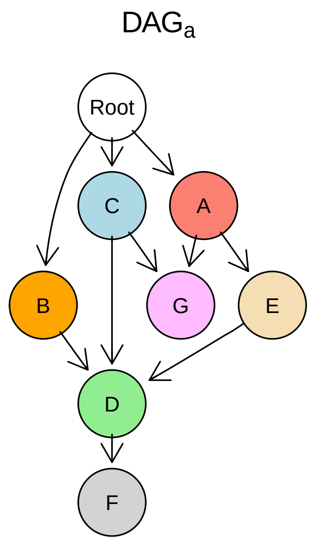

ID	Accessible Genot.	Discrep. DAG_a
S7_206	15	4

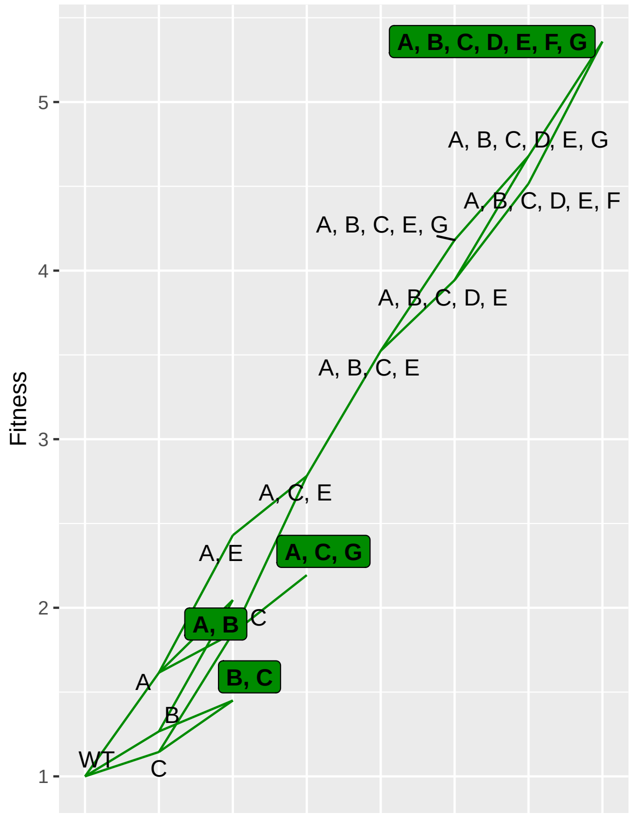

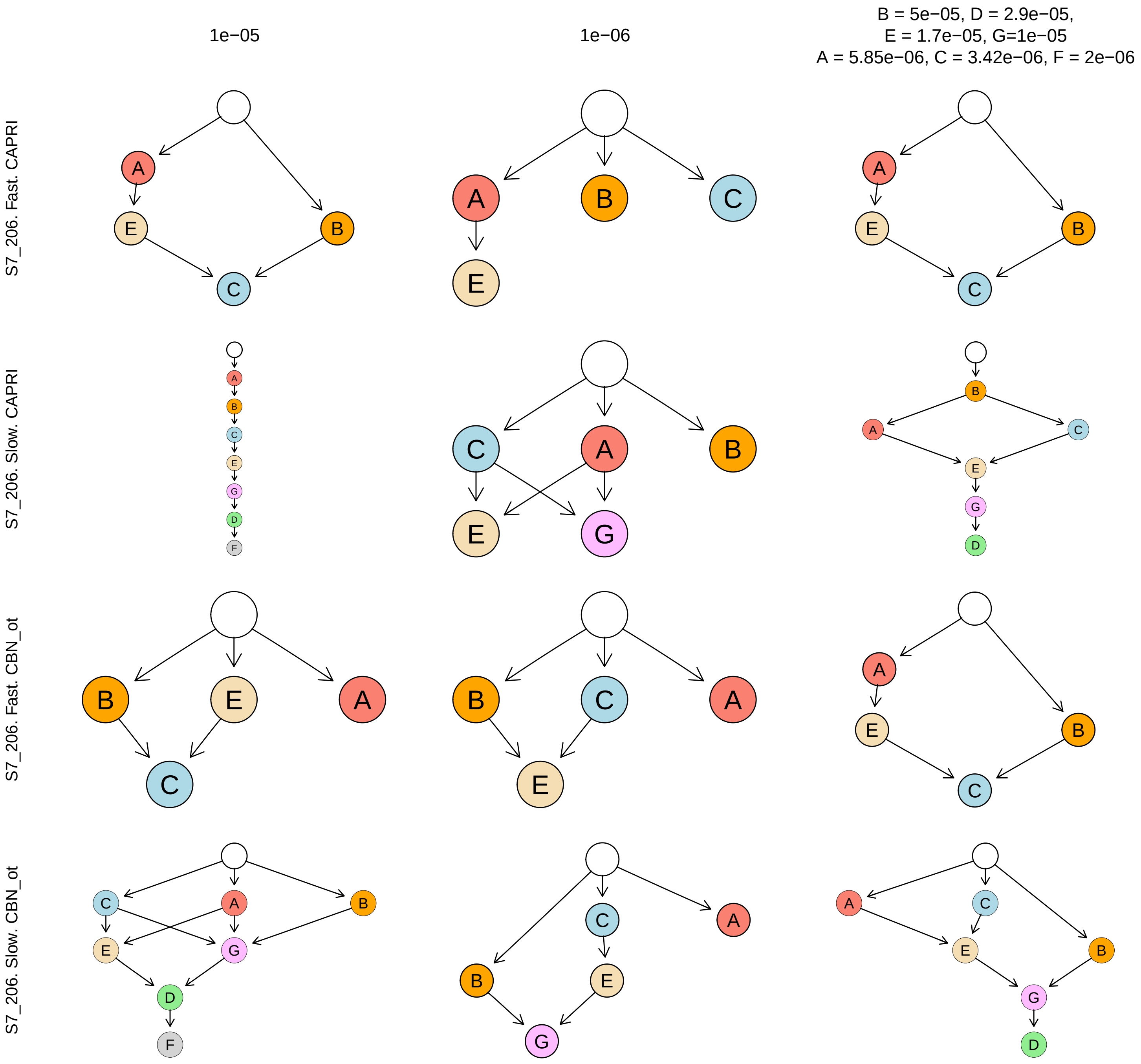

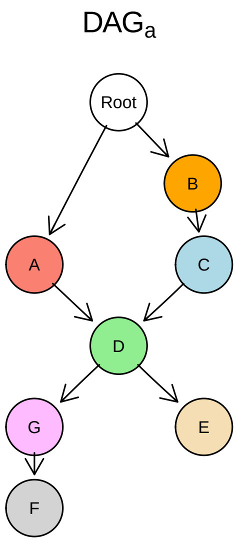

ID	Accessible Genot.	Discrep. DAG_a
S7_207	10	1

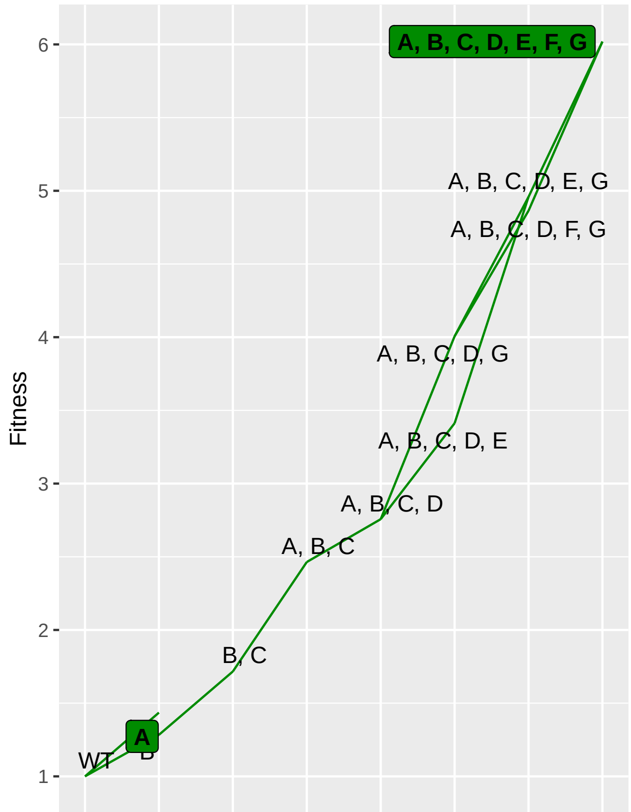

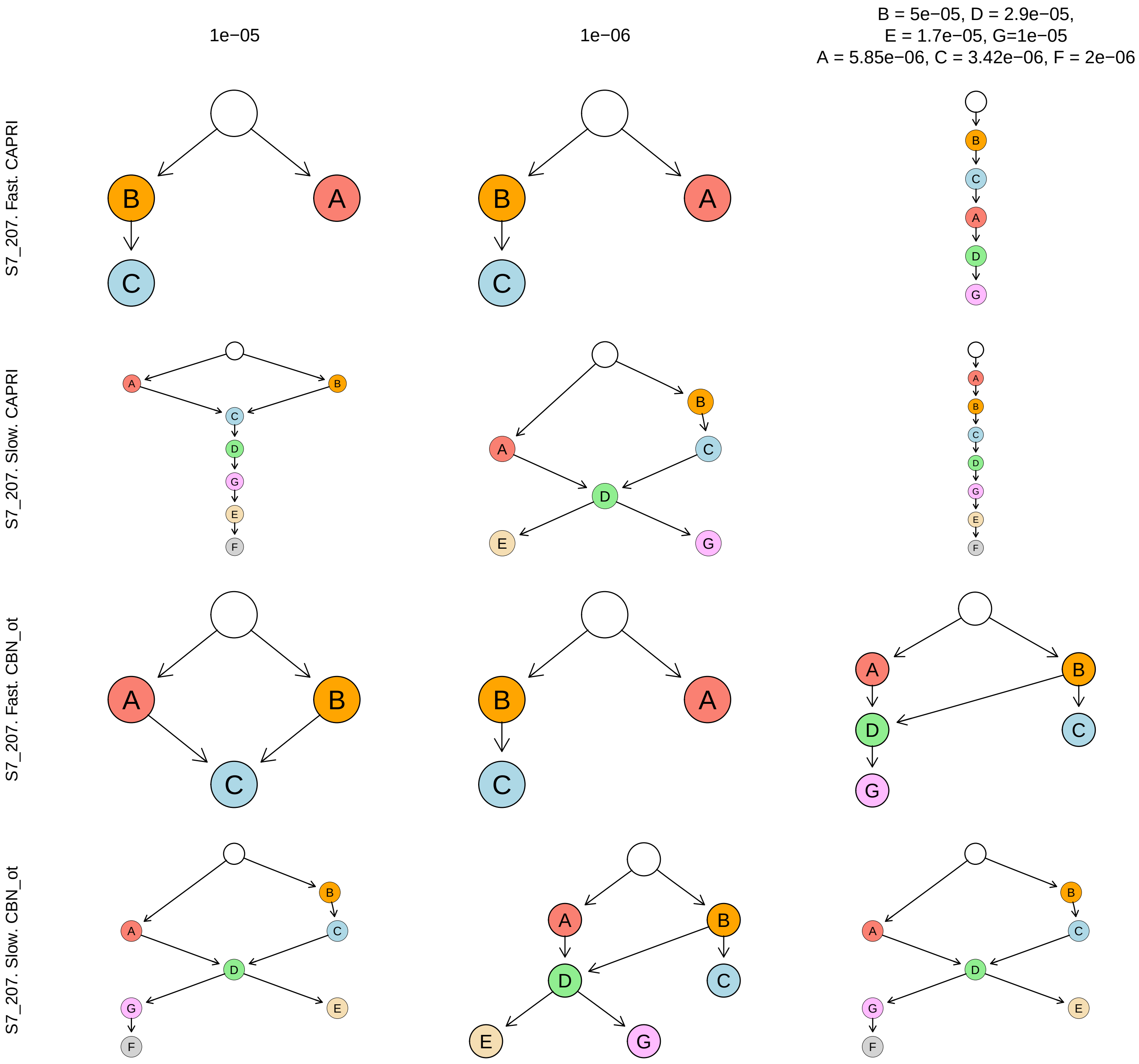

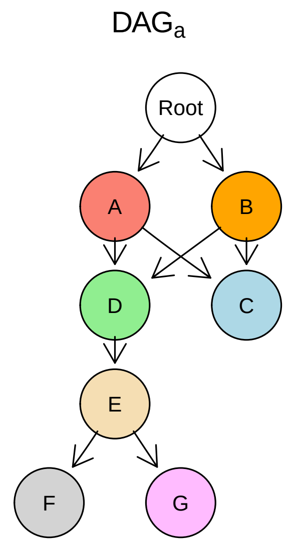

ID	Accessible Genot.	Discrep. DAG_a
S7_208	13	1

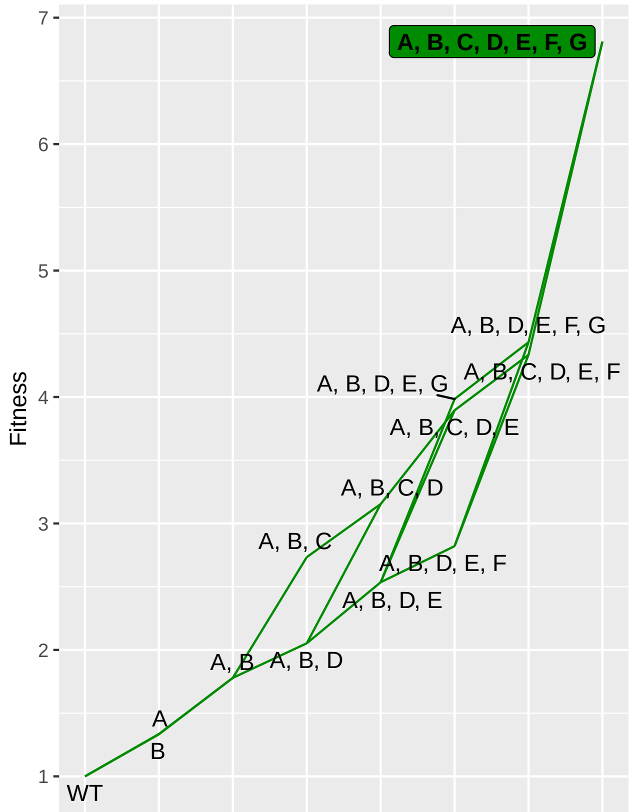

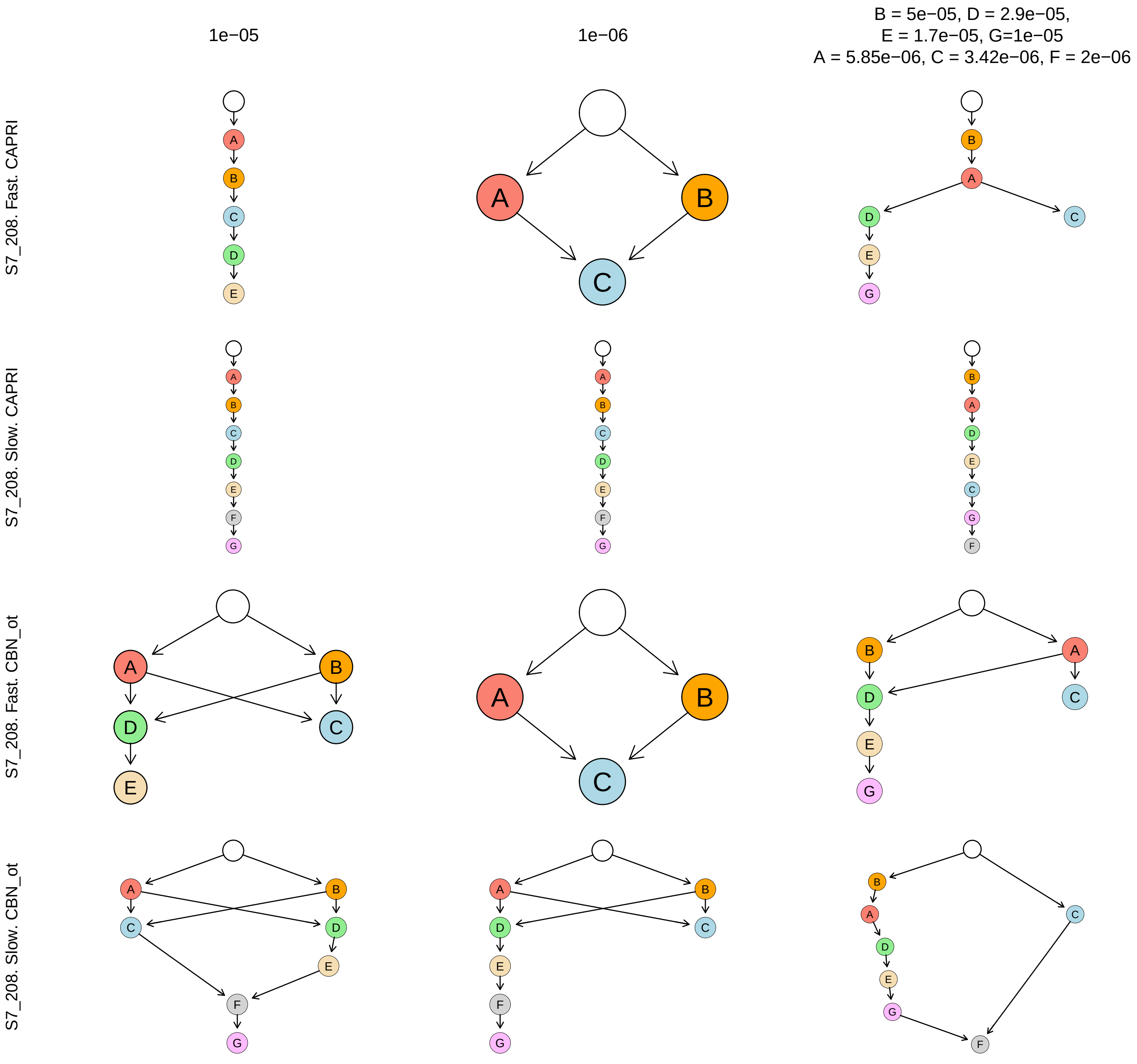

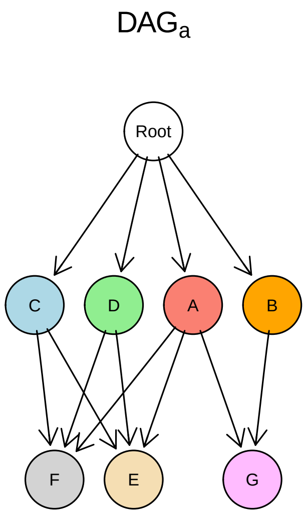

ID	Accessible Genot.	Discrep. DAG_a
S7_209	15	15

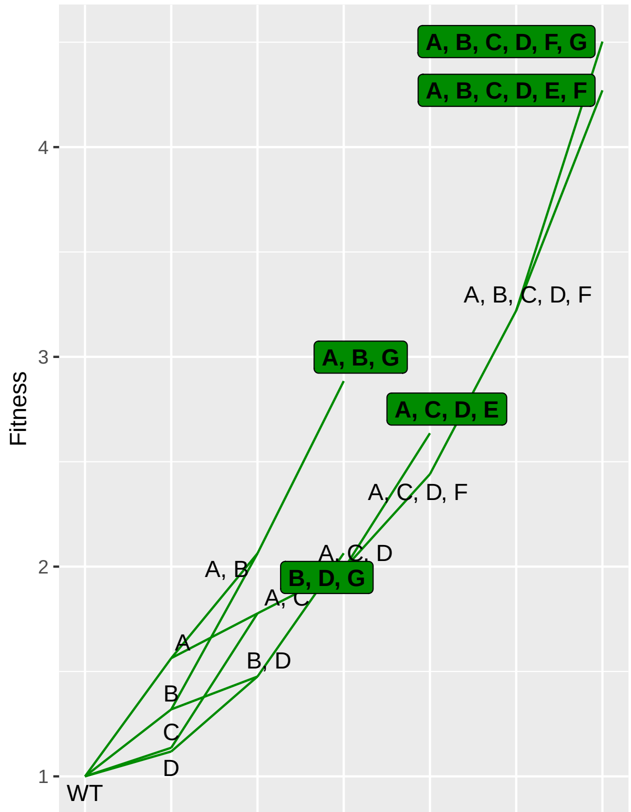

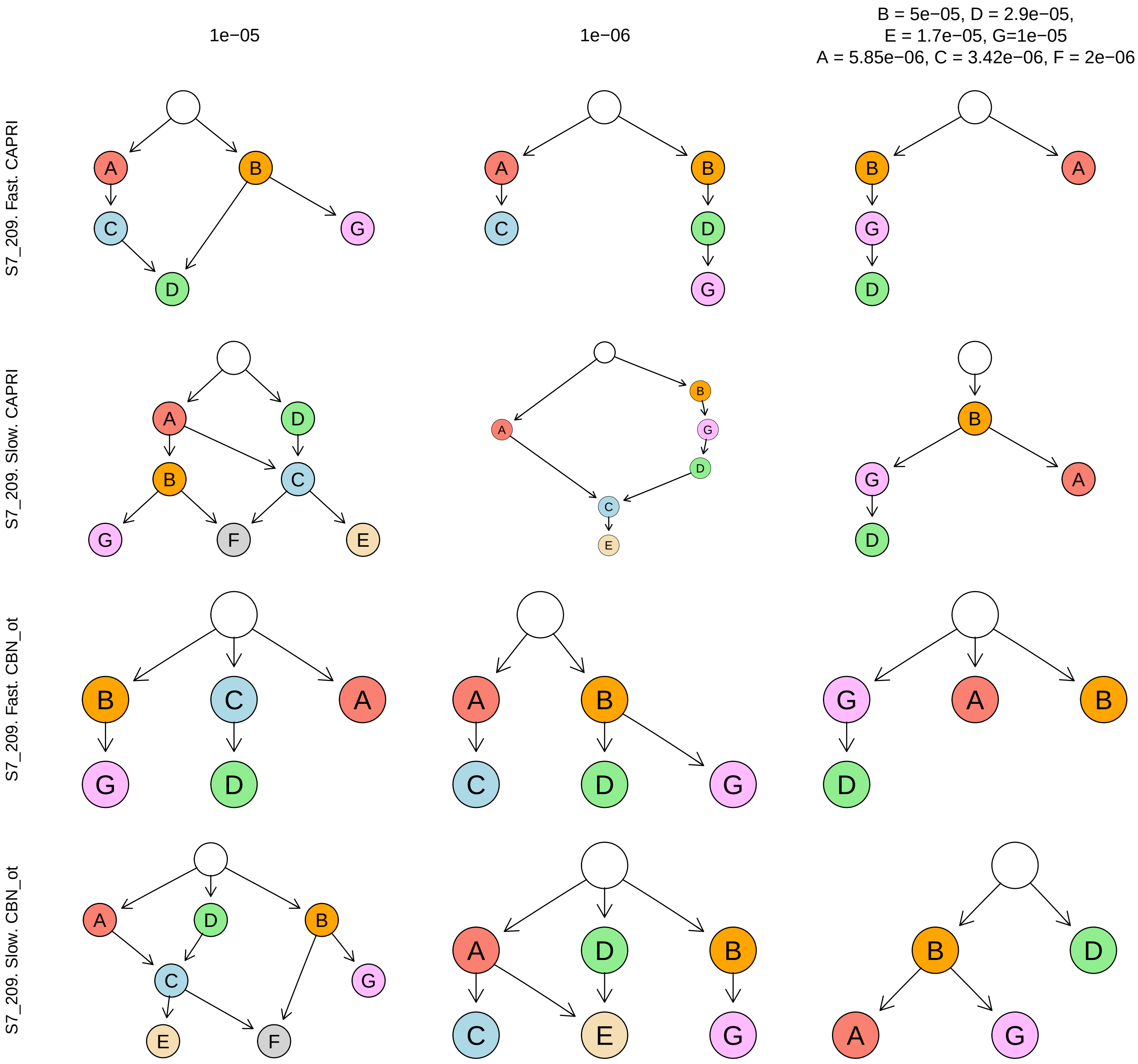

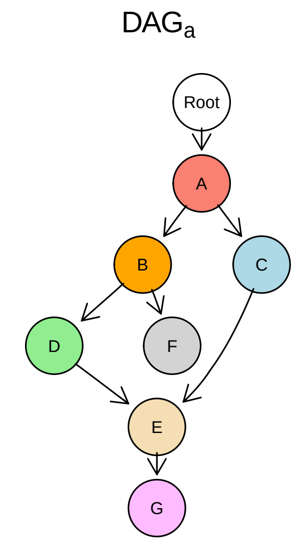

ID	Accessible Genot.	Discrep. DAG_a
S7_210	13	3

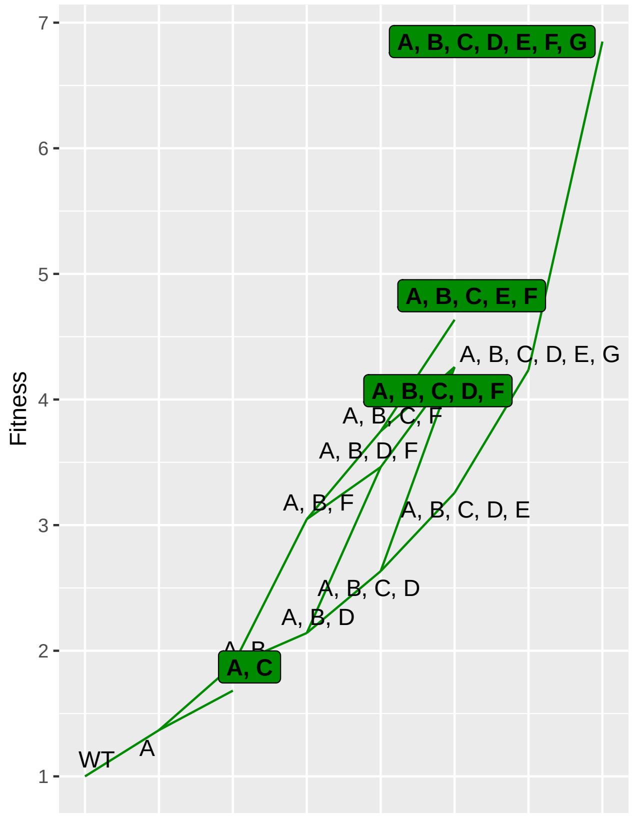

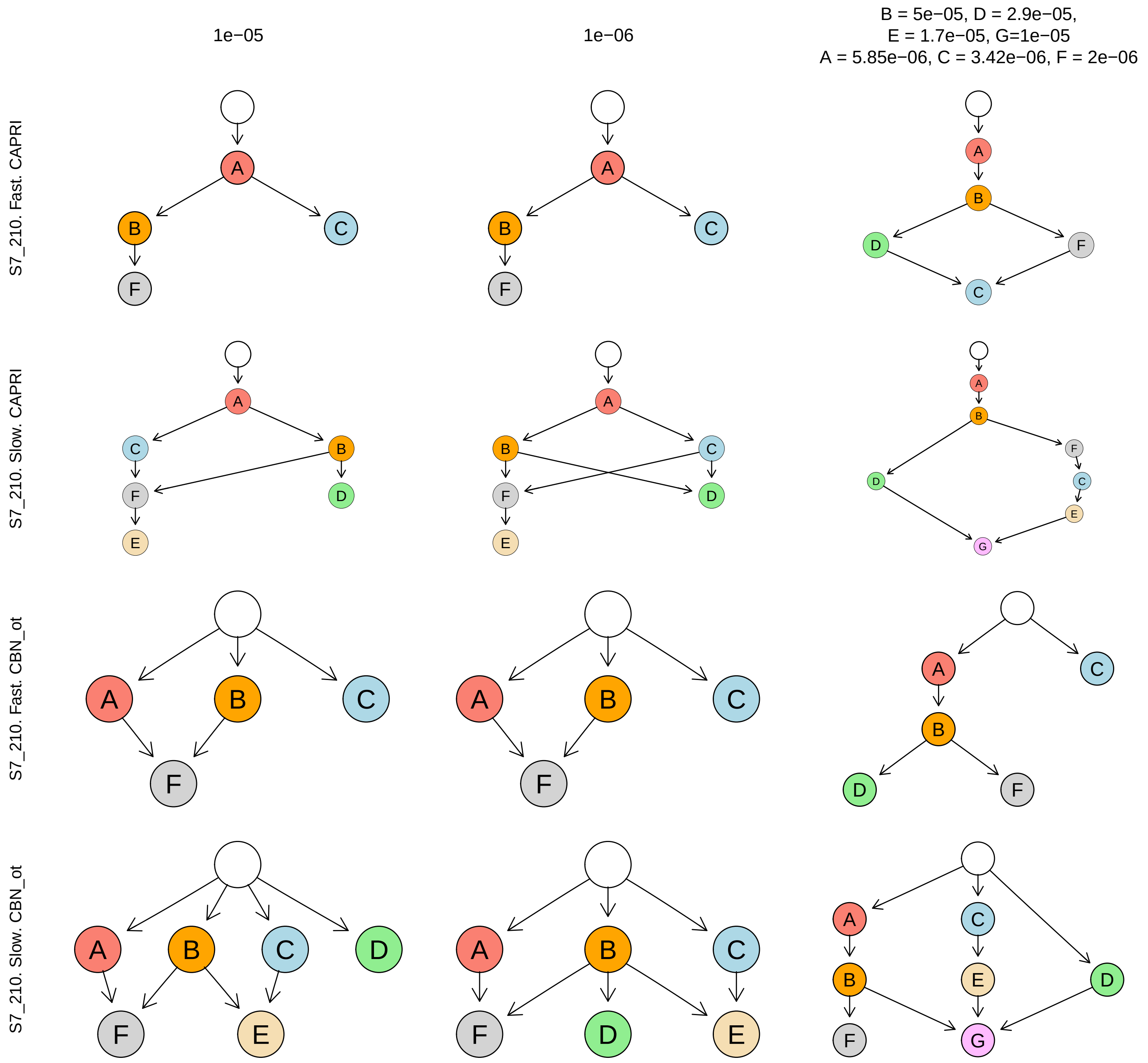

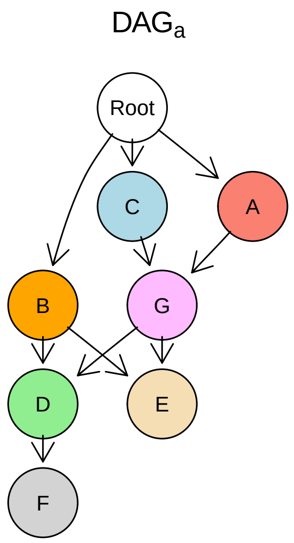

ID	Accessible Genot.	Discrep. DAG_a
S7_211	13	3

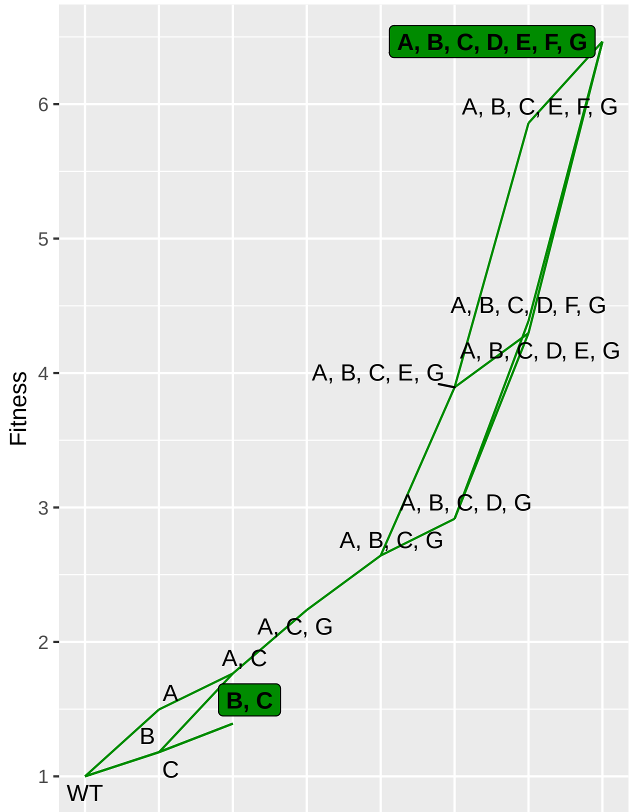

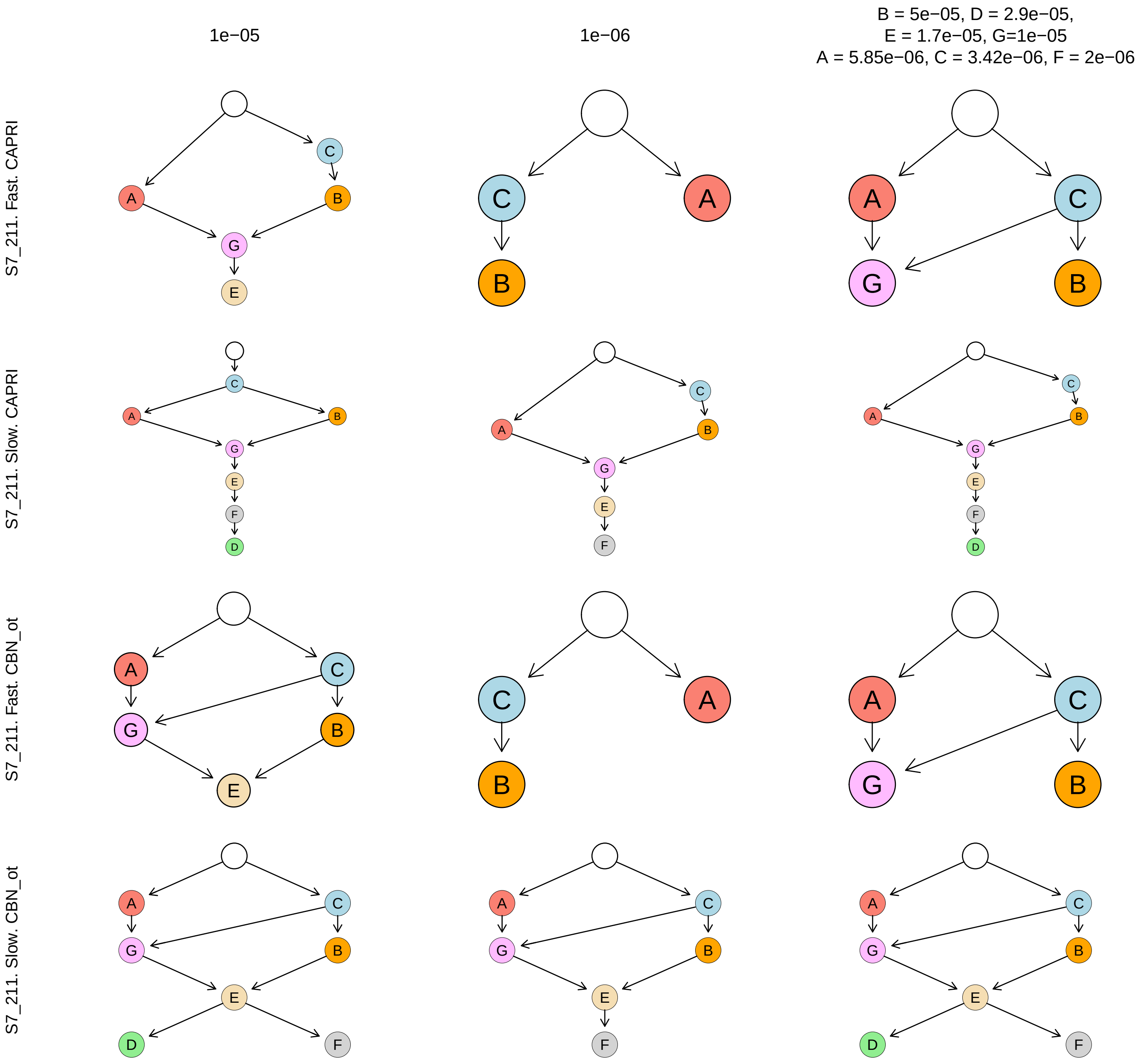

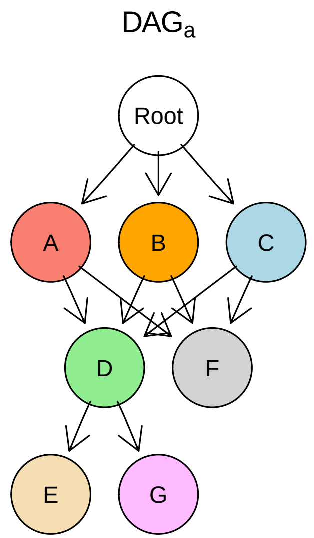

ID	Accessible Genot.	Discrep. DAG_a
S7_212	14	4

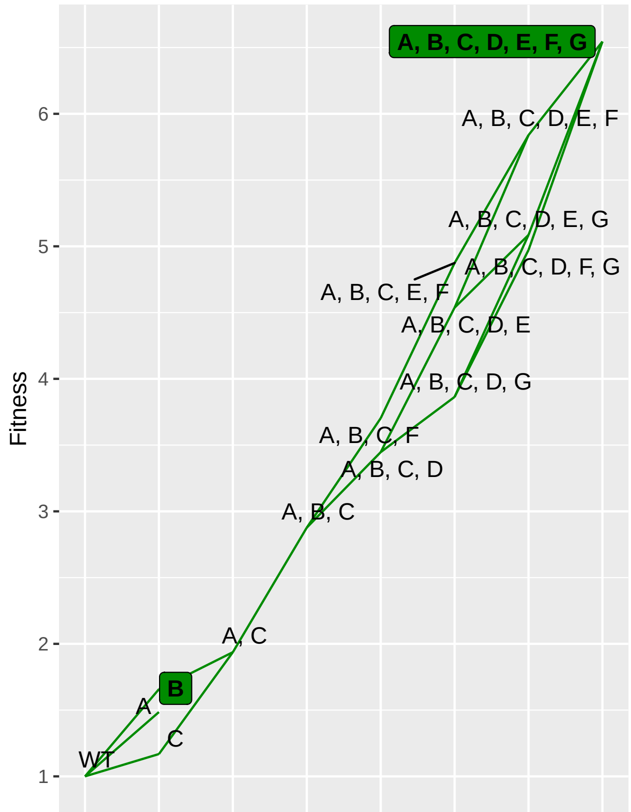

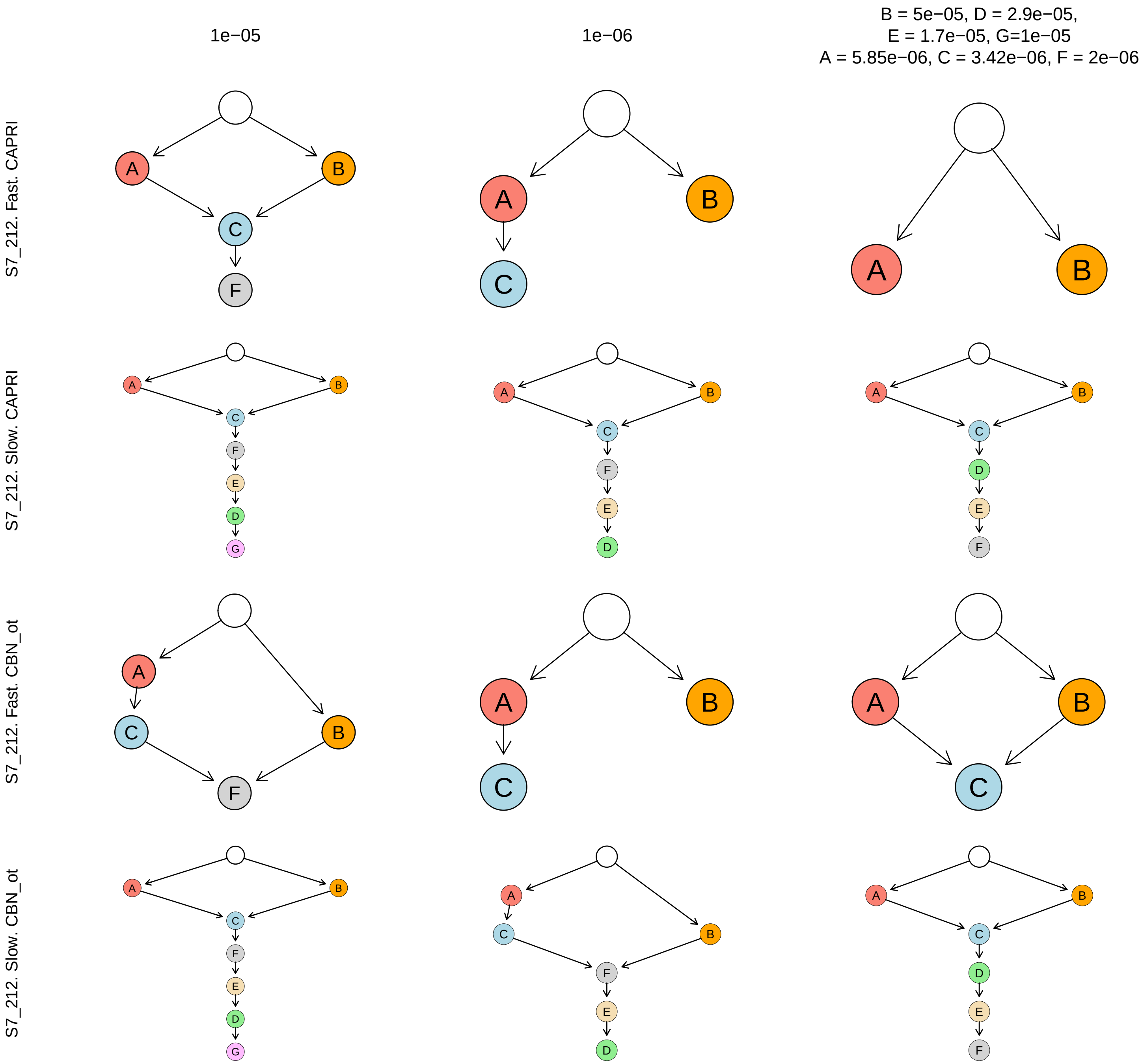

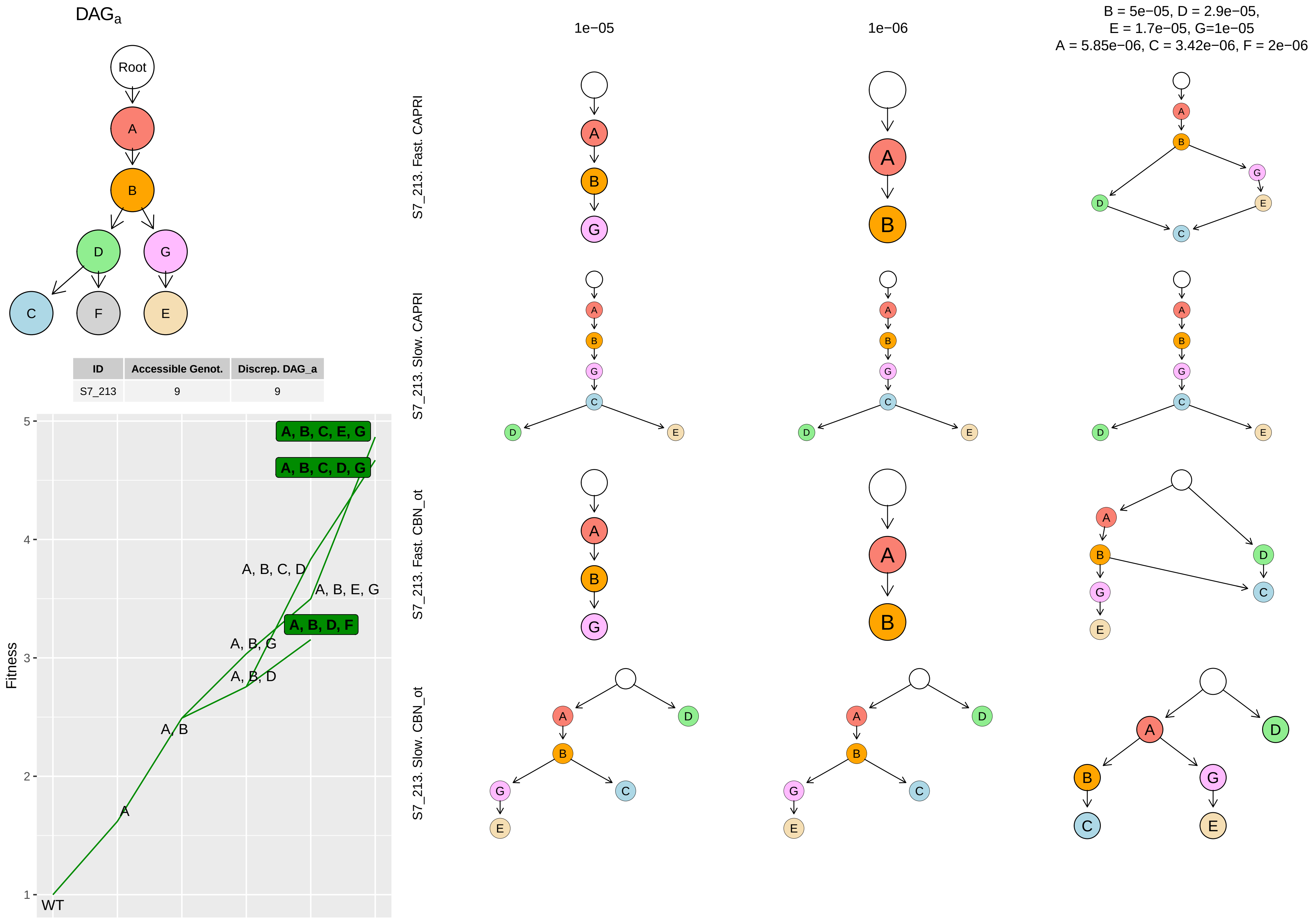

DAG$_a$
Root
A
B
D
G
C
F
E
ID
Accessible Genot.
Discrep. DAG_a
S7_213
9
9
Fitness
WT
A
A, B
A, B, D
A, B, G
A, B, D, F
A, B, C, D
A, B, E, G
A, B, C, D, G
A, B, C, E, G
1e−05
1e−06
B = 5e−05, D = 2.9e−05,
E = 1.7e−05, G=1e−05
A = 5.85e−06, C = 3.42e−06, F = 2e−06
S7_213. Fast. CAPRI
S7_213. Slow. CAPRI
S7_213. Fast. CBN_ot
S7_213. Slow. CBN_ot

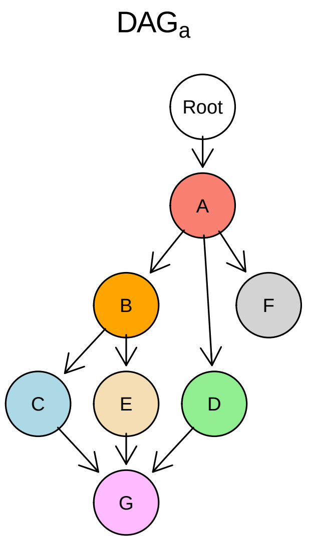

ID	Accessible Genot.	Discrep. DAG_a
S7_214	28	12

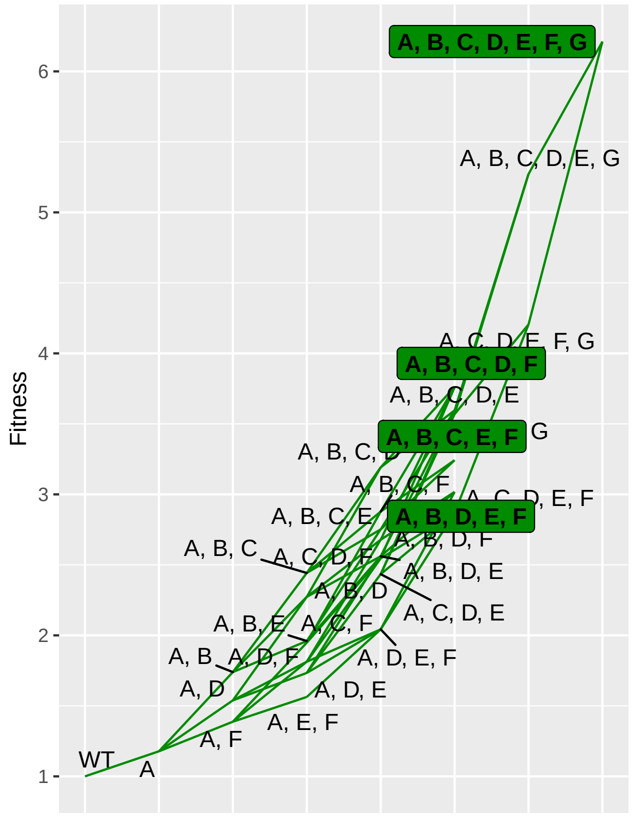

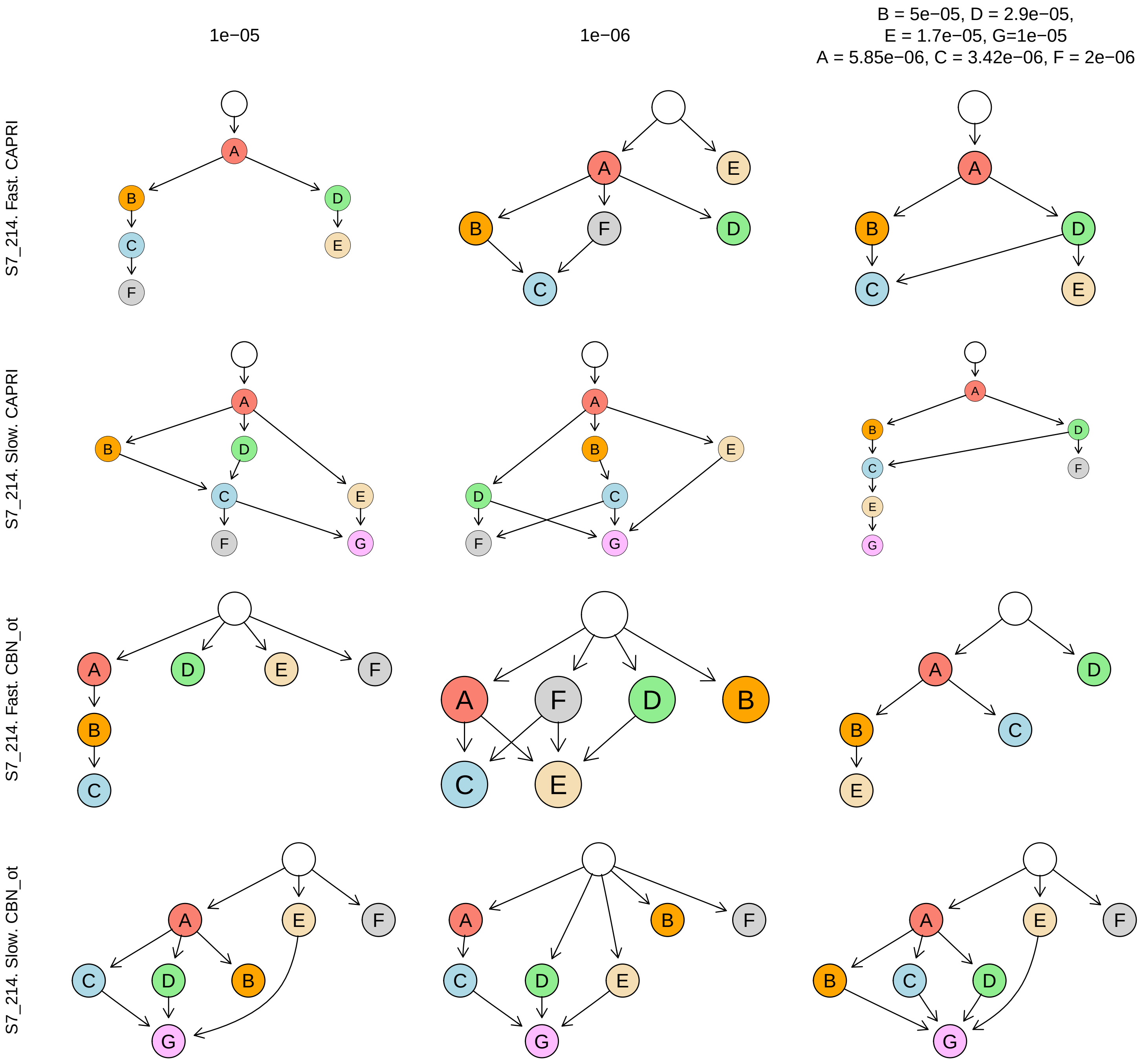

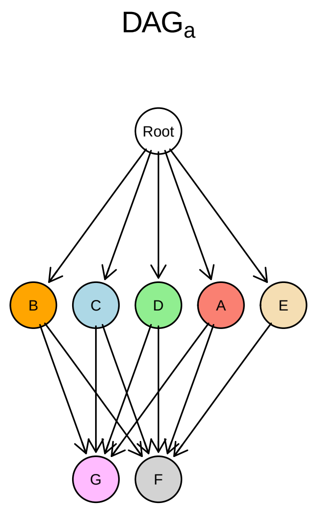

ID	Accessible Genot.	Discrep. DAG_a
S7_215	22	13

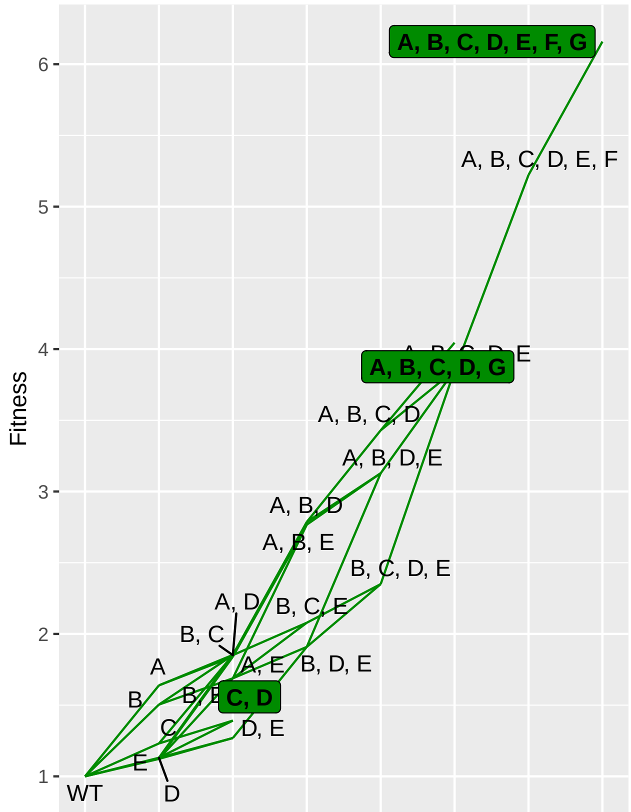

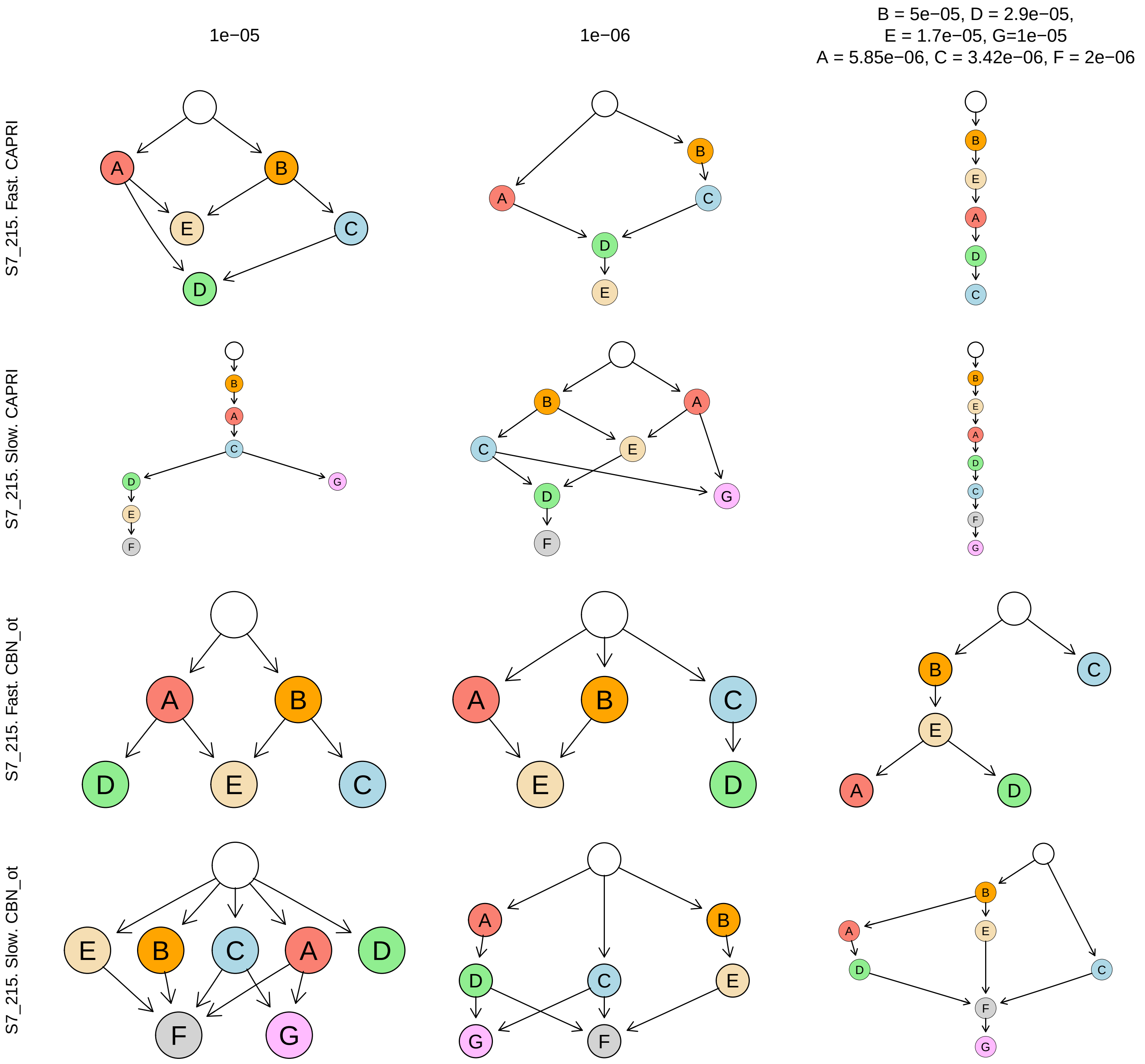

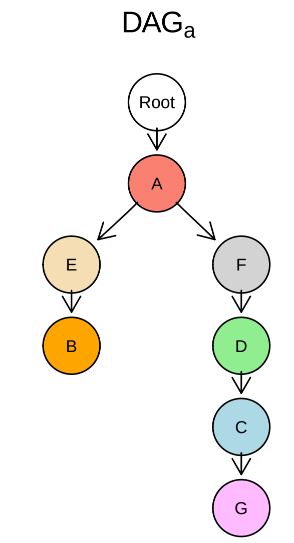

ID	Accessible Genot.	Discrep. DAG_a
S7_216	10	7

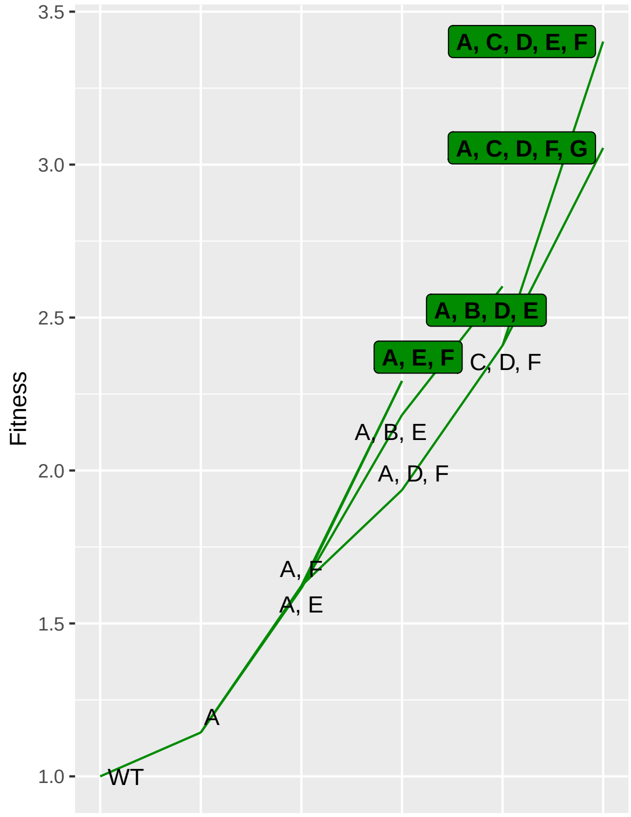

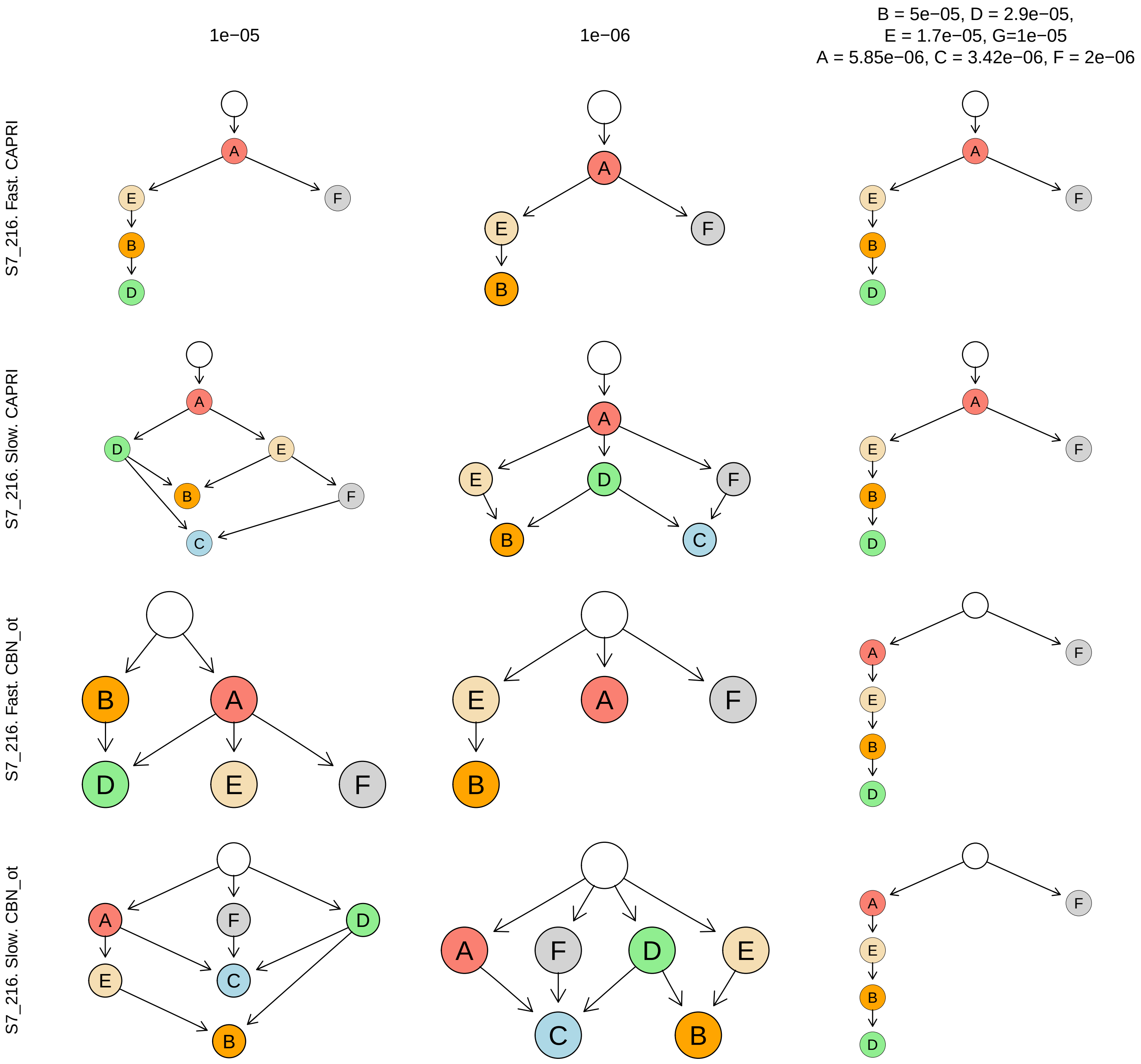

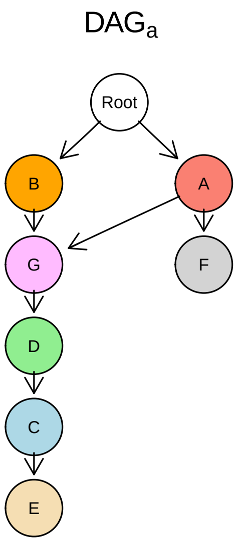

ID	Accessible Genot.	Discrep. DAG_a
S7_217	10	3

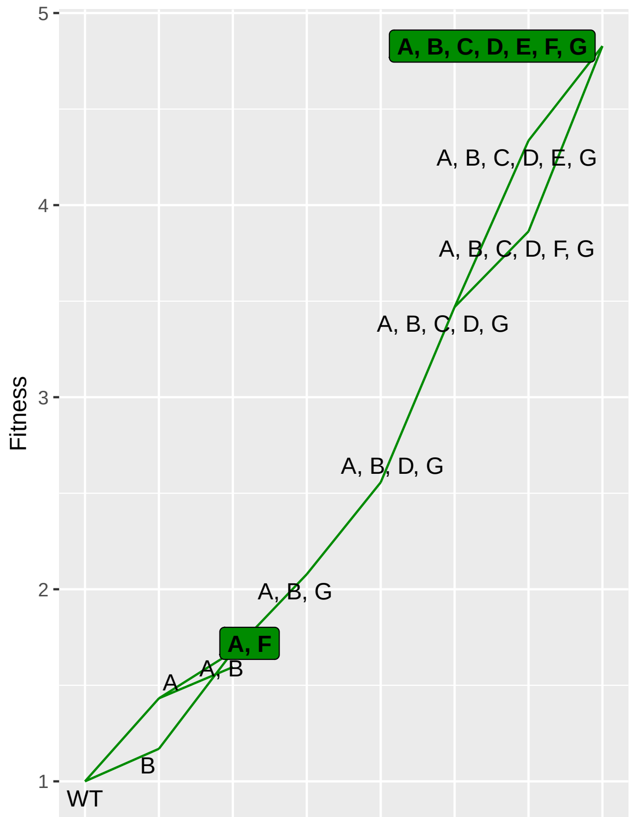

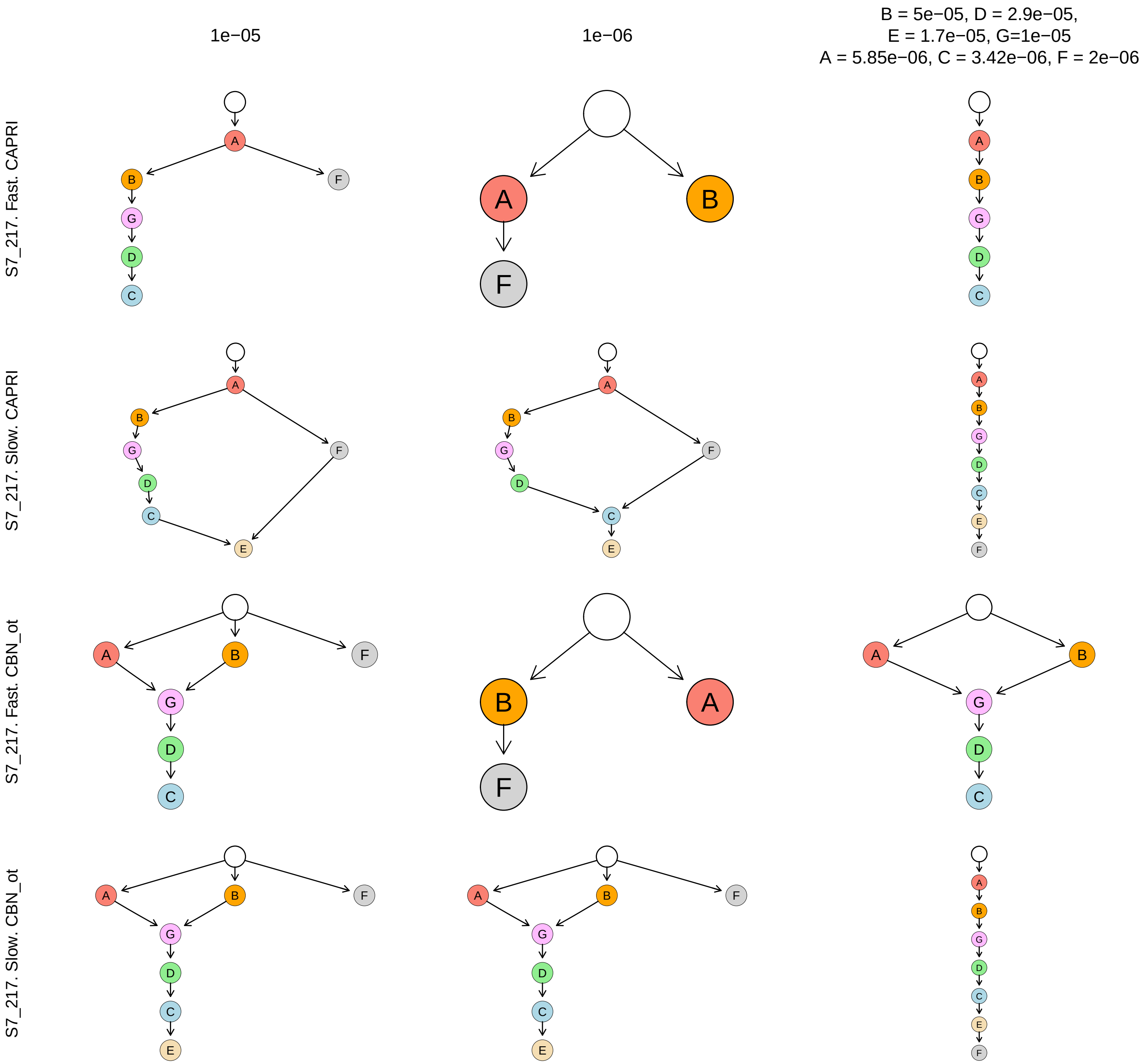

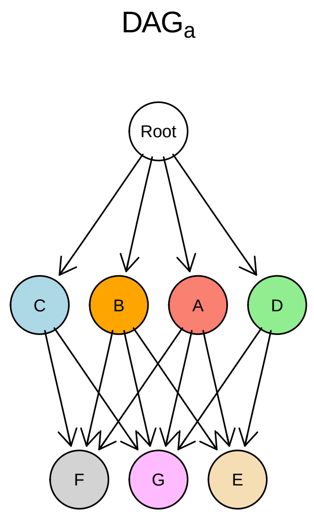

ID	Accessible Genot.	Discrep. DAG_a
S7_218	21	3

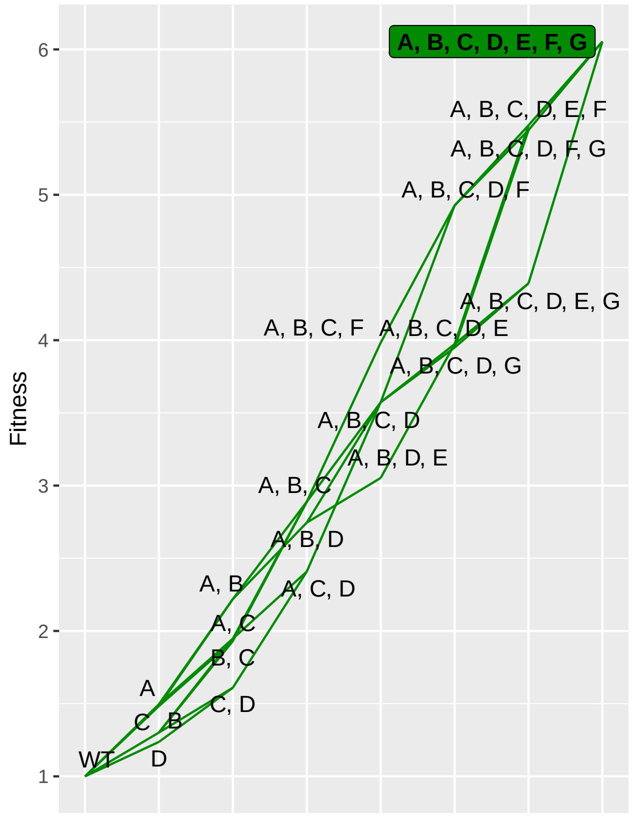

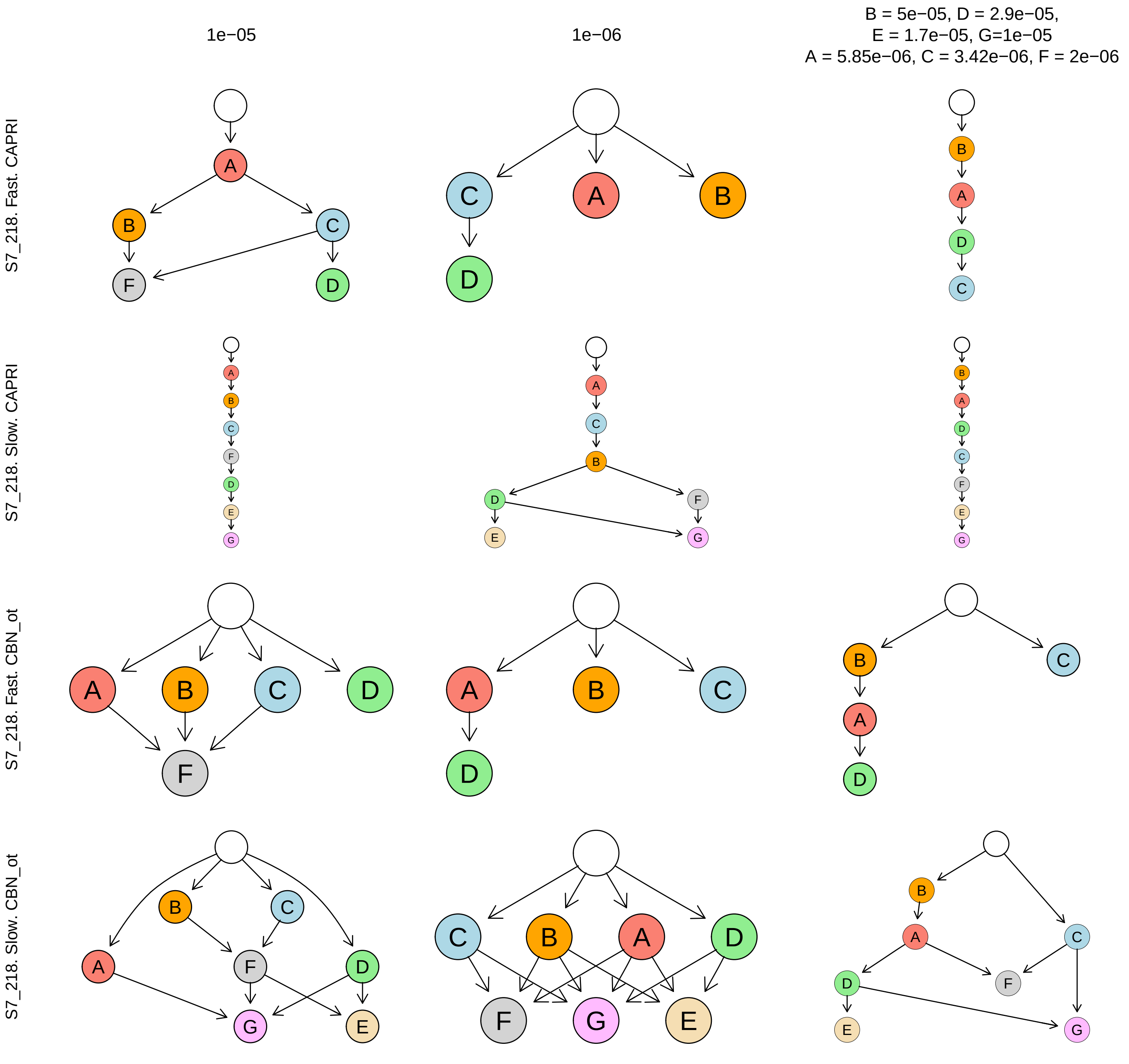

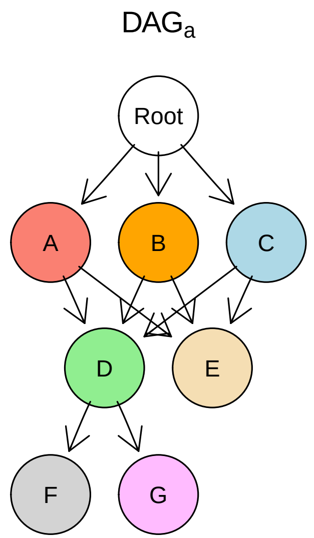

ID	Accessible Genot.	Discrep. DAG_a
S7_219	12	4

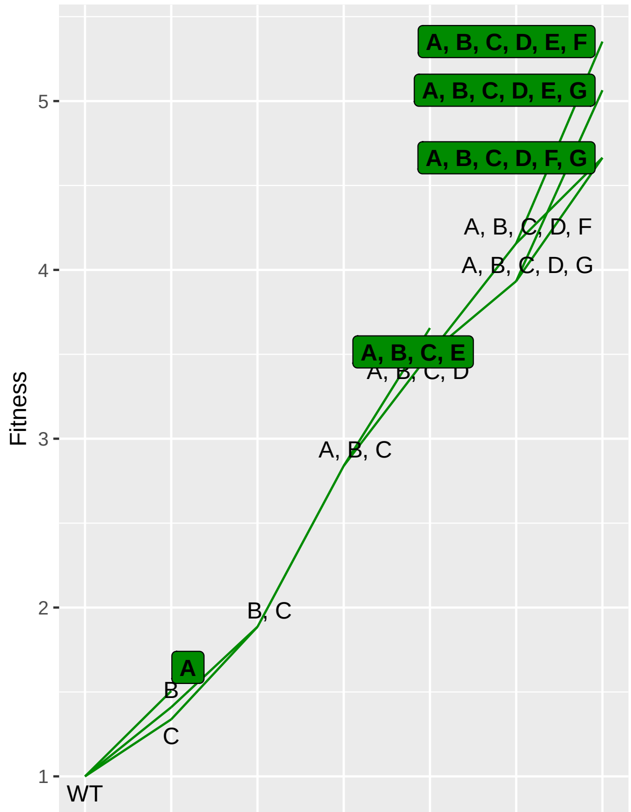

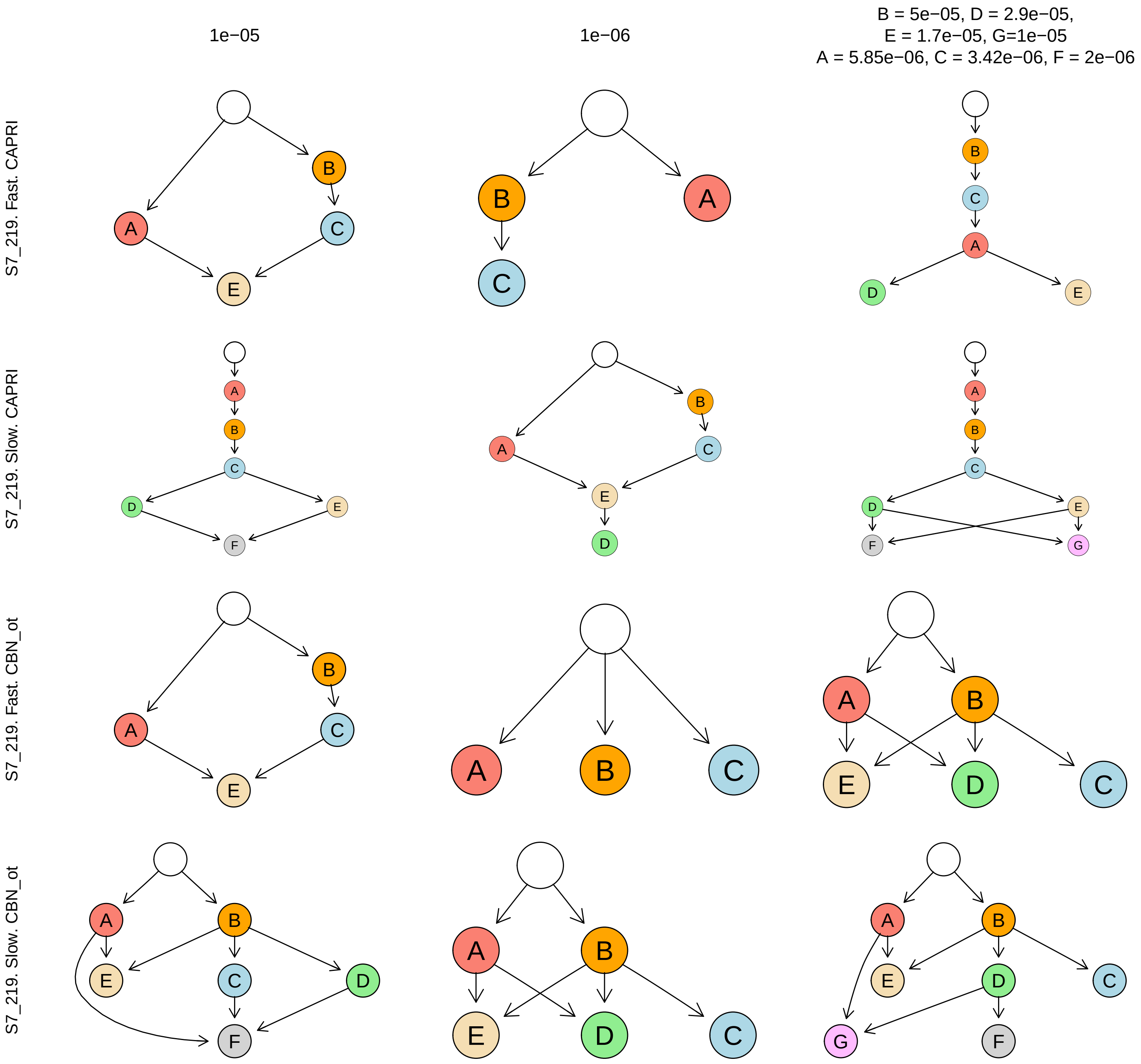

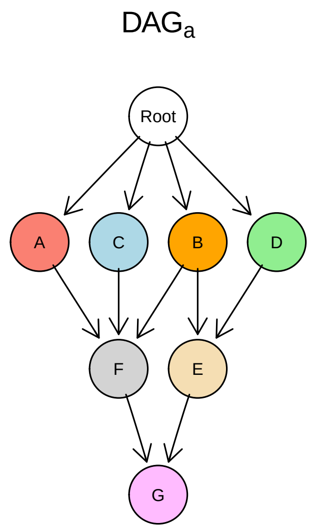

ID	Accessible Genot.	Discrep. DAG_a
S7_220	16	7

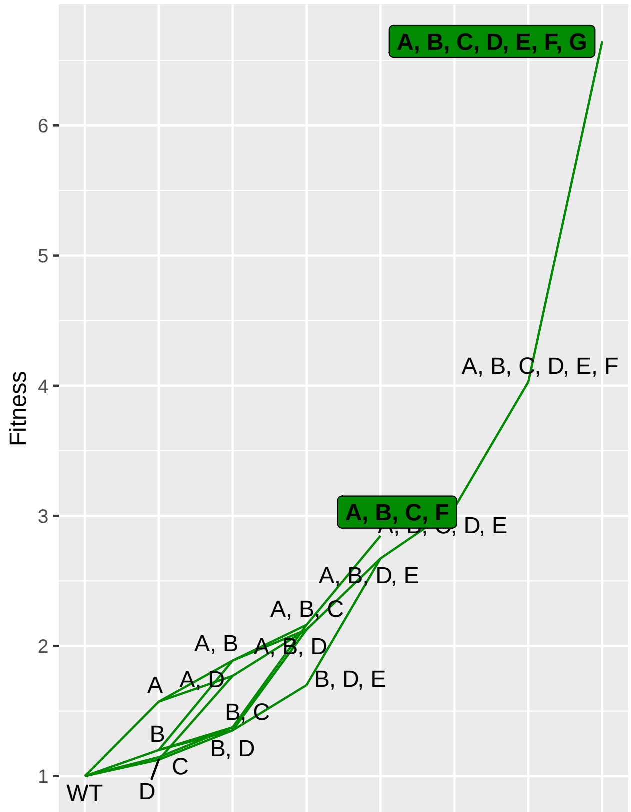

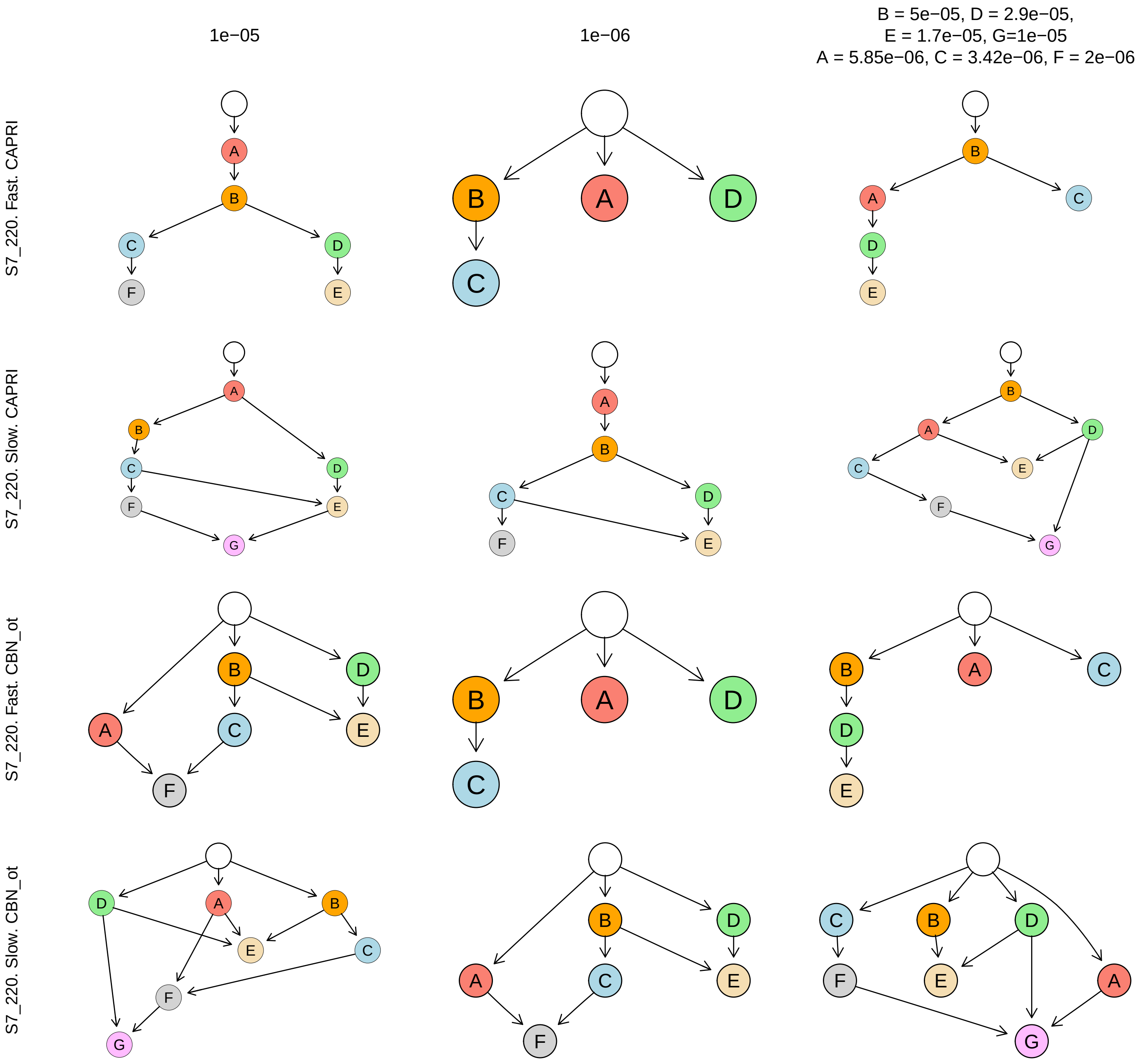

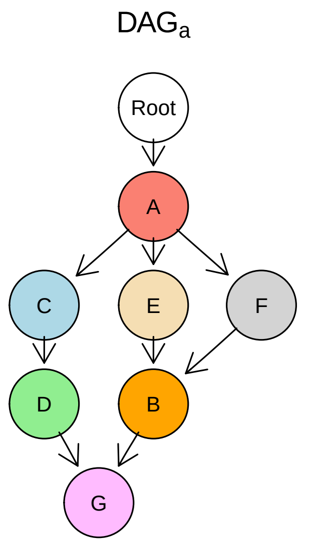

ID	Accessible Genot.	Discrep. DAG_a
S7_221	13	9

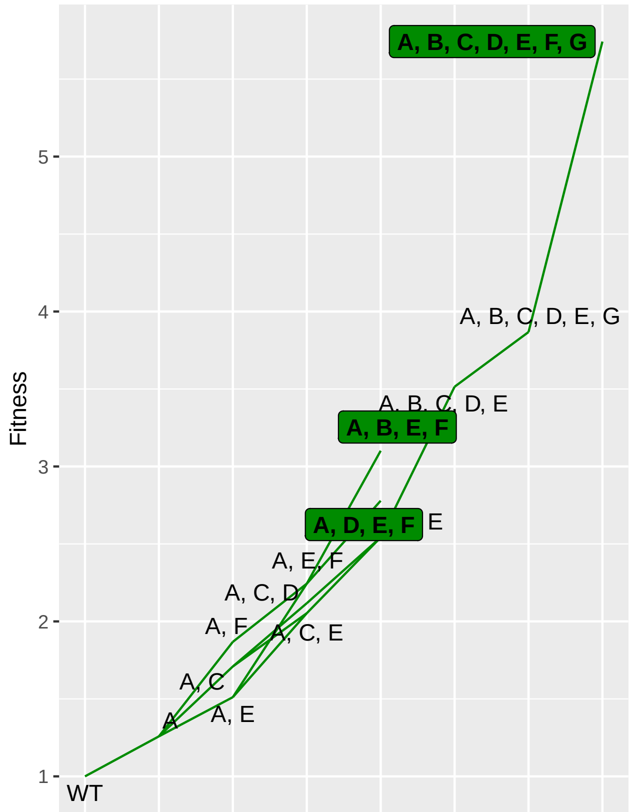

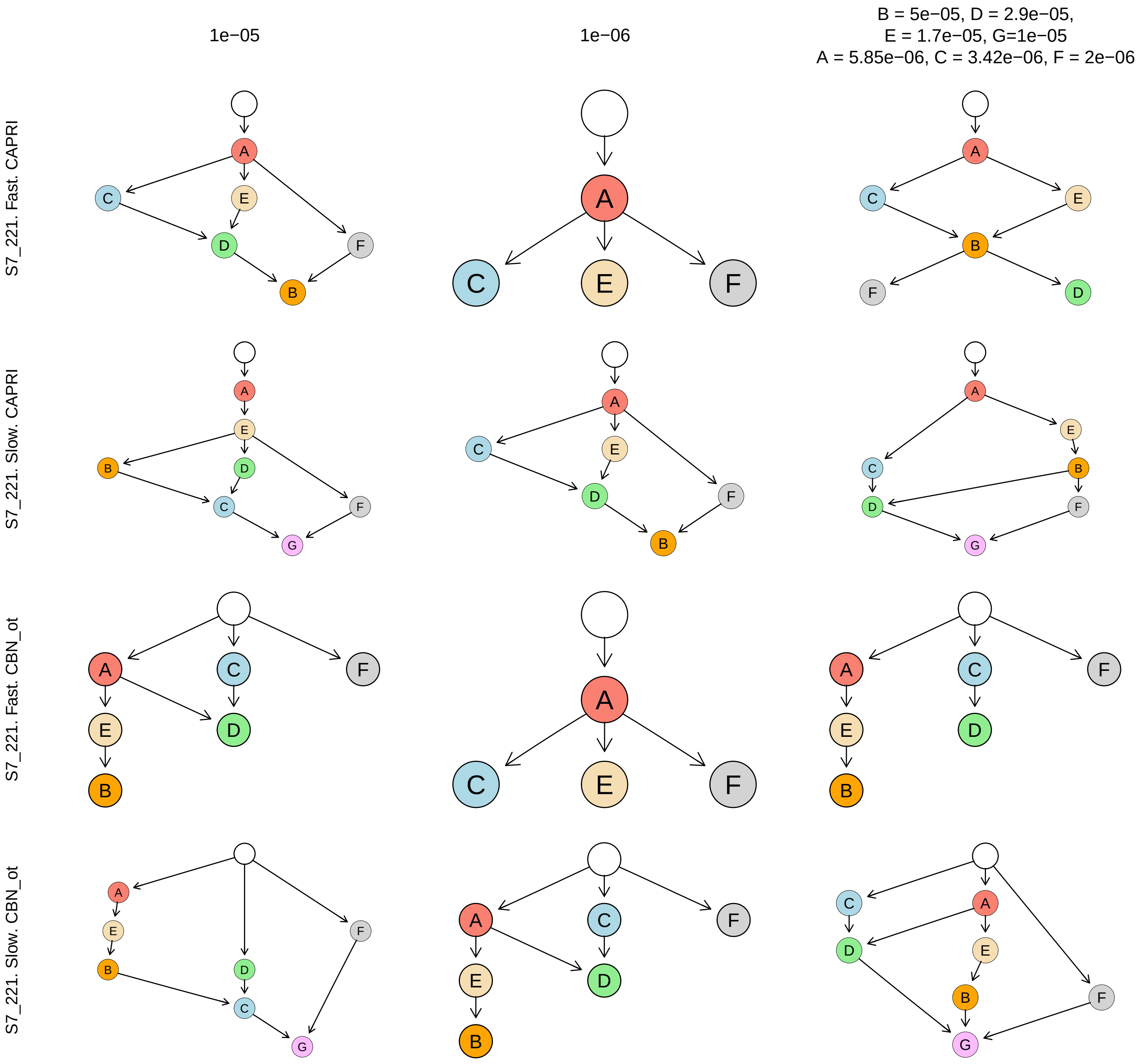

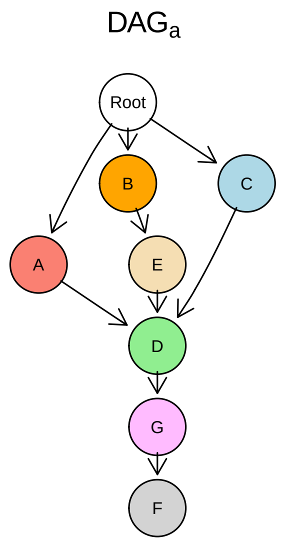

ID	Accessible Genot.	Discrep. DAG_a
S7_222	13	1

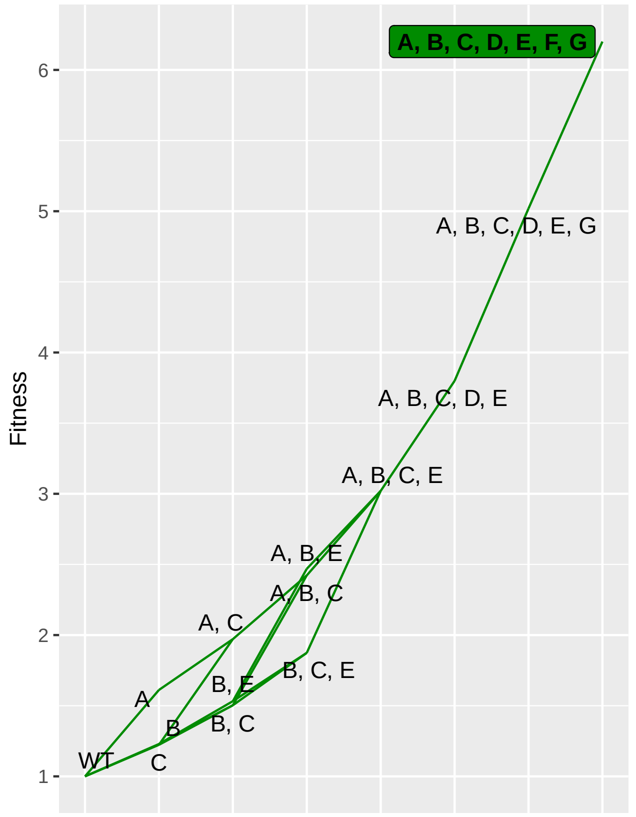

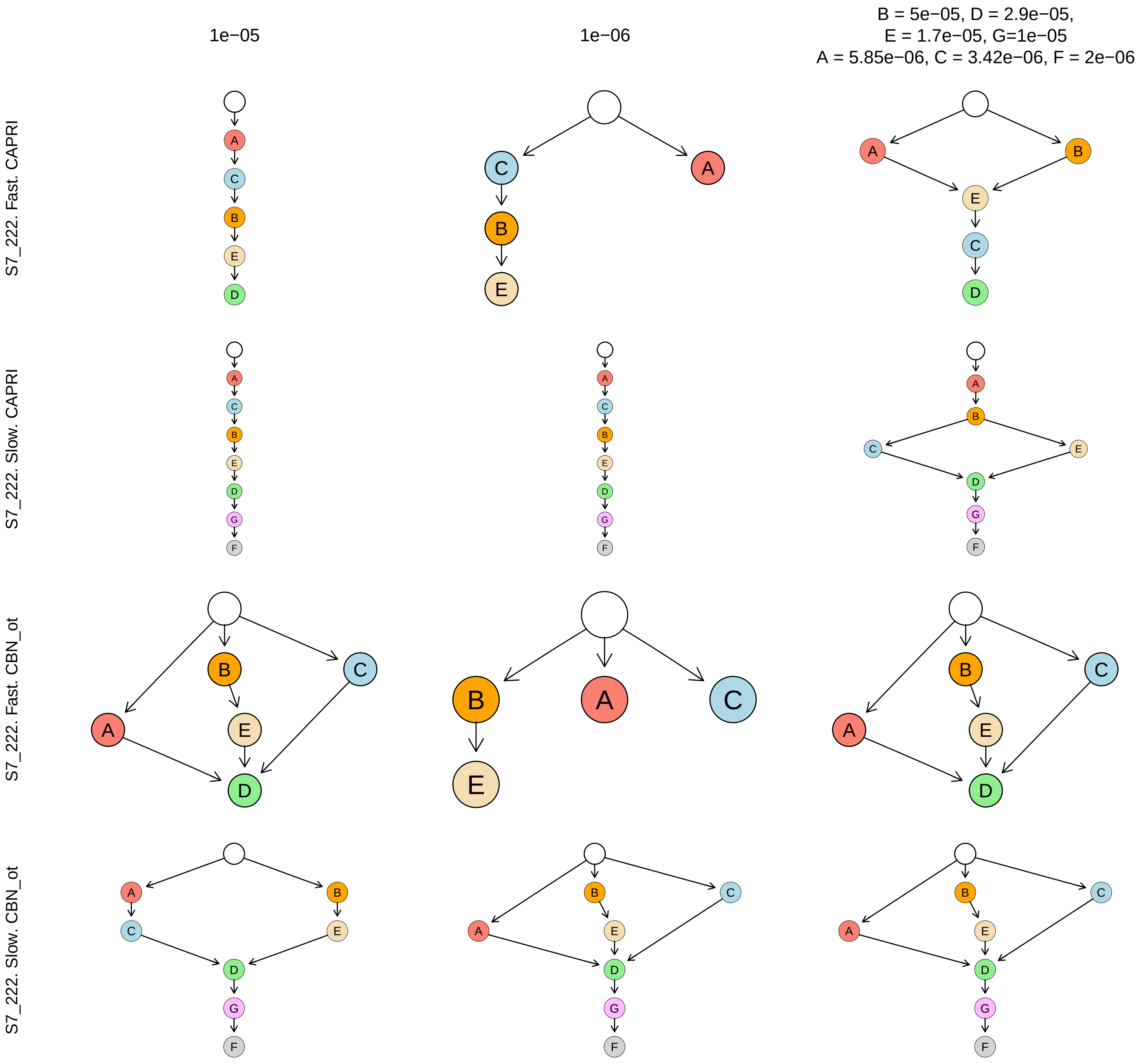

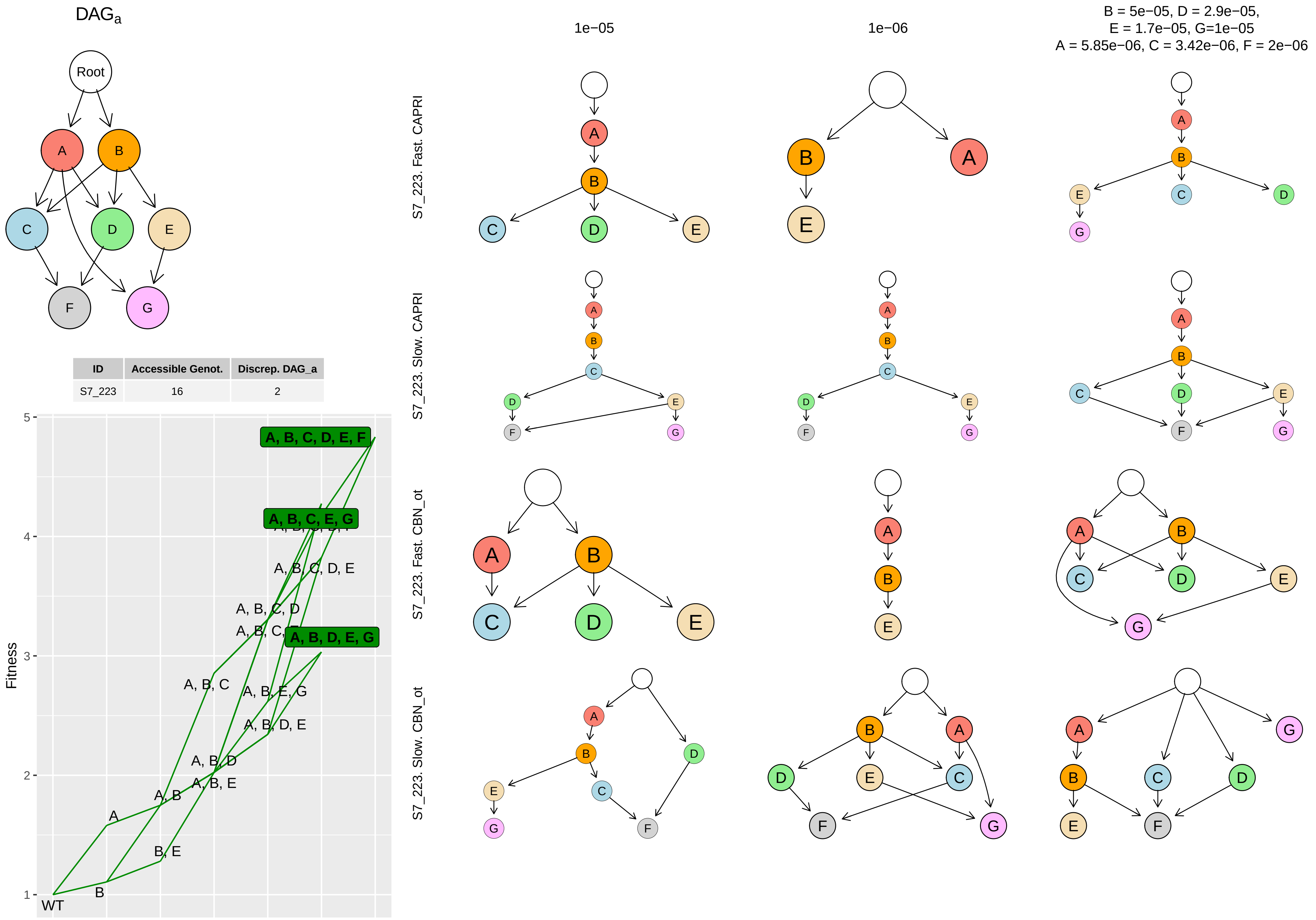

ID	Accessible Genot.	Discrep. DAG_a
S7_223	16	2

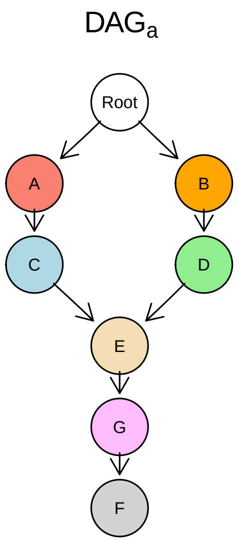

ID	Accessible Genot.	Discrep. DAG_a
S7_224	10	1

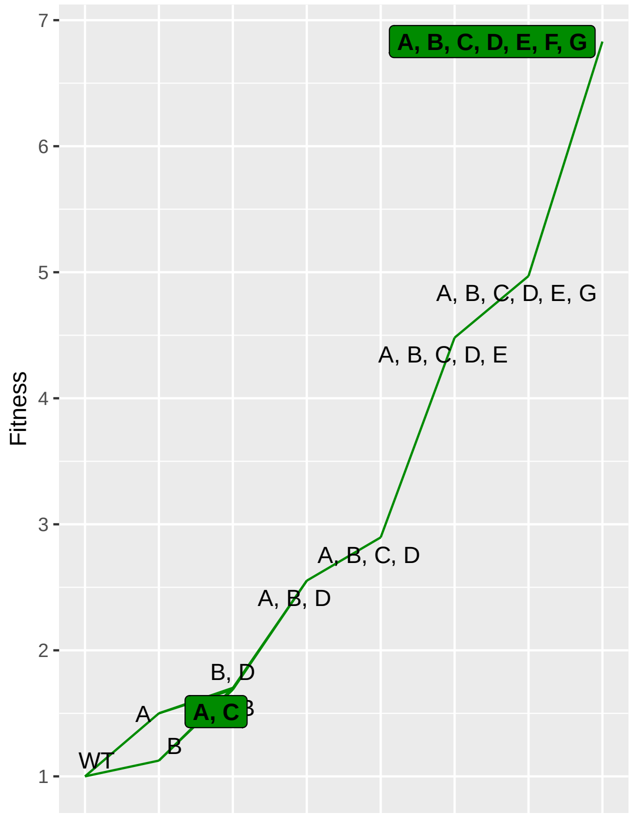

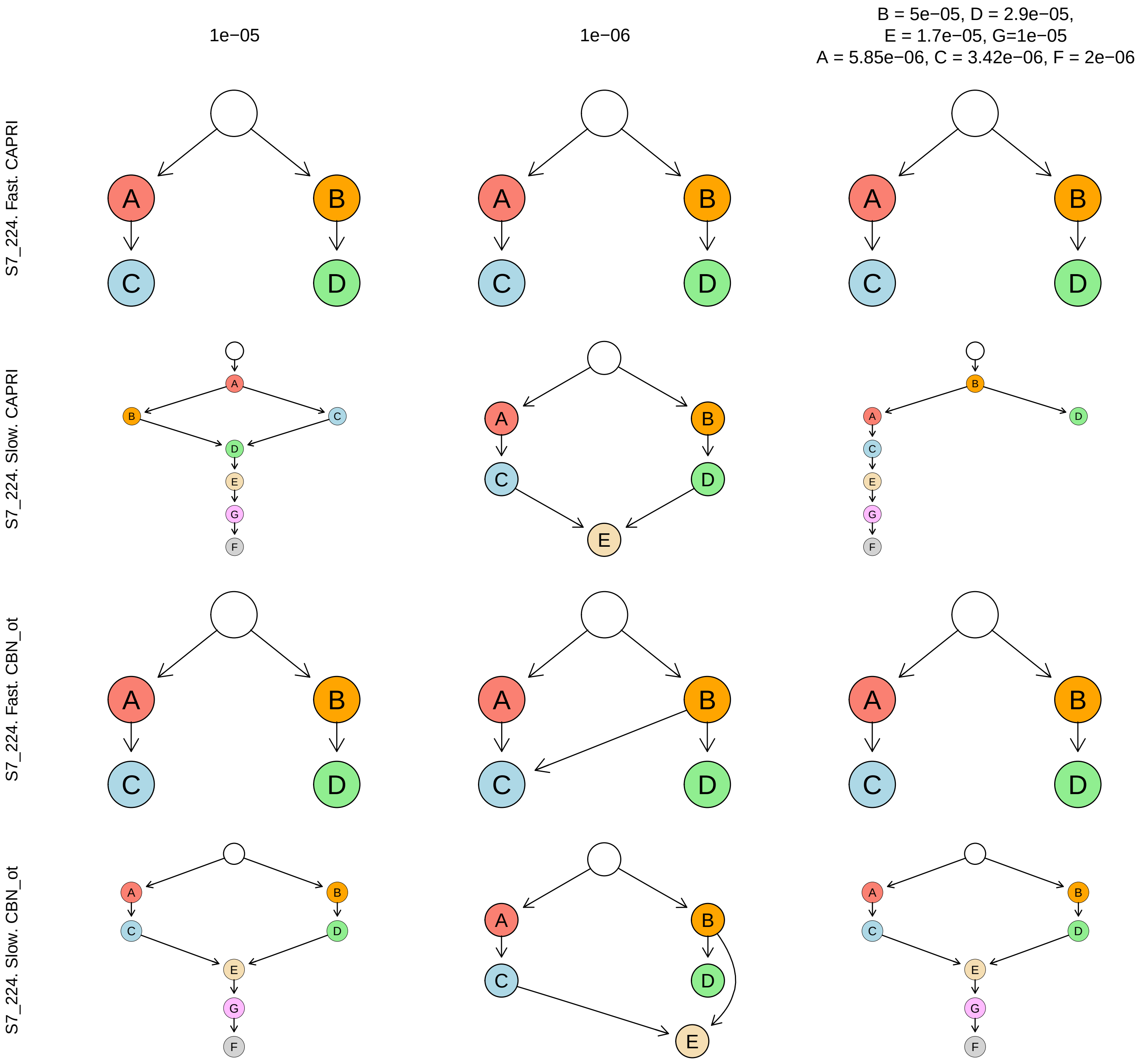

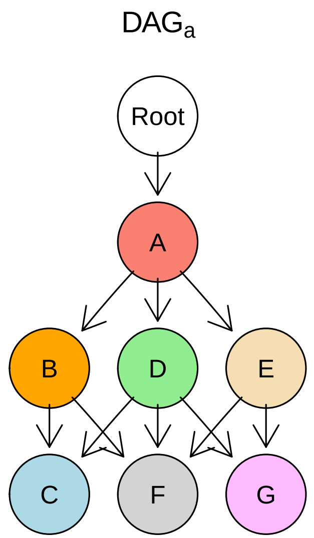

ID	Accessible Genot.	Discrep. DAG_a
S7_225	18	3

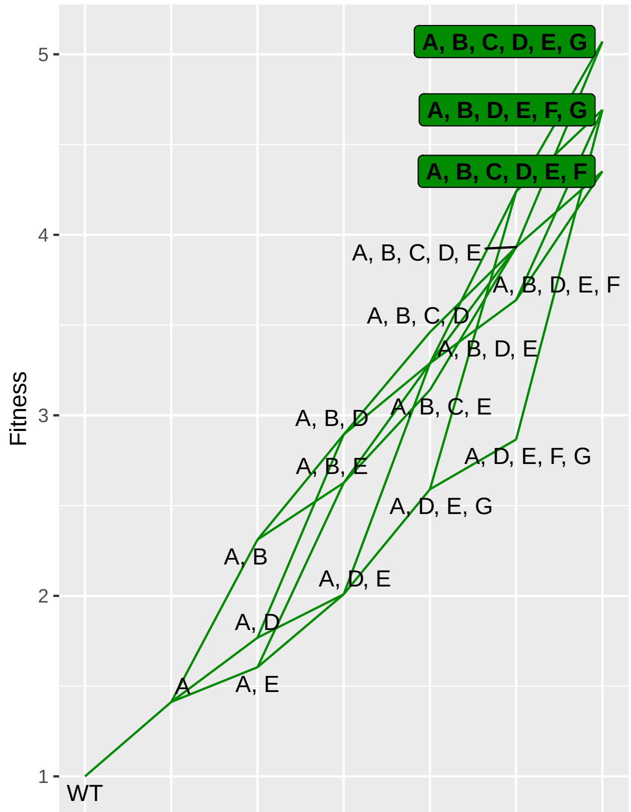

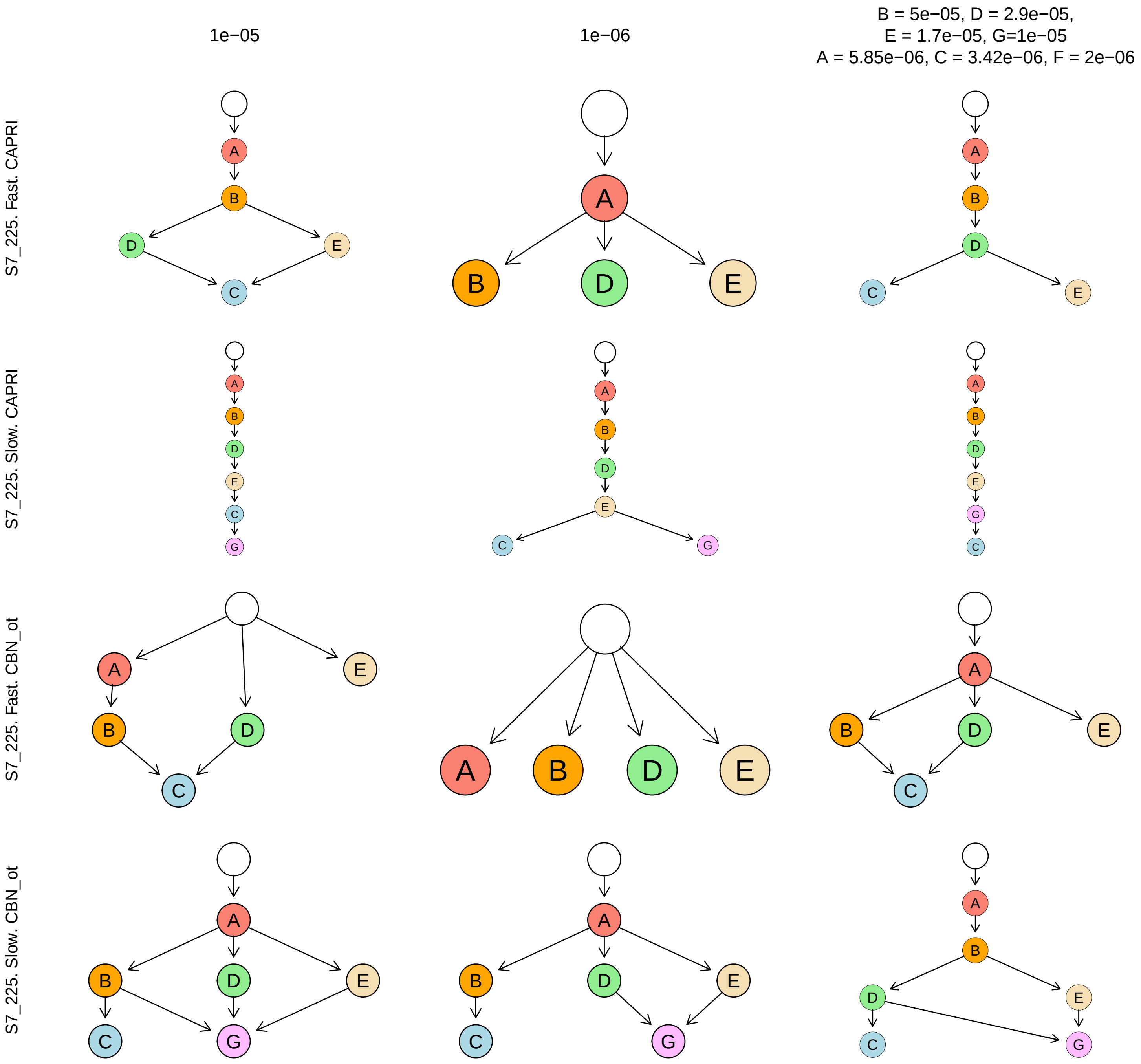

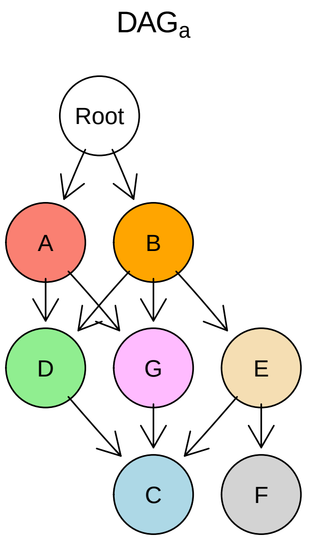

ID	Accessible Genot.	Discrep. DAG_a
S7_226	12	10

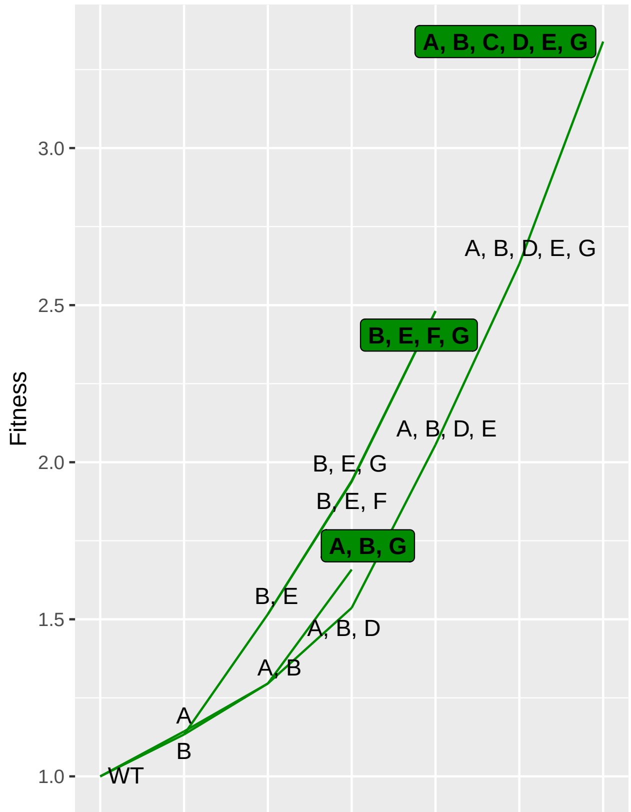

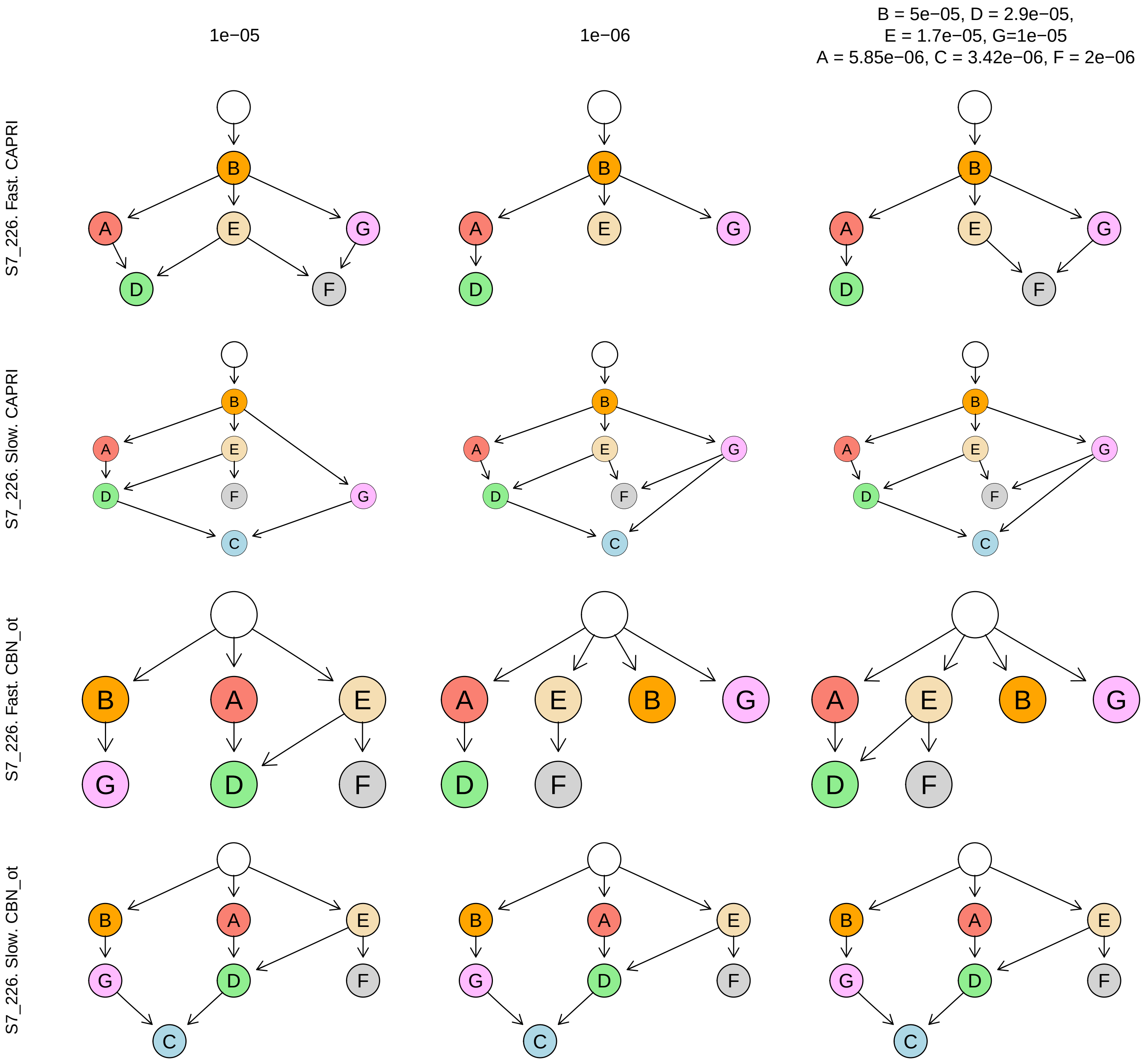

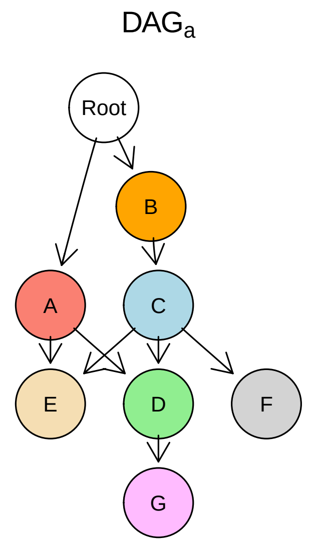

ID	Accessible Genot.	Discrep. DAG_a
S7_227	11	8

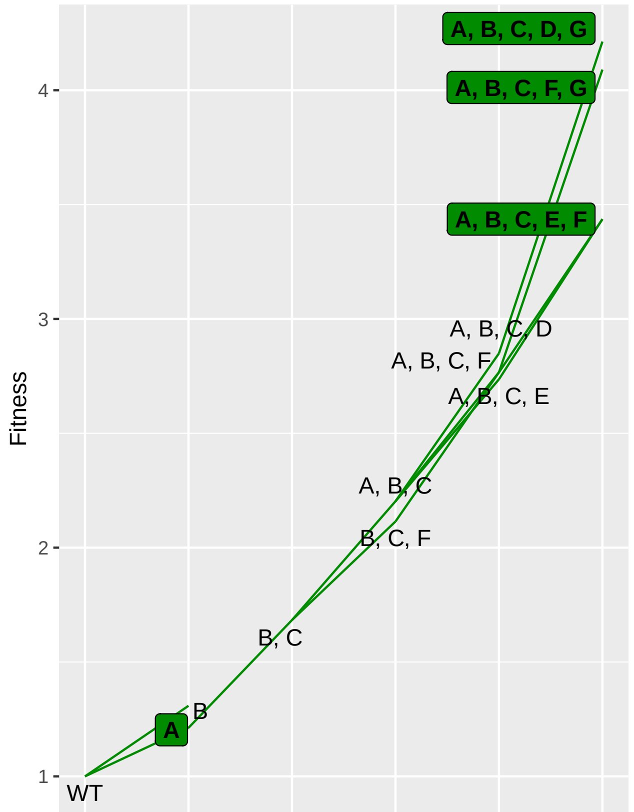

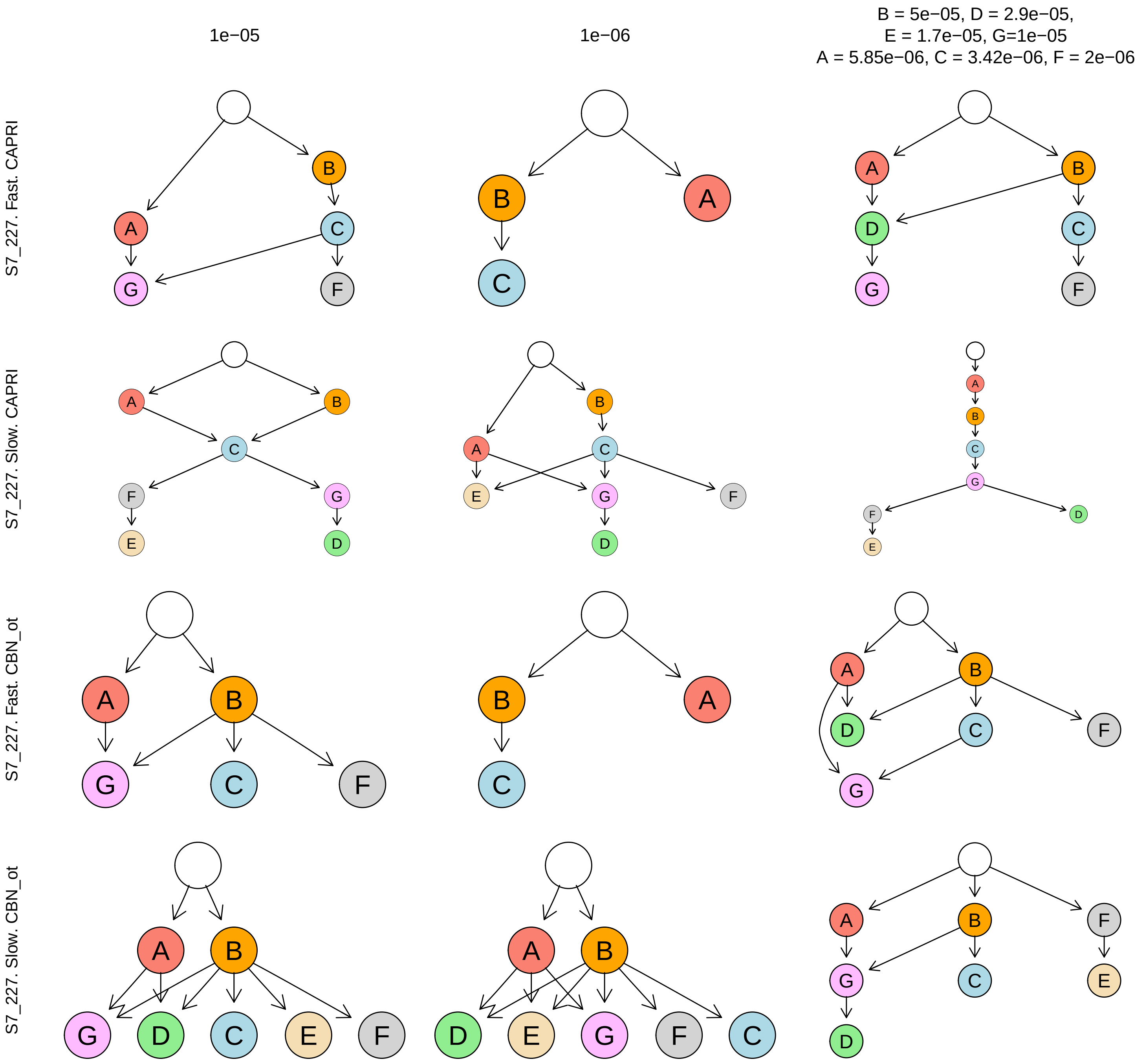

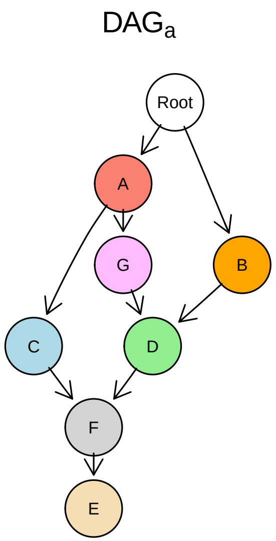

ID	Accessible Genot.	Discrep. DAG_a
S7_228	12	1

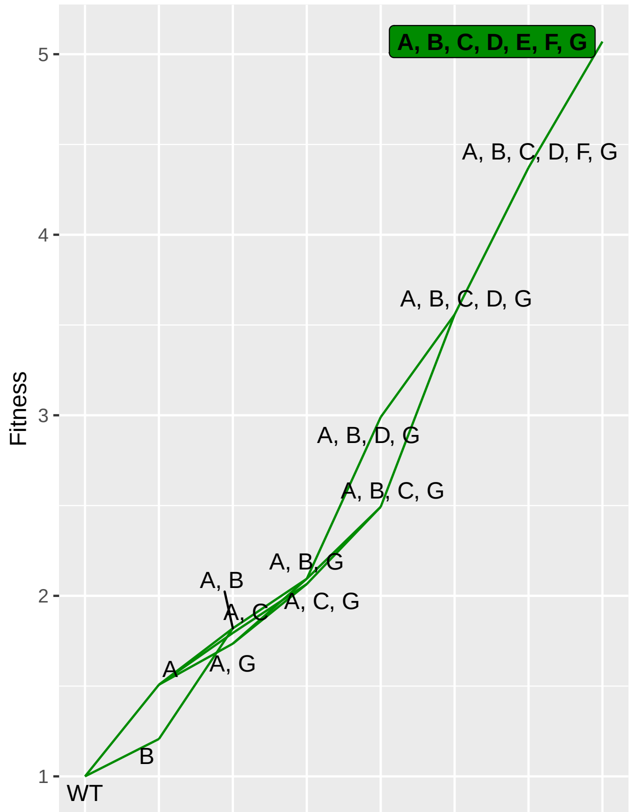

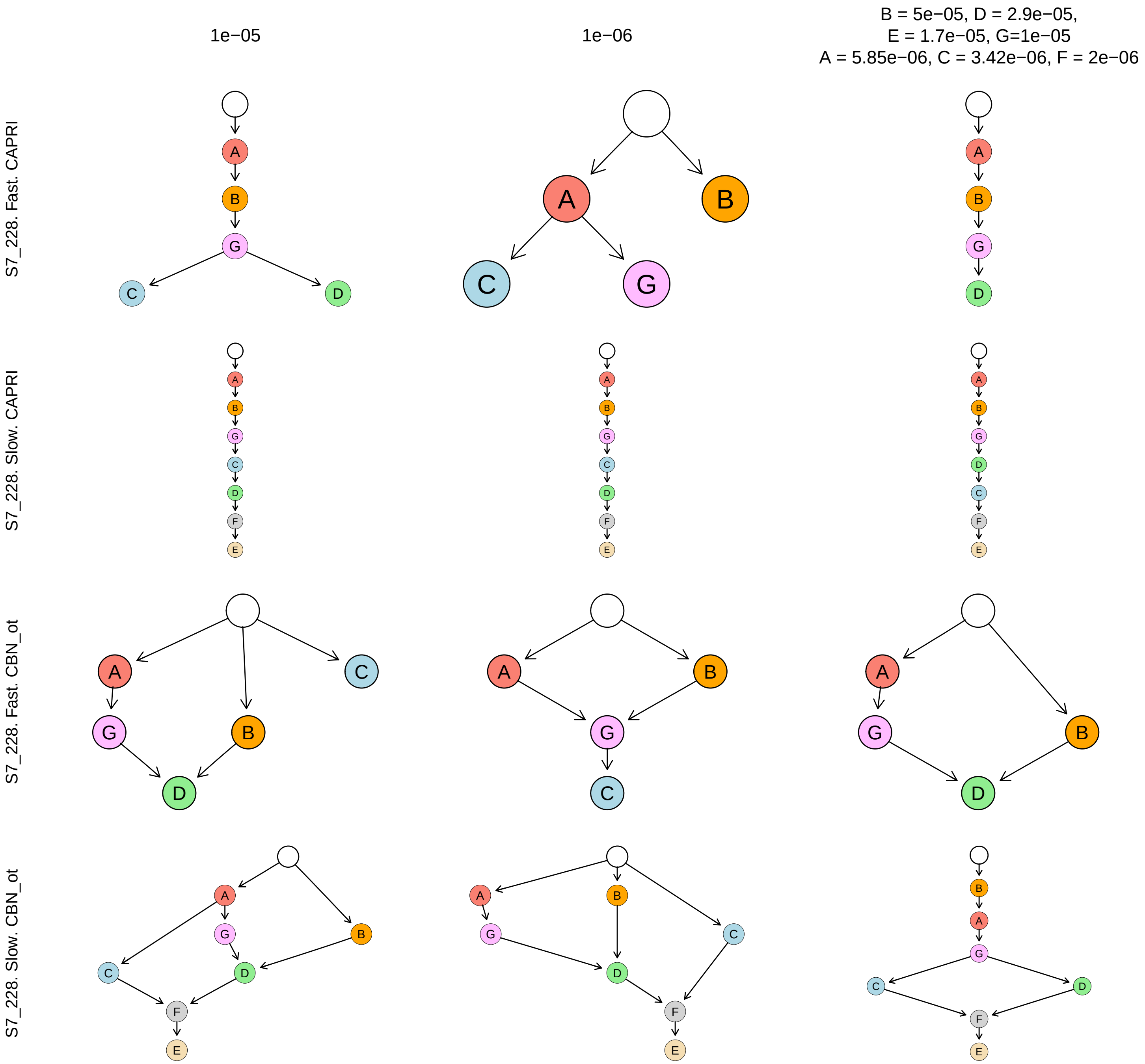

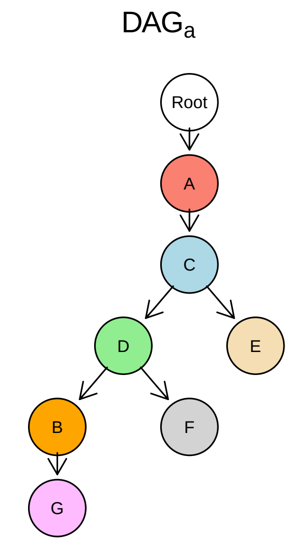

ID	Accessible Genot.	Discrep. DAG_a
S7_229	13	6

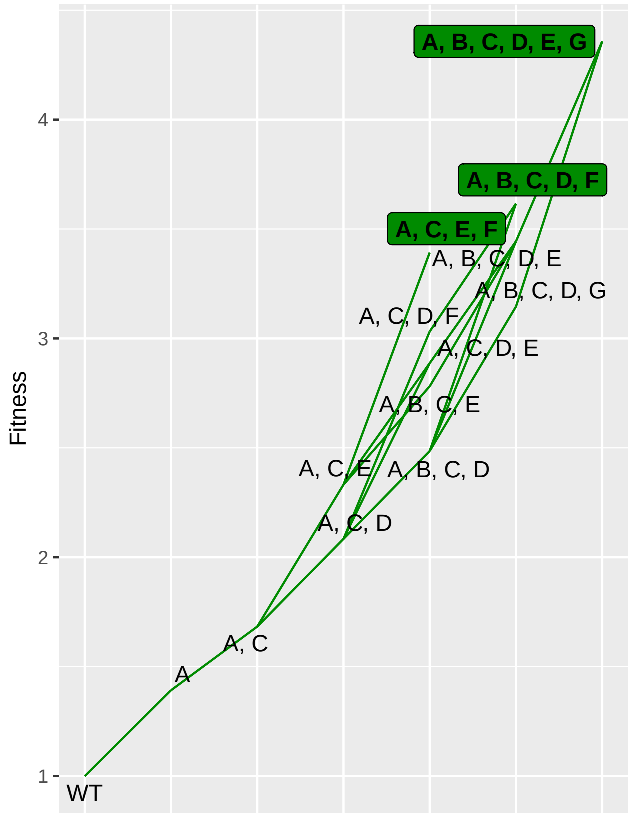

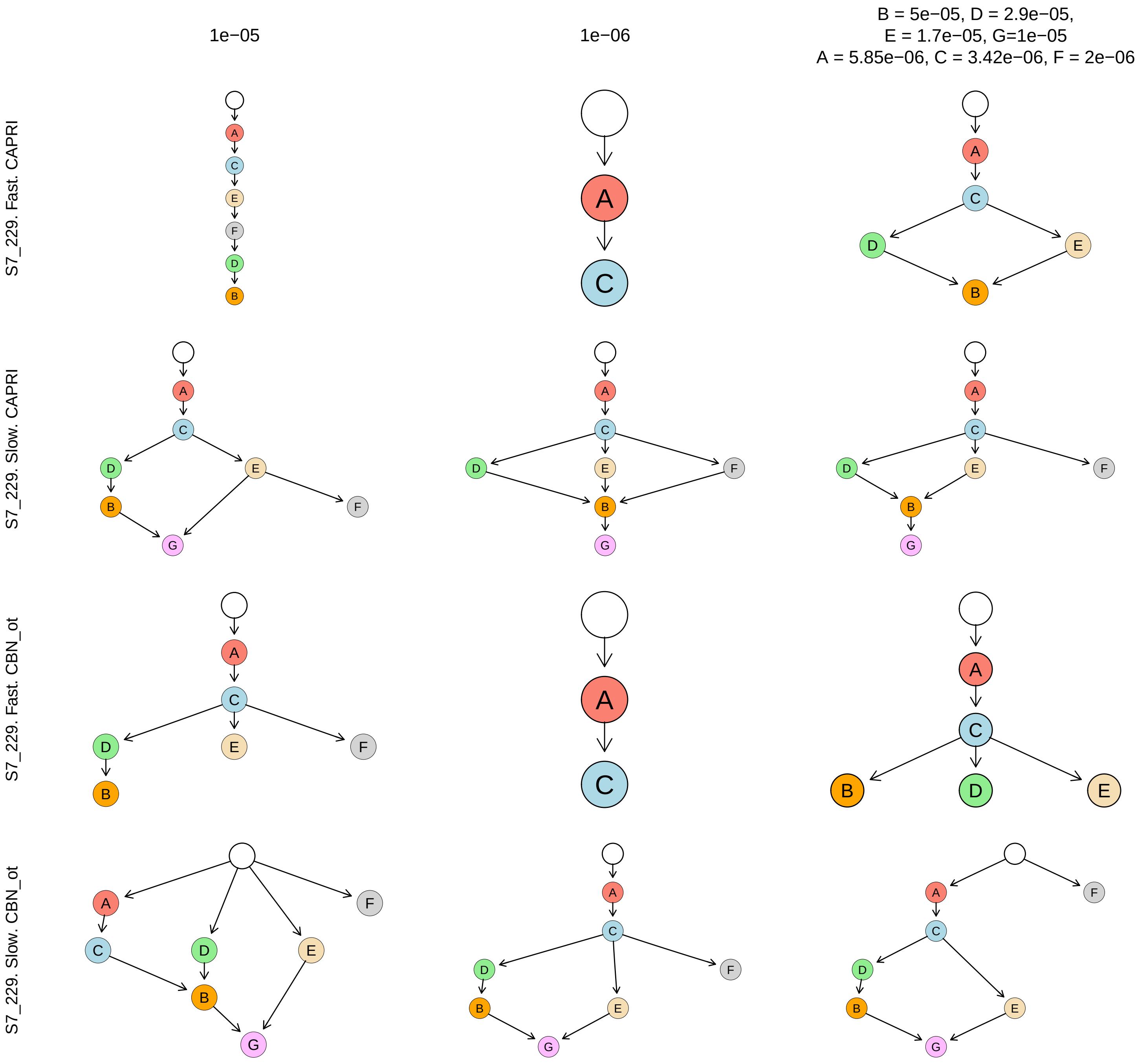

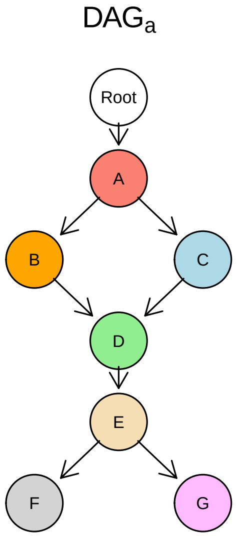

ID	Accessible Genot.	Discrep. DAG_a
S7_230	8	1

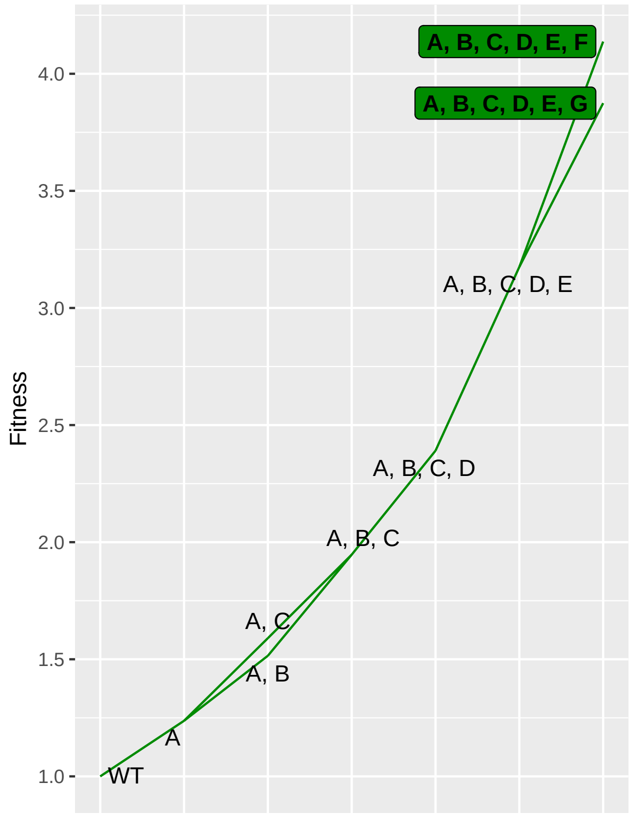

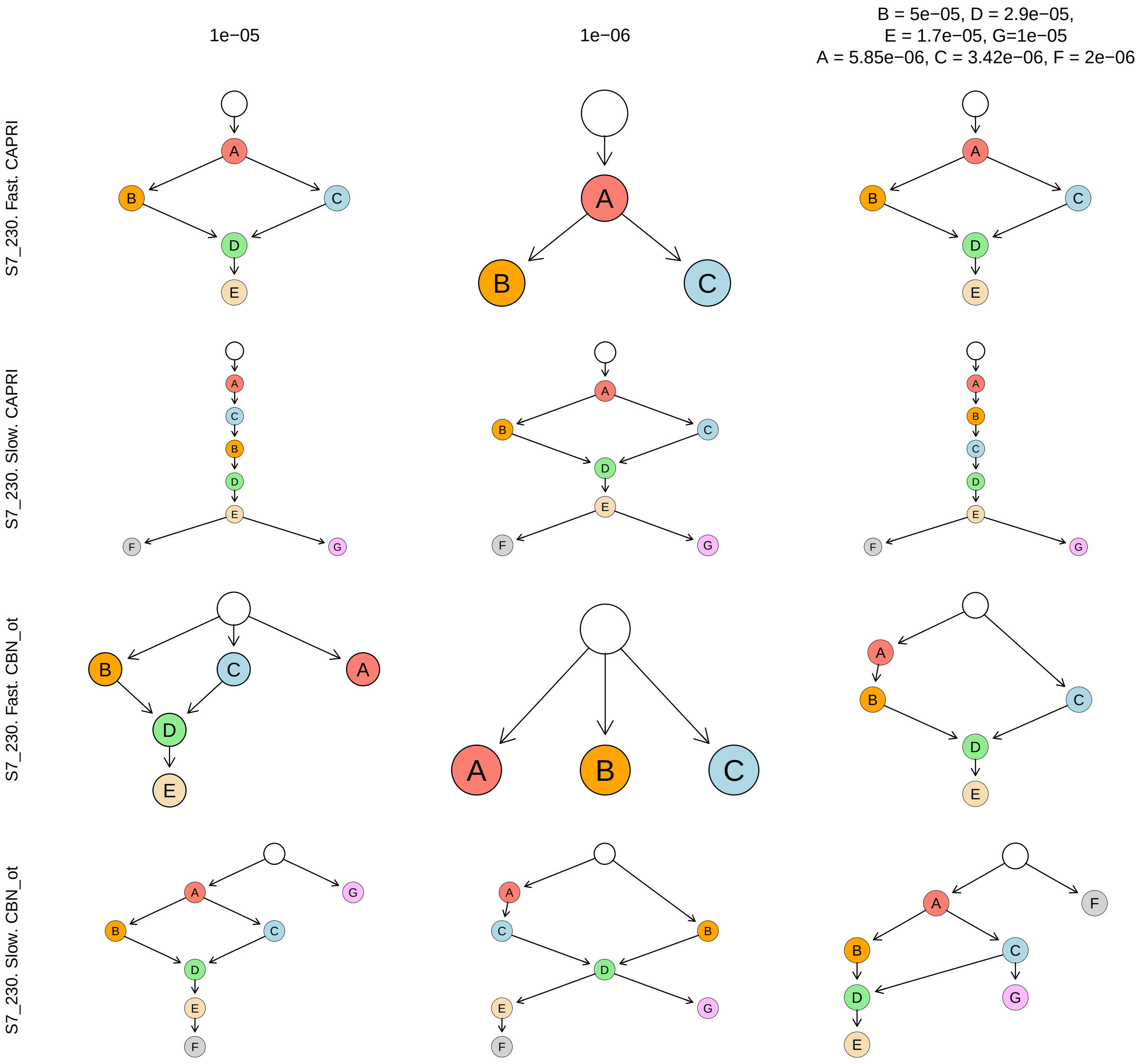

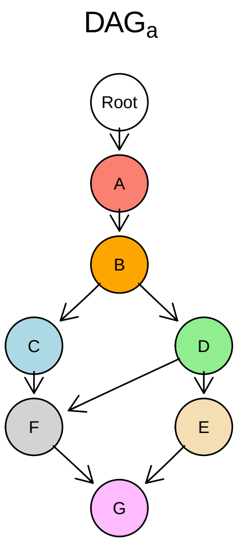

ID	Accessible Genot.	Discrep. DAG_a
S7_231	11	3

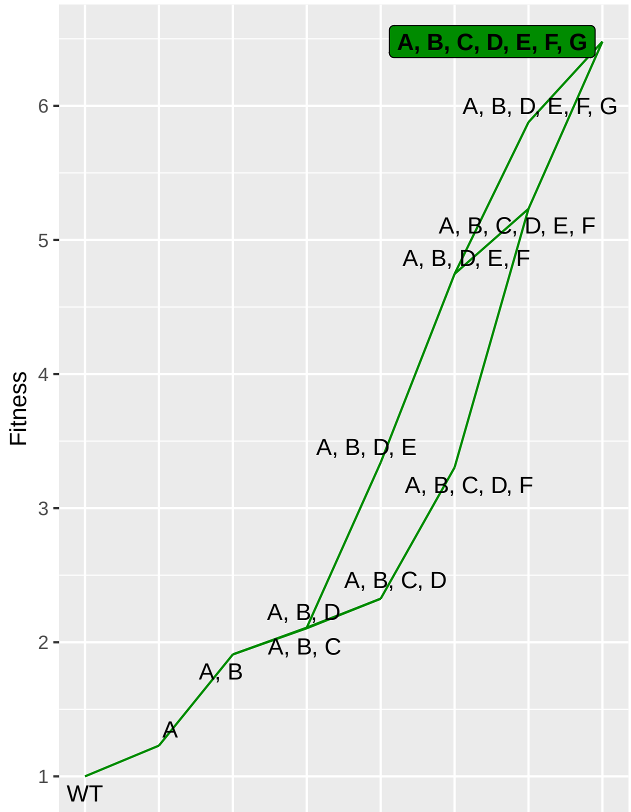

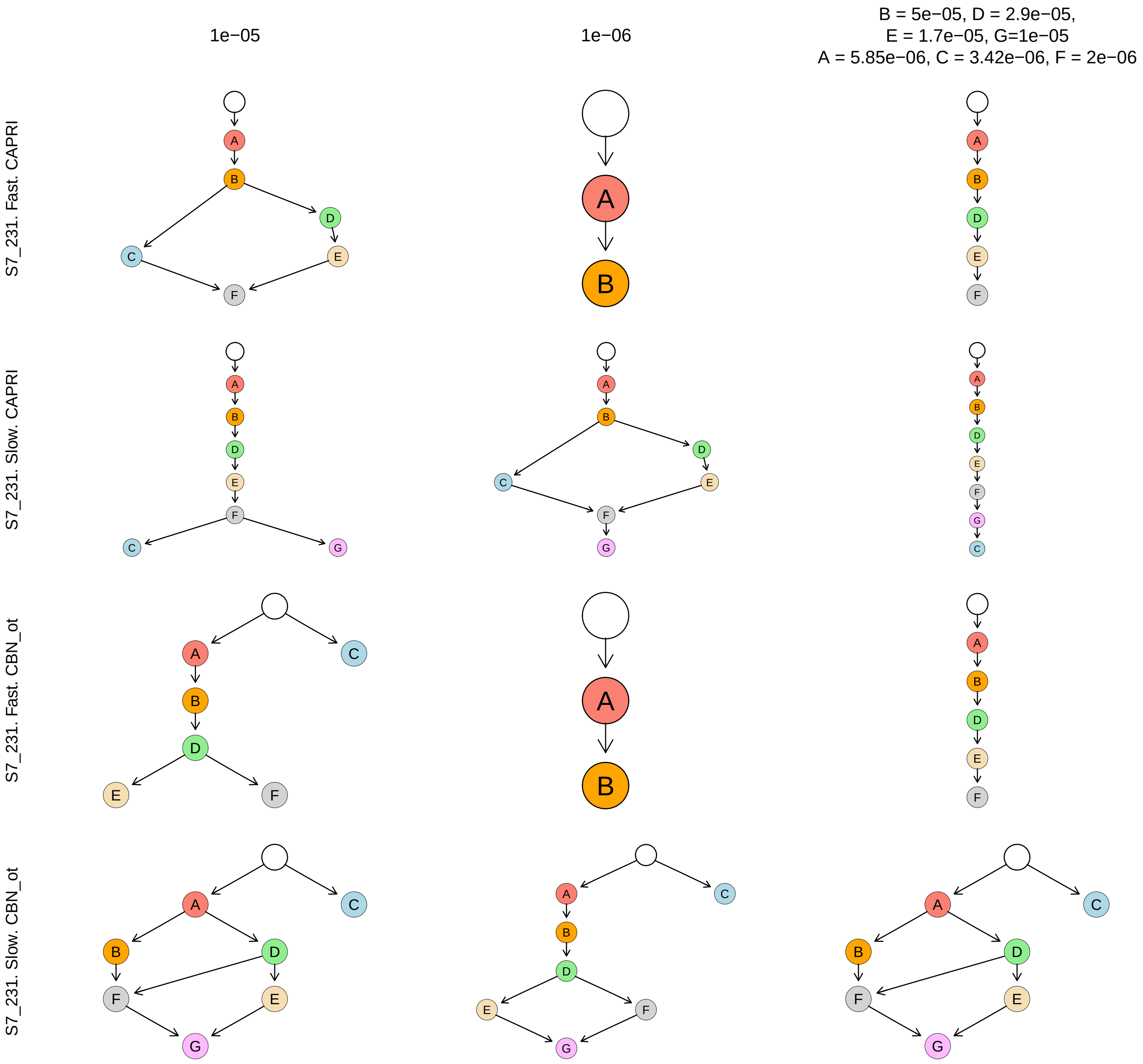

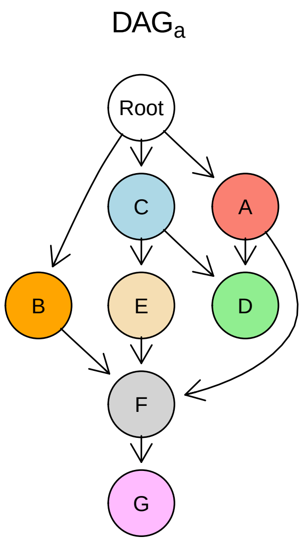

ID	Accessible Genot.	Discrep. DAG_a
S7_232	17	2

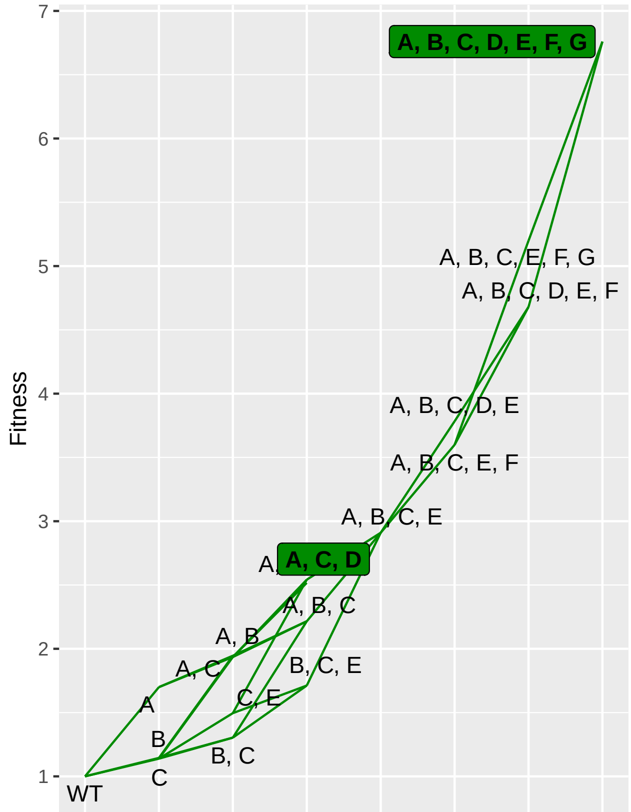

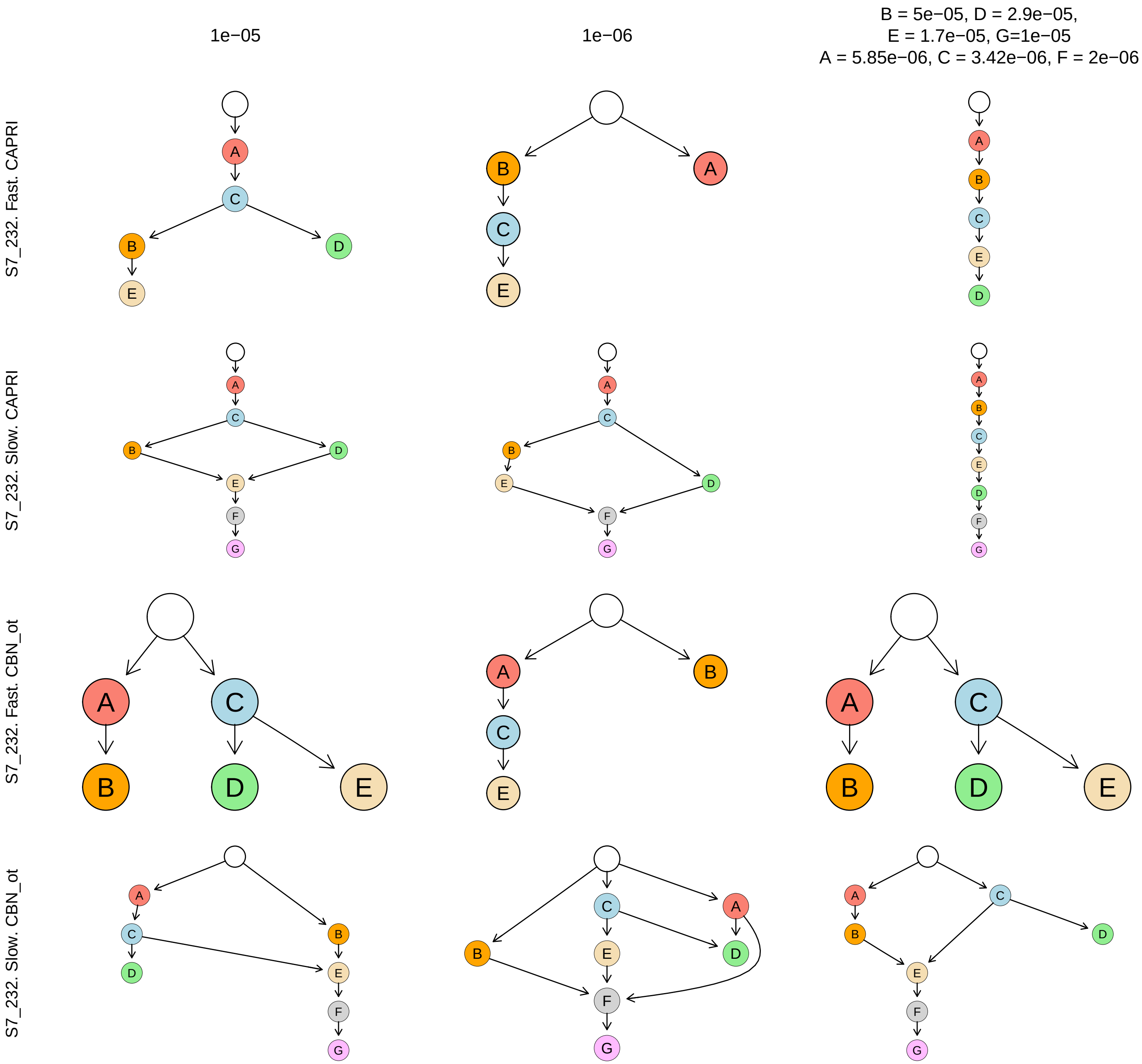

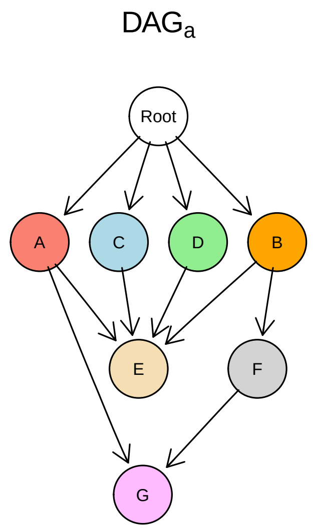

ID	Accessible Genot.	Discrep. DAG_a
S7_233	25	11

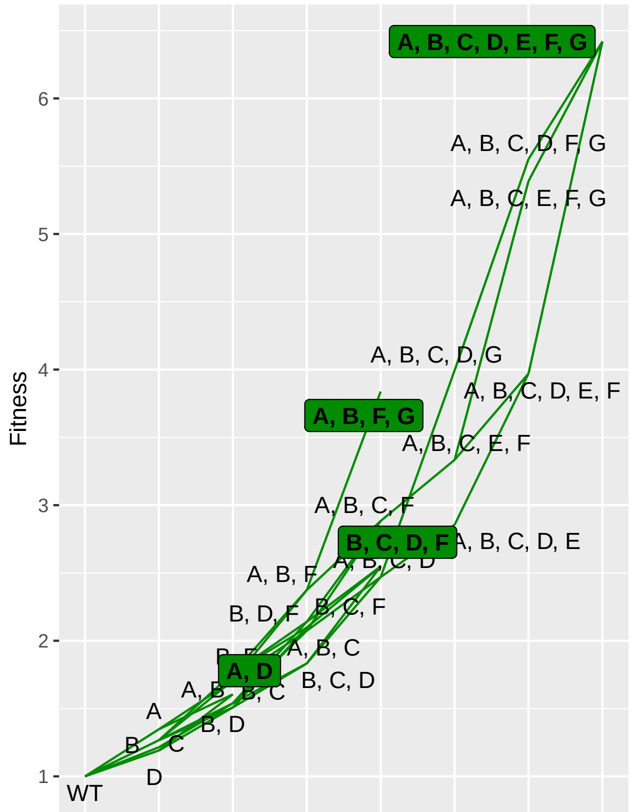

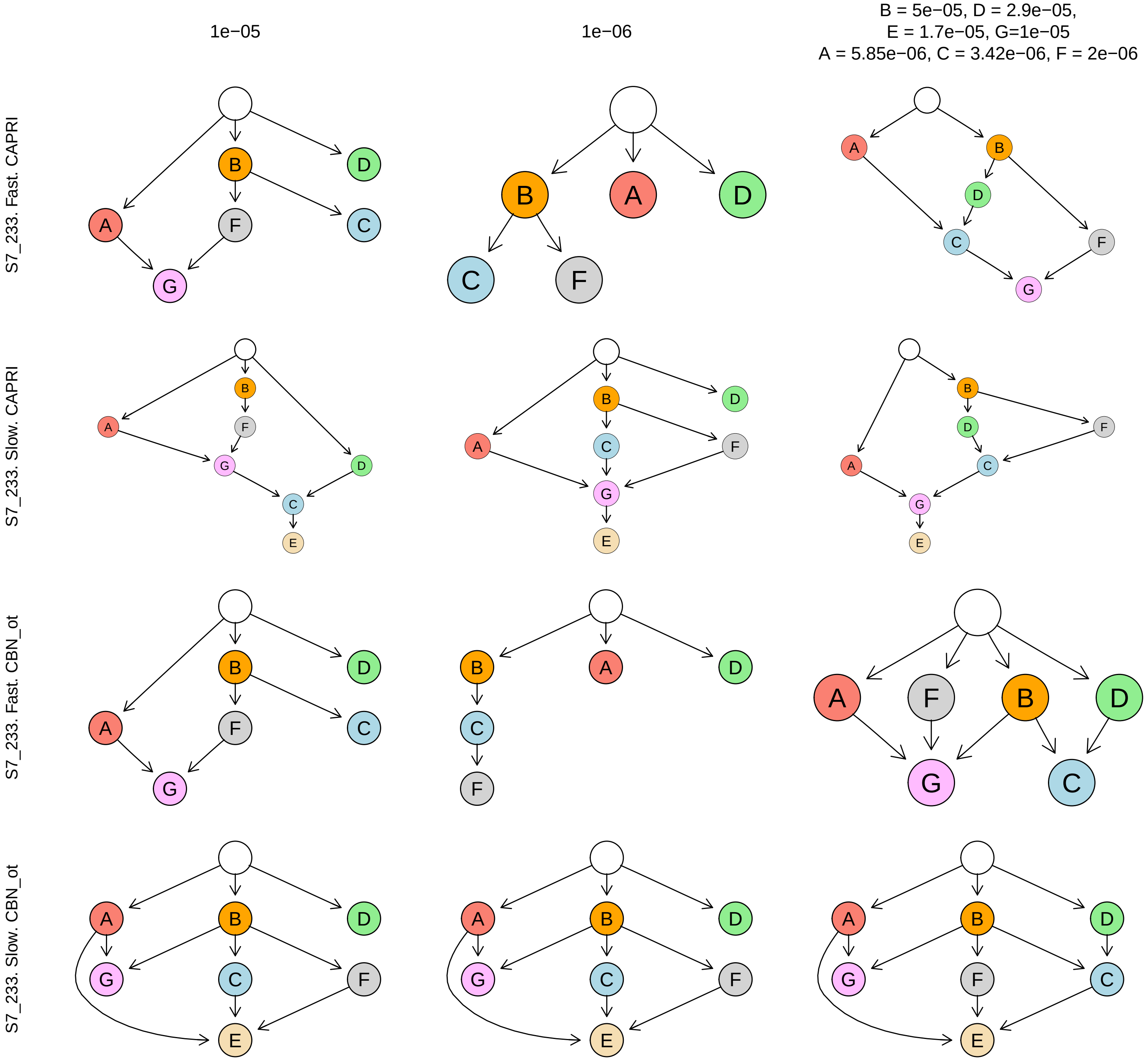

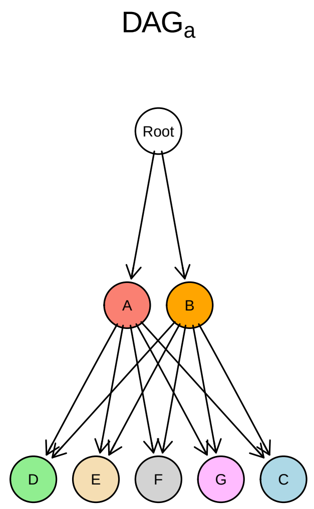

ID	Accessible Genot.	Discrep. DAG_a
S7_234	31	3

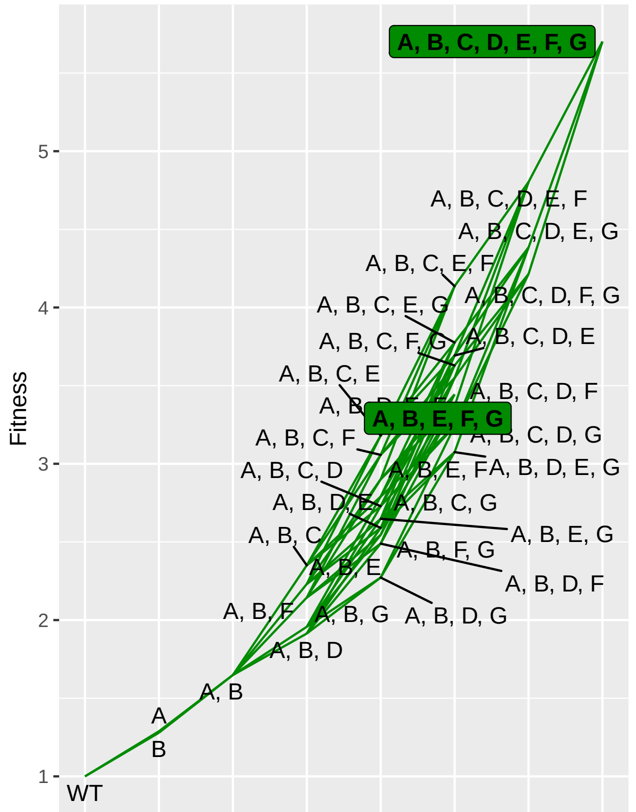

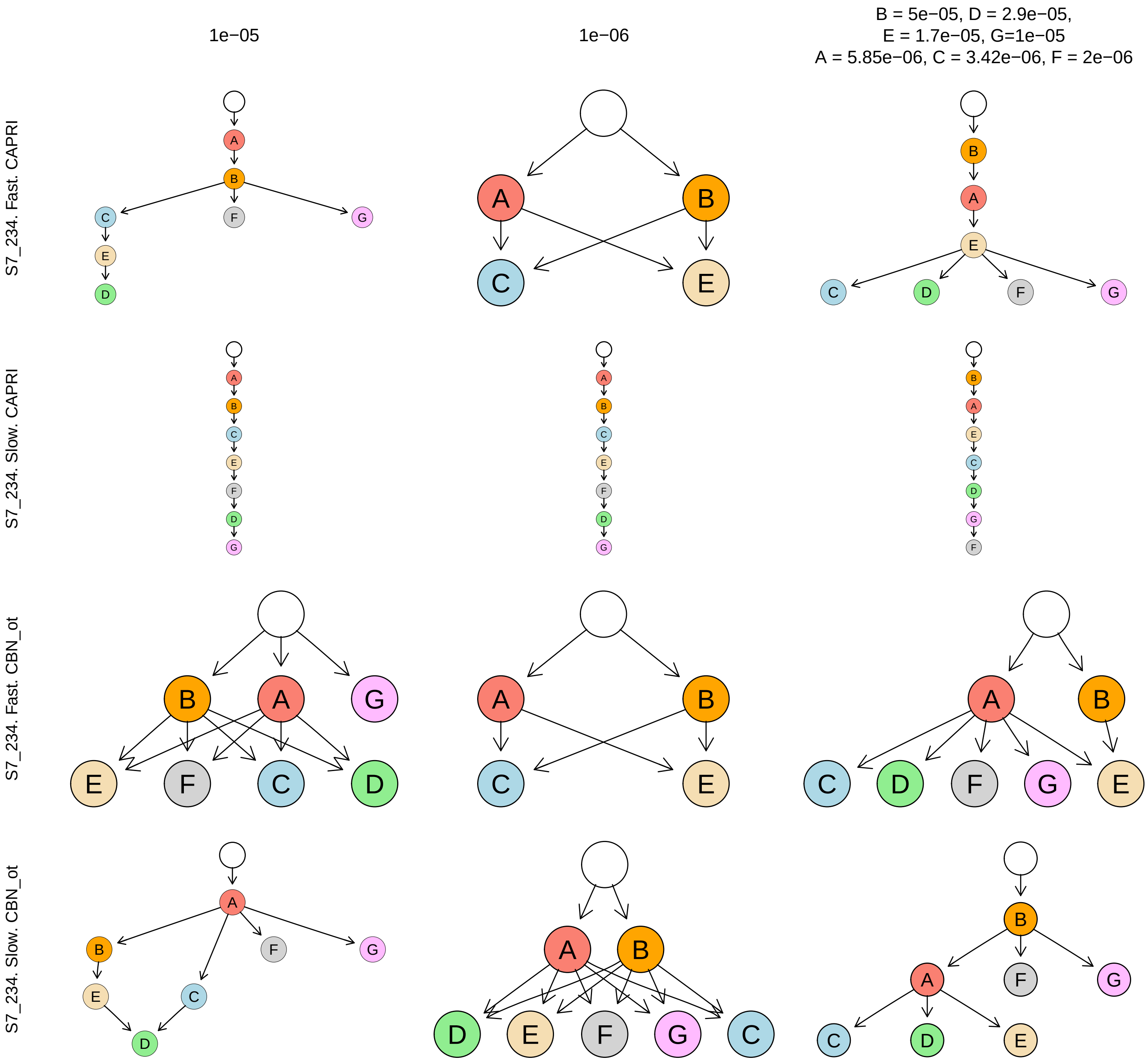

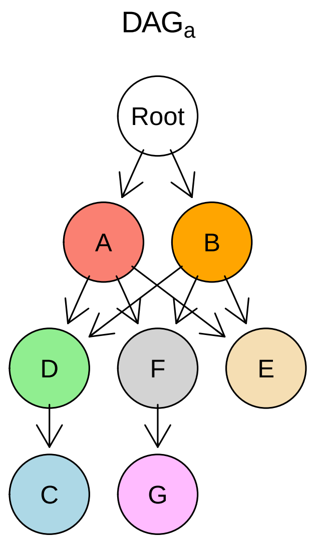

ID	Accessible Genot.	Discrep. DAG_a
S7_235	19	11

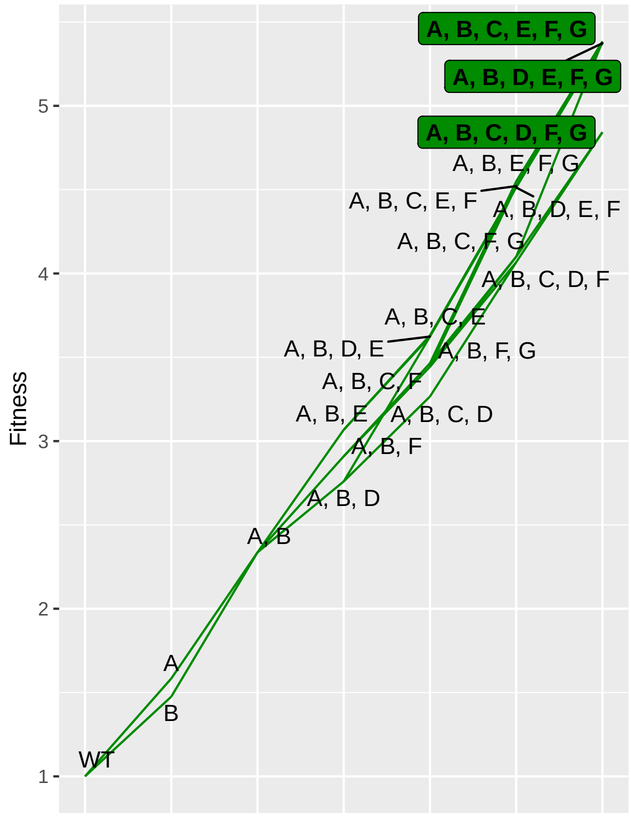

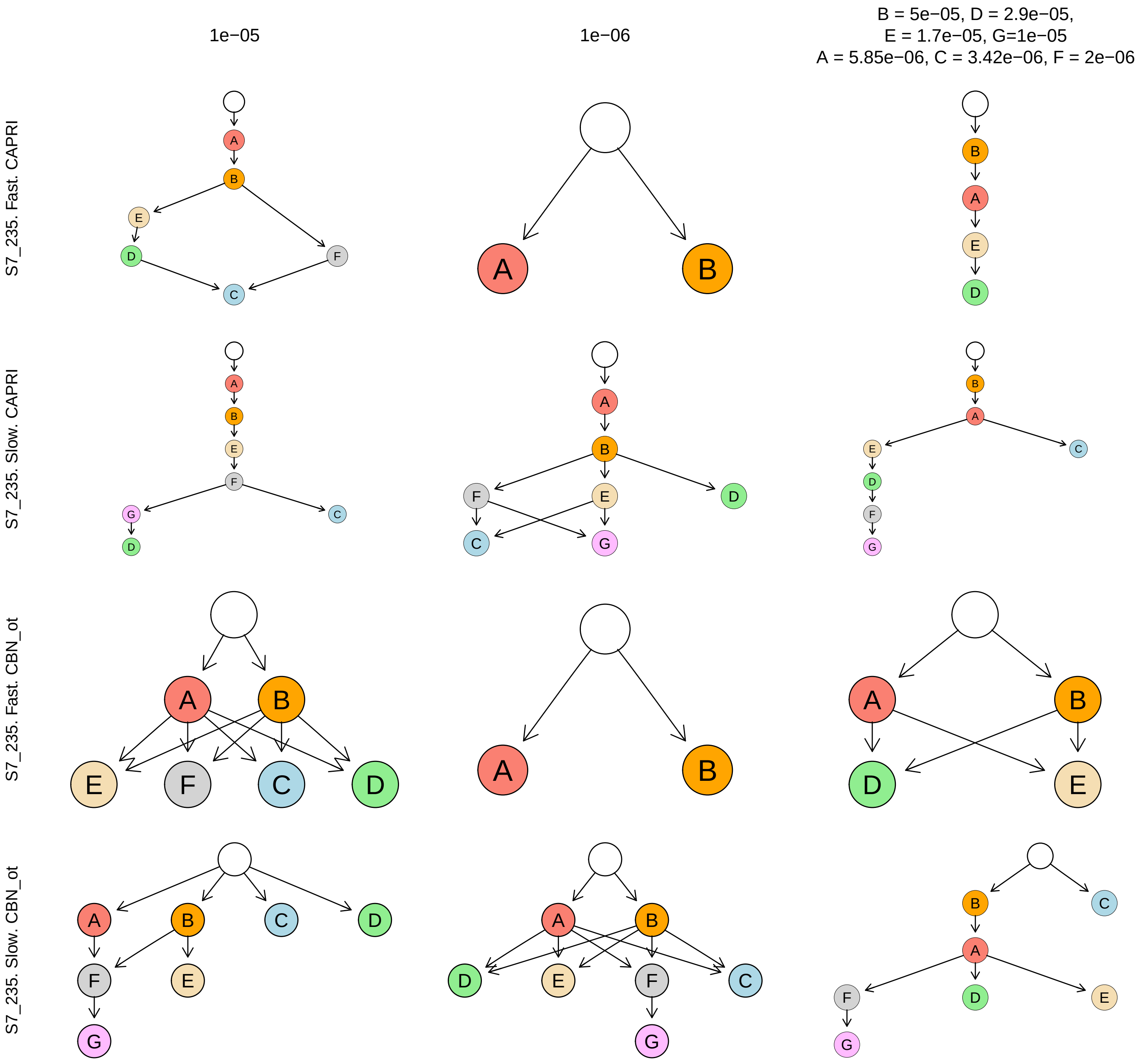

DAG_a

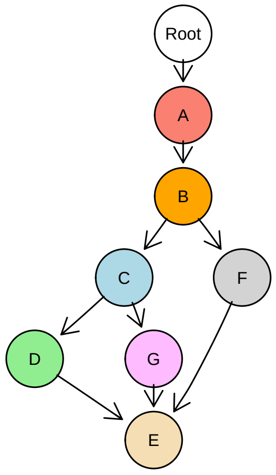

ID	Accessible Genot.	Discrep. DAG_a
S7_236	10	2

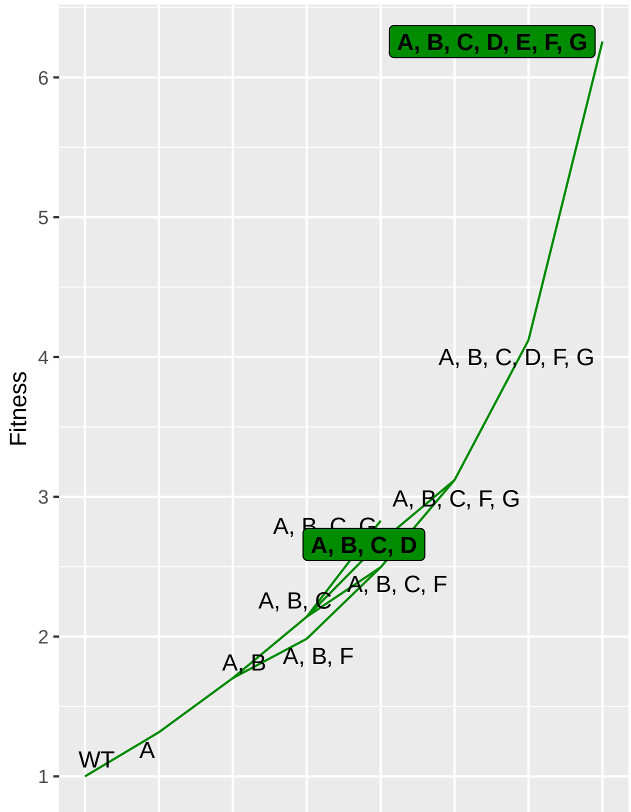

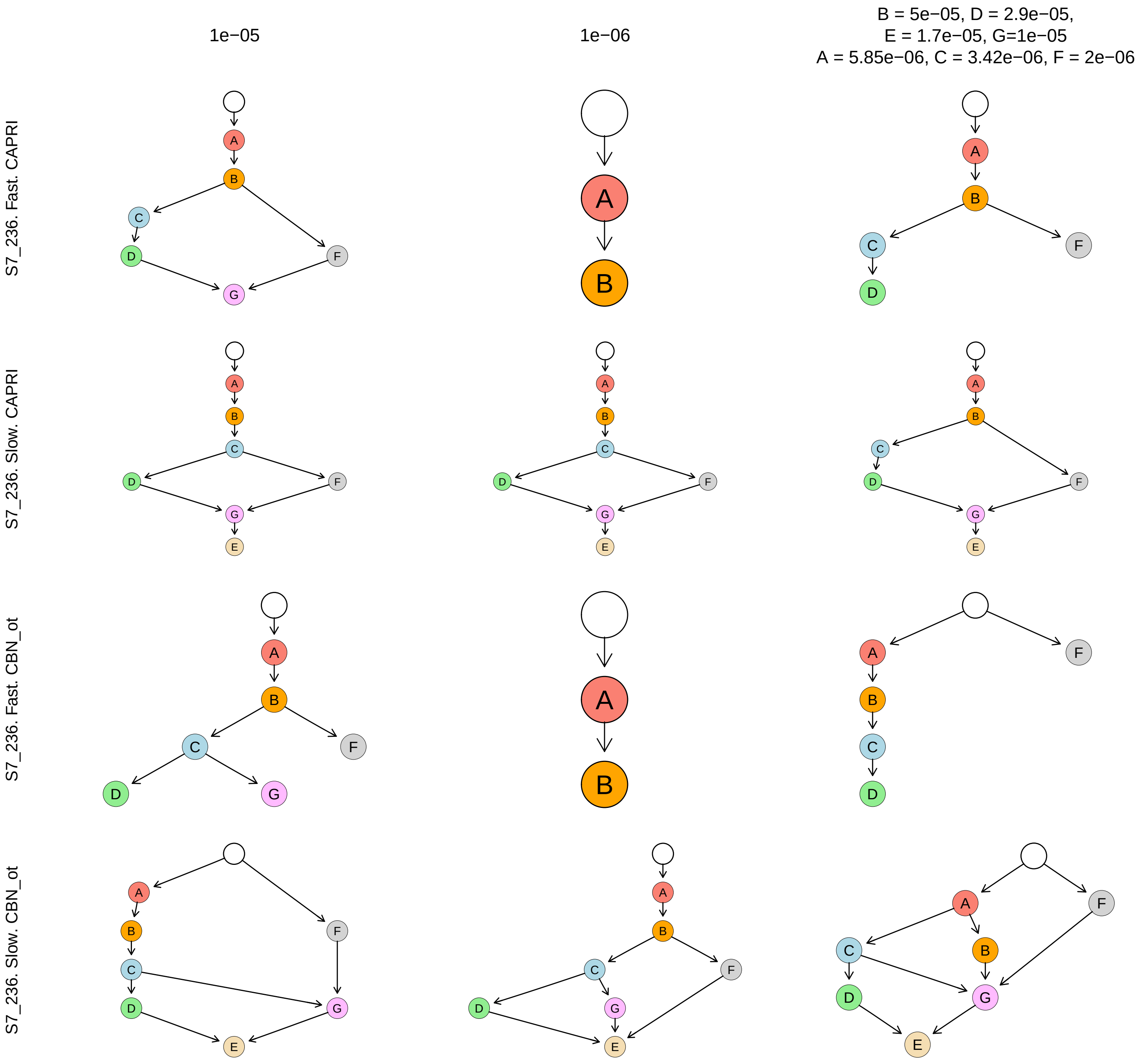

DAG$_a$

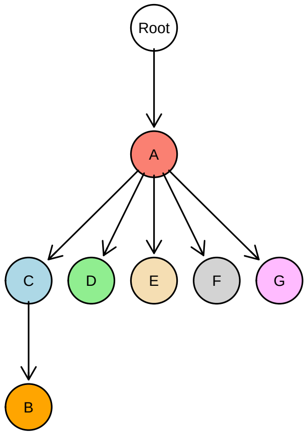

ID	Accessible Genot.	Discrep. DAG_a
S7_237	58	18

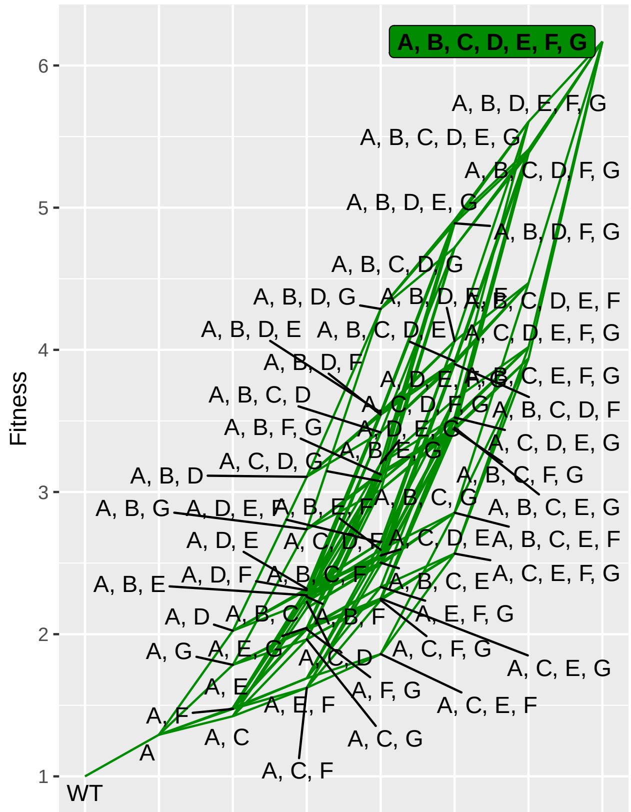

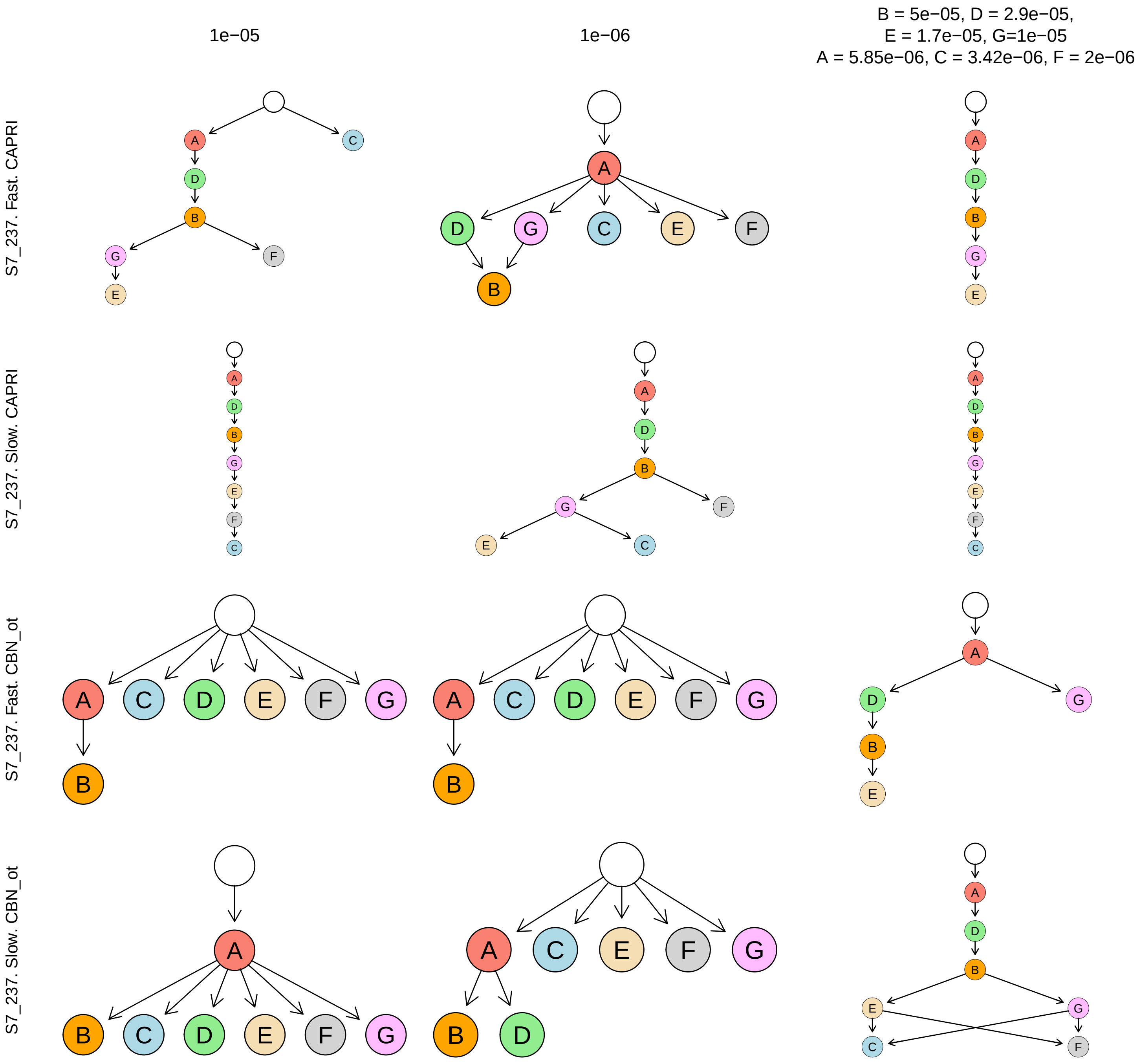

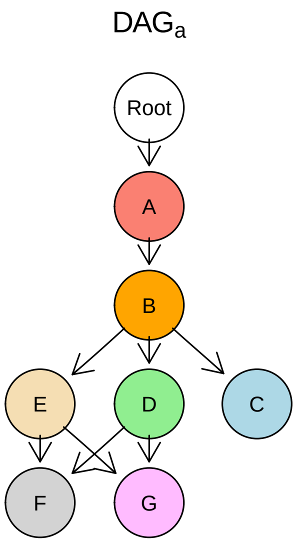

ID	Accessible Genot.	Discrep. DAG_a
S7_238	12	3

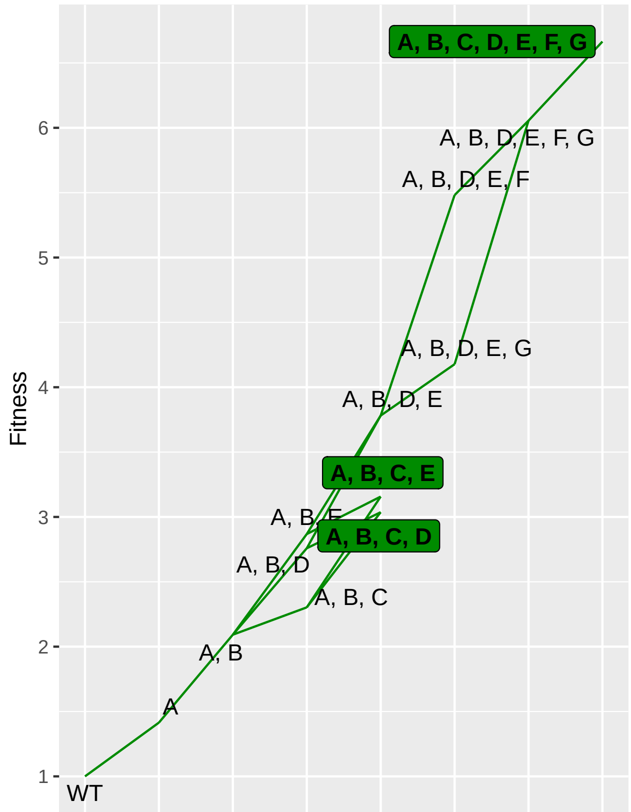

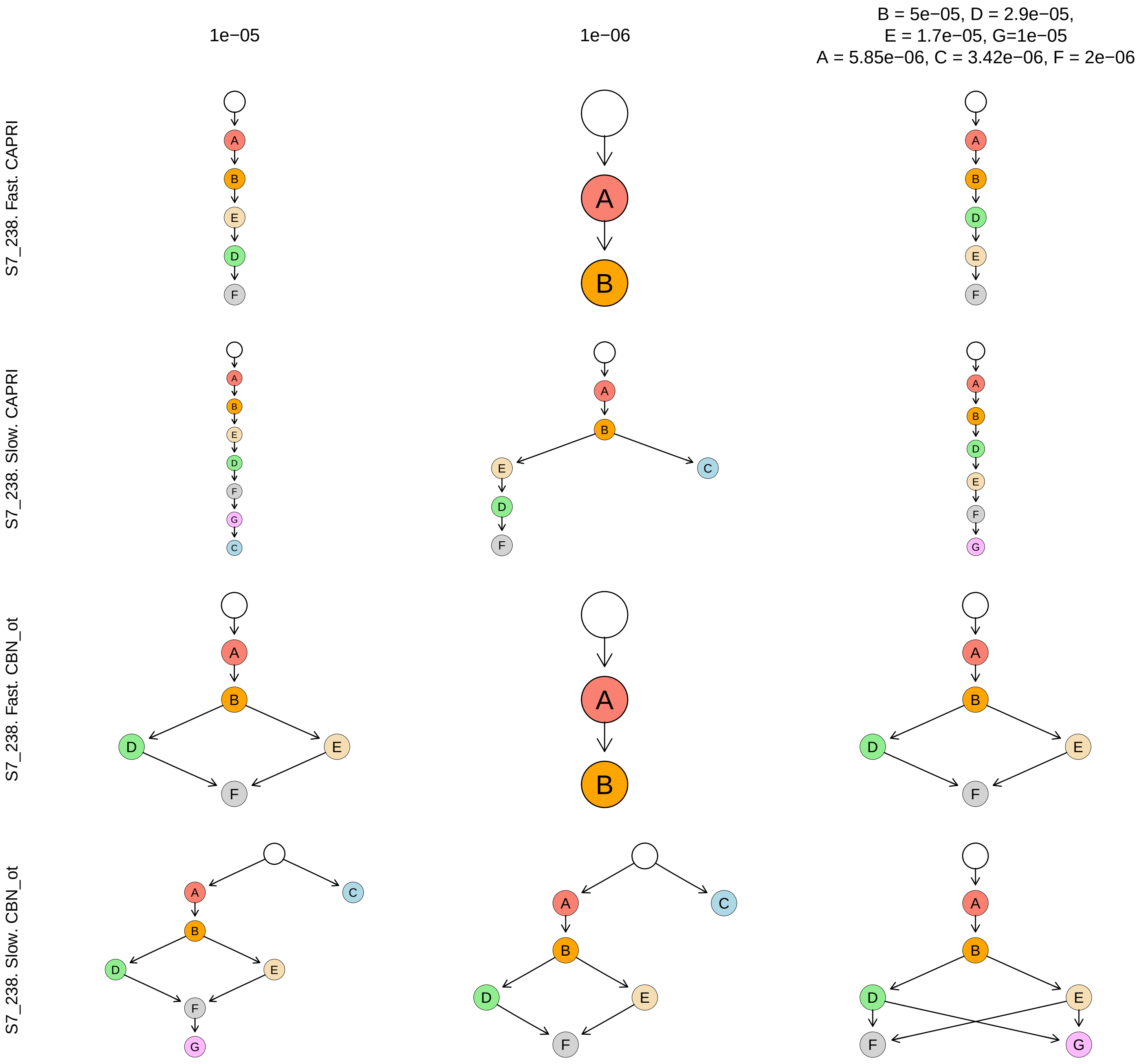

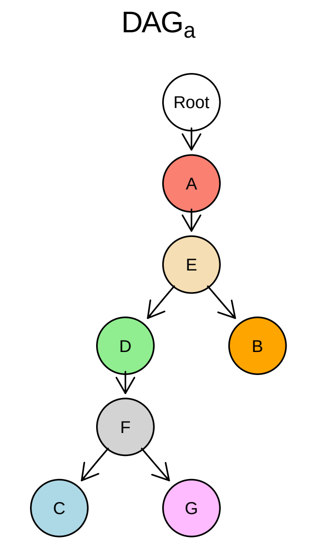

ID	Accessible Genot.	Discrep. DAG_a
S7_239	9	4

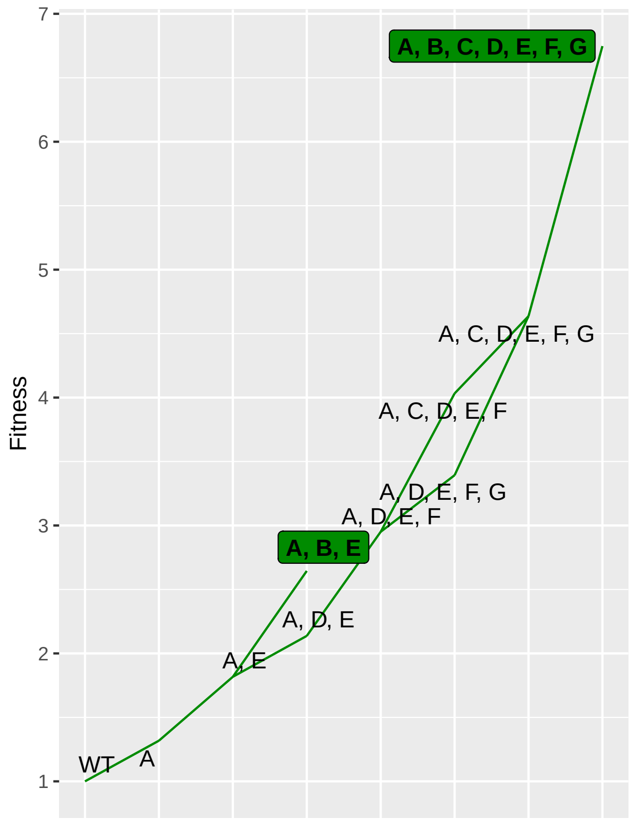

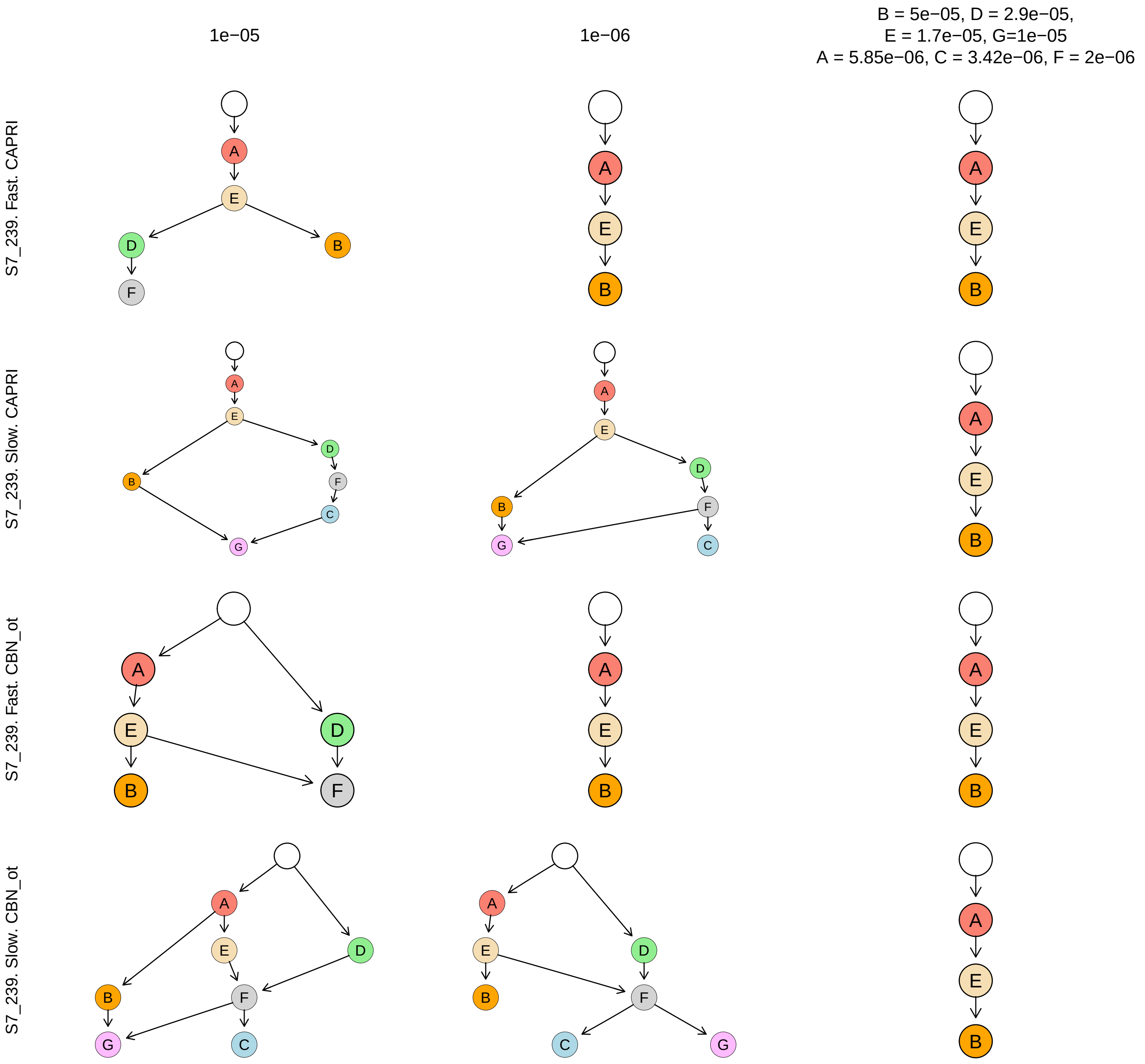

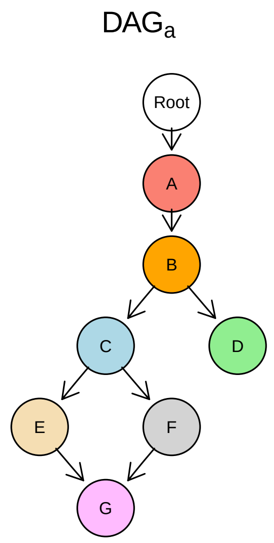

ID	Accessible Genot.	Discrep. DAG_a
S7_240	8	5

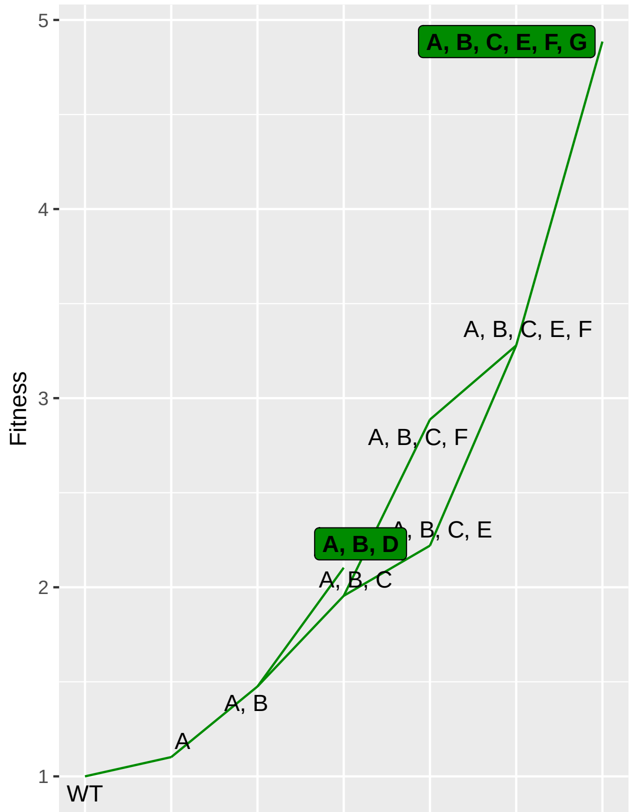

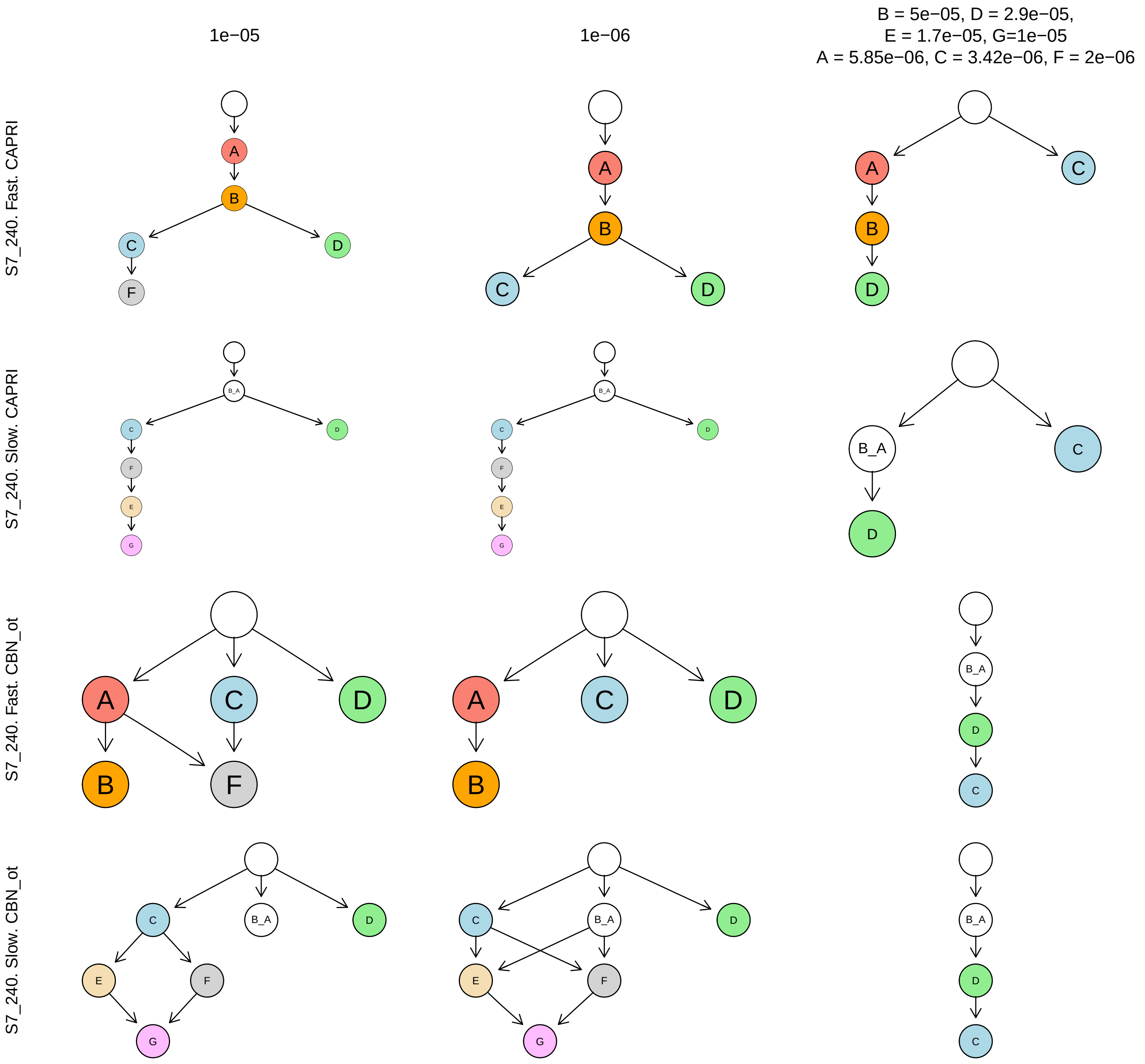

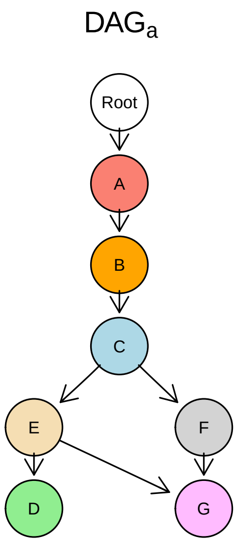

ID	Accessible Genot.	Discrep. DAG_a
S7_241	9	3

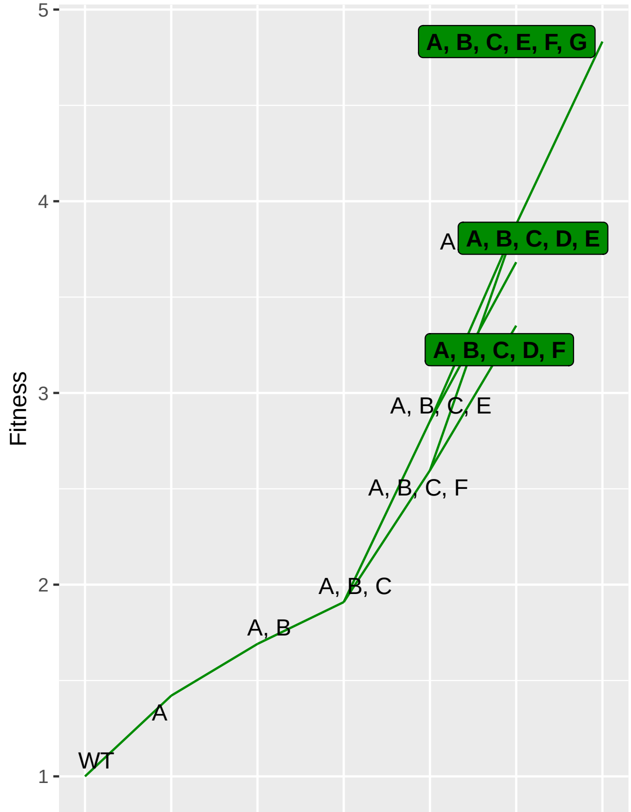

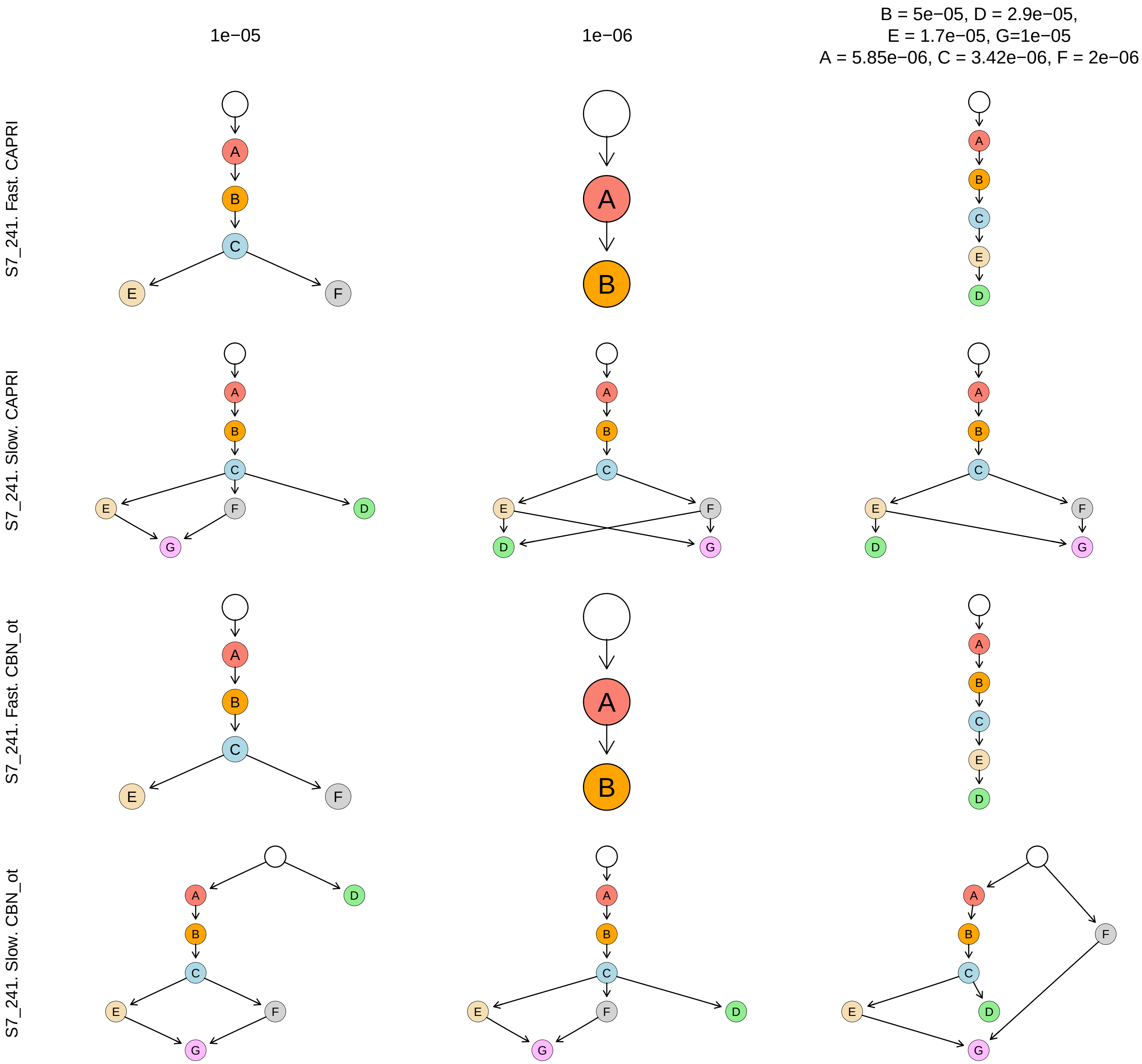

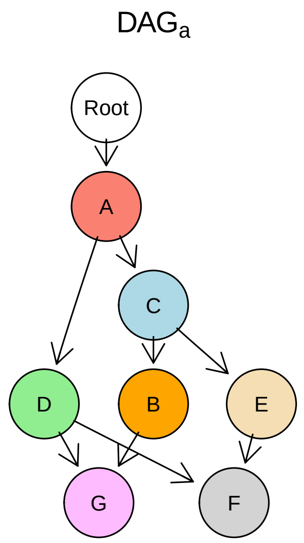

ID	Accessible Genot.	Discrep. DAG_a
S7_242	19	8

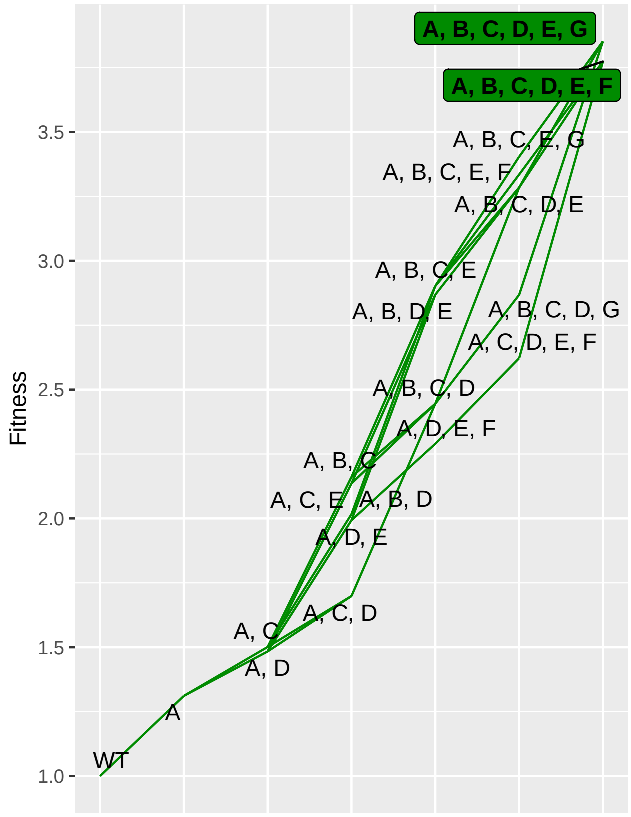

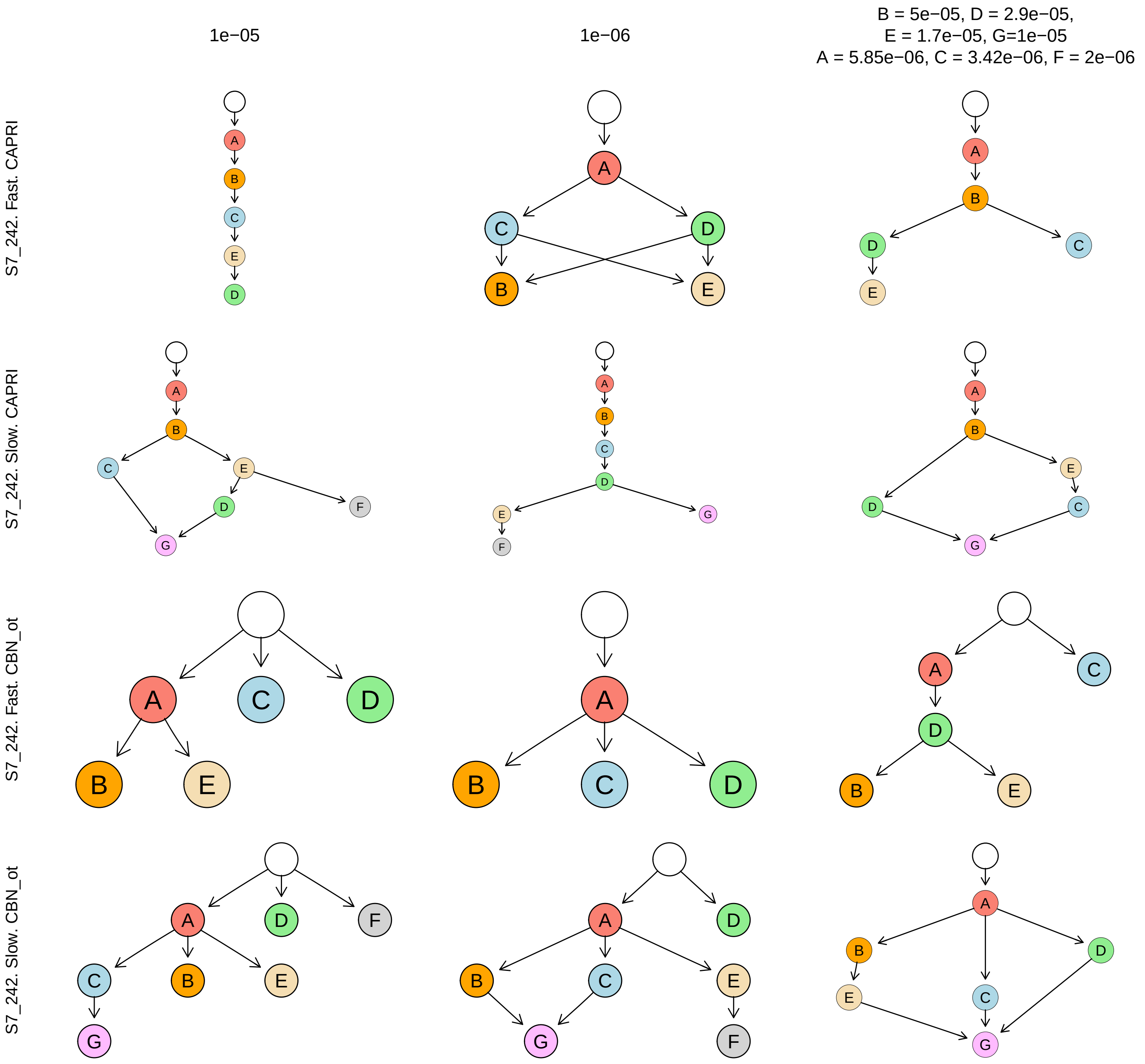

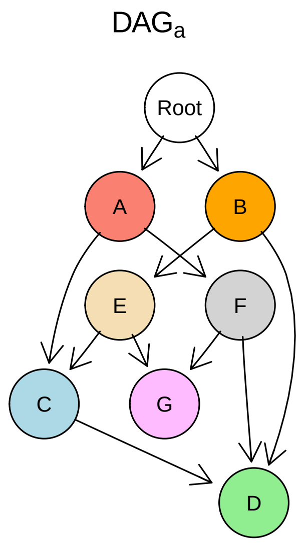

ID	Accessible Genot.	Discrep. DAG_a
S7_243	14	4

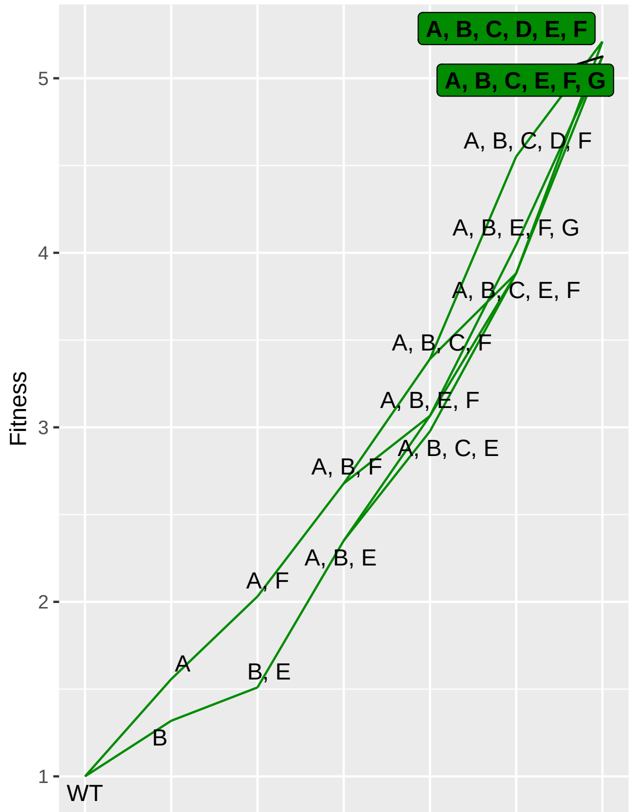

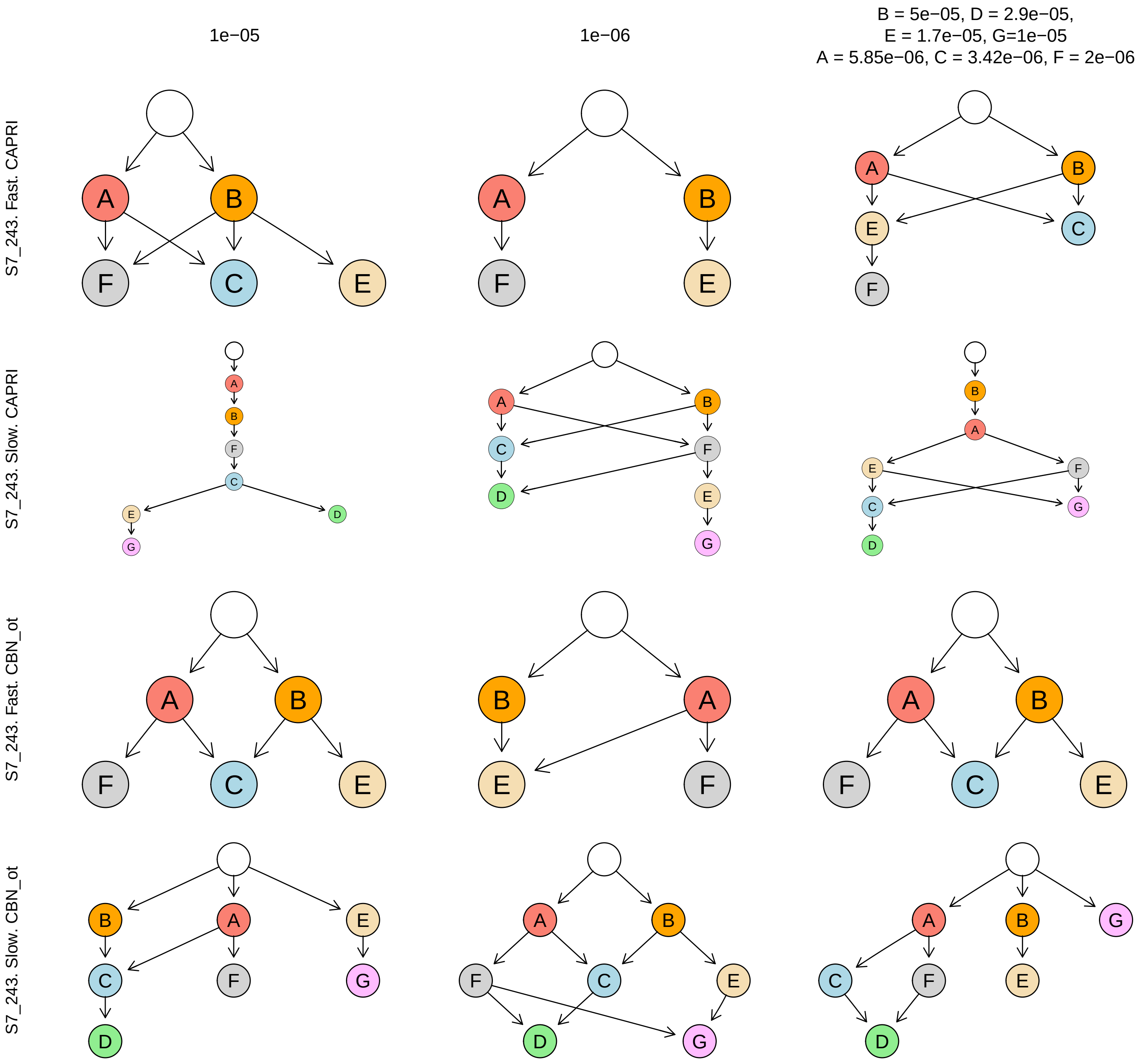

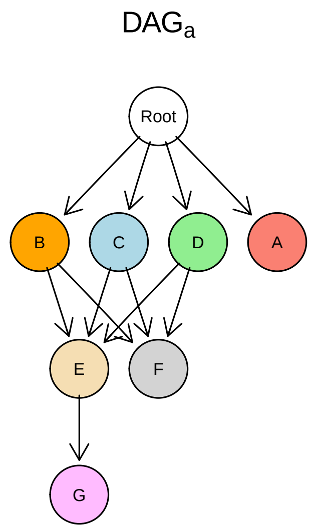

ID	Accessible Genot.	Discrep. DAG_a
S7_244	18	11

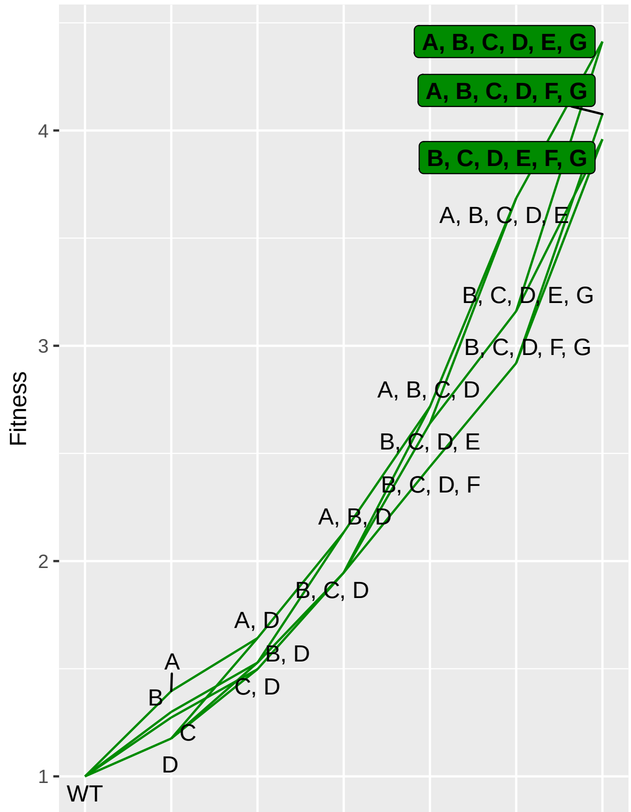

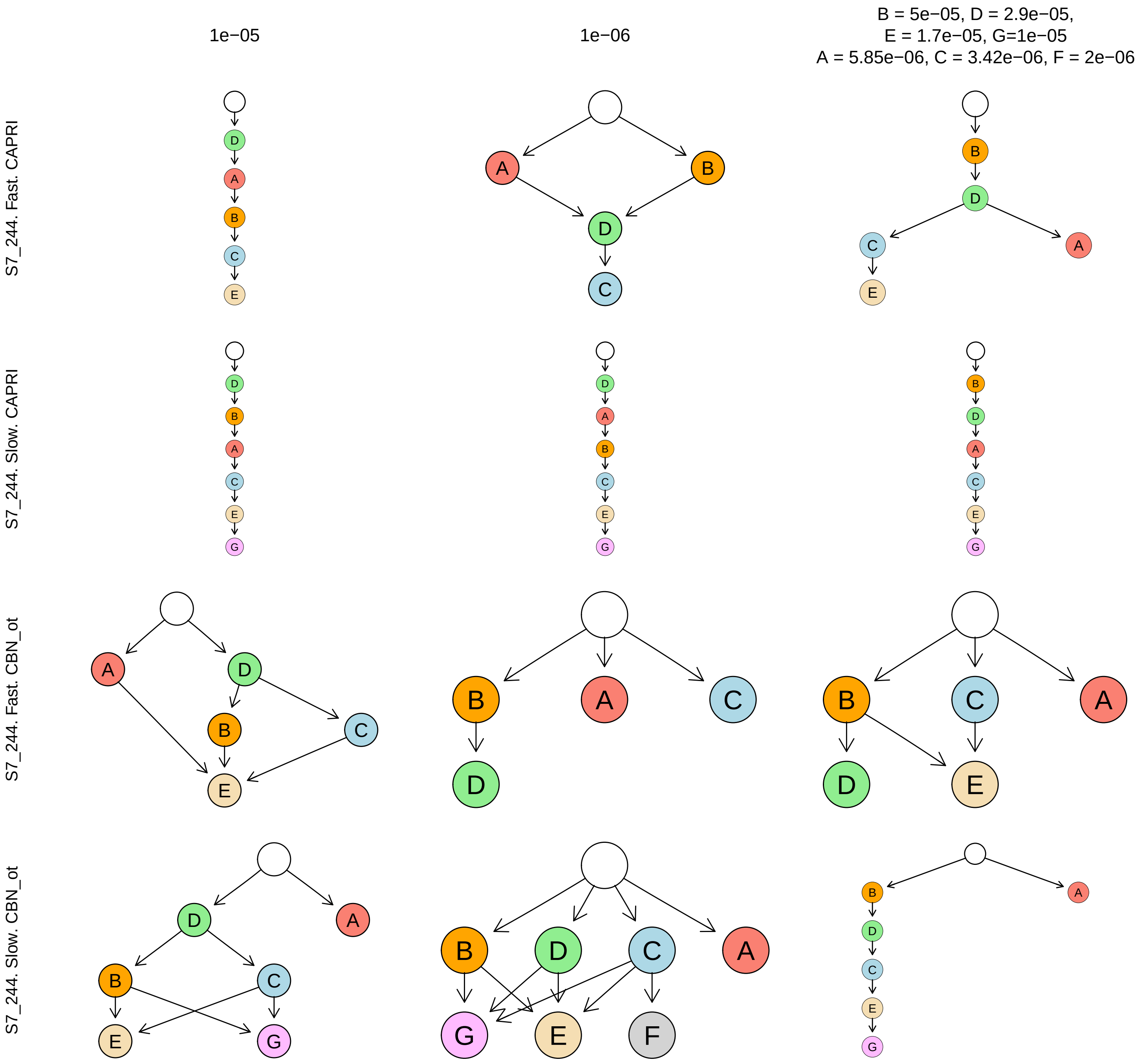

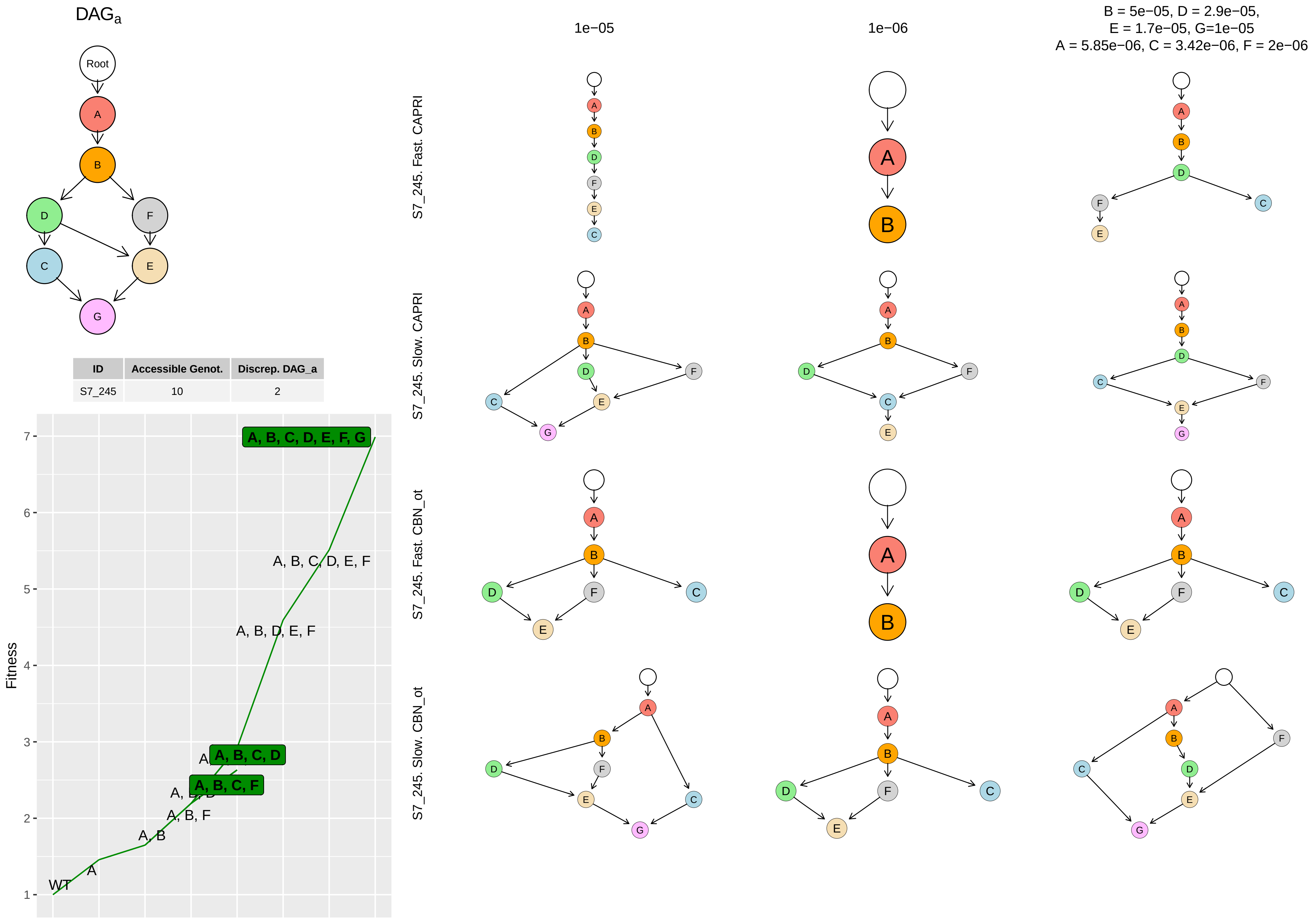

ID	Accessible Genot.	Discrep. DAG_a
S7_245	10	2

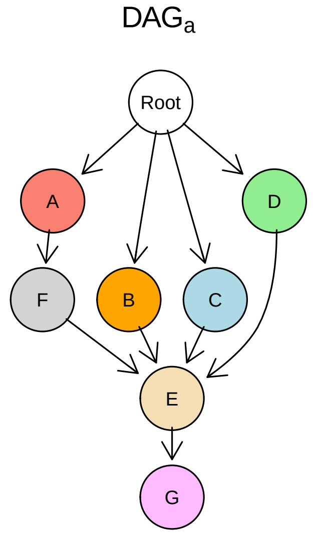

ID	Accessible Genot.	Discrep. DAG_a
S7_246	16	9

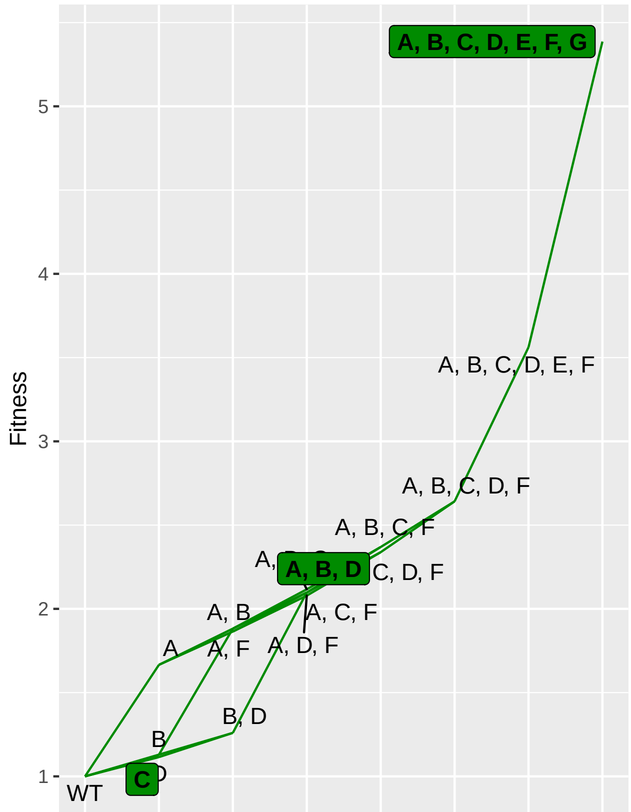

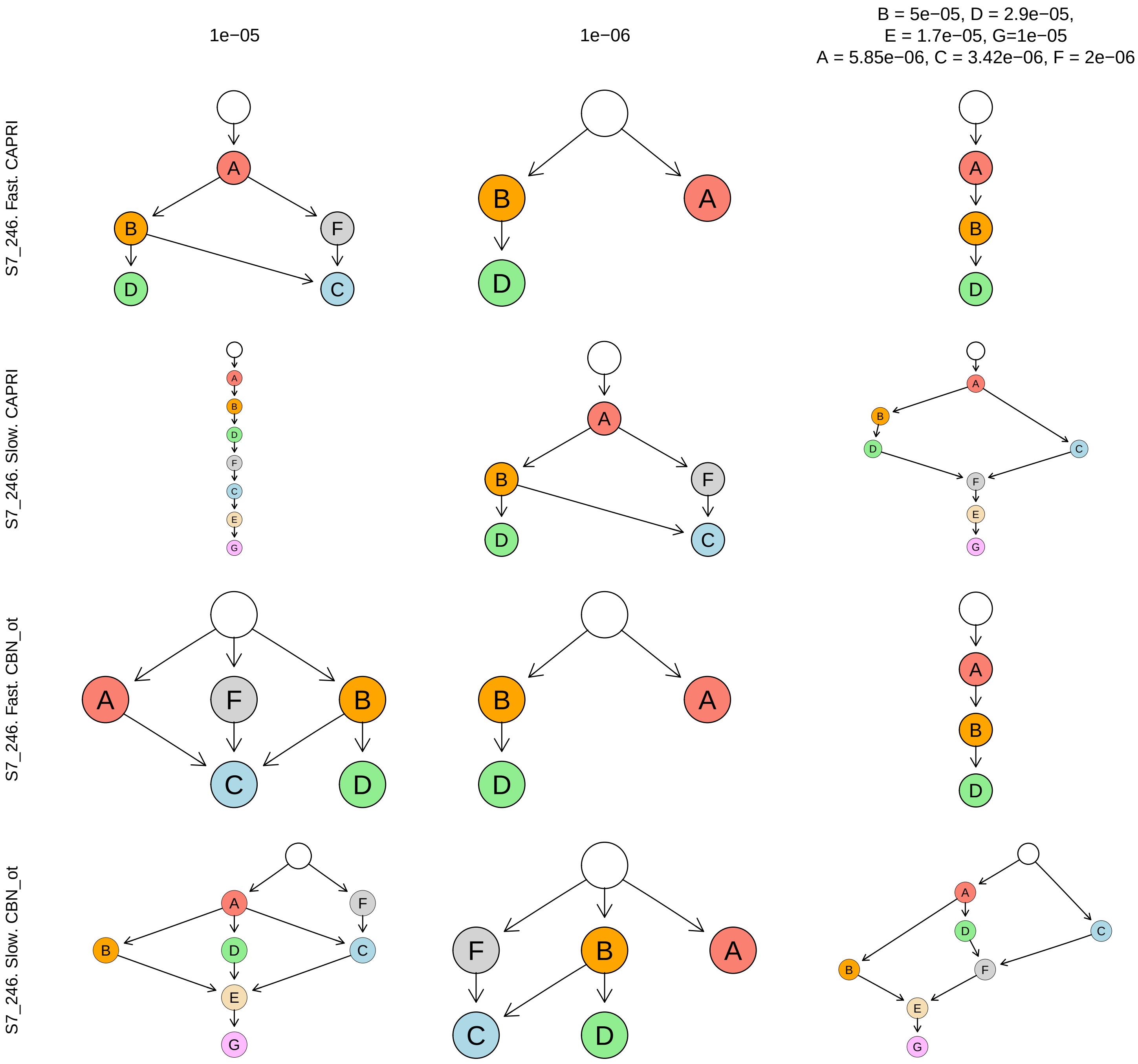

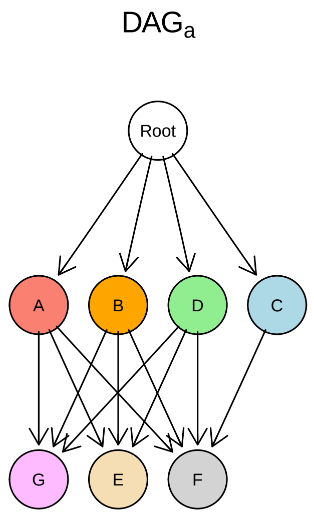

ID	Accessible Genot.	Discrep. DAG_a
S7_247	15	10

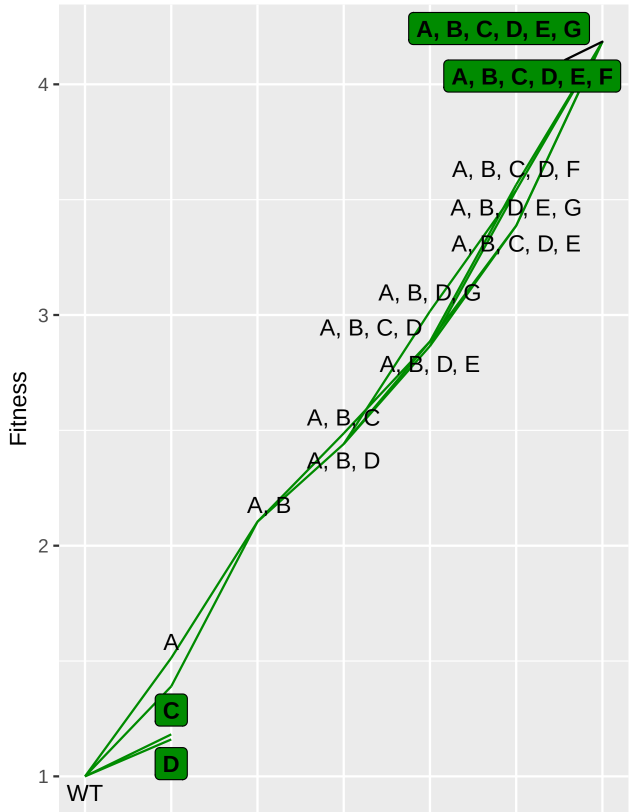

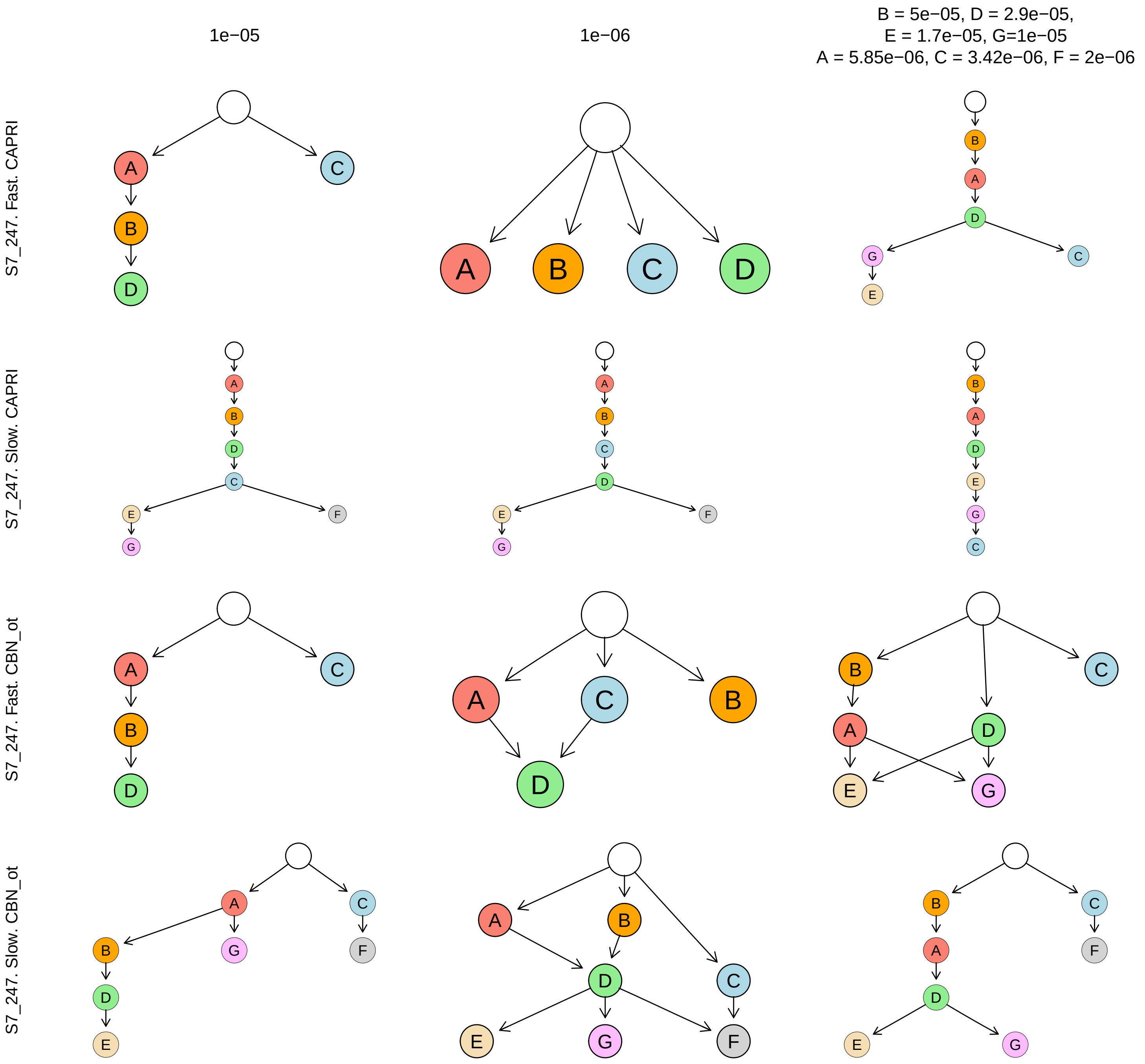

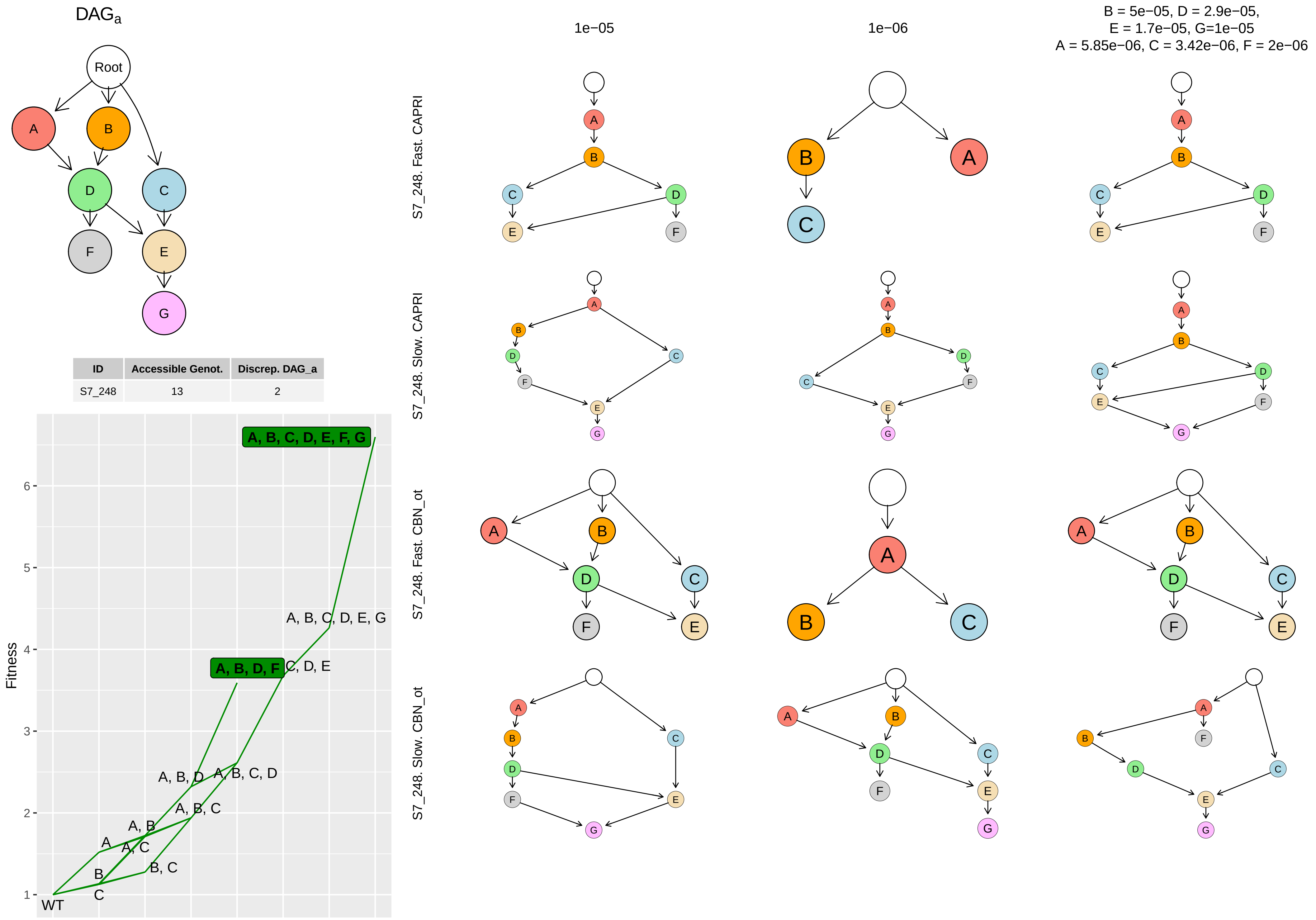

ID	Accessible Genot.	Discrep. DAG_a
S7_248	13	2

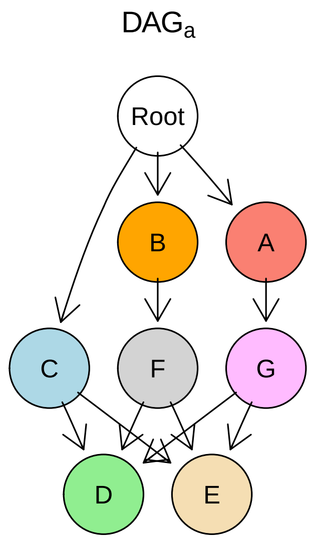

ID	Accessible Genot.	Discrep. DAG_a
S7_249	11	9

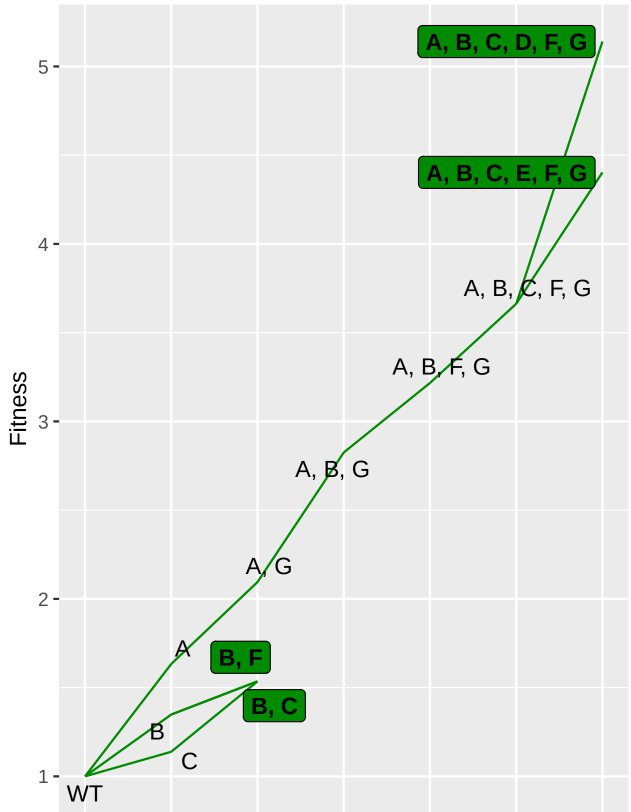

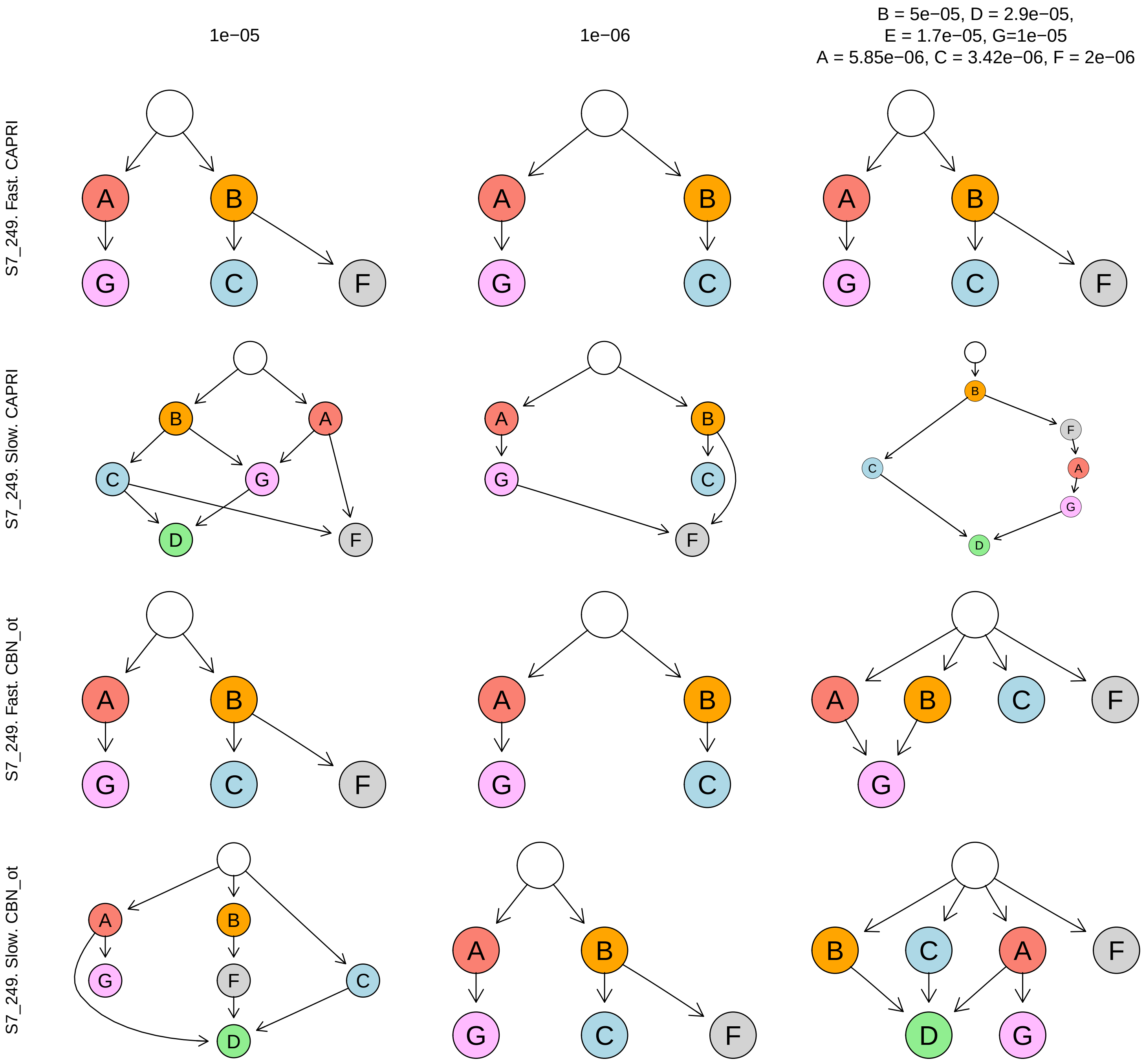

DAG$_a$

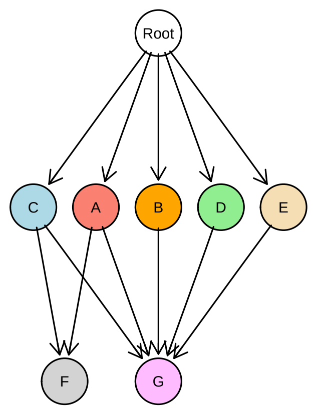

ID	Accessible Genot.	Discrep. DAG_a
S7_250	30	11

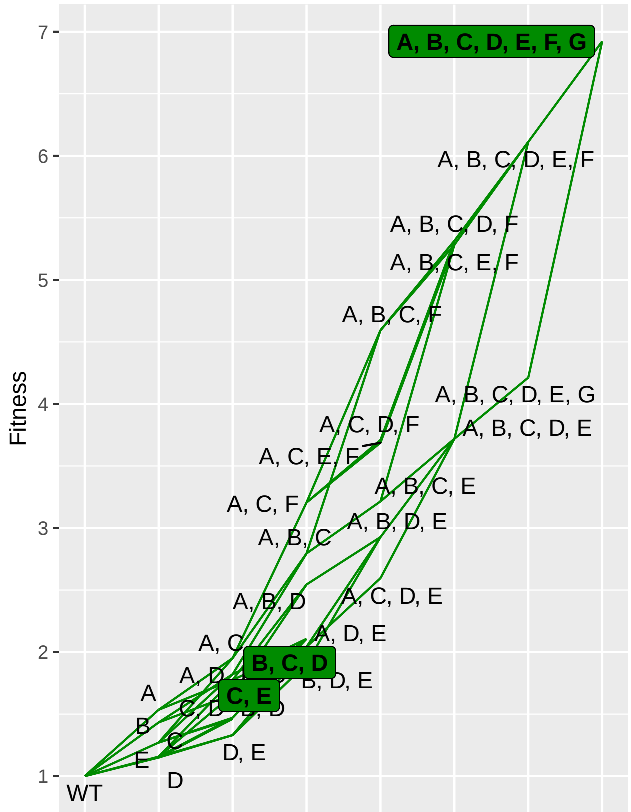

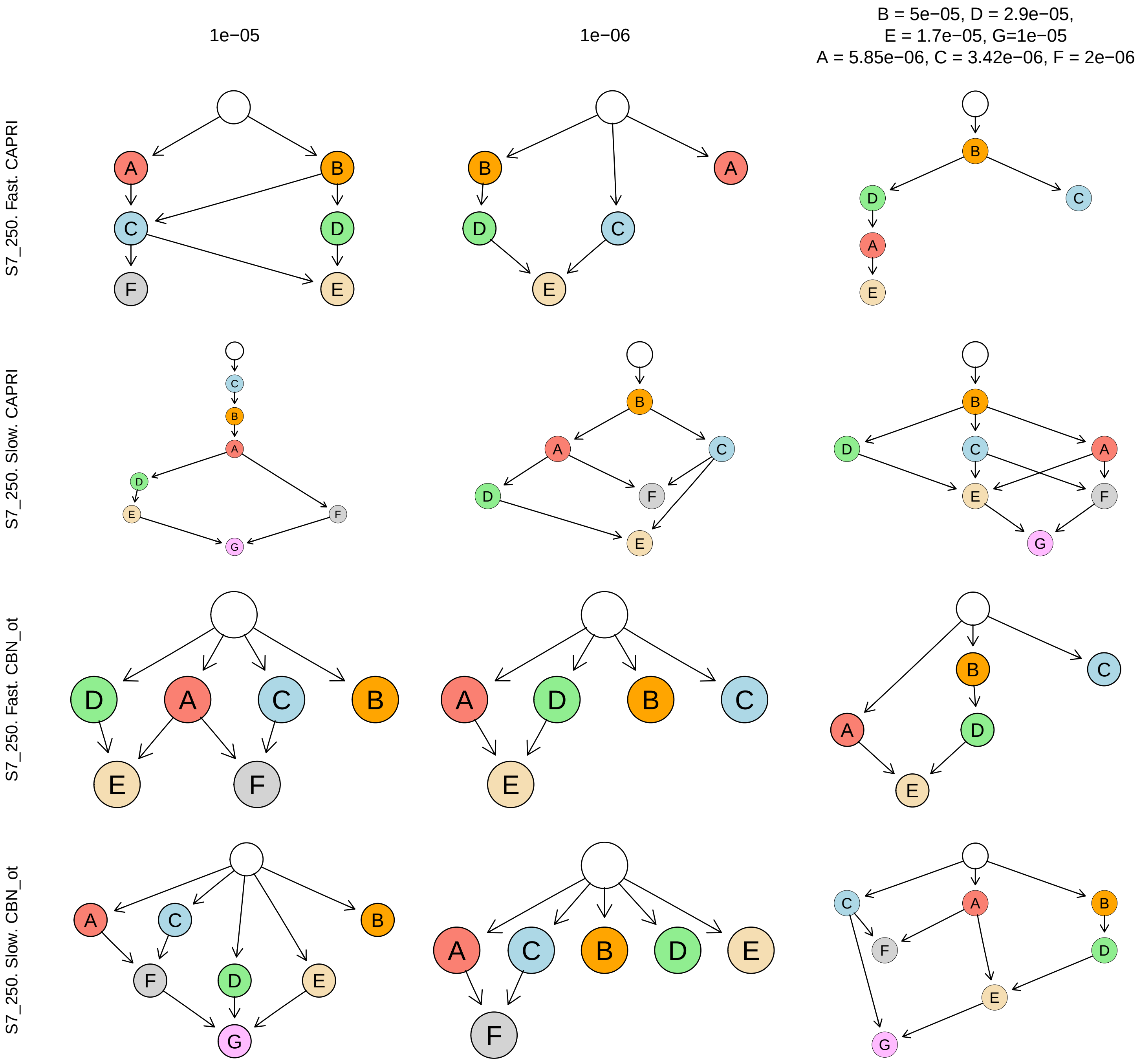

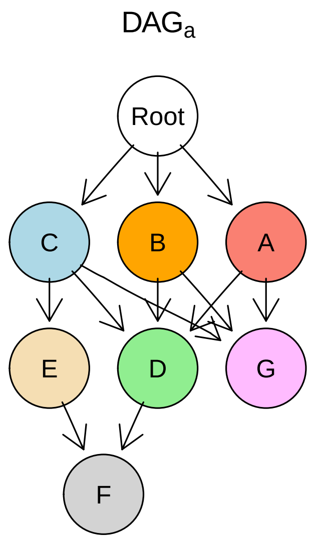

ID	Accessible Genot.	Discrep. DAG_a
S7_251	15	4

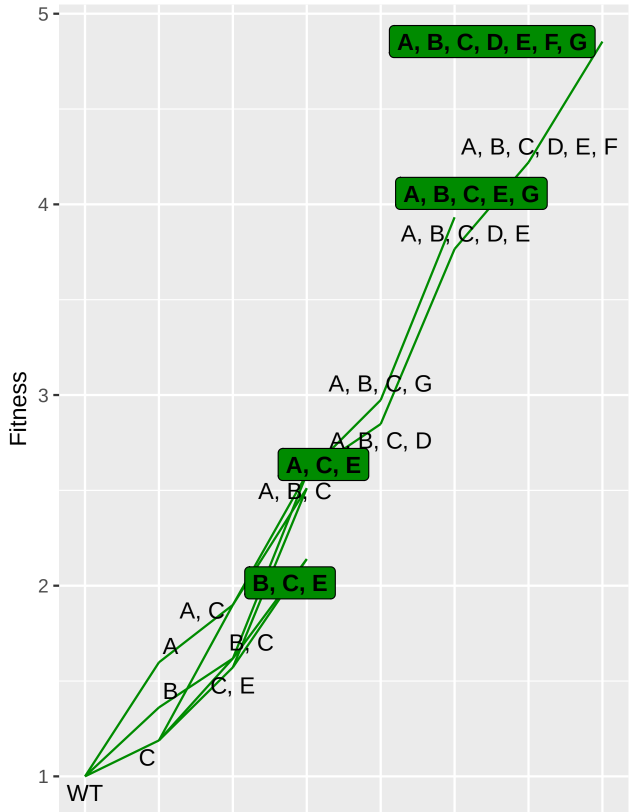

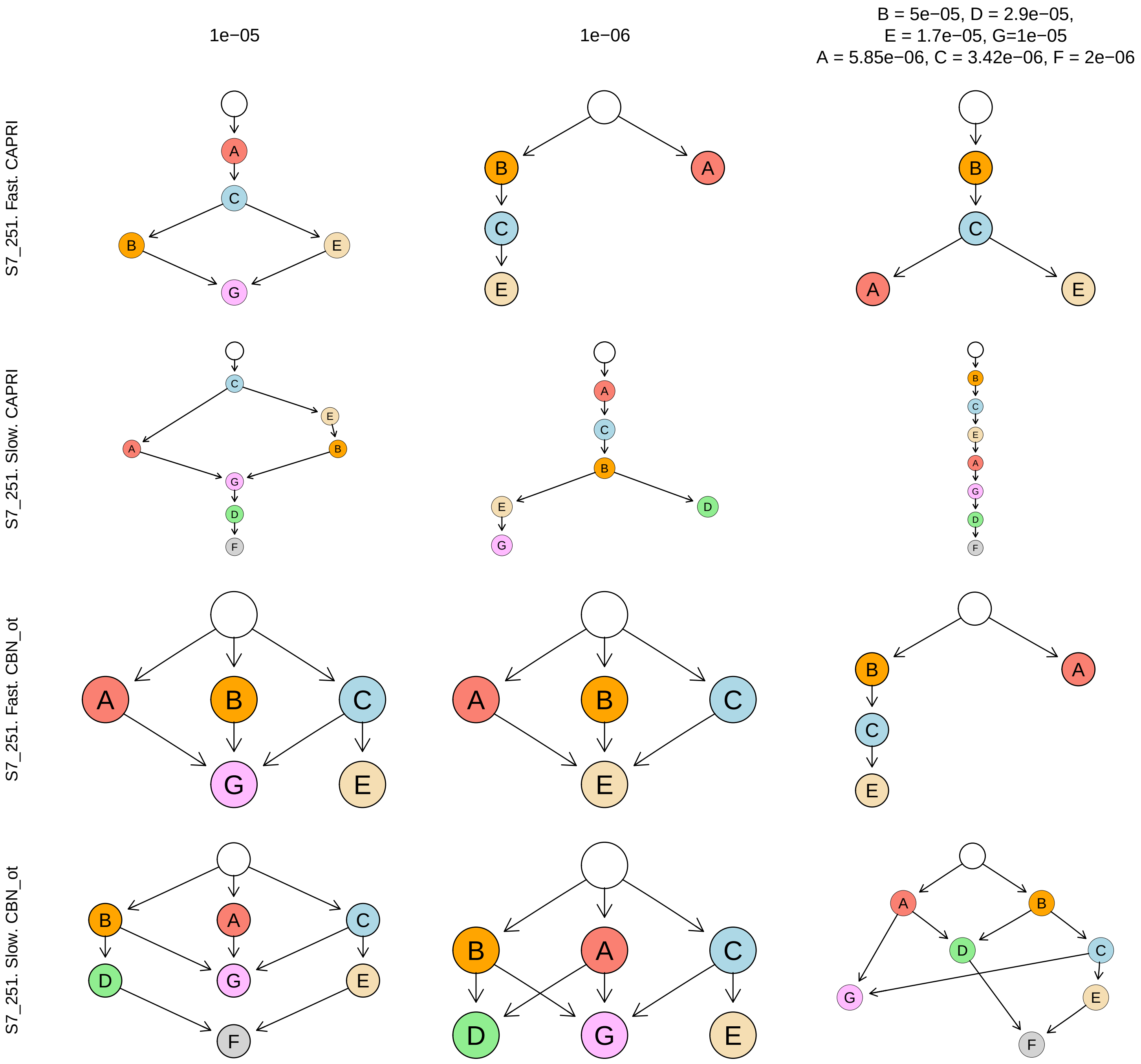

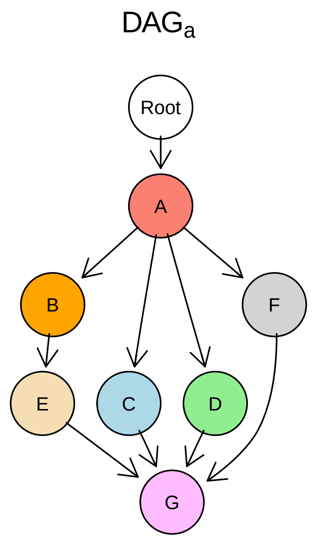

ID	Accessible Genot.	Discrep. DAG_a
S7_252	28	11

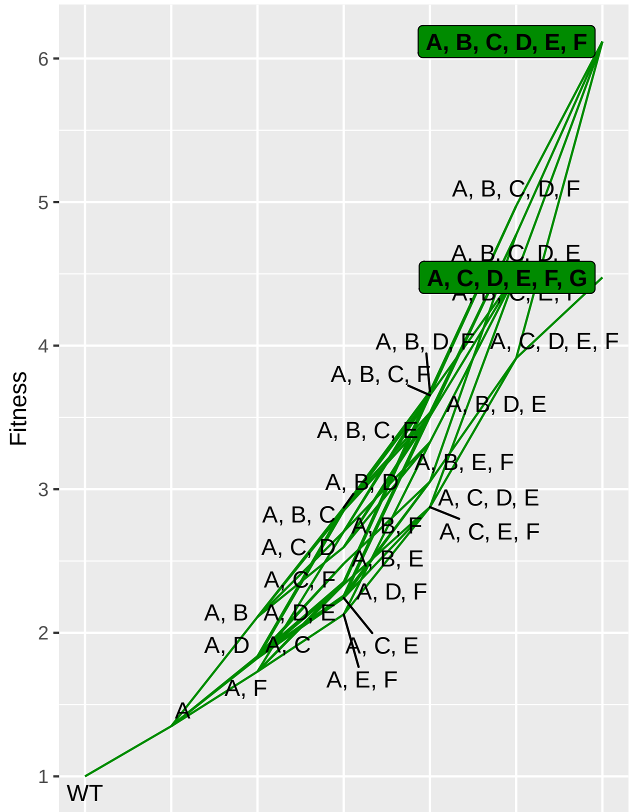

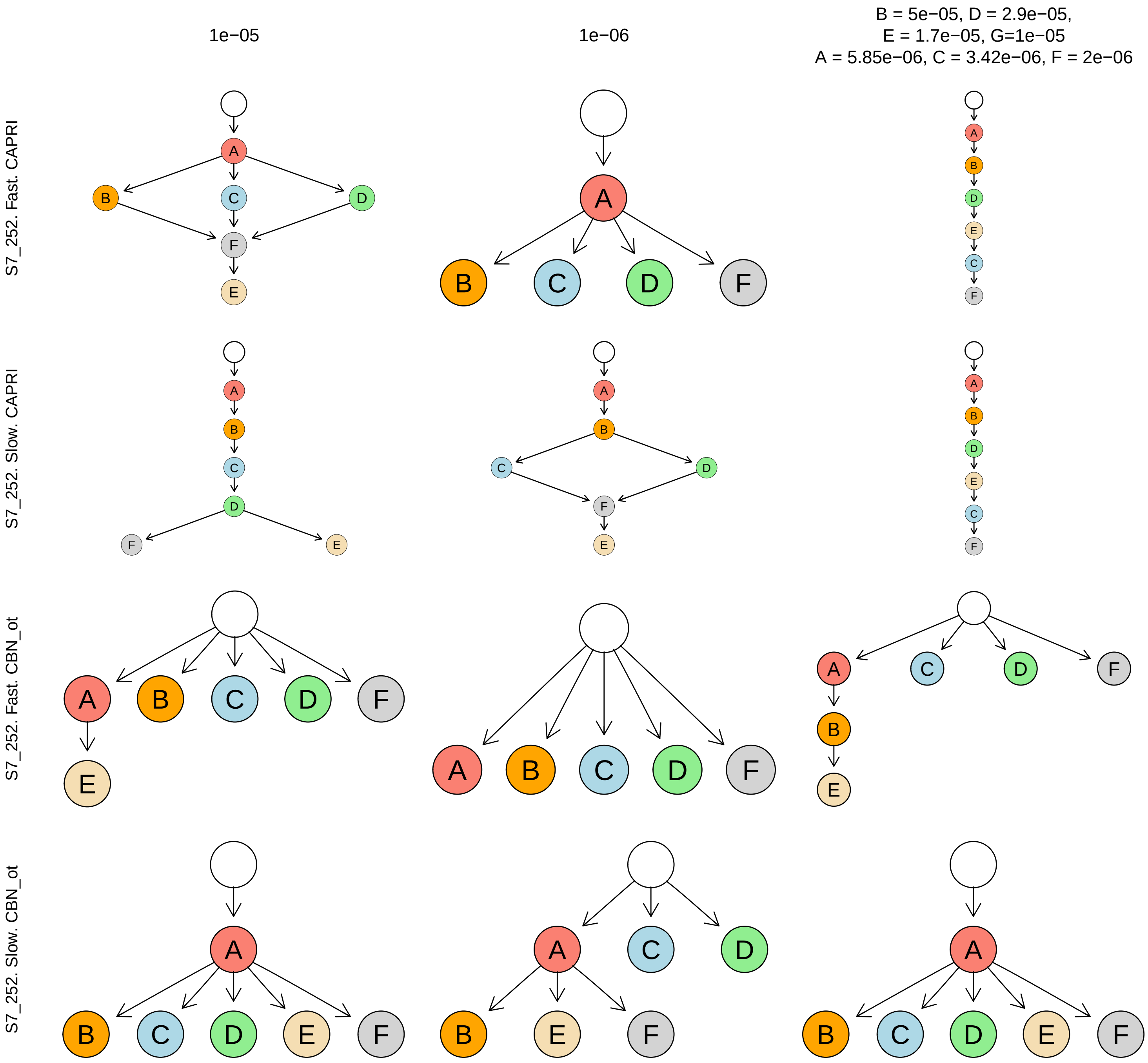

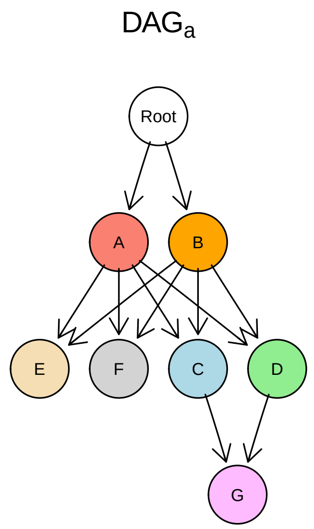

ID	Accessible Genot.	Discrep. DAG_a
S7_253	17	5

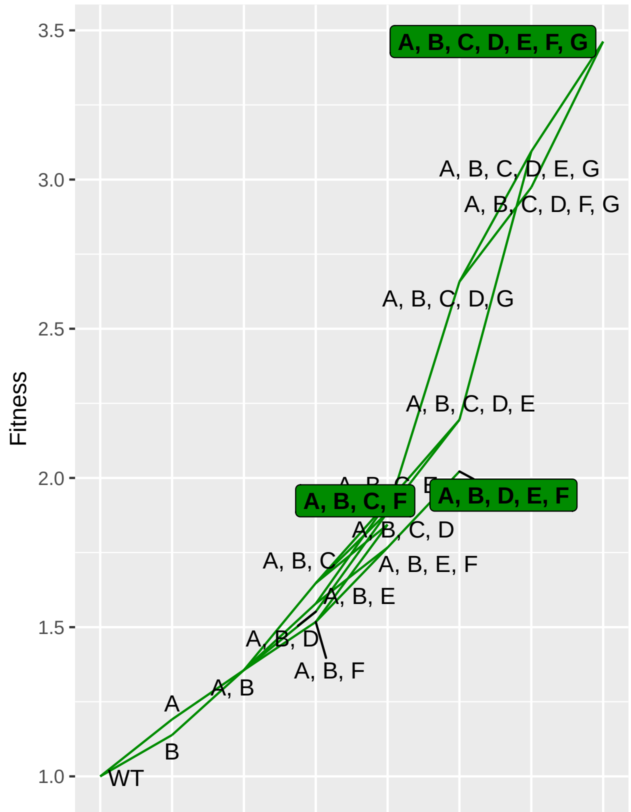

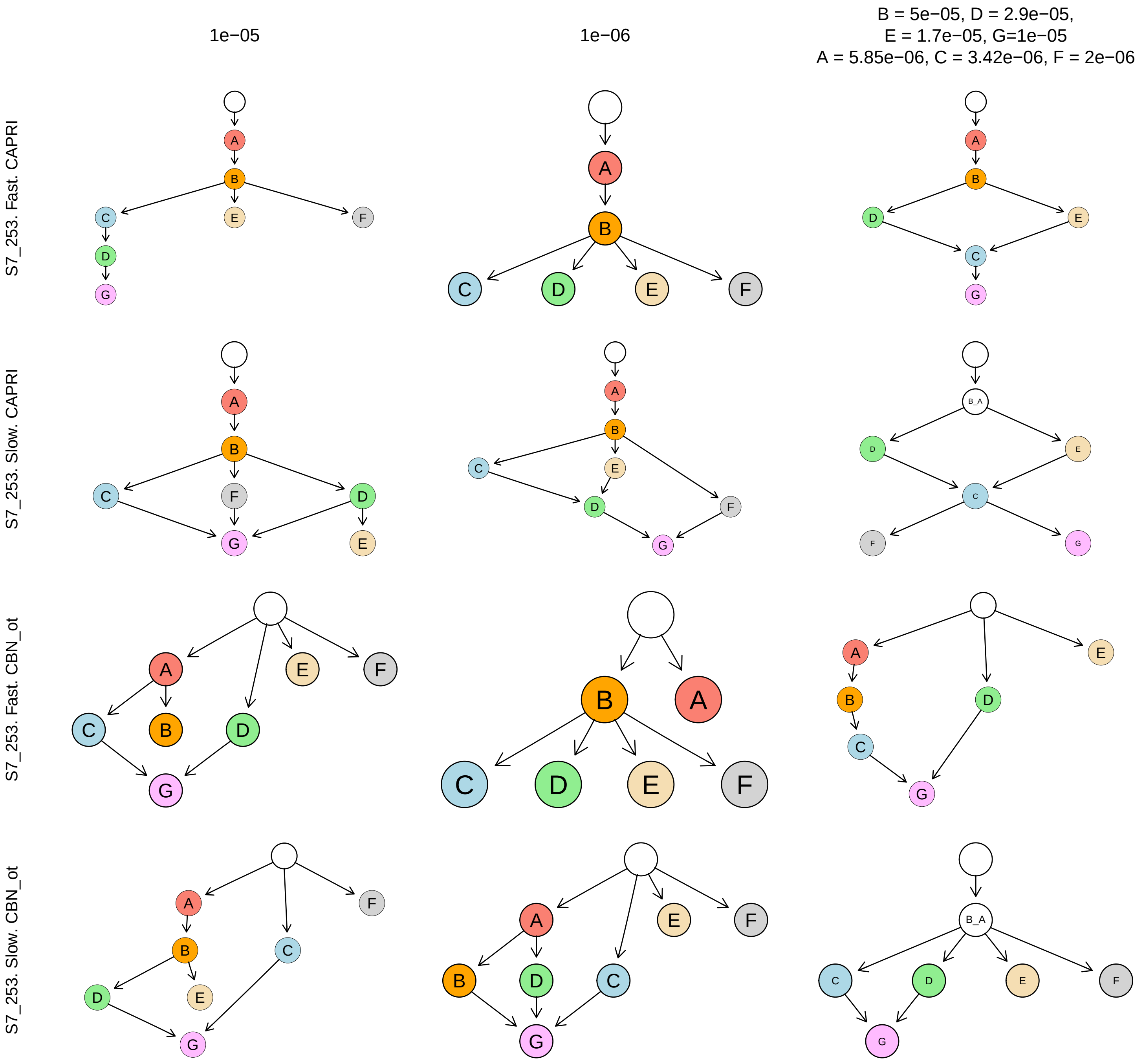

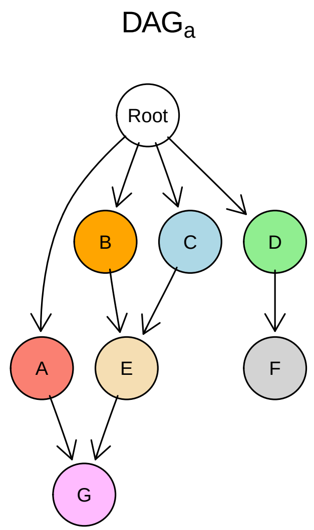

ID	Accessible Genot.	Discrep. DAG_a
S7_254	28	4

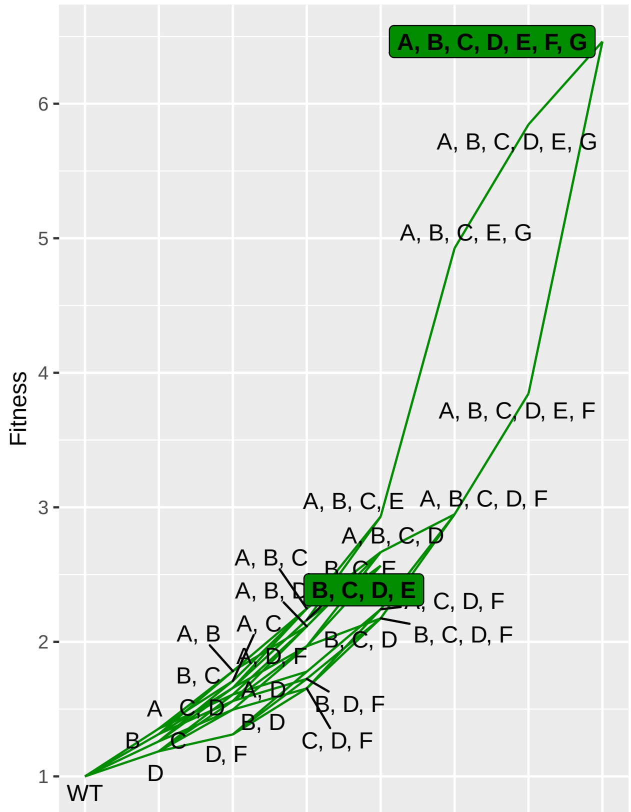

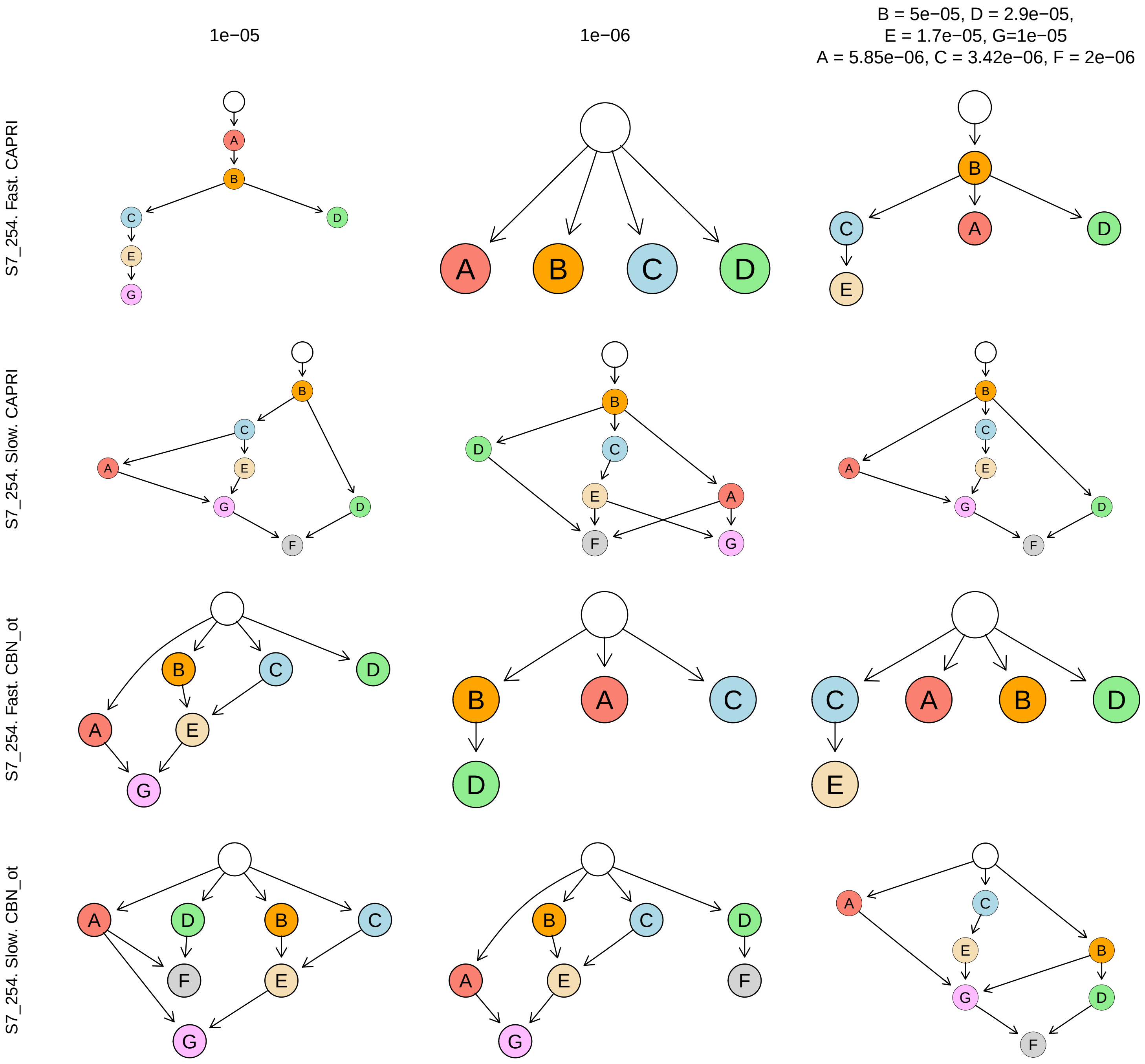

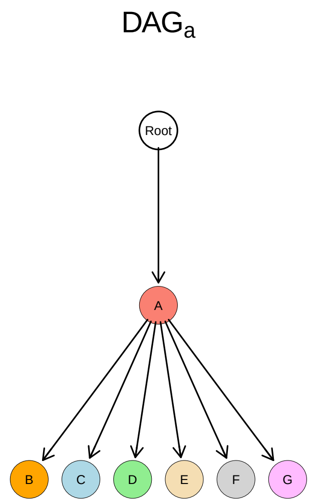

ID	Accessible Genot.	Discrep. DAG_a
S7_255	42	22

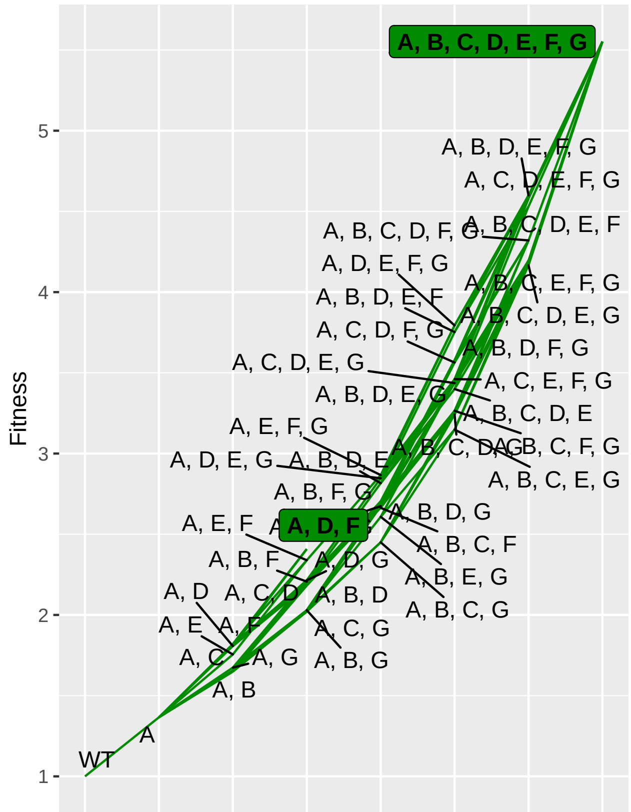

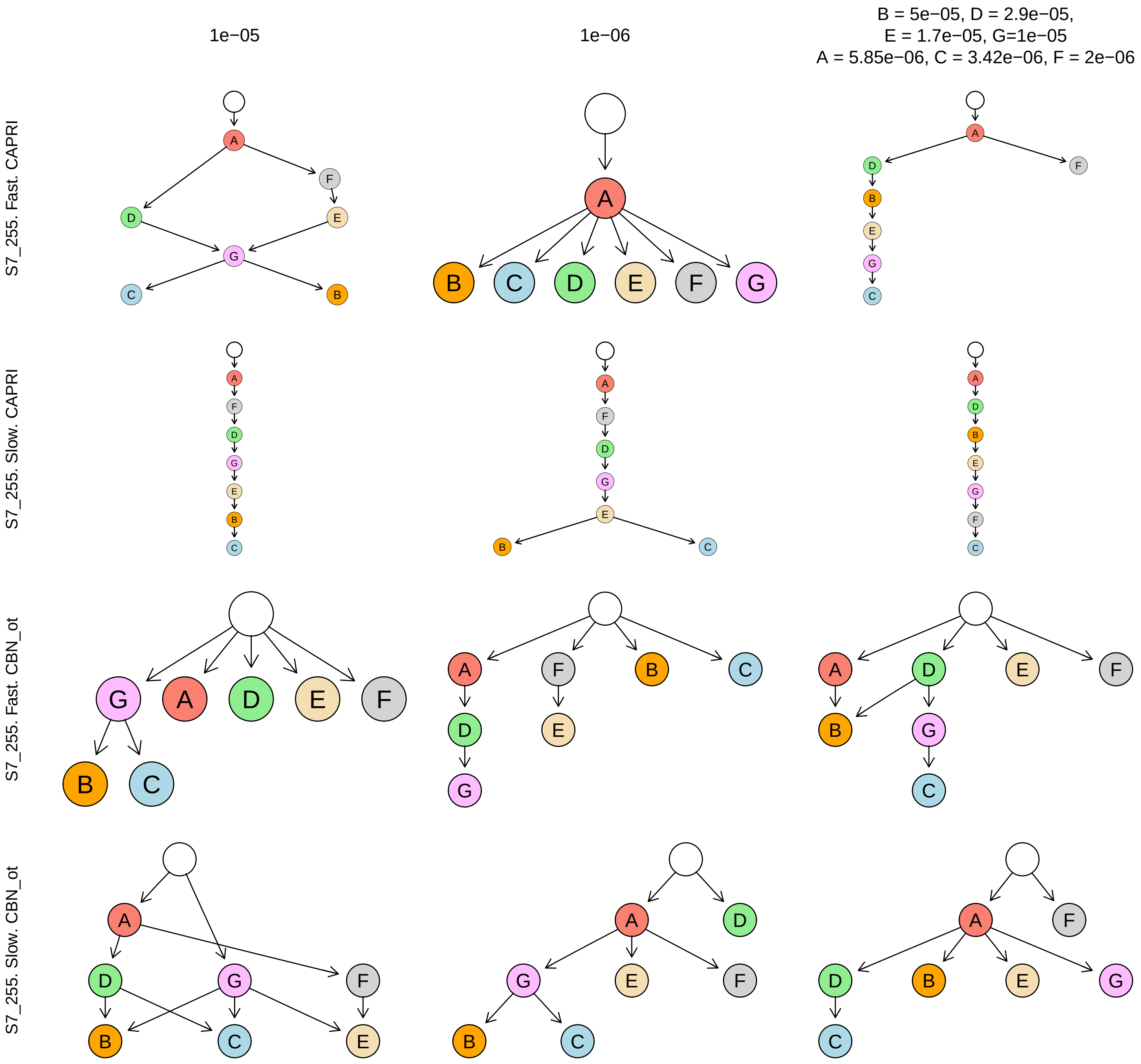

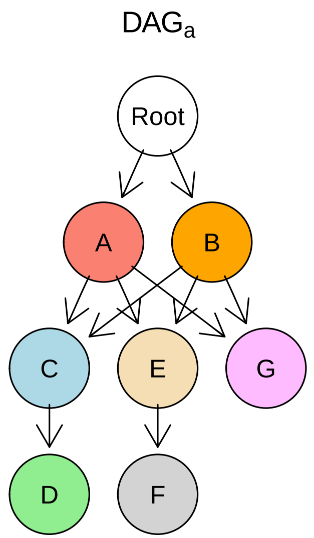

ID	Accessible Genot.	Discrep. DAG_a
S7_256	18	8

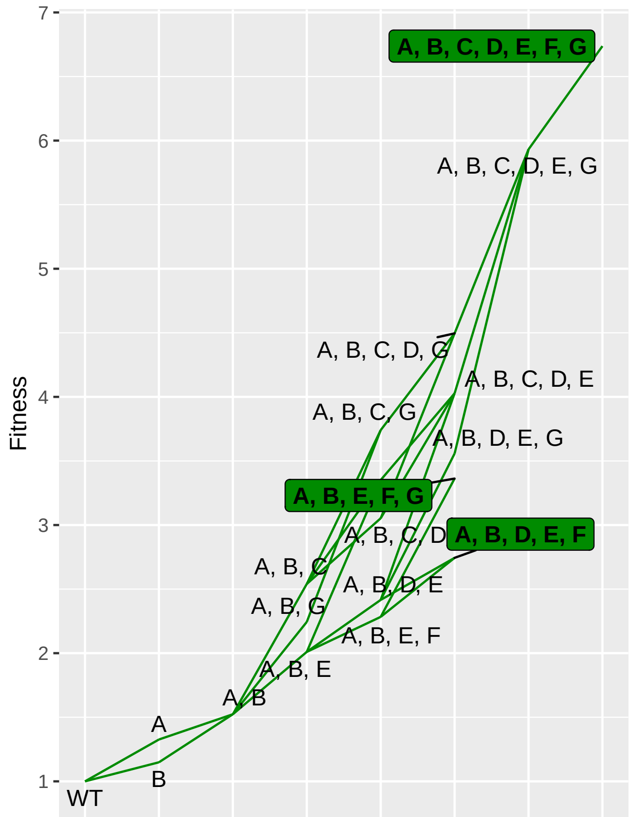

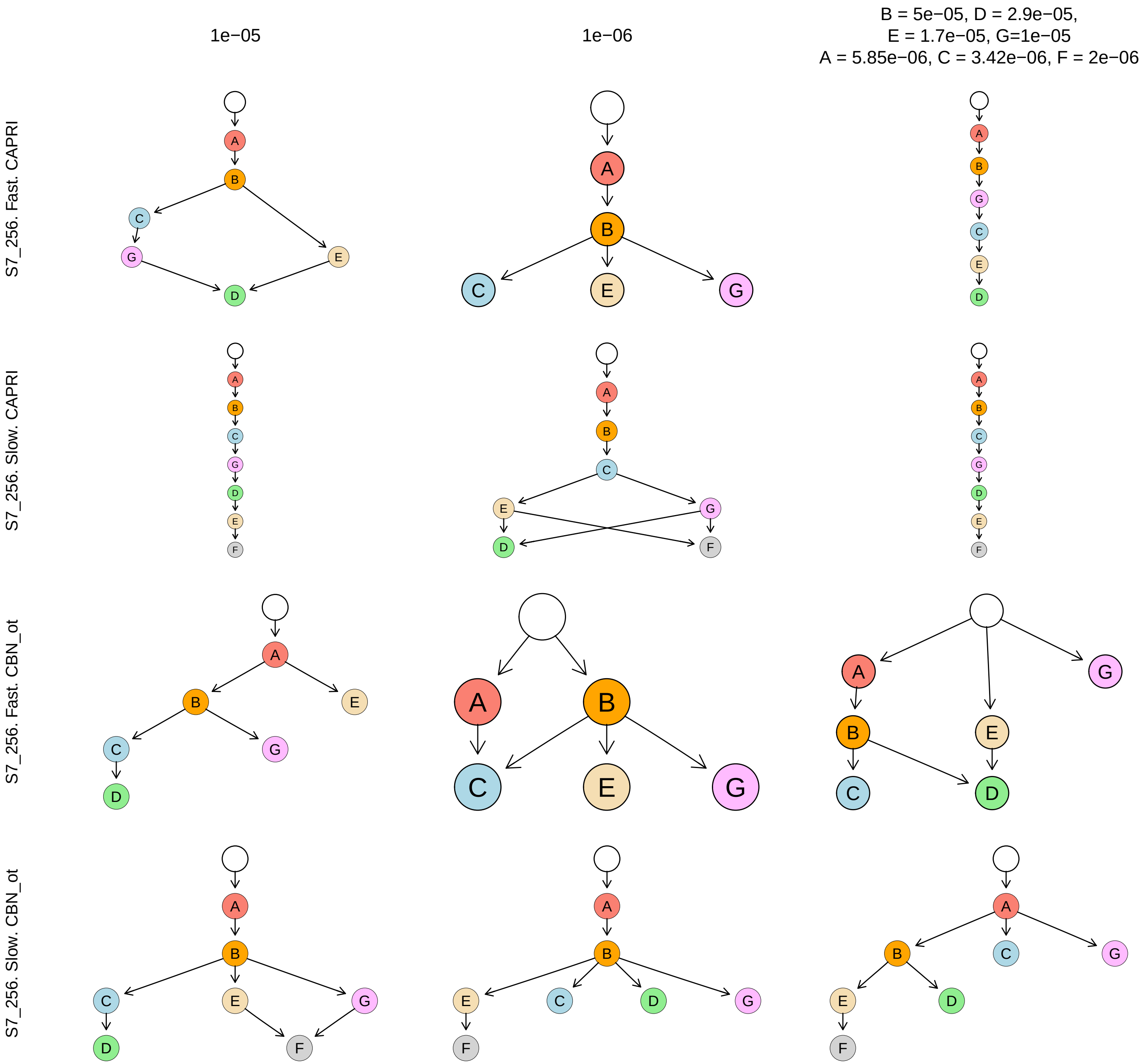

DAG_a

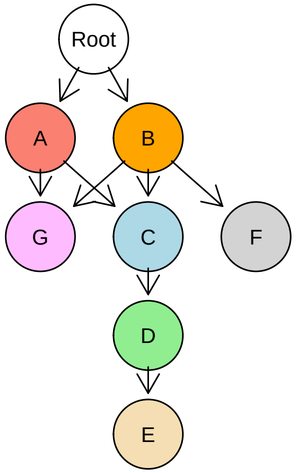

ID	Accessible Genot.	Discrep. DAG_a
S7_257	11	8

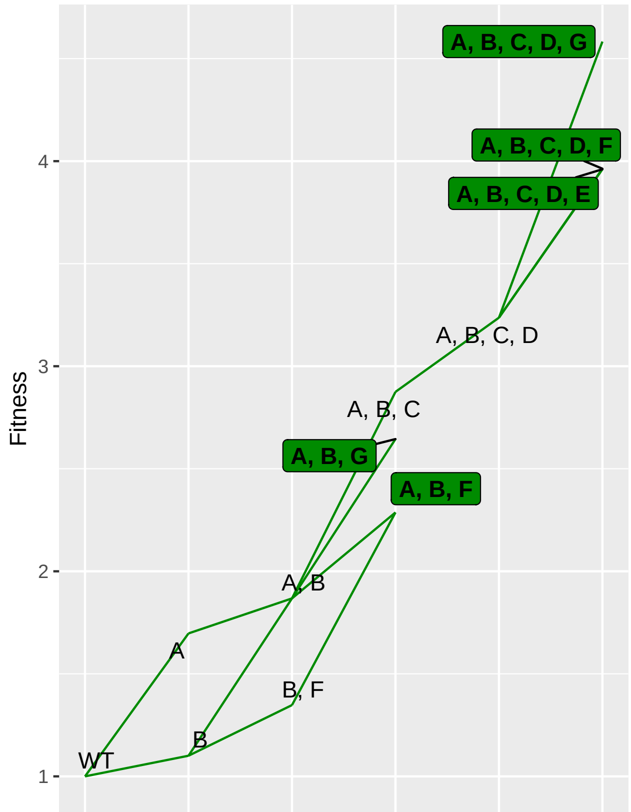

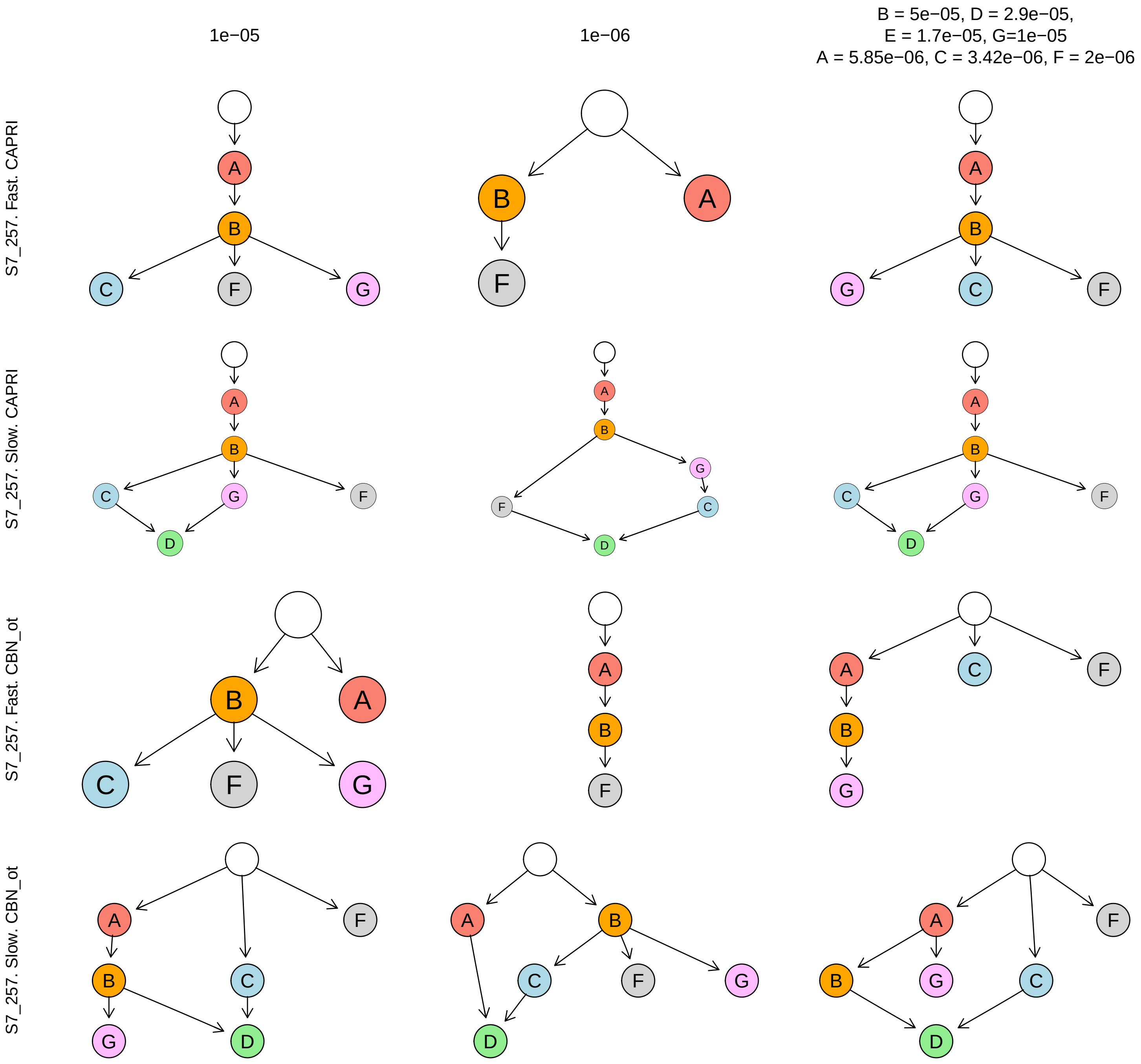

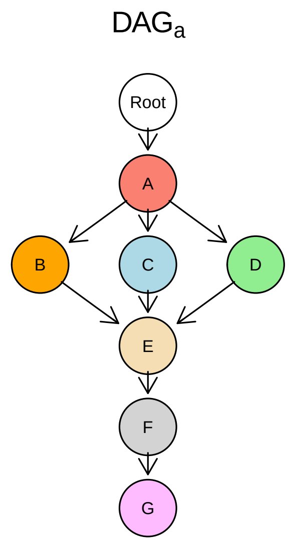

ID	Accessible Genot.	Discrep. DAG_a
S7_258	9	2

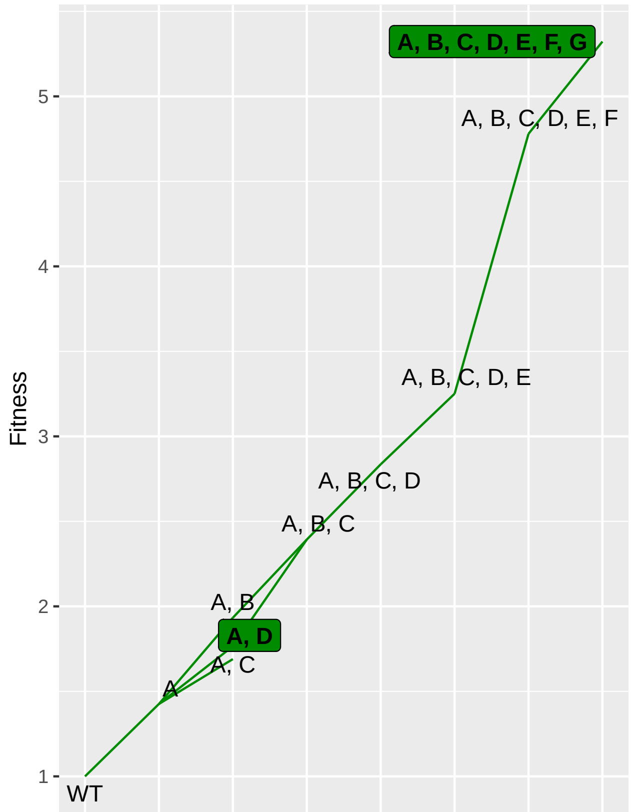

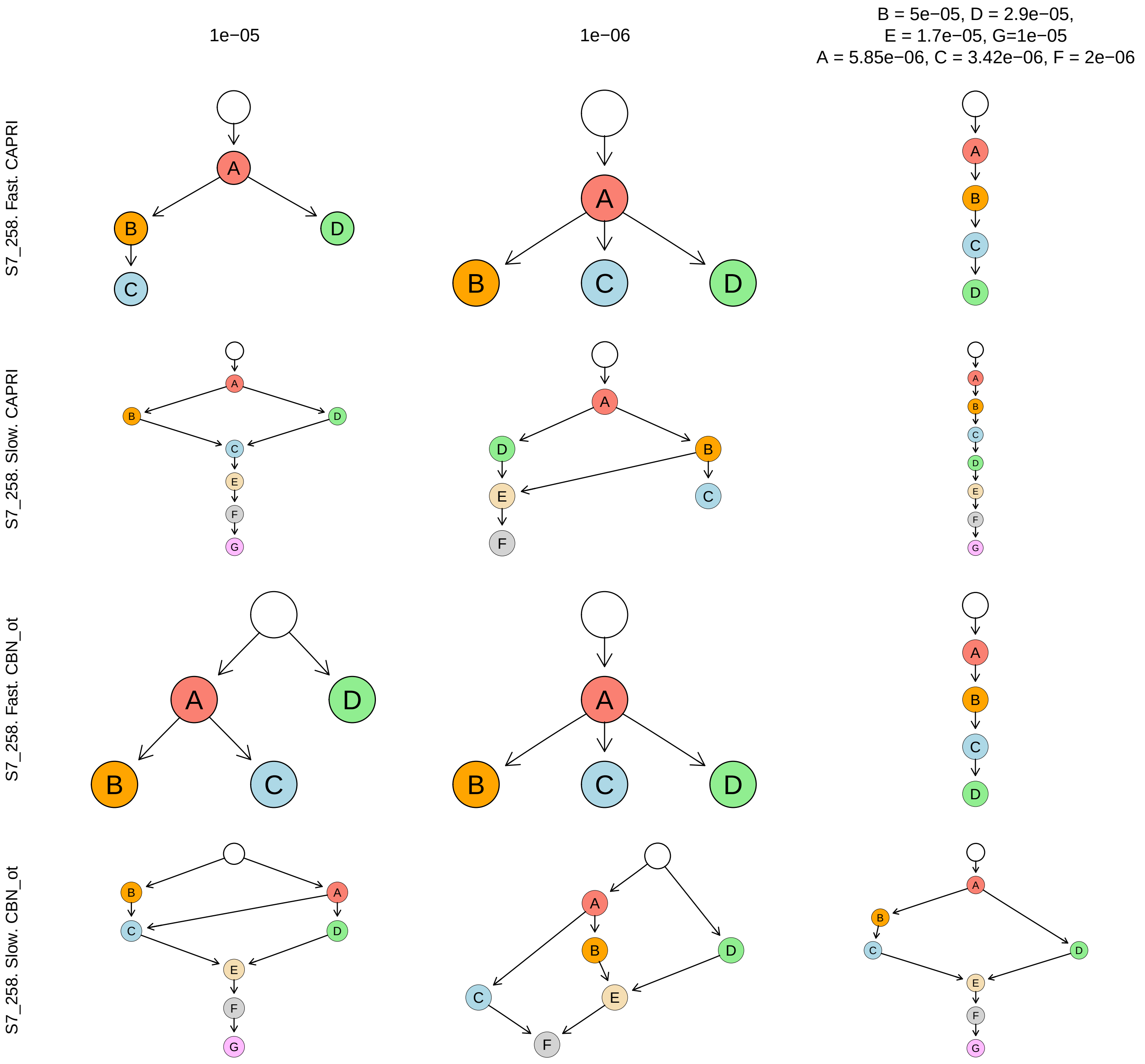

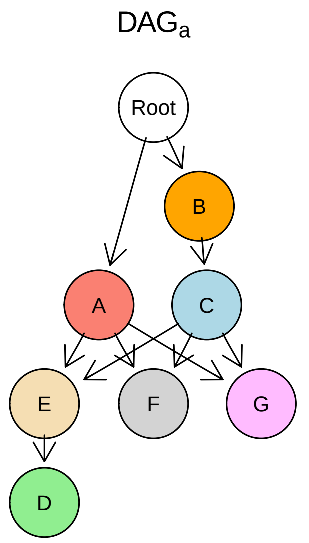

ID	Accessible Genot.	Discrep. DAG_a
S7_259	10	8

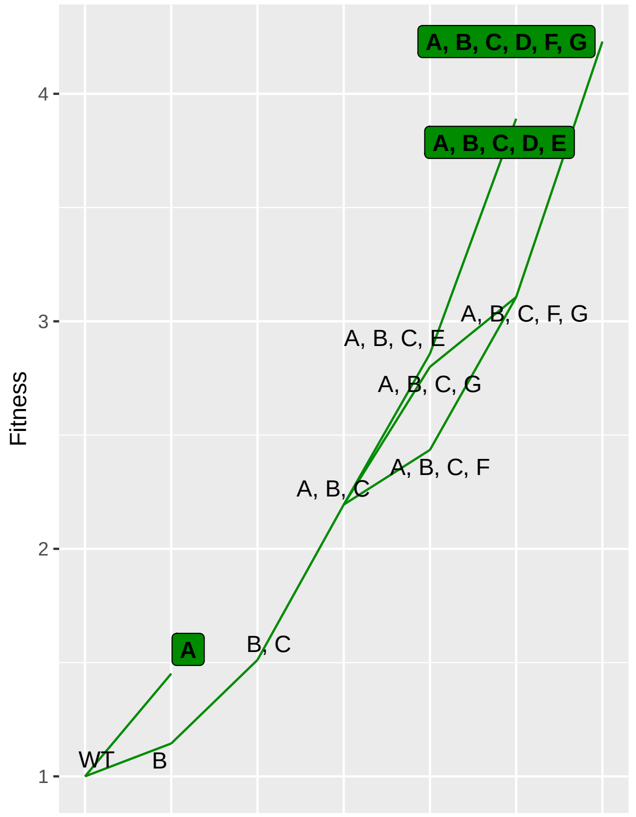

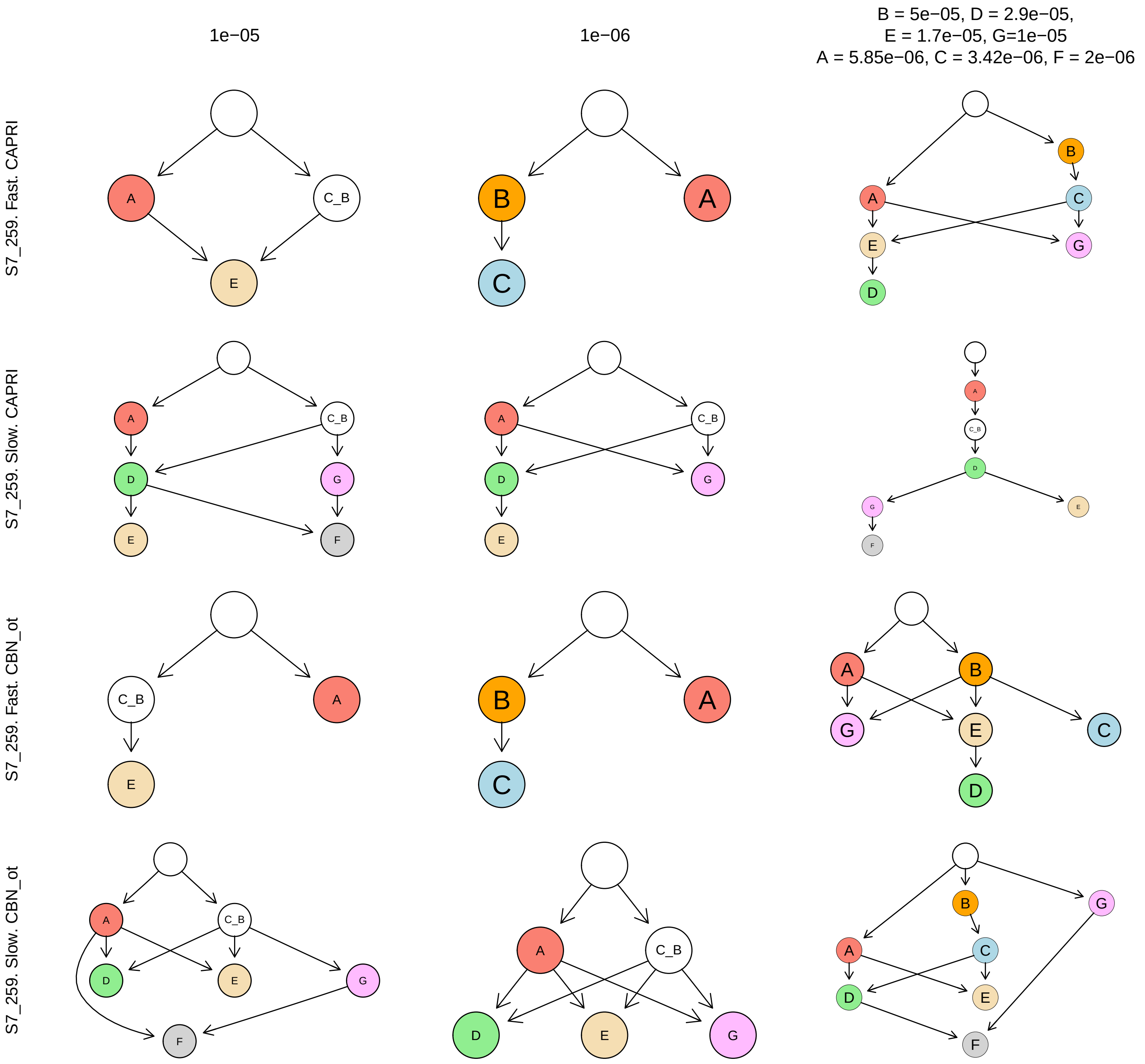

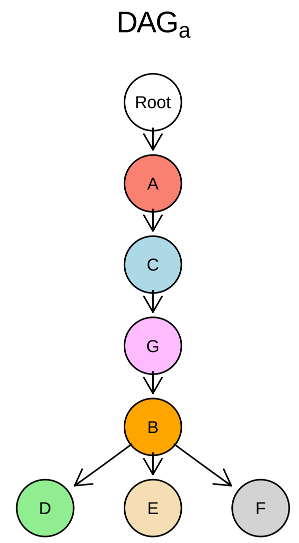

ID	Accessible Genot.	Discrep. DAG_a
S7_260	10	1

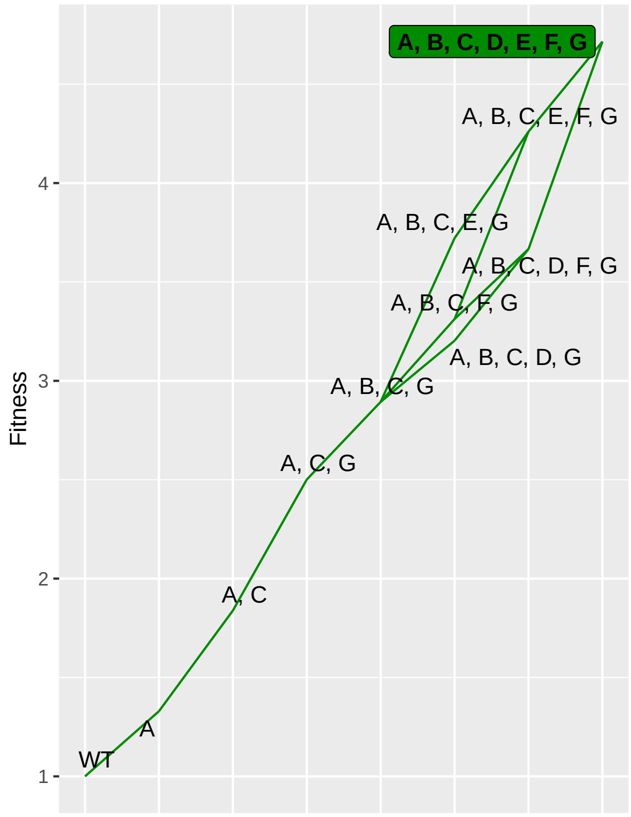

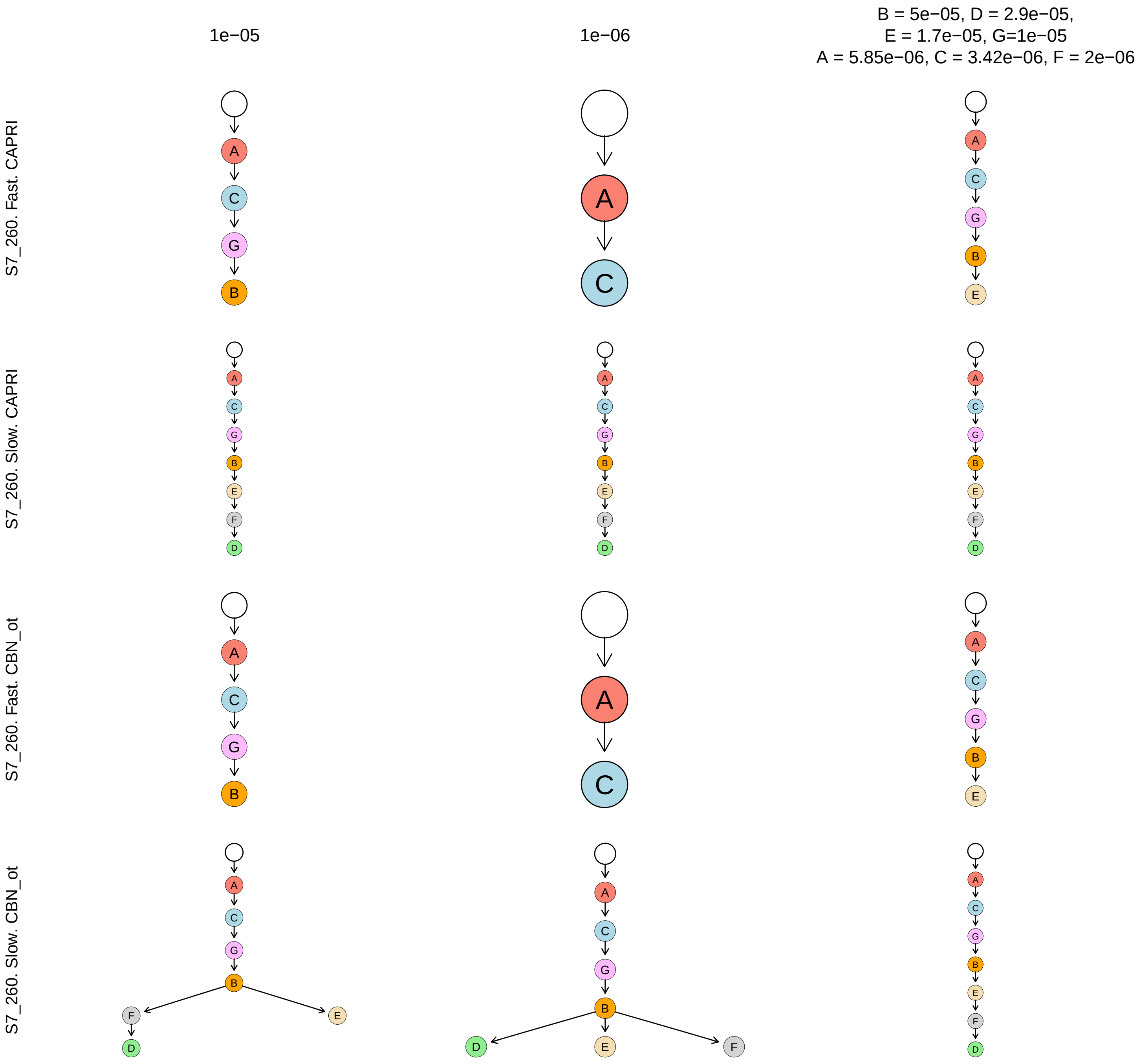

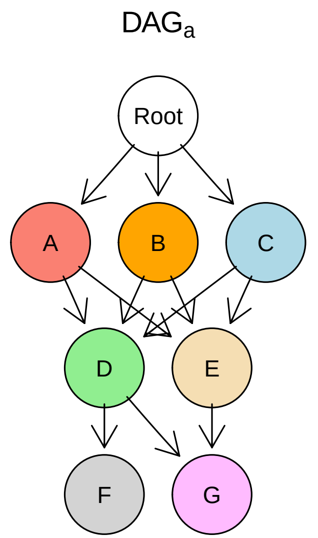

ID	Accessible Genot.	Discrep. DAG_a
S7_261	11	3

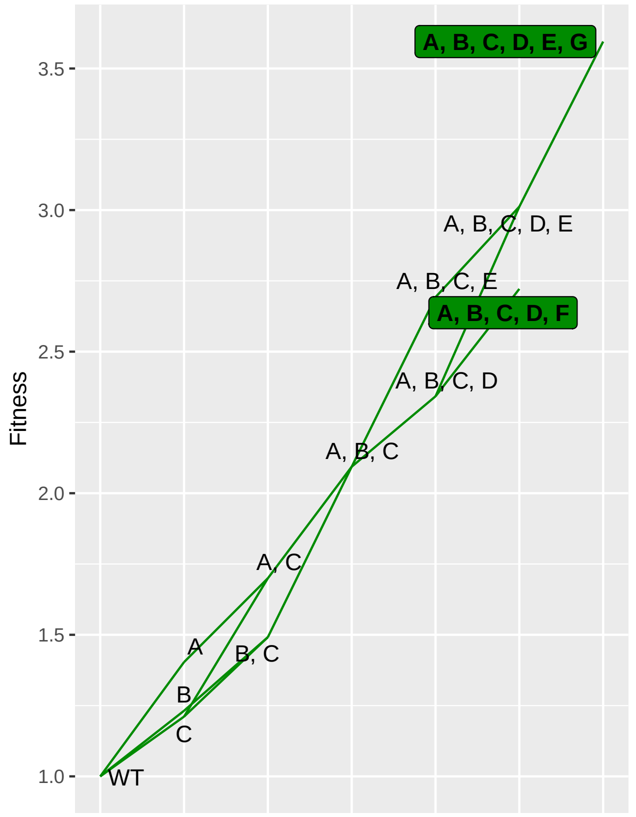

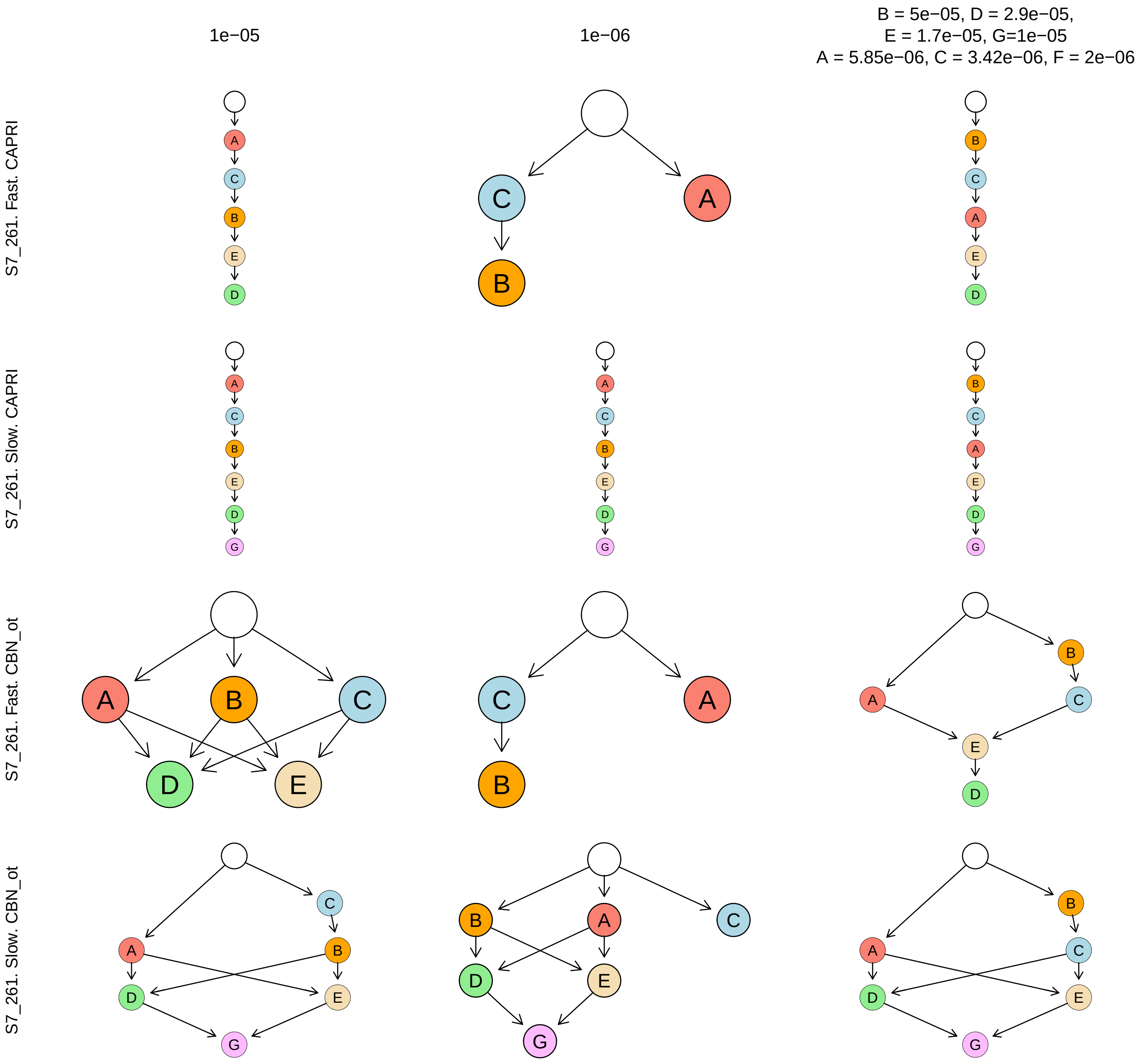

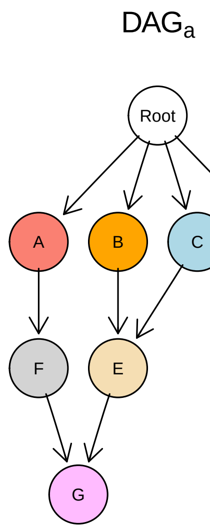

ID	Accessible Genot.	Discrep. DAG_a
S7_262	17	16

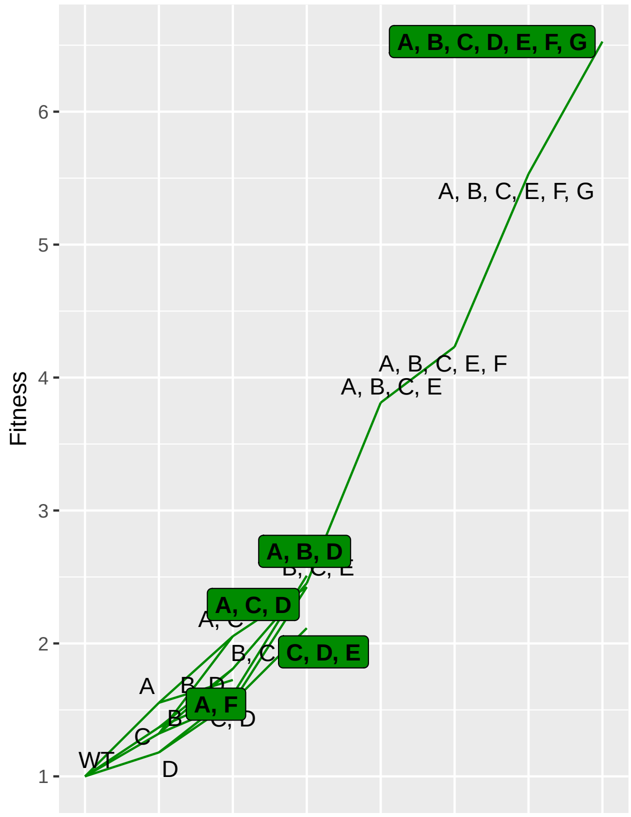

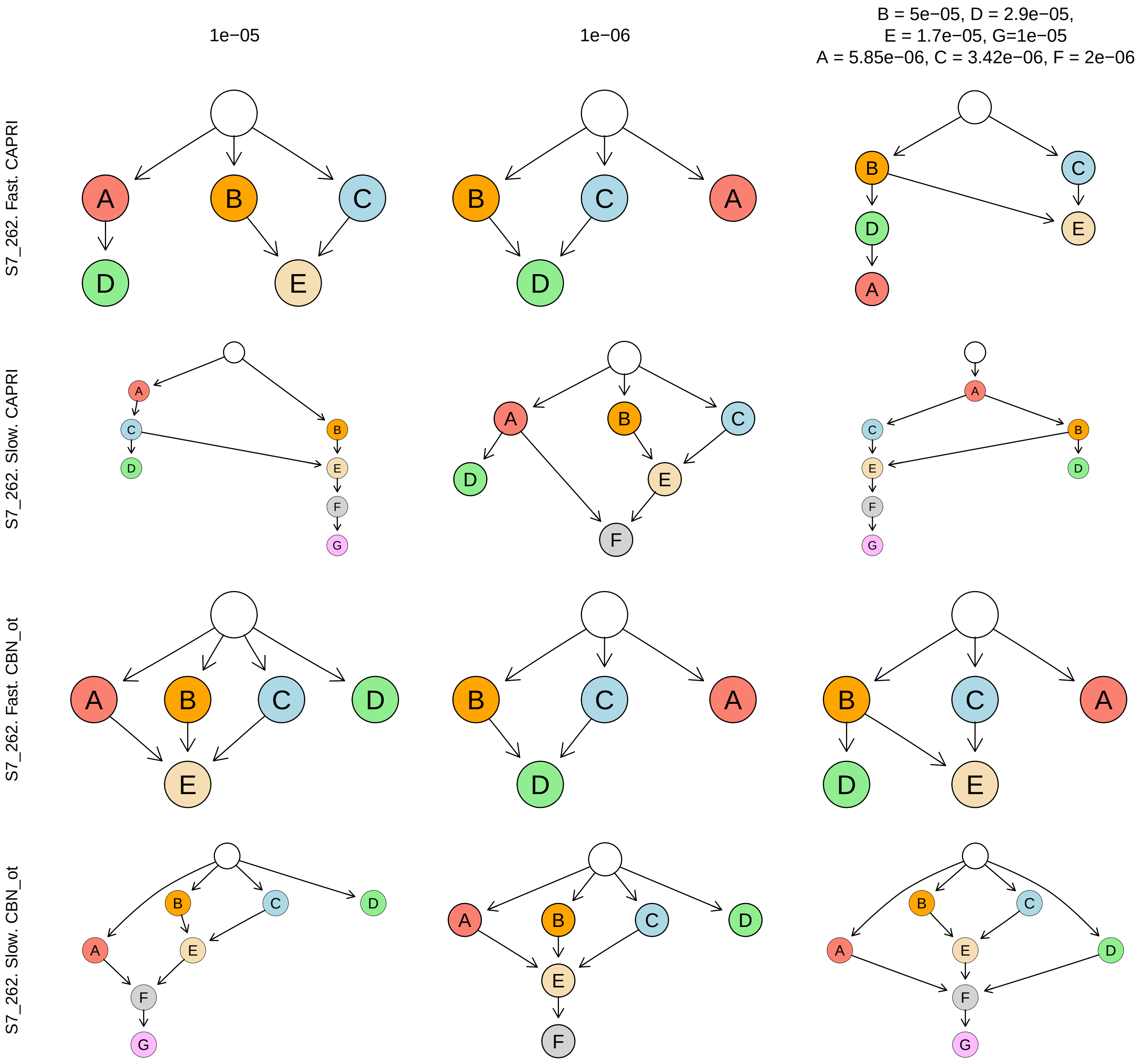

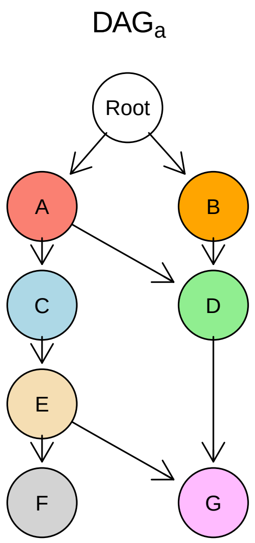

ID	Accessible Genot.	Discrep. DAG_a
S7_263	12	3

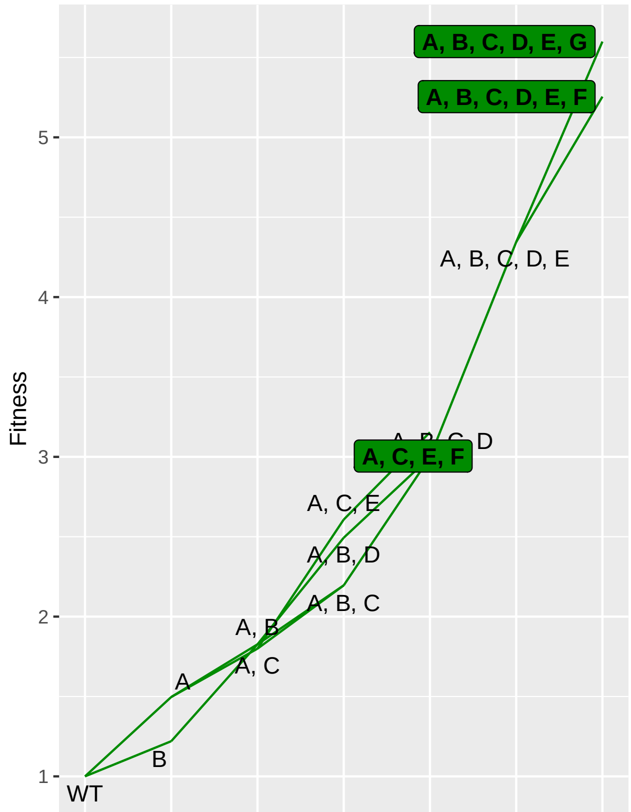

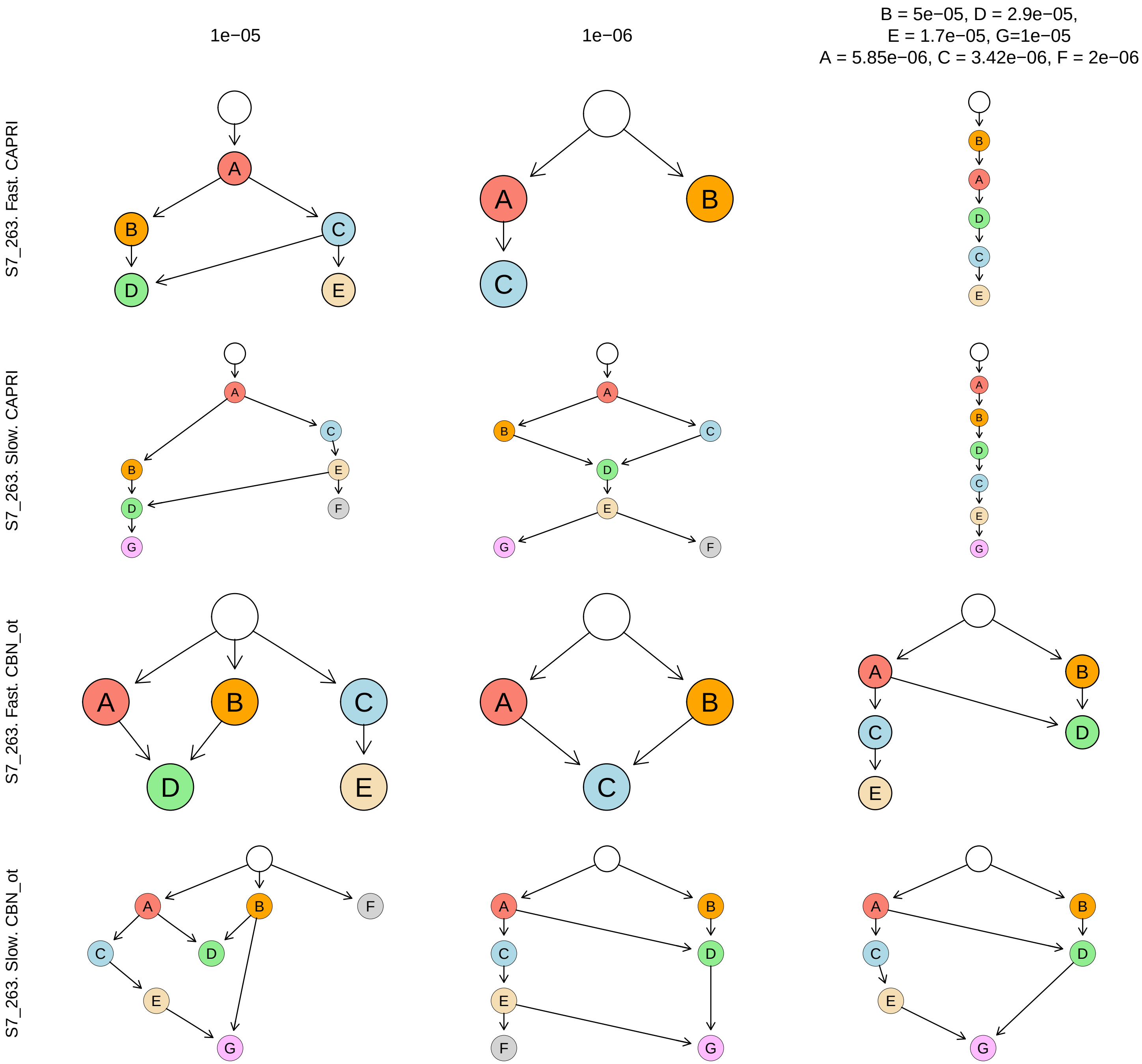

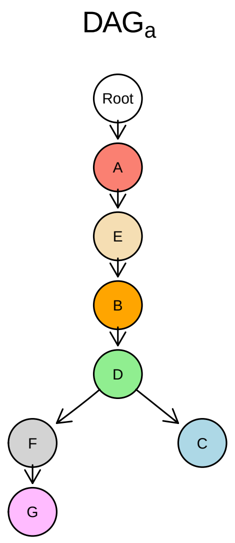

ID	Accessible Genot.	Discrep. DAG_a
S7_264	8	1

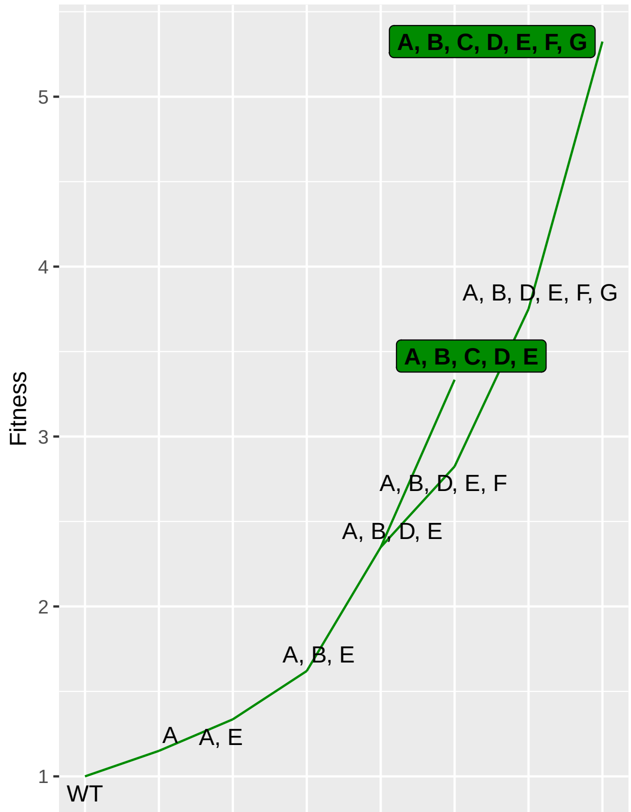

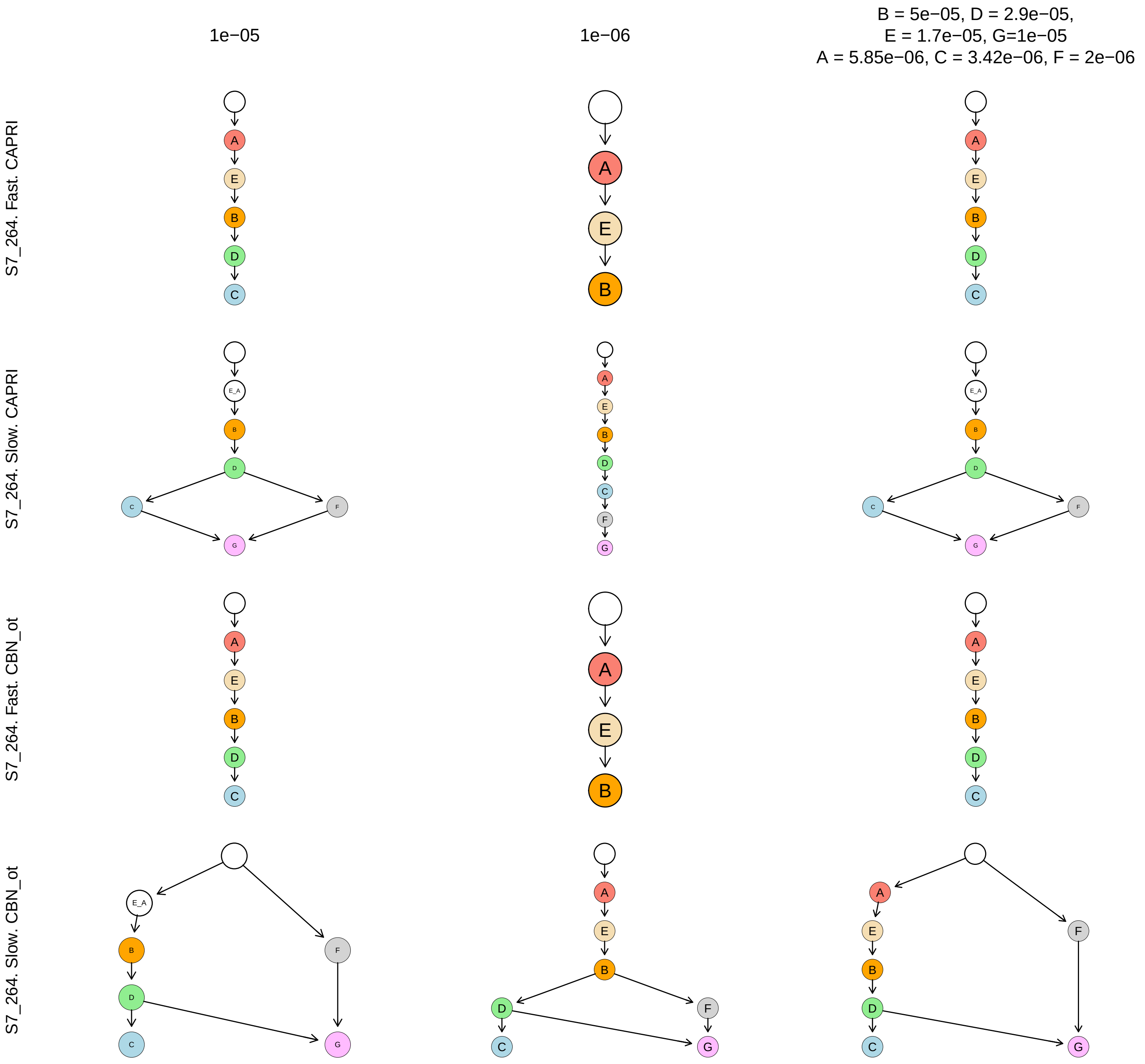

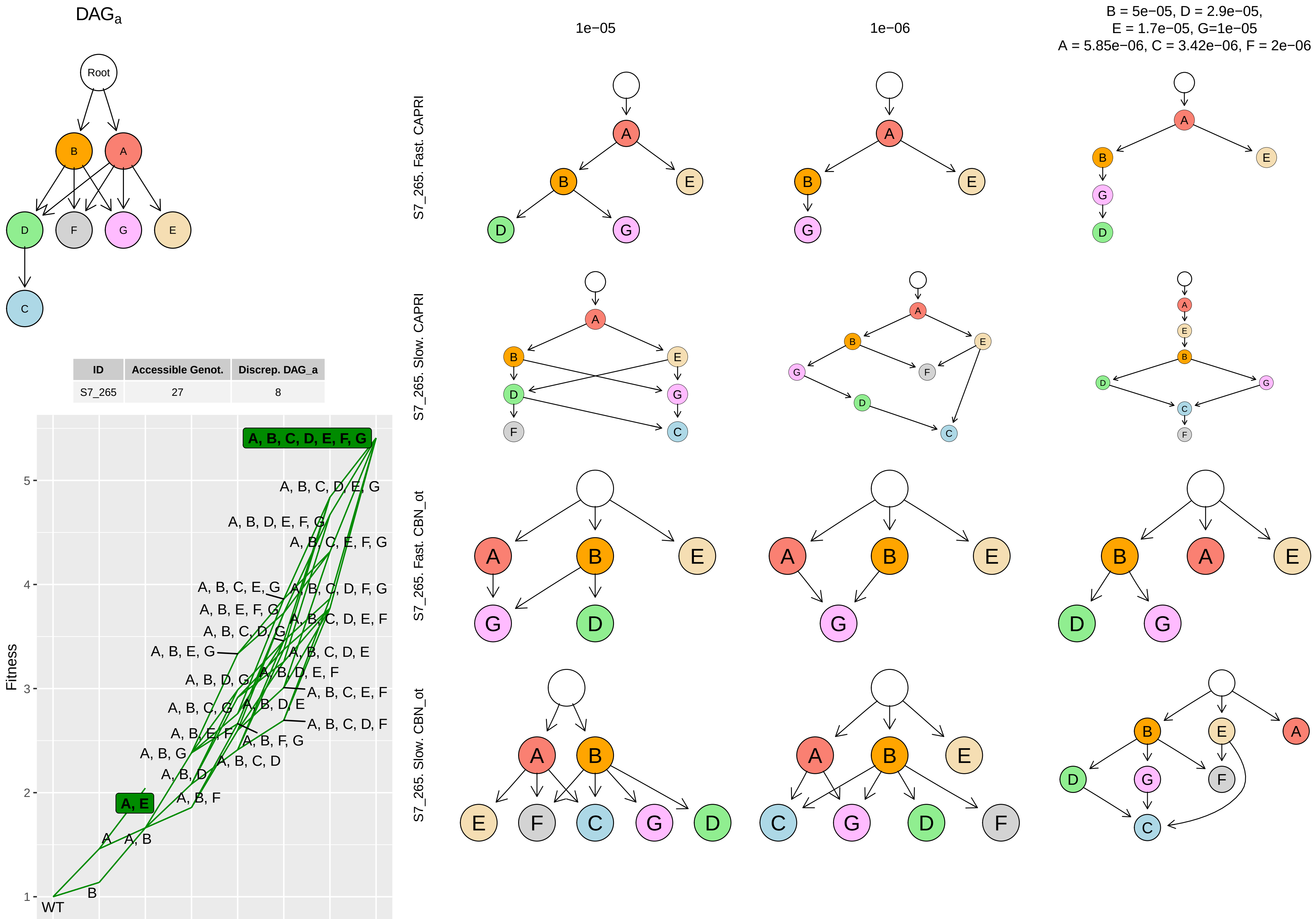

DAGa
Root
1e−05
1e−06
B = 5e−05, D = 2.9e−05,
E = 1.7e−05, G=1e−05
A = 5.85e−06, C = 3.42e−06, F = 2e−06
S7_265. Fast. CAPRI
S7_265. Slow. CAPRI
S7_265. Fast. CBN_ot
S7_265. Slow. CBN_ot
ID
Accessible Genot.
Discrep. DAG_a
S7_265
27
8
Fitness
A, B, C, D, E, F, G
A, B, C, D, E, G
A, B, D, E, F, G
A, B, C, E, F, G
A, B, C, E, G
A, B, C, D, F, G
A, B, E, F, G
A, B, C, D, E, F
A, B, C, D, G
A, B, E, G
A, B, C, D, E
A, B, D, G
A, B, D, E, F
A, B, C, E, F
A, B, C, G
A, B, D, E
A, B, E, F
A, B, C, D, F
A, B, F, G
A, B, G
A, B, C, D
A, B, D
A, B, F
A, E
A
A, B
B
WT

DAG$_a$

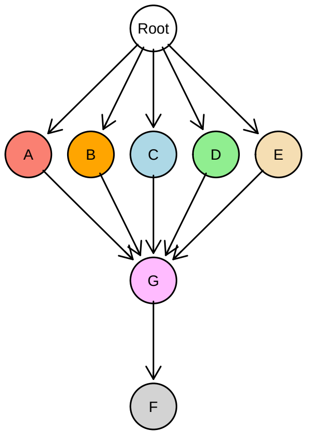

ID	Accessible Genot.	Discrep. DAG_a
S7_266	20	13

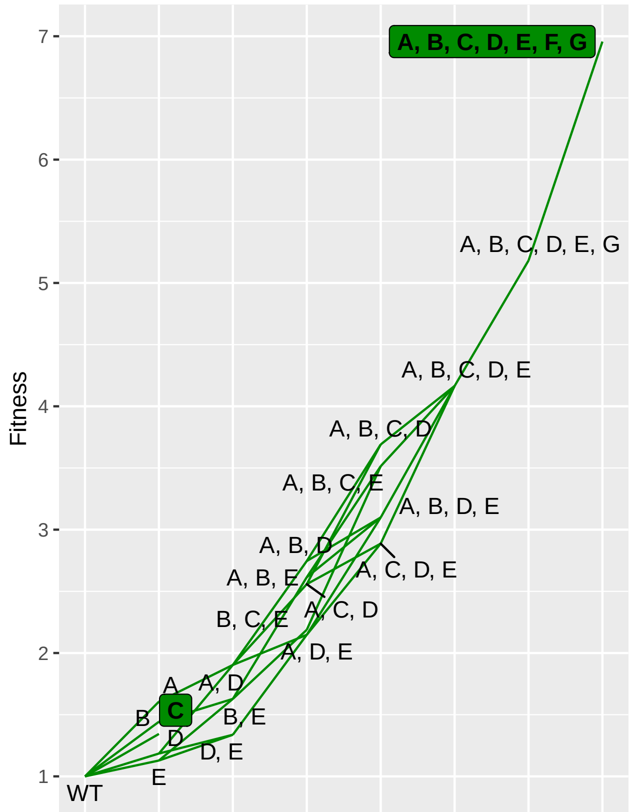

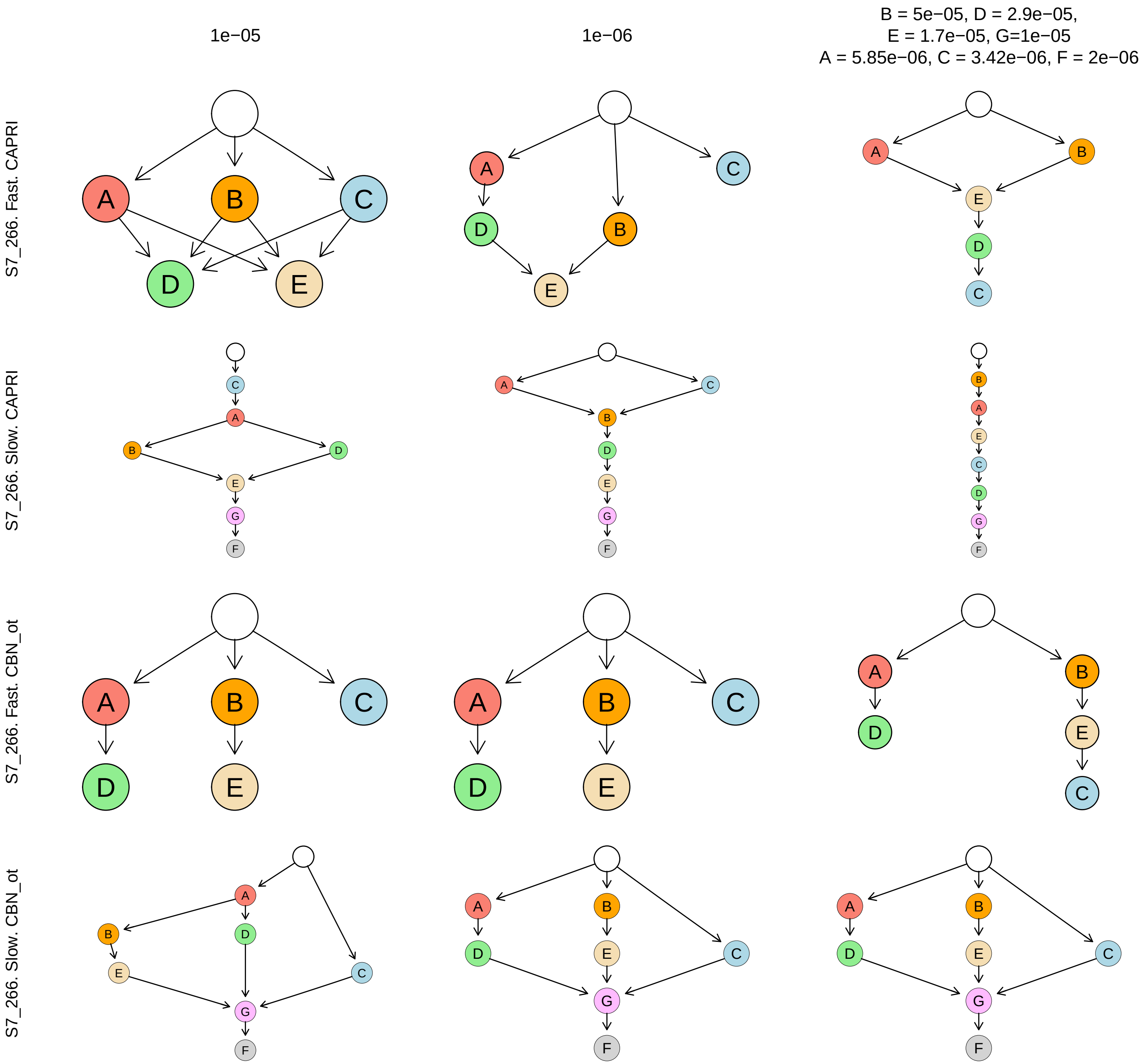

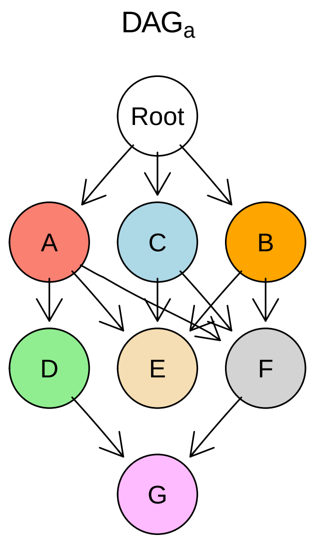

ID	Accessible Genot.	Discrep. DAG_a
S7_267	16	3

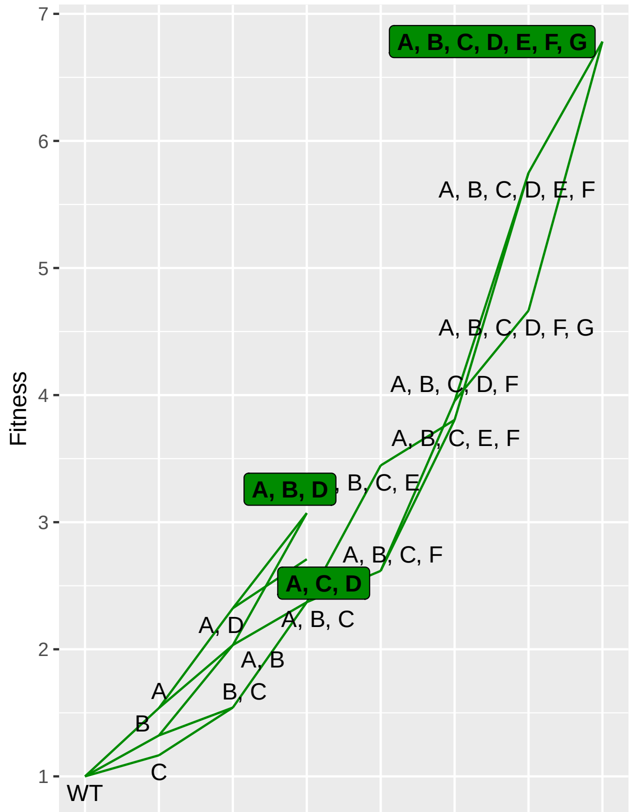

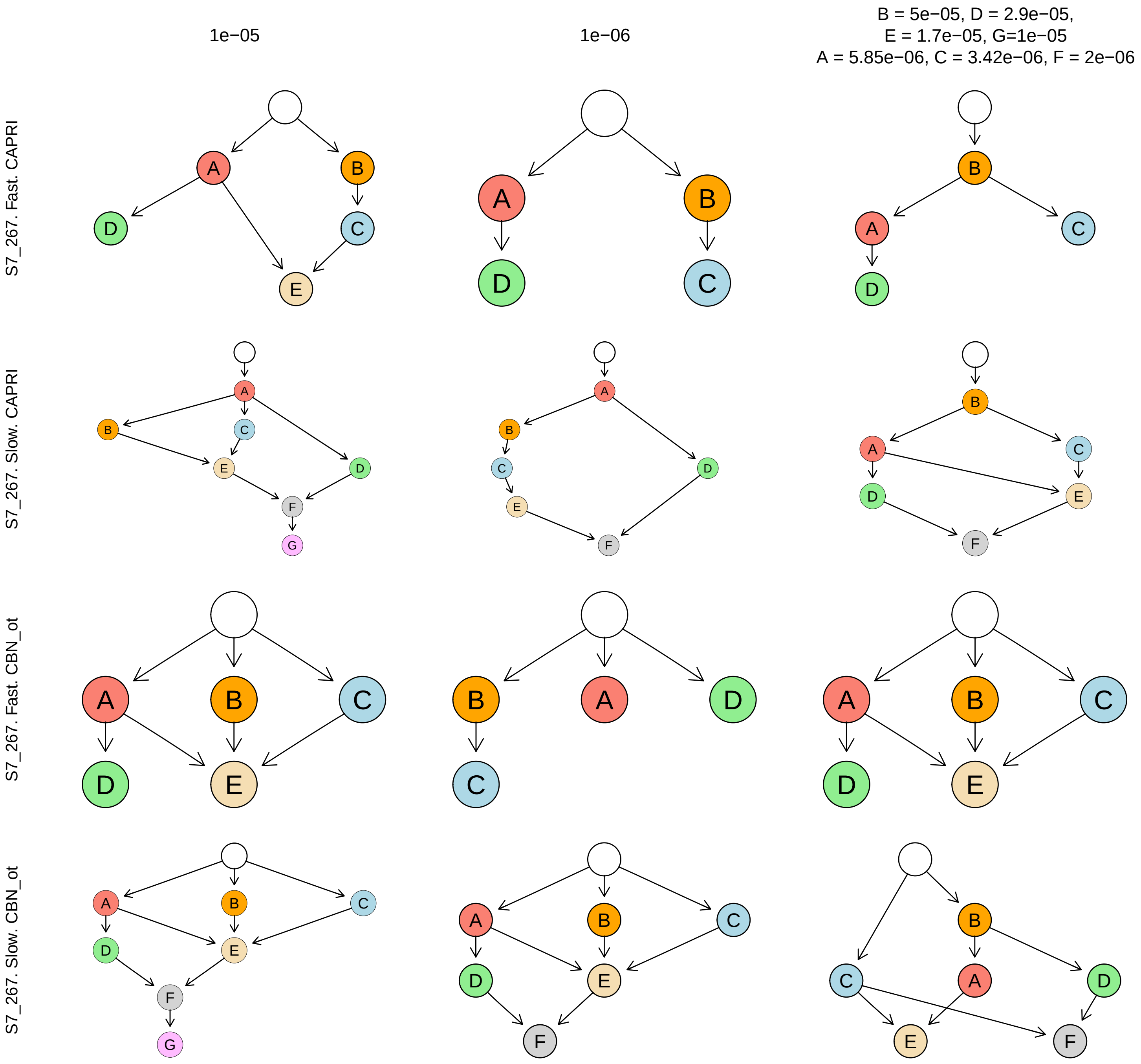

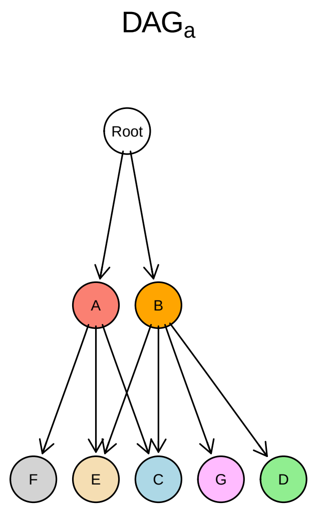

ID	Accessible Genot.	Discrep. DAG_a
S7_268	24	14

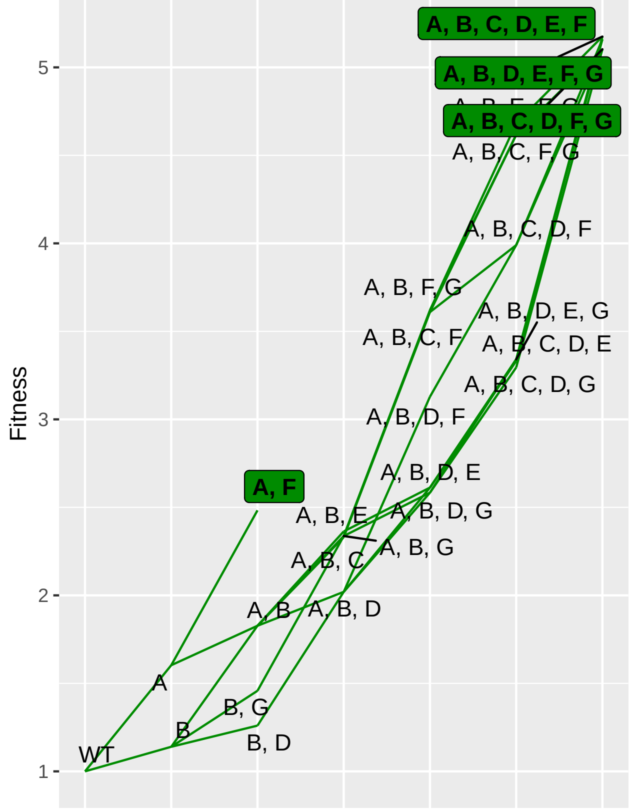

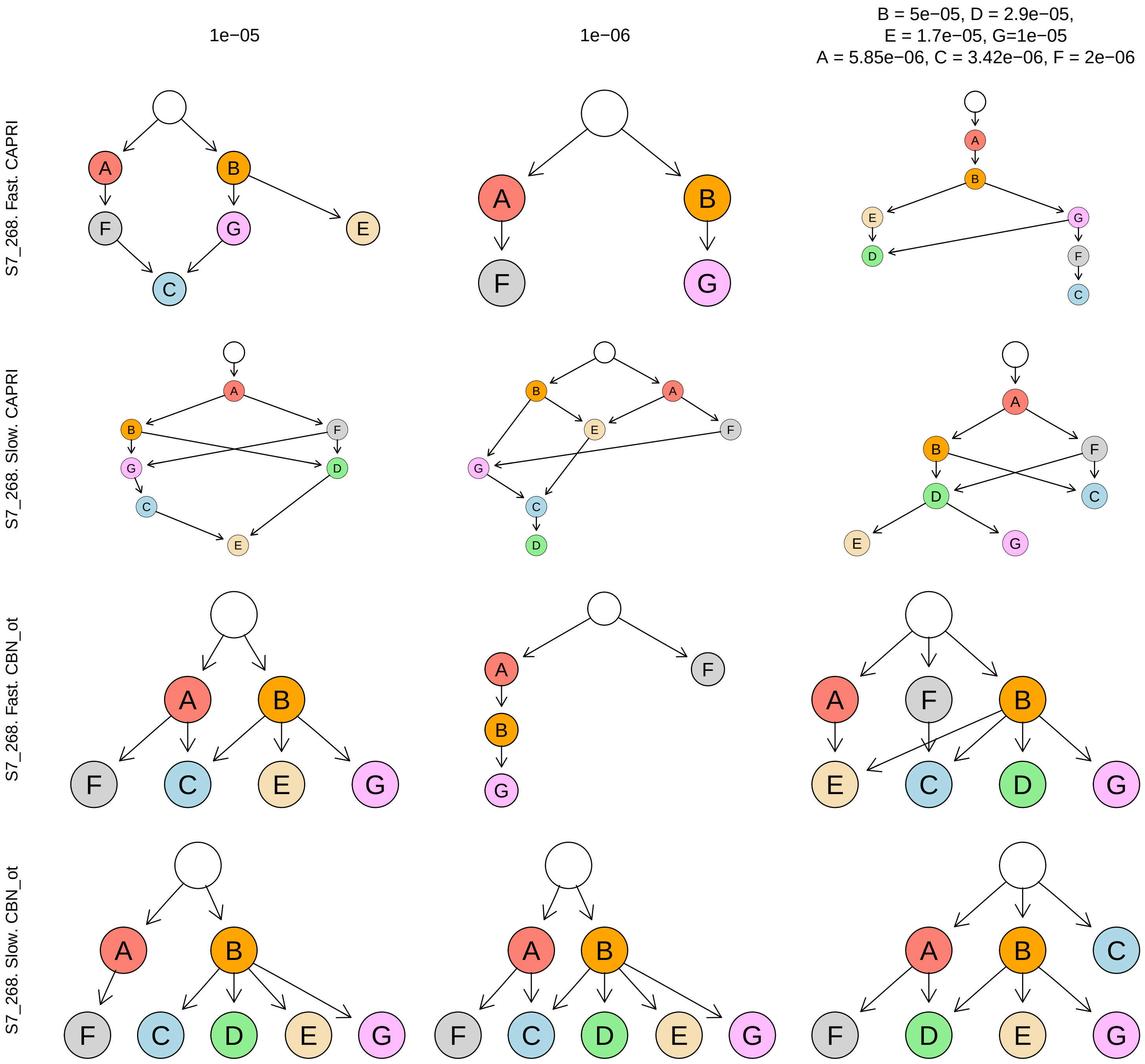

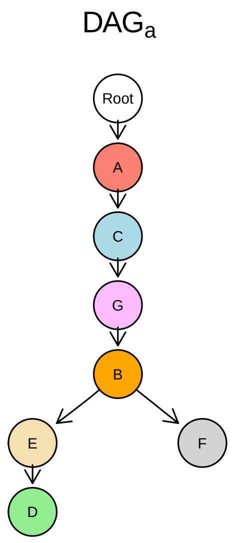

ID	Accessible Genot.	Discrep. DAG_a
S7_269	9	2

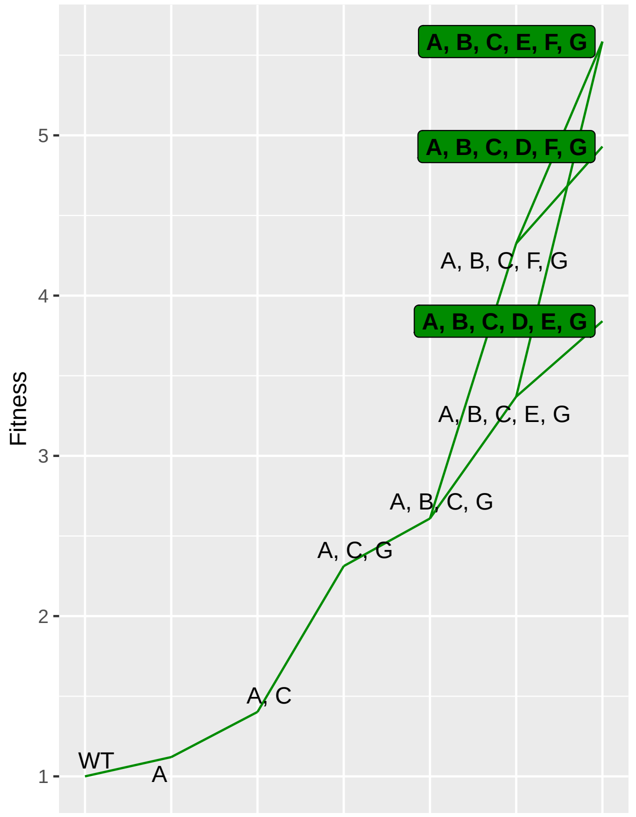

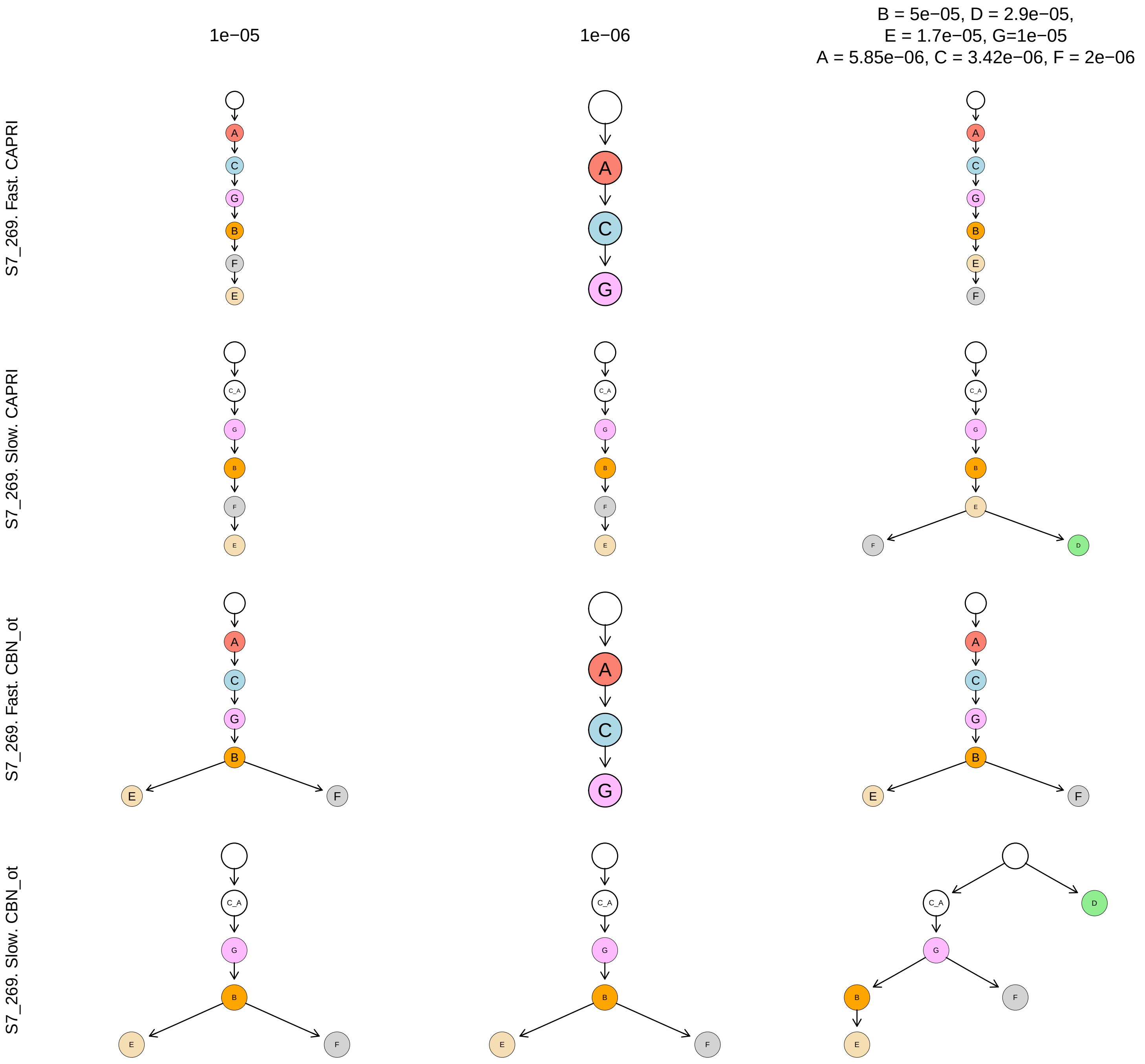

DAG_a

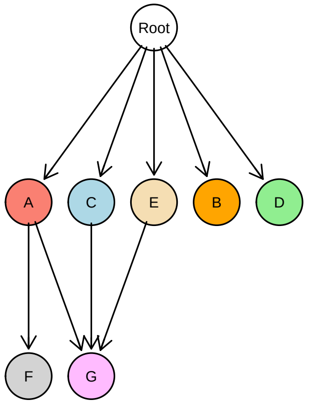

ID	Accessible Genot.	Discrep. DAG_a
S7_270	45	18

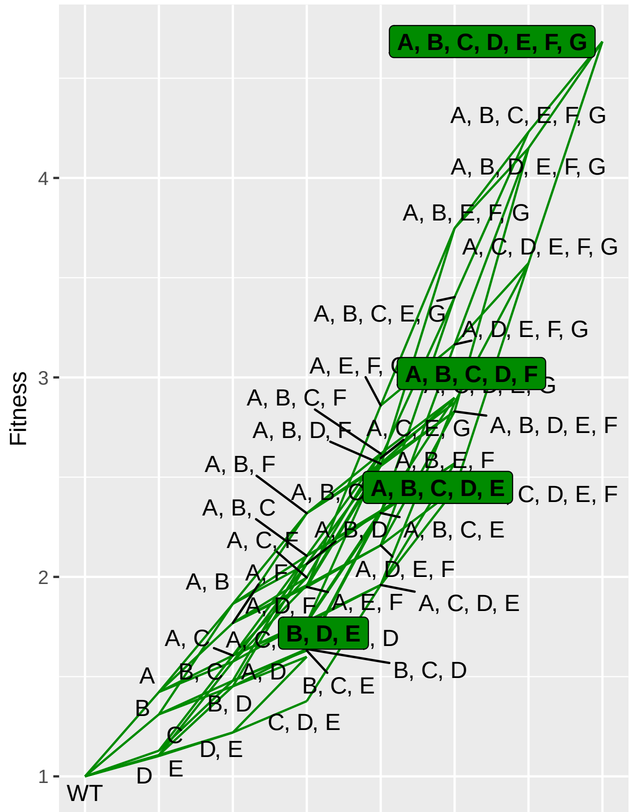

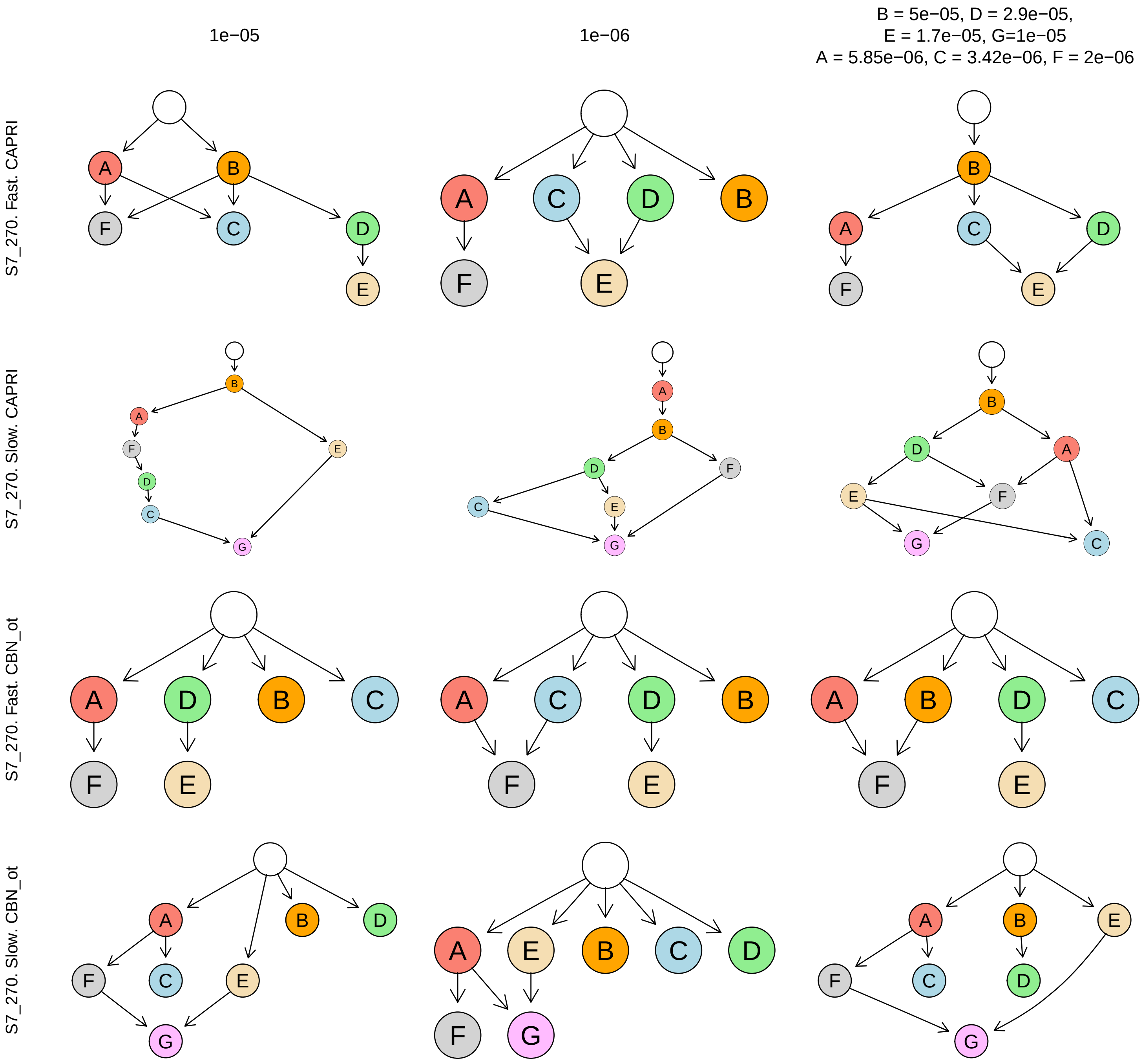

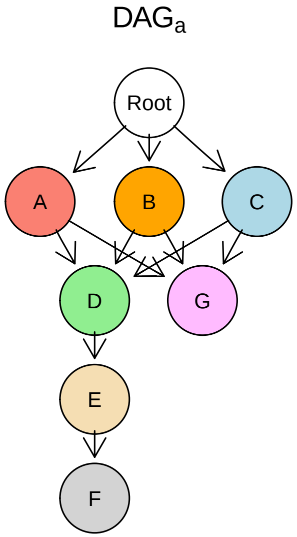

ID	Accessible Genot.	Discrep. DAG_a
S7_271	13	3

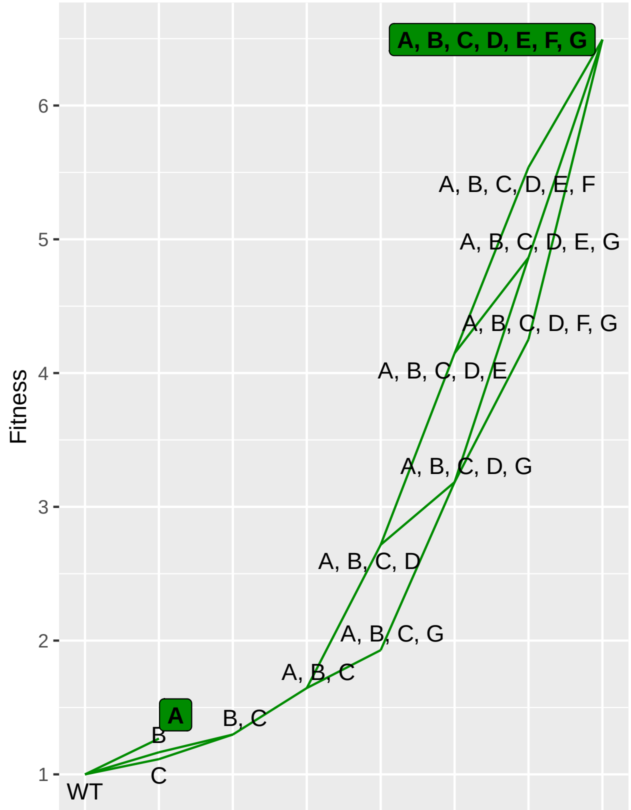

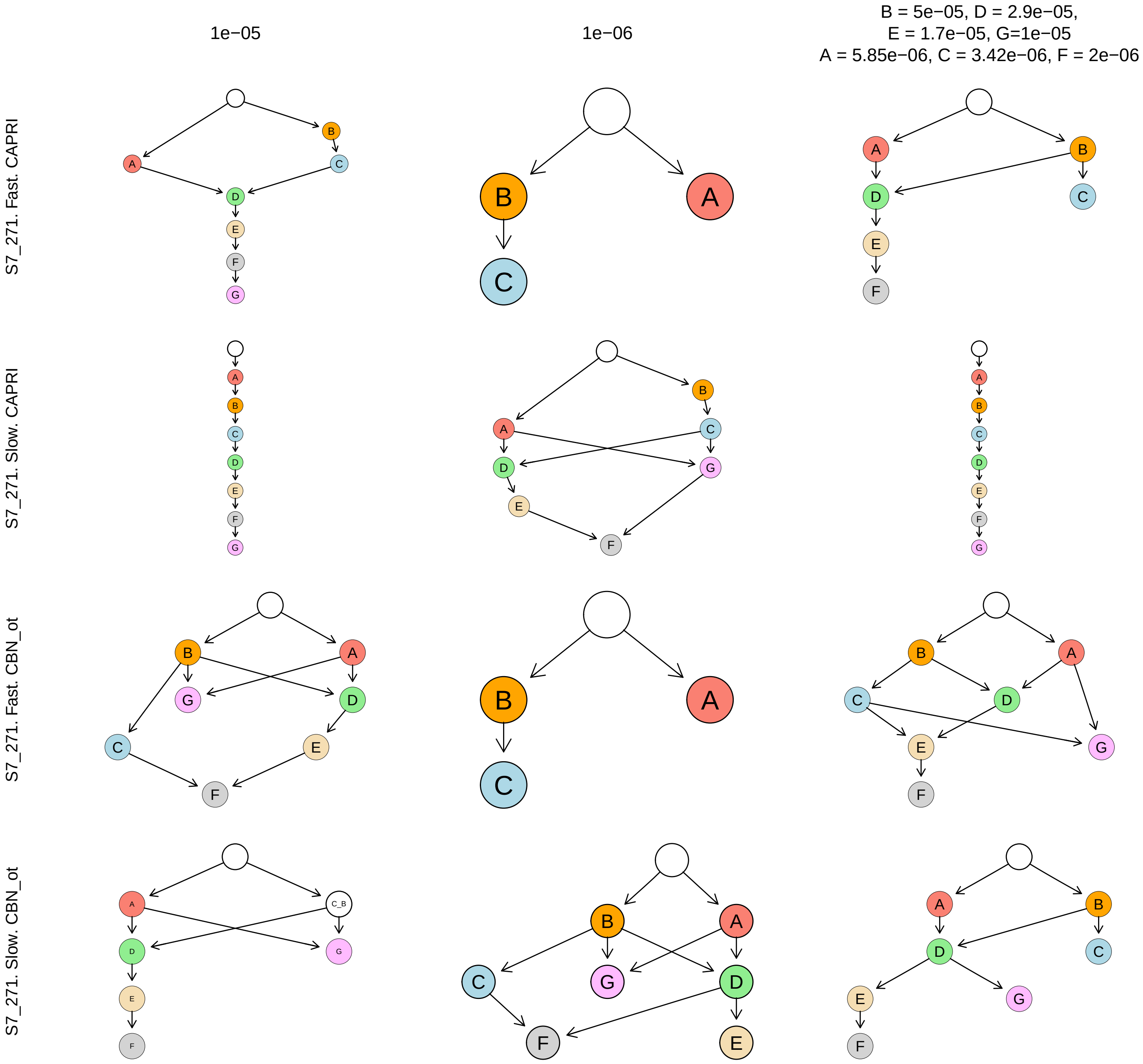

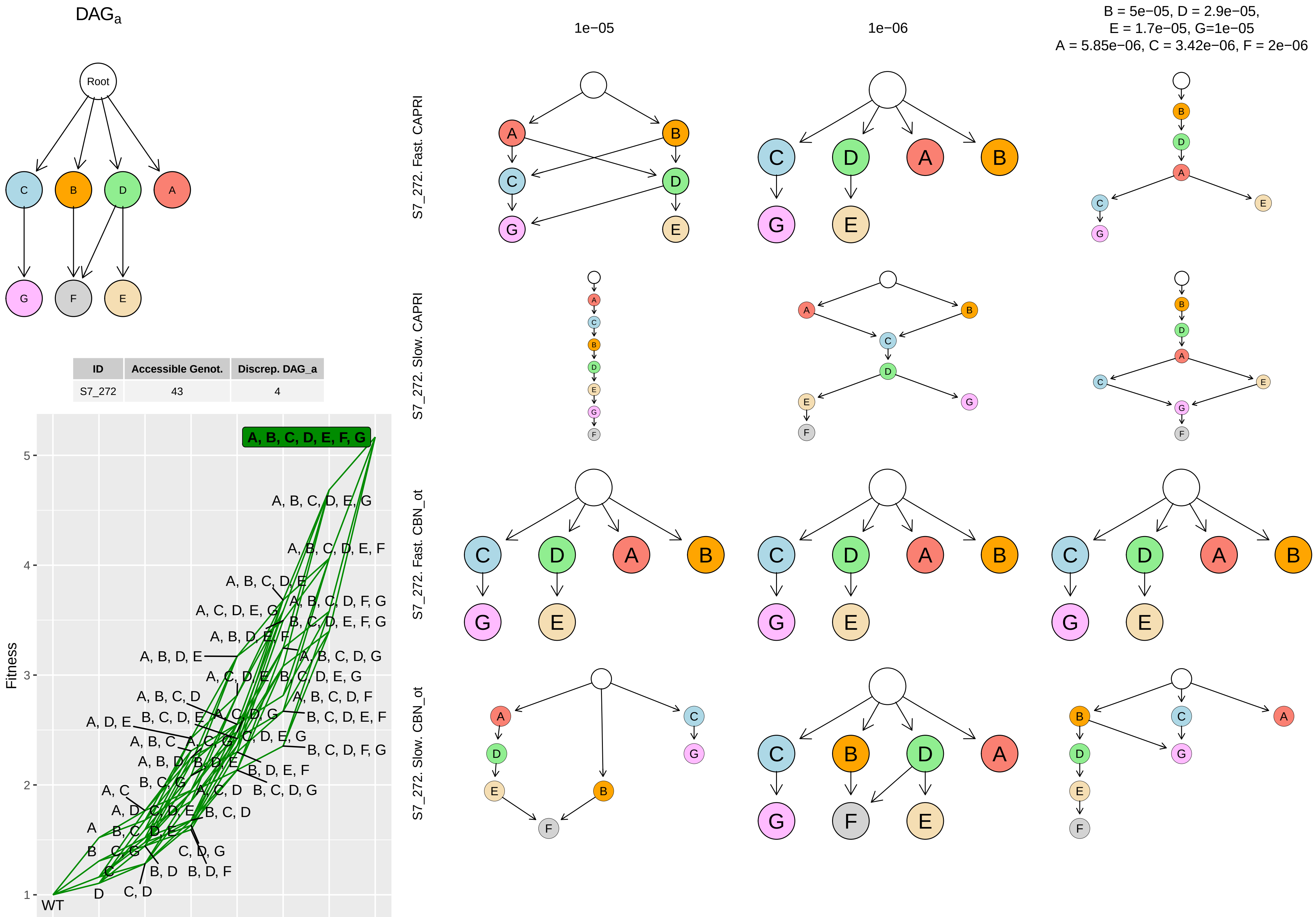

ID	Accessible Genot.	Discrep. DAG_a
S7_272	43	4

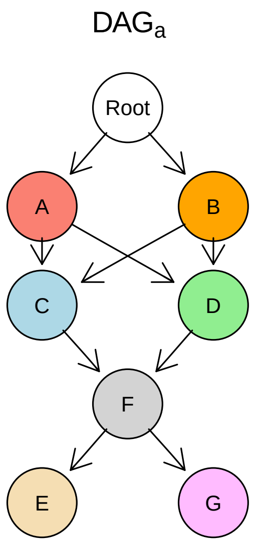

ID	Accessible Genot.	Discrep. DAG_a
S7_273	9	1

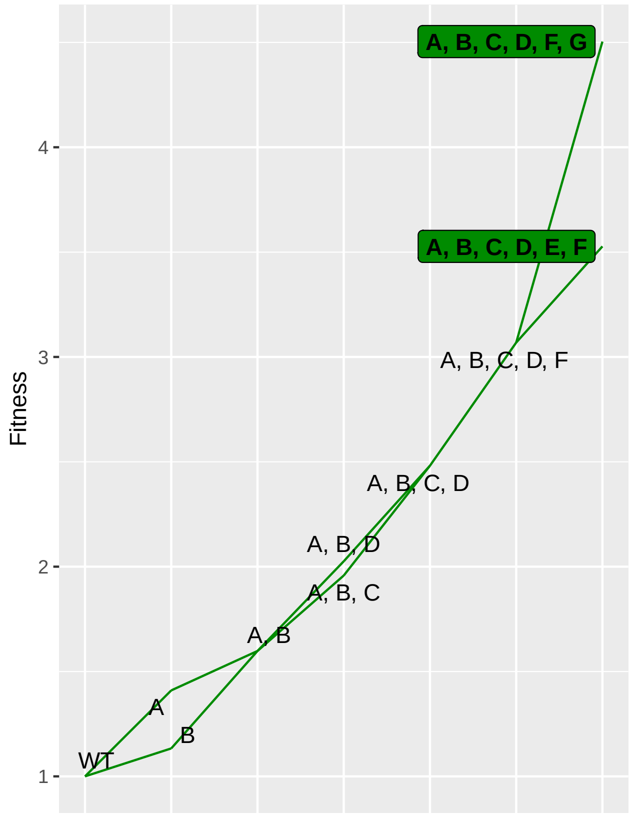

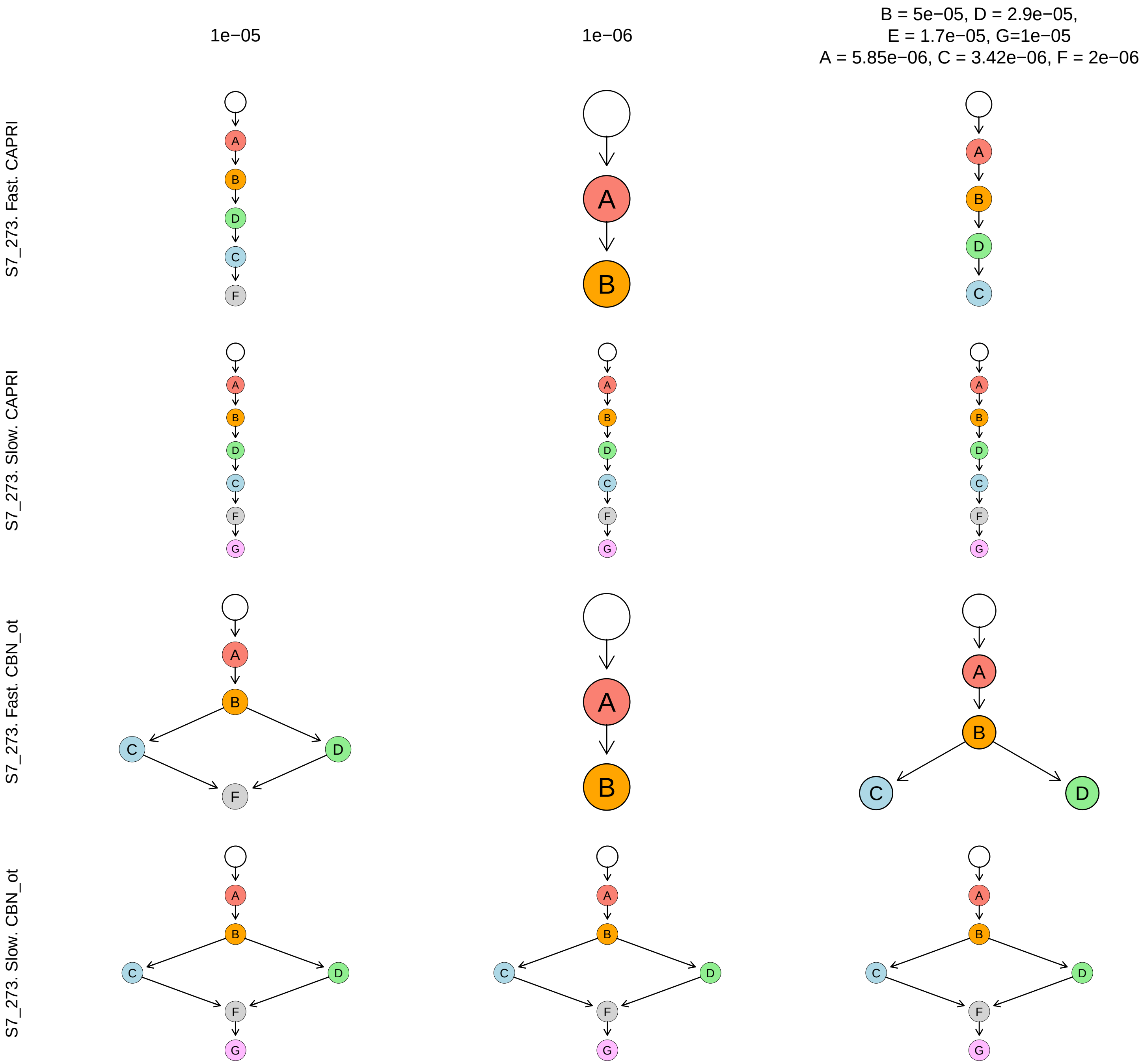

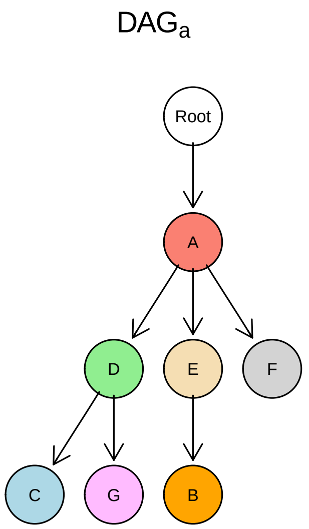

ID	Accessible Genot.	Discrep. DAG_a
S7_274	34	16

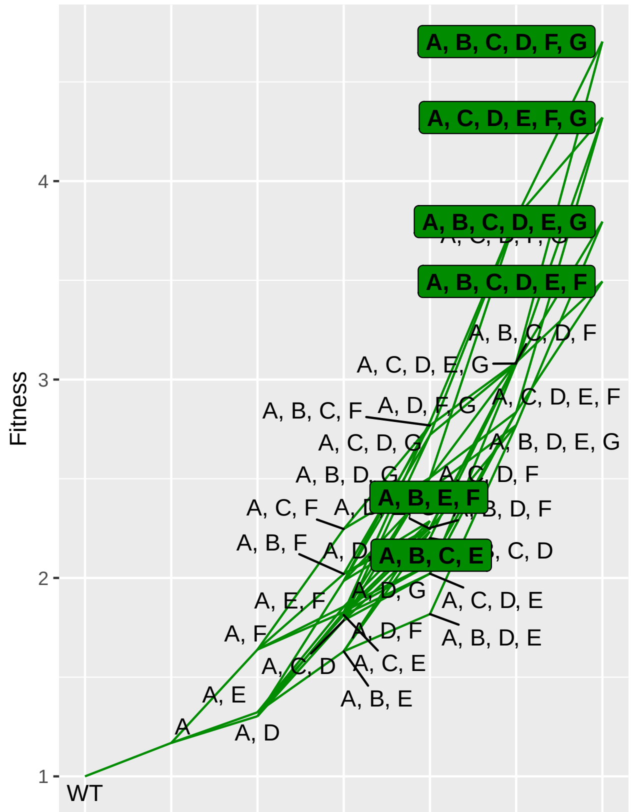

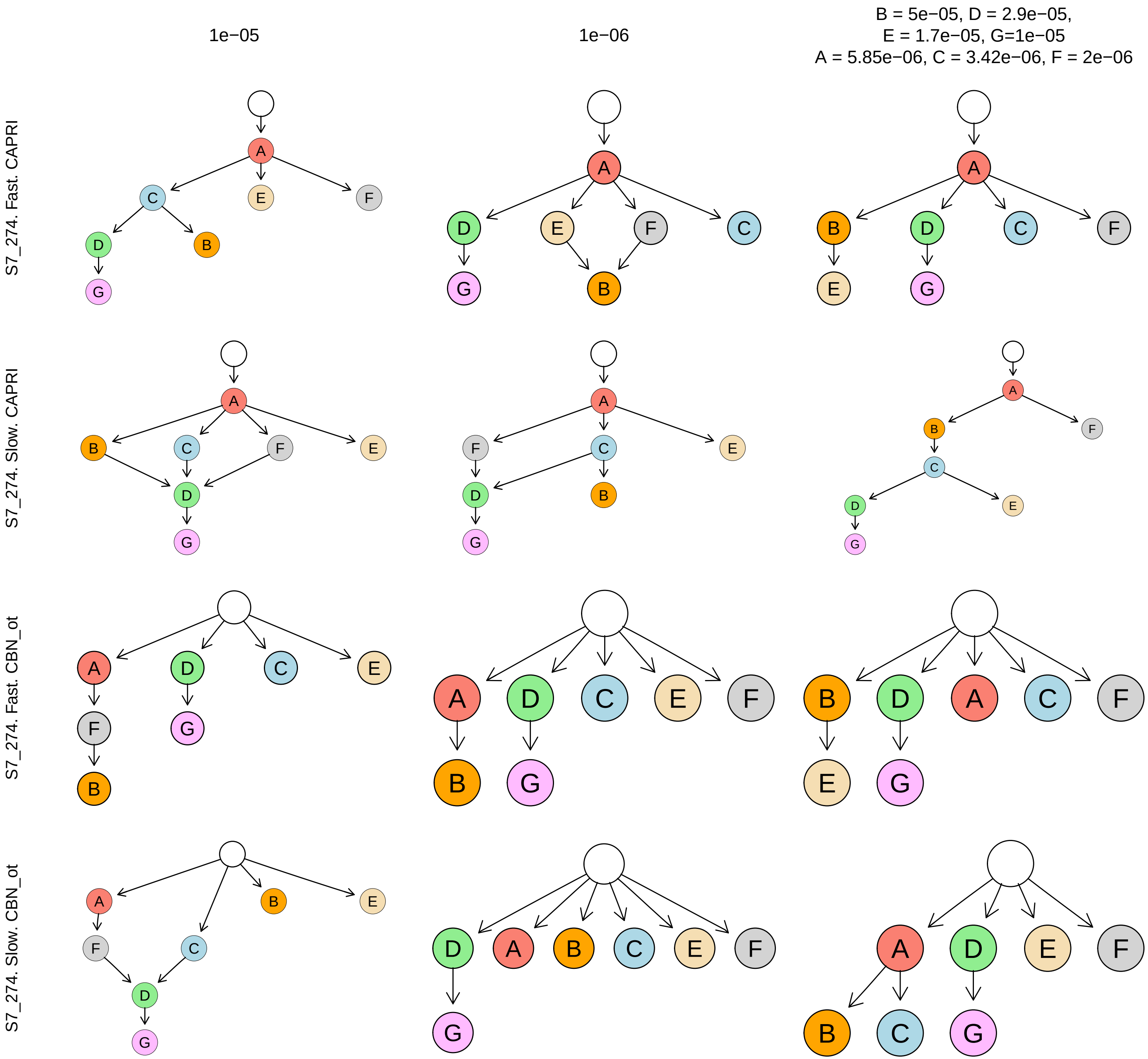

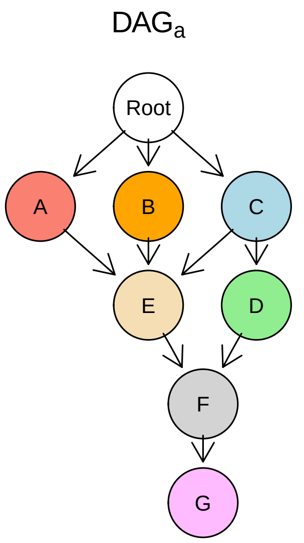

ID	Accessible Genot.	Discrep. DAG_a
S7_275	13	2

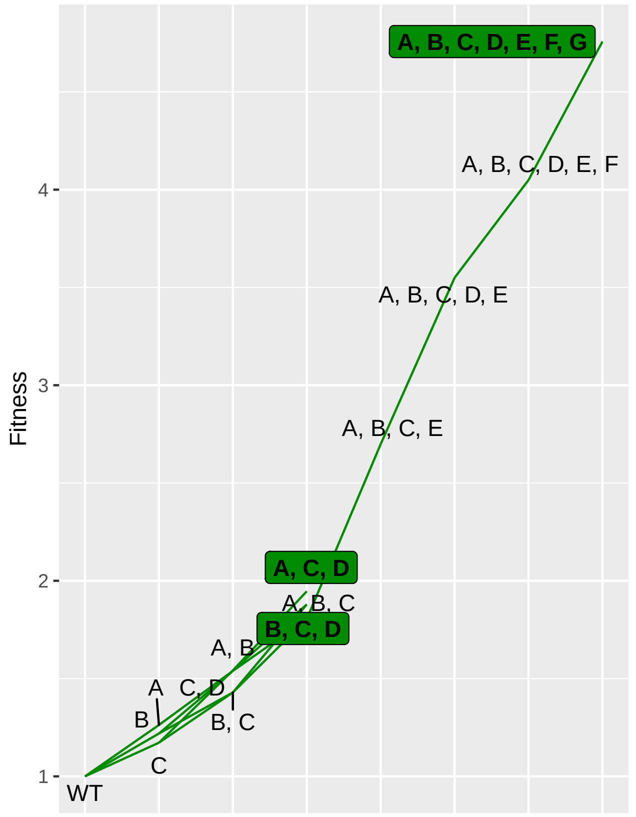

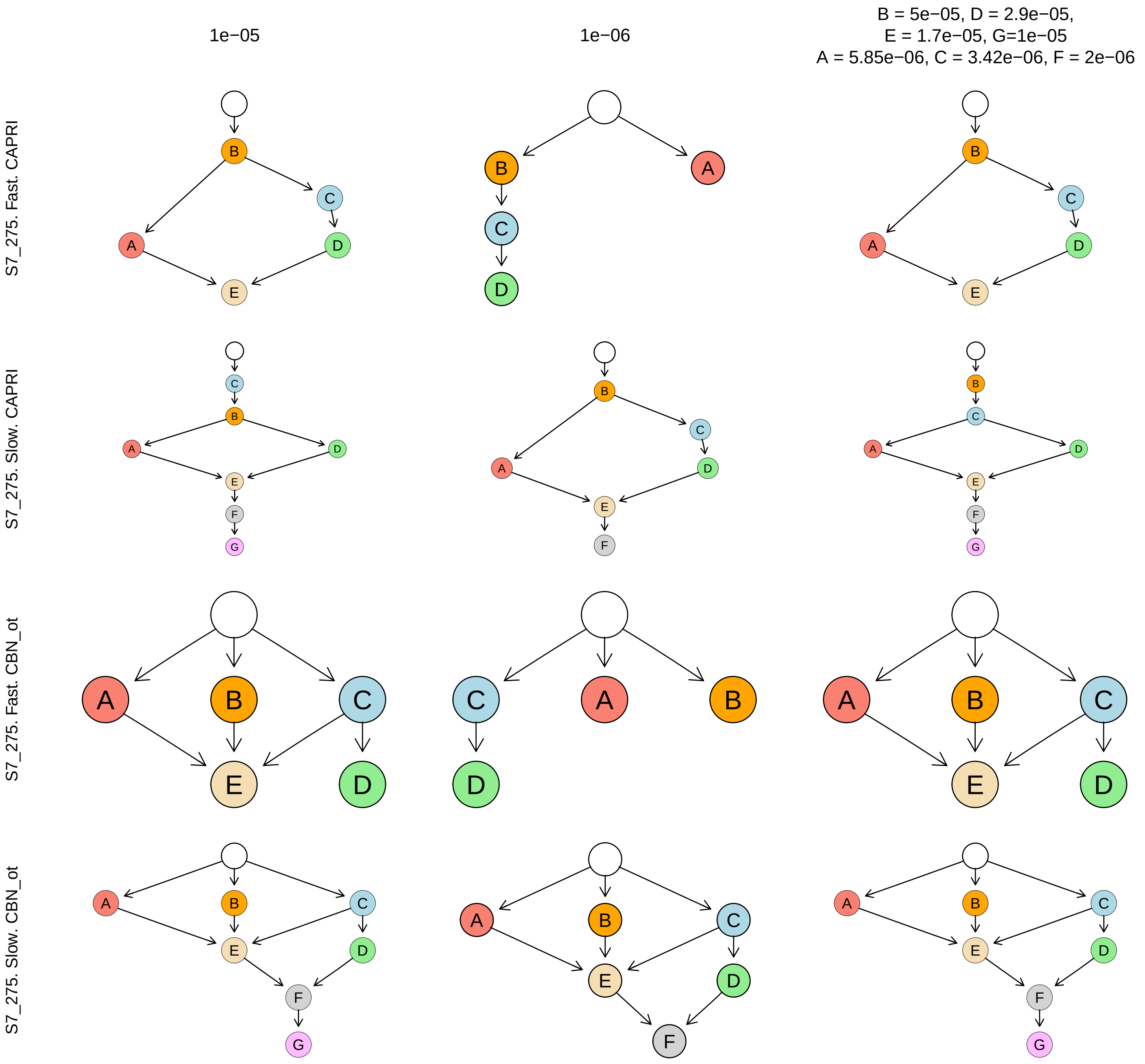

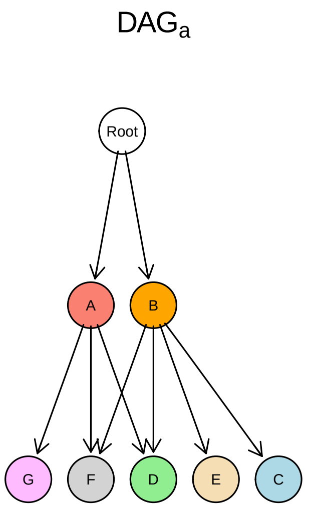

ID	Accessible Genot.	Discrep. DAG_a
S7_276	25	13

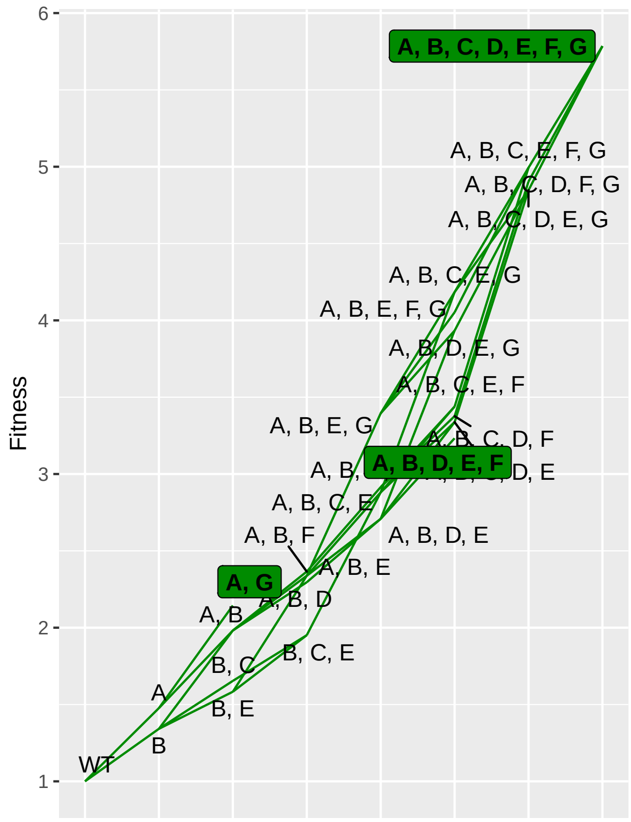

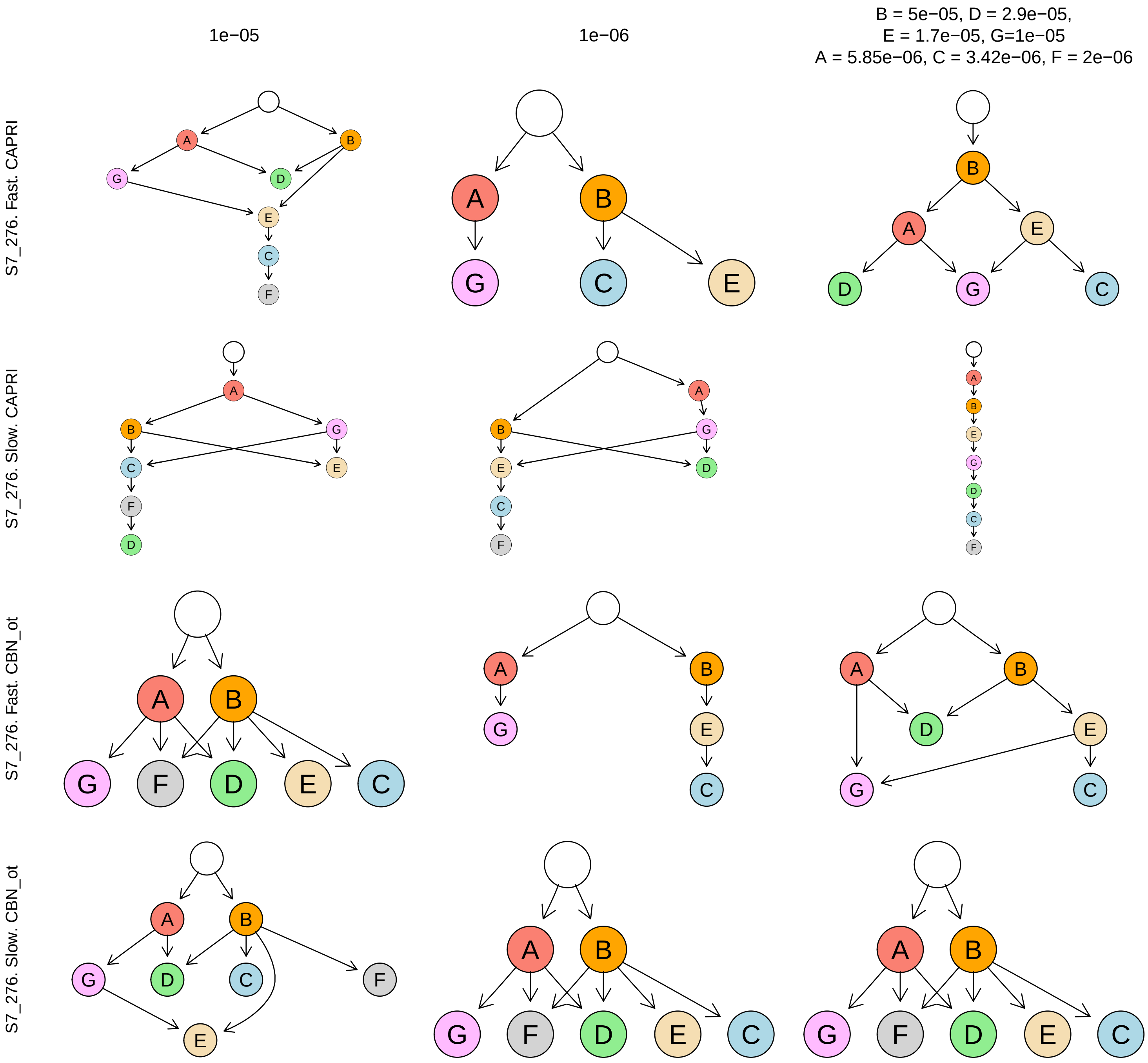

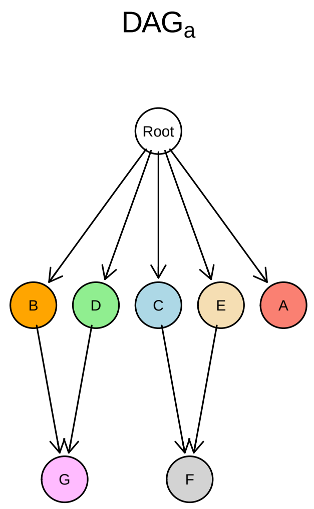

ID	Accessible Genot.	Discrep. DAG_a
S7_277	42	7

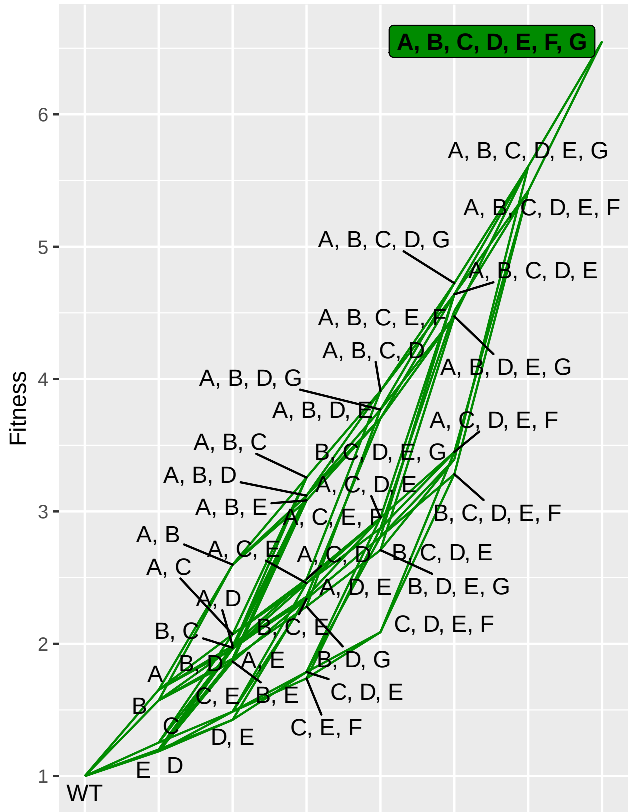

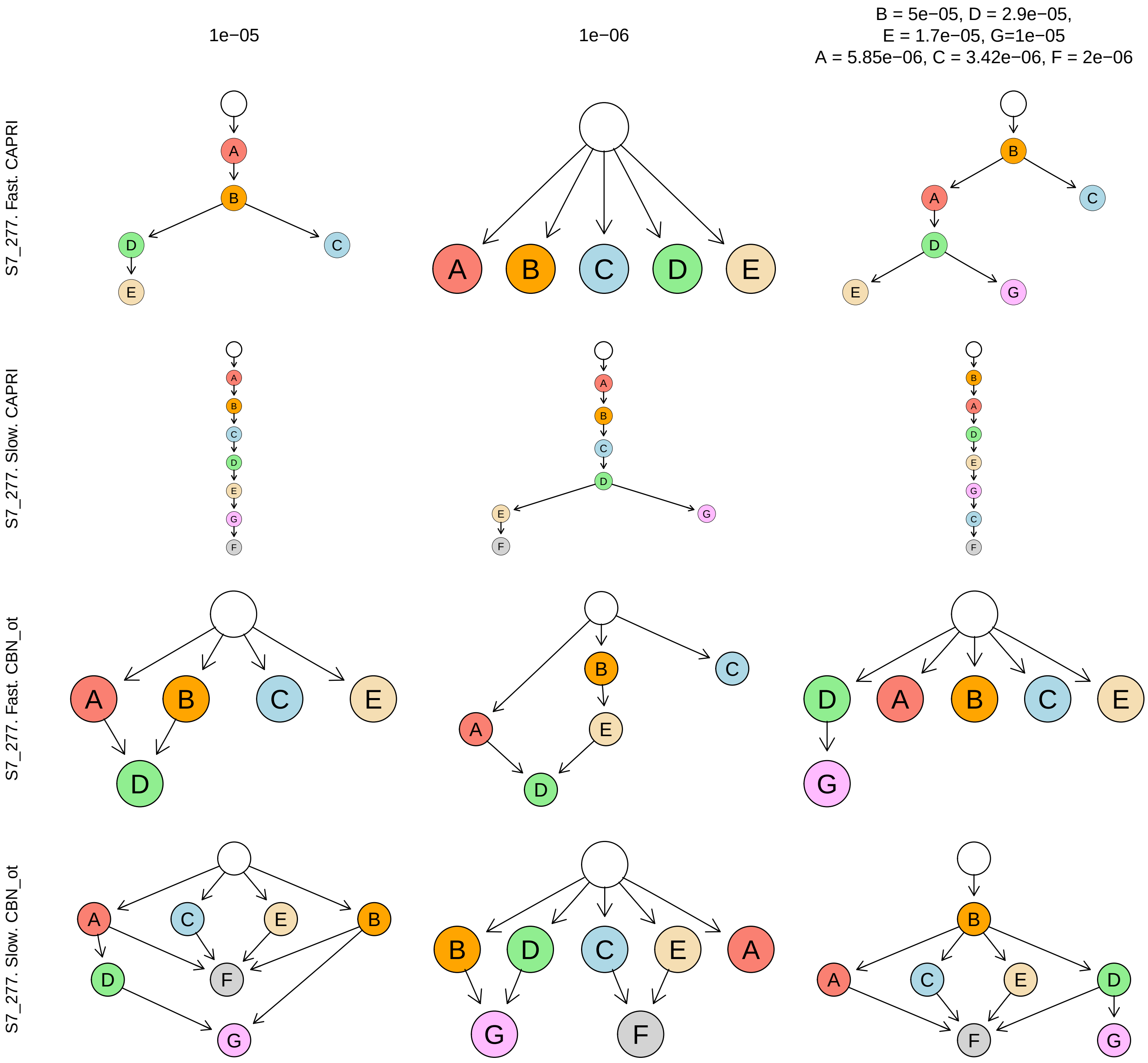

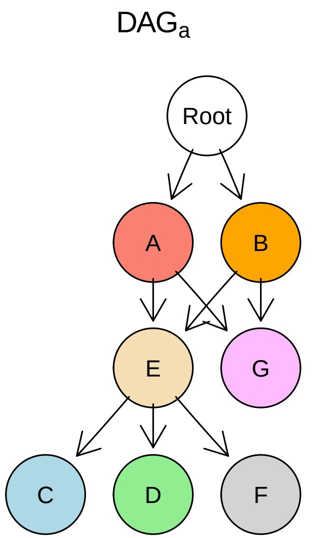

ID	Accessible Genot.	Discrep. DAG_a
S7_278	18	10

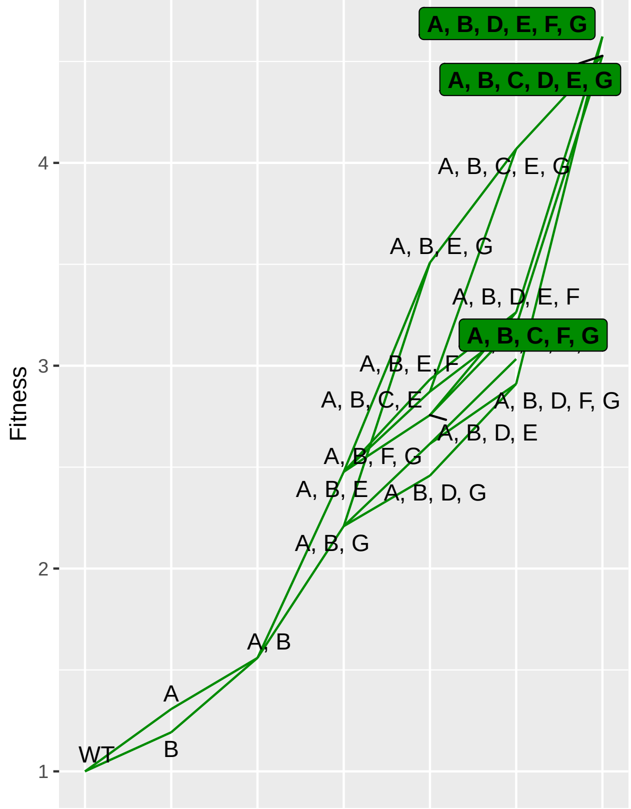

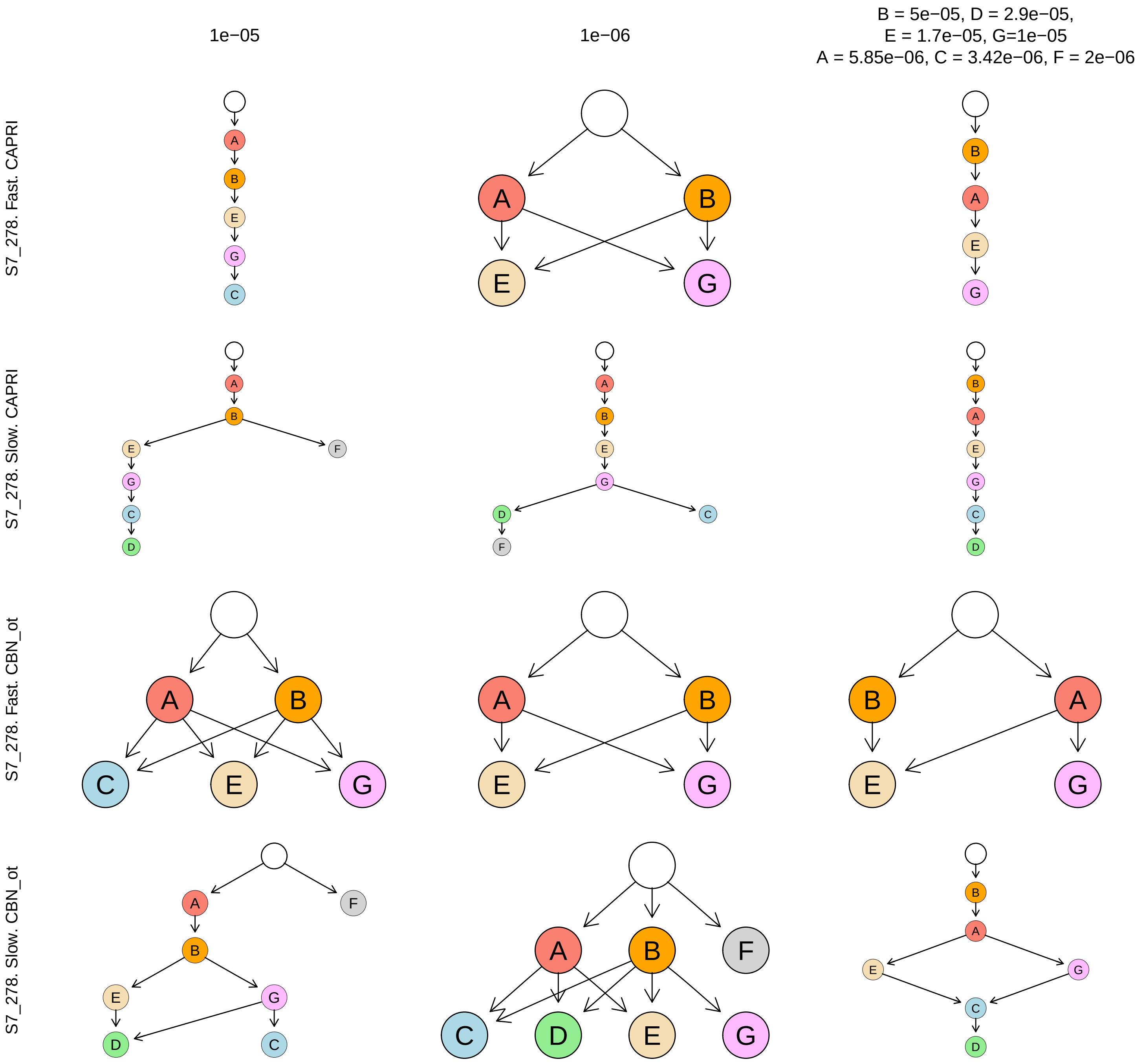

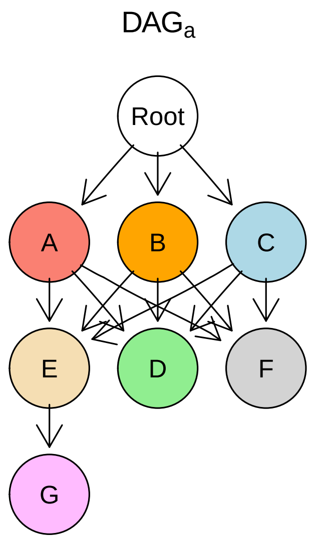

ID	Accessible Genot.	Discrep. DAG_a
S7_279	16	2

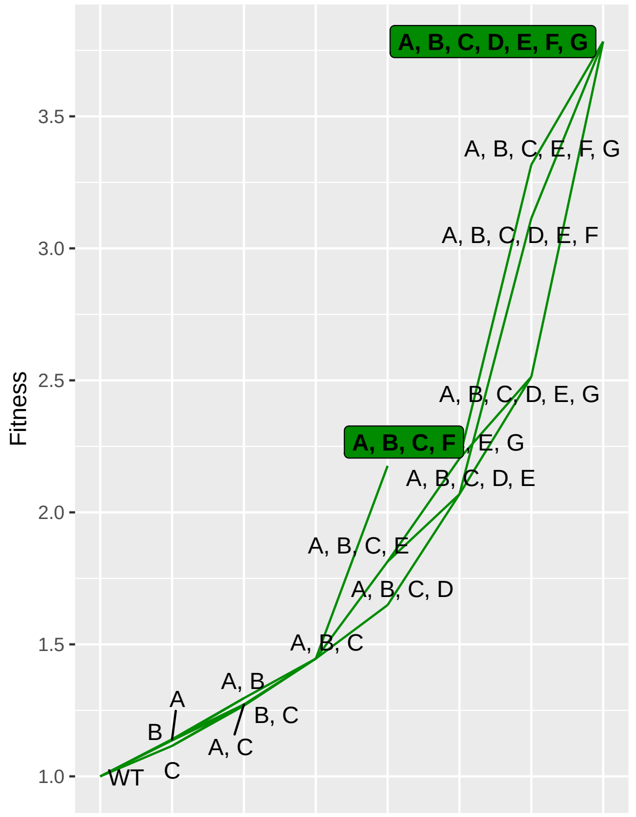

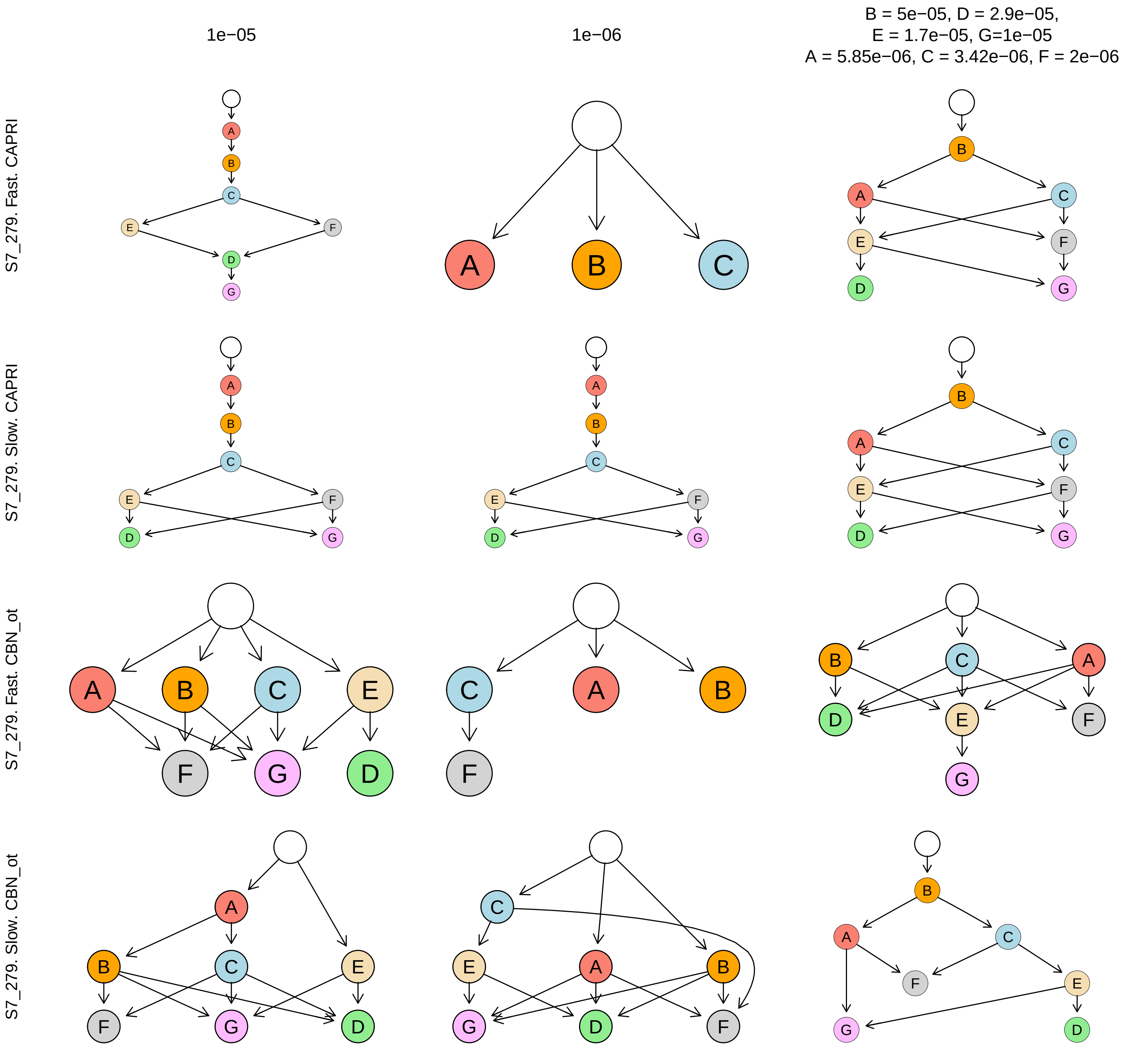

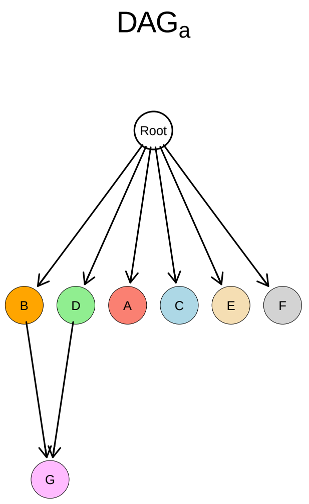

ID	Accessible Genot.	Discrep. DAG_a
S7_280	54	25

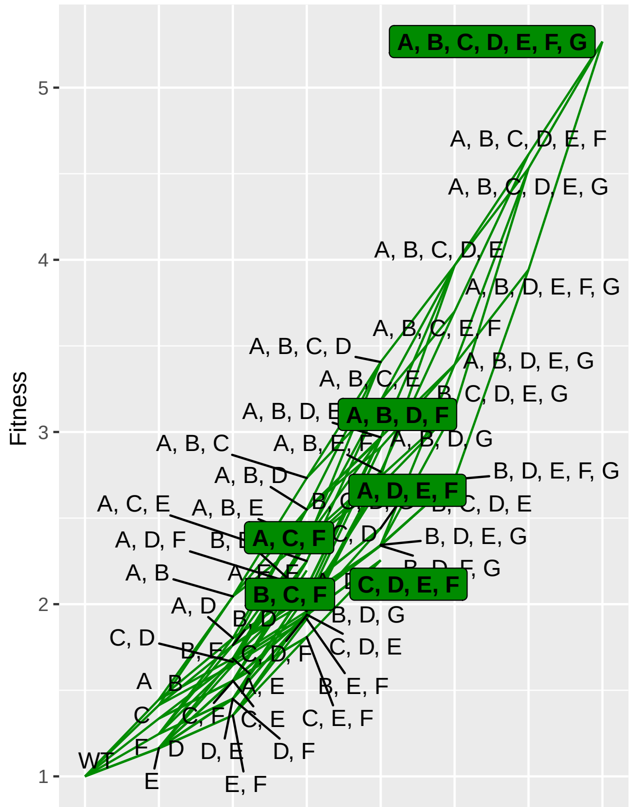

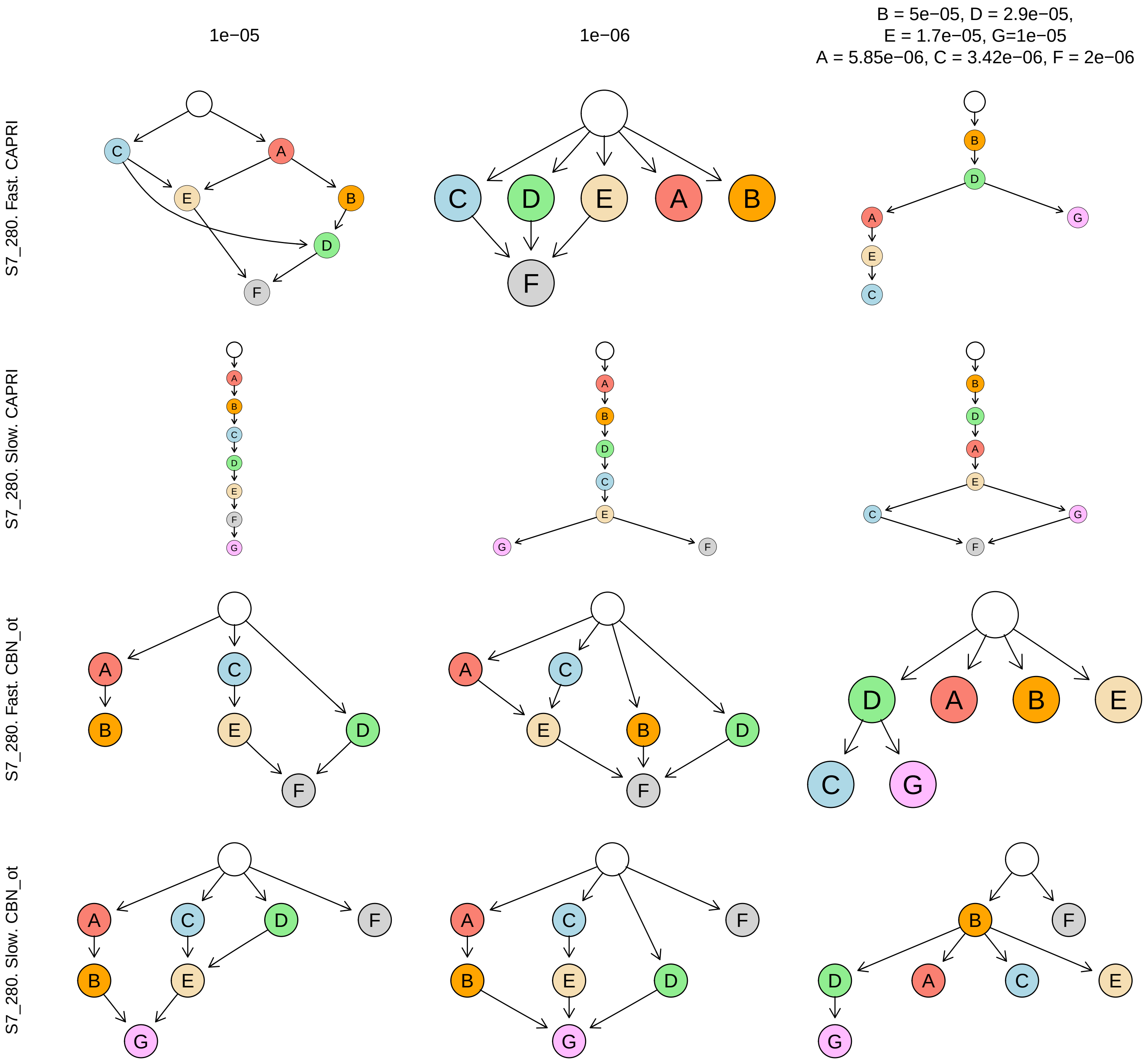

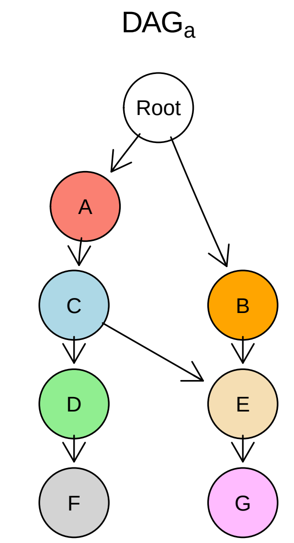

ID	Accessible Genot.	Discrep. DAG_a
S7_281	18	5

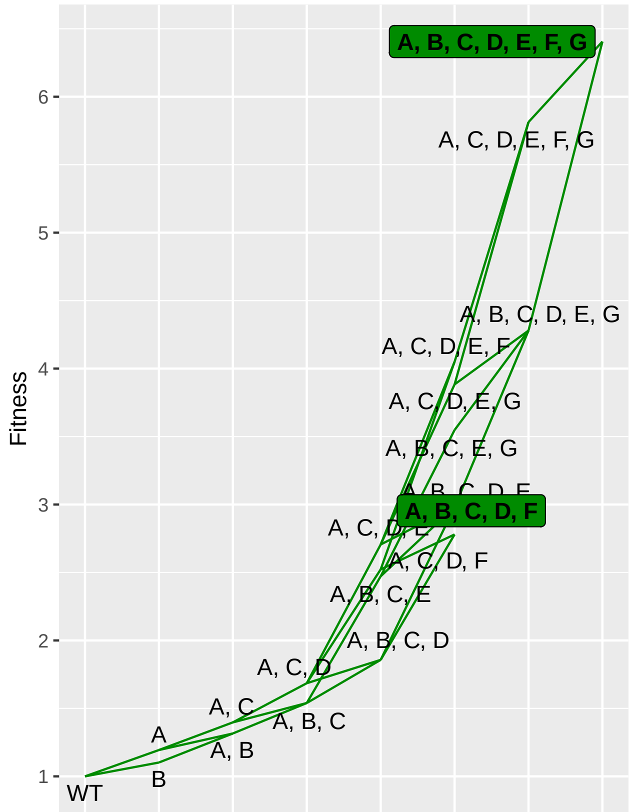

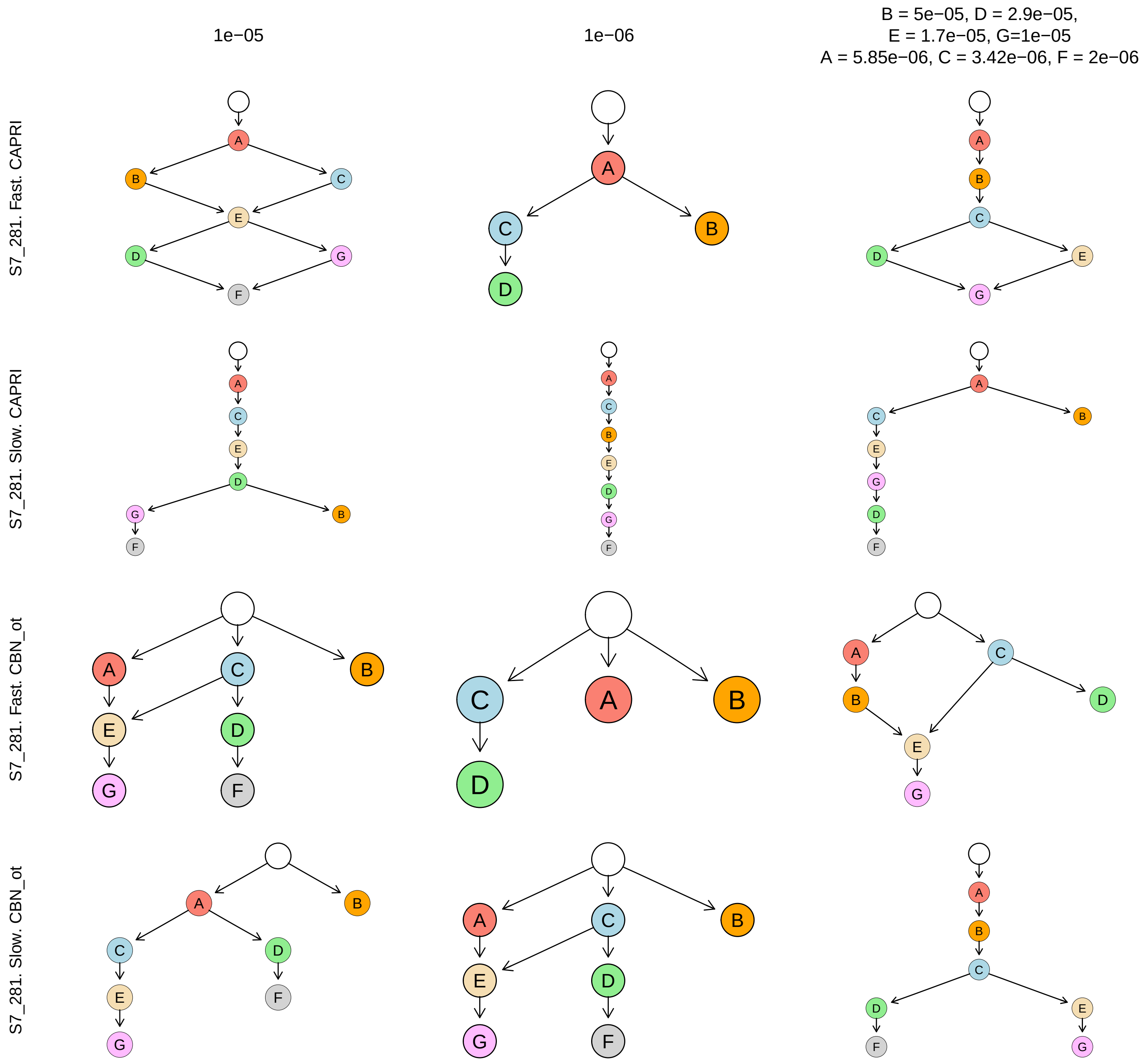

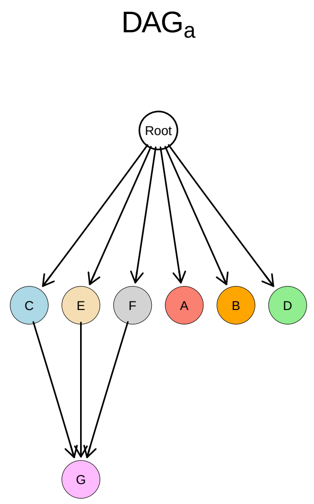

ID	Accessible Genot.	Discrep. DAG_a
S7_282	48	27

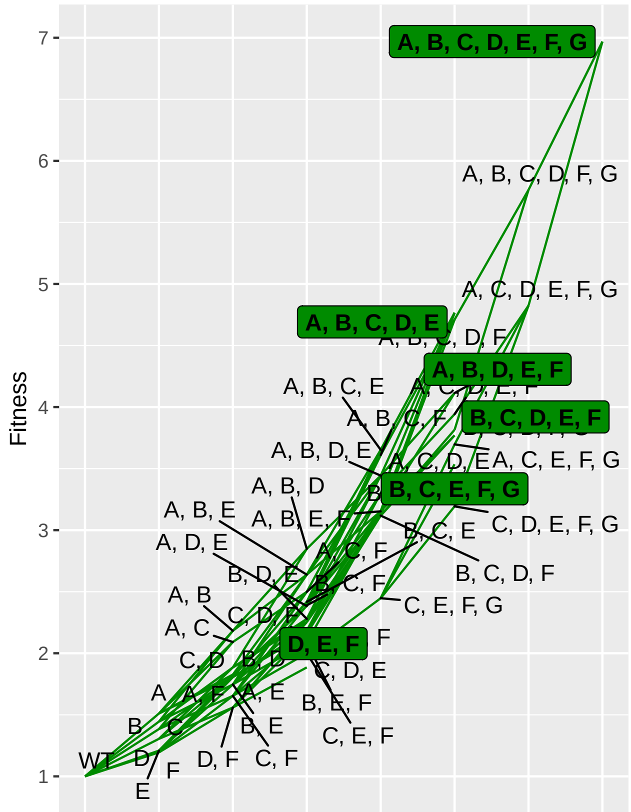

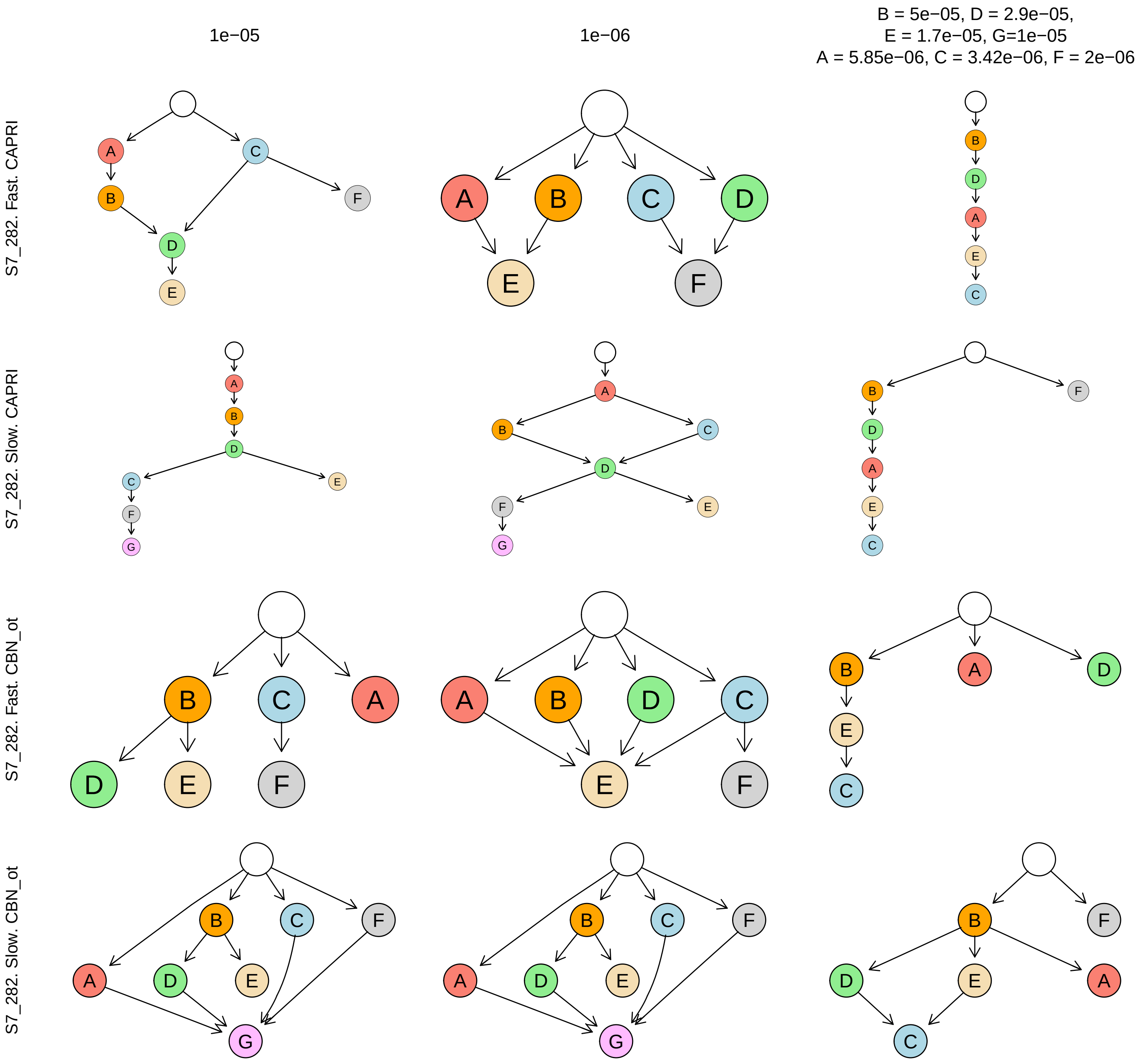

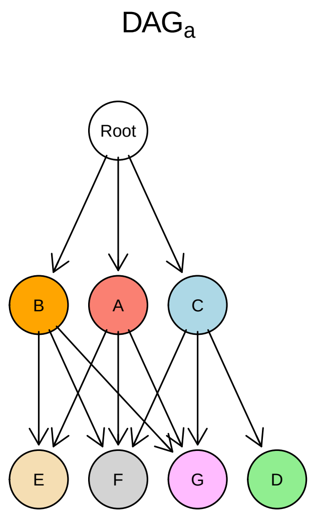

ID	Accessible Genot.	Discrep. DAG_a
S7_283	23	3

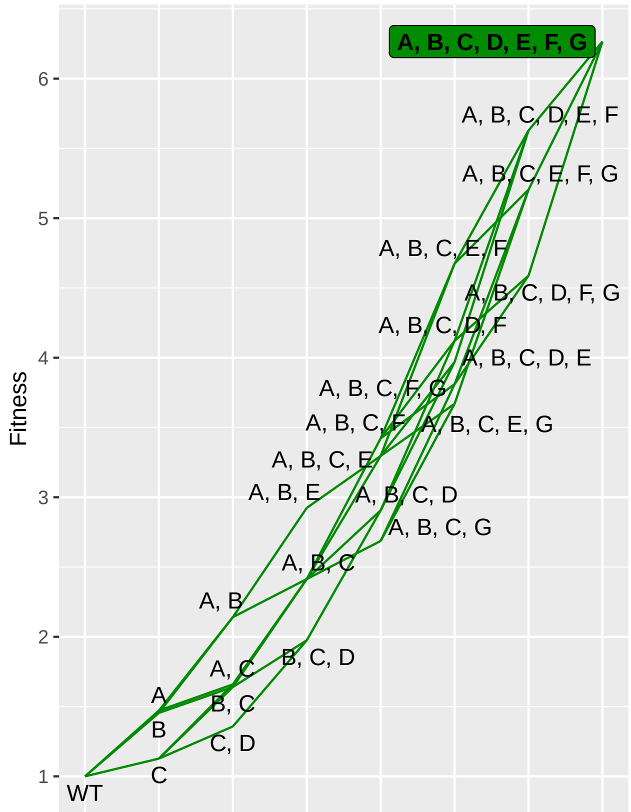

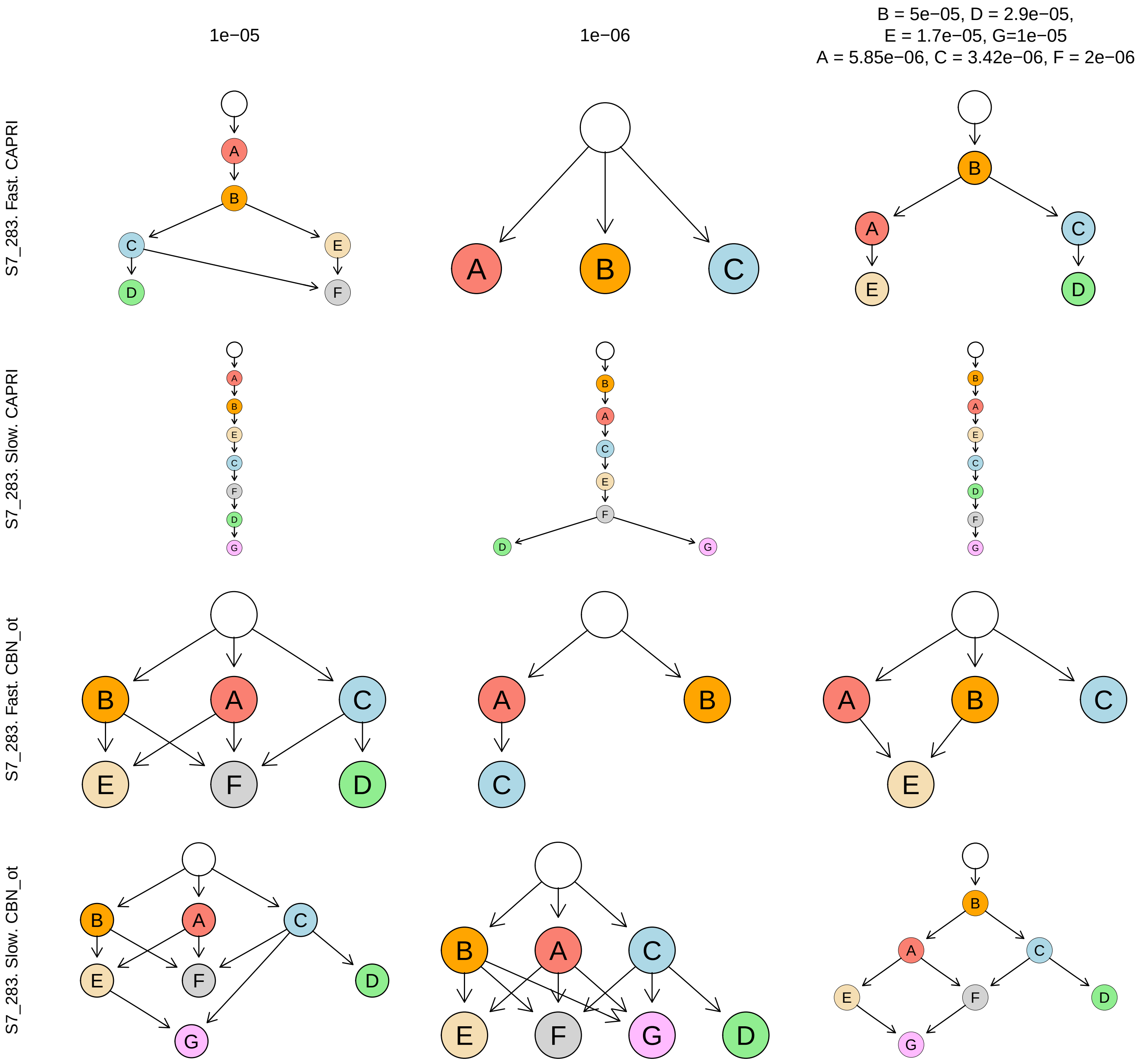

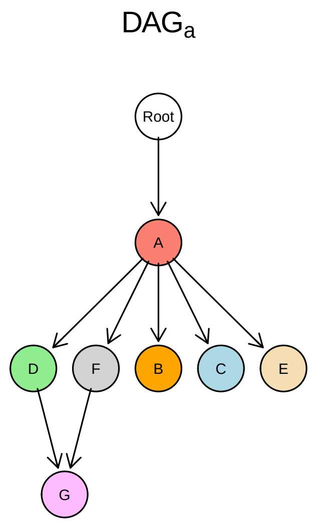

ID	Accessible Genot.	Discrep. DAG_a
S7_284	37	3

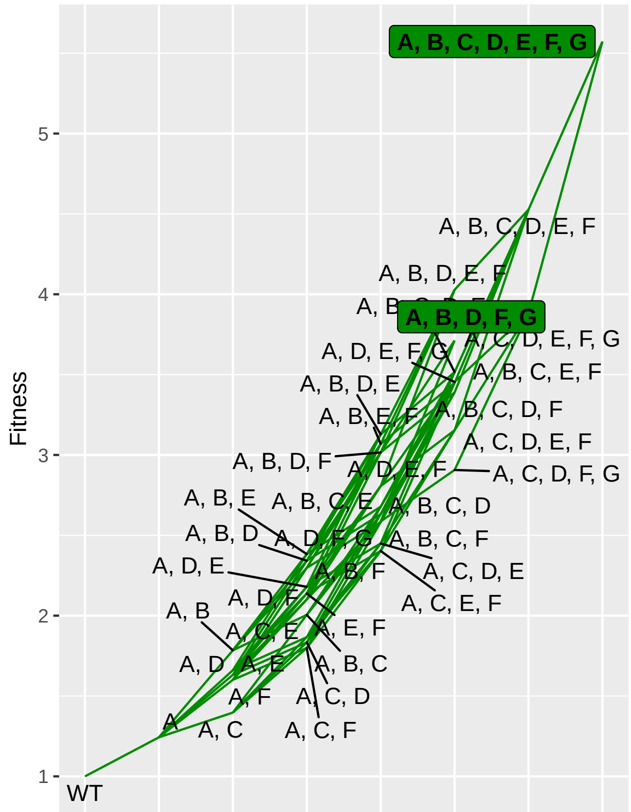

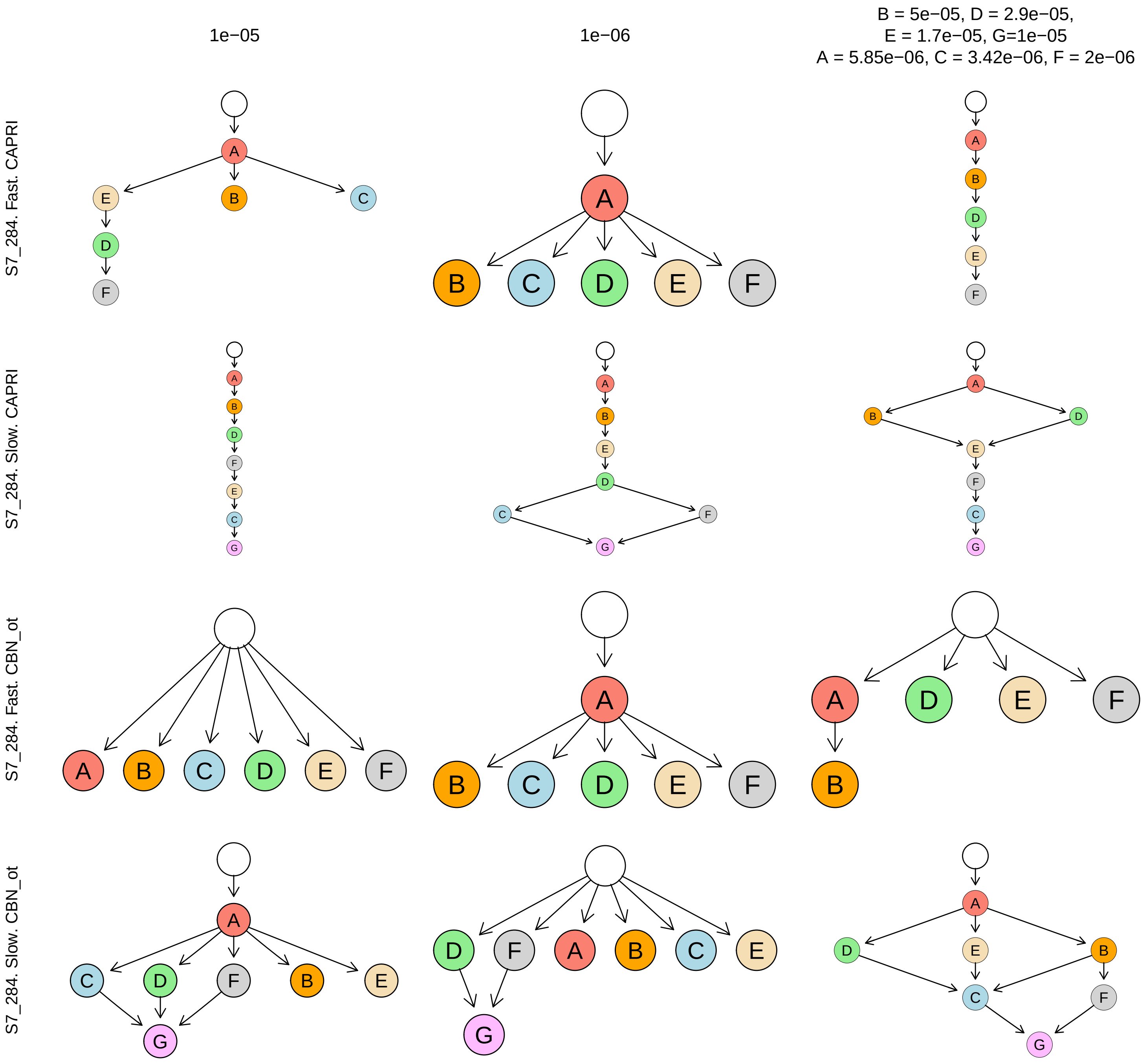

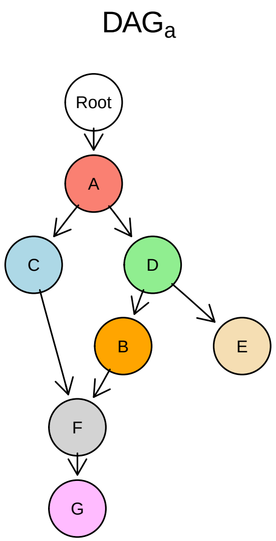

ID	Accessible Genot.	Discrep. DAG_a
S7_285	11	3

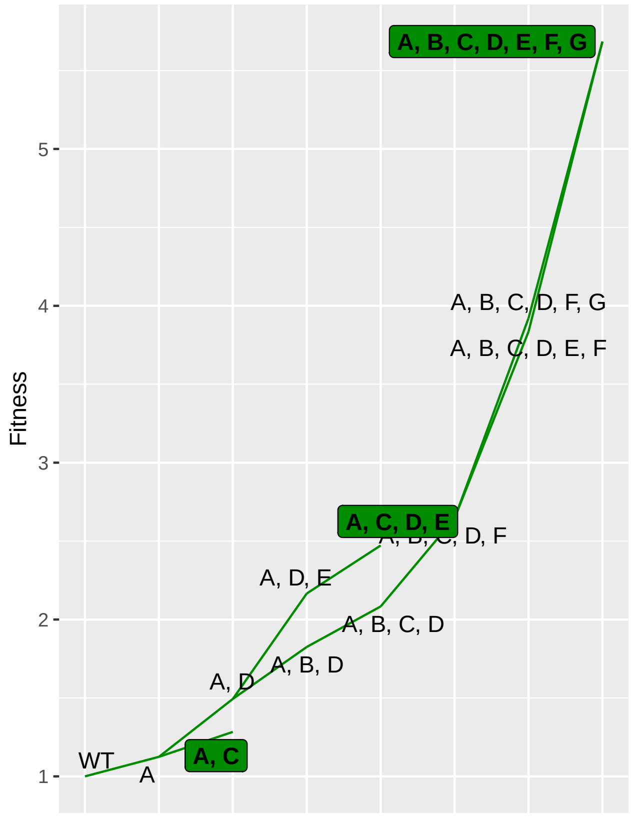

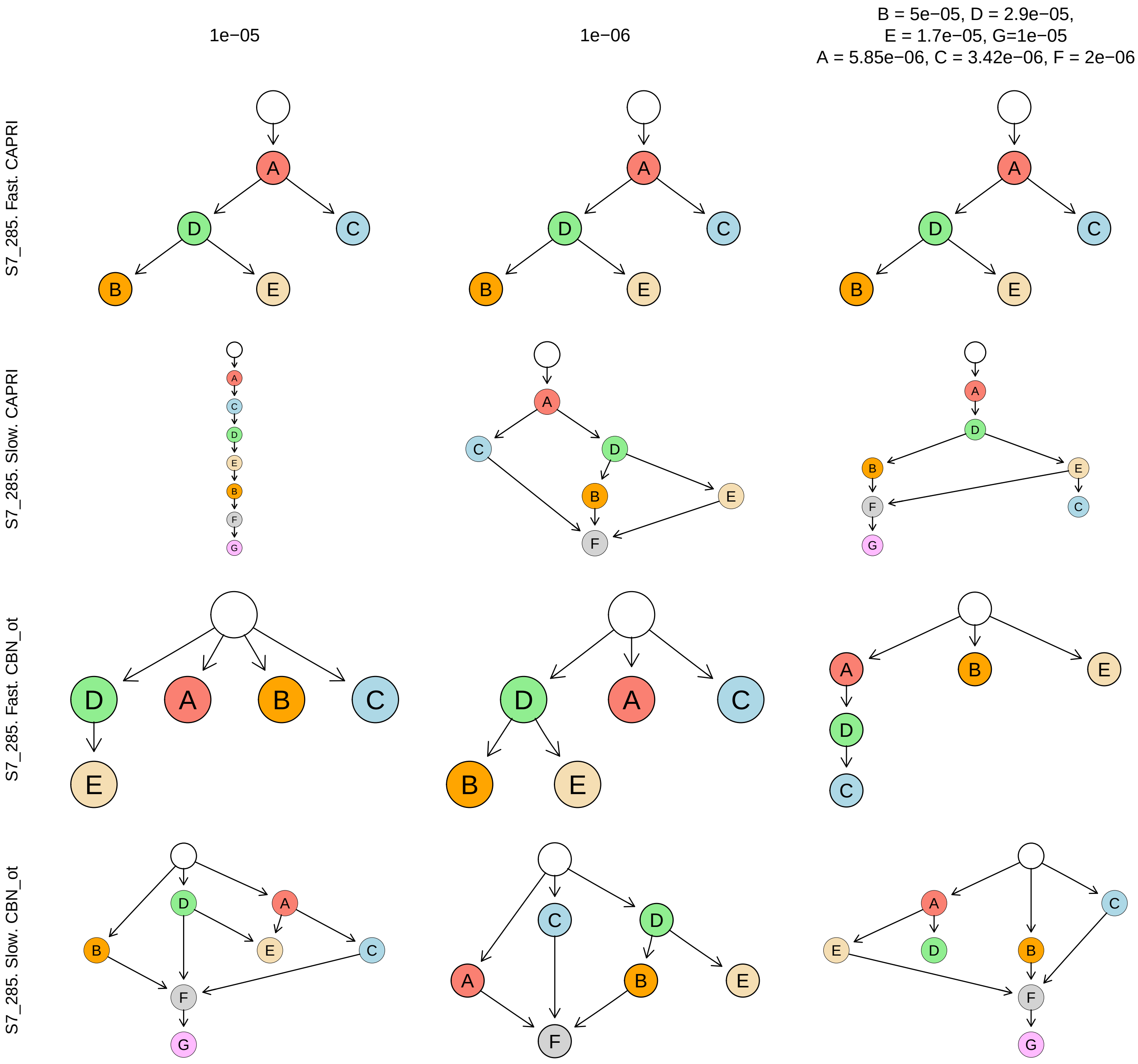

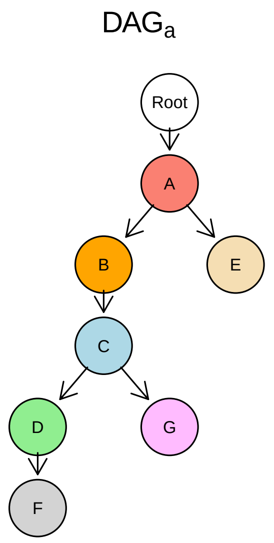

ID	Accessible Genot.	Discrep. DAG_a
S7_286	12	4

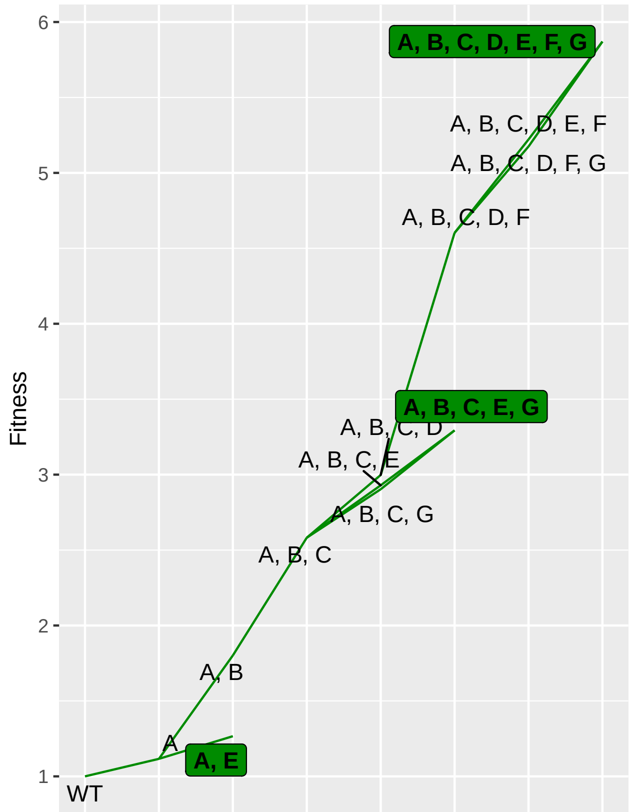

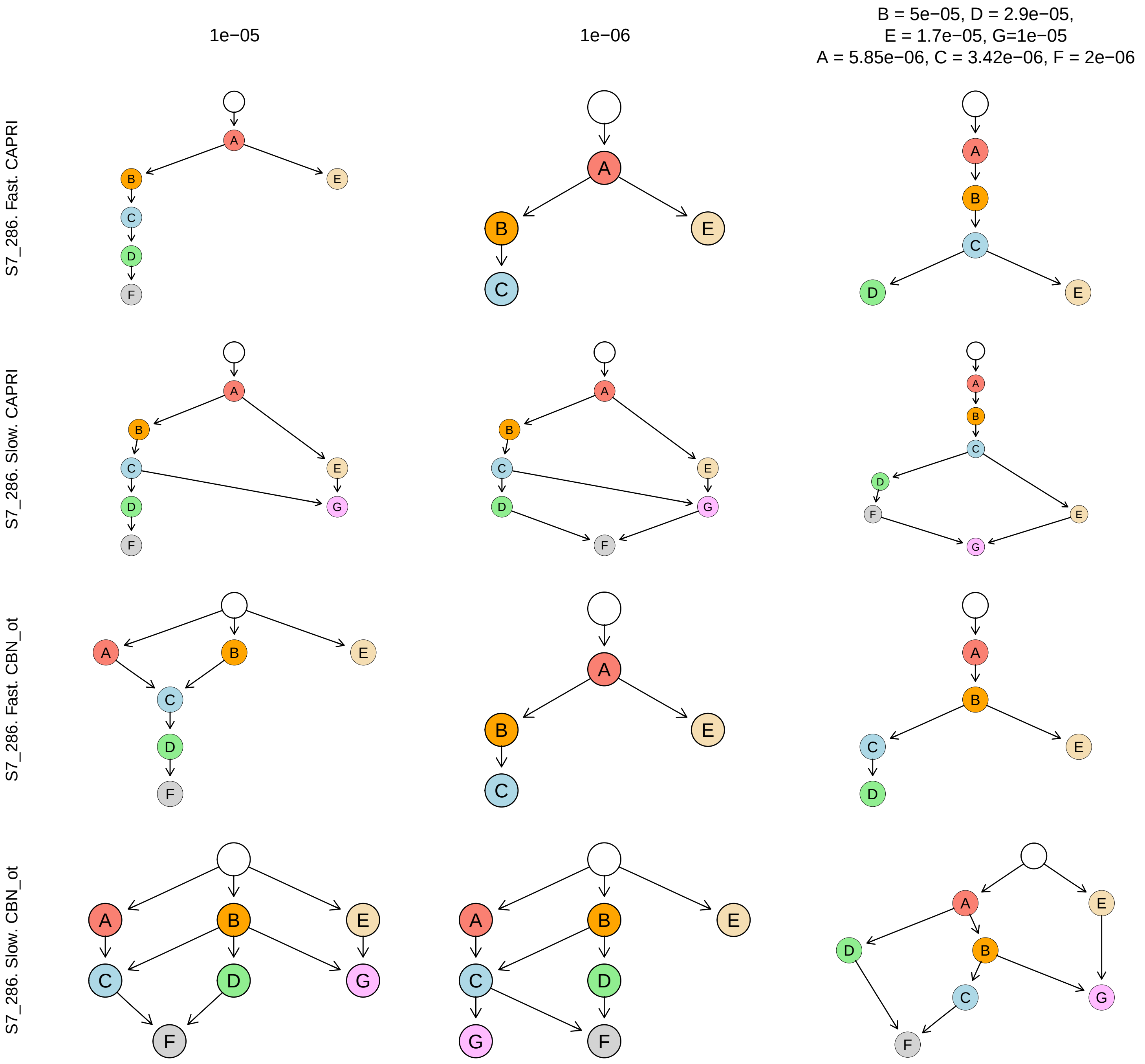

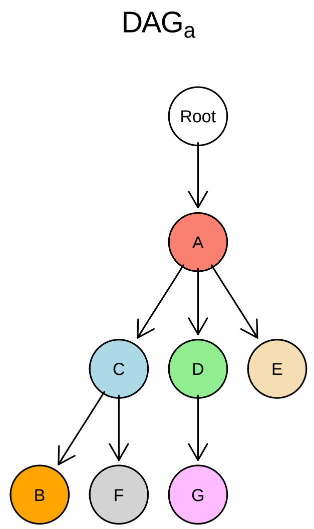

ID	Accessible Genot.	Discrep. DAG_a
S7_287	30	10

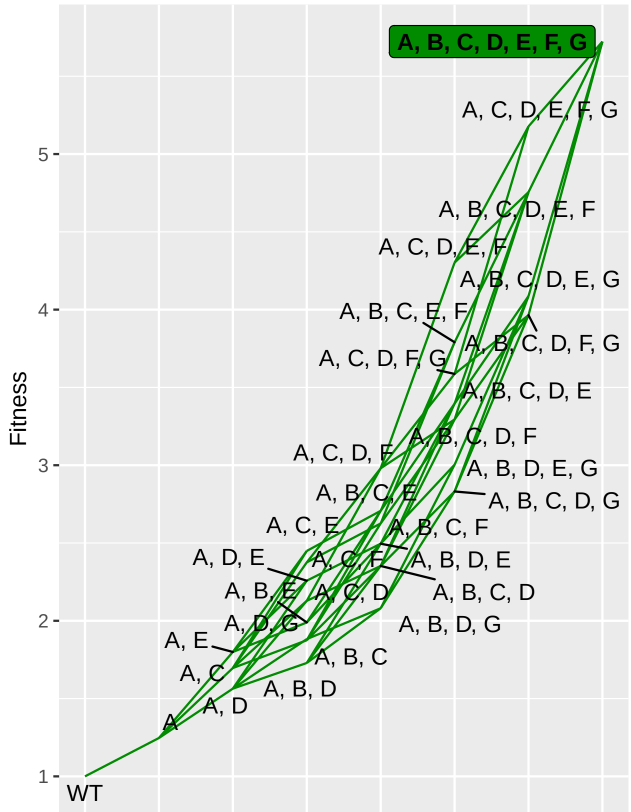

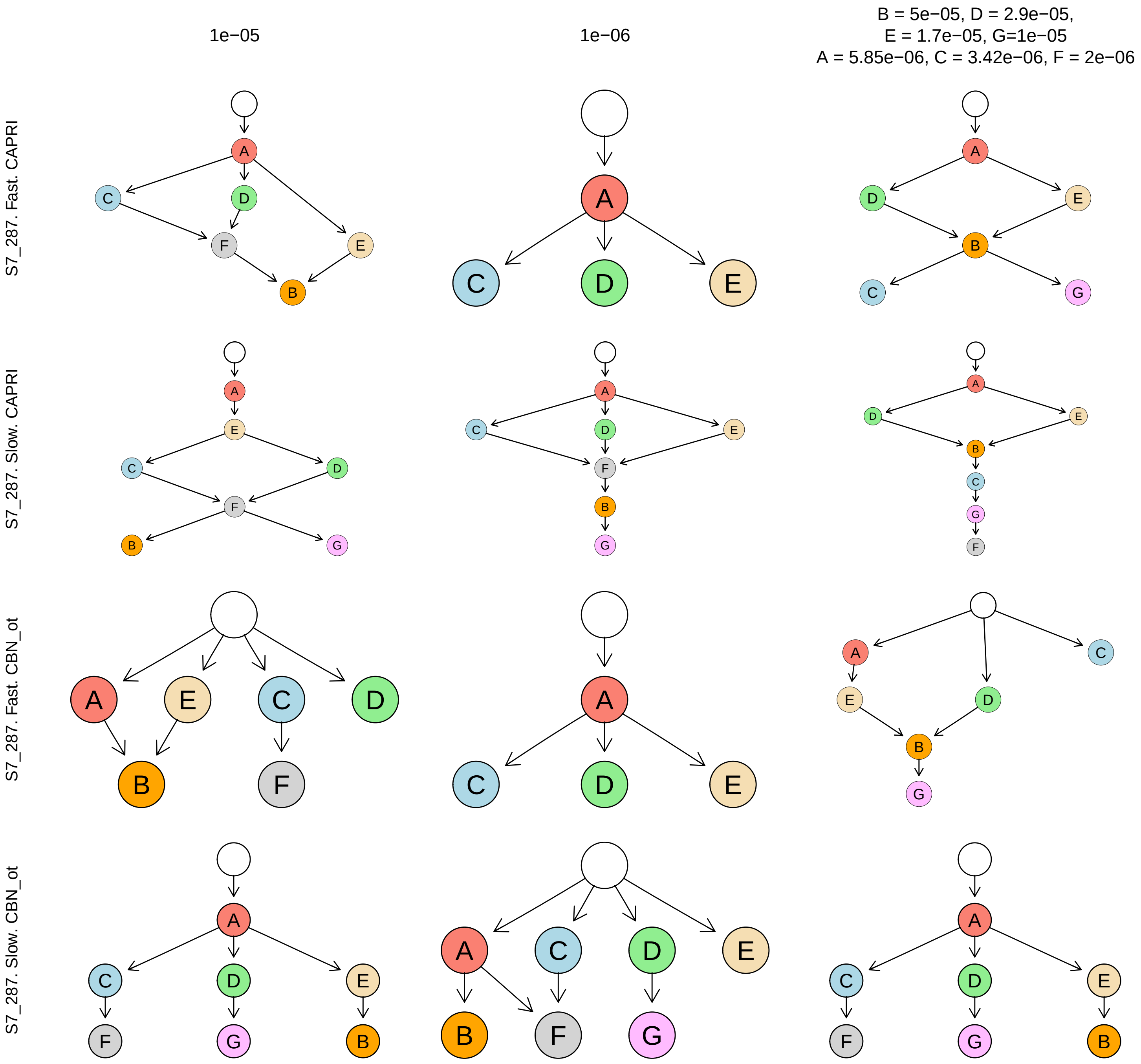

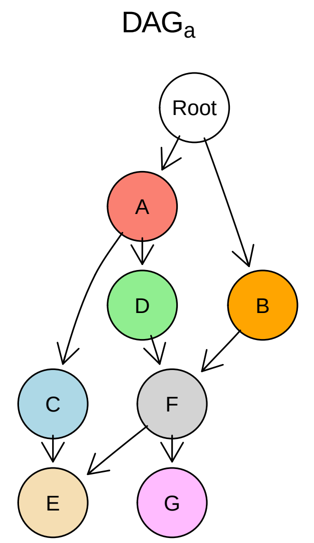

ID	Accessible Genot.	Discrep. DAG_a
S7_288	9	6

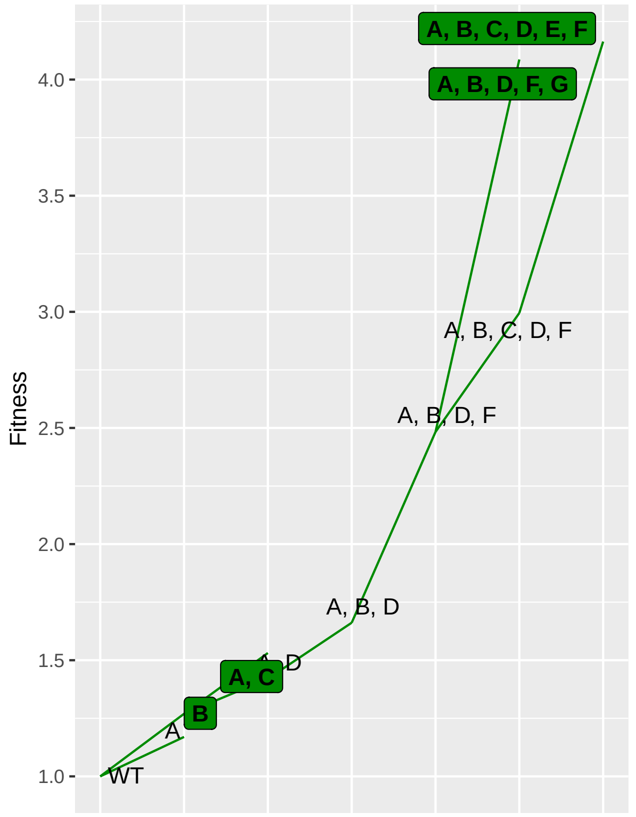

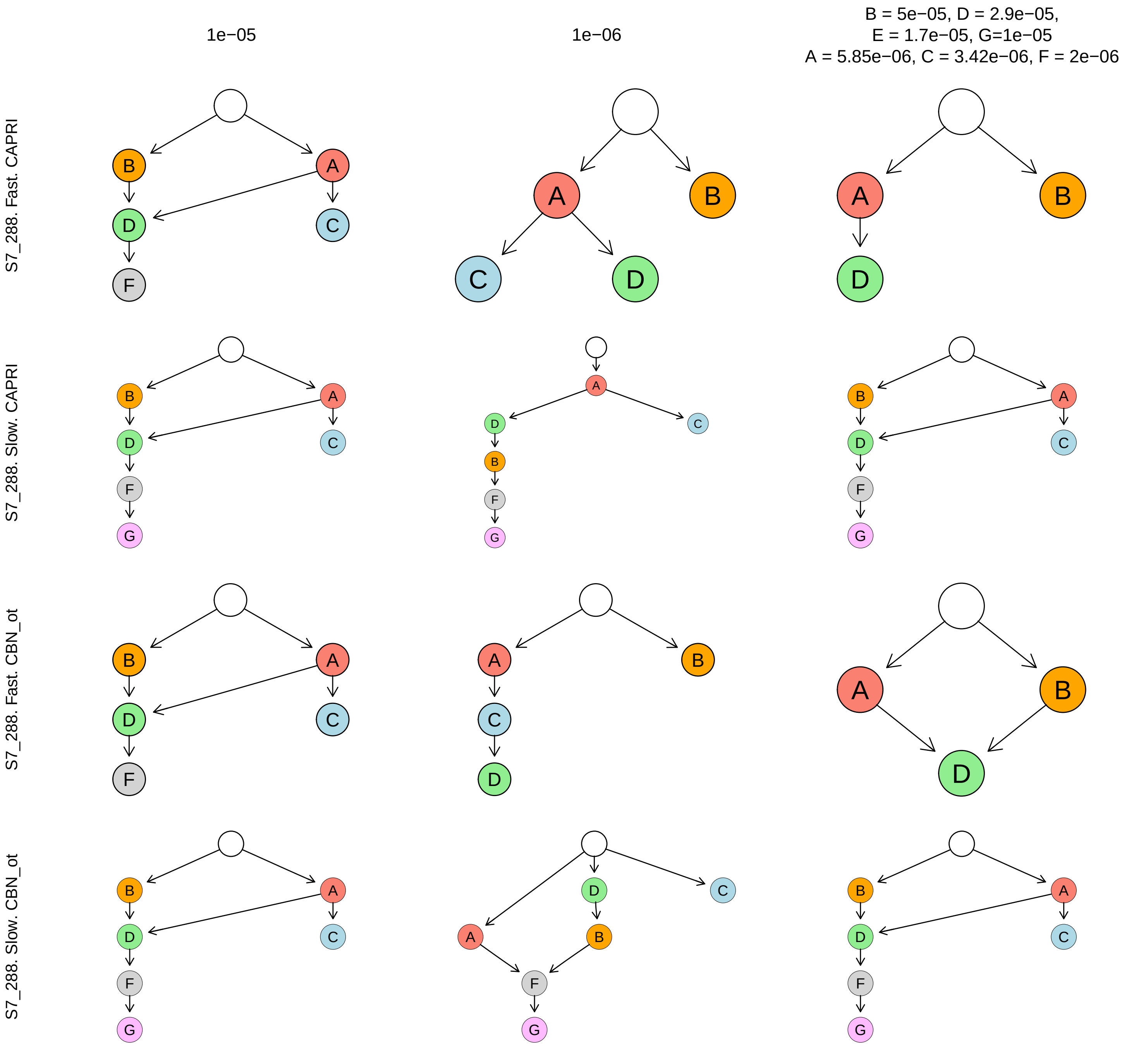

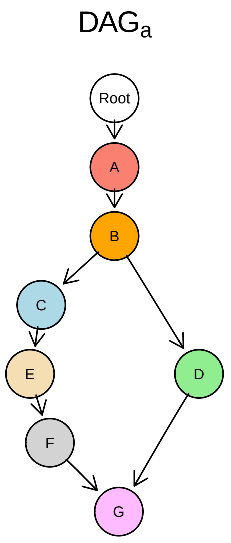

ID	Accessible Genot.	Discrep. DAG_a
S7_289	10	2

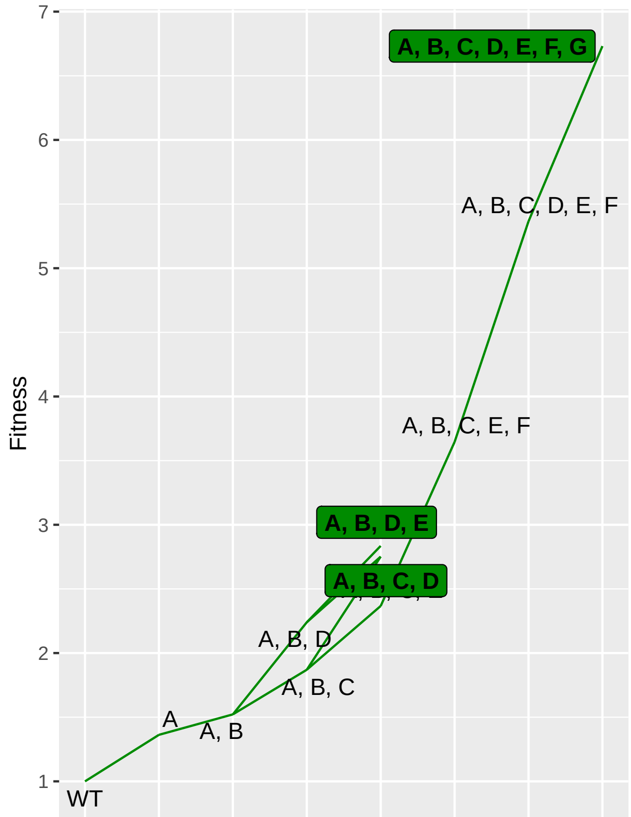

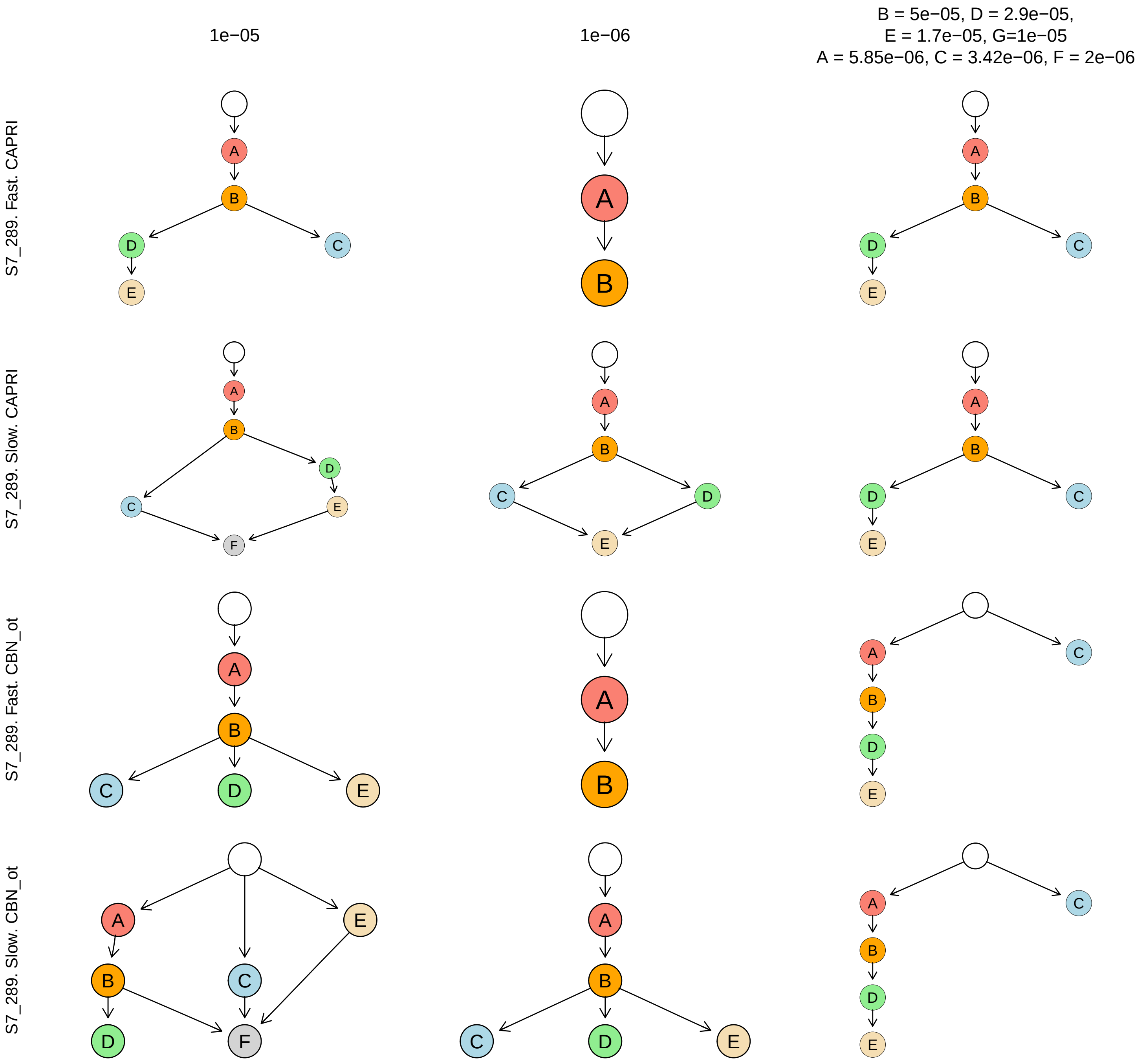

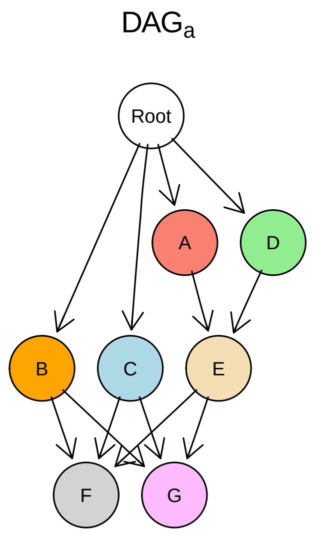

ID	Accessible Genot.	Discrep. DAG_a
S7_290	14	8

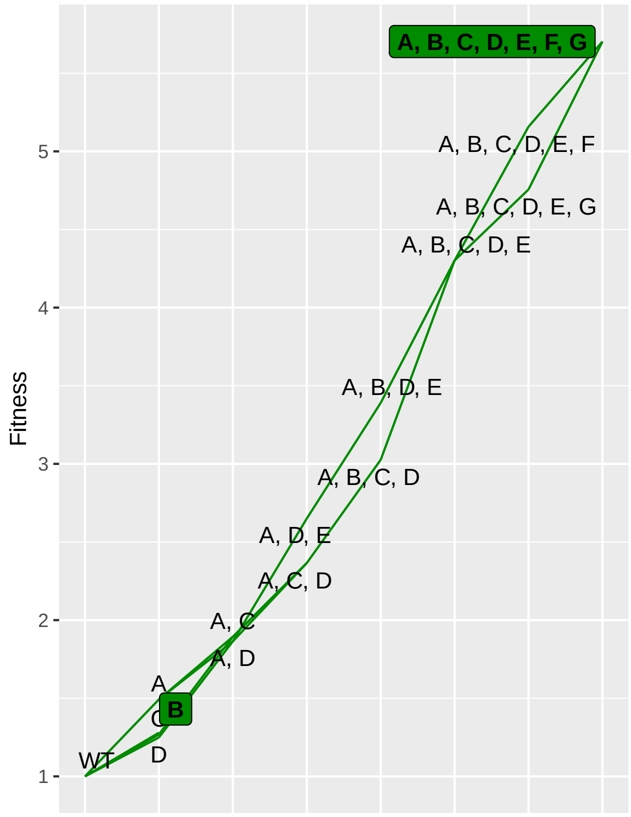

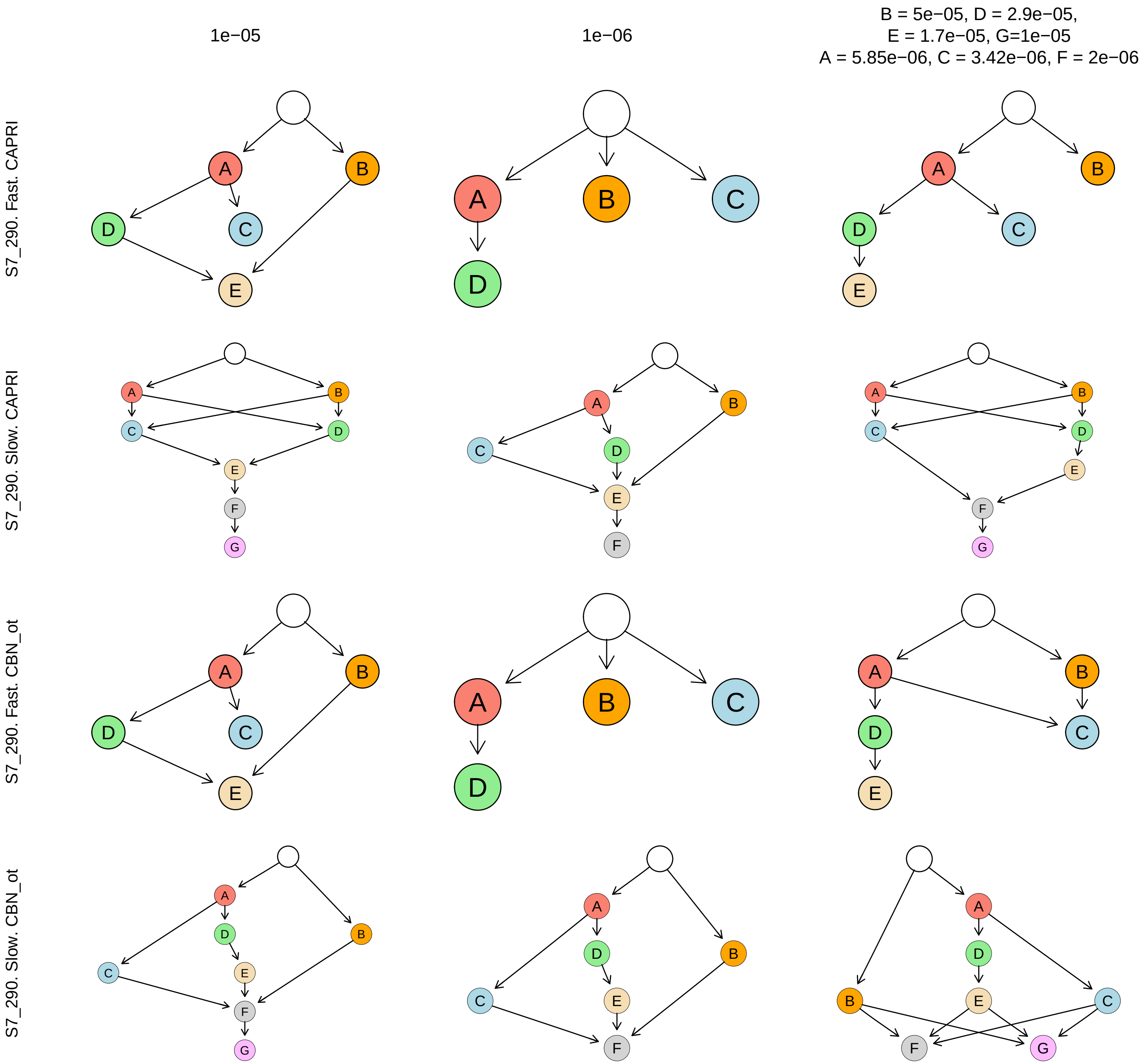

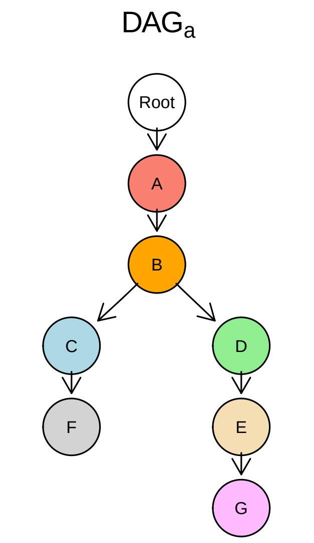

ID	Accessible Genot.	Discrep. DAG_a
S7_291	11	2

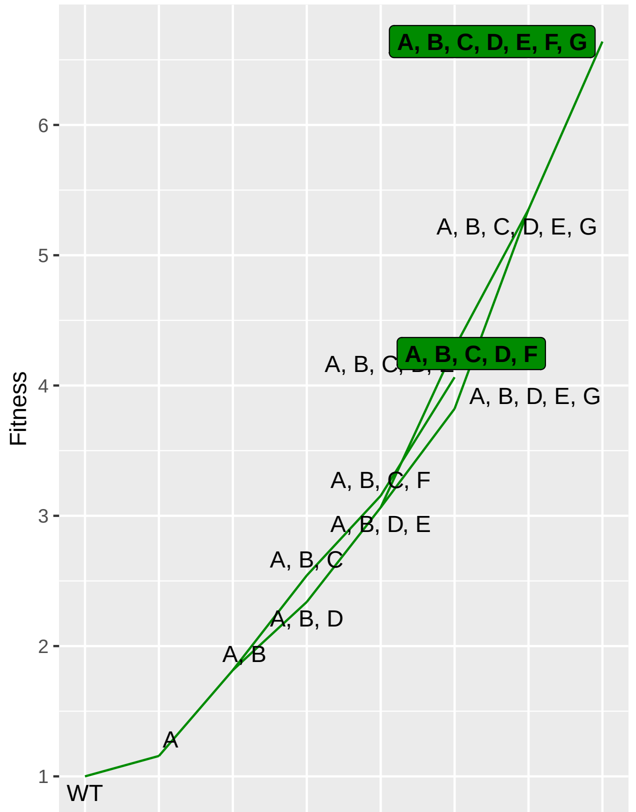

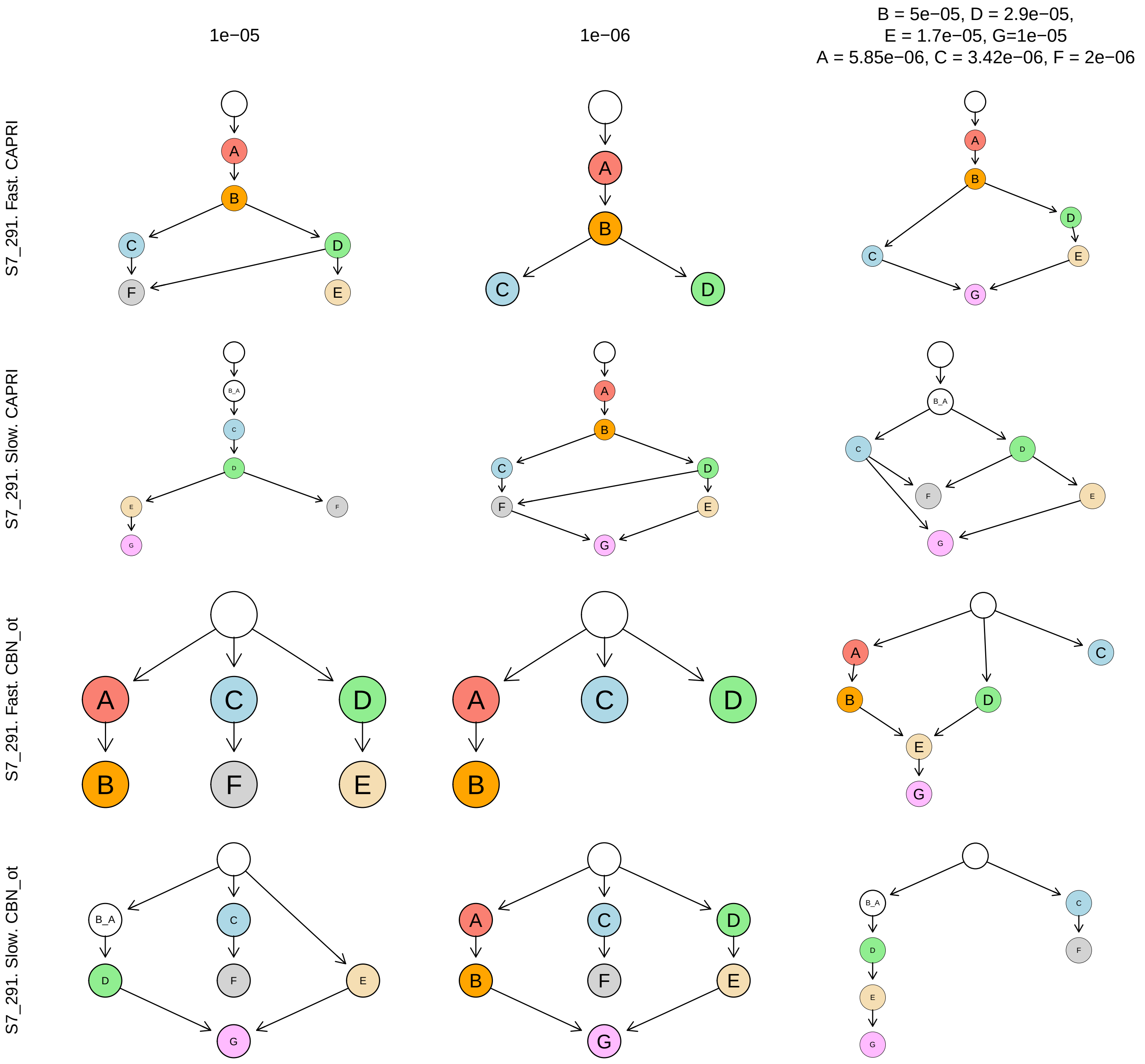

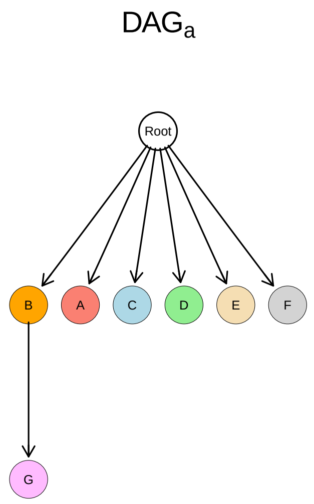

ID	Accessible Genot.	Discrep. DAG_a
S7_292	82	13

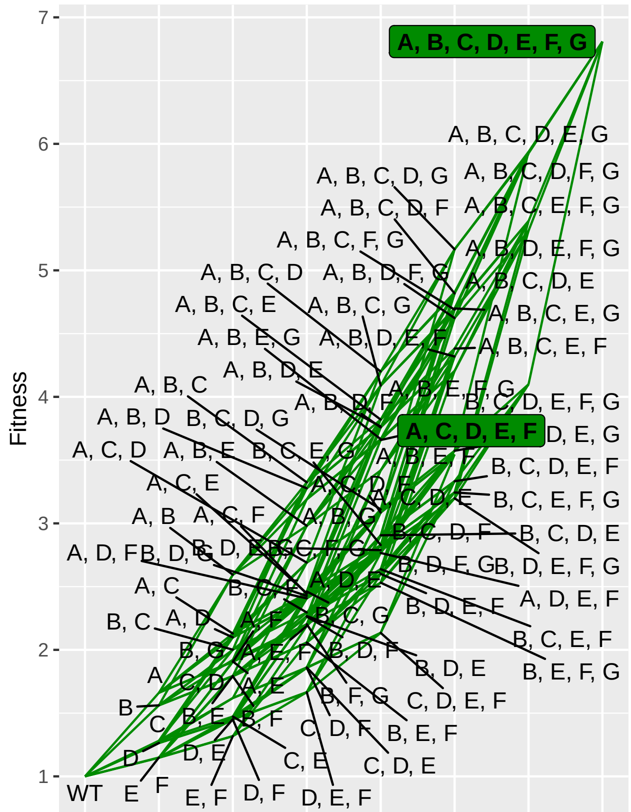

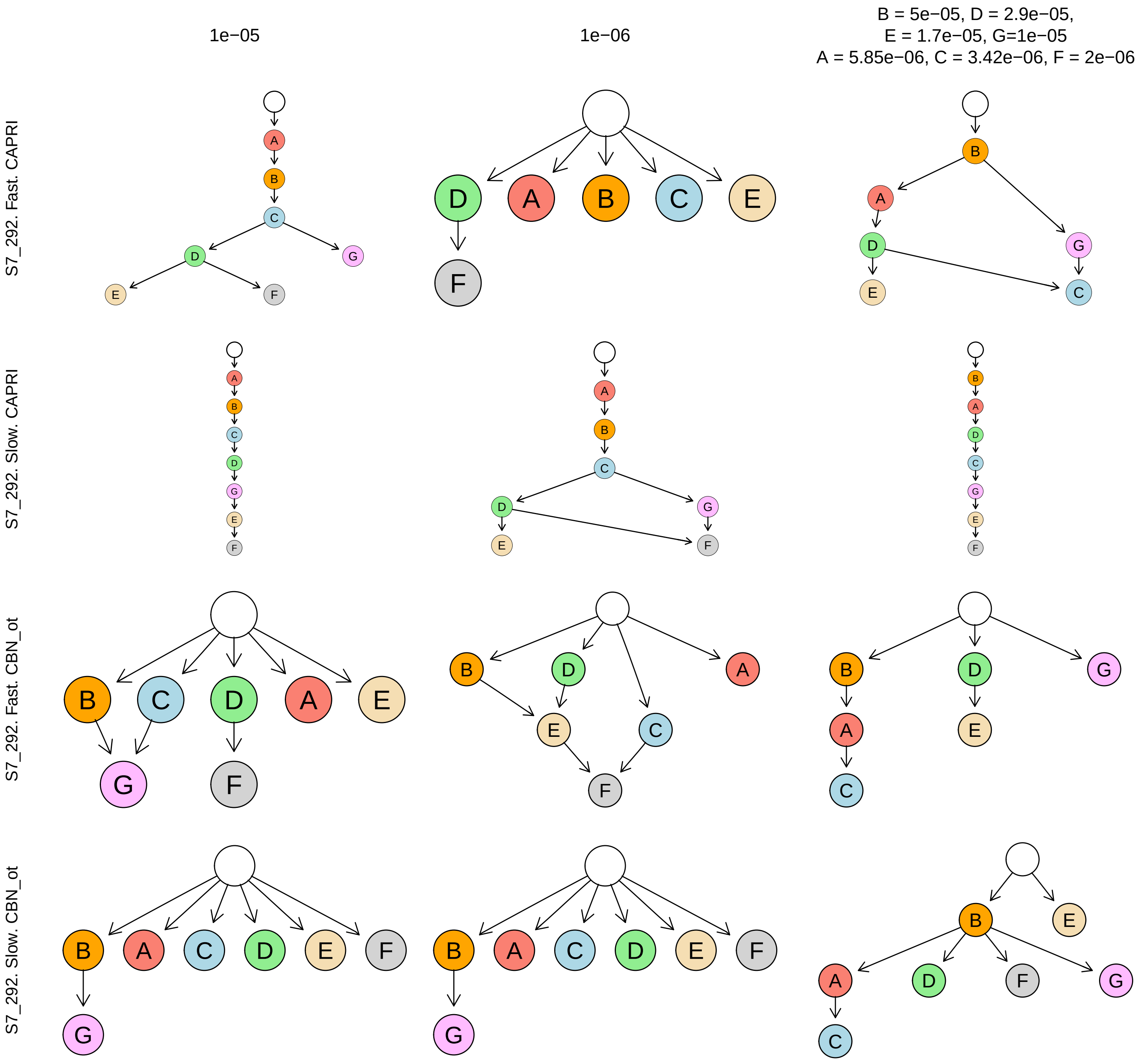

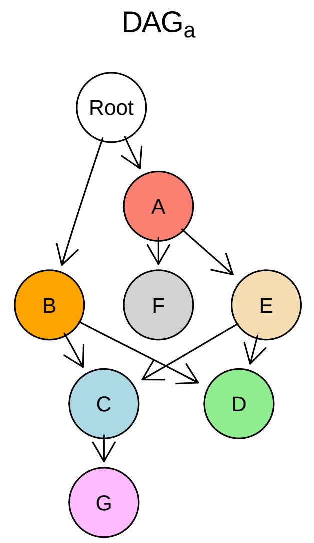

ID	Accessible Genot.	Discrep. DAG_a
S7_293	16	3

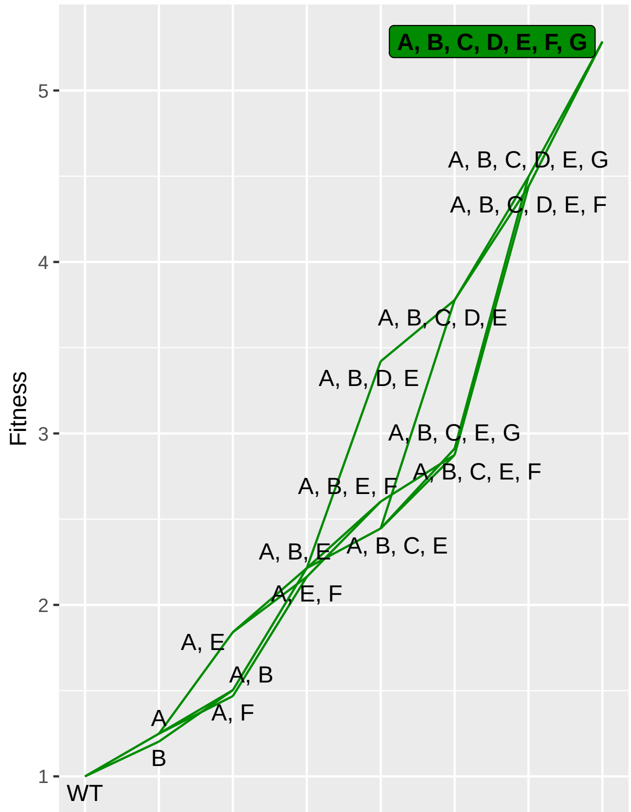

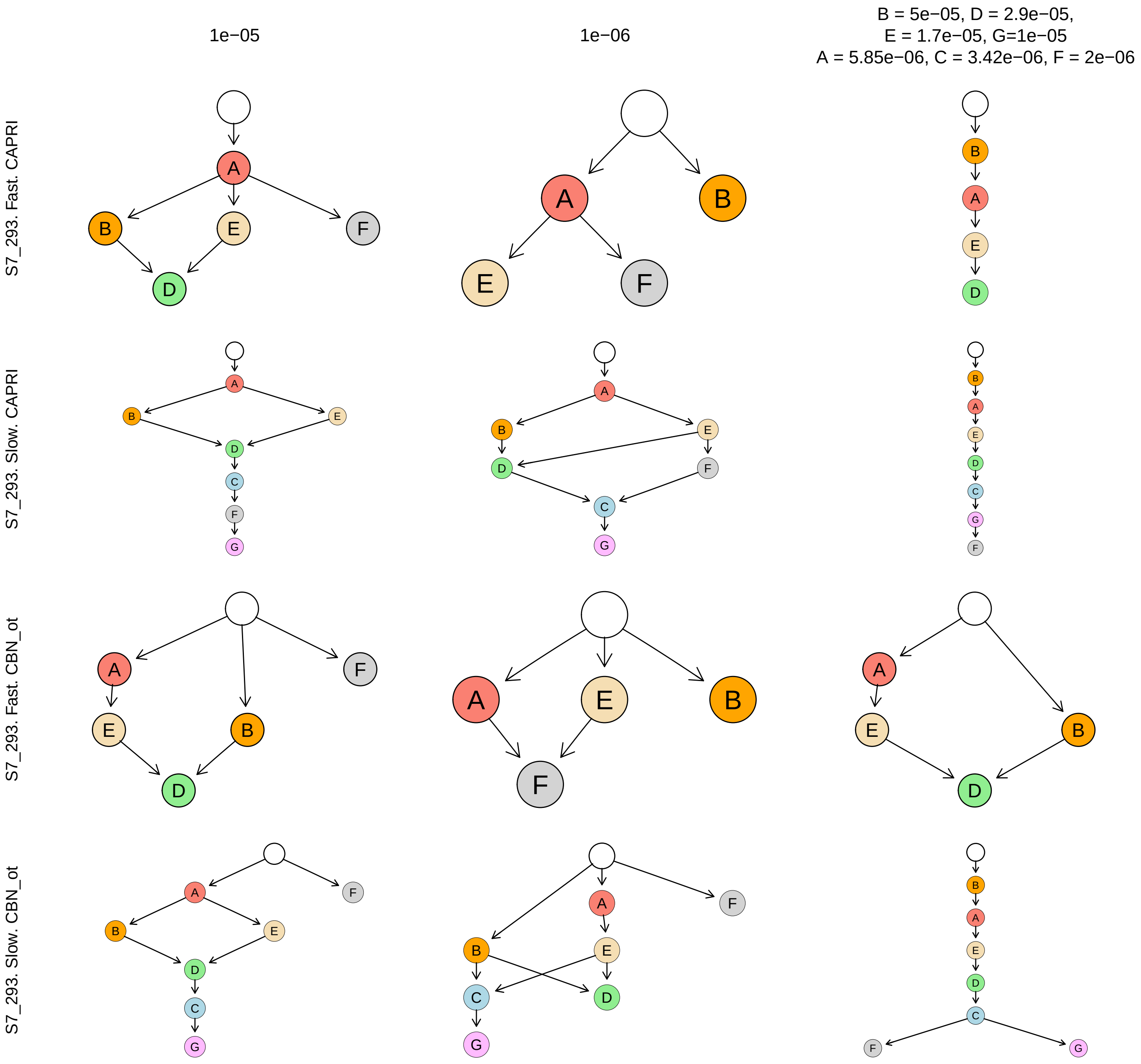

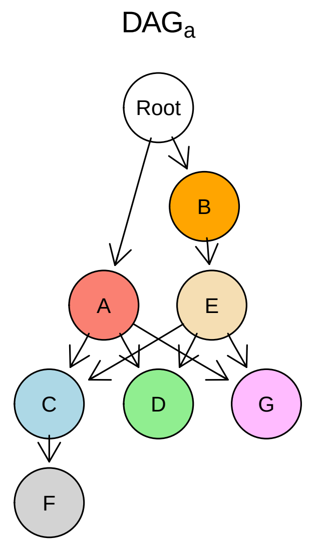

ID	Accessible Genot.	Discrep. DAG_a
S7_294	14	6

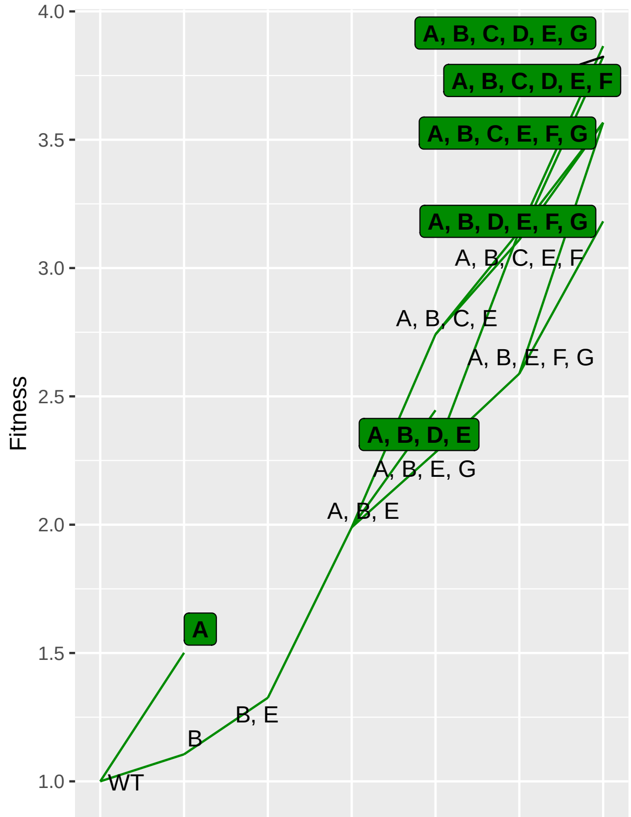

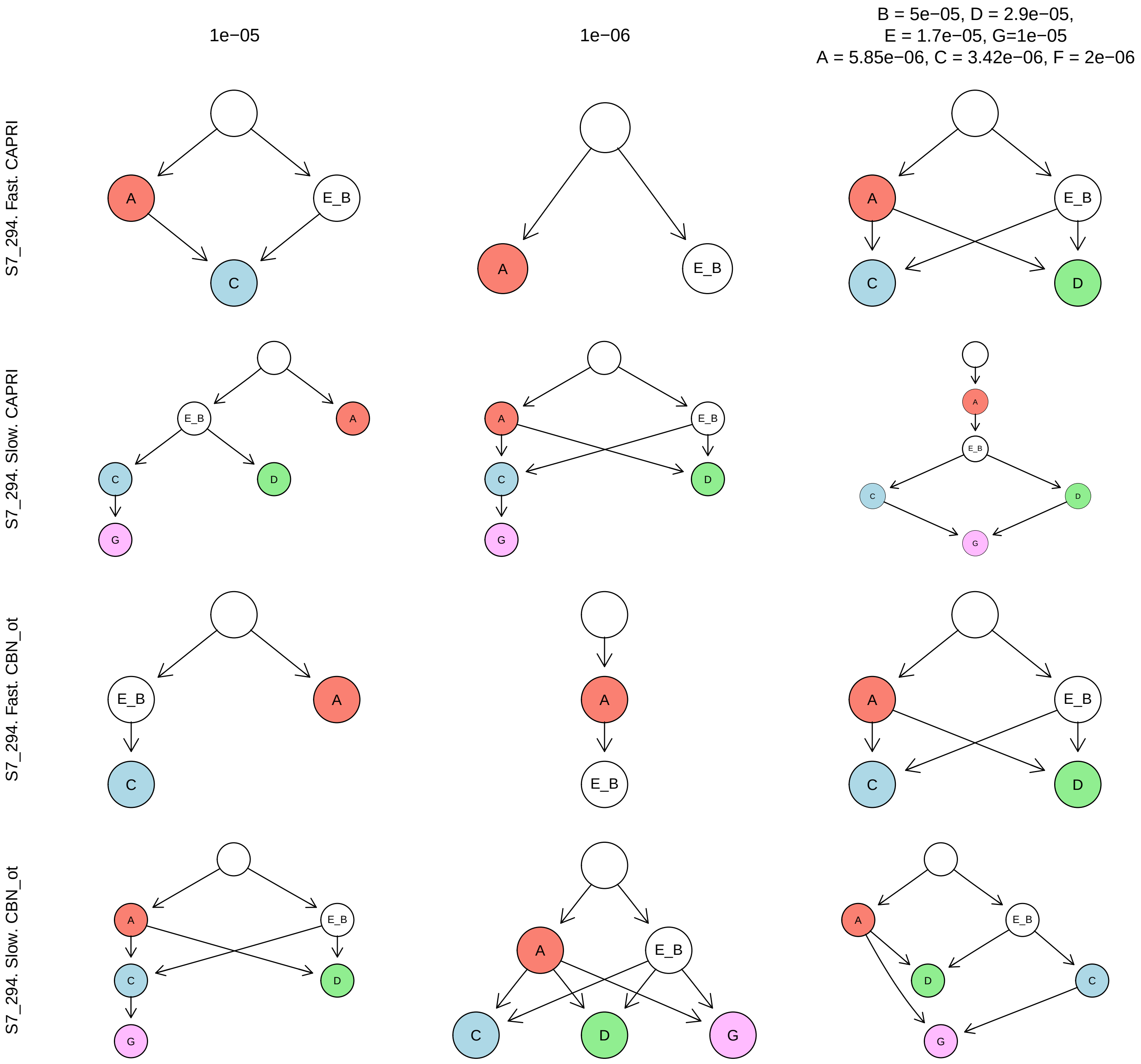

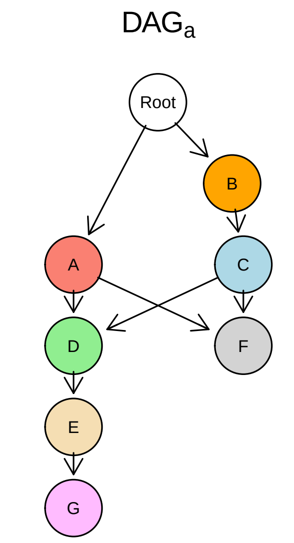

ID	Accessible Genot.	Discrep. DAG_a
S7_295	10	2

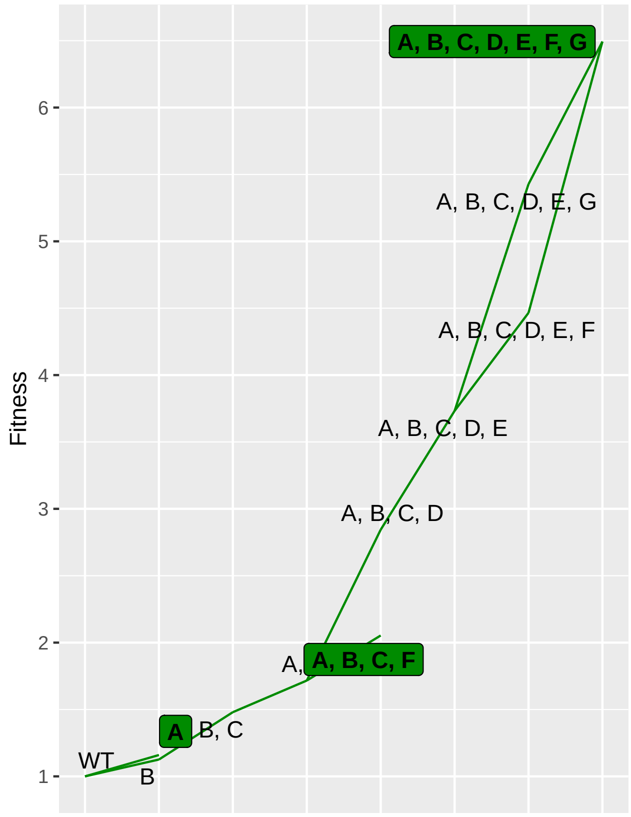

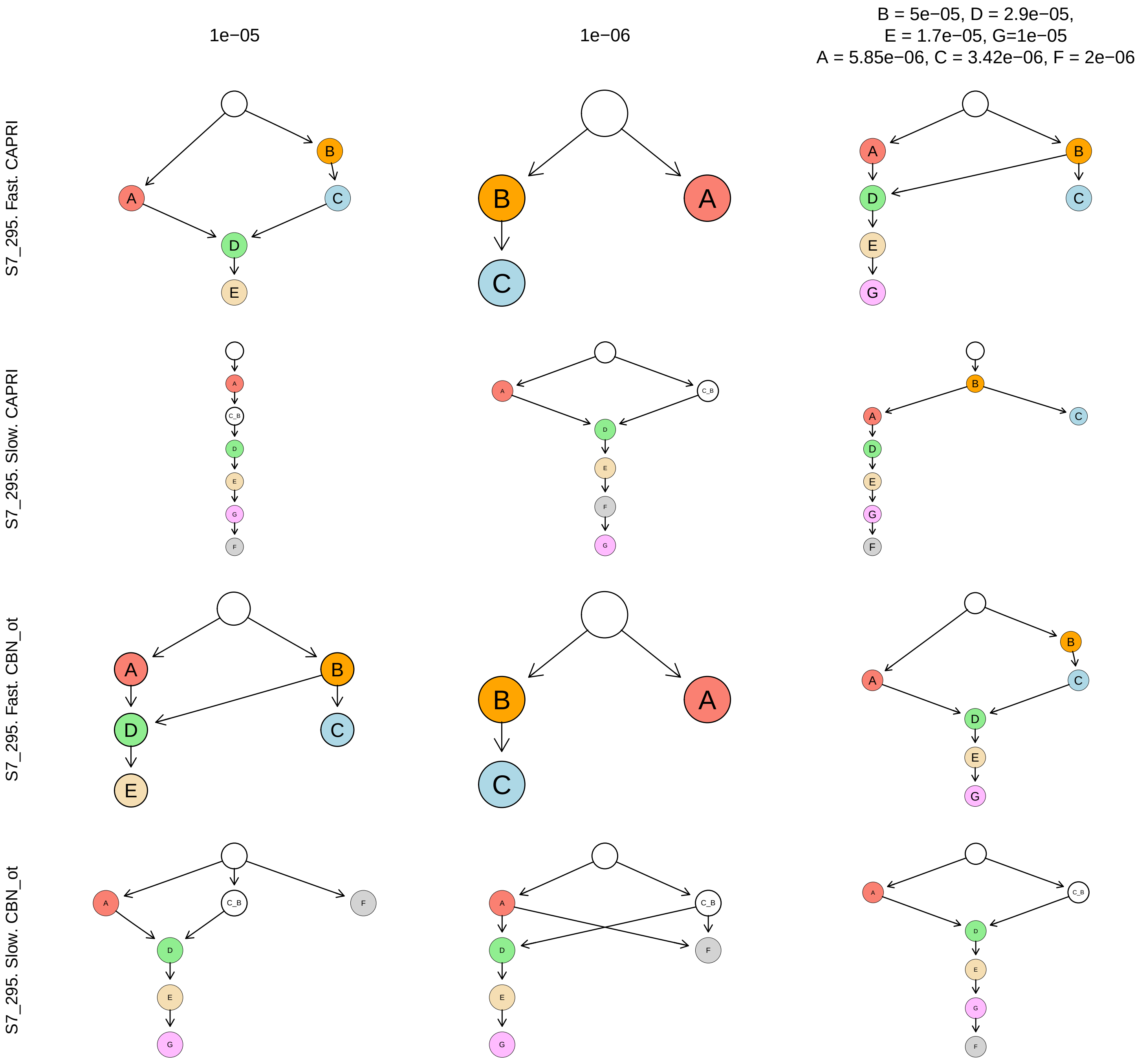

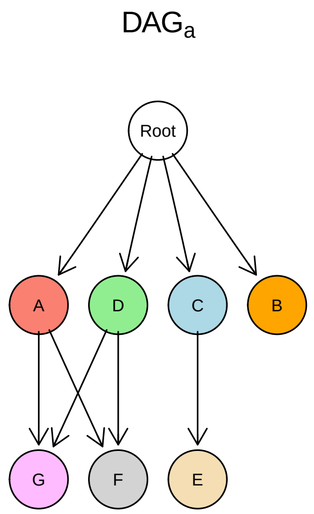

ID	Accessible Genot.	Discrep. DAG_a
S7_296	33	8

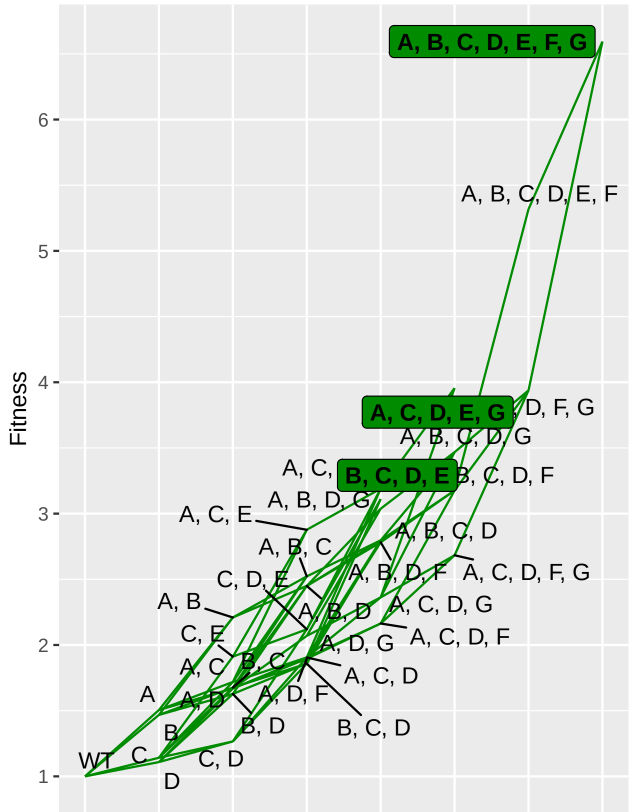

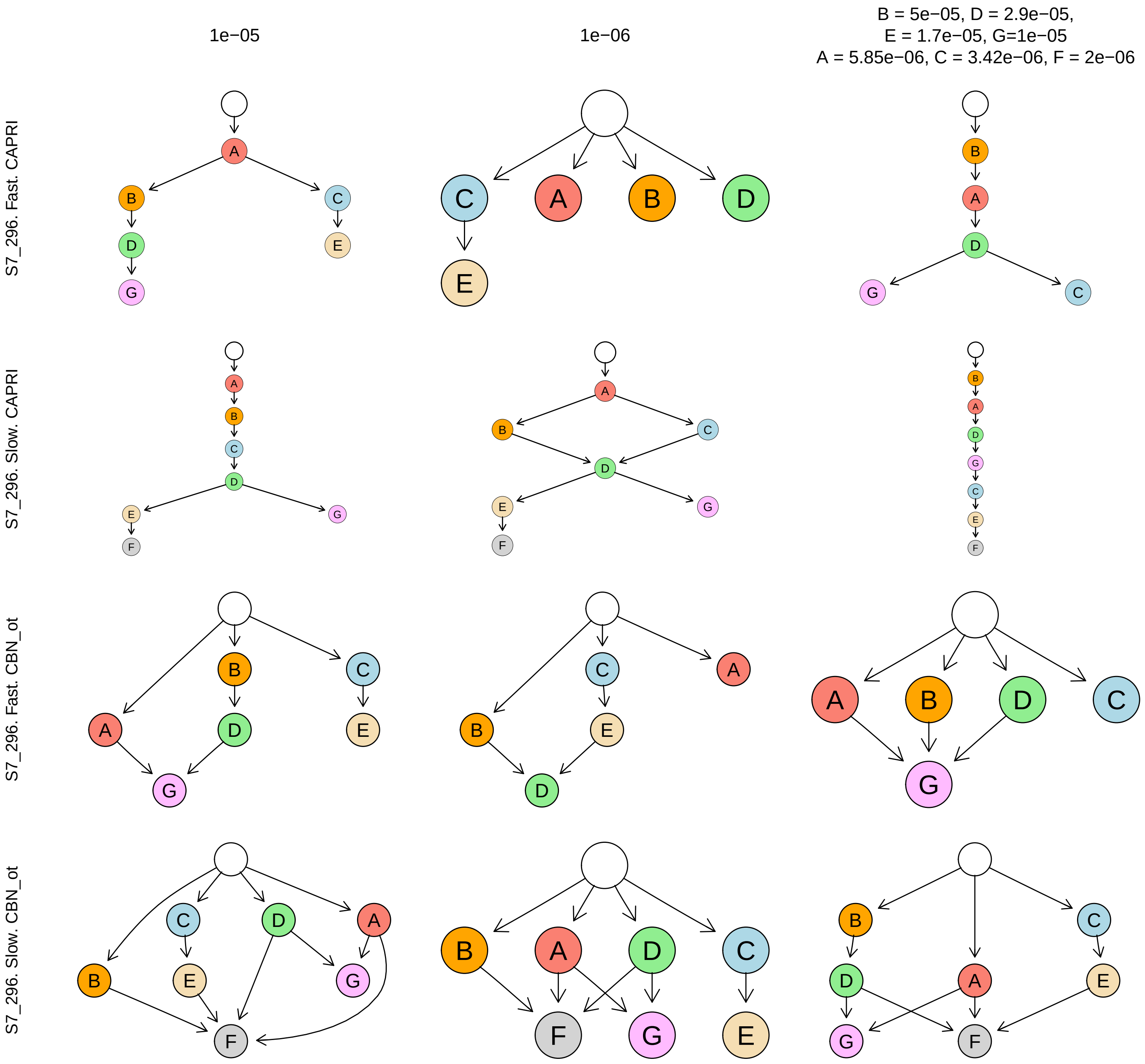

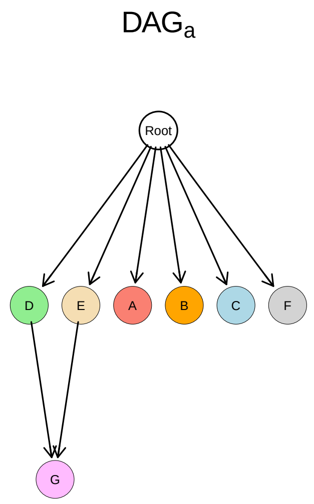

ID	Accessible Genot.	Discrep. DAG_a
S7_297	73	6

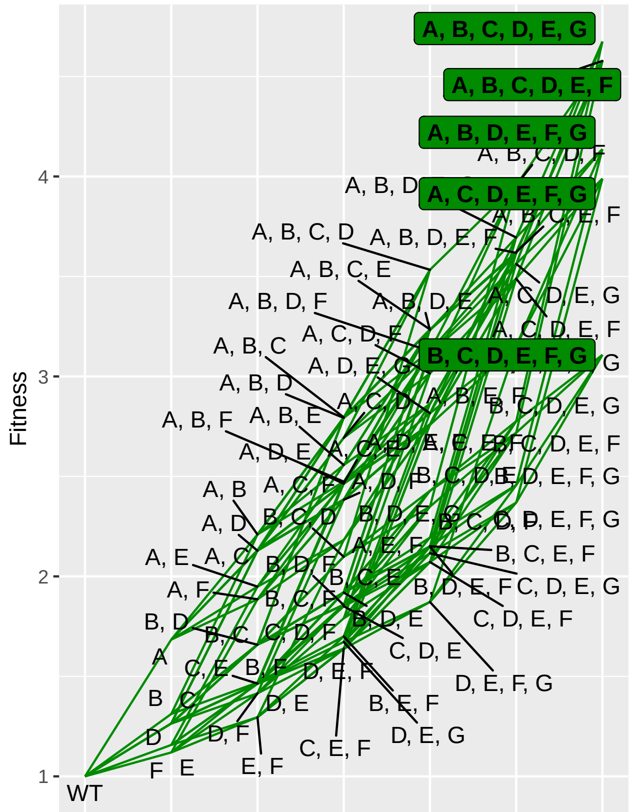

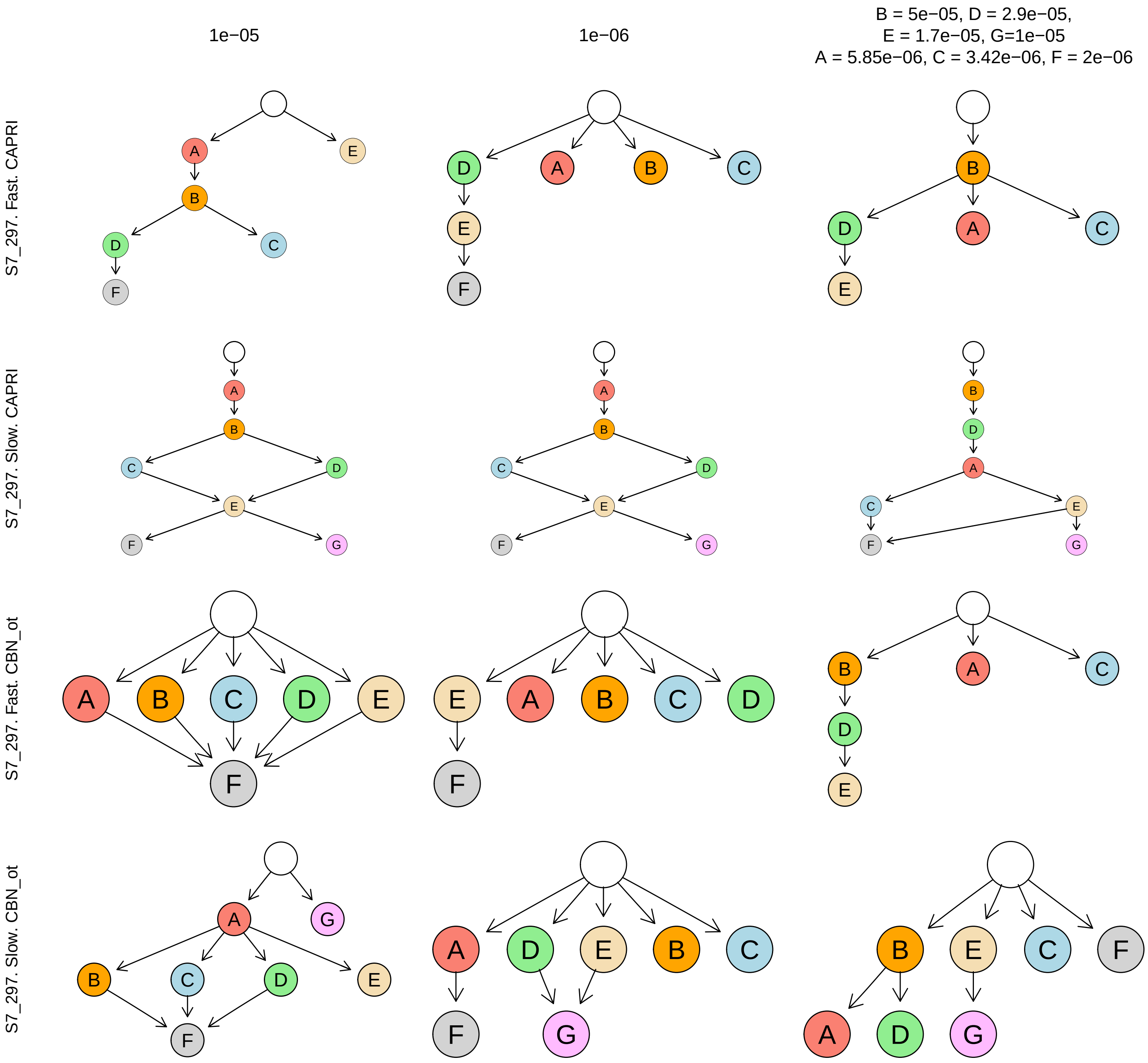

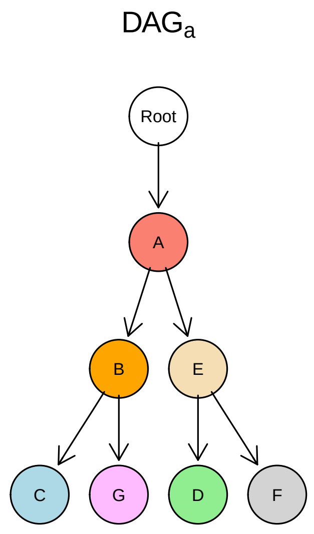

ID	Accessible Genot.	Discrep. DAG_a
S7_298	20	21

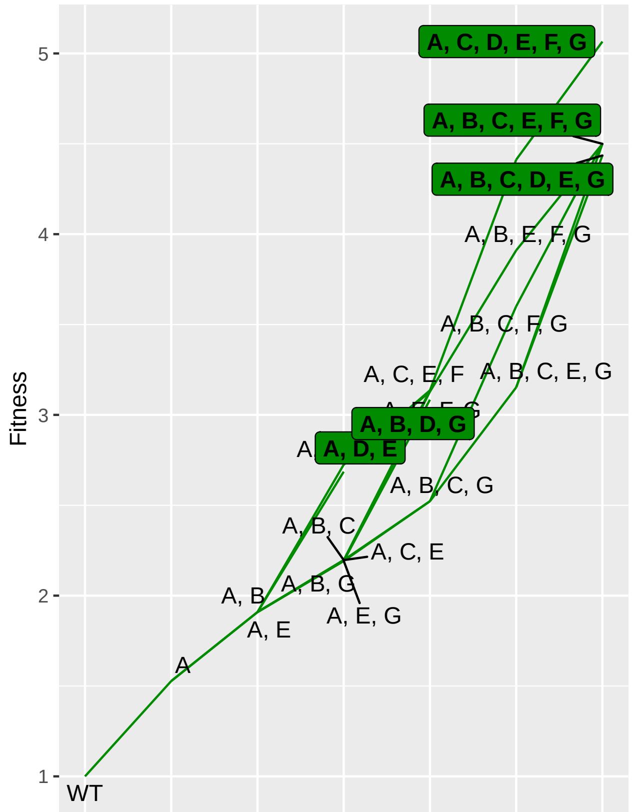

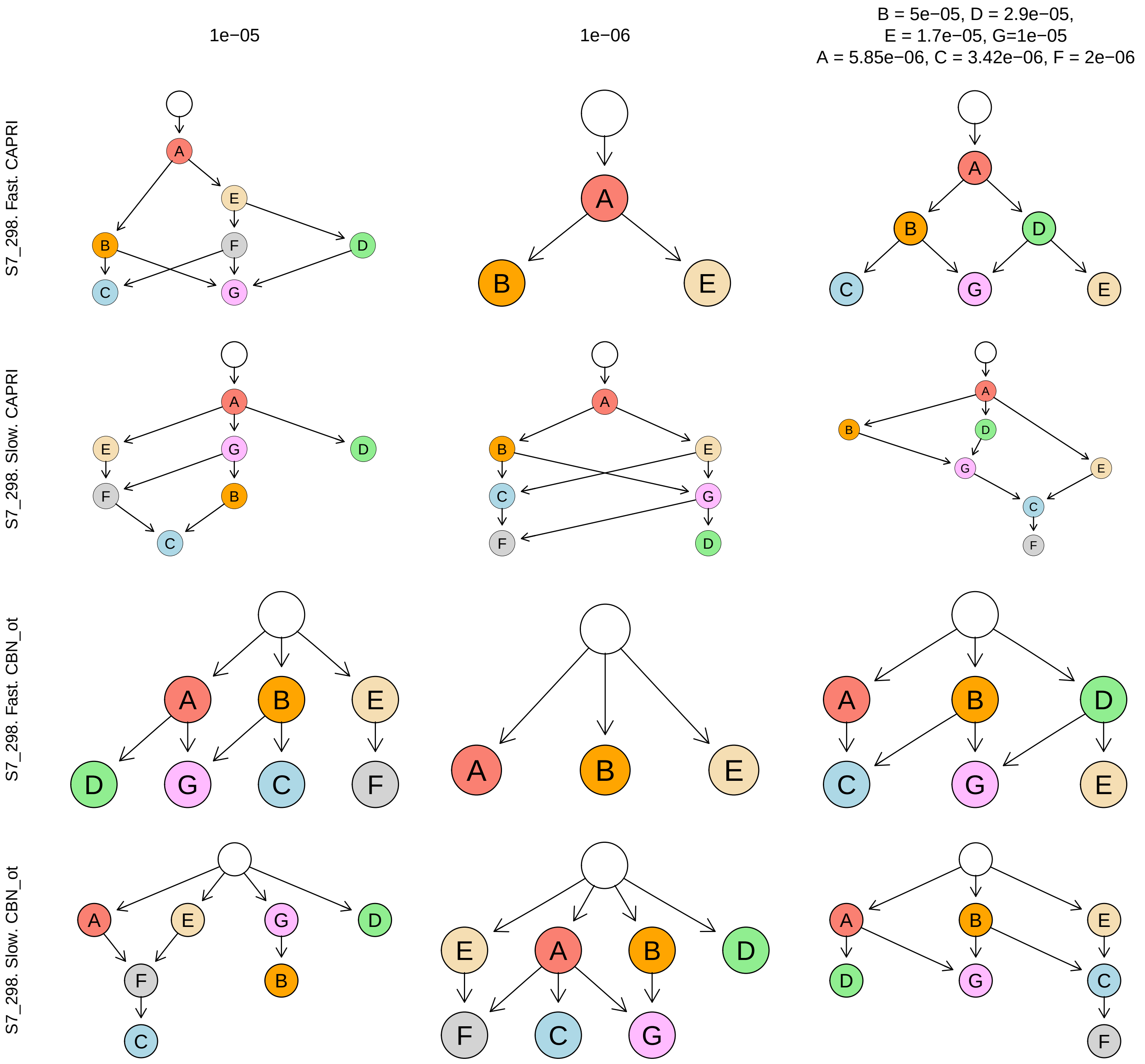

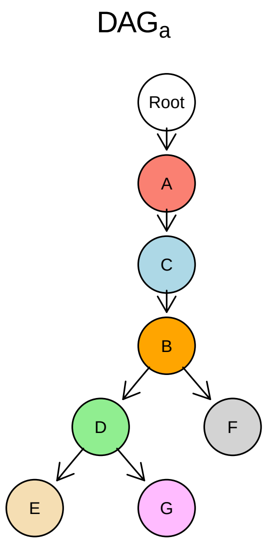

ID	Accessible Genot.	Discrep. DAG_a
S7_299	14	2

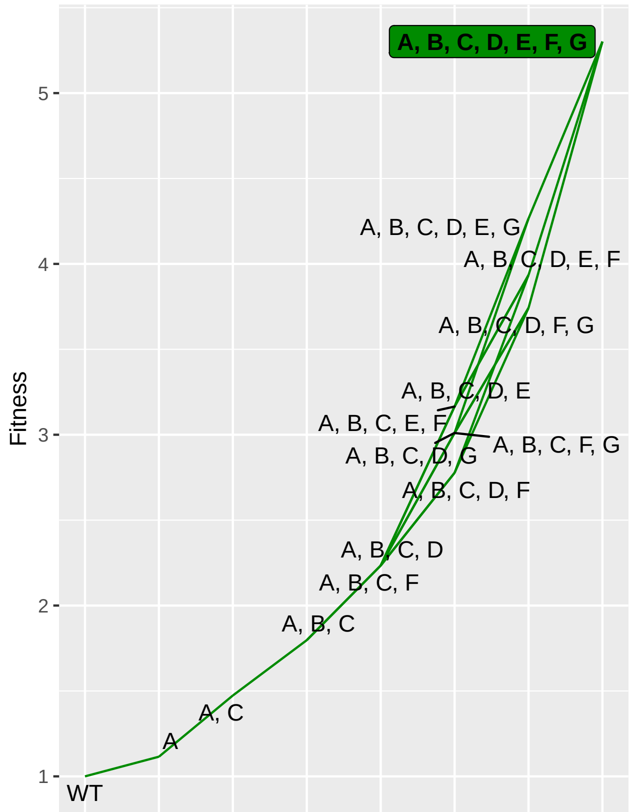

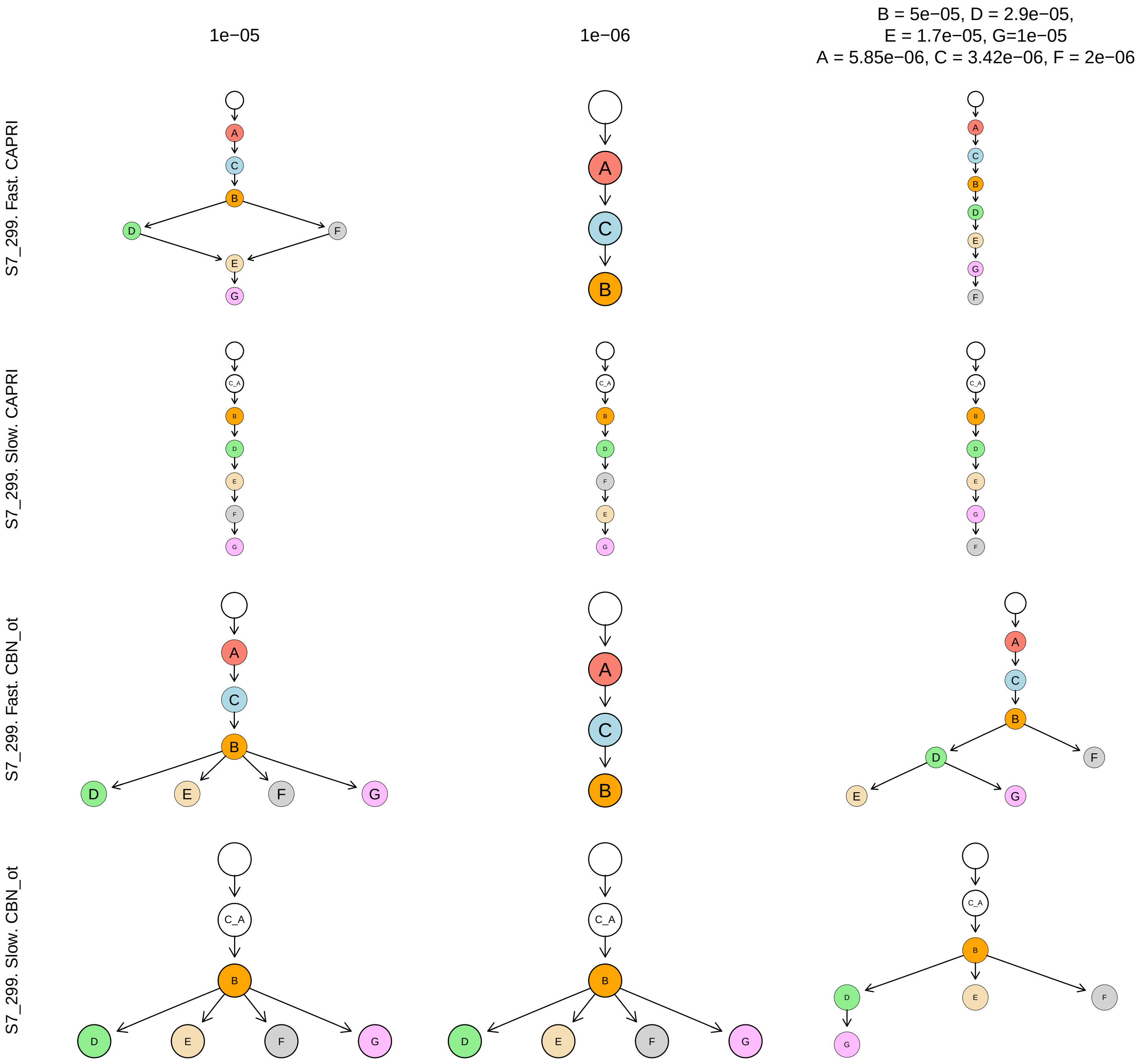

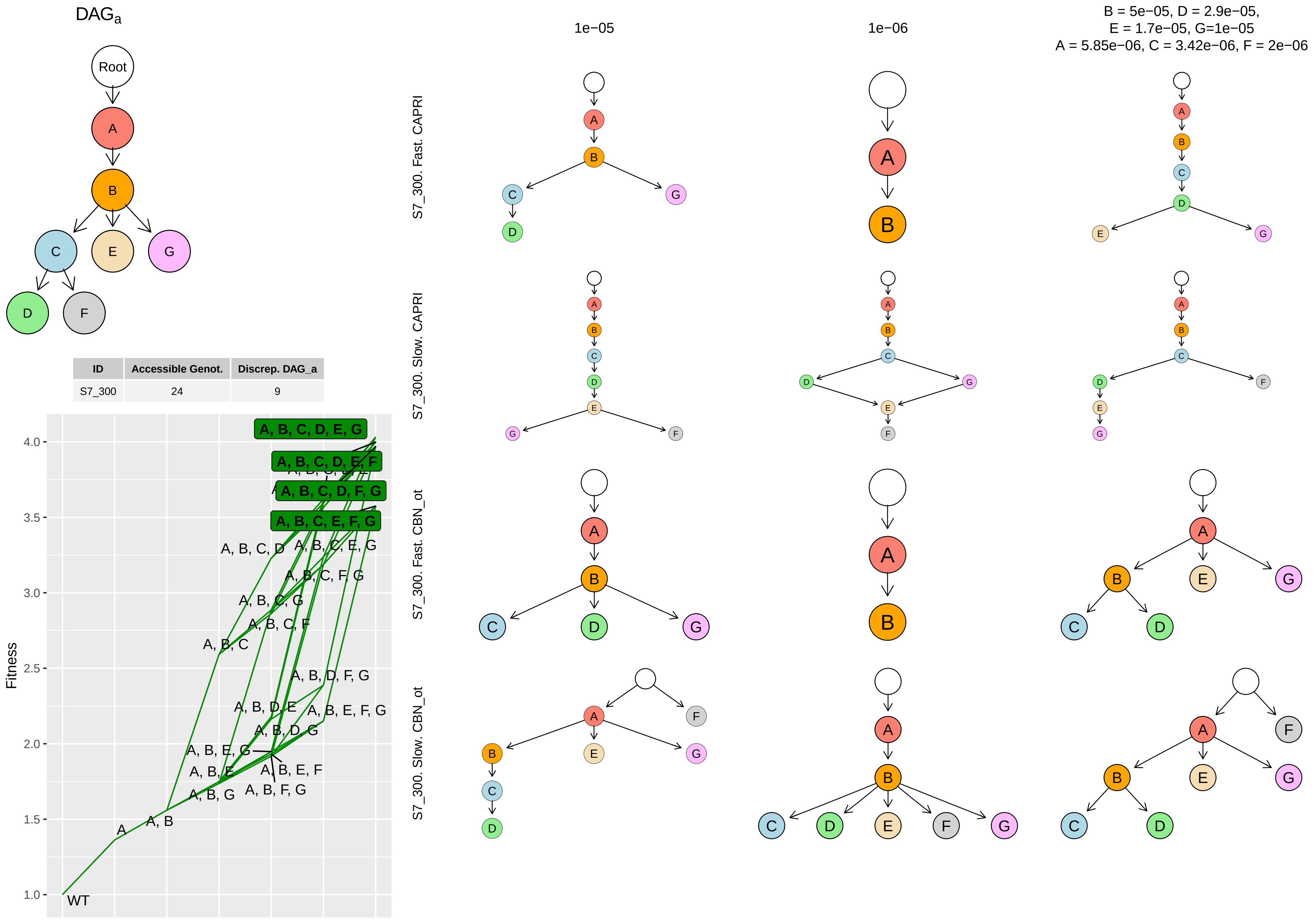

DAGa
Root
A
B
C
E
G
D
F
ID
Accessible Genot.
Discrep. DAG_a
S7_300
24
9
Fitness
4.0
3.5
3.0
2.5
2.0
1.5
1.0
A, B, C, D, E, G
A, B, C, D, E, F
A, B, C, D, F, G
A, B, C, E, F, G
A, B, C, D
A, B, C, E, G
A, B, C, F, G
A, B, C, G
A, B, C, F
A, B, C
A, B, D, F, G
A, B, D, E
A, B, E, F, G
A, B, D, G
A, B, E, G
A, B, E
A, B, E, F
A, B, G
A, B, F, G
A, B
A
WT
1e−05
1e−06
B = 5e−05, D = 2.9e−05,
E = 1.7e−05, G=1e−05
A = 5.85e−06, C = 3.42e−06, F = 2e−06
S7_300. Fast. CAPRI
S7_300. Slow. CAPRI
S7_300. Fast. CBN_ot
S7_300. Slow. CBN_ot

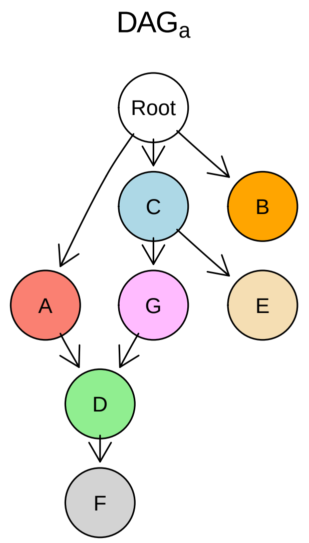

ID	Accessible Genot.	Discrep. DAG_a
S7_301	23	8

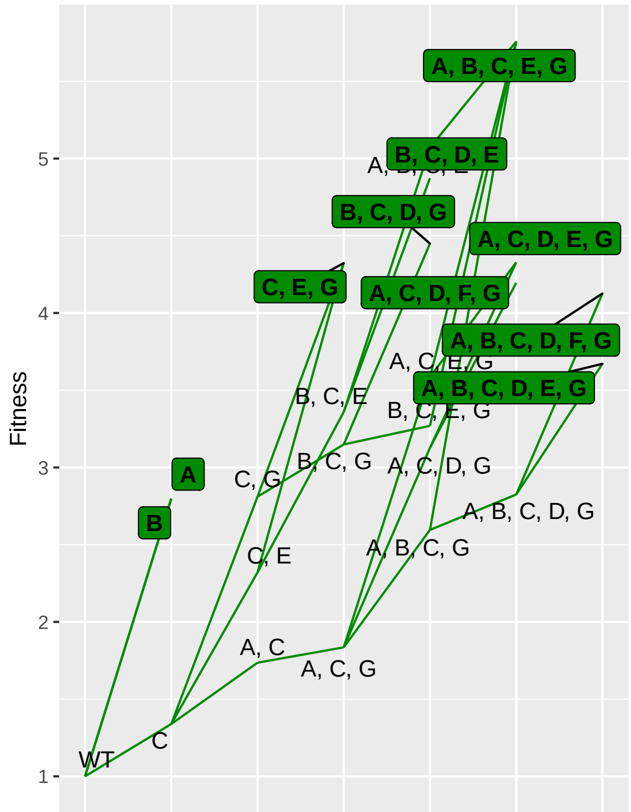

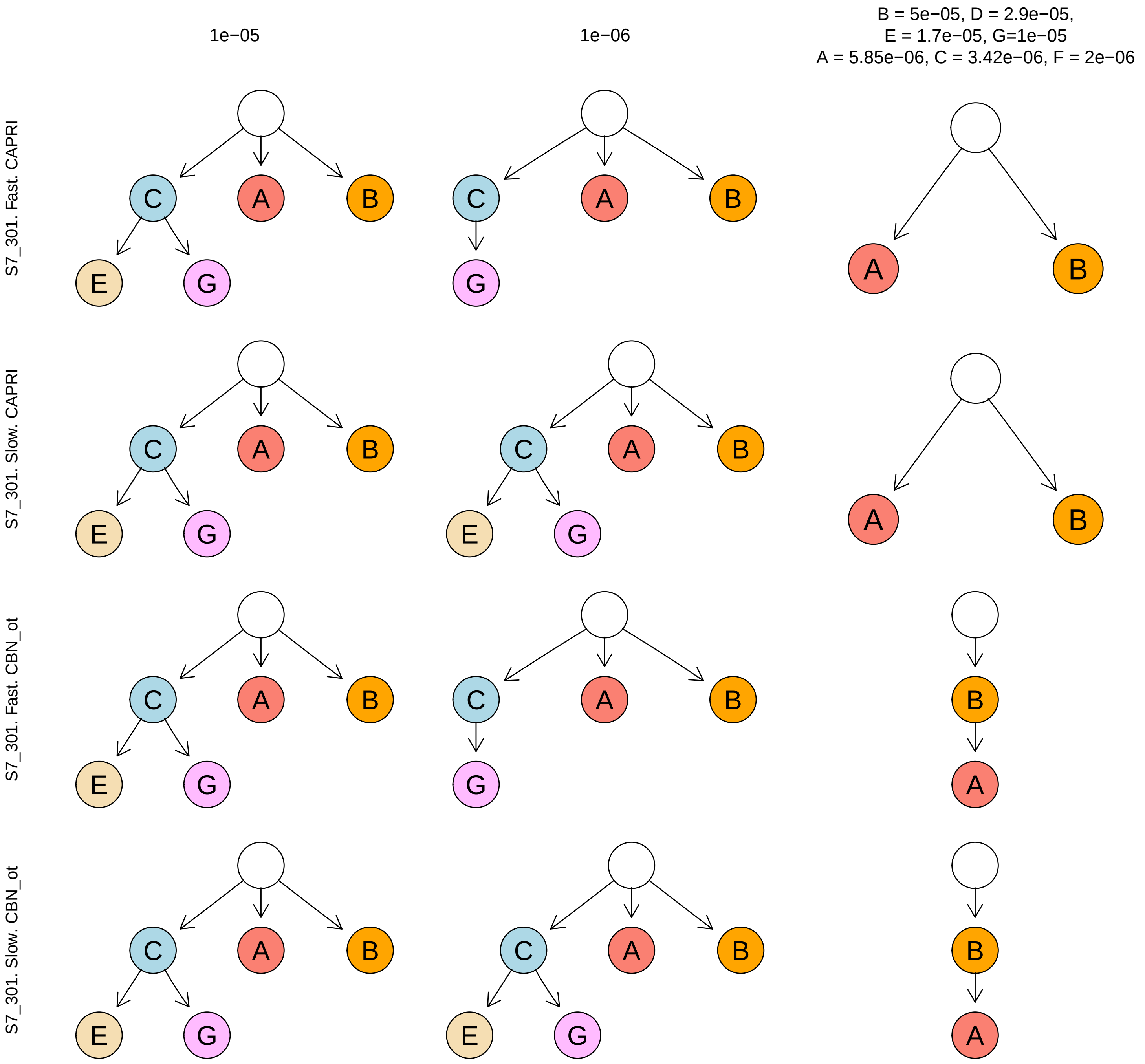

DAG$_a$

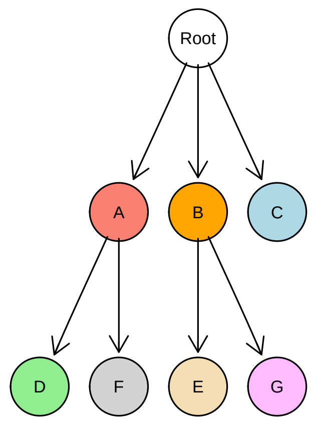

ID	Accessible Genot.	Discrep. DAG_a
S7_302	15	40

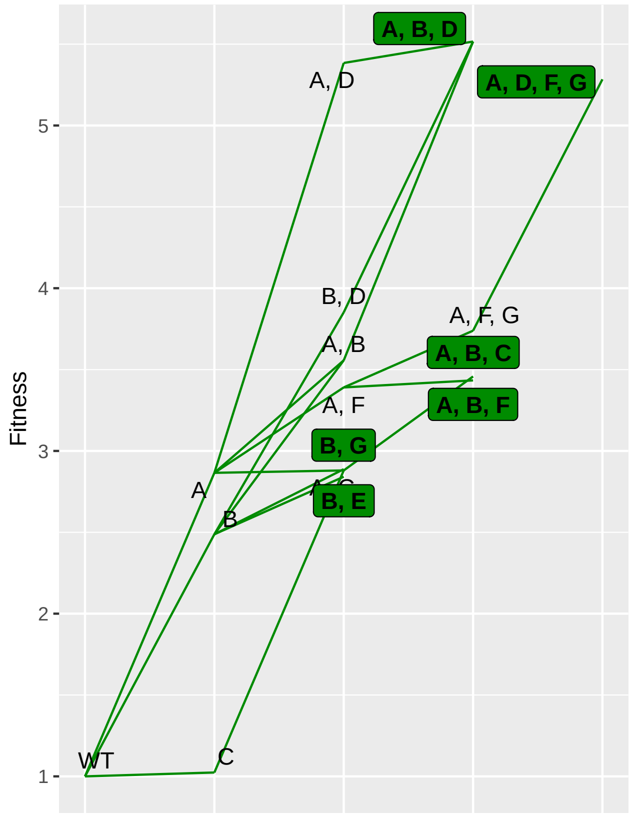

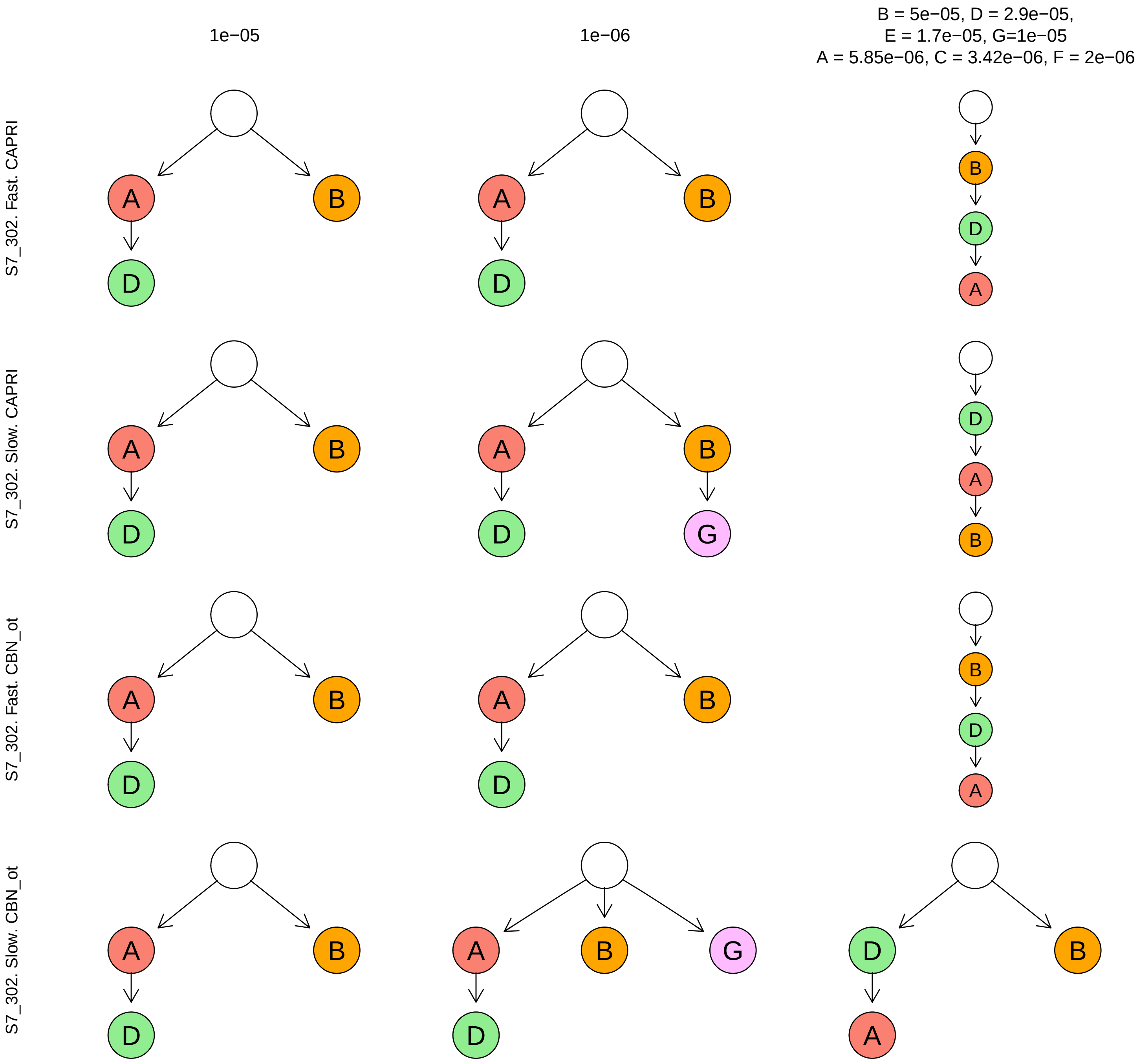

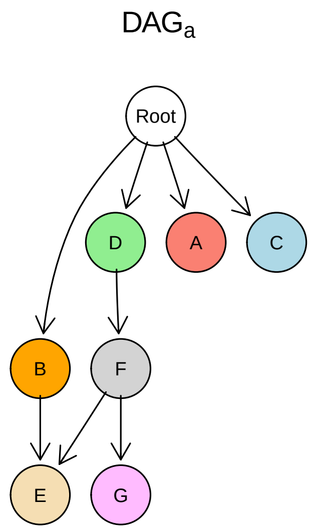

ID	Accessible Genot.	Discrep. DAG_a
S7_303	12	27

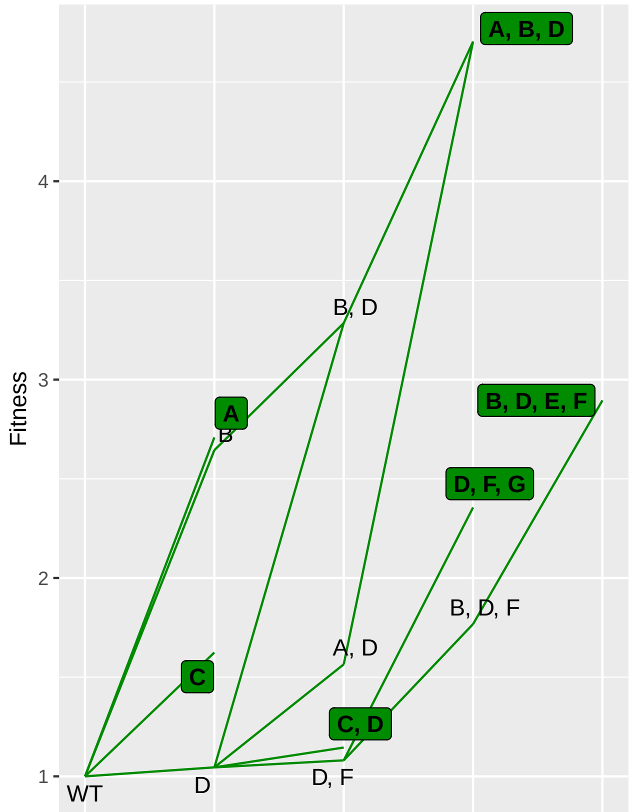

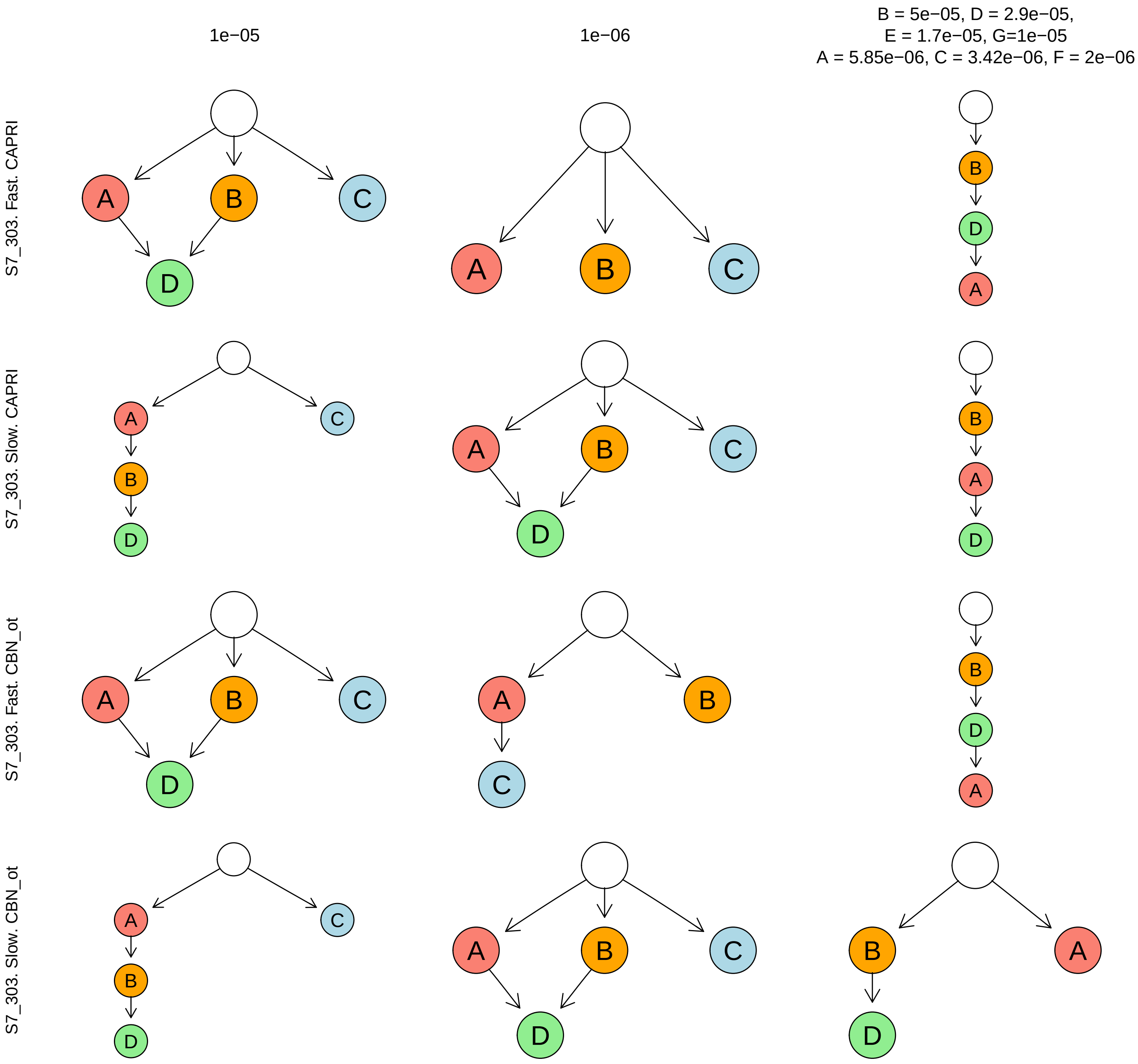

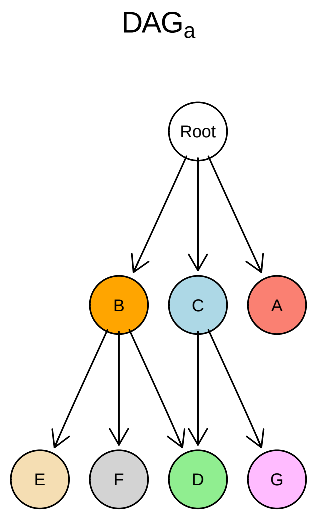

ID	Accessible Genot.	Discrep. DAG_a
S7_304	13	36

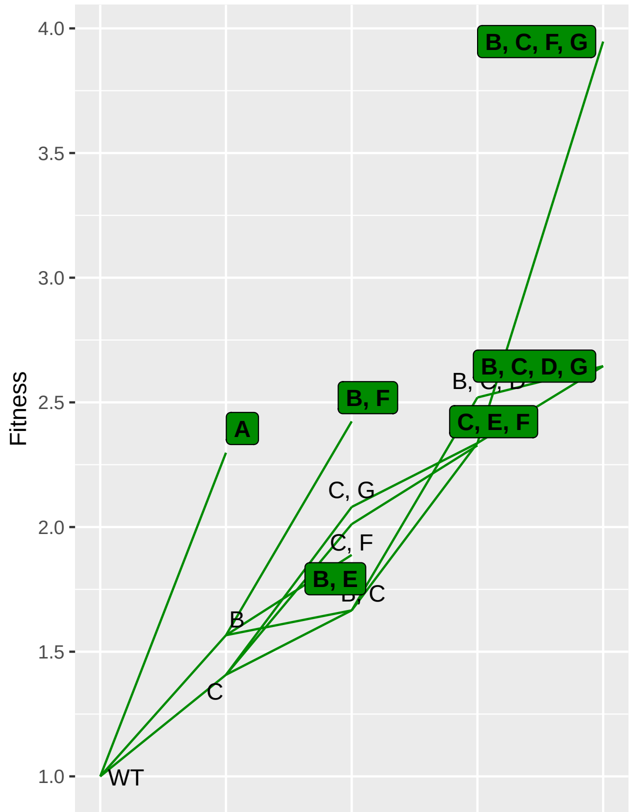

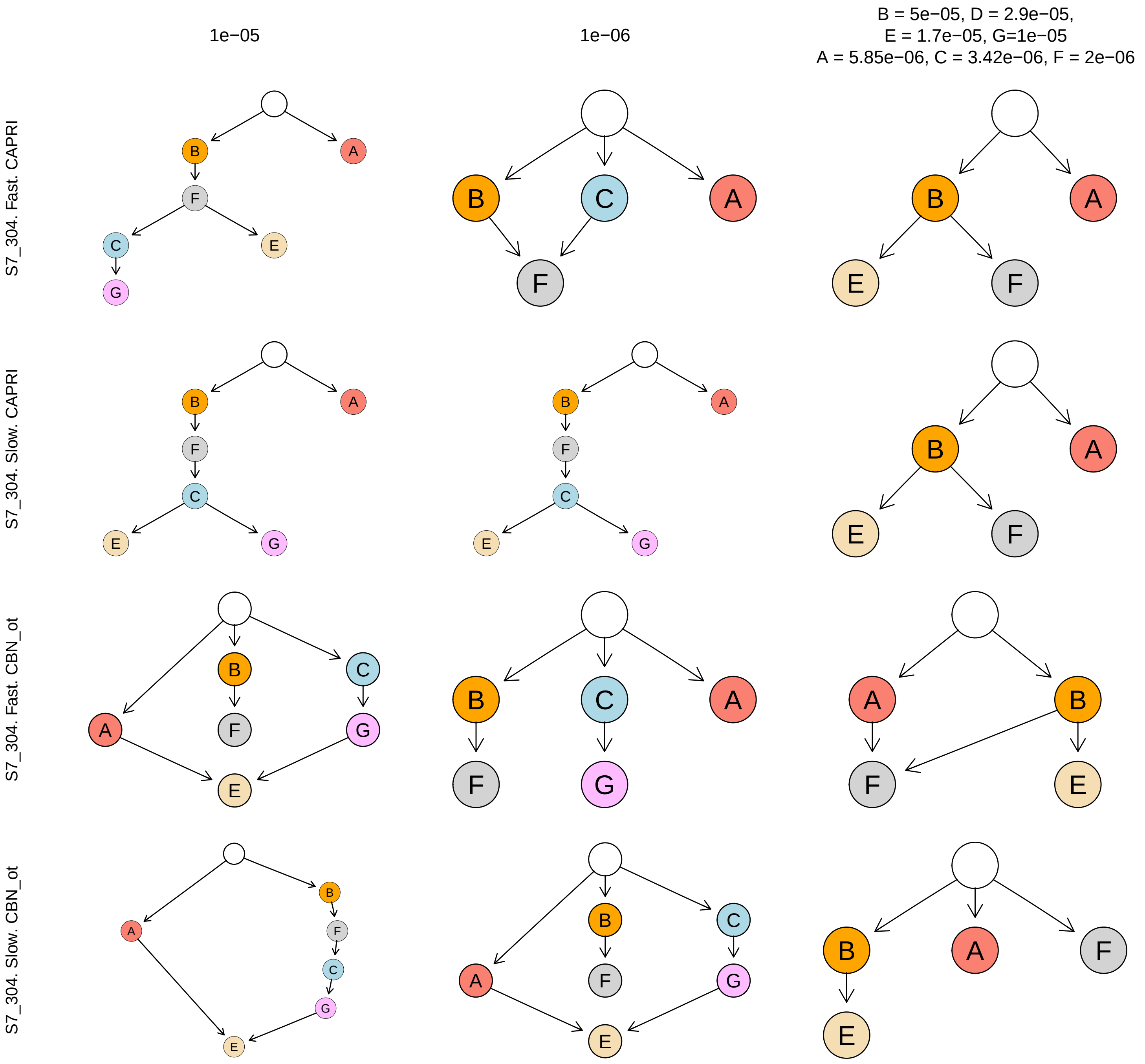

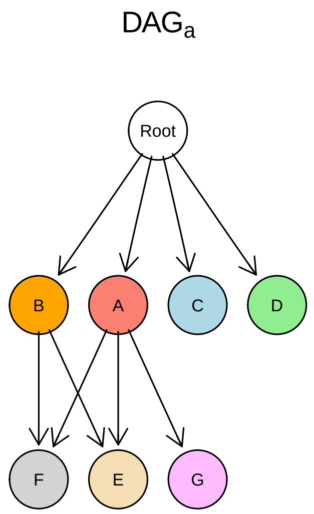

ID	Accessible Genot.	Discrep. DAG_a
S7_305	37	24

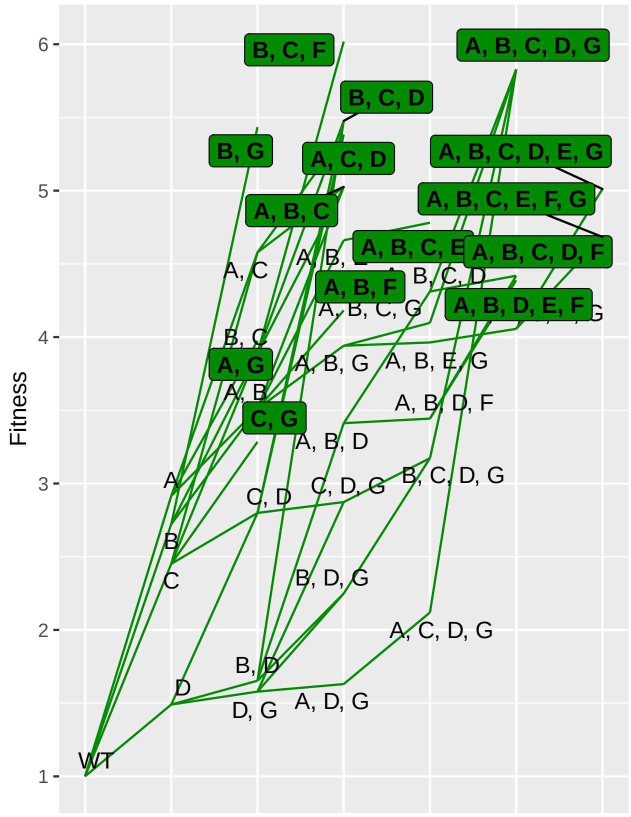

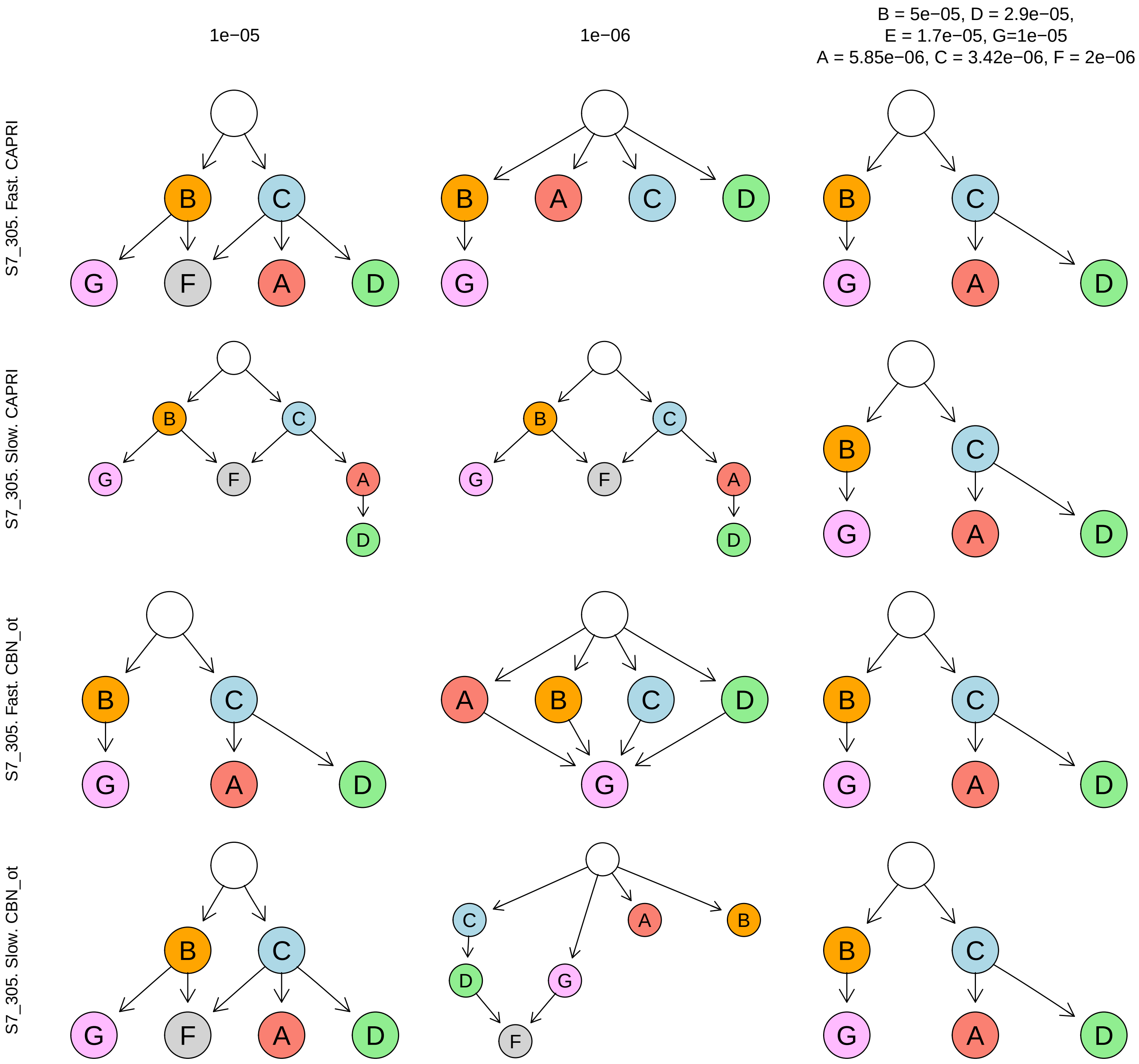

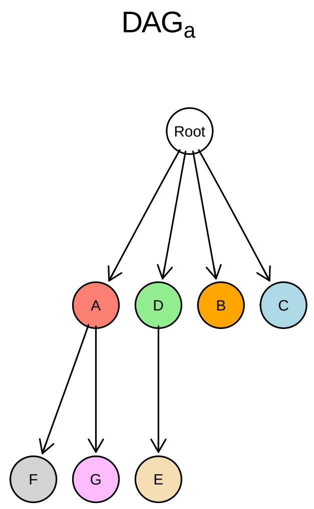

ID	Accessible Genot.	Discrep. DAG_a
S7_306	13	48

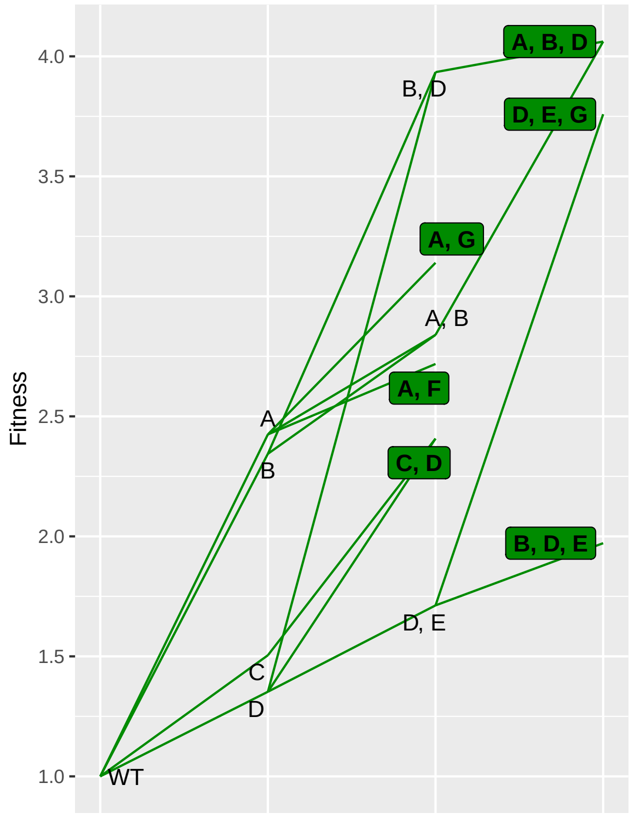

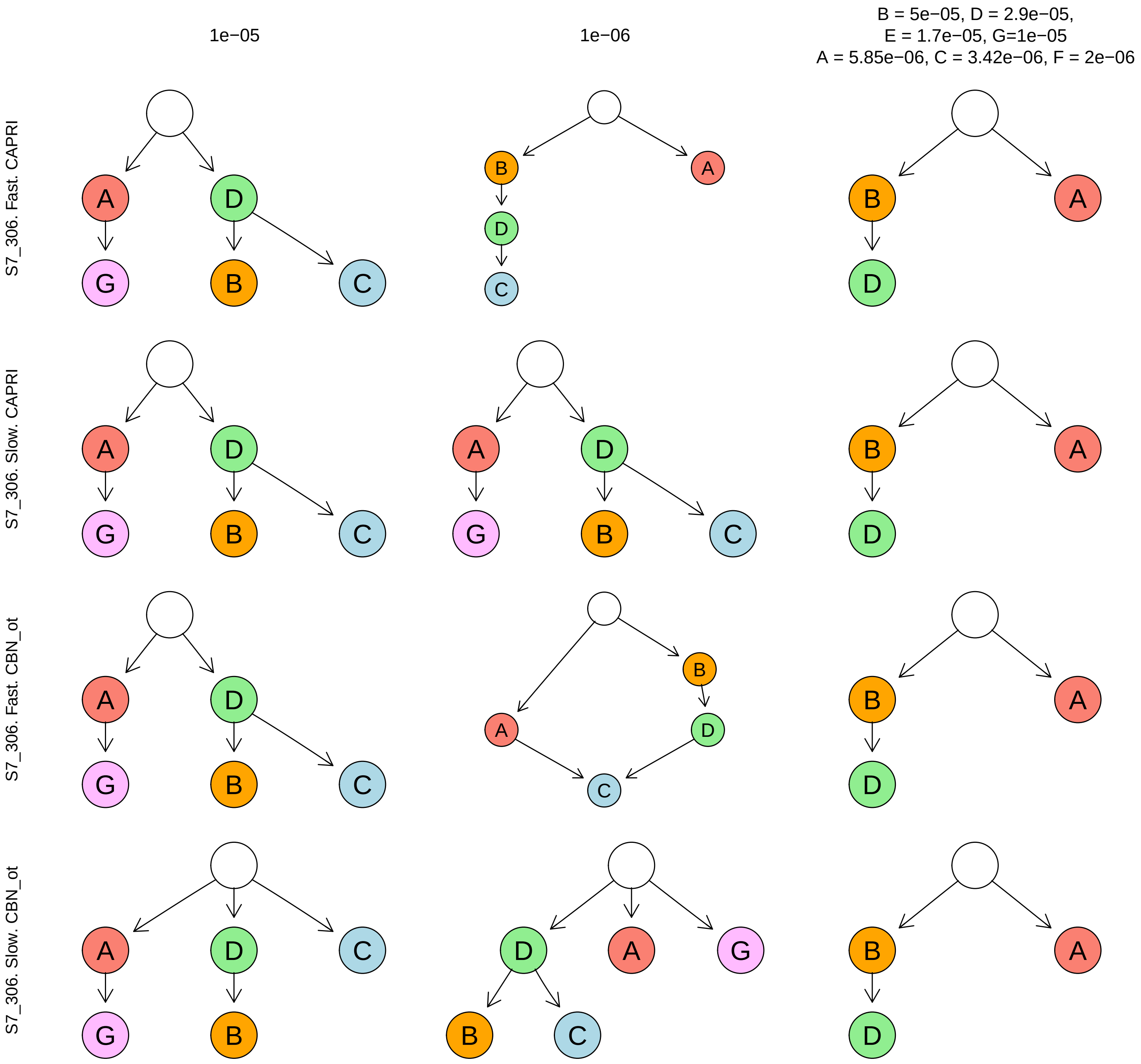

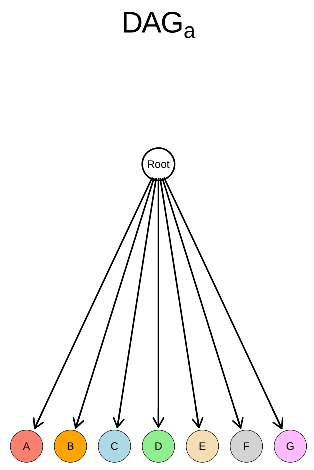

ID	Accessible Genot.	Discrep. DAG_a
S7_307	24	103

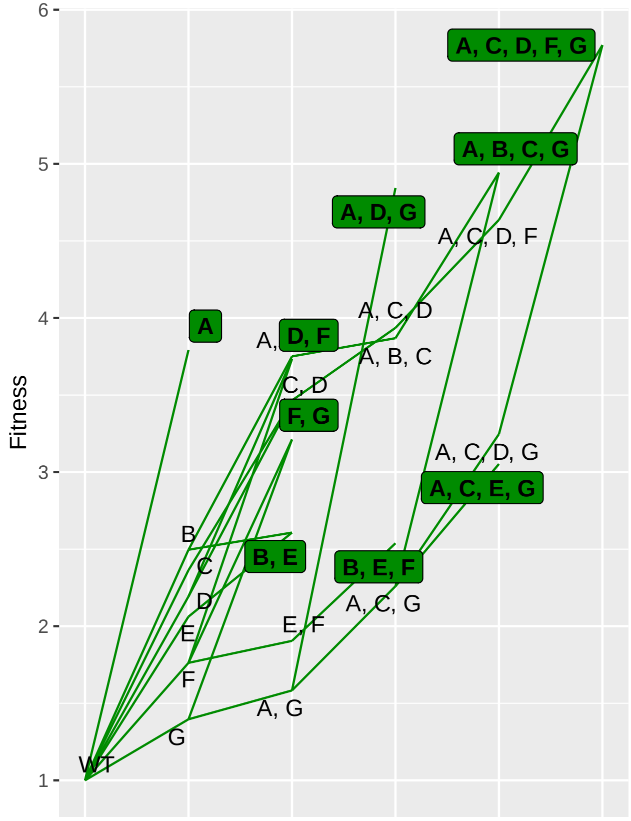

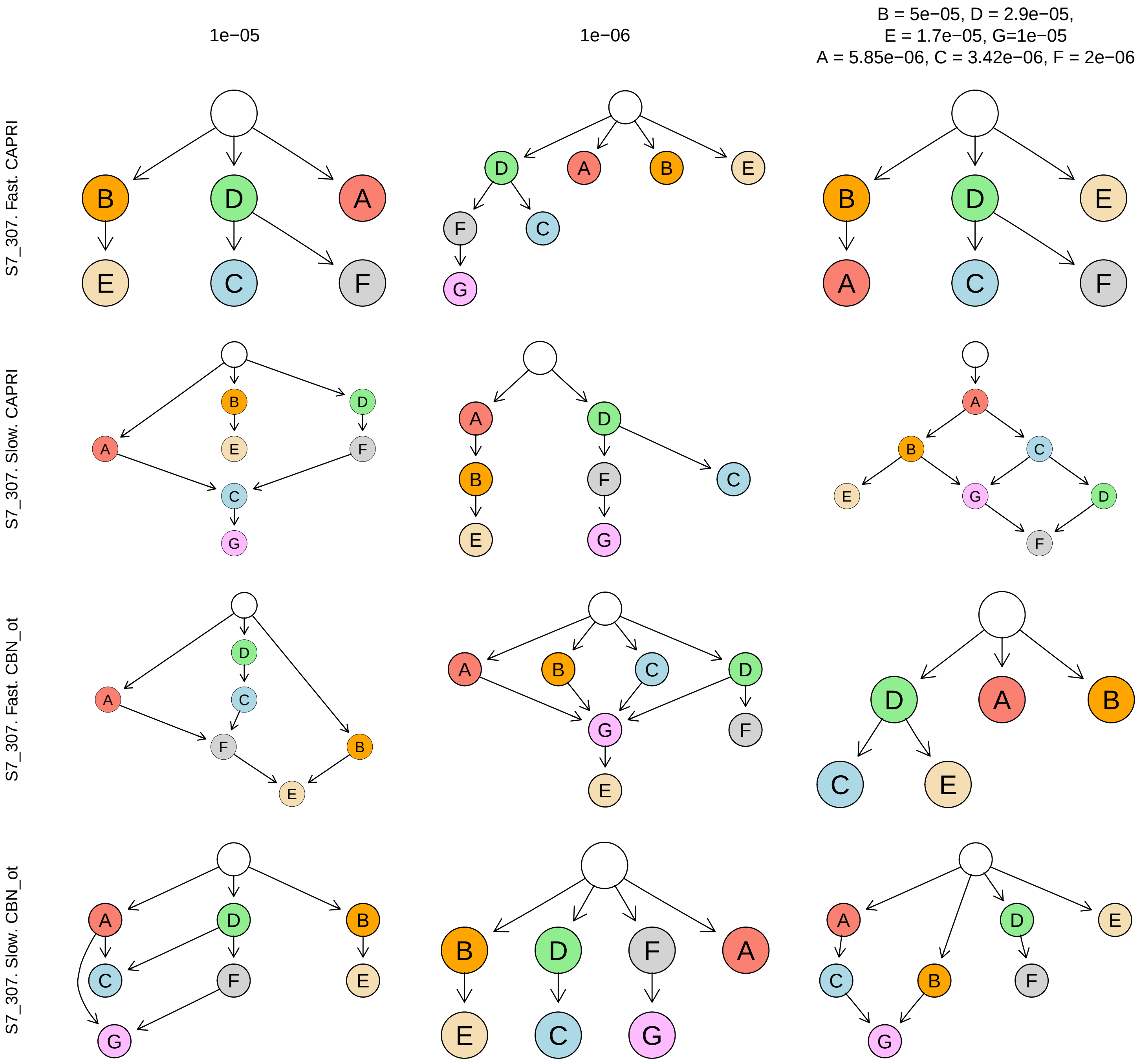

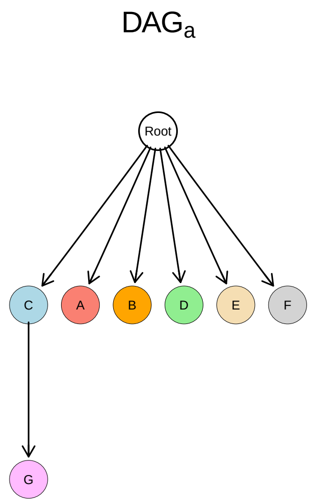

ID	Accessible Genot.	Discrep. DAG_a
S7_308	33	66

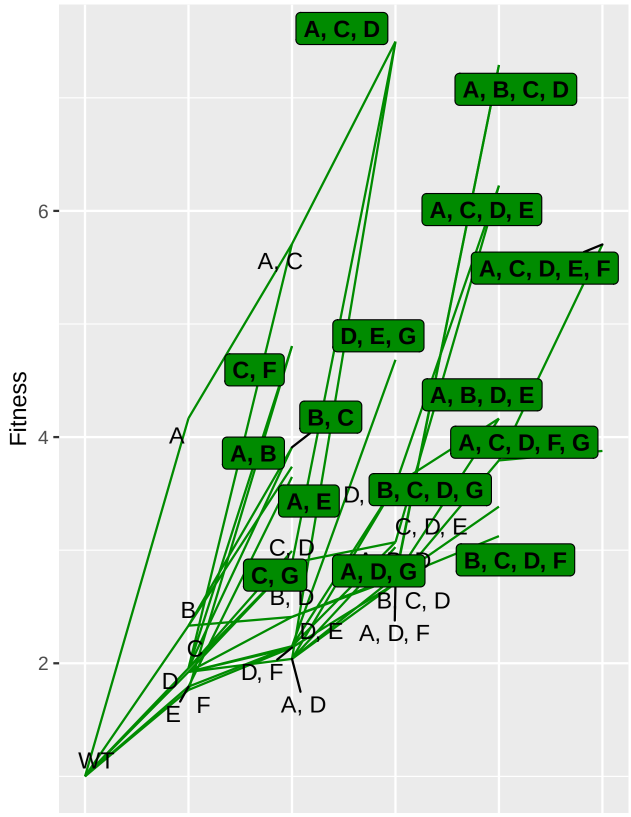

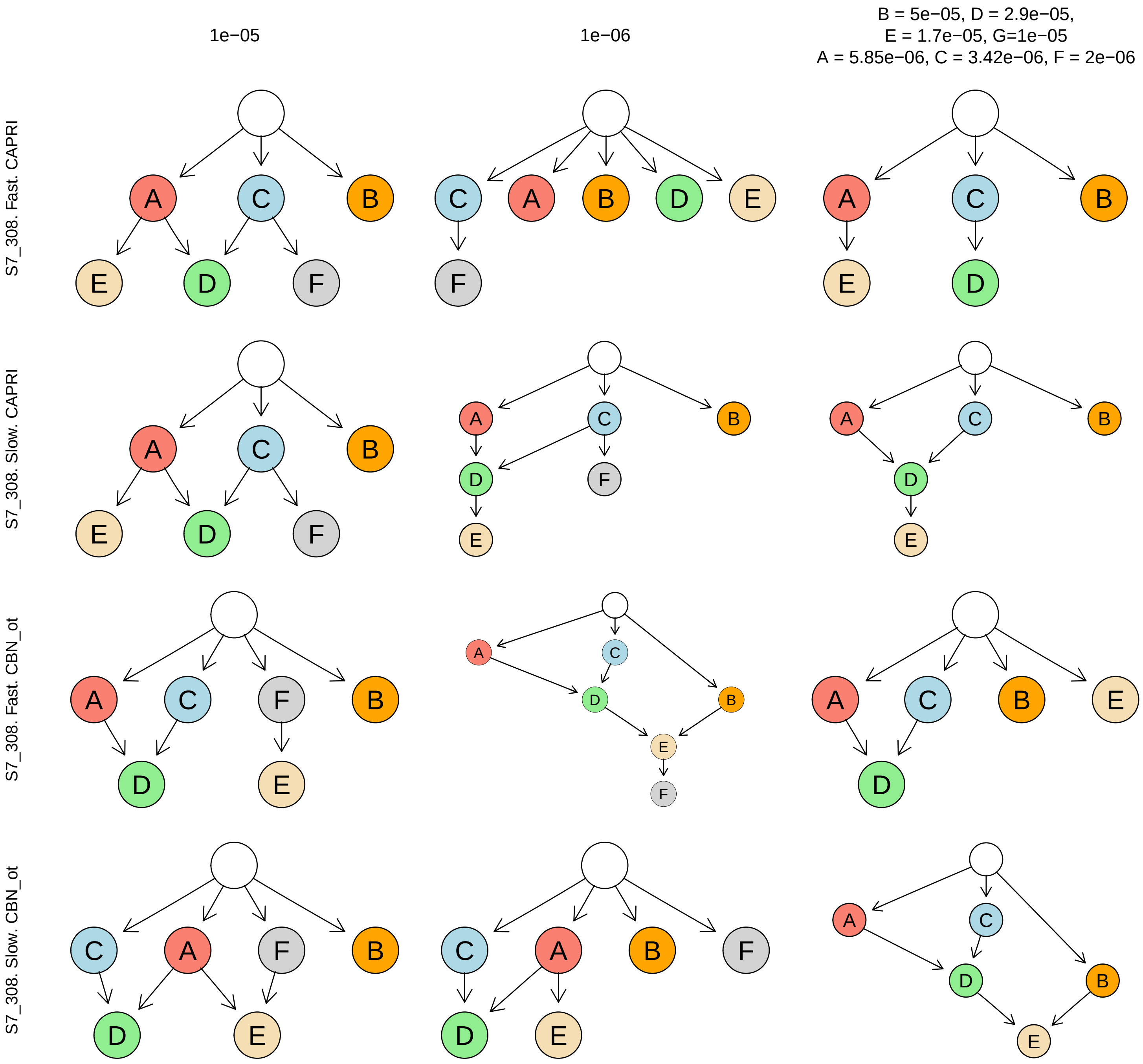

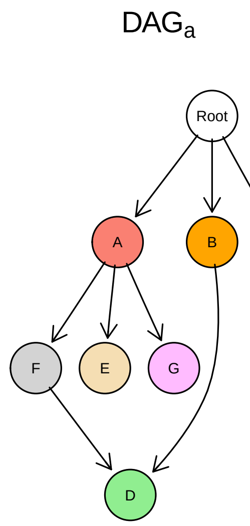

ID	Accessible Genot.	Discrep. DAG_a
S7_309	11	32

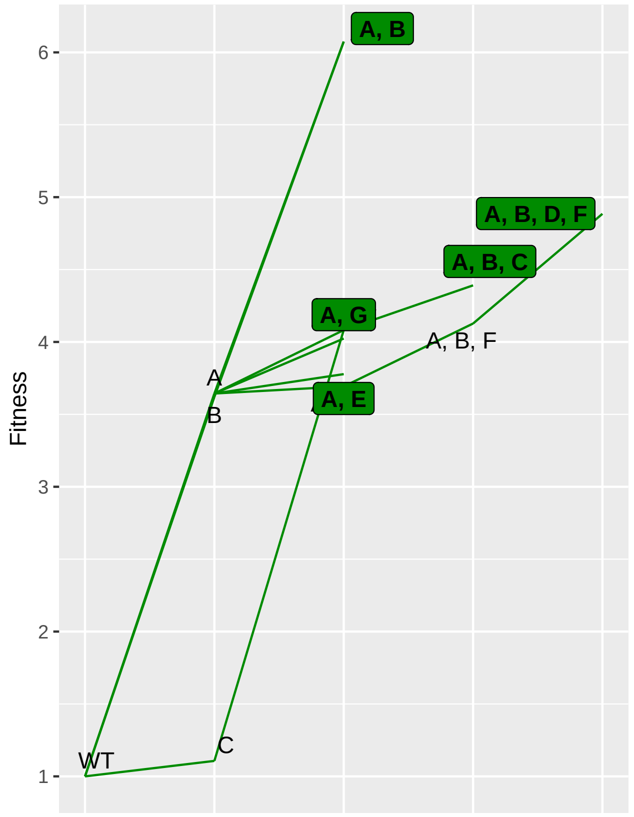

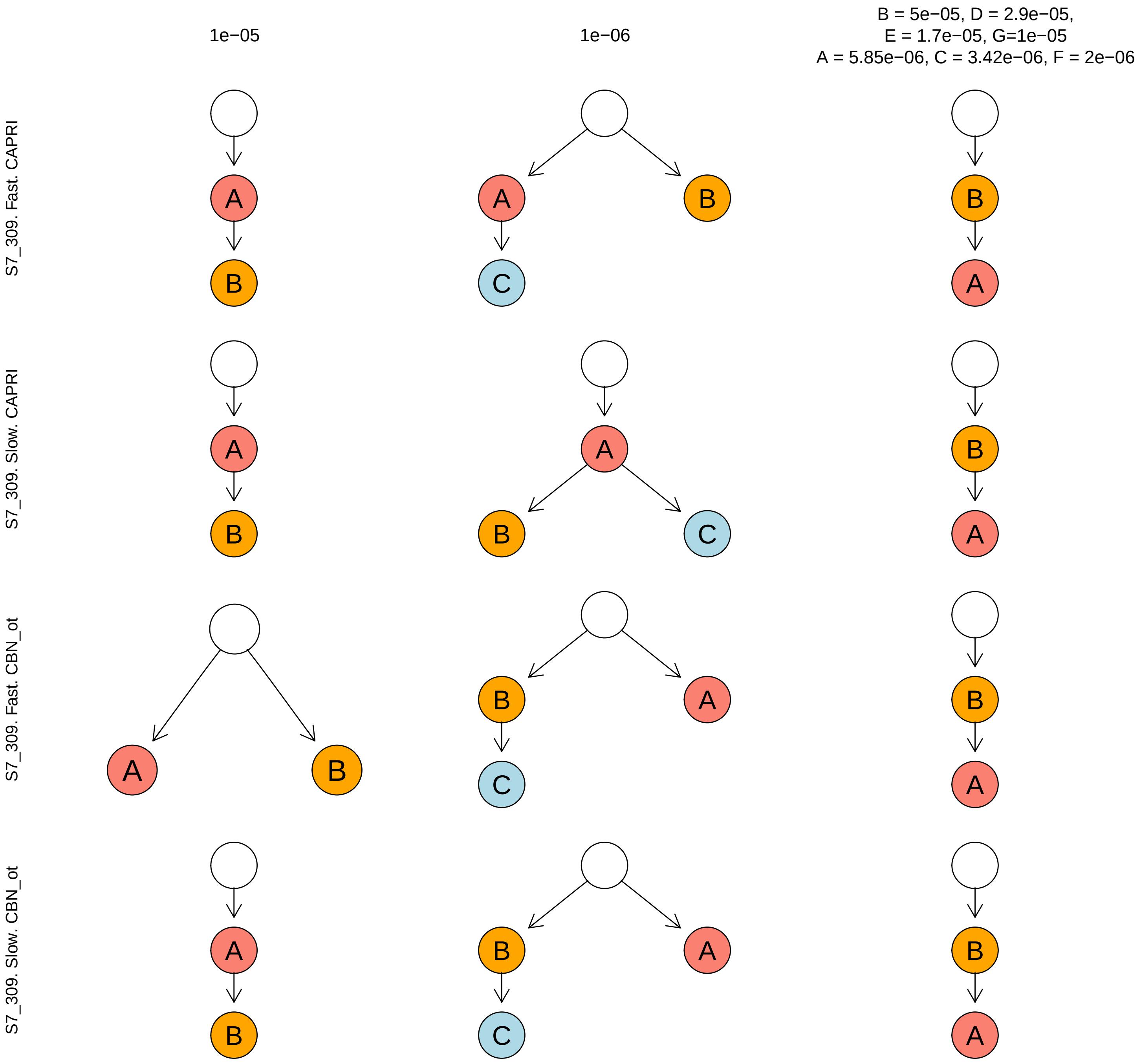

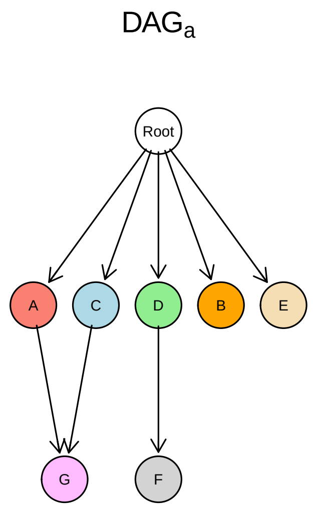

ID	Accessible Genot.	Discrep. DAG_a
S7_310	23	48

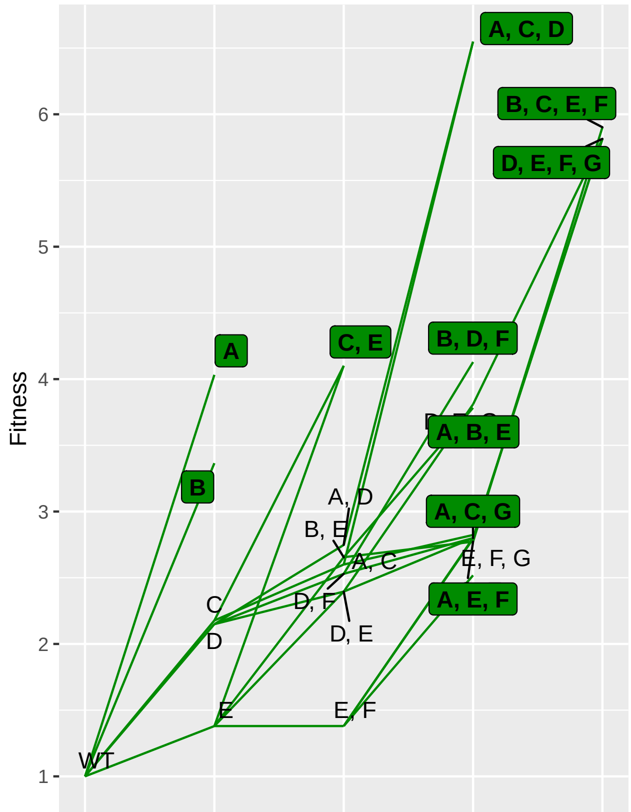

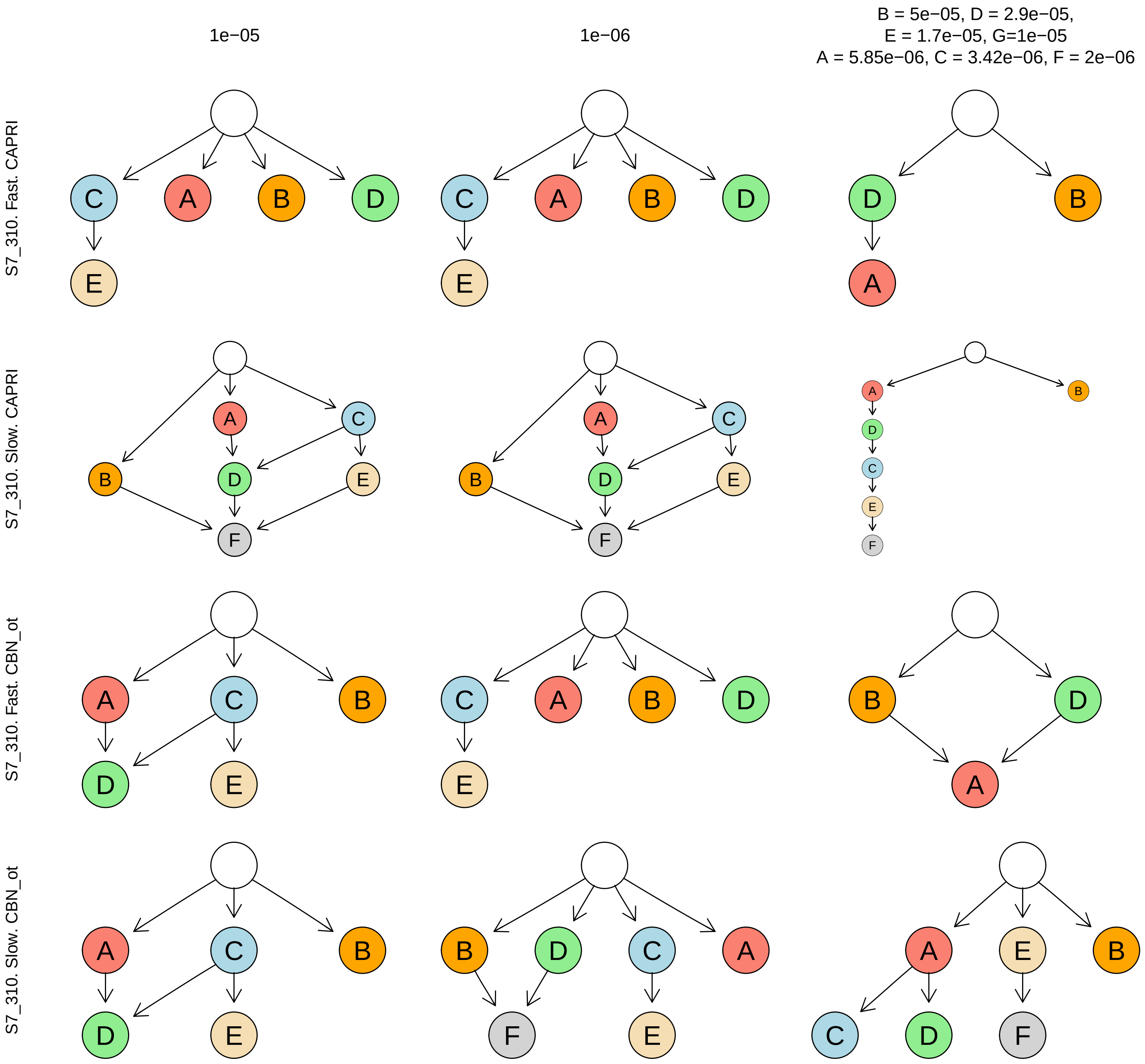

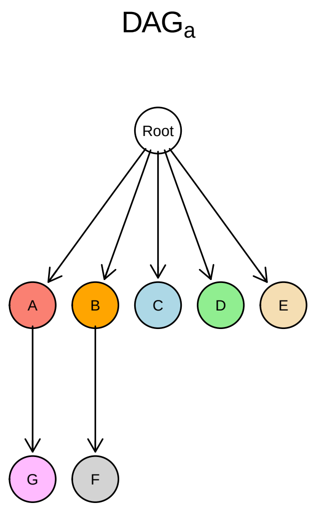

ID	Accessible Genot.	Discrep. DAG_a
S7_311	74	53

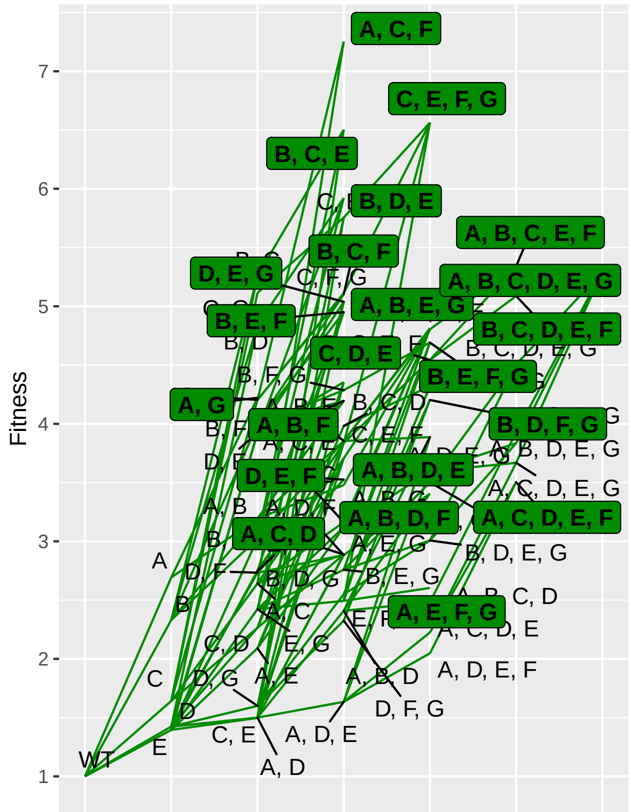

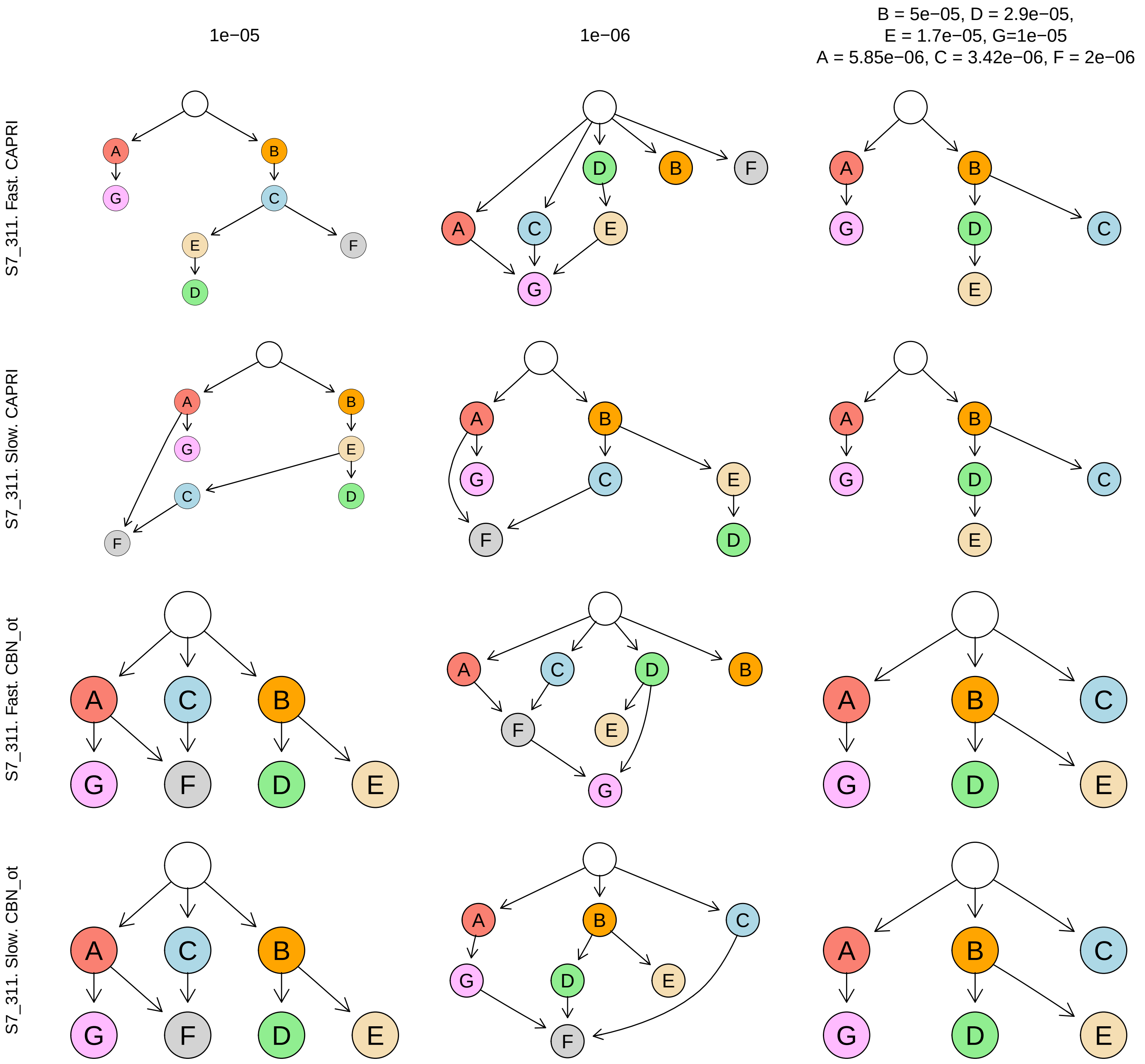

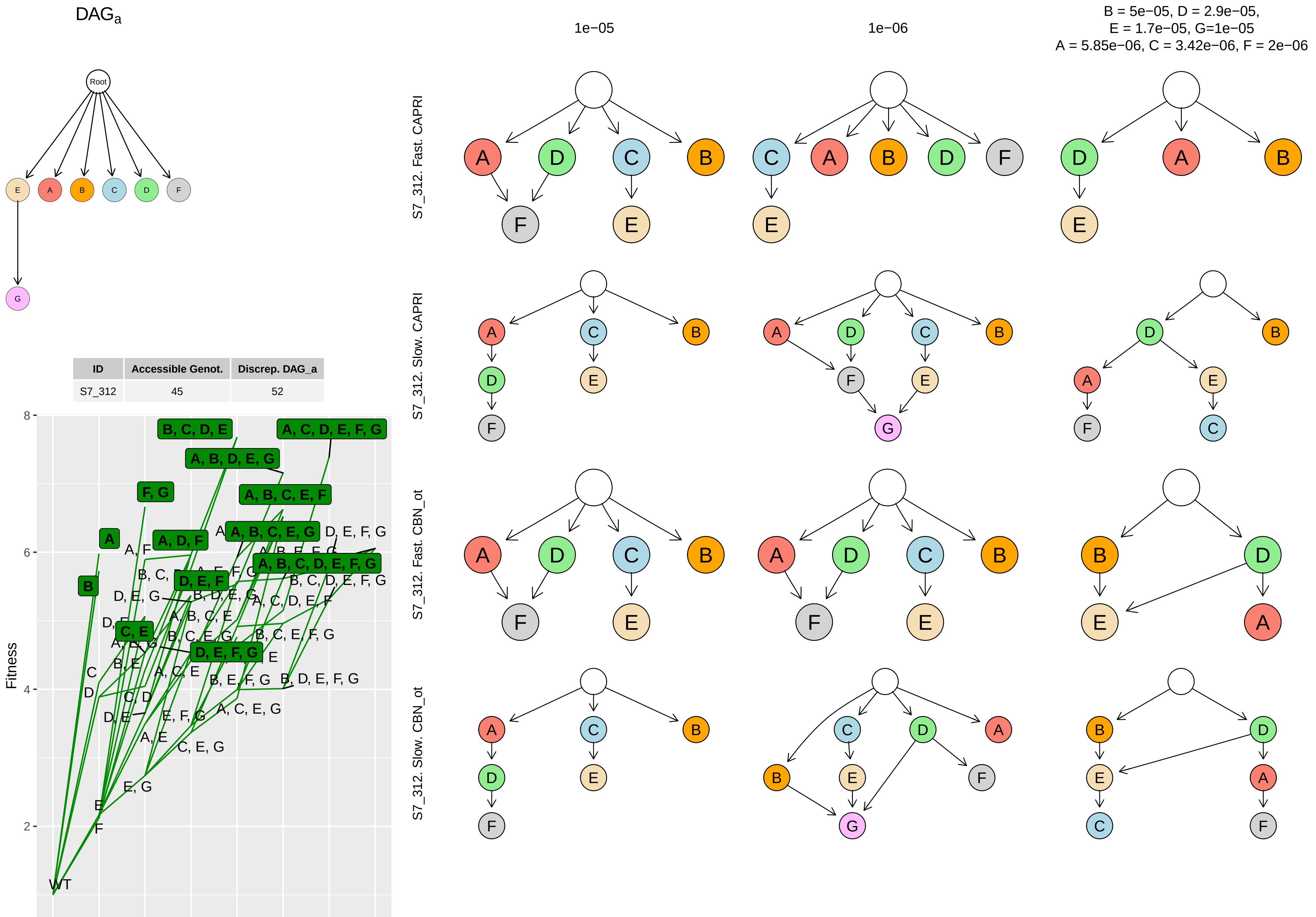

ID	Accessible Genot.	Discrep. DAG_a
S7_312	45	52

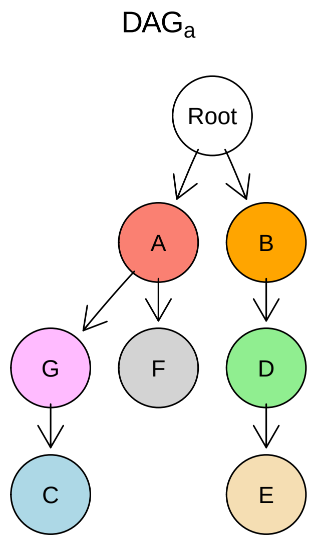

ID	Accessible Genot.	Discrep. DAG_a
S7_313	10	21

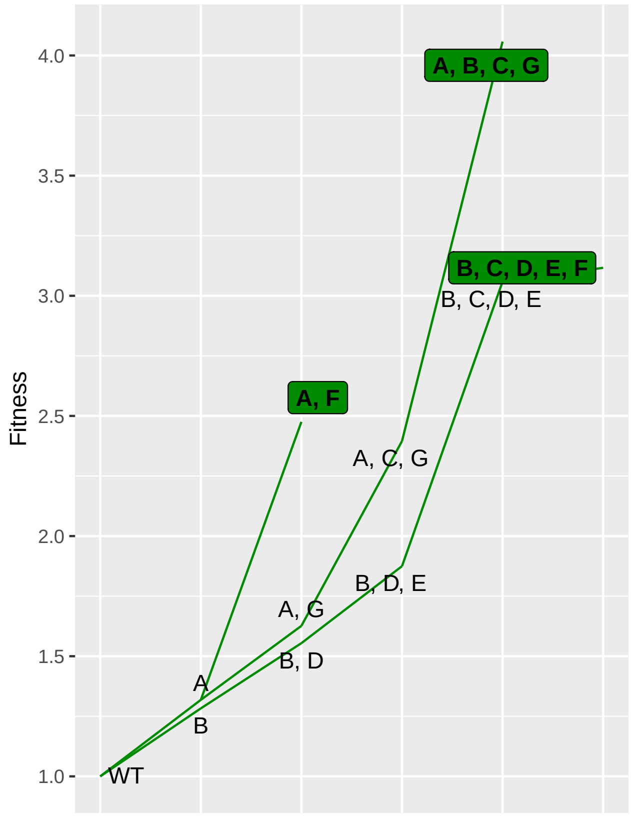

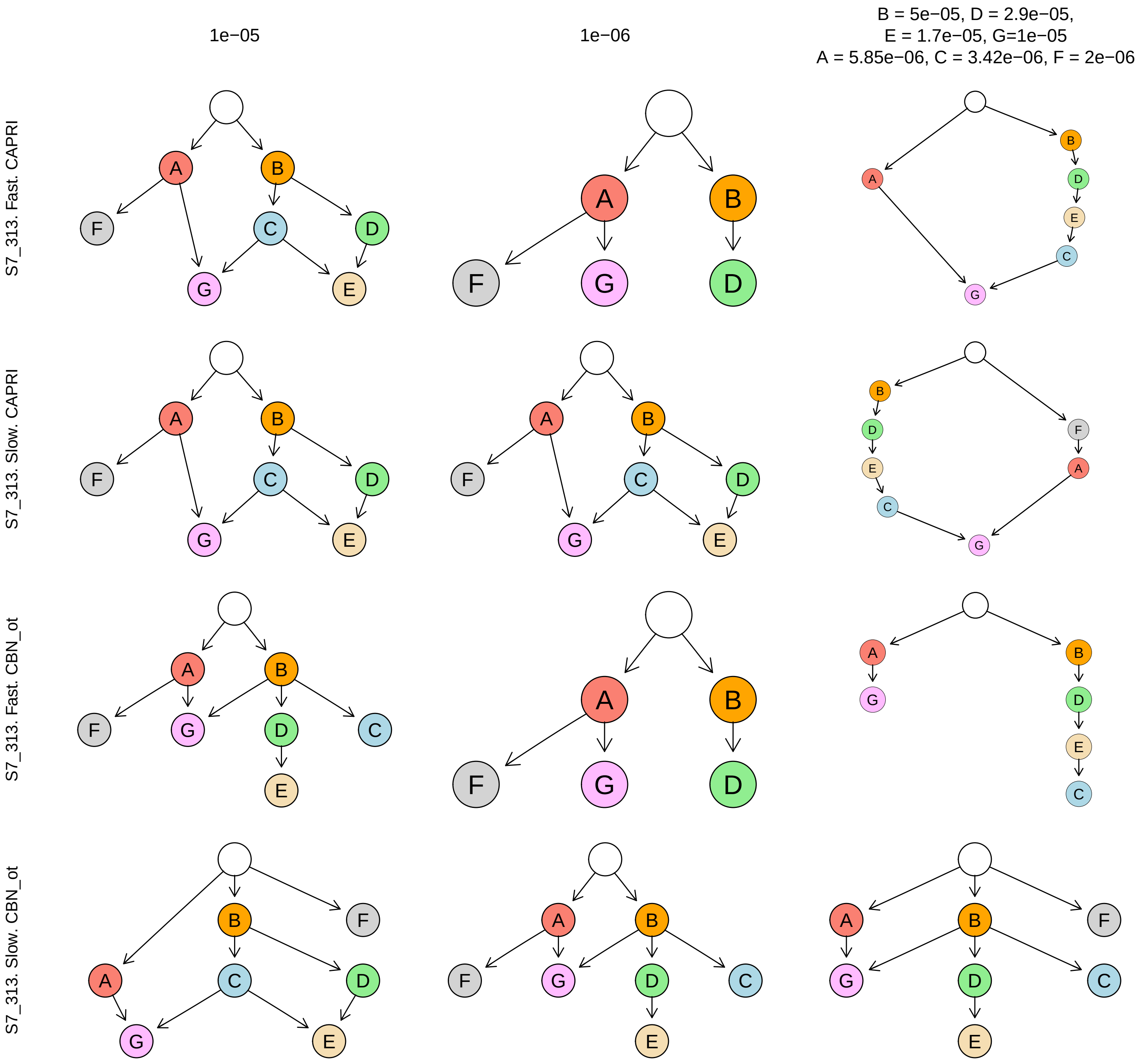

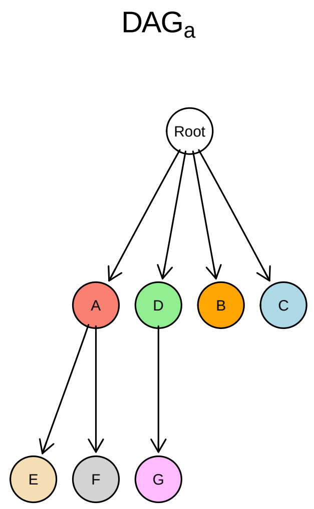

ID	Accessible Genot.	Discrep. DAG_a
S7_314	35	40

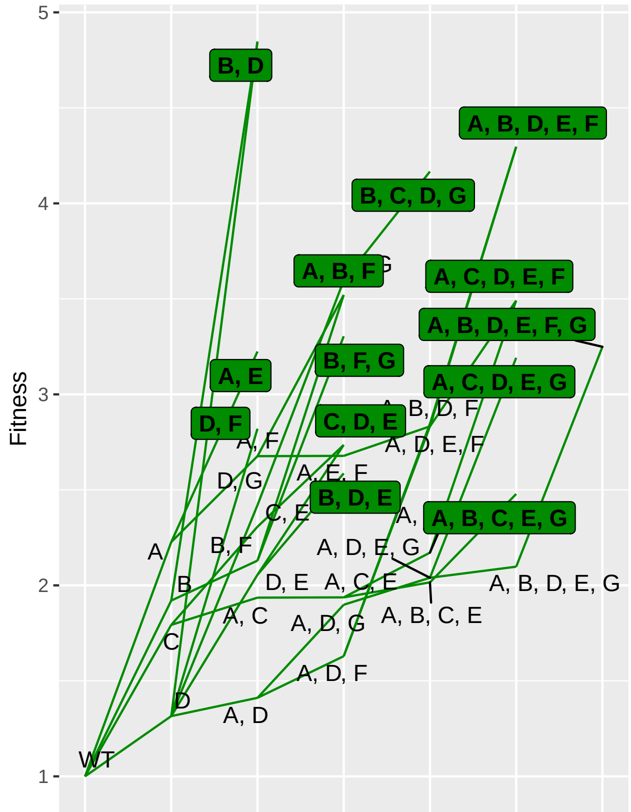

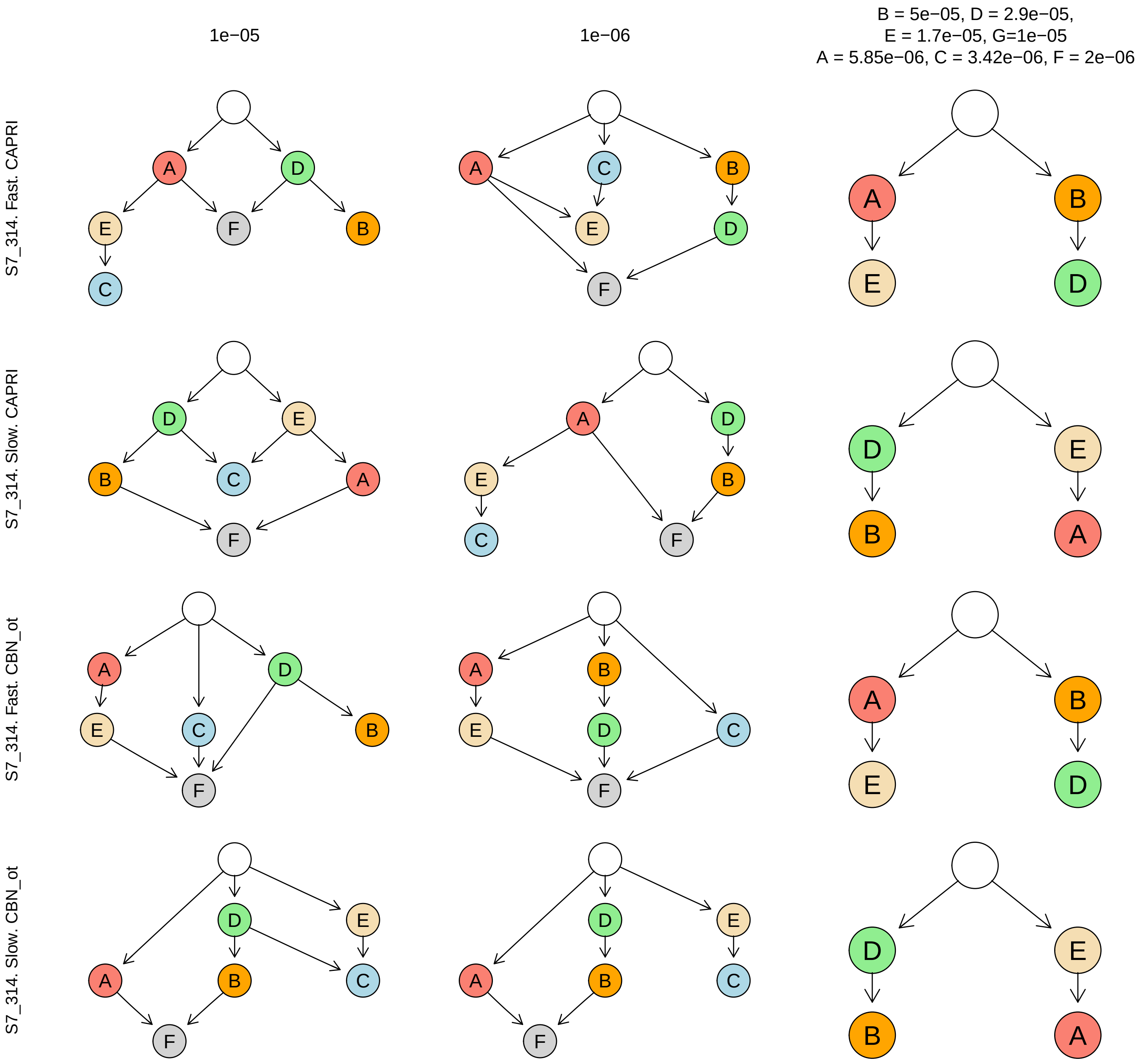

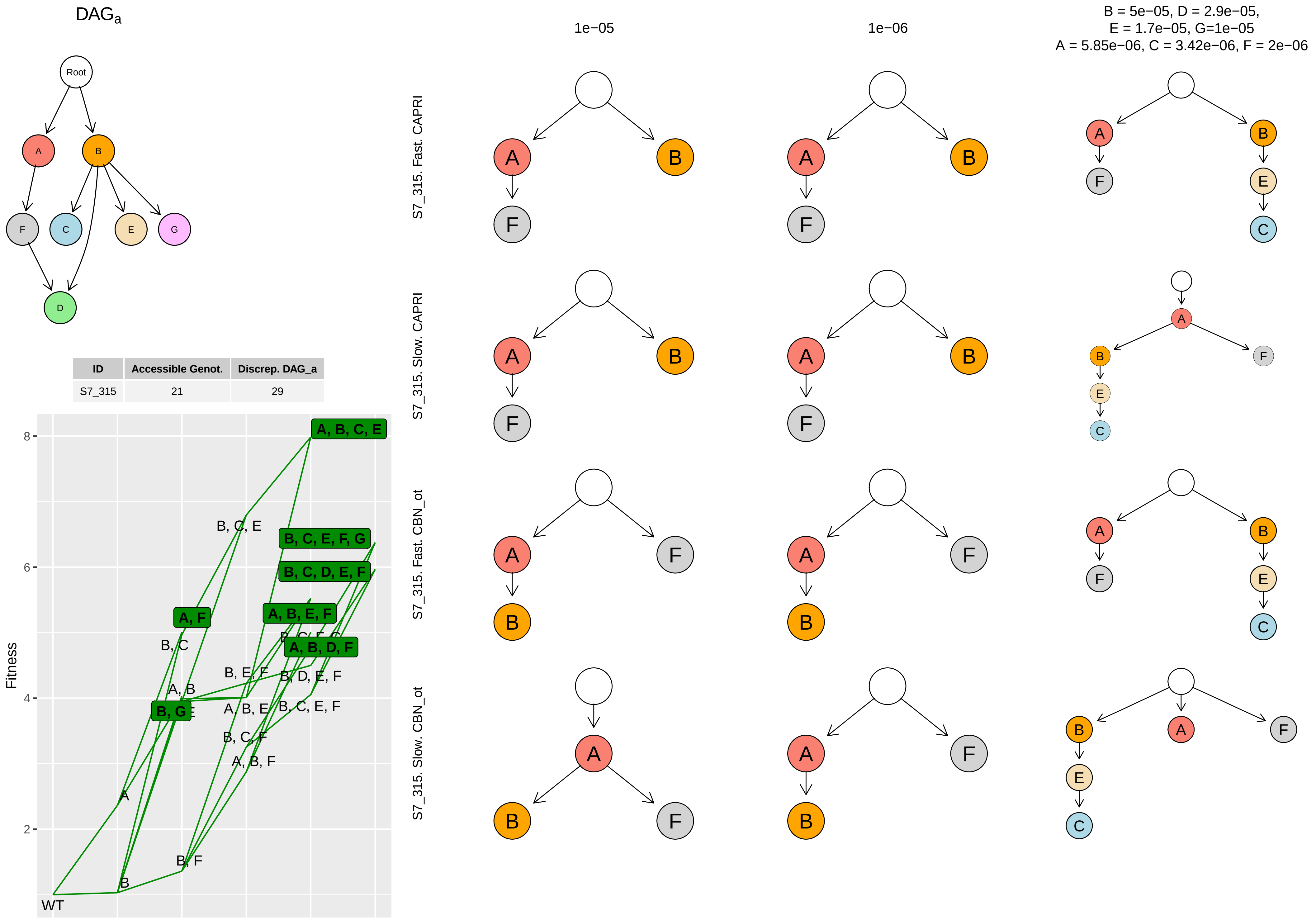

ID	Accessible Genot.	Discrep. DAG_a
S7_315	21	29

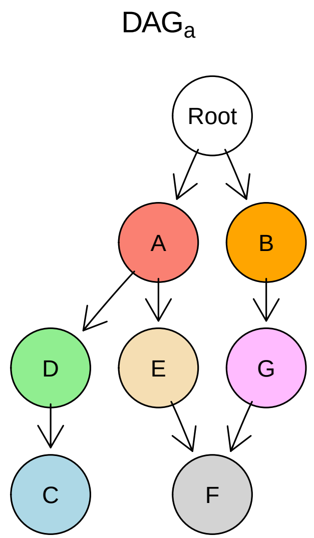

ID	Accessible Genot.	Discrep. DAG_a
S7_316	9	20

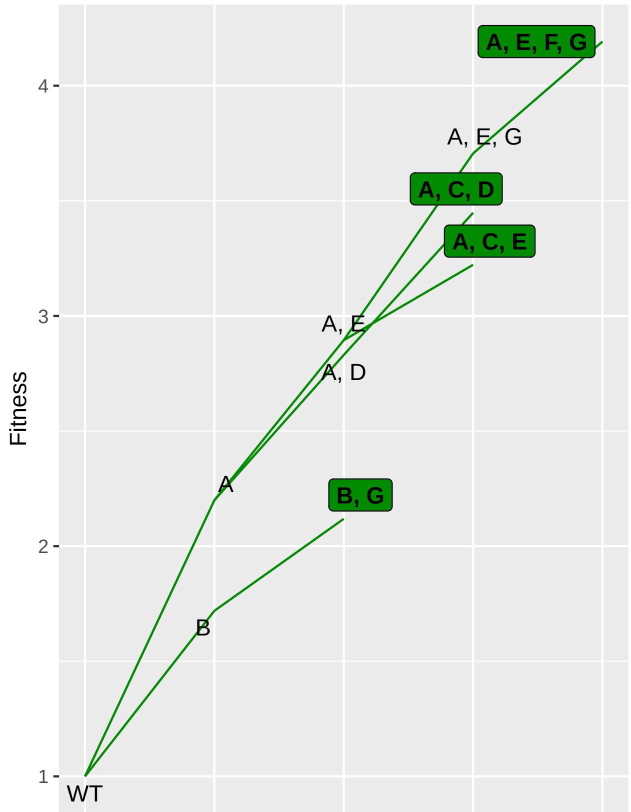

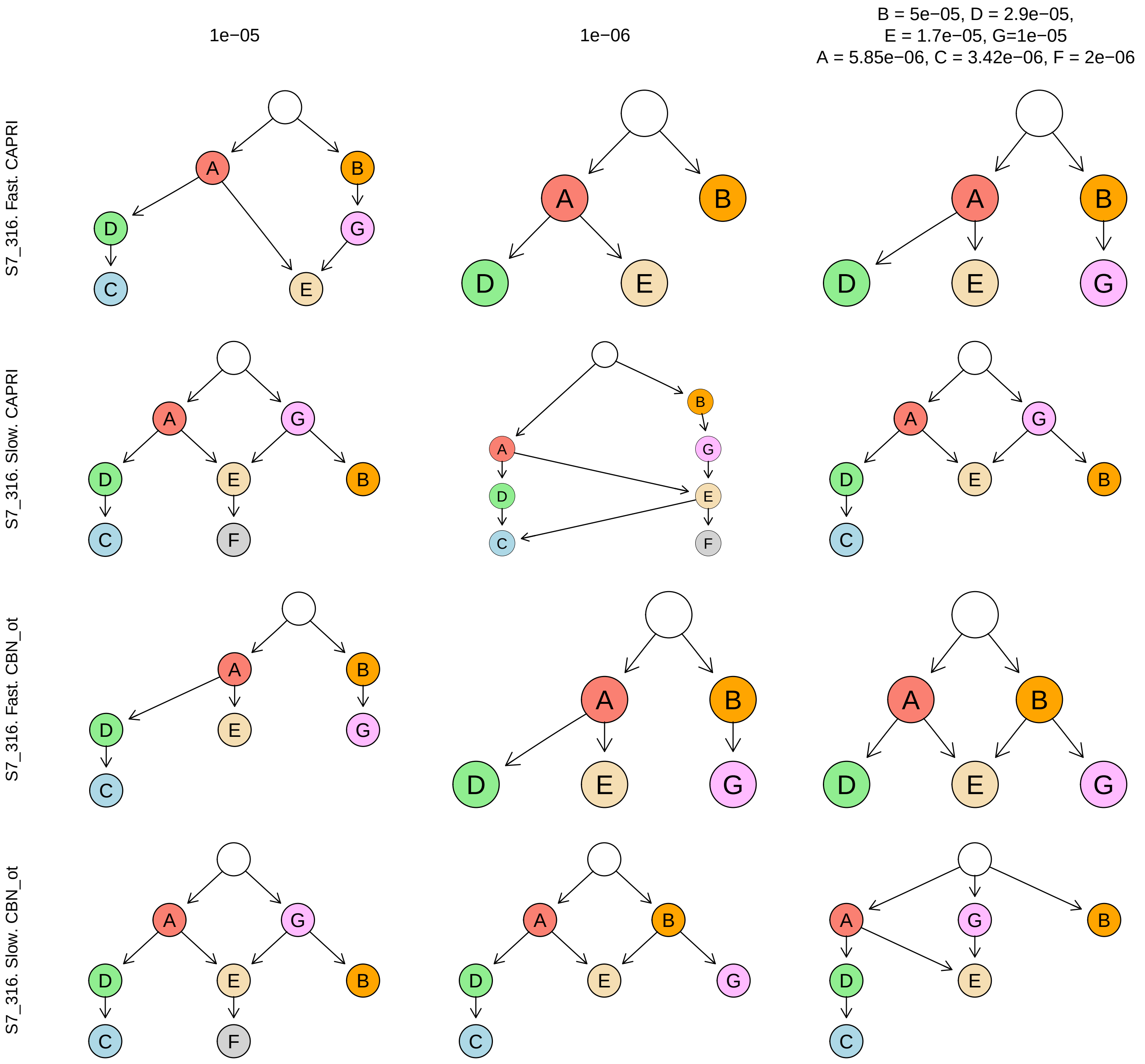

DAG$_a$

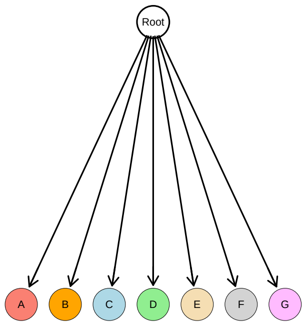

ID	Accessible Genot.	Discrep. DAG_a
S7_317	12	115

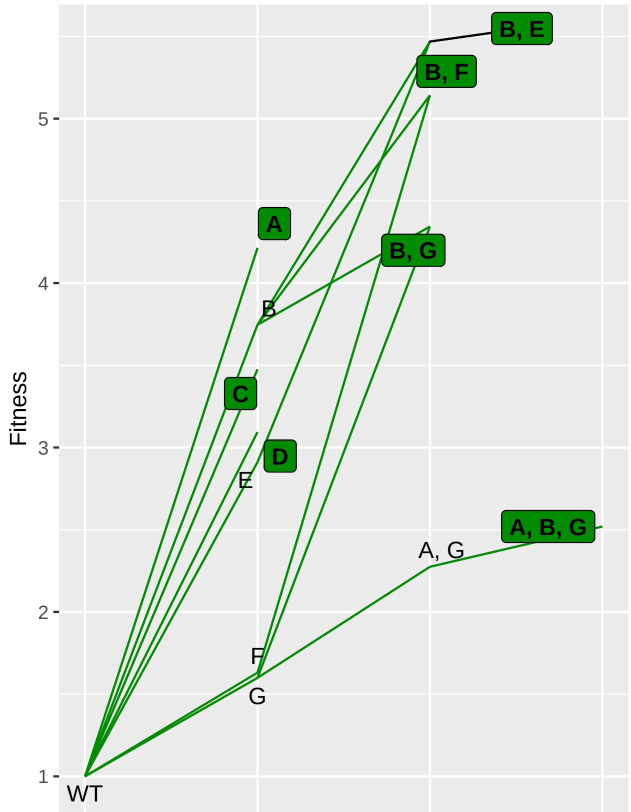

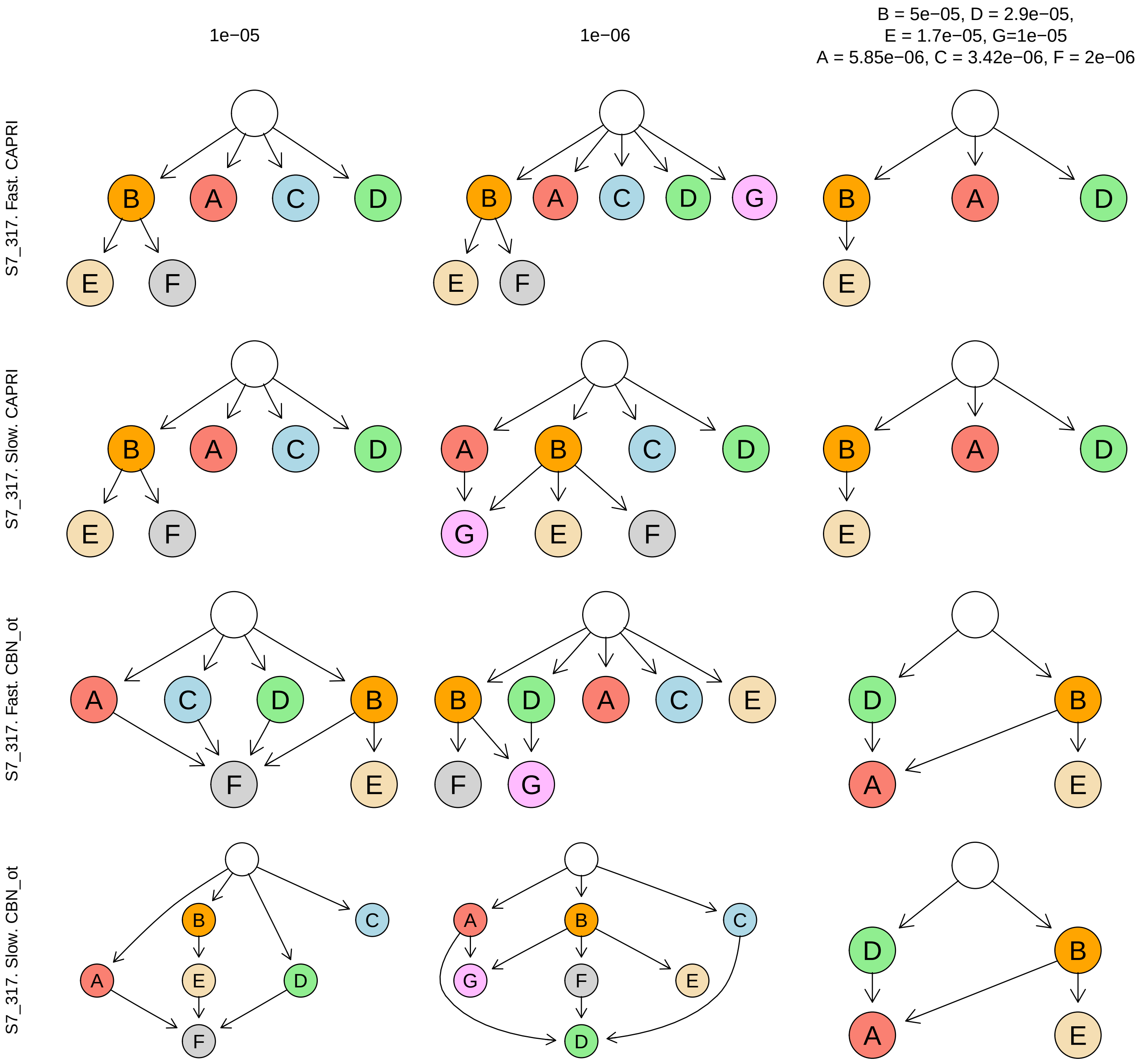

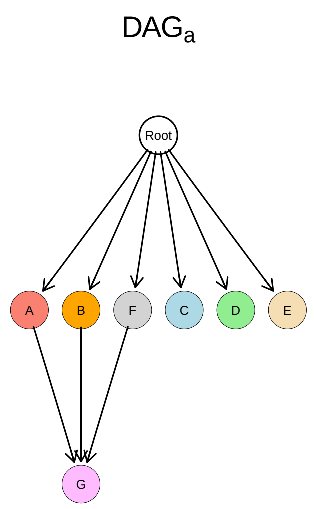

ID	Accessible Genot.	Discrep. DAG_a
S7_318	26	45

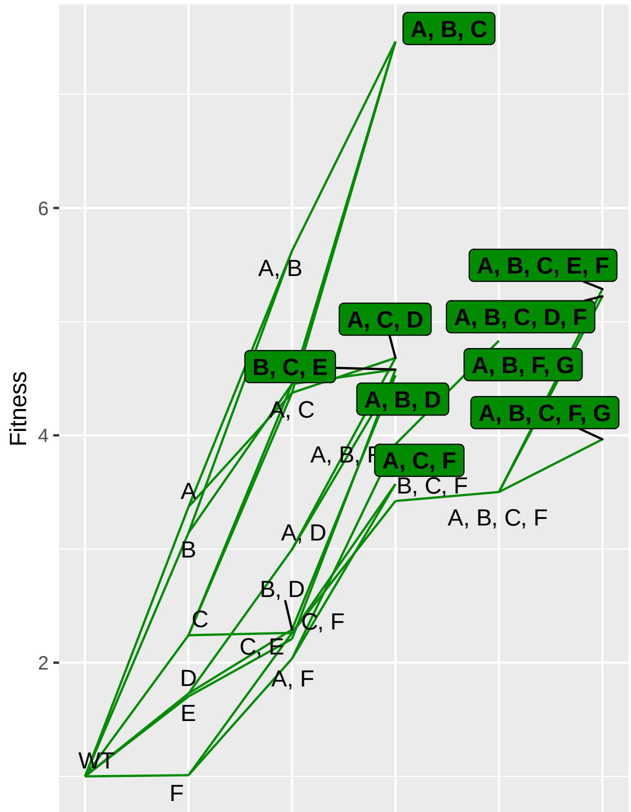

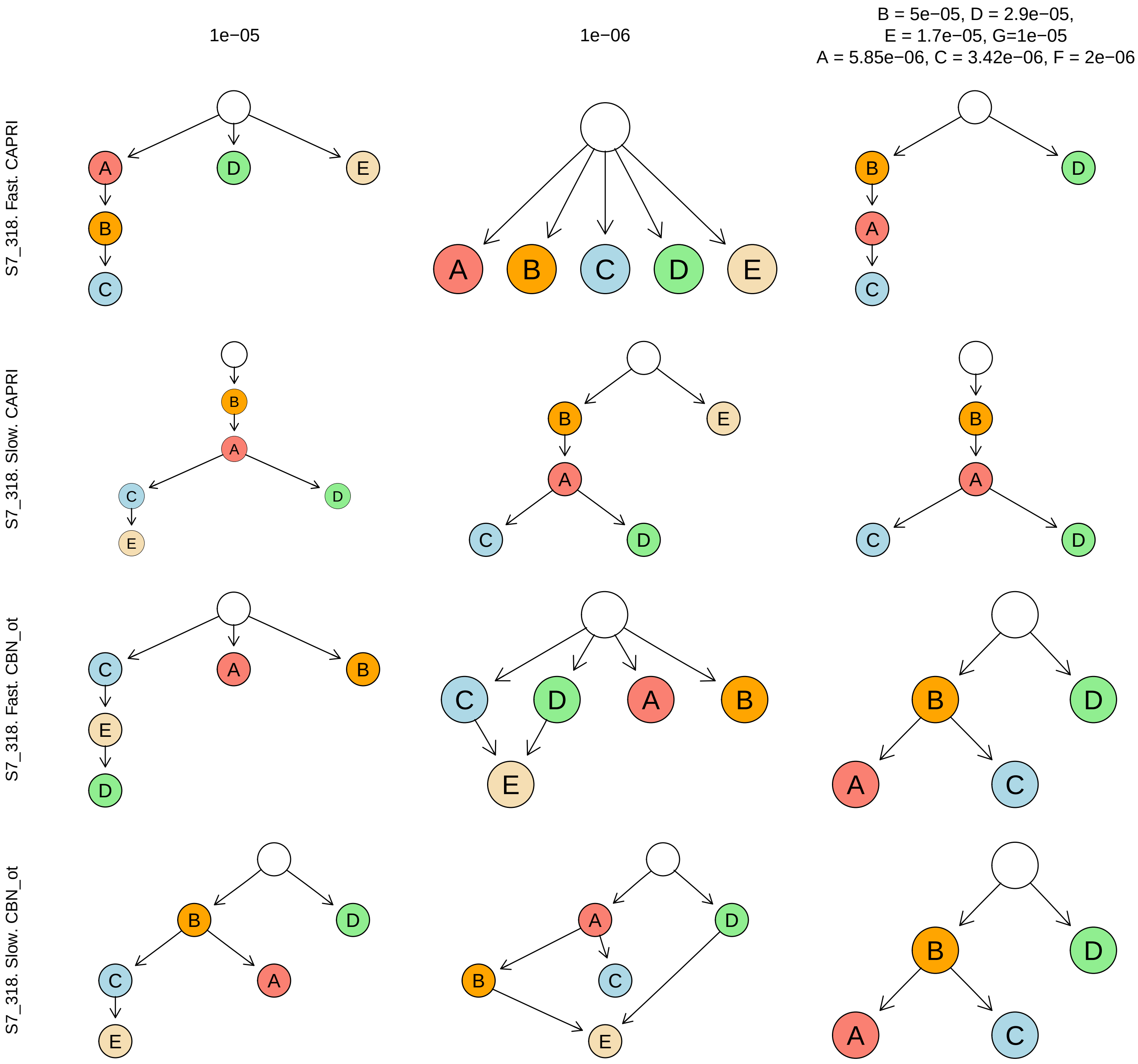

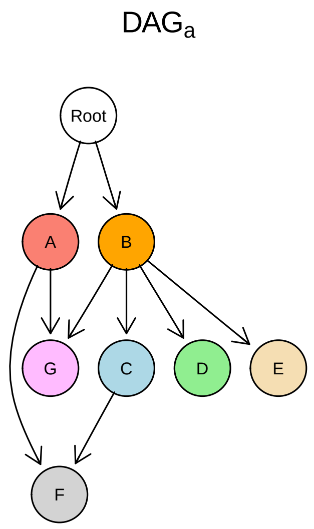

ID	Accessible Genot.	Discrep. DAG_a
S7_319	19	18

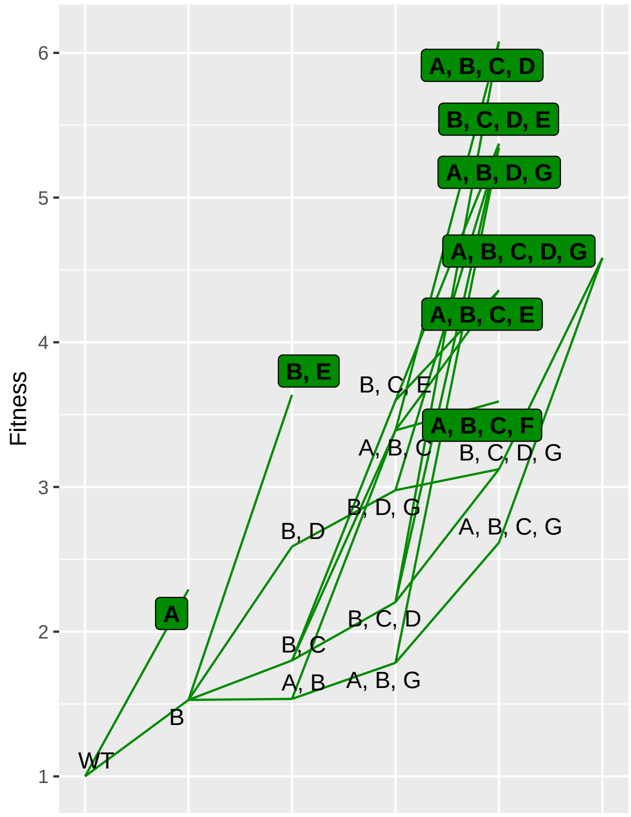

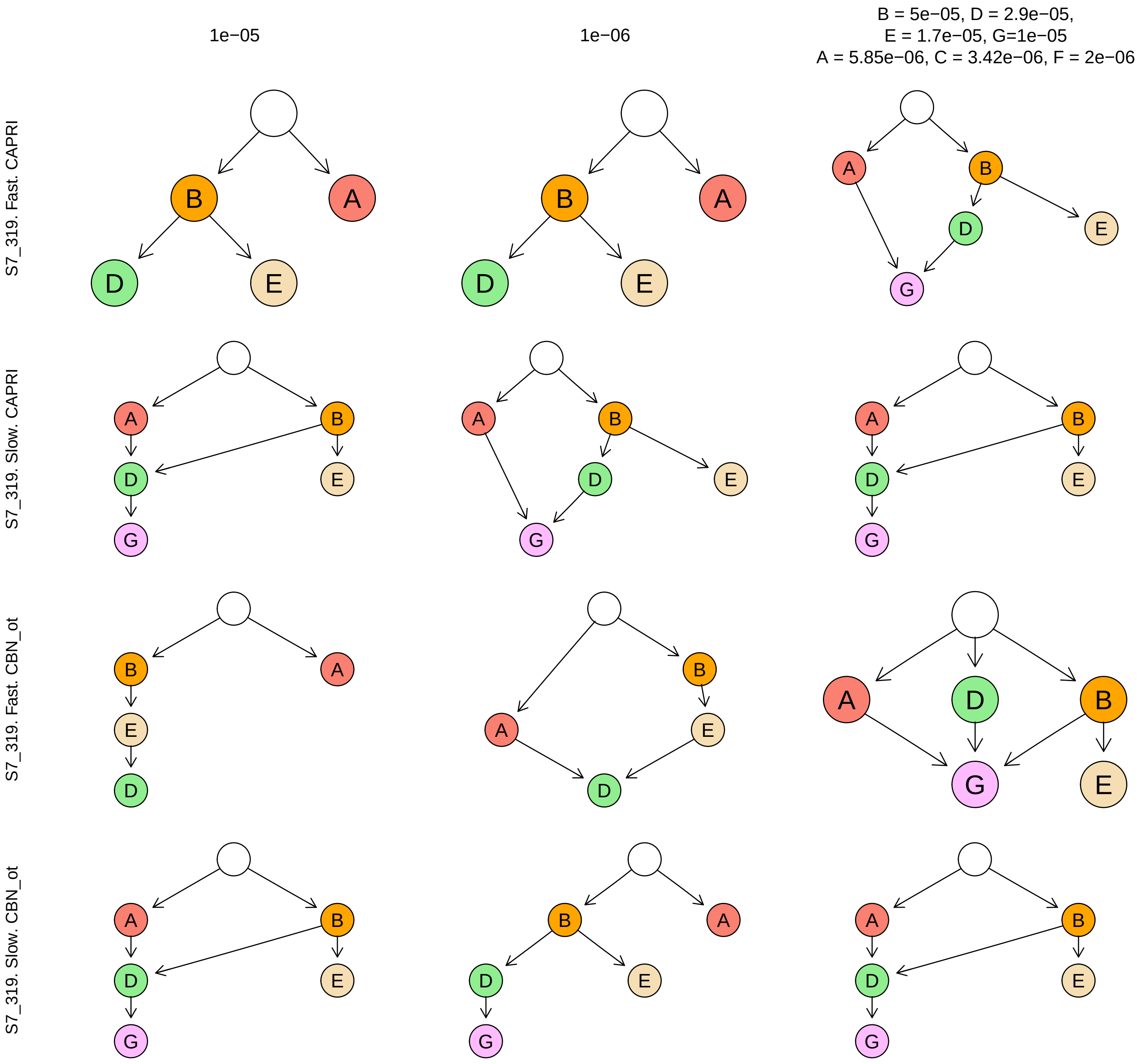

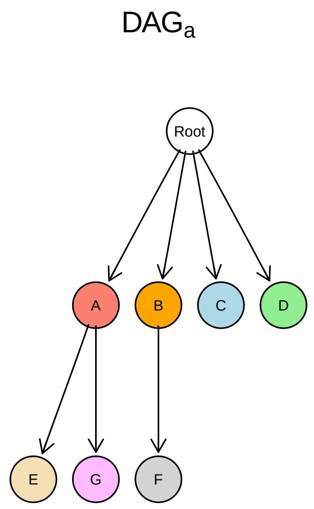

ID	Accessible Genot.	Discrep. DAG_a
S7_320	14	57

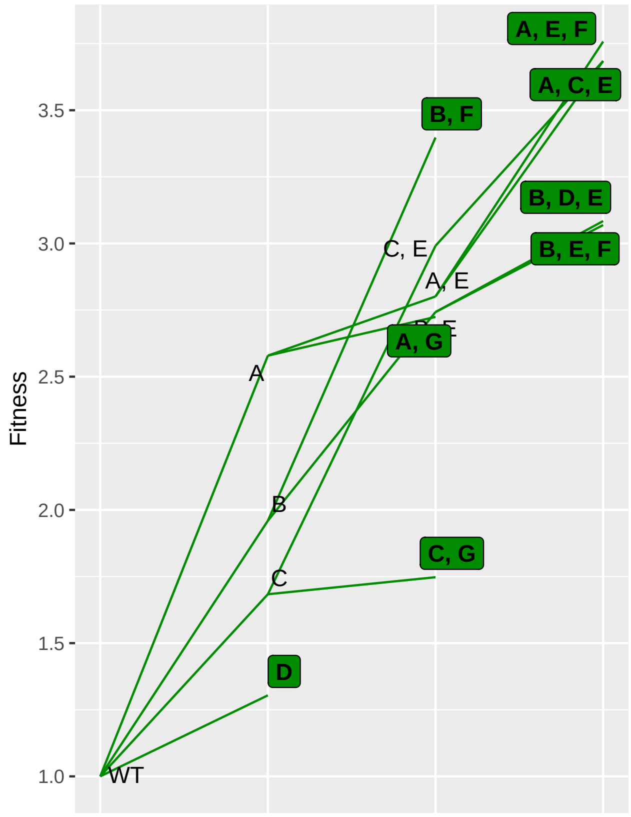

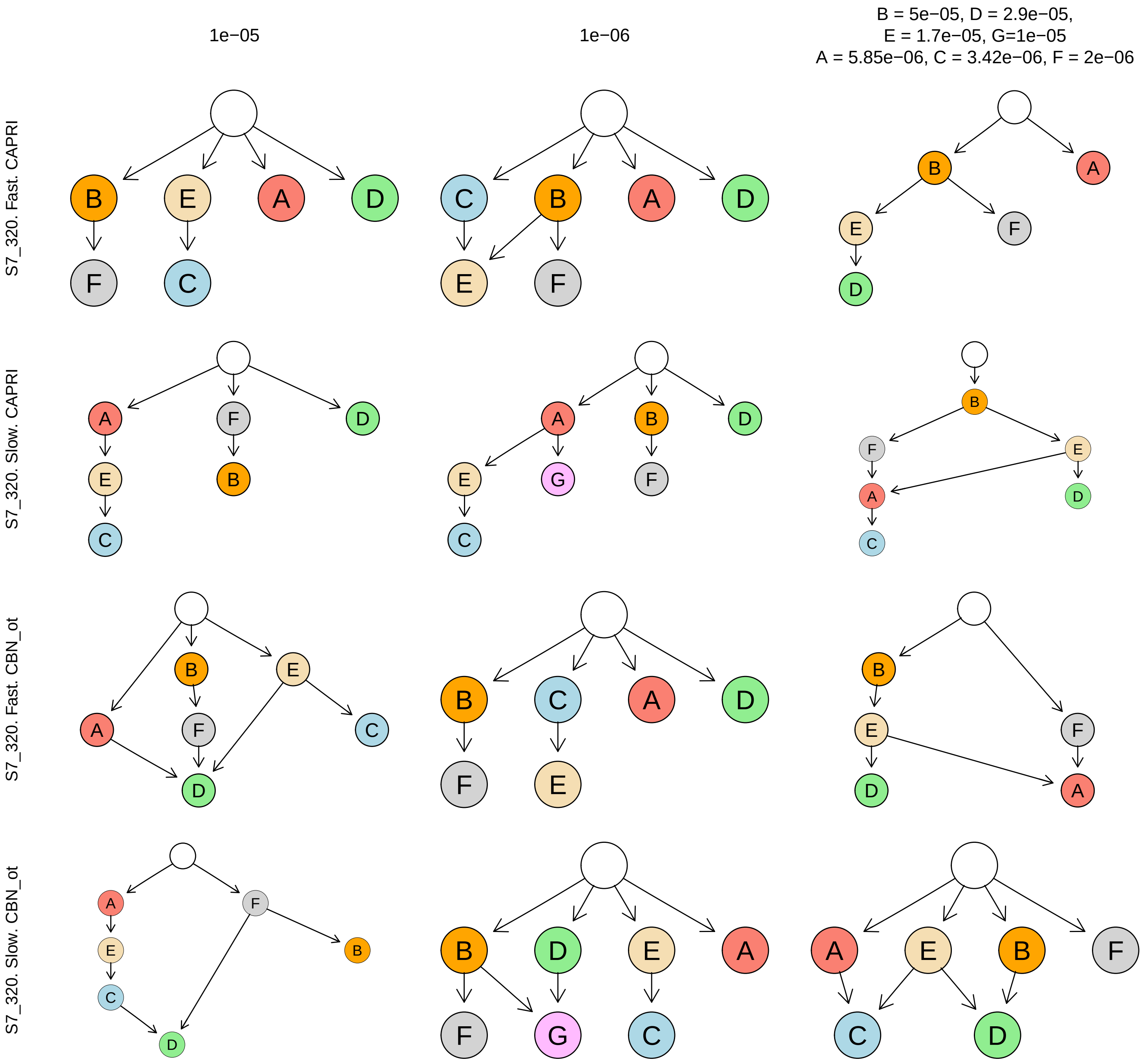

DAG$_a$

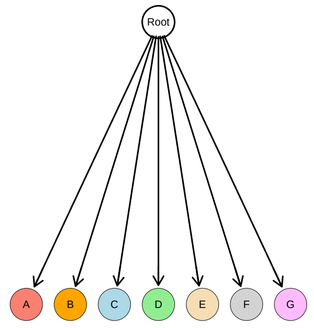

ID	Accessible Genot.	Discrep. DAG_a
S7_321	37	90

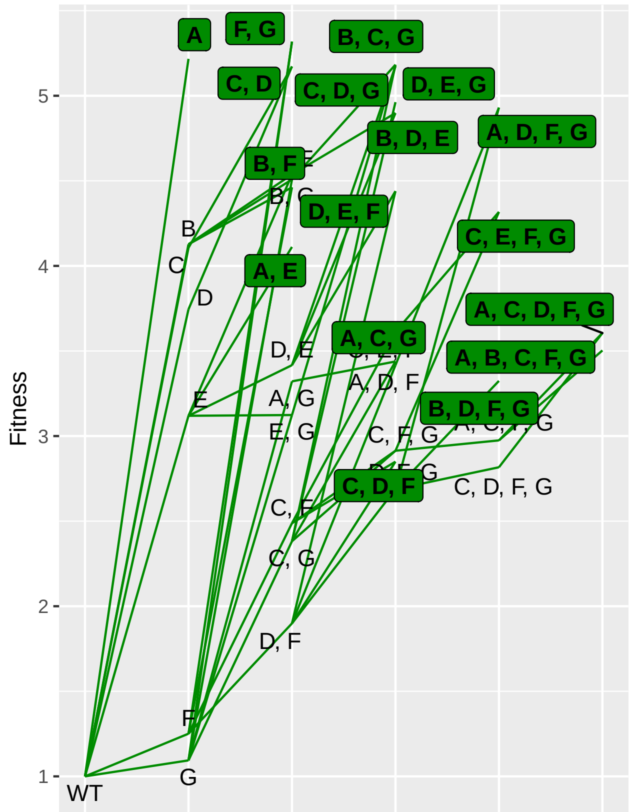

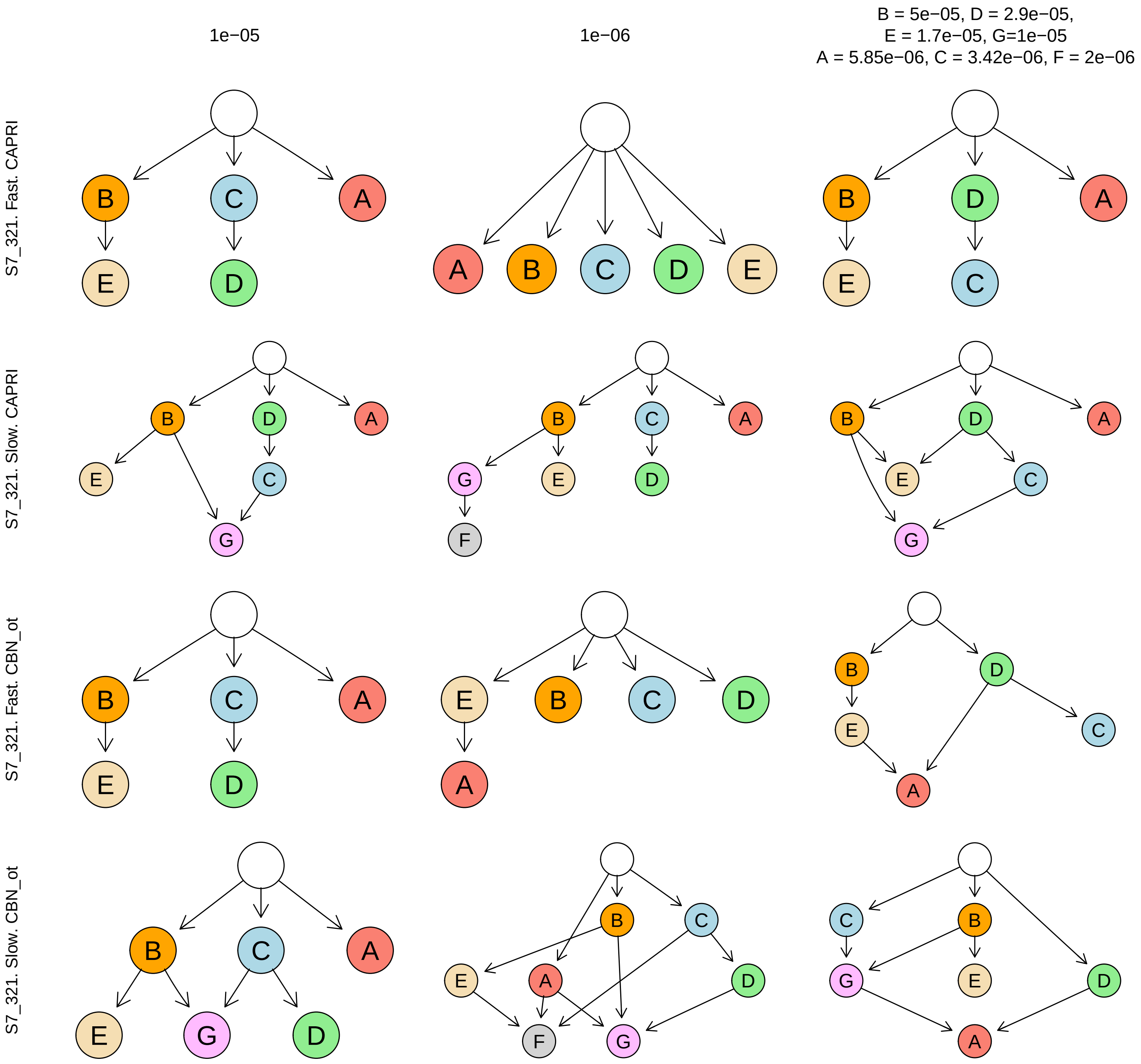

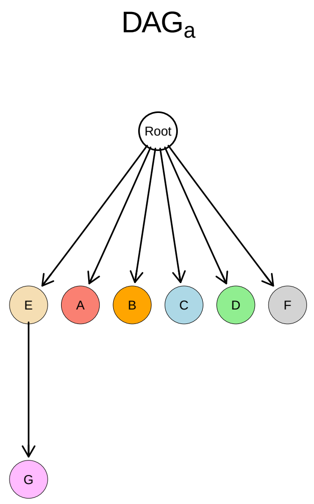

ID	Accessible Genot.	Discrep. DAG_a
S7_322	68	43

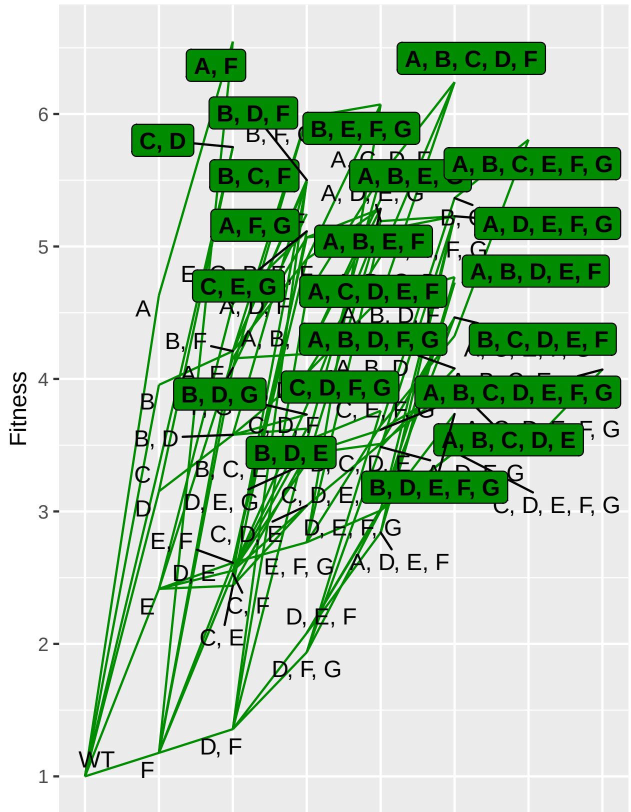

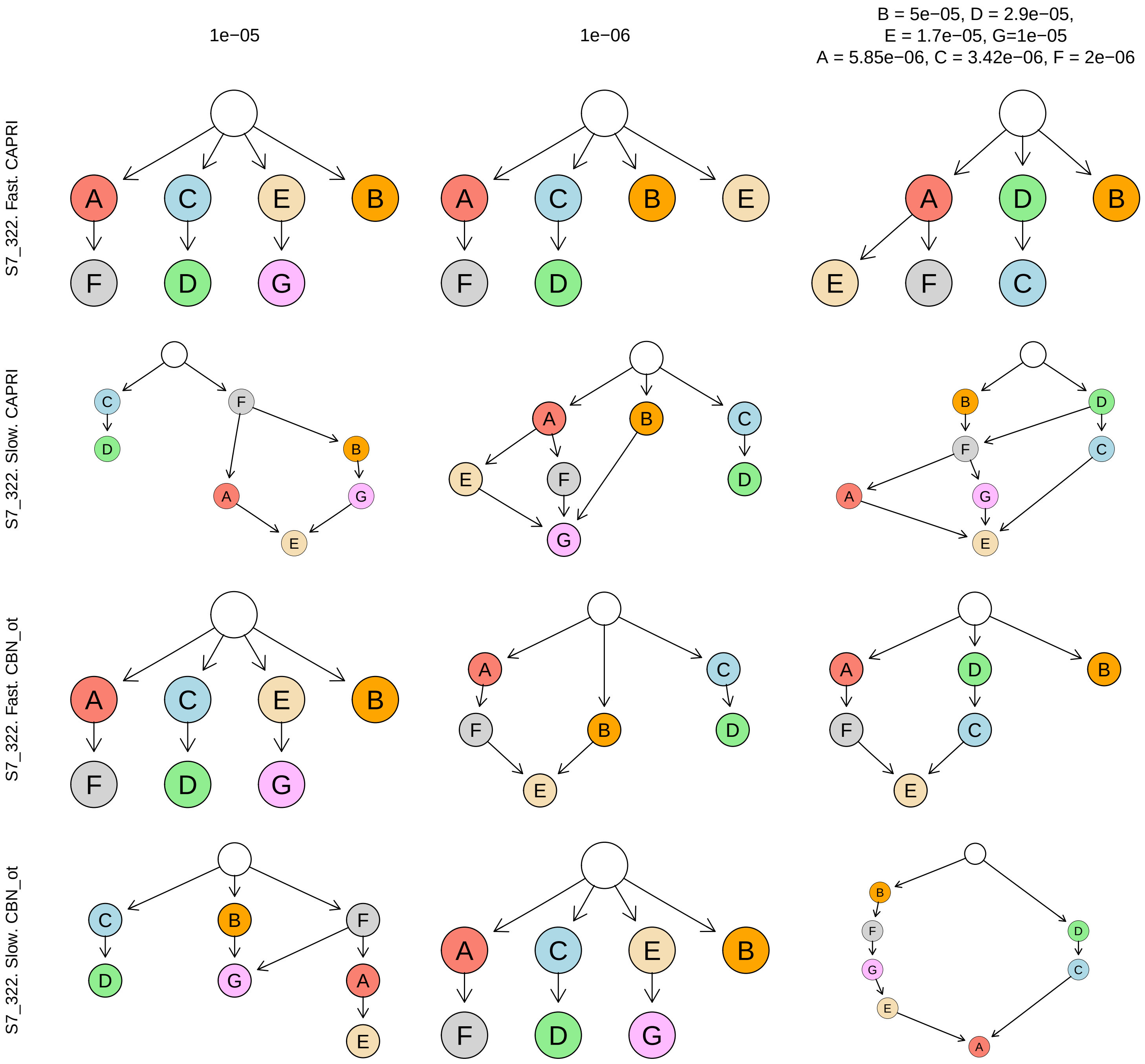

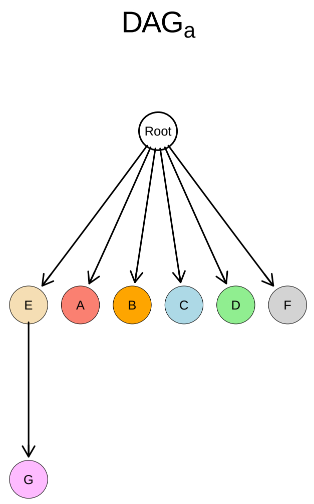

ID	Accessible Genot.	Discrep. DAG_a
S7_323	40	61

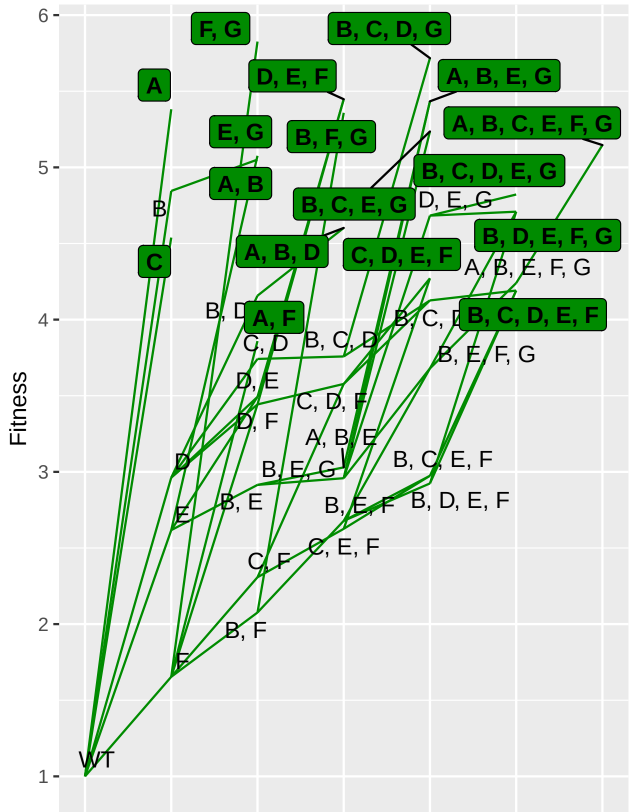

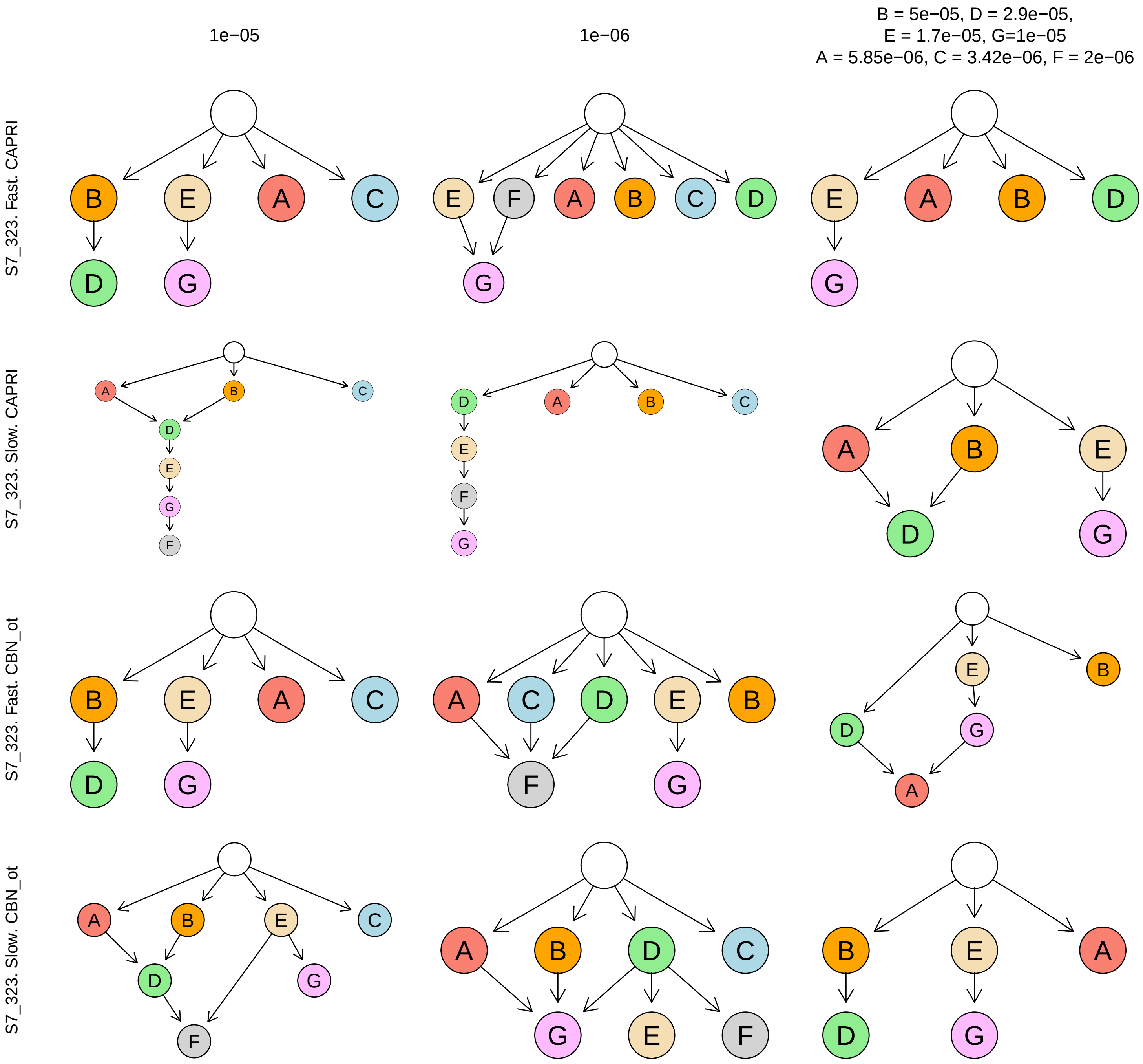

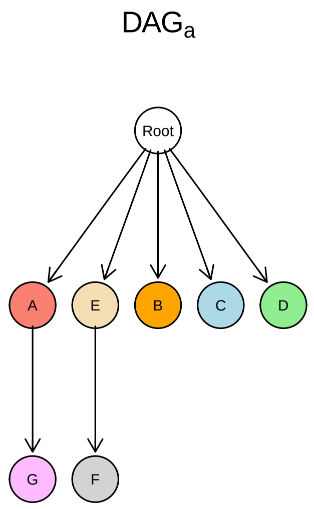

ID	Accessible Genot.	Discrep. DAG_a
S7_324	35	58

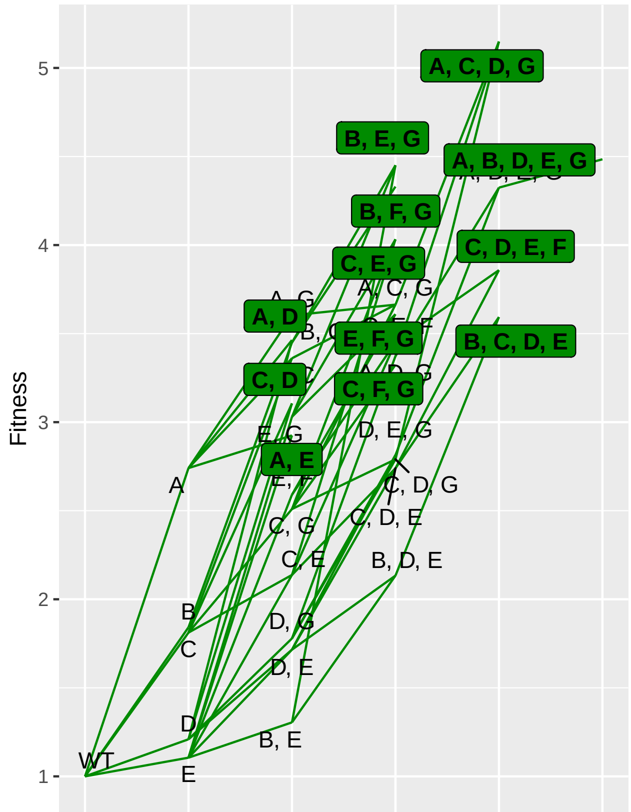

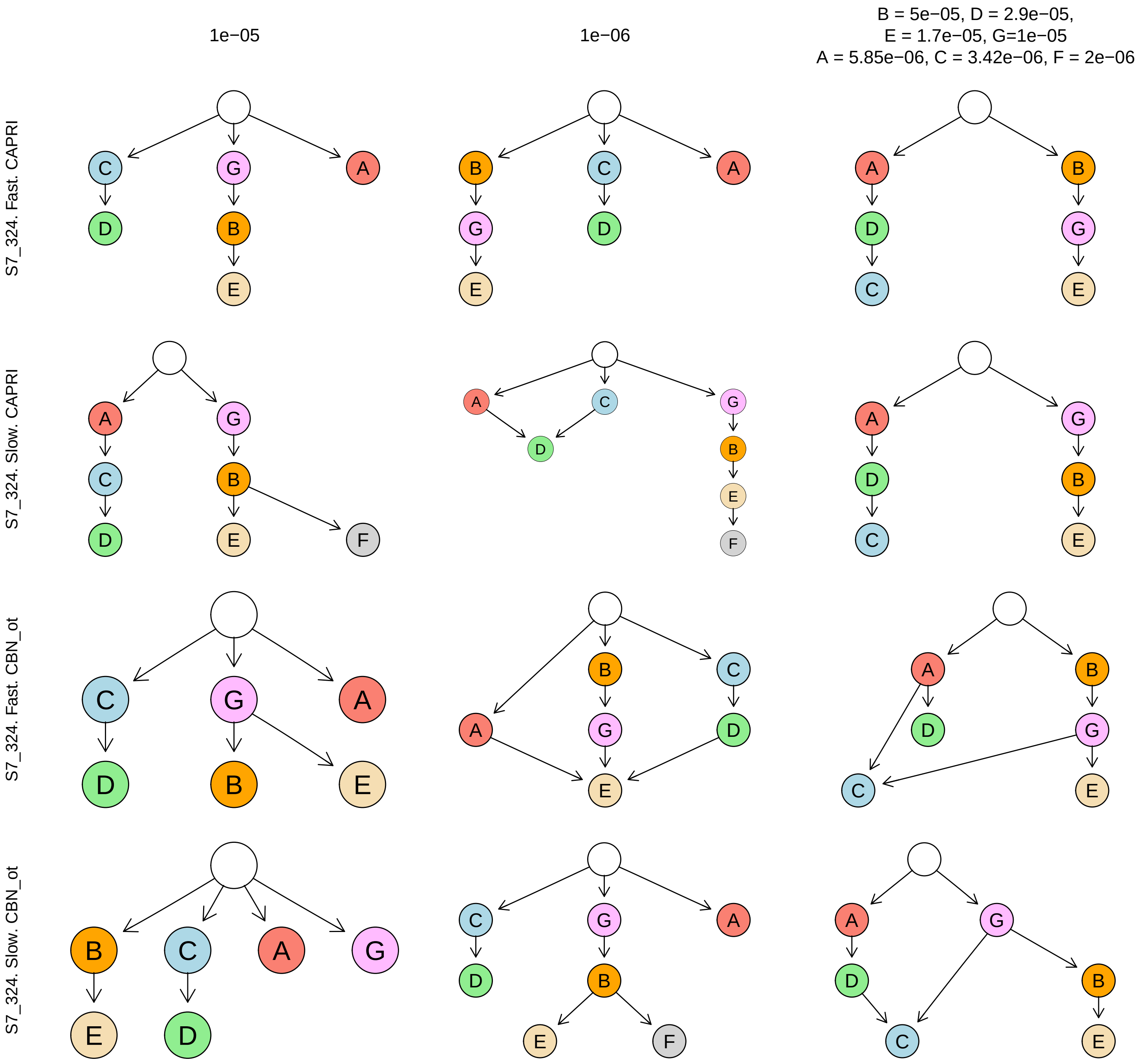

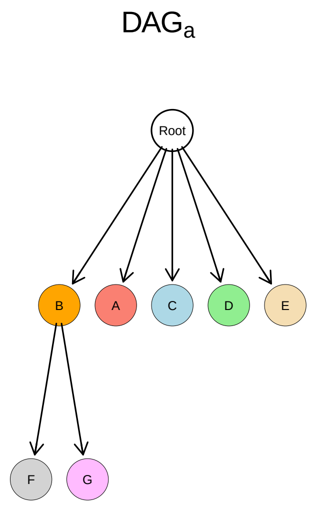

ID	Accessible Genot.	Discrep. DAG_a
S7_325	63	58

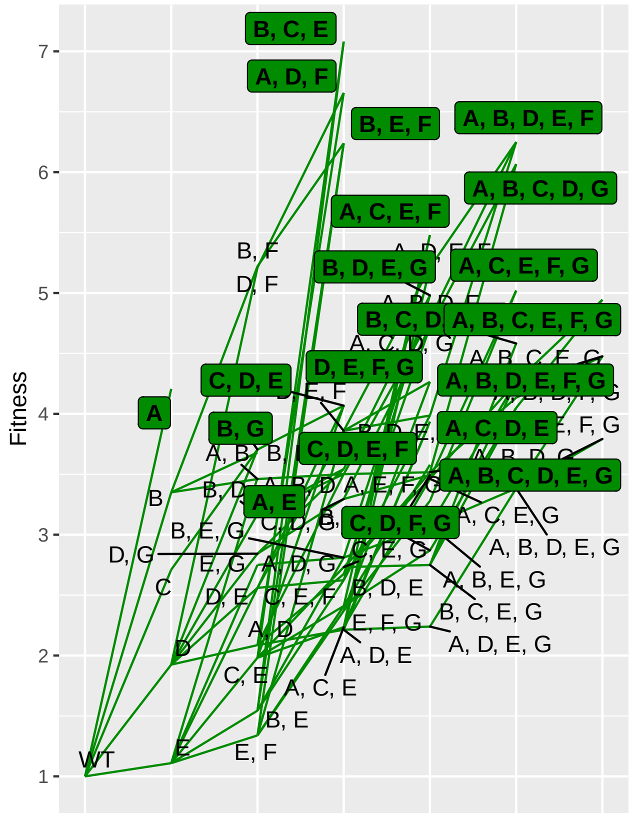

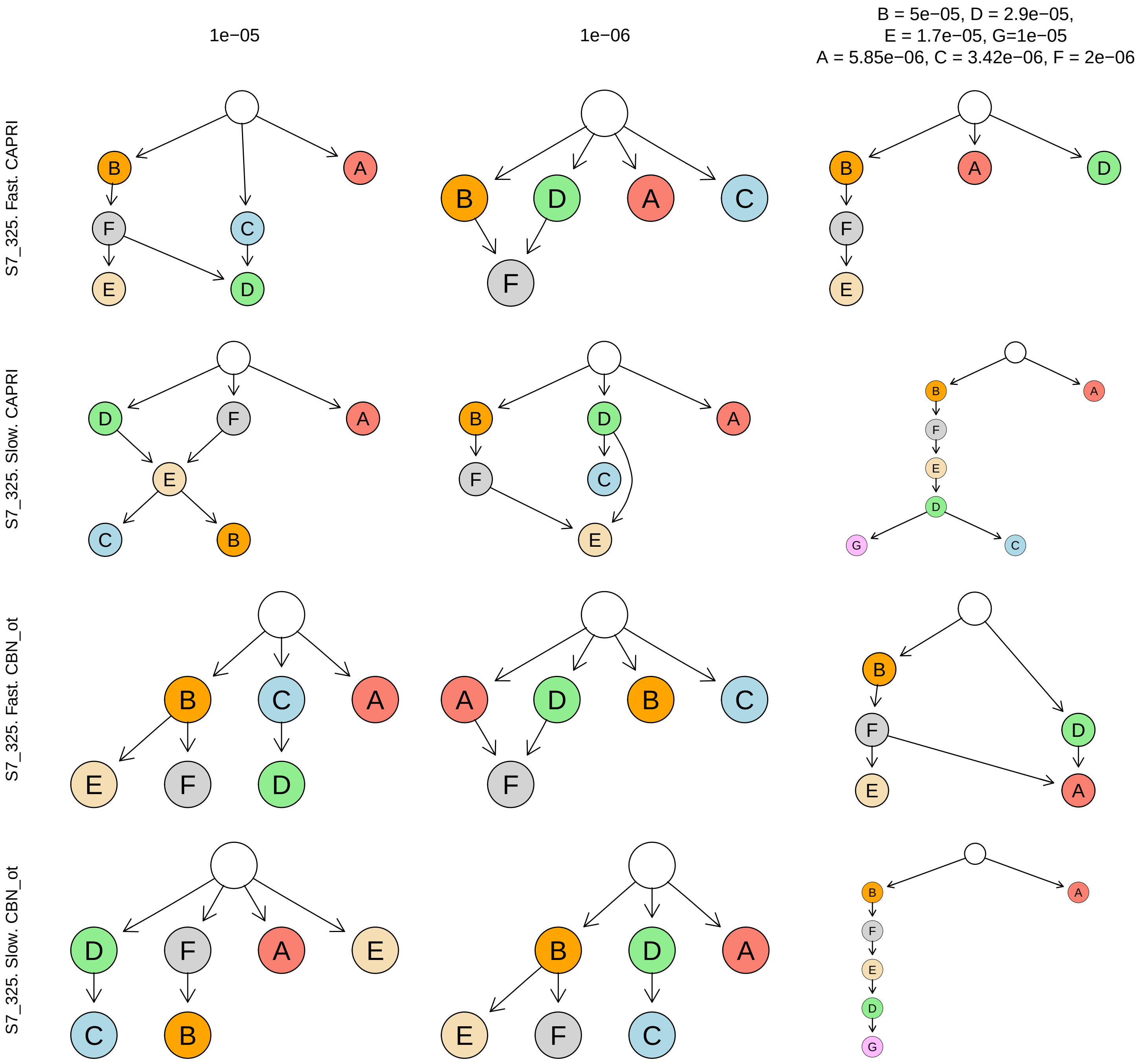

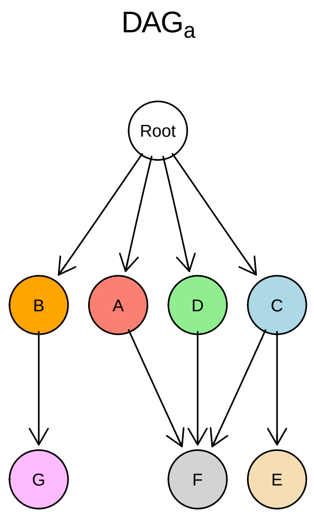

ID	Accessible Genot.	Discrep. DAG_a
S7_326	30	19

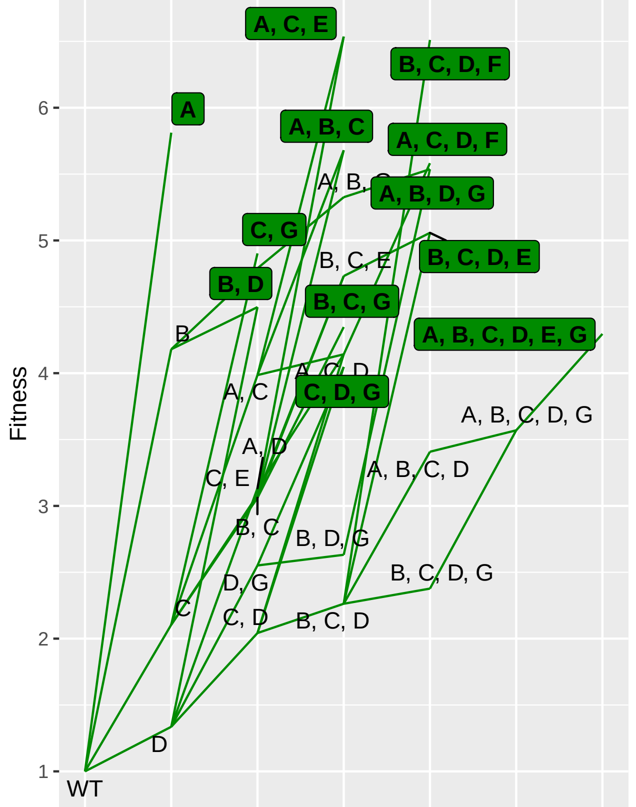

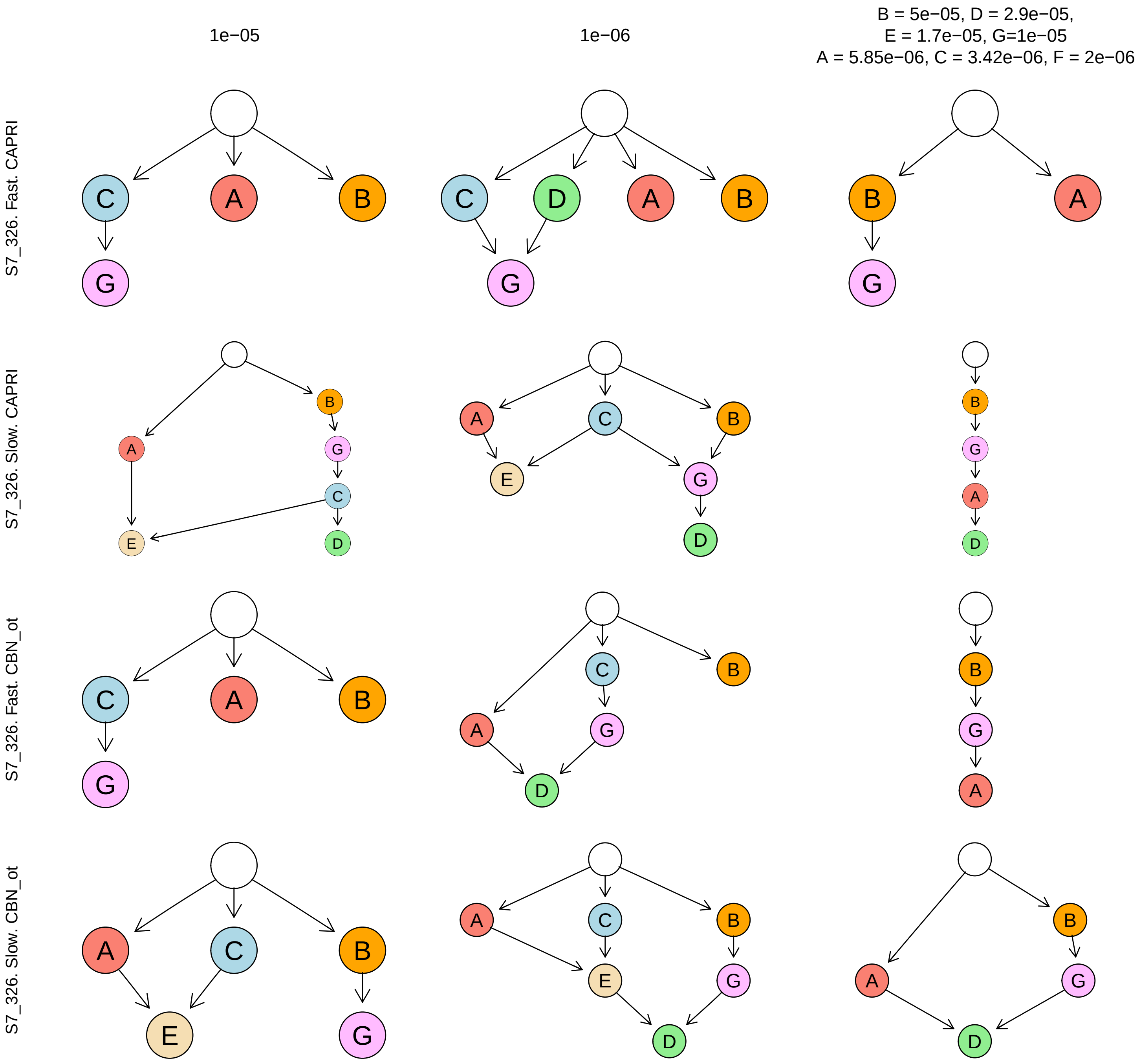

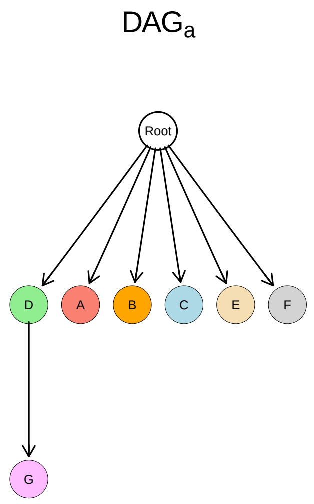

ID	Accessible Genot.	Discrep. DAG_a
S7_327	40	69

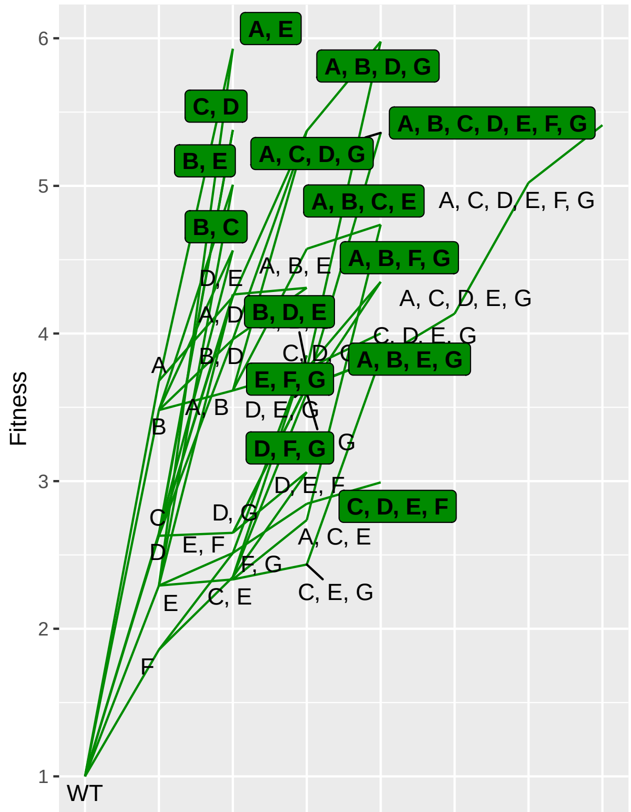

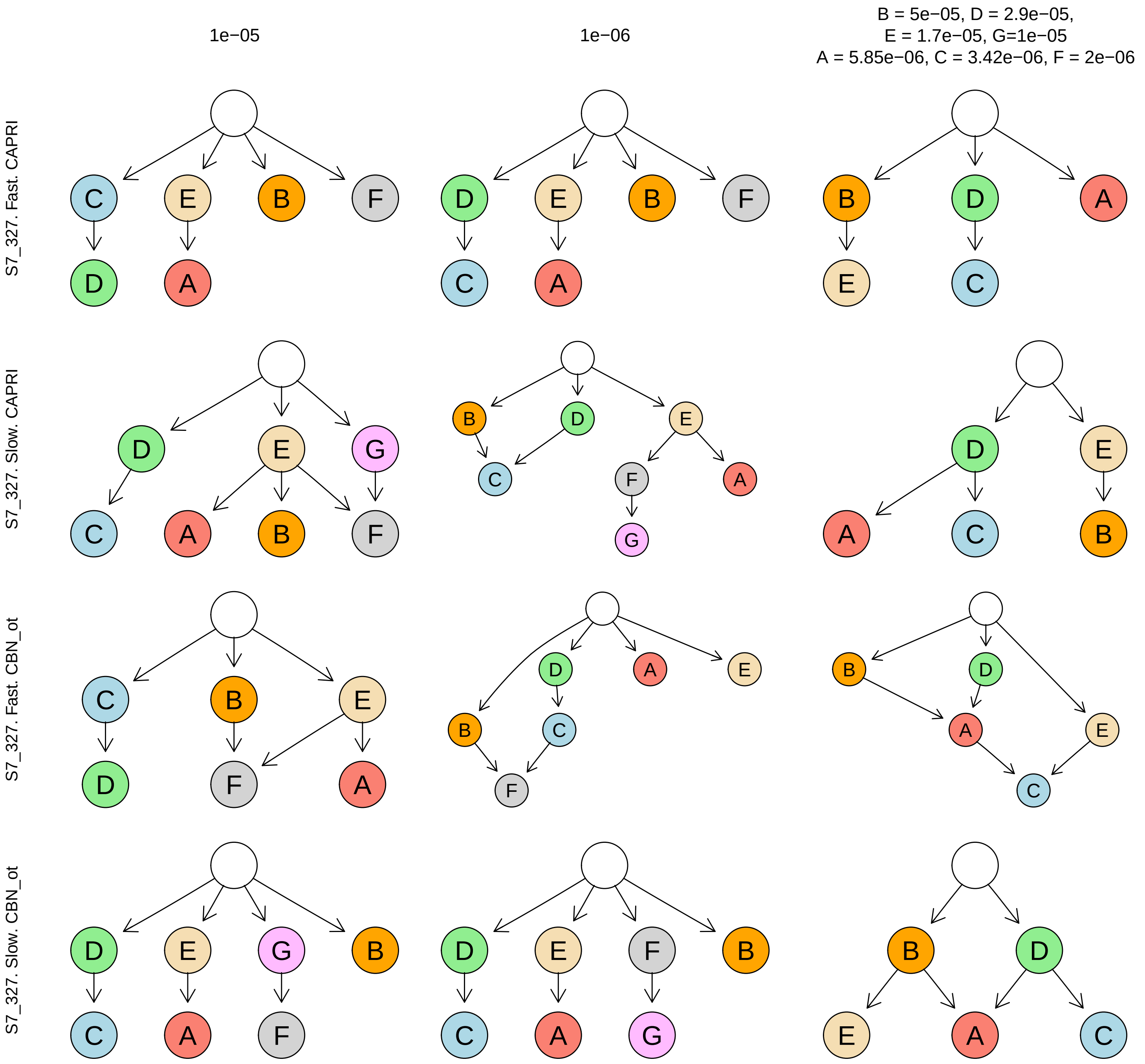

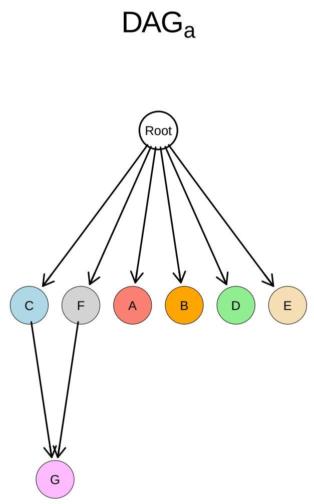

ID	Accessible Genot.	Discrep. DAG_a
S7_328	31	48

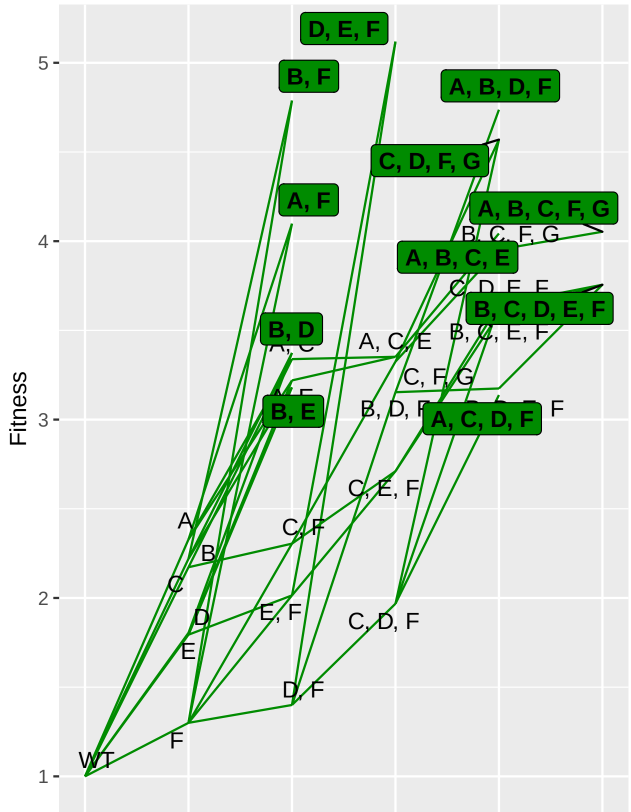

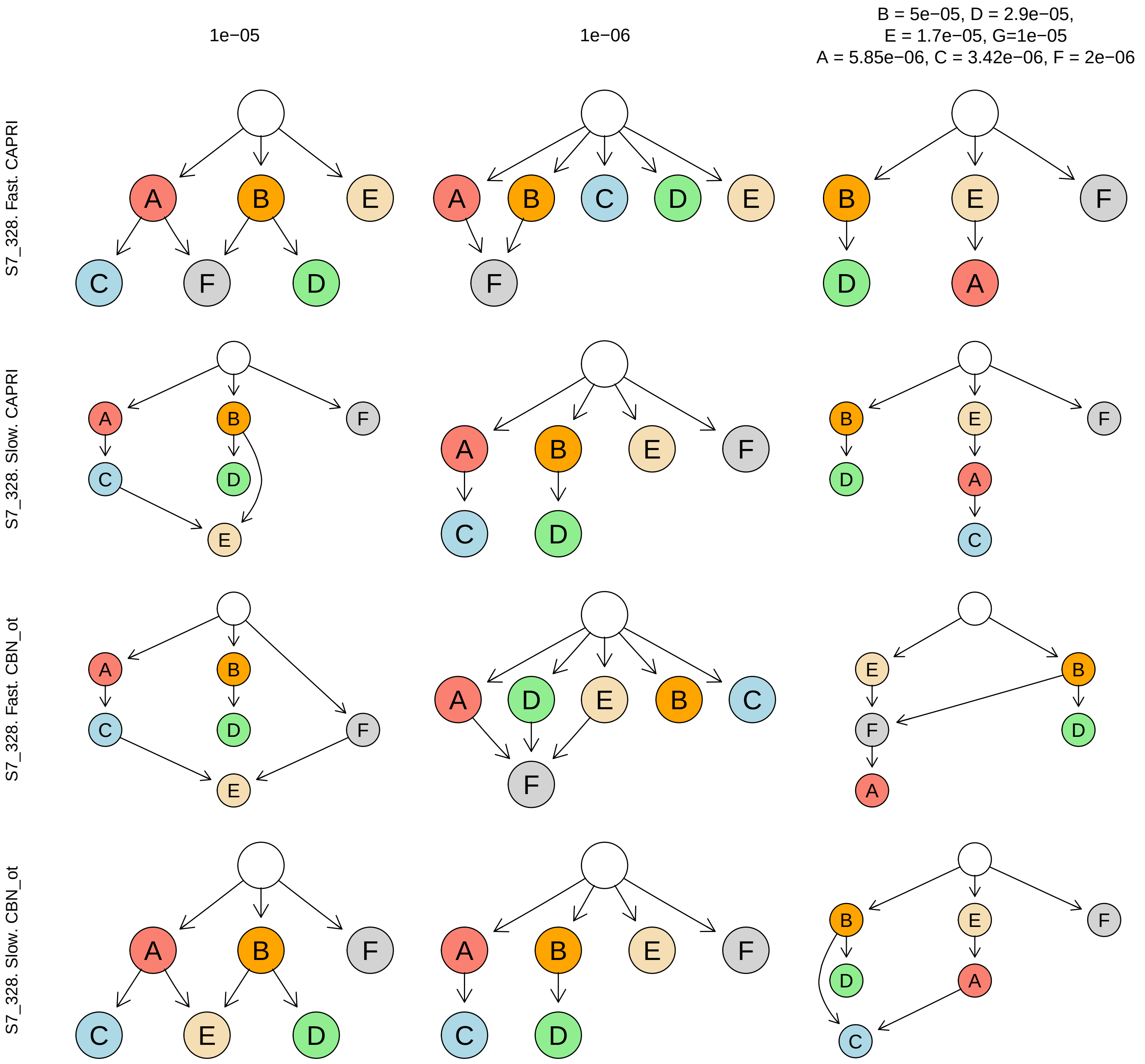

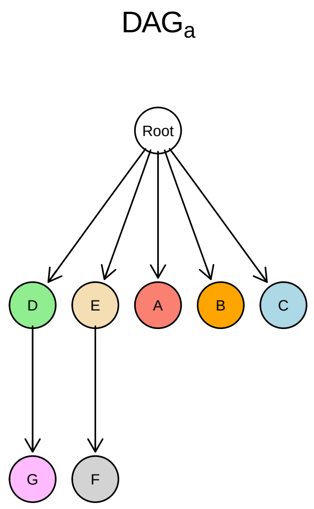

ID	Accessible Genot.	Discrep. DAG_a
S7_329	22	51

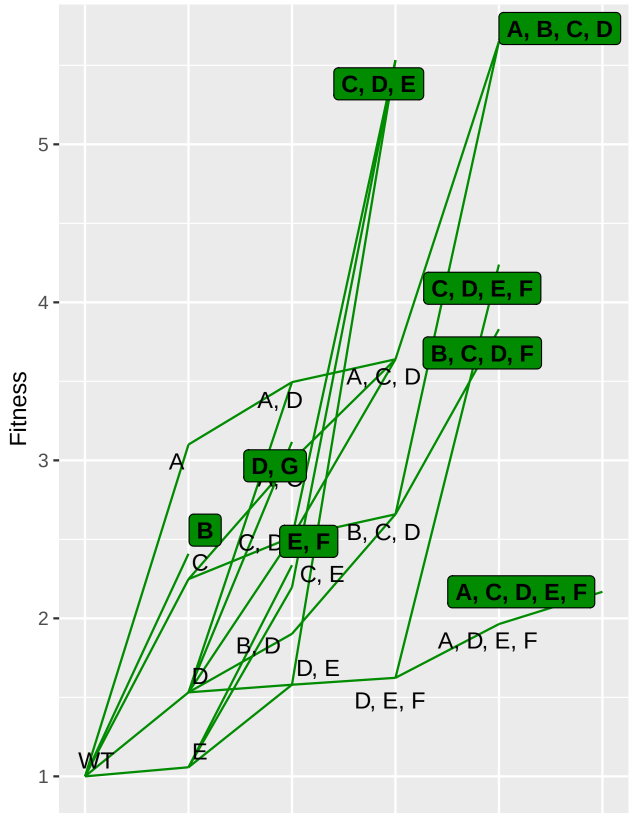

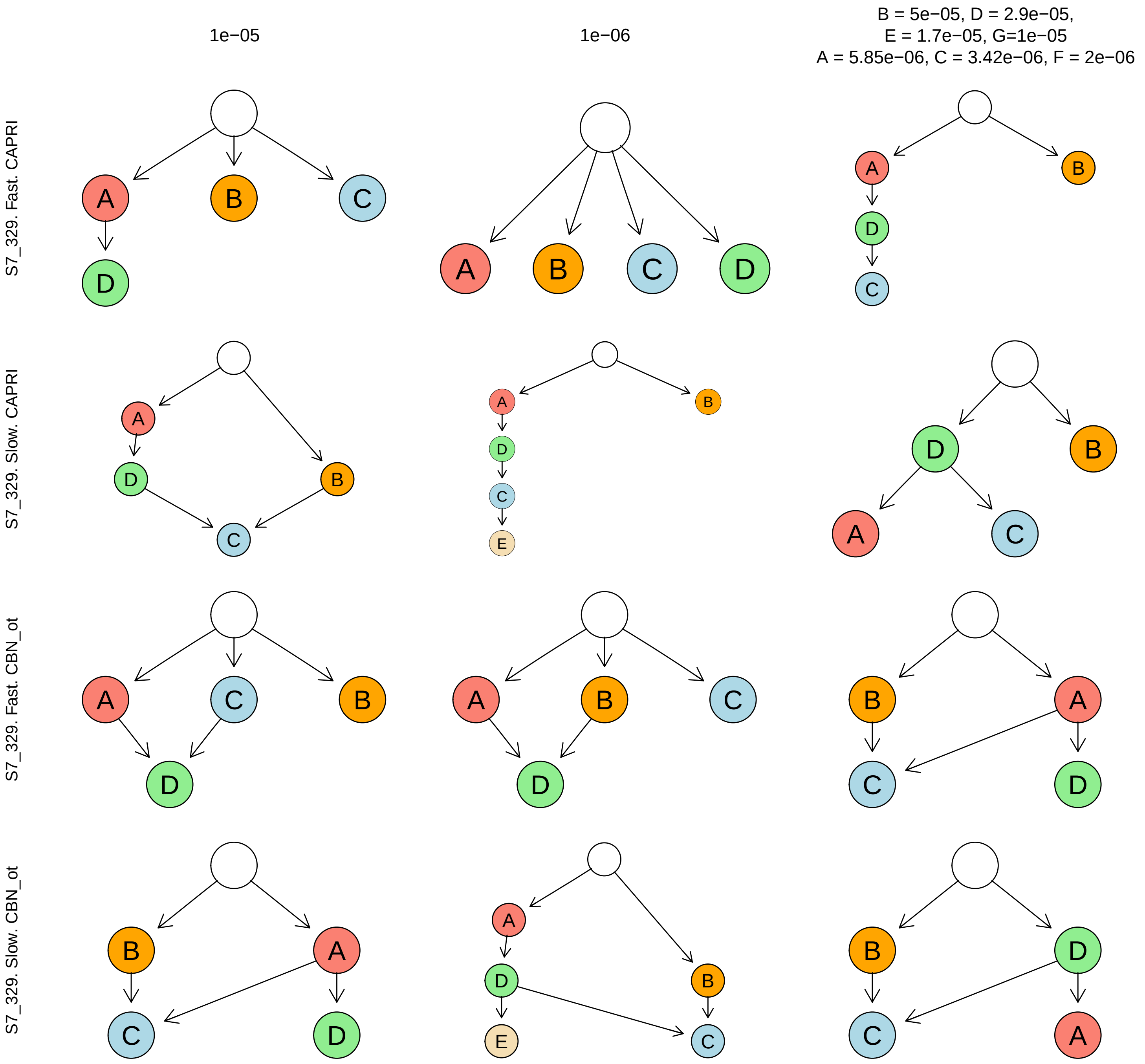

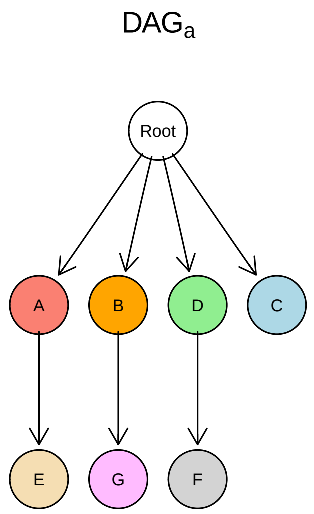

ID	Accessible Genot.	Discrep. DAG_a
S7_330	41	56

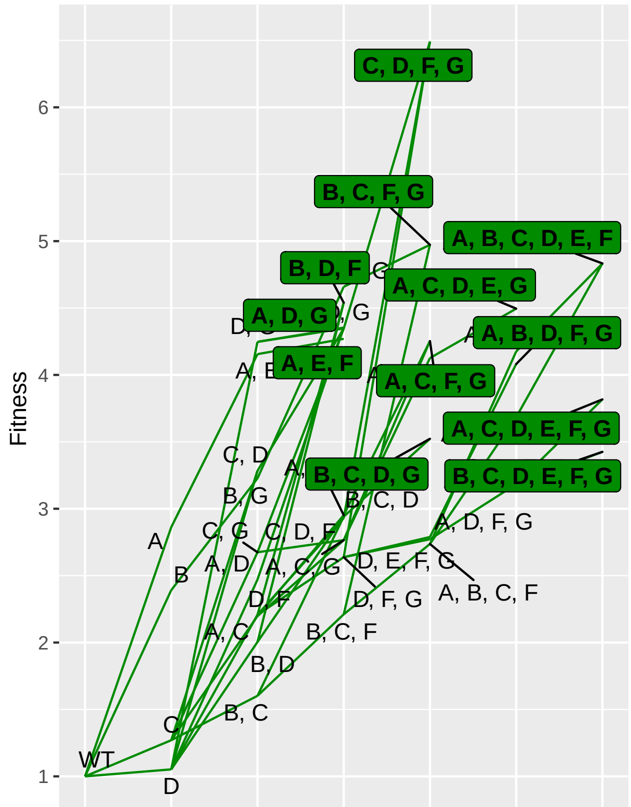

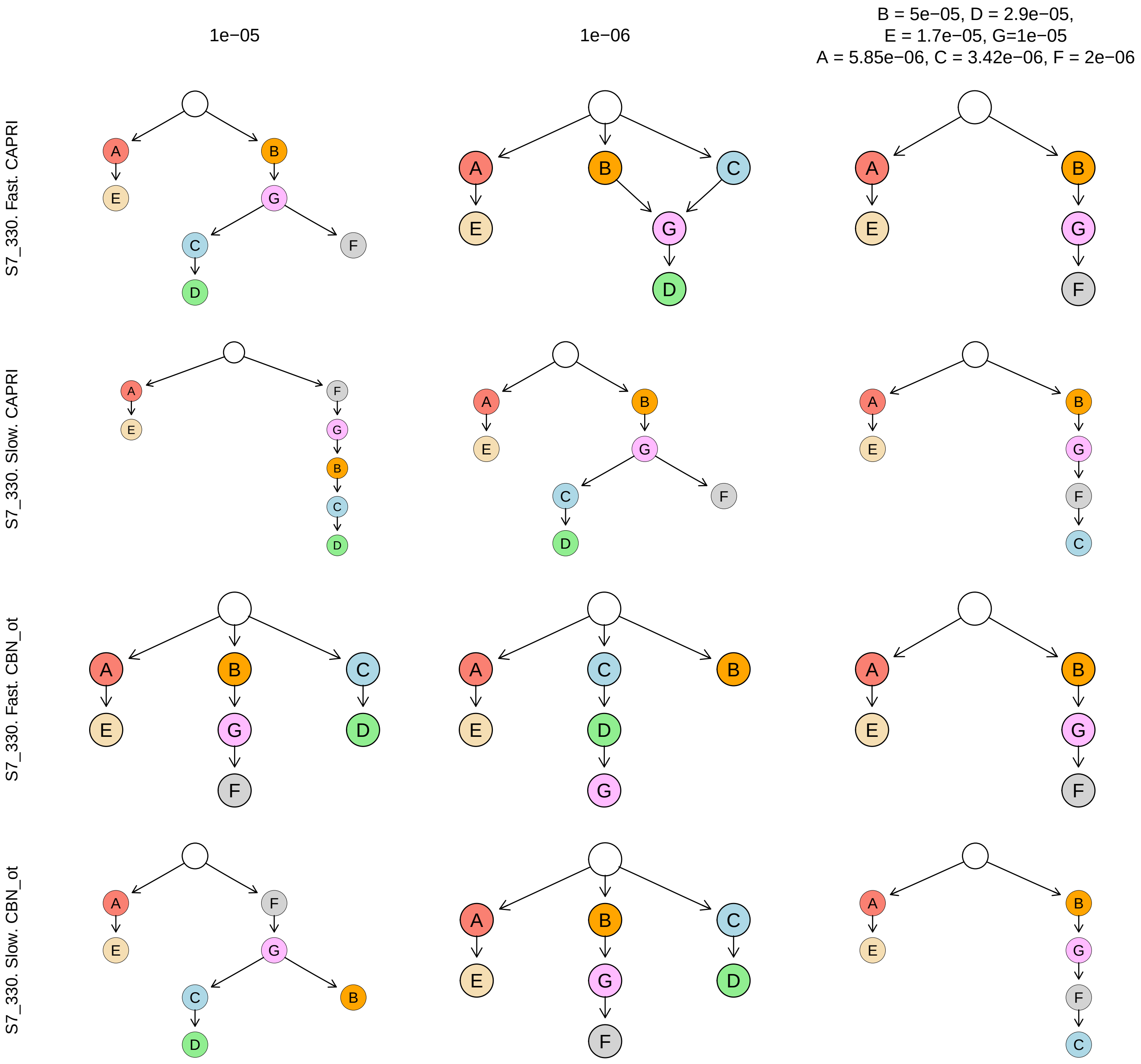

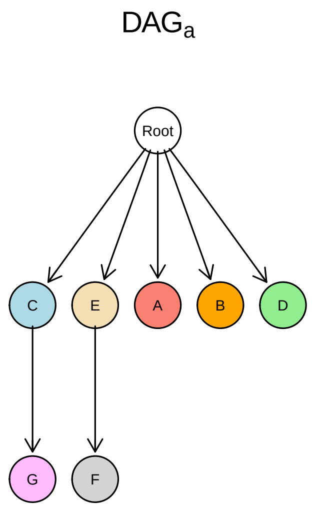

ID	Accessible Genot.	Discrep. DAG_a
S7_331	12	63

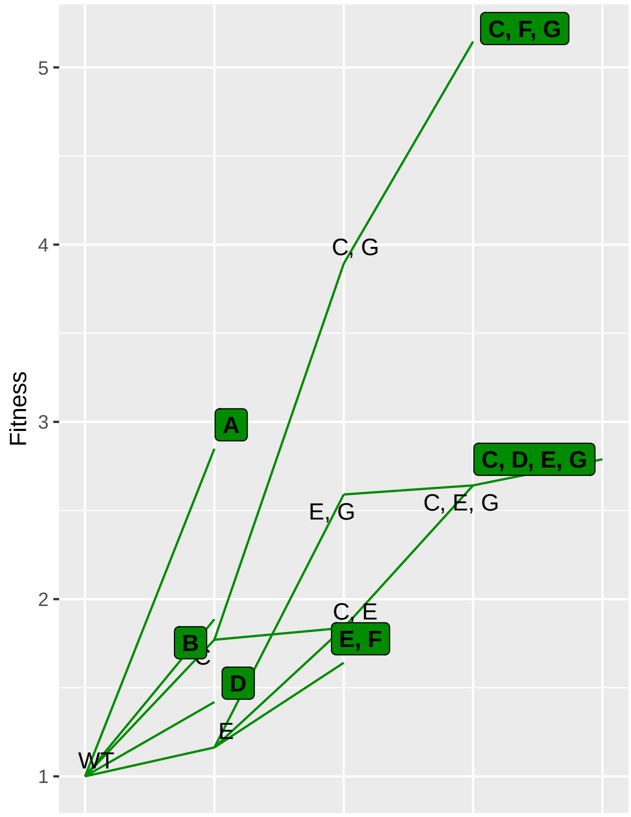

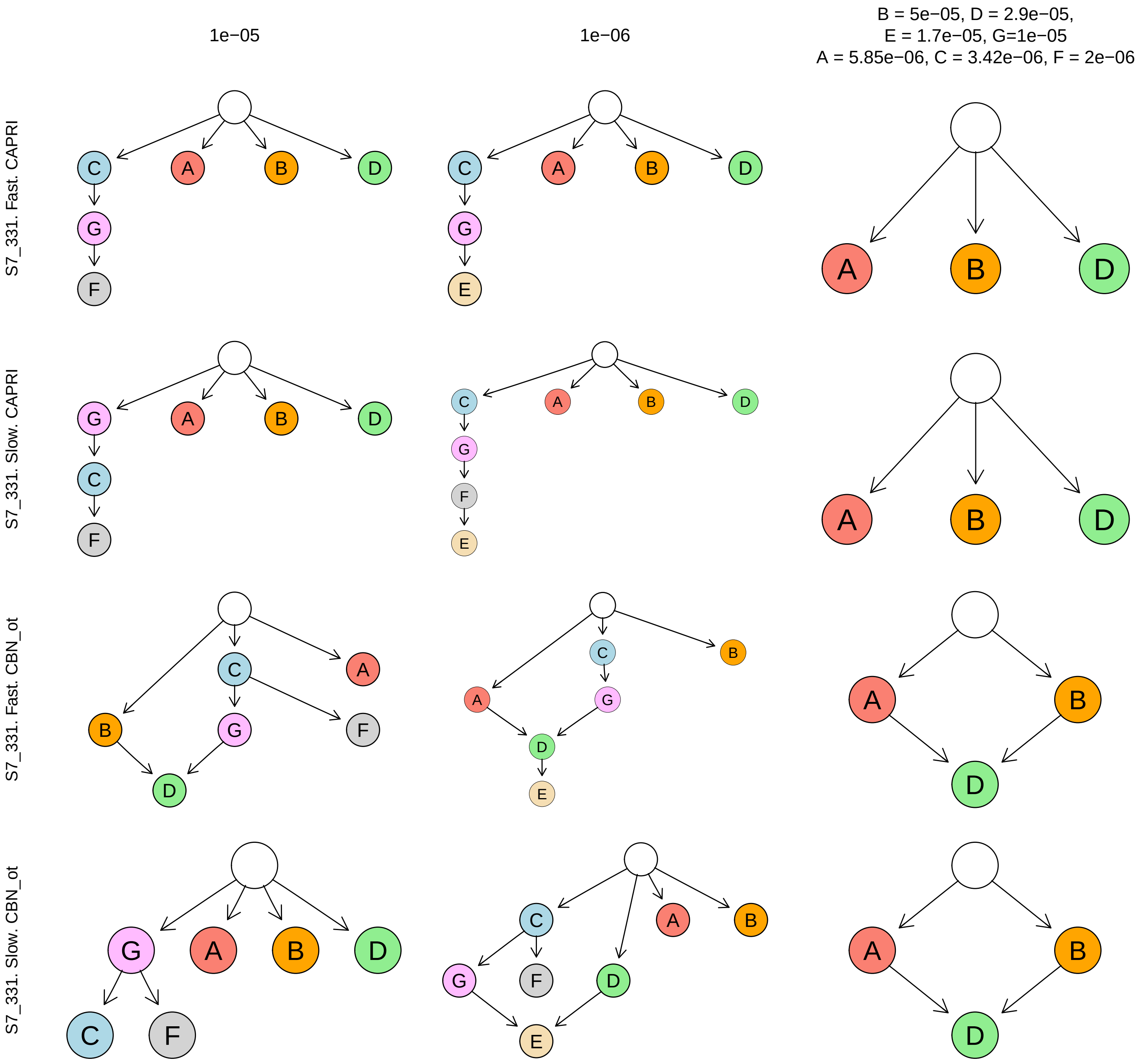

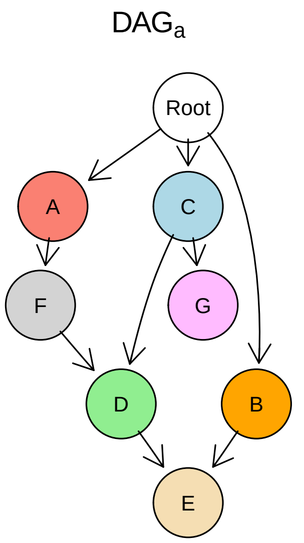

ID	Accessible Genot.	Discrep. DAG_a
S7_332	22	15

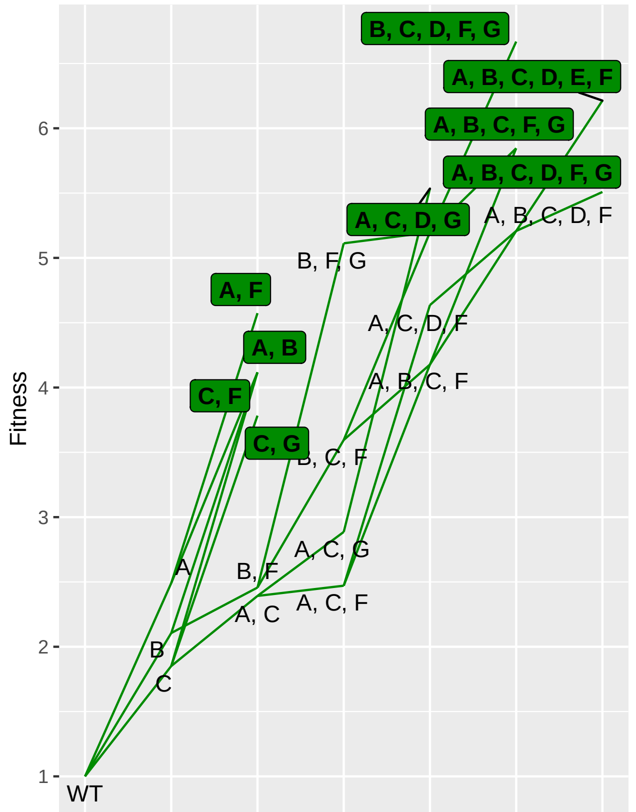

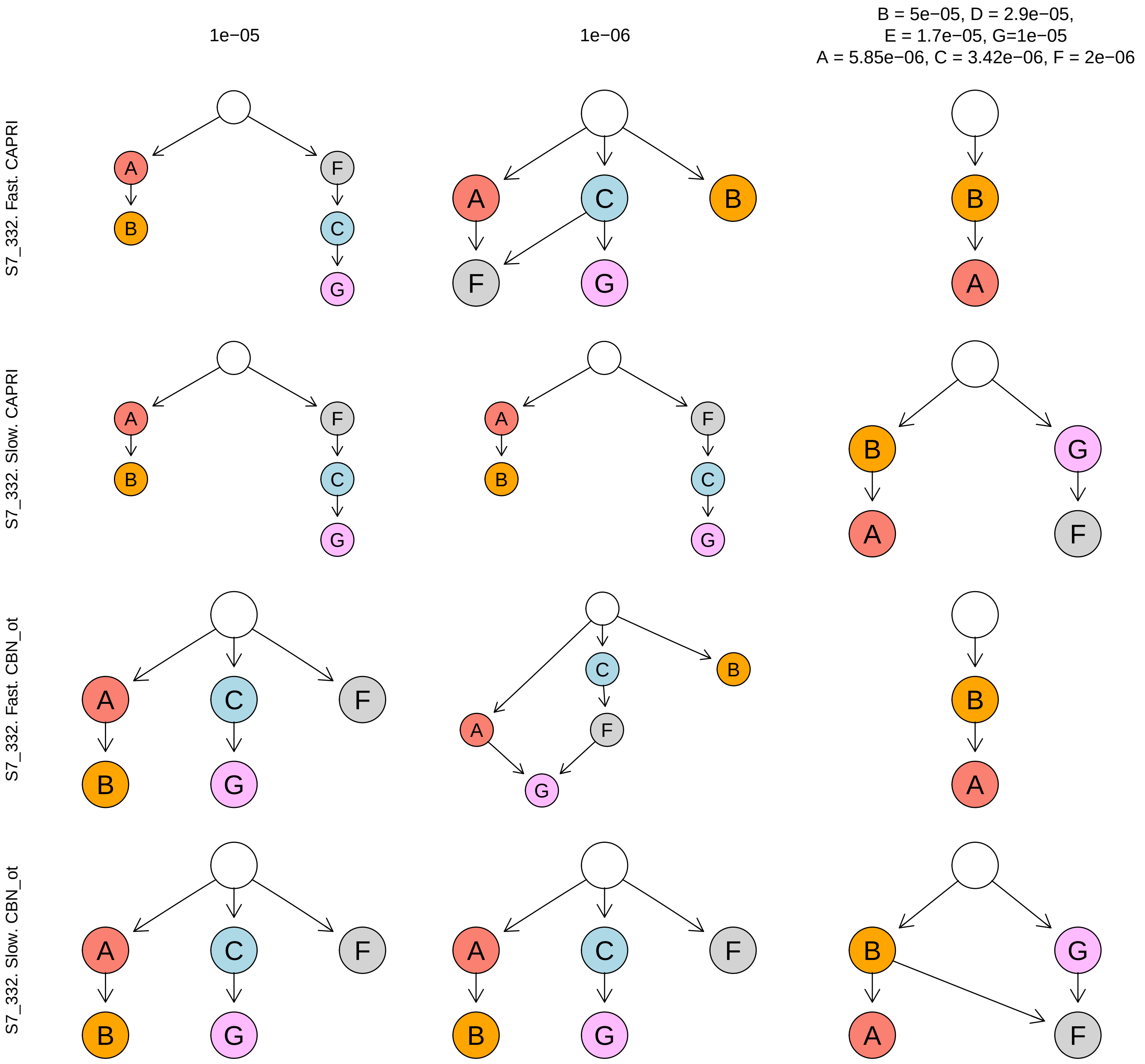

DAG$_a$

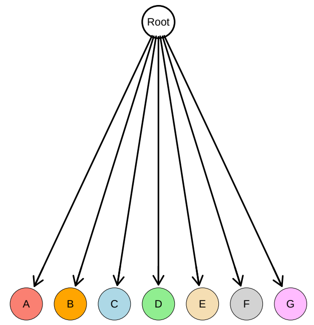

ID	Accessible Genot.	Discrep. DAG_a
S7_333	16	111

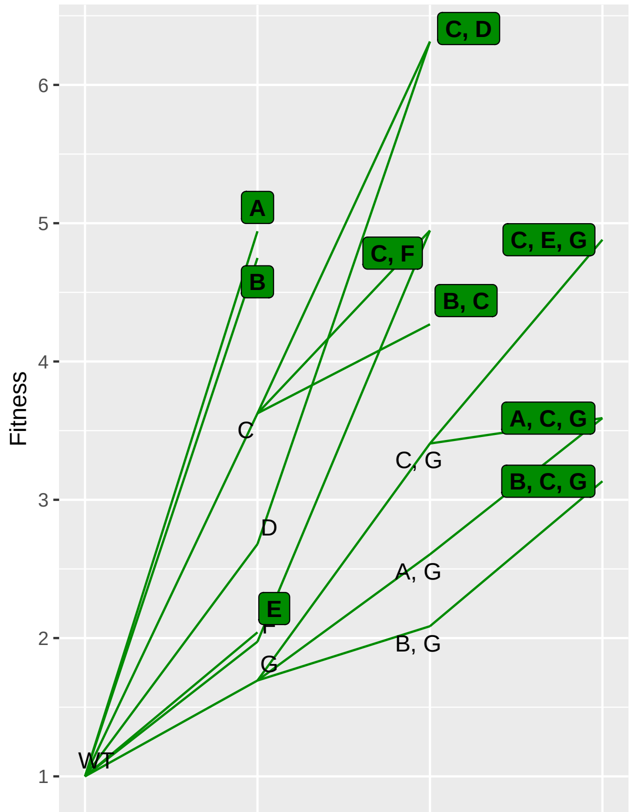

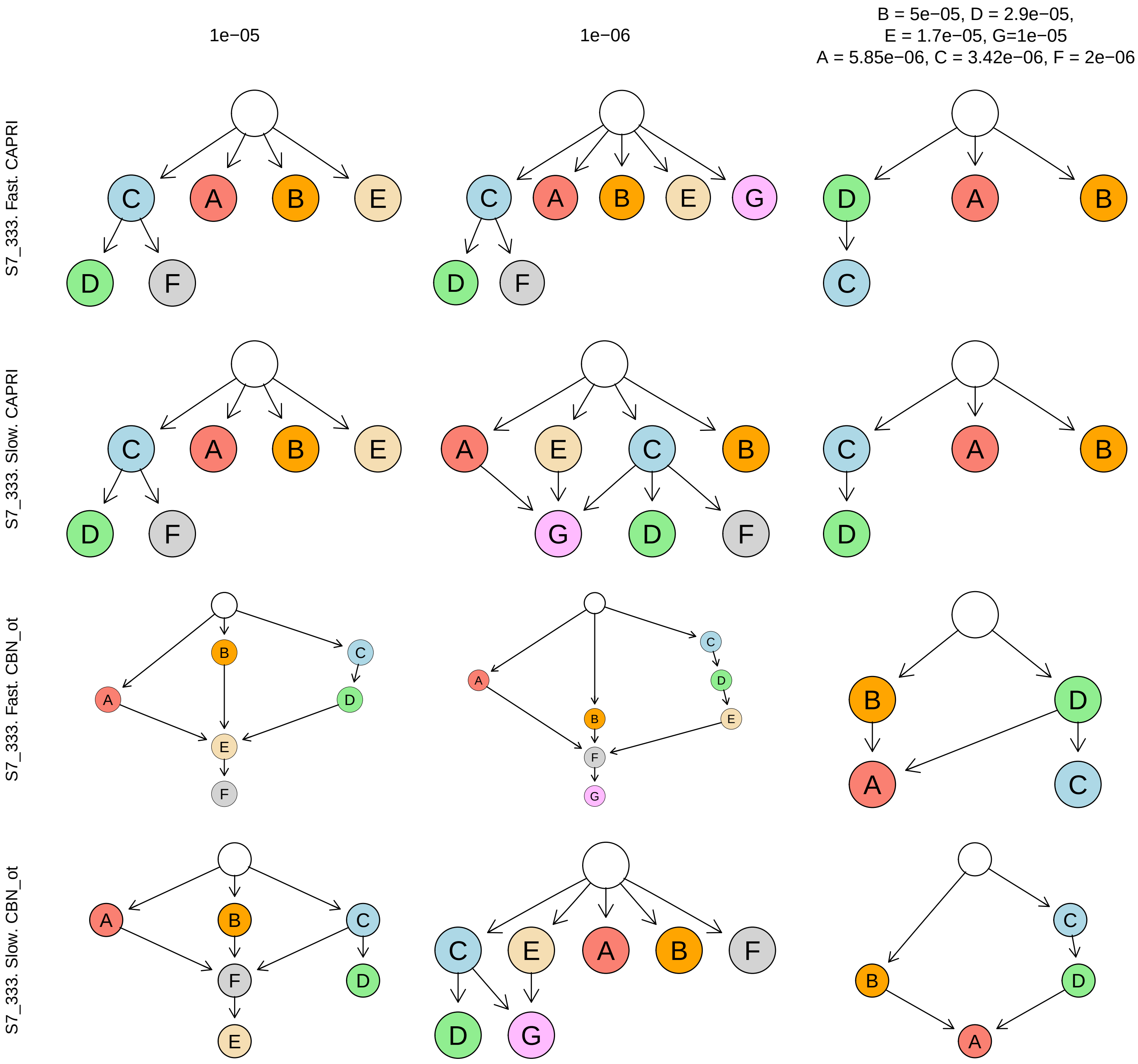

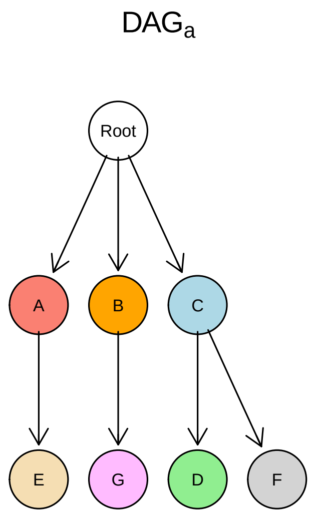

ID	Accessible Genot.	Discrep. DAG_a
S7_334	34	40

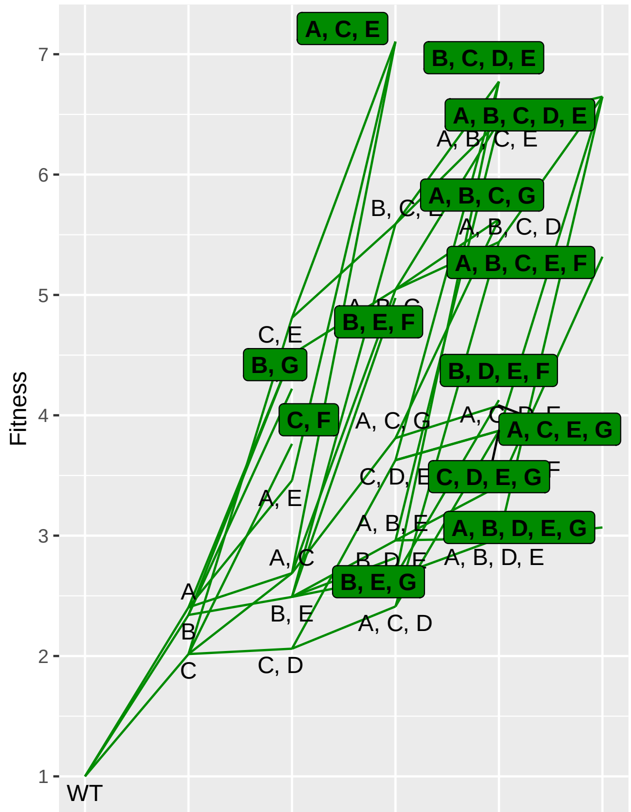

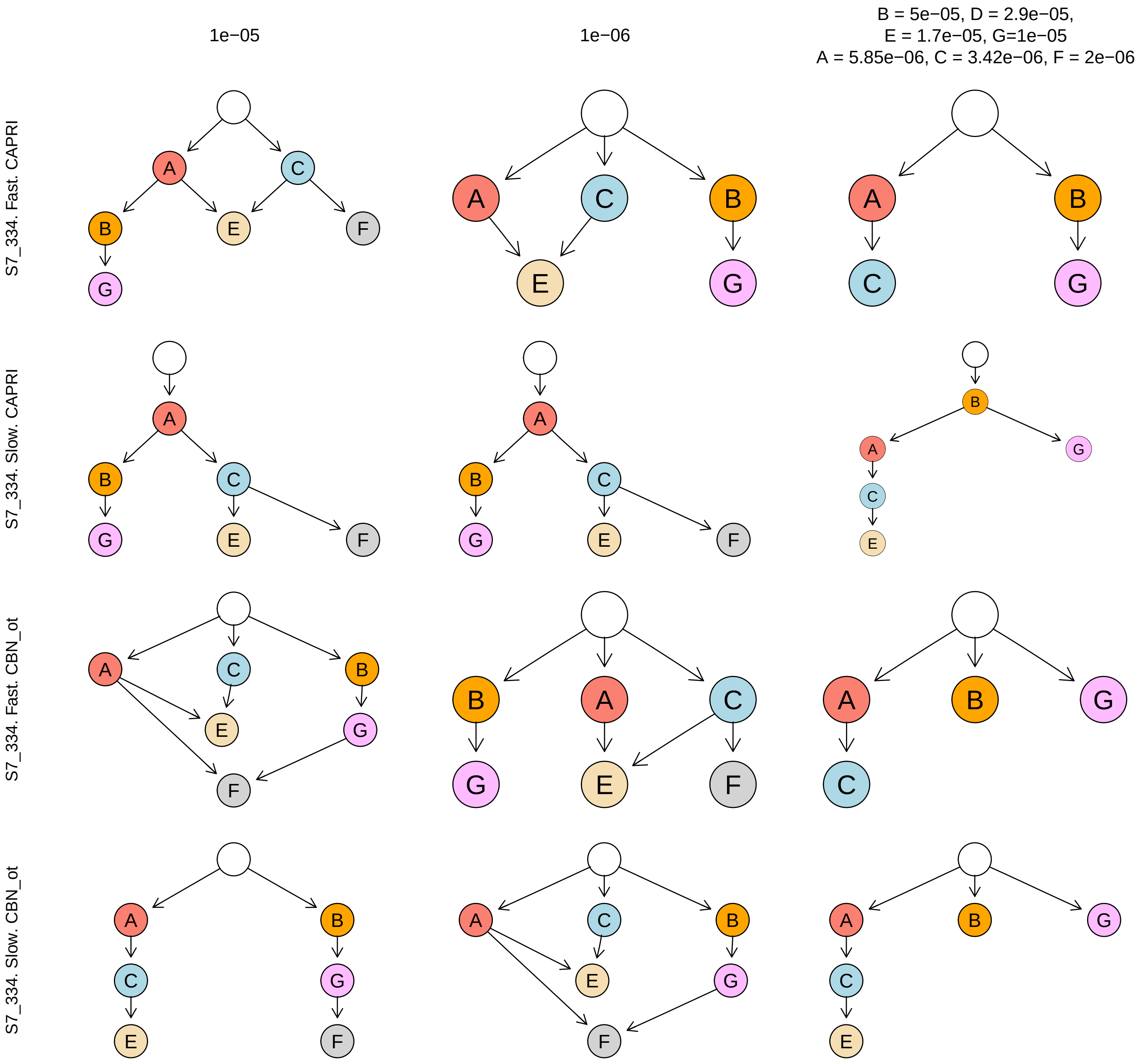

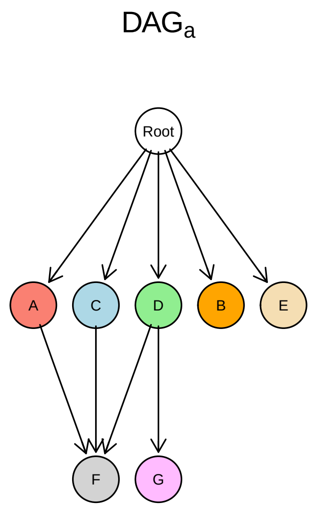

ID	Accessible Genot.	Discrep. DAG_a
S7_335	17	38

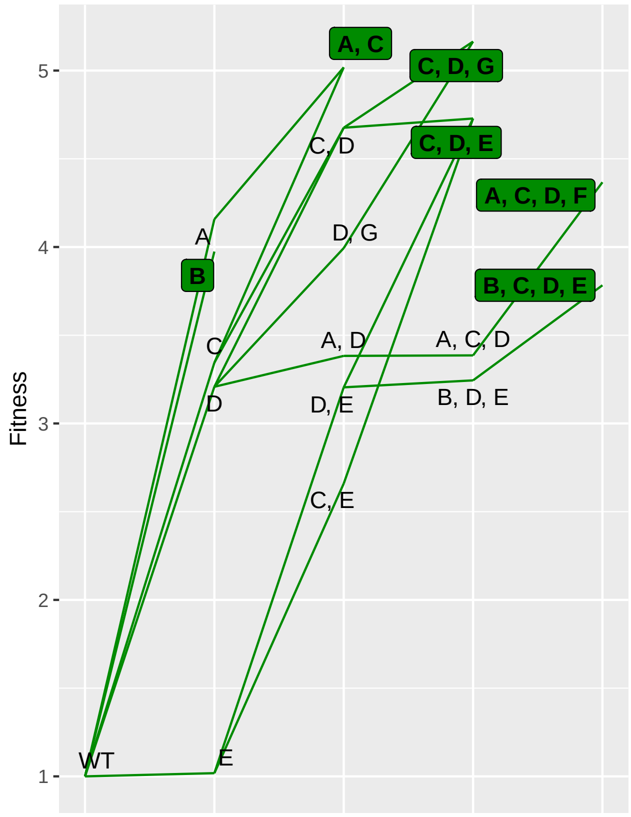

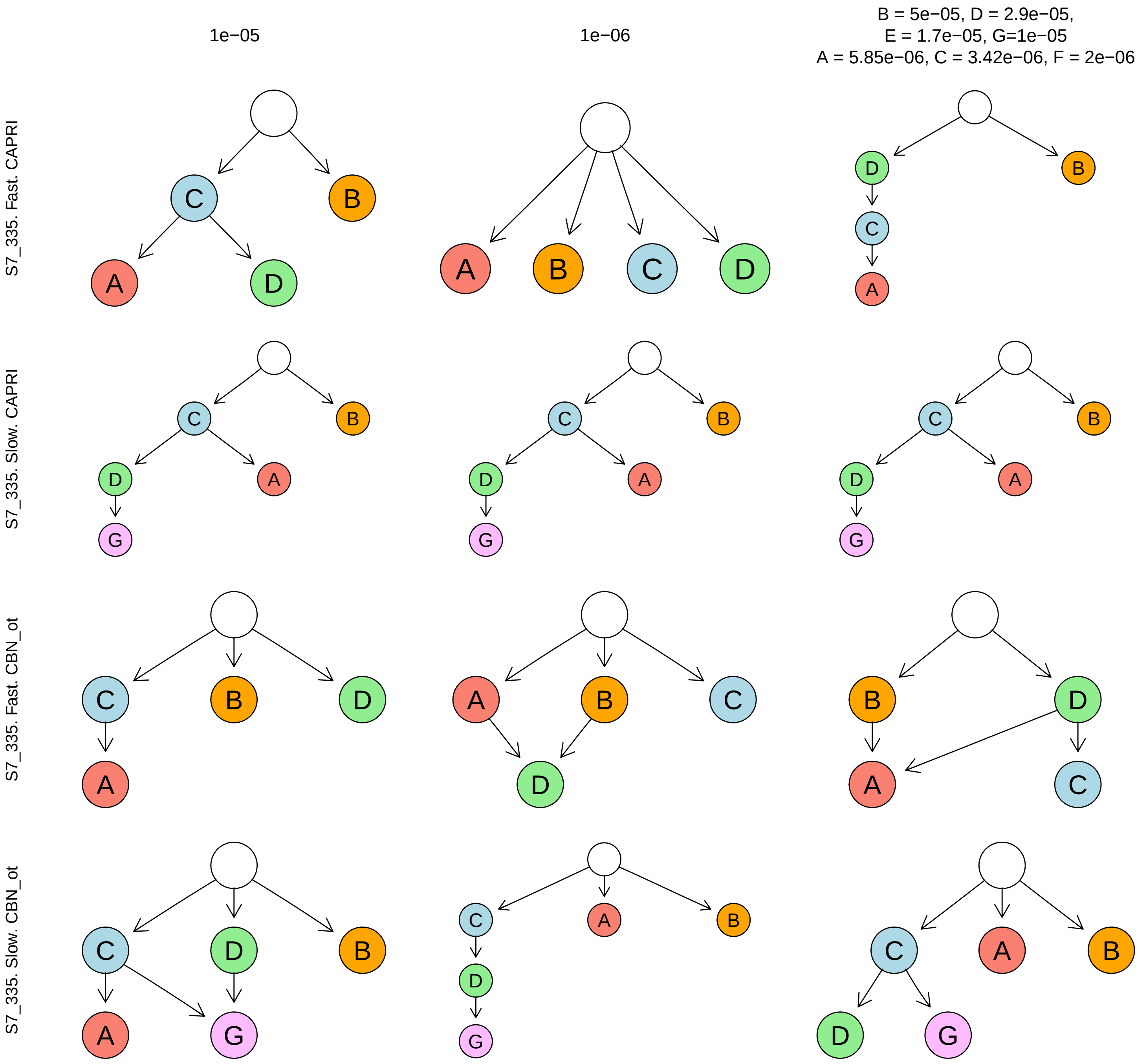

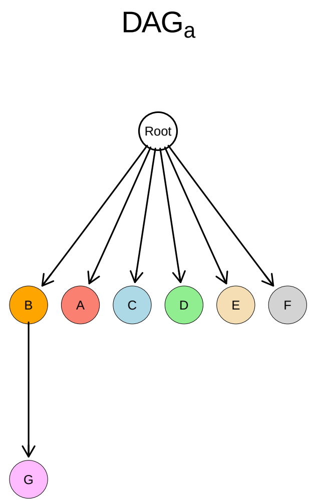

ID	Accessible Genot.	Discrep. DAG_a
S7_336	69	50

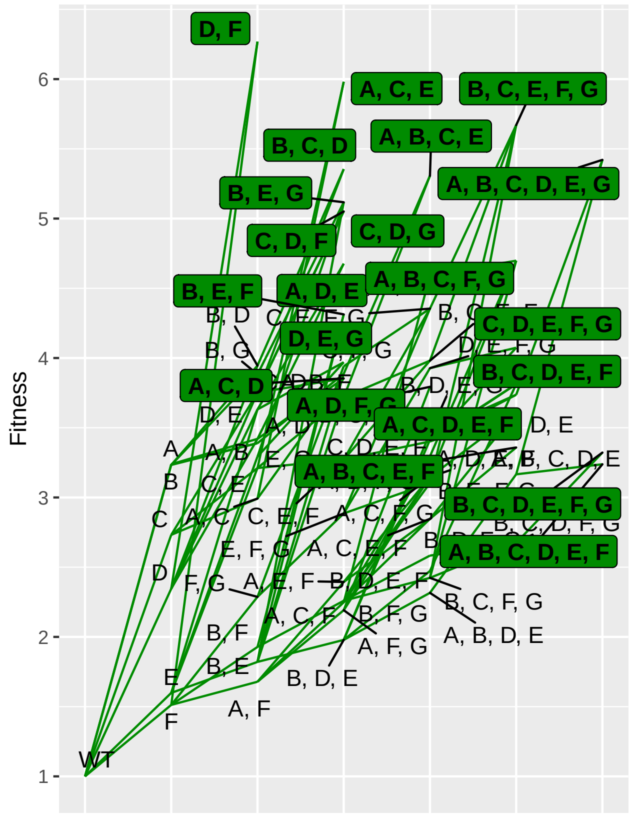

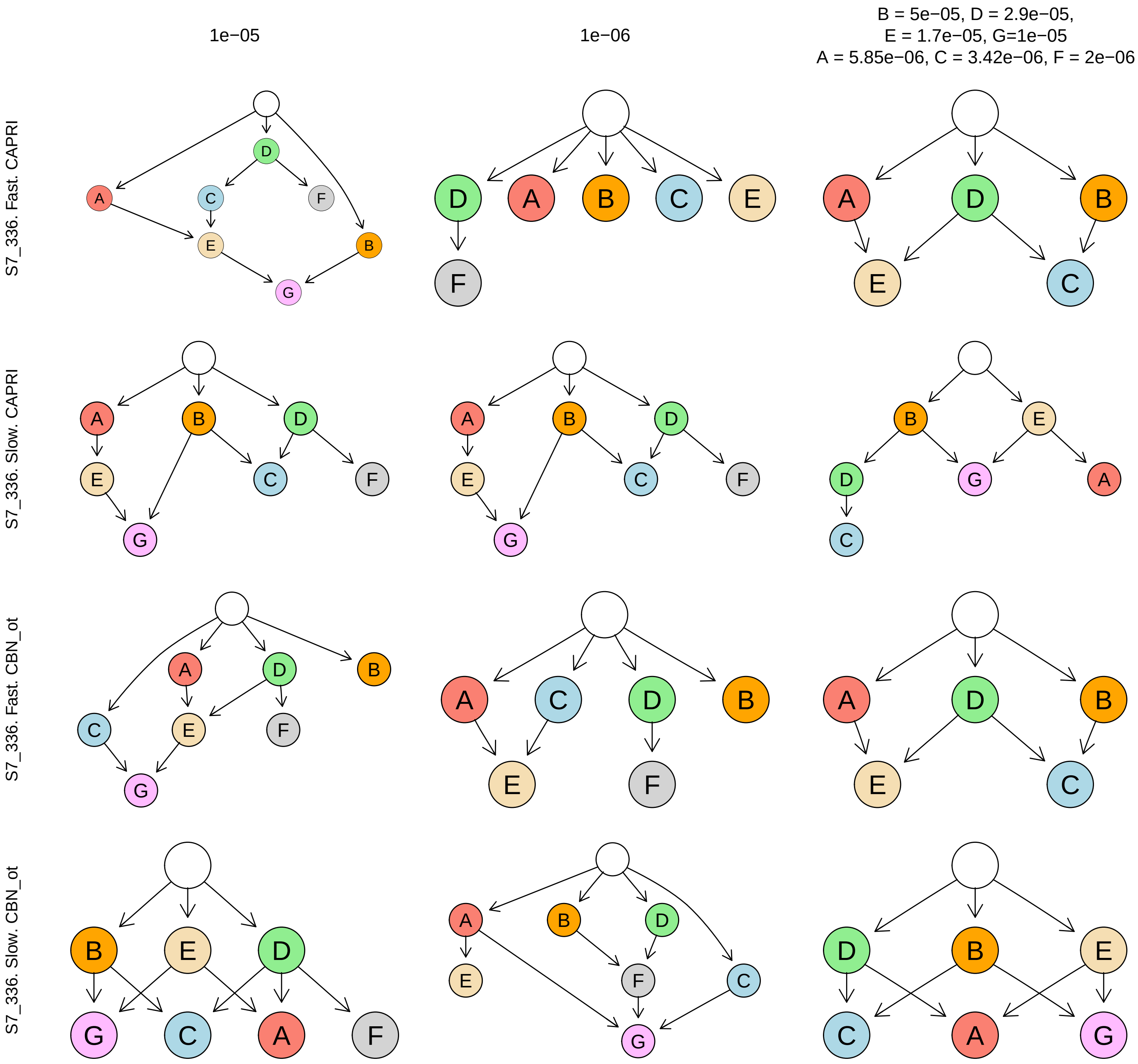

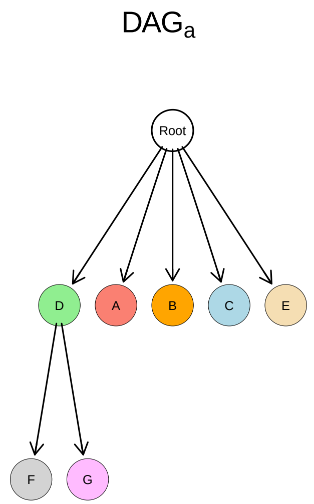

ID	Accessible Genot.	Discrep. DAG_a
S7_337	36	55

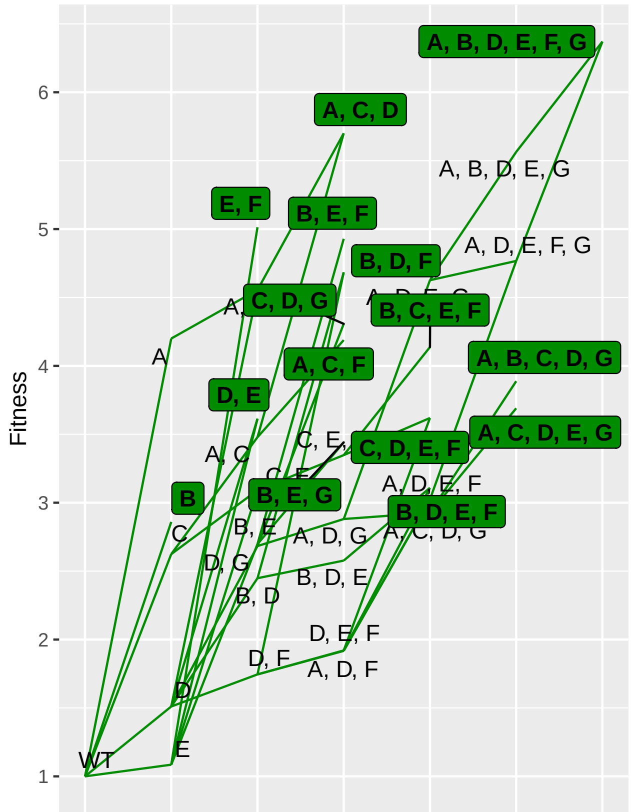

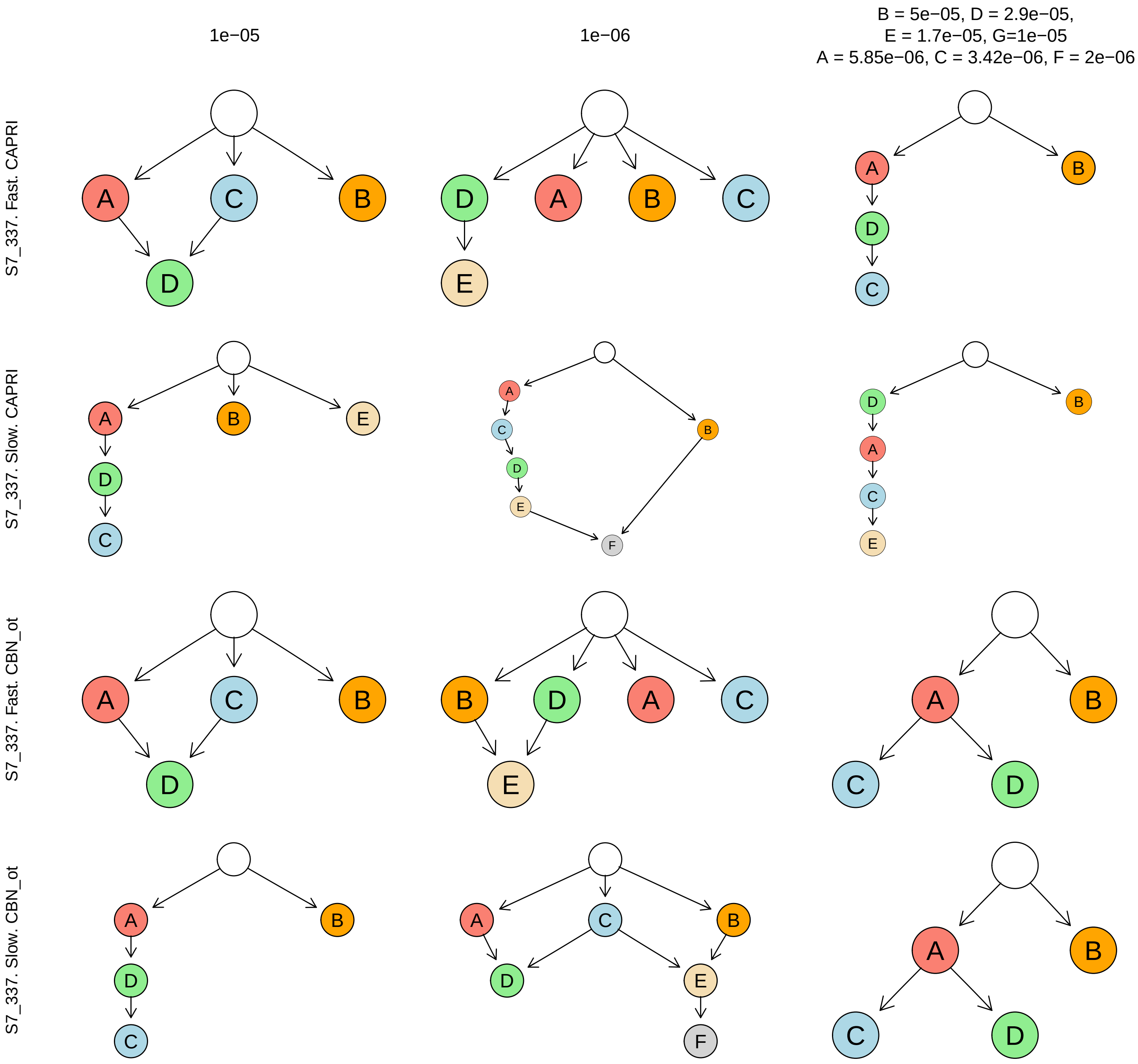

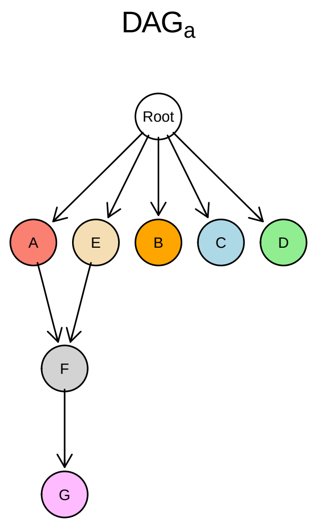

ID	Accessible Genot.	Discrep. DAG_a
S7_338	10	37

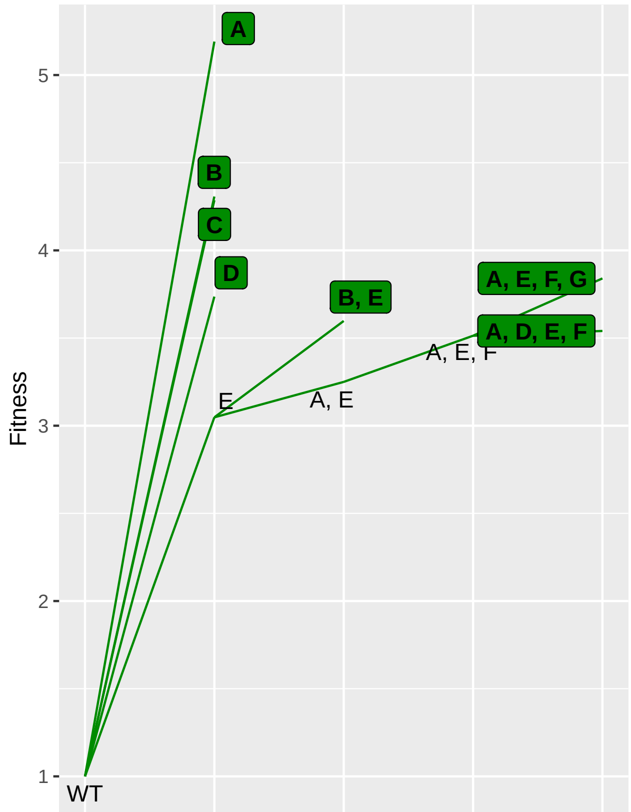

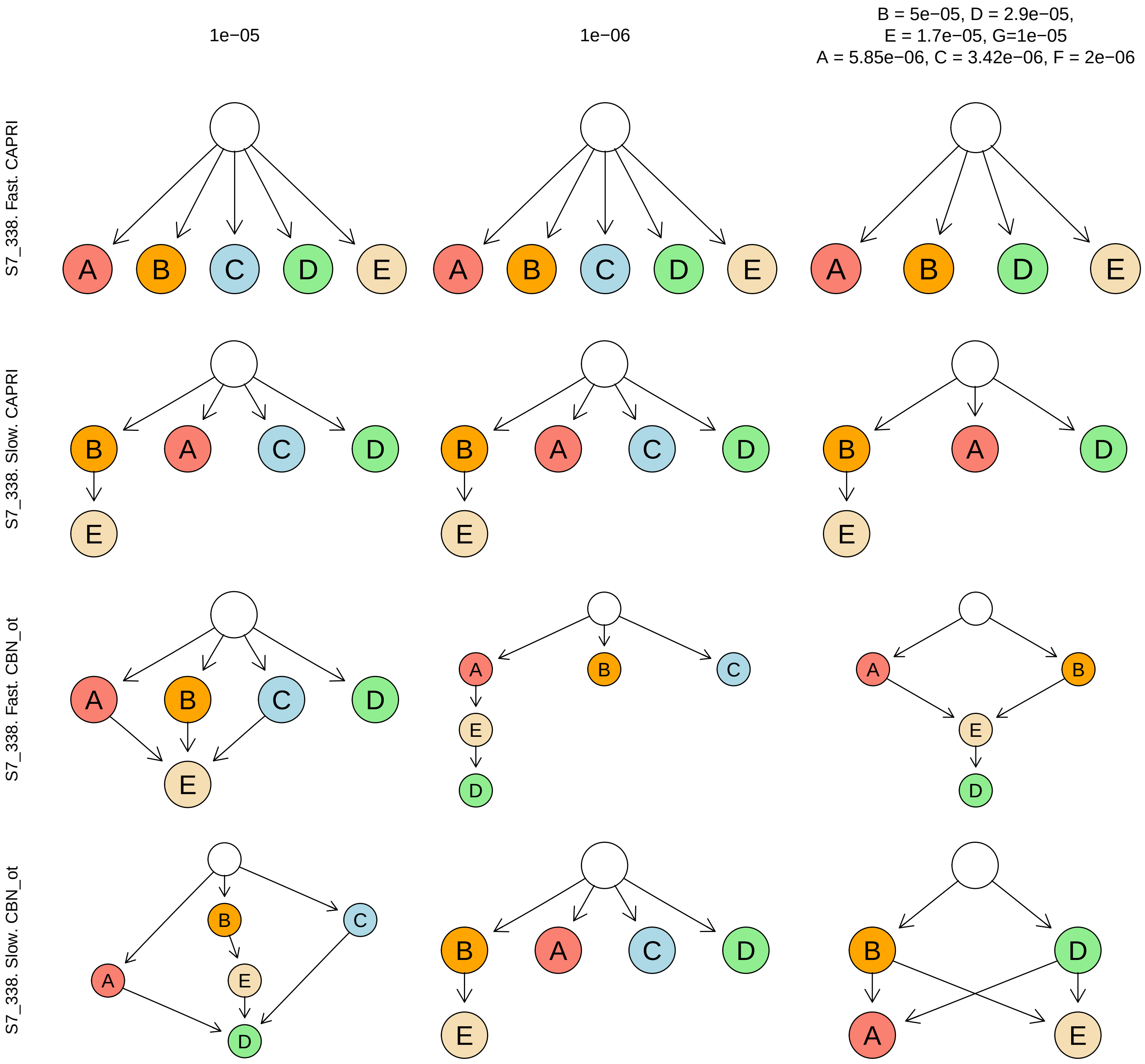

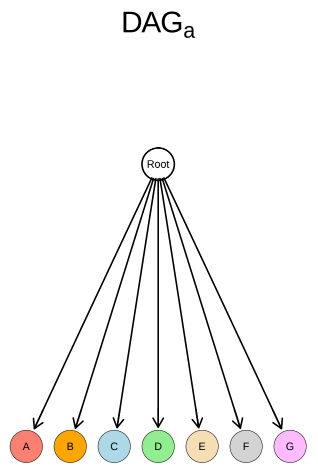

ID	Accessible Genot.	Discrep. DAG_a
S7_339	52	75

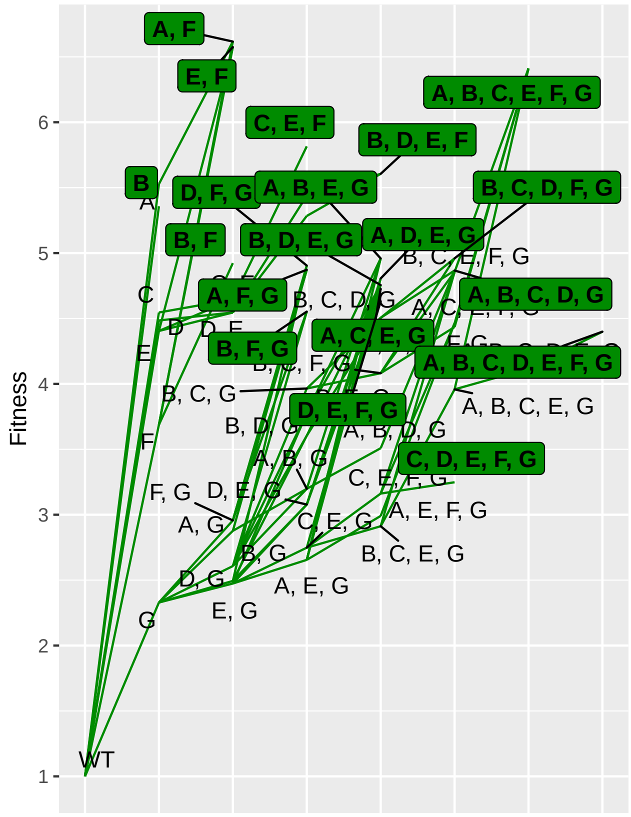

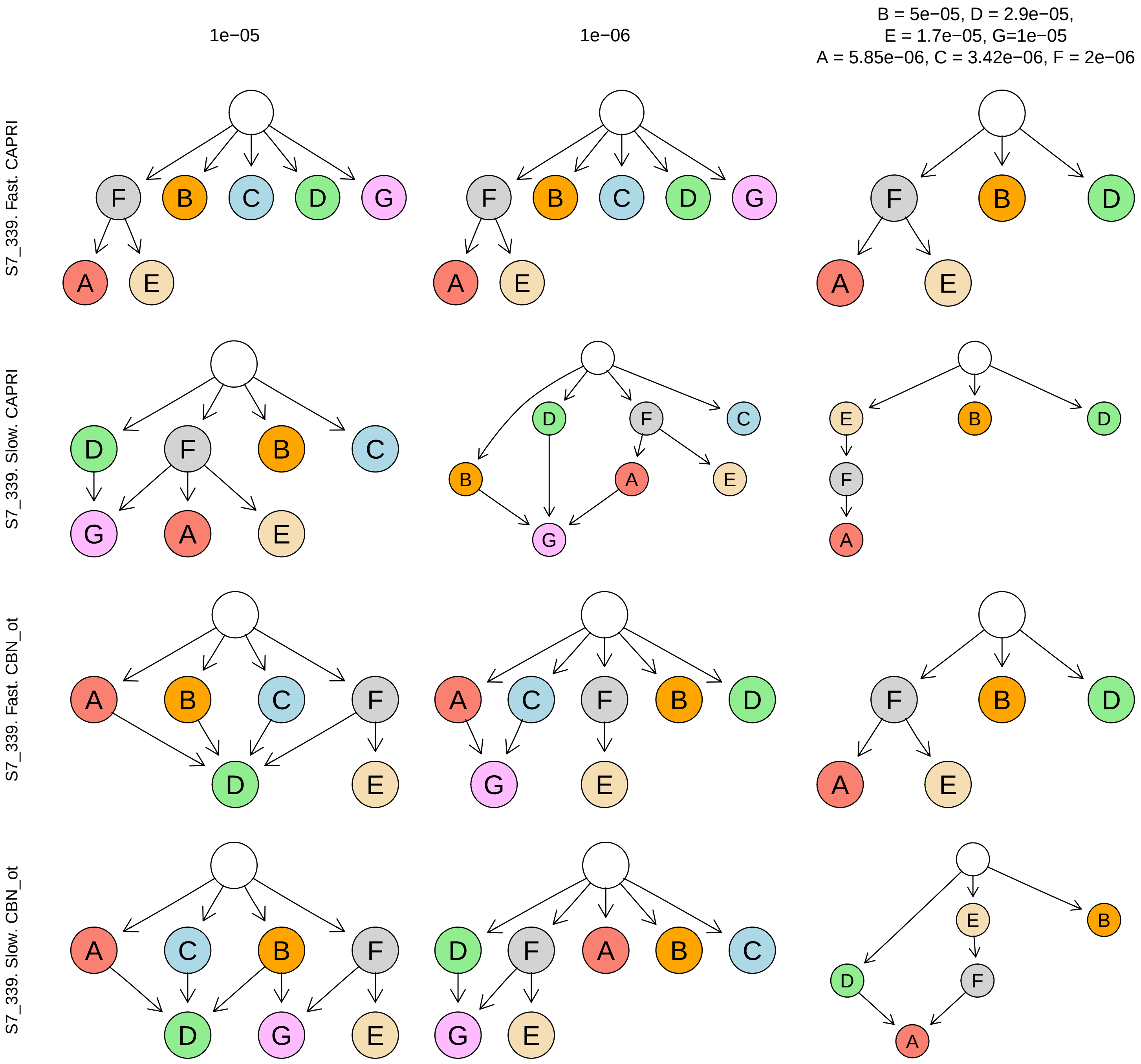

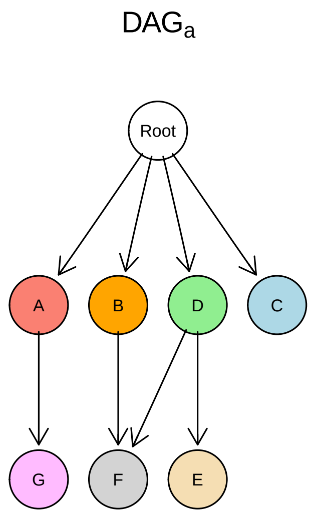

ID	Accessible Genot.	Discrep. DAG_a
S7_340	21	36

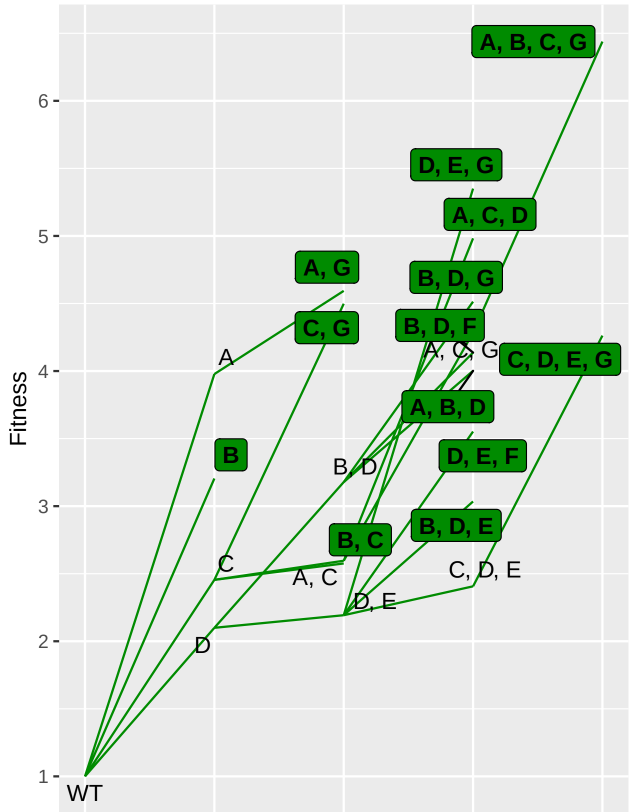

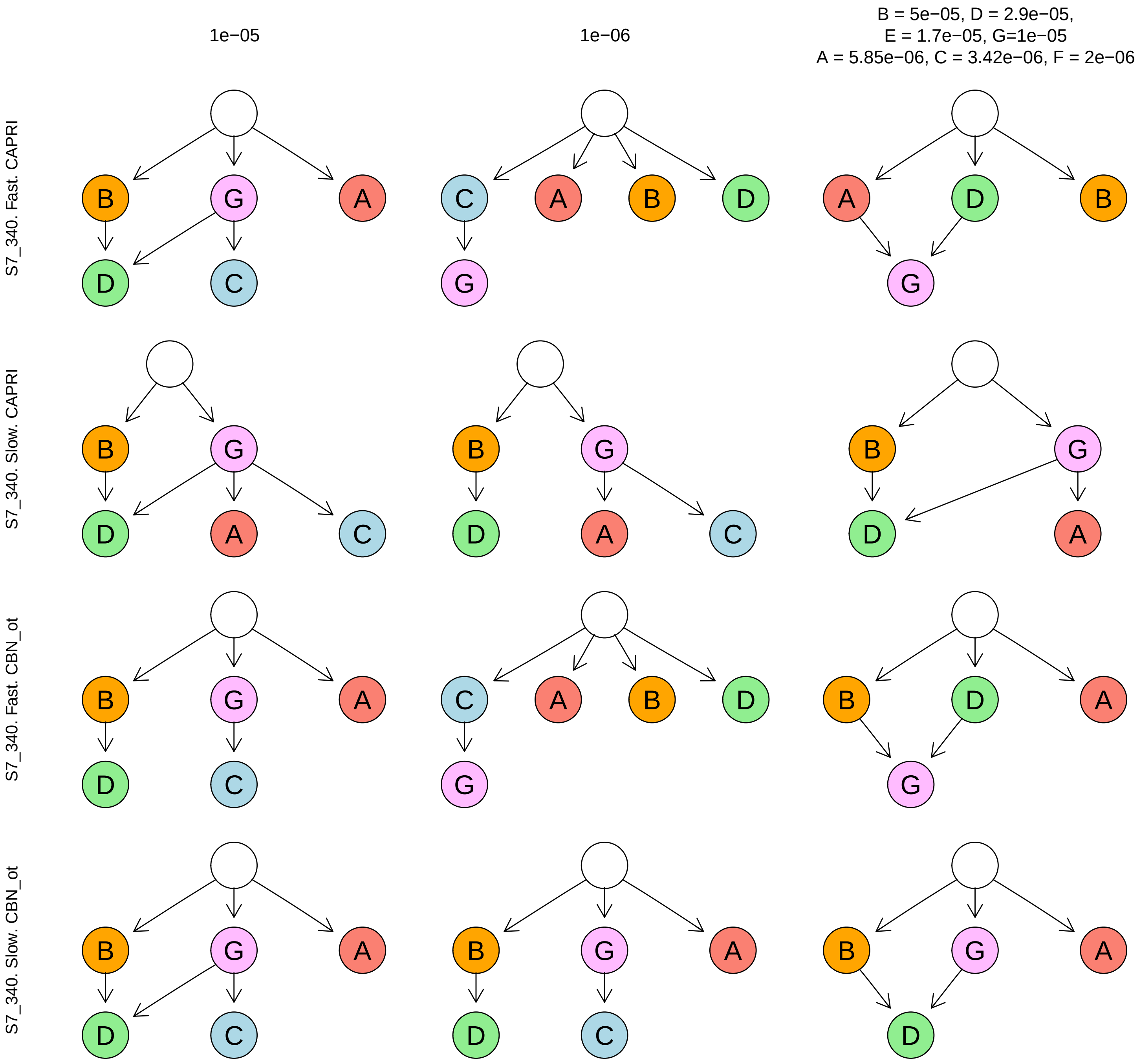

DAG$_a$

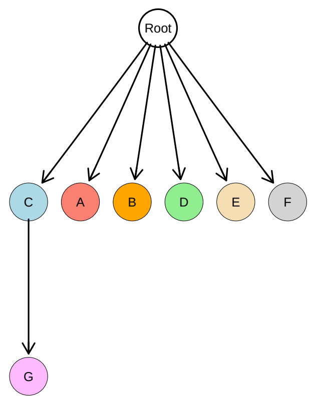

ID	Accessible Genot.	Discrep. DAG_a
S7_341	22	89

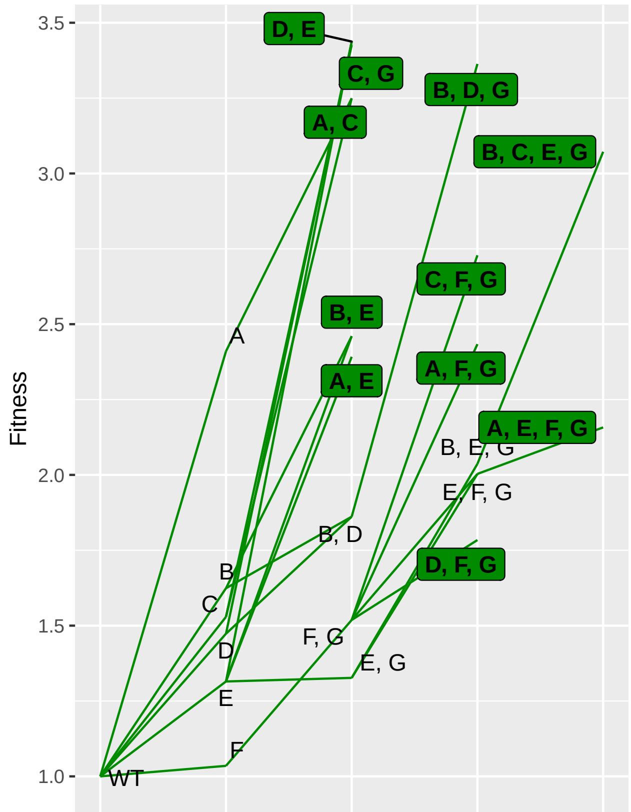

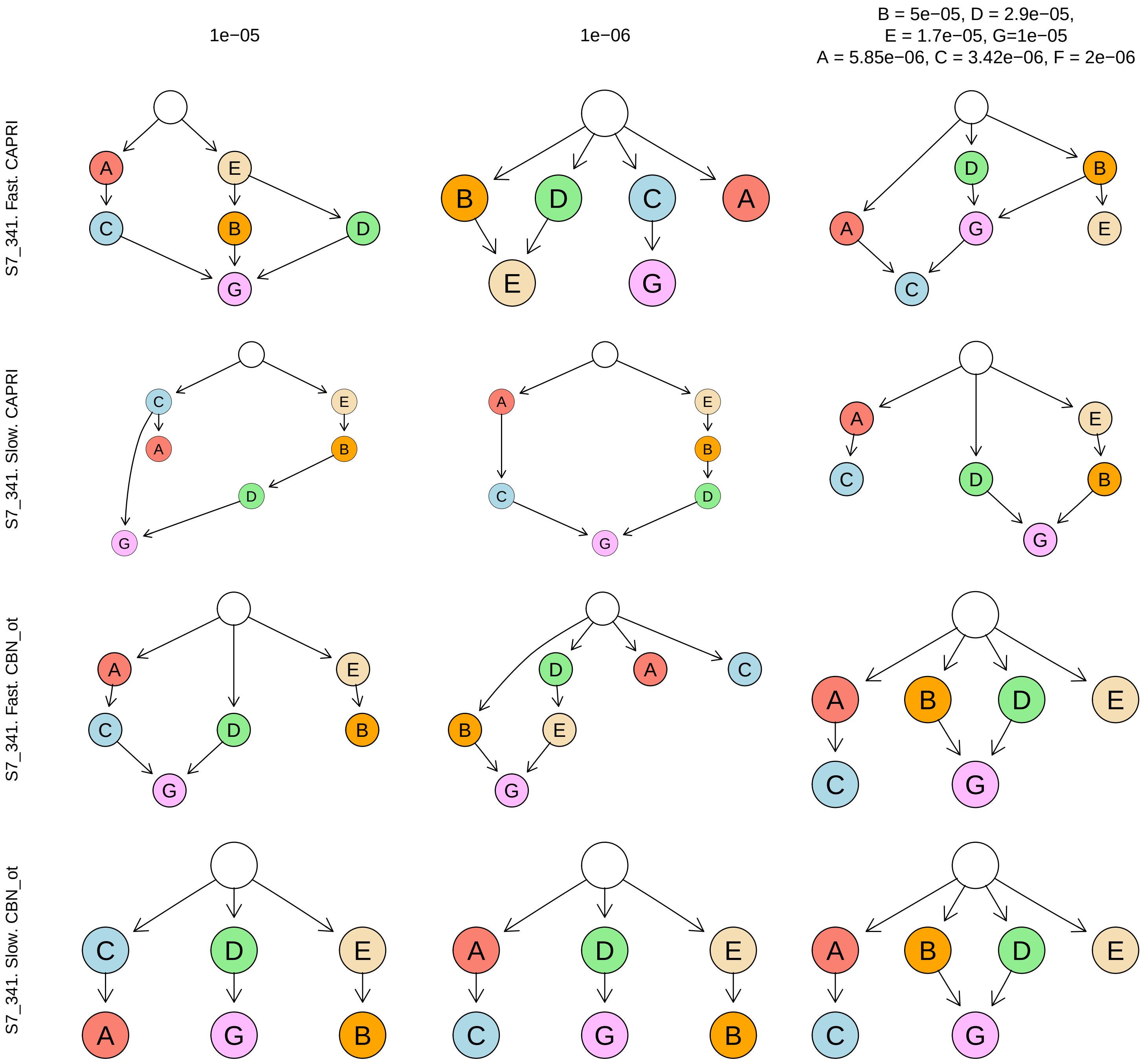

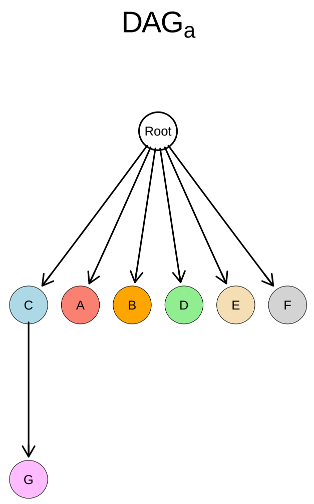

ID	Accessible Genot.	Discrep. DAG_a
S7_342	35	66

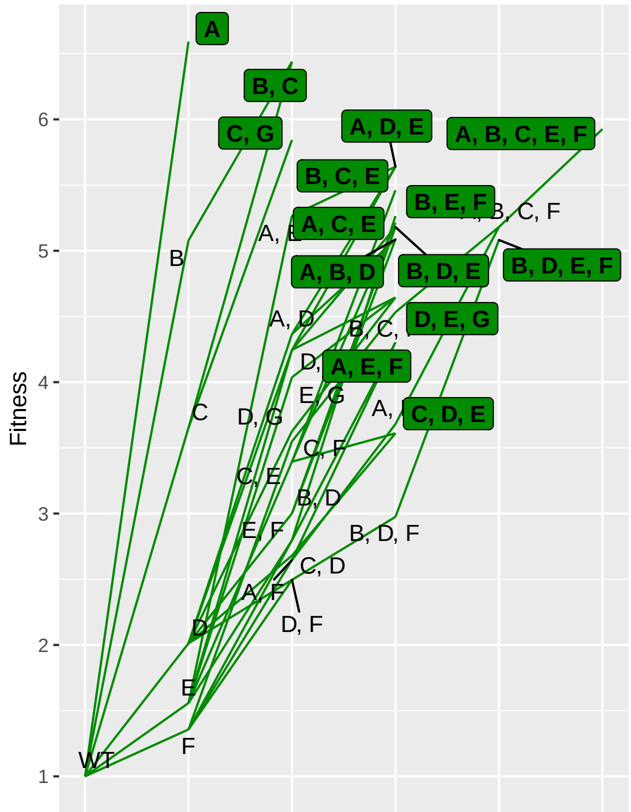

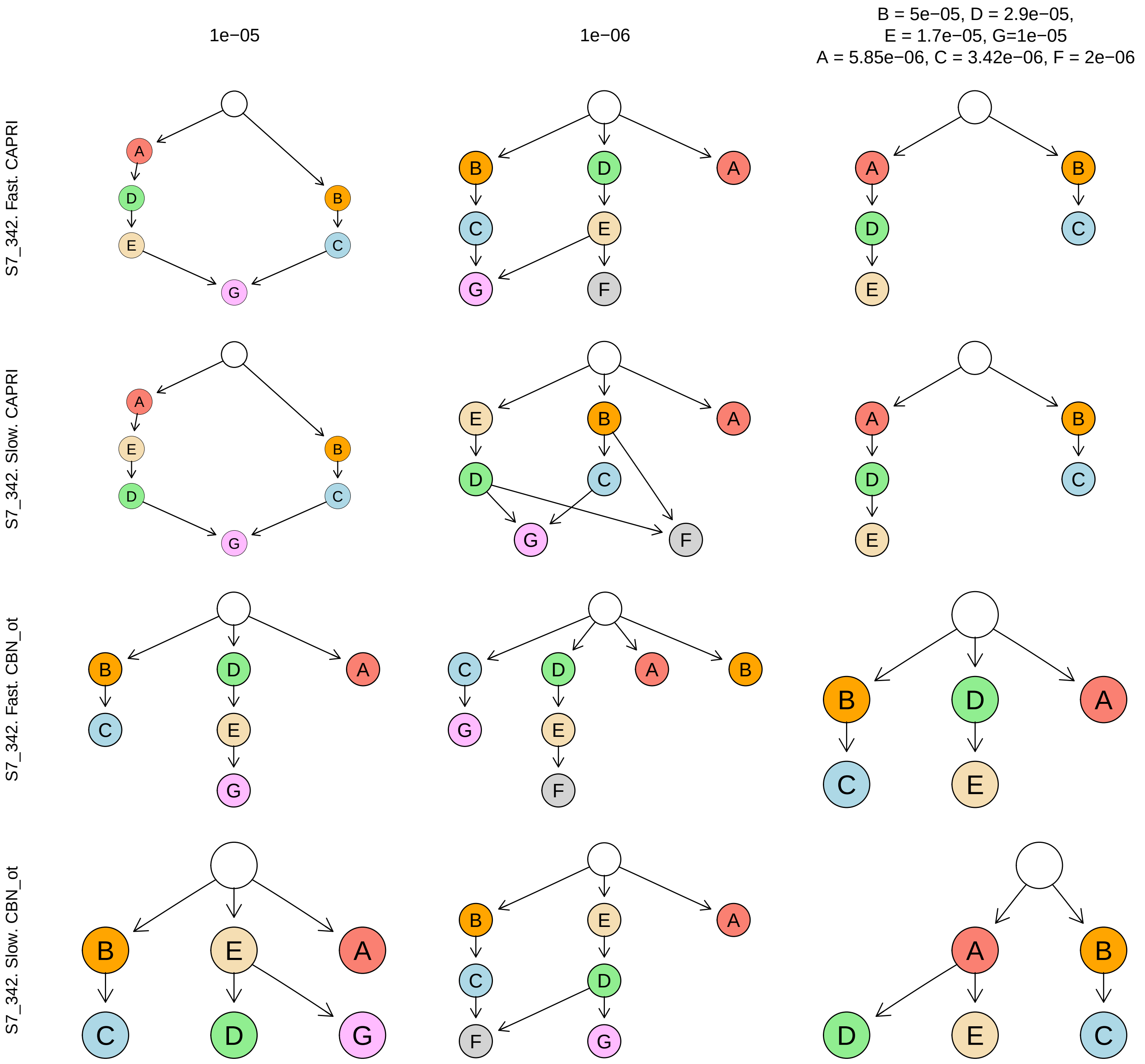

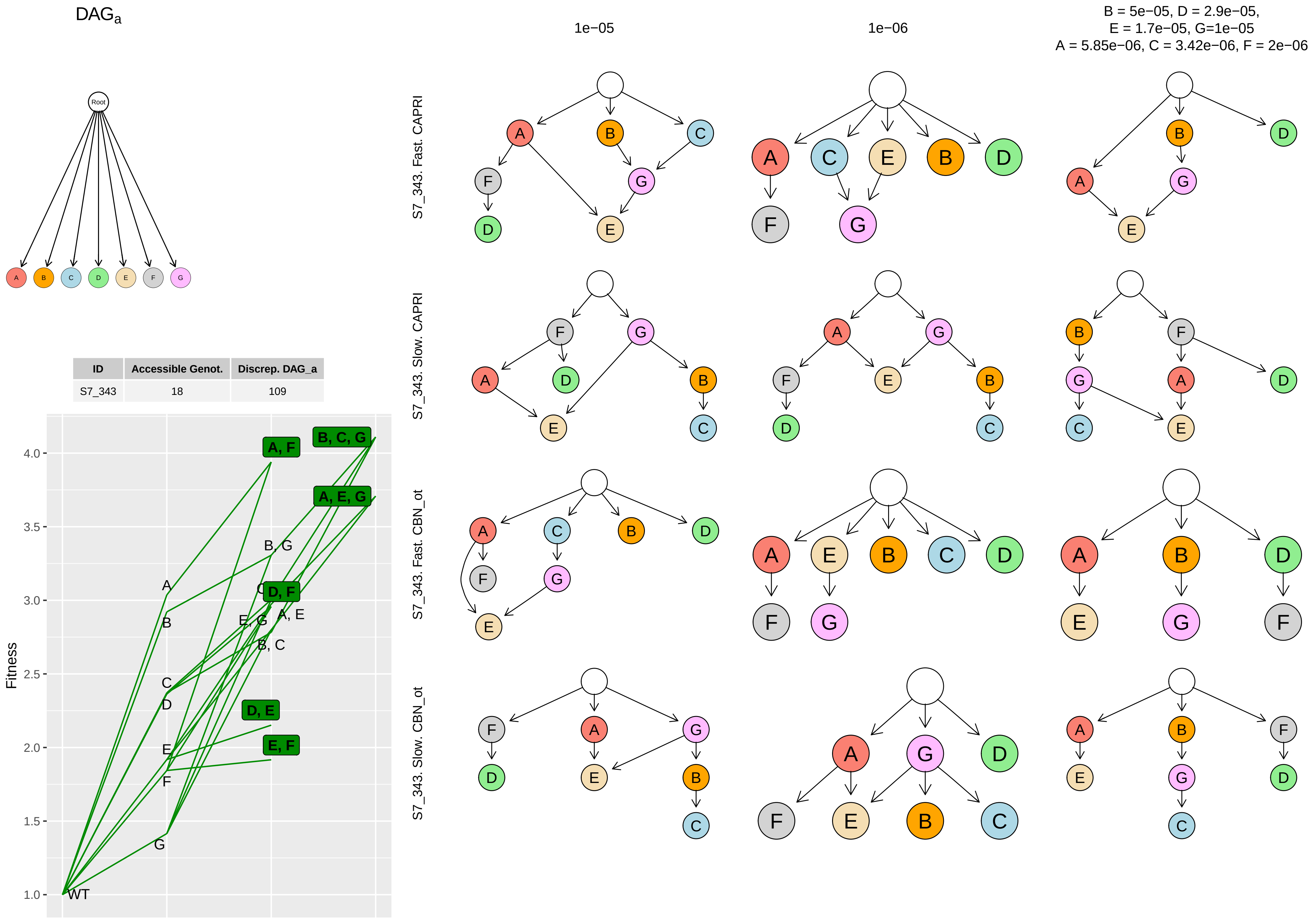

ID	Accessible Genot.	Discrep. DAG_a
S7_343	18	109

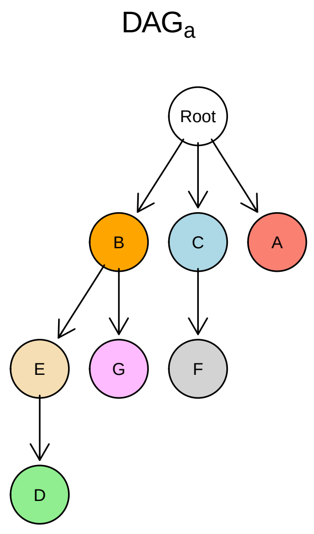

ID	Accessible Genot.	Discrep. DAG_a
S7_344	18	35

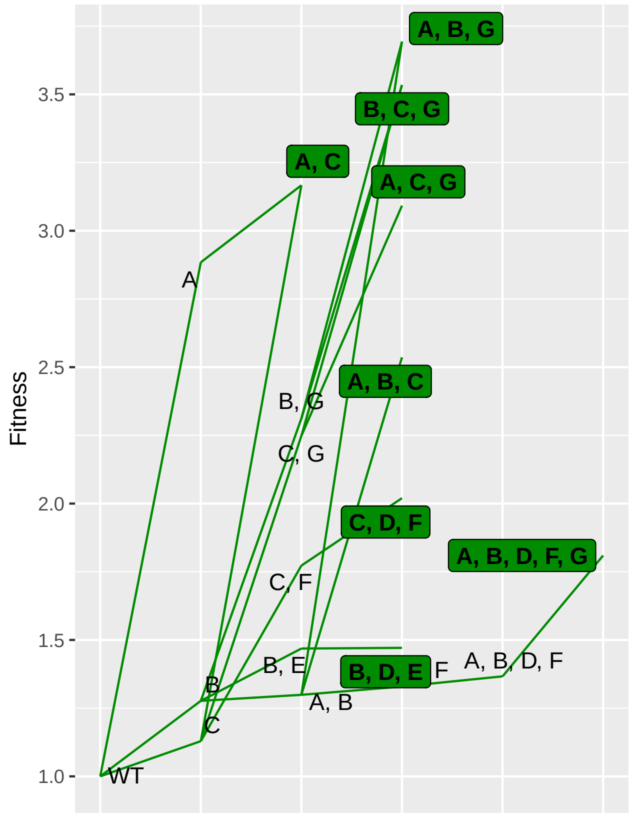

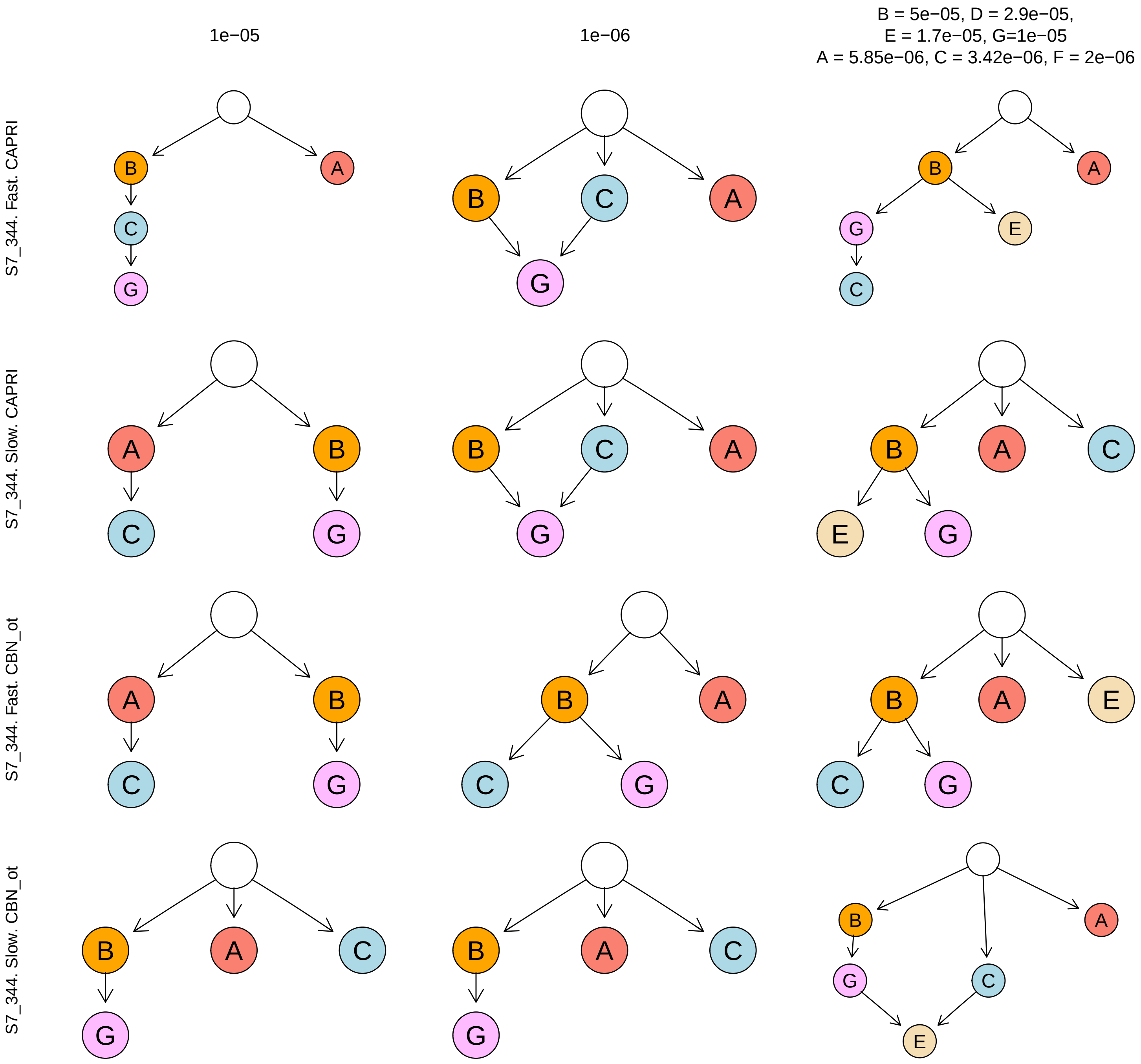

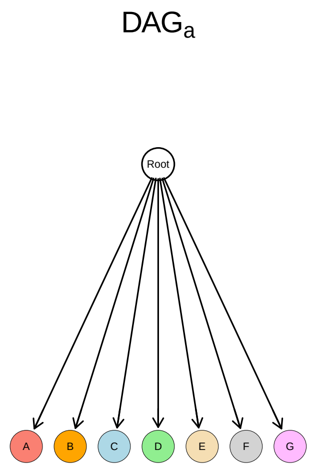

ID	Accessible Genot.	Discrep. DAG_a
S7_345	65	62

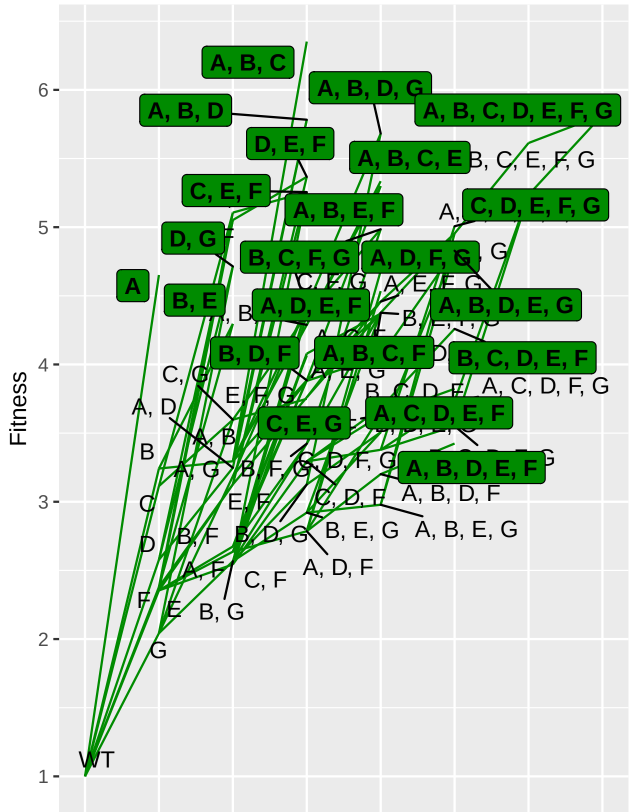

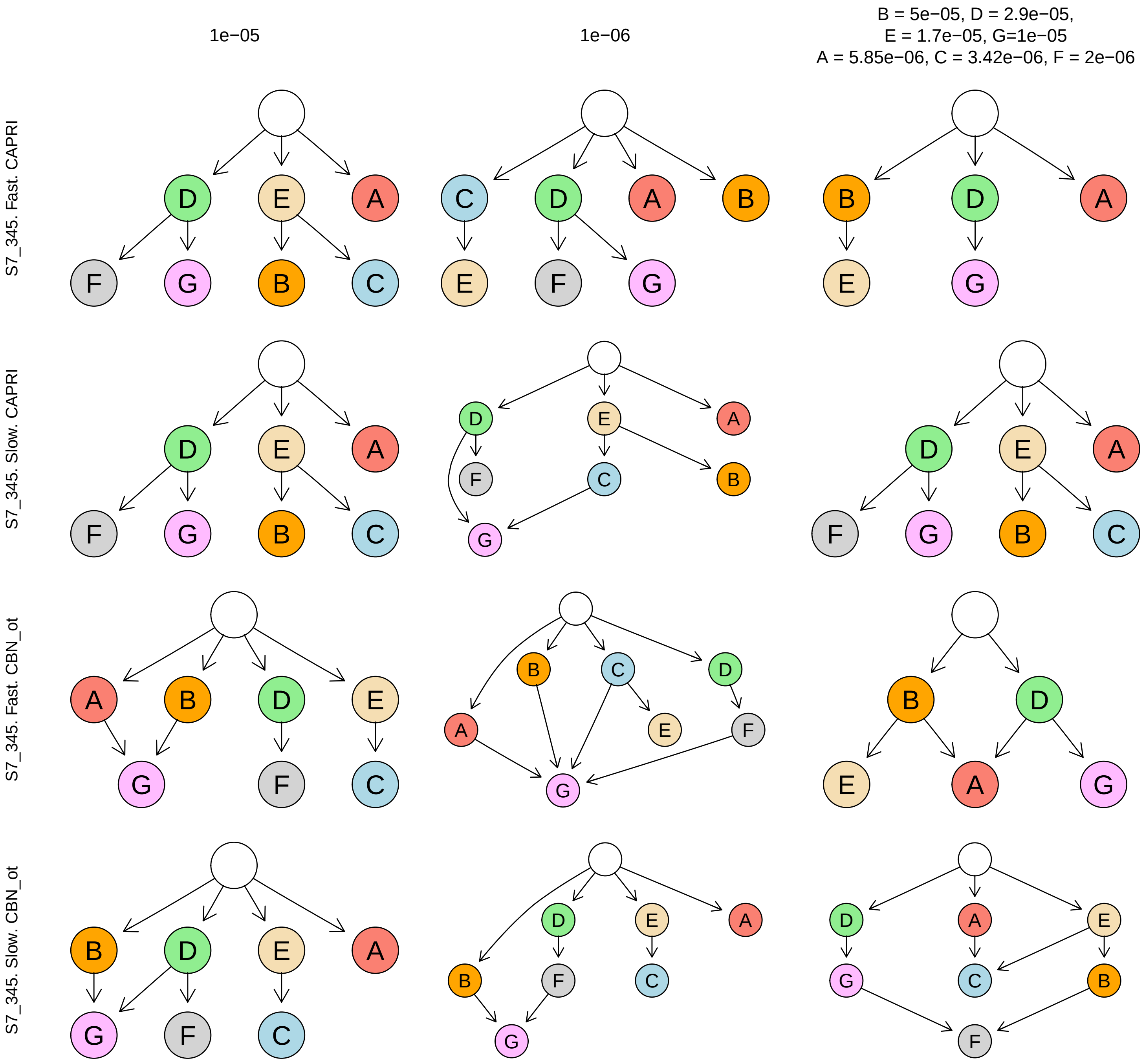

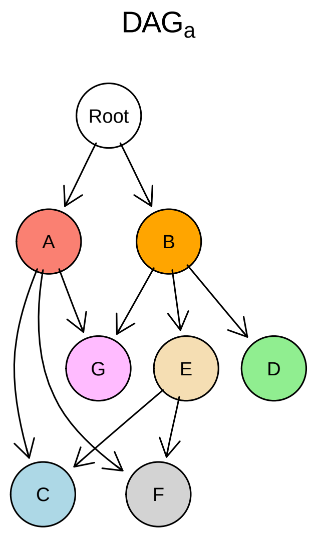

ID	Accessible Genot.	Discrep. DAG_a
S7_346	14	11

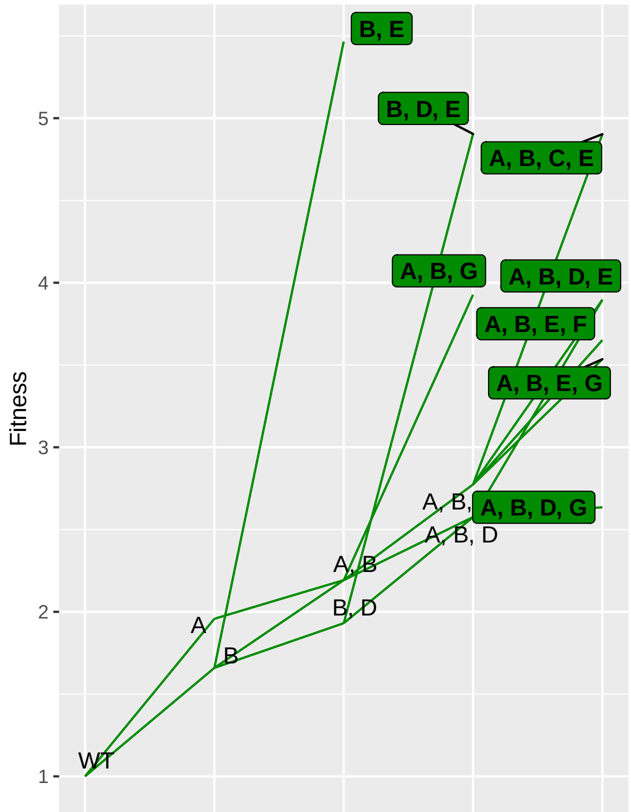

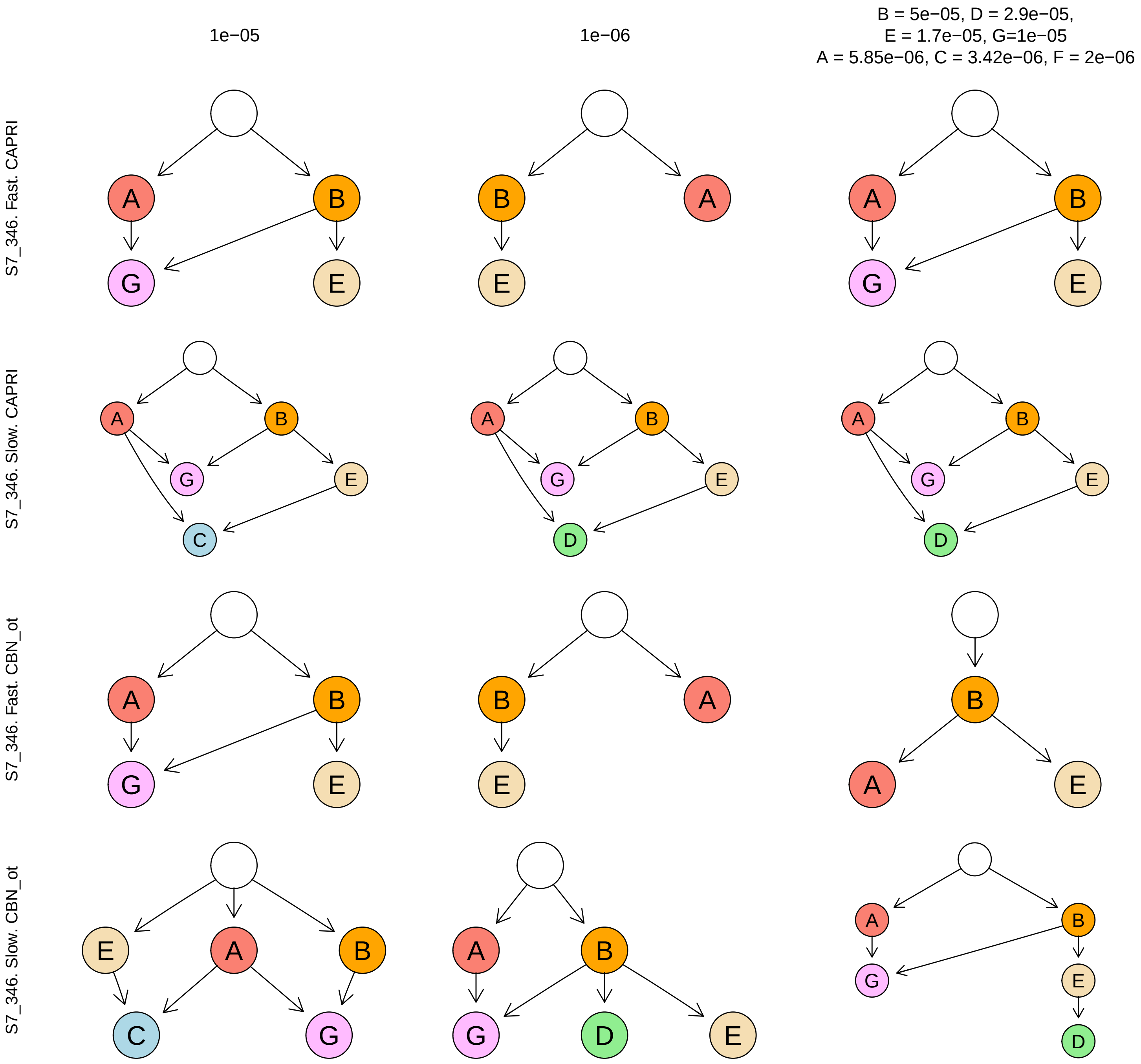

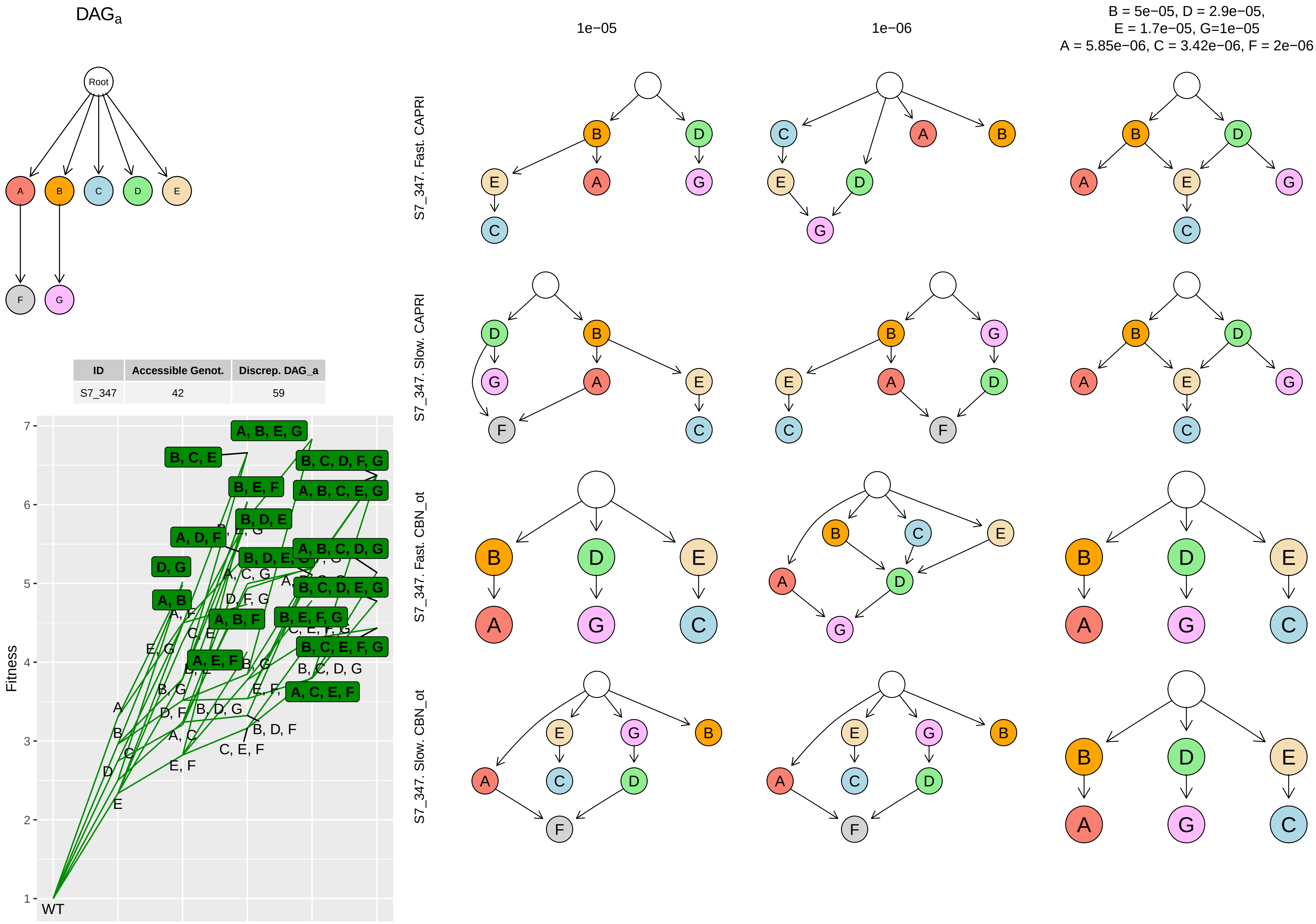

ID	Accessible Genot.	Discrep. DAG_a
S7_347	42	59

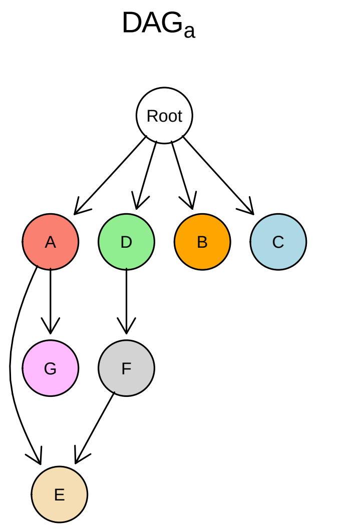

ID	Accessible Genot.	Discrep. DAG_a
S7_348	14	33

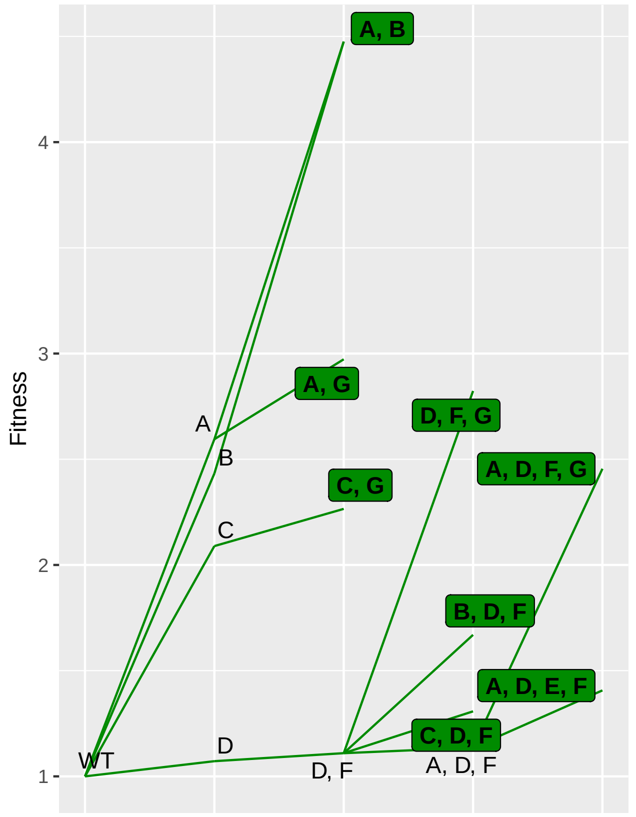

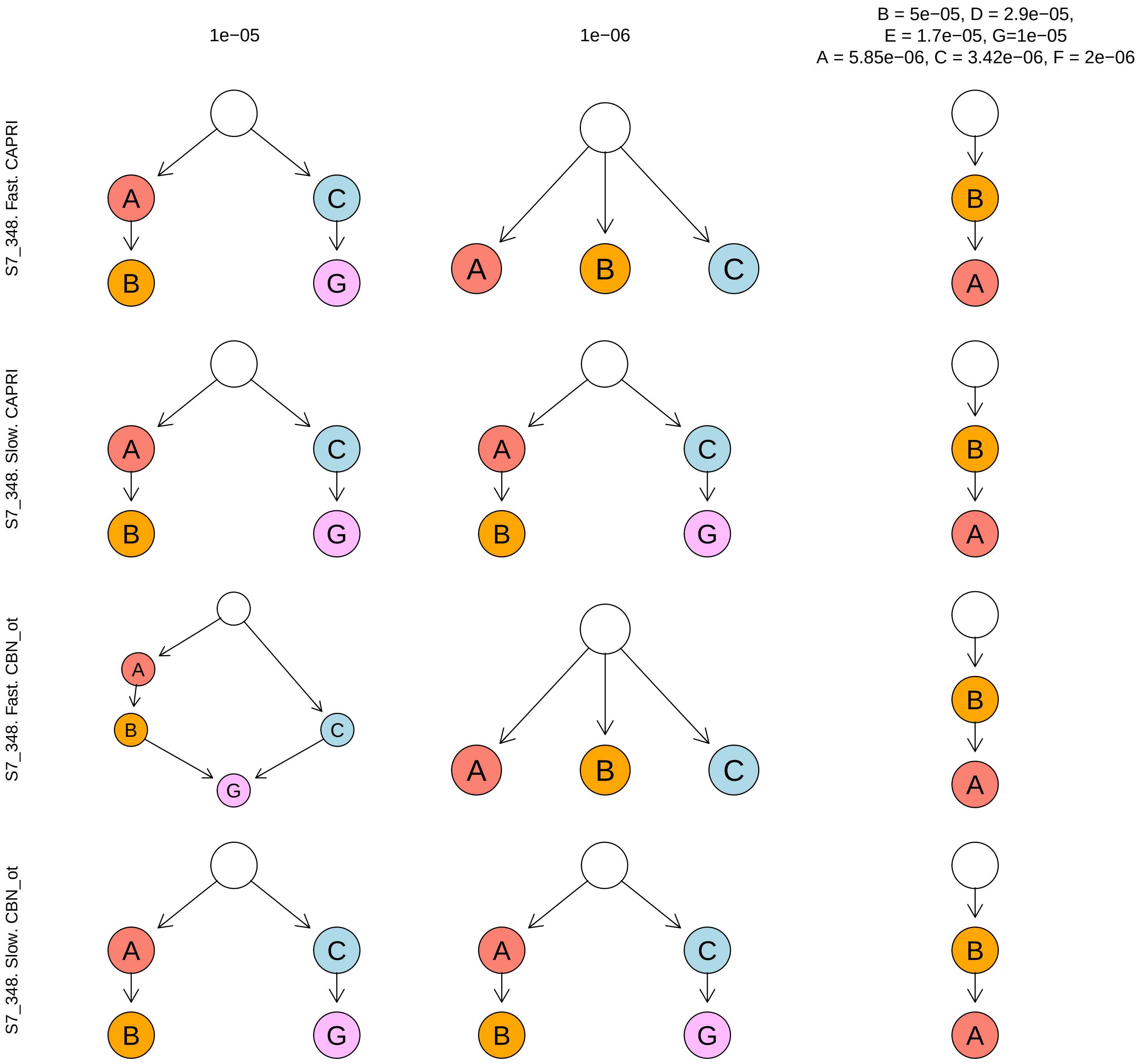

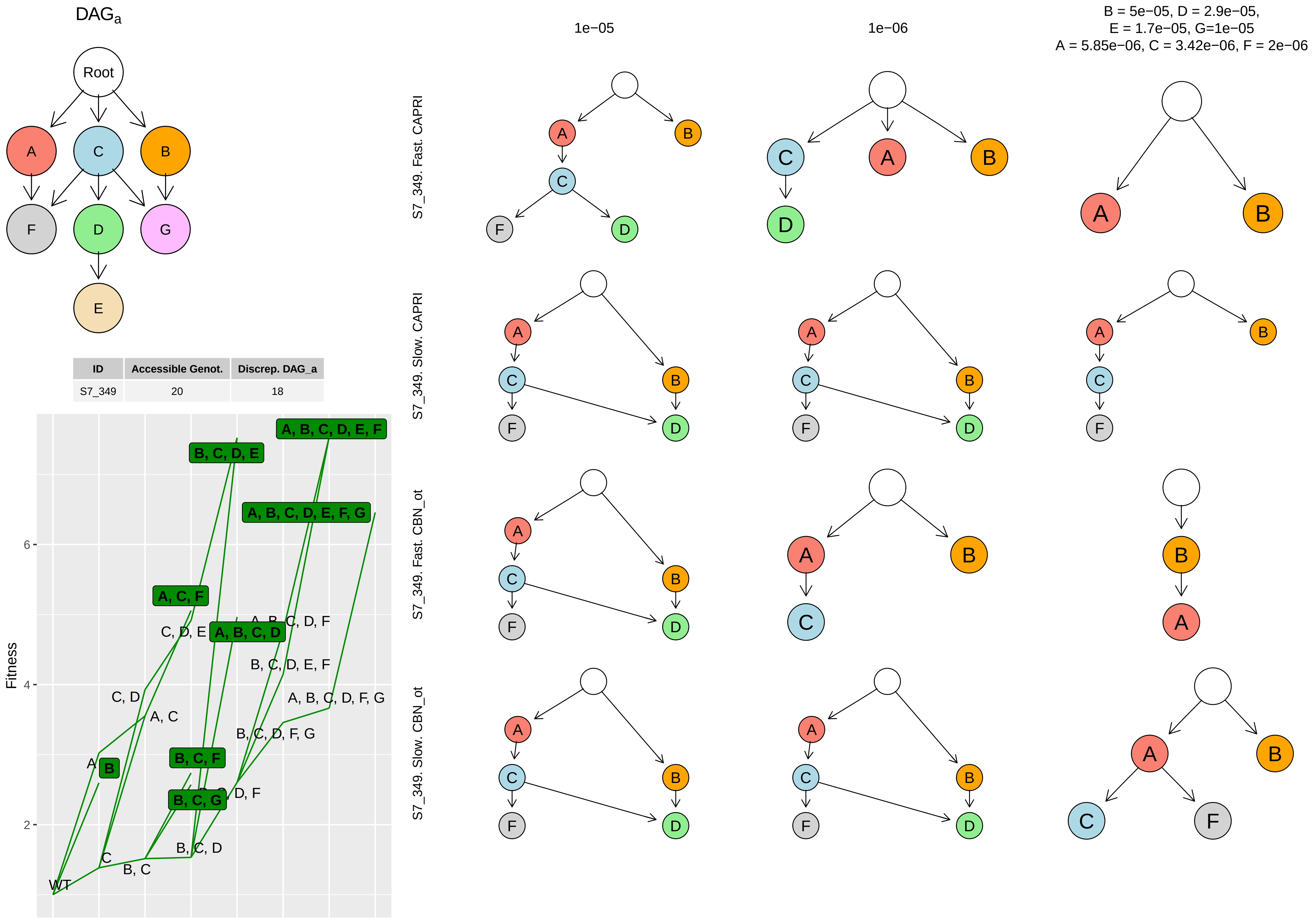

ID	Accessible Genot.	Discrep. DAG_a
S7_349	20	18

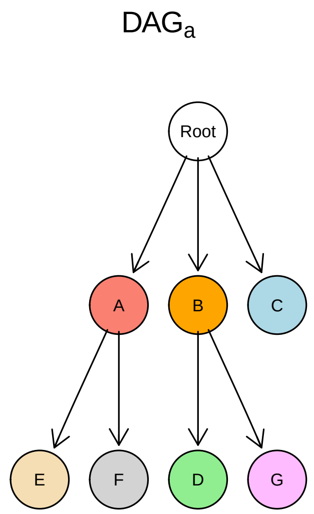

ID	Accessible Genot.	Discrep. DAG_a
S7_350	53	54

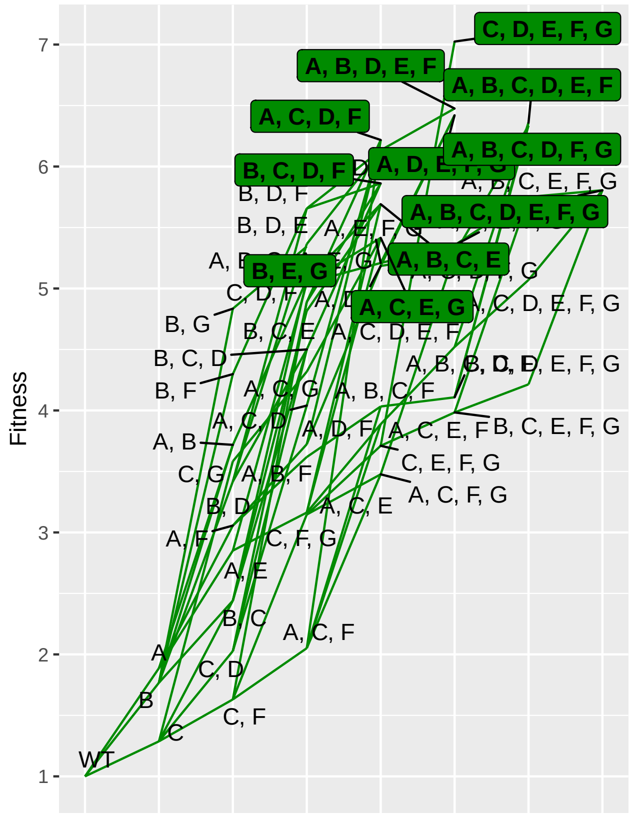

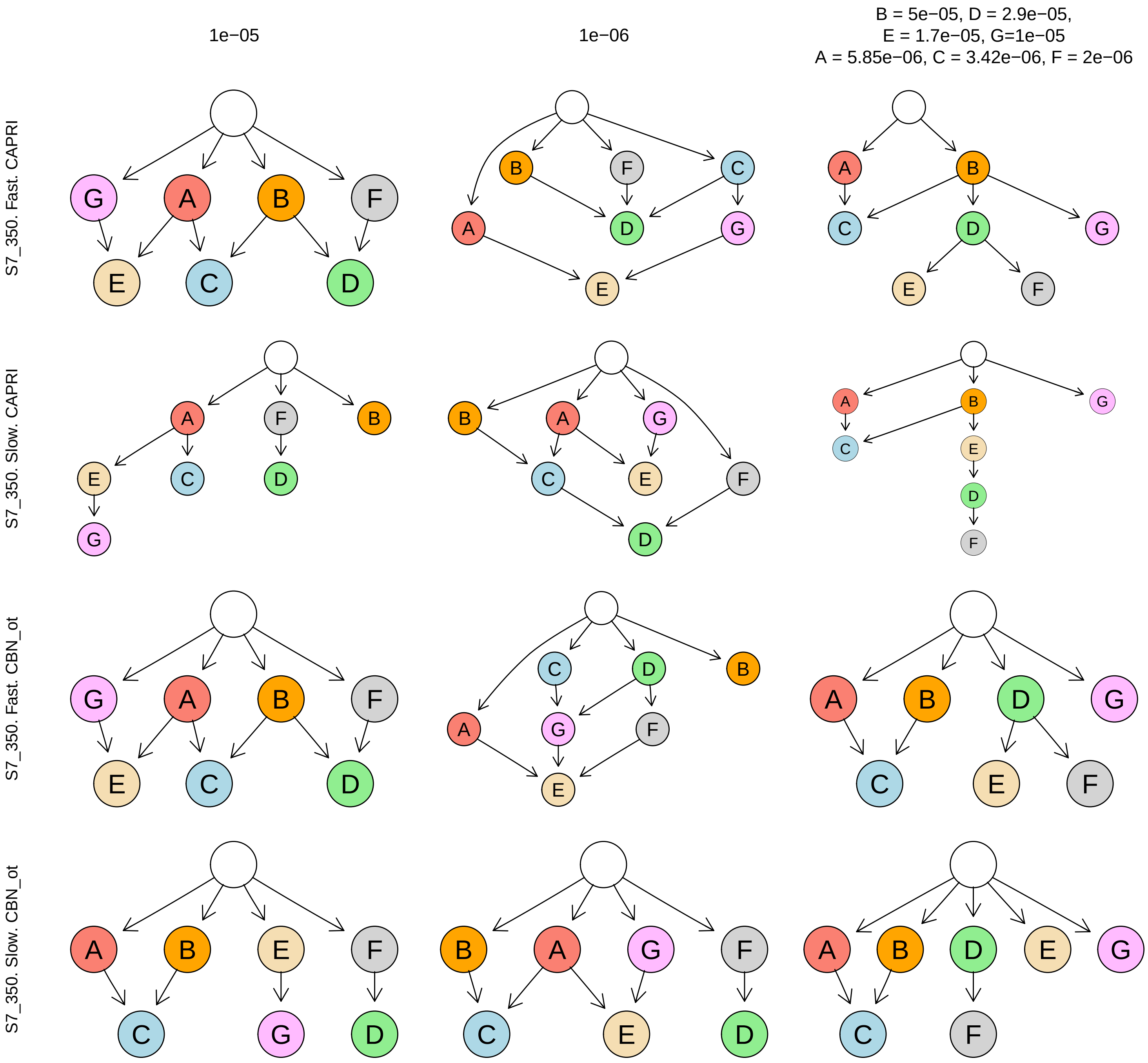

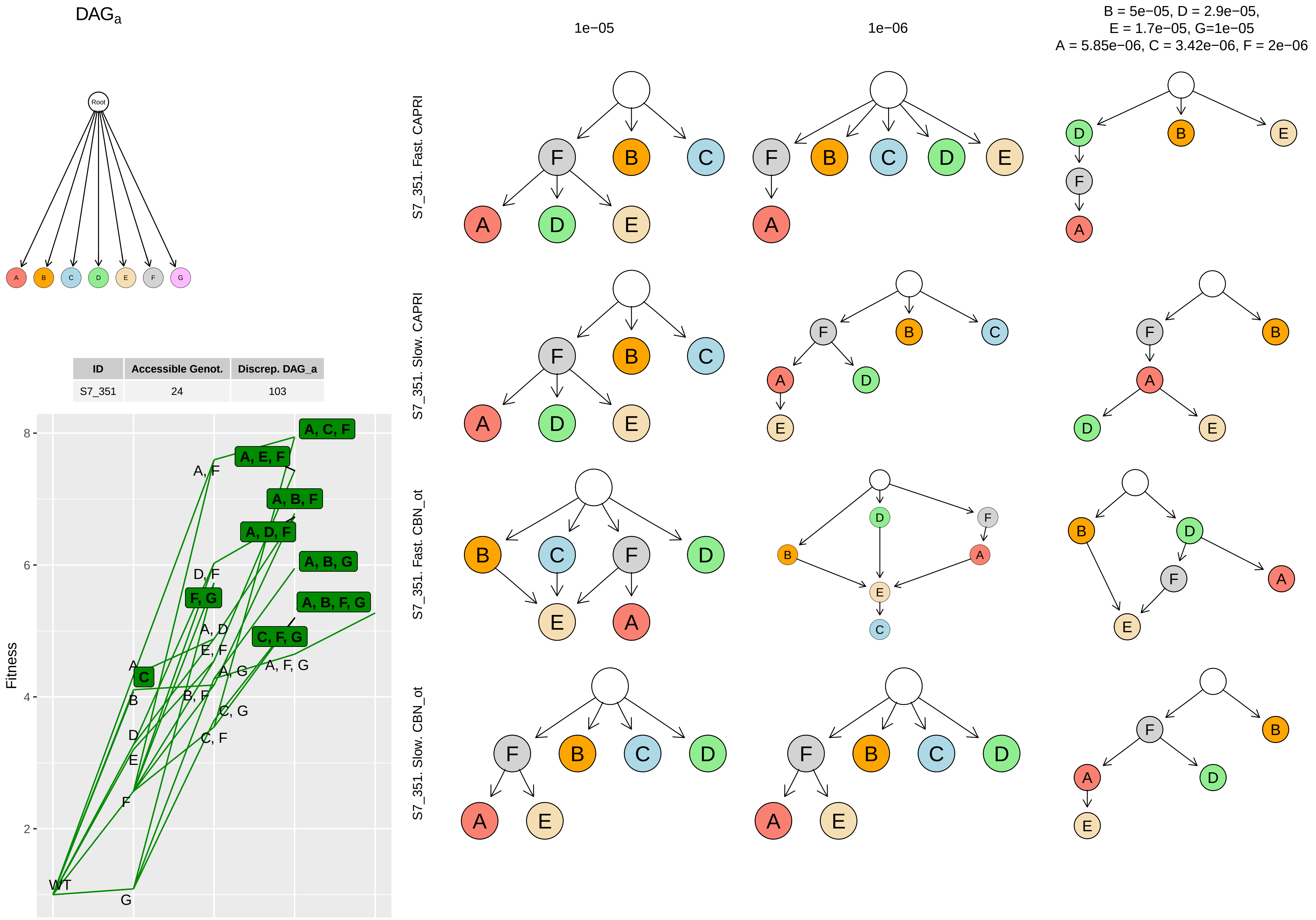

ID	Accessible Genot.	Discrep. DAG_a
S7_351	24	103

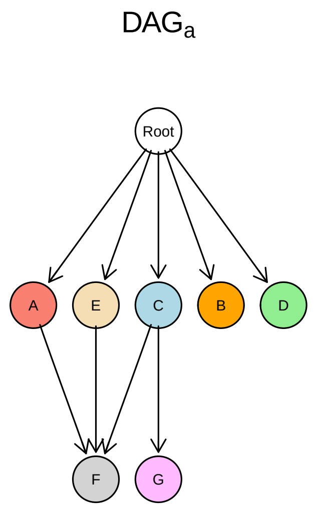

ID	Accessible Genot.	Discrep. DAG_a
S7_352	12	43

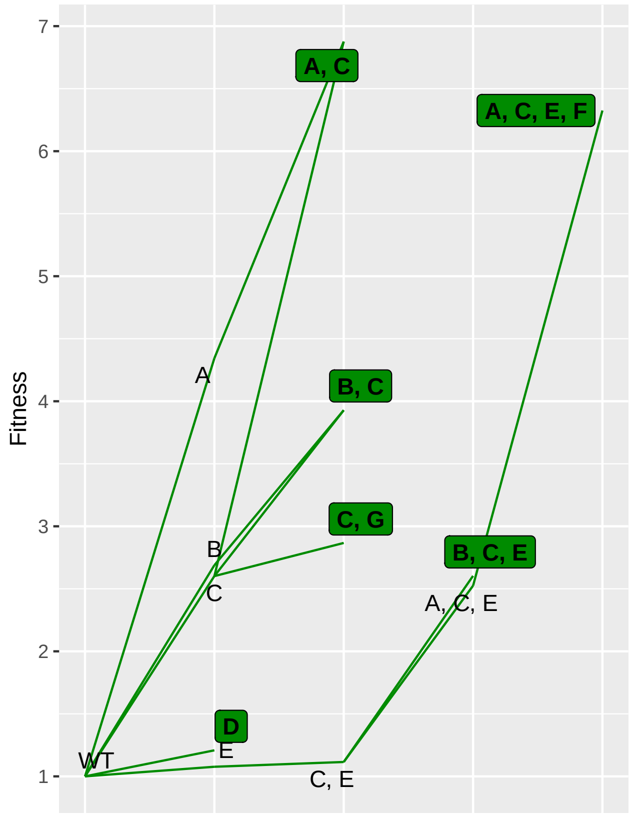

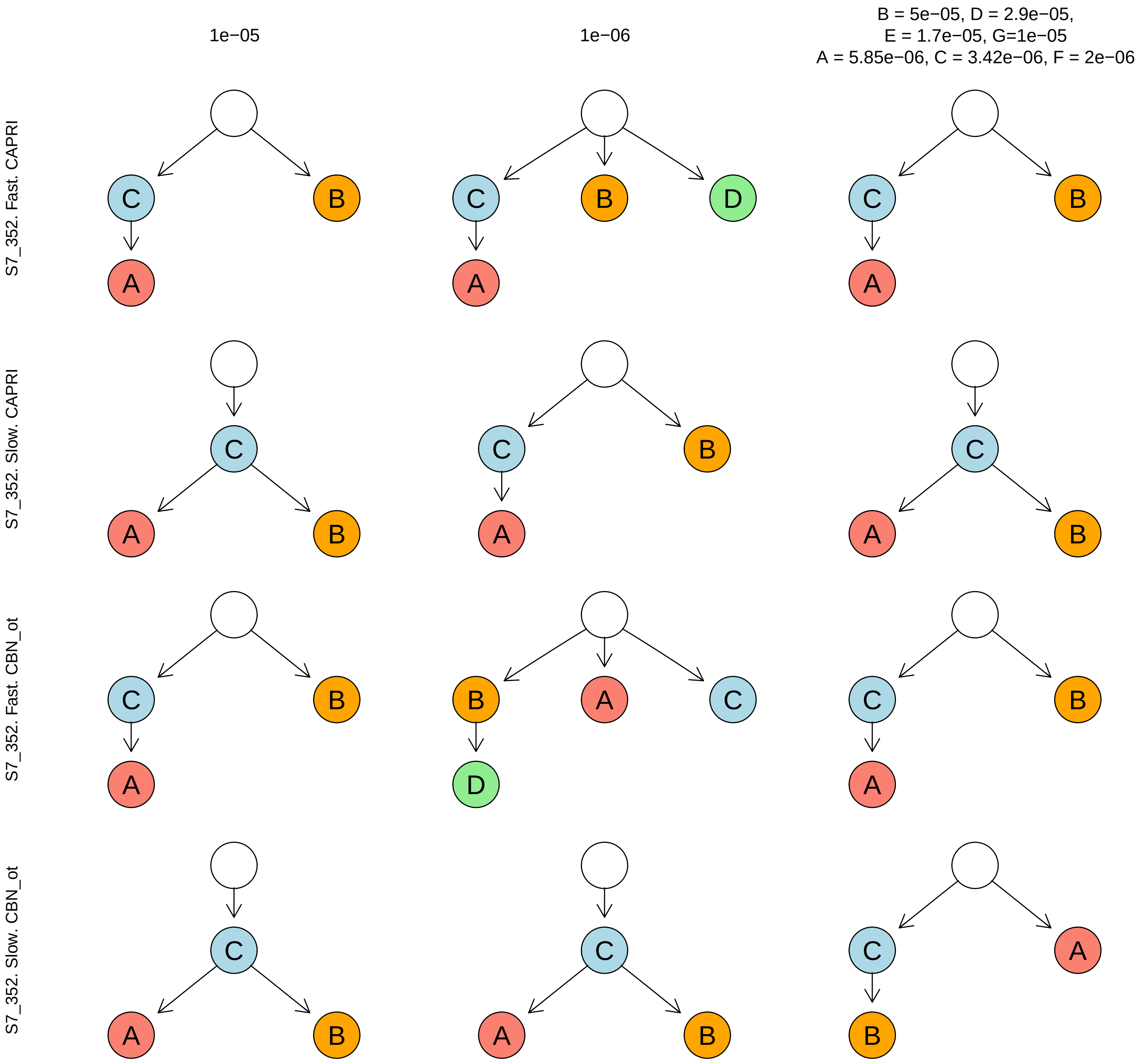

DAG_a

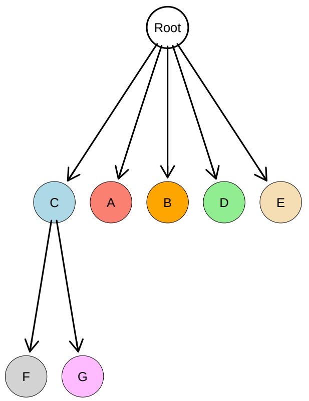

ID	Accessible Genot.	Discrep. DAG_a
S7_353	27	58

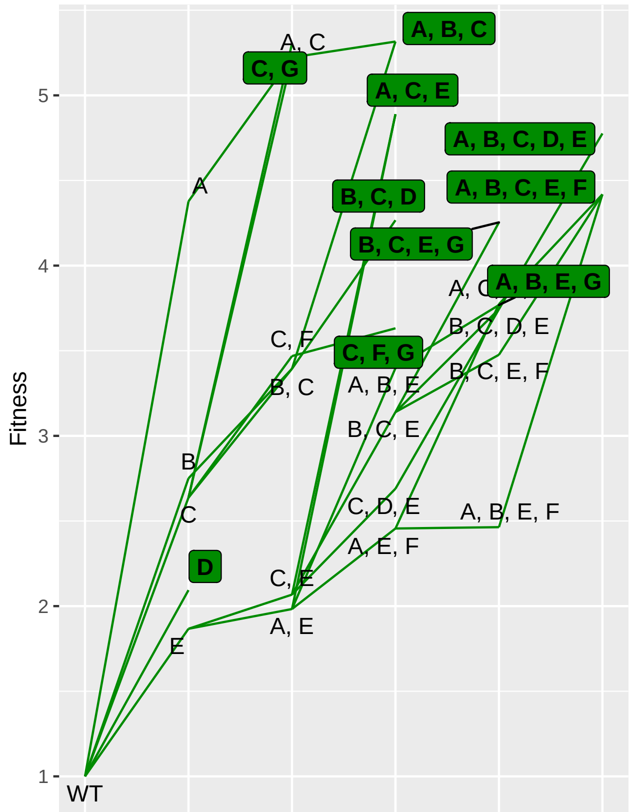

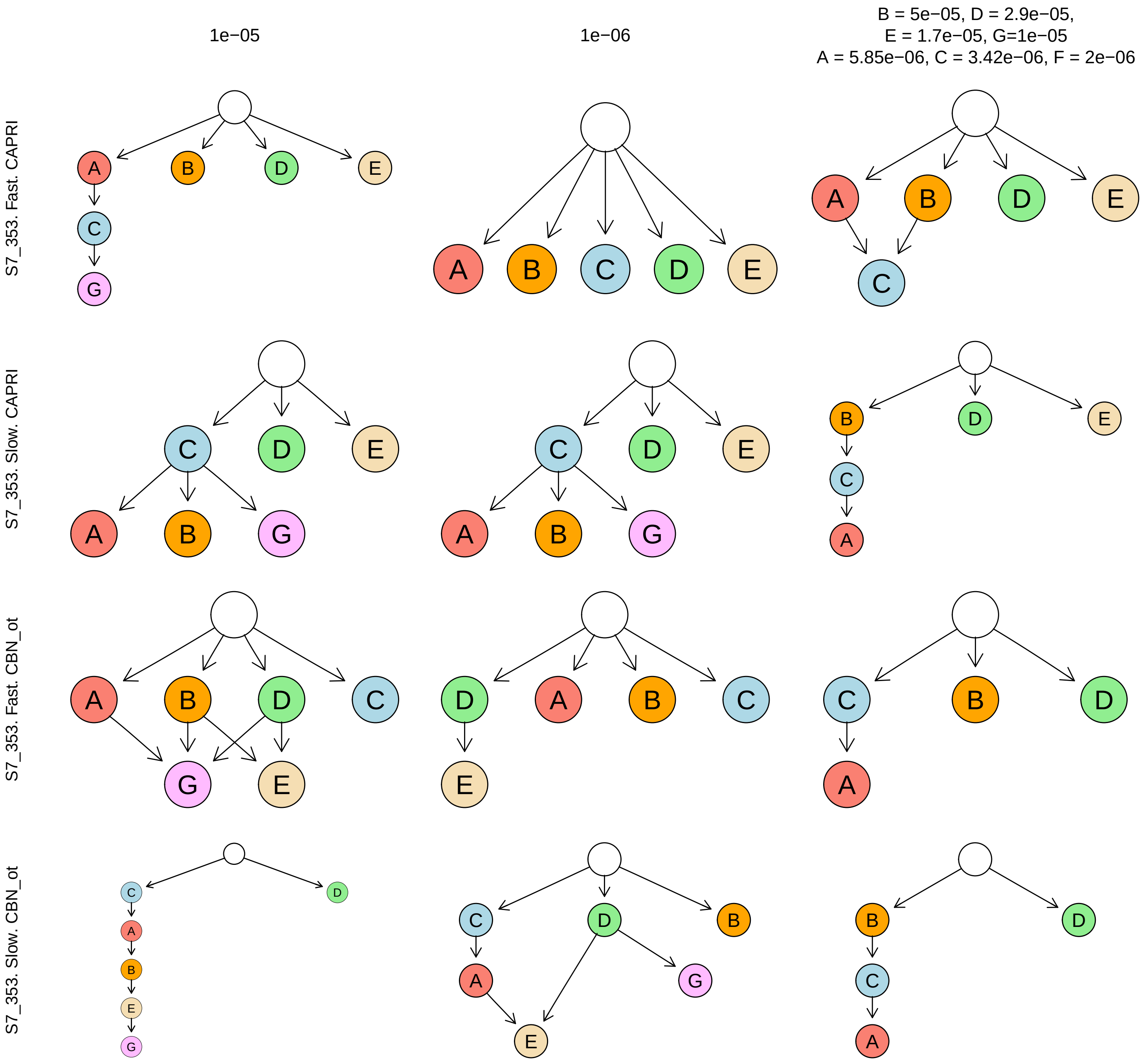

DAG$_a$

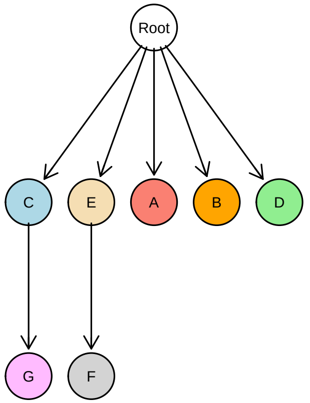

ID	Accessible Genot.	Discrep. DAG_a
S7_354	23	56

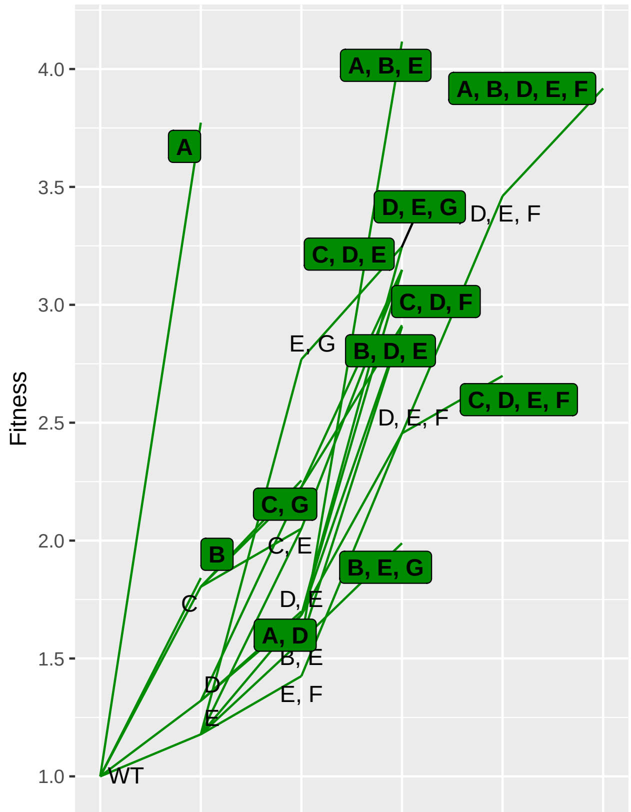

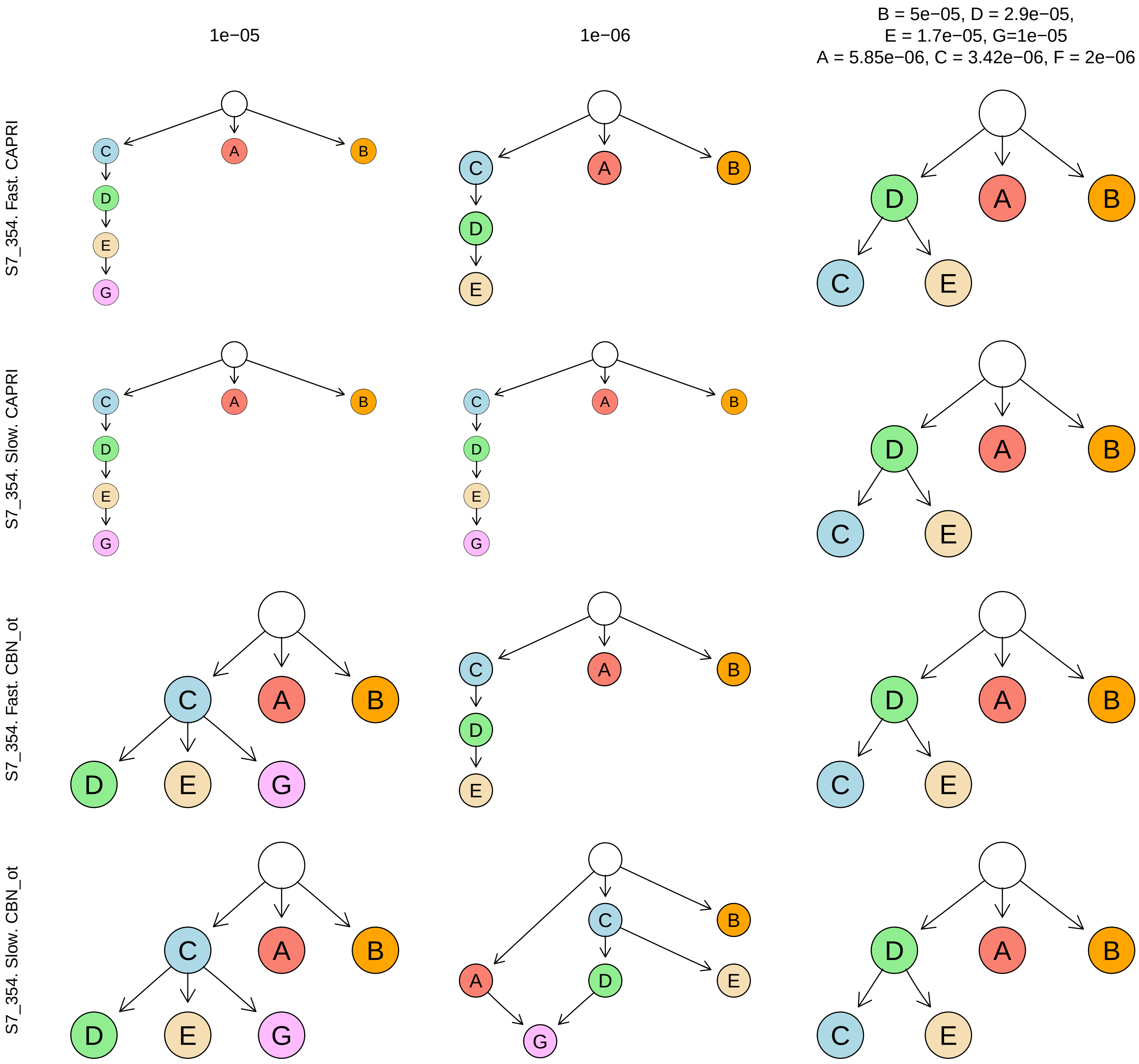

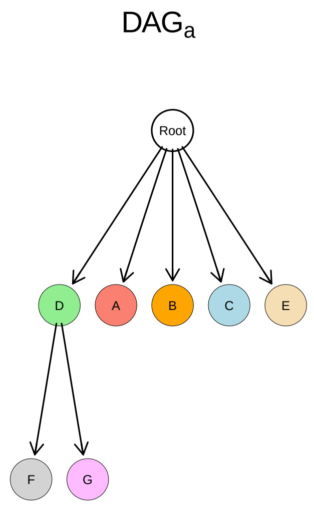

ID	Accessible Genot.	Discrep. DAG_a
S7_355	37	50

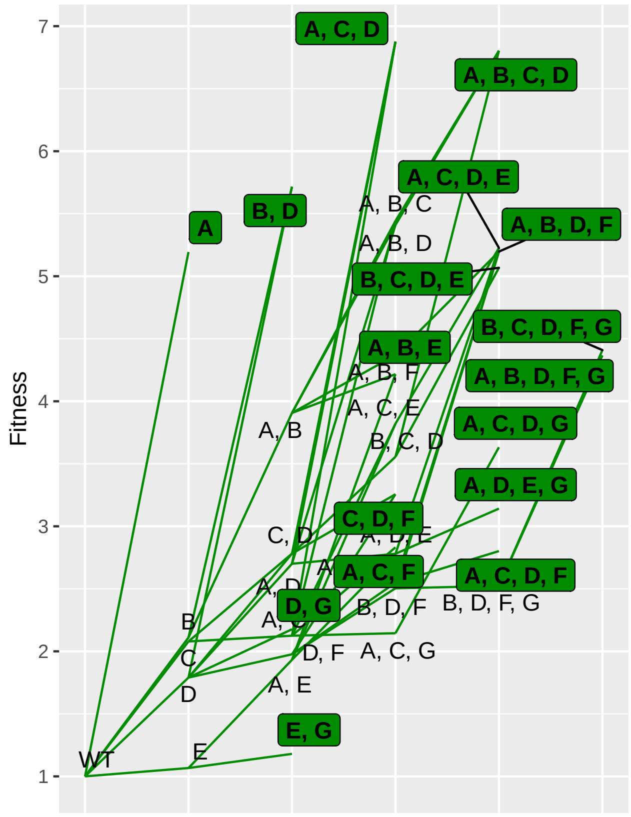

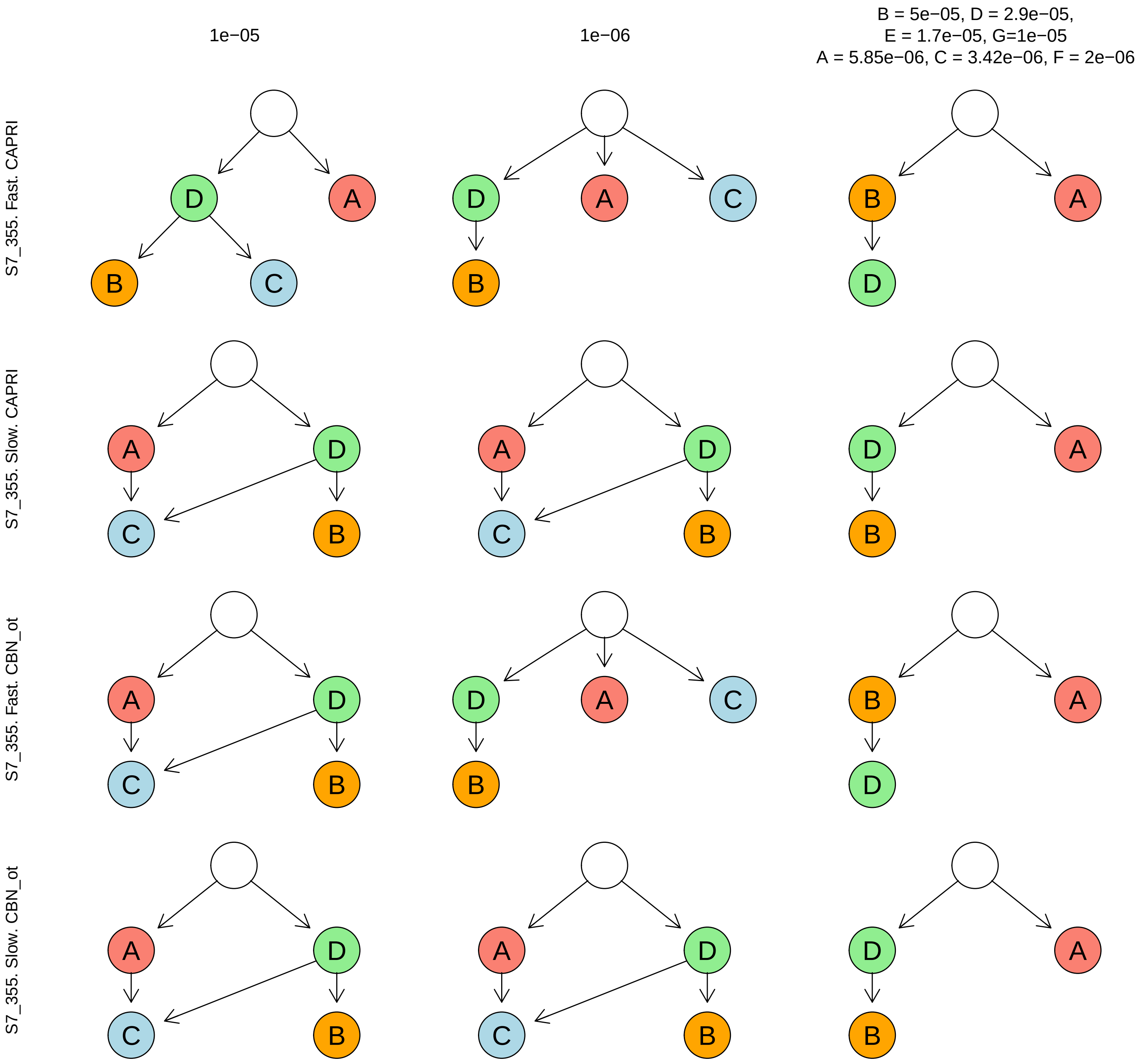

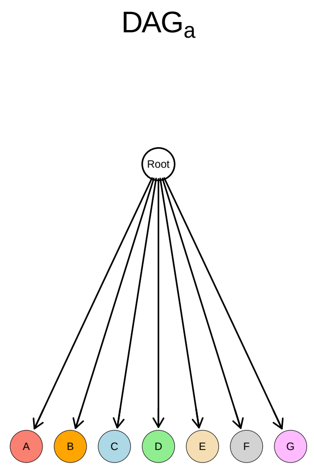

ID	Accessible Genot.	Discrep. DAG_a
S7_356	30	97

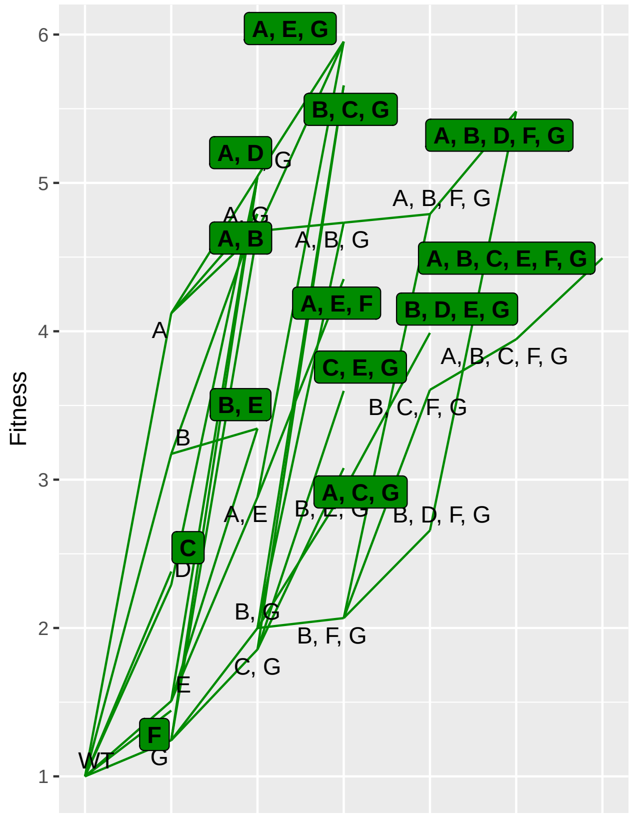

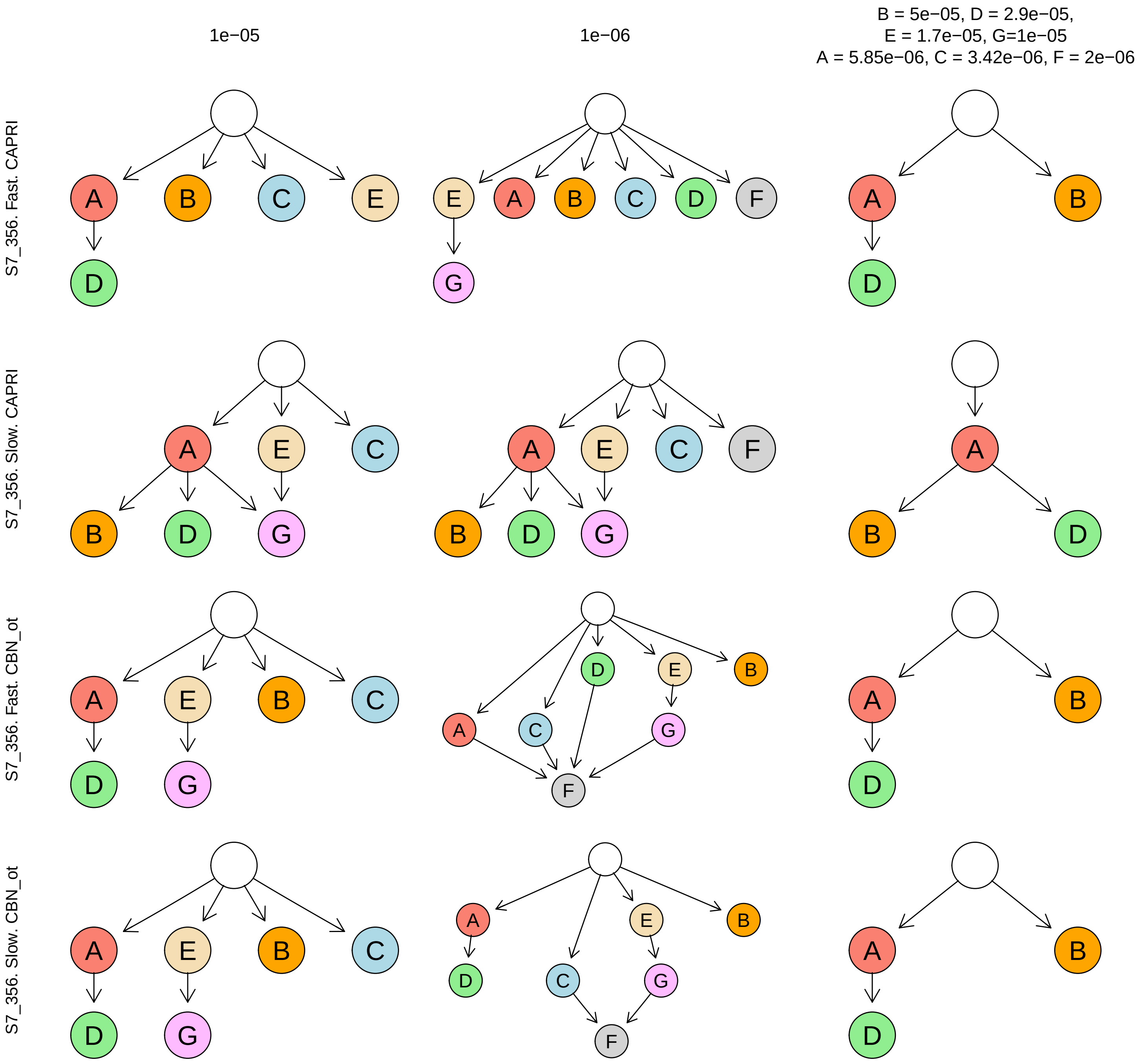

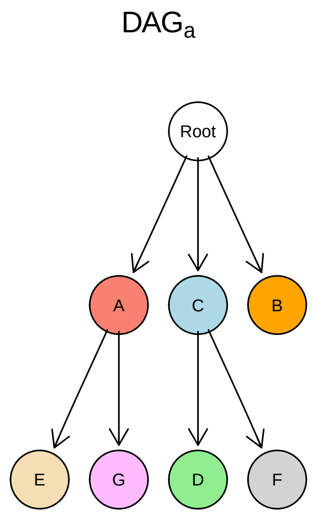

ID	Accessible Genot.	Discrep. DAG_a
S7_357	9	40

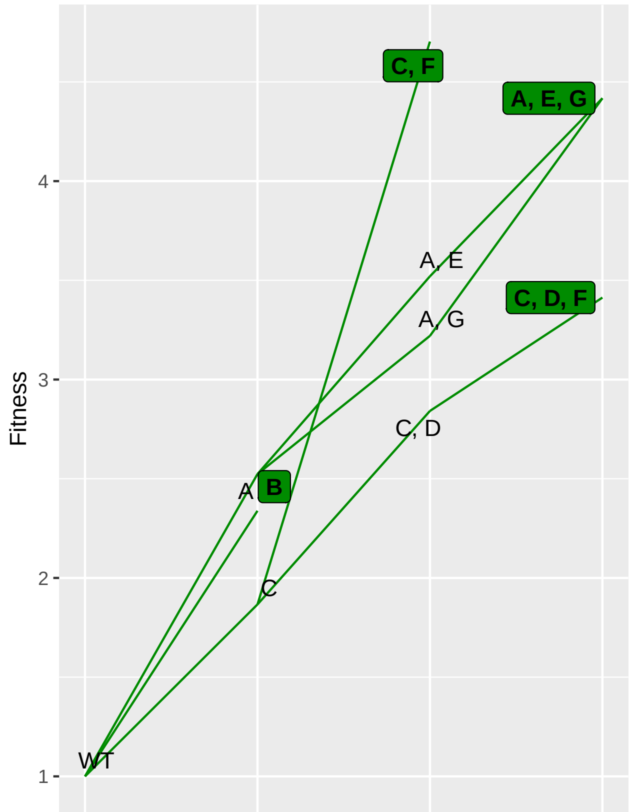

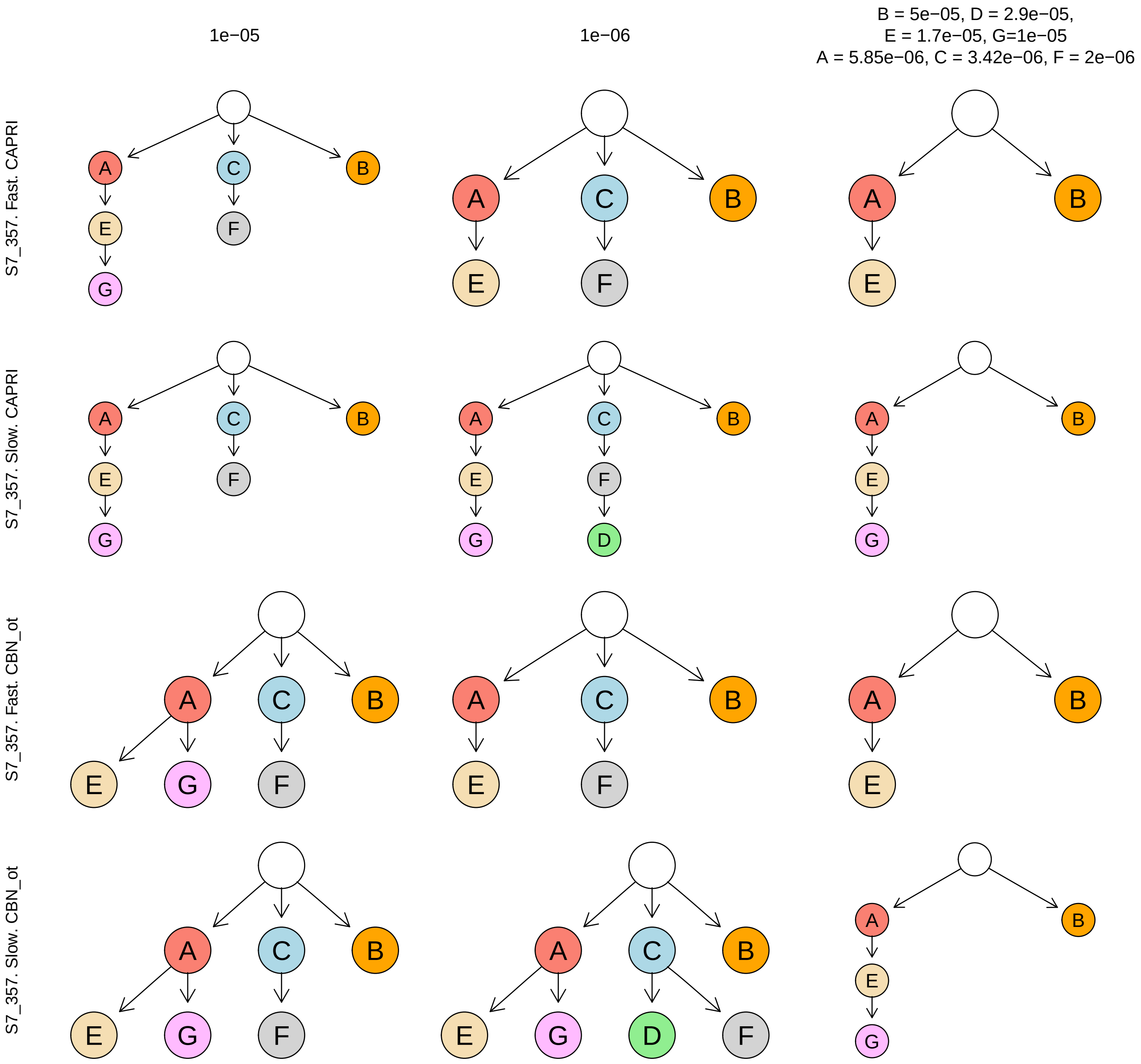

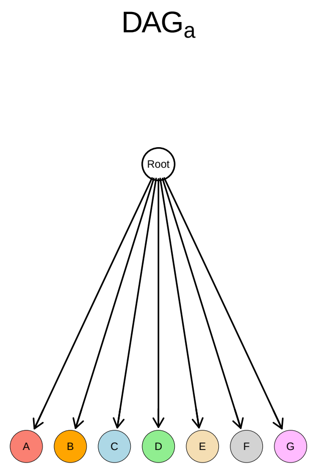

ID	Accessible Genot.	Discrep. DAG_a
S7_358	86	41

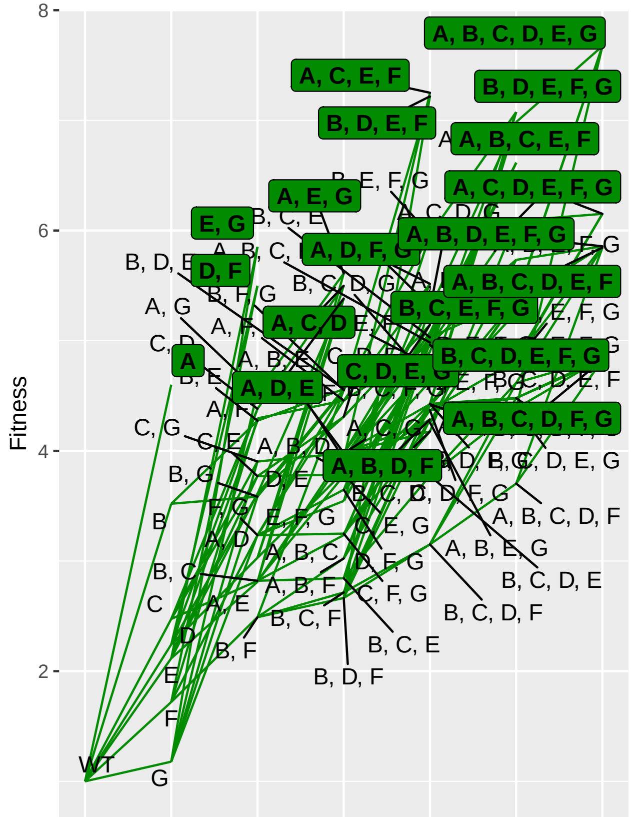

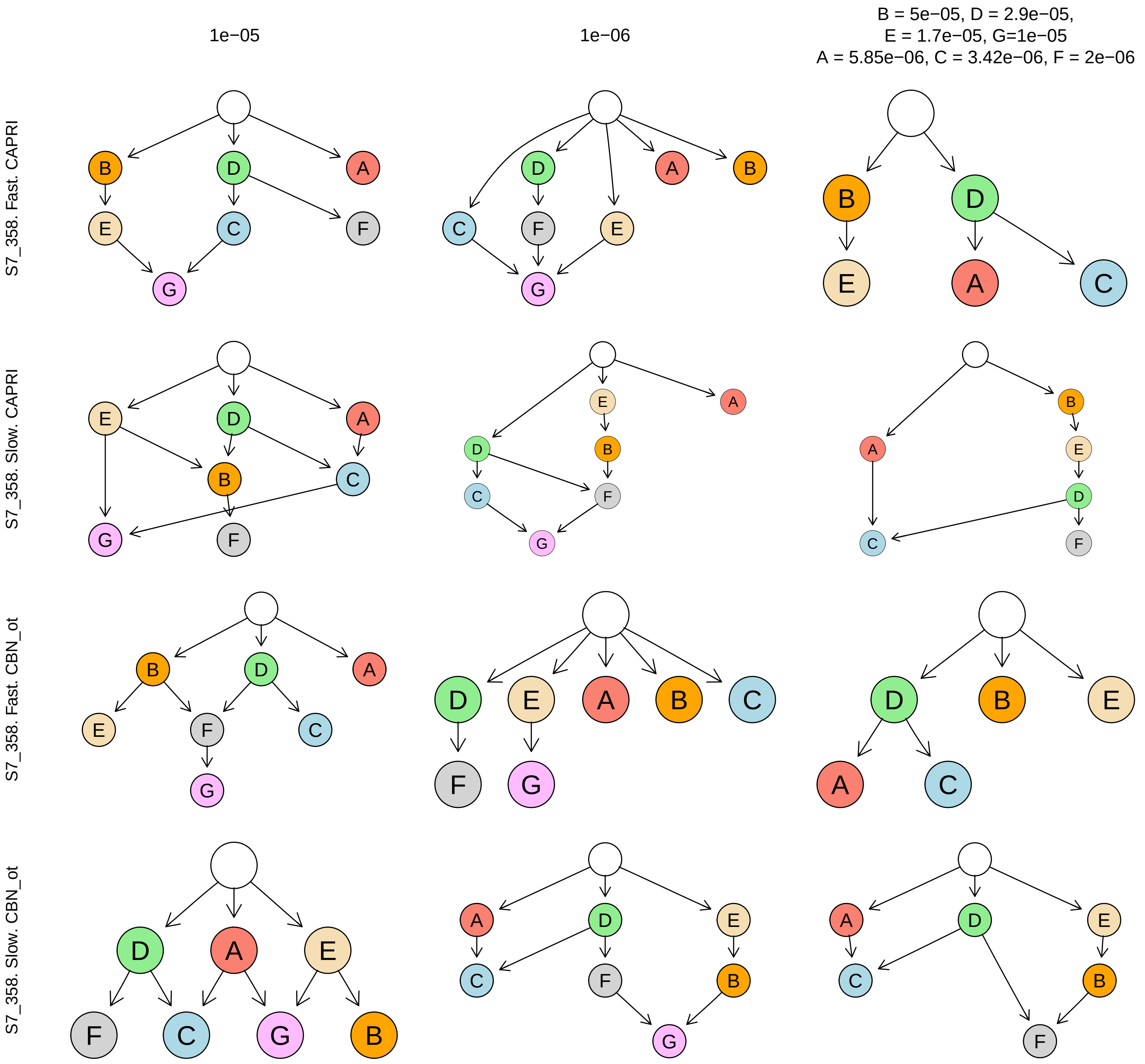

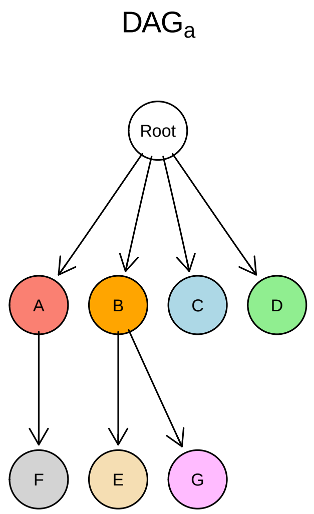

ID	Accessible Genot.	Discrep. DAG_a
S7_359	30	47

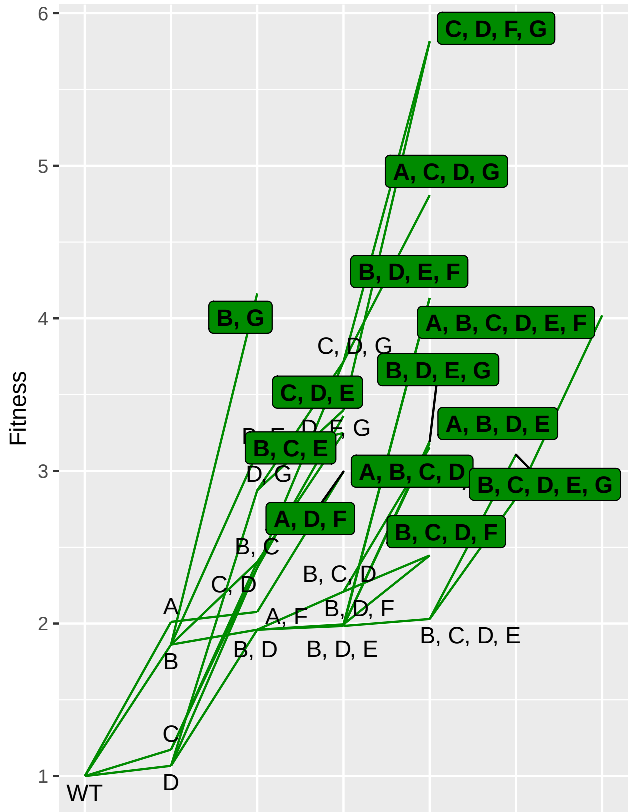

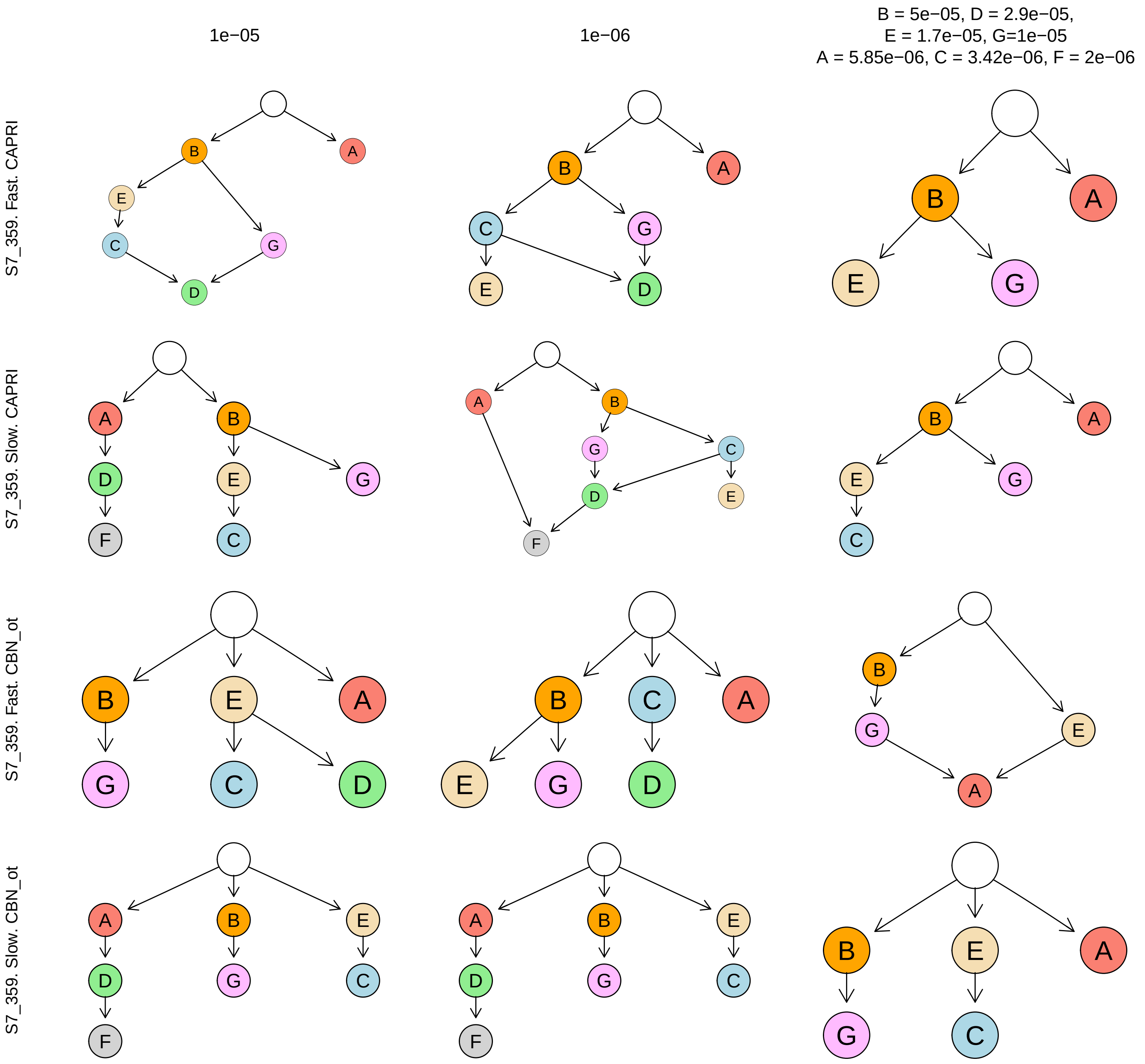

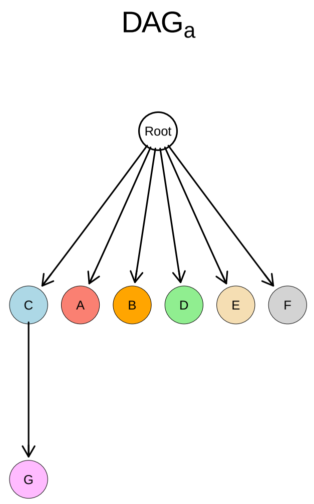

ID	Accessible Genot.	Discrep. DAG_a
S7_360	18	81

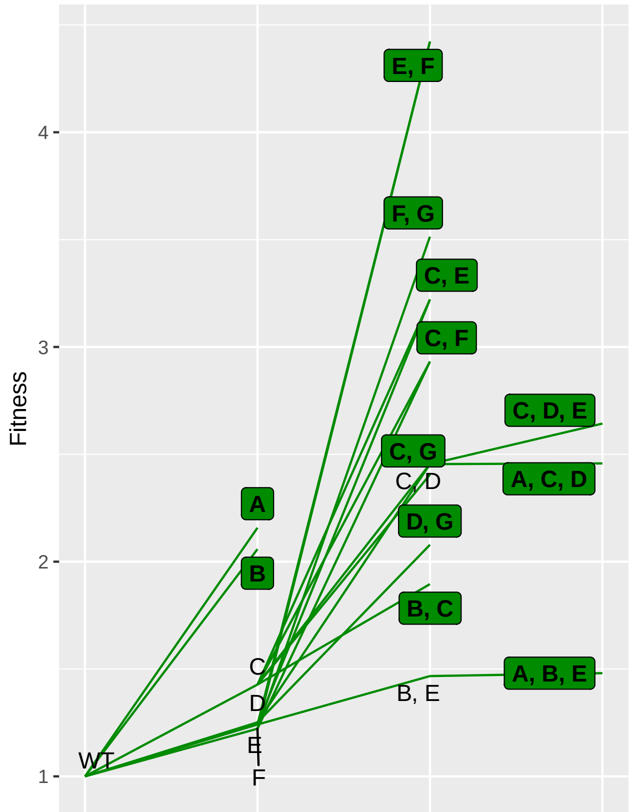

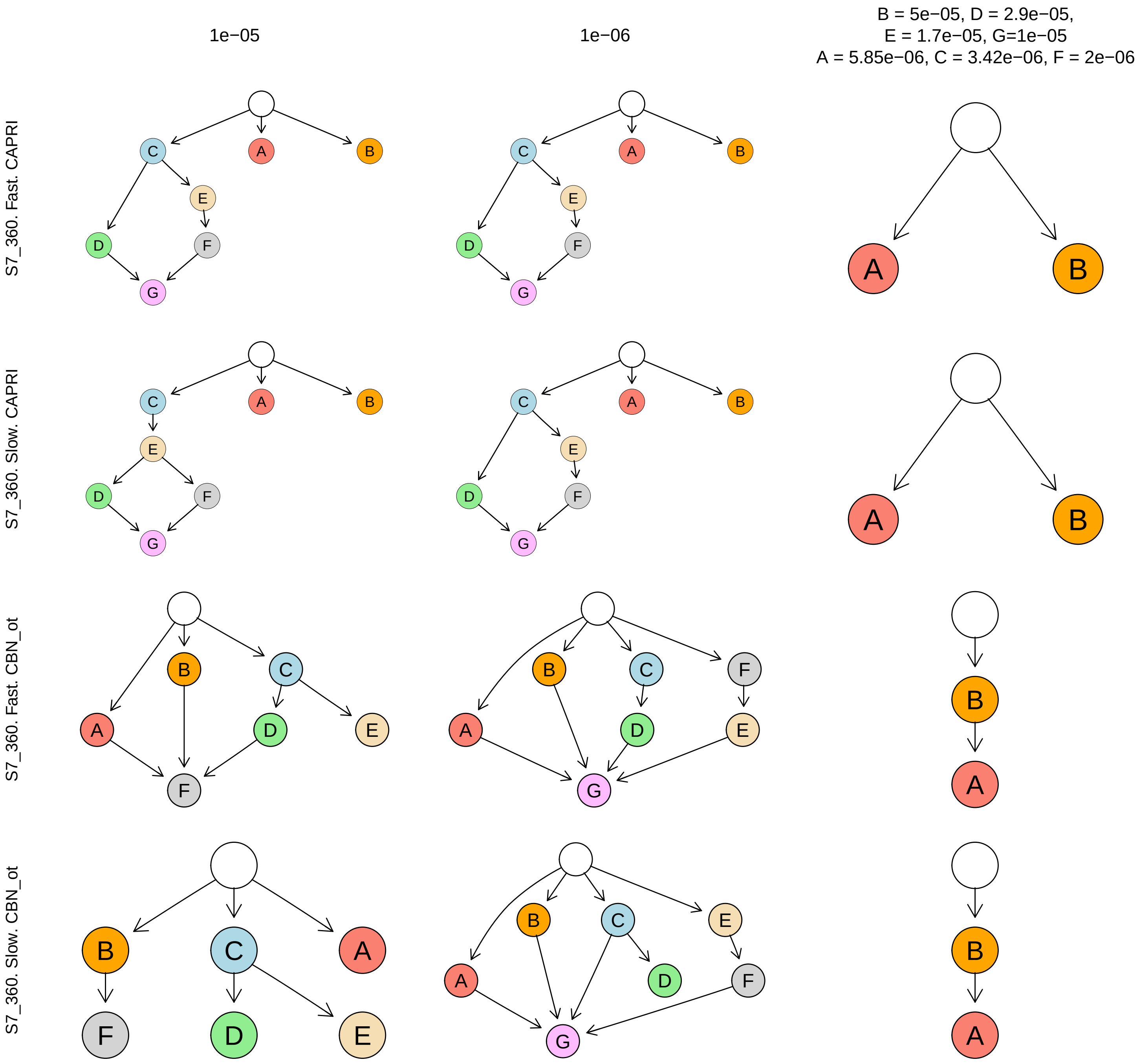

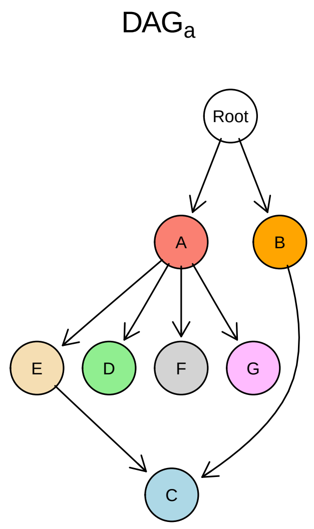

ID	Accessible Genot.	Discrep. DAG_a
S7_361	27	26

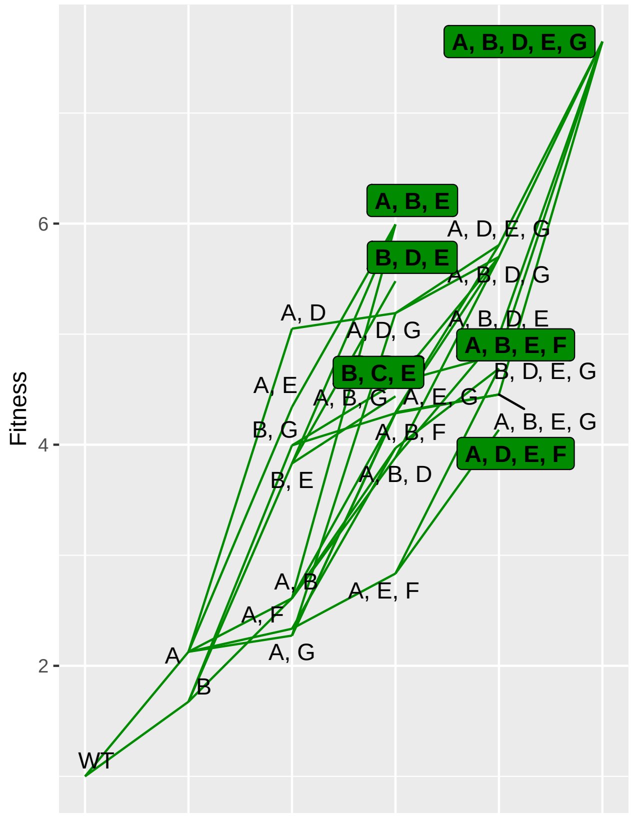

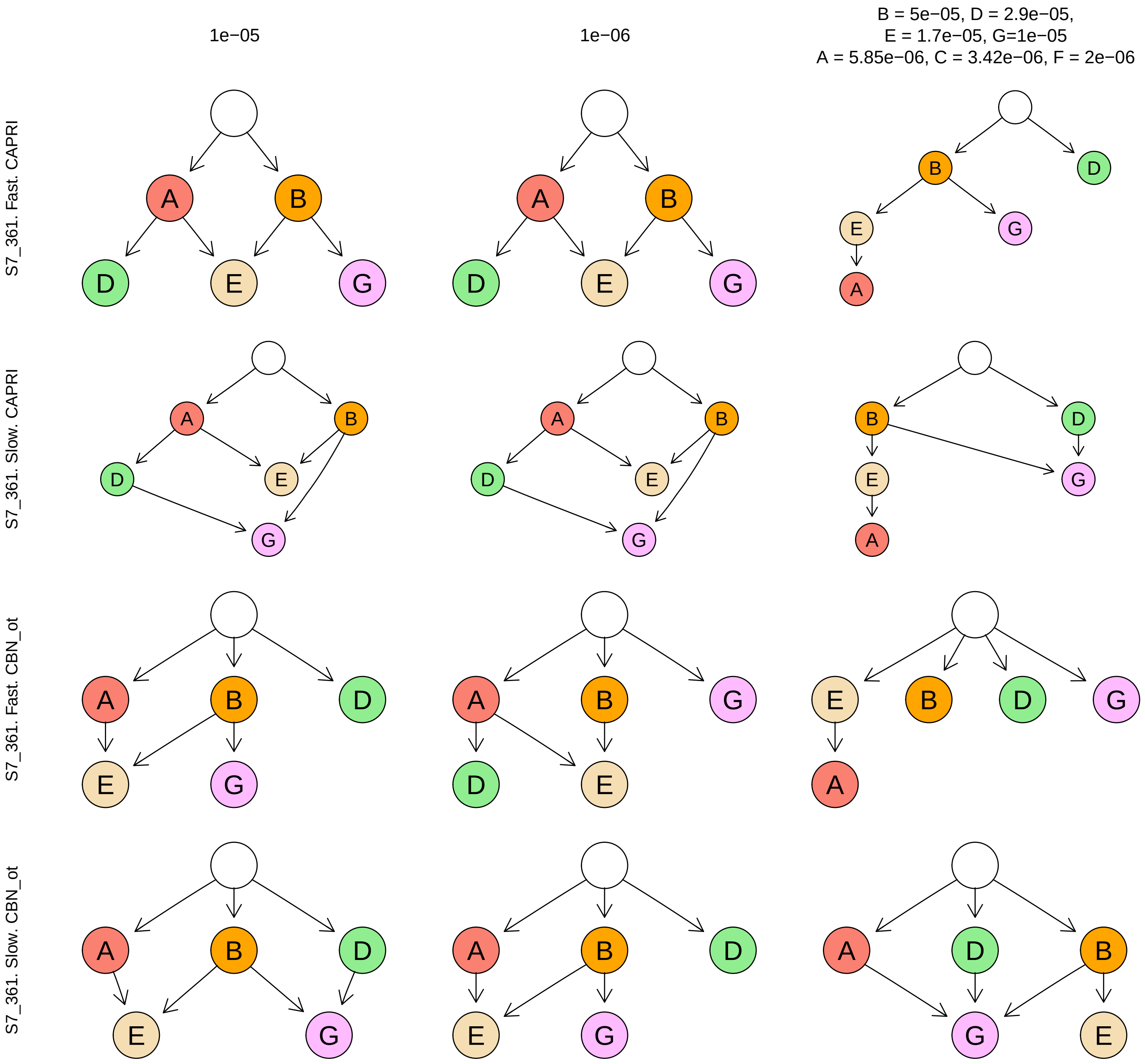

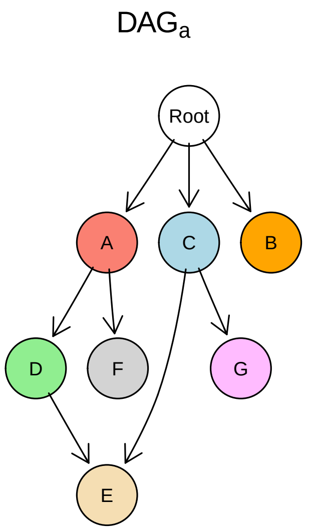

ID	Accessible Genot.	Discrep. DAG_a
S7_362	12	31

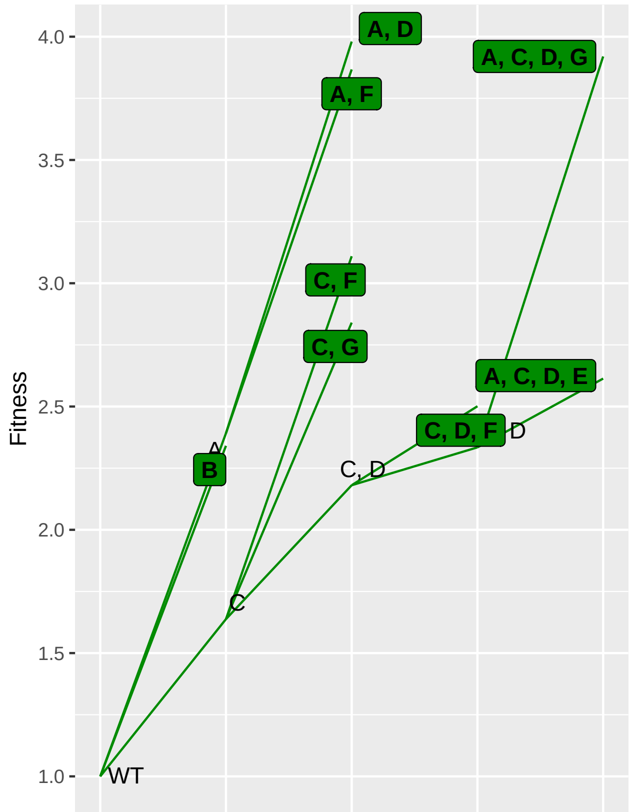

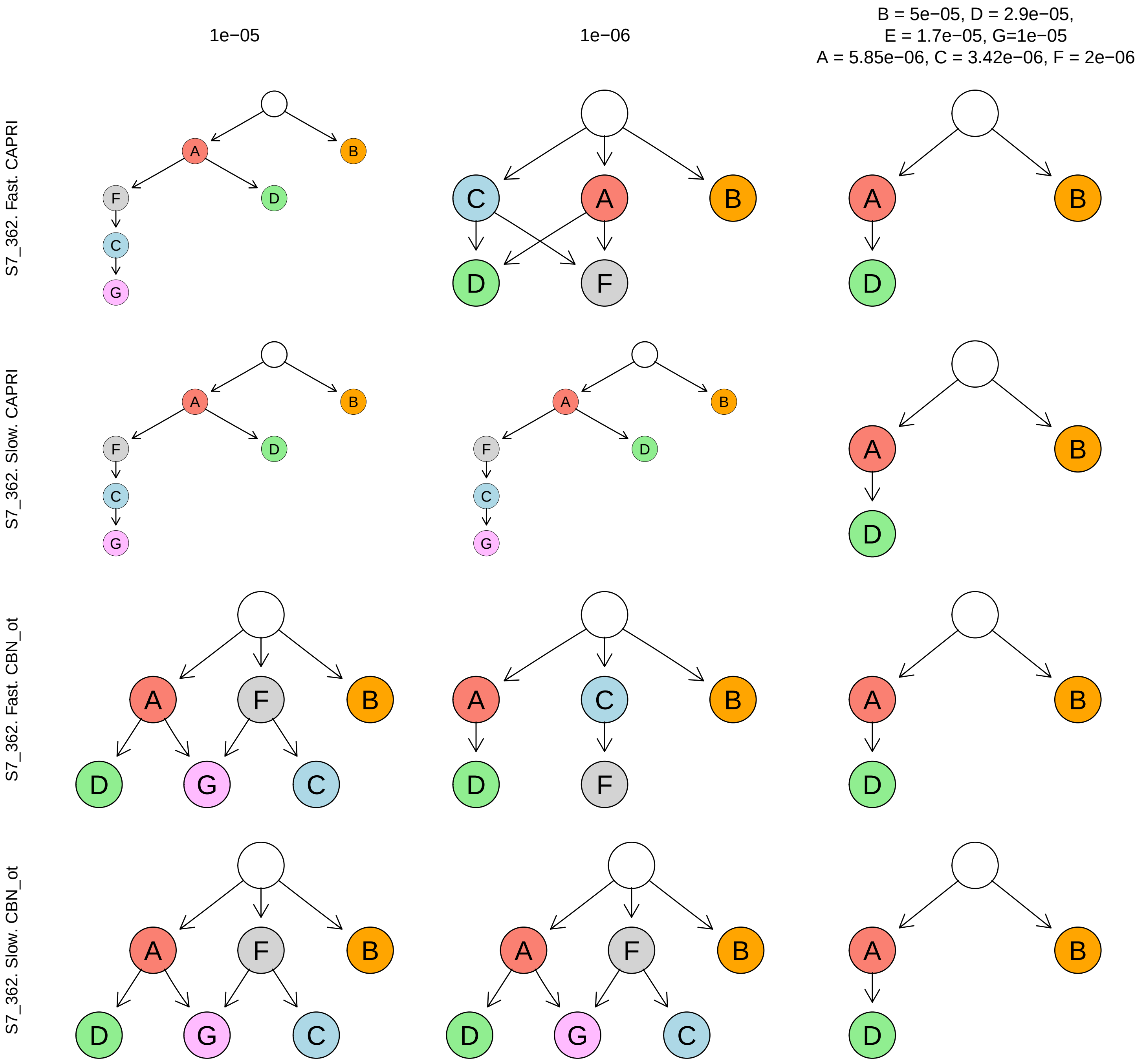

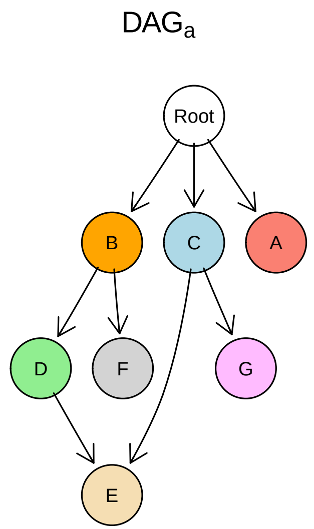

ID	Accessible Genot.	Discrep. DAG_a
S7_363	20	35

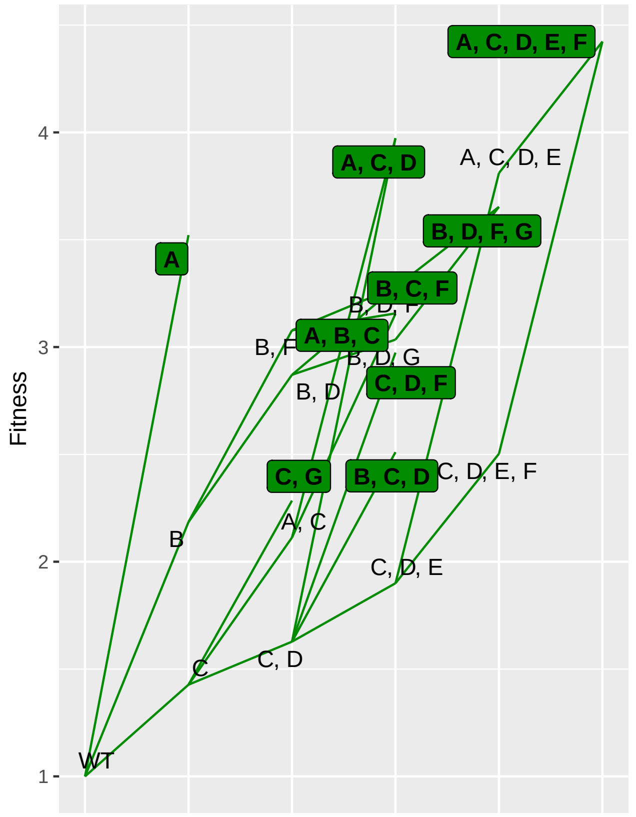

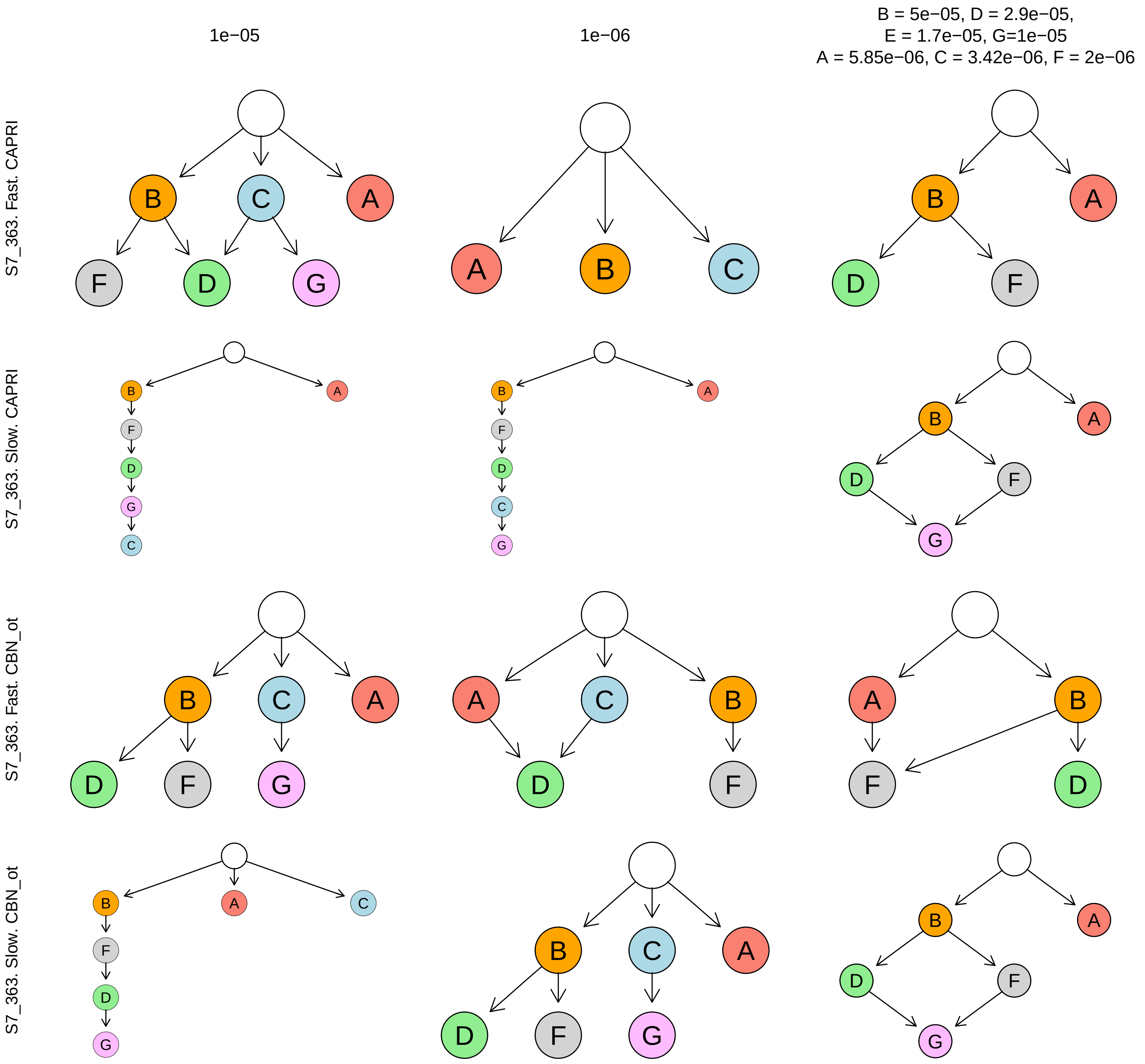

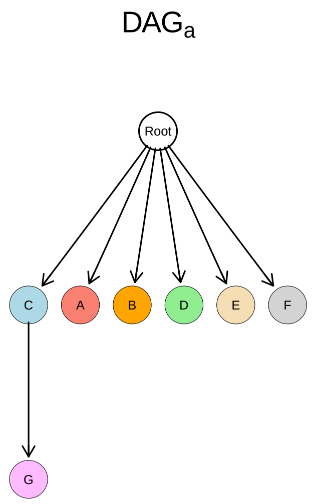

ID	Accessible Genot.	Discrep. DAG_a
S7_364	45	64

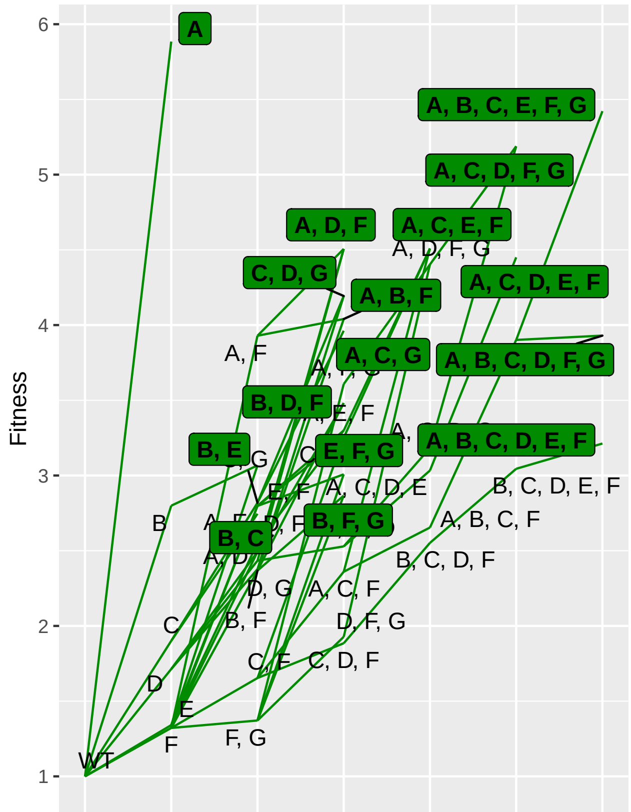

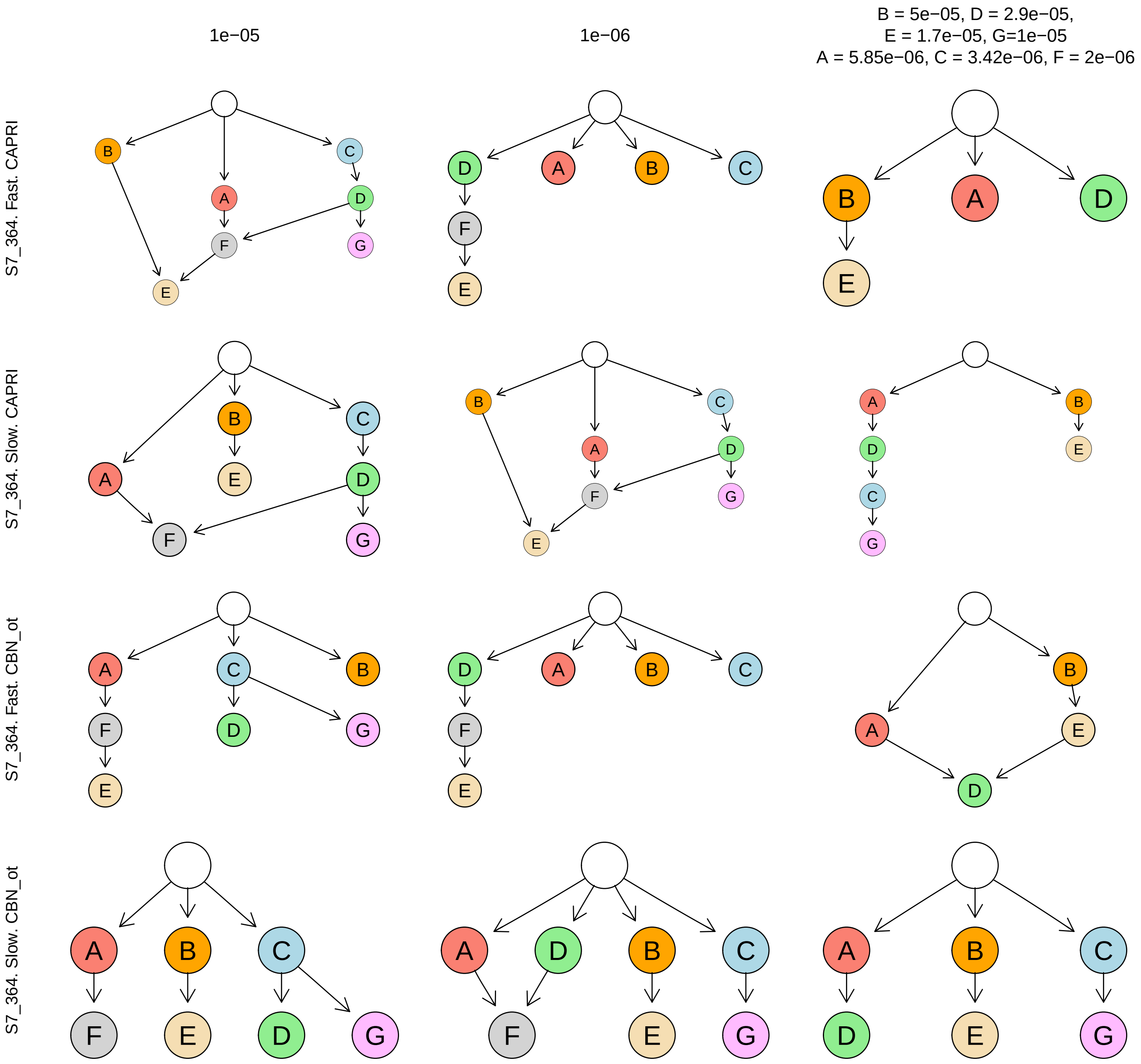

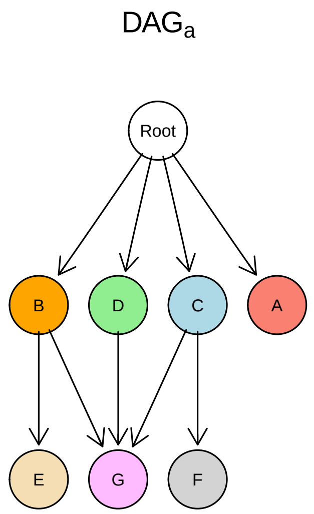

ID	Accessible Genot.	Discrep. DAG_a
S7_365	27	32

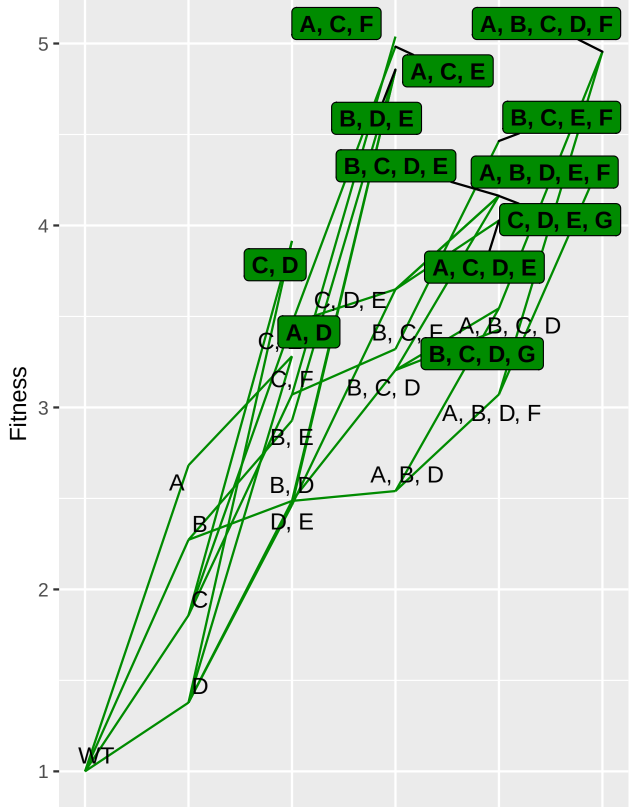

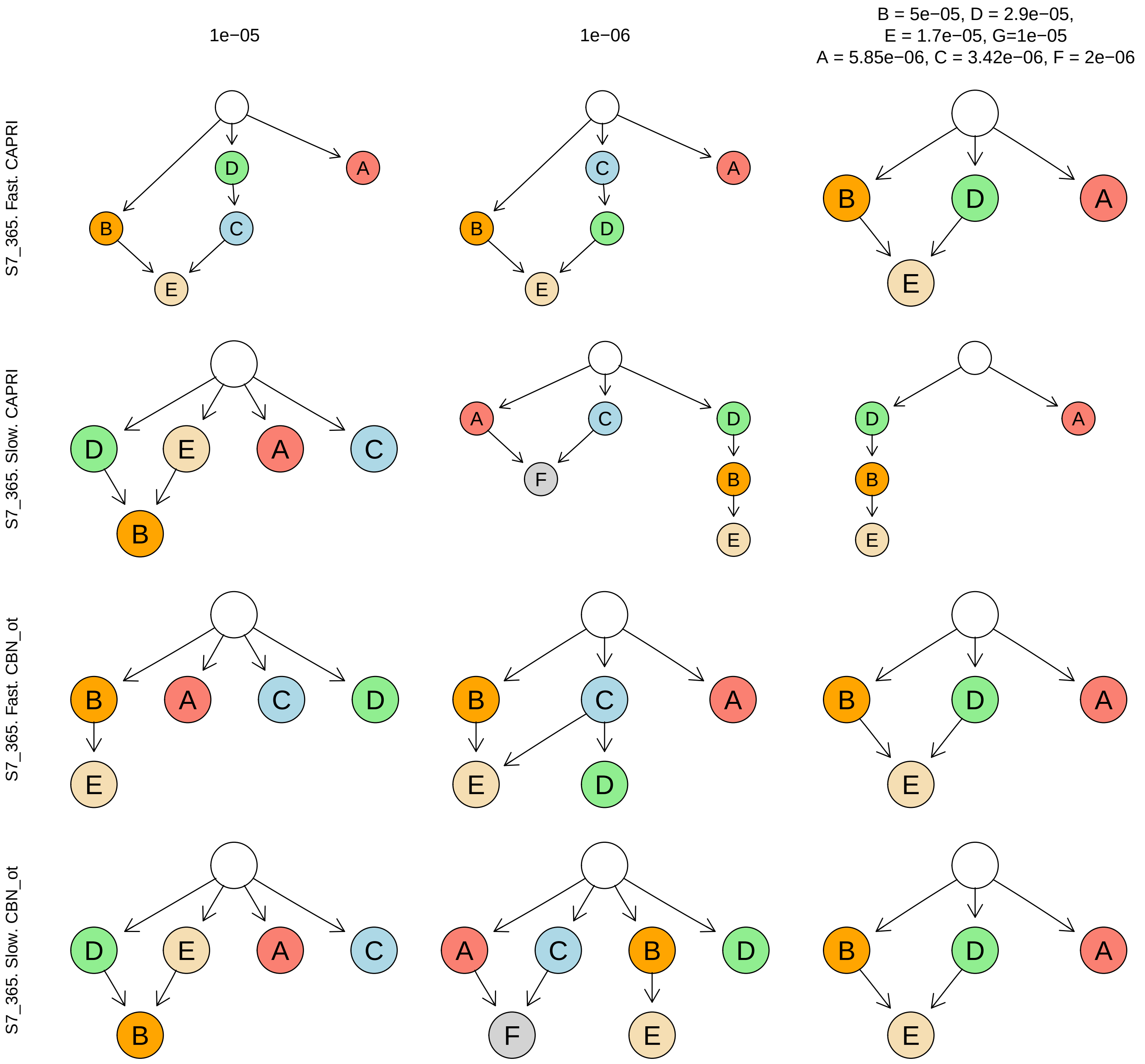

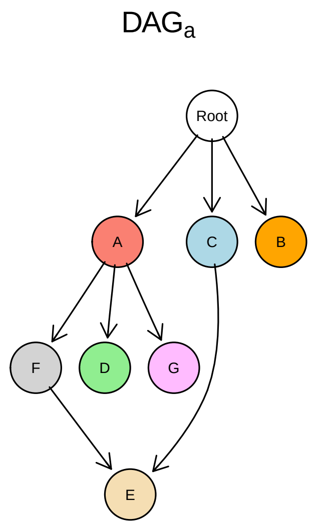

ID	Accessible Genot.	Discrep. DAG_a
S7_366	34	39

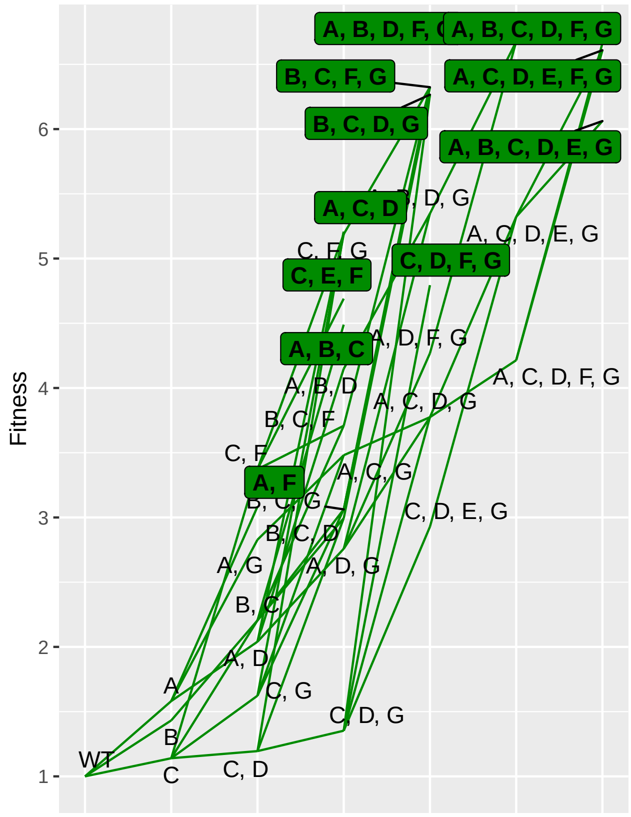

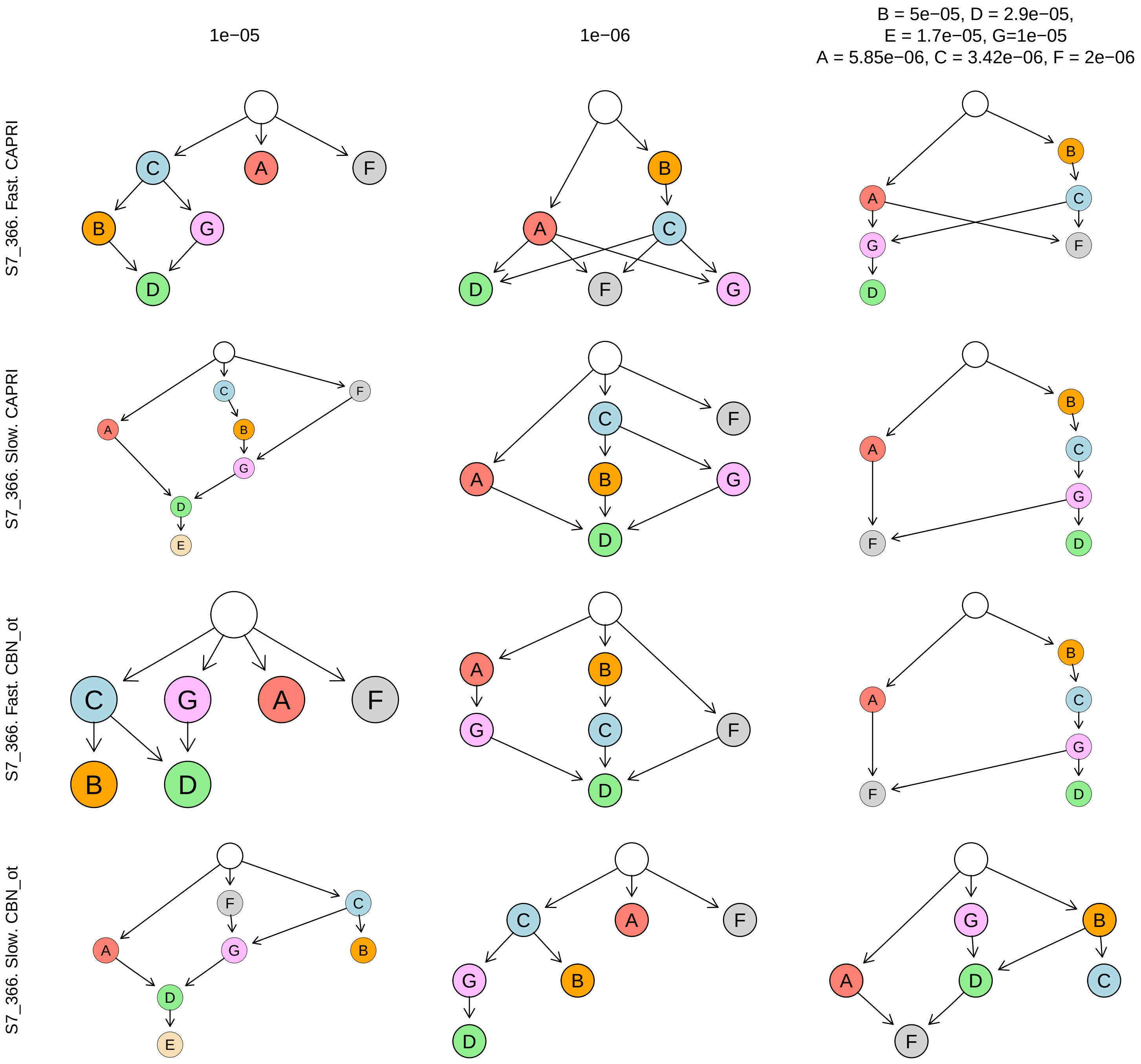

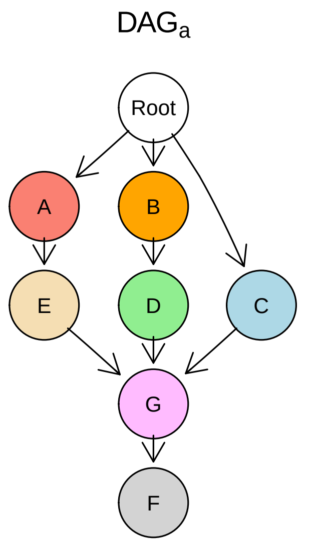

ID	Accessible Genot.	Discrep. DAG_a
S7_367	13	14

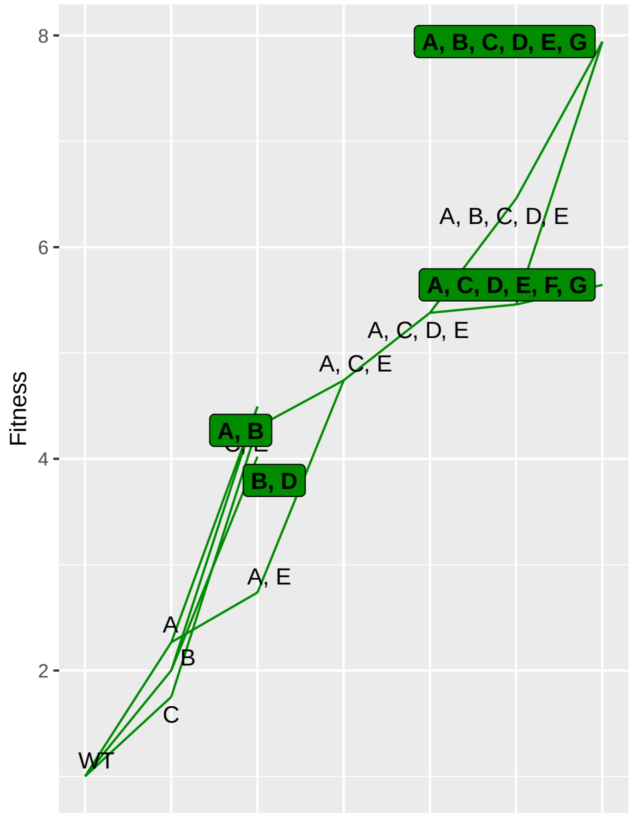

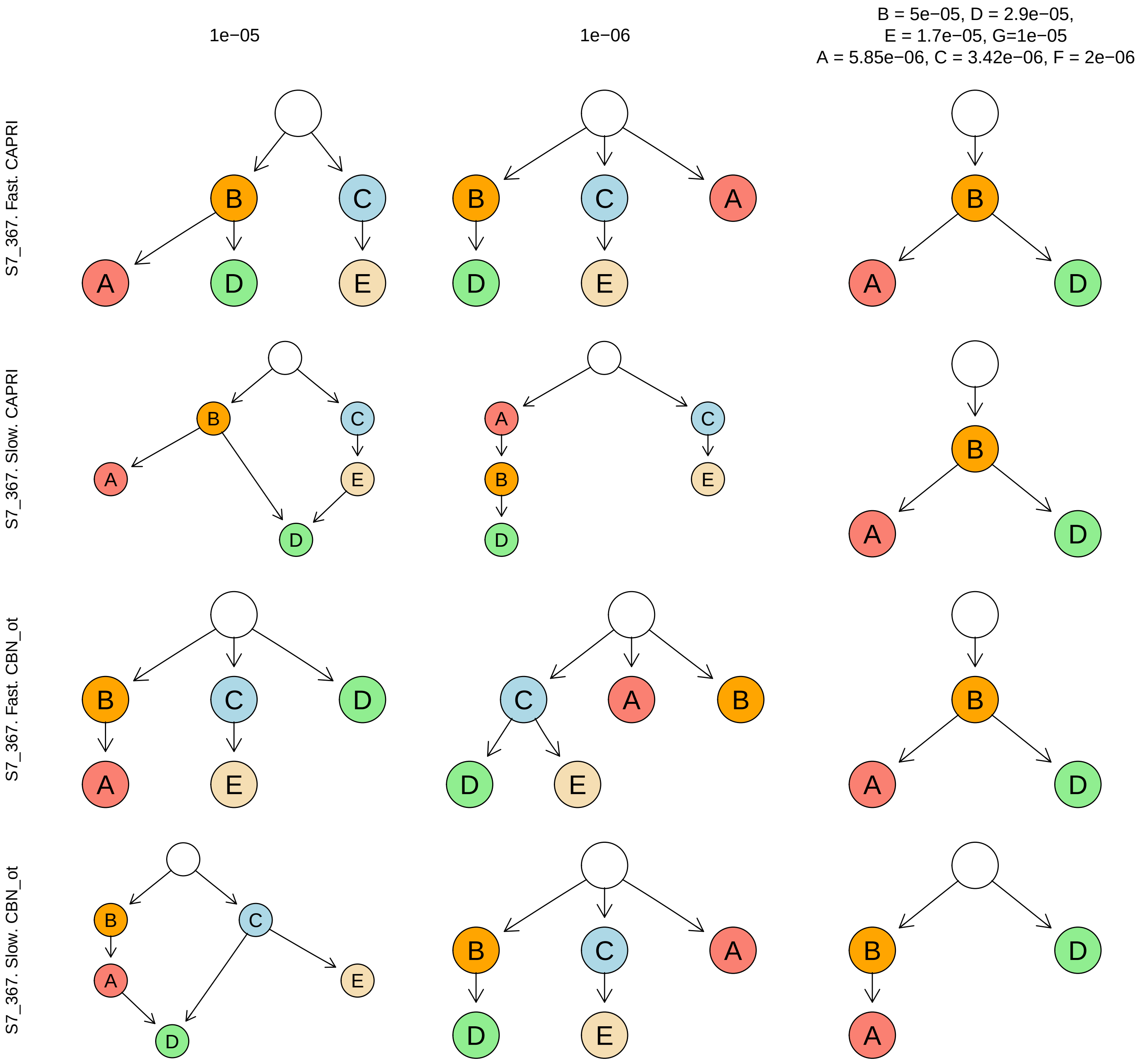

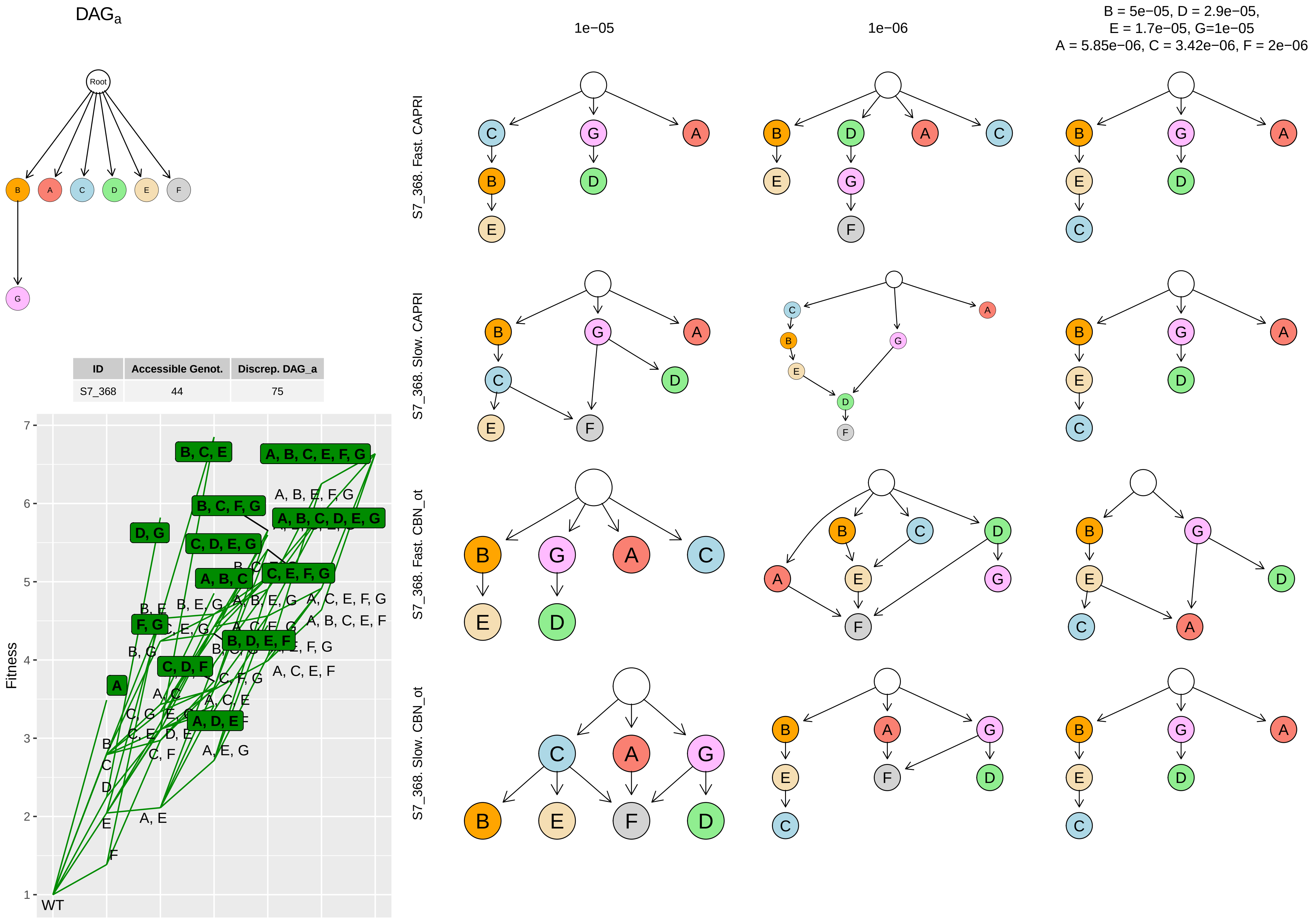

DAG_a
Root
ID | Accessible Genot. | Discrep. DAG_a
S7_368 | 44 | 75
Fitness
WT
1e−05
1e−06
B = 5e−05, D = 2.9e−05,
E = 1.7e−05, G=1e−05
A = 5.85e−06, C = 3.42e−06, F = 2e−06
S7_368. Fast. CAPRI
S7_368. Slow. CAPRI
S7_368. Fast. CBN_ot
S7_368. Slow. CBN_ot

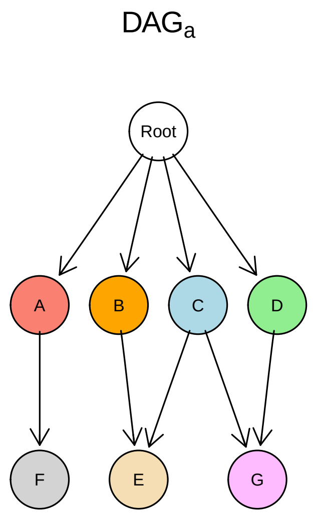

ID	Accessible Genot.	Discrep. DAG_a
S7_369	26	34

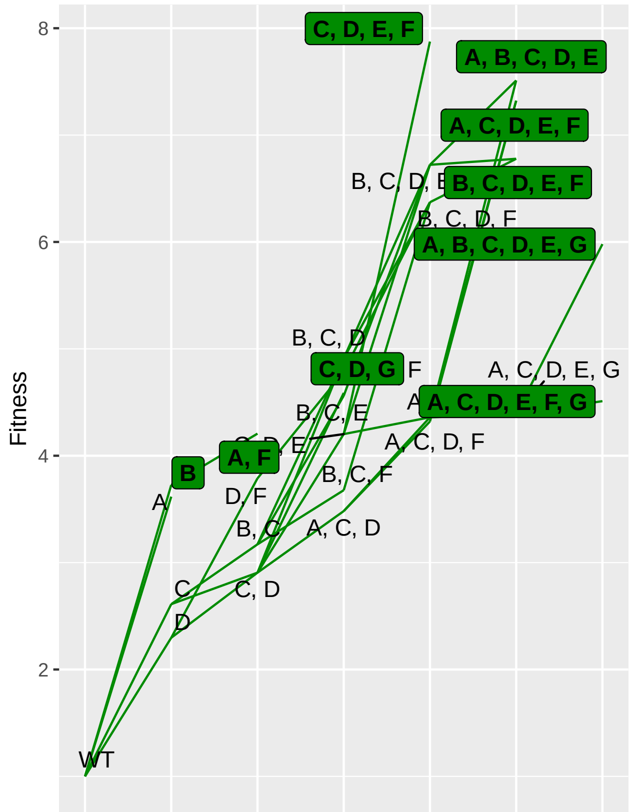

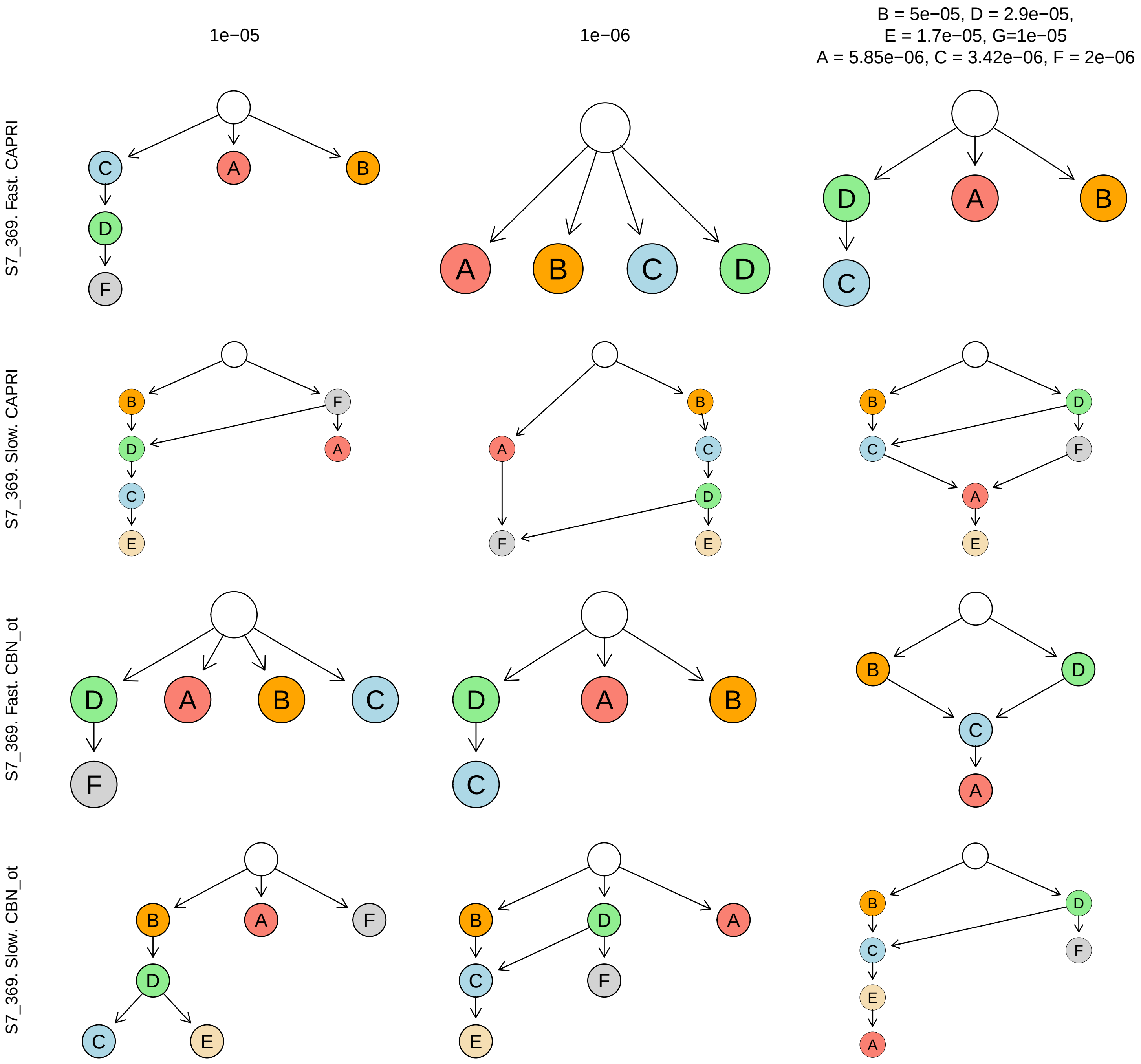

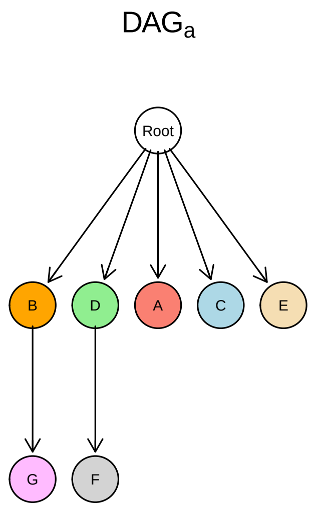

ID	Accessible Genot.	Discrep. DAG_a
S7_370	37	54

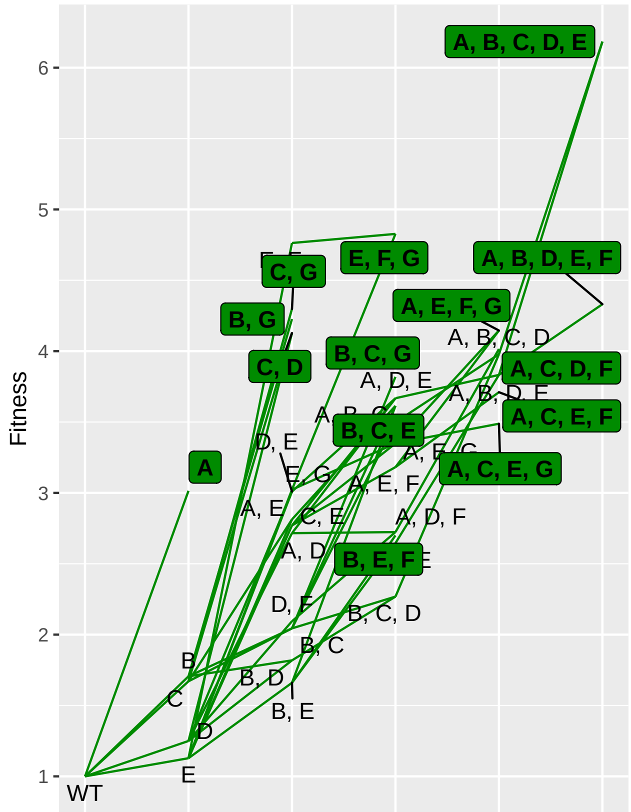

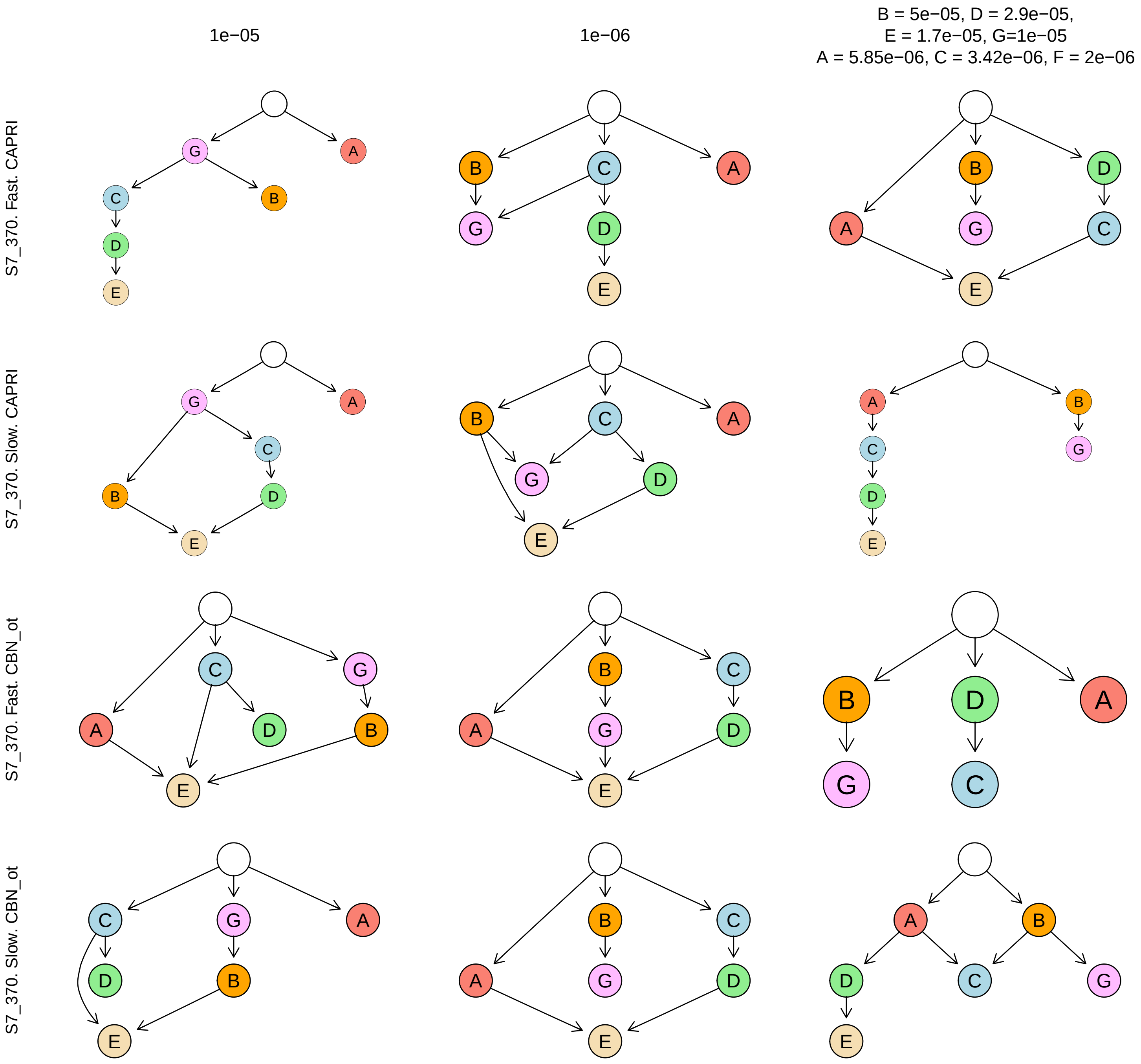

DAG$_a$

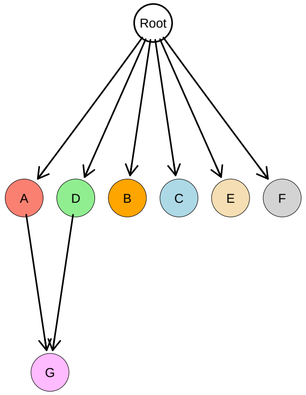

ID	Accessible Genot.	Discrep. DAG_a
S7_371	11	68

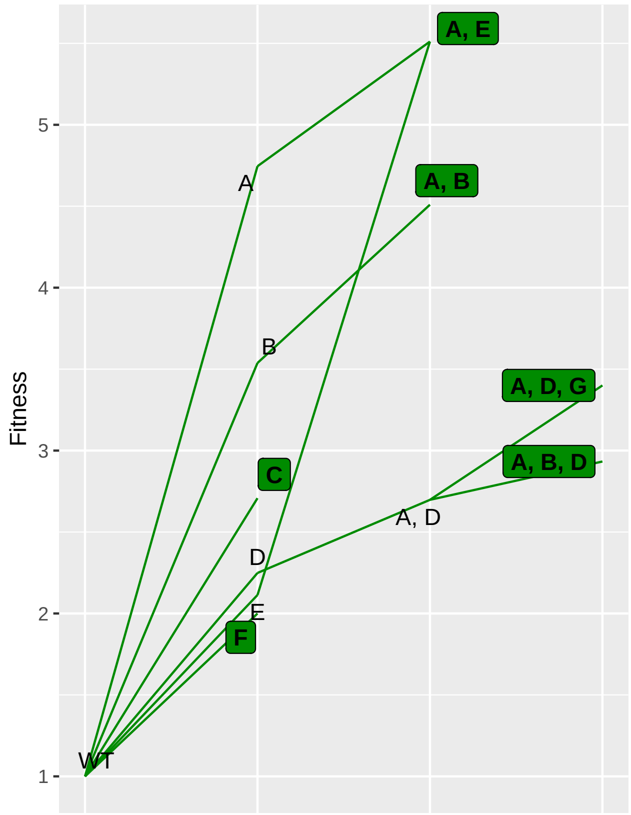

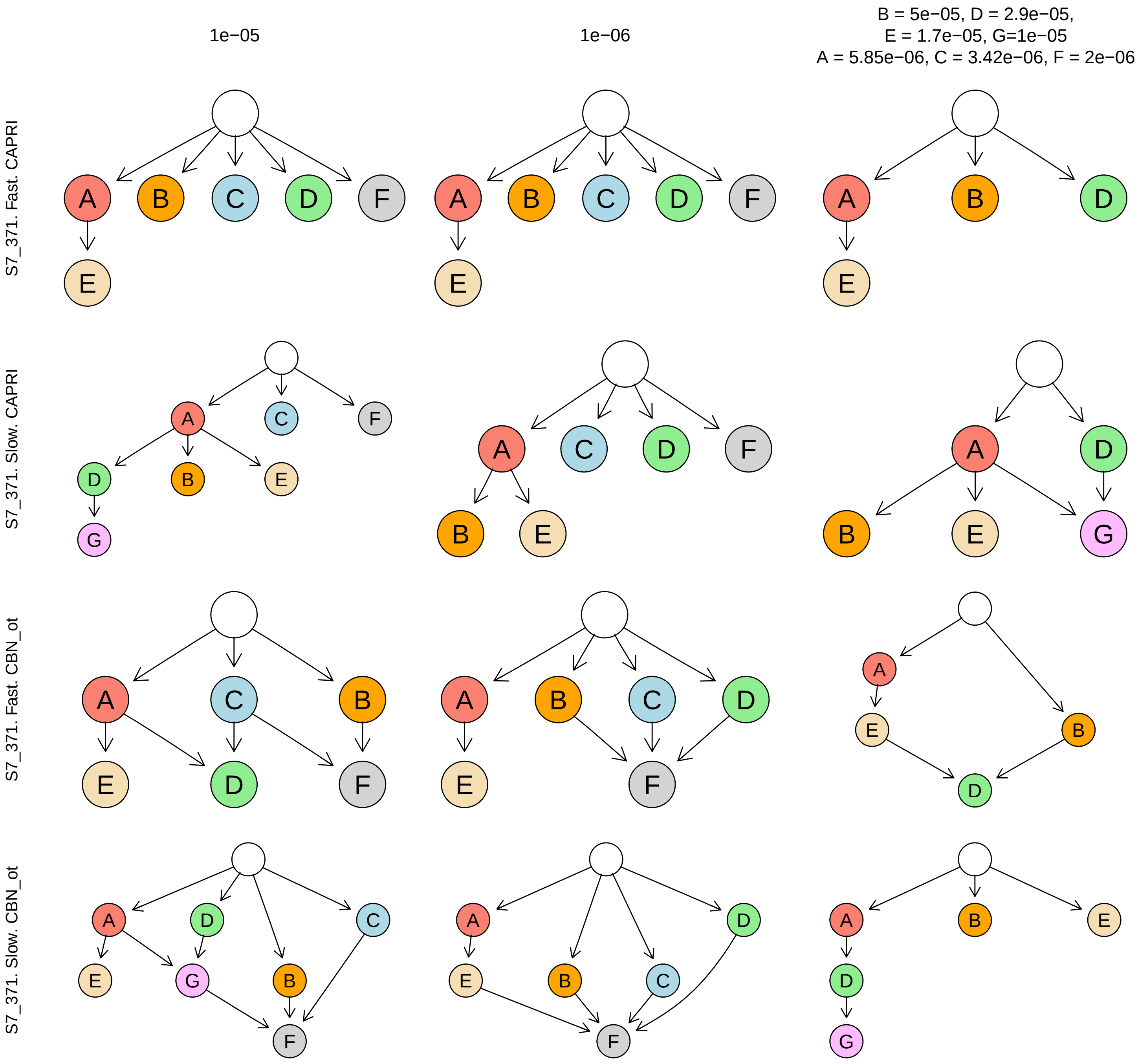

DAG$_a$

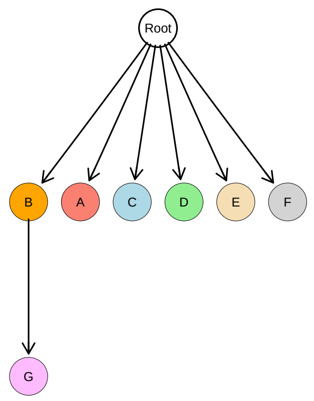

ID	Accessible Genot.	Discrep. DAG_a
S7_372	31	74

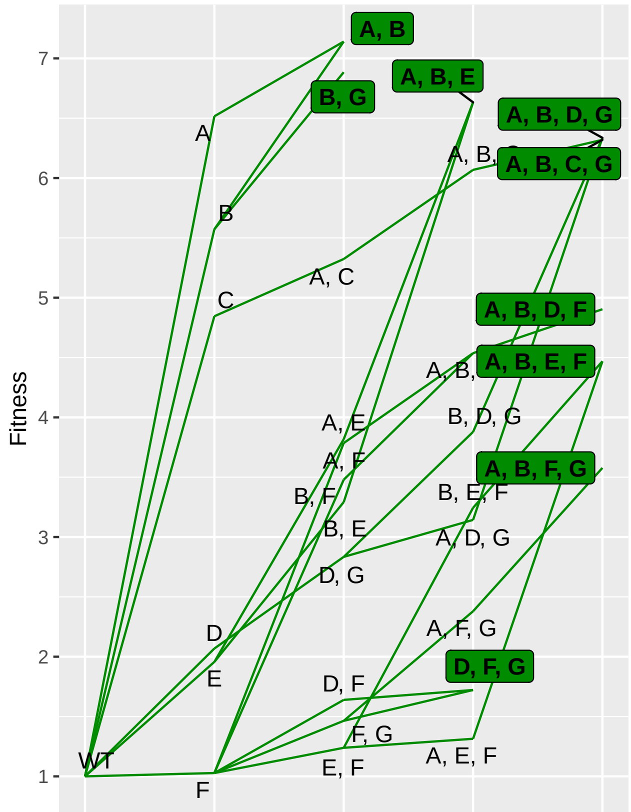

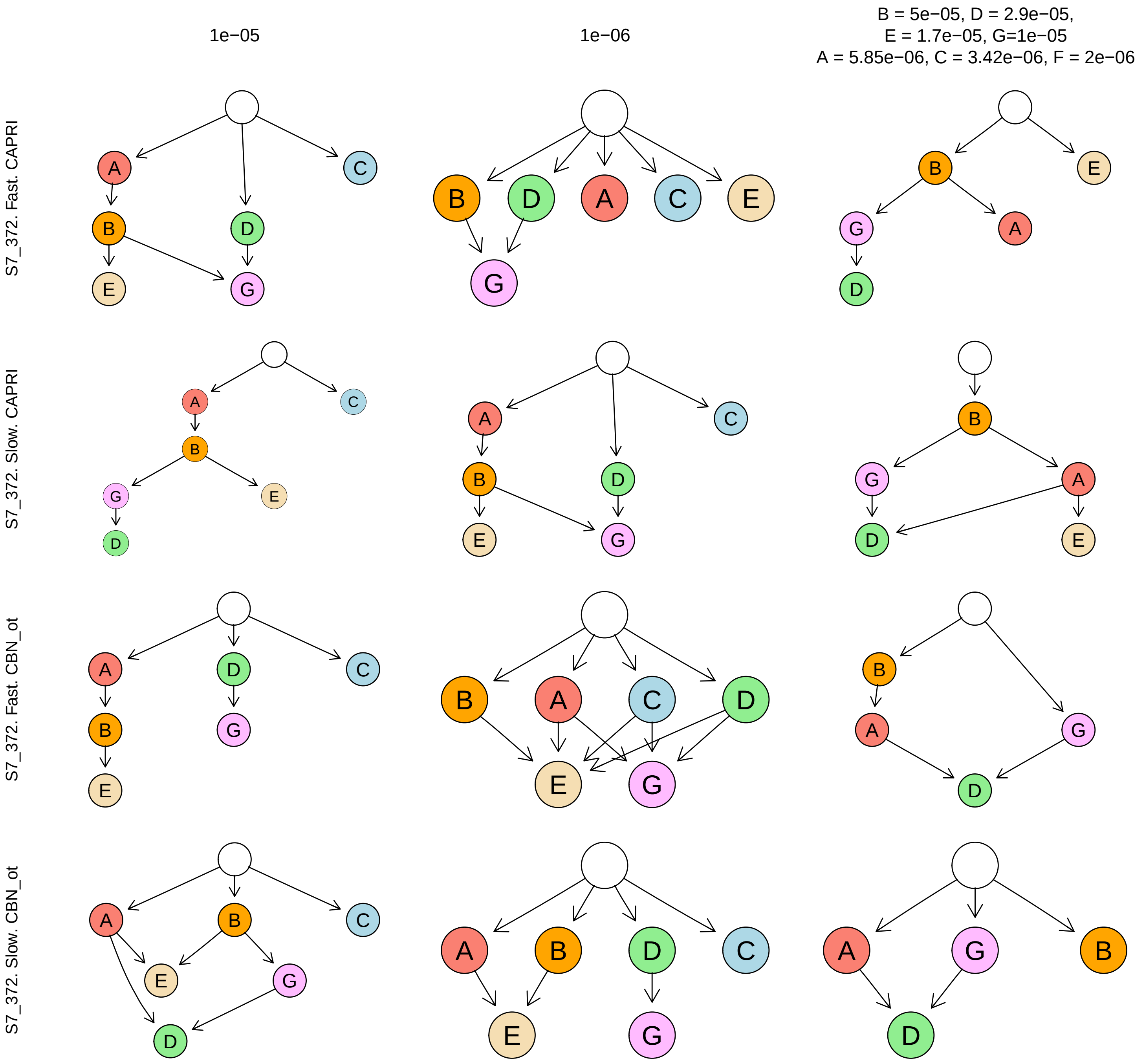

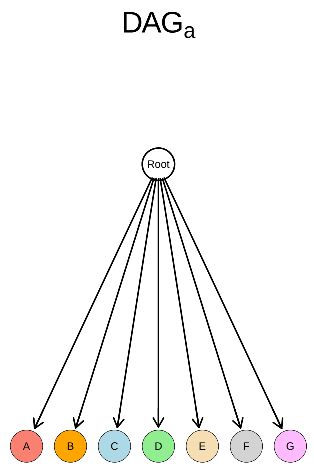

ID	Accessible Genot.	Discrep. DAG_a
S7_373	49	78

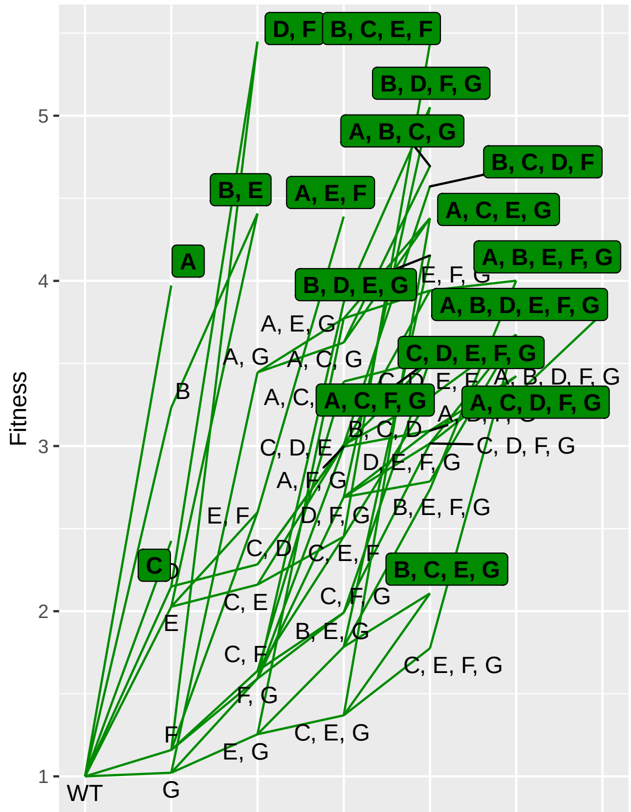

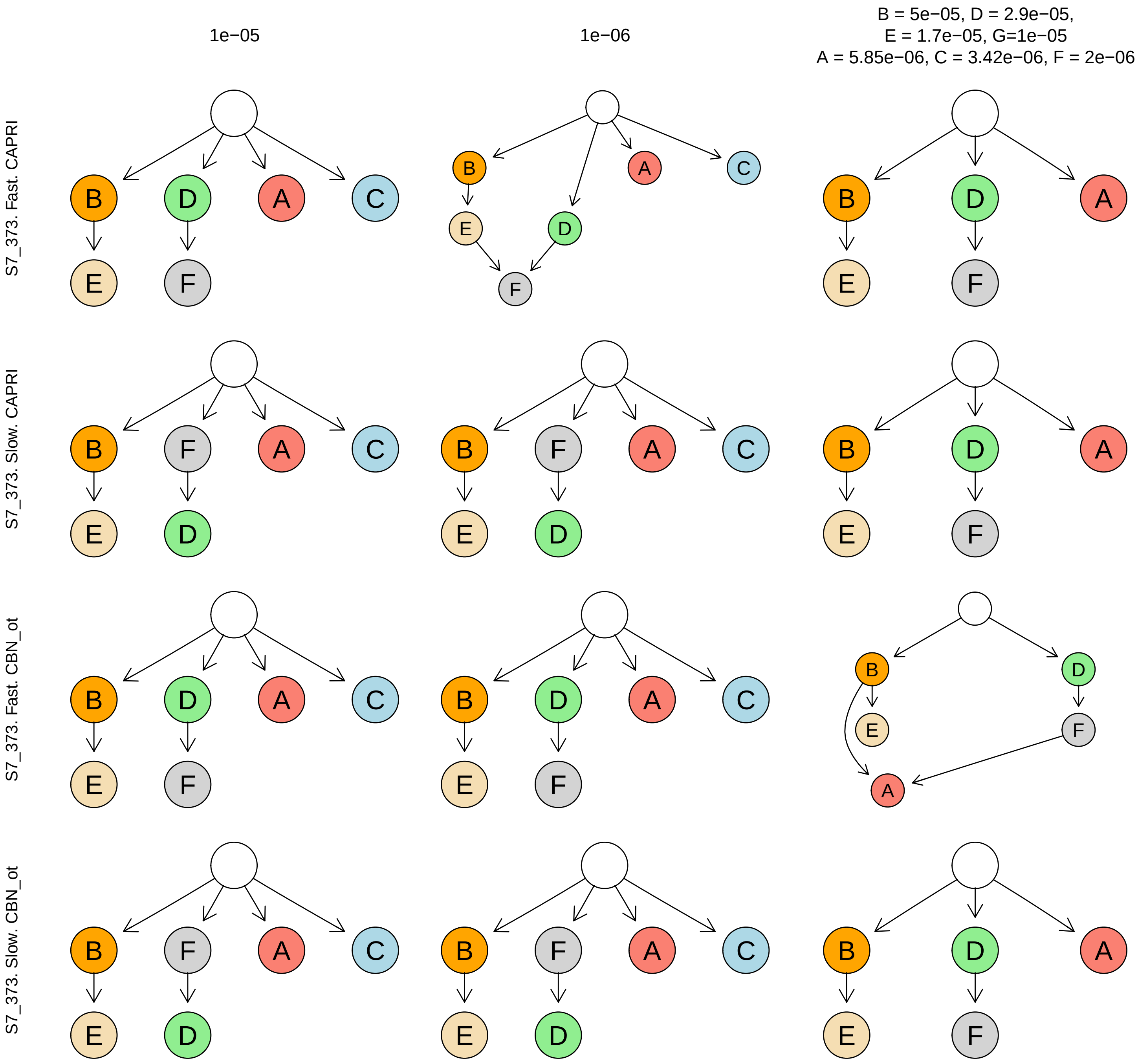

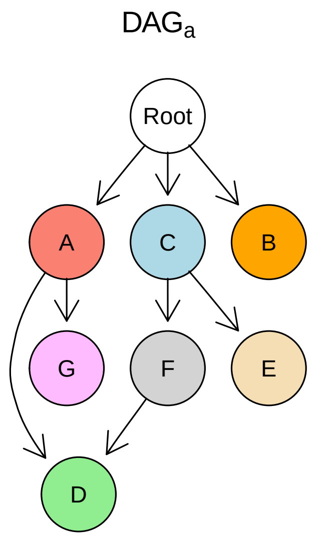

ID	Accessible Genot.	Discrep. DAG_a
S7_374	12	31

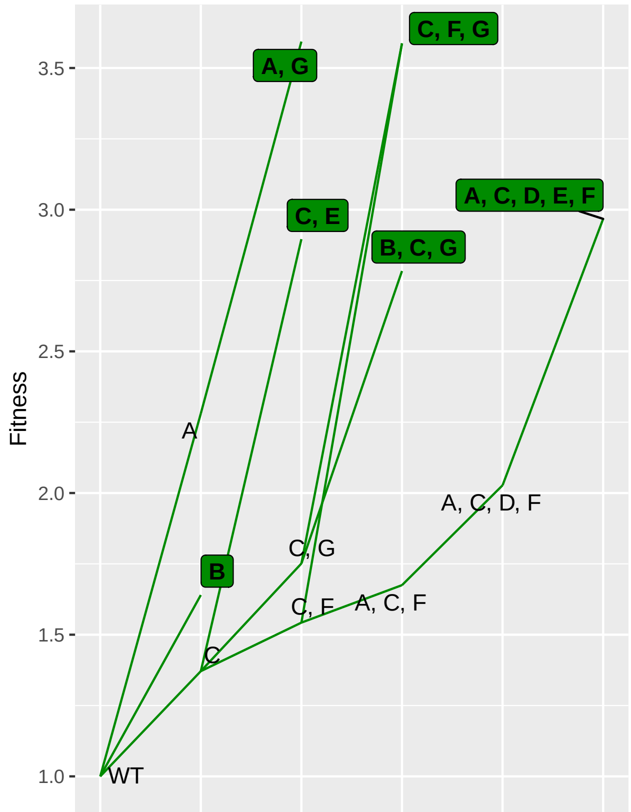

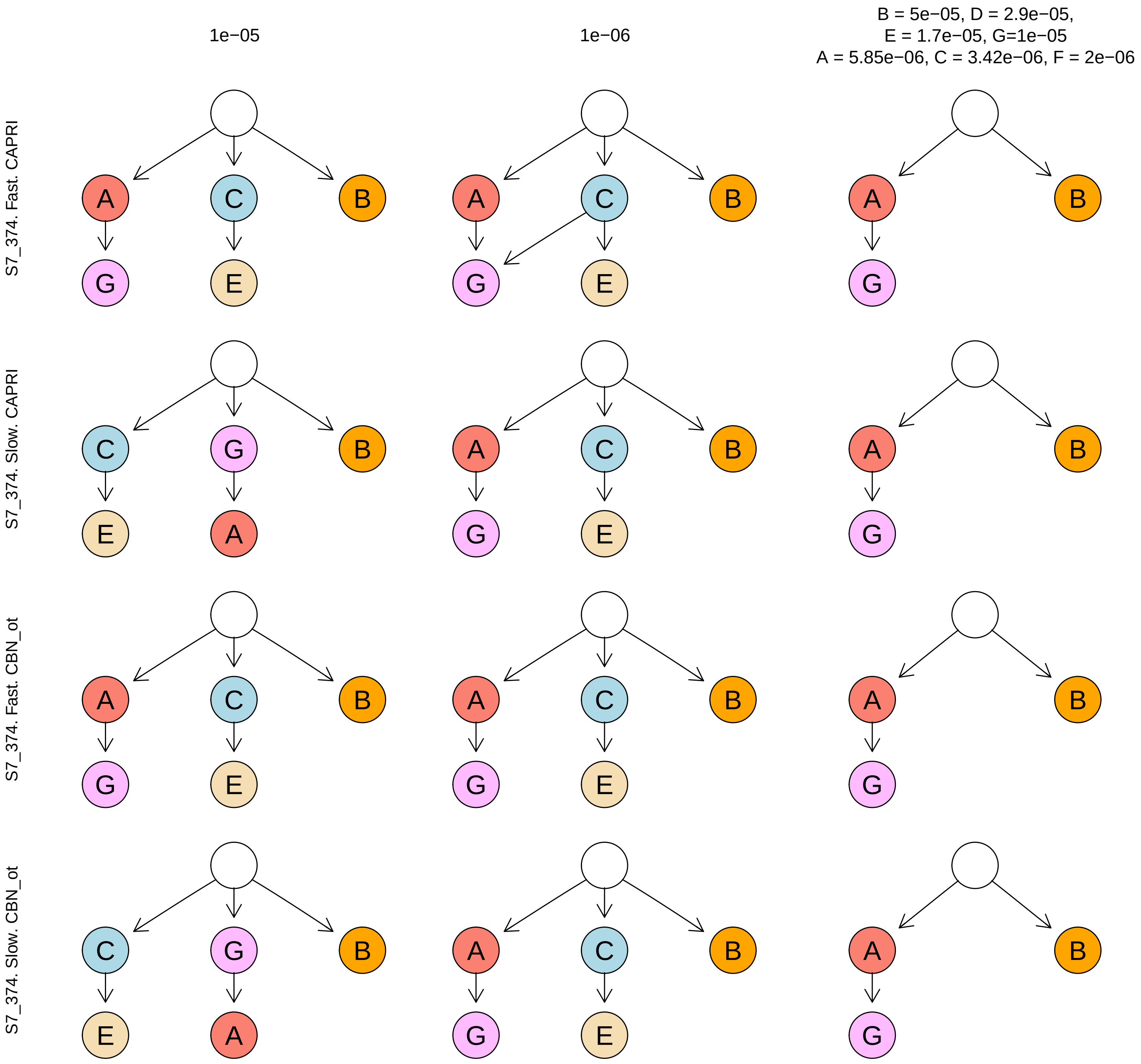

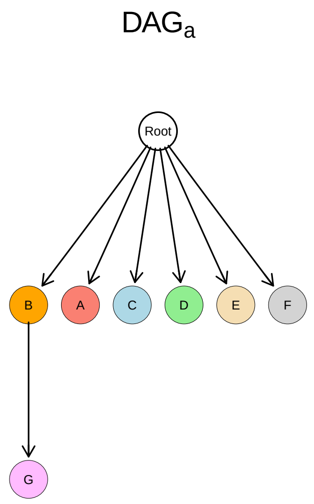

ID	Accessible Genot.	Discrep. DAG_a
S7_375	40	69

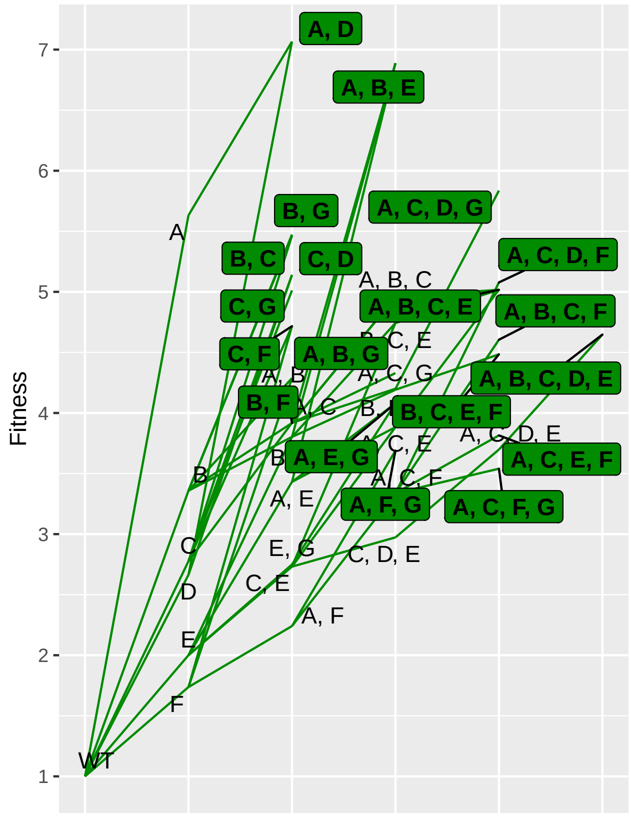

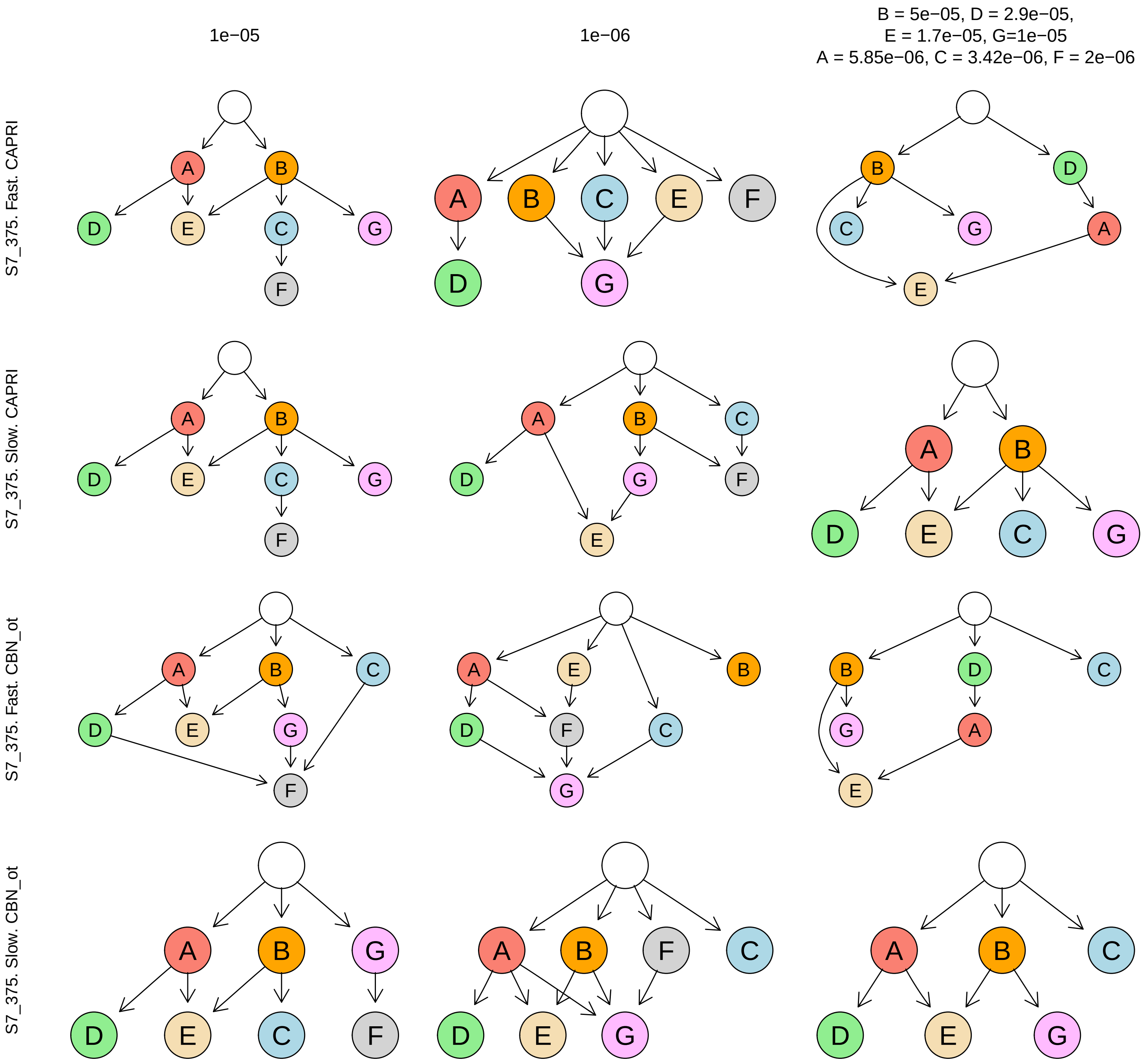

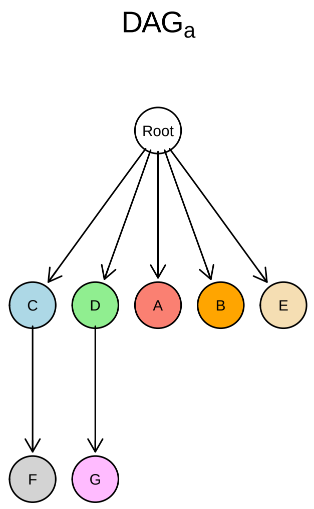

ID	Accessible Genot.	Discrep. DAG_a
S7_376	37	58

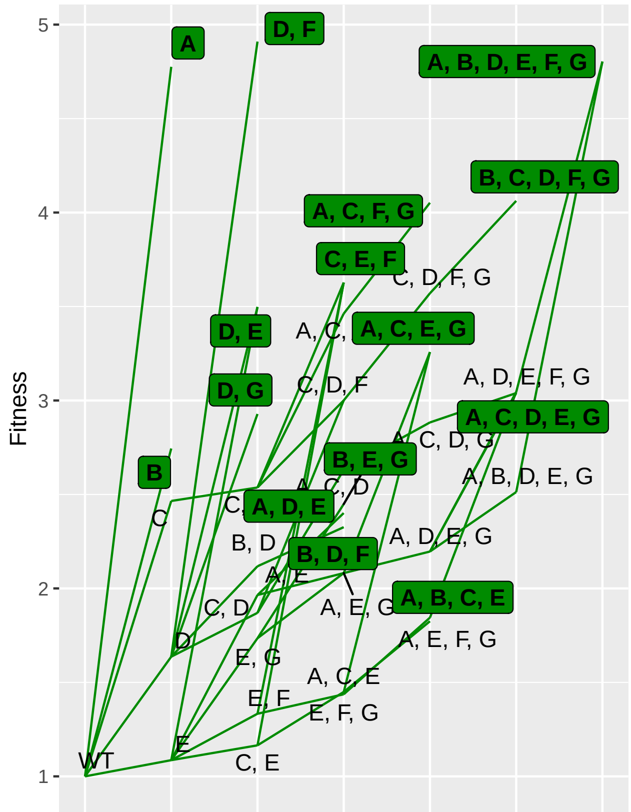

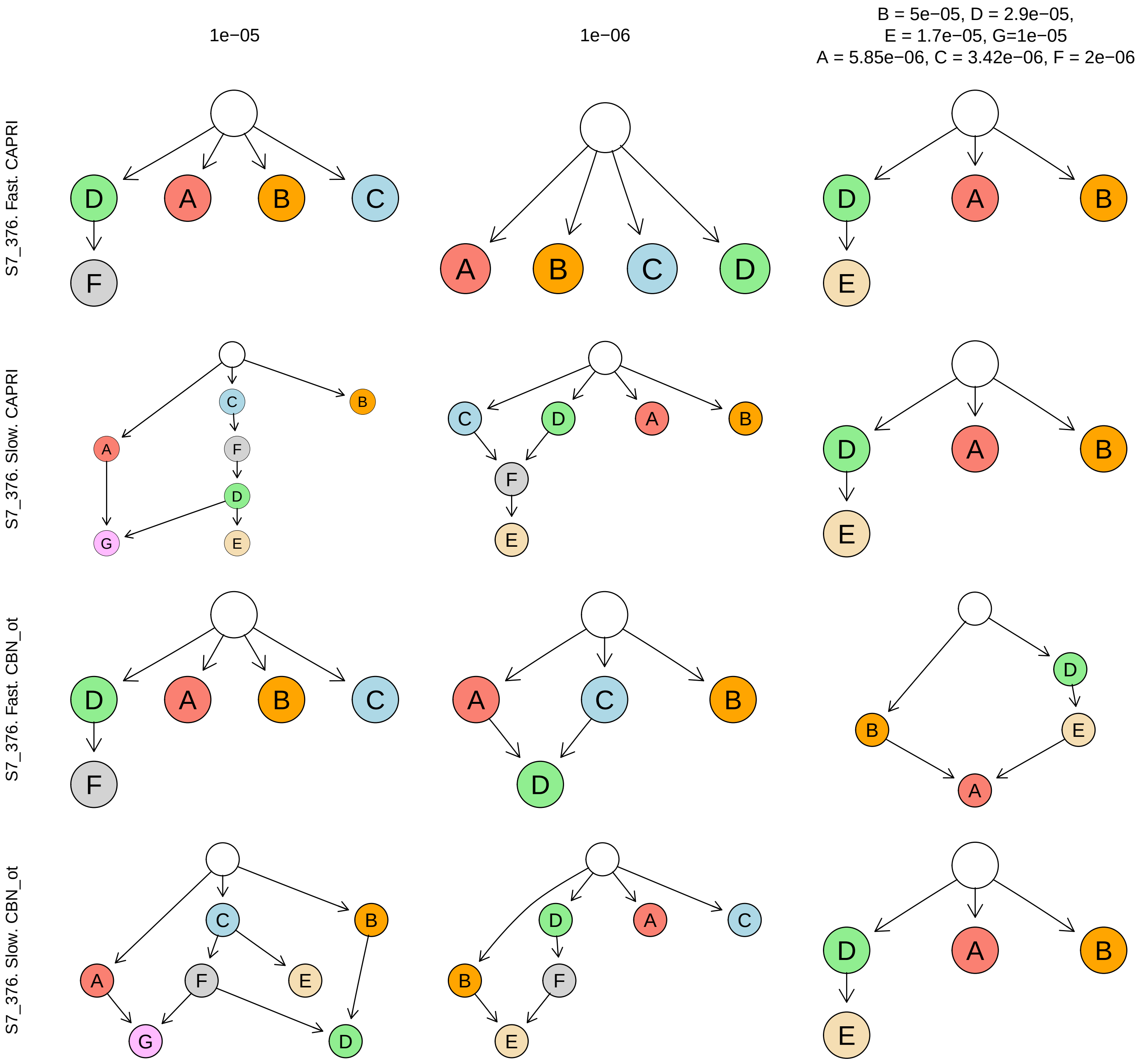

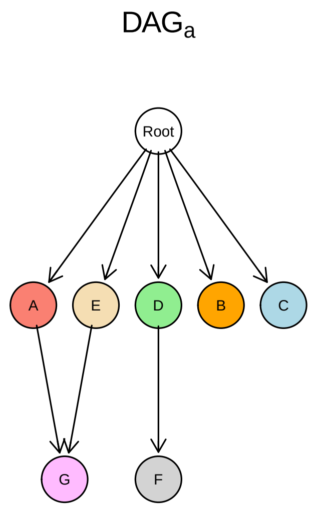

ID	Accessible Genot.	Discrep. DAG_a
S7_377	45	50

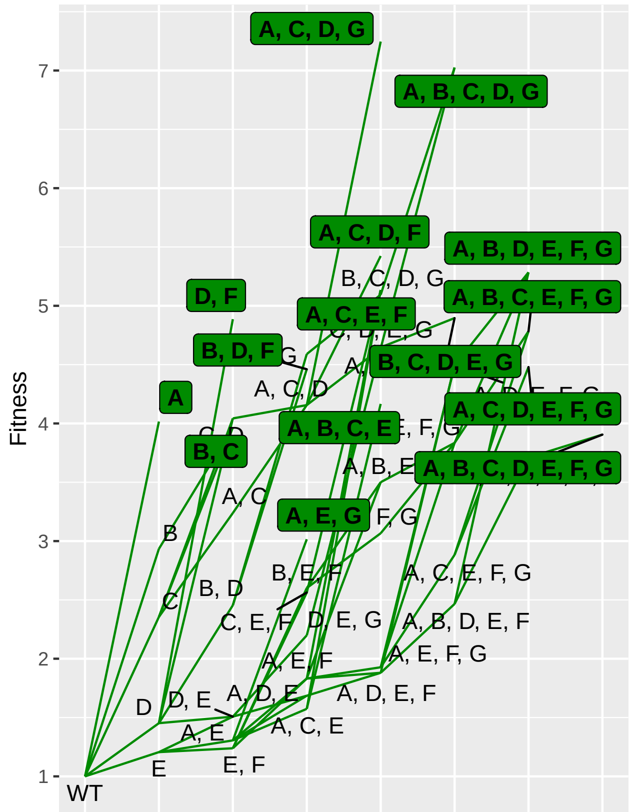

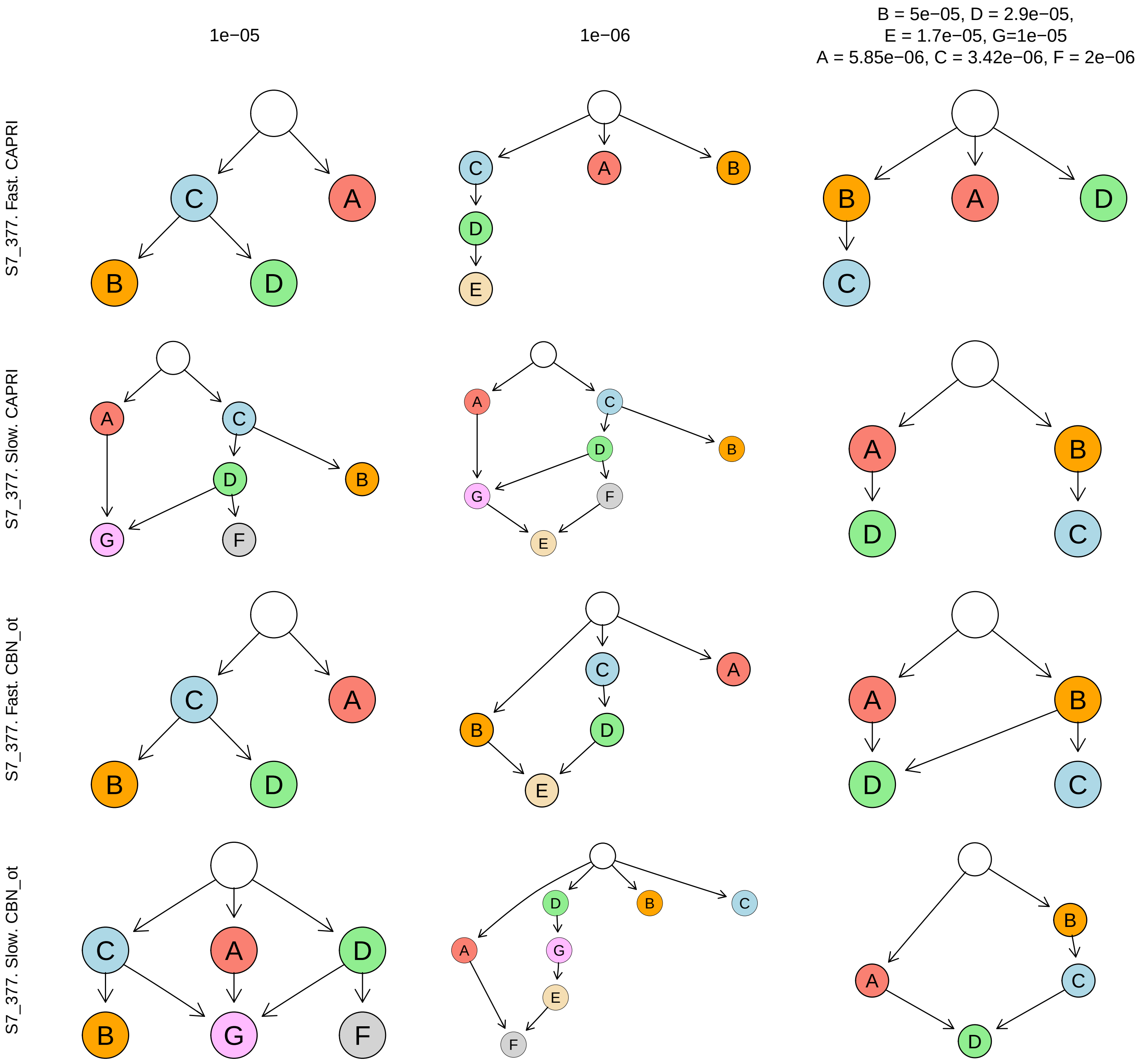

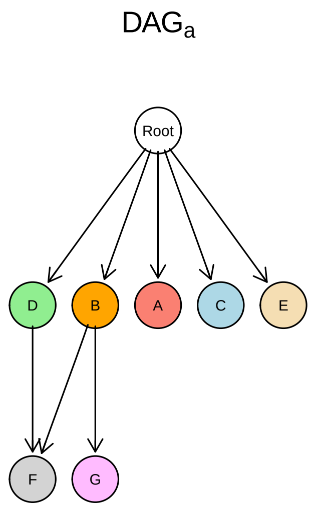

ID	Accessible Genot.	Discrep. DAG_a
S7_378	38	55

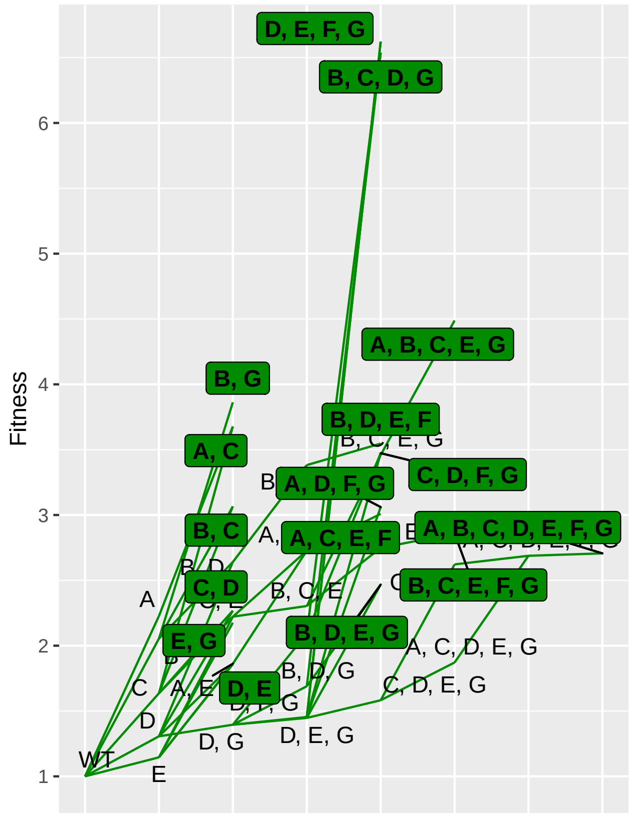

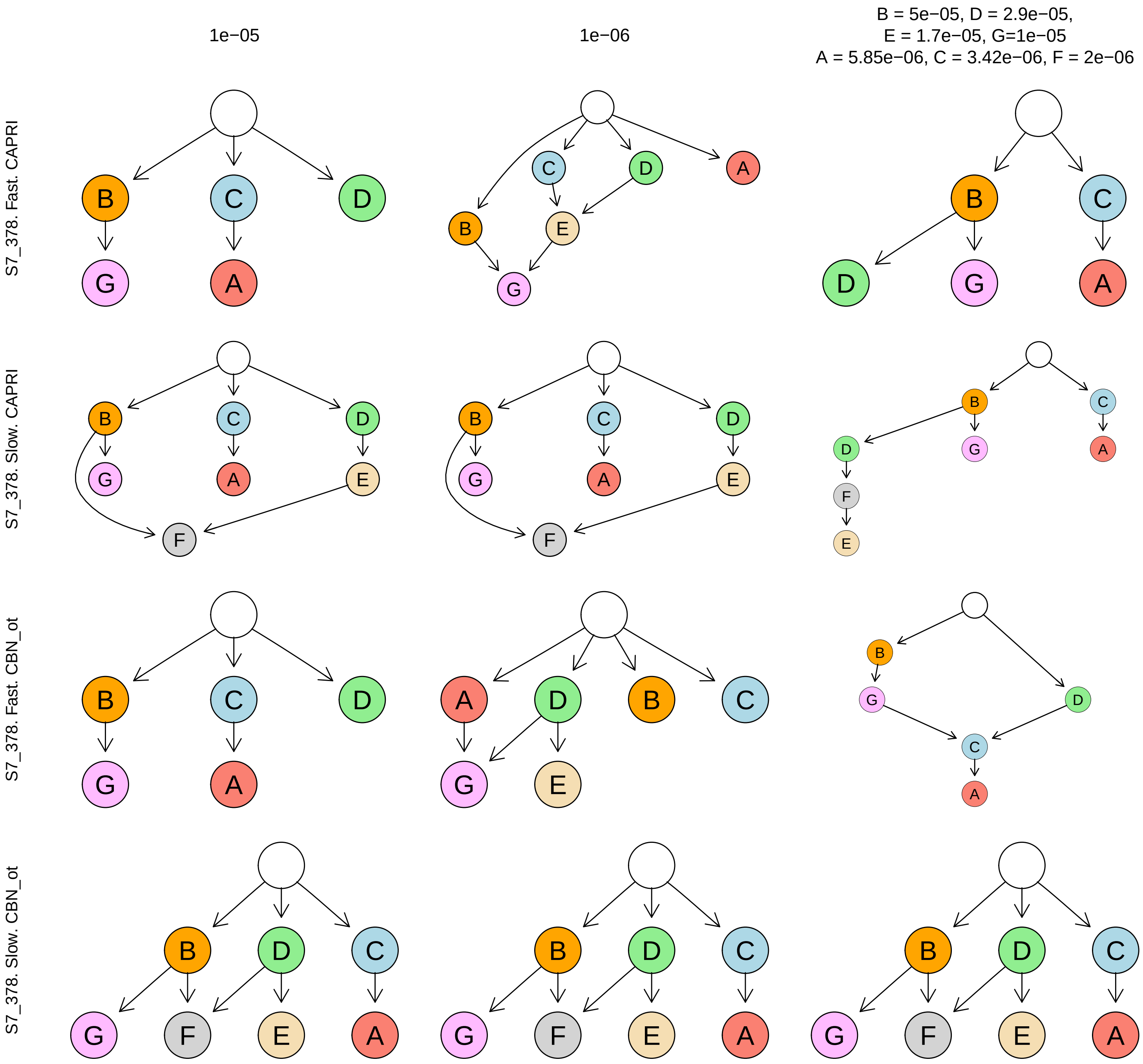

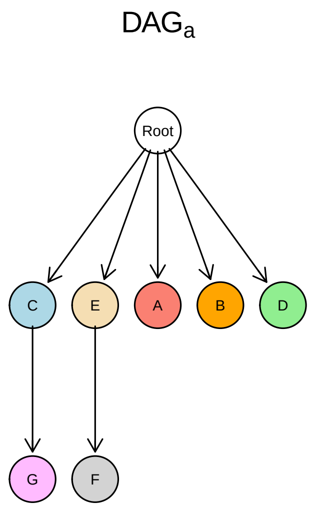

ID	Accessible Genot.	Discrep. DAG_a
S7_379	30	49

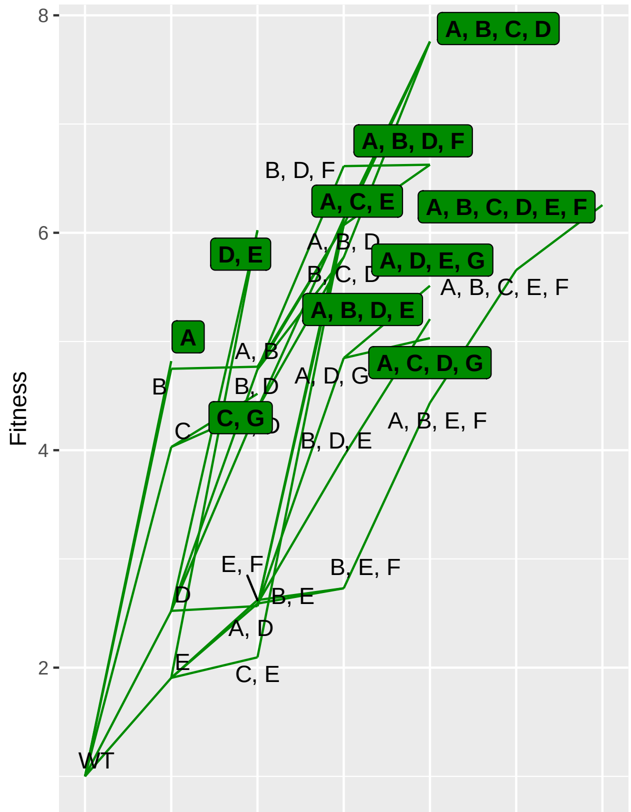

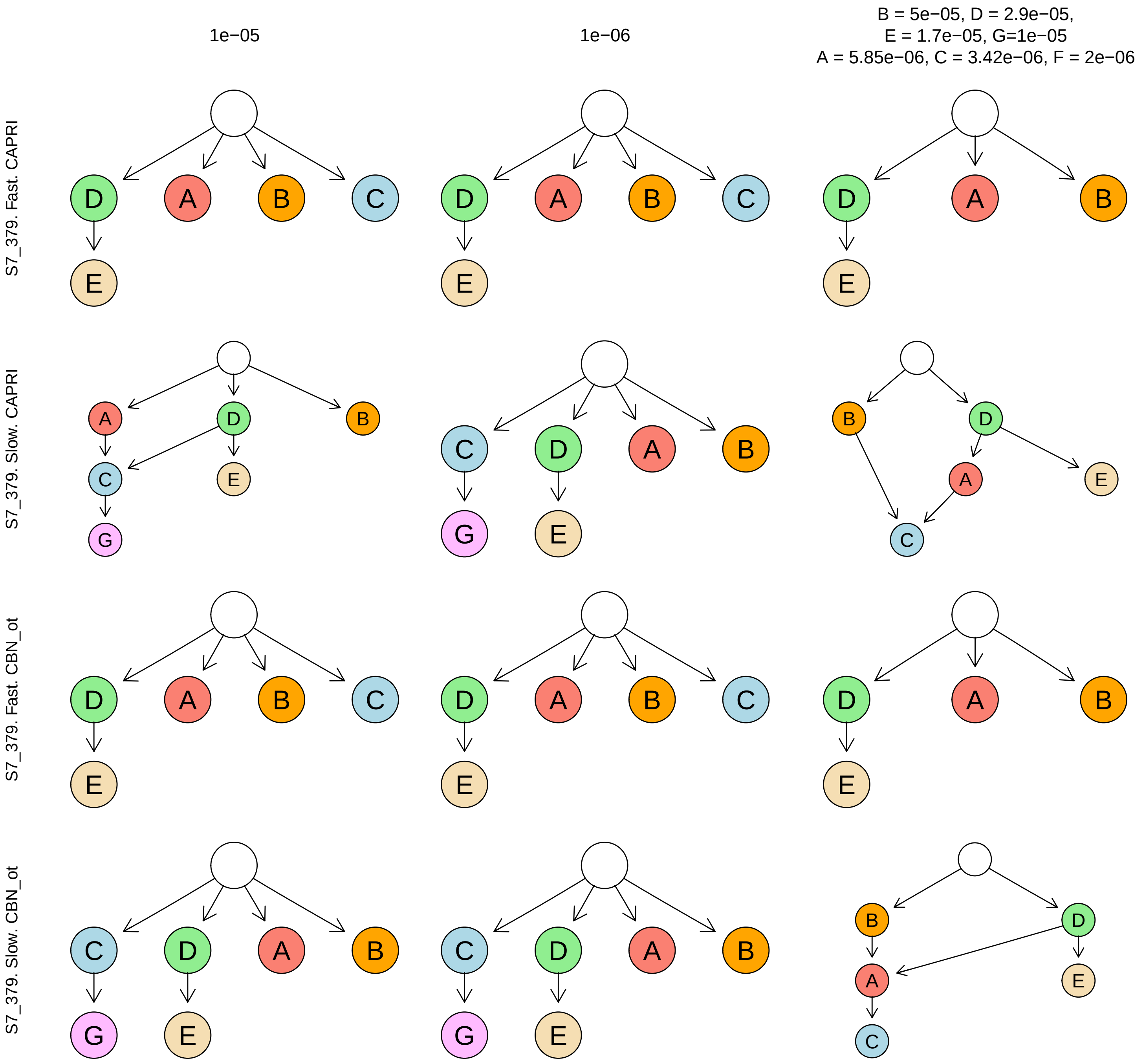

DAG$_a$

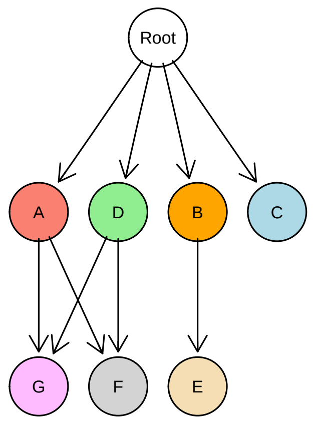

ID	Accessible Genot.	Discrep. DAG_a
S7_380	24	23

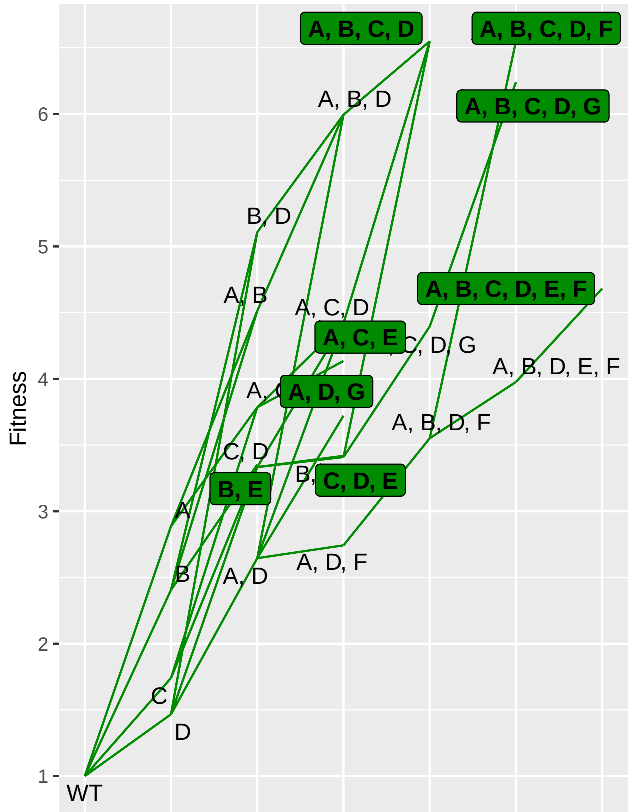

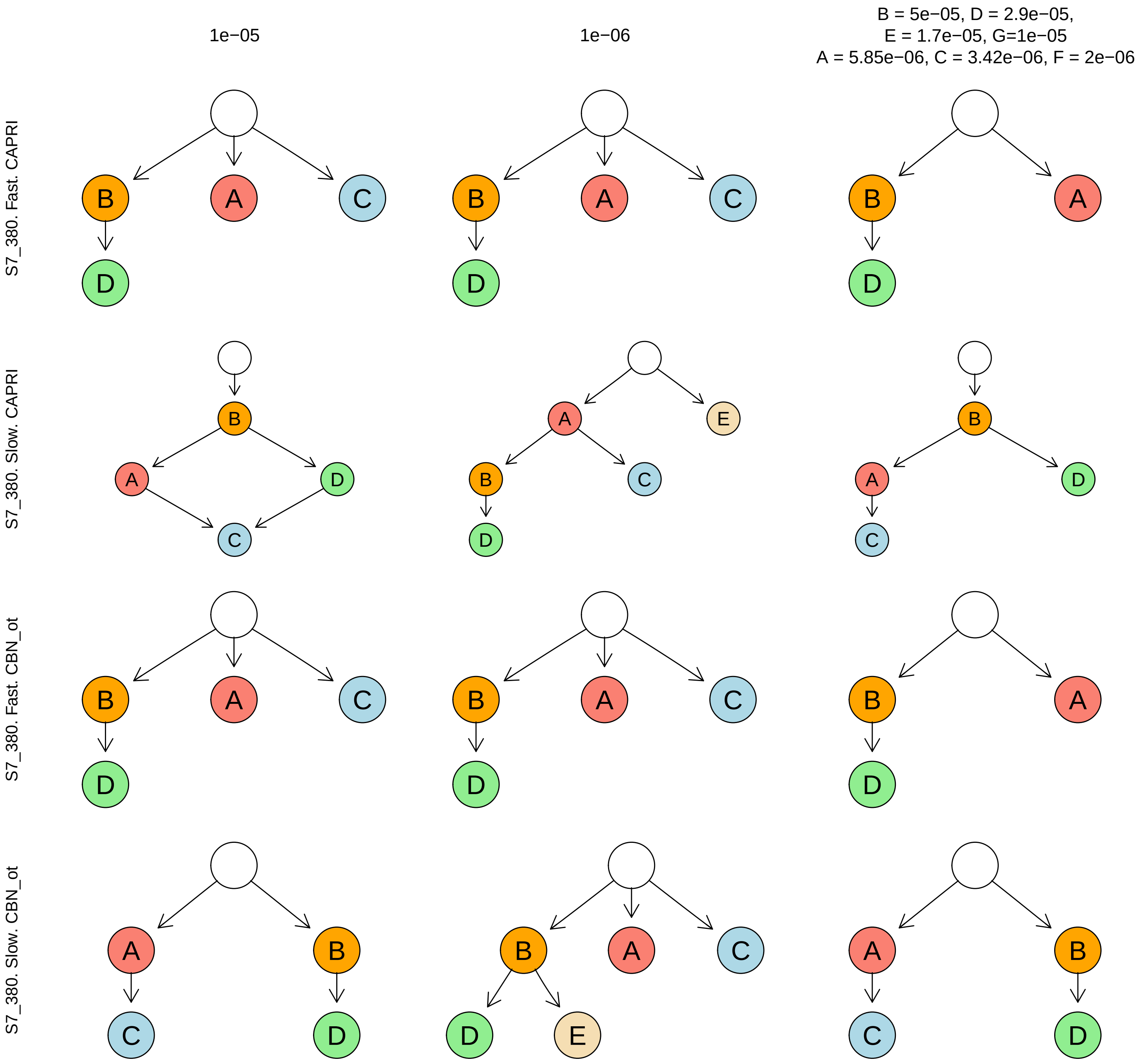

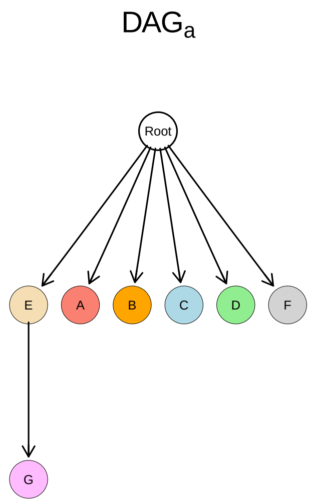

ID	Accessible Genot.	Discrep. DAG_a
S7_381	28	73

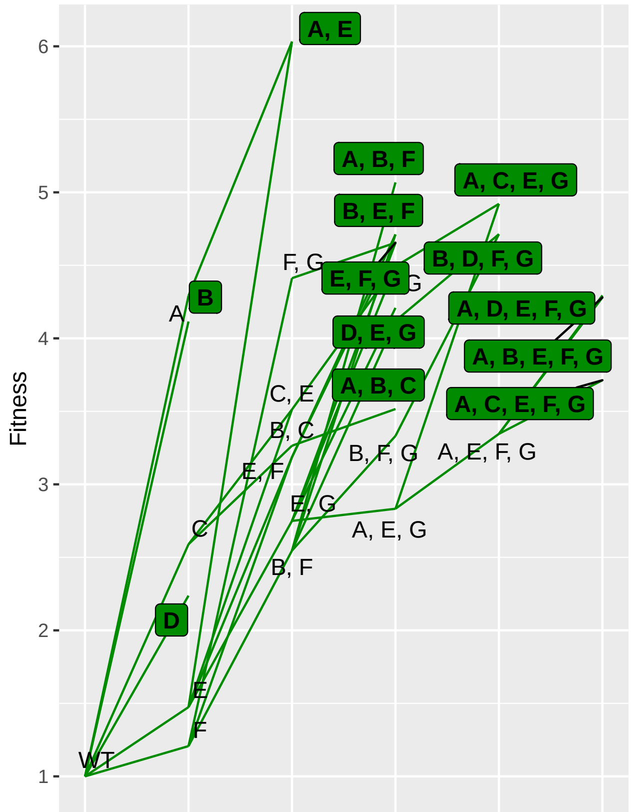

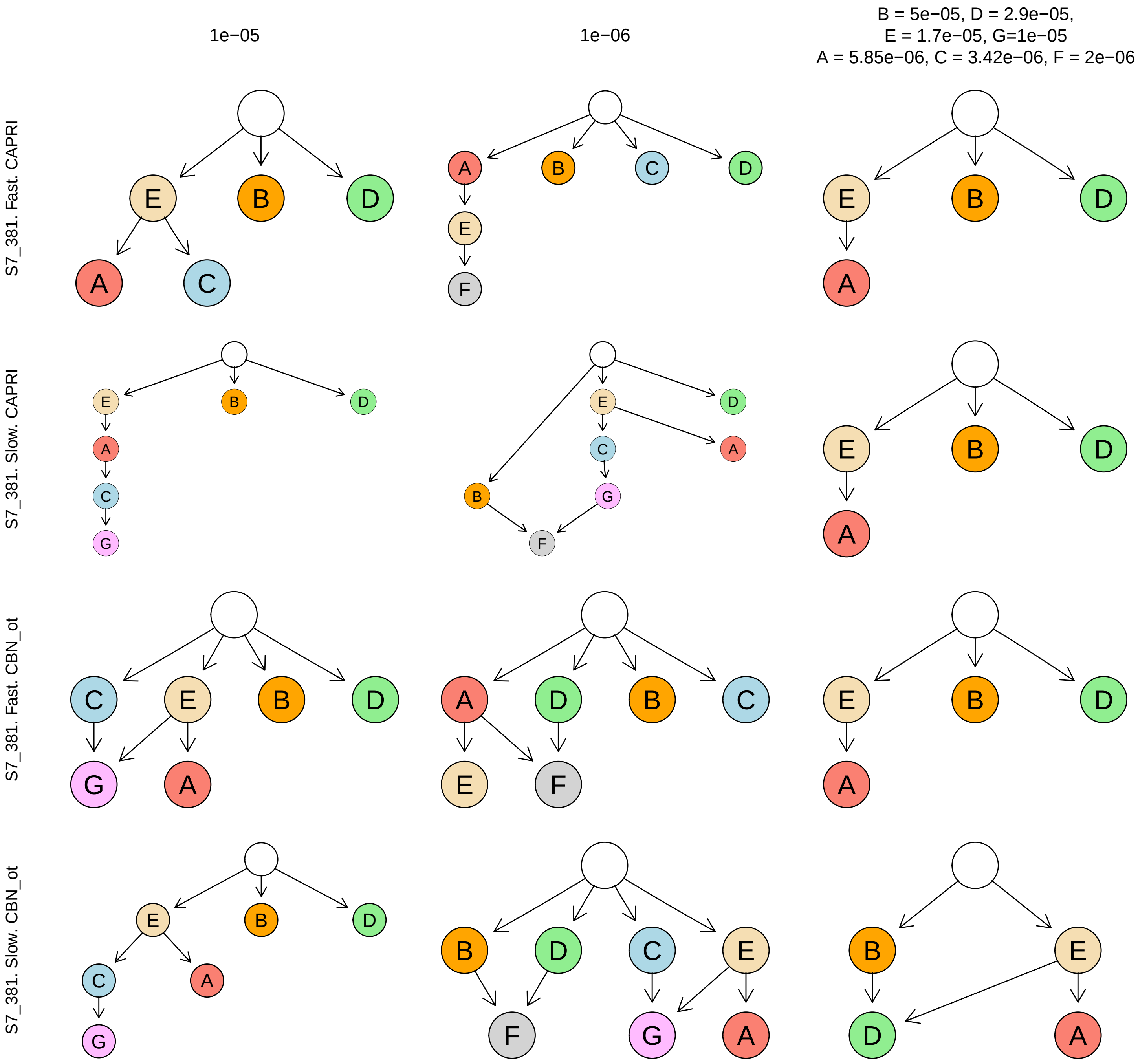

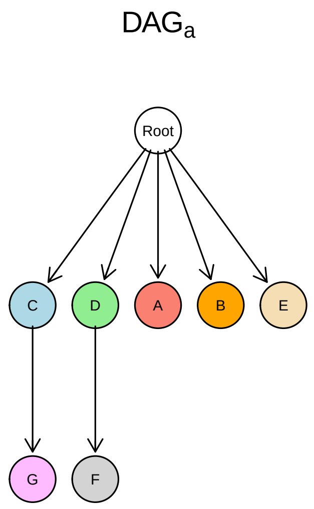

ID	Accessible Genot.	Discrep. DAG_a
S7_382	19	62

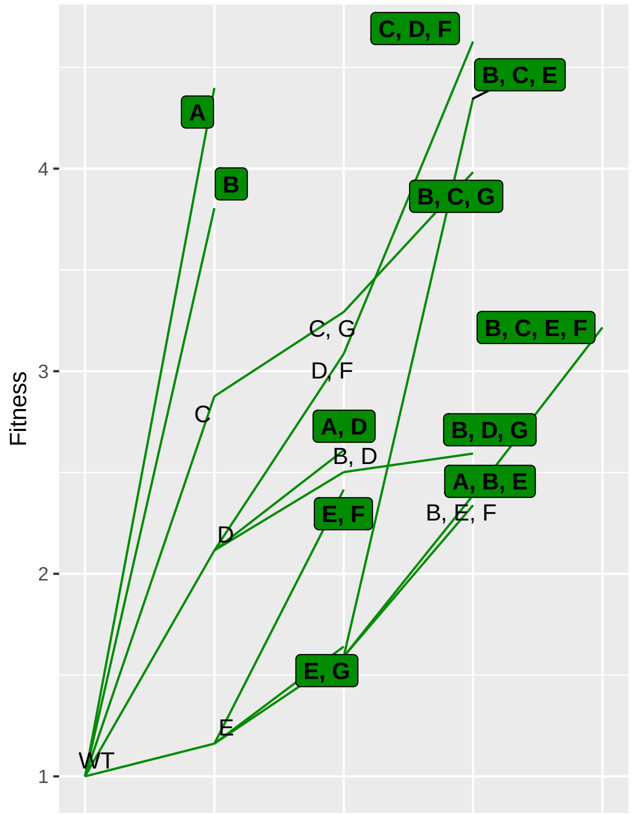

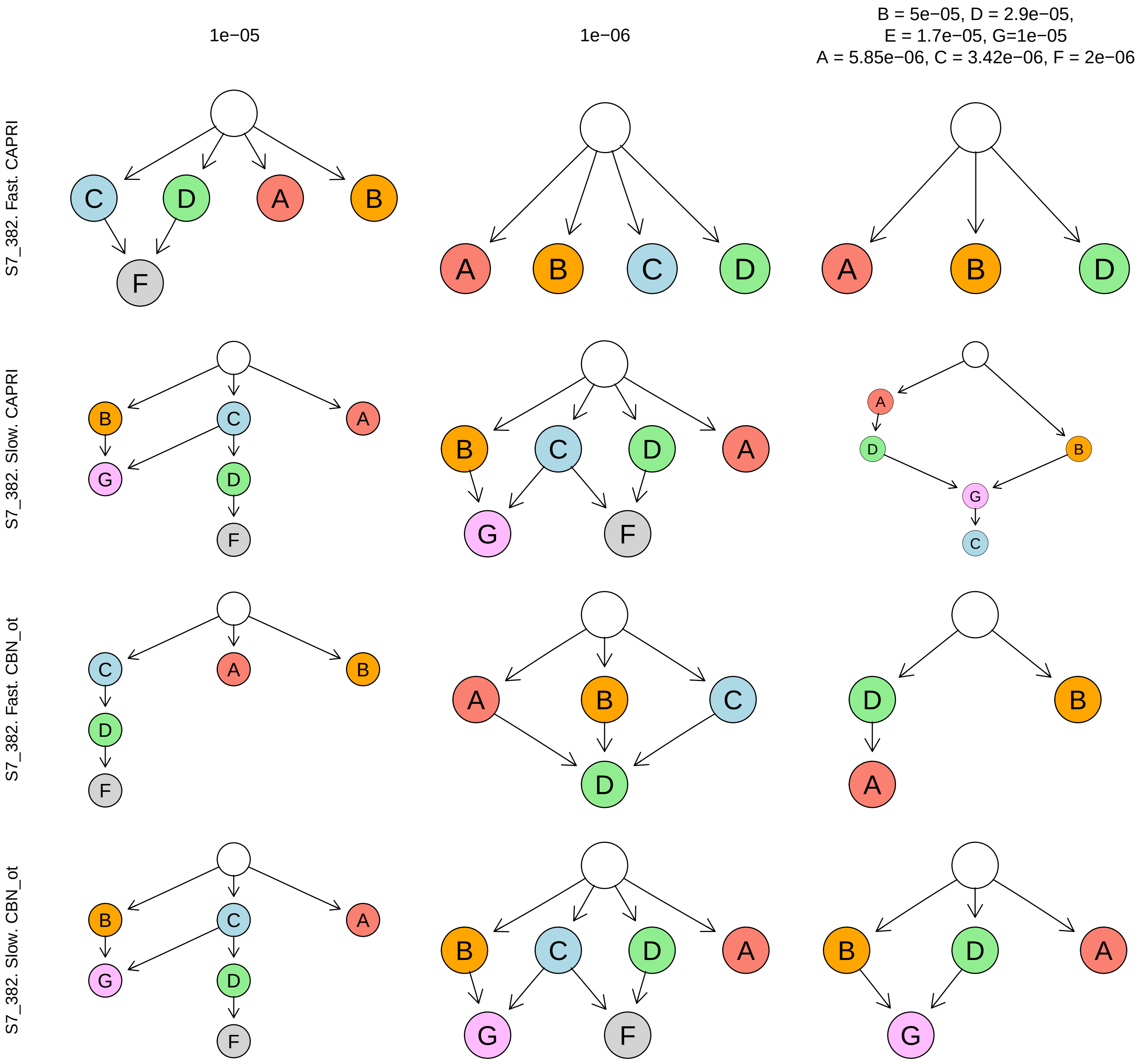

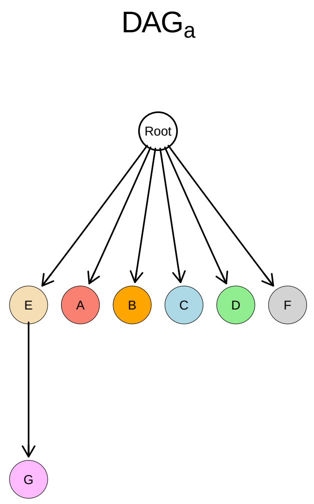

ID	Accessible Genot.	Discrep. DAG_a
S7_383	27	74

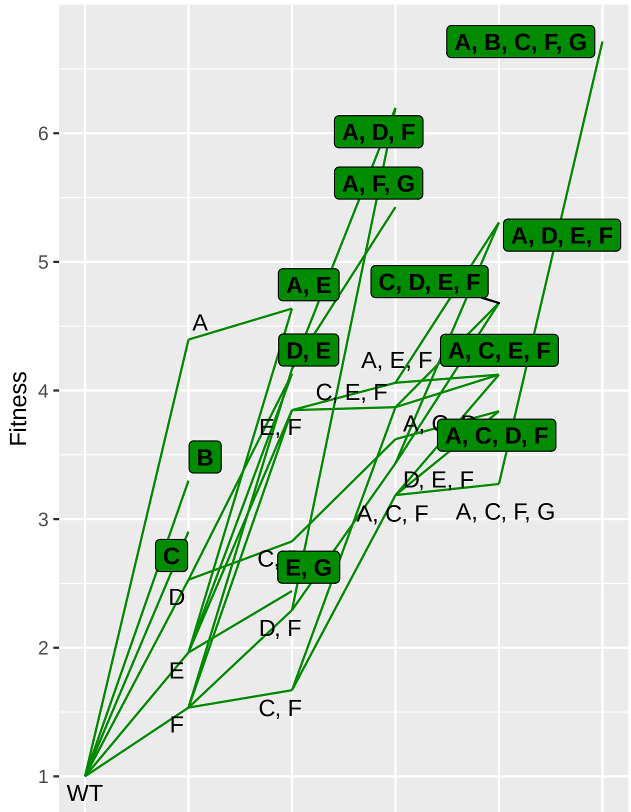

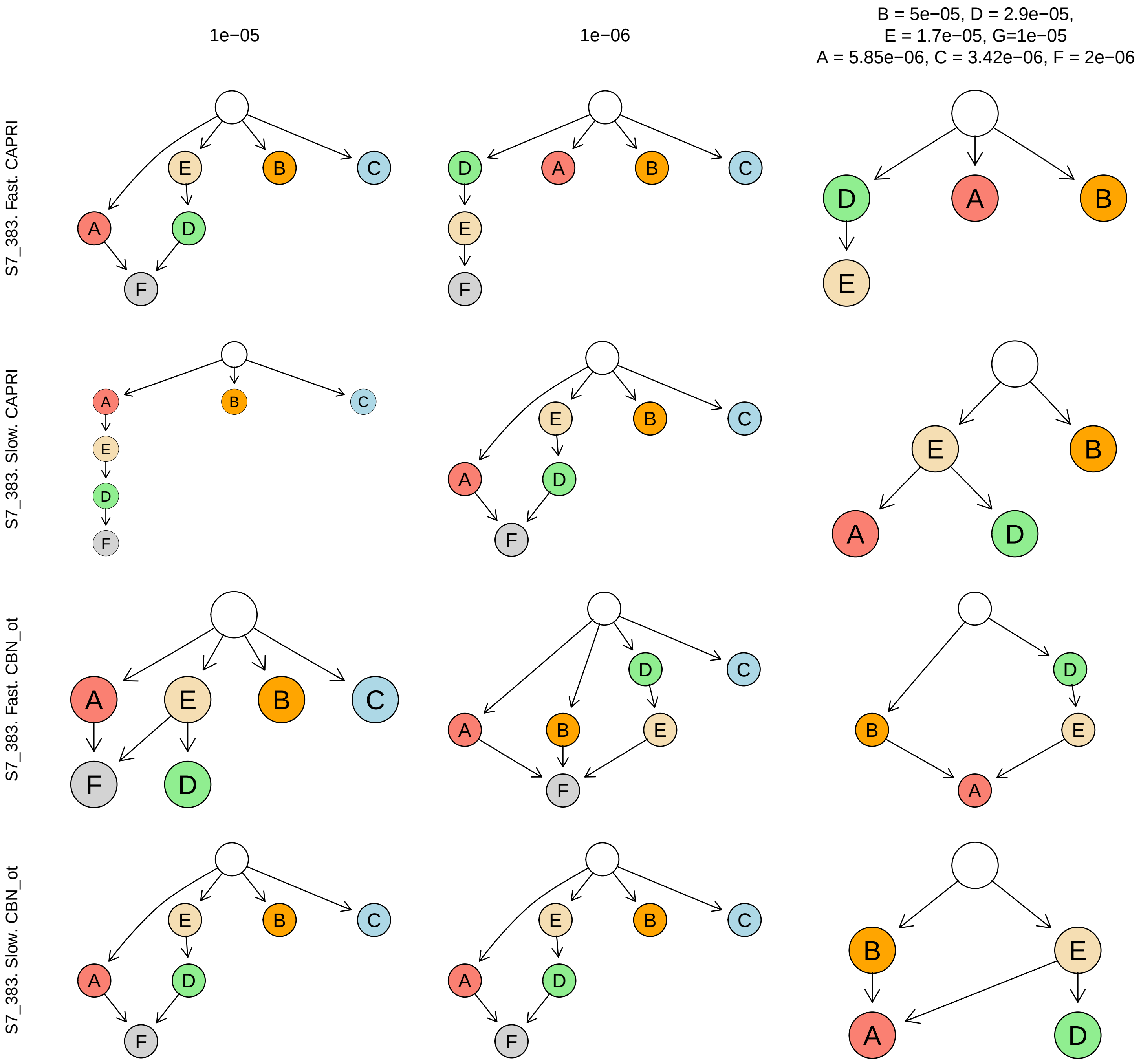

DAG$_a$

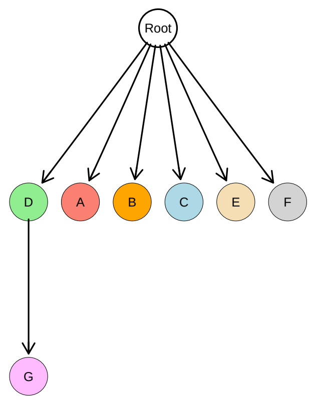

ID	Accessible Genot.	Discrep. DAG_a
S7_384	35	62

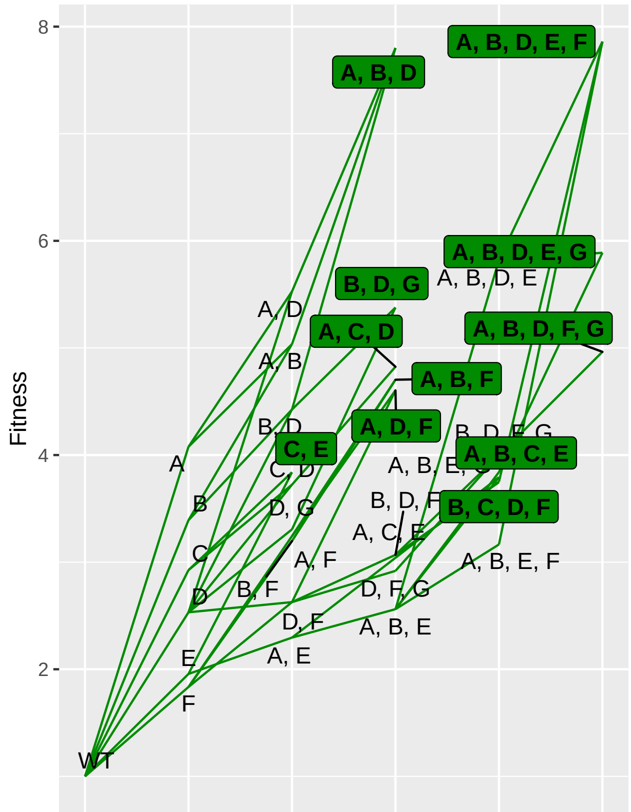

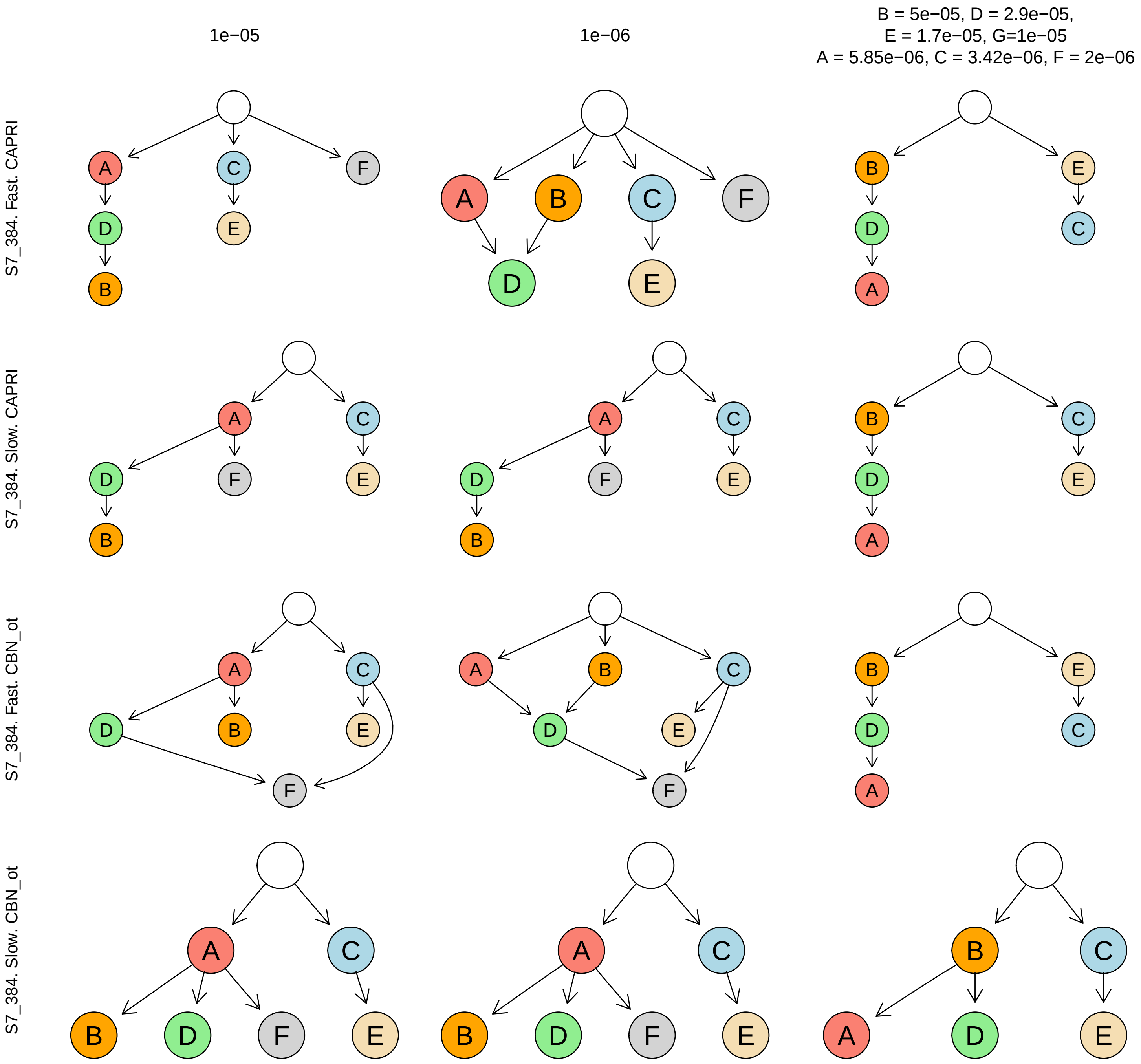

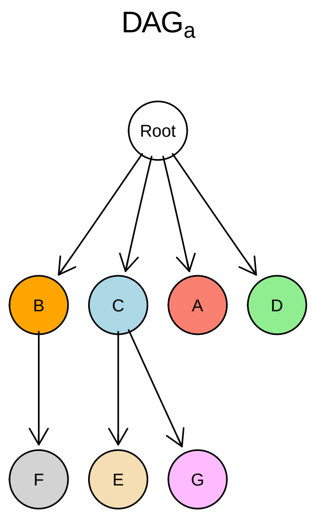

ID	Accessible Genot.	Discrep. DAG_a
S7_385	18	47

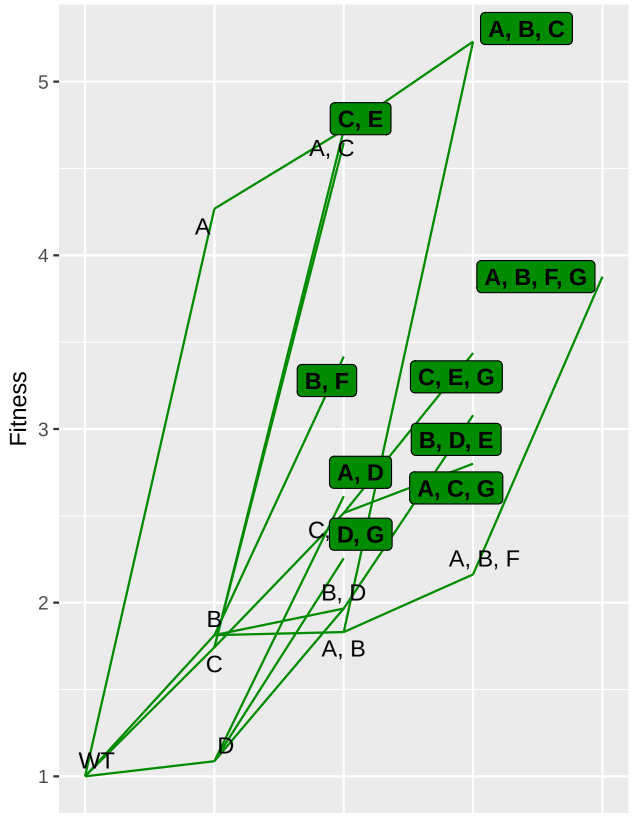

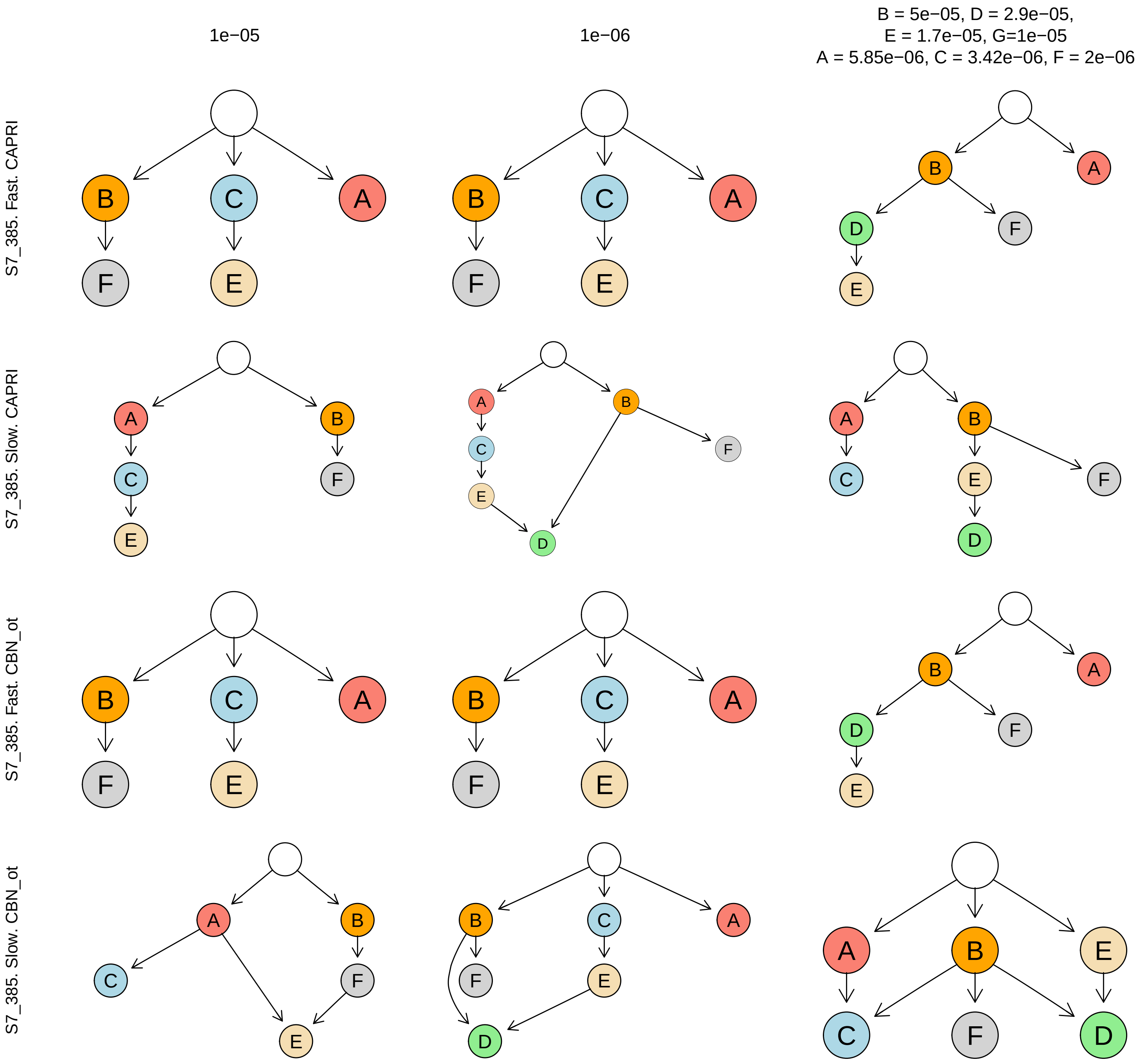

DAG_a

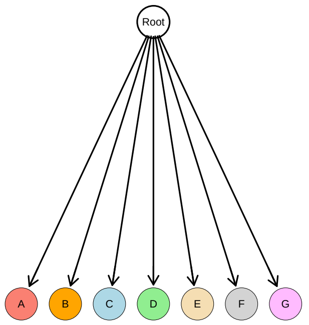

ID	Accessible Genot.	Discrep. DAG_a
S7_386	61	66

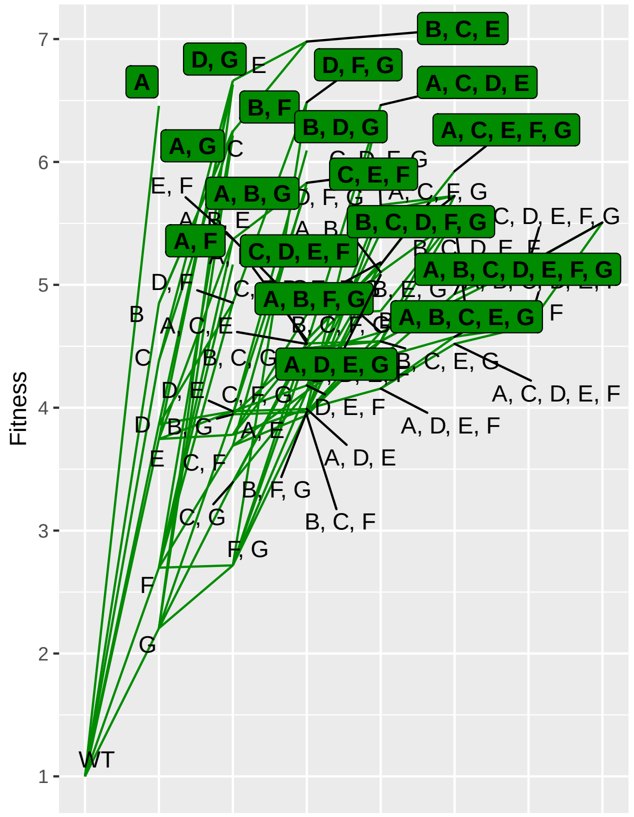

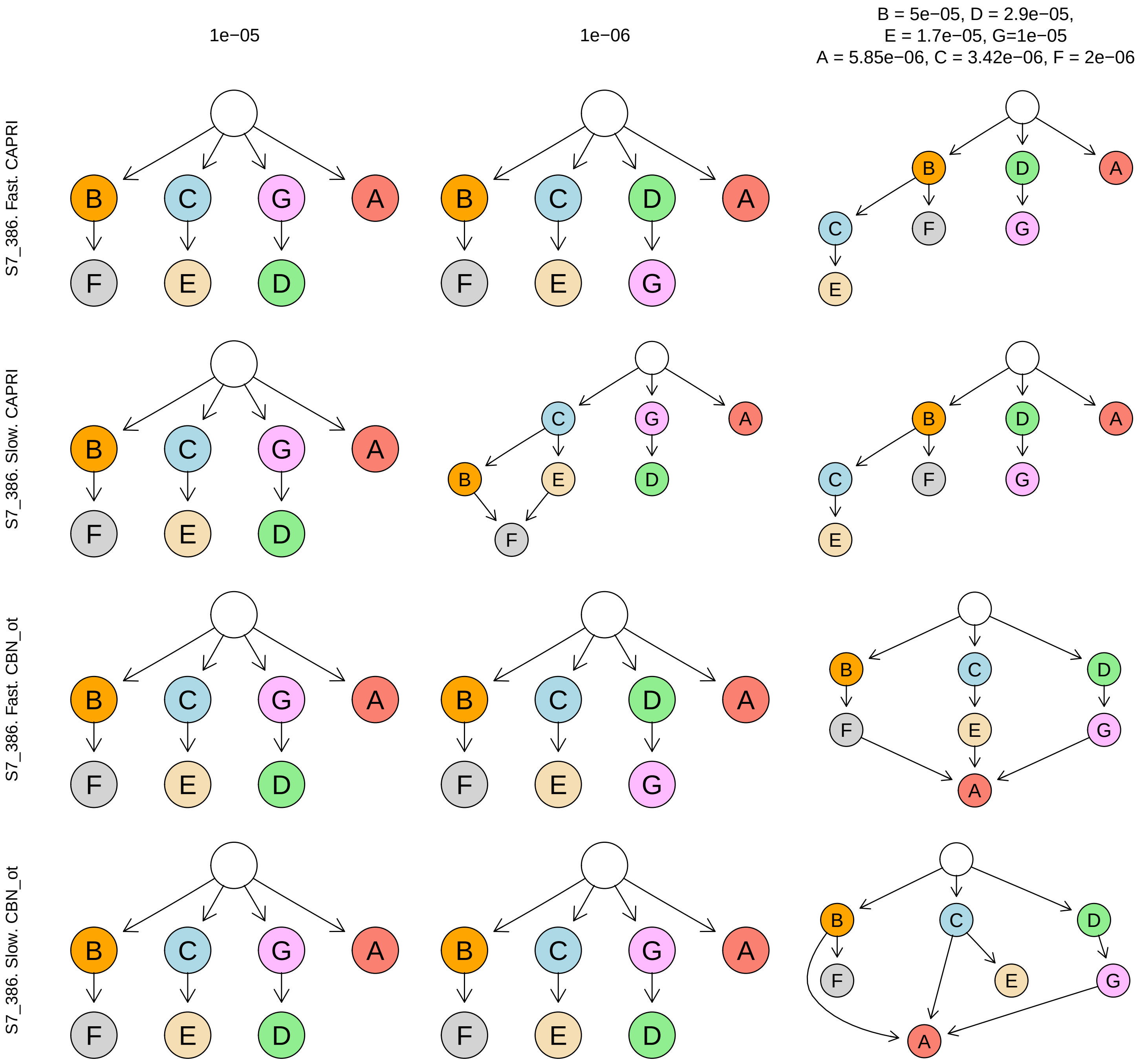

DAG$_a$

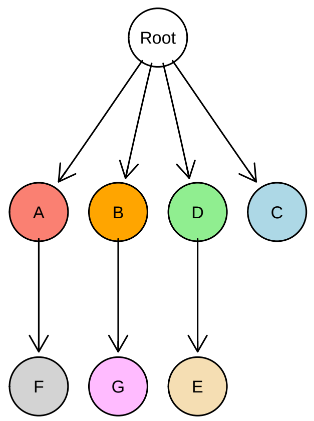

ID	Accessible Genot.	Discrep. DAG_a
S7_387	32	57

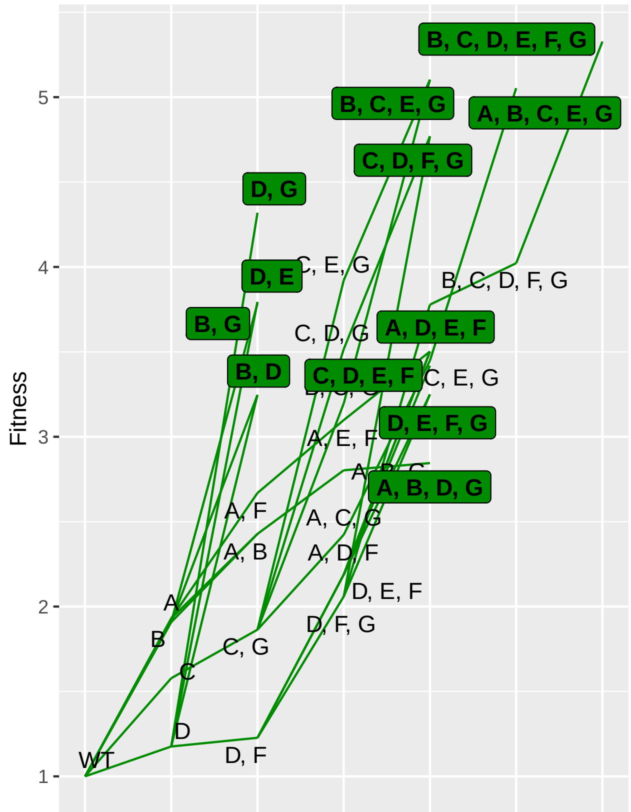

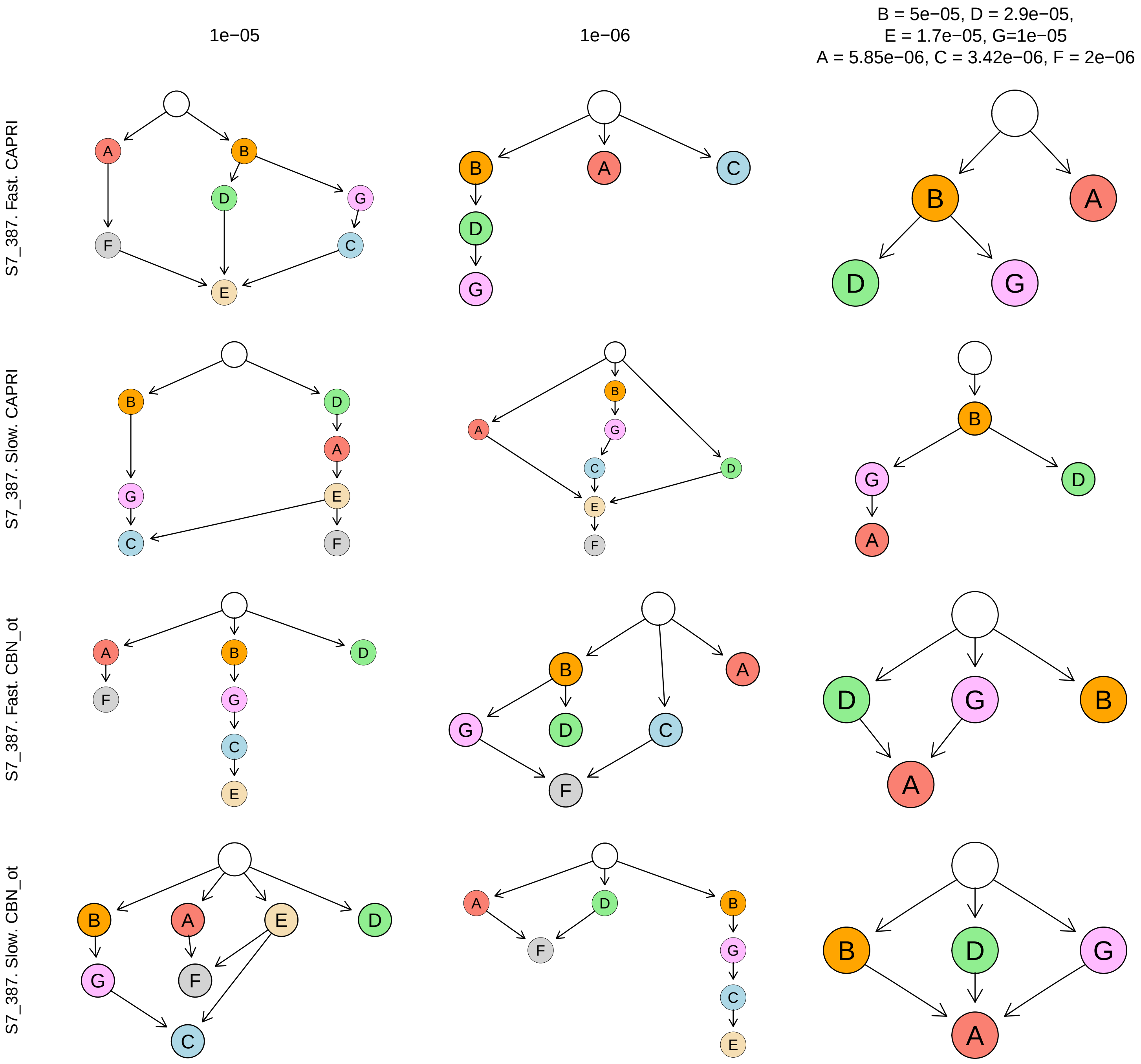

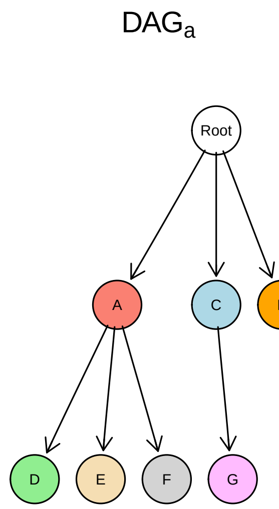

ID	Accessible Genot.	Discrep. DAG_a
S7_388	19	44

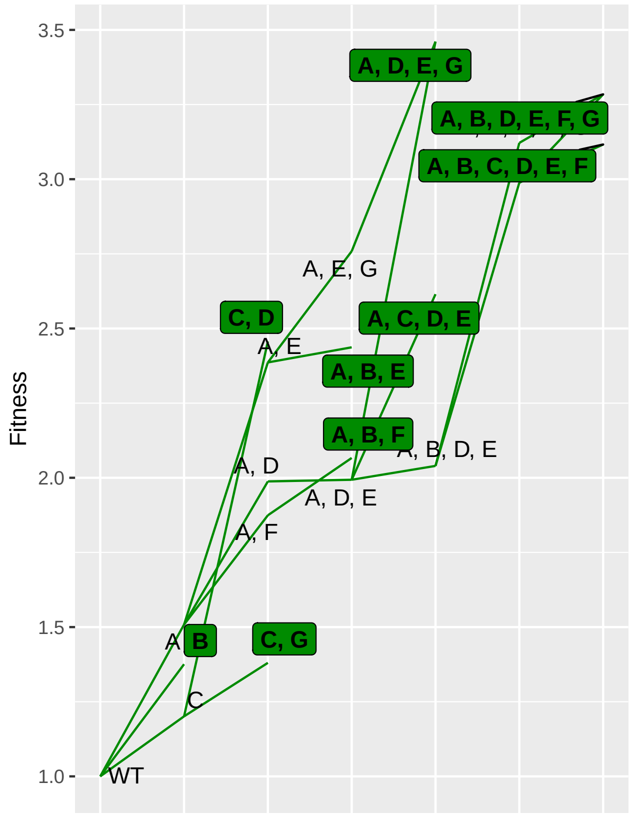

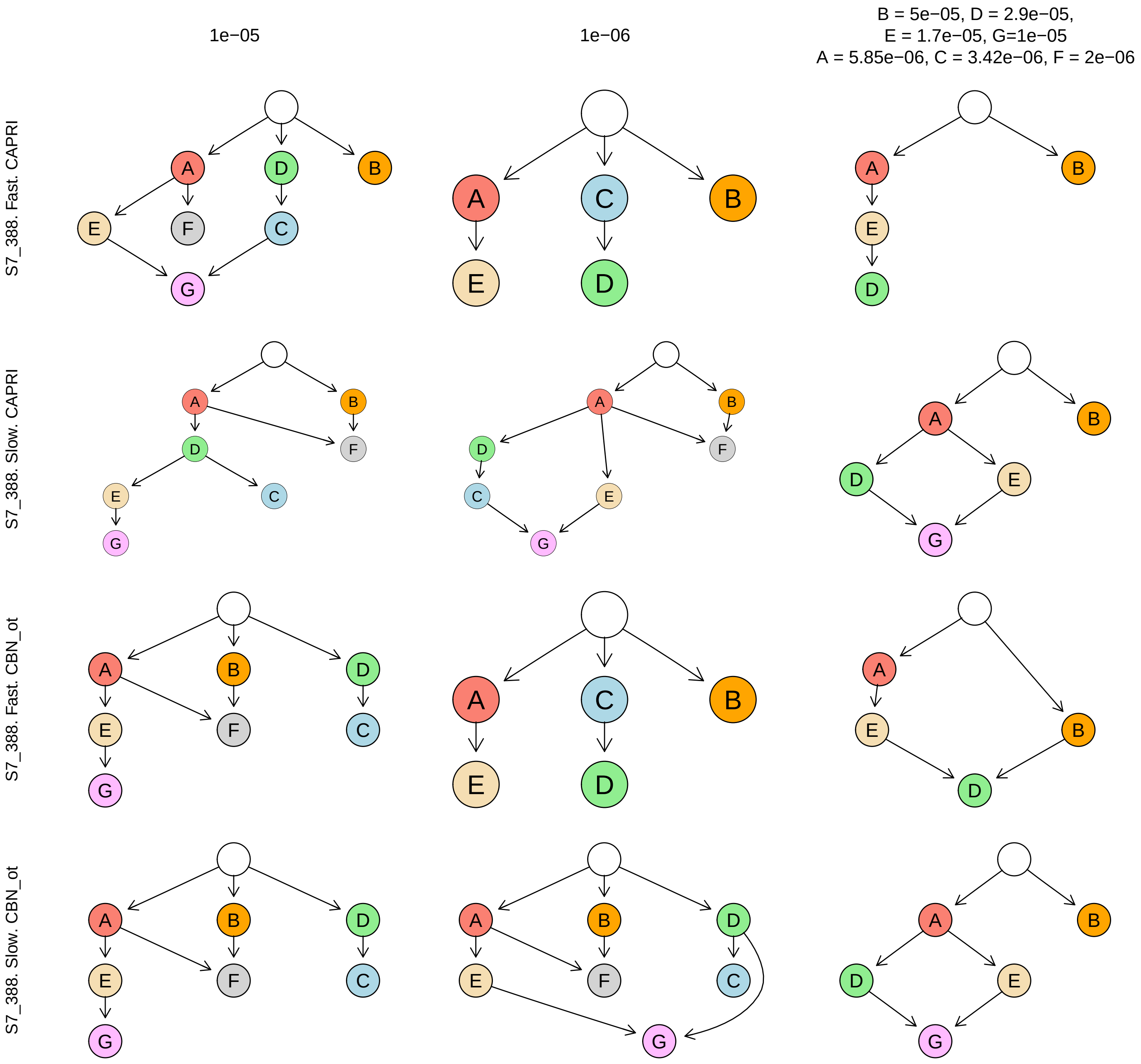

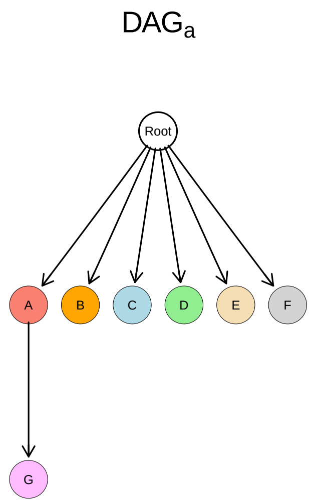

ID	Accessible Genot.	Discrep. DAG_a
S7_389	56	59

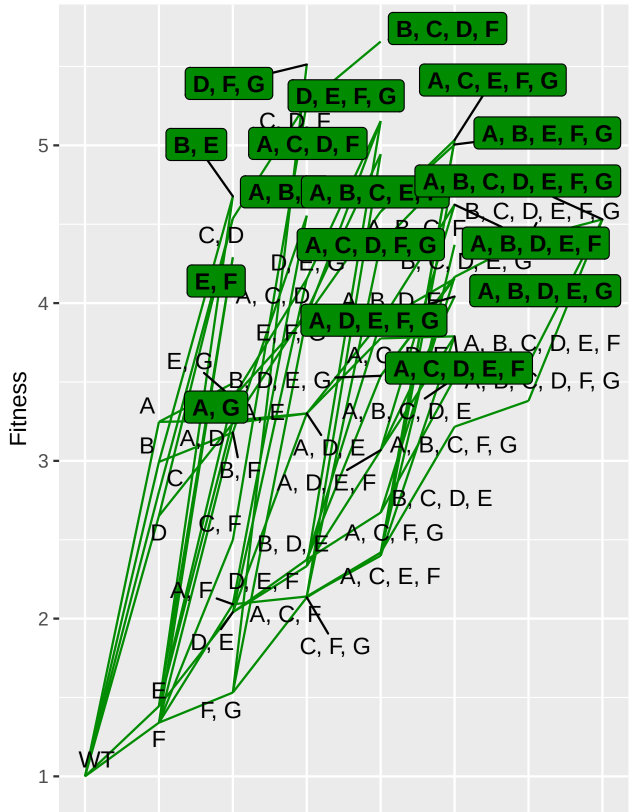

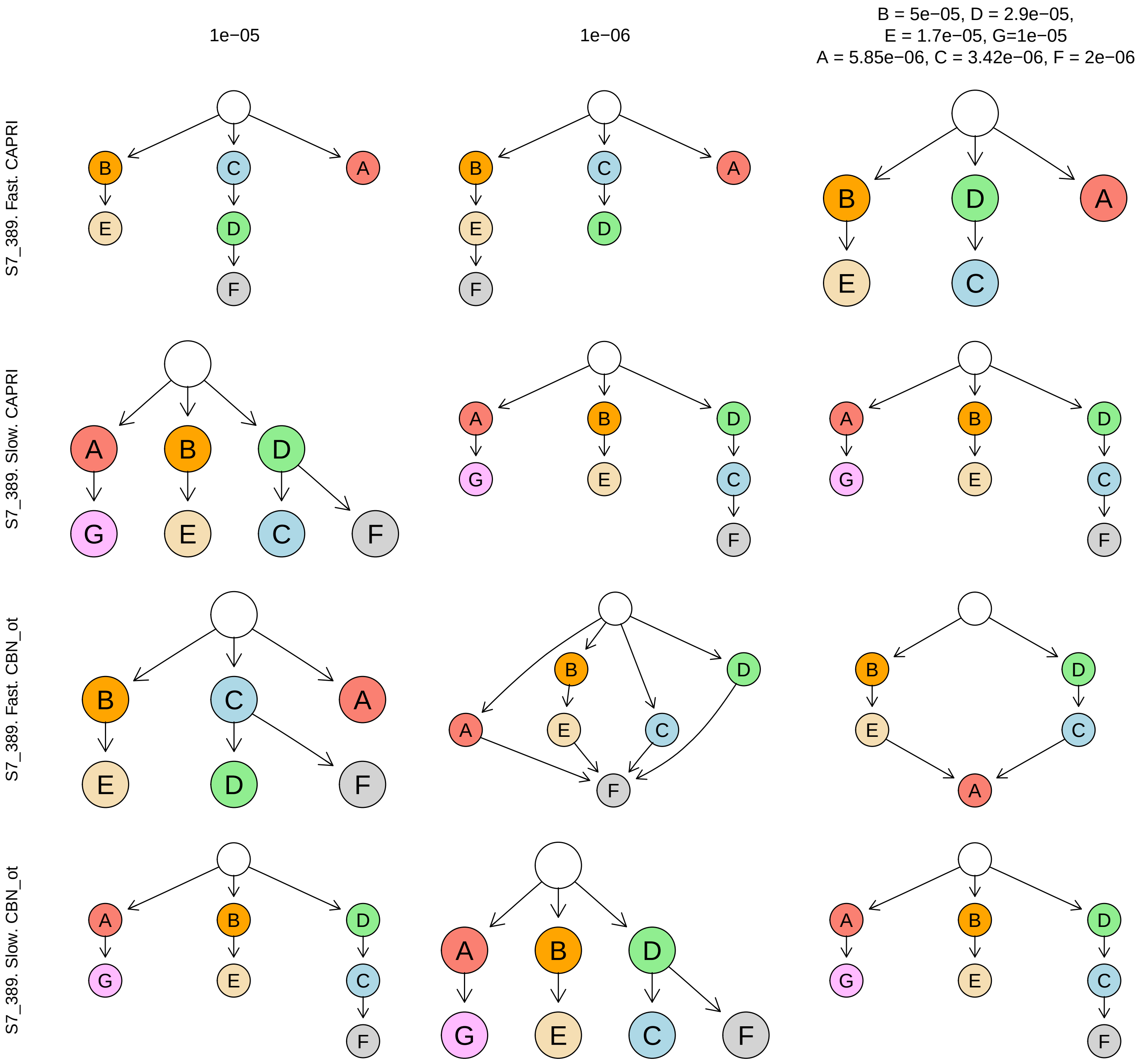

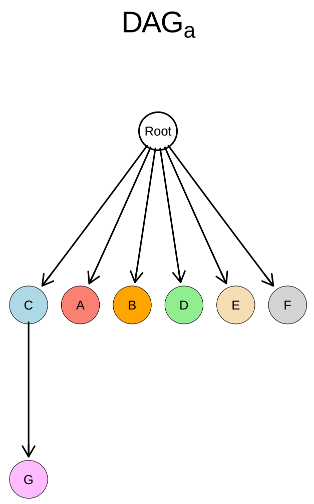

ID	Accessible Genot.	Discrep. DAG_a
S7_390	24	81

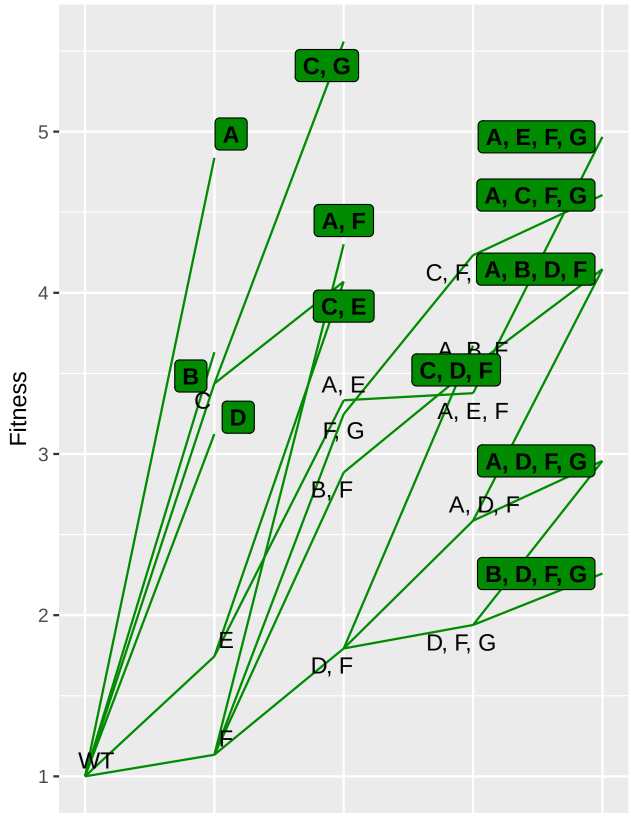

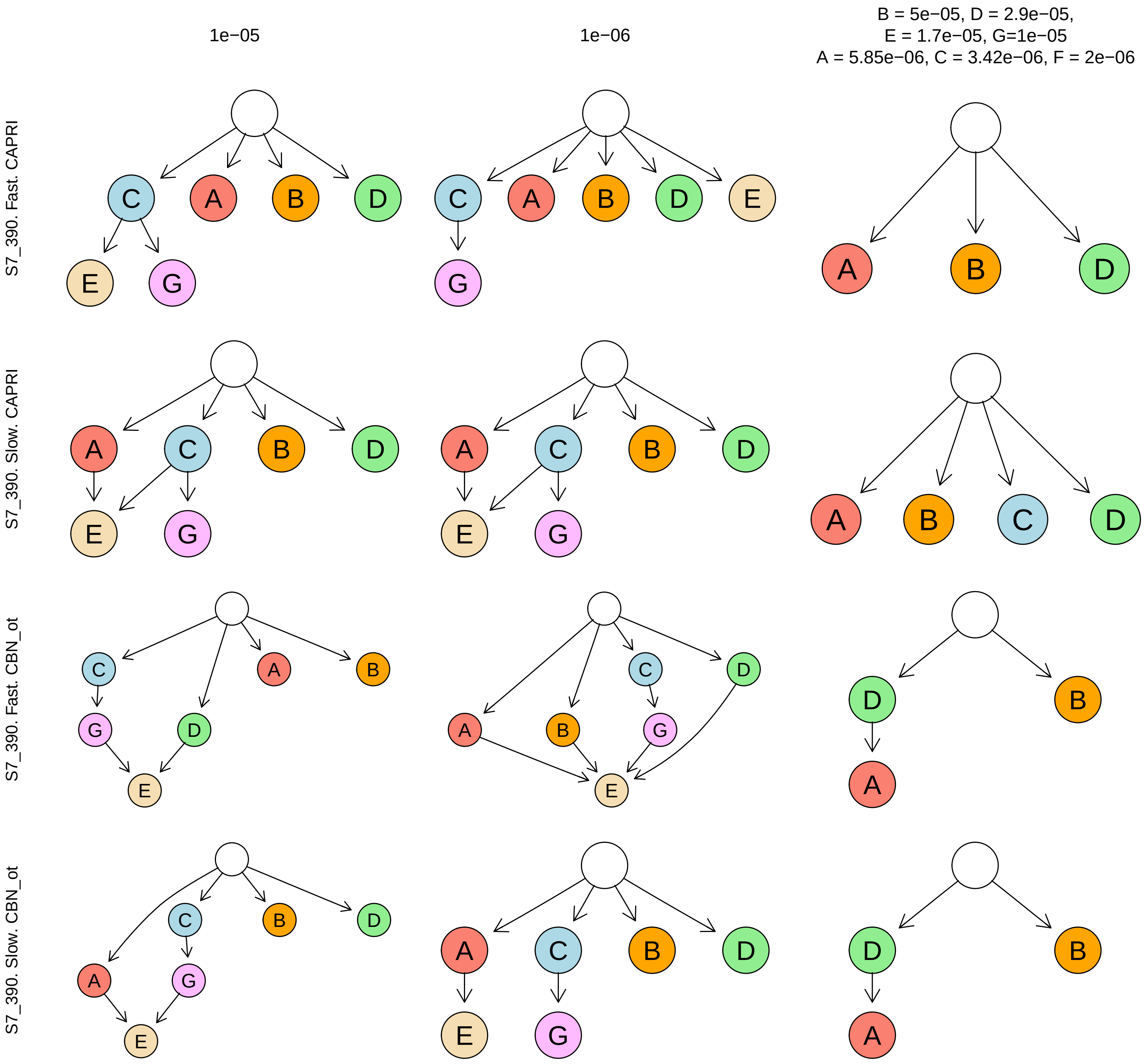

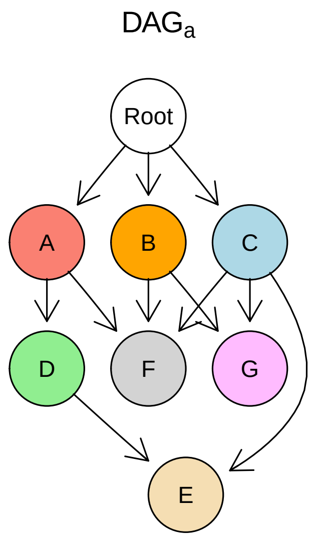

ID	Accessible Genot.	Discrep. DAG_a
S7_391	20	11

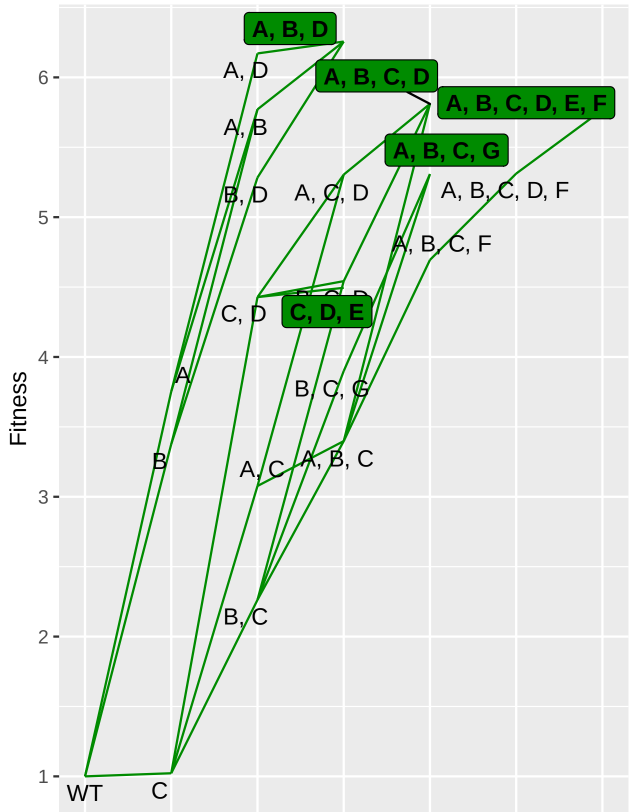

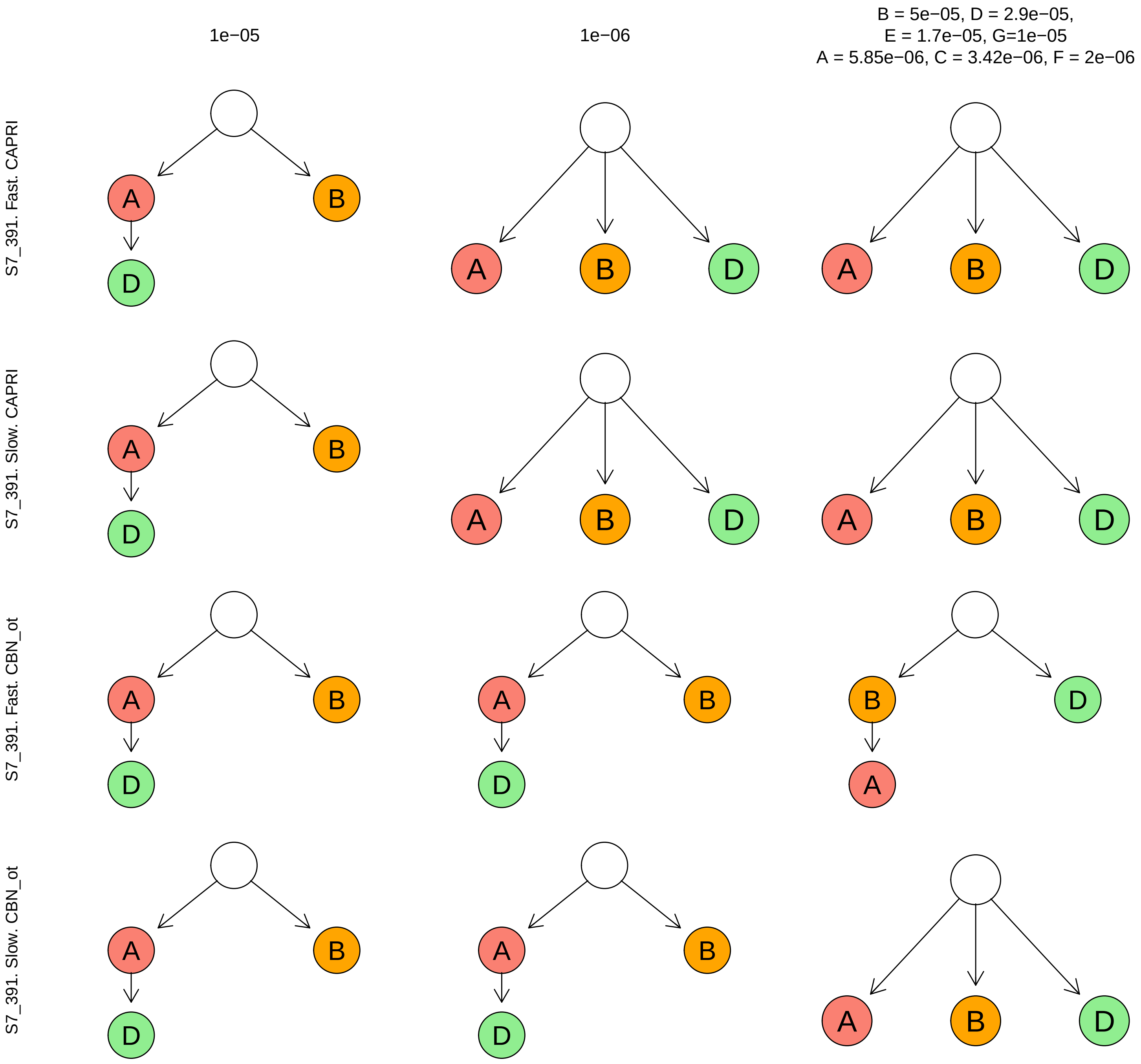

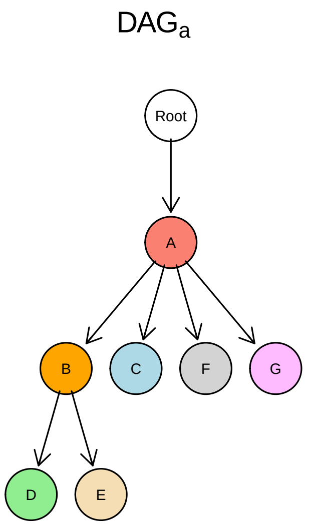

ID	Accessible Genot.	Discrep. DAG_a
S7_392	7	33

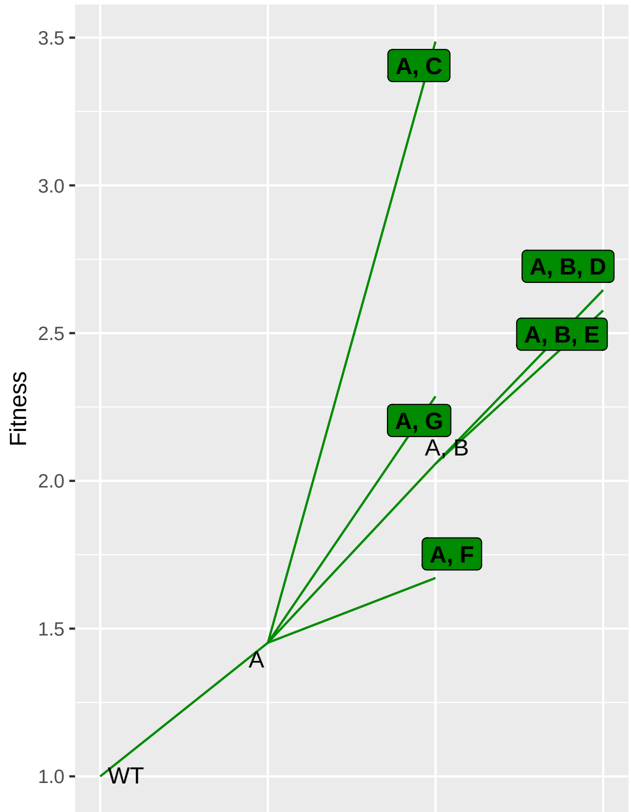

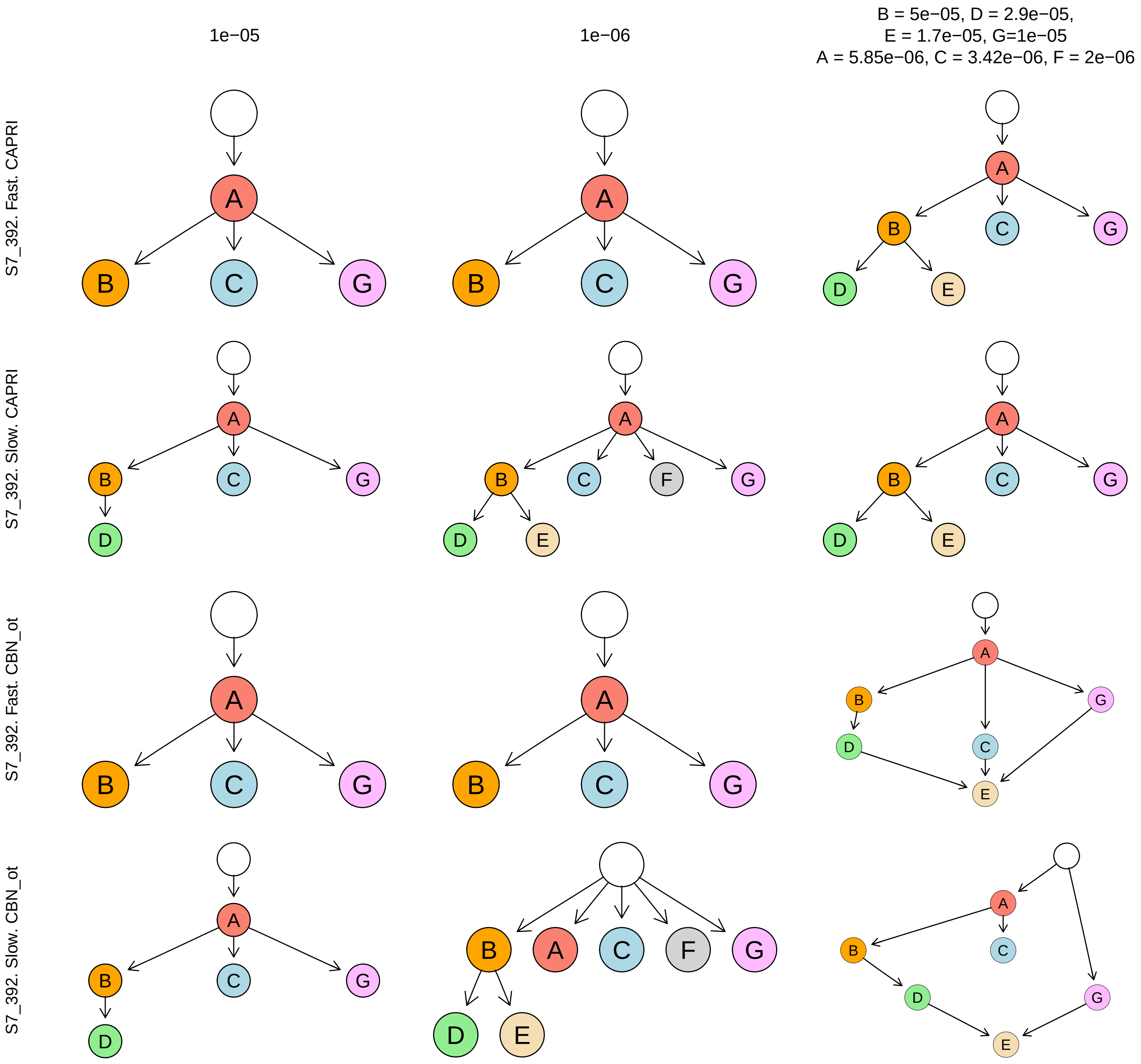

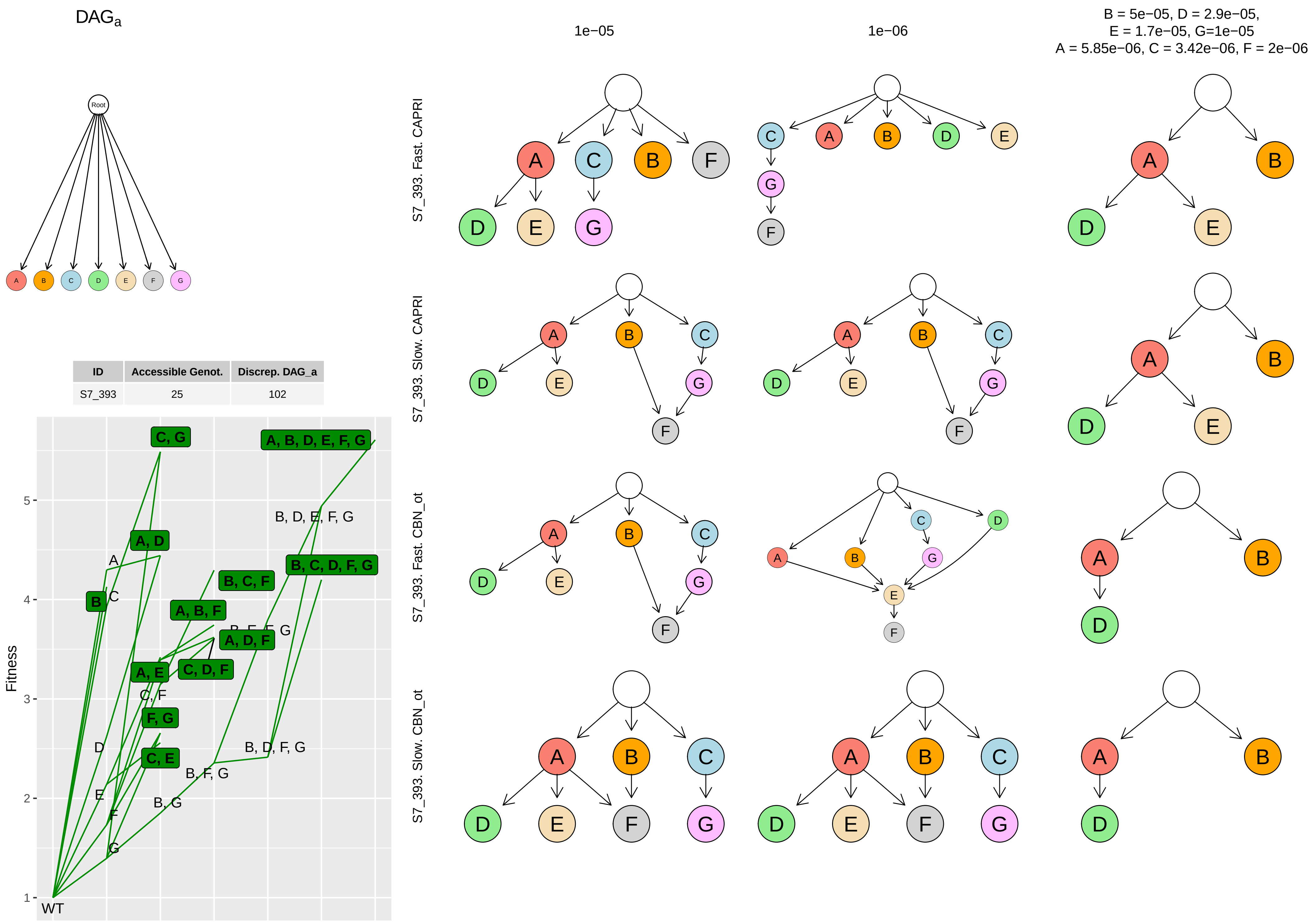

ID	Accessible Genot.	Discrep. DAG_a
S7_393	25	102

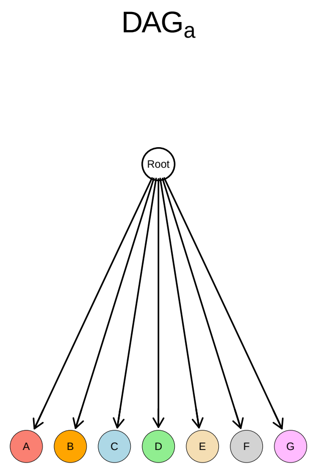

ID	Accessible Genot.	Discrep. DAG_a
S7_394	58	69

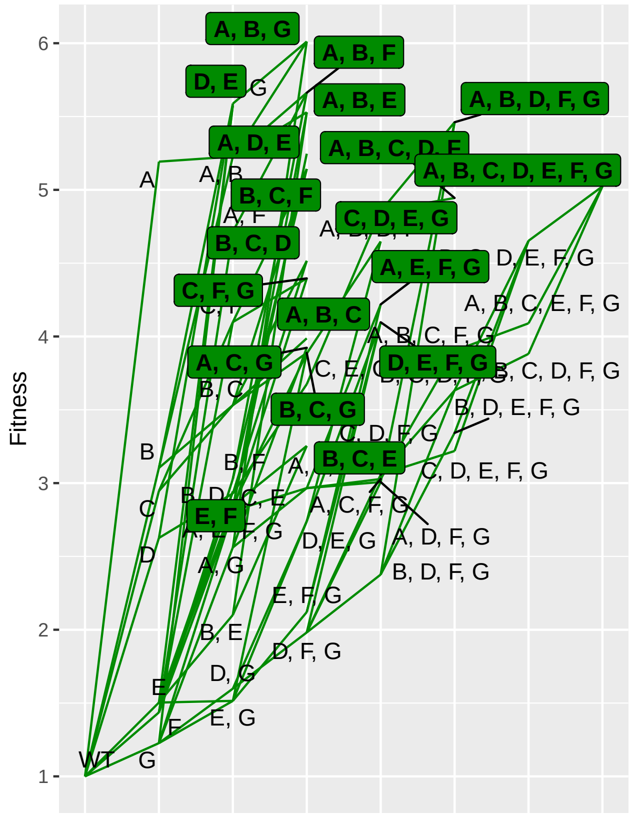

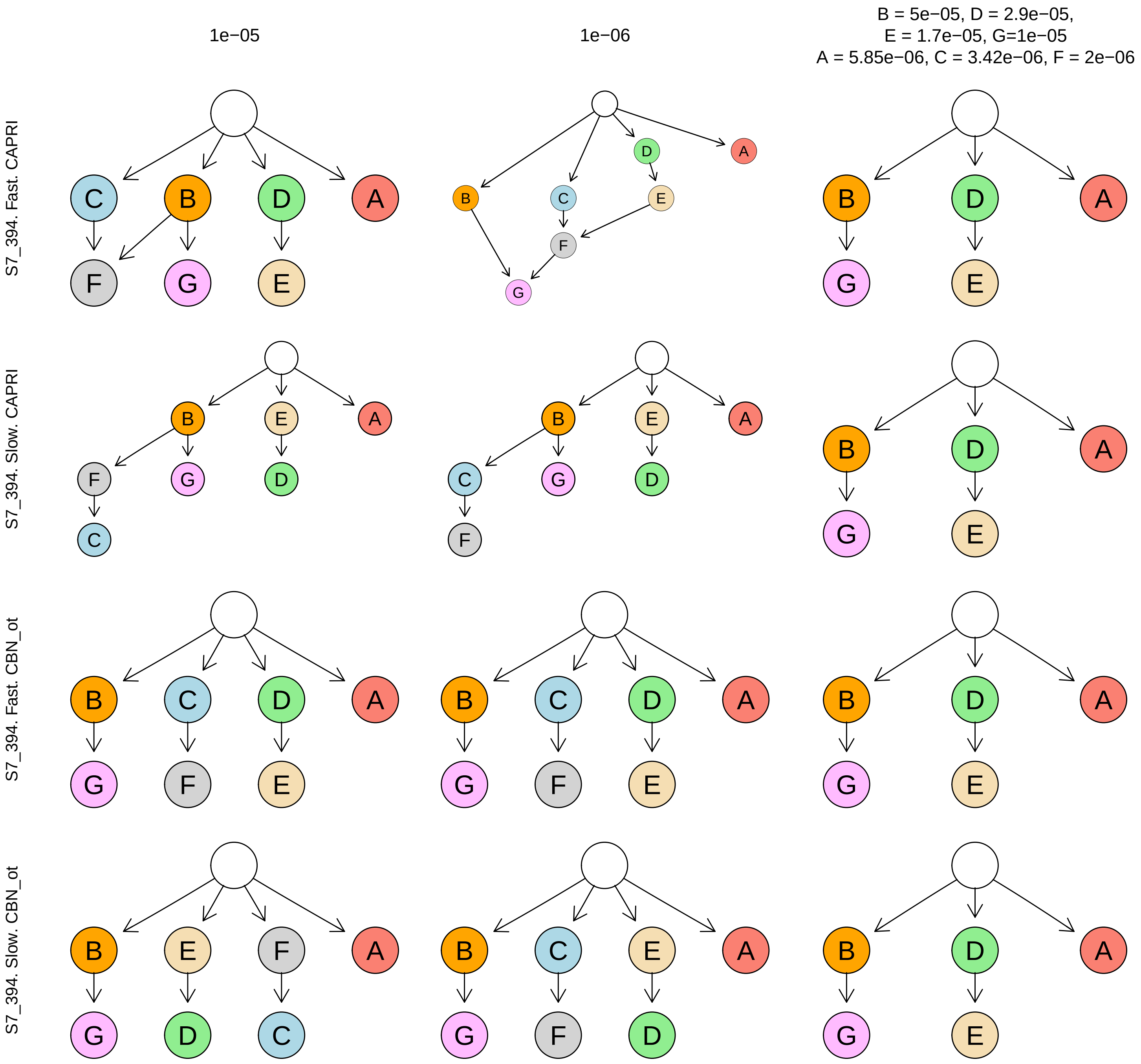

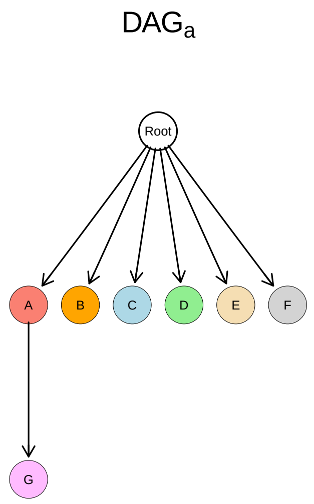

ID	Accessible Genot.	Discrep. DAG_a
S7_395	19	76

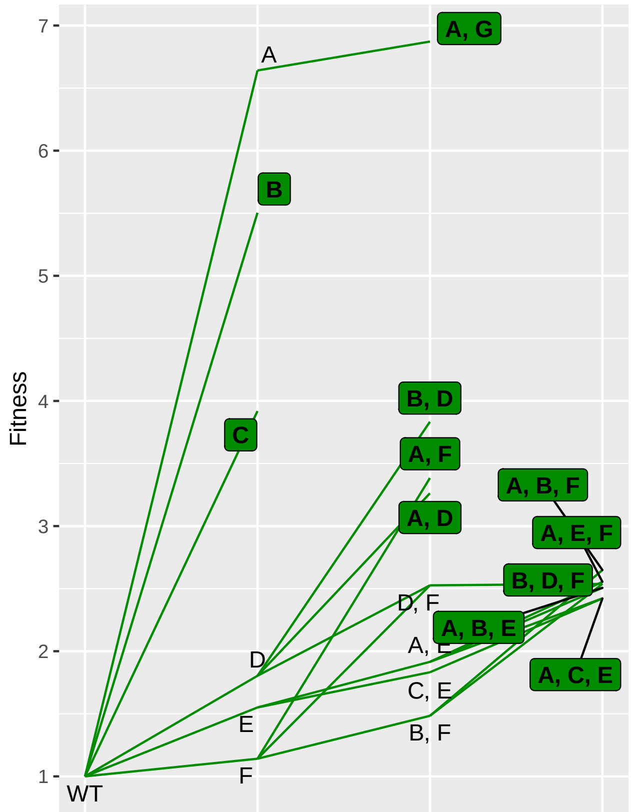

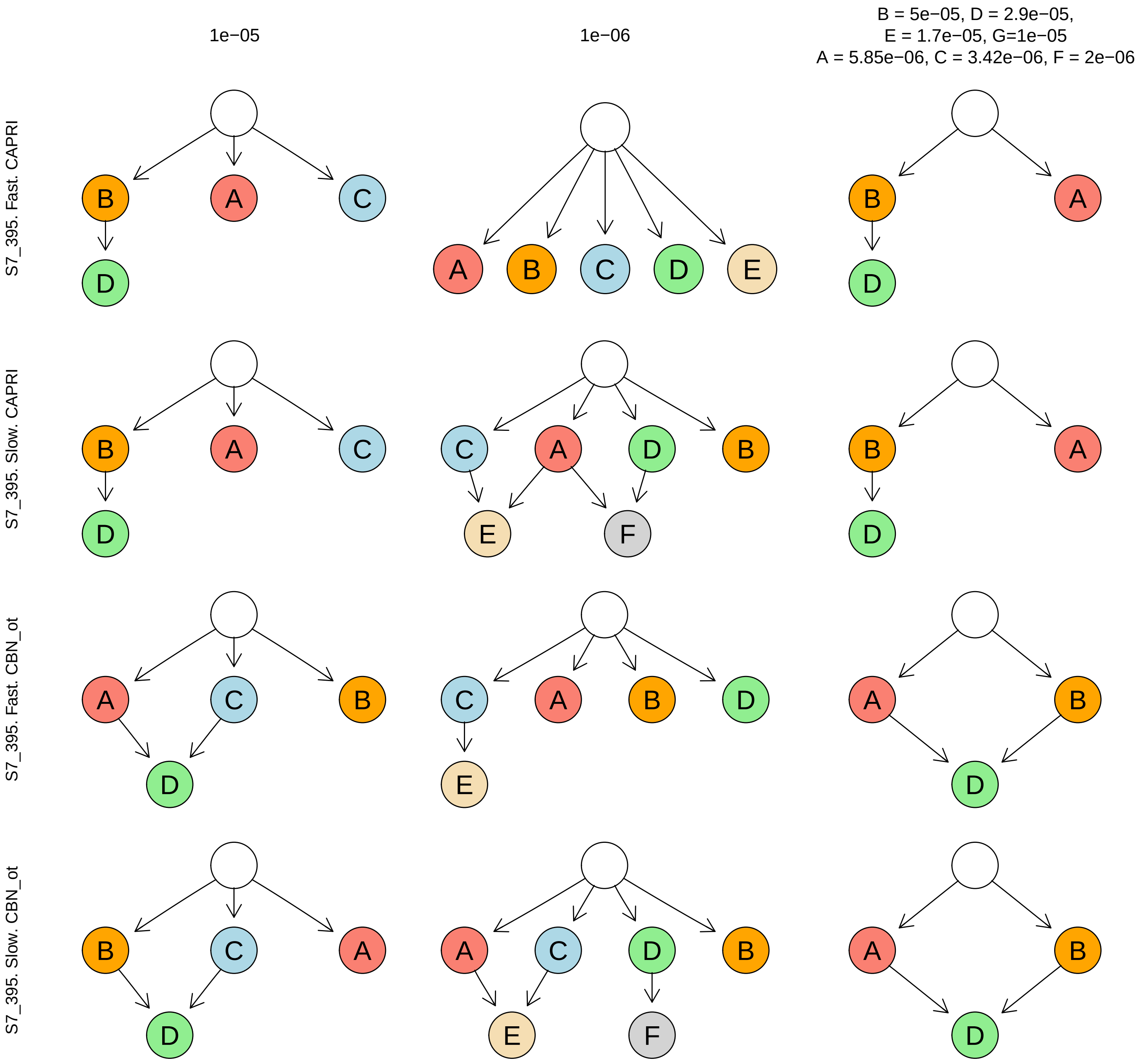

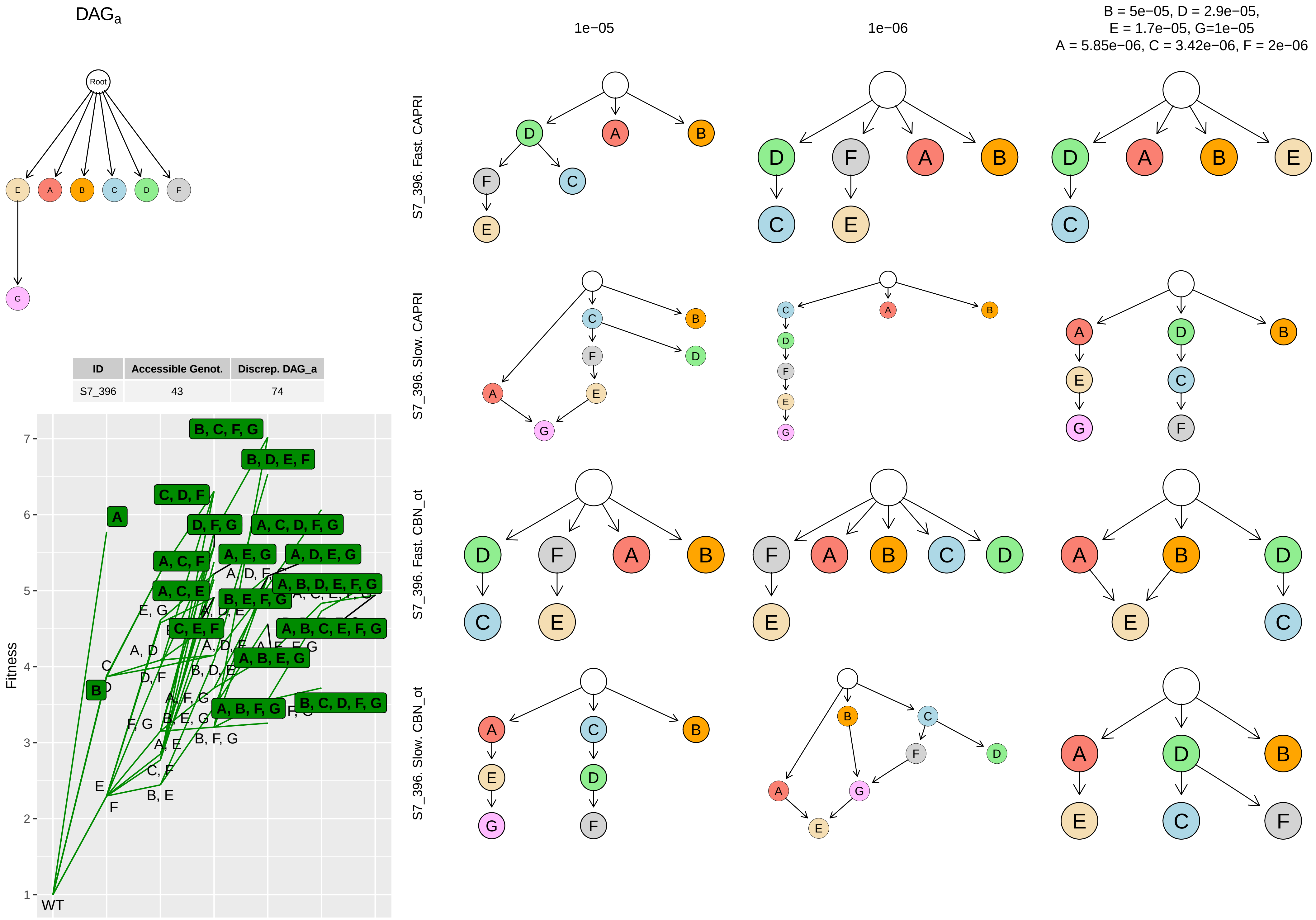

ID	Accessible Genot.	Discrep. DAG_a
S7_396	43	74

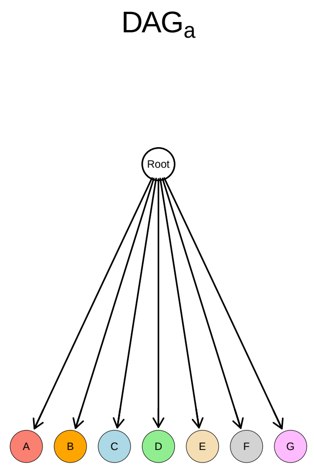

ID	Accessible Genot.	Discrep. DAG_a
S7_397	74	53

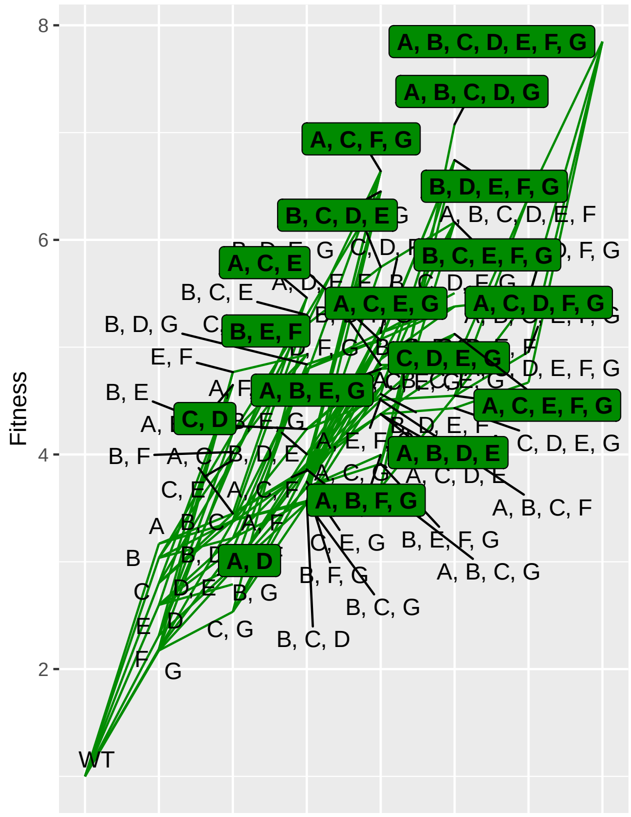

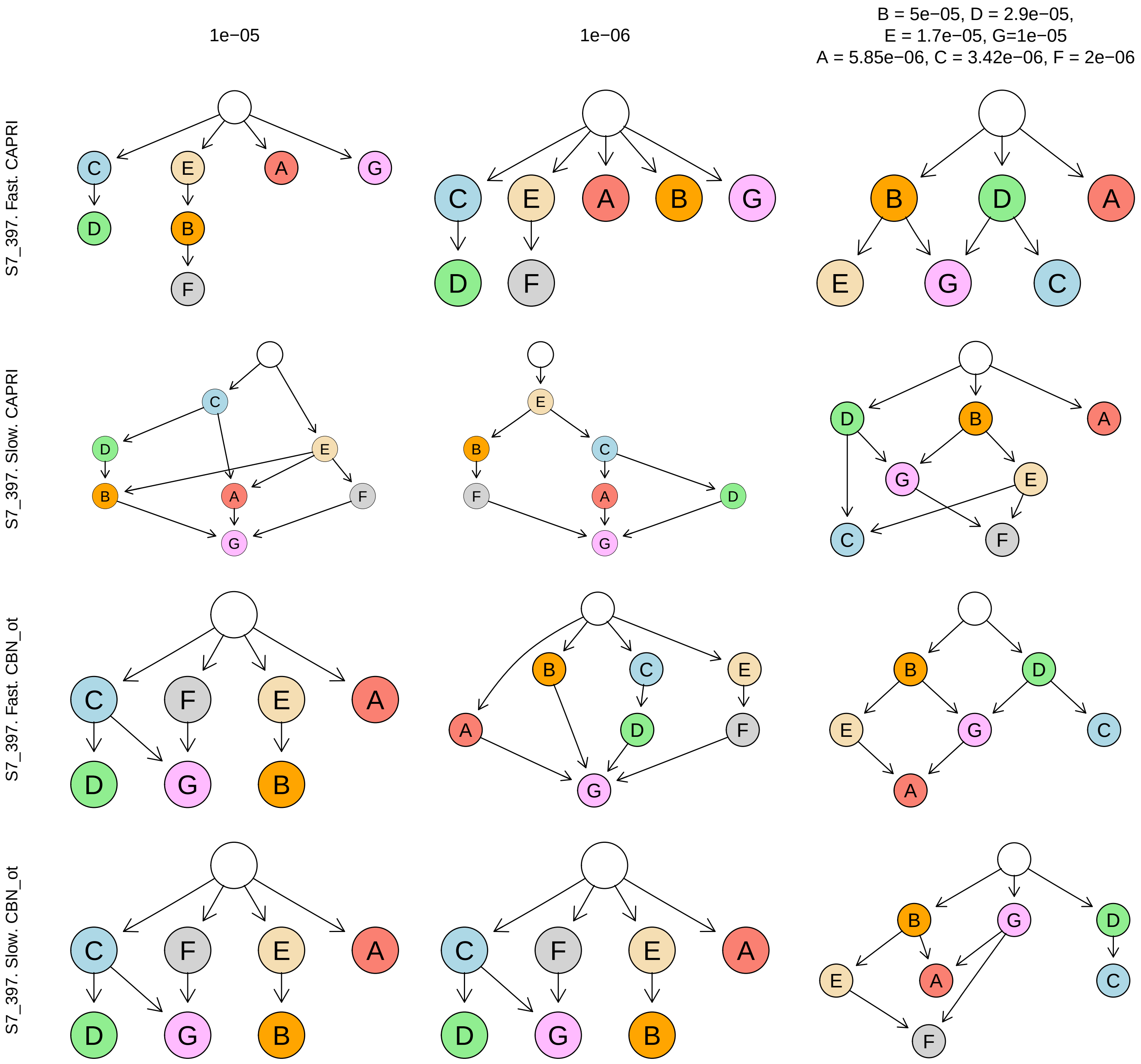

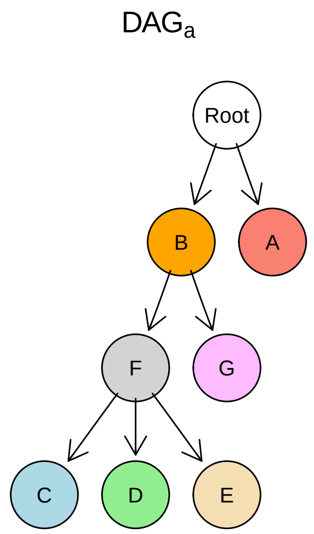

ID	Accessible Genot.	Discrep. DAG_a
S7_398	37	20

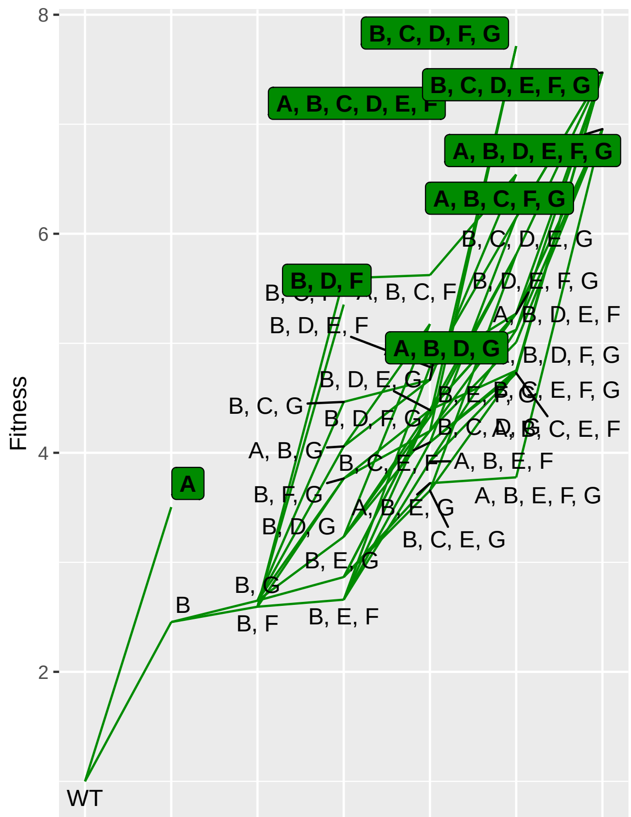

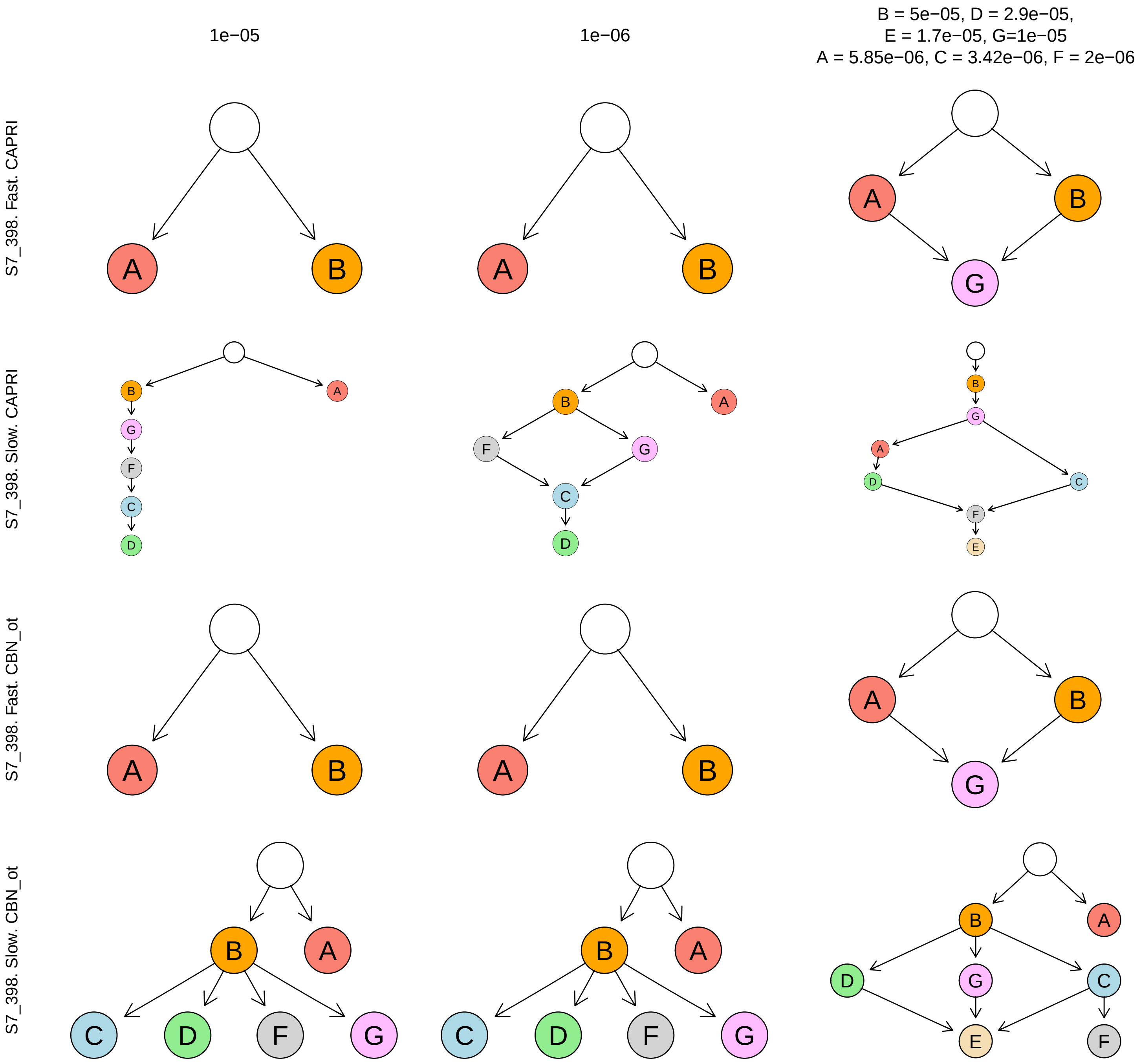

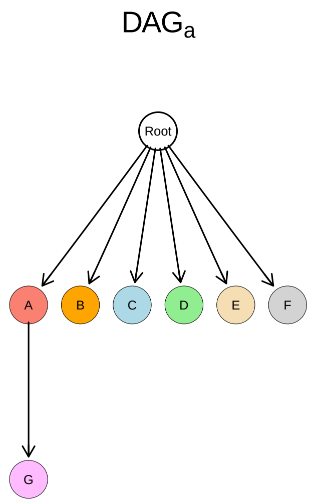

ID	Accessible Genot.	Discrep. DAG_a
S7_399	54	59

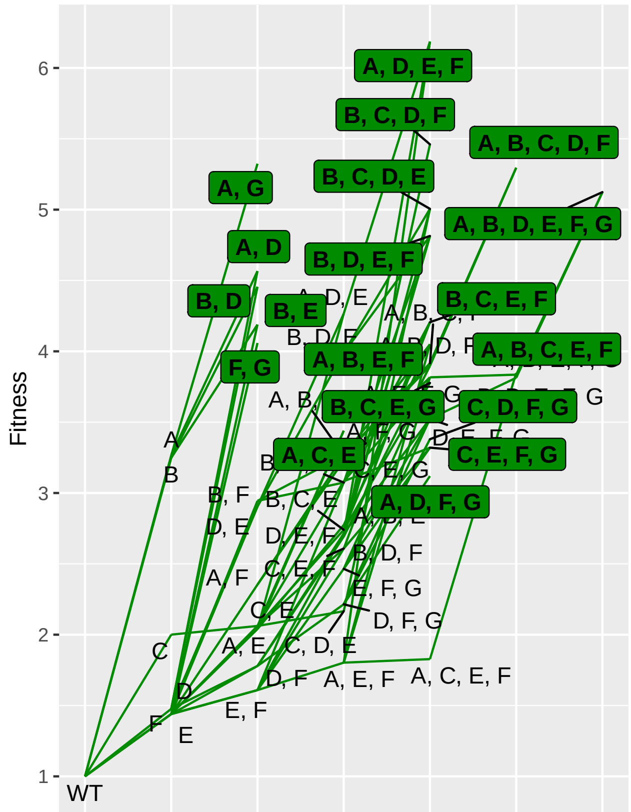

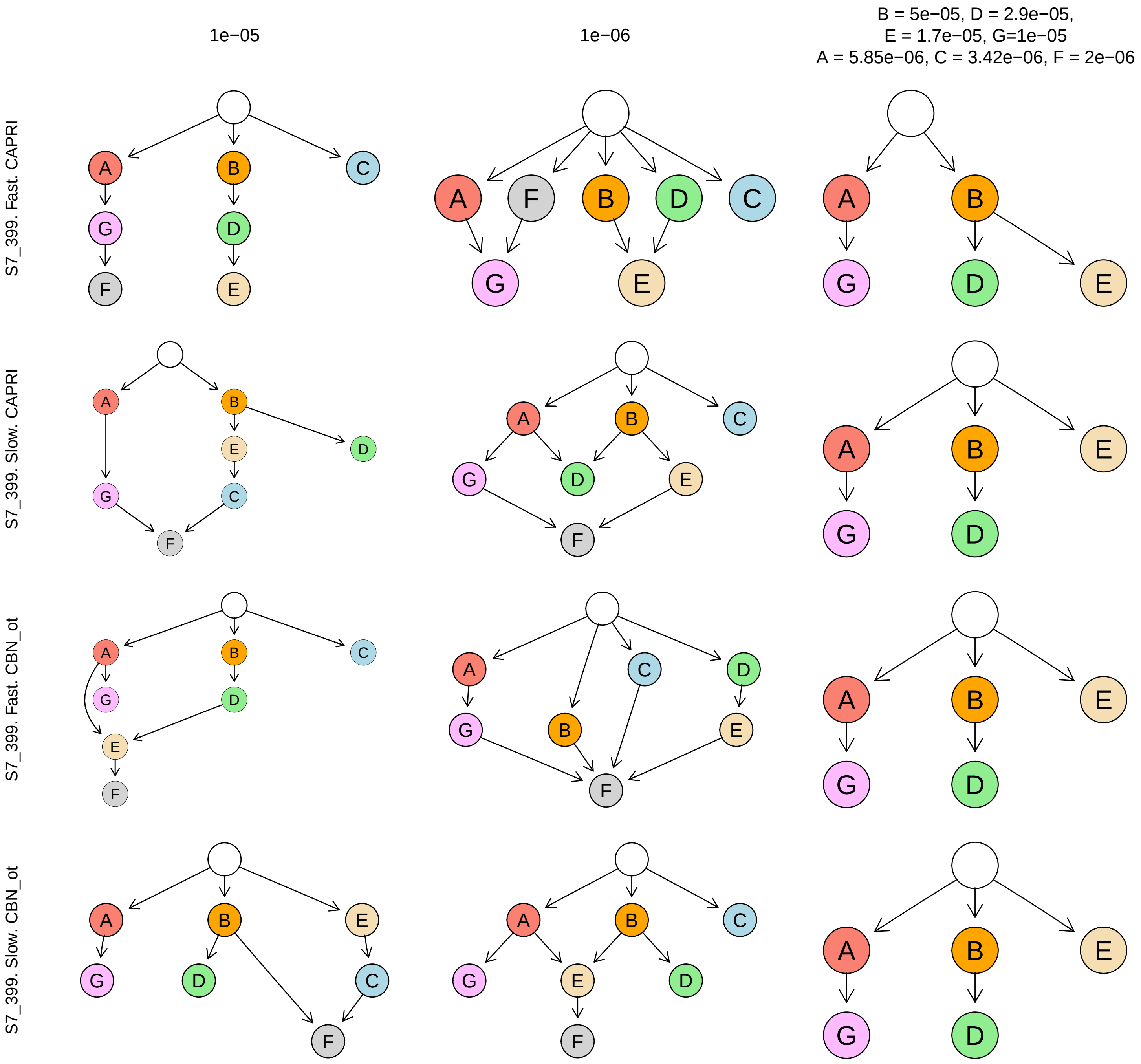

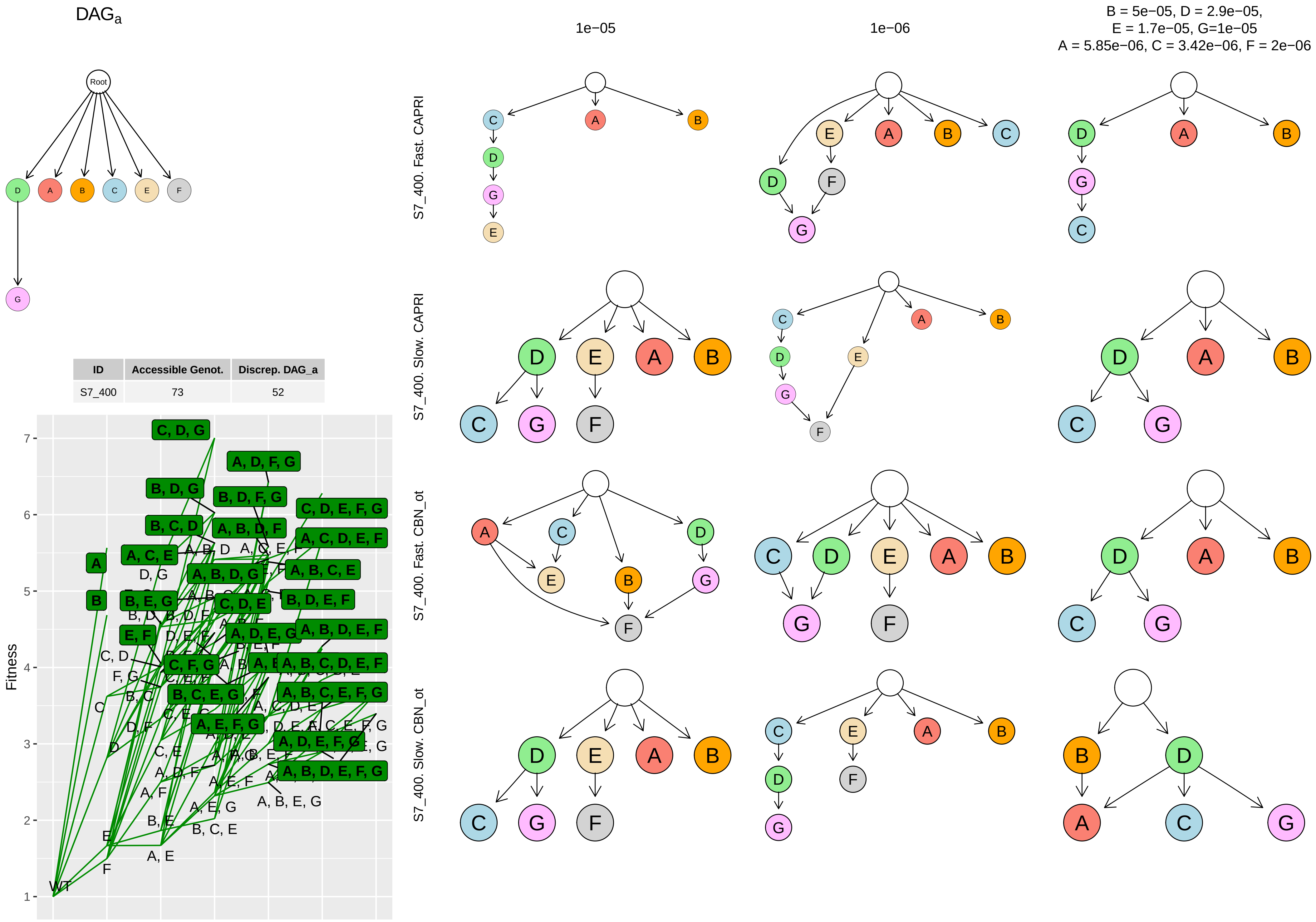

DAG_a
Root
D
A
B
C
E
F
G
ID
Accessible Genot.
Discrep. DAG_a
S7_400
73
52
Fitness
1e−05
1e−06
B = 5e−05, D = 2.9e−05,
E = 1.7e−05, G=1e−05
A = 5.85e−06, C = 3.42e−06, F = 2e−06
S7_400. Fast. CAPRI
S7_400. Slow. CAPRI
S7_400. Fast. CBN_ot
S7_400. Slow. CBN_ot

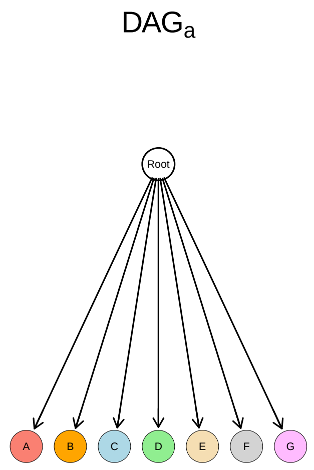

ID	Accessible Genot.	Discrep. DAG_a
S7_401	41	86

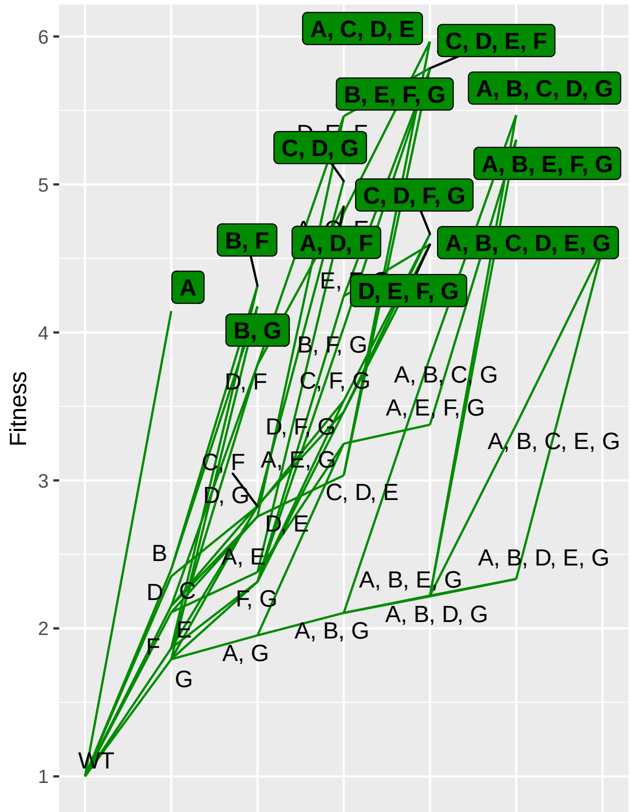

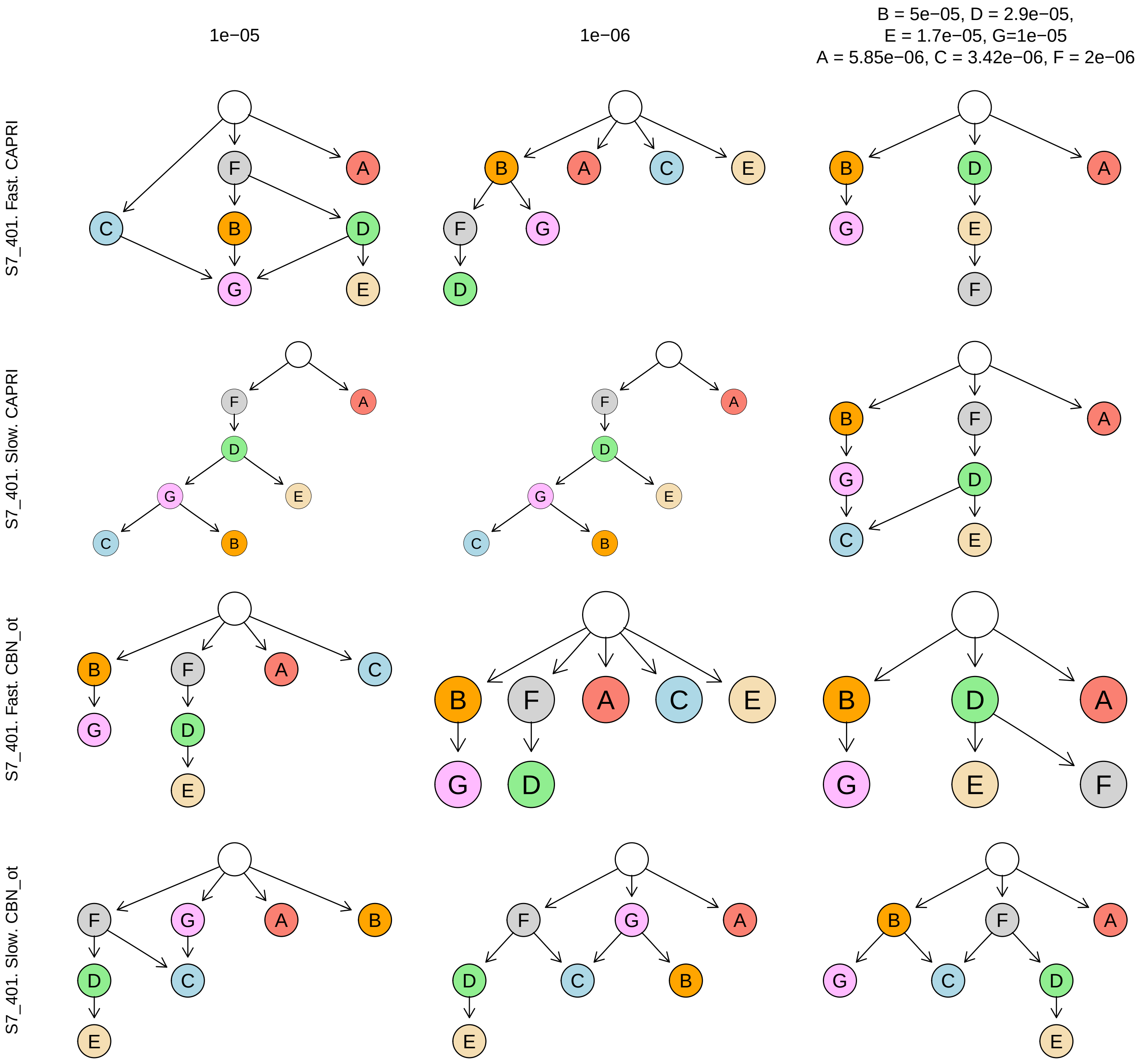

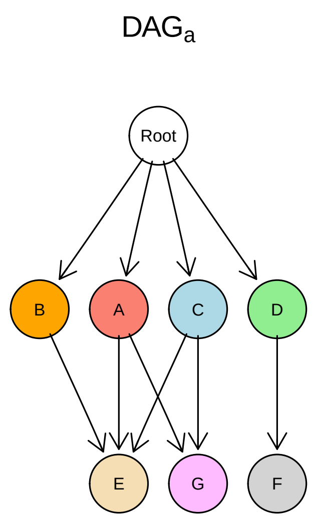

ID	Accessible Genot.	Discrep. DAG_a
S7_402	21	18

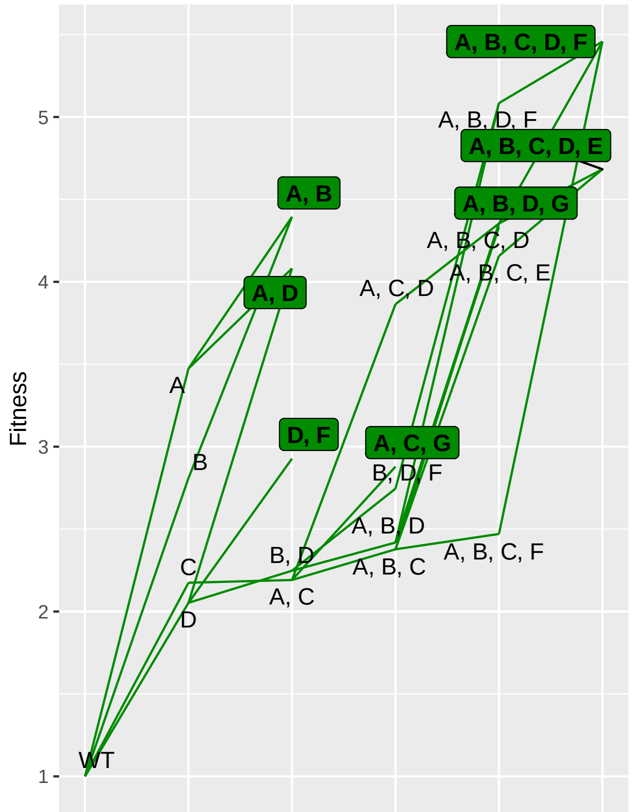

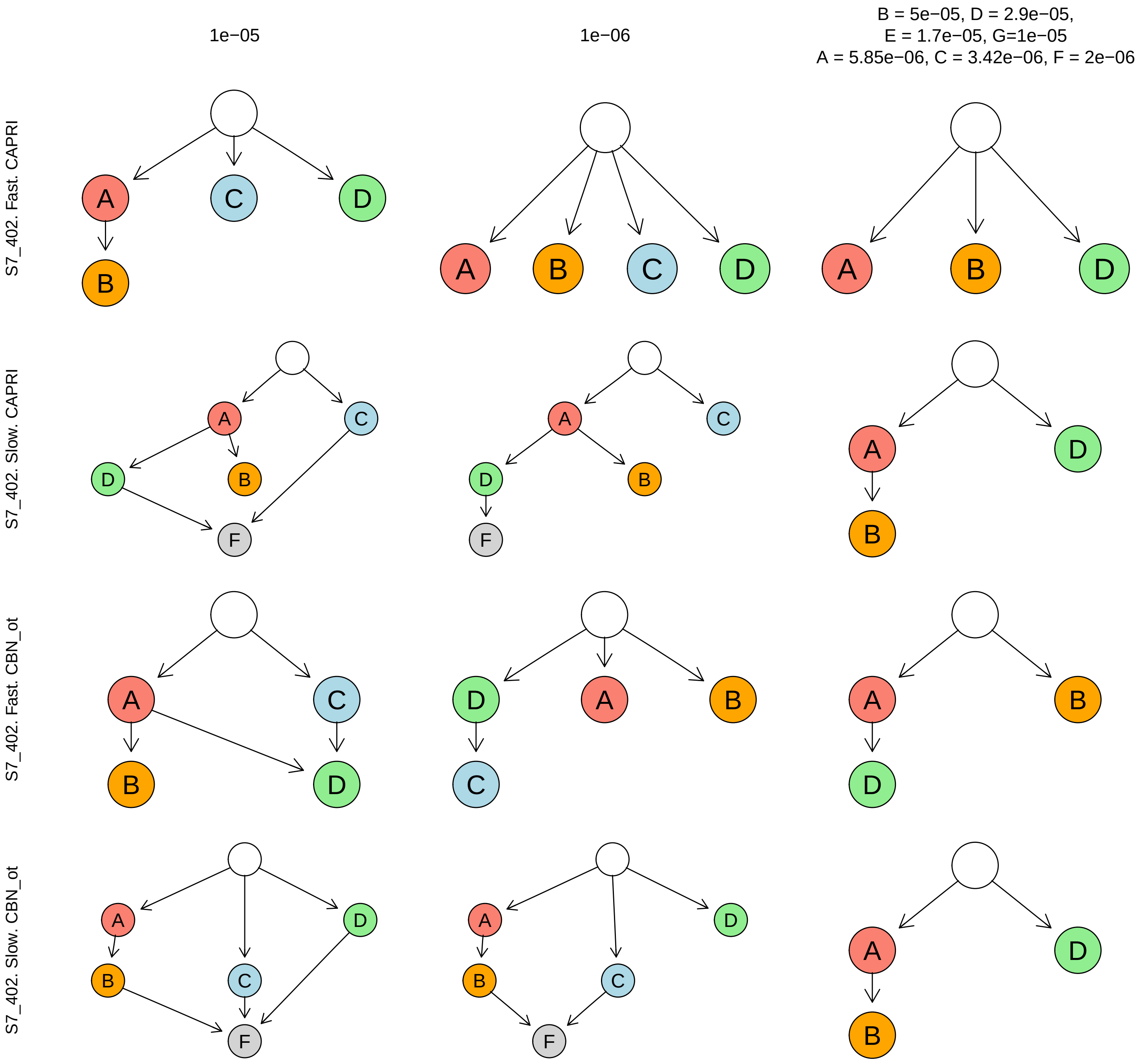

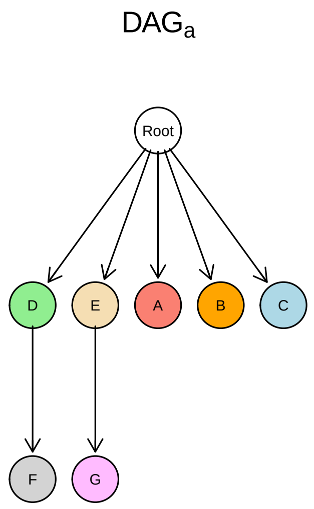

ID	Accessible Genot.	Discrep. DAG_a
S7_403	21	54

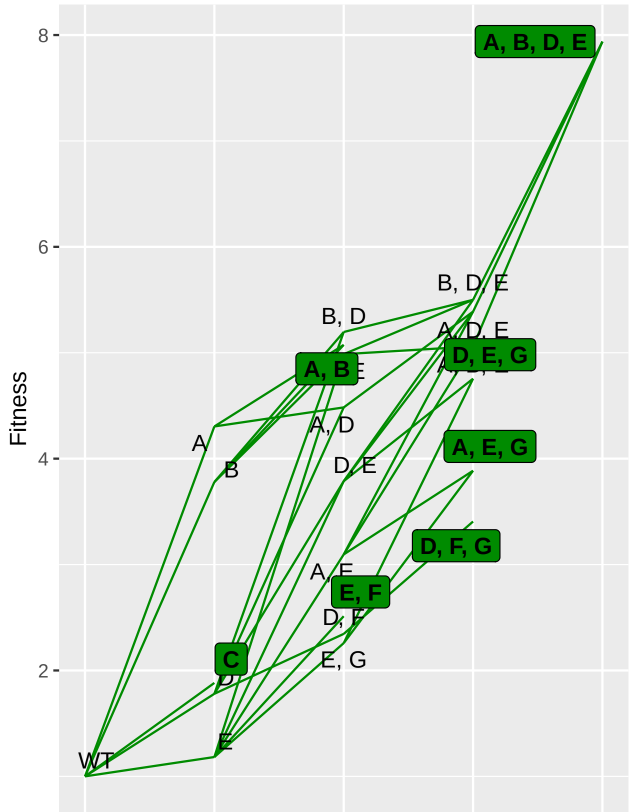

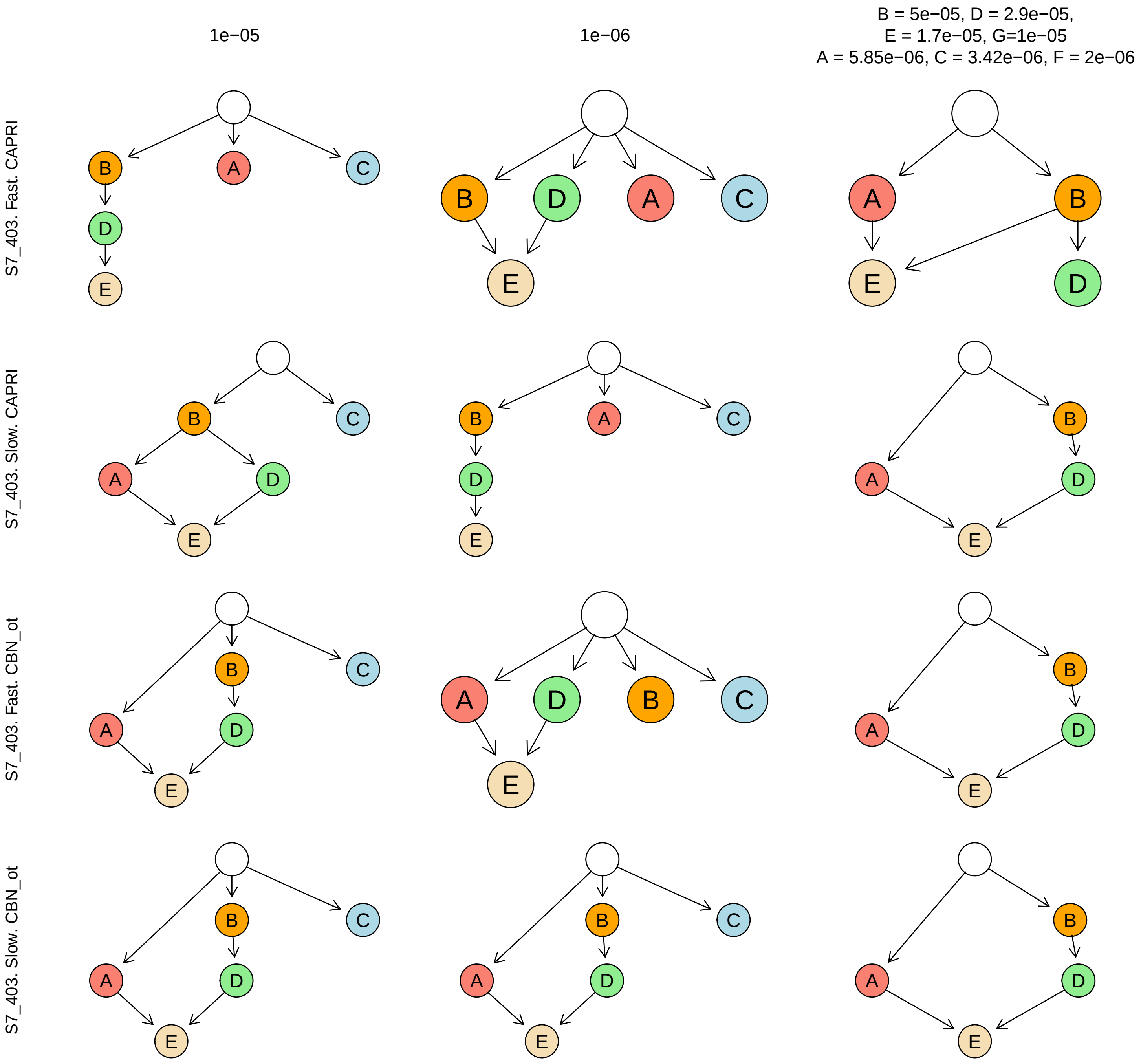

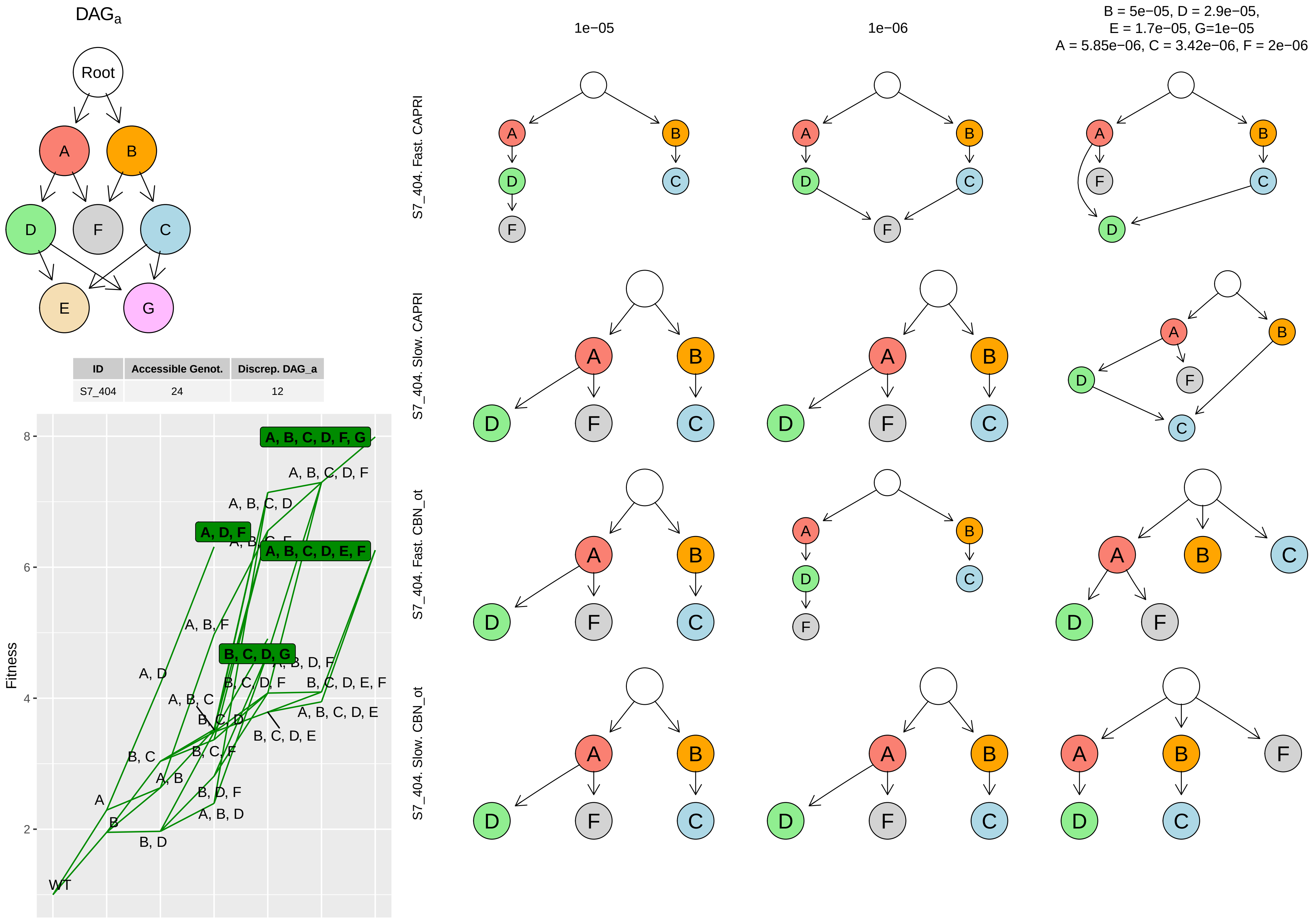

ID	Accessible Genot.	Discrep. DAG_a
S7_404	24	12

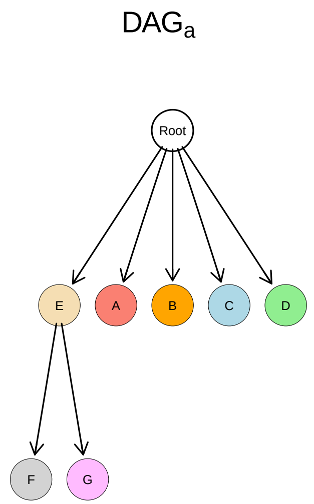

ID	Accessible Genot.	Discrep. DAG_a
S7_405	16	63

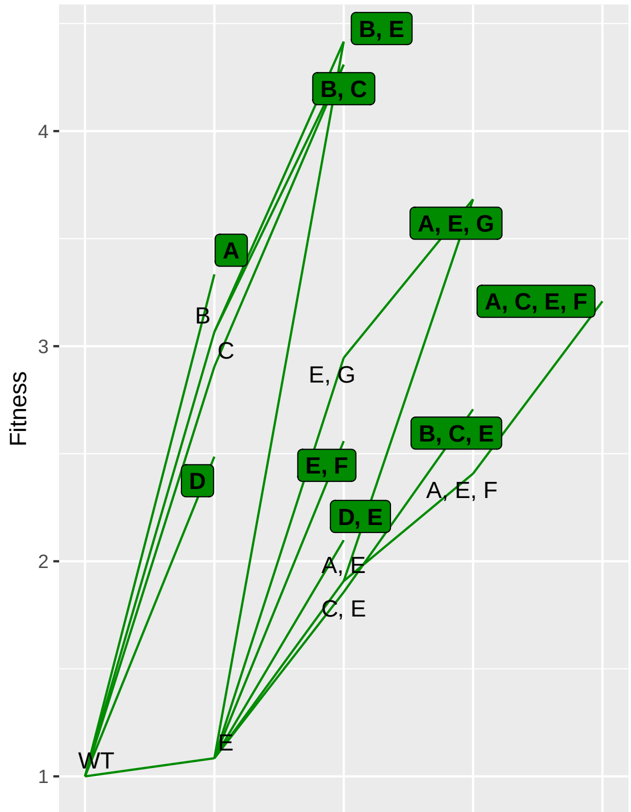

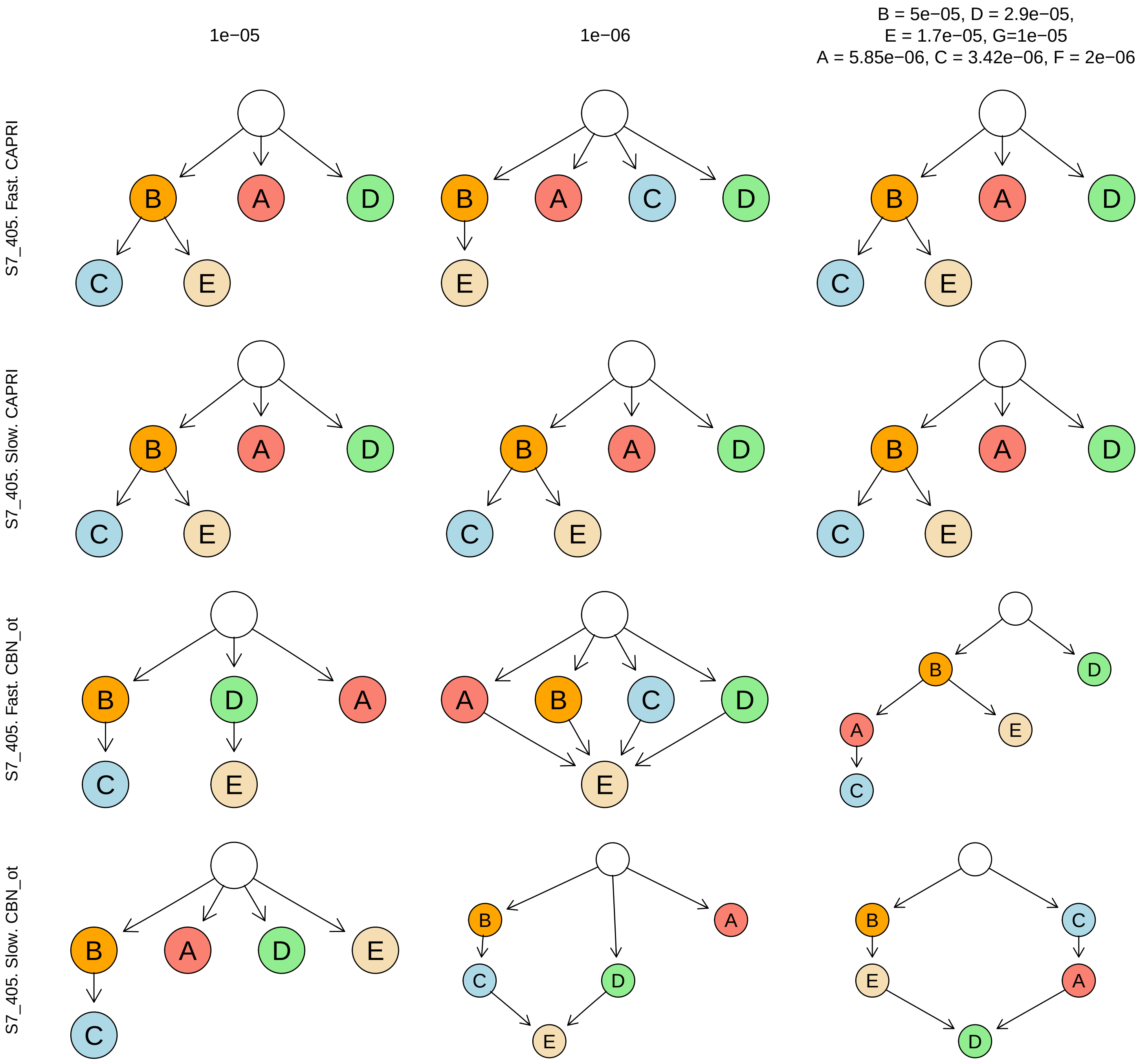

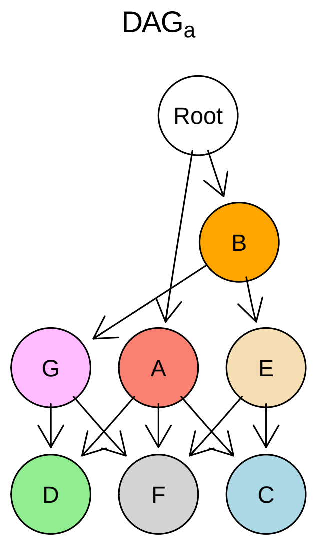

ID	Accessible Genot.	Discrep. DAG_a
S7_406	14	4

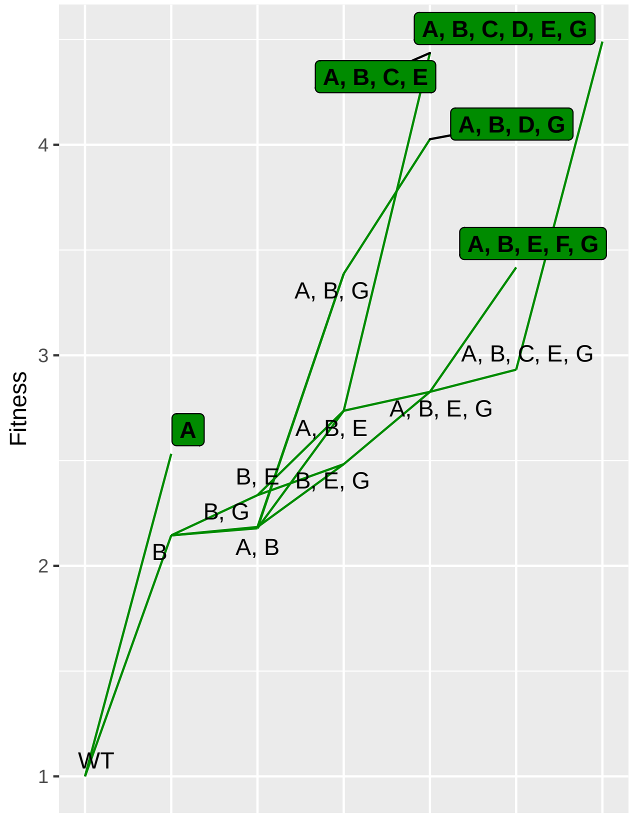

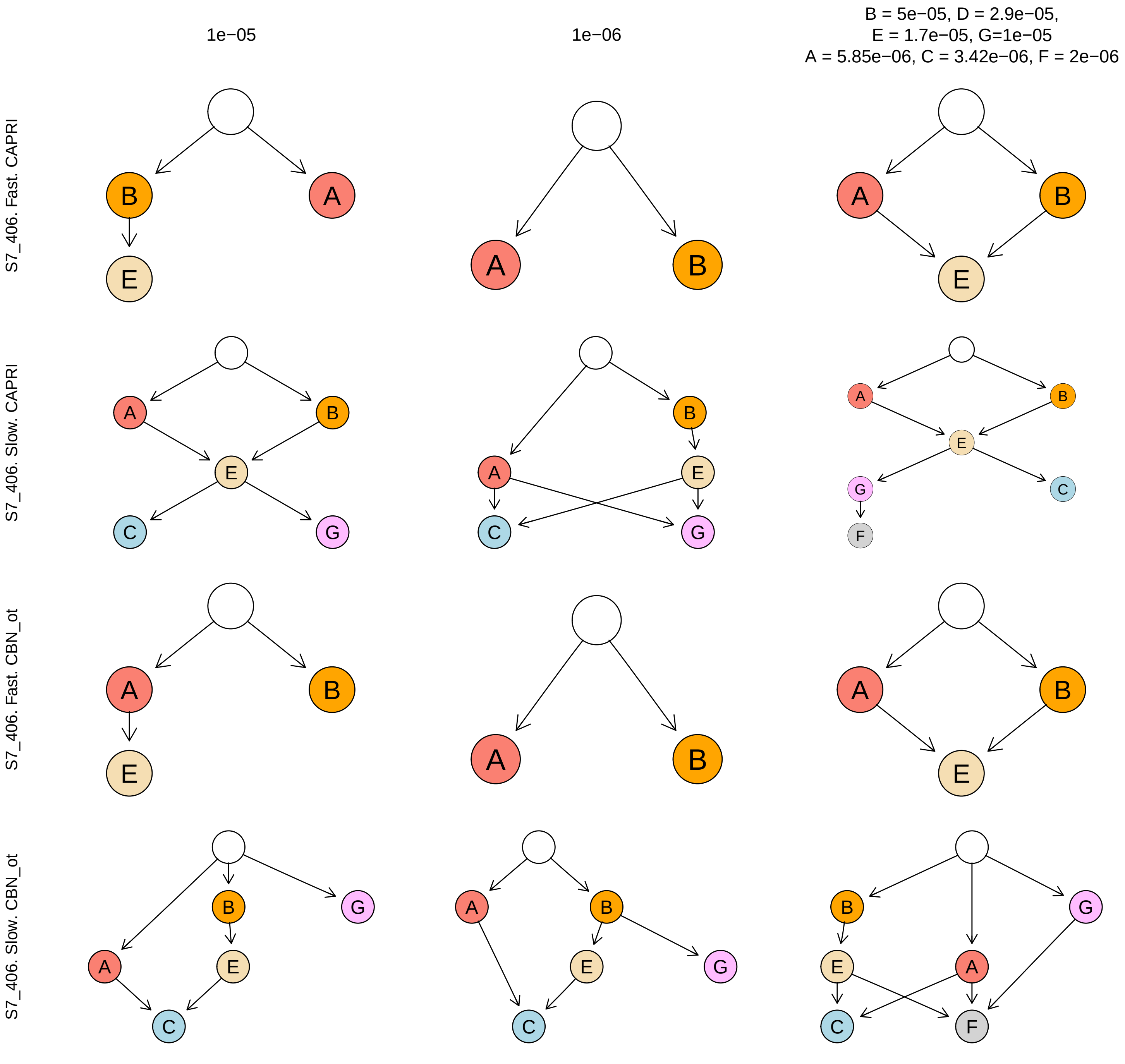

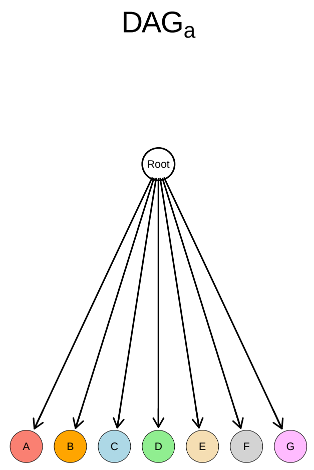

ID	Accessible Genot.	Discrep. DAG_a
S7_407	28	99

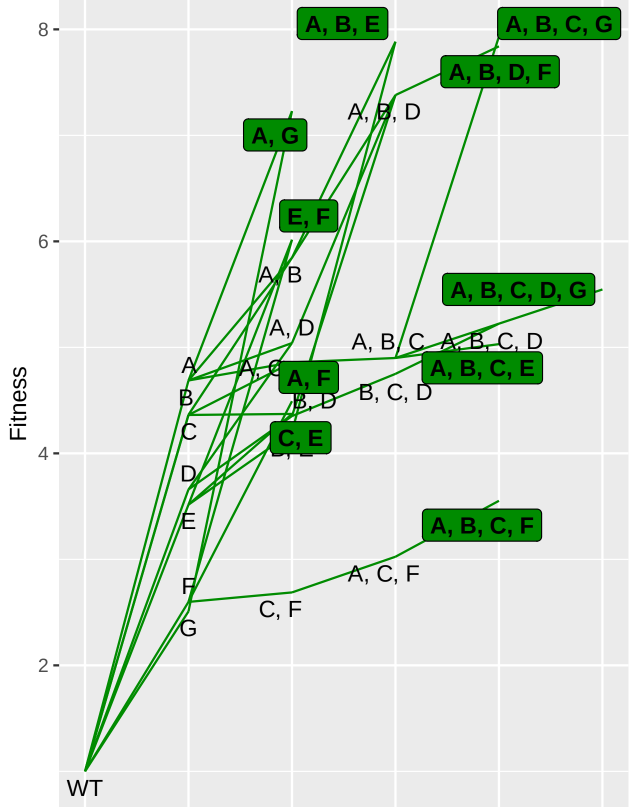

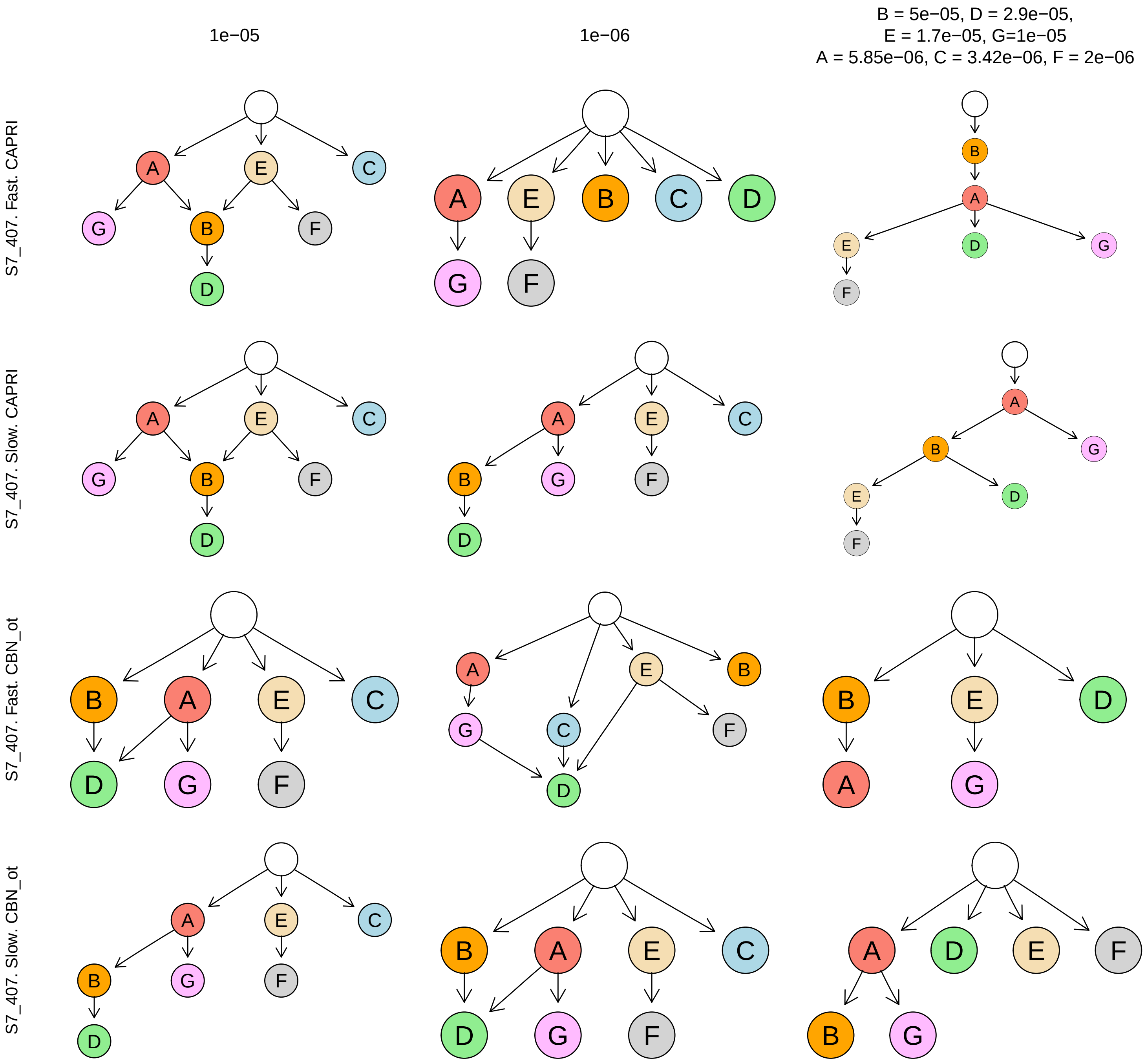

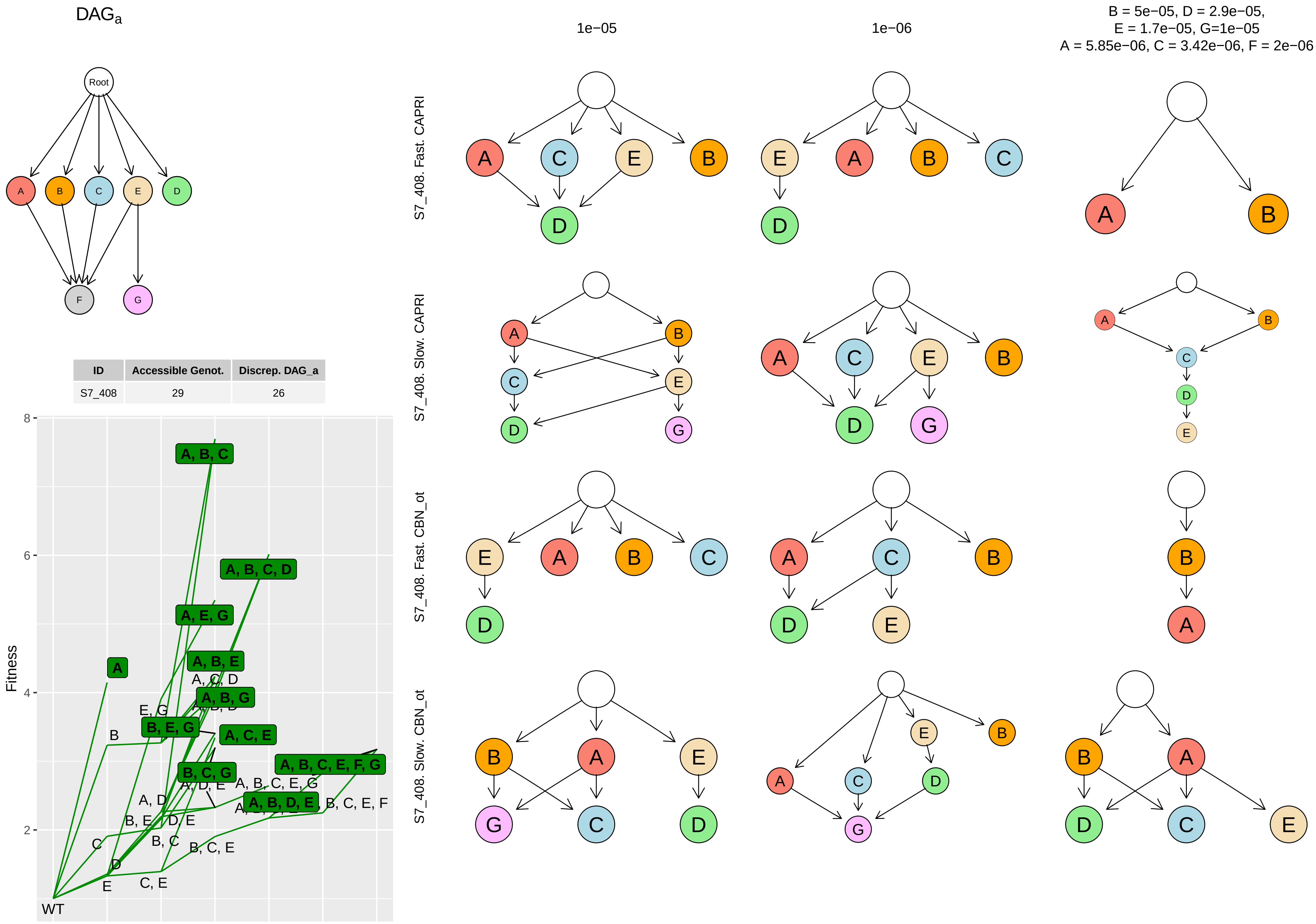

ID	Accessible Genot.	Discrep. DAG_a
S7_408	29	26

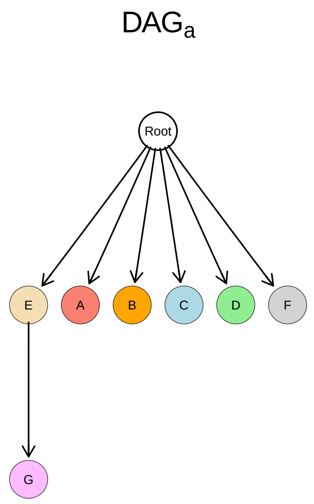

ID	Accessible Genot.	Discrep. DAG_a
S7_409	37	62

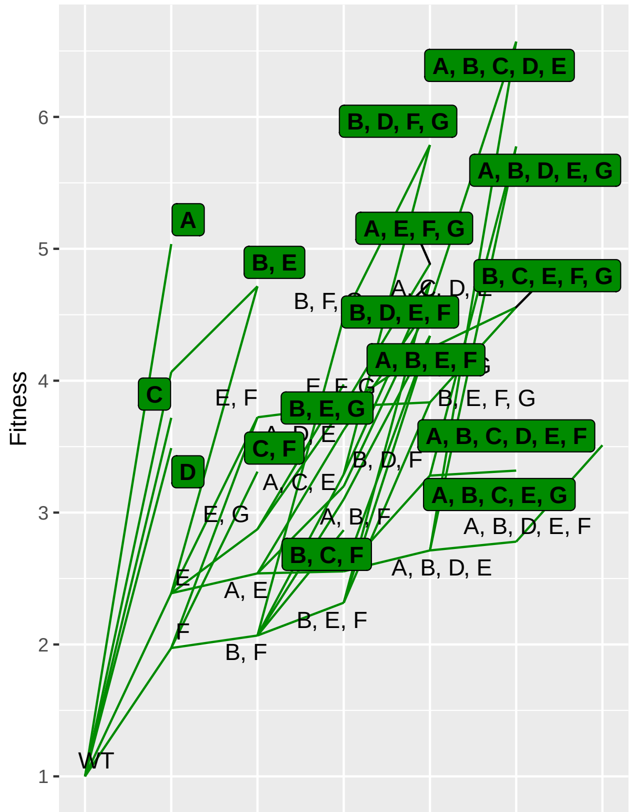

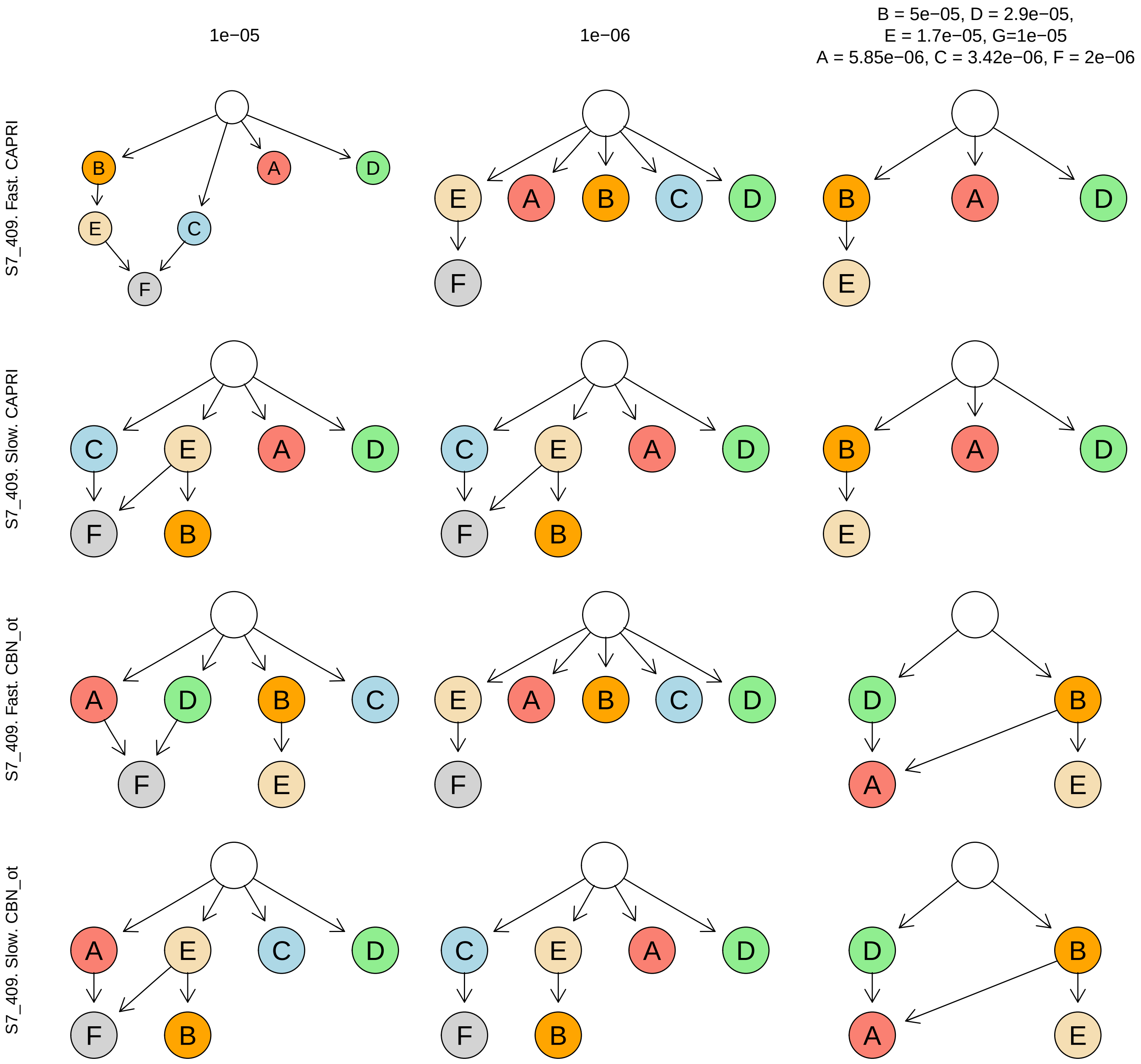

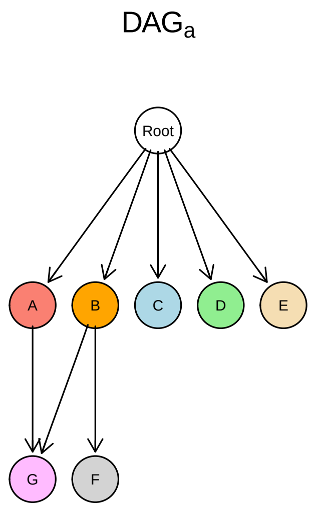

ID	Accessible Genot.	Discrep. DAG_a
S7_410	38	51

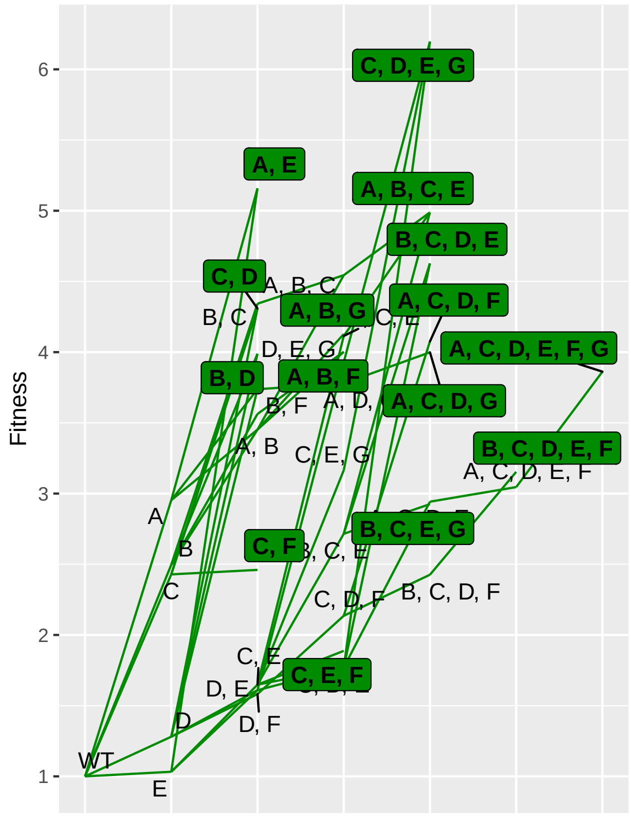

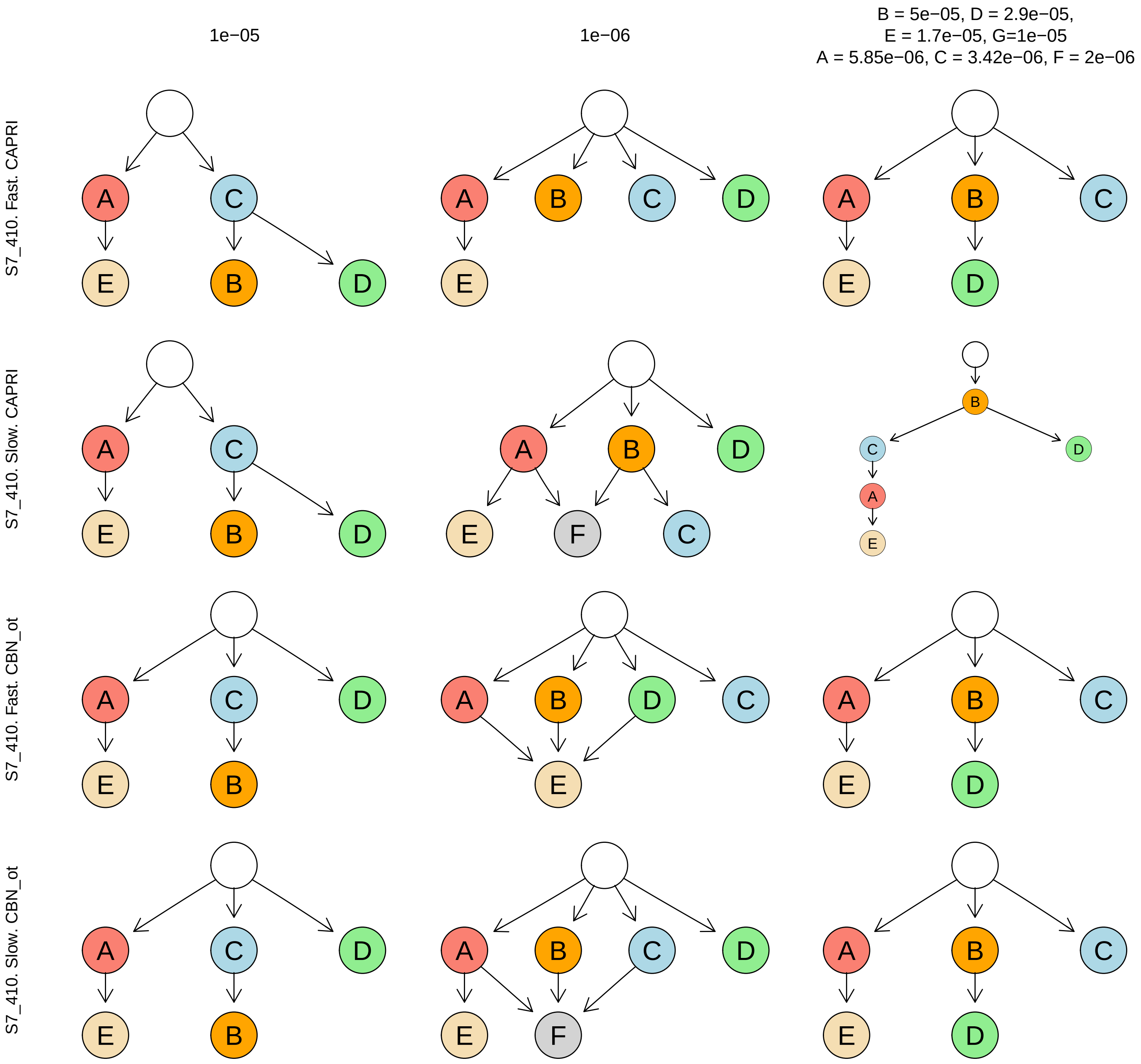

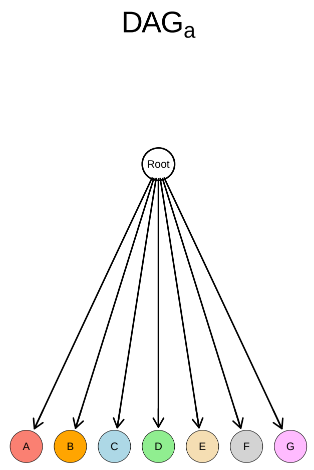

ID	Accessible Genot.	Discrep. DAG_a
S7_411	31	96

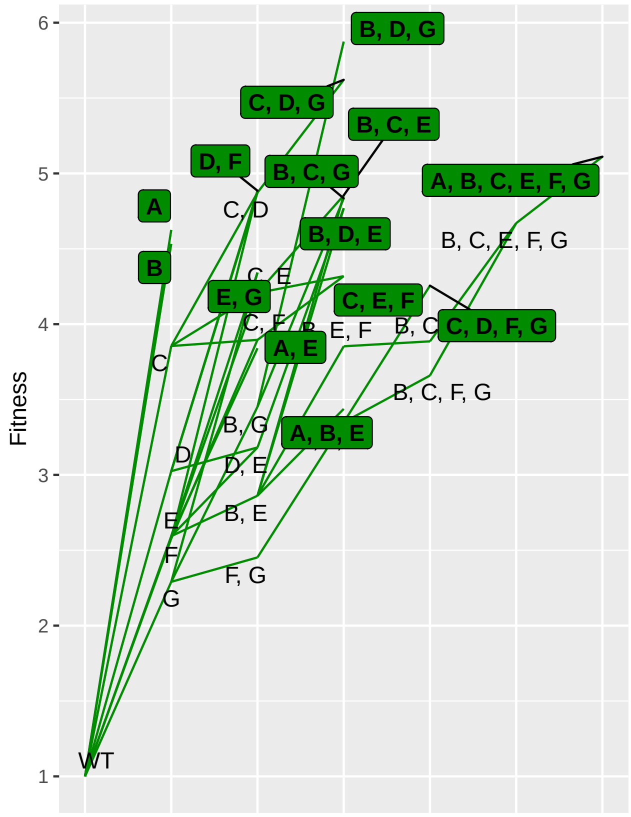

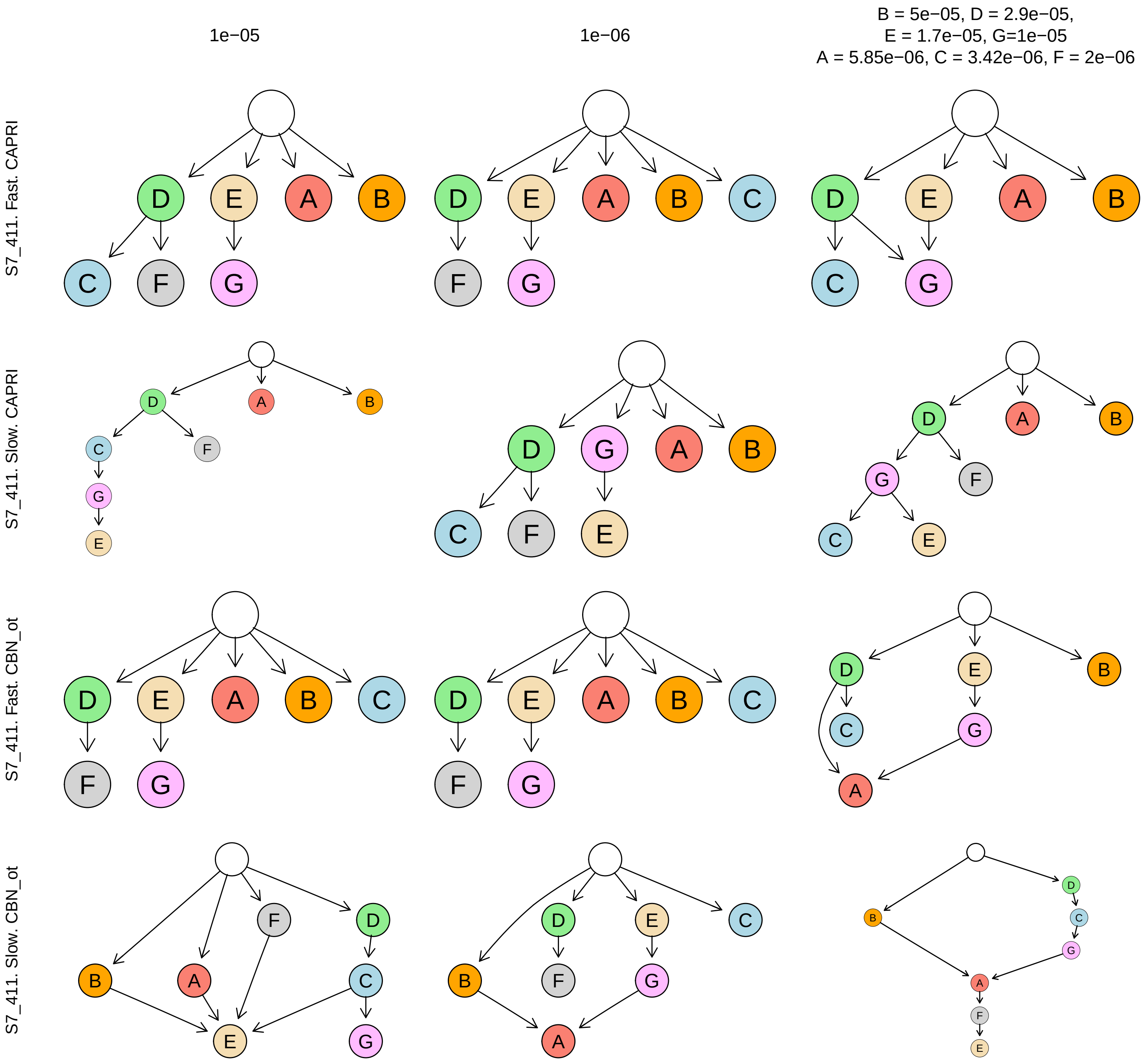

DAG$_a$

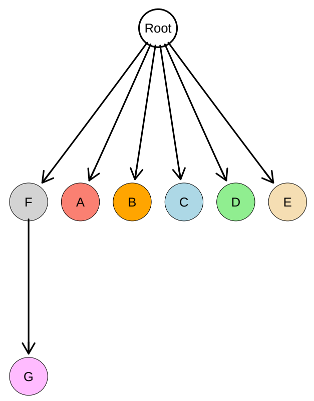

ID	Accessible Genot.	Discrep. DAG_a
S7_412	21	78

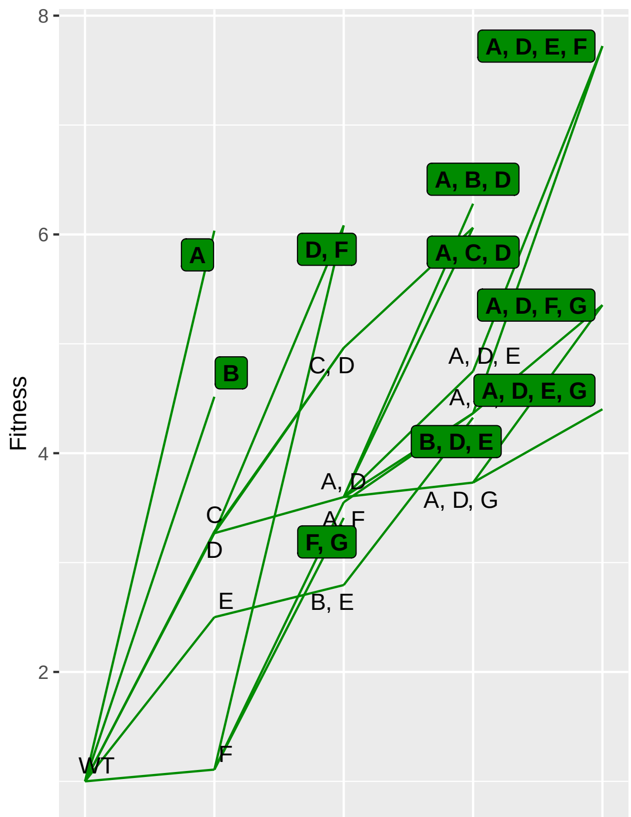

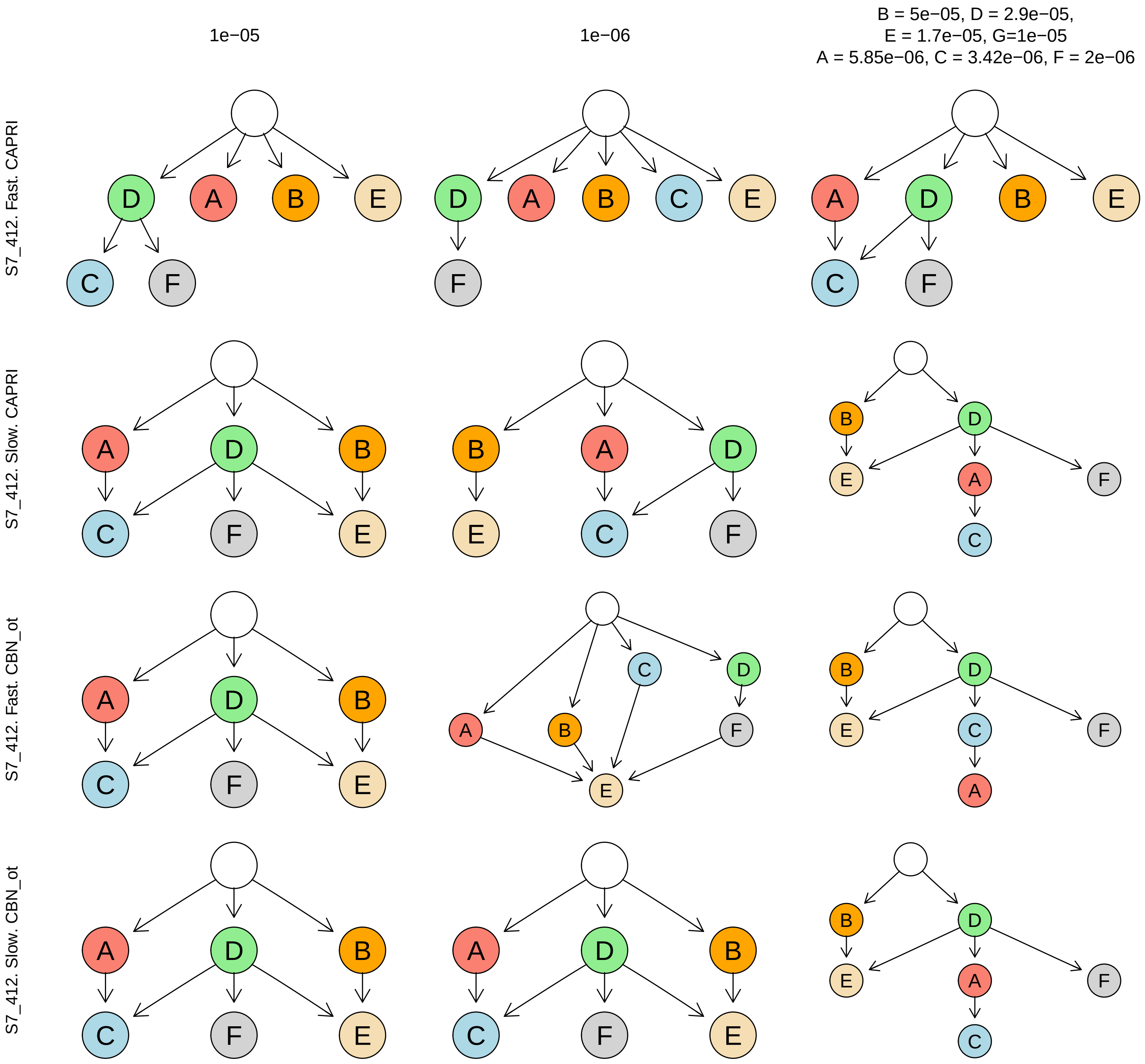

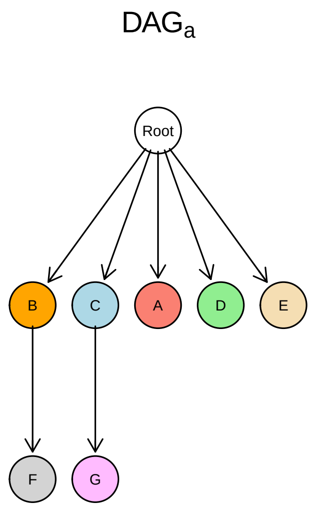

ID	Accessible Genot.	Discrep. DAG_a
S7_413	19	62

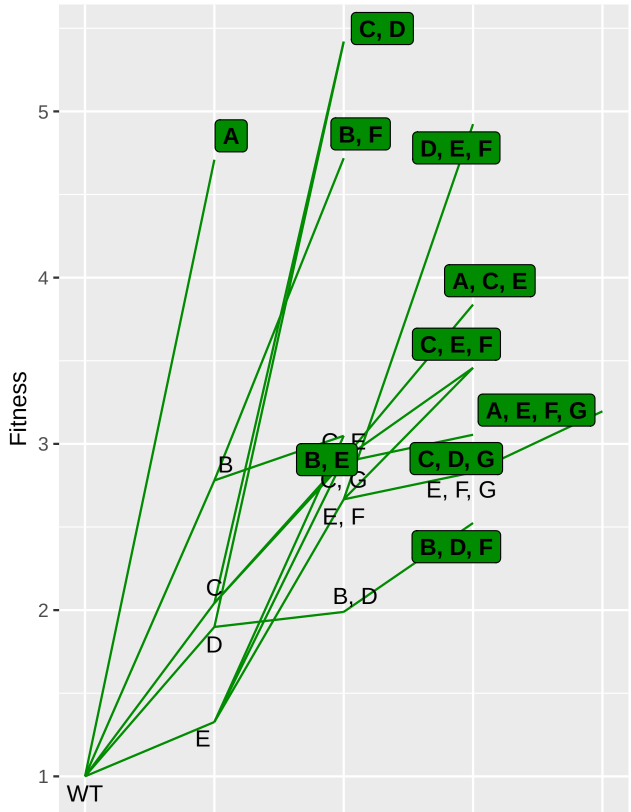

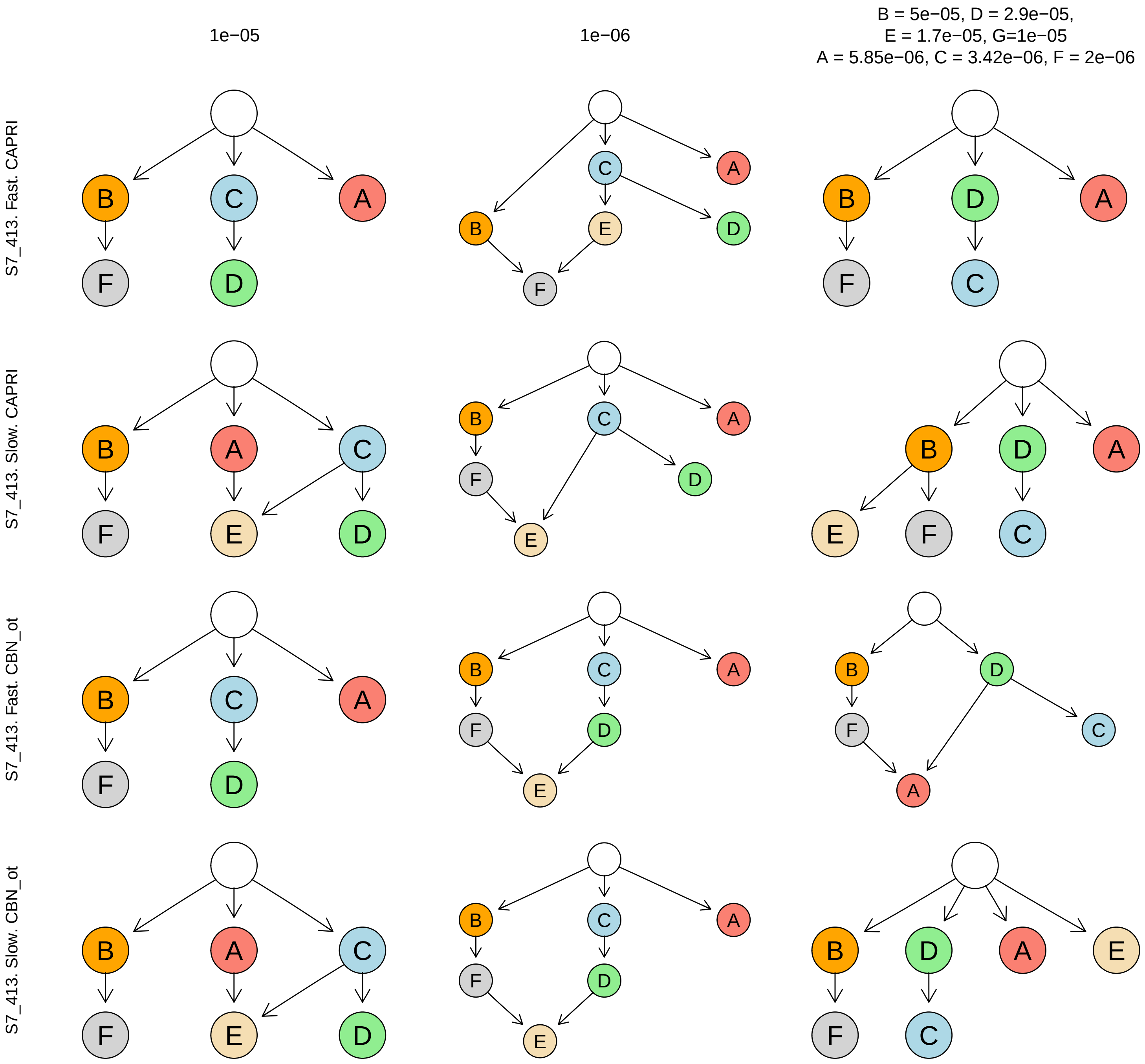

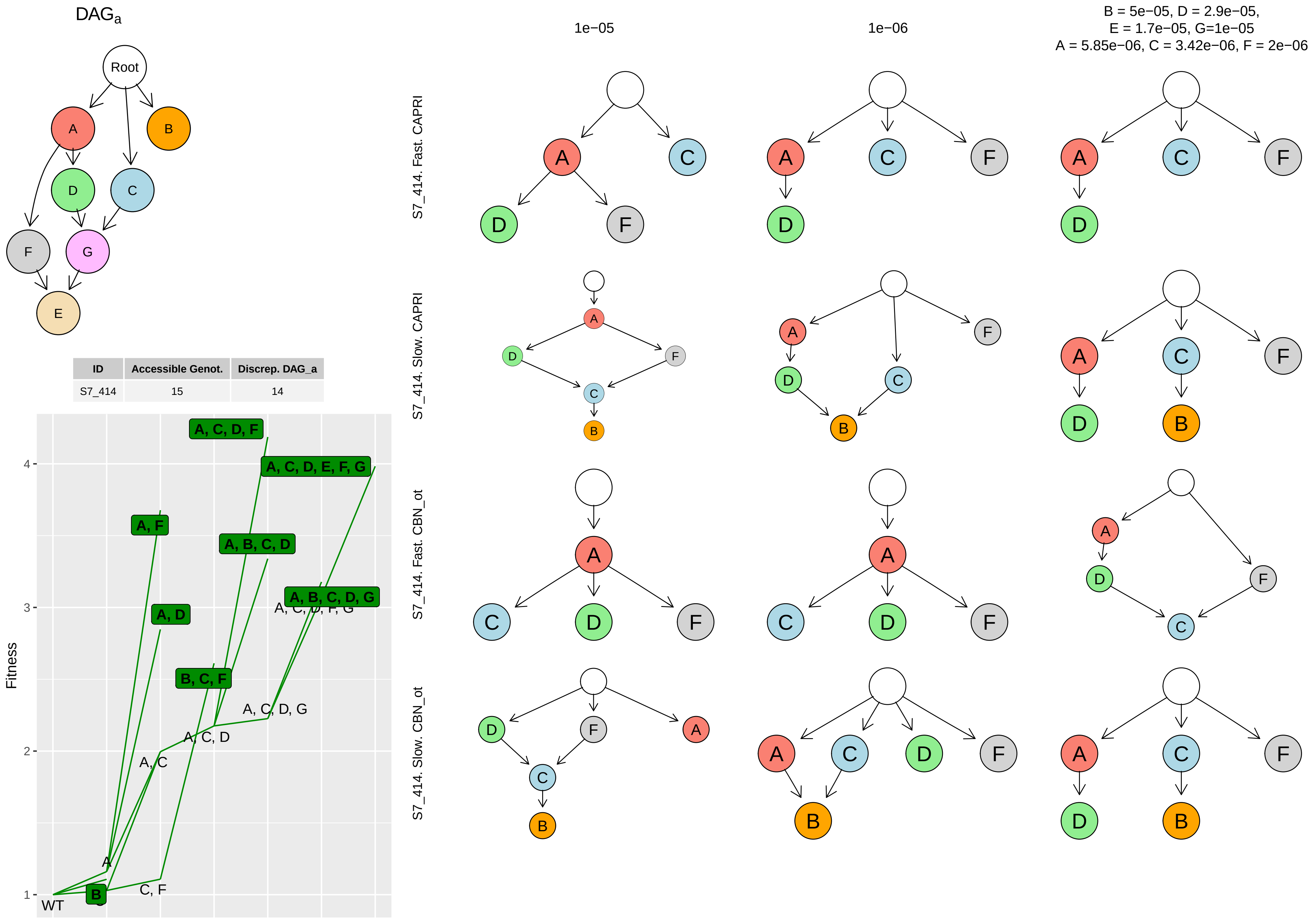

ID	Accessible Genot.	Discrep. DAG_a
S7_414	15	14

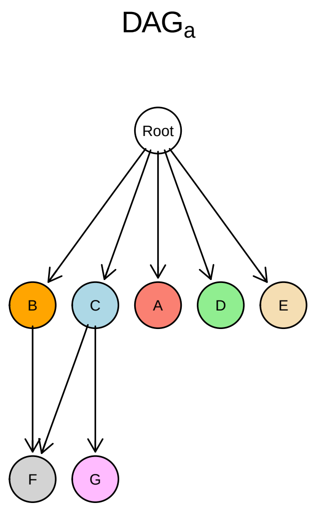

ID	Accessible Genot.	Discrep. DAG_a
S7_415	22	43

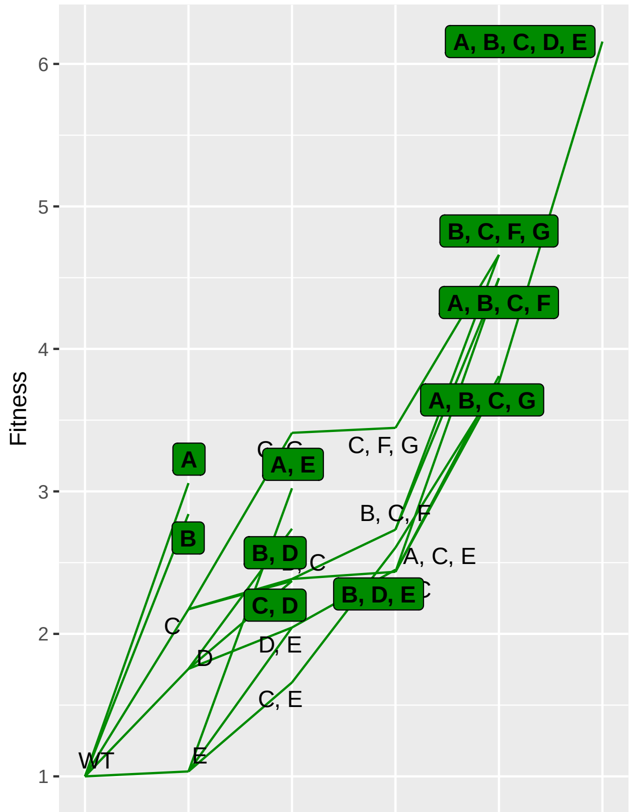

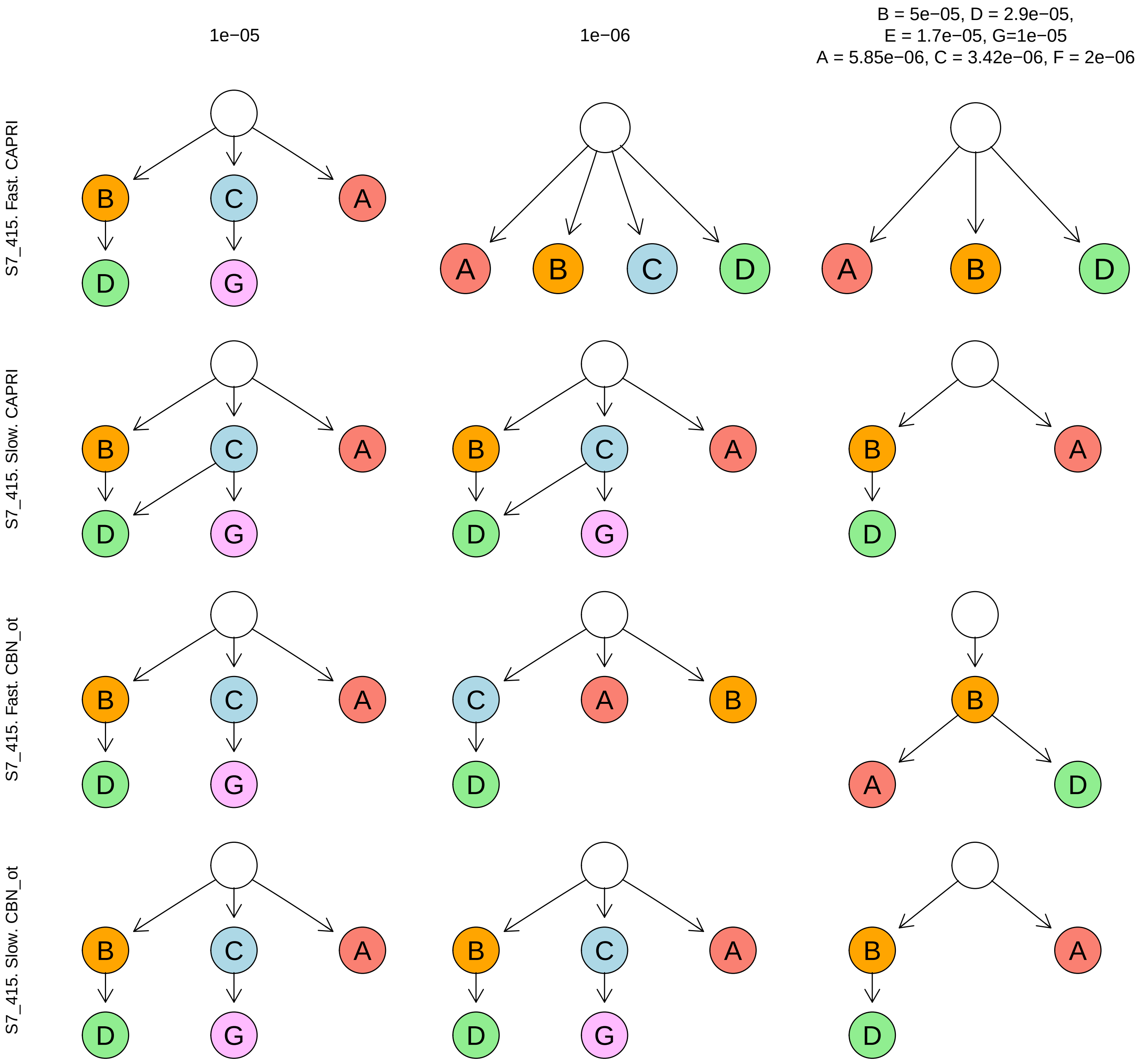

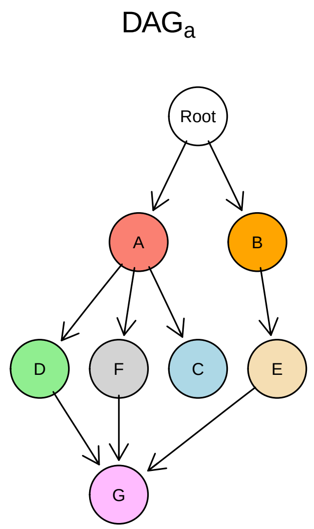

ID	Accessible Genot.	Discrep. DAG_a
S7_416	39	23

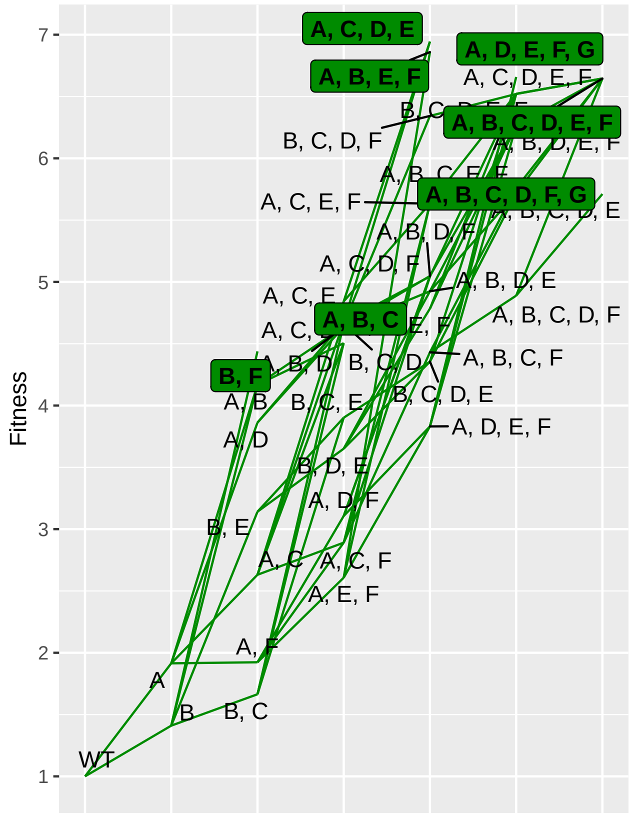

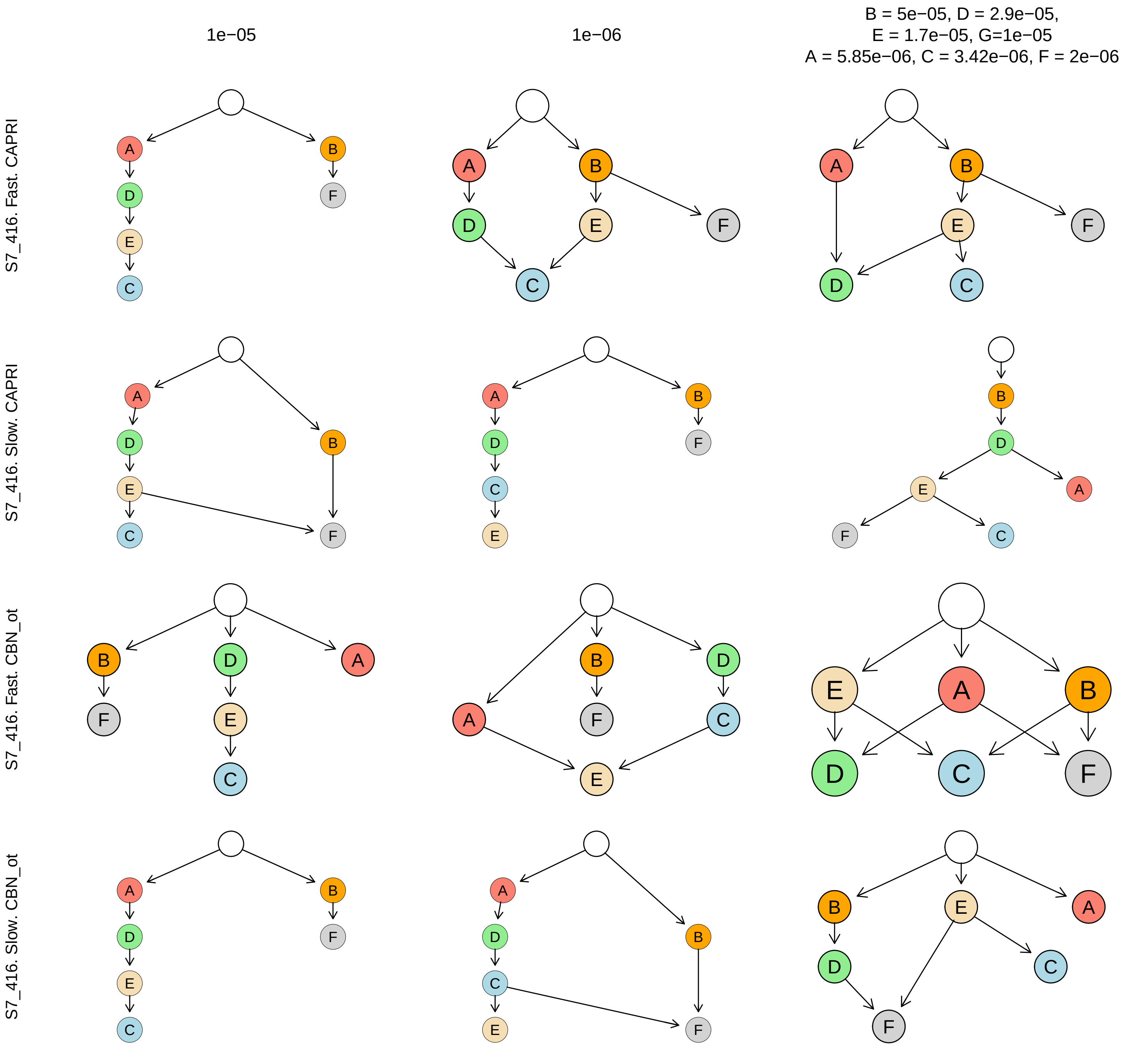

DAG_a

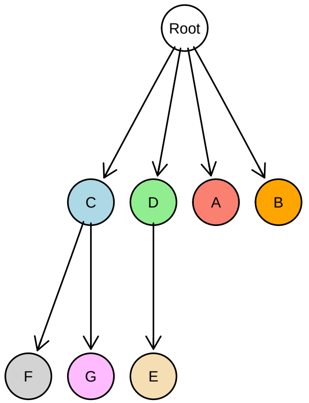

ID	Accessible Genot.	Discrep. DAG_a
S7_417	25	52

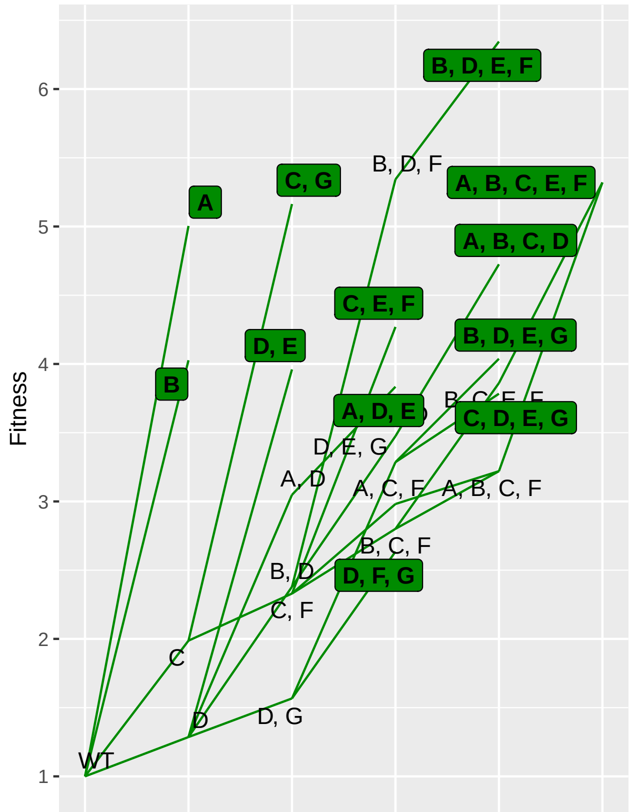

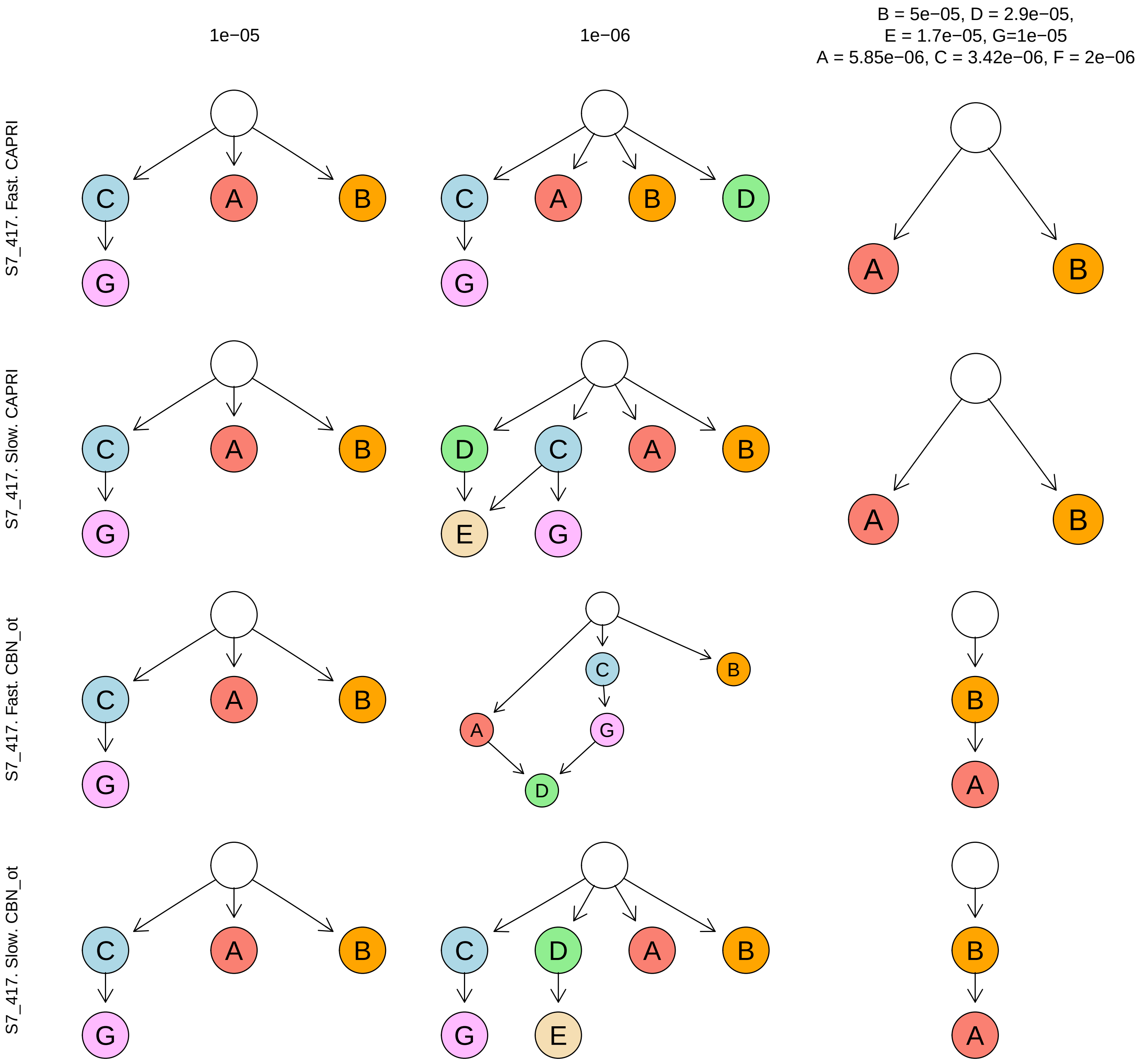

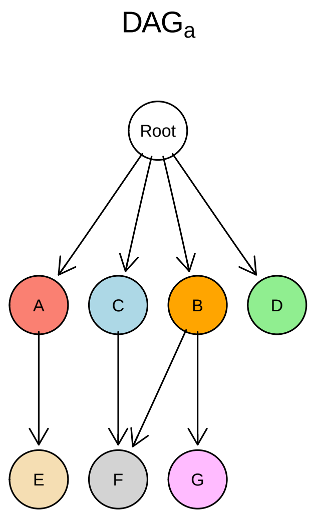

ID	Accessible Genot.	Discrep. DAG_a
S7_418	21	36

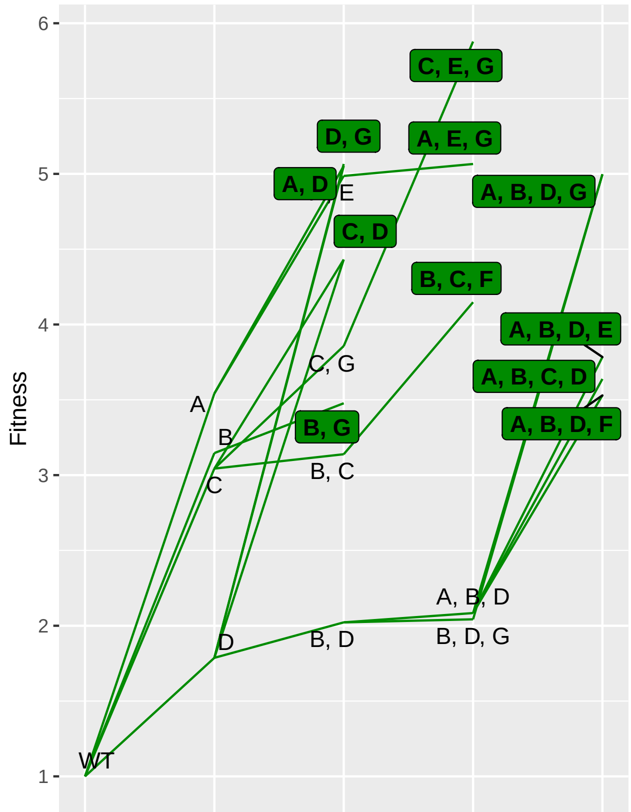

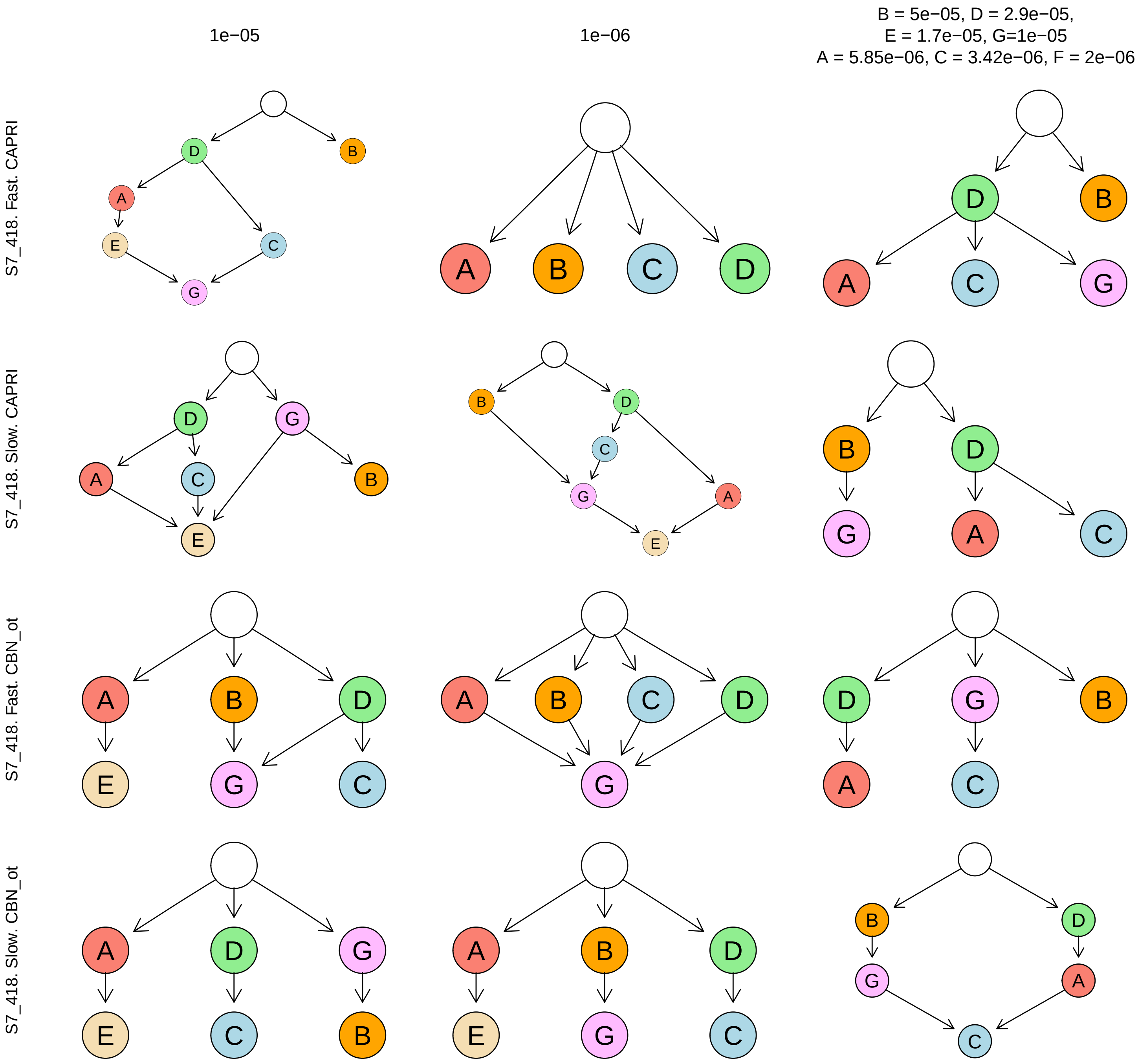

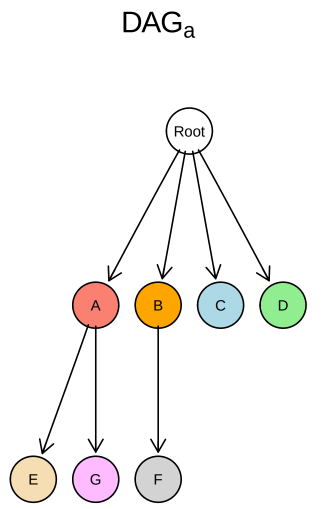

ID	Accessible Genot.	Discrep. DAG_a
S7_419	56	59

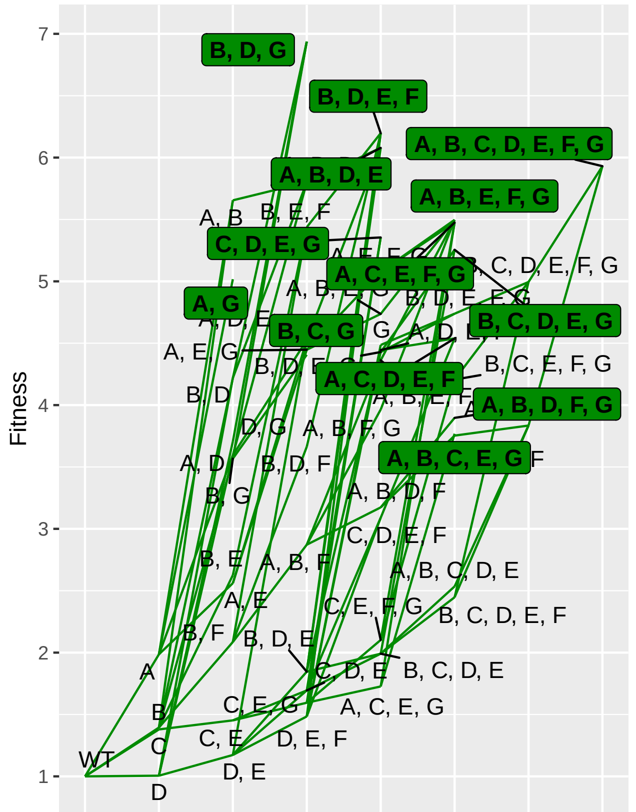

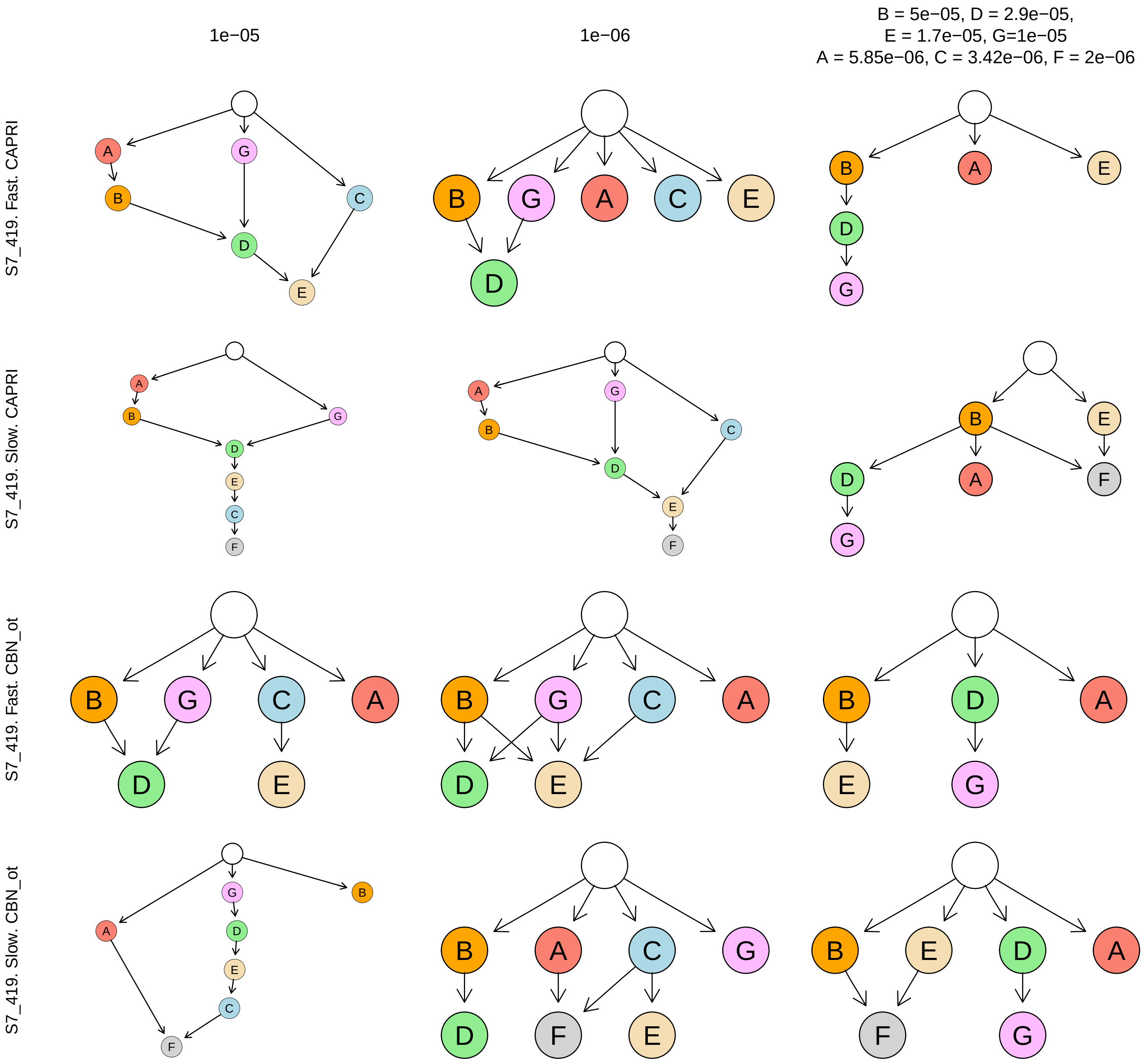

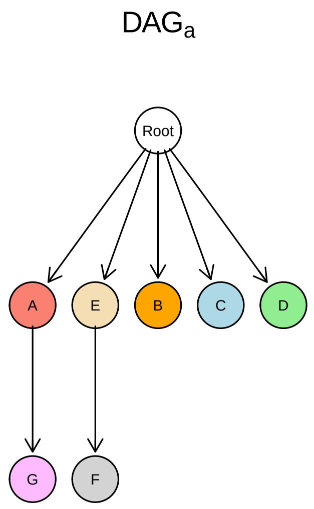

ID	Accessible Genot.	Discrep. DAG_a
S7_420	54	51

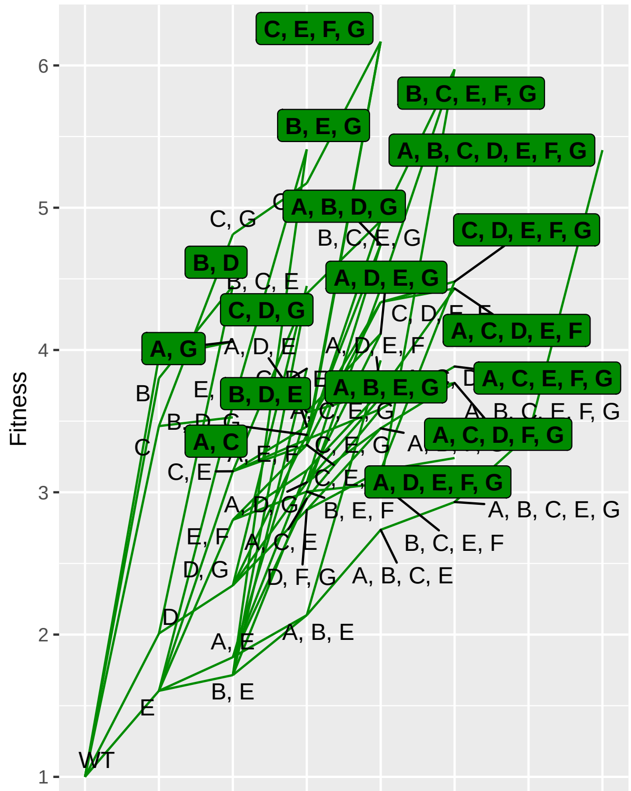

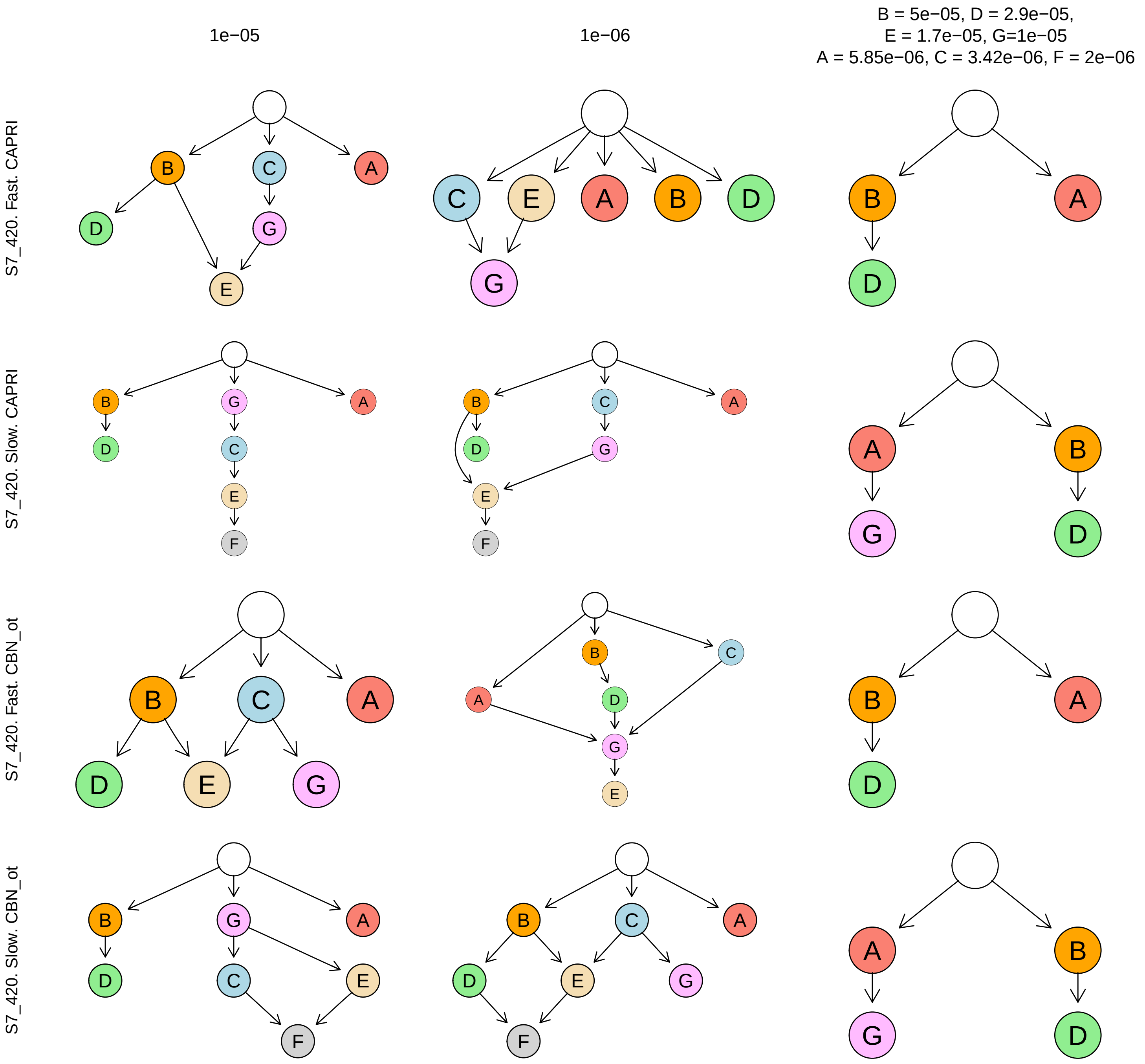

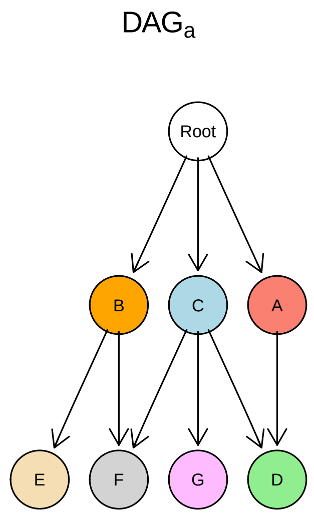

ID	Accessible Genot.	Discrep. DAG_a
S7_421	24	23

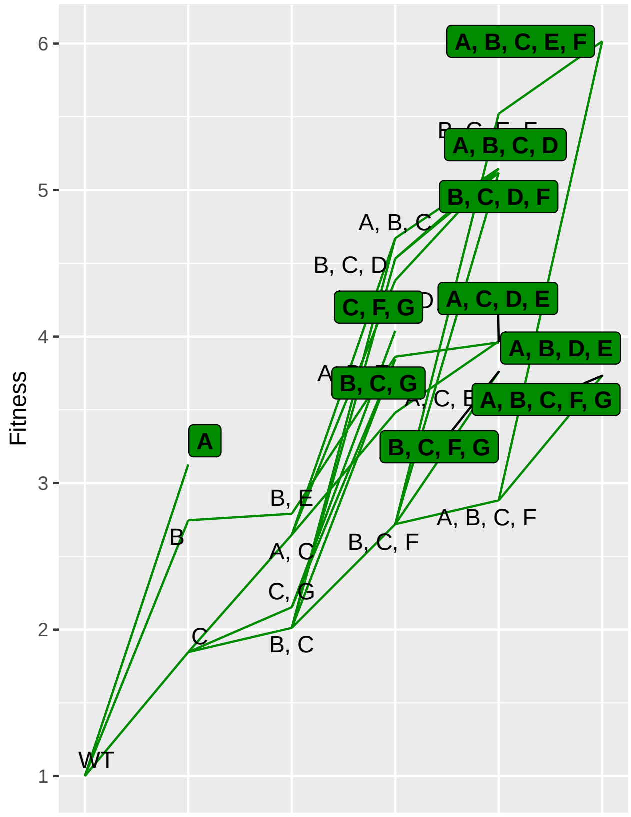

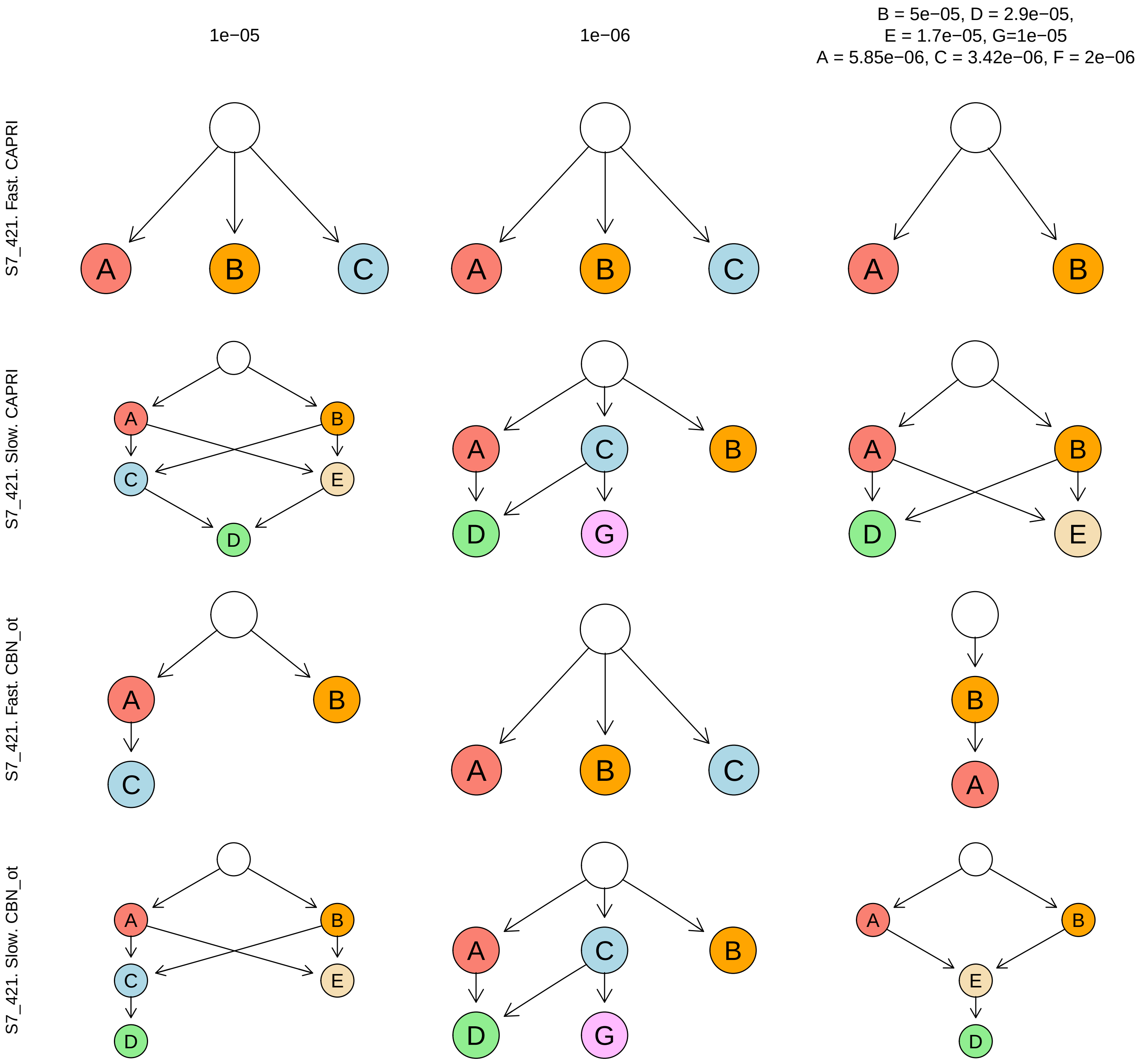

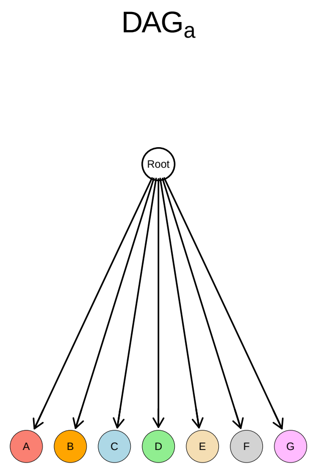

ID	Accessible Genot.	Discrep. DAG_a
S7_422	31	96

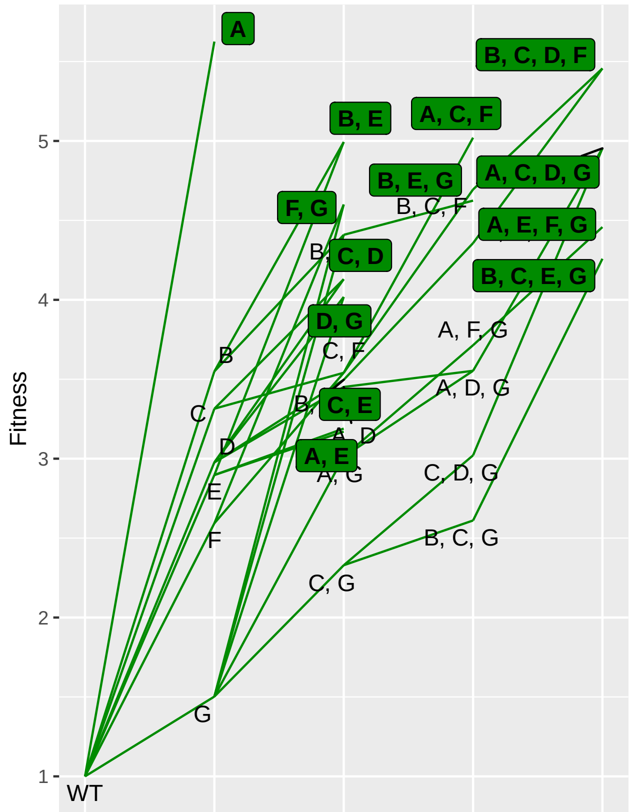

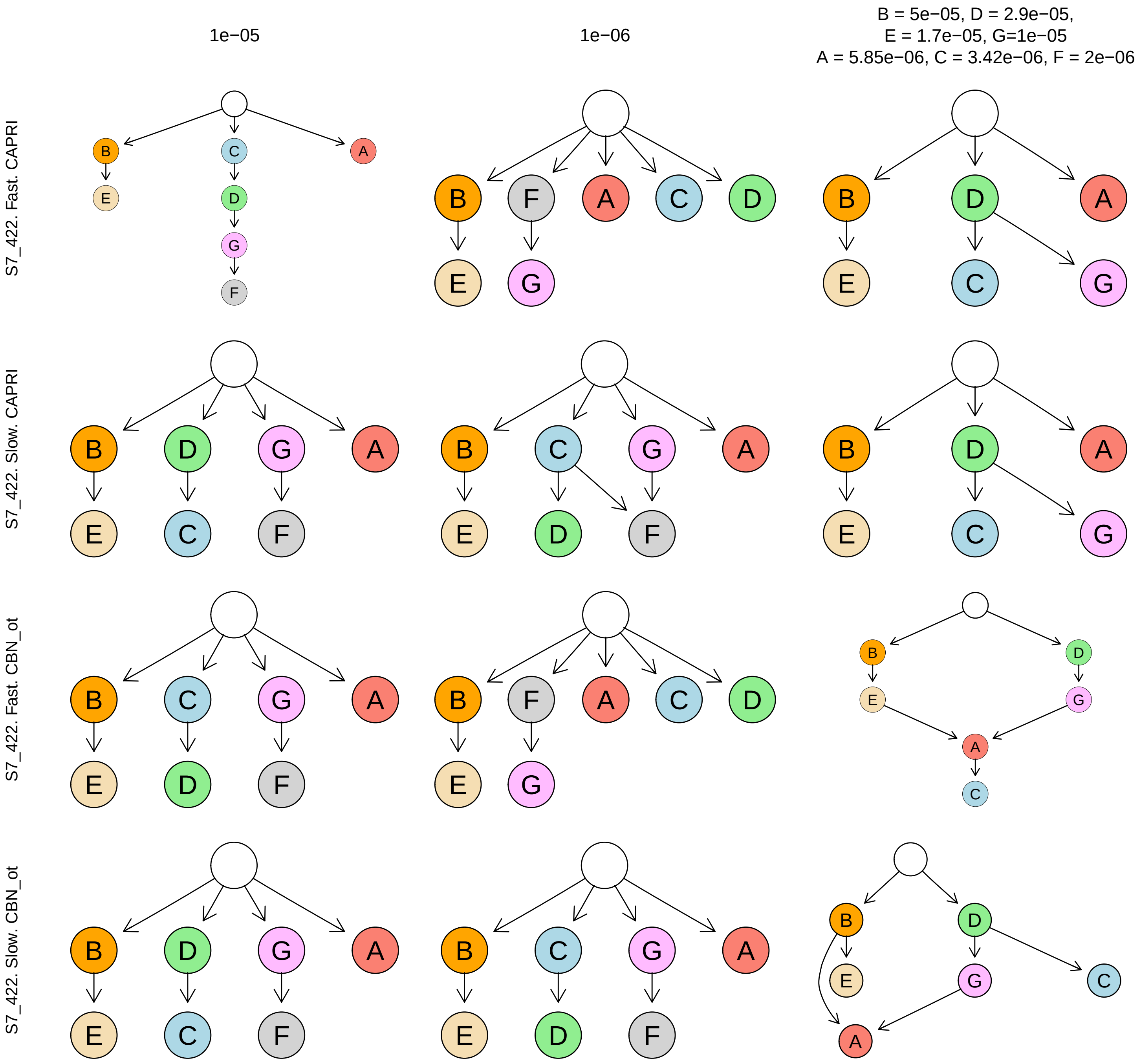

DAG$_a$

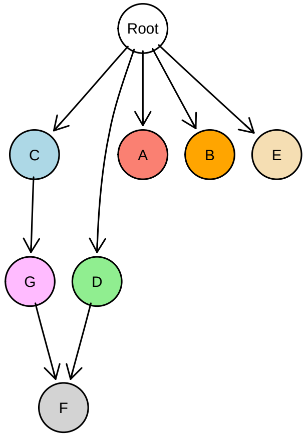

ID	Accessible Genot.	Discrep. DAG_a
S7_423	9	50

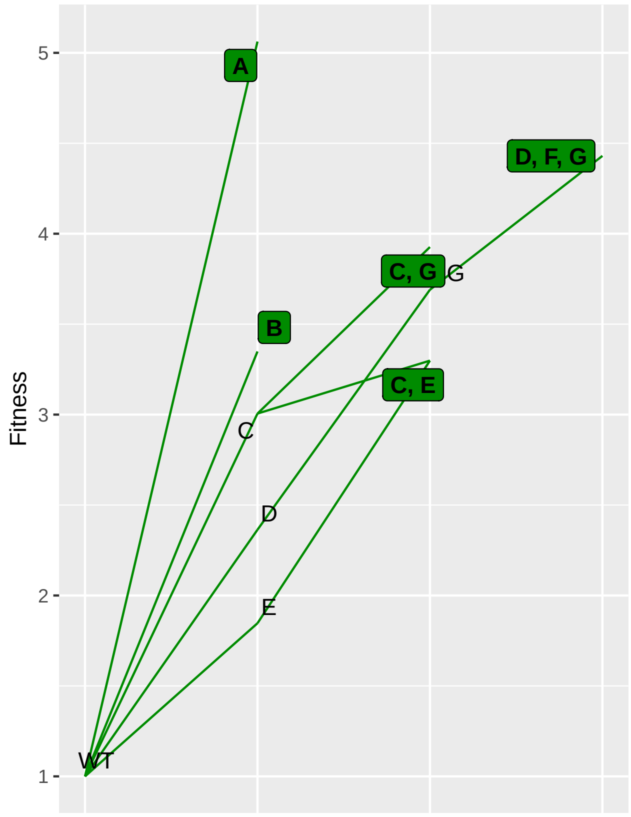

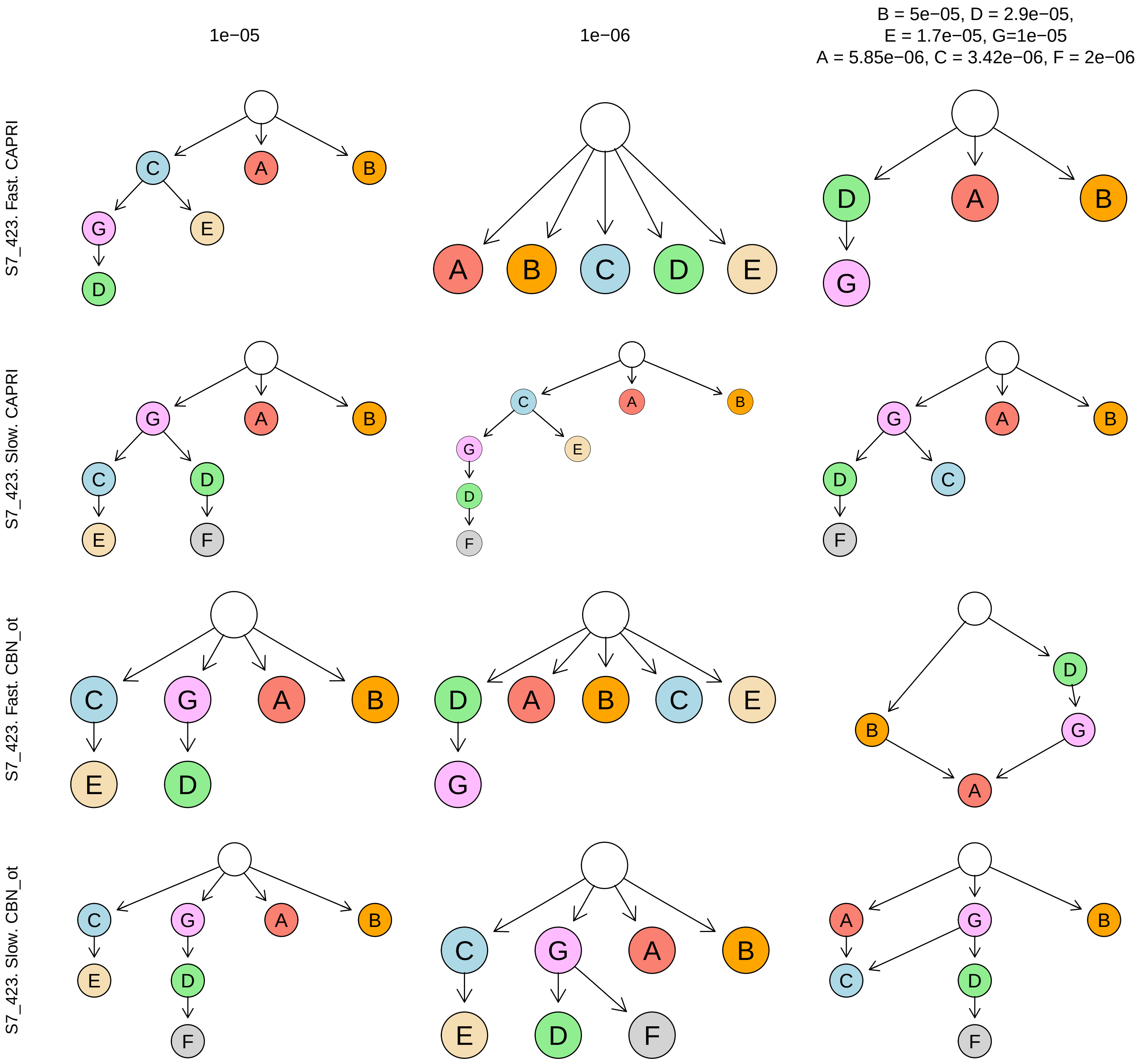

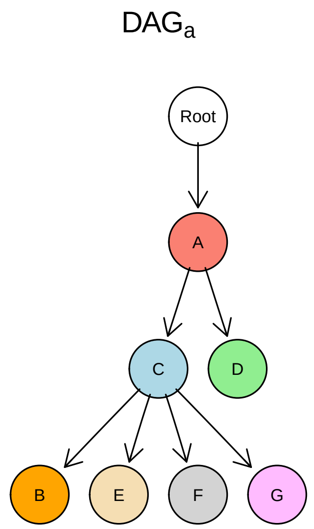

ID	Accessible Genot.	Discrep. DAG_a
S7_424	8	26

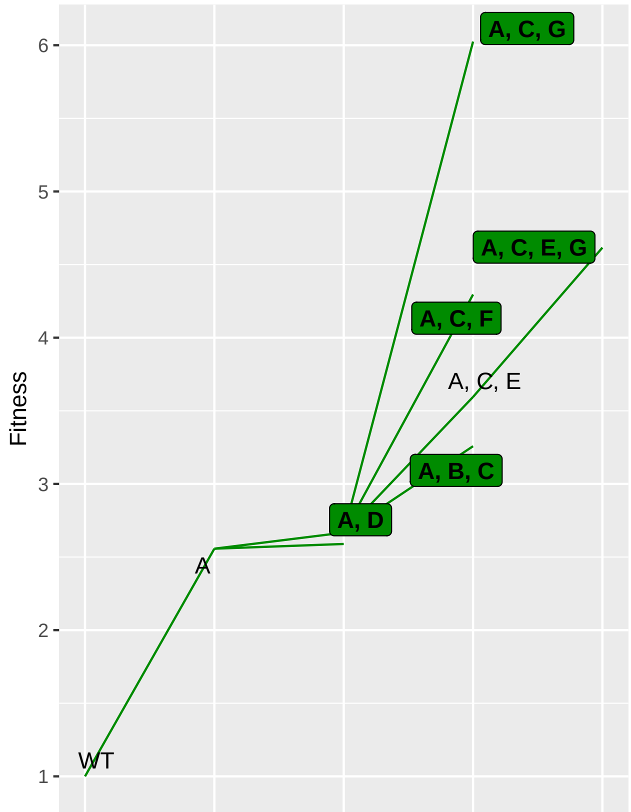

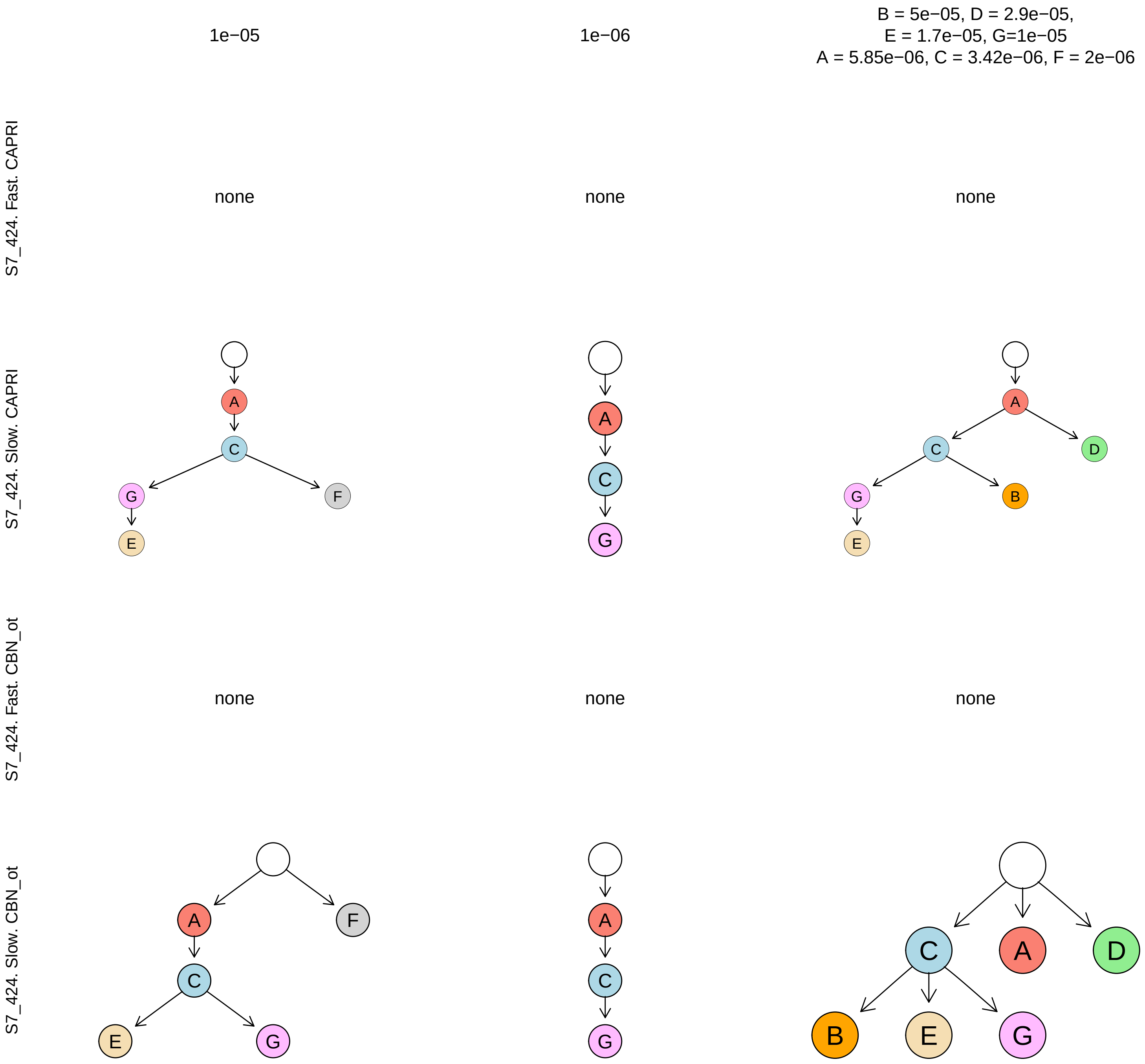

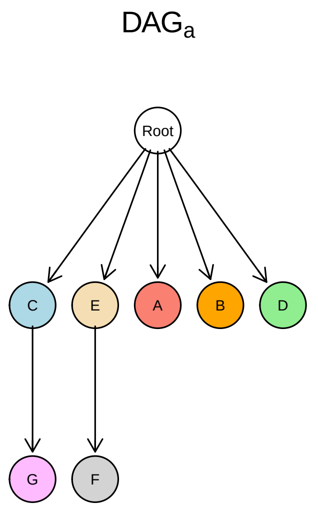

ID	Accessible Genot.	Discrep. DAG_a
S7_425	50	41

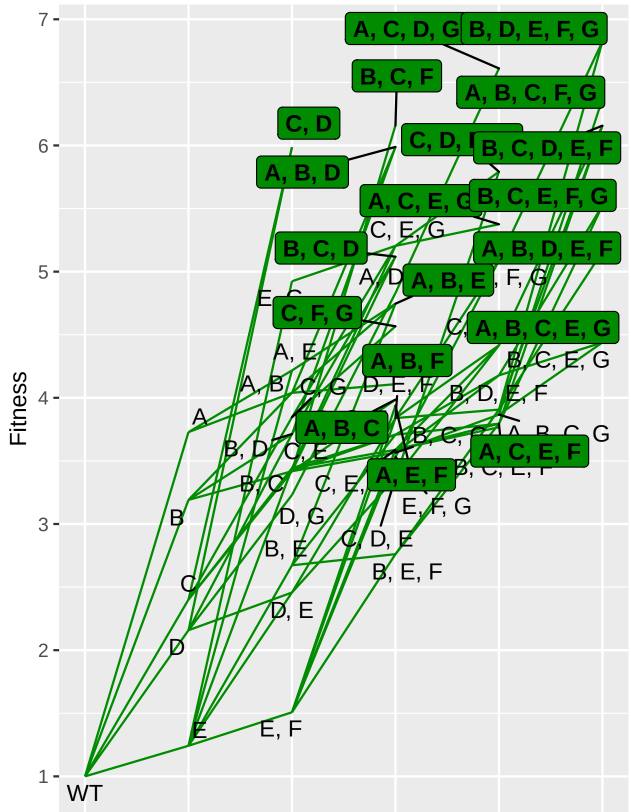

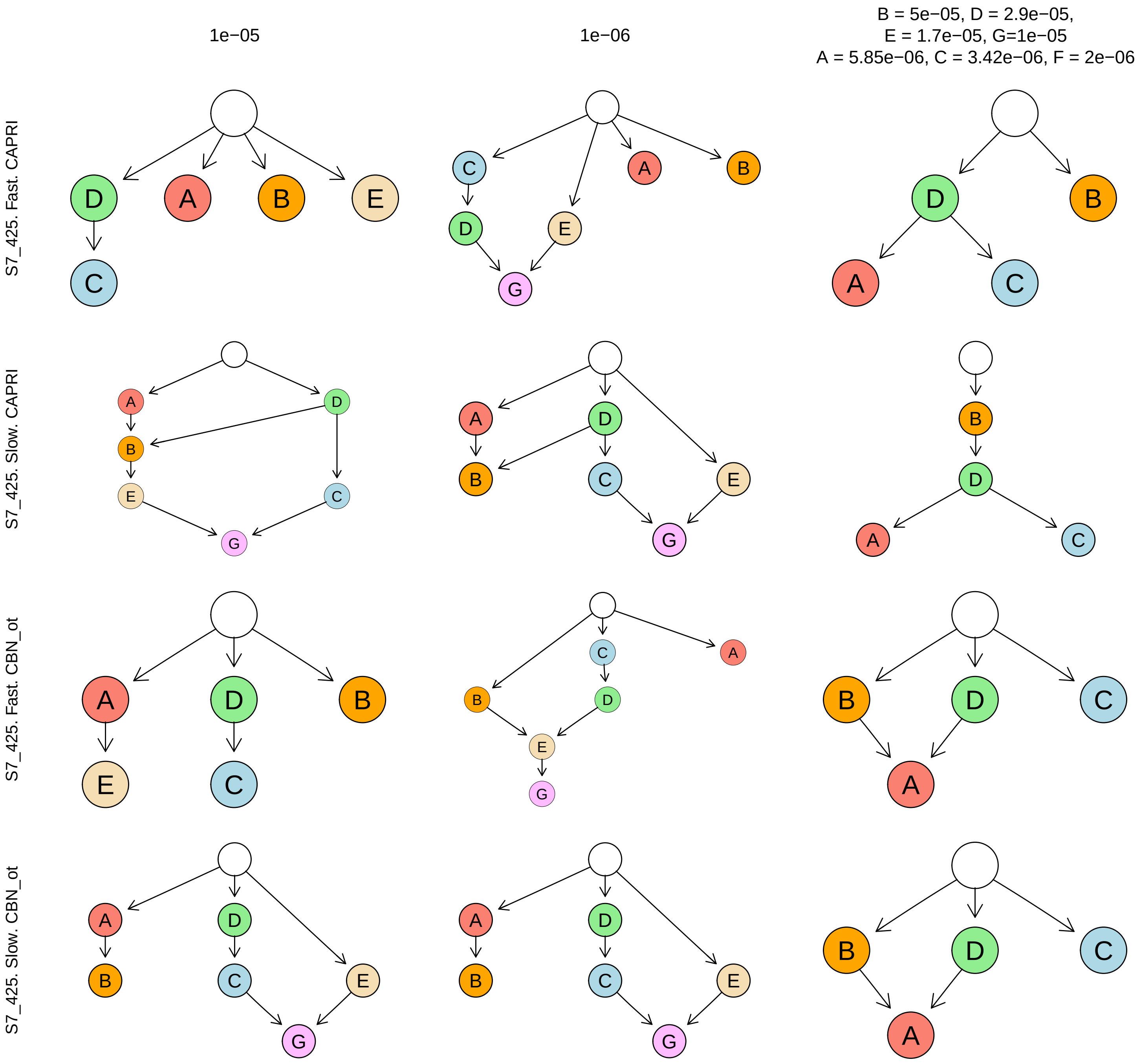

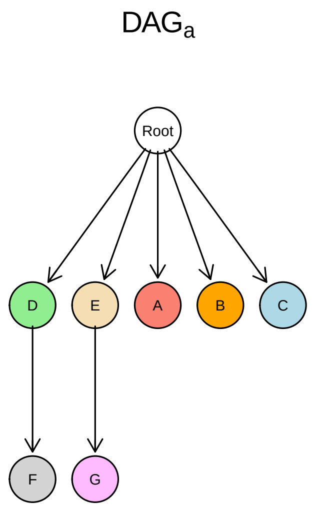

ID	Accessible Genot.	Discrep. DAG_a
S7_426	64	49

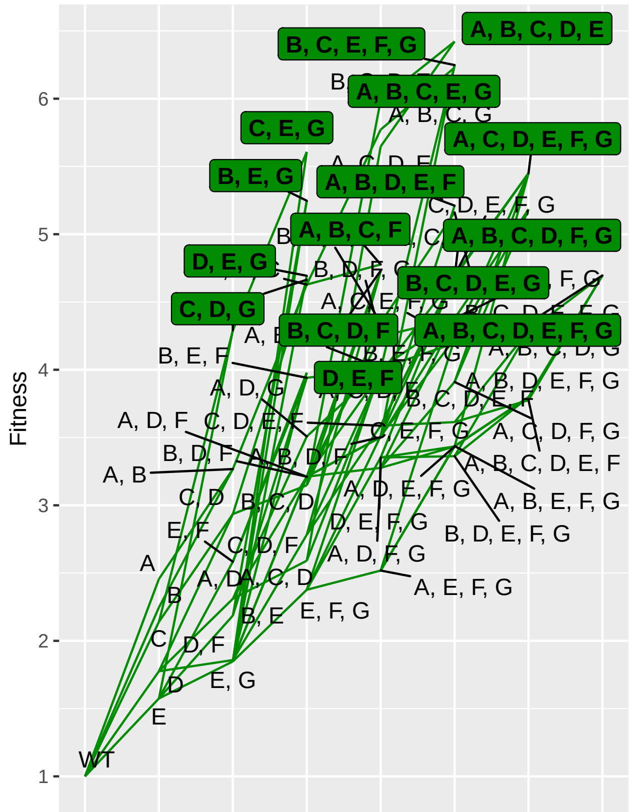

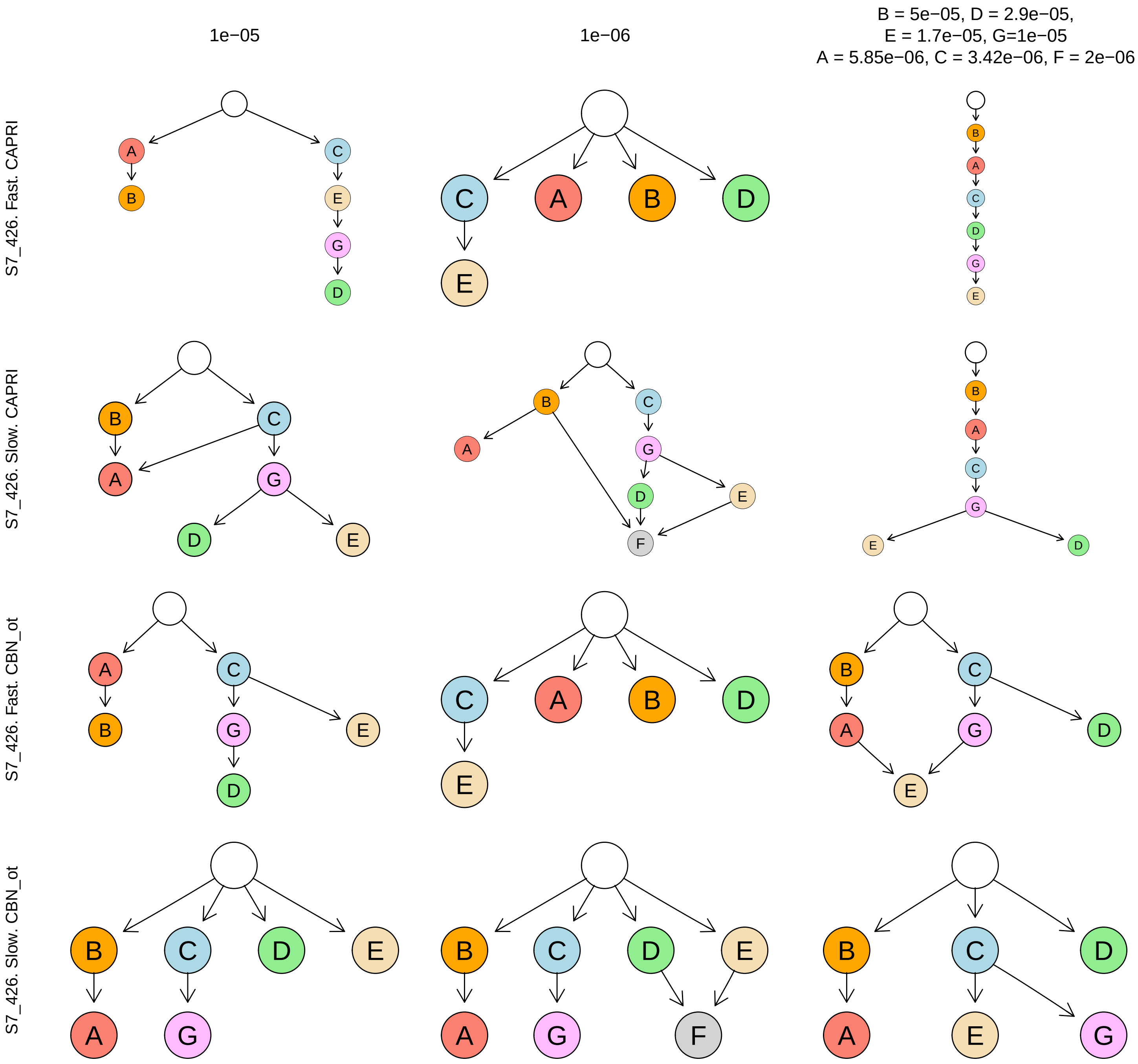

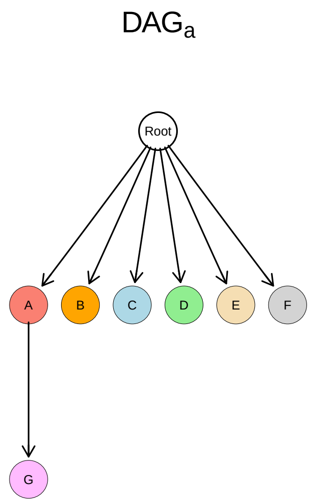

ID	Accessible Genot.	Discrep. DAG_a
S7_427	24	73

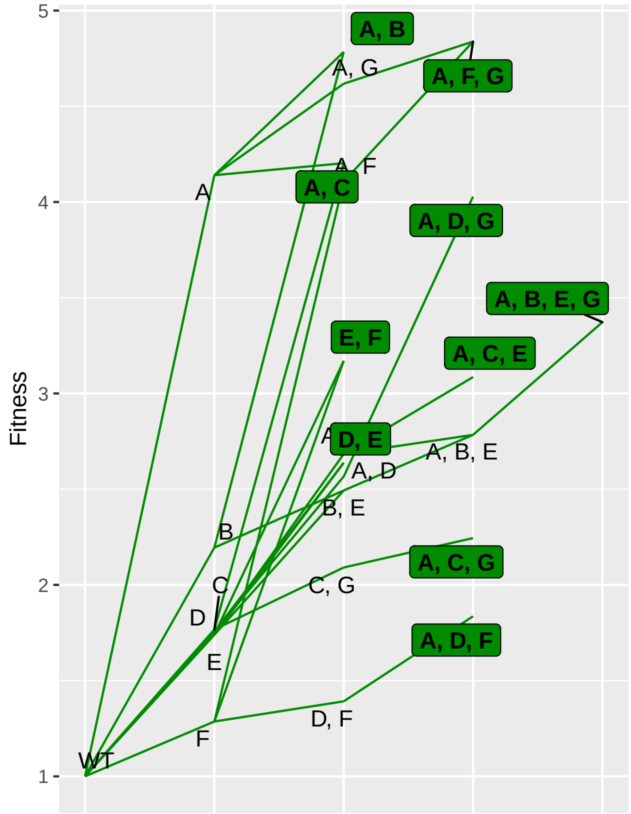

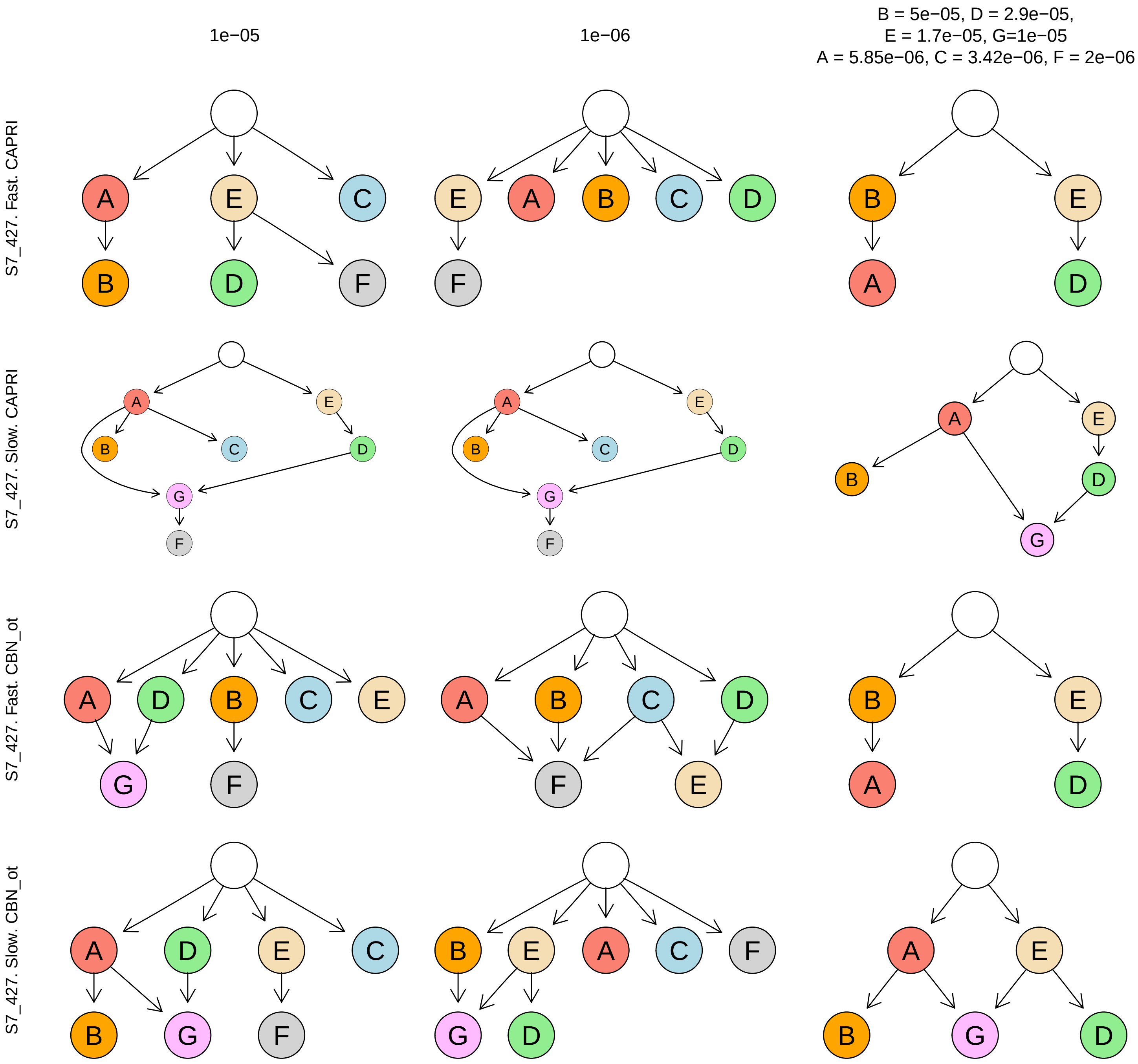

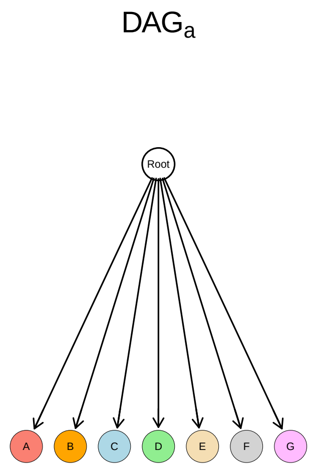

ID	Accessible Genot.	Discrep. DAG_a
S7_428	36	91

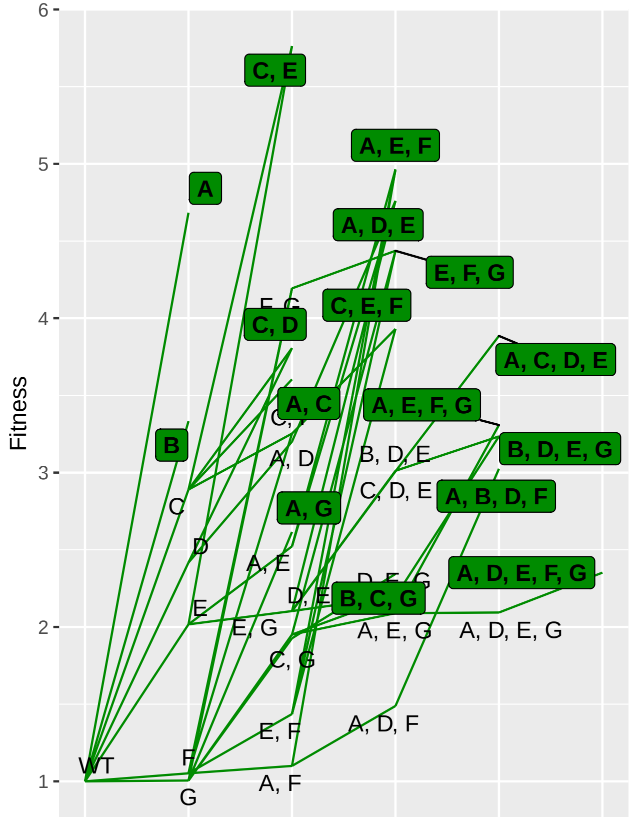

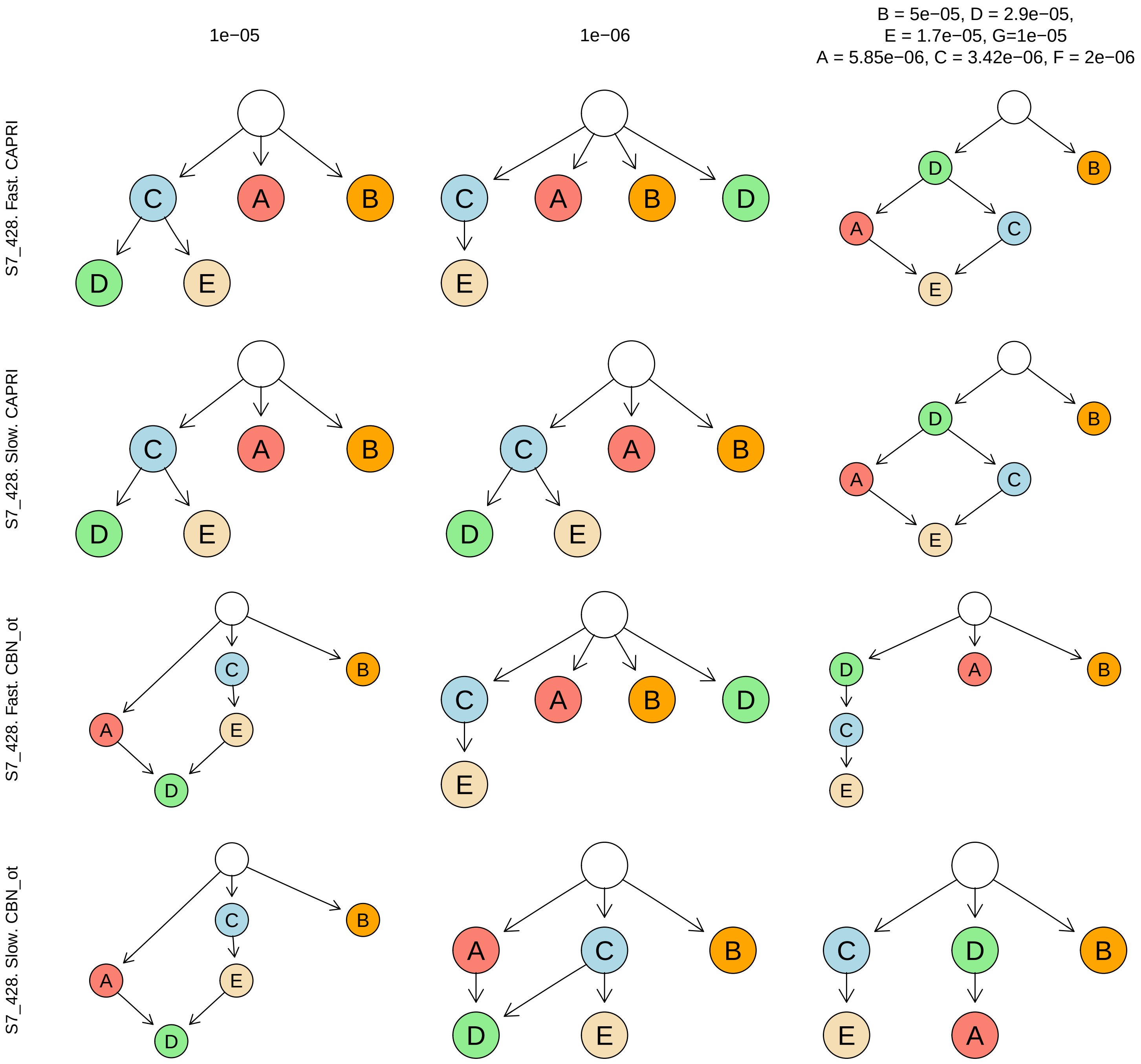

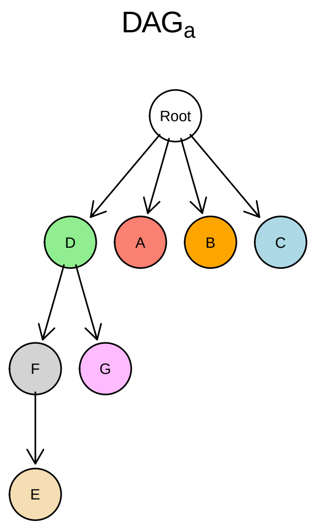

ID	Accessible Genot.	Discrep. DAG_a
S7_429	23	34

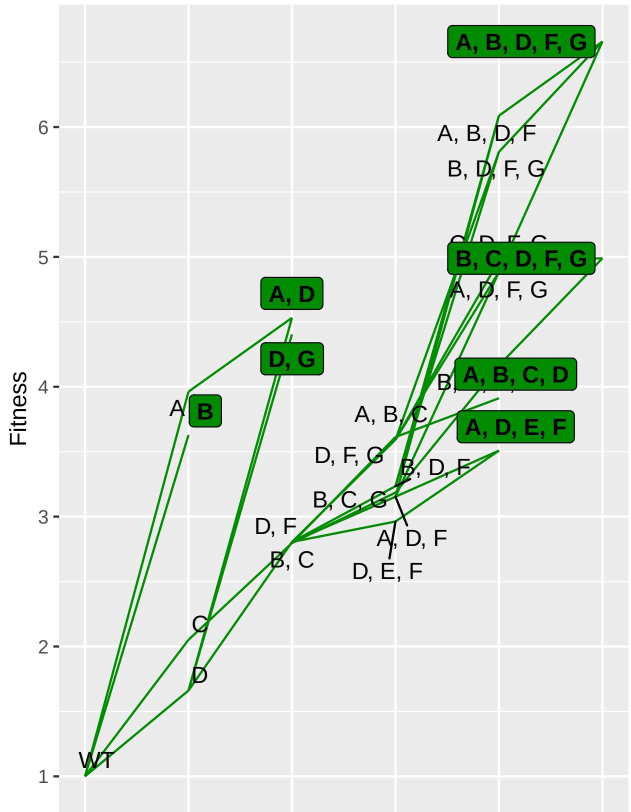

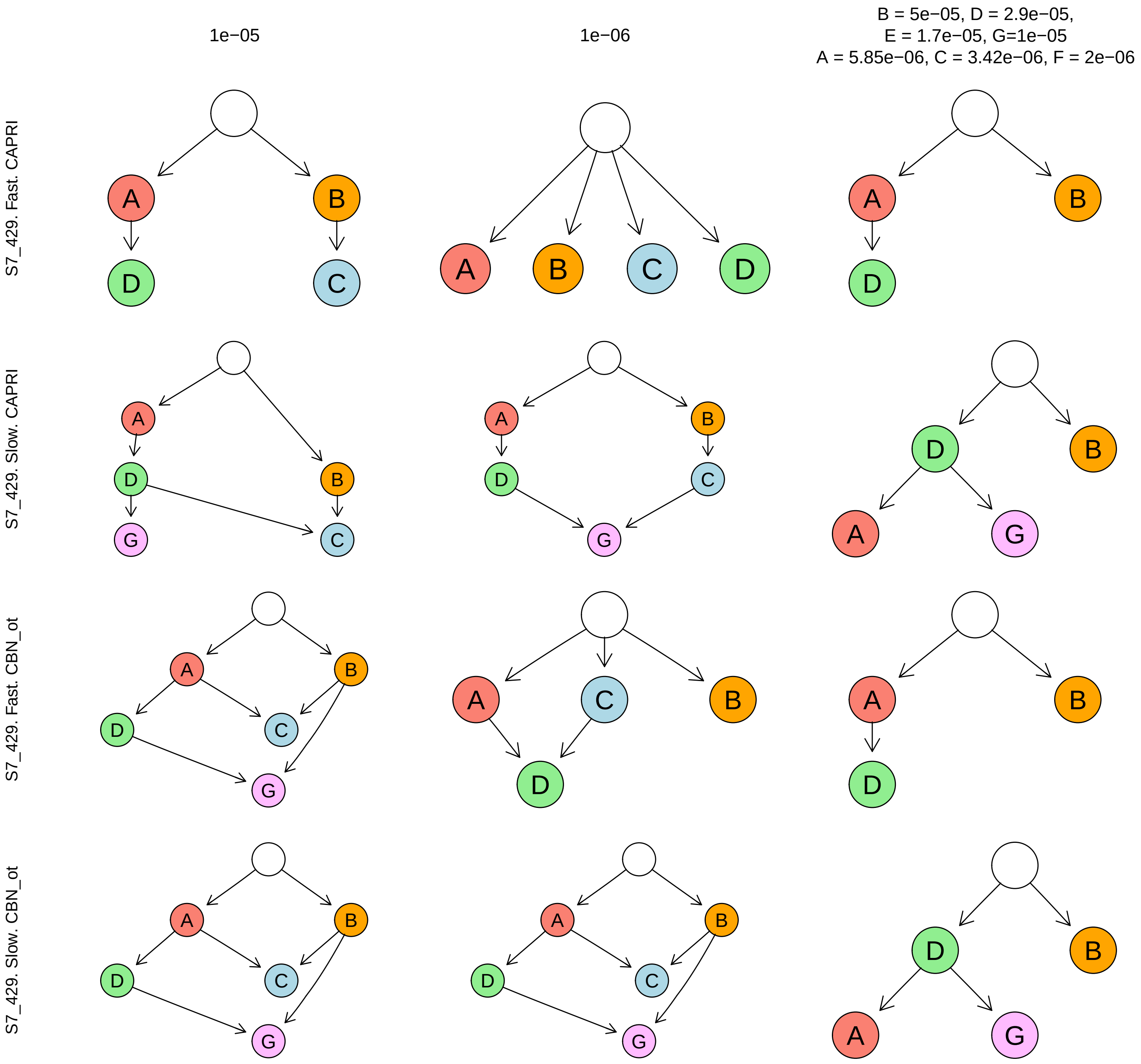

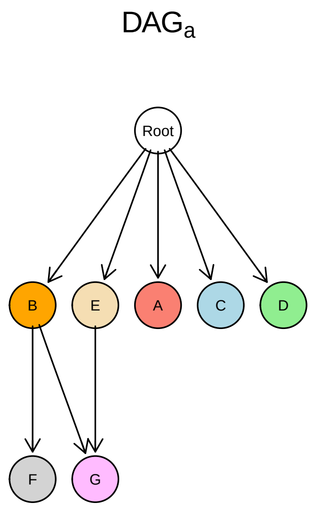

ID	Accessible Genot.	Discrep. DAG_a
S7_430	23	44

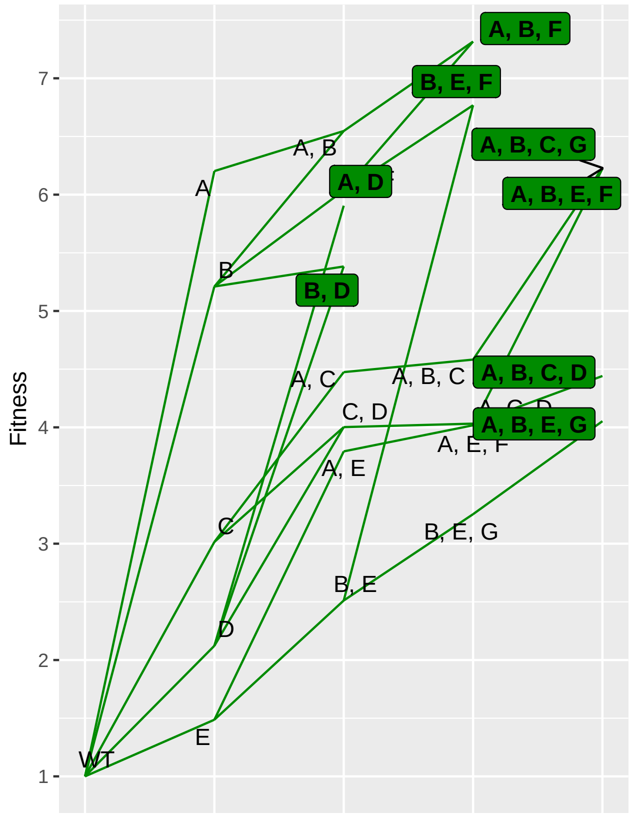

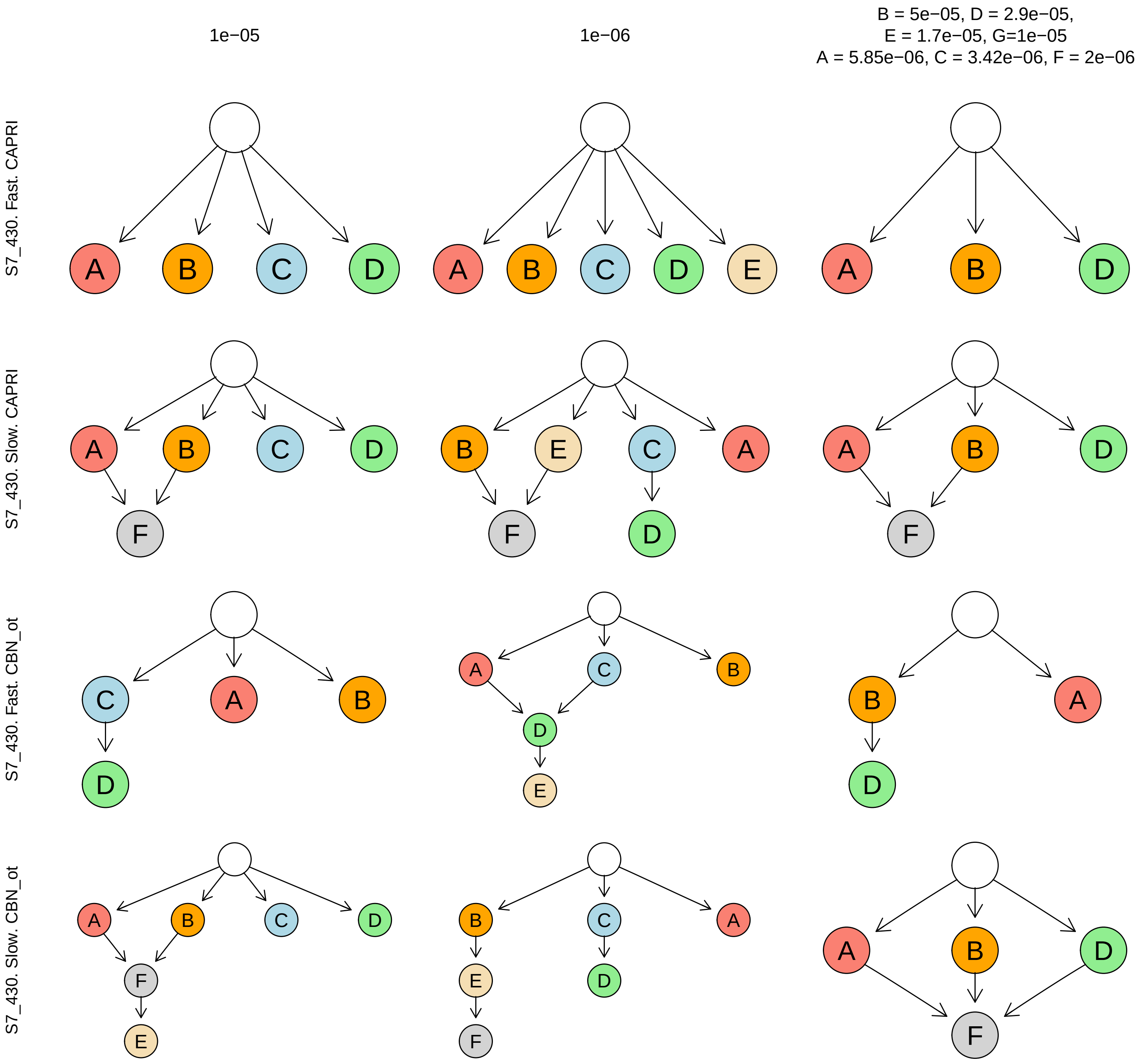

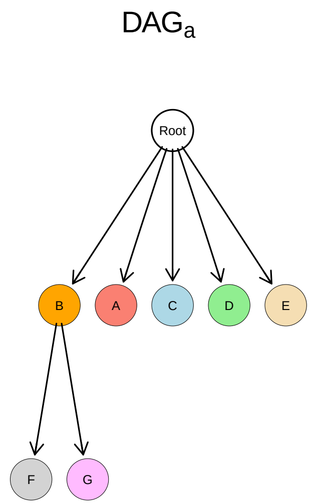

ID	Accessible Genot.	Discrep. DAG_a
S7_431	29	62

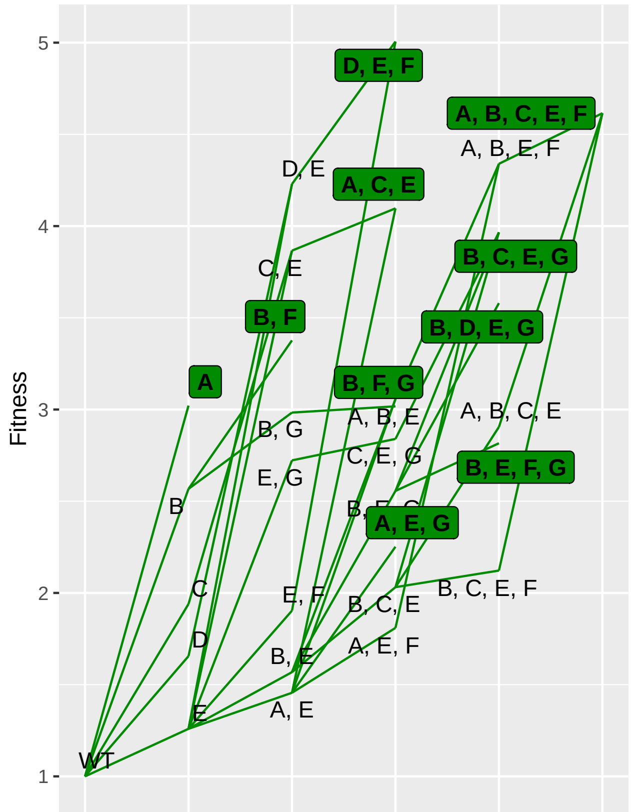

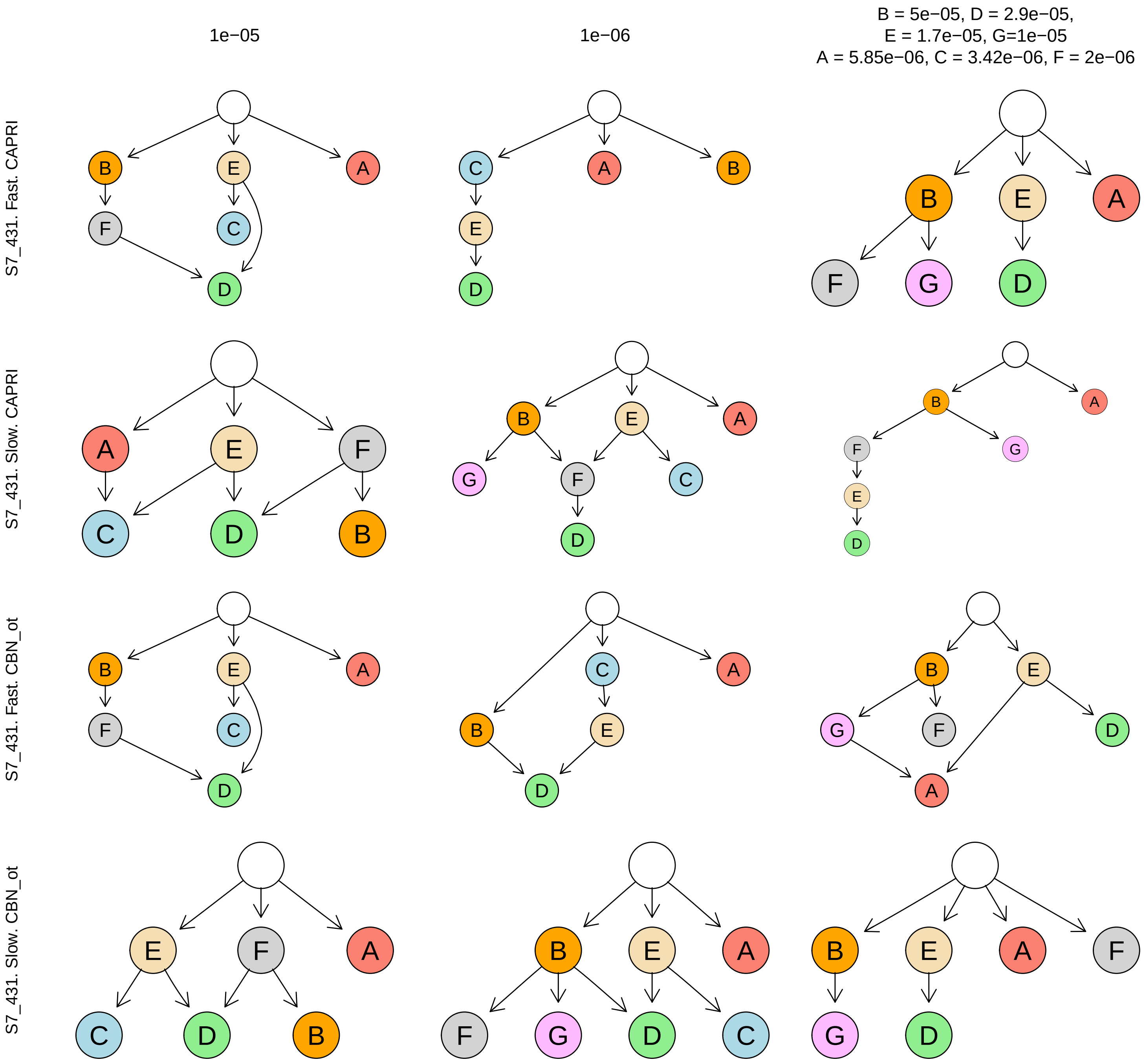

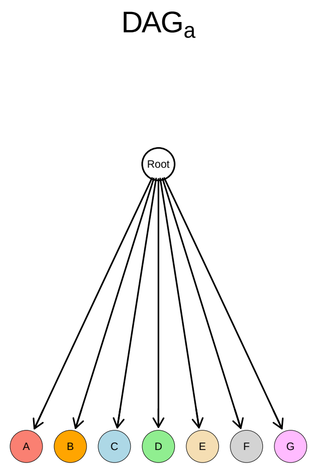

ID	Accessible Genot.	Discrep. DAG_a
S7_432	17	110

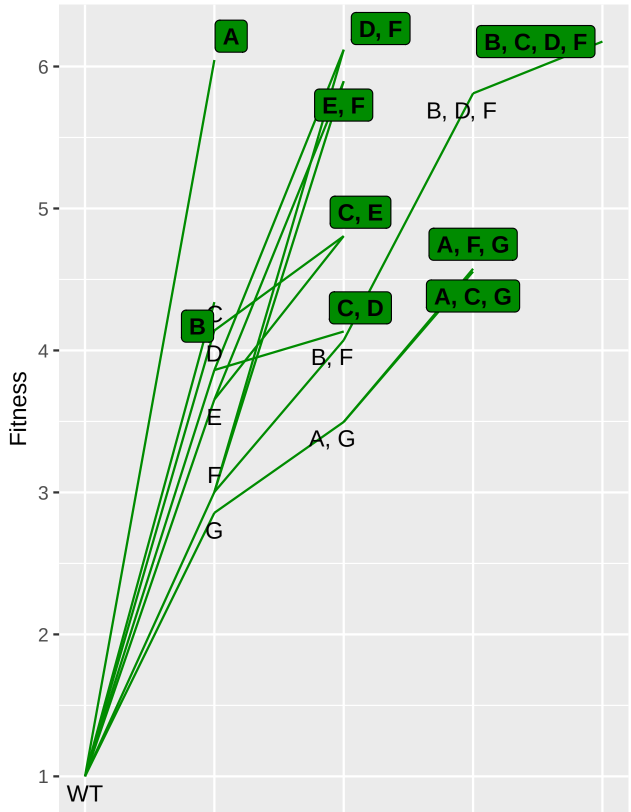

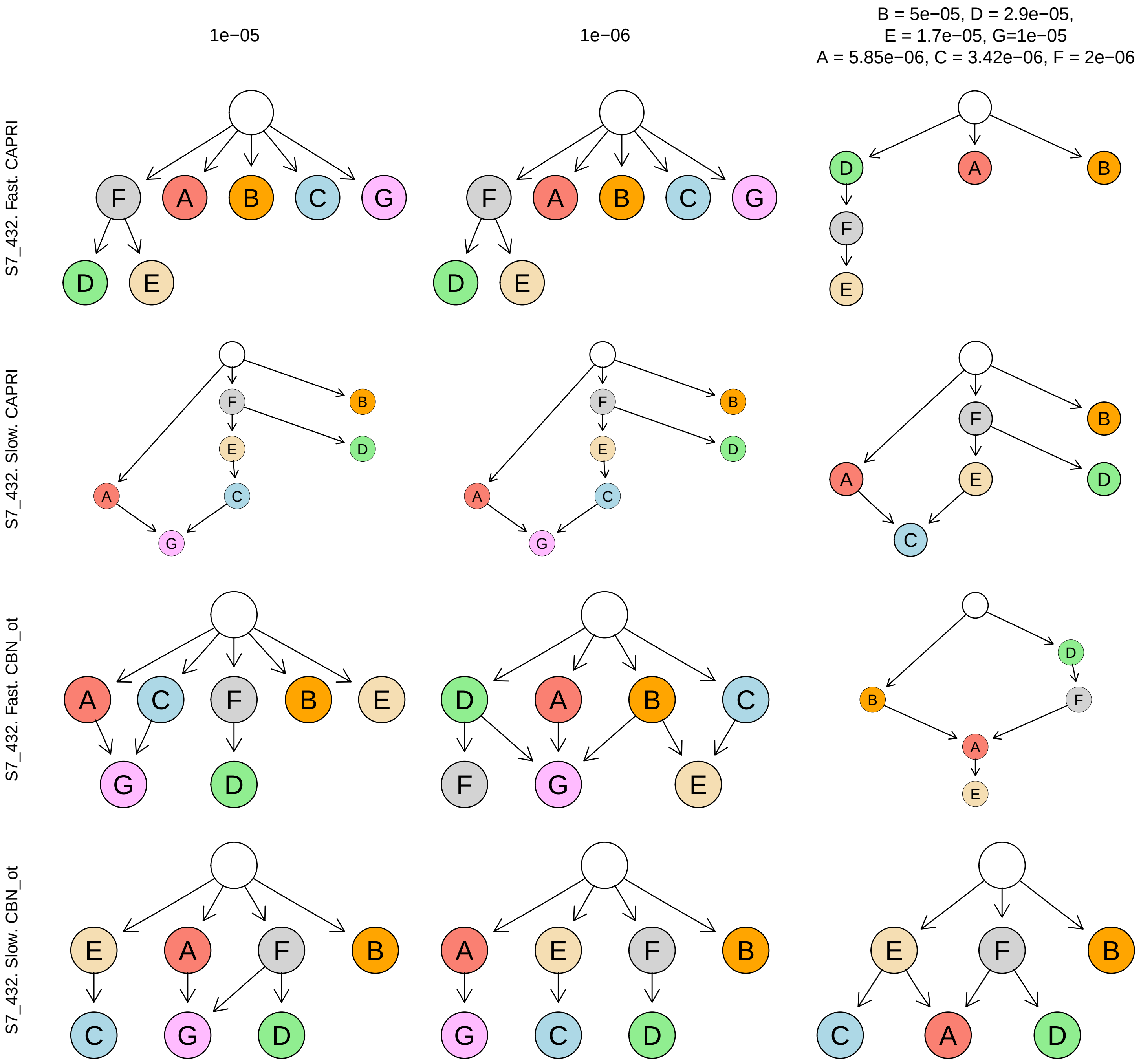

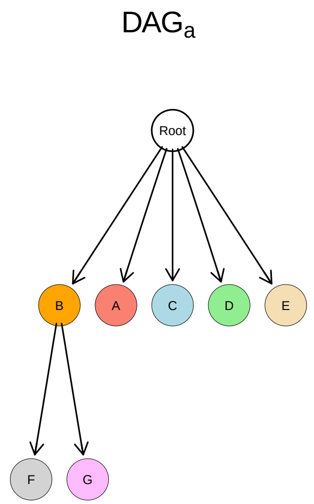

ID	Accessible Genot.	Discrep. DAG_a
S7_433	26	71

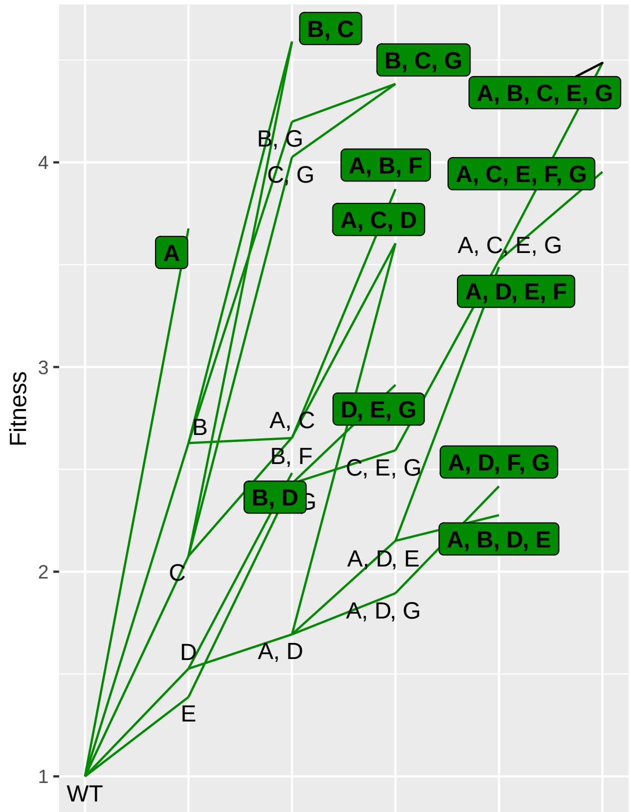

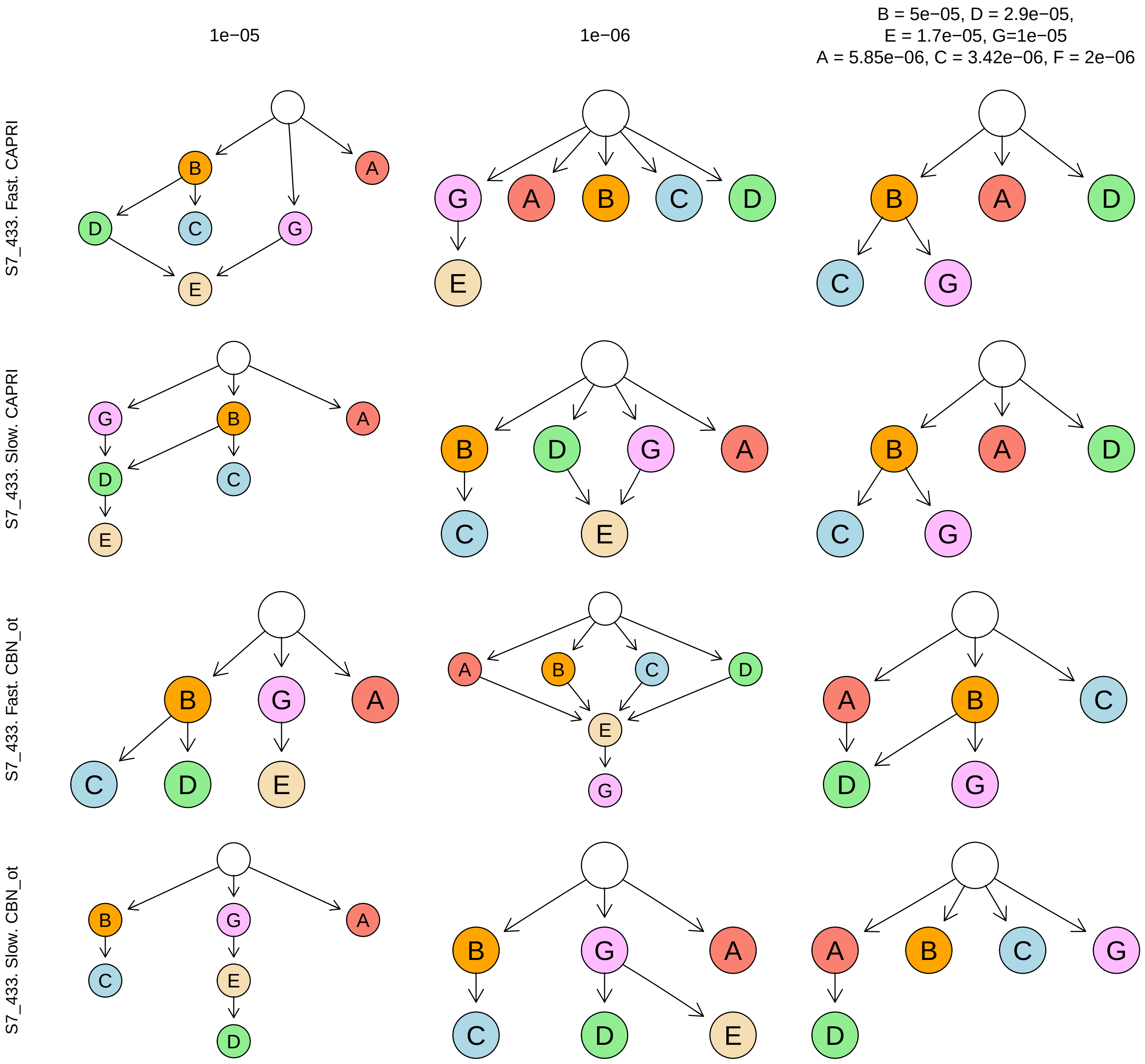

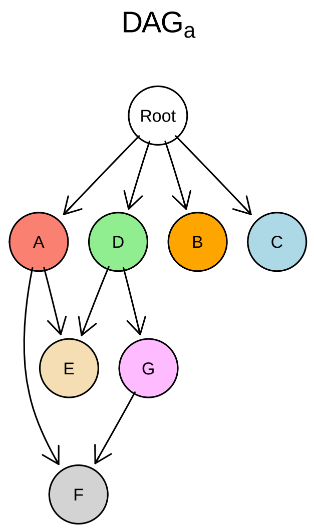

ID	Accessible Genot.	Discrep. DAG_a
S7_434	9	30

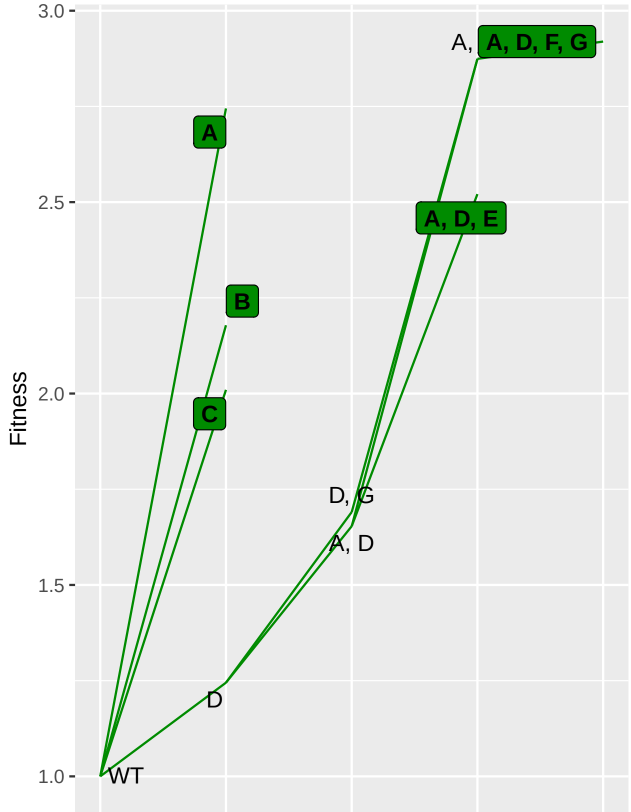

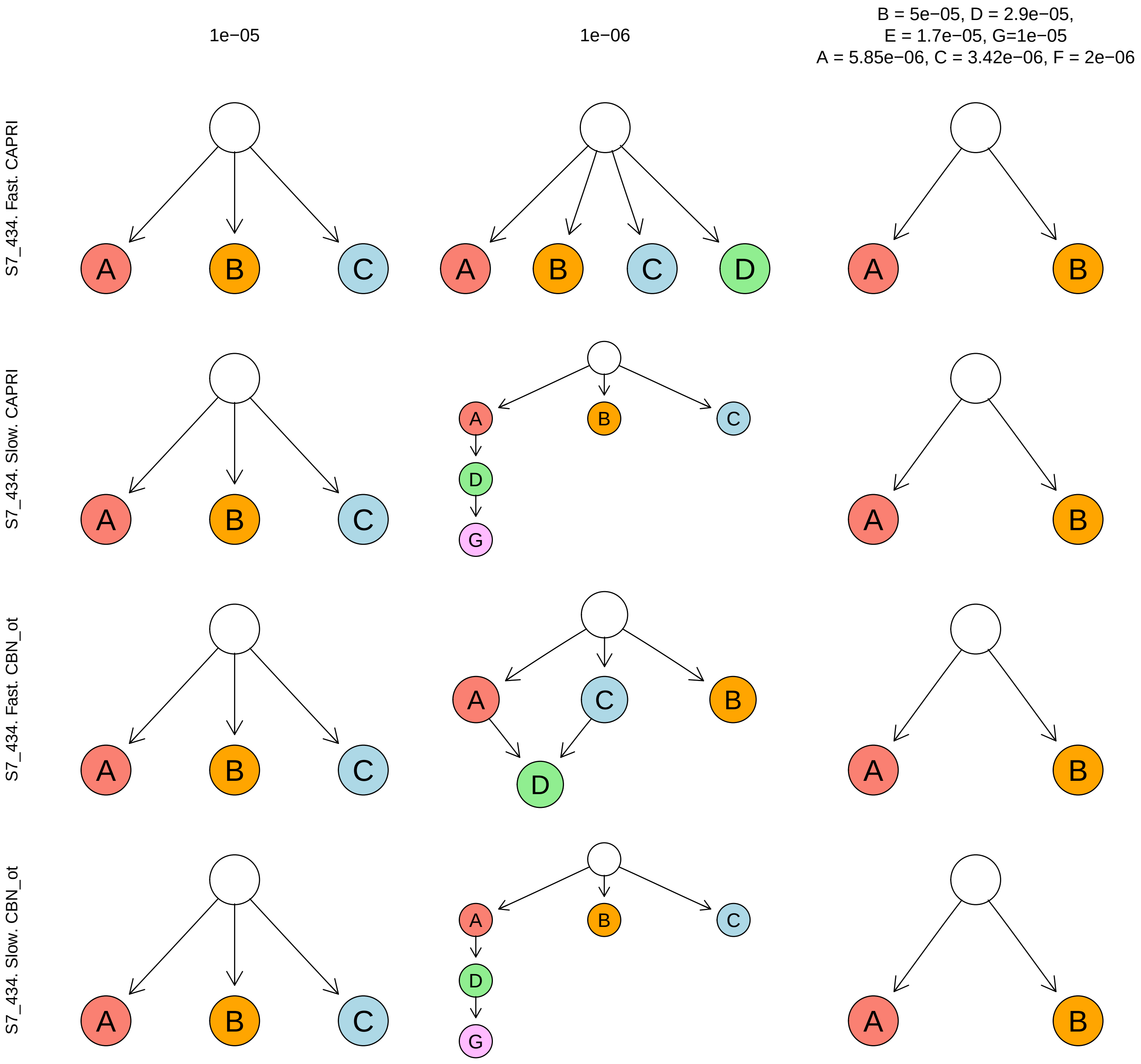

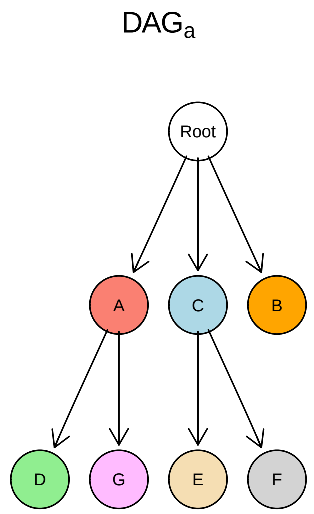

ID	Accessible Genot.	Discrep. DAG_a
S7_435	34	33

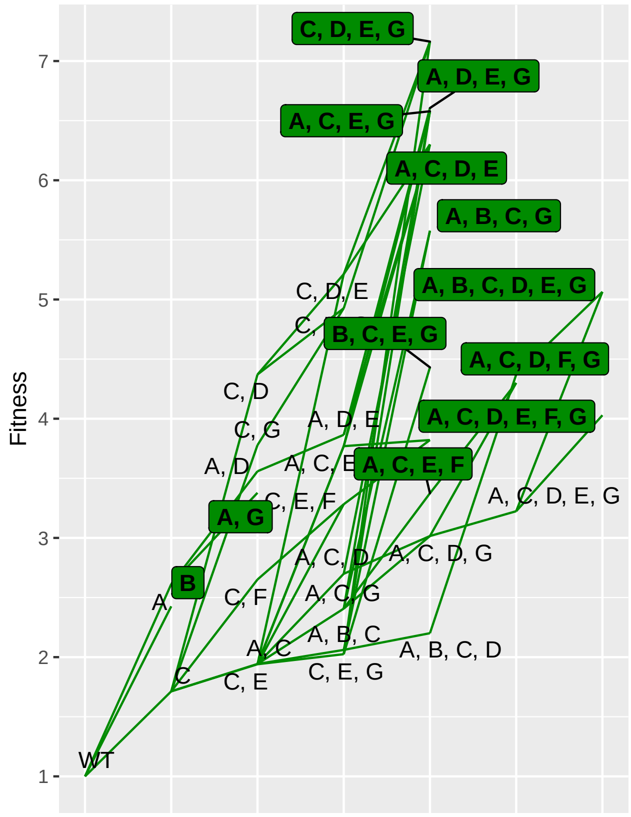

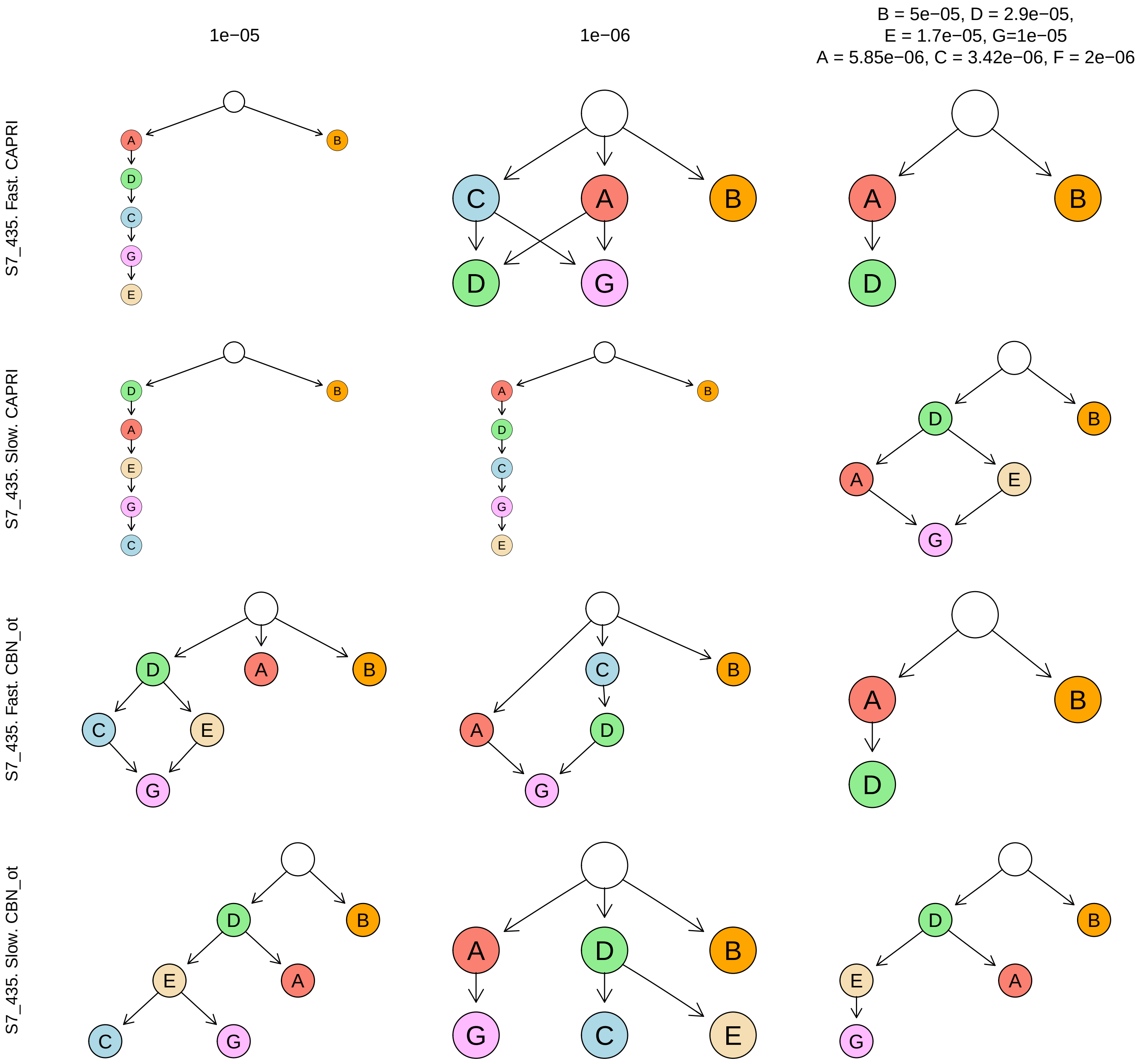

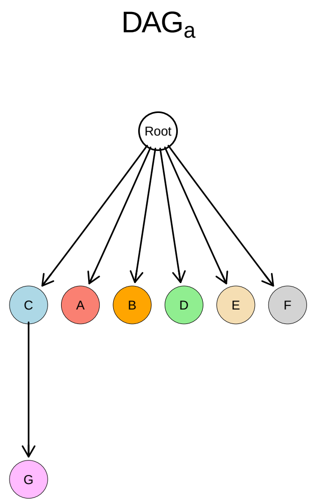

ID	Accessible Genot.	Discrep. DAG_a
S7_436	64	57

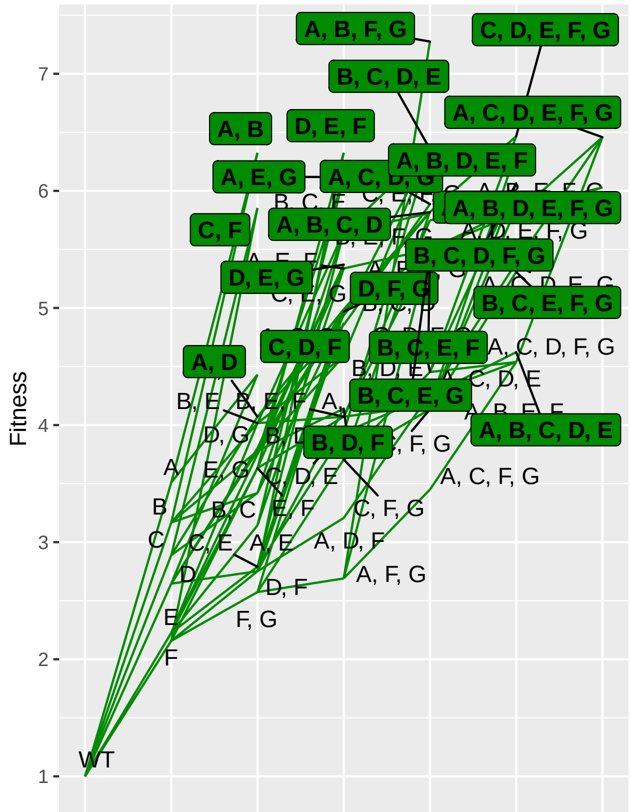

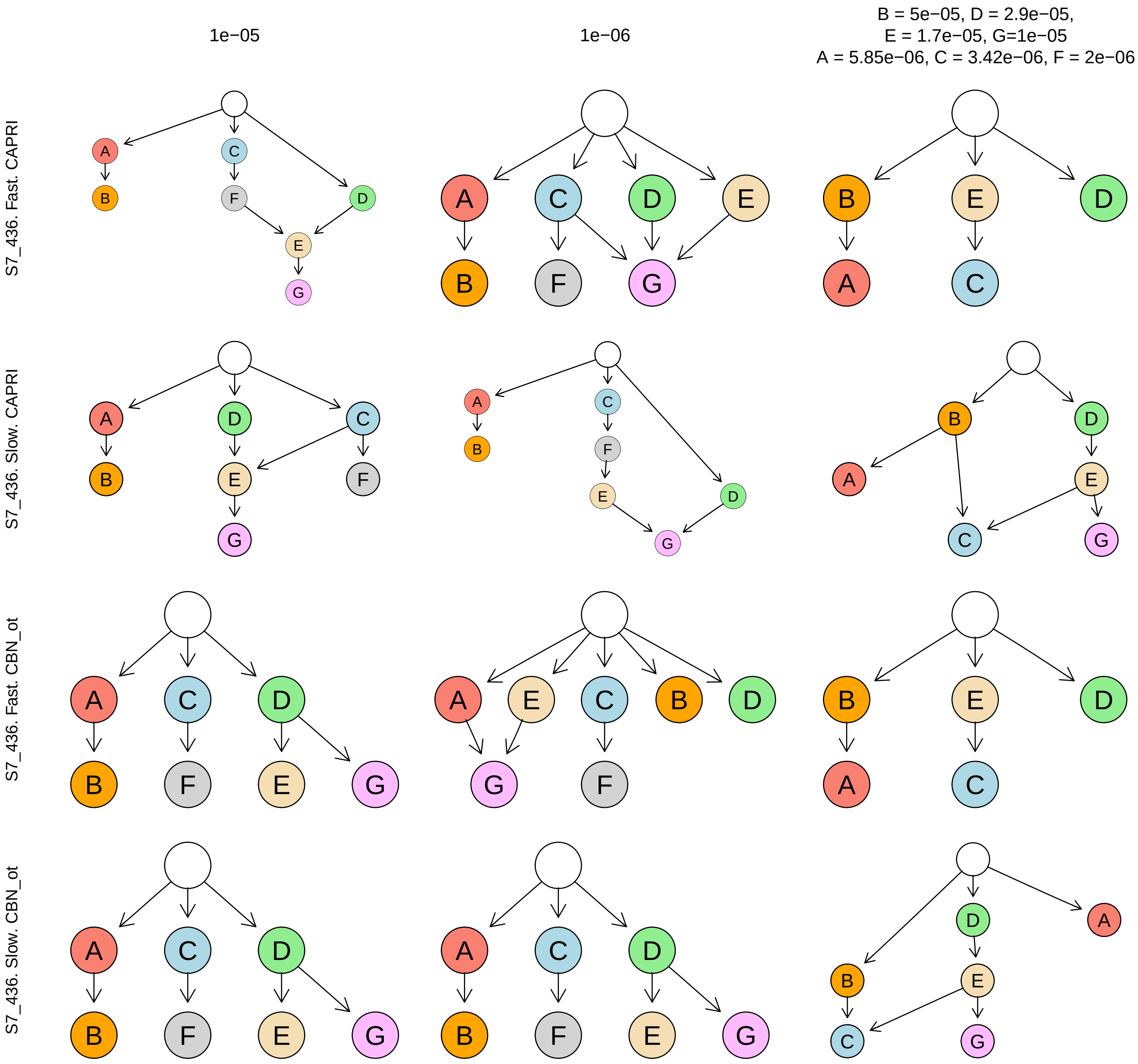

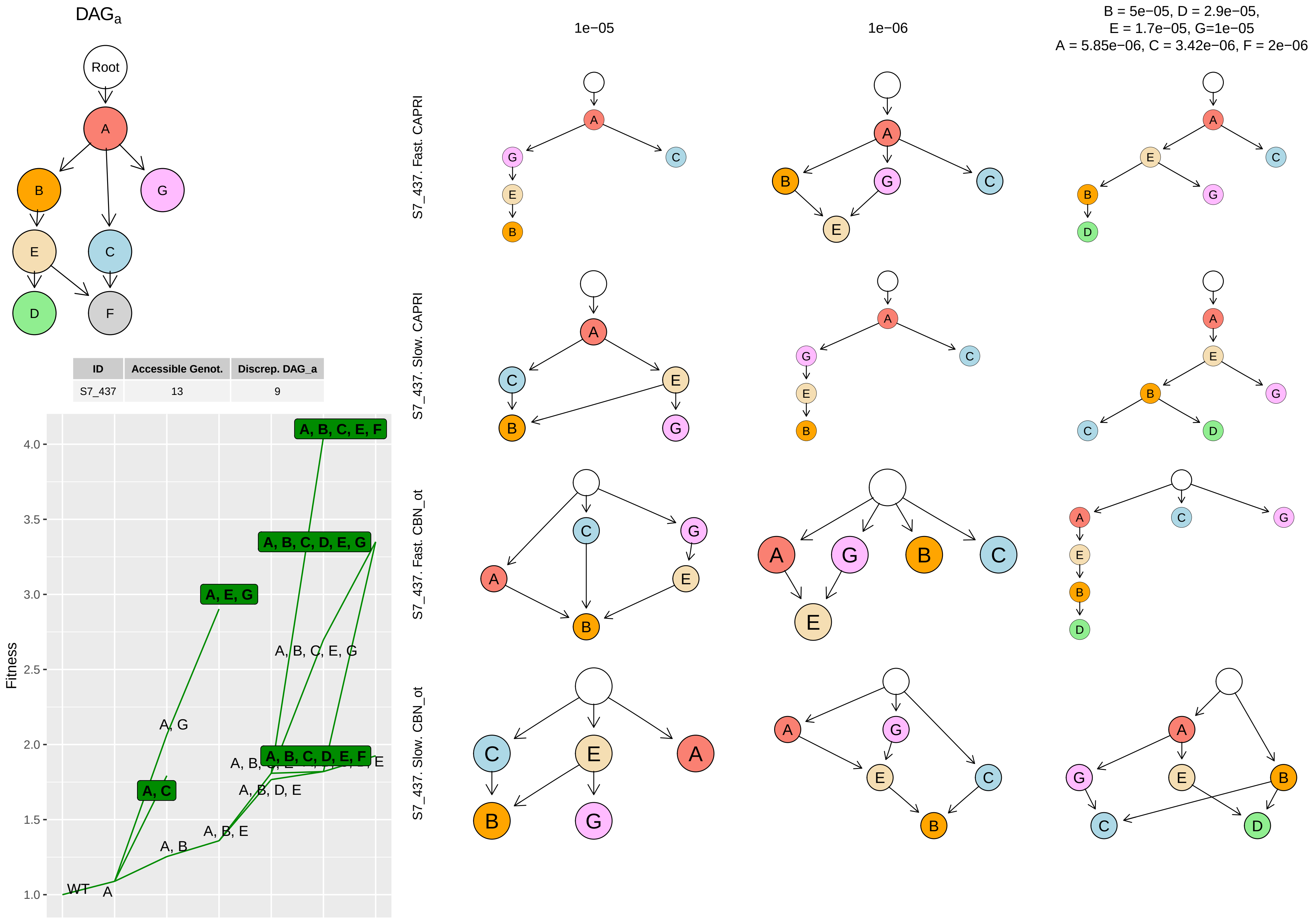

ID	Accessible Genot.	Discrep. DAG_a
S7_437	13	9

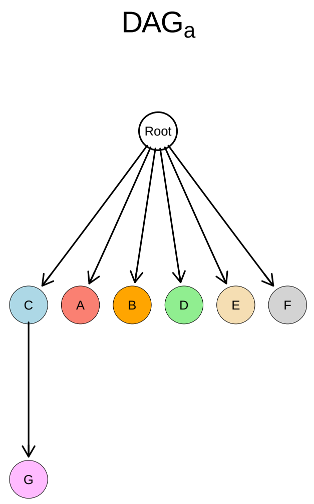

ID	Accessible Genot.	Discrep. DAG_a
S7_438	52	67

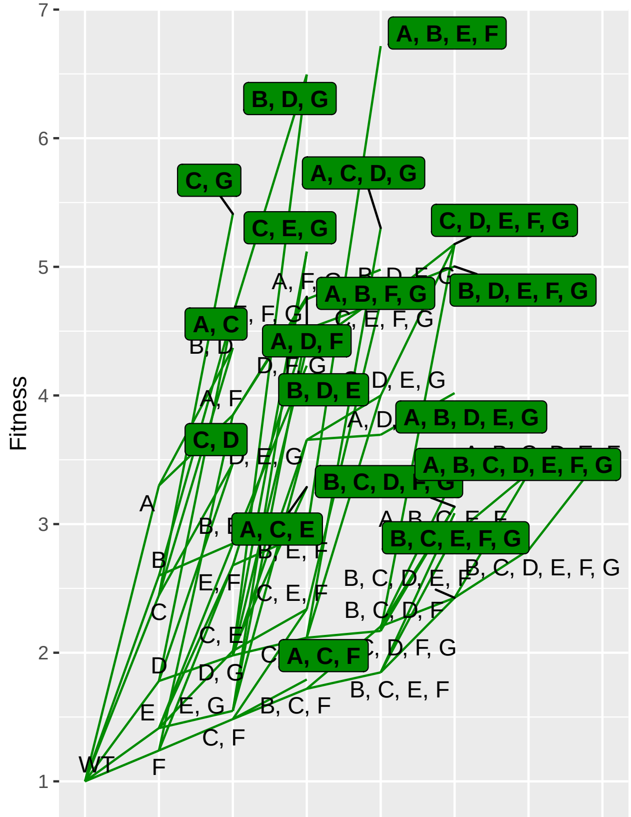

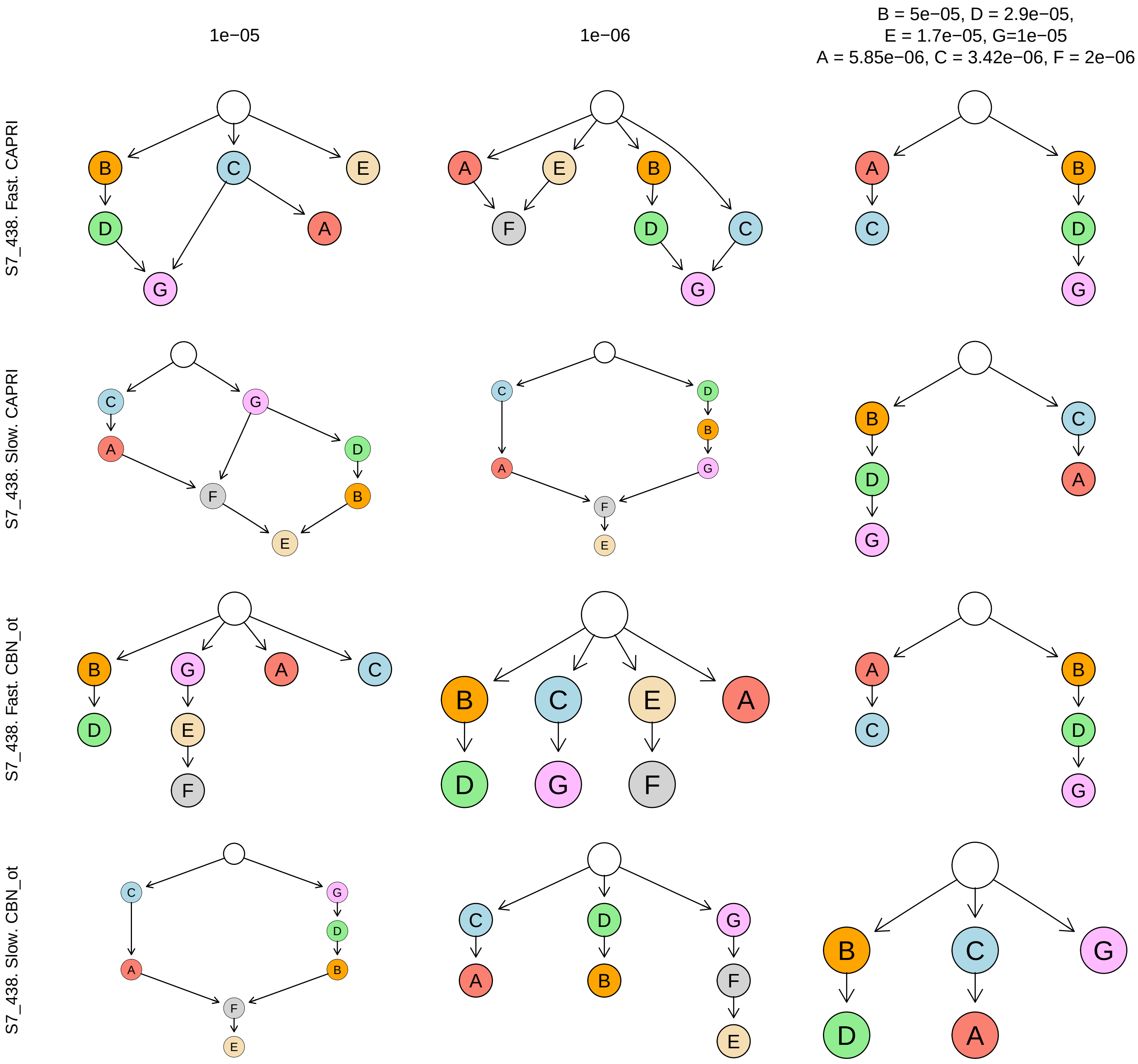

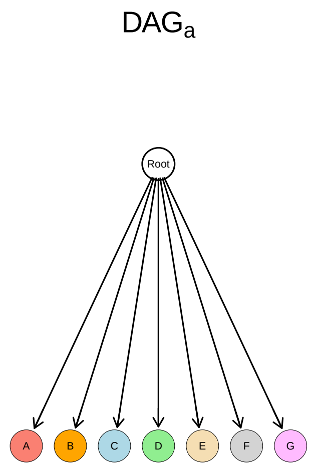

ID	Accessible Genot.	Discrep. DAG_a
S7_439	26	101

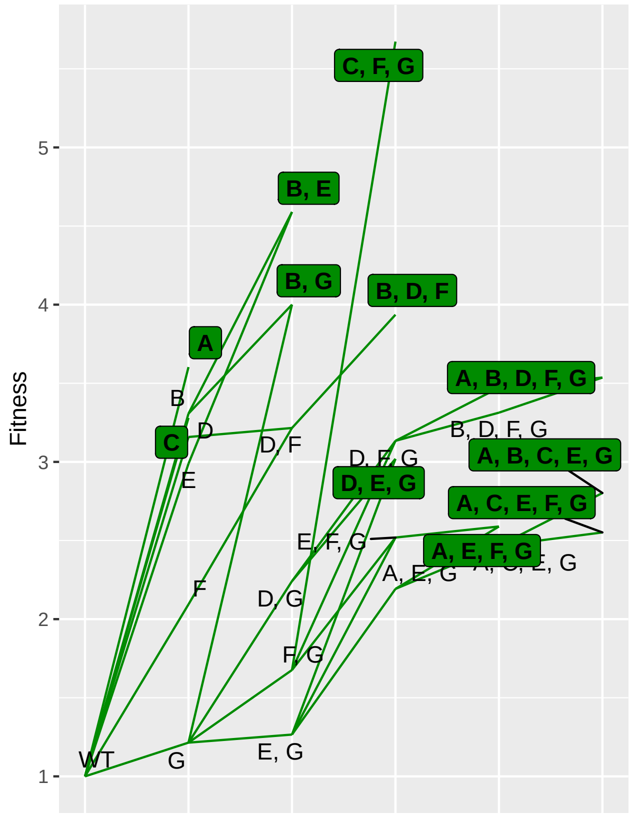

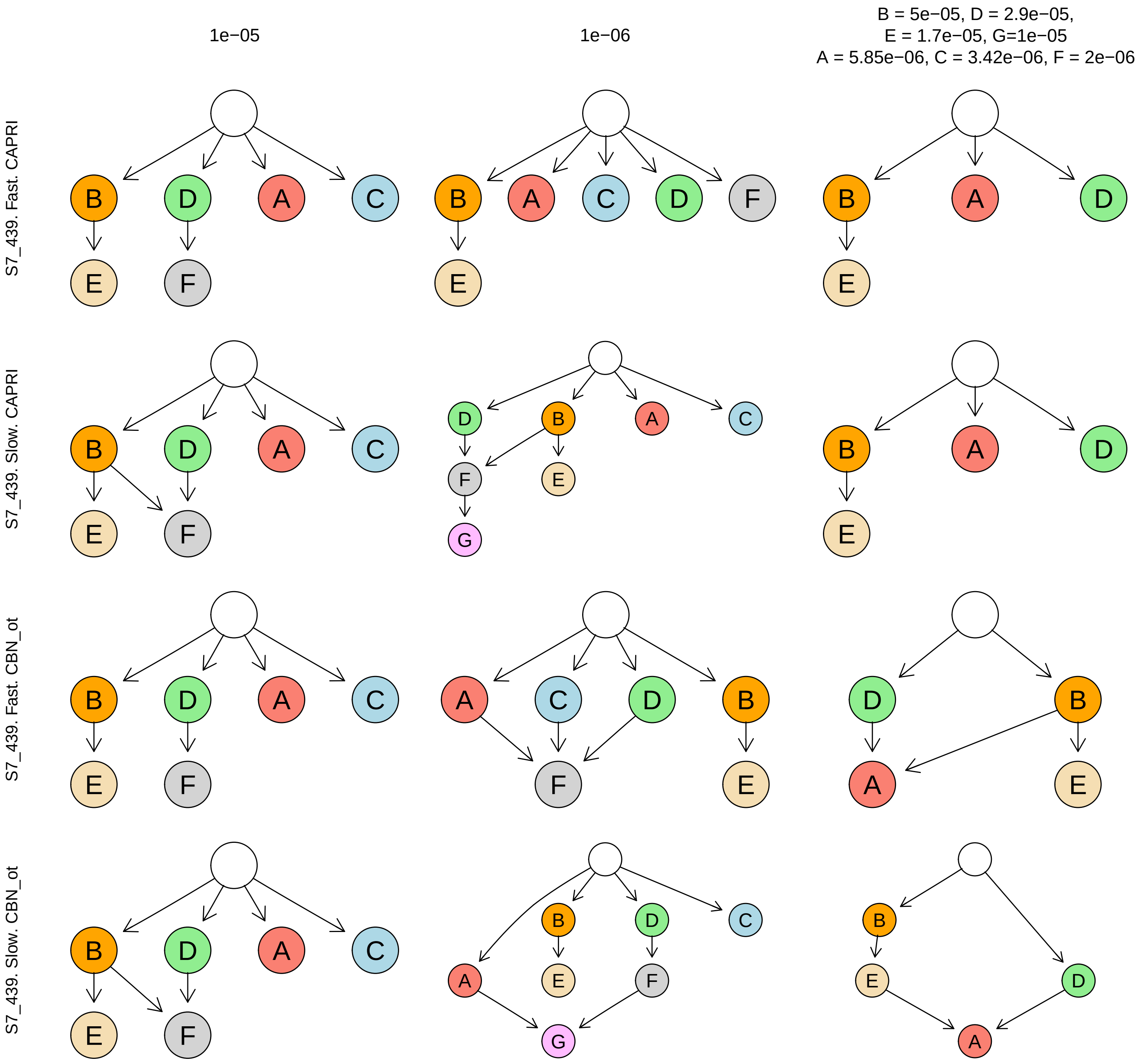

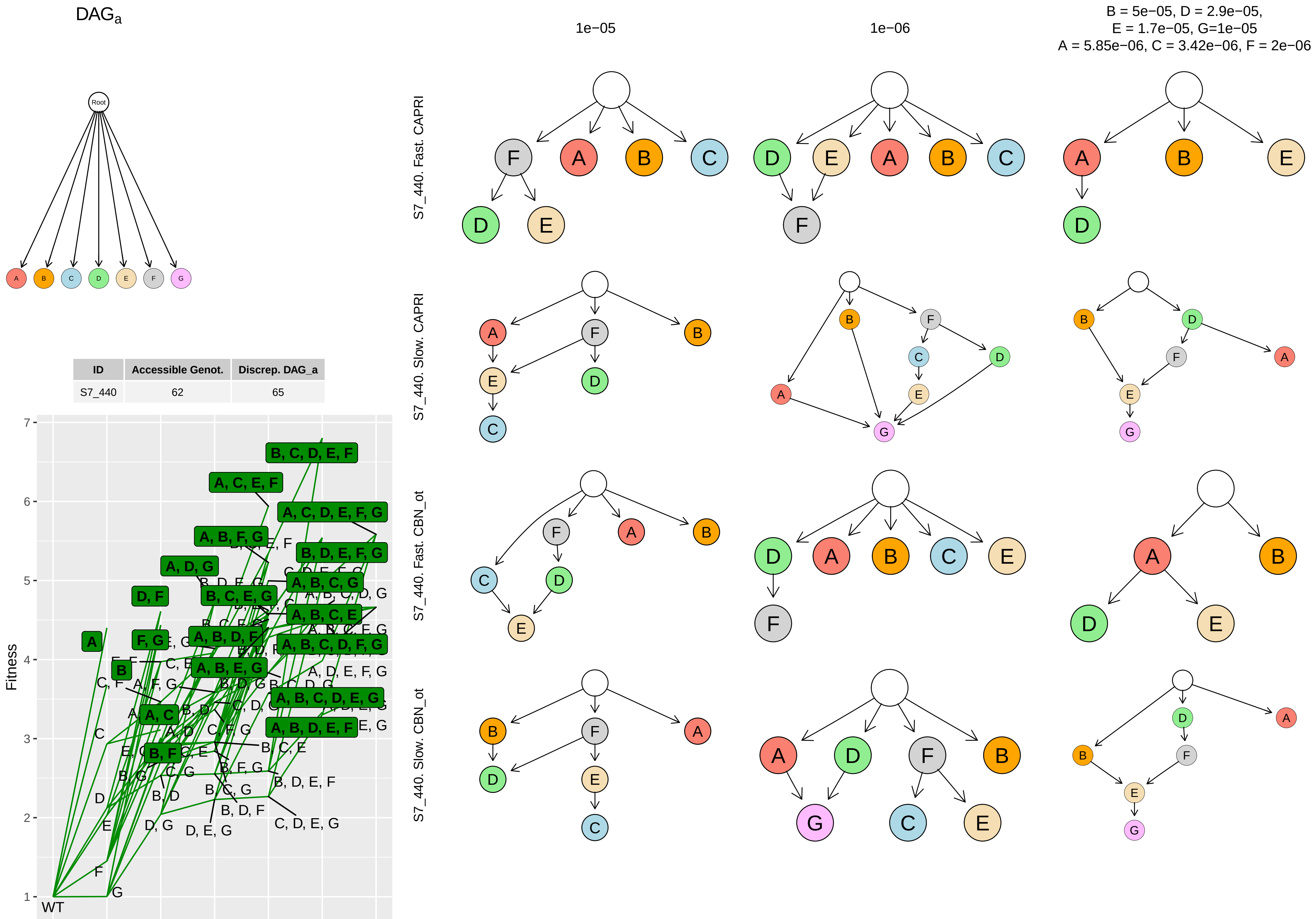
DAGa
1e−05
1e−06
B = 5e−05, D = 2.9e−05,
E = 1.7e−05, G=1e−05
A = 5.85e−06, C = 3.42e−06, F = 2e−06
S7_440. Fast. CAPRI
S7_440. Slow. CAPRI
S7_440. Fast. CBN_ot
S7_440. Slow. CBN_ot
Root
ID
Accessible Genot.
Discrep. DAG_a
S7_440
62
65
Fitness
WT

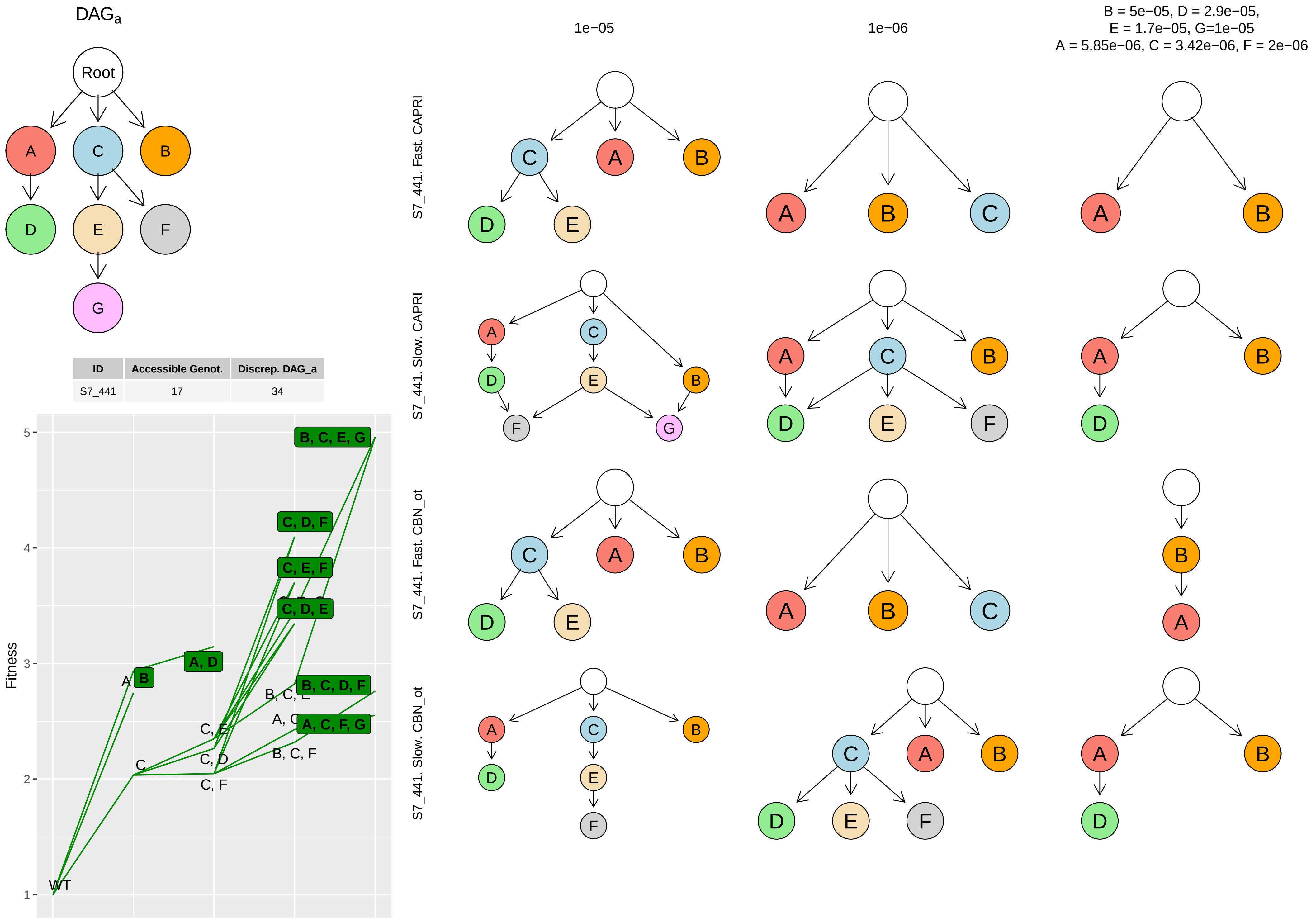

ID	Accessible Genot.	Discrep. DAG_a
S7_441	17	34

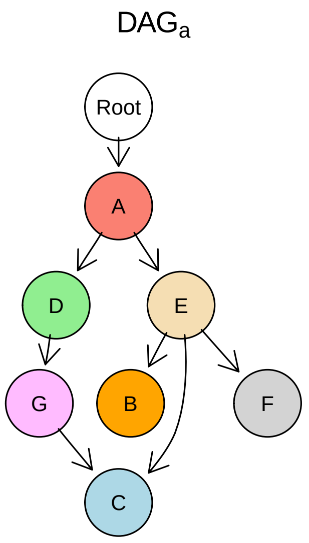

ID	Accessible Genot.	Discrep. DAG_a
S7_442	11	12

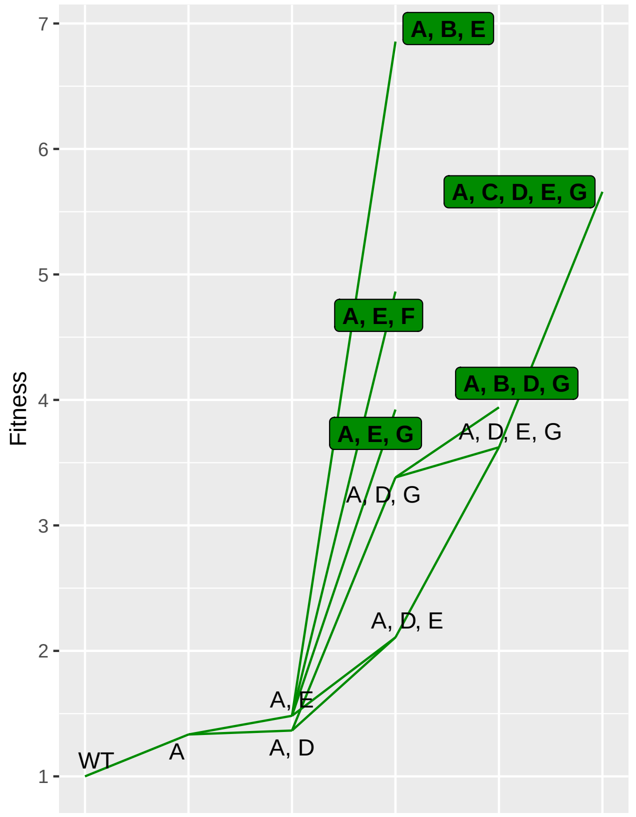

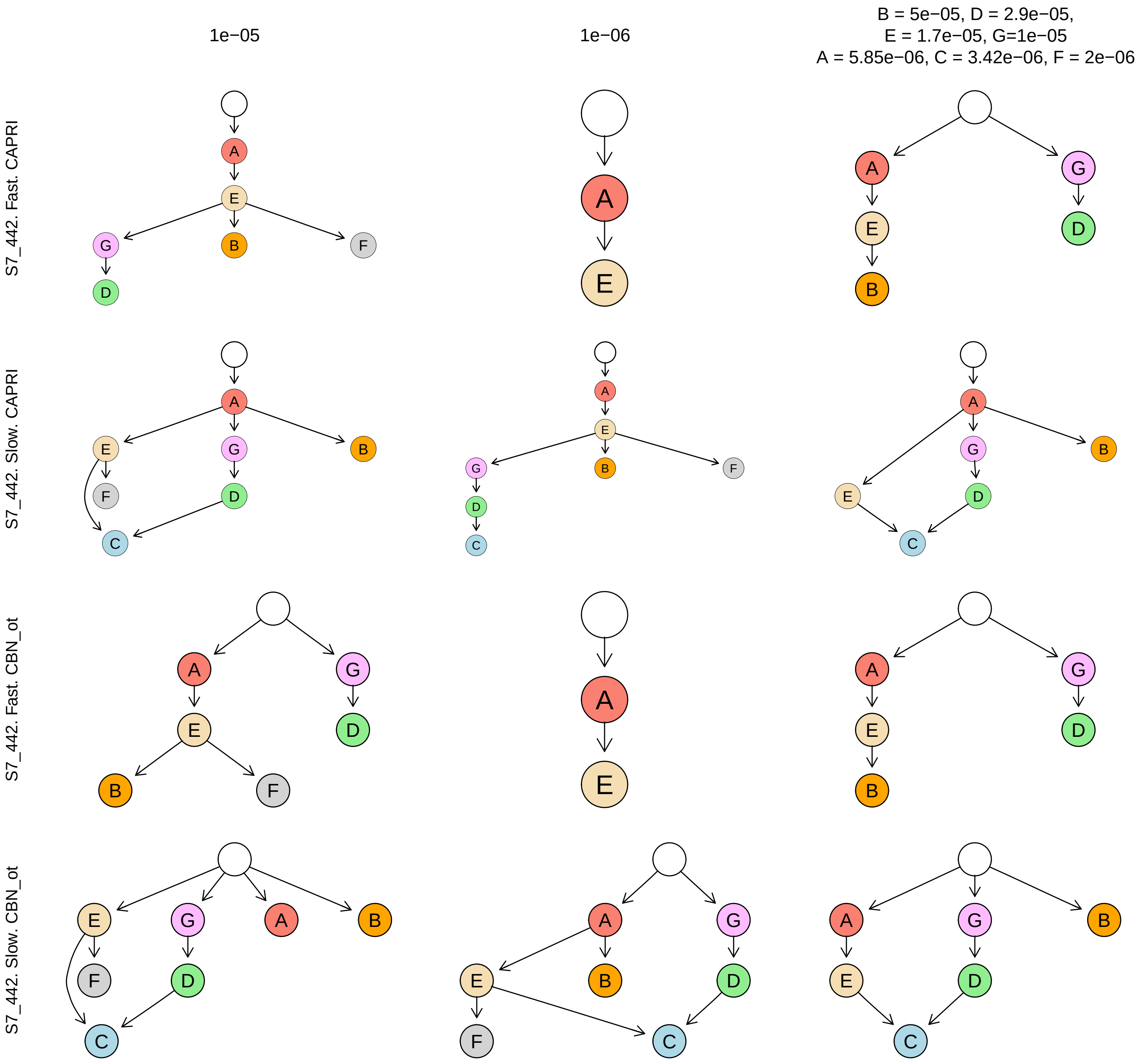

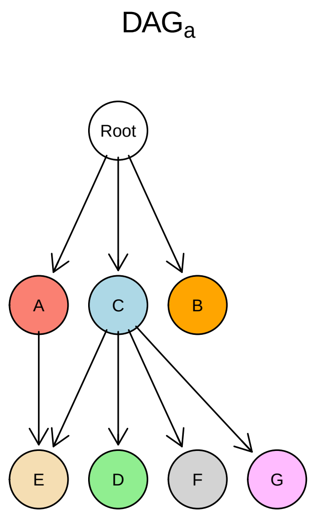

ID	Accessible Genot.	Discrep. DAG_a
S7_443	20	31

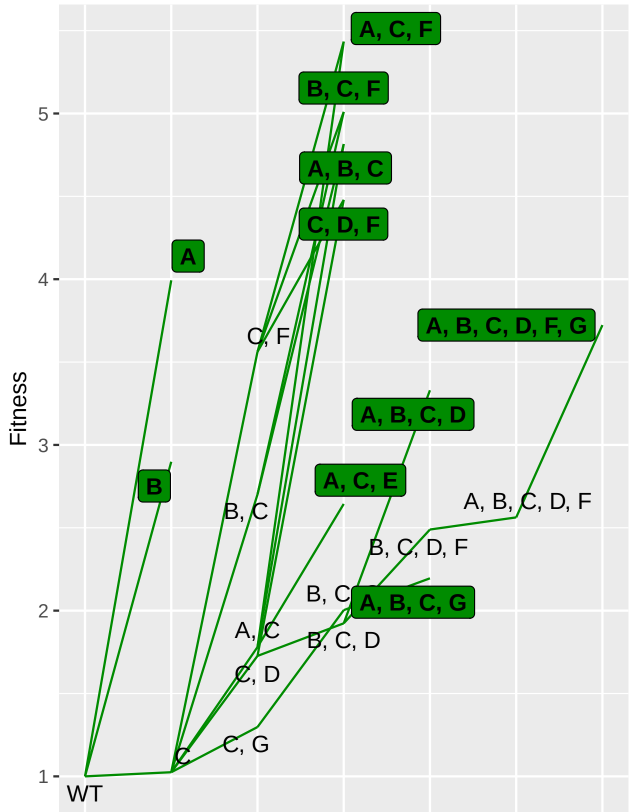

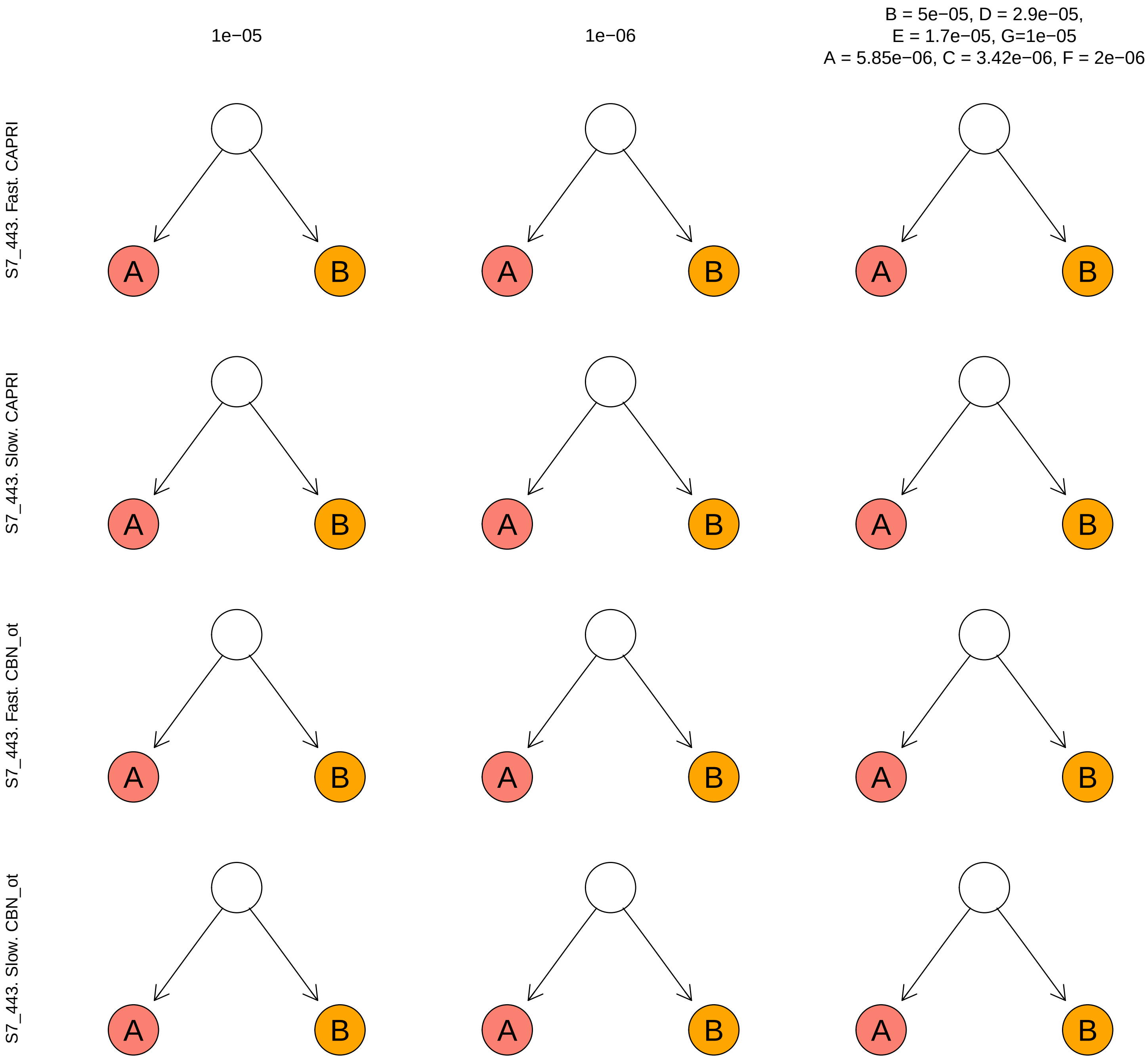

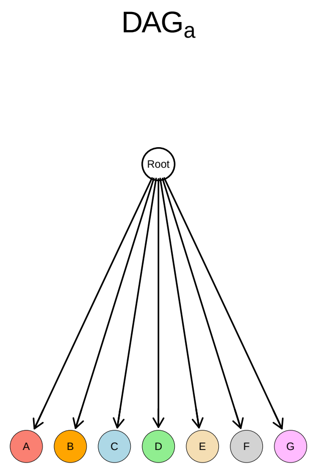

ID	Accessible Genot.	Discrep. DAG_a
S7_444	53	74

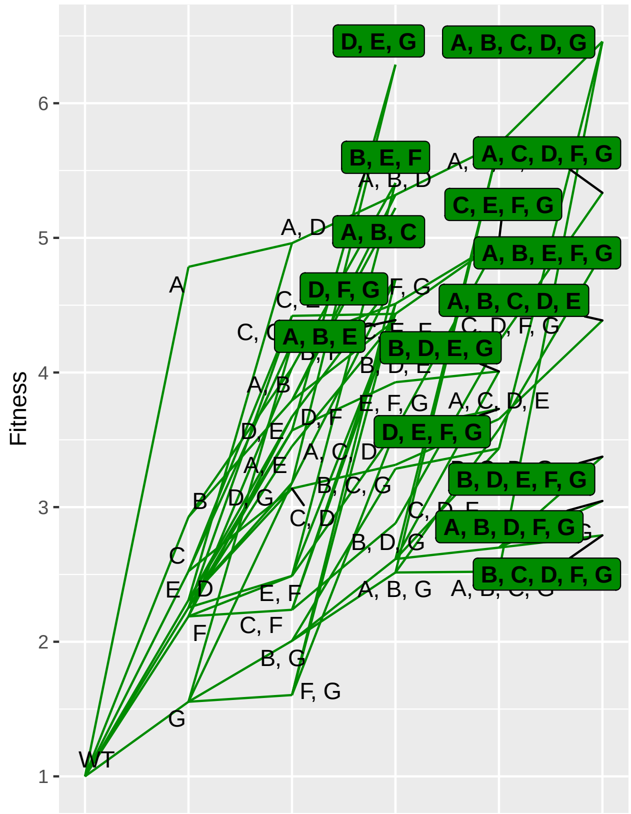

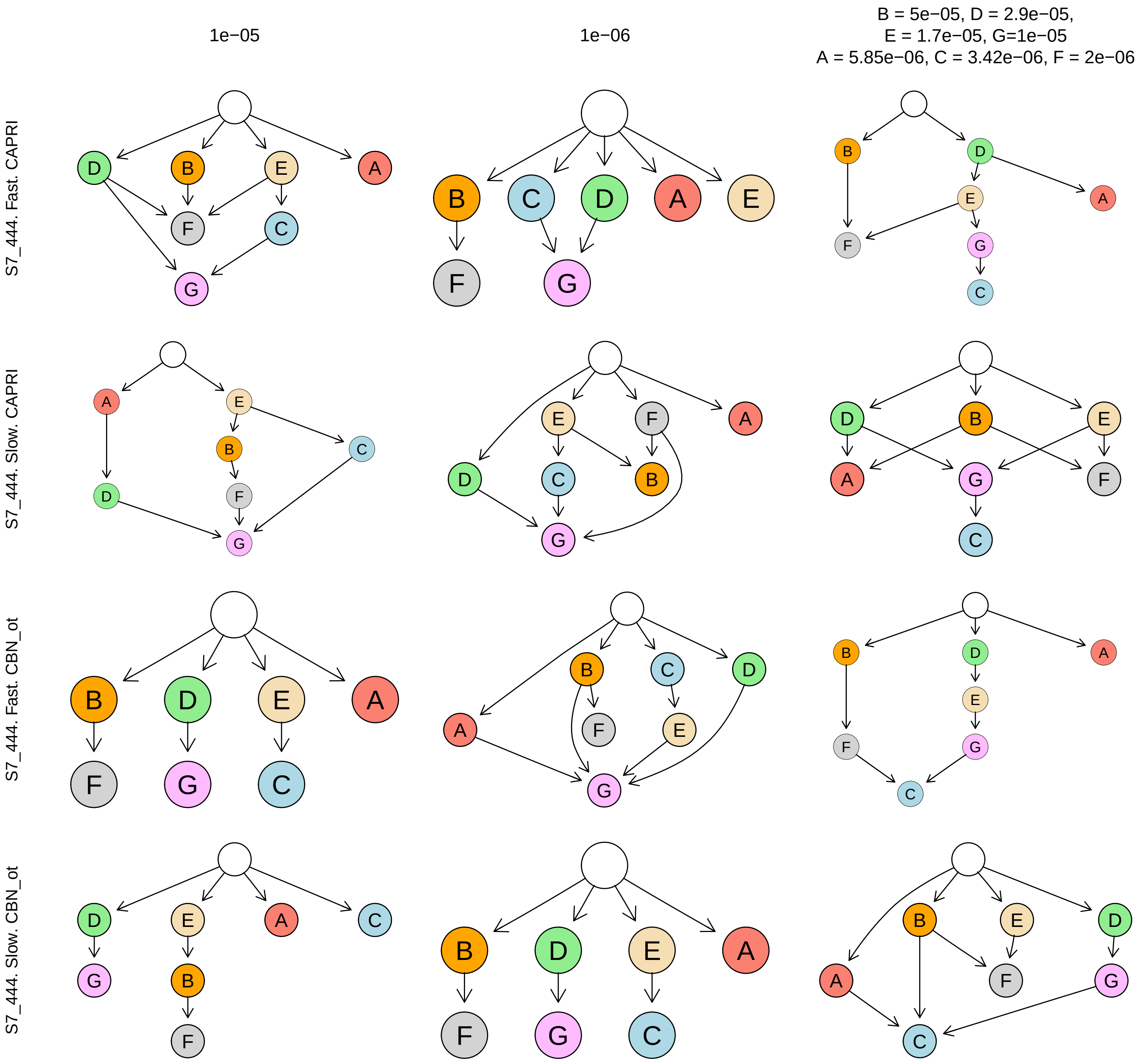

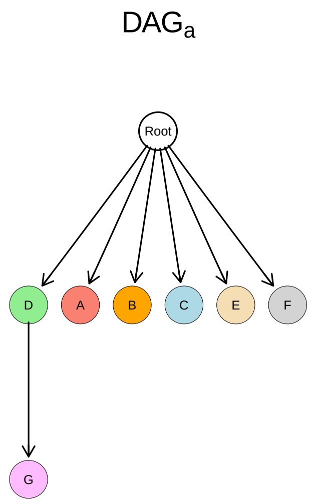

ID	Accessible Genot.	Discrep. DAG_a
S7_445	23	74

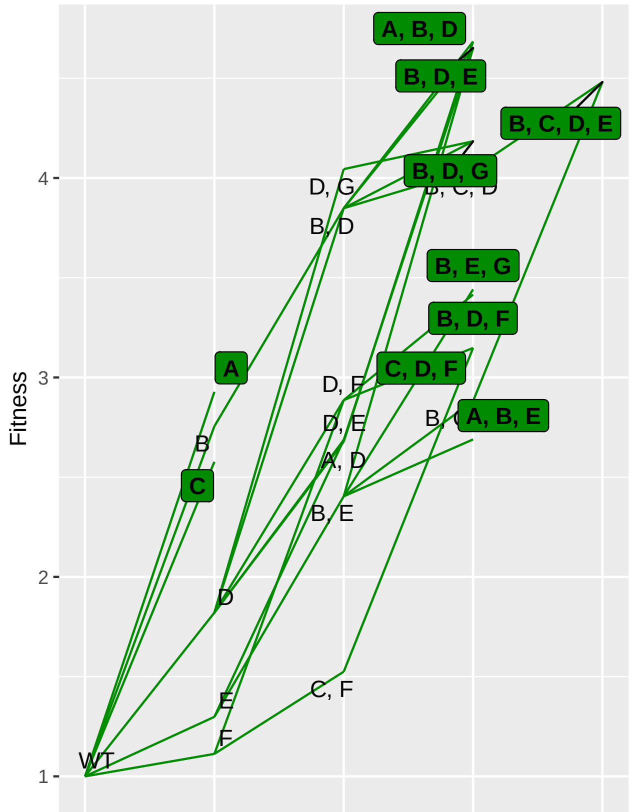

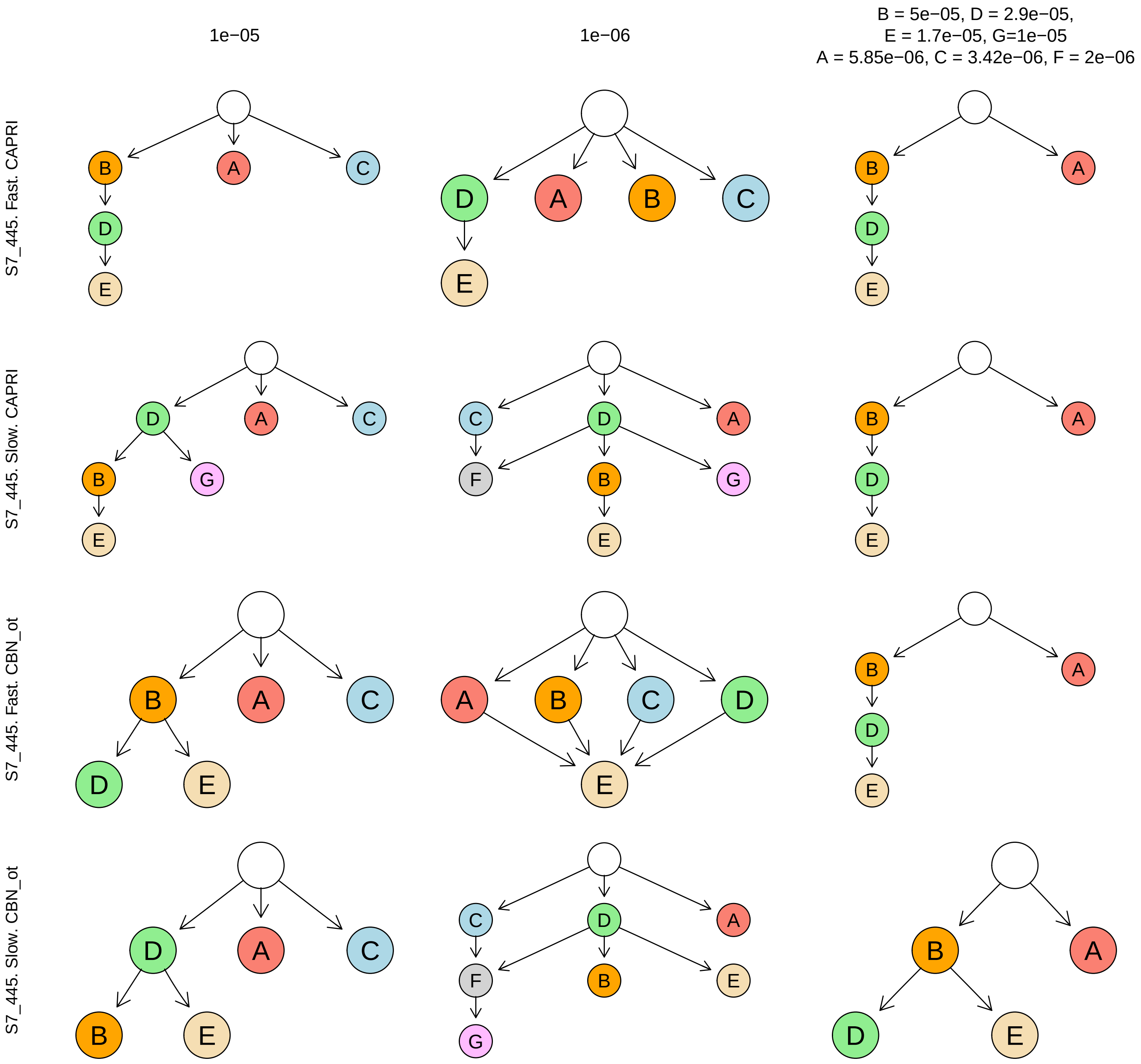

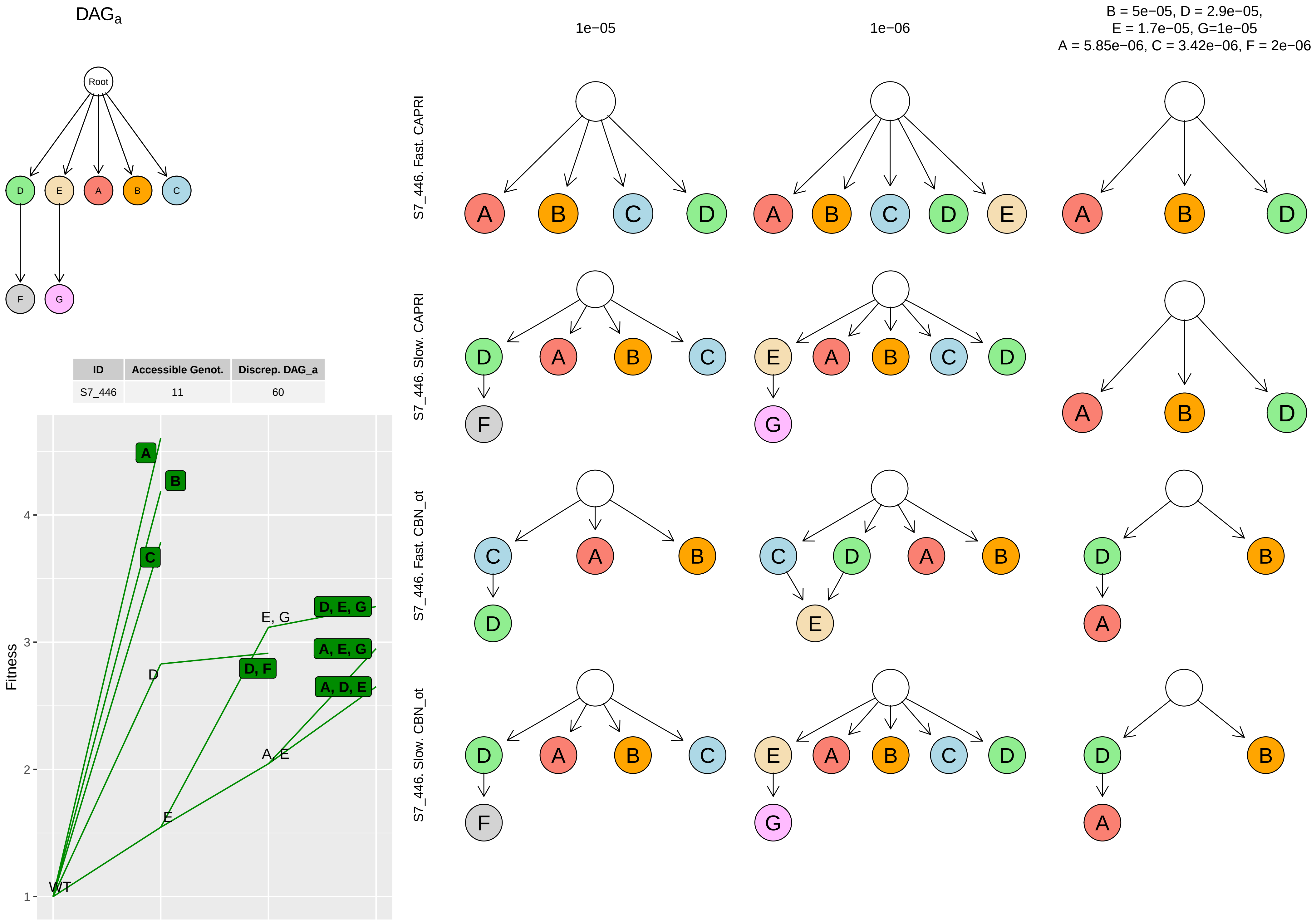

ID	Accessible Genot.	Discrep. DAG_a
S7_446	11	60

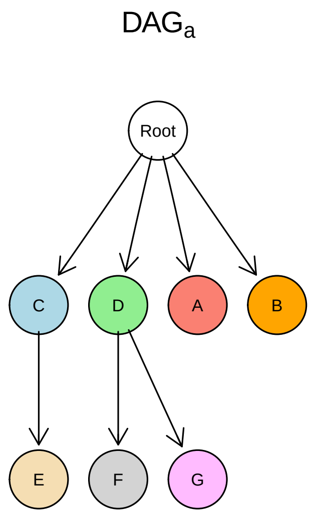

ID	Accessible Genot.	Discrep. DAG_a
S7_447	15	46

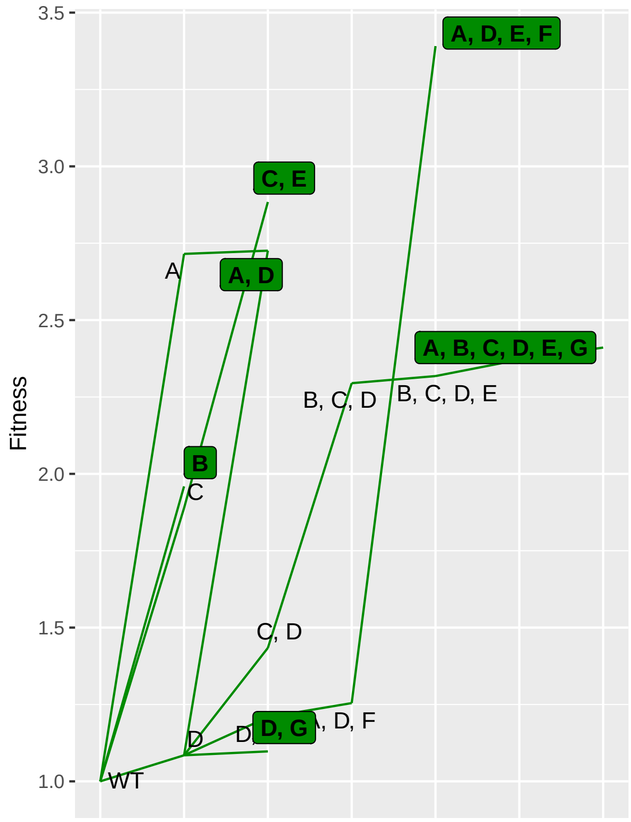

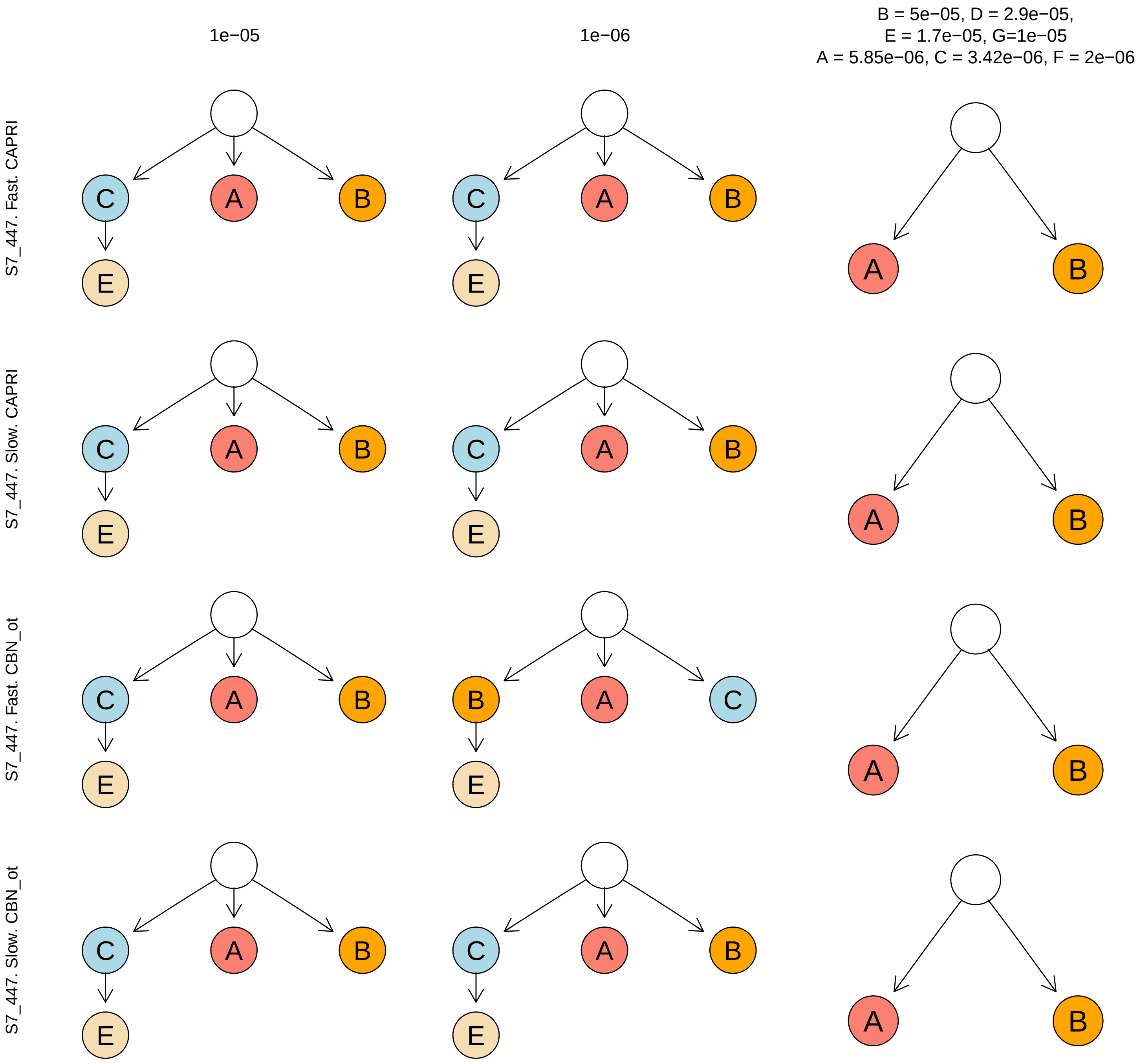

DAG$_a$

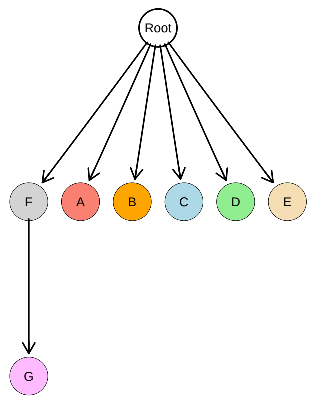

ID	Accessible Genot.	Discrep. DAG_a
S7_448	19	76

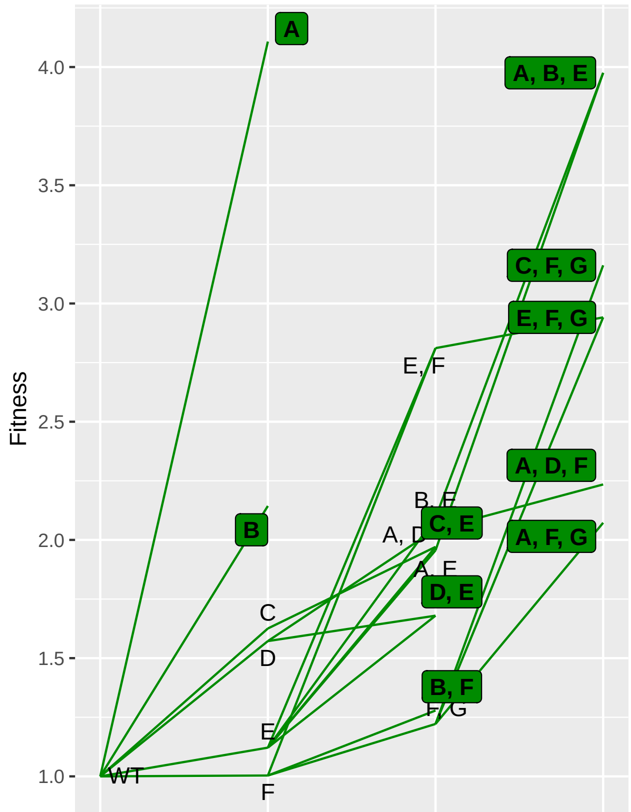

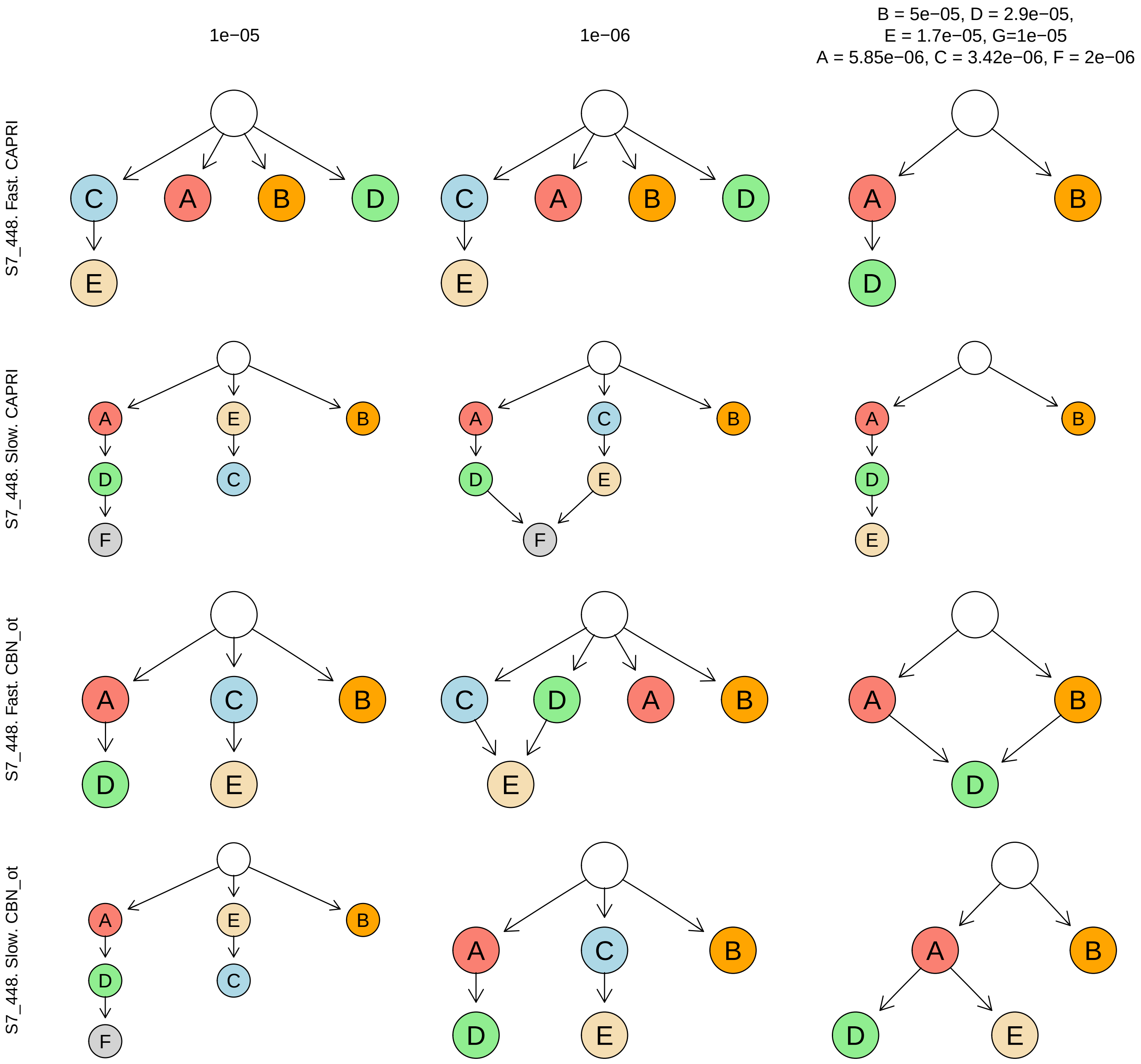

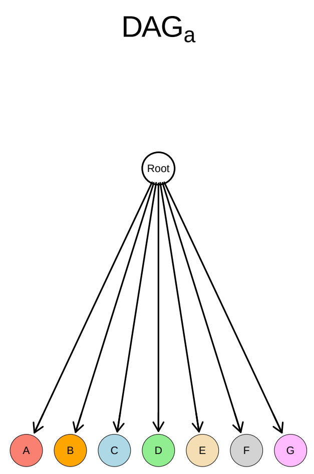

ID	Accessible Genot.	Discrep. DAG_a
S7_449	44	83

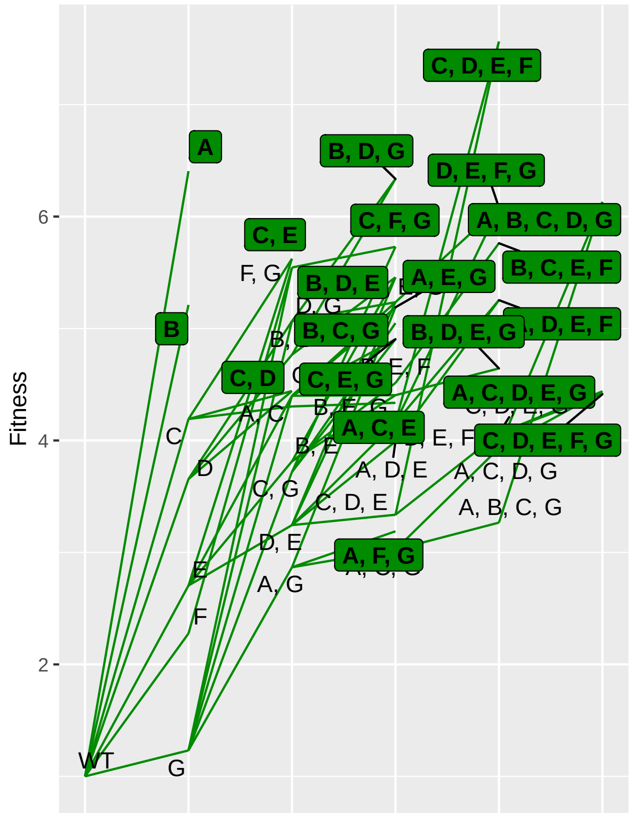

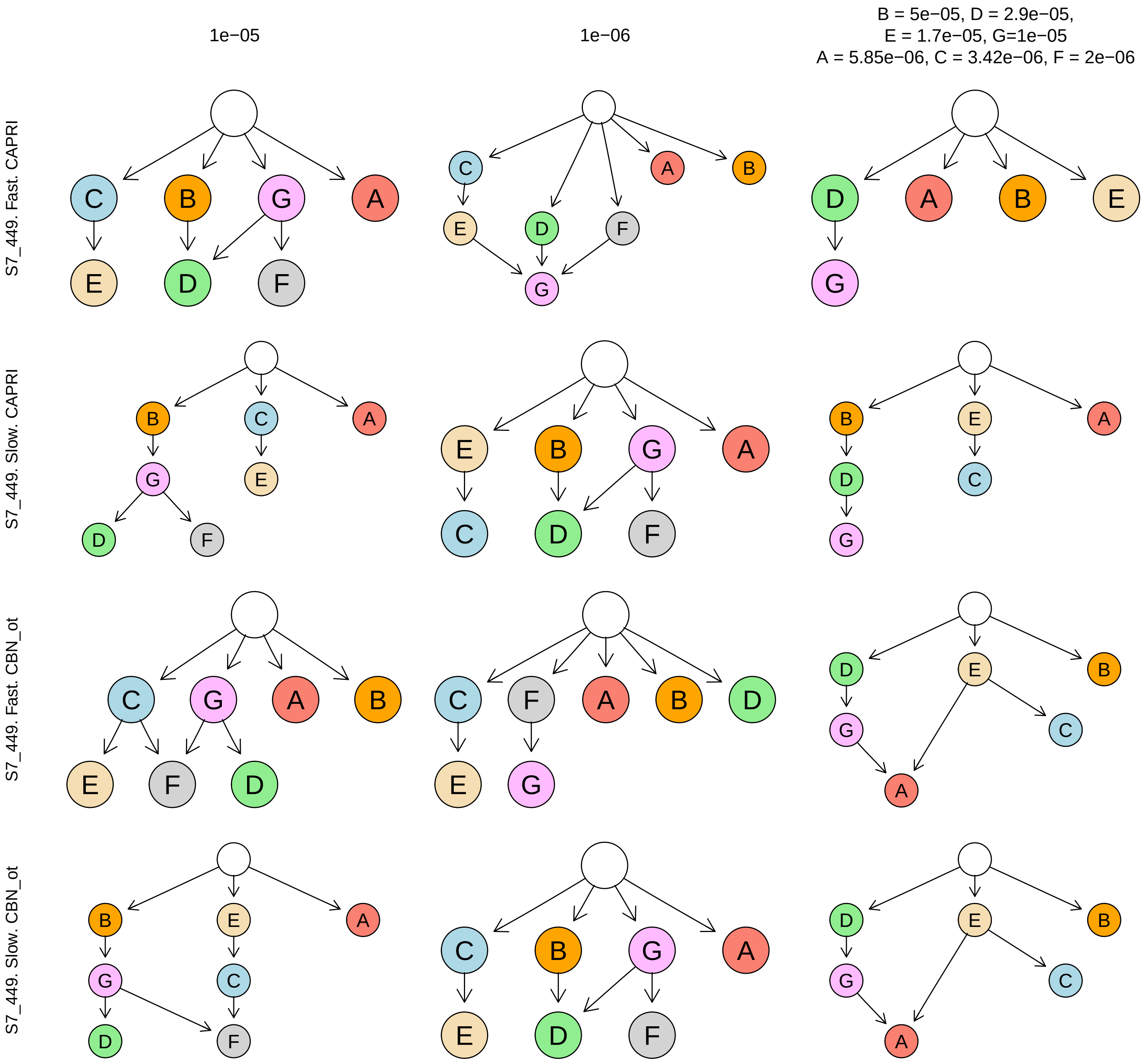

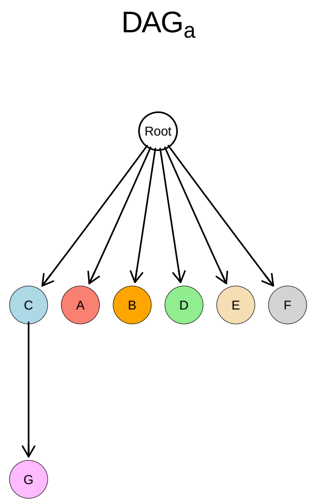

ID	Accessible Genot.	Discrep. DAG_a
S7_450	47	62

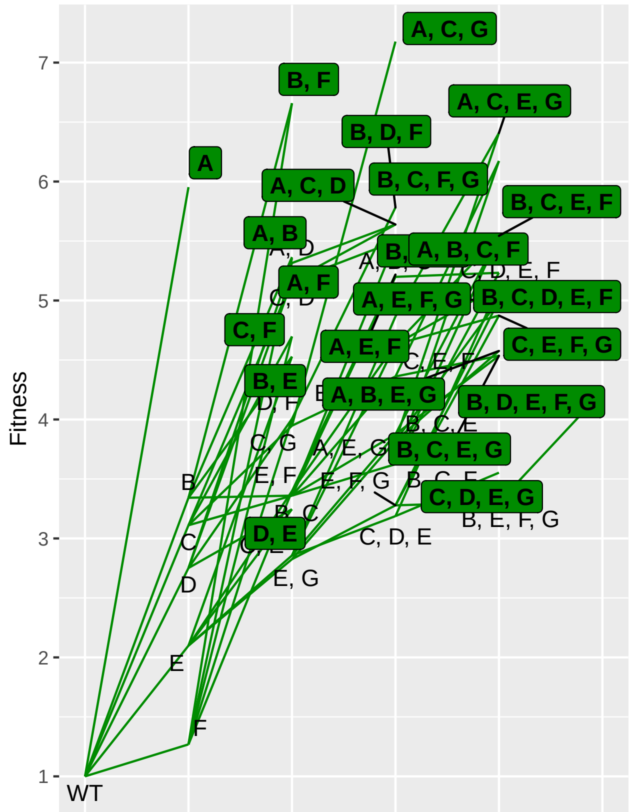

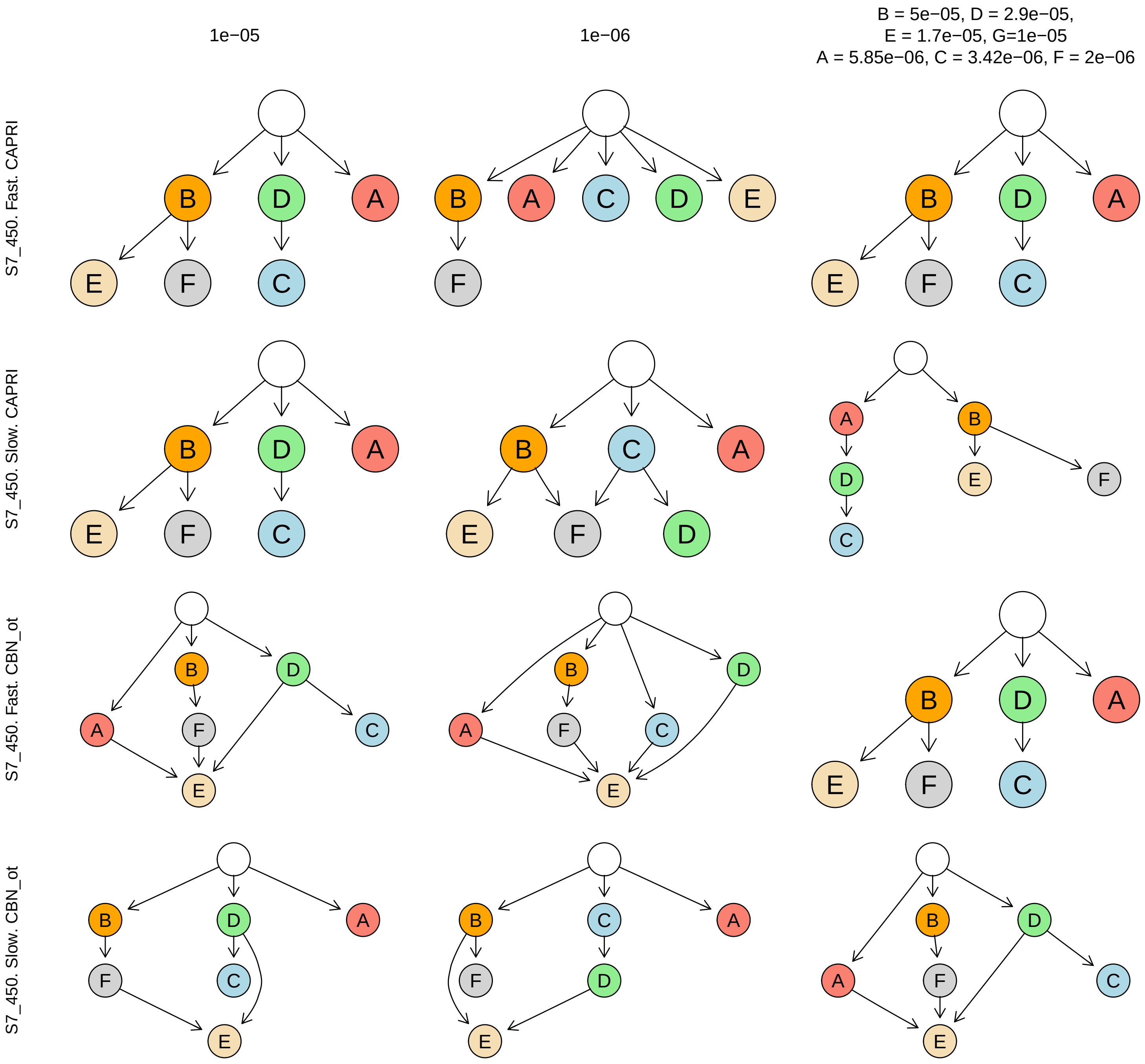

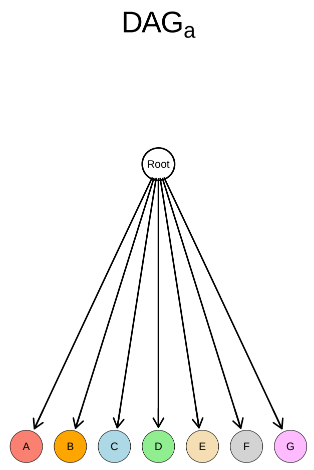

ID	Accessible Genot.	Discrep. DAG_a
S7_451	69	58

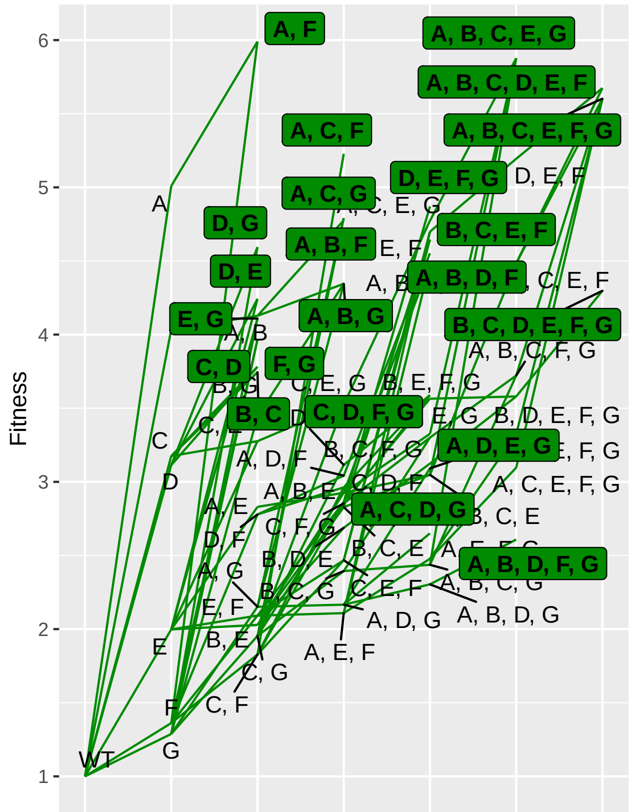

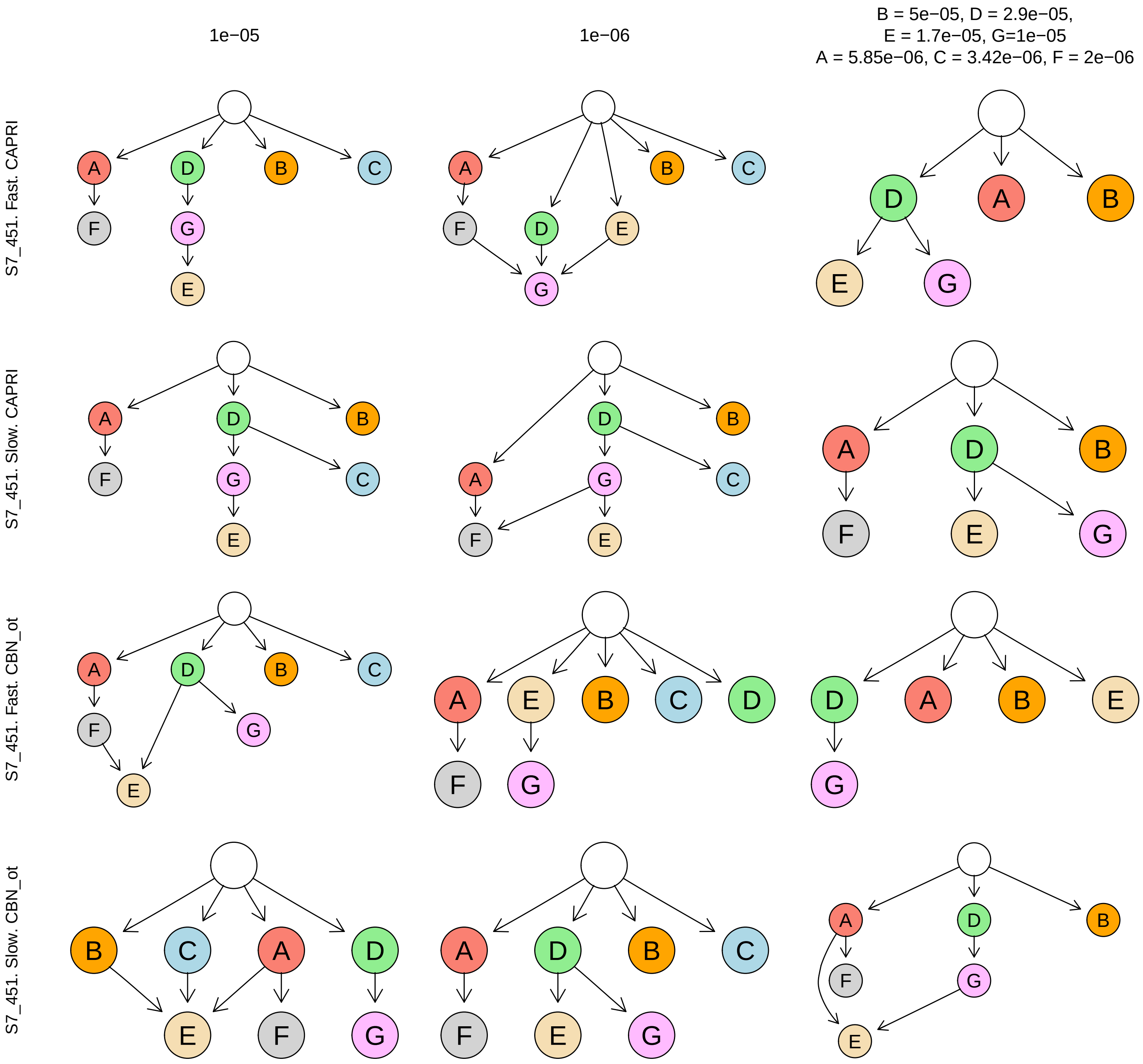

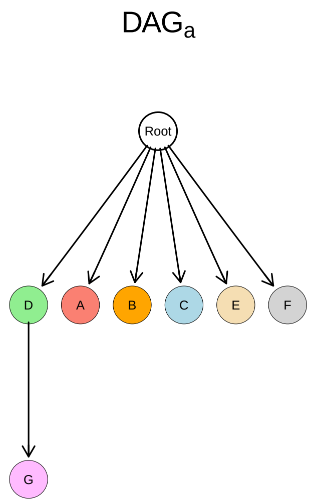

ID	Accessible Genot.	Discrep. DAG_a
S7_452	47	62

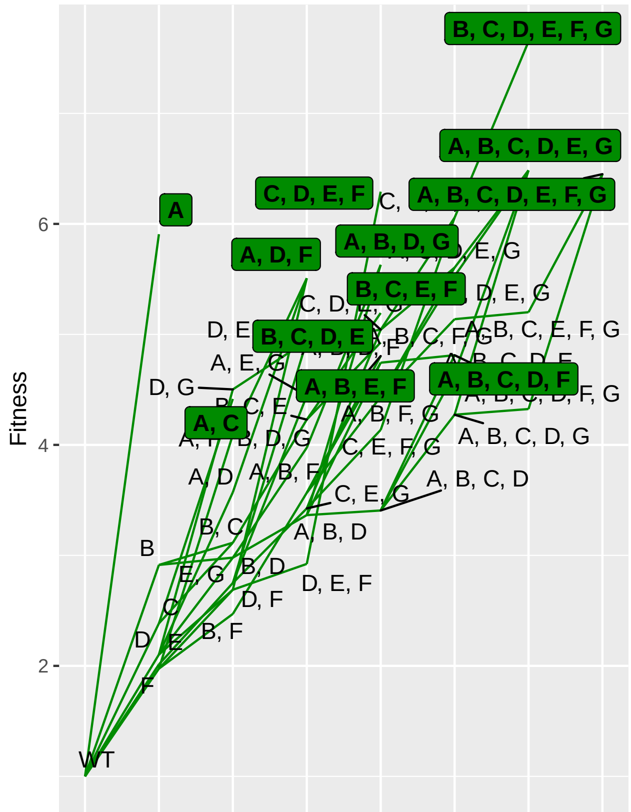

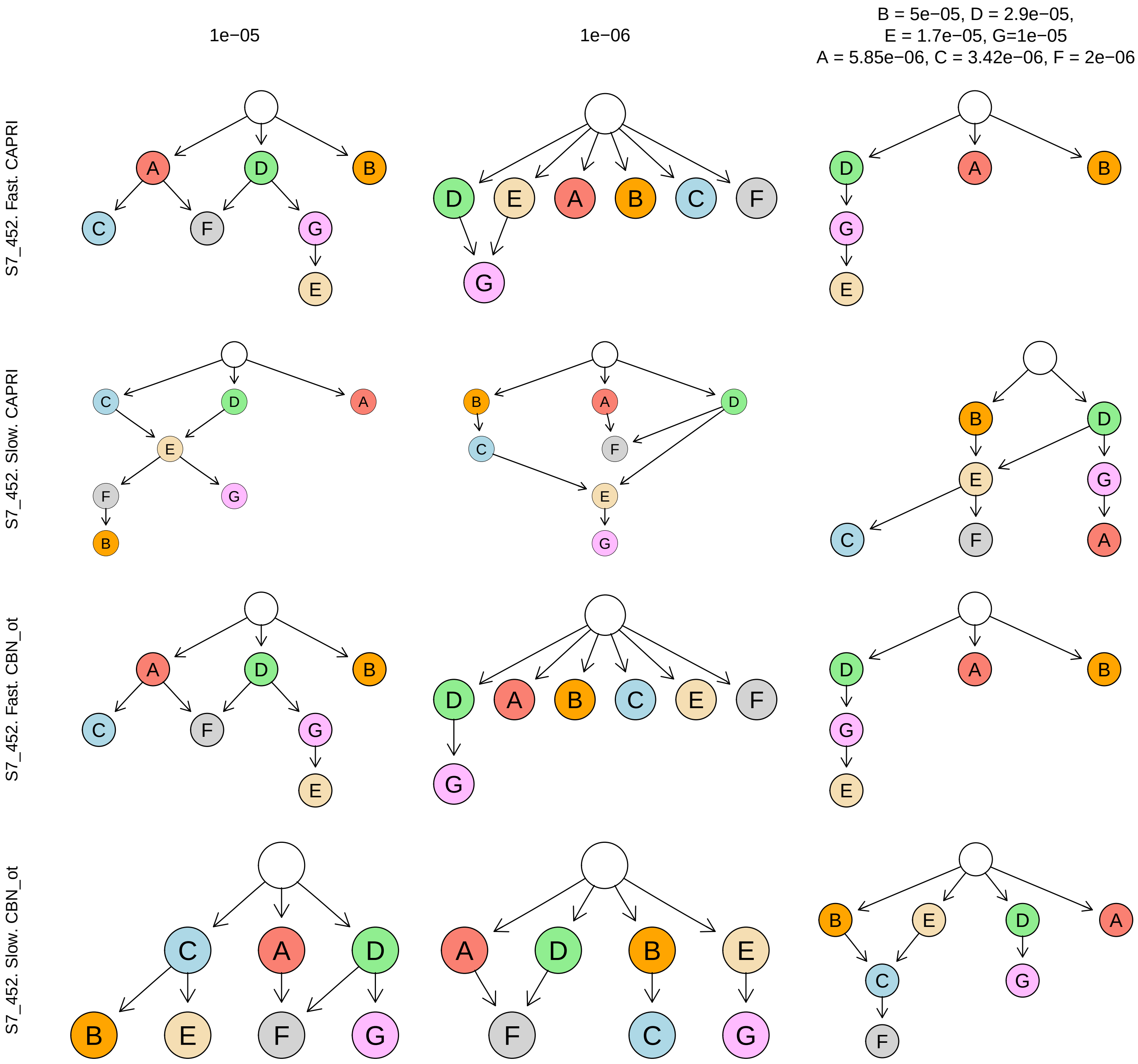

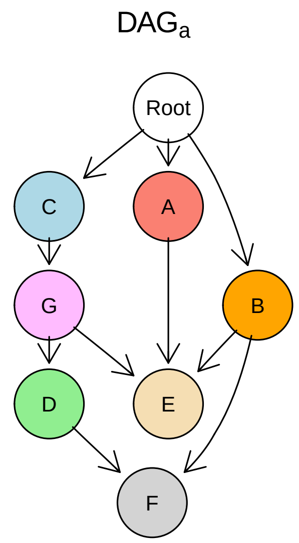

ID	Accessible Genot.	Discrep. DAG_a
S7_453	12	8

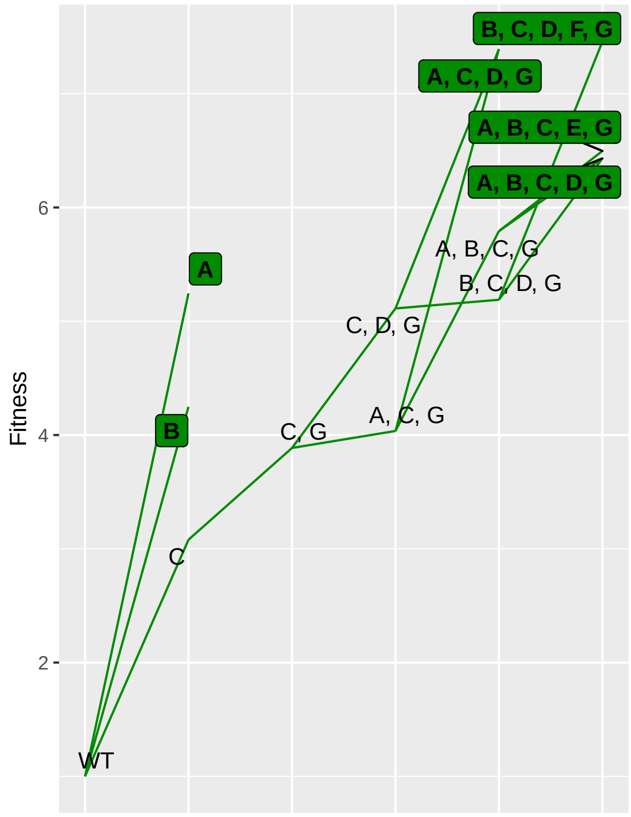

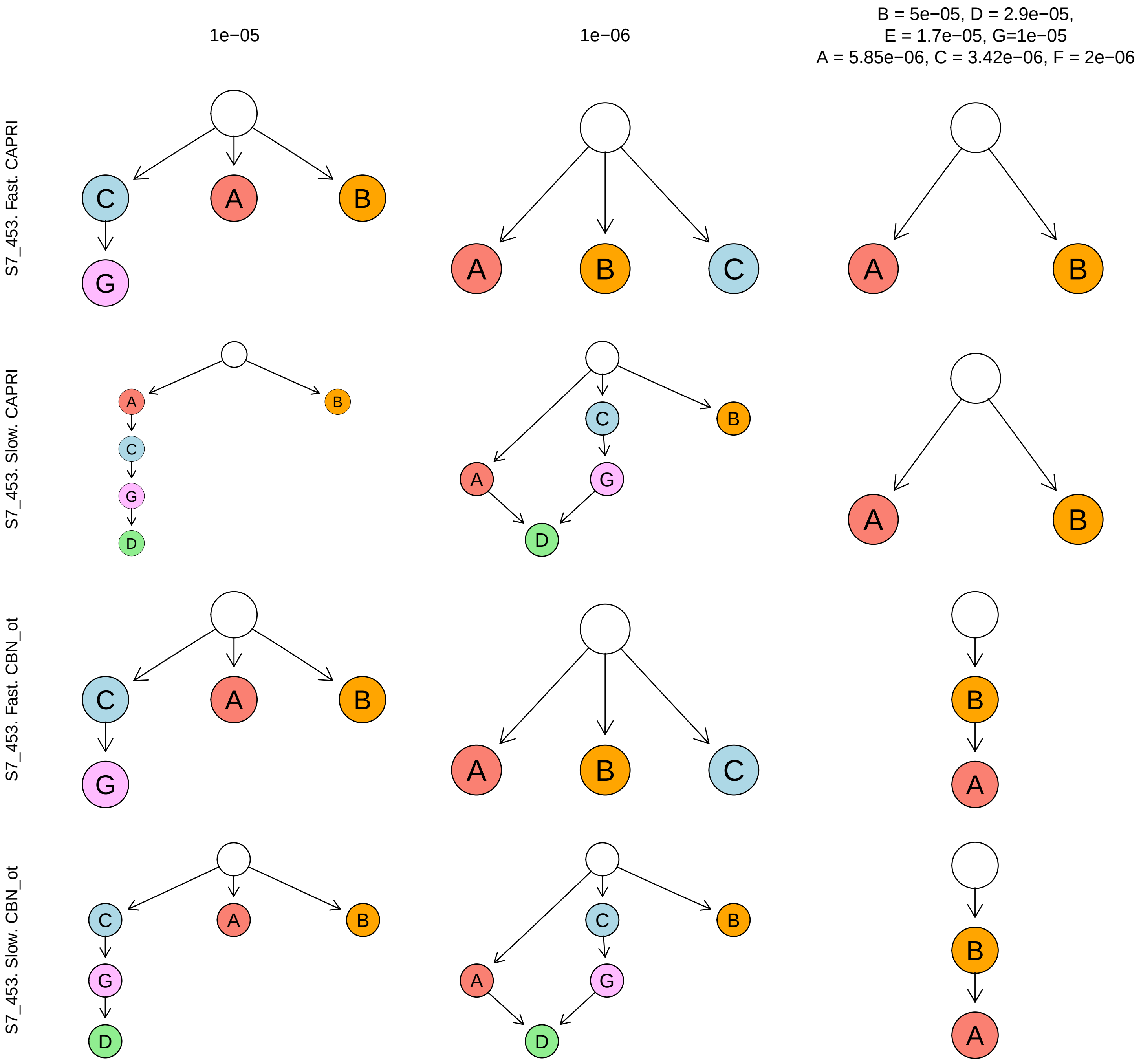

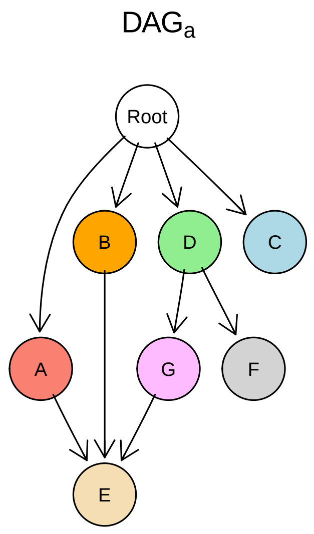

ID	Accessible Genot.	Discrep. DAG_a
S7_454	18	25

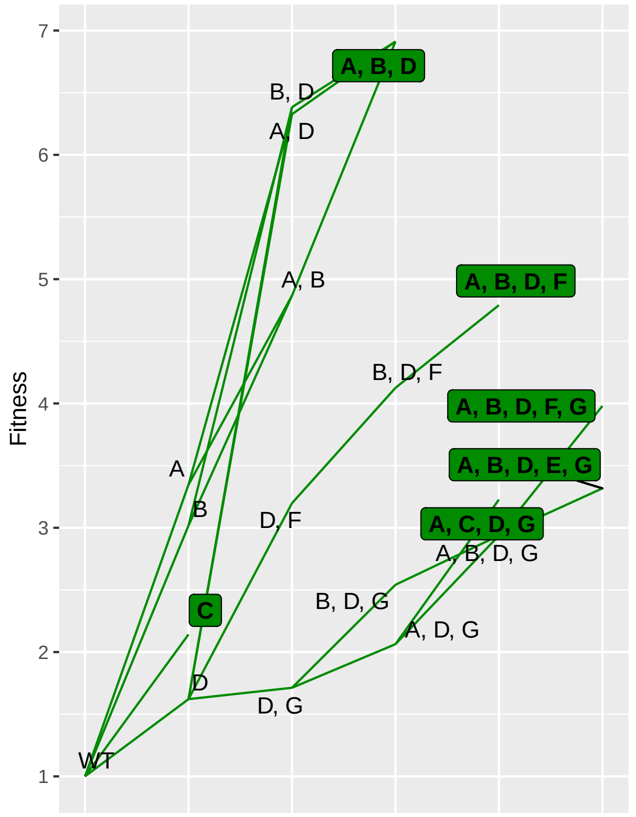

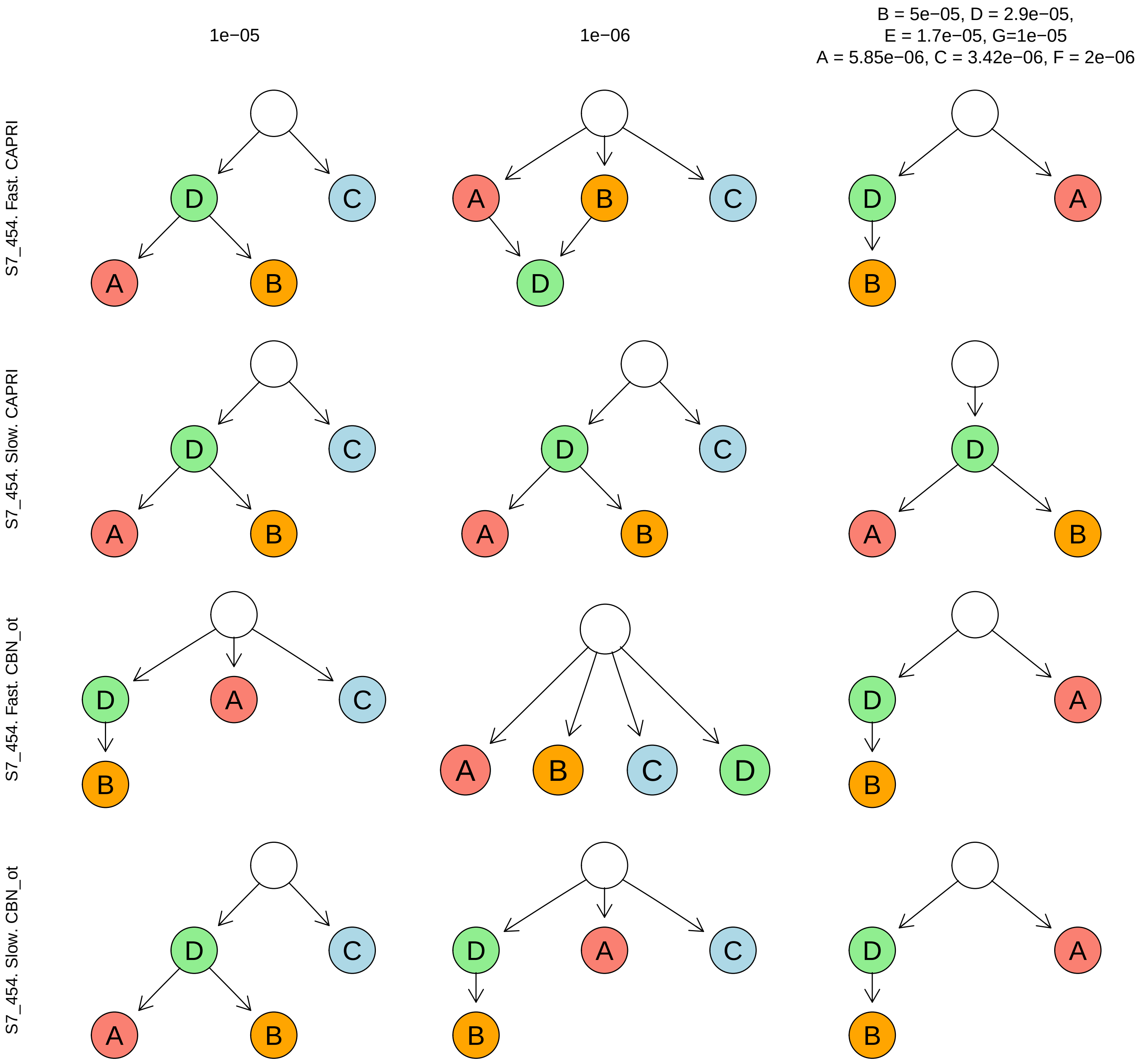

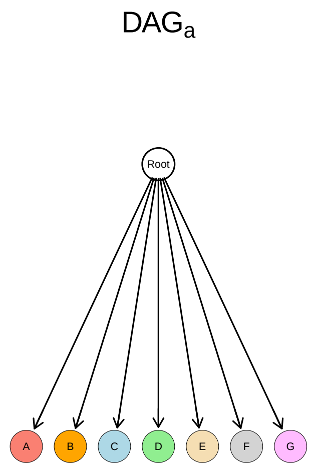

ID	Accessible Genot.	Discrep. DAG_a
S7_455	31	96

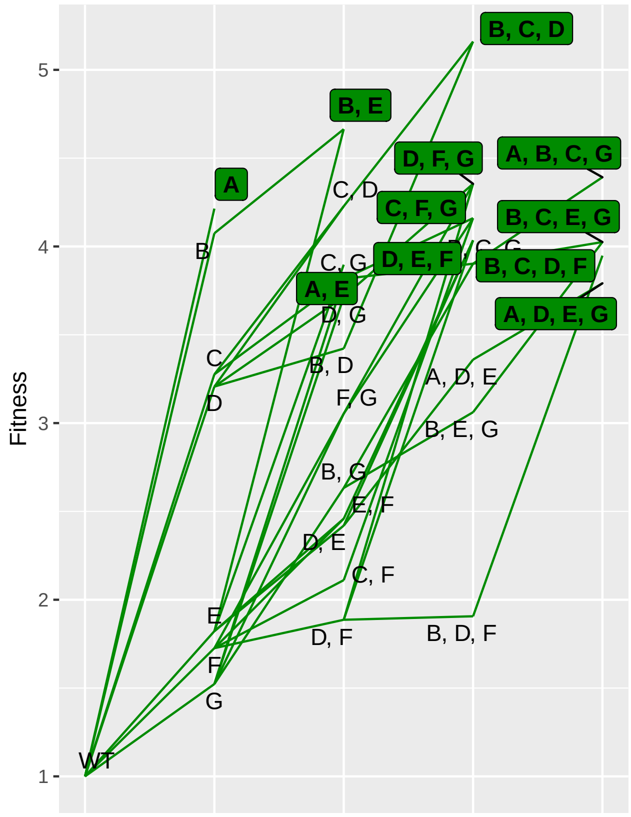

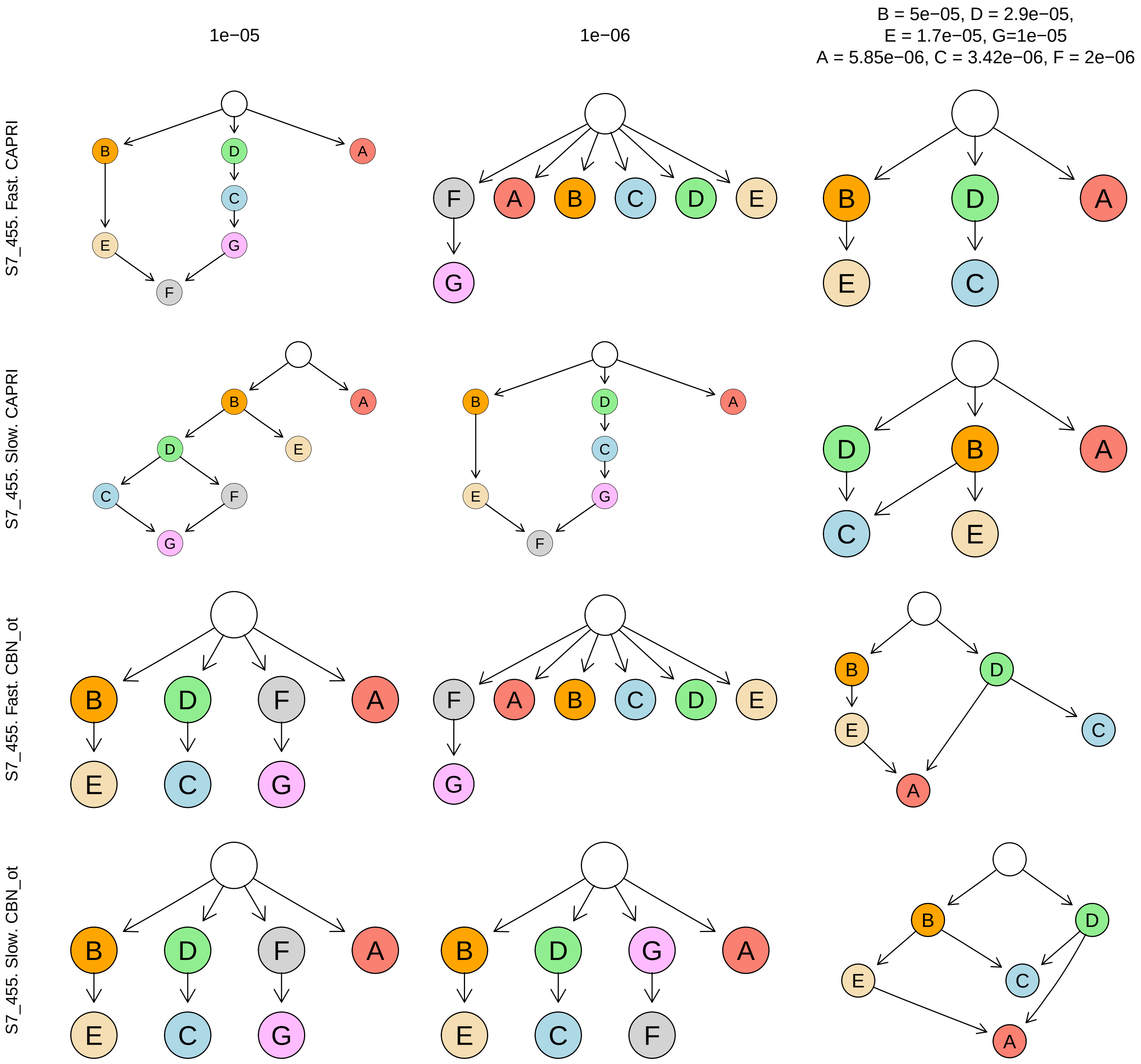

DAG$_a$

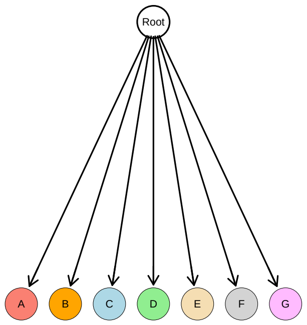

ID	Accessible Genot.	Discrep. DAG_a
S7_456	41	86

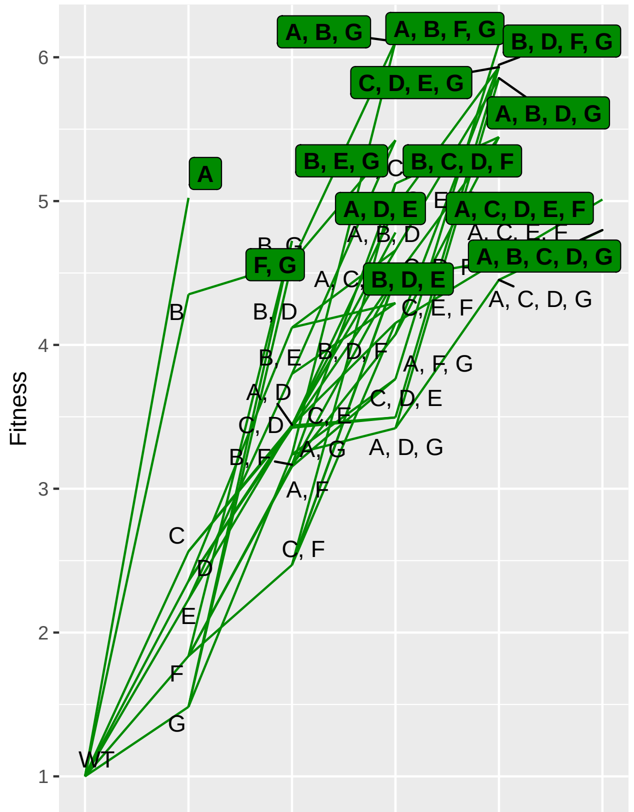

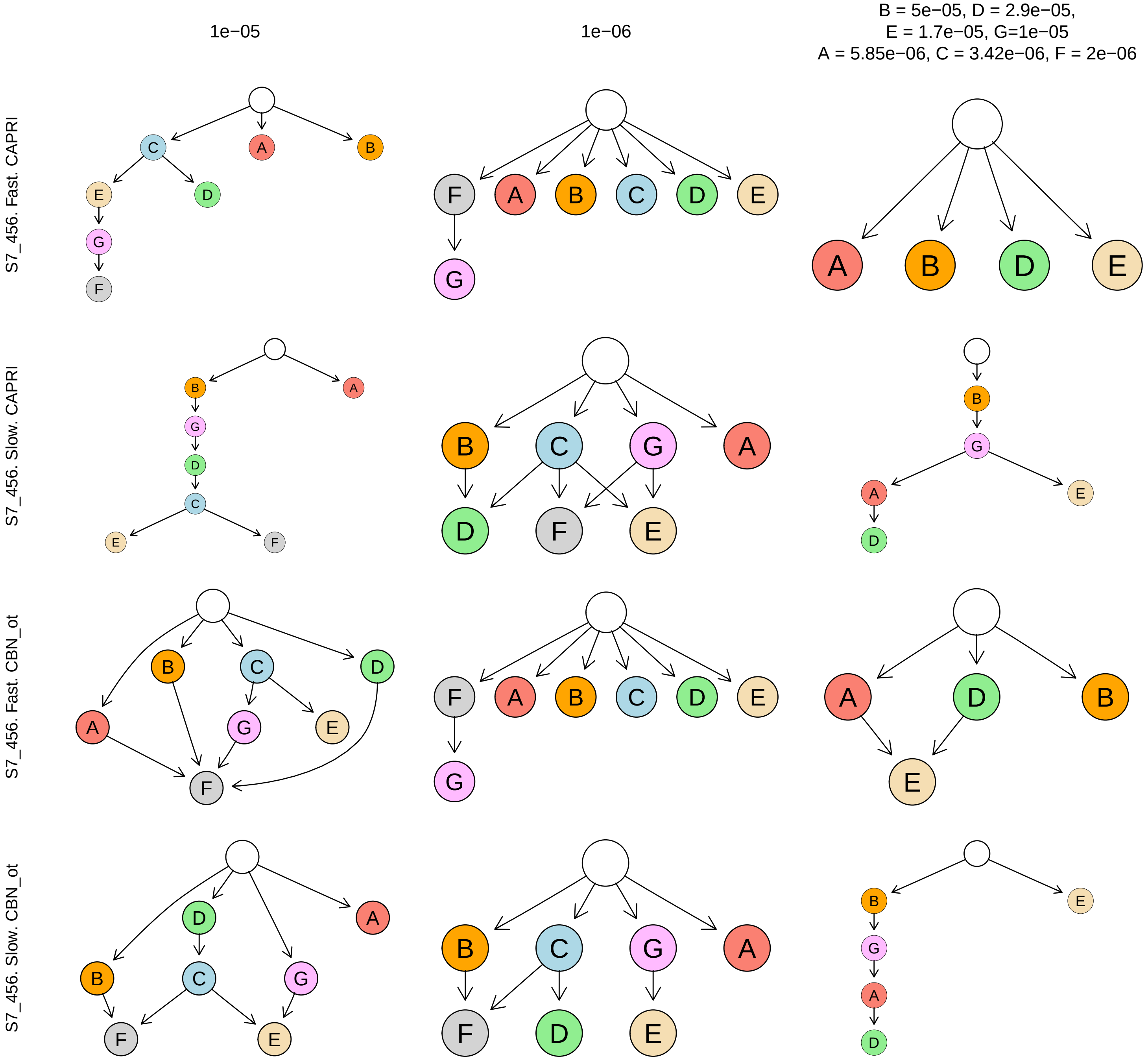

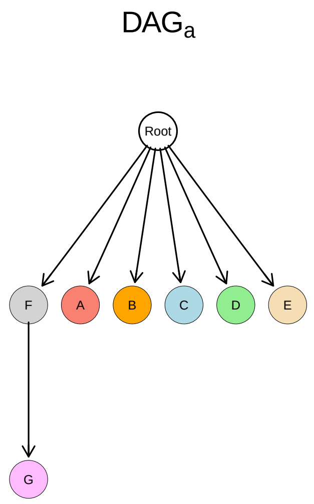

ID	Accessible Genot.	Discrep. DAG_a
S7_457	35	60

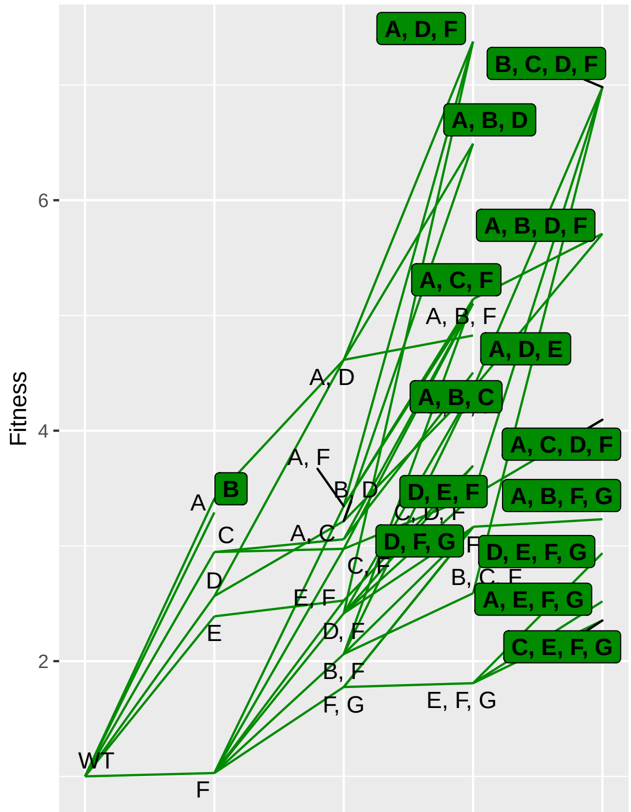

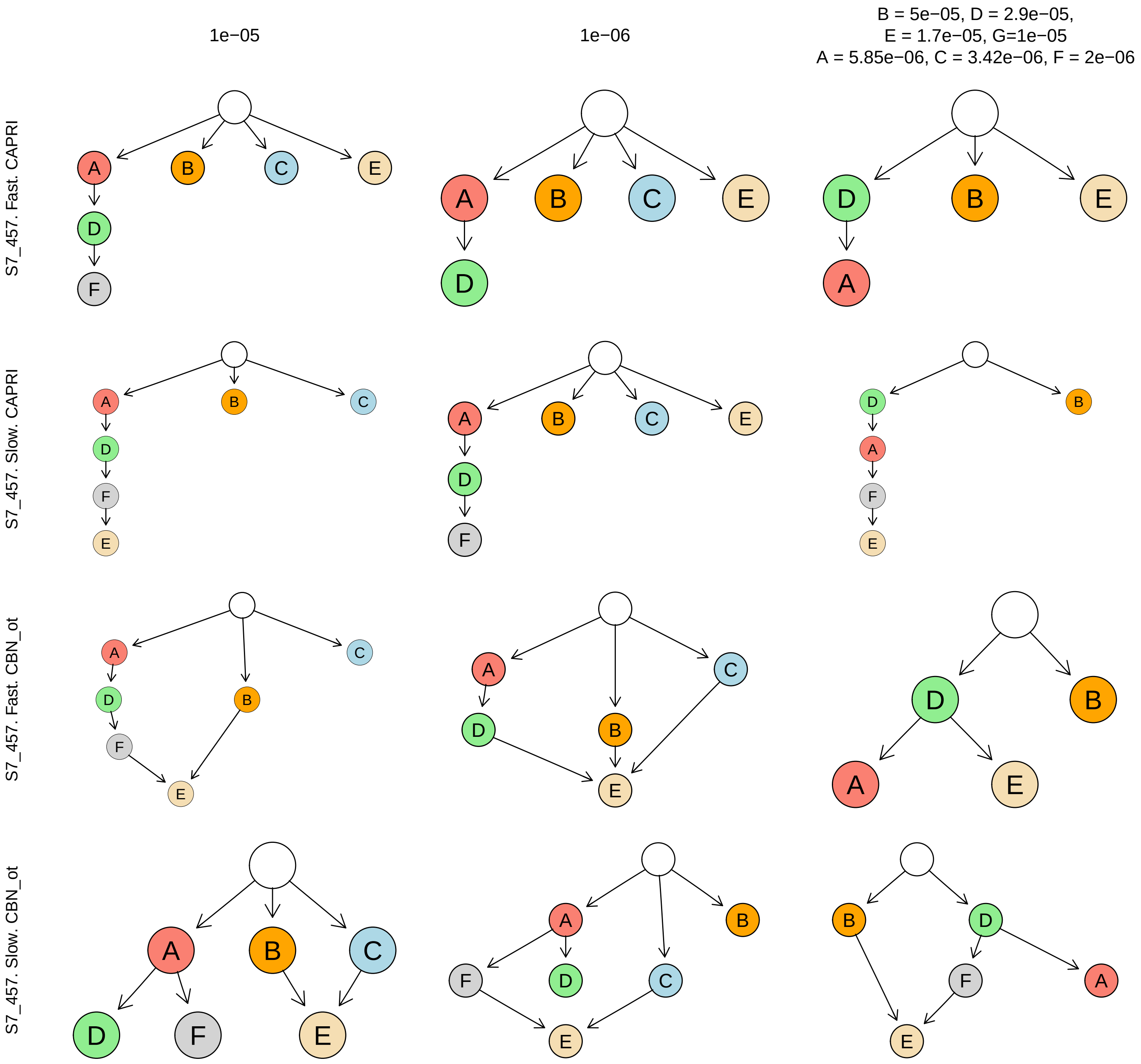

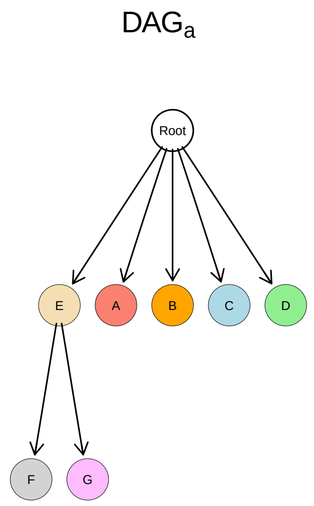

ID	Accessible Genot.	Discrep. DAG_a
S7_458	11	70

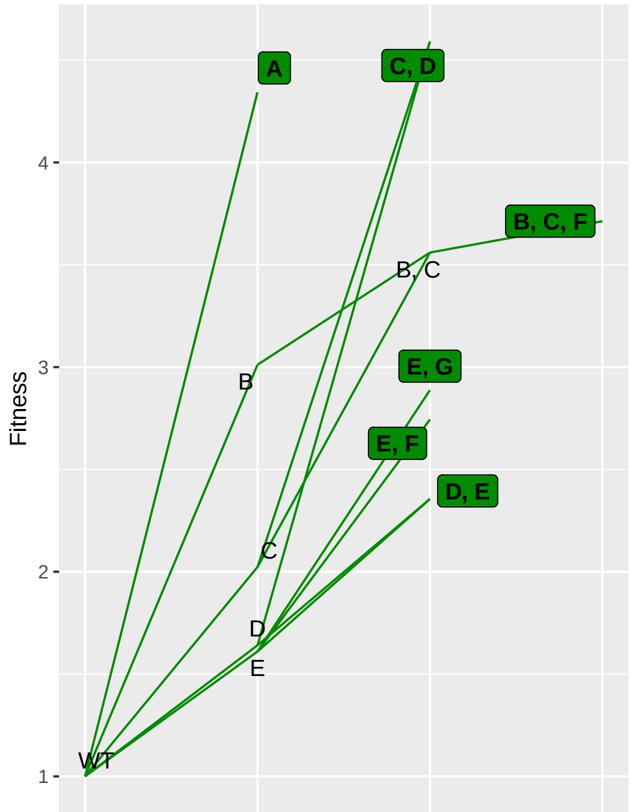

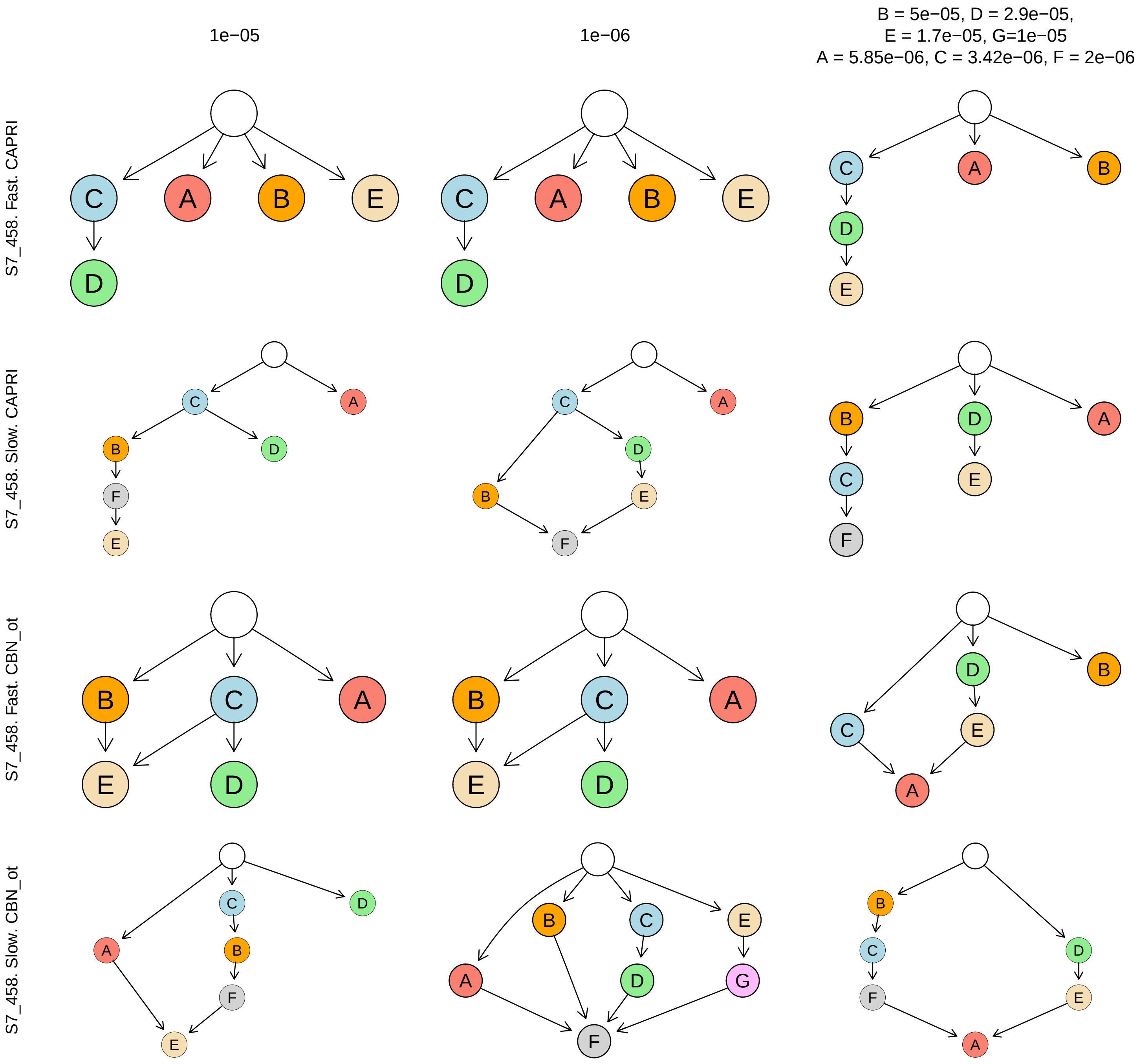

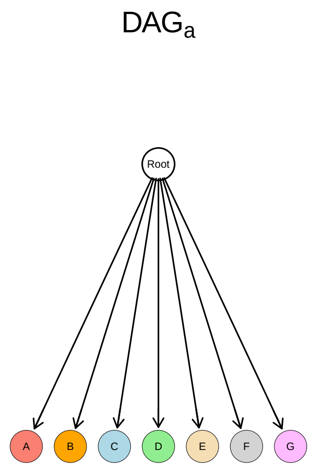

ID	Accessible Genot.	Discrep. DAG_a
S7_459	77	50

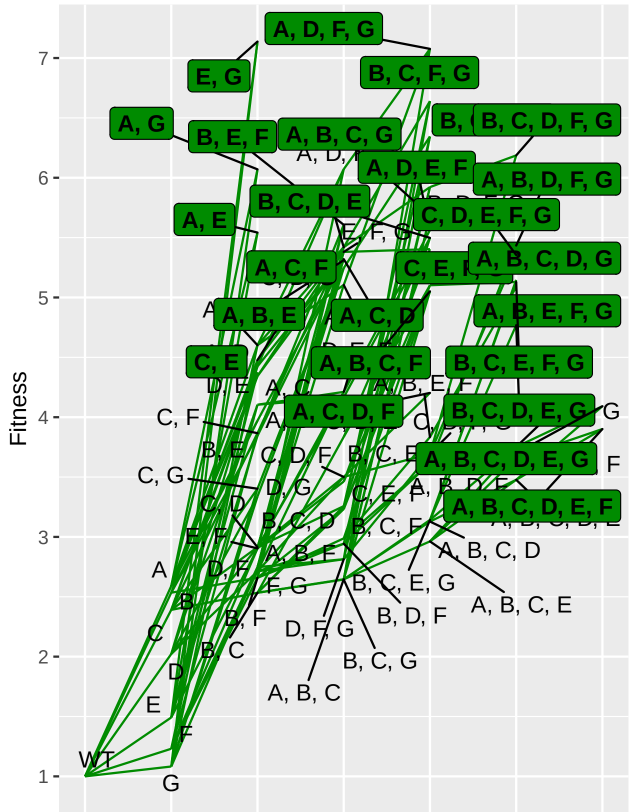

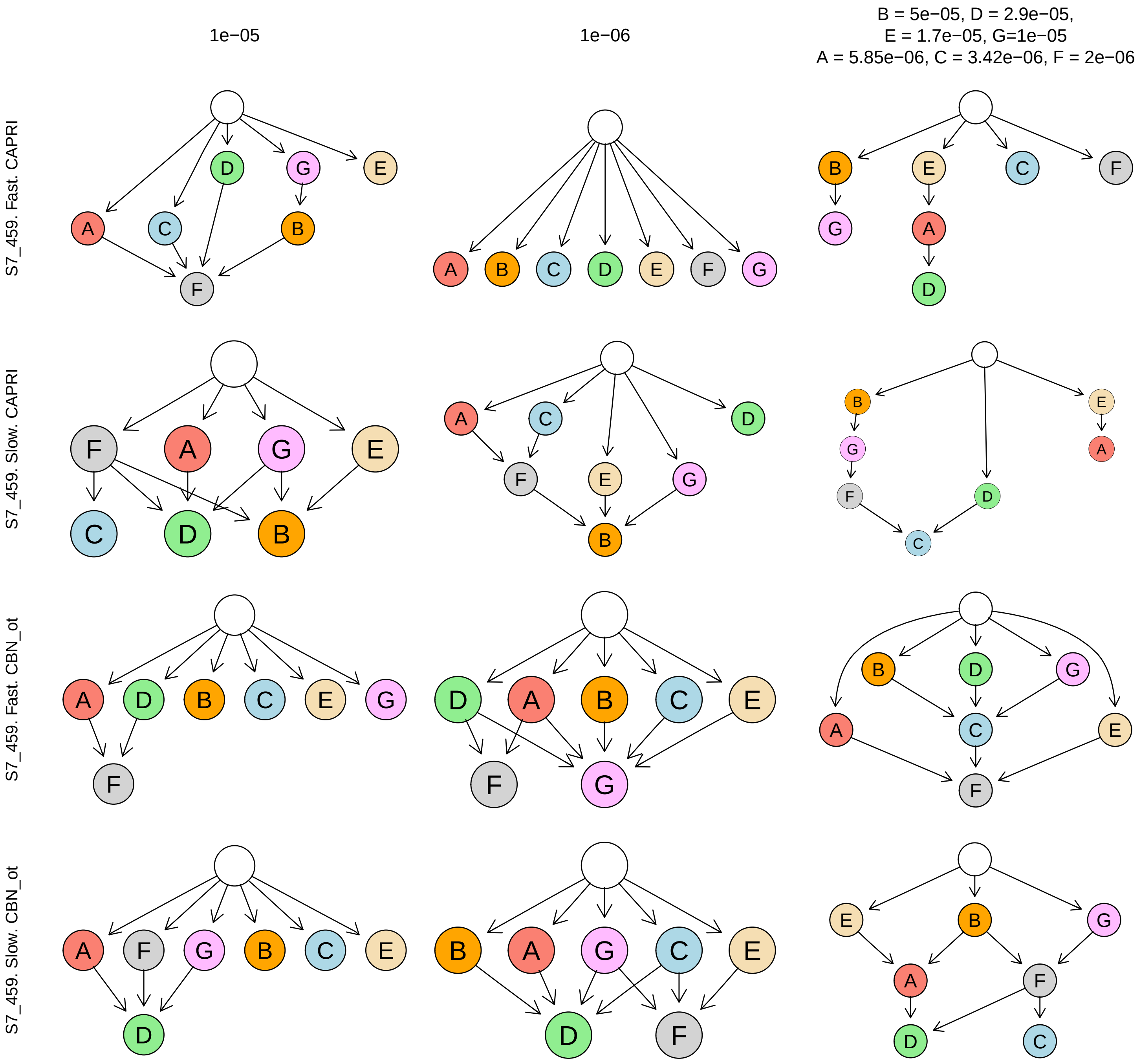

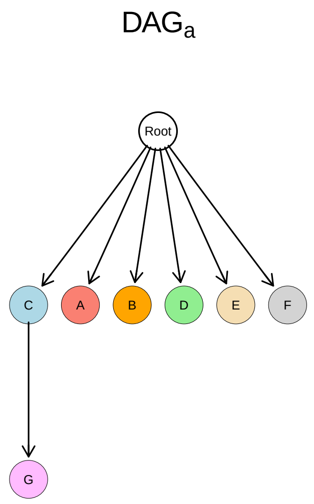

ID	Accessible Genot.	Discrep. DAG_a
S7_460	32	65

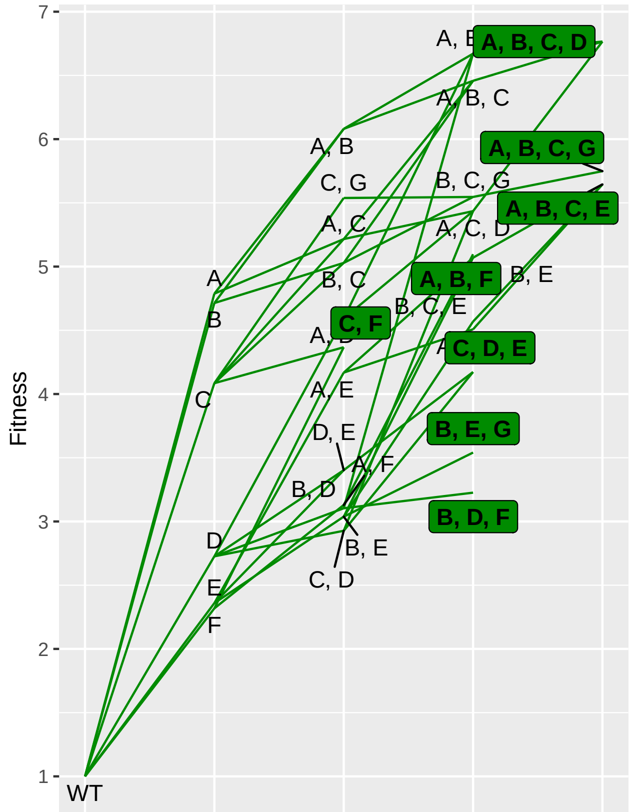

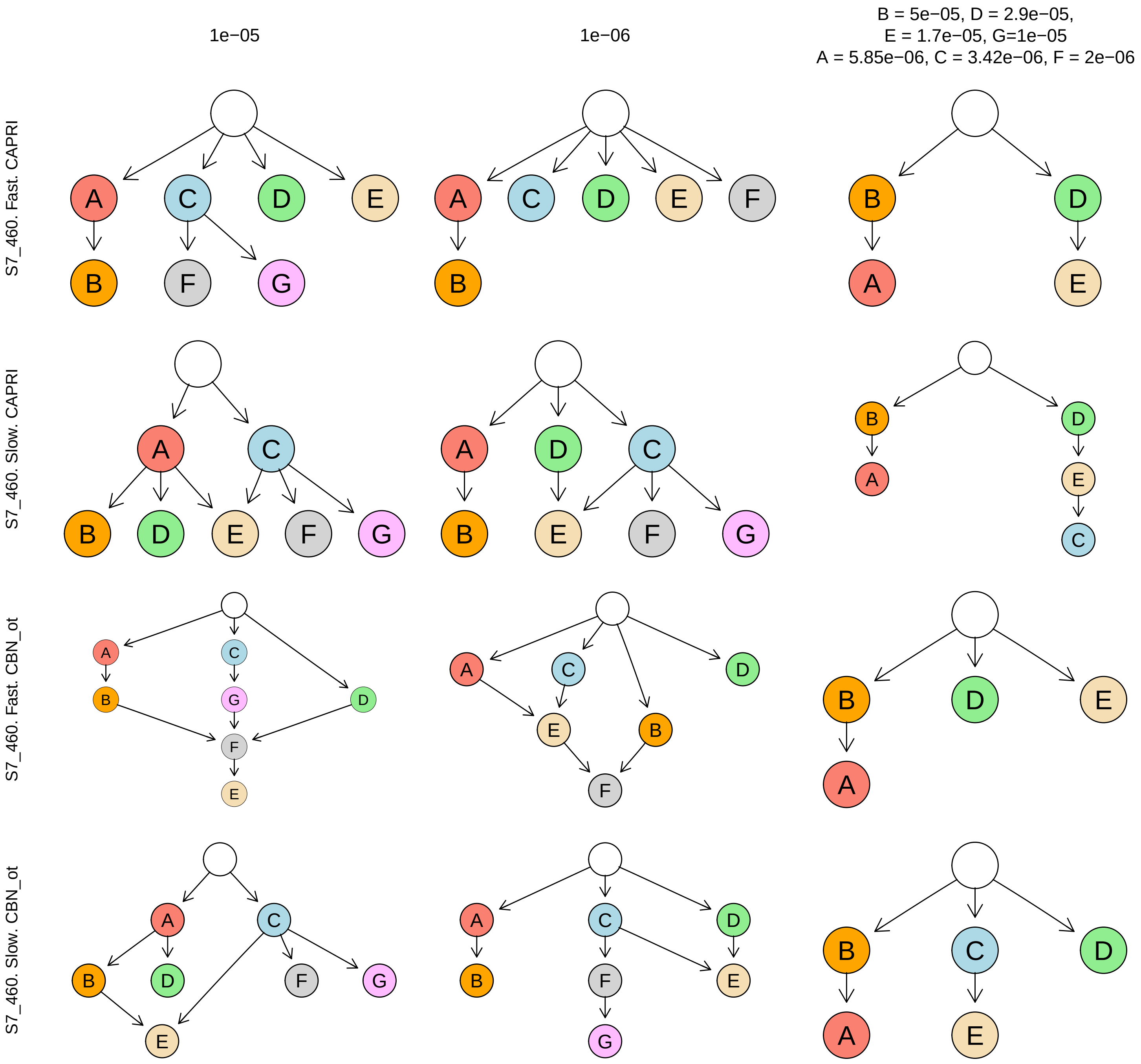

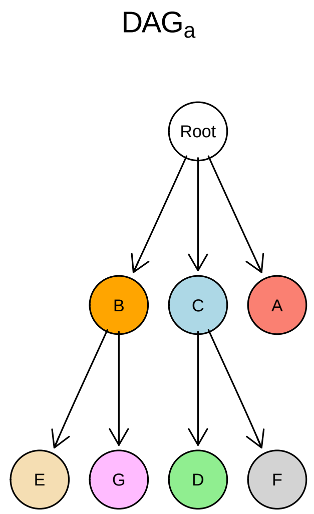

ID	Accessible Genot.	Discrep. DAG_a
S7_461	14	35

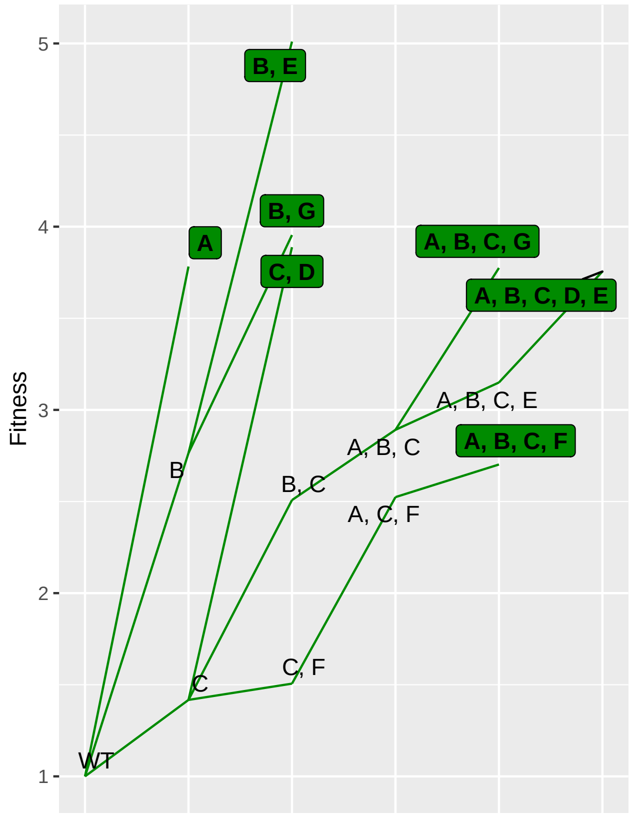

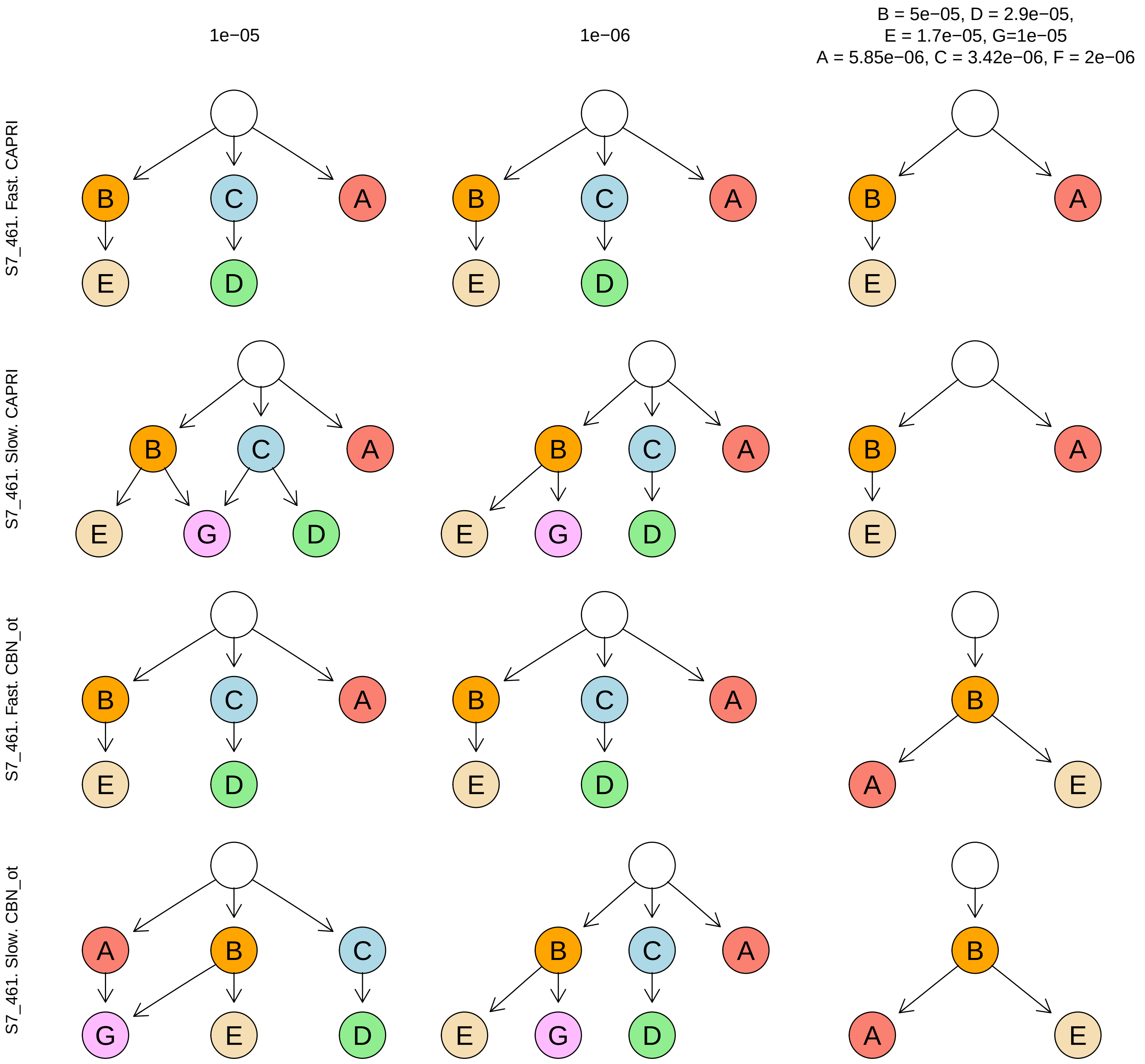

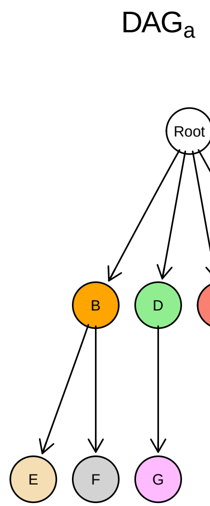

ID	Accessible Genot.	Discrep. DAG_a
S7_462	38	37

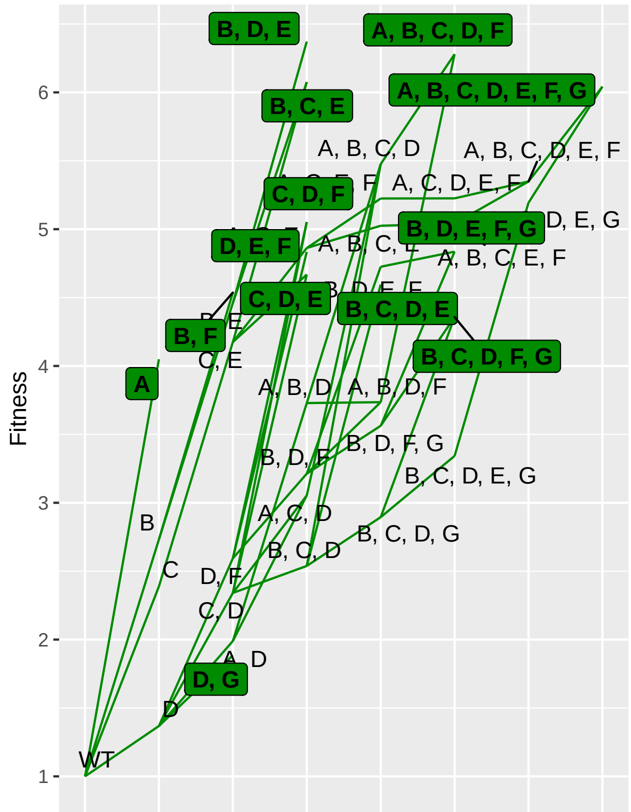

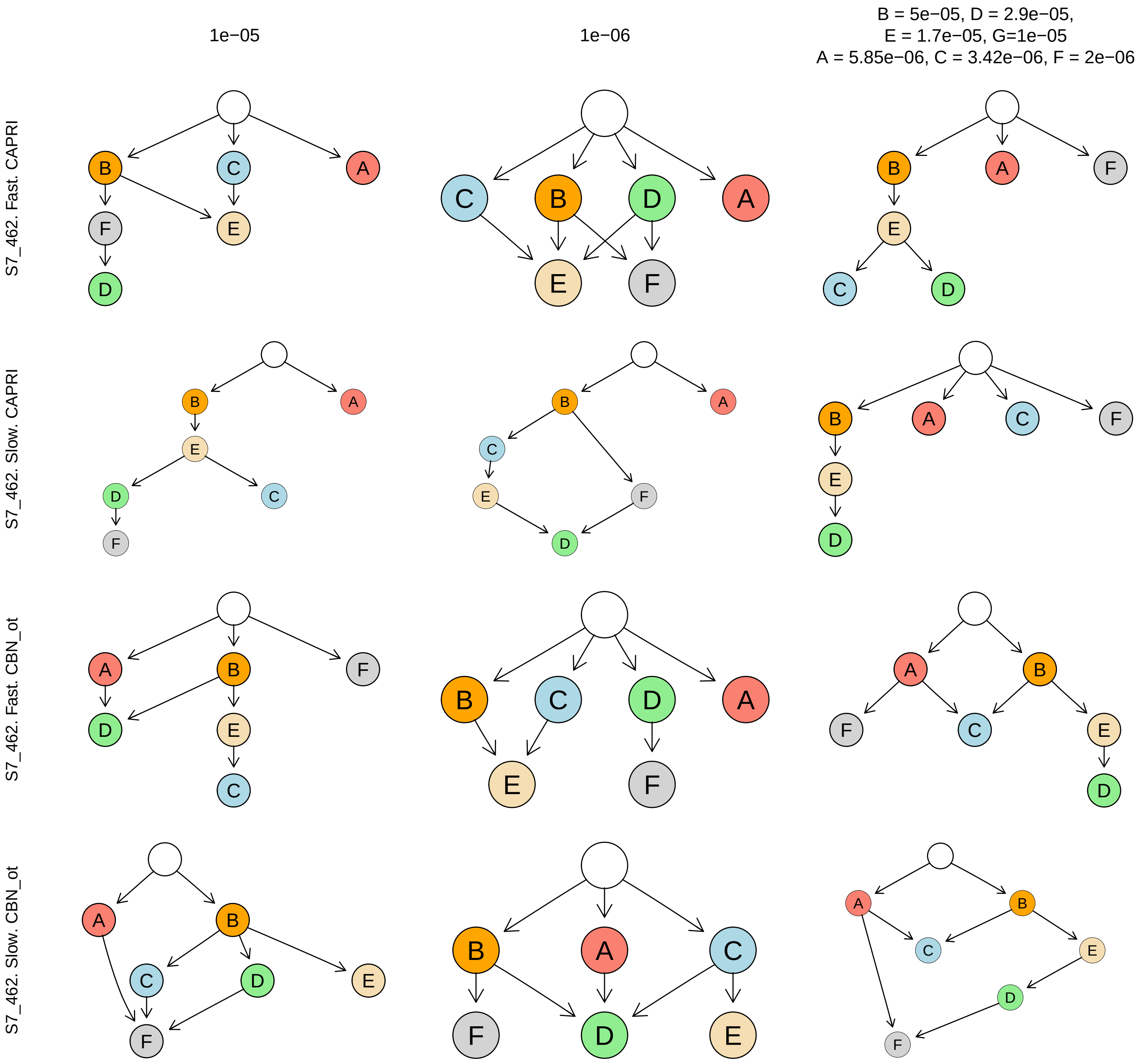

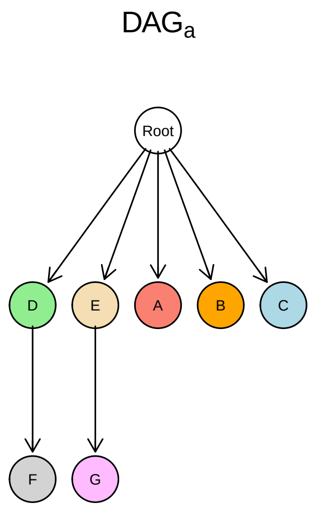

ID	Accessible Genot.	Discrep. DAG_a
S7_463	34	49

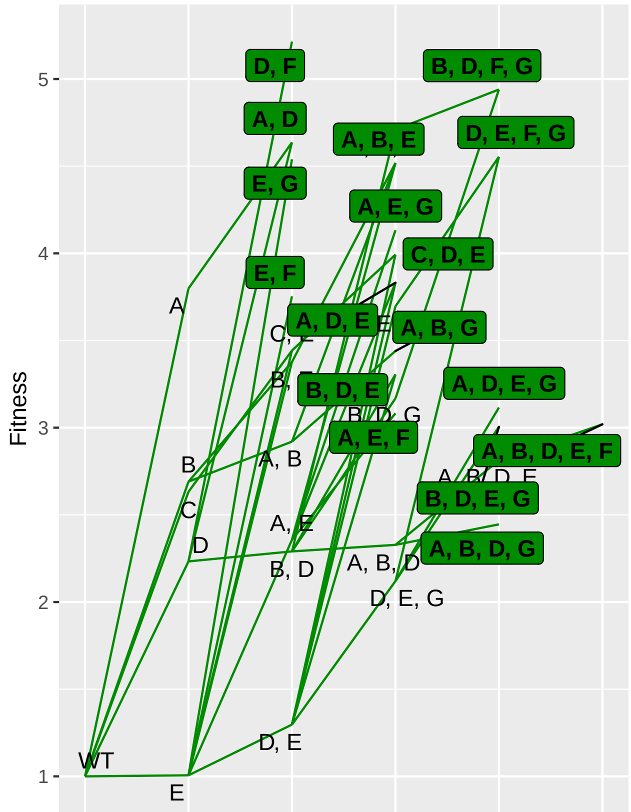

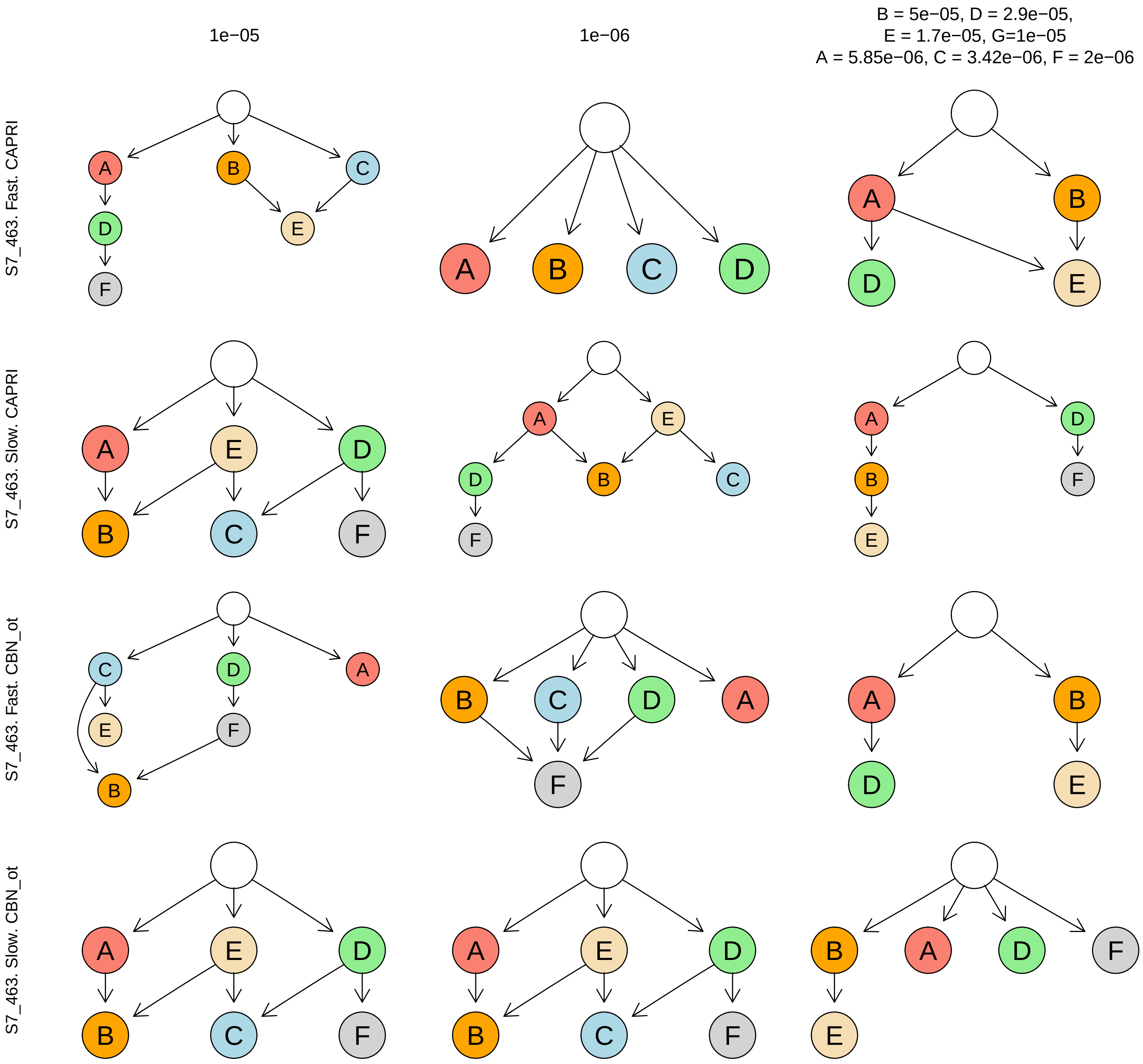

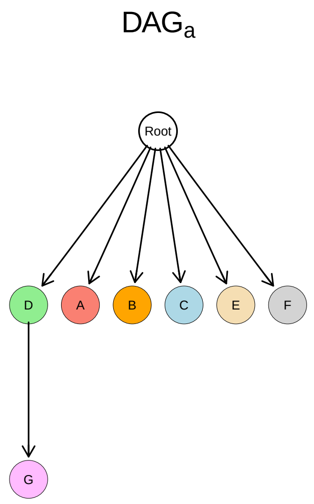

ID	Accessible Genot.	Discrep. DAG_a
S7_464	49	58

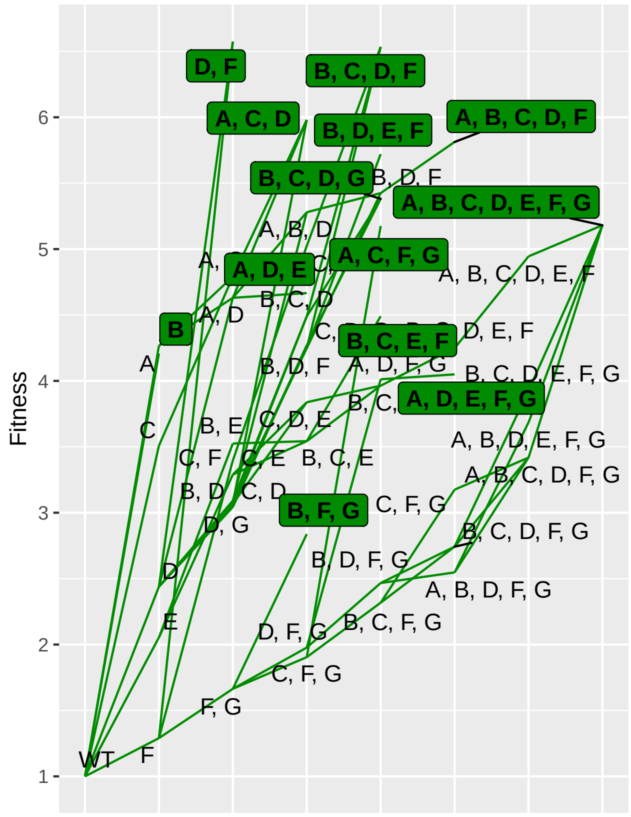

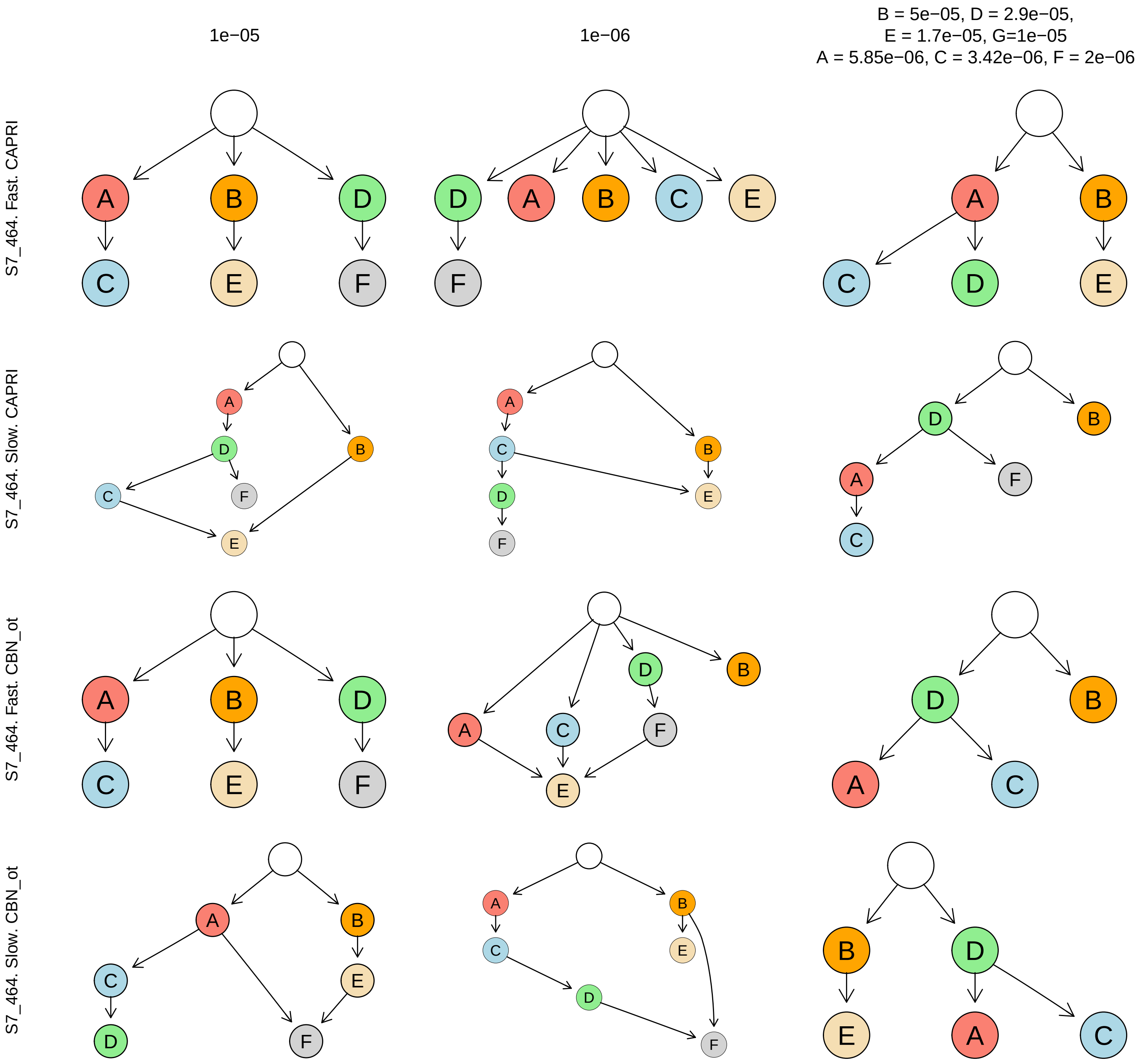

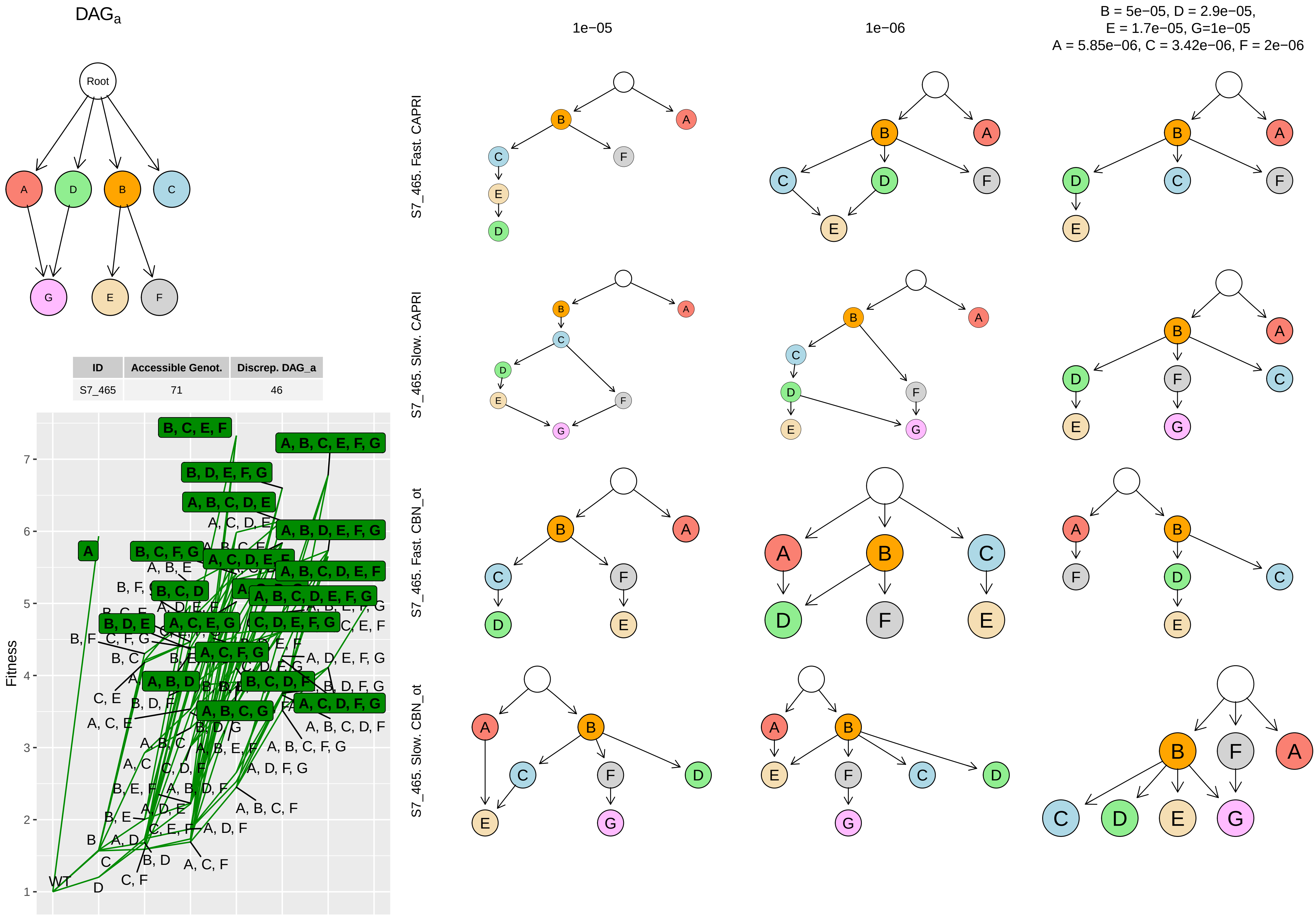

ID	Accessible Genot.	Discrep. DAG_a
S7_465	71	46

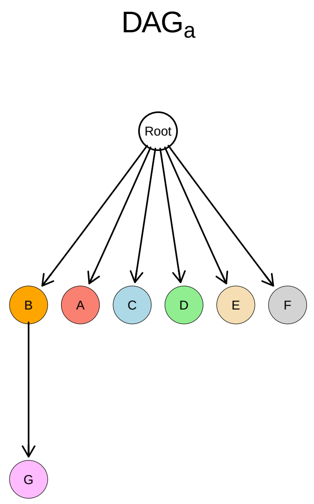

ID	Accessible Genot.	Discrep. DAG_a
S7_466	67	50

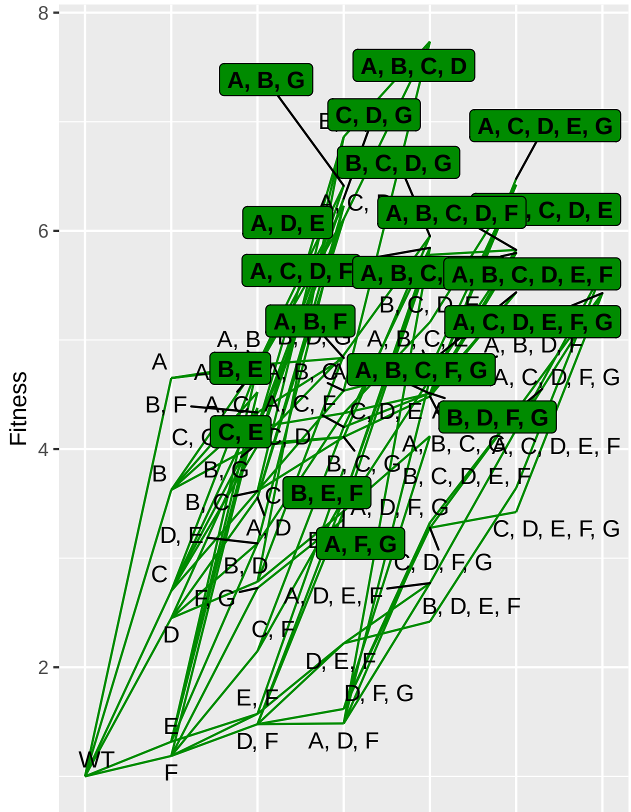

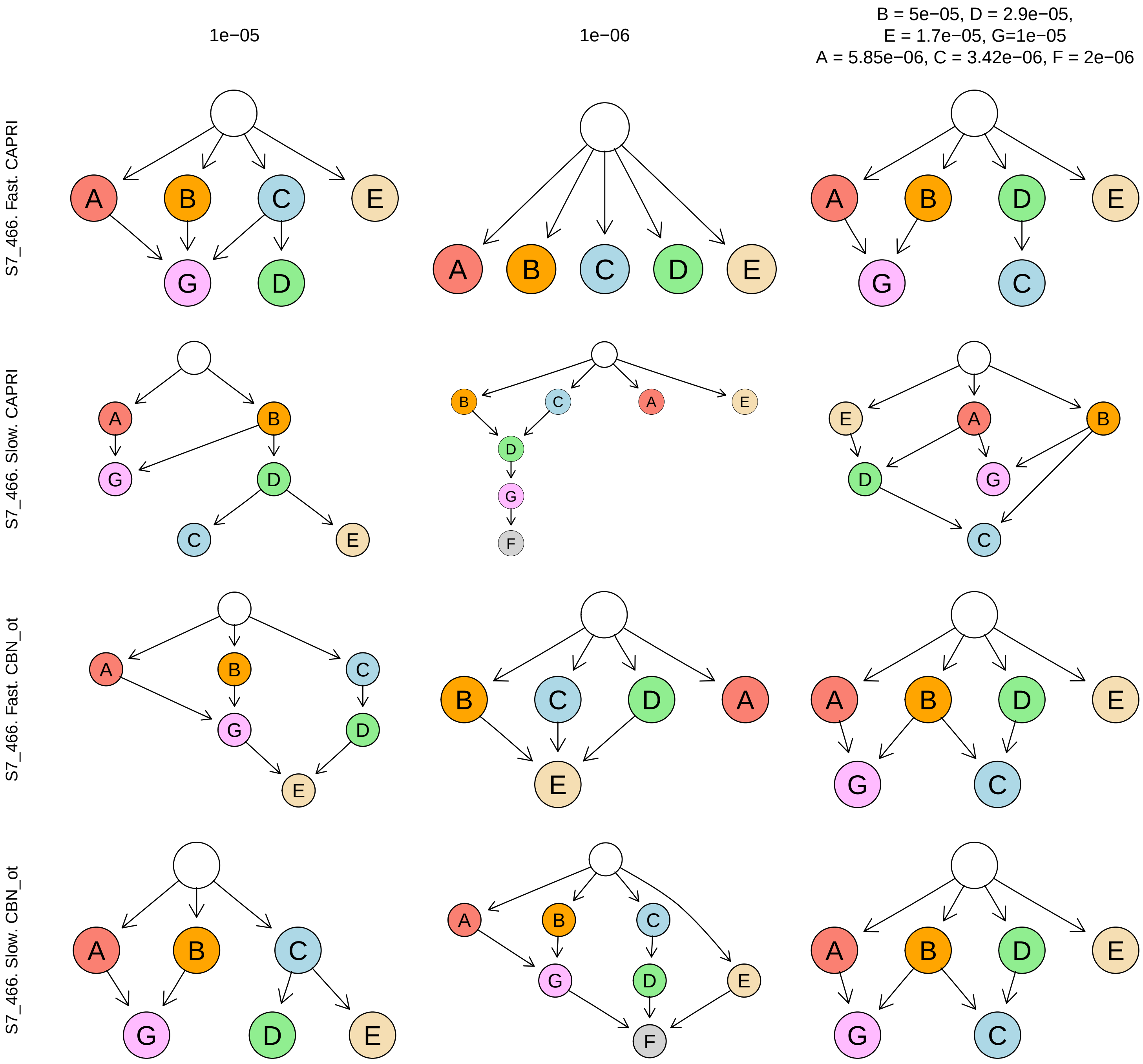

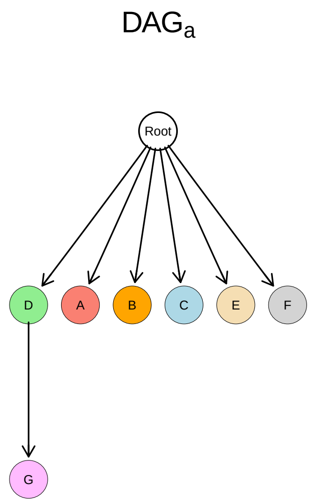

ID	Accessible Genot.	Discrep. DAG_a
S7_467	34	71

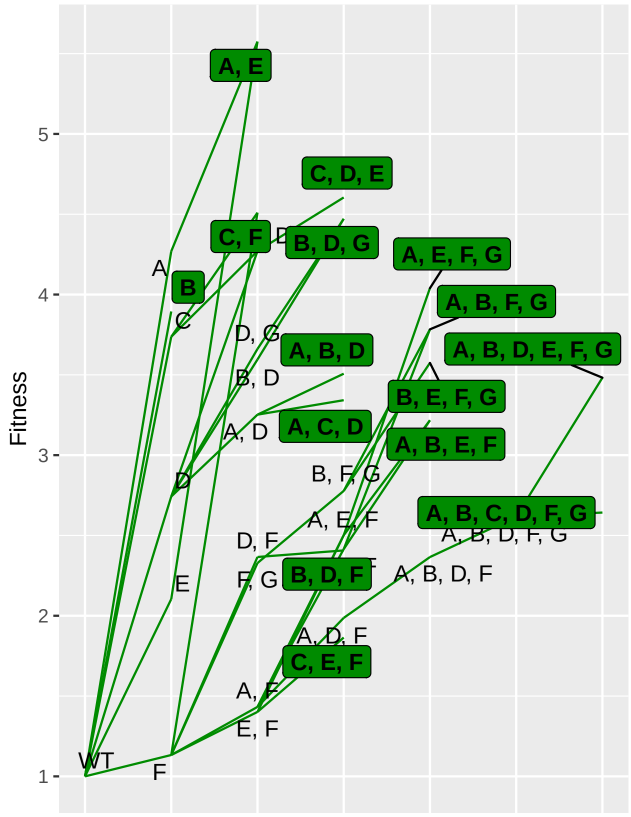

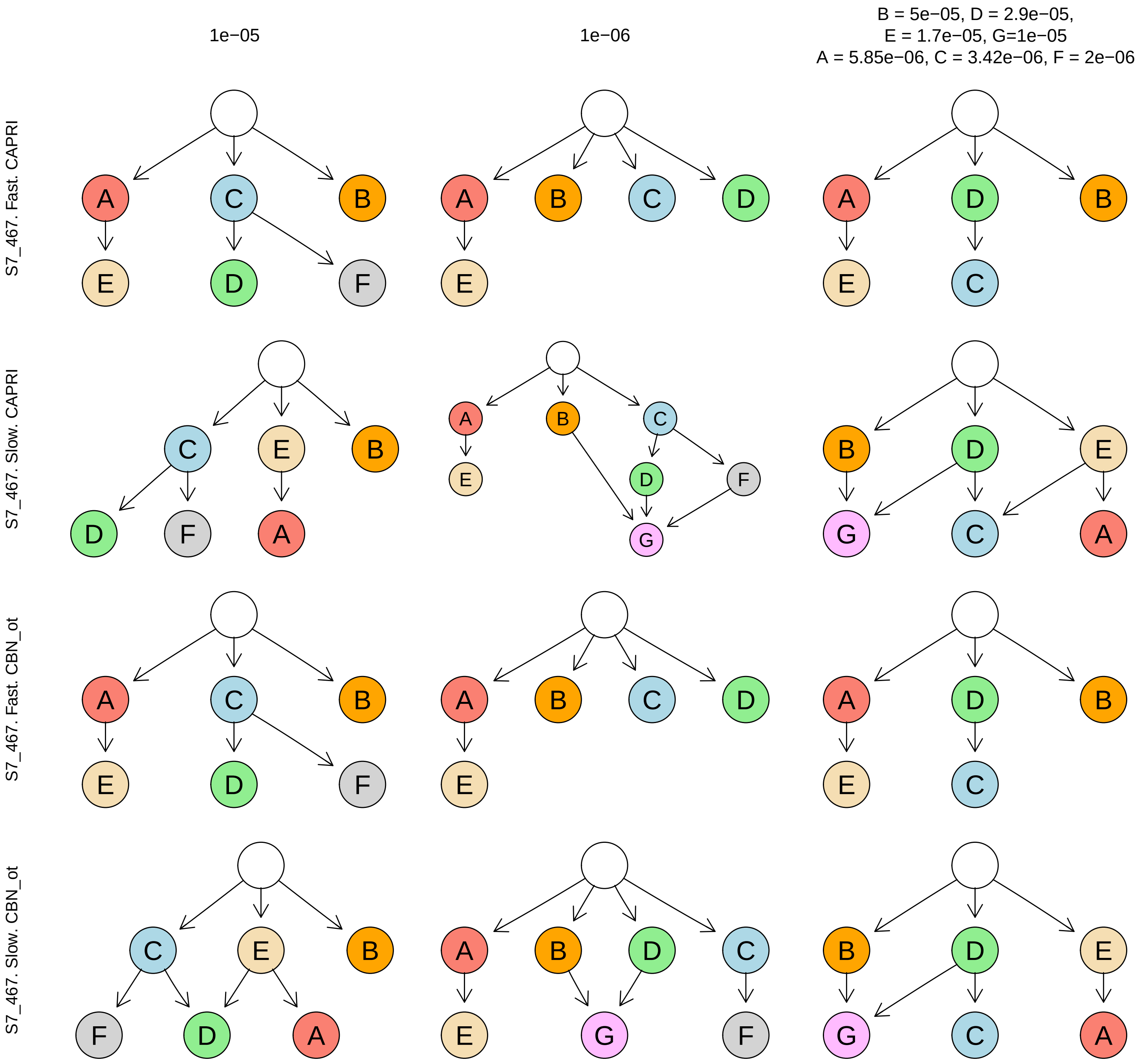

DAG$_a$

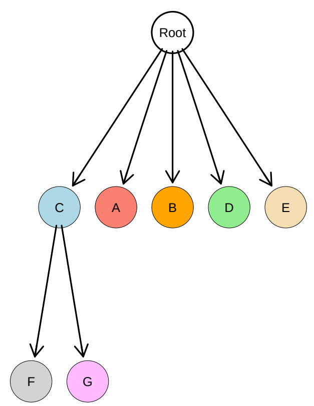

ID	Accessible Genot.	Discrep. DAG_a
S7_468	56	61

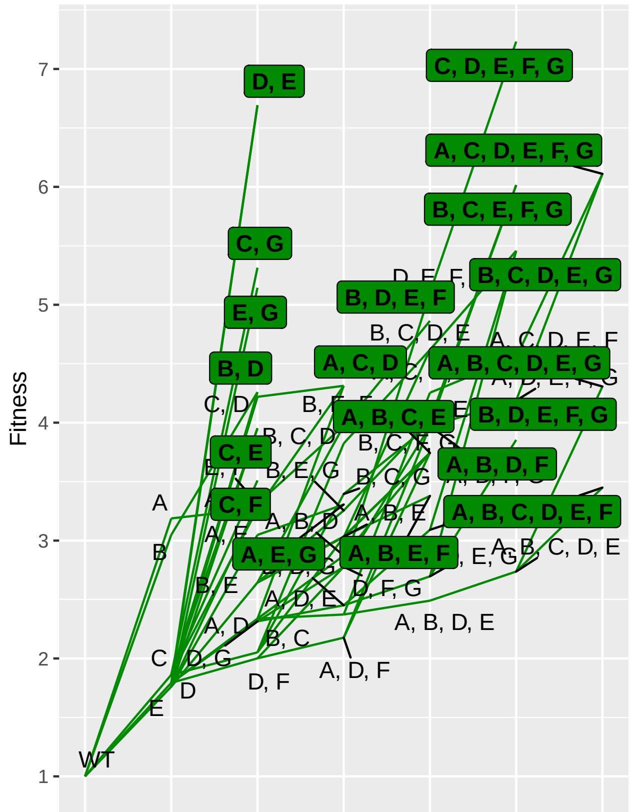

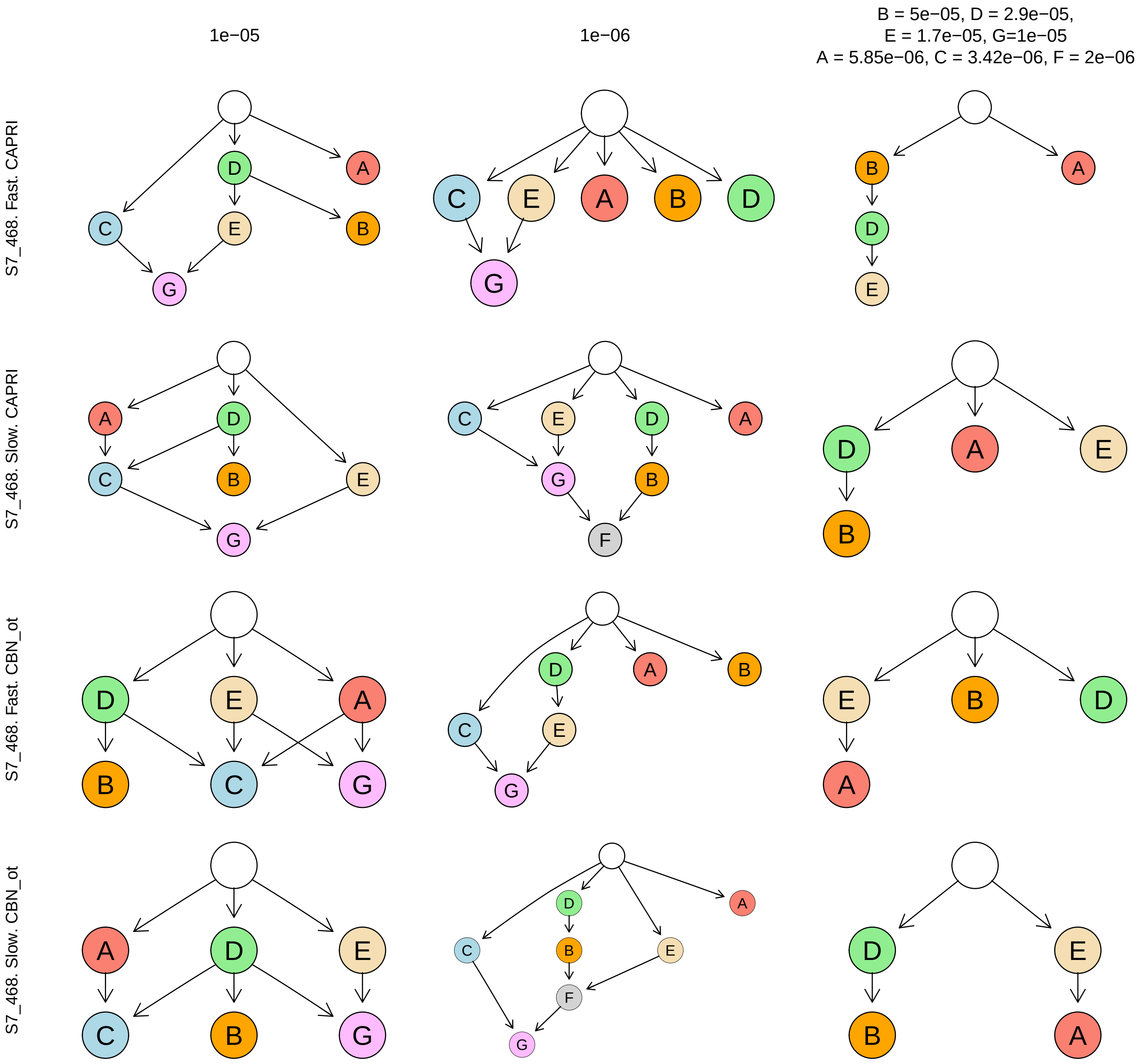

DAG$_a$

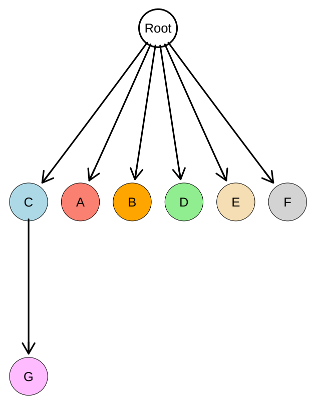

ID	Accessible Genot.	Discrep. DAG_a
S7_469	28	69

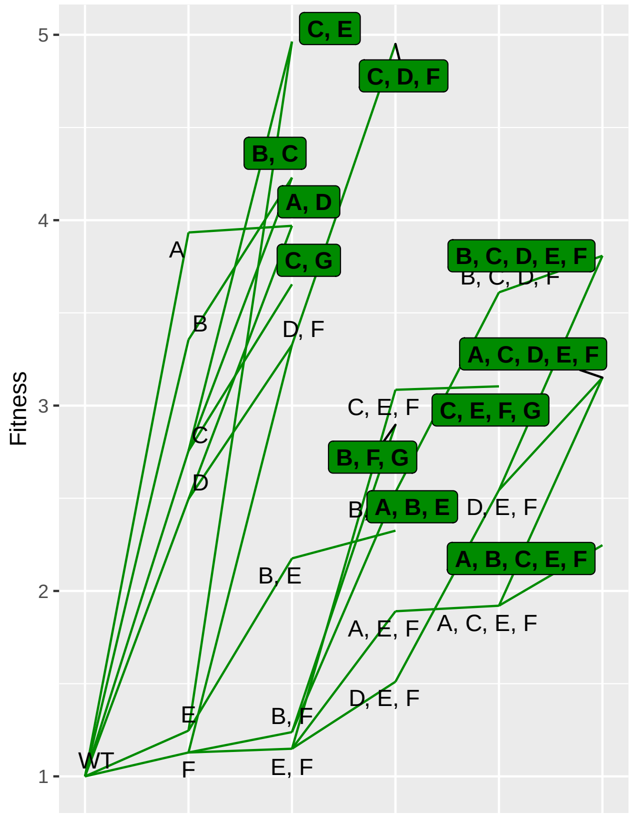

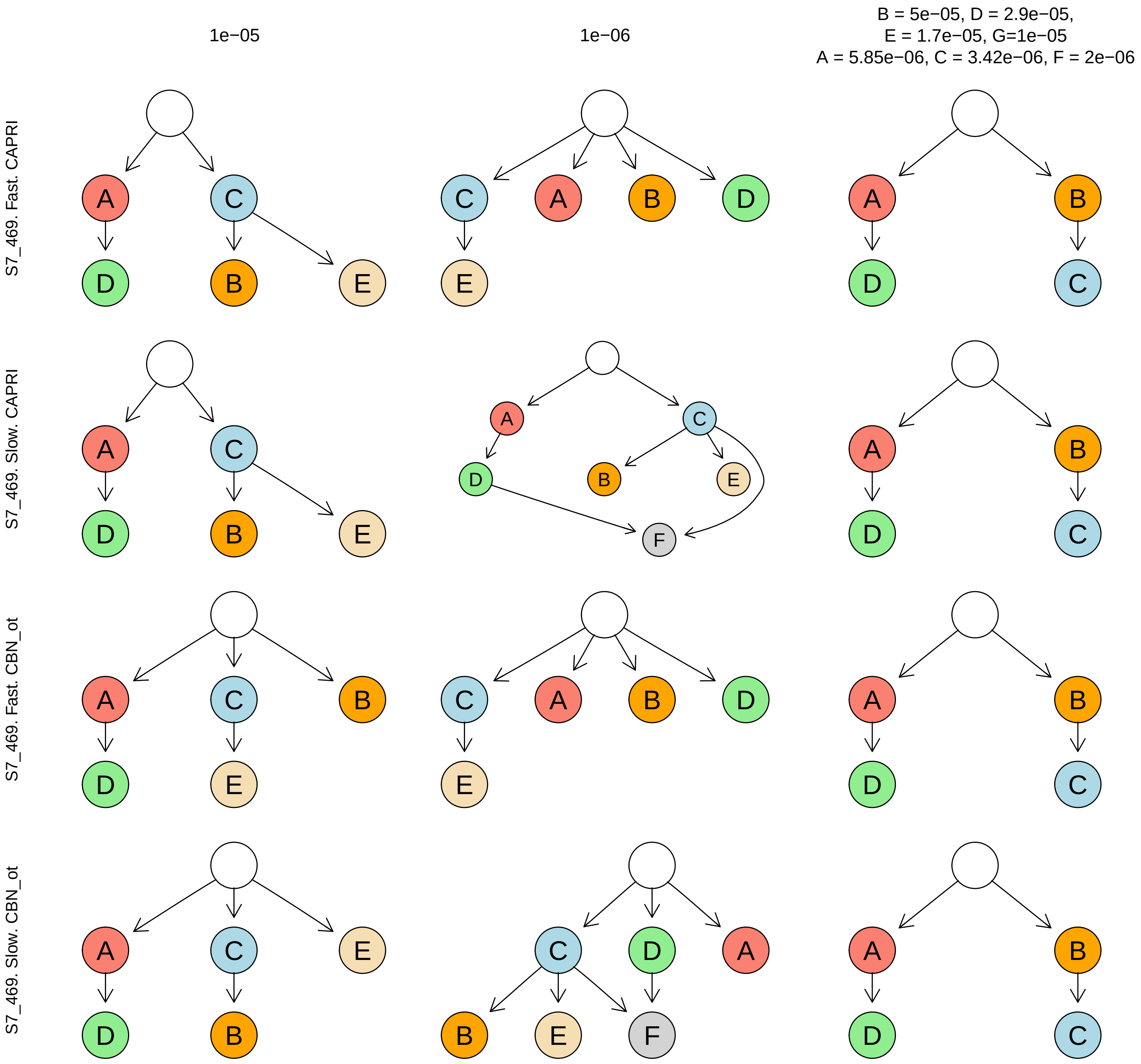

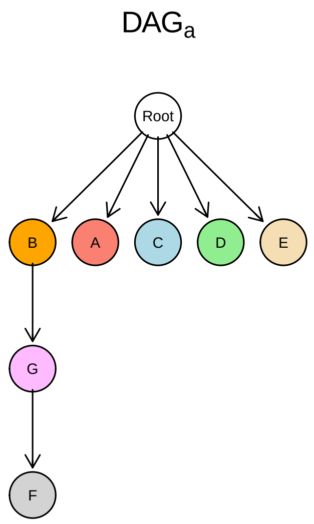

ID	Accessible Genot.	Discrep. DAG_a
S7_470	27	44

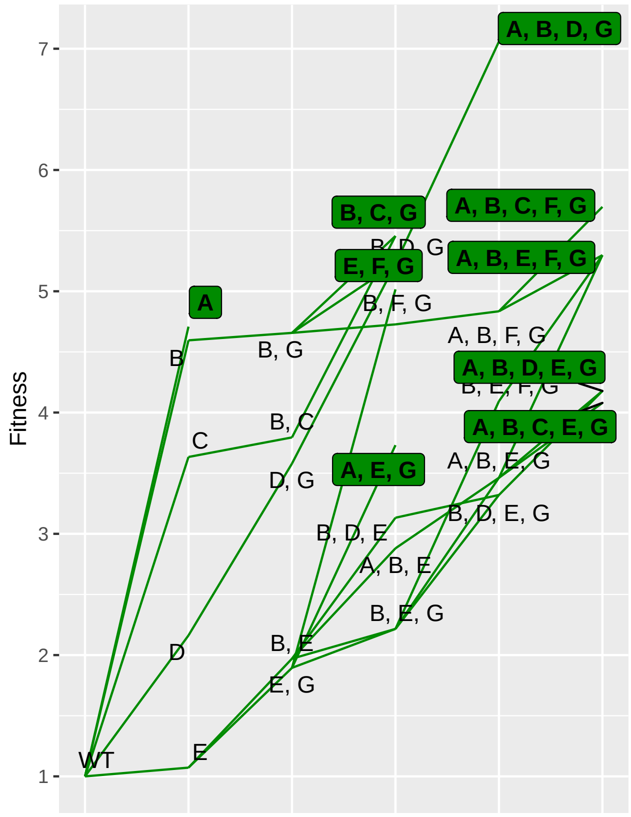

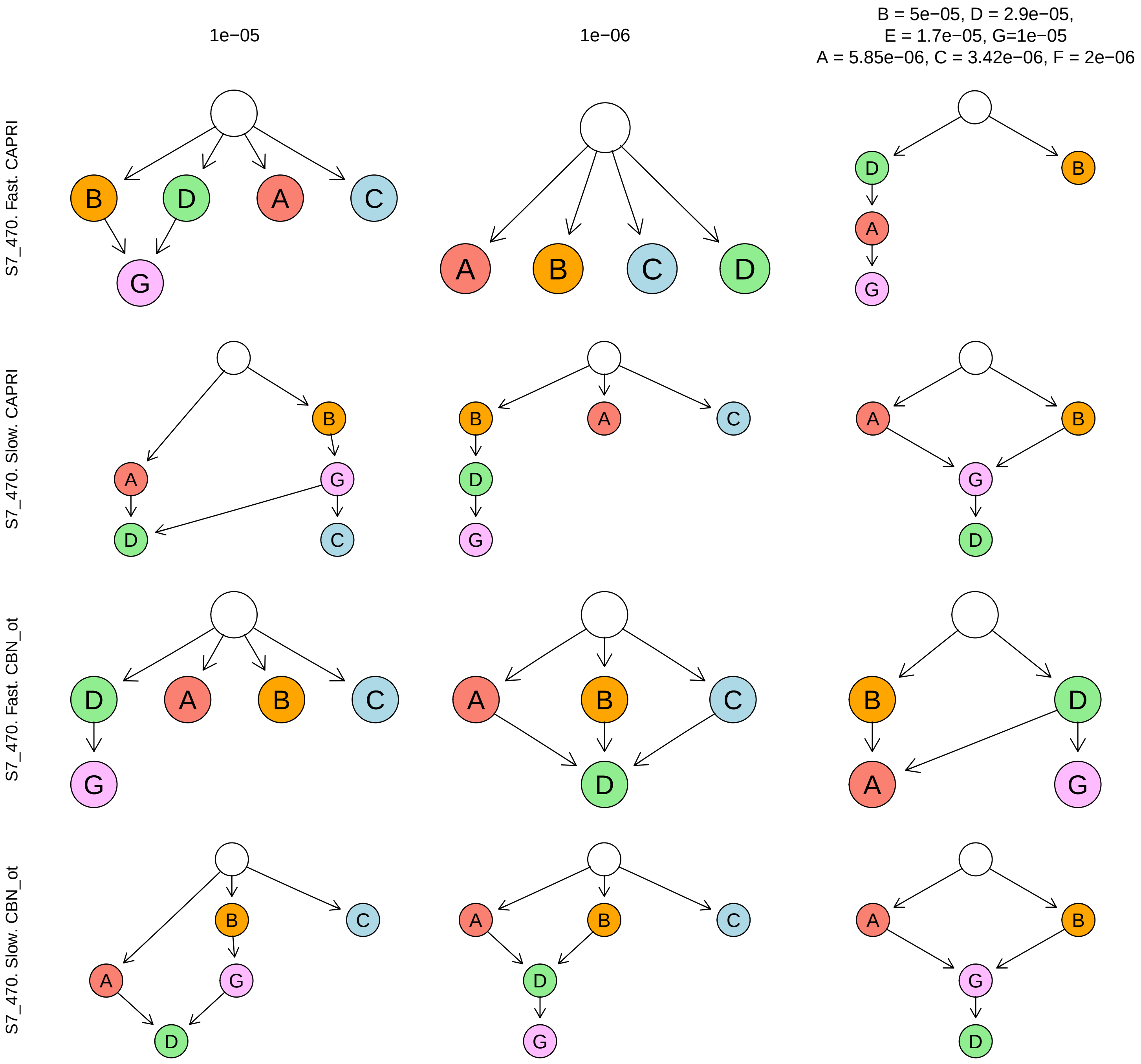

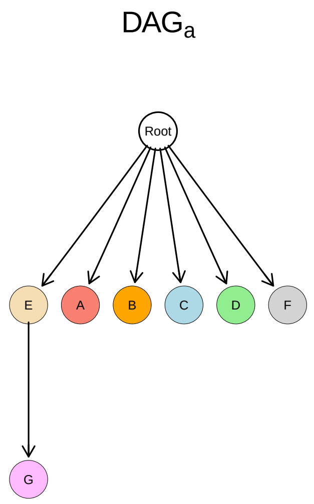

ID	Accessible Genot.	Discrep. DAG_a
S7_471	22	77

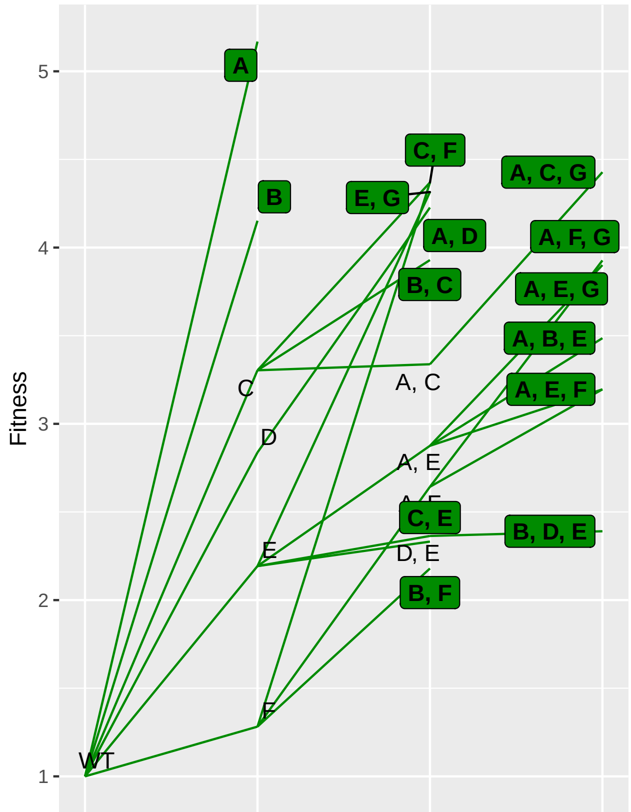

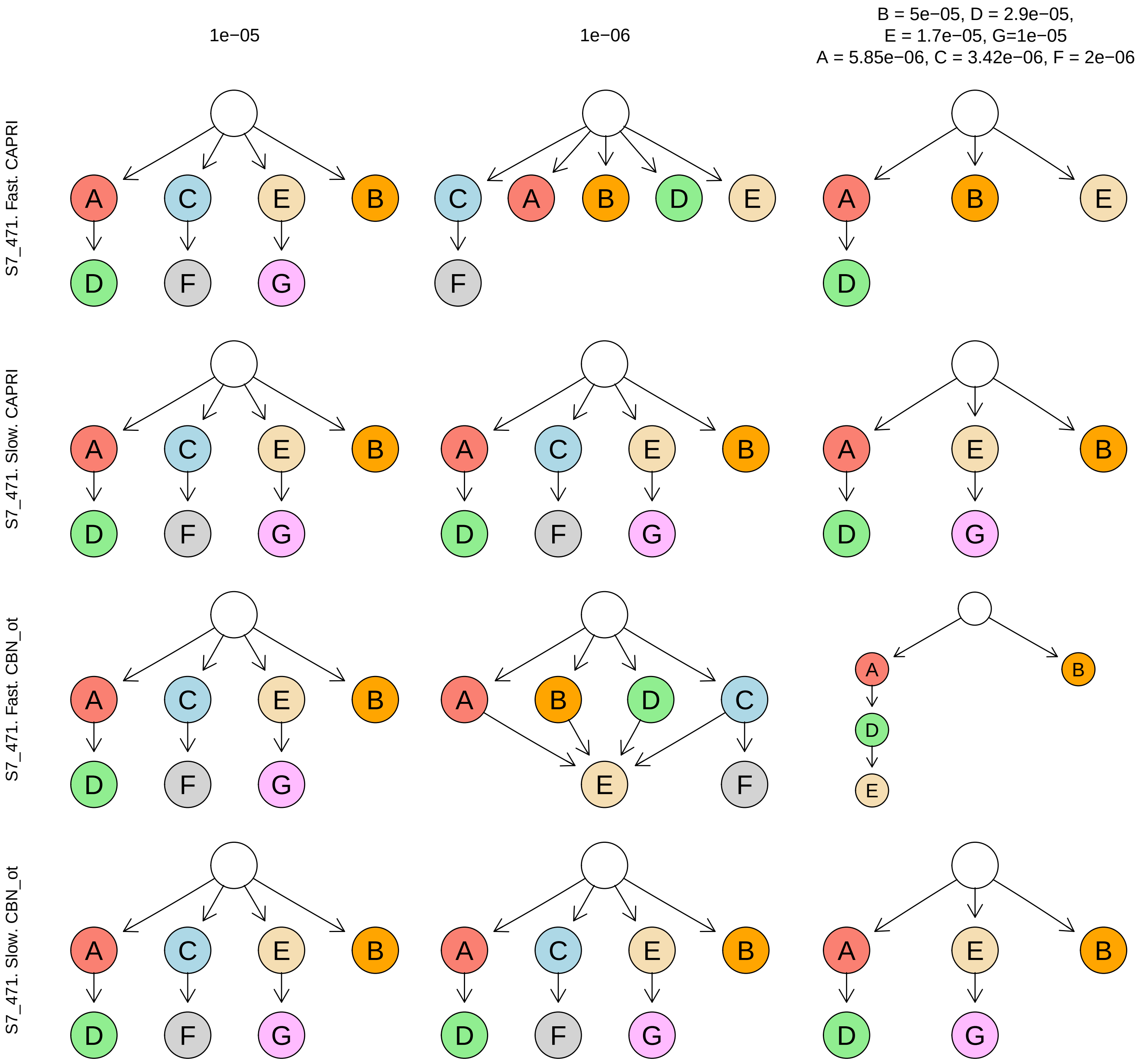

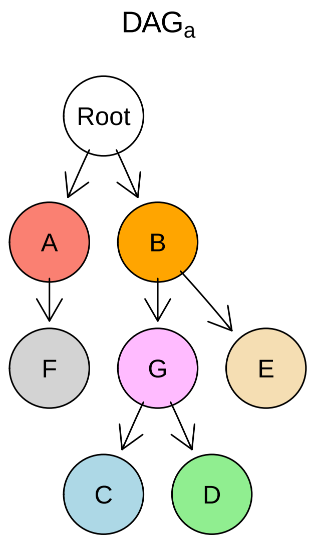

ID	Accessible Genot.	Discrep. DAG_a
S7_472	10	24

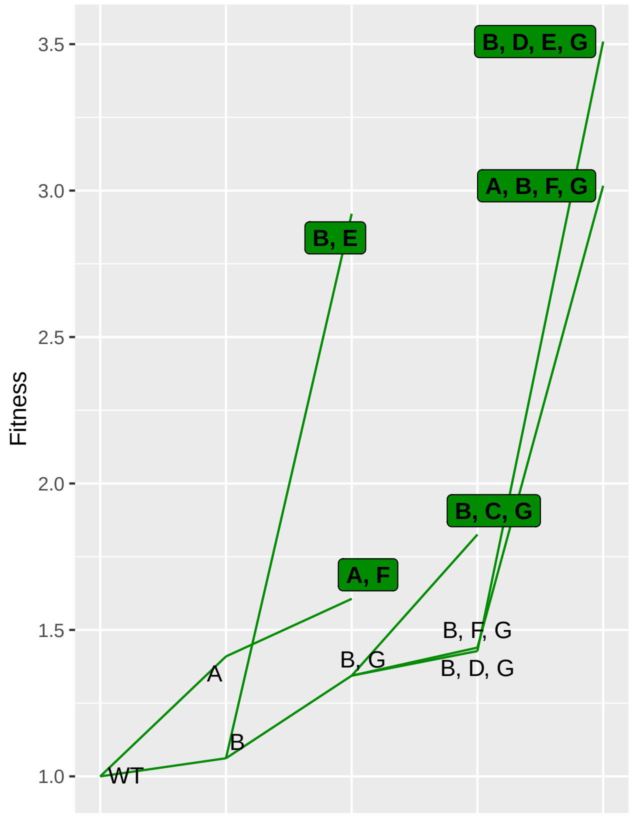

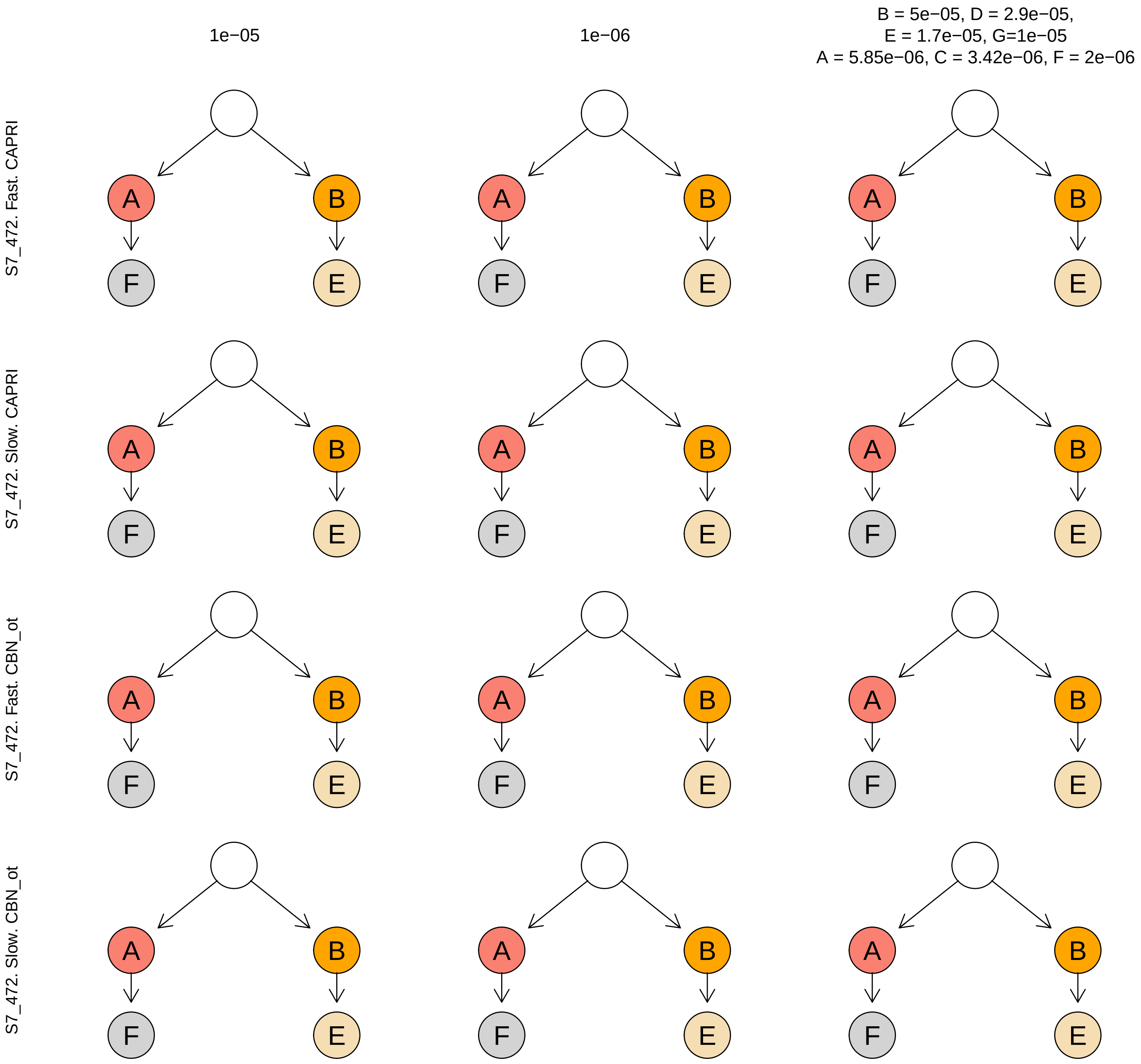

DAG$_a$

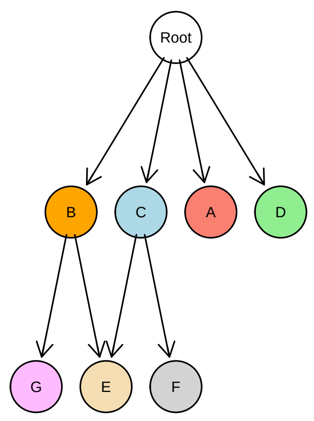

ID	Accessible Genot.	Discrep. DAG_a
S7_473	23	32

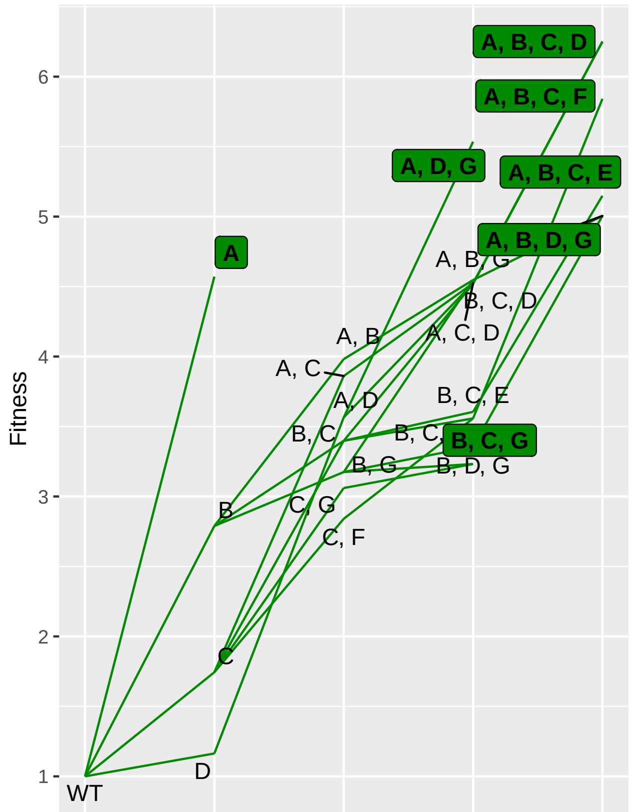

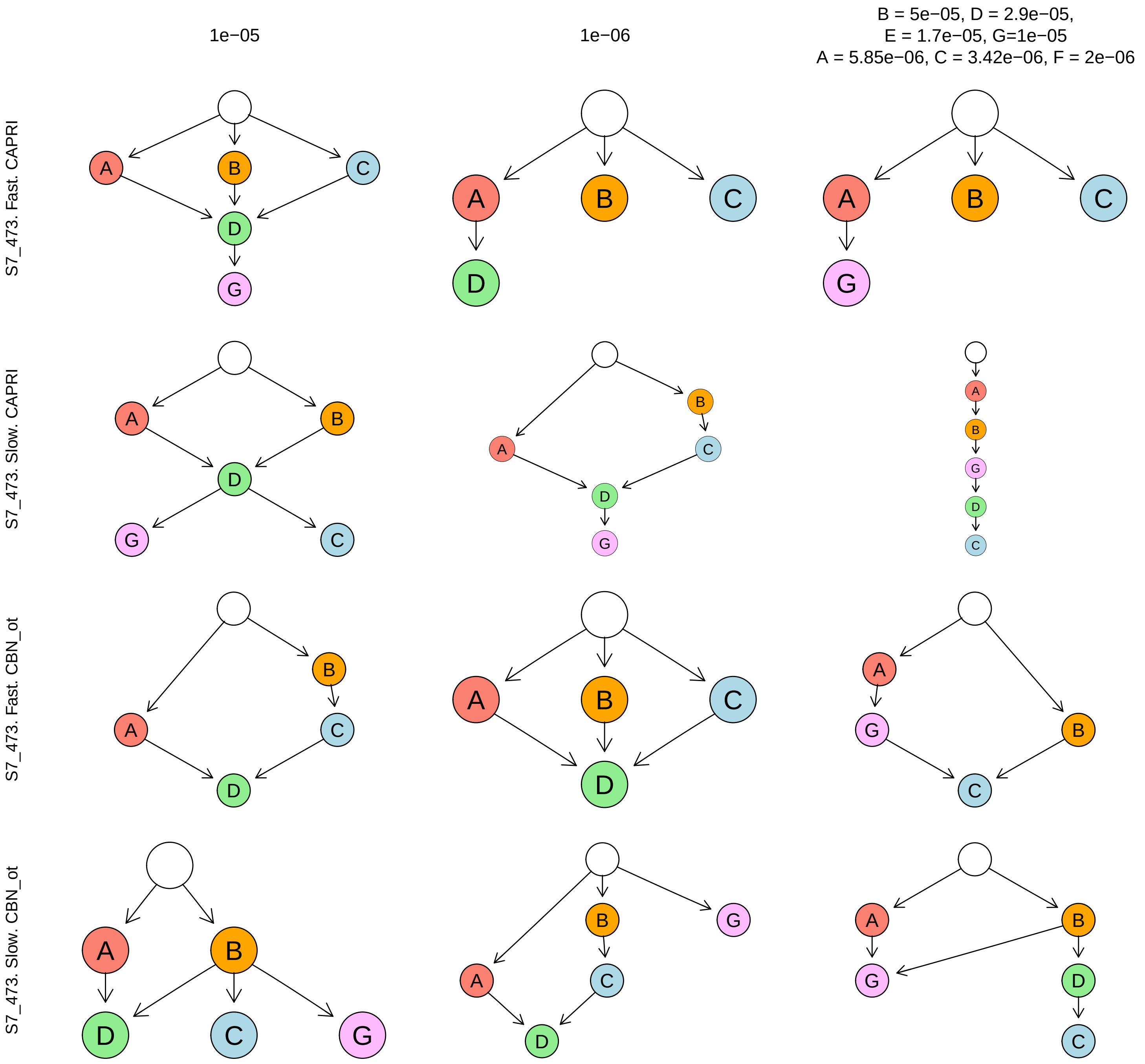

DAG$_a$

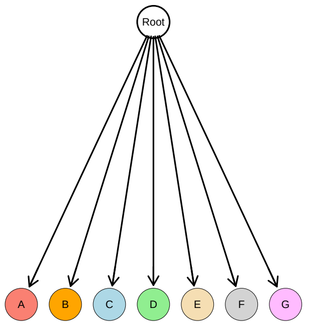

ID	Accessible Genot.	Discrep. DAG_a
S7_474	80	47

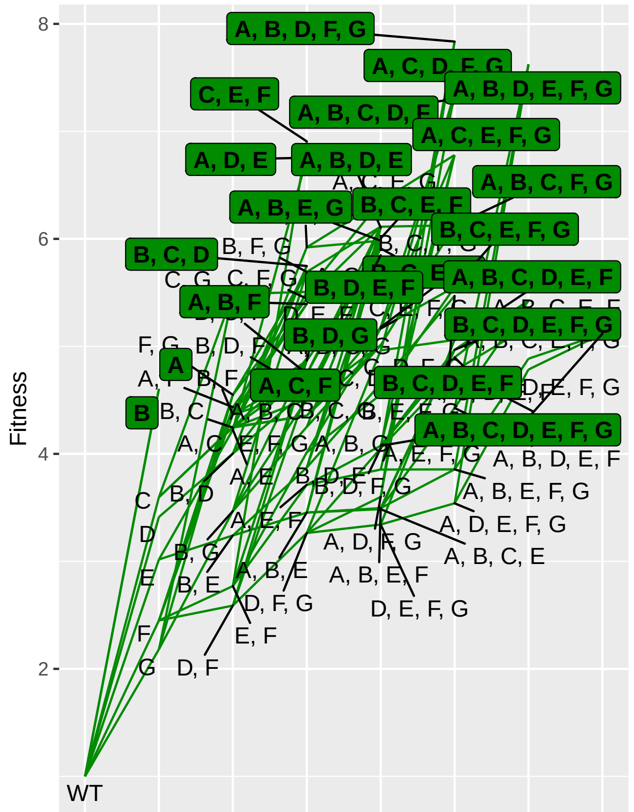

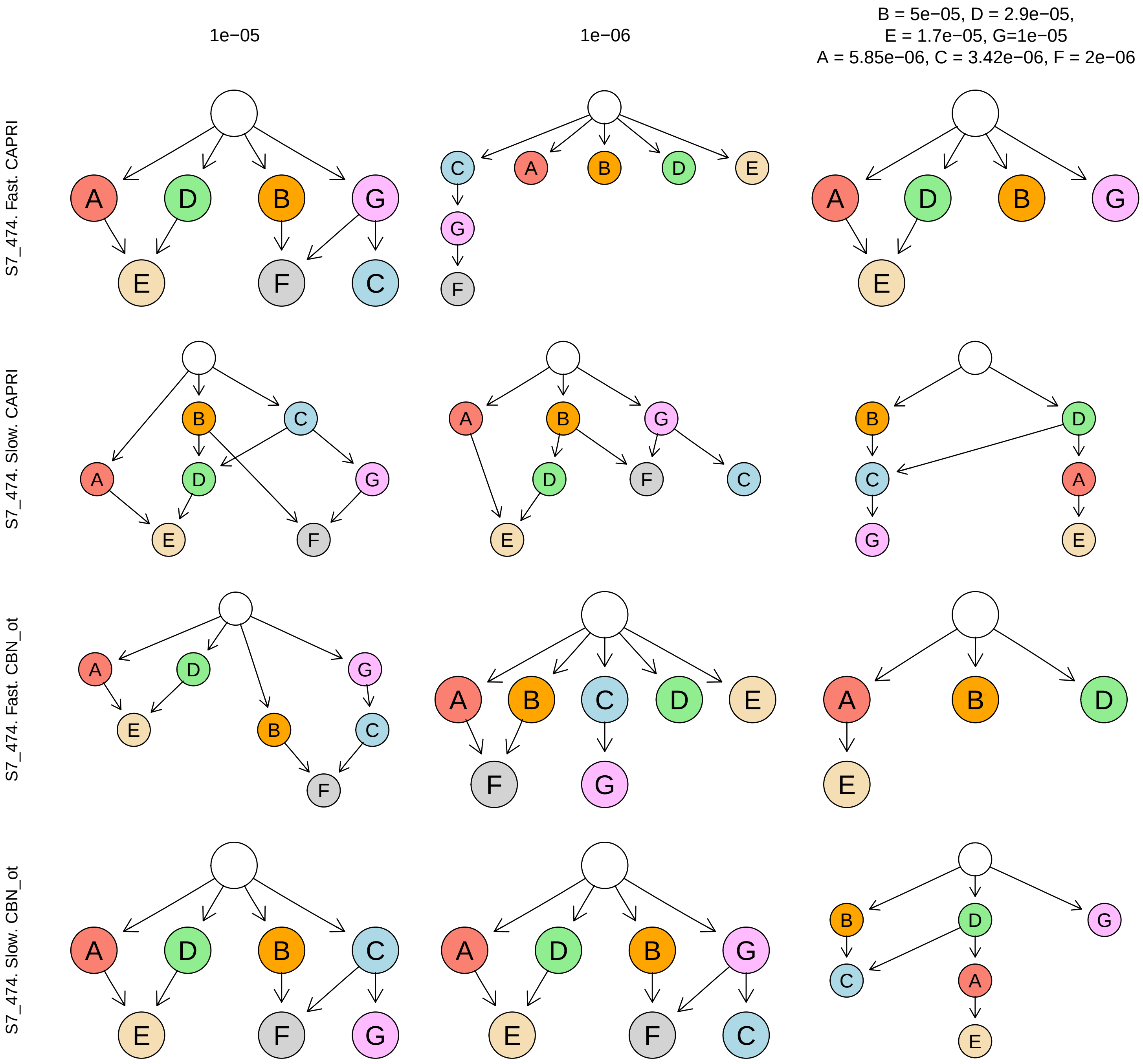

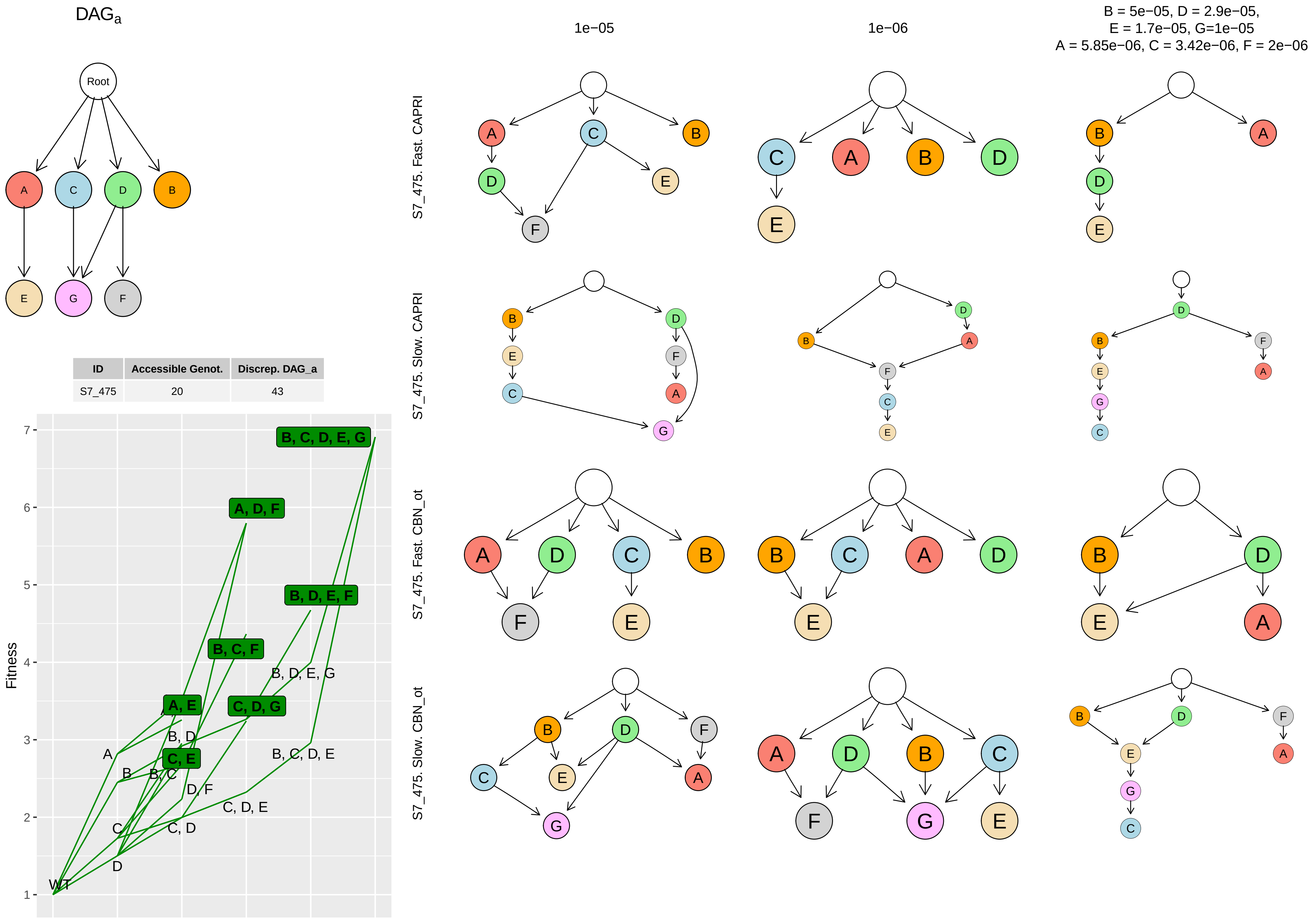

ID	Accessible Genot.	Discrep. DAG_a
S7_475	20	43

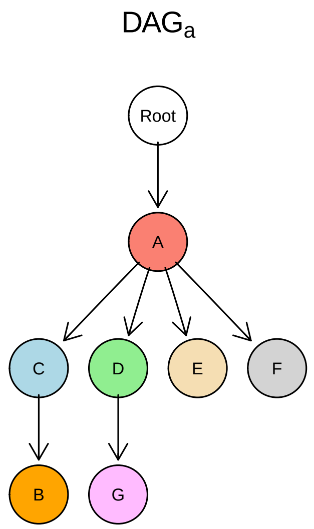

ID	Accessible Genot.	Discrep. DAG_a
S7_476	23	23

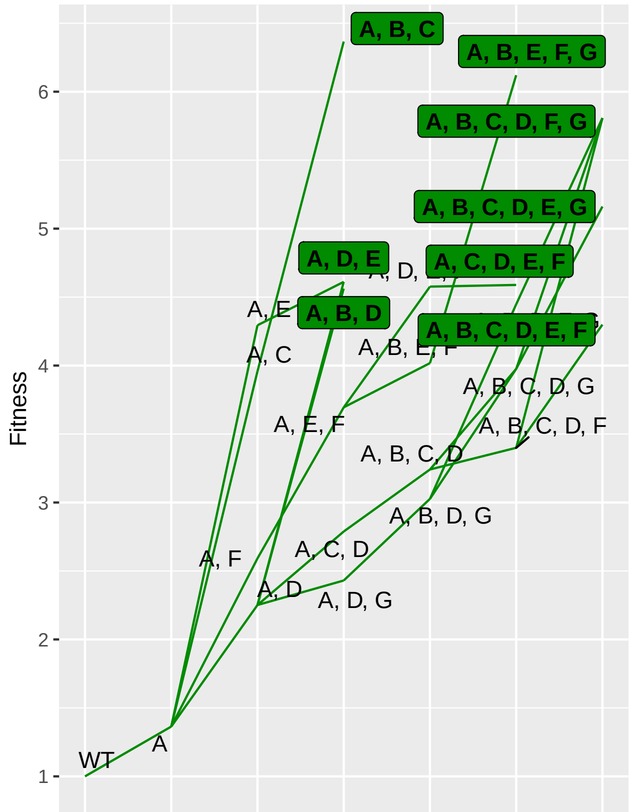

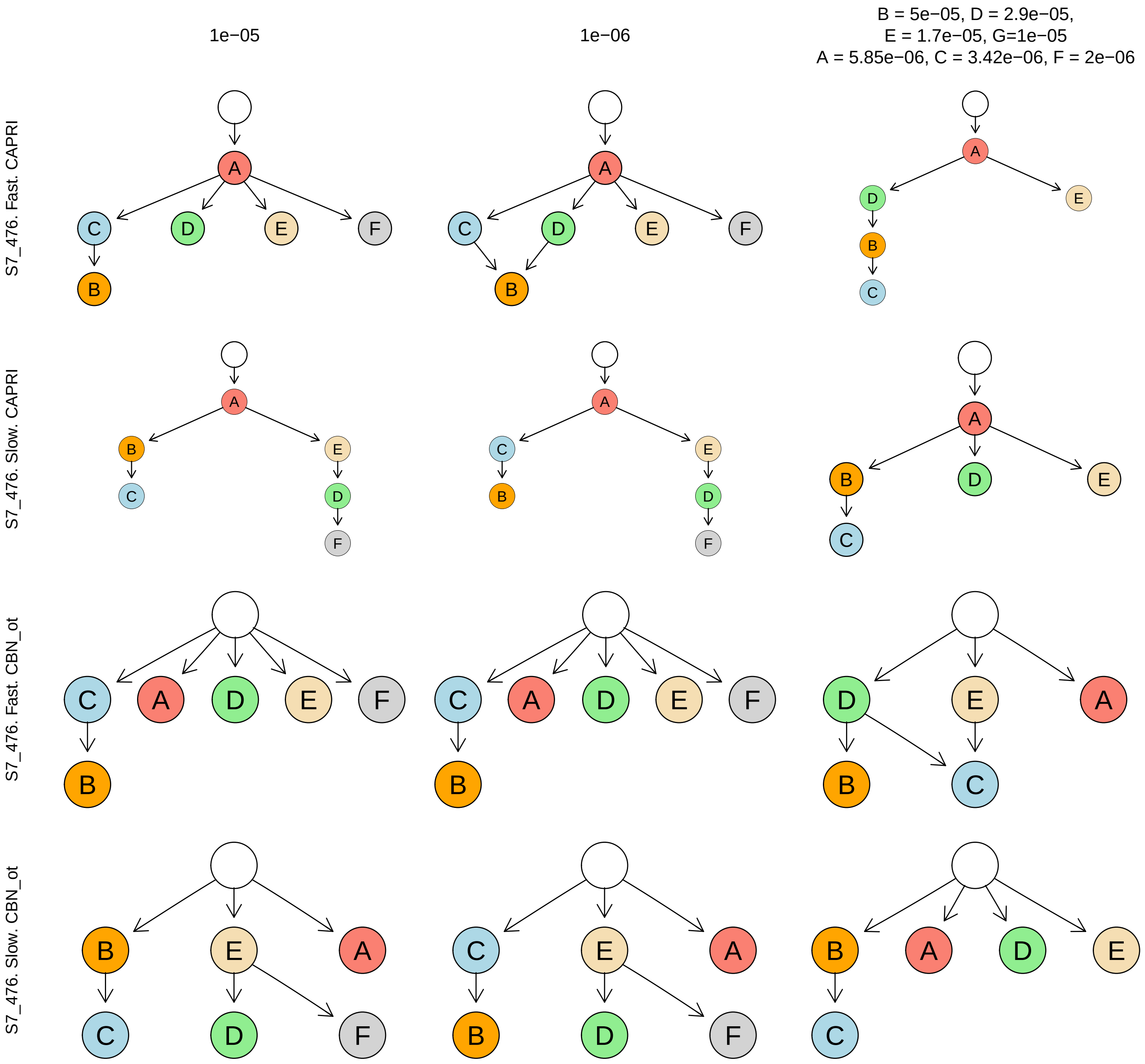

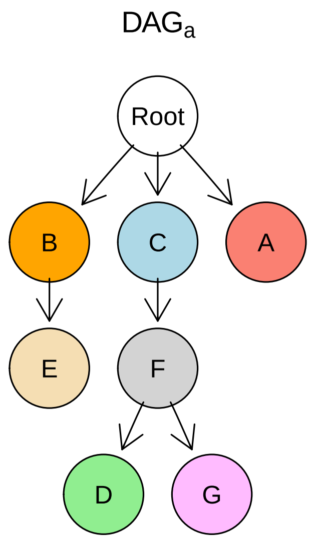

ID	Accessible Genot.	Discrep. DAG_a
S7_477	10	31

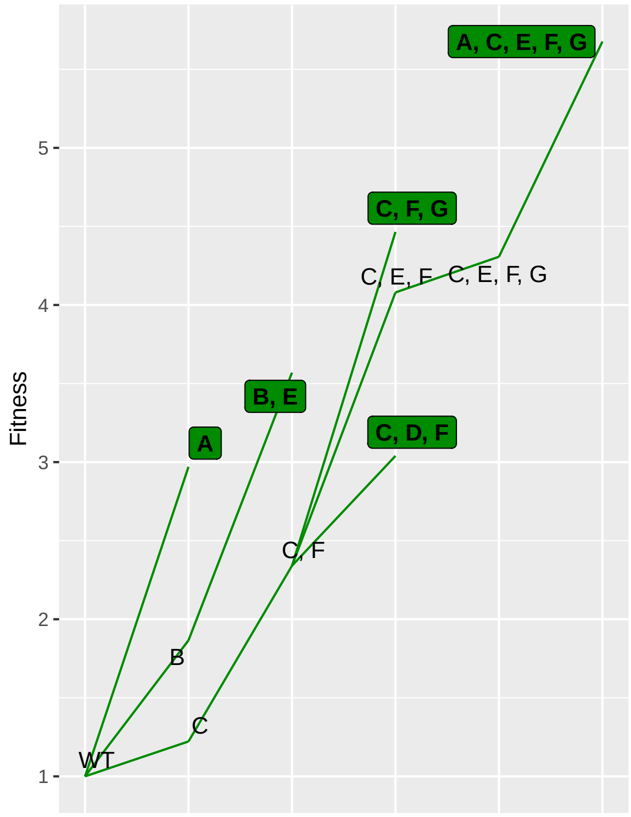

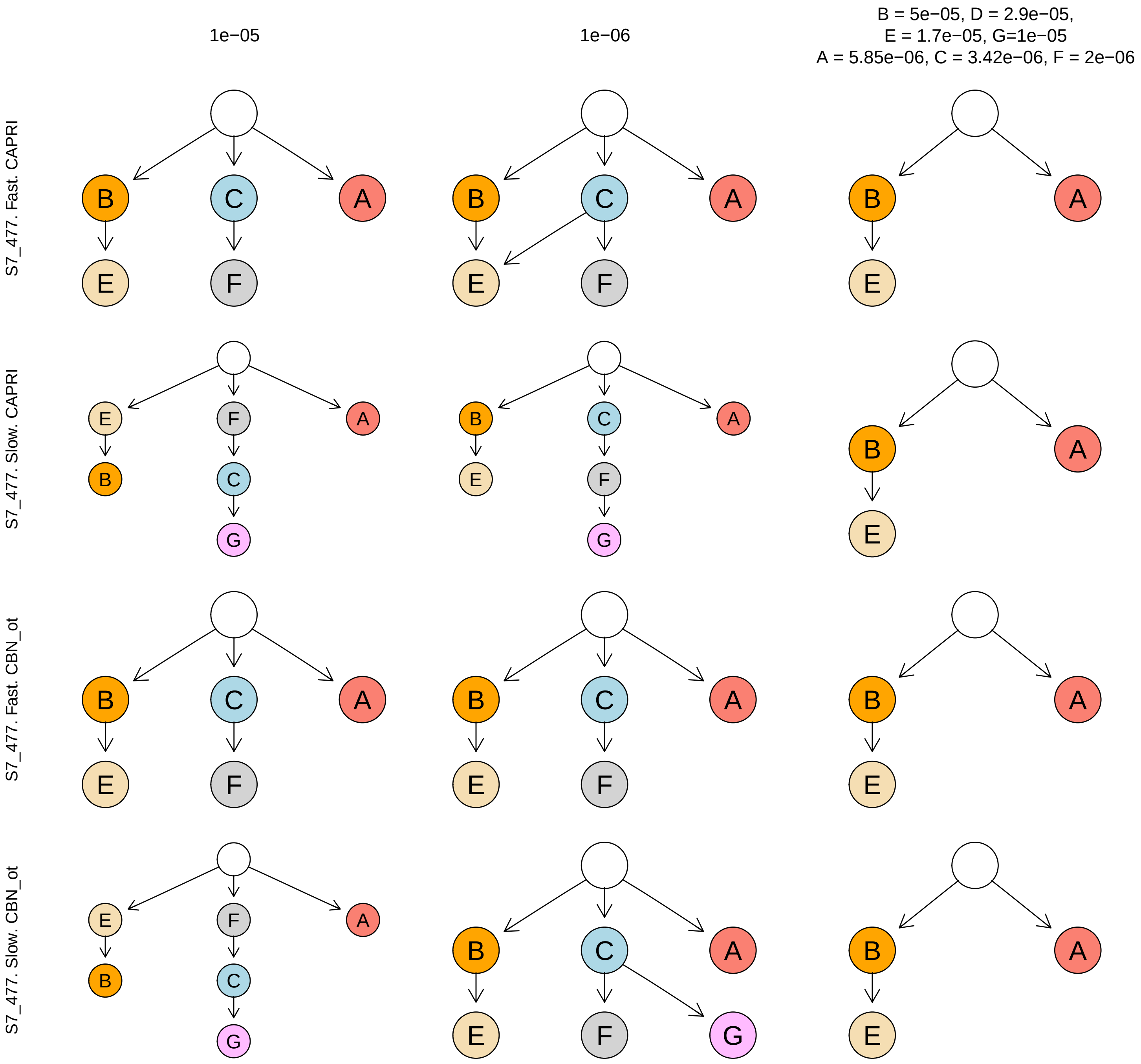

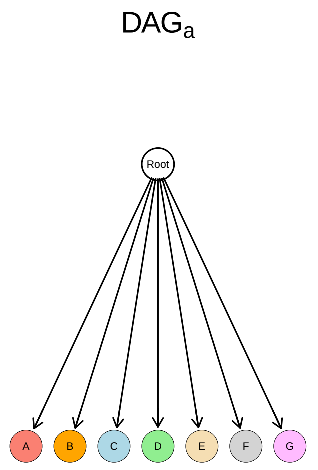

ID	Accessible Genot.	Discrep. DAG_a
S7_478	31	96

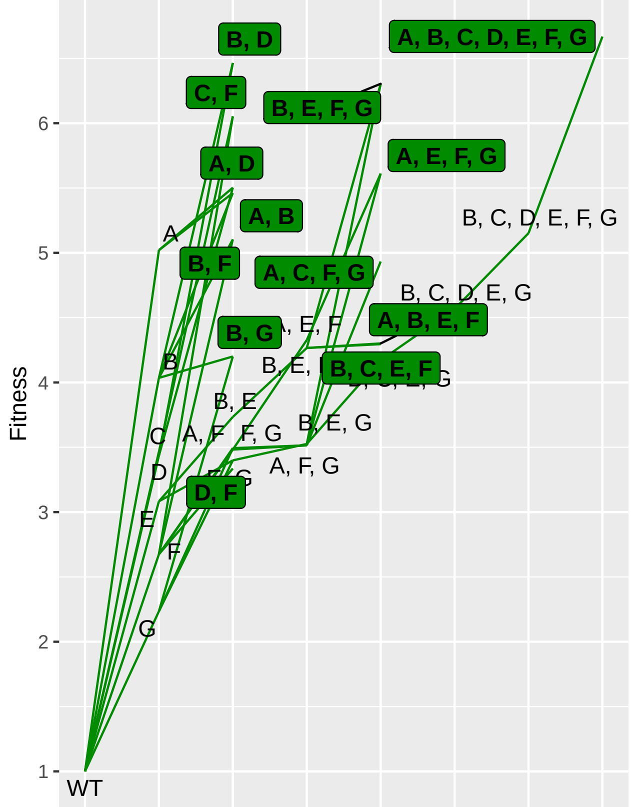

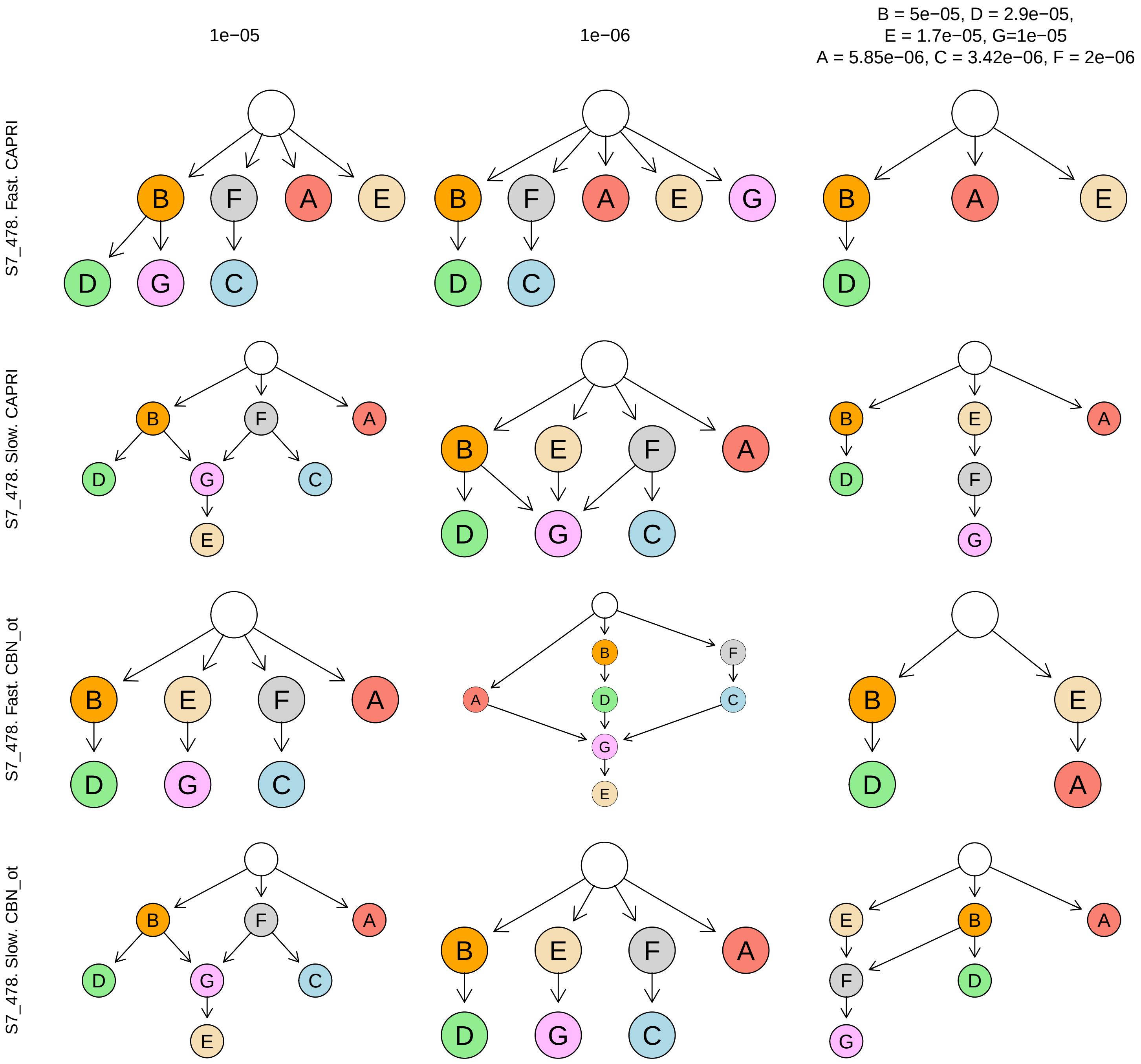

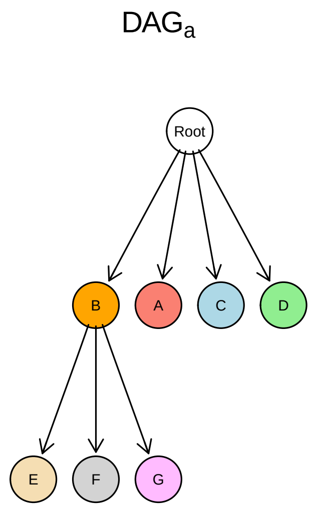

ID	Accessible Genot.	Discrep. DAG_a
S7_479	10	61

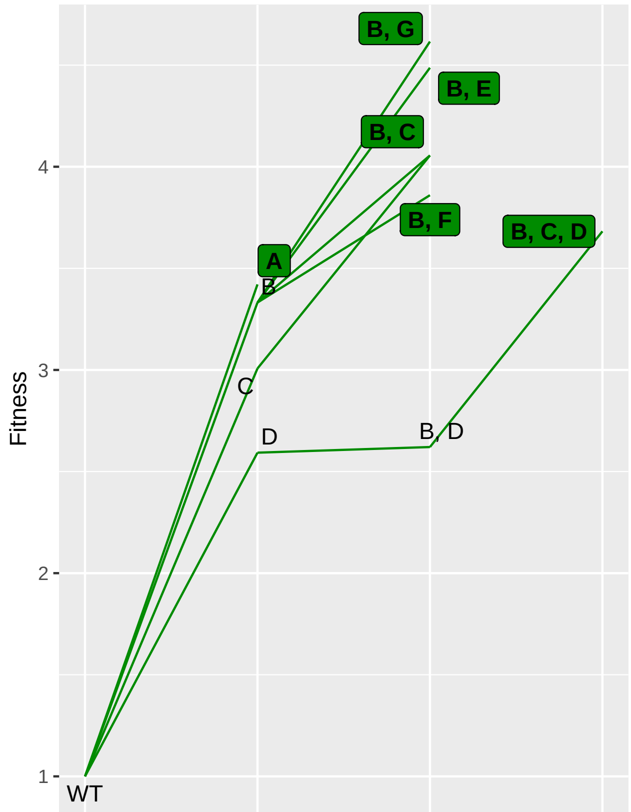

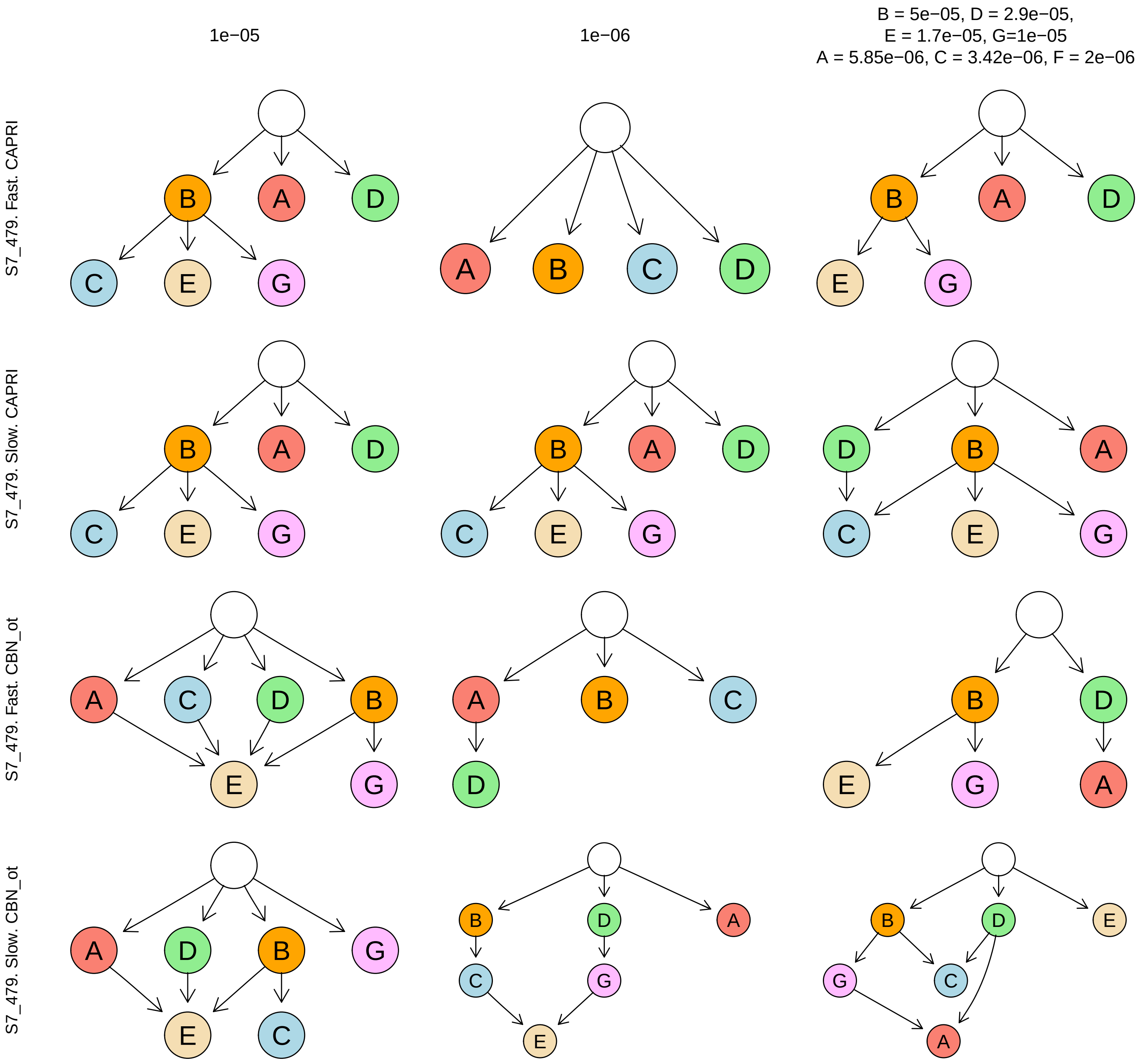

DAG$_a$

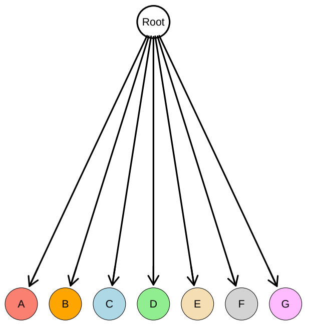

ID	Accessible Genot.	Discrep. DAG_a
S7_480	37	90

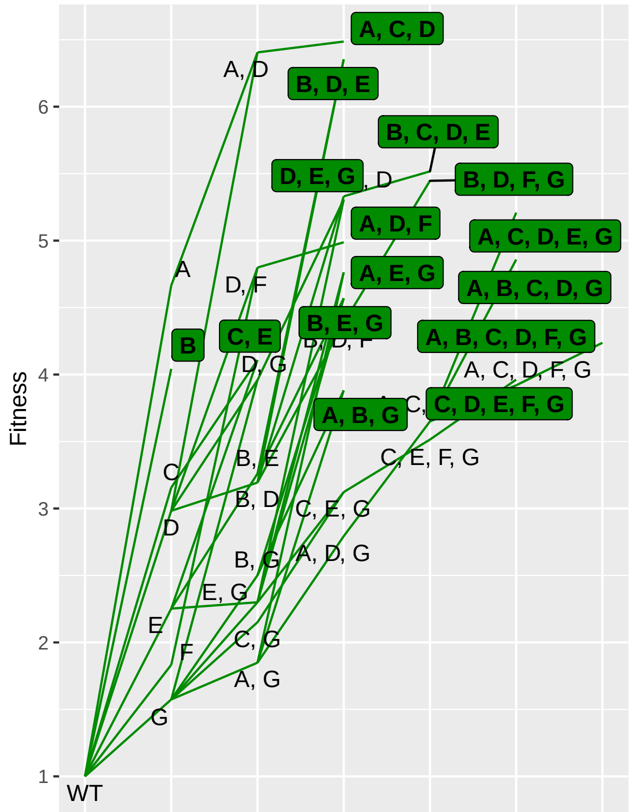

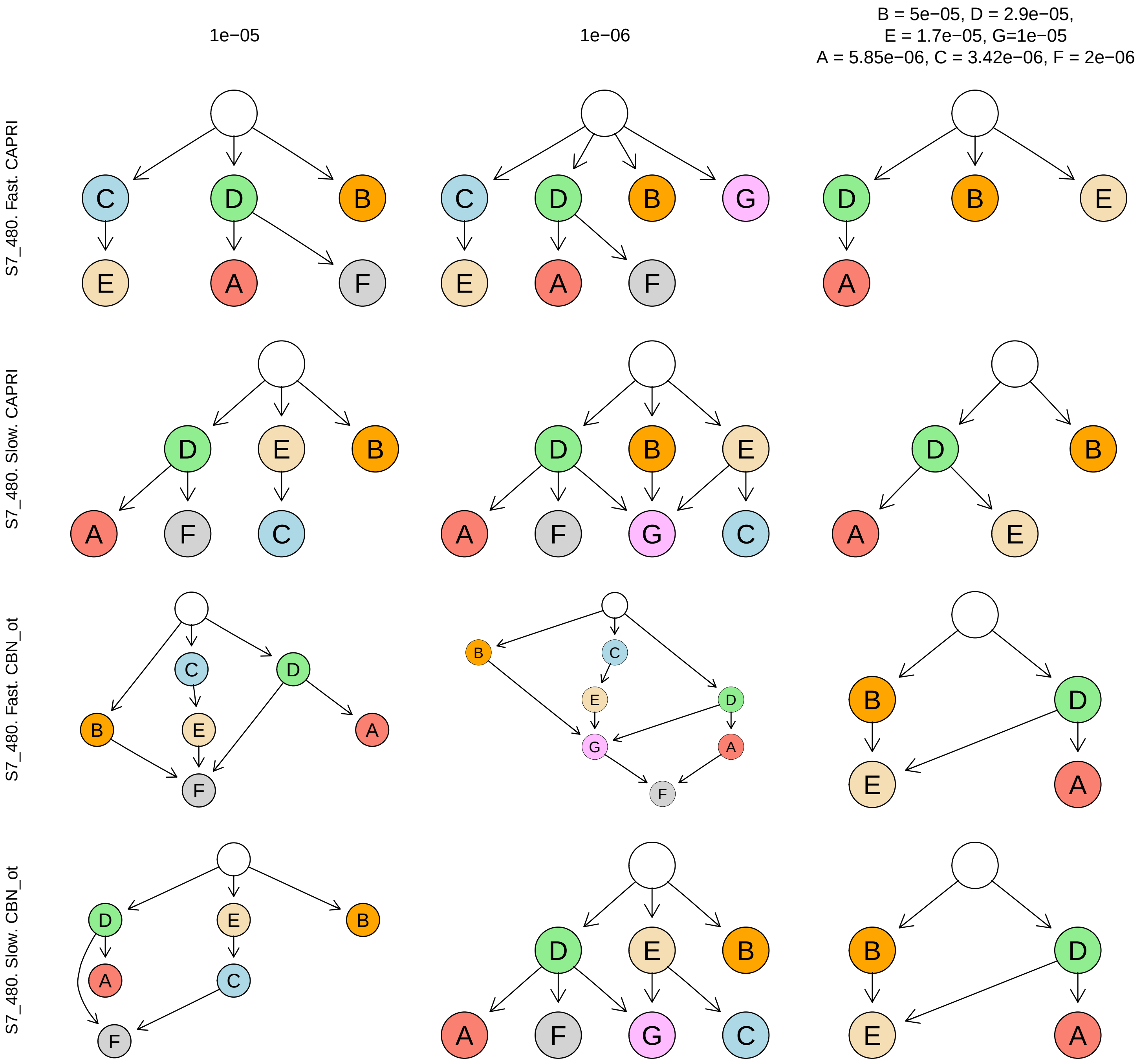

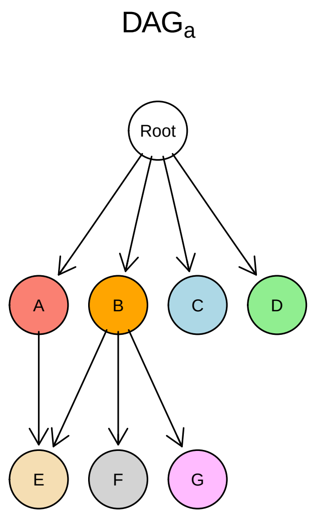

ID	Accessible Genot.	Discrep. DAG_a
S7_481	20	39

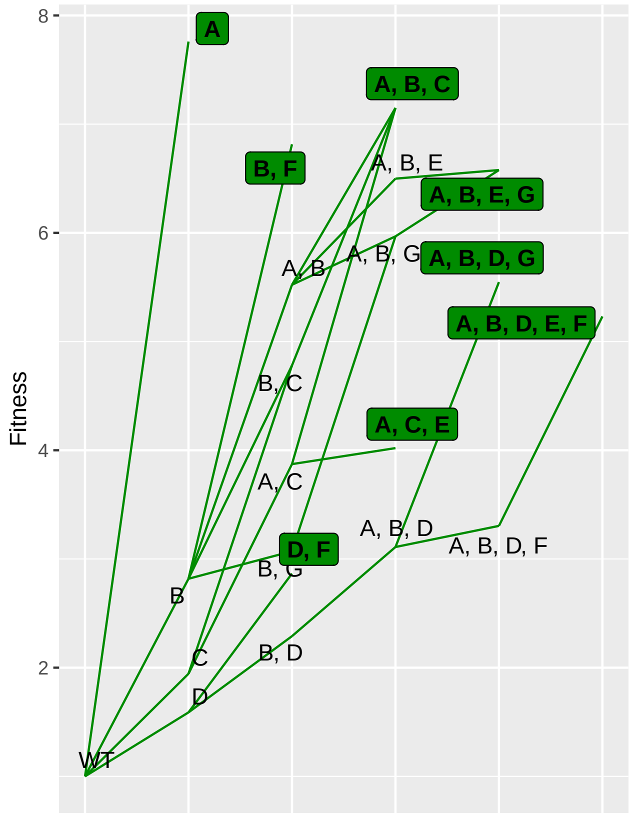

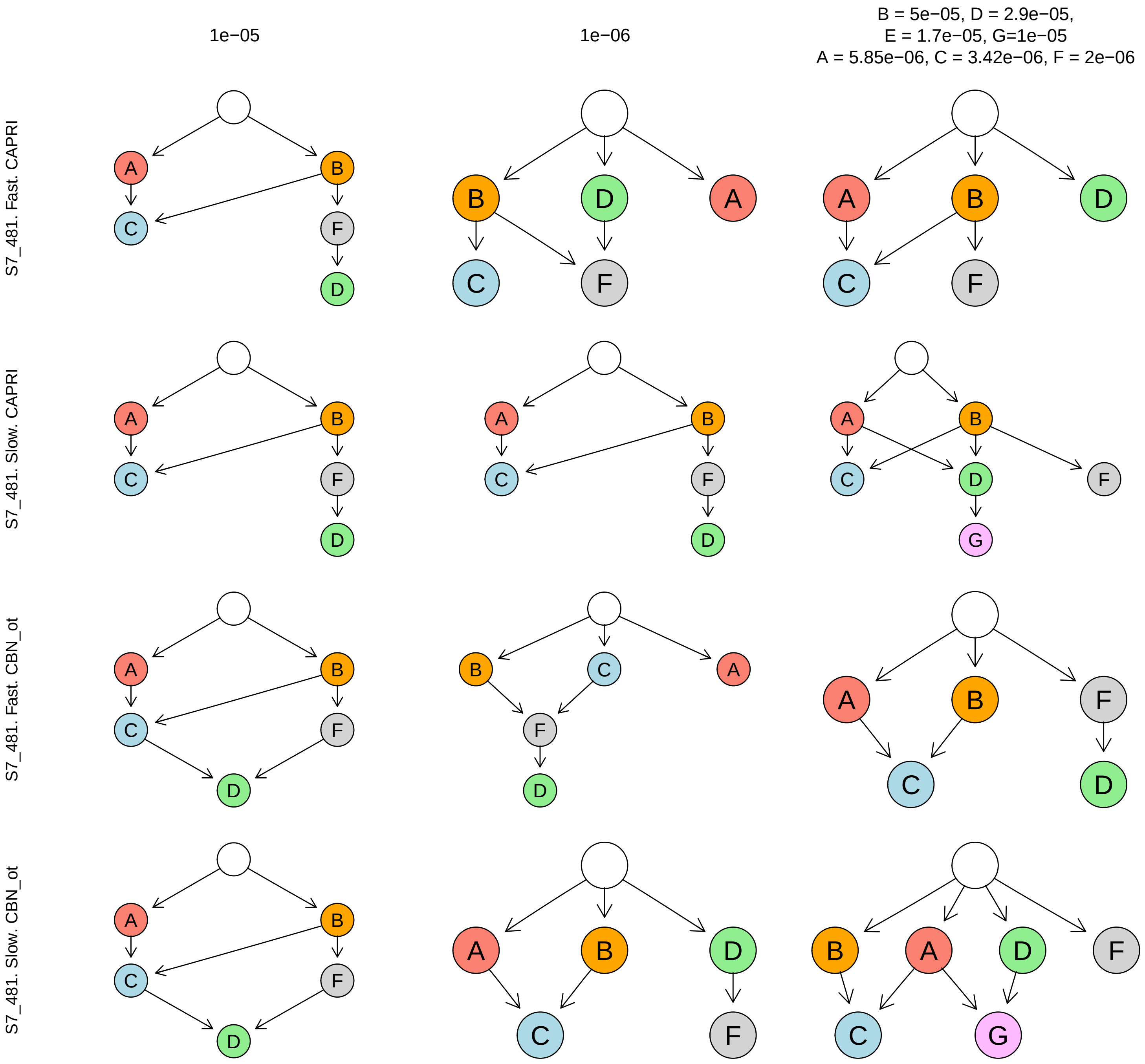

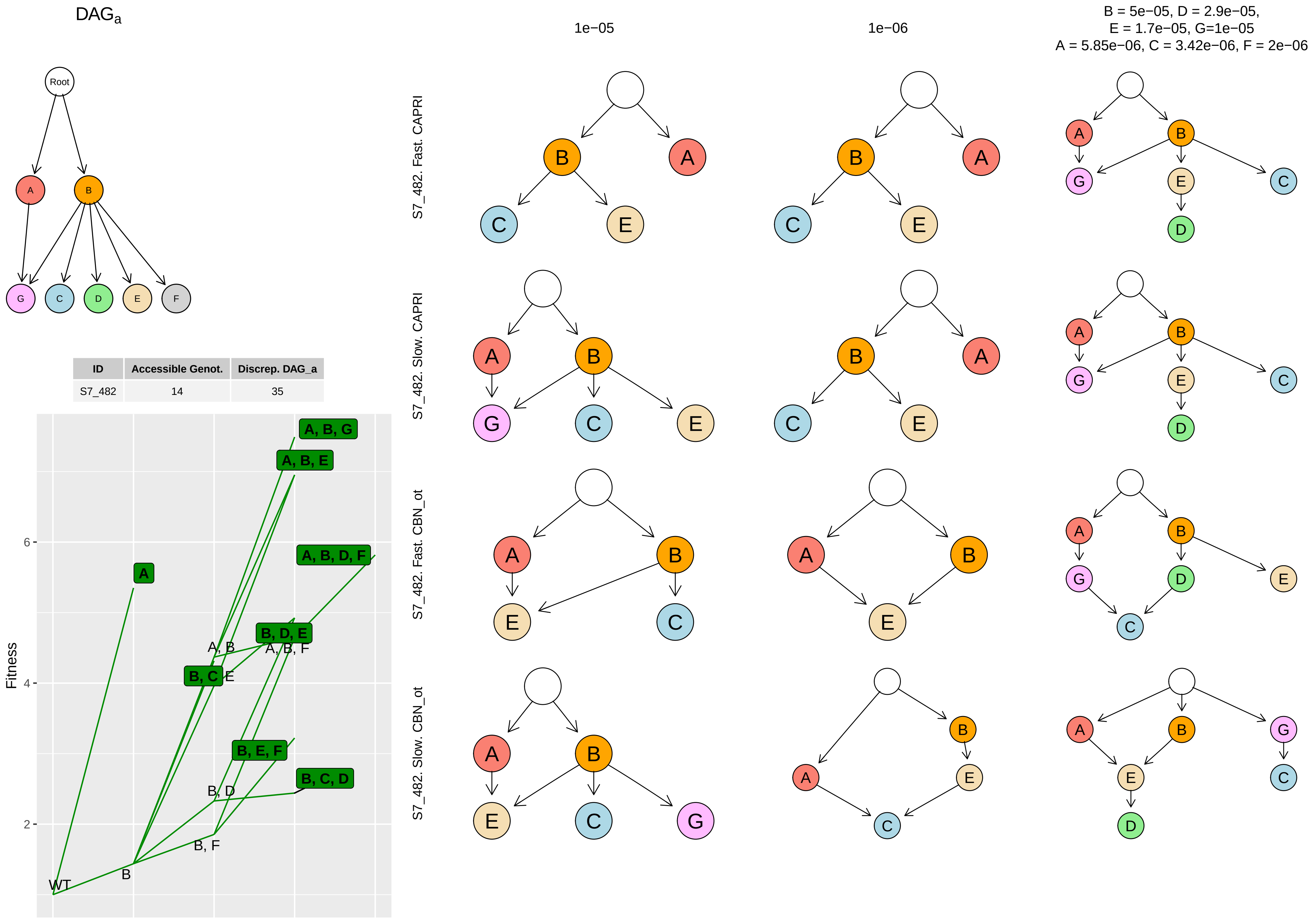

ID	Accessible Genot.	Discrep. DAG_a
S7_482	14	35

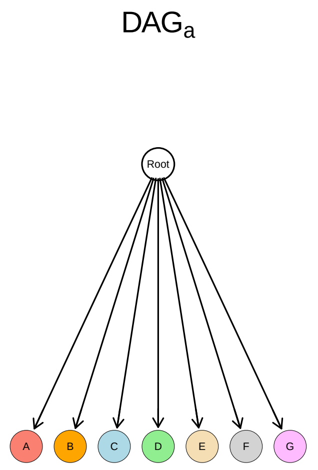

ID	Accessible Genot.	Discrep. DAG_a
S7_483	24	103

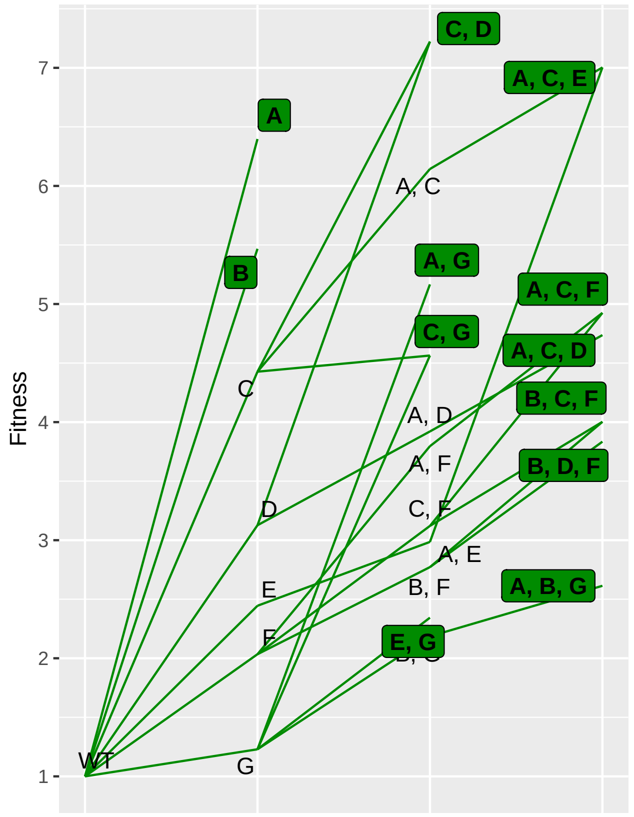

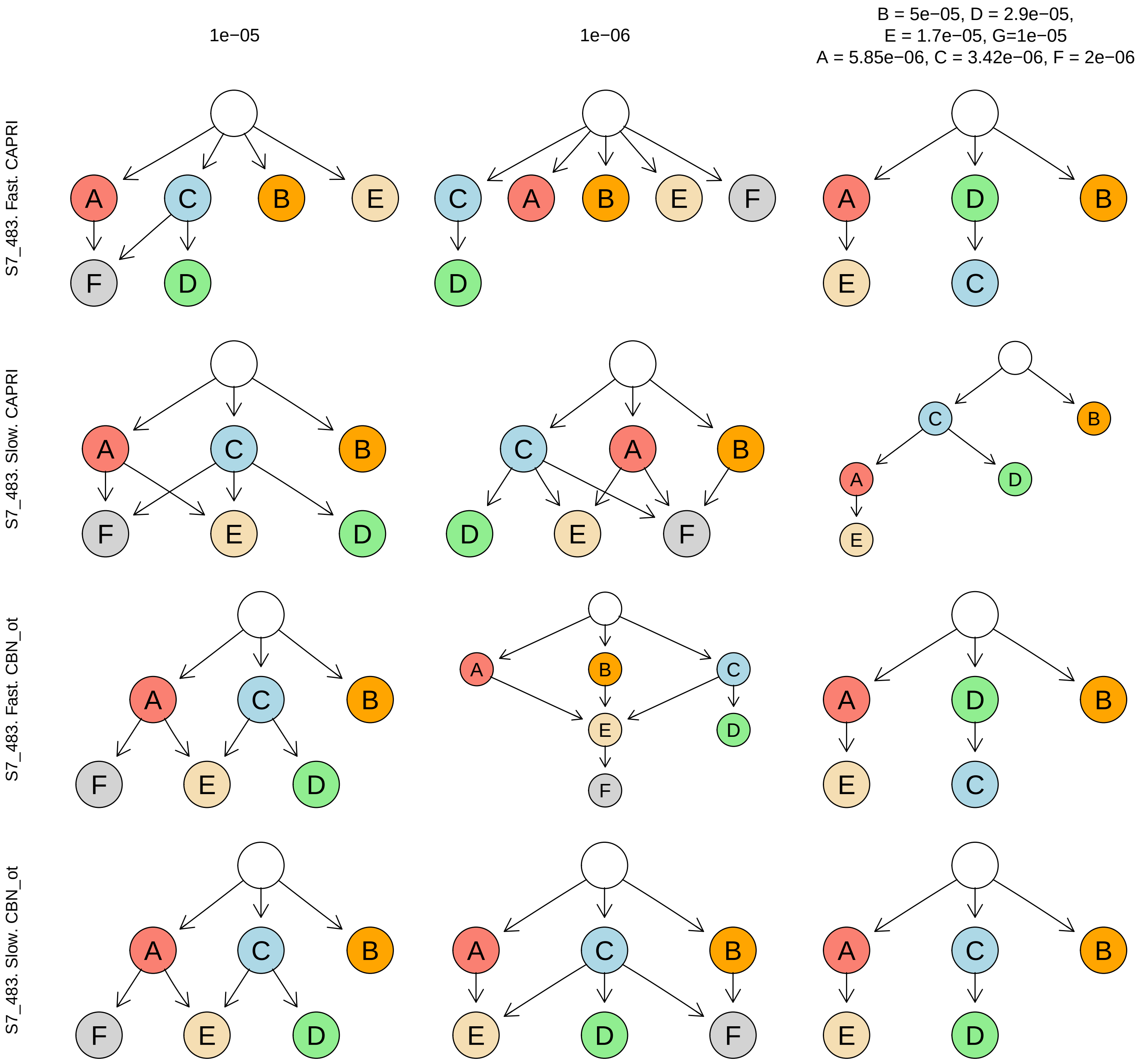

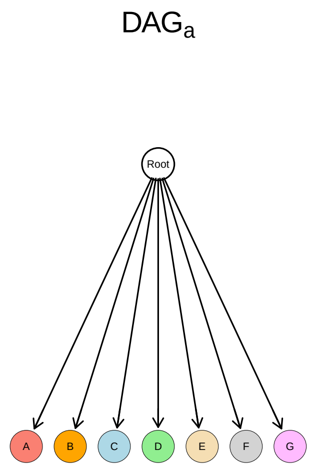

ID	Accessible Genot.	Discrep. DAG_a
S7_484	33	94

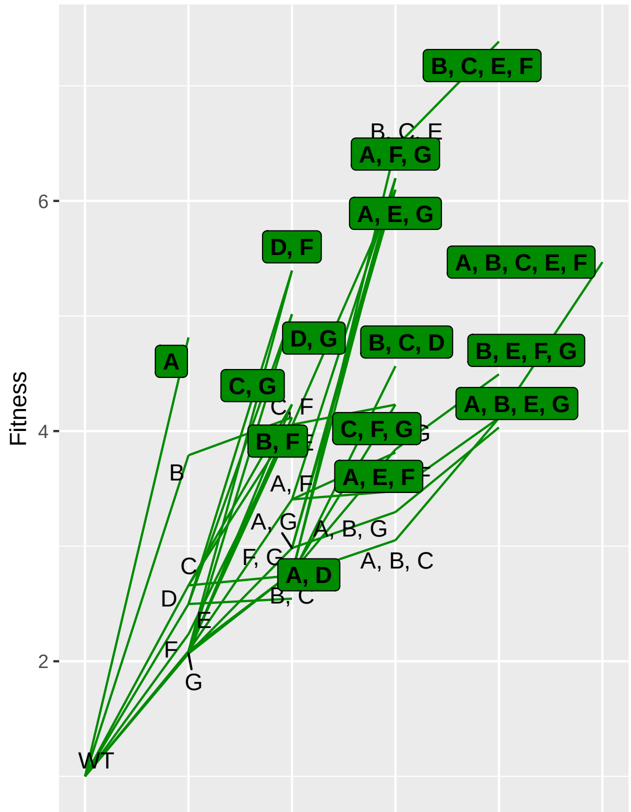

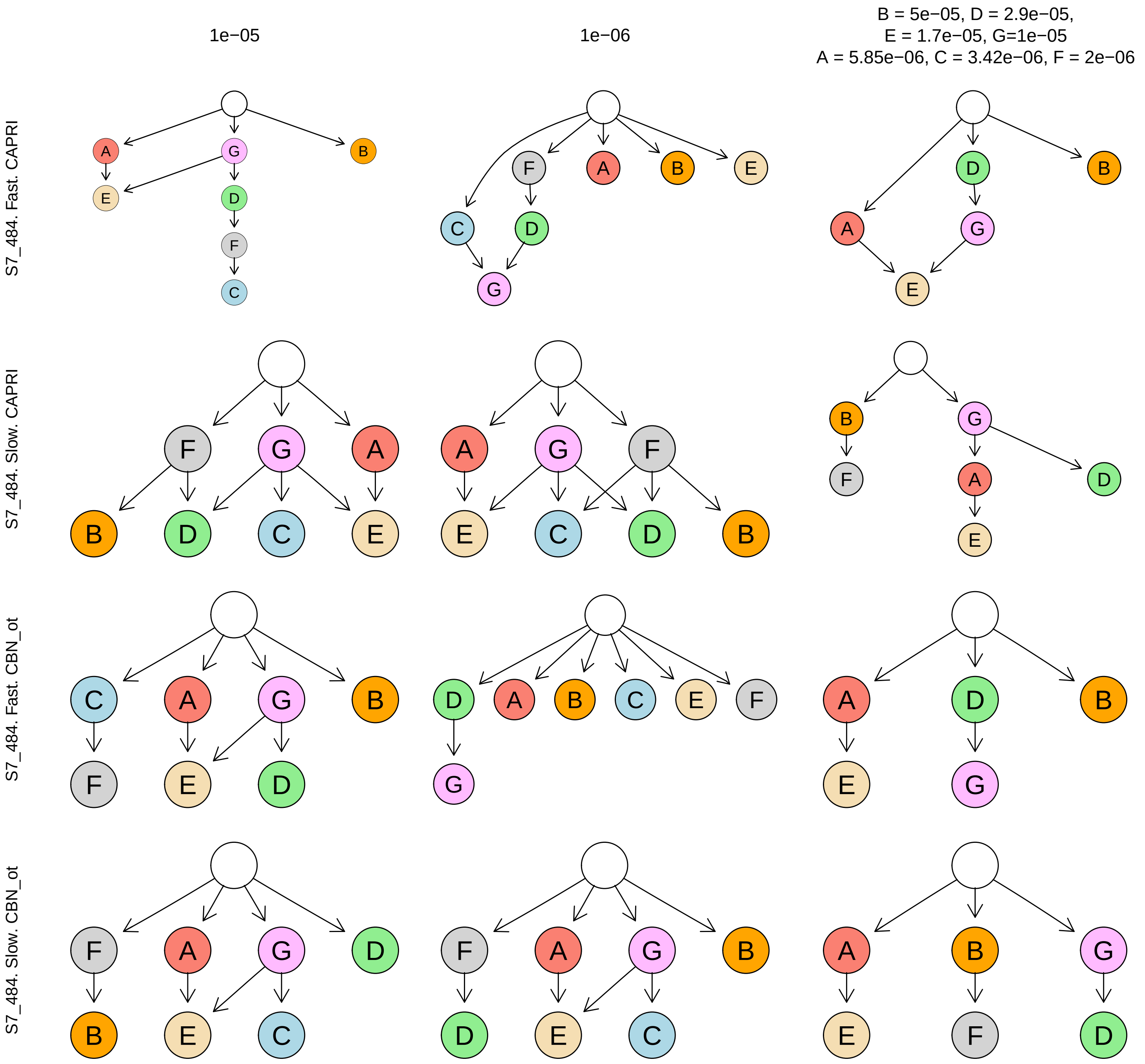

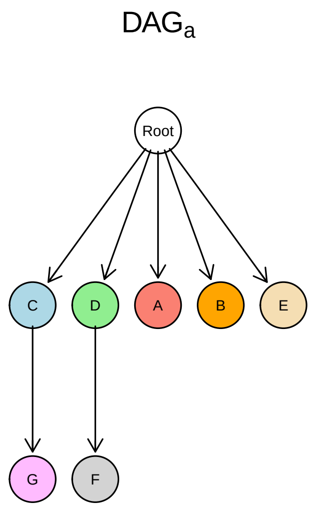

ID	Accessible Genot.	Discrep. DAG_a
S7_485	25	54

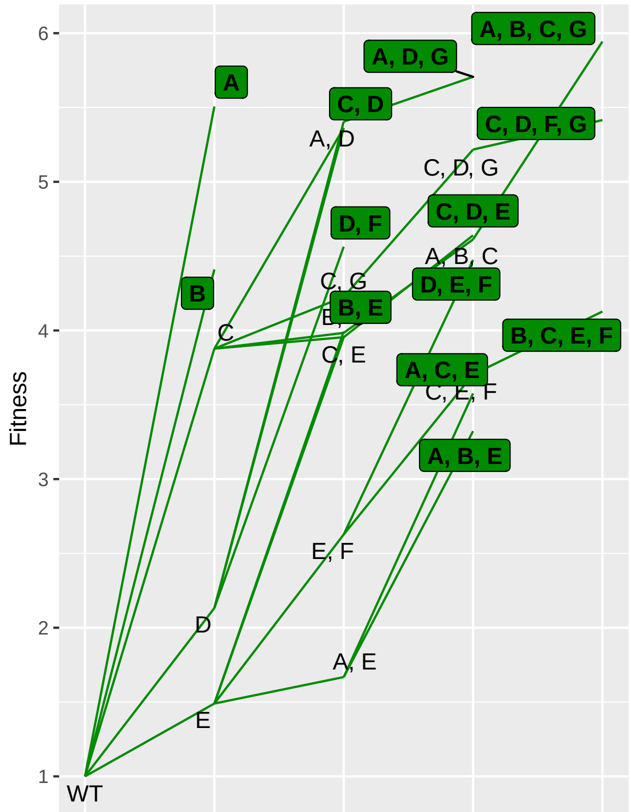

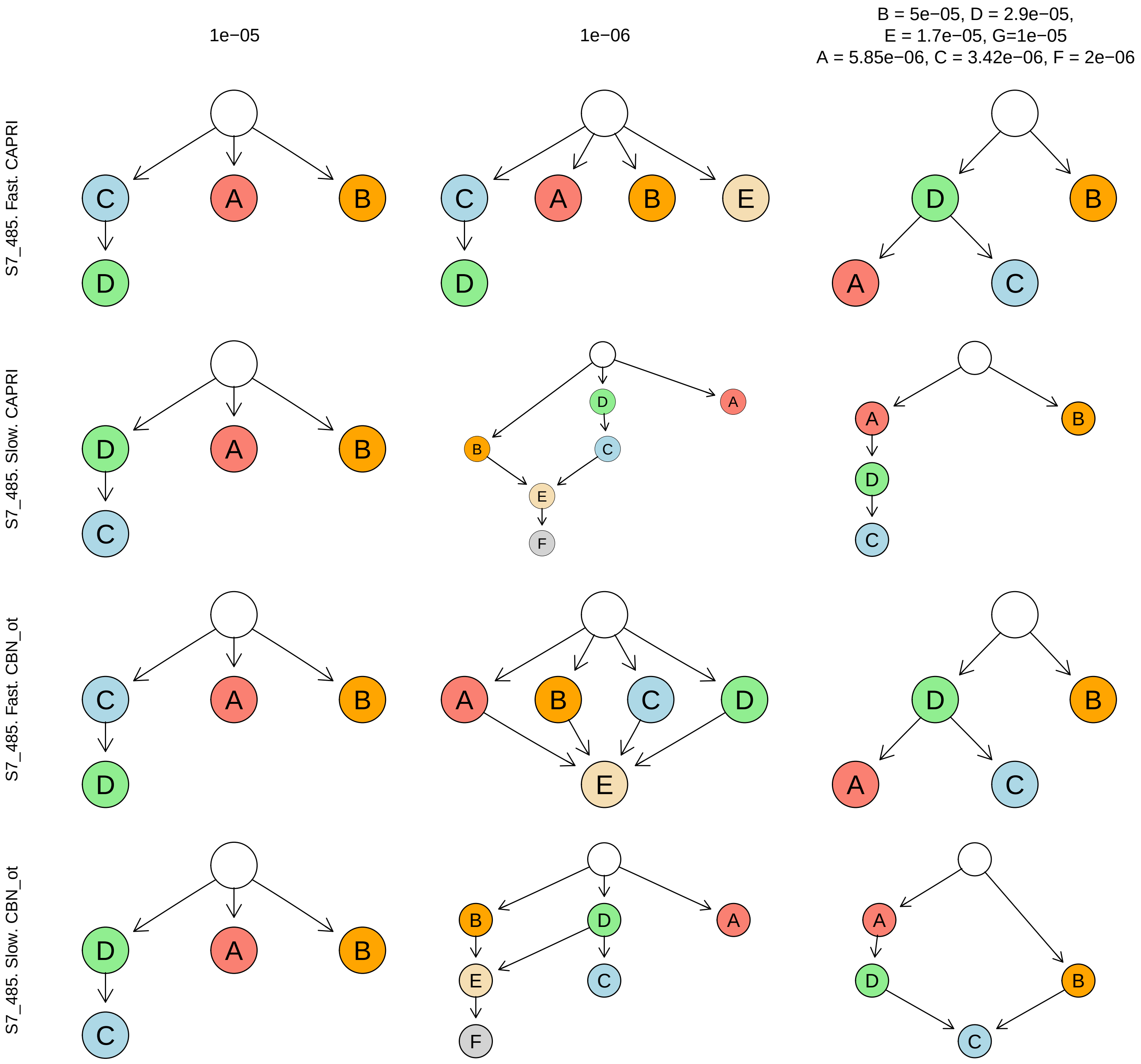

DAG_a

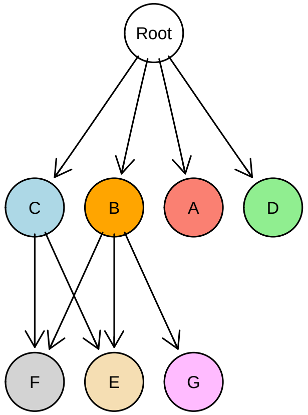

ID	Accessible Genot.	Discrep. DAG_a
S7_486	14	35

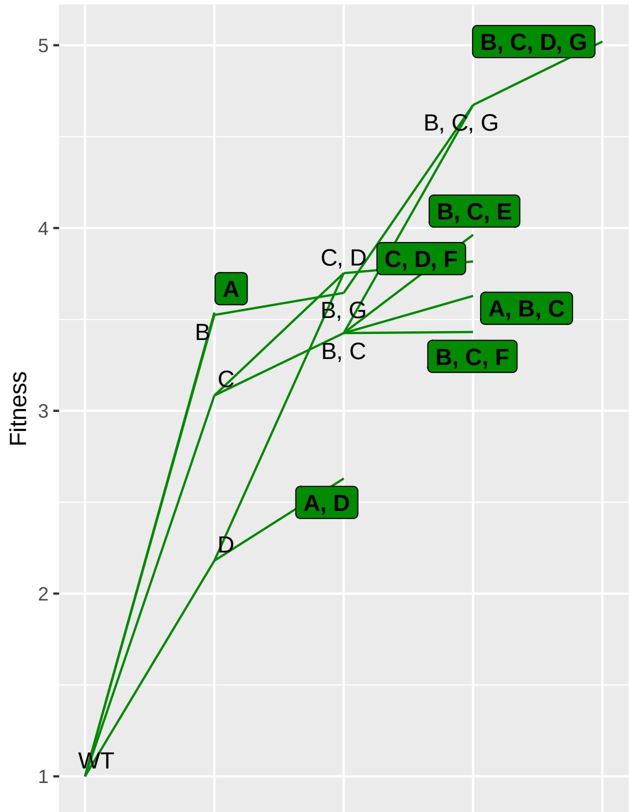

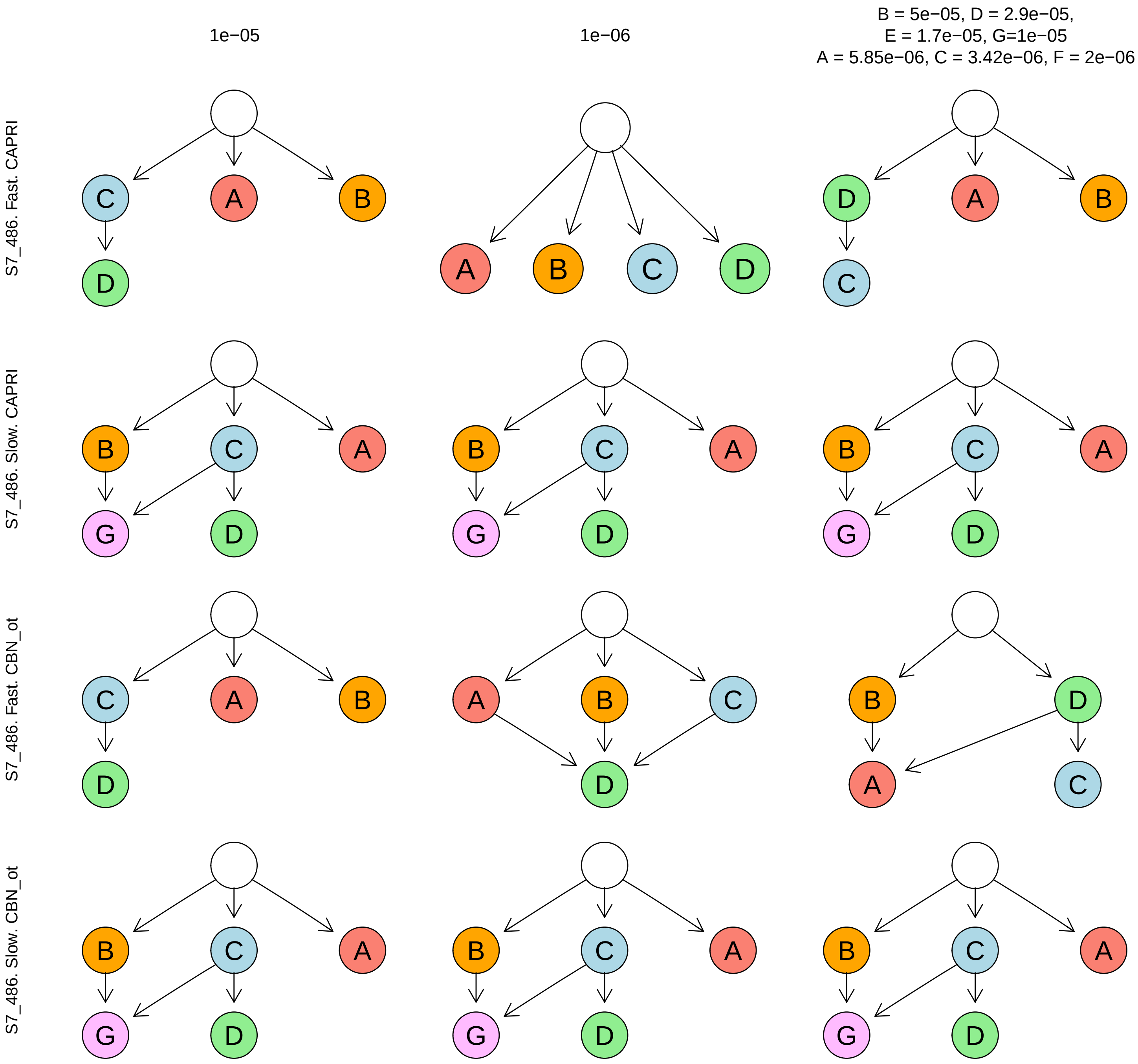

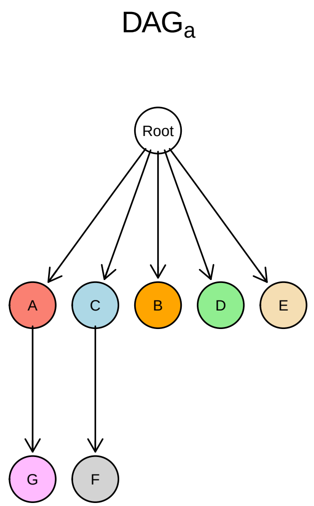

ID	Accessible Genot.	Discrep. DAG_a
S7_487	30	51

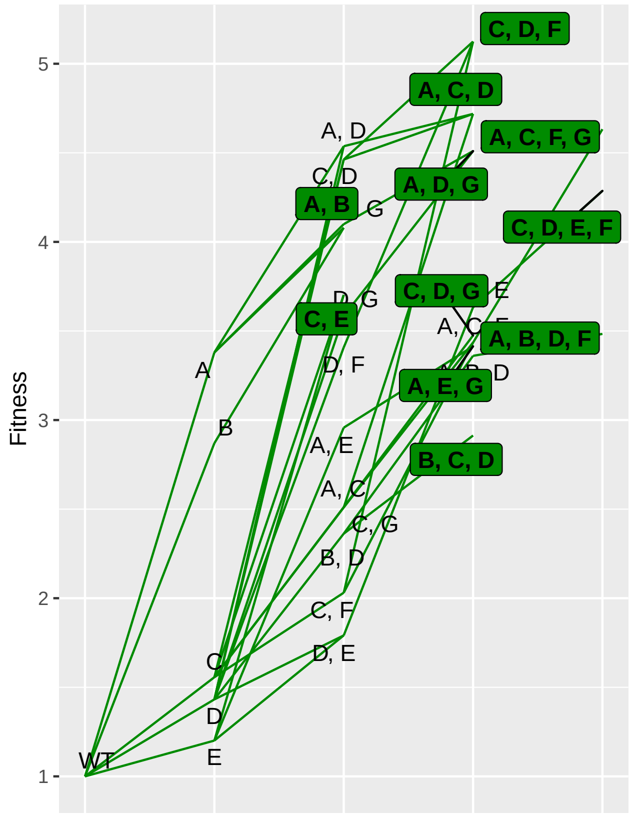

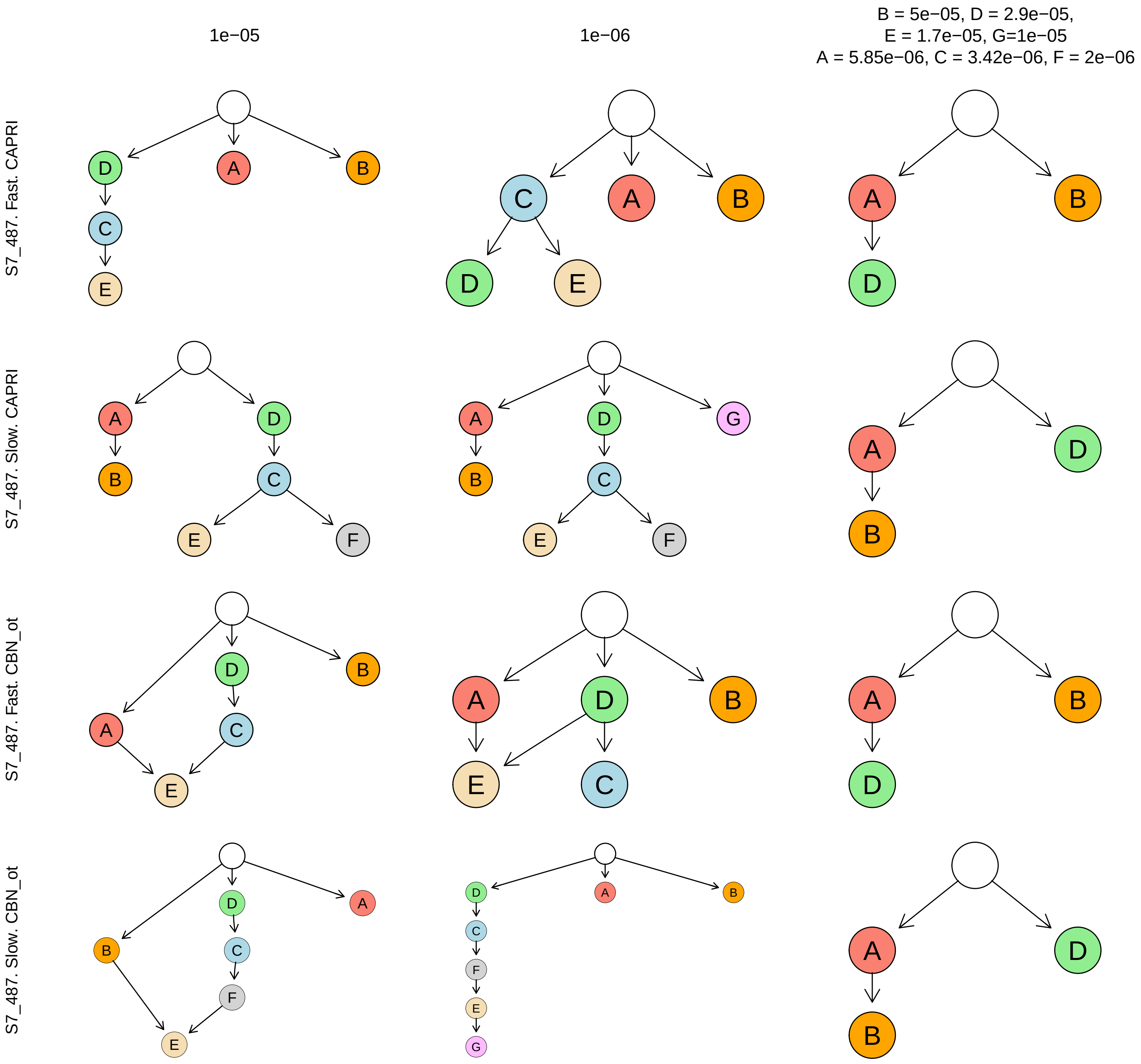

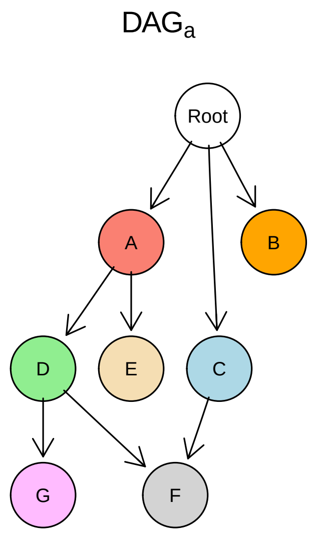

ID	Accessible Genot.	Discrep. DAG_a
S7_488	23	30

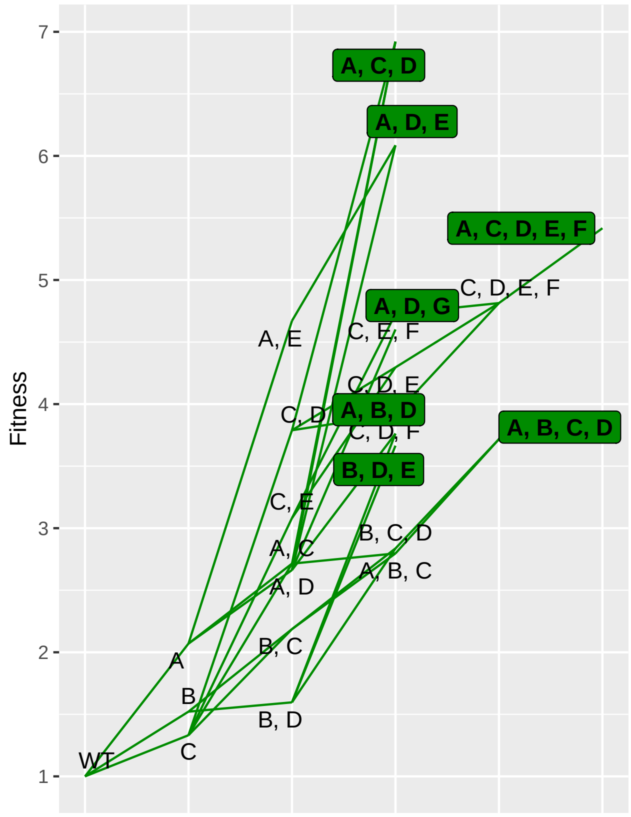

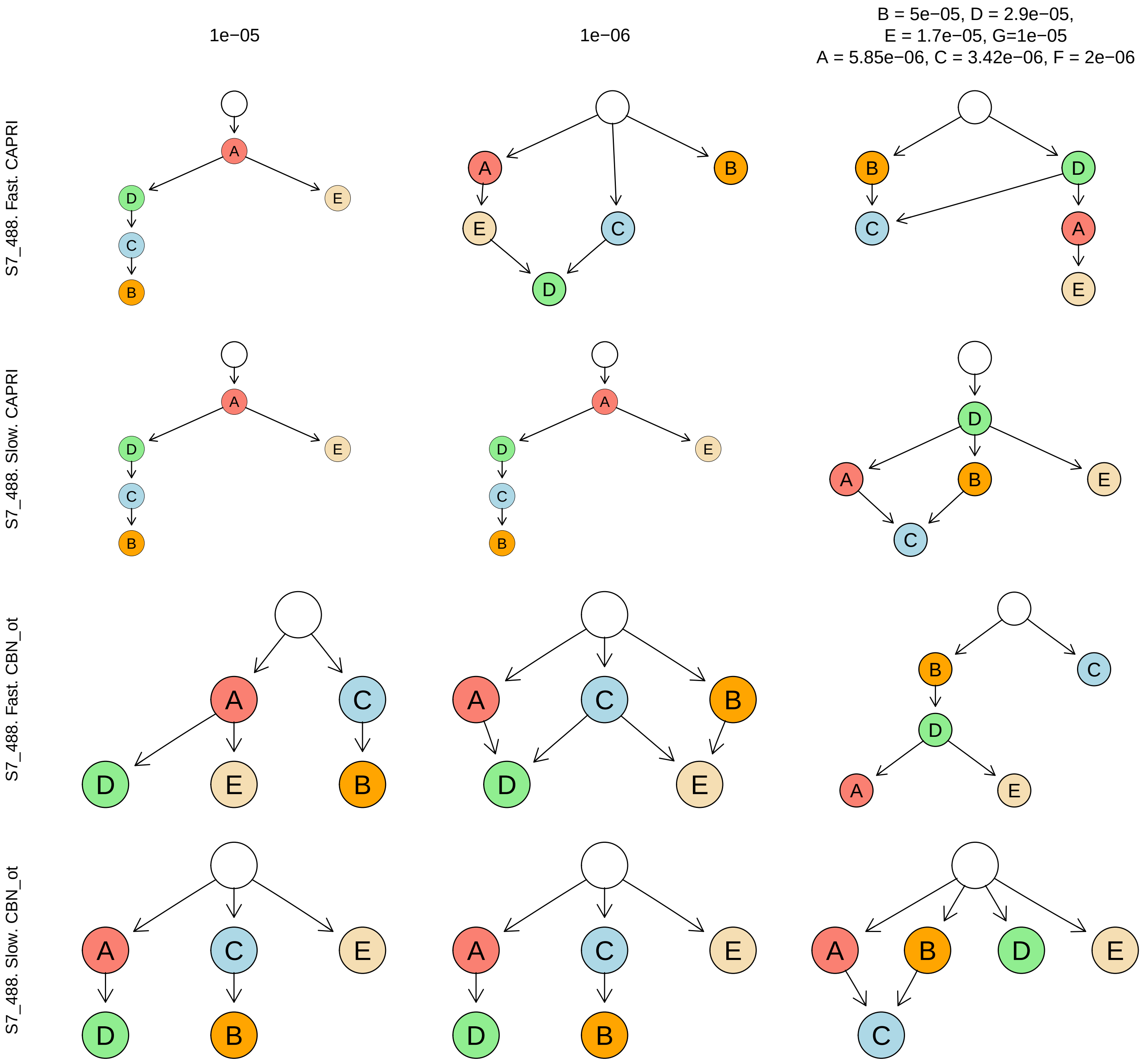

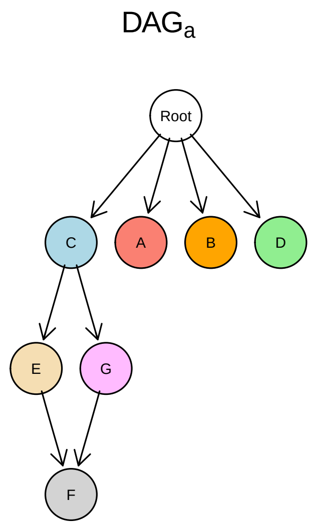

ID	Accessible Genot.	Discrep. DAG_a
S7_489	22	33

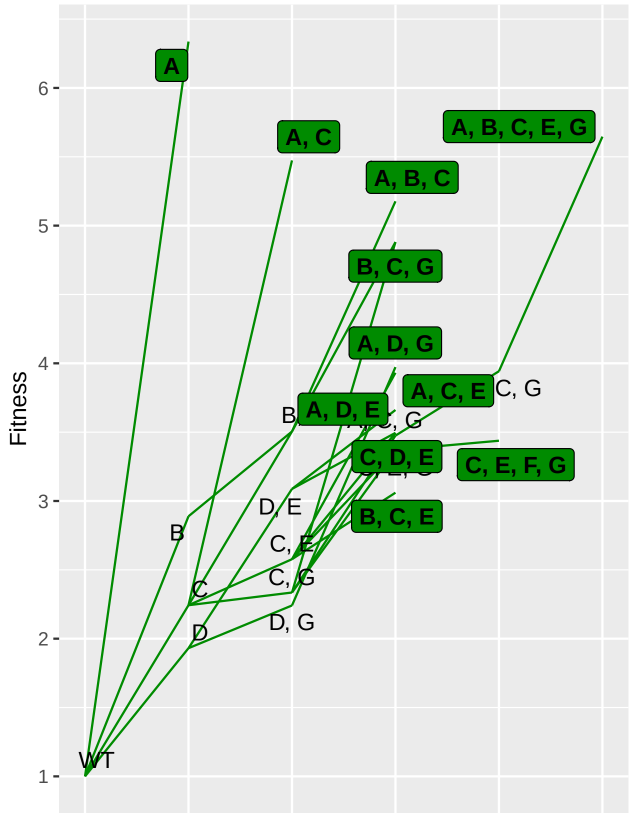

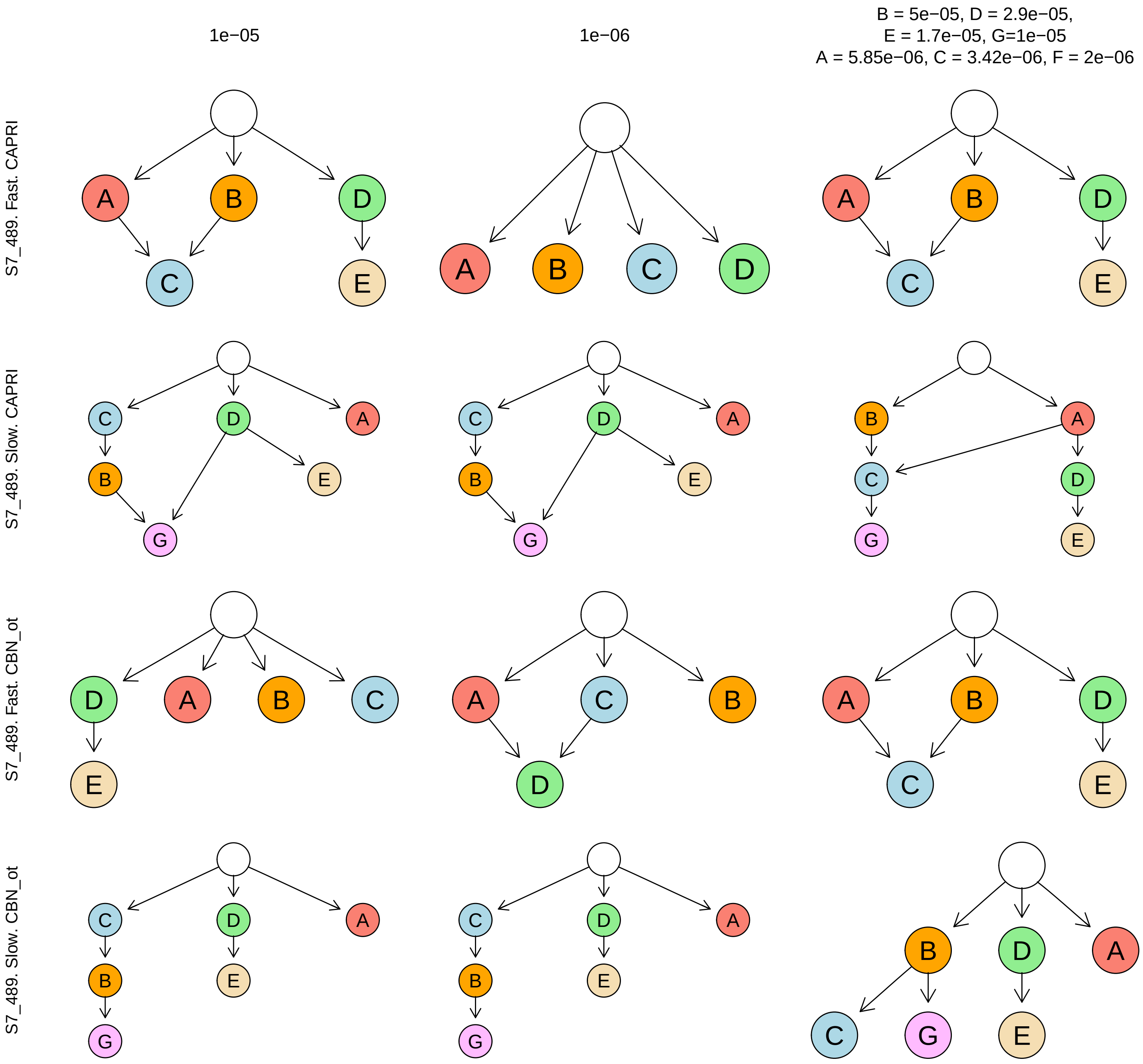

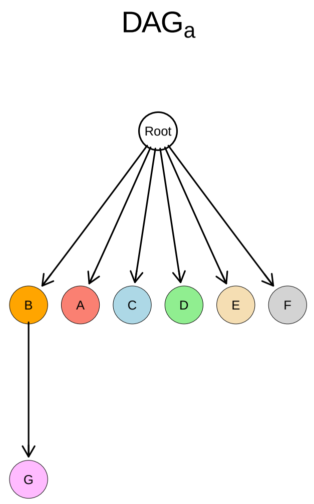

ID	Accessible Genot.	Discrep. DAG_a
S7_490	27	74

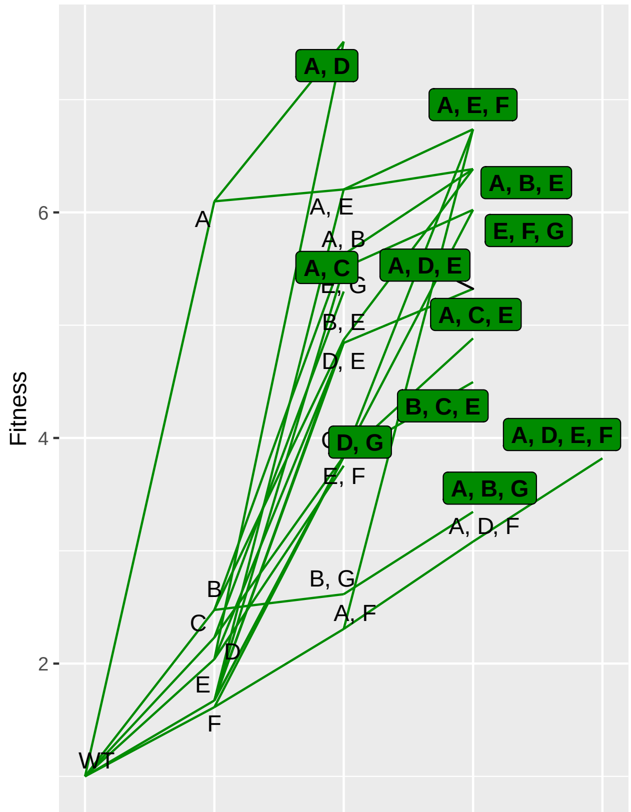

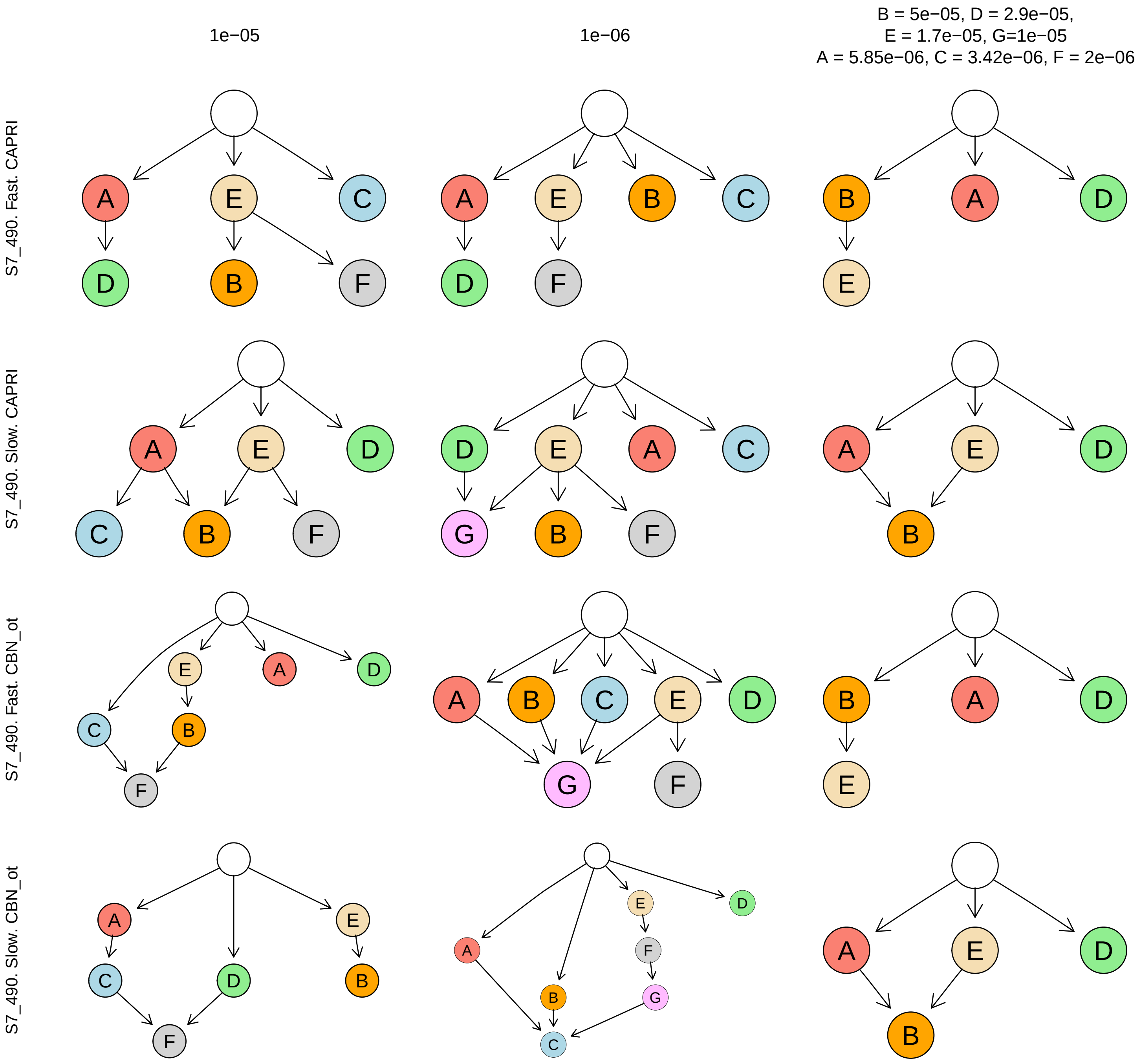

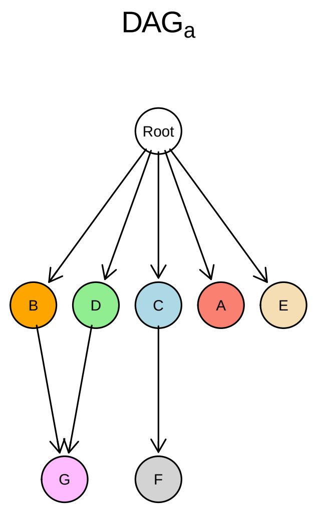

ID	Accessible Genot.	Discrep. DAG_a
S7_491	21	44

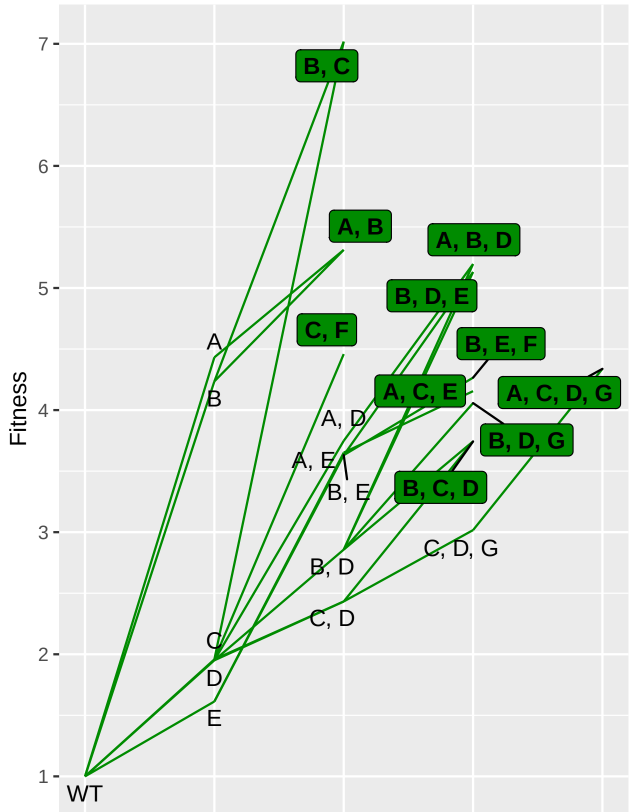

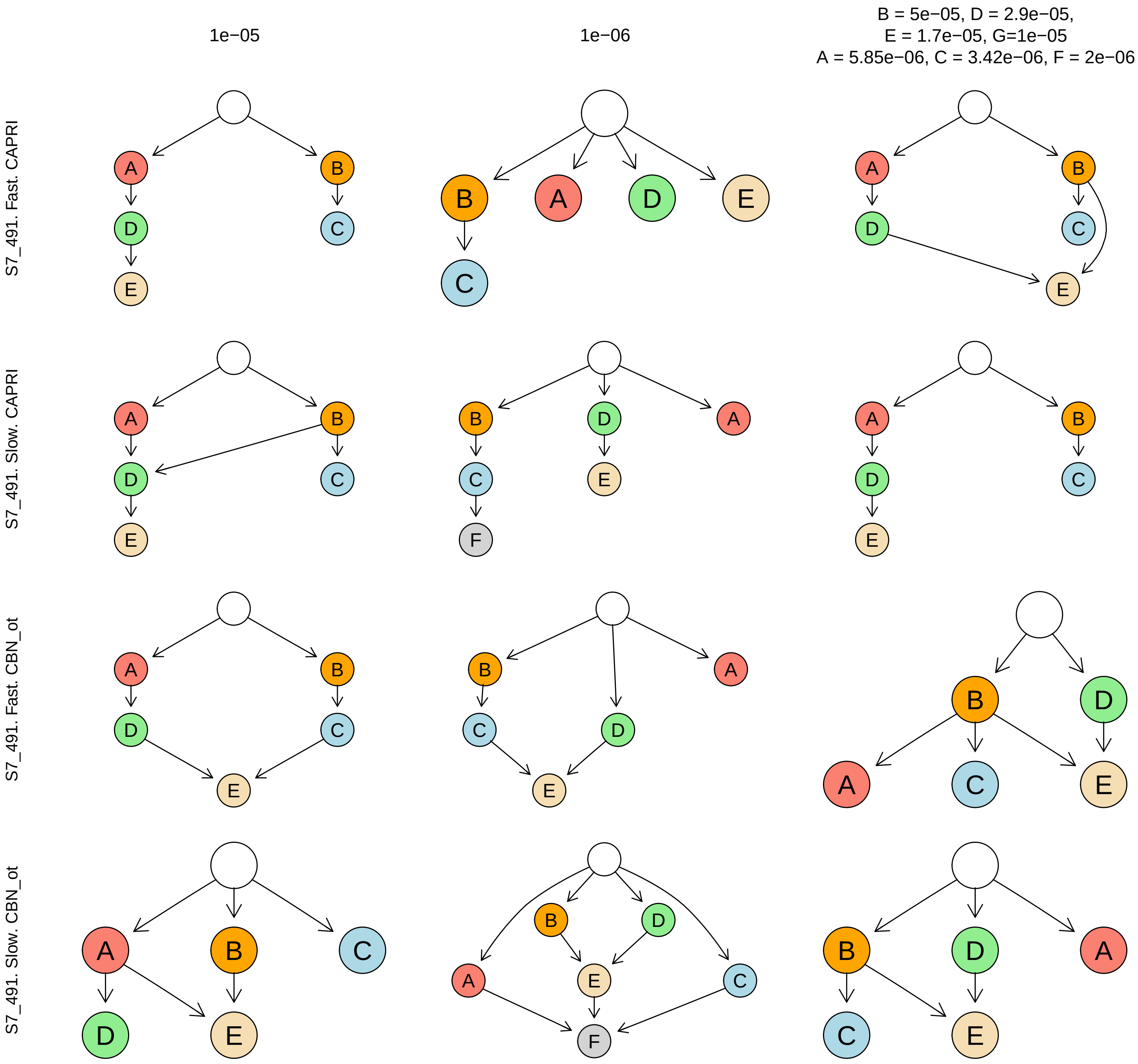

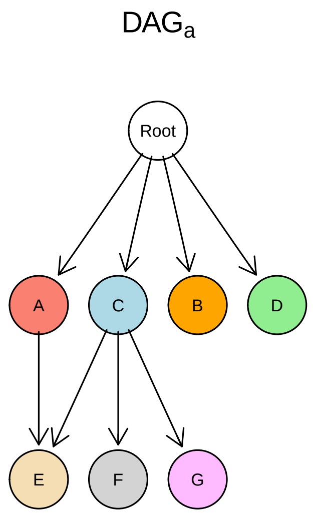

ID	Accessible Genot.	Discrep. DAG_a
S7_492	14	43

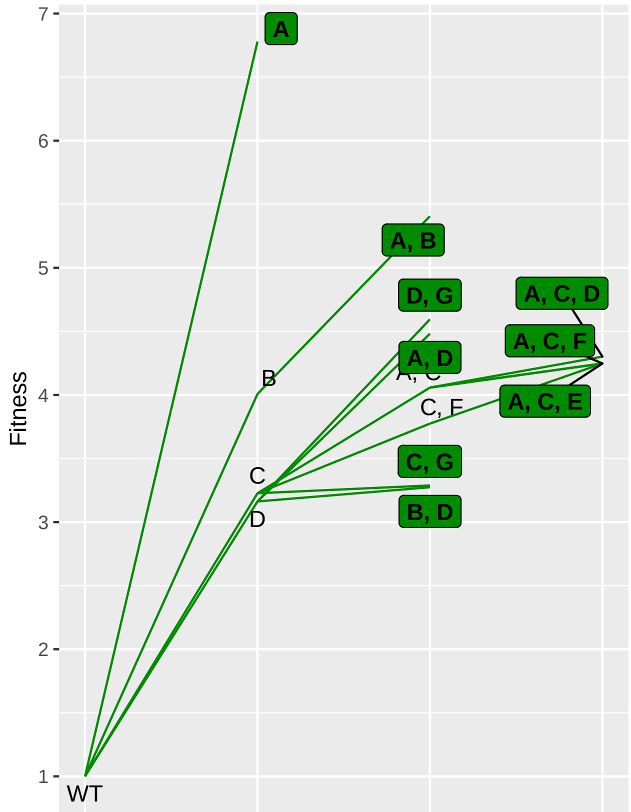

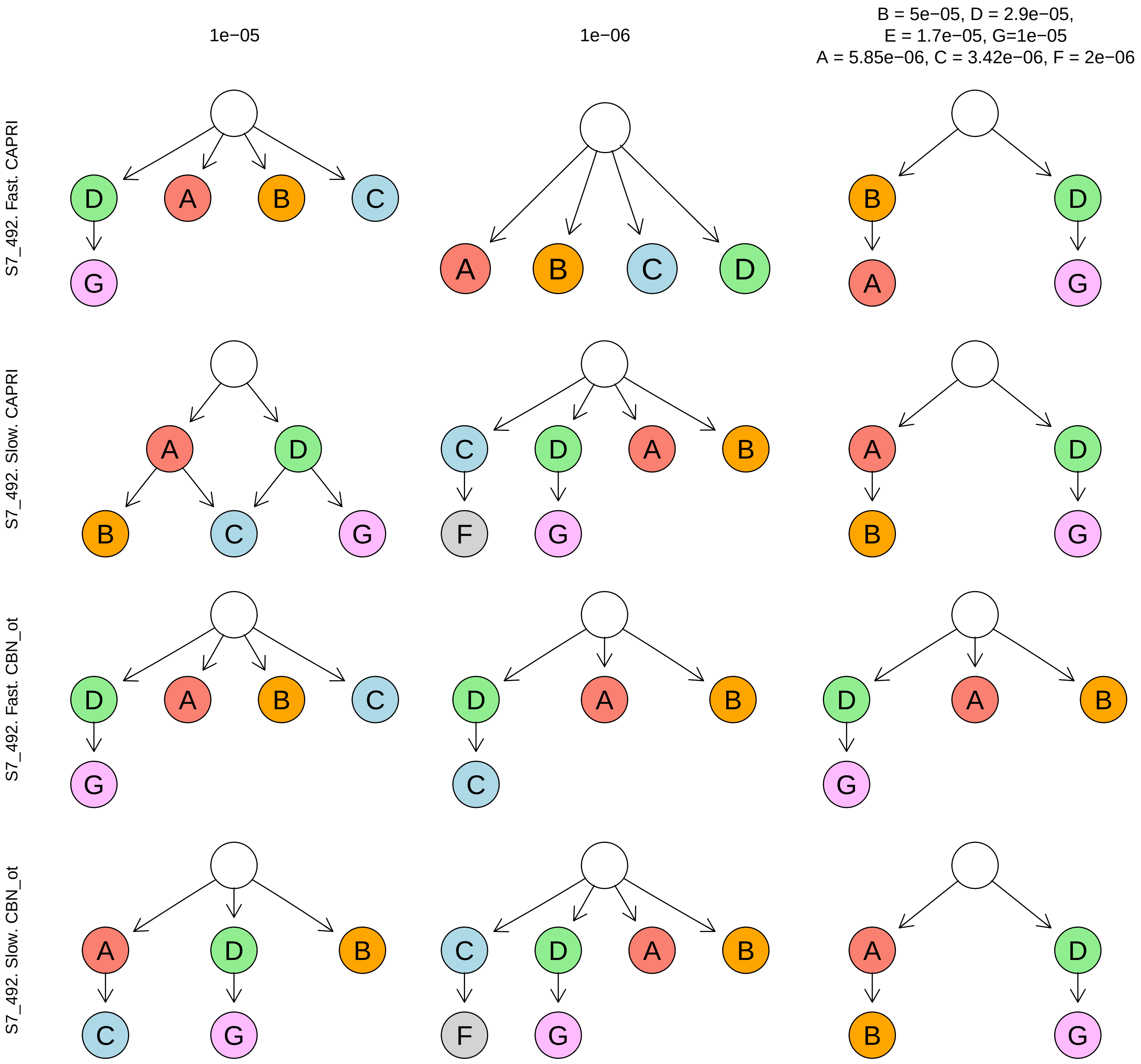

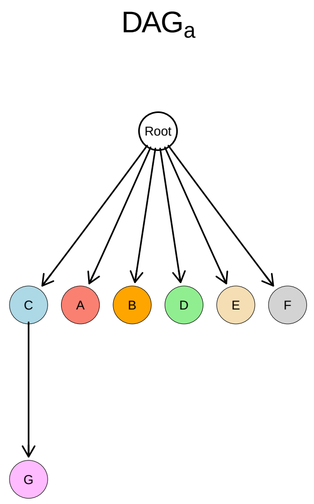

ID	Accessible Genot.	Discrep. DAG_a
S7_493	58	69

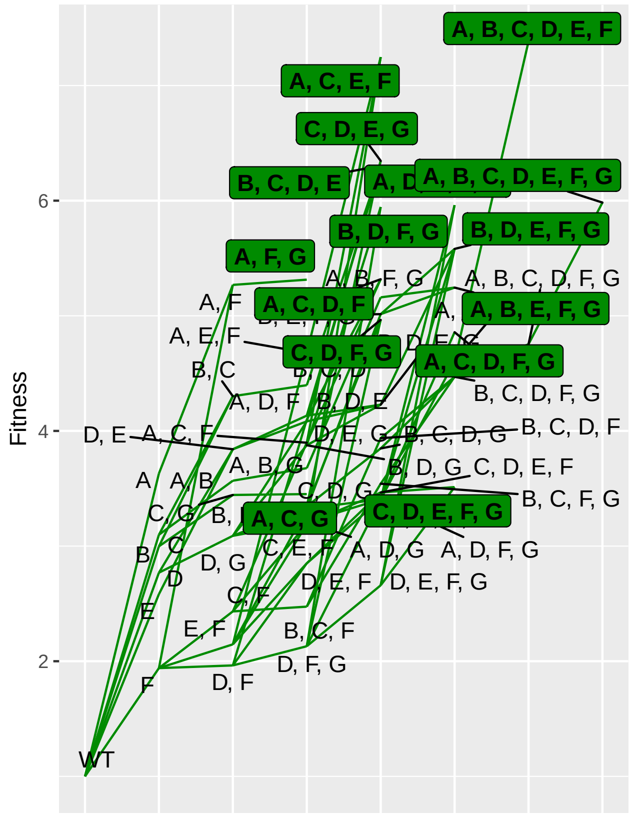

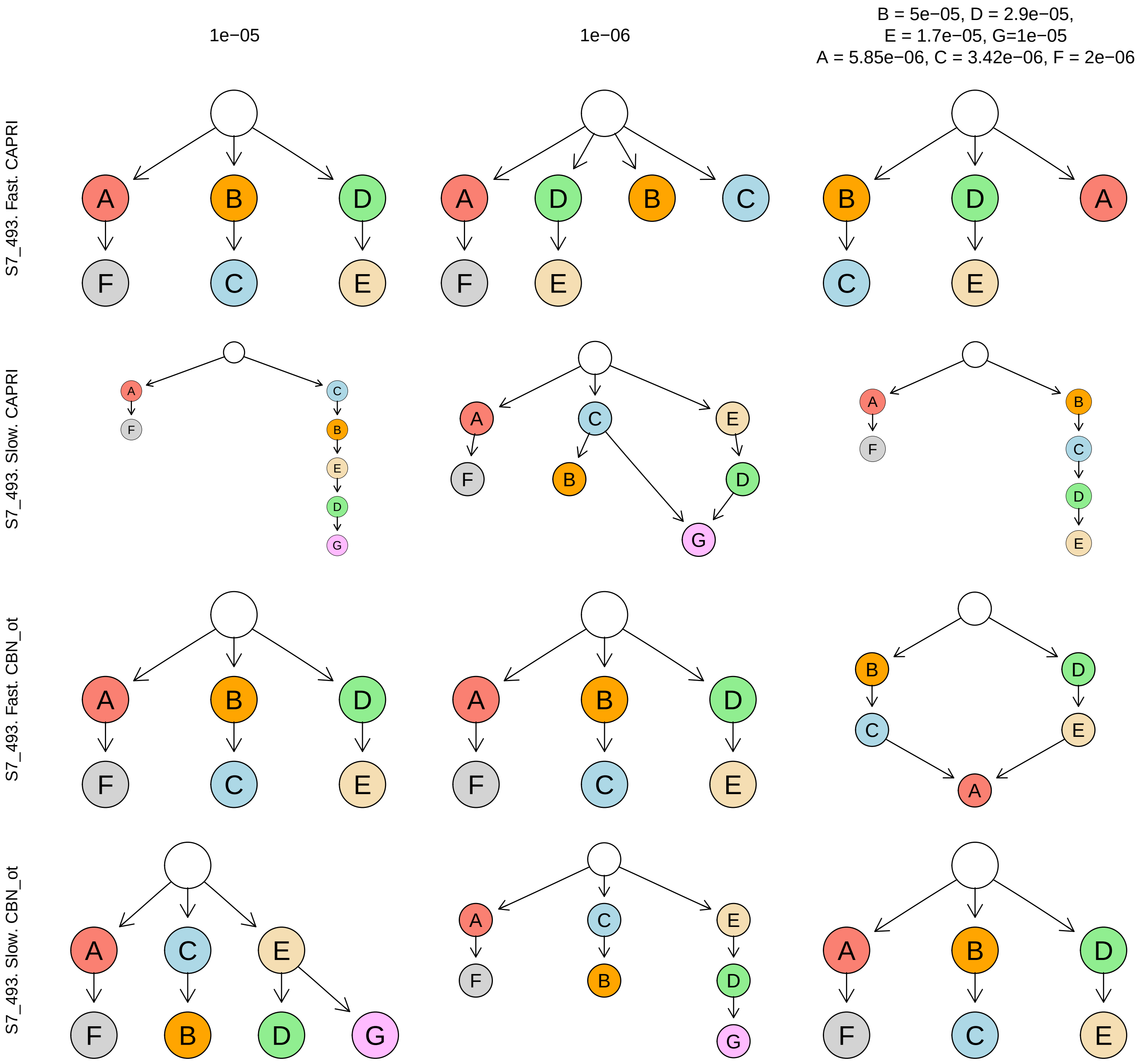

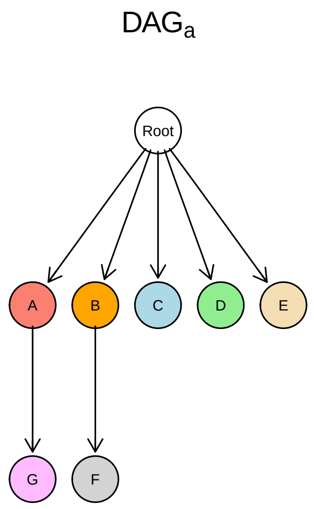

ID	Accessible Genot.	Discrep. DAG_a
S7_494	10	61

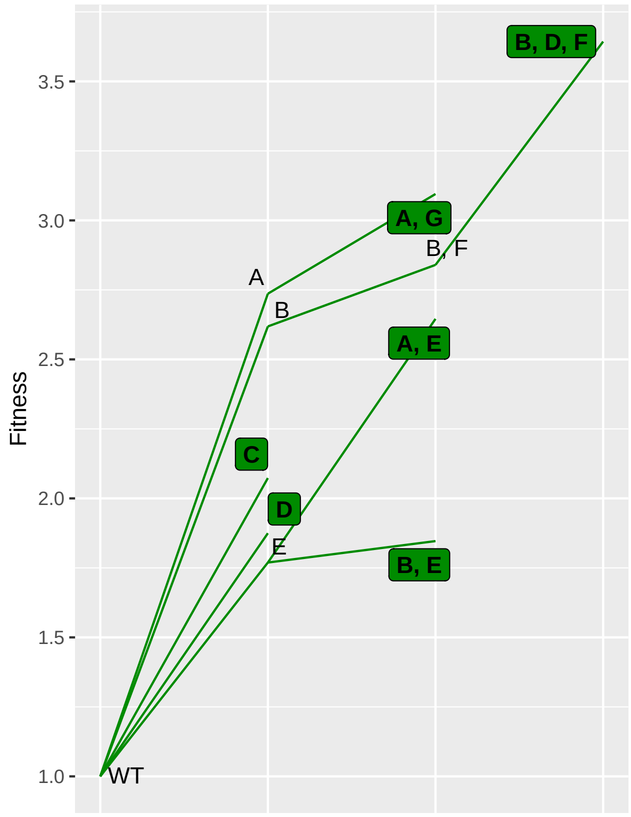

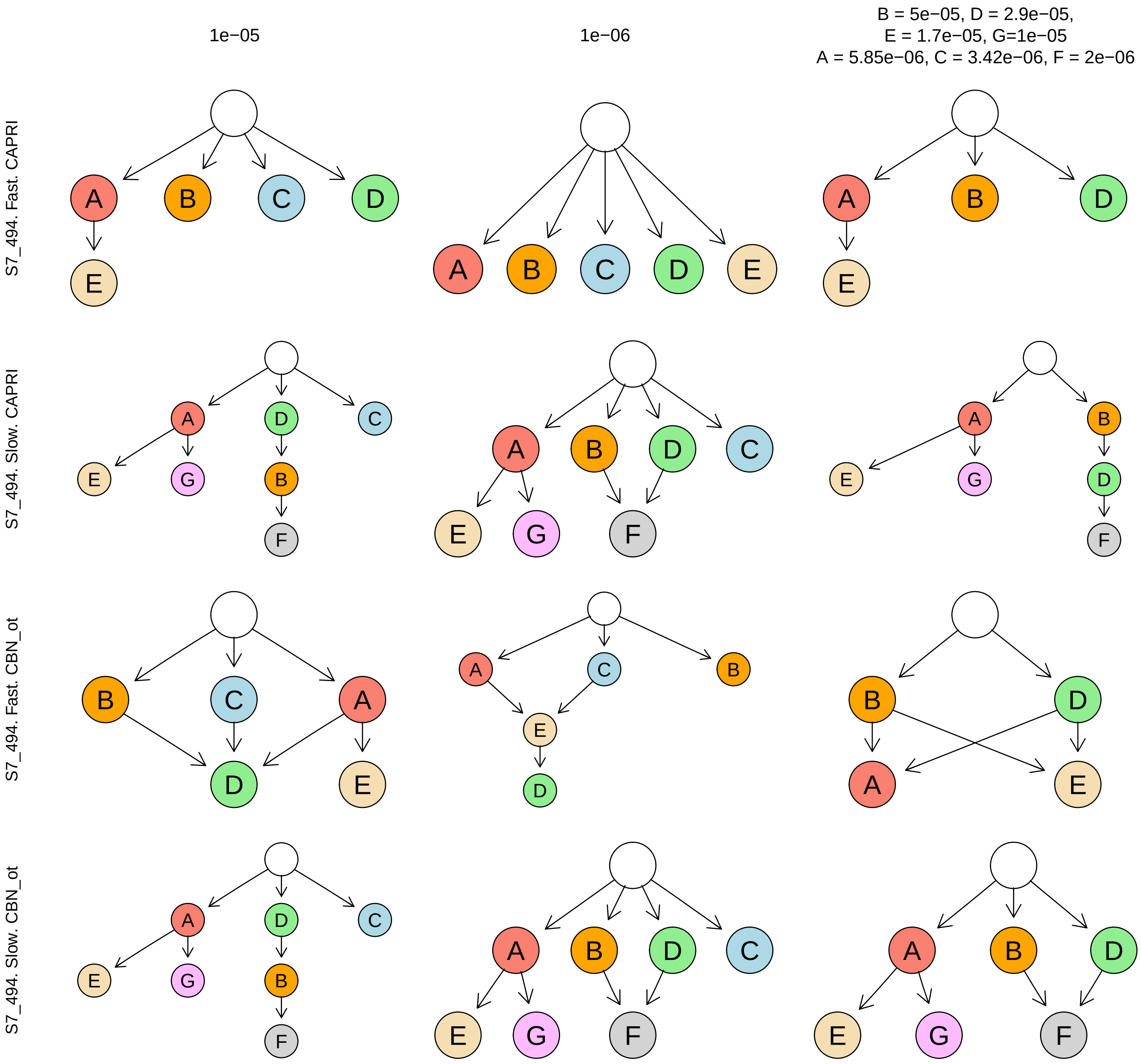

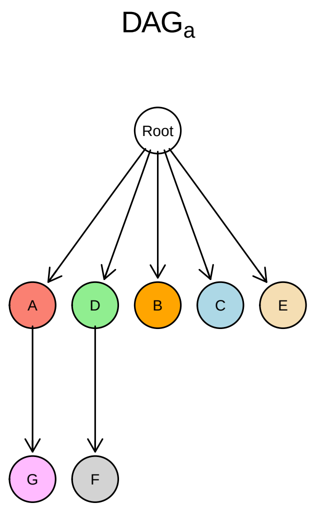

ID	Accessible Genot.	Discrep. DAG_a
S7_495	51	44

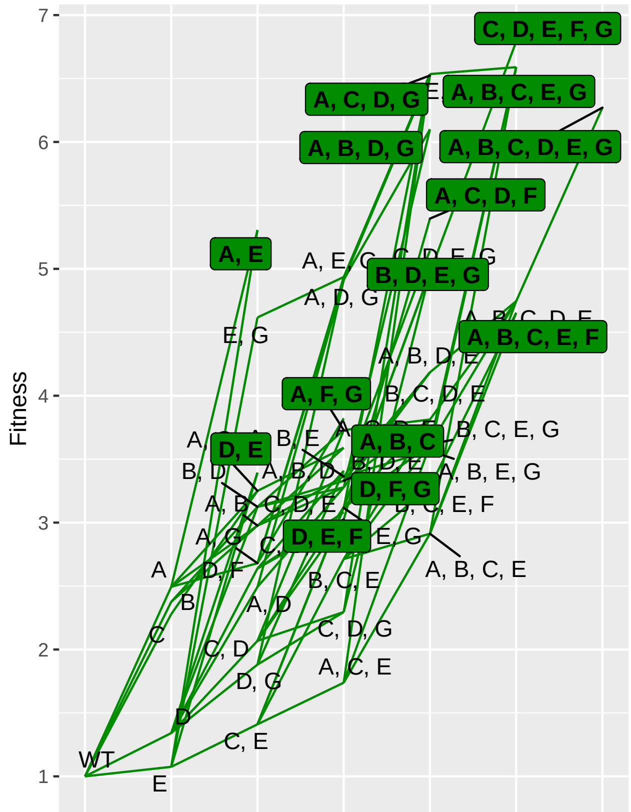

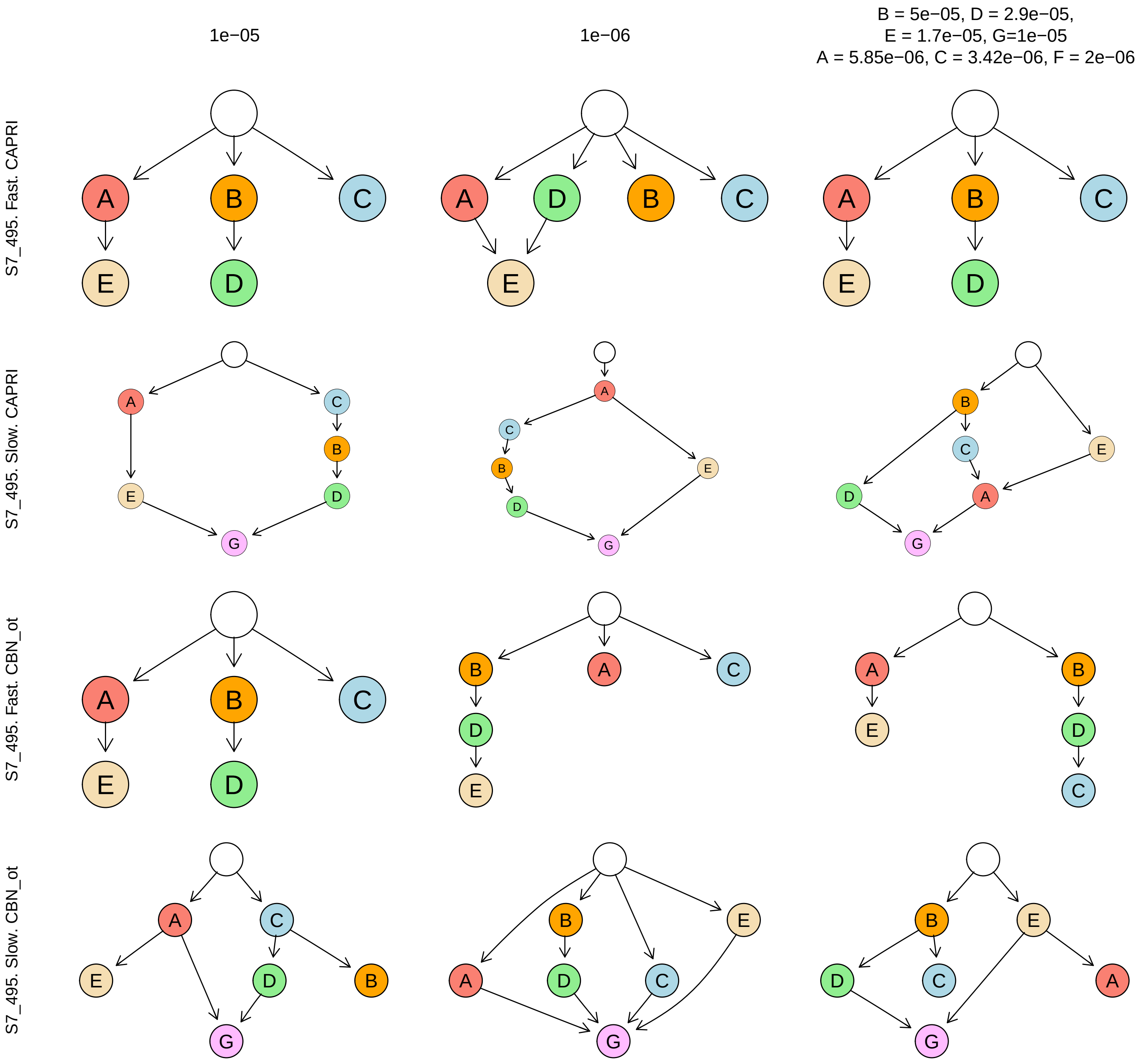

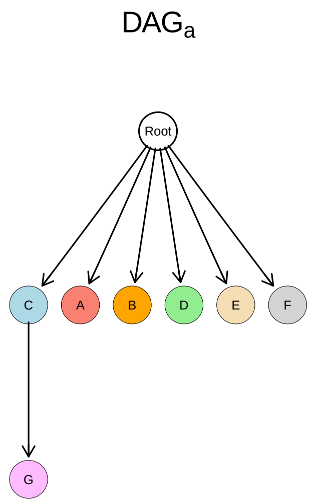

ID	Accessible Genot.	Discrep. DAG_a
S7_496	47	58

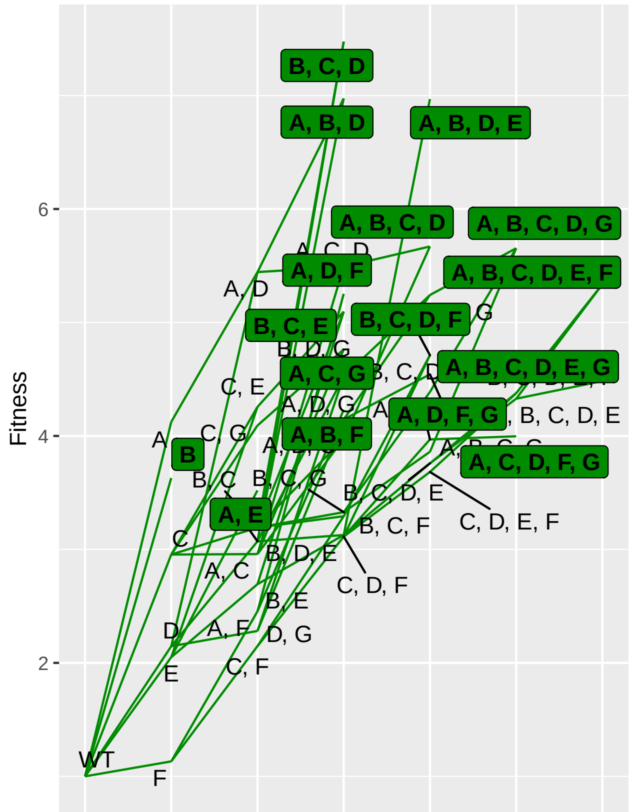

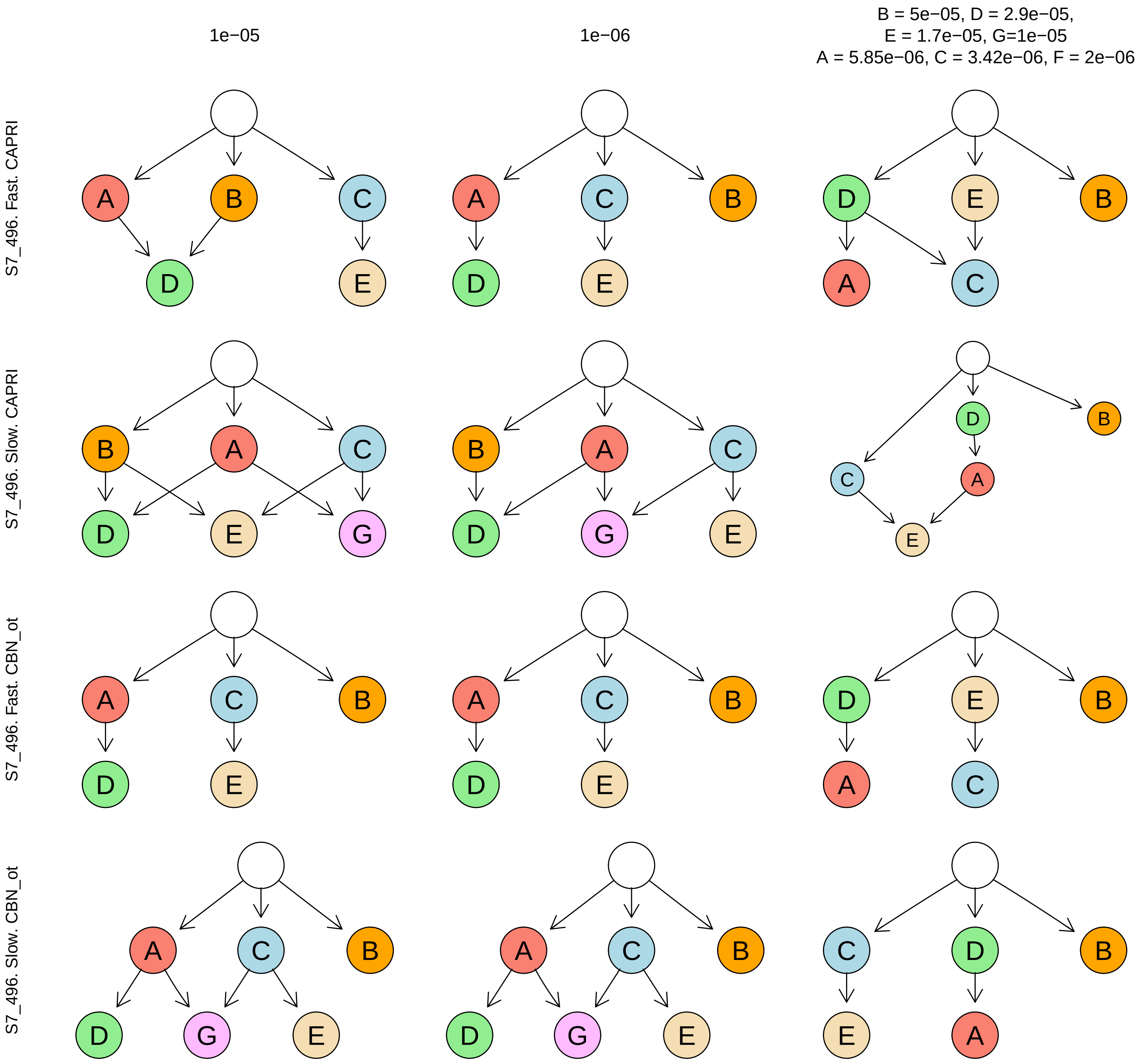

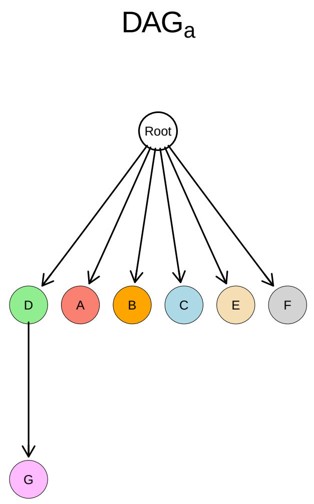

ID	Accessible Genot.	Discrep. DAG_a
S7_497	14	83

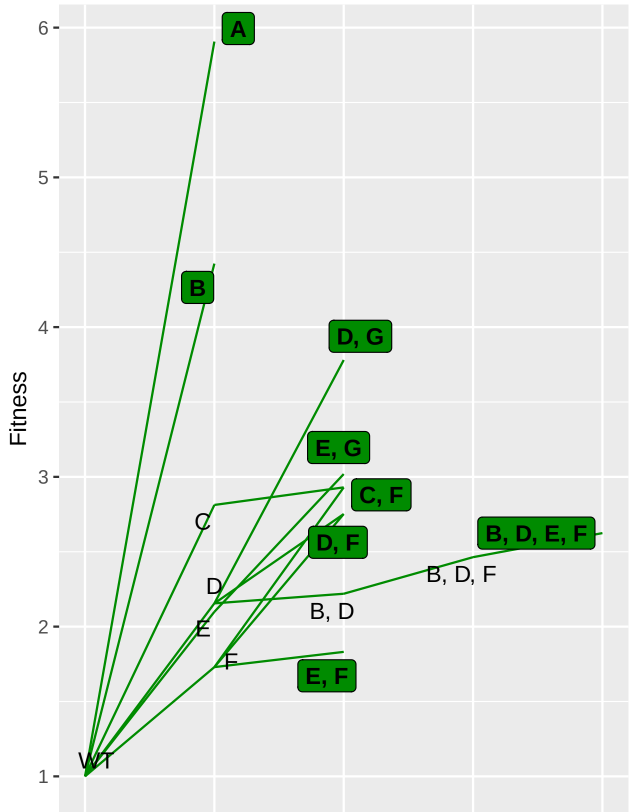

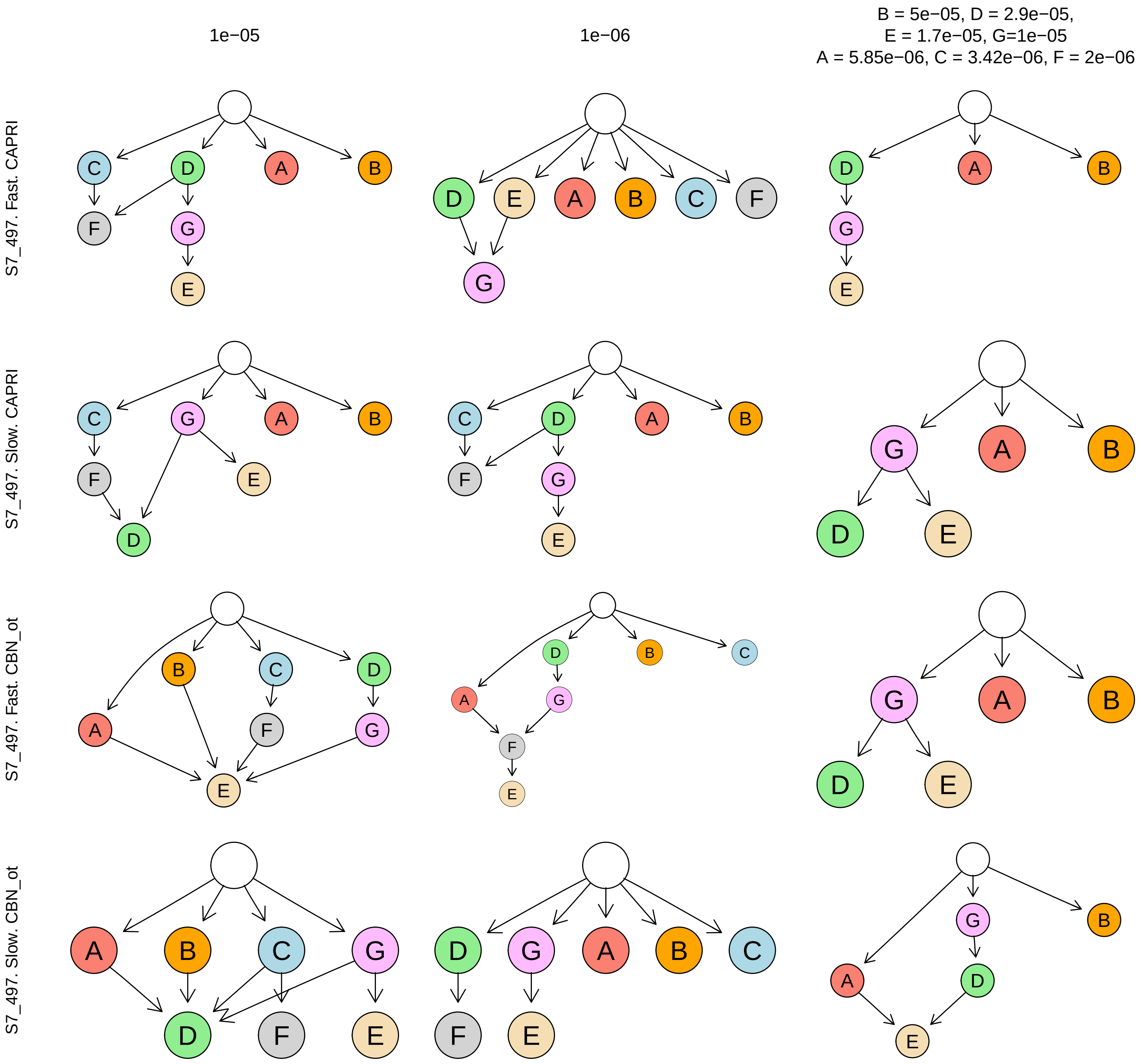

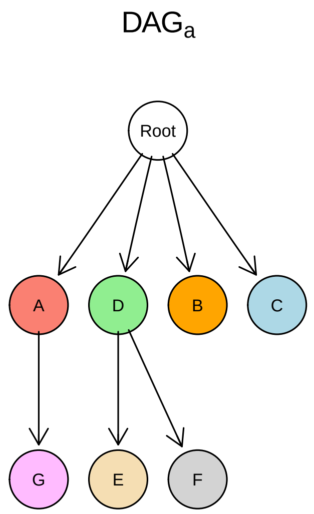

ID	Accessible Genot.	Discrep. DAG_a
S7_498	17	42

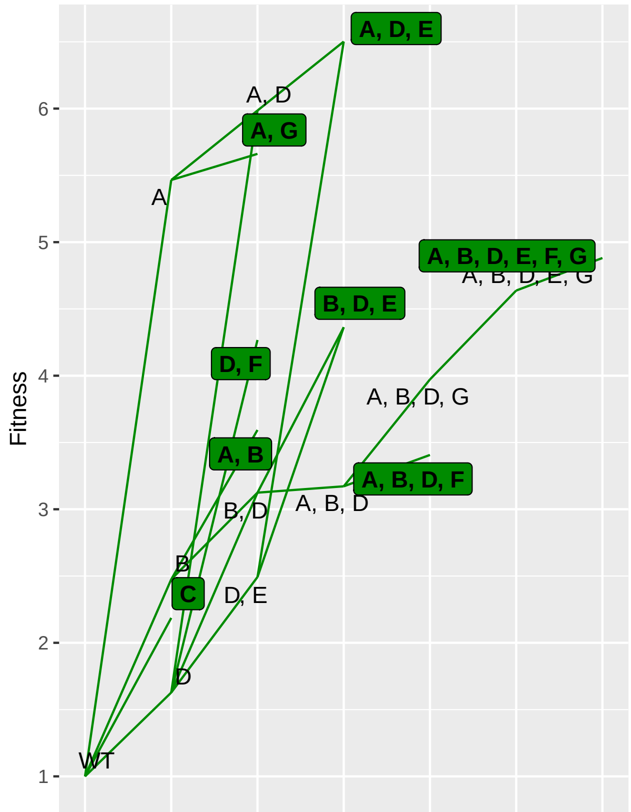

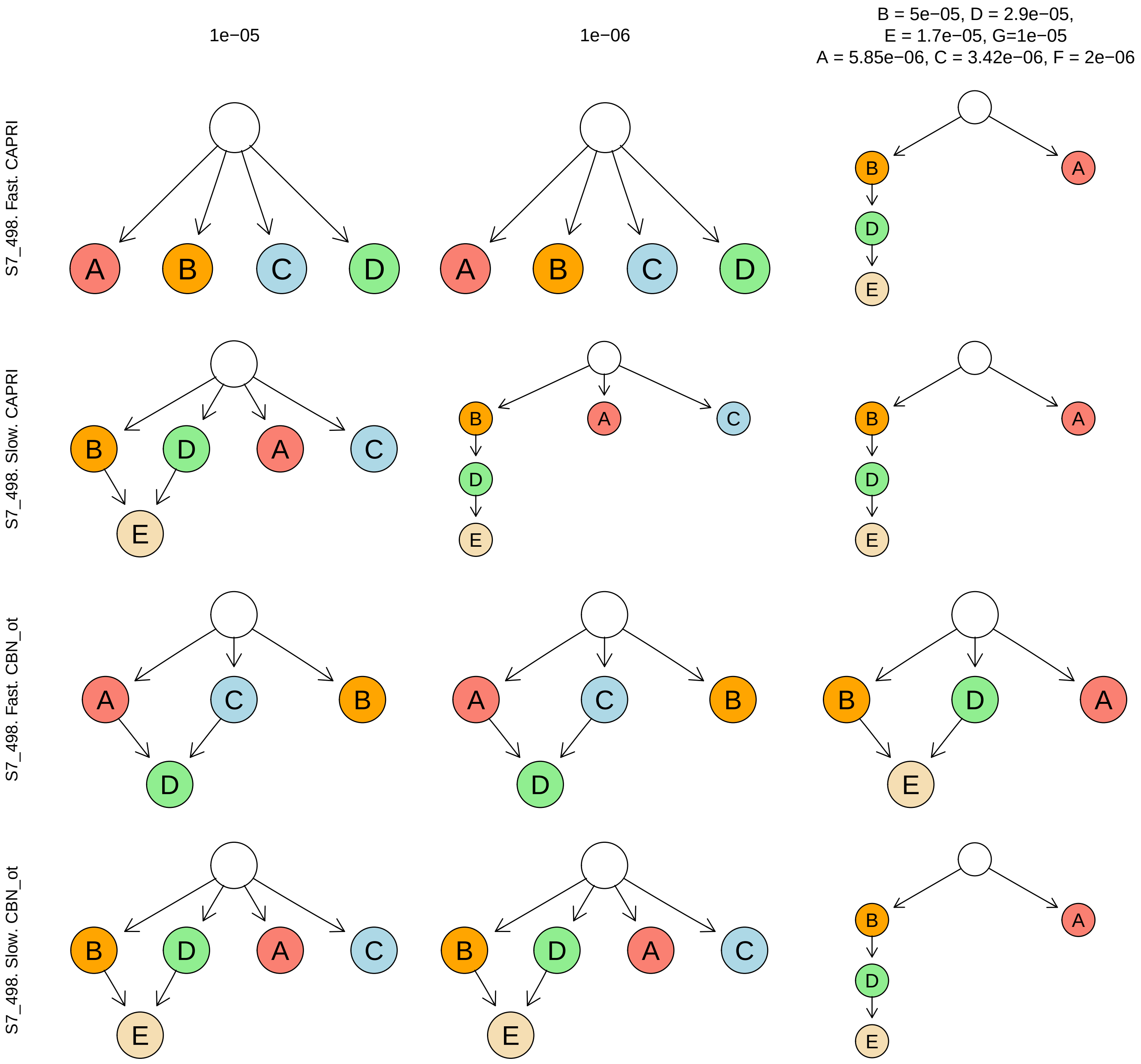

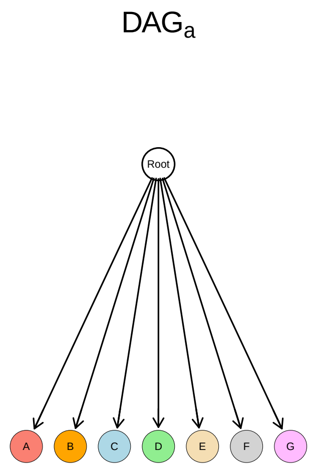

ID	Accessible Genot.	Discrep. DAG_a
S7_499	32	95

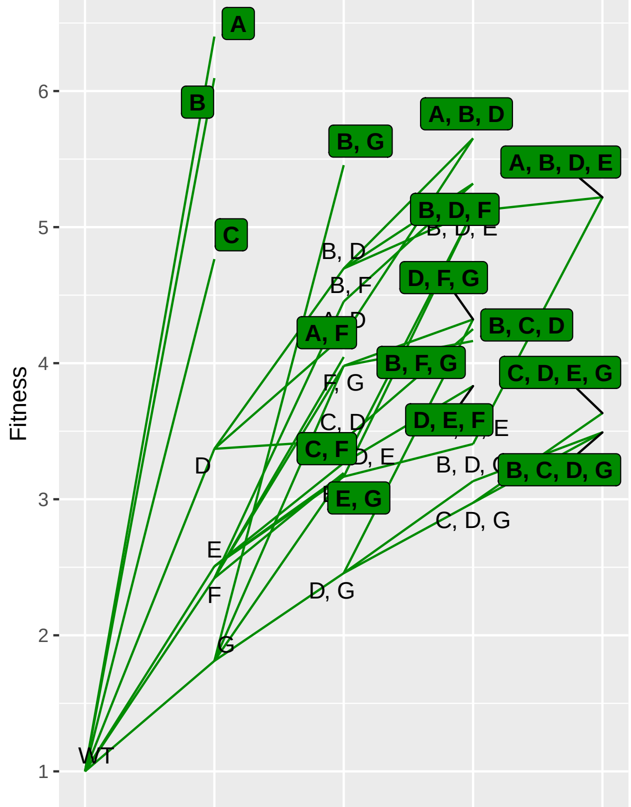

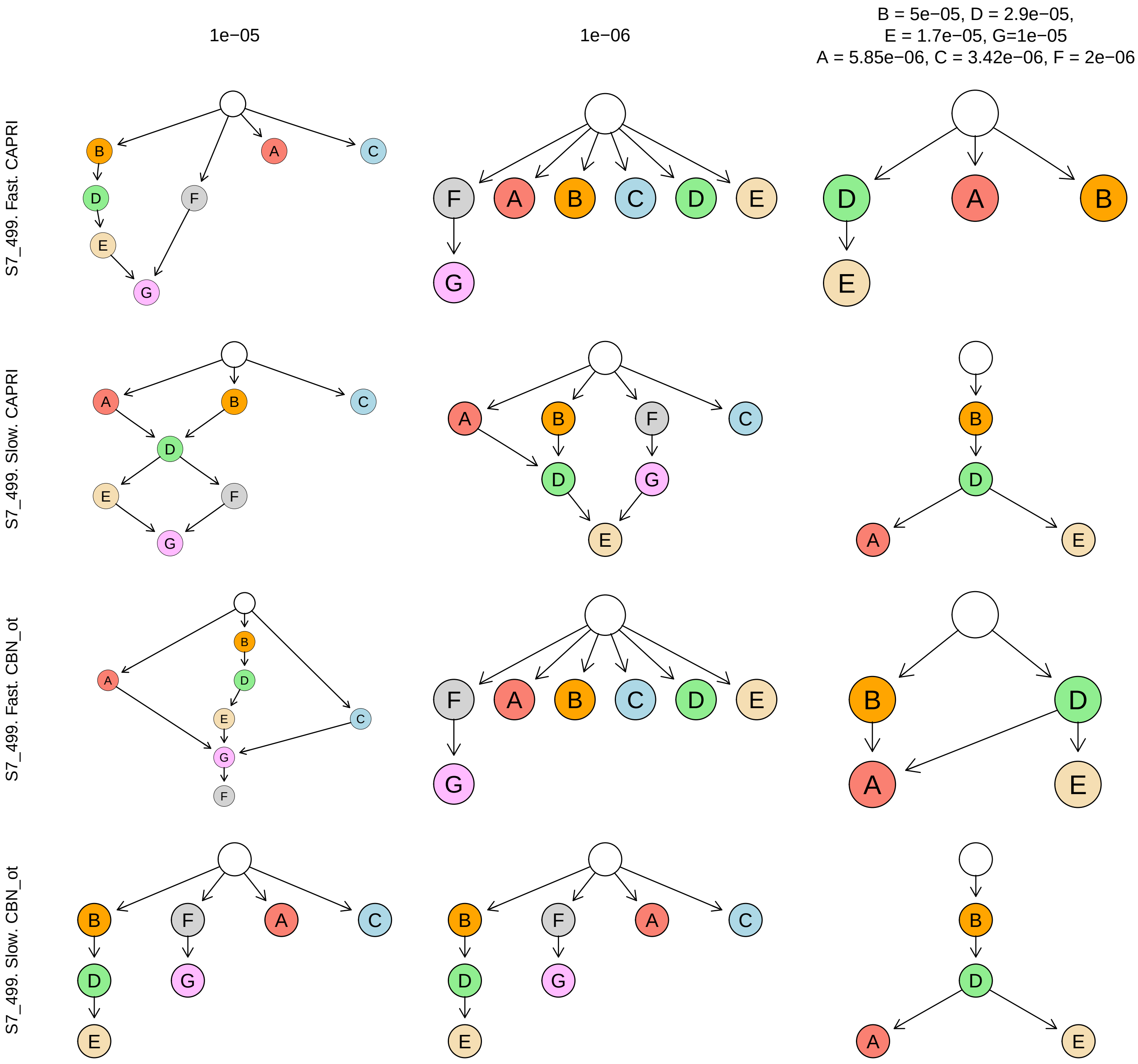

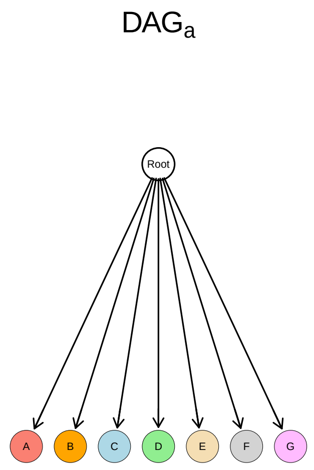

ID	Accessible Genot.	Discrep. DAG_a
S7_500	30	97

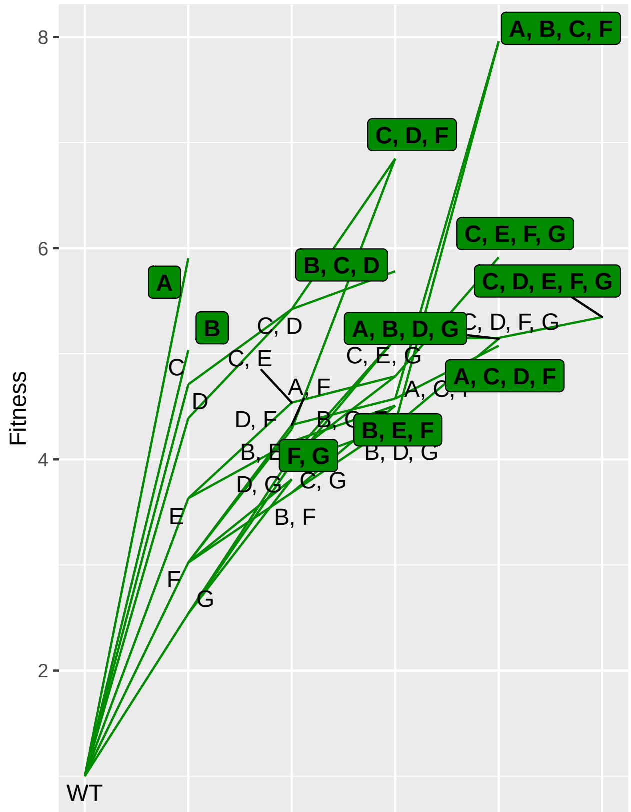

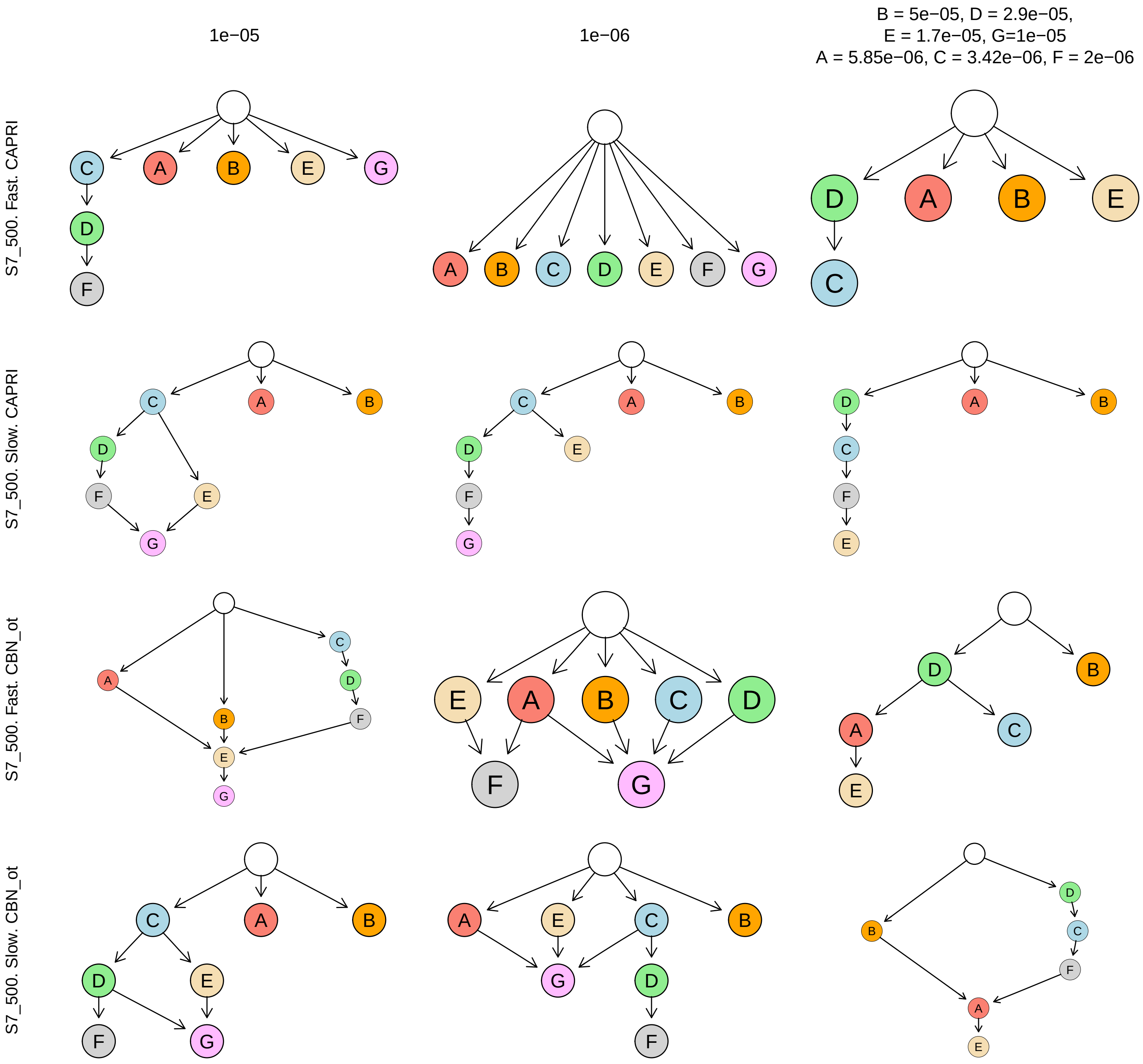

Supplement: Supplementary Data [file plots-landscapes-dags-trans-red.pdf › btx663-suppl_data/plots-landscapes-dags-trans-red.pdf]
